# Derivation of *Escherichia coli* O157:H7 from Its O55:H7 Precursor

**DOI:** 10.1371/journal.pone.0008700

**Published:** 2010-01-14

**Authors:** Zhemin Zhou, Xiaomin Li, Bin Liu, Lothar Beutin, Jianguo Xu, Yan Ren, Lu Feng, Ruiting Lan, Peter R. Reeves, Lei Wang

**Affiliations:** 1 Tianjin Economic-Technological Development Area School of Biological Sciences and Biotechnology, Nankai University, Tianjin, China; 2 Tianjin Research Center for Functional Genomics and Biochip, Tianjin, China; 3 National Reference Laboratory for Escherichia coli, Federal Institute for Risk Assessment, Berlin, Germany; 4 State Key Laboratory for Infectious Disease Prevention and Control, National Institute for Communicable Disease Control and Prevention, Beijing, China; 5 School of Biotechnology and Biomolecular Sciences, University of New South Wales, Sydney, Australia; 6 School of Molecular and Microbial Biosciences, University of Sydney, Sydney, Australia; 7 Tianjin Key Laboratory of Microbial Functional Genomics, Nankai University, Tianjin, China; American Museum of Natural History, United States of America

## Abstract

There are 29 *E. coli* genome sequences available, mostly related to studies of species diversity or mode of pathogenicity, including two genomes of the well-known O157:H7 clone. However, there have been no genome studies of closely related clones aimed at exposing the details of evolutionary change. Here we sequenced the genome of an O55:H7 strain, closely related to the major pathogenic O157:H7 clone, with published genome sequences, and undertook comparative genomic and proteomic analysis. We were able to allocate most differences between the genomes to individual mutations, recombination events, or lateral gene transfer events, in specific lineages. Major differences include a type II secretion system present only in the O55:H7 chromosome, fewer type III secretion system effectors in O55:H7, and 19 phage genomes or phagelike elements in O55:H7 compared to 23 in O157:H7, with only three common to both. Many other changes were found in both O55:H7 and O157:H7 lineages, but in general there has been more change in the O157:H7 lineages. For example, we found 50% more synonymous mutational substitutions in O157:H7 compared to O55:H7. The two strains also diverged at the proteomic level. Mutational synonymous SNPs were used to estimate a divergence time of 400 years using a new clock rate, in contrast to 14,000 to 70,000 years using the traditional clock rates. The same approaches were applied to three closely related extraintestinal pathogenic *E. coli* genomes, and similar levels of mutation and recombination were found. This study revealed for the first time the full range of events involved in the evolution of the O157:H7 clone from its O55:H7 ancestor, and suggested that O157:H7 arose quite recently. Our findings also suggest that *E. coli* has a much lower frequency of recombination relative to mutation than was observed in a comparable study of a *Vibrio cholerae* lineage.

## Introduction

*Escherichia coli* has as its primary niche the large intestine and lower part of the small intestine of mammals, larger birds and reptiles [1], [2], and has been particularly well studied in humans and domestic animals. It is a diverse species with both commensal forms and pathogenic forms, many of the latter falling into well-defined pathovars [3]. Bacterial genomes are generally divided into a core genome, comprising genes that are common to all strains of the species, and other genes, found only in some isolates, that can be referred to as the auxiliary genome, with the total for all known genomes of a species known as the species pan genome. Two recent estimates based on overlapping sets of 17 [4] and 20 [5] genomes, give 2,200 and 1,976 genes respectively in the *E. coli* core genome, out of an average of about 4,700 genes in a genome. Most genes in the auxiliary genome are found in only a small proportion of strains [5], and it is not yet possible to predict the total number of genes in the pan genome, as there is still much *E. coli* diversity to be explored. However in the most recent study [5] the pan genome for 20 strains had reached about 10,000, of which about 8,000 are in the auxiliary genome.

Genome sequence comparisons have also allowed estimation of overall recombination and mutation rates [5]. However these estimates use the coalescent framework model that gives average values for the number of sites changed by recombination and mutation, but the multiple events that mark the evolutionary process are not revealed. It is therefore important to identify directly individual recombination events, in order to study in detail the relative roles of recombination and mutation, as done recently for *V. cholerae* [6].

The O55:H7 and O157:H7 *E. coli* clones have been shown to be closely related [7], [8]. Both are multilocus sequence type 11 [9] and are very suitable for such an analysis. *E. coli* O157:H7 gained international attention as the cause of a multi-state outbreak in the USA in 1982 [10]. It belongs to the enterohemorrhagic *E. coli* (EHEC) pathovar, which carries the etiological agents for bloody diarrhea and the hemolytic uremic syndrome [11], and is the most prominent clone of this pathovar. The phylogenetic analysis by Whittam *et al.* [7] showed that O157:H7 strains are most closely related to enteropathogenic *E. coli* (EPEC) O55:H7 strains. According to their evolutionary model [8], the most recent common ancestor of today's O157:H7 and O55:H7 clones was an *E. coli* O55:H7 strain that contained the locus of enterocyte effacement (LEE) island, and presumably could elicit diarrhea via an attachment-effacement mechanism. One of the descendent lineages gained the pO157 plasmid and Stx2 and Stx1 phages, changed its O antigen, and also lost its ability to ferment sorbitol (SOR-) or to express beta-glucuronidase (GUD-). These changes occurred through a series of transitional steps that finally gave rise to today's O157:H7 clone [8].

In this paper we present the complete nucleotide sequence of *E. coli* O55:H7 strain CB9615, and compare it to available genome sequences of *E. coli* O157:H7. We identify individual recombination and mutation events, and assess the relative contributions of recombination and mutation in generating single nucleotide polymorphisms (SNPs). We were able to allocate most of the differences observed to specific lineages, giving us the frequency of events involved in the derivation of the extant clones from their common ancestor. Some interesting changes in gene content were found, including in type II secretion system (T2SS) genes and some potential virulence genes. The details of the recombination event that replaced the O-antigen gene cluster and deleted the ribitol utilization gene cluster were revealed.

We find that the average rate of mutation (synonymous substitutions only) is nearly 50% faster in the O157:H7 lineage than in the O55:H7 lineage. If we assume that the average clock rate is similar to that estimated for *V. cholerae* [6], we find that the two lineages diverged about 400 years ago, and the two O157:H7 strains EDL933 and Sakai for which the genome sequences are known, diverged much more recently. We then applied the same approaches to a group of extraintestinal pathogenic (ExPEC) *E. coli* genomes that are also closely related to each other, and compared their levels of variation to those in the O55:H7/O157:H7 lineages.

This study gives us a better insight and understanding of the evolution of the O157:H7 clone. A genome scale study of an O55:H7 strain and six O157 strains [12] was published after we had completed our analysis, but there is little overlap in the conclusions, as it was based on sequences of those individual ORFs that are shared by 2 or more of the strains used. It provided detail of the relationships of the strains involved, whereas our study provides detail of the events involved in the major divergence between the O55:H7 and O157:H7 lineages. Where there is overlap the 2 studies are in agreement.

## Results and Discussion

### General Features of the Genome

The genome of *E. coli* O55:H7 strain CB9615 comprises a circular chromosome (5,386,352 bps) and a plasmid (pO55; 66,001 bps) (Table 1). The chromosome contains 5,097 predicted protein-encoding genes (including 69 pseudogenes; Table S1), which is slightly smaller than the genomes of Sakai and EDL933 with 5,253 and 5,324 genes, respectively, but still makes it one of the larger *E. coli* genomes. There are also 100 tRNA genes, and seven rRNA operons in the chromosome.

**Table 1:** General features of the chromosome and plasmid of *E. coli* CB9615.

	Chromosome	pO55	Total
length (bp)	5,386,352	66,001	5,452,353
G+C ratio (%)	50.52	48.87	50.5
open reading frame (ORF)	5028	109	5,137
protein coding region (% of genome size)	87	84.4	87
average ORF length (bp)	923.2	511	914.5
pseudogene	69	-	69
rRNA (16S-23S-5S)	7	0	7
tRNA and tmRNA	101	0	101
ncRNA	52	0	52

### Distinguishing Recombination and Mutation Events

**Figure 1:**
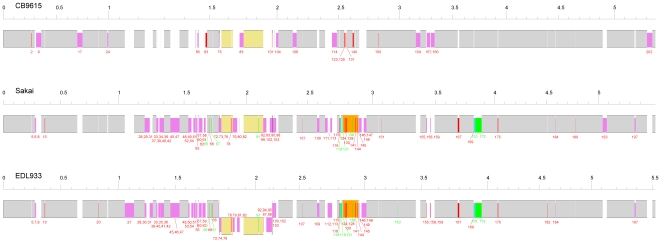
Alignment of the genomes of CB9615, Sakai and EDL933. Scales are Mbp. The grey and yellow shaded regions represent segments present in all strains, with inverted segments shaded in yellow. Purple and cyan boxes within the grey or yellow regions represent the indels of phages or phage-like elements (insertions and deletions respectively) defined in Table S9. Red and green boxes represent other major indels (insertions and deletions respectively) that involve the changes of the gene numbers in Table S10, with the indel numbers also shown as defined in Table S9. The orange boxes indicate the *E. coli* O157:H7 O-antigen segment gained by recombination. Figure S1 is a greatly expanded version of this figure showing individual genes.

**Figure 2:**
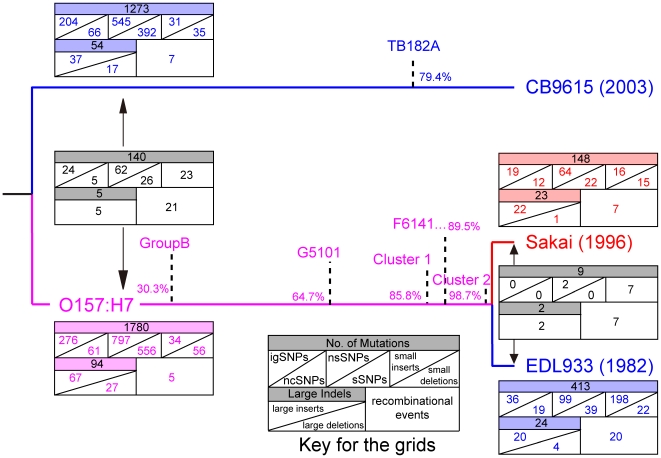
Tree showing the relationships of CB9615 and 2 O157:H7 strains. The tree topography is taken from the alignment of 26 completed genomes (Figure 3) and Whittam [7]. For each lineage the number of mutations (including small indels), recombination events and insertion or deletion events (large indels) are shown in a grid, as specified with the key. Mutations are shown as intergenic, other non-coding, non-synonymous or synonymous SNPs (igSNPs, ncSNPs, nsSNP, sSNPs), small insertions and small deletions or indels if not differentiable. Large indels are separated into insertions or deletions where possible. Events allocated to the divergence between CB9615 and O157:H7, or between Sakai and EDL933, respectively, but not to either lineage, are shown in the grids between the two lineages. The branch point estimates for group B [12] including strain 493-89), G5101 and F6141, and clusters 1 (Strains 14359 and 87-14) and 2 (86-24) are marked with dotted lines on the O157:H7 lineage, and TB182A on the O55:H7 lineage. The distribution of SNPs along that lineage is based on reanalysis of data from Zhang *et al.* [17] and Leopold *et al.* [12].

We compared the CB9615 genome with the two published O157:H7 genomes and generated an alignment as described in Materials and Methods, and shown in Figures 1 and S1. We were also able to define putative recombinant segments, as done previously [6], [13], using the very different distribution of SNPs introduced by recombination from those arising by mutation (see Materials and Methods). These segments are shown in Figure S1 and summaries of both mutational and recombinational changes are shown in Figure 2 and Tables 2 and S2. Previous estimates of the level of recombination in *E. coli* used statistical approaches that give only an average value, as for example the recent estimates by Touchon *et al.* [5] using the coalescent framework model [14], which gives average values for recombination and mutation. It also uses a model for the population which is not really applicable, with McVean *et al.* [14] cautioning that “coalescent estimation of likelihoods assumes that a random sample has been taken from a population of constant size, with random mating, no migration to or from different populations, and no natural selection”. While such methods have been useful in showing that recombination plays a significant role, they are not suited to giving a reliable measure of variation between sub-populations of the species, or detail of individual events during divergence. However it must be pointed out that while the criteria that we use to distinguish segments that we identify as recombinant are mostly easily applied, and the differences easily observed in Figure S1, it is still a hypothesis that the distinctions are due to recombination. We use this distinction in much of what follows and it is important to bear in mind the basis for defining the putative recombinant segments.

**Table 2:** Summary of mutational and recombination changes in the 3 O55:H7/O157:H7 genomes.

Lineage^a^	Mutational SNPs^bc^									Recombination events and recombination related SNPs											
										Recombination events				Recombination related SNPs^c^							
	NS	S	I	NC	ins	del	indel	total	No. genes^d^	No. events	% genome involved	Average and range of divergence (%)^e^	No. Genes	NS	S	I	NC	ins	del	indel	total
CB9615	545	392	204	66	31	35	-	1273	838 (486)	7	0.02%	6.94 (3.08∼8.67)	10	61	44	4	14	0	1	-	124
O157	797	556	276	61	34	56	-	1780	1145 (690)	Total: 5^f^	2.01%	3.24 (3.22∼ 27.91)	92	744	1839	259	557	18	12	-	3429
										Other: 4^g^	0.01%	8.60 (3.23∼27.91)	4	20	17	0	0	0	0	-	37
										Rseg: 1^h^	2.00%	3.22	88	724	1822	259	557	18	12	-	3392
Sakai	64	22	19	12	16	15	-	148	109 (61)	7	0.70%	0.47 (0.12∼8.90)	11	19	10	9	26	0	1	-	65
EDL933	99	39	36	19	198	22	-	413	181 (82)	20	1.56%	1.10 (0.12∼78.63)	56	196	34	148	47	26	3	-	454
O55/O157	62	26	24	5	-	-	23	140	66 (44)	21	0.48%	2.91 (0.53∼24.39)	38	165	253	61	134	-	-	13	626
Sakai/EDL933	2	0	0	0	-	-	7	9	8 (2)	7	1.25%	2.70 (0.69∼9.98)	28	218	54	9	64	-	-	5	350
Total	1569	1035	559	163	279	128	30	3763	1897(1193)	67	-	2.56 (0.12∼78.45)	223	1403	2234	490	842	44	17	18	5048

a The CB9615, EDL933 and Sakai lineages are the strain specific lineages as shown in Figure 1. The O157 lineage is the segment of the O157:H7 lineage prior to divergence of EDL933 and Sakai. Events shown in the O55/O157 and Sakai/EDL933 rows are those allocated to the O55/O157 and Sakai/EDL933 divergence respectively, but not to a specific lineage.

b Excludes SNPs in regions thought to have entered by recombination.

c NS, non-synonymous; S, synonymous; I, intergenic; NC, in non-coding genes; ins, insertion; del, deletion; indel, insertion or deletion (not distinguishable).

d The number in brackets is number of genes carrying at least 1 non-synonymous SNP.

e Covers only recombinant regions longer than 20 bps.

f Includes 3388 SNPs in the large recombinant event involving the O-antigen gene cluster.

g Excludes the 3388 SNPs in the large recombinant event involving the O-antigen gene cluster.

h The 3388 SNPs in the large recombinant event involving the O-antigen gene cluster.

### Allocation of Mutations to Specific Lineages

We were able to allocate mutational differences between the two O157:H7 clones (Sakai and EDL933) to specific lineages using CB9615 as outgroup, but there was no closely related genome to use as an outgroup for the divergence between CB9615 and the O157:H7 common lineage. However most of the mutational differences could be allocated to one or other lineage by the virtual outgroup approach as described in Materials and Methods, using a panel of 23 genomes (Tables 3, S3, and S4). The allocation to lineages is shown in the alignment in Figure S1, and the tree in Figure 2. The tree has 4 branches, for the CB9615 lineage, the joint O157:H7 lineage, and the separate Sakai and EDL933 lineages.

**Table 3:** Principal characteristics of the 28 *Escherichia coli*/*Shigella* strains.

Name^a^	additional information	Serotype	Clinical condition (Pathotype^c^)	GenBank accession	Genome sequence reference
K-12	K-12 MG1655	O16^b^	Commensal	U00096	[67]
	K-12 W3110	O16^b^	Commensal	AP009048	[68]
	K-12 DH10B	O16^b^	Commensal	CP000948	[69]
	K12 BW2952	O16^b^	Commensal	CP001396	[13]
HS		O9	Commensal	CP000802	[4]
ATCC 8739		O146	Commensal	CP000946	
IAI1		O8	Commensal	CU928160	[5]
**CB9615**		**O55:H7**	**Diarrhoea (EPEC)**	**CP001846**	**this work**
EDL933		O157:H7	Diarrhoea (EHEC)	AE005174	[26]
Sakai		O157:H7	Diarrhoea (EHEC)	BA000007	[25]
UMN026		O17:K52:H18	Cystitis (ExPEC)	CU928163	[5]
IAI39		O7:K1	Pyeloneprhitis (ExPEC)	CU928164	[5]
UTI89		O18	Cystitis (ExPEC)	CP000243	[70]
APEC 01		O1	Colisepticemia (ExPEC)	CP000468	[71]
S88		O45:K1:H7	New born meningitis (ExPEC)	CU928161	[5]
CFT073		O6:K2:H1	Pyeloneprhitis (ExPEC)	AE014075	[72]
ED1a		O81	Commensal	CU928162	[5]
536		O6:K15:H31	Pyeloneprhitis (ExPEC)	CP000247	[73]
E2348/69		O127:H6	Diarrhoea (EPEC)	FM180568	[43]
E24377A		O139:H28	Diarrhoea (ETEC)	CP000800	[4]
SMS-3-5		O19:H34	Commensal	CP000970	[74]
SE11		O152:H28	Commensal	AP009240	[75]
B4 Sb227	*S. boydii* 4 227	B4	Shigellosis	CP000036	[76]
B18 BS512	*S. boydii* CDC 3083-94	B18	Shigellosis	CP001063	[77]
SS Ss046	*S. sonnei* 046	Sonnei	Shigellosis	CP000038	[76]
F2a 301	*S. flexneri* 2a 301	F2a	Shigellosis	AE005674	[78]
F2a 2457T	*S. flexneri* 2a 2457T	F2a	Shigellosis	AE014073	[79]
F5b 8401	*S. flexneri* 5b 8401	F5b	Shigellosis	CP000266	[80]
D1 Sd197	*S. dysenteriae* 1 197	D1	Shigellosis	CP000034	[76]

a Name as used in this paper.

b O antigen not expressed in K-12 due to mutation.

c EAEC (Enteroaggregative *E. coli*), EPEC(Enteropathogenic *E.coli*), EHEC (Enterohaemorrhagic *E. coli*), ExPEC (Extraintestinal pathogenic *E. coli*).

There were substantially more SNPs in the recombinant regions than the 3,763 SNPs attributed to mutation. The major recombination event in the O55:H7/O157:H7 lineage introduced 3,392 base changes and the remaining 66 proposed recombination events introduced another 1,656 changes, giving the 67 recombination events considerable weighting if each base change was treated as independent.

Overall we find that there are significantly more mutational synonymous SNPs (sSNPs) in the O157:H7 lineage than in the O55:H7 lineage, and likewise if all classes of mutation are included (Table 2 and Figure 2). It is not possible to speculate if the difference between the lineages is due to different levels of selection, different numbers of generations, or other factors. There were also more recombination events in the O157:H7 lineage than in the O55:H7 lineage, although a higher proportion than for mutation could not be allocated to a specific lineage. However although the ancestor is thought to have been of O55:H7 serotype, the O55:H7 strain as seen today also differs substantially from the common ancestor.

### Divergence Date for O55:H7 and O157:H7

We used the mutation frequency, after excluding presumed recombinant segments, to estimate the date of divergence. Exclusion of recombinant segments had a significant effect, as the divergence between O55:H7 and O157:H7 is 0.17% (Ks: 0.003745) if we include all homologous regions, but reduces to 0.06% (Ks: 0.0008364) after excluding the recombinant regions. If we take the mutation rates traditionally used for these calculations [15], [16] we obtain a divergence date for O55:H7 and O157:H7 about 14–70K years ago (calculation based on 0.0008364 synonymous divergence), rather than 62–312K years based on the 0.003745 overall synonymous divergence. However, a more direct estimate of divergence times for *V. cholerae* showed that such estimates can be 100 fold too high for closely related isolates [6] and if the same applies to *E. coli* we have a divergence time of about 400 years ago.

### Divergence within the O157:H7 and O55:H7 Lineages

The CB9615 genome sequence gives us a good outgroup for allocating the SNPs between the two O157:H7 genomes, and we find that the two branches within the O157:H7 lineage, for Sakai and EDL933, are very short. We were also able to allocate the SNPs found by Zhang *et al.* [17] and Leopold *et al.* [12] along the O157:H7 lineage. Zhang *et al.* [17] had used microarray resequencing of 1,199 genes in 10 different O157:H7 or related O157:H- strains, to estimate where the strains diverged from the Sakai/EDL933 lineage, and generated a tree with 3 branches along the major O157:H7 lineage. Leopold *et al.* [12] sequenced about 3,000 backbone genes to identify 2 subgroups and 3 clusters of O157:H7 strains. Both studies included strain 493-89 and we combined the datasets from these 2 studies. We used our CB9615 data for the bases involved, to locate the branch points on our tree, as shown in Figure 2. Note that the data obtained by Zhang *et al.* [17] was based on a comparison of the *E. coli* K-12 and Sakai genomes, so we have no knowledge of mutations on the branches to the 3 strains but only the locations of the three branch points. For data obtained by Leopold *et al.* [12] we also only mapped the branch points as full genome data are not available. The data show that 30.3% of sequence divergence from the O55 lineage occurred before the divergence of strain 493-89 in group B (Figure 2). The results are in agreement with the study of Wick *et al.* [18], which showed that strain 493-89 is the earliest among the strains to have the O157 antigen, while G5010 is the first to be sorbitol negative, and cluster 1 the first to be beta glucuronidase negative.

### Recombination

The pattern for recombination is more variable than that for mutation. The recombination event that brought the O157 O antigen to the O157:H7 lineage involved 131 kb and was by far the biggest in the two lineages, and also accounts for 67% of the SNPs attributed to recombination. Even without the O-antigen event, the ratio of substitutions due to mutation and recombination still varies substantially among the four branches (Table 2, Figure 2), which is not surprising as the recombinant segments differed both in length and divergence level. However the total length affected in each of the 3 strains, is always under 5% of the genome, although it accounted for about 35% more base changes overall than mutation. When the O-antigen related recombination event is excluded,recombination accounts for only half as many base changes as mutation.

For the segments that have undergone recombination, we have no knowledge of the donor strain, so cannot use such information to identify the incoming sequence. However the virtual outgroup analysis often gave a consistent assessment of the base that had changed (see Materials and Methods and Table S4), and we were able to allocate many of the recombinant events to a specific lineage as shown in Figures 2 and S1.

The O-antigen-related recombination event has been recognized for some time [18], [19], [20]. It covers 131 kb including the *his* operon, the O-antigen gene cluster, the *baeSR* genes, and the galactitol utilization gene cluster, and, in CB9615 only, a ribitol and arabinitol utilization gene cluster. The virtual outgroup analysis did not give a consistent assignment overall, but there were many runs of consecutive SNPs that were allocated either to CB9615 or the O157:H7 lineage. The probable explanation is that this region is subject to frequent recombination due to the presence of the O-antigen gene cluster, as discussed by Milkman et al. [21] and that the whole region has undergone multiple recombination events in both the donor and ancestral strain. However there is strong support for the O55:H7 form being ancestral [18].

Within the large recombinant region there are some indel differences between the O55:H7 and O157:H7 genomes, presumably reflecting differences between donor and recipient, and an internal recombinant region with much higher divergence (G2583_2642–G2583_2648) that includes *papC* and *papD* of a *pap* gene cluster (but not the other genes of a typical pap gene cluster), one putative adhesin gene and one other gene. The ribitol and arabinitol utilization gene cluster, present in CB9615 only, is adjacent to the shared galacticol gene cluster. In *E. coli* generally the galacticol and ribitol/arabinitol pathways are alternatives, with only 13% having both [22], putting CB9615 in the minority group and the O157:H7 genome in one of the major groups. Given the low overall low level of such differences between the 2 lineages, all of these indels are most probably due to the one recombination event.

Sixty five of the recombinant segments are adjacent to or within phage genomes and often appear to be associated with gain of all or part of a phage genome by homologous recombination. These recombinant segments involve about 1–5 kb each and have a high density of SNPs (5–20 or more per kb).

Of the remaining 44 recombinant segments most are either very short (<1 kb) or have only a few SNPs per kb. It is interesting that many genes in these segments encode surface proteins, and it may be that these events are due to selection pressure for change at the exposed parts of the proteins as reported for porins [23]. Others are associated with one of the 7 *rhs* loci in the genome. The *E. coli rhs* loci are a group of 9 loci, one of them reported here for the first time. They have the same pattern of genes, but with significant sequence variation. Not all of them are present in any given strain and often some of those present may be incomplete [24]. They are known as sites of genome rearrangements [24] and are related to type VI secretion system gene clusters, although have not been studied in this context. The O55:H7 and O157:H7 genomes have the *rhsA,C,D,E,F,G* loci and the new one that we name *rhsI*. Some of the recombinant segments observed are associated with deletions or substitutions of parts of the site and the data give an indication for the first time of the rates of change at *rhs* sites.

### Relationship of the O55:H7/O157 Lineage to Other *E. coli*

**Figure 3:**
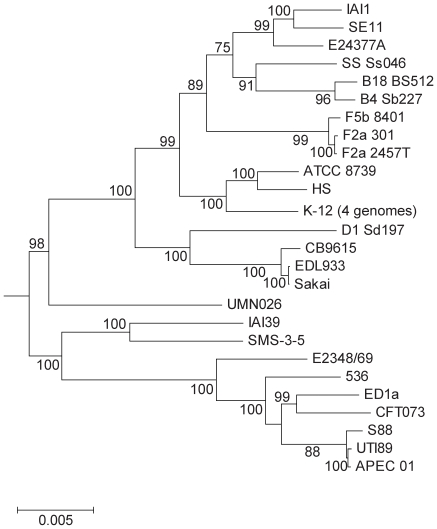
Maximum likelihood phylogenetic tree of 26 *Escherichia coli* and *Shigella* strains. The phylogenetic tree of the *Escherichia* core genome genes was constructed from the concatenated alignments of the 2034 genes in the core genome of the *E. coli/Shigella* genomes. The closely related species, *E. fergusonii* (CU928158), was chosen to root the tree.

In order to put our observations on the balance of changes due to mutation or recombination in context, we constructed a tree for these *E. coli* strains for which the full genome sequence was available (Figure 3). The levels of sequence identity among the genomes ranged from 97% to 99.9% for shared genes. As can be seen, the CB9615 and O157:H7 genomes are among the most similar, with F5 and F2a Flexneri isolates having similar divergence levels, and only the S88, UTI189 and APEC 01 group of ExPEC genomes being more similar. All of the others are much more divergent.

**Figure 4:**
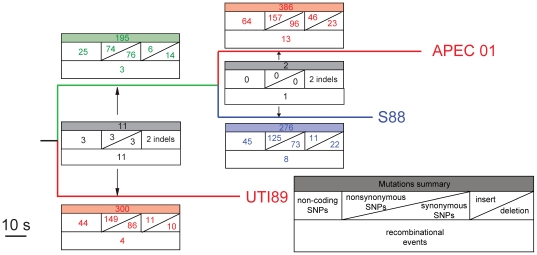
Tree showing the relationships of UTI89, S88 and APEC 01 ExPEC strains. The tree topography is for mutational SNPs as allocated by the virtual outgroup analysis (Tables S7 and S8). For each lineage the number of mutations and recombination events is shown in grids as for Figure 1.

We then undertook an analysis of recombination and mutation for the S88, UTI89 and APEC 01 group (Tables S5, S6, S7, S8, and Figure 4). The omission of recombinant segment SNPs for the phylogenic analysis changed the topology relative to that shown in Figure 3 and by Touchon *et al.* [5]. There is strong support for the topology shown in Figure 4. The difference arises because the previous study [5] did not distinguish between changes due to mutation and those due to recombination, although there was statistical evaluation of the role of recombination in sequence variation. We suggest that the alternate topology observed earlier [5] is due to inclusion of recombinant regions in the analysis. Analysis of the SNPs involved in that part of the tree (data not shown) showed that most supported the new tree presented in Figure 4 with UTI89 the first to diverge. The SNPS that take S88 to be first to diverge in the original tree are in segments covered by putative recombination events 36 and 40 (Table S8), in which S88 appeared to gain DNA from a K-12 like donor, These two segments have 47% of the SNPs in recombinant segments in this 3 strain alignment, which accounts for their effect on the tree.

The current analysis shows about 79% of SNPs being due to recombination, not too different from that found in the O55/O157 comparison (67%). The two groups of isolates are as far apart as is possible in the *E. coli* tree (Figure 3), suggesting that the impact of recombination on gain of SNPs may be consistent in the species. These ratios in *E. coli* are very different from those in the *V. cholerae* lineage studied previously, where recombination contributed about 40 times as many SNPs as mutation. We also attempted to include the CFT073 and ED1a genomes in the analysis of the ExPEC group. However the divergence between the strains was such that it was difficult to consistently distinguish mutational and recombinational SNPs, as the frequency of mutations was higher so that putative recombinant segments were not easily distinguished. It is important to be aware of this limitation as most of the branches in the *E. coli* tree are of similar or greater length.

### Indels

Despite their close relationship, many insertion/deletion events have occurred during divergence of O157:H7 and O55:H7 (Table S9). Many of those present only in the O157:H7 strains were known from a genomic oligoarray study [18], but now that we have the complete sequence for an O55:H7 strain, we have a much better insight into the changes that have taken place in both lineages. We find 725 genes in 54 indels attributed to changes in CB9615, 1,359 genes in 95 indels attributed to changes in the O157:H7 common lineage, plus 318 genes in 23 indels and 343 genes in 24 indels to attributed to changes in Sakai and EDL933 respectively. As discussed above for mutation there are more events in the O157:H7 lineages, but still a substantial number in the O55 lineage.

The major changes in terms of amount of DNA and number of genes involved are due to gain and loss of phages or parts of phage genomes (Figures 1 and S1, and Table S10). There are 19 phages or phage-like elements in CB9615, compared to 24 in the Sakai and EDL933 genomes (Table S11) [25], [26]. Only three of the phages and phage-like elements are shared between CB9615 and the two O157:H7 genomes, and there are 6 cases of a block of genes being shared between phages in the CB9615 and O157:H7 genomes which suggests a high level of recombination within the phages during their divergence. There are also pairs of phages that are integrated at the same site in the O55:H7 and O157:H7 genomes but share no similarity over most of their sequence which indicates that although very different, they share the integration site and probably integration processes. Several important virulence factors, including two types of Shiga toxins and the *ter* and *ure* operons are carried by phages in O157:H7, but absent in CB9615.

The starvation sensing protein RspAB is encoded by genes *rspA* and *rspB* downstream of phage Ep9 and found in CB9615 only. The genes are present at the same location in 18 of the 26 *E. coli* genomes, including in K-12 where they were shown to be involved in repression of homoserine lactone synthesis. Their widespread occurrence at the same locus suggests that they were lost in the O157:H7 lineage. Two cold shock-like protein genes, *cspB* and *cspF*, are also present in CB9615 but absent in both of the O157:H7 genomes. They are shared by 12 of the *E. coli* genomes, but in this case they are not consistently at the same locus and the sequence can vary. So it is not clear if the difference between the lineages is due to a gain or loss event. In both cases it would appear that the O157:H7 strains lack some stress response capacity that is present in CB9615, and such differences can be very important in niche adaptation.

There is a block of 72 kb (block 172, Figure S1 and Table 2) in CB9615 that is absent in the two O157:H7 genomes. It includes the *flu* gene and a gene cluster for a T2SS, both with a potential role in pathogenesis, and a glycolate operon. The *flu* gene encodes an autotransporter which has been shown to induce bacterial aggregation and biofilm formation, and to regulate the diffuse-adhering process in diffuse-adhering DAEC *E. coli* [27], [28]. The distribution of *flu* is unusual: it is present in 5 of the 23 other genomes but at varying locations and is presumably mobile. It also has IS rich segments to either side (positions 3,704K–3,725K combined) which may account for mobility. We have no outgroup information for it at this location and it is not clear if *flu* was present in the common ancestor and therefore not clear if it was gained by CB9615 or lost by the O157:H7 lineage.

The remainder of block 172 (positions 3,725 kb–3,765 kb) is present at the same region in the chromosome in whole or in part in 19 of the 23 other genomes, and thus appears to have a long history in *E. coli*. Presumably the T2SS and glycolate operon were present in the common ancestor and lost in the O157:H7 lineage. This T2SS is in addition to the T2SS encoded by plasmid pO55. Thus the O55:H7 genome includes 2 sets of T2SS genes, whereas the O157:H7 strains carry only the set in the pO157 plasmid. The CB9615 chromosomal T2SS is very similar to that in enterotoxigenic *E. coli* and uropathogenic *E. coli*, but has limited similarity to that in the pO55 and pO157 plasmids. The T2SS on pO157 has been reported recently to promote the adherence and intestinal colonization by EHEC [29], and the T2SS on pO55 is likely to have similar functions. However, the role of the chromosomal T2SS in CB9615 is not known.

Block 172 is adjacent to phage-like element EpLE2 (Table S11), which is over 99% identical in sequence to SpLE3 in Sakai and EDL933. This region (also named OI-122 in EDL933) carries many important virulence factors such as *nleB*, *nleE* and *efa*-1/*lifA*. It is now clear that *efa*-1/*lifA* is truncated in EHEC O157:H7 as it is intact in both CB9615 and E2348. The truncated form retains some of the properties of full-length Efa1 [30], [31], [32]. This region is rich in transposase genes and seems to be dynamic in gene content. However it is not clear what role the changes in block 172 and adjacent phage EpLE2/SpLE3 have had in the evolution of the O57:H7 clone, as most of the changes appear to have involved gene loss.

### The Plasmid

It is well known that O55:H7 is an atypical EPEC that does not contain the EAF plasmid found in typical EPEC strains [33], [34]. It does however carry a plasmid, pO55, which has pO157 as its closest relative, although only 32kb of the 66kb pO55 aligns with pO157 or its close relative pSFO157 [35] (see Figure S2). The largest aligned segment contains the *etpCDEFGHIJKLMNO* gene cluster encoding a functional T2SS in pO157. The pO55 plasmid does not carry most of the other virulence genes present on pO157.

pO55 also carries a *traMJYALEK* gene cluster and separate *traIX* gene cluster that together contain 10 of the 38 genes of the plasmid F *tra* gene cluster [36]. The *tra* genes encode the proteins for assembling the F pilus and for DNA transfer in conjugation. pO157 has only 2 *tra* genes but pSFO157, from a sorbitol-fermenting EHEC O157 isolate, has 17 *tra* genes [35], and is presumably the ancestor of pO157. Note that the strain with pSFO157 would have branched from the main O157:H7 lineage between the Group B and G5101 branch points (Figure 2). It is clear that the defective *tra* gene clusters of pO55, pO157 and pSFO157 are not functional for conjugational transfer, as many of the missing genes are required for conjugation [37]. It appears that the plasmids were inherited from the common ancestor of O157:H7 and O55:H7, but have undergone major structural change. It should be noted that while all three plasmids are defective in conjugation, all but pO157 retain the *oriT* site and so are expected to still be capable of being mobilized for transfer by a complementing plasmid. The common ancestor of these plasmids presumably was transferred to the lineage either by mobilization, or by conjugation involving *tra* genes that have since been lost.

pO55 also encodes a protein that is >80% identical to NleA of EDL933. NleA is one of seven non-LEE proteins (NleA-NleG), that are secreted by, but not encoded on, the LEE encoded T3SS of *Citrobacter rodentium*, which is used as a model for EPEC *E. coli* [38]. All but *nleG* had homologues (2 for *nleB*) in the EDL933 chromosome: *nleA* and *nleF* in O-island 71, *nleB*, *nleC* and *nleD* in O-island 36, and *nleB* and *nleE* in O-island 122. NleA was shown to play an important role in the virulence of EHEC and EPEC [39], [40]. The LEE-encoded T3SS directs translocation of NleA into host cells, where it localizes to the Golgi apparatus [39]. NleA encoded by pO55 has less than 93% similarity to any of the 15 known types of NleA [40], indicating a new NleA type 16. There is also an *nleA* pseudogene in the CB9615 genome, which retains only part of the 5′ end of *nleA* found in the chromosome of O55:H7, perhaps having been functionally replaced by the plasmid encoded *nleA* gene.

In addition to the T2SS and *tra* genes, there are other segments of pO55 and pO157 with shared genes, which include *finO*, *traX*, and many other genes of no known function. All of the shared genes have a level of divergence ranging from ∼50%–95%, which is much higher than the level found on the chromosome as discussed below. However there are reports of such high levels of mutation on the F factor [41]. The current data makes the possibility that such plasmids have a high level of mutation worthy of further study.

### Genomic Comparison of CB9615 and Typical EPEC Strain E2348/69

The genome sequence of E2348/69, one of the well studied typical EPEC strains, has recently been published, and we compared it with that of our atypical EPEC strain. The major difference reported between the 2 types of EPEC is the presence of the adherence factor (EAF) plasmid encoding the bundle forming pilus (BFP) in typical EPEC strains [42]. We found no homologues to any of the BFP genes on the CB9615 chromosome or pO55 plasmid.

Previous comparisons [43] showed that E2348/69 has a smaller repertoire of T3SS effectors than EHEC O157:H7, and we undertook a comparison of the effectors in CB9615, Sakai and E2348/69 (Table S12). We used a data set of T3SS effectors (see Materials and Methods) for BLAST searches and found 56 effectors in CB9615 and, using the same criteria, 62 in Sakai, including all of those reported previously [44] and 35 in E2348/69, Of these 10, 3 and 12 respectively are unique to those strains. There were, 20 present in all three, giving a core of shared effectors, although identity levels are low in some cases. Most interesting are the numbers shared by only two genomes. There are 31 in CB9615 and Sakai, two in CB9615 and E2348/69 and one in Sakai and E2348/69. Clearly the variation correlates with the lineage rather than the pathovar. Both EPEC CB9615 and EHEC O157:H7 strains have more T3SS effectors than EPEC 2348/69, and only two of them are present specifically in both EPEC strains, neither with high-level identity. Also the substantial sequence differences often seen in the shared effectors may also confer differences between typical and atypical EPEC.

The comparison also revealed a number of genes that may contribute to the differences in pathogenesis of typical and atypical EPEC. The *espC* gene [45], [46], encoding an effector delivered into epithelial cells, is present in E2348/69 but absent in CB9615. The *paa* [47] gene encoding the porcine AE-lesion-associated protein is present in CB9615 (and also O157:H7 strains) but absent in E2348/69. There are 2 long polar fimbriae (*lpf*) loci in CB9615 but only one in E2348/69. The second (*lpfA*) locus present in CB9615 is also in O157:H7 as well as many other atypical EPEC [48]. Both *paa* and *lpfA* were found to be significantly associated with atypical EPEC diarrhoeal isolates in a microarray study of pathogenesis related genes [48]. Another protein, EspFu/TccP (Tir cytoskeleton coupling protein), is present in CB9615 and O157:H7 but absent in E2348/69. This was shown previously as a difference between EPEC and EHEC O157:H7 in activating actin polymerization pathways [49]. However, a number of other genes are also found to be associated with atypical EPEC such as *nleB*, *nleE*, *set/ent* and *efa/lifA*, but these genes are present in both CB9615 and E2348/69. Indeed all of the genes discussed here that are in CB9615 are also in the O157:H7 strains.

### Comparative Proteomics of O157:H7 and O55:H7

Proteomic studies in O157:H7 and the typical EPEC strain E2348/69 have shown significant differences in the conditions for expression of pathogenicity as discussed below, and a proteomic comparison of the related O157:H7 EHEC and O55:H7 EPEC strains could shed light on their differences in mode of pathogenesis. Many bacterial genes, including virulence genes, are growth phase regulated. For example, *Salmonella* invasiveness is specifically expressed in the late logarithmic phase [50]. In EPEC, the ability to induce A/E lesions after adherence to Hela cells was observed only for cells in early to mid-logarithmic growth phase. At this stage, the activated EPEC bacteria induce rapid formation of A/E lesions and invade host cells within minutes after infection [51]. In contrast, in EHEC, the expression of pathogenicity genes in stationary phase is much higher than in mid-log phase [52], [53].

The fact that EHEC and EPEC virulence genes are expressed at different growth stages complicates comparison of their regulation. For easy interpretation, we used stationary phase as the reference condition to compare with exponential phase for both O157:H7 and O55 strains. The global pattern of protein expression of O157:H7 and O55:H7 at both stages of growth was analyzed using 2-DE combined with MALDI-TOF MS, and the results are summarized in Table S13. There were 10 and 8 proteins that were either up or down regulated respectively for both strains, suggesting that these proteins are growth phase dependent. One protein of unknown function was regulated in the opposite direction in the 2 strains, with up and down regulation in O55:H7 and O157:H7 respectively. However there were 118 genes differentially expressed under the 2 conditions in only one strain, with 47 up and 31 down regulated in O55, and 37 up and 13 down regulated in O157:H7 in exponential phase. It is interesting that there are fewer genes up or down regulated in O157:H7. Three hundred and fifty proteins had differences of less than 2 fold, and were considered not differentially regulated. Almost all of the proteins expressed differently in the 2 strains fall into a few COG categories, with the majority associated with metabolism. In many cases, as shown in Table S13, different pathways from the same COG category or different genes of the same pathway are up or down regulated (for example, up regulation of *proA* in O55 and *proB* in O157:H7). It is difficult to relate these differences to pathogenicity, but it appears that the adaptations that accompanied the gain of the *stx* genes in the EHEC O157:H7 clone may involve changes in metabolism rather than in expression of known virulence factors.

### Concluding Remarks

We have used genomic sequences to define with reasonable precision the events that occurred during the divergence of the O55:H7 clone (atypical EPEC) and the EHEC O157:H7 clone. We were also able to allocate most of the differences to events in one or other lineage, opening the way to a broader understanding of their relationships, and the origin of the O157:H7 clone. Previous work indicated that the O157:H7 clone originated from an ancestral O55:H7 form. We can identify many of the events that occurred in the making of the current highly pathogenic O157:H7 form from its O55:H7 ancestor, and we also found that many changes had occurred in the O55:H7 lineage since their most recent common ancestor. There have been about 70 recombination events overall since divergence, which introduced many of the SNPs that distinguish the two lineages, although they affect at most 5% of the genome. There were also about 200 insertion or deletion events, many of them small, with the larger ones mostly involving phage genomes, of which there are 9 and 23 (including phage-like elements) in the O55:H7 and O157:H7 genomes respectively, with only three common to both. There have been about 120 indel events in each of the O157:H7 strains and 54 in the O55 strain since ther divergence. Thus although the common ancestor was O55:H7 in serotype, it has undergone considerable change since divergence, but less than in the O157:H7 lineage.

The distribution of putative T3SS effectors was particularly interesting. In addition to those present in all three or unique to one genome, there were 32 in the atypical EPEC CB9615 and EHEC O157:H7, but only two in both EPEC genomes and one in Sakai and E2348/69. This strongly suggests that the distinction seen between typical and atypical EPEC strains will apply to EHEC strains related to them and that the differences observed between E2348/69 and Sakai relate more that than to differences between EPEC and EHEC.

The divergence time of O157:H7 and O55:H7 was calculated based on the number of mutational changes, by excluding regions that have undergone recombination. Using traditional clock rates we get a divergence time of 14K to 70K years ago. We also applied the clock rate estimated earlier for clones in *V. cholerae*, which gave a divergence time of only 400 years. We suggest that the latter is a more realistic estimate, as the rate for closely related isolates is expected to be much higher than the traditional clock rates used for species divergence. However it is important that estimates be made directly of the mutation clock rates for clonal divergence in *E. coli* and other species of bacteria. Regardless of clock rate the proportion of SNPs due to recombination relative to mutation was about 30 fold lower in *E. coli* than in *V. cholerae*. The same approaches were applied to the genome sequences of the closely related ExPEC strains S88, UTI189 and APEC 01, revealing a comparable ratio of mutation and recombination within both groups of *E. coli*. There are, however, limits to our ability to distinguish mutation and recombination, as we only detect recombination events that bring in DNA with a higher level of sequence divergence than that due to mutation. That will have little effect when comparing closely related strains, but when we extended the recombination analysis of ExPEC strains to include the more distantly related CFT073 and ED1a genomes we found that there were segments of intermediate level divergence, and the level of confidence in defining recombinant segments was not as high as for the more closely related strains used for this study. The approach is only reliable for closely related strains, but there should soon be many genomes of closely related strains available in a range of species.

## Materials and Methods

### Bacterial Strains and Genome Sequences

The *E. coli* O55:H7 strain CB9615 was isolated from an infant with diarrhea in Germany in 2003 [54] and confirmed to belong to the same sequence type (ST11) as the O157:H7 clone by multilocus sequence typing [9]. O157:H7 strain EDL933 was obtained from the University of Maryland. The main characteristics of the 28 strains (21 *E. coli* and 7 *Shigella*) with freely available genomes at the time of the study are presented in Table 3. These genomes were used for comparison purposes. The EDL933 and Sakai genome sequences were downloaded from GenBank without correcting for the errors in the 2 genomes recently reported by Leopold *et al.* [12], which has no negative effect on our interpretation except on the terminal branch lengths (number of mutations) for the 2 strains in Figure 2. Note that the major branch to the Sakai/EDL933 divergence is not affected, as the SNPs involved are verified by having the same base in both O157:H7 strains. The ORF sequences from Leopold *et al.* [12] were downloaded as individual entries from GenBank (accession numbers: EU889374–889556, EU889560–889952, EU889956–891224, EU891230–EU892004, EU892010–EU896054, EU896060–EU901119, EU901125–EU906854, FJ197142–197143 and FJ667493–FJ667497).

### Genomic DNA Extraction

A single colony of CB9615 was used to seed a 20 mL overnight culture in 2x Yeast Extract Tryptone (2xYT). After a dilution of 1∶100 to fresh media (100 mL), cells were grown in 2xYT and harvested at mid-log phase, then resuspended in TE buffer and lysed with lysozyme (20 mg/mL) at 37^o^C for 30 minutes, followed by treatment with Proteinase K (20 mg/mL), 10% SDS and RNaseA (10 mg/mL) at 50°C for 60 minutes. Genomic DNA from the sample was isolated by 3 rounds of extraction with phenol∶chloroform∶isoamyl alcohol (25∶24∶1) and twice with chloroform to remove any residual phenol. After centrifugation, the upper phase was precipitated with 2.5 volumes of ethanol. The precipitated DNA was wound onto a sterile glass pipette, washed 3 times in 70% ethanol, air dried and dissolved in 500 µL TE buffer.

### Genome Sequencing

Two pUC118 libraries (inserts 2–3 and 6–8 kb) were generated by mechanical shearing of chromosomal DNA. Double-ended plasmid sequencing reactions were done using an ABI BigDye Terminator V3.1 Cycle Sequencing Kit and an ABI 3730 Automated DNA Analyzer (Applied Biosystems) in Tianjin Biochip Corporation. 68,881 reads were generated providing a 9.47-fold coverage and assembled into 349 contigs using the PHRED, PHRAP and CONSED programs [55]. Linkages among contigs were based on sequences of gap-spanning clones and comparison of the contigs to the published genome sequences of Sakai and EDL933. Sequence gaps were closed by primer walking on linking clones or sequencing PCR products amplified from genome DNA. All repeated DNA regions and low-quality regions were verified by PCR and sequencing of the product. The ribosomal RNA operon sequences were assembled separately by construction of DNaseI shotgun banks. The final genome is based on 70,775 reads. To verify the SNPs in CB9615, the sites were confirmed to have at least 2-fold coverage of ABI 3730 reads.

### Annotation and Analysis

Open reading frames from 30 amino acids in length were predicted using Glimmer 3.0 [56] and verified manually using the annotation of EDL933 and Sakai. Transfer RNA and ribosomal RNA genes were predicted using tRNAscan-SE [57] or by similarity to EDL933 and Sakai rRNA genes. Artemis [58] was used to collate data and facilitate annotation. Function predictions were based on BLASTp similarity searches in the UniProtKB [59], GenBank [60], and Swiss-Prot protein [61] databases, and the clusters of orthologous groups (COG) database [62]. Pseudogenes were detected by BLASTn, comparing the genome sequences of CB9615 with those of EDL933 and Sakai, and the annotation revised manually.

### Generation of an Alignment of the Three Genomes and Identification of Recombinant Regions

The co-linear blocks of the 3 genomes were determined using BLASTn, and the alignment within each of the blocks was based on the *Mauve* method [63], with a seed length set to 11. The aligned regions were then analyzed to identify regions that had undergone recombination. The steps involved are preliminary recognition of recombinant regions as segments with a higher frequency of base changes, and then refining the boundaries of these segments until there are alternate regions proposed to have either undergone only mutation, or undergone recombination. The details of the processes are given in the supplementary methods section of Feng *et al.* [6]. When combined, the putative mutational segments have a near random distribution of base changes. The deviation from randomness is presence of a small excess of base changes closer than predicted, which are presumably due to clustered mutations, as identified by Drake [64]. Regions identified as recombinant have a generally much higher frequency of base changes, as can be seen in Figure S1. The distribution of base changes in recombinant regions is not random when they are combined and often not random even within a recombinant region. The final plot of the 3 genome alignment, as shown in Figure S1, including indels and location of putative recombinant segments, was generated by methods used previously for *Vibrio cholerae* and *E. coli* K12 [6], [13], and collectively known as GA-Plot (Genome Alignment Plot).

### Assignment of Orthologs and Phylogenetic Analysis on *E. coli*/*Shigella* Genomes

To overcome any effect of the different annotation in the assignment of orthologs, the amino acid sequences of CB9615 were used as the reference. We then mapped the reference sequences onto all the *E. coli*/*Shigella* genomes (Table 3) using tBLASTn, with cutoff of at least 80% identity in amino acid sequence and less than 20% difference in protein length. The corresponding locations in the genomes were extracted and translated into amino acids. The set of orthologs was defined by pairwise reciprocal best hits.

The reference phylogenetic tree of the *Escherichia* core genome genes (Figure 3) was constructed from the concatenated alignments of the 2034 genes in the core genome of the *E. coli/Shigella* genomes. We used Tree-puzzle 5.2 [65] to compute the distance matrix between all strains using maximum likelihood under the GTR+gamma (with 8 categories)+I model. The tree was then built from the distance matrix using BioNJ [66]. We made 1000 bootstrap experiments on the concatenated sequences to assess the robustness of the topology.

### Virtual Outgroup Analysis for Assignment of Mutations to a Lineage

Virtual Outgroup analysis [6], [13] was used separately on the CB9615, Sakai and EDL933 set of genomes (Table S3), and the UTI89, APEC 01 and S88 genomes (Tables S7 and S8). These were compared with 26 other *E. coli*/*Shigella* genomes (Table 3) using BLASTn. The regions in the set of genomes under study that included mutation sites were retrieved from all *E. coli* genomes (Table 3), and local alignments were then generated and joined to form a consensus alignment. Tables S3 and S7 list each of the mutation sites for the genomes under study and give the base (if any) present in each of the reference genomes. In most cases where the base involved was present in all or most of the genomes, one of the bases found in our genomes was present in all or most of the outgroup genomes, making it easy to nominate the probable ancestral base and hence the lineage for the mutation. Strain *S. dysenteriae* Sd197 was the most closely related to the O55/O157 strains, and strains CFT073, ED1a and 536 were the most closely related to the ExPEC strains (Figure 3), and in cases of ambiguity these were given priority in allocation. The details and criteria for estimating the level of support are given in the footnotes to Tables S3 and S7.

### Virtual Outgroup Analysis for Assignment of Recombinant Regions to a Lineage

Virtual Outgroup analysis was conducted on the SNPs in recombinant segments of the genomes as for mutational segments, using both sets of genomes to generate Tables S4 and S8. The SNPs for each assigned recombinant region were then assessed as a group, and if 80% or more give a consistent allocation, this was used to allocate the segment to a lineage using criteria described in the footnotes.

### Virtual Outgroup Analysis for Indel Assessment

Many of the indels could be allocated to a lineage by a variation of the virtual outgroup approach used for mutations. The sequence of each block, together with 1kb of sequence flanking the block at each end, was subjected to BLASTn analysis against the genomes used for virtual outgroup analysis. If an outgroup strain included both ends, and if the block or a significant part of it was present at that location, then the indel is reported to be present. If both flanking regions are present as a single segment without the indel block, then the indel reported as absent. The criteria for estimating the level of support are similar to those used for mutational base changes and the details are given in the footnotes to Table S14.

### Bioinformatics Search for Effector Candidates

A data set that contains over 300 proven or predicted effectors was downloaded from the supporting information of Tobe *et al.* [44]. The E2348/69 genome was sequenced after that data set was assembled, and as 3 of the 27 effectors reported for E2348/69 (NleH, Cif and NleB) were not in the original data set, these were added to avoid any bias towards Sakai. The peptide sequences were used to search the genome and protein sequences of *E. coli* O55:H7 CB9615, *E. coli* O157:H7 Sakai and *E. coli* O127:H6 E2348/69 using TBLASTN and BLASTP under default conditions [44]. An E-value of <1e-05 was chosen as a cutoff value for significance. All newly identified effectors were then subjected to PSI-BLAST searches over the NCBI's NR peptide database and the genes in CB9615, to identify more distantly related homologs. Pseudogenes were identified on the grounds of partial matches to much longer homologous coding sequences, and where possible, evidence of frame shifts or truncations was gathered by comparing family members at the nucleotide level.

### Growth Conditions for Proteomic Analysis

*E. coli* O55:H7 strain CB9615 and O157:H7 strain EDL933 were grown to early exponential phase and early stationary phase in 100 mL Dulbecco's Modified Eagle Medium (DMEM, GibcoTM, Invitrogen corporation). Bacterial growth was monitored by measuring light absorbance at 600 nm using a 752 UV visible spectrophotometer at 1-h intervals for 8h. Cells were harvested by centrifugation (8000×g for 10min at 4°C). For each condition, proteins prepared from three independent cultures were pooled for further analysis.

### Protein Extraction for 2-DE

To extract cytosolic proteins, the harvested cells were washed twice in 50 ml washing buffer (0.01 M Tris, 0.25 M sucrose, pH7.2), and then suspended in lysis buffer (8 M urea, 2 M thiourea, 4% CHAPS, 1% DTT, 0.8% ampholine pH3-10) followed by sonication for 5 min using a Ultraschallprozessor UP200S (Hielscher). Insoluble material was removed by centrifugation at 25 000×g for 1 h at 4°C. The supernatant was collected and stored at −80°C until use. Protein concentration was determined by the Bradford method with bovine serum albumin as the standard.

### 2-DE and Image Analysis

Approximately 1 mg protein samples were applied onto 17cm IPG strips (pH 4–7 linear, Bio-rad) using the anoid Ettan IPGphor Cup Loading Manifold (GE Healthcare Bio-Science AB). The first dimension (IEF) was performed in the IPGphor Isoelectric Focusing System (Pharmersham) by stepwise increase of the voltage as follows: 0 V–500 V for 2 h, 500 V for 5 h, 500 V–3500 V for 3 h and finally 3500 V continuing until the total volt-hours reached 45 KVh. After completion of IEF, IPG strips were incubated for 15 min in equilibration solution I (50 mM Tris pH 6.8, 6 M urea, 30% glycerol, 2% SDS, 2% DTT, trace bromophenol blue) and 15 min in equilibration solution II (solution I with 2.5% iodoacetamide instead of DTT). The second dimension was performed on 12% SDS-PAGE gels using Bio-Rad ProteanTM Plus DodecaTM Cell, at 15 mA per gel for 15 min and then at a constant voltage of 250 V, until the dye front reached the bottom of the gel. Low-molecular-weight markers (BBI) were applied next to the acidic end of the IPG strips. Proteins were visualized by staining gels with Coomassie Brilliant Blue G-250 (BBI).

The gel images were acquired by scanning with a UMAX Powerlook 2100XL scanner. Analysis of profiles and statistical analysis of protein spot data were performed with the PDQuestTM 7.3.0 software (Bio-rad). Gels were normalized based on the total spot volume in each gel of the matched set, where the value assigned to a protein spot was calculated as a percentage of the sum of volumes of all spots detected and present in each sample.

### In-gel Digestion

Protein spots were manually excised from Coomassie Brilliant Blue G-250 (BBI) stained gels. Excised gel spots were washed several times with destaining solutions (deionized water for 15 min three times and then with 50% (v/v) acetonitrile containing 25 mM NH_4_HCO_3_ for 15 min three times), then with pure water for 15min three times. The gel spots were dehydrated in 50ul 100% ACN for 20 min at room temperature and rehydrated in 25 mM NH_4_HCO_3_ containing 0.005 ug/ul modified trypsin (Promega). Proteins were digested by trypsin for 16–20 h at 37°C to generate peptides.

### MALDI-TOF Analysis

Peptides were analyzed by MALDI-TOF using a 4700 series Proteomics Analyzer (Applied Biosystems). An internal calibration was obtained with the peptides derived from 700–4000 Da. CHCA(sigma) was used as a matrix. The peak list of the spectra was created by the Peak-to-MASCOT script of the 4700 Explorer software. The samples were analyzed by PMF and comparison to a local database using the mascot algorithm of the GPS software.

### Data Analysis

To identify proteins with significant differences in protein synthesis between early stationary phase and exponential phase, we determined the ratios of the total normalized quantities of corresponding spots in the images from the two samples. Spots with synthesis ratios of >2.0 (up) and <0.5 (down) in all 3 replicates were considered to contain proteins with significant changes in the synthesis rate.

## Supporting Information

Figure S1 Plot of mutations, recombination events, and indels in the genomes of strains CB9615, Sakai, and EDL933. The genomes of CB9615, Sakai, and EDL933 were aligned as described in the “Materials and Methods” section. The whole chromosome is presented in seven pages with 100 KB per row. The maps are best viewed on-screen zoomed in at appropriate magnification or printed at A0 size. The segment inverted in EDL933 relative to CB9615 and Sakai is inverted for presentation to align with CB9615, and the phage genomes at the junctions are not included, but their positions are marked by two gray boxes that contain their names. Top: the genes for CB9615 with annotation. The 7 rrn operons are named A through H as in the GenBank annotation for EDL933. Below, from top to bottom, three bands for the genomes of CB9615, Sakai, and EDL933, respectively, plus two bands for the single nucleotide polymorphisms in the O157 joint lineage and for the O55/O157 divergence, respectively. The top three bands have genome map positions in kilobytes. In each band, large indels shown as red (insertion) or green (absence) blocks and named as in Table S3. Vertical lines mark sites where that genome differs from the others as follows: CB9615, events attributed to the CB9615 lineage; Sakai and EDL933, events attributed to the specific lineage after their divergence; O157, events attributed to the O157 lineage prior to divergence of Sakai and EDL933. The lines are coded as follows: blue, base substitution in gene (half height, synonymous substitutions; full height, non-synonymous substitutions); gray, base substitution in pseudogene or non-coding region; red, base present; green, base absent. An orange line above a group of base difference markers indicates a segment inferred to have undergone recombination. The gray boxes separate sections of the alignment.
(0.71 MB PDF)

Figure S2 Base changes in plasmid genomes. A plot of base changes in the genomes of pO55, pSFO157, and pO157 from both Sakai and EDL933. The base changes in the homologous regions of the plasmids pO55, pSFO157, and pO157 from both Sakai and EDL933, were plotted using the same approach as in Figure S1. Each genome was cut into seven segments and ordered as in pO157 from EDL933.
(0.35 MB PDF)

Table S1 The genes of *E. coli* CB9651. All genes are shown with locus tag, start and end positions, name, and gene product.
(0.44 MB PDF)

Table S2 Single base and small indel differences. A full list of single base and small indel differences between the CB9651, Sakai, and EDL933 genomes, including location, nature of difference, and name of gene affected.
(0.31 MB PDF)

Table S3 Virtual outgroup analysis of mutations in the CB9651, Sakai, and EDL933 genomes. The mutational and large indel differences between the CB9651, Sakai, and EDL933 genomes were analyzed using the virtual outgroup approach, and the mutations or indels allocated to the CB9651, O157, Sakai, or EDL933 lineages. The 23 genomes used for the analysis are shown, with details of the base or bases present in both outgroup genomes and genomes under analysis, and also the final allocation and a measure of support level for that allocation.
(0.21 MB PDF)

Table S4 Virtual outgroup analysis of the recombinant regions in the CB9651, Sakai, and EDL933 genomes.
(0.29 MB PDF)

Table S5 A summary of mutational and recombination changes in three ExPEC genomes. The numbers of bases affected by mutation or recombination, as shown in Table S7, were separated into synonymous, non-synonymous, or non-coding sites and small inserts or deletions.
(0.03 MB PDF)

Table S6 Single base and small indel differences between the UTI89, S88, and APEC 01 genomes. A full list with location, nature of difference, length of indels, and name of gene affected.
(0.18 MB PDF)

Table S7 Virtual outgroup analysis for allocation of mutational single nucleotide polymorphisms and small indels to the UTI189, S88, or APEC 01 lineages. The 23 genomes used for the analysis are shown, with details of the base or bases present in both outgroup genomes and genomes under analysis, and also the final allocation and a measure of support level for that allocation.
(0.08 MB PDF)

Table S8 Virtual outgroup analysis for allocation of recombinant regions to the UTI189, S88, or APEC 01 lineages. The 23 genomes used for the analysis are shown, with details of the base or bases present in both outgroup genomes and genomes under analysis, and also the final allocation and a measure of support level for that allocation.
(0.32 MB PDF)

Table S9 Large Indels. Large indels in affecting one or two of the genomes are shown with the length (bp), if thought to be an insertion or deletion, the strain(s) affected and the gene or genes affected.
(0.02 MB PDF)

Table S10 The orthologs in the *E. coli* CB9651, Sakai, and EDL933 genomes. Genes present in two or three of the genomes are listed with the gene tag numbers, gene name, and product. For those absent in one or two of the genomes due to one of the indel events, the indel number from Table S3 is shown in place of the locus tag.
(0.49 MB PDF)

Table S11 Comparison of the phages in the O55:H7 strain and the two O157:H7 strains.
(0.02 MB PDF)

Table S12 Type III secretory system effectors of CB9651, Sakai, and EDL933. The effectors are shown with their distribution among the genomes and the level of amino acid identity where shared by two or three of the strains.
(0.06 MB PDF)

Table S13 A comparative proteomic analysis of the CB9615 and EDL933 genomes. All proteins with expression levels that differed by twofold or more in one or both of the genomes are shown, with details of the protein involved and the direction of change.
(0.11 MB PDF)

Table S14 Virtual outgroup analysis of the large indels in the CB9651, Sakai, and EDL933 genomes.
(0.02 MB PDF)
