# Supplementary figures and images for: Derivation of Escherichia coli O157:H7 from Its O55:H7 Precursor
Source: PLoS One. 2010 Jan 14;5(1):e8700. doi: 10.1371/journal.pone.0008700 (PMC2806823; doi:10.1371/journal.pone.0008700)

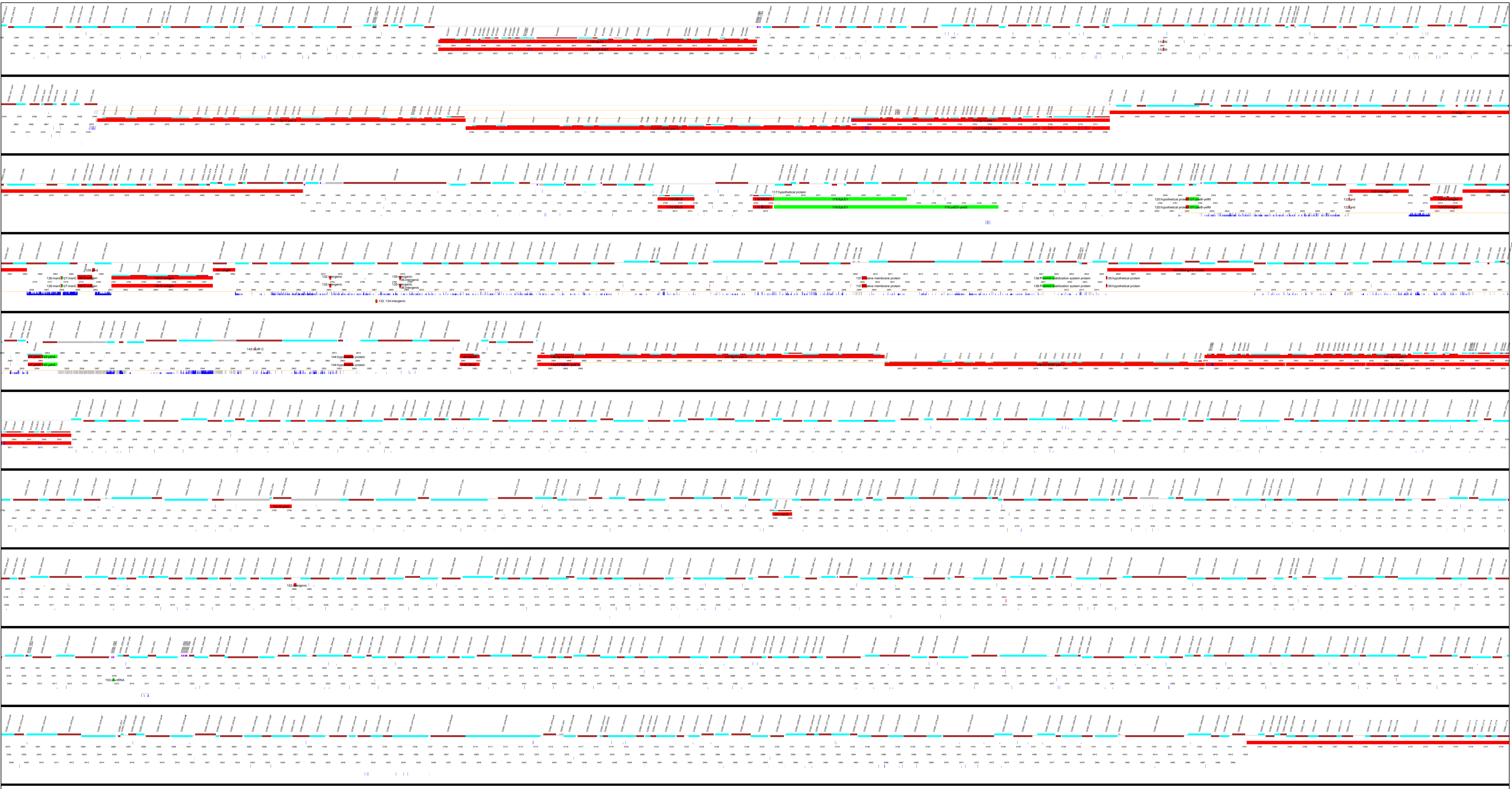



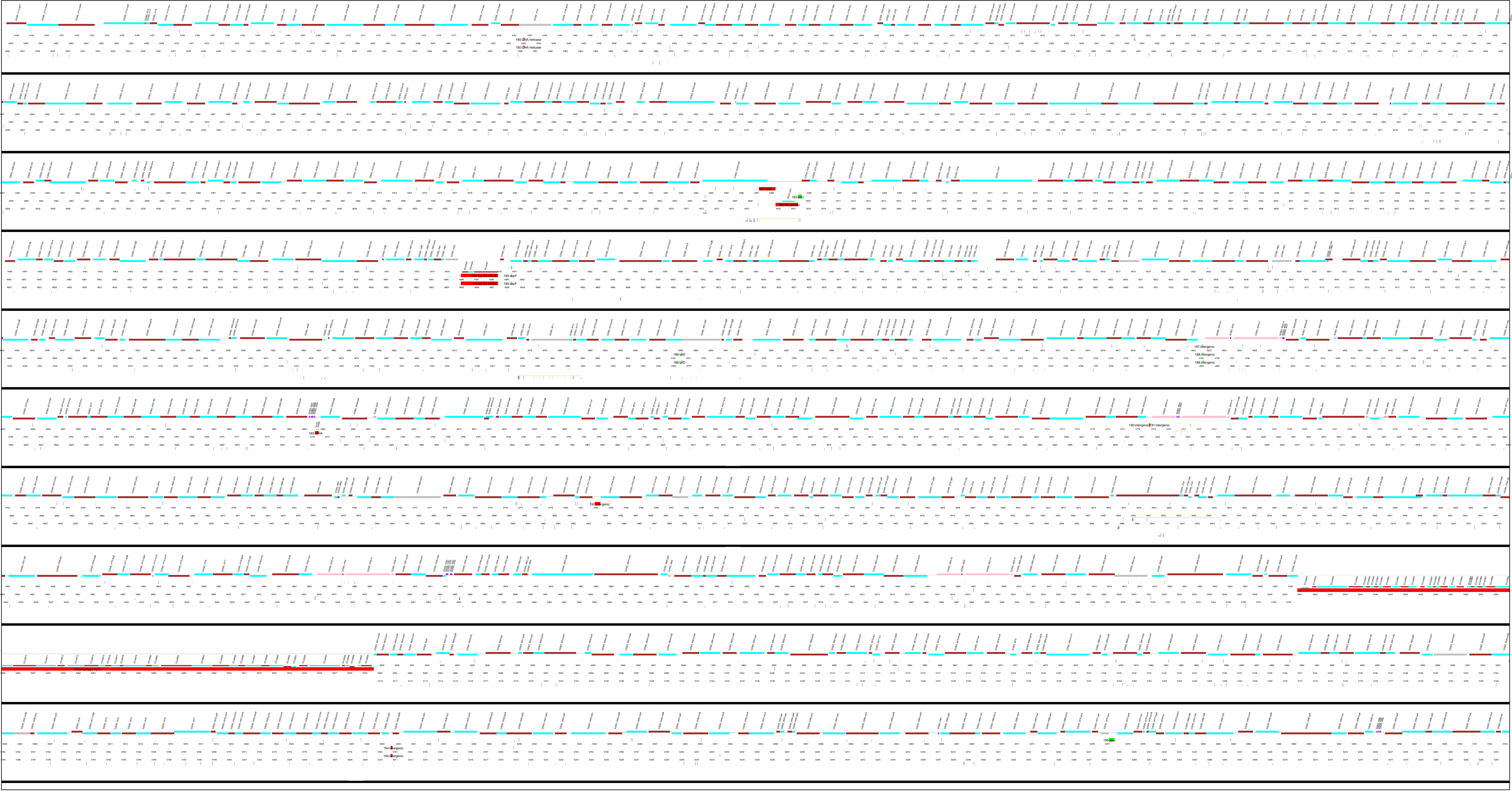

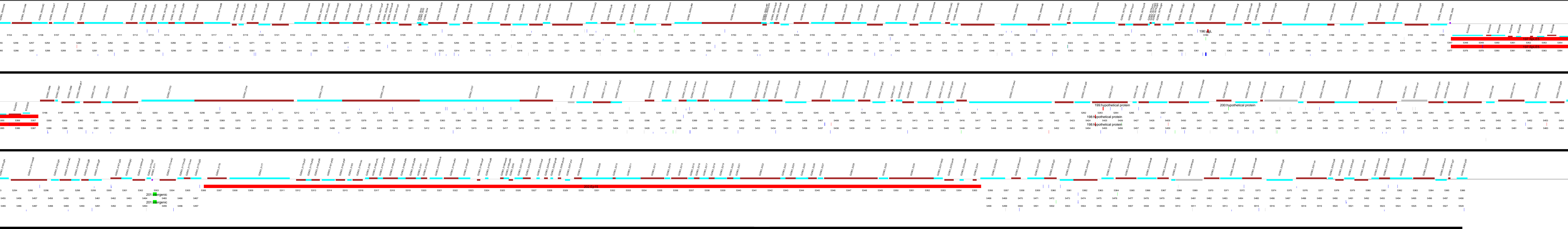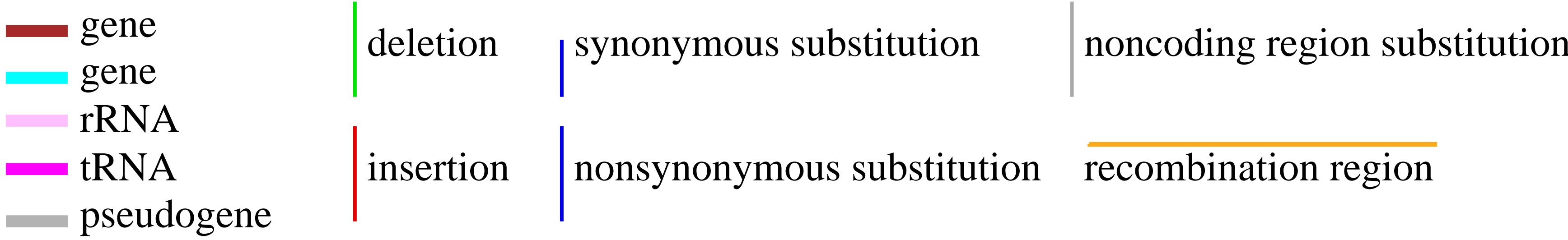

Supplement: Figure S1 — Plot of mutations, recombination events, and indels in the genomes of strains CB9615, Sakai, and EDL933. The genomes of CB9615, Sakai, and EDL933 were aligned as described in the “Materials and Methods” section. The whole chromosome is presented in seven pages with 100 KB per row. The maps are best viewed on-screen zoomed in at appropriate magnification or printed at A0 size. The segment inverted in EDL933 relative to CB9615 and Sakai is inverted for presentation to align with CB9615, and the phage genomes at the junctions are not included, but their positions are marked by two gray boxes that contain their names. Top: the genes for CB9615 with annotation. The 7 rrn operons are named A through H as in the GenBank annotation for EDL933. Below, from top to bottom, three bands for the genomes of CB9615, Sakai, and EDL933, respectively, plus two bands for the single nucleotide polymorphisms in the O157 joint lineage and for the O55/O157 divergence, respectively. The top three bands have genome map positions in kilobytes. In each band, large indels shown as red (insertion) or green (absence) blocks and named as in Table S3. Vertical lines mark sites where that genome differs from the others as follows: CB9615, events attributed to the CB9615 lineage; Sakai and EDL933, events attributed to the specific lineage after their divergence; O157, events attributed to the O157 lineage prior to divergence of Sakai and EDL933. The lines are coded as follows: blue, base substitution in gene (half height, synonymous substitutions; full height, non-synonymous substitutions); gray, base substitution in pseudogene or non-coding region; red, base present; green, base absent. An orange line above a group of base difference markers indicates a segment inferred to have undergone recombination. The gray boxes separate sections of the alignment. (0.71 MB PDF) [file pone.0008700.s001.pdf]
