# Supplementary material for: Derivation of Escherichia coli O157:H7 from Its O55:H7 Precursor
Source: PLoS One. 2010 Jan 14;5(1):e8700. doi: 10.1371/journal.pone.0008700 (PMC2806823; doi:10.1371/journal.pone.0008700)
Supplement: Figure S2 — Base changes in plasmid genomes. A plot of base changes in the genomes of pO55, pSFO157, and pO157 from both Sakai and EDL933. The base changes in the homologous regions of the plasmids pO55, pSFO157, and pO157 from both Sakai and EDL933, were plotted using the same approach as in Figure S1. Each genome was cut into seven segments and ordered as in pO157 from EDL933. (0.35 MB PDF) [file pone.0008700.s002.pdf]

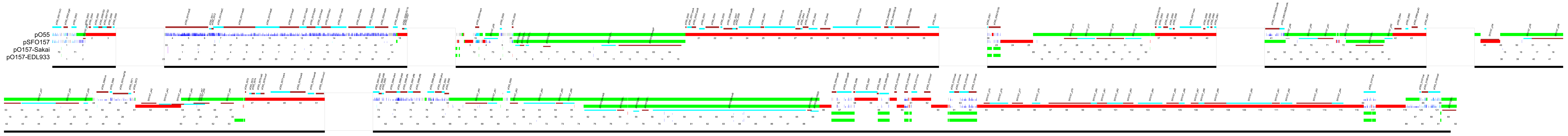

Figure S2. Plot of base changes in the genomes of pO55, pSFO157 and pO157 from both Sakai and EDL933.

The base changes in the homologous regions of the plasmids pO55, pSFO157, and pO157 from both Sakai and EDL933, were plotted using the same approach as in Figure S1. Each genome was cut into 7 segments and ordered as in pO55. pO157 from Sakai which have different start point to others, were changed and the original start point was marked in purple line.
