# Supplementary material for: Derivation of Escherichia coli O157:H7 from Its O55:H7 Precursor
Source: PLoS One. 2010 Jan 14;5(1):e8700. doi: 10.1371/journal.pone.0008700 (PMC2806823; doi:10.1371/journal.pone.0008700)
Supplement: Table S1 — The genes of E. coli CB9651. All genes are shown with locus tag, start and end positions, name, and gene product. (0.44 MB PDF) [file pone.0008700.s003.pdf]

Locus_tag	Type	Start	End	+/-[a]	Gene[b]	Product
G2583_0001	pseudogene	190	255	+	thrL	thr operon leader peptide
G2583_0002	CDS	336	2798	+	thrA	Aspartokinase I, homoserine dehydrogenase I
G2583_0003	CDS	2800	3732	+	thrB	Homoserine kinase
G2583_0004	CDS	3733	5019	+	thrC	Threonine synthase
G2583_0005	CDS	5233	5529	+	yaaX	conserved hypothetical protein
G2583_0006	CDS	5682	6458	-	yaaA	UPF0246 protein yaaA
G2583_0007	CDS	6528	7958	-	yaaJ	inner membrane transport protein
G2583_0008	CDS	8237	9190	+	talB	Transaldolase 1
G2583_0009	CDS	9305	9892	+	mog	Molybdopterin biosynthesis mog protein
G2583_0010	CDS	9927	10493	-	yaaH	membrane protein, GPR1/FUN34/yaaH family
G2583_0012	CDS	10820	11344	+	htgA	Positive regulator for sigma 32 heat shock promoters
G2583_0013	CDS	11381	11785	-	yaaI	UPF0412 protein yaaI precursor
G2583_0014	CDS	12162	14078	+	dnaK	Chaperone protein dnaK
G2583_0015	CDS	14167	15297	+	dnaJ	Chaperone protein DnaJ
G2583_0016	CDS	15401	15610	-	mokC	Gef protein
G2583_0017	CDS	16139	17305	+	nhaA	Sodium/proton antiporter nhaA
G2583_0018	CDS	17365	18270	+	nhaR	DNA-binding transcriptional activator
G2583_0019	CDS	18308	19267	-	-	hypothetical protein
G2583_0020	CDS	19280	21730	-	yehB	Putative usher protein
G2583_0021	CDS	21743	22426	-	yehC	Gram-negative pilus assembly chaperone
G2583_0022	CDS	22476	23009	-	stcA	Putative type-1 fimbrial protein
G2583_0023	CDS	23311	24732	-	-	hypothetical protein
G2583_0024	CDS	25202	25465	-	rpsT	30S ribosomal protein S20
G2583_0025	CDS	25568	25792	+	yaaY	hypothetical protein
G2583_0026	CDS	25800	26741	+	ribF	Riboflavin biosynthesis protein ribF [Includes: Riboflavin kinase
G2583_0027	CDS	26784	29600	+	ileS	Isoleucyl-tRNA synthetase
G2583_0028	CDS	29600	30094	+	lspA	Lipoprotein signal peptidase
G2583_0029	CDS	30182	30631	+	fkpB	FKBP-type 16 kDa peptidyl-prolyl cis-trans isomerase
G2583_0030	CDS	30633	31583	+	ispH	4-hydroxy-3-methylbut-2-enyl diphosphate reductase
G2583_0031	CDS	31649	32563	+	rihC	Non-specific ribonucleoside hydrolase rihC
G2583_0032	CDS	32730	33551	+	dapB	Dihydrodipicolinate reductase
G2583_0033	CDS	34007	35155	+	carA	Carbamoylphosphate synthase small subunit
G2583_0034	CDS	35173	38394	+	carB	Carbamoyl-phosphate synthase large chain
G2583_0035	CDS	38402	38620	-	-	hypothetical protein
G2583_0036	CDS	38655	39050	+	caiF	DNA-binding transcriptional activator CaiF
G2583_0037	CDS	39169	39780	-	caiE	Carnitine operon protein caiE
G2583_0038	CDS	39765	40658	-	caiD	Crotonobetainyl CoA hydratase
G2583_0039	CDS	40659	42227	-	caiC	Putative crotonobetaine/carnitine-CoA ligase
G2583_0040	CDS	42286	43503	-	caiB	Crotonobetainyl-CoA:carnitine CoA-transferase
G2583_0041	CDS	43632	44774	-	caiA	Crotonobetainyl-CoA dehydrogenase
G2583_0042	CDS	44805	46319	-	caiT	L-carnitine/gamma-butyrobetaine antiporter
G2583_0043	CDS	46730	47563	+	fixA	conserved hypothetical protein
G2583_0044	CDS	47578	48519	+	fixB	hypothetical protein
G2583_0045	CDS	48570	49856	+	fixC	PAPS (adenosine 3'-phosphate 5'-phosphosulfate) 3'(2'),5'-
G2583_0046	CDS	49853	50140	+	fixX	Ferredoxin-like protein fixX
G2583_0047	CDS	50198	51529	+	yaaU	Major facilitator family transporter
G2583_0048	CDS	51637	52167	+	kefF	Glutathione-regulated potassium-efflux system ancillary protein
G2583_0049	CDS	52160	54022	+	kefC	Glutathione-regulated potassium-efflux system protein kefC
G2583_0050	CDS	54103	54693	+	folA	Dihydrofolate reductase
G2583_0051	CDS	54779	55012	+	-	putative antitoxin of gyrase inhibiting toxin-antitoxin system
G2583_0052	CDS	55015	55329	+	ccdB	CcdB protein
G2583_0053	CDS	55326	56174	-	apaH	Bis(5'-nucleosyl)-tetraphosphatase, symmetrical
G2583_0054	CDS	56181	56558	-	apaG	ApaG
G2583_0055	CDS	56561	57382	-	ksgA	Dimethyladenosine transferase (S-adenosylmethionine-6-N', N'-
G2583_0056	CDS	57379	58368	-	pdxA	4-hydroxythreonine-4-phosphate dehydrogenase (4-
G2583_0057	CDS	58368	59654	-	surA	Chaperone surA precursor
G2583_0058	CDS	59707	62055	-	imp	LPS-assembly protein precursor
G2583_0059	CDS	62310	63125	+	djlA	putative oxidoreductase Fe-S binding subunit
G2583_0060	CDS	63420	64157	+	yabP	hypothetical protein
G2583_0061	CDS	64575	65234	-	rluA	Ribosomal large subunit pseudouridine synthase A

Locus_tag	Type	Start	End	+/-[a]	Gene[b]	Product
G2583_0062	CDS	65246	68152	-	hepA	RNA polymerase-associated protein rapA
G2583_0063	CDS	68316	70667	-	polB	DNA polymerase II
G2583_0064	CDS	70742	71437	-	araD	L-ribulose-5-phosphate 4-epimerase
G2583_0065	CDS	71637	73139	-	araA	L-arabinose isomerase
G2583_0066	CDS	73150	74850	-	araB	Ribulokinase
G2583_0067	CDS	75138	76067	+	araC	arabinose operon regulatory protein
G2583_0068	CDS	76153	76917	+	yabI	hypothetical protein
G2583_0069	CDS	77004	77702	-	thiQ	Thiamine import ATP-binding protein thiQ
G2583_0070	CDS	77686	79296	-	thiP	Thiamine/thiamine pyrophosphate ABC transporter, permease
G2583_0071	CDS	79272	80267	-	tbpA	Thiamin/thiamin pyrophosphate ABC transporter, periplasmic
G2583_0072	CDS	80627	81196	+	-	hypothetical protein
G2583_0073	CDS	81425	83083	-	sgrR	putative transport protein
G2583_0074	ncRNA	83151	83376	+	-	ncRNA
G2583_0075	CDS	83411	84016	-	leuD	3-isopropylmalate dehydratase small subunit
G2583_0076	CDS	84027	85427	-	leuC	3-isopropylmalate dehydratase large subunit
G2583_0077	CDS	85430	86521	-	leuB	3-isopropylmalate dehydrogenase
G2583_0078	CDS	86521	88092	-	leuA	2-isopropylmalate synthase
G2583_0079	CDS	88185	88271	-	leuL	leader; Amino acid biosynthesis: Leucine
G2583_0080	CDS	88911	89873	+	leuO	leucine transcriptional activator
G2583_0081	CDS	90191	91915	+	ilvI	Acetolactate synthase
G2583_0082	CDS	91888	92409	+	ilvH	Acetolactate synthase III, valine sensitive, small subunit
G2583_0083	CDS	92421	92507	+	fruL	fruR leader peptide
G2583_0084	CDS	92589	93593	+	fruR	Fructose repressor
G2583_0085	CDS	94195	94653	+	mraZ	hypothetical protein
G2583_0086	CDS	94655	95596	+	mraW	S-adenosyl-L-methionine-dependent methyltransferase mraW
G2583_0087	CDS	95593	95958	+	ftsL	Cell division protein ftsL
G2583_0088	CDS	95974	97740	+	ftsI	Peptidoglycan synthetase ftsI precursor
G2583_0089	CDS	97727	99214	+	murE	UDP-N-acetylmuramoyl-L-alanyl-D-glutamate--2, 6-
G2583_0090	CDS	99211	100569	+	murF	UDP-N-acetylmuramoyl-tripeptide--D-alanyl-D-alanine ligase
G2583_0091	CDS	100563	101645	+	mraY	Phospho-N-acetylmuramoyl-pentapeptide-transferase
G2583_0092	CDS	101648	102964	+	murD	UDP-N-acetylmuramoylalanine--D-glutamate ligase
G2583_0093	CDS	102964	104208	+	ftsW	Cell division protein ftsW
G2583_0094	CDS	104205	105272	+	murG	UDP-N-acetylglucosamine--N-acetylmuramyl-(pentapeptide)
G2583_0095	CDS	105326	106801	+	murC	UDP-N-acetylmuramate--L-alanine ligase
G2583_0096	CDS	106794	107714	+	ddlB	D-alanine--D-alanine ligase B
G2583_0097	CDS	107716	108546	+	ftsQ	Cell division protein FtsQ
G2583_0098	CDS	108543	109805	+	ftsA	Cell division protein ftsA
G2583_0099	CDS	109866	111017	+	ftsZ	Cell division protein ftsZ
G2583_0100	CDS	111118	112035	+	lpxC	UDP-3-O-[3-hydroxymyristoyl] N-acetylglucosamine
G2583_0101	CDS	112094	112777	+	secM	Secretion monitor protein
G2583_0102	CDS	112839	115544	+	secA	preprotein translocase subunit SecA
G2583_0103	CDS	115604	116002	+	mutT	7,8-dihydro-8-oxoguanine-triphosphatase
G2583_0104	CDS	116008	116142	-	-	hypothetical protein
G2583_0105	CDS	116093	116290	-	yacG	Uncharacterized protein conserved in bacteria
G2583_0106	CDS	116300	117043	-	yacF	UPF0289 protein yacF
G2583_0107	CDS	117043	117663	-	coaE	Dephospho-CoA kinase
G2583_0108	CDS	117888	118931	+	guaC	GMP reductase
G2583_0109	CDS	118851	118958	-	-	hypothetical protein
G2583_0110	CDS	118966	120168	-	hofC	Type IV pilus assembly protein PilC
G2583_0111	pseudogene	120158	121543	-	hofB	GspE family protein HofB
G2583_0112	CDS	121553	121993	-	ppdD	Prelipin peptidase dependent protein
G2583_0113	CDS	122196	123089	-	nadC	Nicotinate-nucleotide pyrophosphorylase
G2583_0114	CDS	123177	123728	+	ampD	N-acetylmuramoyl-L-alanine amidase
G2583_0115	CDS	123725	124579	+	ampE	regulatory protein AmpE
G2583_0116	CDS	124622	125995	-	aroP	Aromatic amino acid transport protein
G2583_0117	CDS	126536	127300	+	pdhR	transcriptional regulator, GntR family
G2583_0118	CDS	127461	130124	+	aceE	Pyruvate dehydrogenase E1 component
G2583_0119	CDS	130139	132031	+	aceF	Dihydrolipoyllysine-residue acetyltransferase
G2583_0120	CDS	132176	133663	+	lpd	Pyruvate/2-oxoglutarate dehydrogenase complex,
G2583_0121	CDS	133735	135588	-	yacH	hypothetical protein

Locus_tag	Type	Start	End	+/-[a]	Gene[b]	Product
G2583_0122	CDS	135943	138540	+	acnB	Aconitate hydratase 2
G2583_0123	CDS	138716	139078	+	yacL	hypothetical protein
G2583_0124	CDS	139116	139910	-	speD	S-adenosylmethionine decarboxylase proenzyme (AdoMetDC)
G2583_0125	CDS	139926	140792	-	speE	Spermidine synthase
G2583_0126	CDS	140898	141416	-	yacC	hypothetical protein
G2583_0127	CDS	141411	142961	+	cueO	Copper oxidase CueO
G2583_0128	CDS	143163	145553	-	gcd	Quinoprotein glucose dehydrogenase
G2583_0129	CDS	145759	146295	+	hpt	Hypoxanthine phosphoribosyltransferase
G2583_0130	CDS	146336	146998	-	can	Carbonic anhydrase
G2583_0131	CDS	147107	148033	+	yadG	ABC transporter, ATP-binding protein
G2583_0132	CDS	148030	148800	+	yadH	hypothetical protein
G2583_0133	CDS	148905	149345	+	yadI	PTS system IIA component domain protein
G2583_0134	CDS	149409	150638	+	yadE	Polysaccharide deacetylase domain protein
G2583_0135	CDS	150642	151022	-	panD	Aspartate 1-decarboxylase precursor (Aspartate alpha-
G2583_0136	CDS	151296	152192	+	yadD	hypothetical protein
G2583_0137	CDS	152484	153335	-	panC	Pantothenate synthetase
G2583_0138	CDS	153347	154141	-	panB	3-methyl-2-oxobutanoate hydroxymethyltransferase
G2583_0139	CDS	154276	155385	-	yadC	Putative fimbrial protein
G2583_0140	CDS	155397	155987	-	yadK	Putative fimbrial protein
G2583_0141	CDS	156008	156613	-	yadL	Putative fimbrial protein
G2583_0142	CDS	156628	157188	-	yadM	Putative fimbrial protein
G2583_0143	CDS	157190	159790	-	htrE	Probable outer membrane porin protein involved in fimbrial
G2583_0144	CDS	159832	160563	-	ecpD	Gram-negative pili assembly chaperone
G2583_0145	CDS	160644	161249	-	yadN	Putative fimbrial protein
G2583_0146	CDS	161520	162002	-	folK	2-amino-4-hydroxy-6-hydroxymethyldihydropteridine
G2583_0147	CDS	161999	163417	-	pcnB	Poly(A) polymerase
G2583_0148	CDS	163456	164382	-	yadB	Glutamyl-Q tRNA(Asp) synthetase
G2583_0149	CDS	164419	164874	-	dksA	RNA polymerase-binding protein DksA
G2583_0150	CDS	165052	165756	-	sfsA	Sugar fermentation stimulation protein A
G2583_0151	CDS	165771	166310	-	ligT	hypothetical protein
G2583_0152	CDS	166330	168804	+	hrpB	ATP-dependent helicase HrpB
G2583_0153	CDS	169000	171534	+	mrcB	Penicillin-binding protein 1B
G2583_0154	CDS	171754	173997	+	fhuA	Ferrichrome-iron receptor
G2583_0155	CDS	174048	174845	+	fhuC	Ferrichrome transport ATP-binding protein FhuC
G2583_0156	CDS	174845	175735	+	fhuD	Ferrichrome-binding periplasmic protein FhuD
G2583_0157	CDS	175732	177714	+	fhuB	Ferrichrome ABC transporter, permease protein FhuB
G2583_0158	CDS	177749	179029	-	hemL	Glutamate-1-semialdehyde 2,1-aminomutase
G2583_0159	CDS	179254	180675	+	clcA	H(+)/Cl(-) exchange transporter clcA
G2583_0160	CDS	180757	181101	+	yadR	Iron-sulfur cluster insertion protein erpA
G2583_0161	CDS	181148	181771	-	yadS	UPF0126 inner membrane protein yadS
G2583_0162	CDS	181809	182609	-	btuF	Vitamin B12-binding protein precursor
G2583_0163	CDS	182602	183300	-	mtn	MTA/SAH nucleosidase
G2583_0164	CDS	183384	184901	+	dgt	Deoxyguanosinetriphosphate triphosphohydrolase
G2583_0165	CDS	185031	186455	+	degP	Protease do precursor
G2583_0166	CDS	186610	187767	+	cdaR	hypothetical protein
G2583_0167	CDS	187856	188242	-	yaeH	UPF0325 protein yaeH
G2583_0168	CDS	188557	189381	-	dapD	2,3,4,5-tetrahydropyridine-2,6-dicarboxylate N-
G2583_0169	CDS	189412	191907	-	glnD	[Protein-PII] uridylyltransferase
G2583_0170	CDS	192146	192940	-	map	Methionine aminopeptidase
G2583_0171	ncRNA	193146	193281	+	-	ncRNA
G2583_0172	CDS	193308	194033	+	rpsB	30S ribosomal protein S2
G2583_0173	CDS	194291	195142	+	tsf	Elongation factor Ts
G2583_0174	CDS	195289	196014	+	pyrH	UMP kinase
G2583_0175	CDS	196164	196721	+	frr	Ribosome recycling factor
G2583_0176	CDS	196813	198009	+	dxr	Putative ATP-binding component of a transport system
G2583_0177	CDS	198195	198956	+	ispU	Undecaprenyl pyrophosphate synthetase
G2583_0178	CDS	198969	199826	+	cdsA	Phosphatidate cytidylyltransferase
G2583_0179	CDS	199838	201190	+	rseP	Regulator of sigma E protease
G2583_0180	CDS	201220	203652	+	yaeT	Outer membrane protein assembly factor yaeT precursor
G2583_0181	CDS	203774	204259	+	skp	Chaperone protein skp precursor

Locus_tag	Type	Start	End	+/-[a]	Gene[b]	Product
G2583_0182	CDS	204263	205288	+	lpxD	UDP-3-O-[3-hydroxymyristoyl] glucosamine N-acyltransferase
G2583_0183	CDS	205393	205848	+	fabZ	(3R)-hydroxymyristoyl-(acyl carrier protein) dehydratase
G2583_0184	CDS	205852	206640	+	lpxA	Acyl-[acyl-carrier-protein]--UDP-N-acetylglucosamine O-
G2583_0185	CDS	206640	207788	+	lpxB	Lipid-A-disaccharide synthase
G2583_0186	CDS	207785	208381	+	rnhB	Ribonuclease HII
G2583_0187	CDS	208418	211900	+	dnaE	DNA polymerase III subunit alpha
G2583_0188	CDS	211913	212872	+	accA	Acetyl-coenzyme A carboxylase carboxyl transferase subunit
G2583_0189	CDS	212971	215112	+	ldcC	Lysine decarboxylase, constitutive
G2583_0190	CDS	215169	215558	+	yaeR	Putative lactoylglutathione lyase
G2583_0191	CDS	215623	216918	+	tilS	tRNA(Ile)-lysidine synthase (tRNA(Ile)-lysidine synthetase)
G2583_0192	CDS	216971	217231	-	rof	Rof protein
G2583_0193	CDS	217218	217436	-	yaeP	hypothetical protein
G2583_0194	CDS	217584	218129	+	yaeQ	hypothetical protein
G2583_0195	CDS	218126	218548	+	yaeJ	hypothetical protein
G2583_0196	CDS	218562	219272	+	nlpE	Copper homeostasis protein CutF
G2583_0197	CDS	219472	220296	-	yaeF	hypothetical protein
G2583_0198	CDS	220349	222067	-	proS	Prolyl-tRNA synthetase
G2583_0199	CDS	222178	222885	-	yaeB	hypothetical protein
G2583_0200	CDS	222882	223286	-	rcsF	Regulator in colanic acid synthesis
G2583_0201	CDS	223404	224219	-	metQ	D-methionine-binding lipoprotein metQ precursor
G2583_0202	CDS	224259	224912	-	metI	D-methionine transport system permease protein metI
G2583_0203	CDS	224905	225936	-	metN	Methionine import ATP-binding protein metN
G2583_0204	CDS	226124	226699	+	gmhB	D,D-heptose 1,7-bisphosphate phosphatase
G2583_0205	rRNA	227062	228603	+	rrsH	16S ribosomal RNA
G2583_0206	tRNA	228672	228748	+	-	Ile tRNA
G2583_0207	tRNA	228790	228867	+	-	Ala tRNA
G2583_0208	rRNA	229050	231952	+	rrlH	23S ribosomal RNA
G2583_0209	rRNA	232047	232162	+	rrfH	5S ribosomal RNA
G2583_0210	tRNA	232216	232294	+	-	Asp tRNA
G2583_0211	CDS	232456	233259	+	dkgB	2,5-diketo-D-gluconic acid reductase B
G2583_0212	CDS	233256	234170	-	yafC	putative transcriptional regulator LYSR-type
G2583_0213	CDS	234411	235211	+	yafD	hypothetical protein
G2583_0214	CDS	235289	236059	+	yafE	Methyltransferase, UbiE/COQ5 family
G2583_0215	CDS	236107	237465	-	mltD	Membrane-bound lytic murein transglycosylase D precursor
G2583_0216	CDS	237537	238292	-	gloB	Hydroxyacylglutathione hydrolase
G2583_0217	CDS	238308	239048	+	yafS	hypothetical protein
G2583_0218	CDS	239045	239512	-	rnhA	RNase HI
G2583_0219	CDS	239577	240308	+	dnaQ	DNA polymerase III, epsilon subunit
G2583_0220	tRNA	240440	240518	+	-	Asp tRNA
G2583_0221	CDS	240845	241645	+	yafT	hypothetical protein
G2583_0222	CDS	241830	242126	-	-	hypothetical protein
G2583_0223	CDS	242123	242578	-	-	hypothetical protein
G2583_0224	CDS	242575	243471	-	-	hypothetical protein
G2583_0225	CDS	243492	243971	-	yhhZ	hypothetical protein
G2583_0226	CDS	243937	245436	-	ImpA	ImpA domain protein
G2583_0227	CDS	245357	248821	-	-	putative macrophage toxin
G2583_0228	CDS	248900	250312	-	ImpA	ImpA domain protein
G2583_0229	CDS	250317	251060	-	-	Type VI secretion-associated protein, VC_A0118 family
G2583_0230	CDS	251057	253840	-	clpB	type VI secretion ATPase, ClpV1 family
G2583_0231	CDS	253849	254610	-	-	hypothetical protein
G2583_0232	CDS	254615	255946	-	-	Uncharacterized protein conserved in bacteria
G2583_0233	CDS	255949	256473	-	-	Type VI secretion lipoprotein, VC_A0113 family
G2583_0234	CDS	256470	257480	-	-	hypothetical protein
G2583_0235	CDS	257774	258856	-	-	hypothetical protein
G2583_0236	CDS	258820	260670	-	-	hypothetical protein
G2583_0237	CDS	260674	261087	-	-	hypothetical protein
G2583_0238	pseudogene	261094	262569	-	-	hypothetical protein
G2583_0239	CDS	262620	262844	-	-	hypothetical protein
G2583_0240	CDS	262879	263379	-	-	hypothetical protein
G2583_0241	CDS	263806	263949	+	-	hypothetical protein

Locus_tag	Type	Start	End	+/-[a]	Gene[b]	Product
G2583_0242	CDS	264076	264594	+	-	Hcp
G2583_0243	CDS	264627	264764	+	-	hypothetical protein
G2583_0244	CDS	264804	266945	+	VgrG	VgrG
G2583_0245	pseudogene	267021	271253	+	rhsG1	rhsG-1
G2583_0246	CDS	271514	272110	+	-	Ankyrin repeat protein
G2583_0247	pseudogene	272292	273800	+	rhsG2	RhsG-2
G2583_0248	CDS	273803	274378	+	yibG	hypothetical protein
G2583_0249	CDS	274400	274582	+	-	hypothetical protein
G2583_0250	CDS	274726	275016	+	-	Hypothetical membrane protein
G2583_0251	CDS	275124	276260	+	yhhI	ISEc3, transposase
G2583_0252	CDS	276263	278023	+	-	unknown protein associated with Rhs element
G2583_0253	pseudogene	278007	278488	+	ydcD	hypothetical protein
G2583_0254	CDS	278669	279841	+	yncI	Putative transposase yncI
G2583_0255	CDS	279959	280729	-	yafV	Hydrolase, carbon-nitrogen family
G2583_0256	CDS	280883	281356	+	ivy	Inhibitor of vertebrate lysozyme precursor
G2583_0257	CDS	281399	283843	-	fadE	Acyl-coenzyme A dehydrogenase
G2583_0258	CDS	284083	284661	+	lpcA	Phosphoheptose isomerase
G2583_0259	CDS	284766	285533	+	yafJ	Glutamine amidotransferase, class II
G2583_0260	CDS	285504	286244	-	yafK	hypothetical protein
G2583_0261	CDS	286400	286678	-	yafQ	Addiction module toxin, RelE/StbE family
G2583_0262	CDS	286681	286941	-	dinJ	Addiction module antitoxin, RelB/DinJ family
G2583_0263	CDS	287091	287900	+	yafL	NlpC/P60 family protein
G2583_0264	CDS	288076	288372	+	yafM	Putative transposase
G2583_0265	CDS	288806	290545	-	FhiA	Type III secretion protein, FHIPEP family
G2583_0266	CDS	290490	291275	+	mbhA	Putative motility protein
G2583_0267	CDS	291346	292401	+	dinB	DNA polymerase IV
G2583_0268	CDS	292453	292746	+	yafN	Prevent-host-death family protein
G2583_0269	CDS	292749	293147	+	yafO	putative toxin YafO
G2583_0270	CDS	293157	293609	+	yafP	Acetyltransferase, GNAT family
G2583_0271	CDS	293916	294182	+	ykfJ	hypothetical protein
G2583_0272	CDS	294151	294651	+	prfH	Probable peptide chain release factor
G2583_0273	CDS	294708	296165	-	pepD	Aminoacyl-histidine dipeptidase
G2583_0274	CDS	296426	296884	+	gpt	Xanthine phosphoribosyltransferase
G2583_0275	CDS	296976	298220	+	frsA	Esterase frsA
G2583_0276	CDS	298278	298679	+	crl	Sigma factor-binding protein crl
G2583_0277	CDS	298718	299773	-	phoE	Outer membrane protein (porin)
G2583_0278	CDS	300061	301164	+	proB	Glutamate 5-kinase
G2583_0279	CDS	301176	302429	+	proA	Gamma-glutamyl phosphate reductase
G2583_0280	tRNA	302544	302619	+	-	Thr tRNA
G2583_0281	CDS	302634	303794	-	-	Site-specific recombinase, phage integrase family
G2583_0282	CDS	304108	304452	-	-	hypothetical protein
G2583_0283	CDS	304553	305317	-	eaa	hypothetical protein
G2583_0284	CDS	305314	305820	-	-	ORF8
G2583_0285	CDS	305817	306038	-	-	hypothetical C4-type zinc finger protein TraR-family
G2583_0286	CDS	306137	306418	-	-	hypothetical protein
G2583_0287	CDS	306429	306620	-	-	unknown protein encoded by prophage CP-933K
G2583_0288	CDS	306593	306781	-	-	hypothetical protein
G2583_0289	CDS	306772	307452	-	-	Exonuclease
G2583_0290	CDS	307449	308234	-	bet	Bet protein
G2583_0291	CDS	308240	308536	-	gamW	Gam protein
G2583_0292	CDS	308722	308988	-	-	antitermination protein
G2583_0293	CDS	309110	309232	-	ral	Lambda ant-restriction protein
G2583_0294	CDS	309577	310059	+	-	Superinfection exclusion protein B
G2583_0295	CDS	310060	310383	-	-	Antitermination protein
G2583_0296	CDS	310735	311046	-	-	Gene 38 protein
G2583_0297	CDS	311136	311768	-	ymfK	SOS-response transcriptional repressors
G2583_0298	CDS	311872	312087	+	-	gene 40 protein
G2583_0299	CDS	312207	312500	+	-	Phage regulatory protein
G2583_0300	CDS	312533	313432	+	-	Putative replication protein O of bacteriophage
G2583_0301	CDS	313429	314130	+	-	putative replication protein P of bacteriophage BP-933W

Locus_tag	Type	Start	End	+/-[a]	Gene[b]	Product
G2583_0302	CDS	314127	314417	+	-	Ren protein
G2583_0303	CDS	314491	314931	+	ninB	Unknown protein encoded within prophage
G2583_0304	CDS	314928	315455	+	-	Putative DNA N-6-adenine-methyltransferase of bacteriophage
G2583_0305	CDS	315452	315634	+	ninE	NinE protein
G2583_0306	CDS	315794	316399	+	NinG	NinG protein
G2583_0307	CDS	316396	317067	+	-	Serine/threonine-protein phosphatase 1
G2583_0308	CDS	317058	317576	+	-	putative antitermination protein
G2583_5263	tRNA	317640	317714	+	-	Asn tRNA
G2583_5264	tRNA	317720	317795	+	-	Thr tRNA
G2583_5265	tRNA	317799	317873	+	-	Gly tRNA
G2583_0309	CDS	317979	318209	+	-	conserved hypothetical protein
G2583_0310	CDS	318392	320347	+	YjhS	YjhS
G2583_0311	CDS	320610	320786	-	-	hypothetical protein
G2583_0312	CDS	320794	321000	+	-	Putative lysis protein S of prophage CP-933V
G2583_0313	CDS	321000	321497	+	ybcS	Phage-related lysozyme (muraminidase)
G2583_0314	CDS	321482	321958	+	-	Putative Rz endopeptidase from lambdoid prophage DLP12
G2583_0315	CDS	322041	322181	+	-	unknown protein encoded within prophage CP-933R
G2583_0316	CDS	322423	322737	+	-	Putative transcriptional regulator
G2583_0317	CDS	322818	323042	-	ynfO	unknown protein encoded within prophage CP-933R
G2583_0318	CDS	323197	323322	+	-	Prophage Qin DNA packaging protein NU1-like protein
G2583_0319	CDS	323444	323953	+	-	Prophage Qin DNA packaging protein NU1-like protein
G2583_0320	CDS	323889	325853	+	-	Putative terminase large subunit of prophage CP-933O
G2583_0321	CDS	325837	326043	+	-	Head-stabilizing protein
G2583_0322	CDS	326040	327632	+	-	Putative capsid protein of prophage
G2583_0323	CDS	327622	329127	+	-	Head-tail preconnector protein GP5
G2583_0324	CDS	329164	329511	+	-	Head decoration protein
G2583_0325	CDS	329569	330597	+	-	Major head protein
G2583_0326	CDS	330649	331023	+	-	Uncharacterized 13.5 kDa protein in GP7-GP8 intergenic region
G2583_0327	CDS	331016	331369	+	-	Putative head-tail joining protein of prophage
G2583_0328	CDS	331381	331914	+	-	Prophage minor tail protein Z
G2583_0329	CDS	331911	332306	+	-	Minor tail protein U
G2583_0330	CDS	332284	333054	+	-	Putative tail component of prophage CP-933X
G2583_0331	CDS	333070	333492	+	-	Putative tail component of prophage
G2583_0332	CDS	333474	333908	+	-	Minor tail protein T
G2583_0333	CDS	333901	336450	+	-	Phage-related minor tail protein
G2583_0334	CDS	336447	336776	+	-	minor tail protein
G2583_0335	CDS	336776	337474	+	-	Phage-related protein
G2583_0336	CDS	337480	338223	+	-	Tail assembly protein
G2583_0337	CDS	338121	338792	+	-	Tail assembly protein I
G2583_0338	CDS	338853	342266	+	-	Phage-related protein, tail component
G2583_0339	CDS	342337	342936	+	lomK	Enterobacterial Ail/Lom family protein
G2583_0340	CDS	342990	344312	+	-	Putative tail fiber protein
G2583_0341	CDS	344689	345267	+	nleG	hypothetical protein
G2583_0342	CDS	345587	345904	-	-	PotB, trcA, ORF2, ORF3, ORF4 genes,
G2583_0343	CDS	346007	346633	-	-	PotB, trcA, ORF2, ORF3, ORF4 genes,
G2583_0344	CDS	346816	347406	+	IpgB	Putative chaperone protein
G2583_0345	CDS	347651	347782	+	-	Tail fiber assembly protein
G2583_0346	CDS	348095	348235	-	intR	Putative integrase for prophage CP-933R
G2583_0347	CDS	348406	349386	+	-	NleB
G2583_0348	CDS	349463	349741	-	-	hypothetical protein
G2583_0349	CDS	349803	350270	-	gogB	conserved hypothetical protein
G2583_0350	CDS	350472	351164	-	-	conserved hypothetical protein
G2583_0351	CDS	351744	352976	+	-	Integrase protein for prophage CP-933I
G2583_0352	CDS	352980	353396	+	-	unknown protein encoded in prophage CP-933I
G2583_0353	CDS	353369	353986	+	-	unknown protein encoded in prophage CP-933I
G2583_0354	CDS	353986	354444	+	-	unknown protein encoded in prophage CP-933I
G2583_0355	pseudogene	354437	355071	+	-	hypothetical protein
G2583_0356	CDS	355101	355622	+	-	unknown protein encoded in prophage CP-933I
G2583_0357	CDS	355690	356256	+	-	unknown protein encoded in prophage CP-933I
G2583_0358	CDS	356666	356938	-	ogrK	Putative activator encoded in prophage CP-933I

Locus_tag	Type	Start	End	+/-[a]	Gene[b]	Product
G2583_0359	CDS	356944	357495	-	psuI	Putative polarity suppression protein encoded in CP-933I
G2583_0360	CDS	357492	358244	-	sidI	Putative capsid morphogenesis protein encoded in CP-933I
G2583_0361	CDS	358632	358802	+	-	unknown protein encoded in prophage CP-933I
G2583_0362	CDS	359163	359423	+	alpA	Phage DNA binding protein
G2583_0363	CDS	359420	359977	+	-	putative CI repressor
G2583_0364	CDS	359974	360195	+	-	hypothetical protein
G2583_0365	CDS	360195	360518	+	-	unknown protein encoded in prophage CP-933I
G2583_0366	CDS	360475	362865	+	-	Alpha replication protein of prophage CP-933I
G2583_0367	CDS	362998	363954	-	-	unknown protein encoded in prophage CP-933I
G2583_0368	CDS	364629	365528	-	yagP	putative LysR-like transcriptional regulator
G2583_0369	CDS	365627	366349	+	-	Clavaldehyde dehydrogenase
G2583_0370	CDS	366515	366793	+	-	hypothetical protein
G2583_0371	CDS	367068	367217	+	-	hypothetical protein
G2583_0372	CDS	367496	368398	-	-	LysR substrate binding domain protein
G2583_0373	CDS	368566	369702	+	-	Hydrolase of the alpha/beta superfamily
G2583_0374	CDS	369778	370971	+	-	Purine ribonucleoside efflux pump NepI
G2583_0375	pseudogene	371091	372107	-	yagQ	Putative xanthine dehydrogenase accessory factor
G2583_0376	CDS	372117	374315	-	yagR	Putative xanthine dehydrogenase yagR molybdenum-binding
G2583_0377	CDS	374312	375268	-	yagS	FAD binding domain in molybdopterin dehydrogenase
G2583_0378	CDS	375265	375954	-	yagT	Putative xanthine dehydrogenase yagT iron-sulfur-binding
G2583_0379	CDS	376372	376986	+	yagU	Inner membrane protein yagU
G2583_0380	CDS	377234	377563	-	ykgJ	putative ferredoxin
G2583_0381	CDS	377876	378631	-	yagV	hypothetical protein
G2583_0382	CDS	378555	380198	-	yagW	putative receptor
G2583_0383	CDS	380188	382713	-	yagX	putative enzyme
G2583_0384	CDS	382739	383407	-	matC	hypothetical protein
G2583_0385	CDS	383465	384052	-	matB	hypothetical protein
G2583_0386	CDS	384127	384717	-	matA	MatA
G2583_0387	CDS	385122	385346	-	-	hypothetical protein
G2583_0388	CDS	385493	385684	+	ykgL	hypothetical protein
G2583_0389	CDS	385754	385894	-	ykgO	50S ribosomal protein L36
G2583_0390	CDS	385894	386160	-	ykgM	Ribosomal protein L31
G2583_0391	CDS	387096	388238	-	-	Oxidoreductase, FAD/FMN-binding
G2583_0392	CDS	388473	389393	-	ycjY	hypothetical protein
G2583_0393	CDS	389487	390476	+	ycaN	Putative LysR-like transcriptional regulator
G2583_0394	CDS	390764	391120	-	-	hypothetical protein
G2583_0395	CDS	391151	391279	+	-	hypothetical protein
G2583_0396	CDS	391313	391888	-	-	2,5-diketo-D-gluconic acid reductase A
G2583_0397	CDS	392454	396707	+	eaeH	Attaching and effacing protein-like protein precursor
G2583_0398	CDS	396828	397718	-	ykgA	Putative AraC-like transcriptional regulator
G2583_0399	CDS	397919	398803	+	-	Putative dehydrogenase
G2583_0400	CDS	398963	399556	-	ykgB	protein of unknown function DUF417
G2583_0401	CDS	399539	399808	+	-	hypothetical protein
G2583_0402	CDS	399568	399819	-	ykgI	hypothetical protein
G2583_0403	CDS	399913	401238	-	ykgC	Pyridine nucleotide-disulphide oxidoreductase
G2583_0404	CDS	401464	402318	+	ykgD	Hypothetical transcriptional regulator ykgD
G2583_0405	CDS	402845	403564	+	ykgE	Cysteine-rich domain protein
G2583_0406	CDS	403575	405002	+	ykgF	Iron-sulfur cluster binding protein
G2583_0407	CDS	404995	405690	+	ykgG	hypothetical protein
G2583_0408	CDS	405645	405857	-	-	hypothetical protein
G2583_0409	pseudogene	405933	406601	-	ykgH	hypothetical protein
G2583_0410	CDS	406783	409080	-	-	Putative autotransporter
G2583_0411	CDS	409122	409883	-	-	hypothetical protein
G2583_0412	CDS	410037	410831	-	-	hypothetical protein
G2583_0413	pseudogene	411161	411723	-	fimX	Site-specific recombinase, phage integrase family
G2583_0414	CDS	412531	412782	+	-	hypothetical protein
G2583_0415	CDS	412784	414472	-	betA	Choline dehydrogenase
G2583_0416	CDS	414486	415958	-	betB	Betaine aldehyde dehydrogenase
G2583_0417	CDS	415972	416577	-	betI	Transcriptional regulator
G2583_0418	CDS	416688	418721	+	betT	High-affinity choline transport protein

Locus_tag	Type	Start	End	+/-[a]	Gene[b]	Product
G2583_0419	CDS	419229	423278	+	AidA-I	AidA-I adhesin-like protein
G2583_0420	CDS	423411	424508	+	yahA	LuxR-family transcriptional regulator/cyclic diguanylate
G2583_0421	pseudogene	424550	425481	-	yahB	Uncharacterized HTH-type transcriptional regulator yahB
G2583_0422	pseudogene	425573	426090	-	yahC	Uncharacterized protein yahC
G2583_0423	CDS	426348	426953	+	yahD	Ankyrin repeat protein
G2583_0424	CDS	426993	427856	+	yahE	hypothetical protein
G2583_0425	CDS	427846	429393	+	yahF	Bacterial FdrA protein
G2583_0426	CDS	429393	430811	+	yahG	hypothetical protein
G2583_0427	CDS	430830	431294	+	yahH	hypothetical protein
G2583_0428	CDS	431326	432276	+	yahI	Carbamate kinase family protein
G2583_0429	CDS	432286	433668	+	yahJ	Amidohydrolase family protein
G2583_0430	CDS	433937	434377	+	-	hypothetical protein
G2583_0431	CDS	434628	435614	+	-	Sugar ABC transporter, periplasmic sugar-binding protein
G2583_0432	CDS	435663	437147	+	-	Sugar ABC transporter, ATP-binding protein
G2583_0433	CDS	437140	438111	+	-	Putative permease component of transport system, probably
G2583_0434	CDS	438108	439064	+	-	Putative permease component of transport system
G2583_0435	CDS	439151	440200	+	yahK	Oxidoreductase, zinc-binding dehydrogenase family
G2583_0436	CDS	440443	441258	+	yahL	hypothetical protein
G2583_0437	tRNA	441604	441684	+	-	Xaa tRNA
G2583_0438	CDS	441743	441919	+	yahM	hypothetical protein
G2583_0439	CDS	441936	442607	-	yahN	Putative homoserine/threonine efflux protein
G2583_0440	CDS	442754	443029	+	yahO	hypothetical protein
G2583_0441	CDS	443127	444713	-	prpR	Propionate catabolism operon regulatory protein PrpR
G2583_0442	CDS	444952	445842	+	prpB	Methylisocitrate lyase
G2583_0443	CDS	445998	447167	+	prpC	2-methylcitrate synthase
G2583_0444	CDS	447201	448652	+	prpD	2-methylcitrate dehydratase
G2583_0445	CDS	448692	450578	+	prpE	Propionate--CoA ligase
G2583_0446	pseudogene	450583	450820	+	nrdB	mannitol-1-phosphate 5-dehydrogenase
G2583_0447	CDS	451284	452543	+	codB	Cytosine permease
G2583_0448	CDS	452533	453816	+	codA	Cytosine deaminase and related metal-dependent hydrolases
G2583_0449	CDS	453949	454872	-	cynR	DNA-binding transcriptional regulator CynR
G2583_0450	CDS	454958	455617	+	cynT	Carbonic anhydrase 1
G2583_0451	CDS	455648	456118	+	cynS	Cyanate hydratase
G2583_0452	CDS	456151	457305	+	cynX	Cyanate transport
G2583_0453	CDS	457408	458019	-	lacA	Galactoside O-acetyltransferase LacA
G2583_0454	CDS	458085	459338	-	lacY	Lactose permease
G2583_0455	CDS	459390	462464	-	lacZ	Beta-D-galactosidase
G2583_0456	CDS	462587	463678	-	lacI	lac repressor
G2583_0457	CDS	463706	464659	-	-	putative AraC-like transcriptional regulator
G2583_0458	CDS	464679	465467	-	-	Beta-lactamase fold protein
G2583_0459	CDS	465570	466517	-	mhpR	DNA-binding transcriptional activator, 3HPP-binding
G2583_0460	CDS	466594	468258	+	mhpA	3-(3-hydroxy-phenyl)propionate/3-hydroxycinnamic acid
G2583_0461	CDS	468260	469204	+	mhpB	2,3-dihydroxyphenylpropionate/2,3-dihydroxicinnamic acid 1,2-
G2583_0462	CDS	469159	470088	+	mhpC	2-hydroxy-6-oxononadienedioate/2-hydroxy-6-
G2583_0463	CDS	470098	470907	+	mhpD	2-keto-4-pentenoate hydratase
G2583_0464	CDS	470904	471854	+	mhpF	Acetaldehyde dehydrogenase
G2583_0465	CDS	471851	472864	+	mhpE	4-hydroxy-2-oxovalerate aldolase
G2583_0466	CDS	473298	474554	+	mhpT	Putative transport protein
G2583_0467	CDS	474656	475195	+	yaiL	Nucleoprotein/polynucleotide-associated enzyme
G2583_0468	CDS	475320	476153	-	frmB	putative esterase
G2583_0469	CDS	476246	477355	-	frmA	S-(hydroxymethyl)glutathione dehydrogenase
G2583_0470	CDS	477390	477686	-	frmR	regulator protein FrmR
G2583_0471	CDS	477825	478871	-	afuC	Fe(3+) ions import ATP-binding protein fbpC
G2583_0472	CDS	478883	480961	-	afuB	Binding-protein-dependent transport systems inner membrane
G2583_0473	CDS	481030	482061	-	afuA	Periplasmic ferric iron-binding protein
G2583_0474	CDS	482058	483362	-	UhpC	putative permease; hexosephosphate transport
G2583_0475	CDS	483447	484988	-	-	Integral membrane sensor signal transduction histidine kinase
G2583_0476	CDS	484988	485617	-	-	Putative response regulator
G2583_0477	CDS	485920	486882	+	tauA	Taurine transport system periplasmic protein
G2583_0478	CDS	486895	487662	+	tauB	Taurine import ATP-binding protein tauB

Locus_tag	Type	Start	End	+/-[a]	Gene[b]	Product
G2583_0479	CDS	487659	488486	+	tauC	ABC-type nitrate/sulfonate/bicarbonate transpo rt system,
G2583_0480	CDS	488483	489334	+	tauD	Taurine dioxygenase
G2583_0481	CDS	489441	490448	-	hemB	Delta-aminolevulinic acid dehydratase
G2583_0482	CDS	490941	493883	+	yaiU	Putative flagellin structural protein
G2583_0483	CDS	493926	494594	+	yaiV	putative DNA-binding transcriptional regulator
G2583_0484	CDS	494595	495752	-	ampH	Penicillin-binding protein ampH
G2583_0485	CDS	496104	497324	+	sbmA	Inner-membrane transport protein, Microcin 25
G2583_0486	CDS	497337	498431	+	yaiW	hypothetical protein
G2583_0487	CDS	498490	498798	-	yaiY	Inner membrane protein yaiY
G2583_0488	CDS	498926	499270	+	yaiZ	hypothetical protein
G2583_0489	CDS	499294	500388	-	ddlA	D-alanine--D-alanine ligase A
G2583_0490	CDS	500851	501111	+	iraP	Anti-adapter protein iraP
G2583_0491	CDS	501143	502627	+	phoA	Alkaline phosphatase
G2583_0492	CDS	502728	503066	+	psiF	Phosphate starvation-inducible protein
G2583_0493	CDS	503168	504283	+	adrA	MASE2 domain/diguanylate cyclase
G2583_0494	CDS	504300	505109	-	proC	Pyrroline-5-carboxylate reductase
G2583_0495	CDS	505229	505687	+	yaiI	hypothetical protein
G2583_0496	CDS	505870	506394	+	aroL	Shikimate kinase 2
G2583_0497	CDS	506444	506635	+	yaiA	hypothetical protein
G2583_0498	CDS	506893	507570	+	aroM	AroM protein
G2583_0499	CDS	507642	507926	+	yaiE	UPF0345 protein yaiE
G2583_0500	CDS	508184	510349	+	ydbD	hypothetical protein
G2583_0501	CDS	510346	510801	+	-	hypothetical protein
G2583_0502	CDS	510879	511790	-	rdgC	Recombination-associated protein rdgC
G2583_0503	CDS	511915	512823	+	mak	Possible NAGC-like transcriptional regulator
G2583_0504	CDS	512966	514234	-	araJ	protein AraJ
G2583_0505	CDS	514276	517419	-	sbcC	Nuclease SbcCD, C subunit
G2583_0506	CDS	517416	518618	-	sbcD	Nuclease sbcCD subunit D
G2583_0507	CDS	518808	519497	+	phoB	Positive response regulator for pho regulon
G2583_0508	CDS	519555	520850	+	phoR	Signal transduction histidine kinase
G2583_0509	CDS	521257	522576	+	brnQ	Branched-chain amino acid transport system 2 carrier protein
G2583_0510	CDS	522652	524025	+	proY	Proline-specific permease proY
G2583_0511	CDS	524181	525998	+	malZ	Maltodextrin glucosidase
G2583_0512	CDS	526186	527631	+	-	hypothetical protein
G2583_0513	CDS	527652	528233	-	acpH	Acyl carrier protein phosphodiesterase
G2583_0514	CDS	528325	529395	+	queA	S-adenosylmethionine:tRNA ribosyltransferase-isomerase
G2583_0515	CDS	529450	530577	+	tgt	Queuine tRNA-ribosyltransferase
G2583_0516	CDS	530600	530932	+	yajC	UPF0092 membrane protein yajC
G2583_0517	CDS	530960	532807	+	secD	Protein-export membrane protein secD
G2583_0518	CDS	532818	533789	+	secF	Protein-export membrane protein SecF
G2583_0519	CDS	533941	534183	+	-	UPF0156 protein Z0509/ECs0461
G2583_0520	CDS	534176	534457	+	-	Plasmid stabilization system protein, RelE/ParE family
G2583_0521	CDS	534545	534892	+	yajD	hypothetical protein
G2583_0522	CDS	535069	535953	-	tsx	Nucleoside-specific channel-forming protein, Tsx
G2583_0523	CDS	536252	536791	-	yajI	hypothetical protein
G2583_0524	CDS	536942	537391	+	nrdR	ATP-cone domain protein
G2583_0525	CDS	537395	538498	+	ribD	Bifunctional deaminase-reductase, C-terminal:Riboflavin
G2583_0526	CDS	538587	539057	+	ribE	6,7-dimethyl-8-ribityllumazine synthase
G2583_0527	CDS	539077	539496	+	nusB	N utilization substance protein B-like protein
G2583_0528	CDS	539574	540551	+	thiL	Thiamine-monophosphate kinase
G2583_0529	CDS	540529	541044	+	pgpA	Phosphatidylglycerophosphatase A
G2583_0530	CDS	541222	542793	-	-	hypothetical protein
G2583_0531	CDS	543024	543998	-	yajO	Oxidoreductase, aldo/keto reductase family
G2583_0532	CDS	544053	545915	-	dxs	1-deoxy-D-xylulose-5-phosphate synthase
G2583_0533	CDS	545940	546839	-	ispA	Geranyltranstransferase
G2583_0534	CDS	546839	547081	-	xseB	Exodeoxyribonuclease 7 small subunit
G2583_0535	CDS	547287	548735	+	thiI	Thiamine biosynthesis protein thiI
G2583_0536	CDS	548789	549385	-	yajL	4-methyl-5(Beta-hydroxyethyl)-thiazole monophosphate
G2583_0537	CDS	549342	550253	-	panE	2-dehydropantoate 2-reductase
G2583_0538	CDS	550421	550912	+	yajQ	UPF0234 protein yajQ

Locus_tag	Type	Start	End	+/-[a]	Gene[b]	Product
G2583_0539	CDS	551040	552410	-	yajR	Hypothetical transport protein YajR
G2583_0540	CDS	552553	553443	-	cyoE	Protoheme IX farnesyltransferase
G2583_0541	CDS	553455	553784	-	cyoD	Cytochrome O ubiquinol oxidase protein CyoD
G2583_0542	CDS	553784	554398	-	cyoC	Cytochrome o ubiquinol oxidase subunit 3
G2583_0543	CDS	554388	556379	-	cyoB	Ubiquinol oxidase subunit 1 (Ubiquinol oxidase polypeptide I)
G2583_0544	CDS	556401	557348	-	cyoA	Cytochrome o ubiquinol oxidase, subunit II
G2583_0545	CDS	557808	559283	-	ampG	Regulates beta-lactamase synthesis
G2583_0546	CDS	559327	559905	-	yajG	hypothetical protein
G2583_0547	CDS	560210	560527	+	bolA	transcriptional regulator BolA
G2583_0548	CDS	560871	562169	+	tig	Trigger factor
G2583_0549	CDS	562415	563038	+	clpP	ATP-dependent Clp protease proteolytic subunit
G2583_0550	CDS	563164	564438	+	clpX	ATP-dependent Clp protease ATP-binding subunit clpX
G2583_0551	CDS	564581	566980	+	lon	DNA-binding ATP-dependent protease La
G2583_0552	CDS	567189	567461	+	hupB	DNA-binding protein HU-beta
G2583_0553	CDS	567653	569524	+	ppiD	Peptidylprolyl isomerase
G2583_0554	CDS	569675	570046	+	ybaV	Competence protein ComEA
G2583_0555	CDS	570152	570550	+	ybaW	Thioesterase family protein
G2583_0556	CDS	570602	571297	-	queC	Queuosine biosynthesis protein queC
G2583_0557	CDS	571362	573077	-	ybaE	Bacterial extracellular solute-binding protein, family 5
G2583_0558	CDS	573150	573980	+	cof	Predicted hydrolases of the HAD superfamily
G2583_0559	CDS	574006	574590	+	ybaO	Putative transcriptional regulator YbaO
G2583_0560	CDS	574620	576392	+	mdlA	Multidrug resistance, ATP-binding protein mdlA
G2583_0561	CDS	576385	578166	+	mdlB	Multidrug resistance-like ATP-binding protein mdlB
G2583_0562	CDS	578347	578685	+	glnK	Glutamine synthetase regulation protein
G2583_0563	CDS	578715	580001	+	amtB	Ammonia channel precursor
G2583_0564	CDS	580050	580910	-	tesB	Acyl-CoA thioesterase II
G2583_0565	CDS	581128	581700	+	ybaY	Glycoprotein/polysaccharide metabolism precursor
G2583_0566	CDS	581733	582122	-	ybaZ	hypothetical protein
G2583_0567	ncRNA	582207	582304	+	srpB	ncRNA
G2583_0568	CDS	582423	582776	+	ybaA	hypothetical protein
G2583_0569	CDS	582818	584374	-	ylaB	hypothetical protein
G2583_0570	CDS	584532	585041	-	ylaC	hypothetical protein
G2583_0571	CDS	585119	585670	-	maa	Maltose O-acetyltransferase
G2583_0572	CDS	585842	586060	-	hha	Haemolysin expression modulating protein
G2583_0573	CDS	586086	586460	-	ybaJ	hypothetical protein
G2583_0574	CDS	587006	590155	-	acrB	Acriflavine resistance protein B
G2583_0575	CDS	590178	591371	-	acrA	Acriflavine resistance protein A
G2583_0576	CDS	591513	592160	+	acrR	DNA-binding transcriptional repressor
G2583_0577	CDS	592288	595650	+	kefA	Potassium efflux system KefA
G2583_0578	CDS	595689	595853	-	ybaM	hypothetical protein
G2583_0579	CDS	595867	596394	-	priC	Primosomal replication protein N''
G2583_0580	CDS	596464	596841	+	ybaN	Inner membrane protein ybaN
G2583_0581	CDS	596994	597545	+	apt	Adenine phosphoribosyltransferase
G2583_0582	CDS	597674	599605	+	dnaX	DNA polymerase III, tau subunit
G2583_0583	CDS	599658	599987	+	ybaB	UPF0133 protein ybaB
G2583_0584	CDS	599987	600592	+	recR	Recombination protein recR
G2583_0585	CDS	600702	602576	+	htpG	Chaperone protein htpG
G2583_0586	CDS	602697	603401	+	adk	Adenylate kinase
G2583_0587	CDS	603533	604495	+	hemH	Ferrochelatase
G2583_0588	CDS	604492	605451	-	aes	Acetyl esterase
G2583_0589	CDS	605603	606907	+	gsk	Inosine-guanosine kinase
G2583_0590	CDS	607040	608716	-	ybaL	Transporter, monovalent cation:proton antiporter-2 family
G2583_0591	CDS	608954	610174	-	fsr	Fosmidomycin resistance protein
G2583_0592	CDS	610392	612044	+	ushA	UDP-sugar hydrolase/5'-nucleotidase
G2583_0593	CDS	612081	612560	-	ybaK	hypothetical protein
G2583_0594	ncRNA	612683	612765	+	sroB	ncRNA
G2583_0595	CDS	612764	613558	-	ybaP	GumN family protein
G2583_0596	CDS	613642	614037	+	ybaQ	Addiction module antidote protein, HigA family
G2583_0597	CDS	614252	616756	-	copA	Copper-transporting P-type ATPase
G2583_0598	CDS	617018	617950	+	ybaS	Glutaminase 1

Locus_tag	Type	Start	End	+/-[a]	Gene[b]	Product
G2583_0599	CDS	617953	619245	+	ybaT	Amino acid permease family protein
G2583_0600	CDS	619583	620938	+	-	Putative outer membrane export protein
G2583_0601	CDS	621041	641818	+	-	hypothetical protein
G2583_0602	CDS	641822	643984	+	-	Putative cytoplasmic membrane export protein
G2583_0603	CDS	643981	645156	+	-	Membrane spanning export protein
G2583_0604	CDS	645153	645560	+	cueR	DNA-binding transcriptional activator of copper-responsive
G2583_0605	CDS	645764	646186	-	-	hypothetical protein
G2583_0606	CDS	646271	647287	-	-	hypothetical protein
G2583_0607	CDS	647654	648016	+	-	hypothetical protein
G2583_0608	CDS	648195	648653	-	ybbJ	Nodulation efficiency family protein
G2583_0609	CDS	648650	649567	-	qmcA	putative protease
G2583_0610	CDS	649713	650390	+	ybbL	ABC transporter, ATP-binding protein
G2583_0611	CDS	650350	651156	+	ybbM	Putative metal resistance protein
G2583_0612	CDS	651219	652109	-	ybbN	putative thioredoxin-like protein
G2583_0613	CDS	652134	652943	-	ybbO	Oxidoreductase, short chain dehydrogenase/reductase family
G2583_0614	CDS	652933	653559	-	tesA	Acyl-CoA thioesterase I
G2583_0615	CDS	653527	654213	+	ybbA	Uncharacterized ABC transporter ATP-binding protein ybbA
G2583_0616	CDS	654210	656624	+	ybbP	putative oxidoreductase
G2583_0617	pseudogene	657054	661251	+	rhsD	RHS Repeat family protein
G2583_0618	CDS	661232	661492	+	ybbD	hypothetical protein
G2583_0620	CDS	661531	661680	+	-	hypothetical protein
G2583_0621	CDS	661675	661827	-	-	hypothetical protein
G2583_0622	CDS	662236	662643	-	ylbG	hypothetical protein
G2583_0623	CDS	662723	663817	-	ybbB	tRNA 2-selenouridine synthase
G2583_0624	CDS	663886	664812	-	allS	DNA-binding transcriptional activator of the allD operon
G2583_0625	CDS	665042	665524	+	allA	Ureidoglycolate hydrolase
G2583_0626	CDS	665602	666417	+	allR	Putative regulator
G2583_0627	CDS	666507	668288	+	gcl	Glyoxylate carboligase
G2583_0628	CDS	668301	669077	+	hyi	Glyoxylate-induced protein
G2583_0629	CDS	669178	670056	+	glxR	2-hydroxy-3-oxopropionate reductase
G2583_0630	CDS	670088	670366	+	ybbV	hypothetical protein
G2583_0631	CDS	670225	671616	+	ybbW	Cytosine/uracil/thiamine/allantoin permeases
G2583_0632	CDS	671653	673014	+	allB	Dihydroorotase and related cyclic amidohydrolases
G2583_0633	CDS	673065	674372	+	ybbY	Putative purine permease ybbY
G2583_0634	CDS	674394	675539	+	glxK	Glycerate kinase
G2583_0635	CDS	675767	676552	-	ylbA	hypothetical protein
G2583_0636	CDS	676563	677798	-	allC	Allantoate amidohydrolase
G2583_0637	CDS	677820	678869	-	allD	Ureidoglycolate dehydrogenase
G2583_0638	CDS	679186	680853	+	fdrA	Bacterial FdrA protein
G2583_0639	CDS	680863	682122	+	ylbE	hypothetical protein
G2583_0640	CDS	682133	682948	+	ylbF	putative carboxylase
G2583_0641	CDS	682945	683838	+	ybcF	Carbamate kinase
G2583_0642	CDS	683977	685044	-	purK	Phosphoribosylaminoimidazole carboxylase, ATPase subunit
G2583_0643	CDS	685041	685550	-	purE	Phosphoribosylaminoimidazole carboxylase catalytic subunit
G2583_0644	CDS	685668	686390	-	lpxH	UDP-2,3-diacylglucosamine hydrolase
G2583_0645	CDS	686393	686887	-	ppiB	Peptidyl-prolyl cis-trans isomerase
G2583_0646	CDS	687061	688446	+	cysS	Cysteinyl-tRNA synthetase
G2583_0647	CDS	688482	689003	-	ybcI	hypothetical protein
G2583_0648	CDS	689111	689323	-	ybcJ	hypothetical protein
G2583_0649	CDS	689325	690191	-	folD	Bifunctional protein folD [Includes: Methylenetetrahydrofolate
G2583_0650	CDS	690638	691213	+	sfmA	Putative fimbrial-like protein
G2583_0651	CDS	691433	692125	+	sfmC	Chaperone protein FimC-like protein
G2583_0652	CDS	692156	694765	+	sfmD	Outer membrane usher protein SfmD
G2583_0653	CDS	694778	695785	+	sfmH	Involved in fimbrial asembly precursor
G2583_0654	CDS	695796	696311	+	sfmF	Putative fimbrial protein
G2583_0655	CDS	696314	697009	-	fimZ	Fimbrial Z protein
G2583_0656	tRNA	697188	697266	+	-	Arg tRNA
G2583_0657	CDS	697281	698444	-	intD	Prophage DLP12 integrase
G2583_0658	CDS	698643	698921	-	-	Hypothetical phage protein
G2583_0659	CDS	698969	699187	-	-	Hypothetical phage protein

Locus_tag	Type	Start	End	+/-[a]	Gene[b]	Product
G2583_0660	CDS	699286	699567	-	-	Unknown protein encoded within prophage
G2583_0661	CDS	699578	699769	-	-	protein of unknown function DUF1382
G2583_0662	CDS	699721	700047	+	-	hypothetical protein
G2583_0663	CDS	699921	700601	-	ybcC	Putative exonuclease encoded by prophage CP-933K
G2583_0664	CDS	700598	701383	-	betW	Bet protein
G2583_0665	CDS	701389	701685	-	gam	Host-nuclease inhibitor protein gam
G2583_0666	CDS	701761	701967	-	kil	Prophage Kil protein
G2583_0667	CDS	702448	702825	-	-	Hypothetical phage associated protein
G2583_0668	CDS	702803	703864	-	-	hypothetical protein
G2583_0669	CDS	703945	704559	-	-	Putative repressor protein
G2583_0670	CDS	704739	704969	+	-	hypothetical protein
G2583_0671	CDS	705039	705578	+	-	hypothetical protein
G2583_0672	CDS	705575	706594	+	-	Phage replication protein O
G2583_0673	CDS	706591	707292	+	-	replication protein P
G2583_0674	CDS	707289	707591	+	renD	Ren protein
G2583_0675	CDS	707659	707991	+	emrE	EmrE SMR transporter
G2583_0676	CDS	708248	709774	+	ybcK	DLP12 prophage; predicted recombinase
G2583_0677	CDS	710239	710790	+	ybcL	Putative phosphatidylethanolamine-binding protein
G2583_0678	CDS	710800	711597	+	ybcM	DLP12 prophage; predicted DNA-binding transcriptional
G2583_0679	CDS	711714	711815	+	ylcH	hypothetical protein
G2583_0680	CDS	711812	712267	+	ybcN	hypothetical protein
G2583_0681	CDS	712267	712437	+	ninE	NinE protein
G2583_0682	CDS	712430	712720	+	ybcO	hypothetical protein
G2583_0683	CDS	712717	713079	+	rusA	Crossover junction endodeoxyribonuclease rusA
G2583_0684	CDS	713076	713216	+	ylcG	hypothetical protein
G2583_0685	CDS	713302	713685	+	ybcQ	Phage antitermination Q type 1 family
G2583_0686	CDS	713874	714956	-	nmpC	Outer membrane porin protein C
G2583_0687	CDS	715501	715761	+	essD	hypothetical protein
G2583_0688	CDS	715761	716258	+	ybcS	Lysozyme
G2583_0689	CDS	716243	716716	+	rzpD	Putative Rz endopeptidase from lambdoid prophage DLP12
G2583_0690	CDS	716748	717041	-	borD	Lambdoid prophage DLP12 Bor-like protein
G2583_0691	CDS	717986	718531	+	nohB	Bacteriophage DNA packaging protein
G2583_0692	CDS	718506	720431	+	-	Bacteriophage tail assembly protein
G2583_0693	CDS	720428	720634	+	-	Lambda prophage-derived head-to-tail joining protein W
G2583_0694	CDS	720631	722232	+	-	Putative capsid structural protein of prophage
G2583_0695	CDS	722213	723532	+	-	Minor capsid protein C
G2583_0696	CDS	723536	723874	+	-	Putative head-DNA stabilization protein of prophage
G2583_0697	CDS	723903	724955	+	-	phage major capsid protein E
G2583_0698	CDS	724958	725392	+	-	Putative DNA packaging protein of prophage
G2583_0699	CDS	725404	725757	+	-	Phage Head-Tail Attachment
G2583_0700	CDS	725769	726347	+	-	prophage minor tail protein Z
G2583_0701	CDS	726344	726739	+	-	Permeases of the major facilitator superfamily
G2583_0702	CDS	726717	727487	+	-	Putative tail component of prophage
G2583_0703	CDS	727503	727925	+	-	phage minor tail protein G
G2583_0704	CDS	727907	728341	+	-	Minor tail protein T
G2583_0705	CDS	728334	730913	+	-	Minor tail protein H
G2583_0706	CDS	730910	731239	+	-	Minor tail protein M
G2583_0707	CDS	731239	731937	+	-	Phage-related protein
G2583_0708	CDS	731943	732686	+	-	Putative tail fiber component K of prophage
G2583_0709	CDS	732584	733225	+	-	Putative tail component of prophage CP-933K
G2583_0710	CDS	733286	736699	+	-	Host specificity protein J
G2583_0711	CDS	736769	737368	+	-	hypothetical protein
G2583_0712	CDS	737427	740261	+	ydfN	PPE-repeat proteins
G2583_0713	CDS	740261	740845	+	ynaC	Tail fiber assembly protein
G2583_0714	CDS	740900	741568	-	ybcY	unknown protein encoded by prophage CP-933X
G2583_0715	CDS	741625	741930	+	ylcE	tail fiber assembly protein
G2583_0716	CDS	742114	743598	-	-	Putative protease encoded within prophage CP-933X
G2583_0717	CDS	743785	744738	-	ompT	Protease VII
G2583_0718	CDS	745237	745821	+	-	Helix-turn-helix domain protein
G2583_0719	CDS	745846	746283	-	yjaB	Hypothetical acetyltransferase YjaB

Locus_tag	Type	Start	End	+/-[a]	Gene[b]	Product
G2583_0720	CDS	746726	747487	-	envY	envelope protein; thermoregulation of porin biosynthesis
G2583_0721	CDS	747670	748560	-	ybcH	hypothetical protein
G2583_0722	CDS	748561	751533	-	nfrA	Bacteriophage N4 adsorption protein A
G2583_0723	CDS	751520	753757	-	nfrB	Bacteriophage N4 adsorption protein B
G2583_0724	CDS	754023	755159	-	yhhI	ISEc4, transposase
G2583_0725	CDS	755374	755595	-	-	hypothetical protein
G2583_0726	CDS	755622	756956	-	-	YD repeat
G2583_0727	CDS	757125	757532	-	-	hypothetical protein
G2583_0728	CDS	757550	762400	-	rhsI	Rhs core protein with extension
G2583_0729	pseudogene	762420	762881	-	-	conserved hypothetical protein
G2583_0730	CDS	762909	764810	-	-	Type VI secretion system Vgr family protein
G2583_0731	CDS	765404	766852	-	cusS	Sensor kinase cusS
G2583_0732	CDS	766842	767525	-	cusR	DNA-binding response regulator in two-component regulatory
G2583_0733	CDS	767682	769064	+	cusC	Cation efflux system protein cusC precursor
G2583_0734	CDS	769088	769420	+	cusF	Cation efflux system protein cusF precursor
G2583_0735	CDS	769436	770659	+	cusB	Cation efflux system protein CusB
G2583_0736	CDS	770671	773814	+	cusA	Cation efflux system protein cusA
G2583_0737	CDS	773916	775292	+	pheP	Phenylalanine-specific permease
G2583_0738	CDS	775360	776607	-	ybdG	Transporter, small conductance mechanosensitive ion channel
G2583_0739	CDS	776715	777368	-	nfsB	Oxygen-insensitive NAD(P)H nitroreductase
G2583_0740	CDS	777462	777830	-	ybdF	hypothetical protein
G2583_0741	CDS	777895	778143	-	ybdJ	putative membrane protein YbdJ
G2583_0742	CDS	778209	779327	-	ybdK	Carboxylate-amine ligase ybdK
G2583_0743	CDS	779622	779918	+	mokC2	Hok/Gef family protein
G2583_0744	CDS	780170	780421	+	hokE	Hok/Gef family protein
G2583_0745	CDS	780543	781313	-	entD	4'-phosphopantetheinyl transferase entD
G2583_0746	CDS	781338	783578	-	fepA	Ferrienterobactin receptor
G2583_0747	CDS	783821	785023	+	fes	Enterochelin esterase
G2583_0748	CDS	785026	785244	+	ybdZ	MbtH-like protein
G2583_0749	CDS	785241	789122	+	entF	Enterobactin synthetase component F
G2583_0750	CDS	789338	790471	+	fepE	Ferric enterobactin transport protein fepE
G2583_0751	CDS	790468	791283	-	fepC	Ferric enterobactin transport ATP-binding protein
G2583_0752	CDS	791280	792272	-	fepG	Ferric enterobactin transport protein
G2583_0753	CDS	792269	793273	-	fepD	ABC-type Fe3+-siderophore transport system, permease
G2583_0754	CDS	793384	794634	+	entS	Enterobactin exporter entS
G2583_0755	CDS	794638	795594	-	fepB	Ferrienterobactin ABC transporter, ferrienterobactin-binding
G2583_0756	CDS	795783	796958	+	entC	Isochorismate synthase entC
G2583_0757	CDS	796968	798578	+	entE	Enterobactin synthetase component E (Enterochelin synthase
G2583_0758	CDS	798592	799449	+	entB	Isochorismatase
G2583_0759	CDS	799449	800195	+	entA	2,3-dihydroxybenzoate-2,3-dehydrogenase
G2583_0760	CDS	800198	800611	+	ybdB	Esterase ybdB
G2583_0761	CDS	800792	802897	+	cstA	Carbon starvation protein A
G2583_0762	CDS	803079	803276	+	ybdD	hypothetical protein
G2583_0763	CDS	803286	804374	-	ybdH	Alcohol dehydrogenase, iron-containing
G2583_0764	CDS	804483	805643	+	ybdL	Aminotransferase, classes I and II
G2583_0765	CDS	805644	806273	-	ybdM	Immunoglobulin-binding regulator family protein
G2583_0766	CDS	806246	807466	-	ybdN	Phosphoadenosine phosphosulfate reductase family protein
G2583_0767	CDS	807613	808515	-	ybdO	putative transcriptional regulator LYSR-type
G2583_0768	CDS	808725	809525	-	dsbG	Thiol:disulfide interchange protein DsbG
G2583_0769	CDS	809843	810406	+	ahpC	Alkyl hydroperoxide reductase subunit C
G2583_0770	CDS	810505	812100	+	ahpF	Alkyl hydroperoxide reductase subunit F
G2583_0771	CDS	812221	812649	-	uspG	Universal stress protein UspG
G2583_0772	CDS	812870	814108	+	ybdR	Oxidoreductase, zinc-binding dehydrogenase family
G2583_0773	CDS	814112	814300	-	-	hypothetical protein
G2583_0774	CDS	814339	814749	-	rnk	Regulator of nucleoside diphosphate kinase
G2583_0775	CDS	814979	815785	-	rna	Ribonuclease I
G2583_0776	CDS	815899	817362	-	citT	Citrate carrier
G2583_0777	CDS	817413	818291	-	citG	2-(5''-triphosphoribosyl)-3'-dephosphocoenzyme-A synthase
G2583_0778	CDS	818266	818817	-	citX	Apo-citrate lyase phosphoribosyl-dephospho-CoA transferase
G2583_0779	CDS	818821	820353	-	citF	Citrate lyase, alpha subunit

Locus_tag	Type	Start	End	+/-[a]	Gene[b]	Product
G2583_0780	CDS	820364	821287	-	citE	Citrate lyase beta chain
G2583_0781	CDS	821269	821565	-	citD	Citrate lyase acyl carrier protein
G2583_0782	CDS	821580	822725	-	citC	Citrate lyase synthetase
G2583_0783	CDS	823018	824676	+	dpiB	Sensor histidine kinase DpiB
G2583_0784	pseudogene	824645	825324	+	dpiA	transcriptional regulatory protein DpiA
G2583_0785	CDS	825365	826750	-	dcuC	C4-dicarboxylate transporter
G2583_0786	CDS	827339	827899	+	pagP	Antimicrobial peptide resistance and lipid A acylation protein
G2583_0787	CDS	828074	828283	+	cspE	hypothetical protein
G2583_0788	CDS	828337	828720	-	crcB	camphor resistance protein CrcB
G2583_0789	CDS	828813	829601	+	ybeM	UPF0012 hydrolase ybeM
G2583_0790	CDS	829730	829933	+	tatE	Sec-independent protein translocase protein tatE
G2583_0791	CDS	830034	830999	-	lipA	Lipoyl synthase
G2583_0792	CDS	831208	832161	-	ybeF	putative DNA-binding transcriptional regulator
G2583_0793	CDS	832421	833062	-	lipB	Octanoyltransferase
G2583_0794	CDS	833163	833426	-	ybeD	Putative cytoplasmic protein
G2583_0795	CDS	833536	834747	-	dacA	D-alanyl-D-alanine carboxypeptidase dacA precursor
G2583_0796	CDS	834887	835975	-	rlpA	Rare lipoprotein A precursor
G2583_0797	CDS	835986	837098	-	mrdB	rod shape-determining protein RodA
G2583_0798	CDS	837101	839002	-	mrdA	Penicillin-binding protein 2
G2583_0799	CDS	839033	839500	-	ybeA	UPF0247 protein ybeA
G2583_0800	CDS	839504	839821	-	ybeB	hypothetical protein
G2583_0801	CDS	840081	840692	-	cobC	Alpha-ribazole phosphatase
G2583_0802	CDS	840716	841357	-	nadD	Nicotinate-nucleotide adenylyltransferase
G2583_0803	CDS	841359	842390	-	holA	DNA polymerase III, delta subunit
G2583_0804	CDS	842390	842971	-	rlpB	LPS-assembly lipoprotein rlpB precursor
G2583_0805	CDS	842986	845568	-	leuS	Leucyl-tRNA synthetase
G2583_0806	CDS	845804	846286	+	ybeL	hypothetical protein
G2583_0807	CDS	846356	847333	-	ybeQ	FOG: TPR repeat, SEL1 subfamily
G2583_0808	CDS	847498	848205	+	ybeR	hypothetical protein
G2583_0809	CDS	848202	849629	+	djlB	DnaJ domain protein
G2583_0810	CDS	849639	850193	-	ybeT	Sel1 domain protein repeat-containing protein
G2583_0811	CDS	850295	851002	+	ybeU	putative tRNA ligase
G2583_0812	pseudogene	851062	852450	+	ybeV	DnaJ domain protein
G2583_0813	CDS	852510	854180	-	hscC	DnaK family protein HscC
G2583_0814	CDS	854264	855199	-	rihA	Pyrimidine-specific ribonucleoside hydrolase rihA
G2583_0815	CDS	855317	856042	-	gltL	Glutamate/aspartate transport ATP-binding protein gltL
G2583_0816	CDS	856042	856716	-	gltK	Glutamate/aspartate transport system permease protein gltK
G2583_0817	CDS	856716	857456	-	gltJ	Glutamate/aspartate ABC transporter, permease protein GltJ
G2583_0818	ncRNA	857468	857630	-	sroC	ncRNA
G2583_0819	CDS	857626	858609	-	gltI	Glutamate/aspartate periplasmic binding protein
G2583_0820	CDS	858931	860469	-	lnt	Apolipoprotein N-acyltransferase
G2583_0821	CDS	860494	861372	-	ybeX	Magnesium and cobalt efflux protein corC
G2583_0822	CDS	861462	861929	-	ybeY	conserved hypothetical protein
G2583_0823	CDS	861926	863005	-	ybeZ	PhoH family protein
G2583_0824	CDS	863119	864543	-	miaB	UPF0004 protein yleA
G2583_0825	CDS	864689	865864	+	ubiF	2-octaprenyl-3-methyl-6-methoxy-1,4-benzoquinol hydroxylase
G2583_0827	tRNA	866052	866128	-	-	Gln tRNA
G2583_0828	tRNA	866164	866240	-	-	Gln tRNA
G2583_0829	tRNA	866287	866365	-	-	Met tRNA
G2583_0831	tRNA	866379	866455	-	-	Gln tRNA
G2583_0832	tRNA	866489	866563	-	-	Gln tRNA
G2583_0833	tRNA	866586	866672	-	-	Leu tRNA
G2583_0834	tRNA	866681	866759	-	-	Met tRNA
G2583_0835	CDS	867138	868802	-	asnB	Asparagine synthase
G2583_0836	CDS	869059	869811	-	nagD	UMP phosphatase
G2583_0837	CDS	869859	871079	-	nagC	N-acetylglucosamine repressor
G2583_0838	CDS	871088	872236	-	nagA	N-acetylglucosamine-6-phosphate deacetylase
G2583_0839	CDS	872296	873096	-	nagB	Glucosamine-6-phosphate deaminase
G2583_0840	CDS	873429	875375	+	nagE	PTS system, N-acetylglucosamine-specific IICBA component
G2583_0841	CDS	875578	877242	+	glnS	Glutaminyl-tRNA synthetase

Locus_tag	Type	Start	End	+/-[a]	Gene[b]	Product
G2583_0842	CDS	877821	879227	+	ybfM	Outer membrane porin, OprD family
G2583_0843	CDS	879277	879603	+	ybfN	hypothetical protein
G2583_0844	CDS	879687	880133	-	fur	Ferric uptake regulation protein
G2583_0845	CDS	880422	880952	-	fldA	Flavodoxin
G2583_0846	CDS	881092	881454	-	ybfE	CopG domain protein DNA-binding domain protein
G2583_0847	CDS	881525	882289	-	ybfF	Esterase YbfF
G2583_0848	CDS	882474	883019	+	seqA	Negative modulator of initiation of replication
G2583_0849	CDS	883045	884685	+	pgm	Phosphoglucomutase
G2583_0850	CDS	884742	886061	-	potE	Putrescine-ornithine antiporter
G2583_0851	CDS	886058	888265	-	speF	Ornithine decarboxylase, inducible
G2583_0852	CDS	888946	889623	-	kdpE	KDP operon transcriptional regulatory protein KdpE
G2583_0853	CDS	889620	892304	-	kdpD	sensor protein KdpD
G2583_0854	CDS	892297	892869	-	kdpC	Potassium-transporting ATPase C chain
G2583_0855	CDS	892878	894926	-	kdpB	Potassium-transporting ATPase B chain
G2583_0856	CDS	894949	896622	-	kdpA	Potassium-transporting ATPase A chain
G2583_0857	CDS	896622	896825	-	kdpF	K+-transporting ATPase, F subunit
G2583_0858	CDS	897024	897230	+	ybfA	hypothetical protein
G2583_0859	CDS	897473	901672	+	rhsC	rhsC
G2583_0860	CDS	901669	902238	+	ybfC	hypothetical protein
G2583_0861	CDS	903826	904962	+	yhhI	Transposase IS4 family protein
G2583_0862	CDS	905111	905620	+	ybgA	hypothetical protein
G2583_0863	CDS	905617	907035	+	phr	Deoxyribodipyrimidine photolyase
G2583_0864	CDS	907185	908666	-	ybgH	Amino acid/peptide transporter
G2583_0865	CDS	908937	909680	+	ybgI	NIF3 family protein
G2583_0866	CDS	909703	910359	+	ybgJ	putative carboxylase
G2583_0867	CDS	910353	911285	+	ybgK	Putative carboxylase
G2583_0868	CDS	911275	912009	+	ybgL	UPF0271 protein ybgL
G2583_0869	CDS	912045	912836	+	nei	Endonuclease VIII (DNA glycosylase/AP lyase Nei) (DNA-
G2583_0870	CDS	912833	913924	-	abrB	Putative transport protein
G2583_0871	CDS	914028	915089	-	ybgO	hypothetical protein
G2583_0872	CDS	915086	915817	-	ybgP	Periplasmic pilus chaperone family protein
G2583_0873	CDS	915832	918282	-	ybgQ	outer membrane protein
G2583_0874	CDS	918341	918922	-	ybgD	Putative fimbrial-like protein
G2583_0875	CDS	919294	920577	-	gltA	Citrate (Si)-synthase
G2583_0876	CDS	921286	921675	+	sdhC	Succinate dehydrogenase cytochrome b-556 subunit
G2583_0877	CDS	921669	922016	+	sdhD	Succinate dehydrogenase hydrophobic membrane anchor
G2583_0878	CDS	922016	923782	+	sdhA	Succinate dehydrogenase flavoprotein subunit
G2583_0879	CDS	923798	924514	+	sdhB	Succinate dehydrogenase, iron-sulfur subunit
G2583_0880	CDS	924573	924833	+	-	hypothetical protein
G2583_0881	CDS	924815	927616	+	sucA	2-oxoglutarate dehydrogenase E1 component
G2583_0882	CDS	927631	928848	+	sucB	Dihydrolipoyllysine-residue succinyltransferase, E2 component
G2583_0883	CDS	928942	930108	+	sucC	Succinyl-CoA ligase [ADP-forming] subunit beta
G2583_0884	CDS	930108	930977	+	sucD	Succinyl-CoA ligase [ADP-forming] subunit alpha
G2583_0885	CDS	931227	931373	+	-	conserved domain protein
G2583_0886	CDS	931644	932549	+	-	putative LysR-like transcriptional regulator
G2583_0887	CDS	932583	933083	-	cobO	Cob(I)yrinic acid a,c-diamide adenosyltransferase
G2583_0888	CDS	933194	934846	-	fumA	Hydro-lyase, Fe-S type, tartrate/fumarate family
G2583_0889	CDS	934954	936174	-	-	Transporter, dicarboxylate/amino acid:cation family
G2583_0890	CDS	936335	936652	-	-	hypothetical protein
G2583_0891	CDS	936649	938019	-	-	hypothetical protein
G2583_0892	CDS	938023	939264	-	-	Putative methylaspartate ammonia-lyase
G2583_0893	CDS	939264	940709	-	mutE	Methylaspartate mutase, E subunit
G2583_0894	CDS	940728	942116	-	-	Putative glutamate mutase mutL
G2583_0895	CDS	942116	942628	-	mamA	Methylaspartate mutase S chain
G2583_0896	CDS	942752	942919	-	-	hypothetical protein
G2583_0897	CDS	943113	943535	-	-	hypothetical protein
G2583_0898	pseudogene	943617	944560	-	-	hypothetical protein
G2583_0899	CDS	945363	946934	+	cydA	Cytochrome d ubiquinol oxidase subunit 1
G2583_0900	CDS	946950	948089	+	cydB	Cytochrome d ubiquinol oxidase subunit 2
G2583_0901	CDS	948104	948217	+	ybgT	hypothetical protein

Locus_tag	Type	Start	End	+/-[a]	Gene[b]	Product
G2583_0902	CDS	948217	948510	+	ybgE	hypothetical protein
G2583_0903	CDS	948660	949064	+	ybgC	Putative esterase YbgC
G2583_0904	CDS	949061	949753	+	tolQ	Inner membrane protein
G2583_0905	CDS	949757	950185	+	tolR	hypothetical protein
G2583_0906	CDS	950250	951515	+	tolA	TolA colicin import membrane protein
G2583_0907	CDS	951645	952940	+	tolB	Protein tolB precursor
G2583_0908	CDS	952975	953496	+	pal	Peptidoglycan-associated lipoprotein precursor
G2583_0909	CDS	953506	954297	+	ybgF	Tol-pal system protein YbgF
G2583_0910	tRNA	954461	954538	+	-	Lys tRNA
G2583_0911	tRNA	954573	954648	+	-	Val tRNA
G2583_0912	tRNA	954651	954726	+	-	Lys tRNA
G2583_0913	tRNA	954778	954853	+	-	Val tRNA
G2583_0914	tRNA	954857	954932	+	-	Lys tRNA
G2583_0914	tRNA	955079	955154	+	-	Lys tRNA
G2583_5266	tRNA	955188	955263	+	-	Lys tRNA
G2583_0916	CDS	955541	956584	+	nadA	Quinolinate synthetase A
G2583_0917	CDS	956622	957341	+	pnuC	Nicotinamide mononucleotide transporter PnuC
G2583_0918	CDS	957338	958279	-	zitB	Zinc transporter zitB
G2583_0919	CDS	958393	958773	-	ybgS	hypothetical protein
G2583_0920	CDS	959090	960142	+	aroG	Phospho-2-dehydro-3-deoxyheptonate aldolase, Phe-sensitive
G2583_0921	CDS	960308	961060	-	gpmA	2,3-bisphosphoglycerate-dependent phosphoglycerate mutase
G2583_0922	CDS	961262	962302	-	galM	Galactose mutarotase
G2583_0923	CDS	962296	963444	-	galK	Galactokinase
G2583_0924	CDS	963448	964494	-	galT	Galactose-1-phosphate uridylyltransferase
G2583_0925	CDS	964504	965532	-	galE	UDP-galactose-4-epimerase
G2583_0926	CDS	965782	967254	-	modF	ABC transporter, ATP-binding protein
G2583_0927	CDS	967322	968110	-	modE	transcriptional regulator ModE
G2583_0928	CDS	968239	968388	+	ybhT	hypothetical protein
G2583_0929	CDS	968555	969328	+	modA	Molybdate ABC transporter, periplasmic molybdate-binding
G2583_0930	CDS	969328	970017	+	modB	ABC-type molybdate transport system, permease component
G2583_0931	CDS	970020	971078	+	modC	Molybdenum import ATP-binding protein modC
G2583_0932	CDS	971079	971999	-	ybhA	Phosphatase YbhA
G2583_0933	CDS	972052	973047	+	pgl	6-phosphogluconolactonase
G2583_0934	CDS	973088	974104	-	ybhD	putative transcriptional regulator LYSR-type
G2583_0935	CDS	974225	975277	+	ybhH	hypothetical protein
G2583_0936	CDS	975353	976786	+	ybhI	Anion transporter
G2583_0937	CDS	976969	979230	+	ybhJ	aconitase family protein
G2583_0938	CDS	979371	980654	-	ybhC	Pectinesterase
G2583_0939	CDS	980789	981859	-	intE	Phage integrase family
G2583_0940	CDS	981837	982055	-	-	Excisionase
G2583_0941	CDS	982095	982262	-	-	unknown protein encoded by prophage CP-933K
G2583_0942	CDS	982505	983107	+	-	unknown protein encoded by prophage CP-933K
G2583_0943	CDS	983318	983539	-	-	hypothetical C4-type zinc finger protein TraR-family
G2583_0944	CDS	983638	983919	-	-	hypothetical protein
G2583_0945	CDS	983930	984121	-	-	unknown protein encoded by prophage CP-933K
G2583_0946	CDS	984273	984953	-	-	Exonuclease
G2583_0947	CDS	984950	985693	-	bet	Bacteriophage recombination protein
G2583_0948	CDS	985742	986038	-	gamW	Gam protein
G2583_0949	CDS	986224	986460	-	CIII	antitermination protein
G2583_0950	CDS	986461	986829	-	-	Lambda prophage-derived protein ea10
G2583_0951	CDS	987025	987474	-	-	Gene 34 protein
G2583_0952	CDS	987759	988103	-	-	N protein
G2583_0953	CDS	988760	989056	-	-	Hypothetical protein
G2583_0954	CDS	989229	989882	-	-	CI protein
G2583_0955	CDS	989930	990214	+	CRO	hypothetical protein
G2583_0956	CDS	990356	990655	+	-	regulatory protein CII
G2583_0957	CDS	990688	991587	+	-	hypothetical protein
G2583_0958	CDS	991584	992285	+	-	putative replication protein P of bacteriophage BP-933W
G2583_0959	CDS	992282	992572	+	-	Ren protein
G2583_0960	CDS	992646	993086	+	ninB	Unknown protein encoded within prophage

Locus_tag	Type	Start	End	+/-[a]	Gene[b]	Product
G2583_0961	CDS	993023	993265	+	ninE	NinE protein
G2583_0962	CDS	993220	993432	+	NinF	NinF protein
G2583_0963	CDS	993422	994045	+	NinG	NinG protein
G2583_0964	CDS	994042	994707	+	-	Serine/threonine-protein phosphatase 1
G2583_0965	CDS	994919	995878	-	-	Putative outer membrane protein
G2583_0966	CDS	996353	997042	+	-	Putative antiterminator Q protein of prophage CP-933K
G2583_0967	CDS	997213	997971	+	-	unknown protein encoded by prophage CP-933K
G2583_0968	CDS	998057	998215	+	-	unknown protein encoded by prophage CP-933K
G2583_0969	CDS	998792	999052	+	-	Putative holin protein
G2583_0970	CDS	999052	999549	+	ybcS	putative lysozyme protein R of prophage CP-933K
G2583_0971	CDS	999546	1000013	+	-	Bacteriophage lysis protein
G2583_0972	CDS	1000001	1000153	+	-	hypothetical protein
G2583_0973	CDS	1000449	1000838	+	-	hypothetical protein
G2583_0974	CDS	1000828	1001319	+	-	unknown protein encoded by prophage CP-933K
G2583_0975	CDS	1001319	1003421	+	-	Phage terminase large subunit
G2583_0976	CDS	1003418	1003630	+	-	hypothetical protein
G2583_0977	CDS	1003558	1005138	+	-	Putative capsid protein
G2583_0978	pseudogene	1004987	1007110	+	clpP	Clp protease domain protein
G2583_0979	CDS	1007152	1007520	+	-	hypothetical protein
G2583_0980	CDS	1007513	1007788	+	-	unknown protein encoded by prophage CP-933K
G2583_0981	CDS	1007800	1008378	+	-	prophage minor tail protein Z
G2583_0982	CDS	1008375	1008776	+	-	putative tail component of prophage CP-933K
G2583_0983	CDS	1008781	1009530	+	-	Putative tail component of prophage CP-933K
G2583_0984	CDS	1009579	1009977	+	-	ATPase components of ABC transporters with duplicated
G2583_0985	CDS	1009986	1010315	+	-	Putative tail component of prophage
G2583_0986	CDS	1010287	1013352	+	-	Putative tail component of prophage CP-933K
G2583_0987	CDS	1013352	1013681	+	-	putative minor tail protein
G2583_0988	CDS	1013691	1014389	+	-	putative minor tail protein
G2583_0989	CDS	1014386	1015138	+	-	Putative tail fiber component K of prophage
G2583_0990	CDS	1015036	1015683	+	-	Putative tail component of prophage CP-933K
G2583_0991	CDS	1015744	1019157	+	-	Host specificity protein J
G2583_0992	CDS	1019227	1019826	+	-	Enterobacterial Ail/Lom family protein
G2583_0993	CDS	1019885	1021204	+	-	hypothetical protein
G2583_0994	CDS	1021167	1021475	+	-	putative prophage tail fibre C-terminus family protein
G2583_0995	CDS	1021652	1022632	+	nleB	NleB
G2583_0996	CDS	1022693	1023685	+	nleC	Non-LEE encoded type III effector C
G2583_0997	CDS	1024296	1024343	+	-	hypothetical protein
G2583_0998	CDS	1024512	1025393	+	nleH	non-LEE-encoded type III effector H
G2583_0999	CDS	1025428	1025562	+	-	hypothetical protein
G2583_1000	CDS	1025624	1026322	+	nleD	hypothetical protein
G2583_1001	CDS	1026829	1027305	-	ybhB	UPF0098 protein ybhB
G2583_1002	CDS	1027364	1028653	-	bioA	Adenosylmethionine-8-amino-7-oxononanoate transaminase
G2583_1003	CDS	1028740	1029780	+	bioB	Biotin synthase
G2583_1004	CDS	1029777	1030931	+	bioF	8-amino-7-oxononanoate synthase
G2583_1005	CDS	1030918	1031673	+	bioC	Biotin biosynthesis protein BioC
G2583_1006	CDS	1031666	1032343	+	bioD	Dethiobiotin synthase
G2583_1007	CDS	1032922	1034943	+	uvrB	UvrABC system protein B
G2583_1008	CDS	1035134	1036042	-	ybhK	hypothetical protein
G2583_1009	CDS	1036439	1037428	+	moaA	Molybdenum cofactor biosynthesis protein A
G2583_1010	CDS	1037450	1037962	+	moaB	Molybdenum cofactor biosynthesis protein B
G2583_1011	CDS	1037965	1038450	+	moaC	Molybdenum cofactor biosynthesis protein C
G2583_1012	CDS	1038443	1038688	+	moaD	Molybdopterin biosynthesis
G2583_1013	CDS	1038690	1039142	+	moaE	Molybdopterin converting factor, subunit 2
G2583_1014	CDS	1039278	1039982	+	ybhL	hypothetical protein
G2583_1015	CDS	1040190	1040903	+	ybhM	hypothetical protein
G2583_1016	CDS	1040939	1041895	-	ybhN	hypothetical protein
G2583_1017	CDS	1041895	1043136	-	ybhO	Putative cardiolipin synthetase ybhO
G2583_1018	CDS	1043133	1043894	-	ybhP	hypothetical protein
G2583_1019	CDS	1044027	1044437	+	ybhQ	Inner membrane protein ybhQ
G2583_1020	CDS	1044399	1045505	-	ybhR	hypothetical protein

Locus_tag	Type	Start	End	+/-[a]	Gene[b]	Product
G2583_1021	CDS	1045516	1046649	-	ybhS	ABC-2 type transporter, permease protein
G2583_1022	CDS	1046642	1048393	-	ybhF	Putative ATP-binding component of a transport system
G2583_1023	CDS	1048371	1049369	-	ybhG	UPF0194 membrane protein ybhG precursor
G2583_1024	CDS	1049369	1050052	-	ybiH	Hypothetical transcriptional regulator ybiH
G2583_1025	CDS	1050269	1051636	+	rhlE	ATP-dependent RNA helicase RhlE
G2583_1026	CDS	1052244	1054256	+	sopA	hypothetical protein
G2583_1027	CDS	1054402	1056690	+	dinG	ATP-dependent DNA helicase DinG
G2583_1028	CDS	1056718	1057680	+	ybiB	Glycosyl transferase family protein
G2583_1029	CDS	1057821	1058906	+	ybiC	Uncharacterized oxidoreductase ybiC
G2583_1030	CDS	1059135	1059395	-	ybiJ	hypothetical protein
G2583_1031	CDS	1059660	1059926	-	ybiI	C4-type zinc finger protein, DksA/TraR family
G2583_1032	CDS	1060000	1060677	-	ybiX	Putative enzyme
G2583_1033	CDS	1060719	1063001	-	fiu	Catecholate siderophore receptor fiu precursor
G2583_1034	CDS	1063266	1063670	-	ybiM	hypothetical protein
G2583_1035	CDS	1063721	1064728	+	ybiN	hypothetical protein
G2583_1036	CDS	1064725	1066950	-	ybiO	Transporter, small conductance mechanosensitive ion channel
G2583_1037	CDS	1067067	1067789	-	glnQ	Glutamine ABC transporter, ATP-binding protein
G2583_1038	CDS	1067786	1068445	-	glnP	Glutamine transport system permease protein glnP
G2583_1039	CDS	1068583	1069329	-	glnH	hypothetical protein
G2583_1040	CDS	1069733	1070236	-	dps	Global regulator, starvation conditions
G2583_1041	CDS	1070535	1071422	-	rhtA	Inner membrane transporter rhtA
G2583_1042	CDS	1071775	1072290	+	ompX	Outer membrane protein X precursor
G2583_1043	CDS	1072339	1073922	-	ybiP	Sulfatase family protein
G2583_1044	CDS	1074158	1074265	+	yliL	hypothetical protein
G2583_1045	CDS	1074508	1074975	+	mntR	manganese transport regulator MntR
G2583_1046	CDS	1074972	1076090	+	ybiR	Citrate transporter family protein
G2583_1047	CDS	1076148	1077068	-	ybiS	hypothetical protein
G2583_1048	CDS	1077287	1078879	+	ybiT	Uncharacterized ABC transporter ATP-binding protein ybiT
G2583_1049	CDS	1079047	1080312	-	ybiU	hypothetical protein
G2583_1050	CDS	1080464	1081279	-	ybiV	Sugar phosphatase SupH
G2583_1051	CDS	1081425	1083857	-	ybiW	Pyruvate-formate lyase
G2583_1052	CDS	1083863	1084762	-	ybiY	Glycyl-radical enzyme activating protein family
G2583_1053	CDS	1084893	1085555	+	fsaA	Fructose-6-phosphate aldolase 1
G2583_1054	CDS	1085734	1086483	-	moeB	Molybdopterin biosynthesis
G2583_1055	CDS	1086483	1087718	-	moeA	Molybdopterin biosynthesis protein MoeA
G2583_1056	CDS	1087922	1088887	+	iaaA	Putative asparaginase
G2583_1057	CDS	1088874	1090745	+	gsiA	Glutathione ABC transporter, ATP-binding protein GsiA
G2583_1058	CDS	1090765	1092303	+	gsiB	Glutathione-binding protein gsiB precursor
G2583_1059	CDS	1092321	1093241	+	gsiC	Glutathione transport system permease protein gsiC
G2583_1060	CDS	1093244	1094155	+	gsiD	Glutathione transport system permease protein gsiD
G2583_1061	CDS	1094333	1096681	+	yliE	cyclic diguanylate phosphodiesterase (EAL) domain protein
G2583_1062	CDS	1096689	1098017	+	yliF	Uncharacterized membrane protein yliF
G2583_1063	CDS	1098087	1098416	-	-	Helix-turn-helix DNA-binding domain protein
G2583_1064	CDS	1098406	1098792	-	-	hypothetical protein
G2583_1065	CDS	1099018	1100343	-	yliG	UPF0004 protein yliG
G2583_1066	CDS	1100556	1100939	+	bssR	Biofilm regulator bssR
G2583_1067	CDS	1101050	1102165	+	yliI	Glucose / sorbosone dehydrogenase protein
G2583_1068	CDS	1102162	1102794	-	yliJ	putative transferase
G2583_1069	CDS	1103025	1104236	+	dacC	Penicillin-binding protein 6
G2583_1070	CDS	1104283	1105041	-	deoR	Deoxyribose operon repressor
G2583_1071	CDS	1105099	1105695	-	ybjG	Undecaprenyl-diphosphatase
G2583_1072	CDS	1105980	1107212	+	cmr	Multidrug translocase MdfA
G2583_1073	CDS	1107253	1107537	-	ybjH	hypothetical protein
G2583_1074	CDS	1107623	1108438	-	ybjI	Cof-like hydrolase
G2583_1075	CDS	1108438	1109646	-	ybjJ	putative DEOR-type transcriptional regulator
G2583_1076	CDS	1109730	1110266	+	ybjK	putative DEOR-type transcriptional regulator
G2583_1077	ncRNA	1110283	1110361	-	rybB	ncRNA
G2583_1078	CDS	1110441	1112126	-	ybjL	Putative transport protein ybjL
G2583_1079	CDS	1112396	1112773	+	ybjM	Inner membrane protein ybjM
G2583_1080	CDS	1112803	1113060	-	grxA	Glutaredoxin 1

Locus_tag	Type	Start	End	+/-[a]	Gene[b]	Product
G2583_1081	CDS	1113220	1113507	+	ybjC	hypothetical protein
G2583_1082	CDS	1113491	1114213	+	nfsA	Oxygen-insensitive NADPH nitroreductase
G2583_1083	CDS	1114274	1115176	+	rimK	Ribosomal protein S6 modification protein
G2583_1084	CDS	1115264	1115740	+	ybjN	Putative sensory transduction regulator
G2583_1085	CDS	1115941	1117203	+	potF	Putrescine ABC transporter, periplasmic putrescine-binding
G2583_1086	CDS	1117298	1118431	+	potG	Putrescine transport ATP-binding protein PotG
G2583_1087	CDS	1118441	1119394	+	potH	Putrescine transport protein
G2583_1088	CDS	1119391	1120236	+	potI	Putrescine ABC transporter, permease protein PotI
G2583_1089	CDS	1120296	1120784	+	ybjO	Inner membrane protein ybjO
G2583_1090	CDS	1120825	1121952	+	rumB	23S rRNA (uracil-5-)-methyltransferase rumB (23S rRNA(M- 5-
G2583_1091	CDS	1122232	1123575	+	ulaA	Ascorbate-specific PTS system enzyme IIC
G2583_1092	CDS	1123597	1123917	+	-	hypothetical protein
G2583_1093	CDS	1123929	1125410	+	-	putative sulfatase
G2583_1094	CDS	1125461	1126243	-	artJ	Arginine 3rd transport system periplasmic binding protein
G2583_1095	CDS	1126483	1127151	-	artM	Arginine transport system permease protein artM
G2583_1096	CDS	1127151	1127867	-	artQ	Arginine ABC transporter, permease protein ArtQ
G2583_1097	CDS	1127874	1128605	-	artI	Arginine ABC transporter, periplasmic arginine-binding protein
G2583_1098	CDS	1128623	1129351	-	artP	Arginine transport ATP-binding protein ArtP
G2583_1099	CDS	1129569	1130090	-	ybjP	Putative lipoprotein YbjP
G2583_1100	CDS	1130098	1130280	-	-	hypothetical protein
G2583_1101	pseudogene	1130708	1132507	+	-	hypothetical protein
G2583_1102	CDS	1132475	1132663	-	-	hypothetical protein
G2583_1103	CDS	1132718	1133041	+	ybjQ	UPF0145 protein ybjQ
G2583_1104	CDS	1133038	1133868	+	ybjR	N-acetylmuramoyl-L-alanine amidase AmiD
G2583_1105	CDS	1133865	1134914	-	ybjS	NAD dependent epimerase/dehydratase family
G2583_1106	CDS	1134977	1136407	-	ybjT	NAD dependent epimerase/dehydratase family protein
G2583_1107	CDS	1136418	1137419	-	ltaE	Low specificity L-threonine aldolase
G2583_1108	CDS	1137456	1139174	-	poxB	Pyruvate dehydrogenase
G2583_1109	CDS	1139307	1140275	-	hcr	NADH oxidoreductase hcr
G2583_1110	CDS	1140287	1141939	-	hcp	Hydroxylamine reductase
G2583_1111	CDS	1142083	1142982	-	ybjE	Putative surface protein
G2583_1112	CDS	1143440	1144135	-	aqpZ	Aquaporin Z
G2583_1113	CDS	1144561	1146219	+	ybjD	hypothetical protein
G2583_1114	CDS	1146216	1147208	-	ybjX	putative enzyme
G2583_1115	CDS	1147323	1148438	+	macA	macrolide transporter subunit MacA
G2583_1116	CDS	1148435	1150381	+	macB	Macrolide-specific ABC-type efflux carrier protein MacB
G2583_1117	CDS	1150454	1150708	-	cspD	Cold shock proteins
G2583_1118	CDS	1151001	1151321	+	clpS	ATP-dependent Clp protease adapter protein clpS
G2583_1119	CDS	1151352	1153628	+	clpA	ATP-dependent clp protease ATP-binding subunit clpA
G2583_1120	tRNA	1153972	1154061	-	-	Ser tRNA
G2583_1121	CDS	1154314	1154532	-	infA	hypothetical protein
G2583_1122	CDS	1154817	1155521	-	aat	Leucyl/phenylalanyl-tRNA--protein transferase
G2583_1123	CDS	1155563	1157284	-	cydC	ABC transporter, CydDC cysteine exporter (CydDC-E) family,
G2583_1124	CDS	1157285	1159051	-	cydD	ABC transporter, CydDC cysteine exporter (CydDC-E) family,
G2583_1125	CDS	1159174	1160139	-	trxB	Thioredoxin reductase
G2583_1126	CDS	1160684	1161178	+	lrp	Leucine-responsive regulatory protein
G2583_1127	CDS	1161313	1165341	+	ftsK	DNA translocase FtsK
G2583_1128	CDS	1165493	1166107	+	lolA	Outer-membrane lipoprotein carrier protein precursor
G2583_1129	CDS	1166118	1167461	+	rarA	Replication-associated recombination protein A
G2583_1130	CDS	1167552	1168844	+	serS	Seryl-tRNA synthetase
G2583_1131	CDS	1169083	1171527	+	dmsA	Anaerobic dimethyl sulfoxide reductase, A subunit
G2583_1132	CDS	1171538	1172155	+	dmsB	Anaerobic dimethyl sulfoxide reductase chain B
G2583_1133	CDS	1172157	1173020	+	dmsC	Anaerobic dimethyl sulfoxide reductase subunit C
G2583_1134	CDS	1173056	1173682	-	ycaC	Protein ycaC
G2583_1135	CDS	1173997	1175145	+	ycaD	putative MFS family transporter protein
G2583_1136	CDS	1175163	1176785	+	ycaM	Amino acid permease family protein
G2583_1137	CDS	1176995	1177735	-	pflA	Pyruvate formate lyase-activating enzyme 1
G2583_1138	CDS	1177927	1180209	-	pflB	Formate acetyltransferase
G2583_1139	CDS	1180264	1181121	-	focA	FocA formate FNT transporter
G2583_1140	CDS	1181527	1183296	-	ycaO	hypothetical protein

Locus_tag	Type	Start	End	+/-[a]	Gene[b]	Product
G2583_1141	CDS	1183417	1184109	+	ycaP	hypothetical protein
G2583_1142	CDS	1184308	1185396	+	serC	Phosphoserine aminotransferase
G2583_1143	CDS	1185467	1186750	+	aroA	3-phosphoshikimate 1-carboxyvinyltransferase
G2583_1144	CDS	1186895	1187683	+	ycaL	Peptidase, M48B family
G2583_1145	CDS	1187856	1188539	+	cmk	Cytidylate kinase
G2583_1146	CDS	1188650	1190323	+	rpsA	30S ribosomal protein S1
G2583_1147	CDS	1190483	1190767	+	ihfB	Integration host factor subunit beta
G2583_1148	CDS	1190974	1193238	+	ycaI	DNA internalization-related competence protein ComEC/Rec2
G2583_1149	CDS	1193275	1195023	+	msbA	Lipid A export ATP-binding/permease protein msbA
G2583_1150	CDS	1195020	1196006	+	lpxK	Tetraacyldisaccharide 4'-kinase
G2583_1151	CDS	1196043	1197275	+	ycaQ	hypothetical protein
G2583_1152	CDS	1197327	1197509	+	ycaR	UPF0434 protein ycaR
G2583_1153	CDS	1197506	1198252	+	kdsB	3-deoxy-manno-octulosonate cytidylyltransferase
G2583_1154	CDS	1198406	1199299	+	ycbJ	hypothetical protein
G2583_1155	CDS	1199276	1200055	-	ycbC	hypothetical protein
G2583_1156	CDS	1200191	1200976	+	smtA	SmtA protein
G2583_1157	CDS	1200973	1202295	+	mukF	Chromosome partition protein mukF
G2583_1158	CDS	1202276	1202980	+	mukE	MukE
G2583_1159	CDS	1202980	1207440	+	mukB	Uncharacterized protein involved in chromosome partitioning
G2583_1160	CDS	1207701	1209548	+	ycbB	Putative peptidoglycan binding domain
G2583_1161	CDS	1209729	1210277	+	ycbK	hypothetical protein
G2583_1162	CDS	1210304	1210951	+	ycbL	Metallo-beta-lactamase family protein
G2583_1163	CDS	1211002	1212192	-	aspC	Aspartate transaminase
G2583_1164	CDS	1212376	1213464	-	ompF	Outer membrane protein F
G2583_1165	CDS	1214066	1215466	-	asnS	Asparaginyl-tRNA synthetase
G2583_1166	CDS	1215635	1216837	-	pncB	Nicotinate phosphoribosyltransferase
G2583_1167	CDS	1217103	1219715	+	pepN	Aminopeptidase N
G2583_1168	CDS	1219758	1220525	-	ssuB	ABC transporter, ATP-binding protein
G2583_1169	CDS	1220522	1221313	-	ssuC	Putative sulfonate ABC transporter, permease protein
G2583_1170	CDS	1221325	1222470	-	ssuD	Alkanesulfonate monooxygenase
G2583_1171	CDS	1222467	1223426	-	ssuA	ABC transporter, periplasmic substrate-binding protein, aliphatic
G2583_1172	CDS	1223419	1223994	-	ssuE	NAD(P)H-dependent FMN reductase
G2583_1173	CDS	1224337	1224885	+	ycbQ	Putative fimbrial-like protein
G2583_1174	CDS	1224968	1225669	+	ycbR	Putative chaperone
G2583_1175	pseudogene	1225694	1228293	+	ycbS	PapC-like porin protein involved in fimbrial biogenesis
G2583_1176	CDS	1228284	1229354	+	ycbT	Putative fimbrial protein
G2583_1177	CDS	1229400	1229909	+	ycbU	Uncharacterized fimbrial-like protein ycbU precursor
G2583_1178	CDS	1229869	1230432	+	ycbV	Putative fimbrial-like protein
G2583_1179	CDS	1230398	1231135	+	ycbF	hypothetical protein
G2583_1180	CDS	1231246	1232256	+	pyrD	Dihydroorotate dehydrogenase
G2583_1181	CDS	1232394	1232972	+	ycbW	hypothetical protein
G2583_1182	CDS	1232969	1234078	-	ycbX	MOSC domain protein
G2583_1183	CDS	1234322	1236430	+	rlmL	Putative RNA methylase family UPF0020
G2583_1184	CDS	1236442	1238349	+	uup	ABC transporter, ATP-binding protein
G2583_1185	CDS	1238479	1239732	+	pqiA	Paraquat-inducible protein A
G2583_1186	CDS	1239737	1241377	+	pqiB	Paraquat-inducible protein B
G2583_1187	CDS	1241374	1241937	+	ymbA	putative lipoprotein
G2583_1188	CDS	1242193	1242360	+	rmf	Ribosome modulation factor
G2583_1189	CDS	1242430	1242948	-	fabA	3-hydroxydecanoyl-ACP dehydratase
G2583_1190	CDS	1243017	1244777	-	ycbZ	Peptidase, S16 (Lon protease) family
G2583_1191	CDS	1244963	1245415	+	ycbG	UPF0268 protein ycbG
G2583_1192	CDS	1245491	1246555	-	ompA	OmpA domain protein transmembrane region-containing protein
G2583_1193	CDS	1246888	1247397	-	sulA	Suppressor of lon
G2583_1194	CDS	1247616	1248245	+	sxy	conserved protein
G2583_1195	CDS	1248208	1250370	-	yccS	hypothetical protein
G2583_1196	CDS	1250380	1250826	-	yccF	hypothetical protein
G2583_1197	CDS	1250949	1253003	+	helD	DNA helicase IV
G2583_1198	CDS	1253035	1253493	-	mgsA	Methylglyoxal synthase
G2583_1199	CDS	1253589	1254251	-	yccT	UPF0319 protein yccT precursor
G2583_1200	CDS	1254424	1254837	+	yccU	hypothetical protein

Locus_tag	Type	Start	End	+/-[a]	Gene[b]	Product
G2583_1201	CDS	1254882	1255250	-	hspQ	Heat shock protein hspQ
G2583_1202	CDS	1255257	1256447	-	yccW	putative oxidoreductase
G2583_1203	CDS	1256542	1256820	+	yccX	Acylphosphatase
G2583_1204	CDS	1256817	1257146	-	yccK	Sulfurtransferase tusE
G2583_1205	CDS	1257237	1257896	-	yccA	Inner membrane protein yccA
G2583_1206	tRNA	1258103	1258191	-	-	Ser tRNA
G2583_1207	CDS	1258616	1259734	+	hyaA	Nickel-dependent hydrogenase 1, small subunit
G2583_1208	CDS	1259731	1261524	+	hyaB	Hydrogenase-1 large subunit
G2583_1209	CDS	1261543	1262250	+	hyaC	Probable Ni/Fe-hydrogenase 1 B-type cytochrome subunit
G2583_1210	CDS	1262247	1262834	+	hyaD	hydrogenase 1 maturation protease
G2583_1211	CDS	1262831	1263229	+	hyaE	Hydrogenase-1 expression protein HyaE
G2583_1212	CDS	1263226	1264083	+	hyaF	Hydrogenase-1 operon protein HyaF
G2583_1213	CDS	1264217	1265761	+	appC	Cytochrome bd-II oxidase, subunit I
G2583_1214	CDS	1265773	1266909	+	appB	Cytochrome bd-II oxidase, subunit II
G2583_1215	CDS	1267064	1268398	+	appA	Phosphoanhydride phosphorylase
G2583_1216	CDS	1268518	1270698	-	etk	Tyrosine-protein kinase etk
G2583_1217	CDS	1270718	1271164	-	etp	Putative phosphatase
G2583_1218	CDS	1271152	1272291	-	gfcE	Putative polysaccharide export protein gfcE precursor
G2583_1219	CDS	1272337	1274433	-	gfcD	Group 4 capsule (G4C) polysaccharide, lipoprotein YmcA
G2583_1220	CDS	1274433	1275179	-	gfcC	Group 4 capsule (G4C) polysaccharide, YmcB
G2583_1221	CDS	1275176	1275820	-	gfcB	Group 4 capsule (G4C) polysaccharide, lipoprotein YmcC
G2583_1222	CDS	1275927	1276250	-	ymcD	putative inner membrane protein
G2583_1223	CDS	1276674	1276886	-	cspH	Cold shock-like protein cspH
G2583_1224	CDS	1277172	1277384	+	cspG	Cold shock-like protein cspG
G2583_1225	CDS	1277558	1277788	+	ymcE	cold shock gene
G2583_1226	CDS	1278000	1279073	-	yccM	4Fe-4S binding domain protein
G2583_1227	CDS	1279145	1281889	-	torS	hybrid sensory histidine kinase TorS
G2583_1228	CDS	1281972	1283000	+	torT	Periplasmic protein torT precursor
G2583_1229	CDS	1282973	1283665	-	torR	TorCAD operon transcriptional regulatory protein torR
G2583_1230	CDS	1283795	1284967	+	torC	Cytochrome c-type protein TorC
G2583_1231	CDS	1284967	1287513	+	torA	Trimethylamine-N-oxide reductase 1 precursor
G2583_1232	CDS	1287510	1288109	+	torD	Chaperone protein torD
G2583_1233	CDS	1288263	1288568	-	cbpM	Chaperone modulatory protein cbpM
G2583_1234	CDS	1288568	1289488	-	cbpA	Curved DNA-binding protein
G2583_1235	CDS	1289748	1291049	+	yccE	conserved hypothetical protein
G2583_1236	CDS	1291340	1292581	+	agp	Glucose-1-phosphatase
G2583_1237	CDS	1292619	1292846	-	yccJ	hypothetical protein
G2583_1238	CDS	1292867	1293463	-	wrbA	Flavoprotein wrbA
G2583_1239	CDS	1294092	1295486	-	rutG	Putative purine permease ycdG
G2583_1240	CDS	1295441	1295935	-	rutF	Flavin reductase domain protein FMN-binding
G2583_1241	CDS	1295946	1296536	-	rutE	Putative NADH dehydrogenase/NAD(P)H nitroreductase rutE
G2583_1242	CDS	1296546	1297346	-	rutD	putative acetyltransferase
G2583_1243	CDS	1297354	1297740	-	rutC	hypothetical protein
G2583_1244	CDS	1297752	1298486	-	rutB	Putative synthetase
G2583_1245	CDS	1298444	1299592	-	rutA	Putative monooxygenase rutA
G2583_1246	CDS	1299823	1300461	+	rutR	putative tet operon regulator
G2583_1247	CDS	1300501	1304463	-	putA	Bifunctional protein PutA
G2583_1248	CDS	1304886	1306394	+	putP	Sodium/proline symporter
G2583_1249	CDS	1306550	1307893	-	-	hypothetical protein
G2583_1250	CDS	1308455	1308601	+	-	hypothetical protein
G2583_1251	CDS	1308892	1309038	-	-	hypothetical protein
G2583_1252	CDS	1309065	1309904	+	ycdN	Ferrous iron permease efeU
G2583_1253	CDS	1309962	1311089	+	ycdO	UPF0409 protein ycdO precursor
G2583_1254	CDS	1311095	1312366	+	ycdB	Tat-translocated enzyme
G2583_1255	CDS	1312629	1313693	+	phoH	hypothetical protein
G2583_1256	CDS	1313743	1314156	-	pgaD	Biofilm PGA synthesis protein pgaD
G2583_1257	CDS	1314158	1315483	-	pgaC	Biofilm PGA synthesis N-glycosyltransferase pgaC
G2583_1258	pseudogene	1315476	1317493	-	pgaB	Biofilm PGA synthesis lipoprotein pgaB
G2583_1259	CDS	1317502	1319925	-	pgaA	Biofilm PGA synthesis protein pgaA precursor
G2583_1260	pseudogene	1320512	1321869	+	ycdT	diguanylate cyclase (GGDEF) domain protein

Locus_tag	Type	Start	End	+/-[a]	Gene[b]	Product
G2583_1261	CDS	1321967	1323577	+	rtn	Rtn-like protein
G2583_1262	CDS	1323679	1324170	-	fidL	hypothetical protein
G2583_1263	CDS	1324175	1324987	-	marT	hypothetical protein
G2583_1264	CDS	1325666	1326457	+	-	2-deoxy-D-gluconate 3-dehydrogenase
G2583_1265	CDS	1326603	1327292	-	-	Putative chaperone protein
G2583_1266	CDS	1327289	1328623	-	mrkD	hypothetical protein
G2583_1267	CDS	1328639	1331161	-	mrkC	Putative outer membrane usher protein
G2583_1268	CDS	1331214	1331936	-	-	Gram-negative pili assembly chaperone
G2583_1269	CDS	1332001	1332561	-	mrkA	Fimbrial protein
G2583_1270	CDS	1332604	1333227	-	-	hypothetical protein
G2583_1271	CDS	1333496	1333606	-	-	hypothetical protein
G2583_1272	CDS	1334068	1334187	-	-	hypothetical protein
G2583_1273	CDS	1334618	1338430	+	-	Hemagglutinin/hemolysin-related protein
G2583_1274	CDS	1338519	1340138	+	-	Putative outer membrane transporter of ShlA/HecA/FhaA
G2583_1275	CDS	1340154	1340522	+	-	Probable holo-[acyl-carrier-protein] synthase 2
G2583_1276	CDS	1340542	1341297	+	-	NAD dependent epimerase/dehydratase family
G2583_1277	CDS	1341301	1341849	+	cylZ	Putative fatty acyl chain dehydrase
G2583_1278	CDS	1341871	1342152	+	-	Putative acyl-carrier protein
G2583_1279	CDS	1342188	1343348	+	gcvT	Putative aminomethyltransferase
G2583_1280	CDS	1343422	1345980	+	fabF	Putative beta-ketoacyl-[acyl carrier protein] synthase
G2583_1281	CDS	1345985	1347208	+	olmA	Beta-ketoacyl synthase, C-domain protein
G2583_1282	CDS	1347205	1348158	+	-	hypothetical protein
G2583_1283	CDS	1348169	1348876	+	-	Lipoprotein-releasing system ATP-binding protein LolD
G2583_1284	CDS	1348860	1349516	+	-	hypothetical protein
G2583_1285	CDS	1349517	1350827	+	-	hypothetical protein
G2583_1286	CDS	1350836	1351624	+	-	hypothetical protein
G2583_1287	CDS	1351621	1353009	+	-	hypothetical protein
G2583_1288	CDS	1353020	1353964	+	-	Malonyl CoA-acyl carrier protein transacylase
G2583_1289	CDS	1353961	1354947	+	ycdU	hypothetical protein
G2583_1290	tRNA	1355153	1355228	-	-	Ser tRNA
G2583_1291	CDS	1355422	1356399	+	ghrA	Putative dehydrogenase
G2583_1292	CDS	1356454	1357191	+	ycdX	Putative hydrolase ycdX
G2583_1293	CDS	1357215	1357769	+	ycdY	Chaperone, TorD family
G2583_1294	CDS	1357823	1358362	+	ycdZ	hypothetical protein
G2583_1295	CDS	1358426	1359259	-	csgG	Curli production assembly/transport component csgG precursor
G2583_1296	CDS	1359286	1359702	-	csgF	Curli production assembly/transport component csgF precursor
G2583_1297	CDS	1359727	1360116	-	csgE	Curli production assembly/transport component csgE precursor
G2583_1298	CDS	1360121	1360771	-	csgD	CsgD
G2583_1299	CDS	1361183	1361431	-	-	hypothetical protein
G2583_1300	CDS	1361498	1361980	+	csgB	Minor curlin subunit
G2583_1301	CDS	1362021	1362479	+	csgA	Major curlin subunit precursor
G2583_1302	CDS	1362538	1362870	+	csgC	CsgC
G2583_1303	CDS	1362991	1363302	+	ymdA	hypothetical protein
G2583_1304	CDS	1363397	1363930	+	ymdB	UPF0189 protein ymdB
G2583_1305	CDS	1363872	1365353	+	ymdC	Putative synthase
G2583_1306	CDS	1365361	1366518	-	mdoC	Glucans biosynthesis protein C
G2583_1307	CDS	1366911	1368446	+	mdoG	Glucans biosynthesis protein G precursor
G2583_1308	CDS	1368409	1370982	+	mdoH	Periplasmic glucans biosynthesis protein MdoH
G2583_1309	CDS	1371155	1371382	+	yceK	hypothetical protein
G2583_1310	CDS	1371383	1371760	-	msyB	Acidic protein MsyB
G2583_1311	CDS	1371787	1371885	-	-	hypothetical protein
G2583_1312	CDS	1371840	1373066	-	mdtG	Multidrug resistance protein mdtG
G2583_1313	CDS	1373238	1374158	-	lpxL	Heat shock protein
G2583_1314	CDS	1374383	1375435	+	yceA	UPF0176 protein yceA
G2583_1315	CDS	1375477	1376052	-	yceI	hypothetical protein
G2583_1316	CDS	1376056	1376622	-	yceJ	Nickel-dependent hydrogenase b-type cytochrome subunit
G2583_1317	CDS	1376883	1377023	-	yceO	hypothetical protein
G2583_1318	CDS	1377044	1378162	-	solA	N-methyl-L-tryptophan oxidase
G2583_1319	CDS	1378277	1378531	-	bssS	hypothetical protein
G2583_1320	CDS	1378821	1379066	-	dinI	DinI

Locus_tag	Type	Start	End	+/-[a]	Gene[b]	Product
G2583_1321	CDS	1379140	1380186	-	pyrC	Dihydroorotase
G2583_1322	CDS	1380292	1380852	-	yceB	Putative lipoprotein yceB
G2583_1323	CDS	1380986	1381633	-	grxB	Glutaredoxin-2
G2583_1324	CDS	1381697	1382905	-	mdtH	Multidrug resistance protein mdtH
G2583_1325	CDS	1383141	1383725	+	rimJ	Ribosomal-protein-alanine acetyltransferase
G2583_1326	CDS	1383736	1384383	+	yceH	UPF0502 protein yceH
G2583_1327	CDS	1384385	1385308	+	MviM	Oxidoreductase family, NAD-binding
G2583_1328	CDS	1385418	1386953	+	MviN	Virulence factor mviN-like protein
G2583_1329	CDS	1386993	1387409	-	flgN	Flagellar biosynthesis/type III secretory path way chaperone
G2583_1330	CDS	1387414	1387707	-	flgM	Negative regulator of flagellin synthesis
G2583_1331	CDS	1387783	1388442	-	flgA	Flagellar biosynthesis
G2583_1332	CDS	1388597	1389013	+	flgB	Flagellar basal-body rod protein FlgB
G2583_1333	CDS	1389017	1389421	+	flgC	Cell-proximal portion of basal-body rod
G2583_1334	CDS	1389433	1390128	+	flgD	Flagellar hook protein FlgE
G2583_1335	CDS	1390153	1391358	+	flgE	Flagellar hook protein FlgE
G2583_1336	CDS	1391378	1392133	+	flgF	Flagellar basal-body rod protein FlgF
G2583_1337	CDS	1392271	1393053	+	flgG	Flagellar basal-body rod protein flgG
G2583_1338	CDS	1393106	1393804	+	flgH	Flagellar L-ring protein precursor
G2583_1339	CDS	1393813	1394913	+	flgI	flagellar basal body P-ring protein
G2583_1340	CDS	1394913	1395854	+	flgJ	Peptidoglycan hydrolase flgJ
G2583_1341	CDS	1395920	1397563	+	flgK	Flagellar hook-associated protein FlgK
G2583_1342	CDS	1397575	1398528	+	flgL	Flagellar hook-associated protein 3
G2583_1343	CDS	1398723	1401908	-	rne	Ribonuclease, Rne/Rng family
G2583_1344	CDS	1401989	1402363	+	yceQ	hypothetical protein
G2583_1345	CDS	1402481	1403440	+	rluC	Ribosomal large subunit pseudouridine synthase C
G2583_1346	CDS	1403552	1404175	-	yceF	Septum formation protein Maf
G2583_1347	ncRNA	1404130	1404298	+	sraB	ncRNA
G2583_1348	CDS	1404335	1404856	+	yceD	hypothetical protein
G2583_1349	CDS	1404908	1405081	+	rpmF	50S ribosomal protein L32
G2583_1350	CDS	1405162	1406232	+	plsX	fatty acid/phospholipid synthesis protein
G2583_1351	CDS	1406300	1407253	+	fabH	3-oxoacyl-[acyl-carrier-protein] synthase 3
G2583_1352	CDS	1407269	1408198	+	fabD	Malonyl CoA-acyl carrier protein transacylase
G2583_1353	CDS	1408211	1408945	+	fabG	3-oxoacyl-[acyl-carrier-protein] reductase
G2583_1354	CDS	1409156	1409392	+	acpP	Acyl carrier protein
G2583_1355	CDS	1409480	1410721	+	fabF	3-oxoacyl-[acyl-carrier-protein] synthase 2
G2583_1356	CDS	1410841	1411650	+	pabC	Aminodeoxychorismate lyase
G2583_1357	CDS	1411653	1412675	+	yceG	Putative thymidylate kinase
G2583_1358	CDS	1412665	1413306	+	tmk	Thymidylate kinase
G2583_1359	CDS	1413303	1414307	+	holB	DNA polymerase III, delta' subunit
G2583_1360	CDS	1414318	1415115	+	ycfH	Hydrolase, TatD family
G2583_1361	CDS	1415410	1416843	+	ptsG	hypothetical protein
G2583_1362	CDS	1416903	1419083	-	fhuE	Outer membrane receptor for ferric iron uptake
G2583_1363	CDS	1419399	1419776	+	hinT	hypothetical protein
G2583_1364	CDS	1419779	1420156	+	ycfL	Putative outer membrane lipoprotein YcfL
G2583_1365	CDS	1420170	1420811	+	ycfM	hypothetical protein
G2583_1366	CDS	1420792	1421616	+	thiK	Thiamine kinase
G2583_1367	CDS	1421627	1422652	+	nagZ	Beta-hexosaminidase
G2583_1368	CDS	1422675	1423217	+	ycfP	hypothetical protein
G2583_1369	CDS	1423617	1424921	+	ndh	NADH dehydrogenase
G2583_1370	CDS	1425148	1425687	+	ycfJ	Surface antigen domain protein
G2583_1371	CDS	1425749	1426459	-	ycfQ	hypothetical protein
G2583_1372	CDS	1426622	1426879	+	bhsA	Putative outer membrane protein
G2583_1373	CDS	1426962	1427924	-	ycfS	hypothetical protein
G2583_1374	CDS	1428068	1431514	-	mfd	transcription-repair coupling factor
G2583_1375	CDS	1431642	1432715	-	ycfT	hypothetical protein
G2583_1376	CDS	1432977	1434176	+	lolC	outer membrane-specific lipoprotein transporter subunit LolC
G2583_1377	CDS	1434169	1434870	+	lolD	ABC transporter, ATP-binding protein
G2583_1378	CDS	1434870	1436114	+	lolE	Lipoprotein releasing system, transmembrane protein LolE
G2583_1379	CDS	1436143	1437054	+	nagK	N-acetyl-D-glucosamine kinase
G2583_1380	CDS	1437070	1437891	+	cobB	NAD-dependent deacetylase

Locus_tag	Type	Start	End	+/-[a]	Gene[b]	Product
G2583_1381	CDS	1438047	1439093	-	potD	Spermidine/putrescine-binding periplasmic protein precursor
G2583_1382	CDS	1439090	1439884	-	potC	Spermidine/putrescine transport system permease
G2583_1383	CDS	1439881	1440738	-	potB	Spermidine/putrescine ABC transporter, permease protein PotB
G2583_1384	CDS	1440722	1441858	-	potA	Spermidine/putrescine import ATP-binding protein potA
G2583_1385	CDS	1442108	1443334	+	pepT	Peptidase T
G2583_1386	CDS	1443383	1444513	-	ycfD	YcfD protein
G2583_1387	CDS	1444732	1445982	+	-	Site-specific recombinase, phage integrase family
G2583_1388	CDS	1446348	1446536	+	-	putative DNA binding protein
G2583_1389	CDS	1446403	1446573	-	-	unknown protein encoded by prophage CP-933C
G2583_1390	CDS	1446594	1447538	+	-	conserved hypothetical protein
G2583_1391	CDS	1447531	1447743	+	-	hypothetical protein
G2583_1392	pseudogene	1447733	1448198	+	-	hypothetical protein
G2583_1393	CDS	1447965	1448198	+	-	hypothetical protein
G2583_1394	CDS	1448191	1448424	+	-	hypothetical protein
G2583_1395	CDS	1448430	1448729	+	-	hypothetical protein
G2583_1396	CDS	1448726	1450126	+	YabA	Bacteriophage P4 DNA primase
G2583_1397	CDS	1450327	1450578	+	-	hypothetical bacteriophage protein
G2583_1398	CDS	1450575	1450985	+	-	Single-stranded DNA-binding protein
G2583_1399	CDS	1451871	1452077	+	-	hypothetical protein
G2583_1400	CDS	1452077	1453132	+	-	Major capsid protein
G2583_1401	CDS	1453145	1453480	+	-	Capsid protein small subunit
G2583_1402	CDS	1453493	1453906	+	-	hypothetical protein
G2583_1403	CDS	1454127	1454654	+	-	Phage DNA packaging protein NU1-like protein
G2583_1404	CDS	1454910	1455191	+	-	Hypothetical bacteriophage protein
G2583_1405	CDS	1455793	1457253	-	phoQ	sensor protein PhoQ
G2583_1406	CDS	1457253	1457924	-	phoP	DNA-binding response regulator in two-component regulatory
G2583_1407	CDS	1458093	1459463	-	purB	Adenylosuccinate lyase
G2583_1408	CDS	1459467	1460108	-	hflD	hypothetical protein
G2583_1409	CDS	1460144	1461250	-	mnmA	Predicted tRNA(5-methylaminomethyl-2-thiouridylate)
G2583_1410	CDS	1461304	1461765	-	nudJ	Phosphatase nudJ
G2583_1411	CDS	1461775	1462428	-	rluE	Ribosomal large subunit pseudouridine synthase E
G2583_1412	CDS	1462600	1463850	+	icd	Isocitrate dehydrogenase [NADP]
G2583_1413	CDS	1463953	1464276	-	elbA	Anti-adapter protein iraM
G2583_1414	CDS	1464972	1465376	-	ycgX	hypothetical protein
G2583_1415	CDS	1465597	1466328	-	ycgE	transcriptional regulator mlrA
G2583_1416	CDS	1466533	1467009	-	ycgF	BLUF domain/cyclic diguanylate phosphodiesterase (EAL)
G2583_1417	CDS	1467006	1467818	-	ycgF	BLUF domain/cyclic diguanylate phosphodiesterase (EAL)
G2583_1418	CDS	1468054	1468290	+	ycgZ	hypothetical protein
G2583_1419	CDS	1468267	1468440	-	-	hypothetical protein
G2583_1420	CDS	1468333	1468605	+	ymgA	hypothetical protein
G2583_1421	CDS	1468634	1468900	+	ymgB	hypothetical protein
G2583_1422	CDS	1469013	1469261	+	ymgC	hypothetical protein
G2583_1423	CDS	1469597	1471120	+	ycgG	Cyclic diguanylate phosphodiesterase (EAL) domain protein
G2583_1424	CDS	1471252	1471470	+	ymgF	hypothetical protein
G2583_1425	CDS	1471587	1471706	+	-	hypothetical protein
G2583_1426	CDS	1471870	1472847	+	-	Porin, autotransporter (AT) family
G2583_1427	CDS	1472896	1474512	+	-	Porin, autotransporter (AT) family
G2583_1428	CDS	1474568	1474903	-	ymgD	hypothetical protein
G2583_1429	CDS	1474907	1475251	-	-	hypothetical protein
G2583_1430	CDS	1475253	1475426	-	ymgI	hypothetical protein
G2583_1431	CDS	1475527	1475712	+	ymgJ	hypothetical protein
G2583_1432	CDS	1475857	1479135	+	ypjA	Putative ATP-binding component of a transport system
G2583_1433	CDS	1479507	1479773	-	minE	Cell division topological specificity factor
G2583_1434	CDS	1479777	1480589	-	minD	Septum site-determining protein minD
G2583_1435	CDS	1480613	1481308	-	minC	Septum site-determining protein minC
G2583_1436	CDS	1481828	1482196	+	ycgJ	Putative fels-1 Prophage Protein
G2583_1437	CDS	1482299	1482700	-	ycgK	Bacterial pre-peptidase C-terminal domain protein
G2583_1438	CDS	1482729	1482929	+	-	hypothetical protein
G2583_1439	CDS	1482908	1483234	+	ycgL	hypothetical protein
G2583_1440	CDS	1483306	1483965	+	ycgM	Fumarylacetoacetate hydrolase family protein

Locus_tag	Type	Start	End	+/-[a]	Gene[b]	Product
G2583_1441	CDS	1484042	1484503	+	ycgN	conserved hypothetical protein
G2583_1442	CDS	1484710	1485627	-	hlyE	Hemolysin E
G2583_1443	ncRNA	1485856	1485934	+	-	ncRNA
G2583_1444	CDS	1485994	1486413	+	umuD	DNA polymerase V subunit UmuD
G2583_1445	CDS	1486413	1487681	+	umuC	DNA polymerase V subunit UmuC
G2583_1446	CDS	1487727	1488257	-	dsbB	Disulfide bond formation protein B
G2583_1447	CDS	1488403	1489944	-	nhaB	Na(+)/H(+) antiporter nhaB
G2583_1448	CDS	1490166	1490885	+	fadR	Fatty acid metabolism regulator protein
G2583_1449	CDS	1490937	1492469	-	ycgB	SpoVR family protein
G2583_1450	CDS	1492800	1494098	+	dadA	D-amino acid dehydrogenase small subunit
G2583_1451	CDS	1494108	1495178	+	dadX	Alanine racemase, catabolic
G2583_1452	CDS	1495347	1496789	-	ipaH	hypothetical protein
G2583_1453	CDS	1497181	1497336	+	-	hypothetical protein
G2583_1454	CDS	1497439	1499175	-	cvrA	potassium/proton antiporter
G2583_1455	CDS	1499271	1500185	-	ldcA	Muramoyltetrapeptide carboxypeptidase
G2583_1456	CDS	1500285	1500896	+	emtA	Membrane-bound lytic murein transglycosylase E
G2583_1457	CDS	1500898	1501632	-	ycgR	hypothetical protein
G2583_1458	CDS	1501833	1502087	+	ymgE	hypothetical protein
G2583_1459	CDS	1502137	1504107	-	prrA	Putative TonB dependent outer membrane receptor
G2583_1460	CDS	1504133	1504987	-	modD	Putative pyrophosphorylase modD
G2583_1461	CDS	1504984	1505796	-	-	Putative methyltransferase
G2583_1462	CDS	1505806	1506564	-	-	Putative iron compound ABC transporter, ATP-binding protein
G2583_1463	CDS	1506561	1507541	-	-	Putative iron compound ABC transporter, permease protein
G2583_1464	CDS	1507541	1508563	-	-	Putative iron compound ABC transporter, periplasmic-binding
G2583_1465	CDS	1508671	1508871	-	-	hypothetical protein
G2583_1466	CDS	1508899	1510356	-	treA	putative trehalase
G2583_1467	CDS	1510218	1510583	-	treA	trehalase
G2583_1468	CDS	1510903	1512324	-	dhaM	Putative PTS system enzyme I
G2583_1469	CDS	1512332	1512964	-	dhaL	Putative dihydroxyacetone kinase
G2583_1470	CDS	1512975	1514075	-	dhaK	Dihydroxyacetone kinase, N-terminal domain
G2583_1471	CDS	1514273	1516192	+	dhaR	PTS-dependent dihydroxyacetone kinase operon regulatory
G2583_1472	CDS	1516292	1519177	-	ycgV	putative outer membrane autotransporter
G2583_1473	CDS	1519522	1519725	+	-	hypothetical protein
G2583_1474	CDS	1520021	1521112	-	ychF	GTP-dependent nucleic acid-binding protein engD
G2583_1475	CDS	1521229	1521813	-	pth	Peptidyl-tRNA hydrolase
G2583_1476	CDS	1522091	1522369	+	ychH	predicted inner membrane protein
G2583_1477	CDS	1522424	1524103	-	ychM	Putative sulfate transporter ychM
G2583_1478	CDS	1524228	1525175	-	prs	Ribose-phosphate pyrophosphokinase
G2583_1479	CDS	1525326	1526177	-	ispE	4-diphosphocytidyl-2-C-methyl-D-erythritol kinase (CMK) (4-
G2583_1480	CDS	1526177	1526800	-	lolB	Outer membrane lipoprotein LolB
G2583_1481	CDS	1527014	1528270	+	hemA	Glutamyl-tRNA reductase
G2583_1482	CDS	1528312	1529394	+	prfA	Peptide chain release factor 1
G2583_1483	CDS	1529394	1530227	+	prmC	Protein-(Glutamine-N5) methyltransferase, release factor-
G2583_1484	CDS	1530224	1530616	+	ychQ	Putative Invasion gene expression up-regulator SirB
G2583_1485	CDS	1530620	1531429	+	ychA	putative transcriptional regulator
G2583_1486	CDS	1531465	1532319	+	kdsA	2-dehydro-3-deoxyphosphooctonate aldolase
G2583_1487	CDS	1532467	1532601	-	ldrB	hypothetical protein
G2583_1488	CDS	1533515	1534615	-	chaA	Calcium/proton antiporter
G2583_1489	CDS	1534885	1535115	+	chaB	Cation transport regulator chaB
G2583_1490	CDS	1535252	1535971	+	chaC	Cation transport regulator
G2583_1491	CDS	1536015	1536368	-	ychN	hypothetical protein
G2583_1492	CDS	1536518	1537948	+	ychO	Putative invasin
G2583_1493	CDS	1537949	1538599	-	narL	Nitrate/nitrite response regulator protein narL
G2583_1494	CDS	1538592	1540388	-	narX	Nitrate/nitrite sensor protein NarX
G2583_1495	CDS	1540414	1540632	+	-	hypothetical protein
G2583_1496	CDS	1540727	1542118	+	narK	Nitrite extrusion protein 1
G2583_1497	CDS	1542511	1546254	+	narG	Nitrate reductase, alpha subunit
G2583_1498	CDS	1546251	1547789	+	narH	Nitrate reductase, beta subunit
G2583_1499	CDS	1547786	1548496	+	narJ	Respiratory nitrate reductase 1 delta chain
G2583_1500	CDS	1548496	1549173	+	narI	Respiratory nitrate reductase, gamma subunit

Locus_tag	Type	Start	End	+/-[a]	Gene[b]	Product
G2583_1502	ncRNA	1549357	1549527	-	-	ncRNA
G2583_1503	tRNA	1549534	1549620	-	-	Tyr tRNA
G2583_1504	ncRNA	1549654	1549823	-	-	ncRNA
G2583_1505	tRNA	1549828	1549914	-	-	Tyr tRNA
G2583_1506	CDS	1550073	1550915	-	purU	Formyltetrahydrofolate deformylase
G2583_1507	CDS	1550965	1551423	-	ychJ	SEC-C motif domain protein
G2583_1508	CDS	1551497	1552441	+	rssA	Phospholipase, patatin family
G2583_1509	CDS	1552533	1553546	+	rssB	response regulator of RpoS
G2583_1510	CDS	1553748	1554656	+	galU	UTP--glucose-1-phosphate uridylyltransferase
G2583_1511	CDS	1554800	1555213	-	hns	DNA-binding protein H-NS
G2583_1512	CDS	1555817	1556434	+	tdk	Thymidine kinase
G2583_1513	CDS	1556735	1559410	-	adhE	Aldehyde-alcohol dehydrogenase
G2583_1514	CDS	1559887	1560534	+	ychE	Integral membrane protein, MarC family
G2583_1515	CDS	1561227	1562903	+	oppA	Periplasmic oligopeptide-binding protein
G2583_1516	CDS	1562989	1563909	+	oppB	Oligopeptide transport system permease protein oppB
G2583_1517	CDS	1563924	1564832	+	oppC	Oligopeptide transport system permease protein oppC
G2583_1518	CDS	1564844	1565857	+	oppD	Oligopeptide ABC transporter, ATP-binding protein OppD
G2583_1519	CDS	1565854	1566858	+	oppF	Oligopeptide ABC transporter, ATP-binding protein OppF
G2583_1520	CDS	1566911	1567240	-	yciU	hypothetical protein
G2583_1521	CDS	1567275	1568735	-	cls	Cardiolipin synthetase
G2583_1522	CDS	1568767	1569051	+	yciY	hypothetical protein
G2583_1523	CDS	1569106	1570359	-	kch	Potassium channel protein kch
G2583_1524	CDS	1570660	1570956	-	yciI	Uncharacterized protein conserved in bacteria
G2583_1525	CDS	1571180	1571899	+	tonB	outer membrane receptor-mediated transport energizer protein
G2583_1526	CDS	1571939	1572337	-	yciA	Acyl-CoA thioester hydrolase yciA
G2583_1527	CDS	1572442	1572981	-	yciB	Probable intracellular septation protein
G2583_1528	CDS	1573011	1573754	-	yciC	UPF0259 membrane protein yciC
G2583_1529	CDS	1574111	1574749	+	ompW	Outer membrane protein W
G2583_1530	CDS	1574630	1575913	-	intE	Putative integrase for prophage CP-933O
G2583_1531	CDS	1575891	1576139	-	xisE	Excisionase
G2583_1532	CDS	1576204	1578654	-	exoO	putative exonuclease
G2583_1533	CDS	1578747	1578947	-	ydfD	unknown protein encoded by prophage CP-933O
G2583_1534	CDS	1578935	1579228	-	dicB	Putative regulator of cell division encoded by prophage CP-
G2583_1535	ncRNA	1579298	1579348	-	dicF4	ncRNA
G2583_1536	CDS	1579682	1579915	+	-	unknown protein encoded by prophage CP-933O
G2583_1537	CDS	1579893	1580300	-	-	unknown protein encoded by prophage CP-933O
G2583_1538	CDS	1580323	1580541	-	ydfC	unknown protein encoded by prophage CP-933O
G2583_1539	CDS	1580614	1580970	-	-	hypothetical protein
G2583_1540	CDS	1581178	1581585	-	dicA	Similar to DicA, regulator of DicB encoded by prophage CP-
G2583_1541	CDS	1581662	1581889	+	dicC	DNA-binding transcriptional regulator DicC
G2583_1542	CDS	1581873	1582424	+	-	unknown protein encoded by prophage CP-933O
G2583_1543	CDS	1582396	1583436	+	-	unknown protein encoded by prophage CP-933O
G2583_1544	CDS	1583225	1583890	+	-	unknown protein encoded by prophage CP-933O
G2583_1545	CDS	1583924	1584658	+	-	hypothetical protein EcolO15_16731
G2583_1546	CDS	1584788	1585099	+	papH	putative fimbrial minor pilin protein precursor
G2583_1547	CDS	1585518	1586276	+	paa	Porcine attaching-effacing associated protein
G2583_1548	CDS	1586555	1586767	+	gef	Putative killer protein encoded by prophage CP-933O
G2583_1549	CDS	1586989	1587246	+	rem	hypothetical protein
G2583_1550	CDS	1587316	1587594	+	-	hypothetical protein
G2583_1551	CDS	1587596	1588651	+	-	hypothetical protein
G2583_1552	CDS	1588652	1589017	+	-	Holliday junction resolvase
G2583_1553	CDS	1589014	1589703	+	-	Antitermination protein Q
G2583_5267	tRNA	1589899	1589974	+	-	Met tRNA
G2583_5268	tRNA	1590065	1590141	+	-	Arg tRNA
G2583_1554	CDS	1590556	1591056	-	-	hypothetical protein
G2583_1555	CDS	1591008	1591193	+	-	hypothetical protein
G2583_1556	CDS	1591222	1593072	+	-	hypothetical protein
G2583_1557	CDS	1593351	1593512	-	-	hypothetical protein
G2583_1558	CDS	1593520	1593726	+	-	Putative lysis protein S of prophage CP-933V
G2583_1559	CDS	1593726	1594223	+	ybcS	lysozyme-like protein

Locus_tag	Type	Start	End	+/-[a]	Gene[b]	Product
G2583_1560	CDS	1594208	1594687	+	ybcT	Bacteriophage lysis protein
G2583_1561	CDS	1594770	1594910	+	-	hypothetical protein
G2583_1562	CDS	1595152	1595466	+	-	Putative transcriptional regulator
G2583_1563	CDS	1595548	1595772	-	-	unknown protein encoded within prophage CP-933R
G2583_1564	CDS	1595814	1596344	+	-	unknown protein encoded by prophage CP-933N
G2583_1565	CDS	1596460	1597023	+	-	Phage terminase, small subunit
G2583_1566	CDS	1597020	1598681	+	-	Phage terminase-like protein, large subunit
G2583_1567	CDS	1598745	1598864	+	-	Phage head maturation protease
G2583_1568	CDS	1598885	1599232	+	insN	unknown protein encoded by IS911 within prophage CP-933L
G2583_1569	CDS	1599445	1600098	+	-	putative transposase
G2583_1570	CDS	1600201	1601943	+	-	Phage head maturation protease
G2583_1571	CDS	1601988	1602209	+	-	conserved hypothetical protein
G2583_1572	CDS	1604736	1605062	+	-	conserved hypothetical protein
G2583_1573	CDS	1605073	1605423	+	-	Bacteriophage head-tail adaptor
G2583_1574	CDS	1605420	1605866	+	-	Phage protein, HK97 gp10 family
G2583_1575	CDS	1605863	1606207	+	-	unknown protein encoded by prophage CP-933N
G2583_1576	CDS	1606274	1606990	+	-	Putative major tail subunit
G2583_1577	CDS	1606996	1607370	+	-	Phage tail assembly chaperone
G2583_1578	CDS	1607394	1607675	+	-	putative tail protein
G2583_1579	CDS	1607727	1610993	+	-	putative tail length tape measure protein
G2583_1580	CDS	1610986	1611327	+	-	minor tail protein
G2583_1581	CDS	1611327	1612025	+	-	Phage-related protein
G2583_1582	CDS	1611898	1612779	+	-	Putative tail fiber component
G2583_1583	CDS	1612677	1613354	+	-	putative tail assembly protein
G2583_1584	CDS	1613595	1617071	+	-	putative host specificity protein
G2583_1585	CDS	1617153	1618199	-	insF	IS911 transposase orfB
G2583_1586	CDS	1618019	1618366	-	insN	unknown protein encoded by IS911 within prophage CP-933L
G2583_1587	CDS	1618496	1619023	+	-	Opacity protein and related surface antigens
G2583_1588	CDS	1619082	1620401	+	-	hypothetical protein
G2583_1589	CDS	1620893	1621435	+	-	conserved hypothetical protein
G2583_1590	CDS	1621584	1621991	-	-	NleA8-2 protein
G2583_1591	CDS	1622647	1623387	-	-	putative integrase
G2583_1592	CDS	1623813	1624724	+	nleH	Non-LEE-encoded type III effector H
G2583_1593	CDS	1624790	1625359	+	nleF	hypothetical protein
G2583_1594	CDS	1625693	1626040	-	-	Putative transposase within CP-933O
G2583_1595	CDS	1627643	1627759	+	yciD	Putative outer membrane protein
G2583_1596	CDS	1627819	1628325	-	yciE	YciE protein
G2583_1597	CDS	1628371	1628892	-	yciF	YciF protein
G2583_1598	CDS	1628957	1629160	-	yciG	hypothetical protein
G2583_1599	CDS	1629517	1630323	-	trpA	Tryptophan synthase alpha chain
G2583_1600	CDS	1630323	1631516	-	trpB	Tryptophan synthase beta chain
G2583_1601	CDS	1631528	1632889	-	trpC	bifunctional indole-3-glycerol phosphate
G2583_1602	CDS	1632890	1634485	-	trpD	Anthranilate synthase, component II
G2583_1603	CDS	1634485	1636047	-	trpE	anthranilate synthase component I
G2583_1604	CDS	1636139	1636183	-	trpL	trp operon leader peptide
G2583_1605	CDS	1636321	1637202	+	trpH	Putative phosphoesterase
G2583_1606	CDS	1637163	1637819	+	yciO	hypothetical protein
G2583_1607	CDS	1637847	1639430	+	-	conserved hypothetical protein
G2583_1608	CDS	1639643	1640515	+	rluB	Ribosomal large subunit pseudouridine synthase B
G2583_1609	CDS	1640555	1641145	-	btuR	Cob(I)yrinic acid a,c-diamide adenosyltransferase
G2583_1610	CDS	1641142	1641900	-	yciK	Oxidoreductase, short chain dehydrogenase/reductase family
G2583_1611	CDS	1642120	1643169	+	sohB	Peptidase, S49 (Protease IV) family
G2583_1612	CDS	1643205	1643456	-	yciN	hypothetical protein
G2583_1613	CDS	1643836	1646433	+	topA	DNA topoisomerase I
G2583_1614	CDS	1646643	1647617	+	cysB	transcriptional regulator CysB
G2583_1615	CDS	1647936	1648076	+	ymiA	hypothetical protein
G2583_1616	CDS	1648067	1648246	+	yciX	hypothetical protein
G2583_1617	CDS	1648619	1651294	+	acnA	Aconitate hydrase 1
G2583_1618	CDS	1651358	1651948	-	ribA	GTP cyclohydrolase-2
G2583_1619	CDS	1652118	1652882	+	pgpB	Phosphatidylglycerophosphatase B

Locus_tag	Type	Start	End	+/-[a]	Gene[b]	Product
G2583_1620	CDS	1653031	1653339	+	yciS	hypothetical protein
G2583_1621	CDS	1653346	1654515	+	yciM	hypothetical protein
G2583_1622	CDS	1654708	1655445	+	pyrF	Orotidine 5'-phosphate decarboxylase
G2583_1623	CDS	1655442	1655771	+	yciH	Putative translation initation factor SUI1
G2583_1624	CDS	1655897	1656115	-	osmB	hypothetical protein
G2583_1625	CDS	1656384	1657133	-	yciT	putative DEOR-type transcriptional regulator
G2583_1626	CDS	1657223	1657405	-	yciZ	UPF0509 protein yciZ
G2583_1627	CDS	1657544	1659529	-	gmr	sensory box-containing diguanylate cyclase/cyclic diguanylate
G2583_1628	CDS	1659764	1661698	-	rnb	Exoribonuclease 2
G2583_1629	CDS	1661766	1662893	-	yciW	Putative oxidoreductase
G2583_1630	CDS	1663037	1663825	-	fabI	Enoyl-[acyl-carrier-protein] reductase [NADH]
G2583_1631	CDS	1664162	1664548	-	-	hypothetical protein
G2583_1632	CDS	1664303	1664869	+	eefR	Putative transcriptional repressor
G2583_1633	CDS	1665018	1666139	+	eefA	Acriflavine resistance protein A
G2583_1634	CDS	1666139	1669228	+	-	putative multidrug-efflux transport protein
G2583_1635	CDS	1669251	1670624	+	eefC	Multidrug efflux outer membrane protein EefC
G2583_1636	CDS	1670633	1671796	+	eefD	Multidrug efflux transport protein EefD
G2583_1637	CDS	1671848	1672654	-	sapF	Peptide transport system, ATP-binding protein SapF
G2583_1638	CDS	1672656	1673648	-	sapD	Peptide transport system ATP-binding protein sapD
G2583_1639	CDS	1673648	1674538	-	sapC	Peptide ABC transporter, permease protein SapC
G2583_1640	CDS	1674525	1675490	-	sapB	Peptide transport system permease protein sapB
G2583_1641	CDS	1675487	1677130	-	sapA	peptide transport periplasmic protein
G2583_1642	CDS	1677443	1677688	-	ymjA	hypothetical protein
G2583_1643	CDS	1677822	1679207	-	puuP	Putative amino acid/amine transport protein
G2583_1644	CDS	1679510	1681006	-	puuA	Putative glutamine synthetase
G2583_1645	CDS	1681128	1681904	+	puuD	Gamma-glutamyl-gamma-aminobutyrate hydrolase
G2583_1646	CDS	1681931	1682488	+	puuR	DNA-binding transcriptional repressor
G2583_1647	CDS	1682638	1684125	+	puuC	Gamma-glutamyl-gamma-aminobutyraldehyde dehydrogenase
G2583_1648	CDS	1684127	1685407	+	puuB	Gamma-glutamylputrescine oxidoreductase
G2583_1649	CDS	1685445	1686710	+	puuE	4-aminobutyrate transaminase
G2583_1650	CDS	1686841	1687818	-	pspF	Psp operon transcriptional activator
G2583_1651	CDS	1687985	1688653	+	pspA	Phage shock protein
G2583_1652	CDS	1688707	1688931	+	pspB	Phage shock protein B
G2583_1653	CDS	1688931	1689290	+	pspC	Phage shock protein C
G2583_1654	CDS	1689299	1689520	+	pspD	Phage shock protein
G2583_1655	CDS	1689595	1689909	+	pspE	Phage shock protein E
G2583_1656	pseudogene	1690119	1691797	+	ycjM	Alpha amylase family protein
G2583_1657	CDS	1691811	1693103	+	ycjN	Sugar-binding periplasmic protein
G2583_1658	CDS	1693124	1694005	+	ycjO	Putative sugar ABC transporter, permease protein
G2583_1659	CDS	1693992	1694834	+	ycjP	Binding-protein-dependent transport system inner membrane
G2583_1660	CDS	1694865	1695917	+	ycjQ	Oxidoreductase, zinc-binding dehydrogenase family
G2583_1661	CDS	1695935	1696723	+	ycjR	AP endonuclease, family 2
G2583_1662	CDS	1696733	1697788	+	ycjS	Gfo/idh/mocA family
G2583_1663	CDS	1697785	1700052	+	ycjT	glycosyl hydrolase, family 65
G2583_1664	CDS	1700049	1700708	+	ycjU	Beta-phosphoglucomutase
G2583_1665	CDS	1700722	1701804	+	ycjV	Uncharacterized ABC transporter ATP-binding protein ycjV
G2583_1666	CDS	1701849	1702754	+	ompG	Outer membrane protein G
G2583_1667	CDS	1702865	1703863	-	ycjW	putative LACI-type transcriptional regulator
G2583_1668	CDS	1704018	1705415	+	ycjX	putative enzyme
G2583_1669	CDS	1705412	1706473	+	ycjF	UPF0283 membrane protein ycjF
G2583_1670	CDS	1706621	1708162	+	tyrR	DNA-binding transcriptional dual regulator, tyrosine-binding
G2583_1671	CDS	1708206	1708712	-	tpx	Thiol peroxidase
G2583_1672	CDS	1708831	1709796	+	ycjG	Mandelate racemase/muconate lactonizing enzyme family
G2583_1673	CDS	1709771	1710559	-	mpaA	murein peptide amidase A
G2583_1674	CDS	1710790	1711440	-	ymjC	NmrA family protein
G2583_1675	CDS	1711449	1712381	-	-	hypothetical protein
G2583_1676	CDS	1712381	1713313	-	ycjY	hypothetical protein
G2583_1677	CDS	1713439	1714338	+	ycjZ	putative transcriptional regulator LYSR-type
G2583_1678	CDS	1714653	1716287	+	mppA	Putative transport periplasmic protein
G2583_1679	CDS	1716338	1717369	-	ynaI	MscS family inner membrane protein ynaI

Locus_tag	Type	Start	End	+/-[a]	Gene[b]	Product
G2583_1680	CDS	1717613	1717870	+	ynaJ	hypothetical protein
G2583_1681	CDS	1717920	1718870	-	uspE	Universal stress protein E
G2583_1682	CDS	1719022	1719774	-	fnr	Fumarate and nitrate reduction regulatory protein
G2583_1683	CDS	1719969	1720484	-	ogt	Methylated-DNA-[protein]-cysteine S-methyltransferase
G2583_1684	CDS	1720495	1722027	-	abgT	Aminobenzoyl-glutamate transport protein
G2583_1685	CDS	1722058	1723503	-	abgB	Aminobenzoyl-glutamate utilization protein B
G2583_1686	CDS	1723503	1724813	-	abgA	Aminobenzoyl-glutamate utilization protein A
G2583_1687	CDS	1724989	1725897	+	abgR	putative DNA-binding transcriptional regulator
G2583_1688	ncRNA	1725878	1726057	+	-	ncRNA
G2583_1689	CDS	1726227	1726790	+	ydaL	Smr domain protein
G2583_1690	CDS	1726811	1728103	-	ydaM	hypothetical protein
G2583_1691	CDS	1728298	1729281	+	ydaN	Zinc transport protein zntB
G2583_1692	ncRNA	1729611	1729685	+	-	ncRNA
G2583_1693	CDS	1729759	1731132	+	dbpA	ATP-independent RNA helicase DbpA
G2583_1694	CDS	1731261	1732196	-	ttcA	C32 tRNA thiolase
G2583_1695	CDS	1732248	1733483	-	intR	Putative integrase for prophage CP-933R
G2583_1696	CDS	1733485	1733724	-	ydaQ	hypothetical protein
G2583_1697	CDS	1733800	1734060	-	ydaC	hypothetical protein
G2583_1698	CDS	1734026	1734175	-	lar	Restriction alleviation and modification enhancement protein
G2583_1699	CDS	1734231	1735040	-	recT	Recombinase, DNA renaturation protein encoded by prophage
G2583_1700	CDS	1735033	1737705	-	recE	Putative exodeoxyribonuclease VIII of prophage CP-933R
G2583_1701	CDS	1737805	1738080	-	racC	Putative bacteriophage protein
G2583_1702	CDS	1738155	1738331	-	ydaE	Putative bacteriophage protein
G2583_1703	CDS	1738325	1738558	-	kilR	Putative cell division inhibitor protein
G2583_1704	CDS	1738955	1739107	-	-	conserved hypothetical protein
G2583_1705	CDS	1739425	1739901	-	racR	Rac prophage repressor
G2583_1706	CDS	1740025	1740282	+	ydaS	putative tail fiber protein
G2583_1707	CDS	1740344	1740766	+	ydaT	conserved hypothetical protein
G2583_1708	CDS	1740844	1741632	+	ydaU	hypothetical protein
G2583_1709	CDS	1741639	1742379	+	ydaV	Putative DNA replication factor encoded by prophage CP-933R
G2583_1710	CDS	1742405	1743175	+	ydaW	conserved hypothetical protein
G2583_1711	CDS	1743191	1743586	+	renP	hypothetical protein
G2583_1712	CDS	1743583	1744227	+	-	unknown protein encoded by cryptic prophage CP-933P
G2583_1713	CDS	1744298	1744447	+	-	conserved hypothetical protein
G2583_1714	CDS	1744436	1744591	-	-	hypothetical protein
G2583_1715	CDS	1744636	1744848	+	mokP	Putative cell killing protein encoded within cryptic prophage CP-
G2583_1716	CDS	1745200	1745337	+	-	Putative bacteriophage cohesive ends
G2583_1717	CDS	1745409	1746008	+	-	hypothetical protein
G2583_1718	CDS	1746295	1746849	+	-	Putative antitermination protein
G2583_5269	tRNA	1746989	1747064	+	-	Met tRNA
G2583_5270	tRNA	1747074	1747150	+	-	Arg tRNA
G2583_5271	tRNA	1747164	1747240	+	-	Arg tRNA
G2583_1719	CDS	1747170	1747520	-	-	unknown protein encoded by prophage CP-933O
G2583_1720	CDS	1747771	1749717	+	-	YjhS
G2583_1721	CDS	1750075	1750347	+	-	hypothetical protein
G2583_1722	CDS	1750424	1750639	+	-	Putative lysis protein S of prophage CP-933V
G2583_1723	CDS	1750644	1751177	+	-	putative endolysin
G2583_1724	CDS	1751146	1751262	+	-	putative endolysin
G2583_1725	CDS	1751452	1752021	+	-	Anti-repressor protein Ant
G2583_1726	CDS	1752178	1752645	+	-	Endopeptidase
G2583_1727	CDS	1752633	1752785	+	-	hypothetical protein
G2583_1728	CDS	1752864	1753151	+	-	hypothetical protein
G2583_1729	CDS	1753665	1754141	+	-	conserved hypothetical protein
G2583_1730	CDS	1754138	1756261	+	-	Phage terminase large subunit
G2583_1731	CDS	1756234	1756470	+	-	hypothetical protein
G2583_1732	CDS	1757986	1759941	+	-	Peptidase S14, ClpP
G2583_1733	CDS	1760029	1760355	+	-	unknown protein encoded within prophage CP-933U
G2583_1734	CDS	1760348	1760629	+	-	hypothetical protein
G2583_1735	CDS	1760626	1761255	+	-	Putative tail fiber component Z
G2583_1736	CDS	1761268	1761666	+	-	putative tail fiber component U of prophage CP-933U

Locus_tag	Type	Start	End	+/-[a]	Gene[b]	Product
G2583_1737	CDS	1761674	1762426	+	-	hypothetical protein
G2583_1738	CDS	1762440	1762862	+	-	Minor tail protein G
G2583_1739	CDS	1762889	1763197	+	-	Minor tail protein T
G2583_1740	CDS	1763232	1765886	+	-	Minor tail protein H
G2583_1741	CDS	1765883	1766212	+	-	putative tail component of prophage CP-933O
G2583_1742	CDS	1766212	1766910	+	-	Phage-related protein
G2583_1743	CDS	1766846	1767664	+	-	Putative tail fiber component
G2583_1744	CDS	1767562	1768242	+	-	putative tail assembly protein
G2583_1745	CDS	1768490	1771963	+	-	Phage-related protein, tail component
G2583_1746	CDS	1772031	1772630	+	-	Putative outer membrane protein Lom of prophage CP-933O
G2583_1747	CDS	1772689	1773924	+	-	putative tail fiber protein
G2583_1748	CDS	1774308	1774883	+	-	unknown protein encoded by prophage CP-933R
G2583_1749	CDS	1774956	1775585	+	-	unknown protein encoded by prophage CP-933R
G2583_1750	CDS	1775667	1776308	+	-	unknown protein encoded by prophage CP-933R
G2583_1751	CDS	1777249	1777755	-	uspF	Universal stress protein F
G2583_1752	CDS	1777824	1778957	-	ompN	Outer membrane protein N
G2583_1753	ncRNA	1779182	1779303	+	-	ncRNA
G2583_1754	CDS	1779323	1782847	-	ydbK	Pyruvate-flavodoxin oxidoreductase
G2583_1755	CDS	1783121	1783387	+	ydbJ	hypothetical protein
G2583_1756	CDS	1783384	1783806	-	hslJ	Heat shock protein HslJ
G2583_1757	CDS	1783917	1784906	-	ldhA	D-lactate dehydrogenase
G2583_1758	CDS	1785114	1787753	+	ydbH	hypothetical protein
G2583_1759	CDS	1787750	1787935	+	ynbE	hypothetical protein
G2583_1760	CDS	1787937	1788266	+	ydbL	hypothetical protein
G2583_1761	CDS	1788605	1788724	+	-	hypothetical protein
G2583_1762	CDS	1788679	1791624	+	-	putative BigA-like protein
G2583_1763	CDS	1791844	1794303	+	-	putative outer membrane protein
G2583_1764	CDS	1794523	1795383	+	ydbC	Oxidoreductase, aldo/keto reductase family
G2583_1765	CDS	1795441	1797753	+	ydbD	hypothetical protein
G2583_1766	CDS	1797918	1798529	+	ynbA	Phosphatidylglycerophosphate synthase
G2583_1767	CDS	1798529	1799425	+	ynbB	Phosphatidate cytidylyltransferase
G2583_1768	CDS	1799441	1801198	+	ynbC	hypothetical protein
G2583_1769	CDS	1801212	1802504	+	ynbD	hypothetical protein
G2583_1770	CDS	1802555	1803160	-	azoR	FMN-dependent NADH-azoreductase
G2583_1771	CDS	1803361	1807263	+	hrpA	HrpA-like helicases
G2583_1772	CDS	1807409	1807834	+	-	hypothetical protein
G2583_1773	CDS	1807809	1808177	+	-	hypothetical protein
G2583_1774	CDS	1808164	1808595	+	-	hypothetical protein
G2583_1775	CDS	1808720	1809097	+	-	hypothetical protein
G2583_1776	CDS	1809213	1810013	+	ydcF	hypothetical protein
G2583_1777	CDS	1810210	1811649	+	aldA	Aldehyde dehydrogenase A
G2583_1778	CDS	1811691	1812692	-	gapC	Glyceraldehyde-3-phosphate dehydrogenase C
G2583_1779	CDS	1812845	1813411	+	cybB	Nickel-dependent hydrogenase, b-type cytochrome subunit
G2583_1780	ncRNA	1813422	1813485	-	-	ncRNA
G2583_1781	CDS	1813656	1813829	+	ydcA	hypothetical protein
G2583_1782	CDS	1813941	1814108	-	mokB	hypothetical protein
G2583_1783	CDS	1814449	1816089	+	trg	Methyl-accepting chemotaxis protein III, ribose sensor receptor
G2583_1784	CDS	1816127	1817191	-	ydcI	putative transcriptional regulator LYSR-type
G2583_1785	CDS	1817267	1818610	+	ydcJ	hypothetical protein
G2583_1786	CDS	1818835	1820490	+	mdoD	Glucans biosynthesis protein D precursor
G2583_1787	CDS	1820411	1820614	-	-	hypothetical protein
G2583_1788	CDS	1820630	1820854	+	ydcH	hypothetical protein
G2583_1789	CDS	1820917	1821453	+	rimL	Ribosomal-protein-serine acetyltransferase
G2583_1790	CDS	1821448	1822428	-	ydcK	hypothetical protein
G2583_1791	CDS	1822552	1823544	+	tehA	Tellurite resistance protein tehA
G2583_1792	CDS	1823541	1824134	+	tehB	Tellurite resistance protein TehB
G2583_1793	CDS	1824438	1825106	+	ydcL	Uncharacterized lipoprotein ydcL precursor
G2583_1794	CDS	1825141	1826370	-	ydcO	hypothetical protein
G2583_1795	CDS	1826405	1826941	+	ydcN	DNA-binding protein
G2583_1796	CDS	1826972	1828975	+	ydcP	Peptidase, U32 family

Locus_tag	Type	Start	End	+/-[a]	Gene[b]	Product
G2583_1797	CDS	1829067	1829297	-	yncJ	hypothetical protein
G2583_1798	CDS	1829483	1829680	-	-	hypothetical protein
G2583_1799	CDS	1829720	1830157	+	ydcQ	Predicted DNA-binding transcriptional regulator
G2583_1800	CDS	1830236	1831642	+	ydcR	multi modular; putative transcriptional regulator; also putative
G2583_1801	CDS	1831887	1833032	+	ydcS	ABC transporter, periplasmic substrate-binding protein
G2583_1802	CDS	1833050	1834063	+	ydcT	ABC transporter, ATP-binding protein
G2583_1803	CDS	1834064	1835005	+	ydcU	ABC transporter, permease protein
G2583_1804	CDS	1834995	1835813	+	ydcV	Putative transport system permease protein
G2583_1805	CDS	1835803	1837227	+	ydcW	Gamma-aminobutyraldehyde dehydrogenase
G2583_1806	CDS	1837566	1837787	+	ydcX	hypothetical protein
G2583_1807	CDS	1837873	1838106	+	ydcY	hypothetical protein
G2583_1808	CDS	1838107	1838556	-	ydcZ	hypothetical protein
G2583_1809	CDS	1838553	1839134	-	yncA	Acetyltransferase, GNAT family
G2583_1810	CDS	1839252	1840289	+	yncB	Putative NADP-dependent oxidoreductase yncB
G2583_1811	CDS	1840430	1841152	+	yncC	putative DNA-binding transcriptional regulator
G2583_1812	CDS	1841188	1843290	-	yncD	TonB-dependent receptor
G2583_1813	CDS	1843532	1844593	+	yncE	Uncharacterized conserved protein
G2583_1814	CDS	1844708	1846258	-	ansP	L-asparagine permease
G2583_1815	CDS	1846474	1847091	+	yncG	putative transferase
G2583_1816	CDS	1847167	1847379	+	yncH	conserved hypothetical protein
G2583_1817	CDS	1847792	1847938	-	-	hypothetical protein
G2583_1818	CDS	1848164	1850308	+	vgrE	unknown protein associated with Rhs element
G2583_1819	pseudogene	1850375	1854577	+	rhsE	protein rhsE
G2583_1820	CDS	1854774	1854983	+	-	DsORF-e4
G2583_1821	CDS	1855050	1855199	+	-	unknown protein associated with Rhs element
G2583_1822	pseudogene	1855491	1856652	+	ydcC	H repeat-associated protein
G2583_1823	CDS	1856712	1856978	+	pptA	Probable tautomerase ydcE
G2583_1824	CDS	1856982	1857551	-	yddH	hypothetical protein
G2583_1825	CDS	1857724	1858569	+	nhoA	Putative N-hydroxyarylamine O-acetyltransferase
G2583_1826	CDS	1858665	1859558	-	yddE	Uncharacterized isomerase yddE
G2583_1827	CDS	1859637	1860317	-	narV	Nitrate reductase 2, gamma subunit
G2583_1828	CDS	1860314	1861009	-	narW	Nitrate reductase molybdenum cofactor assembly chaperone 2
G2583_1829	CDS	1861009	1862553	-	narY	Nitrate reductase 2, beta subunit
G2583_1830	CDS	1862550	1866290	-	narZ	Nitrate reductase 2, alpha subunit
G2583_1831	CDS	1866372	1867760	-	narU	Nitrite extrusion protein 2
G2583_1832	CDS	1868066	1869325	-	yddJ	Leucine-rich repeat protein
G2583_1833	CDS	1869388	1870056	-	-	hypothetical protein
G2583_1834	CDS	1870143	1870646	-	-	hypothetical protein
G2583_1835	CDS	1870815	1871915	-	-	Gram-negative porin family
G2583_1836	CDS	1872174	1873055	-	yddG	hypothetical protein
G2583_1837	pseudogene	1873287	1876334	+	fdnG	nitrate-inducible formate dehydrogenase-N alpha subunit
G2583_1838	CDS	1876347	1877231	+	fdnH	Formate dehydrogenase, nitrate-inducible, iron-sulfur subunit
G2583_1839	CDS	1877224	1877877	+	fdnI	Formate dehydrogenase-N gamma subunit
G2583_1840	CDS	1877927	1878289	-	yddM	hypothetical protein
G2583_1841	CDS	1878357	1879367	-	adhP	Zn-dependent alcohol dehydrogenases
G2583_1842	CDS	1879501	1881225	-	maeA	NAD-dependent malic enzyme
G2583_1843	CDS	1881355	1881492	-	sra	Stationary-phase-induced ribosome-associated protein
G2583_1844	CDS	1881594	1881809	-	bdm	biofilm-dependent modulation protein
G2583_1845	CDS	1882154	1882585	+	osmC	Peroxiredoxin OsmC
G2583_1846	CDS	1882641	1883567	-	ddpF	Putative ABC transport system ATP-binding protein
G2583_1847	CDS	1883560	1884546	-	ddpD	ABC transporter, ATP-binding protein
G2583_1848	CDS	1884543	1885439	-	ddpC	Inner membrane ABC transporter permease protein YddQ
G2583_1849	CDS	1885436	1886458	-	ddpB	Binding-protein-dependent transport systems inner membrane
G2583_1850	CDS	1886460	1888010	-	ddpA	Putative ABC transporter periplasmic-binding protein yddS
G2583_1851	CDS	1888024	1888605	-	ddpX	D-alanyl-D-alanine dipeptidase
G2583_1852	CDS	1888863	1891286	-	dos	FOG: PAS/PAC domain
G2583_1853	CDS	1891287	1892669	-	yddV	Diguanylate cyclase yddV
G2583_1854	CDS	1893046	1894365	-	yddW	Uncharacterized lipoprotein yddW precursor
G2583_1855	CDS	1894496	1896031	-	gadC	Probable glutamate/gamma-aminobutyrate antiporter
G2583_1856	CDS	1896187	1897587	-	gadB	Glutamate decarboxylase beta

Locus_tag	Type	Start	End	+/-[a]	Gene[b]	Product
G2583_1857	CDS	1897949	1900744	-	pqqL	Putative peptidase
G2583_1858	CDS	1900789	1903161	-	yddB	TonB-dependent receptor
G2583_1859	CDS	1903199	1904884	-	yddA	Inner membrane ABC transporter ATP-binding protein
G2583_1860	CDS	1905175	1906347	-	ydeM	Putative enzyme
G2583_1861	CDS	1906384	1908099	-	ydeN	Putative sulfatase
G2583_1862	CDS	1908470	1909231	-	ydeO	transcriptional regulator YdeO
G2583_1863	CDS	1909305	1909502	-	-	Two-component-system connector protein yneN
G2583_1864	CDS	1909750	1912029	-	ydeP	putative oxidoreductase major subunit
G2583_1865	CDS	1912363	1913277	-	ydeQ	transcriptional regulator BolA
G2583_1866	CDS	1913337	1913840	-	ydeR	Uncharacterized fimbrial-like protein ydeR precursor
G2583_1867	CDS	1913853	1914383	-	ydeS	biofilm-dependent modulation protein
G2583_1868	CDS	1914397	1917048	-	fimD	Fimbrial usher family protein
G2583_1869	CDS	1917090	1917809	-	FimC	P pilus assembly protein, chaperone PapD
G2583_1870	CDS	1918161	1918724	-	fmlA	Putative Fml fimbriae subunit
G2583_1871	CDS	1919158	1919412	-	yneL	Putative ARAC-type regulatory protein
G2583_1872	CDS	1919699	1921021	-	hipA	regulator with hipB
G2583_1873	CDS	1921021	1921287	-	hipB	transcriptional regulator HipB
G2583_1874	CDS	1921498	1922898	-	ydeU	Outer membrane autotransporter barrel domain protein
G2583_1875	CDS	1922895	1926926	-	ydeK	hypothetical protein
G2583_1876	CDS	1927457	1929049	-	lsrK	Carbohydrate kinase FGGY
G2583_1877	CDS	1929128	1930081	-	lsrR	putative transcriptional regulator, sorC family
G2583_1878	CDS	1930330	1931865	+	lsrA	Autoinducer-2 ABC transporter, ATP-binding protein LsrA
G2583_1879	CDS	1931859	1932887	+	lsrC	Autoinducer-2 ABC transporter, permease protein LsrC
G2583_1880	CDS	1932887	1933879	+	lsrD	Autoinducer-2 ABC transporter, permease protein LsrD
G2583_1881	CDS	1933891	1934913	+	lsrB	Autoinducer-2 ABC transporter, periplasmic autoinducer-2-
G2583_1882	CDS	1934940	1935815	+	lsrF	Uncharacterized aldolase yneB
G2583_1883	CDS	1935839	1936129	+	lsrG	autoinducer-2 (AI-2) modifying protein LsrG
G2583_1884	CDS	1936186	1936944	+	tam	Trans-aconitate 2-methyltransferase
G2583_1885	CDS	1936948	1937913	-	yneE	hypothetical protein
G2583_1886	CDS	1938059	1939510	-	uxaB	Altronate oxidoreductase
G2583_1887	CDS	1939737	1941155	-	yneF	Diguanylate cyclase (GGDEF) domain protein
G2583_1888	CDS	1941294	1941653	-	yneG	hypothetical protein
G2583_1889	CDS	1941653	1942579	-	yneH	Glutaminase 2
G2583_1890	CDS	1942643	1944055	-	yneI	NAD-dependent aldehyde dehydrogenases
G2583_1891	CDS	1944132	1945013	+	yneJ	putative transcriptional regulator LYSR-type
G2583_1892	pseudogene	1945091	1946206	+	yneK	hypothetical protein
G2583_1893	CDS	1946357	1947547	+	ydeA	Probable sugar efflux transporter
G2583_1894	CDS	1947572	1948237	-	marC	MarC
G2583_1895	CDS	1948449	1948883	+	marR	MarR
G2583_1896	CDS	1948903	1949286	+	marA	Multiple antibiotic resistance protein marA
G2583_1897	CDS	1949318	1949536	+	marB	Multiple antibiotic resistance protein MarB
G2583_1898	CDS	1949567	1950466	-	eamA	Transporter, 10 TMS drug/metabolite exporter (DME) family
G2583_1899	CDS	1950661	1951848	+	ydeE	Transporter, major facilitator family
G2583_1900	CDS	1951889	1952284	-	-	Rhodanese domain protein
G2583_1901	CDS	1952367	1953350	+	ftrA	Transcriptional activator FtrA
G2583_1902	CDS	1953772	1954662	-	ydeH	Diguanylate cyclase
G2583_1903	CDS	1954917	1955309	-	ydeI	hypothetical protein
G2583_1904	CDS	1955585	1956103	+	ydeJ	Competence/damage-inducible protein CinA C-terminal domain
G2583_1905	CDS	1956148	1958193	-	dcp	Peptidyl-dipeptidase Dcp
G2583_1906	CDS	1958330	1959076	+	ydfG	L-allo-threonine dehydrogenase, NAD(P)-binding protein
G2583_1907	CDS	1959165	1959851	+	ydfH	Hypothetical transcriptional regulator ydfH
G2583_1908	CDS	1960028	1960231	+	ydfZ	Putative selenoprotein ydfZ
G2583_1909	CDS	1960267	1961727	-	ydfI	Mannitol dehydrogenase family protein
G2583_1910	CDS	1961834	1963099	-	ydfJ	Inner membrane metabolite transport protein ydfJ
G2583_1911	CDS	1963138	1963473	+	-	unknown protein encoded within CP-933O
G2583_1912	CDS	1963523	1964731	-	tnpA	Transposase and inactivated derivatives
G2583_1913	CDS	1964881	1965531	-	-	unknown protein encoded by prophage CP-933O
G2583_1914	CDS	1965516	1965863	-	-	hypothetical protein
G2583_1915	CDS	1965805	1965957	-	-	conserved hypothetical protein
G2583_1916	CDS	1966242	1966817	-	-	hypothetical protein

Locus_tag	Type	Start	End	+/-[a]	Gene[b]	Product
G2583_1917	CDS	1967201	1968514	-	-	putative tail fiber protein encoded by prophage CP-933R
G2583_1918	CDS	1968579	1969178	-	-	Enterobacterial Ail/Lom family protein
G2583_1919	CDS	1969245	1969553	-	-	Host specificity protein J
G2583_1920	CDS	1969589	1972624	-	-	Host specificity protein
G2583_1921	CDS	1973254	1974072	-	-	Putative tail fiber component K of prophage
G2583_1922	CDS	1974002	1974700	-	-	minor tail protein
G2583_1923	CDS	1974700	1975029	-	-	Minor tail family protein
G2583_1924	CDS	1975026	1977605	-	-	putative tail component of prophage CP-933O
G2583_1925	CDS	1977586	1977999	-	-	hypothetical protein
G2583_1926	CDS	1978026	1978457	-	-	phage minor tail protein G
G2583_1927	CDS	1978471	1979223	-	-	Bacterial surface proteins containing Ig-like domains
G2583_1928	CDS	1979231	1979626	-	-	hypothetical protein
G2583_1929	CDS	1979623	1980156	-	-	hypothetical protein
G2583_1930	CDS	1980171	1980524	-	-	hypothetical protein
G2583_1931	CDS	1980517	1980900	-	-	conserved hypothetical protein
G2583_1932	CDS	1980952	1981980	-	-	phage major capsid protein E
G2583_1933	CDS	1982038	1982385	-	-	Bacteriophage lambda head decoration protein D
G2583_1934	CDS	1982422	1983927	-	-	Periplasmic serine proteases (ClpP class)
G2583_1935	CDS	1983917	1985509	-	-	Bacteriophage capsid protein
G2583_1936	CDS	1985506	1985712	-	-	putative head completion protein
G2583_1937	CDS	1985696	1987660	-	-	Putative terminase large subunit of prophage CP-933O
G2583_1938	CDS	1987596	1988105	-	-	Prophage Qin DNA packaging protein NU1-like protein
G2583_1939	CDS	1988227	1988334	-	nohA	conserved hypothetical protein
G2583_1940	CDS	1988500	1988727	+	-	conserved hypothetical protein
G2583_1941	CDS	1989090	1989557	-	-	Putative endopeptidase Rz
G2583_1942	CDS	1989711	1990280	-	antU	Antirepressor protein
G2583_1943	CDS	1990551	1991084	-	-	putative endolysin
G2583_1944	CDS	1991135	1991479	-	-	unknown protein encoded by cryptic prophage CP-933P
G2583_1945	CDS	1991484	1991699	-	-	Putative lysis protein S of prophage CP-933V
G2583_1946	CDS	1991775	1992200	-	-	hypothetical protein
G2583_1947	CDS	1992082	1992264	-	-	hypothetical protein
G2583_1948	CDS	1992412	1994349	-	-	hypothetical protein
G2583_1949	CDS	1994378	1994563	-	-	hypothetical protein
G2583_1950	CDS	1994515	1995015	+	-	hypothetical protein
G2583_1951	CDS	1995428	1996249	-	-	DnaJ-class molecular chaperone with C-terminal Zn finger
G2583_1952	CDS	1996246	1996620	-	-	Putative endonuclease of cryptic prophage CP-933M
G2583_1953	CDS	1996633	1997679	-	-	conserved hypothetical protein
G2583_1954	CDS	1997681	1998040	-	-	hypothetical protein
G2583_1955	CDS	1998127	1998339	-	-	Putative cell killing protein encoded within cryptic prophage CP-
G2583_1956	CDS	1998749	1999618	-	-	Eaa protein
G2583_1957	CDS	1999629	1999892	-	-	unknown protein encoded within prophage CP-933R
G2583_1958	CDS	1999894	2000112	-	-	hypothetical protein
G2583_1959	CDS	2000145	2000357	-	-	unknown protein encoded within prophage CP-933R
G2583_1960	CDS	2000408	2000584	-	-	Putative bacteriophage protein
G2583_1961	CDS	2000784	2001209	-	-	Unknown protein encoded within prophage
G2583_1962	CDS	2001250	2002215	-	-	conserved hypothetical protein
G2583_1963	CDS	2002240	2002665	-	-	hypothetical protein
G2583_1964	CDS	2002662	2002877	-	-	hypothetical protein
G2583_1965	CDS	2002927	2003643	+	-	SOS-response transcriptional repressors (RecA-mediated
G2583_1966	CDS	2003831	2004061	+	ydaF	unknown protein encoded by prophage CP-933N
G2583_1967	CDS	2004176	2004925	-	-	unknown protein encoded by prophage CP-933N
G2583_1968	CDS	2005144	2005428	+	dicB	Putative regulator of cell division encoded by prophage CP-
G2583_1969	CDS	2005425	2005628	+	-	conserved hypothetical protein
G2583_1970	CDS	2005708	2008179	+	ydfE	Exonuclease family protein
G2583_1971	CDS	2008251	2008502	+	xisP	Putative phage excisionase protein
G2583_1972	CDS	2008522	2009817	+	intQ	Integrase family protein
G2583_1973	CDS	2009843	2009947	-	YdfJ	Inner membrane metabolite transport protein ydfJ
G2583_1974	CDS	2010005	2011024	-	rspB	Starvation sensing protein RspB
G2583_1975	CDS	2011036	2012250	-	rspA	Starvation-sensing protein rspA
G2583_1976	CDS	2012456	2012782	-	ynfA	UPF0060 membrane protein ynfA

Locus_tag	Type	Start	End	+/-[a]	Gene[b]	Product
G2583_1977	CDS	2012917	2013258	+	ynfB	UPF0482 protein ynfB precursor
G2583_1978	CDS	2013293	2013853	+	speG	Spermidine N(1)-acetyltransferase
G2583_1979	CDS	2013856	2014602	-	ynfC	hypothetical protein
G2583_1980	CDS	2014671	2014979	+	ynfD	hypothetical protein
G2583_1981	CDS	2015178	2017604	+	ynfE	Anaerobic dimethyl sulfoxide reductase, A subunit YnfE
G2583_1982	CDS	2017692	2020088	+	ynfF	Anaerobic dimethyl sulfoxide reductase, A subunit, DmsA/YnfE
G2583_1983	CDS	2020099	2020716	+	ynfG	Probable anaerobic dimethyl sulfoxide reductase chain ynfG
G2583_1984	CDS	2020718	2021572	+	ynfH	Oxidoreductase, membrane subunit
G2583_1985	CDS	2021615	2022229	+	dmsD	Twin-arginine leader-binding protein dmsD
G2583_1986	CDS	2022423	2023679	+	clcB	Putative chloride channel
G2583_1987	CDS	2023632	2024327	-	ynfK	Putative dethiobiotin synthetase
G2583_1988	CDS	2024452	2025672	-	dgsA	putative NAGC-like transcriptional regulator
G2583_1989	CDS	2025807	2026700	-	ynfL	putative transcriptional regulator LYSR-type
G2583_1990	CDS	2026807	2028060	+	ynfM	Major facilitator family transporter
G2583_1991	CDS	2028478	2028792	+	asr	acid shock protein precursor
G2583_1992	CDS	2029068	2029889	+	ydgD	V8-like Glu-specific endopeptidase
G2583_1993	CDS	2029928	2030257	-	mdtI	multidrug resistance protein MdtI
G2583_1994	CDS	2030244	2030609	-	mdtJ	Spermidine export protein mdtJ
G2583_1995	CDS	2031021	2032055	+	tqsA	Putative permease, PerM family
G2583_1996	CDS	2032080	2033468	-	pntB	NAD(P) transhydrogenase subunit beta
G2583_1997	CDS	2033479	2035065	-	pntA	NAD(P) transhydrogenase, alpha subunit
G2583_1998	CDS	2035535	2036479	+	ydgH	predicted protein
G2583_1999	CDS	2036665	2038047	+	ydgI	Arginine/ornithine antiporter
G2583_2000	CDS	2038084	2038806	+	folM	Dihydrofolate reductase folM
G2583_2001	CDS	2038803	2039138	-	ydgC	Inner membrane protein ydgC
G2583_2002	CDS	2039258	2039986	+	rstA	DNA-binding response regulator in two-component regulatory
G2583_2003	CDS	2039990	2041291	+	rstB	Sensor histidine kinase RstB
G2583_2004	CDS	2041355	2042296	+	tus	DNA replication terminus site-binding protein
G2583_2005	CDS	2042293	2043696	-	fumC	Fumarate hydratase class II
G2583_2006	CDS	2043839	2045485	-	fumA	Fumarate hydratase class I, aerobic
G2583_2007	CDS	2045684	2046859	+	manA	Mannose-6-phosphate isomerase, class I
G2583_2008	CDS	2046960	2048468	+	ydgA	hypothetical protein
G2583_2009	pseudogene	2048513	2049777	-	uidC	membrane-associated protein
G2583_2010	CDS	2049816	2051189	-	uidB	Glucuronide permease uidB
G2583_2011	CDS	2051186	2052997	-	uidA	Beta-glucuronidase
G2583_2012	CDS	2053388	2053978	-	uidR	repressor for uid operon
G2583_2013	CDS	2054207	2054974	-	hdhA	7-alpha-hydroxysteroid dehydrogenase
G2583_2014	CDS	2055086	2056114	-	malI	Maltose regulon regulatory protein MalI
G2583_2015	CDS	2056289	2057881	+	malX	PTS system, maltose and glucose-specific IIABC component
G2583_2016	CDS	2057891	2059063	+	malY	Maltose regulon modulator MalY
G2583_2017	CDS	2059167	2060168	+	add	Adenosine deaminase
G2583_2018	CDS	2060204	2061244	-	ydgJ	Oxidoreductase, NAD-binding
G2583_2019	CDS	2061487	2061612	+	blr	Beta-lactam resistance protein
G2583_2020	CDS	2061885	2062100	+	cnu	OriC-binding nucleoid-associated protein
G2583_2021	CDS	2062162	2062626	+	ydgK	hypothetical protein
G2583_2022	CDS	2062703	2063284	+	rsxA	Electron transport complex protein rnfA
G2583_2023	CDS	2063284	2063862	+	rsxB	Electron transport complex protein rnfB
G2583_2024	CDS	2063855	2066173	+	rsxC	Electron transport complex protein RnfC
G2583_2025	CDS	2066174	2067232	+	rsxD	Electron transport complex protein rnfD
G2583_2026	CDS	2067236	2067856	+	rsxG	Electron transport complex protein rnfG
G2583_2027	CDS	2067860	2068555	+	rsxE	Predicted NADH:ubiquinone oxidoreductase, subu nit RnfE
G2583_2028	CDS	2068555	2069190	+	nth	Endonuclease III (DNA-(apurinic or apyrimidinic site) lyase)
G2583_2029	CDS	2069801	2071303	+	tppB	Tripeptide permease tppB
G2583_2030	CDS	2071409	2072014	+	gst	Glutathione S-transferase
G2583_2031	CDS	2072058	2072921	-	pdxY	Pyridoxamine kinase
G2583_2032	CDS	2072980	2074266	-	tyrS	Tyrosyl-tRNA synthetase
G2583_2033	CDS	2074383	2075039	-	pdxH	Pyridoxine/pyridoxamine 5'-phosphate oxidase
G2583_2034	CDS	2075098	2075427	-	ydhA	predicted lipoprotein
G2583_2035	CDS	2075525	2076634	-	anmK	Anhydro-N-acetylmuramic acid kinase
G2583_2036	CDS	2076907	2077374	+	slyB	Outer membrane lipoprotein slyB precursor

Locus_tag	Type	Start	End	+/-[a]	Gene[b]	Product
G2583_2037	CDS	2077421	2077855	-	slyA	Transcriptional regulators
G2583_2038	CDS	2078056	2078292	+	ydhI	hypothetical protein
G2583_2039	CDS	2078253	2079152	+	ydhJ	hypothetical protein
G2583_2040	CDS	2079152	2081164	+	ydhK	Uncharacterized transporter ydhK
G2583_2041	CDS	2081165	2081686	-	sodC	Superoxide dismutase [Cu-Zn]
G2583_2042	CDS	2081767	2082663	-	ydhF	Oxidoreductase ydhF
G2583_2043	CDS	2082712	2083089	-	ydhL	hypothetical protein
G2583_2044	CDS	2083054	2083653	+	ydhM	hypothetical protein
G2583_2045	CDS	2083690	2084787	+	nemA	N-ethylmaleimide reductase
G2583_2046	CDS	2084868	2085275	+	gloA	Lactoylglutathione lyase
G2583_2047	CDS	2085378	2086025	+	rnt	Ribonuclease T
G2583_2048	CDS	2086118	2090734	+	lhr	DEAD/DEAH box helicase family protein Lhr
G2583_2049	CDS	2090785	2091132	-	grxD	Glutaredoxin-4
G2583_2050	CDS	2091467	2092282	+	ydhO	NlpC/P60 family protein
G2583_2051	CDS	2092410	2092991	+	sodB	Superoxide dismutase [Fe]
G2583_2052	CDS	2093137	2094306	-	ydhP	predicted transporter
G2583_2053	CDS	2094861	2095886	+	purR	DNA-binding transcriptional repressor PurR
G2583_2054	CDS	2095883	2096815	-	ydhB	putative DNA-binding transcriptional regulator
G2583_2055	CDS	2096928	2098139	+	ydhC	Drug resistance transporter, Bcr/CflA subfamily
G2583_2056	CDS	2098430	2099578	+	cfa	Cyclopropane-fatty-acyl-phospholipid synthase
G2583_2057	CDS	2099618	2100259	-	ribC	Riboflavin synthase alpha chain
G2583_2058	CDS	2100474	2101847	+	mdtK	Multidrug resistance protein mdtK
G2583_2059	CDS	2101888	2103144	-	ydhQ	hypothetical protein
G2583_2060	tRNA	2103451	2103529	+	-	Val tRNA
G2583_2061	tRNA	2103532	2103610	+	-	Val tRNA
G2583_2062	CDS	2103717	2104022	+	ydhR	Protein ydhR
G2583_2063	CDS	2104148	2105752	+	ydhS	hypothetical protein
G2583_2064	CDS	2105764	2106576	-	ydhT	hypothetical protein
G2583_2065	CDS	2106580	2107365	-	ydhU	Nickel-dependent hydrogenase, b-type cytochrome subunit
G2583_2066	CDS	2107362	2108081	-	ydhX	Uncharacterized ferredoxin-like protein ydhX
G2583_2067	CDS	2108094	2108741	-	ydhW	hypothetical protein
G2583_2068	CDS	2108745	2110847	-	ydhV	hypothetical protein
G2583_2069	CDS	2110868	2111494	-	ydhY	Uncharacterized ferredoxin-like protein ydhY
G2583_2070	CDS	2111950	2112159	-	ydhZ	hypothetical protein
G2583_2071	CDS	2112500	2114128	+	pykF	Pyruvate kinase I
G2583_2072	CDS	2114439	2114675	+	lpp	Major outer membrane lipoprotein precursor
G2583_2073	CDS	2114738	2115742	-	ynhG	LysM domain/ErfK/YbiS/YcfS/YnhG family protein
G2583_2074	CDS	2115891	2116307	-	sufE	Cysteine desulfuration protein sufE
G2583_2075	CDS	2116320	2117540	-	sufS	Cysteine desulfurase
G2583_2076	CDS	2117537	2118808	-	sufD	FeS assembly protein SufD
G2583_2077	CDS	2118783	2119529	-	sufC	FeS assembly ATPase SufC
G2583_2078	CDS	2119539	2121065	-	sufB	cysteine desulfurase activator complex subunit SufB
G2583_2079	CDS	2121035	2121403	-	sufA	FeS assembly scaffold SufA
G2583_2080	ncRNA	2121730	2121797	+	-	ncRNA
G2583_2081	CDS	2121952	2122221	-	ydiH	hypothetical protein
G2583_2082	CDS	2122240	2122650	-	ydiI	Esterase YdiI
G2583_2083	CDS	2122647	2125703	-	ydiJ	Oxidoreductase, FAD-binding
G2583_2084	CDS	2126092	2127204	+	ydiK	Inner membrane protein YdiK
G2583_2085	ncRNA	2127390	2127497	+	rprA	ncRNA
G2583_2086	CDS	2127606	2127989	+	ydiL	hypothetical protein
G2583_2087	CDS	2128089	2129294	+	ydiM	Major facilitator family transporter
G2583_2088	CDS	2129515	2130786	+	ydiN	Transporter, major facilitator family
G2583_2089	CDS	2130798	2131664	+	ydiB	Quinate/shikimate dehydrogenase
G2583_2090	CDS	2131695	2132453	+	aroD	3-dehydroquinate dehydratase
G2583_2091	CDS	2132598	2134193	+	ydiF	Propionate CoA-transferase
G2583_2092	CDS	2134207	2135358	+	ydiO	Acyl-coA dehydrogenase
G2583_2093	CDS	2135401	2136312	-	ydiP	putative ARAC-type regulatory protein
G2583_2094	CDS	2136628	2137392	+	ydiQ	camphor resistance protein CrcB
G2583_2095	CDS	2137412	2138350	+	ydiR	iron donor protein CyaY
G2583_2096	CDS	2138406	2139695	+	ydiS	putative sulfate transport protein CysZ

Locus_tag	Type	Start	End	+/-[a]	Gene[b]	Product
G2583_2097	CDS	2139692	2139985	+	ydiT	Iron-sulfur cluster-binding protein
G2583_2098	CDS	2139988	2141688	+	fadK	Putative ligase/synthetase
G2583_2099	CDS	2141745	2144123	-	pps	Phosphoenolpyruvate synthase
G2583_2100	CDS	2144456	2145289	+	ydiA	Putative phosphotransferase ydiA
G2583_2101	CDS	2145446	2146492	+	aroH	Phospho-2-dehydro-3-deoxyheptonate aldolase, Trp-sensitive
G2583_2102	CDS	2146597	2146815	+	ydiE	hypothetical protein
G2583_2103	CDS	2146819	2148255	-	ydiU	UPF0061 protein ydiU
G2583_2104	CDS	2148318	2149031	-	ydiV	hypothetical protein
G2583_2105	CDS	2149278	2149742	-	nlpC	Lipoprotein, NlpC/P60 family
G2583_2106	CDS	2149820	2150569	-	btuD	Vitamin B12 import ATP-binding protein btuD
G2583_2107	CDS	2150569	2151120	-	btuE	Vitamin B12 transport periplasmic protein btuE
G2583_2108	CDS	2151183	2152163	-	btuC	Vitamin B12 import system permease protein btuC
G2583_2109	CDS	2152353	2152748	-	-	hypothetical protein
G2583_2110	CDS	2152759	2153733	-	intT	integrase
G2583_2111	CDS	2153783	2154127	-	cI	gpC
G2583_2112	CDS	2154188	2154466	+	-	hypothetical phage DNA-binding protein
G2583_2113	CDS	2154481	2154819	+	-	unknown protein encoded by prophage CP-933T
G2583_2114	CDS	2154830	2155117	+	-	hypothetical protein
G2583_2115	CDS	2155129	2155371	+	-	hypothetical protein
G2583_2116	CDS	2155575	2155985	+	-	hypothetical protein
G2583_2117	CDS	2156009	2156212	+	-	hypothetical protein
G2583_2118	CDS	2156209	2156475	+	-	hypothetical protein
G2583_2119	CDS	2156472	2156771	+	-	conserved hypothetical protein
G2583_2120	CDS	2156783	2157400	+	-	hypothetical protein
G2583_2121	CDS	2157397	2157786	+	-	hypothetical protein
G2583_2122	CDS	2157783	2160623	+	-	putative phage replication protein
G2583_2123	CDS	2160700	2161659	+	-	Molecular chaperone
G2583_2124	CDS	2161664	2161975	+	-	hypothetical protein
G2583_2125	CDS	2162039	2162371	+	clpX	ATP-dependent protease Clp, ATPase subunit
G2583_2126	CDS	2162368	2162706	+	-	hypothetical protein
G2583_2127	CDS	2163198	2164244	-	gpq	Putative phage protein
G2583_2128	CDS	2164244	2165995	-	gpP	Putative phage protein gpP
G2583_2129	CDS	2166150	2166986	+	-	putative capsid scaffolding protein
G2583_2130	CDS	2167010	2168062	+	gpn	Predicted major capsid protein
G2583_2131	CDS	2168108	2168908	+	gpm	Putative phage terminase
G2583_2132	CDS	2169011	2169505	+	gpl	phage head completion protein
G2583_2133	CDS	2169505	2169705	+	-	Probable phage tail protein
G2583_2134	CDS	2169708	2170031	+	-	Phosphotransferase system IIC components,
G2583_2135	CDS	2170028	2170420	+	-	Phage capsid scaffolding protein
G2583_2136	CDS	2170417	2170824	+	-	hypothetical protein
G2583_2137	CDS	2170962	2171429	+	-	Predicted tail completion phage protein
G2583_2138	CDS	2171413	2172057	+	gps	Predicted tail completion phage protein
G2583_2139	CDS	2172054	2172635	+	-	Phage P2 baseplate assembly protein gpV
G2583_2140	CDS	2172632	2172982	+	-	Phage baseplate assembly protein
G2583_2141	CDS	2172986	2173882	+	-	probable phage baseplate assembly protein
G2583_2142	CDS	2173875	2174483	+	gpI	phage tail protein I
G2583_2143	CDS	2174480	2175835	+	-	DNA inversion product
G2583_2144	CDS	2176674	2177045	+	-	Alternative bacteriophage tail fiber C-terminus
G2583_2145	CDS	2177052	2177663	-	tfaE	Tail fiber assembly protein
G2583_2146	CDS	2177663	2178121	-	-	Sc/SvM1 protein
G2583_2147	CDS	2178132	2178617	-	ycfK	putative tail fiber protein
G2583_2148	CDS	2178632	2179231	+	pin	DNA-invertase
G2583_2149	CDS	2179258	2179752	-	-	Phage protein U
G2583_2150	CDS	2179759	2182566	-	-	Phage-related tail protein
G2583_2151	CDS	2182553	2182789	-	-	Putative phage tail protein
G2583_2152	CDS	2182717	2183091	-	-	ABC-type antimicrobial peptide transport syste m, ATPase
G2583_2153	CDS	2183147	2183659	-	-	Putative tail fiber component of prophage CP-933T
G2583_2154	CDS	2183659	2184843	-	-	Phage tail sheath protein FI
G2583_2155	CDS	2185001	2186110	+	gpd	Phage protein D
G2583_2156	CDS	2186467	2186970	+	-	hypothetical protein

Locus_tag	Type	Start	End	+/-[a]	Gene[b]	Product
G2583_2157	CDS	2187120	2187380	+	-	putative phage transcriptional activator, Ogr/Delta
G2583_2158	CDS	2187571	2187711	+	-	conserved hypothetical protein
G2583_2159	CDS	2188015	2188314	-	ihfA	Integration host factor subunit alpha
G2583_2160	CDS	2188319	2190706	-	pheT	Phenylalanyl-tRNA synthetase beta chain
G2583_2161	CDS	2190721	2191704	-	pheS	Phenylalanyl-tRNA synthetase alpha chain
G2583_2162	CDS	2191987	2192031	-	pheM	phenylalanyl-tRNA synthetase (pheST) operon leader peptide
G2583_2163	CDS	2192154	2192510	-	rplT	50S ribosomal protein L20
G2583_2164	CDS	2192563	2192760	-	rpmI	50S ribosomal protein L35
G2583_2165	CDS	2192857	2193291	-	infC	Translation initiation factor IF-3
G2583_2166	CDS	2193403	2195331	-	thrS	Threonyl-tRNA synthetase
G2583_2167	CDS	2196198	2197751	+	arpA	Ankyrin repeat protein B
G2583_2168	CDS	2198086	2198844	-	ydiY	hypothetical protein
G2583_2169	CDS	2199128	2200060	+	pfkB	6-phosphofructokinase II
G2583_2170	CDS	2200161	2200451	+	ydiZ	hypothetical protein
G2583_2171	CDS	2200557	2201417	+	yniA	hypothetical protein
G2583_2172	CDS	2201458	2201994	-	yniB	hypothetical protein
G2583_2173	CDS	2202141	2202809	+	yniC	Phosphatase yniC
G2583_2174	CDS	2202960	2203562	+	ydjM	Inner membrane protein ydjM precursor
G2583_2175	CDS	2203695	2205086	+	ydjN	Sodium:dicarboxylate symporter precursor
G2583_2176	CDS	2205090	2205905	-	ydjO	hypothetical protein
G2583_2177	CDS	2206182	2206445	-	cedA	Cell division activator cedA
G2583_2178	CDS	2206628	2208889	+	katE	Catalase HPII
G2583_2179	CDS	2208936	2209694	-	chbG	UPF0249 protein chbG
G2583_2180	CDS	2209707	2211059	-	chbF	Phospho-beta-glucosidase
G2583_2181	CDS	2211165	2212007	-	chbR	ChbR
G2583_2182	CDS	2212015	2212365	-	chbA	N,N'-diacetylchitobiose-specific phosphotransferase enzyme IIA
G2583_2183	CDS	2212416	2213774	-	chbC	N,N'-diacetylchitobiose permease IIC component
G2583_2184	CDS	2213859	2214179	-	chbB	PTS system, lactose/cellobiose family IIB subunit precursor
G2583_2185	CDS	2214479	2214817	-	osmE	Osmotically-inducible lipoprotein E precursor
G2583_2186	CDS	2215019	2215846	+	nadE	NAD synthase
G2583_2187	CDS	2216076	2216963	+	cho	Excinuclease cho
G2583_2188	CDS	2216923	2217561	-	ves	Uncharacterized protein conserved in bacteria
G2583_2189	CDS	2217701	2218186	-	spy	Spy
G2583_2190	CDS	2218516	2219484	-	astE	Succinylglutamate desuccinylase
G2583_2191	CDS	2219477	2220820	-	astB	N-succinylarginine dihydrolase
G2583_2192	CDS	2220817	2222295	-	astD	N-succinylglutamate 5-semialdehyde dehydrogenase
G2583_2193	CDS	2222292	2223326	-	astA	Arginine N-succinyltransferase
G2583_2194	CDS	2223323	2224543	-	astC	Succinylornithine transaminase
G2583_2195	CDS	2224989	2225795	+	xthA	Exodeoxyribonuclease III
G2583_2196	CDS	2225914	2226672	+	ydjX	hypothetical protein
G2583_2197	CDS	2226515	2227354	+	ydjY	hypothetical protein
G2583_2198	CDS	2227369	2228076	+	ydjZ	hypothetical protein
G2583_2199	CDS	2228076	2228624	+	ynjA	Carboxymuconolactone decarboxylase family protein
G2583_2200	CDS	2228631	2229800	+	ynjB	hypothetical protein
G2583_2201	CDS	2229773	2231308	+	ynjC	ABC transporter, permease protein
G2583_2202	CDS	2231308	2231961	+	ynjD	Putative ATP-binding component of a transport system
G2583_2203	CDS	2232013	2233335	+	ynjE	Putative thiosulfate sulfurtransferase YnjE
G2583_2204	CDS	2233344	2233970	-	ynjF	Putative cytochrome oxidase
G2583_2205	CDS	2234051	2234458	+	nudG	CTP pyrophosphohydrolase
G2583_2206	CDS	2234424	2234696	-	ynjH	hypothetical protein
G2583_2207	CDS	2234932	2236275	+	gdhA	NADP-specific glutamate dehydrogenase
G2583_2208	CDS	2236392	2237432	-	ynjI	hypothetical protein
G2583_2209	CDS	2237560	2239521	-	topB	DNA topoisomerase III
G2583_2210	CDS	2239526	2240569	-	selD	Selenide, water dikinase
G2583_2211	CDS	2240686	2241237	-	ydjA	Protein ydjA
G2583_2212	CDS	2241398	2243254	+	sppA	Protease 4
G2583_2213	CDS	2243277	2244080	+	-	hypothetical protein
G2583_2214	CDS	2244212	2245228	+	ansA	L-asparaginase 1
G2583_2215	CDS	2245239	2245880	+	pncA	hypothetical protein
G2583_2216	CDS	2245973	2247331	-	ydjE	Inner membrane metabolite transport protein ydjE

Locus_tag	Type	Start	End	+/-[a]	Gene[b]	Product
G2583_2217	CDS	2247449	2248207	-	ydjF	transcriptional regulator, DeoR family
G2583_2218	CDS	2248344	2249324	-	ydjG	Oxidoreductase, aldo/keto reductase family
G2583_2219	CDS	2249334	2250266	-	ydjH	Kinase, pfkB family
G2583_2220	CDS	2250271	2251107	-	ydjI	Fructose-bisphosphate aldolase, class II family
G2583_2221	CDS	2251128	2252171	-	ydjJ	Sorbitol dehydrogenase
G2583_2222	CDS	2252188	2252826	-	ydjK	major facilitator family transporter
G2583_2223	CDS	2252816	2253892	-	ydjL	Oxidoreductase, zinc-binding dehydrogenase family
G2583_2224	CDS	2254262	2254579	-	yeaC	hypothetical protein
G2583_2225	CDS	2254576	2254989	-	msrB	Methionine sulfoxide reductase B
G2583_2226	CDS	2255331	2256326	+	gapA	Glyceraldehyde-3-phosphate dehydrogenase A
G2583_2227	CDS	2256389	2257294	+	yeaD	Aldose 1-epimerase family protein
G2583_2228	CDS	2257345	2258199	-	yeaE	Oxidoreductase, aldo/keto reductase family
G2583_2229	CDS	2258289	2259035	-	mipA	MltA-interacting protein precursor
G2583_2230	CDS	2259471	2261405	+	yeaG	hypothetical protein
G2583_2231	CDS	2261518	2262801	+	yeaH	UPF0229 protein yeaH
G2583_2232	CDS	2263080	2264423	+	yeaI	Diguanylate cyclase (GGDEF) domain protein
G2583_2233	CDS	2264604	2266094	+	yeaJ	hypothetical protein
G2583_2234	CDS	2266137	2266640	+	yeaK	hypothetical protein
G2583_2235	CDS	2266641	2266793	-	-	hypothetical protein
G2583_2236	CDS	2266915	2267361	+	yeaL	Predicted membrane protein
G2583_2237	CDS	2267318	2268139	-	yeaM	Putative AraC-type regulatory protein
G2583_2238	CDS	2268236	2269417	+	yeaN	inner membrane transport protein yeaN
G2583_2239	CDS	2269451	2269819	+	yeaO	hypothetical protein
G2583_2240	CDS	2269841	2270095	-	yoaF	hypothetical protein
G2583_2241	CDS	2270149	2271303	+	yeaP	GAF domain/diguanylate cyclase (GGDEF) domain protein
G2583_2242	CDS	2271570	2271818	-	yeaQ	hypothetical protein
G2583_2243	CDS	2271965	2272147	-	yoaG	Protein yoaG
G2583_2244	CDS	2272151	2272510	-	yeaR	hypothetical protein
G2583_2245	CDS	2272683	2273321	-	leuE	Leucine efflux protein
G2583_2246	CDS	2273448	2274392	-	yeaT	putative transcriptional regulator LYSR-type
G2583_2247	CDS	2274474	2275559	+	yeaU	Tartrate dehydrogenase/decarboxylase
G2583_2248	CDS	2275810	2277420	+	yeaV	Transporter, betaine/carnitine/choline transporter (BCCT) family
G2583_2249	CDS	2277452	2278576	+	yeaW	Putative dioxygenase subunit alpha yeaW
G2583_2250	CDS	2278632	2279597	+	yeaX	Putative diogenase beta subunit
G2583_2251	CDS	2279651	2280778	-	rnd	Ribonuclease D
G2583_2252	ncRNA	2280804	2280889	-	-	ncRNA
G2583_2253	CDS	2280848	2282533	-	fadD	Long-chain-fatty-acid--CoA ligase
G2583_2254	CDS	2282738	2283319	-	yeaY	Outer membrane protein Slp
G2583_2255	CDS	2283359	2284054	-	yeaZ	Glycoprotease family protein
G2583_2256	CDS	2284112	2286022	-	yoaA	Helicase c2
G2583_2257	CDS	2286106	2286498	+	yoaB	hypothetical protein
G2583_2258	CDS	2286860	2287219	+	yoaC	hypothetical protein
G2583_2259	CDS	2287339	2287518	-	yoaH	UPF0181 protein yoaH
G2583_2260	CDS	2287592	2288953	+	pabB	p-aminobenzoate synthetase, component I
G2583_2261	CDS	2288957	2289535	+	nudL	Uncharacterized Nudix hydrolase nudL
G2583_2262	CDS	2289719	2291083	+	sdaA	L-serine ammonia-lyase 1
G2583_2263	CDS	2291184	2292812	+	yoaD	hypothetical protein
G2583_2264	CDS	2292816	2294372	-	yoaE	UPF0053 inner membrane protein yoaE
G2583_2265	CDS	2294360	2294614	-	-	putative cytoplasmic protein
G2583_2266	CDS	2294835	2295806	+	manX	Putative PTS system, mannose-specific component IIAB
G2583_2267	CDS	2295869	2296669	+	manY	PTS enzyme IIC, mannose-specific
G2583_2268	CDS	2296673	2297533	+	manZ	Mannose permease IID component
G2583_2269	CDS	2297588	2298046	+	yobD	UPF0266 membrane protein yobD
G2583_2270	CDS	2298422	2299042	+	yebN	hypothetical protein
G2583_2271	CDS	2299039	2299848	-	rrmA	23S rRNA methyltransferase A
G2583_2272	CDS	2300014	2300223	-	cspC	hypothetical protein
G2583_2273	CDS	2300236	2300379	-	yobF	hypothetical protein
G2583_2274	CDS	2301048	2301335	-	yebO	hypothetical protein
G2583_2275	CDS	2301410	2301553	-	mgrB	hypothetical protein
G2583_2276	CDS	2301713	2301952	+	yobH	hypothetical protein

Locus_tag	Type	Start	End	+/-[a]	Gene[b]	Product
G2583_2277	CDS	2302096	2302887	-	kdgR	putative regulator
G2583_2278	CDS	2302953	2304437	+	yebQ	Putative transport protein
G2583_2279	CDS	2304483	2305364	-	htpX	Probable protease htpX
G2583_2280	CDS	2305556	2307604	-	prc	C-terminal processing peptidase
G2583_2281	CDS	2307624	2308322	-	proQ	ProP effector
G2583_2282	CDS	2308419	2308970	-	yebR	GAF domain protein
G2583_2283	CDS	2309046	2310329	+	yebS	hypothetical protein
G2583_2284	CDS	2310292	2312931	+	yebT	Mce-related protein
G2583_2285	CDS	2312931	2314451	+	rsmF	Ribosomal RNA small subunit methyltransferase F
G2583_2286	CDS	2314554	2314805	+	yebV	hypothetical protein
G2583_2287	CDS	2314826	2315101	+	yebW	hypothetical protein
G2583_2288	CDS	2315102	2315761	-	pphA	Protein phosphatase 1 modulates phosphoproteins, signals
G2583_2289	ncRNA	2315855	2316103	+	-	ncRNA
G2583_2290	ncRNA	2315893	2315992	-	-	ncRNA
G2583_2291	CDS	2316154	2316495	-	yebY	hypothetical protein
G2583_2292	CDS	2316508	2317380	-	yebZ	Copper resistance protein D
G2583_2293	CDS	2317384	2317758	-	yobA	Copper resistance protein CopC
G2583_2294	CDS	2317897	2318127	+	holE	DNA polymerase III subunit theta
G2583_2295	CDS	2318229	2318885	+	yobB	Hydrolase, carbon-nitrogen family
G2583_2296	CDS	2318909	2319571	+	exoX	Exodeoxyribonuclease 10
G2583_2297	CDS	2319568	2321628	-	ptrB	Oligopeptidase B
G2583_2298	CDS	2321837	2322496	-	yebE	hypothetical protein
G2583_2299	CDS	2322823	2323191	-	yebF	hypothetical protein
G2583_2300	CDS	2323246	2323536	-	yebG	hypothetical protein
G2583_2301	CDS	2323670	2324848	+	purT	Phosphoribosylglycinamide formyltransferase 2
G2583_2302	CDS	2324904	2325545	-	eda	KDPG and KHG aldolase
G2583_2303	CDS	2325582	2327393	-	edd	Phosphogluconate dehydratase
G2583_2304	CDS	2327628	2329103	-	zwf	Glucose-6-phosphate 1-dehydrogenase
G2583_2305	CDS	2329441	2330310	+	yebK	DNA-binding transcriptional regulator HexR
G2583_2306	CDS	2330438	2331880	+	pykA	Pyruvate kinase II
G2583_2307	CDS	2332011	2332982	-	lpxM	Lipid A biosynthesis (KDO)2-(Lauroyl)-lipid IVA acyltransferase
G2583_2308	CDS	2333102	2334424	-	yebA	Uncharacterized metalloprotease yebA
G2583_2309	CDS	2334440	2335387	-	znuA	Putative adhesin
G2583_2310	CDS	2335451	2336206	+	znuC	Zinc import ATP-binding protein znuC
G2583_2311	CDS	2336203	2336988	+	znuB	Zinc uptake ABC transporter, permease protein ZnuB
G2583_2312	CDS	2337234	2338244	-	ruvB	Holliday junction ATP-dependent DNA helicase ruvB
G2583_2313	CDS	2338253	2338864	-	ruvA	Holliday junction ATP-dependent DNA helicase ruvA
G2583_2314	CDS	2339139	2339741	+	yebB	hypothetical protein
G2583_2315	CDS	2339743	2340264	-	ruvC	Crossover junction endodeoxyribonuclease ruvC
G2583_2316	CDS	2340299	2341039	-	yebC	UPF0082 protein yebC
G2583_2317	CDS	2341068	2341577	-	nudB	dATP pyrophosphohydrolase
G2583_2318	CDS	2341638	2343410	-	aspS	Aspartyl-tRNA synthetase
G2583_2319	CDS	2343720	2344286	+	yecD	hypothetical protein
G2583_2320	CDS	2344283	2345101	+	yecE	hypothetical protein
G2583_2321	CDS	2345196	2345549	+	yecN	hypothetical protein
G2583_2322	CDS	2345590	2346333	+	cmoA	tRNA (cmo5U34)-methyltransferase
G2583_2323	CDS	2346330	2347301	+	cmoB	tRNA (mo5U34)-methyltransferase
G2583_2324	CDS	2347466	2349913	-	torZ	Trimethylamine N-oxide reductase III, subunit TorZ
G2583_2325	CDS	2349920	2351020	-	torY	Cytochrome c-type protein torY
G2583_2326	CDS	2351408	2352154	-	cutC	Copper homeostasis protein cutC
G2583_2327	CDS	2352168	2352740	-	yecM	hypothetical protein
G2583_2328	CDS	2352950	2354683	+	argS	Arginyl-tRNA synthetase
G2583_2329	CDS	2354860	2355348	+	yecT	hypothetical protein
G2583_2330	CDS	2355468	2355860	-	flhE	hypothetical protein
G2583_2331	CDS	2355860	2357938	-	flhA	Flagellar biosynthesis protein flhA
G2583_2332	CDS	2357931	2359079	-	flhB	Flagellar biosynthetic protein FlhB
G2583_2333	CDS	2359281	2359925	-	cheZ	Chemotaxis phosphatase CheZ
G2583_2334	CDS	2359936	2360325	-	cheY	Chemotaxis protein cheY
G2583_2335	CDS	2360340	2361389	-	cheB	Chemotaxis response regulator protein-glutamate
G2583_2336	CDS	2361392	2362252	-	cheR	Chemotaxis protein methyltransferase CheR

Locus_tag	Type	Start	End	+/-[a]	Gene[b]	Product
G2583_2337	CDS	2362271	2363872	-	tap	Methyl-accepting chemotaxis protein IV
G2583_2338	CDS	2363918	2365579	-	tar	Methyl-accepting chemotaxis protein II
G2583_2339	ncRNA	2365627	2365702	+	-	ncRNA
G2583_2340	CDS	2365722	2366225	-	cheW	Chemotaxis protein cheW
G2583_2341	CDS	2366246	2368210	-	cheA	Sensory transducer kinase between chemo-signal receptors
G2583_2342	CDS	2368215	2369141	-	motB	Chemotaxis protein motB
G2583_2343	CDS	2369138	2370025	-	motA	Chemotaxis protein MotA
G2583_2344	CDS	2370153	2370731	-	flhC	Flagellar transcriptional activator flhC
G2583_2345	CDS	2370734	2371093	-	flhD	Transcriptional activator
G2583_2346	CDS	2371864	2372292	+	uspC	Universal stress protein C
G2583_2347	CDS	2372299	2373723	-	otsA	Alpha,alpha-trehalose-phosphate synthase [UDP-forming]
G2583_2348	CDS	2373698	2374498	-	otsB	Trehalose-phosphatase
G2583_2349	CDS	2374665	2375654	-	araH	ABC-type arabinose transport system, permease component
G2583_2350	CDS	2375666	2377180	-	araG	Arabinose import ATP-binding protein araG
G2583_2351	CDS	2377250	2378239	-	araF	L-arabinose-binding periplasmic protein
G2583_2352	CDS	2379036	2379539	+	ftnB	Ferritin-like protein 2
G2583_2353	CDS	2379619	2379870	-	yecJ	hypothetical protein
G2583_2354	CDS	2379622	2379894	+	-	hypothetical protein
G2583_2355	CDS	2380334	2380657	+	yecR	hypothetical protein
G2583_2356	CDS	2380828	2381325	+	ftnA	Ferritin-1
G2583_2357	CDS	2381362	2381601	-	yecH	hypothetical protein
G2583_2358	CDS	2381793	2383004	+	tyrP	Tyrosine-specific transport protein
G2583_2359	CDS	2383066	2383731	-	yecA	SEC-C domain protein
G2583_2360	tRNA	2383927	2384013	-	-	Leu tRNA
G2583_2361	tRNA	2384026	2384099	-	-	Cys tRNA
G2583_2362	tRNA	2384153	2384228	-	-	Gly tRNA
G2583_2363	CDS	2384380	2384928	-	pgsA	CDP-diacylglycerol--glycerol-3-phosphate 3-
G2583_2364	CDS	2384985	2386817	-	uvrC	UvrABC system protein C
G2583_2365	CDS	2386814	2387470	-	uvrY	Response regulator uvrY
G2583_2366	CDS	2387929	2388153	+	yecF	hypothetical protein
G2583_2367	CDS	2388221	2388943	-	sdiA	DNA-binding transcriptional activator
G2583_2368	CDS	2389173	2389925	-	yecC	Amino acid ABC transporter, ATP-binding protein
G2583_2369	CDS	2389922	2390590	-	yecS	putative transport system permease protein
G2583_2370	CDS	2390605	2391591	-	dcyD	Putative 1-aminocyclopropane-1-carboxylate deaminase
G2583_2371	CDS	2391696	2392496	-	fliY	Cystine-binding periplasmic protein
G2583_2372	CDS	2392584	2393171	-	fliZ	protein FliZ
G2583_2373	CDS	2393181	2393900	-	fliA	RNA polymerase sigma factor for flagellar operon
G2583_2374	CDS	2394220	2395977	-	fliC	Flagellin
G2583_2375	CDS	2396225	2397649	+	fliD	Flagellar capping protein
G2583_2376	CDS	2397674	2398084	+	fliS	Flagellar protein FliS
G2583_2377	CDS	2398084	2398449	+	fliT	Flagellar protein fliT
G2583_2378	CDS	2398527	2400014	+	amyA	Alpha-amylase, cytoplasmic
G2583_2379	CDS	2400048	2400464	-	yedD	hypothetical protein
G2583_2380	CDS	2400648	2401853	+	yedE	predicted inner membrane protein
G2583_2381	CDS	2401850	2402083	+	yedF	UPF0033 protein yedF
G2583_2382	CDS	2402192	2402860	+	yedK	hypothetical protein
G2583_2383	CDS	2402971	2403450	+	yedL	Acetyltransferase, GNAT family
G2583_2384	CDS	2403588	2404727	-	yedM	putative secreted protein
G2583_2385	CDS	2405109	2405408	+	yedL	acetyltransferase, gnat family
G2583_2386	CDS	2405648	2406685	-	yedM	putative secreted protein
G2583_2387	CDS	2406863	2407114	+	int	Putative lambdoid prophage defective integrase
G2583_2388	CDS	2407034	2407348	-	fliE	Flagellar hook-basal body complex protein fliE
G2583_2389	CDS	2407563	2409221	+	fliF	Flagellar M-ring protein FliF precursor
G2583_2390	CDS	2409214	2410209	+	fliG	Flagellar motor switch protein fliG
G2583_2391	CDS	2410202	2410888	+	fliH	Flagellar biosynthesis
G2583_2392	CDS	2410888	2412261	+	fliI	Flagellum-specific ATP synthase
G2583_2393	CDS	2412280	2412723	+	fliJ	Flagellar fliJ protein
G2583_2394	CDS	2412720	2413847	+	fliK	Flagellar hook-length control protein Flik
G2583_2395	CDS	2413952	2414416	+	fliL	Flagellar fliL protein
G2583_2396	CDS	2414421	2415425	+	fliM	Flagellar motor switch protein FliM

Locus_tag	Type	Start	End	+/-[a]	Gene[b]	Product
G2583_2397	CDS	2415422	2415835	+	fliN	Flagellar motor switch protein FliN
G2583_2398	CDS	2415838	2416203	+	fliO	Flagellar biogenesis protein
G2583_2399	CDS	2416203	2416940	+	fliP	Flagellar biosynthetic protein fliP precursor
G2583_2400	CDS	2416950	2417219	+	fliQ	hypothetical protein
G2583_2401	CDS	2417227	2418012	+	fliR	Flagellar biosynthetic protein FliR
G2583_2402	CDS	2418302	2418925	+	rcsA	Colanic acid capsular biosynthesis activation protein A
G2583_2403	CDS	2418969	2419157	-	dsrB	putative oxidoreductase Fe-S binding subunit
G2583_2404	CDS	2419305	2419547	+	yodD	hypothetical protein
G2583_2405	ncRNA	2419561	2419647	-	dsrA	ncRNA
G2583_2406	CDS	2419845	2420660	+	yedP	Putative mannosyl-3-phosphoglycerate phosphatase
G2583_2407	CDS	2420657	2422351	-	yedQ	Cellulose synthesis regulatory protein
G2583_2408	CDS	2422522	2422704	-	yodC	hypothetical protein
G2583_2409	CDS	2422783	2423700	-	yedI	putative membrane protein
G2583_2410	CDS	2423873	2424793	+	yedA	Uncharacterized inner membrane transporter yedA
G2583_2411	CDS	2424782	2425252	-	vsr	Very short patch repair protein
G2583_2412	CDS	2425233	2426651	-	dcm	DNA-cytosine methyltransferase
G2583_2413	CDS	2426718	2427413	-	yedJ	Putative metal-dependent phosphohydrolase
G2583_2414	CDS	2427453	2427818	-	yedR	hypothetical protein
G2583_2415	CDS	2428383	2429570	+	ompS	Outer membrane protein
G2583_2416	CDS	2429651	2430001	+	-	hypothetical protein
G2583_2417	CDS	2430162	2431013	+	hchA	Chaperone protein hchA
G2583_2418	CDS	2431121	2432479	-	yedV	Heavy metal sensor histidine kinase
G2583_2419	CDS	2432479	2433261	-	yedW	Heavy metal response regulator
G2583_2420	CDS	2433283	2433696	+	yedX	Transthyretin-like protein precursor
G2583_2421	CDS	2433804	2434808	+	yedY	UPF0190 protein yedY precursor
G2583_2422	CDS	2434809	2435444	+	yedZ	UPF0191 membrane protein yedZ
G2583_2423	CDS	2435701	2436351	+	yodA	Ribulose-phosphate 3-epimerase precursor
G2583_2424	CDS	2436461	2436610	+	-	hypothetical protein
G2583_2425	CDS	2436664	2437224	+	yodB	putative cytochrome
G2583_2426	CDS	2437248	2437490	+	-	hypothetical protein
G2583_2427	CDS	2437794	2438153	+	-	unknown protein encoded within prophage CP-933U
G2583_2428	CDS	2438361	2439014	+	-	unknown protein encoded within prophage CP-933U
G2583_2429	CDS	2439339	2440211	+	-	Tir-cytoskeleton coupling protein
G2583_2430	CDS	2440608	2441636	-	-	Tail fiber protein
G2583_2431	CDS	2441987	2442586	-	-	Enterobacterial Ail/Lom family protein
G2583_2432	CDS	2442654	2446130	-	-	Putative host specificity protein
G2583_2433	CDS	2445805	2446791	+	-	Superoxide dismutase [Cu-Zn]
G2583_2434	CDS	2446822	2447028	-	-	hypothetical protein
G2583_2435	CDS	2447560	2448300	-	-	Cell wall-associated hydrolases (invasion-associated proteins)
G2583_2436	CDS	2448306	2449004	-	-	Phage-related protein
G2583_2437	CDS	2449004	2449333	-	-	Putative tail component of prophage CP-933R
G2583_2438	CDS	2449330	2451909	-	-	putative tail component of prophage CP-933O
G2583_2439	CDS	2451890	2452303	-	-	Phage tail assembly protein T
G2583_2440	CDS	2452330	2452833	-	-	hypothetical protein
G2583_2441	CDS	2452775	2453527	-	-	hypothetical protein
G2583_2442	CDS	2453535	2453930	-	-	Putative tail component of prophage
G2583_2443	CDS	2453927	2454460	-	-	prophage minor tail protein Z
G2583_2444	CDS	2454476	2454829	-	-	Phage Head-Tail Attachment
G2583_2445	CDS	2454822	2455205	-	-	conserved hypothetical protein
G2583_2446	CDS	2455257	2456285	-	-	phage major capsid protein E
G2583_2447	CDS	2456343	2456690	-	-	Bacteriophage lambda head decoration protein D
G2583_2448	CDS	2456727	2458232	-	-	Periplasmic serine proteases (ClpP class)
G2583_2449	CDS	2458222	2459814	-	-	Bacteriophage capsid protein
G2583_2450	CDS	2459811	2460017	-	-	putative head completion protein
G2583_2451	CDS	2460001	2461965	-	-	Putative terminase large subunit of prophage CP-933O
G2583_2452	CDS	2461901	2462410	-	-	Prophage Qin DNA packaging protein NU1-like protein
G2583_2453	CDS	2463134	2463271	-	-	hypothetical protein
G2583_2454	CDS	2463688	2463882	-	-	hypothetical protein
G2583_2455	CDS	2463879	2464457	-	-	putative endopeptidase
G2583_2456	CDS	2464473	2464688	-	-	conserved hypothetical protein

Locus_tag	Type	Start	End	+/-[a]	Gene[b]	Product
G2583_2457	CDS	2465016	2465549	-	-	Phage-related lysozyme (muraminidase)
G2583_2458	CDS	2465592	2466581	-	-	hypothetical protein
G2583_2459	CDS	2466586	2466861	-	-	Putative holin protein
G2583_2460	CDS	2467085	2468935	-	-	YjhS
G2583_5272	tRNA	2469425	2469501	-	-	Arg tRNA
G2583_5273	tRNA	2469599	2469674	-	-	Met tRNA
G2583_2461	CDS	2469703	2470416	-	-	Putative envelope protein encoded within prophage CP-933N
G2583_2462	CDS	2471037	2471861	-	-	CAAX amino terminal protease family
G2583_2463	CDS	2472008	2472379	-	ybcQ	Phage antitermination Q type 1 family
G2583_2464	CDS	2472369	2472740	-	rusA	Crossover junction endodeoxyribonuclease RusA
G2583_2465	CDS	2472753	2473802	-	-	hypothetical protein
G2583_2466	CDS	2473804	2474082	-	-	hypothetical protein
G2583_2467	CDS	2474152	2474409	-	rem	hypothetical protein
G2583_2468	CDS	2474567	2475658	-	-	Helix-turn-helix domain protein
G2583_2469	CDS	2475651	2476301	-	-	hypothetical protein
G2583_2470	CDS	2476467	2476622	-	-	Putative cell killing protein encoded within cryptic prophage CP-
G2583_2471	CDS	2476994	2478025	-	-	conserved hypothetical protein
G2583_2472	CDS	2478394	2478801	-	-	LygF
G2583_2473	CDS	2478842	2479807	-	-	Phage O protein family
G2583_2474	CDS	2479788	2480309	-	ydfX	hypothetical protein
G2583_2475	CDS	2480293	2480520	-	-	hypothetical protein
G2583_2476	CDS	2480547	2481005	+	dicA	Similar to DicA, regulator of DicB encoded by prophage
G2583_2477	CDS	2481042	2481350	+	ydfA	hypothetical protein
G2583_2478	CDS	2481464	2482213	-	-	unknown protein encoded by prophage CP-933N
G2583_2479	CDS	2482416	2482709	+	DicB	Putative regulator of cell division encoded by prophage CP-
G2583_2480	CDS	2482706	2482894	+	-	hypothetical protein
G2583_2481	CDS	2482987	2485404	+	recE	Exodeoxyribonuclease VIII
G2583_2482	CDS	2485463	2485666	+	-	hypothetical protein
G2583_2483	CDS	2485666	2486691	+	intU	Site-specific recombinase, phage integrase family
G2583_2484	tRNA	2486743	2486834	-	-	Ser tRNA
G2583_2485	CDS	2486888	2487724	+	mtfA	hypothetical protein
G2583_2486	tRNA	2487824	2487901	+	-	Asn tRNA
G2583_2487	pseudogene	2488187	2489323	+	yeeJ	Putative invasin
G2583_2488	CDS	2489387	2496196	+	-	putative factor
G2583_2489	CDS	2496458	2497510	-	-	hypothetical protein
G2583_2490	CDS	2497825	2499141	+	shiA	Shikimate transporter
G2583_2491	CDS	2499243	2500697	+	amn	AMP nucleosidase
G2583_2492	CDS	2501040	2501756	+	yeeN	UPF0082 protein yeeN
G2583_2493	tRNA	2502205	2502282	-	-	Asn tRNA
G2583_2494	pseudogene	2502382	2502705	-	yeeO	MATE efflux family protein
G2583_2495	CDS	2502580	2504022	-	yeeO	MATE efflux family protein
G2583_2496	tRNA	2504029	2504106	+	-	Asn tRNA
G2583_2497	CDS	2504143	2505093	-	cbl	transcriptional regulator Cbl
G2583_2498	CDS	2505195	2506112	-	nac	Nitrogen assimilation regulatory protein Nac
G2583_2499	tRNA	2506437	2506514	+	-	Asn tRNA
G2583_2500	CDS	2506569	2507501	-	erfK	Conserved protein with NAD(P)-binding Rossmann-fold domain
G2583_2501	CDS	2507566	2508645	-	cobT	Nicotinate-nucleotide--dimethylbenzimidazole
G2583_2502	CDS	2508657	2509400	-	cobS	cobalamin synthase
G2583_2503	CDS	2509397	2509942	-	cobU	cobalamin synthase
G2583_2504	CDS	2511602	2513761	+	yoeE	Putative outer membrane receptor for iron compound or colicin
G2583_2505	CDS	2514132	2514770	-	ibrB	putative ParB-like nuclease
G2583_2506	CDS	2514755	2515261	-	ibrA	Immunoglobulin-binding regulator A-like protein
G2583_2507	CDS	2515197	2515544	+	insN	unknown protein encoded by IS911 within prophage CP-933L
G2583_2508	CDS	2515757	2516410	+	-	putative transposase
G2583_2509	CDS	2517416	2517691	+	-	Putative transposase subunit
G2583_2510	CDS	2517918	2518262	-	-	hypothetical protein
G2583_2511	CDS	2518721	2518867	-	-	hypothetical protein
G2583_2512	CDS	2518895	2520046	+	-	hypothetical protein
G2583_2513	CDS	2520130	2521002	+	yfjP	hypothetical protein
G2583_2514	CDS	2521220	2523616	+	-	hypothetical protein

Locus_tag	Type	Start	End	+/-[a]	Gene[b]	Product
G2583_2515	CDS	2523613	2524518	+	-	hypothetical protein
G2583_2516	CDS	2524515	2525585	+	-	putative esterase
G2583_2517	CDS	2525613	2526404	+	-	hypothetical protein
G2583_2518	CDS	2526420	2526830	+	-	hypothetical protein
G2583_2519	CDS	2527051	2527872	+	yafZ	conserved hypothetical protein
G2583_2520	CDS	2527954	2528433	+	yafX	Antirestriction protein
G2583_2521	CDS	2528448	2528924	+	yeeS	DNA repair protein, RadC family
G2583_2522	CDS	2528987	2529208	+	yeeT	hypothetical protein
G2583_2523	CDS	2529282	2529650	+	yeeU	Putative structural protein
G2583_2524	pseudogene	2529739	2530112	+	yeeV1	hypothetical protein
G2583_2525	CDS	2530109	2530303	+	yeeW	hypothetical protein
G2583_2526	CDS	2530744	2531100	+	yoeF	hypothetical protein
G2583_2527	CDS	2531201	2531596	-	yeeX	hypothetical protein
G2583_2528	CDS	2531702	2532760	-	yeeA	hypothetical protein
G2583_2529	CDS	2532959	2533432	-	sbmC	DNA gyrase inhibitory protein
G2583_2530	CDS	2533551	2534723	-	dacD	Serine-type D-Ala-D-Ala carboxypeptidase
G2583_2531	CDS	2534926	2536353	+	sbcB	Exonuclease I
G2583_2532	CDS	2536396	2536623	-	yeeD	hypothetical protein
G2583_2533	CDS	2536637	2537695	-	yeeE	Membrane protein, YeeE/YedE family
G2583_2534	CDS	2537874	2539238	-	yeeF	Hypothetical transport protein YeeF
G2583_2535	CDS	2539499	2540449	-	yeeY	Predicted DNA-binding transcriptional regulator
G2583_2536	CDS	2540474	2541298	-	yeeZ	NAD dependent epimerase/dehydratase family protein
G2583_2537	CDS	2541381	2541635	-	yoeB	Toxin yoeB
G2583_2538	CDS	2541632	2541883	-	yefM	Antitoxin yefM
G2583_2539	CDS	2542167	2542217	+	hisL	his operon leader peptide
G2583_2540	CDS	2542363	2543262	+	hisG	ATP phosphoribosyltransferase
G2583_2541	CDS	2543268	2544572	+	hisD	Bifunctional histidinal dehydrogenase and histidinol
G2583_2542	CDS	2544569	2545639	+	hisC	Histidinol-phosphate aminotransferase
G2583_2543	CDS	2545639	2546706	+	hisB	Fused histidinol-phosphatase/imidazoleglycerol-phosphate
G2583_2544	CDS	2546706	2547296	+	hisH	Imidazole glycerol phosphate synthase
G2583_2545	CDS	2547296	2548033	+	hisA	1-(5-phosphoribosyl)-5-[(5-
G2583_2546	CDS	2548015	2548791	+	hisF	Imidazole glycerol phosphate synthase subunit hisF
G2583_2547	CDS	2548785	2549396	+	hisI	Fused phosphoribosyl-AMP cyclohydrolase/phosphoribosyl-
G2583_2548	CDS	2549493	2550530	-	wzz	O-antigen chain length determinant Wzz
G2583_2549	CDS	2550620	2551786	-	ugd	UDP-glucose-6-dehydrogenase
G2583_2550	CDS	2552477	2553100	-	ydcC	Transposase IS4 family protein
G2583_2551	CDS	2553701	2554867	-	wbdK	Putative pyridoxamine 5-phosphate-dependent dehydrase
G2583_2552	CDS	2554864	2555787	-	wbcJ	WbdJ
G2583_2553	CDS	2555899	2557305	-	gnd	6-phosphogluconate dehydrogenase, decarboxylating
G2583_2554	CDS	2557389	2558138	-	wbgP	Putative glycosyltransferase WbgP
G2583_2555	CDS	2558149	2558946	-	wbgO	Putative glycosyltransferase WbgO
G2583_2556	CDS	2558943	2560220	-	wzx	O-antigen flippase Wzx
G2583_2557	CDS	2560238	2561215	-	wxy	O-antigen polymerase Wzy
G2583_2558	CDS	2561227	2562099	-	wbgN	Putative fucosyltransferase WbgN
G2583_2559	CDS	2562096	2563478	-	manB	Phosphomannomutase ManB
G2583_2560	CDS	2563471	2564928	-	manC	Mannose-1-P guanosyltransferase ManC
G2583_2561	CDS	2564921	2565415	-	wcaH	GDP-mannose mannosyl hydrolase
G2583_2562	CDS	2565567	2566685	-	gmd	GDP-mannose dehydratase Gmd
G2583_2563	CDS	2566706	2567623	-	wbgM	Putative galactosyltransferase WbgM
G2583_2564	CDS	2568181	2569074	-	galF	UDP-glucose pyrophosphorylase GalF
G2583_2565	CDS	2569316	2570311	-	gne	UDP-N-acetylglucosamine 4-epimerase
G2583_2566	CDS	2570469	2571863	-	wcaM	Predicted colanic acid biosynthesis protein WcaM
G2583_2567	CDS	2571874	2573094	-	wcaL	Predicted glycosyl transferase WcaL
G2583_2568	CDS	2573091	2574371	-	wcaK	Predicted pyruvyl transferase WcaK
G2583_2569	CDS	2574442	2575920	-	wzxC	Colanic acid exporter WzxC
G2583_2570	CDS	2575922	2577316	-	wcaJ	Predicted UDP-glucose lipid carrier transferase WcaJ
G2583_2571	CDS	2577452	2578822	-	cpsG	Phosphomannomutase CpsG
G2583_2572	CDS	2578924	2580360	-	cpsB	Mannose-1-phosphate guanyltransferase CpsB
G2583_2573	CDS	2580363	2581586	-	wcaI	Predicted glycosyl transferase WcaI
G2583_2574	CDS	2581583	2582065	-	gmm	GDP-mannose mannosyl hydrolase

Locus_tag	Type	Start	End	+/-[a]	Gene[b]	Product
G2583_2575	CDS	2582065	2583030	-	fcl	Bifunctional GDP-fucose synthetase
G2583_2576	CDS	2583033	2584154	-	gmd	GDP-mannose 4,6-dehydratase
G2583_2577	CDS	2584181	2584729	-	wcaF	Predicted acyl transferase WcaF
G2583_2578	CDS	2584745	2585491	-	wcaE	Predicted glycosyl transferase WcaE
G2583_2579	CDS	2585502	2586719	-	wcaD	Putative colanic acid polymerase
G2583_2580	CDS	2586694	2587911	-	wcaC	Predicted glycosyl transferase WcaC
G2583_2581	CDS	2587908	2588396	-	wcaB	Predicted acyl transferase WcaB
G2583_2582	CDS	2588399	2589238	-	wcaA	Predicted glycosyl transferase WcaA
G2583_2583	CDS	2589331	2591493	-	wzc	Protein-tyrosine kinase Wzc
G2583_2584	CDS	2591496	2591939	-	wzb	Low molecular weight protein-tyrosine-phosphatase wzb
G2583_2585	CDS	2591945	2593048	-	wza	Putative polysaccharide export protein wza precursor
G2583_2586	CDS	2593677	2595326	+	yegH	Putative transport protein
G2583_2587	CDS	2595401	2595739	+	-	hypothetical protein
G2583_2588	CDS	2595729	2596019	+	-	Putative DNA-binding protein
G2583_2589	CDS	2596072	2597925	-	asmA	Predicted assembly protein AsmA
G2583_2590	CDS	2597947	2598528	-	dcd	Deoxycytidine triphosphate deaminase
G2583_2591	CDS	2598620	2599261	-	udk	Uridine/cytidine kinase
G2583_2592	CDS	2599579	2602896	+	yegE	Predicted diguanylate cyclase
G2583_2593	CDS	2602997	2603845	-	alkA	3-methyl-adenine DNA glycosylase II
G2583_2594	CDS	2603979	2605331	+	yegD	Predicted chaperone
G2583_2595	CDS	2605344	2607284	-	yegI	conserved predicted protein
G2583_2596	CDS	2607281	2608042	-	yegK	conserved predicted protein
G2583_2597	CDS	2608039	2608698	-	yegL	Uncharacterized protein encoded in toxicity protection region of
G2583_2598	ncRNA	2608845	2608993	+	-	ncRNA
G2583_2599	ncRNA	2609179	2609321	+	-	ncRNA
G2583_2600	CDS	2609585	2610832	+	mdtA	hypothetical protein
G2583_2601	CDS	2610832	2613954	+	mdtB	Multidrug resistance protein mdtB
G2583_2602	CDS	2613955	2617032	+	mdtC	Multidrug resistance protein mdtC
G2583_2603	CDS	2617033	2618448	+	mdtD	Multidrug resistance protein mdtD
G2583_2604	CDS	2618445	2619848	+	baeS	Sensor histidine kinase BaeS
G2583_2605	CDS	2619845	2620567	+	baeR	DNA-binding response regulator in two-component regulatory
G2583_2606	CDS	2620719	2621090	+	yegP	hypothetical protein
G2583_2607	CDS	2621334	2621585	+	-	Putative addiction module antidote protein, CC2985 family
G2583_2608	CDS	2621587	2621883	+	-	Plasmid stabilization system protein, RelE/ParE family
G2583_2609	CDS	2621986	2623347	+	yegQ	hypothetical protein
G2583_2610	ncRNA	2623430	2623510	+	-	ncRNA
G2583_2611	CDS	2623540	2623689	-	-	hypothetical protein
G2583_2612	CDS	2623677	2624054	-	yegR	hypothetical protein
G2583_2613	CDS	2624400	2625299	+	yegS	Lipid kinase yegS
G2583_2614	CDS	2625372	2626019	-	-	hypothetical protein
G2583_2615	CDS	2626064	2627341	-	rbtT	D-arabitol membrane transporter
G2583_2616	CDS	2627410	2628873	-	-	hypothetical protein
G2583_2617	CDS	2628887	2630254	-	-	Arabitol dehydrogenase
G2583_2618	CDS	2630462	2631403	+	-	D-arabitol repressor
G2583_2619	CDS	2631405	2632436	-	-	hypothetical protein
G2583_2620	CDS	2632596	2633345	+	-	Ribitol dehydrogenase
G2583_2621	CDS	2633356	2634960	+	rtlK	Ribitol kinase
G2583_2622	CDS	2635021	2635800	-	gatR	Galactitol utilization operon repressor
G2583_2623	CDS	2635900	2636940	-	gatD	Galactitol-1-phosphate 5-dehydrogenase
G2583_2624	CDS	2636988	2638343	-	gatC	PTS system, galactitol-specific IIC component
G2583_2625	CDS	2638347	2638631	-	gatB	Galactitol-specific phosphotransferase enzyme IIB component
G2583_2626	CDS	2638662	2639114	-	gatA	Galactitol-specific phosphotransferase enzyme IIA component
G2583_2627	CDS	2639124	2640386	-	gatZ	Tagatose 6-phosphate kinase
G2583_2628	CDS	2640415	2641269	-	gatY	Class II aldolase, tagatose bisphosphate family
G2583_2629	CDS	2641576	2642628	-	fbaB	Fructose-bisphosphate aldolase class 1
G2583_2630	CDS	2642885	2644162	+	yegT	Nucleoside transporter
G2583_2631	CDS	2644159	2645163	+	yegU	ADP-ribosylglycohydrolase family protein
G2583_2632	CDS	2645160	2646125	+	yegV	Kinase, PfkB family
G2583_2633	CDS	2646099	2646845	-	yegW	transcriptional regulator, GntR family
G2583_2634	CDS	2646897	2647724	-	yegX	hypothetical protein

Locus_tag	Type	Start	End	+/-[a]	Gene[b]	Product
G2583_2635	CDS	2647780	2648580	-	thiD	Hydroxymethylpyrimidine/phosphomethylpyrimidin e kinase
G2583_2636	CDS	2648577	2649365	-	thiM	Hydroxyethylthiazole kinase
G2583_2637	CDS	2649699	2649938	+	-	hypothetical protein
G2583_2638	CDS	2650502	2650621	-	-	hypothetical protein
G2583_2639	CDS	2650990	2651337	+	-	hypothetical protein
G2583_2640	CDS	2651347	2651661	+	-	hypothetical protein
G2583_2641	CDS	2651771	2652043	-	rcnR	hypothetical protein
G2583_2642	CDS	2652164	2652988	+	rcnA	Nickel/cobalt efflux system rcnA
G2583_2643	CDS	2653027	2653545	+	yohN	hypothetical protein
G2583_2644	CDS	2653627	2654661	-	yehA	predicted fimbrial-like adhesin protein
G2583_2645	pseudogene	2654677	2657157	-	yehB	fimbrial usher protein
G2583_2646	pseudogene	2657173	2657879	-	yehC	gram-negative pilus assembly chaperone
G2583_2647	CDS	2657927	2658469	-	yehD	Fimbrial protein
G2583_2648	CDS	2658763	2659044	-	yehE	hypothetical protein
G2583_2649	CDS	2659306	2660415	-	mrp	Putative ATPase
G2583_2650	CDS	2660547	2662580	+	metG	Methionyl-tRNA synthetase
G2583_2651	CDS	2662721	2665012	+	molR_A	putative molybdate metabolism regulator
G2583_2652	pseudogene	2665013	2666521	+	molR_B	putative molybdate metabolism regulator
G2583_2653	CDS	2666534	2669566	+	molR_C	interrupted molybdate metabolism regulator
G2583_2654	CDS	2669576	2673208	+	yehI	Molybdate metabolism regulator MolR-like protein
G2583_2655	CDS	2673269	2673586	+	yehK	hypothetical protein
G2583_2656	CDS	2674151	2675305	+	yehL	hypothetical protein
G2583_2657	CDS	2675316	2677595	+	yehM	hypothetical protein
G2583_2658	CDS	2677588	2678724	+	yehP	hypothetical protein
G2583_2659	CDS	2678721	2680724	+	yehQ	hypothetical protein
G2583_2660	CDS	2680837	2681310	+	yehR	Hypothetical lipoprotein YehR
G2583_2661	CDS	2681352	2681822	-	yehS	hypothetical protein
G2583_2662	CDS	2681869	2682603	-	yehT	putative two-component response-regulatory protein YehT
G2583_2663	CDS	2682585	2684270	-	yehU	Sensor histidine kinase
G2583_2664	CDS	2684492	2685223	+	mlrA	DNA-binding transcriptional regulator
G2583_2665	CDS	2685371	2686102	-	yehW	ABC transporter, quaternary amine uptake (QAT) family,
G2583_2666	CDS	2686107	2687033	-	yehX	ABC transporter, quaternary amine uptake (QAT) family, ATP-
G2583_2667	CDS	2687026	2688183	-	yehY	ABC transporter, quaternary amine uptake (QAT) family,
G2583_2668	CDS	2688190	2689107	-	osmF	Substrate-binding region of ABC-type glycine betaine transport
G2583_2669	CDS	2689359	2691656	-	bglX	Periplasmic beta-glucosidase
G2583_2670	CDS	2691852	2693567	+	dld	D-lactate dehydrogenase
G2583_2671	CDS	2693605	2694546	-	pbpG	Penicillin-binding protein 7
G2583_2672	CDS	2694711	2695322	-	yohC	hypothetical protein
G2583_2673	CDS	2695432	2696046	+	yohD	hypothetical protein
G2583_2674	CDS	2696176	2696937	-	yohF	Putative 3-oxoacyl-[acyl-carrier-protein] reductase
G2583_2675	CDS	2696990	2698426	-	yohG	Multidrug resistance outer membrane protein mdtQ precursor
G2583_2676	CDS	2698770	2699093	-	-	hypothetical protein
G2583_2677	CDS	2699121	2700071	-	dusC	tRNA-dihydrouridine synthase C
G2583_2678	CDS	2700231	2701424	-	-	FAD dependent oxidoreductase
G2583_2679	CDS	2701439	2702086	-	maiA	Putative glutathione-S-transferase
G2583_2680	CDS	2702092	2702793	-	-	Fumarylacetoacetate hydrolase family protein
G2583_2681	CDS	2702808	2703836	-	gtdA	Gentisate 1,2-dioxygenase
G2583_2682	CDS	2703848	2705206	-	-	Putative transporter
G2583_2683	CDS	2705285	2706241	+	-	putative regulator
G2583_2684	CDS	2706372	2706770	+	yohJ	UPF0299 membrane protein yohJ
G2583_2685	CDS	2706767	2707462	+	yohK	Putative seritonin transporter
G2583_2686	CDS	2707592	2708476	+	cdd	Cytidine deaminase
G2583_2687	CDS	2708626	2709345	+	sanA	hypothetical protein
G2583_2688	CDS	2709348	2709587	+	yeiS	hypothetical protein
G2583_2689	CDS	2709781	2711019	+	yeiT	Uncharacterized oxidoreductase yeiT
G2583_2690	CDS	2711013	2712248	+	yeiA	putative oxidoreductase
G2583_2691	CDS	2712491	2713501	-	mglC	Galactose ABC transporter, permease protein
G2583_2692	CDS	2713517	2715037	-	mglA	Galactose/methyl galactoside import ATP-binding protein mglA
G2583_2693	CDS	2715098	2716096	-	mglB	Galactose ABC transporter, periplasmic galactose-binding
G2583_2694	CDS	2716376	2717416	-	galS	DNA-binding transcriptional repressor

Locus_tag	Type	Start	End	+/-[a]	Gene[b]	Product
G2583_2695	CDS	2717558	2718715	-	yeiB	hypothetical protein
G2583_2696	CDS	2718732	2719400	-	folE	GTP cyclohydrolase 1
G2583_2697	CDS	2719658	2720494	+	yeiG	S-formylglutathione hydrolase yeiG
G2583_2698	CDS	2720526	2722505	-	cirA	Colicin I receptor
G2583_2699	CDS	2722797	2724266	-	lysP	Lysine-specific permease
G2583_2700	CDS	2724471	2725352	-	yeiE	putative DNA-binding transcriptional regulator
G2583_2701	CDS	2725451	2726500	+	yeiH	UPF0324 inner membrane protein yeiH
G2583_2702	CDS	2726574	2727431	+	nfo	Endonuclease 4
G2583_2703	CDS	2727434	2728522	+	yeiI	Kinase, pfkB family
G2583_2704	CDS	2728578	2729828	-	nupX	Nucleoside permease
G2583_2705	CDS	2729928	2730869	-	rihB	Pyrimidine-specific ribonucleoside hydrolase rihB
G2583_2706	CDS	2730999	2731697	+	yeiL	DNA-binding transcriptional activator of stationary phase
G2583_2707	CDS	2731768	2733018	-	yeiM	Nucleoside transporter, NupC family
G2583_2708	CDS	2733112	2734050	-	yeiN	Indigoidine synthase A like protein
G2583_2709	CDS	2734038	2734979	-	yeiC	Kinase, pfkB family protein
G2583_2710	CDS	2735403	2737094	-	fruA	fructose-specific PTS system IIBC component
G2583_2711	CDS	2737111	2738049	-	fruK	1-phosphofructokinase
G2583_2712	CDS	2738049	2739179	-	fruB	Pts system mannitol-specific eiicba component
G2583_2713	CDS	2739547	2740728	+	setB	Sugar efflux transporter B
G2583_2714	CDS	2741134	2741706	+	yeiP	Elongation factor P family protein
G2583_2715	CDS	2741929	2743395	+	yeiQ	Mannitol dehydrogenase family protein
G2583_2716	CDS	2743513	2744499	+	yeiR	CobW/P47K family protein
G2583_2717	CDS	2744502	2745251	+	yeiU	hypothetical protein
G2583_2718	CDS	2745664	2746230	+	spr	Lipoprotein spr precursor
G2583_2719	CDS	2746411	2747967	+	rtn	Putative cyclic diguanylate phosphodiesterase
G2583_2720	CDS	2748049	2749863	+	yejA	ABC transporter, periplasmic solute-binding protein
G2583_2721	CDS	2749864	2750958	+	yejB	Inner membrane ABC transporter permease protein yejB
G2583_2722	CDS	2750958	2751983	+	yejE	ABC transporter, permease protein
G2583_2723	CDS	2751985	2753574	+	yejF	ABC transporter, ATP-binding protein
G2583_2724	CDS	2753578	2753922	-	yejG	hypothetical protein
G2583_2725	CDS	2754255	2755445	-	bcr	Bicyclomycin resistance protein
G2583_2726	CDS	2755473	2756168	-	rsuA	Pseudouridine synthase
G2583_2727	CDS	2756317	2758077	+	yejH	Putative helicase
G2583_2728	CDS	2758202	2758486	+	rplY	50S ribosomal protein L25
G2583_2729	CDS	2758625	2759632	-	yejK	Nucleoid-associated protein ndpA
G2583_2730	CDS	2759814	2760041	+	yejL	UPF0352 protein yejL
G2583_2731	CDS	2760061	2761821	+	yejM	Inner membrane protein yejM
G2583_2732	tRNA	2761895	2761973	+	-	Pro tRNA
G2583_2733	CDS	2762073	2764664	-	yejO	Putative autotransporter, IS5K-containing
G2583_2734	CDS	2764984	2765631	+	narP	Nitrate/nitrite response regulator NarP
G2583_2735	CDS	2765666	2766718	-	ccmH	Cytochrome c-type biogenesis family protein
G2583_2736	CDS	2766715	2767272	-	ccmG	Thiol:disulfide interchange protein dsbE
G2583_2737	CDS	2767269	2769212	-	ccmF	Cytochrome c-type biogenesis protein CcmF
G2583_2738	CDS	2769209	2769688	-	ccmE	Cytochrome c-type biogenesis protein ccmE
G2583_2739	CDS	2769685	2769855	-	ccmD	heme exporter protein D
G2583_2740	CDS	2769891	2770628	-	ccmC	Heme exporter protein C
G2583_2741	CDS	2770670	2771332	-	ccmB	Heme exporter protein B
G2583_2742	CDS	2771329	2771952	-	ccmA	Cytochrome c biogenesis ATP-binding export protein ccmA
G2583_2743	CDS	2771965	2772567	-	napC	Cytochrome c-type protein napC
G2583_2744	CDS	2772577	2773026	-	napB	Cytochrome c-type protein
G2583_2745	CDS	2773023	2773886	-	napH	Polyferredoxin
G2583_2746	CDS	2773873	2774568	-	napG	Ferredoxin-type protein napG precursor
G2583_2747	CDS	2774575	2777061	-	napA	Periplasmic nitrate reductase precursor
G2583_2748	CDS	2777058	2777321	-	napD	Periplasmic nitrate reductase
G2583_2749	CDS	2777311	2777805	-	napF	Ferredoxin-type protein napF
G2583_2750	CDS	2778212	2778700	+	eco	Ecotin precursor
G2583_2751	CDS	2778850	2780496	-	mqo	Malate:quinone oxidoreductase
G2583_2752	CDS	2780714	2782357	-	yojI	ABC transporter ATP-binding protein
G2583_2753	CDS	2782433	2783083	-	alkB	alkylated DNA repair protein
G2583_2754	CDS	2783083	2784147	-	ada	O6-methylguanine-DNA methyltransferase; transcription

Locus_tag	Type	Start	End	+/-[a]	Gene[b]	Product
G2583_2755	CDS	2784221	2785276	-	apbE	Thiamine biosynthesis lipoprotein apbE precursor
G2583_2756	CDS	2785388	2786491	-	ompC	Outer membrane protein C precursor
G2583_2757	ncRNA	2786825	2786918	+	micF	ncRNA
G2583_2758	CDS	2787230	2789902	+	rcsD	Sensor histidine kinase YojN
G2583_2759	CDS	2789919	2790569	+	rcsB	Capsular synthesis regulator component B
G2583_2760	CDS	2790769	2793618	-	rcsC	Sensor histidine kinase/response regulator RcsC
G2583_2761	CDS	2793893	2794669	-	yfaP	hypothetical protein
G2583_2762	pseudogene	2794674	2796323	-	yfaQ	hypothetical protein
G2583_2763	pseudogene	2796324	2797721	-	yfaSR	alpha-2-macroglobulin family protein
G2583_2764	CDS	2797711	2797842	+	-	hypothetical protein
G2583_2765	CDS	2797951	2799147	+	ISSfl2	ISSfl2 ORF
G2583_2766	pseudogene	2799142	2802378	-	yfaS	alpha-2-macroglobulin family protein
G2583_2767	CDS	2802312	2802962	-	yfaT	hypothetical protein
G2583_2768	CDS	2802932	2804620	-	yfaA	hypothetical protein
G2583_2769	CDS	2804769	2807396	-	gyrA	DNA gyrase, A subunit
G2583_2770	CDS	2807543	2808265	+	ubiG	3-demethylubiquinone-9 3-methyltransferase
G2583_2771	CDS	2808406	2812158	-	yfaL	Putative ATP-binding component of a transport system
G2583_2772	CDS	2812854	2815139	+	nrdA	Ribonucleoside-diphosphate reductase, alpha subunit
G2583_2773	CDS	2815327	2816496	+	nrdB	Ribonucleoside-diphosphate reductase 1, beta subunit, B2
G2583_2774	CDS	2816496	2816750	+	yfaE	Ferredoxin
G2583_2775	CDS	2816804	2817454	-	inaA	pH-inducible protein involved in stress response
G2583_2776	pseudogene	2817535	2818725	-	-	transporter, major facilitator family
G2583_2777	CDS	2818877	2819755	+	yfaH	putative regulator
G2583_2778	CDS	2820219	2821289	+	-	hypothetical protein
G2583_2779	CDS	2821513	2822589	-	glpQ	Glycerophosphoryl diester phosphodiesterase
G2583_2780	CDS	2822594	2823952	-	glpT	Glycerol-3-phosphate transporter
G2583_2781	CDS	2824225	2825853	+	glpA	Anaerobic glycerol-3-phosphate dehydrogenase subunit A
G2583_2782	CDS	2825843	2827102	+	glpB	Anaerobic glycerol-3-phosphate dehydrogenase subunit B
G2583_2783	CDS	2827099	2828289	+	glpC	Anaerobic glycerol-3-phosphate dehydrogenase subunit C
G2583_2784	CDS	2828482	2829408	+	yfaD	hypothetical protein
G2583_2785	CDS	2829449	2830252	-	yfaU	putative aldolase
G2583_2786	CDS	2830270	2831598	-	yfaV	Putative transport protein
G2583_2787	CDS	2831616	2832833	-	yfaW	Putative racemase
G2583_2788	CDS	2832836	2833618	-	yfaX	putative regulator
G2583_2789	CDS	2833838	2835040	-	yfaY	CinA family protein
G2583_2790	CDS	2835140	2835703	-	yfaZ	hypothetical protein
G2583_2791	CDS	2835961	2836386	+	nudI	Nucleoside triphosphatase nudI
G2583_2792	CDS	2836425	2837027	-	ais	protein induced by aluminum
G2583_2793	CDS	2837317	2838474	+	arnB	UDP-4-amino-4-deoxy-L-arabinose--oxoglutarate
G2583_2794	CDS	2838478	2839446	+	arnC	Undecaprenyl-phosphate 4-deoxy-4-formamido-L-arabinose
G2583_2795	CDS	2839446	2841428	+	arnA	Bifunctional polymyxin resistance protein arnA [Includes: UDP-
G2583_2796	CDS	2841425	2842315	+	yfbH	hypothetical protein
G2583_2797	CDS	2842315	2843967	+	arnT	Undecaprenyl phosphate-alpha-4-amino-4-deoxy-L-arabinose
G2583_2798	CDS	2843964	2844299	+	yfbW	Sucrose-6 phosphate hydrolase
G2583_2799	CDS	2844299	2844685	+	yfbJ	putative transport/receptor protein
G2583_2800	CDS	2844679	2844975	-	pmrD	Polymyxin resistance protein B
G2583_2801	CDS	2845055	2846410	-	menE	O-succinylbenzoate-CoA ligase
G2583_2802	CDS	2846407	2847369	-	menC	o-succinylbenzoate synthase (OSB synthase) (OSBS) (4- (2'-
G2583_2803	CDS	2847369	2848226	-	menB	Naphthoate synthase
G2583_2804	CDS	2848241	2848999	-	yfbB	2-succinyl-6-hydroxy-2,4-cyclohexadiene-1-carboxylate
G2583_2805	CDS	2848996	2850666	-	menD	2-succinyl-5-enolpyruvyl-6-hydroxy-3-cyclohexene-1-
G2583_2806	CDS	2850755	2852050	-	menF	Isochorismate synthase, menaquinone-specific
G2583_2807	CDS	2852129	2852434	-	elaB	regulatory protein AmpE
G2583_2808	CDS	2852489	2852950	-	elaA	Acetyltransferase, GNAT family
G2583_2809	CDS	2852997	2853932	+	rbn	ribonuclease Z
G2583_2810	pseudogene	2854120	2855334	+	elaD	putative sulfatase / phosphatase
G2583_2811	CDS	2855983	2856363	+	yfbL	Peptidase, M28 family
G2583_2812	CDS	2856466	2856969	+	yfbM	hypothetical protein
G2583_2813	CDS	2857036	2858493	-	nuoN	NADH-quinone oxidoreductase subunit N
G2583_2814	CDS	2858500	2860029	-	nuoM	NADH-quinone oxidoreductase subunit M

Locus_tag	Type	Start	End	+/-[a]	Gene[b]	Product
G2583_2815	CDS	2860260	2862101	-	nuoL	NADH-quinone oxidoreductase, L subunit
G2583_2816	CDS	2862098	2862400	-	nuoK	NADH-quinone oxidoreductase subunit K
G2583_2817	CDS	2862397	2862951	-	nuoJ	NADH-quinone oxidoreductase subunit J
G2583_2818	CDS	2862963	2863505	-	nuoI	NADH-quinone oxidoreductase subunit I
G2583_2819	CDS	2863520	2864497	-	nuoH	NADH-quinone oxidoreductase subunit H
G2583_2820	CDS	2864494	2867226	-	nuoG	NADH-quinone oxidoreductase
G2583_2821	CDS	2867273	2868610	-	nuoF	NADH-quinone oxidoreductase, F subunit
G2583_2822	CDS	2868607	2869107	-	nuoE	NADH-quinone oxidoreductase, E subunit
G2583_2823	CDS	2869110	2870912	-	nuoC	NADH-quinone oxidoreductase, C/D subunit
G2583_2824	CDS	2871006	2871668	-	nuoB	NADH-quinone oxidoreductase subunit B
G2583_2825	CDS	2871684	2872127	-	nuoA	NADH-quinone oxidoreductase subunit A
G2583_2826	CDS	2872758	2873696	-	lrhA	Probable HTH-type transcriptional regulator lrhA
G2583_2827	CDS	2874616	2875833	+	yfbQ	Uncharacterized aminotransferase yfbQ
G2583_2828	CDS	2875917	2876516	+	yfbR	5'-nucleotidase yfbR
G2583_2829	CDS	2876575	2878407	-	yfbS	Putative transport protein
G2583_2830	CDS	2878494	2879144	-	yfbT	Sugar-phosphatase, YfbT
G2583_2831	CDS	2879155	2879649	-	yfbU	UPF0304 protein yfbU
G2583_2832	CDS	2879732	2880187	-	yfbV	UPF0208 membrane protein yfbV
G2583_2833	CDS	2880525	2881727	+	ackA	Acetate kinase
G2583_2834	CDS	2881802	2883946	+	pta	Phosphate acetyltransferase
G2583_2835	CDS	2884136	2885656	+	yfcC	C4-dicarboxylate anaerobic carrier protein
G2583_2836	CDS	2885689	2886231	-	yfcD	Uncharacterized Nudix hydrolase yfcD
G2583_2837	CDS	2886289	2886843	-	yfcE	Phosphodiesterase yfcE
G2583_2838	CDS	2886896	2887540	-	yfcF	Glutathione S-transferase domain protein
G2583_2839	CDS	2887676	2888323	+	yfcG	Glutathione S-transferase
G2583_2840	CDS	2888380	2888742	+	folX	D-erythro-7,8-dihydroneopterin triphosphate epimerase
G2583_2841	CDS	2888763	2889656	+	yfcH	NAD-binding domain 4 protein
G2583_2842	CDS	2889704	2890594	-	yfcI	hypothetical protein
G2583_2843	CDS	2890791	2891564	-	hisP	ATP-binding component of histidine transport
G2583_2844	CDS	2891572	2892288	-	hisM	Histidine transport system permease protein hisM
G2583_2845	CDS	2892285	2892971	-	hisQ	ABC-type arginine transport system, permease component
G2583_2846	CDS	2893061	2893843	-	hisJ	Histidine-binding periplasmic protein precursor
G2583_2847	CDS	2894064	2894846	-	argT	Lysine-arginine-ornithine-binding periplasmic protein precursor
G2583_2848	CDS	2895112	2895681	-	ubiX	3-octaprenyl-4-hydroxybenzoate carboxy-lyase
G2583_2849	CDS	2895776	2897293	-	purF	Amidophosphoribosyltransferase
G2583_2850	CDS	2897330	2897818	-	cvpA	CvpA protein
G2583_2851	CDS	2898441	2899103	-	dedD	Sporulation and cell division repeat protein
G2583_2852	CDS	2899093	2900361	-	folC	Tetrahydrofolate synthase/dihydrofolate synthase
G2583_2853	CDS	2900431	2901345	-	accD	Acetyl-coenzyme A carboxylase carboxyl transferase subunit
G2583_2854	CDS	2901501	2902160	-	dedA	hypothetical protein
G2583_2855	CDS	2902243	2903055	-	truA	tRNA pseudouridine synthase A
G2583_2856	CDS	2903055	2904068	-	usg	USG-1 protein
G2583_2857	CDS	2904134	2905270	-	pdxB	Erythronate-4-phosphate dehydrogenase
G2583_2858	CDS	2905369	2906364	+	flk	Cell division protein
G2583_2859	CDS	2906361	2907539	-	yfcJ	Permeases of the major facilitator superfamily
G2583_2860	CDS	2907814	2909034	-	fabB	3-oxoacyl-[acyl-carrier-protein] synthase 1
G2583_2861	CDS	2909193	2911199	+	mnmC	UPF0209 protein yfcK
G2583_2862	CDS	2911320	2911598	-	yfcL	hypothetical protein
G2583_2863	CDS	2911632	2912180	-	yfcM	putative transporting ATPase
G2583_2864	CDS	2912180	2912989	-	yfcA	Inner membrane protein yfcA
G2583_2865	CDS	2912989	2913813	-	mepA	Penicillin-insensitive murein endopeptidase precursor
G2583_2866	CDS	2913817	2914902	-	aroC	Chorismate synthase
G2583_2867	CDS	2914937	2916202	-	prmB	Putative adenine-specific methylase
G2583_2868	CDS	2916035	2916586	+	yfcN	UPF0115 protein yfcN
G2583_2869	CDS	2916757	2917599	-	yfcO	hypothetical protein
G2583_2870	CDS	2917601	2918149	-	yfcP	Fimbrial protein
G2583_2871	CDS	2918119	2918589	-	yfcQ	Fimbrial subunit
G2583_2872	CDS	2918586	2919179	-	yfcR	Putative minor fimbrial subunit
G2583_2873	CDS	2919097	2919855	-	yfcS	Chaperone protein PapD
G2583_2874	CDS	2919878	2922517	-	yfcU	Fimbrial usher family protein

Locus_tag	Type	Start	End	+/-[a]	Gene[b]	Product
G2583_2875	CDS	2922599	2923162	-	yfcV	Fimbrial protein
G2583_2876	CDS	2923808	2924293	-	sixA	Phosphohistidine phosphatase SixA
G2583_2877	CDS	2924496	2926640	-	fadJ	Fatty oxidation complex, alpha subunit FadJ
G2583_2878	CDS	2926640	2927950	-	fadI	3-ketoacyl-CoA thiolase
G2583_2879	CDS	2928130	2928438	-	yfcZ	hypothetical protein
G2583_2880	CDS	2928780	2930126	+	fadL	Transport of long-chain fatty acids
G2583_2881	CDS	2930491	2931522	+	yfdF	hypothetical protein
G2583_2882	CDS	2931917	2932672	-	vacJ	Lipoprotein, VacJ family
G2583_2883	CDS	2932966	2933898	+	yfdC	putative transport
G2583_2884	tRNA	2933997	2934049	+	-	Arg tRNA
G2583_2885	CDS	2934174	2935367	+	intS	putative prophage integrase
G2583_2886	CDS	2935542	2936678	-	-	Putative prophage DNA injection protein
G2583_2887	CDS	2936688	2937368	-	-	Phage DNA transfer protein
G2583_2888	CDS	2937355	2937822	-	-	hypothetical protein
G2583_2889	CDS	2937822	2938391	-	-	Gene 9 protein
G2583_2890	CDS	2938374	2939069	+	-	hypothetical protein
G2583_2891	CDS	2939739	2941151	-	-	hypothetical protein
G2583_2892	CDS	2941338	2941514	-	-	hypothetical protein
G2583_2893	CDS	2941817	2942443	+	-	Resolvase domain protein
G2583_2894	CDS	2943041	2944288	-	-	Oligosaccharide:H+ symporter
G2583_2895	CDS	2944360	2945274	-	cscK	Fructokinase
G2583_2896	CDS	2945490	2946923	+	-	Sucrose-6-phosphate hydrolase
G2583_2897	CDS	2946931	2947989	-	-	Sugar binding transcriptional regulator, LacI family
G2583_2898	CDS	2948169	2948387	+	dsdX	Putative uncharacterized dsdX-like protein
G2583_2899	CDS	2948507	2949535	+	dsdA	D-serine ammonia-lyase
G2583_2900	CDS	2949841	2951379	-	emrY	Multidrug resistance protein Y
G2583_2901	CDS	2951379	2952542	-	emrK	Drug resistance MFS transporter, membrane fusion protein
G2583_2902	CDS	2952958	2953572	+	evgA	Positive transcription regulator evgA
G2583_2903	CDS	2953577	2957170	+	evgS	hybrid sensory histidine kinase in two-component regulatory
G2583_2904	CDS	2957226	2958410	-	yfdE	hypothetical protein
G2583_2905	CDS	2958445	2959389	-	yfdV	Uncharacterized transporter yfdV
G2583_2906	CDS	2959459	2961153	-	oxc	Thiamine pyrophosphate-dependent enzyme
G2583_2907	CDS	2961207	2962457	-	frc	Formyl-coenzyme A transferase
G2583_2908	CDS	2962970	2963605	-	yfdX	Protein yfdX precursor
G2583_2909	CDS	2963949	2964176	+	ypdI	hypothetical protein
G2583_2910	CDS	2964253	2964495	-	yfdY	hypothetical protein
G2583_2911	CDS	2964848	2965768	+	lpxP	hypothetical protein
G2583_2912	CDS	2966260	2967498	-	yfdZ	Aminotransferase, classes I and II
G2583_2913	CDS	2967875	2969572	+	ypdA	Inner membrane protein ypdA
G2583_2914	CDS	2969587	2970321	+	ypdB	Uncharacterized response regulatory protein ypdB
G2583_2915	CDS	2970334	2971191	+	ypdC	helix-turn-helix- domain containing protein AraC type
G2583_2916	CDS	2971194	2973689	-	fryA	Putative phosphoenolpyruvate-protein phosphotransferase
G2583_2917	CDS	2973714	2974751	-	ypdE	Aminopeptidase
G2583_2918	CDS	2974751	2975836	-	ypdF	Aminopeptidase YpdF
G2583_2919	CDS	2975851	2977098	-	fryC	Fructose-like permease IIC component
G2583_2920	CDS	2977120	2977446	-	fryB	Fructose-like phosphotransferase enzyme IIB component 1
G2583_2921	CDS	2977665	2978630	-	glk	Glucokinase
G2583_2922	CDS	2978834	2980090	+	yfeO	Putative ion-transport protein yfeO
G2583_2923	CDS	2980205	2980531	+	ypeC	conserved hypothetical protein
G2583_2924	CDS	2980380	2980613	+	-	hypothetical protein
G2583_2925	CDS	2980671	2981909	-	mntH	Manganese transport protein mntH
G2583_2926	CDS	2982245	2983447	+	nupC	Nucleoside transporter NupC
G2583_2927	CDS	2983497	2985725	-	yfeA	hypothetical protein
G2583_2928	tRNA	2985893	2985970	-	-	Ala tRNA
G2583_2929	tRNA	2986009	2986084	-	-	Ala tRNA
G2583_2930	CDS	2986305	2986664	+	yfeC	hypothetical protein
G2583_2931	CDS	2986666	2987058	+	yfeD	hypothetical protein
G2583_2932	CDS	2987098	2988306	-	ydcM	IS605 family transposase orfB
G2583_2933	CDS	2988314	2988856	+	-	Putative transposase TnA
G2583_2934	CDS	2988857	2990272	-	gltX	Glutamyl-tRNA synthetase

Locus_tag	Type	Start	End	+/-[a]	Gene[b]	Product
G2583_2935	tRNA	2990531	2990606	+	-	Val tRNA
G2583_2936	tRNA	2990650	2990727	+	-	Val tRNA
G2583_2937	tRNA	2990772	2990849	+	-	Val tRNA
G2583_2938	tRNA	2990853	2990928	+	-	Lys tRNA
G2583_2939	CDS	2991048	2991389	+	flxA	hypothetical protein
G2583_2940	CDS	2991380	2992306	-	yfeR	transcriptional regulator, LysR family protein
G2583_2941	CDS	2992396	2993394	+	yfeH	putative cytochrome oxidase
G2583_2942	CDS	2993391	2993609	-	ypeB	hypothetical protein
G2583_2943	CDS	2993611	2995626	-	ligA	DNA ligase
G2583_2944	CDS	2995697	2996695	-	zipA	Cell division protein ZipA
G2583_2945	CDS	2996925	2997686	+	cysZ	putative sulfate transport protein CysZ
G2583_2946	CDS	2997871	2998842	+	cysK	Cysteine synthase A (O-acetylserine sulfhydrylase A) (CSase
G2583_2947	CDS	2999226	2999483	+	ptsH	PTS system, phosphocarrier protein
G2583_2948	CDS	2999528	3001255	+	ptsI	Phosphoenolpyruvate-protein phosphotransferase
G2583_2949	CDS	3001296	3001805	+	crr	Glucose-specific phosphotransferase enzyme IIA component
G2583_2950	CDS	3001848	3002699	-	pdxK	pyridoxal kinase
G2583_2951	CDS	3002804	3003136	+	yfeK	hypothetical protein
G2583_2952	CDS	3003174	3004085	-	cysM	Cysteine synthase
G2583_2953	CDS	3004219	3005316	-	cysA	Sulfate/thiosulfate import ATP-binding protein cysA
G2583_2954	CDS	3005306	3006181	-	cysW	Sulfate ABC transporter, permease protein CysW
G2583_2955	CDS	3006181	3007014	-	cysU	Sulfate ABC transporter, permease protein CysT
G2583_2956	CDS	3007014	3008030	-	cysP	Sulfate/thiosulfate ABC transporter, periplasmic
G2583_2957	CDS	3008188	3008979	-	ucpA	Oxidoreductase ucpA
G2583_2958	CDS	3009108	3009965	-	yfeT	hypothetical protein
G2583_2959	CDS	3010129	3011025	+	murQ	Predicted sugar phosphate isomerase
G2583_2960	CDS	3011029	3012453	+	murP	Phosphoenolpyruvate-dependent sugar phosphotransferase
G2583_2961	CDS	3012458	3013762	+	yfeW	Putative hydrolase/beta lactamase fusion protein
G2583_2962	CDS	3013820	3014746	-	yfeX	hypothetical protein
G2583_2963	CDS	3014815	3015390	-	yfeY	hypothetical protein
G2583_2964	CDS	3015451	3015906	-	yfeZ	hypothetical protein
G2583_2965	CDS	3015887	3016423	-	ypeA	Acetyltransferase ypeA
G2583_2966	CDS	3016526	3017395	+	amiA	N-acetylmuramoyl-L-alanine amidase
G2583_2967	CDS	3017399	3018298	+	hemF	Coproporphyrinogen III oxidase, aerobic
G2583_2968	CDS	3018304	3019356	-	eutR	AraC-type DNA-binding domain-containing protei ns
G2583_2969	CDS	3019402	3019908	-	eutK	Ethanolamine utilization protein EutK
G2583_2970	CDS	3019915	3020574	-	eutL	Putative ethanolamine utilization protein EutL
G2583_2971	CDS	3020584	3021471	-	eutC	Ethanolamine ammonia-lyase light chain
G2583_2972	CDS	3021492	3022853	-	eutB	Ethanolamine ammonia-lyase heavy chain
G2583_2973	CDS	3022865	3024268	-	eutA	Ethanolamine utilization protein EutA
G2583_2974	CDS	3024265	3025491	-	eutH	EutH
G2583_2975	CDS	3025591	3026805	-	eutG	EutG
G2583_2976	CDS	3026768	3027604	-	eutJ	Ethanolamine utilization protein EutJ
G2583_2977	CDS	3027615	3029018	-	eutE	Ethanolamine utilization
G2583_2978	CDS	3029030	3029317	-	eutN	Ethanolamine utilization protein
G2583_2979	CDS	3029424	3029717	-	eutM	Detox protein
G2583_2980	CDS	3029756	3030772	-	eutD	Ethanolamine utilization protein EutD
G2583_2981	CDS	3030769	3031500	-	eutT	Ethanolamine utilization cobalamin adenosyltransferase
G2583_2982	CDS	3031497	3032198	-	eutQ	Ethanolamine utilization protein EutQ
G2583_2983	CDS	3032173	3032652	-	eutP	hypothetical protein
G2583_2984	CDS	3032665	3033072	-	eutS	hypothetical protein
G2583_2985	CDS	3033293	3035572	-	maeB	Malate dehydrogenase (Oxaloacetate-decarboxylating)
G2583_2986	CDS	3035861	3036811	+	talA	Transaldolase A
G2583_2987	CDS	3036831	3038834	+	tktB	Transketolase 2
G2583_2988	CDS	3038929	3039972	-	ypfG	hypothetical protein
G2583_2989	CDS	3040098	3040673	-	nudK	GDP-mannose pyrophosphatase nudK
G2583_2990	CDS	3040741	3042720	-	aegA	putative oxidoreductase Fe-S binding subunit
G2583_2991	CDS	3042926	3044626	+	narQ	Nitrate/nitrite sensor histidine kinase NarQ
G2583_2992	CDS	3044790	3047903	+	acrD	Probable aminoglycoside efflux pump
G2583_2993	CDS	3048442	3048798	+	yffB	ArsC family protein
G2583_2994	CDS	3048802	3049929	+	dapE	Succinyl-diaminopimelate desuccinylase

Locus_tag	Type	Start	End	+/-[a]	Gene[b]	Product
G2583_2995	CDS	3049957	3050157	+	ypfN	UPF0370 protein ypfN
G2583_2996	CDS	3050238	3050936	-	ypfH	Esterase, AB hydrolase 2 family
G2583_2997	CDS	3051010	3053025	-	ypfI	hypothetical protein
G2583_2998	CDS	3053040	3053903	-	ypfJ	hypothetical protein
G2583_2999	CDS	3054071	3054784	-	purC	Phosphoribosylaminoimidazole-succinocarboxamide synthase
G2583_3000	CDS	3054997	3056034	-	nlpB	Lipoprotein-34
G2583_3001	CDS	3056048	3056926	-	dapA	Dihydrodipicolinate synthase
G2583_3002	CDS	3057072	3057644	+	gcvR	Glycine cleavage system transcriptional repressor
G2583_3003	CDS	3057644	3058114	+	bcp	Putative peroxiredoxin bcp
G2583_3004	CDS	3058328	3058984	+	hyfA	Hydrogenase 4 Fe-S subunit
G2583_3005	CDS	3058984	3061002	+	hyfB	Hydrogenase 4 membrane subunit
G2583_3006	CDS	3060992	3061960	+	hyfC	Hydrogenase 4 membrane subunit
G2583_3007	CDS	3061977	3063416	+	hyfD	Hydrogenase-4 component D
G2583_3008	CDS	3063428	3064072	+	hyfE	Hydrogenase-4 component E
G2583_3009	CDS	3064077	3065657	+	hyfF	Hydrogenase 4 membrane subunit
G2583_3010	CDS	3065647	3067362	+	hyfG	Hydrogenase-4, G subunit
G2583_3011	CDS	3067372	3067917	+	hyfH	Iron-sulfur cluster-binding protein
G2583_3012	CDS	3067914	3068672	+	hyfI	Hydrogenase-4, I subunit
G2583_3013	CDS	3068602	3069078	+	hyfJ	putative protein processing element
G2583_3014	CDS	3069108	3071120	+	hyfR	Hydrogenase-4 transcriptional regulator
G2583_3015	CDS	3071142	3071990	+	focB	Formate/nitrite transporter
G2583_3016	CDS	3072028	3073089	-	yfgO	Putative permease perM
G2583_3017	CDS	3073302	3074765	+	yfgC	Peptidase, M48 family
G2583_3018	CDS	3074786	3075145	+	yfgD	Arsenate reductase
G2583_3019	CDS	3075283	3076029	-	hda	DnaA-homolog protein hda
G2583_3020	CDS	3076079	3077368	-	uraA	Uracil permease
G2583_3021	CDS	3077454	3078080	-	upp	Uracil phosphoribosyltransferase
G2583_3022	CDS	3078405	3079442	+	purM	Phosphoribosylformylglycinamidine cyclo-ligase
G2583_3023	CDS	3079442	3080080	+	purN	Phosphoribosylglycinamide formyltransferase 1
G2583_3024	CDS	3080251	3082317	+	ppk	Polyphosphate kinase
G2583_3025	CDS	3082322	3083863	+	ppx	exopolyphosphatase
G2583_3026	CDS	3083902	3086145	-	yfgF	Putative cytochrome C-type biogenesis protein
G2583_3027	CDS	3086327	3086479	-	-	hypothetical protein
G2583_3028	CDS	3086497	3086688	+	yfgG	hypothetical protein
G2583_3029	CDS	3086999	3087517	+	yfgH	Uncharacterized lipoprotein yfgH precursor
G2583_3030	CDS	3087533	3088072	+	yfgI	hypothetical protein
G2583_3031	CDS	3088165	3089742	-	guaA	GMP synthase [glutamine-hydrolyzing]
G2583_3032	CDS	3089811	3091277	-	guaB	Inosine-5'-monophosphate dehydrogenase
G2583_3033	CDS	3091439	3092809	+	xseA	Exodeoxyribonuclease 7 large subunit
G2583_3034	CDS	3092806	3093021	-	yfgJ	hypothetical protein
G2583_3035	CDS	3093090	3094562	-	der	GTP-binding protein engA
G2583_3036	CDS	3094680	3095858	-	yfgL	Outer membrane assembly lipoprotein YfgL
G2583_3037	CDS	3095869	3096489	-	yfgM	UPF0070 protein yfgM
G2583_3038	CDS	3096507	3097781	-	hisS	Histidyl-tRNA synthetase
G2583_3039	ncRNA	3097801	3097892	-	-	ncRNA
G2583_3040	CDS	3097892	3099010	-	ispG	4-hydroxy-3-methylbut-2-en-1-yl diphosphate synthase (1-
G2583_3041	CDS	3099037	3100050	-	yfgA	Helix-turn-helix DNA-binding domain protein
G2583_3042	CDS	3100335	3101489	-	yfgB	Radical SAM enzyme, Cfr family
G2583_3043	CDS	3101639	3102070	-	ndk	Nucleoside diphosphate kinase
G2583_3044	CDS	3102211	3103065	-	-	Putative polyferredoxin
G2583_3045	CDS	3103065	3103886	-	ynfH	Putative dimethyl sulfoxide reductase subunit C
G2583_3046	CDS	3103879	3104508	-	-	Dimethylsulfoxide reductase, chain B
G2583_3047	CDS	3104505	3106886	-	dmsA	putative anaerobic dimethyl sulfoxide reductase chain A
G2583_3048	CDS	3107050	3109362	-	pbpC	Penicillin-binding protein 1C
G2583_3049	CDS	3109363	3114324	-	yfhM	Alpha-2-macroglobulin domain protein
G2583_3050	CDS	3114531	3115376	+	sseA	Putative thiosulfate sulfurtransferase
G2583_3051	ncRNA	3115573	3115877	+	-	ncRNA
G2583_3052	CDS	3115876	3116661	-	sseB	Enhanced serine sensitivity
G2583_3053	CDS	3116795	3118078	-	pepB	Peptidase B
G2583_3054	CDS	3118256	3118456	-	iscX	hypothetical protein

Locus_tag	Type	Start	End	+/-[a]	Gene[b]	Product
G2583_3055	CDS	3118468	3118803	-	fdx	2Fe-2S ferredoxin
G2583_3056	CDS	3118805	3120655	-	hscA	Chaperone protein hscA
G2583_3057	CDS	3120672	3121187	-	hscB	Fe-S protein assembly co-chaperone HscB
G2583_3058	CDS	3121283	3121606	-	iscA	Iron-binding protein iscA
G2583_3059	CDS	3121623	3122009	-	iscU	NifU-like protein
G2583_3060	CDS	3122037	3123275	-	iscS	Cysteine desulfurase
G2583_3061	CDS	3123363	3123851	-	iscR	DNA-binding transcriptional repressor
G2583_3062	CDS	3124152	3124889	-	trmJ	tRNA (cytidine/uridine-2'-O-)-methyltransferase trmJ
G2583_3063	CDS	3125008	3125811	+	suhB	Inositol-1-monophosphatase
G2583_3064	CDS	3125929	3126810	+	yfhR	putative enzyme (3.4.-)
G2583_3065	CDS	3127001	3128281	+	csiE	Transcriptional antiterminator
G2583_3066	CDS	3128273	3129412	-	hcaT	3-phenylpropionic acid transporter
G2583_3067	CDS	3129572	3130465	-	hcaR	Hca operon transcriptional activator
G2583_3068	CDS	3130598	3131959	+	hcaE	3-phenylpropionate/cinnamic acid dioxygenase subunit alpha
G2583_3069	CDS	3131956	3132474	+	hcaF	3-phenylpropionate/cinnamic acid dioxygenase subunit beta
G2583_3070	CDS	3132474	3132794	+	hcaC	3-phenylpropionate/cinnamic acid dioxygenase ferredoxin
G2583_3071	CDS	3132791	3133603	+	hcaB	3-phenylpropionate-dihydrodiol/cinnamic acid-dihydrodiol
G2583_3072	CDS	3133613	3134815	+	hcaD	3-phenylpropionate/cinnamic acid dioxygenase ferredoxin--
G2583_3073	CDS	3134867	3135334	+	yphA	Predicted inner membrane protein
G2583_3074	CDS	3135382	3136254	-	yphB	Aldose 1-epimerase family protein
G2583_3075	CDS	3136266	3137360	-	yphC	Hypothetical zinc-type alcohol dehydrogenase-like protein yphC
G2583_3076	CDS	3137393	3138391	-	yphD	Putative transport system permease protein
G2583_3077	CDS	3138416	3139927	-	yphE	Putative sugar ABC transporter, ATP-binding protein
G2583_3078	CDS	3139950	3140933	-	yphF	Periplasmic binding protein/LacI transcriptional regulator
G2583_3079	CDS	3141030	3144404	-	yphG	hypothetical protein
G2583_3080	CDS	3144423	3145622	+	yphH	ROK family protein
G2583_3081	CDS	3145685	3146938	-	glyA	Serine hydroxymethyltransferase
G2583_3082	CDS	3147266	3148456	+	hmp	Flavohemoprotein
G2583_3083	CDS	3148501	3148839	-	glnB	Nitrogen regulatory protein P-II 1
G2583_3084	CDS	3148900	3150234	-	yfhA	putative 2-component transcriptional regulator
G2583_3085	CDS	3150224	3150937	-	yfhG	hypothetical protein
G2583_3086	CDS	3151102	3152592	-	yfhK	putative 2-component sensor protein
G2583_3087	ncRNA	3152623	3152771	+	-	ncRNA
G2583_3088	CDS	3153105	3156992	-	purL	Phosphoribosylformylglycinamidine synthase
G2583_3089	CDS	3157249	3158805	+	yfhD	Predicted soluble lytic transglycosylase fused to an ABC-type
G2583_3090	CDS	3158802	3159338	-	tadA	tRNA-specific adenosine deaminase
G2583_3091	CDS	3159363	3159998	-	yfhB	HAD hydrolase YhfB
G2583_3092	CDS	3160207	3161055	+	yfhH	hypothetical protein
G2583_3093	CDS	3161922	3162341	+	-	hypothetical protein
G2583_3094	CDS	3162414	3162998	-	pinE	DNA invertase from prophage CP-933H
G2583_3095	CDS	3162992	3163390	+	yfdL	putative tail fiber protein
G2583_3096	CDS	3163394	3163813	+	yfdK	tail fiber assembly protein
G2583_3097	CDS	3163785	3164390	-	-	hypothetical protein
G2583_3098	CDS	3164387	3165232	-	-	Phage-related tail fibre protein
G2583_3099	CDS	3165232	3165912	-	-	putative bacteriophage protein
G2583_3100	CDS	3165909	3167108	-	-	phage Mu protein gp 47
G2583_3101	CDS	3167108	3167461	-	-	putative bacteriophage protein
G2583_3102	CDS	3167461	3168213	-	-	Phage P2 baseplate assembly protein gpV
G2583_3103	CDS	3169012	3169359	-	-	Putative secreted protein precursor
G2583_3104	CDS	3169362	3170426	-	-	hypothetical protein
G2583_3105	CDS	3170429	3170731	-	-	Putative bacteriophage protein
G2583_3106	CDS	3170731	3171228	-	-	putative bacteriophage protein
G2583_3107	CDS	3171318	3173306	-	-	Putative bacteriophage protein
G2583_3108	CDS	3173484	3173936	-	-	Putative bacteriophage protein
G2583_3109	CDS	3173940	3174380	-	-	Putative bacteriophage protein
G2583_3110	CDS	3174391	3175536	-	-	Hypothetical prophage protein
G2583_3111	CDS	3175540	3176088	-	-	Putative bacteriophage protein
G2583_3112	CDS	3176078	3176392	-	-	Putative bacteriophage protein
G2583_3113	CDS	3176454	3177008	-	-	Putative bacteriophage protein
G2583_3114	CDS	3177005	3177412	-	-	hypothetical protein

Locus_tag	Type	Start	End	+/-[a]	Gene[b]	Product
G2583_3115	CDS	3177378	3177746	-	-	Putative bacteriophage protein
G2583_3116	CDS	3177787	3178728	-	-	Putative bacteriophage protein
G2583_3117	CDS	3178740	3179246	-	-	Putative bacteriophage protein
G2583_3118	CDS	3179250	3180470	-	-	Uncharacterized protein conserved in bacteria
G2583_3119	CDS	3180485	3181015	-	-	phage Mu protein gp30
G2583_3120	CDS	3181110	3182576	-	-	Uncharacterized protein conserved in bacteria
G2583_3121	CDS	3182576	3184198	-	-	hypothetical protein
G2583_3122	CDS	3184201	3184773	-	-	Phage terminase, small subunit
G2583_3123	CDS	3184835	3185359	-	-	conserved hypothetical protein
G2583_3124	CDS	3185343	3185819	-	-	Lysozyme
G2583_3125	CDS	3185823	3186164	-	-	phage holin, lambda family
G2583_3126	CDS	3186610	3186774	-	-	hypothetical protein
G2583_3127	CDS	3186983	3187405	-	-	Transcriptional regulator
G2583_3128	CDS	3187690	3189876	-	-	Predicted P-loop ATPase and inactivated derivatives
G2583_3129	CDS	3190162	3190836	+	-	hypothetical protein
G2583_3130	CDS	3191470	3191619	+	-	hypothetical protein
G2583_3131	CDS	3191616	3192518	+	-	hypothetical protein
G2583_3132	CDS	3192521	3193822	+	-	Bbp38
G2583_3133	CDS	3193838	3194386	+	-	hypothetical protein
G2583_3134	CDS	3194439	3195068	-	-	hypothetical protein
G2583_3135	CDS	3195115	3197178	+	dpoL	DNA polymerase I - 3""-5"" exonuclease and polymerase
G2583_3136	CDS	3197779	3198330	+	-	hypothetical protein
G2583_3137	CDS	3198373	3198642	+	-	Phage associated protein
G2583_3138	CDS	3198648	3199358	-	-	hypothetical protein
G2583_3139	CDS	3199397	3200788	+	-	Superfamily II DNA/RNA helicases, SNF2 family
G2583_3140	CDS	3200785	3200985	+	-	hypothetical protein
G2583_3141	CDS	3200982	3202379	-	-	Integrase
G2583_3142	CDS	3202594	3202854	+	yfhL	hypothetical protein
G2583_3143	CDS	3203125	3203277	-	-	hypothetical protein
G2583_3144	CDS	3203550	3203930	-	acpS	Holo-[acyl-carrier-protein] synthase
G2583_3145	CDS	3203930	3204661	-	pdxJ	Pyridoxine 5'-phosphate synthase
G2583_3146	CDS	3204673	3205401	-	recO	DNA repair protein recO
G2583_3147	CDS	3205413	3206318	-	era	GTP-binding protein era
G2583_3148	CDS	3206315	3206995	-	rnc	Ribonuclease 3
G2583_3149	CDS	3207053	3207217	+	-	hypothetical protein
G2583_3150	CDS	3207268	3208242	-	lepB	Signal peptidase I
G2583_3151	CDS	3208258	3210057	-	lepA	GTP-binding protein lepA
G2583_3152	CDS	3210255	3210734	-	rseC	Sigma-E factor regulatory protein RseC
G2583_3153	CDS	3210731	3211687	-	rseB	Sigma-E factor regulatory protein rseB precursor
G2583_3154	CDS	3211687	3212325	-	rseA	Sigma-E factor negative regulatory protein
G2583_3155	CDS	3212358	3212933	-	rpoE	DNA-directed RNA polymerase specialized sigma subunit,
G2583_3156	CDS	3213341	3214963	+	nadB	L-aspartate oxidase
G2583_3157	CDS	3214948	3215805	-	yfiC	Putative enzyme
G2583_3158	CDS	3215817	3217151	+	srmB	ATP-dependent RNA helicase SrmB
G2583_3159	CDS	3217184	3218110	-	yfiE	putative transcriptional regulator LYSR-type
G2583_3160	CDS	3218168	3218755	+	eamB	Cysteine/O-acetylserine efflux protein
G2583_3161	CDS	3218811	3219194	-	yfiD	Autonomous glycyl radical cofactor
G2583_3162	CDS	3219499	3220188	+	ung	Uracil-DNA glycosylase
G2583_3163	CDS	3220236	3221273	-	yfiF	rRNA methylases
G2583_3164	CDS	3221263	3221466	-	-	hypothetical protein
G2583_3165	CDS	3221480	3221899	+	trxC	Thioredoxin-2
G2583_3166	CDS	3221968	3222666	+	yfiP	DTW domain protein
G2583_3167	CDS	3222698	3225358	+	yfiQ	CoA binding domain/acetyltransferase domain protein
G2583_3168	CDS	3225469	3226827	+	pssA	CDP-diacylglycerol--serine O-phosphatidyltransferase
G2583_3169	CDS	3226873	3227196	+	yfiM	hypothetical protein
G2583_3170	CDS	3227193	3228491	-	kgtP	Alpha-ketoglutarate permease
G2583_3171	rRNA	3228814	3228929	-	rrfG	5S ribosomal RNA
G2583_3172	rRNA	3229024	3231926	-	rrlG	23S ribosomal RNA
G2583_3173	tRNA	3232109	3232186	-	-	Glu tRNA
G2583_3174	rRNA	3232271	3233812	-	rrsG	16S ribosomal RNA

Locus_tag	Type	Start	End	+/-[a]	Gene[b]	Product
G2583_3175	CDS	3234255	3236840	-	-	Heat shock protein
G2583_3176	CDS	3236958	3237689	-	yfiH	hypothetical protein
G2583_3177	CDS	3237686	3238666	-	rluD	Ribosomal large subunit pseudouridine synthase D
G2583_3178	CDS	3238801	3239538	+	yfiO	predicted lipoprotein
G2583_3179	CDS	3239568	3239774	+	-	hypothetical protein
G2583_3180	CDS	3239809	3240150	+	raiA	Ribosome-associated inhibitor A
G2583_3181	CDS	3240400	3241560	+	pheA	P-protein [Includes: Chorismate mutase (CM)
G2583_3182	CDS	3241603	3242724	-	tyrA	Chorismate mutase/prephenate dehydrogenase
G2583_3183	CDS	3242735	3243805	-	aroF	Phospho-2-dehydro-3-deoxyheptonate aldolase, Tyr-sensitive
G2583_3184	CDS	3243976	3244380	+	yfiL	hypothetical protein
G2583_3185	CDS	3244530	3245048	+	yfiR	hypothetical protein
G2583_3186	CDS	3245038	3246264	+	yfiN	GGDEF domain protein
G2583_3187	CDS	3246280	3246762	+	yfiB	OmpA family protein
G2583_3188	CDS	3246839	3247186	-	rplS	50S ribosomal protein L19
G2583_3189	CDS	3247228	3247995	-	trmD	tRNA (guanine-N(1)-)-methyltransferase
G2583_3190	CDS	3248026	3248577	-	rimM	16S rRNA-processing protein rimM
G2583_3191	CDS	3248593	3248841	-	rpsP	30S ribosomal subunit protein S16
G2583_3192	CDS	3248978	3250339	-	ffh	Signal recognition particle protein
G2583_3193	CDS	3250431	3251297	+	ypjD	hypothetical protein
G2583_3194	CDS	3251343	3252605	+	yfjD	CBS/transporter associated domain protein
G2583_3195	CDS	3252660	3253253	-	grpE	heat shock protein GrpE
G2583_3196	CDS	3253376	3254254	+	nadK	Probable inorganic polyphosphate/ATP-NAD kinase
G2583_3197	CDS	3254340	3256001	+	recN	recombination and repair protein
G2583_3198	CDS	3256150	3256491	+	smpA	Lipoprotein, SmpA/OmlA family
G2583_3199	CDS	3256553	3256843	-	yfjF	hypothetical protein
G2583_3200	CDS	3256833	3257309	-	yfjG	Polyketide cyclase/dehydrase family protein
G2583_3201	CDS	3257441	3257923	+	smpB	SsrA-binding protein
G2583_3202	tmRNA	3258138	3258500	+	-	tmRNA
G2583_3203	CDS	3258769	3259017	+	-	DinI-like protein Z3916/ECs3483
G2583_3204	CDS	3259519	3260109	-	IpgB	Putative chaperone protein
G2583_3205	CDS	3260291	3260851	+	-	PotB, trcA, ORF2, ORF3, ORF4 genes,
G2583_3206	pseudogene	3261015	3261725	+	-	conserved hypothetical protein
G2583_3207	CDS	3261654	3262226	-	-	hypothetical protein
G2583_3208	CDS	3262608	3263921	-	-	putative tail fiber protein encoded by prophage CP-933R
G2583_3209	CDS	3263986	3264609	-	-	Opacity protein and related surface antigens
G2583_3210	CDS	3264679	3268155	-	-	Phage-related protein, tail component
G2583_3211	CDS	3268402	3269082	-	-	putative tail assembly protein
G2583_3212	CDS	3268980	3269756	-	-	Cell wall-associated hydrolases (invasion-associated proteins)
G2583_3213	CDS	3269772	3270776	-	ant	Antirepressor protein
G2583_3214	CDS	3270766	3270939	-	-	hypothetical protein
G2583_3215	CDS	3271384	3272082	-	-	putative tail fiber component L of prophage CP-933U
G2583_3216	CDS	3272082	3272423	-	-	Putative minor tail protein
G2583_3217	CDS	3272416	3275658	-	-	Phage-related minor tail protein
G2583_3218	CDS	3275706	3275987	-	-	Gp14
G2583_3219	CDS	3276011	3276433	-	-	Putative tail assembly chaperone encoded by prophage CP-
G2583_3220	CDS	3276400	3277116	-	-	Putative tail component of cryptic prophage CP-933P
G2583_3221	CDS	3277183	3277527	-	-	Gp11
G2583_3222	CDS	3277524	3277970	-	-	Phage protein, HK97 gp10 family
G2583_3223	CDS	3277967	3278317	-	-	Bacteriophage head-tail adaptor
G2583_3224	CDS	3278328	3278654	-	-	unknown protein encoded by cryptic prophage CP-933M
G2583_3225	CDS	3278651	3280036	-	-	Portal protein (GP3)
G2583_3226	CDS	3280033	3281397	-	-	Portal protein (GP3)
G2583_3227	CDS	3281343	3281564	-	-	hypothetical protein
G2583_3228	CDS	3281609	3283546	-	-	Phage head maturation protease
G2583_3229	CDS	3283610	3285271	-	-	Phage terminase-like protein, large subunit
G2583_3230	CDS	3285268	3285831	-	-	unknown protein encoded by prophage CP-933N
G2583_3231	CDS	3286122	3286487	-	-	unknown protein encoded by prophage CP-933N
G2583_3232	CDS	3286529	3286729	+	-	conserved hypothetical protein
G2583_3233	CDS	3286862	3287203	-	tonB	tonB-like membrane protein encoded within prophage CP-933N
G2583_3234	CDS	3287535	3287759	-	-	conserved hypothetical protein

Locus_tag	Type	Start	End	+/-[a]	Gene[b]	Product
G2583_3235	CDS	3287756	3288250	-	-	Putative endopeptidase Rz
G2583_3236	CDS	3288404	3288973	-	antU	Antirepressor protein
G2583_3237	CDS	3289244	3289777	-	-	putative endolysin
G2583_3238	CDS	3289828	3290172	-	-	hypothetical protein
G2583_3239	CDS	3290177	3290452	-	-	putative holin protein of prophage CP-933U
G2583_3240	CDS	3290758	3291105	+	insN	unknown protein encoded by IS911 within prophage CP-933L
G2583_3241	CDS	3291318	3291971	+	-	putative transposase
G2583_3242	CDS	3292088	3293938	-	-	YjhS
G2583_3243	CDS	3294179	3294697	+	-	hypothetical protein
G2583_3244	CDS	3294510	3295004	-	-	unknown protein encoded within prophage CP-933R
G2583_5274	tRNA	3295111	3295187	-	-	Arg tRNA
G2583_5275	tRNA	3295201	3295277	-	-	Arg tRNA
G2583_5276	tRNA	3295287	3295362	-	-	Met tRNA
G2583_3245	CDS	3295403	3295555	-	-	DNA modification methylase
G2583_3246	CDS	3295804	3296238	-	ybcQ	Antitermination protein Q
G2583_3247	CDS	3296231	3296425	-	ninH	hypothetical protein
G2583_3248	CDS	3296422	3297027	-	NinG	NinG protein
G2583_3249	CDS	3297027	3297755	-	roi	hypothetical protein
G2583_3250	CDS	3297824	3298528	-	ant	Putative antirepressor
G2583_3251	CDS	3298806	3298988	-	ninE	NinE protein
G2583_3252	CDS	3298985	3299512	-	-	Putative DNA N-6-adenine-methyltransferase of bacteriophage
G2583_3253	CDS	3299509	3299949	-	ninB	hypothetical protein
G2583_3254	CDS	3300023	3300313	-	-	Ren protein
G2583_3255	CDS	3300310	3301011	-	-	P protein
G2583_3256	CDS	3301008	3301946	-	-	phage replication protein O
G2583_3257	CDS	3301979	3302275	-	-	CII protein
G2583_3258	CDS	3302385	3302570	-	cro	Cro
G2583_3259	CDS	3302651	3303301	+	ymfK	P22 repressor protein c2
G2583_3260	CDS	3303586	3303921	+	-	hypothetical lipoprotein
G2583_3261	CDS	3303918	3304262	+	-	hypothetical protein
G2583_3262	CDS	3304396	3304866	+	-	Gp45
G2583_3263	CDS	3305016	3305384	+	-	Lambda prophage-derived protein ea10
G2583_3264	CDS	3305457	3305621	+	-	Lambda phage regulatory protein CIII
G2583_3265	CDS	3305590	3305754	+	kilW	putative Kil protein of bacteriophage BP-933W
G2583_3266	CDS	3306111	3306896	+	bet	Recombination protein Bet
G2583_3267	CDS	3306893	3307573	+	-	Putative exonuclease encoded by prophage CP-933K
G2583_3268	CDS	3307564	3307752	+	-	hypothetical protein
G2583_3269	CDS	3307725	3307916	+	-	hypothetical protein
G2583_3270	CDS	3307927	3308208	+	-	hypothetical protein
G2583_3271	CDS	3308307	3308528	+	-	C4-type zinc finger protein
G2583_3272	CDS	3308525	3309343	+	-	Enterohemolysin 2
G2583_3273	CDS	3309340	3309843	+	-	Valyl-tRNA synthetase
G2583_3274	CDS	3309931	3310173	+	-	hypothetical protein
G2583_3275	CDS	3310177	3310323	+	-	Putative bacteriophage protein
G2583_3276	CDS	3310496	3311680	+	-	hypothetical protein
G2583_3277	CDS	3312173	3313096	+	-	hypothetical protein
G2583_3278	CDS	3313387	3314616	+	intA	phage integrase family protein
G2583_3279	CDS	3315239	3316870	-	-	hypothetical protein
G2583_3280	CDS	3316939	3317553	-	-	hypothetical protein
G2583_3281	CDS	3317550	3318521	-	-	hypothetical protein
G2583_3282	CDS	3318496	3318663	-	-	hypothetical protein
G2583_3283	CDS	3318656	3320044	-	-	hypothetical protein
G2583_3284	CDS	3320630	3320818	+	intA	Prophage integrase
G2583_3285	CDS	3321014	3322207	+	-	conserved hypothetical protein
G2583_3286	CDS	3322846	3325167	+	-	Hypothetical purine NTPase
G2583_3287	CDS	3326354	3326755	+	-	hypothetical protein
G2583_3288	CDS	3326760	3327992	+	-	hypothetical protein
G2583_3289	CDS	3328303	3329004	+	-	hypothetical protein
G2583_3290	CDS	3329001	3329375	+	-	hypothetical protein
G2583_3291	CDS	3329451	3330695	-	-	hypothetical protein

Locus_tag	Type	Start	End	+/-[a]	Gene[b]	Product
G2583_3292	CDS	3331091	3331561	-	-	hypothetical protein
G2583_3293	CDS	3331602	3332000	-	-	hypothetical protein
G2583_3294	CDS	3332001	3333632	-	-	Conserved DNA-binding protein
G2583_3295	CDS	3333629	3334954	-	-	Site-specific recombinase, phage integrase family protein
G2583_3296	CDS	3334944	3336149	-	-	Putative site specific recombinase
G2583_3297	CDS	3336472	3336720	-	alpA	Putative DNA binding protein
G2583_3298	CDS	3336778	3337629	-	-	hypothetical protein
G2583_3299	pseudogene	3338057	3342654	-	ypjA	Outer membrane autotransporter barrel domain protein
G2583_3300	CDS	3342993	3343136	-	-	Putative DNA-invertase from prophage CP4-44
G2583_3301	CDS	3343590	3344939	-	ypjB	hypothetical protein
G2583_3302	CDS	3345447	3345599	-	-	hypothetical protein
G2583_3303	tRNA	3345691	3345766	-	-	Met tRNA
G2583_3304	CDS	3346327	3348579	+	ygaR	hypothetical protein
G2583_3305	CDS	3348916	3349893	+	csiD	alpha amylase family protein
G2583_3306	CDS	3349847	3351181	+	ygaF	hypothetical protein
G2583_3307	CDS	3351204	3352652	+	gabD	Succinate-semialdehyde dehydrogenase (NAD(P)(+))
G2583_3308	CDS	3352666	3353946	+	gabT	4-aminobutyrate transaminase
G2583_3309	CDS	3354184	3355584	+	gabP	GABA permease
G2583_3310	CDS	3355605	3356267	+	csiR	DNA-binding transcriptional regulator CsiR
G2583_3311	CDS	3356268	3356717	-	ygaU	Phospholipid-binding protein
G2583_3312	CDS	3356801	3356959	-	yqaE	UPF0057 membrane protein yqaE
G2583_3313	CDS	3357142	3357441	+	ygaV	hypothetical protein
G2583_3314	CDS	3357451	3357969	+	ygaP	hypothetical protein
G2583_3315	CDS	3358022	3358426	-	stpA	DNA-binding protein H-NS
G2583_3316	CDS	3359094	3359543	+	ygaW	hypothetical protein
G2583_3317	CDS	3359580	3359924	-	ygaC	hypothetical protein
G2583_3318	CDS	3360064	3360405	+	ygaM	hypothetical protein
G2583_3319	CDS	3360555	3361889	-	-	transcriptional regulator, GntR family
G2583_3320	CDS	3361977	3362408	+	-	Carboxymuconolactone decarboxylase family protein
G2583_3321	CDS	3362617	3362862	+	nrdH	Glutaredoxin-like protein nrdH
G2583_3322	CDS	3362859	3363269	+	nrdI	ribonucleotide reductase stimulatory protein
G2583_3323	CDS	3363242	3365386	+	nrdE	Ribonucleoside-diphosphate reductase, alpha subunit
G2583_3324	CDS	3365396	3366355	+	nrdF	Ribonucleoside-diphosphate reductase 2 subunit beta
G2583_3325	CDS	3366711	3367913	+	proV	ATP-binding component of transport system
G2583_3326	CDS	3367906	3368970	+	proW	Glycine betaine/L-proline ABC transporter, permease protein
G2583_3327	CDS	3369027	3370019	+	proX	Glycine betaine-binding periplasmic protein precursor
G2583_3328	CDS	3370211	3371395	+	ygaX	Putative transport protein
G2583_3329	CDS	3371519	3372256	+	ygaZ	Transporter, branched chain amino acid exporter (LIV-E) family
G2583_3330	CDS	3372246	3372581	+	ygaH	predicted inner membrane protein
G2583_3331	CDS	3372672	3373202	+	mprA	transcriptional repressor MprA
G2583_3332	CDS	3373329	3374501	+	emrA	Multidrug resistance protein A
G2583_3333	CDS	3374518	3376056	+	emrB	Probably membrane translocase
G2583_3334	CDS	3376120	3376635	-	luxS	S-ribosylhomocysteine lyase
G2583_3335	ncRNA	3376703	3376777	+	sraD	ncRNA
G2583_3336	CDS	3376785	3378341	-	gshA	Glutamate--cysteine ligase
G2583_3337	CDS	3378414	3378842	-	yqaA	hypothetical protein
G2583_3338	CDS	3378839	3379405	-	yqaB	HAD-superfamily hydrolase, subfamily IA
G2583_3339	tRNA	3379685	3379763	-	-	Arg tRNA
G2583_3340	tRNA	3379864	3379942	-	-	Arg tRNA
G2583_3341	tRNA	3380003	3380081	-	-	Arg tRNA
G2583_3342	tRNA	3380144	3380222	-	-	Arg tRNA
G2583_3343	tRNA	3380224	3380318	-	-	Ser tRNA
G2583_3344	CDS	3380633	3380818	-	csrA	Carbon storage regulator-like protein
G2583_3345	CDS	3381053	3383683	-	alaS	Alanyl-tRNA synthetase
G2583_3346	CDS	3383811	3384311	-	recX	regulatory protein RecX
G2583_3347	CDS	3384379	3385440	-	recA	RecA/GFP fusion protein
G2583_3348	CDS	3385520	3386017	-	ygaD	Competence/damage-inducible protein CinA domain protein
G2583_3349	CDS	3386162	3387247	-	mltB	Membrane-bound lytic murein transglycosylase B
G2583_3350	CDS	3387502	3388065	+	srlA	Glucitol/sorbitol permease IIC component
G2583_3351	CDS	3388062	3389021	+	srlE	Phosphotransferase system sorbitol-specific component IIBC

Locus_tag	Type	Start	End	+/-[a]	Gene[b]	Product
G2583_3352	CDS	3389032	3389403	+	srlB	sorbitol-6-phosphate dehydrogenase
G2583_3353	CDS	3389407	3390186	+	srlD	Sorbitol-6-phosphate 2-dehydrogenase
G2583_3354	CDS	3390292	3390651	+	gutM	Glucitol operon activator protein
G2583_3355	CDS	3390718	3391491	+	srlR	Glucitol operon repressor
G2583_3356	CDS	3391484	3392449	+	gutQ	D-arabinose 5-phosphate isomerase
G2583_3357	CDS	3392446	3393960	-	norR	Anaerobic nitric oxide reductase transcription regulator norR
G2583_3358	CDS	3394147	3395586	+	norV	Uncharacterized flavoproteins
G2583_3359	CDS	3395583	3396716	+	norW	Nitric oxide reductase FlRd-NAD(+) reductase
G2583_3360	CDS	3396844	3399144	-	hypF	Carbamoyltransferase HypF
G2583_3361	CDS	3399249	3399776	-	hydN	Electron transport protein hydN
G2583_3362	CDS	3399925	3400977	-	ascG	transcriptional regulator AscG
G2583_3363	CDS	3401195	3402652	+	ascF	PTS system enzyme II ABC (Asc), cryptic, transports specific
G2583_3364	CDS	3402661	3404085	+	ascB	6-phospho-beta-glucosidase
G2583_3365	CDS	3404209	3404679	-	hycI	Hydrogenase 3 maturation protease
G2583_3366	CDS	3404672	3405082	-	hycH	Formate hydrogenlyase maturation protein hycH
G2583_3367	CDS	3405079	3405846	-	hycG	Formate hydrogenlyase, subunit G
G2583_3368	CDS	3405846	3406388	-	hycF	Formate hydrogenlyase, subunit F
G2583_3369	CDS	3406398	3408128	-	hycE	Formate hydrogenlyase, subunit E
G2583_3370	CDS	3408125	3409048	-	hycD	Membrane-spanning protein of hydrogenase 3
G2583_3371	CDS	3409051	3410877	-	hycC	Formate hydrogenlyase, subunit C
G2583_3372	CDS	3410874	3411485	-	hycB	Formate hydrogenlyase, subunit B
G2583_3373	CDS	3411610	3412071	-	hycA	Formate hydrogenlyase regulatory protein hycA
G2583_3374	CDS	3412105	3412242	-	-	hypothetical protein
G2583_3375	CDS	3412271	3412633	+	hypA	Hydrogenase nickel insertion protein HypA
G2583_3376	CDS	3412637	3413509	+	hypB	Hydrogenase isoenzymes nickel incorporation protein hypB
G2583_3377	CDS	3413500	3413772	+	hypC	Hydrogenase isoenzymes formation protein hypC
G2583_3378	CDS	3413772	3414893	+	hypD	Hydrogenase expression/formation protein HypD
G2583_3379	CDS	3414890	3415900	+	hypE	Hydrogenase maturation factor
G2583_3380	CDS	3415974	3418052	+	fhlA	Formate hydrogenlyase transcriptional activator
G2583_3381	CDS	3418089	3418442	-	ygbA	hypothetical protein
G2583_3382	CDS	3418519	3418653	-	-	hypothetical protein
G2583_3383	CDS	3418729	3421290	+	mutS	DNA mismatch repair protein mutS
G2583_3384	CDS	3421396	3422052	+	pphB	O218 protein
G2583_3385	CDS	3422093	3422329	-	kpdD	4-hydroxybenzoate decarboxylase, subunit D
G2583_3386	CDS	3422340	3423767	-	kpdC	YclC protein
G2583_3387	CDS	3423767	3424360	-	padI	Probable aromatic acid decarboxylase
G2583_3388	CDS	3424474	3424914	+	-	putative regulator
G2583_3389	CDS	3425034	3426026	-	rpoS	RNA polymerase sigma factor
G2583_3390	CDS	3426089	3427228	-	nlpD	Lipoprotein
G2583_3391	CDS	3427368	3427994	-	pcm	Protein-L-isoaspartate O-methyltransferase
G2583_3392	CDS	3427988	3428749	-	surE	Multifunctional protein surE [Includes: 5'/3'-nucleotidase
G2583_3393	CDS	3428730	3429779	-	truD	tRNA pseudouridine synthase D
G2583_3394	CDS	3429776	3430255	-	ispF	2-C-methyl-D-erythritol 2,4-cyclodiphosphate synthase
G2583_3395	CDS	3430255	3430965	-	ispD	2-C-methyl-D-erythritol 4-phosphate cytidylyltransferase
G2583_3396	CDS	3430984	3431295	-	ftsB	Cell division protein ftsB-like protein
G2583_3397	CDS	3431489	3431812	-	ygbE	hypothetical protein
G2583_3398	CDS	3431862	3432467	-	cysC	Adenylyl-sulfate kinase
G2583_3399	CDS	3432467	3433894	-	cysN	Sulfate adenylyltransferase subunit 1
G2583_3400	CDS	3433896	3434804	-	cysD	Sulfate adenylyltransferase subunit 2
G2583_3401	CDS	3435056	3436093	+	iap	Alkaline phosphatase isozyme conversion peptidase
G2583_3402	CDS	3436483	3436776	-	ygbF	CRISPR-associated protein Cas2
G2583_3403	CDS	3436773	3437696	-	ygbT	CRISPR-associated protein Cas1
G2583_3404	CDS	3437693	3438343	-	cse	CRISPR-associated protein, Cse3 family
G2583_3405	CDS	3438325	3439071	-	ygcI	CRISPR-associated protein Cas5
G2583_3406	CDS	3439082	3440137	-	ygcJ	CRISPR-associated protein, Cse4 family
G2583_3407	CDS	3440149	3440685	-	cse	CRISPR-associated protein, Cse2 family
G2583_3408	CDS	3440682	3442244	-	ygcL	CRISPR-associated protein, Cse1 family
G2583_3409	CDS	3442342	3445041	-	ygcB	CRISPR-associated helicase Cas3
G2583_3410	CDS	3445233	3445520	-	small	Small toxic membrane polypeptide
G2583_3411	ncRNA	3445569	3445643	-	-	ncRNA

Locus_tag	Type	Start	End	+/-[a]	Gene[b]	Product
G2583_3412	CDS	3445649	3446383	-	cysH	Phosphoadenosine phosphosulfate reductase
G2583_3413	CDS	3446457	3448169	-	cysI	Sulfite reductase [NADPH] hemoprotein beta-component
G2583_3414	CDS	3448169	3449968	-	cysJ	Sulfite reductase [NADPH] flavoprotein alpha-component
G2583_3415	CDS	3450200	3450649	+	sscR	Putative 6-pyruvoyl tetrahydrobiopterin synthase
G2583_3416	CDS	3450697	3451998	+	ygcN	hypothetical protein
G2583_3417	CDS	3451953	3452249	+	ygcO	hypothetical protein
G2583_3418	CDS	3452266	3452841	+	ygcP	Glycerol-3-phosphate responsive antiterminator
G2583_3419	pseudogene	3452989	3453849	-	ygcQ	Electron transfer flavoprotein
G2583_3420	CDS	3453846	3454631	-	ygcR	Electron transfer flavoprotein
G2583_3421	CDS	3454603	3456012	-	ygcS	Permease (major facilitator superfamily)
G2583_3422	CDS	3456034	3457488	-	ygcU	Uncharacterized flavoprotein ygcU
G2583_3423	CDS	3457558	3458418	-	ygcW	Hypothetical oxidoreductase ygcW
G2583_3424	CDS	3458662	3459792	+	yqcE	putative transport protein
G2583_3425	CDS	3459966	3461444	+	ygcE	Carbohydrate kinase, FGGY family protein
G2583_3426	CDS	3462632	3463303	-	ygcF	hypothetical protein
G2583_3427	CDS	3463584	3464078	+	LemA	LemA family protein
G2583_3428	CDS	3464092	3464985	+	-	hypothetical protein
G2583_3429	CDS	3465000	3466148	+	-	hypothetical protein
G2583_3430	CDS	3466154	3467059	+	ygcG	hypothetical protein
G2583_3431	CDS	3467119	3468417	-	eno	Enolase
G2583_3432	CDS	3468505	3470142	-	pyrG	CTP synthase
G2583_3433	CDS	3470370	3471161	-	mazG	Nucleoside triphosphate pyrophosphohydrolase
G2583_3434	CDS	3471232	3471567	-	chpA	PemK-like protein 1
G2583_3435	CDS	3471567	3471815	-	chpR	PemI-like protein 1
G2583_3436	CDS	3471893	3474127	-	relA	GTP diphosphokinase
G2583_3437	CDS	3474175	3475476	-	rumA	23S rRNA (uracil-5-)-methyltransferase rumA (23S rRNA(M- 5-
G2583_3438	CDS	3475533	3478289	+	barA	Signal transduction histidine-protein kinase barA
G2583_3439	CDS	3478522	3479862	-	gudD	Glucarate dehydratase
G2583_3440	CDS	3479883	3481223	-	gudX	Glucarate dehydratase
G2583_3441	CDS	3481225	3482577	-	gudP	Glucarate permease
G2583_3442	CDS	3483012	3483461	-	yqcA	flavodoxin
G2583_3443	CDS	3483479	3484261	-	truC	tRNA pseudouridine synthase C
G2583_3444	CDS	3484261	3484590	-	yqcC	hypothetical protein
G2583_3445	ncRNA	3484633	3484992	-	csrB	ncRNA
G2583_3446	CDS	3485212	3485757	-	syd	SecY interacting protein Syd
G2583_3447	CDS	3485825	3486673	+	queF	NADPH-dependent 7-cyano-7-deazaguanine reductase (7-
G2583_3448	CDS	3486785	3488149	+	ygdH	hypothetical protein
G2583_3449	CDS	3488706	3489995	+	sdaC	Serine transporter family protein
G2583_3450	CDS	3490053	3491420	+	sdaB	L-serine ammonia-lyase 2
G2583_3451	CDS	3491442	3492287	+	xni	Uncharacterized exonuclease xni
G2583_3452	CDS	3492342	3493493	-	fucO	Lactaldehyde reductase
G2583_3453	CDS	3493518	3494165	-	fucA	L-fuculose phosphate aldolase
G2583_3454	CDS	3494712	3496028	+	fucP	L-fucose:H+ symporter permease
G2583_3455	CDS	3496061	3497836	+	fucI	L-fucose isomerase
G2583_3456	CDS	3497915	3499363	+	fucK	L-fuculokinase
G2583_3457	CDS	3499365	3499787	+	fucU	Fucose operon fucU protein
G2583_3458	CDS	3499845	3500576	+	fucR	L-fucose operon activator
G2583_3459	CDS	3500620	3501618	-	ygdE	Putative RNA 2'-O-ribose methyltransferase ygdE
G2583_3460	CDS	3501712	3502107	-	ygdD	UPF0382 inner membrane protein ygdD
G2583_3461	CDS	3502126	3503043	-	gcvA	Glycine cleavage system transcriptional activator
G2583_3462	ncRNA	3503172	3503318	+	gcvB	ncRNA
G2583_3463	CDS	3503394	3503624	-	ygdI	hypothetical protein
G2583_3464	CDS	3503813	3505018	+	csdA	Cysteine desulfurase, catalytic subunit CsdA
G2583_3465	CDS	3505018	3505461	+	csdE	Uncharacterized sufE-like protein ygdK
G2583_3466	CDS	3505512	3506318	-	ygdL	Putative enzyme
G2583_3467	CDS	3506557	3507855	-	mltA	Membrane-bound lytic murein transglycosylase A
G2583_3468	tRNA	3507863	3507939	+	-	Met tRNA
G2583_3469	tRNA	3507973	3508049	+	-	Met tRNA
G2583_3470	tRNA	3508083	3508159	+	-	Met tRNA
G2583_3471	CDS	3508233	3509576	-	amiC	Putative amidase

Locus_tag	Type	Start	End	+/-[a]	Gene[b]	Product
G2583_3472	CDS	3509718	3511049	+	argA	Amino-acid acetyltransferase
G2583_3473	CDS	3511111	3512937	-	recD	Exodeoxyribonuclease V, alpha subunit
G2583_3474	CDS	3512937	3516479	-	recB	Exodeoxyribonuclease V, beta subunit
G2583_3475	CDS	3516472	3519360	-	ptrA	Protease III
G2583_3476	CDS	3519536	3522904	-	recC	Exodeoxyribonuclease V, gamma subunit
G2583_3477	CDS	3522917	3523240	-	ppdC	Prepilin peptidase-dependent protein C
G2583_3478	CDS	3523225	3523632	-	ygdB	hypothetical protein
G2583_3479	CDS	3523629	3524192	-	ppdB	Prepilin peptidase dependent protein B
G2583_3480	CDS	3524183	3524653	-	ppdA	Prepilin peptidase-dependent protein A
G2583_3481	CDS	3524837	3525631	-	thyA	Thymidylate synthase
G2583_3482	CDS	3525638	3526513	-	lgt	Prolipoprotein diacylglyceryl transferase
G2583_3483	CDS	3526664	3528910	-	ptsP	Phosphoenolpyruvate-protein phosphotransferase
G2583_3484	CDS	3528923	3529453	-	nudH	RNA pyrophosphohydrolase ((Di)nucleoside polyphosphate
G2583_3485	CDS	3529806	3529952	-	ygdT	hypothetical protein
G2583_3486	CDS	3530138	3530827	+	mutH	DNA mismatch repair protein mutH
G2583_3487	CDS	3530896	3531609	+	ygdQ	UPF0053 inner membrane protein ygdQ
G2583_3488	CDS	3531747	3531965	+	ygdR	Uncharacterized lipoprotein ygdR precursor
G2583_3489	CDS	3532073	3533113	+	tas	predicted oxidoreductase, NADP(H)-dependent aldo-keto
G2583_3490	CDS	3533145	3534338	-	lplT	hypothetical protein
G2583_3491	CDS	3534331	3536490	-	aas	2-acyl-glycerophospho-ethanolamine acyltransferase
G2583_3492	ncRNA	3536578	3536665	-	-	ncRNA
G2583_3493	ncRNA	3536781	3536862	-	-	ncRNA
G2583_3494	CDS	3537076	3538107	+	galR	DNA-binding transcriptional repressor
G2583_3495	CDS	3538114	3539376	-	lysA	Diaminopimelate decarboxylase
G2583_3496	CDS	3539498	3540433	+	lysR	DNA-binding transcriptional regulator LysR
G2583_3497	CDS	3540420	3541112	-	ygeA	Putative resistance proteins
G2583_3498	CDS	3541241	3542659	-	araE	Arabinose-proton symporter
G2583_3499	CDS	3542974	3543735	-	kduD	2-deoxy-D-gluconate 3-dehydrogenase
G2583_3500	CDS	3543765	3544601	-	kduI	4-deoxy-L-threo-5-hexosulose-uronate ketol-isomerase
G2583_3501	CDS	3544888	3546069	-	yqeF	Acetyl-CoA acetyltransferase
G2583_3502	CDS	3546324	3547553	+	yqeG	putative transporter protein
G2583_3503	CDS	3547953	3548645	+	yqeH	hypothetical protein
G2583_3504	CDS	3548979	3549788	+	yqeI	putative sensory transducer
G2583_3505	CDS	3549781	3550263	+	yqeJ	hypothetical protein
G2583_3506	CDS	3550412	3550849	-	yqeK	hypothetical protein
G2583_3507	CDS	3550977	3551213	-	-	hypothetical protein
G2583_3508	CDS	3551267	3551758	+	ygeF	hypothetical protein
G2583_3509	CDS	3551984	3552475	+	ygeG	Tetratricopeptide repeat protein
G2583_3510	pseudogene	3552809	3553099	+	ygeH	transcriptional regulatory protein
G2583_3511	CDS	3553066	3553233	-	-	flagellum-specific ATP synthase
G2583_3512	CDS	3553238	3555298	-	EivA	Type III secretion apparatus protein
G2583_3513	CDS	3555305	3556429	-	EivE	Putative secreted protein
G2583_3514	CDS	3556434	3558137	-	EivG	Putative type III secretion apparatus protein
G2583_3515	CDS	3558134	3558883	-	invF	Putative type III secretion apparatus regulatory protein
G2583_3516	CDS	3559229	3559408	-	-	hypothetical protein
G2583_3517	pseudogene	3559475	3561096	-	-	hypothetical protein
G2583_3518	tRNA	3561317	3561392	-	-	Gly tRNA
G2583_3519	CDS	3561470	3562225	-	ygeR	Hypothetical lipoprotein YgeR
G2583_3520	CDS	3562641	3564938	+	xdhA	Xanthine dehydrogenase
G2583_3521	CDS	3564949	3565827	+	xdhB	Xanthine dehydrogenase FAD-binding subunit
G2583_3522	CDS	3565824	3566303	+	xdhC	Xanthine dehydrogenase iron-sulfur-binding subunit
G2583_3523	CDS	3566343	3568121	-	ygeV	Putative transcriptional regulator
G2583_3524	CDS	3568597	3569787	+	ygeW	hypothetical protein
G2583_3525	CDS	3569845	3571041	+	ygeX	Putative diaminopropionate ammonia-lyase
G2583_3526	CDS	3571099	3572310	+	ygeY	peptidase
G2583_3527	CDS	3572351	3573748	+	hyuA	D-phenylhydantoinase
G2583_3528	CDS	3573796	3574728	+	yqeA	Carbamate kinase arcC-like protein
G2583_3529	CDS	3574769	3576394	-	yqeB	Putative xanthine dehydrogenase accessory factor
G2583_3530	CDS	3576442	3577212	-	yqeC	Probable selenium-dependent hydroxylase accessory protein
G2583_3531	CDS	3577315	3577893	+	ygfJ	Molybdenum hydroxylase accessory protein, YgfJ family

Locus_tag	Type	Start	End	+/-[a]	Gene[b]	Product
G2583_3532	CDS	3578215	3581313	+	ygfK	putative oxidoreductase, Fe-S subunit
G2583_3533	CDS	3581316	3582644	+	ssnA	Cytosine deaminase and related metal-dependent hydrolases
G2583_3534	CDS	3582694	3583473	+	ygfM	hypothetical protein
G2583_3535	CDS	3583470	3586340	+	xdhD	Probable hypoxanthine oxidase xdhD
G2583_3536	CDS	3586448	3587905	+	ygfO	Putative purine permease ygfO
G2583_3537	CDS	3587920	3589239	+	guaD	guanine deaminase
G2583_3538	CDS	3589275	3590642	+	ygfQ	hypothetical protein
G2583_3539	CDS	3590678	3591166	-	ygfS	Putative electron transport protein ygfS
G2583_3540	CDS	3591166	3593100	-	ygfT	putative oxidoreductase Fe-S binding subunit
G2583_3541	CDS	3593452	3594969	+	ygfU	Putative xanthine permease
G2583_3542	CDS	3595219	3595767	+	idi	Isopentenyl-diphosphate Delta-isomerase
G2583_3543	CDS	3595810	3597327	-	lysS	Lysyl-tRNA synthetase
G2583_3544	pseudogene	3597337	3598218	-	prfB	Peptide chain release factor 2
G2583_3545	CDS	3598526	3600259	-	recJ	Single-stranded-DNA-specific exonuclease RecJ
G2583_3546	CDS	3600265	3600975	-	dsbC	Thiol:disulfide interchange protein dsbC precursor
G2583_3547	CDS	3601000	3601896	-	xerD	Tyrosine recombinase xerD
G2583_3548	CDS	3602008	3602529	+	fldB	Flavodoxin-2
G2583_3549	CDS	3602569	3602976	-	ygfX	hypothetical protein
G2583_3550	CDS	3602957	3603223	-	ygfY	UPF0350 protein ygfY
G2583_3551	CDS	3603466	3604446	+	ygfZ	tRNA-modifying protein ygfZ
G2583_3552	CDS	3604523	3605182	-	yqfA	UPF0073 inner membrane protein yqfA
G2583_3553	CDS	3605346	3605657	-	yqfB	Uncharacterized protein conserved in bacteria
G2583_3554	CDS	3605696	3607135	+	bglA	6-phospho-beta-glucosidase BglA
G2583_3555	CDS	3607301	3610174	-	gcvP	Glycine dehydrogenase [decarboxylating]
G2583_3556	CDS	3610292	3610681	-	gcvH	Glycine cleavage system H protein
G2583_3557	CDS	3610705	3611799	-	gcvT	Glycine cleavage complex protein T, aminomethyltransferase,
G2583_3558	CDS	3612247	3613449	-	visC	Ubiquinone biosynthesis hydroxylase, UbiH/UbiF/VisC/COQ6
G2583_3559	CDS	3613473	3614651	-	ubiH	2-octaprenyl-6-methoxyphenol 4-monoxygenase
G2583_3560	CDS	3614648	3615973	-	pepP	Xaa-Pro aminopeptidase
G2583_3561	CDS	3615999	3616577	-	ygfB	UPF0149 protein ygfB
G2583_3562	CDS	3616745	3617074	+	zapA	Z-ring-associated protein
G2583_3563	ncRNA	3617116	3617299	+	ssrS	ncRNA
G2583_3564	CDS	3617320	3617922	+	ygfA	Putative ligase
G2583_3565	ncRNA	3617948	3618098	+	-	ncRNA
G2583_3566	CDS	3618095	3618325	-	-	hypothetical protein
G2583_3567	ncRNA	3618341	3618491	+	-	ncRNA
G2583_3568	CDS	3618704	3619936	-	serA	D-3-phosphoglycerate dehydrogenase
G2583_3569	CDS	3620191	3620850	-	rpiA	Ribose 5-phosphate isomerase
G2583_3570	CDS	3621233	3622126	+	argP	Chromosome initiation inhibitor
G2583_3571	CDS	3622330	3624474	+	scpA	Methylmalonyl-CoA mutase
G2583_3572	CDS	3624467	3625462	+	argK	LAO/AO transport system kinase
G2583_3573	CDS	3625473	3626258	+	scpB	Methylmalonyl-CoA decarboxylase
G2583_3574	CDS	3626282	3627760	+	scpC	Succinate CoA transferase
G2583_3575	pseudogene	3627757	3628653	-	ygfI	Uncharacterized HTH-type transcriptional regulator ygfI
G2583_3576	CDS	3628820	3629560	-	yggE	hypothetical protein
G2583_3577	CDS	3629653	3630288	-	argO	arginine exporter protein
G2583_3578	CDS	3630427	3631287	-	mscS	Small-conductance mechanosensitive channel
G2583_3579	CDS	3631645	3632724	-	fbaA	Fructose-bisphosphate aldolase
G2583_3580	CDS	3632939	3634102	-	pgk	Phosphoglycerate kinase
G2583_3581	CDS	3634152	3635171	-	epd	D-erythrose-4-phosphate dehydrogenase
G2583_3582	CDS	3635543	3635974	+	-	hypothetical protein
G2583_3583	CDS	3635997	3636575	+	-	hypothetical protein
G2583_3584	CDS	3636576	3637283	+	-	hypothetical protein
G2583_3585	CDS	3637265	3637948	+	-	ABC transporter, ATP-binding protein
G2583_3586	CDS	3637909	3638619	+	-	putative ATP-binding protein of ABC transport system
G2583_3587	CDS	3638591	3639304	-	yggC	putative fructose transport system kinase
G2583_3588	CDS	3639301	3639810	-	yggD	Mannitol operon repressor
G2583_3589	CDS	3639832	3640797	-	yggF	Fructose-1,6-bisphosphatase, class II
G2583_3590	CDS	3640794	3642071	-	yggP	L-sorbose 1-phosphate reductase
G2583_3591	CDS	3642086	3643474	-	cmtA	PTS system mannitol-specific EIICB component (EIICB-Mtl)

Locus_tag	Type	Start	End	+/-[a]	Gene[b]	Product
G2583_3592	CDS	3643502	3643945	-	cmtB	hypothetical protein
G2583_3593	CDS	3644259	3646250	-	tktA	transketolase
G2583_3594	CDS	3646402	3647286	+	yggG	hypothetical protein
G2583_3595	CDS	3647492	3648412	-	speB	agmatinase
G2583_3596	CDS	3648548	3649279	-	-	hypothetical protein
G2583_3597	CDS	3649425	3651401	-	speA	Biosynthetic arginine decarboxylase
G2583_3598	CDS	3651410	3651556	-	yqgB	hypothetical protein
G2583_3599	CDS	3651677	3651892	+	yqgC	hypothetical protein
G2583_3600	CDS	3651889	3652155	-	yqgD	hypothetical protein
G2583_3601	CDS	3652196	3653350	+	metK	S-adenosylmethionine synthetase
G2583_3602	CDS	3653787	3655181	+	galP	Galactose-proton symporter
G2583_3603	CDS	3655258	3655755	+	yggI	hypothetical protein
G2583_3604	CDS	3655850	3656557	+	endA	Endonuclease I
G2583_3605	CDS	3656637	3657368	+	rsmE	hypothetical protein
G2583_3606	CDS	3657381	3658328	+	gshB	Glutathione synthetase
G2583_3607	CDS	3658440	3659003	+	yqgE	UPF0301 protein yqgE
G2583_3608	CDS	3659003	3659419	+	yqgF	Putative Holliday junction resolvase
G2583_3609	CDS	3659610	3660635	-	yggR	Twitching motility family protein
G2583_3610	CDS	3660608	3661312	+	yggS	UPF0001 protein yggS
G2583_3611	CDS	3661330	3661896	+	yggT	putative resistance protein
G2583_3612	CDS	3661893	3662183	+	yggU	conserved hypothetical protein
G2583_3613	CDS	3662191	3662784	+	rdgB	Nucleoside-triphosphatase rdgB
G2583_3614	CDS	3662777	3663913	+	yggW	Putative oxygen-independent coproporphyrinogen III oxidase
G2583_3615	CDS	3664065	3665072	-	yggM	putative alpha helix chain
G2583_3616	CDS	3665189	3666235	-	ansB	L-asparaginase 2
G2583_3617	CDS	3666411	3667130	-	yggN	hypothetical protein
G2583_3618	CDS	3667314	3667640	-	yggL	hypothetical protein
G2583_3619	CDS	3667640	3668359	-	trmI	tRNA (Guanine-N(7)-)-methyltransferase
G2583_3620	CDS	3668520	3669572	+	mutY	A/G-specific adenine glycosylase
G2583_3621	CDS	3669600	3669875	+	yggX	Probable Fe(2+)-trafficking protein
G2583_3622	CDS	3669937	3671019	+	mltC	Membrane-bound lytic murein transglycosylase C precursor
G2583_3623	CDS	3671173	3672477	+	nupG	Transport of nucleosides, permease protein
G2583_3624	CDS	3672527	3674722	-	speC	Ornithine decarboxylase isozyme
G2583_3625	CDS	3675060	3675767	+	yqgA	putative transport protein
G2583_3626	tRNA	3675872	3675949	+	-	Phe tRNA
G2583_3627	CDS	3676146	3677411	+	intB	Site-specific recombinase, phage integrase family protein
G2583_3628	CDS	3677528	3678313	-	-	unknown protein encoded by ISEc8
G2583_3629	CDS	3678402	3679076	+	ISSfl4	ISSfl3 OrfA
G2583_3630	CDS	3679073	3679420	+	ISSfl4	ISSfl4 ORF2
G2583_3631	CDS	3679440	3680888	+	ISSfl4	Transposase and inactivated derivatives
G2583_3632	CDS	3680689	3681633	-	-	DNA helicase II
G2583_3633	CDS	3681742	3682383	-	PagC	PagC-like membrane protein
G2583_3634	CDS	3682864	3683064	-	-	hypothetical protein
G2583_3635	pseudogene	3683215	3684483	+	-	ISEc13 transposase
G2583_3636	CDS	3684836	3685063	+	-	ST51 protein
G2583_3637	CDS	3685271	3686920	+	ospD	Ent protein
G2583_3638	pseudogene	3687528	3688517	+	-	NleB
G2583_3639	CDS	3688566	3689240	+	NleE	ST47 protein
G2583_3640	CDS	3689999	3690463	+	-	Putative transposase
G2583_3641	CDS	3691115	3700786	+	lifA	EHEC factor for adherence
G2583_3642	CDS	3700958	3701353	+	-	Transposase and inactivated derivatives
G2583_3643	CDS	3701440	3701865	+	-	Transposase and inactivated derivatives
G2583_3644	CDS	3701862	3702212	+	-	IS66 family transposase orfB
G2583_3645	CDS	3702425	3703828	+	-	Transposase and inactivated derivatives
G2583_3646	CDS	3703848	3704105	+	-	IS3
G2583_3647	CDS	3704250	3704447	-	yagK	hypothetical protein
G2583_3648	CDS	3704692	3705117	-	-	hypothetical protein
G2583_3649	CDS	3705114	3705497	-	-	DNA-directed RNA polymerase, beta subunit/140 kD subunit
G2583_3650	CDS	3705665	3705874	-	-	hypothetical protein
G2583_3651	CDS	3705871	3706197	-	yagK	hypothetical protein

Locus_tag	Type	Start	End	+/-[a]	Gene[b]	Product
G2583_3652	CDS	3706397	3706591	+	-	hypothetical protein
G2583_3653	CDS	3706840	3707028	-	-	hypothetical protein
G2583_3654	CDS	3707743	3707949	+	-	Predicted transcriptional regulator
G2583_3655	CDS	3708044	3708646	+	-	hypothetical protein
G2583_3656	CDS	3708936	3709232	+	-	ORFa1203
G2583_3657	CDS	3709177	3710064	+	-	IS629 transposase orfB
G2583_3658	CDS	3710650	3710778	+	-	hypothetical protein
G2583_3659	CDS	3711105	3711977	+	-	hypothetical protein
G2583_3660	CDS	3712076	3712948	+	yfjP	hypothetical protein
G2583_3661	CDS	3713320	3716133	+	flu	Type V secretory pathway, adhesin AidA
G2583_3662	CDS	3716165	3716512	+	insN	unknown protein encoded by IS911 within prophage CP-933L
G2583_3663	CDS	3716725	3717378	+	-	putative transposase
G2583_3664	CDS	3717463	3717657	+	ykfF	hypothetical protein
G2583_3665	CDS	3717812	3718630	+	-	hypothetical protein
G2583_3666	CDS	3718721	3719206	+	yfjX	hypothetical protein
G2583_3667	CDS	3719221	3719697	+	yeeS	DNA repair protein, RadC family
G2583_3668	CDS	3719760	3719981	+	yeeT	hypothetical protein
G2583_3669	CDS	3720055	3720423	+	yeeU	Putative structural protein
G2583_3670	CDS	3720512	3720775	+	yeeV	hypothetical protein
G2583_3671	CDS	3720885	3721148	+	yeeW	hypothetical protein
G2583_3672	CDS	3721263	3722804	+	tnpA	Transposase for ISEc12
G2583_3673	CDS	3722816	3723565	+	tnpA	TnpA
G2583_3674	CDS	3723717	3723959	+	-	hypothetical protein
G2583_3675	CDS	3723926	3724168	+	-	hypothetical protein
G2583_3676	CDS	3724253	3725095	+	-	hypothetical protein
G2583_3677	CDS	3725376	3725912	-	yghD	Putative general secretion pathway protein M-type yghD
G2583_3678	CDS	3725914	3727092	-	gspL	Type II secretory pathway, component PulL
G2583_3679	CDS	3727089	3728066	-	gspK	General secretion pathway protein K
G2583_3680	CDS	3728063	3728632	-	gspJ	General secretion pathway protein GspJ
G2583_3681	CDS	3728665	3729036	-	gspI	General secretion pathway protein GspI
G2583_3682	CDS	3729033	3729596	-	gspH	General secretion pathway protein H
G2583_3683	CDS	3729600	3730055	-	gspG	General secretion pathway protein G
G2583_3684	CDS	3730072	3731295	-	gspF	Hypothetical type II secretion protein
G2583_3685	CDS	3731295	3732788	-	gspE	Type II secretory pathway, ATPase PulE/Tfp pil us assembly
G2583_3686	CDS	3732788	3734848	-	gspD	General secretion pathway protein D
G2583_3687	CDS	3734878	3735837	-	gspC	General secretion pathway protein C
G2583_3688	CDS	3735855	3736265	-	yghG	Hypothetical lipoprotein
G2583_3689	CDS	3736331	3737266	-	pppA	Putative prepilin peptidase A
G2583_3690	CDS	3737467	3742026	-	yghJ	Putative lipoprotein acfD-like protein precursor
G2583_3691	CDS	3742177	3742359	+	-	hypothetical protein
G2583_3692	CDS	3742374	3742496	-	-	hypothetical protein
G2583_3693	CDS	3742511	3744193	-	glcA	Glycolate permease glcA
G2583_3694	CDS	3744548	3746719	-	glcB	Malate synthase G
G2583_3695	CDS	3746741	3747145	-	glcG	hypothetical protein
G2583_3696	CDS	3747150	3748373	-	glcF	Glycolate oxidase iron-sulfur subunit
G2583_3697	CDS	3748384	3749436	-	glcE	Glycolate oxidase, subunit GlcE
G2583_3698	CDS	3749436	3750935	-	glcD	Glycolate oxidase subunit glcD
G2583_3699	CDS	3751186	3751950	+	glcC	Glc operon transcriptional activator
G2583_3700	CDS	3751957	3753099	-	yghO	hypothetical protein
G2583_3701	CDS	3753503	3755191	+	-	AMP-binding enzyme
G2583_3702	CDS	3755188	3756102	+	-	hypothetical protein
G2583_3703	CDS	3756155	3756382	+	-	Phosphopantetheine-binding
G2583_3704	CDS	3756382	3757554	+	-	7-keto-8-aminopelargonate synthetase and relat ed enzymes
G2583_3705	CDS	3757589	3758668	-	-	Permease YjgP/YjgQ family protein precursor
G2583_3706	CDS	3758665	3759735	-	-	Permease YjgP/YjgQ family protein precursor
G2583_3707	CDS	3759766	3760326	-	-	Protein ytfJ-like
G2583_3708	CDS	3760338	3761162	-	-	hypothetical protein
G2583_3709	CDS	3761162	3762511	-	yghQ	Putative polysaccharide biosynthesis protein
G2583_3710	CDS	3762557	3763315	-	yghR	Thymidylate kinase
G2583_3711	CDS	3763347	3764060	-	yghS	Uncharacterized ATP-binding protein yghS

Locus_tag	Type	Start	End	+/-[a]	Gene[b]	Product
G2583_3712	CDS	3764234	3764668	+	yghT	Uncharacterized ATP-binding protein yghT
G2583_3713	CDS	3764975	3766474	-	pitB	Probable low-affinity inorganic phosphate transporter 2
G2583_3714	CDS	3766766	3768625	-	gsp	Glutathionylspermidine amidase/glutathionylspermidine
G2583_3715	CDS	3768782	3769696	+	yghU	putative glutathione S-transferase YghU
G2583_3716	CDS	3769819	3770067	-	hybG	Hydrogenase-2 operon protein hybG
G2583_3717	CDS	3770080	3770421	-	hybF	Probable hydrogenase nickel incorporation protein hybF
G2583_3718	CDS	3770414	3770902	-	hybE	Hydrogenase-2 operon protein hybE
G2583_3719	CDS	3770895	3771389	-	hybD	predicted maturation element for hydrogenase 2
G2583_3720	CDS	3771389	3773092	-	hybC	Hydrogenase-2 large chain precursor
G2583_3721	CDS	3773089	3774267	-	hybB	Probable Ni/Fe-hydrogenase 2 b-type cytochrome subunit
G2583_3722	CDS	3774257	3775243	-	hybA	Hydrogenase-2 operon protein hybA precursor
G2583_3723	CDS	3775246	3776364	-	hybO	Hydrogenase-2 small chain precursor
G2583_3724	CDS	3776553	3776840	-	yghW	hypothetical protein
G2583_3725	CDS	3776959	3777885	-	yghYX	Putative enzyme
G2583_3726	CDS	3778003	3779043	+	yghZ	aldo-keto reductase
G2583_3727	CDS	3779083	3779577	-	yqhA	UPF0114 protein yqhA
G2583_3728	CDS	3779768	3780652	+	yghA	Uncharacterized oxidoreductase yghA
G2583_3729	CDS	3780924	3781349	-	exbD	Biopolymer transport protein exbD
G2583_3730	CDS	3781356	3782090	-	exbB	Biopolymer transport protein exbB
G2583_3731	CDS	3782083	3782238	-	-	hypothetical protein
G2583_3732	CDS	3782342	3783529	+	metC	Cystathionine beta-lyase
G2583_3733	CDS	3783669	3784328	+	yghB	Inner membrane protein yghB
G2583_3734	CDS	3784368	3785495	-	yqhC	Putative ARAC-type regulatory protein
G2583_3735	CDS	3785461	3786624	+	yqhD	Alcohol dehydrogenase yqhD
G2583_3736	CDS	3786729	3787556	+	dkgA	2,5-diketo-D-gluconic acid reductase A
G2583_3737	CDS	3787756	3788682	+	yqhG	hypothetical protein
G2583_3738	CDS	3788731	3788988	+	yqhH	predicted outer membrane lipoprotein
G2583_3739	CDS	3789031	3791250	-	ygiQ	UPF0313 protein ygiQ
G2583_3740	CDS	3791361	3792773	-	sufI	repressor protein for FtsI
G2583_3741	CDS	3792848	3793585	-	plsC	1-acyl-sn-glycerol-3-phosphate acyltransferase
G2583_3742	CDS	3793819	3796077	-	parC	DNA topoisomerase 4 subunit A
G2583_3743	CDS	3796215	3797822	-	ygiS	Bacterial extracellular solute-binding protein, family 5
G2583_3744	CDS	3797931	3798413	-	ygiV	hypothetical protein
G2583_3745	CDS	3798466	3798858	-	ygiW	Protein ygiW
G2583_3746	CDS	3799010	3799669	+	qseB	DNA-binding response regulator in two-component regulatory
G2583_3747	CDS	3799666	3801015	+	qseC	sensor protein QseC
G2583_3748	CDS	3801125	3801706	+	mdaB	Modulator of drug activity B
G2583_3749	CDS	3801737	3802051	+	ygiN	Protein ygiN
G2583_3750	CDS	3802096	3802983	-	-	Iron transport system regulatory protein FitR
G2583_3751	CDS	3802980	3803990	-	-	Putative iron compound-binding protein of ABC transporter
G2583_3752	CDS	3803938	3804987	-	-	Putative iron compound permease protein of ABC transporter
G2583_3753	CDS	3804984	3805967	-	-	Iron ABC transporter, permease protein FitC
G2583_3754	CDS	3805964	3806773	-	FepC	Iron ABC transporter, ATP binding protein FitB
G2583_3755	CDS	3807147	3809288	+	-	TonB-dependent outer membrane ferric coprogen receptor FitA
G2583_3756	CDS	3809352	3811244	-	parE	DNA topoisomerase IV, B subunit
G2583_3757	CDS	3811273	3811854	-	yqiA	Esterase yqiA
G2583_3758	CDS	3811854	3812681	-	cpdA	cyclic 3',5'-adenosine monophosphate phosphodiesterase
G2583_3759	CDS	3812706	3813128	-	yqiB	hypothetical protein
G2583_3760	CDS	3813129	3813758	-	nudF	ADP-ribose pyrophosphatase
G2583_3761	CDS	3813924	3815444	+	tolC	Outer membrane protein TolC
G2583_3762	CDS	3815444	3815704	+	ygiA	hypothetical protein
G2583_3763	CDS	3815592	3816263	+	ygiB	UPF0441 protein ygiB
G2583_3764	CDS	3816269	3817429	+	ygiC	putative synthetase/amidase
G2583_3765	CDS	3817467	3818282	-	ygiD	Aromatic ring-opening dioxygenase
G2583_3766	CDS	3818398	3819171	+	zupT	Zinc transporter zupT
G2583_3767	CDS	3819229	3819399	-	-	hypothetical protein
G2583_3768	CDS	3819661	3820314	-	ribB	3,4-dihydroxy-2-butanone 4-phosphate synthase
G2583_3769	CDS	3820652	3820987	+	yqiC	hypothetical protein
G2583_3770	CDS	3821022	3821228	-	glgS	Glycogen synthesis protein glgS
G2583_3771	CDS	3821491	3822105	+	yqiJ	Putative oxidoreductase

Locus_tag	Type	Start	End	+/-[a]	Gene[b]	Product
G2583_3772	CDS	3822147	3823808	+	yqiK	SPFH/band 7 domain protein
G2583_3773	ncRNA	3824032	3824181	-	-	ncRNA
G2583_3774	CDS	3824143	3824535	+	-	hypothetical protein
G2583_3775	ncRNA	3824408	3824557	-	-	ncRNA
G2583_3776	CDS	3824602	3826035	-	rfaE	Bifunctional protein hldE [Includes: D-beta-D-heptose 7-
G2583_3777	CDS	3826083	3828923	-	glnE	bifunctional glutamine-synthetase
G2583_3778	CDS	3828946	3830247	-	ygiF	Adenylate cyclase
G2583_3779	CDS	3830489	3831109	+	htrG	putative signal transduction protein
G2583_3780	CDS	3831173	3832411	+	cca	TRNA nucleotidyltransferase
G2583_3781	CDS	3832592	3833413	-	bacA	Putative undecaprenol kinase
G2583_3782	CDS	3833504	3833875	-	folB	Dihydroneopterin aldolase
G2583_3783	CDS	3833978	3834595	+	ygiH	UPF0078 membrane protein ygiH
G2583_3784	CDS	3834608	3835540	-	ttdR	transcriptional activator TtdR
G2583_3785	CDS	3835747	3836658	+	ttdA	L(+)-tartrate dehydratase subunit alpha
G2583_3786	CDS	3836655	3837260	+	ttdB	L(+)-tartrate dehydratase subunit beta
G2583_3787	CDS	3837309	3838772	+	ttdT	L-tartrate/succinate antiporter
G2583_3788	CDS	3838815	3839828	-	ygjD	Probable O-sialoglycoprotein endopeptidase
G2583_3789	CDS	3840066	3840281	+	rpsU	Ribosomal protein S21
G2583_3790	CDS	3840392	3842137	+	dnaG	DNA primase
G2583_3791	CDS	3842332	3844173	+	rpoD	RNA polymerase sigma factor RpoD
G2583_3792	CDS	3844252	3844758	-	mug	G/U mismatch-specific DNA glycosylase
G2583_3793	tRNA	3844882	3844959	+	-	Met tRNA
G2583_3794	CDS	3845012	3845776	-	yqjH	Siderophore-interacting protein
G2583_3795	CDS	3846064	3846687	+	yqjI	transcriptional regulator, PadR family
G2583_3796	CDS	3846841	3848361	-	aer	Aerotaxis receptor
G2583_3797	CDS	3848752	3850158	+	ygjG	Ornithine/acetylornithine aminotransferase
G2583_3798	CDS	3850200	3850532	-	ygjH	T-RNA-binding domain protein
G2583_3799	CDS	3850751	3851734	+	ebgR	DNA-binding transcriptional repressor
G2583_3800	CDS	3851918	3855010	+	ebgA	Evolved beta-D-galactosidase, alpha subunit
G2583_3801	CDS	3855007	3855456	+	ebgC	Evolved beta-galactosidase subunit beta
G2583_3802	CDS	3855519	3856952	+	ygjI	Amino acid permease family protein
G2583_3803	CDS	3857086	3858156	+	ygjJ	hypothetical protein
G2583_3804	CDS	3858173	3860524	+	ygjK	predicted glycosyl hydrolase
G2583_3805	CDS	3861050	3863068	+	fadH	2,4-dienoyl-CoA reductase
G2583_3806	CDS	3863113	3863529	-	ygjM	Helix-turn-helix DNA-binding domain protein
G2583_3807	CDS	3863526	3863840	-	ygjN	hypothetical protein
G2583_3808	CDS	3864124	3865260	-	rlmG	Putative enzyme
G2583_3809	CDS	3865309	3865848	+	ygjP	hypothetical protein
G2583_3810	CDS	3865925	3866617	+	ygjQ	Putative SanA protein
G2583_3811	CDS	3866696	3867682	+	ygjR	Oxidoreductase, NAD binding
G2583_3812	CDS	3867966	3868931	+	alx	Inner membrane protein alx
G2583_3813	CDS	3869330	3870574	+	sstT	Sodium:serine/threonine symporter
G2583_3814	CDS	3870579	3871130	-	ygjV	Putative inner membrane protein
G2583_3815	CDS	3871213	3872700	-	uxaA	Altronate dehydratase
G2583_3816	CDS	3872715	3874127	-	uxaC	Uronate isomerase
G2583_3817	CDS	3874490	3875908	+	exuT	Hexuronate transporter
G2583_3818	CDS	3876038	3876814	+	exuR	Exu regulon transcriptional regulator
G2583_3819	CDS	3877159	3877821	+	yqjA	hypothetical protein
G2583_3820	CDS	3877825	3878208	+	yqjB	hypothetical protein
G2583_3821	CDS	3878340	3878723	+	yqjC	hypothetical protein
G2583_3822	CDS	3878761	3879066	+	yqjD	hypothetical protein
G2583_3823	CDS	3879069	3879473	+	yqjE	ORF_o157
G2583_3824	CDS	3879463	3879762	+	yqjK	hypothetical protein
G2583_3825	CDS	3879858	3880340	+	yqjF	Putative inner membrane protein YqjF
G2583_3826	CDS	3880410	3881396	+	yqjG	putative transferase
G2583_3827	CDS	3881688	3882053	+	yhaH	putative inner membrane protein YhaH
G2583_3828	CDS	3882295	3882651	+	yhaI	Inner membrane protein yhaI
G2583_3829	CDS	3882702	3883598	-	yhaJ	putative transcriptional regulator LYSR-type
G2583_3830	CDS	3883703	3884404	+	yhaK	Pirin-like protein yhaK
G2583_3831	CDS	3884421	3884591	+	yhaL	hypothetical protein

Locus_tag	Type	Start	End	+/-[a]	Gene[b]	Product
G2583_3832	CDS	3884725	3886035	-	yhaM	UPF0597 protein yhaM
G2583_3833	CDS	3886063	3887394	-	yhaO	putative transport system permease protein
G2583_3834	CDS	3887669	3889039	-	tdcG	L-serine ammonia-lyase TdcG
G2583_3835	CDS	3889105	3889557	-	tdcF	TdcF protein
G2583_3836	CDS	3889508	3891802	-	tdcE	Keto-acid formate acetyltransferase
G2583_3837	CDS	3891836	3893056	-	tdcD	Putative kinase
G2583_3838	CDS	3893070	3894401	-	tdcC	Threonine/serine transporter tdcC (H(+)/threonine-serine
G2583_3839	CDS	3894423	3895412	-	tdcB	Threonine dehydratase catabolic
G2583_3840	CDS	3895511	3896449	-	tdcA	DNA-binding transcriptional activator
G2583_3841	CDS	3896638	3896982	+	tdcR	Threonine dehydratase operon activator protein
G2583_3842	CDS	3897217	3897777	+	yhaB	hypothetical protein
G2583_3843	CDS	3897799	3898986	+	yhaC	hypothetical protein
G2583_3844	CDS	3899047	3899574	+	-	hypothetical protein
G2583_3845	ncRNA	3899115	3899409	-	rnpB	ncRNA
G2583_3846	CDS	3899502	3900728	-	garK	hypothetical protein
G2583_3847	CDS	3900744	3901634	-	garR	2-hydroxy-3-oxopropionate reductase
G2583_3848	CDS	3901664	3902434	-	garL	2-dehydro-3-deoxyglucarate aldolase
G2583_3849	CDS	3902450	3903784	-	garP	Probable galactarate transporter
G2583_3850	CDS	3904159	3905730	+	garD	Galactarate dehydratase
G2583_3851	CDS	3905879	3906214	+	sohA	HtrA suppressor protein
G2583_3852	CDS	3906214	3906678	+	yhaV	hypothetical protein
G2583_3853	CDS	3906733	3907542	-	agaR	Putative aga operon transcriptional repressor
G2583_3854	CDS	3907791	3909071	+	kbaZ	D-tagatose-bisphosphate aldolase, class II, non-catalytic
G2583_3855	CDS	3909058	3909567	+	agaV	N-acetylgalactosamine-specific PTS system enzyme IIB
G2583_3856	CDS	3909578	3910357	+	agaW	Putative phosphotransferase system enzyme subunit
G2583_3857	CDS	3910347	3911225	+	agaE	PTS system N-acetylgalactosamine-specific, IID component
G2583_3858	CDS	3911243	3911677	+	agaF	PTS system, mannose/sorbose-specific, IIA component
G2583_3859	CDS	3911653	3912807	+	agaA	N-acetylgalactosamine-6-phosphate deacetylase
G2583_3860	CDS	3913158	3914312	+	agaS	Putative sugar isomerase, AgaS family
G2583_3861	CDS	3914325	3915185	+	kbaY	Tagatose-1,6-bisphosphate aldolase kbaY
G2583_3862	CDS	3915352	3915828	+	agaB	PTS system, D galactosamine-specific, IIB component
G2583_3863	CDS	3915867	3916670	+	agaC	N-acetylgalactosamine permease IIC component 1
G2583_3864	CDS	3916660	3917451	+	agaD	N-acetylglucosamine transport enzyme IID component 1
G2583_3865	pseudogene	3917452	3918208	+	agaI	putative galactosamine-6-phosphate isomerase
G2583_3866	CDS	3918609	3919193	+	yraH	Putative type 1 fimbrial protein
G2583_3867	CDS	3919273	3919968	+	yraI	Gram-negative pili assembly chaperone protein
G2583_3868	CDS	3919922	3922513	+	yraJ	Fimbrial usher family protein
G2583_3869	CDS	3922326	3923615	+	yraK	Putative fimbrial protein
G2583_3870	CDS	3923658	3924518	-	yraL	UPF0011 protein yraL
G2583_3871	CDS	3924583	3926619	+	yraM	hypothetical protein
G2583_3872	CDS	3926577	3926972	+	yraN	UPF0102 protein yraN
G2583_3873	CDS	3926992	3927582	+	diaA	DnaA initiator-associating protein diaA
G2583_3874	CDS	3927592	3928167	+	yraP	hypothetical protein
G2583_3875	CDS	3928281	3929321	-	yraQ	Putative permease
G2583_3876	CDS	3929394	3930074	-	yraR	hypothetical protein
G2583_3877	CDS	3930115	3930675	+	yhbO	hypothetical protein
G2583_3878	CDS	3930655	3931098	-	yhbP	UPF0306 protein yhbP
G2583_3879	CDS	3931149	3931451	+	yhbQ	UPF0213 protein yhbQ
G2583_3880	CDS	3931438	3931941	-	yhbS	Uncharacterized acetyltransferase yhbS
G2583_3881	CDS	3931935	3932459	-	yhbT	hypothetical protein
G2583_3882	CDS	3932668	3933663	+	yhbU	Peptidase, U32 family
G2583_3883	CDS	3933654	3934550	+	yhbV	hypothetical protein
G2583_3884	CDS	3934631	3935638	+	yhbW	Monooxygenase, luciferase family
G2583_3885	CDS	3935755	3936915	-	mtr	Tryptophan-specific transport protein
G2583_3886	CDS	3937153	3939042	-	deaD	Cold-shock DEAD-box protein A
G2583_3887	CDS	3939222	3940106	-	nlpI	Lipoprotein nlpI precursor
G2583_3888	CDS	3940215	3942419	-	pnp	Polynucleotide phosphorylase/polyadenylase
G2583_3889	ncRNA	3942409	3942580	+	-	ncRNA
G2583_3890	CDS	3942597	3942866	-	rpsO	30S ribosomal protein S15
G2583_3891	CDS	3943015	3943959	-	truB	tRNA pseudouridine synthase B

Locus_tag	Type	Start	End	+/-[a]	Gene[b]	Product
G2583_3892	CDS	3943959	3944360	-	rbfA	Ribosome-binding factor A
G2583_3893	CDS	3944525	3947197	-	infB	Translation initiation factor IF-2
G2583_3894	CDS	3947222	3948709	-	nusA	Transcription elongation protein nusA
G2583_3895	CDS	3948737	3949195	-	yhbC	hypothetical protein
G2583_3896	tRNA	3949396	3949472	-	-	Met tRNA
G2583_3897	CDS	3949820	3951163	+	argG	Argininosuccinate synthase
G2583_3898	CDS	3951171	3952829	-	yhbX	Outer-membrane protein yhbX
G2583_3899	tRNA	3953254	3953342	-	-	Leu tRNA
G2583_3900	CDS	3953356	3953688	-	secG	Preprotein translocase IISP family, auxillary membrane
G2583_3901	CDS	3953916	3955253	-	glmM	Phosphoglucosamine mutase
G2583_3902	CDS	3955246	3956094	-	folP	7,8-dihydropteroate synthase
G2583_3903	CDS	3956184	3958118	-	hflB	Cell division protease ftsH
G2583_3904	CDS	3958218	3958847	-	rrmJ	Ribosomal RNA large subunit methyltransferase J (rRNA
G2583_3905	CDS	3958973	3959266	+	yhbY	hypothetical protein
G2583_3906	CDS	3959422	3959898	-	greA	Transcription elongation factor greA
G2583_3907	CDS	3960146	3961579	+	dacB	D-alanyl-D-alanine carboxypeptidase/D-alanyl-D-alanine-
G2583_3908	CDS	3961619	3962791	-	obgE	GTP-binding protein Obg/CgtA
G2583_3909	CDS	3962807	3963772	-	yhbE	Uncharacterized inner membrane transporter yhbE
G2583_3910	CDS	3963899	3964156	-	rpmA	50S ribosomal protein L27
G2583_3911	CDS	3964177	3964488	-	rplU	50S ribosomal protein L21
G2583_3912	CDS	3964747	3965718	+	ispB	hypothetical protein
G2583_3913	CDS	3965947	3966225	+	sfsB	Sugar fermentation stimulation protein B
G2583_3914	CDS	3966273	3967532	-	murA	UDP-N-acetylglucosamine 1-carboxyvinyltransferase
G2583_3915	CDS	3967587	3967856	-	yrbA	Predicted transcriptional regulator, BolA superfamily
G2583_3916	CDS	3968001	3968390	-	yrbB	hypothetical protein
G2583_3917	CDS	3968294	3968929	-	yrbC	Toluene tolerance protein Ttg2D
G2583_3918	CDS	3968948	3969499	-	yrbD	hypothetical protein
G2583_3919	CDS	3969504	3970286	-	yrbE	UPF0393 inner membrane protein yrbE
G2583_3920	CDS	3970294	3971103	-	yrbF	Uncharacterized ABC transporter ATP-binding protein yrbF
G2583_3921	CDS	3971313	3972290	+	yrbG	Sodium/calcium exchanger protein
G2583_3922	CDS	3972304	3973290	+	kdsD	Arabinose 5-phosphate isomerase
G2583_3923	CDS	3973311	3973877	+	kdsC	3-deoxy-D-manno-octulosonate 8-phosphate phosphatase
G2583_3924	CDS	3973874	3974449	+	yrbK	hypothetical protein
G2583_3925	CDS	3974418	3974975	+	lptA	Protein yhbN precursor
G2583_3926	CDS	3974982	3975707	+	lptB	Uncharacterized ABC transporter ATP-binding protein yhbG
G2583_3927	CDS	3975755	3977188	+	rpoN	RNA polymerase sigma-54 factor
G2583_3928	CDS	3977211	3977498	+	hpf	Probable sigma(54) modulation protein
G2583_3929	CDS	3977616	3978107	+	ptsN	PTS IIA-like nitrogen-regulatory protein PtsN
G2583_3930	CDS	3978153	3979007	+	yhbJ	UPF0042 protein yhbJ
G2583_3931	CDS	3979004	3979276	+	npr	Phosphocarrier protein NPr
G2583_3932	CDS	3979490	3980122	+	yrbL	hypothetical protein
G2583_3933	CDS	3980119	3980847	-	mtgA	Monofunctional biosynthetic peptidoglycan transglycosylase
G2583_3934	CDS	3980844	3981506	-	elbB	Sigma cross-reacting protein 27A
G2583_3935	ncRNA	3981615	3981722	+	-	ncRNA
G2583_3936	CDS	3981727	3984063	-	arcB	Aerobic respiration control sensor protein arcB
G2583_3937	CDS	3984159	3985088	-	yhcC	hypothetical protein
G2583_3938	CDS	3985669	3990222	+	gltB	Glutamate synthase (NADPH), large subunit
G2583_3939	CDS	3990235	3991653	+	gltD	Glutamate synthase (NADPH), small subunit
G2583_3940	CDS	3991837	3992964	+	yhcG	conserved hypothetical protein
G2583_3941	CDS	3993024	3993488	-	yhcH	hypothetical protein
G2583_3942	CDS	3993485	3994360	-	nanK	N-acetylmannosamine kinase
G2583_3943	CDS	3994357	3995046	-	nanE	Putative N-acetylmannosamine-6-phosphate 2-epimerase
G2583_3944	CDS	3995094	3996584	-	nanT	Sialic acid transporter
G2583_3945	CDS	3996693	3997586	-	nanA	N-acetylneuraminate lyase
G2583_3946	CDS	3997708	3998499	-	nanR	Transcriptional regulators
G2583_3947	CDS	3998879	4000246	+	dcuD	Putative C4-dicarboxylate carrier protein
G2583_3948	CDS	4000289	4000786	-	sspB	Stringent starvation protein B
G2583_3949	CDS	4000792	4001430	-	sspA	Stringent starvation protein A
G2583_3950	CDS	4001825	4002217	-	rpsI	30S ribosomal protein S9
G2583_3951	CDS	4002233	4002661	-	rplM	50S ribosomal protein L13

Locus_tag	Type	Start	End	+/-[a]	Gene[b]	Product
G2583_3952	CDS	4002880	4004007	-	yhcM	hypothetical protein
G2583_3953	CDS	4004195	4004599	+	yhcB	hypothetical protein
G2583_3954	CDS	4004753	4006120	+	degQ	Serine peptidase DegQ
G2583_3955	CDS	4006210	4007277	+	degS	Protease degS precursor
G2583_3956	CDS	4007341	4008345	-	mdh	Malate dehydrogenase
G2583_3957	CDS	4008714	4009184	+	argR	Arginine repressor
G2583_3958	CDS	4009500	4009814	+	yhcN	hypothetical protein
G2583_3959	CDS	4009870	4010142	-	yhcO	hypothetical protein
G2583_3960	CDS	4010234	4012201	-	aaeB	p-hydroxybenzoic acid efflux pump subunit aaeB
G2583_3961	CDS	4012207	4013139	-	aaeA	p-hydroxybenzoic acid efflux pump subunit aaeA
G2583_3962	CDS	4013147	4013419	-	aaeX	hypothetical protein
G2583_3963	CDS	4013533	4014462	+	aaeR	putative DNA-binding transcriptional regulator
G2583_3964	CDS	4014590	4016035	-	tldD	suppresses inhibitory activity of CsrA
G2583_3965	CDS	4016191	4019991	-	yhdP	hypothetical protein
G2583_3966	CDS	4020059	4021528	-	rng	Ribonuclease G
G2583_3967	CDS	4021518	4022111	-	yhdE	Maf-like protein yhdE
G2583_3968	CDS	4022120	4022608	-	mreD	Rod shape-determining protein
G2583_3969	CDS	4022608	4023711	-	mreC	Rod shape-determining protein MreC
G2583_3970	CDS	4023777	4024880	-	mreB	Rod shape-determining protein mreB
G2583_3971	CDS	4025125	4027065	-	csrD	Cyclic diguanylate phosphodiesterase (EAL) domain
G2583_3972	CDS	4027217	4028191	+	yhdH	Quinone oxidoreductase, YhdH family
G2583_3973	CDS	4028249	4028350	-	-	hypothetical protein
G2583_3974	CDS	4029169	4029639	+	accB	acetyl-CoA carboxylase, biotin carboxyl carrier protein
G2583_3975	CDS	4029650	4030999	+	accC	Acetyl CoA carboxylase, biotin carboxylase subunit
G2583_3976	CDS	4031108	4031350	+	yhdT	hypothetical protein
G2583_3977	CDS	4031340	4032791	+	panF	Sodium/pantothenate symporter
G2583_3978	CDS	4032803	4033684	+	prmA	Ribosomal protein L11 methyltransferase
G2583_3979	CDS	4034013	4034978	+	dusB	tRNA-dihydrouridine synthase B
G2583_3980	CDS	4035004	4035300	+	fis	DNA-binding protein fis
G2583_3981	CDS	4035385	4036269	+	yhdJ	DNA methylase, N4/N6-methyltransferase family
G2583_3982	CDS	4036353	4036532	+	yhdU	hypothetical protein
G2583_3983	CDS	4036535	4037197	-	envR	Probable acrEF/envCD operon repressor
G2583_3984	CDS	4037596	4038753	+	acrE	Acriflavine resistance protein E
G2583_3985	CDS	4038765	4041869	+	acrF	Transporter, hydrophobe/amphiphile efflux-1 (HAE1) family
G2583_3986	CDS	4042122	4042343	+	yhdV	hypothetical protein
G2583_3987	CDS	4042774	4043799	+	yhdW	Putative amino-acid ABC transporter-binding protein yhdW
G2583_3988	CDS	4043867	4045048	+	yhdX	Amino acid ABC transporter, permease protein,
G2583_3989	pseudogene	4045058	4046161	+	yhdY	general L-amino acid transport system permease protein AapM
G2583_3990	CDS	4046169	4046927	+	yhdZ	Amino acid ABC transporter, ATP-binding protein
G2583_3991	rRNA	4047158	4047273	-	rrfD	5S ribosomal RNA
G2583_3992	tRNA	4047312	4047389	-	-	Thr tRNA
G2583_3993	rRNA	4047403	4047518	-	rrfD	5S ribosomal RNA
G2583_3994	rRNA	4047613	4050515	-	rrlD	23S ribosomal RNA
G2583_3995	tRNA	4050698	4050775	-	-	Ala tRNA
G2583_3996	tRNA	4050817	4050893	-	-	Ile tRNA
G2583_3997	rRNA	4050962	4052503	-	rrsD	16S ribosomal RNA
G2583_3998	CDS	4052649	4053530	+	yrdA	hypothetical protein
G2583_3999	CDS	4053506	4053763	-	yrdB	hypothetical protein
G2583_4000	CDS	4053760	4054578	-	aroE	Shikimate dehydrogenase
G2583_4001	CDS	4054583	4055200	-	rimN	Sua5/YciO/YrdC/YwlC family protein
G2583_4002	CDS	4055160	4055702	-	yrdD	predicted DNA topoisomerase
G2583_4003	CDS	4055731	4056204	-	smg	hypothetical protein
G2583_4004	CDS	4056176	4057300	-	smf	hypothetical protein
G2583_4005	CDS	4057430	4057939	+	def	Peptide deformylase
G2583_4006	CDS	4057954	4058901	+	fmt	Methionyl-tRNA formyltransferase
G2583_4007	CDS	4058947	4060236	+	rsmB	Ribosomal RNA small subunit methyltransferase B (rRNA
G2583_4008	CDS	4060258	4061634	+	trkA	Trk system potassium uptake protein trkA (K(+)-uptake protein
G2583_4009	CDS	4061764	4062174	+	mscL	Large-conductance mechanosensitive channel
G2583_4010	CDS	4062445	4062870	-	zntR	zinc-responsive transcriptional regulator
G2583_4011	CDS	4062881	4063249	-	yhdN	hypothetical protein

Locus_tag	Type	Start	End	+/-[a]	Gene[b]	Product
G2583_4012	CDS	4063356	4063739	-	rplQ	50S ribosomal protein L17
G2583_4013	CDS	4063780	4064769	-	rpoA	DNA-directed RNA polymerase subunit alpha
G2583_4014	CDS	4064795	4065415	-	rpsD	30S ribosomal protein S4
G2583_4015	CDS	4065449	4065838	-	rpsK	30S ribosomal protein S11
G2583_4016	CDS	4065855	4066211	-	rpsM	30S ribosomal protein S13
G2583_4017	CDS	4066358	4066474	-	rpmJ	ribosomal protein L36
G2583_4018	CDS	4066506	4067837	-	secY	Preprotein translocase subunit secY
G2583_4019	CDS	4067845	4068279	-	rplO	50S ribosomal protein L15
G2583_4020	CDS	4068283	4068462	-	rpmD	50S ribosomal protein L30
G2583_4021	CDS	4068466	4068969	-	rpsE	30S ribosomal protein S5
G2583_4022	CDS	4068984	4069337	-	rplR	50S ribosomal protein L18
G2583_4023	CDS	4069347	4069880	-	rplF	50S ribosomal protein L6
G2583_4024	CDS	4069893	4070285	-	rpsH	30S ribosomal protein S8
G2583_4025	CDS	4070319	4070624	-	rpsN	30S ribosomal protein S14
G2583_4026	CDS	4070639	4071178	-	rplE	50S ribosomal protein L5
G2583_4027	CDS	4071193	4071507	-	rplX	50S ribosomal protein L24
G2583_4028	CDS	4071518	4071889	-	rplN	50S ribosomal protein L14
G2583_4029	CDS	4072054	4072308	-	rpsQ	30S ribosomal protein S17
G2583_4030	CDS	4072308	4072499	-	rpmC	50S ribosomal protein L29
G2583_4031	CDS	4072499	4072909	-	rplP	50S ribosomal protein L16
G2583_4032	CDS	4072922	4073623	-	rpsC	30S ribosomal protein S3
G2583_4033	CDS	4073641	4073973	-	rplV	50S ribosomal protein L22
G2583_4034	CDS	4073988	4074266	-	rpsS	30S ribosomal protein S19
G2583_4035	CDS	4074283	4075104	-	rplB	50S ribosomal protein L2
G2583_4036	CDS	4075122	4075424	-	rplW	50S ribosomal protein L23
G2583_4037	CDS	4075421	4076026	-	rplD	50S ribosomal protein L4
G2583_4038	CDS	4076037	4076666	-	rplC	50S ribosomal protein L3
G2583_4039	CDS	4076699	4077010	-	rpsJ	Ribosomal protein S10
G2583_4040	CDS	4077155	4077340	+	-	hypothetical protein
G2583_4041	CDS	4077389	4077856	+	gspO	Peptidase, A24 (Type IV prepilin peptidase) family
G2583_4042	CDS	4077853	4078329	-	bfr	Bacterioferritin
G2583_4043	CDS	4078402	4078596	-	bfd	Bacterioferritin-associated ferredoxin
G2583_4044	CDS	4078779	4079963	-	tufA	Elongation factor Tu 1
G2583_4045	CDS	4080034	4082148	-	fusA	Elongation factor G
G2583_4046	CDS	4082245	4082715	-	rpsG	30S ribosomal protein S7
G2583_4047	CDS	4082812	4083186	-	rpsL	Ribosomal protein S12
G2583_4048	CDS	4083312	4083599	-	yheL	sulfur relay protein TusD/DsrH
G2583_4049	CDS	4083607	4083966	-	yheM	hypothetical protein
G2583_4050	CDS	4083966	4084352	-	yheN	Sulfurtransferase tusD
G2583_4051	CDS	4084352	4085086	-	yheO	hypothetical protein
G2583_4052	CDS	4085241	4086053	-	fkpA	FKBP-type peptidyl-prolyl cis-trans isomerase fkpA precursor
G2583_4053	CDS	4086274	4086492	+	slyX	slyX protein
G2583_4054	CDS	4086541	4087131	-	slyD	FKBP-type peptidyl-prolyl cis-trans isomerase SlyD
G2583_4055	CDS	4087226	4087426	-	yheV	hypothetical protein
G2583_4056	CDS	4087436	4089241	-	kefB	Glutathione-regulated potassium-efflux system protein kefB
G2583_4057	CDS	4089241	4089795	-	kefG	Glutathione-regulated potassium-efflux system ancillary protein
G2583_4058	CDS	4089923	4091836	+	yheS	Uncharacterized ABC transporter ATP-binding protein yheS
G2583_4059	CDS	4091836	4092858	+	yheT	Hydrolase, alpha/beta fold family
G2583_4060	CDS	4092852	4093070	+	yheU	UPF0270 protein yheU
G2583_4061	CDS	4093124	4093993	+	prkB	Phosphoribulokinase/uridine kinase family protein
G2583_4062	CDS	4094048	4094452	-	yhfA	Protein yhfA
G2583_4063	CDS	4094754	4095386	+	crp	Catabolite gene activator
G2583_4064	CDS	4095425	4097527	+	yhfK	integral membrane protein, YccS/YhfK family
G2583_4065	CDS	4097594	4098814	-	argD	Acetylornithine/succinyldiaminopimelate aminotransferase
G2583_4066	CDS	4098900	4099463	-	pabA	Aminodeoxychorismate synthase, component II
G2583_4067	CDS	4099495	4100097	-	fic	Cell filamentation protein Fic
G2583_4068	CDS	4100087	4100254	-	yhfG	hypothetical protein
G2583_4069	CDS	4100359	4100931	-	ppiA	Peptidyl-prolyl cis-trans isomerase
G2583_4070	CDS	4101202	4102383	+	tsgA	hypothetical protein
G2583_4071	CDS	4102645	4105188	+	nirB	Nitrite reductase [NAD(P)H], large subunit

Locus_tag	Type	Start	End	+/-[a]	Gene[b]	Product
G2583_4072	CDS	4105185	4105511	+	nirD	Nitrite reductase [NAD(P)H] large subunit
G2583_4073	CDS	4105637	4106443	+	nirC	Response regulator receiver protein
G2583_4074	CDS	4106462	4107835	+	cysG	Siroheme synthase [Includes: Uroporphyrinogen-III C-
G2583_4075	CDS	4108090	4108257	+	yhfL	putative lipoprotein
G2583_4076	CDS	4108502	4109890	+	frlA	Amino acid transporters
G2583_4077	CDS	4109890	4110933	+	frlB	SIS family protein
G2583_4078	CDS	4110984	4111811	+	frlC	predicted isomerase
G2583_4079	pseudogene	4111811	4112596	+	-	hypothetical protein
G2583_4080	CDS	4112630	4113427	+	frlR	GntR-family transcriptional regulator FrlR
G2583_4081	CDS	4113558	4114562	-	trpS	Tryptophanyl-tRNA synthetase
G2583_4082	CDS	4114555	4115313	-	gph	Phosphoglycolate phosphatase
G2583_4083	CDS	4115306	4115983	-	rpe	Ribulose-phosphate 3-epimerase
G2583_4084	CDS	4116001	4116837	-	dam	DNA adenine methylase
G2583_4085	CDS	4116944	4118230	-	damX	Uncharacterized protein conserved in bacteria
G2583_4086	CDS	4118322	4119410	-	aroB	3-dehydroquinate synthase
G2583_4087	CDS	4119467	4120144	-	aroK	Shikimate kinase
G2583_4088	CDS	4120389	4121627	-	hofQ	predicted fimbrial transporter
G2583_4089	CDS	4121539	4122264	-	hofP	hypothetical protein
G2583_4090	CDS	4122248	4122787	-	hofN	PilN family protein
G2583_4091	CDS	4122787	4123593	-	hofM	hypothetical protein
G2583_4092	CDS	4123662	4126238	+	mrcA	Penicillin-binding protein 1A
G2583_4093	CDS	4126403	4126963	-	nudE	ADP compounds hydrolase NudE
G2583_4094	CDS	4127283	4129418	+	yrfF	hypothetical protein
G2583_4095	CDS	4129438	4130151	+	yrfG	Putative phosphatase
G2583_4096	CDS	4130162	4130563	+	hslR	Heat shock protein 15
G2583_4097	CDS	4130582	4131466	+	hslO	Disulfide bond chaperones of the HSP33 family
G2583_4098	CDS	4131529	4133253	-	yhgE	putative transport
G2583_4099	CDS	4133329	4133460	-	-	hypothetical protein
G2583_4100	CDS	4133632	4135254	+	pck	Phosphoenolpyruvate carboxykinase [ATP]
G2583_4101	CDS	4135330	4136682	-	envZ	Osmolarity sensor protein envZ
G2583_4102	CDS	4136679	4137398	-	ompR	hypothetical protein
G2583_4103	CDS	4137626	4138102	+	greB	Transcription elongation factor greB
G2583_4104	CDS	4138199	4140520	+	yhgF	protein yhgF
G2583_4105	CDS	4140958	4141185	+	feoA	Ferrous iron transport protein A
G2583_4106	CDS	4141202	4143523	+	feoB	Ferrous iron transport protein B
G2583_4107	CDS	4143523	4143759	+	feoC	conserved hypothetical protein
G2583_4108	CDS	4143962	4144840	+	yhgA	hypothetical protein
G2583_4109	CDS	4144867	4145637	-	bioH	Carboxylesterase bioH
G2583_4110	CDS	4145675	4146358	+	gntX	gluconate periplasmic binding protein
G2583_4111	CDS	4146417	4146992	+	gntY	Fe/S biogenesis protein nfuA
G2583_4112	CDS	4147353	4148669	+	gntT	High-affinity gluconate transporter
G2583_4113	CDS	4148714	4150798	-	malQ	4-alpha-glucanotransferase
G2583_4114	CDS	4150808	4153201	-	malP	Maltodextrin phosphorylase
G2583_4115	CDS	4153813	4156518	+	malT	transcriptional regulator MalT
G2583_4116	CDS	4156515	4156643	+	-	hypothetical protein
G2583_4117	CDS	4156579	4156863	+	-	hypothetical protein
G2583_4118	CDS	4156854	4157339	+	-	Acetyltransferase, gnat family
G2583_4119	CDS	4157389	4158420	-	rtcA	RNA 3'-terminal phosphate cyclase
G2583_4120	CDS	4158421	4159647	-	rtcB	RtcB protein
G2583_4121	CDS	4159835	4161433	+	rtcR	Sigma-54 dependent transcriptional regulator RtcR
G2583_4122	CDS	4161415	4162173	-	glpR	Glycerol-3-phosphate regulon repressor
G2583_4123	CDS	4162190	4163020	-	glpG	Rhomboid protease glpG
G2583_4124	CDS	4163065	4163391	-	glpE	Thiosulfate sulfurtransferase glpE
G2583_4125	CDS	4163581	4165086	+	glpD	Glycerol-3-phosphate dehydrogenase
G2583_4126	CDS	4165301	4165903	-	-	hypothetical protein
G2583_4127	CDS	4165903	4166895	-	-	hypothetical protein
G2583_4128	CDS	4166921	4168438	-	yzgL	Hypothetical membrane protein
G2583_4129	CDS	4168555	4171002	-	glgP	Glycogen phosphorylase
G2583_4130	CDS	4171021	4172454	-	glgA	Glycogen synthase
G2583_4131	CDS	4172454	4173749	-	glgC	Glucose-1-phosphate adenylyltransferase

Locus_tag	Type	Start	End	+/-[a]	Gene[b]	Product
G2583_4132	CDS	4173767	4175740	-	glgX	Glycogen debranching enzyme
G2583_4133	CDS	4175737	4177923	-	glgB	1,4-alpha-glucan-branching enzyme
G2583_4134	CDS	4178196	4179299	-	asd	Aspartate-semialdehyde dehydrogenase
G2583_4135	CDS	4179491	4180084	+	yhgN	UPF0056 inner membrane protein yhgN
G2583_4136	CDS	4180130	4181425	-	-	Putative DNA processing protein
G2583_4137	pseudogene	4181332	4183427	-	-	putative ATP-dependent DNA helicase
G2583_4138	CDS	4183567	4184907	-	gntU	H+/gluconate symporter and related permeases
G2583_4139	CDS	4184911	4185438	-	gntK	Thermoresistant gluconokinase
G2583_4140	CDS	4185577	4186572	-	gntR	regulator of gluconate operon
G2583_4141	CDS	4186796	4187491	-	yhhW	hypothetical protein
G2583_4142	CDS	4187614	4188651	-	yhhX	Oxidoreductase, NAD-binding
G2583_4143	ncRNA	4188770	4188852	-	-	ncRNA
G2583_4144	CDS	4188986	4189474	+	yhhY	Acetyltransferase, GNAT family
G2583_4145	CDS	4189685	4190533	+	-	hypothetical protein
G2583_4146	CDS	4190546	4190962	-	-	hypothetical protein
G2583_4147	CDS	4191331	4193076	-	ggt	Gamma-glutamyltranspeptidase
G2583_4148	CDS	4193196	4193636	+	yhhA	hypothetical protein
G2583_4149	CDS	4193623	4194366	-	ugpQ	Glycerophosphodiester phosphodiesterase, cytosolic
G2583_4150	CDS	4194363	4195433	-	ugpC	sn-glycerol-3-phosphate import ATP-binding protein ugpC
G2583_4151	CDS	4195435	4196280	-	ugpE	sn-glycerol-3-phosphate transport system permease protein
G2583_4152	CDS	4196277	4197164	-	ugpA	sn-glycerol-3-phosphate transport system permease protein
G2583_4153	CDS	4197262	4198578	-	ugpB	sn-glycerol-3-phosphate-binding periplasmic protein ugpB
G2583_4154	CDS	4198938	4199684	-	-	hypothetical protein
G2583_4155	CDS	4199803	4200516	-	livF	ATP-binding component of leucine transport
G2583_4156	CDS	4200518	4201285	-	livG	High-affinity branched-chain amino acid transport ATP-binding
G2583_4157	CDS	4201282	4202559	-	livM	High-affinity branched-chain amino acid ABC transporter,
G2583_4158	CDS	4202556	4203482	-	livH	High-affinity branched-chain amino acid transport system
G2583_4159	CDS	4203530	4204693	-	livK	High-affinity branched-chain amino acid ABC transporter,
G2583_4160	CDS	4205063	4205446	+	yhhK	Acetyltransferase, GNAT family
G2583_4161	CDS	4205443	4205970	-	-	hypothetical protein
G2583_4162	CDS	4205977	4206243	-	-	hypothetical protein
G2583_4163	CDS	4206393	4207553	-	livJ	High-affinity amino acid transport protein, periplasmic binding
G2583_4164	CDS	4207767	4208621	-	rpoH	RNA polymerase sigma factor
G2583_4165	CDS	4208866	4209924	-	ftsX	Cell division protein ftsX
G2583_4166	CDS	4209917	4210585	-	ftsE	Cell division ATP-binding protein ftsE
G2583_4167	CDS	4210588	4212084	-	ftsY	Cell division protein FtsY
G2583_4168	CDS	4212234	4212830	+	rsmD	16S rRNA methyltransferase RsmD
G2583_4169	CDS	4212820	4213089	+	yhhL	hypothetical protein
G2583_4170	CDS	4213092	4213451	-	yhhM	putative receptor
G2583_4171	CDS	4213592	4214218	+	yhhN	Uncharacterized membrane protein yhhN
G2583_4172	CDS	4214292	4216490	+	zntA	Cadmium-translocating P-type ATPase
G2583_4173	CDS	4216592	4216837	-	sirA	Sulfurtransferase tusA
G2583_4174	CDS	4217058	4217723	+	yhhQ	hypothetical protein
G2583_4175	CDS	4217796	4218353	+	dcrB	hypothetical protein
G2583_4176	CDS	4218357	4219607	-	yhhS	Transporter, major facilitator family
G2583_4177	CDS	4219661	4220755	+	yhhT	hypothetical protein
G2583_4178	CDS	4221135	4221524	+	-	hypothetical protein
G2583_4179	CDS	4221593	4222651	+	-	O-methyltransferase, family 2
G2583_4180	CDS	4222692	4223414	+	-	Beta-ketoacyl synthase domain protein
G2583_4181	CDS	4223411	4224232	+	-	Putative phospholipid biosynthesis acyltransferase
G2583_4182	CDS	4224207	4224464	+	-	Acyl carrier protein
G2583_4183	CDS	4224476	4224727	+	acyl	acyl carrier protein
G2583_4184	CDS	4224732	4225313	+	-	DNA gyrase subunit B
G2583_4185	CDS	4225310	4226671	+	-	Putative surfactin synthetase
G2583_4186	CDS	4226658	4227011	+	-	hypothetical protein
G2583_4187	CDS	4227002	4228678	+	-	Glycosyl transferase, family 2
G2583_4188	CDS	4228682	4229104	+	-	Thioesterase superfamily protein
G2583_4189	CDS	4229101	4229706	+	-	Outer membrane lipoprotein carrier protein LolA
G2583_4190	CDS	4229675	4231993	+	-	hypothetical protein
G2583_4191	CDS	4231990	4232574	+	-	hypothetical protein

Locus_tag	Type	Start	End	+/-[a]	Gene[b]	Product
G2583_4192	CDS	4232576	4233745	+	-	3-oxoacyl-(acyl carrier protein) synthase I
G2583_4193	CDS	4233742	4234206	+	-	FabA-like domain protein
G2583_4194	CDS	4234206	4234937	+	-	Putative 3-oxoacyl-(Acyl-carrier-protein) reductase
G2583_4195	CDS	4234934	4236163	+	-	Beta-ketoacyl synthase
G2583_4196	CDS	4236165	4236752	+	acpT	4'-phosphopantetheinyl transferase acpT
G2583_4197	CDS	4236863	4238437	+	nikA	Nickel ABC transporter, periplasmic nickel-binding protein NikA
G2583_4198	CDS	4238437	4239381	+	nikB	Nickel transport system permease protein nikB
G2583_4199	CDS	4239378	4240211	+	nikC	Nickel transport system permease protein nikC
G2583_4200	CDS	4240211	4240975	+	nikD	Nickel import ATP-binding protein nikD
G2583_4201	CDS	4240972	4241778	+	nikE	Nickel import ATP-binding protein nikE
G2583_4202	CDS	4241784	4242185	+	nikR	Nickel-responsive regulator
G2583_4203	CDS	4242375	4243130	+	-	putative regulator
G2583_4204	CDS	4243155	4243628	+	-	PEP-dependent sugar transporting PTS family, IIA component
G2583_4205	CDS	4243625	4243906	+	-	PEP-dependent sugar transporting PTS family, IIB component
G2583_4206	CDS	4243953	4245341	+	-	PEP-dependent sugar transporting PTS family, IIC component
G2583_4207	CDS	4245334	4246842	+	-	Carbohydrate kinase, FGGY family
G2583_4208	CDS	4246832	4247101	+	-	Phosphocarrier, HPr family
G2583_4209	CDS	4247133	4247993	+	gatY	Fructose-bisphosphate aldolase, class II
G2583_4210	CDS	4248043	4248402	-	hicB	HicB family protein
G2583_4211	CDS	4248399	4248674	-	HicA	HicA-like protein
G2583_4212	CDS	4248747	4249874	-	yhhJ	hypothetical protein
G2583_4213	CDS	4249871	4252606	-	rbbA	ABC transporter, ATP binding protein
G2583_4214	CDS	4252603	4253670	-	yhiI	Auxiliary transport protein, membrane fusion protein (MFP)
G2583_4215	CDS	4254036	4255658	-	yhiJ	hypothetical protein
G2583_4216	CDS	4255920	4257593	-	yhiL	hypothetical protein
G2583_4217	CDS	4257815	4258966	+	yhiM	Inner membrane protein yhiM
G2583_4218	CDS	4259284	4260486	-	yhiN	Pyridine nucleotide-disulfide oxidoreductase family protein
G2583_4219	CDS	4260718	4262217	+	pitA	Low-affinity inorganic phosphate transporter 1
G2583_4220	CDS	4262288	4262623	-	uspB	Universal stress protein B
G2583_4221	CDS	4263014	4263448	+	uspA	Universal stress protein A
G2583_4222	CDS	4263765	4265234	+	yhiP	Hypothetical transporter YhiP
G2583_4223	CDS	4265283	4266035	-	yhiQ	UPF0341 protein yhiQ
G2583_4224	CDS	4266043	4268085	-	prlC	Oligopeptidase A
G2583_4225	CDS	4268288	4269130	+	yhiR	DNA utilization protein YhiR
G2583_4226	CDS	4269202	4270554	+	gor	Glutathione-disulfide reductase
G2583_4227	CDS	4271435	4271788	+	arsR	Arsenical resistance operon repressor
G2583_4228	CDS	4271842	4273131	+	arsB	Arsenical pump membrane protein
G2583_4229	CDS	4273144	4273569	+	arsC	Arsenate reductase
G2583_4230	CDS	4273681	4273788	+	-	hypothetical protein
G2583_4231	CDS	4274196	4275419	+	yhiS	hypothetical protein
G2583_4232	CDS	4275633	4276232	+	slp	Outer membrane lipoprotein, Slp family
G2583_4233	CDS	4276388	4276918	+	dctR	hypothetical protein
G2583_4234	CDS	4276979	4278007	-	chuS	Hemin transport protein HmuS
G2583_4235	CDS	4278056	4280038	-	chuA	Outer membrane heme/hemoglobin receptor ChuA
G2583_4236	CDS	4280314	4280598	-	-	hypothetical protein
G2583_4237	CDS	4280721	4281635	+	chuT	Putative periplasmic binding protein
G2583_4238	CDS	4281655	4282992	+	chuW	Putative coproporphyrinogen III oxidase
G2583_4239	CDS	4283005	4283499	+	chuX	hypothetical protein
G2583_4240	CDS	4283499	4284122	+	chuY	hypothetical protein
G2583_4241	CDS	4284171	4285163	+	chuU	Putative permease of iron compound ABC transport system
G2583_4242	CDS	4285160	4285930	+	shuV	Hemin import ATP-binding protein hmuV
G2583_4243	CDS	4285982	4286641	-	yhiD	putative Mg2+ transporter-C (MgtC) family protein
G2583_4244	CDS	4286693	4287031	-	hdeB	acid-resistance protein
G2583_4245	CDS	4287135	4287467	-	hdeA	Chaperone-like protein hdeA precursor
G2583_4246	CDS	4287722	4288294	+	hdeD	acid-resistance membrane protein
G2583_4247	CDS	4289093	4289620	+	gadE	hypothetical protein
G2583_4248	CDS	4289959	4291116	+	mdtE	Multidrug resistance protein mdtE precursor
G2583_4249	CDS	4291141	4294254	+	mdtF	Multidrug resistance protein mdtF
G2583_4250	CDS	4294617	4295345	-	gadW	putative ARAC-type regulatory protein
G2583_4251	ncRNA	4295588	4295701	+	-	ncRNA

Locus_tag	Type	Start	End	+/-[a]	Gene[b]	Product
G2583_4252	CDS	4295713	4296537	-	gadX	DNA-binding transcriptional dual regulator
G2583_4253	CDS	4296908	4298308	-	gadA	Glutamate decarboxylase alpha
G2583_4254	CDS	4298519	4299916	-	yhjA	Di-haem cytochrome c peroxidase family protein
G2583_4255	CDS	4300321	4301970	+	treF	Cytoplasmic trehalase
G2583_4256	CDS	4302021	4302623	-	yhjB	Putative HTH-type transcriptional regulator yhjB
G2583_4257	CDS	4303071	4304042	+	yhjC	putative transcriptional regulator LYSR-type
G2583_4258	CDS	4304091	4305104	+	yhjD	Putative ribonuclease
G2583_4259	CDS	4305516	4306838	+	yhjE	Inner membrane metabolite transport protein yhjE
G2583_4260	CDS	4307020	4309095	-	yhjG	hypothetical protein
G2583_4261	CDS	4309150	4309920	-	yhjH	Cyclic diguanylate phosphodiesterase
G2583_4262	CDS	4310149	4311078	+	kdgK	Ketodeoxygluconokinase
G2583_4263	CDS	4311174	4312670	-	yhjJ	Predicted Zn-dependent peptidases
G2583_4264	CDS	4312891	4314177	-	dctA	C4-dicarboxylate transport protein
G2583_4265	CDS	4314360	4316348	-	yhjK	Putative diguanylate cyclase
G2583_4266	CDS	4316430	4319903	-	bcsC	Cellulose synthase operon protein C
G2583_4267	CDS	4319885	4320991	-	bcsZ	Endoglucanase precursor
G2583_4268	CDS	4320998	4323337	-	bcsB	Cyclic di-GMP binding protein
G2583_4269	CDS	4323348	4325966	-	bcsA	Cellulose synthase catalytic subunit [UDP-forming]
G2583_4270	CDS	4325963	4326715	-	yhjQ	ATPases involved in chromosome partitioning
G2583_4271	CDS	4326727	4326915	-	yhjR	hypothetical protein
G2583_4272	CDS	4327188	4328759	+	bcsE	Putative protease
G2583_4273	CDS	4328756	4328947	+	bcsF	hypothetical protein
G2583_4274	CDS	4328944	4330623	+	bcsG	hypothetical protein
G2583_4275	CDS	4330710	4330952	-	ldrD	hypothetical protein
G2583_4276	CDS	4331293	4332564	+	yhjV	Serine transporter family protein
G2583_4277	CDS	4332594	4333598	-	dppF	ABC-type oligopeptide transport system, ATPase component
G2583_4278	CDS	4333595	4334578	-	dppD	Dipeptide transport ATP-binding protein dppD
G2583_4279	CDS	4334589	4335491	-	dppC	Dipeptide transport system permease protein dppC
G2583_4280	CDS	4335501	4336520	-	dppB	Dipeptide transport system permease protein dppB
G2583_4281	CDS	4336671	4338278	-	dppA	Dipeptide ABC transporter, periplasmic dipeptide-binding
G2583_4282	tRNA	4339022	4339100	-	-	Pro tRNA
G2583_4283	CDS	4339191	4340882	-	eptB	hypothetical protein
G2583_4284	CDS	4341137	4341667	-	lpfE	putative fimbrial subunit
G2583_4285	CDS	4341672	4342727	-	lpfD	putative fimbrial protein
G2583_4286	CDS	4342742	4345315	-	fimD	PapC-like porin protein involved in fimbrial biogenesis
G2583_4287	CDS	4345344	4346042	-	lpfB	Putative fimbrial chaperone
G2583_4288	CDS	4346095	4346631	-	lpfA	Putative major fimbrial subunit
G2583_4289	CDS	4346963	4348165	-	yhjX	Inner membrane protein YhjX
G2583_4290	CDS	4348394	4349098	-	yhjY	Putative lipase
G2583_4291	CDS	4349250	4349813	+	tag	3-methyladenine DNA glycosylase I
G2583_4292	CDS	4349810	4350250	+	yiaC	Acetyltransferase, GNAT family
G2583_4293	CDS	4350219	4352552	-	bisC	Biotin sulfoxide reductase
G2583_4294	CDS	4352705	4353364	+	yiaD	Inner membrane lipoprotein yiaD precursor
G2583_4295	CDS	4353468	4354442	+	ghrB	2-ketogluconate reductase
G2583_4296	CDS	4354492	4355322	-	yiaF	hypothetical protein
G2583_4297	CDS	4355636	4355926	+	yiaG	putative transcriptional regulator
G2583_4298	CDS	4356207	4356419	+	cspA	Cold shock protein cspA
G2583_4299	CDS	4356607	4356819	-	hokA	small toxic polypeptide
G2583_4300	CDS	4357082	4359151	-	glyS	Glycyl-tRNA synthetase beta subunit
G2583_4301	CDS	4359161	4360072	-	glyQ	Glycyl-tRNA synthetase alpha subunit
G2583_4302	CDS	4360167	4360466	-	ysaB	Uncharacterized lipoprotein ysaB precursor
G2583_4303	CDS	4360641	4361636	+	wecH	Acyltransferase family protein
G2583_4304	CDS	4361678	4362118	-	yiaA	Inner membrane protein yiaA
G2583_4305	CDS	4362161	4362514	-	yiaB	hypothetical protein
G2583_4306	CDS	4362671	4364125	-	xylB	Xylulokinase
G2583_4307	CDS	4364197	4365519	-	xylA	Xylose isomerase
G2583_4308	CDS	4365885	4366877	+	xylF	Xylose binding protein transport system
G2583_4309	CDS	4366955	4368496	+	xylG	Xylose import ATP-binding protein xylG
G2583_4310	CDS	4368474	4369655	+	xylH	Xylose transport system permease protein xylH
G2583_4311	CDS	4369733	4370911	+	xylR	Xylose operon regulatory protein

Locus_tag	Type	Start	End	+/-[a]	Gene[b]	Product
G2583_4312	CDS	4371019	4371843	-	bax	hypothetical protein
G2583_4313	CDS	4372163	4374193	+	malS	Alpha-amylase, periplasmic
G2583_4314	CDS	4374371	4375624	+	avtA	Alanine-alpha-ketoisovalerate (Or valine-pyruvate)
G2583_4315	CDS	4375776	4376249	-	ysaA	4Fe-4S binding domain protein
G2583_4316	CDS	4376493	4377308	-	-	AraC-type DNA-binding domain-containing protei ns
G2583_4317	CDS	4377534	4378934	+	-	Sugar transporter, glycoside-pentoside-hexuronide family
G2583_4318	CDS	4378945	4380915	+	-	hypothetical protein
G2583_4319	CDS	4381045	4381785	-	yiaT	Putative outer membrane protein yiaT precursor
G2583_4320	CDS	4381909	4382883	+	yiaU	Transcriptional regulator
G2583_4321	CDS	4382880	4384016	-	yiaV	Auxiliary transport protein, membrane fusion protein family
G2583_4322	CDS	4384022	4384345	-	yiaW	Inner membrane protein yiaW
G2583_4323	CDS	4384890	4386518	-	aldB	Aldehyde dehydrogenase B
G2583_4324	CDS	4386536	4387831	-	-	Fic family protein
G2583_4325	CDS	4387961	4389112	-	yiaY	Alcohol dehydrogenase II
G2583_4326	CDS	4389302	4391146	-	selB	Selenocysteine-specific translation elongation factor
G2583_4327	CDS	4391143	4392534	-	selA	L-seryl-tRNA(Sec) selenium transferase (Selenocysteine
G2583_4328	CDS	4392632	4393240	-	yibF	Glutathione S-transferase
G2583_4329	CDS	4393469	4397746	+	rhsA	rhsA
G2583_4330	CDS	4398528	4399052	+	yibJ	Rhs family protein
G2583_4331	CDS	4399064	4399525	+	yibG	hypothetical protein
G2583_4332	CDS	4400232	4400669	+	yibV	hypothetical protein
G2583_4333	CDS	4401128	4402264	-	yibH	Inner membrane protein yibH
G2583_4334	CDS	4402267	4402629	-	yibI	hypothetical protein
G2583_4335	CDS	4403166	4405085	+	mtlA	PTS system, mannitol-specific IIABC component
G2583_4336	CDS	4405180	4406328	+	mtlD	Mannitol-1-phosphate dehydrogenase
G2583_4337	CDS	4406328	4406915	+	mtlR	Mannitol operon repressor
G2583_4338	CDS	4407421	4407783	+	yibL	conserved hypothetical protein
G2583_4339	CDS	4408097	4408255	-	-	hypothetical protein
G2583_4340	CDS	4408428	4409009	+	-	putative lipoprotein
G2583_4341	CDS	4409053	4413819	+	-	Putative adhesin
G2583_4342	CDS	4414187	4415842	+	lldP	L-lactate permease
G2583_4343	CDS	4415842	4416618	+	lldR	Putative L-lactate dehydrogenase operon regulatory protein
G2583_4344	CDS	4416615	4417805	+	lldD	L-lactate dehydrogenase [cytochrome]
G2583_4345	CDS	4417991	4418464	+	yibK	RNA methyltransferase, TrmH family, group 2
G2583_4346	CDS	4418517	4419338	-	cysE	Serine acetyltransferase
G2583_4347	CDS	4419418	4420437	-	gpsA	Glycerol-3-phosphate dehydrogenase [NAD(P)+] (NAD(P)H-
G2583_4348	CDS	4420437	4420904	-	secB	Protein-export protein secB
G2583_4349	CDS	4420967	4421218	-	grxC	Glutaredoxin-3
G2583_4350	CDS	4421360	4421791	-	yibN	hypothetical protein
G2583_4351	CDS	4422036	4423580	+	gpmM	Putative 2,3-bisphosphoglycerate-independent
G2583_4352	CDS	4423590	4424873	+	envC	hypothetical protein
G2583_4353	CDS	4424877	4425836	+	yibQ	Uncharacterized protein conserved in bacteria
G2583_4354	CDS	4425842	4426858	-	yibD	Putative regulator
G2583_4355	CDS	4427097	4428122	-	tdh	L-threonine 3-dehydrogenase
G2583_4356	CDS	4428132	4429328	-	kbl	2-amino-3-ketobutyrate CoA ligase
G2583_4357	CDS	4429603	4430475	-	htrL	Involved in lipopolysaccharide biosynthesis
G2583_4358	CDS	4430764	4431696	+	rfaD	ADP-L-glycero-D-manno-heptose-6-epimerase
G2583_4359	CDS	4431706	4432752	+	rfaF	Lipopolysaccharide heptosyltransferase II
G2583_4360	CDS	4432756	4433748	+	rfaC	Lipopolysaccharide heptosyltransferase I
G2583_4361	CDS	4433745	4434953	+	waaL	Lipid A-core:surface polymer ligase WaaL
G2583_4362	CDS	4434990	4436132	-	waaD	Lipopolysaccharide 1,2-N-acetylglucosaminetransferase
G2583_4363	CDS	4436141	4437154	-	rfaJ	Lipopolysaccharide 1,2-glucosyltransferase
G2583_4364	CDS	4437179	4437886	-	rfaY	lipopolysaccharide core biosynthesis protein
G2583_4365	CDS	4437912	4438919	-	rfaI	Lipopolysaccharide 1,3-galactosyltransferase
G2583_4366	CDS	4438962	4439768	-	rfaP	Lipopolysaccharide core biosynthesis protein RfaP
G2583_4367	CDS	4439752	4440876	-	rfaG	Lipopolysaccharide core biosynthesis protein RfaG
G2583_4368	CDS	4440873	4441931	-	rfaQ	Lipopolysaccharide core biosynthesis protein
G2583_4369	CDS	4442344	4443621	+	waaA	Kdo transferase WaaA
G2583_4370	CDS	4443629	4444108	+	coaD	Phosphopantetheine adenylyltransferase
G2583_4371	CDS	4444147	4444956	-	mutM	Formamidopyrimidine-DNA glycosylase (Fapy-DNA

Locus_tag	Type	Start	End	+/-[a]	Gene[b]	Product
G2583_4372	CDS	4445054	4445221	-	rpmG	50S ribosomal protein L33
G2583_4373	CDS	4445242	4445478	-	rpmB	50S ribosomal protein L28
G2583_4374	CDS	4445695	4446369	-	yicR	DNA repair proteins
G2583_4375	CDS	4446535	4447755	+	dfp	Dfp
G2583_4376	CDS	4447733	4448191	+	dut	Deoxyuridine 5'-triphosphate nucleotidohydrolase
G2583_4377	CDS	4448298	4448894	+	slmA	HTH-type protein slmA
G2583_4378	CDS	4448931	4449572	-	pyrE	Orotate phosphoribosyltransferase
G2583_4379	CDS	4449638	4450354	-	rph	Ribonuclease PH
G2583_4380	CDS	4450481	4451344	+	yicC	Uncharacterized stress-induced protein
G2583_4381	CDS	4451565	4452389	+	dinD	Pyridoxine biosynthesis enzyme
G2583_4382	CDS	4452627	4453298	+	yicG	hypothetical protein
G2583_4383	CDS	4453295	4454977	-	ligB	DNA ligase B
G2583_4384	CDS	4455235	4455858	+	gmk	Guanylate kinase
G2583_4385	CDS	4455913	4456188	+	rpoZ	DNA-directed RNA polymerase, subunit K/omega
G2583_4386	CDS	4456207	4458315	+	spoT	Guanosine-3',5'-bis(diphosphate) 3'-pyrophosphohydrolase
G2583_4387	CDS	4458322	4459011	+	trmH	tRNA guanosine-2'-O-methyltransferase
G2583_4388	CDS	4459017	4461098	+	recG	ATP-dependent DNA helicase recG
G2583_4389	CDS	4461083	4461949	-	-	hypothetical protein
G2583_4390	CDS	4461952	4463157	-	gltS	Glutamate transport protein
G2583_4391	CDS	4463437	4464828	+	yicE	Putative purine permease yicE
G2583_4392	CDS	4464949	4466658	+	yicH	hypothetical protein
G2583_4393	CDS	4466711	4469029	-	yicI	Alpha-xylosidase
G2583_4394	CDS	4469039	4470421	-	yicJ	Sugar transporter, glycoside-pentoside-hexuronide
G2583_4395	tRNA	4470714	4470808	+	-	Sec tRNA
G2583_4396	CDS	4470932	4472185	+	intL	CP4-like integrase
G2583_4397	CDS	4472284	4472631	+	insN	unknown protein encoded by IS911 within prophage CP-933L
G2583_4398	CDS	4472844	4473497	+	-	putative transposase
G2583_4399	CDS	4473494	4473658	+	-	hypothetical protein
G2583_4400	CDS	4473748	4474122	+	-	unknown protein encoded within prophage CP-933L
G2583_4401	CDS	4474119	4474610	+	-	unknown protein encoded within prophage CP-933L
G2583_4402	CDS	4474604	4474819	+	-	hypothetical protein
G2583_4403	pseudogene	4474904	4475746	+	-	hypothetical protein
G2583_4404	CDS	4476081	4476314	+	-	hypothetical protein
G2583_4405	CDS	4476749	4477513	-	espF	espF
G2583_4406	CDS	4477598	4477876	-	-	hypothetical protein
G2583_4407	CDS	4477882	4478103	-	EscF	Type III secretion apparatus needle protein
G2583_4408	CDS	4478139	4478546	-	-	hypothetical protein
G2583_4409	CDS	4478553	4479491	-	espB	Secreted protein EspB
G2583_4410	CDS	4479512	4480636	-	espD	secreted protein EspD
G2583_4411	CDS	4480649	4481227	-	espA	espA
G2583_4412	CDS	4481286	4482341	-	sepL	SepL
G2583_4413	CDS	4482484	4483704	+	EscD	Pas
G2583_4414	CDS	4483968	4486772	-	eae	Gamma intimin
G2583_4415	CDS	4486832	4487302	-	CesT	Tir chaperone
G2583_4416	CDS	4487440	4489110	-	tir	Translocated intimin receptor Tir
G2583_4417	CDS	4489534	4490145	-	ipgB	hypothetical protein
G2583_4418	CDS	4490411	4490794	+	-	hypothetical protein
G2583_4419	CDS	4490992	4491498	-	-	hypothetical protein
G2583_4420	CDS	4491529	4492446	-	SepQ	sepQ
G2583_4421	CDS	4492409	4492825	-	-	hypothetical protein
G2583_4422	CDS	4492818	4493195	-	-	hypothetical protein
G2583_4423	CDS	4493198	4494538	-	escN	escN
G2583_4424	CDS	4494522	4496549	-	escV	escV
G2583_4425	CDS	4496546	4496899	-	-	hypothetical protein
G2583_4426	CDS	4497084	4497383	+	sepZ	SepZ
G2583_4427	CDS	4497416	4497844	+	-	hypothetical protein
G2583_4428	CDS	4497847	4498419	+	EscJ	EscJ
G2583_4429	CDS	4498425	4498880	+	SepD	hypothetical protein
G2583_4430	CDS	4498880	4500418	+	EscC	escC
G2583_4431	CDS	4500432	4500887	+	cesD	Type III secretion low calcium response chaperone LcrH/SycD

Locus_tag	Type	Start	End	+/-[a]	Gene[b]	Product
G2583_4432	CDS	4501271	4501684	-	-	hypothetical protein
G2583_4433	CDS	4501739	4502110	-	-	hypothetical protein
G2583_4434	CDS	4502288	4502764	+	-	transglycosylase SLT domain
G2583_4435	CDS	4502761	4503798	-	escU	secretion system apparatus protein SsaU
G2583_4436	CDS	4503791	4504567	-	EscT	escT
G2583_4437	CDS	4504567	4504839	-	EscS	EscS
G2583_4438	CDS	4504836	4505489	-	escR	Type III secretion system EscR protein
G2583_4439	CDS	4505494	4506189	-	-	hypothetical protein
G2583_4440	CDS	4506134	4506733	-	-	hypothetical protein
G2583_4441	CDS	4506730	4507053	-	-	hypothetical protein
G2583_4442	CDS	4507057	4507275	-	-	type III secretion system protein, YseE family
G2583_4443	CDS	4507290	4507679	-	-	hypothetical protein
G2583_4444	CDS	4508913	4510109	+	EspG	hypothetical protein
G2583_4445	CDS	4510237	4511055	+	-	hypothetical protein
G2583_4446	CDS	4511369	4511611	-	-	conserved hypothetical protein
G2583_4447	CDS	4511827	4512021	-	-	hypothetical protein
G2583_4448	CDS	4512075	4512998	+	yicL	YicL
G2583_4449	CDS	4513002	4513820	-	nlpA	Lipoprotein 28 precursor
G2583_4450	CDS	4513973	4514335	+	yicS	hypothetical protein
G2583_4451	CDS	4514376	4515614	-	nepI	Major facilitator family transporter
G2583_4452	CDS	4515800	4516153	+	-	hypothetical protein
G2583_4453	CDS	4516137	4516460	+	-	Putative DNA-binding protein
G2583_4454	CDS	4516576	4517055	-	yicN	hypothetical protein
G2583_4455	pseudogene	4517081	4518414	-	yicO	Inorganic anion transporter, sulfate permease (SulP) family
G2583_4456	CDS	4518589	4520355	+	ade	Adenine deaminase
G2583_4457	CDS	4520401	4521792	-	uhpT	Hexose phosphate transport protein
G2583_4458	CDS	4521930	4523252	-	uhpC	Regulator of uhpT
G2583_4459	CDS	4523259	4524764	-	uhpB	sensory histidine kinase UhpB
G2583_4460	CDS	4524761	4525351	-	uhpA	transcriptional regulatory protein UhpA
G2583_4461	CDS	4525513	4526946	-	-	hypothetical protein
G2583_4462	CDS	4527226	4527708	-	-	hypothetical protein
G2583_4463	pseudogene	4527226	4528521	-	-	hypothetical protein
G2583_4464	CDS	4528772	4529062	-	ilvN	Acetolactate synthase isozyme 1 small subunit
G2583_4465	CDS	4529066	4530754	-	ilvB	Acetolactate synthase, large subunit, isozyme I
G2583_4467	CDS	4530860	4530958	-	ivbL	ilvBN operon leader peptide
G2583_4468	CDS	4531886	4533076	+	emrD	Multidrug resistance protein D
G2583_4469	CDS	4533084	4533581	-	yidF	putative transcriptional regulator
G2583_4470	CDS	4533578	4533940	-	yidG	Inner membrane protein yidG
G2583_4471	CDS	4533930	4534277	-	yidH	Inner membrane protein yidH
G2583_4472	CDS	4534386	4534835	+	yidI	hypothetical protein
G2583_4473	CDS	4534882	4536375	-	yidJ	Sulfatase
G2583_4474	CDS	4536372	4538087	-	yidK	Transporter, solute:sodium symporter (SSS) family
G2583_4475	CDS	4538224	4539147	+	yidL	putative ARAC-type regulatory protein
G2583_4476	CDS	4539144	4540466	-	glvA	Maltose-6'-phosphate glucosidase
G2583_4477	CDS	4540466	4542088	-	glvC	PTS system arbutin-like IIC component
G2583_4478	CDS	4542377	4543093	+	yidP	transcriptional regulator, GntR family
G2583_4479	CDS	4543090	4544751	-	yidE	Putative transport protein yidE
G2583_4480	CDS	4544947	4545375	-	ibpB	Small heat shock protein ibpB
G2583_4481	CDS	4545487	4545900	-	ibpA	Small heat shock protein ibpA
G2583_4482	CDS	4546131	4546538	+	yidQ	hypothetical protein
G2583_4483	CDS	4546540	4547790	-	yidR	hypothetical protein
G2583_4484	CDS	4547837	4548919	+	cbrA	conserved hypothetical protein
G2583_4485	CDS	4549165	4549821	+	yidX	Putative replicase
G2583_4486	CDS	4549867	4550679	-	yidA	Phosphatase yidA
G2583_4487	CDS	4550794	4551201	-	yidB	hypothetical protein
G2583_4488	CDS	4551432	4553846	-	gyrB	DNA gyrase subunit B
G2583_4489	CDS	4553875	4554948	-	recF	DNA replication and repair protein recF
G2583_4490	CDS	4554948	4556048	-	dnaN	DNA polymerase III subunit beta
G2583_4491	CDS	4556053	4557456	-	dnaA	Chromosomal replication initiator protein dnaA
G2583_4492	CDS	4558063	4558203	+	rpmH	hypothetical protein

Locus_tag	Type	Start	End	+/-[a]	Gene[b]	Product
G2583_4493	CDS	4558220	4558579	+	rnpA	Ribonuclease P protein component
G2583_4494	CDS	4558803	4560449	+	yidC	Preprotein translocase subunit YidC
G2583_4495	CDS	4560555	4561919	+	mnmE	tRNA modification GTPase trmE
G2583_4496	CDS	4562069	4564240	-	-	ShET2 enterotoxin, N-region family
G2583_4497	CDS	4564345	4564449	-	-	hypothetical protein
G2583_4498	CDS	4564640	4564714	+	tnaL	tryptophanase leader peptide
G2583_4499	CDS	4564920	4566350	+	tnaA	Tryptophanase
G2583_4500	CDS	4566441	4567688	+	tnaB	Low affinity tryptophan permease
G2583_4501	CDS	4567820	4568995	+	mdtL	Multidrug resistance protein mdtL
G2583_4502	CDS	4568970	4569929	+	yidZ	DNA-binding transcriptional regulator YidZ
G2583_4503	CDS	4570074	4570835	+	yieE	hypothetical protein
G2583_4504	CDS	4570857	4571423	+	yieF	hypothetical protein
G2583_4505	CDS	4571477	4572814	-	yieG	Putative membrane transport protein
G2583_4506	CDS	4572980	4573645	+	yieH	HAD hydrolase, IA family
G2583_4507	CDS	4573782	4576190	-	-	hypothetical protein
G2583_4508	CDS	4576491	4577162	+	-	hypothetical protein
G2583_4509	CDS	4577208	4577678	+	-	hypothetical protein
G2583_4510	CDS	4577777	4577956	-	-	hypothetical protein
G2583_4511	pseudogene	4578135	4580634	-	-	hypothetical protein
G2583_4512	CDS	4580615	4580821	-	-	hypothetical protein
G2583_4513	CDS	4580887	4581102	-	-	hypothetical protein
G2583_4514	CDS	4581059	4581784	-	phoU	Phosphate transport system regulatory protein PhoU
G2583_4515	CDS	4581799	4582572	-	pstB	Phosphate import ATP-binding protein pstB
G2583_4516	CDS	4582755	4583645	-	pstA	Phosphate ABC transporter, permease protein PstA
G2583_4517	CDS	4583645	4584604	-	pstC	Phosphate transport system permease protein pstC
G2583_4518	CDS	4584691	4585731	-	pstS	Phosphate ABC transporter, periplasmic phosphate-binding
G2583_4519	CDS	4585943	4587025	-	lpfD	Fimbrial family protein
G2583_4520	CDS	4587053	4588144	-	lpfD	Putative fimbrial protein
G2583_4521	pseudogene	4588156	4590690	-	lpfC	fimbrial usher protein
G2583_4522	CDS	4590712	4590873	-	stgB	Putative fimbrial chaperone
G2583_4523	CDS	4591014	4591397	-	stgB	Putative fimbrial chaperone
G2583_4524	CDS	4591500	4592102	-	lpfA	Fimbrial protein
G2583_4525	CDS	4592802	4594631	-	glmS	Glucosamine--fructose-6-phosphate aminotransferase
G2583_4526	CDS	4594793	4596163	-	glmU	Bifunctional protein glmU [Includes: UDP-N-acetylglucosamine
G2583_4527	CDS	4596515	4596934	-	atpC	ATP synthase epsilon chain
G2583_4528	CDS	4596955	4598337	-	atpD	ATP synthase subunit beta
G2583_4529	CDS	4598364	4599227	-	atpG	ATP synthase gamma chain
G2583_4530	CDS	4599278	4600819	-	atpA	ATP synthase subunit alpha
G2583_4531	CDS	4600832	4601365	-	atpH	ATP synthase delta chain
G2583_4532	CDS	4601380	4601850	-	atpF	ATP synthase B chain
G2583_4533	CDS	4601912	4602151	-	atpE	ATP synthase C chain
G2583_4534	CDS	4602198	4603013	-	atpB	ATP synthase A chain
G2583_4535	CDS	4603022	4603414	-	atpI	Membrane-bound ATP synthase
G2583_4536	CDS	4604019	4604642	-	gidB	Ribosomal RNA small subunit methyltransferase G
G2583_4537	CDS	4604706	4606595	-	mnmG	tRNA uridine 5-carboxymethylaminomethyl modification enzyme
G2583_4538	CDS	4606974	4607417	-	mioC	flavodoxin
G2583_4539	CDS	4607507	4607965	-	asnC	DNA-binding transcriptional regulator AsnC
G2583_4540	CDS	4608117	4609109	+	asnA	Aspartate--ammonia ligase
G2583_4541	CDS	4609114	4610565	-	viaA	hypothetical protein
G2583_4542	CDS	4610559	4612079	-	ravA	ATPase ravA
G2583_4543	CDS	4612278	4614146	+	kup	Low affinity potassium transport system protein kup
G2583_4544	CDS	4614301	4614732	+	rbsD	D-ribose high-affinity transport system
G2583_4545	CDS	4614740	4616245	+	rbsA	Ribose import ATP-binding protein rbsA
G2583_4546	CDS	4616250	4617215	+	rbsC	Ribose transport system permease protein rbsC
G2583_4547	CDS	4617240	4618130	+	rbsB	D-ribose-binding periplasmic protein
G2583_4548	CDS	4618241	4619185	+	rbsK	Ribokinase
G2583_4549	CDS	4619189	4620181	+	rbsR	Regulator for rbs operon
G2583_4550	CDS	4620147	4621574	-	hsrA	Drug resistance MFS transporter, drug:H+ antiporter-1 (DHA2)
G2583_4551	CDS	4621597	4622289	-	yieP	hypothetical protein
G2583_4552	rRNA	4622773	4624314	+	rrsC	16S ribosomal RNA

Locus_tag	Type	Start	End	+/-[a]	Gene[b]	Product
G2583_4553	tRNA	4624399	4624476	+	-	Glu tRNA
G2583_4554	rRNA	4624669	4627572	+	rrlC	23S ribosomal RNA
G2583_4555	rRNA	4627667	4627782	+	rrfC	5S ribosomal RNA
G2583_4556	tRNA	4627836	4627914	+	-	Asp tRNA
G2583_4557	tRNA	4627921	4627998	+	-	Trp tRNA
G2583_4558	CDS	4628093	4628932	-	hdfR	transcriptional regulator HdfR
G2583_4559	CDS	4629051	4629389	+	yifE	UPF0438 protein yifE
G2583_4560	CDS	4629414	4630964	-	yifB	Putative 2-component regulator
G2583_4561	CDS	4631287	4631385	+	ilvL	ilvGMEDA operon leader peptide
G2583_4562	CDS	4631525	4633171	+	ilvG	Acetolactate synthase, large subunit, isozyme II
G2583_4563	CDS	4633168	4633431	+	ilvM	Acetolactate synthase isozyme 2 small subunit
G2583_4564	CDS	4633451	4634380	+	ilvE	Branched-chain-amino-acid aminotransferase
G2583_4565	CDS	4634445	4636295	+	ilvD	Dihydroxy-acid dehydratase
G2583_4566	CDS	4636298	4637842	+	ilvA	Threonine dehydratase biosynthetic
G2583_4567	CDS	4637894	4638787	-	ilvY	DNA-binding transcriptional regulator IlvY
G2583_4568	CDS	4638937	4640412	+	ilvC	Ketol-acid reductoisomerase
G2583_4569	CDS	4640458	4640739	-	ppiC	Peptidyl-prolyl cis-trans isomerase C
G2583_4570	CDS	4640938	4641387	-	yifO	hypothetical protein
G2583_4571	CDS	4641604	4643625	+	rep	ATP-dependent DNA helicase Rep
G2583_4572	CDS	4643672	4645156	-	gpp	Guanosine-5'-triphosphate,3'-diphosphate pyrophosphatase
G2583_4573	CDS	4645292	4646557	-	rhlB	ATP-dependent RNA helicase RhlB
G2583_4574	CDS	4646634	4647017	+	trxA	thioredoxin TrxA
G2583_4575	CDS	4647158	4647259	+	rhoL	rho operon leader peptide
G2583_4576	CDS	4647344	4648603	+	rho	Transcription termination factor rho
G2583_4577	CDS	4648613	4648831	+	-	hypothetical protein
G2583_4578	CDS	4648843	4649946	+	rfe	Undecaprenyl-phosphate alpha-N-acetylglucosaminyl 1-
G2583_4579	CDS	4649955	4651004	+	wzzE	Lipopolysaccharide biosynthesis protein
G2583_4580	CDS	4651018	4652190	+	rffE	UDP-N-acetylglucosamine 2-epimerase
G2583_4581	CDS	4652187	4653449	+	rffD	UDP-N-acetyl-D-mannosamine dehydrogenase
G2583_4582	CDS	4653449	4654516	+	rffG	dTDP-glucose 4,6-dehydratase
G2583_4583	CDS	4654535	4655416	+	rffH	Glucose-1-phosphate thymidylyltransferase
G2583_4584	CDS	4655394	4656068	+	rffC	TDP-D-fucosamine acetyltransferase
G2583_4585	CDS	4656073	4657203	+	rffA	TDP-4-oxo-6-deoxy-D-glucose transaminase
G2583_4586	CDS	4657205	4658455	+	wzxE	Polysaccharide biosynthesis protein
G2583_4587	CDS	4658452	4659531	+	rffT	TDP-Fuc4NAc:lipid II Fuc4Nac transferase
G2583_4588	CDS	4659528	4660880	+	wzyE	Putative ECA polymerase
G2583_4589	CDS	4660883	4661623	+	rffM	Probable UDP-N-acetyl-D-mannosaminuronic acid transferase
G2583_4590	CDS	4661814	4663199	+	yifK	Probable transport protein yifK
G2583_4591	tRNA	4663302	4663378	+	-	Arg tRNA
G2583_4592	tRNA	4663436	4663513	+	-	His tRNA
G2583_4593	tRNA	4663532	4663620	+	-	Leu tRNA
G2583_4594	tRNA	4663662	4663724	+	-	Pro tRNA
G2583_4595	CDS	4663885	4665120	+	aslB	Arylsulfatase-activating protein AslB
G2583_4596	CDS	4665288	4666943	-	aslA	Arylsulfatase precursor
G2583_4597	ncRNA	4667368	4667539	+	-	ncRNA
G2583_4598	CDS	4667622	4668818	-	hemY	putative protoheme IX biogenesis protein
G2583_4599	CDS	4668821	4670002	-	hemX	Uroporphyrinogen III methylase
G2583_4600	CDS	4670024	4670764	-	hemD	Uroporphyrinogen-III synthase
G2583_4601	CDS	4670761	4671723	-	hemC	Porphobilinogen deaminase
G2583_4602	CDS	4672089	4674635	+	cyaA	Adenylate cyclase
G2583_4603	CDS	4674675	4674995	-	cyaY	iron donor protein CyaY
G2583_4604	CDS	4674786	4675271	+	yzcX	hypothetical protein
G2583_4605	CDS	4675458	4675661	+	yifL	Uncharacterized lipoprotein yifL precursor
G2583_4606	CDS	4675698	4676522	+	dapF	Diaminopimelate epimerase
G2583_4607	CDS	4676519	4677226	+	yigA	hypothetical protein
G2583_4608	CDS	4677223	4678119	+	xerC	Tyrosine recombinase xerC
G2583_4609	CDS	4678119	4678835	+	yigB	HAD-superfamily hydrolase
G2583_4610	CDS	4678919	4681081	+	uvrD	DNA helicase II
G2583_4611	CDS	4681126	4682028	-	orf	hypothetical protein
G2583_4612	CDS	4682111	4682875	-	yigE	hypothetical protein

Locus_tag	Type	Start	End	+/-[a]	Gene[b]	Product
G2583_4613	CDS	4683245	4684195	+	corA	Magnesium transport protein corA
G2583_4614	CDS	4684237	4684728	-	-	putative membrane protein
G2583_4615	CDS	4684725	4685573	-	-	hypothetical protein
G2583_4616	CDS	4685709	4685906	+	-	hypothetical protein
G2583_4617	CDS	4685931	4686317	-	-	hypothetical protein
G2583_4618	CDS	4686518	4686832	+	-	Hypothetical membrane protein
G2583_4619	CDS	4686879	4687766	-	rarD	hypothetical protein
G2583_4620	CDS	4687818	4688285	-	yigI	Thioesterase family protein
G2583_4621	CDS	4688450	4689319	+	pldA	Phospholipase A1 precursor
G2583_4622	CDS	4689446	4691281	+	recQ	ATP-dependent DNA helicase RecQ
G2583_4623	CDS	4691345	4691965	+	rhtC	Threonine efflux protein
G2583_4624	CDS	4692027	4692647	-	rhtB	Homoserine/homoserine lactone efflux protein
G2583_4625	CDS	4692758	4693780	+	pldB	Lysophospholipase L2
G2583_4626	CDS	4693671	4694588	+	yigL	Putative hydrolase
G2583_4627	CDS	4694664	4695563	+	yigM	Uncharacterized membrane protein yigM
G2583_4628	CDS	4695451	4696404	-	metR	regulator for metE and metH
G2583_4629	CDS	4696640	4698901	+	metE	5-methyltetrahydropteroyltriglutamate-homocysteine S-
G2583_4630	CDS	4698941	4699822	-	ysgA	Putative enzyme
G2583_4631	CDS	4700018	4700779	+	udp	Uridine phosphorylase
G2583_4632	CDS	4700920	4702347	+	rmuC	DNA recombination protein rmuC
G2583_4633	CDS	4702442	4703197	+	ubiE	Ubiquinone/menaquinone biosynthesis methyltransferase ubiE
G2583_4634	CDS	4703211	4703816	+	yigP	hypothetical protein
G2583_4635	CDS	4703813	4705453	+	ubiB	Probable ubiquinone biosynthesis protein ubiB
G2583_4636	CDS	4705490	4705801	+	tatA	Sec-independent twin-arginine translocase subunit TatA
G2583_4637	CDS	4705805	4706320	+	tatB	sec-independent translocase
G2583_4638	CDS	4706323	4707099	+	tatC	TatABCE protein translocation system subunit
G2583_4639	CDS	4707129	4707923	+	tatD	MttC
G2583_4640	CDS	4707920	4708408	-	rfaH	transcriptional activator RfaH
G2583_4641	CDS	4708575	4710068	+	ubiD	3-octaprenyl-4-hydroxybenzoate carboxy-lyase
G2583_4642	CDS	4710114	4710815	+	fre	Oxidoreductase
G2583_4643	CDS	4711007	4712170	-	fadA	Beta-ketoadipyl CoA thiolase
G2583_4644	CDS	4712180	4714369	-	fadB	Fused 3-hydroxybutyryl-CoA epimerase/delta(3)-cis-delta(2)-
G2583_4645	CDS	4714559	4715890	+	pepQ	Xaa-Pro dipeptidase
G2583_4646	CDS	4715887	4716504	+	yigZ	hypothetical protein
G2583_4647	CDS	4716543	4717994	+	trkH	Trk system potassium uptake protein trkH
G2583_4648	CDS	4718006	4718551	+	hemG	Protoporphyrinogen oxidase
G2583_4649	rRNA	4718929	4720470	+	rrsA	16S ribosomal RNA
G2583_4650	tRNA	4720539	4720615	+	-	Ile tRNA
G2583_4651	tRNA	4720657	4720734	+	-	Ala tRNA
G2583_4652	rRNA	4720917	4723819	+	rrlA	23S ribosomal RNA
G2583_4653	rRNA	4723914	4724029	+	rrfA	5S ribosomal RNA
G2583_4654	CDS	4724130	4724657	-	mobB	Molybdopterin-guanine dinucleotide biosynthesis protein B
G2583_4655	CDS	4724639	4725223	-	mobA	Molybdopterin-guanine dinucleotide biosynthesis protein A
G2583_4656	CDS	4725293	4725562	+	yihD	hypothetical protein
G2583_4657	CDS	4725639	4726625	+	rdoA	predicted kinase
G2583_4658	CDS	4726642	4727268	+	dsbA	Thiol:disulfide interchange protein dsbA precursor
G2583_4659	CDS	4727381	4728853	+	yihF	Putative GTP-binding protein
G2583_4660	CDS	4728894	4729826	-	yihG	Acyltransferase domain protein
G2583_4661	CDS	4729946	4730188	-	-	hypothetical protein
G2583_4662	CDS	4730190	4732976	+	polA	DNA polymerase I
G2583_4663	ncRNA	4733114	4733232	+	spf	ncRNA
G2583_4664	CDS	4733358	4733990	-	yihA	Probable GTP-binding protein engB
G2583_4665	ncRNA	4734261	4734514	+	-	ncRNA
G2583_4666	CDS	4734572	4735078	+	yihI	UPF0241 protein yihI
G2583_4667	CDS	4735267	4736640	+	hemN	Oxygen-independent coproporphyrinogen III oxidase
G2583_4668	CDS	4737052	4738470	-	glnG	Nitrogen regulation protein NR
G2583_4669	CDS	4738473	4739522	-	glnL	Signal transduction histidine kinase, nitrogen specific
G2583_4670	CDS	4739696	4741105	-	glnA	Glutamine synthetase
G2583_4671	CDS	4741478	4743301	+	typA	GTP-binding protein typA/bipA
G2583_4672	CDS	4743518	4744228	+	yihL	putative transcriptional regulator

Locus_tag	Type	Start	End	+/-[a]	Gene[b]	Product
G2583_4673	CDS	4744236	4745216	+	yihM	AP endonuclease, family 2
G2583_4674	CDS	4745318	4746583	+	yihN	Transporter, major facilitator family
G2583_4675	CDS	4746674	4747432	-	ompL	hypothetical protein
G2583_4676	CDS	4747433	4748842	-	yihO	Sugar (Glycoside-Pentoside-Hexuronide) transporter family
G2583_4677	CDS	4748876	4750261	-	yihP	Sugar transporter family protein
G2583_4678	CDS	4750307	4752343	-	yihQ	Glycosyl hydrolase, family 31
G2583_4679	CDS	4752451	4753326	-	-	hypothetical protein
G2583_4680	CDS	4753387	4754289	-	yihR	Aldose-1-epimerase family protein
G2583_4681	CDS	4754356	4755597	-	yihS	N-acylglucosamine 2-epimerase
G2583_4682	CDS	4755613	4756491	-	yihT	Uncharacterized aldolase yihT
G2583_4683	CDS	4756515	4757411	-	yihU	3-hydroxyisobutyrate dehydrogenase family
G2583_4684	CDS	4757572	4758474	+	yihV	Kinase, PfkB family
G2583_4685	CDS	4758484	4759293	+	yihW	Putative DEOR-type transcriptional activator
G2583_4686	CDS	4759392	4759991	+	yihX	Phosphatase YihX
G2583_4687	CDS	4759985	4760857	+	yihY	tRNA-processing ribonuclease BN
G2583_4688	CDS	4760854	4761291	+	dtd	D-tyrosyl-tRNA(Tyr) deacylase
G2583_4689	CDS	4761288	4762277	+	yiiD	putative acetyltransferase
G2583_4690	CDS	4762688	4764502	+	-	hypothetical protein
G2583_4691	CDS	4764675	4764893	-	-	hypothetical protein
G2583_4692	CDS	4764895	4765011	-	-	hypothetical protein
G2583_4693	CDS	4765169	4765414	+	yiiE	Ribbon-helix-helix protein, copG family
G2583_4694	CDS	4765631	4765873	+	yiiF	hypothetical protein
G2583_4695	CDS	4766203	4767132	-	fdhE	protein AraJ
G2583_4696	CDS	4767129	4767764	-	fdoI	Formate dehydrogenase, cytochrome b556(fdo) subunit
G2583_4697	CDS	4767761	4768663	-	fdoH	Formate dehydrogenase-O, iron-sulfur subunit
G2583_4698	pseudogene	4768676	4771726	-	fdoG	formate dehydrogenase-O major subunit
G2583_4699	CDS	4771920	4772753	+	fdhD	ApaG
G2583_4700	CDS	4772906	4773961	+	yiiG	hypothetical protein
G2583_4701	CDS	4774011	4775759	-	frvR	Putative PTS system, IIA component
G2583_4702	CDS	4775759	4776823	-	frvX	Aminopeptidase
G2583_4703	CDS	4776813	4778270	-	frvB	Phosphotransferase system, fructose-specific I IC component
G2583_4704	CDS	4778275	4778721	-	frvA	PTS system, fructose family, IIA component
G2583_4705	CDS	4779199	4780593	+	rafY	Glycoporin RafY
G2583_4706	CDS	4780634	4780948	-	rhaM	L-rhamnose 1-epimerase
G2583_4707	CDS	4780958	4781782	-	rhaD	Rhamnulose-1-phosphate aldolase
G2583_4708	CDS	4782325	4783584	-	rhaA	L-rhamnose isomerase
G2583_4709	CDS	4783581	4785050	-	rhaB	Rhamnulokinase
G2583_4710	CDS	4785338	4786174	+	rhaS	positive regulator for rhaBAD operon
G2583_4711	CDS	4786158	4787096	+	rhaR	positive regulator for rhaRS operon
G2583_4712	pseudogene	4787093	4788127	-	rhaT	rhamnose transport
G2583_4713	CDS	4788406	4789032	+	sodA	Superoxide dismutase
G2583_4714	CDS	4789292	4790275	+	kdgT	2-keto-3-deoxygluconate permease
G2583_4715	CDS	4790424	4791098	+	yiiM	MOSC domain protein
G2583_4716	CDS	4791204	4792577	-	cpxA	two-component sensor protein
G2583_4717	CDS	4792574	4793272	-	cpxR	DNA-binding response regulator in two-component regulatory
G2583_4718	CDS	4793419	4793922	+	cpxP	P pilus assembly/Cpx signaling pathway, periplasmic
G2583_4719	CDS	4794071	4794973	+	fieF	Ferrous-iron efflux pump fieF
G2583_4720	CDS	4795154	4796116	+	pfkA	6-phosphofructokinase isozyme 1
G2583_4721	CDS	4796225	4796407	-	-	hypothetical protein
G2583_4722	CDS	4796435	4797424	+	sbp	Sulfate-binding protein
G2583_4723	CDS	4797531	4798286	+	cdh	CDP-diacylglycerol pyrophosphatase
G2583_4724	CDS	4798341	4799108	-	tpiA	Triosephosphate isomerase
G2583_4725	CDS	4799216	4799815	-	yiiQ	hypothetical protein
G2583_4726	CDS	4799916	4800356	+	yiiR	hypothetical protein
G2583_4727	CDS	4800568	4800867	+	yiiS	hypothetical protein
G2583_4728	CDS	4800894	4801322	+	uspD	Universal stress protein D
G2583_4729	CDS	4801327	4802073	-	fpr	Ferredoxin--NADP(+) reductase
G2583_4730	CDS	4802170	4803180	-	glpX	Fructose-1,6-bisphosphatase, class II
G2583_4731	CDS	4803410	4804939	-	glpK	Glycerol kinase
G2583_4732	CDS	4804941	4805786	-	glpF	glycerol uptake facilitator protein

Locus_tag	Type	Start	End	+/-[a]	Gene[b]	Product
G2583_4733	CDS	4806212	4806457	+	yiiU	Cell division protein zapB
G2583_4734	CDS	4806496	4807212	-	-	hypothetical protein
G2583_4735	CDS	4807245	4807931	-	-	hypothetical protein
G2583_4736	CDS	4807979	4808464	-	rraA	Regulator of ribonuclease activity A
G2583_4737	CDS	4808557	4809495	-	menA	1,4-dihydroxy-2-naphthoate octaprenyltransferase
G2583_4738	CDS	4809550	4810881	-	hslU	ATP-dependent hsl protease ATP-binding subunit hslU
G2583_4739	CDS	4810891	4811421	-	hslV	ATP-dependent protease hslV
G2583_4740	CDS	4811514	4812473	-	ftsN	Cell division protein FtsN
G2583_4741	CDS	4812565	4813590	-	cytR	Transcriptional regulators
G2583_4742	CDS	4813746	4816016	-	priA	Primosomal protein N'
G2583_4743	CDS	4816147	4816359	+	rpmE	50S ribosomal protein L31
G2583_4744	CDS	4816519	4820703	+	rhsF	RhsH core protein with extension
G2583_4745	CDS	4820705	4820941	+	-	hypothetical protein
G2583_4746	CDS	4820994	4821272	+	insB	hypothetical protein
G2583_4747	CDS	4821305	4821490	-	-	IS1N transposase
G2583_4748	CDS	4821712	4822005	+	-	hypothetical protein
G2583_4749	CDS	4821934	4822542	-	yiiX	hypothetical protein
G2583_4750	CDS	4822602	4822919	-	metJ	MetJ
G2583_4751	CDS	4823196	4824356	+	metB	O-succinylhomoserine (Thiol)-lyase
G2583_4752	CDS	4824359	4826791	+	metL	Aspartokinase/homoserine dehydrogenase II
G2583_4753	CDS	4827140	4828030	+	metF	5,10-methylenetetrahydrofolate reductase
G2583_4754	CDS	4828359	4830539	+	katG	Peroxidase/catalase HPI
G2583_4755	CDS	4830600	4831538	+	yijE	Hypothetical transport protein yijE
G2583_4756	CDS	4831565	4832182	-	yijF	hypothetical protein
G2583_4757	CDS	4832456	4833559	-	gldA	Glycerol dehydrogenase,
G2583_4758	CDS	4833570	4834232	-	fsaB	Fructose-6-phosphate aldolase 2
G2583_4759	CDS	4834244	4836745	-	ptsA	PEP-protein phosphotransferase system enzyme I
G2583_4760	CDS	4836381	4836845	+	yijI	hypothetical protein
G2583_4761	CDS	4837054	4838133	+	frwC	PTS system, fructose-like-2 IIC component
G2583_4762	CDS	4838148	4838468	+	frwB	PTS system fructose-like IIB component 1
G2583_4763	CDS	4838519	4840816	+	pflD	Formate C-acetyltransferase 2
G2583_4764	CDS	4840782	4841660	+	pflC	Glycyl-radical enzyme activating protein family
G2583_4765	CDS	4841662	4842003	+	frwD	Predicted enzyme IIB component of PTS
G2583_4766	CDS	4841990	4842841	-	yijO	putative ARAC-type regulatory protein
G2583_4767	CDS	4843056	4844789	-	yijP	Membrane protein
G2583_4768	CDS	4844972	4847623	-	ppc	Phosphoenolpyruvate carboxylase
G2583_4769	CDS	4847975	4849126	-	argE	Acetylornithine deacetylase
G2583_4770	CDS	4849280	4850284	+	argC	N-acetyl-gamma-glutamyl-phosphate reductase
G2583_4771	CDS	4850292	4851068	+	argB	Acetylglutamate kinase
G2583_4772	CDS	4851129	4852502	+	argH	Argininosuccinate lyase
G2583_4773	ncRNA	4852564	4852673	-	oxyS	ncRNA
G2583_4774	CDS	4852769	4853686	+	oxyR	Transcriptional regulator
G2583_4775	CDS	4853669	4855069	-	sthA	Soluble pyridine nucleotide transhydrogenase (STH)
G2583_4776	CDS	4855399	4856565	+	-	Hippuricase
G2583_4777	CDS	4856608	4857924	+	-	Major facilitator superfamily
G2583_4778	CDS	4857974	4858678	+	fabR	Transcriptional regulator
G2583_4779	CDS	4858678	4859037	+	yijD	Inner membrane protein yijD
G2583_4780	CDS	4859077	4860177	-	trmA	tRNA (uracil-5-)-methyltransferase (tRNA(M-5-U54)-
G2583_4781	CDS	4860546	4862390	+	btuB	Vitamin B12 transporter btuB precursor
G2583_4782	CDS	4862335	4863192	+	murI	Glutamate racemase
G2583_4783	rRNA	4863569	4865110	+	rrsB	16S ribosomal RNA
G2583_4784	tRNA	4865195	4865272	+	-	Glu tRNA
G2583_4785	rRNA	4865465	4868367	+	rrlB	23S ribosomal RNA
G2583_4786	rRNA	4868462	4868577	+	rrfB	5S ribosomal RNA
G2583_4787	CDS	4868714	4869742	+	murB	UDP-N-acetylenolpyruvoylglucosamine reductase
G2583_4788	CDS	4869739	4870704	+	birA	Bifunctional protein BirA
G2583_4789	CDS	4870733	4871839	-	coaA	Pantothenate kinase
G2583_4790	CDS	4871870	4872025	-	-	hypothetical protein
G2583_4791	tRNA	4872044	4872121	+	-	Thr tRNA
G2583_4792	tRNA	4872129	4872213	+	-	Tyr tRNA

Locus_tag	Type	Start	End	+/-[a]	Gene[b]	Product
G2583_4793	tRNA	4872330	4872404	+	-	Gly tRNA
G2583_4794	tRNA	4872410	4872487	+	-	Thr tRNA
G2583_4795	CDS	4872601	4873785	+	tufB	Elongation factor Tu 2
G2583_4796	CDS	4874015	4874398	+	secE	Preprotein translocase
G2583_4797	CDS	4874400	4874945	+	nusG	Transcription antitermination protein nusG
G2583_4798	CDS	4875104	4875532	+	rplK	50S ribosomal protein L11
G2583_4799	CDS	4875536	4876240	+	rplA	50S ribosomal protein L1
G2583_4800	CDS	4876532	4877029	+	rplJ	50S ribosomal protein L10
G2583_4801	CDS	4877096	4877461	+	rplL	50S ribosomal protein L7/L12
G2583_4802	ncRNA	4877466	4877527	+	-	ncRNA
G2583_4803	CDS	4877781	4881809	+	rpoB	DNA-directed RNA polymerase subunit beta
G2583_4804	CDS	4881886	4886109	+	rpoC	DNA-directed RNA polymerase subunit beta'
G2583_4805	CDS	4886322	4886765	+	yjaZ	Heat shock protein C
G2583_4806	ncRNA	4886792	4886952	-	-	ncRNA
G2583_4807	CDS	4887200	4888333	-	thiH	Thiazole biosynthesis protein ThiH
G2583_4808	CDS	4888330	4889100	-	thiG	Thiamin biosynthesis, thiazole moiety
G2583_4809	CDS	4889102	4889302	-	thiS	Thiamine biosynthesis protein ThiS
G2583_4810	CDS	4889286	4890041	-	thiF	Thiazole biosynthesis adenylyltransferase ThiF
G2583_4811	CDS	4890034	4890669	-	thiE	Thiamine-phosphate pyrophosphorylase
G2583_4812	CDS	4890669	4892564	-	thiC	Thiamine biosynthesis protein thiC
G2583_4813	CDS	4892797	4893273	-	rsd	Regulator of sigma D
G2583_4814	CDS	4893368	4894141	+	nudC	NADH pyrophosphatase
G2583_4815	CDS	4894181	4895245	+	hemE	Uroporphyrinogen decarboxylase
G2583_4816	CDS	4895255	4895926	+	nfi	Endonuclease V
G2583_4817	CDS	4895969	4896559	+	yjaG	hypothetical protein
G2583_4818	CDS	4896746	4897018	+	hupA	DNA-binding protein HU-alpha
G2583_4819	CDS	4897031	4897726	+	yjaH	hypothetical protein
G2583_4820	CDS	4897728	4898294	-	zraP	Zinc resistance-associated protein
G2583_4821	CDS	4898391	4899767	+	zraS	sensor protein ZraS
G2583_4822	CDS	4899764	4901089	+	zraR	transcriptional regulatory protein ZraR
G2583_4823	CDS	4901086	4902375	-	purD	Phosphoribosylamine--glycine ligase
G2583_4824	CDS	4902387	4903976	-	purH	IMP cyclohydrolase /
G2583_4825	rRNA	4904593	4906134	+	rrsE	16S ribosomal RNA
G2583_4826	tRNA	4906219	4906296	+	-	Glu tRNA
G2583_4827	rRNA	4906479	4909381	+	rrlE	23S ribosomal RNA
G2583_4828	rRNA	4909476	4909591	+	rrfE	5S ribosomal RNA
G2583_4829	CDS	4909708	4910151	-	-	hypothetical protein
G2583_4830	CDS	4910308	4911237	+	metA	Homoserine O-succinyltransferase
G2583_4831	CDS	4911506	4913107	+	aceB	Malate synthase A
G2583_4832	CDS	4913132	4914451	+	aceA	Isocitrate lyase
G2583_4833	CDS	4914635	4916371	+	aceK	Isocitrate dehydrogenase kinase/phosphatase
G2583_4834	pseudogene	4916340	4918525	-	arp	ShET2 enterotoxin, N- region family
G2583_4835	CDS	4918842	4919666	-	iclR	Repressor of aceBA operon
G2583_4836	CDS	4919865	4923548	+	metH	Methionine synthase
G2583_4837	CDS	4923768	4925399	+	yjbB	hypothetical protein
G2583_4838	CDS	4925490	4926179	-	pepE	Peptidase E
G2583_4839	CDS	4926287	4926442	+	-	hypothetical protein
G2583_4840	CDS	4926562	4927803	-	sorE	Putative L-sorbose-1-P-reductase
G2583_4841	CDS	4927843	4928667	-	sorM	PTS system, mannose/fructose/sorbose family, IID component
G2583_4842	CDS	4928678	4929475	-	sorA	PTS system, mannose/fructose/sorbose family, IIC component
G2583_4843	CDS	4929541	4930035	-	sorB	Putative sorbose PTS component
G2583_4844	CDS	4930035	4930442	-	sorF	PTS system, mannose/fructose/sorbose family, IIA component
G2583_4845	CDS	4930452	4931258	-	sorD	Sorbitol-6-phosphate 2-dehydrogenase
G2583_4846	CDS	4931328	4932296	-	-	Putative transcriptional regulator of sorbose uptake and
G2583_4847	CDS	4932623	4933495	+	rluF	Ribosomal large subunit pseudouridine synthase F
G2583_4848	CDS	4933496	4933768	-	pagB	hypothetical protein
G2583_4849	CDS	4934021	4935370	-	lysC	Lysine-sensitive aspartokinase 3
G2583_4850	CDS	4935895	4937544	+	pgi	Glucose-6-phosphate isomerase
G2583_4851	CDS	4938057	4938299	+	yjbE	hypothetical protein
G2583_4852	CDS	4938383	4939051	+	yjbF	hypothetical protein

Locus_tag	Type	Start	End	+/-[a]	Gene[b]	Product
G2583_4853	CDS	4939048	4939785	+	yjbG	hypothetical protein
G2583_4854	CDS	4939785	4941881	+	yjbH	hypothetical protein
G2583_4855	CDS	4942476	4942886	+	psiE	phosphate-starvation-inducible protein PsiE
G2583_4856	CDS	4942930	4944405	-	xylE	D-xylose-proton symporter
G2583_4857	CDS	4944777	4945667	-	malG	Part of maltose permease, inner membrane
G2583_4858	CDS	4945682	4947226	-	malF	Maltose transport system permease protein malF
G2583_4859	CDS	4947380	4948570	-	malE	Maltose-binding periplasmic protein precursor
G2583_4860	CDS	4948935	4950050	+	malK	Maltose/maltodextrin import ATP-binding protein malK
G2583_4861	CDS	4950122	4951462	+	lamB	Maltoporin precursor
G2583_4862	CDS	4951613	4952533	+	malM	Maltose operon periplasmic protein precursor
G2583_4863	CDS	4952762	4954342	+	yjbI	hypothetical protein
G2583_4864	CDS	4954565	4955062	+	ubiC	Chorismate--pyruvate lyase
G2583_4865	CDS	4955075	4955947	+	ubiA	4-hydroxybenzoate octaprenyltransferase
G2583_4866	CDS	4956102	4958585	-	plsB	Glycerol-3-phosphate acyltransferase
G2583_4867	CDS	4958696	4959064	+	dgkA	Diacylglycerol kinase
G2583_4868	CDS	4959174	4959782	+	lexA	LexA repressor
G2583_4869	CDS	4959801	4961180	+	dinF	MATE efflux family protein
G2583_4870	CDS	4961296	4961505	+	yjbJ	UPF0337 protein yjbJ
G2583_4871	CDS	4961547	4962062	-	zur	Putative zinc uptake regulation protein
G2583_4872	CDS	4962381	4963337	+	yjbM	hypothetical protein
G2583_4873	CDS	4963699	4964736	+	dusA	tRNA-dihydrouridine synthase A
G2583_4874	CDS	4964870	4965112	+	pspG	hypothetical protein
G2583_4875	CDS	4965278	4966261	-	qor	Quinone oxidoreductase
G2583_4876	CDS	4966344	4967759	+	dnaB	Replicative DNA helicase
G2583_4877	CDS	4967812	4968891	+	alr	Alanine racemase, biosynthetic
G2583_4878	CDS	4969144	4970337	+	tyrB	Aromatic-amino-acid transaminase
G2583_4879	CDS	4970676	4971074	-	-	hypothetical protein
G2583_4880	CDS	4971436	4972149	+	aphA	Diadenosine tetraphosphatase
G2583_4881	CDS	4972260	4972676	+	yjbQ	UPF0047 protein yjbQ
G2583_4882	CDS	4972680	4973036	+	yjbR	hypothetical protein
G2583_4883	CDS	4973071	4975893	-	uvrA	UvrABC system protein A
G2583_4884	CDS	4976148	4976684	+	ssb	Single-stranded DNA-binding protein
G2583_4885	CDS	4976783	4977133	-	yjcB	hypothetical protein
G2583_4886	CDS	4977493	4979079	+	yjcC	Cyclic diguanylate phosphodiesterase (EAL) domain protein
G2583_4887	CDS	4979082	4979405	-	soxS	DNA-binding transcriptional dual regulator
G2583_4888	CDS	4979491	4979955	+	soxR	Redox-sensitive transcriptional activator soxR
G2583_4889	CDS	4980501	4981850	+	yjcD	Inorganic anion transporter, sulfate permease (SulP) family
G2583_4890	CDS	4982001	4983650	+	yjcE	Na+/H+ antiporter
G2583_4891	CDS	4983804	4985096	-	yjcF	hypothetical protein
G2583_4892	CDS	4985277	4986926	-	actP	Cation/acetate symporter actP
G2583_4893	CDS	4986923	4987237	-	yjcH	hypothetical protein
G2583_4894	CDS	4987438	4989396	-	acs	Acetyl-coenzyme A synthetase
G2583_4895	CDS	4989789	4991225	+	nrfA	Cytochrome c-552 precursor
G2583_4896	CDS	4991264	4991836	+	nrfB	NrfB, formate-dependent nitrite reductase
G2583_4897	CDS	4991833	4992504	+	nrfC	formate-dependent nitrite reductase; Fe-S centers
G2583_4898	CDS	4992501	4993457	+	nrfD	NrfD protein
G2583_4899	CDS	4993537	4995195	+	nrfE	Cytochrome c-type biogenesis protein NrfE
G2583_4900	CDS	4995188	4995571	+	nrfF	Formate-dependent nitrite reductase complex subunit nrfF
G2583_4901	CDS	4995568	4996164	+	nrfG	Formate-dependent nitrite reductase complex NrfG subunit
G2583_4902	CDS	4996506	4997819	+	gltP	Sodium:dicarboxylate symporter
G2583_4903	CDS	4997997	4998686	-	yjcO	hypothetical protein
G2583_4904	pseudogene	4998780	5000927	-	fdhF	selenopolypeptide subunit of formate dehydrogenase H
G2583_4905	CDS	5001125	5002591	-	mdtP	Multidrug resistance outer membrane protein mdtP precursor
G2583_4906	CDS	5002588	5004639	-	mdtO	Multidrug resistance protein mdtO
G2583_4907	pseudogene	5004639	5005670	-	mdtN	multidrug resistance protein MdtN
G2583_4908	CDS	5005689	5005964	-	ytcA	hypothetical protein
G2583_4909	CDS	5006173	5008170	-	yjcS	hypothetical protein
G2583_4910	CDS	5008431	5009156	+	-	Putative transcriptional regulator
G2583_4911	CDS	5009153	5010154	-	PfkB	PfkB domain protein
G2583_4912	CDS	5010151	5011011	-	-	Fructose-bisphosphate aldolase

Locus_tag	Type	Start	End	+/-[a]	Gene[b]	Product
G2583_4913	CDS	5011026	5011709	-	-	hypothetical protein
G2583_4914	CDS	5011764	5012705	-	-	Putative periplasmic ribose-binding protein of ABC transport
G2583_4915	CDS	5012724	5013698	-	-	Putative permease of ribose ABC transport system
G2583_4916	CDS	5013695	5015248	-	-	Ribose import ATP-binding protein rbsA 2
G2583_4917	CDS	5015233	5017590	+	-	Integral membrane sensor hybrid histidine kinase
G2583_4918	CDS	5017659	5017988	+	yjdP	hypothetical protein
G2583_4919	CDS	5018065	5018823	-	phnP	Phosphonate utilization protein
G2583_4920	CDS	5018825	5019259	-	phnO	predicted acyltransferase with acyl-CoA N-acyltransferase
G2583_4921	CDS	5019246	5019803	-	phnN	Phosphonate metabolism protein/1,5-bisphosphokinase (PRPP-
G2583_4922	CDS	5019803	5020939	-	phnM	Phosphonate metabolism protein PhnM
G2583_4923	CDS	5020936	5021616	-	phnL	Phosphonate C-P lyase system protein PhnL
G2583_4924	CDS	5021727	5022485	-	phnK	Phosphonate C-P lyase system protein PhnK
G2583_4925	CDS	5022482	5023327	-	phnJ	PhnJ protein
G2583_4926	CDS	5023320	5024384	-	phnI	Phosphonate metabolism protein PhnI
G2583_4927	CDS	5024384	5024968	-	phnH	Bacterial phosphonate metabolism protein PhnH
G2583_4928	CDS	5024965	5025417	-	phnG	Phosphonate C-P lyase system protein PhnG
G2583_4929	CDS	5025418	5026143	-	phnF	Phosphonates metabolism transcriptional regulator PhnF
G2583_4930	CDS	5026164	5026994	-	phnE	Phosphonate ABC transporter, permease protein
G2583_4931	CDS	5027049	5028065	-	phnD	Phosphonate ABC transporter, periplasmic phosphonate-
G2583_4932	CDS	5028090	5028878	-	phnC	Phosphonates import ATP-binding protein phnC
G2583_4933	CDS	5029011	5029454	-	yjdN	Putative phnB protein
G2583_4934	CDS	5029613	5030026	-	yjdM	Alkylphosphonate utilization operon protein PhnA
G2583_4935	CDS	5030350	5032578	+	yjdA	hypothetical protein
G2583_4936	CDS	5032575	5033453	+	yjcZ	hypothetical protein
G2583_4937	CDS	5033717	5035219	+	proP	Proline/betaine transporter
G2583_4938	CDS	5035396	5036496	-	basS	Sensor histidine kinase BasS
G2583_4939	CDS	5036497	5037165	-	basR	DNA-binding response regulator BasR
G2583_4940	CDS	5037162	5038835	-	eptA	Putative sulfatase
G2583_4941	CDS	5038909	5040246	-	adiC	Putative amino acid permease
G2583_4942	CDS	5040383	5041144	-	adiY	putative ARAC-type regulatory protein
G2583_4943	CDS	5041469	5043739	-	adiA	Biodegradative arginine decarboxylase
G2583_4944	CDS	5043935	5044843	-	melR	Melibiose operon regulatory protein
G2583_4945	CDS	5045126	5046481	+	melA	Alpha-galactosidase
G2583_4946	CDS	5046596	5048005	+	melB	Melibiose carrier protein
G2583_4947	CDS	5048144	5048773	-	yjdF	hypothetical protein
G2583_4948	CDS	5048896	5050542	-	fumB	Fumarase B
G2583_4949	CDS	5050620	5051960	-	dcuB	Anaerobic C4-dicarboxylate transporter DcuB
G2583_4950	CDS	5052531	5053250	-	dcuR	DNA-binding response regulator in two-component regulatory
G2583_4951	CDS	5053247	5054878	-	dcuS	sensory histidine kinase DcuS
G2583_4952	CDS	5055059	5055289	+	yjdI	hypothetical protein
G2583_4953	CDS	5055301	5055573	+	yjdJ	hypothetical protein
G2583_4954	CDS	5055800	5056096	+	yjdK	hypothetical protein
G2583_4955	CDS	5056124	5056297	+	yjdO	hypothetical protein
G2583_4956	CDS	5056416	5057933	-	lysU	Lysyl-tRNA synthetase, heat inducible
G2583_4957	CDS	5058170	5059627	-	yjdL	Amino acid/peptide transporter
G2583_4958	CDS	5059686	5061833	-	cadA	Lysine decarboxylase, inducible
G2583_4959	CDS	5061913	5063247	-	cadB	Probable cadaverine/lysine antiporter
G2583_4960	CDS	5063613	5065151	-	cadC	Transcriptional activator cadC
G2583_4961	tRNA	5065767	5065844	-	-	Phe tRNA
G2583_4962	CDS	5065950	5066549	-	yjdC	YjdC
G2583_4963	CDS	5066562	5068259	-	dipZ	Thiol:disulfide interchange protein DsbD
G2583_4964	CDS	5068235	5068573	-	cutA	Divalent-cation tolerance protein cutA
G2583_4965	CDS	5068689	5069897	-	dcuA	Anaerobic C4-dicarboxylate transporter dcuA
G2583_4966	CDS	5070108	5071589	-	aspA	aspartate ammonia-lyase
G2583_4967	CDS	5071881	5072357	+	fxsA	FxsA
G2583_4968	CDS	5072373	5073629	-	yjeH	Amino acid-polyamine-organocation (APC) permease family
G2583_4969	CDS	5073905	5074198	+	groS	hypothetical protein
G2583_4970	CDS	5074242	5075888	+	groL	60 kDa chaperonin 1
G2583_4971	CDS	5075993	5076379	+	yjeI	hypothetical protein
G2583_4972	pseudogene	5076572	5077099	-	yjeJ	conserved hypothetical protein

Locus_tag	Type	Start	End	+/-[a]	Gene[b]	Product
G2583_4973	CDS	5077320	5078348	-	yjeK	KamA family protein
G2583_4974	CDS	5078390	5078956	+	efp	Elongation factor P
G2583_4975	CDS	5079008	5079133	+	ecnA	Predicted small secreted protein
G2583_4976	CDS	5079244	5079390	+	ecnB	Entericidin B
G2583_4977	CDS	5079416	5079883	+	sugE	SugE
G2583_4978	CDS	5079880	5080413	-	blc	Outer membrane lipoprotein blc precursor
G2583_4979	CDS	5080501	5081634	-	ampC	Beta-lactamase
G2583_4980	CDS	5081697	5082056	-	frdD	Fumarate reductase subunit D
G2583_4981	CDS	5082067	5082462	-	frdC	Fumarate reductase subunit C
G2583_4982	CDS	5082473	5083207	-	frdB	Fumarate reductase iron-sulfur subunit
G2583_4983	CDS	5083200	5085008	-	frdA	Fumarate reductase, anaerobic, flavoprotein subunit
G2583_4984	CDS	5085333	5086310	+	poxA	Putative lysyl-tRNA synthetase
G2583_4985	CDS	5086487	5088031	+	yjeM	Hypothetical transporter YjeM
G2583_4986	CDS	5088182	5091505	-	yjeP	Mechanosensitive ion channel family protein
G2583_4987	CDS	5091527	5092495	-	psd	Phosphatidylserine decarboxylase proenzyme
G2583_4988	CDS	5092592	5093644	-	rsgA	Probable GTPase engC precursor
G2583_4989	CDS	5093739	5094284	+	orn	Oligoribonuclease
G2583_4990	tRNA	5094495	5094570	+	-	Gly tRNA
G2583_4991	tRNA	5094606	5094683	+	-	Gly tRNA
G2583_4992	tRNA	5094717	5094794	+	-	Gly tRNA
G2583_4993	CDS	5095063	5096202	-	yjeS	hypothetical protein
G2583_4994	CDS	5096201	5097748	+	yjeF	hypothetical protein
G2583_4995	CDS	5097720	5098181	+	yjeE	UPF0079 ATP-binding protein yjeE
G2583_4996	CDS	5098200	5099537	+	amiB	N-acetylmuramoyl-L-alanine amidase AmiB
G2583_4997	CDS	5099547	5101394	+	mutL	DNA mismatch repair protein MutL
G2583_4998	CDS	5101387	5102337	+	miaA	tRNA delta(2)-isopentenylpyrophosphate transferase
G2583_4999	CDS	5102423	5102731	+	hfq	RNA-binding protein Hfq
G2583_5000	CDS	5102808	5104088	+	hflX	GTP-binding protein HflX
G2583_5001	CDS	5104174	5105433	+	hflK	FtsH protease regulator HflK
G2583_5002	CDS	5105436	5106440	+	hflC	FtsH protease regulator HflC
G2583_5003	CDS	5106522	5106719	+	yjeT	hypothetical protein
G2583_5004	CDS	5106823	5108121	+	purA	Adenylosuccinate synthetase
G2583_5005	CDS	5108326	5108751	+	nsrR	transcriptional repressor NsrR
G2583_5006	CDS	5108790	5111231	+	rnr	Putative enzyme
G2583_5007	CDS	5111412	5112143	+	rlmB	23S rRNA (guanosine-2'-O-)-methyltransferase rlmB
G2583_5008	CDS	5112270	5112671	+	yjfI	hypothetical protein
G2583_5009	CDS	5112690	5113388	+	yjfJ	PspA/IM30 family protein
G2583_5010	CDS	5113439	5114098	+	yjfK	hypothetical protein
G2583_5011	CDS	5114116	5114514	+	yjfL	hypothetical protein
G2583_5012	CDS	5114524	5115162	+	yjfM	hypothetical protein
G2583_5013	CDS	5115165	5116328	+	yjfC	Glutathionylspermidine synthase domain protein
G2583_5014	CDS	5116397	5118037	+	aidB	Putative acyl coenzyme A dehydrogenase
G2583_5015	CDS	5118154	5118456	-	yjfN	hypothetical protein
G2583_5016	CDS	5118578	5118907	-	yjfO	hypothetical protein
G2583_5017	CDS	5119089	5119838	+	yjfP	Esterase yjfP
G2583_5018	CDS	5119835	5120590	-	ulaR	putative DEOR-type transcriptional regulator
G2583_5019	CDS	5120698	5121768	-	ulaG	Predicted Zn-dependent hydrolases of the beta-lactamase fold
G2583_5020	CDS	5122117	5123514	+	ulaA	ascorbate-specific PTS system enzyme IIC
G2583_5021	CDS	5123530	5123835	+	ulaB	Ascorbate-specific phosphotransferase enzyme IIB component
G2583_5022	CDS	5123845	5124309	+	ulaC	Ascorbate-specific phosphotransferase enzyme IIA component
G2583_5023	CDS	5124323	5124973	+	ulaD	Putative hexulose-6-phosphate synthase
G2583_5024	CDS	5124983	5125837	+	ulaE	L-ribulose-5-phosphate 3-epimerase ulaE
G2583_5025	CDS	5125789	5126523	+	ulaF	L-ribulose-5-phosphate 4-epimerase ulaF
G2583_5026	CDS	5126652	5126927	-	yjfY	UPF0379 protein yjfY precursor
G2583_5027	CDS	5127254	5127649	+	rpsF	Ribosomal protein S6
G2583_5028	CDS	5127656	5127970	+	priB	Primosomal replication protein n
G2583_5029	CDS	5127975	5128202	+	rpsR	30S ribosomal protein S18
G2583_5030	CDS	5128244	5128693	+	rplI	50S ribosomal protein L9
G2583_5031	CDS	5128764	5129558	-	yjfZ	hypothetical protein
G2583_5033	CDS	5129998	5130180	-	-	hypothetical protein

Locus_tag	Type	Start	End	+/-[a]	Gene[b]	Product
G2583_5034	CDS	5130181	5130612	-	-	Transposase, family
G2583_5035	CDS	5130620	5131828	+	ydcM	IS605 family transposase orfB
G2583_5036	CDS	5131963	5132637	-	ytfB	hypothetical protein
G2583_5037	CDS	5132819	5133439	+	fklB	Peptidyl-prolyl cis-trans isomerase
G2583_5038	CDS	5133748	5135160	+	cycA	D-serine/D-alanine/glycine transporter
G2583_5039	CDS	5135205	5135867	-	ytfE	Regulator of cell morphogenesis and NO signaling
G2583_5040	CDS	5135975	5136949	-	ytfF	Putative transmembrane subunit
G2583_5041	CDS	5137048	5137908	-	ytfG	NmrA family protein
G2583_5042	CDS	5137907	5138377	+	ytfH	hypothetical protein
G2583_5043	CDS	5138495	5140438	-	cpdB	2',3'-cyclic-nucleotide 2'-phosphodiesterase
G2583_5044	CDS	5140628	5141368	+	cysQ	PAPS (adenosine 3'-phosphate 5'-phosphosulfate) 3'(2'),5'-
G2583_5045	CDS	5141580	5142518	+	ytfI	hypothetical protein
G2583_5046	CDS	5142581	5143135	-	ytfJ	hypothetical protein
G2583_5047	CDS	5143421	5143666	+	ytfK	hypothetical protein
G2583_5048	CDS	5143745	5145088	-	ytfL	UPF0053 inner membrane protein ytfL
G2583_5049	CDS	5145411	5146049	-	msrA	Peptide methionine sulfoxide reductase msrA (Protein-
G2583_5050	CDS	5146255	5147988	+	ytfM	hypothetical protein
G2583_5051	CDS	5147985	5151764	+	ytfN	hypothetical protein
G2583_5052	CDS	5151767	5152108	+	ytfP	UPF0131 protein ytfP
G2583_5053	CDS	5151849	5152118	-	yzfA	Ile repressor (ileR)
G2583_5054	CDS	5152314	5152571	+	chpS	PemI protein 2
G2583_5055	CDS	5152565	5152915	+	chpB	PemK protein 2
G2583_5056	CDS	5152995	5153525	-	ppa	Inorganic pyrophosphatase
G2583_5057	CDS	5153835	5154791	+	ytfQ	Putative sugar ABC transporter, periplasmic sugar-binding
G2583_5058	CDS	5154931	5156433	+	ytfR	Uncharacterized ABC transporter ATP-binding protein ytfR
G2583_5059	CDS	5156444	5157469	+	ytfT	Putative transport system permease protein
G2583_5060	CDS	5157456	5158451	+	yjfF	Putative sugar ABC transporter, permease protein
G2583_5061	CDS	5158484	5159482	-	fbp	Fructose-1,6-bisphosphatase
G2583_5062	CDS	5159658	5161031	+	mpl	UDP-N-acetylmuramate:L-alanyl-gamma-D-glutamyl-meso-
G2583_5063	CDS	5161187	5161738	-	yjgA	UPF0307 protein yjgA
G2583_5064	CDS	5161832	5163184	+	pmbA	Putative peptide maturation protein
G2583_5065	CDS	5163452	5163754	+	cybC	Soluble cytochrome b562 precursor
G2583_5066	CDS	5163799	5164263	-	nrdG	Anaerobic ribonucleoside-triphosphate reductase-activating
G2583_5067	CDS	5164453	5166591	-	nrdD	Anaerobic ribonucleoside-triphosphate reductase
G2583_5068	CDS	5166985	5168640	-	treC	Trehalase 6-P hydrolase
G2583_5069	CDS	5168690	5170111	-	treB	PTS system, trehalose-specific IIBC component
G2583_5070	CDS	5170230	5171177	-	treR	Trehalose operon repressor
G2583_5071	CDS	5171182	5171415	+	-	hypothetical protein
G2583_5072	CDS	5171556	5174252	+	mgtA	Magnesium-transporting ATPase, P-type 1
G2583_5073	CDS	5174458	5174883	-	yjgF	hypothetical protein
G2583_5074	CDS	5174917	5175378	-	pyrI	Aspartate carbamoyltransferase regulatory chain
G2583_5075	CDS	5175391	5176326	-	pyrB	Aspartate carbamoyltransferase
G2583_5076	CDS	5176330	5176464	-	pyrL	PyrBI operon leader peptide
G2583_5077	CDS	5176444	5176566	+	yjgG	hypothetical protein
G2583_5078	CDS	5176594	5176776	+	-	hypothetical protein
G2583_5079	CDS	5176745	5177140	-	yjgH	Endoribonuclease L-PSP family protein
G2583_5080	CDS	5177271	5177984	-	yjgI	Oxidoreductase, short chain dehydrogenase/reductase family
G2583_5081	CDS	5178055	5178648	+	yjgJ	hypothetical protein
G2583_5082	CDS	5178784	5179245	+	yjgK	hypothetical protein
G2583_5083	CDS	5179368	5181248	+	yjgL	hypothetical protein
G2583_5084	CDS	5181305	5182309	-	argI	Ornithine carbamoyltransferase
G2583_5085	CDS	5182471	5182887	+	rraB	hypothetical protein
G2583_5086	CDS	5182933	5183436	-	yjgM	Acetyltransferase, GNAT family
G2583_5087	CDS	5183602	5184825	+	yjgN	hypothetical protein
G2583_5088	CDS	5184880	5187735	-	valS	Valyl-tRNA synthetase
G2583_5089	CDS	5187735	5188178	-	holC	DNA polymerase III, chi subunit
G2583_5090	CDS	5188532	5190043	-	pepA	Cytosol aminopeptidase
G2583_5091	CDS	5190310	5191410	+	yjgP	Inner membrane protein yjgP
G2583_5092	CDS	5191407	5192492	+	yjgQ	Inner membrane protein yjgQ
G2583_5093	CDS	5192653	5194155	-	yjgR	hypothetical protein

Locus_tag	Type	Start	End	+/-[a]	Gene[b]	Product
G2583_5094	CDS	5194285	5195346	-	yjgB	Alcohol dehydrogenase
G2583_5095	tRNA	5195499	5195585	+	-	Leu tRNA
G2583_5096	CDS	5195707	5196612	+	-	Prophage P4 integrase
G2583_5097	CDS	5196672	5196839	+	-	hypothetical protein
G2583_5098	CDS	5197072	5197956	-	-	hypothetical protein
G2583_5099	CDS	5197910	5198236	-	yjhT	N-acetylneuraminate epimerase 2
G2583_5100	CDS	5198464	5199567	-	-	hypothetical protein
G2583_5101	CDS	5199571	5200278	-	-	hypothetical protein
G2583_5102	CDS	5200285	5201937	-	-	hypothetical protein
G2583_5103	CDS	5202149	5205508	+	-	hypothetical protein
G2583_5104	CDS	5205508	5211822	+	-	hypothetical protein
G2583_5105	CDS	5212046	5214904	+	-	Helicase family protein
G2583_5106	CDS	5214907	5219841	+	-	hypothetical protein
G2583_5107	CDS	5219841	5226182	+	-	DEAD/DEAH box helicase domain protein
G2583_5108	CDS	5226179	5228293	+	-	ATP-dependent DNA helicase, UvrD/REP family
G2583_5109	pseudogene	5229246	5229664	-	-	hypothetical protein
G2583_5110	CDS	5229787	5230767	-	yjhS	hypothetical protein
G2583_5111	CDS	5230832	5232046	-	yjhT	N-acetylneuraminate-epimerase precursor
G2583_5112	CDS	5231958	5232683	-	nanC	hypothetical protein
G2583_5113	CDS	5234130	5234732	+	fimB	Type 1 fimbriae regulatory protein fimB
G2583_5114	CDS	5235210	5235806	+	fimE	Type 1 fimbriae regulatory protein FimE
G2583_5115	CDS	5236287	5236835	+	fimA	Major type 1 subunit fimbrin
G2583_5116	CDS	5236792	5237439	+	fimI	FimI fimbrial protein
G2583_5117	CDS	5237476	5238201	+	fimC	Chaperone protein FimC
G2583_5118	CDS	5238268	5240904	+	fimD	Export and assembly outer membrane protein of type 1 fimbriae
G2583_5119	CDS	5240914	5241444	+	fimF	Fimbrial protein FimF
G2583_5120	CDS	5241457	5241960	+	fimG	FimG
G2583_5121	CDS	5241980	5242882	+	fimH	Adhesin
G2583_5122	CDS	5243056	5244399	-	gntP	High-affinity gluconate transporter
G2583_5123	CDS	5244739	5245923	+	uxuA	D-mannonate dehydratase
G2583_5124	CDS	5246004	5247464	+	uxuB	Fructuronate reductase
G2583_5125	CDS	5247679	5248452	+	uxuR	DNA-binding transcriptional repressor
G2583_5126	CDS	5248593	5249423	-	yjiC	hypothetical protein
G2583_5127	CDS	5249757	5249900	+	-	hypothetical protein
G2583_5128	CDS	5250087	5250488	+	yjiD	Anti-adapter protein iraD
G2583_5129	CDS	5250481	5251224	-	yjiE	transcriptional regulator, LysR family
G2583_5130	CDS	5251457	5252629	-	iadA	Beta-aspartyl peptidase
G2583_5131	CDS	5252642	5253103	-	yjiG	putative membrane protein
G2583_5132	CDS	5253100	5253795	-	yjiH	hypothetical protein
G2583_5133	CDS	5253931	5254584	+	yjiI	Putative uncharacterized protein yjiI
G2583_5134	CDS	5254741	5259981	-	yeeJ	Putative invasin
G2583_5135	CDS	5260192	5261370	-	yjiJ	Transporter, major facilitator family
G2583_5136	CDS	5261438	5262427	-	yjiK	SdiA-regulated protein
G2583_5137	CDS	5262546	5264759	-	-	hypothetical protein
G2583_5138	CDS	5265071	5265337	-	-	hypothetical protein
G2583_5139	CDS	5265454	5266101	-	yjiL	(R)-2-hydroxyglutaryl-CoA dehydratase activator
G2583_5140	CDS	5266111	5267283	-	yjiM	hypothetical protein
G2583_5141	CDS	5267378	5268658	-	yjiN	conserved hypothetical protein
G2583_5142	CDS	5268699	5269931	-	mdtM	Multidrug resistance protein mdtM
G2583_5143	CDS	5270398	5271330	+	yjiP	hypothetical protein
G2583_5144	CDS	5271573	5272985	-	yjiR	transcriptional regulator, GntR family/aminotransferase, classes
G2583_5145	CDS	5273162	5273326	+	yjiS	YjiS
G2583_5146	pseudogene	5273423	5275516	+	-	hypothetical protein
G2583_5147	CDS	5275563	5275904	-	yjiW	HSP20-like domain protein
G2583_5148	CDS	5276125	5277879	-	hsdS	Putative type I restriction-modification system, S subunit
G2583_5149	CDS	5277879	5279360	-	hsdM	Type I restriction-modification system, M subunit
G2583_5150	CDS	5279415	5281847	-	hsdR	Type I restriction-modification system, R subunit
G2583_5151	CDS	5282125	5282409	+	-	protein of unknown function DUF262
G2583_5152	pseudogene	5282125	5283776	+	orf	protein of unknown function DUF262
G2583_5153	CDS	5283845	5284801	-	yjiA	Putative GTPase

Locus_tag	Type	Start	End	+/-[a]	Gene[b]	Product
G2583_5154	CDS	5284812	5285015	-	yjiX	hypothetical protein
G2583_5155	CDS	5285065	5287230	-	yjiY	Carbon starvation family protein
G2583_5156	CDS	5287508	5288050	-	-	hypothetical protein
G2583_5157	CDS	5288279	5289943	+	tsr	Methyl-accepting chemotaxis protein I
G2583_5158	CDS	5289992	5291353	-	yjjL	Transporter, major facilitator family
G2583_5159	CDS	5291568	5292482	-	yjjM	transcriptional regulator, GntR family
G2583_5160	CDS	5292606	5293643	+	yjjN	Putative oxidoreductase
G2583_5161	CDS	5293780	5296071	-	mdoB	Phosphoglycerol transferase I
G2583_5162	CDS	5296325	5296822	-	yjjA	hypothetical protein
G2583_5163	CDS	5296868	5297605	-	dnaC	DNA replication protein DnaC
G2583_5164	CDS	5297608	5298147	-	dnaT	Primosomal protein 1
G2583_5165	CDS	5298254	5298727	-	yjjB	Uncharacterized conserved protein
G2583_5166	CDS	5298718	5299551	-	yjjP	putative structural protein
G2583_5167	CDS	5300109	5300834	+	yjjQ	hypothetical protein
G2583_5168	CDS	5300792	5301469	+	bglJ	transcriptional regulator, LuxR family
G2583_5169	CDS	5301507	5302295	-	fhuF	Ferric iron reductase protein FhuF
G2583_5170	CDS	5302397	5302672	+	yjjZ	hypothetical protein
G2583_5171	tRNA	5302710	5302798	-	-	Leu tRNA
G2583_5277	tRNA	5302832	5302918	-	-	Leu tRNA
G2583_5278	tRNA	5302947	5303033	-	-	Leu tRNA
G2583_5172	CDS	5303223	5304254	-	rsmC	16S RNA G1207 methylase RsmC
G2583_5173	CDS	5304357	5304770	+	holD	DNA polymerase III, psi subunit
G2583_5174	CDS	5304739	5305185	+	rimI	Ribosomal-protein-alanine acetyltransferase
G2583_5175	CDS	5305200	5305877	+	yjjG	5'-nucleotidase yjjG
G2583_5176	CDS	5306263	5307486	+	-	Phage integrase
G2583_5177	CDS	5307669	5311496	+	-	hypothetical protein
G2583_5178	CDS	5311893	5312513	-	yfdT	hypothetical protein
G2583_5179	CDS	5312513	5312875	-	yfdS	hypothetical protein
G2583_5180	CDS	5312866	5313402	-	yfdR	hypothetical protein
G2583_5181	CDS	5313530	5314354	-	yfdQ	hypothetical protein
G2583_5182	CDS	5314420	5315013	-	yfdP	hypothetical protein
G2583_5183	CDS	5315151	5315429	-	-	hypothetical protein
G2583_5184	CDS	5315485	5316132	-	rpc	Putative regulatory protein
G2583_5185	CDS	5316275	5316535	+	-	hypothetical protein
G2583_5186	CDS	5316528	5317079	+	ymfL	hypothetical protein
G2583_5187	CDS	5317076	5317414	+	ymfM	e14 prophage predicted protein
G2583_5188	CDS	5317424	5318365	+	yfdO	Unknown protein encoded by cryptic prophage
G2583_5189	CDS	5318362	5318856	+	yfdN	hypothetical protein
G2583_5190	CDS	5318856	5319509	+	yfdM	putative DNA adenine methylase
G2583_5191	CDS	5319506	5319832	+	-	hypothetical protein
G2583_5192	CDS	5319829	5320224	+	-	Holliday junction resolvase
G2583_5193	CDS	5320387	5321202	+	KilA-N	KilA-N domain family
G2583_5194	CDS	5321282	5322199	+	ydfU	hypothetical protein
G2583_5195	CDS	5322213	5322965	+	ydfT	Antitermination protein Q
G2583_5196	CDS	5323388	5323600	-	cspF	Cold shock-like protein cspF
G2583_5197	CDS	5323901	5324116	+	cspB	Cold shock-like protein cspB
G2583_5198	CDS	5324869	5325084	+	essQ	Lysis protein S-like protein from lambdoid prophage Qin
G2583_5199	CDS	5325089	5325433	+	ydfR	hypothetical protein
G2583_5200	CDS	5325399	5325671	-	-	hypothetical protein
G2583_5201	CDS	5325777	5326310	+	ydfQ	phage lysozyme
G2583_5202	CDS	5326307	5326798	+	ydfP	hypothetical protein
G2583_5203	CDS	5327167	5327379	+	cspI	Cold shock-like protein cspI
G2583_5204	CDS	5327651	5327881	+	ynfN	hypothetical protein
G2583_5205	CDS	5328054	5328227	+	gnsB	GnsB protein
G2583_5206	CDS	5328523	5328729	+	ybcW	conserved hypothetical protein
G2583_5207	CDS	5328983	5329303	-	ylcI	hypothetical protein
G2583_5208	CDS	5329563	5330111	+	nohA	Prophage Qin DNA packaging protein NU1-like protein
G2583_5209	CDS	5330041	5332011	+	-	Putative DNA packaging protein of prophage
G2583_5210	CDS	5331995	5332201	+	-	Head-stabilizing protein
G2583_5211	CDS	5332198	5333790	+	-	Putative capsid protein of prophage

Locus_tag	Type	Start	End	+/-[a]	Gene[b]	Product
G2583_5212	CDS	5333780	5335285	+	-	Head-tail preconnector protein GP5
G2583_5213	CDS	5335322	5335669	+	-	Head decoration protein
G2583_5214	CDS	5335727	5336755	+	-	Major head protein
G2583_5215	CDS	5336807	5337181	+	-	Uncharacterized protein
G2583_5216	CDS	5337174	5337527	+	-	Putative head-tail joining protein of prophage
G2583_5217	CDS	5337539	5338117	+	-	Prophage minor tail protein Z
G2583_5218	CDS	5338114	5338509	+	-	Permeases of the major facilitator superfamily
G2583_5219	CDS	5338487	5339257	+	-	Putative tail component of prophage
G2583_5220	CDS	5339273	5339695	+	-	Putative tail component of prophage
G2583_5221	CDS	5339677	5340111	+	-	Minor tail protein T
G2583_5222	CDS	5340104	5342665	+	-	Putative tail length tape measure protein
G2583_5223	CDS	5342662	5342991	+	-	Minor tail protein
G2583_5224	CDS	5342991	5343689	+	-	Phage-related protein
G2583_5225	CDS	5343640	5344437	+	-	Putative tail fiber component K of prophage
G2583_5226	CDS	5344335	5344976	+	-	Putative tail component of prophage CP-933K
G2583_5227	CDS	5345037	5345387	+	-	hypothetical protein
G2583_5228	CDS	5345454	5348852	+	-	Phage-related protein, tail component
G2583_5229	CDS	5348919	5349518	+	-	putative membrane protein precursor
G2583_5230	CDS	5349583	5352411	+	-	PPE-repeat proteins
G2583_5231	CDS	5352411	5352986	+	tfaQ	Tail fiber assembly protein from lambdoid prophage Qin
G2583_5232	CDS	5353084	5353674	-	pinR	Putative DNA-invertase from lambdoid prophage Rac
G2583_5233	CDS	5354053	5354319	-	ydfK	hypothetical protein
G2583_5234	CDS	5354895	5355143	-	-	hypothetical protein
G2583_5235	CDS	5355375	5356949	+	prfC	Peptide chain release factor RF-3
G2583_5236	CDS	5357342	5357947	+	osmY	Osmotically inducible protein Y
G2583_5237	CDS	5358357	5359430	+	yjjU	hypothetical protein
G2583_5238	CDS	5359427	5360209	+	yjjV	Hydrolase, TatD family
G2583_5239	CDS	5360423	5361286	-	yjjW	Putative activating enzyme
G2583_5240	CDS	5361258	5362808	-	yjjI	hypothetical protein
G2583_5241	CDS	5363066	5363845	+	deoC	Deoxyribose-phosphate aldolase
G2583_5242	CDS	5363972	5365294	+	deoA	Thymidine phosphorylase
G2583_5243	CDS	5365346	5366569	+	deoB	Phosphopentomutase
G2583_5244	CDS	5366626	5367345	+	deoD	Purine nucleoside phosphorylase
G2583_5245	CDS	5367506	5367769	-	-	Helix-turn-helix domain protein
G2583_5246	pseudogene	5367801	5369489	-	lplA	protein smp precursor
G2583_5247	CDS	5369595	5370563	+	serB	Phosphoserine phosphatase
G2583_5248	CDS	5370612	5371994	+	radA	Predicted ATP-dependent serine protease
G2583_5249	CDS	5372015	5373247	+	nadR	bifunctional DNA-binding transcriptional repressor and NMN
G2583_5250	CDS	5373555	5375222	-	yjjK	Uncharacterized ABC transporter ATP-binding protein yjjK
G2583_5251	CDS	5375433	5377370	+	slt	Soluble lytic murein transglycosylase
G2583_5252	CDS	5377460	5377786	+	trpR	Trp operon repressor
G2583_5253	CDS	5377933	5378454	-	yjjX	conserved hypothetical protein
G2583_5254	CDS	5378497	5379144	+	ytjC	Probable phosphoglycerate mutase gpmB
G2583_5255	CDS	5379141	5380010	-	rob	Right origin-binding protein
G2583_5256	CDS	5380221	5380694	+	creA	hypothetical protein
G2583_5257	CDS	5380707	5381396	+	creB	DNA-binding response regulator CreB
G2583_5258	CDS	5381396	5382820	+	creC	sensory histidine kinase CreC
G2583_5259	CDS	5382878	5384230	+	creD	Inner membrane protein CreD
G2583_5260	CDS	5384290	5385006	-	arcA	Aerobic respiration control protein arcA
G2583_5261	CDS	5385102	5385242	+	yjjY	hypothetical protein
G2583_5262	CDS	5385642	5386328	+	yjtD	RNA methyltransferase, TrmH family, group 1

[a]+/-, orientation as annotated. [b]Gene name from K12 MG1655 and EDL933 genome annotation.
