# Supplementary material for: Derivation of Escherichia coli O157:H7 from Its O55:H7 Precursor
Source: PLoS One. 2010 Jan 14;5(1):e8700. doi: 10.1371/journal.pone.0008700 (PMC2806823; doi:10.1371/journal.pone.0008700)
Supplement: Table S2 — Single base and small indel differences. A full list of single base and small indel differences between the CB9651, Sakai, and EDL933 genomes, including location, nature of difference, and name of gene affected. (0.31 MB PDF) [file pone.0008700.s004.pdf]

lineage[a]	site[b]	mutation type[c]	recomb	gene	Gene name	Type
CB9615	68	i		intergenic		
O157	230	ins-18		G2583_0001	thrL	pseudogene
CB9615	1772	s		G2583_0002	thrA	CDS
O157	3135	s		G2583_0003	thrB	CDS
CB9615	3160	ns		G2583_0003	thrB	CDS
O157	11682	ns		G2583_0013	yaaI	CDS
CB9615	13083	s		G2583_0014	dnaK	CDS
CB9615	15216	s		G2583_0015	dnaJ	CDS
CB9615	15594	ns		G2583_0016	mokC	CDS
CB9615	15595	ns		G2583_0016	mokC	CDS
CB9615	16562	ns		G2583_0017	nhaA	CDS
O157	17613	s		G2583_0018	nhaR	CDS
O157	17693	ns		G2583_0018	nhaR	CDS
O157	18711	ns		G2583_0019	-	CDS
Sakai	18870	del		G2583_0019	-	CDS
O157	18994	ins		G2583_0019	-	CDS
O157	23608	s		G2583_0023	-	CDS
CB9615	24215	ns		G2583_0023	-	CDS
CB9615	24797	i		intergenic		
CB9615	24966	i		intergenic		
O157	27142	ns		G2583_0027	ileS	CDS
CB9615	29411	s		G2583_0027	ileS	CDS
O157	33077	s		G2583_0032	dapB	CDS
CB9615	35214	s		G2583_0034	carB	CDS
CB9615	36586	s		G2583_0034	carB	CDS
CB9615	37875	s		G2583_0034	carB	CDS
O157	39899	ns		G2583_0038	caiD	CDS
O157	41181	ns		G2583_0039	caiC	CDS
O157	45916	ns		G2583_0042	caiT	CDS
CB9615	46532	del-5		intergenic		
CB9615	46568	i		intergenic		
CB9615	47781	s		G2583_0044	fixB	CDS
CB9615	47895	s		G2583_0044	fixB	CDS
O157	48272	s		G2583_0044	fixB	CDS
CB9615	48524	i		intergenic		
CB9615	48749	s		G2583_0045	fixC	CDS
O157	49130	ns		G2583_0045	fixC	CDS
O157	51720	ns		G2583_0048	kefF	CDS
O157	53695	ns		G2583_0049	kefC	CDS
O157	53715	s		G2583_0049	kefC	CDS
O157	54375	ns		G2583_0050	folA	CDS
O157	61460	ns		G2583_0058	imp	CDS
CB9615	61782	del-6		G2583_0058	imp	CDS
O157	61916	ns		G2583_0058	imp	CDS
CB9615	62434	ns		G2583_0059	djlA	CDS

lineage[a]	site[b]	mutation type[c]	recomb	gene	Gene name	Type
O157	63127	s		G2583_0059	djlA	CDS
O55/O157	64021	ns		G2583_0060	yabP	CDS
CB9615	64037	del-15		G2583_0060	yabP	CDS
O157	68716	ns		G2583_0063	polB	CDS
O157	68978	s		G2583_0063	polB	CDS
CB9615	70349	ns		G2583_0063	polB	CDS
CB9615	72621	s		G2583_0065	araA	CDS
O157	77773	ns		G2583_0070	thiP	CDS
O157	80651	i		intergenic		
O157	81668	ns		G2583_0073	sgrR	CDS
CB9615	84216	s		G2583_0076	leuC	CDS
O157	84308	s		G2583_0076	leuC	CDS
CB9615	88405	i		intergenic		
CB9615	88632	i		intergenic		
O157	88701	i		intergenic		
CB9615	88938	ns		G2583_0080	leuO	CDS
O157	92154	ns		G2583_0082	ilvH	CDS
CB9615	97089	s		G2583_0088	ftsI	CDS
CB9615	97345	ns		G2583_0088	ftsI	CDS
CB9615	98791	s		G2583_0089	murE	CDS
O157	99098	ns		G2583_0089	murE	CDS
O157	104256	ns		G2583_0094	murG	CDS
CB9615	105290	i		intergenic		
O157	107438	ns		G2583_0096	ddlB	CDS
CB9615	111523	ns		G2583_0100	lpxC	CDS
O157	112158	ins		G2583_0101	secM	CDS
CB9615	113450	s		G2583_0102	secA	CDS
CB9615	113690	s		G2583_0102	secA	CDS
CB9615	120536	nc		G2583_0111	hofB	pseudogene
CB9615	120839	nc		G2583_0111	hofB	pseudogene
CB9615	124154	ns		G2583_0115	ampE	CDS
CB9615	124225	s		G2583_0115	ampE	CDS
O157	125601	ns		G2583_0116	aroP	CDS
O157	126145	i		intergenic		
O157	130378	s		G2583_0119	aceF	CDS
O157	130479	ns		G2583_0119	aceF	CDS
O157	130485	ns		G2583_0119	aceF	CDS
CB9615	133723	ins		intergenic		
O157	137723	s		G2583_0122	acnB	CDS
CB9615	137832	ns		G2583_0122	acnB	CDS
CB9615	140722	ns		G2583_0125	speE	CDS
CB9615	140745	s		G2583_0125	speE	CDS
O157	142584	ns		G2583_0127	cueO	CDS
O157	142879	s		G2583_0127	cueO	CDS
O157	142935	ns		G2583_0127	cueO	CDS

lineage[a]	site[b]	mutation type[c]	recomb	gene	Gene name	Type
CB9615	143250	s		G2583_0128	gcd	CDS
O157	149664	ns		G2583_0134	yadE	CDS
CB9615	150418	ns		G2583_0134	yadE	CDS
CB9615	151169	i		intergenic		
O157	152504	ins-7		intergenic		
O157	153464	s		G2583_0138	panB	CDS
O55/O157	155342	ns		G2583_0139	yadC	CDS
O55/O157	155668	ns		G2583_0140	yadK	CDS
O157	157299	ns		G2583_0143	htrE	CDS
O157	157731	ns		G2583_0143	htrE	CDS
CB9615	157787	s		G2583_0143	htrE	CDS
EDL933	158811	ns		G2583_0143	htrE	CDS
O55/O157	159379	ns		G2583_0143	htrE	CDS
O157	159972	ns		G2583_0144	ecpD	CDS
CB9615	160118	ns		G2583_0144	ecpD	CDS
Sakai	160672	i		intergenic		
CB9615	160916	ns		G2583_0145	yadN	CDS
O55/O157	161322	i		intergenic		
O55/O157	161496	indel		intergenic		
CB9615	162437	s		G2583_0147	pcnB	CDS
O157	164723	s		G2583_0149	dksA	CDS
O157	165836	ns		G2583_0151	ligT	CDS
CB9615	168865	i		intergenic		
CB9615	171141	s		G2583_0153	mrcB	CDS
O157	171729	i		intergenic		
O157	171915	ns		G2583_0154	fhuA	CDS
CB9615	172434	s		G2583_0154	fhuA	CDS
CB9615	172823	ns		G2583_0154	fhuA	CDS
CB9615	173010	s		G2583_0154	fhuA	CDS
O157	174080	i		intergenic		
CB9615	175920	s		G2583_0157	fhuB	CDS
CB9615	176796	s		G2583_0157	fhuB	CDS
O157	178365	s		G2583_0158	hemL	CDS
O157	180160	s		G2583_0159	clcA	CDS
O157	181573	ns		G2583_0161	yadS	CDS
O157	182330	ns		G2583_0162	btuF	CDS
O157	184717	s		G2583_0164	dgt	CDS
CB9615	186669	s		G2583_0166	cdaR	CDS
O157	188771	ns		G2583_0168	dapD	CDS
CB9615	192082	i		intergenic		
EDL933	193444	s		G2583_0172	rpsB	CDS
CB9615	194111	i		intergenic		
O157	203763	del		intergenic		
Sakai	205552	ns		G2583_0183	fabZ	CDS
O157	206371	ns		G2583_0184	lpxA	CDS

lineage[a]	site[b]	mutation type[c]	recomb	gene	Gene name	Type
O157	207004	ns		G2583_0185	lpxB	CDS
CB9615	207476	s		G2583_0185	lpxB	CDS
CB9615	209270	ns		G2583_0187	dnaE	CDS
O157	215798	ns		G2583_0191	tilS	CDS
O157	216648	s		G2583_0191	tilS	CDS
O157	216680	ns		G2583_0191	tilS	CDS
O157	216899	ns		G2583_0191	tilS	CDS
CB9615	217696	ns		G2583_0194	yaeQ	CDS
O157	218091	ns		G2583_0194	yaeQ	CDS
O157	218572	del-12		G2583_0195	yaeJ	CDS
O157	220106	ns		G2583_0197	yaeF	CDS
CB9615	220361	s		G2583_0198	proS	CDS
O157	222346	s		G2583_0199	yaeB	CDS
CB9615	225362	ns		G2583_0203	metN	CDS
CB9615	226620	ns		G2583_0204	gmhB	CDS
O157	226827	i		intergenic		
CB9615	226834	del		intergenic		
CB9615	226836	i		intergenic		
CB9615	226868	i		intergenic		
O157	227246	nc		G2583_0205	rrs	rRNA
O157	229735	nc		G2583_0208	rrl	rRNA
Sakai	230035	nc		G2583_0208	rrl	rRNA
CB9615	230268	nc		G2583_0208	rrl	rRNA
CB9615	230277	nc		G2583_0208	rrl	rRNA
O157	232097	nc		G2583_0209	rrf	rRNA
CB9615	232171	i		intergenic		
O157	235029	s		G2583_0213	yafD	CDS
O157	235432	ns		G2583_0214	yafE	CDS
O157	238495	ns		G2583_0217	yafS	CDS
O157	239591	i		intergenic		
O157	240618	ins		intergenic		
O55/O157	241801	i		intergenic		
CB9615	242655	ns		G2583_0224	-	CDS
O157	243292	ns		G2583_0224	-	CDS
O157	243714	ns		G2583_0225	yhhZ	CDS
O157	249997	del		G2583_0228	ImpA	CDS
CB9615	252262	ins-12		G2583_0230	clpB	CDS
CB9615	252743	s		G2583_0230	clpB	CDS
O157	253307	ns		G2583_0230	clpB	CDS
O157	254058	s		G2583_0231	-	CDS
O157	254587	ns		G2583_0231	-	CDS
O157	255200	ns		G2583_0232	-	CDS
CB9615	255682	ns		G2583_0232	-	CDS
CB9615	257039	ns		G2583_0234	-	CDS
CB9615	257682	del		intergenic		

lineage[a]	site[b]	mutation type[c]	recomb	gene	Gene name	Type
O157	259555	s		G2583_0236	-	CDS
O157	261137	ns		G2583_0237	-	CDS
O157	261237	nc		G2583_0238	-	pseudogene
CB9615	261680	nc		G2583_0238	-	pseudogene
CB9615	261984	nc		G2583_0238	-	pseudogene
O157	263163	ns		G2583_0240	-	CDS
CB9615	263595	i		intergenic		
O157	263672	i		intergenic		
O157	263701	i		intergenic		
O157	263921	ns		G2583_0241	-	CDS
Sakai	264928	ns		G2583_0244	VgrG	CDS
O157	264970	ns		G2583_0244	VgrG	CDS
CB9615	265088	s		G2583_0244	VgrG	CDS
O157	265423	ns		G2583_0244	VgrG	CDS
CB9615	266558	s		G2583_0244	VgrG	CDS
O157	267092	nc		G2583_0245	rhsD	pseudogene
CB9615	267119	nc		G2583_0245	rhsD	pseudogene
CB9615	267128	nc		G2583_0245	rhsD	pseudogene
O157	268213	nc		G2583_0245	rhsD	pseudogene
O157	269117	nc		G2583_0245	rhsD	pseudogene
CB9615	269127	nc		G2583_0245	rhsD	pseudogene
O157	269551	nc		G2583_0245	rhsD	pseudogene
O55/O157	272486	nc	rec	G2583_0247	rhsH	pseudogene
O55/O157	272489	nc	rec	G2583_0247	rhsH	pseudogene
O55/O157	272507	nc	rec	G2583_0247	rhsH	pseudogene
O55/O157	272519	nc	rec	G2583_0247	rhsH	pseudogene
O55/O157	272540	nc	rec	G2583_0247	rhsH	pseudogene
O55/O157	272550	nc	rec	G2583_0247	rhsH	pseudogene
O55/O157	272570	nc	rec	G2583_0247	rhsH	pseudogene
O55/O157	272573	nc	rec	G2583_0247	rhsH	pseudogene
O55/O157	272576	nc	rec	G2583_0247	rhsH	pseudogene
O55/O157	272591	nc	rec	G2583_0247	rhsH	pseudogene
O55/O157	272693	nc	rec	G2583_0247	rhsH	pseudogene
O55/O157	272711	nc	rec	G2583_0247	rhsH	pseudogene
O55/O157	272723	nc	rec	G2583_0247	rhsH	pseudogene
O55/O157	272726	nc	rec	G2583_0247	rhsH	pseudogene
O55/O157	272736	nc	rec	G2583_0247	rhsH	pseudogene
O55/O157	272738	nc	rec	G2583_0247	rhsH	pseudogene
O55/O157	272739	nc	rec	G2583_0247	rhsH	pseudogene
O55/O157	272740	nc	rec	G2583_0247	rhsH	pseudogene
O55/O157	272741	nc	rec	G2583_0247	rhsH	pseudogene
O55/O157	272744	nc	rec	G2583_0247	rhsH	pseudogene
O55/O157	272745	nc	rec	G2583_0247	rhsH	pseudogene
O55/O157	272747	nc	rec	G2583_0247	rhsH	pseudogene
O55/O157	272759	nc	rec	G2583_0247	rhsH	pseudogene

lineage[a]	site[b]	mutation type[c]	recomb	gene	Gene name	Type
O55/O157	272771	nc	rec	G2583_0247	rhsH	pseudogene
O55/O157	272810	nc	rec	G2583_0247	rhsH	pseudogene
O55/O157	272834	nc	rec	G2583_0247	rhsH	pseudogene
O55/O157	272867	nc	rec	G2583_0247	rhsH	pseudogene
O55/O157	272933	nc	rec	G2583_0247	rhsH	pseudogene
Sakai	270484	nc	rec	G2583_0247	rhsH	pseudogene
Sakai	270502	nc	rec	G2583_0247	rhsH	pseudogene
Sakai	270545	nc	rec	G2583_0247	rhsH	pseudogene
Sakai	270562	nc	rec	G2583_0247	rhsH	pseudogene
Sakai	270577	nc	rec	G2583_0247	rhsH	pseudogene
Sakai	270579	nc	rec	G2583_0247	rhsH	pseudogene
Sakai	270584	nc	rec	G2583_0247	rhsH	pseudogene
Sakai	270592	nc	rec	G2583_0247	rhsH	pseudogene
Sakai	270594	nc	rec	G2583_0247	rhsH	pseudogene
Sakai	270595	nc	rec	G2583_0247	rhsH	pseudogene
Sakai	270596	nc	rec	G2583_0247	rhsH	pseudogene
Sakai	270598	nc	rec	G2583_0247	rhsH	pseudogene
Sakai	270627	nc	rec	G2583_0247	rhsH	pseudogene
Sakai	270646	nc	rec	G2583_0247	rhsH	pseudogene
Sakai	270666	nc	rec	G2583_0247	rhsH	pseudogene
Sakai	270668	nc	rec	G2583_0247	rhsH	pseudogene
Sakai	270674	nc	rec	G2583_0247	rhsH	pseudogene
O157	272354	ns		G2583_0250	-	CDS
CB9615	274856	ns		G2583_0250	-	CDS
O157	272851	ns		G2583_0251	yhhI	CDS
O157	273176	s		G2583_0251	yhhI	CDS
O157	273923	ns		G2583_0252	rhsD	CDS
CB9615	276826	s		G2583_0252	rhsD	CDS
O157	274506	ns		G2583_0252	rhsD	CDS
CB9615	277259	s		G2583_0252	rhsD	CDS
O157	274975	s		G2583_0252	rhsD	CDS
O157	280350	ns		G2583_0257	fadE	CDS
CB9615	282980	s		G2583_0257	fadE	CDS
CB9615	283888	i		intergenic		
O157	284183	del-2		G2583_0261	yafQ	CDS
O157	284481	i		intergenic		
O157	284949	s		G2583_0263	yafL	CDS
O157	285478	i		intergenic		
O157	285598	i		intergenic		
CB9615	288917	s		G2583_0265	FhiA	CDS
O157	287783	s		G2583_0265	FhiA	CDS
O157	289210	ns		G2583_0267	dinB	CDS
CB9615	293727	i		intergenic		
CB9615	294876	s		G2583_0273	pepD	CDS
O157	294920	s		G2583_0275	frsA	CDS

lineage[a]	site[b]	mutation type[c]	recomb	gene	Gene name	Type
O157	295149	ns		G2583_0275	frsA	CDS
CB9615	302241	ns		G2583_0279	proA	CDS
CB9615	302620	i		intergenic		
O55/O157	351852	ns		G2583_0351	-	CDS
O55/O157	352160	s		G2583_0351	-	CDS
O55/O157	352655	s		G2583_0351	-	CDS
O55/O157	353019	ns		G2583_0352	-	CDS
O55/O157	354030	ns		G2583_0354	-	CDS
O55/O157	354909	indel		G2583_0355	-	pseudogene
O55/O157	354927	nc		G2583_0355	-	pseudogene
O55/O157	355063	nc		G2583_0355	-	pseudogene
O55/O157	355069	nc		G2583_0355	-	pseudogene
O55/O157	355077	i		intergenic		
O55/O157	355165	ns		G2583_0356	-	CDS
O55/O157	355596	ns		G2583_0356	-	CDS
O55/O157	314615	indel		G2583_0356	-	CDS
O55/O157	355719	s		G2583_0357	-	CDS
O55/O157	356069	ns		G2583_0357	-	CDS
O55/O157	356460	i		intergenic		
O55/O157	357897	s		G2583_0360	sidI	CDS
O55/O157	317349	indel-15		intergenic		
O55/O157	358376	i		intergenic		
O55/O157	358942	i		intergenic		
O55/O157	359857	ns		G2583_0363	-	CDS
CB9615	361724	ns		G2583_0366	-	CDS
O55/O157	363783	ns		G2583_0367	-	CDS
O55/O157	363874	s		G2583_0367	-	CDS
O55/O157	323350	indel		intergenic		
O55/O157	364419	i		intergenic		
O55/O157	366418	i		intergenic		
O55/O157	325514	indel		intergenic		
O157	327035	ns		G2583_0372	-	CDS
CB9615	368572	ns		G2583_0373	-	CDS
O157	327992	s		G2583_0373	-	CDS
O157	329623	ns		G2583_0374	-	CDS
O157	329641	ns		G2583_0374	-	CDS
CB9615	370778	ns		G2583_0374	-	CDS
CB9615	371592	ins		G2583_0375	yagQ	pseudogene
O157	331010	nc		G2583_0375	yagQ	pseudogene
CB9615	372009	nc		G2583_0375	yagQ	pseudogene
O157	331468	s		G2583_0376	yagR	CDS
O157	331675	ns		G2583_0376	yagR	CDS
O157	332184	ns		G2583_0376	yagR	CDS
CB9615	373580	s		G2583_0376	yagR	CDS
O157	333584	ns		G2583_0377	yagS	CDS

lineage[a]	site[b]	mutation type[c]	recomb	gene	Gene name	Type
CB9615	375238	ns		G2583_0377	yagS	CDS
CB9615	377140	i		intergenic		
O157	337018	ns		G2583_0381	yagV	CDS
Sakai	338461	s		G2583_0382	yagW	CDS
CB9615	379728	s		G2583_0382	yagW	CDS
O157	342459	i		intergenic		
CB9615	383951	s		G2583_0385	matB	CDS
CB9615	383993	s		G2583_0385	matB	CDS
CB9615	384694	s		G2583_0386	matA	CDS
O157	343853	ins		intergenic		
O157	344143	i		intergenic		
Sakai	345586	ns		ECs0328	-	CDS
CB9615	386535	i		intergenic		
EDL933	348032	ins		intergenic		
O157	348047	del		intergenic		
EDL933	349752	ins		intergenic		
O157	349878	i		intergenic		
CB9615	389309	ns		G2583_0392	ycjY	CDS
O157	351109	ns		G2583_0393	ycaN	CDS
O157	351209	ns		G2583_0393	ycaN	CDS
O55/O157	390700	i		intergenic		
O55/O157	391163	ns		G2583_0395	-	CDS
CB9615	391626	ns		G2583_0396	-	CDS
CB9615	393151	ns		G2583_0397	eaeH	CDS
CB9615	394296	ns		G2583_0397	eaeH	CDS
O157	357832	ns		G2583_0397	eaeH	CDS
O157	358198	i		intergenic		
O157	358636	ns		G2583_0398	ykgA	CDS
O157	360481	ns		G2583_0400	ykgB	CDS
CB9615	399018	ns		G2583_0400	ykgB	CDS
O157	360985	ns		G2583_0400	ykgB	CDS
O157	361040	ns		G2583_0401	-	CDS
CB9615	399792	ns		G2583_0401-0402	ykgI	CDS
O157	363999	i		intergenic		
O157	364187	i		intergenic		
CB9615	403579	ns		G2583_0406	ykgF	CDS
O157	366985	ns		G2583_0407	ykgG	CDS
O157	367851	nc		G2583_0409	ykgH	pseudogene
O157	371105	ns		G2583_0412	-	CDS
O157	371331	i		intergenic		
CB9615	411165	nc		G2583_0413	fimX	pseudogene
O55/O157	412020	i	rec	intergenic		
O55/O157	412048	i	rec	intergenic		
O55/O157	412101	i	rec	intergenic		
O55/O157	412121	i	rec	intergenic		

lineage[a]	site[b]	mutation type[c]	recomb	gene	Gene name	Type
Sakai	372674	ins		intergenic		
O55/O157	412242	indel	rec	intergenic		
O55/O157	412309	indel	rec	intergenic		
Sakai	372766	i		intergenic		
O55/O157	412337	i	rec	intergenic		
O55/O157	412358	i	rec	intergenic		
Sakai	372814	i		intergenic		
O55/O157	412388	i	rec	intergenic		
O157	374824	s		G2583_0415	betA	CDS
Sakai	375317	ns		G2583_0416	betB	CDS
CB9615	415679	ns		G2583_0416	betB	CDS
CB9615	416071	s		G2583_0417	betI	CDS
O157	377002	s		G2583_0417	betI	CDS
CB9615	419077	i		intergenic		
O157	384735	ns		G2583_0420	yahA	CDS
CB9615	424356	ns		G2583_0420	yahA	CDS
O157	385197	nc		G2583_0421	yahB	pseudogene
CB9615	425321	nc		G2583_0421	yahB	pseudogene
O157	385959	i		intergenic		
CB9615	425989	ins-10		G2583_0422	yahC	pseudogene
O157	389262	ns		G2583_0425	yahF	CDS
O157	389921	ns		G2583_0426	yahG	CDS
O157	390964	s		G2583_0426	yahG	CDS
O157	392225	s		G2583_0428	yahI	CDS
CB9615	431898	s		G2583_0428	yahI	CDS
CB9615	432970	ns		G2583_0429	yahJ	CDS
CB9615	433721	i		intergenic		
CB9615	435536	ns		G2583_0431	-	CDS
O157	396350	ns		G2583_0432	-	CDS
O157	398898	ns		G2583_0434	-	CDS
O157	399826	s		G2583_0435	yahK	CDS
O157	401607	ns		G2583_0436	yahL	CDS
O157	401721	i		intergenic		
CB9615	441386	i		intergenic		
O157	402100	nc		G2583_0437	-	tRNA
CB9615	441945	s		G2583_0439	yahN	CDS
CB9615	442217	ns		G2583_0439	yahN	CDS
CB9615	442641	i		intergenic		
O157	407749	s		G2583_0444	prpD	CDS
O157	410978	ns		G2583_0445	prpE	CDS
O55/O157	450717	nc	rec	G2583_0446	nrdB	pseudogene
EDL933	411352	nc		G2583_0446	nrdB	pseudogene
O55/O157	450735	nc	rec	G2583_0446	nrdB	pseudogene
Sakai	411352	nc		G2583_0446	nrdB	pseudogene
O55/O157	450737	nc	rec	G2583_0446	nrdB	pseudogene

lineage[a]	site[b]	mutation type[c]	recomb	gene	Gene name	Type
O55/O157	450738	nc	rec	G2583_0446	nrdB	pseudogene
O157	411933	s		G2583_0447	codB	CDS
O157	412363	ns		G2583_0447	codB	CDS
O157	413574	ns		G2583_0448	codA	CDS
O157	414499	i		intergenic		
O157	415011	ns		G2583_0449	cynR	CDS
Sakai	415750	ns		G2583_0450	cynT	CDS
O157	421370	ns		G2583_0455	lacZ	CDS
O157	421747	s		G2583_0455	lacZ	CDS
O157	422932	s		G2583_0455	lacZ	CDS
EDL933	423051	s	rec	G2583_0455	lacZ	CDS
EDL933	423122	i	rec	intergenic		
EDL933	423143	i	rec	intergenic		
EDL933	423149	i	rec	intergenic		
EDL933	423183	i	rec	intergenic		
EDL933	423233	s	rec	G2583_0456	lacI	CDS
CB9615	465651	s		G2583_0459	mhpR	CDS
O157	427692	ns		G2583_0460	mhpA	CDS
O157	429392	s		G2583_0461	mhpB	CDS
O157	430636	s		G2583_0462	mhpC	CDS
Sakai	431214	ns		G2583_0463	mhpD	CDS
CB9615	471397	ns		G2583_0464	mhpF	CDS
CB9615	471930	ns		G2583_0465	mhpE	CDS
O157	435465	ns		G2583_0467	yaiL	CDS
O157	435873	ns		G2583_0468	frmB	CDS
O157	438024	i		intergenic		
CB9615	479501	s		G2583_0472	afuB	CDS
CB9615	479849	s		G2583_0472	afuB	CDS
O157	440654	ns		G2583_0472	afuB	CDS
CB9615	484172	s		G2583_0475	-	CDS
CB9615	485002	s		G2583_0476	-	CDS
CB9615	485360	s		G2583_0476	-	CDS
O157	446906	ns		G2583_0477	tauA	CDS
CB9615	488042	s		G2583_0479	tauC	CDS
O157	450782	i		intergenic		
O157	450929	i		intergenic		
O157	451279	ns		G2583_0482	yaiU	CDS
EDL933	451346	s		G2583_0482	yaiU	CDS
O157	452182	ns		G2583_0482	yaiU	CDS
O157	452571	s		G2583_0482	yaiU	CDS
CB9615	494242	ns		G2583_0483	yaiV	CDS
CB9615	495868	i		intergenic		
O157	456729	s		G2583_0485	sbmA	CDS
CB9615	497548	ns		G2583_0486	yaiW	CDS
CB9615	497748	s		G2583_0486	yaiW	CDS

lineage[a]	site[b]	mutation type[c]	recomb	gene	Gene name	Type
CB9615	499863	ns		G2583_0489	ddlA	CDS
O157	461082	i		intergenic		
O157	462752	s		G2583_0491	phoA	CDS
CB9615	503681	s		G2583_0493	adrA	CDS
O157	467555	s		G2583_0498	aroM	CDS
CB9615	507474	s		G2583_0498	aroM	CDS
O157	467930	i		intergenic		
Sakai	469824	ns		G2583_0500	ydbD	CDS
O157	471093	ins		G2583_0501	-	CDS
O157	471125	i		intergenic		
Sakai	474603	ns		G2583_0505	sbcC	CDS
O157	474608	ns		G2583_0505	sbcC	CDS
O157	476521	ns		G2583_0505	sbcC	CDS
CB9615	516795	ns		G2583_0505	sbcC	CDS
CB9615	517616	ns		G2583_0506	sbcD	CDS
CB9615	519775	ns		G2583_0508	phoR	CDS
CB9615	520247	s		G2583_0508	phoR	CDS
O157	480831	ns		G2583_0508	phoR	CDS
CB9615	525160	ns		G2583_0511	malZ	CDS
CB9615	528257	del		intergenic		
O157	490047	s		G2583_0515	tgt	CDS
O55/O157	533850	indel		intergenic		
CB9615	540196	ns		G2583_0528	thiL	CDS
O157	501877	ns		G2583_0530	-	CDS
CB9615	544052	i		intergenic		
O157	506173	s		G2583_0532	dxs	CDS
O157	506245	i		intergenic		
O157	508776	ns		G2583_0535	thiI	CDS
O157	509818	ns		G2583_0537	panE	CDS
CB9615	552748	s		G2583_0540	cyoE	CDS
O157	513363	s		G2583_0540	cyoE	CDS
O157	513827	s		G2583_0541	cyoD	CDS
CB9615	555531	s		G2583_0543	cyoB	CDS
O157	516322	s		G2583_0543	cyoB	CDS
O157	516850	ns		G2583_0544	cyoA	CDS
Sakai	517475	s		G2583_0544	cyoA	CDS
O157	517703	i		intergenic		
O157	517886	i		intergenic		
O157	522313	s		G2583_0548	tig	CDS
CB9615	562235	ins		intergenic		
O157	522905	s		G2583_0549	clpP	CDS
O157	525668	s		G2583_0551	lon	CDS
O157	525900	ns		G2583_0551	lon	CDS
O157	526859	s		G2583_0551	lon	CDS
CB9615	569037	ns		G2583_0553	ppiD	CDS

lineage[a]	site[b]	mutation type[c]	recomb	gene	Gene name	Type
EDL933	529982	ins		intergenic		
O157	533962	ns		G2583_0558	cof	CDS
CB9615	574961	s		G2583_0560	mdlA	CDS
CB9615	576982	ns		G2583_0561	mdlB	CDS
CB9615	577776	s		G2583_0561	mdlB	CDS
O157	539520	s		G2583_0563	amtB	CDS
O157	540081	s		G2583_0563	amtB	CDS
CB9615	580569	s		G2583_0564	tesB	CDS
O157	541360	i		intergenic		
CB9615	581277	s		G2583_0565	ybaY	CDS
CB9615	582089	ns		G2583_0566	ybaZ	CDS
O157	545398	i		intergenic		
CB9615	585114	ins		intergenic		
O157	545673	ns		G2583_0571	maa	CDS
O157	546138	i		intergenic		
CB9615	586838	i		intergenic		
O157	548438	s		G2583_0574	acrB	CDS
O157	550084	ns		G2583_0574	acrB	CDS
O157	552072	ns		G2583_0576	acrR	CDS
O157	553182	s		G2583_0577	kefA	CDS
O157	556554	ns		G2583_0579	priC	CDS
CB9615	596874	i		intergenic		
O157	557954	i		intergenic		
O157	561782	ns		G2583_0585	htpG	CDS
CB9615	602866	ns		G2583_0586	adk	CDS
CB9615	604611	ns		G2583_0588	aes	CDS
O157	565364	ns		G2583_0588	aes	CDS
O157	569170	del		intergenic		
O157	572853	ns		G2583_0593	ybaK	CDS
O157	575527	s		G2583_0597	copA	CDS
O157	579211	ns		G2583_0599	ybaT	CDS
O157	579703	i		intergenic		
CB9615	620423	ns		G2583_0600	-	CDS
O157	581100	s		G2583_0600	-	CDS
O157	584229	s		G2583_0601	-	CDS
CB9615	624651	ns		G2583_0601	-	CDS
O157	585736	ns		G2583_0601	-	CDS
O157	585942	s		G2583_0601	-	CDS
O157	586158	s		G2583_0601	-	CDS
CB9615	627784	s		G2583_0601	-	CDS
O157	589040	ns		G2583_0601	-	CDS
O157	590150	ns		G2583_0601	-	CDS
O157	590515	ns		G2583_0601	-	CDS
O157	590905	ns		G2583_0601	-	CDS
O157	591849	s		G2583_0601	-	CDS

lineage[a]	site[b]	mutation type[c]	recomb	gene	Gene name	Type
O157	591957	s		G2583_0601	-	CDS
CB9615	632912	ns		G2583_0601	-	CDS
O157	593478	s		G2583_0601	-	CDS
O157	595901	ns		G2583_0601	-	CDS
O157	598277	ns		G2583_0601	-	CDS
EDL933	598781	ns		G2583_0601	-	CDS
O157	600475	ns		G2583_0601	-	CDS
O157	600749	ns		G2583_0601	-	CDS
CB9615	640777	s		G2583_0601	-	CDS
CB9615	643932	ns		G2583_0602	-	CDS
CB9615	647451	i		intergenic		
O157	613491	s		G2583_0614	tesA	CDS
O157	615323	ns		G2583_0616	ybbP	CDS
O157	616762	i		intergenic		
Sakai	616828	i	rec	intergenic		
Sakai	616829	i	rec	intergenic		
Sakai	616890	i	rec	intergenic		
CB9615	657107	nc		G2583_0617	-	pseudogene
CB9615	657395	nc		G2583_0617	-	pseudogene
CB9615	657780	ins		G2583_0617	-	pseudogene
O157	618213	nc		G2583_0617	-	pseudogene
CB9615	658750	nc		G2583_0617	-	pseudogene
EDL933	619400	nc		G2583_0617	-	pseudogene
Sakai	619723	nc	rec	G2583_0617	-	pseudogene
Sakai	619813	nc	rec	G2583_0617	-	pseudogene
O55/O157	660075	nc	rec	G2583_0617	-	pseudogene
O55/O157	660078	nc	rec	G2583_0617	-	pseudogene
O55/O157	660081	nc	rec	G2583_0617	-	pseudogene
O55/O157	660083	nc	rec	G2583_0617	-	pseudogene
O55/O157	660086	nc	rec	G2583_0617	-	pseudogene
O55/O157	660087	nc	rec	G2583_0617	-	pseudogene
O55/O157	660096	nc	rec	G2583_0617	-	pseudogene
O55/O157	660102	nc	rec	G2583_0617	-	pseudogene
O55/O157	660105	nc	rec	G2583_0617	-	pseudogene
O55/O157	660107	nc	rec	G2583_0617	-	pseudogene
O55/O157	660123	nc	rec	G2583_0617	-	pseudogene
O55/O157	660141	nc	rec	G2583_0617	-	pseudogene
O55/O157	660162	nc	rec	G2583_0617	-	pseudogene
O55/O157	660165	nc	rec	G2583_0617	-	pseudogene
O55/O157	660174	nc	rec	G2583_0617	-	pseudogene
O55/O157	660178	nc	rec	G2583_0617	-	pseudogene
O55/O157	660192	nc	rec	G2583_0617	-	pseudogene
O55/O157	660198	nc	rec	G2583_0617	-	pseudogene
O55/O157	660207	nc	rec	G2583_0617	-	pseudogene
O55/O157	660241	nc	rec	G2583_0617	-	pseudogene

lineage[a]	site[b]	mutation type[c]	recomb	gene	Gene name	Type
O55/O157	660255	nc	rec	G2583_0617	-	pseudogene
O55/O157	660256	nc	rec	G2583_0617	-	pseudogene
O55/O157	660261	nc	rec	G2583_0617	-	pseudogene
O55/O157	660267	nc	rec	G2583_0617	-	pseudogene
O55/O157	660273	nc	rec	G2583_0617	-	pseudogene
O55/O157	660282	nc	rec	G2583_0617	-	pseudogene
O55/O157	660294	nc	rec	G2583_0617	-	pseudogene
O55/O157	660306	nc	rec	G2583_0617	-	pseudogene
O55/O157	660311	nc	rec	G2583_0617	-	pseudogene
O55/O157	660315	nc	rec	G2583_0617	-	pseudogene
O55/O157	660330	nc	rec	G2583_0617	-	pseudogene
O55/O157	660334	nc	rec	G2583_0617	-	pseudogene
O55/O157	660339	nc	rec	G2583_0617	-	pseudogene
O55/O157	660345	nc	rec	G2583_0617	-	pseudogene
O55/O157	660351	nc	rec	G2583_0617	-	pseudogene
O55/O157	660354	nc	rec	G2583_0617	-	pseudogene
O55/O157	660384	nc	rec	G2583_0617	-	pseudogene
O55/O157	660502	nc	rec	G2583_0617	-	pseudogene
O55/O157	660514	nc	rec	G2583_0617	-	pseudogene
O157	621511	i		intergenic		
O157	624356	s		G2583_0624	allS	CDS
CB9615	664765	s		G2583_0624	allS	CDS
O157	624939	i		intergenic		
CB9615	665007	i		intergenic		
O157	626494	i		intergenic		
O157	626496	i		intergenic		
O157	628605	ns		G2583_0628	hyi	CDS
O157	632543	ns		G2583_0632	allB	CDS
Sakai	632644	ns		G2583_0632	allB	CDS
CB9615	673092	ns		G2583_0633	ybbY	CDS
O157	634437	s		G2583_0634	glxK	CDS
O157	637417	s		G2583_0636	allC	CDS
O157	637672	ns		G2583_0636	allC	CDS
O157	641846	ns		G2583_0639	ylbE	CDS
O157	643644	ns		G2583_0641	ybcF	CDS
O157	643984	ns		G2583_0642	purK	CDS
O157	644714	ns		G2583_0642	purK	CDS
O157	645862	s		G2583_0644	lpxH	CDS
CB9615	686819	s		G2583_0645	ppiB	CDS
CB9615	688431	s		G2583_0646	cysS	CDS
Sakai	648904	s		G2583_0647	ybcI	CDS
O157	650331	del-9		intergenic		
CB9615	690361	i		intergenic		
O157	651644	s		G2583_0651	sfmC	CDS
CB9615	691836	ns		G2583_0651	sfmC	CDS

lineage[a]	site[b]	mutation type[c]	recomb	gene	Gene name	Type
O157	652297	ns		G2583_0652	sfmD	CDS
CB9615	692684	ns		G2583_0652	sfmD	CDS
CB9615	693275	ns		G2583_0652	sfmD	CDS
CB9615	696482	s		G2583_0655	fimZ	CDS
CB9615	696808	ns		G2583_0655	fimZ	CDS
CB9615	750817	s		G2583_0722	nfrA	CDS
O157	661587	s		G2583_0722	nfrA	CDS
O157	661812	s		G2583_0722	nfrA	CDS
O157	662515	s		G2583_0723	nfrB	CDS
CB9615	754142	ns		G2583_0724	yhhI	CDS
O157	664909	ns		G2583_0724	yhhI	CDS
O157	665627	s		G2583_0724	yhhI	CDS
CB9615	755901	ns		G2583_0726	-	CDS
CB9615	756288	ns		G2583_0726	-	CDS
O157	667651	i		intergenic		
EDL933	667568	ins		G2583_0727	-	CDS
O55/O157	757306	ns		G2583_0727	-	CDS
O55/O157	757436	ns		G2583_0727	-	CDS
O55/O157	757561	ns		G2583_0728	-	CDS
CB9615	759261	ns		G2583_0728	-	CDS
O157	670048	s		G2583_0728	-	CDS
CB9615	759482	s		G2583_0728	-	CDS
O157	670647	ns		G2583_0728	-	CDS
O157	670745	ns		G2583_0728	-	CDS
CB9615	761058	ns		G2583_0728	-	CDS
CB9615	762767	nc		G2583_0729	-	pseudogene
CB9615	763612	ns		G2583_0730	-	CDS
CB9615	765307	i		intergenic		
CB9615	768773	s		G2583_0733	cusC	CDS
CB9615	769240	s		G2583_0734	cusF	CDS
EDL933	680741	ns		G2583_0735	cusB	CDS
CB9615	770955	s		G2583_0736	cusA	CDS
CB9615	771395	ns		G2583_0736	cusA	CDS
O157	682092	del-6		G2583_0736	cusA	CDS
CB9615	772873	ns		G2583_0736	cusA	CDS
O157	684162	ns		G2583_0736	cusA	CDS
CB9615	774965	s		G2583_0737	pheP	CDS
CB9615	776713	i		intergenic		
O157	696262	s		G2583_0749	entF	CDS
O157	697011	ns		G2583_0749	entF	CDS
O157	697087	s		G2583_0749	entF	CDS
EDL933	696914	s		G2583_0749	entF	CDS
CB9615	786962	s		G2583_0749	entF	CDS
O157	699238	s		G2583_0749	entF	CDS
EDL933	699494	i		intergenic		

lineage[a]	site[b]	mutation type[c]	recomb	gene	Gene name	Type
EDL933	700558	s		G2583_0750	fepE	CDS
O157	702532	s		G2583_0752	fepG	CDS
O157	705845	ns		G2583_0755	fepB	CDS
O157	710900	ns		G2583_0760	ybdB	CDS
O55/O157	802986	i		intergenic		
O157	714341	s		G2583_0763	ybdH	CDS
CB9615	804688	ns		G2583_0764	ybdL	CDS
O157	717679	s		G2583_0766	ybdN	CDS
CB9615	807588	i		intergenic		
O157	723657	s		G2583_0772	ybdR	CDS
CB9615	816974	ns		G2583_0776	citT	CDS
O157	727629	s		G2583_0776	citT	CDS
O157	727975	i		intergenic		
CB9615	817704	s		G2583_0777	citG	CDS
CB9615	818339	ns		G2583_0778	citX	CDS
O157	730066	ns		G2583_0779	citF	CDS
O157	730645	ns		G2583_0779	citF	CDS
Sakai	730801	ns		G2583_0779	citF	CDS
O157	730987	s		G2583_0780	citE	CDS
O157	731710	ns		G2583_0780	citE	CDS
O157	732424	ns		G2583_0782	citC	CDS
O157	733443	i		intergenic		
O157	733807	ns		G2583_0783	dpiB	CDS
CB9615	824795	nc	rec	G2583_0784	dpiA	pseudogene
CB9615	824813	nc	rec	G2583_0784	dpiA	pseudogene
CB9615	824867	nc	rec	G2583_0784	dpiA	pseudogene
CB9615	824868	nc	rec	G2583_0784	dpiA	pseudogene
CB9615	824869	nc	rec	G2583_0784	dpiA	pseudogene
CB9615	824869	del	rec	G2583_0784	dpiA	pseudogene
O157	736937	s		G2583_0785	dcuC	CDS
CB9615	827006	i		intergenic		
O157	737698	i		intergenic		
O157	737842	i		intergenic		
CB9615	827310	i		intergenic		
O157	738521	i		intergenic		
EDL933	738685	del		intergenic		
O157	739778	s		G2583_0789	ybeM	CDS
Sakai	745039	ns		G2583_0795	dacA	CDS
CB9615	835047	ns		G2583_0796	rlpA	CDS
O157	752015	ns		G2583_0803	holA	CDS
O157	752996	s		G2583_0804	rlpB	CDS
CB9615	844264	s		G2583_0805	leuS	CDS
O157	757752	ns		G2583_0807	ybeQ	CDS
O157	760739	ns		G2583_0810	ybeT	CDS
CB9615	850331	ns		G2583_0811	ybeU	CDS

lineage[a]	site[b]	mutation type[c]	recomb	gene	Gene name	Type
CB9615	851059	i		intergenic		
CB9615	851562	nc		G2583_0812	ybeV	pseudogene
CB9615	852623	ns		G2583_0813	hscC	CDS
O157	764010	ns		G2583_0813	hscC	CDS
O157	770408	ns		G2583_0820	lnt	CDS
CB9615	860210	ns		G2583_0820	lnt	CDS
O157	772229	ns		G2583_0822	ybeY	CDS
O157	772916	s		G2583_0823	ybeZ	CDS
CB9615	865961	i		intergenic		
O157	779527	i		intergenic		
O157	780083	s		G2583_0836	nagD	CDS
O157	783818	i		intergenic		
CB9615	873700	ns		G2583_0840	nagE	CDS
O157	786941	s		G2583_0841	glnS	CDS
O157	788796	del		G2583_0842	ybfM	CDS
CB9615	878266	ns		G2583_0842	ybfM	CDS
Sakai	789194	s		G2583_0842	ybfM	CDS
EDL933	790462	ins		intergenic		
EDL933	790526	ins		intergenic		
O157	791773	s		G2583_0846	ybfE	CDS
O157	792567	ns		G2583_0847	ybfF	CDS
O157	794606	ns		G2583_0849	pgm	CDS
O157	794988	s		G2583_0849	pgm	CDS
CB9615	886808	s		G2583_0851	speF	CDS
CB9615	888855	i		intergenic		
O157	802184	ns		G2583_0853	kdpD	CDS
O157	802852	s		G2583_0854	kdpC	CDS
O157	802855	s		G2583_0854	kdpC	CDS
O157	807079	s		G2583_0856	kdpA	CDS
CB9615	896878	i		intergenic		
CB9615	897278	i		intergenic		
O157	808007	del		intergenic		
O157	808068	s		G2583_0859	-	CDS
O157	808161	s		G2583_0859	-	CDS
O157	808211	ns		G2583_0859	-	CDS
CB9615	898304	ns		G2583_0859	-	CDS
EDL933	809606	s		G2583_0859	-	CDS
CB9615	899511	ns		G2583_0859	-	CDS
O55/O157	900304	s	rec	G2583_0859	-	CDS
O55/O157	900361	s	rec	G2583_0859	-	CDS
O55/O157	900364	s	rec	G2583_0859	-	CDS
O55/O157	900423	ns	rec	G2583_0859	-	CDS
O55/O157	900580	s	rec	G2583_0859	-	CDS
O55/O157	900595	s	rec	G2583_0859	-	CDS
O55/O157	900597	ns	rec	G2583_0859	-	CDS

lineage[a]	site[b]	mutation type[c]	recomb	gene	Gene name	Type
O55/O157	900649	s	rec	G2583_0859	-	CDS
O55/O157	900655	s	rec	G2583_0859	-	CDS
O55/O157	900940	s	rec	G2583_0859	rhsC	CDS
O55/O157	900949	s	rec	G2583_0859	rhsC	CDS
O55/O157	900951	ns	rec	G2583_0859	rhsC	CDS
O55/O157	900961	s	rec	G2583_0859	rhsC	CDS
O55/O157	900988	s	rec	G2583_0859	rhsC	CDS
O55/O157	901016	ns	rec	G2583_0859	rhsC	CDS
O55/O157	901021	s	rec	G2583_0859	rhsC	CDS
O55/O157	901036	s	rec	G2583_0859	rhsC	CDS
O55/O157	901051	s	rec	G2583_0859	rhsC	CDS
O157	812228	ins		G2583_0860	ybfC	CDS
CB9615	901936	ns		G2583_0860	ybfC	CDS
Sakai	812703	s		G2583_0860	ybfC	CDS
O157	813195	i		intergenic		
CB9615	902798	i		intergenic		
CB9615	902830	ins		intergenic		
O157	814208	i		intergenic		
O55/O157	903884	ns	rec	G2583_0861	yhhI	CDS
O55/O157	903930	s	rec	G2583_0861	yhhI	CDS
O55/O157	904195	ns	rec	G2583_0861	yhhI	CDS
O55/O157	904288	ns	rec	G2583_0861	yhhI	CDS
O55/O157	904289	ns	rec	G2583_0861	yhhI	CDS
O55/O157	904293	s	rec	G2583_0861	yhhI	CDS
O55/O157	904308	ns	rec	G2583_0861	yhhI	CDS
O55/O157	904350	s	rec	G2583_0861	yhhI	CDS
O55/O157	904500	ns	rec	G2583_0861	yhhI	CDS
O55/O157	904551	ns	rec	G2583_0861	yhhI	CDS
O55/O157	904657	ns	rec	G2583_0861	yhhI	CDS
O55/O157	904703	ns	rec	G2583_0861	yhhI	CDS
O55/O157	904789	ns	rec	G2583_0861	yhhI	CDS
O55/O157	904794	s	rec	G2583_0861	yhhI	CDS
O157	816243	ns		G2583_0863	phr	CDS
CB9615	908396	ns		G2583_0864	ybgH	CDS
O157	822101	s		G2583_0868	ybgL	CDS
O157	823452	ns		G2583_0870	abrB	CDS
CB9615	914500	ns		G2583_0871	ybgO	CDS
O157	825854	ns		G2583_0872	ybgP	CDS
O157	826657	ns		G2583_0873	ybgQ	CDS
CB9615	916869	ns		G2583_0873	ybgQ	CDS
O157	827559	s		G2583_0873	ybgQ	CDS
O157	827934	s		G2583_0873	ybgQ	CDS
O157	833994	s		G2583_0878	sdhA	CDS
CB9615	925663	s		G2583_0881	sucA	CDS
CB9615	933088	del		intergenic		

lineage[a]	site[b]	mutation type[c]	recomb	gene	Gene name	Type
CB9615	935650	s		G2583_0889	-	CDS
CB9615	935664	ns		G2583_0889	-	CDS
O157	853320	i		intergenic		
CB9615	942938	i		intergenic		
CB9615	943839	nc		G2583_0898	-	pseudogene
CB9615	943855	del		G2583_0898	-	pseudogene
O157	854873	nc		G2583_0898	-	pseudogene
CB9615	946770	ns		G2583_0899	cydA	CDS
CB9615	951871	ns		G2583_0907	tolB	CDS
O157	865136	nc		G2583_0911	-	tRNA
O157	865341	nc		G2583_0913	-	tRNA
CB9615	955899	ns		G2583_0916	nadA	CDS
CB9615	956193	ns		G2583_0916	nadA	CDS
O157	867563	s		G2583_0917	pnuC	CDS
EDL933	870326	del-6		G2583_0918	zitB	CDS
CB9615	959012	ins		intergenic		
CB9615	961804	ns		G2583_0922	galM	CDS
CB9615	963826	s		G2583_0924	galT	CDS
O157	875910	s		G2583_0925	galE	CDS
O157	876026	i		intergenic		
O157	879561	s		G2583_0929	modA	CDS
CB9615	969444	s		G2583_0930	modB	CDS
O157	881973	ns		G2583_0932	ybhA	CDS
O157	883849	s		G2583_0934	ybhD	CDS
EDL933	887390	ins		intergenic		
CB9615	976003	s		G2583_0936	ybhI	CDS
CB9615	977223	s		G2583_0937	ybhJ	CDS
O157	888327	s		G2583_0937	ybhJ	CDS
CB9615	978266	ns		G2583_0937	ybhJ	CDS
O157	888915	ns		G2583_0937	ybhJ	CDS
O157	889133	ns		G2583_0937	ybhJ	CDS
CB9615	980592	s		G2583_0938	ybhC	CDS
EDL933	893627	ins		G2583_0939	intE	CDS
O157	892201	del-4		G2583_0939	intE	CDS
Sakai	892952	s		G2583_0942	-	CDS
O157	893023	ns		G2583_0942	-	CDS
O55/O157	983399	s	rec	G2583_0943	-	CDS
O55/O157	983406	ns	rec	G2583_0943	-	CDS
O55/O157	983408	s	rec	G2583_0943	-	CDS
O55/O157	983417	s	rec	G2583_0943	-	CDS
O55/O157	983472	ns	rec	G2583_0943	-	CDS
O55/O157	983579	i	rec	intergenic		
O55/O157	983668	s	rec	G2583_0944	-	CDS
O55/O157	983777	ns	rec	G2583_0944	-	CDS
EDL933	896580	s		G2583_0946	-	CDS

lineage[a]	site[b]	mutation type[c]	recomb	gene	Gene name	Type
CB9615	985295	s		G2583_0947	bet	CDS
CB9615	985600	ns		G2583_0947	bet	CDS
O157	896277	s		G2583_0962	NinF	CDS
O157	896389	ns		G2583_0962	NinF	CDS
O157	896669	ns		G2583_0963	NinG	CDS
EDL933	898725	del		G2583_0964	-	CDS
EDL933	898803	del		G2583_0964	-	CDS
EDL933	898821	del		G2583_0964	-	CDS
CB9615	995731	ns		G2583_0965	-	CDS
O157	898910	i		intergenic		
CB9615	995981	i		intergenic		
O157	899053	del		intergenic		
CB9615	996158	del		intergenic		
Sakai/EDL933	901704	indel		intergenic		
CB9615	997114	ins-2		intergenic		
O157	902073	ns		G2583_0970	ybcS	CDS
O157	902570	ns		G2583_0971	-	CDS
CB9615	1000575	ns		G2583_0973	-	CDS
CB9615	1002051	ns		G2583_0975	-	CDS
CB9615	1004630	ns		G2583_0977	-	CDS
O157	907623	ns		G2583_0977	-	CDS
O157	907629	ins		G2583_0977	-	CDS
O157	908248	nc		G2583_0978	clpP	pseudogene
CB9615	1006082	nc		G2583_0978	clpP	pseudogene
O157	910873	ns		G2583_0981	-	CDS
O157	912752	s		G2583_0984	-	CDS
CB9615	1011066	s		G2583_0986	-	CDS
CB9615	1012126	ns		G2583_0986	-	CDS
O157	915220	ns		G2583_0986	-	CDS
CB9615	1013262	ns		G2583_0986	-	CDS
CB9615	1013575	ns		G2583_0987	-	CDS
O55/O157	1013897	s	rec	G2583_0988	-	CDS
O55/O157	1013910	ns	rec	G2583_0988	-	CDS
O55/O157	1014101	s	rec	G2583_0988	-	CDS
O55/O157	1014122	s	rec	G2583_0988	-	CDS
O55/O157	1014137	s	rec	G2583_0988	-	CDS
O55/O157	1014155	s	rec	G2583_0988	-	CDS
O55/O157	1014167	s	rec	G2583_0988	-	CDS
O55/O157	1014227	s	rec	G2583_0988	-	CDS
O55/O157	1014254	s	rec	G2583_0988	-	CDS
O55/O157	1014278	s	rec	G2583_0988	-	CDS
O55/O157	1014320	s	rec	G2583_0988	-	CDS
O55/O157	1014323	s	rec	G2583_0988	-	CDS
O55/O157	1014332	s	rec	G2583_0988	-	CDS
O55/O157	1014342	ns	rec	G2583_0988	-	CDS

lineage[a]	site[b]	mutation type[c]	recomb	gene	Gene name	Type
O55/O157	1014383	s	rec	G2583_0988	-	CDS
O55/O157	1014388	s	rec	G2583_0988-0989	-	CDS
O55/O157	1014401	ns	rec	G2583_0989	-	CDS
O55/O157	1014469	s	rec	G2583_0989	-	CDS
O55/O157	1014472	ns	rec	G2583_0989	-	CDS
O55/O157	1014481	s	rec	G2583_0989	-	CDS
O55/O157	1014496	s	rec	G2583_0989	-	CDS
O55/O157	1014538	s	rec	G2583_0989	-	CDS
O55/O157	1014550	s	rec	G2583_0989	-	CDS
O55/O157	1014551	ns	rec	G2583_0989	-	CDS
O55/O157	1014561	ns	rec	G2583_0989	-	CDS
O55/O157	1014568	s	rec	G2583_0989	-	CDS
O55/O157	1014688	s	rec	G2583_0989	-	CDS
O55/O157	1014689	ns	rec	G2583_0989	-	CDS
O55/O157	1014690	ns	rec	G2583_0989	-	CDS
O55/O157	1014721	s	rec	G2583_0989	-	CDS
O55/O157	1014781	s	rec	G2583_0989	-	CDS
O55/O157	1014836	ns	rec	G2583_0989	-	CDS
O55/O157	1014899	ns	rec	G2583_0989	-	CDS
O55/O157	1014901	ns	rec	G2583_0989	-	CDS
O55/O157	1014904	s	rec	G2583_0989	-	CDS
O55/O157	1014907	s	rec	G2583_0989	-	CDS
O55/O157	1014919	s	rec	G2583_0989	-	CDS
O55/O157	1014976	s	rec	G2583_0989	-	CDS
O55/O157	1015080	ns	rec	G2583_0989-0990	-	CDS
O55/O157	1015104	ns	rec	G2583_0989-0990	-	CDS
O55/O157	1015217	ns	rec	G2583_0990	-	CDS
O55/O157	1015260	s	rec	G2583_0990	-	CDS
O55/O157	1015261	ns	rec	G2583_0990	-	CDS
O55/O157	1015293	s	rec	G2583_0990	-	CDS
O55/O157	1015326	s	rec	G2583_0990	-	CDS
O55/O157	1015521	s	rec	G2583_0990	-	CDS
O55/O157	1015524	s	rec	G2583_0990	-	CDS
O55/O157	1015527	s	rec	G2583_0990	-	CDS
O55/O157	1015554	s	rec	G2583_0990	-	CDS
O55/O157	1015581	s	rec	G2583_0990	-	CDS
O55/O157	1015608	s	rec	G2583_0990	-	CDS
O55/O157	1015617	s	rec	G2583_0990	-	CDS
O55/O157	1015620	s	rec	G2583_0990	-	CDS
O55/O157	1015647	s	rec	G2583_0990	-	CDS
O55/O157	1015680	s	rec	G2583_0990	-	CDS
O55/O157	1015686	i	rec	intergenic		
O55/O157	1015691	i	rec	intergenic		
O55/O157	1015712	i	rec	intergenic		
O55/O157	1015730	i	rec	intergenic		

lineage[a]	site[b]	mutation type[c]	recomb	gene	Gene name	Type
O55/O157	1015737	i	rec	intergenic		
O55/O157	1015741	i	rec	intergenic		
O55/O157	1015755	s	rec	G2583_0991	-	CDS
O55/O157	1015800	s	rec	G2583_0991	-	CDS
O55/O157	1015803	s	rec	G2583_0991	-	CDS
O55/O157	1015804	ns	rec	G2583_0991	-	CDS
O55/O157	1015806	ns	rec	G2583_0991	-	CDS
O55/O157	1015810	ns	rec	G2583_0991	-	CDS
O55/O157	1015818	s	rec	G2583_0991	-	CDS
O55/O157	1015824	s	rec	G2583_0991	-	CDS
O55/O157	1015827	s	rec	G2583_0991	-	CDS
CB9615	1015896	s		G2583_0991	-	CDS
EDL933	920975	ins		G2583_0991	-	CDS
EDL933	920981	ins		G2583_0991	-	CDS
EDL933	920987	ins		G2583_0991	-	CDS
CB9615	1016982	s		G2583_0991	-	CDS
EDL933	921815	ns	rec	G2583_0991	-	CDS
EDL933	921816	ns	rec	G2583_0991	-	CDS
EDL933	921821	ns	rec	G2583_0991	-	CDS
EDL933	921844	ns	rec	G2583_0991	-	CDS
EDL933	921883	ns	rec	G2583_0991	-	CDS
EDL933	921987	s	rec	G2583_0991	-	CDS
O55/O157	1018440	s	rec	G2583_0991	-	CDS
O55/O157	1018443	s	rec	G2583_0991	-	CDS
O55/O157	1018569	s	rec	G2583_0991	-	CDS
O55/O157	1018571	ns	rec	G2583_0991	-	CDS
O55/O157	1018575	s	rec	G2583_0991	-	CDS
O55/O157	1018651	ns	rec	G2583_0991	-	CDS
O55/O157	1018668	s	rec	G2583_0991	-	CDS
O55/O157	1019133	s	rec	G2583_0991	-	CDS
O55/O157	1019151	s	rec	G2583_0991	-	CDS
O55/O157	1019161	i	rec	intergenic		
O55/O157	1019162	i	rec	intergenic		
O55/O157	1019172	i	rec	intergenic		
O55/O157	1019173	i	rec	intergenic		
O55/O157	1019177	indel	rec	intergenic		
O55/O157	1019182	i	rec	intergenic		
O55/O157	1019193	i	rec	intergenic		
O55/O157	1019214	i	rec	intergenic		
O55/O157	1019217	i	rec	intergenic		
O55/O157	1019236	ns	rec	G2583_0992	-	CDS
O55/O157	1019240	ns	rec	G2583_0992	-	CDS
O55/O157	1019244	s	rec	G2583_0992	-	CDS
O55/O157	1019247	s	rec	G2583_0992	-	CDS
O55/O157	1019253	ns	rec	G2583_0992	-	CDS

lineage[a]	site[b]	mutation type[c]	recomb	gene	Gene name	Type
O55/O157	1019274	s	rec	G2583_0992	-	CDS
O55/O157	1019285	ns	rec	G2583_0992	-	CDS
O55/O157	1019298	s	rec	G2583_0992	-	CDS
O55/O157	1019304	s	rec	G2583_0992	-	CDS
O55/O157	1019325	s	rec	G2583_0992	-	CDS
O55/O157	1019328	s	rec	G2583_0992	-	CDS
O55/O157	1019341	s	rec	G2583_0992	-	CDS
O55/O157	1019342	s	rec	G2583_0992	-	CDS
O55/O157	1019343	s	rec	G2583_0992	-	CDS
O55/O157	1019347	ns	rec	G2583_0992	-	CDS
O55/O157	1019351	ns	rec	G2583_0992	-	CDS
O55/O157	1019422	ns	rec	G2583_0992	-	CDS
O55/O157	1019424	ns	rec	G2583_0992	-	CDS
O55/O157	1019430	s	rec	G2583_0992	-	CDS
O55/O157	1019436	s	rec	G2583_0992	-	CDS
O55/O157	1019480	ns	rec	G2583_0992	-	CDS
O55/O157	1019493	s	rec	G2583_0992	-	CDS
O55/O157	1019498	ns	rec	G2583_0992	-	CDS
O55/O157	1019529	s	rec	G2583_0992	-	CDS
O55/O157	1019544	s	rec	G2583_0992	-	CDS
O55/O157	1019562	s	rec	G2583_0992	-	CDS
O55/O157	1019581	ns	rec	G2583_0992	-	CDS
O55/O157	1019607	s	rec	G2583_0992	-	CDS
O55/O157	1019620	ns	rec	G2583_0992	-	CDS
O55/O157	1019622	ns	rec	G2583_0992	-	CDS
O55/O157	1019775	s	rec	G2583_0992	-	CDS
O55/O157	1019778	s	rec	G2583_0992	-	CDS
O55/O157	1019820	s	rec	G2583_0992	-	CDS
O55/O157	1019823	s	rec	G2583_0992	-	CDS
O55/O157	1019831	indel-5	rec	intergenic		
O55/O157	1019844	i	rec	intergenic		
O55/O157	1019884	i	rec	intergenic		
O55/O157	1019888	ns	rec	G2583_0993	-	CDS
O55/O157	1019889	ns	rec	G2583_0993	-	CDS
O55/O157	1019896	indel-3	rec	G2583_0993	-	CDS
O55/O157	1019900	ns	rec	G2583_0993	-	CDS
O55/O157	1019902	ns	rec	G2583_0993	-	CDS
O55/O157	1019905	s	rec	G2583_0993	-	CDS
O55/O157	1019911	s	rec	G2583_0993	-	CDS
O55/O157	1019914	s	rec	G2583_0993	-	CDS
O55/O157	1019923	s	rec	G2583_0993	-	CDS
O55/O157	1019926	s	rec	G2583_0993	-	CDS
O55/O157	1019927	ns	rec	G2583_0993	-	CDS
O55/O157	1019929	ns	rec	G2583_0993	-	CDS
O55/O157	1019938	s	rec	G2583_0993	-	CDS

lineage[a]	site[b]	mutation type[c]	recomb	gene	Gene name	Type
O55/O157	1019939	ns	rec	G2583_0993	-	CDS
O55/O157	1019942	ns	rec	G2583_0993	-	CDS
O55/O157	1019974	s	rec	G2583_0993	-	CDS
O55/O157	1019976	ns	rec	G2583_0993	-	CDS
O55/O157	1019982	ns	rec	G2583_0993	-	CDS
O55/O157	1020025	s	rec	G2583_0993	-	CDS
O55/O157	1020028	s	rec	G2583_0993	-	CDS
O55/O157	1020031	s	rec	G2583_0993	-	CDS
O55/O157	1020049	s	rec	G2583_0993	-	CDS
O55/O157	1020055	s	rec	G2583_0993	-	CDS
O55/O157	1020072	ns	rec	G2583_0993	-	CDS
O55/O157	1020085	s	rec	G2583_0993	-	CDS
O55/O157	1020097	s	rec	G2583_0993	-	CDS
O55/O157	1020116	ns	rec	G2583_0993	-	CDS
O55/O157	1020139	s	rec	G2583_0993	-	CDS
O55/O157	1020196	s	rec	G2583_0993	-	CDS
O55/O157	1020202	s	rec	G2583_0993	-	CDS
O55/O157	1020213	ns	rec	G2583_0993	-	CDS
O55/O157	1020226	s	rec	G2583_0993	-	CDS
O55/O157	1020228	ns	rec	G2583_0993	-	CDS
O55/O157	1020229	ns	rec	G2583_0993	-	CDS
O55/O157	1020241	s	rec	G2583_0993	-	CDS
O55/O157	1020268	s	rec	G2583_0993	-	CDS
O55/O157	1020271	s	rec	G2583_0993	-	CDS
O55/O157	1020280	s	rec	G2583_0993	-	CDS
O55/O157	1020308	ns	rec	G2583_0993	-	CDS
O55/O157	1020313	s	rec	G2583_0993	-	CDS
O55/O157	1020318	ns	rec	G2583_0993	-	CDS
O55/O157	1020325	s	rec	G2583_0993	-	CDS
O55/O157	1020337	s	rec	G2583_0993	-	CDS
O55/O157	1020348	ns	rec	G2583_0993	-	CDS
O55/O157	1020352	ns	rec	G2583_0993	-	CDS
O55/O157	1020394	s	rec	G2583_0993	-	CDS
O55/O157	1020401	ns	rec	G2583_0993	-	CDS
O55/O157	1020412	ns	rec	G2583_0993	-	CDS
O55/O157	1020427	s	rec	G2583_0993	-	CDS
O55/O157	1020430	s	rec	G2583_0993	-	CDS
O55/O157	1020435	ns	rec	G2583_0993	-	CDS
O55/O157	1020462	ns	rec	G2583_0993	-	CDS
O55/O157	1020473	ns	rec	G2583_0993	-	CDS
O55/O157	1020478	ns	rec	G2583_0993	-	CDS
O55/O157	1020481	s	rec	G2583_0993	-	CDS
O55/O157	1020484	s	rec	G2583_0993	-	CDS
O55/O157	1020492	ns	rec	G2583_0993	-	CDS
O55/O157	1020542	ns	rec	G2583_0993	-	CDS

lineage[a]	site[b]	mutation type[c]	recomb	gene	Gene name	Type
O55/O157	1020562	s	rec	G2583_0993	-	CDS
O55/O157	1020598	s	rec	G2583_0993	-	CDS
O55/O157	1020613	ns	rec	G2583_0993	-	CDS
O55/O157	1020623	s	rec	G2583_0993	-	CDS
O55/O157	1020628	s	rec	G2583_0993	-	CDS
O55/O157	1020643	s	rec	G2583_0993	-	CDS
O55/O157	1020652	s	rec	G2583_0993	-	CDS
O55/O157	1020666	ns	rec	G2583_0993	-	CDS
O55/O157	1020670	s	rec	G2583_0993	-	CDS
O55/O157	1020697	s	rec	G2583_0993	-	CDS
O55/O157	1020703	s	rec	G2583_0993	-	CDS
O55/O157	1020712	s	rec	G2583_0993	-	CDS
O55/O157	1020715	s	rec	G2583_0993	-	CDS
O55/O157	1020724	s	rec	G2583_0993	-	CDS
O55/O157	1020727	s	rec	G2583_0993	-	CDS
O55/O157	1020729	ns	rec	G2583_0993	-	CDS
O55/O157	1020730	ns	rec	G2583_0993	-	CDS
O55/O157	1020742	s	rec	G2583_0993	-	CDS
O55/O157	1020756	ns	rec	G2583_0993	-	CDS
O55/O157	1020760	s	rec	G2583_0993	-	CDS
O55/O157	1020764	ns	rec	G2583_0993	-	CDS
O55/O157	1020766	ns	rec	G2583_0993	-	CDS
O55/O157	1020778	s	rec	G2583_0993	-	CDS
Sakai	923765	s	rec	G2583_0993	-	CDS
Sakai	923798	s	rec	G2583_0993	-	CDS
Sakai	923803	ns	rec	G2583_0993	-	CDS
Sakai	923805	ns	rec	G2583_0993	-	CDS
Sakai	923819	s	rec	G2583_0993	-	CDS
EDL933	925482	s		G2583_0993	-	CDS
O55/O157	1020868	s	rec	G2583_0993	-	CDS
O55/O157	1020871	s	rec	G2583_0993	-	CDS
Sakai	923841	ns	rec	G2583_0993	-	CDS
Sakai	923846	s	rec	G2583_0993	-	CDS
Sakai	923855	s	rec	G2583_0993	-	CDS
Sakai	923858	s	rec	G2583_0993	-	CDS
Sakai	923873	s	rec	G2583_0993	-	CDS
Sakai	923903	s	rec	G2583_0993	-	CDS
O55/O157	1020949	s	rec	G2583_0993	-	CDS
Sakai	923919	ns	rec	G2583_0993	-	CDS
O55/O157	1020979	s	rec	G2583_0993	-	CDS
O55/O157	1020982	s	rec	G2583_0993	-	CDS
O55/O157	1020985	s	rec	G2583_0993	-	CDS
O55/O157	1020998	ns	rec	G2583_0993	-	CDS
O55/O157	1020999	ns	rec	G2583_0993	-	CDS
O55/O157	1021003	ns	rec	G2583_0993	-	CDS

lineage[a]	site[b]	mutation type[c]	recomb	gene	Gene name	Type
O55/O157	1021022	ns	rec	G2583_0993	-	CDS
O55/O157	1021106	ns	rec	G2583_0993	-	CDS
O55/O157	1021129	s	rec	G2583_0993	-	CDS
O55/O157	1021144	s	rec	G2583_0993	-	CDS
O55/O157	1021162	s	rec	G2583_0993	-	CDS
O55/O157	1021195	ns	rec	G2583_0993-0994	-	CDS
O55/O157	1021211	s	rec	G2583_0994	-	CDS
O55/O157	1021212	ns	rec	G2583_0994	-	CDS
O55/O157	1021214	ns	rec	G2583_0994	-	CDS
O55/O157	1021215	s	rec	G2583_0994	-	CDS
O55/O157	1021217	s	rec	G2583_0994	-	CDS
O55/O157	1021219	ns	rec	G2583_0994	-	CDS
O55/O157	1021223	s	rec	G2583_0994	-	CDS
O55/O157	1021233	ns	rec	G2583_0994	-	CDS
O55/O157	1021244	s	rec	G2583_0994	-	CDS
O55/O157	1021246	ns	rec	G2583_0994	-	CDS
O55/O157	1021263	ns	rec	G2583_0994	-	CDS
O55/O157	1021269	ns	rec	G2583_0994	-	CDS
O55/O157	1021274	s	rec	G2583_0994	-	CDS
O55/O157	1021275	ns	rec	G2583_0994	-	CDS
O55/O157	1021276	ns	rec	G2583_0994	-	CDS
O55/O157	1021279	ns	rec	G2583_0994	-	CDS
O55/O157	1021293	s	rec	G2583_0994	-	CDS
O55/O157	1021298	s	rec	G2583_0994	-	CDS
O55/O157	1021304	s	rec	G2583_0994	-	CDS
O55/O157	1021316	s	rec	G2583_0994	-	CDS
O55/O157	1021337	s	rec	G2583_0994	-	CDS
O55/O157	1021354	ns	rec	G2583_0994	-	CDS
O55/O157	1021367	s	rec	G2583_0994	-	CDS
O55/O157	1021388	s	rec	G2583_0994	-	CDS
O157	927433	ins		intergenic		
CB9615	1025582	i		intergenic		
O157	928637	ns		G2583_1000	nleD	CDS
O157	929080	ns		G2583_1000	nleD	CDS
CB9615	1026181	s		G2583_1000	nleD	CDS
O157	929177	ns		G2583_1000	nleD	CDS
CB9615	1026718	i		intergenic		
EDL933	931402	ins		intergenic		
O157	932040	ns		G2583_1003	bioB	CDS
O157	933875	s		G2583_1004	bioF	CDS
O157	935246	ns		G2583_1006	bioD	CDS
EDL933	937001	ins		intergenic		
Sakai	935573	i		intergenic		
O157	939207	i		intergenic		
CB9615	1037023	s		G2583_1009	moaA	CDS

lineage[a]	site[b]	mutation type[c]	recomb	gene	Gene name	Type
CB9615	1038537	ns		G2583_1012	moaD	CDS
O157	941892	ns		G2583_1013	moaE	CDS
O157	941948	ns		G2583_1013	moaE	CDS
O157	949422	s		G2583_1021	ybhS	CDS
CB9615	1048669	ns		G2583_1023	ybhG	CDS
CB9615	1051769	i		intergenic		
O157	954756	i		intergenic		
CB9615	1053247	ns		G2583_1026	sopA	CDS
CB9615	1054190	ns		G2583_1026	sopA	CDS
CB9615	1054323	i		intergenic		
CB9615	1054946	ns		G2583_1027	dinG	CDS
O157	958598	s		G2583_1027	dinG	CDS
EDL933	962356	ins		intergenic		
CB9615	1058153	s		G2583_1029	ybiC	CDS
O157	961276	s		G2583_1029	ybiC	CDS
EDL933	963209	s		G2583_1029	ybiC	CDS
EDL933	963601	ins		intergenic		
O157	961981	i		intergenic		
O157	962912	ins-2		intergenic		
O157	962926	i		intergenic		
O157	963066	ins-9		G2583_1032	ybiX	CDS
O157	963843	s		G2583_1033	fiu	CDS
O157	964831	s		G2583_1033	fiu	CDS
O157	965032	s		G2583_1033	fiu	CDS
O157	967383	ns		G2583_1035	ybiN	CDS
O157	967469	s		G2583_1035	ybiN	CDS
O157	969118	ns		G2583_1036	ybiO	CDS
EDL933	973215	ins		intergenic		
EDL933	974119	i		intergenic		
CB9615	1071861	s		G2583_1042	ompX	CDS
EDL933	978220	s		G2583_1043	ybiP	CDS
CB9615	1074877	ns		G2583_1045	mntR	CDS
O157	978253	ns		G2583_1046	ybiR	CDS
EDL933	981745	ins		intergenic		
O157	980189	i		intergenic		
CB9615	1078504	ns		G2583_1048	ybiT	CDS
CB9615	1078786	s		G2583_1048	ybiT	CDS
O157	982480	ns		G2583_1049	ybiU	CDS
EDL933	985052	ins		intergenic		
O157	985536	ns		G2583_1051	ybiW	CDS
O157	986309	del		G2583_1051	ybiW	CDS
CB9615	1084204	ns		G2583_1052	ybiY	CDS
O157	987457	ns		G2583_1052	ybiY	CDS
CB9615	1084813	i		intergenic		
CB9615	1086319	s		G2583_1054	moeB	CDS

lineage[a]	site[b]	mutation type[c]	recomb	gene	Gene name	Type
CB9615	1087266	s		G2583_1055	moeA	CDS
O157	991247	ns		G2583_1056	iaaA	CDS
CB9615	1088417	ns		G2583_1056	iaaA	CDS
CB9615	1088690	ns		G2583_1056	iaaA	CDS
CB9615	1089463	ns		G2583_1057	gsiA	CDS
O157	992822	s		G2583_1057	gsiA	CDS
O157	995579	ns		G2583_1059	gsiC	CDS
CB9615	1093284	ns		G2583_1060	gsiD	CDS
O157	997719	ns		G2583_1061	yliE	CDS
O157	998270	ns		G2583_1061	yliE	CDS
O157	999350	ns		G2583_1061	yliE	CDS
O157	1000811	s		G2583_1062	yliF	CDS
CB9615	1099295	ns		G2583_1065	yliG	CDS
CB9615	1108324	ns		G2583_1074	ybjI	CDS
CB9615	1108788	ns		G2583_1075	ybjJ	CDS
CB9615	1110546	s		G2583_1078	ybjL	CDS
O157	1015134	i		intergenic		
EDL933	1016923	i		intergenic		
EDL933	1017032	ins		intergenic		
CB9615	1113392	ns		G2583_1081	ybjC	CDS
CB9615	1113428	ns		G2583_1081	ybjC	CDS
CB9615	1114264	i		intergenic		
O157	1018191	i		intergenic		
CB9615	1115829	i		intergenic		
O157	1020868	s		G2583_1086	potG	CDS
O157	1021692	ns		G2583_1087	potH	CDS
O157	1022959	ns		G2583_1088	potI	CDS
O157	1023105	s		G2583_1088	potI	CDS
CB9615	1120325	ns		G2583_1089	ybjO	CDS
O157	1025210	i		intergenic		
CB9615	1123758	ns		G2583_1092	-	CDS
O157	1027219	ns		G2583_1093	-	CDS
O157	1027830	ns		G2583_1093	-	CDS
O157	1029023	s		G2583_1094	artJ	CDS
O157	1030347	s		G2583_1096	artQ	CDS
O157	1032910	ns		G2583_1099	ybjP	CDS
EDL933	1035050	ins		intergenic		
CB9615	1130628	i		intergenic		
O157	1033693	nc		G2583_1101	-	pseudogene
CB9615	1131140	nc		G2583_1101	-	pseudogene
CB9615	1131296	nc		G2583_1101	-	pseudogene
CB9615	1131422	nc		G2583_1101	-	pseudogene
CB9615	1131759	nc		G2583_1101	-	pseudogene
O157	1034910	ins-7		G2583_1101	-	pseudogene
O157	1037575	s		G2583_1105	ybjS	CDS

lineage[a]	site[b]	mutation type[c]	recomb	gene	Gene name	Type
O157	1039857	ns		G2583_1107	ltaE	CDS
CB9615	1139385	s		G2583_1109	hcr	CDS
O157	1042561	s		G2583_1109	hcr	CDS
CB9615	1139855	ns		G2583_1109	hcr	CDS
CB9615	1140088	ns		G2583_1109	hcr	CDS
CB9615	1140396	ns		G2583_1110	hcp	CDS
O157	1043658	s		G2583_1110	hcp	CDS
O157	1045975	i		intergenic		
CB9615	1143104	i		intergenic		
EDL933	1047831	i		intergenic		
EDL933	1047833	i		intergenic		
EDL933	1047834	i		intergenic		
CB9615	1143375	i		intergenic		
O157	1047805	s		G2583_1113	ybjD	CDS
EDL933	1053619	ns		G2583_1116	macB	CDS
EDL933	1053625	ns		G2583_1116	macB	CDS
CB9615	1153896	del		intergenic		
O157	1057224	i		intergenic		
O157	1058900	ns		G2583_1123	cydC	CDS
O157	1059792	ns		G2583_1123	cydC	CDS
O157	1060753	s		G2583_1124	cydD	CDS
CB9615	1158058	ns		G2583_1124	cydD	CDS
CB9615	1158847	ns		G2583_1124	cydD	CDS
CB9615	1160395	i		intergenic		
O157	1064616	ns		G2583_1127	ftsK	CDS
O157	1066322	s		G2583_1127	ftsK	CDS
O157	1068309	ns		G2583_1127	ftsK	CDS
CB9615	1165450	i		intergenic		
CB9615	1166262	ns		G2583_1129	rarA	CDS
EDL933	1160369	ns		G2583_1130	serS	CDS
CB9615	1168985	i		intergenic		
O157	1074617	ns		G2583_1132	dmsB	CDS
O157	1075240	s		G2583_1133	dmsC	CDS
CB9615	1178467	s		G2583_1138	pflB	CDS
CB9615	1180804	s		G2583_1139	focA	CDS
CB9615	1183760	ns		G2583_1141	ycaP	CDS
O157	1087342	ns		G2583_1142	serC	CDS
CB9615	1185822	ns		G2583_1143	aroA	CDS
CB9615	1186891	i		intergenic		
CB9615	1187559	ns		G2583_1144	ycaL	CDS
O157	1095098	s		G2583_1148	ycaI	CDS
O157	1095795	ns		G2583_1148	ycaI	CDS
CB9615	1193541	s		G2583_1149	msbA	CDS
O157	1099686	ns		G2583_1151	ycaQ	CDS
O157	1101693	s		G2583_1154	ycbJ	CDS

lineage[a]	site[b]	mutation type[c]	recomb	gene	Gene name	Type
CB9615	1198848	ns		G2583_1154	ycbJ	CDS
CB9615	1201044	s		G2583_1157	mukF	CDS
CB9615	1204963	ns		G2583_1159	mukB	CDS
CB9615	1206013	ns		G2583_1159	mukB	CDS
O157	1109237	s		G2583_1159	mukB	CDS
O157	1111344	ns		G2583_1160	ycbB	CDS
EDL933	1212328	ins		intergenic		
O157	1116845	ins		intergenic		
CB9615	1214573	s		G2583_1165	asnS	CDS
CB9615	1215788	ns		G2583_1166	pncB	CDS
O157	1119462	s		G2583_1166	pncB	CDS
CB9615	1218645	ns		G2583_1167	pepN	CDS
O157	1122663	s		G2583_1167	pepN	CDS
O157	1124089	ns		G2583_1169	ssuC	CDS
O157	1124352	ns		G2583_1170	ssuD	CDS
O157	1127116	i		intergenic		
CB9615	1224147	del-4		intergenic		
CB9615	1225775	nc		G2583_1175	ycbS	pseudogene
O157	1130668	nc		G2583_1175	ycbS	pseudogene
O157	1132256	ns		G2583_1176	ycbT	CDS
CB9615	1229308	ns		G2583_1176	ycbT	CDS
CB9615	1229795	s		G2583_1177	ycbU	CDS
O157	1133470	s		G2583_1179	ycbF	CDS
O157	1133596	ns		G2583_1179	ycbF	CDS
O157	1133863	del		G2583_1179	ycbF	CDS
CB9615	1233217	ns		G2583_1182	ycbX	CDS
CB9615	1234198	i		intergenic		
O157	1138109	s		G2583_1183	rlmL	CDS
O157	1140211	s		G2583_1184	uup	CDS
CB9615	1238514	s		G2583_1185	pqiA	CDS
O157	1143210	ns		G2583_1186	pqiB	CDS
CB9615	1246780	i		intergenic		
O157	1150523	i		intergenic		
O157	1150795	ns		G2583_1194	sxy	CDS
EDL933	1240814	s		G2583_1195	yccS	CDS
CB9615	1251306	ns		G2583_1197	helD	CDS
CB9615	1256702	ns		G2583_1203	yccX	CDS
O157	1160684	ns		G2583_1205	yccA	CDS
CB9615	1257704	s		G2583_1205	yccA	CDS
CB9615	1257905	i		intergenic		
O157	1161047	i		intergenic		
EDL933	1250462	ins		intergenic		
EDL933	1253627	ns		ECs1057	-	CDS
EDL933	1253654	ns		ECs1057	-	CDS
EDL933	1255086	ins		ECs1060	-	CDS

lineage[a]	site[b]	mutation type[c]	recomb	gene	Gene name	Type
Sakai/EDL933	1175577	ns	rec	ECs1082	-	CDS
Sakai/EDL933	1175578	ns	rec	ECs1082	-	CDS
Sakai/EDL933	1175581	ns	rec	ECs1082	-	CDS
Sakai/EDL933	1175596	ns	rec	ECs1082	-	CDS
Sakai/EDL933	1175605	ns	rec	ECs1082	-	CDS
Sakai/EDL933	1175611	ns	rec	ECs1082	-	CDS
Sakai/EDL933	1175614	ns	rec	ECs1082	-	CDS
Sakai/EDL933	1175668	ns	rec	ECs1082	-	CDS
Sakai/EDL933	1175671	ns	rec	ECs1082	-	CDS
Sakai/EDL933	1175680	ns	rec	ECs1082	-	CDS
Sakai/EDL933	1175683	ns	rec	ECs1082	-	CDS
Sakai/EDL933	1175689	ns	rec	ECs1082	-	CDS
Sakai/EDL933	1175692	ns	rec	ECs1082	-	CDS
Sakai/EDL933	1175732	ns	rec	ECs1082	-	CDS
Sakai/EDL933	1175740	ns	rec	ECs1082	-	CDS
Sakai/EDL933	1175756	ns	rec	ECs1082	-	CDS
Sakai/EDL933	1175757	ns	rec	ECs1082	-	CDS
Sakai/EDL933	1175764	ns	rec	ECs1082	-	CDS
Sakai/EDL933	1175767	ns	rec	ECs1082	-	CDS
Sakai/EDL933	1175776	ns	rec	ECs1082	-	CDS
Sakai/EDL933	1175800	ns	rec	ECs1082	-	CDS
Sakai/EDL933	1175804	ns	rec	ECs1082	-	CDS
Sakai/EDL933	1175817	ns	rec	ECs1082	-	CDS
Sakai/EDL933	1175824	ns	rec	ECs1082	-	CDS
Sakai/EDL933	1175825	ns	rec	ECs1082	-	CDS
Sakai/EDL933	1175830	ns	rec	ECs1082	-	CDS
Sakai/EDL933	1175833	ns	rec	ECs1082	-	CDS
Sakai/EDL933	1175836	ns	rec	ECs1082	-	CDS
Sakai/EDL933	1175841	ns	rec	ECs1082	-	CDS
Sakai/EDL933	1175842	ns	rec	ECs1082	-	CDS
Sakai/EDL933	1175851	ns	rec	ECs1082	-	CDS
Sakai/EDL933	1175857	ns	rec	ECs1082	-	CDS
Sakai/EDL933	1175860	ns	rec	ECs1082	-	CDS
Sakai/EDL933	1175862	ns	rec	ECs1082	-	CDS
Sakai/EDL933	1175863	ns	rec	ECs1082	-	CDS
Sakai/EDL933	1175966	ns	rec	ECs1082	-	CDS
Sakai/EDL933	1176041	ns	rec	ECs1082	-	CDS
Sakai/EDL933	1176051	ns	rec	ECs1082	-	CDS
Sakai/EDL933	1176175	ns	rec	ECs1082	-	CDS
Sakai/EDL933	1176316	ns	rec	ECs1082	-	CDS
Sakai/EDL933	1176375	ns	rec	ECs1083	-	CDS
Sakai/EDL933	1176509	ns	rec	ECs1083	-	CDS
Sakai/EDL933	1176517	ns	rec	ECs1083	-	CDS
Sakai/EDL933	1176523	ns	rec	ECs1083	-	CDS
Sakai/EDL933	1176532	ns	rec	ECs1083	-	CDS

lineage[a]	site[b]	mutation type[c]	recomb	gene	Gene name	Type
Sakai/EDL933	1176535	ns	rec	ECs1083	-	CDS
Sakai/EDL933	1176568	ns	rec	ECs1083	-	CDS
Sakai/EDL933	1176580	ns	rec	ECs1083	-	CDS
Sakai/EDL933	1176606	ns	rec	ECs1083	-	CDS
Sakai/EDL933	1176607	ns	rec	ECs1083	-	CDS
Sakai/EDL933	1176608	ns	rec	ECs1083	-	CDS
Sakai/EDL933	1176613	ns	rec	ECs1083	-	CDS
Sakai/EDL933	1176616	ns	rec	ECs1083	-	CDS
Sakai/EDL933	1176619	ns	rec	ECs1083	-	CDS
Sakai/EDL933	1176622	ns	rec	ECs1083	-	CDS
Sakai/EDL933	1176646	ns	rec	ECs1083	-	CDS
Sakai/EDL933	1176649	ns	rec	ECs1083	-	CDS
Sakai/EDL933	1176652	ns	rec	ECs1083	-	CDS
Sakai/EDL933	1176654	ns	rec	ECs1083	-	CDS
Sakai/EDL933	1176661	ns	rec	ECs1083	-	CDS
Sakai/EDL933	1176664	ns	rec	ECs1083	-	CDS
Sakai/EDL933	1176665	ns	rec	ECs1083	-	CDS
Sakai/EDL933	1176667	ns	rec	ECs1083	-	CDS
Sakai/EDL933	1176676	ns	rec	ECs1083	-	CDS
Sakai/EDL933	1176682	ns	rec	ECs1083	-	CDS
CB9615	1258254	i		intergenic		
CB9615	1259582	ns		G2583_1207	hyaA	CDS
CB9615	1263377	ns		G2583_1212	hyaF	CDS
O157	1222017	ns		G2583_1216	etk	CDS
CB9615	1270175	ns		G2583_1216	etk	CDS
CB9615	1274993	ns		G2583_1220	gfcC	CDS
CB9615	1275427	ns		G2583_1221	gfcB	CDS
CB9615	1276488	i		intergenic		
O157	1230443	del		intergenic		
CB9615	1278057	s		G2583_1226	yccM	CDS
O157	1238521	s		G2583_1231	torA	CDS
O157	1239117	s		G2583_1231	torA	CDS
O157	1239925	ns		G2583_1231	torA	CDS
O157	1240061	ns		G2583_1231	torA	CDS
CB9615	1289246	s		G2583_1234	cbpA	CDS
O157	1242359	i		intergenic		
O157	1243278	ins		G2583_1235	yccE	CDS
O157	1243622	ns		G2583_1235	yccE	CDS
O157	1245860	ns		G2583_1238	wrbA	CDS
O157	1246019	ns		G2583_1238	wrbA	CDS
Sakai/EDL933	1251647	ns	rec	ECs1172	-	CDS
Sakai/EDL933	1251664	ns	rec	ECs1172-1173	-	CDS
Sakai/EDL933	1251670	ns	rec	ECs1172-1173	-	CDS
Sakai/EDL933	1251693	s	rec	ECs1173	-	CDS
Sakai/EDL933	1251696	s	rec	ECs1173	-	CDS

lineage[a]	site[b]	mutation type[c]	recomb	gene	Gene name	Type
Sakai/EDL933	1251714	s	rec	ECs1173	-	CDS
Sakai/EDL933	1251738	s	rec	ECs1173	-	CDS
Sakai/EDL933	1251746	s	rec	ECs1173	-	CDS
Sakai/EDL933	1251809	ns	rec	ECs1173	-	CDS
Sakai/EDL933	1251810	s	rec	ECs1173	-	CDS
Sakai/EDL933	1251843	ns	rec	ECs1174	-	CDS
Sakai/EDL933	1251844	ns	rec	ECs1174	-	CDS
Sakai/EDL933	1251858	ns	rec	ECs1174	-	CDS
Sakai/EDL933	1251946	ns	rec	ECs1174	-	CDS
Sakai/EDL933	1251955	ns	rec	ECs1174	-	CDS
Sakai/EDL933	1251986	s	rec	ECs1174	-	CDS
Sakai/EDL933	1251989	s	rec	ECs1174	-	CDS
Sakai/EDL933	1252067	s	rec	ECs1174	-	CDS
Sakai/EDL933	1252142	s	rec	ECs1174	-	CDS
Sakai/EDL933	1252181	s	rec	ECs1174	-	CDS
Sakai/EDL933	1252218	ns	rec	ECs1174	-	CDS
Sakai/EDL933	1252247	s	rec	ECs1174	-	CDS
Sakai/EDL933	1252289	s	rec	ECs1174	-	CDS
Sakai/EDL933	1252292	s	rec	ECs1174	-	CDS
Sakai/EDL933	1252331	s	rec	ECs1174	-	CDS
Sakai/EDL933	1252334	s	rec	ECs1174	-	CDS
Sakai/EDL933	1252385	s	rec	ECs1174	-	CDS
Sakai/EDL933	1252391	s	rec	ECs1174	-	CDS
Sakai/EDL933	1252400	s	rec	ECs1174	-	CDS
Sakai/EDL933	1252411	ns	rec	ECs1174	-	CDS
Sakai/EDL933	1252421	s	rec	ECs1174	-	CDS
Sakai/EDL933	1252451	s	rec	ECs1174	-	CDS
Sakai/EDL933	1252460	s	rec	ECs1174	-	CDS
Sakai/EDL933	1252487	s	rec	ECs1174	-	CDS
Sakai/EDL933	1252522	s	rec	ECs1175	-	CDS
Sakai/EDL933	1252534	s	rec	ECs1175	-	CDS
Sakai/EDL933	1252539	ns	rec	ECs1175	-	CDS
Sakai/EDL933	1252546	s	rec	ECs1175	-	CDS
Sakai/EDL933	1252565	ns	rec	ECs1175	-	CDS
Sakai/EDL933	1252570	s	rec	ECs1175	-	CDS
Sakai/EDL933	1252603	s	rec	ECs1175	-	CDS
Sakai/EDL933	1252690	s	rec	ECs1175	-	CDS
Sakai/EDL933	1252738	s	rec	ECs1175	-	CDS
Sakai/EDL933	1252756	s	rec	ECs1175	-	CDS
Sakai/EDL933	1252816	s	rec	ECs1175	-	CDS
Sakai/EDL933	1252846	s	rec	ECs1175	-	CDS
Sakai/EDL933	1252879	s	rec	ECs1175	-	CDS
Sakai/EDL933	1252882	s	rec	ECs1175	-	CDS
Sakai/EDL933	1252969	s	rec	ECs1175	-	CDS
Sakai/EDL933	1252990	s	rec	ECs1175	-	CDS

lineage[a]	site[b]	mutation type[c]	recomb	gene	Gene name	Type
Sakai/EDL933	1253041	s	rec	ECs1175	-	CDS
Sakai/EDL933	1253160	s	rec	ECs1175	-	CDS
Sakai/EDL933	1253275	s	rec	ECs1175	-	CDS
Sakai/EDL933	1253305	i	rec	intergenic		
Sakai/EDL933	1253382	ns	rec	ECs1176	-	CDS
Sakai/EDL933	1253463	ns	rec	ECs1176	-	CDS
Sakai/EDL933	1253466	ns	rec	ECs1176	-	CDS
Sakai/EDL933	1253490	ns	rec	ECs1176	-	CDS
Sakai/EDL933	1253497	s	rec	ECs1176	-	CDS
Sakai/EDL933	1253558	ns	rec	ECs1176	-	CDS
Sakai/EDL933	1253619	indel	rec	ECs1176	-	CDS
Sakai/EDL933	1253621	ns	rec	ECs1176	-	CDS
Sakai/EDL933	1253650	ns	rec	ECs1176	-	CDS
Sakai/EDL933	1253673	s	rec	ECs1176	-	CDS
Sakai/EDL933	1253711	ns	rec	ECs1176-1177	-	CDS
Sakai/EDL933	1253741	s	rec	ECs1177	-	CDS
Sakai/EDL933	1253744	s	rec	ECs1177	-	CDS
Sakai/EDL933	1253823	ns	rec	ECs1177-1178	-	CDS
Sakai/EDL933	1253845	ns	rec	ECs1177-1178	-	CDS
Sakai/EDL933	1254019	i	rec	intergenic		
Sakai/EDL933	1254025	i	rec	intergenic		
Sakai/EDL933	1254036	s	rec	ECs1179	-	CDS
Sakai/EDL933	1254116	ns	rec	ECs1179	-	CDS
Sakai/EDL933	1254245	ns	rec	ECs1179	-	CDS
Sakai/EDL933	1264454	ns	rec	ECs1200	-	CDS
Sakai/EDL933	1264456	ns	rec	ECs1200	-	CDS
Sakai/EDL933	1264472	ns	rec	ECs1200	-	CDS
Sakai/EDL933	1264475	ns	rec	ECs1200	-	CDS
Sakai/EDL933	1264658	ns	rec	ECs1200	-	CDS
Sakai/EDL933	1264769	ns	rec	ECs1200	-	CDS
Sakai/EDL933	1264770	ns	rec	ECs1200	-	CDS
Sakai/EDL933	1264808	ns	rec	ECs1200	-	CDS
Sakai/EDL933	1264907	ns	rec	ECs1200	-	CDS
Sakai/EDL933	1264919	ns	rec	ECs1200	-	CDS
Sakai/EDL933	1264934	ns	rec	ECs1200	-	CDS
Sakai/EDL933	1264944	ns	rec	ECs1200	-	CDS
Sakai/EDL933	1264990	ns	rec	ECs1201	-	CDS
Sakai/EDL933	1264993	ns	rec	ECs1201	-	CDS
EDL933	1357447	i		intergenic		
EDL933	1379630	ns		ECs1241	-	CDS
EDL933	1380292	ns		ECs1242	-	CDS
EDL933	1383978	ns		ECs1242	-	CDS
Sakai/EDL933	1304915	ns		ECs1244	-	CDS
Sakai/EDL933	1306660	ns	rec	ECs1247	-	CDS
Sakai/EDL933	1306676	s	rec	ECs1247	-	CDS

lineage[a]	site[b]	mutation type[c]	recomb	gene	Gene name	Type
Sakai/EDL933	1306755	ns	rec	ECs1247	-	CDS
Sakai/EDL933	1306770	ns	rec	ECs1247	-	CDS
Sakai/EDL933	1306813	indel	rec	ECs1248	-	CDS
Sakai/EDL933	1306826	ns	rec	ECs1248	-	CDS
Sakai/EDL933	1306833	ns	rec	ECs1248	-	CDS
Sakai/EDL933	1306839	ns	rec	ECs1248	-	CDS
Sakai/EDL933	1306861	s	rec	ECs1248	-	CDS
Sakai/EDL933	1307002	ns	rec	ECs1248	-	CDS
Sakai/EDL933	1307004	ns	rec	ECs1248-1249	-	CDS
Sakai/EDL933	1307008	ns	rec	ECs1248-1249	-	CDS
Sakai/EDL933	1307010	ns	rec	ECs1248-1249	-	CDS
Sakai/EDL933	1307291	ns	rec	ECs1250	-	CDS
Sakai/EDL933	1307292	ns	rec	ECs1250	-	CDS
Sakai/EDL933	1307305	ns	rec	ECs1250	-	CDS
Sakai/EDL933	1307306	ns	rec	ECs1250	-	CDS
Sakai/EDL933	1307314	ns	rec	ECs1250	-	CDS
Sakai/EDL933	1307317	ns	rec	ECs1250	-	CDS
Sakai/EDL933	1307319	ns	rec	ECs1250	-	CDS
Sakai/EDL933	1307325	ns	rec	ECs1250	-	CDS
Sakai/EDL933	1307335	ns	rec	ECs1250	-	CDS
Sakai/EDL933	1307338	ns	rec	ECs1250	-	CDS
Sakai/EDL933	1307343	ns	rec	ECs1250	-	CDS
Sakai/EDL933	1307344	ns	rec	ECs1250	-	CDS
Sakai/EDL933	1307346	ns	rec	ECs1250	-	CDS
Sakai/EDL933	1307347	ns	rec	ECs1250	-	CDS
Sakai/EDL933	1307350	ns	rec	ECs1250	-	CDS
Sakai/EDL933	1307353	ns	rec	ECs1250	-	CDS
Sakai/EDL933	1307354	ns	rec	ECs1250	-	CDS
Sakai/EDL933	1307359	ns	rec	ECs1250	-	CDS
Sakai/EDL933	1307370	ns	rec	ECs1250	-	CDS
Sakai/EDL933	1307371	ns	rec	ECs1250	-	CDS
Sakai/EDL933	1307383	ns	rec	ECs1250	-	CDS
EDL933	1391910	ns		ECs1251	-	CDS
O157	1309105	del		intergenic		
O157	1310870	ns		G2583_1240	rutF	CDS
CB9615	1296179	ns		G2583_1241	rutE	CDS
O157	1313206	ns		G2583_1244	rutB	CDS
O157	1313399	s		G2583_1244	rutB	CDS
O157	1313591	s		G2583_1244	rutB	CDS
O157	1314825	ns		G2583_1245	rutA	CDS
CB9615	1301611	s		G2583_1247	putA	CDS
Sakai	1317211	ns		G2583_1247	putA	CDS
CB9615	1302302	ns		G2583_1247	putA	CDS
CB9615	1304300	ns		G2583_1247	putA	CDS
CB9615	1304828	i		intergenic		

lineage[a]	site[b]	mutation type[c]	recomb	gene	Gene name	Type
CB9615	1305638	s		G2583_1248	putP	CDS
CB9615	1305866	s		G2583_1248	putP	CDS
O157	1321549	s		G2583_1248	putP	CDS
CB9615	1306455	ins-7		intergenic		
CB9615	1307974	i		intergenic		
O157	1323368	ins		intergenic		
CB9615	1309018	s		G2583_1251	-	CDS
O157	1327295	ns		G2583_1254	ycdB	CDS
O157	1327875	i		intergenic		
O157	1330622	ns		G2583_1257	pgaC	CDS
CB9615	1316705	del		G2583_1258	pgaB	pseudogene
CB9615	1317032	nc		G2583_1258	pgaB	pseudogene
O157	1332703	nc		G2583_1258	pgaB	pseudogene
O157	1335468	i		intergenic		
CB9615	1320380	i		intergenic		
CB9615	1320900	nc		G2583_1260	ycdT	pseudogene
CB9615	1320994	del		G2583_1260	ycdT	pseudogene
O55/O157	1323925	ns		G2583_1262	fidL	CDS
O55/O157	1325205	i		intergenic		
O55/O157	1326226	s		G2583_1264	ygcW	CDS
O55/O157	1327265	ns		G2583_1265	-	CDS
O55/O157	1328035	ns		G2583_1266	mrkD	CDS
O55/O157	1333298	i		intergenic		
O55/O157	1337596	s		G2583_1273	-	CDS
O55/O157	1338704	ns		G2583_1274	-	CDS
O55/O157	1338821	ns		G2583_1274	-	CDS
O55/O157	1339221	ns		G2583_1274	-	CDS
O55/O157	1341649	ns		G2583_1277	cylZ	CDS
O55/O157	1342740	ns		G2583_1279	gcvT	CDS
O55/O157	1343015	s		G2583_1279	gcvT	CDS
EDL933	1442315	ns		G2583_1279	gcvT	CDS
O55/O157	1345873	ns		G2583_1280	fabF	CDS
O55/O157	1349411	s		G2583_1284	-	CDS
O55/O157	1351716	s		G2583_1287	-	CDS
O55/O157	1352529	s		G2583_1287	-	CDS
CB9615	1354119	s		G2583_1289	ycdU	CDS
CB9615	1354282	ns		G2583_1289	ycdU	CDS
CB9615	1354521	ns		G2583_1289	ycdU	CDS
EDL933	1462501	i		intergenic		
EDL933	1466649	ns		ECs1307	-	CDS
EDL933	1467619	ns		ECs1308	-	CDS
EDL933	1467701	s		ECs1309	-	CDS
Sakai	1383967	ns		ECs1309	-	CDS
EDL933	1481615	ins		ECs1332	-	CDS
EDL933	1485047	ns		ECs1337	-	CDS

lineage[a]	site[b]	mutation type[c]	recomb	gene	Gene name	Type
Sakai	1404441	i		intergenic		
Sakai/EDL933	1408882	indel		ECs1347	-	CDS
Sakai/EDL933	1409533	ns		ECs1348	-	CDS
EDL933	1495971	i		intergenic		
EDL933	1501974	i		intergenic		
Sakai	1418991	s		ECs1360	-	CDS
EDL933	1505129	ins		ECs1361	-	CDS
EDL933	1507006	i		intergenic		
EDL933	1507913	ins		intergenic		
Sakai/EDL933	1426613	indel		ECs1370	-	CDS
EDL933	1516291	ns		ECs1380	-	CDS
Sakai/EDL933	1437442	indel		ECs1385	-	CDS
EDL933	1525470	ns		ECs1393	-	CDS
EDL933	1527633	i		intergenic		
EDL933	1537072	ns		ECs1403	-	CDS
EDL933	1537210	ns		ECs1403	-	CDS
EDL933	1537727	ns	rec	ECs1404	-	CDS
EDL933	1537728	ns	rec	ECs1404	-	CDS
EDL933	1537729	ns	rec	ECs1404	-	CDS
EDL933	1537732	ns	rec	ECs1404	-	CDS
EDL933	1537733	ns	rec	ECs1404	-	CDS
EDL933	1537735	ns	rec	ECs1404	-	CDS
EDL933	1537738	ns	rec	ECs1404	-	CDS
EDL933	1537739	ns	rec	ECs1404	-	CDS
EDL933	1537740	ns	rec	ECs1404	-	CDS
EDL933	1537741	ns	rec	ECs1404	-	CDS
EDL933	1537742	ns	rec	ECs1404	-	CDS
EDL933	1537743	ns	rec	ECs1404	-	CDS
EDL933	1537744	ns	rec	ECs1404	-	CDS
EDL933	1537745	ns	rec	ECs1404	-	CDS
EDL933	1537746	i	rec	intergenic		
EDL933	1537747	i	rec	intergenic		
EDL933	1537748	i	rec	intergenic		
EDL933	1537749	i	rec	intergenic		
EDL933	1537750	i	rec	intergenic		
EDL933	1537751	i	rec	intergenic		
EDL933	1537753	i	rec	intergenic		
EDL933	1537754	i	rec	intergenic		
EDL933	1537755	i	rec	intergenic		
EDL933	1537756	i	rec	intergenic		
EDL933	1537757	i	rec	intergenic		
EDL933	1537758	i	rec	intergenic		
EDL933	1537759	i	rec	intergenic		
EDL933	1537760	i	rec	intergenic		
EDL933	1537763	i	rec	intergenic		

lineage[a]	site[b]	mutation type[c]	recomb	gene	Gene name	Type
EDL933	1537764	i	rec	intergenic		
EDL933	1537765	i	rec	intergenic		
EDL933	1537766	i	rec	intergenic		
EDL933	1537767	i	rec	intergenic		
EDL933	1537768	i	rec	intergenic		
EDL933	1537769	i	rec	intergenic		
EDL933	1537770	i	rec	intergenic		
EDL933	1537772	i	rec	intergenic		
EDL933	1537773	i	rec	intergenic		
EDL933	1537774	i	rec	intergenic		
EDL933	1537776	i	rec	intergenic		
EDL933	1537777	i	rec	intergenic		
EDL933	1537778	i	rec	intergenic		
EDL933	1537779	i	rec	intergenic		
EDL933	1537780	i	rec	intergenic		
EDL933	1537783	i	rec	intergenic		
EDL933	1537785	i	rec	intergenic		
EDL933	1537786	i	rec	intergenic		
EDL933	1537787	i	rec	intergenic		
EDL933	1537788	i	rec	intergenic		
EDL933	1537789	i	rec	intergenic		
EDL933	1537790	i	rec	intergenic		
EDL933	1537791	i	rec	intergenic		
EDL933	1537792	i	rec	intergenic		
EDL933	1537793	i	rec	intergenic		
EDL933	1537794	i	rec	intergenic		
EDL933	1537795	i	rec	intergenic		
EDL933	1537796	i	rec	intergenic		
EDL933	1537797	i	rec	intergenic		
EDL933	1537799	i	rec	intergenic		
EDL933	1537800	i	rec	intergenic		
EDL933	1537802	i	rec	intergenic		
EDL933	1537803	i	rec	intergenic		
EDL933	1537805	i	rec	intergenic		
EDL933	1537806	i	rec	intergenic		
EDL933	1537807	i	rec	intergenic		
EDL933	1537809	i	rec	intergenic		
EDL933	1537810	i	rec	intergenic		
EDL933	1537812	i	rec	intergenic		
EDL933	1537814	i	rec	intergenic		
EDL933	1537816	i	rec	intergenic		
EDL933	1537818	i	rec	intergenic		
EDL933	1537819	i	rec	intergenic		
EDL933	1537820	i	rec	intergenic		
EDL933	1537821	i	rec	intergenic		

lineage[a]	site[b]	mutation type[c]	recomb	gene	Gene name	Type
EDL933	1537822	i	rec	intergenic		
EDL933	1537823	i	rec	intergenic		
EDL933	1537824	i	rec	intergenic		
EDL933	1537826	i	rec	intergenic		
EDL933	1537827	i	rec	intergenic		
EDL933	1537828	i	rec	intergenic		
EDL933	1537829	i	rec	intergenic		
EDL933	1537830	i	rec	intergenic		
EDL933	1537832	i	rec	intergenic		
EDL933	1537833	i	rec	intergenic		
EDL933	1537834	i	rec	intergenic		
EDL933	1537835	i	rec	intergenic		
EDL933	1537836	i	rec	intergenic		
EDL933	1537837	i	rec	intergenic		
EDL933	1537840	i	rec	intergenic		
EDL933	1537841	i	rec	intergenic		
EDL933	1537842	i	rec	intergenic		
EDL933	1537843	i	rec	intergenic		
EDL933	1537893	i		intergenic		
O157	1456933	i		intergenic		
O157	1457156	ns		G2583_1291	ghrA	CDS
CB9615	1355672	ns		G2583_1291	ghrA	CDS
O157	1457566	ns		G2583_1291	ghrA	CDS
O157	1462537	i		intergenic		
EDL933	1547749	ins		intergenic		
CB9615	1371194	ns		G2583_1309	yceK	CDS
O157	1474522	ns		G2583_1312	mdtG	CDS
CB9615	1374367	i		intergenic		
O157	1477504	s		G2583_1315	yceI	CDS
CB9615	1376344	s		G2583_1316	yceJ	CDS
CB9615	1376363	ns		G2583_1316	yceJ	CDS
CB9615	1378271	i		intergenic		
CB9615	1378863	s		G2583_1320	dinI	CDS
O157	1480670	i		intergenic		
CB9615	1381340	s		G2583_1323	grxB	CDS
CB9615	1385077	s		G2583_1327	yceM	CDS
O157	1487937	s		G2583_1328	yceN	CDS
CB9615	1386770	s		G2583_1328	yceN	CDS
O157	1489450	s		G2583_1331	flgA	CDS
O157	1490050	i		intergenic		
CB9615	1392294	s		G2583_1337	flgG	CDS
O157	1494084	s		G2583_1337	flgG	CDS
CB9615	1393193	s		G2583_1338	flgH	CDS
CB9615	1393561	s		G2583_1338	flgH	CDS
CB9615	1396548	ns		G2583_1341	flgK	CDS

lineage[a]	site[b]	mutation type[c]	recomb	gene	Gene name	Type
CB9615	1396667	ns		G2583_1341	flgK	CDS
CB9615	1396708	s		G2583_1341	flgK	CDS
O157	1500144	i		intergenic		
Sakai	1501902	s		G2583_1343	rne	CDS
CB9615	1403192	ns		G2583_1345	rluC	CDS
CB9615	1403319	ns		G2583_1345	rluC	CDS
O157	1505031	del-11		intergenic		
Sakai	1505266	ns		G2583_1346	yceF	CDS
CB9615	1403981	s		G2583_1346	yceF	CDS
CB9615	1408747	s		G2583_1353	fabG	CDS
O157	1510369	ns		G2583_1353	fabG	CDS
O157	1510808	s		G2583_1354	acpP	CDS
CB9615	1410989	ns		G2583_1356	pabC	CDS
O157	1512902	ns		G2583_1356	pabC	CDS
CB9615	1411746	ns		G2583_1357	yceG	CDS
O157	1514408	ns		G2583_1358	tmk	CDS
EDL933	1601792	ins		intergenic		
CB9615	1419009	del-9		G2583_1362	fhuE	CDS
O157	1520731	i		intergenic		
EDL933	1605834	ins-7		intergenic		
O157	1520747	ins-12		intergenic		
EDL933	1605879	ins		intergenic		
CB9615	1420962	s		G2583_1366	thiK	CDS
O157	1524001	ns		G2583_1367	nagZ	CDS
EDL933	1610100	ins		intergenic		
CB9615	1424402	s		G2583_1369	ndh	CDS
CB9615	1426328	s		G2583_1371	ycfQ	CDS
CB9615	1427164	ns		G2583_1373	ycfS	CDS
O157	1530771	s		G2583_1374	mfd	CDS
O157	1531911	s		G2583_1374	mfd	CDS
CB9615	1431302	s		G2583_1374	mfd	CDS
O157	1533239	ns		G2583_1375	ycfT	CDS
CB9615	1431989	ns		G2583_1375	ycfT	CDS
O157	1536286	s		G2583_1377	lolD	CDS
CB9615	1434947	s		G2583_1378	lolE	CDS
Sakai	1537161	s		G2583_1378	lolE	CDS
O157	1538337	ns		G2583_1379	nagK	CDS
O157	1539725	ns		G2583_1381	potD	CDS
O157	1540899	s		G2583_1382	potC	CDS
CB9615	1439902	s		G2583_1383	potB	CDS
Sakai	1545192	i		intergenic		
EDL933	1632320	ns		ECs1510	-	CDS
EDL933	1632848	ns		ECs1511	-	CDS
EDL933	1636139	ns		ECs1519	-	CDS
Sakai/EDL933	1552060	ns	rec	ECs1522	-	CDS

lineage[a]	site[b]	mutation type[c]	recomb	gene	Gene name	Type
Sakai/EDL933	1552061	ns	rec	ECs1522	-	CDS
Sakai/EDL933	1552064	ns	rec	ECs1522	-	CDS
Sakai/EDL933	1552079	ns	rec	ECs1522	-	CDS
Sakai/EDL933	1552088	ns	rec	ECs1522	-	CDS
Sakai/EDL933	1552094	ns	rec	ECs1522	-	CDS
Sakai/EDL933	1552097	ns	rec	ECs1522	-	CDS
Sakai/EDL933	1552151	ns	rec	ECs1522	-	CDS
Sakai/EDL933	1552154	ns	rec	ECs1522	-	CDS
Sakai/EDL933	1552163	ns	rec	ECs1522	-	CDS
Sakai/EDL933	1552166	ns	rec	ECs1522	-	CDS
Sakai/EDL933	1552172	ns	rec	ECs1522	-	CDS
Sakai/EDL933	1552175	ns	rec	ECs1522	-	CDS
Sakai/EDL933	1552215	ns	rec	ECs1522	-	CDS
Sakai/EDL933	1552223	ns	rec	ECs1522	-	CDS
Sakai/EDL933	1552239	ns	rec	ECs1522	-	CDS
Sakai/EDL933	1552240	ns	rec	ECs1522	-	CDS
Sakai/EDL933	1552247	ns	rec	ECs1522	-	CDS
Sakai/EDL933	1552250	ns	rec	ECs1522	-	CDS
Sakai/EDL933	1552259	ns	rec	ECs1522	-	CDS
Sakai/EDL933	1552283	ns	rec	ECs1522	-	CDS
Sakai/EDL933	1552287	ns	rec	ECs1522	-	CDS
Sakai/EDL933	1552300	ns	rec	ECs1522	-	CDS
Sakai/EDL933	1552307	ns	rec	ECs1522	-	CDS
Sakai/EDL933	1552308	ns	rec	ECs1522	-	CDS
Sakai/EDL933	1552313	ns	rec	ECs1522	-	CDS
Sakai/EDL933	1552316	ns	rec	ECs1522	-	CDS
Sakai/EDL933	1552319	ns	rec	ECs1522	-	CDS
Sakai/EDL933	1552324	ns	rec	ECs1522	-	CDS
Sakai/EDL933	1552325	ns	rec	ECs1522	-	CDS
Sakai/EDL933	1552334	ns	rec	ECs1522	-	CDS
Sakai/EDL933	1552340	ns	rec	ECs1522	-	CDS
Sakai/EDL933	1552343	ns	rec	ECs1522	-	CDS
Sakai/EDL933	1552345	ns	rec	ECs1522	-	CDS
Sakai/EDL933	1552346	ns	rec	ECs1522	-	CDS
Sakai/EDL933	1552449	ns	rec	ECs1522	-	CDS
Sakai/EDL933	1552524	ns	rec	ECs1522	-	CDS
Sakai/EDL933	1552534	ns	rec	ECs1522	-	CDS
Sakai/EDL933	1552658	ns	rec	ECs1522	-	CDS
Sakai/EDL933	1552799	ns	rec	ECs1522	-	CDS
Sakai/EDL933	1552858	ns	rec	ECs1523	-	CDS
Sakai/EDL933	1552992	ns	rec	ECs1523	-	CDS
Sakai/EDL933	1553000	ns	rec	ECs1523	-	CDS
Sakai/EDL933	1553006	ns	rec	ECs1523	-	CDS
Sakai/EDL933	1553015	ns	rec	ECs1523	-	CDS
Sakai/EDL933	1553018	ns	rec	ECs1523	-	CDS

lineage[a]	site[b]	mutation type[c]	recomb	gene	Gene name	Type
Sakai/EDL933	1553051	ns	rec	ECs1523	-	CDS
Sakai/EDL933	1553063	ns	rec	ECs1523	-	CDS
Sakai/EDL933	1571409	ns	rec	ECs1550	-	CDS
CB9615	1439906	del		intergenic		
EDL933	1658523	ns		ECs1552	-	CDS
Sakai	1572531	ins		ECs1552-1553	-	CDS
Sakai/EDL933	1576601	indel		ECs1556	-	CDS
Sakai/EDL933	1663238	indel		ECs1560	-	CDS
EDL933	1663828	ns		ECs1560	-	CDS
Sakai/EDL933	1666646	indel		ECs1561	-	CDS
EDL933	1668330	ns		ECs1561	-	CDS
EDL933	1668336	ns		ECs1561	-	CDS
EDL933	1668373	ns		ECs1561	-	CDS
O157	1590628	ns		G2583_1383	-	CDS
Sakai	1592092	i		intergenic		
CB9615	1442704	s		G2583_1385	pepT	CDS
O55/O157	1445562	s	rec	G2583_1387	-	CDS
O55/O157	1445568	s	rec	G2583_1387	-	CDS
O55/O157	1445571	s	rec	G2583_1387	-	CDS
O55/O157	1445589	s	rec	G2583_1387	-	CDS
O55/O157	1445592	s	rec	G2583_1387	-	CDS
O55/O157	1445604	s	rec	G2583_1387	-	CDS
O55/O157	1445629	ns	rec	G2583_1387	-	CDS
O55/O157	1445664	s	rec	G2583_1387	-	CDS
O55/O157	1445691	s	rec	G2583_1387	-	CDS
O55/O157	1445692	ns	rec	G2583_1387	-	CDS
O55/O157	1445694	ns	rec	G2583_1387	-	CDS
O55/O157	1445700	s	rec	G2583_1387	-	CDS
O55/O157	1445709	s	rec	G2583_1387	-	CDS
O55/O157	1445712	s	rec	G2583_1387	-	CDS
O55/O157	1445724	s	rec	G2583_1387	-	CDS
O55/O157	1445733	s	rec	G2583_1387	-	CDS
O55/O157	1445754	s	rec	G2583_1387	-	CDS
O55/O157	1445781	s	rec	G2583_1387	-	CDS
O55/O157	1445802	s	rec	G2583_1387	-	CDS
O55/O157	1445808	s	rec	G2583_1387	-	CDS
O55/O157	1445811	s	rec	G2583_1387	-	CDS
O55/O157	1445886	s	rec	G2583_1387	-	CDS
O55/O157	1445990	indel	rec	intergenic		
O55/O157	1446004	i	rec	intergenic		
O55/O157	1446042	i	rec	intergenic		
O55/O157	1446067	i	rec	intergenic		
O55/O157	1446179	i	rec	intergenic		
O55/O157	1446234	i	rec	intergenic		
O55/O157	1446356	s	rec	G2583_1388	-	CDS

lineage[a]	site[b]	mutation type[c]	recomb	gene	Gene name	Type
O55/O157	1446552	ns	rec	G2583_1389	-	CDS
O55/O157	1446647	s	rec	G2583_1390	-	CDS
O55/O157	1446652	ns	rec	G2583_1390	-	CDS
O55/O157	1446658	ns	rec	G2583_1390	-	CDS
O55/O157	1446680	s	rec	G2583_1390	-	CDS
O55/O157	1446738	ns	rec	G2583_1390	-	CDS
O55/O157	1446740	ns	rec	G2583_1390	-	CDS
O55/O157	1446746	s	rec	G2583_1390	-	CDS
O55/O157	1446748	ns	rec	G2583_1390	-	CDS
O55/O157	1446804	ns	rec	G2583_1390	-	CDS
O55/O157	1446841	ns	rec	G2583_1390	-	CDS
O55/O157	1446846	ns	rec	G2583_1390	-	CDS
O55/O157	1446893	s	rec	G2583_1390	-	CDS
O55/O157	1447055	ns	rec	G2583_1390	-	CDS
Sakai	1599400	ins		ECs1582	-	CDS
EDL933	1683393	del		ECs1582	-	CDS
Sakai	1599494	del		intergenic		
Sakai	1599684	ins		ECs1583	-	CDS
Sakai	1599754	ins		intergenic		
EDL933	1688625	ns		ECs1592	-	CDS
Sakai	1607790	ns		ECs1598	-	CDS
EDL933	1692998	ns		ECs1599	-	CDS
O157	1610142	ns		G2583_1405	phoQ	CDS
O157	1610519	ns		G2583_1405	phoQ	CDS
EDL933	1696596	s		G2583_1407	purB	CDS
CB9615	1458735	s		G2583_1407	purB	CDS
CB9615	1459722	s		G2583_1408	hflD	CDS
O157	1615966	s		G2583_1410	nudJ	CDS
CB9615	1461967	s		G2583_1411	rluE	CDS
O55/O157	1463703	s	rec	G2583_1412	icd	CDS
O55/O157	1463754	s	rec	G2583_1412	icd	CDS
O55/O157	1463802	s	rec	G2583_1412	icd	CDS
O55/O157	1463820	s	rec	G2583_1412	icd	CDS
O55/O157	1463829	s	rec	G2583_1412	icd	CDS
O55/O157	1463844	s	rec	G2583_1412	icd	CDS
EDL933	1704396	ins		ECs1611	-	CDS
EDL933	1704550	ins		ECs1612	-	CDS
EDL933	1715423	ns	rec	ECs1630	-	CDS
EDL933	1715588	ns	rec	ECs1630	-	CDS
EDL933	1715669	ns	rec	ECs1630	-	CDS
EDL933	1715681	ns	rec	ECs1630	-	CDS
EDL933	1715750	ns	rec	ECs1630	-	CDS
EDL933	1715792	ns	rec	ECs1630	-	CDS
EDL933	1715849	ns	rec	ECs1630	-	CDS
EDL933	1715993	ns	rec	ECs1630	-	CDS

lineage[a]	site[b]	mutation type[c]	recomb	gene	Gene name	Type
EDL933	1716017	ns	rec	ECs1630	-	CDS
EDL933	1716239	ns	rec	ECs1630	-	CDS
Sakai	1633414	ns		ECs1632	-	CDS
EDL933	1719535	ns		ECs1633	-	CDS
EDL933	1719899	s		ECs1634	-	CDS
EDL933	1719900	ins		ECs1634	-	CDS
EDL933	1719949	i		intergenic		
EDL933	1719951	i		intergenic		
Sakai	1637604	ns		ECs1637	-	CDS
Sakai/EDL933	1649126	ns	rec	ECs1649	-	CDS
Sakai/EDL933	1649147	ns	rec	ECs1649	-	CDS
Sakai/EDL933	1649165	ns	rec	ECs1649	-	CDS
Sakai/EDL933	1649246	ns	rec	ECs1649	-	CDS
Sakai/EDL933	1649333	ns	rec	ECs1649	-	CDS
Sakai/EDL933	1649356	ns	rec	ECs1649	-	CDS
Sakai/EDL933	1649420	ns	rec	ECs1649	-	CDS
Sakai/EDL933	1649438	ns	rec	ECs1649	-	CDS
Sakai/EDL933	1649444	ns	rec	ECs1649	-	CDS
EDL933	1741635	ns		ECs1650	-	CDS
Sakai	1657028	ns		ECs1658	-	CDS
EDL933	1749013	ins		ECs1658	-	CDS
EDL933	1749070	ns		ECs1659	-	CDS
EDL933	1749082	ins		ECs1659	-	CDS
EDL933	1749092	ins		ECs1659	-	CDS
EDL933	1749152	ns	rec	ECs1659	-	CDS
EDL933	1749161	ns	rec	ECs1659	-	CDS
EDL933	1749195	ns	rec	ECs1659	-	CDS
EDL933	1749196	ns	rec	ECs1659	-	CDS
EDL933	1749199	ins-2	rec	ECs1659	-	CDS
EDL933	1749206	ns	rec	ECs1659	-	CDS
EDL933	1749212	ns	rec	ECs1659	-	CDS
EDL933	1749216	ins	rec	ECs1659	-	CDS
EDL933	1749226	ns	rec	ECs1659	-	CDS
EDL933	1749239	ins	rec	ECs1659	-	CDS
EDL933	1749244	ns	rec	ECs1659	-	CDS
EDL933	1749245	ns	rec	ECs1659	-	CDS
EDL933	1749249	ins	rec	ECs1659	-	CDS
EDL933	1749250	ns	rec	ECs1659	-	CDS
EDL933	1749268	ins	rec	ECs1659	-	CDS
EDL933	1749290	ns	rec	ECs1659	-	CDS
EDL933	1749293	ns	rec	ECs1659	-	CDS
EDL933	1749294	ns	rec	ECs1659	-	CDS
EDL933	1749295	ns	rec	ECs1659	-	CDS
EDL933	1749312	ns	rec	ECs1659	-	CDS
EDL933	1749333	ns	rec	ECs1659	-	CDS

lineage[a]	site[b]	mutation type[c]	recomb	gene	Gene name	Type
EDL933	1749341	ns	rec	ECs1659	-	CDS
EDL933	1749343	ns	rec	ECs1659	-	CDS
EDL933	1754044	i		intergenic		
EDL933	1754081	ins		intergenic		
EDL933	1755379	ns		ECs1666	-	CDS
EDL933	1755494	ns		ECs1666	-	CDS
Sakai	1664318	ns		ECs1666	-	CDS
CB9615	1478774	ns		G2583_1432	ypjA	CDS
O157	1668969	i		intergenic		
EDL933	1760503	ins		G2583_1438	-	CDS
CB9615	1485267	ns		G2583_1442	hlyE	CDS
O157	1675796	s		G2583_1447	nhaB	CDS
CB9615	1492633	i		intergenic		
CB9615	1492634	ins		intergenic		
CB9615	1493359	ns		G2583_1450	dadA	CDS
Sakai	1681338	ns		G2583_1451	dadX	CDS
Sakai	1681339	ns		G2583_1451	dadX	CDS
Sakai	1681342	ns		G2583_1451	dadX	CDS
Sakai	1681360	ins		G2583_1451	dadX	CDS
Sakai	1681748	del		G2583_1452	ipaH	CDS
EDL933	1773155	del		G2583_1453	-	CDS
O157	1687920	ns		G2583_1459	prrA	CDS
O157	1688820	ns		G2583_1459	prrA	CDS
CB9615	1503339	ns		G2583_1459	prrA	CDS
O157	1690889	ns		G2583_1461	-	CDS
CB9615	1510826	i		intergenic		
O157	1696836	ns		G2583_1468	dhaM	CDS
CB9615	1512932	ns		G2583_1469	dhaL	CDS
O55/O157	1513217	ns		G2583_1470	dhaK	CDS
O157	1702338	i		intergenic		
O157	1703964	s		G2583_1475	pth	CDS
EDL933	1797852	s		G2583_1477	ychM	CDS
EDL933	1802448	ns		G2583_1482	prfA	CDS
O157	1714840	ns		G2583_1486	kdsA	CDS
O157	1715760	del-6		intergenic		
EDL933	1807802	ins		intergenic		
O157	1723107	ns		G2583_1495	-	CDS
O157	1723540	s		G2583_1496	narK	CDS
O157	1723961	ns		G2583_1496	narK	CDS
O157	1724806	i		intergenic		
CB9615	1542317	i		intergenic		
CB9615	1542331	i		intergenic		
EDL933	1818849	s		G2583_1497	narG	CDS
CB9615	1546652	s		G2583_1498	narH	CDS
O157	1729903	s		G2583_1498	narH	CDS

lineage[a]	site[b]	mutation type[c]	recomb	gene	Gene name	Type
CB9615	1549440	nc	rec	G2583_1501-1502	-	pseudogene
CB9615	1549452	nc	rec	G2583_1501-1502	-	pseudogene
CB9615	1549473	nc	rec	G2583_1501-1502	-	pseudogene
CB9615	1549494	nc	rec	G2583_1501-1502	-	pseudogene
CB9615	1549506	nc	rec	G2583_1501-1502	-	pseudogene
O157	1732419	ns		G2583_1501-1504	ychS	CDS
CB9615	1552984	ns		G2583_1509	rssB	CDS
CB9615	1554968	s		G2583_1511	hns	CDS
CB9615	1559499	i		intergenic		
O157	1742449	i		intergenic		
EDL933	1835258	ins		intergenic		
CB9615	1560684	i		intergenic		
CB9615	1561112	i		intergenic		
CB9615	1561322	s		G2583_1515	oppA	CDS
O157	1746954	s		G2583_1517	oppC	CDS
CB9615	1566220	ns		G2583_1519	oppF	CDS
O157	1750924	ns		G2583_1521	cls	CDS
EDL933	1843596	ins		G2583_1522	yciY	CDS
CB9615	1569663	ns		G2583_1523	kch	CDS
EDL933	1845763	ins		intergenic		
CB9615	1571179	i		intergenic		
CB9615	1571207	ns		G2583_1525	tonB	CDS
CB9615	1571265	ns		G2583_1525	tonB	CDS
O157	1754756	ns		G2583_1526	yciA	CDS
O157	1757422	ns		G2583_1529-1530	ompW - intE	CDS
CB9615	1574689	ns		G2583_1529-1530	ompW - intE	CDS
CB9615	1574765	del-13		G2583_1530	intE	CDS
CB9615	1574800	ins		G2583_1530	intE	CDS
O157	1757699	s		G2583_1530	intE	CDS
CB9615	1575107	s		G2583_1530	intE	CDS
CB9615	1575186	ns		G2583_1530	intE	CDS
CB9615	1575371	s		G2583_1530	intE	CDS
O157	1758846	s		G2583_1531	xisE	CDS
CB9615	1576227	ns	rec	G2583_1532	exoO	CDS
CB9615	1576244	ns	rec	G2583_1532	exoO	CDS
CB9615	1576245	ns	rec	G2583_1532	exoO	CDS
CB9615	1576273	s	rec	G2583_1532	exoO	CDS
CB9615	1576279	s	rec	G2583_1532	exoO	CDS
CB9615	1576282	s	rec	G2583_1532	exoO	CDS
CB9615	1576288	s	rec	G2583_1532	exoO	CDS
CB9615	1576309	s	rec	G2583_1532	exoO	CDS
CB9615	1576315	s	rec	G2583_1532	exoO	CDS
CB9615	1576335	ns	rec	G2583_1532	exoO	CDS
CB9615	1576379	ns	rec	G2583_1532	exoO	CDS
CB9615	1576384	s	rec	G2583_1532	exoO	CDS

lineage[a]	site[b]	mutation type[c]	recomb	gene	Gene name	Type
CB9615	1576402	s	rec	G2583_1532	exoO	CDS
CB9615	1576408	s	rec	G2583_1532	exoO	CDS
CB9615	1576432	s	rec	G2583_1532	exoO	CDS
CB9615	1576450	s	rec	G2583_1532	exoO	CDS
CB9615	1576456	s	rec	G2583_1532	exoO	CDS
CB9615	1576477	s	rec	G2583_1532	exoO	CDS
CB9615	1576513	s	rec	G2583_1532	exoO	CDS
CB9615	1576515	s	rec	G2583_1532	exoO	CDS
CB9615	1576558	s	rec	G2583_1532	exoO	CDS
CB9615	1576567	s	rec	G2583_1532	exoO	CDS
CB9615	1576579	s	rec	G2583_1532	exoO	CDS
CB9615	1576595	ns	rec	G2583_1532	exoO	CDS
CB9615	1576596	ns	rec	G2583_1532	exoO	CDS
CB9615	1576600	s	rec	G2583_1532	exoO	CDS
CB9615	1576612	s	rec	G2583_1532	exoO	CDS
CB9615	1576624	ns	rec	G2583_1532	exoO	CDS
CB9615	1576636	s	rec	G2583_1532	exoO	CDS
CB9615	1576642	s	rec	G2583_1532	exoO	CDS
CB9615	1576651	s	rec	G2583_1532	exoO	CDS
CB9615	1576690	s	rec	G2583_1532	exoO	CDS
CB9615	1576702	s	rec	G2583_1532	exoO	CDS
CB9615	1576714	s	rec	G2583_1532	exoO	CDS
CB9615	1576747	s	rec	G2583_1532	exoO	CDS
CB9615	1576753	ns	rec	G2583_1532	exoO	CDS
CB9615	1576754	ns	rec	G2583_1532	exoO	CDS
CB9615	1576755	ns	rec	G2583_1532	exoO	CDS
CB9615	1576756	ns	rec	G2583_1532	exoO	CDS
CB9615	1576758	ns	rec	G2583_1532	exoO	CDS
CB9615	1576761	ns	rec	G2583_1532	exoO	CDS
CB9615	1576762	s	rec	G2583_1532	exoO	CDS
CB9615	1576779	ns	rec	G2583_1532	exoO	CDS
CB9615	1576780	ns	rec	G2583_1532	exoO	CDS
CB9615	1576782	ns	rec	G2583_1532	exoO	CDS
CB9615	1576783	ns	rec	G2583_1532	exoO	CDS
CB9615	1576791	ns	rec	G2583_1532	exoO	CDS
CB9615	1576796	ns	rec	G2583_1532	exoO	CDS
CB9615	1576805	ns	rec	G2583_1532	exoO	CDS
CB9615	1576808	ns	rec	G2583_1532	exoO	CDS
CB9615	1576815	ns	rec	G2583_1532	exoO	CDS
CB9615	1576818	ns	rec	G2583_1532	exoO	CDS
CB9615	1576832	ns	rec	G2583_1532	exoO	CDS
CB9615	1576840	ns	rec	G2583_1532	exoO	CDS
CB9615	1576841	ns	rec	G2583_1532	exoO	CDS
CB9615	1576843	s	rec	G2583_1532	exoO	CDS
CB9615	1576859	ns	rec	G2583_1532	exoO	CDS

lineage[a]	site[b]	mutation type[c]	recomb	gene	Gene name	Type
CB9615	1576876	ns	rec	G2583_1532	exoO	CDS
CB9615	1576877	ns	rec	G2583_1532	exoO	CDS
CB9615	1576883	ns	rec	G2583_1532	exoO	CDS
CB9615	1576887	ns	rec	G2583_1532	exoO	CDS
CB9615	1576888	s	rec	G2583_1532	exoO	CDS
CB9615	1576911	ns	rec	G2583_1532	exoO	CDS
CB9615	1576925	ns	rec	G2583_1532	exoO	CDS
CB9615	1576926	ns	rec	G2583_1532	exoO	CDS
CB9615	1576932	ns	rec	G2583_1532	exoO	CDS
CB9615	1576946	ns	rec	G2583_1532	exoO	CDS
CB9615	1577036	ns	rec	G2583_1532	exoO	CDS
CB9615	1577037	ns	rec	G2583_1532	exoO	CDS
CB9615	1577050	s	rec	G2583_1532	exoO	CDS
CB9615	1577056	s	rec	G2583_1532	exoO	CDS
CB9615	1577062	s	rec	G2583_1532	exoO	CDS
CB9615	1577087	ns	rec	G2583_1532	exoO	CDS
CB9615	1577088	ns	rec	G2583_1532	exoO	CDS
CB9615	1577091	ns	rec	G2583_1532	exoO	CDS
CB9615	1577460	ns		G2583_1532	exoO	CDS
CB9615	1578358	s		G2583_1532	exoO	CDS
O157	1761585	s		G2583_1533	ydfD	CDS
CB9615	1579211	s		G2583_1534	dicB	CDS
CB9615	1579236	i		intergenic		
CB9615	1579238	i		intergenic		
O157	1763664	del		G2583_1539	-	CDS
O55/O157	1581496	s		G2583_1540	dicA	CDS
O157	1764356	i		intergenic		
O157	1765092	s		G2583_1542	-	CDS
O157	1765093	ns		G2583_1542	-	CDS
O157	1766676	ns		G2583_1545	-	CDS
O157	1769362	ns		G2583_1548	gef	CDS
CB9615	1586959	ins		intergenic		
O157	1769693	ns		G2583_1549	rem	CDS
O157	1769754	ns		G2583_1549	rem	CDS
CB9615	1587098	ns		G2583_1549	rem	CDS
CB9615	1587102	ns		G2583_1549	rem	CDS
O157	1769827	ns		G2583_1549	rem	CDS
CB9615	1587129	ns		G2583_1549	rem	CDS
O55/O157	1587638	ns		G2583_1551	-	CDS
EDL933	1862309	ins		G2583_1551	-	CDS
O157	1770409	s		G2583_1551	-	CDS
CB9615	1588294	s		G2583_1551	-	CDS
CB9615	1588339	s		G2583_1551	-	CDS
Sakai/EDL933	1796779	nc	rec	ECs1806	-	pseudogene
Sakai/EDL933	1796785	nc	rec	ECs1806	-	pseudogene

lineage[a]	site[b]	mutation type[c]	recomb	gene	Gene name	Type
Sakai/EDL933	1796787	nc	rec	ECs1806	-	pseudogene
Sakai/EDL933	1796800	nc	rec	ECs1806	-	pseudogene
Sakai/EDL933	1796821	nc	rec	ECs1806	-	pseudogene
Sakai/EDL933	1796824	nc	rec	ECs1806	-	pseudogene
Sakai/EDL933	1796825	nc	rec	ECs1806	-	pseudogene
Sakai/EDL933	1796826	nc	rec	ECs1806	-	pseudogene
Sakai/EDL933	1796827	nc	rec	ECs1806	-	pseudogene
Sakai/EDL933	1796832	nc	rec	ECs1806	-	pseudogene
Sakai/EDL933	1796839	nc	rec	ECs1806	-	pseudogene
Sakai/EDL933	1796842	nc	rec	ECs1806	-	pseudogene
Sakai/EDL933	1796875	nc	rec	ECs1806	-	pseudogene
Sakai/EDL933	1796883	nc	rec	ECs1806	-	pseudogene
Sakai/EDL933	1796891	nc	rec	ECs1806	-	pseudogene
Sakai/EDL933	1796893	nc	rec	ECs1806	-	pseudogene
Sakai/EDL933	1796915	nc	rec	ECs1806	-	pseudogene
Sakai/EDL933	1796926	nc	rec	ECs1806	-	pseudogene
Sakai/EDL933	1796933	nc	rec	ECs1806	-	pseudogene
Sakai/EDL933	1796937	nc	rec	ECs1806	-	pseudogene
Sakai/EDL933	1796953	nc	rec	ECs1806	-	pseudogene
Sakai/EDL933	1796989	nc	rec	ECs1806	-	pseudogene
Sakai/EDL933	1797010	nc	rec	ECs1806	-	pseudogene
Sakai/EDL933	1797013	nc	rec	ECs1806	-	pseudogene
Sakai/EDL933	1797031	nc	rec	ECs1806	-	pseudogene
Sakai/EDL933	1797043	nc	rec	ECs1806	-	pseudogene
Sakai/EDL933	1797046	nc	rec	ECs1806	-	pseudogene
Sakai/EDL933	1797048	nc	rec	ECs1806	-	pseudogene
Sakai/EDL933	1797050	nc	rec	ECs1806	-	pseudogene
Sakai/EDL933	1797055	nc	rec	ECs1806	-	pseudogene
Sakai/EDL933	1797064	nc	rec	ECs1806	-	pseudogene
Sakai/EDL933	1797065	nc	rec	ECs1806	-	pseudogene
Sakai/EDL933	1797066	nc	rec	ECs1806	-	pseudogene
Sakai/EDL933	1797067	nc	rec	ECs1806	-	pseudogene
Sakai/EDL933	1797070	nc	rec	ECs1806	-	pseudogene
Sakai/EDL933	1797073	nc	rec	ECs1806	-	pseudogene
Sakai/EDL933	1797094	nc	rec	ECs1806	-	pseudogene
Sakai/EDL933	1797097	nc	rec	ECs1806	-	pseudogene
Sakai/EDL933	1797106	nc	rec	ECs1806	-	pseudogene
Sakai/EDL933	1797115	nc	rec	ECs1806	-	pseudogene
Sakai/EDL933	1797122	nc	rec	ECs1806	-	pseudogene
Sakai/EDL933	1797130	nc	rec	ECs1806	-	pseudogene
Sakai/EDL933	1797157	nc	rec	ECs1806	-	pseudogene
Sakai/EDL933	1797166	nc	rec	ECs1806	-	pseudogene
Sakai/EDL933	1797175	nc	rec	ECs1806	-	pseudogene
Sakai/EDL933	1797184	nc	rec	ECs1806	-	pseudogene
Sakai/EDL933	1797188	nc	rec	ECs1806	-	pseudogene

lineage[a]	site[b]	mutation type[c]	recomb	gene	Gene name	Type
Sakai/EDL933	1797196	nc	rec	ECs1806	-	pseudogene
Sakai/EDL933	1797223	nc	rec	ECs1806	-	pseudogene
Sakai/EDL933	1797233	nc	rec	ECs1806	-	pseudogene
Sakai/EDL933	1797235	nc	rec	ECs1806	-	pseudogene
Sakai/EDL933	1797244	nc	rec	ECs1806	-	pseudogene
Sakai/EDL933	1797247	nc	rec	ECs1806	-	pseudogene
Sakai/EDL933	1797265	nc	rec	ECs1806	-	pseudogene
Sakai/EDL933	1797307	nc	rec	ECs1806	-	pseudogene
Sakai/EDL933	1797313	nc	rec	ECs1806	-	pseudogene
Sakai/EDL933	1797373	nc	rec	ECs1806	-	pseudogene
Sakai/EDL933	1797376	nc	rec	ECs1806	-	pseudogene
Sakai/EDL933	1797377	nc	rec	ECs1806	-	pseudogene
Sakai/EDL933	1797379	nc	rec	ECs1806	-	pseudogene
Sakai/EDL933	1797381	nc	rec	ECs1806	-	pseudogene
Sakai/EDL933	1797382	nc	rec	ECs1806	-	pseudogene
Sakai/EDL933	1797385	nc	rec	ECs1806	-	pseudogene
EDL933	2289690	nc		ECs1806	-	pseudogene
Sakai	1809380	s		ECs1820	-	CDS
CB9615	1628142	ns		G2583_1596	yciE	CDS
O157	1816270	i		intergenic		
O157	1817110	i		intergenic		
O157	1817628	s		G2583_1599	trpA	CDS
CB9615	1630095	ns		G2583_1599	trpA	CDS
O157	1818104	ns		G2583_1599	trpA	CDS
EDL933	2264476	ins		G2583_1603	trpE	CDS
O157	1823642	ns		G2583_1603	trpE	CDS
CB9615	1635957	ns		G2583_1603	trpE	CDS
O157	1824156	i		intergenic		
O157	1824237	i		intergenic		
O157	1825597	s		G2583_1606	yciO	CDS
CB9615	1638617	ns		G2583_1607	-	CDS
O157	1829004	ns		G2583_1609	btuR	CDS
O157	1830902	s		G2583_1611	sohB	CDS
CB9615	1646614	i		intergenic		
O157	1834948	s		G2583_1614	cysB	CDS
O157	1835167	s		G2583_1614	cysB	CDS
CB9615	1648691	s		G2583_1617	acnA	CDS
EDL933	2247204	ins		intergenic		
O157	1840051	s		G2583_1619	pgpB	CDS
CB9615	1655198	ns		G2583_1622	pyrF	CDS
EDL933	2242049	ins		intergenic		
O157	1845248	ns		G2583_1626	yciZ	CDS
O55/O157	1658246	s		G2583_1627	gmr	CDS
O157	1846543	s		G2583_1627	gmr	CDS
O157	1846588	s		G2583_1627	gmr	CDS

lineage[a]	site[b]	mutation type[c]	recomb	gene	Gene name	Type
CB9615	1660014	ns		G2583_1628	rnb	CDS
O157	1849924	ns		G2583_1629	yciW	CDS
EDL933	2236241	ins		intergenic		
O157	1854374	del-6		G2583_1634	acrB	CDS
O157	1855245	s		G2583_1634	acrB	CDS
CB9615	1667461	s		G2583_1634	acrB	CDS
CB9615	1668269	ns		G2583_1634	acrB	CDS
O157	1857017	ns		G2583_1634	acrB	CDS
O157	1857124	ns		G2583_1634	acrB	CDS
CB9615	1669577	s		G2583_1635	eefC	CDS
EDL933	2228993	ins		G2583_1635	eefC	CDS
EDL933	2227971	ins		G2583_1636	eefD	CDS
CB9615	1674365	s		G2583_1639	sapC	CDS
O157	1863935	ns		G2583_1641	sapA	CDS
CB9615	1676620	ns		G2583_1641	sapA	CDS
CB9615	1677185	i		intergenic		
EDL933	2222023	ins		intergenic		
EDL933	2221451	ins		intergenic		
O157	1867323	i		intergenic		
CB9615	1681028	i		intergenic		
O157	1869052	ns		G2583_1645	puuD	CDS
O157	1869324	ns		G2583_1645	puuD	CDS
O157	1869679	s		G2583_1645	puuD	CDS
O157	1872868	ns		G2583_1648	puuB	CDS
O55/O157	1686425	s		G2583_1649	puuE	CDS
CB9615	1689339	ns		G2583_1654	pspD	CDS
EDL933	2209244	ins		intergenic		
CB9615	1690852	nc		G2583_1656	ycjM	pseudogene
CB9615	1691023	nc		G2583_1656	ycjM	pseudogene
O157	1879802	ns		G2583_1657	ycjN	CDS
O157	1880351	ns		G2583_1657	ycjN	CDS
O157	1880878	ns		G2583_1657	ycjN	CDS
CB9615	1693449	ns		G2583_1658	ycjO	CDS
CB9615	1693893	ns		G2583_1658	ycjO	CDS
O157	1881947	ns		G2583_1659	ycjP	CDS
O157	1882879	s		G2583_1660	ycjQ	CDS
O157	1883983	ns		G2583_1661	ycjR	CDS
O157	1884344	ns		G2583_1661	ycjR	CDS
CB9615	1697890	ns		G2583_1663	ycjT	CDS
CB9615	1701972	ns		G2583_1666	ompG	CDS
EDL933	2196477	ins		intergenic		
CB9615	1703127	ns		G2583_1667	ycjW	CDS
CB9615	1704503	s		G2583_1668	ycjX	CDS
CB9615	1709284	ns		G2583_1672	ycjG	CDS
O157	1897288	ns		G2583_1672	ycjG	CDS

lineage[a]	site[b]	mutation type[c]	recomb	gene	Gene name	Type
CB9615	1712035	ins-2		G2583_1675	-	CDS
EDL933	2184672	ins		intergenic		
O157	1914551	s		G2583_1687	abgR	CDS
O157	1916392	s		G2583_1690	ydaM	CDS
O55/O157	1732193	ns	rec	G2583_1694	ttcA	CDS
O55/O157	1732251	s	rec	G2583_1695	intR	CDS
O55/O157	1732350	s	rec	G2583_1695	intR	CDS
O55/O157	1732365	s	rec	G2583_1695	intR	CDS
O55/O157	1732467	s	rec	G2583_1695	intR	CDS
O55/O157	1732470	s	rec	G2583_1695	intR	CDS
O55/O157	1732473	s	rec	G2583_1695	intR	CDS
O55/O157	1732503	s	rec	G2583_1695	intR	CDS
O55/O157	1732521	s	rec	G2583_1695	intR	CDS
O55/O157	1732584	s	rec	G2583_1695	intR	CDS
O157	1923856	s		G2583_1699	recT	CDS
O157	1923899	s		G2583_1699	recT	CDS
CB9615	1735969	ns	rec	G2583_1700	recE	CDS
CB9615	1735991	ns	rec	G2583_1700	recE	CDS
CB9615	1736000	ns	rec	G2583_1700	recE	CDS
CB9615	1736038	s	rec	G2583_1700	recE	CDS
CB9615	1736044	s	rec	G2583_1700	recE	CDS
CB9615	1736056	ns	rec	G2583_1700	recE	CDS
CB9615	1736057	ns	rec	G2583_1700	recE	CDS
CB9615	1736059	s	rec	G2583_1700	recE	CDS
CB9615	1736062	s	rec	G2583_1700	recE	CDS
CB9615	1736065	ns	rec	G2583_1700	recE	CDS
CB9615	1736066	ns	rec	G2583_1700	recE	CDS
CB9615	1736071	s	rec	G2583_1700	recE	CDS
CB9615	1736080	ns	rec	G2583_1700	recE	CDS
CB9615	1736081	ns	rec	G2583_1700	recE	CDS
CB9615	1736087	ns	rec	G2583_1700	recE	CDS
CB9615	1736090	ns	rec	G2583_1700	recE	CDS
CB9615	1736108	ns	rec	G2583_1700	recE	CDS
EDL933	2157772	ins		ECs1941	-	CDS
EDL933	2157723	ns		ECs1941	-	CDS
EDL933	2155153	ns		ECs1946	-	CDS
EDL933	2146918	ins		ECs1958	-	CDS
EDL933	2145293	i	rec	intergenic		
EDL933	2145291	i	rec	intergenic		
EDL933	2145289	ins	rec	intergenic		
EDL933	2145284	i	rec	intergenic		
EDL933	2145158	ns	rec	ECs1961	-	CDS
EDL933	2144616	ns	rec	ECs1961	-	CDS
EDL933	2144405	ns	rec	ECs1961	-	CDS
EDL933	2144352	ns	rec	ECs1961	-	CDS

lineage[a]	site[b]	mutation type[c]	recomb	gene	Gene name	Type
EDL933	2144286	ns	rec	ECs1961	-	CDS
EDL933	2144051	ns	rec	ECs1961	-	CDS
EDL933	2144013	ns	rec	ECs1961	-	CDS
EDL933	2143825	ns	rec	ECs1961	-	CDS
EDL933	2143747	ns	rec	ECs1961	-	CDS
EDL933	2143614	ns	rec	ECs1961	-	CDS
EDL933	2143593	ns	rec	ECs1961	-	CDS
EDL933	2143483	ns	rec	ECs1961	-	CDS
EDL933	2143346	ns	rec	ECs1961	-	CDS
EDL933	2143322	ns	rec	ECs1961	-	CDS
EDL933	2143318	ns	rec	ECs1961	-	CDS
EDL933	2120440	ns	rec	ECs1991	-	CDS
EDL933	2120419	ns	rec	ECs1991	-	CDS
EDL933	2120401	ns	rec	ECs1991	-	CDS
EDL933	2120362	ns	rec	ECs1991	-	CDS
EDL933	2120333	ns	rec	ECs1991	-	CDS
EDL933	2120290	ns	rec	ECs1991	-	CDS
EDL933	2120233	ns	rec	ECs1991	-	CDS
EDL933	2120210	ns	rec	ECs1991	-	CDS
EDL933	2120146	ns	rec	ECs1991	-	CDS
EDL933	2120128	ns	rec	ECs1991	-	CDS
EDL933	2120122	ns	rec	ECs1991	-	CDS
EDL933	2119968	i	rec	intergenic		
EDL933	2119966	i	rec	intergenic		
EDL933	2119961	i	rec	intergenic		
EDL933	2119960	i	rec	intergenic		
EDL933	2119872	i	rec	intergenic		
EDL933	2119854	i	rec	intergenic		
EDL933	2119803	ns	rec	ECs1992	-	CDS
EDL933	2119800	ns	rec	ECs1992	-	CDS
EDL933	2119617	ns	rec	ECs1992	-	CDS
EDL933	2119530	ns	rec	ECs1992	-	CDS
EDL933	2119377	ns	rec	ECs1992	-	CDS
CB9615	1773669	s	rec	G2583_1747	-	CDS
CB9615	1773693	s	rec	G2583_1747	-	CDS
CB9615	1773699	s	rec	G2583_1747	-	CDS
CB9615	1773702	s	rec	G2583_1747	-	CDS
CB9615	1773705	s	rec	G2583_1747	-	CDS
CB9615	1773742	ns	rec	G2583_1747	-	CDS
O157	1969616	ns		G2583_1747	-	CDS
O157	1969701	s		G2583_1747	-	CDS
O157	1969949	i		intergenic		
CB9615	1774764	ns		G2583_1748	-	CDS
CB9615	1774765	ns		G2583_1748	-	CDS
O55/O157	1775389	ns		G2583_1749	-	CDS

lineage[a]	site[b]	mutation type[c]	recomb	gene	Gene name	Type
O55/O157	1775484	ns		G2583_1749	-	CDS
O55/O157	1775603	i		intergenic		
O55/O157	1776128	s		G2583_1750	-	CDS
O55/O157	1776310	i		intergenic		
O55/O157	1776395	i	rec	intergenic		
O55/O157	1776397	i	rec	intergenic		
O55/O157	1776406	i	rec	intergenic		
O55/O157	1776411	i	rec	intergenic		
O55/O157	1776413	i	rec	intergenic		
O55/O157	1776417	indel	rec	intergenic		
O55/O157	1776427	i	rec	intergenic		
O55/O157	1776433	i	rec	intergenic		
O55/O157	1776434	i	rec	intergenic		
O55/O157	1776435	i	rec	intergenic		
O55/O157	1776831	indel-13		intergenic		
O157	1975875	s		G2583_1754	ydbK	CDS
O157	1976735	ns		G2583_1754	ydbK	CDS
EDL933	2109214	ins		intergenic		
EDL933	2108013	ins		intergenic		
CB9615	1787006	s		G2583_1758	ydbH	CDS
CB9615	1788160	ns		G2583_1760	ydbL	CDS
O55/O157	1983970	indel		intergenic		
O55/O157	1789062	s		G2583_1762	-	CDS
EDL933	2103929	ins		G2583_1762	-	CDS
O55/O157	1789102	ns		G2583_1762	-	CDS
O55/O157	1789131	s		G2583_1762	-	CDS
O55/O157	1789483	ns		G2583_1762	-	CDS
O55/O157	1790383	ns		G2583_1762	-	CDS
O55/O157	1791108	s		G2583_1762	-	CDS
Sakai	1987879	ns		G2583_1763	entS	CDS
O55/O157	1792369	ns		G2583_1763	entS	CDS
O157	1990992	ns		G2583_1765	ydbD	CDS
CB9615	1796309	ns		G2583_1765	ydbD	CDS
CB9615	1796758	s		G2583_1765	ydbD	CDS
CB9615	1797653	ns		G2583_1765	ydbD	CDS
CB9615	1798123	ns		G2583_1766	ynbA	CDS
O157	1997244	ns		G2583_1769	ynbD	CDS
CB9615	1802230	ns		G2583_1769	ynbD	CDS
O157	1998914	s		G2583_1771	hrpA	CDS
O157	1999181	s		G2583_1771	hrpA	CDS
O157	2001300	ns		G2583_1771	hrpA	CDS
O157	2004345	s		G2583_1775	-	CDS
O157	2004346	ns		G2583_1775	-	CDS
O157	2005705	i		intergenic		
CB9615	1812976	ns		G2583_1779	cybB	CDS

lineage[a]	site[b]	mutation type[c]	recomb	gene	Gene name	Type
CB9615	1813669	ns		G2583_1781	ydcA	CDS
O157	2009219	ns		G2583_1781	ydcA	CDS
EDL933	2075503	ins		G2583_1785	ydcJ	CDS
EDL933	2074922	ins		G2583_1785	ydcJ	CDS
EDL933	2074322	ins		intergenic		
O157	2016281	ns		G2583_1788	ydcH	CDS
CB9615	1820817	ns		G2583_1788	ydcH	CDS
O157	2016743	ns		G2583_1789	rimL	CDS
CB9615	1821814	s		G2583_1790	ydcK	CDS
EDL933	2068776	ins		intergenic		
EDL933	2068689	ins		intergenic		
CB9615	1825601	ns		G2583_1794	ydcO	CDS
O157	2023761	s		G2583_1796	ydcP	CDS
O157	2025633	s		G2583_1799	ydcQ	CDS
CB9615	1833726	ns		G2583_1802	ydcT	CDS
O157	2030050	ns		G2583_1803	ydcU	CDS
CB9615	1835749	del-8		G2583_1804	ydcV	CDS
O157	2032185	ns		G2583_1805	ydcW	CDS
O157	2033176	ns		G2583_1806	ydcX	CDS
O157	2034031	ns		G2583_1808	ydcZ	CDS
CB9615	1844594	i		intergenic		
CB9615	1845392	s		G2583_1814	ansP	CDS
Sakai	2041566	s		G2583_1814	ansP	CDS
O157	2041968	i		intergenic		
CB9615	1847799	ns		G2583_1817	-	CDS
O157	2044028	s		G2583_1818	vgrE	CDS
O157	2045155	ns		G2583_1818	vgrE	CDS
EDL933	2042310	nc	rec	G2583_1819	-	pseudogene
Sakai	2046254	nc		G2583_1819	-	pseudogene
EDL933	2042207	nc	rec	G2583_1819	-	pseudogene
EDL933	2042172	nc	rec	G2583_1819	-	pseudogene
EDL933	2042135	nc	rec	G2583_1819	-	pseudogene
EDL933	2042093	nc	rec	G2583_1819	-	pseudogene
EDL933	2042092	nc	rec	G2583_1819	-	pseudogene
EDL933	2042091	del-2	rec	G2583_1819	-	pseudogene
EDL933	2042088	nc	rec	G2583_1819	-	pseudogene
EDL933	2042086	nc	rec	G2583_1819	-	pseudogene
EDL933	2042082	nc	rec	G2583_1819	-	pseudogene
EDL933	2042081	nc	rec	G2583_1819	-	pseudogene
EDL933	2042076	nc	rec	G2583_1819	-	pseudogene
EDL933	2042074	ins	rec	G2583_1819	-	pseudogene
EDL933	2042016	ins	rec	G2583_1819	-	pseudogene
EDL933	2042003	ins	rec	G2583_1819	-	pseudogene
EDL933	2041991	ins	rec	G2583_1819	-	pseudogene
EDL933	2041987	ins	rec	G2583_1819	-	pseudogene

lineage[a]	site[b]	mutation type[c]	recomb	gene	Gene name	Type
EDL933	2041979	nc	rec	G2583_1819	-	pseudogene
EDL933	2041977	ins	rec	G2583_1819	-	pseudogene
EDL933	2041953	nc	rec	G2583_1819	-	pseudogene
EDL933	2041944	nc	rec	G2583_1819	-	pseudogene
EDL933	2041924	nc	rec	G2583_1819	-	pseudogene
EDL933	2041886	nc	rec	G2583_1819	-	pseudogene
EDL933	2041866	nc	rec	G2583_1819	-	pseudogene
EDL933	2041843	nc	rec	G2583_1819	-	pseudogene
EDL933	2041831	nc	rec	G2583_1819	-	pseudogene
EDL933	2041830	nc	rec	G2583_1819	-	pseudogene
EDL933	2041827	nc	rec	G2583_1819	-	pseudogene
O55/O157	1851307	nc	rec	G2583_1819	-	pseudogene
O55/O157	1851318	nc	rec	G2583_1819	-	pseudogene
O55/O157	1851330	nc	rec	G2583_1819	-	pseudogene
O55/O157	1851421	nc	rec	G2583_1819	-	pseudogene
O55/O157	1851438	nc	rec	G2583_1819	-	pseudogene
O55/O157	1851448	nc	rec	G2583_1819	-	pseudogene
O55/O157	1851485	nc	rec	G2583_1819	-	pseudogene
O55/O157	1851487	nc	rec	G2583_1819	-	pseudogene
O55/O157	1851505	nc	rec	G2583_1819	-	pseudogene
O55/O157	1851688	nc	rec	G2583_1819	-	pseudogene
O55/O157	1851727	nc	rec	G2583_1819	-	pseudogene
O55/O157	1851742	nc	rec	G2583_1819	-	pseudogene
O55/O157	1851943	nc	rec	G2583_1819	-	pseudogene
O55/O157	1851952	nc	rec	G2583_1819	-	pseudogene
O55/O157	1852006	nc	rec	G2583_1819	-	pseudogene
O55/O157	1852048	nc	rec	G2583_1819	-	pseudogene
O55/O157	1852066	nc	rec	G2583_1819	-	pseudogene
O55/O157	1852069	nc	rec	G2583_1819	-	pseudogene
O55/O157	1852070	nc	rec	G2583_1819	-	pseudogene
O55/O157	1852090	nc	rec	G2583_1819	-	pseudogene
O55/O157	1852099	nc	rec	G2583_1819	-	pseudogene
O55/O157	1852107	nc	rec	G2583_1819	-	pseudogene
O55/O157	1852140	nc	rec	G2583_1819	-	pseudogene
O55/O157	1852216	nc	rec	G2583_1819	-	pseudogene
O55/O157	1852261	nc	rec	G2583_1819	-	pseudogene
O55/O157	1852303	nc	rec	G2583_1819	-	pseudogene
O55/O157	1852306	nc	rec	G2583_1819	-	pseudogene
O55/O157	1852312	nc	rec	G2583_1819	-	pseudogene
EDL933	2040519	del-3		G2583_1819	-	pseudogene
EDL933	2040449	del		G2583_1819	-	pseudogene
EDL933	2040435	nc		G2583_1819	-	pseudogene
EDL933	2040428	del		G2583_1819	-	pseudogene
EDL933	2040426	nc		G2583_1819	-	pseudogene
EDL933	2040423	del		G2583_1819	-	pseudogene

lineage[a]	site[b]	mutation type[c]	recomb	gene	Gene name	Type
EDL933	2040419	del		G2583_1819	-	pseudogene
EDL933	2040403	ins		G2583_1819	-	pseudogene
EDL933	2040391	ins		G2583_1819	-	pseudogene
EDL933	2040378	ins		G2583_1819	-	pseudogene
O157	2048579	nc		G2583_1819	-	pseudogene
O157	2049629	nc		G2583_1819	-	pseudogene
Sakai	2049909	nc		G2583_1819	-	pseudogene
Sakai	2049911	nc		G2583_1819	-	pseudogene
Sakai	2049912	nc		G2583_1819	-	pseudogene
O157	2050790	i		intergenic		
O157	2051703	nc		G2583_1822	ydcC	pseudogene
CB9615	1856299	nc		G2583_1822	ydcC	pseudogene
O157	2051940	del		G2583_1822	ydcC	pseudogene
CB9615	1856776	ns		G2583_1823	pptA	CDS
O157	2052966	ns		G2583_1824	yddH	CDS
O157	2053926	del		G2583_1825	nhoA	CDS
CB9615	1861732	s		G2583_1829	narY	CDS
O157	2058259	ns		G2583_1830	narZ	CDS
CB9615	1863921	s		G2583_1830	narZ	CDS
O157	2060459	s		G2583_1830	narZ	CDS
O157	2062153	ns		G2583_1831	narU	CDS
O157	2064500	ns		G2583_1832	yddJ	CDS
CB9615	1870552	ns		G2583_1834	-	CDS
CB9615	1870598	ns		G2583_1834	-	CDS
CB9615	1870717	i		intergenic		
CB9615	1871038	ns		G2583_1835	-	CDS
O157	2067489	i		intergenic		
O157	2067510	i		intergenic		
CB9615	1871991	i		intergenic		
O157	2067605	i		intergenic		
O157	2069089	nc		G2583_1837	fdnG	pseudogene
CB9615	1874145	nc		G2583_1837	fdnG	pseudogene
O157	2071783	nc		G2583_1837	fdnG	pseudogene
Sakai	2074660	ns		G2583_1841	adhP	CDS
CB9615	1879371	i		intergenic		
O157	2075820	ns		G2583_1842	maeA	CDS
EDL933	2010690	ins		intergenic		
CB9615	1882216	s		G2583_1845	osmC	CDS
O157	2079029	ns		G2583_1846	ddpF	CDS
O157	2079647	ns		G2583_1847	ddpD	CDS
CB9615	1884831	s		G2583_1848	ddpC	CDS
O157	2082137	del		G2583_1849	ddpB	CDS
O157	2084563	i		intergenic		
O157	2085515	ns		G2583_1852	dos	CDS
O157	2085751	del-10		G2583_1852	dos	CDS

lineage[a]	site[b]	mutation type[c]	recomb	gene	Gene name	Type
CB9615	1891521	s		G2583_1853	yddV	CDS
CB9615	1893302	ns		G2583_1854	yddW	CDS
O157	2091724	ns		G2583_1855	gadC	CDS
O157	2093428	del		intergenic		
CB9615	1897751	i		intergenic		
O157	2094506	s		G2583_1857	pqqL	CDS
O157	2095350	ns		G2583_1857	pqqL	CDS
CB9615	1900706	s		G2583_1857	pqqL	CDS
O157	2100054	ns		G2583_1859	yddA	CDS
O157	2100062	s		G2583_1859	yddA	CDS
CB9615	1904900	i		intergenic		
CB9615	1905233	ns		G2583_1860	ydeM	CDS
O157	2103917	i		intergenic		
O157	2105396	i		intergenic		
CB9615	1911433	s		G2583_1864	ydeP	CDS
CB9615	1912162	i		intergenic		
O157	2109492	ns		G2583_1866	ydeR	CDS
CB9615	1915332	ns		G2583_1868	fimD	CDS
O157	2111112	ns		G2583_1868	fimD	CDS
Sakai	2111156	ns		G2583_1868	fimD	CDS
CB9615	1917088	i		intergenic		
CB9615	1918516	ns		G2583_1870	fmlA	CDS
CB9615	1918897	i		intergenic		
Sakai	2115344	i		intergenic		
CB9615	1919658	ins		intergenic		
O157	2115869	ns		G2583_1872	hipA	CDS
CB9615	1921520	ns		G2583_1874	ydeU	CDS
CB9615	1923343	ns		G2583_1875	ydeK	CDS
CB9615	1923497	ns		G2583_1875	ydeK	CDS
O157	2119322	ns		G2583_1875	ydeK	CDS
O157	2122287	i		intergenic		
O157	2125595	ns		G2583_1878	lsrA	CDS
CB9615	1934653	ns		G2583_1881	lsrB	CDS
O157	2130616	ns		G2583_1882	lsrF	CDS
O157	2131739	ns		G2583_1884	tam	CDS
O157	2133007	i		intergenic		
Sakai	2133132	ins		intergenic		
CB9615	1938314	s		G2583_1886	uxaB	CDS
CB9615	1939752	s		G2583_1887	yneF	CDS
CB9615	1939775	ns		G2583_1887	yneF	CDS
EDL933	1952349	ins		G2583_1887	yneF	CDS
O157	2136461	ns		G2583_1888	yneG	CDS
O157	2136467	s		G2583_1888	yneG	CDS
CB9615	1941610	ns		G2583_1888	yneG	CDS
O157	2138222	ns		G2583_1890	yneI	CDS

lineage[a]	site[b]	mutation type[c]	recomb	gene	Gene name	Type
O157	2138359	s		G2583_1890	yneI	CDS
CB9615	1944146	s		G2583_1891	yneJ	CDS
CB9615	1944290	s		G2583_1891	yneJ	CDS
Sakai	2140670	nc		G2583_1892	yneK	pseudogene
O157	2141148	nc		G2583_1892	yneK	pseudogene
O157	2141241	nc		G2583_1892	yneK	pseudogene
EDL933	1947035	ins		intergenic		
O157	2142663	ns		G2583_1894	marC	CDS
O157	2145176	ns		G2583_1898	eamA	CDS
O55/O157	1951871	i		intergenic		
CB9615	1953251	s		G2583_1901	ftrA	CDS
CB9615	1954261	s		G2583_1902	ydeH	CDS
O157	2149562	ns		G2583_1902	ydeH	CDS
O157	2150030	s		G2583_1903	ydeI	CDS
CB9615	1956112	i		intergenic		
CB9615	1956928	s		G2583_1905	dcp	CDS
CB9615	1960234	i		intergenic		
CB9615	1961209	s		G2583_1909	ydfI	CDS
CB9615	1961836	ns		G2583_1910	ydfJ	CDS
O157	2157556	ns		G2583_1910	ydfJ	CDS
EDL933	1929919	ins		G2583_1911	-	CDS
EDL933	1928838	ns		ECs2155	-	CDS
Sakai/EDL933	2160964	ns	rec	ECs2157	-	CDS
Sakai/EDL933	2160982	ns	rec	ECs2157	-	CDS
Sakai/EDL933	2160991	ns	rec	ECs2157	-	CDS
Sakai/EDL933	2161003	ns	rec	ECs2157	-	CDS
Sakai/EDL933	2161015	ns	rec	ECs2157	-	CDS
Sakai/EDL933	2161038	ns	rec	ECs2157	-	CDS
Sakai/EDL933	2161066	ns	rec	ECs2157	-	CDS
Sakai/EDL933	2161083	ns	rec	ECs2157	-	CDS
Sakai/EDL933	2161092	s	rec	ECs2157	-	CDS
Sakai/EDL933	2161142	ns	rec	ECs2158	-	CDS
Sakai/EDL933	2161219	ns	rec	ECs2158	-	CDS
Sakai/EDL933	2161230	ns	rec	ECs2158	-	CDS
Sakai/EDL933	2161256	s	rec	ECs2158	-	CDS
Sakai/EDL933	2161259	ns	rec	ECs2158	-	CDS
Sakai/EDL933	2161269	ns	rec	ECs2158	-	CDS
Sakai/EDL933	2161285	ns	rec	ECs2158	-	CDS
Sakai/EDL933	2161305	ns	rec	ECs2158	-	CDS
Sakai/EDL933	2161307	ns	rec	ECs2158	-	CDS
Sakai/EDL933	2161308	ns	rec	ECs2158	-	CDS
Sakai/EDL933	2161322	s	rec	ECs2158	-	CDS
Sakai/EDL933	2161325	s	rec	ECs2158	-	CDS
Sakai/EDL933	2161328	s	rec	ECs2158	-	CDS
Sakai/EDL933	2161334	s	rec	ECs2158	-	CDS

lineage[a]	site[b]	mutation type[c]	recomb	gene	Gene name	Type
Sakai/EDL933	2161358	s	rec	ECs2158	-	CDS
Sakai/EDL933	2188677	i	rec	intergenic		
Sakai/EDL933	2188678	i	rec	intergenic		
Sakai/EDL933	2188896	i	rec	intergenic		
Sakai/EDL933	2189067	ns	rec	ECs2190	-	CDS
Sakai/EDL933	2189164	ns	rec	ECs2190	-	CDS
Sakai/EDL933	2189182	indel	rec	ECs2190	-	CDS
Sakai/EDL933	2189535	nc	rec	argO5	-	tRNA
Sakai/EDL933	2190252	ns	rec	ECs2191	-	CDS
Sakai/EDL933	2190267	ns	rec	ECs2191	-	CDS
Sakai/EDL933	2190347	ns	rec	ECs2191	-	CDS
Sakai/EDL933	2192509	ns	rec	ECs2195	-	CDS
Sakai/EDL933	2193209	ns	rec	ECs2196	-	CDS
Sakai/EDL933	2193225	ns	rec	ECs2196	-	CDS
Sakai/EDL933	2193226	indel	rec	ECs2196	-	CDS
Sakai/EDL933	2193877	indel	rec	intergenic		
Sakai/EDL933	2193903	i	rec	intergenic		
Sakai/EDL933	2194158	i	rec	intergenic		
Sakai/EDL933	2194215	i	rec	intergenic		
EDL933	1886140	ins		ECs2204	-	CDS
EDL933	1884950	ns		ECs2206	-	CDS
EDL933	1881454	ns		ECs2213	-	CDS
EDL933	1877194	ns		ECs2222	-	CDS
EDL933	1877164	ns		ECs2222	-	CDS
EDL933	1876613	ins		ECs2224	-	CDS
EDL933	1876215	ins		ECs2224	-	CDS
EDL933	1875666	ins		ECs2224	-	CDS
EDL933	1875215	ins		ECs2224	-	CDS
EDL933	1875170	ins		ECs2224	-	CDS
EDL933	1875164	ins		ECs2224	-	CDS
EDL933	1874498	ins		G2583_1913	-	CDS
O55/O157	1964910	ns		G2583_1913	-	CDS
EDL933	1873080	ins		G2583_1916	-	CDS
O157	2211399	ns		G2583_1916	-	CDS
EDL933	1872584	ins		G2583_1916	-	CDS
O157	2211554	ins		intergenic		
CB9615	1966939	i		intergenic		
EDL933	2306602	s	rec	ECs2251	-	CDS
EDL933	2306626	s	rec	ECs2251	-	CDS
EDL933	2306662	s	rec	ECs2251	-	CDS
EDL933	2306692	s	rec	ECs2251	-	CDS
EDL933	2306695	s	rec	ECs2251	-	CDS
EDL933	2306704	s	rec	ECs2251	-	CDS
EDL933	2306710	s	rec	ECs2251	-	CDS
EDL933	2306722	s	rec	ECs2251	-	CDS

lineage[a]	site[b]	mutation type[c]	recomb	gene	Gene name	Type
EDL933	2306727	ns	rec	ECs2251	-	CDS
EDL933	2306743	s	rec	ECs2251	-	CDS
EDL933	2306749	s	rec	ECs2251	-	CDS
EDL933	2306760	s	rec	ECs2251	-	CDS
EDL933	2306780	ns	rec	ECs2251	-	CDS
EDL933	2306831	ns	rec	ECs2251	-	CDS
CB9615	1989147	s		G2583_1941	-	CDS
O157	2234921	ns		G2583_1941	-	CDS
O55/O157	1990458	i	rec	intergenic		
O55/O157	1990549	i	rec	intergenic		
O55/O157	1990620	s	rec	G2583_1943	-	CDS
O55/O157	1990626	s	rec	G2583_1943	-	CDS
O55/O157	1990641	s	rec	G2583_1943	-	CDS
O55/O157	1990695	s	rec	G2583_1943	-	CDS
O55/O157	1990707	s	rec	G2583_1943	-	CDS
O55/O157	1990743	s	rec	G2583_1943	-	CDS
O55/O157	1990770	s	rec	G2583_1943	-	CDS
O55/O157	1990797	s	rec	G2583_1943	-	CDS
O55/O157	1990912	ns	rec	G2583_1943	-	CDS
O55/O157	1991021	ns	rec	G2583_1943	-	CDS
O55/O157	1991025	s	rec	G2583_1943	-	CDS
O55/O157	1991031	ns	rec	G2583_1943	-	CDS
O55/O157	1991032	ns	rec	G2583_1943	-	CDS
O55/O157	1991225	ns	rec	G2583_1944	-	CDS
O55/O157	1991227	ns	rec	G2583_1944	-	CDS
EDL933	1991231	s		G2583_1944	-	CDS
O55/O157	1991231	s	rec	G2583_1944	-	CDS
Sakai	1991231	s		G2583_1944	-	CDS
EDL933	2311936	s		G2583_1944	-	CDS
O55/O157	1991339	s	rec	G2583_1944	-	CDS
O55/O157	1991481	i	rec	intergenic		
O55/O157	1991508	s	rec	G2583_1945	-	CDS
O55/O157	1991514	s	rec	G2583_1945	-	CDS
O55/O157	1991518	ns	rec	G2583_1945	-	CDS
O55/O157	1991520	s	rec	G2583_1945	-	CDS
O55/O157	1991523	s	rec	G2583_1945	-	CDS
O55/O157	1991538	s	rec	G2583_1945	-	CDS
O55/O157	1991547	s	rec	G2583_1945	-	CDS
O55/O157	1991556	s	rec	G2583_1945	-	CDS
O55/O157	1991580	s	rec	G2583_1945	-	CDS
O55/O157	1991673	s	rec	G2583_1945	-	CDS
EDL933	2315689	ins-2		ECs2266	-	CDS
EDL933	2324754	i		intergenic		
Sakai	2249978	ns		ECs2283	-	CDS
CB9615	2012313	i		intergenic		

lineage[a]	site[b]	mutation type[c]	recomb	gene	Gene name	Type
O157	2255206	ns		G2583_1976	ynfA	CDS
O157	2258375	ns		G2583_1981	ynfE	CDS
CB9615	2017353	ns		G2583_1981	ynfE	CDS
CB9615	2017683	i		intergenic		
CB9615	2017806	ns		G2583_1982	ynfF	CDS
O157	2261242	ns		G2583_1982	ynfF	CDS
CB9615	2021482	s		G2583_1984	ynfH	CDS
CB9615	2021834	ns		G2583_1985	dmsD	CDS
EDL933	2346304	i		intergenic		
O157	2271124	i		intergenic		
Sakai	2272248	del		G2583_1992	ydgD	CDS
CB9615	2029777	ns		G2583_1992	ydgD	CDS
O157	2273726	ns		G2583_1995	tqsA	CDS
O157	2277665	ns		G2583_1997	pntA	CDS
O157	2278458	s		G2583_1998	ydgH	CDS
CB9615	2036980	ns		G2583_1999	ydgI	CDS
O157	2282037	ns		G2583_2002	rstA	CDS
Sakai	2283012	ns		G2583_2003	rstB	CDS
EDL933	2359335	ins		G2583_2004	tus	CDS
O157	2289099	s		G2583_2007	manA	CDS
O157	2290138	s		G2583_2008	ydgA	CDS
O157	2294915	ns		G2583_2011	uidA	CDS
O157	2295006	ins-2		G2583_2011	uidA	CDS
O157	2295600	ns		G2583_2011	uidA	CDS
O157	2295934	i		intergenic		
O157	2296491	s		G2583_2012	uidR	CDS
CB9615	2054336	s		G2583_2013	hdhA	CDS
CB9615	2059604	ns		G2583_2017	add	CDS
EDL933	2379344	ins		intergenic		
CB9615	2061406	i		intergenic		
O157	2307940	ns		G2583_2024	rsxC	CDS
CB9615	2065771	ns		G2583_2024	rsxC	CDS
EDL933	2383815	ns		G2583_2024	rsxC	CDS
EDL933	2383829	s		G2583_2024	rsxC	CDS
EDL933	2383835	s		G2583_2024	rsxC	CDS
O157	2310653	s		G2583_2027	rsxE	CDS
O157	2311553	ns		G2583_2028	nth	CDS
O157	2312057	i		intergenic		
O157	2312150	i		intergenic		
CB9615	2072702	ns		G2583_2031	pdxY	CDS
EDL933	2392168	ins		intergenic		
O157	2317755	i		intergenic		
O157	2318157	i		intergenic		
CB9615	2076155	s		G2583_2035	anmK	CDS
O157	2319096	ns		G2583_2035	anmK	CDS

lineage[a]	site[b]	mutation type[c]	recomb	gene	Gene name	Type
CB9615	2076470	s		G2583_2035	anmK	CDS
EDL933	2394763	ins		intergenic		
O157	2320152	s		G2583_2037	slyA	CDS
O157	2320694	i		intergenic		
O157	2323563	ns		G2583_2040	ydhK	CDS
O157	2324571	ns		G2583_2042	ydhF	CDS
O157	2328300	s		G2583_2047	rnt	CDS
CB9615	2086448	ns		G2583_2048	lhr	CDS
O157	2329370	s		G2583_2048	lhr	CDS
O157	2330070	ns		G2583_2048	lhr	CDS
O157	2330900	s		G2583_2048	lhr	CDS
CB9615	2089674	ns		G2583_2048	lhr	CDS
O157	2334471	ns		G2583_2050	ydhO	CDS
O157	2336109	ns		G2583_2052	ydhP	CDS
O157	2336134	ns		G2583_2052	ydhP	CDS
CB9615	2094383	ins		intergenic		
CB9615	2094845	i		intergenic		
O157	2337749	s		G2583_2053	purR	CDS
CB9615	2096607	ns		G2583_2054	ydhB	CDS
O157	2340245	s		G2583_2055	ydhC	CDS
O157	2342030	ns		G2583_2056	cfa	CDS
O157	2342329	ns		G2583_2057	ribC	CDS
CB9615	2100032	ns		G2583_2057	ribC	CDS
EDL933	2418251	ins		intergenic		
EDL933	2418263	ins		intergenic		
O157	2343203	s		G2583_2058	mdtK	CDS
CB9615	2101497	ns		G2583_2058	mdtK	CDS
O157	2344492	ns		G2583_2058	mdtK	CDS
CB9615	2102468	ns		G2583_2059	ydhQ	CDS
CB9615	2102650	s		G2583_2059	ydhQ	CDS
O157	2346310	i		intergenic		
O157	2346625	ns		G2583_2062	ydhR	CDS
O157	2352200	ns		G2583_2068	ydhV	CDS
CB9615	2110600	ns		G2583_2068	ydhV	CDS
O157	2354319	i		intergenic		
O157	2354526	i		intergenic		
O157	2355009	i		intergenic		
CB9615	2114878	ns		G2583_2073	ynhG	CDS
CB9615	2114943	ns		G2583_2073	ynhG	CDS
O157	2357729	s		G2583_2073	ynhG	CDS
O157	2363113	ns		G2583_2078	sufB	CDS
O157	2364173	i		intergenic		
O157	2364465	nc		G2583_2080	-	pseudogene
CB9615	2124543	s		G2583_2083	ydiJ	CDS
O157	2367630	s		G2583_2083	ydiJ	CDS

lineage[a]	site[b]	mutation type[c]	recomb	gene	Gene name	Type
O157	2368236	s		G2583_2083	ydiJ	CDS
CB9615	2129197	del-9		G2583_2087	ydiM	CDS
O157	2373010	s		G2583_2088	ydiN	CDS
O157	2373421	ns		G2583_2088	ydiN	CDS
O157	2375834	s		G2583_2091	ydiF	CDS
CB9615	2133633	ns		G2583_2091	ydiF	CDS
CB9615	2133915	ns		G2583_2091	ydiF	CDS
EDL933	2454223	ins		intergenic		
EDL933	2454229	ins		intergenic		
EDL933	2454259	ins		intergenic		
EDL933	2454271	ins		intergenic		
EDL933	2454284	ins		intergenic		
CB9615	2136873	s		G2583_2094	ydiQ	CDS
CB9615	2137856	ns		G2583_2095	ydiR	CDS
CB9615	2138741	s		G2583_2096	ydiS	CDS
CB9615	2140260	s		G2583_2098	fadK	CDS
O157	2382978	s		G2583_2098	fadK	CDS
CB9615	2141836	ns		G2583_2099	pps	CDS
EDL933	2461084	ns		G2583_2099	pps	CDS
O157	2386002	s		G2583_2099	pps	CDS
O157	2386770	ns		G2583_2099	pps	CDS
O157	2386947	i		intergenic		
O157	2388500	ns		G2583_2101	aroH	CDS
O157	2390081	ns		G2583_2103	ydiU	CDS
O157	2391291	s		G2583_2104	ydiV	CDS
CB9615	2149142	i		intergenic		
CB9615	2151609	s		G2583_2108	btuC	CDS
EDL933	2470125	ins		intergenic		
CB9615	2188835	s		G2583_2160	pheT	CDS
CB9615	2190258	ns		G2583_2160	pheT	CDS
CB9615	2193073	s		G2583_2165	infC	CDS
O157	2402254	s		G2583_2166	thrS	CDS
O157	2402595	i		intergenic		
O157	2402915	i		intergenic		
CB9615	2196051	del		intergenic		
O157	2405057	s		G2583_2168	ydiY	CDS
O157	2405150	s		G2583_2168	ydiY	CDS
O157	2408364	s		G2583_2171	yniA	CDS
EDL933	2483622	s		G2583_2172	yniB	CDS
O157	2408657	s		G2583_2172	yniB	CDS
O157	2409000	i		intergenic		
Sakai	2409083	i		intergenic		
EDL933	2484908	ns		G2583_2173	yniC	CDS
O157	2411105	ns		G2583_2175	ydjN	CDS
O157	2412151	del		G2583_2176	ydjO	CDS

lineage[a]	site[b]	mutation type[c]	recomb	gene	Gene name	Type
CB9615	2208397	s		G2583_2178	katE	CDS
O157	2416466	ns		G2583_2179	chbG	CDS
O157	2419218	s		G2583_2182	chbA	CDS
O157	2419507	ns		G2583_2183	chbC	CDS
CB9615	2213163	s		G2583_2183	chbC	CDS
O157	2421780	i		intergenic		
CB9615	2215212	ns		G2583_2186	nadE	CDS
O157	2422346	ns		G2583_2186	nadE	CDS
O157	2422823	i		intergenic		
Sakai	2425295	del		intergenic		
O157	2425508	s		G2583_2190	astE	CDS
O157	2426710	s		G2583_2191	astB	CDS
O157	2428497	s		G2583_2192	astD	CDS
O157	2428646	ns		G2583_2192	astD	CDS
CB9615	2221818	s		G2583_2192	astD	CDS
O157	2428968	s		G2583_2192	astD	CDS
CB9615	2223336	ns		G2583_2194	astC	CDS
O157	2430802	s		G2583_2194	astC	CDS
O157	2431592	i		intergenic		
O157	2431966	s		G2583_2195	xthA	CDS
O157	2436220	ns		G2583_2200	ynjB	CDS
O157	2437603	ns		G2583_2201	ynjC	CDS
O157	2439117	ns		G2583_2203	ynjE	CDS
O157	2439501	ns		G2583_2203	ynjE	CDS
CB9615	2232945	s		G2583_2203	ynjE	CDS
O157	2444848	ns		G2583_2209	topB	CDS
CB9615	2240127	ns		G2583_2210	selD	CDS
O157	2449258	ns		G2583_2212	sppA	CDS
CB9615	2242841	ns		G2583_2212	sppA	CDS
O157	2449973	ns		G2583_2212	sppA	CDS
CB9615	2245333	ns		G2583_2215	pncA	CDS
EDL933	2529969	ns	rec	ECs2478	-	CDS
EDL933	2530015	ns	rec	ECs2478	-	CDS
EDL933	2530017	ns	rec	ECs2478	-	CDS
EDL933	2530022	ns	rec	ECs2478	-	CDS
O157	2456621	ns		G2583_2218	ydjG	CDS
CB9615	2249632	ns		G2583_2219	ydjH	CDS
O157	2458034	ns		G2583_2219	ydjH	CDS
O157	2458362	ns		G2583_2219	ydjH	CDS
CB9615	2251460	ns		G2583_2221	ydjJ	CDS
O157	2460898	ns		G2583_2222	ydjK	CDS
O157	2463011	i		intergenic		
CB9615	2253996	i		intergenic		
O157	2463095	i		intergenic		
O157	2467385	ns		G2583_2229	mipA	CDS

lineage[a]	site[b]	mutation type[c]	recomb	gene	Gene name	Type
O157	2467507	ns		G2583_2229	mipA	CDS
CB9615	2259638	s		G2583_2230	yeaG	CDS
O157	2471893	i		intergenic		
O157	2474192	ns		G2583_2233	yeaJ	CDS
CB9615	2265975	ns		G2583_2233	yeaJ	CDS
O157	2480166	ns		G2583_2241	yeaP	CDS
O157	2481626	i		intergenic		
O157	2482171	ns		G2583_2245	leuE	CDS
CB9615	2277357	s		G2583_2248	yeaV	CDS
CB9615	2277452	ns		G2583_2249	yeaW	CDS
O157	2487750	ns		G2583_2250	yeaX	CDS
O157	2487838	s		G2583_2250	yeaX	CDS
O157	2488046	ns		G2583_2250	yeaX	CDS
O157	2488189	ns		G2583_2250	yeaX	CDS
O157	2488195	s		G2583_2250	yeaX	CDS
EDL933	2563854	ins		intergenic		
O157	2490115	s		G2583_2253	fadD	CDS
O157	2490201	s		G2583_2253	fadD	CDS
O157	2490378	ns		G2583_2253	fadD	CDS
CB9615	2282267	s		G2583_2253	fadD	CDS
CB9615	2282866	ns		G2583_2254	yeaY	CDS
O157	2493862	s		G2583_2256	yoaA	CDS
O157	2495854	i		intergenic		
O157	2497152	s		G2583_2260	pabB	CDS
O157	2497229	ns		G2583_2260	pabB	CDS
EDL933	2497478	ns		G2583_2260	pabB	CDS
O157	2497478	ns		G2583_2260	pabB	CDS
Sakai	2497478	ns		G2583_2260	pabB	CDS
Sakai	2497693	ns		G2583_2260	pabB	CDS
O157	2497905	s		G2583_2260	pabB	CDS
O157	2500922	ns		G2583_2263	yoaD	CDS
O157	2500955	ns		G2583_2263	yoaD	CDS
EDL933	2579047	ins		intergenic		
EDL933	2579062	ins		intergenic		
O157	2504704	s		G2583_2266	manX	CDS
O157	2504889	i		intergenic		
CB9615	2298152	i		intergenic		
O157	2509603	del		intergenic		
CB9615	2300616	i		intergenic		
O157	2509845	ins		intergenic		
O157	2510150	s		G2583_2274	yebO	CDS
CB9615	2301418	ns		G2583_2275	mgrB	CDS
O157	2510655	i		intergenic		
O157	2510858	ns		G2583_2276	yobH	CDS
EDL933	2586240	ins		intergenic		

lineage[a]	site[b]	mutation type[c]	recomb	gene	Gene name	Type
EDL933	2587381	s		G2583_2278	yebQ	CDS
EDL933	2589737	i		intergenic		
O157	2521259	s		G2583_2284	yebT	CDS
O157	2521611	ns		G2583_2284	yebT	CDS
CB9615	2313885	ns		G2583_2285	rsmF	CDS
CB9615	2314782	ns		G2583_2286	yebV	CDS
CB9615	2317964	ns		G2583_2294	holE	CDS
CB9615	2318692	ns		G2583_2295	yobB	CDS
O157	2528063	ns		G2583_2296	exoX	CDS
O157	2528914	ns		G2583_2297	ptrB	CDS
CB9615	2320307	ns		G2583_2297	ptrB	CDS
O157	2529900	s		G2583_2297	ptrB	CDS
CB9615	2321220	ns		G2583_2297	ptrB	CDS
O157	2530416	s		G2583_2297	ptrB	CDS
EDL933	2607040	ins		intergenic		
CB9615	2323458	ns		G2583_2300	yebG	CDS
O157	2532606	i		intergenic		
O157	2532635	i		intergenic		
O157	2538112	s		G2583_2304	zwf	CDS
O157	2538120	ns		G2583_2304	zwf	CDS
CB9615	2329216	i		intergenic		
O157	2539816	ns		G2583_2306	pykA	CDS
CB9615	2334479	ns		G2583_2309	znuA	CDS
O157	2543574	s		G2583_2309	znuA	CDS
CB9615	2334850	ns		G2583_2309	znuA	CDS
O157	2544439	i		intergenic		
EDL933	2619755	ns		G2583_2310	znuC	CDS
CB9615	2335606	s		G2583_2310	znuC	CDS
O157	2545924	ns		G2583_2311	znuB	CDS
O157	2547985	i		intergenic		
O157	2548222	ns		G2583_2314	yebB	CDS
CB9615	2341266	s		G2583_2317	nudB	CDS
O157	2551489	ns		G2583_2318	aspS	CDS
O157	2551649	s		G2583_2318	aspS	CDS
O157	2552681	i		intergenic		
O157	2553794	ns		G2583_2320	yecE	CDS
CB9615	2346001	s		G2583_2322	cmoA	CDS
O157	2557968	ns		G2583_2324	torZ	CDS
O157	2558154	ns		G2583_2324	torZ	CDS
O157	2559990	ns		G2583_2325	torY	CDS
O157	2560114	i		intergenic		
EDL933	2635515	ins		intergenic		
EDL933	2635526	ins		intergenic		
EDL933	2635548	ins		intergenic		
O157	2560927	s		G2583_2326	cutC	CDS

lineage[a]	site[b]	mutation type[c]	recomb	gene	Gene name	Type
O157	2561473	ns		G2583_2327	yecM	CDS
O157	2562746	ns		G2583_2328	argS	CDS
O157	2566210	s		G2583_2331	flhA	CDS
O157	2567148	ns		G2583_2332	flhB	CDS
CB9615	2358858	s		G2583_2332	flhB	CDS
CB9615	2360624	ns		G2583_2335	cheB	CDS
CB9615	2361613	s		G2583_2336	cheR	CDS
O157	2571940	s		G2583_2337	tap	CDS
O157	2573289	ns		G2583_2338	tar	CDS
CB9615	2365408	ns		G2583_2338	tar	CDS
CB9615	2370148	ins		intergenic		
O157	2579896	ns		G2583_2345	flhD	CDS
CB9615	2371619	i		intergenic		
CB9615	2371655	i		intergenic		
O157	2580804	i		intergenic		
O157	2581344	ns		G2583_2347	otsA	CDS
O157	2581533	ns		G2583_2347	otsA	CDS
O157	2584040	ns		G2583_2349	araH	CDS
O157	2584402	s		G2583_2349	araH	CDS
O157	2584432	ns		G2583_2349	araH	CDS
EDL933	2660874	ns		G2583_2350	araG	CDS
O157	2586270	i		intergenic		
O157	2587311	i		intergenic		
O157	2587780	i		intergenic		
CB9615	2380218	ins		intergenic		
EDL933	2669770	ns		ECs2619	-	CDS
EDL933	2670185	ns		ECs2619	-	CDS
EDL933	2674223	ns		ECs2630	-	CDS
EDL933	2674230	ns		ECs2630	-	CDS
EDL933	2674426	ns		ECs2632	-	CDS
EDL933	2676082	ns		ECs2633	-	CDS
EDL933	2676453	ns		ECs2633	-	CDS
Sakai	2604364	ns		ECs2637	-	CDS
EDL933	2683208	ns		ECs2641	-	CDS
CB9615	2384842	s		G2583_2363	pgsA	CDS
O157	2618227	ns		G2583_2366	yecF	CDS
O157	2619260	i		intergenic		
CB9615	2389485	s		G2583_2368	yecC	CDS
O157	2620059	s		G2583_2368	yecC	CDS
O157	2620304	s		G2583_2369	yecS	CDS
O157	2621009	ns		G2583_2370	dcyD	CDS
Sakai	2621341	del		G2583_2370	dcyD	CDS
O157	2624217	i		intergenic		
O157	2624273	i		intergenic		
CB9615	2396466	ins-9		G2583_2375	fliD	CDS

lineage[a]	site[b]	mutation type[c]	recomb	gene	Gene name	Type
CB9615	2396678	ns		G2583_2375	fliD	CDS
O157	2626896	s		G2583_2375	fliD	CDS
CB9615	2397084	ns		G2583_2375	fliD	CDS
CB9615	2397311	s		G2583_2375	fliD	CDS
CB9615	2400966	ns		G2583_2380	yedE	CDS
Sakai	2632861	ns		G2583_2382	yedK	CDS
O157	2633914	ns		G2583_2384	yedM	CDS
O157	2634233	s		G2583_2384	yedM	CDS
CB9615	2404684	ns		G2583_2384	yedM	CDS
CB9615	2405372	s		G2583_2385	yedL	CDS
O157	2635774	i		intergenic		
CB9615	2405650	ns		G2583_2386	yedM	CDS
O157	2636559	ns		G2583_2386	yedM	CDS
O157	2636652	ns		G2583_2386	yedM	CDS
O157	2636957	i		intergenic		
O157	2638382	ns		G2583_2389	fliF	CDS
CB9615	2411099	ns		G2583_2392	fliI	CDS
Sakai	2642468	ns		G2583_2392	fliI	CDS
O157	2643500	ns		G2583_2394	fliK	CDS
CB9615	2413610	s		G2583_2394	fliK	CDS
EDL933	2719369	ins		intergenic		
CB9615	2418038	i		intergenic		
CB9615	2420583	ns		G2583_2406	yedP	CDS
CB9615	2420891	s		G2583_2407	yedQ	CDS
EDL933	2727876	ins		intergenic		
EDL933	2728490	s		G2583_2409	yedI	CDS
O157	2653334	ns		G2583_2409	yedI	CDS
CB9615	2426366	ns		G2583_2412	dcm	CDS
O157	2657412	ns		G2583_2413	yedJ	CDS
O157	2657768	del		G2583_2414	yedR	CDS
O157	2658005	ns		G2583_2414	yedR	CDS
CB9615	2428072	del		intergenic		
CB9615	2428231	i		intergenic		
CB9615	2428235	i		intergenic		
O157	2658480	i		intergenic		
CB9615	2428279	del		intergenic		
CB9615	2428290	i		intergenic		
CB9615	2428411	ns		G2583_2415	ompS	CDS
O157	2659202	del		G2583_2415	ompS	CDS
O157	2659204	ns		G2583_2415	ompS	CDS
O157	2659420	ns		G2583_2415	ompS	CDS
O157	2661521	ns		G2583_2418	yedV	CDS
EDL933	2738701	ins		intergenic		
O157	2665745	i		intergenic		
CB9615	2436942	s		G2583_2425	yodB	CDS

lineage[a]	site[b]	mutation type[c]	recomb	gene	Gene name	Type
O157	2667354	ns		G2583_2425	yodB	CDS
EDL933	2742716	ins		G2583_2426	-	CDS
O157	2667880	i		intergenic		
EDL933	2743162	ins		intergenic		
O55/O157	2439173	i		intergenic		
EDL933	2745061	s	rec	G2583_2429	-	CDS
EDL933	2745100	ns	rec	G2583_2429	-	CDS
EDL933	2745170	ns	rec	G2583_2429	-	CDS
EDL933	2745254	ns	rec	G2583_2429	-	CDS
EDL933	2745256	ns	rec	G2583_2429	-	CDS
EDL933	2745258	ns	rec	G2583_2429	-	CDS
EDL933	2745287	s	rec	G2583_2429	-	CDS
EDL933	2745311	ns	rec	G2583_2429	-	CDS
EDL933	2745351	ns	rec	G2583_2429	-	CDS
EDL933	2745395	ns	rec	G2583_2429	-	CDS
EDL933	2745397	ns	rec	G2583_2429	-	CDS
EDL933	2745428	s	rec	G2583_2429	-	CDS
CB9615	2440023	ns	rec	G2583_2429	-	CDS
EDL933	2745492	ns	rec	G2583_2429	-	CDS
Sakai	2670319	ns	rec	G2583_2429	-	CDS
Sakai	2670321	ns	rec	G2583_2429	-	CDS
Sakai	2670352	s	rec	G2583_2429	-	CDS
CB9615	2440164	ns	rec	G2583_2429	-	CDS
Sakai	2694827	ns	rec	ECs2746	-	CDS
Sakai	2694831	ns	rec	ECs2746	-	CDS
Sakai	2694855	ns	rec	ECs2746	-	CDS
Sakai	2694992	ns	rec	ECs2746	-	CDS
Sakai	2695102	s	rec	ECs2746	-	CDS
EDL933	2771924	ns	rec	ECs2746	-	CDS
EDL933	2772135	s	rec	ECs2746	-	CDS
EDL933	2772139	ns	rec	ECs2746	-	CDS
EDL933	2772141	s	rec	ECs2746	-	CDS
EDL933	2772147	ns	rec	ECs2746	-	CDS
EDL933	2772149	ns	rec	ECs2746	-	CDS
EDL933	2772155	ns	rec	ECs2746	-	CDS
EDL933	2772165	ns	rec	ECs2746	-	CDS
EDL933	2772166	ns	rec	ECs2746	-	CDS
EDL933	2772171	ns	rec	ECs2746	-	CDS
EDL933	2772225	ns	rec	ECs2746	-	CDS
EDL933	2772278	ns	rec	ECs2746	-	CDS
EDL933	2772301	ns	rec	ECs2746	-	CDS
EDL933	2772339	ns	rec	ECs2746	-	CDS
EDL933	2772342	ns	rec	ECs2746	-	CDS
EDL933	2772354	ns	rec	ECs2746	-	CDS
EDL933	2774442	i		intergenic		

lineage[a]	site[b]	mutation type[c]	recomb	gene	Gene name	Type
EDL933	2776138	ns		ECs2752	-	CDS
EDL933	2783156	ns	rec	ECs2766	-	CDS
EDL933	2783160	ns	rec	ECs2766	-	CDS
EDL933	2783599	ns	rec	ECs2767	-	CDS
EDL933	2783613	ns	rec	ECs2767	-	CDS
EDL933	2783720	i	rec	intergenic		
EDL933	2783810	i	rec	intergenic		
EDL933	2784259	ns	rec	ECs2768	-	CDS
EDL933	2784411	ns	rec	ECs2769	-	CDS
EDL933	2784412	ins	rec	ECs2769	-	CDS
EDL933	2784445	ns	rec	ECs2769	-	CDS
EDL933	2784515	ins	rec	intergenic		
EDL933	2785340	ns	rec	ECs2770	-	CDS
EDL933	2786266	ns	rec	ECs2770	-	CDS
EDL933	2786274	ns	rec	ECs2770	-	CDS
EDL933	2786278	ns	rec	ECs2770	-	CDS
EDL933	2786762	ns	rec	ECs2770-2771	-	CDS
EDL933	2786770	ins	rec	ECs2770-2771	-	CDS
EDL933	2786796	ns	rec	ECs2771	-	CDS
EDL933	2787097	i	rec	intergenic		
CB9615	2486675	ns	rec	G2583_2483	intU	CDS
CB9615	2486709	i	rec	intergenic		
CB9615	2486720	i	rec	intergenic		
CB9615	2486724	i	rec	intergenic		
CB9615	2486725	i	rec	intergenic		
CB9615	2489321	nc		G2583_2487	yeeJ	pseudogene
O157	2717174	s		G2583_2488	-	CDS
EDL933	2793711	s		G2583_2488	-	CDS
Sakai	2717449	ins		G2583_2488	-	CDS
CB9615	2495641	s		G2583_2488	-	CDS
O157	2723059	del		intergenic		
O157	2724885	s		G2583_2491	amn	CDS
O157	2724930	s		G2583_2491	amn	CDS
O157	2726331	i		intergenic		
CB9615	2502116	i		intergenic		
CB9615	2502580	ns		G2583_2494-2495	yeeO	CDS
O157	2728024	ns		G2583_2495	yeeO	CDS
O157	2728096	ns		G2583_2495	yeeO	CDS
O157	2729029	ns		G2583_2495	yeeO	CDS
CB9615	2505411	s		G2583_2498	nac	CDS
O157	2732519	ns		G2583_2500	erfK	CDS
CB9615	2509919	s		G2583_2503	cobU	CDS
EDL933	2812486	ns		ECs2790	-	CDS
O157	2738154	i		intergenic		
O157	2738242	i		intergenic		

lineage[a]	site[b]	mutation type[c]	recomb	gene	Gene name	Type
CB9615	2510715	i		intergenic		
O157	2738596	i		intergenic		
EDL933	2815514	ins		intergenic		
CB9615	2511537	i		intergenic		
CB9615	2511777	ns		G2583_2504	yoeE	CDS
O157	2740459	s		G2583_2504	yoeE	CDS
EDL933	2818757	ns	rec	ECs2795	-	CDS
EDL933	2818781	ns	rec	ECs2795	-	CDS
EDL933	2818793	ns	rec	ECs2795	-	CDS
EDL933	2818805	ns	rec	ECs2795	-	CDS
EDL933	2818838	ns	rec	ECs2795	-	CDS
CB9615	2523379	ns		G2583_2514	-	CDS
CB9615	2524635	ns		G2583_2516	-	CDS
Sakai	2745758	ns		G2583_2516	-	CDS
O55/O157	2528100	ns	rec	G2583_2520	yafX	CDS
O55/O157	2528205	ns	rec	G2583_2520	yafX	CDS
O55/O157	2528220	ns	rec	G2583_2520	yafX	CDS
O55/O157	2528271	ns	rec	G2583_2520	yafX	CDS
O55/O157	2528322	ns	rec	G2583_2520	yafX	CDS
O55/O157	2528404	ns	rec	G2583_2520	yafX	CDS
CB9615	2530024	nc		G2583_2524	yeeV1	pseudogene
O157	2750723	i		intergenic		
EDL933	2821100	ins		intergenic		
O157	2752965	ns		G2583_2528	yeeA	CDS
CB9615	2533359	ns		G2583_2529	sbmC	CDS
EDL933	2825288	ins		intergenic		
O157	2756165	s		G2583_2531	sbcB	CDS
CB9615	2536701	ns		G2583_2533	yeeE	CDS
CB9615	2537453	s		G2583_2533	yeeE	CDS
EDL933	2828286	i		intergenic		
EDL933	2828290	i		intergenic		
O157	2761240	s	rec	G2583_2536	yeeZ	CDS
O157	2761255	s	rec	G2583_2536	yeeZ	CDS
O157	2761258	s	rec	G2583_2536	yeeZ	CDS
O157	2761303	s	rec	G2583_2536	yeeZ	CDS
O157	2761318	s	rec	G2583_2536	yeeZ	CDS
O157	2761402	s	rec	G2583_2536	yeeZ	CDS
O157	2761968	i	rec	intergenic		
O157	2762084	ns	rec	G2583_2539	hisL	CDS
O157	2762086	ns	rec	G2583_2539	hisL	CDS
O157	2762191	i	rec	intergenic		
O157	2762319	s	rec	G2583_2540	hisG	CDS
O157	2762352	s	rec	G2583_2540	hisG	CDS
O157	2762442	s	rec	G2583_2540	hisG	CDS
O157	2762460	s	rec	G2583_2540	hisG	CDS

lineage[a]	site[b]	mutation type[c]	recomb	gene	Gene name	Type
O157	2762511	s	rec	G2583_2540	hisG	CDS
O157	2762529	s	rec	G2583_2540	hisG	CDS
O157	2762571	s	rec	G2583_2540	hisG	CDS
O157	2762598	ns	rec	G2583_2540	hisG	CDS
O157	2762607	s	rec	G2583_2540	hisG	CDS
O157	2762613	s	rec	G2583_2540	hisG	CDS
O157	2762721	s	rec	G2583_2540	hisG	CDS
O157	2762748	s	rec	G2583_2540	hisG	CDS
O157	2762760	s	rec	G2583_2540	hisG	CDS
O157	2762793	s	rec	G2583_2540	hisG	CDS
O157	2762823	s	rec	G2583_2540	hisG	CDS
O157	2762844	s	rec	G2583_2540	hisG	CDS
O157	2762847	s	rec	G2583_2540	hisG	CDS
O157	2762871	s	rec	G2583_2540	hisG	CDS
O157	2762892	s	rec	G2583_2540	hisG	CDS
O157	2762916	s	rec	G2583_2540	hisG	CDS
O157	2762962	ns	rec	G2583_2540	hisG	CDS
O157	2762967	s	rec	G2583_2540	hisG	CDS
O157	2763033	s	rec	G2583_2540	hisG	CDS
O157	2763039	s	rec	G2583_2540	hisG	CDS
O157	2763204	ns	rec	G2583_2541	hisD	CDS
O157	2763286	ns	rec	G2583_2541	hisD	CDS
O157	2763323	s	rec	G2583_2541	hisD	CDS
O157	2763356	s	rec	G2583_2541	hisD	CDS
O157	2763363	ns	rec	G2583_2541	hisD	CDS
O157	2763390	ns	rec	G2583_2541	hisD	CDS
O157	2763479	s	rec	G2583_2541	hisD	CDS
O157	2763506	s	rec	G2583_2541	hisD	CDS
O157	2763533	s	rec	G2583_2541	hisD	CDS
O157	2763542	s	rec	G2583_2541	hisD	CDS
O157	2763545	s	rec	G2583_2541	hisD	CDS
O157	2763599	s	rec	G2583_2541	hisD	CDS
O157	2763614	s	rec	G2583_2541	hisD	CDS
O157	2763636	s	rec	G2583_2541	hisD	CDS
O157	2763665	s	rec	G2583_2541	hisD	CDS
O157	2763668	s	rec	G2583_2541	hisD	CDS
O157	2763674	s	rec	G2583_2541	hisD	CDS
O157	2763692	s	rec	G2583_2541	hisD	CDS
O157	2763737	s	rec	G2583_2541	hisD	CDS
O157	2763764	s	rec	G2583_2541	hisD	CDS
O157	2763788	s	rec	G2583_2541	hisD	CDS
O157	2763791	s	rec	G2583_2541	hisD	CDS
O157	2763809	s	rec	G2583_2541	hisD	CDS
O157	2763818	s	rec	G2583_2541	hisD	CDS
O157	2763824	s	rec	G2583_2541	hisD	CDS

lineage[a]	site[b]	mutation type[c]	recomb	gene	Gene name	Type
O157	2763879	s	rec	G2583_2541	hisD	CDS
O157	2763899	s	rec	G2583_2541	hisD	CDS
O157	2763902	s	rec	G2583_2541	hisD	CDS
O157	2763908	s	rec	G2583_2541	hisD	CDS
O157	2763932	s	rec	G2583_2541	hisD	CDS
O157	2763935	s	rec	G2583_2541	hisD	CDS
O157	2763959	s	rec	G2583_2541	hisD	CDS
O157	2763977	s	rec	G2583_2541	hisD	CDS
O157	2763980	s	rec	G2583_2541	hisD	CDS
O157	2763982	ns	rec	G2583_2541	hisD	CDS
O157	2763983	ns	rec	G2583_2541	hisD	CDS
O157	2763997	ns	rec	G2583_2541	hisD	CDS
O157	2763999	ns	rec	G2583_2541	hisD	CDS
O157	2764000	ns	rec	G2583_2541	hisD	CDS
O157	2764001	ns	rec	G2583_2541	hisD	CDS
O157	2764004	s	rec	G2583_2541	hisD	CDS
O157	2764013	s	rec	G2583_2541	hisD	CDS
O157	2764028	s	rec	G2583_2541	hisD	CDS
O157	2764034	s	rec	G2583_2541	hisD	CDS
O157	2764058	s	rec	G2583_2541	hisD	CDS
O157	2764069	ns	rec	G2583_2541	hisD	CDS
O157	2764079	s	rec	G2583_2541	hisD	CDS
O157	2764112	s	rec	G2583_2541	hisD	CDS
O157	2764151	s	rec	G2583_2541	hisD	CDS
O157	2764172	ns	rec	G2583_2541	hisD	CDS
O157	2764226	s	rec	G2583_2541	hisD	CDS
O157	2764235	s	rec	G2583_2541	hisD	CDS
O157	2764253	s	rec	G2583_2541	hisD	CDS
O157	2764262	s	rec	G2583_2541	hisD	CDS
O157	2764265	s	rec	G2583_2541	hisD	CDS
O157	2764268	s	rec	G2583_2541	hisD	CDS
O157	2764280	s	rec	G2583_2541	hisD	CDS
O157	2764307	s	rec	G2583_2541	hisD	CDS
O157	2764313	s	rec	G2583_2541	hisD	CDS
O157	2764328	s	rec	G2583_2541	hisD	CDS
O157	2764354	ns	rec	G2583_2541	hisD	CDS
O157	2764366	ns	rec	G2583_2541	hisD	CDS
O157	2764382	s	rec	G2583_2541	hisD	CDS
O157	2764418	s	rec	G2583_2541	hisD	CDS
O157	2764549	s	rec	G2583_2542	hisC	CDS
O157	2764579	s	rec	G2583_2542	hisC	CDS
O157	2764586	ns	rec	G2583_2542	hisC	CDS
O157	2764636	s	rec	G2583_2542	hisC	CDS
O157	2764669	s	rec	G2583_2542	hisC	CDS
O157	2764690	s	rec	G2583_2542	hisC	CDS

lineage[a]	site[b]	mutation type[c]	recomb	gene	Gene name	Type
O157	2764756	s	rec	G2583_2542	hisC	CDS
O157	2764839	ns	rec	G2583_2542	hisC	CDS
O157	2764855	ns	rec	G2583_2542	hisC	CDS
O157	2764873	s	rec	G2583_2542	hisC	CDS
O157	2764936	s	rec	G2583_2542	hisC	CDS
O157	2764987	s	rec	G2583_2542	hisC	CDS
O157	2765005	s	rec	G2583_2542	hisC	CDS
O157	2765026	s	rec	G2583_2542	hisC	CDS
O157	2765038	s	rec	G2583_2542	hisC	CDS
O157	2765063	ns	rec	G2583_2542	hisC	CDS
O157	2765131	s	rec	G2583_2542	hisC	CDS
O157	2765134	s	rec	G2583_2542	hisC	CDS
O157	2765168	s	rec	G2583_2542	hisC	CDS
O157	2765182	s	rec	G2583_2542	hisC	CDS
O157	2765245	s	rec	G2583_2542	hisC	CDS
O157	2765248	s	rec	G2583_2542	hisC	CDS
O157	2765249	ns	rec	G2583_2542	hisC	CDS
O157	2765269	s	rec	G2583_2542	hisC	CDS
O157	2765342	ns	rec	G2583_2542	hisC	CDS
O157	2765386	s	rec	G2583_2542	hisC	CDS
O157	2765532	s	rec	G2583_2542	hisC	CDS
O157	2765607	s	rec	G2583_2543	hisB	CDS
O157	2765647	ns	rec	G2583_2543	hisB	CDS
O157	2765649	ns	rec	G2583_2543	hisB	CDS
O157	2765679	s	rec	G2583_2543	hisB	CDS
O157	2765694	s	rec	G2583_2543	hisB	CDS
O157	2765700	s	rec	G2583_2543	hisB	CDS
O157	2765709	s	rec	G2583_2543	hisB	CDS
O157	2765739	s	rec	G2583_2543	hisB	CDS
O157	2765769	s	rec	G2583_2543	hisB	CDS
O157	2765787	s	rec	G2583_2543	hisB	CDS
O157	2765847	s	rec	G2583_2543	hisB	CDS
O157	2765898	s	rec	G2583_2543	hisB	CDS
O157	2765904	s	rec	G2583_2543	hisB	CDS
O157	2765940	s	rec	G2583_2543	hisB	CDS
O157	2765943	s	rec	G2583_2543	hisB	CDS
O157	2765946	s	rec	G2583_2543	hisB	CDS
O157	2765976	s	rec	G2583_2543	hisB	CDS
O157	2766015	s	rec	G2583_2543	hisB	CDS
O157	2766021	s	rec	G2583_2543	hisB	CDS
O157	2766023	ns	rec	G2583_2543	hisB	CDS
O157	2766027	s	rec	G2583_2543	hisB	CDS
O157	2766039	s	rec	G2583_2543	hisB	CDS
O157	2766042	s	rec	G2583_2543	hisB	CDS
O157	2766051	s	rec	G2583_2543	hisB	CDS

lineage[a]	site[b]	mutation type[c]	recomb	gene	Gene name	Type
O157	2766054	s	rec	G2583_2543	hisB	CDS
O157	2766069	s	rec	G2583_2543	hisB	CDS
O157	2766078	s	rec	G2583_2543	hisB	CDS
O157	2766096	s	rec	G2583_2543	hisB	CDS
O157	2766117	s	rec	G2583_2543	hisB	CDS
O157	2766177	s	rec	G2583_2543	hisB	CDS
O157	2766294	s	rec	G2583_2543	hisB	CDS
O157	2766312	s	rec	G2583_2543	hisB	CDS
O157	2766339	s	rec	G2583_2543	hisB	CDS
O157	2766342	s	rec	G2583_2543	hisB	CDS
O157	2766378	s	rec	G2583_2543	hisB	CDS
O157	2766384	s	rec	G2583_2543	hisB	CDS
O157	2766396	s	rec	G2583_2543	hisB	CDS
O157	2766420	s	rec	G2583_2543	hisB	CDS
O157	2766450	s	rec	G2583_2543	hisB	CDS
O157	2766468	s	rec	G2583_2543	hisB	CDS
O157	2766471	s	rec	G2583_2543	hisB	CDS
O157	2766501	s	rec	G2583_2543	hisB	CDS
O157	2766528	s	rec	G2583_2543	hisB	CDS
O157	2766537	s	rec	G2583_2543	hisB	CDS
O157	2766619	ns	rec	G2583_2544	hisH	CDS
O157	2766665	s	rec	G2583_2544	hisH	CDS
O157	2766704	s	rec	G2583_2544	hisH	CDS
O157	2766848	s	rec	G2583_2544	hisH	CDS
O157	2766860	s	rec	G2583_2544	hisH	CDS
O157	2766881	s	rec	G2583_2544	hisH	CDS
O157	2766896	s	rec	G2583_2544	hisH	CDS
O157	2766956	s	rec	G2583_2544	hisH	CDS
O157	2767004	s	rec	G2583_2544	hisH	CDS
O157	2767068	ns	rec	G2583_2544	hisH	CDS
O157	2767085	s	rec	G2583_2544	hisH	CDS
O157	2767091	s	rec	G2583_2544	hisH	CDS
O157	2767133	s	rec	G2583_2544	hisH	CDS
O157	2767216	s	rec	G2583_2545	hisA	CDS
O157	2767225	s	rec	G2583_2545	hisA	CDS
O157	2767252	s	rec	G2583_2545	hisA	CDS
O157	2767288	s	rec	G2583_2545	hisA	CDS
O157	2767306	s	rec	G2583_2545	hisA	CDS
O157	2767309	s	rec	G2583_2545	hisA	CDS
O157	2767325	s	rec	G2583_2545	hisA	CDS
O157	2767331	s	rec	G2583_2545	hisA	CDS
O157	2767399	s	rec	G2583_2545	hisA	CDS
O157	2767432	s	rec	G2583_2545	hisA	CDS
O157	2767471	s	rec	G2583_2545	hisA	CDS
O157	2767474	s	rec	G2583_2545	hisA	CDS

lineage[a]	site[b]	mutation type[c]	recomb	gene	Gene name	Type
O157	2767501	s	rec	G2583_2545	hisA	CDS
O157	2767521	ns	rec	G2583_2545	hisA	CDS
O157	2767543	s	rec	G2583_2545	hisA	CDS
O157	2767642	s	rec	G2583_2545	hisA	CDS
O157	2767771	s	rec	G2583_2545	hisA	CDS
O157	2767792	s	rec	G2583_2545	hisA	CDS
O157	2767801	s	rec	G2583_2545	hisA	CDS
O157	2767907	ns	rec	G2583_2545	hisA	CDS
O157	2767923	ns	rec	G2583_2545-2546	hisAF	CDS
O157	2767932	s	rec	G2583_2546	hisF	CDS
O157	2767947	s	rec	G2583_2546	hisF	CDS
O157	2767962	s	rec	G2583_2546	hisF	CDS
O157	2768004	s	rec	G2583_2546	hisF	CDS
O157	2768007	s	rec	G2583_2546	hisF	CDS
O157	2768028	s	rec	G2583_2546	hisF	CDS
O157	2768031	s	rec	G2583_2546	hisF	CDS
O157	2768100	s	rec	G2583_2546	hisF	CDS
O157	2768124	s	rec	G2583_2546	hisF	CDS
O157	2768139	s	rec	G2583_2546	hisF	CDS
O157	2768142	s	rec	G2583_2546	hisF	CDS
O157	2768220	s	rec	G2583_2546	hisF	CDS
O157	2768235	s	rec	G2583_2546	hisF	CDS
O157	2768265	s	rec	G2583_2546	hisF	CDS
O157	2768292	s	rec	G2583_2546	hisF	CDS
O157	2768311	ns	rec	G2583_2546	hisF	CDS
O157	2768520	s	rec	G2583_2546	hisF	CDS
O157	2768526	s	rec	G2583_2546	hisF	CDS
O157	2768565	s	rec	G2583_2546	hisF	CDS
O157	2768589	s	rec	G2583_2546	hisF	CDS
O157	2768723	s	rec	G2583_2547	hisI	CDS
O157	2768732	s	rec	G2583_2547	hisI	CDS
O157	2768765	s	rec	G2583_2547	hisI	CDS
O157	2768807	s	rec	G2583_2547	hisI	CDS
O157	2768910	ns	rec	G2583_2547	hisI	CDS
O157	2768934	s	rec	G2583_2547	hisI	CDS
O157	2768940	ns	rec	G2583_2547	hisI	CDS
O157	2768942	ns	rec	G2583_2547	hisI	CDS
O157	2768954	s	rec	G2583_2547	hisI	CDS
O157	2768960	s	rec	G2583_2547	hisI	CDS
O157	2768963	s	rec	G2583_2547	hisI	CDS
O157	2769003	ns	rec	G2583_2547	hisI	CDS
O157	2769023	s	rec	G2583_2547	hisI	CDS
O157	2769039	s	rec	G2583_2547	hisI	CDS
O157	2769074	s	rec	G2583_2547	hisI	CDS
O157	2769077	s	rec	G2583_2547	hisI	CDS

lineage[a]	site[b]	mutation type[c]	recomb	gene	Gene name	Type
O157	2769164	s	rec	G2583_2547	hisI	CDS
O157	2769197	s	rec	G2583_2547	hisI	CDS
O157	2769203	s	rec	G2583_2547	hisI	CDS
O157	2769219	s	rec	G2583_2547	hisI	CDS
O157	2769224	s	rec	G2583_2547	hisI	CDS
O157	2769225	s	rec	G2583_2547	hisI	CDS
O157	2769241	ns	rec	G2583_2547	hisI	CDS
O157	2769242	ns	rec	G2583_2547	hisI	CDS
O157	2769254	s	rec	G2583_2547	hisI	CDS
O157	2769281	s	rec	G2583_2547	hisI	CDS
O157	2769299	i	rec	intergenic		
O157	2769314	i	rec	intergenic		
O157	2769319	i	rec	intergenic		
O157	2769351	i	rec	intergenic		
O157	2769365	i	rec	intergenic		
O157	2769414	s	rec	G2583_2548	cld	CDS
O157	2769425	s	rec	G2583_2548	cld	CDS
O157	2769492	s	rec	G2583_2548	cld	CDS
O157	2769531	s	rec	G2583_2548	cld	CDS
O157	2769561	s	rec	G2583_2548	cld	CDS
O157	2769568	ns	rec	G2583_2548	cld	CDS
O157	2769573	s	rec	G2583_2548	cld	CDS
O157	2769581	s	rec	G2583_2548	cld	CDS
O157	2769594	s	rec	G2583_2548	cld	CDS
O157	2769696	ns	rec	G2583_2548	cld	CDS
O157	2769698	ns	rec	G2583_2548	cld	CDS
O157	2769703	del-3	rec	G2583_2548	cld	CDS
O157	2769711	s	rec	G2583_2548	cld	CDS
O157	2769716	ns	rec	G2583_2548	cld	CDS
O157	2769717	s	rec	G2583_2548	cld	CDS
O157	2769720	s	rec	G2583_2548	cld	CDS
O157	2769723	s	rec	G2583_2548	cld	CDS
O157	2769728	ns	rec	G2583_2548	cld	CDS
O157	2769783	ns	rec	G2583_2548	cld	CDS
O157	2769804	s	rec	G2583_2548	cld	CDS
O157	2769856	ns	rec	G2583_2548	cld	CDS
O157	2770053	s	rec	G2583_2548	cld	CDS
O157	2770080	s	rec	G2583_2548	cld	CDS
O157	2770092	s	rec	G2583_2548	cld	CDS
O157	2770136	ns	rec	G2583_2548	cld	CDS
O157	2770161	s	rec	G2583_2548	cld	CDS
O157	2770224	s	rec	G2583_2548	cld	CDS
O157	2770236	s	rec	G2583_2548	cld	CDS
O157	2770322	ns	rec	G2583_2548	cld	CDS
O157	2770377	s	rec	G2583_2548	cld	CDS

lineage[a]	site[b]	mutation type[c]	recomb	gene	Gene name	Type
O157	2770382	ns	rec	G2583_2548	cld	CDS
O157	2770384	del	rec	G2583_2548	cld	CDS
O157	2770386	ns	rec	G2583_2548	cld	CDS
O157	2770389	ns	rec	G2583_2548	cld	CDS
O157	2770390	ns	rec	G2583_2548	cld	CDS
O157	2770396	ns	rec	G2583_2548	cld	CDS
O157	2770397	ns	rec	G2583_2548	cld	CDS
O157	2770416	ns	rec	G2583_2548	cld	CDS
O157	2770418	ns	rec	G2583_2548	cld	CDS
O157	2770419	ns	rec	G2583_2548	cld	CDS
O157	2770437	i	rec	intergenic		
O157	2770459	i	rec	intergenic		
O157	2770461	i	rec	intergenic		
O157	2770462	i	rec	intergenic		
O157	2770466	i	rec	intergenic		
O157	2770471	i	rec	intergenic		
O157	2770476	i	rec	intergenic		
O157	2770487	i	rec	intergenic		
O157	2770492	i	rec	intergenic		
O157	2770505	i	rec	intergenic		
O157	2770516	s	rec	G2583_2549	ugd	CDS
O157	2770540	s	rec	G2583_2549	ugd	CDS
O157	2770558	ns	rec	G2583_2549	ugd	CDS
O157	2770651	s	rec	G2583_2549	ugd	CDS
O157	2770681	s	rec	G2583_2549	ugd	CDS
O157	2770690	s	rec	G2583_2549	ugd	CDS
O157	2770708	s	rec	G2583_2549	ugd	CDS
O157	2770780	s	rec	G2583_2549	ugd	CDS
O157	2770792	s	rec	G2583_2549	ugd	CDS
O157	2770825	s	rec	G2583_2549	ugd	CDS
O157	2770840	s	rec	G2583_2549	ugd	CDS
O157	2770882	s	rec	G2583_2549	ugd	CDS
O157	2770918	s	rec	G2583_2549	ugd	CDS
O157	2770945	s	rec	G2583_2549	ugd	CDS
O157	2770969	s	rec	G2583_2549	ugd	CDS
O157	2770972	s	rec	G2583_2549	ugd	CDS
O157	2770993	s	rec	G2583_2549	ugd	CDS
O157	2770996	s	rec	G2583_2549	ugd	CDS
O157	2770999	s	rec	G2583_2549	ugd	CDS
O157	2771002	ns	rec	G2583_2549	ugd	CDS
O157	2771004	ns	rec	G2583_2549	ugd	CDS
O157	2771050	s	rec	G2583_2549	ugd	CDS
O157	2771065	s	rec	G2583_2549	ugd	CDS
O157	2771104	s	rec	G2583_2549	ugd	CDS
O157	2771107	s	rec	G2583_2549	ugd	CDS

lineage[a]	site[b]	mutation type[c]	recomb	gene	Gene name	Type
O157	2771110	s	rec	G2583_2549	ugd	CDS
O157	2771116	s	rec	G2583_2549	ugd	CDS
O157	2771125	s	rec	G2583_2549	ugd	CDS
O157	2771158	s	rec	G2583_2549	ugd	CDS
O157	2771185	s	rec	G2583_2549	ugd	CDS
O157	2771200	s	rec	G2583_2549	ugd	CDS
O157	2771212	s	rec	G2583_2549	ugd	CDS
O157	2771218	s	rec	G2583_2549	ugd	CDS
O157	2771221	s	rec	G2583_2549	ugd	CDS
O157	2771233	s	rec	G2583_2549	ugd	CDS
O157	2771245	s	rec	G2583_2549	ugd	CDS
O157	2771617	s	rec	G2583_2549	ugd	CDS
O157	2771638	s	rec	G2583_2549	ugd	CDS
O157	2771722	i	rec	intergenic		
O157	2771776	i	rec	intergenic		
O157	2771805	i	rec	intergenic		
O157	2771806	i	rec	intergenic		
O157	2771838	i	rec	intergenic		
O157	2771843	i	rec	intergenic		
O157	2771851	i	rec	intergenic		
O157	2771870	i	rec	intergenic		
EDL933	2844710	ns		ECs2833	-	CDS
EDL933	2845009	ins		intergenic		
O157	2787598	ns	rec	G2583_2564	galF	CDS
O157	2787599	ns	rec	G2583_2564	galF	CDS
O157	2787600	ns	rec	G2583_2564	galF	CDS
O157	2787601	ns	rec	G2583_2564	galF	CDS
O157	2787604	s	rec	G2583_2564	galF	CDS
O157	2787605	s	rec	G2583_2564	galF	CDS
O157	2787607	s	rec	G2583_2564	galF	CDS
O157	2787608	ns	rec	G2583_2564	galF	CDS
O157	2787610	ns	rec	G2583_2564	galF	CDS
O157	2787614	s	rec	G2583_2564	galF	CDS
O157	2787618	ns	rec	G2583_2564	galF	CDS
O157	2787619	ns	rec	G2583_2564	galF	CDS
O157	2787620	s	rec	G2583_2564	galF	CDS
O157	2787624	ns	rec	G2583_2564	galF	CDS
O157	2787626	s	rec	G2583_2564	galF	CDS
O157	2787629	s	rec	G2583_2564	galF	CDS
O157	2787635	s	rec	G2583_2564	galF	CDS
O157	2787641	s	rec	G2583_2564	galF	CDS
O157	2787644	s	rec	G2583_2564	galF	CDS
O157	2787646	s	rec	G2583_2564	galF	CDS
O157	2787653	s	rec	G2583_2564	galF	CDS
O157	2787656	s	rec	G2583_2564	galF	CDS

lineage[a]	site[b]	mutation type[c]	recomb	gene	Gene name	Type
O157	2787668	s	rec	G2583_2564	galF	CDS
O157	2787698	s	rec	G2583_2564	galF	CDS
O157	2787707	s	rec	G2583_2564	galF	CDS
O157	2787713	s	rec	G2583_2564	galF	CDS
O157	2787728	s	rec	G2583_2564	galF	CDS
O157	2787740	s	rec	G2583_2564	galF	CDS
O157	2787749	s	rec	G2583_2564	galF	CDS
O157	2787755	s	rec	G2583_2564	galF	CDS
O157	2787758	s	rec	G2583_2564	galF	CDS
O157	2787764	s	rec	G2583_2564	galF	CDS
O157	2787785	s	rec	G2583_2564	galF	CDS
O157	2787800	s	rec	G2583_2564	galF	CDS
O157	2787806	s	rec	G2583_2564	galF	CDS
O157	2787812	s	rec	G2583_2564	galF	CDS
O157	2787845	s	rec	G2583_2564	galF	CDS
O157	2787860	s	rec	G2583_2564	galF	CDS
O157	2787893	s	rec	G2583_2564	galF	CDS
O157	2787926	s	rec	G2583_2564	galF	CDS
O157	2787980	s	rec	G2583_2564	galF	CDS
O157	2788010	s	rec	G2583_2564	galF	CDS
O157	2788013	s	rec	G2583_2564	galF	CDS
O157	2788055	s	rec	G2583_2564	galF	CDS
O157	2788058	s	rec	G2583_2564	galF	CDS
O157	2788060	s	rec	G2583_2564	galF	CDS
O157	2788705	ins	rec	intergenic		
O157	2789221	s	rec	G2583_2565	gne	CDS
O157	2789254	s	rec	G2583_2565	gne	CDS
O157	2789308	s	rec	G2583_2565	gne	CDS
O157	2789320	s	rec	G2583_2565	gne	CDS
O157	2789344	s	rec	G2583_2565	gne	CDS
O157	2789530	s	rec	G2583_2565	gne	CDS
O157	2789969	s	rec	G2583_2566	wcaM	CDS
O157	2789973	ns	rec	G2583_2566	wcaM	CDS
O157	2789975	s	rec	G2583_2566	wcaM	CDS
O157	2790019	ns	rec	G2583_2566	wcaM	CDS
O157	2790032	s	rec	G2583_2566	wcaM	CDS
O157	2790038	s	rec	G2583_2566	wcaM	CDS
O157	2790068	ns	rec	G2583_2566	wcaM	CDS
O157	2790069	ns	rec	G2583_2566	wcaM	CDS
O157	2790086	s	rec	G2583_2566	wcaM	CDS
O157	2790104	s	rec	G2583_2566	wcaM	CDS
O157	2790113	s	rec	G2583_2566	wcaM	CDS
O157	2790118	ns	rec	G2583_2566	wcaM	CDS
O157	2790145	s	rec	G2583_2566	wcaM	CDS
O157	2790149	s	rec	G2583_2566	wcaM	CDS

lineage[a]	site[b]	mutation type[c]	recomb	gene	Gene name	Type
O157	2790155	s	rec	G2583_2566	wcaM	CDS
O157	2790179	s	rec	G2583_2566	wcaM	CDS
O157	2790182	s	rec	G2583_2566	wcaM	CDS
O157	2790197	s	rec	G2583_2566	wcaM	CDS
O157	2790209	s	rec	G2583_2566	wcaM	CDS
O157	2790220	ns	rec	G2583_2566	wcaM	CDS
O157	2790221	s	rec	G2583_2566	wcaM	CDS
O157	2790257	s	rec	G2583_2566	wcaM	CDS
O157	2790287	s	rec	G2583_2566	wcaM	CDS
O157	2790299	ns	rec	G2583_2566	wcaM	CDS
O157	2790395	s	rec	G2583_2566	wcaM	CDS
O157	2790398	s	rec	G2583_2566	wcaM	CDS
O157	2790432	ns	rec	G2583_2566	wcaM	CDS
O157	2790445	ns	rec	G2583_2566	wcaM	CDS
O157	2790491	s	rec	G2583_2566	wcaM	CDS
O157	2790515	s	rec	G2583_2566	wcaM	CDS
O157	2790530	s	rec	G2583_2566	wcaM	CDS
O157	2790551	s	rec	G2583_2566	wcaM	CDS
O157	2790566	s	rec	G2583_2566	wcaM	CDS
O157	2790571	ns	rec	G2583_2566	wcaM	CDS
O157	2790575	s	rec	G2583_2566	wcaM	CDS
O157	2790578	s	rec	G2583_2566	wcaM	CDS
O157	2790599	s	rec	G2583_2566	wcaM	CDS
O157	2790668	ns	rec	G2583_2566	wcaM	CDS
O157	2790670	ns	rec	G2583_2566	wcaM	CDS
O157	2790680	s	rec	G2583_2566	wcaM	CDS
O157	2790701	s	rec	G2583_2566	wcaM	CDS
O157	2790728	s	rec	G2583_2566	wcaM	CDS
O157	2790749	s	rec	G2583_2566	wcaM	CDS
O157	2790797	ns	rec	G2583_2566	wcaM	CDS
O157	2790799	ns	rec	G2583_2566	wcaM	CDS
O157	2790800	s	rec	G2583_2566	wcaM	CDS
O157	2790803	s	rec	G2583_2566	wcaM	CDS
O157	2790872	s	rec	G2583_2566	wcaM	CDS
O157	2790893	s	rec	G2583_2566	wcaM	CDS
O157	2790914	s	rec	G2583_2566	wcaM	CDS
O157	2790917	s	rec	G2583_2566	wcaM	CDS
O157	2790949	ns	rec	G2583_2566	wcaM	CDS
O157	2791004	s	rec	G2583_2566	wcaM	CDS
O157	2791040	s	rec	G2583_2566	wcaM	CDS
O157	2791043	s	rec	G2583_2566	wcaM	CDS
O157	2791049	s	rec	G2583_2566	wcaM	CDS
O157	2791052	s	rec	G2583_2566	wcaM	CDS
O157	2791080	ns	rec	G2583_2566	wcaM	CDS
O157	2791082	ns	rec	G2583_2566	wcaM	CDS

lineage[a]	site[b]	mutation type[c]	recomb	gene	Gene name	Type
O157	2791088	s	rec	G2583_2566	wcaM	CDS
O157	2791100	s	rec	G2583_2566	wcaM	CDS
O157	2791151	s	rec	G2583_2566	wcaM	CDS
O157	2791154	s	rec	G2583_2566	wcaM	CDS
O157	2791166	s	rec	G2583_2566	wcaM	CDS
O157	2791169	s	rec	G2583_2566	wcaM	CDS
O157	2791187	s	rec	G2583_2566	wcaM	CDS
O157	2791214	s	rec	G2583_2566	wcaM	CDS
O157	2791277	i	rec	intergenic		
O157	2791350	s	rec	G2583_2567	wcaL	CDS
O157	2791365	s	rec	G2583_2567	wcaL	CDS
O157	2791385	s	rec	G2583_2567	wcaL	CDS
O157	2791412	ns	rec	G2583_2567	wcaL	CDS
O157	2791419	s	rec	G2583_2567	wcaL	CDS
O157	2791425	s	rec	G2583_2567	wcaL	CDS
O157	2791431	s	rec	G2583_2567	wcaL	CDS
O157	2791470	s	rec	G2583_2567	wcaL	CDS
O157	2791473	s	rec	G2583_2567	wcaL	CDS
O157	2791484	s	rec	G2583_2567	wcaL	CDS
O157	2791488	s	rec	G2583_2567	wcaL	CDS
O157	2791539	s	rec	G2583_2567	wcaL	CDS
O157	2791545	s	rec	G2583_2567	wcaL	CDS
O157	2791548	s	rec	G2583_2567	wcaL	CDS
O157	2791560	s	rec	G2583_2567	wcaL	CDS
O157	2791581	s	rec	G2583_2567	wcaL	CDS
O157	2791584	s	rec	G2583_2567	wcaL	CDS
O157	2791587	s	rec	G2583_2567	wcaL	CDS
O157	2791617	s	rec	G2583_2567	wcaL	CDS
O157	2791627	ns	rec	G2583_2567	wcaL	CDS
O157	2791662	ns	rec	G2583_2567	wcaL	CDS
O157	2791665	s	rec	G2583_2567	wcaL	CDS
O157	2791698	s	rec	G2583_2567	wcaL	CDS
O157	2791754	ns	rec	G2583_2567	wcaL	CDS
O157	2791773	s	rec	G2583_2567	wcaL	CDS
O157	2791785	s	rec	G2583_2567	wcaL	CDS
O157	2791800	s	rec	G2583_2567	wcaL	CDS
O157	2791809	s	rec	G2583_2567	wcaL	CDS
O157	2791830	s	rec	G2583_2567	wcaL	CDS
O157	2791833	s	rec	G2583_2567	wcaL	CDS
O157	2791836	s	rec	G2583_2567	wcaL	CDS
O157	2791845	s	rec	G2583_2567	wcaL	CDS
O157	2791863	s	rec	G2583_2567	wcaL	CDS
O157	2791878	s	rec	G2583_2567	wcaL	CDS
O157	2791887	s	rec	G2583_2567	wcaL	CDS
O157	2791890	s	rec	G2583_2567	wcaL	CDS

lineage[a]	site[b]	mutation type[c]	recomb	gene	Gene name	Type
O157	2791968	s	rec	G2583_2567	wcaL	CDS
O157	2791980	s	rec	G2583_2567	wcaL	CDS
O157	2791986	s	rec	G2583_2567	wcaL	CDS
O157	2791989	s	rec	G2583_2567	wcaL	CDS
O157	2791992	s	rec	G2583_2567	wcaL	CDS
O157	2792001	s	rec	G2583_2567	wcaL	CDS
O157	2792031	s	rec	G2583_2567	wcaL	CDS
O157	2792043	s	rec	G2583_2567	wcaL	CDS
O157	2792052	s	rec	G2583_2567	wcaL	CDS
O157	2792061	s	rec	G2583_2567	wcaL	CDS
O157	2792080	ns	rec	G2583_2567	wcaL	CDS
O157	2792100	s	rec	G2583_2567	wcaL	CDS
O157	2792121	s	rec	G2583_2567	wcaL	CDS
O157	2792124	s	rec	G2583_2567	wcaL	CDS
O157	2792127	s	rec	G2583_2567	wcaL	CDS
O157	2792133	s	rec	G2583_2567	wcaL	CDS
O157	2792142	s	rec	G2583_2567	wcaL	CDS
O157	2792145	s	rec	G2583_2567	wcaL	CDS
O157	2792154	ns	rec	G2583_2567	wcaL	CDS
O157	2792155	ns	rec	G2583_2567	wcaL	CDS
O157	2792159	ns	rec	G2583_2567	wcaL	CDS
O157	2792165	ns	rec	G2583_2567	wcaL	CDS
O157	2792167	ns	rec	G2583_2567	wcaL	CDS
O157	2792204	ns	rec	G2583_2567	wcaL	CDS
O157	2792259	s	rec	G2583_2567	wcaL	CDS
O157	2792268	s	rec	G2583_2567	wcaL	CDS
O157	2792271	s	rec	G2583_2567	wcaL	CDS
O157	2792277	s	rec	G2583_2567	wcaL	CDS
O157	2792283	s	rec	G2583_2567	wcaL	CDS
O157	2792358	s	rec	G2583_2567	wcaL	CDS
O157	2792368	ns	rec	G2583_2567	wcaL	CDS
O157	2792379	ns	rec	G2583_2567	wcaL	CDS
O157	2792381	ns	rec	G2583_2567	wcaL	CDS
O157	2792385	s	rec	G2583_2567	wcaL	CDS
O157	2792399	s	rec	G2583_2567	wcaL	CDS
O157	2792406	s	rec	G2583_2567	wcaL	CDS
O157	2792415	s	rec	G2583_2567	wcaL	CDS
O157	2792421	s	rec	G2583_2567	wcaL	CDS
O157	2792439	s	rec	G2583_2567	wcaL	CDS
O157	2792442	s	rec	G2583_2567	wcaL	CDS
O157	2792448	s	rec	G2583_2567	wcaL	CDS
O157	2792451	s	rec	G2583_2567	wcaL	CDS
O157	2792454	s	rec	G2583_2567	wcaL	CDS
O157	2792466	s	rec	G2583_2567	wcaL	CDS
O157	2792486	s	rec	G2583_2567	wcaL	CDS

lineage[a]	site[b]	mutation type[c]	recomb	gene	Gene name	Type
O157	2792490	s	rec	G2583_2567	wcaL	CDS
O157	2792511	ns	rec	G2583_2568	wcaK	CDS
O157	2792525	s	rec	G2583_2568	wcaK	CDS
O157	2792528	s	rec	G2583_2568	wcaK	CDS
O157	2792531	s	rec	G2583_2568	wcaK	CDS
O157	2792534	s	rec	G2583_2568	wcaK	CDS
O157	2792537	s	rec	G2583_2568	wcaK	CDS
O157	2792564	s	rec	G2583_2568	wcaK	CDS
O157	2792583	ns	rec	G2583_2568	wcaK	CDS
O157	2792591	s	rec	G2583_2568	wcaK	CDS
O157	2792609	s	rec	G2583_2568	wcaK	CDS
O157	2792641	s	rec	G2583_2568	wcaK	CDS
O157	2792651	s	rec	G2583_2568	wcaK	CDS
O157	2792653	s	rec	G2583_2568	wcaK	CDS
O157	2792672	s	rec	G2583_2568	wcaK	CDS
O157	2792681	s	rec	G2583_2568	wcaK	CDS
O157	2792684	s	rec	G2583_2568	wcaK	CDS
O157	2792687	s	rec	G2583_2568	wcaK	CDS
O157	2792693	s	rec	G2583_2568	wcaK	CDS
O157	2792696	s	rec	G2583_2568	wcaK	CDS
O157	2792704	ns	rec	G2583_2568	wcaK	CDS
O157	2792723	s	rec	G2583_2568	wcaK	CDS
O157	2792738	s	rec	G2583_2568	wcaK	CDS
O157	2792747	s	rec	G2583_2568	wcaK	CDS
O157	2792753	s	rec	G2583_2568	wcaK	CDS
O157	2792759	s	rec	G2583_2568	wcaK	CDS
O157	2792807	s	rec	G2583_2568	wcaK	CDS
O157	2792837	s	rec	G2583_2568	wcaK	CDS
O157	2792858	s	rec	G2583_2568	wcaK	CDS
O157	2792891	s	rec	G2583_2568	wcaK	CDS
O157	2792900	s	rec	G2583_2568	wcaK	CDS
O157	2792933	s	rec	G2583_2568	wcaK	CDS
O157	2792970	ns	rec	G2583_2568	wcaK	CDS
O157	2793015	ns	rec	G2583_2568	wcaK	CDS
O157	2793020	s	rec	G2583_2568	wcaK	CDS
O157	2793134	s	rec	G2583_2568	wcaK	CDS
O157	2793215	s	rec	G2583_2568	wcaK	CDS
O157	2793217	s	rec	G2583_2568	wcaK	CDS
O157	2793221	s	rec	G2583_2568	wcaK	CDS
O157	2793241	s	rec	G2583_2568	wcaK	CDS
O157	2793247	s	rec	G2583_2568	wcaK	CDS
O157	2793251	s	rec	G2583_2568	wcaK	CDS
O157	2793353	s	rec	G2583_2568	wcaK	CDS
O157	2793365	s	rec	G2583_2568	wcaK	CDS
O157	2793383	s	rec	G2583_2568	wcaK	CDS

lineage[a]	site[b]	mutation type[c]	recomb	gene	Gene name	Type
O157	2793386	s	rec	G2583_2568	wcaK	CDS
O157	2793443	s	rec	G2583_2568	wcaK	CDS
O157	2793445	s	rec	G2583_2568	wcaK	CDS
O157	2793485	s	rec	G2583_2568	wcaK	CDS
O157	2793527	s	rec	G2583_2568	wcaK	CDS
O157	2793533	s	rec	G2583_2568	wcaK	CDS
O157	2793602	s	rec	G2583_2568	wcaK	CDS
O157	2793617	s	rec	G2583_2568	wcaK	CDS
O157	2793685	ns	rec	G2583_2568	wcaK	CDS
O157	2793696	ns	rec	G2583_2568	wcaK	CDS
O157	2793815	del-14	rec	intergenic		
O157	2793944	i	rec	intergenic		
O157	2793959	i	rec	intergenic		
O157	2793961	i	rec	intergenic		
O157	2793963	i	rec	intergenic		
O157	2793981	ns	rec	G2583_2569	wzxC	CDS
O157	2794012	s	rec	G2583_2569	wzxC	CDS
O157	2794060	s	rec	G2583_2569	wzxC	CDS
O157	2794131	ns	rec	G2583_2569	wzxC	CDS
O157	2794132	ns	rec	G2583_2569	wzxC	CDS
O157	2794134	ns	rec	G2583_2569	wzxC	CDS
O157	2794142	ns	rec	G2583_2569	wzxC	CDS
O157	2794183	s	rec	G2583_2569	wzxC	CDS
O157	2794189	s	rec	G2583_2569	wzxC	CDS
O157	2794222	s	rec	G2583_2569	wzxC	CDS
O157	2794225	s	rec	G2583_2569	wzxC	CDS
O157	2794269	ns	rec	G2583_2569	wzxC	CDS
O157	2794324	s	rec	G2583_2569	wzxC	CDS
O157	2794351	s	rec	G2583_2569	wzxC	CDS
O157	2794390	s	rec	G2583_2569	wzxC	CDS
O157	2794426	s	rec	G2583_2569	wzxC	CDS
O157	2794429	s	rec	G2583_2569	wzxC	CDS
O157	2794438	s	rec	G2583_2569	wzxC	CDS
O157	2794441	s	rec	G2583_2569	wzxC	CDS
O157	2794447	ns	rec	G2583_2569	wzxC	CDS
O157	2794449	ns	rec	G2583_2569	wzxC	CDS
O157	2794489	s	rec	G2583_2569	wzxC	CDS
O157	2794492	s	rec	G2583_2569	wzxC	CDS
O157	2794498	s	rec	G2583_2569	wzxC	CDS
O157	2794609	s	rec	G2583_2569	wzxC	CDS
O157	2794627	s	rec	G2583_2569	wzxC	CDS
O157	2794630	s	rec	G2583_2569	wzxC	CDS
O157	2794645	s	rec	G2583_2569	wzxC	CDS
O157	2794657	s	rec	G2583_2569	wzxC	CDS
O157	2794666	s	rec	G2583_2569	wzxC	CDS

lineage[a]	site[b]	mutation type[c]	recomb	gene	Gene name	Type
O157	2794708	s	rec	G2583_2569	wzxC	CDS
O157	2794720	ns	rec	G2583_2569	wzxC	CDS
O157	2794722	ns	rec	G2583_2569	wzxC	CDS
O157	2794765	s	rec	G2583_2569	wzxC	CDS
O157	2794822	s	rec	G2583_2569	wzxC	CDS
O157	2794825	s	rec	G2583_2569	wzxC	CDS
O157	2794828	s	rec	G2583_2569	wzxC	CDS
O157	2794852	s	rec	G2583_2569	wzxC	CDS
O157	2794897	s	rec	G2583_2569	wzxC	CDS
O157	2794987	s	rec	G2583_2569	wzxC	CDS
O157	2795035	s	rec	G2583_2569	wzxC	CDS
O157	2795071	s	rec	G2583_2569	wzxC	CDS
O157	2795104	s	rec	G2583_2569	wzxC	CDS
O157	2795122	s	rec	G2583_2569	wzxC	CDS
O157	2795130	ns	rec	G2583_2569	wzxC	CDS
O157	2795167	s	rec	G2583_2569	wzxC	CDS
O157	2795248	s	rec	G2583_2569	wzxC	CDS
O157	2795257	s	rec	G2583_2569	wzxC	CDS
O157	2795308	s	rec	G2583_2569	wzxC	CDS
O157	2795311	s	rec	G2583_2569	wzxC	CDS
O157	2795316	s	rec	G2583_2569	wzxC	CDS
O157	2795317	s	rec	G2583_2569	wzxC	CDS
O157	2795367	s	rec	G2583_2569	wzxC	CDS
O157	2795468	s	rec	G2583_2570	wcaJ	CDS
O157	2795471	s	rec	G2583_2570	wcaJ	CDS
O157	2795477	s	rec	G2583_2570	wcaJ	CDS
O157	2795636	s	rec	G2583_2570	wcaJ	CDS
O157	2795663	s	rec	G2583_2570	wcaJ	CDS
O157	2795690	s	rec	G2583_2570	wcaJ	CDS
O157	2795699	s	rec	G2583_2570	wcaJ	CDS
O157	2795735	s	rec	G2583_2570	wcaJ	CDS
O157	2795771	s	rec	G2583_2570	wcaJ	CDS
O157	2795777	s	rec	G2583_2570	wcaJ	CDS
O157	2795819	s	rec	G2583_2570	wcaJ	CDS
O157	2795822	s	rec	G2583_2570	wcaJ	CDS
O157	2795879	s	rec	G2583_2570	wcaJ	CDS
O157	2795885	s	rec	G2583_2570	wcaJ	CDS
O157	2795957	s	rec	G2583_2570	wcaJ	CDS
O157	2796056	s	rec	G2583_2570	wcaJ	CDS
O157	2796071	s	rec	G2583_2570	wcaJ	CDS
O157	2796089	ns	rec	G2583_2570	wcaJ	CDS
O157	2796091	ns	rec	G2583_2570	wcaJ	CDS
O157	2796101	s	rec	G2583_2570	wcaJ	CDS
O157	2796107	s	rec	G2583_2570	wcaJ	CDS
O157	2796122	s	rec	G2583_2570	wcaJ	CDS

lineage[a]	site[b]	mutation type[c]	recomb	gene	Gene name	Type
O157	2796130	ns	rec	G2583_2570	wcaJ	CDS
Sakai	2796191	s		G2583_2570	wcaJ	CDS
O157	2796200	s	rec	G2583_2570	wcaJ	CDS
O157	2796233	s	rec	G2583_2570	wcaJ	CDS
O157	2796239	s	rec	G2583_2570	wcaJ	CDS
O157	2796263	s	rec	G2583_2570	wcaJ	CDS
O157	2796266	s	rec	G2583_2570	wcaJ	CDS
O157	2796294	ns	rec	G2583_2570	wcaJ	CDS
O157	2796302	s	rec	G2583_2570	wcaJ	CDS
O157	2796308	s	rec	G2583_2570	wcaJ	CDS
O157	2796311	s	rec	G2583_2570	wcaJ	CDS
O157	2796314	s	rec	G2583_2570	wcaJ	CDS
O157	2796344	s	rec	G2583_2570	wcaJ	CDS
O157	2796371	s	rec	G2583_2570	wcaJ	CDS
O157	2796392	s	rec	G2583_2570	wcaJ	CDS
O157	2796395	s	rec	G2583_2570	wcaJ	CDS
O157	2796404	s	rec	G2583_2570	wcaJ	CDS
O157	2796479	s	rec	G2583_2570	wcaJ	CDS
O157	2796504	ns	rec	G2583_2570	wcaJ	CDS
O157	2796521	s	rec	G2583_2570	wcaJ	CDS
O157	2796589	s	rec	G2583_2570	wcaJ	CDS
O157	2796715	s	rec	G2583_2570	wcaJ	CDS
O157	2796743	s	rec	G2583_2570	wcaJ	CDS
O157	2796852	i	rec	intergenic		
O157	2796956	s	rec	G2583_2571	cpsG	CDS
O157	2797004	s	rec	G2583_2571	cpsG	CDS
O157	2797076	s	rec	G2583_2571	cpsG	CDS
O157	2797085	s	rec	G2583_2571	cpsG	CDS
O157	2797098	ns	rec	G2583_2571	cpsG	CDS
O157	2797109	ns	rec	G2583_2571	cpsG	CDS
O157	2797141	ns	rec	G2583_2571	cpsG	CDS
O157	2797160	s	rec	G2583_2571	cpsG	CDS
O157	2797166	s	rec	G2583_2571	cpsG	CDS
O157	2797184	s	rec	G2583_2571	cpsG	CDS
O157	2797187	ns	rec	G2583_2571	cpsG	CDS
O157	2797188	ns	rec	G2583_2571	cpsG	CDS
O157	2797262	s	rec	G2583_2571	cpsG	CDS
O157	2797265	s	rec	G2583_2571	cpsG	CDS
O157	2797313	s	rec	G2583_2571	cpsG	CDS
O157	2797355	s	rec	G2583_2571	cpsG	CDS
O157	2797361	s	rec	G2583_2571	cpsG	CDS
O157	2797388	s	rec	G2583_2571	cpsG	CDS
O157	2797403	s	rec	G2583_2571	cpsG	CDS
O157	2797460	s	rec	G2583_2571	cpsG	CDS
O157	2797481	s	rec	G2583_2571	cpsG	CDS

lineage[a]	site[b]	mutation type[c]	recomb	gene	Gene name	Type
O157	2797535	s	rec	G2583_2571	cpsG	CDS
O157	2797562	s	rec	G2583_2571	cpsG	CDS
O157	2797580	s	rec	G2583_2571	cpsG	CDS
O157	2797589	s	rec	G2583_2571	cpsG	CDS
O157	2797603	s	rec	G2583_2571	cpsG	CDS
O157	2797604	s	rec	G2583_2571	cpsG	CDS
O157	2797607	s	rec	G2583_2571	cpsG	CDS
O157	2797637	s	rec	G2583_2571	cpsG	CDS
O157	2797649	s	rec	G2583_2571	cpsG	CDS
O157	2797667	s	rec	G2583_2571	cpsG	CDS
O157	2797697	s	rec	G2583_2571	cpsG	CDS
O157	2797721	s	rec	G2583_2571	cpsG	CDS
O157	2797760	s	rec	G2583_2571	cpsG	CDS
O157	2797928	s	rec	G2583_2571	cpsG	CDS
O157	2797940	s	rec	G2583_2571	cpsG	CDS
O157	2797952	s	rec	G2583_2571	cpsG	CDS
O157	2797964	s	rec	G2583_2571	cpsG	CDS
O157	2797976	s	rec	G2583_2571	cpsG	CDS
O157	2798060	s	rec	G2583_2571	cpsG	CDS
O157	2798063	s	rec	G2583_2571	cpsG	CDS
O157	2798087	s	rec	G2583_2571	cpsG	CDS
O157	2798106	ns	rec	G2583_2571	cpsG	CDS
O157	2798120	s	rec	G2583_2571	cpsG	CDS
O157	2798124	ns	rec	G2583_2571	cpsG	CDS
O157	2798138	s	rec	G2583_2571	cpsG	CDS
O157	2798174	s	rec	G2583_2571	cpsG	CDS
O157	2798183	s	rec	G2583_2571	cpsG	CDS
O157	2798192	s	rec	G2583_2571	cpsG	CDS
O157	2798218	s	rec	G2583_2571	cpsG	CDS
O157	2798222	s	rec	G2583_2571	cpsG	CDS
O157	2798446	i	rec	intergenic		
O157	2798451	i	rec	intergenic		
O157	2798454	i	rec	intergenic		
O157	2798455	i	rec	intergenic		
O157	2798456	s	rec	G2583_2572	cpsB	CDS
O157	2798567	s	rec	G2583_2572	cpsB	CDS
O157	2798594	s	rec	G2583_2572	cpsB	CDS
O157	2798630	s	rec	G2583_2572	cpsB	CDS
O157	2798633	s	rec	G2583_2572	cpsB	CDS
O157	2798651	s	rec	G2583_2572	cpsB	CDS
O157	2798684	s	rec	G2583_2572	cpsB	CDS
O157	2798780	s	rec	G2583_2572	cpsB	CDS
O157	2798786	s	rec	G2583_2572	cpsB	CDS
O157	2798885	s	rec	G2583_2572	cpsB	CDS
O157	2798995	ns	rec	G2583_2572	cpsB	CDS

lineage[a]	site[b]	mutation type[c]	recomb	gene	Gene name	Type
O157	2799002	s	rec	G2583_2572	cpsB	CDS
O157	2799041	s	rec	G2583_2572	cpsB	CDS
O157	2799047	s	rec	G2583_2572	cpsB	CDS
O157	2799050	s	rec	G2583_2572	cpsB	CDS
O157	2799101	s	rec	G2583_2572	cpsB	CDS
O157	2799143	s	rec	G2583_2572	cpsB	CDS
O157	2799152	ns	rec	G2583_2572	cpsB	CDS
O157	2799164	s	rec	G2583_2572	cpsB	CDS
O157	2799184	ns	rec	G2583_2572	cpsB	CDS
O157	2799215	s	rec	G2583_2572	cpsB	CDS
O157	2799221	s	rec	G2583_2572	cpsB	CDS
O157	2799266	s	rec	G2583_2572	cpsB	CDS
O157	2799281	s	rec	G2583_2572	cpsB	CDS
O157	2799338	s	rec	G2583_2572	cpsB	CDS
O157	2799392	s	rec	G2583_2572	cpsB	CDS
O157	2799434	s	rec	G2583_2572	cpsB	CDS
O157	2799452	s	rec	G2583_2572	cpsB	CDS
O157	2799488	s	rec	G2583_2572	cpsB	CDS
O157	2799491	s	rec	G2583_2572	cpsB	CDS
O157	2799506	s	rec	G2583_2572	cpsB	CDS
O157	2799512	s	rec	G2583_2572	cpsB	CDS
O157	2799547	s	rec	G2583_2572	cpsB	CDS
O157	2799548	s	rec	G2583_2572	cpsB	CDS
O157	2799560	s	rec	G2583_2572	cpsB	CDS
O157	2799567	ns	rec	G2583_2572	cpsB	CDS
O157	2799620	s	rec	G2583_2572	cpsB	CDS
O157	2799656	s	rec	G2583_2572	cpsB	CDS
O157	2799680	s	rec	G2583_2572	cpsB	CDS
O157	2799689	s	rec	G2583_2572	cpsB	CDS
O157	2799725	s	rec	G2583_2572	cpsB	CDS
O157	2799812	s	rec	G2583_2572	cpsB	CDS
O157	2799845	s	rec	G2583_2572	cpsB	CDS
O157	2800027	s	rec	G2583_2573	wcaI	CDS
O157	2800081	s	rec	G2583_2573	wcaI	CDS
O157	2800090	s	rec	G2583_2573	wcaI	CDS
O157	2800102	s	rec	G2583_2573	wcaI	CDS
O157	2800105	s	rec	G2583_2573	wcaI	CDS
O157	2800129	s	rec	G2583_2573	wcaI	CDS
O157	2800249	s	rec	G2583_2573	wcaI	CDS
O157	2800265	ns	rec	G2583_2573	wcaI	CDS
O157	2800294	s	rec	G2583_2573	wcaI	CDS
O157	2800348	s	rec	G2583_2573	wcaI	CDS
O157	2800363	s	rec	G2583_2573	wcaI	CDS
O157	2800472	ns	rec	G2583_2573	wcaI	CDS
O157	2800474	s	rec	G2583_2573	wcaI	CDS

lineage[a]	site[b]	mutation type[c]	recomb	gene	Gene name	Type
O157	2800480	s	rec	G2583_2573	wcaI	CDS
O157	2800498	s	rec	G2583_2573	wcaI	CDS
O157	2800504	s	rec	G2583_2573	wcaI	CDS
O157	2800534	s	rec	G2583_2573	wcaI	CDS
O157	2800588	s	rec	G2583_2573	wcaI	CDS
O157	2800690	s	rec	G2583_2573	wcaI	CDS
O157	2800693	s	rec	G2583_2573	wcaI	CDS
O157	2800975	s	rec	G2583_2573	wcaI	CDS
O157	2801044	s	rec	G2583_2573	wcaI	CDS
O157	2801053	s	rec	G2583_2573	wcaI	CDS
O157	2801250	ns	rec	G2583_2574	gmm	CDS
O157	2801259	s	rec	G2583_2574	gmm	CDS
O157	2801262	s	rec	G2583_2574	gmm	CDS
O157	2801373	s	rec	G2583_2574	gmm	CDS
O157	2801425	ns	rec	G2583_2574	gmm	CDS
O157	2801532	s	rec	G2583_2574	gmm	CDS
O157	2801550	s	rec	G2583_2574	gmm	CDS
O157	2801735	s	rec	G2583_2575	fcl	CDS
O157	2801741	s	rec	G2583_2575	fcl	CDS
O157	2801840	s	rec	G2583_2575	fcl	CDS
O157	2801861	s	rec	G2583_2575	fcl	CDS
O157	2801876	s	rec	G2583_2575	fcl	CDS
O157	2801891	s	rec	G2583_2575	fcl	CDS
O157	2801936	s	rec	G2583_2575	fcl	CDS
O157	2801945	s	rec	G2583_2575	fcl	CDS
O157	2801975	s	rec	G2583_2575	fcl	CDS
O157	2802017	s	rec	G2583_2575	fcl	CDS
O157	2802032	s	rec	G2583_2575	fcl	CDS
O157	2802041	s	rec	G2583_2575	fcl	CDS
O157	2802053	s	rec	G2583_2575	fcl	CDS
O157	2802056	s	rec	G2583_2575	fcl	CDS
O157	2802059	s	rec	G2583_2575	fcl	CDS
O157	2802062	s	rec	G2583_2575	fcl	CDS
O157	2802083	s	rec	G2583_2575	fcl	CDS
O157	2802101	s	rec	G2583_2575	fcl	CDS
O157	2802116	s	rec	G2583_2575	fcl	CDS
O157	2802182	s	rec	G2583_2575	fcl	CDS
O157	2802236	s	rec	G2583_2575	fcl	CDS
O157	2802320	s	rec	G2583_2575	fcl	CDS
O157	2802374	s	rec	G2583_2575	fcl	CDS
O157	2802398	ns	rec	G2583_2575	fcl	CDS
O157	2802400	ns	rec	G2583_2575	fcl	CDS
O157	2802425	s	rec	G2583_2575	fcl	CDS
O157	2802555	ns	rec	G2583_2575	fcl	CDS
O157	2802563	i	rec	intergenic		

lineage[a]	site[b]	mutation type[c]	recomb	gene	Gene name	Type
O157	2802742	s	rec	G2583_2576	gmd	CDS
O157	2802763	s	rec	G2583_2576	gmd	CDS
O157	2802784	s	rec	G2583_2576	gmd	CDS
O157	2802796	s	rec	G2583_2576	gmd	CDS
O157	2802832	s	rec	G2583_2576	gmd	CDS
O157	2802847	s	rec	G2583_2576	gmd	CDS
O157	2802910	s	rec	G2583_2576	gmd	CDS
O157	2802931	s	rec	G2583_2576	gmd	CDS
O157	2802934	s	rec	G2583_2576	gmd	CDS
O157	2802949	s	rec	G2583_2576	gmd	CDS
O157	2802952	s	rec	G2583_2576	gmd	CDS
O157	2803171	s	rec	G2583_2576	gmd	CDS
O157	2803180	s	rec	G2583_2576	gmd	CDS
O157	2803228	s	rec	G2583_2576	gmd	CDS
O157	2803246	s	rec	G2583_2576	gmd	CDS
O157	2803369	s	rec	G2583_2576	gmd	CDS
O157	2803378	s	rec	G2583_2576	gmd	CDS
O157	2803468	s	rec	G2583_2576	gmd	CDS
O157	2803582	s	rec	G2583_2576	gmd	CDS
O157	2803703	ins-2	rec	intergenic		
O157	2803735	ns	rec	G2583_2577	wcaF	CDS
O157	2803744	ns	rec	G2583_2577	wcaF	CDS
O157	2803853	s	rec	G2583_2577	wcaF	CDS
O157	2803937	s	rec	G2583_2577	wcaF	CDS
O157	2804015	ns	rec	G2583_2577	wcaF	CDS
O157	2804031	ns	rec	G2583_2577	wcaF	CDS
O157	2804132	s	rec	G2583_2577	wcaF	CDS
O157	2804173	ns	rec	G2583_2577	wcaF	CDS
O157	2804249	s	rec	G2583_2577	wcaF	CDS
O157	2804321	s	rec	G2583_2578	wcaE	CDS
O157	2804345	s	rec	G2583_2578	wcaE	CDS
O157	2804351	s	rec	G2583_2578	wcaE	CDS
O157	2804372	s	rec	G2583_2578	wcaE	CDS
O157	2804390	s	rec	G2583_2578	wcaE	CDS
O157	2804402	s	rec	G2583_2578	wcaE	CDS
O157	2804438	s	rec	G2583_2578	wcaE	CDS
O157	2804474	s	rec	G2583_2578	wcaE	CDS
O157	2804483	s	rec	G2583_2578	wcaE	CDS
O157	2804501	s	rec	G2583_2578	wcaE	CDS
O157	2804513	s	rec	G2583_2578	wcaE	CDS
O157	2804516	s	rec	G2583_2578	wcaE	CDS
O157	2804558	s	rec	G2583_2578	wcaE	CDS
O157	2804618	s	rec	G2583_2578	wcaE	CDS
O157	2804725	ns	rec	G2583_2578	wcaE	CDS
O157	2804861	ns	rec	G2583_2578	wcaE	CDS

lineage[a]	site[b]	mutation type[c]	recomb	gene	Gene name	Type
O157	2804885	s	rec	G2583_2578	wcaE	CDS
O157	2804888	s	rec	G2583_2578	wcaE	CDS
O157	2804894	s	rec	G2583_2578	wcaE	CDS
O157	2804936	ns	rec	G2583_2578	wcaE	CDS
O157	2804966	s	rec	G2583_2578	wcaE	CDS
O157	2804985	ns	rec	G2583_2578	wcaE	CDS
O157	2805106	ns	rec	G2583_2579	wcaD	CDS
O157	2805237	s	rec	G2583_2579	wcaD	CDS
O157	2805246	s	rec	G2583_2579	wcaD	CDS
O157	2805420	s	rec	G2583_2579	wcaD	CDS
O157	2805501	s	rec	G2583_2579	wcaD	CDS
O157	2805651	s	rec	G2583_2579	wcaD	CDS
O157	2805714	s	rec	G2583_2579	wcaD	CDS
O157	2805765	s	rec	G2583_2579	wcaD	CDS
O157	2805966	s	rec	G2583_2579	wcaD	CDS
O157	2805981	s	rec	G2583_2579	wcaD	CDS
O157	2805999	s	rec	G2583_2579	wcaD	CDS
O157	2806028	s	rec	G2583_2579	wcaD	CDS
O157	2806070	ns	rec	G2583_2579	wcaD	CDS
O157	2806181	ns	rec	G2583_2579	wcaD	CDS
O157	2806199	s	rec	G2583_2579	wcaD	CDS
O157	2806206	s	rec	G2583_2579	wcaD	CDS
O157	2806288	s	rec	G2583_2580	wcaC	CDS
O157	2806329	ns	rec	G2583_2580	wcaC	CDS
O157	2806360	s	rec	G2583_2580	wcaC	CDS
O157	2806375	ns	rec	G2583_2580	wcaC	CDS
O157	2806413	s	rec	G2583_2580	wcaC	CDS
O157	2806420	s	rec	G2583_2580	wcaC	CDS
O157	2806462	s	rec	G2583_2580	wcaC	CDS
O157	2806467	s	rec	G2583_2580	wcaC	CDS
O157	2806513	s	rec	G2583_2580	wcaC	CDS
O157	2806630	s	rec	G2583_2580	wcaC	CDS
O157	2806657	s	rec	G2583_2580	wcaC	CDS
O157	2806660	s	rec	G2583_2580	wcaC	CDS
O157	2806678	s	rec	G2583_2580	wcaC	CDS
O157	2806705	s	rec	G2583_2580	wcaC	CDS
O157	2806753	s	rec	G2583_2580	wcaC	CDS
O157	2806768	s	rec	G2583_2580	wcaC	CDS
O157	2806786	s	rec	G2583_2580	wcaC	CDS
O157	2806789	s	rec	G2583_2580	wcaC	CDS
O157	2806814	ns	rec	G2583_2580	wcaC	CDS
O157	2806825	s	rec	G2583_2580	wcaC	CDS
O157	2806837	s	rec	G2583_2580	wcaC	CDS
O157	2806927	s	rec	G2583_2580	wcaC	CDS
O157	2806930	s	rec	G2583_2580	wcaC	CDS

lineage[a]	site[b]	mutation type[c]	recomb	gene	Gene name	Type
O157	2806954	s	rec	G2583_2580	wcaC	CDS
O157	2806985	ns	rec	G2583_2580	wcaC	CDS
O157	2806999	s	rec	G2583_2580	wcaC	CDS
O157	2807023	s	rec	G2583_2580	wcaC	CDS
O157	2807041	s	rec	G2583_2580	wcaC	CDS
O157	2807047	s	rec	G2583_2580	wcaC	CDS
O157	2807049	s	rec	G2583_2580	wcaC	CDS
O157	2807061	s	rec	G2583_2580	wcaC	CDS
O157	2807067	ns	rec	G2583_2580	wcaC	CDS
O157	2807089	s	rec	G2583_2580	wcaC	CDS
O157	2807122	s	rec	G2583_2580	wcaC	CDS
O157	2807124	s	rec	G2583_2580	wcaC	CDS
O157	2807164	s	rec	G2583_2580	wcaC	CDS
O157	2807173	s	rec	G2583_2580	wcaC	CDS
O157	2807401	s	rec	G2583_2580	wcaC	CDS
O157	2807591	ns	rec	G2583_2581	wcaB	CDS
O157	2807643	s	rec	G2583_2581	wcaB	CDS
O157	2807661	s	rec	G2583_2581	wcaB	CDS
O157	2807694	s	rec	G2583_2581	wcaB	CDS
O157	2807814	s	rec	G2583_2581	wcaB	CDS
O157	2807826	s	rec	G2583_2581	wcaB	CDS
O157	2807862	s	rec	G2583_2581	wcaB	CDS
O157	2807865	s	rec	G2583_2581	wcaB	CDS
O157	2807877	s	rec	G2583_2581	wcaB	CDS
O157	2807916	s	rec	G2583_2581	wcaB	CDS
O157	2807931	i	rec	intergenic		
O157	2808011	s	rec	G2583_2582	wcaA	CDS
O157	2808056	s	rec	G2583_2582	wcaA	CDS
O157	2808062	s	rec	G2583_2582	wcaA	CDS
O157	2808068	s	rec	G2583_2582	wcaA	CDS
O157	2808197	s	rec	G2583_2582	wcaA	CDS
O157	2808389	s	rec	G2583_2582	wcaA	CDS
O157	2808404	s	rec	G2583_2582	wcaA	CDS
O157	2808467	s	rec	G2583_2582	wcaA	CDS
O157	2808476	s	rec	G2583_2582	wcaA	CDS
O157	2808491	s	rec	G2583_2582	wcaA	CDS
O157	2808533	s	rec	G2583_2582	wcaA	CDS
O157	2808584	s	rec	G2583_2582	wcaA	CDS
O157	2808587	s	rec	G2583_2582	wcaA	CDS
O157	2808780	i	rec	intergenic		
O157	2808861	i	rec	intergenic		
O157	2808970	s	rec	G2583_2583	wzc	CDS
O157	2809120	s	rec	G2583_2583	wzc	CDS
O157	2809139	ns	rec	G2583_2583	wzc	CDS
O157	2809168	s	rec	G2583_2583	wzc	CDS

lineage[a]	site[b]	mutation type[c]	recomb	gene	Gene name	Type
O157	2809192	s	rec	G2583_2583	wzc	CDS
O157	2809246	s	rec	G2583_2583	wzc	CDS
O157	2809657	s	rec	G2583_2583	wzc	CDS
O157	2809672	s	rec	G2583_2583	wzc	CDS
O157	2809702	s	rec	G2583_2583	wzc	CDS
O157	2809723	s	rec	G2583_2583	wzc	CDS
O157	2809828	s	rec	G2583_2583	wzc	CDS
O157	2809915	s	rec	G2583_2583	wzc	CDS
O157	2809924	s	rec	G2583_2583	wzc	CDS
O157	2809951	s	rec	G2583_2583	wzc	CDS
O157	2810011	s	rec	G2583_2583	wzc	CDS
O157	2810224	s	rec	G2583_2583	wzc	CDS
O157	2810491	ns	rec	G2583_2583	wzc	CDS
O157	2810530	s	rec	G2583_2583	wzc	CDS
O157	2810551	ns	rec	G2583_2583	wzc	CDS
O157	2810553	ns	rec	G2583_2583	wzc	CDS
O157	2810585	ns	rec	G2583_2583	wzc	CDS
O157	2810599	s	rec	G2583_2583	wzc	CDS
O157	2810611	s	rec	G2583_2583	wzc	CDS
O157	2810626	s	rec	G2583_2583	wzc	CDS
O157	2810635	s	rec	G2583_2583	wzc	CDS
O157	2810713	s	rec	G2583_2583	wzc	CDS
O157	2810899	s	rec	G2583_2583	wzc	CDS
O157	2810905	s	rec	G2583_2583	wzc	CDS
O157	2810961	s	rec	G2583_2583	wzc	CDS
O157	2811387	s	rec	G2583_2584	wzb	CDS
O157	2811644	s	rec	G2583_2585	wza	CDS
O157	2811680	s	rec	G2583_2585	wza	CDS
O157	2811709	s	rec	G2583_2585	wza	CDS
O157	2811728	s	rec	G2583_2585	wza	CDS
O157	2811758	s	rec	G2583_2585	wza	CDS
O157	2811764	s	rec	G2583_2585	wza	CDS
O157	2811776	s	rec	G2583_2585	wza	CDS
O157	2811782	s	rec	G2583_2585	wza	CDS
O157	2811902	s	rec	G2583_2585	wza	CDS
O157	2811905	s	rec	G2583_2585	wza	CDS
O157	2811911	s	rec	G2583_2585	wza	CDS
O157	2811934	s	rec	G2583_2585	wza	CDS
O157	2811950	s	rec	G2583_2585	wza	CDS
O157	2812031	s	rec	G2583_2585	wza	CDS
O157	2812058	s	rec	G2583_2585	wza	CDS
O157	2812073	s	rec	G2583_2585	wza	CDS
O157	2812088	s	rec	G2583_2585	wza	CDS
O157	2812091	s	rec	G2583_2585	wza	CDS
O157	2812103	s	rec	G2583_2585	wza	CDS

lineage[a]	site[b]	mutation type[c]	recomb	gene	Gene name	Type
O157	2812109	s	rec	G2583_2585	wza	CDS
O157	2812208	s	rec	G2583_2585	wza	CDS
O157	2812211	s	rec	G2583_2585	wza	CDS
O157	2812238	s	rec	G2583_2585	wza	CDS
O157	2812244	s	rec	G2583_2585	wza	CDS
O157	2812340	s	rec	G2583_2585	wza	CDS
O157	2812349	s	rec	G2583_2585	wza	CDS
O157	2812385	s	rec	G2583_2585	wza	CDS
O157	2812852	i	rec	intergenic		
O157	2812863	i	rec	intergenic		
O157	2812864	i	rec	intergenic		
O157	2812871	i	rec	intergenic		
O157	2813118	i	rec	intergenic		
EDL933	2883374	ins		intergenic		
O157	2813954	s	rec	G2583_2586	yegH	CDS
O157	2814008	s	rec	G2583_2586	yegH	CDS
O157	2814011	s	rec	G2583_2586	yegH	CDS
O157	2814062	s	rec	G2583_2586	yegH	CDS
O157	2814242	s	rec	G2583_2586	yegH	CDS
O157	2814245	s	rec	G2583_2586	yegH	CDS
O157	2814293	s	rec	G2583_2586	yegH	CDS
O157	2814296	s	rec	G2583_2586	yegH	CDS
O157	2814299	s	rec	G2583_2586	yegH	CDS
O157	2814308	s	rec	G2583_2586	yegH	CDS
O157	2814345	s	rec	G2583_2586	yegH	CDS
O157	2814353	s	rec	G2583_2586	yegH	CDS
O157	2814356	s	rec	G2583_2586	yegH	CDS
O157	2814389	s	rec	G2583_2586	yegH	CDS
O157	2814464	s	rec	G2583_2586	yegH	CDS
O157	2814707	s	rec	G2583_2586	yegH	CDS
O157	2814716	s	rec	G2583_2586	yegH	CDS
O157	2815436	s	rec	G2583_2588	-	CDS
O157	2815457	s	rec	G2583_2588	-	CDS
O157	2815509	s	rec	G2583_2588	-	CDS
O157	2815511	s	rec	G2583_2588	-	CDS
O157	2815530	ns	rec	G2583_2588	-	CDS
O157	2815583	i	rec	intergenic		
O157	2815594	i	rec	intergenic		
O157	2815595	i	rec	intergenic		
O157	2815798	ns	rec	G2583_2589	asmA	CDS
O157	2815810	ns	rec	G2583_2589	asmA	CDS
O157	2815811	ns	rec	G2583_2589	asmA	CDS
O157	2815816	s	rec	G2583_2589	asmA	CDS
O157	2815828	s	rec	G2583_2589	asmA	CDS
O157	2815831	ns	rec	G2583_2589	asmA	CDS

lineage[a]	site[b]	mutation type[c]	recomb	gene	Gene name	Type
O157	2815833	ns	rec	G2583_2589	asmA	CDS
O157	2815834	s	rec	G2583_2589	asmA	CDS
O157	2815837	s	rec	G2583_2589	asmA	CDS
O157	2815867	ns	rec	G2583_2589	asmA	CDS
O157	2815870	s	rec	G2583_2589	asmA	CDS
O157	2815883	ns	rec	G2583_2589	asmA	CDS
O157	2815885	s	rec	G2583_2589	asmA	CDS
O157	2815888	ns	rec	G2583_2589	asmA	CDS
O157	2815889	ns	rec	G2583_2589	asmA	CDS
O157	2815894	ns	rec	G2583_2589	asmA	CDS
O157	2815896	ns	rec	G2583_2589	asmA	CDS
O157	2815908	ns	rec	G2583_2589	asmA	CDS
O157	2815909	s	rec	G2583_2589	asmA	CDS
O157	2816086	s	rec	G2583_2589	asmA	CDS
O157	2816091	ns	rec	G2583_2589	asmA	CDS
O157	2816172	s	rec	G2583_2589	asmA	CDS
O157	2816224	s	rec	G2583_2589	asmA	CDS
O157	2816332	s	rec	G2583_2589	asmA	CDS
O157	2816482	s	rec	G2583_2589	asmA	CDS
O157	2816491	s	rec	G2583_2589	asmA	CDS
O157	2816530	s	rec	G2583_2589	asmA	CDS
O157	2816554	s	rec	G2583_2589	asmA	CDS
O157	2816605	s	rec	G2583_2589	asmA	CDS
O157	2816708	ns	rec	G2583_2589	asmA	CDS
O157	2816770	s	rec	G2583_2589	asmA	CDS
O157	2816896	s	rec	G2583_2589	asmA	CDS
O157	2817529	s	rec	G2583_2590	dcd	CDS
O157	2817787	s	rec	G2583_2590	dcd	CDS
O157	2817831	ns	rec	G2583_2590	dcd	CDS
O157	2817832	s	rec	G2583_2590	dcd	CDS
O157	2817835	s	rec	G2583_2590	dcd	CDS
O157	2817958	s	rec	G2583_2590	dcd	CDS
O157	2818152	i	rec	intergenic		
EDL933	2889112	ins		intergenic		
EDL933	2889119	ins		intergenic		
O157	2818966	i	rec	intergenic		
O157	2818967	i	rec	intergenic		
O157	2818978	i	rec	intergenic		
O157	2818989	i	rec	intergenic		
O157	2819199	s	rec	G2583_2592	yegE	CDS
O157	2819310	s	rec	G2583_2592	yegE	CDS
O157	2819313	s	rec	G2583_2592	yegE	CDS
O157	2819328	s	rec	G2583_2592	yegE	CDS
O157	2819391	ns	rec	G2583_2592	yegE	CDS
O157	2819570	ins	rec	G2583_2592	yegE	CDS

lineage[a]	site[b]	mutation type[c]	recomb	gene	Gene name	Type
O157	2819752	s	rec	G2583_2592	yegE	CDS
O157	2819836	s	rec	G2583_2592	yegE	CDS
O157	2820022	s	rec	G2583_2592	yegE	CDS
O157	2820034	s	rec	G2583_2592	yegE	CDS
Sakai	2820047	ins		G2583_2592	yegE	CDS
O157	2820050	s	rec	G2583_2592	yegE	CDS
O157	2820062	s	rec	G2583_2592	yegE	CDS
O157	2820155	s	rec	G2583_2592	yegE	CDS
O157	2820299	s	rec	G2583_2592	yegE	CDS
O157	2820383	s	rec	G2583_2592	yegE	CDS
O157	2820401	s	rec	G2583_2592	yegE	CDS
O157	2820410	s	rec	G2583_2592	yegE	CDS
O157	2820452	s	rec	G2583_2592	yegE	CDS
O157	2820453	s	rec	G2583_2592	yegE	CDS
O157	2820512	s	rec	G2583_2592	yegE	CDS
O157	2820569	s	rec	G2583_2592	yegE	CDS
O157	2820572	s	rec	G2583_2592	yegE	CDS
O157	2820590	s	rec	G2583_2592	yegE	CDS
O157	2820596	s	rec	G2583_2592	yegE	CDS
O157	2820605	s	rec	G2583_2592	yegE	CDS
O157	2820620	s	rec	G2583_2592	yegE	CDS
O157	2820710	s	rec	G2583_2592	yegE	CDS
O157	2820713	s	rec	G2583_2592	yegE	CDS
O157	2820722	s	rec	G2583_2592	yegE	CDS
O157	2820737	s	rec	G2583_2592	yegE	CDS
O157	2820740	s	rec	G2583_2592	yegE	CDS
O157	2820743	s	rec	G2583_2592	yegE	CDS
O157	2821037	s	rec	G2583_2592	yegE	CDS
O157	2821091	s	rec	G2583_2592	yegE	CDS
O157	2821094	s	rec	G2583_2592	yegE	CDS
O157	2821100	s	rec	G2583_2592	yegE	CDS
O157	2821190	s	rec	G2583_2592	yegE	CDS
O157	2821208	s	rec	G2583_2592	yegE	CDS
O157	2821905	ns	rec	G2583_2592	yegE	CDS
O157	2822063	s	rec	G2583_2592	yegE	CDS
O157	2822075	ns	rec	G2583_2592	yegE	CDS
O157	2822110	ns	rec	G2583_2592	yegE	CDS
O157	2822117	s	rec	G2583_2592	yegE	CDS
O157	2822123	s	rec	G2583_2592	yegE	CDS
O157	2822126	s	rec	G2583_2592	yegE	CDS
O157	2822127	ns	rec	G2583_2592	yegE	CDS
O157	2822132	s	rec	G2583_2592	yegE	CDS
O157	2822352	ns	rec	G2583_2592	yegE	CDS
O157	2822369	ns	rec	G2583_2592	yegE	CDS
O157	2822403	ns	rec	G2583_2592	yegE	CDS

lineage[a]	site[b]	mutation type[c]	recomb	gene	Gene name	Type
O157	2822518	i	rec	intergenic		
O157	2822548	s	rec	G2583_2593	alkA	CDS
O157	2822707	s	rec	G2583_2593	alkA	CDS
O157	2822713	s	rec	G2583_2593	alkA	CDS
O157	2822770	s	rec	G2583_2593	alkA	CDS
O157	2822773	s	rec	G2583_2593	alkA	CDS
O157	2822776	s	rec	G2583_2593	alkA	CDS
O157	2822779	s	rec	G2583_2593	alkA	CDS
O157	2822863	s	rec	G2583_2593	alkA	CDS
O157	2822890	s	rec	G2583_2593	alkA	CDS
O157	2822962	s	rec	G2583_2593	alkA	CDS
O157	2823034	s	rec	G2583_2593	alkA	CDS
O157	2823054	ns	rec	G2583_2593	alkA	CDS
O157	2823092	ns	rec	G2583_2593	alkA	CDS
O157	2823138	ns	rec	G2583_2593	alkA	CDS
O157	2823166	s	rec	G2583_2593	alkA	CDS
O157	2823175	s	rec	G2583_2593	alkA	CDS
O157	2823185	ns	rec	G2583_2593	alkA	CDS
O157	2823284	ns	rec	G2583_2593	alkA	CDS
O157	2823285	ns	rec	G2583_2593	alkA	CDS
O157	2823289	s	rec	G2583_2593	alkA	CDS
O157	2823307	s	rec	G2583_2593	alkA	CDS
O157	2823554	ns	rec	G2583_2594	yegD	CDS
O157	2823556	ns	rec	G2583_2594	yegD	CDS
O157	2823709	s	rec	G2583_2594	yegD	CDS
O157	2823736	s	rec	G2583_2594	yegD	CDS
O157	2823814	s	rec	G2583_2594	yegD	CDS
O157	2823940	s	rec	G2583_2594	yegD	CDS
O157	2823943	s	rec	G2583_2594	yegD	CDS
O157	2823956	ns	rec	G2583_2594	yegD	CDS
O157	2823976	s	rec	G2583_2594	yegD	CDS
O157	2824003	s	rec	G2583_2594	yegD	CDS
O157	2824022	ns	rec	G2583_2594	yegD	CDS
O157	2824033	s	rec	G2583_2594	yegD	CDS
O157	2824066	s	rec	G2583_2594	yegD	CDS
O157	2824069	s	rec	G2583_2594	yegD	CDS
O157	2824071	ns	rec	G2583_2594	yegD	CDS
O157	2824075	s	rec	G2583_2594	yegD	CDS
O157	2824105	s	rec	G2583_2594	yegD	CDS
O157	2824123	s	rec	G2583_2594	yegD	CDS
O157	2824126	s	rec	G2583_2594	yegD	CDS
O157	2824129	s	rec	G2583_2594	yegD	CDS
O157	2824147	s	rec	G2583_2594	yegD	CDS
O157	2824243	s	rec	G2583_2594	yegD	CDS
O157	2824288	s	rec	G2583_2594	yegD	CDS

lineage[a]	site[b]	mutation type[c]	recomb	gene	Gene name	Type
O157	2824312	s	rec	G2583_2594	yegD	CDS
O157	2824313	s	rec	G2583_2594	yegD	CDS
O157	2824315	s	rec	G2583_2594	yegD	CDS
O157	2824321	s	rec	G2583_2594	yegD	CDS
O157	2824323	ns	rec	G2583_2594	yegD	CDS
O157	2824487	s	rec	G2583_2594	yegD	CDS
O157	2824531	s	rec	G2583_2594	yegD	CDS
O157	2824555	s	rec	G2583_2594	yegD	CDS
O157	2824671	ns	rec	G2583_2594	yegD	CDS
O157	2824690	s	rec	G2583_2594	yegD	CDS
O157	2824693	s	rec	G2583_2594	yegD	CDS
O157	2824738	s	rec	G2583_2594	yegD	CDS
O157	2825014	ns	rec	G2583_2595	yegI	CDS
O157	2825048	ns	rec	G2583_2595	yegI	CDS
O157	2825050	ns	rec	G2583_2595	yegI	CDS
O157	2825066	ns	rec	G2583_2595	yegI	CDS
O157	2825153	s	rec	G2583_2595	yegI	CDS
O157	2825156	s	rec	G2583_2595	yegI	CDS
O157	2825168	s	rec	G2583_2595	yegI	CDS
O157	2825180	s	rec	G2583_2595	yegI	CDS
O157	2825198	s	rec	G2583_2595	yegI	CDS
O157	2825201	s	rec	G2583_2595	yegI	CDS
O157	2825246	s	rec	G2583_2595	yegI	CDS
O157	2825324	s	rec	G2583_2595	yegI	CDS
O157	2825342	s	rec	G2583_2595	yegI	CDS
O157	2825348	s	rec	G2583_2595	yegI	CDS
O157	2825351	s	rec	G2583_2595	yegI	CDS
O157	2825398	ns	rec	G2583_2595	yegI	CDS
O157	2825517	ns	rec	G2583_2595	yegI	CDS
O157	2825608	s	rec	G2583_2595	yegI	CDS
O157	2825617	ns	rec	G2583_2595	yegI	CDS
O157	2825638	ns	rec	G2583_2595	yegI	CDS
O157	2825722	ns	rec	G2583_2595	yegI	CDS
O157	2825783	ns	rec	G2583_2595	yegI	CDS
O157	2825785	ns	rec	G2583_2595	yegI	CDS
O157	2825845	ns	rec	G2583_2595	yegI	CDS
O157	2825861	s	rec	G2583_2595	yegI	CDS
O157	2825871	ns	rec	G2583_2595	yegI	CDS
O157	2825876	s	rec	G2583_2595	yegI	CDS
O157	2825924	ns	rec	G2583_2595	yegI	CDS
O157	2825925	ns	rec	G2583_2595	yegI	CDS
O157	2825936	s	rec	G2583_2595	yegI	CDS
O157	2825951	s	rec	G2583_2595	yegI	CDS
O157	2825964	ns	rec	G2583_2595	yegI	CDS
O157	2825972	s	rec	G2583_2595	yegI	CDS

lineage[a]	site[b]	mutation type[c]	recomb	gene	Gene name	Type
O157	2825987	s	rec	G2583_2595	yegI	CDS
O157	2825990	s	rec	G2583_2595	yegI	CDS
O157	2826251	s	rec	G2583_2595	yegI	CDS
O157	2826401	s	rec	G2583_2595	yegI	CDS
O157	2826452	s	rec	G2583_2595	yegI	CDS
O157	2826491	s	rec	G2583_2595	yegI	CDS
O157	2826516	ns	rec	G2583_2595	yegI	CDS
O157	2826628	s	rec	G2583_2595	yegI	CDS
O157	2826635	s	rec	G2583_2595	yegI	CDS
O157	2826720	ns	rec	G2583_2595	yegI	CDS
O157	2826728	ns	rec	G2583_2595	yegI	CDS
O157	2826730	ns	rec	G2583_2595	yegI	CDS
O157	2826746	s	rec	G2583_2595	yegI	CDS
O157	2826752	s	rec	G2583_2595	yegI	CDS
O157	2826755	s	rec	G2583_2595	yegI	CDS
O157	2826840	ns	rec	G2583_2596	yegK	CDS
O157	2826877	s	rec	G2583_2596	yegK	CDS
O157	2826883	s	rec	G2583_2596	yegK	CDS
O157	2826922	s	rec	G2583_2596	yegK	CDS
O157	2826946	s	rec	G2583_2596	yegK	CDS
O157	2826958	s	rec	G2583_2596	yegK	CDS
O157	2826993	s	rec	G2583_2596	yegK	CDS
O157	2827015	s	rec	G2583_2596	yegK	CDS
O157	2827046	ns	rec	G2583_2596	yegK	CDS
O157	2827085	ns	rec	G2583_2596	yegK	CDS
O157	2827273	s	rec	G2583_2596	yegK	CDS
O157	2827330	s	rec	G2583_2596	yegK	CDS
O157	2827446	ns	rec	G2583_2596	yegK	CDS
O157	2827447	ns	rec	G2583_2596	yegK	CDS
O157	2827449	ns	rec	G2583_2596	yegK	CDS
O157	2827450	s	rec	G2583_2596	yegK	CDS
O157	2827453	ns	rec	G2583_2596	yegK	CDS
O157	2827455	ns	rec	G2583_2596	yegK	CDS
O157	2827457	ns	rec	G2583_2596	yegK	CDS
O157	2827458	ns	rec	G2583_2596	yegK	CDS
O157	2827461	s	rec	G2583_2596	yegK	CDS
O157	2827608	s	rec	G2583_2597	yegL	CDS
O157	2827650	s	rec	G2583_2597	yegL	CDS
O157	2827677	s	rec	G2583_2597	yegL	CDS
O157	2827698	ns	rec	G2583_2597	yegL	CDS
O157	2827700	ns	rec	G2583_2597	yegL	CDS
O157	2827713	s	rec	G2583_2597	yegL	CDS
O157	2827716	s	rec	G2583_2597	yegL	CDS
O157	2827725	s	rec	G2583_2597	yegL	CDS
O157	2827731	s	rec	G2583_2597	yegL	CDS

lineage[a]	site[b]	mutation type[c]	recomb	gene	Gene name	Type
O157	2827737	ns	rec	G2583_2597	yegL	CDS
O157	2827959	s	rec	G2583_2597	yegL	CDS
EDL933	2898460	ins		intergenic		
O157	2828393	nc	rec	G2583_2598	-	pseudogene
O157	2828395	nc	rec	G2583_2598	-	pseudogene
O157	2828429	nc	rec	G2583_2598	-	pseudogene
O157	2828515	nc	rec	G2583_2598	-	pseudogene
O157	2828523	nc	rec	G2583_2598	-	pseudogene
O157	2828568	i	rec	intergenic		
O157	2828603	i	rec	intergenic		
O157	2828606	i	rec	intergenic		
O157	2828609	i	rec	intergenic		
O157	2828622	i	rec	intergenic		
O157	2828625	i	rec	intergenic		
O157	2828626	i	rec	intergenic		
O157	2828628	i	rec	intergenic		
O157	2828630	i	rec	intergenic		
O157	2828632	i	rec	intergenic		
O157	2828645	i	rec	intergenic		
O157	2828646	i	rec	intergenic		
O157	2828712	i	rec	intergenic		
O157	2828721	nc	rec	G2583_2599	-	pseudogene
O157	2828725	nc	rec	G2583_2599	-	pseudogene
O157	2828727	nc	rec	G2583_2599	-	pseudogene
O157	2828792	nc	rec	G2583_2599	-	pseudogene
O157	2828795	nc	rec	G2583_2599	-	pseudogene
O157	2828799	nc	rec	G2583_2599	-	pseudogene
O157	2828804	nc	rec	G2583_2599	-	pseudogene
O157	2828807	nc	rec	G2583_2599	-	pseudogene
O157	2828830	nc	rec	G2583_2599	-	pseudogene
O157	2828831	nc	rec	G2583_2599	-	pseudogene
O157	2828832	nc	rec	G2583_2599	-	pseudogene
O157	2828833	nc	rec	G2583_2599	-	pseudogene
O157	2828860	i	rec	intergenic		
O157	2828931	i	rec	intergenic		
O157	2828934	i	rec	intergenic		
O157	2828936	i	rec	intergenic		
O157	2828937	i	rec	intergenic		
O157	2828950	i	rec	intergenic		
O157	2828953	i	rec	intergenic		
O157	2828954	i	rec	intergenic		
O157	2828956	i	rec	intergenic		
O157	2828957	i	rec	intergenic		
O157	2828974	i	rec	intergenic		
O157	2828978	i	rec	intergenic		

lineage[a]	site[b]	mutation type[c]	recomb	gene	Gene name	Type
O157	2828983	i	rec	intergenic		
EDL933	2899359	ins		intergenic		
EDL933	2899514	i		intergenic		
O157	2829494	s	rec	G2583_2600	mdtA	CDS
O157	2829633	ns	rec	G2583_2600	mdtA	CDS
O157	2829722	s	rec	G2583_2600	mdtA	CDS
O157	2829782	s	rec	G2583_2600	mdtA	CDS
O157	2830424	s	rec	G2583_2600	mdtA	CDS
O157	2830427	s	rec	G2583_2600	mdtA	CDS
O157	2830702	s	rec	G2583_2601	mdtB	CDS
O157	2830724	ns	rec	G2583_2601	mdtB	CDS
O157	2830852	s	rec	G2583_2601	mdtB	CDS
O157	2830951	s	rec	G2583_2601	mdtB	CDS
O157	2831002	s	rec	G2583_2601	mdtB	CDS
O157	2831026	s	rec	G2583_2601	mdtB	CDS
O157	2831047	s	rec	G2583_2601	mdtB	CDS
O157	2831150	ns	rec	G2583_2601	mdtB	CDS
O157	2831164	s	rec	G2583_2601	mdtB	CDS
O157	2831371	s	rec	G2583_2601	mdtB	CDS
O157	2831383	s	rec	G2583_2601	mdtB	CDS
O157	2831398	s	rec	G2583_2601	mdtB	CDS
O157	2831416	s	rec	G2583_2601	mdtB	CDS
O157	2831419	s	rec	G2583_2601	mdtB	CDS
O157	2831425	s	rec	G2583_2601	mdtB	CDS
O157	2831434	s	rec	G2583_2601	mdtB	CDS
O157	2831437	s	rec	G2583_2601	mdtB	CDS
O157	2831443	s	rec	G2583_2601	mdtB	CDS
O157	2831453	s	rec	G2583_2601	mdtB	CDS
O157	2831455	s	rec	G2583_2601	mdtB	CDS
O157	2831458	s	rec	G2583_2601	mdtB	CDS
O157	2831506	s	rec	G2583_2601	mdtB	CDS
O157	2831551	s	rec	G2583_2601	mdtB	CDS
O157	2831654	s	rec	G2583_2601	mdtB	CDS
O157	2831719	s	rec	G2583_2601	mdtB	CDS
O157	2831761	s	rec	G2583_2601	mdtB	CDS
O157	2831815	s	rec	G2583_2601	mdtB	CDS
O157	2832806	ns	rec	G2583_2601	mdtB	CDS
O157	2832904	s	rec	G2583_2601	mdtB	CDS
O157	2832940	s	rec	G2583_2601	mdtB	CDS
O157	2832979	s	rec	G2583_2601	mdtB	CDS
O157	2832982	s	rec	G2583_2601	mdtB	CDS
O157	2832997	s	rec	G2583_2601	mdtB	CDS
O157	2833015	s	rec	G2583_2601	mdtB	CDS
O157	2833018	s	rec	G2583_2601	mdtB	CDS
O157	2833070	s	rec	G2583_2601	mdtB	CDS

lineage[a]	site[b]	mutation type[c]	recomb	gene	Gene name	Type
O157	2833088	ns	rec	G2583_2601	mdtB	CDS
O157	2833093	s	rec	G2583_2601	mdtB	CDS
O157	2833153	s	rec	G2583_2601	mdtB	CDS
O157	2833165	s	rec	G2583_2601	mdtB	CDS
O157	2833198	s	rec	G2583_2601	mdtB	CDS
O157	2833213	s	rec	G2583_2601	mdtB	CDS
O157	2833249	s	rec	G2583_2601	mdtB	CDS
O157	2833275	ns	rec	G2583_2601	mdtB	CDS
O157	2833396	s	rec	G2583_2601	mdtB	CDS
O157	2833672	s	rec	G2583_2601	mdtB	CDS
O157	2833876	s	rec	G2583_2602	mdtC	CDS
O157	2834245	s	rec	G2583_2602	mdtC	CDS
O157	2834279	s	rec	G2583_2602	mdtC	CDS
O157	2834332	s	rec	G2583_2602	mdtC	CDS
O157	2834435	ns	rec	G2583_2602	mdtC	CDS
O157	2834665	s	rec	G2583_2602	mdtC	CDS
O157	2834680	s	rec	G2583_2602	mdtC	CDS
O157	2834681	s	rec	G2583_2602	mdtC	CDS
O157	2834710	s	rec	G2583_2602	mdtC	CDS
O157	2834779	s	rec	G2583_2602	mdtC	CDS
O157	2834866	s	rec	G2583_2602	mdtC	CDS
O157	2834968	s	rec	G2583_2602	mdtC	CDS
O157	2835142	s	rec	G2583_2602	mdtC	CDS
O157	2835167	s	rec	G2583_2602	mdtC	CDS
O157	2835190	s	rec	G2583_2602	mdtC	CDS
O157	2835193	s	rec	G2583_2602	mdtC	CDS
O157	2835205	s	rec	G2583_2602	mdtC	CDS
O157	2835244	s	rec	G2583_2602	mdtC	CDS
O157	2835253	s	rec	G2583_2602	mdtC	CDS
O157	2835259	s	rec	G2583_2602	mdtC	CDS
O157	2835260	s	rec	G2583_2602	mdtC	CDS
O157	2835273	ns	rec	G2583_2602	mdtC	CDS
O157	2835307	s	rec	G2583_2602	mdtC	CDS
O157	2835322	s	rec	G2583_2602	mdtC	CDS
O157	2835346	s	rec	G2583_2602	mdtC	CDS
O157	2835364	s	rec	G2583_2602	mdtC	CDS
O157	2835367	s	rec	G2583_2602	mdtC	CDS
O157	2835373	s	rec	G2583_2602	mdtC	CDS
O157	2835410	ns	rec	G2583_2602	mdtC	CDS
O157	2835416	s	rec	G2583_2602	mdtC	CDS
O157	2835433	s	rec	G2583_2602	mdtC	CDS
O157	2835434	s	rec	G2583_2602	mdtC	CDS
O157	2835448	s	rec	G2583_2602	mdtC	CDS
O157	2835460	s	rec	G2583_2602	mdtC	CDS
O157	2835490	s	rec	G2583_2602	mdtC	CDS

lineage[a]	site[b]	mutation type[c]	recomb	gene	Gene name	Type
O157	2835550	s	rec	G2583_2602	mdtC	CDS
O157	2835553	s	rec	G2583_2602	mdtC	CDS
O157	2835592	s	rec	G2583_2602	mdtC	CDS
O157	2835610	s	rec	G2583_2602	mdtC	CDS
O157	2835616	s	rec	G2583_2602	mdtC	CDS
O157	2835628	s	rec	G2583_2602	mdtC	CDS
O157	2835634	s	rec	G2583_2602	mdtC	CDS
O157	2835685	s	rec	G2583_2602	mdtC	CDS
O157	2835700	s	rec	G2583_2602	mdtC	CDS
O157	2835709	s	rec	G2583_2602	mdtC	CDS
O157	2835712	s	rec	G2583_2602	mdtC	CDS
O157	2835799	s	rec	G2583_2602	mdtC	CDS
O157	2835802	s	rec	G2583_2602	mdtC	CDS
O157	2836105	s	rec	G2583_2602	mdtC	CDS
O157	2836123	s	rec	G2583_2602	mdtC	CDS
O157	2836126	s	rec	G2583_2602	mdtC	CDS
O157	2836135	ns	rec	G2583_2602	mdtC	CDS
O157	2836138	s	rec	G2583_2602	mdtC	CDS
O157	2836150	s	rec	G2583_2602	mdtC	CDS
O157	2836165	s	rec	G2583_2602	mdtC	CDS
O157	2836183	s	rec	G2583_2602	mdtC	CDS
O157	2836189	s	rec	G2583_2602	mdtC	CDS
O157	2836192	s	rec	G2583_2602	mdtC	CDS
O157	2836234	s	rec	G2583_2602	mdtC	CDS
O157	2836252	s	rec	G2583_2602	mdtC	CDS
O157	2836279	s	rec	G2583_2602	mdtC	CDS
O157	2836285	s	rec	G2583_2602	mdtC	CDS
O157	2836303	s	rec	G2583_2602	mdtC	CDS
O157	2836309	s	rec	G2583_2602	mdtC	CDS
O157	2836315	s	rec	G2583_2602	mdtC	CDS
O157	2836318	s	rec	G2583_2602	mdtC	CDS
O157	2836339	s	rec	G2583_2602	mdtC	CDS
O157	2836342	s	rec	G2583_2602	mdtC	CDS
O157	2836393	s	rec	G2583_2602	mdtC	CDS
O157	2836396	s	rec	G2583_2602	mdtC	CDS
O157	2836468	s	rec	G2583_2602	mdtC	CDS
O157	2836486	s	rec	G2583_2602	mdtC	CDS
O157	2836501	s	rec	G2583_2602	mdtC	CDS
O157	2836567	s	rec	G2583_2602	mdtC	CDS
O157	2836603	s	rec	G2583_2602	mdtC	CDS
O157	2836738	s	rec	G2583_2602	mdtC	CDS
O157	2836750	s	rec	G2583_2602	mdtC	CDS
O157	2836763	s	rec	G2583_2602	mdtC	CDS
O157	2836792	s	rec	G2583_2602	mdtC	CDS
O157	2836798	s	rec	G2583_2602	mdtC	CDS

lineage[a]	site[b]	mutation type[c]	recomb	gene	Gene name	Type
O157	2836831	s	rec	G2583_2602	mdtC	CDS
O157	2836837	s	rec	G2583_2602	mdtC	CDS
O157	2836852	s	rec	G2583_2602	mdtC	CDS
O157	2836874	ns	rec	G2583_2602	mdtC	CDS
O157	2836984	s	rec	G2583_2603	mdtD	CDS
O157	2837047	s	rec	G2583_2603	mdtD	CDS
O157	2837086	s	rec	G2583_2603	mdtD	CDS
O157	2837140	s	rec	G2583_2603	mdtD	CDS
O157	2837143	s	rec	G2583_2603	mdtD	CDS
O157	2837254	s	rec	G2583_2603	mdtD	CDS
O157	2837258	s	rec	G2583_2603	mdtD	CDS
O157	2837260	s	rec	G2583_2603	mdtD	CDS
O157	2837440	s	rec	G2583_2603	mdtD	CDS
O157	2837441	s	rec	G2583_2603	mdtD	CDS
O157	2837449	s	rec	G2583_2603	mdtD	CDS
O157	2837579	ns	rec	G2583_2603	mdtD	CDS
O157	2837676	ns	rec	G2583_2603	mdtD	CDS
O157	2837677	ns	rec	G2583_2603	mdtD	CDS
O157	2837680	s	rec	G2583_2603	mdtD	CDS
O157	2837833	s	rec	G2583_2603	mdtD	CDS
O157	2837842	s	rec	G2583_2603	mdtD	CDS
O157	2837848	s	rec	G2583_2603	mdtD	CDS
O157	2838052	s	rec	G2583_2603	mdtD	CDS
O157	2838173	ns	rec	G2583_2603	mdtD	CDS
O157	2838188	ns	rec	G2583_2603	mdtD	CDS
O157	2838324	s	rec	G2583_2604	baeS	CDS
O157	2838327	s	rec	G2583_2604	baeS	CDS
O157	2838507	s	rec	G2583_2604	baeS	CDS
O157	2838609	s	rec	G2583_2604	baeS	CDS
O157	2838624	s	rec	G2583_2604	baeS	CDS
O157	2838654	s	rec	G2583_2604	baeS	CDS
O157	2838846	s	rec	G2583_2604	baeS	CDS
O157	2838852	s	rec	G2583_2604	baeS	CDS
O157	2838855	s	rec	G2583_2604	baeS	CDS
O157	2838870	s	rec	G2583_2604	baeS	CDS
O157	2838874	s	rec	G2583_2604	baeS	CDS
O157	2838876	s	rec	G2583_2604	baeS	CDS
O157	2838877	s	rec	G2583_2604	baeS	CDS
O157	2838882	s	rec	G2583_2604	baeS	CDS
O157	2838888	s	rec	G2583_2604	baeS	CDS
O157	2838894	s	rec	G2583_2604	baeS	CDS
O157	2838897	s	rec	G2583_2604	baeS	CDS
O157	2838900	s	rec	G2583_2604	baeS	CDS
O157	2838903	s	rec	G2583_2604	baeS	CDS
O157	2838936	s	rec	G2583_2604	baeS	CDS

lineage[a]	site[b]	mutation type[c]	recomb	gene	Gene name	Type
O157	2838945	s	rec	G2583_2604	baeS	CDS
O157	2839008	s	rec	G2583_2604	baeS	CDS
O157	2839011	s	rec	G2583_2604	baeS	CDS
O157	2839194	s	rec	G2583_2604	baeS	CDS
O157	2839275	s	rec	G2583_2604	baeS	CDS
O157	2839278	s	rec	G2583_2604	baeS	CDS
O157	2839299	s	rec	G2583_2604	baeS	CDS
O157	2839344	s	rec	G2583_2604	baeS	CDS
O157	2839395	s	rec	G2583_2604	baeS	CDS
O157	2839452	s	rec	G2583_2604	baeS	CDS
O157	2839464	s	rec	G2583_2604	baeS	CDS
O157	2839524	s	rec	G2583_2604	baeS	CDS
O157	2839563	s	rec	G2583_2604	baeS	CDS
O157	2839588	s	rec	G2583_2604	baeS	CDS
O157	2839638	s	rec	G2583_2604	baeS	CDS
O157	2839851	ns	rec	G2583_2605	baeR	CDS
O157	2839856	s	rec	G2583_2605	baeR	CDS
O157	2839868	s	rec	G2583_2605	baeR	CDS
O157	2839871	s	rec	G2583_2605	baeR	CDS
O157	2839907	s	rec	G2583_2605	baeR	CDS
O157	2839917	s	rec	G2583_2605	baeR	CDS
O157	2839973	s	rec	G2583_2605	baeR	CDS
O157	2840003	s	rec	G2583_2605	baeR	CDS
O157	2840006	s	rec	G2583_2605	baeR	CDS
O157	2840027	s	rec	G2583_2605	baeR	CDS
O157	2840030	s	rec	G2583_2605	baeR	CDS
O157	2840033	s	rec	G2583_2605	baeR	CDS
O157	2840036	s	rec	G2583_2605	baeR	CDS
O157	2840069	s	rec	G2583_2605	baeR	CDS
O157	2840105	s	rec	G2583_2605	baeR	CDS
O157	2840135	s	rec	G2583_2605	baeR	CDS
O157	2840216	s	rec	G2583_2605	baeR	CDS
O157	2840306	s	rec	G2583_2605	baeR	CDS
O157	2840393	s	rec	G2583_2605	baeR	CDS
O157	2840467	i	rec	intergenic		
O157	2840660	ns	rec	G2583_2606	yegP	CDS
O157	2840700	s	rec	G2583_2606	yegP	CDS
O157	2840724	s	rec	G2583_2606	yegP	CDS
O157	2840874	s	rec	G2583_2606	yegP	CDS
O157	2840990	ins-12	rec	intergenic		
O157	2841005	i	rec	intergenic		
O157	2841008	i	rec	intergenic		
O157	2841010	i	rec	intergenic		
O157	2841011	i	rec	intergenic		
O157	2841243	s	rec	G2583_2609	yegQ	CDS

lineage[a]	site[b]	mutation type[c]	recomb	gene	Gene name	Type
O157	2841444	s	rec	G2583_2609	yegQ	CDS
O157	2841453	s	rec	G2583_2609	yegQ	CDS
O157	2841468	s	rec	G2583_2609	yegQ	CDS
O157	2841504	s	rec	G2583_2609	yegQ	CDS
O157	2841561	s	rec	G2583_2609	yegQ	CDS
O157	2841663	s	rec	G2583_2609	yegQ	CDS
O157	2841732	ns	rec	G2583_2609	yegQ	CDS
O157	2841834	s	rec	G2583_2609	yegQ	CDS
O157	2841936	s	rec	G2583_2609	yegQ	CDS
O157	2841972	s	rec	G2583_2609	yegQ	CDS
O157	2841978	s	rec	G2583_2609	yegQ	CDS
O157	2842017	s	rec	G2583_2609	yegQ	CDS
O157	2842020	s	rec	G2583_2609	yegQ	CDS
O157	2842056	s	rec	G2583_2609	yegQ	CDS
O157	2842059	s	rec	G2583_2609	yegQ	CDS
O157	2842077	s	rec	G2583_2609	yegQ	CDS
O157	2842440	s	rec	G2583_2609	yegQ	CDS
O157	2842452	s	rec	G2583_2609	yegQ	CDS
O157	2842551	nc	rec	G2583_2610	-	pseudogene
O157	2842656	i	rec	intergenic		
O157	2842658	ns	rec	G2583_2611	-	CDS
O157	2842681	s	rec	G2583_2611	-	CDS
O157	2842716	ns	rec	G2583_2611	-	CDS
O157	2842735	ns	rec	G2583_2611	-	CDS
O157	2842749	ns	rec	G2583_2611	-	CDS
O157	2842755	s	rec	G2583_2611	-	CDS
O157	2842770	ns	rec	G2583_2611	-	CDS
O157	2843019	s	rec	G2583_2612	yegR	CDS
O157	2843030	ns	rec	G2583_2612	yegR	CDS
O157	2843106	ns	rec	G2583_2612	yegR	CDS
O157	2843111	ns	rec	G2583_2612	yegR	CDS
O157	2843212	i	rec	intergenic		
O157	2843272	i	rec	intergenic		
O157	2843297	i	rec	intergenic		
O157	2843308	i	rec	intergenic		
O157	2843322	i	rec	intergenic		
O157	2843354	i	rec	intergenic		
O157	2843361	i	rec	intergenic		
O157	2843372	i	rec	intergenic		
O157	2843408	i	rec	intergenic		
O157	2843426	i	rec	intergenic		
O157	2843432	i	rec	intergenic		
O157	2843489	i	rec	intergenic		
O157	2843571	s	rec	G2583_2613	yegS	CDS
O157	2843576	s	rec	G2583_2613	yegS	CDS

lineage[a]	site[b]	mutation type[c]	recomb	gene	Gene name	Type
O157	2843766	ns	rec	G2583_2613	yegS	CDS
O157	2843852	s	rec	G2583_2613	yegS	CDS
O157	2843942	s	rec	G2583_2613	yegS	CDS
O157	2844405	s	rec	G2583_2613	yegS	CDS
O157	2844407	s	rec	G2583_2613	yegS	CDS
O157	2844543	s	rec	G2583_2622	gatR	CDS
O157	2844549	ns	rec	G2583_2622	gatR	CDS
O157	2844558	s	rec	G2583_2622	gatR	CDS
O157	2845035	s	rec	G2583_2622	gatR	CDS
O157	2845089	s	rec	G2583_2622	gatR	CDS
O157	2845122	s	rec	G2583_2622	gatR	CDS
O157	2845445	ns	rec	G2583_2623	gatD	CDS
O157	2845638	s	rec	G2583_2623	gatD	CDS
O157	2845647	s	rec	G2583_2623	gatD	CDS
O157	2845689	s	rec	G2583_2623	gatD	CDS
O157	2845740	s	rec	G2583_2623	gatD	CDS
O157	2845754	ns	rec	G2583_2623	gatD	CDS
O157	2845848	s	rec	G2583_2623	gatD	CDS
O157	2845986	s	rec	G2583_2623	gatD	CDS
O157	2846043	s	rec	G2583_2623	gatD	CDS
O157	2846132	s	rec	G2583_2623	gatD	CDS
O157	2846190	s	rec	G2583_2623	gatD	CDS
O157	2846428	i	rec	intergenic		
O157	2846481	ns	rec	G2583_2624	gatC	CDS
O157	2846537	s	rec	G2583_2624	gatC	CDS
O157	2846710	ns	rec	G2583_2624	gatC	CDS
O157	2846798	s	rec	G2583_2624	gatC	CDS
O157	2846834	s	rec	G2583_2624	gatC	CDS
O157	2846846	s	rec	G2583_2624	gatC	CDS
O157	2846849	s	rec	G2583_2624	gatC	CDS
O157	2846879	s	rec	G2583_2624	gatC	CDS
O157	2847044	s	rec	G2583_2624	gatC	CDS
O157	2847053	s	rec	G2583_2624	gatC	CDS
O157	2847056	s	rec	G2583_2624	gatC	CDS
O157	2847102	ns	rec	G2583_2624	gatC	CDS
O157	2847209	s	rec	G2583_2624	gatC	CDS
O157	2847230	s	rec	G2583_2624	gatC	CDS
O157	2847233	s	rec	G2583_2624	gatC	CDS
O157	2847293	s	rec	G2583_2624	gatC	CDS
O157	2847377	s	rec	G2583_2624	gatC	CDS
O157	2847655	s	rec	G2583_2624	gatC	CDS
O157	2847926	s	rec	G2583_2625	gatB	CDS
O157	2847929	s	rec	G2583_2625	gatB	CDS
O157	2847932	ns	rec	G2583_2625	gatB	CDS
O157	2847933	ns	rec	G2583_2625	gatB	CDS

lineage[a]	site[b]	mutation type[c]	recomb	gene	Gene name	Type
O157	2847938	s	rec	G2583_2625	gatB	CDS
O157	2847941	s	rec	G2583_2625	gatB	CDS
O157	2847944	s	rec	G2583_2625	gatB	CDS
O157	2847959	s	rec	G2583_2625	gatB	CDS
O157	2848294	ns	rec	G2583_2626	gatA	CDS
O157	2848359	ns	rec	G2583_2626	gatA	CDS
O157	2848883	s	rec	G2583_2627	gatZ	CDS
O157	2848892	s	rec	G2583_2627	gatZ	CDS
O157	2848997	s	rec	G2583_2627	gatZ	CDS
O157	2849117	s	rec	G2583_2627	gatZ	CDS
O157	2849228	s	rec	G2583_2627	gatZ	CDS
O157	2849568	ns	rec	G2583_2627	gatZ	CDS
O157	2849603	s	rec	G2583_2627	gatZ	CDS
O157	2849606	s	rec	G2583_2627	gatZ	CDS
O157	2849621	s	rec	G2583_2627	gatZ	CDS
O157	2849624	s	rec	G2583_2627	gatZ	CDS
O157	2849636	s	rec	G2583_2627	gatZ	CDS
O157	2849639	s	rec	G2583_2627	gatZ	CDS
O157	2849656	ns	rec	G2583_2627	gatZ	CDS
O157	2850513	s	rec	G2583_2628	gatY	CDS
O157	2850531	ns	rec	G2583_2628	gatY	CDS
O157	2850560	ns	rec	G2583_2628	gatY	CDS
O157	2850693	s	rec	G2583_2628	gatY	CDS
O157	2850708	s	rec	G2583_2628	gatY	CDS
O157	2850752	i	rec	intergenic		
O157	2850762	i	rec	intergenic		
O157	2850781	i	rec	intergenic		
O157	2850792	i	rec	intergenic		
O157	2850845	i	rec	intergenic		
O157	2850846	i	rec	intergenic		
O157	2850848	i	rec	intergenic		
O157	2850854	i	rec	intergenic		
O157	2850882	ins	rec	intergenic		
EDL933	2850892	i		intergenic		
O157	2850892	i	rec	intergenic		
Sakai	2850892	i		intergenic		
O157	2850895	i	rec	intergenic		
O157	2850896	i	rec	intergenic		
O157	2850908	i	rec	intergenic		
O157	2850916	i	rec	intergenic		
O157	2850917	i	rec	intergenic		
O157	2850927	i	rec	intergenic		
O157	2850936	i	rec	intergenic		
O157	2850949	i	rec	intergenic		
O157	2850968	i	rec	intergenic		

lineage[a]	site[b]	mutation type[c]	recomb	gene	Gene name	Type
O157	2851003	i	rec	intergenic		
O157	2851012	i	rec	intergenic		
O157	2851037	i	rec	intergenic		
O157	2851040	del-9	rec	intergenic		
O157	2851630	s	rec	G2583_2629	fbaB	CDS
O157	2851705	s	rec	G2583_2629	fbaB	CDS
O157	2851711	s	rec	G2583_2629	fbaB	CDS
O157	2851729	s	rec	G2583_2629	fbaB	CDS
O157	2851732	s	rec	G2583_2629	fbaB	CDS
O157	2851752	s	rec	G2583_2629	fbaB	CDS
O157	2851768	s	rec	G2583_2629	fbaB	CDS
O157	2852008	s	rec	G2583_2629	fbaB	CDS
O157	2852189	i	rec	intergenic		
O157	2852190	i	rec	intergenic		
EDL933	2922483	ins		intergenic		
O157	2852311	i	rec	intergenic		
O157	2852366	ns	rec	G2583_2630	yegT	CDS
O157	2852432	s	rec	G2583_2630	yegT	CDS
O157	2852539	s	rec	G2583_2630	yegT	CDS
O157	2852582	ns	rec	G2583_2630	yegT	CDS
O157	2852592	ns	rec	G2583_2630	yegT	CDS
O157	2852608	s	rec	G2583_2630	yegT	CDS
O157	2852722	s	rec	G2583_2630	yegT	CDS
O157	2852776	s	rec	G2583_2630	yegT	CDS
O157	2852908	s	rec	G2583_2630	yegT	CDS
O157	2853121	s	rec	G2583_2630	yegT	CDS
O157	2853151	s	rec	G2583_2630	yegT	CDS
O157	2853191	ns	rec	G2583_2630	yegT	CDS
O157	2853271	s	rec	G2583_2630	yegT	CDS
O157	2853280	s	rec	G2583_2630	yegT	CDS
O157	2853361	s	rec	G2583_2630	yegT	CDS
O157	2853370	s	rec	G2583_2630	yegT	CDS
O157	2853547	s	rec	G2583_2630	yegT	CDS
O157	2853771	s	rec	G2583_2631	yegU	CDS
O157	2853960	s	rec	G2583_2631	yegU	CDS
O157	2853963	s	rec	G2583_2631	yegU	CDS
O157	2854067	ns	rec	G2583_2631	yegU	CDS
O157	2854068	ns	rec	G2583_2631	yegU	CDS
O157	2854087	ns	rec	G2583_2631	yegU	CDS
O157	2854107	s	rec	G2583_2631	yegU	CDS
O157	2854140	s	rec	G2583_2631	yegU	CDS
O157	2854176	s	rec	G2583_2631	yegU	CDS
O157	2854213	s	rec	G2583_2631	yegU	CDS
O157	2854250	ns	rec	G2583_2631	yegU	CDS
O157	2854254	s	rec	G2583_2631	yegU	CDS

lineage[a]	site[b]	mutation type[c]	recomb	gene	Gene name	Type
O157	2854257	s	rec	G2583_2631	yegU	CDS
O157	2854278	s	rec	G2583_2631	yegU	CDS
O157	2854281	s	rec	G2583_2631	yegU	CDS
O157	2854431	s	rec	G2583_2631	yegU	CDS
O157	2854434	s	rec	G2583_2631	yegU	CDS
O157	2854467	s	rec	G2583_2631	yegU	CDS
O157	2854482	s	rec	G2583_2631	yegU	CDS
O157	2854497	s	rec	G2583_2631	yegU	CDS
O157	2854500	s	rec	G2583_2631	yegU	CDS
O157	2854519	ns	rec	G2583_2631	yegU	CDS
O157	2854551	s	rec	G2583_2631	yegU	CDS
O157	2854552	s	rec	G2583_2631	yegU	CDS
O157	2854605	s	rec	G2583_2631	yegU	CDS
O157	2854611	s	rec	G2583_2631	yegU	CDS
O157	2854625	ns	rec	G2583_2631	yegU	CDS
O157	2854628	ns	rec	G2583_2631	yegU	CDS
O157	2854685	s	rec	G2583_2632	yegV	CDS
O157	2854691	s	rec	G2583_2632	yegV	CDS
O157	2854692	ns	rec	G2583_2632	yegV	CDS
O157	2854694	ns	rec	G2583_2632	yegV	CDS
O157	2854703	s	rec	G2583_2632	yegV	CDS
O157	2854712	s	rec	G2583_2632	yegV	CDS
O157	2854844	s	rec	G2583_2632	yegV	CDS
O157	2854865	s	rec	G2583_2632	yegV	CDS
O157	2854868	s	rec	G2583_2632	yegV	CDS
O157	2854907	s	rec	G2583_2632	yegV	CDS
O157	2854932	ns	rec	G2583_2632	yegV	CDS
O157	2854952	s	rec	G2583_2632	yegV	CDS
O157	2855186	ns	rec	G2583_2632	yegV	CDS
O157	2855204	s	rec	G2583_2632	yegV	CDS
O157	2855210	s	rec	G2583_2632	yegV	CDS
O157	2855216	s	rec	G2583_2632	yegV	CDS
O157	2855219	s	rec	G2583_2632	yegV	CDS
O157	2855220	s	rec	G2583_2632	yegV	CDS
O157	2855222	s	rec	G2583_2632	yegV	CDS
O157	2855228	s	rec	G2583_2632	yegV	CDS
O157	2855249	s	rec	G2583_2632	yegV	CDS
O157	2855282	ns	rec	G2583_2632	yegV	CDS
O157	2855285	s	rec	G2583_2632	yegV	CDS
O157	2855288	s	rec	G2583_2632	yegV	CDS
O157	2855291	s	rec	G2583_2632	yegV	CDS
O157	2855294	s	rec	G2583_2632	yegV	CDS
O157	2855320	ns	rec	G2583_2632	yegV	CDS
O157	2855322	ns	rec	G2583_2632	yegV	CDS
O157	2855324	ns	rec	G2583_2632	yegV	CDS

lineage[a]	site[b]	mutation type[c]	recomb	gene	Gene name	Type
O157	2855328	s	rec	G2583_2632	yegV	CDS
O157	2855333	s	rec	G2583_2632	yegV	CDS
O157	2855334	ns	rec	G2583_2632	yegV	CDS
O157	2855336	ns	rec	G2583_2632	yegV	CDS
O157	2855351	s	rec	G2583_2632	yegV	CDS
O157	2855354	s	rec	G2583_2632	yegV	CDS
O157	2855357	s	rec	G2583_2632	yegV	CDS
O157	2855370	ns	rec	G2583_2632	yegV	CDS
O157	2855373	ns	rec	G2583_2632	yegV	CDS
O157	2855381	s	rec	G2583_2632	yegV	CDS
O157	2855388	ns	rec	G2583_2632	yegV	CDS
O157	2855389	ns	rec	G2583_2632	yegV	CDS
O157	2855402	s	rec	G2583_2632	yegV	CDS
O157	2855411	s	rec	G2583_2632	yegV	CDS
O157	2855420	s	rec	G2583_2632	yegV	CDS
O157	2855426	s	rec	G2583_2632	yegV	CDS
O157	2855435	s	rec	G2583_2632	yegV	CDS
O157	2855438	s	rec	G2583_2632	yegV	CDS
O157	2855441	s	rec	G2583_2632	yegV	CDS
O157	2855456	s	rec	G2583_2632	yegV	CDS
O157	2855463	s	rec	G2583_2632	yegV	CDS
O157	2855468	s	rec	G2583_2632	yegV	CDS
O157	2855622	s	rec	G2583_2633	yegW	CDS
O157	2855745	s	rec	G2583_2633	yegW	CDS
O157	2855748	s	rec	G2583_2633	yegW	CDS
O157	2855760	s	rec	G2583_2633	yegW	CDS
O157	2855778	s	rec	G2583_2633	yegW	CDS
O157	2856090	s	rec	G2583_2633	yegW	CDS
O157	2856412	ns	rec	G2583_2634	yegX	CDS
O157	2856414	s	rec	G2583_2634	yegX	CDS
O157	2856443	ns	rec	G2583_2634	yegX	CDS
O157	2856450	s	rec	G2583_2634	yegX	CDS
O157	2856465	s	rec	G2583_2634	yegX	CDS
O157	2856489	s	rec	G2583_2634	yegX	CDS
O157	2856516	s	rec	G2583_2634	yegX	CDS
O157	2856576	s	rec	G2583_2634	yegX	CDS
O157	2856608	ns	rec	G2583_2634	yegX	CDS
O157	2856618	s	rec	G2583_2634	yegX	CDS
O157	2856659	s	rec	G2583_2634	yegX	CDS
O157	2856888	s	rec	G2583_2634	yegX	CDS
O157	2856894	s	rec	G2583_2634	yegX	CDS
O157	2856900	s	rec	G2583_2634	yegX	CDS
O157	2856918	s	rec	G2583_2634	yegX	CDS
O157	2857005	ns	rec	G2583_2634	yegX	CDS
O157	2857006	ns	rec	G2583_2634	yegX	CDS

lineage[a]	site[b]	mutation type[c]	recomb	gene	Gene name	Type
O157	2857057	ns	rec	G2583_2634	yegX	CDS
O157	2857101	s	rec	G2583_2634	yegX	CDS
O157	2857105	ns	rec	G2583_2634	yegX	CDS
O157	2857145	s	rec	G2583_2634	yegX	CDS
O157	2857154	ns	rec	G2583_2634	yegX	CDS
O157	2857170	s	rec	G2583_2634	yegX	CDS
O157	2857176	s	rec	G2583_2634	yegX	CDS
O157	2857215	i	rec	intergenic		
O157	2857516	s	rec	G2583_2635	thiD	CDS
O157	2857543	s	rec	G2583_2635	thiD	CDS
O157	2857560	ns	rec	G2583_2635	thiD	CDS
O157	2857618	s	rec	G2583_2635	thiD	CDS
O157	2857675	s	rec	G2583_2635	thiD	CDS
O157	2857681	ns	rec	G2583_2635	thiD	CDS
O157	2857682	ns	rec	G2583_2635	thiD	CDS
O157	2857687	s	rec	G2583_2635	thiD	CDS
O157	2857705	s	rec	G2583_2635	thiD	CDS
O157	2857714	s	rec	G2583_2635	thiD	CDS
O157	2857828	s	rec	G2583_2635	thiD	CDS
O157	2857924	s	rec	G2583_2635	thiD	CDS
O157	2858185	ns	rec	G2583_2636	thiM	CDS
O157	2858205	s	rec	G2583_2636	thiM	CDS
O157	2858288	ns	rec	G2583_2636	thiM	CDS
O157	2858293	ns	rec	G2583_2636	thiM	CDS
O157	2858295	s	rec	G2583_2636	thiM	CDS
O157	2858315	ns	rec	G2583_2636	thiM	CDS
O157	2858379	s	rec	G2583_2636	thiM	CDS
O157	2858383	ns	rec	G2583_2636	thiM	CDS
O157	2858388	s	rec	G2583_2636	thiM	CDS
O157	2858415	s	rec	G2583_2636	thiM	CDS
O157	2858419	ns	rec	G2583_2636	thiM	CDS
O157	2858508	s	rec	G2583_2636	thiM	CDS
O157	2858553	s	rec	G2583_2636	thiM	CDS
O157	2858583	s	rec	G2583_2636	thiM	CDS
O157	2858616	s	rec	G2583_2636	thiM	CDS
O157	2858705	ns	rec	G2583_2636	thiM	CDS
O157	2858757	s	rec	G2583_2636	thiM	CDS
O157	2858795	ns	rec	G2583_2636	thiM	CDS
O157	2858941	i	rec	intergenic		
O157	2858990	i	rec	intergenic		
O157	2859001	i	rec	intergenic		
O157	2859003	i	rec	intergenic		
O157	2859023	i	rec	intergenic		
O157	2859688	i	rec	intergenic		
O157	2859789	i	rec	intergenic		

lineage[a]	site[b]	mutation type[c]	recomb	gene	Gene name	Type
O157	2860132	del	rec	intergenic		
O157	2860167	i	rec	intergenic		
O157	2860331	i	rec	intergenic		
O157	2860435	i	rec	intergenic		
O157	2860451	i	rec	intergenic		
O157	2861051	s	rec	G2583_2640	-	CDS
O157	2861203	i	rec	intergenic		
O157	2861292	ns	rec	G2583_2641	rcnR	CDS
O157	2861320	s	rec	G2583_2641	rcnR	CDS
O157	2861329	s	rec	G2583_2641	rcnR	CDS
O157	2861419	s	rec	G2583_2641	rcnR	CDS
O157	2861473	s	rec	G2583_2641	rcnR	CDS
O157	2861570	i	rec	intergenic		
O157	2861994	s	rec	G2583_2642	rcnA	CDS
O157	2862001	ins-9	rec	G2583_2642	rcnA	CDS
O157	2862015	s	rec	G2583_2642	rcnA	CDS
O157	2862018	s	rec	G2583_2642	rcnA	CDS
O157	2862027	ins	rec	G2583_2642	rcnA	CDS
EDL933	2932247	ins-8		G2583_2642	rcnA	CDS
Sakai	2862027	del-4		G2583_2642	rcnA	CDS
O157	2862030	s	rec	G2583_2642	rcnA	CDS
O157	2862036	ns	rec	G2583_2642	rcnA	CDS
O157	2862037	ns	rec	G2583_2642	rcnA	CDS
O157	2862039	ns	rec	G2583_2642	rcnA	CDS
O157	2862040	ns	rec	G2583_2642	rcnA	CDS
O157	2862051	s	rec	G2583_2642	rcnA	CDS
O157	2862053	ns	rec	G2583_2642	rcnA	CDS
O157	2862069	s	rec	G2583_2642	rcnA	CDS
O157	2862079	ins-9	rec	G2583_2642	rcnA	CDS
O157	2862175	s	rec	G2583_2642	rcnA	CDS
O157	2862189	s	rec	G2583_2642	rcnA	CDS
O157	2862201	s	rec	G2583_2642	rcnA	CDS
O157	2862291	s	rec	G2583_2642	rcnA	CDS
O157	2862294	s	rec	G2583_2642	rcnA	CDS
O157	2862300	s	rec	G2583_2642	rcnA	CDS
O157	2862301	s	rec	G2583_2642	rcnA	CDS
O157	2862312	s	rec	G2583_2642	rcnA	CDS
O157	2862315	s	rec	G2583_2642	rcnA	CDS
O157	2862327	s	rec	G2583_2642	rcnA	CDS
O157	2862330	s	rec	G2583_2642	rcnA	CDS
O157	2862339	s	rec	G2583_2642	rcnA	CDS
O157	2862345	s	rec	G2583_2642	rcnA	CDS
O157	2862348	s	rec	G2583_2642	rcnA	CDS
O157	2862351	s	rec	G2583_2642	rcnA	CDS
O157	2862414	s	rec	G2583_2642	rcnA	CDS

lineage[a]	site[b]	mutation type[c]	recomb	gene	Gene name	Type
O157	2862420	s	rec	G2583_2642	rcnA	CDS
O157	2862429	s	rec	G2583_2642	rcnA	CDS
O157	2862441	s	rec	G2583_2642	rcnA	CDS
O157	2862444	s	rec	G2583_2642	rcnA	CDS
O157	2862453	s	rec	G2583_2642	rcnA	CDS
O157	2862459	s	rec	G2583_2642	rcnA	CDS
O157	2862462	s	rec	G2583_2642	rcnA	CDS
O157	2862466	s	rec	G2583_2642	rcnA	CDS
O157	2862472	i	rec	intergenic		
O157	2862473	i	rec	intergenic		
O157	2862479	i	rec	intergenic		
O157	2862480	i	rec	intergenic		
O157	2862481	i	rec	intergenic		
O157	2862483	i	rec	intergenic		
O157	2862488	del-3	rec	intergenic		
O157	2862489	i	rec	intergenic		
O157	2862491	i	rec	intergenic		
O157	2862496	ins-2	rec	intergenic		
O157	2862500	i	rec	intergenic		
O157	2862527	ns	rec	G2583_2643	yohN	CDS
O157	2862528	ns	rec	G2583_2643	yohN	CDS
O157	2862536	ns	rec	G2583_2643	yohN	CDS
O157	2862540	ns	rec	G2583_2643	yohN	CDS
O157	2862541	ns	rec	G2583_2643	yohN	CDS
O157	2862542	ns	rec	G2583_2643	yohN	CDS
O157	2862543	ns	rec	G2583_2643	yohN	CDS
O157	2862548	ns	rec	G2583_2643	yohN	CDS
O157	2862551	ns	rec	G2583_2643	yohN	CDS
O157	2862563	ns	rec	G2583_2643	yohN	CDS
O157	2862564	ns	rec	G2583_2643	yohN	CDS
O157	2862565	ns	rec	G2583_2643	yohN	CDS
O157	2862566	ns	rec	G2583_2643	yohN	CDS
O157	2862643	ns	rec	G2583_2643	yohN	CDS
O157	2862808	s	rec	G2583_2643	yohN	CDS
O157	2862834	ns	rec	G2583_2643	yohN	CDS
O157	2862844	s	rec	G2583_2643	yohN	CDS
O157	2862847	s	rec	G2583_2643	yohN	CDS
O157	2862853	s	rec	G2583_2643	yohN	CDS
O157	2862868	s	rec	G2583_2643	yohN	CDS
O157	2862901	s	rec	G2583_2643	yohN	CDS
O157	2862916	s	rec	G2583_2643	yohN	CDS
O157	2862919	s	rec	G2583_2643	yohN	CDS
O157	2862922	s	rec	G2583_2643	yohN	CDS
O157	2862928	s	rec	G2583_2643	yohN	CDS
O157	2862931	s	rec	G2583_2643	yohN	CDS

lineage[a]	site[b]	mutation type[c]	recomb	gene	Gene name	Type
O157	2863041	i	rec	intergenic		
O157	2863042	i	rec	intergenic		
O157	2863043	i	rec	intergenic		
O157	2863044	i	rec	intergenic		
O157	2863045	i	rec	intergenic		
O157	2863053	i	rec	intergenic		
O157	2863068	i	rec	intergenic		
O157	2863075	i	rec	intergenic		
O157	2863096	i	rec	intergenic		
O157	2863112	s	rec	G2583_2644	yehA	CDS
O157	2863132	s	rec	G2583_2644	yehA	CDS
O157	2863142	s	rec	G2583_2644	yehA	CDS
O157	2863166	ns	rec	G2583_2644	yehA	CDS
O157	2863170	ns	rec	G2583_2644	yehA	CDS
O157	2863171	ns	rec	G2583_2644	yehA	CDS
O157	2863175	s	rec	G2583_2644	yehA	CDS
O157	2863178	s	rec	G2583_2644	yehA	CDS
O157	2863181	s	rec	G2583_2644	yehA	CDS
O157	2864173	nc	rec	G2583_2645	yehB	pseudogene
O157	2864217	nc	rec	G2583_2645	yehB	pseudogene
O157	2864231	nc	rec	G2583_2645	yehB	pseudogene
O157	2864249	nc	rec	G2583_2645	yehB	pseudogene
O157	2864258	nc	rec	G2583_2645	yehB	pseudogene
O157	2864279	nc	rec	G2583_2645	yehB	pseudogene
O157	2864286	nc	rec	G2583_2645	yehB	pseudogene
O157	2864301	nc	rec	G2583_2645	yehB	pseudogene
O157	2864307	nc	rec	G2583_2645	yehB	pseudogene
O157	2864316	nc	rec	G2583_2645	yehB	pseudogene
O157	2864319	nc	rec	G2583_2645	yehB	pseudogene
O157	2864352	nc	rec	G2583_2645	yehB	pseudogene
O157	2864355	nc	rec	G2583_2645	yehB	pseudogene
O157	2864361	nc	rec	G2583_2645	yehB	pseudogene
O157	2864363	nc	rec	G2583_2645	yehB	pseudogene
O157	2864370	nc	rec	G2583_2645	yehB	pseudogene
O157	2864391	nc	rec	G2583_2645	yehB	pseudogene
O157	2864411	nc	rec	G2583_2645	yehB	pseudogene
O157	2864418	nc	rec	G2583_2645	yehB	pseudogene
O157	2864421	nc	rec	G2583_2645	yehB	pseudogene
O157	2864439	nc	rec	G2583_2645	yehB	pseudogene
O157	2864444	nc	rec	G2583_2645	yehB	pseudogene
O157	2864445	nc	rec	G2583_2645	yehB	pseudogene
O157	2864451	nc	rec	G2583_2645	yehB	pseudogene
O157	2864457	nc	rec	G2583_2645	yehB	pseudogene
O157	2864463	nc	rec	G2583_2645	yehB	pseudogene
O157	2864471	nc	rec	G2583_2645	yehB	pseudogene

lineage[a]	site[b]	mutation type[c]	recomb	gene	Gene name	Type
O157	2864498	nc	rec	G2583_2645	yehB	pseudogene
O157	2864528	nc	rec	G2583_2645	yehB	pseudogene
O157	2864538	nc	rec	G2583_2645	yehB	pseudogene
O157	2864547	nc	rec	G2583_2645	yehB	pseudogene
O157	2864555	nc	rec	G2583_2645	yehB	pseudogene
O157	2864565	nc	rec	G2583_2645	yehB	pseudogene
O157	2864598	nc	rec	G2583_2645	yehB	pseudogene
O157	2864604	nc	rec	G2583_2645	yehB	pseudogene
O157	2864612	nc	rec	G2583_2645	yehB	pseudogene
O157	2864661	nc	rec	G2583_2645	yehB	pseudogene
O157	2864670	nc	rec	G2583_2645	yehB	pseudogene
O157	2864673	nc	rec	G2583_2645	yehB	pseudogene
O157	2864675	nc	rec	G2583_2645	yehB	pseudogene
O157	2864676	nc	rec	G2583_2645	yehB	pseudogene
O157	2864697	nc	rec	G2583_2645	yehB	pseudogene
O157	2864698	nc	rec	G2583_2645	yehB	pseudogene
O157	2864727	nc	rec	G2583_2645	yehB	pseudogene
O157	2864742	nc	rec	G2583_2645	yehB	pseudogene
O157	2864745	nc	rec	G2583_2645	yehB	pseudogene
O157	2864746	nc	rec	G2583_2645	yehB	pseudogene
O157	2864751	nc	rec	G2583_2645	yehB	pseudogene
O157	2864755	nc	rec	G2583_2645	yehB	pseudogene
O157	2864763	nc	rec	G2583_2645	yehB	pseudogene
O157	2864769	nc	rec	G2583_2645	yehB	pseudogene
O157	2864772	nc	rec	G2583_2645	yehB	pseudogene
O157	2864805	nc	rec	G2583_2645	yehB	pseudogene
O157	2864808	nc	rec	G2583_2645	yehB	pseudogene
O157	2864810	nc	rec	G2583_2645	yehB	pseudogene
O157	2864816	nc	rec	G2583_2645	yehB	pseudogene
O157	2864823	nc	rec	G2583_2645	yehB	pseudogene
O157	2864826	nc	rec	G2583_2645	yehB	pseudogene
O157	2864835	nc	rec	G2583_2645	yehB	pseudogene
O157	2864838	nc	rec	G2583_2645	yehB	pseudogene
O157	2864839	nc	rec	G2583_2645	yehB	pseudogene
O157	2864844	nc	rec	G2583_2645	yehB	pseudogene
O157	2864847	nc	rec	G2583_2645	yehB	pseudogene
O157	2864853	nc	rec	G2583_2645	yehB	pseudogene
O157	2864862	nc	rec	G2583_2645	yehB	pseudogene
O157	2864868	nc	rec	G2583_2645	yehB	pseudogene
O157	2864874	nc	rec	G2583_2645	yehB	pseudogene
O157	2864880	nc	rec	G2583_2645	yehB	pseudogene
O157	2864895	nc	rec	G2583_2645	yehB	pseudogene
O157	2864925	nc	rec	G2583_2645	yehB	pseudogene
O157	2864928	nc	rec	G2583_2645	yehB	pseudogene
O157	2864936	nc	rec	G2583_2645	yehB	pseudogene

lineage[a]	site[b]	mutation type[c]	recomb	gene	Gene name	Type
O157	2864946	nc	rec	G2583_2645	yehB	pseudogene
O157	2864952	nc	rec	G2583_2645	yehB	pseudogene
O157	2864982	nc	rec	G2583_2645	yehB	pseudogene
O157	2864994	nc	rec	G2583_2645	yehB	pseudogene
O157	2864995	nc	rec	G2583_2645	yehB	pseudogene
O157	2864996	nc	rec	G2583_2645	yehB	pseudogene
O157	2864997	nc	rec	G2583_2645	yehB	pseudogene
O157	2865000	nc	rec	G2583_2645	yehB	pseudogene
O157	2865012	nc	rec	G2583_2645	yehB	pseudogene
O157	2865045	nc	rec	G2583_2645	yehB	pseudogene
O157	2865049	nc	rec	G2583_2645	yehB	pseudogene
O157	2865050	nc	rec	G2583_2645	yehB	pseudogene
O157	2865054	nc	rec	G2583_2645	yehB	pseudogene
O157	2865068	nc	rec	G2583_2645	yehB	pseudogene
O157	2865069	nc	rec	G2583_2645	yehB	pseudogene
O157	2865078	nc	rec	G2583_2645	yehB	pseudogene
O157	2865093	nc	rec	G2583_2645	yehB	pseudogene
O157	2865108	nc	rec	G2583_2645	yehB	pseudogene
O157	2865117	nc	rec	G2583_2645	yehB	pseudogene
O157	2865135	nc	rec	G2583_2645	yehB	pseudogene
O157	2865141	nc	rec	G2583_2645	yehB	pseudogene
O157	2865159	nc	rec	G2583_2645	yehB	pseudogene
O157	2865168	nc	rec	G2583_2645	yehB	pseudogene
O157	2865219	nc	rec	G2583_2645	yehB	pseudogene
O157	2865236	nc	rec	G2583_2645	yehB	pseudogene
O157	2865243	nc	rec	G2583_2645	yehB	pseudogene
O157	2865261	nc	rec	G2583_2645	yehB	pseudogene
O157	2865264	nc	rec	G2583_2645	yehB	pseudogene
O157	2865270	nc	rec	G2583_2645	yehB	pseudogene
O157	2865273	nc	rec	G2583_2645	yehB	pseudogene
O157	2865275	nc	rec	G2583_2645	yehB	pseudogene
O157	2865279	nc	rec	G2583_2645	yehB	pseudogene
O157	2865288	nc	rec	G2583_2645	yehB	pseudogene
O157	2865291	nc	rec	G2583_2645	yehB	pseudogene
O157	2865313	nc	rec	G2583_2645	yehB	pseudogene
O157	2865333	nc	rec	G2583_2645	yehB	pseudogene
O157	2865336	nc	rec	G2583_2645	yehB	pseudogene
O157	2865339	nc	rec	G2583_2645	yehB	pseudogene
O157	2865350	nc	rec	G2583_2645	yehB	pseudogene
O157	2865360	nc	rec	G2583_2645	yehB	pseudogene
O157	2865381	nc	rec	G2583_2645	yehB	pseudogene
O157	2865390	nc	rec	G2583_2645	yehB	pseudogene
O157	2865396	nc	rec	G2583_2645	yehB	pseudogene
O157	2865399	nc	rec	G2583_2645	yehB	pseudogene
O157	2865408	nc	rec	G2583_2645	yehB	pseudogene

lineage[a]	site[b]	mutation type[c]	recomb	gene	Gene name	Type
O157	2865414	nc	rec	G2583_2645	yehB	pseudogene
O157	2865420	nc	rec	G2583_2645	yehB	pseudogene
O157	2865423	nc	rec	G2583_2645	yehB	pseudogene
O157	2865426	nc	rec	G2583_2645	yehB	pseudogene
O157	2865435	nc	rec	G2583_2645	yehB	pseudogene
O157	2865441	nc	rec	G2583_2645	yehB	pseudogene
O157	2865444	nc	rec	G2583_2645	yehB	pseudogene
O157	2865450	nc	rec	G2583_2645	yehB	pseudogene
O157	2865453	nc	rec	G2583_2645	yehB	pseudogene
O157	2865456	nc	rec	G2583_2645	yehB	pseudogene
O157	2865462	nc	rec	G2583_2645	yehB	pseudogene
O157	2865465	nc	rec	G2583_2645	yehB	pseudogene
O157	2865468	nc	rec	G2583_2645	yehB	pseudogene
O157	2865471	nc	rec	G2583_2645	yehB	pseudogene
O157	2865473	nc	rec	G2583_2645	yehB	pseudogene
O157	2865474	nc	rec	G2583_2645	yehB	pseudogene
O157	2865477	nc	rec	G2583_2645	yehB	pseudogene
O157	2865480	nc	rec	G2583_2645	yehB	pseudogene
O157	2865483	nc	rec	G2583_2645	yehB	pseudogene
O157	2865486	nc	rec	G2583_2645	yehB	pseudogene
O157	2865495	nc	rec	G2583_2645	yehB	pseudogene
O157	2865498	nc	rec	G2583_2645	yehB	pseudogene
O157	2865506	nc	rec	G2583_2645	yehB	pseudogene
O157	2865510	nc	rec	G2583_2645	yehB	pseudogene
O157	2865528	nc	rec	G2583_2645	yehB	pseudogene
O157	2865573	nc	rec	G2583_2645	yehB	pseudogene
O157	2865582	nc	rec	G2583_2645	yehB	pseudogene
O157	2865594	nc	rec	G2583_2645	yehB	pseudogene
O157	2865600	nc	rec	G2583_2645	yehB	pseudogene
O157	2865603	nc	rec	G2583_2645	yehB	pseudogene
O157	2865621	nc	rec	G2583_2645	yehB	pseudogene
O157	2865624	nc	rec	G2583_2645	yehB	pseudogene
O157	2865627	nc	rec	G2583_2645	yehB	pseudogene
O157	2865630	nc	rec	G2583_2645	yehB	pseudogene
O157	2865645	nc	rec	G2583_2645	yehB	pseudogene
O157	2865648	nc	rec	G2583_2645	yehB	pseudogene
O157	2865651	nc	rec	G2583_2645	yehB	pseudogene
O157	2865654	nc	rec	G2583_2645	yehB	pseudogene
O157	2865672	nc	rec	G2583_2645	yehB	pseudogene
O157	2865675	nc	rec	G2583_2645	yehB	pseudogene
O157	2865678	nc	rec	G2583_2645	yehB	pseudogene
O157	2865681	nc	rec	G2583_2645	yehB	pseudogene
O157	2865684	nc	rec	G2583_2645	yehB	pseudogene
O157	2865687	nc	rec	G2583_2645	yehB	pseudogene
O157	2865690	nc	rec	G2583_2645	yehB	pseudogene

lineage[a]	site[b]	mutation type[c]	recomb	gene	Gene name	Type
O157	2865693	nc	rec	G2583_2645	yehB	pseudogene
O157	2865696	nc	rec	G2583_2645	yehB	pseudogene
O157	2865699	nc	rec	G2583_2645	yehB	pseudogene
O157	2865702	nc	rec	G2583_2645	yehB	pseudogene
O157	2865717	nc	rec	G2583_2645	yehB	pseudogene
O157	2865720	nc	rec	G2583_2645	yehB	pseudogene
O157	2865723	nc	rec	G2583_2645	yehB	pseudogene
O157	2865726	nc	rec	G2583_2645	yehB	pseudogene
O157	2865729	nc	rec	G2583_2645	yehB	pseudogene
O157	2865732	nc	rec	G2583_2645	yehB	pseudogene
O157	2865744	nc	rec	G2583_2645	yehB	pseudogene
O157	2865745	nc	rec	G2583_2645	yehB	pseudogene
O157	2865752	nc	rec	G2583_2645	yehB	pseudogene
O157	2865759	nc	rec	G2583_2645	yehB	pseudogene
O157	2865762	nc	rec	G2583_2645	yehB	pseudogene
O157	2865765	nc	rec	G2583_2645	yehB	pseudogene
O157	2865769	nc	rec	G2583_2645	yehB	pseudogene
O157	2865786	nc	rec	G2583_2645	yehB	pseudogene
O157	2865795	nc	rec	G2583_2645	yehB	pseudogene
O157	2865815	nc	rec	G2583_2645	yehB	pseudogene
O157	2865828	nc	rec	G2583_2645	yehB	pseudogene
O157	2865840	nc	rec	G2583_2645	yehB	pseudogene
O157	2865849	nc	rec	G2583_2645	yehB	pseudogene
O157	2865861	nc	rec	G2583_2645	yehB	pseudogene
O157	2865870	nc	rec	G2583_2645	yehB	pseudogene
O157	2865894	nc	rec	G2583_2645	yehB	pseudogene
O157	2865900	nc	rec	G2583_2645	yehB	pseudogene
O157	2865903	nc	rec	G2583_2645	yehB	pseudogene
O157	2865906	nc	rec	G2583_2645	yehB	pseudogene
O157	2865907	nc	rec	G2583_2645	yehB	pseudogene
O157	2865918	nc	rec	G2583_2645	yehB	pseudogene
O157	2865927	nc	rec	G2583_2645	yehB	pseudogene
O157	2865934	nc	rec	G2583_2645	yehB	pseudogene
O157	2865935	nc	rec	G2583_2645	yehB	pseudogene
O157	2865939	nc	rec	G2583_2645	yehB	pseudogene
O157	2865954	nc	rec	G2583_2645	yehB	pseudogene
O157	2865956	nc	rec	G2583_2645	yehB	pseudogene
O157	2865957	nc	rec	G2583_2645	yehB	pseudogene
O157	2865960	nc	rec	G2583_2645	yehB	pseudogene
O157	2865966	nc	rec	G2583_2645	yehB	pseudogene
O157	2865974	nc	rec	G2583_2645	yehB	pseudogene
O157	2865978	nc	rec	G2583_2645	yehB	pseudogene
O157	2865980	nc	rec	G2583_2645	yehB	pseudogene
O157	2865988	nc	rec	G2583_2645	yehB	pseudogene
O157	2865990	nc	rec	G2583_2645	yehB	pseudogene

lineage[a]	site[b]	mutation type[c]	recomb	gene	Gene name	Type
O157	2866002	nc	rec	G2583_2645	yehB	pseudogene
O157	2866005	nc	rec	G2583_2645	yehB	pseudogene
O157	2866018	nc	rec	G2583_2645	yehB	pseudogene
O157	2866020	nc	rec	G2583_2645	yehB	pseudogene
O157	2866023	nc	rec	G2583_2645	yehB	pseudogene
O157	2866029	nc	rec	G2583_2645	yehB	pseudogene
O157	2866034	nc	rec	G2583_2645	yehB	pseudogene
O157	2866035	nc	rec	G2583_2645	yehB	pseudogene
O157	2866041	nc	rec	G2583_2645	yehB	pseudogene
O157	2866044	nc	rec	G2583_2645	yehB	pseudogene
O157	2866056	nc	rec	G2583_2645	yehB	pseudogene
O157	2866065	nc	rec	G2583_2645	yehB	pseudogene
O157	2866071	nc	rec	G2583_2645	yehB	pseudogene
O157	2866077	nc	rec	G2583_2645	yehB	pseudogene
O157	2866085	nc	rec	G2583_2645	yehB	pseudogene
O157	2866089	nc	rec	G2583_2645	yehB	pseudogene
O157	2866101	nc	rec	G2583_2645	yehB	pseudogene
O157	2866107	nc	rec	G2583_2645	yehB	pseudogene
O157	2866110	nc	rec	G2583_2645	yehB	pseudogene
O157	2866122	nc	rec	G2583_2645	yehB	pseudogene
O157	2866128	nc	rec	G2583_2645	yehB	pseudogene
O157	2866155	nc	rec	G2583_2645	yehB	pseudogene
O157	2866158	nc	rec	G2583_2645	yehB	pseudogene
O157	2866167	nc	rec	G2583_2645	yehB	pseudogene
O157	2866182	nc	rec	G2583_2645	yehB	pseudogene
O157	2866185	nc	rec	G2583_2645	yehB	pseudogene
O157	2866203	nc	rec	G2583_2645	yehB	pseudogene
O157	2866206	nc	rec	G2583_2645	yehB	pseudogene
O157	2866212	nc	rec	G2583_2645	yehB	pseudogene
O157	2866215	nc	rec	G2583_2645	yehB	pseudogene
O157	2866219	nc	rec	G2583_2645	yehB	pseudogene
O157	2866227	nc	rec	G2583_2645	yehB	pseudogene
O157	2866233	nc	rec	G2583_2645	yehB	pseudogene
O157	2866242	nc	rec	G2583_2645	yehB	pseudogene
O157	2866248	nc	rec	G2583_2645	yehB	pseudogene
O157	2866252	nc	rec	G2583_2645	yehB	pseudogene
O157	2866253	nc	rec	G2583_2645	yehB	pseudogene
O157	2866256	nc	rec	G2583_2645	yehB	pseudogene
O157	2866260	nc	rec	G2583_2645	yehB	pseudogene
O157	2866262	nc	rec	G2583_2645	yehB	pseudogene
O157	2866271	nc	rec	G2583_2645	yehB	pseudogene
O157	2866275	nc	rec	G2583_2645	yehB	pseudogene
O157	2866286	nc	rec	G2583_2645	yehB	pseudogene
O157	2866287	nc	rec	G2583_2645	yehB	pseudogene
O157	2866293	nc	rec	G2583_2645	yehB	pseudogene

lineage[a]	site[b]	mutation type[c]	recomb	gene	Gene name	Type
O157	2866296	nc	rec	G2583_2645	yehB	pseudogene
O157	2866326	nc	rec	G2583_2645	yehB	pseudogene
O157	2866329	nc	rec	G2583_2645	yehB	pseudogene
O157	2866332	nc	rec	G2583_2645	yehB	pseudogene
O157	2866333	nc	rec	G2583_2645	yehB	pseudogene
O157	2866337	nc	rec	G2583_2645	yehB	pseudogene
O157	2866338	nc	rec	G2583_2645	yehB	pseudogene
O157	2866341	nc	rec	G2583_2645	yehB	pseudogene
O157	2866344	nc	rec	G2583_2645	yehB	pseudogene
O157	2866356	nc	rec	G2583_2645	yehB	pseudogene
O157	2866359	nc	rec	G2583_2645	yehB	pseudogene
O157	2866371	nc	rec	G2583_2645	yehB	pseudogene
O157	2866373	nc	rec	G2583_2645	yehB	pseudogene
O157	2866376	nc	rec	G2583_2645	yehB	pseudogene
O157	2866380	nc	rec	G2583_2645	yehB	pseudogene
O157	2866382	nc	rec	G2583_2645	yehB	pseudogene
O157	2866383	nc	rec	G2583_2645	yehB	pseudogene
O157	2866385	nc	rec	G2583_2645	yehB	pseudogene
O157	2866386	nc	rec	G2583_2645	yehB	pseudogene
O157	2866388	nc	rec	G2583_2645	yehB	pseudogene
O157	2866389	nc	rec	G2583_2645	yehB	pseudogene
O157	2866392	nc	rec	G2583_2645	yehB	pseudogene
O157	2866398	nc	rec	G2583_2645	yehB	pseudogene
O157	2866401	nc	rec	G2583_2645	yehB	pseudogene
O157	2866407	nc	rec	G2583_2645	yehB	pseudogene
O157	2866413	nc	rec	G2583_2645	yehB	pseudogene
O157	2866416	nc	rec	G2583_2645	yehB	pseudogene
O157	2866431	nc	rec	G2583_2645	yehB	pseudogene
O157	2866434	nc	rec	G2583_2645	yehB	pseudogene
O157	2866443	nc	rec	G2583_2645	yehB	pseudogene
O157	2866461	nc	rec	G2583_2645	yehB	pseudogene
O157	2866467	nc	rec	G2583_2645	yehB	pseudogene
O157	2866476	nc	rec	G2583_2645	yehB	pseudogene
O157	2866482	nc	rec	G2583_2645	yehB	pseudogene
O157	2866485	nc	rec	G2583_2645	yehB	pseudogene
O157	2866503	nc	rec	G2583_2645	yehB	pseudogene
O157	2866505	nc	rec	G2583_2645	yehB	pseudogene
O157	2866511	nc	rec	G2583_2645	yehB	pseudogene
O157	2866512	nc	rec	G2583_2645	yehB	pseudogene
O157	2866515	nc	rec	G2583_2645	yehB	pseudogene
O157	2866520	nc	rec	G2583_2645	yehB	pseudogene
O157	2866521	nc	rec	G2583_2645	yehB	pseudogene
O157	2866522	nc	rec	G2583_2645	yehB	pseudogene
O157	2866533	nc	rec	G2583_2645	yehB	pseudogene
O157	2866536	nc	rec	G2583_2645	yehB	pseudogene

lineage[a]	site[b]	mutation type[c]	recomb	gene	Gene name	Type
O157	2866551	nc	rec	G2583_2645	yehB	pseudogene
O157	2866566	nc	rec	G2583_2645	yehB	pseudogene
O157	2866574	nc	rec	G2583_2645	yehB	pseudogene
O157	2866575	nc	rec	G2583_2645	yehB	pseudogene
O157	2866599	nc	rec	G2583_2645	yehB	pseudogene
O157	2866602	nc	rec	G2583_2645	yehB	pseudogene
O157	2866617	nc	rec	G2583_2645	yehB	pseudogene
O157	2866680	nc	rec	G2583_2646	yehC	pseudogene
O157	2866698	nc	rec	G2583_2646	yehC	pseudogene
O157	2866700	nc	rec	G2583_2646	yehC	pseudogene
O157	2866705	nc	rec	G2583_2646	yehC	pseudogene
O157	2866706	nc	rec	G2583_2646	yehC	pseudogene
O157	2866710	nc	rec	G2583_2646	yehC	pseudogene
O157	2866720	nc	rec	G2583_2646	yehC	pseudogene
O157	2866725	nc	rec	G2583_2646	yehC	pseudogene
O157	2866726	nc	rec	G2583_2646	yehC	pseudogene
O157	2866729	nc	rec	G2583_2646	yehC	pseudogene
O157	2866735	nc	rec	G2583_2646	yehC	pseudogene
O157	2866747	nc	rec	G2583_2646	yehC	pseudogene
O157	2866748	nc	rec	G2583_2646	yehC	pseudogene
O157	2866749	nc	rec	G2583_2646	yehC	pseudogene
O157	2866752	nc	rec	G2583_2646	yehC	pseudogene
O157	2866761	nc	rec	G2583_2646	yehC	pseudogene
O157	2866764	nc	rec	G2583_2646	yehC	pseudogene
O157	2866776	nc	rec	G2583_2646	yehC	pseudogene
O157	2866782	nc	rec	G2583_2646	yehC	pseudogene
O157	2866785	nc	rec	G2583_2646	yehC	pseudogene
O157	2866794	nc	rec	G2583_2646	yehC	pseudogene
O157	2866797	nc	rec	G2583_2646	yehC	pseudogene
O157	2866803	nc	rec	G2583_2646	yehC	pseudogene
O157	2866812	nc	rec	G2583_2646	yehC	pseudogene
O157	2866821	nc	rec	G2583_2646	yehC	pseudogene
O157	2866823	nc	rec	G2583_2646	yehC	pseudogene
O157	2866824	nc	rec	G2583_2646	yehC	pseudogene
O157	2866839	nc	rec	G2583_2646	yehC	pseudogene
O157	2866842	nc	rec	G2583_2646	yehC	pseudogene
O157	2866844	nc	rec	G2583_2646	yehC	pseudogene
O157	2866845	nc	rec	G2583_2646	yehC	pseudogene
O157	2866846	nc	rec	G2583_2646	yehC	pseudogene
O157	2866847	nc	rec	G2583_2646	yehC	pseudogene
O157	2866851	nc	rec	G2583_2646	yehC	pseudogene
O157	2866854	nc	rec	G2583_2646	yehC	pseudogene
O157	2866857	nc	rec	G2583_2646	yehC	pseudogene
O157	2866861	nc	rec	G2583_2646	yehC	pseudogene
O157	2866863	nc	rec	G2583_2646	yehC	pseudogene

lineage[a]	site[b]	mutation type[c]	recomb	gene	Gene name	Type
O157	2866866	nc	rec	G2583_2646	yehC	pseudogene
O157	2866867	nc	rec	G2583_2646	yehC	pseudogene
O157	2866872	nc	rec	G2583_2646	yehC	pseudogene
O157	2866881	nc	rec	G2583_2646	yehC	pseudogene
O157	2866886	nc	rec	G2583_2646	yehC	pseudogene
O157	2866908	nc	rec	G2583_2646	yehC	pseudogene
O157	2866911	nc	rec	G2583_2646	yehC	pseudogene
O157	2866914	nc	rec	G2583_2646	yehC	pseudogene
O157	2866919	ins	rec	G2583_2646	yehC	pseudogene
O157	2866923	nc	rec	G2583_2646	yehC	pseudogene
O157	2866926	nc	rec	G2583_2646	yehC	pseudogene
O157	2866944	nc	rec	G2583_2646	yehC	pseudogene
O157	2866950	nc	rec	G2583_2646	yehC	pseudogene
O157	2866953	nc	rec	G2583_2646	yehC	pseudogene
O157	2866955	nc	rec	G2583_2646	yehC	pseudogene
O157	2866956	nc	rec	G2583_2646	yehC	pseudogene
O157	2866965	nc	rec	G2583_2646	yehC	pseudogene
O157	2866971	nc	rec	G2583_2646	yehC	pseudogene
O157	2866973	nc	rec	G2583_2646	yehC	pseudogene
O157	2866980	nc	rec	G2583_2646	yehC	pseudogene
O157	2866986	nc	rec	G2583_2646	yehC	pseudogene
O157	2867007	nc	rec	G2583_2646	yehC	pseudogene
O157	2867019	nc	rec	G2583_2646	yehC	pseudogene
O157	2867025	nc	rec	G2583_2646	yehC	pseudogene
O157	2867032	nc	rec	G2583_2646	yehC	pseudogene
O157	2867037	nc	rec	G2583_2646	yehC	pseudogene
O157	2867039	nc	rec	G2583_2646	yehC	pseudogene
O157	2867046	nc	rec	G2583_2646	yehC	pseudogene
O157	2867052	nc	rec	G2583_2646	yehC	pseudogene
O157	2867058	nc	rec	G2583_2646	yehC	pseudogene
O157	2867066	nc	rec	G2583_2646	yehC	pseudogene
O157	2867070	nc	rec	G2583_2646	yehC	pseudogene
O157	2867076	nc	rec	G2583_2646	yehC	pseudogene
O157	2867082	nc	rec	G2583_2646	yehC	pseudogene
O157	2867086	nc	rec	G2583_2646	yehC	pseudogene
O157	2867090	nc	rec	G2583_2646	yehC	pseudogene
O157	2867094	nc	rec	G2583_2646	yehC	pseudogene
O157	2867095	nc	rec	G2583_2646	yehC	pseudogene
O157	2867097	nc	rec	G2583_2646	yehC	pseudogene
O157	2867130	nc	rec	G2583_2646	yehC	pseudogene
O157	2867139	nc	rec	G2583_2646	yehC	pseudogene
O157	2867142	nc	rec	G2583_2646	yehC	pseudogene
O157	2867144	nc	rec	G2583_2646	yehC	pseudogene
O157	2867145	nc	rec	G2583_2646	yehC	pseudogene
O157	2867151	nc	rec	G2583_2646	yehC	pseudogene

lineage[a]	site[b]	mutation type[c]	recomb	gene	Gene name	Type
O157	2867177	nc	rec	G2583_2646	yehC	pseudogene
O157	2867187	nc	rec	G2583_2646	yehC	pseudogene
O157	2867199	nc	rec	G2583_2646	yehC	pseudogene
O157	2867207	nc	rec	G2583_2646	yehC	pseudogene
O157	2867208	nc	rec	G2583_2646	yehC	pseudogene
O157	2867211	nc	rec	G2583_2646	yehC	pseudogene
O157	2867217	nc	rec	G2583_2646	yehC	pseudogene
O157	2867220	nc	rec	G2583_2646	yehC	pseudogene
O157	2867226	nc	rec	G2583_2646	yehC	pseudogene
O157	2867228	nc	rec	G2583_2646	yehC	pseudogene
O157	2867229	nc	rec	G2583_2646	yehC	pseudogene
O157	2867244	nc	rec	G2583_2646	yehC	pseudogene
O157	2867247	nc	rec	G2583_2646	yehC	pseudogene
O157	2867250	nc	rec	G2583_2646	yehC	pseudogene
O157	2867257	nc	rec	G2583_2646	yehC	pseudogene
O157	2867259	nc	rec	G2583_2646	yehC	pseudogene
O157	2867265	nc	rec	G2583_2646	yehC	pseudogene
O157	2867277	nc	rec	G2583_2646	yehC	pseudogene
Sakai	2867278	nc		G2583_2646	yehC	pseudogene
O157	2867280	nc	rec	G2583_2646	yehC	pseudogene
O157	2867283	nc	rec	G2583_2646	yehC	pseudogene
O157	2867285	nc	rec	G2583_2646	yehC	pseudogene
O157	2867286	nc	rec	G2583_2646	yehC	pseudogene
O157	2867288	nc	rec	G2583_2646	yehC	pseudogene
O157	2867296	nc	rec	G2583_2646	yehC	pseudogene
O157	2867298	nc	rec	G2583_2646	yehC	pseudogene
O157	2867316	nc	rec	G2583_2646	yehC	pseudogene
O157	2867339	nc	rec	G2583_2646	yehC	pseudogene
O157	2867343	nc	rec	G2583_2646	yehC	pseudogene
O157	2867345	nc	rec	G2583_2646	yehC	pseudogene
O157	2867346	nc	rec	G2583_2646	yehC	pseudogene
O157	2867347	ins-2	rec	G2583_2646	yehC	pseudogene
O157	2867349	nc	rec	G2583_2646	yehC	pseudogene
O157	2867359	nc	rec	G2583_2646	yehC	pseudogene
O157	2867360	i	rec	intergenic		
O157	2867361	i	rec	intergenic		
O157	2867364	i	rec	intergenic		
O157	2867373	i	rec	intergenic		
O157	2867376	i	rec	intergenic		
O157	2867378	i	rec	intergenic		
O157	2867380	i	rec	intergenic		
O157	2867381	i	rec	intergenic		
O157	2867385	i	rec	intergenic		
O157	2867387	i	rec	intergenic		
O157	2867392	ins-4	rec	intergenic		

lineage[a]	site[b]	mutation type[c]	recomb	gene	Gene name	Type
O157	2867397	i	rec	intergenic		
O157	2867398	i	rec	intergenic		
O157	2867399	i	rec	intergenic		
O157	2867400	i	rec	intergenic		
O157	2867407	i	rec	intergenic		
O157	2867408	i	rec	intergenic		
O157	2867411	ins	rec	intergenic		
O157	2867421	s	rec	G2583_2647	yehD	CDS
O157	2867424	s	rec	G2583_2647	yehD	CDS
O157	2867438	s	rec	G2583_2647	yehD	CDS
O157	2867439	s	rec	G2583_2647	yehD	CDS
O157	2867445	ns	rec	G2583_2647	yehD	CDS
O157	2867446	ns	rec	G2583_2647	yehD	CDS
O157	2867447	ns	rec	G2583_2647	yehD	CDS
O157	2867448	s	rec	G2583_2647	yehD	CDS
O157	2867451	ns	rec	G2583_2647	yehD	CDS
O157	2867452	ns	rec	G2583_2647	yehD	CDS
O157	2867453	ns	rec	G2583_2647	yehD	CDS
O157	2867457	s	rec	G2583_2647	yehD	CDS
O157	2867461	ns	rec	G2583_2647	yehD	CDS
O157	2867462	ns	rec	G2583_2647	yehD	CDS
O157	2867463	s	rec	G2583_2647	yehD	CDS
O157	2867466	ns	rec	G2583_2647	yehD	CDS
O157	2867467	ns	rec	G2583_2647	yehD	CDS
O157	2867468	ns	rec	G2583_2647	yehD	CDS
O157	2867469	ns	rec	G2583_2647	yehD	CDS
O157	2867470	ns	rec	G2583_2647	yehD	CDS
O157	2867472	s	rec	G2583_2647	yehD	CDS
O157	2867475	s	rec	G2583_2647	yehD	CDS
O157	2867480	s	rec	G2583_2647	yehD	CDS
O157	2867481	s	rec	G2583_2647	yehD	CDS
O157	2867487	s	rec	G2583_2647	yehD	CDS
O157	2867496	s	rec	G2583_2647	yehD	CDS
O157	2867505	s	rec	G2583_2647	yehD	CDS
O157	2867508	s	rec	G2583_2647	yehD	CDS
O157	2867517	s	rec	G2583_2647	yehD	CDS
O157	2867520	ns	rec	G2583_2647	yehD	CDS
O157	2867523	s	rec	G2583_2647	yehD	CDS
O157	2867526	s	rec	G2583_2647	yehD	CDS
O157	2867538	s	rec	G2583_2647	yehD	CDS
O157	2867550	s	rec	G2583_2647	yehD	CDS
O157	2867556	s	rec	G2583_2647	yehD	CDS
O157	2867561	ns	rec	G2583_2647	yehD	CDS
O157	2867568	s	rec	G2583_2647	yehD	CDS
O157	2867571	s	rec	G2583_2647	yehD	CDS

lineage[a]	site[b]	mutation type[c]	recomb	gene	Gene name	Type
O157	2867574	s	rec	G2583_2647	yehD	CDS
O157	2867578	ns	rec	G2583_2647	yehD	CDS
O157	2867583	s	rec	G2583_2647	yehD	CDS
O157	2867586	s	rec	G2583_2647	yehD	CDS
O157	2867601	s	rec	G2583_2647	yehD	CDS
O157	2867604	s	rec	G2583_2647	yehD	CDS
O157	2867607	s	rec	G2583_2647	yehD	CDS
O157	2867610	s	rec	G2583_2647	yehD	CDS
O157	2867613	s	rec	G2583_2647	yehD	CDS
O157	2867616	s	rec	G2583_2647	yehD	CDS
O157	2867624	ns	rec	G2583_2647	yehD	CDS
O157	2867628	s	rec	G2583_2647	yehD	CDS
O157	2867637	s	rec	G2583_2647	yehD	CDS
O157	2867644	ns	rec	G2583_2647	yehD	CDS
O157	2867645	ns	rec	G2583_2647	yehD	CDS
O157	2867646	s	rec	G2583_2647	yehD	CDS
O157	2867652	ns	rec	G2583_2647	yehD	CDS
O157	2867661	s	rec	G2583_2647	yehD	CDS
O157	2867670	s	rec	G2583_2647	yehD	CDS
O157	2867679	s	rec	G2583_2647	yehD	CDS
O157	2867683	ns	rec	G2583_2647	yehD	CDS
O157	2867687	ns	rec	G2583_2647	yehD	CDS
O157	2867688	ns	rec	G2583_2647	yehD	CDS
O157	2867689	ns	rec	G2583_2647	yehD	CDS
O157	2867690	ns	rec	G2583_2647	yehD	CDS
O157	2867691	s	rec	G2583_2647	yehD	CDS
O157	2867697	s	rec	G2583_2647	yehD	CDS
O157	2867700	ns	rec	G2583_2647	yehD	CDS
O157	2867701	ns	rec	G2583_2647	yehD	CDS
O157	2867709	s	rec	G2583_2647	yehD	CDS
O157	2867711	s	rec	G2583_2647	yehD	CDS
O157	2867715	s	rec	G2583_2647	yehD	CDS
O157	2867730	s	rec	G2583_2647	yehD	CDS
O157	2867733	s	rec	G2583_2647	yehD	CDS
O157	2867736	ns	rec	G2583_2647	yehD	CDS
O157	2867737	ns	rec	G2583_2647	yehD	CDS
O157	2867745	s	rec	G2583_2647	yehD	CDS
O157	2867748	s	rec	G2583_2647	yehD	CDS
O157	2867757	s	rec	G2583_2647	yehD	CDS
O157	2867766	ns	rec	G2583_2647	yehD	CDS
O157	2867772	s	rec	G2583_2647	yehD	CDS
O157	2867781	s	rec	G2583_2647	yehD	CDS
O157	2867805	s	rec	G2583_2647	yehD	CDS
O157	2867817	s	rec	G2583_2647	yehD	CDS
O157	2867820	s	rec	G2583_2647	yehD	CDS

lineage[a]	site[b]	mutation type[c]	recomb	gene	Gene name	Type
O157	2867823	ns	rec	G2583_2647	yehD	CDS
O157	2867824	ns	rec	G2583_2647	yehD	CDS
O157	2867826	s	rec	G2583_2647	yehD	CDS
O157	2867830	ns	rec	G2583_2647	yehD	CDS
O157	2867835	s	rec	G2583_2647	yehD	CDS
O157	2867838	s	rec	G2583_2647	yehD	CDS
O157	2867853	s	rec	G2583_2647	yehD	CDS
O157	2867862	s	rec	G2583_2647	yehD	CDS
O157	2867868	s	rec	G2583_2647	yehD	CDS
O157	2867880	s	rec	G2583_2647	yehD	CDS
O157	2867883	ns	rec	G2583_2647	yehD	CDS
O157	2867886	s	rec	G2583_2647	yehD	CDS
O157	2867892	s	rec	G2583_2647	yehD	CDS
O157	2867895	ns	rec	G2583_2647	yehD	CDS
O157	2867897	ns	rec	G2583_2647	yehD	CDS
O157	2867898	s	rec	G2583_2647	yehD	CDS
O157	2867904	s	rec	G2583_2647	yehD	CDS
O157	2867913	ns	rec	G2583_2647	yehD	CDS
O157	2867915	ns	rec	G2583_2647	yehD	CDS
O157	2867922	ns	rec	G2583_2647	yehD	CDS
O157	2867947	ns	rec	G2583_2647	yehD	CDS
O157	2867955	i	rec	intergenic		
O157	2867958	i	rec	intergenic		
O157	2867959	i	rec	intergenic		
O157	2867960	i	rec	intergenic		
O157	2867969	del	rec	intergenic		
O157	2867972	i	rec	intergenic		
O157	2867981	i	rec	intergenic		
O157	2867982	i	rec	intergenic		
O157	2867983	i	rec	intergenic		
O157	2868031	i	rec	intergenic		
O157	2868059	i	rec	intergenic		
O157	2868080	i	rec	intergenic		
O157	2868082	i	rec	intergenic		
O157	2868105	i	rec	intergenic		
O157	2868108	i	rec	intergenic		
O157	2868109	i	rec	intergenic		
O157	2868110	i	rec	intergenic		
O157	2868111	i	rec	intergenic		
O157	2868112	i	rec	intergenic		
O157	2868115	i	rec	intergenic		
O157	2868116	i	rec	intergenic		
O157	2868120	i	rec	intergenic		
O157	2868125	i	rec	intergenic		
O157	2868127	i	rec	intergenic		

lineage[a]	site[b]	mutation type[c]	recomb	gene	Gene name	Type
O157	2868128	i	rec	intergenic		
O157	2868129	i	rec	intergenic		
O157	2868131	i	rec	intergenic		
O157	2868132	i	rec	intergenic		
O157	2868133	i	rec	intergenic		
O157	2868142	i	rec	intergenic		
O157	2868143	i	rec	intergenic		
O157	2868147	i	rec	intergenic		
O157	2868151	i	rec	intergenic		
O157	2868152	i	rec	intergenic		
O157	2868153	i	rec	intergenic		
O157	2868160	i	rec	intergenic		
O157	2868161	i	rec	intergenic		
O157	2868162	i	rec	intergenic		
O157	2868166	i	rec	intergenic		
O157	2868167	i	rec	intergenic		
O157	2868170	i	rec	intergenic		
O157	2868171	i	rec	intergenic		
O157	2868174	i	rec	intergenic		
O157	2868177	del	rec	intergenic		
O157	2868187	i	rec	intergenic		
O157	2868196	i	rec	intergenic		
O157	2868200	i	rec	intergenic		
O157	2868201	i	rec	intergenic		
O157	2868210	i	rec	intergenic		
O157	2868217	i	rec	intergenic		
O157	2868223	i	rec	intergenic		
O157	2868224	i	rec	intergenic		
O157	2868225	i	rec	intergenic		
O157	2868226	i	rec	intergenic		
O157	2868233	i	rec	intergenic		
O157	2868234	i	rec	intergenic		
O157	2868235	i	rec	intergenic		
O157	2868236	i	rec	intergenic		
O157	2868241	i	rec	intergenic		
O157	2868271	ns	rec	G2583_2648	yehE	CDS
O157	2868272	ns	rec	G2583_2648	yehE	CDS
O157	2868273	s	rec	G2583_2648	yehE	CDS
O157	2868280	ns	rec	G2583_2648	yehE	CDS
O157	2868281	ns	rec	G2583_2648	yehE	CDS
O157	2868282	ns	rec	G2583_2648	yehE	CDS
O157	2868284	ns	rec	G2583_2648	yehE	CDS
O157	2868286	ns	rec	G2583_2648	yehE	CDS
O157	2868291	ns	rec	G2583_2648	yehE	CDS
O157	2868292	ns	rec	G2583_2648	yehE	CDS

lineage[a]	site[b]	mutation type[c]	recomb	gene	Gene name	Type
O157	2868295	ns	rec	G2583_2648	yehE	CDS
O157	2868297	s	rec	G2583_2648	yehE	CDS
O157	2868312	s	rec	G2583_2648	yehE	CDS
O157	2868315	s	rec	G2583_2648	yehE	CDS
O157	2868321	ns	rec	G2583_2648	yehE	CDS
O157	2868322	ns	rec	G2583_2648	yehE	CDS
O157	2868324	ns	rec	G2583_2648	yehE	CDS
O157	2868326	ns	rec	G2583_2648	yehE	CDS
O157	2868327	s	rec	G2583_2648	yehE	CDS
O157	2868334	ns	rec	G2583_2648	yehE	CDS
O157	2868336	ns	rec	G2583_2648	yehE	CDS
O157	2868337	ns	rec	G2583_2648	yehE	CDS
O157	2868341	ns	rec	G2583_2648	yehE	CDS
O157	2868342	ns	rec	G2583_2648	yehE	CDS
O157	2868343	ns	rec	G2583_2648	yehE	CDS
O157	2868344	ns	rec	G2583_2648	yehE	CDS
O157	2868348	ns	rec	G2583_2648	yehE	CDS
O157	2868351	s	rec	G2583_2648	yehE	CDS
O157	2868354	ns	rec	G2583_2648	yehE	CDS
O157	2868355	ns	rec	G2583_2648	yehE	CDS
O157	2868356	ns	rec	G2583_2648	yehE	CDS
O157	2868357	ns	rec	G2583_2648	yehE	CDS
O157	2868359	ns	rec	G2583_2648	yehE	CDS
O157	2868360	ns	rec	G2583_2648	yehE	CDS
O157	2868361	ns	rec	G2583_2648	yehE	CDS
O157	2868362	ns	rec	G2583_2648	yehE	CDS
O157	2868363	s	rec	G2583_2648	yehE	CDS
O157	2868369	s	rec	G2583_2648	yehE	CDS
O157	2868376	ns	rec	G2583_2648	yehE	CDS
O157	2868378	s	rec	G2583_2648	yehE	CDS
O157	2868381	s	rec	G2583_2648	yehE	CDS
O157	2868385	ns	rec	G2583_2648	yehE	CDS
O157	2868387	ns	rec	G2583_2648	yehE	CDS
O157	2868391	ns	rec	G2583_2648	yehE	CDS
O157	2868399	ns	rec	G2583_2648	yehE	CDS
O157	2868401	ns	rec	G2583_2648	yehE	CDS
O157	2868403	ns	rec	G2583_2648	yehE	CDS
O157	2868417	s	rec	G2583_2648	yehE	CDS
O157	2868422	ns	rec	G2583_2648	yehE	CDS
O157	2868426	s	rec	G2583_2648	yehE	CDS
O157	2868429	s	rec	G2583_2648	yehE	CDS
O157	2868438	s	rec	G2583_2648	yehE	CDS
O157	2868441	s	rec	G2583_2648	yehE	CDS
O157	2868450	ns	rec	G2583_2648	yehE	CDS
O157	2868452	ns	rec	G2583_2648	yehE	CDS

lineage[a]	site[b]	mutation type[c]	recomb	gene	Gene name	Type
O157	2868456	ns	rec	G2583_2648	yehE	CDS
O157	2868459	s	rec	G2583_2648	yehE	CDS
O157	2868474	s	rec	G2583_2648	yehE	CDS
O157	2868477	s	rec	G2583_2648	yehE	CDS
O157	2868480	s	rec	G2583_2648	yehE	CDS
O157	2868482	s	rec	G2583_2648	yehE	CDS
O157	2868489	s	rec	G2583_2648	yehE	CDS
O157	2868500	ns	rec	G2583_2648	yehE	CDS
O157	2868502	ns	rec	G2583_2648	yehE	CDS
O157	2868503	ns	rec	G2583_2648	yehE	CDS
O157	2868512	s	rec	G2583_2648	yehE	CDS
O157	2868558	del	rec	intergenic		
O157	2868561	i	rec	intergenic		
O157	2868566	i	rec	intergenic		
O157	2868580	i	rec	intergenic		
O157	2868598	i	rec	intergenic		
O157	2868607	ins	rec	intergenic		
O157	2868613	i	rec	intergenic		
O157	2868623	i	rec	intergenic		
O157	2868626	i	rec	intergenic		
O157	2868627	i	rec	intergenic		
O157	2868630	i	rec	intergenic		
O157	2868633	i	rec	intergenic		
O157	2868634	i	rec	intergenic		
O157	2868637	i	rec	intergenic		
O157	2868640	i	rec	intergenic		
O157	2868649	i	rec	intergenic		
O157	2868657	i	rec	intergenic		
O157	2868668	i	rec	intergenic		
O157	2868672	i	rec	intergenic		
O157	2868676	i	rec	intergenic		
O157	2868678	i	rec	intergenic		
O157	2868741	i	rec	intergenic		
O157	2868768	i	rec	intergenic		
O157	2868948	s	rec	G2583_2649	mrp	CDS
O157	2868951	s	rec	G2583_2649	mrp	CDS
O157	2868975	s	rec	G2583_2649	mrp	CDS
O157	2868981	s	rec	G2583_2649	mrp	CDS
O157	2868990	s	rec	G2583_2649	mrp	CDS
O157	2868993	s	rec	G2583_2649	mrp	CDS
O157	2869020	s	rec	G2583_2649	mrp	CDS
O157	2870369	s	rec	G2583_2650	metG	CDS
O157	2870540	s	rec	G2583_2650	metG	CDS
O157	2870575	s	rec	G2583_2650	metG	CDS
O157	2870707	s	rec	G2583_2650	metG	CDS

lineage[a]	site[b]	mutation type[c]	recomb	gene	Gene name	Type
O157	2870794	s	rec	G2583_2650	metG	CDS
O157	2870938	s	rec	G2583_2650	metG	CDS
O157	2870950	s	rec	G2583_2650	metG	CDS
O157	2870990	s	rec	G2583_2650	metG	CDS
O157	2871001	s	rec	G2583_2650	metG	CDS
O157	2871013	s	rec	G2583_2650	metG	CDS
O157	2871022	s	rec	G2583_2650	metG	CDS
O157	2871034	s	rec	G2583_2650	metG	CDS
O157	2871355	s	rec	G2583_2650	metG	CDS
O157	2871364	s	rec	G2583_2650	metG	CDS
O157	2871424	s	rec	G2583_2650	metG	CDS
O157	2871445	s	rec	G2583_2650	metG	CDS
O157	2871457	s	rec	G2583_2650	metG	CDS
O157	2871466	s	rec	G2583_2650	metG	CDS
O157	2871499	s	rec	G2583_2650	metG	CDS
O157	2871508	s	rec	G2583_2650	metG	CDS
O157	2871529	s	rec	G2583_2650	metG	CDS
O157	2871532	s	rec	G2583_2650	metG	CDS
O157	2871535	s	rec	G2583_2650	metG	CDS
O157	2871547	s	rec	G2583_2650	metG	CDS
O157	2871590	s	rec	G2583_2650	metG	CDS
O157	2871598	s	rec	G2583_2650	metG	CDS
O157	2871613	s	rec	G2583_2650	metG	CDS
O157	2871643	s	rec	G2583_2650	metG	CDS
O157	2871667	s	rec	G2583_2650	metG	CDS
O157	2871688	s	rec	G2583_2650	metG	CDS
O157	2871700	s	rec	G2583_2650	metG	CDS
O157	2871703	s	rec	G2583_2650	metG	CDS
O157	2871712	s	rec	G2583_2650	metG	CDS
O157	2871721	s	rec	G2583_2650	metG	CDS
O157	2871760	s	rec	G2583_2650	metG	CDS
O157	2871826	s	rec	G2583_2650	metG	CDS
O157	2871886	s	rec	G2583_2650	metG	CDS
O157	2871892	s	rec	G2583_2650	metG	CDS
O157	2872066	i	rec	intergenic		
O157	2872069	i	rec	intergenic		
O157	2872082	i	rec	intergenic		
O157	2872127	i	rec	intergenic		
O157	2872151	i	rec	intergenic		
O157	2872337	ns	rec	G2583_2651	molR_A	CDS
O157	2872341	s	rec	G2583_2651	molR_A	CDS
O157	2872349	ns	rec	G2583_2651	molR_A	CDS
O157	2872357	ns	rec	G2583_2651	molR_A	CDS
O157	2872359	ns	rec	G2583_2651	molR_A	CDS
O157	2872362	s	rec	G2583_2651	molR_A	CDS

lineage[a]	site[b]	mutation type[c]	recomb	gene	Gene name	Type
O157	2872368	s	rec	G2583_2651	molR_A	CDS
O157	2872374	s	rec	G2583_2651	molR_A	CDS
O157	2872434	s	rec	G2583_2651	molR_A	CDS
O157	2872488	s	rec	G2583_2651	molR_A	CDS
O157	2872506	s	rec	G2583_2651	molR_A	CDS
O157	2872509	s	rec	G2583_2651	molR_A	CDS
O157	2872514	ns	rec	G2583_2651	molR_A	CDS
O157	2872516	ns	rec	G2583_2651	molR_A	CDS
O157	2872533	s	rec	G2583_2651	molR_A	CDS
O157	2872623	s	rec	G2583_2651	molR_A	CDS
O157	2872662	s	rec	G2583_2651	molR_A	CDS
O157	2872697	ns	rec	G2583_2651	molR_A	CDS
O157	2872702	ns	rec	G2583_2651	molR_A	CDS
O157	2872706	ns	rec	G2583_2651	molR_A	CDS
O157	2872709	ns	rec	G2583_2651	molR_A	CDS
O157	2872725	s	rec	G2583_2651	molR_A	CDS
O157	2872743	s	rec	G2583_2651	molR_A	CDS
O157	2872761	s	rec	G2583_2651	molR_A	CDS
O157	2872777	ns	rec	G2583_2651	molR_A	CDS
O157	2872824	s	rec	G2583_2651	molR_A	CDS
O157	2872838	ns	rec	G2583_2651	molR_A	CDS
O157	2872843	ns	rec	G2583_2651	molR_A	CDS
O157	2872845	ns	rec	G2583_2651	molR_A	CDS
O157	2872848	s	rec	G2583_2651	molR_A	CDS
O157	2872857	s	rec	G2583_2651	molR_A	CDS
O157	2872859	ns	rec	G2583_2651	molR_A	CDS
O157	2872892	ns	rec	G2583_2651	molR_A	CDS
O157	2872909	ns	rec	G2583_2651	molR_A	CDS
O157	2872931	ns	rec	G2583_2651	molR_A	CDS
O157	2873005	ns	rec	G2583_2651	molR_A	CDS
O157	2873007	ns	rec	G2583_2651	molR_A	CDS
O157	2873010	s	rec	G2583_2651	molR_A	CDS
O157	2873016	s	rec	G2583_2651	molR_A	CDS
O157	2873017	ns	rec	G2583_2651	molR_A	CDS
O157	2873028	s	rec	G2583_2651	molR_A	CDS
O157	2873035	ns	rec	G2583_2651	molR_A	CDS
O157	2873076	s	rec	G2583_2651	molR_A	CDS
O157	2873080	ns	rec	G2583_2651	molR_A	CDS
O157	2873088	ns	rec	G2583_2651	molR_A	CDS
O157	2873090	ns	rec	G2583_2651	molR_A	CDS
O157	2873091	ns	rec	G2583_2651	molR_A	CDS
O157	2873097	s	rec	G2583_2651	molR_A	CDS
O157	2873115	s	rec	G2583_2651	molR_A	CDS
O157	2873124	ns	rec	G2583_2651	molR_A	CDS
O157	2873136	ns	rec	G2583_2651	molR_A	CDS

lineage[a]	site[b]	mutation type[c]	recomb	gene	Gene name	Type
O157	2873137	ns	rec	G2583_2651	molR_A	CDS
O157	2873140	ns	rec	G2583_2651	molR_A	CDS
O157	2873143	ns	rec	G2583_2651	molR_A	CDS
O157	2873147	ns	rec	G2583_2651	molR_A	CDS
O157	2873155	ns	rec	G2583_2651	molR_A	CDS
O157	2873156	ns	rec	G2583_2651	molR_A	CDS
O157	2873160	s	rec	G2583_2651	molR_A	CDS
O157	2873164	ns	rec	G2583_2651	molR_A	CDS
O157	2873165	ns	rec	G2583_2651	molR_A	CDS
O157	2873166	ns	rec	G2583_2651	molR_A	CDS
O157	2873168	ns	rec	G2583_2651	molR_A	CDS
O157	2873169	ns	rec	G2583_2651	molR_A	CDS
O157	2873170	ns	rec	G2583_2651	molR_A	CDS
O157	2873171	ns	rec	G2583_2651	molR_A	CDS
O157	2873181	s	rec	G2583_2651	molR_A	CDS
O157	2873183	ns	rec	G2583_2651	molR_A	CDS
O157	2873184	ns	rec	G2583_2651	molR_A	CDS
O157	2873194	s	rec	G2583_2651	molR_A	CDS
O157	2873196	s	rec	G2583_2651	molR_A	CDS
O157	2873200	ns	rec	G2583_2651	molR_A	CDS
O157	2873201	ns	rec	G2583_2651	molR_A	CDS
O157	2873208	s	rec	G2583_2651	molR_A	CDS
O157	2873211	s	rec	G2583_2651	molR_A	CDS
O157	2873214	ins-9	rec	G2583_2651	molR_A	CDS
O157	2873223	ns	rec	G2583_2651	molR_A	CDS
O157	2873232	s	rec	G2583_2651	molR_A	CDS
O157	2873234	ns	rec	G2583_2651	molR_A	CDS
O157	2873239	s	rec	G2583_2651	molR_A	CDS
O157	2873253	s	rec	G2583_2651	molR_A	CDS
O157	2873254	ns	rec	G2583_2651	molR_A	CDS
O157	2873259	s	rec	G2583_2651	molR_A	CDS
O157	2873274	s	rec	G2583_2651	molR_A	CDS
O157	2873280	s	rec	G2583_2651	molR_A	CDS
O157	2873281	ns	rec	G2583_2651	molR_A	CDS
O157	2873284	ns	rec	G2583_2651	molR_A	CDS
O157	2873286	ns	rec	G2583_2651	molR_A	CDS
O157	2873287	ns	rec	G2583_2651	molR_A	CDS
O157	2873289	ns	rec	G2583_2651	molR_A	CDS
O157	2873291	ns	rec	G2583_2651	molR_A	CDS
O157	2873292	ns	rec	G2583_2651	molR_A	CDS
O157	2873296	ns	rec	G2583_2651	molR_A	CDS
O157	2873299	ns	rec	G2583_2651	molR_A	CDS
O157	2873301	ns	rec	G2583_2651	molR_A	CDS
O157	2873303	ns	rec	G2583_2651	molR_A	CDS
O157	2873304	ns	rec	G2583_2651	molR_A	CDS

lineage[a]	site[b]	mutation type[c]	recomb	gene	Gene name	Type
O157	2873307	ns	rec	G2583_2651	molR_A	CDS
O157	2873308	ns	rec	G2583_2651	molR_A	CDS
O157	2873309	ns	rec	G2583_2651	molR_A	CDS
O157	2873313	s	rec	G2583_2651	molR_A	CDS
O157	2873314	ns	rec	G2583_2651	molR_A	CDS
O157	2873316	ns	rec	G2583_2651	molR_A	CDS
O157	2873319	s	rec	G2583_2651	molR_A	CDS
O157	2873323	ns	rec	G2583_2651	molR_A	CDS
O157	2873324	ns	rec	G2583_2651	molR_A	CDS
O157	2873325	ns	rec	G2583_2651	molR_A	CDS
O157	2873327	ns	rec	G2583_2651	molR_A	CDS
O157	2873330	del-3	rec	G2583_2651	molR_A	CDS
O157	2873332	ns	rec	G2583_2651	molR_A	CDS
O157	2873333	ns	rec	G2583_2651	molR_A	CDS
O157	2873335	ns	rec	G2583_2651	molR_A	CDS
O157	2873337	ns	rec	G2583_2651	molR_A	CDS
O157	2873338	ns	rec	G2583_2651	molR_A	CDS
O157	2873344	ns	rec	G2583_2651	molR_A	CDS
O157	2873411	ns	rec	G2583_2651	molR_A	CDS
O157	2873415	s	rec	G2583_2651	molR_A	CDS
O157	2873445	s	rec	G2583_2651	molR_A	CDS
O157	2873449	ns	rec	G2583_2651	molR_A	CDS
O157	2873451	ns	rec	G2583_2651	molR_A	CDS
O157	2873457	s	rec	G2583_2651	molR_A	CDS
O157	2873478	s	rec	G2583_2651	molR_A	CDS
O157	2873509	ns	rec	G2583_2651	molR_A	CDS
O157	2873514	ns	rec	G2583_2651	molR_A	CDS
O157	2873547	s	rec	G2583_2651	molR_A	CDS
O157	2873571	ns	rec	G2583_2651	molR_A	CDS
O157	2873595	s	rec	G2583_2651	molR_A	CDS
O157	2873627	ns	rec	G2583_2651	molR_A	CDS
O157	2873629	ns	rec	G2583_2651	molR_A	CDS
O157	2873634	s	rec	G2583_2651	molR_A	CDS
O157	2873685	s	rec	G2583_2651	molR_A	CDS
O157	2873686	ns	rec	G2583_2651	molR_A	CDS
O157	2873688	ns	rec	G2583_2651	molR_A	CDS
O157	2873691	s	rec	G2583_2651	molR_A	CDS
O157	2873694	s	rec	G2583_2651	molR_A	CDS
O157	2873698	ns	rec	G2583_2651	molR_A	CDS
O157	2873700	ns	rec	G2583_2651	molR_A	CDS
O157	2873702	ns	rec	G2583_2651	molR_A	CDS
O157	2873703	ns	rec	G2583_2651	molR_A	CDS
O157	2873706	ns	rec	G2583_2651	molR_A	CDS
O157	2873707	ns	rec	G2583_2651	molR_A	CDS
O157	2873718	ns	rec	G2583_2651	molR_A	CDS

lineage[a]	site[b]	mutation type[c]	recomb	gene	Gene name	Type
O157	2873719	ns	rec	G2583_2651	molR_A	CDS
O157	2873720	ns	rec	G2583_2651	molR_A	CDS
O157	2873721	ns	rec	G2583_2651	molR_A	CDS
O157	2873723	ns	rec	G2583_2651	molR_A	CDS
O157	2873724	ns	rec	G2583_2651	molR_A	CDS
O157	2873727	s	rec	G2583_2651	molR_A	CDS
O157	2873730	s	rec	G2583_2651	molR_A	CDS
O157	2873737	s	rec	G2583_2651	molR_A	CDS
O157	2873739	s	rec	G2583_2651	molR_A	CDS
O157	2873742	s	rec	G2583_2651	molR_A	CDS
O157	2873745	s	rec	G2583_2651	molR_A	CDS
O157	2873751	s	rec	G2583_2651	molR_A	CDS
O157	2873752	ns	rec	G2583_2651	molR_A	CDS
O157	2873758	ns	rec	G2583_2651	molR_A	CDS
O157	2873760	ns	rec	G2583_2651	molR_A	CDS
O157	2873763	s	rec	G2583_2651	molR_A	CDS
O157	2873764	ns	rec	G2583_2651	molR_A	CDS
O157	2873766	ns	rec	G2583_2651	molR_A	CDS
O157	2873770	ns	rec	G2583_2651	molR_A	CDS
O157	2873775	s	rec	G2583_2651	molR_A	CDS
O157	2873780	ns	rec	G2583_2651	molR_A	CDS
O157	2873781	ns	rec	G2583_2651	molR_A	CDS
O157	2873787	s	rec	G2583_2651	molR_A	CDS
O157	2873788	ns	rec	G2583_2651	molR_A	CDS
O157	2873790	ns	rec	G2583_2651	molR_A	CDS
O157	2873791	ns	rec	G2583_2651	molR_A	CDS
O157	2873792	ns	rec	G2583_2651	molR_A	CDS
O157	2873794	ns	rec	G2583_2651	molR_A	CDS
O157	2873796	ns	rec	G2583_2651	molR_A	CDS
O157	2873798	ns	rec	G2583_2651	molR_A	CDS
O157	2873799	ns	rec	G2583_2651	molR_A	CDS
O157	2873801	ns	rec	G2583_2651	molR_A	CDS
O157	2873806	ns	rec	G2583_2651	molR_A	CDS
O157	2873809	ns	rec	G2583_2651	molR_A	CDS
O157	2873811	ins-4	rec	G2583_2651	molR_A	CDS
O157	2873816	ns	rec	G2583_2651	molR_A	CDS
O157	2873820	ns	rec	G2583_2651	molR_A	CDS
O157	2873821	ns	rec	G2583_2651	molR_A	CDS
O157	2873823	ns	rec	G2583_2651	molR_A	CDS
O157	2873827	s	rec	G2583_2651	molR_A	CDS
O157	2873827	del	rec	G2583_2651	molR_A	CDS
O157	2873832	s	rec	G2583_2651	molR_A	CDS
O157	2873835	s	rec	G2583_2651	molR_A	CDS
O157	2873839	ns	rec	G2583_2651	molR_A	CDS
O157	2873841	ns	rec	G2583_2651	molR_A	CDS

lineage[a]	site[b]	mutation type[c]	recomb	gene	Gene name	Type
O157	2873847	s	rec	G2583_2651	molR_A	CDS
O157	2873848	ns	rec	G2583_2651	molR_A	CDS
O157	2873851	ns	rec	G2583_2651	molR_A	CDS
O157	2873853	ns	rec	G2583_2651	molR_A	CDS
O157	2873859	s	rec	G2583_2651	molR_A	CDS
O157	2873862	s	rec	G2583_2651	molR_A	CDS
O157	2873871	s	rec	G2583_2651	molR_A	CDS
O157	2873877	s	rec	G2583_2651	molR_A	CDS
O157	2873879	ns	rec	G2583_2651	molR_A	CDS
O157	2873880	ns	rec	G2583_2651	molR_A	CDS
O157	2873883	s	rec	G2583_2651	molR_A	CDS
O157	2873884	ns	rec	G2583_2651	molR_A	CDS
O157	2873891	ns	rec	G2583_2651	molR_A	CDS
O157	2873903	ns	rec	G2583_2651	molR_A	CDS
O157	2873905	ns	rec	G2583_2651	molR_A	CDS
O157	2873906	ns	rec	G2583_2651	molR_A	CDS
O157	2873907	ns	rec	G2583_2651	molR_A	CDS
O157	2873910	ns	rec	G2583_2651	molR_A	CDS
O157	2873913	s	rec	G2583_2651	molR_A	CDS
O157	2873916	s	rec	G2583_2651	molR_A	CDS
O157	2873917	ns	rec	G2583_2651	molR_A	CDS
O157	2873925	s	rec	G2583_2651	molR_A	CDS
O157	2873928	s	rec	G2583_2651	molR_A	CDS
O157	2873937	s	rec	G2583_2651	molR_A	CDS
O157	2873940	s	rec	G2583_2651	molR_A	CDS
O157	2873946	ns	rec	G2583_2651	molR_A	CDS
O157	2873947	s	rec	G2583_2651	molR_A	CDS
O157	2873949	s	rec	G2583_2651	molR_A	CDS
O157	2873956	ns	rec	G2583_2651	molR_A	CDS
O157	2873958	ns	rec	G2583_2651	molR_A	CDS
O157	2873959	ns	rec	G2583_2651	molR_A	CDS
O157	2873966	ns	rec	G2583_2651	molR_A	CDS
O157	2873968	ns	rec	G2583_2651	molR_A	CDS
O157	2873970	ns	rec	G2583_2651	molR_A	CDS
O157	2873971	ns	rec	G2583_2651	molR_A	CDS
O157	2873975	ns	rec	G2583_2651	molR_A	CDS
O157	2873977	ns	rec	G2583_2651	molR_A	CDS
O157	2873979	ns	rec	G2583_2651	molR_A	CDS
O157	2873980	ns	rec	G2583_2651	molR_A	CDS
O157	2873982	ns	rec	G2583_2651	molR_A	CDS
O157	2873983	ns	rec	G2583_2651	molR_A	CDS
O157	2873986	ns	rec	G2583_2651	molR_A	CDS
O157	2873988	ns	rec	G2583_2651	molR_A	CDS
O157	2873996	ns	rec	G2583_2651	molR_A	CDS
O157	2873997	ns	rec	G2583_2651	molR_A	CDS

lineage[a]	site[b]	mutation type[c]	recomb	gene	Gene name	Type
O157	2873998	ns	rec	G2583_2651	molR_A	CDS
O157	2873999	ns	rec	G2583_2651	molR_A	CDS
O157	2874000	ns	rec	G2583_2651	molR_A	CDS
O157	2874001	ns	rec	G2583_2651	molR_A	CDS
O157	2874002	ns	rec	G2583_2651	molR_A	CDS
O157	2874003	ns	rec	G2583_2651	molR_A	CDS
O157	2874006	s	rec	G2583_2651	molR_A	CDS
O157	2874015	s	rec	G2583_2651	molR_A	CDS
O157	2874025	s	rec	G2583_2651	molR_A	CDS
O157	2874028	s	rec	G2583_2651	molR_A	CDS
O157	2874036	s	rec	G2583_2651	molR_A	CDS
O157	2874051	s	rec	G2583_2651	molR_A	CDS
O157	2874054	ns	rec	G2583_2651	molR_A	CDS
O157	2874060	s	rec	G2583_2651	molR_A	CDS
O157	2874065	ns	rec	G2583_2651	molR_A	CDS
O157	2874067	ns	rec	G2583_2651	molR_A	CDS
O157	2874069	ns	rec	G2583_2651	molR_A	CDS
O157	2874075	s	rec	G2583_2651	molR_A	CDS
O157	2874084	s	rec	G2583_2651	molR_A	CDS
O157	2874085	ns	rec	G2583_2651	molR_A	CDS
O157	2874102	s	rec	G2583_2651	molR_A	CDS
O157	2874103	ns	rec	G2583_2651	molR_A	CDS
O157	2874104	ns	rec	G2583_2651	molR_A	CDS
O157	2874108	s	rec	G2583_2651	molR_A	CDS
O157	2874111	s	rec	G2583_2651	molR_A	CDS
O157	2874118	s	rec	G2583_2651	molR_A	CDS
O157	2874120	s	rec	G2583_2651	molR_A	CDS
O157	2874123	s	rec	G2583_2651	molR_A	CDS
O157	2874126	s	rec	G2583_2651	molR_A	CDS
O157	2874135	s	rec	G2583_2651	molR_A	CDS
O157	2874141	s	rec	G2583_2651	molR_A	CDS
O157	2874147	s	rec	G2583_2651	molR_A	CDS
O157	2874153	s	rec	G2583_2651	molR_A	CDS
O157	2874157	ns	rec	G2583_2651	molR_A	CDS
O157	2874161	ns	rec	G2583_2651	molR_A	CDS
O157	2874162	ns	rec	G2583_2651	molR_A	CDS
O157	2874165	s	rec	G2583_2651	molR_A	CDS
O157	2874168	ns	rec	G2583_2651	molR_A	CDS
O157	2874169	ns	rec	G2583_2651	molR_A	CDS
O157	2874170	ns	rec	G2583_2651	molR_A	CDS
O157	2874171	ns	rec	G2583_2651	molR_A	CDS
O157	2874174	s	rec	G2583_2651	molR_A	CDS
O157	2874177	s	rec	G2583_2651	molR_A	CDS
O157	2874184	ns	rec	G2583_2651	molR_A	CDS
O157	2874185	ns	rec	G2583_2651	molR_A	CDS

lineage[a]	site[b]	mutation type[c]	recomb	gene	Gene name	Type
O157	2874187	ns	rec	G2583_2651	molR_A	CDS
O157	2874189	ns	rec	G2583_2651	molR_A	CDS
O157	2874198	s	rec	G2583_2651	molR_A	CDS
O157	2874199	ns	rec	G2583_2651	molR_A	CDS
O157	2874210	s	rec	G2583_2651	molR_A	CDS
O157	2874211	ns	rec	G2583_2651	molR_A	CDS
O157	2874213	ns	rec	G2583_2651	molR_A	CDS
O157	2874219	s	rec	G2583_2651	molR_A	CDS
O157	2874225	s	rec	G2583_2651	molR_A	CDS
O157	2874232	ns	rec	G2583_2651	molR_A	CDS
O157	2874238	s	rec	G2583_2651	molR_A	CDS
O157	2874246	s	rec	G2583_2651	molR_A	CDS
O157	2874251	ns	rec	G2583_2651	molR_A	CDS
O157	2874252	ns	rec	G2583_2651	molR_A	CDS
O157	2874254	ns	rec	G2583_2651	molR_A	CDS
O157	2874258	s	rec	G2583_2651	molR_A	CDS
O157	2874264	ns	rec	G2583_2651	molR_A	CDS
O157	2874265	ns	rec	G2583_2651	molR_A	CDS
O157	2874267	ns	rec	G2583_2651	molR_A	CDS
O157	2874271	ns	rec	G2583_2651	molR_A	CDS
O157	2874274	ns	rec	G2583_2651	molR_A	CDS
O157	2874279	s	rec	G2583_2651	molR_A	CDS
O157	2874280	ns	rec	G2583_2651	molR_A	CDS
O157	2874282	ns	rec	G2583_2651	molR_A	CDS
O157	2874285	s	rec	G2583_2651	molR_A	CDS
O157	2874287	ns	rec	G2583_2651	molR_A	CDS
O157	2874297	s	rec	G2583_2651	molR_A	CDS
O157	2874306	s	rec	G2583_2651	molR_A	CDS
O157	2874315	s	rec	G2583_2651	molR_A	CDS
O157	2874317	ns	rec	G2583_2651	molR_A	CDS
O157	2874318	ns	rec	G2583_2651	molR_A	CDS
O157	2874333	ns	rec	G2583_2651	molR_A	CDS
O157	2874334	ns	rec	G2583_2651	molR_A	CDS
O157	2874339	s	rec	G2583_2651	molR_A	CDS
O157	2874340	ns	rec	G2583_2651	molR_A	CDS
O157	2874342	ns	rec	G2583_2651	molR_A	CDS
O157	2874348	s	rec	G2583_2651	molR_A	CDS
O157	2874349	ns	rec	G2583_2651	molR_A	CDS
O157	2874350	ns	rec	G2583_2651	molR_A	CDS
O157	2874351	ns	rec	G2583_2651	molR_A	CDS
O157	2874354	s	rec	G2583_2651	molR_A	CDS
O157	2874360	s	rec	G2583_2651	molR_A	CDS
O157	2874361	ns	rec	G2583_2651	molR_A	CDS
O157	2874363	ns	rec	G2583_2651	molR_A	CDS
O157	2874364	ns	rec	G2583_2651	molR_A	CDS

lineage[a]	site[b]	mutation type[c]	recomb	gene	Gene name	Type
O157	2874367	ns	rec	G2583_2651	molR_A	CDS
O157	2874369	ns	rec	G2583_2651	molR_A	CDS
O157	2874372	s	rec	G2583_2651	molR_A	CDS
O157	2874393	s	rec	G2583_2651	molR_A	CDS
O157	2874396	s	rec	G2583_2651	molR_A	CDS
O157	2874400	ns	rec	G2583_2651	molR_A	CDS
O157	2874405	s	rec	G2583_2651	molR_A	CDS
O157	2874415	ns	rec	G2583_2651	molR_A	CDS
O157	2874416	ns	rec	G2583_2651	molR_A	CDS
O157	2874417	ns	rec	G2583_2651	molR_A	CDS
O157	2874418	ns	rec	G2583_2651	molR_A	CDS
O157	2874420	ns	rec	G2583_2651	molR_A	CDS
O157	2874421	ns	rec	G2583_2651	molR_A	CDS
O157	2874424	ns	rec	G2583_2651	molR_A	CDS
O157	2874429	s	rec	G2583_2651	molR_A	CDS
O157	2874432	ns	rec	G2583_2651	molR_A	CDS
O157	2874434	ns	rec	G2583_2651	molR_A	CDS
O157	2874447	s	rec	G2583_2651	molR_A	CDS
O157	2874450	s	rec	G2583_2651	molR_A	CDS
O157	2874452	ns	rec	G2583_2651	molR_A	CDS
O157	2874453	ns	rec	G2583_2651	molR_A	CDS
O157	2874459	s	rec	G2583_2651	molR_A	CDS
O157	2874465	ns	rec	G2583_2651	molR_A	CDS
O157	2874498	s	rec	G2583_2651	molR_A	CDS
O157	2874502	ns	rec	G2583_2651	molR_A	CDS
O157	2874543	nc	rec	G2583_2652	molR_B	pseudogene
O157	2874546	nc	rec	G2583_2652	molR_B	pseudogene
O157	2874573	nc	rec	G2583_2652	molR_B	pseudogene
O157	2874576	nc	rec	G2583_2652	molR_B	pseudogene
O157	2874610	nc	rec	G2583_2652	molR_B	pseudogene
O157	2874615	nc	rec	G2583_2652	molR_B	pseudogene
O157	2874627	nc	rec	G2583_2652	molR_B	pseudogene
O157	2874655	nc	rec	G2583_2652	molR_B	pseudogene
O157	2874684	nc	rec	G2583_2652	molR_B	pseudogene
O157	2874688	nc	rec	G2583_2652	molR_B	pseudogene
O157	2874699	nc	rec	G2583_2652	molR_B	pseudogene
O157	2874712	nc	rec	G2583_2652	molR_B	pseudogene
O157	2874728	nc	rec	G2583_2652	molR_B	pseudogene
O157	2874729	nc	rec	G2583_2652	molR_B	pseudogene
O157	2874735	nc	rec	G2583_2652	molR_B	pseudogene
O157	2874737	nc	rec	G2583_2652	molR_B	pseudogene
O157	2874741	nc	rec	G2583_2652	molR_B	pseudogene
O157	2874743	nc	rec	G2583_2652	molR_B	pseudogene
O157	2874768	nc	rec	G2583_2652	molR_B	pseudogene
O157	2874783	nc	rec	G2583_2652	molR_B	pseudogene

lineage[a]	site[b]	mutation type[c]	recomb	gene	Gene name	Type
O157	2874786	nc	rec	G2583_2652	molR_B	pseudogene
O157	2874787	nc	rec	G2583_2652	molR_B	pseudogene
O157	2874789	nc	rec	G2583_2652	molR_B	pseudogene
O157	2874792	nc	rec	G2583_2652	molR_B	pseudogene
O157	2874804	nc	rec	G2583_2652	molR_B	pseudogene
O157	2874822	nc	rec	G2583_2652	molR_B	pseudogene
O157	2874837	nc	rec	G2583_2652	molR_B	pseudogene
O157	2874843	nc	rec	G2583_2652	molR_B	pseudogene
O157	2874850	nc	rec	G2583_2652	molR_B	pseudogene
O157	2874862	nc	rec	G2583_2652	molR_B	pseudogene
O157	2874877	nc	rec	G2583_2652	molR_B	pseudogene
O157	2874888	nc	rec	G2583_2652	molR_B	pseudogene
O157	2874903	nc	rec	G2583_2652	molR_B	pseudogene
O157	2874906	nc	rec	G2583_2652	molR_B	pseudogene
O157	2874924	nc	rec	G2583_2652	molR_B	pseudogene
O157	2874930	nc	rec	G2583_2652	molR_B	pseudogene
O157	2874932	nc	rec	G2583_2652	molR_B	pseudogene
O157	2874945	nc	rec	G2583_2652	molR_B	pseudogene
O157	2874951	nc	rec	G2583_2652	molR_B	pseudogene
O157	2874952	nc	rec	G2583_2652	molR_B	pseudogene
O157	2874953	nc	rec	G2583_2652	molR_B	pseudogene
O157	2874957	nc	rec	G2583_2652	molR_B	pseudogene
O157	2874960	nc	rec	G2583_2652	molR_B	pseudogene
O157	2874966	nc	rec	G2583_2652	molR_B	pseudogene
O157	2874967	nc	rec	G2583_2652	molR_B	pseudogene
O157	2874975	nc	rec	G2583_2652	molR_B	pseudogene
O157	2874979	nc	rec	G2583_2652	molR_B	pseudogene
O157	2874993	nc	rec	G2583_2652	molR_B	pseudogene
O157	2874998	nc	rec	G2583_2652	molR_B	pseudogene
O157	2875002	nc	rec	G2583_2652	molR_B	pseudogene
O157	2875012	nc	rec	G2583_2652	molR_B	pseudogene
O157	2875013	nc	rec	G2583_2652	molR_B	pseudogene
O157	2875014	nc	rec	G2583_2652	molR_B	pseudogene
O157	2875033	nc	rec	G2583_2652	molR_B	pseudogene
O157	2875038	nc	rec	G2583_2652	molR_B	pseudogene
O157	2875040	nc	rec	G2583_2652	molR_B	pseudogene
O157	2875047	nc	rec	G2583_2652	molR_B	pseudogene
O157	2875050	nc	rec	G2583_2652	molR_B	pseudogene
O157	2875053	nc	rec	G2583_2652	molR_B	pseudogene
O157	2875056	nc	rec	G2583_2652	molR_B	pseudogene
O157	2875062	nc	rec	G2583_2652	molR_B	pseudogene
O157	2875065	nc	rec	G2583_2652	molR_B	pseudogene
O157	2875068	nc	rec	G2583_2652	molR_B	pseudogene
O157	2875074	nc	rec	G2583_2652	molR_B	pseudogene
O157	2875080	nc	rec	G2583_2652	molR_B	pseudogene

lineage[a]	site[b]	mutation type[c]	recomb	gene	Gene name	Type
O157	2875092	nc	rec	G2583_2652	molR_B	pseudogene
O157	2875101	nc	rec	G2583_2652	molR_B	pseudogene
O157	2875104	nc	rec	G2583_2652	molR_B	pseudogene
O157	2875254	nc	rec	G2583_2652	molR_B	pseudogene
O157	2875260	nc	rec	G2583_2652	molR_B	pseudogene
O157	2875262	nc	rec	G2583_2652	molR_B	pseudogene
O157	2875275	nc	rec	G2583_2652	molR_B	pseudogene
O157	2875308	nc	rec	G2583_2652	molR_B	pseudogene
O157	2875311	nc	rec	G2583_2652	molR_B	pseudogene
O157	2875314	nc	rec	G2583_2652	molR_B	pseudogene
O157	2875317	nc	rec	G2583_2652	molR_B	pseudogene
O157	2875329	nc	rec	G2583_2652	molR_B	pseudogene
O157	2875333	nc	rec	G2583_2652	molR_B	pseudogene
O157	2875334	nc	rec	G2583_2652	molR_B	pseudogene
O157	2875335	nc	rec	G2583_2652	molR_B	pseudogene
O157	2875557	nc	rec	G2583_2652	molR_B	pseudogene
O157	2875575	nc	rec	G2583_2652	molR_B	pseudogene
O157	2875576	nc	rec	G2583_2652	molR_B	pseudogene
O157	2875613	nc	rec	G2583_2652	molR_B	pseudogene
O157	2875650	nc	rec	G2583_2652	molR_B	pseudogene
O157	2875653	nc	rec	G2583_2652	molR_B	pseudogene
O157	2875659	nc	rec	G2583_2652	molR_B	pseudogene
O157	2875662	nc	rec	G2583_2652	molR_B	pseudogene
O157	2875677	nc	rec	G2583_2652	molR_B	pseudogene
O157	2875687	nc	rec	G2583_2652	molR_B	pseudogene
O157	2875688	nc	rec	G2583_2652	molR_B	pseudogene
O157	2875689	nc	rec	G2583_2652	molR_B	pseudogene
O157	2875692	nc	rec	G2583_2652	molR_B	pseudogene
O157	2875696	nc	rec	G2583_2652	molR_B	pseudogene
O157	2875699	nc	rec	G2583_2652	molR_B	pseudogene
O157	2875708	nc	rec	G2583_2652	molR_B	pseudogene
O157	2875710	nc	rec	G2583_2652	molR_B	pseudogene
O157	2875716	nc	rec	G2583_2652	molR_B	pseudogene
O157	2875719	nc	rec	G2583_2652	molR_B	pseudogene
O157	2875788	nc	rec	G2583_2652	molR_B	pseudogene
O157	2875793	nc	rec	G2583_2652	molR_B	pseudogene
O157	2875804	nc	rec	G2583_2652	molR_B	pseudogene
O157	2875806	nc	rec	G2583_2652	molR_B	pseudogene
O157	2875813	nc	rec	G2583_2652	molR_B	pseudogene
O157	2875818	nc	rec	G2583_2652	molR_B	pseudogene
O157	2875820	nc	rec	G2583_2652	molR_B	pseudogene
O157	2875836	nc	rec	G2583_2652	molR_B	pseudogene
O157	2875860	nc	rec	G2583_2652	molR_B	pseudogene
O157	2875873	nc	rec	G2583_2652	molR_B	pseudogene
O157	2875880	nc	rec	G2583_2652	molR_B	pseudogene

lineage[a]	site[b]	mutation type[c]	recomb	gene	Gene name	Type
O157	2875883	nc	rec	G2583_2652	molR_B	pseudogene
O157	2875884	nc	rec	G2583_2652	molR_B	pseudogene
O157	2875887	nc	rec	G2583_2652	molR_B	pseudogene
O157	2875890	nc	rec	G2583_2652	molR_B	pseudogene
O157	2875909	nc	rec	G2583_2652	molR_B	pseudogene
O157	2875911	nc	rec	G2583_2652	molR_B	pseudogene
O157	2875914	nc	rec	G2583_2652	molR_B	pseudogene
O157	2875915	nc	rec	G2583_2652	molR_B	pseudogene
O157	2875926	nc	rec	G2583_2652	molR_B	pseudogene
O157	2875943	nc	rec	G2583_2652	molR_B	pseudogene
O157	2875954	nc	rec	G2583_2652	molR_B	pseudogene
O157	2876023	i	rec	intergenic		
O157	2876086	ns	rec	G2583_2653	molR_C	CDS
O157	2876095	ns	rec	G2583_2653	molR_C	CDS
O157	2876103	s	rec	G2583_2653	molR_C	CDS
O157	2876112	s	rec	G2583_2653	molR_C	CDS
O157	2876121	s	rec	G2583_2653	molR_C	CDS
O157	2876124	s	rec	G2583_2653	molR_C	CDS
O157	2876154	s	rec	G2583_2653	molR_C	CDS
O157	2876158	ns	rec	G2583_2653	molR_C	CDS
O157	2876169	s	rec	G2583_2653	molR_C	CDS
O157	2876178	s	rec	G2583_2653	molR_C	CDS
O157	2876180	ns	rec	G2583_2653	molR_C	CDS
O157	2876255	ns	rec	G2583_2653	molR_C	CDS
O157	2876303	ns	rec	G2583_2653	molR_C	CDS
O157	2876348	ns	rec	G2583_2653	molR_C	CDS
O157	2877138	s	rec	G2583_2653	molR_C	CDS
O157	2877166	ns	rec	G2583_2653	molR_C	CDS
O157	2877169	ns	rec	G2583_2653	molR_C	CDS
O157	2877265	s	rec	G2583_2653	molR_C	CDS
O157	2877273	ns	rec	G2583_2653	molR_C	CDS
O157	2877322	s	rec	G2583_2653	molR_C	CDS
O157	2877358	ns	rec	G2583_2653	molR_C	CDS
O157	2877595	s	rec	G2583_2653	molR_C	CDS
O157	2877630	ns	rec	G2583_2653	molR_C	CDS
O157	2877634	s	rec	G2583_2653	molR_C	CDS
O157	2877639	ns	rec	G2583_2653	molR_C	CDS
O157	2877640	ns	rec	G2583_2653	molR_C	CDS
O157	2877679	s	rec	G2583_2653	molR_C	CDS
O157	2877770	ns	rec	G2583_2653	molR_C	CDS
O157	2877775	s	rec	G2583_2653	molR_C	CDS
O157	2877781	s	rec	G2583_2653	molR_C	CDS
O157	2877790	s	rec	G2583_2653	molR_C	CDS
O157	2877793	s	rec	G2583_2653	molR_C	CDS
O157	2877814	s	rec	G2583_2653	molR_C	CDS

lineage[a]	site[b]	mutation type[c]	recomb	gene	Gene name	Type
O157	2877820	s	rec	G2583_2653	molR_C	CDS
O157	2877829	s	rec	G2583_2653	molR_C	CDS
O157	2877838	s	rec	G2583_2653	molR_C	CDS
O157	2877874	s	rec	G2583_2653	molR_C	CDS
O157	2877880	s	rec	G2583_2653	molR_C	CDS
O157	2877940	s	rec	G2583_2653	molR_C	CDS
O157	2877958	s	rec	G2583_2653	molR_C	CDS
O157	2877964	s	rec	G2583_2653	molR_C	CDS
O157	2877985	s	rec	G2583_2653	molR_C	CDS
O157	2878000	s	rec	G2583_2653	molR_C	CDS
O157	2878006	ns	rec	G2583_2653	molR_C	CDS
O157	2878017	ns	rec	G2583_2653	molR_C	CDS
O157	2878024	s	rec	G2583_2653	molR_C	CDS
O157	2878030	s	rec	G2583_2653	molR_C	CDS
O157	2878039	s	rec	G2583_2653	molR_C	CDS
O157	2878042	s	rec	G2583_2653	molR_C	CDS
O157	2878047	ns	rec	G2583_2653	molR_C	CDS
O157	2878048	ns	rec	G2583_2653	molR_C	CDS
O157	2878079	ns	rec	G2583_2653	molR_C	CDS
O157	2878118	ns	rec	G2583_2653	molR_C	CDS
O157	2878158	ns	rec	G2583_2653	molR_C	CDS
O157	2878171	s	rec	G2583_2653	molR_C	CDS
O157	2878173	ns	rec	G2583_2653	molR_C	CDS
O157	2878177	s	rec	G2583_2653	molR_C	CDS
O157	2878261	s	rec	G2583_2653	molR_C	CDS
O157	2878565	ns	rec	G2583_2653	molR_C	CDS
O157	2878979	ns	rec	G2583_2653	molR_C	CDS
O157	2879644	s	rec	G2583_2654	yehI	CDS
O157	2879810	ns	rec	G2583_2654	yehI	CDS
O157	2879817	ns	rec	G2583_2654	yehI	CDS
O157	2879840	ns	rec	G2583_2654	yehI	CDS
O157	2879852	ns	rec	G2583_2654	yehI	CDS
O157	2879870	ns	rec	G2583_2654	yehI	CDS
O157	2879881	s	rec	G2583_2654	yehI	CDS
O157	2879884	s	rec	G2583_2654	yehI	CDS
O157	2879893	s	rec	G2583_2654	yehI	CDS
O157	2879918	s	rec	G2583_2654	yehI	CDS
O157	2879941	s	rec	G2583_2654	yehI	CDS
O157	2879949	ns	rec	G2583_2654	yehI	CDS
O157	2879959	s	rec	G2583_2654	yehI	CDS
O157	2879960	s	rec	G2583_2654	yehI	CDS
O157	2879962	s	rec	G2583_2654	yehI	CDS
O157	2879965	s	rec	G2583_2654	yehI	CDS
O157	2880025	s	rec	G2583_2654	yehI	CDS
O157	2880037	s	rec	G2583_2654	yehI	CDS

lineage[a]	site[b]	mutation type[c]	recomb	gene	Gene name	Type
O157	2880118	s	rec	G2583_2654	yehI	CDS
O157	2880130	s	rec	G2583_2654	yehI	CDS
O157	2880133	s	rec	G2583_2654	yehI	CDS
O157	2880136	ns	rec	G2583_2654	yehI	CDS
O157	2880192	ns	rec	G2583_2654	yehI	CDS
O157	2880196	s	rec	G2583_2654	yehI	CDS
O157	2880203	ns	rec	G2583_2654	yehI	CDS
O157	2880204	ns	rec	G2583_2654	yehI	CDS
O157	2880224	s	rec	G2583_2654	yehI	CDS
O157	2880232	s	rec	G2583_2654	yehI	CDS
O157	2880238	ns	rec	G2583_2654	yehI	CDS
O157	2880249	ns	rec	G2583_2654	yehI	CDS
O157	2880255	ns	rec	G2583_2654	yehI	CDS
O157	2880259	s	rec	G2583_2654	yehI	CDS
O157	2880366	ns	rec	G2583_2654	yehI	CDS
O157	2880379	s	rec	G2583_2654	yehI	CDS
O157	2880408	ns	rec	G2583_2654	yehI	CDS
O157	2880422	ns	rec	G2583_2654	yehI	CDS
O157	2880487	s	rec	G2583_2654	yehI	CDS
O157	2880536	s	rec	G2583_2654	yehI	CDS
O157	2880553	s	rec	G2583_2654	yehI	CDS
O157	2880799	s	rec	G2583_2654	yehI	CDS
O157	2880826	s	rec	G2583_2654	yehI	CDS
O157	2880841	s	rec	G2583_2654	yehI	CDS
O157	2880868	s	rec	G2583_2654	yehI	CDS
O157	2880890	ns	rec	G2583_2654	yehI	CDS
O157	2880943	s	rec	G2583_2654	yehI	CDS
O157	2880996	ns	rec	G2583_2654	yehI	CDS
O157	2881072	s	rec	G2583_2654	yehI	CDS
O157	2881079	ns	rec	G2583_2654	yehI	CDS
O157	2881081	ns	rec	G2583_2654	yehI	CDS
O157	2881090	ns	rec	G2583_2654	yehI	CDS
O157	2881099	s	rec	G2583_2654	yehI	CDS
O157	2881123	s	rec	G2583_2654	yehI	CDS
O157	2881132	ns	rec	G2583_2654	yehI	CDS
O157	2881135	s	rec	G2583_2654	yehI	CDS
O157	2881144	s	rec	G2583_2654	yehI	CDS
O157	2881150	s	rec	G2583_2654	yehI	CDS
O157	2881156	s	rec	G2583_2654	yehI	CDS
O157	2881159	s	rec	G2583_2654	yehI	CDS
O157	2881162	s	rec	G2583_2654	yehI	CDS
O157	2881165	s	rec	G2583_2654	yehI	CDS
O157	2881168	s	rec	G2583_2654	yehI	CDS
O157	2881174	s	rec	G2583_2654	yehI	CDS
O157	2881177	s	rec	G2583_2654	yehI	CDS

lineage[a]	site[b]	mutation type[c]	recomb	gene	Gene name	Type
O157	2881178	ns	rec	G2583_2654	yehI	CDS
O157	2881180	ns	rec	G2583_2654	yehI	CDS
O157	2881204	ns	rec	G2583_2654	yehI	CDS
O157	2881207	s	rec	G2583_2654	yehI	CDS
O157	2881210	s	rec	G2583_2654	yehI	CDS
O157	2881222	s	rec	G2583_2654	yehI	CDS
O157	2881228	ns	rec	G2583_2654	yehI	CDS
O157	2881274	ns	rec	G2583_2654	yehI	CDS
O157	2881275	ns	rec	G2583_2654	yehI	CDS
O157	2881283	ns	rec	G2583_2654	yehI	CDS
O157	2881294	s	rec	G2583_2654	yehI	CDS
O157	2881312	s	rec	G2583_2654	yehI	CDS
O157	2881429	s	rec	G2583_2654	yehI	CDS
O157	2881534	s	rec	G2583_2654	yehI	CDS
O157	2881543	s	rec	G2583_2654	yehI	CDS
O157	2881546	s	rec	G2583_2654	yehI	CDS
O157	2881568	del-3	rec	G2583_2654	yehI	CDS
O157	2881593	ns	rec	G2583_2654	yehI	CDS
O157	2881594	ns	rec	G2583_2654	yehI	CDS
O157	2881597	s	rec	G2583_2654	yehI	CDS
O157	2881603	s	rec	G2583_2654	yehI	CDS
O157	2881606	s	rec	G2583_2654	yehI	CDS
O157	2881613	s	rec	G2583_2654	yehI	CDS
O157	2881652	ns	rec	G2583_2654	yehI	CDS
O157	2881657	s	rec	G2583_2654	yehI	CDS
O157	2881666	s	rec	G2583_2654	yehI	CDS
O157	2881673	ns	rec	G2583_2654	yehI	CDS
O157	2881674	ns	rec	G2583_2654	yehI	CDS
O157	2881675	ns	rec	G2583_2654	yehI	CDS
O157	2881676	ns	rec	G2583_2654	yehI	CDS
O157	2881678	ns	rec	G2583_2654	yehI	CDS
O157	2881687	ns	rec	G2583_2654	yehI	CDS
O157	2881693	s	rec	G2583_2654	yehI	CDS
O157	2881711	s	rec	G2583_2654	yehI	CDS
O157	2881773	ns	rec	G2583_2654	yehI	CDS
O157	2881795	s	rec	G2583_2654	yehI	CDS
O157	2881819	ns	rec	G2583_2654	yehI	CDS
O157	2881846	s	rec	G2583_2654	yehI	CDS
O157	2882072	ns	rec	G2583_2654	yehI	CDS
O157	2882080	s	rec	G2583_2654	yehI	CDS
O157	2882169	ns	rec	G2583_2654	yehI	CDS
O157	2882185	s	rec	G2583_2654	yehI	CDS
O157	2882194	s	rec	G2583_2654	yehI	CDS
O157	2882203	s	rec	G2583_2654	yehI	CDS
O157	2882212	s	rec	G2583_2654	yehI	CDS

lineage[a]	site[b]	mutation type[c]	recomb	gene	Gene name	Type
O157	2882246	ns	rec	G2583_2654	yehI	CDS
O157	2882318	ns	rec	G2583_2654	yehI	CDS
O157	2882347	s	rec	G2583_2654	yehI	CDS
O157	2882394	ns	rec	G2583_2654	yehI	CDS
O157	2882396	ns	rec	G2583_2654	yehI	CDS
O157	2882440	s	rec	G2583_2654	yehI	CDS
O157	2882534	s	rec	G2583_2654	yehI	CDS
O157	2882580	ns	rec	G2583_2654	yehI	CDS
O157	2882582	ns	rec	G2583_2654	yehI	CDS
O157	2882606	ns	rec	G2583_2654	yehI	CDS
O157	2882649	ins	rec	G2583_2654	yehI	CDS
O157	2882685	i	rec	intergenic		
O157	2882854	ns	rec	G2583_2655	yehK	CDS
O157	2882856	ns	rec	G2583_2655	yehK	CDS
O157	2883046	i	rec	intergenic		
O157	2883071	i	rec	intergenic		
O157	2883831	i	rec	intergenic		
O157	2883961	ins	rec	intergenic		
O157	2884151	i	rec	intergenic		
O157	2885217	s		G2583_2656	yehL	CDS
O157	2885926	s		G2583_2657	yehM	CDS
O157	2886896	ns		G2583_2657	yehM	CDS
O157	2887410	ns		G2583_2657	yehM	CDS
O157	2887489	s		G2583_2657	yehM	CDS
O157	2887818	ns		G2583_2658	yehP	CDS
CB9615	2680283	ns		G2583_2659	yehQ	CDS
EDL933	2961114	ns		ECs2932	-	CDS
Sakai	2891902	ns		ECs2933	-	CDS
CB9615	2681224	ns		G2583_2660	yehR	CDS
CB9615	2682624	s		G2583_2663	yehU	CDS
O157	2894159	ns		G2583_2663	yehU	CDS
O157	2895652	i		intergenic		
EDL933	2967471	ns	rec	ECs2941	-	CDS
EDL933	2967473	ns	rec	ECs2941	-	CDS
EDL933	2967474	ns	rec	ECs2941	-	CDS
EDL933	2967744	ns	rec	ECs2941	-	CDS
EDL933	2967789	ns	rec	ECs2941	-	CDS
EDL933	2967816	ins	rec	ECs2941	-	CDS
EDL933	2967817	s	rec	ECs2941	-	CDS
EDL933	2967909	s	rec	ECs2941	-	CDS
EDL933	2967951	s	rec	ECs2941	-	CDS
EDL933	2967955	ns	rec	ECs2941	-	CDS
EDL933	2967956	ns	rec	ECs2941	-	CDS
EDL933	2967963	s	rec	ECs2941	-	CDS
EDL933	2967984	ns	rec	ECs2941	-	CDS

lineage[a]	site[b]	mutation type[c]	recomb	gene	Gene name	Type
EDL933	2968117	ns	rec	ECs2941	-	CDS
EDL933	2968149	ns	rec	ECs2941	-	CDS
EDL933	2968237	ns	rec	ECs2941	-	CDS
EDL933	2968388	ns	rec	ECs2941	-	CDS
EDL933	2968654	ns	rec	ECs2942	-	CDS
EDL933	2968678	ns	rec	ECs2942	-	CDS
EDL933	2968765	ns	rec	ECs2942	-	CDS
EDL933	2968894	s	rec	ECs2942	-	CDS
EDL933	2968933	s	rec	ECs2942	-	CDS
EDL933	2968951	s	rec	ECs2942	-	CDS
EDL933	2990240	i	rec	intergenic		
EDL933	2990247	i	rec	intergenic		
EDL933	2990257	i	rec	intergenic		
EDL933	2990261	i	rec	intergenic		
EDL933	2990274	i	rec	intergenic		
EDL933	2990281	ins	rec	intergenic		
EDL933	2990345	i	rec	intergenic		
EDL933	2990377	i	rec	intergenic		
EDL933	2990520	ns	rec	ECs2965-2966	-	CDS
EDL933	2990657	s	rec	ECs2966	-	CDS
EDL933	2990693	ns	rec	ECs2966	-	CDS
EDL933	2990711	ns	rec	ECs2966	-	CDS
EDL933	2990723	ns	rec	ECs2966	-	CDS
EDL933	2990726	ns	rec	ECs2966	-	CDS
EDL933	2990729	ns	rec	ECs2966	-	CDS
EDL933	2990733	ns	rec	ECs2966	-	CDS
EDL933	2990735	ns	rec	ECs2966	-	CDS
EDL933	2990779	ns	rec	ECs2966	-	CDS
EDL933	2990780	ns	rec	ECs2966	-	CDS
EDL933	2990781	ns	rec	ECs2966	-	CDS
EDL933	2990783	ns	rec	ECs2966	-	CDS
EDL933	2990786	ns	rec	ECs2966	-	CDS
EDL933	2990789	ns	rec	ECs2966	-	CDS
EDL933	2991336	ns	rec	ECs2967	-	CDS
EDL933	2991733	i	rec	intergenic		
EDL933	2991742	i	rec	intergenic		
EDL933	2995440	i	rec	intergenic		
EDL933	2995546	i	rec	intergenic		
EDL933	2995550	i	rec	intergenic		
EDL933	2995555	i	rec	intergenic		
EDL933	2995562	i	rec	intergenic		
EDL933	2995569	i	rec	intergenic		
EDL933	2995575	i	rec	intergenic		
EDL933	2995589	i	rec	intergenic		
EDL933	2995593	i	rec	intergenic		

lineage[a]	site[b]	mutation type[c]	recomb	gene	Gene name	Type
EDL933	2995594	i	rec	intergenic		
EDL933	2995596	i	rec	intergenic		
EDL933	2995598	i	rec	intergenic		
EDL933	2995602	i	rec	intergenic		
EDL933	2995611	i	rec	intergenic		
EDL933	2995613	i	rec	intergenic		
EDL933	2995614	i	rec	intergenic		
EDL933	2995615	i	rec	intergenic		
EDL933	2995617	i	rec	intergenic		
EDL933	2997577	ns	rec	ECs2975	-	CDS
EDL933	2998210	ns	rec	ECs2977	-	CDS
EDL933	2999850	ns	rec	ECs2981	-	CDS
EDL933	2999956	ns	rec	ECs2981	-	CDS
EDL933	2999998	ns	rec	ECs2982	-	CDS
EDL933	3000304	ns	rec	ECs2982	-	CDS
EDL933	3000480	ins	rec	ECs2983	-	CDS
EDL933	3000716	ns	rec	ECs2983	-	CDS
EDL933	3000894	i	rec	intergenic		
EDL933	3000900	i	rec	intergenic		
EDL933	3000910	i	rec	intergenic		
EDL933	3000912	i	rec	intergenic		
EDL933	3000922	i	rec	intergenic		
EDL933	3001094	ns	rec	ECs2984	-	CDS
EDL933	3001404	ns	rec	ECs2985	-	CDS
EDL933	3001730	ns	rec	ECs2986	-	CDS
EDL933	3001900	ns	rec	ECs2986	-	CDS
EDL933	3002123	ns	rec	ECs2986	-	CDS
EDL933	3002606	ns	rec	ECs2987	-	CDS
EDL933	3002943	ns	rec	ECs2987	-	CDS
EDL933	3003917	i	rec	intergenic		
EDL933	3004295	ns	rec	ECs2990	-	CDS
EDL933	3004308	ns	rec	ECs2990	-	CDS
EDL933	3004865	ns	rec	ECs2991	-	CDS
EDL933	3004878	ins	rec	ECs2991	-	CDS
EDL933	3005393	ns	rec	ECs2992	-	CDS
EDL933	3006567	ins	rec	ECs2995	-	CDS
EDL933	3007820	ns	rec	ECs2997-2998	-	CDS
EDL933	3007962	ns	rec	ECs2998-2999	-	CDS
EDL933	3008087	ns	rec	ECs2999-3000	-	CDS
EDL933	3008391	ns	rec	ECs3001	-	CDS
EDL933	3008986	ns	rec	ECs3001	-	CDS
EDL933	3009737	ns	rec	ECs3002	-	CDS
EDL933	3010456	ns	rec	ECs3005	-	CDS
EDL933	3010459	ns	rec	ECs3005	-	CDS
EDL933	3010509	i	rec	intergenic		

lineage[a]	site[b]	mutation type[c]	recomb	gene	Gene name	Type
EDL933	3010557	i	rec	intergenic		
EDL933	3010570	ns	rec	ECs3006	-	CDS
EDL933	3010611	ns	rec	ECs3006	-	CDS
EDL933	3010629	ns	rec	ECs3006	-	CDS
EDL933	3010639	ns	rec	ECs3006	-	CDS
EDL933	3010645	ns	rec	ECs3006	-	CDS
EDL933	3011882	ns	rec	ECs3008	-	CDS
EDL933	3012893	ns	rec	ECs3010	-	CDS
EDL933	3013049	ns	rec	ECs3011	-	CDS
O157	2944126	ns		G2583_2664	mlrA	CDS
O157	2945204	s		G2583_2665	yehW	CDS
O157	2946097	s		G2583_2666	yehX	CDS
O157	2946884	s		G2583_2667	yehY	CDS
O157	2947090	s		G2583_2667	yehY	CDS
O157	2947671	ns		G2583_2668	osmF	CDS
O157	2949320	ns		G2583_2669	bglX	CDS
O157	2951226	ns		G2583_2670	dld	CDS
Sakai	2954379	ns		G2583_2672	yohC	CDS
O157	2954807	ns		G2583_2673	yohD	CDS
O157	2956842	s		G2583_2675	yohG	CDS
EDL933	3029502	del		G2583_2676	-	CDS
EDL933	3029797	ns		G2583_2677	dusC	CDS
O157	2961481	ns		G2583_2680	-	CDS
CB9615	2702853	s		G2583_2681	gtdA	CDS
O157	2963486	ns		G2583_2682	-	CDS
EDL933	3035541	ns		G2583_2682	-	CDS
O157	2964565	ns		G2583_2683	-	CDS
O157	2965013	s		G2583_2683	-	CDS
O157	2967804	i		intergenic		
CB9615	2710116	s		G2583_2689	yeiT	CDS
CB9615	2710575	s		G2583_2689	yeiT	CDS
O157	2971595	i		intergenic		
EDL933	3042907	ins		intergenic		
CB9615	2713639	ns		G2583_2692	mglA	CDS
Sakai	2974286	i		intergenic		
O157	2975504	del		intergenic		
EDL933	3046860	ins		intergenic		
CB9615	2717627	s		G2583_2695	yeiB	CDS
CB9615	2717984	s		G2583_2695	yeiB	CDS
O157	2977922	s		G2583_2695	yeiB	CDS
CB9615	2722860	s		G2583_2699	lysP	CDS
O157	2988888	s		G2583_2704	nupX	CDS
O157	2989231	ns		G2583_2705	rihB	CDS
O157	2992981	s		G2583_2708	yeiN	CDS
CB9615	2733841	s		G2583_2708	yeiN	CDS

lineage[a]	site[b]	mutation type[c]	recomb	gene	Gene name	Type
O157	2994900	s		G2583_2710	fruA	CDS
CB9615	2741548	ns		G2583_2714	yeiP	CDS
O157	3002581	ns		G2583_2715	yeiQ	CDS
CB9615	2745239	s		G2583_2717	yeiU	CDS
O157	3004830	i		intergenic		
O157	3005531	i		intergenic		
EDL933	3076899	ins		intergenic		
O157	3007656	s		G2583_2720	yejA	CDS
O157	3008692	ns		G2583_2720	yejA	CDS
O157	3008913	s		G2583_2720	yejA	CDS
CB9615	2750259	ns		G2583_2721	yejB	CDS
CB9615	2750480	ns		G2583_2721	yejB	CDS
CB9615	2750652	s		G2583_2721	yejB	CDS
O157	3012972	ns		G2583_2724	yejG	CDS
CB9615	2758080	i		intergenic		
O157	3017596	ns		G2583_2728	rplY	CDS
CB9615	2761038	s		G2583_2731	yejM	CDS
O157	3021132	i		intergenic		
O157	3021297	i		intergenic		
O157	3022574	ns		G2583_2733	yejO	CDS
CB9615	2763372	s		G2583_2733	yejO	CDS
CB9615	2763800	ns		G2583_2733	yejO	CDS
O157	3026664	s		G2583_2737	ccmF	CDS
CB9615	2769093	s		G2583_2737	ccmF	CDS
O157	3032970	s		G2583_2745	napH	CDS
O157	3033422	ns		G2583_2746	napG	CDS
CB9615	2776008	ns		G2583_2747	napA	CDS
O157	3036534	s		G2583_2748	napD	CDS
CB9615	2778229	s		G2583_2750	eco	CDS
O157	3040123	ns		G2583_2752	yojI	CDS
EDL933	3115208	s		G2583_2755	apbE	CDS
CB9615	2786336	s		G2583_2756	ompC	CDS
CB9615	2787328	s		G2583_2758	rcsD	CDS
O157	3052087	s		G2583_2760	rcsC	CDS
CB9615	2794107	ns		G2583_2761	yfaP	CDS
O157	3053374	s		G2583_2761	yfaP	CDS
O157	3054296	nc		G2583_2762	yfaQ	pseudogene
O157	3054400	nc		G2583_2762	yfaQ	pseudogene
CB9615	2795498	nc		G2583_2762	yfaQ	pseudogene
CB9615	2800777	nc		G2583_2766	yfaS	pseudogene
CB9615	2800837	nc		G2583_2766	yfaS	pseudogene
O157	3062397	del		G2583_2768	yfaA	CDS
O157	3064399	s		G2583_2769	gyrA	CDS
O157	3068185	ns		G2583_2771	yfaL	CDS
O157	3069776	s		G2583_2771	yfaL	CDS

lineage[a]	site[b]	mutation type[c]	recomb	gene	Gene name	Type
O157	3070402	i		intergenic		
Sakai	3071058	s		G2583_2772	nrdA	CDS
CB9615	2815789	ns		G2583_2773	nrdB	CDS
CB9615	2817852	nc		G2583_2776	-	pseudogene
CB9615	2818960	s		G2583_2777	yfaH	CDS
O157	3078076	ns		G2583_2778	-	CDS
CB9615	2824138	i		intergenic		
O157	3081966	i		intergenic		
CB9615	2827682	ns		G2583_2783	glpC	CDS
CB9615	2829254	ns		G2583_2784	yfaD	CDS
O157	3088027	del		G2583_2785	yfaU	CDS
O157	3088239	ns		G2583_2786	yfaV	CDS
CB9615	2831622	s		G2583_2787	yfaW	CDS
O157	3090881	s		G2583_2787	yfaW	CDS
CB9615	2835844	i		intergenic		
O157	3096465	s		G2583_2793	arnB	CDS
O157	3097793	s		G2583_2794	arnC	CDS
CB9615	2838888	s		G2583_2794	arnC	CDS
O157	3099391	s		G2583_2795	arnA	CDS
CB9615	2844947	ns		G2583_2800	pmrD	CDS
O157	3104427	ns		G2583_2801	menE	CDS
Sakai	3104535	ns		G2583_2801	menE	CDS
O157	3105313	s		G2583_2801	menE	CDS
O157	3105362	ns		G2583_2801	menE	CDS
O157	3112634	ns		G2583_2809	rbn	CDS
CB9615	2854331	nc		G2583_2810	elaD	pseudogene
O157	3113601	nc		G2583_2810	elaD	pseudogene
CB9615	2854769	nc		G2583_2810	elaD	pseudogene
O157	3114041	ins-9		G2583_2810	elaD	pseudogene
CB9615	2854935	nc	rec	G2583_2810	elaD	pseudogene
CB9615	2854936	nc	rec	G2583_2810	elaD	pseudogene
CB9615	2854938	nc	rec	G2583_2810	elaD	pseudogene
CB9615	2854941	nc	rec	G2583_2810	elaD	pseudogene
O157	3114413	nc		G2583_2810	elaD	pseudogene
CB9615	2855917	i		intergenic		
CB9615	2860078	i		intergenic		
CB9615	2860437	s		G2583_2815	nuoL	CDS
CB9615	2862431	ns		G2583_2817	nuoJ	CDS
EDL933	3193281	s		G2583_2819	nuoH	CDS
O157	3124715	s		G2583_2820	nuoG	CDS
O157	3126514	s		G2583_2821	nuoF	CDS
CB9615	2874438	i		intergenic		
O157	3133563	i		intergenic		
CB9615	2875345	ns		G2583_2827	yfbQ	CDS
O157	3134715	ns		G2583_2827	yfbQ	CDS

lineage[a]	site[b]	mutation type[c]	recomb	gene	Gene name	Type
CB9615	2877862	s		G2583_2829	yfbS	CDS
O157	3137373	s		G2583_2829	yfbS	CDS
O157	3138049	s		G2583_2830	yfbT	CDS
Sakai	3139077	ins		G2583_2832	yfbV	CDS
CB9615	2881452	s		G2583_2833	ackA	CDS
O157	3141268	s		G2583_2834	pta	CDS
CB9615	2883023	ns		G2583_2834	pta	CDS
CB9615	2883148	s		G2583_2834	pta	CDS
O157	3145135	s		G2583_2836	yfcD	CDS
CB9615	2886331	s		G2583_2837	yfcE	CDS
CB9615	2886962	s		G2583_2838	yfcF	CDS
O157	3148281	ns		G2583_2841	yfcH	CDS
O157	3149385	ns		G2583_2842	yfcI	CDS
O157	3150027	s		G2583_2843	hisP	CDS
O157	3151705	ns		G2583_2845	hisQ	CDS
CB9615	2893786	ns		G2583_2846	hisJ	CDS
O157	3154447	s		G2583_2848	ubiX	CDS
EDL933	3226930	ins		intergenic		
O157	3157188	i		intergenic		
CB9615	2898274	i		intergenic		
O157	3157296	i		intergenic		
CB9615	2898365	i		intergenic		
CB9615	2898989	ns		G2583_2851	dedD	CDS
CB9615	2899321	s		G2583_2852	folC	CDS
O157	3159034	ns		G2583_2852	folC	CDS
O157	3160750	s		G2583_2854	dedA	CDS
O157	3160813	s		G2583_2854	dedA	CDS
O157	3162697	s		G2583_2856	usg	CDS
O157	3162969	ns		G2583_2856	usg	CDS
O157	3164529	ns		G2583_2858	flk	CDS
CB9615	2906928	s		G2583_2859	yfcJ	CDS
CB9615	2907464	ns		G2583_2859	yfcJ	CDS
CB9615	2908744	s		G2583_2860	fabB	CDS
O157	3172360	ns		G2583_2865	mepA	CDS
CB9615	2914753	s		G2583_2866	aroC	CDS
O157	3177232	ns		G2583_2871	yfcQ	CDS
O55/O157	2918934	s		G2583_2872	yfcR	CDS
O157	3178295	s		G2583_2873	yfcS	CDS
O157	3178299	ns		G2583_2873	yfcS	CDS
O157	3179215	ns		G2583_2874	yfcU	CDS
CB9615	2920321	s		G2583_2874	yfcU	CDS
CB9615	2920930	ns		G2583_2874	yfcU	CDS
CB9615	2921624	s		G2583_2874	yfcU	CDS
O157	3181965	s		G2583_2875	yfcV	CDS
O157	3182373	i		intergenic		

lineage[a]	site[b]	mutation type[c]	recomb	gene	Gene name	Type
O55/O157	2923576	i		intergenic		
CB9615	2923726	i		intergenic		
O157	3183685	s		G2583_2877	fadJ	CDS
O157	3184174	s		G2583_2877	fadJ	CDS
CB9615	2925279	ns		G2583_2877	fadJ	CDS
O157	3184895	ns		G2583_2877	fadJ	CDS
O157	3185787	s		G2583_2878	fadI	CDS
CB9615	2927725	ns		G2583_2878	fadI	CDS
O157	3190466	i		intergenic		
O157	3190467	i		intergenic		
O157	3190468	i		intergenic		
O157	3193091	i		intergenic		
CB9615	2935314	ns		G2583_2885	intS	CDS
O55/O157	2937582	ns		G2583_2888	-	CDS
CB9615	2937887	s		G2583_2889	-	CDS
O55/O157	2940866	ns		G2583_2891	-	CDS
EDL933	3270066	i		intergenic		
EDL933	3270094	i		intergenic		
EDL933	3270095	i		intergenic		
CB9615	2944601	ns		G2583_2895	cscK	CDS
EDL933	3274062	ins		G2583_2895	cscK	CDS
EDL933	3274100	ins		G2583_2895	cscK	CDS
EDL933	3274135	ins		G2583_2895	cscK	CDS
O157	3204972	s		G2583_2896	-	CDS
CB9615	2946740	s		G2583_2896	-	CDS
CB9615	2948484	i		intergenic		
CB9615	2949533	ns		G2583_2899	dsdA	CDS
O157	3209001	ns		G2583_2900	emrY	CDS
CB9615	2950267	s		G2583_2900	emrY	CDS
O157	3209571	ns		G2583_2900	emrY	CDS
O157	3210003	ns		G2583_2900	emrY	CDS
CB9615	2951469	ns		G2583_2901	emrK	CDS
O157	3211415	ns		G2583_2901	emrK	CDS
O157	3211574	i		intergenic		
CB9615	2957581	ns		G2583_2904	yfdE	CDS
O157	3217124	del-4		G2583_2904	yfdE	CDS
CB9615	2962985	s		G2583_2908	yfdX	CDS
O157	3221960	s		G2583_2908	yfdX	CDS
O157	3223188	s		G2583_2910	yfdY	CDS
EDL933	3293475	ins		intergenic		
CB9615	2965198	s		G2583_2911	lpxP	CDS
CB9615	2965915	i		intergenic		
O157	3225030	i		intergenic		
CB9615	2968369	s		G2583_2913	ypdA	CDS
O157	3231110	ns		G2583_2916	fryA	CDS

lineage[a]	site[b]	mutation type[c]	recomb	gene	Gene name	Type
O157	3233895	ns		G2583_2918	ypdF	CDS
CB9615	2975370	ns		G2583_2918	ypdF	CDS
CB9615	2977625	i		intergenic		
O157	3241222	s		G2583_2926	nupC	CDS
O157	3241832	ns		G2583_2926	nupC	CDS
CB9615	2986844	ns		G2583_2931	yfeD	CDS
O55/O157	2987890	s	rec	G2583_2932	ydcM	CDS
O55/O157	2987894	ns	rec	G2583_2932	ydcM	CDS
O55/O157	2988052	ns	rec	G2583_2932	ydcM	CDS
O55/O157	2988115	s	rec	G2583_2932	ydcM	CDS
O55/O157	2988268	s	rec	G2583_2932	ydcM	CDS
O55/O157	2988329	ns	rec	G2583_2933	-	CDS
O55/O157	2988334	ns	rec	G2583_2933	-	CDS
O55/O157	2988352	s	rec	G2583_2933	-	CDS
O55/O157	2988355	s	rec	G2583_2933	-	CDS
Sakai	3249294	del		intergenic		
O157	3250420	s		G2583_2940	yfeR	CDS
O157	3250682	ns		G2583_2940	yfeR	CDS
O157	3253275	s		G2583_2943	ligA	CDS
EDL933	3325086	del-12		G2583_2944	zipA	CDS
EDL933	3325106	ns		G2583_2944	zipA	CDS
O157	3258948	s		G2583_2948	ptsI	CDS
CB9615	3000279	ns		G2583_2948	ptsI	CDS
O157	3259260	s		G2583_2948	ptsI	CDS
CB9615	3000817	s		G2583_2948	ptsI	CDS
O157	3260604	ns		G2583_2949	crr	CDS
EDL933	3332114	s		G2583_2952	cysM	CDS
O157	3262513	ns		G2583_2952	cysM	CDS
CB9615	3004101	i		intergenic		
CB9615	3004715	ns		G2583_2953	cysA	CDS
O157	3269816	s		G2583_2959	murQ	CDS
O157	3272219	ns		G2583_2961	yfeW	CDS
O157	3274130	ns		G2583_2963	yfeY	CDS
O157	3276796	ns		G2583_2967	hemF	CDS
O157	3278459	ns		G2583_2969	eutK	CDS
Sakai	3280156	ns		G2583_2971	eutC	CDS
CB9615	3024955	s		G2583_2974	eutH	CDS
O157	3284361	s		G2583_2974	eutH	CDS
CB9615	3026506	s		G2583_2975	eutG	CDS
O157	3286008	ns		G2583_2976	eutJ	CDS
O157	3286610	s		G2583_2977	eutE	CDS
CB9615	3028423	ns		G2583_2977	eutE	CDS
CB9615	3028720	ns		G2583_2977	eutE	CDS
O157	3294196	s		G2583_2985	maeB	CDS
O157	3295368	s		G2583_2986	talA	CDS

lineage[a]	site[b]	mutation type[c]	recomb	gene	Gene name	Type
CB9615	3037397	s		G2583_2987	tktB	CDS
O157	3296437	s		G2583_2987	tktB	CDS
O157	3296635	s		G2583_2987	tktB	CDS
CB9615	3040104	ns		G2583_2989	nudK	CDS
CB9615	3040646	ns		G2583_2989	nudK	CDS
O157	3302045	ns		G2583_2991	narQ	CDS
Sakai	3304097	ns		G2583_2992	acrD	CDS
O157	3304521	ns		G2583_2992	acrD	CDS
O157	3307260	i		intergenic		
EDL933	3377329	ns		G2583_2993	yffB	CDS
O157	3307585	ns		G2583_2993	yffB	CDS
O157	3307707	ns		G2583_2993	yffB	CDS
O157	3310710	s		G2583_2997	ypfI	CDS
Sakai	3311013	s		G2583_2997	ypfI	CDS
O157	3311217	s		G2583_2997	ypfI	CDS
CB9615	3053792	s		G2583_2998	ypfJ	CDS
CB9615	3054572	s		G2583_2999	purC	CDS
CB9615	3055812	ns		G2583_3000	nlpB	CDS
O157	3314926	ns		G2583_3000	nlpB	CDS
O157	3314943	ns		G2583_3000	nlpB	CDS
CB9615	3058312	i		intergenic		
O157	3318095	s		G2583_3005	hyfB	CDS
CB9615	3059774	ns		G2583_3005	hyfB	CDS
O157	3318896	s		G2583_3005	hyfB	CDS
O157	3321357	ns		G2583_3007	hyfD	CDS
CB9615	3062763	ns		G2583_3007	hyfD	CDS
CB9615	3063544	del-6		G2583_3008	hyfE	CDS
CB9615	3066628	ns		G2583_3010	hyfG	CDS
O157	3327497	ns		G2583_3012	hyfI	CDS
CB9615	3068611	ns		G2583_3012-3013	hyfIJ	CDS
O157	3328117	s		G2583_3014	hyfR	CDS
EDL933	3399850	ins		G2583_3014	hyfR	CDS
EDL933	3399858	ins		G2583_3014	hyfR	CDS
O157	3332703	ns		G2583_3017	yfgC	CDS
CB9615	3076460	s		G2583_3020	uraA	CDS
O157	3338734	ns		G2583_3023	purN	CDS
CB9615	3080249	i		intergenic		
CB9615	3080310	s		G2583_3024	ppk	CDS
CB9615	3087305	ns		G2583_3029	yfgH	CDS
CB9615	3091074	s		G2583_3032	guaB	CDS
EDL933	3420165	i		intergenic		
O157	3354039	ns		G2583_3036	yfgL	CDS
O157	3355385	ns		G2583_3037	yfgM	CDS
CB9615	3098785	ns		G2583_3040	ispG	CDS
O55/O157	3102696	ns		G2583_3044	-	CDS

lineage[a]	site[b]	mutation type[c]	recomb	gene	Gene name	Type
O55/O157	3102846	ns		G2583_3044	-	CDS
O55/O157	3102916	ns		G2583_3044	-	CDS
O55/O157	3104750	ns		G2583_3047	dmsA	CDS
O55/O157	3105262	ns		G2583_3047	dmsA	CDS
O55/O157	3105533	ns		G2583_3047	dmsA	CDS
O55/O157	3106970	i		intergenic		
CB9615	3113828	ns		G2583_3049	yfhM	CDS
CB9615	3113869	ns		G2583_3049	yfhM	CDS
EDL933	3445308	ins		G2583_3052	sseB	CDS
CB9615	3119046	ns		G2583_3056	hscA	CDS
CB9615	3119807	s		G2583_3056	hscA	CDS
CB9615	3119967	ns		G2583_3056	hscA	CDS
O157	3380175	i		intergenic		
CB9615	3123303	i		intergenic		
CB9615	3124446	s		G2583_3062	trmJ	CDS
CB9615	3125631	s		G2583_3063	suhB	CDS
O157	3385418	s		G2583_3064	yfhR	CDS
O157	3388387	i		intergenic		
EDL933	3458316	ins		intergenic		
O157	3389485	i		intergenic		
O157	3390792	s		G2583_3068	hcaE	CDS
O157	3391262	s		G2583_3069	hcaF	CDS
CB9615	3133865	ns		G2583_3072	hcaD	CDS
CB9615	3133963	s		G2583_3072	hcaD	CDS
O157	3395694	ns		G2583_3075	yphC	CDS
O157	3396073	ns		G2583_3075	yphC	CDS
O157	3396091	ns		G2583_3075	yphC	CDS
O157	3397051	s		G2583_3076	yphD	CDS
O157	3397262	ns		G2583_3076	yphD	CDS
O157	3398211	ns		G2583_3077	yphE	CDS
CB9615	3142104	s		G2583_3079	yphG	CDS
O157	3401183	s		G2583_3079	yphG	CDS
O157	3401445	ns		G2583_3079	yphG	CDS
O157	3402265	ns		G2583_3079	yphG	CDS
CB9615	3143736	s		G2583_3079	yphG	CDS
CB9615	3144788	s		G2583_3080	yphH	CDS
O157	3403834	s		G2583_3080	yphH	CDS
CB9615	3145958	ns		G2583_3081	glyA	CDS
CB9615	3146660	s		G2583_3081	glyA	CDS
CB9615	3147613	s		G2583_3082	hmp	CDS
CB9615	3150181	s		G2583_3084	yfhA	CDS
CB9615	3152738	nc		G2583_3087	-	pseudogene
O157	3414001	s		G2583_3088	purL	CDS
CB9615	3157425	s		G2583_3089	yfhD	CDS
O157	3417358	ns		G2583_3089	yfhD	CDS

lineage[a]	site[b]	mutation type[c]	recomb	gene	Gene name	Type
EDL933	3490196	ins		intergenic		
O157	3430702	i		intergenic		
O157	3434492	s		G2583_3158	srmB	CDS
O157	3436799	i		intergenic		
Sakai	3437590	ins		G2583_3162	ung	CDS
CB9615	3221386	s		G2583_3164	-	CDS
CB9615	3221956	i		intergenic		
Sakai	3441546	ins		G2583_3167	yfiQ	CDS
O157	3442864	i		intergenic		
O157	3445691	s		G2583_3170	kgtP	CDS
EDL933	3516081	ins		intergenic		
O157	3446373	nc		G2583_3171	rrf	rRNA
CB9615	3229212	nc		G2583_3172	rrl	rRNA
EDL933	3517536	ins		G2583_3172	rrl	rRNA
EDL933	3517877	ins		G2583_3172	rrl	rRNA
CB9615	3230699	nc		G2583_3172	rrl	rRNA
EDL933	3230699	nc		G2583_3172	rrl	rRNA
Sakai	3448153	nc	rec	G2583_3172	rrl	rRNA
Sakai	3448157	nc	rec	G2583_3172	rrl	rRNA
CB9615	3230708	nc		G2583_3172	rrl	rRNA
EDL933	3230708	nc		G2583_3172	rrl	rRNA
Sakai	3448162	nc	rec	G2583_3172	rrl	rRNA
EDL933	3448735	nc		G2583_3172	rrl	rRNA
O157	3448735	nc		G2583_3172	rrl	rRNA
Sakai	3448735	nc	rec	G2583_3172	rrl	rRNA
EDL933	3518669	ins		G2583_3172	rrl	rRNA
EDL933	3518682	ins		G2583_3172	rrl	rRNA
Sakai	3449117	nc	rec	G2583_3172	rrl	rRNA
EDL933	3519167	ins		G2583_3172	rrl	rRNA
O157	3449401	ins-18		intergenic		
Sakai	3449416	i	rec	intergenic		
O55/O157	3231947	indel-8		intergenic		
CB9615	3231959	i		intergenic		
Sakai	3449652	i	rec	intergenic		
EDL933	3519566	i		intergenic		
EDL933	3519567	i		intergenic		
EDL933	3520082	nc	rec	G2583_3174	rrs	rRNA
EDL933	3520097	nc	rec	G2583_3174	rrs	rRNA
EDL933	3520098	nc	rec	G2583_3174	rrs	rRNA
EDL933	3520099	nc	rec	G2583_3174	rrs	rRNA
EDL933	3520100	nc	rec	G2583_3174	rrs	rRNA
EDL933	3520101	nc	rec	G2583_3174	rrs	rRNA
EDL933	3520110	nc	rec	G2583_3174	rrs	rRNA
EDL933	3520114	nc	rec	G2583_3174	rrs	rRNA
EDL933	3520119	ins	rec	G2583_3174	rrs	rRNA

lineage[a]	site[b]	mutation type[c]	recomb	gene	Gene name	Type
EDL933	3520356	ins	rec	G2583_3174	rrs	rRNA
EDL933	3520424	ins	rec	G2583_3174	rrs	rRNA
EDL933	3520532	ins	rec	G2583_3174	rrs	rRNA
EDL933	3520725	ins	rec	G2583_3174	rrs	rRNA
CB9615	3233603	nc		G2583_3174	rrs	rRNA
EDL933	3521156	ins		intergenic		
CB9615	3233856	i		intergenic		
O157	3451460	i		intergenic		
CB9615	3234003	i		intergenic		
O157	3452810	s		G2583_3175	clpB	CDS
CB9615	3240480	ns		G2583_3181	pheA	CDS
O157	3462631	ns		G2583_3186	yfiN	CDS
O157	3464218	ns		G2583_3187	yfiB	CDS
CB9615	3247118	s		G2583_3188	rplS	CDS
O157	3466754	s		G2583_3192	ffh	CDS
CB9615	3250353	i		intergenic		
O157	3469372	ns		G2583_3194	yfjD	CDS
CB9615	3251951	s		G2583_3194	yfjD	CDS
O157	3471077	ns		G2583_3196	nadK	CDS
CB9615	3254051	ns		G2583_3196	nadK	CDS
O157	3472844	s		G2583_3197	recN	CDS
CB9615	3256022	i		intergenic		
O157	3474160	ns		G2583_3199	yfjF	CDS
O157	3474265	ns		G2583_3199	yfjF	CDS
O157	3475410	i		intergenic		
CB9615	3258124	i		intergenic		
O55/O157	3259976	ns	rec	G2583_3204	lpgB	CDS
O55/O157	3259977	ns	rec	G2583_3204	lpgB	CDS
O55/O157	3259981	s	rec	G2583_3204	lpgB	CDS
O55/O157	3259993	ns	rec	G2583_3204	lpgB	CDS
O55/O157	3260007	ns	rec	G2583_3204	lpgB	CDS
O55/O157	3260027	ns	rec	G2583_3204	lpgB	CDS
O55/O157	3260050	s	rec	G2583_3204	lpgB	CDS
O55/O157	3260087	ns	rec	G2583_3204	lpgB	CDS
O55/O157	3260147	i	rec	intergenic		
O55/O157	3260155	i	rec	intergenic		
O55/O157	3260198	indel	rec	intergenic		
O55/O157	3260205	i	rec	intergenic		
O55/O157	3260235	i	rec	intergenic		
O55/O157	3260243	i	rec	intergenic		
O55/O157	3260320	s	rec	G2583_3205	-	CDS
O55/O157	3260362	s	rec	G2583_3205	-	CDS
O55/O157	3260375	ns	rec	G2583_3205	-	CDS
O55/O157	3260460	ns	rec	G2583_3205	-	CDS
O55/O157	3260513	ns	rec	G2583_3205	-	CDS

lineage[a]	site[b]	mutation type[c]	recomb	gene	Gene name	Type
O55/O157	3260605	s	rec	G2583_3205	-	CDS
O55/O157	3260626	s	rec	G2583_3205	-	CDS
O55/O157	3260813	indel	rec	G2583_3205	-	CDS
O55/O157	3260816	ns	rec	G2583_3205	-	CDS
O55/O157	3260919	i	rec	intergenic		
O55/O157	3260949	i	rec	intergenic		
O55/O157	3260967	indel-4	rec	intergenic		
O55/O157	3261009	i	rec	intergenic		
O55/O157	3261021	nc	rec	G2583_3206	-	pseudogene
O55/O157	3261088	nc	rec	G2583_3206	-	pseudogene
O55/O157	3261102	nc	rec	G2583_3206	-	pseudogene
O55/O157	3261240	nc	rec	G2583_3206	-	pseudogene
O55/O157	3261308	nc	rec	G2583_3206	-	pseudogene
O55/O157	3261349	nc	rec	G2583_3206	-	pseudogene
O55/O157	3261416	nc	rec	G2583_3206	-	pseudogene
O55/O157	3261435	nc	rec	G2583_3206	-	pseudogene
O55/O157	3261442	nc	rec	G2583_3206	-	pseudogene
O55/O157	3261454	nc	rec	G2583_3206	-	pseudogene
O55/O157	3261459	nc	rec	G2583_3206	-	pseudogene
O55/O157	3261465	nc	rec	G2583_3206	-	pseudogene
O55/O157	3261472	nc	rec	G2583_3206	-	pseudogene
O55/O157	3261476	nc	rec	G2583_3206	-	pseudogene
O55/O157	3261479	nc	rec	G2583_3206	-	pseudogene
O55/O157	3261483	nc	rec	G2583_3206	-	pseudogene
O55/O157	3261486	nc	rec	G2583_3206	-	pseudogene
O55/O157	3261495	nc	rec	G2583_3206	-	pseudogene
O55/O157	3261516	nc	rec	G2583_3206	-	pseudogene
O55/O157	3261529	nc	rec	G2583_3206	-	pseudogene
O55/O157	3261532	indel-3	rec	G2583_3206	-	CDS
O55/O157	3261545	nc	rec	G2583_3206	-	pseudogene
O55/O157	3261565	nc	rec	G2583_3206	-	pseudogene
O55/O157	3261572	nc	rec	G2583_3206	-	pseudogene
O55/O157	3261596	nc	rec	G2583_3206	-	pseudogene
O55/O157	3261627	nc	rec	G2583_3206	-	pseudogene
Sakai	3484256	ns	rec	G2583_3242	-	CDS
Sakai	3484260	ns	rec	G2583_3242	-	CDS
EDL933	3551606	ns		G2583_3242	-	CDS
O55/O157	3292159	ns	rec	G2583_3242	-	CDS
Sakai	3484362	ns	rec	G2583_3242	-	CDS
O55/O157	3292221	ns	rec	G2583_3242	-	CDS
EDL933	3551743	ns		G2583_3242	-	CDS
O55/O157	3292596	ns	rec	G2583_3242	-	CDS
EDL933	3552289	ins		G2583_3242	-	CDS
Sakai	3484989	ns	rec	G2583_3242	-	CDS
O55/O157	3293060	s	rec	G2583_3242	-	CDS

lineage[a]	site[b]	mutation type[c]	recomb	gene	Gene name	Type
O55/O157	3293072	s	rec	G2583_3242	-	CDS
O55/O157	3293116	ns	rec	G2583_3242	-	CDS
O55/O157	3293118	ns	rec	G2583_3242	-	CDS
O55/O157	3293135	s	rec	G2583_3242	-	CDS
O55/O157	3293153	ns	rec	G2583_3242	-	CDS
O55/O157	3293155	ns	rec	G2583_3242	-	CDS
O55/O157	3293174	ns	rec	G2583_3242	-	CDS
O55/O157	3293175	ns	rec	G2583_3242	-	CDS
O55/O157	3293176	ns	rec	G2583_3242	-	CDS
O55/O157	3293212	ns	rec	G2583_3242	-	CDS
O157	3485969	ns		G2583_3242	-	CDS
Sakai	3490148	ns	rec	ECs3504	-	CDS
Sakai	3490449	del-6	rec	intergenic		
Sakai	3491733	ns	rec	G2583_3291	-	CDS
Sakai	3491734	ns	rec	G2583_3291	-	CDS
O55/O157	3329609	ns		G2583_3291	-	CDS
EDL933	3559317	ins		G2583_3291	-	CDS
EDL933	3559344	ins		G2583_3291	-	CDS
O55/O157	3330853	i		intergenic		
EDL933	3560499	ins		intergenic		
O55/O157	3331099	ns		G2583_3292	-	CDS
EDL933	3561379	ins		G2583_3293	-	CDS
EDL933	3561587	ins		G2583_3294	-	CDS
EDL933	3561786	ins		G2583_3294	-	CDS
O55/O157	3332519	ns		G2583_3294	-	CDS
O55/O157	3332606	ns		G2583_3294	-	CDS
O55/O157	3332674	ns		G2583_3294	-	CDS
O55/O157	3334462	ns		G2583_3295	-	CDS
O55/O157	3334651	ns		G2583_3295	-	CDS
O55/O157	3334724	s		G2583_3295	-	CDS
EDL933	3564608	ins-2		G2583_3296	-	CDS
EDL933	3564684	ins		G2583_3296	-	CDS
O55/O157	3336184	i		intergenic		
O55/O157	3336522	ns		G2583_3297	alpA	CDS
O55/O157	3337895	indel		intergenic		
CB9615	3339057	ins-12		G2583_3299	ypjA	pseudogene
CB9615	3339874	del		G2583_3299	ypjA	pseudogene
CB9615	3340946	nc		G2583_3299	ypjA	pseudogene
O157	3506470	s		G2583_3301	ypjB	CDS
CB9615	3344441	ns		G2583_3301	ypjB	CDS
CB9615	3345494	ns		G2583_3302	-	CDS
CB9615	3345979	i		intergenic		
CB9615	3346865	ns		G2583_3304	ygaR	CDS
CB9615	3347295	s		G2583_3304	ygaR	CDS
O157	3509803	s		G2583_3304	ygaR	CDS

lineage[a]	site[b]	mutation type[c]	recomb	gene	Gene name	Type
O157	3509804	ns		G2583_3304	ygaR	CDS
O157	3509819	ns		G2583_3304	ygaR	CDS
O157	3510176	ns		G2583_3304	ygaR	CDS
CB9615	3348697	i		intergenic		
O157	3511047	i		intergenic		
O157	3511215	ns		G2583_3305	csiD	CDS
CB9615	3350871	ns		G2583_3306	ygaF	CDS
O157	3519014	i		intergenic		
O157	3519241	i		intergenic		
CB9615	3357112	i		intergenic		
O157	3519880	ns		G2583_3314	ygaP	CDS
CB9615	3358865	i		intergenic		
CB9615	3358907	i		intergenic		
CB9615	3359030	i		intergenic		
CB9615	3360359	ns		G2583_3318	ygaM	CDS
CB9615	3360519	i		intergenic		
CB9615	3361222	ns		G2583_3319	-	CDS
CB9615	3366030	ns		G2583_3324	nrdF	CDS
O157	3528591	ns		G2583_3324	nrdF	CDS
O157	3529453	ns		G2583_3325	proV	CDS
O157	3529723	ns		G2583_3325	proV	CDS
O157	3533864	s		G2583_3329	ygaZ	CDS
CB9615	3374376	ns		G2583_3332	emrA	CDS
EDL933	3606613	s		G2583_3336	gshA	CDS
CB9615	3377564	ns		G2583_3336	gshA	CDS
CB9615	3377805	s		G2583_3336	gshA	CDS
O157	3541678	i		intergenic		
CB9615	3379643	i		intergenic		
EDL933	3609798	ins		G2583_3343	-	tRNA
EDL933	3610668	ins		G2583_3345	alaS	CDS
CB9615	3381806	s		G2583_3345	alaS	CDS
O157	3544256	s		G2583_3345	alaS	CDS
EDL933	3614571	ns		G2583_3347	recA	CDS
CB9615	3385787	s		G2583_3348	ygaD	CDS
Sakai	3549660	del-5		intergenic		
CB9615	3387429	i		intergenic		
CB9615	3387453	i		intergenic		
O157	3550124	del		G2583_3350	srlA	CDS
O157	3550404	ins-2		G2583_3351	srlE	CDS
EDL933	3620383	ins		G2583_3355	srlR	CDS
O157	3557741	s		G2583_3359	norW	CDS
CB9615	3399544	ns		G2583_3361	hydN	CDS
CB9615	3401015	i		intergenic		
CB9615	3401096	i		intergenic		
CB9615	3402118	s		G2583_3363	ascF	CDS

lineage[a]	site[b]	mutation type[c]	recomb	gene	Gene name	Type
O157	3564998	ns		G2583_3364	ascB	CDS
O157	3565101	s		G2583_3364	ascB	CDS
CB9615	3403539	s		G2583_3364	ascB	CDS
CB9615	3403798	ns		G2583_3364	ascB	CDS
O157	3570574	s		G2583_3370	hycD	CDS
CB9615	3408904	s		G2583_3370	hycD	CDS
EDL933	3638477	ns		G2583_3371	hycC	CDS
CB9615	3409557	ns		G2583_3371	hycC	CDS
CB9615	3411487	i		intergenic		
O157	3574988	ns		G2583_3376	hypB	CDS
O157	3576088	ns		G2583_3378	hypD	CDS
O157	3577400	ns		G2583_3379	hypE	CDS
O157	3577442	ns		G2583_3379	hypE	CDS
CB9615	3422941	ns		G2583_3386	kpdC	CDS
O157	3586076	s		G2583_3387	padI	CDS
O157	3586558	ns		G2583_3388	-	CDS
O157	3587532	s		G2583_3389	rpoS	CDS
EDL933	3655312	ns		G2583_3389	rpoS	CDS
EDL933	3655316	ns		G2583_3389	rpoS	CDS
O157	3588380	s		G2583_3390	nlpD	CDS
CB9615	3429411	s		G2583_3393	truD	CDS
O157	3592455	ns		G2583_3395	ispD	CDS
O157	3595801	s		G2583_3399	cysN	CDS
O157	3596582	ns		G2583_3400	cysD	CDS
O157	3598937	ns		G2583_3403	ygbT	CDS
CB9615	3437303	ns		G2583_3403	ygbT	CDS
CB9615	3438424	s		G2583_3405	ygcI	CDS
O157	3601712	s		G2583_3406	ygcJ	CDS
O157	3602384	ns		G2583_3407	cse	CDS
CB9615	3440341	s		G2583_3407	cse	CDS
O157	3604022	ns		G2583_3408	ygcL	CDS
CB9615	3443601	ns		G2583_3409	ygcB	CDS
O157	3607226	i		intergenic		
CB9615	3446825	ns		G2583_3413	cysI	CDS
O157	3609450	s		G2583_3413	cysI	CDS
CB9615	3452155	ns		G2583_3417	ygcO	CDS
O157	3616969	del		G2583_3421	ygcS	CDS
CB9615	3455322	ns		G2583_3421	ygcS	CDS
CB9615	3455764	s		G2583_3421	ygcS	CDS
CB9615	3456041	ns		G2583_3422	ygcU	CDS
CB9615	3457182	ns		G2583_3422	ygcU	CDS
CB9615	3457645	ns		G2583_3423	ygcW	CDS
CB9615	3459792	ns		G2583_3424	yqcE	CDS
O157	3621850	i		intergenic		
O157	3622700	ns		G2583_3425	ygcE	CDS

lineage[a]	site[b]	mutation type[c]	recomb	gene	Gene name	Type
CB9615	3461846	i		intergenic		
O157	3623898	del-2		intergenic		
CB9615	3461916	i		intergenic		
O157	3626293	s		G2583_3428	-	CDS
CB9615	3464730	s		G2583_3428	-	CDS
O157	3626688	ns		G2583_3428	-	CDS
CB9615	3468885	ns		G2583_3432	pyrG	CDS
Sakai	3637135	ns		G2583_3437	rumA	CDS
CB9615	3480539	ns		G2583_3440	gudX	CDS
O157	3642927	ns		G2583_3440	gudX	CDS
O157	3643158	ns		G2583_3441	gudP	CDS
CB9615	3481664	ns		G2583_3441	gudP	CDS
O157	3645412	ns		G2583_3443	truC	CDS
CB9615	3485893	ns		G2583_3447	queF	CDS
CB9615	3487207	s		G2583_3448	ygdH	CDS
O157	3656572	i		intergenic		
O157	3659882	s		G2583_3456	fucK	CDS
O157	3661237	ns		G2583_3456	fucK	CDS
CB9615	3501682	del		intergenic		
O157	3667951	ns		G2583_3466	ygdL	CDS
CB9615	3507805	s		G2583_3467	mltA	CDS
O157	3674715	s		G2583_3473	recD	CDS
O157	3675231	ns		G2583_3474	recB	CDS
O157	3676423	ns		G2583_3474	recB	CDS
O157	3676880	s		G2583_3474	recB	CDS
O157	3677574	ns		G2583_3474	recB	CDS
O157	3678664	ns		G2583_3475	ptrA	CDS
CB9615	3517207	s		G2583_3475	ptrA	CDS
CB9615	3517947	ns		G2583_3475	ptrA	CDS
O157	3680760	s		G2583_3475	ptrA	CDS
O157	3681585	ns		G2583_3476	recC	CDS
O157	3684014	ns		G2583_3476	recC	CDS
O157	3684856	s		G2583_3477	ppdC	CDS
O157	3690295	s		G2583_3483	ptsP	CDS
O157	3694856	ns		G2583_3489	tas	CDS
CB9615	3535080	ns		G2583_3491	aas	CDS
CB9615	3535667	ns		G2583_3491	aas	CDS
EDL933	3766085	ins		intergenic		
O157	3700229	ns		G2583_3495	lysA	CDS
CB9615	3538874	ns		G2583_3495	lysA	CDS
O157	3700922	ns		G2583_3495	lysA	CDS
CB9615	3539965	s		G2583_3496	lysR	CDS
O157	3706765	i		intergenic		
CB9615	3547870	i		intergenic		
O157	3710057	ns		G2583_3503	yqeH	CDS

lineage[a]	site[b]	mutation type[c]	recomb	gene	Gene name	Type
O157	3711187	s		G2583_3504	yqeI	CDS
O157	3711725	ins		G2583_3505	yqeJ	CDS
O157	3712943	ns		G2583_3507	-	CDS
EDL933	3781741	ins		intergenic		
EDL933	3784341	ns		ECs3711	-	CDS
EDL933	3787702	ins		ECs3718	-	CDS
EDL933	3787760	ins		ECs3718	-	CDS
EDL933	3787779	ins		ECs3718	-	CDS
EDL933	3787815	ins		ECs3718	-	CDS
EDL933	3787871	ins		ECs3719	-	CDS
EDL933	3788848	i		intergenic		
O157	3729082	ns		G2583_3510-3511	-	CDS
CB9615	3553579	ns		G2583_3512	EivA	CDS
CB9615	3553801	ns		G2583_3512	EivA	CDS
CB9615	3555318	del-7		G2583_3513	EivE	CDS
CB9615	3555319	ns	rec	G2583_3513	EivE	CDS
CB9615	3555320	s	rec	G2583_3513	EivE	CDS
CB9615	3555325	ns	rec	G2583_3513	EivE	CDS
CB9615	3555327	ns	rec	G2583_3513	EivE	CDS
CB9615	3555421	ns		G2583_3513	EivE	CDS
CB9615	3555639	ns		G2583_3513	EivE	CDS
O157	3732315	ns		G2583_3513	EivE	CDS
CB9615	3556461	s		G2583_3514	EivG	CDS
CB9615	3558046	ns		G2583_3514	EivG	CDS
O157	3734395	ns		G2583_3515	invF	CDS
CB9615	3559935	del		G2583_3517	-	pseudogene
O157	3736522	del-5		G2583_3517	-	pseudogene
EDL933	3804912	ins		G2583_3519	ygeR	CDS
O157	3738559	i		intergenic		
O157	3739042	ns		G2583_3520	xdhA	CDS
O157	3739366	ns		G2583_3520	xdhA	CDS
CB9615	3563791	ns		G2583_3520	xdhA	CDS
O157	3739828	ns		G2583_3520	xdhA	CDS
CB9615	3565245	s		G2583_3521	xdhB	CDS
O157	3741887	ns		G2583_3522	xdhC	CDS
O157	3741927	s		G2583_3522	xdhC	CDS
O157	3743928	ns		G2583_3523	ygeV	CDS
O157	3743999	ns		G2583_3523	ygeV	CDS
O157	3744290	i		intergenic		
CB9615	3568681	ns		G2583_3524	ygeW	CDS
Sakai	3744736	s		G2583_3524	ygeW	CDS
CB9615	3576801	ns		G2583_3530	yqeC	CDS
Sakai	3752833	ns		G2583_3530	yqeC	CDS
EDL933	3821432	ins		intergenic		
EDL933	3821482	ins		intergenic		

lineage[a]	site[b]	mutation type[c]	recomb	gene	Gene name	Type
CB9615	3579827	ns		G2583_3532	ygfK	CDS
O157	3757549	ns		G2583_3533	ssnA	CDS
CB9615	3587846	ns		G2583_3536	ygfO	CDS
O157	3767078	ns		G2583_3539	ygfS	CDS
CB9615	3591256	s		G2583_3540	ygfT	CDS
O157	3769367	i		intergenic		
O157	3769926	ns		G2583_3541	ygfU	CDS
O157	3772260	ns		G2583_3543	lysS	CDS
O157	3774902	s		G2583_3545	recJ	CDS
O157	3777224	ns		G2583_3547	xerD	CDS
O157	3777482	s		G2583_3547	xerD	CDS
CB9615	3603794	ns		G2583_3551	ygfZ	CDS
CB9615	3604302	s		G2583_3551	ygfZ	CDS
CB9615	3606795	ns		G2583_3554	bglA	CDS
O157	3783456	s		G2583_3555	gcvP	CDS
CB9615	3607726	ns		G2583_3555	gcvP	CDS
O157	3786417	s		G2583_3556	gcvH	CDS
O157	3787155	ns		G2583_3557	gcvT	CDS
O157	3787680	ns		G2583_3557	gcvT	CDS
O157	3788978	s		G2583_3558	visC	CDS
O157	3792372	s		G2583_3561	ygfB	CDS
CB9615	3618675	i		intergenic		
O157	3796111	i		intergenic		
CB9615	3620119	i		intergenic		
O157	3797071	i		intergenic		
O157	3798707	ns		G2583_3571	scpA	CDS
O157	3800106	ns		G2583_3571	scpA	CDS
O157	3800518	ns		G2583_3572	argK	CDS
Sakai	3800637	ns		G2583_3572	argK	CDS
O157	3803527	ns		G2583_3574	scpC	CDS
O157	3805598	i		intergenic		
O157	3805904	s		G2583_3577	argO	CDS
O157	3809571	s		G2583_3580	pgk	CDS
CB9615	3637022	s		G2583_3584	-	CDS
CB9615	3637038	ns		G2583_3584	-	CDS
CB9615	3638234	ns		G2583_3586	-	CDS
CB9615	3639104	s		G2583_3587	yggC	CDS
CB9615	3639118	ns		G2583_3587	yggC	CDS
O157	3815794	ns		G2583_3588	yggD	CDS
CB9615	3640947	s		G2583_3590	yggP	CDS
CB9615	3640959	s		G2583_3590	yggP	CDS
O157	3817060	s		G2583_3590	yggP	CDS
O157	3820158	i		intergenic		
O157	3820933	s		G2583_3593	tktA	CDS
O157	3824349	s		G2583_3595	speB	CDS

lineage[a]	site[b]	mutation type[c]	recomb	gene	Gene name	Type
O157	3826627	s		G2583_3597	speA	CDS
CB9615	3651252	s		G2583_3597	speA	CDS
O157	3829681	i		intergenic		
O157	3829808	ns		G2583_3602	galP	CDS
O157	3830235	s		G2583_3602	galP	CDS
CB9615	3654811	ns		G2583_3602	galP	CDS
O157	3831481	s		G2583_3603	yggI	CDS
O157	3831641	ns		G2583_3603	yggI	CDS
CB9615	3656287	s		G2583_3604	endA	CDS
EDL933	3902787	ins		intergenic		
EDL933	3902857	ins		intergenic		
EDL933	3902862	ins		intergenic		
EDL933	3902864	ins		intergenic		
O157	3840793	ns		G2583_3615	yggM	CDS
CB9615	3670729	ns		G2583_3622	mltC	CDS
O157	3848246	s		G2583_3623	nupG	CDS
O157	3848435	s		G2583_3623	nupG	CDS
CB9615	3672686	s		G2583_3624	speC	CDS
CB9615	3673035	ns		G2583_3624	speC	CDS
EDL933	3921252	ns		G2583_3628	-	CDS
O157	3854773	s	rec	G2583_3629	ISSfl4	CDS
O157	3854777	ns	rec	G2583_3629	ISSfl4	CDS
O157	3854780	s	rec	G2583_3629	ISSfl4	CDS
O157	3854781	s	rec	G2583_3629	ISSfl4	CDS
O157	3854788	s	rec	G2583_3629	ISSfl4	CDS
O157	3854800	s	rec	G2583_3629	ISSfl4	CDS
O157	3854801	ns	rec	G2583_3629	ISSfl4	CDS
O157	3854804	s	rec	G2583_3629	ISSfl4	CDS
O157	3854806	s	rec	G2583_3629	ISSfl4	CDS
O157	3854811	ns	rec	G2583_3629	ISSfl4	CDS
O157	3854812	ns	rec	G2583_3629	ISSfl4	CDS
O157	3854815	s	rec	G2583_3629	ISSfl4	CDS
CB9615	3678824	s		G2583_3629	ISSfl4	CDS
O157	3855548	ns		G2583_3631	ISSfl4	CDS
O157	3855919	s		G2583_3631	ISSfl4	CDS
CB9615	3682316	ins		G2583_3633	PagC	CDS
EDL933	3926642	ins		G2583_3635	-	pseudogene
O157	3859844	nc		G2583_3635	-	pseudogene
CB9615	3684322	nc		G2583_3635	-	pseudogene
O157	3862467	ns		G2583_3637	ospD	CDS
CB9615	3687827	nc		G2583_3638	-	pseudogene
CB9615	3688151	nc		G2583_3638	-	pseudogene
O157	3864589	nc		G2583_3638	-	pseudogene
CB9615	3690706	i		intergenic		
O157	3867936	ns		G2583_3641	-	CDS

lineage[a]	site[b]	mutation type[c]	recomb	gene	Gene name	Type
EDL933	3935780	ns		G2583_3641	-	CDS
O157	3868500	del		G2583_3641	-	CDS
EDL933	3937956	ns		ECs3864	-	CDS
Sakai	3871025	ns		ECs3865	-	CDS
O157	3874355	ns		G2583_3645	-	CDS
CB9615	3703890	ns		G2583_3646	-	CDS
EDL933	3942531	i		intergenic		
CB9615	3765449	s		G2583_3713	pitB	CDS
CB9615	3766955	ns		G2583_3714	gsp	CDS
CB9615	3767789	ns		G2583_3714	gsp	CDS
CB9615	3768550	ns		G2583_3714	gsp	CDS
O157	3881065	ns		G2583_3718	hybE	CDS
CB9615	3772270	ns		G2583_3720	hybC	CDS
O157	3884025	ns		G2583_3721	hybB	CDS
Sakai	3885057	s		G2583_3722	hybA	CDS
O157	3890991	i		intergenic		
O157	3891514	s		G2583_3729	exbD	CDS
O157	3894868	s		G2583_3734	yqhC	CDS
O157	3897228	ns		G2583_3736	dkgA	CDS
EDL933	3965305	ins		intergenic		
O157	3898234	del-4		G2583_3737	yqhG	CDS
CB9615	3789268	s		G2583_3739	ygiQ	CDS
CB9615	3789305	ns		G2583_3739	ygiQ	CDS
CB9615	3790417	s		G2583_3739	ygiQ	CDS
CB9615	3792348	s		G2583_3740	sufI	CDS
O157	3903293	s		G2583_3741	plsC	CDS
Sakai	3910430	del		G2583_3747	qseC	CDS
O157	3913803	ns		G2583_3751	-	CDS
O157	3914619	s		G2583_3752	-	CDS
O157	3915743	s		G2583_3753	-	CDS
CB9615	3806129	s		G2583_3754	FepC	CDS
O157	3917269	i		intergenic		
CB9615	3807029	i		intergenic		
O157	3919301	ns		G2583_3755	-	CDS
O157	3922268	s		G2583_3758	cpdA	CDS
EDL933	3992087	s		G2583_3761	tolC	CDS
O157	3925035	ns		G2583_3761	tolC	CDS
O157	3926802	s		G2583_3764	ygiC	CDS
O157	3931151	ns		G2583_3769	yqiC	CDS
O157	3931427	s		G2583_3770	glgS	CDS
O157	3931641	i		intergenic		
O157	3931652	i		intergenic		
O157	3931685	i		intergenic		
CB9615	3821791	ns		G2583_3771	yqiJ	CDS
O157	3934014	ns		G2583_3772	yqiK	CDS

lineage[a]	site[b]	mutation type[c]	recomb	gene	Gene name	Type
CB9615	3825647	ns		G2583_3776	rfaE	CDS
O157	3936197	ns		G2583_3776	rfaE	CDS
O157	3937593	ns		G2583_3777	glnE	CDS
CB9615	3827899	ns		G2583_3777	glnE	CDS
O157	3938599	ns		G2583_3777	glnE	CDS
CB9615	3829522	s		G2583_3778	ygiF	CDS
O157	3942169	ns		G2583_3780	cca	CDS
O157	3942753	i		intergenic		
CB9615	3834606	i		intergenic		
O157	3949305	ns		G2583_3788	ygjD	CDS
O157	3954859	ns		G2583_3792	mug	CDS
CB9615	3846017	i		intergenic		
O157	3956835	ns		G2583_3795	yqjI	CDS
Sakai	3957788	ns		G2583_3796	aer	CDS
CB9615	3848892	s		G2583_3797	ygjG	CDS
EDL933	4029119	ns		G2583_3799	ebgR	CDS
CB9615	3853328	s		G2583_3800	ebgA	CDS
O157	3964851	ns		G2583_3800	ebgA	CDS
O157	3965583	ns		G2583_3801	ebgC	CDS
O157	3967442	s		G2583_3803	ygjJ	CDS
CB9615	3858887	ns		G2583_3804	ygjK	CDS
O157	3970071	s		G2583_3804	ygjK	CDS
CB9615	3860431	s		G2583_3804	ygjK	CDS
O157	3970734	ns		G2583_3804	ygjK	CDS
CB9615	3864199	s		G2583_3808	rlmG	CDS
CB9615	3865140	ns		G2583_3808	rlmG	CDS
O157	3976786	ns		G2583_3810	ygjQ	CDS
CB9615	3866680	i		intergenic		
O157	3981552	s		G2583_3815	uxaA	CDS
CB9615	3872384	ns		G2583_3815	uxaA	CDS
O157	3983286	ns		G2583_3816	uxaC	CDS
O157	3983614	s		G2583_3816	uxaC	CDS
EDL933	4051784	ins		intergenic		
O157	3991636	ns		G2583_3826	yqjG	CDS
CB9615	3881607	i		intergenic		
O157	3992419	del		intergenic		
O157	3996822	ns		G2583_3833	yhaO	CDS
O157	3997842	i		intergenic		
O157	3997944	s		G2583_3834	tdcG	CDS
O157	3998133	ns		G2583_3834	tdcG	CDS
O157	4000385	ns		G2583_3836	tdcE	CDS
CB9615	3891826	i		intergenic		
CB9615	3892223	s		G2583_3837	tdcD	CDS
O157	4002739	ns		G2583_3837	tdcD	CDS
CB9615	3893033	s		G2583_3837	tdcD	CDS

lineage[a]	site[b]	mutation type[c]	recomb	gene	Gene name	Type
CB9615	3893769	s		G2583_3838	tdcC	CDS
O157	4006809	i		intergenic		
O157	4006837	i		intergenic		
O157	4007236	i		intergenic		
O157	4008218	ns		G2583_3843	yhaC	CDS
EDL933	4075542	ns		G2583_3843	yhaC	CDS
CB9615	3898133	ns		G2583_3843	yhaC	CDS
EDL933	4076397	ns		G2583_3843	yhaC	CDS
O157	4009251	i		intergenic		
CB9615	3899242	ns		G2583_3844-3845	-	CDS
CB9615	3907655	i		intergenic		
CB9615	3908041	ns		G2583_3854	kbaZ	CDS
O157	4019884	ns		G2583_3855	agaV	CDS
CB9615	3910124	ns		G2583_3856	agaW	CDS
O157	4025196	s		G2583_3861	kbaY	CDS
O157	4026246	ns		G2583_3863	agaC	CDS
CB9615	3916856	ns		G2583_3864	agaD	CDS
CB9615	3917600	ins		G2583_3865	agaI	pseudogene
O157	4032948	ns		G2583_3869	-	CDS
CB9615	3924525	i		intergenic		
O157	4040274	s		G2583_3875	yraQ	CDS
CB9615	3930061	ns		G2583_3876	yraR	CDS
O157	4042722	ns		G2583_3879	yhbQ	CDS
O157	4046817	ns		G2583_3884	yhbW	CDS
CB9615	3935603	s		G2583_3884	yhbW	CDS
CB9615	3936139	s		G2583_3885	mtr	CDS
O157	4047846	s		G2583_3885	mtr	CDS
CB9615	3941892	s		G2583_3888	pnp	CDS
O157	4053943	ns		G2583_3890	rpsO	CDS
O157	4056310	s		G2583_3893	infB	CDS
O157	4060069	s		G2583_3895	yhbC	CDS
O157	4062106	s		G2583_3897	argG	CDS
O157	4062586	ns		G2583_3898	yhbX	CDS
O157	4065106	i		intergenic		
O157	4065371	ns		G2583_3901	glmM	CDS
CB9615	3954069	s		G2583_3901	glmM	CDS
O157	4067597	ns		G2583_3903	hflB	CDS
O157	4076866	ns		G2583_3912	ispB	CDS
O157	4077127	i		intergenic		
O157	4077165	i		intergenic		
Sakai	4077481	ns		G2583_3913	sfsB	CDS
CB9615	3967415	ns		G2583_3914	murA	CDS
CB9615	3967433	s		G2583_3914	murA	CDS
O157	4081542	s		G2583_3919	yrbE	CDS
O157	4083988	ns		G2583_3922	kdsD	CDS

lineage[a]	site[b]	mutation type[c]	recomb	gene	Gene name	Type
O157	4086506	s		G2583_3926	lptB	CDS
O157	4086858	s		G2583_3926	lptB	CDS
O157	4087954	s		G2583_3927	rpoN	CDS
CB9615	3977087	ns		G2583_3927	rpoN	CDS
CB9615	3980613	ns		G2583_3933	mtgA	CDS
O157	4091967	ns		G2583_3933	mtgA	CDS
EDL933	4160087	ins		intergenic		
O157	4092951	nc		G2583_3935	-	pseudogene
O157	4093286	ns		G2583_3936	arcB	CDS
CB9615	3985323	i		intergenic		
CB9615	3985393	i		intergenic		
EDL933	4164506	ns		G2583_3938	gltB	CDS
O157	4097903	ns		G2583_3938	gltB	CDS
Sakai	4100596	ns		G2583_3938	gltB	CDS
O157	4105064	s		G2583_3942	nanK	CDS
O157	4105320	ns		G2583_3942	nanK	CDS
O157	4106520	s		G2583_3944	nanT	CDS
O157	4110204	del-3		G2583_3947	dcuD	CDS
O157	4110244	ns		G2583_3947	dcuD	CDS
O157	4110958	del-6		G2583_3947	dcuD	CDS
O157	4111331	del-2		G2583_3947	dcuD	CDS
O157	4113276	s		G2583_3950	rpsI	CDS
CB9615	4003038	ns		G2583_3952	yhcM	CDS
O157	4114838	s		G2583_3952	yhcM	CDS
CB9615	4004276	ns		G2583_3953	yhcB	CDS
O157	4116906	ns		G2583_3954	degQ	CDS
CB9615	4007407	s		G2583_3956	mdh	CDS
O157	4119805	i		intergenic		
O157	4120502	i		intergenic		
CB9615	4009361	ins-2		intergenic		
EDL933	4188637	ins		intergenic		
CB9615	4010292	ns		G2583_3960	aaeB	CDS
O157	4121550	ns		G2583_3960	aaeB	CDS
CB9615	4011587	s		G2583_3960	aaeB	CDS
O157	4123421	ns		G2583_3960	aaeB	CDS
O157	4124170	del-3		G2583_3961	aaeA	CDS
O157	4124478	ns		G2583_3962	aaeX	CDS
O157	4125741	ins-6		intergenic		
O157	4126692	ns		G2583_3964	tldD	CDS
O157	4133899	ns		G2583_3969	mreC	CDS
CB9615	4025174	ns		G2583_3971	csrD	CDS
O157	4139812	i		intergenic		
CB9615	4029379	ns		G2583_3974	accB	CDS
O157	4141540	ns		G2583_3975	accC	CDS
O157	4142288	i		intergenic		

lineage[a]	site[b]	mutation type[c]	recomb	gene	Gene name	Type
O157	4143190	s		G2583_3977	panF	CDS
O157	4143433	s		G2583_3977	panF	CDS
CB9615	4033165	s		G2583_3978	prmA	CDS
O157	4150626	ns		G2583_3985	acrF	CDS
O55/O157	4040626	indel-8		G2583_3985	acrF	CDS
O157	4153853	i		intergenic		
O157	4156633	nc		G2583_3989	yhdY	pseudogene
O157	4156718	nc		G2583_3989	yhdY	pseudogene
CB9615	4045524	nc		G2583_3989	yhdY	pseudogene
O157	4156905	nc		G2583_3989	yhdY	pseudogene
O157	4157289	nc		G2583_3989	yhdY	pseudogene
CB9615	4047394	i		intergenic		
CB9615	4047395	i		intergenic		
EDL933	4226034	ins		intergenic		
Sakai	4160560	nc		G2583_3994	rrl	rRNA
Sakai	4160569	nc		G2583_3994	rrl	rRNA
EDL933	4227835	ins		G2583_3994	rrl	rRNA
Sakai	4161142	nc		G2583_3994	rrl	rRNA
EDL933	4228455	del-12		G2583_3994	rrl	rRNA
EDL933	4228456	ins-14		G2583_3994	rrl	rRNA
EDL933	4228654	nc		G2583_3994	rrl	rRNA
O157	4161524	nc		G2583_3994	rrl	rRNA
EDL933	4228868	nc		G2583_3994	rrl	rRNA
EDL933	4228873	nc		G2583_3994	rrl	rRNA
EDL933	4229960	nc	rec	G2583_3997	rrs	rRNA
EDL933	4229975	nc	rec	G2583_3997	rrs	rRNA
EDL933	4229976	nc	rec	G2583_3997	rrs	rRNA
EDL933	4229977	nc	rec	G2583_3997	rrs	rRNA
EDL933	4229978	nc	rec	G2583_3997	rrs	rRNA
EDL933	4229979	nc	rec	G2583_3997	rrs	rRNA
EDL933	4229988	nc	rec	G2583_3997	rrs	rRNA
EDL933	4229992	nc	rec	G2583_3997	rrs	rRNA
EDL933	4229996	nc	rec	G2583_3997	rrs	rRNA
O157	4163631	nc		G2583_3997	rrs	rRNA
O157	4163997	ns		G2583_3998	yrdA	CDS
O157	4164849	s		G2583_3999	yrdB	CDS
CB9615	4056533	s		G2583_4004	smf	CDS
CB9615	4056888	ns		G2583_4004	smf	CDS
CB9615	4057093	ns		G2583_4004	smf	CDS
CB9615	4057929	ns		G2583_4005	def	CDS
Sakai	4170227	ns		G2583_4007	rsmB	CDS
O157	4170778	ns		G2583_4007	rsmB	CDS
Sakai	4172246	s		G2583_4008	trkA	CDS
CB9615	4061703	i		intergenic		
CB9615	4063470	s		G2583_4012	rplQ	CDS

lineage[a]	site[b]	mutation type[c]	recomb	gene	Gene name	Type
O157	4179942	s		G2583_4021	rpsE	CDS
O157	4187549	s		G2583_4038	rplC	CDS
O157	4190210	s		G2583_4044	tufA	CDS
O157	4192284	s		G2583_4045	fusA	CDS
O157	4194601	ns		G2583_4048	yheL	CDS
O157	4195033	ns		G2583_4049	yheM	CDS
O157	4195353	ns		G2583_4050	yheN	CDS
CB9615	4085339	ns		G2583_4052	fkpA	CDS
EDL933	4085339	ns		G2583_4052	fkpA	CDS
Sakai	4085339	ns		G2583_4052	fkpA	CDS
O157	4197435	i		intergenic		
CB9615	4086910	ns		G2583_4054	slyD	CDS
O157	4198737	ns		G2583_4056	kefB	CDS
CB9615	4088589	ns		G2583_4056	kefB	CDS
O157	4200690	s		G2583_4057	kefG	CDS
CB9615	4090409	ns		G2583_4058	yheS	CDS
CB9615	4091034	ns		G2583_4058	yheS	CDS
O157	4204857	s		G2583_4061	prkB	CDS
O157	4207271	ns		G2583_4064	yhfK	CDS
CB9615	4099995	ns		G2583_4067	fic	CDS
O157	4212761	s		G2583_4070	tsgA	CDS
CB9615	4101599	ns		G2583_4070	tsgA	CDS
CB9615	4102152	s		G2583_4070	tsgA	CDS
Sakai	4213984	ns		G2583_4071	nirB	CDS
CB9615	4109661	ns		G2583_4076	frlA	CDS
O157	4221007	ns		G2583_4076	frlA	CDS
CB9615	4110445	ns		G2583_4077	frlB	CDS
O157	4223512	nc		G2583_4079	-	pseudogene
O157	4224678	s		G2583_4080	frlR	CDS
CB9615	4114478	ns		G2583_4081	trpS	CDS
O157	4232974	ns		G2583_4089	hofP	CDS
O157	4233965	s		G2583_4090	hofN	CDS
O157	4234194	del		G2583_4091	hofM	CDS
O157	4237354	s		G2583_4092	mrcA	CDS
O157	4239608	ns		G2583_4094	yrfF	CDS
O157	4244359	ns		G2583_4098	yhgE	CDS
O157	4246730	ns		G2583_4101	envZ	CDS
O157	4250491	ns		G2583_4104	yhgF	CDS
O157	4250541	s		G2583_4104	yhgF	CDS
O157	4252233	i		intergenic		
Sakai	4253956	ns		G2583_4106	feoB	CDS
CB9615	4145419	s		G2583_4109	bioH	CDS
O157	4258834	s		G2583_4112	gntT	CDS
O157	4261820	ns		G2583_4113	malQ	CDS
O157	4262127	ns		G2583_4113	malQ	CDS

lineage[a]	site[b]	mutation type[c]	recomb	gene	Gene name	Type
O157	4262902	s		G2583_4114	malP	CDS
O157	4269534	s		G2583_4119	rtcA	CDS
O157	4269943	ns		G2583_4120	rtcB	CDS
CB9615	4158807	ns		G2583_4120	rtcB	CDS
O157	4271823	s		G2583_4121	rtcR	CDS
CB9615	4163991	s		G2583_4125	glpD	CDS
CB9615	4164972	s		G2583_4125	glpD	CDS
CB9615	4165486	ns		G2583_4126	-	CDS
CB9615	4167562	ns		G2583_4128	yzgL	CDS
CB9615	4167770	s		G2583_4128	yzgL	CDS
O157	4279897	del		intergenic		
O157	4280655	ns		G2583_4129	glgP	CDS
O157	4292642	ns		G2583_4136	-	CDS
CB9615	4181462	nc		G2583_4137	-	pseudogene
CB9615	4181805	nc		G2583_4137	-	pseudogene
O157	4294165	nc		G2583_4137	-	pseudogene
CB9615	4186806	ns		G2583_4141	yhhW	CDS
O157	4299067	s		G2583_4142	yhhX	CDS
CB9615	4188464	ns		G2583_4142	yhhX	CDS
CB9615	4188778	ins		G2583_4143	-	pseudogene
CB9615	4188862	i		intergenic		
CB9615	4189151	ns		G2583_4144	yhhY	CDS
O55/O157	4190193	ns		G2583_4145	-	CDS
O55/O157	4190500	indel		G2583_4145	-	CDS
O55/O157	4190626	ns		G2583_4146	-	CDS
O55/O157	4191169	i		intergenic		
EDL933	4369934	ins		intergenic		
O157	4303023	ns		G2583_4147	ggt	CDS
CB9615	4194804	s		G2583_4150	ugpC	CDS
O157	4306654	ns		G2583_4150	ugpC	CDS
O157	4307830	s		G2583_4152	ugpA	CDS
CB9615	4197149	ns		G2583_4152	ugpA	CDS
EDL933	4375820	ins		intergenic		
O157	4315671	s		G2583_4159	livK	CDS
O157	4316371	i		intergenic		
EDL933	4384250	ns		G2583_4161	-	CDS
O157	4320461	s		G2583_4165	ftsX	CDS
O157	4321246	ns		G2583_4165	ftsX	CDS
CB9615	4211248	ns		G2583_4167	ftsY	CDS
EDL933	4392388	ns		G2583_4171	yhhN	CDS
CB9615	4214641	ns		G2583_4172	zntA	CDS
CB9615	4214826	ns		G2583_4172	zntA	CDS
CB9615	4215147	ns		G2583_4172	zntA	CDS
CB9615	4215551	s		G2583_4172	zntA	CDS
CB9615	4215769	ns		G2583_4172	zntA	CDS

lineage[a]	site[b]	mutation type[c]	recomb	gene	Gene name	Type
CB9615	4215904	ns		G2583_4172	zntA	CDS
O157	4329552	s		G2583_4175	dcrB	CDS
CB9615	4218701	ns		G2583_4176	yhhS	CDS
O157	4330514	ns		G2583_4176	yhhS	CDS
O157	4330923	ns		G2583_4176	yhhS	CDS
Sakai	4333454	del		G2583_4179	-	CDS
Sakai	4333526	ins		G2583_4179	-	CDS
Sakai	4333539	ins-3		G2583_4179	-	CDS
Sakai	4333553	s		G2583_4179	-	CDS
O157	4334477	ns		G2583_4180	-	CDS
O157	4335972	ns		G2583_4183	acyl	CDS
O157	4339435	ns		G2583_4187	-	CDS
O157	4344939	ns		G2583_4192	-	CDS
O157	4345465	s		G2583_4193	-	CDS
CB9615	4234616	s		G2583_4194	fabG	CDS
O157	4348937	s		G2583_4197	nikA	CDS
O157	4349045	s		G2583_4197	nikA	CDS
CB9615	4237716	ns		G2583_4197	nikA	CDS
O157	4350614	ns		G2583_4198	nikB	CDS
O157	4352543	ns		G2583_4201	nikE	CDS
O157	4354638	s		G2583_4204	-	CDS
O157	4354899	s		G2583_4204	-	CDS
CB9615	4245017	s		G2583_4206	-	CDS
CB9615	4245392	ns		G2583_4207	-	CDS
CB9615	4246978	s		G2583_4208	-	CDS
EDL933	4425759	ins		intergenic		
O157	4359924	ns		G2583_4211	HicA	CDS
CB9615	4250830	ns		G2583_4213	rbbA	CDS
O157	4365549	ns		G2583_4215	yhiJ	CDS
O157	4365645	ns		G2583_4215	yhiJ	CDS
O157	4366308	ns		G2583_4215	yhiJ	CDS
O157	4366680	ns		G2583_4215	yhiJ	CDS
CB9615	4255512	s		G2583_4215	yhiJ	CDS
EDL933	4436235	ins		intergenic		
O157	4369026	i		intergenic		
O157	4370237	s		G2583_4217	yhiM	CDS
CB9615	4259408	ns		G2583_4218	yhiN	CDS
EDL933	4439345	ins		intergenic		
EDL933	4441586	ins		intergenic		
CB9615	4263599	i		intergenic		
O157	4375053	i		intergenic		
O157	4375356	s		G2583_4222	yhiP	CDS
O157	4375603	ns		G2583_4222	yhiP	CDS
O157	4378727	s		G2583_4224	prlC	CDS
O157	4382189	i		intergenic		

lineage[a]	site[b]	mutation type[c]	recomb	gene	Gene name	Type
O157	4385186	ns		G2583_4230	-	CDS
O157	4385236	i		intergenic		
EDL933	4452734	ins		intergenic		
CB9615	4274138	i		intergenic		
O157	4386699	del		G2583_4231	yhiS	CDS
EDL933	4454210	ins		intergenic		
CB9615	4277769	ns		G2583_4234	chuS	CDS
O157	4390671	ns		G2583_4235	chuA	CDS
O157	4391980	ns		G2583_4236	-	CDS
O157	4395620	s		G2583_4241	chuU	CDS
O157	4397023	s		G2583_4242	shuV	CDS
O157	4397175	ns		G2583_4242	shuV	CDS
O157	4398078	i		intergenic		
CB9615	4286968	ns		G2583_4244	hdeB	CDS
O157	4398425	ns		G2583_4244	hdeB	CDS
CB9615	4287992	ns		G2583_4246	hdeD	CDS
CB9615	4288987	i		intergenic		
O157	4402351	ns		G2583_4248	mdtE	CDS
CB9615	4295920	s		G2583_4252	gadX	CDS
O157	4407409	s		G2583_4252	gadX	CDS
O157	4408856	s		G2583_4253	gadA	CDS
O157	4413669	ns		G2583_4256	yhjB	CDS
CB9615	4302797	i		intergenic		
CB9615	4305311	i		intergenic		
O157	4418157	ns		G2583_4259	yhjE	CDS
CB9615	4309952	i		intergenic		
O157	4424348	ns		G2583_4264	dctA	CDS
O157	4426243	ns		G2583_4265	yhjK	CDS
O157	4426670	ns		G2583_4265	yhjK	CDS
O157	4428520	ns		G2583_4266	bcsC	CDS
O157	4428751	ns		G2583_4266	bcsC	CDS
CB9615	4318922	ns		G2583_4266	bcsC	CDS
O157	4430782	ns		G2583_4266	bcsC	CDS
O157	4430902	del-10		G2583_4266	bcsC	CDS
O157	4432024	s		G2583_4267	bcsZ	CDS
O157	4432369	s		G2583_4267	bcsZ	CDS
O157	4432500	ins-18		G2583_4268	bcsB	CDS
O157	4436826	ns		G2583_4269	bcsA	CDS
O157	4437936	ns		G2583_4270	yhjQ	CDS
O157	4438106	ns		G2583_4270	yhjQ	CDS
O157	4438234	ns		G2583_4271	yhjR	CDS
O157	4442074	i		intergenic		
O157	4443246	ns		G2583_4276	yhjV	CDS
O157	4444663	ns		G2583_4277	dppF	CDS
EDL933	4512050	s		G2583_4277	dppF	CDS

lineage[a]	site[b]	mutation type[c]	recomb	gene	Gene name	Type
O157	4446912	i		intergenic		
O157	4447835	s		G2583_4280	dppB	CDS
O157	4449503	s		G2583_4281	dppA	CDS
Sakai	4450419	i		intergenic		
Sakai	4451098	ns		G2583_4283	eptB	CDS
CB9615	4340244	s		G2583_4283	eptB	CDS
EDL933	4519633	ins		intergenic		
EDL933	4519738	ins		intergenic		
O55/O157	4341161	s		G2583_4284	lpfE	CDS
O55/O157	4341929	ns		G2583_4285	lpfD	CDS
O55/O157	4342108	ns		G2583_4285	lpfD	CDS
O55/O157	4342307	ns		G2583_4285	lpfD	CDS
O55/O157	4342371	ns		G2583_4285	lpfD	CDS
O157	4455438	ns		G2583_4286	fimD	CDS
O157	4455630	ns		G2583_4286	fimD	CDS
O55/O157	4346215	ns		G2583_4288	lpfA	CDS
CB9615	4346771	ins		intergenic		
O157	4458200	i		intergenic		
CB9615	4346824	i		intergenic		
O157	4458252	i		intergenic		
O157	4458298	i		intergenic		
O157	4458335	ins		intergenic		
O157	4458476	ns		G2583_4289	yhjX	CDS
CB9615	4352263	ns		G2583_4293	bisC	CDS
CB9615	4352387	s		G2583_4293	bisC	CDS
O157	4465177	s		G2583_4295	ghrB	CDS
CB9615	4355806	s		G2583_4297	yiaG	CDS
CB9615	4356714	ns		G2583_4299	hokA	CDS
O157	4472769	ns		G2583_4303	wecH	CDS
O157	4478173	s		G2583_4308	xylF	CDS
O157	4480525	s		G2583_4310	xylH	CDS
O157	4480615	s		G2583_4310	xylH	CDS
O157	4483037	ns		G2583_4312	bax	CDS
O157	4483743	s		G2583_4313	malS	CDS
CB9615	4374461	ns		G2583_4314	avtA	CDS
CB9615	4375323	ns		G2583_4314	avtA	CDS
CB9615	4376835	s		G2583_4316	-	CDS
O157	4488646	ns		G2583_4316	-	CDS
CB9615	4377842	s		G2583_4317	-	CDS
O157	4489389	ns		G2583_4317	-	CDS
O157	4491515	s		G2583_4318	-	CDS
CB9615	4381450	s		G2583_4319	yiaT	CDS
O157	4493515	ns		G2583_4320	yiaU	CDS
O157	4497520	ns		G2583_4323	aldB	CDS
O157	4498049	ns		G2583_4324	-	CDS

lineage[a]	site[b]	mutation type[c]	recomb	gene	Gene name	Type
Sakai	4500657	i		intergenic		
O157	4500663	del		intergenic		
CB9615	4390049	s		G2583_4326	selB	CDS
O157	4501631	ns		G2583_4326	selB	CDS
CB9615	4391540	ns		G2583_4327	selA	CDS
O157	4504901	s	rec	G2583_4329	rhsB	CDS
O157	4504943	s	rec	G2583_4329	rhsB	CDS
O157	4505009	s	rec	G2583_4329	rhsB	CDS
Sakai	4505017	ns		G2583_4329	rhsB	CDS
O157	4505036	s	rec	G2583_4329	rhsB	CDS
O157	4505042	s	rec	G2583_4329	rhsB	CDS
O157	4505086	ns	rec	G2583_4329	rhsB	CDS
CB9615	4394300	ns		G2583_4329	rhsB	CDS
CB9615	4395507	ns		G2583_4329	rhsB	CDS
O55/O157	4396300	s	rec	G2583_4329	rhsB	CDS
O55/O157	4396357	s	rec	G2583_4329	rhsB	CDS
O55/O157	4396360	s	rec	G2583_4329	rhsB	CDS
O55/O157	4396419	ns	rec	G2583_4329	rhsB	CDS
O55/O157	4396576	s	rec	G2583_4329	rhsB	CDS
O55/O157	4396591	s	rec	G2583_4329	rhsB	CDS
O55/O157	4396593	ns	rec	G2583_4329	rhsB	CDS
O55/O157	4396645	s	rec	G2583_4329	rhsB	CDS
O55/O157	4396651	s	rec	G2583_4329	rhsB	CDS
O55/O157	4396699	s	rec	G2583_4329	rhsB	CDS
O55/O157	4396843	s	rec	G2583_4329	rhsB	CDS
O55/O157	4396848	ns	rec	G2583_4329	rhsB	CDS
O55/O157	4396860	ns	rec	G2583_4329	rhsB	CDS
O55/O157	4396876	s	rec	G2583_4329	rhsB	CDS
O55/O157	4397110	ns	rec	G2583_4329	rhsB	CDS
EDL933	4575772	ns		G2583_4329	rhsB	CDS
O55/O157	4398299	i	rec	intergenic		
O55/O157	4398300	i	rec	intergenic		
O55/O157	4398338	i	rec	intergenic		
O55/O157	4398346	i	rec	intergenic		
O55/O157	4398368	i	rec	intergenic		
O55/O157	4398401	i	rec	intergenic		
O55/O157	4398463	i	rec	intergenic		
O55/O157	4398464	i	rec	intergenic		
O55/O157	4398470	i	rec	intergenic		
O157	4509941	i		intergenic		
CB9615	4400096	i		intergenic		
O157	4510466	s		G2583_4332	yibV	CDS
O157	4510621	ins		G2583_4332	yibV	CDS
CB9615	4400664	ns		G2583_4332	yibV	CDS
CB9615	4404604	ins-6		G2583_4335	mtlA	CDS

lineage[a]	site[b]	mutation type[c]	recomb	gene	Gene name	Type
CB9615	4406735	s		G2583_4337	mtlR	CDS
O157	4518251	ns		G2583_4339	-	CDS
O157	4523544	ns		G2583_4341	-	CDS
O157	4526398	ns		G2583_4343	lldR	CDS
O157	4534545	s		G2583_4352	envC	CDS
O157	4534683	s		G2583_4352	envC	CDS
CB9615	4427826	s		G2583_4355	tdh	CDS
Sakai	4538018	ns		G2583_4355	tdh	CDS
EDL933	4609523	ins		intergenic		
O157	4543120	s		G2583_4360	rfaC	CDS
O157	4543685	ns		G2583_4360	rfaC	CDS
CB9615	4434958	ins		intergenic		
O157	4547226	i		intergenic		
O157	4547453	s		G2583_4364	rfaY	CDS
O157	4547914	ns		G2583_4364	rfaY	CDS
O157	4556350	s		G2583_4374	yicR	CDS
O157	4558841	s		G2583_4377	slmA	CDS
CB9615	4451003	ns		G2583_4380	yicC	CDS
O157	4564096	ns		G2583_4383	ligB	CDS
O157	4569728	s		G2583_4388	recG	CDS
O157	4570712	s		G2583_4388	recG	CDS
O157	4573161	s		G2583_4390	gltS	CDS
CB9615	4465106	ns		G2583_4392	yicH	CDS
EDL933	4645780	ns		G2583_4393	yicI	CDS
O157	4577526	ns		G2583_4393	yicI	CDS
O157	4578359	s		G2583_4393	yicI	CDS
O157	4578821	s		G2583_4393	yicI	CDS
CB9615	4469664	ns		G2583_4394	yicJ	CDS
O157	4580637	i		intergenic		
O157	4582645	ins-3		G2583_4397	insN	CDS
CB9615	4475455	nc		G2583_4403	-	pseudogene
Sakai	4588247	ns		ECs4547	-	CDS
O157	4588998	i		intergenic		
O157	4589115	i		intergenic		
CB9615	4476633	i		intergenic		
CB9615	4476805	ns		G2583_4405	espF	CDS
O55/O157	4476813	indel-13		G2583_4405	espF	CDS
CB9615	4476857	s		G2583_4405	espF	CDS
CB9615	4478763	s		G2583_4409	espB	CDS
CB9615	4479788	s		G2583_4410	espD	CDS
O55/O157	4480858	ns		G2583_4411	espA	CDS
O55/O157	4484015	ns		G2583_4414	eae	CDS
O55/O157	4484066	ns		G2583_4414	eae	CDS
O55/O157	4600397	indel-6		G2583_4416	tir	CDS
O55/O157	4489376	i		intergenic		

lineage[a]	site[b]	mutation type[c]	recomb	gene	Gene name	Type
O157	4604475	ns		G2583_4420	SepQ	CDS
O157	4608284	s		G2583_4424	escV	CDS
CB9615	4496576	s		G2583_4425	-	CDS
O157	4610762	s		G2583_4428	EscJ	CDS
CB9615	4498936	s		G2583_4430	EscC	CDS
CB9615	4499965	s		G2583_4430	EscC	CDS
O157	4612578	s		G2583_4430	EscC	CDS
O157	4614822	s		G2583_4434	-	CDS
CB9615	4502473	s		G2583_4434	-	CDS
O157	4616686	s		G2583_4436	EscT	CDS
Sakai	4617900	ns		G2583_4438	escR	CDS
CB9615	4506287	s		G2583_4440	-	CDS
Sakai	4619636	ns		G2583_4442	-	CDS
Sakai	4620815	i		intergenic		
CB9615	4508362	del-11		intergenic		
O157	4622761	s		G2583_4445	-	CDS
O157	4622776	s		G2583_4445	-	CDS
CB9615	4511885	ns		G2583_4447	-	CDS
CB9615	4512866	s		G2583_4448	yicL	CDS
O157	4625706	ns		G2583_4449	nlpA	CDS
O157	4626391	i		intergenic		
CB9615	4514883	s		G2583_4451	nepI	CDS
O157	4628210	i		intergenic		
CB9615	4522953	s		G2583_4458	uhpC	CDS
O157	4635950	ns		G2583_4459	uhpB	CDS
O157	4636144	s		G2583_4459	uhpB	CDS
O157	4636789	s		G2583_4459	uhpB	CDS
O55/O157	4524941	s		G2583_4460	uhpA	CDS
O157	4639100	ns		G2583_4461	-	CDS
O157	4639140	ns		G2583_4461	-	CDS
CB9615	4526999	ins-2		intergenic		
CB9615	4527252	ns		G2583_4462-4463	-	CDS
O157	4640095	ns		G2583_4462-4463	-	CDS
O157	4640747	nc		G2583_4463	-	pseudogene
O157	4641102	i		intergenic		
O157	4641219	i		intergenic		
O157	4643460	ns		G2583_4466-4467	ivbL	CDS
O157	4644320	i		intergenic		
O157	4647222	ns		G2583_4472	yidI	CDS
O157	4650236	ns		G2583_4474	yidK	CDS
CB9615	4538937	s		G2583_4475	yidL	CDS
Sakai	4652077	ns		G2583_4476	glvA	CDS
Sakai	4652078	s		G2583_4476	glvA	CDS
O157	4653763	s		G2583_4477	glvC	CDS
CB9615	4541755	ns		G2583_4477	glvC	CDS

lineage[a]	site[b]	mutation type[c]	recomb	gene	Gene name	Type
CB9615	4542302	del		intergenic		
O157	4654972	s		G2583_4478	yidP	CDS
O157	4660184	ns		G2583_4483	yidR	CDS
CB9615	4549647	ns		G2583_4485	yidX	CDS
O157	4664304	s		G2583_4488	gyrB	CDS
O157	4671008	ns		G2583_4493	rnpA	CDS
EDL933	4743419	del		intergenic		
EDL933	4743436	del		intergenic		
EDL933	4743441	i		intergenic		
O157	4674802	del-2		G2583_4496	-	CDS
O157	4675085	ns		G2583_4496	-	CDS
O55/O157	4563020	ns		G2583_4496	-	CDS
O55/O157	4564220	s		G2583_4496	-	CDS
O55/O157	4564396	s		G2583_4497	-	CDS
O55/O157	4564463	i		intergenic		
O157	4679462	ns		G2583_4500	tnaB	CDS
O157	4680564	ns		G2583_4501	mdtL	CDS
O157	4683441	s		G2583_4504	yieF	CDS
O157	4686702	ns		G2583_4507	-	CDS
O157	4687603	s		G2583_4507	-	CDS
O157	4689103	ns		G2583_4508	-	CDS
O55/O157	4577237	ns	rec	G2583_4509	-	CDS
O55/O157	4577248	ns	rec	G2583_4509	-	CDS
O55/O157	4577277	ns	rec	G2583_4509	-	CDS
O55/O157	4577316	ns	rec	G2583_4509	-	CDS
O55/O157	4577595	ns	rec	G2583_4509	-	CDS
Sakai	4690503	i		intergenic		
O55/O157	4578264	nc	rec	G2583_4511	-	pseudogene
O55/O157	4578502	nc	rec	G2583_4511	-	pseudogene
O55/O157	4578596	indel	rec	G2583_4511	-	pseudogene
O55/O157	4578636	nc	rec	G2583_4511	-	pseudogene
O55/O157	4578726	nc	rec	G2583_4511	-	pseudogene
O55/O157	4578902	nc	rec	G2583_4511	-	pseudogene
O55/O157	4578936	nc	rec	G2583_4511	-	pseudogene
O55/O157	4579334	nc	rec	G2583_4511	-	pseudogene
O55/O157	4579526	nc	rec	G2583_4511	-	pseudogene
O55/O157	4579865	nc	rec	G2583_4511	-	pseudogene
O55/O157	4580312	nc	rec	G2583_4511	-	pseudogene
O55/O157	4580555	indel	rec	G2583_4511	-	pseudogene
O55/O157	4580825	i	rec	intergenic		
O55/O157	4580862	i	rec	intergenic		
O55/O157	4580886	i	rec	intergenic		
O55/O157	4580923	ns	rec	G2583_4513	-	CDS
O55/O157	4581403	s	rec	G2583_4514	phoU	CDS
O55/O157	4587361	ns		G2583_4520	lpfD	CDS

lineage[a]	site[b]	mutation type[c]	recomb	gene	Gene name	Type
O55/O157	4588117	s		G2583_4520	lpfD	CDS
O55/O157	4588686	nc		G2583_4521	lpfC	pseudogene
O55/O157	4589045	nc		G2583_4521	lpfC	pseudogene
O157	4701796	nc		G2583_4521	lpfC	pseudogene
EDL933	4772701	s		G2583_4523	stgB	CDS
O55/O157	4703897	indel		intergenic		
O55/O157	4591950	s		G2583_4524	lpfA	CDS
Sakai	4705415	ns		G2583_4525	glmS	CDS
O157	4709196	ns		G2583_4527	atpC	CDS
O157	4710217	s		G2583_4528	atpD	CDS
CB9615	4599020	ns		G2583_4529	atpG	CDS
CB9615	4599056	ns		G2583_4529	atpG	CDS
O157	4713571	s		G2583_4531	atpH	CDS
Sakai	4714723	ns		G2583_4534	atpB	CDS
CB9615	4604524	ns		G2583_4536	gidB	CDS
O157	4717164	i		intergenic		
O157	4719219	i		intergenic		
O157	4722625	s		G2583_4541	viaA	CDS
O157	4723890	s		G2583_4542	ravA	CDS
CB9615	4611994	ns		G2583_4542	ravA	CDS
O157	4724624	i		intergenic		
O157	4724628	i		intergenic		
O157	4727841	ns		G2583_4545	rbsA	CDS
CB9615	4616599	ns		G2583_4546	rbsC	CDS
O157	4730036	s		G2583_4547	rbsB	CDS
CB9615	4618739	ns		G2583_4548	rbsK	CDS
CB9615	4621645	s		G2583_4551	yieP	CDS
EDL933	4805134	del		G2583_4552	rrs	rRNA
EDL933	4805653	nc		G2583_4552	rrs	rRNA
O157	4736808	i		intergenic		
Sakai	4737077	i		intergenic		
CB9615	4624599	i		intergenic		
EDL933	4624599	i		intergenic		
Sakai	4624599	i		intergenic		
EDL933	4806264	nc		G2583_4554	rrl	rRNA
EDL933	4806269	nc		G2583_4554	rrl	rRNA
O157	4737503	nc		G2583_4554	rrl	rRNA
O55/O157	4625208	indel-14		G2583_4554	rrl	rRNA
O55/O157	4625209	indel-12		G2583_4554	rrl	rRNA
CB9615	4626386	ins		G2583_4554	rrl	rRNA
O157	4740157	nc		G2583_4555	rrf	rRNA
Sakai	4740362	del		G2583_4556	-	tRNA
CB9615	4629998	ns		G2583_4560	yifB	CDS
O157	4742586	s		G2583_4560	yifB	CDS
EDL933	4815862	ins		intergenic		

lineage[a]	site[b]	mutation type[c]	recomb	gene	Gene name	Type
CB9615	4637035	s		G2583_4566	ilvA	CDS
EDL933	4818677	ins		G2583_4566	ilvA	CDS
EDL933	4818685	ins		G2583_4566	ilvA	CDS
EDL933	4818689	ins		G2583_4566	ilvA	CDS
CB9615	4638572	s		G2583_4567	ilvY	CDS
O157	4751367	i		intergenic		
O157	4753270	i		intergenic		
CB9615	4641660	s		G2583_4571	rep	CDS
CB9615	4644979	ns		G2583_4572	gpp	CDS
O157	4757621	s		G2583_4572	gpp	CDS
O157	4757979	ns		G2583_4573	rhlB	CDS
CB9615	4652627	s		G2583_4581	rffD	CDS
CB9615	4653801	ns		G2583_4582	rffG	CDS
O157	4766939	ns		G2583_4582	rffG	CDS
O157	4769453	ns		G2583_4585	rffA	CDS
O157	4770002	s		G2583_4586	wzxE	CDS
O157	4770353	ns		G2583_4586	wzxE	CDS
O157	4771600	ns		G2583_4587	rffT	CDS
O157	4771724	ns		G2583_4587	rffT	CDS
CB9615	4661744	i		intergenic		
O157	4775743	i		intergenic		
EDL933	4846627	ins		intergenic		
CB9615	4665815	ns		G2583_4596	aslA	CDS
CB9615	4666276	ns		G2583_4596	aslA	CDS
O157	4779437	ns		G2583_4596	aslA	CDS
EDL933	4848587	ins		intergenic		
O157	4781580	ins-18		G2583_4599	hemX	CDS
CB9615	4671430	ns		G2583_4601	hemC	CDS
EDL933	4853567	ins		intergenic		
O157	4787456	ns		G2583_4603	cyaY	CDS
O157	4796982	ns		G2583_4614	-	CDS
CB9615	4685240	ns		G2583_4615	-	CDS
EDL933	4868062	ins		G2583_4618	-	CDS
O157	4799496	ns		G2583_4618	-	CDS
O157	4801051	i		intergenic		
CB9615	4692255	s		G2583_4624	rhtB	CDS
O157	4805677	s		G2583_4625	pldB	CDS
CB9615	4693751	ns		G2583_4625-4626	pldB - yigL	CDS
O157	4806588	ns		G2583_4626	yigL	CDS
CB9615	4696138	s		G2583_4628	metR	CDS
O157	4809964	s		G2583_4629	metE	CDS
O157	4810439	ns		G2583_4629	metE	CDS
O157	4810988	ns		G2583_4629	metE	CDS
O157	4818901	s		G2583_4637	tatB	CDS
O157	4824725	s		G2583_4643	fadA	CDS

lineage[a]	site[b]	mutation type[c]	recomb	gene	Gene name	Type
CB9615	4714416	i		intergenic		
O157	4827989	ns		G2583_4645	pepQ	CDS
CB9615	4715910	s		G2583_4646	yigZ	CDS
EDL933	4898620	s		G2583_4647	trkH	CDS
O157	4831321	i		intergenic		
EDL933	4900562	nc		G2583_4649	rrs	rRNA
CB9615	4719331	nc		G2583_4649	rrs	rRNA
Sakai	4833075	nc	rec	G2583_4649	rrs	rRNA
Sakai	4833206	i	rec	intergenic		
Sakai	4833207	i	rec	intergenic		
Sakai	4833571	i	rec	intergenic		
Sakai	4833572	i	rec	intergenic		
Sakai	4833997	nc	rec	G2583_4652	rrl	rRNA
EDL933	4902764	nc		G2583_4652	rrl	rRNA
O55/O157	4721456	indel-14		G2583_4652	rrl	rRNA
O55/O157	4721457	indel-12		G2583_4652	rrl	rRNA
CB9615	4724037	i		intergenic		
CB9615	4724086	del		intergenic		
O157	4836930	s		G2583_4654	mobB	CDS
O157	4837543	ns		G2583_4655	mobA	CDS
O157	4844012	s		G2583_4662	polA	CDS
CB9615	4732626	ns		G2583_4662	polA	CDS
O157	4847602	s		G2583_4666	yihI	CDS
CB9615	4736541	s		G2583_4667	hemN	CDS
O157	4851097	s		G2583_4668	glnG	CDS
O157	4854958	s		G2583_4671	typA	CDS
O157	4857689	s		G2583_4673	yihM	CDS
CB9615	4746293	ns		G2583_4674	yihN	CDS
CB9615	4746715	ns		G2583_4675	ompL	CDS
CB9615	4748877	del-3		G2583_4677	yihP	CDS
O157	4862840	s		G2583_4677	yihP	CDS
O157	4864493	s		G2583_4678	yihQ	CDS
CB9615	4753302	ns		G2583_4679	-	CDS
CB9615	4757462	del		intergenic		
O157	4870245	i		intergenic		
O157	4871067	ns		G2583_4684	yihV	CDS
O157	4876312	ns		G2583_4690	-	CDS
O157	4877355	i		intergenic		
CB9615	4765975	i		intergenic		
O157	4879930	s		G2583_4696	fdoI	CDS
O157	4882975	nc		G2583_4698	fdoG	pseudogene
O157	4885836	ns		G2583_4700	yiiG	CDS
O157	4886787	s		G2583_4701	frvR	CDS
O157	4887830	ns		G2583_4701	frvR	CDS
CB9615	4776461	del-6		G2583_4702	frvX	CDS

lineage[a]	site[b]	mutation type[c]	recomb	gene	Gene name	Type
CB9615	4776712	ns		G2583_4702	frvX	CDS
CB9615	4776776	ns		G2583_4702	frvX	CDS
CB9615	4777074	s		G2583_4703	frvB	CDS
O157	4889943	del-12		G2583_4703	frvB	CDS
CB9615	4778371	s		G2583_4704	frvA	CDS
O157	4891246	ns		G2583_4704	frvA	CDS
EDL933	4960143	s		G2583_4704	frvA	CDS
O157	4892004	ns		G2583_4705	rafY	CDS
O157	4893124	ns		G2583_4705	rafY	CDS
CB9615	4780665	ns		G2583_4706	rhaM	CDS
CB9615	4780780	ns		G2583_4706	rhaM	CDS
CB9615	4781673	ns		G2583_4707	rhaD	CDS
O157	4897986	s		G2583_4710	rhaS	CDS
CB9615	4787518	nc		G2583_4712	rhaT	pseudogene
O157	4901210	s		G2583_4713	sodA	CDS
O157	4901282	s		G2583_4713	sodA	CDS
O157	4901520	i		intergenic		
O157	4902883	s		G2583_4715	yiiM	CDS
EDL933	4972990	ns		G2583_4716	cpxA	CDS
O157	4906028	s		G2583_4718	cpxP	CDS
O157	4907301	s		G2583_4719	fieF	CDS
O157	4909420	ns		G2583_4722	sbp	CDS
EDL933	4978364	ns		G2583_4722	sbp	CDS
CB9615	4800334	ns		G2583_4726	yiiR	CDS
O157	4913321	s		G2583_4728	uspD	CDS
CB9615	4802739	ns		G2583_4730	glpX	CDS
O157	4915241	ns		G2583_4730	glpX	CDS
O157	4918684	s		G2583_4733	yiiU	CDS
O157	4919484	s		G2583_4734	-	CDS
O157	4928376	i		intergenic		
O157	4928936	s	rec	G2583_4744	rhsB	CDS
O157	4929002	s	rec	G2583_4744	rhsB	CDS
O157	4929010	ns	rec	G2583_4744	rhsB	CDS
O157	4929029	s	rec	G2583_4744	rhsB	CDS
O157	4929035	s	rec	G2583_4744	rhsB	CDS
O157	4929079	ns	rec	G2583_4744	rhsB	CDS
EDL933	4998103	ins		G2583_4744	rhsB	CDS
CB9615	4817350	ns		G2583_4744	rhsB	CDS
EDL933	4998719	ns	rec	G2583_4744	rhsB	CDS
EDL933	4998724	s	rec	G2583_4744	rhsB	CDS
EDL933	4998734	ins	rec	G2583_4744	rhsB	CDS
EDL933	4998756	ns	rec	G2583_4744	rhsB	CDS
EDL933	4999680	ns	rec	G2583_4744	rhsB	CDS
O55/O157	4819350	s	rec	G2583_4744	rhsB	CDS
O55/O157	4819407	s	rec	G2583_4744	rhsB	CDS

lineage[a]	site[b]	mutation type[c]	recomb	gene	Gene name	Type
O55/O157	4819410	s	rec	G2583_4744	rhsB	CDS
O55/O157	4819469	ns	rec	G2583_4744	rhsB	CDS
O55/O157	4819626	s	rec	G2583_4744	rhsB	CDS
O55/O157	4819641	s	rec	G2583_4744	rhsB	CDS
O55/O157	4819643	ns	rec	G2583_4744	rhsB	CDS
CB9615	4820063	ns		G2583_4744	rhsB	CDS
O157	4932964	ns		G2583_4744	rhsB	CDS
EDL933	5004050	i	rec	intergenic		
EDL933	5004529	s	rec	G2583_4751	metB	CDS
O157	4935796	s		G2583_4751	metB	CDS
CB9615	4823757	ns		G2583_4751	metB	CDS
O157	4936980	s		G2583_4752	metL	CDS
O157	4938288	s		G2583_4752	metL	CDS
O157	4939449	i		intergenic		
O157	4939502	ns		G2583_4753	metF	CDS
CB9615	4828448	s		G2583_4754	katG	CDS
CB9615	4829724	ns		G2583_4754	katG	CDS
CB9615	4831762	ns		G2583_4756	yijF	CDS
O157	4945738	s		G2583_4757	gldA	CDS
O157	4950150	ns		G2583_4761	frwC	CDS
O157	4952636	ns		G2583_4763	pflD	CDS
O157	4954297	ns		G2583_4765	frwD	CDS
O157	4955502	ns		G2583_4767	yijP	CDS
O157	4958068	s		G2583_4768	ppc	CDS
CB9615	4849469	ns		G2583_4770	argC	CDS
O157	4962486	s		G2583_4770	argC	CDS
EDL933	5032559	s		G2583_4772	argH	CDS
O157	4964826	ns		G2583_4772	argH	CDS
CB9615	4853031	ns		G2583_4774	oxyR	CDS
O157	4966334	ns		G2583_4775	sthA	CDS
CB9615	4855289	i		intergenic		
O157	4967663	i		intergenic		
O157	4968734	s		G2583_4776	-	CDS
O157	4973232	ns		G2583_4781	btuB	CDS
EDL933	5044458	i	rec	intergenic		
EDL933	5044499	i	rec	intergenic		
EDL933	5044505	i	rec	intergenic		
EDL933	5044509	i	rec	intergenic		
EDL933	5044511	i	rec	intergenic		
EDL933	5044512	del	rec	intergenic		
EDL933	5044516	i	rec	intergenic		
EDL933	5044521	i	rec	intergenic		
EDL933	5044523	i	rec	intergenic		
EDL933	5044524	i	rec	intergenic		
EDL933	5044528	del-2	rec	intergenic		

lineage[a]	site[b]	mutation type[c]	recomb	gene	Gene name	Type
EDL933	5044529	i	rec	intergenic		
EDL933	5044533	i	rec	intergenic		
EDL933	5044534	i	rec	intergenic		
EDL933	5044537	i	rec	intergenic		
EDL933	5044538	i	rec	intergenic		
EDL933	5044539	i	rec	intergenic		
EDL933	5044542	i	rec	intergenic		
EDL933	5044543	i	rec	intergenic		
EDL933	5044679	i	rec	intergenic		
O157	4977348	nc		G2583_4783	rrs	rRNA
O157	4977483	i		intergenic		
CB9615	4865163	i		intergenic		
CB9615	4865334	i		intergenic		
CB9615	4865601	nc		G2583_4785	rrl	rRNA
CB9615	4865606	nc		G2583_4785	rrl	rRNA
EDL933	5046849	nc		G2583_4785	rrl	rRNA
O157	4978178	nc		G2583_4785	rrl	rRNA
O55/O157	4866005	indel-12		G2583_4785	rrl	rRNA
O55/O157	4866016	indel-14		G2583_4785	rrl	rRNA
O157	4980832	nc		G2583_4786	rrf	rRNA
CB9615	4868556	nc		G2583_4786	rrf	rRNA
EDL933	5052468	s		G2583_4789	coaA	CDS
EDL933	5052789	del-17		G2583_4789	coaA	CDS
EDL933	5054039	s	rec	G2583_4795	tufB	CDS
EDL933	5054048	s	rec	G2583_4795	tufB	CDS
EDL933	5054051	s	rec	G2583_4795	tufB	CDS
EDL933	5054055	ns	rec	G2583_4795	tufB	CDS
EDL933	5054066	s	rec	G2583_4795	tufB	CDS
EDL933	5054070	s	rec	G2583_4795	tufB	CDS
CB9615	4873168	s		G2583_4795	tufB	CDS
O157	4985546	s		G2583_4795	tufB	CDS
O157	4999070	ns		G2583_4805	yjaZ	CDS
O157	5001585	s		G2583_4809	thiS	CDS
CB9615	4891021	ns		G2583_4812	thiC	CDS
O157	5006606	s		G2583_4815	hemE	CDS
O157	5007108	ns		G2583_4815	hemE	CDS
O157	5008885	ns		G2583_4817	yjaG	CDS
O157	5009583	ns		G2583_4819	yjaH	CDS
O157	5009945	s		G2583_4819	yjaH	CDS
O157	5012872	ns		G2583_4822	zraR	CDS
CB9615	4903215	s		G2583_4824	purH	CDS
O157	5016799	i		intergenic		
EDL933	5086700	nc	rec	G2583_4825	rrs	rRNA
EDL933	5086704	nc	rec	G2583_4825	rrs	rRNA
EDL933	5086708	nc	rec	G2583_4825	rrs	rRNA

lineage[a]	site[b]	mutation type[c]	recomb	gene	Gene name	Type
EDL933	5086717	nc	rec	G2583_4825	rrs	rRNA
EDL933	5086718	nc	rec	G2583_4825	rrs	rRNA
EDL933	5086719	nc	rec	G2583_4825	rrs	rRNA
EDL933	5086720	nc	rec	G2583_4825	rrs	rRNA
EDL933	5086721	nc	rec	G2583_4825	rrs	rRNA
EDL933	5086736	nc	rec	G2583_4825	rrs	rRNA
EDL933	5018374	nc		G2583_4825	rrs	rRNA
O157	5018374	nc		G2583_4825	rrs	rRNA
Sakai	5018374	nc		G2583_4825	rrs	rRNA
O157	5018509	i		intergenic		
EDL933	5018779	i		intergenic		
O157	5018779	i		intergenic		
Sakai	5018779	i		intergenic		
CB9615	4906446	i		intergenic		
O157	5018811	ins-18		intergenic		
O55/O157	4906451	indel-8		intergenic		
O157	5019204	nc		G2583_4827	rrl	rRNA
Sakai	5019389	ins-14		G2583_4827	rrl	rRNA
Sakai	5019402	del-12		G2583_4827	rrl	rRNA
EDL933	5088734	nc		G2583_4827	rrl	rRNA
CB9615	4907989	nc		G2583_4827	rrl	rRNA
O157	5023716	i		intergenic		
O157	5024166	ns		G2583_4831	aceB	CDS
CB9615	4913123	ins-10		G2583_4832	aceA	CDS
O157	5028109	s		G2583_4833	aceK	CDS
CB9615	4917270	nc		G2583_4834	arp	pseudogene
CB9615	4917280	del		G2583_4834	arp	pseudogene
O157	5029898	nc		G2583_4834	arp	pseudogene
CB9615	4918082	nc		G2583_4834	arp	pseudogene
O157	5030718	nc		G2583_4834	arp	pseudogene
CB9615	4918467	nc		G2583_4834	arp	pseudogene
O157	5031093	i		intergenic		
EDL933	5099867	ins		intergenic		
CB9615	4919667	del		intergenic		
CB9615	4919690	i		intergenic		
O157	5036147	ns		G2583_4837	yjbB	CDS
O157	5037395	ns		G2583_4837	yjbB	CDS
CB9615	4926188	i		intergenic		
O157	5081419	s		G2583_4844	sorF	CDS
O157	5083289	ns		G2583_4846	-	CDS
CB9615	4932874	s		G2583_4847	rluF	CDS
CB9615	4935166	ns		G2583_4849	lysC	CDS
O157	5089027	i		intergenic		
O157	5090702	ns		G2583_4853	yjbG	CDS
O157	5091692	ns		G2583_4854	yjbH	CDS

lineage[a]	site[b]	mutation type[c]	recomb	gene	Gene name	Type
O157	5093259	i		intergenic		
CB9615	4942298	i		intergenic		
CB9615	4944661	i		intergenic		
O157	5097893	ns		G2583_4858	malF	CDS
O157	5099962	i		intergenic		
O157	5103794	i		intergenic		
CB9615	4952672	i		intergenic		
Sakai	5106168	ns		G2583_4864	ubiC	CDS
O157	5111653	ns		G2583_4869	dinF	CDS
O157	5111998	ns		G2583_4869	dinF	CDS
CB9615	4961064	s		G2583_4869	dinF	CDS
CB9615	4961624	ns		G2583_4871	zur	CDS
O157	5112844	s		G2583_4871	zur	CDS
CB9615	4962679	ns		G2583_4872	yjbM	CDS
Sakai	5114397	ins		G2583_4872	yjbM	CDS
CB9615	4963389	i		intergenic		
CB9615	4965275	i		intergenic		
EDL933	5149793	s		G2583_4877	alr	CDS
O157	5121942	ns		G2583_4879	-	CDS
CB9615	4972424	s		G2583_4881	yjbQ	CDS
O157	5128634	s		G2583_4886	yjcC	CDS
CB9615	4977688	ns		G2583_4886	yjcC	CDS
CB9615	4977692	ns		G2583_4886	yjcC	CDS
O157	5129239	ns		G2583_4886	yjcC	CDS
O157	5129585	s		G2583_4886	yjcC	CDS
O157	5129966	s		G2583_4886	yjcC	CDS
CB9615	4981185	ns		G2583_4889	yjcD	CDS
CB9615	4983408	ns		G2583_4890	yjcE	CDS
O157	5135368	ns		G2583_4891	yjcF	CDS
O157	5135792	s		G2583_4891	yjcF	CDS
CB9615	4984805	ns		G2583_4891	yjcF	CDS
O157	5136147	ns		G2583_4891	yjcF	CDS
O157	5137163	s		G2583_4892	actP	CDS
O157	5137547	s		G2583_4892	actP	CDS
CB9615	4989224	ns		G2583_4894	acs	CDS
O157	5140813	i		intergenic		
CB9615	4992682	ns		G2583_4898	nrfD	CDS
O157	5143833	ns		G2583_4898	nrfD	CDS
O157	5143897	s		G2583_4898	nrfD	CDS
CB9615	4992917	s		G2583_4898	nrfD	CDS
O157	5145755	s		G2583_4899	nrfE	CDS
CB9615	4997948	i		intergenic		
O157	5150197	nc		G2583_4904	fdhF	pseudogene
O157	5154019	ns		G2583_4906	mdtO	CDS
CB9615	5003754	ns		G2583_4906	mdtO	CDS

lineage[a]	site[b]	mutation type[c]	recomb	gene	Gene name	Type
CB9615	5004933	nc		G2583_4907	mdtN	pseudogene
O157	5158150	ns		G2583_4909	yjcS	CDS
CB9615	5007295	s		G2583_4909	yjcS	CDS
CB9615	5009318	s		G2583_4911	PfkB	CDS
O157	5160539	ns		G2583_4911	PfkB	CDS
O157	5161881	s		G2583_4912	-	CDS
O157	5163907	ns		G2583_4915	-	CDS
O157	5165067	ns		G2583_4916	-	CDS
O157	5165695	s		G2583_4916	-	CDS
O157	5167109	ins-9		G2583_4917	-	CDS
O157	5168127	ns		G2583_4917	-	CDS
O157	5168910	ns		G2583_4918	yjdP	CDS
CB9615	5017795	ns		G2583_4918	yjdP	CDS
O157	5170377	ins		G2583_4920	phnO	CDS
O157	5171650	ns		G2583_4922	phnM	CDS
CB9615	5022664	ns		G2583_4925	phnJ	CDS
O157	5174015	s		G2583_4925	phnJ	CDS
O157	5175333	s		G2583_4926	phnI	CDS
CB9615	5025121	s		G2583_4928	phnG	CDS
CB9615	5027617	ns		G2583_4931	phnD	CDS
O157	5178885	s		G2583_4931	phnD	CDS
CB9615	5032653	ns		G2583_4936	yjcZ	CDS
Sakai	5188884	ns		G2583_4940	eptA	CDS
CB9615	5037696	s		G2583_4940	eptA	CDS
CB9615	5037986	ns		G2583_4940	eptA	CDS
O157	5189869	s		G2583_4940	eptA	CDS
O157	5191662	s		G2583_4942	adiY	CDS
O157	5192313	s		G2583_4942	adiY	CDS
CB9615	5045438	ns		G2583_4945	melA	CDS
CB9615	5045536	ns		G2583_4945	melA	CDS
O157	5200162	ns		G2583_4948	fumB	CDS
O157	5200942	ns		G2583_4948	fumB	CDS
O157	5201064	ns		G2583_4948	fumB	CDS
CB9615	5050071	ns		G2583_4948	fumB	CDS
EDL933	5232540	ns		G2583_4949	dcuB	CDS
O157	5203708	i		intergenic		
CB9615	5055429	ns		G2583_4953	yjdJ	CDS
O157	5207335	i		intergenic		
CB9615	5059740	s		G2583_4958	cadA	CDS
CB9615	5061372	s		G2583_4958	cadA	CDS
CB9615	5064876	s		G2583_4960	cadC	CDS
O157	5217021	nc		G2583_4961	-	tRNA
CB9615	5066021	ns		G2583_4962	yjdC	CDS
O157	5218707	ns		G2583_4963	dipZ	CDS
O157	5218749	ns		G2583_4963	dipZ	CDS

lineage[a]	site[b]	mutation type[c]	recomb	gene	Gene name	Type
O157	5219396	ns		G2583_4963	dipZ	CDS
O157	5224725	ns		G2583_4968	yjeH	CDS
O157	5227086	s		G2583_4970	groL	CDS
O157	5227092	s		G2583_4970	groL	CDS
O157	5231058	ns		G2583_4977	sugE	CDS
CB9615	5081156	ns		G2583_4979	ampC	CDS
O157	5234120	s		G2583_4982	frdB	CDS
O157	5234975	ns		G2583_4983	frdA	CDS
O157	5235900	s		G2583_4983	frdA	CDS
CB9615	5084832	ns		G2583_4983	frdA	CDS
O157	5239210	s		G2583_4985	yjeM	CDS
CB9615	5094438	i		intergenic		
O157	5246821	s		G2583_4993	yjeS	CDS
O157	5248084	s		G2583_4994	yjeF	CDS
O157	5250352	ns		G2583_4996	amiB	CDS
O157	5252285	s		G2583_4997	mutL	CDS
O157	5252774	s		G2583_4997	mutL	CDS
O157	5252841	ns		G2583_4997	mutL	CDS
O157	5253752	ns		G2583_4998	miaA	CDS
O157	5257352	s		G2583_5002	hflC	CDS
EDL933	5289877	ins		intergenic		
O157	5261225	s		G2583_5006	rnr	CDS
O157	5262270	ns		G2583_5006	rnr	CDS
CB9615	5112762	ns		G2583_5009	yjfJ	CDS
CB9615	5113491	ns		G2583_5010	yjfK	CDS
O157	5265360	ns		G2583_5010	yjfK	CDS
EDL933	5299595	s		G2583_5014	aidB	CDS
O157	5271154	ns		G2583_5017	yjfP	CDS
O157	5271255	ns		G2583_5017	yjfP	CDS
CB9615	5119958	s		G2583_5018	ulaR	CDS
O157	5274770	ns		G2583_5020	ulaA	CDS
EDL933	5307104	s		G2583_5024	ulaE	CDS
EDL933	5309620	ns		G2583_5029	rpsR	CDS
O157	5282664	ns		G2583_5035	ydcM	CDS
O157	5282945	ns		G2583_5035	ydcM	CDS
CB9615	5131507	ns		G2583_5035	ydcM	CDS
O157	5283352	s		G2583_5035	ydcM	CDS
O157	5283861	ns		G2583_5036	ytfB	CDS
O157	5284141	s		G2583_5036	ytfB	CDS
O157	5286135	s		G2583_5038	cycA	CDS
EDL933	5317485	i		intergenic		
CB9615	5135921	i		intergenic		
O157	5288578	i		intergenic		
CB9615	5137189	s		G2583_5041	ytfG	CDS
CB9615	5140559	i		intergenic		

lineage[a]	site[b]	mutation type[c]	recomb	gene	Gene name	Type
CB9615	5140709	ns		G2583_5044	cysQ	CDS
CB9615	5141284	s		G2583_5044	cysQ	CDS
O157	5293138	i		intergenic		
Sakai	5293337	s		G2583_5045	ytfI	CDS
CB9615	5143702	del-17		intergenic		
O157	5299877	s		G2583_5051	ytfN	CDS
EDL933	5300432	ns		G2583_5051	ytfN	CDS
O157	5300432	ns		G2583_5051	ytfN	CDS
Sakai	5300432	ns		G2583_5051	ytfN	CDS
CB9615	5149452	ns		G2583_5051	ytfN	CDS
O157	5303128	s		G2583_5051	ytfN	CDS
O157	5303294	s		G2583_5051	ytfN	CDS
CB9615	5154071	s		G2583_5057	ytfQ	CDS
O157	5307634	ns		G2583_5058	ytfR	CDS
O157	5310581	ns		G2583_5061	fbp	CDS
Sakai	5311572	ns		G2583_5062	mpl	CDS
O157	5311630	s		G2583_5062	mpl	CDS
CB9615	5164331	i		intergenic		
O157	5318359	i		intergenic		
CB9615	5167333	s		G2583_5068	treC	CDS
CB9615	5167675	s		G2583_5068	treC	CDS
O157	5319301	ns		G2583_5068	treC	CDS
CB9615	5169276	ns		G2583_5069	treB	CDS
O157	5321883	ns		G2583_5070	treR	CDS
EDL933	5352814	del		G2583_5071	-	CDS
EDL933	5352840	ns		G2583_5071	-	CDS
CB9615	5175296	ns		G2583_5074	pyrI	CDS
O157	5328187	i		intergenic		
O157	5330104	ns		G2583_5081	yjgJ	CDS
O157	5330335	i		intergenic		
CB9615	5179489	ns		G2583_5083	yjgL	CDS
O157	5331428	ns		G2583_5083	yjgL	CDS
O157	5331593	ns	rec	G2583_5083	yjgL	CDS
O157	5331599	ns	rec	G2583_5083	yjgL	CDS
O157	5331610	ns	rec	G2583_5083	yjgL	CDS
Sakai	5331610	del-18		G2583_5083	yjgL	CDS
O157	5331610	ns	rec	G2583_5083	yjgL	CDS
O157	5331610	ns	rec	G2583_5083	yjgL	CDS
O157	5331610	ns	rec	G2583_5083	yjgL	CDS
O157	5331611	ns	rec	G2583_5083	yjgL	CDS
O157	5331612	ns	rec	G2583_5083	yjgL	CDS
O157	5331613	ns	rec	G2583_5083	yjgL	CDS
O157	5331617	ns	rec	G2583_5083	yjgL	CDS
O157	5331629	ns	rec	G2583_5083	yjgL	CDS
O157	5331630	ns	rec	G2583_5083	yjgL	CDS

lineage[a]	site[b]	mutation type[c]	recomb	gene	Gene name	Type
O157	5331631	ns	rec	G2583_5083	yjgL	CDS
O157	5332754	i		intergenic		
CB9615	5184038	ns		G2583_5087	yjgN	CDS
O157	5335551	ns		G2583_5087	yjgN	CDS
O157	5338146	s		G2583_5088	valS	CDS
Sakai	5344115	i		intergenic		
CB9615	5195096	ns		G2583_5094	yjgB	CDS
EDL933	5379093	ns		ECs5242	-	CDS
EDL933	5379098	ns		ECs5242	-	CDS
EDL933	5379101	ns		ECs5242	-	CDS
EDL933	5385484	ns		ECs5251	-	CDS
O157	5357913	ns		G2583_5096	-	CDS
O157	5357951	s		G2583_5096	-	CDS
O157	5359174	ns		G2583_5098	-	CDS
O157	5360362	ns		G2583_5100	-	CDS
O157	5360787	s		G2583_5100	-	CDS
O157	5361933	s		G2583_5101	-	CDS
CB9615	5202015	i		intergenic		
CB9615	5207132	ns		G2583_5104	-	CDS
CB9615	5210475	s		G2583_5104	-	CDS
CB9615	5213308	s		G2583_5105	-	CDS
O157	5376424	s		G2583_5105	-	CDS
CB9615	5216001	s		G2583_5106	-	CDS
CB9615	5218098	ns		G2583_5106	-	CDS
CB9615	5221461	ns		G2583_5107	-	CDS
CB9615	5221567	ns		G2583_5107	-	CDS
O157	5383688	ns		G2583_5107	-	CDS
CB9615	5222654	s		G2583_5107	-	CDS
CB9615	5223426	ns		G2583_5107	-	CDS
CB9615	5223874	ns		G2583_5107	-	CDS
CB9615	5225623	ns		G2583_5107	-	CDS
CB9615	5228495	i		intergenic		
O157	5394737	i		intergenic		
CB9615	5233141	i		intergenic		
O157	5395824	i		intergenic		
O157	5396395	s		G2583_5113	fimB	CDS
O157	5397208	s		G2583_5114	fimE	CDS
O157	5397653	del-16		intergenic		
CB9615	5236058	i		intergenic		
CB9615	5236106	i		intergenic		
O157	5397827	i		intergenic		
Sakai	5398304	ns		G2583_5115	fimA	CDS
CB9615	5236754	s		G2583_5115	fimA	CDS
CB9615	5236978	ns		G2583_5116	fimI	CDS
O157	5398698	ns		G2583_5116	fimI	CDS

lineage[a]	site[b]	mutation type[c]	recomb	gene	Gene name	Type
O157	5398749	ns		G2583_5116	fimI	CDS
CB9615	5238564	s		G2583_5118	fimD	CDS
O157	5401773	ns		G2583_5118	fimD	CDS
CB9615	5241300	s		G2583_5119	fimF	CDS
O157	5404166	ns		G2583_5121	fimH	CDS
EDL933	5437265	ns		G2583_5123	uxuA	CDS
EDL933	5437301	ns		G2583_5123	uxuA	CDS
O157	5407638	ns		G2583_5123	uxuA	CDS
EDL933	5437694	ins		intergenic		
CB9615	5247933	s		G2583_5125	uxuR	CDS
O157	5412547	ns		G2583_5129	yjiE	CDS
CB9615	5252977	ns		G2583_5131	yjiG	CDS
CB9615	5254071	s		G2583_5133	yjiI	CDS
O157	5415953	del		G2583_5133	yjiI	CDS
CB9615	5255263	s		G2583_5134	yeeJ	CDS
O157	5421522	ins		G2583_5134	yeeJ	CDS
O157	5424816	ns		G2583_5137	-	CDS
CB9615	5263664	ns		G2583_5137	-	CDS
EDL933	5456090	del-12		G2583_5137	-	CDS
CB9615	5264393	ns		G2583_5137	-	CDS
O157	5427094	i		intergenic		
CB9615	5265456	ns		G2583_5139	yjiL	CDS
Sakai	5428580	s		G2583_5140	yjiM	CDS
O157	5428887	ns		G2583_5140	yjiM	CDS
EDL933	5459043	ins		intergenic		
O157	5429478	del-9		G2583_5141	yjiN	CDS
O157	5430083	ns		G2583_5141	yjiN	CDS
O157	5431702	i		intergenic		
O157	5433469	ns		G2583_5144	yjiR	CDS
O157	5434437	ns		G2583_5144	yjiR	CDS
O157	5435888	nc		G2583_5146	-	pseudogene
O157	5435955	del		G2583_5146	-	pseudogene
CB9615	5274410	nc		G2583_5146	-	pseudogene
CB9615	5275058	nc		G2583_5146	-	pseudogene
CB9615	5276035	i		intergenic		
CB9615	5276927	ns		G2583_5148	hsdS	CDS
CB9615	5280327	s		G2583_5150	hsdR	CDS
O157	5442652	s		G2583_5150	hsdR	CDS
O157	5442889	s		G2583_5150	hsdR	CDS
CB9615	5281820	ns		G2583_5150	hsdR	CDS
O157	5444004	ns		G2583_5151-5152	-	CDS
CB9615	5282850	nc		G2583_5152	orf	pseudogene
CB9615	5283013	nc		G2583_5152	orf	pseudogene
O55/O157	5287498	i		intergenic		
EDL933	5481736	ns		G2583_5158	yjjL	CDS

lineage[a]	site[b]	mutation type[c]	recomb	gene	Gene name	Type
EDL933	5483083	ins		intergenic		
O157	5456480	s		G2583_5161	mdoB	CDS
CB9615	5295608	ns		G2583_5161	mdoB	CDS
O157	5459145	s		G2583_5163	dnaC	CDS
O157	5460660	ns		G2583_5166	yjjP	CDS
CB9615	5299069	s		G2583_5166	yjjP	CDS
CB9615	5300139	ns		G2583_5167	yjjQ	CDS
CB9615	5300156	s		G2583_5167	yjjQ	CDS
O157	5464108	i		intergenic		
O157	5465597	ns		G2583_5172	rsmC	CDS
CB9615	5305771	ns		G2583_5175	yjjG	CDS
O157	5469081	i		intergenic		
O157	5469953	ns		G2583_5236	osmY	CDS
CB9615	5359368	ns		G2583_5237	yjjU	CDS
CB9615	5359719	ns		G2583_5238	yjjV	CDS
EDL933	5502279	del		G2583_5238	yjjV	CDS
CB9615	5360458	ns		G2583_5239	yjjW	CDS
Sakai	5473682	ns		G2583_5240	yjjI	CDS
EDL933	5503677	ns		G2583_5240	yjjI	CDS
EDL933	5503678	s		G2583_5240	yjjI	CDS
Sakai	5476180	del		G2583_5242	deoA	CDS
CB9615	5367951	nc		G2583_5246	lplA	pseudogene
O157	5480943	nc		G2583_5246	lplA	pseudogene
O157	5483438	s		G2583_5248	radA	CDS
CB9615	5372126	ns		G2583_5249	nadR	CDS
O157	5485170	ns		G2583_5249	nadR	CDS
O157	5485573	i		intergenic		
CB9615	5375474	s		G2583_5251	slt	CDS
EDL933	5520694	ns		G2583_5254	ytjC	CDS
EDL933	5520736	s		G2583_5254	ytjC	CDS
O157	5493143	s		G2583_5257	creB	CDS
CB9615	5381292	ns		G2583_5257	creB	CDS
CB9615	5381880	ns		G2583_5258	creC	CDS
O157	5498209	ns		G2583_5262	yjtD	CDS

[a] O157 allocated to the shared lineage to the common ancestor of EDL933 and Sakai; O55/O157 allocated to the SNPs at the divergence of CB9615 and O157:H7; Sakai/EDL933 allocated to the SNPs at the divergence of Sakai and EDL933.

[b] For indels the base indicated is the base before the insertion or deletion.

[c] ins, insertion; del, deletion; indel, insertion or deletion; i, intergenic; nc, in non-coding genes; ns, non-synonymous; s, synonymous. number after "ins" or "del" or "indel" is number of bases if greater than 1
