# Supplementary material for: Derivation of Escherichia coli O157:H7 from Its O55:H7 Precursor
Source: PLoS One. 2010 Jan 14;5(1):e8700. doi: 10.1371/journal.pone.0008700 (PMC2806823; doi:10.1371/journal.pone.0008700)
Supplement: Table S3 — Virtual outgroup analysis of mutations in the CB9651, Sakai, and EDL933 genomes. The mutational and large indel differences between the CB9651, Sakai, and EDL933 genomes were analyzed using the virtual outgroup approach, and the mutations or indels allocated to the CB9651, O157, Sakai, or EDL933 lineages. The 23 genomes used for the analysis are shown, with details of the base or bases present in both outgroup genomes and genomes under analysis, and also the final allocation and a measure of support level for that allocation. (0.21 MB PDF) [file pone.0008700.s005.pdf]

O55 and O157 genome site details										Outgroup Strain Details[g]																						
CB9615 base[a]	CB9615 Site[b]	Sakai base[a]	Sakai Site[b]	EDL933 base[a]	EDL933 Site[b]	type[c]	Inferred ancestral base[d]	Lineage inferred to mutate[e]	Support level[f]	D1 Sd197	K-12	HS	ATCC 8739	UMN026	IAI39	SMS 3-5	E2348/69	536	ED1a	CFT073	S88	APEC O1	UTI89	E24377A	IAI1	SE11	F5 8401	F2a 2457T	F2a 301	SS Ss046	B4 Sb227	B18 BS512
t	68	a	68	a	68	i	a	CB9615	++++	a	a	a	a	a	a	a	a	a		a	a	a	a	a	a	a	a	a	a	a	a	a
-	229	c	230	c	230	ins	-	O157	++++	-	-	-	-	-	-	-	-	-		-	-	-	-	-	-	-	-	-	-	-	-	-
a	1772	g	1790	g	1790	s	g	CB9615	++++	g	g	g	g	g	g	g	g	g	g	g	g	g	g	g	g	g	g	g	g	g	g	g
g	3117	t	3135	t	3135	s	g	O157	++++	g	g	g	g	g	g	g	g	g	g	g	g	g	g	g	g	g	g	g	g	g	g	g
c	3160	a	3178	a	3178	ns	a	CB9615	++++	a	a	a	a	a	a	a	a	a	a	a	a	a	a	a	a	a	a	a	a	a	a	a
t	11664	g	11682	g	11682	ns	t	O157	++++	t	t	t	t	t	t	t	t	t	t	t	t	t	t	t	t	t	t	t	t	t		t
t	13083	c	13101	c	13101	s	c	CB9615	++++	c	c	c	c	c	c	c	c	c	c	c	c	c	c	c	c	c	c	c	c	c	t	c
t	15216	c	15234	c	15234	s	c	CB9615	++++	c	c	c	c	c	c	c	c	c	c	c	c	c	c	c	c	c	c	c	c	c	c	c
g	15594	c	15612	c	15612	ns	c	CB9615	++++	c	c	c	c	c	c	c	c	c	c	c	c	c	c	c	c	c	c	c	c	c	c	c
a	15595	t	15613	t	15613	ns	t	CB9615	++++	t	t	t	t	t	t	t	t	t	t	t	t	t	t	t	t	t	t	t	t	t	t	t
a	16562	g	16580	g	16580	ns	g	CB9615	++++	g	g	g	g	g	g	g	g	g	g	g	g	g	g	g	g	g	g	g	g	g	g	g
c	17595	t	17613	t	17613	s	c	O157	++++	c	c	c	c	c	c	c	c	c	c	c	c	c	c	c	c	c	c	c	c	c	c	c
c	17675	t	17693	t	17693	ns	c	O157	++++	c	c	c	c	c	c	c	c	c	c	c	c	c	c	c	c	c	c	c	c	c	c	c
c	18693	a	18711	a	18711	ns	c	O157	++++	c		c	c	c	c									c	c	c				c	c	c
g	18853	-	18870	g	18871	del	g	Sakai	++++	g		g	g	g	g									g	g	g				g		
-	18976	t	18994	t	18995	ins	-	O157	+++			-	-	-	-									-	-	-				t		
a	23590	g	23608	g	23609	s	a	O157	+++			a	a	a	a									a	a	a				g		
g	24215	a	24233	a	24234	ns	a	CB9615	++++			a	a	a	a									a	a	a				a		
g	24797	a	24815	a	24816	i	a	CB9615	++++			a	a	a	a									a	a	a				a		
a	24966	c	24984	c	24985	i	c	CB9615	++++	c	c	c	c	c	c									c	c	c	c	c	c	c		
g	27124	a	27142	a	27143	ns	g	O157	++++	g	g	g	g	g	g	g	g	g	g	g	g	g	g	g	g	g	g	g	g	g	g	g
t	29411	a	29429	a	29430	s	a	CB9615	++++	a	a	a	a	a	a	a	a	a	a	a	a	a	a	a	a	a	a	a	a	a	a	a
t	33059	a	33077	a	33078	s	t	O157	++++	t	t	t	t	t	t	t	t	t	t	t	t	t	t	t	t	t	t	t	t	t	t	t
g	35214	t	35232	t	35233	s	t	CB9615	++++	t	t	t	t	t	t	t	t	t	t	t	t	t	t	t	t	t	t	t	t	t	t	t
t	36586	c	36604	c	36605	s	c	CB9615	++++	c	c	c	c	c	c	c	c	c	c	c	c	c	c	c	c	c	c	c	c	c	c	c
a	37875	g	37893	g	37894	s	g	CB9615	++	g	g	g	g	g	g	g	g	g	g	g	g	g	g	g	g	g	a	a	a	g	g	g
g	39881	t	39899	t	39900	ns	g	O157	++++	g	g	g	g	g	g	g	g	g	g	g	g	g	g	g	g	g	g	g	g	g		
t	41163	g	41181	g	41182	ns	t	O157	++++	t	t	t	t	t	t	t	t	t	t	t	t	t	t	t	t	t	t	t	t	t		
t	45898	a	45916	a	45917	ns	t	O157	++++	t	t	t	t	t	t	t	t	t	t	t	t	t	t	t	t	t	t	t	t	t		
-	46532	5	46551	5	46552	del	5	CB9615	++	5	-	-	-	-	-	-	-	5	5	-	5	5	5	5	5	5	-	-	-	5		
a	46568	g	46591	g	46592	i	g	CB9615	++++	g	g	g	g	g	g	g	g	g	g	g	g	g	g	g	g	g	g	g	g	g		
t	47781	c	47804	c	47805	s	c	CB9615	++++	c	c	c	c	c	c	c	c	c	c	c	c	c	c	c	c	c	c	c	c	c		
a	47895	g	47918	g	47919	s	g	CB9615	++++	g	g	g	g	g	g	g	g	g	g	g	g	g	g	g	g	g	g	g	g	g		
g	48249	a	48272	a	48273	s	g	O157	++++	g	g	g	g	g	g	g	g	g	g	g	g	g	g	g	g	g	g	g	g	g		
t	48524	c	48547	c	48548	i	c	CB9615	++++	c	c	c	c	c	c	c	c	c	c	c	c	c	c	c	c	c	c	c	c	c		
a	48749	t	48772	t	48773	s	t	CB9615	++	t	t	t	t	a	t	t	t	t	t	t	t	t	t	t	t	t	a	a	a	t		
g	49107	a	49130	a	49131	ns	g	O157	++++	g	g	g	g	g	g	g	g	g	g	g	g	g	g	g	g	g	g	g	g	g		
c	51697	t	51720	t	51721	ns	c	O157	++++	c	c	c	c	c	c	c	c	c	c	c	c	c	c	c	c	c	c	c	c	c	c	c
c	53672	t	53695	t	53696	ns	c	O157	++++	c	c	c	c	c	c	c	c	c	c	c	c	c	c	c	c	c	c	c	c	c	c	c
t	53692	c	53715	c	53716	s	t	O157	++++	t	t	t	t	t	t	t	t	t	t	t	t	t	t	t	t	t	t	t	t	t	t	t
t	54352	a	54375	a	54376	ns	t	O157	++++	t	t	t	t	t	t	t	t	t	t	t	t	t	t	t	t	t	t	t	t	t	t	t
g	61437	a	61460	a	61461	ns	g	O157	++++	g	g	g	g	g	g	g	g	g	g	g	g	g	g	g	g	g	g	g	g	g	g	g
-	61782	6	61806	6	61807	del	6	CB9615	++++	6	6	6	6	6	6	6	6	6	6	6	6	6	6	6	6	6	6	6	6	6	6	6
c	61887	t	61916	t	61917	ns	c	O157	++++	c	c	c	c	c	c	c	c	c	c	c	c	c	c	c	c	c	c	c	c	c	c	c
a	62434	c	62463	c	62464	ns	c	CB9615	++++	c	c	c	c	c	c	c	c	c	c	c	c	c	c	c	c	c	c	c	c	c	c	c
g	63098	t	63127	t	63128	s	g	O157	++++	g	g	g	g	g	g	g	g	g	g	g	g	g	g	g	g	g	g	g	g	g	g	g
t	64021	c	64050	c	64051	ns	?	O55/O157	+/-		a			a																		
-	64037	15	64067	15	64068	del	15	CB9615	+		15			-																		
c	68672	a	68716	a	68717	ns	c	O157	++++	c	c	c	c	c	c	c	c	c	c	c	c	c	c	c	c	c	c	c	c	c	c	c
c	68934	t	68978	t	68979	s	c	O157	++	c	c	c	c	t	t	t	c	c	c	c	c	c	c	c	c	c	c	c	c	c	c	c
t	70349	c	70393	c	70394	ns	c	CB9615	++++	c	c	c	c	c	c	c	c	c	c	c	c	c	c	c	c	c	c	c	c	c	c	c
a	72621	g	72665	g	72666	s	g	CB9615	++++	g	g	g	g	g	g	g	g	g	g	g	g	g	g	g	g	g	g	g	g	g	g	g
g	77729	c	77773	c	77774	ns	g	O157	++++	g	g	g	g	g	g	g	g	g	g	g	g	g	g	g	g	g	g	g	g	g	g	g
c	80607	t	80651	t	80652	i	c	O157	+					c	c	c																
g	81624	a	81668	a	81669	ns	g	O157	++++	g	g	g	g	g	g	g	g	g	g	g	g	g	g	g	g	g	g	g	g	g	g	g
t	84216	c	84260	c	84261	s	c	CB9615	++	c	c	t	t	c	c	c	c	c	c	c	c	c	c	c	c	c	c	c	c	c	c	c
c	84264	t	84308	t	84309	s	c	O157	++++	c	c	c	c	c	c	c	c	c	c	c	c	c	c	c	c	c	c	c	c	c	c	c
a	88405	g	88449	g	88450	i	g	CB9615	++++		g	g	g	g	g	g	g	g	g	g	g	g	g	g	g	g	g	g	g	g	g	g
c	88632	t	88676	t	88677	i	t	CB9615	+		t	t	t	c	c	c	c	c	c	c	c	c	c	t	t	t	t	t	t	t		t
c	88657	t	88701	t	88702	i	c	O157	++++		c	c	c	c	c	c	c	c	c	c	c	c	c	c	c	c	c	c	c	c		c
a	88938	g	88982	g	88983	ns	g	CB9615	++++		g	g	g	g	g	g	g	g	g	g	g	g	g	g	g	g	g	g	g	g	g	g
c	92110	t	92154	t	92155	ns	c	O157	++++	c	c	c	c	c	c	c	c	c	c	c	c	c	c	c	c	c	c	c	c	c	c	c
a	97089	g	97133	g	97134	s	g	CB9615	++++	g	g	g	g	g	g	g	g	g	g	g	g	g	g	g	g	g	g	g	g	g	g	g
a	97345	g	97389	g	97390	ns	g	CB9615	++++	g	g	g	g	g	g	g	g	g	g	g	g	g	g	g	g	g	g	g	g	g	g	g
a	98791	g	98835	g	98836	s	g	CB9615	++++	g	g	g	g	g	g	g	g	g	g	g	g	g	g	g	g	g	g	g	g	g	g	g
g	99054	a	99098	a	99099	ns	g	O157	++++	g	g	g	g	g	g	g	g	g	g	g	g	g	g	g	g	g	g	g	g	g	g	g
g	104212	c	104256	c	104257	ns	g	O157	++++	g	g	g	g	g	g	g	g	g	g	g	g	g	g	g	g	g	g	g	g	g	g	g
t	105290	g	105334	g	105335	i	g	CB9615	++++	g	g	g	g	g	g	g	g	g	g	g	g	g	g	g	g	g	g	g	g	g	g	g
a	107394	g	107438	g	107439	ns	a	O157	++++	a	a	a	a	a	a	a	a	a	a	a	a	a	a	a	a	a	a	a	a	a	a	a
a	111523	g	111567	g	111568	ns	g	CB9615	++++	g	g	g	g	g	g	g	g	g	g	g	g	g	g	g	g	g	g	g	g	g	g	g
-	112113	t	112158	t	112159	ins	-	O157	++	-	t	t	t	t	t	t	-	t	t	t	t	t	t	t	t	t	t	t	t	t	t	t
t	113450	c	113495	c	113496	s	c	CB9615	++++	c	c	c	c	c	c	c	c	c	c	c	c	c	c	c	c	c	c	c	c	c	c	c
g	113690	a	113735	a	113736	s	a	CB9615	++++	a	a	a	a	a	a	a	a	a	a	a	a	a	a	a	a	a	a	a	a	a	a	a
t	120536	c	120581	c	120582	nc	c	CB9615	++++	c	c	c	c	c	c	c	c	c	c	c	c	c	c	c	c	c	c	c	c	c	c	c
a	120839	g	120884	g	120885	nc	g	CB9615	++++	g	g	g	g	g	g	g	g	g	g	g	g	g	g	g	g	g				g	g	g
c	124154	t	124199	t	124200	ns	t	CB9615	++++	t	t	t	t	t	t	t	t	t	t	t	t	t	t	t	t	t	t	t	t	t	t	t
c	124225	t	124270	t	124271	s	t	CB9615	++	t	t	t	t	t	c	t	t	t	t	t	t	t	t	t	t	t	c	c	c	t	t	t
t	125556	g	125601	g	125602	ns	t	O157	++++	t	t	t	t	t	t	t	t	t	t	t	t	t	t	t	t	t	t	t	t	t	t	t
t	126100	a	126145	a	126146	i	t	O157	++++	t	t	t	t	t	t		t							t	t	t	t	t	t	t	t	t

O55 and O157 genome site details										Outgroup Strain Details[g]																						
CB9615 base[a]	CB9615 Site[b]	Sakai base[a]	Sakai Site[b]	EDL933 base[a]	EDL933 Site[b]	type[c]	Inferred ancestral base[d]	Lineage inferred to mutate[e]	Support level[f]	D1 Sd197	K-12	HS	ATCC 8739	UMN026	IAI39	SMS 3-5	E2348/69	536	ED1a	CFT073	S88	APEC O1	UTI89	E24377A	IAI1	SE11	F5 8401	F2a 2457T	F2a 301	SS Ss046	B4 Sb227	B18 BS512
c	130333	t	130378	t	130379	s	c	O157	++++	c	c	c	c	c	t	c	c	c	c	c	c	c	c	c	c	c	c	c	c	c	c	c
c	130434	t	130479	t	130480	ns	c	O157	++++	c	c	c	c	c	c	c	c	c	c	c	c	c	c	c	c	c	c	c	c	c	c	c
c	130440	a	130485	a	130486	ns	c	O157	++++	c	c	c	c	c	c	c	c	c	c	c	c	c	c	c	c	c	c	c	c	c	c	c
t	133723	-	133767	-	133768	ins	-	CB9615	++	-	-	-	-	-	-	-	-	t	t	t	t	t	t	-	-	-	-	-	-	-	-	-
g	137679	a	137723	a	137724	s	g	O157	++++	g	g	g	c	g	g	g	g	g	g	g	g	g	g	g	g	g	g	g	g	g	g	g
a	137832	g	137876	g	137877	ns	g	CB9615	++++	g	g	g	g	g	g	g	g	g	g	g	g	g	g	g	g	g	g	g	g	g	g	g
g	140722	a	140766	a	140767	ns	a	CB9615	++++	a	a	a	a	a	a	a	a	a	a	a	a	a	a	a	a	a	a	a	a	a	a	a
t	140745	c	140789	c	140790	s	c	CB9615	++++	c	c	c	c	c	c	c	c	c	c	c	c	c	c	c	c	c	c	c	c	c	c	c
c	142540	t	142584	t	142585	ns	c	O157	++++	c	c	c	c	c	c	c	c	c	c	c	c	c	c	c	c	c	c	c	c	c	c	c
a	142835	c	142879	c	142880	s	a	O157	++++	a	a	a	a	a	a	a	a	a	a	a	a	a	a	a	a	a	a	a	a	a	a	a
a	142891	g	142935	g	142936	ns	a	O157	++++	a	a	a	a	a	a	a	a	a	a	a	a	a	a	a	a	a	a	a	a	a	a	a
t	143250	c	143294	c	143295	s	c	CB9615	++++	c	c	c	c	c	c	c	c	c	t	c	c	c	c	c	c	c	c	c	c	c	c	
g	149620	a	149664	a	149665	ns	g	O157	++++	g	g	g	g	g	g	g	g	g	g	g	g	g	g	g	g	g	g	g	g	g	g	g
c	150418	a	150462	a	150463	ns	a	CB9615	++++	a	a	a	a	a	a	a	a	a	a	a	a	a	a	a	a	a	a	a	a	a	a	a
a	151169	c	151213	c	151214	i	c	CB9615	++++	c	c	c	c	c	c	c	c	c	c	c	c	c	c	c	c	c	c	c	c	c	c	c
-	152459	c	152504	c	152505	ins	-	O157	+																					-	-	-
a	153413	c	153464	c	153465	s	a	O157	++++	a	a	a	a	a	a	a	a	a	a	a	a	a	a	a	a	a	a	a	a	a	a	a
g	155342	a	155393	a	155394	ns	?	O55/O157	+/-																							
a	155668	g	155719	g	155720	ns	?	O55/O157	+/-																							
a	157248	t	157299	t	157300	ns	a	O157	+				a																			
g	157680	a	157731	a	157732	ns	g	O157	++++				g	g		g		g	g	g	g	g	g		g							
c	157787	t	157838	t	157839	s	t	CB9615	+				t	g		a		g	g	g	g	g	g		a							
t	158759	t	158810	g	158811	ns	t	EDL933	+++		t		t	c	t	c	c	c	c	c	c	c	c		t							
g	159379	a	159430	a	159431	ns	?	O55/O157	+/-				t																			
c	159921	t	159972	t	159973	ns	c	O157	+				c																			
a	160118	g	160169	g	160170	ns	g	CB9615	+				g																			
c	160621	t	160672	c	160673	i	c	Sakai	+				c																			
g	160916	t	160967	t	160968	ns	t	CB9615	+				t																			
g	161322	t	161373	t	161374	i	?	O55/O157	+/-				a																			
-	161444	a	161496	a	161497	indel	?	O55/O157	+/-																							
a	162437	g	162489	g	162490	s	g	CB9615	++++	g	g	g	g	g	g	g	g	g	g	g	g	g	g	g	g	g	g	g	g	g	g	g
g	164671	a	164723	a	164724	s	g	O157	++++	g	g	g	g	g	g	g	g	g	g	g	g	g	g	g	g	g	g	g	g	g	g	g
g	165784	a	165836	a	165837	ns	g	O157	++++	g	g	g	g	g	g	g	g	g	g	g	g	g	g	g	g	g	g	g	g	g	g	g
a	168865	t	168917	t	168918	i	t	CB9615	++++	t	t	t	t											t	t	t	t	t	t	t	t	t
t	171141	c	171193	c	171194	s	c	CB9615	++	c	c	c	c	c	c	c	t	t	t	t	t	t	t	c	c	c	c	c	c	c	c	c
c	171677	a	171729	a	171730	i	c	O157	++++	c	c	c	c	c	c	c	c	c	c	c	c	c	c	c	c	c	c	c	c	c	c	c
c	171863	t	171915	t	171916	ns	c	O157	++++	c	c	c	c	c	c	c	c	c	c	c	c	c	c	c	c	c	c	c	c	c	c	c
a	172434	g	172486	g	172487	s	g	CB9615	++++	g	g	g	g	g	g	g	g	g	g	g	g	g	g	g	g	g	g	g	g	g	g	g
t	172823	c	172875	c	172876	ns	c	CB9615	++++	c	c		c					c			c	c	c	c	c	c	c	c	c			
t	173010	c	173062	c	173063	s	c	CB9615	++	c	c		c	t	c	t		c			c	c	c	c	c	c	c	c	c			
c	174028	t	174080	t	174081	i	c	O157	++++	c	c	c	c	c	c	c	c	c	c	c	c	c	c	c	c	c	c	c	c	c	c	c
a	175920	g	175972	g	175973	s	g	CB9615	++++	g	g	g	g	g	g	g	g	g	g	g	g	g	g	g	g	g	g	g	g	g	g	t
t	176796	c	176848	c	176849	s	c	CB9615	++++	c	c	c	c	c	c	c	c	c	c	c	c	c	c	c	t	c	c	c	c	c	c	c
c	178313	t	178365	t	178366	s	c	O157	++	c	c	t	t	c	c	c	c	c	c	c	c	c	c	t	t	t	c	c	c	t	c	c
c	180108	t	180160	t	180161	s	c	O157	++	c	c	c	c	t	t	c	c	t	t	t	t	t	t	c	c	c	c	c	c	c	c	c
c	181521	t	181573	t	181574	ns	c	O157	++++	c	c	c	c	c	c	c	c	c	c	c	c	c	c	c	c	c	c	c	c	c	c	c
c	182278	t	182330	t	182331	ns	c	O157	++++	c	c	c	c	c	c	c	c	c	c	c	c	c	c	c	c	c	c	c	c	c	c	c
c	184665	t	184717	t	184718	s	c	O157	++++	c	c	c	c	c	c	c	c	c	c	c	c	c	c	c	c	c	c	c	c	c	c	c
t	186669	c	186721	c	186722	s	c	CB9615	++++	c	c	c	c	c	c	c	c	c	c	c	c	c	c	c	c	c	c	c	c	c	c	c
t	188719	g	188771	g	188772	ns	t	O157	++++	t	t	t	t	t	t	t	t	t	t	t	t	t	t	t	t	t	t	t	t	t	t	t
g	192082	c	192134	c	192135	i	c	CB9615	++++	c	c	c	c	c	c	c	c	c	c	c	c	c	c	c	c	c	c	c	c	c	c	c
g	193391	g	193443	a	193444	s	g	EDL933	++++	g	g	g	g	g	g	g	g	g	g	g	g	g	g	g	g	g	g	g	g	g	g	g
t	194111	a	194163	a	194164	i	a	CB9615	++++	a	a	a	a	a			a							a	a	a	a	a	a	a	a	a
g	203712	-	203763	-	203764	del	g	O157	++++	g	g	g	g	g	g	g	g	g	g	g	g	g	g	g	g	g	g	g	g	g	g	g
t	205501	c	205552	t	205553	ns	t	Sakai	++++	t	t	t	t	t	t	t	t	t	t	t	t	t	t	t	t	t	t	t	t	t	t	t
a	206320	g	206371	g	206372	ns	a	O157	++++	a	a	a	a	a	a	a	a	a	a	a	a	a	a	a	a	a	a	a	a	a	a	a
c	206953	t	207004	t	207005	ns	c	O157	++++	c	c	c	c	c	c	c	c	c	c	c	c	c	c	c	c	c	c	c	c	c	c	c
t	207476	c	207527	c	207528	s	c	CB9615	++++	c	c	c	c	a	c	c	c	a	a	a	a	a	a	c	c	c	c	c	c	c	c	c
a	209270	g	209321	g	209322	ns	g	CB9615	++++	g	g	g	g	g	g	g	g	g	g	g	g	g	g	g	g	g	g	g	g	g	g	g
t	215747	g	215798	g	215799	ns	t	O157	++++	t	t	t	t	t	t	t	t	t	t	t	t	t	t	t	t	t	t	t	t	t	t	t
g	216597	a	216648	a	216649	s	g	O157	++++	g	g	g	g	g	g	g	g	g	g	g	g	g	g	g	g	g	g	g	g	g	g	g
t	216629	a	216680	a	216681	ns	t	O157	++++	t	t	t	t	t	t	t	t	t	t	t	t	t	t	t	t	t	t	t	t	t	t	t
t	216848	g	216899	g	216900	ns	t	O157	++++	t	t	t	t	t	t	t	t	t	t	t	t	t	t	t	t	t	t	t	t	t	t	t
t	217696	a	217747	a	217748	ns	a	CB9615	++++	a	a	a	a	a	a	a	a	a	a	a	a	a	a	a	a	a	a	a	a	a	a	a
c	218040	t	218091	t	218092	ns	c	O157	++++	c	c	c	c	c	c	c	c	c	c	c	c	c	c	c	c	c	c	c	c	c	c	c
12	218522	-	218572	-	218573	del	12	O157	++++	12	12	12	12	12	12	12	12	12	12	12	12	12	12	12	12	12	12	12	12	12	12	12
c	220067	t	220106	t	220107	ns	c	O157	++++	c	c	c	c	c	c	c	c	c	c	c	c	c	c	c	c	c	c	c	c	c	c	c
t	220361	c	220400	c	220401	s	c	CB9615	++++	c	c	c	c	c	c	c	c	c	c	c	c	c	c	c	c	c	c	c	c	c	c	c
t	222307	c	222346	c	222347	s	t	O157	++	t	c	c	c	c	c	c	c	c	c	c	c	c	c	c	c	c	c	c	c	c	c	c
g	225362	a	225401	a	225402	ns	a	CB9615	++++	a	a	a	a	a	a	a	a	a	a	a	a	a	a	a	a	a	a	a	a	a	a	a
g	226620	a	226659	a	226660	ns	a	CB9615	++++	a	a	a	a	a	a	a	a	a	a	a	a	a	a	a	a	a	a	a	a	a	a	a
g	226788	a	226827	a	226828	i	g	O157	++++	g	g	g	g	g	g	g	g	g	g	g	g	g	g	g	g	g	g	g	g	g	g	g
-	226834	a	226874	a	226875	del	a	CB9615	++	a	-	-	-	-	-	-	-	-	-	-	-	-	-	a	-	-	a	a	a	a	-	-
t	226836	c	226876	c	226877	i	c	CB9615	++++	c	c	c	c	c	c	c	c	c	c	c	c	c	c	c	c	c	c	c	c	c	c	c
g	226868	a	226908	a	226909	i	a	CB9615	++++	a	a	a	a	a	a	a	a	a	a	a	a	a	a	a	a	a	a	a	a	a	a	a
g	227206	a	227246	a	227247	nc	g	O157	++++	g	g	g	g	g	g	g	g	g	g	g	g	g	g	g	g	g	g	g	g	g	g	g
t	229695	c	229735	c	229736	nc	t	O157	++++	t	t	t	t	t	t	t	t	t	t	t	t	t	t	t	t	t	t	t	t	t	t	t
c	229995	t	230035	c	230036	nc	c	Sakai	++++	c	c	c	c	c	c	c	c	c	c	c	c	c	c	c	c	c	c	c	c	c	c	c
g	230268	a	230308	a	230309	nc	a	CB9615	++	a	a	g	a	a	a	g	c	c	c	a	c	a	c	g	g	a	g	g	g	g	g	a

O55 and O157 genome site details										Outgroup Strain Details[g]																						
CB9615 base[a]	CB9615 Site[b]	Sakai base[a]	Sakai Site[b]	EDL933 base[a]	EDL933 Site[b]	type[c]	Inferred ancestral base[d]	Lineage inferred to mutate[e]	Support level[f]	D1 Sd197	K-12	HS	ATCC 8739	UMN026	IAI39	SMS 3-5	E2348/69	536	ED1a	CFT073	S88	APEC O1	UTI89	E24377A	IAI1	SE11	F5 8401	F2a 2457T	F2a 301	SS Ss046	B4 Sb227	B18 BS512
c	230277	t	230317	t	230318	nc	t	CB9615	++	t	t	c	t	t	t	c	g	g	g	t	g	t	g	c	c	t	t	t	t	c	t	t
g	232057	t	232097	t	232098	nc	g	O157	++	g	g	t	g	t	t	t	t	t	t	t	g	g	g	t	t	t	g	g	g	g	g	g
t	232171	a	232211	a	232212	i	a	CB9615	++	a	a	a	t	a	a	a	a	t	t	t	t	t	t	a	a	a	a	a	a	a	a	a
c	234989	t	235029	t	235030	s	c	O157	++++	c	c	c	c	c	c	c	c	c	c	c	c	c	c	c	c	c	c	c	c	c	c	c
g	235392	t	235432	t	235433	ns	g	O157	++++	g		g		g	g	g	g	g	g	g	g	g	g	g	g	g	g	g	g			
c	238455	t	238495	t	238496	ns	c	O157	++++	c	c	c	c	c	c	c	c	c	c	c	c	c	c	c	c	c	c	c	c	c	c	c
c	239551	t	239591	t	239592	i	c	O157	++++	c	c	c	c	c	c	c	c	c	c	c	c	c	c	c	t	c	c	c	c	c	c	c
-	240577	t	240618	t	240619	ins	-	O157	++	-	-	-		-				-	-		-	-	-	-	-	t				-	-	t
g	241801	a	241842	a	241843	i	?	O55/O157	+/-																							
g	242655	a	242696	a	242697	ns	a	CB9615	++	a																						
a	243251	t	243292	t	243293	ns	a	O157	++	a																						
t	243673	a	243714	a	243715	ns	t	O157	+++	t				t				t	t		t	t	t									
c	249929	-	249997	-	249998	del	c	O157	++++			c		c				c	c		c	c	c	c	c	c				c		
12	252262	-	252329	-	252330	ins	-	CB9615	+			12						-	-		-	-	-	-	12	12				12		
t	252743	g	252799	g	252800	s	g	CB9615	++++			g		c				g	g		g	g	g	g	g	g				g		
t	253251	g	253307	g	253308	ns	t	O157	++++			t		t				t	t		t	t	t	t	t	t				t		
c	254002	t	254058	t	254059	s	c	O157	++++			c		c				c	c		c	c	c	c	c	c				c		
c	254531	t	254587	t	254588	ns	c	O157	++++			c		c				c	c		c	c	c	c	c	c				c		
c	255144	t	255200	t	255201	ns	c	O157	+++			c		c										c	c	c				c		
a	255682	g	255738	g	255739	ns	g	CB9615	++++			g		g				g	g		g	g	g	g	g	g				g		
g	257039	t	257095	t	257096	ns	t	CB9615	++++			t		t				t	t		t	t	t	t	t	t				t		
-	257682	c	257739	c	257740	del	c	CB9615	++++			c		c				c	c		c	c	c	c	c	c				c		
t	259498	c	259555	c	259556	s	t	O157	+++			t												t	t	t				t		
g	261080	a	261137	a	261138	ns	g	O157	+++			g												g	g	g				g		
g	261180	a	261237	a	261238	nc	g	O157	++++			g		g				g	g		g	g	g	g	g	g				g		
t	261680	c	261737	c	261738	nc	c	CB9615	++++			c		c				c	c		c	c	c	c	c	c				c		
a	261984	c	262041	c	262042	nc	c	CB9615	++++			c		c				c	c		c	c	c	c	c	c				c		
g	263106	t	263163	t	263164	ns	g	O157	+++			g												g	g	g				g		
t	263595	c	263652	c	263653	i	c	CB9615	+++			c												c	c	c				c		
a	263615	g	263672	g	263673	i	a	O157	+++			a												a	a	a				a		
a	263644	g	263701	g	263702	i	a	O157	+			a												a	-	a				g		
t	263864	c	263921	c	263922	ns	t	O157	+++			t												t	t	t				t		
c	264871	t	264928	c	264929	ns	c	Sakai	+++			c	c											c	c	c				c		
a	264913	c	264970	c	264971	ns	a	O157	+++			a	a											a	c	a				a		
a	265088	g	265145	g	265146	s	g	CB9615	+++			g	g											g	g	g				a		
g	265366	t	265423	t	265424	ns	g	O157	+++			g	g											g	g	g				g		
c	266558	a	266615	a	266616	s	a	CB9615	+++			a	a											a	a	a				a		
a	267035	g	267092	g	267093	nc	a	O157	+		a	g	a											g	g	g				g	a	a
a	267119	c	267176	c	267177	nc	c	CB9615	+		c	a	c											a	a	t				a	c	c
t	267128	c	267185	c	267186	nc	c	CB9615	+		c	t	c											t	t	c				t	c	c
a	268156	t	268213	t	268214	nc	a	O157	++++	a	a	a	a											a	a	a				a	a	a
c	269060	t	269117	t	269118	nc	c	O157	++++	c	c	c	c											c	c	t				c	c	c
a	269127	g	269184	g	269185	nc	g	CB9615	++	g	g	a	a											a	a	a				a	g	a
c	269494	t	269551	t	269552	nc	c	O157	++++	c	c	c	c											c	c	c				c	c	c
a	274826	g	272354	g	272355	ns	a	O157	+				a												a							
c	274856	t	272384	t	272385	ns	t	CB9615	+				t												t							
g	275323	c	272851	c	272852	ns	g	O157	++++	g	g	g	g	g				g	g		g	g	g	g	g	g				g		g
t	275648	g	273176	g	273177	s	t	O157	++++	t	t	c	t	t										t	t	t				a		t
c	276395	t	273923	t	273924	ns	c	O157	++	c	c	t	c											c	c	t				c	c	c
t	276826	c	274354	c	274355	s	c	CB9615	+++	c	a	c	a											a	a	a				c	c	c
a	276978	g	274506	g	274507	ns	a	O157	++++	a	a	a	a											a	a	a				a	a	a
t	277259	c	274787	c	274788	s	c	CB9615	+	a	g	c	g											a	a	a				c	a	a
c	277447	t	274975	t	274976	s	c	O157	++++	c	c	c	c											c	c	c				c	c	c
c	282822	a	280350	a	280351	ns	c	O157	++++	c	c	c	c	c	c	c	c	c	c	c	c	c	c	c	c	c	c	c	c	c	c	c
t	282980	c	280508	c	280509	s	c	CB9615	++++	c	c	c	c	c	c	c	c	c	c	c	c	c	c	c	c	c	c	c	c	c	c	c
g	283888	a	281416	a	281417	i	a	CB9615	++++	a	a	a	a	a	a	a	a	a	a	a	a	a	a	a	a	a	a	a	a	a	a	a
ta	286656	-	284183	-	284184	del	ta	O157	++++	ta	ta	ta	ta		ta	ta								ta	ta	ta				ta		
g	286955	a	284481	a	284482	i	g	O157	++++	g	g	g	g		g	g								g	g	g				g		
g	287423	a	284949	a	284950	s	g	O157	++++	g	t	g	a	g	g	g	g	g	g	g	g	g	g	g	g	g	g	g	g	g		g
g	287952	a	285478	a	285479	i	g	O157	++++	g	g		g	g	g	g		g	g	g	g	g	g				g	g	g	g		g
c	288072	t	285598	t	285599	i	c	O157	++++	c	c		c	c	c	c		c	c		c	c	c				c	c	c	c		c
t	288917	c	286355	c	286356	s	c	CB9615	+		c	c	c	c	c	c	c	c	c	c	c	c	c	c	c	c	t	t	t	c		
g	290345	a	287783	a	287784	s	g	O157	++++		g	g	g	g	g	g	g	g	g	g	g	g	g	g	g	g	g	g	g	g		
g	291772	a	289210	a	289211	ns	g	O157	++++	g	g	g	g	g	g	g	g	g	g	g	g	g	g	g	g	g	g	g	g	g		g
t	293727	a	291165	a	291166	i	a	CB9615	++++	a	a	a		a	a	a	a	a	a	a	a	a	a	a	a	a	a	a	a	a		a
t	294876	c	292314	c	292315	s	c	CB9615	++++	c	c	c	c	c	c	c	c	c	c	c	c	c	c	c	c	c	c	c	c	c	c	c
g	297482	a	294920	a	294921	s	g	O157	++	g	g	g	g	g	g	g	a	a	g	g	g	g	g	g	g	g	g	g	g	g	g	g
a	297711	t	295149	t	295150	ns	a	O157	++++	a	a	a	a	a	a	a	a	a	a	a	a	a	a	a	a	a	a	a	a	a	a	a
a	302241	g	299679	g	299680	ns	g	CB9615	++++	g	g	g	g	g	g	g	g	g	g	g	g	g	g	g	g	g	g	g	g	g	g	g
c	302620	t	300058	t	300059	i	t	CB9615	++++		t	t	t	t	t	t	t	t	t	t	t	t	t	t	t	t	t	t	t	t	t	t
c	351852	t	310863	t	310864	ns	?	O55/O157	+/-																							
t	352160	c	311171	c	311172	s	?	O55/O157	+/-																							
g	352655	a	311666	a	311667	s	?	O55/O157	+/-																							
a	353019	g	312030	g	312031	ns	?	O55/O157	+/-																							
a	354030	g	313041	g	313042	ns	?	O55/O157	+/-																							
t	354909	-	313919	-	313920	indel	?	O55/O157	+/-																							
a	354927	g	313937	g	313938	nc	?	O55/O157	+/-																							
g	355063	a	314073	a	314074	nc	?	O55/O157	+/-																							
t	355069	a	314079	a	314080	nc	?	O55/O157	+/-																							

O55 and O157 genome site details										Outgroup Strain Details[g]																						
CB9615 base[a]	CB9615 Site[b]	Sakai base[a]	Sakai Site[b]	EDL933 base[a]	EDL933 Site[b]	type[c]	Inferred ancestral base[d]	Lineage inferred to mutate[e]	Support level[f]	D1 Sd197	K-12	HS	ATCC 8739	UMN026	IAI39	SMS 3-5	E2348/69	536	ED1a	CFT073	S88	APEC O1	UTI89	E24377A	IAI1	SE11	F5 8401	F2a 2457T	F2a 301	SS Ss046	B4 Sb227	B18 BS512
t	355077	c	314087	c	314088	i	?	O55/O157	+/-																							
c	355165	t	314175	t	314176	ns	?	O55/O157	+/-																							
g	355596	t	314606	t	314607	ns	?	O55/O157	+/-																							
-	355604	a	314615	a	314616	indel	?	O55/O157	+/-																							
c	355719	t	314730	t	314731	s	?	O55/O157	+/-																							
t	356069	g	315080	g	315081	ns	?	O55/O157	+/-																							
c	356460	t	315471	t	315472	i	?	O55/O157	+/-																							
t	357897	c	316908	c	316909	s	?	O55/O157	+/-																							
-	358337	t	317349	t	317350	indel	?	O55/O157	+/-																							
t	358376	g	317402	g	317403	i	?	O55/O157	+/-																							
g	358942	t	317968	t	317969	i	?	O55/O157	+/-																							
a	359857	c	318883	c	318884	ns	?	O55/O157	+/-																							
a	361724	c	320750	c	320751	ns	c	CB9615	+				c			c															c	
t	363783	c	322809	c	322810	ns	?	O55/O157	+/-																							
a	363874	g	322900	g	322901	s	?	O55/O157	+/-																							
-	364323	a	323350	a	323351	indel	?	O55/O157	+/-																							
g	364419	t	323446	t	323447	i	?	O55/O157	+/-																							
t	366418	c	325445	c	325446	i	?	O55/O157	+/-																							
-	366486	t	325514	t	325515	indel	?	O55/O157	+/-																							
c	368007	t	327035	t	327036	ns	c	O157	+					c																		
t	368572	g	327600	g	327601	ns	g	CB9615	+					g																		
c	368964	t	327992	t	327993	s	c	O157	+					c																		
c	370595	t	329623	t	329624	ns	c	O157	+					c																		
t	370613	g	329641	g	329642	ns	t	O157	+					t																		
t	370778	c	329806	c	329807	ns	c	CB9615	+					c																		
c	371592	-	330619	-	330620	ins	-	CB9615	++++		-		-	-										-	-	-						
a	371983	c	331010	c	331011	nc	a	O157	++++		a		a	a										a	a	a						
t	372009	c	331036	c	331037	nc	c	CB9615	++++		c		c	c										c	c	c						
c	372441	t	331468	t	331469	s	c	O157	++++		c		c	c										c	c	c						
c	372648	t	331675	t	331676	ns	c	O157	++++		c		c	c										c	c	c						
c	373157	t	332184	t	332185	ns	c	O157	++++		c		c	c										c	c	c						
a	373580	g	332607	g	332608	s	g	CB9615	++++		g		g	g										g	g	g						
c	374557	t	333584	t	333585	ns	c	O157	++++		c		c	c										c	c	c						
g	375238	t	334265	t	334266	ns	t	CB9615	++++		t		t	t										t	t	t						
t	377140	c	336167	c	336168	i	c	CB9615	++++		c		c	c	c	c	c	c		c	c	c	c	c	c	c						
c	377991	t	337018	t	337019	ns	c	O157	++++		c		c	c	c	c	c	c		c	c	c	c	c	c	c						
t	379434	a	338461	t	338462	s	t	Sakai	++++		t		t	t	t	t	t	t	t	t	t	t	t	t	t	t					t	
a	379728	g	338755	g	338756	s	g	CB9615	++++		g		g	g	g	g	g	g	g	g	g	g	g	g	g	g					g	
a	383432	t	342459	t	342460	i	a	O157	++++		a		a	a	a	a	a	a	a	a	a	a	a	a	a	a					a	
c	383951	t	342978	t	342979	s	t	CB9615	+		c		c	t	t	t	t	t	t	t	t	t	t	c	c	c					c	
a	383993	c	343020	c	343021	s	c	CB9615	++++		c	c	c	c	c	c	c	c	c	c	c	c	c	c	c	c					c	
a	384694	g	343721	g	343722	s	g	CB9615	+		a	g	a	g	g	g	g	g	g	g	g	g	g	a	g	g					a	
-	384825	t	343853	t	343854	ins	-	O157	++++		-	-	-	-		-	-	-	-	-	-	-	-	-	-	-					-	
a	385115	g	344143	g	344144	i	a	O157	++++		a	a	a	a		a	a	a	a	a	a	a	a	a	a	a					a	
-	386328	a	345586	c	345587	ns	c	Sakai	++++	c				c				c	c	c	c			c						c		c
a	386535	g	348013	g	348014	i	g	CB9615	++++		g	g	g	a	g	g	g	g	g	g	g	g	g	g	g	g					g	
-	386552	-	348030	t	348032	ins	-	EDL933	++++		-	-	-	-	-	-	-	-	-	-	-	-	-	-	-	-					-	
a	386570	-	348047	-	348049	del	a	O157	++++		a	a	a	a	a	a	a	a	a	a	a	a	a	a	a	a					a	
-	388272	-	349749	a	349752	ins	-	EDL933	++++					-	-	-	-	-		-	-	-	-									
g	388401	a	349878	a	349881	i	g	O157	++++					g	g	g	g	g		g	g	g	g									
g	389309	a	350786	a	350789	ns	a	CB9615	++++					a	a	a	a	a		a	a	a	a									
c	389632	t	351109	t	351112	ns	c	O157	++++					c	c	c	c	c		c	c	c	c									
g	389732	c	351209	c	351212	ns	g	O157	++++					g	g	g	g	g		g	g	g	g									
t	390700	g	352177	g	352180	i	?	O55/O157	+/-																							
g	391163	a	352640	a	352643	ns	?	O55/O157	+/-																							
a	391626	t	353103	t	353106	ns	t	CB9615	++++					t	t	t	t	t		t	t	t	t									
a	393151	g	354628	g	354631	ns	g	CB9615	++++		g	g	g	g	g	g	g	g		g	g	g	g	g	g	g					g	
t	394296	c	355773	c	355776	ns	c	CB9615	++++			c	c	c	c	c	c	c		c	c	c	c	c	c	c						
a	396355	c	357832	c	357835	ns	a	O157	++++			a	a	a	a	a	a	a		a	a	a	a	a	a	a						
a	396721	g	358198	g	358201	i	a	O157	++++			a	a	a	a	a	-	-		-	-	-	-	a	a	a						
a	397159	g	358636	g	358639	ns	a	O157	++++		a	a	a	a	a	a	a	a		a	a	a	a	a	a	a						
g	399004	c	360481	c	360484	ns	g	O157	++++		g	g	g	g	g	g	g	g	g	g	g	g	g	g	g	g				g		g
c	399018	t	360495	t	360498	ns	t	CB9615	++++		t	t	t	t	t	t	t	t	t	t	t	t	t	t	t	t				t		t
t	399508	c	360985	c	360988	ns	t	O157	++++		t	t	t	t	t	t	t	t	t	t	t	t	t	t	t	t				a		t
c	399563	t	361040	t	361043	ns	c	O157	++++		c	c	c	c	c	c	c	c	c	c	c	c	c	c	c	c				c		c
g	399792	a	361269	a	361272	ns	a	CB9615	++++		a	a	a	a	a	a	a	a	a	a	a	a	a	a	a	a				a		a
g	402522	a	363999	a	364002	i	g	O157	+		g	g	g	g	a	g	a	g	g	g	g	g	g	g	g	g	g	g	g	g		g
t	402710	c	364187	c	364190	i	t	O157	++++		t	t	t	t	t	t	t	t	t	t	t	t	t	t	t	t	t	t	t	t		t
t	403579	c	365056	c	365059	ns	c	CB9615	++++		c	c	c	c	c	c	c	c	c	c	c	c	c	c	c	c	c	c	c	c		c
a	405508	c	366985	c	366988	ns	a	O157	++++		a	a	a	a	a	a	a	a	a	a	a	a	a	a	a	a	a	a	a	a		a
g	406374	t	367851	t	367854	nc	g	O157	++++		a	g	a	g				g	g	g	g	g	g	g	g	g	g	g	g			
g	410665	t	371105	t	371108	ns	g	O157	+++			g						g		g	g	g	g			g						
g	410891	t	371331	t	371334	i	g	O157	+++			g						g		g	g	g	g			g						
a	411165	c	371605	c	371608	nc	c	CB9615	+++			c						c		c	c	c	c			c						
-	412233	t	372674	-	372676	ins	-	Sakai	+++			-						-		-	-	-	-			-						
a	412327	g	372766	a	372768	i	a	Sakai	+++			a						a		a	a	a	a			a						
g	412375	t	372814	g	372816	i	g	Sakai	+++			g						g		g	g	g	g			g						
g	414385	a	374824	a	374826	s	g	O157	++++		g	g	g	g	g	g	g	g		g	g	g	g	g	g	g	g					
c	414878	a	375317	c	375319	ns	c	Sakai	++++		c	c	c	c	c	c	c	c		c	c	c	c	c	c	c	c					

O55 and O157 genome site details											Outgroup Strain Details[g]																					
CB9615 base[a]	CB9615 Site[b]	Sakai base[a]	Sakai Site[b]	EDL933 base[a]	EDL933 Site[b]	type[c]	Inferred ancestral base[d]	Lineage inferred to mutate[e]	Support level[f]	D1 Sd197	K-12	HS	ATCC 8739	UMN026	IAI39	SMS 3-5	E2348/69	536	ED1a	CFT073	S88	APEC O1	UTI89	E24377A	IAI1	SE11	F5 8401	F2a 2457T	F2a 301	SS Ss046	B4 Sb227	B18 BS512
c	415679	t	376118	t	376120	ns	t	CB9615	++++		t	t	t	t	t	t	t	t		t	t	t	t	t	t	t	t					
c	416071	t	376510	t	376512	s	t	CB9615	++++		t	t	t	t	t	t	t	t		t	t	t	t	t	t	t	t					
c	416563	t	377002	t	377004	s	c	O157	++++		c	c	c	c	c	c	c	c		c	c	c	c	c	c	c	c					
g	419077	a	379516	a	379518	i	a	CB9615	+			-												a	-							
a	424296	g	384735	g	384737	ns	a	O157	++++		a	a	a	a	a	a	a	a		a	a	a	a	a	a	a						
a	424356	g	384795	g	384797	ns	g	CB9615	++++		g	g	g	g	g	g	g	g		g	g	g	g	g	g	g						
c	424758	a	385197	a	385199	nc	c	O157	++++		c	c	c	c	c	c	c	c		c	c	c	c	c	c	c						
t	425321	c	385760	c	385762	nc	c	CB9615	++++		c	c	c	c	c	c	c	c		c	c	c	c	c	c	c						
g	425520	a	385959	a	385961	i	g	O157	++++		g	g	g	g	g	g	g	g		g	g	g	g	g	g	g						
t	425989	-	386427	-	386429	ins	-	CB9615	++++		-	-	-	-	-	-	-	-		-	-	-	-	-	-	-						
g	428833	a	389262	a	389264	ns	g	O157	++++		g	g	g	g	g	g	g	g	g	g	g	g	g	g	g	g				g		
c	429492	t	389921	t	389923	ns	c	O157	++++		c	c	c	c	c	c	c	c	c	c	c	c	c	c	c	c				c		
t	430535	g	390964	g	390966	s	t	O157	+++		c	c	c	t	c	c	t	t	t	c	c	c	c	c	c	c				c		
c	431796	t	392225	t	392227	s	c	O157	++++		c	c	c	c	c	c	c	c	c	c	c	c	c	c	c	c				c		
t	431898	c	392327	c	392329	s	c	CB9615	++++		c	c	c	c	c	c	c	c	c	c	c	c	c	c	c	c				c		
t	432970	c	393399	c	393401	ns	c	CB9615	+		c	c	c	c	c	g	c	t	t	c	c	c	c	c	c	c				c		
a	433721	g	394150	g	394152	i	g	CB9615	++++		g	g	g	g	g	g	g	g	g	g	g	g	g	g	g	g				g		
g	435536	c	395965	c	395967	ns	c	CB9615	++++			c		c	c				c		c	c	c	c	c	c						
c	435921	t	396350	t	396352	ns	c	O157	++++			c		c	c				c		c	c	c	c	c	c						
c	438469	t	398898	t	398900	ns	c	O157	++++			c		c	c				c		c	c	c	c	c	c						
c	439397	t	399826	t	399828	s	c	O157	++++		c	c	c	c	c	c	c	c	c	c	c	c	c	c	c	c				c		
t	441178	g	401607	g	401609	ns	t	O157	++++		t	t	t	t	t	t								t	t	t				t		
c	441292	t	401721	t	401723	i	c	O157	++++		c	c	c	c	c	c	c	c	c	c	c	c	c	c	c	c				c		
c	441386	t	401815	t	401817	i	t	CB9615	++++		t	t	t	t	t	t	t	t	t	t	t	t	t	t	t	t				t		
t	441671	c	402100	c	402102	nc	t	O157	++++		t	t	t	t	t	t	t	t	t	t	t	t	t	t	t	t				t		
t	441945	c	402374	c	402376	s	c	CB9615	++++		c	c	c	t	c	c	c	c	c	c	c	c	c	c	c	c				c		
t	442217	c	402646	c	402648	ns	c	CB9615	++++		c	c	c	c	c	c	c	c	c	c	c	c	c	c	c	c				c		
g	442641	t	403070	t	403072	i	t	CB9615	++++		t	t	t	t	t	t	t	t	t	t	t	t	t	t	t	t						
c	447320	t	407749	t	407751	s	c	O157	++++		c	c	c	c	c	c	c	c	c	c	c	c	c	c	c	c						
g	450549	a	410978	a	410980	ns	g	O157	++++		g	g	g	g	g	g		g	g	g	g	g	g	g	g	g						
t	450735	a	411352	m	411354	nc	t	EDL933	IUPAC				t	t				c														
a	451316	g	411933	g	411935	s	a	O157	++++		a	a	a	a	a	a	a	a	a	a	a	a	a	a	a	a						
g	451746	t	412363	t	412365	ns	g	O157	++++		g	g	g	g	g	g	g	g	g	g	g	g	g	g	g	g						
a	452957	t	413574	t	413576	ns	a	O157	++++		a	a	a	a	a	a	a	a	a	a	a	a	a	a	a	a						
c	453882	a	414499	a	414501	i	c	O157	++++		c		c	c	c	c	c		c	c	c	c	c									
g	454394	a	415011	a	415013	ns	g	O157	++++		g	g	g	g	g	g								g	g	g						
t	455133	g	415750	t	415752	ns	t	Sakai	++++		t	t	t	t	t	t								t	t	t						
c	460753	a	421370	a	421372	ns	c	O157	++++	c	c	c	c	c	c	c	c	c	c	c	c	c	c	c	c	c				c		
c	461130	t	421747	t	421749	s	c	O157	++++	c	c	c	c	c	c	c	c	c	c	c	c	c	c	c	c	c				c		
t	462315	c	422932	c	422934	s	t	O157	++++	t	t	t	t	t	t	t	t	t	t	t	t	t	t	t	c	t				t		
t	465651	c	426268	c	426270	s	c	CB9615	++++		c	c	c	c	c	c			c					c	c	c				c		
a	467075	t	427692	t	427694	ns	a	O157	++++		a	a	a	a	a	a			a					a	a	a				a		
g	468775	a	429392	a	429394	s	g	O157	++++		g	g	g	g	t	g			t					g	g	g				g		
t	470019	g	430636	g	430638	s	t	O157	++++		t	t	t	t	t	t			t					t	t	t				t		
g	470597	c	431214	g	431216	ns	g	Sakai	++++		g	g	g	g	g	g			g					g	g	g				g		
t	471397	c	432014	c	432016	ns	c	CB9615	++++		c	c	c	c	c	c			c					c	c	c				c		
t	471930	a	432547	a	432549	ns	a	CB9615	++++		a	a	a	a	a	a			a					a	a	a				a		
g	475151	a	435465	a	435467	ns	g	O157	++++		g	g	g	g	g	g	g	g	g	g	g	g	g	g	g	g				g		
c	475559	g	435873	g	435875	ns	c	O157	++++		c	c	c	c	c	c	c	c	c	c	c	c	c	c	c	c				c		c
c	477710	a	438024	a	438026	i	c	O157	++++		c	c	c	c	a	c	c	c	c	c	c	c	c	c	c	c				c		c
a	479501	g	439815	g	439817	s	g	CB9615	+										g													
a	479849	g	440163	g	440165	s	g	CB9615	+										g													
t	480340	c	440654	c	440656	ns	t	O157	+										t													
a	484172	g	444486	g	444488	s	g	CB9615	++	g									g													
a	485002	g	445316	g	445318	s	g	CB9615	++	g									g													
t	485360	c	445674	c	445676	s	c	CB9615	++	c									c													
g	486592	t	446906	t	446908	ns	g	O157	++++	g	g	g	g	g	g	g	g	g	g	g	g	g	g	g	g	g	g	g	g	g	g	g
a	488042	g	448356	g	448358	s	g	CB9615	++++	g	g	g	g	g	g	g	g	g	g	g	g	g	g	g	g	g	g	g	g	g	g	g
a	490468	t	450782	t	450784	i	a	O157	++++	a	a	a	a	g	g	g	g	g	g	g	g	g	g	a	a	a	a	a	a	a	a	a
c	490615	t	450929	t	450931	i	c	O157	++	c	g	g	g	c	g	g	g	g		g	g	g	g	g	g	g	t	g	g	t	t	t
g	490965	a	451279	a	451281	ns	g	O157	++++		g	g	g	g	g	g	g	g		g	g	g	g	g	g	g	g	g	g	g		
c	491030	c	451344	t	451346	s	t	EDL933	-		t		c				c	t		t	t	t	t	t	c	t	c	c	c			
g	491868	a	452182	a	452184	ns	g	O157	+				g				g								g							
g	492257	a	452571	a	452573	s	g	O157	++++	t			t	g	g	g	t	g		g	g	g	g	g	t	g				g		g
t	494242	a	454556	a	454558	ns	a	CB9615	++++	a	a	a	a	a	a	a	a	a		a	a	a	a	a	a	a	a	a	a	a	a	a
g	495868	a	456182	a	456184	i	a	CB9615	++++	a	a	a	a	a	a	a	a	a	a	a	a	a	a	a	a	a	a	a	a	a	a	a
c	496415	t	456729	t	456731	s	c	O157	++++	c	c	c	c	c	c	c	c	c	c	c	c	c	c	c	c	c	c	c	c	c	c	c
t	497548	c	457862	c	457864	ns	c	CB9615	++++	c	c	c	c	c	c	c	c	c	c	c	c	c	c	c	c	c	c	c	c	c	c	c
c	497748	t	458062	t	458064	s	t	CB9615	++++	t	t	t	t	t	t	t	t	t	t	t	t	t	t	t	t	t	t	t	t	t	t	t
c	499863	a	460177	a	460179	ns	a	CB9615	++++	a	a	a	a	a	a	a	a	a	a	a	a	a	a	a	a	a	a	a	a	a	a	a
t	500768	c	461082	c	461084	i	t	O157	++++	t	t	t	t	t	t	t	t	t	t	t	t	t	t	t	t	t	t	t	t	t	t	t
c	502438	t	462752	t	462754	s	c	O157	++	c	c	t	c	c	c	c	c	c	c	c	c	c	c	c	c	t	t	t	t	t	c	c
t	503681	c	463995	c	463997	s	c	CB9615	++++	c	c	c	c	c	c	c	c	c	c	c	c	c	c	c	c	c	c	c	c	c	c	c
c	507241	a	467555	a	467557	s	c	O157	++++	c	c	c	c	c	c	c	c	c	c	c	c	c	c	c	c	c	c	c	c	c	c	c
t	507474	c	467788	c	467790	s	c	CB9615	++++	c	c	c	c	c	c	c	c	c	c	c	c	c	c	c	c	c	c	c	c	c	c	c
g	507616	a	467930	a	467932	i	g	O157	++++	g	g	g	a	g	g	g	g	g	g	g	g	g	g	g	g	g	g	g	g	g	g	g
c	509510	t	469824	c	469826	ns	c	Sakai	++	c																						
-	510778	a	471093	a	471095	ins	-	O157	++++	-	-	-	-	-	-	-	-	-	-	-	-	-	-	-	-	-	-	-	-	-	-	-
t	510810	c	471125	c	471127	i	t	O157	++	t	c	c	c	c	c	c	c	c	c	c	c	c	c	c	c	c	c	c	c	c	c	c

O55 and O157 genome site details										Outgroup Strain Details[g]																						
CB9615 base[a]	CB9615 Site[b]	Sakai base[a]	Sakai Site[b]	EDL933 base[a]	EDL933 Site[b]	type[c]	Inferred ancestral base[d]	Lineage inferred to mutate[e]	Support level[f]	D1 Sd197	K-12	HS	ATCC 8739	UMN026	IAI39	SMS 3-5	E2348/69	536	ED1a	CFT073	S88	APEC O1	UTI89	E24377A	IAI1	SE11	F5 8401	F2a 2457T	F2a 301	SS Ss046	B4 Sb227	B18 BS512
a	514288	c	474603	a	474605	ns	a	Sakai	++++	a	a	a	a	a	a	a	a	a	a	a	a	a	a	a	a	a	a	a	a	a	a	a
c	514293	t	474608	t	474610	ns	c	O157	++	c	t	t	t	c	c	c	c	c	c	c	c	c	c	t	t	t	t	t	t	t	t	t
g	516206	a	476521	a	476523	ns	g	O157	++++	g	g	g	g	g	g	g	g	g	g	g	g	g	g	g	g	g	g	g	g	g	g	g
a	516795	c	477110	c	477112	ns	c	CB9615	++++	c	c	c	c	c	c	c	c	c	t	c	c	c	c	c	c	c	c	c	c	c	c	c
t	517616	c	477931	c	477933	ns	c	CB9615	++++	c	c	c	c	c	c	c	c	c	c	c	c	c	c	c	c	c	c	c	c	c	c	c
t	519775	c	480090	c	480092	ns	c	CB9615	++++	c	c	c	c	c	c	c	c	c	c	c	c	c	c	c	c	c	c	c	c	c	c	c
a	520247	g	480562	g	480564	s	g	CB9615	++++	g	g	g	g	g	g	g	g	g	g	g	g	g	g	g	g	g	g	g	g	g	g	g
c	520516	t	480831	t	480833	ns	c	O157	++++	c	c	c	c	c	c	c	c	c	c	c	c	c	c	c	c	c	c	c	c	c	c	c
a	525160	c	485475	c	485477	ns	c	CB9615	++++	c	c	c	c	c	c	c	c	c	c	c	c	c	c	c	c	c	c	c	c	c	c	c
-	528257	g	488573	g	488575	del	g	CB9615	++++	g	g	g	g	g	g	g	g	g	g	g	g	g	g	g	g	g	g	g	g	g	g	g
g	529731	a	490047	a	490049	s	g	O157	++++	g	g	g	g	g	g	g	g	g	g	g	g	g	g	g	g	g	g	g	g	g	g	g
t	533850	-	494165	-	494167	indel	?	O55/O157	+/-						t	-																
g	540196	a	500511	a	500513	ns	a	CB9615	++++	a	a	a	a	a	a	a	a	a	a	a	a	a	a	a	a	a	a	a	a	a	a	a
c	541562	t	501877	t	501879	ns	c	O157	++	c				c																		
g	544052	a	504367	a	504369	i	a	CB9615	++++	a	a	a	a	a	a	a	a	a	a	a	a	a	a	a	a	a	a	a	a	a	a	a
a	545858	g	506173	g	506175	s	a	O157	++++	a	a	a	a	a	a	a	a	a	a	a	a	a	a	a	a	a	a	a	a	a	a	a
t	545930	c	506245	c	506247	i	t	O157	++++	t	t	t	t	t	t	t	t	t	t	t	t	t	t	t	t	t	t	t	t	t	t	t
a	548461	g	508776	g	508778	ns	a	O157	++++	a	a	a	a	a	a	a	a	a	a	a	a	a	a	a	a	a	a	a	a	a	a	a
c	549503	t	509818	t	509820	ns	c	O157	++++	c	c	c	c	c	c	c	c	c	c	c	c	c	c	c	c	c	c	c	c	c	c	c
c	552748	t	513063	t	513065	s	t	CB9615	++++		t	t	t	t	t	t	t	t	t	t	t	t	t	t	t	t	t	t	t	t	t	t
t	553048	g	513363	g	513365	s	t	O157	++++		a	t	t	t	a	c	a	a	a	a	a	a	a	t	t	t	t	t	t	c	t	t
c	553512	t	513827	t	513829	s	c	O157	++++		c	c	c	c	c	c	c	c	c	c	c	c	c	c	c	c	c	c	c	c	c	c
t	555531	g	515846	g	515848	s	g	CB9615	++++		g	g	g	g	g	g	g	g	g	g	g	g	g	g	g	g	g	g	g	g	g	g
g	556007	a	516322	a	516324	s	g	O157	++++		g	g	g	g	g	g	g	g	g	g	g	g	g	g	g	g	g	g	g	g	g	g
c	556535	t	516850	t	516852	ns	c	O157	++++		c	c	c	c	c	c	c	c	c	c	c	c	c	c	c	c	c	c	c	c	c	c
a	557160	c	517475	a	517477	s	a	Sakai	++++		a	a	a	a	a	a	a	a	a	a	a	a	a	a	a	a				a	a	a
g	557388	a	517703	a	517705	i	g	O157	++++		g	g	g	g	g	g	g	g	g	g	g	g	g	g	g	g				g	g	g
c	557571	t	517886	t	517888	i	c	O157	+		c	c	c	c	c	c	t	t	t	t	t	t	t	c	c	c	c	c	c	c	c	c
t	561998	a	522313	a	522315	s	t	O157	++++	t	t	t	t	t	t	t	t	t	t	t	t	t	t	t	t	t	t	t	t	t	t	t
t	562235	-	522549	-	522551	ins	-	CB9615	++	-	t	t	t	t	t	t	t	t	t	t	t	t	t	t	t	t	t	t	t	t	t	t
g	562591	t	522905	t	522907	s	g	O157	++++	g	g	g	g	g	g	g	g	g	g	g	g	g	g	g	g	g	g	g	g	g	g	g
a	565354	g	525668	g	525670	s	a	O157	++++	a	a	a	a	a	a	a	a	a	a	a	a	a	a	a	a	a	a	a	a	a	a	a
g	565586	a	525900	a	525902	ns	g	O157	++++	g	g	g	g	g	g	g	g	g	g	g	g	g	g	g	g	g	g	g	g	g	g	g
g	566545	t	526859	t	526861	s	g	O157	++++	g	g	g	g	g	g	g	g	g	g	g	g	g	g	g	g	g	g	g	g	g	g	g
t	569037	g	529351	g	529353	ns	g	CB9615	++++	g	g	g	g	g	g	g	g	g	g	g	g	g	g	g	g	g	g	g	g	g	g	g
-	569665	-	529979	a	529982	ins	-	EDL933	++++	-	-	-	-	-	-	-	-	-	-	-	-	-	-	-	-	-	-	-	-	-	-	-
t	573648	c	533962	c	533965	ns	t	O157	++++	t	t	t	t	t	t	t	t	t	t	t	t	t	t	t	t	t	t	t	t	t	t	t
g	574961	t	535275	t	535278	s	t	CB9615	++++	t	t	t	t	t	t	t	t	t	t	t	t	t	t	t	t	t	t	t	t	t	t	t
a	576982	c	537296	c	537299	ns	c	CB9615	++++	c	t	c	c	c	c	c	c	c	c	c	c	c	c	c	c	c	c	c	c	c	c	c
t	577776	c	538090	c	538093	s	c	CB9615	++	c	c	c	t	c	c	c	t	t	c	c	t	t	t	t	c	t	t	t	t	c	t	t
g	579206	t	539520	t	539523	s	g	O157	++++	a	g	g	g	g	g	g	g	g	g	g	g	g	g	g	g	a	g	g	g	g	g	g
c	579767	t	540081	t	540084	s	c	O157	++	c	c	c	c	c	c	c	c	c	c	c	c	c	c	t	c	t	c	c	c	t	c	c
c	580569	a	540883	a	540886	s	a	CB9615	++++	a	a	a	a	a	a	a	a	a	a	a	a	a	a	a	a	a	a	a	a	a	a	a
c	581046	a	541360	a	541363	i	c	O157	++++	c	c	c	c	c	c	c	c	c	c	c	c	c	c	c	c	c	c	c	c	c	c	c
a	581277	c	541591	c	541594	s	c	CB9615	++++	c	c	c	c	c	c	c	c	c	c	c	c	c	c	c	c	c	c	c	c	c	c	c
t	582089	c	542403	c	542406	ns	c	CB9615	++++	c	c	c	c	c	c	c	c	c	c	c	c	c	c	c	c	c	c	c	c	c	c	c
a	585084	g	545398	g	545401	i	a	O157	++	a	a	a	a	a	g	g	a	g	g	g	g	g	g	a	a	g	a	a	a	a	a	a
a	585114	-	545427	-	545430	ins	-	CB9615	++++	-	-	-	-	-	-	-	-	-	-	-	-	-	-	-	-	-	-	-	-	-	-	-
g	585360	a	545673	a	545676	ns	g	O157	++++	g	g	g	g	g	g	g	g	g	g	g	g	g	g	g	g	g	g	g	g	g	g	g
t	585825	c	546138	c	546141	i	t	O157	++	t	c	c	c	c	c	c	c	c	c	c	c	c	c	c	c	c	c	c	c	c	c	c
t	586838	c	547151	c	547154	i	c	CB9615	++++	c	c	c	c	c	c	c	c	c	c	c	c	c	c	c	c	c	c	c	c	c	c	c
t	588125	g	548438	g	548441	s	t	O157	++++	t	t	t	t	t	t	t	t	t	t	t	t	t	t	t	t	t	t	t	t	t	t	t
c	589771	t	550084	t	550087	ns	c	O157	++++	c	c	c	c	c	c	c	c	c	c	c	c	c	c	c	c	c	c	c	c	c	c	c
g	591759	a	552072	a	552075	ns	g	O157	++++	g	g	g	g	g	g	g	g	g	g	g	g	g	g	g	g	g	g	g	g	g	g	g
t	592869	g	553182	g	553185	s	t	O157	++++	t	a	a	a	t	t	t	t	a	t	t	a	a	a	a	t	a	t	t	t	t	t	t
c	596241	t	556554	t	556557	ns	c	O157	++++	c	c	c	c	c	c	c	c	c	c	c	c	c	c	c	c	c	c	c	c	c	c	c
t	596874	c	557187	c	557190	i	c	CB9615	++++	c	c	c	c	c	c	c	c	c	c	c	c	c	c	c	c	c	c	c	c	c	c	c
t	597641	a	557954	a	557957	i	t	O157	++++	t	t	t	t	t	t	t	t	t	t	t	t	t	t	t	t	t	t	t	t	t	t	t
c	601469	t	561782	t	561785	ns	c	O157	++++	c	c	c	c	c	c	c	c	c	c	c	c	c	c	c	c	c	c	c	c	c	c	c
t	602866	c	563179	c	563182	ns	c	CB9615	++++	c	c	c	c	c	c	c	c	c	c	c	c	c	c	c	c	c	c	c	c	c	c	c
a	604611	g	564924	g	564927	ns	g	CB9615	++++	g	g	g	g	g	g	g	g	g	g	g	g	g	g	g	g	g	g	g	g	g	g	g
g	605051	a	565364	a	565367	ns	g	O157	++++	g	g	g	g	g	g	g	g	g	g	g	g	g	g	g	g	g	g	g	g	g	g	g
a	608858	-	569170	-	569173	del	a	O157	++++	a	a	a	a	a		a	a	a	a	a	a	a	a	a	a	a	a	a	a	a	a	a
a	612541	g	572853	g	572856	ns	a	O157	++++	a	a	a	a	a		a	a	a	a	a	a	a	a	a	a	a	a	a	a	a	a	a
g	615215	a	575527	a	575530	s	g	O157	++++	g	g	g	g	g		g	g	g	g	g	g	g	g	g	g	g	g	g	g	g	g	g
t	618899	g	579211	g	579214	ns	t	O157	++++	t	t	t	t	t		t	t	t	t	t	t	t	t	t	t	t	t	t	t	t	t	t
c	619391	t	579703	t	579706	i	c	O157	+	g				c																		
g	620423	a	580735	a	580738	ns	a	CB9615	++	a				a																		
g	620788	a	581100	a	581103	s	g	O157	++	g				g																		
c	623917	t	584229	t	584232	i	c	O157	++	c				c																		
t	624651	c	584963	c	584966	i	c	CB9615	++	c				c																		
g	625424	t	585736	t	585739	i	g	O157	++	g				g																		
c	625630	t	585942	t	585945	i	c	O157	++	c				c																		
t	625846	g	586158	g	586161	i	t	O157	++	t				c																		
a	627784	g	588096	g	588099	i	g	CB9615	++	g				g																		
c	628728	t	589040	t	589043	i	c	O157	++	c				c																		
c	630138	t	590150	t	590153	i	c	O157	++	c				c																		
c	630503	t	590515	t	590518	i	c	O157	++	c				c																		
a	630893	g	590905	g	590908	i	a	O157	++	a				a																		

O55 and O157 genome site details											Outgroup Strain Details[g]																						
CB9615 base[a]	CB9615 Site[b]	Sakai base[a]	Sakai Site[b]	EDL933 base[a]	EDL933 Site[b]	type[c]	Inferred ancestral base[d]	Lineage inferred to mutate[e]	Support level[f]		D1 Sd197	K-12	HS	ATCC 8739	UMN026	IAI39	SMS 3-5	E2348/69	536	ED1a	CFT073	S88	APEC O1	UTI89	E24377A	IAI1	SE11	F5 8401	F2a 2457T	F2a 301	SS Ss046	B4 Sb227	B18 BS512
c	631837	t	591849	t	591852	i	c	O157	++		c				c																		
c	631945	t	591957	t	591960	i	c	O157	++		c				c																		
a	632912	g	592924	g	592927	i	g	CB9615	++		g				g																		
t	633466	g	593478	g	593481	i	t	O157	++		t				t																		
c	635889	t	595901	t	595904	i	c	O157	++		c				c																		
g	638274	a	598277	a	597971	ns	g	O157	++		g				g																		
c	639084	c	599087	t	598781	ns	c	EDL933	+						c																		
a	640472	t	600475	t	600169	ns	a	O157	++		a				a																		
t	640746	g	600749	g	600443	ns	t	O157	++		t				t																		
c	640777	t	600780	t	600474	s	t	CB9615	++		t				t																		
t	643932	c	603935	c	603629	ns	c	CB9615	++		c				c																		
a	647451	g	607454	g	607148	i	g	CB9615	++++		g				g	g	g	g				g	g	g									
g	653488	a	613491	a	613185	s	g	O157	++++		g	g	g	g	g		g	g	g	g	g	g	g	g	g	g	g	a	g	g	g	g	g
g	655320	t	615323	t	615017	ns	g	O157	++++		g	g	g	g	g		g	g	g	g	g	g	g	g	g	g	g	g	g	g	g	g	g
a	656759	g	616762	g	616456	i	a	O157	++++			a													a	a	a					a	a
t	657107	c	617110	c	616804	nc	c	CB9615	++++		c	c	t	c											c	c	c				c	c	c
c	657395	t	617398	t	617092	nc	t	CB9615	++		t	c	c	c											c	c	c				c	c	c
g	657780	-	617782	-	617476	ins	-	CB9615	++++		-	-	-	-											-	-	-				-	-	-
g	658211	a	618213	a	617907	nc	g	O157	++++		c	g	g	g											g	g	g				g	c	c
t	658750	c	618752	c	618446	nc	c	CB9615	++++		c	c	c	c											c	c	c				c	c	c
t	659704	t	619706	c	619400	nc	c	EDL933	-		c	c	c	c											c	c	c				c	c	c
t	661509	g	621511	g	621205	i	t	O157	+			t													g	g	t					t	t
c	664354	t	624356	t	624050	s	c	O157	++++		c	c	c	c	c		c	c	c	c	c	c	c	c	c	c	c	c	c	c	c	c	c
c	664765	t	624767	t	624461	s	t	CB9615	++++		t	t	t	t	t		t	t	t	t	t	t	t	t	t	t	t	t	t	t	t	t	t
c	664937	g	624939	g	624633	i	c	O157	++++		c	c	c	c	c		c	c	c	c	c	c	c	c	c	c	c	c	c	c	c	c	c
t	665007	c	625009	c	624703	i	c	CB9615	++		c	g	g	g	g		g	g	g	g	g	g	g	g	a	a	c	g	g	g		g	g
a	666492	g	626494	g	626188	i	a	O157	++++		a	a	a	a	a		a	a	a	a	a	a	a	a	a	a	a	a	a	g		a	a
a	666494	g	626496	g	626190	i	a	O157	++++		a	a	a	a	a		a	a	a	a	a	a	a	a	a	a	a	a	a	a		a	g
a	668603	t	628605	t	628299	ns	a	O157	++++			a	a	a	a		a	a	a		a	a	a	a	a	a	a				a		
c	672541	t	632543	t	632237	ns	c	O157	++++			c	c	c	c	c	c	c	c	c	c	c	c	c	c	c	c				c		
a	672642	t	632644	a	632338	ns	a	Sakai	++++			a	a	a	a	a	a	a	a	a	a	a	a	a	a	a	a				a		
a	673092	c	633094	c	632788	ns	c	CB9615	++++			c	c	c	c	c	c	c	c	c	c	c	c	c	c	c	c				c		
a	674435	g	634437	g	634131	s	a	O157	++++			a	a	a	a	a	a	a	a	a	a	a	a	a	a	a	a						
g	677415	a	637417	a	637111	s	g	O157	++++			g	g	g	g	g	g	g	g	g	g	g	g	g	g	g	g	g	g	g			
a	677670	t	637672	t	637366	ns	a	O157	++++			a	a	a	a	a	a	a	a	a	a	a	a	a	a	a	a	a	a	a			
g	681844	a	641846	a	641540	ns	g	O157	++++		g	g	g	g	g	g	g	g	g	g	g	g	g	g	g	g	g				g	g	g
c	683642	t	643644	t	643338	ns	c	O157	++++		c	c	c	c	c	c	c	c	c	c	c	c	c	c	c	c	c	c	c	c	c	c	c
t	683982	a	643984	a	643678	ns	t	O157	++++		t	c	t	t	t	t	t	t	t	t	t	t	t	t	t	t	c	t	t	t	t	t	t
a	684712	c	644714	c	644408	ns	a	O157	++		a	a	a	a	a	a	a	a	a	a	a	a	a	a	c	a	c	a	a	a	a	a	a
g	685860	a	645862	a	645556	s	g	O157	++++		g	g	g	g	g	g	g	g	g	g	g	g	g	g	g	g	g	g	g	g	g	g	g
a	686819	t	646821	t	646515	s	t	CB9615	++++		t	t	t	t	t	t	t	t	t	t	t	t	t	t	t	t	t	t	t	t	t	t	t
t	688431	c	648433	c	648127	s	c	CB9615	++++		c	c	c	c	c	c	c	c	c	c	c	c	c	c	c	c	c	c	c	c	c	c	c
c	688902	t	648904	c	648598	s	t	Sakai	-		t	c	c	c	c	c	c	c	c	c	c	c	c	c	c	c	c	c	c	c	c	c	c
9	690330	-	650331	-	650025	del	9	O157	++		9	-	9	-	-	-	-								9	9	9	9	9	9	-		
t	690361	c	650354	c	650048	i	c	CB9615	++++		c	c	c	c	c	c	c								c	c	c	c	c	c	c		
c	691651	t	651644	t	651338	s	c	O157	++++		c	c	c	c	c	c	c								c	c	c	c	c	c			
t	691836	g	651829	g	651523	ns	g	CB9615	++++			g	g	g	g	g	g								g	g	g	g	g	g			
c	692304	t	652297	t	651991	ns	c	O157	++++			c	c	c	c	c	c								c	c	c	c	c	c			
g	692684	a	652677	a	652371	ns	a	CB9615	++++			a	a	a	a	a	a								a	a	a						
a	693275	g	653268	g	652962	ns	g	CB9615	++++			g	g	g	g	g	g								g	g	g				g		
t	696482	c	656475	c	656169	s	c	CB9615	++++			c	c	c	c	c	t								c	c	c	c			c	c	c
g	696808	t	656801	t	656495	ns	t	CB9615	++++			t	t	t	t	t	t								t	t	t	t	t	t	t	t	t
t	750817	c	661395	c	661089	s	c	CB9615	++++		c	c		c	c	c	c	c	c	c	c	c	c	c	c	c	c	c	c	c	t		
a	751009	g	661587	g	661281	s	a	O157	++++		a	a		a	a	a	a	a	a	a	a	a	a	a	a	a	a	a	a	a	a		
c	751234	t	661812	t	661506	s	c	O157	++++		c	c		c	c	c	c	c	c	c	c	c	c	c	c	c	c	c	c	c	c		
g	751937	a	662515	a	662209	s	g	O157	++++		g	g		g	g	g	g	g	g	g	g	g	g	g	g	g	g	g	g	g	g		
a	754142	c	664720	c	664414	ns	c	CB9615	++++			c	c	c	c				c	c		c	c	c	c	c	c				c		
g	754331	a	664909	a	664603	ns	g	O157	++++			g	g	g	g				g	g		g	g	g	g	g	g				g		g
a	755049	g	665627	g	665321	s	a	O157	++++		a	a	a	a	a				a	a		a	a	a	a	a	a				a		a
a	755901	c	666479	c	666173	ns	c	CB9615	+																c		c						
c	756288	g	666866	g	666560	ns	g	CB9615	+++					g											g	g	g				g		
c	757073	t	667651	t	667345	i	c	O157	+++					c											c	c	c				c		
-	757295	-	667873	a	667568	ins	?	EDL933	+/-																								
t	757306	c	667884	c	667579	ns	?	O55/O157	+/-																								
c	757436	a	668014	a	667709	ns	?	O55/O157	+/-																								
t	757561	a	668139	a	667834	ns	?	O55/O157	+/-																								
g	759261	t	669839	t	669534	ns	t	CB9615	+++					t											t	t	t				t		
c	759470	a	670048	a	669743	s	c	O157	+++					c											c	c	c				c		
a	759482	t	670060	t	669755	s	t	CB9615	+++					t											t	t	t				t		
c	760069	g	670647	g	670342	ns	c	O157	+++					c											c	c	g				c		
t	760167	g	670745	g	670527	ns	t	O157	+++					t											t	t	t				t		
t	761058	c	671636	c	671418	ns	c	CB9615	+++					c											c	c	c				c		
a	762767	c	673345	c	673127	nc	c	CB9615	+++				c	c											c	c	c				c		
a	763612	g	674190	g	673972	ns	g	CB9615	++++				g	g	g				g			g	g	g	g	g	g				g		
g	765307	a	675885	a	675667	i	a	CB9615	+++				a	a											a	a	a				a		
t	768773	g	679351	g	679133	s	g	CB9615	++++			g	g	g	g	g	g	g	g	g	g	g	g	g	g	g	g	g	g	g	g		
t	769240	c	679818	c	679600	s	c	CB9615	++++			c	t	c	c	c	c	c	c	c	c	c	c	c	c	c	c	c	c	c	c		
c	770381	c	680959	a	680741	ns	c	EDL933	++++			c	c	c	c	c	c	c	c	c	c	c	c	c	c	c	c	c	c	c	c	c	c
a	770955	c	681533	c	681315	s	c	CB9615	++++			c	c	c	c	c	c	c	c	c	c	c	c	c	c	c	c	c	c	c	c	c	c

O55 and O157 genome site details											Outgroup Strain Details[g]																						
CB9615 base[a]	CB9615 Site[b]	Sakai base[a]	Sakai Site[b]	EDL933 base[a]	EDL933 Site[b]	type[c]	Inferred ancestral base[d]	Lineage inferred to mutate[e]		Support level[f]	D1 Sd197	K-12	HS	ATCC 8739	UMN026	IAI39	SMS 3-5	E2348/69	536	ED1a	CFT073	S88	APEC O1	UTI89	E24377A	IAI1	SE11	F5 8401	F2a 2457T	F2a 301	SS Ss046	B4 Sb227	B18 BS512
g	771395	a	681973	a	681755	ns	a	CB9615		++++		a	a	a	a	a	a	a	a	a	a	a	a	a	a	a	a	a	a	a	a	a	a
6	771515	-	682092	-	681874	del	6	O157		++++		6	6	6	6	6	6	6	6	6	6	6	6	6	6	6	6	6	6	6	6	6	6
g	772873	c	683445	c	683227	ns	c	CB9615		++++	c	c	c	c	c	c	c	c	c	c	c	c	c	c	c	c	c	c	c	c	c	c	c
t	773590	c	684162	c	683944	ns	t	O157		++++	t	t	t	t	t	t	t	t	t	t	t	t	t	t	t	t	t	t	t	t	t	t	t
a	774965	g	685537	g	685319	s	g	CB9615		++++	g	g	g	g	g	g	g	g	g	g	g	g	g	g	g	g	g	g	g	g	g	g	g
g	776713	a	687285	a	687067	i	a	CB9615		++++	a	a	a	a	a	a	a	a	a	a	a	a	a	a	a	a	a	a	a	a	a	a	a
c	785690	t	696262	t	696044	s	c	O157		++	c	c	c	c	a	c	a	a	a	a	a	a	a	a	t	t	t	c	c	c	t	c	c
g	786439	a	697011	a	696793	ns	g	O157		++++	g	g	g	g	g	g	g	g	g	g	g	g	g	g	g	g	g	g	g	g	g	g	g
g	786515	t	697087	t	696869	s	g	O157		++++	g	g	g	g	g	g	g	g	g	g	g	g	g	g	g	g	g	g	g	g	g	g	g
g	786560	g	697132	a	696914	s	g	EDL933		++++	g	g	g	g	g	g	g	g	g	g	g	g	g	g	g	g	g	g	g	g	g	g	g
t	786962	c	697534	c	697316	s	c	CB9615		++++	c	c	c	c	c	c	c	c	c	c	c	c	c	c	c	c	c	c	c	c	c	c	c
g	788666	a	699238	a	699020	s	g	O157		++++	g	g	g	g	g	g	g	g	g	g	g	g	g	g	g	g	g	g			g	g	g
a	789140	a	699712	c	699494	i	a	EDL933		++++	a	a	a	a	a	a	a	a	a			a	a	a	a	a	a	a	a	a	a	a	a
t	790204	t	700776	g	700558	s	t	EDL933		++++	t	t	t	t	t	t	t	t	t	t	t	t	t	t	t	t	t	t	t	t	t	t	t
g	791960	a	702532	a	702314	s	g	O157		++++	g	g	g	g	g	g	g	g	g	g	g	g	g	g	g	g	g	g	g	g	g	g	g
c	795273	t	705845	t	705627	ns	c	O157		++++	c	c	c	c	c	c	c	c	c	c	c	c	c	c	c	c	c	c	c	c	c	c	c
c	800328	t	710900	t	710682	ns	c	O157		++++	c	c	c	c	a	c	c	c	c	c	c	c	c	c	c	c	c	c	c	c	c	c	c
t	802986	g	713558	g	713340	i	?	O55/O157		+/-	a	a	a	a	a										a	a	a	a	a	a	a	a	a
c	803769	a	714341	a	714123	s	c	O157		++++	c	c	c	c	c	c	c	c	c	c	c	c	c	c	c	c	c	c	c	c	c	c	c
g	804688	t	715260	t	715042	ns	t	CB9615		++++	t	t	t	t	t	t	t	t	t	t	t	t	t	t	t	t	t	t	t	t	t	t	t
c	807107	t	717679	t	717461	s	c	O157		++	c	t	t	t	t	c	t	c	c	c	c	c	c	c	c	c	c	c	c	c	c	c	c
c	807588	t	718160	t	717942	i	t	CB9615		++++	t	t	t	t	t	t	t	t	t	t	t	t	t	t	t	t	t	t	t	t		t	t
c	813085	t	723657	t	723439	s	c	O157		++++		c	c	c											c	c	c	c	c	c	c	c	c
t	816974	a	727546	a	727328	ns	a	CB9615		++++		a	a	a	a	a	a	a	a	a	a	a	a	a	a	a	a	a	a	a	a	a	a
g	817057	a	727629	a	727411	s	g	O157		++++		g	g	g	g	g	g	g	g	g	g	g	g	g	g	g	g	g	g	g	g	g	g
g	817403	t	727975	t	727757	i	g	O157		++++		g	g	g	g	g	g	g	g	g	g	g	g	g	g	g	g	g	g	g	g	g	g
t	817704	c	728276	c	728058	s	c	CB9615		++++		c	c	c	c	c	c	c	c	c	c	c	c	c	c	c	c	c	c	c	c	c	c
t	818339	g	728911	g	728693	ns	g	CB9615		++++		g	g	g	g	g	g	g	g	g	g	g	g	g	g	g	g	g	g	g	g	g	g
c	819494	a	730066	a	729848	ns	c	O157		++++		c	c	c	c	c	c	c	c	c	c	c	c	c	c	c	c	c	c	c	c	c	c
g	820073	a	730645	a	730427	ns	g	O157		++++		g	g	g	g	g	g	g	g	g	g	g	g	g	g	g	g	g	g	g	g	g	g
c	820229	a	730801	c	730583	ns	c	Sakai		++++		c	c	c	c	c	c	c	c	c	c	c	c	c	c	c	c	c	c	c	c	c	c
g	820415	a	730987	a	730769	s	g	O157		++++		g	g	g	g	g	g	g	g	g	g	g	g	g	g	g	g	g	g	g	a	g	g
g	821138	c	731710	c	731492	ns	g	O157		++++		g	g	g	g	g	g	g	g	g	g	g	g	g	g	g	g	g	g	g	g	g	g
g	821852	a	732424	a	732206	ns	g	O157		++++		g	g	g	g	g	g	g	g	g	g	g	g	g	g	g	g	g	g	g	g	g	g
g	822871	a	733443	a	733225	i	g	O157		++++		g	g	g	g	g	g	g	g	g	g	g	g	g	g	g	g	g	g	g	g	-	g
c	823235	a	733807	a	733589	ns	c	O157		++++		c	c	c	c	c	c	c	c	c	c	c	c	c	c	c	c				c	c	c
c	826364	t	736937	t	736719	s	c	O157		++++		c	c	c	c	c	c	c	c	c	c	c	c	c	c	c	c	c	c	c	c	c	c
c	827006	t	737579	t	737361	i	t	CB9615		+		t	t	c	c	t	t		t	t	t	t	t	t	t	t	t	t	t	t		t	t
a	827125	g	737698	g	737480	i	a	O157		++++		a	a	a	a	a	a		a	a	a	a	a	a	a	a	a	a	a	a		a	a
a	827269	g	737842	g	737624	i	a	O157		++++		a	a	a	a	a	a	g	a	a	a	a	a	a	a	a	a	a	a	a		a	a
t	827310	g	737883	g	737665	i	g	CB9615		++++		g	g	g	g	g	g	g	g	g	g	g	g	g	g	g	g	g	g	g		g	g
t	827948	c	738521	c	738303	i	t	O157		++	t	t	t	t	t	t	t	t	t	t	t	t	t	t	t	t	t	c	c	c	t	t	t
t	828331	t	738904	-	738685	del	t	EDL933		++++	t	t	t	t	t	t	t	t	t	t	t	t	t	t	t	t	t	t	t	t	t	t	t
c	829205	t	739778	t	739559	s	c	O157		++++	a	c	c	c	a	a	a	a	a	a	a	a	a	a	c	c	c	a	a	a	c	c	c
t	834466	a	745039	t	744820	ns	t	Sakai		++++	t	t	t	t	t	t	t	t	t	t	t	t	t	t	t	t	t	t	t	t	t	t	t
a	835047	t	745620	t	745401	ns	t	CB9615		++++	t	t	t	t	t	t	t	t	t	t	t	t	t	t	t	t	t	t	t	t	t	t	t
c	841442	t	752015	t	751796	ns	c	O157		++++	c	c	c	c	c	c	c	c	c	c	c	c	c	c	c	c	c	c	c	c	c	c	c
c	842423	t	752996	t	752777	s	c	O157		++++	c	c	c	c	c	c	c	c	c	c	c	c	c	c	c	c	c	c	c	c	c	c	c
t	844264	c	754837	c	754618	s	c	CB9615		++	c	c	c	c	c	t	t	c	c	c	c	c	c	c	a	c	c	c	c	c	c	c	c
c	847179	t	757752	t	757533	ns	c	O157		++++	c	c		c	c		c								c	c	c	c	c	c	c	c	c
c	850166	t	760739	t	760520	ns	c	O157		++++		c		c	c		c								c	c	c	c	c	c	c		
c	850331	t	760904	t	760685	ns	t	CB9615		++++		t		t	t		t								t	t	t	t	t	t	t	t	t
t	851059	c	761632	c	761413	i	c	CB9615		++++		c		c	c		c								c	c	c	c	c	c	c		c
t	851562	g	762135	g	761916	nc	g	CB9615		++++		g		g	g		g								g	g	g	g	g	g	g		g
t	852623	c	763196	c	762977	ns	c	CB9615		++++		c		c	c		c								c	c	c	c	c	c	c	c	c
c	853437	t	764010	t	763791	ns	c	O157		++++		c		c	c		c								t	c	c	c	c	c	c	c	c
t	859835	c	770408	c	770189	ns	t	O157		++++	t	t	t	t	t	t	t	t	t	t	t	t	t	t	t	t	t	t	t	t	t	t	t
t	860210	c	770783	c	770564	ns	c	CB9615		++++	c	c	c	c	c	c	c	c	c	c	c	c	c	c	c	c	c	c	c	c	c	c	c
c	861656	t	772229	t	772010	ns	c	O157		++++	c	c	c	c	c	c	c	c	c	c	c	c	c	c	c	c	c	c	c	c	c	c	c
g	862343	a	772916	a	772697	s	g	O157		++++	g	g	g	g	g	g	g	g	g	g	g	g	g	g	g	g	g	g	g	g	g	g	g
c	865961	t	776534	t	776315	i	t	CB9615		++++	t	t	t	t	t	t	t	t	t	t	t	t	t	t	t	t	t	t	t	t	t	t	t
g	868989	a	779527	a	779308	i	g	O157		++++	g	g	g	g	g	g	g	g	g	g	g	g	g	g	g	g	g	g	g	g	g	g	g
c	869545	a	780083	a	779864	s	c	O157		++++	c	c	c	c	c	c	c	c	c	c	c	c	c	c	c	c	c	c	c	c	c	c	c
g	873280	t	783818	t	783599	i	g	O157		++++	g	g	g	g	g	g	g	g	g	g	g	g	g	g	g	g	g	g	g	g	g	g	g
c	873700	a	784238	a	784019	ns	a	CB9615		++++	a	a	a	a	a	a	a	a	a	a	a	a	a	a	a	a	a	a	a	a	a	a	a
c	876403	t	786941	t	786722	s	c	O157		++++	c	c	c	c	c	c	c	c	c	c	c	c	c	c	c	c	c	c	c	c	c	c	c
g	878259	-	788796	-	788577	del	g	O157		++++	g	g	g	g	g	g	g	g	g	g	g	g	g	g	g	g	g	g	g	g	g	g	g
t	878266	c	788803	c	788584	ns	c	CB9615		++++	c	c	c	c	c	c	c	c	c	c	c	c	c	c	c	c	c	c	c	c	c	c	c
c	878657	t	789194	c	788975	s	c	Sakai		++++	c	c	c	c	c	c	c	c	c	c	c	c	c	c	c	c	c	c	c	c	c	c	c
-	880143	-	790680	g	790462	ins	-	EDL933		++++	-	-	-	-	-	-	-	-	-	-	-	-	-	-	-	-	-	-	-	-	-	-	-
-	880206	-	790743	g	790526	ins	-	EDL933		++++	-	-	-	-	-	-	-	-	-	-	-	-	-	-	-	-	-	-	-	-	-	-	-
a	881236	t	791773	t	791556	s	a	O157		++++	a	a	a	a	a	a	a	a	a	a	a	a	a	a	a	a	a	a	a	a	a	a	a
c	882030	g	792567	g	792350	ns	c	O157		++++	c	c	c	c	c	c	c	c	c	c	c	c	c	c	c	c	c	c	c	c	c	c	c
a	884069	c	794606	c	794389	ns	a	O157		++++	a	a	a	a	a	a	a	a	a	a	a	a	a	a	a	a	a	a	a	a	a	a	a
c	884451	t	794988	t	794771	s	c	O157		++++	c	c	c	c	c	c	c	c	c	c	c	c	c	c	c	c	c	c	c	c	c	c	c
a	886808	g	797345	g	797128	s	g	CB9615		++++	t	g	t	g	t	g	g	g	g	g	g	g	g	g	g	g	g	g	g	g	g	g	t
t	888855	c	799392	c	799175	i	c	CB9615		++++			c						c	c	c	c	c	c	c	c	c	c	c	c	c		
c	891647	t	802184	t	801967	ns	c	O157		++++	c	c	c	c	c	c	c	c	c	c	c	c	c	c	c	c	c	c	c	c	c	c	c
c	892315	t	802852	t	802635	s	c	O157		++++	c	c	c	c	c	c	c	c	c	c	c	c	c	c	c	c	c	c	c	c	c	c	c

O55 and O157 genome site details										Outgroup Strain Details[g]																						
CB9615 base[a]	CB9615 Site[b]	Sakai base[a]	Sakai Site[b]	EDL933 base[a]	EDL933 Site[b]	type[c]	Inferred ancestral base[d]	Lineage inferred to mutate[e]	Support level[f]	D1 Sd197	K-12	HS	ATCC 8739	UMN026	IAI39	SMS 3-5	E2348/69	536	ED1a	CFT073	S88	APEC O1	UTI89	E24377A	IAI1	SE11	F5 8401	F2a 2457T	F2a 301	SS Ss046	B4 Sb227	B18 BS512
c	892318	t	802855	t	802638	s	c	O157	++	c	c	c	c	c	c	c	t	t	t	t	t	t	t	c	c	c	t	t	t	c	c	c
c	896542	t	807079	t	806862	s	c	O157	++++	c	c	c	c	c	c	c	c	c	c	c	c	c	c	c	c	c	c	c	c	c	c	c
a	896878	g	807415	g	807198	i	g	CB9615	++	g	a	a	a	g	g	g	g	g	g	g	g	g	g	a	a	a	g	g	g	g	g	g
t	897278	g	807815	g	807598	i	g	CB9615	++++	g	g	g	g	g	g	g	a	g	g	g	g	g	g	g	g	g				g	g	g
a	897471	-	808007	-	807790	del	a	O157	++++	a	a	a	a											a	a	a				-	-	-
c	897532	t	808068	t	807851	i	c	O157	++	c	c	c	t											c	c	t				c	t	t
g	897625	c	808161	c	807944	i	g	O157	++	g	g	g	g											g	g	c				c	g	c
c	897675	g	808211	g	807994	i	c	O157	++	c	g	c	g											c	c	g				c	g	g
t	898304	c	808840	c	808623	i	c	CB9615	++++	c	c	c	c											c	c	c				c	c	c
c	899287	c	809823	t	809606	i	c	EDL933	++++	c	c	c	c											c	c	c				c	c	c
g	899511	a	810047	a	809830	i	a	CB9615	++++	a	a	a	a											a	a	a				a	a	a
-	901691	t	812228	t	813882	ins	-	O157	++++		-	-	-											-	-	-						
g	901936	t	812473	t	814127	ns	t	CB9615	++++		t	t	t											t	t	t						
c	902166	t	812703	c	814357	s	c	Sakai	++++		c	c	c											c	c	c						
a	902658	g	813195	g	814849	i	a	O157	+				a																			
a	902798	c	813335	c	814989	i	c	CB9615	+				c																			
a	902830	-	813366	-	815020	ins	-	CB9615	+				-																			
a	903672	g	814208	g	815862	i	a	O157	+				a																			
g	905707	c	816243	c	817897	ns	g	O157	++++	g	g	g	g	g	g	g	g	g	g	g	g	g	g	g	g	g	g	g	g	g	g	g
t	908396	c	818932	c	820586	ns	c	CB9615	++++	c	c	c	c	c	c	c	c	c	c	c	c	c	c	c	c	c	c	c	c	c	c	c
c	911565	t	822101	t	823755	s	c	O157	++++	c	c	c	c	c	c	c	c	c	c	t	c	c	c	c	c	c	c	c	c	c	c	c
t	912916	g	823452	g	825106	ns	t	O157	++++	t	t	t	t	t	t	t	t	t	t	t	t	t	t	t	t	t	t	t	t	t	t	t
g	914500	t	825036	t	826690	ns	t	CB9615	++++	t	t	t	t	t										t	t	t	t	t	t	t		t
t	915318	g	825854	g	827508	ns	t	O157	++++	t	t	t	t	t										t	t	t	t	t	t	t		t
c	916121	t	826657	t	828311	ns	c	O157	++++	c	c	c	c	c										c	c	c	c	c	c	c		c
t	916869	c	827405	c	829059	ns	c	CB9615	++++	c	c	c	c	c										c	c	c	c	c	c	c		c
a	917023	g	827559	g	829213	s	a	O157	++++	a	a	a	a	a										a	a	a	a	a	a	a		a
c	917398	t	827934	t	829588	s	c	O157	++	c	c	t	t	t										t	t	t	t	t	t	t		t
g	923458	t	833994	t	835648	s	g	O157	++++	g	g	g	g	g	g	g	g	g	g	g	g	g	g	g	g	g	g	g	g	g	g	g
t	925663	c	836199	c	837853	s	c	CB9615	++++	c	c	c	c	c	c	c	c	c	c	c	c	c	c	c	c	c	c	c	c	c	c	c
-	933088	c	843625	c	845279	del	c	CB9615	+					c	c	c																
a	935650	g	846247	g	847901	s	g	CB9615	+					g	g	g																
t	935664	c	846261	c	847915	ns	c	CB9615	+					c	c	c																
a	942723	g	853320	g	854974	i	a	O157	+++	a				a	a	a	a															
a	942938	t	853535	t	855189	i	t	CB9615	+++	t				t	t	t	t															
t	943839	c	854436	c	856090	nc	c	CB9615	+++	c				c	c	c	c															
-	943855	t	854453	t	856107	del	t	CB9615	+++	t				t	t	t	t															
c	944275	t	854873	t	856527	nc	c	O157	+++	c				c	c	c	c															
a	946770	g	857368	g	859022	ns	g	CB9615	++++	g	g	g	g	g	g	g	g	g	g	g	g	g	g	g	g	g	g	g	g	g	g	g
a	951871	g	862388	g	864042	ns	g	CB9615	++++	g	g	g	g	g	g	g	g	g	g	g	g	g	g	g	g	g	g	g	g	g	g	g
t	954619	g	865136	g	866790	nc	t	O157	++++			t	t	t	t	t	t	t	t	t	t	t	t					t				t
t	954824	g	865341	g	866995	nc	t	O157	++++		t	t	t	t	t	t	t	t	t	t	t	t	t	t	t	t		t				t
t	955899	c	866307	c	867961	ns	c	CB9615	++++	c	c	c	c	c	c	c	c	c	c	c	c	c	c	c	c	c	c	c	c	c	c	c
c	956193	a	866601	a	868255	ns	a	CB9615	++++	a	a	a	a	a	a	a	a	a	a	a	a	a	a	a	a	a	a	a	a	a	a	a
g	957155	t	867563	t	869217	s	g	O157	++++	g	g	g	g	g	g	g	a	g	g	g	g	g	g	g	g	g	g	g	g	g	g	g
6	958265	6	868673	-	870326	del	6	EDL933	++++	6	6	6	6	6	6	6	6	6	6		6	6	6	6	6	6	6	6	6	6	6	6
c	959012	-	869419	-	871067	ins	-	CB9615	++++	-	-	-	-	-	-	-	-	-	-	-	-	-	-	-	-	-	-	-	-	-	-	-
a	961804	g	872211	g	873859	ns	g	CB9615	++++	g	g	g	g	g	g	g	g	g	g	g	g	g	g	g	g	g	g	g	g	a	g	g
t	963826	c	874233	c	875881	s	c	CB9615	++	c	c	c	c	c	t	t	t	t	c	t	t	t	t	c	c	c	c	c	c	c	c	c
g	965503	a	875910	a	877558	s	g	O157	++++	g	g	g	g	g	g	g	g	g	g	g	g	g	g	g	g	g	g	g	g	g	g	g
g	965619	a	876026	a	877674	i	g	O157	++	g	a	a	a	a	a	a	a	a	a	a	a	a	a	a	a	a	a	a	a	a	a	a
c	969154	t	879561	t	881209	s	c	O157	++++	c	c	c	c	c	c	c	c	c	c	c	c	c	c	c	c	c	c	c	c	c	c	c
t	969444	c	879851	c	881499	s	c	CB9615	++++	c	c	c	c	c	c	c	c	c	c	c	c	c	c	c	c	c	c	c	c	c	c	c
g	971566	t	881973	t	883621	ns	g	O157	++++	g	g	g	g	g	g	g	g	g	g	g	g	g	g	g	g	g	g	g	g		g	g
g	973442	a	883849	a	885497	s	g	O157	++++	g	g	g	g	g	g	g	g	g	g	g	g	g	g	g	g	g	g	g	g	g	g	g
-	975334	-	885741	a	887390	ins	-	EDL933	++++		-	-	-	-		-	-	-	-	-	-	-	-	-	-	-	-	-	-			-
t	976003	c	886410	c	888059	s	c	CB9615	++++		c	c	c	c		c	c	c	c	c	c	c	c	c	c	c	c	c	c			c
a	977223	g	887630	g	889279	s	g	CB9615	++++		g	g	g	g	g	g	g	g	g	g	g	g	g	g	g	g	g	g	g	g	g	g
c	977920	t	888327	t	889976	s	c	O157	++++		c	c	c	c	c	c	c	c	c	c	c	c	c	c	c	c	c	c	c	c	c	c
g	978266	a	888673	a	890322	ns	a	CB9615	++++		a	a	a	a	a	a	a	a	a	a	a	a	a	a	a	a	a	a	a	a	a	a
a	978508	g	888915	g	890564	ns	a	O157	++++		a	a	a	a	a	a	a	a	a	a	a	a	a	a	a	a	a	a	a	a	a	a
a	978726	c	889133	c	890782	ns	a	O157	++++		a	a	a	a	a	a	a	a	a	a	a	a	a	a	a	a				a	a	a
a	980592	g	890999	g	892648	s	g	CB9615	++++		g	g	g	g	g	g	g	g	g	g	g	g	g	g	g	g	g	g	g	g	g	g
-	981570	-	891977	a	893627	ins	-	EDL933	++++				-	-	-		-							-	-		-	-	-	-		
4	981795	-	892201	-	893851	del	4	O157	+++				4	4			4							4	4							
g	982549	a	892952	g	894602	s	g	Sakai	+															g						g		
c	982620	t	893023	t	894673	ns	c	O157	+															c						c		
t	984527	t	894930	g	896580	s	g	EDL933	-	g	g		g	g	g			g	g	g	g	g	g	g	g	g						
a	985295	g	895698	g	897348	s	g	CB9615	++++	g			g	g	g			g	g	g	g	g	g	g	g	g						
t	985600	c	896003	c	897653	ns	c	CB9615	++++	c			c	c	c			c	c	c	c	c	c	c	c	c						
a	993306	g	896277	g	897927	s	a	O157	+								a															
g	993418	a	896389	a	898039	ns	g	O157	+								g															
a	993698	c	896669	c	898319	ns	a	O157	+										a													
c	994105	c	897076	-	898725	del	?	EDL933	+/-																							
g	994184	g	897155	-	898803	del	?	EDL933	+/-																							
t	994203	t	897174	-	898821	del	?	EDL933	+/-																							
t	995731	c	898702	c	900349	ns	c	CB9615	+								c															
a	995939	g	898910	g	900557	i	a	O157	+								a															
t	995981	c	898952	c	900599	i	c	CB9615	+								c															

O55 and O157 genome site details										Outgroup Strain Details[g]																						
CB9615 base[a]	CB9615 Site[b]	Sakai base[a]	Sakai Site[b]	EDL933 base[a]	EDL933 Site[b]	type[c]	Inferred ancestral base[d]	Lineage inferred to mutate[e]	Support level[f]	D1 Sd197	K-12	HS	ATCC 8739	UMN026	IAI39	SMS 3-5	E2348/69	536	ED1a	CFT073	S88	APEC O1	UTI89	E24377A	IAI1	SE11	F5 8401	F2a 2457T	F2a 301	SS Ss046	B4 Sb227	B18 BS512
t	996083	-	899053	-	900700	del	t	O157	+								t															
-	996158	g	899129	g	900776	del	g	CB9615	+								g															
-	997085	-	900056	t	901704	indel	?	Sakai/EDL933	+/-								-					t										
t	997114	-	900084	-	901732	ins	-	CB9615	+								-					-										
t	999104	c	902073	c	903721	ns	t	O157	++++		t			t	t		t	t		t	t	t	t	t	t	t	t	t		t		
t	999601	a	902570	a	904218	ns	t	O157	++++	t	t			t	t		t	t		t	t	t	t	t	t	t	t	t	t	t		
a	1000575	g	903544	g	905192	ns	g	CB9615	+++					a	g		g				c	c	g				g					
c	1002051	t	905020	t	906668	ns	t	CB9615	+++					t	t		t				t	t	t									
g	1004630	c	907599	c	909247	ns	c	CB9615	+++					c	c		c				c	c	c									
g	1004654	a	907623	a	909271	ns	g	O157	+++					g	g		g				g	g	a									
-	1004659	t	907629	t	909277	ins	-	O157	+++					-	-		-				-	-	-									
c	1005278	a	908248	a	909896	nc	c	O157	+++					c	c		c				c	c	c									
t	1006082	c	909052	c	910700	nc	c	CB9615	+++					c	c		c				c	c	c									
t	1007903	g	910873	g	912521	ns	t	O157	++++					t	t		t				t	t	t				t	t	t			
c	1009782	t	912752	t	914400	s	c	O157	++++					c	c		c				c	c	c				c	c	c		c	
t	1011066	c	914036	c	915684	s	c	CB9615	++++					c	c		c				c	c	c				c	c	c		c	
c	1012126	a	915096	a	916744	ns	a	CB9615	++++					c	a		a				a	a	a				a	a	a		a	
a	1012250	g	915220	g	916868	ns	a	O157	++++					a	a		a				a	a	a				a	a	a		a	
t	1013262	g	916232	g	917880	ns	g	CB9615	++++					g	g		g				g	g	g				g	g	g	g	g	
a	1013575	c	916545	c	918193	ns	c	CB9615	++++				c	c	c		c	c		c	c	c	c		c	c	c	c	c	c	c	
a	1015896	g	918866	g	920514	s	g	CB9615	+				g	g	g	a	g	g	g	g	a	a	g		g	g	g	g	g	g	a	g
-	1016356	-	919326	a	920975	ins	-	EDL933	++++				-	-	-	-	-	-	-	-	-	-	-		-	-	-	-	-	-	-	-
-	1016361	-	919331	c	920981	ins	-	EDL933	++++				-	-	-	-	-	-	-	-	-	-	-		-	-	-	-	-	-	-	-
-	1016366	-	919336	t	920987	ins	-	EDL933	++++				-	-	-	-	-	-	-	-	-	-	-		-	-	-	-	-	-	-	-
a	1016982	g	919952	g	921603	s	g	CB9615	+			g	g	g	g	a	a	g	c	g	g	g	g		g	g	g	g	g	g	g	g
t	1020862	t	923831	c	925482	s	?	EDL933	+/-								t														c	
-	1024463	g	927433	g	929084	ins	-	O157	+								-															
a	1025582	t	928552	t	930203	i	t	CB9615	+								t															
c	1025667	g	928637	g	930288	ns	c	O157	+								c															
c	1026110	g	929080	g	930731	ns	c	O157	+								c															
a	1026181	g	929151	g	930802	s	g	CB9615	+								g															
g	1026207	a	929177	a	930828	ns	g	O157	+								g															
t	1026718	c	929688	c	931339	i	c	CB9615	++++	c			g	c	c		c							c	t			c	c	c		
-	1026780	-	929750	t	931402	ins	-	EDL933	++++	-	-	-	-	-	-	-	-	-	-	-	-	-	-	-	-	-	-	-	-	-	-	-
c	1029070	t	932040	t	933692	ns	c	O157	++++	c	c	c	c	c	c	c	c	c	c	c	c	c	c	c	c	c	c	c	c	c	c	c
c	1030905	t	933875	t	935527	s	c	O157	++++	c	c	c	c	c	c	c	c	c	c	c	c	c	c	c	c	c	c	c	c	c	c	c
t	1032276	g	935246	g	936898	ns	t	O157	++++	t	t	t	t	t	t	t	t	t	t	t	t	t	t	t	t	t	t	t	t	t	t	t
-	1032378	-	935348	g	937001	ins	-	EDL933	++++	-	-	-	-	-	-	-	-	-	-	-	-	-	-	-	-	-	-	-	-	-	-	-
c	1032603	a	935573	c	937226	i	c	Sakai	++++	c	c	c	c	c	c	c	c	c	c	c	c	c	c	c	c	c	c	c	c	c	c	c
t	1036237	g	939207	g	940860	i	t	O157	++++	t	t	t	t	t	t	t	t	t	t	t	t	t	t	t	t	t	t	t	t	t	t	t
g	1037023	a	939993	a	941646	s	a	CB9615	++++	a	a	a	a	a	a	a	a	a	a	a	a	a	a	a	a	a	a	a	a	a	a	a
a	1038537	g	941507	g	943160	ns	g	CB9615	++++	g	g	g	g	g	g	g	g	g	g	g	g	g	g	g	g	g	g	g	g	g	g	g
g	1038922	t	941892	t	943545	ns	g	O157	++++	g	g	g	g	g	g	g	g	g	g	g	g	g	g	g	g	g	g	g	g	g	g	g
g	1038978	a	941948	a	943601	ns	g	O157	++++	g	g	g	g	g	g	g	g	g	g	g	g	g	g	g	g	g	g	g	g	g	g	g
c	1046452	t	949422	t	951075	s	c	O157	++++	c	c	c	c	c	c	c	c	c	c	c	c	c	c	c	c	c	c	c	c	c	c	c
c	1048669	g	951639	g	953292	ns	g	CB9615	++++	g	g	g	g	g	g	g	g	g	g	g	g	g	g	g	g	g	g	g	g	g	g	g
a	1051769	g	954739	g	956392	i	g	CB9615	++	g					g																	
t	1051786	c	954756	c	956409	i	t	O157	++	t					c																	
t	1053247	c	956217	c	957870	ns	c	CB9615	+						c																	
a	1054190	g	957160	g	958813	ns	g	CB9615	++	g					g																	
a	1054323	c	957293	c	958946	i	c	CB9615	++	c					c																	
g	1054946	a	957916	a	959569	ns	a	CB9615	++++	a	a	a	a	a	a	a	a	a	a	a	a	a	a	a	a	a	a	a	a	a	a	a
g	1055628	t	958598	t	960251	s	g	O157	++++	g	g	g	g	g	g	g	g	g	g	g	g	g	g	g	g	g	g	g	g	g	g	g
-	1057732	-	960702	t	962356	ins	-	EDL933	++++	-	-	-	-	-	-	-	-	-	-	-	-	-	-	-	-	-	-	-	-	-	-	-
c	1058153	t	961123	t	962777	s	t	CB9615	++	t	c	c	c	t	t	c	t	c	c	c	c	c	c	c	c	c	t	t	t	c	t	t
c	1058306	g	961276	g	962930	s	c	O157	++++	c	c	c	c	c	c	c	c	c	c	c	c	c	c	c	c	c	c	c	c	c	c	c
g	1058585	g	961555	t	963209	s	g	EDL933	++++	g	g	g	g	g	g	g	g	g	g	g	g	g	g	g	g	g	g	g	g	g	g	g
-	1058976	-	961946	c	963601	ins	-	EDL933	++++	-	-	-	-	-	-	-	-	-	-	-	-	-	-	-	-	-	-	-	-	-	-	-
t	1059011	a	961981	a	963636	i	t	O157	++++	t	t	t	t	t	t	t	t	t	t	t	t	t	t	t	t	t	t	t	t	t	t	t
-	1059941	t	962912	t	964567	ins	-	O157	++++	-	-	-	-	-	-	-	-	-	-	-	-	-	-	-	-	-	-	-	-	-	-	-
c	1059954	t	962926	t	964581	i	c	O157	++++	c	c	c	c	c	c	c	c	c	c	c	c	c	c	c	c	c	c	c	c	c	c	c
-	1060093	a	963066	a	964721	ins	-	O157	++++	-	-	-	-	-	-	-	-	-	-	-	-	-	-	-	-	-	-	-	-	-	-	-
g	1060862	t	963843	t	965498	s	g	O157	++++	g	g	g	g	g	g	g	g	g	g	g	g	g	g	g	g	g	g	g	g	g	g	g
c	1061850	t	964831	t	966486	s	c	O157	++++	c	c	c	c	c	c	c	c	c	t	c	c	c	c	c	c	c	c	c	c	c	c	c
g	1062051	a	965032	a	966687	s	g	O157	++++	g	g	g	g	g	g	g	g	g	g	g	g	g	g	g	g	g	g	g	g	g	g	g
c	1064402	t	967383	t	969038	ns	c	O157	++++	c	c	c	c	c	c	c	c	c	c	c	c	c	c	c	c	c	c	c	c	c	c	c
a	1064488	g	967469	g	969124	s	a	O157	++	a	a	a	a	a	a	a	a	g	g	a	g	g	g	a	a	a	a	a	a	a	a	a
t	1066137	c	969118	c	970773	ns	t	O157	++++	t	t	t	t	t	t	t	t	t	t	t	t	t	t	t	t	t	t	t	t	t	t	t
-	1068578	-	971559	a	973215	ins	-	EDL933	++++	-	-	-	-	-	-	-	-	-	-	-	-	-	-	-	-	-	-	-	-	-	-	-
c	1069482	c	972463	a	974119	i	c	EDL933	++++	c	c	c	c	c	c	c	c	c	c	c	c	c	c	c	c	c	c	c	c	c	c	c
a	1071861	t	974842	t	976498	s	t	CB9615	++++	t	t	t	t	t	t	t	t	t	t	t	t	t	t	t	t	t	t	t	t	t	t	t
g	1073583	g	976564	a	978220	s	g	EDL933	++++	g	g	g	g	g	g	g	g	g	g	g	g	g	g	g	g	t	g	g	g	g	g	g
a	1074877	g	977858	g	979514	ns	g	CB9615	++++	g	g	g	g	g	g	g	g	g	g	g	g	g	g	g	g	g	g	g	g	g	g	g
g	1075272	a	978253	a	979909	ns	g	O157	++++	g	g	g	g	g	g	g	g	g	g	g	g	g	g	g	g	g	g	g	g	g	g	g
-	1077107	-	980088	a	981745	ins	-	EDL933	++++	-	-	-	-	-	-	-	-	-	-	-	-	-	-	-	-	-	-	-	-	-	-	-
c	1077208	t	980189	t	981846	i	c	O157	++++	c	c	c	c	c	c	c	c	c	c	c	c	c	c	c	c	c	c	c	c	c	c	c
g	1078504	c	981485	c	983142	ns	c	CB9615	++++	c	c	c	c	c	c	c	c	c	c	c	c	c	c	t	t	t	t	t	t	t	t	t
t	1078786	c	981767	c	983424	s	c	CB9615	++++	c	c	c	c	c	c	c	c	c	c	c	c	c	c	c	c	c	c	c	c	c	c	c
c	1079499	t	982480	t	984137	ns	c	O157	++++	c	c	c		c	c	c								c	c	c	c	c	c	c	c	c

O55 and O157 genome site details										Outgroup Strain Details[g]																						
CB9615 base[a]	CB9615 Site[b]	Sakai base[a]	Sakai Site[b]	EDL933 base[a]	EDL933 Site[b]	type[c]	Inferred ancestral base[d]	Lineage inferred to mutate[e]	Support level[f]	D1 Sd197	K-12	HS	ATCC 8739	UMN026	IAI39	SMS 3-5	E2348/69	536	ED1a	CFT073	S88	APEC O1	UTI89	E24377A	IAI1	SE11	F5 8401	F2a 2457T	F2a 301	SS Ss046	B4 Sb227	B18 BS512
-	1080413	-	983394	a	985052	ins	-	EDL933	++++	-	-	-		-	-	-								-	-	-	-	-	-	-	-	-
c	1082555	t	985536	t	987194	ns	c	O157	++++	c	c	c	c	c	c	c	c	c	c	c	c	c	c	c	c	c	c	c	c	c	c	c
c	1083329	-	986309	-	987967	del	c	O157	++++	c	c	c	c	c	c	c	c	c	c	c	c	c	c	c	c	c	c	c	c	c	c	c
t	1084204	c	987184	c	988842	ns	c	CB9615	++++	c	c	c	c	c	c	c	c	c	c	c	c	c	c	c	c	c	c	c	c	c	c	c
c	1084477	a	987457	a	989115	ns	c	O157	++++	c	c	c	c	c	c	c	c	c	c	c	c	c	c	c	c	c	c	c	c	c	c	c
g	1084813	a	987793	a	989451	i	a	CB9615	++	a	a	a	a	a	a	a	g	a	a	a	a	a	a	a	a	a	a	a	a	a	g	g
t	1086319	g	989299	g	990957	s	g	CB9615	++++	g	g	g	g	g	g	g	g	g	g	g	g	g	g	g	g	g	g	g	g	g	g	g
t	1087266	c	990246	c	991904	s	c	CB9615	++++	c	c	c	c	c	c	c	c	c	c	c	c	c	c	c	c	c	c	c	c	c	c	c
c	1088267	t	991247	t	992905	ns	c	O157	++++	c	c	c	c	c	c	c	c	c	c	c	c	c	c	c	c	c	c	c	c	c	c	c
t	1088417	c	991397	c	993055	ns	c	CB9615	++++	c	c	c	c	c	c	c	c	c	c	c	c	c	c	c	c	c	c	c	c	c	c	c
a	1088690	g	991670	g	993328	ns	g	CB9615	++++	g	g	g	g	g	g	g	g	g	g	g	g	g	g	g	g	g	g	g	g	g	g	g
t	1089463	c	992443	c	994101	ns	c	CB9615	++++	c	c	c	c	c	c	c	c	c	c	c	c	c	c	c	c	c	c	c	c	c	c	c
c	1089842	t	992822	t	994480	s	c	O157	++++	c	c	c	c	c	c	c	c	c	c	c	c	c	c	c	c	c	c	c	c	c	c	c
c	1092599	g	995579	g	997237	ns	c	O157	++++	c	c	c	c	c	c	c	c	c	c	c	c	c	c	c	c	c	c	c	c	c	c	c
t	1093284	c	996264	c	997922	ns	c	CB9615	++++	c	c	c	c	c	c	c	c	c	c	c	c	c	c	c	c	c	c	c	c	c	c	c
g	1094739	a	997719	a	999377	ns	g	O157	++++		g	g	g	g	g	g	g	g	g	g	g	g	g	g	g	g	g	g	g	g	g	g
g	1095290	a	998270	a	999928	ns	g	O157	++++		g	g	g	g	g	g	g	g	g	g	g	g	g	g	g	g	g	g	g			g
g	1096370	t	999350	t	1001008	ns	g	O157	++++		g	g	g	g	g	g	g	g	g	g	g	g	g	g	g	g	g	g	g	g	g	g
g	1097831	a	1000811	a	1002469	s	g	O157	++++		g	g	g	g	g	g	g	g	g	g	g	g	g	g	g	g	g	g	g	g	g	g
g	1099295	t	1002275	t	1003933	ns	t	CB9615	++++		t	t	t	t	t	t	t	t	t	t	t	t	t	t	t	t	t	t	t	t	t	t
t	1108324	g	1011304	g	1012962	ns	g	CB9615	++++	g	g	g	g	g	g	g	g	g	g	g	g	g	g	g	g	g	g	g	g	g	g	g
c	1108788	t	1011768	t	1013426	ns	t	CB9615	++++	t	t	t	t	t	t	t	t	t	t	t	t	t	t	t	t	t	t	t	t	t	t	t
a	1110546	g	1013526	g	1015184	s	g	CB9615	++++	g	g	g	g	g	g	g	g	g	g	g	g	g	g	g	g	g	g	g	g	g	g	g
g	1112154	a	1015134	a	1016792	i	g	O157	++++	g	g	g	g	g	g	g	g	g	g	g	g	g	g	g	g	g	g	g	g	g	g	g
c	1112285	c	1015265	t	1016923	i	c	EDL933	++++	c	c	c	c	c	c	c	c	c	c	c	c	c	c	c	c	c	c	c	c	c	c	c
-	1112393	-	1015373	c	1017032	ins	-	EDL933	++++	-	-	-	-	-	-	-	-	-	-	-	-	-	-	-	-	-	-	-	-	-	-	-
t	1113392	c	1016372	c	1018031	ns	c	CB9615	++++	c	c	c	c	c	c	c	c	c	c	c	c	c	c	c	c	c	c	c	c	c	c	c
t	1113428	g	1016408	g	1018067	ns	g	CB9615	++++	g	g	g	g	g	g	g	g	g	g	g	g	g	g	g	g	g	g	g	g	g	g	g
a	1114264	g	1017244	g	1018903	i	g	CB9615	++++	g	g	g	g	g	g	g	g	g	g	g	g	g	g	g	g	g	g	g	g	g	g	g
g	1115211	t	1018191	t	1019850	i	g	O157	++++	g	g	g	g	g	g	g	g	g	g	g	g	g	g	g	g	g	g	g	g	g	g	g
t	1115829	c	1018809	c	1020468	i	c	CB9615	++++	c	c	c	c	c	c	c	c	c	c	c	c	c	c	c	c	c	c	c	c	c	c	c
g	1117888	a	1020868	a	1022527	s	g	O157	++++	g	g	g	g	g	g	g	g	g	g	g	g	g	g	g	g	g	g	g	g	g	g	g
a	1118712	g	1021692	g	1023351	ns	a	O157	++++	a	a	a	a	a	a	a	a	a	a	a	a	a	a	a	a	a	a	a	a	a	a	a
a	1119979	g	1022959	g	1024618	ns	a	O157	++++	a	a	a	a	a	a	a	a	a	a	a	a	a	a	a	a	a	a	a	a	a	a	a
g	1120125	a	1023105	a	1024764	s	g	O157	++++	g	g	t	t	g	g	g	g	g	g	g	g	g	g	t	t	t	g	g	g	t	t	t
t	1120325	g	1023305	g	1024964	ns	g	CB9615	++++	g	g	g	g	g	g	g	g	g	g	g	g	g	g	g	g	g	g	g	g	g	g	g
t	1122230	g	1025210	g	1026869	i	t	O157	+					t																		
g	1123758	t	1026738	t	1028397	ns	t	CB9615	+					t																		
g	1124239	a	1027219	a	1028878	ns	g	O157	++	g				g																		
g	1124850	a	1027830	a	1029489	ns	g	O157	++	g				g																		
c	1126043	t	1029023	t	1030682	s	c	O157	++++	c	c	c	c	c	c	c	c	c	c	c	c	c	c	c	c	c	c	c	c	c	c	c
g	1127367	a	1030347	a	1032006	s	g	O157	++++	g	g	g	g	g	g	g	g	g	g	g	g	g	g	g	g	g	g	g	g	g	g	g
c	1129930	t	1032910	t	1034569	ns	c	O157	++++	c	c	c	c	c	c	c	c	c	c	c	c	c	c	c	c	c	c	c	c	c	c	c
-	1130410	-	1033390	g	1035050	ins	-	EDL933	++++	-				-	-	-											-	-	-	-		
t	1130628	c	1033608	c	1035268	i	c	CB9615	++	c				c	c	c											t	t	t	t		
t	1130713	g	1033693	g	1035353	nc	t	O157	+++	a				a	a	a											t	t	t	t		
t	1131140	c	1034120	c	1035780	nc	c	CB9615	++++	c				c	c	c											c	c	c	c		
c	1131296	t	1034276	t	1035936	nc	t	CB9615	++++	t				t	t	t											t	t	t	t		
c	1131422	a	1034402	a	1036062	nc	a	CB9615	++++	a				a	a	a											a	a	a	a		
g	1131759	t	1034739	t	1036399	nc	t	CB9615	++++	t				t	t	t											t	t	t	t		
-	1131929	7	1034910	7	1036570	ins	-	O157	+++	-				-	-	-											7	-	-	-		
c	1134588	t	1037575	t	1039235	s	c	O157	++++	c	c	c	c	c	c	c	c	c	c	c	c	c	c	c	c	c	c	c	c	c	c	c
t	1136870	g	1039857	g	1041517	ns	t	O157	++++	t	t	t	t	t	t	t	t	t	t	t	t	t	t	t	t	t	t	t	t	t	t	t
t	1139385	c	1042372	c	1044032	s	c	CB9615	++++	c	c	c	c	c	c	c	t	c	c	c	c	c	c	c	c	c	c	c	c	c	c	c
c	1139574	a	1042561	a	1044221	s	c	O157	++++	c	c	c	t	c	c	c	c	c	c	c	c	c	c	c	c	c	c	c	c	c	c	c
t	1139855	c	1042842	c	1044502	ns	c	CB9615	++++	c	c	c	c	c	c	c	c	c	c	c	c	c	c	c	c	c	c	c	c	c	c	c
a	1140088	g	1043075	g	1044735	ns	g	CB9615	++++	g	g	g	g	g	g	g	g	g	g	g	g	g	g	g	g	g	g	g	g	g	g	g
t	1140396	g	1043383	g	1045043	ns	g	CB9615	++++	g	g	g	g	g	g	g	g	g	g	g	g	g	g	g	g	g	g	g	g	g	g	g
c	1140671	t	1043658	t	1045318	s	c	O157	++++	c	c	c	c	c	c	c	c	c	c	c	c	c	c	c	c	c	c	c	c	c	c	c
c	1142988	a	1045975	a	1047635	i	c	O157	++	c	c	c	c	c	c	c	a	a	c	a	c	c	c	c	c	c	t	t	t	c	c	c
t	1143104	c	1046091	c	1047751	i	c	CB9615	++++	c								c			c	c	c	c	c		c	c	c			
g	1143184	g	1046171	t	1047831	i	g	EDL933	++++	g								g			g	g	g	g	g		g	g	g			
g	1143186	g	1046173	t	1047833	i	g	EDL933	+++	g								g			g	g	g	g	g		a	a	a			
g	1143187	g	1046174	t	1047834	i	g	EDL933	++++	g								g			g	g	g	g	g		g	g	g			
t	1143375	c	1046362	c	1048022	i	c	CB9615	++++									c			c	c	c	c	c		c	c	c			
c	1144818	t	1047805	t	1049465	s	c	O157	++++	c	c	c	c	c	c	c	c	c	c	c	c	c	c	c	c	c	c	c	c	c	c	c
g	1148972	g	1051959	t	1053619	ns	g	EDL933	++++	g	g	g	g	g	g	g	g	g	g	g	g	g	g	g	g	g	g	g	g	g	g	g
g	1148978	g	1051965	c	1053625	ns	g	EDL933	++++	g	g	g	g	g	g	g	g	g	g	g	g	g	g	g	g	g	g	g	g	g	g	g
-	1153896	c	1056884	c	1058544	del	c	CB9615	++	c	c	-	-					-	-	c				c	c	-	c	c	c	c	c	c
a	1154236	g	1057224	g	1146447	i	a	O157	++++	a	a	a	a	a	a	a	a	a		a				a	a	a	a	a	a	a	a	a
a	1155912	t	1058900	t	1148123	ns	a	O157	++++	a	a	a	a	a	a	a	a	a	a	a	a	a	a	a	a	a	a	a	a	a	a	a
t	1156804	g	1059792	g	1149015	ns	t	O157	++++	t	t	t	t	t	t	t	t	t	t	t	t	t	t	t	t	t	t	t	t	t	t	t
c	1157765	a	1060753	a	1149976	s	c	O157	++++	c	c	c	c	c	c	c	c	c	c	c	c	c	c	c	c	c	c	c	c	c	c	c
a	1158058	g	1061046	g	1150269	ns	g	CB9615	++++	g	g	g	g	g	g	g	g	g	g	g	g	g	g	g	g	g	g	g	g	g	g	g
t	1158847	g	1061835	g	1151058	ns	g	CB9615	++++	g	g	g	g	g	g	g	g	g	g	g	g	g	g	g	g	g	g	g	g	g	g	g
a	1160395	g	1063383	g	1152606	i	g	CB9615	++++	g	g	g	g	g	g	g	g	g	a	g	g	g	g	g	g	g	g	g	g	g	g	g
t	1161628	a	1064616	a	1153839	ns	t	O157	++++	t	t	t	t	t	t	t	t	t	t	t	t	t	t	t	t	t	t	t	t	t	t	t
c	1163334	t	1066322	t	1155545	s	c	O157	++++	c	c	c	c	c	c	c	c	c	c	c	c	c	c	c	c	c	c	c	c	c	c	c
g	1165321	t	1068309	t	1157532	ns	g	O157	++++	g	g	g	g	g	g	g	g	g	g	g	g	g	g	g	g	g	g	g	g	g	g	g

O55 and O157 genome site details										Outgroup Strain Details[g]																						
CB9615 base[a]	CB9615 Site[b]	Sakai base[a]	Sakai Site[b]	EDL933 base[a]	EDL933 Site[b]	type[c]	Inferred ancestral base[d]	Lineage inferred to mutate[e]	Support level[f]	D1 Sd197	K-12	HS	ATCC 8739	UMN026	IAI39	SMS 3-5	E2348/69	536	ED1a	CFT073	S88	APEC O1	UTI89	E24377A	IAI1	SE11	F5 8401	F2a 2457T	F2a 301	SS Ss046	B4 Sb227	B18 BS512
t	1165450	g	1068438	g	1157661	i	g	CB9615	++++	g	g	g	g	g	g	g	g	g	g	g	g	g	g	g	g	g	g	g	g	g	g	g
t	1166262	c	1069250	c	1158473	ns	c	CB9615	++++	c	c	c	c	c	c	c	c	c	c	c	c	c	c	c	c	c	c	c	c	c	c	c
a	1168158	a	1071146	g	1160369	ns	a	EDL933	++++	a	a	a	a	a	a	a	a	a	a	a	a	a	a	a	a	a	a	a	a	a	a	a
c	1168985	t	1071973	t	1161196	i	t	CB9615	++++	t	t	t	t	t	t	t	t	t	t	t	t	t	t	t	t	t	t	t	t	t	t	t
c	1171629	t	1074617	t	1163840	ns	c	O157	++++	c	c	c	c	c	c	c	c	c	c	c	c	c	c	c	c	c	c	c	c	c	c	c
c	1172252	t	1075240	t	1164463	s	c	O157	++++	c	c	c	c	c	c	c	c	c	c	c	c	c	c	c	c	c	c	c	c	c	c	c
t	1178467	c	1081455	c	1170678	s	c	CB9615	++++	c	c	c	c	c	c	c	c	c	c	c	c	c	c	c	c	c	c	c	c	c	c	c
a	1180804	g	1083792	g	1173015	s	g	CB9615	++++	g	g	g	g	g	g	g	g	g	g	g	g	g	g	g	g	g	g	g	g	g	g	g
g	1183760	a	1086748	a	1175971	ns	a	CB9615	++++	a	a	a	a	a	a	a	a	a	a	a	a	a	a	a	a	a	a	a	a	a	a	a
c	1184354	t	1087342	t	1176565	ns	c	O157	++++	c	c	c	c	c	c	c	c	c	c	c	c	c	c	c	c	c	c	c	c	c	c	c
t	1185822	c	1088810	c	1178033	ns	c	CB9615	++++	c	c	c	c	c	c	c	c	c	c	c	c	c	c	c	c	c	c	c	c	c	c	c
g	1186891	a	1089879	a	1179102	i	a	CB9615	++++		a	a	a	a	a	a	a	a	a	a	a	a	a	a	a	a	a	a	a	a	a	a
a	1187559	t	1090547	t	1179770	ns	t	CB9615	++++	t	t	t	t	t	t	t	t	t	t	t	t	t	t	t	t	t	t	t	t	t	t	t
a	1192110	t	1095098	t	1184321	s	a	O157	++++	a	a	a	a	a	a	a	a	a	a	a	a	a	a	a	a	a	a	a	a	a	a	a
a	1192807	c	1095795	c	1185018	ns	a	O157	++++	a	a	a	a	a	a	a	a	a	a	a	a	a	a	a	a	a	a	a	a	a	a	a
t	1193541	c	1096529	c	1185752	s	c	CB9615	++++	c	c	c	c	c	c	c	c	c	c	c	c	c	c	c	c	c	c	c	c	c	c	c
t	1196698	a	1099686	a	1188909	ns	t	O157	++++	t	t	t	t	t	t	t	t	t	t	t	t	t	t	t	t	t	t	t	t	t	t	t
g	1198705	a	1101693	a	1190916	s	g	O157	++++	g	g	g	g	g	g	g	g	g	g	g	g	g	g	g	g	g	g	g	g	g	g	g
t	1198848	a	1101836	a	1191059	ns	a	CB9615	++++	a	a	a	a	a	a	a	a	a	a	a	a	a	a	a	a	a	a	a	a	a	a	a
a	1201044	g	1104032	g	1193255	s	g	CB9615	++++	g	g	g	g	g	g	g	g	g	g	g	g	g	g	g	g	g	g	g	g	g	g	g
g	1204963	c	1107951	c	1197174	ns	c	CB9615	++++	c	c	c	c	c	c	c	c	c	c	c	c	c	c	c	c	c	c	c	c	c	c	c
a	1206013	g	1109001	g	1198224	ns	g	CB9615	++++	g	g	g	g	g	g	g	g	g	g	g	g	g	g	g	g	g	g	g	g	g	g	g
c	1206249	t	1109237	t	1198460	s	c	O157	++++	c	c	c	c	c	c	c	c	c	c	c	c	c	c	c	c	c	c	c	c	c	c	c
c	1208356	t	1111344	t	1200567	ns	c	O157	++++	c	c	c	c	c	c	c	c	c	c	c	c	c	c	c	c	c	c	c	c	c	c	c
-	1212328	a	1115317	a	1204540	ins	?	EDL933	+/-																							
-	1213855	a	1116845	a	1206068	ins	-	O157	++++	-	-	-	-	-	-	-	-	a	-	-	-	-	-	-	-	-	-	-	-	-	-	-
a	1214573	t	1117563	t	1206786	s	t	CB9615	++++	t	t	t	t	t	t	t	t	t	t	t	t	t	t	t	t	t	t	t	t	t	t	t
g	1215788	t	1118778	t	1208001	ns	t	CB9615	++++	t	t	t	t	t	t	t	t	t	t	t	t	t	t	t	t	t	t	t	t	t	t	t
t	1216472	c	1119462	c	1208685	s	t	O157	++++	t	t	t	t	t	t	t	t	t	c	t	t	t	t	t	t	t	t	t	t	t	t	t
t	1218645	g	1121635	g	1210858	ns	g	CB9615	++++	g	g	g	g	g	g	g	g	g	g	g	g	g	g	g	g	g	g	g	g	g	g	g
c	1219673	a	1122663	a	1211886	s	c	O157	++++	c	c	c	c	c	c	c	c	c	c	c	c	c	c	c	c	c	c	c	c	c	c	c
c	1221099	a	1124089	a	1213312	ns	c	O157	++++	c	c	c	c	c	c	c	c	c	c	c	c	c	c	c	c	c	c	c	c	c	c	c
g	1221362	t	1124352	t	1213575	ns	g	O157	++++	g	g	g	g	g	g	g	g	g	g	g	g	g	g	g	g	g	g	g	g	g	g	g
c	1224126	t	1127116	t	1216339	i	c	O157	++++		c	c	c		c	g								c	c	c	c			c	c	c
-	1224147	4	1127138	4	1216361	del	4	CB9615	++++		4	4	4		4	4								4	4	4	4			4	4	4
c	1225775	t	1128769	t	1217992	nc	t	CB9615	++++		t	t	t		t	t								t	t	t	t	t	t	t		t
c	1227674	t	1130668	t	1219891	nc	c	O157	++++	c	c	c	c		c	c								c	c	c	c	c	c	c		c
c	1229262	t	1132256	t	1221479	ns	c	O157	++++	c	c	c	c		c	c								c	c	c	c	c	c	c		c
t	1229308	c	1132302	c	1221525	ns	c	CB9615	++++	c	c	c	c		c	c								c	c	c	c	c	c	c		c
t	1229795	c	1132789	c	1222012	s	c	CB9615	++++	c	c	c	c		c	c								c	c	c	c	c	c	c		c
c	1230476	t	1133470	t	1222693	s	c	O157	++++	c	c	c	c		c	c								c	c	c	c	c	c	c	c	c
a	1230602	c	1133596	c	1222819	ns	a	O157	++++	a	a	a	a		a	a								a	a	a	a	a	a	a	a	a
t	1230870	-	1133863	-	1223086	del	t	O157	++++	t	t	t	t		t	t								t	t	t	t	t	t	t	t	t
t	1233217	c	1136210	c	1225433	ns	c	CB9615	++++	c	c	c	c	c	c	c	c	c	c	c	c	c	c	c	c	c	c	c	c	c	c	c
t	1234198	c	1137191	c	1226414	i	c	CB9615	++++	c	c	c	c	c										c	c	c	c	c	c	c	c	c
a	1235116	t	1138109	t	1227332	s	a	O157	++++	a	a	a	a	a	a	a	a	a	a	a	a	a	a	a	a	a	a	a	a	a	a	a
c	1237218	t	1140211	t	1229434	s	c	O157	++++	c	c	c	c	c	c	c	c	c	c	c	c	c	c	c	c	c	c	c	c	c	c	c
a	1238514	g	1141507	g	1230730	s	g	CB9615	++++	g	g	g	g	g	g	g	g	g	g	g	g	g	g	g	g	g	g	g	g	g	g	g
a	1240217	g	1143210	g	1232433	ns	a	O157	++++	a	a	a	a	a	a	a	c	a	c	c	c	c	c	a	a	a	a	a	a	a	a	a
g	1246780	a	1149773	a	1238996	i	a	CB9615	++++	a	a	a	a	a	a	a	a	a	a	a	a	a	a	a	a	a	a	a	a	a	a	a
c	1247530	g	1150523	g	1239746	i	c	O157	++++	c	c	c	c	c	c	c	c	c	c	c	c	c	c	c	c	c	c	c	c	c	c	c
c	1247802	t	1150795	t	1240018	ns	c	O157	++++	c	c	c	c	c	c	c	c	c	c	c	c	c	c	c	c	c	c	c	c	c	c	c
c	1248598	c	1151591	t	1240814	s	c	EDL933	++++	c	c	c	c	c	c	c	c	c	c	c	c	c	c	c	c	c	c	c	c	c	c	c
a	1251306	c	1154299	c	1243522	ns	c	CB9615	++++	c	c	c	c	c	c	c	c	c	c	c	c	c	c	c	c	c	c	c	c	c	c	c
t	1256702	a	1159695	a	1248918	ns	a	CB9615	++++	a	a	a	a	a	a	a	a	a	a	a	a	a	a	a	a	a	a	a	a	a	a	a
g	1257691	a	1160684	a	1249907	ns	g	O157	++++	g	g	g	g	g	g	g	g	g	g	g	g	g	g	g	g	g	g	g	g	g	g	g
a	1257704	g	1160697	g	1249920	s	g	CB9615	++++	g	g	g	g	g	g	g	g	g	g	g	g	g	g	g	g	g	g	g	g	g	g	g
a	1257905	t	1160898	t	1250121	i	t	CB9615	++++	t	t	t	t	t	t	t	t	t	t	t	t	t	t	t	t	t	t	t	t	t	t	t
a	1258054	g	1161047	g	1250270	i	a	O157	++++	a	a	a	a	a	a	a	a	a	a	a	a	a	a	a	a	a	a	a	a	a	a	a
-	1258162	-	1161238	g	1250462	ins	-	EDL933	+										-													
-	1258162	t	1164403	K	1253627	ns	t	EDL933	++++		t		t	t			t		t		t	t	t	t	t							t
-	1258162	t	1164430	Y	1253654	ns	t	EDL933	++++		c		c	c			c		c		c	c	c	c	c							c
-	1258162	-	1165861	g	1255086	ins	-	EDL933	+		-		-	-		-	-		g	g	g	g	g	-	-	-						g
a	1258254	g	1210897	g	1295714	i	g	CB9615	++++	g	g	g	g	g	g	g	g	g	g	g	g	g	g	g	g	g	g	g	g	g	g	g
t	1259582	a	1212225	a	1297042	ns	a	CB9615	++++	a	a	a	a	a	a	a	a	a	a	a	a	a	a	a	a	a	a	a	a	a	a	a
t	1263377	c	1216020	c	1300837	ns	c	CB9615	++++	c	c	c	c	c	c	c	c	c	c	c	c	c	c	c	c	c	c	c	c	c	c	c
a	1269374	g	1222017	g	1306834	ns	a	O157	++	a	g	g	g				g							g	g	g	g	g	g	g	g	g
g	1270175	a	1222818	a	1307635	ns	a	CB9615	++++	a	a	a	a				a							a	a	a	a	a	a	a	a	a
t	1274993	c	1227636	c	1312453	ns	c	CB9615	++++	c	c	c	c				c							c	c	c	c	c	c	c	c	c
t	1275427	c	1228070	c	1312887	ns	c	CB9615	++++	c	c	c	c				c							c	c	c	c	c	c	c	c	c
a	1276488	g	1229131	g	1313948	i	g	CB9615	++++	g	g	g	g				g							g	g	g	g	g	g	g	g	g
a	1277801	-	1230443	-	1315260	del	a	O157	++++		a	a	a	a	a	a	a	a	a	a	a	a	a	a	a	a	a	a	a	a	a	a
t	1278057	c	1230699	c	1315516	s	c	CB9615	++++		c	c	c	c	c	c	c	c	c	c	c	c	c	c	c	c	c	c	c	c	c	c
c	1285879	t	1238521	t	1323338	s	c	O157	++++	c	c	c	c	c	c	c	c	c	c	c	c	c	c	c	c	c	c	c	c	c	c	c
g	1286475	a	1239117	a	1323934	s	g	O157	++++	g	g	g	g	g	g	g	g	g	g	g	g	g	g	g	g	g	g	g	g	g	g	g
t	1287283	c	1239925	c	1324742	ns	t	O157	++++	t	t	t	t	t	t	t	t	t	t	t	t	t	t	t	t	t	t	t	t	t	t	t
a	1287419	g	1240061	g	1324878	ns	a	O157	++++	a	a	a	a	a	a	a	a	a	a	a	a	a	a	a	a	a	a	a	a		a	a
a	1289246	g	1241888	g	1326705	s	g	CB9615	++++	g	g	g	g	g	g	g	g	g	g	g	g	g	g	g	g	g	g	g	g	g	g	g
c	1289717	t	1242359	t	1327176	i	c	O157	++++		c	c	c	c	c	c								c	c	c	c	c	c	c	c	

O55 and O157 genome site details										Outgroup Strain Details[g]																						
CB9615 base[a]	CB9615 Site[b]	Sakai base[a]	Sakai Site[b]	EDL933 base[a]	EDL933 Site[b]	type[c]	Inferred ancestral base[d]	Lineage inferred to mutate[e]	Support level[f]	D1 Sd197	K-12	HS	ATCC 8739	UMN026	IAI39	SMS 3-5	E2348/69	536	ED1a	CFT073	S88	APEC O1	UTI89	E24377A	IAI1	SE11	F5 8401	F2a 2457T	F2a 301	SS Ss046	B4 Sb227	B18 BS512
-	1290680	a	1243278	a	1328095	ins	-	O157	++++	-	-	-	-	-	-	-								-	-	-	-	-	-	-		-
a	1291024	g	1243622	g	1328439	ns	a	O157	++++	a	a	a	a	a	a	a	a	a	a	a	a	a	a	a	a	a	a	a	a	a		a
c	1293262	t	1245860	t	1330677	ns	c	O157	++++	c	c	c	c	c	c	c	c	c	c	c	c	c	c	c	c	c	c	c	c	c		
t	1293421	c	1246019	c	1330836	ns	t	O157	++++	t	t	t	t	t	t	t	t	t	c	t	t	t	t	t	t	t	t	t	t	t		
-	1293426	a	1273436	c	1357447	i	a	EDL933	+	g				g			g		a		g	g	g								g	g
-	1293426	t	1295619	c	1379630	ns	t	EDL933	++	t									t													
-	1293426	c	1296281	t	1380292	ns	c	EDL933	++	c									c													
-	1293426	g	1299967	a	1383978	ns	g	EDL933	++	g									g													
-	1293426	c	1304915	a	1388926	ns	?	Sakai/EDL933	+/-																							
-	1293426	a	1308169	c	1391910	ns	a	EDL933	+										a													
t	1293800	-	1309105	-	1392877	del	t	O157	++++	t	t	t	t	t	t	t	t	t	t	t	t	t	t	t	t	t	t	t	t	t		
g	1295631	t	1310870	t	1394642	ns	g	O157	++++	g	g	g	g	g	g	g	g	g	g	g	g	g	g	g	g	g	g	g	g	g		
c	1296179	t	1311418	t	1395190	ns	t	CB9615	++++	t	t	t	t	t	t	t	t	t	t	t	t	t	t	t	t	t	t	t	t	t		
c	1297967	t	1313206	t	1396978	ns	c	O157	++++	c	c	c	c	c	c	c	c	c	c	c	c	c	c	c	c	c	c	c	c	c		
c	1298160	g	1313399	g	1397171	s	c	O157	++++	c	c	c	c	c	c	c	c	c	c	c	c	c	c	c	c	c	c	c	c	c		
c	1298352	t	1313591	t	1397363	s	c	O157	++++	c	c	c	c	c	c	c	c	c	c	c	c	c	c	c	c	c	c	c	c	c		
c	1299586	t	1314825	t	1398597	ns	c	O157	++++	c	c	c	c	c	c	c	c	c	c	c	c	c	c	c	c	c	c	c	c	c	c	c
a	1301611	g	1316850	g	1400622	s	g	CB9615	++++	g	g	g	g	g	g	g	g	g	g	g	g	g	g	g	g	g				g	g	g
a	1301972	t	1317211	a	1400983	ns	a	Sakai	++++	a	a	a	a	a	a	a	a	a	a	a	a	a	a	a	a	a				a	a	a
g	1302302	a	1317541	a	1401313	ns	a	CB9615	++++	a	a	a	a	a	a	a	a	a	a	a	a	a	a	a	a	a				a	a	a
a	1304300	g	1319539	g	1403311	ns	g	CB9615	++++	g	g	g	g	g	g	g	g	g	g	g	g	g	g	g	g	g				g	g	g
a	1304828	g	1320067	g	1403839	i	g	CB9615	++++	g	g	g	g	g	g	g	g	g	g	g	g	g	g	g	g	g				g	g	g
g	1305638	a	1320877	a	1404649	s	a	CB9615	++	a	g	a	a	g	a	g	g	g	g	g	g	g	g	g	g	g				g	g	g
t	1305866	c	1321105	c	1404877	s	c	CB9615	++++	c	c	c	c	c	c	c	c	c	c	c	c	c	c	c	c	c				c	c	c
g	1306310	a	1321549	a	1405321	s	g	O157	++++	g	g	g	g	g	g	g	g	g	g	g	g	g	g	g	g	g				g	g	g
g	1306455	-	1321693	-	1405465	ins	-	CB9615	++	-				-																		
c	1307974	g	1323206	g	1406978	i	g	CB9615	++	g				g																		
-	1308135	t	1323368	t	1407140	ins	-	O157	++	-				-																		
a	1309018	g	1324251	g	1408023	s	g	CB9615	++++		g	g	g	g	g	g	g	g	g	g	g	g	g	g	g	g				g	g	
t	1312062	g	1327295	g	1411067	ns	t	O157	++++		t	t	t	t	t	t	t	t	t	t	t	t	t	t	t	t	t	t	t	t	t	
t	1312561	a	1327875	a	1411647	i	t	O157	++++		t	t	t	t	t	t	t	t	t	t	t	t	t	t	t	t	t	t	t	t	t	
t	1315308	c	1330622	c	1414394	ns	t	O157	++++		t	t	t		t			t		t	t	t	t	t		t	t					
-	1316705	t	1332020	t	1415792	del	t	CB9615	++++		t	t	t		t			t		t	t	t	t	t		t	t					
g	1317032	a	1332347	a	1416119	nc	a	CB9615	++++		a	a	a		a			a		a	a	a	a	a		a	a					
c	1317388	t	1332703	t	1416475	nc	c	O157	++++		c	c	c		c			c		c	c	c	c	c		c	c					
a	1320153	g	1335468	g	1419240	i	a	O157	++++		a	a	a		a			a		a	a	a	a	a		a	a					
t	1320380	c	1335695	c	1419467	i	c	CB9615	+		c	c	c		t			t		t	t	t	t	c		c	c					
t	1320900	c	1336215	c	1419987	nc	c	CB9615	++++		c	c	c		c			c		c	c	c	c	c		c	c					
-	1320994	g	1336310	g	1420082	del	g	CB9615	++++		g	g	g		g			g		g	g	g	g	g		g	g					
c	1323925	t	1339241	t	1423013	ns	?	O55/O157	+/-																							
a	1325205	g	1340521	g	1424293	i	?	O55/O157	+/-																							
a	1326226	g	1341542	g	1425314	s	?	O55/O157	+/-																							
c	1327265	t	1342581	t	1426353	ns	?	O55/O157	+/-																							
c	1328035	t	1343351	t	1427123	ns	?	O55/O157	+/-																							
a	1333298	c	1348614	c	1432386	i	?	O55/O157	+/-																							
t	1337596	c	1352912	c	1436684	s	?	O55/O157	+/-																							
a	1338704	t	1354020	t	1437792	ns	?	O55/O157	+/-																							
g	1338821	t	1354137	t	1437909	ns	?	O55/O157	+/-																							
g	1339221	t	1354537	t	1438309	ns	?	O55/O157	+/-																							
c	1341649	t	1356965	t	1440737	ns	?	O55/O157	+/-																							
a	1342740	c	1358056	c	1441828	ns	?	O55/O157	+/-																							
c	1343015	a	1358331	a	1442103	s	?	O55/O157	+/-																							
c	1343227	c	1358543	t	1442315	ns	?	EDL933	+/-																							
a	1345873	g	1361189	g	1444961	ns	?	O55/O157	+/-																							
g	1349411	a	1364727	a	1448499	s	?	O55/O157	+/-																							
c	1351716	t	1367032	t	1450804	s	?	O55/O157	+/-																							
g	1352529	a	1367845	a	1451617	s	?	O55/O157	+/-																							
a	1354119	g	1369435	g	1453207	s	g	CB9615	++++		g	g	g											g		g	g	g	g			
a	1354282	g	1369598	g	1453370	ns	g	CB9615	++++		g	g	g											g		g	g	g	g			
a	1354521	t	1369837	t	1453609	ns	t	CB9615	++++		t	t	t											t		t	t	t	t			
-	1355153	g	1378729	R	1462501	i	g	EDL933	+						g	g			g													
-	1355153	a	1382877	t	1466649	ns	a	EDL933	++++	a				a				a	a	a	a			a						a		a
-	1355153	g	1383847	a	1467619	ns	g	EDL933	++++	g				g				g	g	g	g			g						g		g
-	1355153	c	1383929	a	1467701	s	c	EDL933	++++	c				c				c	c	t	c			c						c		c
-	1355153	g	1383967	t	1467739	ns	t	Sakai	++++	t				t				t	t	a	t			t						t		t
-	1355153	-	1397842	g	1481615	ins	-	EDL933	+										-													
-	1355153	c	1401274	Y	1485047	ns	c	EDL933	+++			g		c		g	g	c	c	c	g		c								c	c
-	1355153	g	1404441	a	1488214	i	a	Sakai	+											a											a	a
-	1355153	c	1408882	-	1492654	indel	?	Sakai/EDL933	+/-																							
-	1355153	a	1409533	g	1493305	ns	?	Sakai/EDL933	+/-																							
-	1355153	a	1412199	R	1495971	i	a	EDL933	++++																							
-	1355153	a	1418202	R	1501974	i	a	EDL933	+++					a					a	a											a	a
-	1355153	t	1418991	c	1502763	s	c	Sakai	+++					c		c			c	c											c	c
-	1355153	-	1421356	c	1505129	ins	-	EDL933	+										-												-	-
-	1355153	g	1423233	R	1507006	i	g	EDL933	+++						g				g								g	g	g			
-	1355153	-	1424139	W	1507913	ins	-	EDL933	+++										-								-	-	-			
-	1355153	t	1426613	-	1510386	indel	?	Sakai/EDL933	+/-																							
-	1355153	c	1432518	Y	1516291	ns	c	EDL933	++++					c	c	c	c	c	c	c	c	c	c	c			c	c	c	c	c	c
-	1355153	a	1437442	-	1521214	indel	?	Sakai/EDL933	+/-																							

O55 and O157 genome site details										Outgroup Strain Details[g]																							
CB9615 base[a]	CB9615 Site[b]	Sakai base[a]	Sakai Site[b]	EDL933 base[a]	EDL933 Site[b]	type[c]	Inferred ancestral base[d]	Lineage inferred to mutate[e]	Support level[f]	D1 Sd197	K-12	HS	ATCC 8739	UMN026	IAI39	SMS 3-5	E2348/69	536	ED1a	CFT073	S88	APEC O1	UTI89	E24377A	IAI1	SE11	F5 8401	F2a 2457T	F2a 301	SS Ss046	B4 Sb227	B18 BS512
-	1355153	c	1441698	Y	1525470	ns	c	EDL933	++++			c		c		c	c	c	c	c	c		c								c	c
-	1355153	c	1443861	Y	1527633	i	c	EDL933	++++			c		c	c	c	c	c			c	c	c	c								
-	1355153	c	1453300	Y	1537072	ns	c	EDL933	++++	c	c			c	c	c	c	c	c	c	c	c	c	c				c	c			c
-	1355153	c	1453438	Y	1537210	ns	c	EDL933	++++	c	c			c	c	c	c	c	c	c	c	c	c	c				c	c			c
-	1355153	a	1454121	R	1537893	i	a	EDL933	+	t	t			t	t	t	a	t	a	t	t	t	t	t				t	t			t
t	1355368	g	1456933	g	1542018	i	t	O157	++++	t	t	t	t	t	t	t	t	t	t	t	t	t	t	t	t	t	t	t	t	t	t	t
t	1355591	c	1457156	c	1542241	ns	t	O157	++++	t	t	t	t	t	t	t	t	t	t	t	t	t	t	t	t	t	t	t	t	t	t	t
c	1355672	t	1457237	t	1542322	ns	t	CB9615	++++	t	t	t	t	t	t	t	t	t	t	t	t	t	t	t	t	t	t	t	t	t	t	t
c	1356001	t	1457566	t	1542651	ns	c	O157	++++	c	c	c	c	c	c	c	c	c	c	c	c	c	c	c	c	c	c	c	c	c	c	c
a	1360972	g	1462537	g	1547622	i	a	O157	++++		a	a	a	a	a	a	a	a	-	a	a	a	a	a	a	a	a	a	a	a	a	
-	1361098	-	1462663	t	1547749	ins	-	EDL933	++++		-	-	-	-	-	-	-	-	-	-	-	-	-	-	-	-	-	-	-	-	-	
g	1371194	a	1472759	a	1557845	ns	a	CB9615	++++	a	a	a	a	a	a	a	a	a	a	a	a	a	a	a	a	a				a	a	a
a	1372957	t	1474522	t	1559608	ns	a	O157	++++	a	a	a	a	a	a	a	a	a	a	a	a	a	a	a	a	a	a	a	a	a	a	a
c	1374367	g	1475932	g	1561018	i	g	CB9615	++++	g	g	g	g	g	g	g	g	g	g	g	g	g	g	g	g	g	g	g	g	g	g	g
g	1375939	a	1477504	a	1562590	s	g	O157	++++	g	g	g	g	g	g	g	g	g	g	g	g	g	g	g	g	g	g	g	g	g	g	g
c	1376344	t	1477909	t	1562995	s	t	CB9615	++++	t	t	t	t	t	t	t	t	t	t	t	t	t	t	t	t	t	t	t	t	t	t	t
t	1376363	c	1477928	c	1563014	ns	c	CB9615	++++	c	c	c	c	c	c	c	c	c	c	c	c	c	c	c	c	c	c	c	c	c	c	c
a	1378271	g	1479836	g	1564922	i	g	CB9615	++++	g	g	g	g	g	g	g	g	g	g	g	g	g	g	g	g	g	g	g	g	g	g	g
t	1378863	c	1480428	c	1565514	s	c	CB9615	++++	c	c	c	c	c	c	c	c	c	c	c	c	c	c	c	c	c	c	c	c	c	c	c
c	1379105	t	1480670	t	1565756	i	c	O157	++	c	t	t	t	t	t	c	t	t	t	t	t	t	t	t	t	t	t	t	t	t	t	t
a	1381340	g	1482905	g	1567991	s	g	CB9615	++++	g	g	g	g	g	g	g	g	g	g	g	g	g	g	g	g	g	g	g	g	g	g	g
t	1385077	g	1486642	g	1571728	s	g	CB9615	++++	g	g	g	g	g	g	g	g	g	g	g	g	g	g	g	g	g	g	g	g	g	g	g
c	1386372	t	1487937	t	1573023	s	c	O157	++++	c	c	c	c	c	c	c	c	c	c	c	c	c	c	c	c	c	c	c	c	c	c	c
a	1386770	g	1488335	g	1573421	s	g	CB9615	++++	g	g	g	g	g	g	g	g	g	g	g	g	g	g	g	g	g	g	g	g	g	g	g
c	1387885	t	1489450	t	1574536	s	c	O157	++++	c	c	c	c	c	c	c	c	c	c	c	c	c	c	c	c	c	c	c	c	c	c	c
g	1388485	a	1490050	a	1575136	i	g	O157	++++	g	g	g	g	g	g	g	g	g	g	g	g	g	g	g	g	g	g	g	g	g	c	c
t	1392294	c	1493859	c	1578945	s	c	CB9615	++++	c	c	c	c	c	c	c	c	c	c	c	c	c	c	c	c	c	c	c	c	c	c	c
g	1392519	a	1494084	a	1579170	s	g	O157	++++	g	g	g	g	g	g	g	g	g	g	g	g	g	g	g	g	g	g	g	g	g	g	g
t	1393193	c	1494758	c	1579844	s	c	CB9615	++++	c	c	c	c	c	c	c	c	c	c	c	c	c	c	c	c	c	c	c	c	c	c	c
c	1393561	t	1495126	t	1580212	s	t	CB9615	++++	t	t	t	t	t	t	t	t	t	t	t	t	t	t	t	t	t	t	t	t	t	t	t
g	1396548	a	1498113	a	1583199	ns	a	CB9615	++++	a	a	a	a	a	a	a	a	a	a	a	a	a	a	a	a	a	a	a	a	a	a	a
t	1396667	a	1498232	a	1583318	ns	a	CB9615	++++	a	a	a	a	a	a	a	a	a	a	a	a	a	a	a	a	a	a	a	a	a	a	a
a	1396708	g	1498273	g	1583359	s	g	CB9615	++++	g	g	g	g	g	g	g	g	g	t	g	g	g	g	g	g	g	g	g	g	g	g	g
t	1398579	a	1500144	a	1585230	i	t	O157	++	t	t	t	t	t	t	t	t	t	c	t	t	t	t	t	t	t	t	t	t	t	a	a
c	1400337	t	1501902	c	1586988	s	c	Sakai	++++	c	c	a	a	a	c	c	c	c	c	c	c	c	c	c	a	a	a	a	a	a	c	c
g	1403192	a	1504757	a	1589843	ns	a	CB9615	++++	a	a	a	a	a	a	a	a	a	a	a	a	a	a	a	a	a	a	a	a	a	a	a
c	1403319	t	1504884	t	1589970	ns	t	CB9615	++++	t	t	t	t	t	t	t	t	t	t	t	t	t	t	t	t	t	t	t	t	t	t	t
11	1403467	-	1505031	-	1590117	del	11	O157	++++	11	11	11	11	11	11	-	11	11	11	11	11	11	11	11	11	11	11	11	11	11	11	11
t	1403712	c	1505266	t	1590352	ns	t	Sakai	++++	t	t	t	t	t	t	t	t	t	t	t	t	t	t	t	t	t	t	t	t	t	t	t
c	1403981	t	1505535	t	1590621	s	t	CB9615	++++	t	t	t	t	t	t	t	c	t	t	t	t	t	t	t	t	t	t	t	t	t	t	t
c	1408747	t	1510301	t	1595387	s	t	CB9615	++++	t	t	t	t	t	t	t	t	t	t	t	t	t	t	t	t	t	t	t	t	t	t	t
c	1408815	t	1510369	t	1595455	ns	c	O157	++++	c	c	c	c	c	c	c	c	c	c	c	c	c	c	c	c	c	c	c	c	c	c	c
g	1409254	a	1510808	a	1595894	s	g	O157	++++	g	g	g	g	g	g	g	g	g	g	g	g	g	g	g	g	g	g	g	g	g	g	g
a	1410989	g	1512543	g	1597629	ns	g	CB9615	++++	g	g	g	g	g	g	g	g	g	g	g	g	g	g	g	g	g	g	g	g	g	g	g
t	1411348	g	1512902	g	1597988	ns	t	O157	++++	t	t	t	t	t	t	t	t	t	t	t	t	t	t	t	t	t	t	t	t	t	t	t
t	1411746	a	1513300	a	1598386	ns	a	CB9615	++++	a	a	a	a	a	a	a	a	a	a	a	a	a	a	a	a	a	a	a	a	a	a	a
g	1412854	a	1514408	a	1599494	ns	g	O157	++++	g	g	g	g	g	g	g	g	g	g	g	g	g	g	g	g	g	g	g	g	g	g	g
-	1415151	-	1516705	a	1601792	ins	-	EDL933	++++	-	-	-	-	-	-	-	-	-	-	-	-	-	-	-	-	-	-	-	-	-	-	-
-	1419009	9	1520564	9	1605651	del	9	CB9615	++++	9	9	9	9	9	9	9	9	9	9	9	9	9	9	9	9	9	9	9	9	9	9	9
t	1419168	c	1520731	c	1605818	i	t	O157	++	t	t	t	t	c	t	t	c	-	-	-	-	-	-	t	t	t	c	c	c	t	t	t
-	1419183	-	1520746	c	1605834	ins	-	EDL933	++++	-	-	-	-	-	-	-	-	-	-	-	-	-	-	-	-	-	-	-	-	-	-	-
-	1419183	12	1520747	12	1605841	ins	-	O157	++	-	-	-	-	12	-	-	12	-	12	12	12	12	12	-	-	-	12	12	12	-	-	-
-	1419209	-	1520784	g	1605879	ins	-	EDL933	++++	-	-	-	-	-	-	-	-	-	-	-	-	-	-	-	-	-	-	-	-	-	-	-
t	1420962	g	1522537	g	1607632	s	g	CB9615	++++	g	g	g	g	g	g	g	g	g	g	g	g	g	g	g	g	g	g	g	g	g	g	g
c	1422426	t	1524001	t	1609096	ns	c	O157	++++	c	c	c	c	c	c	c	c	c	c	c	c	c	c	c	c	c	c	c	c	c	c	c
-	1423429	-	1525004	t	1610100	ins	-	EDL933	++++	-	-	-	-	-	-	-	-	-	-	-	-	-	-	-	-	-	-	-	-	-	-	-
t	1424402	c	1525977	c	1611073	s	c	CB9615	++	c	c	c	c	c	c	c	c	c	c	c	c	c	c	c	c	c	c	c	c	t	t	t
t	1426328	c	1527903	c	1612999	s	c	CB9615	++++	c	c	c	c	c	c	c	c	c	c	c	c	c	c	c	c	c	c	c	c	c	c	c
t	1427164	g	1528739	g	1613835	ns	g	CB9615	++++	g	g	g	g	g	g	g	g	g	g	g	g	g	g	g	g	g	g	g	g	g	g	g
c	1429196	t	1530771	t	1615867	s	c	O157	++++	c	c	c	c	c	c	c	c	c	c	c	c	c	c	c	c	c	c	c	c	c	c	c
a	1430336	g	1531911	g	1617007	s	a	O157	++	a	a	a	a	a	a	a	g	a	a	g	a	a	a	a	a	a	a	a	a	a	a	a
t	1431302	c	1532877	c	1617973	s	c	CB9615	++++	c	c	c	c	c	c	c	c	c	c	c	c	c	c	c	c	c	c	c	c	c	c	c
g	1431664	a	1533239	a	1618335	ns	g	O157	++++	g	g	g	g	g	g	g	g	g	g	g	g	g	g	g	g	g	g	g	g	g	g	g
t	1431989	a	1533564	a	1618660	ns	a	CB9615	++++	a	a	a	a	a	a	a	a	a	a	a	a	a	a	a	a	a	a	a	a	a	a	a
c	1434711	a	1536286	a	1621382	s	c	O157	++++	c	c	c	c	c	c	c	c	c	c	c	c	c	c	c	c	c	c	c	c	c	c	c
t	1434947	c	1536522	c	1621618	s	c	CB9615	++++	c	c	c	c	c	c	c	c	c	c	c	c	c	c	c	c	c	c	c	c	c	c	c
c	1435586	t	1537161	c	1622257	s	c	Sakai	++++	c	c	c	c	c	c	c	c	c	c	c	c	c	c	c	c	c	c	c	c	c	c	c
a	1436762	c	1538337	c	1623433	ns	a	O157	++++	a	a	a	a	a	a	a	a	a	a	a	a	a	a	a	a	a	a	a	a	a	a	a
t	1438150	g	1539725	g	1624821	ns	t	O157	++++	t	t	t	t	t	t	t	t	t	t	t	t	t	t	t	t	t	t	t	t	t	t	t
c	1439324	t	1540899	t	1625995	s	c	O157	++++	c	c	c	c	c	c	c	c	c	c	c	c	c	c	c	c	c	c	c	c	c	c	c
g	1439902	a	1541477	a	1626573	s	a	CB9615	++++	a	a	a	a	a	g	a	a	a	a	a	a	a	a	a	a	a	a	a	a	a	a	a
-	1439906	t	1545192	c	1630288	i	c	Sakai	+		c																					
-	1439906	c	1547224	Y	1632320	ns	c	EDL933	+					c		c	t		t					t								
-	1439906	g	1547752	R	1632848	ns	g	EDL933	+										g											g		
-	1439906	t	1551043	Y	1636139	ns	t	EDL933	++++	c				c		c	c		c	c	c	c	c	c		c	c	c	c	c	c	c
-	1439906	t	1572104	-	1658242	del	t	CB9615	+++							-	t		t			t									t	t
-	1439906	g	1572385	s	1658523	ns	g	EDL933	+++							g	g		g			g									g	g
-	1439906	c	1572531	-	1658668	ins	-	Sakai	+++						-	-	-		-			-									-	-
-	1439906	t	1576601	-	1660575	indel	?	Sakai/EDL933	+/-																							

O55 and O157 genome site details										Outgroup Strain Details[g]																						
CB9615 base[a]	CB9615 Site[b]	Sakai base[a]	Sakai Site[b]	EDL933 base[a]	EDL933 Site[b]	type[c]	Inferred ancestral base[d]	Lineage inferred to mutate[e]	Support level[f]	D1 Sd197	K-12	HS	ATCC 8739	UMN026	IAI39	SMS 3-5	E2348/69	536	ED1a	CFT073	S88	APEC O1	UTI89	E24377A	IAI1	SE11	F5 8401	F2a 2457T	F2a 301	SS Ss046	B4 Sb227	B18 BS512
-	1439906	-	1579263	a	1663238	indel	?	Sakai/EDL933	+/-																							
-	1439906	a	1579853	M	1663828	ns	a	EDL933	++++																							
-	1439906	-	1582670	c	1666646	indel	?	Sakai/EDL933	+/-																							
-	1439906	t	1584354	k	1668330	ns	t	EDL933	++++																							
-	1439906	t	1584360	k	1668336	ns	t	EDL933	++++																							
-	1439906	a	1584397	M	1668373	ns	a	EDL933	++++																							
t	1440630	c	1590628	c	1674604	i	t	O157	++++	t	t	t	t	t	t	t	t	t	t	t	t	t	t	t	t	t	t	t	t	t	t	t
g	1442094	a	1592092	g	1676068	i	g	Sakai	++++	g	g	g	g	g	g	g	g	g	g	g	g	g	g	g	g	g	g	g	g	g	g	g
t	1442704	c	1592702	c	1676678	s	c	CB9615	++++	c	c	c	c	c	c	c	c	c	c	c	c	c	c	c	c	c	c	c	c	c	c	c
-	1447060	a	1599400	-	1683375	ins	-	Sakai	+										-													-
-	1447060	g	1599419	-	1683393	del	g	EDL933	+										g													g
-	1447060	-	1599494	c	1683469	del	c	Sakai	+										c													c
-	1447060	c	1599684	-	1683658	ins	-	Sakai	+										-													-
-	1447060	c	1599754	-	1683727	ins	-	Sakai	+										-													-
-	1447060	a	1604652	R	1688625	ns	a	EDL933	+++						a	a			a								a	a	a			
-	1447060	c	1607790	a	1691763	ns	a	Sakai	+++						a	a			a								a	a	a			
-	1447060	g	1609025	R	1692998	ns	g	EDL933	++++						g	g			g	g							g	g	g		g	
c	1455894	t	1610142	t	1694115	ns	c	O157	++++	c	c	c	c	c	c	c	c	c	c	c	c	c	c	c	c	c	c	c	c	c	c	c
c	1456271	t	1610519	t	1694492	ns	c	O157	++++	c	c	c	c	c	c	c	c	c	c	c	c	c	c	c	c	c	c	c	c	c	c	c
g	1458375	g	1612623	t	1696596	s	g	EDL933	++++	g	g	g	g	g	g	g	g	g	g	g	g	g	g	g	g	g	g	g	g	g	g	g
t	1458735	c	1612983	c	1696956	s	c	CB9615	++++	c	c	c	c	c	c	c	c	c	c	c	c	c	c	c	c	c	c	c	c	c	c	c
t	1459722	c	1613970	c	1697943	s	c	CB9615	++++	c	c	c	c	c	c	c	c	c	c	c	c	c	c	c	c	c	c	c	c	c	c	c
a	1461718	c	1615966	c	1699939	s	a	O157	+++	a	g	g	g	g	g	g	g	g	g	g	g	g	g	g	g	g	g	g	g	a	a	a
g	1461967	a	1616215	a	1700188	s	a	CB9615	++++	a	a	a	a	a	a	a	a	a	a	a	a	a	a	a	a	a	a	a	a	a	a	a
-	1463903	-	1620422	c	1704396	ins	-	EDL933	++++					-			-	-	-	-	-	-	-	-	-	-						
-	1463903	-	1620575	c	1704550	ins	-	EDL933	++++					-			-	-	-	-	-	-	-	-	-	-						
-	1463903	t	1633414	g	1717389	ns	g	Sakai	++++		t		g	g	g			g		g					g	g				g		
-	1463903	a	1635560	W	1719535	ns	a	EDL933	++++		a		a	a	a			c		a					a	a				a		
-	1463903	a	1635924	m	1719899	s	a	EDL933	++++		a		g	a	a			a		a					a	a				a		
-	1463903	-	1635924	t	1719900	ins	-	EDL933	++++		-		-	-	-			-		-					-	-				-		
-	1463903	c	1635973	y	1719949	i	c	EDL933	+++		c		c	c	c			c		c					t	c				t		
-	1463903	g	1635975	k	1719951	i	g	EDL933	+		g		g	t	t			t		t					t	t				t		
-	1463903	g	1637604	a	1721580	ns	a	Sakai	+++				t	a	a			a		a					t	t				a		
-	1463903	g	1649850	a	1741635	ns	g	EDL933	+		g	g	g	g	a	a	g				g	a	g	g	g	g				g	g	g
-	1463903	a	1657028	c	1748813	ns	c	Sakai	++++	c				c				c	c	c	c			c						c		c
-	1463903	-	1657227	a	1749013	ins	-	EDL933	++++	-				-				-	-	-	-			-						-		-
-	1463903	c	1657284	y	1749070	ns	c	EDL933	++++	c				c				c	c	c	c			c						c		c
-	1463903	-	1657295	a	1749082	ins	-	EDL933	++++	-				-				-	-	-	-			-						-		-
-	1463903	-	1657304	g	1749092	ins	-	EDL933	++++	-				-				-	-	-	-			-						-		-
-	1463903	t	1662250	k	1754044	i	t	EDL933	+													t										
-	1463903	-	1662286	g	1754081	ins	-	EDL933	+													-										
-	1463903	a	1663973	R	1755379	ns	a	EDL933	++++					g	g	a	g	g	a	g	g	g	g	a			a	a	a	a	a	a
-	1463903	g	1664088	R	1755494	ns	g	EDL933	++++					g	g	g	g	g	g	g	g	g	g	g			g	g	g	g	g	g
-	1463903	a	1664318	g	1755724	ns	g	Sakai	++++					g	g	g	g	g	g	g	g	g	g	g			g	g	g	g	g	g
g	1478774	c	1665033	c	1756439	ns	c	CB9615	++++	c	c		c	c		c								c	c						c	c
c	1482710	t	1668969	t	1760375	i	c	O157	++++	c	c	c	c	c	c	c	c	c	c	c	c	c	c	c	c	c	c	c	c	c	c	c
-	1482837	-	1669096	g	1760503	ins	g	EDL933	-	g	g	-	-	g	g	g	g	g	g	g	g	g	g	-	-	-	-	-	-	-	-	-
a	1485267	c	1671526	c	1762933	ns	c	CB9615	++++		c	c	c	c	c	c								c	c	c	c	c	c	c		
c	1489537	t	1675796	t	1767203	s	c	O157	++++	c	c	c	c	c	c	c	c	c	c	c	c	c	c	c	c	c	c	c	c	c	c	c
a	1492633	g	1678925	g	1770332	i	g	CB9615	++++	g	g	g	g	g	g	g	g	g	g	g	g	g	g	g	g	g	g	g	g	g	g	g
t	1492634	-	1678925	-	1770332	ins	-	CB9615	++++	-	-	-	-	-	-	-	-	-	-	-	-	-	-	-	-	-	-	-	-	-	-	-
t	1493359	c	1679650	c	1771057	ns	c	CB9615	++++	c	c	c	c	c	c	c	c	c	c	c	c	c	c	c	c	c	c	c	c	c	c	c
g	1495047	c	1681338	g	1772745	ns	g	Sakai	++	g	g	g	g	g	g	g	c	c	c	c	c	c	c	g	g	g	g	g	g	g	g	g
c	1495048	g	1681339	c	1772746	ns	c	Sakai	++	c	c	c	c	c	c	c	g	g	g	g	g	g	g	c	c	c	c	c	c	c	c	c
g	1495051	a	1681342	g	1772749	ns	g	Sakai	++++	g	g	g	g	g	g	g	-	-	-	-	-	-	-	g	g	g	g	g	g	g	g	g
-	1495068	g	1681360	-	1772766	ins	-	Sakai	++++	-	-	-	-	-	-	-	-	-	-	-	-	-	-	-	-	-	-	-	-	-	-	-
g	1495457	-	1681748	g	1773155	del	g	Sakai	+++	g				g	g	g											g	g	g			
c	1497326	c	1681749	-	1773155	del	c	EDL933	+++	c				c	c	c											c	c	c			
t	1502184	c	1687920	c	1779326	ns	t	O157	++++	t				t	t	t	t	t	t	t	t	t	t				t	t	t			
a	1503084	t	1688820	t	1780226	ns	a	O157	++++	a				a	a	a	a	a	a	a	a	a	a				a	a	a			
a	1503339	t	1689075	t	1780481	ns	t	CB9615	++	t				t	t	t	t	t	t	t	t	t	t				a	a	a			
c	1505153	t	1690889	t	1782295	ns	c	O157	++++	c				c	c	c	c	c	c	c	c	c	c				c	c	c			
c	1510826	t	1696562	t	1787968	i	t	CB9615	++++	t	t	t	t	t	t	t	t	t	t	t	t	t	t	t	t	t	t	t	t		t	t
c	1511100	g	1696836	g	1788242	ns	c	O157	++	c	t	t	t	t	t	t	t	t	t	t	t	t	t	t	t	t	t	t	t		t	t
a	1512932	c	1698668	c	1790074	ns	c	CB9615	++++		c	c	c	c	c	c	c	c	c	c	c	c	c	c	c	c	c	c	c		c	c
t	1513217	c	1698953	c	1790359	ns	?	O55/O157	+/-		g	g	g	g	g	g	g	g	g	g	g	g	g	g	g	g	g	g	g		g	g
a	1519756	g	1702338	g	1793744	i	a	O157	++++	a	a	a	a	a										a	a	a	a	a	a	a		a
a	1521382	g	1703964	g	1795370	s	a	O157	++++	a	a	a	a	a	a	a	a	a	a	a	a	a	a	a	a	a	a	a	a	a	a	a
a	1523864	a	1706446	g	1797852	s	a	EDL933	++++	a	a	a	a	a	a	a	a	a	a	a	a	a	a	a	a	a	a	a	a	a	a	a
g	1528460	g	1711042	c	1802448	ns	g	EDL933	++++	g	g	g	g	g	g	g	g	g	g	g	g	g	g	g	g	g	g	g	g	g	g	g
a	1532258	g	1714840	g	1806246	ns	a	O157	++++	a	a	a	a	a	a	a	a	a	a	a	a	a	a	a	a	a	a	a	a	a	a	a
6	1533179	-	1715760	-	1807695	del	6	O157	++++	6	6	6	6	6	6	6	6	6	6	6	6	6	6	6	6	6	6	6	6	6	6	6
-	1533290	-	1715866	g	1807802	ins	-	EDL933	++++	-	-	-	-	-	-	-	-	-	-	-	-	-	-	-	-	-	-	-	-	-	-	-
c	1540531	a	1723107	a	1815043	ns	c	O157	++++	c	c	c	c	c	c	c	c	c	c	c	c	c	c	c	c	c	c	c	c	c	c	c
c	1540964	t	1723540	t	1815476	s	c	O157	++++	c	c	c	c	c	c	c	c	c	c	c	c	c	c	c	c	c	c	c	c	c	c	c
t	1541385	g	1723961	g	1815897	ns	t	O157	++++	t	t	t	t	t	t	t	t	t	t	t	t	t	t	t	t	t	t	t	t	t	t	t
g	1542230	a	1724806	a	1816742	i	g	O157	++++	g	g	g	g	g	g	g	g	g	g	g	g	g	g	g	g	g	g	g	g	g	g	g
a	1542317	g	1724893	g	1816829	i	g	CB9615	++++	g	g	g	g	g	g	g	g	g	g	g	g	g	g	g	g	g	g	g	g	g	g	g
t	1542331	c	1724907	c	1816843	i	c	CB9615	++	c	t	t	t	t	c	c	t	t	t	t	t	t	t	c	t	t	c	c	c	t	t	t

O55 and O157 genome site details											Outgroup Strain Details[g]																					
CB9615 base[a]	CB9615 Site[b]	Sakai base[a]	Sakai Site[b]	EDL933 base[a]	EDL933 Site[b]	type[c]	Inferred ancestral base[d]	Lineage inferred to mutate[e]	Support level[f]	D1 Sd197	K-12	HS	ATCC 8739	UMN026	IAI39	SMS 3-5	E2348/69	536	ED1a	CFT073	S88	APEC O1	UTI89	E24377A	IAI1	SE11	F5 8401	F2a 2457T	F2a 301	SS Ss046	B4 Sb227	B18 BS512
c	1544337	c	1726913	t	1818849	s	t	EDL933	-	t	t	t	t	c	t	c	c	c	c	c	c	c	c	t	t	t	t	t	t	t	t	t
a	1546652	g	1729228	g	1821164	s	g	CB9615	++++	g	g	g	g	g	g	g	g	g	g	g	g	g	g	g	g	g	g	g	g	g	g	g
g	1547327	c	1729903	c	1821839	s	g	O157	++++	g	g	g	g	g	g	g	g	g	g	g	g	g	g	g	g	g	g	g	g	g	g	g
g	1549665	a	1732419	a	1824355	ns	g	O157	++++		g	g	g	g		g								g	g	g						
t	1552984	c	1735738	c	1827674	ns	c	CB9615	++++	c	c	c	c	c	c	c	c	c	c	c	c	c	c	c	c	c	c	c	c	c	c	c
t	1554968	a	1737722	a	1829658	s	a	CB9615	++++	a	a	a	a	a	a	a	a	a	a	a	a	a	a	a	a	a	a	a	a	a	a	a
a	1559499	g	1742253	g	1834189	i	g	CB9615	++++	g	t	t	t	g	g	g	g	g	g	g	g	g	g	t	t	t	g	g	g	t	t	t
c	1559695	a	1742449	a	1834385	i	c	O157	++++	c	c	c	c	c	c	c	c	c	c	c	c	c	c	c	c	c	c	c	c	c	c	c
-	1560567	-	1743321	c	1835258	ins	-	EDL933	++++	-	-	-	-	-	-	-	-	-	-	-	-	-	-	-	-	-	-	-	-	-	-	-
t	1560684	c	1743438	c	1835375	i	c	CB9615	++++	c	c	c	c	c	c	c	c	c	c	c	c	c	c	c	c	c	c	c	c	c	c	
t	1561112	g	1743866	g	1835803	i	g	CB9615	++++	g	g	g	g	g	g	g	g	g	g	g	g	g	g	g	g	g	g	g	g	g	g	g
t	1561322	g	1744076	g	1836013	s	g	CB9615	++++	g	g	g	g	g	g	g	g	g	g	g	g	g	g	g	g	g	g	g	g	g	g	g
c	1564200	t	1746954	t	1838891	s	c	O157	++++	c	c	c	c	c	c	c	c	c	c	c	c	c	c	c	c	c	c	c	c	c	c	c
g	1566220	a	1748974	a	1840911	ns	a	CB9615	++++	a	a	a	a	a	a	a	a	a	a	a	a	a	a	a	a	a	a	a	a	a	a	a
c	1568170	t	1750924	t	1842861	ns	c	O157	++++	c	c	c	c	c	c	c	c	c	c	c	c	c	c	c	c	c	c	c	c	c	c	c
-	1568904	-	1751658	g	1843596	ins	-	EDL933	++++	-	-	-	-	-	-	-	-	-	-	-	-	-	-	-	-	-	-	-	-	-	-	-
t	1569663	c	1752417	c	1844355	ns	c	CB9615	++++	c	c	c	c	c	c	c	c	c	c	c	c	c	c	c	c	c	a	a	a	c	c	c
-	1571070	-	1753824	g	1845763	ins	-	EDL933	++++	-	-	-	-	-	-	-	-	-	-	-	-	-	-	-	-	-	-	-	-	-	-	-
g	1571179	a	1753933	a	1845872	i	a	CB9615	++++	a	a	a	a	a	a	a	a	a	a	a	a	a	a	a	a	a	a	a	a	a	a	a
t	1571207	c	1753961	c	1845900	ns	c	CB9615	++++	c	c	c	c	c	c	c	c	c	c	c	c	c	c	c	c	c	c	c	c	c	c	c
t	1571265	a	1754019	a	1845958	ns	a	CB9615	++++	a	a	a	a	a	a	a	a	a	a	a	a	a	a	a	a	a	a	a	a	a	a	a
t	1572002	g	1754756	g	1846695	ns	t	O157	++++	t	t	t	t	t	t	t	t	t	t	t	t	t	t	t	t	t	t	t	t	t	t	t
t	1574668	c	1757422	c	1849361	ns	t	O157	++	t	t	t	t	t	t	t	c	t	c	t	c	c	c	t	t	t	t	t	t	c	t	c
g	1574689	a	1757443	a	1849382	ns	a	CB9615	++++	a	a	a	a	a	a	a	a	a	a	a	a	a	a	g	a	a	a	a	a	a	a	a
-	1574765	13	1757520	13	1849459	del	13	CB9615	+++								13		13		13	13	13	13						-		13
t	1574800	-	1757566	-	1849505	ins	-	CB9615	++++								-		-		-	-	-	-						-		-
t	1574933	a	1757699	a	1849638	s	t	O157	+								a		a		t	t	t	t						t		a
t	1575107	c	1757873	c	1849812	s	c	CB9615	+								c		c		t	t	t	c								c
c	1575186	a	1757952	a	1849891	ns	a	CB9615	+++								a		a		a	a	a	a								a
a	1575371	c	1758137	c	1850076	s	c	CB9615	+++								c		c		c	c	c	c								c
a	1576080	t	1758846	t	1850785	s	a	O157	+								t		t		a	a	a	a								a
a	1577460	c	1760247	c	1852186	ns	c	CB9615	++++				c	c			c		c		c	c	c	c	c							c
t	1578358	g	1761145	g	1853084	s	g	CB9615	+		g		g	g			t		g		g	g	g	g	g							t
c	1578798	a	1761585	a	1853524	s	c	O157	++++		c		c	c		c	c			c	c	c	c	c	c	c						
t	1579211	a	1761998	a	1853937	s	a	CB9615	++++		a		a	a		a	a		a	a	a	a	a	a	a	a						
a	1579236	g	1762023	g	1853962	i	g	CB9615	++++		g		g	g		g	g		-	g	g	g	g	g	g	g						
c	1579238	t	1762025	t	1853964	i	t	CB9615	++++		t		t	t		t	t		t	t	t	t	t	t	t	t						
t	1580963	-	1763664	-	1855603	del	t	O157	+													t										
c	1581496	g	1764197	g	1856136	s	?	O55/O157	+/-		g		g	c										g	g		c	c	c	c		c
g	1581655	t	1764356	t	1856295	i	g	O157	+		t		t	g										g	t		g	g	g	g		g
t	1582391	g	1765092	g	1857031	s	t	O157	+++					t		t	t							t								
t	1582392	g	1765093	g	1857032	ns	t	O157	+++					t		t	t							t								
g	1583975	t	1766676	t	1858615	ns	g	O157	++++		g			g		g	g		-			g		g		g						
c	1586661	t	1769362	t	1861301	ns	c	O157	++++	c	c		c	c		c	c		c			c		c	c		c	c	c	c	c	c
g	1586959	-	1769659	-	1861598	ins	-	CB9615	++++	-	-		-	-		-	-		-					-	-		-	-	-	-	-	-
a	1586993	g	1769693	g	1861632	ns	a	O157	++++	a	a		a	a		a	a		a					a	a		a	a	a	a	a	a
t	1587054	g	1769754	g	1861693	ns	t	O157	++++	t	t		t	t		t	t		t					t	t		t	t	t	t	t	t
t	1587098	a	1769798	a	1861737	ns	a	CB9615	++++	a	-		-	-		a	-		-					-	-		a	a	a	a	a	a
t	1587102	a	1769802	a	1861741	ns	a	CB9615	++++	a	-		-	-		a	-		-					-	-		a	a	a	a	a	a
t	1587127	c	1769827	c	1861766	ns	t	O157	+	a	a		a	a		c	a		a					a	a		a	a	a	t		t
c	1587129	t	1769829	t	1861768	ns	t	CB9615	++++	t	t		t	t		t	t		t					t	t		t	t	t	t		t
t	1587638	g	1770338	g	1862277	ns	?	O55/O157	+/-	c	c		c	c		c	c		c	c	c	c	c	c	c	c	c	c	c	c	c	c
-	1587669	-	1770369	t	1862309	ins	-	EDL933	++++	-	-		-	-		-	-		-	-	-	-	-	-	-	-	-	-	-	-	-	-
a	1587709	g	1770409	g	1862349	s	a	O157	++++	a	a		a	a		a	a		a	a	a	a	a	a	a	a	a	a	a	a	a	a
g	1588294	a	1770994	a	1862934	s	a	CB9615	++	a	a		a	a		g	a		a	a	g	g	g	a	a	g	a	a	a	a	a	g
a	1588339	t	1771039	t	1862979	s	t	CB9615	++	t	t		t	t		a	t		t	t	t	t	t	t	t	a	t	t	t	a	t	a
-	1627512	c	1797487	t	2289690	nc	c	EDL933	+			c	c	c	c	c	c	c	t	c	c	c	c		c	c	c	c	c	c	c	t
-	1627512	t	1809380	g	2277797	s	g	Sakai	++++	g				g				g	g	g	g			g						g		g
a	1628142	g	1816061	g	2271116	ns	g	CB9615	++++	g	g	g	g	g	g	g		g		g	g	g	g	g	g	g	g	g	g			
t	1628351	c	1816270	c	2270907	i	t	O157	++++		t	t	t	t	t	t		t		t	t	t	t	t	t	t	t	t	t			
c	1629191	t	1817110	t	2270067	i	c	O157	++++		c	c	c	c	c	c		c	c	c	c	c	c	c	c	c	c	c	c			
a	1629709	c	1817628	c	2269549	s	a	O157	++++	a	a	a	a	a	a	a	a	a	a	a	a	a	a	a	a	a	a	a	a	a	a	a
g	1630095	t	1818014	t	2269163	ns	t	CB9615	++++	t	t	t	t	t	t	t	t	t	t	t	t	t	t	t	t	t	t	t	t	t	t	t
c	1630185	t	1818104	t	2269073	ns	c	O157	++++	c	c	c	c	c	c	c	c	c	c	c	c	c	c	c	c	c	c	c	c	c	c	c
-	1634781	-	1822700	t	2264476	ins	?	EDL933	+/-																							
t	1635723	c	1823642	c	2263534	ns	t	O157	++++	t	t	t	t	t	t	t	t	t	t	t	t	t	t	t	t	t	t	t	t	t	t	t
t	1635957	c	1823876	c	2263300	ns	c	CB9615	++	c	c	c	c	t	c	c	c	c	c	c	c	c	c	t	c	c	t	t	t	t	c	c
a	1636237	g	1824156	g	2263020	i	a	O157	++++	a	a	a	a	a	a	a	a	a	a	a	a	a	a	a	a	a	a	a	a	a	a	a
g	1636318	a	1824237	a	2262939	i	g	O157	++++	g	g	g	g	g	g	g	g	g	g	g	g	g	g	g	g	g	g	g	g	g	g	g
t	1637678	c	1825597	c	2261579	s	t	O157	++++	t	t	t	t	t	t	t	t	t	t	t	t	t	t	t	t	t	t	t	t	t	t	t
a	1638617	c	1826536	c	2260640	ns	c	CB9615	++++		c	c	c	c	c	c	c	c	c	c	c	c	c								c	c
c	1641085	t	1829004	t	2258172	ns	c	O157	++++	c	c	c	c	c	c	c	c	c	c	c	c	c	c	c	c	c	c	c	c	c	c	c
a	1642983	g	1830902	g	2256274	s	a	O157	++++	a	a	a	a	a	a	a	a	a	a	a	a	a	a	a	a	a	a	a	a	a	a	a
c	1646614	t	1834533	t	2252643	i	t	CB9615	++++	t	t	t	t	t	t	t	t	t	t	t	t	t	t	t	t	t	t	t	t	t	t	t
c	1647029	t	1834948	t	2252228	s	c	O157	++++	c	c	c	c	c	c	c	c	c	c	c	c	c	c	c	c	c	c	c	c	c	c	c
c	1647248	t	1835167	t	2252009	s	c	O157	++++	c	c	c	c	c	c	c	c	c	c	c	c	c	c	c	c	c	c	c	c	c	c	c
t	1648691	c	1836610	c	2250566	s	c	CB9615	++	c	c	c	c	c	c	t	c	t	t	c	c	c	c	c	c	c	c	c	c	c	c	c
-	1652052	-	1839971	c	2247204	ins	?	EDL933	+/-																							
c	1652132	a	1840051	a	2247124	s	c	O157	++++	c	c	c	c	c	c	c	c	c	c	c	c	c	c	c	c	c	c	c	c	c	c	c

O55 and O157 genome site details										Outgroup Strain Details[g]																						
CB9615 base[a]	CB9615 Site[b]	Sakai base[a]	Sakai Site[b]	EDL933 base[a]	EDL933 Site[b]	type[c]	Inferred ancestral base[d]	Lineage inferred to mutate[e]	Support level[f]	D1 Sd197	K-12	HS	ATCC 8739	UMN026	IAI39	SMS 3-5	E2348/69	536	ED1a	CFT073	S88	APEC O1	UTI89	E24377A	IAI1	SE11	F5 8401	F2a 2457T	F2a 301	SS Ss046	B4 Sb227	B18 BS512
t	1655198	a	1843117	a	2244058	ns	a	CB9615	++++	a	a	a	a	a	a	a	a	a	a	a	a	a	a	a	a	a	a	a	a	a	a	a
-	1657206	-	1845125	a	2242049	ins	?	EDL933	+/-																							
c	1657329	t	1845248	t	2241926	ns	c	O157	++++	c	c	c	c	c	c	c	c	c	c	c	c	c	c	c	c	c	c	c	c	c	c	c
t	1658246	g	1846165	g	2241009	s	?	O55/O157	+/-	c	c	c	c	c	c	c	c	c	c	c	c	c	c	c	c	c	c	c	c	c	c	c
g	1658624	a	1846543	a	2240631	s	g	O157	++++	g	g	g	g	g	g	g	g	g	g	g	g	g	g	g	g	g	g	g	g	g	g	g
c	1658669	t	1846588	t	2240586	s	c	O157	++	c	c	c	c	c	t	t	c	c	c	c	c	c	c	c	c	c	c	c	c	c	c	c
a	1660014	g	1847933	g	2239241	ns	g	CB9615	++++	g	g	g	g	g	g	g	g	g	g	g	g	g	g	g	g	g	g	g	g	g	g	g
t	1662005	g	1849924	g	2237250	ns	t	O157	++	t	t	t	c	c	c	g	g	g	g	g	g	g	g	g	g	t	t	t	t	g	g	g
-	1663013	-	1850932	c	2236241	ins	?	EDL933	+/-																							
6	1666456	-	1854374	-	2232799	del	6	O157	++++	6					6	6	6	6	6	6	6	6	6									
a	1667332	t	1855245	t	2231928	s	a	O157	++++	a					a	a	a	a	a	a	a	a	a									
t	1667461	c	1855374	c	2231799	s	c	CB9615	++++	c					c	c	c	c	c	c	c	c	c									
t	1668269	g	1856182	g	2230991	ns	g	CB9615	++++	g					g	g	g	g	g	g	g	g	g									
g	1669104	t	1857017	t	2230156	ns	g	O157	++++	g					g	g	g	g	g	g	g	g	g									
a	1669211	g	1857124	g	2230049	ns	a	O157	++++	a					a	a	a	a	a	a	a	a	a									
t	1669577	g	1857490	g	2229683	s	g	CB9615	++++	g					g	g	g	g	g	g	g	g	g									
-	1670266	-	1858179	g	2228993	ins	?	EDL933	+/-																							
-	1671287	-	1859200	c	2227971	ins	?	EDL933	+/-																							
g	1674365	a	1862278	a	2224893	s	a	CB9615	++++	a	a	a	a	a	a	a	a	a	a	a	a	a	a	a	a	a	a	a	a	a	a	a
g	1676022	a	1863935	a	2223236	ns	g	O157	++++	g	g	g	g	g	g	g	g	g	g	g	g	g	g	g	g	g	g	g	g	g	g	g
t	1676620	g	1864533	g	2222638	ns	g	CB9615	++++	g	g	g	g	g	g	g	g	g	g	g	g	g	g	g	g	g	g	g	g	g	g	g
g	1677185	t	1865098	t	2222073	i	t	CB9615	++	t	g	g	g	g	g	g	g	g	g	g	g	g	g	g	g	g	g	g	g	g	g	g
-	1677234	-	1865147	c	2222023	ins	?	EDL933	+/-																							
-	1677805	-	1865718	K	2221451	ins	-	EDL933	IUPAC																							
c	1679410	t	1867323	t	2219846	i	c	O157	++++	c	c	c	c	c	c	c								c	c	c	c	c	c	c	c	c
g	1681028	a	1868941	a	2218228	i	a	CB9615	++++	a	a	a	a	a	a	a								a	a	a	a	a	a	a	a	a
c	1681139	a	1869052	a	2218117	ns	c	O157	++++	c	c	c	c	c	c	c								c	c	c	c	c	c	c	c	c
g	1681411	a	1869324	a	2217845	ns	g	O157	++++	g	g	g	g	g	g	g								g	g	g	g	g	g	g	g	g
c	1681766	t	1869679	t	2217490	s	c	O157	++++	c	c	c	c	c	c	c								c	c	c	c	c	c	c	c	c
t	1684955	g	1872868	g	2214301	ns	t	O157	++++	t	t	t	t	t	t	t								t	t	t	t	t	t	t	t	t
g	1686425	a	1874338	a	2212831	s	?	O55/O157	+/-	c	c	c	c	c	c	c								c	c	c	c	c	c	c	c	c
t	1689339	c	1877252	c	2209917	ns	c	CB9615	++	c	c	c	c	c	c	c	c	c	c	c	c	c	c	c	c	c	t	t	t	c	c	t
-	1690011	-	1877924	c	2209244	ins	?	EDL933	+/-																							
a	1690852	g	1878765	g	2208403	nc	g	CB9615	++++	g	g	g	g		g	g	g	g	g	g	g	g	g	g	g	g	g	g	g	g		
a	1691023	g	1878936	g	2208232	nc	g	CB9615	++++	g	g	g	g		g	g	g	g	g	g	g	g	g	g	g	g	g	g	g	g		
a	1691889	c	1879802	c	2207366	ns	a	O157	++++		a	a	a		a	a	a	a	a	a	a	a	a	a	a	a	a	a	a	a		
c	1692438	t	1880351	t	2206817	ns	c	O157	+		c	c	c		c	c	c	c	c	c	c	c	c	c	c	c	t	t	t	c		
c	1692965	a	1880878	a	2206290	ns	c	O157	++++		c	c	c		t	t	c	c	c	c	c	c	c	c	c	c	c	c	c	c		
t	1693449	g	1881362	g	2205806	ns	g	CB9615	++++		g	g	g		g	g	g	g	g	g	g	g	g	g	g	g	g	g	g	g		
g	1693893	t	1881806	t	2205362	ns	t	CB9615	++++		t	t	t		t	t	t	t	t	t	t	t	t	t	t	t	t	t	t	t		
t	1694034	c	1881947	c	2205221	ns	t	O157	++++		t	t	t		t	t	t	t	t	t	t	t	t	t	t	t	t	t	t	t		
c	1694966	t	1882879	t	2204289	s	c	O157	++++		c	c	c		c	c	c	c	c	c	c	c	c	c	c	c	c	c	c	c		
g	1696070	t	1883983	t	2203185	ns	g	O157	++++		g	g	g		g	g	g	g	g	g	g	g	g	g	g	g	g	g	g	g		
t	1696431	g	1884344	g	2202824	ns	t	O157	++++		t	t	t		t	t	t	t	t	t	t	t	t	t	t	t	t	t	t	t		
c	1697890	a	1885803	a	2201365	ns	a	CB9615	++++		a	a	a		a	a	a	a	a	a	a	a	a	a	a	a	a	a	a	a		
t	1701972	g	1889885	g	2197283	ns	g	CB9615	++++		g	g	g		g	g	g	g	g	g	g	g	g	g	g	g	g	g	g			g
-	1702777	-	1890690	N	2196477	ins	-	EDL933	IUPAC																							
c	1703127	t	1891040	t	2196127	ns	t	CB9615	++++		t	t	t		t	t	t	t	t	t	t	t	t	t	t	t	t	t	t	t		t
c	1704503	t	1892416	t	2194751	s	t	CB9615	++++	t	t	t	t	t	t	t	t	t	t	t	t	t	t	t	t	t	t	t	t	t	t	t
a	1709284	c	1897197	c	2189970	ns	c	CB9615	++++	c	c	c	c	c	c	c	c	c	c	c	c	c	c	c	c	c	c	c	c	c	c	c
g	1709375	a	1897288	a	2189879	ns	g	O157	++++	g	g	g	g	g	g	g	g	g	g	g	g	g	g	g	g	g	g	g	g	g	g	g
c	1712035	-	1899947	-	2187220	ins	-	CB9615	+															-	-	-	c	c	c	-	-	-
-	1714583	-	1902494	c	2184672	ins	?	EDL933	+/-																							
c	1725327	a	1914551	a	2172615	s	c	O157	++++		c	c	c	c	c	c								c	c	c				c	c	c
g	1727168	t	1916392	t	2170774	s	g	O157	++++	g	g	g	g	g	g	g	g	g	g	g	g	g	g	g	g	g	g	g	g	g	g	g
g	1734632	a	1923856	a	2163310	s	g	O157	+++		g											g				g						
g	1734675	t	1923899	t	2163267	s	g	O157	+++		g											g				g						
-	1736115	-	1929393	g	2157772	ins	?	EDL933	+/-																							
-	1736115	g	1929442	Y	2157723	ns	g	EDL933	+													g				g					g	
-	1736115	a	1932012	K	2155153	ns	a	EDL933	+++		a								g			a		t		a				-		t
-	1736115	-	1940246	c	2146918	ins	?	EDL933	+/-																							
a	1773797	c	1969616	c	2118865	ns	a	O157	+								a															
a	1773882	g	1969701	g	2118780	s	a	O157	+								a															
g	1774130	a	1969949	a	2118532	i	g	O157	+								g															
a	1774764	g	1970583	g	2117898	ns	g	CB9615	+								g															
c	1774765	t	1970584	t	2117897	ns	t	CB9615	+								t															
t	1775389	c	1971208	c	2117273	ns	?	O55/O157	+/-																							
a	1775484	g	1971303	g	2117178	ns	?	O55/O157	+/-																							
a	1775603	g	1971422	g	2117059	i	?	O55/O157	+/-																							
c	1776128	t	1971947	t	2116534	s	?	O55/O157	+/-																							
a	1776310	g	1972129	g	2116352	i	?	O55/O157	+/-																							
a	1776831	-	1972302	-	2116179	indel	?	O55/O157	+/-																							
a	1780436	g	1975875	g	2112606	s	a	O157	++++	a	a	a	a	a	a	a	a	a	a	a	a	a	a	a	a	a	a	a	a	a	a	a
c	1781296	t	1976735	t	2111746	ns	c	O157	++++	c	c	c	c	c	c	c	c	c	c	c	c	c	c	c	c	c	c	c	c	c	c	c
-	1783827	-	1979266	t	2109214	ins	?	EDL933	+/-																							
-	1785027	-	1980466	y	2108013	ins	-	EDL933	IUPAC																							
t	1787006	c	1982445	c	2106034	s	c	CB9615	++++	c	c	c	c	c	c	c	c	c	c	c	c	c	c	c	c	c	c	c	c	c	c	c
g	1788160	a	1983599	a	2104880	ns	a	CB9615	++++	a	a	a	a	a	a	a	a	a	a	a	a	a	a	a	a	a	a	a	a	a	a	a
-	1788530	t	1983970	t	2104509	indel	?	O55/O157	+/-																							

O55 and O157 genome site details										Outgroup Strain Details[g]																						
CB9615 base[a]	CB9615 Site[b]	Sakai base[a]	Sakai Site[b]	EDL933 base[a]	EDL933 Site[b]	type[c]	Inferred ancestral base[d]	Lineage inferred to mutate[e]	Support level[f]	D1 Sd197	K-12	HS	ATCC 8739	UMN026	IAI39	SMS 3-5	E2348/69	536	ED1a	CFT073	S88	APEC O1	UTI89	E24377A	IAI1	SE11	F5 8401	F2a 2457T	F2a 301	SS Ss046	B4 Sb227	B18 BS512
a	1789062	t	1984502	t	2103977	s	?	O55/O157	+/-																							
-	1789076	-	1984549	g	2103929	ins	?	EDL933	+/-																							
g	1789102	a	1984632	a	2103846	ns	?	O55/O157	+/-																							
c	1789131	t	1984661	t	2103817	s	?	O55/O157	+/-																							
a	1789483	t	1985013	t	2103465	ns	?	O55/O157	+/-																							
g	1790383	a	1985913	a	2102565	ns	?	O55/O157	+/-																							
c	1791108	t	1986638	t	2101840	s	?	O55/O157	+/-																							
a	1792349	t	1987879	a	2100599	ns	?	Sakai	+/-																							
c	1792369	g	1987899	g	2100579	ns	?	O55/O157	+/-																							
a	1795462	c	1990992	c	2097486	ns	a	O157	++++		a	a	a	a	a	a		a		a	a	a	a	a	a	a						
g	1796309	t	1991839	t	2096639	ns	t	CB9615	++++		t	t	t	t	t	t								t	t	t	t	t	t			
t	1796758	c	1992288	c	2096190	s	c	CB9615	++++		c	c	c	c	c	c								c	c	c	c	c	c			
g	1797653	t	1993183	t	2095295	ns	t	CB9615	++++	t	t	t	t	t	t	t								t	t	t	t	t	t			
t	1798123	c	1993653	c	2094825	ns	c	CB9615	++++	c	c	c	c	c	c	c		c		c	c	c	c	c	c	c	c	c	c		c	c
c	1801714	t	1997244	t	2091234	ns	c	O157	++++	c	c	c	c	c	c	c		c		c	c	c	c	c	c	c	c	c	c	c	c	c
g	1802230	t	1997760	t	2090718	ns	t	CB9615	++++	t	t	t	t	t	t	t		t		t	t	t	t	t	t	t	t	t	t	t	t	t
c	1803384	t	1998914	t	2089564	s	c	O157	++++	c	c	c	c	c	c	c	c	c	c	c	c	c	c	c	c	c	c	c	c	c	c	c
c	1803651	a	1999181	a	2089297	s	c	O157	++++	c	c	c	c	c	c	c	c	c	c	c	c	c	c	c	c	c	c	c	c	c	c	c
a	1805770	g	2001300	g	2087178	ns	a	O157	++++	a	a	a	a	a	a	c	a	a	a	a	a	a	a	a	a	a	a	a	a	a	a	a
t	1808815	g	2004345	g	2084133	s	t	O157	++	t																						
a	1808816	g	2004346	g	2084132	ns	a	O157	++	a																						
a	1810175	g	2005705	g	2082773	i	a	O157	++++		a	a	a	a	a	a	a	a	a	a	a	a	a	a	a	a	a	a	a	a	a	a
a	1812976	c	2008506	c	2079972	ns	c	CB9615	++++	c	c	c	c	c		t	c	c	c	c	c	c	c	c	c	c	c	c	c	c	c	c
t	1813669	c	2009199	c	2079279	ns	c	CB9615	++++	c	c	c	c	c		c	c	c	c	c	c	c	c	c	c	c	c	c	c	c		c
a	1813689	g	2009219	g	2079259	ns	a	O157	++	a	a	a	a	g		g	a	a	a	a	a	a	a	a	a	g	a	a	a	a		a
-	1817444	-	2012974	c	2075503	ins	?	EDL933	+/-																							
-	1818024	-	2013554	c	2074922	ins	?	EDL933	+/-																							
-	1818623	-	2014153	V	2074322	ins	-	EDL933	IUPAC																							
g	1820751	a	2016281	a	2072194	ns	g	O157	++++	g	g	g	g	g		g	g	g	g	g	g	g	g	g	g	g	g	g	g	g	g	g
c	1820817	t	2016347	t	2072128	ns	t	CB9615	++++	t	t	t	t	t		t	t	t	t	t	t	t	t	t	t	t	t	t	t	t	t	t
g	1821213	a	2016743	a	2071732	ns	g	O157	++++	g	g	g	g	g		g	g	g	g	g	g	g	g	g	g	g	g	g	g	g	g	g
a	1821814	g	2017344	g	2071131	s	g	CB9615	++++	g	g	g	g	g		g	g	g	g	g	g	g	g	g	g	g	g	g	g	g	g	g
-	1824168	-	2019698	c	2068776	ins	?	EDL933	+/-																							
-	1824254	-	2019784	t	2068689	ins	?	EDL933	+/-																							
t	1825601	c	2021131	c	2067342	ns	c	CB9615	++++	c	c	c	c	c		c	c	c	c	c	c	c	c	c	c	c				c		
c	1828231	t	2023761	t	2064712	s	c	O157	++++	c	c	c	c	c		t	c	c	c	c	c	c	c	c	c	c				c		
c	1830103	a	2025633	a	2062840	s	c	O157	++++	c	c	c	c	c		c		c	c	c	c	c	c	c	c	c				c		
t	1833726	c	2029256	c	2059217	ns	c	CB9615	++++	c	c	c	c	c		c	c	c	c	c	c	c	c	c	c	c				c		
g	1834520	a	2030050	a	2058423	ns	g	O157	++++	g	g	g	g	g		g	g	g	g	g	g	g	g	g	g	g				g		
-	1835749	8	2031280	8	2057193	del	8	CB9615	++++	8	8	8	8	8		8	8	8	8	8	8	8	8	8	8	8	8	8	8	8		
c	1836647	t	2032185	t	2056288	ns	c	O157	++++	c	c	c	c	c		c	c	c	c	c	c	c	c	c	c	c	c	c	c	c		
g	1837638	t	2033176	t	2055297	ns	g	O157	++++	g	g	g	g	g		g	g	g	g	g	g	g	g	g	g	g	g	g	g	g	g	t
t	1838493	a	2034031	a	2054442	ns	t	O157	++++	t	t	t	t	t		t	t	t	t	t	t	t	t	t	t	t	t	t	t	t	t	t
g	1844594	t	2040132	t	2048341	i	t	CB9615	++++	t	t	t	t	t		t	t	t	t	t	t	t	t	t	t	t				t	t	t
t	1845392	c	2040930	c	2047543	s	c	CB9615	++++	c	c	c	c	c		c	c	c	c	c	c	c	c	c	c	c				c	c	c
a	1846028	c	2041566	a	2046907	s	c	Sakai	-	c	c	a	c	c		c	c	c	c	c	c	c	c	a	a	a				a	a	a
g	1846430	t	2041968	t	2046505	i	g	O157	++++	g	g	g	g	g		g		g	g	g	g	g	g	g	g	g				g	g	g
a	1847799	g	2043337	g	2045136	ns	g	CB9615	++++		g	g	g											g	g	g				g		
g	1848490	a	2044028	a	2044445	s	g	O157	+			a	g	g				g	g	g	g	g	g	g	g	g				a		
c	1849617	a	2045155	a	2043318	ns	c	O157	++++			c	c	c				c	c	c	c	c	c	c	c	c				c		
c	1850716	t	2046254	c	2042219	nc	t	Sakai	-	t	c	c	c											c	c	c				c	c	c
3	1852413	3	2047951	-	2040519	del	3	EDL933	++++	3	3	3	3											3	3	3				3	3	3
g	1852486	g	2048024	-	2040449	del	g	EDL933	++++	g	g	g	g											g	g	g				g	g	g
c	1852500	c	2048038	t	2040435	nc	c	EDL933	++++	c	c	c	c											c	c	c				c	c	c
c	1852508	c	2048046	-	2040428	del	c	EDL933	++++	c	c	c	c											c	c	c				c	c	c
c	1852510	c	2048048	t	2040426	nc	c	EDL933	++++	c	c	c	c											c	c	c				c	c	c
c	1852514	c	2048052	-	2040423	del	c	EDL933	++++	c	c	c	c											c	c	c				c	c	c
c	1852519	c	2048057	-	2040419	del	c	EDL933	++++	c	c	c	c											c	c	c				c	c	c
-	1852534	-	2048072	a	2040403	ins	?	EDL933	+/-																							
-	1852545	-	2048083	a	2040391	ins	?	EDL933	+/-																							
-	1852557	-	2048095	a	2040378	ins	?	EDL933	+/-																							
c	1853041	t	2048579	t	2039894	nc	c	O157	++++	c	c	c	c											c	c	c				c	c	t
c	1854091	t	2049629	t	2038844	nc	c	O157	++	c	t	t	t											c	c	c				t	t	t
g	1854371	t	2049909	g	2038564	nc	g	Sakai	+															g	g	g						
g	1854373	c	2049911	g	2038562	nc	g	Sakai	+															g	g	g						
a	1854374	t	2049912	a	2038561	nc	a	Sakai	+															a	a	a						
g	1855252	a	2050790	a	2037683	i	g	O157	+															g	g	g						
g	1856165	t	2051703	t	2036770	nc	g	O157	+		g	g	g	t										g	t	g				t		c
c	1856299	t	2051837	t	2036636	nc	t	CB9615	++++		t	t	t	t				t	t		t	t	t	t	t	t				t		t
c	1856403	-	2051940	-	2036533	del	c	O157	++++		c	c	c	c				c	c		c	c	c	c	c	c				c		c
a	1856776	t	2052313	t	2036160	ns	t	CB9615	++++		t	t	t		t	t	t							t	t	t				t		
c	1857429	t	2052966	t	2035507	ns	c	O157	++++		c	c	c	c	c	c	c	c	c	c	c	c	c	c	c	c				c		
a	1858390	-	2053926	-	2034547	del	a	O157	++++	a	a	a	a	a	a	a	a	a	a	a	a	a	a	a	a	a				a		
a	1861732	g	2057268	g	2031205	s	g	CB9615	++++	g	g	g	g	g	g	g	g	g	g	g	g	g	g	g	g	g	g	g	g	g	g	g
c	1862723	t	2058259	t	2030214	ns	c	O157	++	c	c	c	c	c	c	c	c	c	c	c	c	c	c	c	c	c	c	c	c	c	t	t
t	1863921	g	2059457	g	2029016	s	g	CB9615	++++	g	g	g	g	g	g	g	g	g	g	g	g	g	g	g	g	g	g	g	g	g	g	g
g	1864923	a	2060459	a	2028014	s	g	O157	++++	g	g	g	g	g	g	g	g	g	g	g	g	g	g	g	g	g	g	g	g	g	c	c
c	1866617	t	2062153	t	2026320	ns	c	O157	++++	c	c	c	c	c	c	c	c	c	c	c	c	c	c	c	c	c	c	c	c	c	c	c
g	1868964	t	2064500	t	2023973	ns	g	O157	++++		g	g	g	g	g	g								g	g	g	g	g	g			

O55 and O157 genome site details										Outgroup Strain Details[g]																						
CB9615 base[a]	CB9615 Site[b]	Sakai base[a]	Sakai Site[b]	EDL933 base[a]	EDL933 Site[b]	type[c]	Inferred ancestral base[d]	Lineage inferred to mutate[e]	Support level[f]	D1 Sd197	K-12	HS	ATCC 8739	UMN026	IAI39	SMS 3-5	E2348/69	536	ED1a	CFT073	S88	APEC O1	UTI89	E24377A	IAI1	SE11	F5 8401	F2a 2457T	F2a 301	SS Ss046	B4 Sb227	B18 BS512
t	1870552	c	2066088	c	2022385	ns	c	CB9615	++++	c				c	c	c											c	c	c	c		
t	1870598	a	2066134	a	2022339	ns	a	CB9615	+++	a				a	a	a											g	g	g	g		
g	1870717	a	2066253	a	2022220	i	a	CB9615	++++	a				a	a	a											a	a	a	a		
t	1871038	c	2066574	c	2021899	ns	c	CB9615	++++	c				c	c	c		c		c	c	c	c			c	c	c	c	c		
c	1871953	t	2067489	t	2020984	i	c	O157	++++	c	c	c	c	c	c	c								c	c	c	c	c	c	c	c	
t	1871974	c	2067510	c	2020963	i	t	O157	++++	t	t	t	t	t	t	t								t	t	t	t	t	t	t	t	
g	1871991	a	2067527	a	2020946	i	a	CB9615	++++	a	a	a	a	a	a	a								a	a	a	a	a	a	a	a	
a	1872069	g	2067605	g	2020868	i	a	O157	++++	a	a	a	a	a	a	a								a	a	a	a	a	a	a	a	
t	1873553	g	2069089	g	2019384	nc	t	O157	++++	t	t	t	t	t	t	t	t	t	t	t	t	t	t	t	t	t	t	t	t	t	t	t
a	1874145	g	2069681	g	2018792	nc	g	CB9615	++	g	g	g	g	g	g	g	g	g	g	g	g	g	g	g	g	g	g	a	a	g	g	g
c	1876247	a	2071783	a	2016690	nc	c	O157	++++	c	c	c	c	c	c	c	c	c	c	c	c	c	c	c	c	c	c	c	c	c	c	c
c	1878946	t	2074660	c	2013813	ns	c	Sakai	++++	c	c	c	c	c	c	c	c	c	c	c	c	c	c	c	c	c	c	c	c	c	c	t
g	1879371	t	2075085	t	2013388	i	t	CB9615	++++	t	t	t	t	t	t	t	t	t	t	t	t	t	t	t	t	t	t	t	t	t	t	t
a	1880106	t	2075820	t	2012653	ns	a	O157	++++	a	a	a	a	a	a	a	a	a	a	a	a	a	a	a	a	a	a	a	a	a	a	a
-	1882068	-	2077782	g	2010690	ins	?	EDL933	+/-																							
t	1882216	a	2077930	a	2010542	s	a	CB9615	++	a	a	a	a	t	a	t	a	a	a	a	a	a	a	a	a	a	a	a	a	a	a	a
c	1883315	t	2079029	t	2009443	ns	c	O157	++++	c	c	c	c	c	c	c								c	c	c	c	c	c	c	c	c
g	1883933	t	2079647	t	2008825	ns	g	O157	++++	g	g	g	g	g	g	g								g	g	g	g	g	g	g	g	g
a	1884831	g	2080545	g	2007927	s	g	CB9615	++++		g	g	g	g	g	g								g	g	g	g	g	g	g	g	g
c	1886424	-	2082137	-	2006335	del	c	O157	++++		c	c	c	c	c	c								c	c	c				c	c	c
c	1888850	a	2084563	a	2003909	i	c	O157	++++		c	c	c	c	c	c	c	c	c	c	c	c	c	c	c	c	c	c	c	c	c	c
t	1889802	g	2085515	g	2002957	ns	t	O157	++++		t	t	t	t	t	t	t	t	t	t	t	t	t	t	t	t				t	t	t
10	1890039	-	2085751	-	2002721	del	10	O157	++++		10	10	10	10	10	10	10	10	10	10	10	10	10	10	10	10				10	10	10
g	1891521	a	2087224	a	2001248	s	a	CB9615	++++		a	a	a	a	a	a								a	a	a				a	a	a
a	1893302	g	2089005	g	1999467	ns	g	CB9615	++++	g	g	g	g	g	g	g	g	g	g	g	g	g	g	g	g	g	g	g	g	g	g	g
g	1896021	a	2091724	a	1996748	ns	g	O157	++++	g	g	g	g	g	g	g	g	g	g	g	g	g	g	g	g	g	g	g	g	g	g	g
c	1897726	-	2093428	-	1995044	del	c	O157	++	c	c	c	c	c	c	c	c	c	c	c	c	c	c	c	c	c	-	-	-	c	c	c
t	1897751	c	2093453	c	1995019	i	c	CB9615	++	c	t	t	t	t	c	t	t	t	t	t	t	t	t	t	t	t	t	t	t	t	t	t
c	1898804	t	2094506	t	1993966	s	c	O157	++++		c	c	c	c	c	c	c	c	c	c	c	c	c	c	c	c	c	c		c	c	c
g	1899648	t	2095350	t	1993122	ns	g	O157	++++		g	g	g	g	g	g	g	g		g	g	g	g	g	g	g	g	g	g	g	g	g
t	1900706	c	2096408	c	1992064	s	c	CB9615	++++		c	t	c	c	c	c	c	c		c	c	c	c	c	c	c	c	c	c	c	c	c
g	1904352	a	2100054	a	1988418	ns	g	O157	++++		g	g	g	g	g	g	g	g		g	g	g	g	g	g	g	g	g	g	g	g	g
g	1904360	a	2100062	a	1988410	s	g	O157	++++		g	g	g	g	g	g	g	g		g	g	g	g	g	g	g	g	g	g	g	g	g
c	1904900	g	2100602	g	1987870	i	g	CB9615	++++		g	g	g	g	g	g	g	g		g	g	g	g	g	g	g	g	g	g	g	g	g
c	1905233	t	2100935	t	1987537	ns	t	CB9615	++++		t	t	t	t	t	t	t	t	t	t	t	t	t	t	t	t	t	t	t	t	t	t
g	1908215	a	2103917	a	1984555	i	g	O157	++++	g	g	g	g	a	g	g	g	g	g	g	g	g	g	g	g	g	g	g	g	g	g	
g	1909694	a	2105396	a	1983076	i	g	O157	++++	g	g	g	g	g	g	g	g	g	g	g	g	g	g	g	g	g	g	g	g	g	g	g
t	1911433	c	2107135	c	1981337	s	c	CB9615	++++	c	c	c	c	c	c	c	c	c	c	c	c	c	c	c	c	c	c	c	c	c	c	c
t	1912162	c	2107864	c	1980608	i	c	CB9615	++++	c	c	c	c	c	c	c	c	c	c	c	c	c	c	c	c	c	c	c	c	c	c	c
g	1913790	t	2109492	t	1978980	ns	g	O157	++++		g	g	g	g		g	a			g		g		g	g	g						
a	1915332	c	2111034	c	1977438	ns	c	CB9615	++++		c	c	c	c	c	c	c			c		c			c	c						
t	1915410	c	2111112	c	1977360	ns	t	O157	++++		t	t	t	t		t	t			t		t			t	t						
c	1915454	t	2111156	c	1977316	ns	c	Sakai	++++		c	c	c	c		c	c			c		c		c	c	c						
a	1917088	g	2112790	g	1975682	i	g	CB9615	++++					g		g	g			g		g	g	g	g	g						
t	1918516	c	2114218	c	1974254	ns	c	CB9615	++++					c		c	c			c	c	c	c		c	c						
a	1918897	c	2114599	c	1973873	i	c	CB9615	++++					c		c	c			c	c	c	c		c	c						
g	1919642	a	2115344	g	1973128	i	g	Sakai	++++	g	g	g	g	g	g	g	g	g	g	g	g	g	g		g	g				g	g	g
t	1919658	-	2115359	-	1973113	ins	-	CB9615	++++	-	-	-	-	-	-	-	t	-	-	-	-	-	-		-	-				-	-	-
g	1920168	a	2115869	a	1972603	ns	g	O157	++++		g	g	g	g	g		g	g	g	g	g	g	g		g	g				g		g
t	1921520	a	2116602	a	1971870	ns	a	CB9615	++++		a	a	a	a										a	a	a						
t	1923343	a	2118425	a	1970047	ns	a	CB9615	++++		a	a	a	a		a									a	a						
c	1923497	t	2118579	t	1969893	ns	t	CB9615	++++		t	t	t	t		t									t	t						
t	1924240	c	2119322	c	1969150	ns	t	O157	++++	t	t	t	t	t		t									t	t				t		
t	1927205	c	2122287	c	1966185	i	t	O157	++++		t	t	t	t		t								t	t	t						
c	1930513	t	2125595	t	1962877	ns	c	O157	++++		c	c	c	c		c								c	c	c	c	c	c	c		
a	1934653	g	2129735	g	1958737	ns	g	CB9615	++++		g	g	g	g	g	g								g	g	g	g	g	g			
g	1935534	a	2130616	a	1957856	ns	g	O157	++++	g	g	g	g	g	g	g								g	g	g	g	g	g			
c	1936657	t	2131739	t	1956733	ns	c	O157	++++	c	c	c	c	c	c	c	c	c		c	c	c	c	c	c	c	c	c	c			c
g	1937925	a	2133007	a	1955465	i	g	O157	++	g	g	g	g	g	a	a	a	a	a	a	g	g	g	g	g	g	g	g	g	g		g
-	1938049	g	2133132	-	1955341	ins	-	Sakai	++++	-	-	-	-	-	-	-	-	-	-	-	-	-	-	-	-	-	-	-	-	-		-
a	1938314	g	2133397	g	1955076	s	g	CB9615	++++	g	g	g	g	g	g	g	g	g	g	g	g	g	g	g	g	g	g	g	g	g		g
g	1939752	a	2134835	a	1953638	s	a	CB9615	++++	a	a	a	a	a	a	a	a	a		a	a	a	a	a	a	a	a	a	a	a	a	a
a	1939775	g	2134858	g	1953615	ns	g	CB9615	++++	g	g	g	g	g	g	g	g	g		g	g	g	g	g	g	g	g	g	g	g	g	g
-	1941040	-	2136123	t	1952349	ins	?	EDL933	+/-																							
c	1941378	a	2136461	a	1952011	ns	c	O157	++++	c	c	c	c	c	c	c	c	c		c	c	c	c	c	c	c	c	c	c	c	c	c
a	1941384	g	2136467	g	1952005	s	a	O157	++++	a	a	a	a	a	a	a	a	a		a	a	a	a	a	a	a	a	a	a	a	a	a
g	1941610	t	2136693	t	1951779	ns	t	CB9615	++++	t	t	t	t	t	t	t	t	t		t	t	t	t	t	t	t	t	t	t	t	t	t
c	1943139	t	2138222	t	1950250	ns	c	O157	++++	c	c	c	c	c	c	c	c	c	c	c	c	c	c	c	c	c	c	c	c	c	c	c
c	1943276	t	2138359	t	1950113	s	c	O157	++++	c	c	c	c	c	c	c	c	c	c	c	c	c	c	c	c	c	c	c	c	c	c	c
g	1944146	a	2139229	a	1949243	s	a	CB9615	++++	a	a	a	a	a	a	a	a	a	a	a	a	a	a	a	a	a				a	a	a
a	1944290	g	2139373	g	1949099	s	g	CB9615	++++	g	g	g	g	g	g	g	g	g	g	g	g	g	g	g	g	g				g	g	g
g	1945587	a	2140670	g	1947802	nc	g	Sakai	++++	g	g	g	g	g	g	g								g	g	g	g	g	g	g	g	g
c	1946065	t	2141148	t	1947324	nc	c	O157	++++		c	c	c	c	c	c								c	c	c	c	c	c	c	c	c
a	1946158	g	2141241	g	1947231	nc	a	O157	++++		a	a	a	a	a	a								a	a	a	a	a	a	a	a	a
-	1946353	-	2141436	t	1947035	ins	?	EDL933	+/-																							
a	1947580	t	2142663	t	1945808	ns	a	O157	++++	a	a	a	a	a	a	a	a	a	a	a	a	a	a	a	a	a	a	a	a	a	a	t
a	1950093	g	2145176	g	1943295	ns	a	O157	++++	a	a	a	a	a	a	a	a	a	a	a	a	a	a	a	a	a	a	a	a			
a	1951871	g	2146954	g	1941517	i	?	O55/O157	+/-						c																	
a	1953251	g	2148334	g	1940137	s	g	CB9615	+						g																	

O55 and O157 genome site details										Outgroup Strain Details[g]																						
CB9615 base[a]	CB9615 Site[b]	Sakai base[a]	Sakai Site[b]	EDL933 base[a]	EDL933 Site[b]	type[c]	Inferred ancestral base[d]	Lineage inferred to mutate[e]	Support level[f]	D1 Sd197	K-12	HS	ATCC 8739	UMN026	IAI39	SMS 3-5	E2348/69	536	ED1a	CFT073	S88	APEC O1	UTI89	E24377A	IAI1	SE11	F5 8401	F2a 2457T	F2a 301	SS Ss046	B4 Sb227	B18 BS512
t	1954261	c	2149344	c	1939127	s	c	CB9615	++++	c	c	c	c	c	c	c	c	c	c	c	c	c	c	c	c	c				c		c
c	1954479	t	2149562	t	1938909	ns	c	O157	++++	c	c	c	c	c	c	c	c	c	c	c	c	c	c	c	c	c				c		c
g	1954947	a	2150030	a	1938441	s	g	O157	++++	g	g	g	g	g	g	g	g	g	g	g	g	g	g	g	g	g				g		g
t	1956112	g	2151195	g	1937276	i	g	CB9615	++++	g	g	g	g	g	g	g	g	g	g	g	g	g	g	g	g	g	g	g	g	g	g	g
a	1956928	g	2152011	g	1936460	s	g	CB9615	++++	g	g	g	g	g	g	g	g	g	g	g	g	g	g	g	g	g	g	g	g	g	g	g
t	1960234	g	2155317	g	1933154	i	g	CB9615	++++	g	g	g	g	g	g	g	g	g	g	g	g	g	g	g	g	g	g	g	g	g	g	g
c	1961209	t	2156292	t	1932179	s	t	CB9615	++++	t	t	t	t	t	t	t	t	t	t	t	t	t	t	t	t	t	t	t	t	t	t	t
a	1961836	g	2156919	g	1931552	ns	g	CB9615	++++	g	g	g	g	g	g	g	g	g	g	g	g	g	g	g	g	g	g	g	g		g	g
c	1962473	t	2157556	t	1930915	ns	c	O157	++++	c	c	c	c	c	c	c	c	c	c	c				c	c	c	c	c	c			
-	1963468	-	2158551	R	1929919	ins	-	EDL933	IUPAC																							
-	1963488	c	2159632	N	1928838	ns	c	EDL933	++++																							
-	1963488	-	2196576	t	1886140	ins	?	EDL933	+/-																							
-	1963488	g	2197766	Y	1884950	ns	g	EDL933	+++					g		g	g		g					g								
-	1963488	c	2201262	R	1881454	ns	c	EDL933	++++		c		c	t		c	c		t	c	c	c	c	g	c	g						g
-	1964343	c	2206835	R	1877194	ns	c	EDL933	++++	c				c				c	c	c	c			c						c		c
-	1964343	c	2206865	R	1877164	ns	c	EDL933	++++	c				c				c	c	c	c			c						c		c
-	1964343	-	2207415	t	1876613	ins	?	EDL933	+/-																							
-	1964343	-	2207812	a	1876215	ins	?	EDL933	+/-																							
-	1964343	-	2208360	W	1875666	ins	-	EDL933	IUPAC																							
-	1964343	-	2208810	a	1875215	ins	?	EDL933	+/-																							
-	1964343	-	2208854	a	1875170	ins	?	EDL933	+/-																							
-	1964343	-	2208859	a	1875164	ins	?	EDL933	+/-																							
-	1964900	-	2209524	t	1874498	ins	?	EDL933	+/-																							
t	1964910	c	2209534	c	1874488	ns	?	O55/O157	+/-																							
-	1966317	-	2210941	Y	1873080	ins	-	EDL933	IUPAC																							
c	1966775	a	2211399	a	1872622	ns	c	O157	+								c															
-	1966812	-	2211436	t	1872584	ins	?	EDL933	+/-																							
-	1966929	g	2211554	g	1872466	ins	-	O157	+								-															
t	1966939	c	2211564	c	1872456	i	c	CB9615	+								c															
g	1989147	t	2234557	t	2309840	s	t	CB9615	+					c		c	t		t													t
c	1989511	t	2234921	t	2310204	ns	c	O157	+++					c			c		c													c
c	1991231	g	2236641	a	2311924	s	g	EDL933	++++					g		g	g		g		g	g									g	g
c	1991243	c	2236653	t	2311936	s	c	EDL933	++++					c		c	c		c		c	c									c	c
-	1991693	-	2240405	c	2315689	ins	-	EDL933	+										-													
-	1991693	a	2249469	g	2324754	i	a	EDL933	+															a								
-	1991693	a	2249978	c	2325263	ns	c	Sakai	+													c										
c	2012313	t	2255007	t	2330292	i	t	CB9615	++	t	c	c	c	c	c	c	c	c	c	c	c	c	c	c	c	c	c	c	c			
c	2012512	g	2255206	g	2330491	ns	c	O157	++++	c	c	c	c	c	c	c	c	c	c	c	c	c	c	c	c	c	c	c	c			
g	2015681	a	2258375	a	2333660	ns	g	O157	++++	g	g	g	g	g	g	g	g	g	g	g	g	g	g	g	g	g	g	g	g	g	g	g
t	2017353	c	2260047	c	2335332	ns	c	CB9615	++++	c	c	c	c	c	c	c	c		c	c	c	c	c	c	c	c	c	c	c	c	c	c
t	2017683	g	2260377	g	2335662	i	g	CB9615	++++	g	g	g	g	g	g	g	g		g	g	g	g	g	g	g	g	g	g	g		g	g
a	2017806	g	2260500	g	2335785	ns	g	CB9615	++++	g	g	g	g	g	g	a	g		g	g	g	g	g	g	g	g	g	g	g		g	g
c	2018548	g	2261242	g	2336527	ns	c	O157	++++	c	c	c	c	c	c	c	c		c	c	c	c	c	c	c	c	c	c	c		c	c
a	2021482	g	2264176	g	2339461	s	g	CB9615	++++	g	g	g	g	g	g	g	g	g	g	g	g	g	g	g	g	g	g	g	g	g	g	g
a	2021834	c	2264528	c	2339813	ns	c	CB9615	++++	c	c	c	c	c	c	c	c	c	c	c	c	c	c	c	c	c	c	c	c	c	c	c
c	2028325	c	2271019	t	2346304	i	c	EDL933	++++	c	c	c	c	c	c	c	c	c	c	c	c	c	c	c	c	c	c	c	c	c	c	c
a	2028430	c	2271124	c	2346409	i	a	O157	++++	a	a	a	a	a	a	a	a	a	a	a	a	a	a	a	a	a	a	a	a	a	a	a
t	2029555	-	2272248	t	2347534	del	t	Sakai	++++	t	t	t	t	t	t	t	t	t	t	t	t	t	t	t	t	t	t	t	t	t	t	t
c	2029777	t	2272470	t	2347756	ns	t	CB9615	++++	t	t	t	t	t	t	t	t	t	t	t	t	t	t	t	t	t	t	t	t	t	t	t
a	2031033	c	2273726	c	2349012	ns	a	O157	++++	a	a	a	a	a	a	a	a	a	a	a	a	a	a	a	a	a	a	a	a	a	a	a
t	2034972	g	2277665	g	2352951	ns	t	O157	++++	t	t	t	t	t	t	t	t	t	t	t	t	t	t	t	t	t	t	t	t	t	t	t
t	2035765	g	2278458	g	2353744	s	t	O157	++++	t	t	t	t	t	t	t	t	t	t	t	t	t	t	t	t	t	t	t	t	t	t	t
a	2036980	g	2279673	g	2354959	ns	g	CB9615	++++	g	g	g	g	g	g	g	g	g	g	g	g	g	g	g	g	g	g	g	g	g	g	g
g	2039344	a	2282037	a	2357323	ns	g	O157	++++	g	g	g	g	g	g	g	g	g	g	g	g	g	g	g	g	g	g	g	g	g	g	g
g	2040319	t	2283012	g	2358298	ns	g	Sakai	++++	g	g	g	g	g	g	g	g	g	g	g	g	g	g	g	g	g	g	g	g	g	g	g
-	2041355	-	2284048	g	2359335	ins	-	EDL933	++++	-	-	-	-	-	-	-	-	-	-	-	-	-	-	-	-	-	-	-	-	-	-	-
g	2046406	a	2289099	a	2364386	s	g	O157	++++	g	g	g	g	g	g	g	g	g	g	g	g	g	g	g	g	g	g	g	g	g	g	g
g	2047445	a	2290138	a	2365425	s	g	O157	++++	g	g	g	g	g	g	g	g	g	g	g	g	g	g	g	g	g	g	g	g	g	g	g
t	2052222	c	2294915	c	2370202	ns	t	O157	++++	t	t	t	t	t	t	t	t	t	t	t	t	t	t	t	t	t	t	t	t	t	t	t
-	2052312	c	2295006	c	2370293	ins	-	O157	++++	-	-	-	-	-	-	-	-	-	-	-	-	-	-	-	-	-	-	-	-	-	-	-
a	2052905	c	2295600	c	2370887	ns	a	O157	++++	a	a	a	a	a	a	a	a	a	a	a	a	a	a	a	a	a	a	a	a	a	a	a
a	2053239	g	2295934	g	2371221	i	a	O157	++++	c	a	a	a	a	a	a	a	a	a	a	a	a	a	a	c	a	a	a	a	a	c	c
c	2053796	g	2296491	g	2371778	s	c	O157	++++	c	c	c	c	c	c	c	c	c	c	c	c	c	c	c	a	c	c	c	c	c	c	c
t	2054336	c	2297031	c	2372318	s	c	CB9615	++	c	c	c	c	t	c	t	t	t	t	t	t	t	t	c	c	c	c	c	c	c	c	c
t	2059604	a	2302299	a	2377586	ns	a	CB9615	++++	a	a	a	a	a	a	a	a	a	a	a	a	a	a	a	a	a	a	a	a	a	a	a
-	2061361	-	2304056	M	2379344	ins	-	EDL933	++++	-	-	-	-	-	-	-	-	-	-	-	-	-	-	-	-	-	-	-	-	-	-	-
g	2061406	a	2304101	a	2379389	i	a	CB9615	++++	a	a	a	a	a	a	a	a	a	a	a	a	a	a	a	a	a	a	a	a	a	a	a
a	2065245	t	2307940	t	2383228	ns	a	O157	++++	a	a	a	a	a	a	a	a	a	a	a	a	a	a	a	a	a	a	a	a	a	a	a
t	2065771	g	2308466	g	2383754	ns	g	CB9615	++++	g	g	g	g	g	g	g	g	g	g	g	g	g	g	g	g	g	g	g	g	g	g	g
g	2065832	g	2308527	a	2383815	ns	g	EDL933	++++	g	g	g	g	g	g	g	g	g	g	g	g	g	g	g	g	g	g	g	g	g	g	g
a	2065846	a	2308541	g	2383829	s	a	EDL933	++	a	a	g	a	a	a	g	g	g	a	g	a	a	a	g	g	g	a	a	a	g	g	g
g	2065852	g	2308547	a	2383835	s	g	EDL933	++	g	g	a	g	g	g	g	g	g	g	g	g	g	g	g	g	g	g	g	g	a	a	g
c	2067958	t	2310653	t	2385845	s	c	O157	++++	c	c	c	c	c	c	c	c	c	c	c	c	c	c	c	c	c	c	c	c	c	c	c
g	2068858	a	2311553	a	2386745	ns	g	O157	++++	g	g	g	g	g	g	g	g	g	g	g	g	g	g	g	g	g	g	g	g	g	g	g
c	2069362	a	2312057	a	2387249	i	c	O157	++++	c	c	c	c	c	c	c	c	c	c	c	c	c	c	c	c	c	c	c	c	c	c	c
a	2069455	g	2312150	g	2387342	i	a	O157	++	a	a	a	a	a	a	a	a	a	a	a	a	a	a	a	a	a	g	g	g	a	a	a
c	2072702	g	2315397	g	2390589	ns	g	CB9615	++++	g	g	g	g	g	g	g	g	g	g	g	g	g	g	g	g	g	g	g	g	g	g	g
-	2074280	-	2316975	g	2392168	ins	-	EDL933	++++	-	-	-	-	-	-	-	-	-	-	-	-	-	-	-	-	-	-	-	-	-	-	-
a	2075060	t	2317755	t	2392948	i	a	O157	++++	a	a	a	a	a	a	a	a	a	a	a	a	a	a	a	a	a	a	a	a	a	a	a

O55 and O157 genome site details										Outgroup Strain Details[g]																						
CB9615 base[a]	CB9615 Site[b]	Sakai base[a]	Sakai Site[b]	EDL933 base[a]	EDL933 Site[b]	type[c]	Inferred ancestral base[d]	Lineage inferred to mutate[e]	Support level[f]	D1 Sd197	K-12	HS	ATCC 8739	UMN026	IAI39	SMS 3-5	E2348/69	536	ED1a	CFT073	S88	APEC O1	UTI89	E24377A	IAI1	SE11	F5 8401	F2a 2457T	F2a 301	SS Ss046	B4 Sb227	B18 BS512
c	2075462	t	2318157	t	2393350	i	c	O157	++++	c	c	c	c	c	c	c	c	c	c	c	c	c	c	c	c	c	c	c	c	c	c	c
a	2076155	g	2318850	g	2394043	s	g	CB9615	++++	g	g	g	g	g	g	g	g	g	g	g	g	g	g	g	g	g	g	g	g	g	g	g
a	2076401	c	2319096	c	2394289	ns	a	O157	++++	a	a	a	a	a	a	a	a	a	a	a	a	a	a	a	a	a	a	a	a	a	a	a
t	2076470	c	2319165	c	2394358	s	c	CB9615	++++	c	c	c	c	c	c	c	c	c	c	c	c	c	c	c	c	c	c	c	c	c	c	c
-	2076874	-	2319569	t	2394763	ins	-	EDL933	++++	-	-	-	-	-	-	-	-	-	-	-	-	-	-	-	-	-	-	-	-	-	-	-
a	2077457	t	2320152	t	2395346	s	a	O157	++++	a	a	a	a	a	a	a	a	a	a	a	a	a	a	a	a	a	a	a	a	a	a	a
c	2077999	g	2320694	g	2395888	i	c	O157	++++	c	c	c	c	c	c	c	c	c	c	c	c	c	c	c	c	c	c	c	c	c	c	c
g	2080868	a	2323563	a	2398757	ns	g	O157	++++	g	g	g	g	g	g	g	g	g	g	g	g	g	g	g	g	g	g	g	g	g	g	g
g	2081876	c	2324571	c	2399765	ns	g	O157	++++	g	g	g	g	g	g	g	g	g	g	g	g	g	g	g	g	g	g	g	g	g	g	g
c	2085605	t	2328300	t	2403494	s	c	O157	++++	c	c	c	c	c	c	c	c	c	c	c	c	c	c	c	c	c	c	c	c	c	c	c
a	2086448	g	2329143	g	2404337	ns	g	CB9615	++++	g	g	g	g	g	g	g								g	g	g	g	g	g	g	g	g
c	2086675	a	2329370	a	2404564	s	c	O157	++++	c	c	c	c	c	c	c								c	c	c	c	c	c	c	c	c
g	2087375	a	2330070	a	2405264	ns	g	O157	++++	g	g	g	g	g	g	g								g	g	g	g	g	g	g	g	g
c	2088205	t	2330900	t	2406094	s	c	O157	++++	c	c	c	c	c	c	c								c	c	c	c	c	c	c	c	c
a	2089674	g	2332369	g	2407563	ns	g	CB9615	++++	g	g	g	g	g	g	g								g	g	g	g	g	g	g	g	g
t	2091776	c	2334471	c	2409665	ns	t	O157	++++	t	t	t	t	t	t	t	t	t	t	t	t	t	t	t	t	t	t	t	t	t	t	t
a	2093414	g	2336109	g	2411303	ns	a	O157	++++	a	a	a	a	a	a	a	a	a	a	a	a	a	a	a	a	a	a	a	a	a	a	a
c	2093439	t	2336134	t	2411328	ns	c	O157	++++	c	c	c	c	c	c	c	c	c	c	c	c	c	c	c	c	c	c	c	c	c	c	c
a	2094383	-	2337077	-	2412271	ins	-	CB9615	++++	-	-	-	-	-	-	-	-	-	-	-	-	-	-	-	-	-	-	-	-	-	-	-
g	2094845	t	2337539	t	2412733	i	t	CB9615	++++	t	t	t	t	t	t	t	t	t	t	t	t	t	t	t	t	t	t	t	t	t	t	t
g	2095055	a	2337749	a	2412943	s	g	O157	++++	g	g	g	g	g	g	g	g	g	g	g	g	g	g	g	g	g	g	g	g	g	g	g
a	2096607	c	2339301	c	2414495	ns	c	CB9615	++++	c	c	c	c	c	c	c	c	c	c	c	c	c	c	c	c	c	c	c	c	c	c	c
c	2097551	t	2340245	t	2415439	s	c	O157	++++	c	c	c	c	c	c	c	c	c	c	c	c	c	c	c	c	c	c	c	c	c	c	c
t	2099336	c	2342030	c	2417224	ns	t	O157	++	t	c	c	t	c	c	c	c	c	c	c	c	c	c	c	c	c	c	c	c	c	c	c
g	2099635	a	2342329	a	2417523	ns	g	O157	++++	g	g	g	g	g	g	g	g	g	g	g	g	g	g	g	g	g	g	g	g	g	g	g
a	2100032	t	2342726	t	2417920	ns	t	CB9615	++++	t	t	t	t	t	t	t	t	t	t	t	t	t	t	t	t	t	t	t	t	t	t	t
-	2100362	-	2343056	a	2418251	ins	-	EDL933	++++	-	-	-	-	-	-	-	-	-	-	-	-	-	-	-	-	-	-	-	-	-	-	-
-	2100373	-	2343067	a	2418263	ins	-	EDL933	++++	-	-	-	-	-	-	-	-	-	-	-	-	-	-	-	-	-	-	-	-	-	-	-
a	2100509	g	2343203	g	2418399	s	a	O157	++++	a	a	a	a	a	a	a	a	a	a	a	a	a	a	a	a	a	a	a	a	a	a	a
a	2101497	c	2344191	c	2419387	ns	c	CB9615	++++	c	c	c	c	c	c	c	c	c	c	c	c	c	c	c	c	c	c	c	c	c	c	c
t	2101798	a	2344492	a	2419688	ns	t	O157	++++	t	t	t	t	t	t	t	t	t	t	t	t	t	t	t	t	t	t	t	t	t	t	t
t	2102468	g	2345162	g	2420358	ns	g	CB9615	++++	g	g	g	g	g	g	g	g	g	g	g	g	g	g	g	g	g	g	g	g	g	g	g
a	2102650	t	2345344	t	2420540	s	t	CB9615	++++	t	t	t	t	t	t	t	t	t	t	t	t	t	t	t	t	t	t	t	t	t	t	t
c	2103616	g	2346310	g	2421506	i	c	O157	++++	c	c	c	c	c	c	c	c	c	c	c	c	c	c	c	c	c	c	c	c	c	c	c
g	2103931	a	2346625	a	2421821	ns	g	O157	++++		g	g	g	g	g	g	g	g	g	g	g	g	g	g	g	g	g	g	g	g	g	g
g	2109506	a	2352200	a	2427396	ns	g	O157	++++		g	g	g	g	g	g	g	g	g	g	g	g	g	g	g	g	g	g	g	g	g	g
a	2110600	g	2353294	g	2428490	ns	g	CB9615	++++		g	g	g	g	g	g	g	g	g	g	g	g	g	g	g	g	g	g	g	g	g	
g	2111625	t	2354319	t	2429515	i	g	O157	++++		g	g	g	g	g	g	g	g	g	g	g	g	g	g	g	g	g	g	g	g	g	
t	2111832	c	2354526	c	2429722	i	t	O157	++++		t	t	t	t	t	t	t	t	t	t	t	t	t	t	t	t	t	t	t	t	c	
t	2112315	g	2355009	g	2430205	i	t	O157	+++	t	c	c	c	t	t	t	c	c	c	c	t	t	t	c	c	c	c	c	c	c	c	c
a	2114878	c	2357572	c	2432768	ns	c	CB9615	++++	c	c	c	c	c	c	c	c	c	c	c	c	c	c	c	c	c	c	c	c	c	c	c
c	2114943	g	2357637	g	2432833	ns	g	CB9615	++++	g	g	g	g	g	g	g	g	g	g	g	g	g	g	g	g	g	g	g	g	g	g	g
c	2115035	t	2357729	t	2432925	s	c	O157	++++	c	c	c	c	c	c	c	c	c	c	c	c	c	c	c	c	c	c	c	c	c	c	c
g	2120419	a	2363113	a	2438309	ns	g	O157	++++	g	g	g	g	g	g	g	g	g	g	g	g	g	g	g	g	g	g	g	g	g	g	g
c	2121479	g	2364173	g	2439369	i	c	O157	++++		c	c	c	c	c	c	c	c	c	c	c	c	c	c	c	c	c	c	c	c	c	c
a	2121771	g	2364465	g	2439661	nc	a	O157	++++	a	a	a	a	a	a	a	a	a	a	a	a	a	a	a	a	a	a	a	a	a	a	a
t	2124543	c	2367237	c	2442433	s	c	CB9615	++++	c	c	c	c	c	c	c	c	c	c	c	c	c	c	c	c	c	c	c	c	c	c	c
g	2124936	a	2367630	a	2442826	s	g	O157	++	g	g	g	g	g	a	a	a	g	g	g	g	g	g	g	g	g	g	g	g	g	g	g
c	2125542	t	2368236	t	2443432	s	c	O157	++++	c	c	c	c	c	c	c	c	c	c	c	c	c	c	c	c	c	c	c	c	c	c	c
-	2129197	9	2371892	9	2447088	del	9	CB9615	++	9	9	9	9	9	9	9	9	9	9	9	9	9	9	9	9	9	9	-	-	9		
t	2130307	c	2373010	c	2448206	s	t	O157	+		t	t	t	t	c	c	c	c	c	c	c	c	c	t	t	t	t	t	t	t	t	t
g	2130718	a	2373421	a	2448617	ns	g	O157	++++	g	g	g	g	g	g	g	g	g	g	g	g	g	g	g	g	g	g	g	g	g	g	g
g	2133131	a	2375834	a	2451030	s	g	O157	++++		g	g	g	g	g	g	g	g	g	g	g	g	g	g	g	g				g	g	g
a	2133633	g	2376336	g	2451532	ns	g	CB9615	++++		g	g	g	g	g	g	g	g	g	g	g	g	g	g	g	g					g	g
a	2133915	g	2376618	g	2451814	ns	g	CB9615	++++		g	g	g	g	g	g	g	g	g	g	g	g	g	g	g	g					g	g
-	2136323	-	2379026	c	2454223	ins	-	EDL933	++++	-	-		-	-	-	-	-	-	-	-	-	-	-	-	-	-	-	-	-		-	-
-	2136328	-	2379031	n	2454229	ins	-	EDL933	++++	-	-		-	-	-	-	-	-	-	-	-	-	-	-	-	-	-	-	-		-	-
-	2136357	-	2379060	g	2454259	ins	-	EDL933	++++	-	-		-	-	-	-	-	-	-	-	-	-	-	-	-	-	-	-	-		-	-
-	2136368	-	2379071	g	2454271	ins	-	EDL933	++++	-	-		-	-	-	-	-	-	-	-	-	-	-	-	-	-	-	-	-		-	-
-	2136380	-	2379083	g	2454284	ins	-	EDL933	++++	-	-		-	-	-	-	-	-	-	-	-	-	-	-	-	-	-	-	-		-	-
c	2136873	t	2379576	t	2454777	s	t	CB9615	++++	t	t		t	t	t	t	t	t	t	t	t	t	t	t	t	t	t	t	t		t	t
a	2137856	g	2380559	g	2455760	ns	g	CB9615	++++	g	g		g	g	g	g	g	g	g	g	g	g	g	g	g	g	g	g	g		g	g
a	2138741	g	2381444	g	2456645	s	g	CB9615	++++	g	g		g	g	g	g	g	g	g	g	g	g	g	g	g	g	g	g	g		g	g
g	2140260	a	2382963	a	2458164	s	a	CB9615	++++	a	a		a	a	a	a	a	a	a	a	a	a	a	a	a	a	a	a	a		a	a
c	2140275	a	2382978	a	2458179	s	c	O157	++++	c	c		c	c	c	c	c	c	c	c	c	c	c	c	c	c	c	c	c		c	c
a	2141836	g	2384539	g	2459740	ns	g	CB9615	++++	g	g	g	g	g	g	g	g	g	g	g	g	g	g	g	g	g	g	g	g	g	g	g
c	2143180	c	2385883	t	2461084	ns	c	EDL933	++++	c	c	c	c	c	c	c	c	c	c	c	c	c	c	c	c	c	c	c	c	c	c	c
g	2143299	a	2386002	a	2461203	s	g	O157	++++	g	g	g	g	g	g	g	g	g	g	g	g	g	g	g	g	g	g	g	g	g	g	g
a	2144067	c	2386770	c	2461971	ns	a	O157	++++	a	a	a	a	a	a	a	a	a	a	a	a	a	a	a	a	a	a	a	a	a	a	a
t	2144244	g	2386947	g	2462148	i	t	O157	++++	t	t	t	t	t	t	t	t	t	t	t	t	t	t	t	t	t	t	t	t	t	t	t
g	2145797	a	2388500	a	2463701	ns	g	O157	++++	g	g	g	g	g	g	g	g	g	g	g	g	g	g	g	g	g	g	g	g	g	g	g
t	2147378	a	2390081	a	2465282	ns	t	O157	++++	t	t	t	t	t	t	t	t	t	t	t	t	t	t	t	t	t	t	t	t	t	t	t
a	2148588	g	2391291	g	2466492	s	a	O157	++++	a	a	a	a	a	a	a	a	a	a	a	a	a	a	a	a	a	a	a	a	a	a	a
c	2149142	t	2391845	t	2467046	i	t	CB9615	++++	t	t	t	t	t	t	t	t	t	t	t	t	t	t	t	t	t	t	t	t	t	t	t
a	2151609	g	2394312	g	2469513	s	g	CB9615	++++	g	g	g	g	g	g	g	g	g	g	g	g	g	g	g	g	g	g	g	g	g	g	g
-	2152220	-	2394923	t	2470125	ins	-	EDL933	++++	-	-	-	-	-	-	-	-	-	-	-	-	-	-	-	-	-	-	-	-	-	-	-
t	2188835	a	2395787	a	2470989	s	a	CB9615	++++	a	a	a	a	a	a	a	a	a	a	a	a	a	a	a	a	a	a	a	a	a	a	a
g	2190258	t	2397210	t	2472412	ns	t	CB9615	++++	t	t	t	t	t	t	t	t	t	t	t	t	t	t	t	t	t	t	t	t	t	t	t
g	2193073	t	2400025	t	2475227	s	t	CB9615	++++	t	t	t	t	t	t	t	t	t	t	t	t	t	t	t	t	t	t	t	t	t	t	t

O55 and O157 genome site details										Outgroup Strain Details[g]																						
CB9615 base[a]	CB9615 Site[b]	Sakai base[a]	Sakai Site[b]	EDL933 base[a]	EDL933 Site[b]	type[c]	Inferred ancestral base[d]	Lineage inferred to mutate[e]	Support level[f]	D1 Sd197	K-12	HS	ATCC 8739	UMN026	IAI39	SMS 3-5	E2348/69	536	ED1a	CFT073	S88	APEC O1	UTI89	E24377A	IAI1	SE11	F5 8401	F2a 2457T	F2a 301	SS Ss046	B4 Sb227	B18 BS512
g	2195302	a	2402254	a	2477456	s	g	O157	++++	g	g	g	g	g	g	g	g	g	g	g	g	g	g	g	g	g	g	g	g	g	g	g
g	2195643	a	2402595	a	2477797	i	g	O157	++++	g	g	g	g	g	g	g	g	g	g	g	g	g	g	g	g	g	g	g	g	g	g	g
g	2195963	c	2402915	c	2478117	i	g	O157	++++		g	g	g	g	g	g	g	g	g	g	g	g	g	g	g	g	g	g	g	g	g	g
-	2196051	a	2403004	a	2478206	del	a	CB9615	+		a	a	a	a	a	a	a	a	a	a	a	a	a	a	a	a	-	-	-	a	a	a
c	2198104	t	2405057	t	2480259	s	c	O157	++++		c	c	c	c	c	c	c	c	c	c	c	c	c	c	c	c	c	c	c	c	c	c
t	2198197	c	2405150	c	2480352	s	t	O157	++++		t	t	t	t	t	t	t	t	t	t	t	t	t	t	t	t	t	t	t	t	t	t
a	2201411	t	2408364	t	2483566	s	a	O157	++++	a	a	a	a	a	a	a	a	a	a	a	a	a	a	a	a	a	a	a	a	a	a	a
c	2201467	c	2408420	t	2483622	s	c	EDL933	++++	c	c	c	c	c	c	c	c	c	c	c	c	c	c	c	c	c	c	c	c	c	c	c
g	2201704	a	2408657	a	2483859	s	g	O157	++++	g	g	g	g	g	g	g	g	g	g	g	g	g	g	g	g	g	g	g	g	g	g	g
t	2202047	c	2409000	c	2484202	i	t	O157	++++	t	t	t	t	t	t	t	t	t	t	t	t	t	t	t	t	t	t	t	t	t	t	t
g	2202130	a	2409083	g	2484285	i	g	Sakai	++++	g	g	g	g	g	g	g	g	g	g	g	g	g	g	g	g	g	g	g	g	g	g	g
g	2202753	g	2409706	t	2484908	ns	g	EDL933	++++	g	g	g	g	g	g	g	g	g	g	g	g	g	g	g	g	g	g	g	g	g	g	g
a	2204152	t	2411105	t	2486307	ns	a	O157	++++	a	a	a	a	a	a	a	a	a	a	a	a	a	a	a	a	a	a	a	a	a	a	a
a	2205199	-	2412151	-	2487353	del	a	O157	++++	a	a	a	a	a	a	a								a	a	a	a	a	a	a	a	a
a	2208397	g	2415349	g	2490551	s	g	CB9615	++++		g	g	g	g	g	g	g	g	g	g	g	g	g	g	g	g	g	g	g	g		
g	2209514	t	2416466	t	2491668	ns	g	O157	++++		g	g	g	g	g	g	g	g	g	g	g	g	g	g	g	g	g	g	g	a		
g	2212266	a	2419218	a	2494420	s	g	O157	++++		g	g	g	g	g	g	g	g	g	g	g	g	g	g	g	g	g	g	g	g	g	g
g	2212555	a	2419507	a	2494709	ns	g	O157	++++		g	g	g	g	g	g	g	g	g	g	g	g	g	g	g	g	g	g	g	g	g	g
a	2213163	g	2420115	g	2495317	s	g	CB9615	++++		g	g	g	g	g	g	g	g	g	g	g	g	g	g	g	g	g	g	g	g		g
c	2214828	a	2421780	a	2496982	i	c	O157	++++	c	c	c	c	c	c	c	c	c	c	c	c	c	c	c	c	c	c	c	c	c	c	c
g	2215212	a	2422164	a	2497366	ns	a	CB9615	++++	a	a	a	a	a	a	a	a	a	a	a	a	a	a	a	a	a	a	a	a	a	a	a
g	2215394	t	2422346	t	2497548	ns	g	O157	++++	g	g	g	g	g	g	g	g	g	g	g	g	g	g	g	g	g	g	g	g	g	g	g
g	2215871	a	2422823	a	2498025	i	g	O157	++++	g	g	g	g	g	g	g	g	g	g	g	g	g	g	g	g	g	g	g	g	g	g	g
a	2218344	-	2425295	a	2500498	del	a	Sakai	++++	a	a	a	a	a	a	a	a	a	a	a	a	a	a	a	a	a	a	a	a	a	a	a
a	2218557	g	2425508	g	2500711	s	a	O157	++++	a	a	a	a	a	a	a	a	a	a	a	a	a	a	a	a	a	a	a	a	a	a	a
a	2219759	c	2426710	c	2501913	s	a	O157	++++	a	a	a	a	a	a	a	a	a	a	a	a	a	a	a	a	a	a	a	a	a	a	a
t	2221546	c	2428497	c	2503700	s	t	O157	++++	t	t	t	t	c	t	t	t	t	t	t	t	t	t	t	t	t	t	t	t	t	t	t
g	2221695	a	2428646	a	2503849	ns	g	O157	++++	g	g	g	g	g	g	g	g	g	g	g	g	g	g	g	g	g	g	g	g	g	g	g
a	2221818	g	2428769	g	2503972	s	g	CB9615	++	g	g	g	g	g	g	g	g	g	g	g	g	g	g	a	a	g	g	g	g	g	g	g
t	2222017	c	2428968	c	2504171	s	t	O157	++++	t	t	t	t	t	c	t	t	t	t	t	t	t	t	t	t	t	t	t	t	t	t	t
t	2223336	c	2430287	c	2505490	ns	c	CB9615	++++	c	c	c	c	c	c	c	c	c	c	c	c	c	c	c	c	c	c	c	c	c	c	c
g	2223851	a	2430802	a	2506005	s	g	O157	++++	g	g	g	g	g	g	g	g	g	g	g	g	g	g	g	g	g	g	g	g	g	g	g
a	2224641	g	2431592	g	2506795	i	a	O157	++++	a	a	a	a	a	a	a	a	a	a	a	a	a	a	a	a	a	a	a	a	a	a	a
c	2225015	t	2431966	t	2507169	s	c	O157	++++	c	c	c	c	c	c	c	c	c	c	c	c	c	c	c	c	c	c	c	c	c	c	c
a	2229269	t	2436220	t	2511423	ns	a	O157	++++	a	a	a	a	a	a	a	a	a	a	a	a	a	a	a	a	a	a	a	a	a	a	a
g	2230652	a	2437603	a	2512806	ns	g	O157	++++	g	g	g	g	g	g	g	g	g	g	g	g	g	g	g	g	g	g	g	g	g	g	g
c	2232166	t	2439117	t	2514320	ns	c	O157	++++	c	c	c	c	c	c	c	c	c	c	c	c	c	c	c	c	c	c	c	c	c	c	c
t	2232550	a	2439501	a	2514704	ns	t	O157	++++	t	t	t	t	t	t	t	t	t	t	t	t	t	t	t	t	t	t	t	t	t	t	t
a	2232945	g	2439896	g	2515099	s	g	CB9615	++++	g	g	g	g	g	g	g	g	g	g	g	g	g	g	g	g	g	g	g	g	g	g	g
g	2237897	a	2444848	a	2520051	ns	g	O157	++++	g	g	g	g	g	g	g	g	g	g	g	g	g	g	g	g	g	g	g	g	g	g	g
g	2240127	a	2447078	a	2522281	ns	a	CB9615	++++	a	a	a	a	a	a	a	a	a	a	a	a	a	a	a	a	a	a	a	a	a	a	a
a	2242307	g	2449258	g	2524461	ns	a	O157	++++	a	a	a	a	a	a	a	a	a	a	a	a	a	a	a	a	a	a	a	a	a	a	a
a	2242841	g	2449792	g	2524995	ns	g	CB9615	++++	g	g	g	g	g	g	g	g	g	g	g	g	g	g	g	g	g	g	g	g	g	g	g
t	2243022	c	2449973	c	2525176	ns	t	O157	++++	t	t	t	t	t	t	t	t	t	t	t	t	t	t	t	t	t	t	t	t	t	t	t
c	2245333	t	2452284	t	2527487	ns	t	CB9615	++++	t	t	t	t	t	t	t	t	t	t	t	t	t	t	t	t	t	t	t	t	t	t	t
g	2248357	a	2456621	a	2531824	ns	g	O157	++++	g	g	g	g	g	g	g	g	g	g	g	g	g	g	g	g	g	g	g	g			
t	2249632	a	2457896	a	2533099	ns	a	CB9615	++++	a	a	a	a	a	a	a	a	a	a	a	a	a	a	a	a	a	a	a	a			
a	2249770	t	2458034	t	2533237	ns	a	O157	++++	a	a	a	a	a	a	a	a	a	a	a	a	a	a	a	a	a	a	a	a			
g	2250098	a	2458362	a	2533565	ns	g	O157	++++		g	g	g	g	g	g	g	g	g	g	g	g	g	g	g	g	g	g	g			
a	2251460	c	2459724	c	2534927	ns	c	CB9615	++++	c	c	c	c	c	c	c	c	c	c	c	c	c	c	c	c	c	c	c	c			
c	2252634	t	2460898	t	2536101	ns	c	O157	++++	c	c	c	c	c	c	c	c	c	c	c	c	c	c	c	c	c	c	c	c			
t	2253969	c	2463011	c	2538214	i	t	O157	++++	t	t	t	t	t	t	t	t	t	t	t	t	t	t	t	t	t	t	t	t			
c	2253996	t	2463038	t	2538241	i	t	CB9615	++++	t	t	t	t	t	t	t	t	t	t	c	t	t	t	t	t	t	t	t	t			
g	2254053	t	2463095	t	2538298	i	g	O157	++++	g	g	g	g	g	g	g	g	g	g	g	g	g	g	g	g	g	g	g	g			
c	2258343	a	2467385	a	2542588	ns	c	O157	++++	c	c	c	c	c	c	c	c	c	c	c	c	c	c	c	c	c	c	c	c	c	c	c
g	2258465	a	2467507	a	2542710	ns	g	O157	++++	g	g	g	g	g	g	g	g	g	g	g	g	g	g	g	g	g	g	g	g	g	g	g
a	2259638	g	2468680	g	2543883	s	g	CB9615	++++		g	g	g	g	g	g	g	g	g	g	g	g	g	g	g	g	g	g		g	g	g
a	2262851	g	2471893	g	2547096	i	a	O157	++++		a	a	a	a	a	a	a	a	a	a	a	a	a	a	a	a				a	a	a
t	2265150	a	2474192	a	2549395	ns	t	O157	++++		t	t	t	t	t	t	t	t	t	t	t	t	t	t	t	t		t	t	t	t	t
a	2265975	g	2475017	g	2550220	ns	g	CB9615	++++	g	g	g	g	g	g	g	g	g	g	g	g	g	g	g	g	g				g		g
g	2271124	t	2480166	t	2555369	ns	g	O157	++++	g	g	g	g	g	g	g	g	g	g	g	g	g	g	g	g	g		g	g	g		
a	2272584	t	2481626	t	2556829	i	a	O157	++++	a	a	a	a	a	a	a	a	a	a	a	a	a	a	a	a	a				a	a	a
c	2273129	t	2482171	t	2557374	ns	c	O157	++++	c	c	c	c	c	c	c	c	c	c	c	c	c	c	c	c	c				c	c	c
a	2277357	g	2486399	g	2561602	s	g	CB9615	++++	g	g	g	g	g	g	g								g	g	g				g	g	g
g	2277452	a	2486494	a	2561697	ns	a	CB9615	++++	a	a	a	a	a	a	a								a	a	a				a	a	a
t	2278708	c	2487750	c	2562953	ns	t	O157	++++	t	t	t	t	t	t	t								t	t	t	t	t	t	t	t	t
c	2278796	t	2487838	t	2563041	s	c	O157	++++	c	c	c	c	c	c	c								c	c	c	c	c	c	c	c	c
a	2279004	c	2488046	c	2563249	ns	a	O157	++++	a	a	a	a	a	a	a								a	a	a	a	a	a	a	a	a
t	2279147	a	2488189	a	2563392	ns	t	O157	++++	t	t	c	t	t	t	c								c	c	t	t	t	t	t	c	c
a	2279153	g	2488195	g	2563398	s	a	O157	++++	a	a	a	a	a	a	a								a	a	a	a	a	a	a	a	a
-	2279608	-	2488650	a	2563854	ins	-	EDL933	++++	-	-	-	-	-	-	-	-	-	-	-	-	-	-	-	-	-	-	-	-	-	-	-
g	2281073	a	2490115	a	2565319	s	g	O157	++++	g	g	g	g	g	g	g	g	g	g	g	g	g	g	g	g	g	g	g	g	g	g	g
g	2281159	a	2490201	a	2565405	s	g	O157	++	g	g	g	g	g	g	g	g	g	g	g	g	g	g	g	g	g	a	a	a	g	g	g
g	2281336	a	2490378	a	2565582	ns	g	O157	++++	g	g	g	g	g	g	g	g	g	g	g	g	g	g	g	g	g	g	g	g	g	g	g
t	2282267	c	2491309	c	2566513	s	c	CB9615	++++	c	c	c	c	c	c	c	c	c	c	c	c	c	c	c	c	c	c	c	c	c	c	c
c	2282866	g	2491908	g	2567112	ns	g	CB9615	++++	g	g	g	g	g	g	g	g	g	g	g	g	g	g	g	g	g	g	g	g	g	g	g
c	2284820	t	2493862	t	2569066	s	c	O157	++++	c	c	c	c	c	c	c	c	t	c	c	c	c	c	c	c	c	c	c	c	c	c	c
g	2286812	a	2495854	a	2571058	i	g	O157	++++	g	g	g	g	g	g	g	g	g	g	g	g	g	g	g	g	g	g	g	g	g	g	g
g	2288110	a	2497152	a	2572356	s	g	O157	++++	g	g	g	g	c	c	g	c	g	g	g	g	g	g	g	g	g	g	g	g	g	g	g

O55 and O157 genome site details											Outgroup Strain Details[g]																						
CB9615 base[a]	CB9615 Site[b]	Sakai base[a]	Sakai Site[b]	EDL933 base[a]	EDL933 Site[b]	type[c]	Inferred ancestral base[d]	Lineage inferred to mutate[e]	Support level[f]	D1 Sd197	K-12	HS	ATCC 8739	UMN026	IAI39	SMS 3-5	E2348/69	536	ED1a	CFT073	S88	APEC O1	UTI89	E24377A	IAI1	SE11	F5 8401	F2a 2457T	F2a 301	SS Ss046	B4 Sb227	B18 BS512
a	2288187	g	2497229	g	2572433	ns	a	O157	++++	a	a	a	a	a	a	a	a	a	a	a	a	a	a	a	a	a	a	a	a	a	a	a
a	2288436	c	2497478	y	2572682	ns	a	EDL933	IUPAC	a	a	a	a	a	a	a	a	a	a	a	a	a	a	a	a	a	a	a	a	a	a	a
a	2288436	c	2497478	y	2572682	ns	a	O157	++++	a	a	a	a	a	a	a	a	a	a	a	a	a	a	a	a	a	a	a	a	a	a	a
g	2288651	t	2497693	g	2572897	ns	g	Sakai	++++	g	g	g	g	a	a	g	g	g	g	g	g	g	g	g	g	g	g	g	g	g	g	g
c	2288863	a	2497905	a	2573109	s	c	O157	++++	c	c	c	c	c	g	g	c	c	c	g	c	c	c	c	c	c	c	c	c	c	c	c
c	2291880	t	2500922	t	2576126	ns	c	O157	++++	c	c	c	c	c	c	c	c	c	c	c	c	c	c	c	c	c	c	c	c	c	c	c
a	2291913	c	2500955	c	2576159	ns	a	O157	++++	a	a	a	a	a	a	a	a	a	a	a	a	a	a	a	a	a	a	a	a	a	a	a
-	2294800	-	2503842	c	2579047	ins	-	EDL933	++++	-	-	-	-	-	-	-	-	-	-	-	-	-	-	-	-	-	-	-	-	-	-	-
-	2294814	-	2503856	c	2579062	ins	-	EDL933	++++	-	-	-	-	-	-	-	-	-	-	-	-	-	-	-	-	-	-	-	-	-	-	-
g	2295662	a	2504704	a	2579910	s	g	O157	++++	g	g	g	g	g	g	g	g	g	g	g	g	g	g	g	g	g	g	g	g	g	g	g
t	2295847	a	2504889	a	2580095	i	t	O157	++++	t	t	t	t	t	t	t	t	t	t	t	t	t	t	t	t	t	t	t	t	t	t	t
a	2298152	g	2507194	g	2582400	i	g	CB9615	++++	g	g	g	g	g	g	g	g	g	g	g	g	g	g	g	g	g	g	g	g	g	g	g
g	2300562	-	2509603	-	2584809	del	g	O157	++++	g	g	g	g	g	g	g	g	g	g	g	g	g	g	g	g	g	g	g	g	g	g	
t	2300616	c	2509657	c	2584863	i	c	CB9615	++++	c	c	c	c	c	c	c	c	c	c	c	c	c	c	c	c	c	c	c	c	c	c	
-	2300803	a	2509845	a	2585051	ins	-	O157	++++	-	-	-	-	-	-	-	a	-	-	-	-	-	-	-	-	-	-	-	-	-	-	-
g	2301108	a	2510150	a	2585356	s	g	O157	++++	g	g	g	g	g	g	g	g	g	g	g	t	t	t	g	g	g	g	g	g	g	g	g
a	2301418	g	2510460	g	2585666	ns	g	CB9615	++++	g	g	g	g	g	g	g	g	g	g	g	g	g	g	g	g	g	g	g	g	g	g	g
c	2301613	t	2510655	t	2585861	i	c	O157	++++	c	c	c	c	c	c	c	c	c	c	c	c	c	c	c	c	c	c	c	c	c	c	c
g	2301816	a	2510858	a	2586064	ns	g	O157	++++	g	g	g	g	g	g	g	g	g	g	g	g	g	g	g	g	g	g	g	g	g	g	g
-	2301991	-	2511033	a	2586240	ins	-	EDL933	++++	-	-	-	-	-	-	-	-	-	-	-	-	-	-	-	-	-	-	-	-	-	-	-
t	2303132	t	2512174	c	2587381	s	t	EDL933	++++	t	t	t	t	t	t	t	t	t	t	t	t	t	t	t	t	t	t	t	t	t	t	t
t	2305488	t	2514530	g	2589737	i	t	EDL933	++++	t	t	t	t	t	t	t	t	t	t	t	t	t	t	t	t	t	t	t	t	t	t	t
c	2312217	t	2521259	t	2596466	s	c	O157	++++	c	c	c	c	c	c	c	c	c	c	c	c	c	c	c	c	c	c	c	c	c	c	c
g	2312569	t	2521611	t	2596818	ns	g	O157	++++	g	g	g	g	g	g	g	g	g	g	g	g	g	g	g	g	g	g	g	g	g	g	g
a	2313885	c	2522927	c	2598134	ns	c	CB9615	++++	c	c	c	c	c	c	c	c	c	c	c	c	c	c	c	c	c	c	c	c	c	c	c
t	2314782	g	2523824	g	2599031	ns	g	CB9615	++++	g	g	g	g	g	g	g	g	g	g	g	g	g	g	g	g	g	g	g	g	g	g	g
g	2317964	c	2527006	c	2602213	ns	c	CB9615	++++	c	c	c	c	c	c	c	c	c	c	c	c	c	c	c	c	c	c	c	c	c	c	c
a	2318692	t	2527734	t	2602941	ns	t	CB9615	++++	t	t	t	t	t	t	t	t	t	t	t	t	t	t	t	t	t	t	t	t	t	t	t
g	2319021	a	2528063	a	2603270	ns	g	O157	++++	g	g	g	g	g	g	g	g	g	g	g	g	g	g	g	g	g	g	g	g	g	g	g
g	2319872	a	2528914	a	2604121	ns	g	O157	++++	g	g	g	g	g	g	g	g	g	g	g	g	g	g	g	g	g	g	g	g	g	g	g
t	2320307	c	2529349	c	2604556	ns	c	CB9615	++++	c	c	c	c	c	c	c	c	c	c	c	c	c	c	c	c	c	c	c	c	c	c	c
g	2320858	t	2529900	t	2605107	s	g	O157	++++	g	g	g	g	g	g	g	g	g	g	g	g	g	g	g	g	g	g	g	g	g	g	g
a	2321220	c	2530262	c	2605469	ns	c	CB9615	++++	c	c	c	c	c	c	c	c	c	c	c	c	c	c	c	c	c	c	c	c	c	c	c
a	2321374	g	2530416	g	2605623	s	a	O157	++++	a	a	a	a	a	a	a	a	a	a	a	a	a	a	a	a	a	a	a	a	a	a	a
-	2322790	-	2531832	t	2607040	ins	-	EDL933	++++	-	-	-	-	-	-	-	-	-	-	-	-	-	-	-	-	-	-	-	-	-	-	-
t	2323458	c	2532500	c	2607708	ns	c	CB9615	++++	c	c	c	c	c	c	c	c	c	c	c	c	c	c	c	c	c	c	c	c	c	c	
t	2323564	c	2532606	c	2607814	i	t	O157	++++	t	t	t	t	t	t	t	t	t	t	t	t	t	t	t	t	t	t	t	t	t	t	t
a	2323593	g	2532635	g	2607843	i	a	O157	++++	a	a	a	a	a	a	a	a	a	a	a	a	a	a	a	a	a	a	a	a	a	a	a
g	2329070	a	2538112	a	2613320	s	g	O157	++++	g	g	g	g	g	g	g	g	g	g	g	g	g	g	g	g	g	g	g	g	g	g	g
g	2329078	a	2538120	a	2613328	ns	g	O157	++++	g	g	g	g	g	g	g	g	g	g	g	g	g	g	g	g	g	g	g	g	g	g	g
c	2329216	a	2538258	a	2613466	i	a	CB9615	++++	a	a	a	a	a	a	a	a	a	a	a	a	a	a	a	a	a	a	a	a	a	a	a
g	2330774	a	2539816	a	2615024	ns	g	O157	++++	g	g	g	g	g	g	g	g	g	g	g	g	g	g	g	g	g	g	g	g	g	g	g
a	2334479	t	2543521	t	2618729	ns	t	CB9615	++++	t	t	t	t	t	t	t	t	t	t	t	t	t	t	t	t	t	t	t	t	t	t	t
g	2334532	a	2543574	a	2618782	s	g	O157	++	g	g	g	g	g	g	g	g	g	g	g	g	g	g	g	g	g	g	g	g	g	a	a
a	2334850	c	2543892	c	2619100	ns	c	CB9615	++++	c	c	c	c	c	c	c	c	c	c	c	c	c	c	c	c	c	c	c	c	c	c	c
t	2335397	c	2544439	c	2619647	i	t	O157	++++	t	t	t	t	t	t	t	t	t	t	t	t	t	t	t	t	t	t	t	t	t	t	t
g	2335505	g	2544547	a	2619755	ns	g	EDL933	++++	g	g	g	g	g	g	g	g	g	g	g	g	g	g	g	g	g	g	g	g	g	g	g
t	2335606	g	2544648	g	2619856	s	g	CB9615	++++	g	g	g	g	g	g	g	g	g	a	g	a	a	a	g	g	g	g	g	g	g	g	g
g	2336882	a	2545924	a	2621132	ns	g	O157	++++	g	g	g	g	g	g	g	g	g	g	g	g	g	g	g	g	g	g	g	g	g	g	g
g	2338943	a	2547985	a	2623193	i	g	O157	++++	g	g	g	g	g	g	g	g	g	g	g	g	g	g	g	g	g	g	g	g	g	g	g
c	2339180	a	2548222	a	2623430	ns	c	O157	++++	c	c	c	c	c	c	c	c	c	c	c	c	c	c	c	c	c	c	c	c	c	c	
a	2341266	t	2550308	t	2625516	s	t	CB9615	++++	t	t	t	t	t	t	t	t	t	t	t	t	t	t	t	t	t	t	t	t	t	t	t
g	2342447	a	2551489	a	2626697	ns	g	O157	++++	g	g	g	g	g	g	g	g	g	g	g	g	g	g	g	g	g	g	g	g	g	g	g
c	2342607	t	2551649	t	2626857	s	c	O157	++++	c	c	c	c	c	c	c	c	c	c	c	c	c	c	c	c	c	c	c	c	c	c	c
a	2343639	c	2552681	c	2627889	i	a	O157	++++	a	a	a	a	a	a	a	a	a	a	a	a	a	a	a	a	a	a	a	a	a	a	a
g	2344752	a	2553794	a	2629002	ns	g	O157	++++		g	g	g	g	g	g	g	g	g	g	g	g	g	g	g	g						
t	2346001	c	2555043	c	2630251	s	c	CB9615	++++	c	c	c	c	c	c	c	c	c	c	c	c	c	c	c	c	c	c	c	c	c	c	c
c	2348926	t	2557968	t	2633176	ns	c	O157	++	c	c	c	c	c	c	c	c	c	c	c	c	c	c	c	c	c	t	t	t	c		c
c	2349112	a	2558154	a	2633362	ns	c	O157	++++	c	c	c	c	c	c	c	c	c	c	c	c	c	c	c	c	c	c	c	c	c		c
g	2350948	t	2559990	t	2635198	ns	g	O157	++++	g	g	g	g	g	g	g	g	g	g	g	g	g	g	g	g	g	g	g	g	g	g	g
t	2351072	c	2560114	c	2635322	i	t	O157	++	t	t	c	c	t	t	t	c	t	t	c	c	c	c	c	c	c	c	c	c	c	c	c
-	2351264	-	2560306	g	2635515	ins	-	EDL933	++++	-	-	-	-	-	-	-	-	-	-	-	-	-	-	-	-	-	-	-	-	-	-	-
-	2351274	-	2560316	t	2635526	ins	-	EDL933	++++	-	-	-	-	-	-	-	-	-	-	-	-	-	-	-	-	-	-	-	-	-	-	-
-	2351295	-	2560337	N	2635548	ins	-	EDL933	++++	-	-	-	-	-	-	-	-	-	-	-	-	-	-	-	-	-	-	-	-	-	-	-
c	2351885	g	2560927	g	2636138	s	c	O157	++++	c	c	c	c	c	c	c	c	c	c	c	c	c	c	c	c	c	c	c	c	c	c	c
c	2352431	t	2561473	t	2636684	ns	c	O157	++++	c	c	c	c	c	c	c	c	c	c	c	c	c	c	c	c	c	c	c	c	c	c	c
g	2353704	a	2562746	a	2637957	ns	g	O157	++++	g	g	g	g	g	g	g	g	g	g	g	g	g	g	g	g	g	g	g	g	g	g	g
c	2357168	a	2566210	a	2641421	s	c	O157	++++	c	c	c	c	c	t	c	t	t	t	c	t	t	t	c	c	c	c	c	c	c	c	c
g	2358106	a	2567148	a	2642359	ns	g	O157	++++		g	g	g	g	g	g	g	g	g	g	g	g	g	g	g	g	g	g	g	g	g	g
a	2358858	c	2567900	c	2643111	s	c	CB9615	++++		c	c	c	c	c	c	c	c	c	c	c	c	c	c	c	c	c	c	c	c	c	c
a	2360624	g	2569666	g	2644877	ns	g	CB9615	++++		g	g	g	g	g	g	g	g	g	g	g	g	g	g	g	g	g	g	g	g		g
a	2361613	g	2570655	g	2645866	s	g	CB9615	++++		g	g	g	g	g	g	g	g	g	g	g	g	g	g	g	g	g	g	g	g	g	g
c	2362898	a	2571940	a	2647151	s	c	O157	++++		c	c	c	c		c								c	c	c	c	c	c	c		c
t	2364247	c	2573289	c	2648500	ns	t	O157	++++	t	t	t	t	t	t	t	t	t	t	t	t	t	t	t	t	t	t	t	t	t	t	t
t	2365408	c	2574450	c	2649661	ns	c	CB9615	++++		c	c	c	c	c	c	c	c	c	c	c	c	c	c	c	c	c	c	c	c		c
a	2370148	-	2579189	-	2654400	ins	-	CB9615	++++	-	-	-	-	-	-	-	-	-	-	-	-	-	-	-	-	-	-	-	-	-	-	
t	2370855	a	2579896	a	2655107	ns	t	O157	++++	t	t	t	t	t	t	t	t	t	t	t	t	t	t	t	t	t	t	t	t	t	t	
c	2371619	a	2580660	a	2655871	i	a	CB9615	++++	a	a	a	a	a	a	a	a	a	a	a	a	a	a	a	a	a	a			a	a	
c	2371655	t	2580696	t	2655907	i	t	CB9615	++++	t	t	t	t	t	t	t	t	t	t	t	t	t	t	t	t	t	t			c	t	

O55 and O157 genome site details										Outgroup Strain Details[g]																						
CB9615 base[a]	CB9615 Site[b]	Sakai base[a]	Sakai Site[b]	EDL933 base[a]	EDL933 Site[b]	type[c]	Inferred ancestral base[d]	Lineage inferred to mutate[e]	Support level[f]	D1 Sd197	K-12	HS	ATCC 8739	UMN026	IAI39	SMS 3-5	E2348/69	536	ED1a	CFT073	S88	APEC O1	UTI89	E24377A	IAI1	SE11	F5 8401	F2a 2457T	F2a 301	SS Ss046	B4 Sb227	B18 BS512
a	2371763	g	2580804	g	2656015	i	a	O157	++++	a	a	a	a	a	a	a	a	a	a	a	a	a	a	a	a	a	a			a	a	
g	2372303	a	2581344	a	2656555	ns	g	O157	++++	g	g	g	g	g	g	g	g	g	g	g	g	g	g	g	g	g	g			g	g	
g	2372492	a	2581533	a	2656744	ns	g	O157	++++	g	g	g	g	g	g	g	g	g	g	g	g	g	g	g	g	g	g			g	g	
a	2374999	g	2584040	g	2659251	ns	a	O157	++++	a	a	a	a	a	a	a	a	a	a	a	a	a	a	a	a	a	a	a	a	a	a	a
g	2375361	a	2584402	a	2659613	s	g	O157	++	g	g	g	g	g	g	g	a	g	a	a	g	g	g	g	g	g	g	g	g	g	g	g
a	2375391	t	2584432	t	2659643	ns	a	O157	++++	a	a	a	a	a	a	a	a	a	a	a	a	a	a	a	a	a	a	a	a	a	a	a
g	2376622	g	2585663	a	2660874	ns	g	EDL933	++++	g	g	g	g	g	g	g	g	g	g	g	g	g	g	g	g	g	g	g	g	g	g	g
g	2377229	a	2586270	a	2661481	i	g	O157	++++	g	g	g	g	g	g	g	g	g	g	g	g	g	g	g	g	g	g	g	g	g	g	g
t	2378270	c	2587311	c	2662522	i	t	O157	++++	t	t	t	t	t	t	t	t	t	t	t	t	t	t	t	t	t	t	t	t	t	t	t
c	2378739	a	2587780	a	2662991	i	c	O157	++++	c	c	c	c	c	c	c	c	c	c	c	c	c	c	c	c	c	c	c	c	c	c	c
c	2380218	-	2589258	-	2664469	ins	-	CB9615	++	-	c	c	c	c	c	c	c	c	c	c	c	c	c	c	c	c	c	c	c	c	c	c
-	2383981	c	2594559	Y	2669770	ns	c	EDL933	++++																							
-	2383981	a	2594974	M	2670185	ns	a	EDL933	++++																							
-	2383981	a	2599012	g	2674223	ns	a	EDL933	+								a						a			a						
-	2383981	g	2599019	n	2674230	ns	g	EDL933	+								g						g			g						
-	2383981	c	2599215	Y	2674426	ns	c	EDL933	+								c						c			c						
-	2383981	c	2600871	M	2676082	ns	c	EDL933	+								c						c			c						
-	2383981	c	2601242	N	2676453	ns	c	EDL933	+								c						c			c						
-	2383981	g	2604364	c	2679575	ns	c	Sakai	++++					c	c	c	c	c	c	c	t	t	c	c			c	c	c	c	c	c
-	2383981	c	2607997	Y	2683208	ns	c	EDL933	+++								c						c			c					c	
g	2384842	a	2615002	a	2690213	s	a	CB9615	++	a	g	g	g	g	g	g	g	g	g	g	g	g	g	g	g	g	g	g	g	g	g	g
a	2388067	c	2618227	c	2693438	ns	a	O157	++++	a	a	a	a	a	a	a	a		a	a	a	a	a	a	a	a	a	a	a	a	a	a
g	2389100	t	2619260	t	2694471	i	g	O157	++++	g	g	g	g	g	g	g	g		g	g	g	g	g	g	g	g	g	g	g	g	g	g
a	2389485	g	2619645	g	2694856	s	g	CB9615	++++	g	g	g	g	g	g	g	g		g	g	g	g	g	g	g	g	g	g	g	g	g	g
c	2389899	t	2620059	t	2695270	s	c	O157	++++	c	c	c	c	c	c	c	c		c	c	c	c	c	c	c	c	c	c	c	c	c	c
c	2390144	g	2620304	g	2695515	s	c	O157	++++	c	c	c	c	c	c	c	c		c	c	c	c	c	c	c	c	c	c	c	c	c	c
g	2390849	a	2621009	a	2696220	ns	g	O157	++++	g	g	g	g	g	g	g	g		g	g	g	g	g	g	g	g	g	g	g	g	g	g
g	2391182	-	2621341	g	2696553	del	g	Sakai	++++	g	g	g	g	g	g	g	g		g	g	g	g	g	g	g	g	g	g	g	g	g	g
g	2394058	a	2624217	a	2699429	i	g	O157	++++	g	g		g	g		g	g	g		g	g	g	g	g	g	g					g	g
a	2394114	g	2624273	g	2699485	i	a	O157	++++	a	a		a	a		a	a	-		a	a	a	a	a	a	a					a	a
9	2396466	-	2626624	-	2701836	ins	-	CB9615	++	-	9	9	9	9	9	9	9	9		9	9	9	9	9	9	9	9	9	9		9	9
a	2396678	g	2626828	g	2702040	ns	g	CB9615	++++	g	g	g	g	g	g	g	g	g		g	g	g	g	g	g	g	g	g	g		g	g
c	2396746	t	2626896	t	2702108	s	c	O157	++++	c	c	c	c	c	c	c	c	c		c	c	c	c	c	c	c	c	c	c		c	c
t	2397084	c	2627234	c	2702446	ns	c	CB9615	++++	c	c	c	c	c	c	c	c	c		c	c	c	c	c	c	c	c	c	c		c	c
t	2397311	c	2627461	c	2702673	s	c	CB9615	++++	c	c	a	c	c	c	c	c	c		c	c	c	c	c	c	c	c	c	c		c	c
a	2400966	c	2631116	c	2706328	ns	c	CB9615	++++	c	c	c	c	c	c	c	c	c	c	c	c	c	c	c	c	c	c	c	c	c	c	c
g	2402711	a	2632861	g	2708073	ns	g	Sakai	++++	g	g		g	a	g	g	g	g	g	g	g	g	g	g	g	g	g	g	g	g	g	g
c	2403764	t	2633914	t	2709126	ns	c	O157	+++		c		c	c		c																
t	2404083	c	2634233	c	2709445	s	t	O157	+		g		g	g		t																
c	2404684	t	2634834	t	2710046	ns	t	CB9615	+++		t		t	t		t																
a	2405372	g	2635522	g	2710734	s	g	CB9615	+		a			g	g	g	g															
a	2405624	g	2635774	g	2710986	i	a	O157	+++		a		a	a		a																
a	2405650	g	2635800	g	2711012	ns	g	CB9615	+++		g		g	g		g																
c	2406409	a	2636559	a	2711771	ns	c	O157	+		t		t	t		c																
t	2406502	a	2636652	a	2711864	ns	t	O157	+++		t		t	t		t																
t	2406807	a	2636957	a	2712169	i	t	O157	+++		t		t	t	g	t	g								t							
g	2408232	t	2638382	t	2713594	ns	g	O157	++++		g	g	g	g	g	g	g	g	g	g	g	g	g	g	g	g	g	g	g	g	g	
c	2411099	g	2641311	g	2716523	ns	g	CB9615	++++		g	g	g	g	g	g	g	g	g	g	g	g	g	g	g	g	g	g	g	g	g	
t	2412256	c	2642468	t	2717680	ns	t	Sakai	++++		t	t	t	t	t	t	t	t	t	t	t	t	t	t	t	t	t	t	t	t	t	
t	2413288	a	2643500	a	2718712	ns	t	O157	++++		t	t	t	t	t	t	t	t	t	t	t	t	t	t	t	t	t	t	t	t	t	
a	2413610	g	2643822	g	2719034	s	g	CB9615	++++		g	g	g	g	g	g	g	g	g	g	g	g	g	g	g	g	g	g	g	g	g	
-	2413944	-	2644156	c	2719369	ins	-	EDL933	++++		-	-	-	-	-	-	-	-	-	-	-	-	-	-	-	-	-	-	-	-	-	
c	2418038	t	2648250	t	2723463	i	t	CB9615	++++		t	t	t	t	t	t	t	t	t	t	t	t	t	t	t	t	t	t	t	t	t	t
a	2420583	g	2650795	g	2726008	ns	g	CB9615	++	g	g	g	a	g	g	a	g	g	g	g	a	a	a	a	a	g	g	g	g	a	g	g
a	2420891	g	2651103	g	2726316	s	g	CB9615	++++	g	g	g	g	g	g	g	g	g	g	g	g	g	g	g	g	g	g	g	g	g	g	g
-	2422450	-	2652662	g	2727876	ins	-	EDL933	++++	-	-	-	-	-	-	-	-	-	-	-	-	-	-	-	g	-	-	-	-	-	-	-
g	2423064	g	2653276	a	2728490	s	g	EDL933	++++	g	g	g	g	g	g	g	g	g	g	g	g	g	g	g	g	g	g	g	g	g	g	g
t	2423122	g	2653334	g	2728548	ns	t	O157	++++	t	t	t	t	t	t	t	t	t	t	t	t	t	t	t	t	t	t	t	t	t	t	t
c	2426366	t	2656578	t	2731792	ns	t	CB9615	++++	t	t	t	t	t	t	t	t	t	t	t	t	t	t	t	t	t	t	t	t	t	t	t
g	2427200	t	2657412	t	2732626	ns	g	O157	++++	g	g	g	g	g	g	g	g	g	g	g	g	g	g	g	g	g	g	g	g	g	g	g
c	2427557	-	2657768	-	2732982	del	c	O157	++++	c	c		c	c	c	c	c	c	c	c	c	c	c	c	c	c	c	c	c	c	c	c
t	2427794	c	2658005	c	2733219	ns	t	O157	++++	t	t		t	t	t	t	t	t	t	t	t	t	t	t	t	t				t	t	t
-	2428072	t	2658284	t	2733498	del	t	CB9615	+		t		t	-	-	t	-	-	t	-	-	-	-	t	t	t				-	t	t
g	2428231	a	2658443	a	2733657	i	a	CB9615	++++		a		a	a	a	a	a	a	a	a	a	a	a	a	a	a				a	a	a
a	2428235	g	2658447	g	2733661	i	g	CB9615	++++		g		g	g	g	g	g	g	g	g	g	g	g	g	g	g				g	g	g
a	2428268	g	2658480	g	2733694	i	a	O157	+		a		a	a	g	a	a	a	a	a	a	a	a	a	a	a				a	g	g
-	2428279	t	2658492	t	2733706	del	t	CB9615	++++		t		t	t	t	t	t	t	t	t	t	t	t	t	t	t				t	t	t
a	2428290	g	2658503	g	2733717	i	g	CB9615	++++		g		g	g	g	g	g	g	g	g	g	g	g	g	g	g				g	g	g
c	2428411	t	2658624	t	2733838	ns	t	CB9615	++++		t		t	t	t	t	t	t	t	t	t	t	t	t	t	t				t	t	t
a	2428990	-	2659202	-	2734416	del	a	O157	+++		a			a											a							
g	2428992	a	2659204	a	2734418	ns	g	O157	+++		g			g											g							
a	2429208	c	2659420	c	2734634	ns	a	O157	++++		a			a		a									a	a				a		
t	2431309	c	2661521	c	2736735	ns	t	O157	++++		t	t	t	t	t	t	t	t	t	t	t	t	t	t	t	t	t	t	t	t	t	t
-	2433274	-	2663486	g	2738701	ins	-	EDL933	++++	-	-	-	-	-	-	-	-	-	-	-	-	-	-	-	-	-	-	-	-	-	-	-
t	2435533	a	2665745	a	2740960	i	t	O157	++++	t	t	t	t	t	t	t	t	t	t	t	t	t	t	t	t	t	t	t	t	t	t	t
t	2436942	g	2667154	g	2742369	s	g	CB9615	++++		g	g	g			g			g					g	g	g				g	g	
c	2437142	t	2667354	t	2742569	ns	c	O157	++++		c	c	c			c			c					c	c	c				c	c	
-	2437288	-	2667500	N	2742716	ins	-	EDL933	++++		-	-	-			-			-					-	-	-				-	-	
g	2437668	a	2667880	a	2743096	i	g	O157	++++		g	g	g	g	g	g	g	g	g	g	g	g	g	g	g	g	g	g	g	g	g	

O55 and O157 genome site details										Outgroup Strain Details[g]																						
CB9615 base[a]	CB9615 Site[b]	Sakai base[a]	Sakai Site[b]	EDL933 base[a]	EDL933 Site[b]	type[c]	Inferred ancestral base[d]	Lineage inferred to mutate[e]	Support level[f]	D1 Sd197	K-12	HS	ATCC 8739	UMN026	IAI39	SMS 3-5	E2348/69	536	ED1a	CFT073	S88	APEC O1	UTI89	E24377A	IAI1	SE11	F5 8401	F2a 2457T	F2a 301	SS Ss046	B4 Sb227	B18 BS512
-	2437733	-	2667945	t	2743162	ins	-	EDL933	++++		-	-	-	-	-	-	-	-	-	-	-	-	-	-	-	-	-	-	-	-	-	
a	2439173	g	2669385	g	2744602	i	?	O55/O157	+/-																							
-	2440190	g	2698078	R	2774442	i	g	EDL933	++++							-	-										-	-	-			
-	2440190	c	2699774	Y	2776138	ns	c	EDL933	+	a	a		a	a		a	a		c	a	a	a	a	a	a	a	a	a	a	a	a	a
t	2489321	c	2714613	c	2790978	nc	c	CB9615	++++	c	c	c	c	c	c	c			c		c			c	c	c				c	t	c
a	2491882	c	2717174	c	2793539	s	a	O157	+					a												a						a
c	2492054	c	2717346	t	2793711	s	c	EDL933	+					c												c						c
-	2492156	g	2717449	-	2793813	ins	-	Sakai	+					-												-						-
c	2495641	t	2720934	t	2797298	s	t	CB9615	++++		t	t	t	t	t	t	t	t	t	t	t	t	t	t	t	t				t		
g	2497767	-	2723059	-	2799423	del	g	O157	++++	g	g	g	g	g	g	g	g	g	g	g	g	g	g	g	g	g				g		
g	2499593	a	2724885	a	2801249	s	g	O157	++++	g	g	g	g	g	g	g	g	g	g	g	g	g	g	g	g	g	g	g	g	g		g
g	2499638	a	2724930	a	2801294	s	g	O157	++	g	g	g	g	g	g	g	g	a	g	a	g	g	a	g	g	g	g	g	g	g		g
a	2501039	t	2726331	t	2802695	i	a	O157	++++		a	a	a	a	a	a	a	a	a	a	a	a	a	a	a	a	a	a	a	a	a	a
a	2502116	c	2727408	c	2803772	i	c	CB9615	++++		c	c	c	c	c	c	c	c	c	c	c	c	c	c	c	c	c	c	c	a	c	-
t	2502580	c	2727872	c	2804236	ns	c	CB9615	++++	c	c	c	c	c	c	c	c	c	c	c	c	c	c	c	c	c	c	c	c	c	c	c
g	2502732	a	2728024	a	2804388	ns	g	O157	++++	g	g	g	g	g	g	g	g	g	g	g	g	g	g	g	g	g	g	g	g	g	g	a
a	2502804	t	2728096	t	2804460	ns	a	O157	++++	a	a	a	a	a	a	a	a	a	a	a	a	a	a	a	a	a	a	a	a	a	a	a
g	2503737	a	2729029	a	2805393	ns	g	O157	++++	g	g	g	g	g	g	g	g	g	g	g	g	g	g	g	g	g	g	g	g	g	g	g
t	2505411	g	2730703	g	2807067	s	g	CB9615	++++	g	g	g	g	g	g	g	g	g	g	g	g	g	g	g	g	g	g	g	g	g		g
g	2507227	a	2732519	a	2808883	ns	g	O157	++++	g	t	g	g	g	g	g	g	g	t	g	g	g	g	g	g	g	g	g	g	g	g	g
a	2509919	g	2735211	g	2811575	s	g	CB9615	++++	g	g	g	t	g	g	g	g	g	g	g	g	g	g	g	g	g	g			g	g	g
-	2510211	c	2736122	Y	2812486	ns	c	EDL933	+++	c				c				c	c	t	c			c						c		t
g	2510412	a	2738154	a	2814518	i	g	O157	++++		g	g			g	g		g	g	g	g	g	g				g					g
c	2510500	t	2738242	t	2814606	i	c	O157	++++		c	c			c	c		c	c	c	c	c	c				c					c
t	2510715	c	2738457	c	2814821	i	c	CB9615	++++		c	c			c	c		c	c	c	c	c	c									
g	2510854	t	2738596	t	2814960	i	g	O157	++++		g	g			g	g		g	g	g	g	g	g									
-	2511407	-	2739149	g	2815514	ins	-	EDL933	++++		-	g			-	-		-	-	-	-	-	-									
c	2511537	t	2739279	t	2815644	i	t	CB9615	+		t				c	t		c	t	c	t	t	c									
c	2511777	g	2739519	g	2815884	ns	g	CB9615	++++		g				g	g		g	g	g			g									
c	2512717	t	2740459	t	2816824	s	c	O157	+++						c	c		c		c			c									
g	2523379	a	2743716	-	2819497	ns	a	CB9615	++++						a	a	a	a		a	a	a	a									
a	2524635	g	2744972	-	2819497	ns	g	CB9615	+++						g	g	a	g		g	g	g	g									
c	2525421	t	2745758	-	2819497	ns	c	Sakai	++++	c					c	c	c	c		c	c	c	c									
t	2530024	g	2750361	g	2820572	nc	g	CB9615	++++	g	g	g		g	g	g	g	g	g	g	g	g	g	g				g	g			g
t	2530386	c	2750723	c	2820934	i	t	O157	++++		t	t			t	t		t	t	t	t	t	t									t
-	2530551	-	2750888	R	2821100	ins	-	EDL933	++++		-	-			-	-		-	-	-	-	-	-									-
t	2532628	c	2752965	c	2823177	ns	t	O157	++++	t	t	t	t	t	t	t	t	t	t	t	t	t	t	t	t	t				t		t
a	2533359	g	2753696	g	2823908	ns	g	CB9615	++++	g	g	g	g	g	g	g	g	g	g	g	g	g	g	g	g	g				g		g
-	2534738	-	2755075	c	2825288	ins	-	EDL933	++++	-	-	-	-	-	-	-	-	-	-	-	-	-	-	-	-	-				-		-
g	2535828	a	2756165	a	2826378	s	g	O157	++++	g	g	g	g	g	g	g	g	g	g	g	g	g	g	g	g	g	g	g	g	g	g	g
a	2536701	g	2757038	g	2827251	ns	g	CB9615	++++	g	g	g	g		g	g			g		g	g		g	g	g	g	g	g	g	g	g
t	2537453	c	2757790	c	2828003	s	c	CB9615	++++	c	c	c	c		c	c			c		c	c		c	c	c	c	c	c	c	c	c
a	2537736	a	2758073	t	2828286	i	a	EDL933	++++	a	a	a	a		a	a			a		a	a		a	a	a	a	a	a	a	a	a
c	2537740	c	2758077	g	2828290	i	c	EDL933	++++	c	c	c	c		c	c			c		c	c		c	c	c	c	c	c	c	c	c
-	2557320	t	2774497	N	2844710	ns	t	EDL933	++++		t	t	t	t										t	t	t				t		t
-	2557320	-	2774795	g	2845009	ins	-	EDL933	++++		-	-	-	-										-	-	-				-		-
g	2576669	a	2796191	g	2866405	s	g	Sakai	++++		g		g	g	g	g	g	g	g	g	g	g	g	g	g	g	g	g	g		g	g
-	2593625	-	2813159	g	2883374	ins	-	EDL933	++++	-	-		-	-	-	-	-	-	-	-	-	-	-	-	-	-	-	-	-	-	-	-
-	2599362	-	2818896	g	2889112	ins	-	EDL933	++++	-	-	-	-	-	-	-	-	-	-	-	-	-	-	-	-	-	-	-	-	-	-	-
-	2599368	-	2818902	g	2889119	ins	-	EDL933	++++	-	-	-	-	-	-	-	-	-	-	-	-	-	-	-	-	-	-	-	-	-	-	-
-	2600511	g	2820047	-	2890263	ins	-	Sakai	++++	-	-	-	-	-	-	-	-	-	-	-	-	-	-	-	-	-	-	-	-	-	-	-
-	2608707	-	2828243	t	2898460	ins	-	EDL933	++++	-	-	-	-	-	-	-	-	-	-	-	-	-	-	-	-	-	-	-	-	-	-	-
-	2609449	-	2829141	a	2899359	ins	-	EDL933	++++		-	-	-	-	-	-		-	-	-	-	-	-	-	-	-				-	-	-
-	2609449	g	2829296	N	2899514	i	g	EDL933	++++		g	g	g	g	g	g	g	g	g	g	g	g	g	g	g	g				g	g	g
t	2641414	c	2850892	N	2921110	i	t	EDL933	IUPAC	t	t	t	t	t	t	t	t	t			t	t	t	t	t	t	t	t	t	t	t	t
-	2642795	-	2852264	a	2922483	ins	-	EDL933	++++	-	-	-	-	-	-	-	-	-		-	-	-	-	-	-	-	-			-	-	-
-	2652549	-	2862027	8	2932247	ins	8	EDL933	-	8	-	-	-	-	8	-	-	-	-	-	-	-	-	-	-	-	8	-	-	-		-
4	2652550	-	2862027	4	2932255	del	4	Sakai	++++	4	4	4	4	4	-	4	4	4	4	4	4	4	4	4	4	4	4	4	4	4		4
g	2657800	a	2867278	g	2937509	nc	g	Sakai	++++		g	g	g	g	g	g	g	g	g	g	g	g	g	g	g	g	g	g	g	g		a
a	2675164	g	2885217	g	2955448	s	g	O157	rec		g	g	g	g	g	g	g	g	g	g	g	g	g	g	g	g		g	g		g	
g	2675873	a	2885926	a	2956157	s	g	O157	++++		g	g	g	g	g	g	g	g	g	g	g	g	g	g	g	g		g	g	g	g	
c	2676843	t	2886896	t	2957127	ns	c	O157	++++	c	c	c	c	c	c	c	c	c	c	c	c	c	c	c	c	c		c	c	c	c	
t	2677357	c	2887410	c	2957641	ns	t	O157	++++	t	t	t	t	t	t	t	t	t	t	t	t	t	t	t	t	t		t	t		t	
g	2677436	a	2887489	a	2957720	s	g	O157	++++	g	g	g	g	g	g	a	g	g	g	g	g	g	g	g	g	g		g	g		g	
a	2677765	g	2887818	g	2958049	ns	g	O157	rec	g	g	g	g	g	g	g	g	g	g	g	g	g	g	g	g	g		a	a		g	
a	2680283	g	2890336	g	2960567	ns	g	CB9615	++++	g	g	g	g	g	g	g	g	g	g	g	g	g	g	g	g	g		g	g	g	g	
-	2680722	t	2890883	c	2961114	ns	t	EDL933	++++					t	t	t	t	t	t	t	t	t	t	t			t	t	t	t	t	t
-	2680722	a	2891902	g	2962133	ns	g	Sakai	++++					g	g	g	g	g	g	g	g	g	g	g			g	g	g	g	g	g
a	2681224	g	2892590	g	2962821	ns	g	CB9615	++++	g	g	g	g	g	g	g	g	g	g	g	g	g	g	g	g	g	g	g	g	g	g	g
t	2682624	g	2893990	g	2964221	s	g	CB9615	++++	g	g	g	g	g	g	g	g	g	g	g	g	g	g	g	g	g	g	g	g	g	g	g
g	2682793	a	2894159	a	2964390	ns	g	O157	++++	g	g	g	g	g	g	g	g	g	g	g	g	g	g	g	g	g	g	g	g	g	g	g
t	2684286	a	2895652	a	2965883	i	t	O157	++++	t	t	t	t	t	t	t	t	t	t	t	t	t	t	t	t	t	t	t	t	t	t	t
g	2684880	a	2944126	a	3015393	ns	g	O157	++++	g	g	g	g	g	g	g	g	g	g	g	g	g	g	g	g	g	g	g	g	g	g	g
g	2685958	a	2945204	a	3016471	s	g	O157	++++	g	g	g	g	g	g	g	g	g	g	g	g	g	g	g	g	g	g	g	g	g	g	g
g	2686851	a	2946097	a	3017364	s	g	O157	++++	g	g	g	g	g	g	g	g	g	g	g	g	g	g	g	g	g	g	g	g	g	g	g
c	2687638	g	2946884	g	3018151	s	c	O157	++++	c	c	c	c	c	c	c	c	c	c	c	c	c	c	c	c	c	c	c	c	c	c	c
g	2687844	a	2947090	a	3018357	s	g	O157	++++	g	g	g	g	g	g	g	g	g	g	g	g	g	g	g	g	g	g	g	g	g	g	g
a	2688425	t	2947671	t	3018938	ns	a	O157	++++	a	a	a	a	a	a	a	a	a	a	a	a	a	a	a	a	a	a	a	a	a	a	a
g	2690074	a	2949320	a	3020587	ns	g	O157	++++	g	g	g	g	g	g	g	g	g	g	g	g	g	g	g	g	g	g	g	g	g	g	g

O55 and O157 genome site details										Outgroup Strain Details[g]																						
CB9615 base[a]	CB9615 Site[b]	Sakai base[a]	Sakai Site[b]	EDL933 base[a]	EDL933 Site[b]	type[c]	Inferred ancestral base[d]	Lineage inferred to mutate[e]	Support level[f]	D1 Sd197	K-12	HS	ATCC 8739	UMN026	IAI39	SMS 3-5	E2348/69	536	ED1a	CFT073	S88	APEC O1	UTI89	E24377A	IAI1	SE11	F5 8401	F2a 2457T	F2a 301	SS Ss046	B4 Sb227	B18 BS512
g	2691980	t	2951226	t	3022493	ns	g	O157	++++	g	g	g	g	g	g	g	g	g	g	g	g	g	g	g	g	g	g	g	g	g	g	g
c	2695133	a	2954379	c	3025646	ns	c	Sakai	++++	c	c	c	c	c	c	c	c	c	c	c	c	c	c	c	c	c	c	c	c	c	c	c
g	2695561	c	2954807	c	3026074	ns	g	O157	++++	g	g	g	g	g	g	g	g	g	g	g	g	g	g	g	g	g	g	g	g	g	g	g
g	2697596	c	2956842	c	3028109	s	g	O157	++++	g	g	g	g	g	g	g	g	g	g	g	g	g	g	g	g	g	g	g	g	g	g	g
g	2698990	g	2958236	-	3029502	del	g	EDL933	++++	g							g	g	g	g	g	g	g								g	g
c	2699285	c	2958531	t	3029797	ns	c	EDL933	++++	c	c	c	c	c	c	c	c	c	c	c	c	c	c	c	c	c	c	c	c	c	c	c
t	2702235	a	2961481	a	3032747	ns	t	O157	+															t								
c	2702853	t	2962099	t	3033365	s	t	CB9615	+															t								
c	2704240	t	2963486	t	3034752	ns	c	O157	+															c								
c	2705029	c	2964275	a	3035541	ns	c	EDL933	+															c								
g	2705319	t	2964565	t	3035831	ns	g	O157	+															g								
a	2705767	c	2965013	c	3036279	s	a	O157	+															a								
c	2708558	t	2967804	t	3039070	i	c	O157	++++	c	c	c	c	c	c	c	c	c	c	c	c	c	c	c	c	c	c	c	c	c	c	c
t	2710116	c	2969362	c	3040628	s	c	CB9615	++	c	c	c	c	t	c	c	t	t	t	t	t	t	t	t	t	t	c	c	c	c	c	c
a	2710575	g	2969821	g	3041087	s	g	CB9615	++++	g	g	g	g	g	g	g	g	g	g	g	g	g	g	g	g	g	g	g	g	g	g	g
t	2712349	c	2971595	c	3042861	i	t	O157	++++		t						t	t	t	t	t	t	t									
-	2712394	-	2971640	t	3042907	ins	-	EDL933	++++		-						-	-	-	-	-	-	-							-	-	-
a	2713639	g	2972885	g	3044152	ns	g	CB9615	++++	g	g	g	g	g	g	g	g	g	g	g	g	g	g	g	g	g	g	g	g	g	g	g
a	2715040	g	2974286	a	3045553	i	a	Sakai	++++	a	a	a	a	a	a	a	a	a	a	a	a	a	a	a	a	a	a	a	a	a	a	a
t	2716259	-	2975504	-	3046771	del	t	O157	++++	t	t	t	t	t	t	t	t	t	t	t	t	t	t	t	t	t	t	t	t	t	t	t
-	2716347	-	2975592	N	3046860	ins	-	EDL933	++++	-	-	-	-	-	-	-	-	-	-	-	-	-	-	-	-	-	-	-	-	-	-	-
a	2717627	c	2976872	c	3048140	s	c	CB9615	++++	c	c	c	c	c	c	c	c	c	c	c	c	c	c	c	c	c	c	c	c	c	c	c
t	2717984	a	2977229	a	3048497	s	a	CB9615	++++	a	a	a	a	a	a	a	a	a	a	a	a	a	a	a	a	a	a	a	a	a	a	a
g	2718677	a	2977922	a	3049190	s	g	O157	++++	g	g	g	g	g	g	g	g	g	g	g	g	g	g	g	g	g	g	g	g	g	g	g
a	2722860	c	2982105	c	3053373	s	c	CB9615	++++	c	c	c	c	c	c	c	c	c	c	c	c	c	c	c	c	c	c	c	c	c	c	c
g	2729643	a	2988888	a	3060156	s	g	O157	++++	g	g	g	g	g	g	g	g	g	g	g	g	g	g	g	g	g	g	g	g	g	g	g
g	2729986	c	2989231	c	3060499	ns	g	O157	++++	g	g	g	g	g	g	g	g	g	g	g	g	g	g	g	g	g	g	g	g	g	g	g
g	2733736	t	2992981	t	3064249	s	g	O157	+		g	g	g	g	g	g	t	t	t	t	t	t	t	g	g	g	g	g	g	t	g	g
a	2733841	g	2993086	g	3064354	s	g	CB9615	+		g	g	g	g	g	g	a	a	a	a	a	a	a	g	a	g	g	g	g	a	g	g
c	2735655	a	2994900	a	3066168	s	c	O157	++++	c	c	c	c	c	c	c	c	c	c	c	c	c	c	c	c	c	c	c	c	c	c	c
a	2741548	g	3000793	g	3072061	ns	g	CB9615	++++	g	g	g	g	g	g	g	g	g	g	g	g	g	g	g	g	g	g	g	g	g	g	g
t	2743336	c	3002581	c	3073849	ns	t	O157	++++	t	t	t	t	t	t	t	t	t	t	t	t	t	t	t	t	t	t	t	t	t	t	t
t	2745239	c	3004484	c	3075752	s	c	CB9615	++++	c	c	c	c	c	c	c	c	c	c	c	c	c	c	c	c	c	c	c	c	c	c	c
a	2745585	t	3004830	t	3076098	i	a	O157	++++	a	a	a	a	a	a	a	a	a	a	a	a	a	a	a	a	a	a	a	a	a	a	a
c	2746286	t	3005531	t	3076799	i	c	O157	++++	c	c	c	c	c	c	c	c	c	c	c	c	c	c	c	c	c	c	c	c	c	c	c
-	2746385	-	3005630	M	3076899	ins	-	EDL933	++++	-	-	-	-	-	-	-	-	-	-	-	-	-	-	-	-	-	-	-	-	-	-	-
g	2748411	t	3007656	t	3078925	s	g	O157	++++	g	g	g	g	g	a	g	a	a	g	g	a	a	a	g	g	g	g	g	g	g	g	g
a	2749447	c	3008692	c	3079961	ns	a	O157	++++	a	a	a	a	a	a	a	a	a	a	a	a	a	a	a	a	a	a	a	a	a	a	a
g	2749668	a	3008913	a	3080182	s	g	O157	++	g	g	g	g	g	g	g	g	g	a	a	g	g	g	g	g	g	g	g	g	g	g	g
a	2750259	t	3009504	t	3080773	ns	t	CB9615	++++	t	t	t	t	t	t	t	t	t	t	t	t	t	t	t	t	t	t	t	t	t	t	t
a	2750480	g	3009725	g	3080994	ns	g	CB9615	++++	g	g	g	g	g	g	g	g	g	g	g	g	g	g	g	g	g	g	g	g	g	g	g
a	2750652	g	3009897	g	3081166	s	g	CB9615	++	g	g	g	c	c	a	g	a	a	a	a	a	a	a	g	g	g	g	g	g	g	g	g
t	2753727	c	3012972	c	3084241	ns	t	O157	++++	t	t	t	t	t	t	t	t	t	t	t	t	t	t	t	t	t	t	t	t	t	t	t
a	2758080	g	3017325	g	3088594	i	g	CB9615	++++	g	g	g	g	g	g	g	g	g	g	g	g	g	g	g	g	g	g	g	g	g	g	g
g	2758351	t	3017596	t	3088865	ns	g	O157	++++	g	g	g	g	g	g	g	g	g	g	g	g	g	g	g	g	g	g	g	g	g	g	g
a	2761038	g	3020283	g	3091552	s	g	CB9615	++++	g	g	g	g	g	g	g	g	g	g	g	g	g	g	c	c	c	g	g	g	g	c	c
g	2761887	a	3021132	a	3092401	i	g	O157	++++	g	g	g	g	g	g	g	g	g	g	g	g	g	g	g	g	g	g	g	g	g	g	g
c	2762052	g	3021297	g	3092566	i	c	O157	++++	c	c	c	c	c	c	c	c	c		c	c	c	c	c	c	c	c	c	-	c	c	c
g	2763329	a	3022574	a	3093843	ns	g	O157	++++	g	g	g	g	g	g	g								g	g	g	g	g	g	g	g	g
a	2763372	g	3022617	g	3093886	s	g	CB9615	++++	g	g	g	g	g	g	g								g	g	g	g	g	g	g	g	g
a	2763800	t	3023045	t	3094314	ns	t	CB9615	++++	t	t	t	t	t	t	t								t	t	t	t	t	t	t	t	t
a	2767419	g	3026664	g	3097933	s	a	O157	++	a	g	g	g	g	g	g	g	g	g	g	g	g	g	g	g	g	g	g	g	g	g	g
t	2769093	g	3028338	g	3099607	s	g	CB9615	++++	g	g	g	g	g	g	g	g	g	g	g	g	g	g	g	g	g	g	g	g	g	g	g
g	2773725	a	3032970	a	3104239	s	g	O157	++++	g	g	g	g	g	g	g	g	g	g	g	g	g	g	g	g	g	g	g	g	g	g	g
g	2774177	a	3033422	a	3104691	ns	g	O157	++++	g	g	g	g	g	g	g	g	g	g	g	g	g	g	g	g	g	g	g	g	g	g	g
c	2776008	g	3035253	g	3106522	ns	g	CB9615	++	g	a	a	a	g	a	a	a	a	a	a	a	a	a	a	a	a	a	a	a	a	a	a
g	2777289	a	3036534	a	3107803	s	g	O157	++++	g	g	g	g	g	g	g	g	g	g	g	g	g	g	g	g	g	g	g	g	g	g	g
c	2778229	t	3037474	t	3108743	s	t	CB9615	++++	t	t	t	t	t	t	t	t	t	t	t	t	t	t	t	t	t	t	t	t	t	t	t
g	2780878	a	3040123	a	3111392	ns	g	O157	++++	g	g	g	g	g	g	g	g	g	g	g	g	g	g	g	g	g	g	g	g	g	g	g
g	2784694	g	3043939	a	3115208	s	g	EDL933	++	g	a	g	g	g	g	g	g	g	g	g	g	g	g	g	g	g	g	g	g	g	g	a
a	2786336	g	3045581	g	3116850	s	g	CB9615	++++	g	g	g	g	g	g	g	g	g	g	g	g	g	g	g	g	g	g	g	g	g	g	g
t	2787328	g	3046573	g	3117842	s	g	CB9615	++++	g	g	g	g	g	g	g	g	g	g	g	g	g	g	g	g	g	g	g	g	g	g	g
t	2792842	c	3052087	c	3123356	s	t	O157	++++	t	t	t	t	t	t	t	t	t	t	t	t	t	t	t	t	t	t	t	t	t	t	t
a	2794107	c	3053352	c	3124621	ns	c	CB9615	++++	c	c	c	c	c	c	c	c	c	c	c	c	c	c	c	c	c	c	c	c	c	c	
g	2794129	a	3053374	a	3124643	s	g	O157	++++	g	g	g	g	g	g	g	g	g	g	g	g	g	g	g	g	g	g	g	g	g	g	
g	2795051	t	3054296	t	3125565	nc	g	O157	++++	g	g	g	g	g	g	g	g	g	g	g	g	g	g	g	g	g	g	g	g	g	g	
g	2795155	a	3054400	a	3125669	nc	g	O157	++++	g	g	g	g	g	g	g	g	g	g	g	g	g	g	g	g	g	g	g	g	g	g	
a	2795498	t	3054743	t	3126012	nc	t	CB9615	++++	t	t	t	t	t	t	t	t	t	t	t	t	t	t	t	t	t	t	t	t	t	t	
a	2800777	g	3058572	g	3129841	nc	g	CB9615	++++	g	g	g	g	g	g	g	g	g	g	g	g	g	g	g	g	g	g	g	g		g	g
g	2800837	t	3058632	t	3129901	nc	t	CB9615	++++	t	t	t	t	t	t	t	t	t	t	t	t	t	t	t	t	t	t	t	t		t	t
c	2804603	-	3062397	-	3133666	del	c	O157	++	c	c	c	c	c	c	c	-	c	c	c	c	c	c	c	c	c	c	c	c	-	c	c
a	2806605	g	3064399	g	3135668	s	a	O157	++++	a	a	a	a	a	a	a	a	a	a	a	a	a	a	a	a	a	a	a	a	a	a	a
c	2810391	a	3068185	a	3139454	ns	c	O157	++++	c	c	c	c	c	c	c	c	c	c	c	t	t	t	c	c	c	c	c	c	c	c	c
g	2811982	a	3069776	a	3141045	s	g	O157	++++	g	g	g	g	g	g	g	g	g	g	g	g	g	g	g	g	g	g	g	g	g		
t	2812608	a	3070402	a	3141671	i	t	O157	++++	t	t	t	t	t	t	t	t	t	t	t	t	t	t	t	t	t	t	t	t	t	t	t
c	2813264	t	3071058	c	3142327	s	c	Sakai	++++	c	c	c	c	c	c	c	c	c	c	c	c	c	c	c	c	c	c	c	c	c	c	c
a	2815789	g	3073583	g	3144852	ns	g	CB9615	++++	g	g	g	g	g	g	g	g	g	g	g	g	g	g	g	g	g	g	g	g	g	g	g
a	2817852	g	3075646	g	3146915	nc	g	CB9615	+++	g				g	g	g																
a	2818960	g	3076754	g	3148023	s	g	CB9615	+++	g				g	g	g																

O55 and O157 genome site details										Outgroup Strain Details[g]																						
CB9615 base[a]	CB9615 Site[b]	Sakai base[a]	Sakai Site[b]	EDL933 base[a]	EDL933 Site[b]	type[c]	Inferred ancestral base[d]	Lineage inferred to mutate[e]	Support level[f]	D1 Sd197	K-12	HS	ATCC 8739	UMN026	IAI39	SMS 3-5	E2348/69	536	ED1a	CFT073	S88	APEC O1	UTI89	E24377A	IAI1	SE11	F5 8401	F2a 2457T	F2a 301	SS Ss046	B4 Sb227	B18 BS512
g	2820282	a	3078076	a	3149345	ns	g	O157	+					g																		
t	2824138	c	3081932	c	3153201	i	c	CB9615	++++	c	c	c	c	c	c	c	c	c	c	c	c	c	c	c	c	c	c	c	c	c	c	c
t	2824172	c	3081966	c	3153235	i	t	O157	++++	t	t	t	t	t	t	t	t	t	t	t	t	t	t	t	t	t	t	t	t	t	t	t
a	2827682	t	3085476	t	3156745	ns	t	CB9615	++++	t	t	t	t	t	t	t	t	t	t	t	t	t	t	t	t	t	t	t	t	t	t	t
a	2829254	g	3087048	g	3158317	ns	g	CB9615	++++	g	g	g	g	g	g	g	g	g	g	g	g	g	g	g	g	g	g	g	g	g	g	g
t	2830234	-	3088027	-	3159296	del	t	O157	++++	t	t	t	t	t	t	t	t	t	t	t	t	t	t							t	t	
a	2830446	g	3088239	g	3159508	ns	a	O157	++++	a	a	a	a	a	a	a	a	a	a	a	a	a	a							a	a	
a	2831622	g	3090728	g	3160684	s	g	CB9615	++++	g	g	g	g		g	g	g	g	g	g	g	g	g							g	g	
g	2831775	a	3090881	a	3160837	s	g	O157	++++	g	g	g	g		g	g	g	g	g	g	g	g	g							g	g	
c	2835844	t	3094950	t	3164906	i	t	CB9615	++++		t	t	t	t	t	t	t	t	t	t	t	t	t	t	t	t	t	t	t	t		
c	2837359	a	3096465	a	3166421	s	c	O157	++++		c	c	c	c	c	c	c	c	c	c	c	c	c	c	c	c	c	c	c	c	c	
g	2838687	a	3097793	a	3167749	s	g	O157	++++	g	g	g	g	g	g	g	g	g	g	g	g	g	g	g	g	g	g	g	g	g	g	
c	2838888	t	3097994	t	3167950	s	t	CB9615	++++	t	t	t	t	t	t	t	t	t	t	t	t	t	t	t	t	t	t	t	t	t	t	
g	2840285	a	3099391	a	3169347	s	g	O157	++++	g	g	g	g	g	g	g	g	g	g	g	g	g	g	g	g	g	g	g	g	g	g	g
a	2844947	g	3104053	g	3174009	ns	g	CB9615	++++	g	g	g	g	g	g	g	g	g	g	g	g	g	g	g	g	g	g	g	g	g	g	g
g	2845321	t	3104427	t	3174383	ns	g	O157	++++	g	g	g	g	g	g	g	g	g	g	g	g	g	g	g	g	g	g	g	g	g	g	g
c	2845429	a	3104535	c	3174491	ns	c	Sakai	++++	c	c	c	c	c	c	c	c	c	c	c	c	c	c	c	c	c	c	c	c	c	c	c
g	2846207	a	3105313	a	3175269	s	g	O157	++++	g	g	g	g	g	g	g	g	g	g	g	g	g	g	g	g	g	g	g	g	g	g	g
c	2846256	t	3105362	t	3175318	ns	c	O157	++++	c	c	c	c	c	c	c	c	c	c	c	c	c	c	c	c	c	c	c	c	c	c	c
g	2853528	a	3112634	a	3182590	ns	g	O157	++++	g	g	g	g	g	g	g	g	g	g	g	g	g	g	g	g	g	g	g	g	g	g	g
c	2854331	t	3113437	t	3183393	nc	t	CB9615	++++	t	t	t	t	t	t	t								t	t	t						t
a	2854495	g	3113601	g	3183557	nc	a	O157	++++	a	a	a	a	a	a	a								a	a	a				a		a
a	2854769	g	3113875	g	3183831	nc	g	CB9615	++++		g	g	g	g	g	g								g	g	g				g		g
-	2854934	c	3114041	c	3183997	ins	-	O157	++++		-	-	-	-	-	-								-	-	-						-
a	2855298	c	3114413	c	3184369	nc	a	O157	++++		a	a	a	a	a	a								a	a	a		a	a			
a	2855917	g	3115032	g	3184988	i	g	CB9615	++++	g			g	g		g						g	g	g	g	g		g	g		g	
t	2860078	c	3119193	c	3189149	i	c	CB9615	++	c							c															
g	2860437	a	3119552	a	3189508	s	a	CB9615	++++	a	a	a	a	a	a	a	a	a	a	a	a	a	a	a	a	a	a	a	a	a	a	a
c	2862431	t	3121546	t	3191502	ns	t	CB9615	++++	t	t	t	t	t	t	t	t	t	t	t	t	t	t	t	t	t	t	t	t	t	t	t
c	2864210	c	3123325	t	3193281	s	c	EDL933	++++	c	c	c	c	c	c	c	c	c	c	c	c	c	c	c	c	c	c	c	c	c	c	c
g	2865600	a	3124715	a	3194671	s	g	O157	++++	g	g	g	g	g	g	g	g	g	g	g	g	g	g	g	g	g	g	g	g	g	g	g
t	2867399	a	3126514	a	3196470	s	t	O157	++++	t	t	t	t	t	t	t	t	t	t	t	t	t	t	t	t	t	t	t	t	t	t	t
c	2874438	t	3133553	t	3203509	i	t	CB9615	++++	t	t	t	t	t	t	t	t	t	t	t	t	t	t	t	t	t	t	t	t	c	t	t
a	2874448	g	3133563	g	3203519	i	a	O157	++	a	a	a	a	a	a	a	a	g	g	g	g	g	g	a	a	a	a	a	a	a	a	a
a	2875345	g	3134460	g	3204416	ns	g	CB9615	++++	g	g	g	g	g	g	g	g	g	g	g	g	g	g	g	g	g	g	g	g	g	g	g
c	2875600	t	3134715	t	3204671	ns	c	O157	++++	c	c	c	c	c	c	c	c	c	c	c	c	c	c	c	c	c	c	c	c	c	c	c
a	2877862	g	3136977	g	3206933	s	g	CB9615	++++	g	g	g	g	g	g	g	g	g	g	g	g	g	g	g	g	g	g	g	g	g	g	g
g	2878258	a	3137373	a	3207329	s	g	O157	++++	g	g	g	g	g	g	g	g	g	g	g	g	g	g	g	g	g	g	g	g	g	g	g
g	2878934	a	3138049	a	3208005	s	g	O157	++++	g	g	g	g	g	g	g	g	g	g	g	g	g	g	g	g	g	g	g	g	g	g	g
-	2879961	g	3139077	-	3209032	ins	-	Sakai	++++	-	-	-	-	-	-	-	-	-	-	-	-	-	-	-	-	-	-	-	-	-	-	-
t	2881452	c	3140568	c	3210523	s	c	CB9615	++++	c	c	c	c	c	c	c	c	c	c	c	c	c	c	c	c	c	c	c	c	c	c	c
g	2882152	a	3141268	a	3211223	s	g	O157	++++	g	g	g	g	g	g	g	g	g	g	g	g	g	g	g	g	g	g	g	g	g	g	g
a	2883023	g	3142139	g	3212094	ns	g	CB9615	++++	g	g	g	g	g	g	g	g	g	g	g	g	g	g	g	g	g	g	g	g	g	g	g
g	2883148	t	3142264	t	3212219	s	t	CB9615	++++	t	t	t	t	t	t	t	t	t	t	t	t	t	t	t	t	t	t	t	t	t	t	t
t	2886019	a	3145135	a	3215090	s	t	O157	++++	t	t	t	t	t	t	t	t	t	t	t	t	t	t	t	t	t	t	t	t	t	t	t
a	2886331	g	3145447	g	3215402	s	g	CB9615	++++	g	g	g	g	g	g	g	g	g	g	g	g	g	g	g	g	g	g	g	g	g	g	g
c	2886962	t	3146078	t	3216033	s	t	CB9615	++	t	t	t	c	c	t	c	c	c	c	c	c	c	c	c	c	c	c	c	c	c	c	c
g	2889165	a	3148281	a	3218236	ns	g	O157	++++	g	g	g	g	g	g	g	g	g	g	g	g	g	g	g	g	g	g	g	g	g	g	g
g	2890269	a	3149385	a	3219340	ns	g	O157	++++	g	g	g	g	g	g	g	g	g	g		g	g	g	g	g	g	g	g	g	g	g	g
a	2890911	t	3150027	t	3219982	s	a	O157	++++	a	a	a	a	a	a	a	a	a	a	a	a	a	a	a	a	a	a	a	a	a	a	a
g	2892589	c	3151705	c	3221660	ns	g	O157	++++	g	g	g	g	g	g	g	g	g	g	g	g	g	g	g	g	g	g	g	g	g	g	g
t	2893786	c	3152902	c	3222857	ns	c	CB9615	++++	c	c	c	c	c	c	c	c	c	c	c	c	c	c	c	c	c	c	c	c	c	c	c
c	2895331	t	3154447	t	3224402	s	c	O157	++++	c	c	c	c	c	c	c	c	c	c	c	c	c	c	c	c	c	c	c	c	c	c	c
-	2897858	-	3156974	g	3226930	ins	-	EDL933	++++	-	-	-	-	-	-	-	-	-	-	-	-	-	-	-	-	-	-	-	-	-	-	-
g	2898254	c	3157188	c	3227144	i	g	O157	+++	g					a		a	g	g	g	g	g	g		c							
c	2898274	t	3157208	t	3227164	i	t	CB9615	++	t					c		c	c	c	c	c	c	c									
g	2898362	a	3157296	a	3227252	i	g	O157	++	g	a	a	a	g	a	g	a	g	g	g	g	g	g	a	a	a	a	a	a	a	a	a
t	2898365	c	3157299	c	3227255	i	c	CB9615	++++	c	c	c	c	c	c	c	c	c	c	c	c	c	c	c	c	c	c	c	c	c	c	c
a	2898989	c	3157923	c	3227879	ns	c	CB9615	++++	c	c	c	c	c	c	c	c	c	c	c	c	c	c	c	c	c	c	c	c	c	c	c
a	2899321	g	3158255	g	3228211	s	g	CB9615	++++	g	g	g	g	g	g	g	g	g	g	g	g	g	g	g	g	g	g	g	g	g	g	g
g	2900100	a	3159034	a	3228990	ns	g	O157	++++	g	g	g	g	g	g	g	g	g	g	g	g	g	g	g	g	g	g	g	g	g	g	g
g	2901816	a	3160750	a	3230706	s	g	O157	++	g	g	g	g	a	g	a	g	g	g	g	g	g	g	a	a	a	g	g	g	a	a	a
c	2901879	g	3160813	g	3230769	s	c	O157	++++	c	c	c	c	c	c	c	c	c	c	c	c	c	c	c	c	c	c	c	c	c	c	c
g	2903763	a	3162697	a	3232653	s	g	O157	++++	g	g	g	g	g	g	g	g	g	g	g	g	g	g	g	g	g	g	g	g	g	g	g
c	2904035	t	3162969	t	3232925	ns	c	O157	++++	c	c	c	c	c	c	c	c	c	c	c	c	c	c	c	c	c	c	c	c	c	c	c
t	2905595	g	3164529	g	3234485	ns	t	O157	++++	t	t	t	t	t	t	t	t	t	t	t	t	t	t	t	t	t	t	t	t	t	t	t
t	2906928	c	3165862	c	3235818	s	c	CB9615	++++		c	c	c	c	c	c	c	c	c	c	c	c	c	t	c	c	c	c	c	c	c	c
a	2907464	g	3166398	g	3236354	ns	g	CB9615	++++	g	g	g	g	g	g	g	g	g	g	g	g	g	g	g	g	g	g	g	g	g	g	g
a	2908744	c	3167678	c	3237634	s	c	CB9615	++++	c	c	c	c	c	c	c	c	c	c	c	c	c	c	t	c	c	c	c	c	c	c	t
a	2913426	g	3172360	g	3242316	ns	a	O157	++++	a	a	a	a	a	a	a	a	a	a	a	a	a	a	a	a	a	a	a	a	a	a	a
t	2914753	a	3173687	a	3243643	s	a	CB9615	++++	a	a	a	a	a	a	a	a	a	a	a	a	a	a	a	a	a	a	a	a	a	a	a
t	2918298	g	3177232	g	3247188	ns	t	O157	++++					t		t									t	t	t	t	t	t		
g	2918934	a	3177868	a	3247824	s	?	O55/O157	+/-					a		g		g	g	g	g	g	g		a	a	a	a	a	a		
g	2919361	a	3178295	a	3248251	s	g	O157	++	g	g	g	g	g	g	g	g	g	g	a	a	a	a	g	g	g	g	g	g	g	g	g
c	2919365	a	3178299	a	3248255	ns	c	O157	++++	c	c	c	c	c	c	c	c	c	c	c	c	c	c	c	c	c	c	c	c	c	c	c
g	2920281	c	3179215	c	3249171	ns	g	O157	++++	g	g	g	g	g	g	g	g	g	g	g	g	g	g	g	g	g	g	g	g	g	g	g
a	2920321	g	3179255	g	3249211	s	g	CB9615	++	g	a	g	g	g	g	g	g	a	g	a	a	a	a	a	g	g	g	g	g	g	a	a
c	2920930	g	3179864	g	3249820	ns	g	CB9615	++++	g	g	g	g	g	g	g	g	g	g	g	g	g	g	g	g	g	g	g	g	g	g	g
a	2921624	g	3180558	g	3250514	s	g	CB9615	++	g	g	g	g	a	g	g	a	g	g	g	g	g	g	g	g	g	g	g	g	g	g	g

O55 and O157 genome site details										Outgroup Strain Details[g]																						
CB9615 base[a]	CB9615 Site[b]	Sakai base[a]	Sakai Site[b]	EDL933 base[a]	EDL933 Site[b]	type[c]	Inferred ancestral base[d]	Lineage inferred to mutate[e]	Support level[f]	D1 Sd197	K-12	HS	ATCC 8739	UMN026	IAI39	SMS 3-5	E2348/69	536	ED1a	CFT073	S88	APEC O1	UTI89	E24377A	IAI1	SE11	F5 8401	F2a 2457T	F2a 301	SS Ss046	B4 Sb227	B18 BS512
g	2923031	a	3181965	a	3251921	s	g	O157	++	g	a	a	a	t	a	a	t	a	a	a				a	a	a	a	a	a	a	a	a
g	2923439	a	3182373	a	3252329	i	g	O157	++	g	a	a	a											a	a	a	a			a	a	a
a	2923576	g	3182510	g	3252466	i	?	O55/O157	+/-																							
t	2923726	g	3182660	g	3252616	i	g	CB9615	++	g																						
t	2924751	a	3183685	a	3253641	s	t	O157	++++	t	t	t	t	t	t	t	t	t	t	t	t	t	t	t	t	t	t	t	t	t	t	t
c	2925240	t	3184174	t	3254130	s	c	O157	++++	c	c	c	c	c	c	c	c	c	c	c	c	c	c	c	c	c	c	c	c	c	c	c
t	2925279	c	3184213	c	3254169	ns	c	CB9615	++++	c	c	c	c	c	c	c	c	c	c	c	c	c	c	c	c	c	c	c	c	c	c	c
a	2925961	t	3184895	t	3254851	ns	a	O157	++++	a	a	a	a	a	a	a	a	a	a	a	a	a	a	a	a	a	a	a	a	a	a	a
t	2926853	c	3185787	c	3255743	s	t	O157	++++	t	t	t	t	t	t	t	t	t	t	t	t	t	t	t	t	t	t	t	t	t	t	t
c	2927725	t	3186659	t	3256615	ns	t	CB9615	++++	t	t	t	t	t	t	t	t	t	t	t	t	t	t	t	t	t	t	t	t	t	t	t
a	2931532	c	3190466	c	3260422	i	a	O157	++	a	g	g	g	g	g	g								g	g	g	g	g	g	g	g	g
g	2931533	t	3190467	t	3260423	i	g	O157	++	g	c	c	c	c	c	c								c	c	c	c	c	c	c	c	c
a	2931534	c	3190468	c	3260424	i	a	O157	++++	a	a	a	a	a	a	a								a	a	a	a	a	a	a	a	a
t	2934157	g	3193091	g	3263047	i	t	O157	++++		t				t		t				t	t	t				t	t	t	t	t	t
t	2935314	c	3194248	c	3264204	ns	c	CB9615	++++		c				c		c				c	c	c							c		
a	2937582	c	3196516	c	3266472	ns	?	O55/O157	+/-																							
g	2937887	a	3196821	a	3266777	s	a	CB9615	+++			a															a	a	a			
g	2940866	a	3199800	a	3269756	ns	?	O55/O157	+/-																							
c	2941176	c	3200110	t	3270066	i	?	EDL933	+/-																							
a	2941204	a	3200138	g	3270094	i	?	EDL933	+/-																							
a	2941205	a	3200139	c	3270095	i	?	EDL933	+/-																							
c	2944601	a	3203535	a	3273491	ns	a	CB9615	++++	a				a			a	a						a	a	a	a					
-	2945171	-	3204105	g	3274062	ins	-	EDL933	++++	-				-			-	-						-	-	-	-			-		
-	2945208	-	3204142	n	3274100	ins	-	EDL933	++++	-				-			-	-						-	-	-	-			-		
-	2945242	-	3204176	c	3274135	ins	-	EDL933	++++	-				-			-	-						-	-	-	-			-		
g	2946038	t	3204972	t	3274931	s	g	O157	++++	g				g			g	g						g	g	g	g	g	g	g		
a	2946740	g	3205674	g	3275633	s	g	CB9615	++++	g				g			g	g						g	g	g	g	g	g	g		
a	2948484	g	3207418	g	3277377	i	g	CB9615	++++	g	g	g	g	g	g	g	g	g	g	g	g	g	g	g	g	g	g	g	g	g		
t	2949533	c	3208467	c	3278426	ns	c	CB9615	++++	c	c	c	c	c	c	c	c	c	c	c	c	c	c	c	c	c	c	c	c	c	c	c
g	2950067	t	3209001	t	3278960	ns	g	O157	++++	g	g	g	g	g	g	g	g	g	g	g	g	g	g	g	g	g	g	g	g	g	g	g
t	2950267	c	3209201	c	3279160	s	c	CB9615	++	c	c	c	c	t	t	t	t	t	t	t	t	t	t	t	t	t	t	t	t	c	c	c
g	2950637	a	3209571	a	3279530	ns	g	O157	++++	g	g	g	g	g	g	g	g	g	g	g	g	g	g	g	g	g	g	g	g	g	g	g
g	2951069	a	3210003	a	3279962	ns	g	O157	++++	g	g	g	g	g	g	g	g	g	g	g	g	g	g	g	g	g	g	g	g	g	g	g
g	2951469	c	3210403	c	3280362	ns	c	CB9615	++++	c	c	c	c	c	c	c	c	c	c	c	c	c	c	c	c	c	c	c	c	c	c	c
g	2952481	a	3211415	a	3281374	ns	g	O157	++	g	g	g	g	g	g	g	g	g	g	g	g	g	g	g	g	g	a	a	a	a	g	a
a	2952640	t	3211574	t	3281533	i	a	O157	++++	a	a	a	a	a	a	a	a	g	a	g	a	a	a	a	a	a	a	a	a	a	a	a
a	2957581	t	3216515	t	3286474	ns	t	CB9615	++++	t	t	t	t	t	t	t	t	t	t	t	t	t	t	t	t	t	t	t	t	t	t	t
4	2958191	-	3217124	-	3287083	del	4	O157	++++	4	4	4	4	4	4	4	4	4	4	4	4	4	4	4	4	4	4	4	4	4	4	4
a	2962985	t	3221915	t	3291874	s	t	CB9615	++++	t	t	t	t	t	t	t	t	t	t	t	t	t	t	t	t	t	t	t	t	t	t	t
t	2963030	c	3221960	c	3291919	s	t	O157	++	t	c	c	c	c	c	c	c	c	c	c	c	c	c	c	c	c	c	c	c	c	c	c
g	2964258	t	3223188	t	3293147	s	g	O157	++++	g	g	g	g	g	g	g	g	g	g	g	g	g	g	g	g	g	g	g	g	g	g	g
-	2964585	-	3223515	g	3293475	ins	-	EDL933	++++	-	-	-	-	-	-	-	-	-	-	-	-	-	-	-	-	-	-	-	-	-	-	-
g	2965198	a	3224128	a	3294088	s	a	CB9615	++++	a	a	a	a	a	a	a	a	a	a	a	a	a	a	a	a	a	a	a	a	a	a	a
c	2965915	t	3224845	t	3294805	i	t	CB9615	++++	t	t	t	t	t	t	t	t	t	t	t	t	t	t	t	t	t	t	t	t	t	t	t
a	2966100	c	3225030	c	3294990	i	a	O157	++++	a	a	a	a	a	a	a	a	a	a	a	a	a	a	a	a	a	a	a	a	a	a	a
a	2968369	g	3227299	g	3297259	s	g	CB9615	++++	g	g	g	g	g	g	g	g	g	g	g	g	g	g	g	g	g				g	g	g
t	2972180	c	3231110	c	3301070	ns	t	O157	++++	t	t	t	t	t	t	t	t	t	t	t	t	t	t	t	t	t	t	t	t	t	t	t
c	2974965	g	3233895	g	3303855	ns	c	O157	++++	c	c	c	c	c	c	c	c	c	c	c	c	c	c	c	c	c	c	c	c	c	c	c
a	2975370	g	3234300	g	3304260	ns	g	CB9615	++++	g	g	g	g	g	g	g	g	g	g	g	g	g	g	g	g	g	g	g	g	g	g	g
t	2977625	g	3236555	g	3306515	i	g	CB9615	++++	g	g	g	g	g	g	g	g	g	g	g	g	g	g	g	g	g	g	g	g	g	g	g
t	2982292	c	3241222	c	3311182	s	t	O157	++++	t	t	t	t	t	t	t	t	t	t	t	t	t	t	t	t	t	t	t	t	t	t	t
a	2982902	t	3241832	t	3311792	ns	a	O157	++++	a	a	a	a	a	a	a	a	a	a	a	a	a	a	a	a	a	a	a	a	a	a	a
t	2986844	a	3245774	a	3315619	ns	a	CB9615	++++	a	a	a	a	a	a	a	a	a	a	a	a	a	a	a	a	a	a	a	a	a	a	a
a	2990365	-	3249294	a	3319140	del	a	Sakai	++++	a	a	a	a	a	a	a	a	a	a	a	a	a	a	a	a	a	a	a	a	a	a	a
g	2991491	a	3250420	a	3320266	s	g	O157	++++	g	g	g	g	g		g	g	g	g	g	g	g	g	g	g	g	g	g	g	g	g	g
c	2991753	t	3250682	t	3320528	ns	c	O157	++++	c	c	c	c	c		c	c	c	c	c	c	c	c	c	c	c	c	c	c	c	c	c
g	2994346	a	3253275	a	3323121	s	g	O157	++++	g	g	g	g	g	g	g	g	g	g	g	g	g	g	g	g	g	g	g	g	g	g	g
12	2996312	12	3255241	-	3325086	del	12	EDL933	-	12	-	-	-	12	12	12	12	12	12	12	12	12	12	-	-	-	-	-	-	-	-	-
g	2996343	g	3255272	a	3325106	ns	g	EDL933	++++	g	g	g	g	g	g	g	g	g	g	g	g	g	g	g	g	g	g	g	g	g	g	g
g	3000019	a	3258948	a	3328782	s	g	O157	++++	g	g	g	g	g	g	g	g	g	g	g	g	g	g	g	g	g	g	g	g	g	g	g
a	3000279	c	3259208	c	3329042	ns	c	CB9615	++++	c	c	c	c	c	c	c	c	c	c	c	c	c	c	c	c	c	c	c	c	c	c	c
g	3000331	a	3259260	a	3329094	s	g	O157	++++	g	g	g	g	g	g	g	g	g	g	g	g	g	g	g	g	g	g	g	g	g	g	g
a	3000817	g	3259746	g	3329580	s	g	CB9615	++++	g	g	g	g	g	g	g	g	g	g	g	g	g	g	g	g	g	g	g	g	g	g	g
t	3001675	a	3260604	a	3330438	ns	t	O157	++++	t	t	t	t	t	t	t	t	t	t	t	t	t	t	t	t	t	t	t	t	t	t	t
c	3003351	c	3262280	a	3332114	s	c	EDL933	++++	c	c	c	c	t	t	t	t	t	t	t	t	t	t	t	t	t	c	c	c	t	c	c
g	3003584	a	3262513	a	3332347	ns	g	O157	++++	g	g	g	g	g	g	g	g	g	g	g	g	g	g	g	g	g	g	g	g	g	g	g
a	3004101	g	3263030	g	3332864	i	g	CB9615	++++	g	g	g	g	g	g	g	g	g	g	g	g	g	g	g	g	g	g	g	g	g	g	g
a	3004715	g	3263644	g	3333478	ns	g	CB9615	++++	g	g	g	g	g	g	g	g	g	g	g	g	g	g	g	g	g	g	g	g	g	g	g
g	3010887	a	3269816	a	3339650	s	g	O157	++++	g	g	g	g	g	g	g	g	g	g	g	g	g	g	g	g	g	g	g	g	g	g	g
t	3013290	a	3272219	a	3342053	ns	t	O157	++++	t	t	t	t	t	t	t								t	t	t				t	t	t
g	3015201	c	3274130	c	3343964	ns	g	O157	++++	g	g	g	g	g	g	g	g	g	g	g	g	g	g	g	g	g	g	g	g	g	g	g
g	3017867	a	3276796	a	3346630	ns	g	O157	++++	g	g	g	g	g	g	g	g	g	g	g	g	g	g	g	g	g	g	g	g	g	g	g
t	3019530	c	3278459	c	3348293	ns	t	O157	++++	t	t	t	t	t	t	t	t	t	t	t	t	t	t	t	t	t	t	t	t	t	t	t
c	3021227	t	3280156	c	3349990	ns	c	Sakai	++++	c	c	c	c	c	c	c	c	c	c	c	c	c	c	c	c	c				c	c	c
a	3024955	g	3283884	g	3353718	s	g	CB9615	++++		g	g	g	g	g	g	g	g	g	g	g	g	g	g	g	g				g		g
t	3025432	a	3284361	a	3354195	s	t	O157	++++		t	t	t	t	t	t	t	t	t	t	t	t	t	t	t	t				t		t
a	3026506	g	3285435	g	3355269	s	g	CB9615	++++		g	g	g	g	g	g	g	g	g	g	g	g	g	g	g	g				g	g	g
g	3027079	a	3286008	a	3355842	ns	g	O157	++++		g	g	g	g	g	g	g	g	g	g	g	g	g	g	g	g				g	g	g
t	3027681	c	3286610	c	3356444	s	t	O157	++++		t	t	t	t	t	t	t	t	t	t	t	t	t	t	t	t				t	t	t

O55 and O157 genome site details										Outgroup Strain Details[g]																							
CB9615 base[a]	CB9615 Site[b]	Sakai base[a]	Sakai Site[b]	EDL933 base[a]	EDL933 Site[b]	type[c]	Inferred ancestral base[d]	Lineage inferred to mutate[e]	Support level[f]	D1 Sd197	K-12	HS	ATCC 8739	UMN026	IAI39	SMS 3-5	E2348/69	536	ED1a	CFT073	S88	APEC O1	UTI89	E24377A	IAI1	SE11	F5 8401	F2a 2457T	F2a 301	SS Ss046	B4 Sb227	B18 BS512
a	3028423	g	3287352	g	3357186	ns	g	CB9615	++++		g	g	g	g	g	g	g	g	g	g	g	g	g	g	g	g				g	g	g
t	3028720	c	3287649	c	3357483	ns	c	CB9615	++++		c	c	c	c	c	c	c	c	c	c	c	c	c	c	c	c				c	c	c
g	3035267	a	3294196	a	3364030	s	g	O157	++	g	g	g	g	g	g	g	g	g	g	g	g	g	g	g	g	g	a	a	a	g	g	g
g	3036439	a	3295368	a	3365202	s	g	O157	++++	g	g	g	g	g	g	g	g	g	g	g	g	g	g	g	g	g	g	g	g	g	g	g
c	3037397	t	3296326	t	3366160	s	t	CB9615	++	t	c	c	c	c	c	c	t	c	c	c	c	c	c	c	c	c	c	c	c	c	t	t
g	3037508	a	3296437	a	3366271	s	g	O157	++++	g	g	g	g	g	g	g	g	g	g	g	g	g	g	g	g	g	g	g	g	g	g	g
c	3037706	t	3296635	t	3366469	s	c	O157	++++	c	c	c	c	c	c	c	c	c	c	c	c	c	c	c	c	c	c	c	c	c	c	c
c	3040104	t	3299033	t	3368867	ns	t	CB9615	++	t	c	c	c	c	c	c	c	c	c	c	c	c	c	c	c	c	c	c	c	c	c	c
t	3040646	c	3299575	c	3369409	ns	c	CB9615	++++	c	c	c	c	c	c	c	c	c	c	c	c	c	c	c	c	c	c	c	c	c	c	c
g	3043116	c	3302045	c	3371879	ns	g	O157	++++	g	g	g	g	g	g	g	g	g	g	g	g	g	g	g	g	g	g	g	g	g	g	g
g	3045168	t	3304097	g	3373931	ns	g	Sakai	++++	g	g	g	g	g	g	g	g	g	g	g	g	g	g	g	g	g	g	g	g	g	g	g
t	3045592	c	3304521	c	3374355	ns	t	O157	++++	t	t	t	t	t	t	t	t	t	t	t	t	t	t	t	t	t	t	t	t	t	t	t
t	3048331	c	3307260	c	3377094	i	t	O157	++++	t	t	t	t	t	t	t	t	t	t	t	t	t	t	t	t	t	t	t	t	t	t	t
t	3048566	t	3307495	g	3377329	ns	t	EDL933	++++	t	t	t	t	t	t	t	t	t	t	t	t	t	t	t	t	t	t	t	t	t	t	t
a	3048656	t	3307585	t	3377419	ns	a	O157	++++	a	a	a	a	a	a	a	a	a	a	a	a	a	a	a	a	a	a	a	a	a	a	a
c	3048778	a	3307707	a	3377541	ns	c	O157	++++	c	c	c	c	c	c	c	c	c	c	c	c	c	c	c	c	c	c	c	c	c	c	c
a	3051781	c	3310710	c	3380544	s	a	O157	++	a	a	c	a	g	g	c	a	g	a	a	g	g	g	c	g	g	g	g	g	g	c	c
c	3052084	t	3311013	c	3380847	s	t	Sakai	-	t	c	c	c	c	c	c	t	c	t	t	t	t	t	c	c	c	c	c	c	c	c	c
c	3052288	a	3311217	a	3381051	s	c	O157	++++	c	c	c	c	c	c	c	c	c	c	c	c	c	c	c	c	c	c	c	c	c	c	c
a	3053792	g	3312721	g	3382555	s	g	CB9615	++++	g	g	g	g	g	g	g	g	g	g	g	g	g	g	g	g	g	g	g	g	g	g	g
t	3054572	c	3313501	c	3383335	s	c	CB9615	++++	c	c	c	c	c	c	c	c	c	c	c	c	c	c	c	c	c	c	c	c	c	c	c
t	3055812	c	3314741	c	3384575	ns	c	CB9615	++++	c	c	c	c	c	c	c	c	c	c	c	c	c	c	c	c	c	c	c	c	c	c	c
t	3055997	g	3314926	g	3384760	ns	t	O157	++++	t	t	t	t	t	t	t	t	t	t	t	t	t	t	t	t	t	t	t	t	t	t	t
t	3056014	a	3314943	a	3384777	ns	t	O157	++++	t	t	t	t	t	t	t	t	t	t	t	t	t	t	t	t	t	t	t	t	t	t	t
g	3058312	a	3317241	a	3387075	i	a	CB9615	++	a	g	g	g	g	g	g								g	g	g	g	g	g	g	g	g
c	3059166	t	3318095	t	3387929	s	c	O157	++++	c	c	c	c	c	c	c								c	c	c	c	c	c	c	c	c
t	3059774	a	3318703	a	3388537	ns	a	CB9615	++++	a	a	a	a	a	a	a								a	a	a	a	a	a	a	a	a
g	3059967	a	3318896	a	3388730	s	g	O157	++++	g	g	g	g	g	g	g								g	g	g	g	g	g	g	g	g
c	3062428	t	3321357	t	3391191	ns	c	O157	++++	c	c	c	c	c	c	c								c	c	c	c	c	c	c	c	c
a	3062763	g	3321692	g	3391526	ns	g	CB9615	++++	g	g	g	g	g	g	g									g	g	g	g	g	g		
-	3063544	6	3322474	6	3392308	del	6	CB9615	++++	6	6	6	6	6	6	6								6	6	6	6	6	6	6	6	6
a	3066628	g	3325563	g	3395397	ns	g	CB9615	++++	g	g	g	g	g	g	g	g	g	g	g	g	g	g	g	g	g	g	g	g	g	g	g
g	3068562	a	3327497	a	3397331	ns	g	O157	++++	g	g	g	g	g	g	g								g	g	g	g	g	g	g	g	g
c	3068611	a	3327546	a	3397380	ns	a	CB9615	++++	a	a	a	a	a	a	a								a	a	a	a	a	a	a	a	a
g	3069182	a	3328117	a	3397951	s	g	O157	++++	g	g	g	g	g	g	g								g	g	g	g	g	g	g	g	g
-	3071080	-	3330015	g	3399850	ins	-	EDL933	++++	-	-	-	-	-	-	-								-	-	-	-	-	-	-	-	-
-	3071087	-	3330022	g	3399858	ins	-	EDL933	++++	-	-	-	-	-	-	-								-	-	-	-	-	-	-	-	-
g	3073768	a	3332703	a	3402539	ns	g	O157	++++	g	g	g	g	g	g	g	g	g	g	g	g	g	g	g	g	g	g	g	g	g	g	g
a	3076460	g	3335395	g	3405231	s	g	CB9615	++	g	g	a	a	g	g	g	g	g	g	g	g	g	g	g	g	g	g	g	g	g	g	g
a	3079799	c	3338734	c	3408570	ns	a	O157	++++	a	a	a	a	a	a	a	a	a	a	a	a	a	a	a	a	a	a	a	a	a	a	a
c	3080249	t	3339184	t	3409020	i	t	CB9615	++++	t	t	t	t	t	t	t	t	t	t	t	t	t	t	t	t	t	t	t	t	t	t	t
t	3080310	c	3339245	c	3409081	s	c	CB9615	++++	a	c	c	c	c	c	c	c	c	c	c	c	c	c	c	c	c	c	c	c	c	c	c
t	3087305	g	3346240	g	3416076	ns	g	CB9615	++++	g	g	g	g	g	g	g	g	g	g	g	g	g	g	g	g	g	g	g	g	g		g
a	3091074	g	3350009	g	3419845	s	g	CB9615	++++	g	g	g	g	g	g	g	g	g	g	g	g	g	g	g	g	g	g	g	g	g	g	g
c	3091394	c	3350329	t	3420165	i	c	EDL933	++++	c	c	c	c	c	c	c	c	c	c	c	c	c	c	c	c	c	c	c	c	c	c	c
a	3095104	g	3354039	g	3423875	ns	a	O157	++++	a	a	a	a	a	a	a	a	a	a	a	a	a	a	a	a	a	a	a	a	a	a	a
c	3096450	t	3355385	t	3425221	ns	c	O157	++++	c	c	c	c	c	c	c	c	c	c	c	c	c	c	c	c	c	c	c	c	c	c	c
g	3098785	t	3357720	t	3427556	ns	t	CB9615	++++	t	t	t	t	t	t	t	t	t	t	t	t	t	t	t	t	t	t	t	t	t	t	t
g	3102696	a	3361631	a	3431467	ns	?	O55/O157	+/-																							
g	3102846	a	3361781	a	3431617	ns	?	O55/O157	+/-																							
c	3102916	a	3361851	a	3431687	ns	?	O55/O157	+/-																							
t	3104750	g	3363685	g	3433521	ns	?	O55/O157	+/-																							
a	3105262	g	3364197	g	3434033	ns	?	O55/O157	+/-																							
t	3105533	c	3364468	c	3434304	ns	?	O55/O157	+/-																							
g	3106970	a	3365905	a	3435741	i	?	O55/O157	+/-																							
a	3113828	g	3372763	g	3442599	ns	g	CB9615	++++	g	g	g	g	g	g	g	g	g	g	g	g	g	g	g	g	g				g	g	g
t	3113869	a	3372804	a	3442640	ns	a	CB9615	++++	a	a	a	a	a	a	a	a	a	a	a	a	a	a	a	a	a				a	a	a
-	3116536	-	3375471	N	3445308	ins	-	EDL933	++++	-	-	-	-	-	-	-	-	-	-	-	-	-	-	-	-	-	-	-	-	-	-	-
a	3119046	g	3377981	g	3447818	ns	g	CB9615	++++	g	g	g	g	g	g	g	g	g	g	g	g	g	g	g	g	g	g	g	g	g	g	g
a	3119807	g	3378742	g	3448579	s	g	CB9615	++	g	g	g	g	g	g	g	a	a	a	g	g	g	g	g	g	g	g	g	g	g	g	g
a	3119967	g	3378902	g	3448739	ns	g	CB9615	++++	g	g	g	g	g	g	g	g	g	g	g	g	g	g	g	g	g	g	g	g	g	g	g
a	3121240	c	3380175	c	3450012	i	a	O157	++++	a	a	a	a	a	a	a	a	a	a	a	a	a	a	a	a	a	a	a	a	a	a	a
a	3123303	g	3382238	g	3452075	i	g	CB9615	++++	g	g	g	g	g	g	g	g	g	g	g	g	g	g	g	g	g	g	g	g	g	g	g
t	3124446	c	3383381	c	3453218	s	c	CB9615	++++	c	c	c	c	c	c	c	c	c	c	c	c	c	c	c	c	c	c	c	c	c	c	c
a	3125631	g	3384566	g	3454403	s	g	CB9615	++++	g	g	g	g	g	g	g	g	g	g	g	g	g	g	g	g	g	g	g	g	g	g	g
g	3126483	a	3385418	a	3455255	s	g	O157	++++	g	g	g	g	g	g	g	g	g	g	a	g	g	g	g	g	g	g	g	g	g	g	g
g	3129452	t	3388387	t	3458224	i	g	O157	++++	g	g	g	g	g	g	g	g	g	g	g	g	g	g	g	g	g	g	g	g	g	g	g
-	3129543	-	3388478	g	3458316	ins	-	EDL933	++++	-	-	-	-	-	-	-								-	-	-	-	-	-	-	-	-
g	3130550	a	3389485	a	3459323	i	g	O157	++++		g	g	g	g	g	g								g	g	g	g	g	g	a	g	
c	3131857	t	3390792	t	3460630	s	c	O157	++++		c	c	c	c	c	c								c	c	c	c	c	c	c	c	
a	3132327	c	3391262	c	3461100	s	a	O157	++++		a	a	a	a	a	a								a	a	a	a	a	a	a	a	
a	3133865	g	3392800	g	3462638	ns	g	CB9615	++++		g	g	g	g	g	g								g	g	g	g	g	g	g	g	
a	3133963	g	3392898	g	3462736	s	g	CB9615	++++		g	g	g	g	g	g								g	g	g	g	g	g	g	g	
g	3136759	c	3395694	c	3465532	ns	g	O157	++++		g	g	g	g	g	g	g	g	g	g	g	g	g	g	g	g	g	g	g	g	g	
g	3137138	a	3396073	a	3465911	ns	g	O157	++++		g	g	g	g	g	g	g	g	g	g	g	g	g	g	g	g	g	g	g	g	g	
g	3137156	a	3396091	a	3465929	ns	g	O157	++++		g	g	g	g	g	g	g	g	g	g	g	g	g	g	g	g	g	g	g	g	g	
c	3138116	t	3397051	t	3466889	s	c	O157	++++		c	c	c	c	c	c	c	c	c	c	c	c	c	c	c	c	c	c	c	c	c	
c	3138327	t	3397262	t	3467100	ns	c	O157	++++		c	c	c	c	c	c	c	c	c	c	c	c	c	c	c	c	c	c	c	c	c	
a	3139276	t	3398211	t	3468049	ns	a	O157	++++		a	a	a	a	a	a	a	a	a	a	a	a	a	a	a	a	a	a	a	a	a	

O55 and O157 genome site details										Outgroup Strain Details[g]																						
CB9615 base[a]	CB9615 Site[b]	Sakai base[a]	Sakai Site[b]	EDL933 base[a]	EDL933 Site[b]	type[c]	Inferred ancestral base[d]	Lineage inferred to mutate[e]	Support level[f]	D1 Sd197	K-12	HS	ATCC 8739	UMN026	IAI39	SMS 3-5	E2348/69	536	ED1a	CFT073	S88	APEC O1	UTI89	E24377A	IAI1	SE11	F5 8401	F2a 2457T	F2a 301	SS Ss046	B4 Sb227	B18 BS512
a	3142104	c	3401039	c	3470877	s	c	CB9615	++++	c	c	c	c	c	c	c	c	c	c	c	c	c	c	c	c	c	c	c	c	c	c	
g	3142248	a	3401183	a	3471021	s	g	O157	++++	g	g	g	g	g	g	g	g	g	g	g	g	g	g	g	g	g	g	g	g	g	g	
g	3142510	a	3401445	a	3471283	ns	g	O157	++++	g	g	g	g	g	g	g	g	g	g	g	g	g	g	g	g	g	g	g	g	g	g	
a	3143330	g	3402265	g	3472103	ns	a	O157	++++	a	a	a	a	a	a	a	a	a	a	a	a	a	a	a	a	a	a	a	a	a	a	
a	3143736	g	3402671	g	3472509	s	g	CB9615	++++	g	g	g	g	g	g	g	g	g	g	g	g	g	g	g	g	g	g	g	g	g	g	
a	3144788	g	3403723	g	3473561	s	g	CB9615	++++	g	g	g	g	g	g	g	g	g	g	g	g	g	g	g	g	g	g	g	g	g	g	g
g	3144899	a	3403834	a	3473672	s	g	O157	++	g	g	g	g	g	g	g	g	g	g	g	g	g	g	g	g	g	a	a	a	g	g	g
t	3145958	g	3404893	g	3474731	ns	g	CB9615	++++	g	g	g	g	g	g	g	g	g	g	g	g	g	g	g	g	g	g	g	g	g	g	g
t	3146660	g	3405595	g	3475433	s	g	CB9615	++++	g	g	a	g	g	g	g	g	g	g	g	g	g	g	g	g	g	g	g	g	g	g	g
a	3147613	g	3406548	g	3476386	s	g	CB9615	++++	g	g	g	g	g	g	g	g	g	g	g	g	g	g	g	g	g	g	g	g	g	g	g
t	3150181	c	3409116	c	3478954	s	c	CB9615	++++	c	c	c	c	c	c	c	c	c	c	c	c	c	c	c	c	c	c	c	c	c	c	c
t	3152738	g	3411673	g	3481511	nc	g	CB9615	++++	g	g	g	g	g	g	g	g	g	g	g	g	g	g	g	g	g	g	g	g	g	g	g
g	3155066	a	3414001	a	3483839	s	g	O157	++++	g	g	g	g	g	g	g	g	g	g	g	g	g	g	g	g	g	g	g	g	g	g	g
t	3157425	c	3416360	c	3486198	s	c	CB9615	++++	c	c	c	c	c	c	c	c	c	c	c	c	c	c	c	c	c	c	c	c	c	c	c
t	3158423	a	3417358	a	3487196	ns	t	O157	++++	t	t	t	t	t	t	t	t	t	t	t	t	t	t	t	t	t	t	t	t	t	t	t
-	3202905	-	3420357	g	3490196	ins	-	EDL933	++++	-	-	-	-	-	-	-	-	-	-	-	-	-	-	-	-	-	-	-	-	-	-	-
c	3213250	t	3430702	t	3500541	i	c	O157	++	c	c	c	c	c	c	c	c	t	t	t	t	t	t	c	c	c	c	c	c	c	c	c
t	3217040	a	3434492	a	3504331	s	t	O157	++++	t	t	t	t	t	t	t	t	t	t	t	t	t	t	t	t	t	t	t	t	t	t	t
g	3219347	a	3436799	a	3506638	i	g	O157	++++	g	g	g	g	g	g	g	g	g	g	g	g	g	g	g	g	g	g	g	g	g	g	g
-	3220137	c	3437590	-	3507428	ins	-	Sakai	++++	-	-	-	-	-	-	-	-	-	-	-	-	-	-	-	-	-	-	-	-	-	-	-
a	3221386	g	3438839	g	3508677	s	g	CB9615	++++	g	g	g	g	g	g	g	g	g	g	g	g	g	g	g	g	g	g	g	g	g	g	g
t	3221956	c	3439409	c	3509247	i	c	CB9615	++++	c	c	c	c	c	c	c	c	c	c	c	c	c	c	c	c	c	c	c	c	c	c	c
-	3224092	a	3441546	-	3511383	ins	-	Sakai	++++	-	-	-	-	-	-	-	-	-	-	-	-	-	-	-	-	-	-	-	-	-	-	-
c	3225410	a	3442864	a	3512701	i	c	O157	++++	c	c	c	c	c	c	c	c	c	c	c	c	c	c	c	c	c	c	c	c	c	c	c
g	3228237	a	3445691	a	3515528	s	g	O157	++++	g	g	g	g	g	g	g	g	g	g	g	g	g	g	g	g	g	g	g	g	g	g	g
-	3228789	-	3446243	a	3516081	ins	-	EDL933	++++	-	-	-	-	-	-	-	-	-	-	-	-	-	-	-	-	-	-	-	-	-	-	-
c	3228919	a	3446373	a	3516211	nc	c	O157	++	c	c	a	a	a	a	c	a	a	a	a	a	a	c	a	c	c	c	c	c	c	c	c
a	3229212	g	3446666	g	3516504	nc	g	CB9615	++++	g	g	g	g	g	g	g	g	g	g	g	g	g	g	g	g	g	g	g	g	g	g	g
-	3230243	-	3447697	g	3517536	ins	-	EDL933	++++	-	-	-	-	-	-	-	-	-	-	-	-	-	-	-	-	-	-	-	-	-	-	-
-	3230583	-	3448037	g	3517877	ins	-	EDL933	++++	-	-	-	-	-	-	-	-	-	-	-	-	-	-	-	-	-	-	-	-	-	-	-
g	3230699	a	3448153	r	3517993	nc	a	EDL933	++	a	g	g	g	g	a	a	c	c	c	g	c	c	c	a	g	a	a	a	a	g	a	a
c	3230708	t	3448162	y	3518002	nc	t	EDL933	++	t	c	c	c	c	t	t	g	g	g	c	g	g	g	t	c	t	c	c	c	c	c	t
a	3231281	g	3448735	R	3518575	nc	a	EDL933	IUPAC	a	a	a	a	a	a	a	a	a	a	a	a	a	a	a	a	a	a	a	a	a	a	a
-	3231374	-	3448828	a	3518669	ins	-	EDL933	++++	-	-	-	-	-	-	-	-	-	-	-	-	-	-	a	-	-	-	-	-	-	-	-
-	3231386	-	3448840	t	3518682	ins	-	EDL933	++++	-	-	-	-	-	-	-	-	-	-	-	-	-	-	t	-	-	-	-	-	-	-	-
-	3231870	-	3449324	g	3519167	ins	-	EDL933	++++	-	-	-	-	-	-	-	-	-	-	-	-	-	-	-	-	-	-	-	-	-	-	-
-	3231946	18	3449401	18	3519244	ins	-	O157	++	-	-	-	18	-	-	18	18	18	18	-	18	18	18	-	18	-	18	18	18	18	18	18
a	3231947	-	3449418	-	3519261	indel	?	O55/O157	+/-																							
a	3231959	g	3449423	g	3519266	i	g	CB9615	++++	g	g	g	g	g	g	g	g	g	g	g	g	g	g	g	g	g	g	g	g	g	g	g
a	3232259	a	3449723	g	3519566	i	g	EDL933	-	g	g	g	g	a	g	g	g	g	g	g	g	a	g	g	g	g	g	g	g	g	g	g
g	3232260	g	3449724	c	3519567	i	c	EDL933	-	c	c	c	c	g	c	c	c	c	c	c	c	g	c	c	c	c	c	c	c	c	c	c
a	3233603	g	3451067	g	3520915	nc	g	CB9615	++++	g	g	g	g	g	g	g	g	g	g	g	t	g	t	g	g	g	g	g	g	g	g	g
-	3233843	-	3451307	g	3521156	ins	-	EDL933	++++	-	-	-	-	-	-	-	-	-	-	-	-	-	-	-	-	-	-	-	-	-	-	-
c	3233856	t	3451320	t	3521169	i	t	CB9615	++++	t	t	t	t	t	t	t	t	t	t	t	t	t	t	t	t	t	t	t	t	t	t	t
a	3233996	g	3451460	g	3521309	i	a	O157	++	a	a	a	a	a	a	a	a	g	a	a	g	a	g	a	a	a	a	a	a	a	a	a
c	3234003	t	3451467	t	3521316	i	t	CB9615	++	t	c	t	t	t	t	t	t	t	t	t	t	t	t	t	t	t	t	t	t	t	t	t
g	3235346	a	3452810	a	3522659	s	g	O157	++++	g	g	g	g	g	g	g	g	g	g	g	g	g	g	g	g	g	g	g	g	g	g	g
t	3240480	g	3457944	g	3527793	ns	g	CB9615	++++	g	a	g	g	g	g	g	g	g	g	g	g	g	g	g	g	g	a	a	a	g	g	g
a	3245167	c	3462631	c	3532480	ns	a	O157	++++	a	a	a	a	a	a	a	a	a	a	a	a	a	a	a	a	a	a	a	a		a	a
a	3246754	c	3464218	c	3534067	ns	a	O157	++++	a	a	a	a	a	g	g	a	a	g	a	a	a	a	a	a	a	a	a	a	a	a	a
c	3247118	a	3464582	a	3534431	s	a	CB9615	++++	a	a	a	a	a	a	a	a	a	a	a	a	a	a	a	a	a	a	a	a	a	a	a
c	3249290	a	3466754	a	3536603	s	c	O157	++++	c	c	c	c	c	c	c	c	c	c	c	c	c	c	c	c	c	c	c	c	c	c	c
a	3250353	g	3467817	g	3537666	i	g	CB9615	++++	g	g	g	g	g	g	g	g	g	g	g	g	g	g	g	g	g	g	g	g	g	g	g
t	3251908	c	3469372	c	3539221	ns	t	O157	++++	t	t	t	t	t	t	t	t	t	t	t	t	t	t	t	t	t	t	t	t	t	t	t
t	3251951	c	3469415	c	3539264	s	c	CB9615	++++	c	c	c	c	c	c	c	c	c	c	c	c	c	c	c	c	c	c	c	c	c	c	c
g	3253613	a	3471077	a	3540926	ns	g	O157	++++	g	g	g	g	g	g	g	g	g	g	g	g	g	g	g	g	g	g	g	g	g	g	g
t	3254051	a	3471515	a	3541364	ns	a	CB9615	++++	a	a	a	a	a	a	a	a	a	a	a	a	a	a	a	a	a	a	a	a	a	a	a
g	3255380	a	3472844	a	3542693	s	g	O157	++++	g	g	g	g	g	g	g	g	g	g	g	g	g	g	g	g	g	g	g	g	g	g	g
c	3256022	t	3473486	t	3543335	i	t	CB9615	++++	t	t	t	t	t	t	t	t	t	t	t	t	t	t	t	t	t	t	t	t	t	t	t
t	3256696	c	3474160	c	3544009	ns	t	O157	++++	t	t	t	t	t	t	t	t	t	t	t	t	t	t	t	t	t	t	t	t	t	t	t
c	3256801	t	3474265	t	3544114	ns	c	O157	++++	c	c	c	c	c	c	c	c	c	c	c	c	c	c	c	c	c	c	c	c	c	c	c
a	3257946	t	3475410	t	3545259	i	a	O157	++++	a	a	a	a	a	c	a	a	a	a	a	a	a	a	a	a	a	a	a	a	a	a	a
c	3258124	t	3475588	t	3545437	i	t	CB9615	++++	t	t	t	t	t	t	t	t	t	t	t	t	t	t	t	t	t	t	t	t	t	t	t
c	3292117	c	3484284	t	3551606	ns	c	EDL933	++++					c			c			c	c	c	c				c	c	c	c		c
g	3292254	g	3484421	a	3551743	ns	g	EDL933	+								g													g		g
-	3292799	-	3484966	t	3552289	ins	-	EDL933	+								-													-		-
g	3293802	a	3485969	a	3553292	ns	g	O157	+++								g													g	g	g
a	3329609	g	3491853	-	3559164	ns	?	O55/O157	+/-																							
-	3329767	-	3492035	g	3559317	ins	?	EDL933	+/-																							
-	3329793	-	3492061	g	3559344	ins	?	EDL933	+/-																							
g	3330853	c	3493121	c	3560404	i	?	O55/O157	+/-																							
-	3330947	-	3493215	c	3560499	ins	?	EDL933	+/-																							
c	3331099	t	3493367	t	3560651	ns	?	O55/O157	+/-																							
-	3331826	-	3494094	t	3561379	ins	?	EDL933	+/-																							
-	3332033	-	3494301	W	3561587	ins	?	EDL933	+/-																							
-	3332231	-	3494499	c	3561786	ins	?	EDL933	+/-																							
a	3332519	c	3494787	c	3562074	ns	?	O55/O157	+/-																							
t	3332606	g	3494874	g	3562161	ns	?	O55/O157	+/-																							
a	3332674	c	3494942	c	3562229	ns	?	O55/O157	+/-																							

CB9615 base[a]	CB9615 Site[b]	Sakai base[a]	Sakai Site[b]	EDL933 base[a]	EDL933 Site[b]	type[c]	Inferred ancestral base[d]	Lineage inferred to mutate[e]	Support level[f]	D1 Sd197	K-12	HS	ATCC 8739	UMN026	IAI39	SMS 3-5	E2348/69	536	ED1a	CFT073	S88	APEC O1	UTI89	E24377A	IAI1	SE11	F5 8401	F2a 2457T	F2a 301	SS Ss046	B4 Sb227	B18 BS512
O55 and O157 genome site details										Outgroup Strain Details[g]																						
t	3334462	c	3496730	c	3564017	ns	?	O55/O157	+/-																							
t	3334651	c	3496919	c	3564206	ns	?	O55/O157	+/-																							
a	3334724	g	3496992	g	3564279	s	?	O55/O157	+/-																							
-	3335052	-	3497320	c	3564608	ins	?	EDL933	+/-																							
-	3335126	-	3497394	t	3564684	ins	?	EDL933	+/-																							
c	3336184	a	3498452	a	3565742	i	?	O55/O157	+/-																							
t	3336522	c	3498790	c	3566080	ns	?	O55/O157	+/-																							
c	3337895	-	3500162	-	3567452	indel	?	O55/O157	+/-					-										c								
12	3339057	-	3501323	-	3568613	ins	-	CB9615	+++	-	-		12	-		-																
-	3339874	c	3502130	c	3569420	del	c	CB9615	+++	c	c		c	c		c																
a	3340946	c	3503202	c	3570492	nc	c	CB9615	++++		c		c	c		c								c	c						c	c
c	3344214	t	3506470	t	3573760	s	c	O157	++++		c		c	c	c	a								c	c					a		
c	3344441	t	3506697	t	3573987	ns	t	CB9615	++++		t	t	t	t	t	t								t	t					t		
g	3345494	a	3507750	a	3575040	ns	a	CB9615	+		a	a	a	g	g	g								a	a		g	g	g	a		g
c	3345979	a	3508235	a	3575525	i	a	CB9615	++++		a	a	a	a	a	a								a	a		a	a	a			a
t	3346865	c	3509121	c	3576411	ns	c	CB9615	++++		c	c	c	c	c	c								c	c		c	c	c	c		c
t	3347295	g	3509551	g	3576841	s	g	CB9615	++++		g	g	g	g	g	g								g	g		g	g	g	g	g	g
a	3347547	c	3509803	c	3577093	s	a	O157	++++		a	a	a	a	a	a								a	a	a	a	a	a	a	a	a
a	3347548	c	3509804	c	3577094	ns	a	O157	++++		a	a	a	a	a	a								a	a	a	a	a	a	a	a	a
c	3347563	t	3509819	t	3577109	ns	c	O157	++++		c	c	c	c	c	c								c	c	c	c	c	c	c	c	c
g	3347920	a	3510176	a	3577466	ns	g	O157	++++		g	g	g	g	g	g								g	g	g	g	g	g	g	g	g
a	3348697	g	3510953	g	3578243	i	g	CB9615	++++		g	g	g	g	g	g								g	g	g	g	g	g	g	g	g
g	3348791	t	3511047	t	3578337	i	g	O157	++++		g	g	g	g	g	g	a	g	g	g	g	g	g	g	g	g	g	g	g	g	g	g
g	3348959	a	3511215	a	3578505	ns	g	O157	++++		g	g	g	g	g	g	g	g	g	g	g	g	g	g	g	g	g	g	g	g	g	g
c	3350871	g	3513127	g	3580417	ns	g	CB9615	++++		g	g	g	g	g	g	g	g	g	g	g	g	g	g	g	g	g	g	g	g	g	g
c	3356758	t	3519014	t	3586304	i	c	O157	++++	c	c	c	c	c	c	c	c	c	c	c	c	c	c	c	c	c	c	c	c	c	c	c
c	3356985	t	3519241	t	3586531	i	c	O157	++++	c	c	c	c	c	c	c	c	c	c	c	c	c	c	c	c	c	c	c	c	c	c	c
c	3357112	t	3519368	t	3586658	i	t	CB9615	++++	t	t	t	t	t	t	t	t	t	t	t	t	t	t	t	t	t	t	t	t	t	t	t
g	3357624	t	3519880	t	3587170	ns	g	O157	++++	g	g	g	g	g	g	g	g	g	g	g	g	g	g	g	g	g	g	g	g	g	g	g
a	3358865	c	3521121	c	3588411	i	c	CB9615	++++	t	t	t	t	t	c	c	c	c	c	c	c	c	c	t	t	t	t	t	t	t	c	c
c	3358907	t	3521163	t	3588453	i	t	CB9615	++++	t	t	t	t	t	c	t	t	t	t	t	t	t	t	t	t	t	t	t	t	t	t	t
t	3359030	c	3521286	c	3588576	i	c	CB9615	++++	c	c	c	c	c	c	c	c	c	c	c	c	c	c	c	c	c	c	c	c	c	c	c
t	3360359	c	3522615	c	3589905	ns	c	CB9615	++++	c	c	c	c	c	c	c	c	c	c	c	c	c	c	c	c	c	c	c	c	c	c	c
t	3360519	a	3522775	a	3590065	i	a	CB9615	++	a																						
t	3361222	c	3523478	c	3590768	ns	c	CB9615	++++	c				c	c	c	c	c	c	c	c	c	c									
c	3366030	t	3528286	t	3595576	ns	t	CB9615	++++	a	t	t	t	a	t	t	a	a	a	a	a	a	a	t	t	t	t	t	t	t	t	t
g	3366335	t	3528591	t	3595881	ns	g	O157	++++	g	g	g	g	g	g	g	g	g	g	g	g	g	g	g	g	g	g	g	g	g	g	g
g	3367197	a	3529453	a	3596743	ns	g	O157	++++	g	g	g	g	g	g	g	g	g	g	g	g	g	g	g	g	g	g	g	g	g	g	g
c	3367467	a	3529723	a	3597013	ns	c	O157	++++	c	c	c	c	c	c	c	c	c	c	c	c	c	c	c	c	c	c	c	c	c	c	c
t	3371608	c	3533864	c	3601154	s	t	O157	++++	t	t	t	t	t	t	t	t	t	t	t	t	t	t	t	t	t	t	t	t	t	t	t
a	3374376	c	3536632	c	3603922	ns	c	CB9615	++++	c	c	c	c	c	c	c	c	c	c	c	c	c	c	c	c	c	c	c	c	c	c	c
g	3377067	g	3539323	t	3606613	s	g	EDL933	++++	g	g	g	g	g	g	g	g	g	g	g	g	g	g	g	g	g	g	g	g	g	g	g
t	3377564	c	3539820	c	3607110	ns	c	CB9615	++++	c	c	c	c	c	c	c	c	c	c	c	c	c	c	c	c	c	c	c	c	c	c	c
a	3377805	g	3540061	g	3607351	s	g	CB9615	++++	g	g	g	g	g	g	g	g	g	a	g	g	g	g	g	g	g	g	g	g	g	g	g
g	3379422	a	3541678	a	3608968	i	g	O157	++++	g	g	g	g	g	g	g	g	g	g	g	g	g	g	g	g	g	g	g	g	g	g	g
a	3379643	g	3541899	g	3609189	i	g	CB9615	++++		g		g	a		g			g	g	g	g	g	g		g				g		g
-	3380251	-	3542507	t	3609798	ins	-	EDL933	++++	-	-	-	-	-	-	-	-	-	-	-	-	-	-	-	-	-	-	-	-	-	-	-
-	3381120	-	3543376	g	3610668	ins	-	EDL933	++++	-	-	-	-	-	-	-	-	-	-	-	-	-	-	-	-	-	-	-	-	-	-	-
a	3381806	g	3544062	g	3611354	s	g	CB9615	++++	g	g	g	g	g	g	g	g	g	g	g	g	g	g	g	g	g	g	g	g	g	g	g
g	3382000	a	3544256	a	3611548	s	g	O157	++++	g	g	g	g	g	g	g	g	g	g	g	g	g	g	g	g	g	g	g	g	g	g	g
c	3385023	c	3547279	t	3614571	ns	c	EDL933	++++	c	c	c	c	c	c	c	c	c	c	c	c	c	c	c	c	c	c	c	c	c	c	c
t	3385787	c	3548043	c	3615335	s	c	CB9615	++++	c	c	c	c	c	c	c	c	c	c	c	c	c	c	c	c	c	c	c	c	c	c	c
5	3387405	-	3549660	5	3616953	del	5	Sakai	++++	5	5	5	5	5	5	5		5	5	5	5	5	5	5	5	5	5	5	5	5	5	5
t	3387429	c	3549680	c	3616977	i	c	CB9615	++++	c	c	c	c	c	c	c		c	c	c	c	c	c	c	c	c	c	c	c	c	c	c
g	3387453	a	3549704	a	3617001	i	a	CB9615	++++	a	a	a	a	a	a	a		a	a	a	a	a	a	a	a	a	a	a	a	a	a	a
t	3387874	-	3550124	-	3617421	del	t	O157	++++	t	t	t	t	t	t	t		t	t	t	t	t	t	t	t	t	t	t	t	t	t	t
-	3388153	c	3550404	c	3617701	ins	-	O157	++++	-	-	-	-	-	-	-		-	-	-	-	-	-	-	-	-	-	-	-	-	-	-
-	3390833	-	3553085	a	3620383	ins	-	EDL933	++++	-	-	-	-	-	-	-		-	-	-	-	-	-	-	-	-	-	-	-	-	-	-
c	3395693	t	3557741	t	3625039	s	c	O157	++++	c	c	c	c	c	c	c	c	c	c	c	c	c	c	c	c	c	c	c	c	c	c	c
c	3399544	t	3561592	t	3628890	ns	t	CB9615	++++	t	t	t	t	t	t	t	t	t	t	t	t	t	t	t	t	t	t	t	t	t	t	t
c	3401015	t	3563063	t	3630361	i	t	CB9615	++++	t	t	t	t	t	t	t		t			t	t	t	t	t	t	t			t	t	t
g	3401096	a	3563144	a	3630442	i	a	CB9615	++	a	g	g	g	g	g	g		g			g	g	g	g	g	g	g			g	g	g
a	3402118	g	3564166	g	3631464	s	g	CB9615	++++		g	g	g	g	g	g		g			g	g	g	g	g	g	g			g	g	g
t	3402950	c	3564998	c	3632296	ns	t	O157	++++	t	t	t	t	t	t	t		t			t	t	t	t	t	t	t			t	t	t
g	3403053	a	3565101	a	3632399	s	g	O157	++++	g	g	g	g	g	g	g		g			g	g	g	g	g	g	g			g	g	g
a	3403539	g	3565587	g	3632885	s	g	CB9615	++++	g	g	g	g	g	g	g		g			g	g	g	g	g	g	g			g	g	g
a	3403798	g	3565846	g	3633144	ns	g	CB9615	++++	g	g	g	g	g	g	g		g			g	g	g	g	g	g	g			g	g	g
g	3408526	a	3570574	a	3637872	s	g	O157	++++	g	g	g	g	g	g	g	g	g	g	g	g	g	g	g	g	g	g	g	g	g	g	g
a	3408904	g	3570952	g	3638250	s	g	CB9615	++++	g	g	g	g	g	g	g	g	g	g	g	g	g	g	g	g	g	g	g	g	g	g	g
c	3409131	c	3571179	t	3638477	ns	c	EDL933	++++	c	c	c	c	c	c	c	c	c	c	c	c	c	c	c	c	c	c	c	c	c	c	c
t	3409557	c	3571605	c	3638903	ns	c	CB9615	++++	c	c	c	c	c	c	c	c	c	c	c	c	c	c	c	c	c	c	c	c	c	c	c
g	3411487	a	3573535	a	3640833	i	a	CB9615	++++	a	a	a	a	a	a	a	a	a	a	a	a	a	a	a	a	a	a	a	a	a		a
c	3412940	t	3574988	t	3642286	ns	c	O157	++++	c	c	c	c	c	c	c	c	c	c	c	c	c	c	c	c	c	c	c	c	c	c	c
a	3414040	c	3576088	c	3643386	ns	a	O157	++++	a	a	a	a	a	a	a	a	a	a	a	a	a	a	a	a	a	a	a	a	a	a	a
g	3415352	a	3577400	a	3644698	ns	g	O157	++++	g	g	g	g	g	g	g	g	g	g	g	g	g	g	g	g	g	g	g	g	g	g	g
g	3415394	a	3577442	a	3644740	ns	g	O157	++++	g	g	g	g	g	g	g	g	g	g	g	g	g	g	g	g	g	g	g	g	g	g	g
c	3422941	g	3584989	g	3652287	ns	g	CB9615	++++	g				g	g	g	g							g	g	g	g	g	g			
g	3424028	a	3586076	a	3653374	s	g	O157	++++	g				g	g	g	g							g	g	g	g	g	g			
g	3424510	a	3586558	a	3653856	ns	g	O157	++++	g				g	g	g	g							g	g	g	g	g	g			

O55 and O157 genome site details										Outgroup Strain Details[g]																							
CB9615 base[a]	CB9615 Site[b]	Sakai base[a]	Sakai Site[b]	EDL933 base[a]	EDL933 Site[b]	type[c]	Inferred ancestral base[d]	Lineage inferred to mutate[e]	Support level[f]	D1 Sd197	K-12	HS	ATCC 8739	UMN026	IAI39	SMS 3-5	E2348/69	536	ED1a	CFT073	S88	APEC O1	UTI89	E24377A	IAI1	SE11	F5 8401	F2a 2457T	F2a 301	SS Ss046	B4 Sb227	B18 BS512
g	3425484	t	3587532	t	3654830	s	g	O157	++++	g	g	g	g	g	g	g	g	g	g	g	g	g	g	g	g	g	g	g	g	g	g	g
c	3425966	c	3588014	g	3655312	ns	c	EDL933	++++	c	c	c	c	c	c	c	c	c	c	c	c	c	c	c	c	c	c	c	c	c	c	c
c	3425970	c	3588018	g	3655316	ns	c	EDL933	++++	c	c	c	c	c	c	c	c	c	c	c	c	c	c	c	c	c	c	c	c	c	c	c
g	3426332	a	3588380	a	3655678	s	g	O157	++++	g	g	g	g	g	g	g	g	g	g	g	g	g	g	g	g	g	g	g	g	g	g	g
c	3429411	t	3591459	t	3658757	s	t	CB9615	++++	t	t	t	t	t	t	t	t	t	t	t	t	t	t	t	t	t	t	t	t	t	t	t
t	3430407	c	3592455	c	3659753	ns	t	O157	++++	t	t	t	t	t	t	t	t	t	t	t	t	t	t	t	t	t	t	t	t	t	t	t
g	3433753	a	3595801	a	3663099	s	g	O157	++++	g	g	g	g	g	g	g	g	g	g	g	g	g	g	g	g	g	g	g	g	g	g	g
t	3434534	g	3596582	g	3663880	ns	t	O157	++++	t	t	t	t	t	t	t	t	t	t	t	t	t	t	t	t	t	t	t	t	t	t	t
c	3436889	t	3598937	t	3666235	ns	c	O157	++++	c	c	c	c	c		c								c	c	c				c	c	c
a	3437303	g	3599351	g	3666649	ns	g	CB9615	++++	g	g	g	g	g		g								g	g	g				g	g	g
t	3438424	c	3600472	c	3667770	s	c	CB9615	++++					c		c								c	c	c				c	c	c
c	3439664	t	3601712	t	3669010	s	c	O157	+++					c		c								c	c	c				c	c	
g	3440336	a	3602384	a	3669682	ns	g	O157	+++					g		g								g	g	g				g	g	
a	3440341	g	3602389	g	3669687	s	g	CB9615	+					a		a								g	g	g				g	g	
c	3441974	g	3604022	g	3671320	ns	c	O157	+++					c		c								c	c	c				c	c	
a	3443601	t	3605649	t	3672947	ns	t	CB9615	+++					t		t								t	t	t				t	t	
g	3445178	a	3607226	a	3674524	i	g	O157	++++					g	g	g		g	g	g	g	g	g	g	g	g	g	g	g	g		g
a	3446825	g	3608873	g	3676171	ns	g	CB9615	++++	g	g	g	g	g	g	g	g	g	g	g	g	g	g	g	g	g	g	g	g	g	g	g
c	3447402	t	3609450	t	3676748	s	c	O157	++++	c	c	c	c	c	c	c	c	c	c	c	c	c	c	c	c	c	c	c	c	c	c	c
g	3452155	a	3614203	a	3681501	ns	a	CB9615	++++	a	a	a	a	a	a	a	a	a	a	a	a	a	a	a	a	a	a	a	a	a	a	a
a	3454922	-	3616969	-	3684267	del	a	O157	++++	a	a	a	a	a	a	a	a	a	a	a	a	a	a	a	a	a	a	a	a	a		a
a	3455322	g	3617369	g	3684667	ns	g	CB9615	++++	g	g	g	g	g	g	g	g	g	g	g	g	g	g	g	g	g	g	g	g	g		g
a	3455764	g	3617811	g	3685109	s	g	CB9615	++	g	g	g	g	a	g	a	g	g	g	g	g	g	g	g	g	g	g	g	g	g		g
c	3456041	t	3618088	t	3685386	ns	t	CB9615	++++	t	t	t	t	t	t	t	t	t	t	t	t	t	t	t	t	t	t	t	t	t		
g	3457182	a	3619229	a	3686527	ns	a	CB9615	++++	a	a	a	a	a	a	a	a	a	a	a	a	a	a	a	a	a				a		
a	3457645	c	3619692	c	3686990	ns	c	CB9615	++++	c	c	c	c	c	c	c	c	c	c	c	c	c	c	c	c	c				c		
a	3459792	g	3621839	g	3689137	ns	g	CB9615	++++	g	g	g	g	g	g	g	g	g	g	g	g	g	g	g	g	g				g		
t	3459803	a	3621850	a	3689148	i	t	O157	++++	t	t	t	t	t	t	t	t	t	t	t	t	t	t	t	t	t				t		
g	3460653	a	3622700	a	3689998	ns	g	O157	++++	g	g	g	g	g	g	g	g	g	g	g	g	g	g	g	g	g				g		
c	3461846	t	3623893	t	3691191	i	t	CB9615	+		t		t	c										t	c	t				c	t	
ga	3461852	-	3623898	-	3691196	del	ga	O157	++++		ga		ga	ga										ga	ga	ga				ga	ga	
a	3461916	g	3623961	g	3691259	i	g	CB9615	++++		g		g	g										g	g	g				g	g	
c	3464370	a	3626293	a	3693591	s	c	O157	++++	c				c		c		c	c	c	c	c	c		t							
g	3464730	a	3626653	a	3693951	s	a	CB9615	++++					a		a		a	a	a	a	a	a		t							
t	3464765	g	3626688	g	3693986	ns	t	O157	++++	t				t		t		t	t	t	t	t	t		t							
a	3468885	g	3630808	g	3698106	ns	g	CB9615	++++	g	g	g	g	g	g	g	g	g	g	g	g	g	g	g	g	g	g	g	g	g	g	g
c	3475212	t	3637135	c	3704433	ns	c	Sakai	++++	c	c	c	c	c	c	c	c	c	c	c	c	c	c	c	c	c	c	c	c	c	c	c
t	3480539	c	3642462	c	3709760	ns	c	CB9615	++++	c	c	c	c	c	c	c	c	c	c	c	c	c	c	c	c	c	c	c	c	c	c	c
g	3481004	a	3642927	a	3710225	ns	g	O157	++++	g	g	g	g	g	g	g	g	g	g	g	g	g	g	g	g	g	g	g	g	g	g	g
g	3481235	a	3643158	a	3710456	ns	g	O157	++++	g	g	g	g	g	g	g	g	g	g	g	g	g	g	g	g	g	g	g	g	g	g	g
t	3481664	c	3643587	c	3710885	ns	c	CB9615	++++	c	c	c	c	c	c	c	c	c	c	c	c	c	c	c	c	c	c	c	c	c	c	c
a	3483489	c	3645412	c	3712710	ns	a	O157	++++	a	a	a	a	a	a	a	a	a	a	a	a	a	a	a	a	a	a	a	a	a	a	a
g	3485893	t	3647816	t	3715114	ns	t	CB9615	++++	t	t	t	t	t	t	t	t	t	t	t	t	t	t	t	t	t	t	t	t	t	t	t
a	3487207	g	3649130	g	3716428	s	g	CB9615	++++	g	g	g	g	g	g	g	g	g	g	g	g	g	g	g	g	g	g	g	g	a	g	g
g	3494649	a	3656572	a	3723870	i	g	O157	++++	g	g	g	g	g	g	g	g	g	g	g	g	g	g	g	g	g	g	g	g	g		
t	3497959	g	3659882	g	3727180	s	t	O157	++++	t	t	t	t	t	t	t	t	t	t	t	t	t	t	t	t	t	t	t	t	t	t	t
a	3499314	t	3661237	t	3728535	ns	a	O157	++++	a	a	a	a	a	a	a	a	a	a	a	a	a	a	a	a	a	a	a	a	a	a	a
-	3501682	t	3663606	t	3730904	del	t	CB9615	++++	t	t	t	t	t	t	t	t	t	t	t	t	t	t	t	t	t	t	t	t	t	t	t
g	3506027	a	3667951	a	3735249	ns	g	O157	++++	g	g	g	g	g	g	g	g	g	g	g	g	g	g	g	g	g	g	g	g	g	g	g
g	3507805	t	3669729	t	3737027	s	t	CB9615	++++	t	t	t	t	t	t	t	t	t	t	t	t	t	t	t	t	t	t	t	t	t	t	t
t	3512791	c	3674715	c	3742013	s	t	O157	++++	t	t	t	t	c	t	t	t	t	t	t	t	t	t	t	t	t	t	t	t	t	t	t
a	3513307	g	3675231	g	3742529	ns	a	O157	++++	a	a	a	a	a	a	a	a	a	a	a	a	a	a	a	a	a	a	a	a	a	a	a
g	3514499	t	3676423	t	3743721	ns	g	O157	++++	g	g	g	g	g	g	g	g	g	g	g	g	g	g	g	g	g	g	g	g	g	g	g
g	3514956	a	3676880	a	3744178	s	g	O157	++++	g	g	g	g	g	g	g	g	g	g	g	g	g	g	g	g	g	g	g	g	g	g	g
g	3515650	c	3677574	c	3744872	ns	g	O157	++++	g	g	g	g	g	g	g	g	g	g	g	g	g	g	g	g	g	g	g	g	g	g	g
g	3516740	a	3678664	a	3745962	ns	g	O157	++++	g	g	g	g	g	g	g	g	g	g	g	g	g	g	g	g	g	g	g	g	g	g	g
t	3517207	g	3679131	g	3746429	s	g	CB9615	++++	g	g	g	g	g	g	g	g	g	g	g	g	g	g	g	g	g	g	g	g	g	g	g
t	3517947	c	3679871	c	3747169	ns	c	CB9615	++++	c	c	c	c	c	c	c	c	c	c	c	c	c	c	c	c	c	c	c	c	c	c	c
c	3518836	t	3680760	t	3748058	s	c	O157	++++	c	c	c	c	c	c	c	c	c	c	c	c	c	c	c	c	c	c	c	c	c	c	c
g	3519661	t	3681585	t	3748883	ns	g	O157	++++	g	g	g	g	g	g	g	g	g	g	g	g	g	g	g	g	g	g	g	g	g	g	g
c	3522090	t	3684014	t	3751312	ns	c	O157	++++	c	c	c	c	c	c	c	c	c	c	c	c	c	c	c	c	c	c	c	c	c	c	c
g	3522932	a	3684856	a	3752154	s	g	O157	++++	g	g	g	g	g	g	g	g	g	g	g	g	g	g	g	g	g	g	g	g	g	g	g
c	3528371	t	3690295	t	3757593	s	c	O157	++++	c	c	c	c	c	c	c	c	c	c	c	c	c	c	c	c	c	c	c	c	c	c	c
a	3532932	c	3694856	c	3762154	ns	a	O157	++++	a	a	a	a	a	a	a	a	a	a	c	a	a	a	a	a	a	a	a	a	a	a	a
a	3535080	g	3697004	g	3764302	ns	g	CB9615	++++	g	g	g	g	g	g	g	g	g	g	g	g	g	g	g	g	g	g	g	g	g	g	g
c	3535667	g	3697591	g	3764889	ns	g	CB9615	++++	g	g	g	g	g	g	g	g	g	g	g	g	g	g	g	g	g	g	g	g	g	g	g
-	3536862	-	3698786	g	3766085	ins	-	EDL933	++++	-	-	-	-	-	-	-	-	-	-	-	-	-	-	-	-	-	-	-	-	-	-	-
t	3538305	g	3700229	g	3767528	ns	t	O157	++++	t	t	t	t	t	t	t	t	t	t	t	t	t	t	t	t	t	t	t	t	t	t	t
c	3538874	t	3700798	t	3768097	ns	t	CB9615	++++	t	t	t	t	t	t	t	t	t	t	t	t	t	t	t	t	t	t	t	t	t	t	t
g	3538998	a	3700922	a	3768221	ns	g	O157	++++	g	g	g	g	g	g	g	g	g	g	g	g	g	g	g	g	g	g	g	g	g	g	g
a	3539965	g	3701889	g	3769188	s	g	CB9615	++++	g	g	g	g	g	g	g	g	g	g	g	g	g	g	g	g	g	g	g	g	g	g	g
a	3544841	g	3706765	g	3774064	i	a	O157	++++		a	a	a	a	a	a	a	a	a	a	a	a	a	a	a	a	a	a	a	a	a	a
t	3547870	c	3709794	c	3777093	i	c	CB9615	++++		c	c	c	c	c	c								c	c	c	c	c	c	c		
c	3548133	a	3710057	a	3777356	ns	c	O157	++++		c	c	c	c	c	c								c	c	c				c		
c	3549263	a	3711187	a	3778486	s	c	O157	++++		c	c	c	c	c									c	c	c				c		
-	3549800	a	3711725	a	3779024	ins	-	O157	++++		-	-	-	-	-									-	-	-				-		
g	3551018	a	3712943	a	3780242	ns	g	O157	+++			g	g	g	g															g		
-	3552516	-	3714441	g	3781741	ins	-	EDL933	-		g	g	g	g	g									g	g	g				g		
-	3553078	c	3717041	t	3784341	ns	c	EDL933	++++		c	c	c	c	c									c	c	c						

O55 and O157 genome site details										Outgroup Strain Details[g]																						
CB9615 base[a]	CB9615 Site[b]	Sakai base[a]	Sakai Site[b]	EDL933 base[a]	EDL933 Site[b]	type[c]	Inferred ancestral base[d]	Lineage inferred to mutate[e]	Support level[f]	D1 Sd197	K-12	HS	ATCC 8739	UMN026	IAI39	SMS 3-5	E2348/69	536	ED1a	CFT073	S88	APEC O1	UTI89	E24377A	IAI1	SE11	F5 8401	F2a 2457T	F2a 301	SS Ss046	B4 Sb227	B18 BS512
-	3553078	-	3720401	g	3787702	ins	-	EDL933	+++			-	-	-										-	-	-						
-	3553078	-	3720458	g	3787760	ins	-	EDL933	+++			-	-	-										-	-	-						
-	3553078	-	3720476	c	3787779	ins	-	EDL933	+++			-	-	-										-	-	-						
-	3553078	-	3720511	c	3787815	ins	-	EDL933	+++			-	-	-										-	-	-						
-	3553078	-	3720566	c	3787871	ins	-	EDL933	+++			-	-	-										-	-	-						
-	3553078	t	3721543	Y	3788848	i	t	EDL933	+++			t	t	t										t	t	t						
a	3553089	t	3729082	t	3796387	ns	a	O157	+					a																		
t	3553579	c	3729572	c	3796877	ns	c	CB9615	+					c																		
c	3553801	t	3729794	t	3797099	ns	t	CB9615	+					t																		
-	3555318	7	3731312	7	3798617	del	7	CB9615	+					7																		
c	3555421	t	3731421	t	3798726	ns	t	CB9615	+					t																		
t	3555639	g	3731639	g	3798944	ns	g	CB9615	+					g																		
c	3556315	t	3732315	t	3799620	ns	c	O157	+					c																		
a	3556461	g	3732461	g	3799766	s	g	CB9615	+					g																		
t	3558046	g	3734046	g	3801351	ns	g	CB9615	+					g																		
c	3558395	a	3734395	a	3801700	ns	c	O157	+					c																		
-	3559935	t	3735936	t	3803241	del	t	CB9615	++++			t	t	t	t									t	t	t				t		
5	3560522	-	3736522	-	3803827	del	5	O157	++++		5	5	5	5	5									5	5	5				5		
-	3561610	-	3737606	M	3804912	ins	-	EDL933	++++		-	-	-	-	-	-	-	-	-	-	-	-	-	-	-	-	-	-	-	-	-	-
t	3562563	c	3738559	c	3805865	i	t	O157	++++		t	t	t	t	t	t	t	t	t	t	t	t	t	t	t	t	t	t	t	t	t	
c	3563046	g	3739042	g	3806348	ns	c	O157	++++		c	c	c	c	c	c	c	c	c	c	c	c	c	c	c	c	c	c	c	c	c	
c	3563370	a	3739366	a	3806672	ns	c	O157	++++		c	c	c	c	c	c	c	c	c	c	c	c	c	c	c	c	c	c	c	c	c	
t	3563791	c	3739787	c	3807093	ns	c	CB9615	++++		c	c	c	c	c	c	c	c	c	c	c	c	c	c	c	c	c	c	c	c	c	
g	3563832	t	3739828	t	3807134	ns	g	O157	++++		g	g	g	g	g	g	g	g	g	g	g	g	g	g	g	g	g	g	g	g	g	
t	3565245	c	3741241	c	3808547	s	c	CB9615	++++		c	c	c	c	c	c	a	a	a	a	a	a	a	c	c	c	c	c	c	c	c	
c	3565891	t	3741887	t	3809193	ns	c	O157	++++		c	c	c	c	c	c	c	c	c	c	c	c	c	c	c	c	c	c	c	c	c	
a	3565931	g	3741927	g	3809233	s	a	O157	+		a	a	a	a	a	a	g	g	g	g	g	g	g	a	a	a	a	a	a	a	a	
g	3567932	a	3743928	a	3811234	ns	g	O157	++++		g	g	g	g	g	g	g	g	g	g	g	g	g	g	g	g				g	g	
c	3568003	t	3743999	t	3811305	ns	c	O157	++++		c	c	c	c	c	c	c	c	c	c	c	c	c	c	c	c				c	c	
c	3568294	t	3744290	t	3811596	i	c	O157	++++		c	c	c	c	c	c	c	c	c	c	c	c	c	c	c	c				c	c	
a	3568681	g	3744677	g	3811983	ns	g	CB9615	++++		g	g	g	g	g	g	g	g	g	g	g	g	g	g	g	g				g	g	
g	3568740	a	3744736	g	3812042	s	g	Sakai	++++		g	g	g	g	g	g	g	g	g	g	g	g	g	g	g	g				g	g	
a	3576801	g	3752797	g	3820103	ns	g	CB9615	++++		g	g	g	g	g	g	g	g	g	g	g	g	g	g	g	g				g	g	
g	3576837	a	3752833	g	3820139	ns	g	Sakai	++++		g	g	g	g	g	g	g	g	g	g	g	g	g	g	g	g				g	g	
-	3578129	-	3754125	g	3821432	ins	-	EDL933	++++		-	-	-	-	-	-	-	-	-	-	-	-	-	-	-	-				-	-	
-	3578178	-	3754174	g	3821482	ins	-	EDL933	++++		-	-	-	-	-	-	-	-	-	-	-	-	-	-	-	-				-	-	
g	3579827	a	3755823	a	3823131	ns	a	CB9615	++++		a	a	a	a	a	a	a	a	a	a	a	a	a	a	a	a				a		
t	3581553	a	3757549	a	3824857	ns	t	O157	++++		t	t	t	t	t	t	t	t	t	t	t	t	t	t	t	t				t		
a	3587846	g	3763842	g	3831150	ns	g	CB9615	++++		g	g	g	g	g	g	g	g	g	g	g	g	g	g	g	g						
c	3591082	a	3767078	a	3834386	ns	c	O157	++++		c	c	c	c	c	c	c	c	c	c	c	c	c	c	c	c				c		
g	3591256	a	3767252	a	3834560	s	a	CB9615	++++		a	a	a	a	a	a	a	a	a	a	a	a	a	a	a	a				a		
g	3593371	a	3769367	a	3836675	i	g	O157	++++		g	g	g	g	g	g	g	g	g	g	g	g	g	g	g	g	g	g	g	g	g	
c	3593930	a	3769926	a	3837234	ns	c	O157	++++		c	c	c	c	c	c	c	c	c	c	c	c	c	c	c	c	c	c	c	c	c	
g	3596264	a	3772260	a	3839568	ns	g	O157	++++	g	g	g	g	g	g	g	g	g	g	g	g	g	g	g	g	g	g	g	g	g	g	g
g	3598906	a	3774902	a	3842210	s	g	O157	++++	g	g	g	g	g	g	g	g	g	g	g	g	g	g	g	g	g	g	g	g	g	g	g
g	3601228	t	3777224	t	3844532	ns	g	O157	++++	g	g	g	g	g	g	g	g	g	g	g	g	g	g	g	g	g	g	g	g	g	g	g
g	3601486	a	3777482	a	3844790	s	g	O157	++++	g	g	g	g	g	g	g	g	g	g	g	g	g	g	g	g	g	g	g	g	g	g	g
t	3603794	c	3779790	c	3847098	ns	c	CB9615	++++	c	c	c	c	c	c	c	c	c	c	c	c	c	c	c	c	c	c	c	c	c	c	c
a	3604302	g	3780298	g	3847606	s	g	CB9615	++	g	a	g	g	g	g	g	g	g	g	g	g	g	g	g	g	g	g	g	g	g	g	g
c	3606795	a	3782791	a	3850099	ns	a	CB9615	++++	a	a	a	a	a	a	a	a	a	a	a	a	a	a	a	a	a	a	a	a	a	a	a
g	3607460	a	3783456	a	3850764	s	g	O157	++++	g	g	g	g	g	g	g	g	g	g	g	g	g	g	g	g	g	g	g	g	g	g	g
a	3607726	g	3783722	g	3851030	ns	g	CB9615	++++	g	g	g	g	g	g	g	g	g	g	g	g	g	g	g	g	g	g	g	g	g	g	g
a	3610421	g	3786417	g	3853725	s	a	O157	++++	a	a	a	a	a	a	a	a	a	a	a	a	a	a	a	a	a	a	a	a	a	a	a
g	3611159	c	3787155	c	3854463	ns	g	O157	++++	g	g	g	g	g	g	g	g	g	g	g	g	g	g	g	g	g	g	g	g	g	g	g
g	3611684	t	3787680	t	3854988	ns	g	O157	++++	g	g	g	g	g	g	g	g	g	g	g	g	g	g	g	g	g	g	g	g	g	g	g
c	3612982	a	3788978	a	3856286	s	c	O157	++++	c	c	c	c	c	c	c	c	c	c	c	c	c	c	c	c	c	c	c	c	c	c	c
g	3616376	a	3792372	a	3859680	s	g	O157	++++	g	g	g	g	g	g	g	g	g	g	g	g	g	g	g	g	g	g	g	g	g	g	g
t	3618675	c	3794671	c	3861979	i	c	CB9615	++++	c	c	c	c	c	c	c	c	c	c	c	c	c	c	c	c	c	c	c	c	c	c	c
g	3620115	t	3796111	t	3863419	i	g	O157	++++	g	g	g	g	g	g	g	g	g	g	g	g	g	g	g	g	g	g	g	g	g	g	g
a	3620119	g	3796115	g	3863423	i	g	CB9615	++++	g	g	g	g	g	g	g	g	g	g	g	g	g	g	g	g	g	g	g	g	a	g	g
c	3621075	t	3797071	t	3864379	i	c	O157	++++		c	c	c		c		c	c	c	c	c	c	c	c	c	c	c	c	c	c	c	c
g	3622711	a	3798707	a	3866015	ns	g	O157	++++	g	g	g	g	g	g	g	g	g	g	g	g	g	g	g	g	g	g	g	g	g	g	g
a	3624110	t	3800106	t	3867414	ns	a	O157	++++	a	a	a	a	a	a	a								a	a	a	a	a	a	a	a	a
g	3624522	a	3800518	a	3867826	ns	g	O157	++++	g	g	g	g	g	g	g								g	g	g	g	g	g	g	g	g
g	3624641	a	3800637	g	3867945	ns	g	Sakai	++++	g	g	g	g	g	g	g								g	g	g	g	g	g	g	g	g
g	3627531	a	3803527	a	3870835	ns	g	O157	++++		g	g	g	g	g	g								g	g	g	g	g	g	g	g	g
c	3629602	t	3805598	t	3872906	i	c	O157	++++	c	c	c	c	c	c	c	c	c	c	c	c	c	c	t	c	c	c	c	c	c	c	c
c	3629908	t	3805904	t	3873212	s	c	O157	++++	c	c	c	c	c	c	c	c	c	c	c	c	c	c	c	c	c	c	c	c	c	c	c
t	3633575	c	3809571	c	3876879	s	t	O157	++++	t	t	t	t	t	t	t	t	t	t	t	t	t	t	t	t	t	t	t	t	t	t	t
t	3637022	g	3813018	g	3880326	s	g	CB9615	++++	g				g	g	g	g	g	g	g	g	g	g	g	g	g	g	g	g	g	g	g
a	3637038	g	3813034	g	3880342	ns	g	CB9615	++++	g				g	g	g	g	g	g	g	g	g	g	g	g	g	g	g	g	g	g	g
g	3638234	a	3814230	a	3881538	ns	a	CB9615	++++	a				a	a	a	a	a	a	a	a	a	a	a	a	a	a	a	a	a	a	a
t	3639104	c	3815100	c	3882408	s	c	CB9615	++	c	c	c	c	c	c	c	c	t	t	c	c	c	c	c	c	c	c	c	c	c	c	c
t	3639118	a	3815114	a	3882422	ns	a	CB9615	++++	a	a	a	a	a	a	a	a	a	a	a	a	a	a	a	a	a	a	a	a	a	a	a
t	3639798	a	3815794	a	3883102	ns	t	O157	++++	t	t	t	t	t	t	t	t	t	t	t	t	t	t	t	t	t	t	t	t		t	t
t	3640947	a	3816943	a	3884251	s	a	CB9615	++++	a	a	a	a	a	a	a	g	g	g	g	g	g	g	a	a	a	a	a	a		a	a
a	3640959	g	3816955	g	3884263	s	g	CB9615	++++	g	g	g	g	g	g	g	g	g	g	g	g	g	g	g	g	g	g	g	g		g	g
c	3641064	g	3817060	g	3884368	s	c	O157	++++	c	c	c	c	c	c	c	c	c	c	c	c	c	c	c	c	c	c	c	c		c	c
t	3644162	c	3820158	c	3887466	i	t	O157	++++	t	t	t	t											t	t	t				t	t	t

O55 and O157 genome site details										Outgroup Strain Details[g]																						
CB9615 base[a]	CB9615 Site[b]	Sakai base[a]	Sakai Site[b]	EDL933 base[a]	EDL933 Site[b]	type[c]	Inferred ancestral base[d]	Lineage inferred to mutate[e]	Support level[f]	D1 Sd197	K-12	HS	ATCC 8739	UMN026	IAI39	SMS 3-5	E2348/69	536	ED1a	CFT073	S88	APEC O1	UTI89	E24377A	IAI1	SE11	F5 8401	F2a 2457T	F2a 301	SS Ss046	B4 Sb227	B18 BS512
g	3644937	a	3820933	a	3888241	s	g	O157	+	t	c	c	c	c	c	c	c	c	c	c	c	c	c	c	c	c	c	c	c	g	c	c
t	3648353	c	3824349	c	3891657	s	t	O157	++++	t	t	t	t	t	t	t	t	t	t	t	t	t	t	t	t	t	t	t	t	t	t	t
c	3650631	t	3826627	t	3893935	s	c	O157	++++	c	c	c	c	c	c	c	c	c	c	c	c	c	c	c	c	c	c	c	c	c	c	c
a	3651252	g	3827248	g	3894556	s	g	CB9615	++++	g	g	g	g	g	g	g	g	g	g	g	g	g	g	g	g	g	g	g	g	g	g	g
g	3653685	a	3829681	a	3896989	i	g	O157	++	g	g	g	g	g	g	g	g	g	g	g	a	a	a	g	g	g	g	g	g	g	g	g
g	3653812	a	3829808	a	3897116	ns	g	O157	++++	g	g	g	g	g	g	g	g	g	g	g	g	g	g	g	g	g	g	g	g	g	g	g
t	3654239	a	3830235	a	3897543	s	t	O157	++++	t	t	t	t	t	t	t	t	t	t	t	t	t	t	t	t	t	t	t	t	t	t	t
c	3654811	t	3830807	t	3898115	ns	t	CB9615	++++	t	t	t	t	t	t	t	t	t	t	t	t	t	t	t	t	t	t	t	t	t	t	t
g	3655485	a	3831481	a	3898789	s	g	O157	++	g	a	g	g	g	g	g	g	g	g	g	g	g	g	a	a	g	a	a	a	a	g	g
c	3655645	a	3831641	a	3898949	ns	c	O157	++++	c	c	c	c	c	c	c	c	c	c	c	c	c	c	c	c	c	c	c	c	c	c	c
a	3656287	c	3832283	c	3899591	s	c	CB9615	++++	c	c	c	c	c	c	c	t	c	c	c	c	c	c	c	c	c	c	c	c	c	c	c
-	3659482	-	3835478	t	3902787	ins	-	EDL933	++++	-	-	-	-		-	-								-	-	-	-	-	-	-	-	-
-	3659551	-	3835547	g	3902857	ins	-	EDL933	++++	-	-	-	-		-	-								-	-	-	-	-	-	-	-	-
-	3659555	-	3835551	c	3902862	ins	-	EDL933	++++	-	-	-	-		-	-								-	-	-	-	-	-	-	-	-
-	3659556	-	3835552	g	3902864	ins	-	EDL933	++++	-	-	-	-		-	-								-	-	-	-	-	-	-	-	-
c	3664706	t	3840793	t	3908105	ns	c	O157	++++	c	c	c	c	c	c	c	c	c	c	c	c	c	c	c	c	c	c	c	c	c	c	c
a	3670729	g	3846816	g	3914128	ns	g	CB9615	++++	g	g	g	g	g	g	g	g	g	g	g	g	g	g	g	g	g	g	g	g	g	g	g
g	3672159	a	3848246	a	3915558	s	g	O157	++++	g	g	g	g	g	g	g	g	g	g	g	g	g	g	g	g	g	g	g	g	g	g	g
c	3672348	t	3848435	t	3915747	s	c	O157	++++	c	c	c	c	c	c	c	c	c	c	c	c	c	c	c	c	c	c	c	c	c	c	c
a	3672686	c	3848773	c	3916085	s	c	CB9615	++++	c	c	c	c	c	c	c	c	c	c	c	c	c	c	c	c	c	c	c	c	c	c	c
c	3673035	t	3849122	t	3916434	ns	t	CB9615	++++	t	t	t	t	t	t	t	t	t	t	t	t	t	t	t	t	t	t	t	t	t	t	t
c	3677853	g	3853940	a	3921252	ns	g	EDL933	++++	g				g			g	g	g	g				g				g	g	g	g	g
t	3678824	c	3854911	c	3922223	s	c	CB9615	+++	c							c										c	c	c			
g	3679461	a	3855548	a	3922860	ns	g	O157	+++	g							g										g	g	g			
c	3679832	t	3855919	t	3923231	s	c	O157	++	c						t	c	c		t				t								
t	3682316	-	3858402	-	3925714	ins	-	CB9615	+								-															
-	3683243	-	3859329	c	3926642	ins	-	EDL933	+++							-	-	-		-			-									
g	3683758	a	3859844	a	3927157	nc	g	O157	+++							g	g	g					g									
c	3684322	a	3860408	a	3927721	nc	a	CB9615	+++							a	a	a		a			a									
a	3686381	c	3862467	c	3929780	ns	a	O157	+								a															
g	3687827	a	3863913	a	3931226	nc	a	CB9615	+								a															
a	3688151	c	3864237	c	3931550	nc	c	CB9615	+								c															
a	3688503	t	3864589	t	3931902	nc	a	O157	+								a															
a	3690706	t	3866792	t	3934105	i	t	CB9615	+								t															
a	3691850	c	3867936	c	3935249	i	a	O157	+								a															
a	3692381	a	3868467	c	3935780	i	a	EDL933	+								a															
t	3692415	-	3868500	-	3935813	del	t	O157	+								t															
-	3693246	c	3870643	a	3937956	ns	c	EDL933	+					c	c	a				c							c	c	c	g	c	a
-	3693246	t	3871025	g	3938338	ns	g	Sakai	++++	g				g				g	g	g	g			g						g		g
t	3703280	c	3874355	c	3941668	ns	t	O157	++++			t		t		t	t		t	t	t		t								t	t
t	3703890	c	3874965	c	3942278	i	c	CB9615	+								c															
-	3704076	c	3875218	Y	3942531	i	c	EDL933	+++					c	c	c	c									c						
a	3765449	g	3875700	g	3943013	s	g	CB9615	++++	g	g	g	g	g	g	g	g	g	g	g	g	g	g	g	g	g		g	g	g	g	g
t	3766955	a	3877206	a	3944519	ns	a	CB9615	+++	g	g	g	g	g	g	g	g	g	a	a	a	a	a	g	g	g	g	g	g	g	g	g
t	3767789	g	3878040	g	3945353	ns	g	CB9615	++++	g	g	g	g	g	g	g	g	g	g	g	g	g	g	g	g	g	g	g	g	g	g	g
g	3768550	a	3878801	a	3946114	ns	a	CB9615	++++	a	a	a	a	a	a	a	a	a	a	a	a	a	a	a	a	a	a	a	a	a	a	a
t	3770814	g	3881065	g	3948378	ns	t	O157	++++	t	t	t	t	t	t	t	t	t	t	t	t	t	t	t	t	t	t	t	t	t	t	t
a	3772270	g	3882521	g	3949834	ns	g	CB9615	++++	g	g	g	g	g	g	g	g	g	g	g	g	g	g	g	g	g	g	g	g	g	g	g
c	3773774	t	3884025	t	3951338	ns	c	O157	++++	c	c	c	c	c	c	c	c	c	c	c	c	c	c	c	c	c	c	c	c	c	c	c
g	3774806	a	3885057	g	3952370	s	g	Sakai	++++	g	g	g	g	a	g	g	g	g	g	g	g	g	g	g	g	g	g	g	g	g	g	g
c	3780740	t	3890991	t	3958304	i	c	O157	++++		c	c	c	c										c	c	c	c	c	c	c	c	c
a	3781263	g	3891514	g	3958827	s	a	O157	++++	a	a	a	a	a	a	a	a	a	a	a	a	a	a	a	a	a	a	a	a	a	a	a
g	3784617	a	3894868	a	3962181	s	g	O157	++++	g	g	g	g	g	g	g	g	g	g	g	g	g	g	g	g	g	g	g	g	g	g	g
g	3786977	t	3897228	t	3964541	ns	g	O157	++++	g	g	g	g	g	g	g	g	g	g	g	g	g	g	g	g	g	g	g	g	g	g	g
-	3787740	-	3897991	g	3965305	ins	-	EDL933	++++		-	-	-	-	-	-	-	-	-	-	-	-	-	-	-	-	-	-	-	-	-	-
4	3787984	-	3898234	-	3965548	del	4	O157	++++		4	4	4	4	4	4	4	4	4	4	4	4	4	4	4	4	4	4	4	4	4	4
t	3789268	c	3899515	c	3966829	s	c	CB9615	++	c	c	c	c	c	c	c	t	c	t	c	c	c	c	c	c	c	c	c	c	c	c	c
a	3789305	g	3899552	g	3966866	ns	g	CB9615	++++	g	g	g	g	g	g	g	g	g	g	g	g	g	g	g	g	g	g	g	g	g	g	g
t	3790417	g	3900664	g	3967978	s	g	CB9615	++++	a	g	g	g	g	g	g	g	g	g	g	g	g	g	g	g	g	g	g	g	g	g	g
a	3792348	g	3902595	g	3969909	s	g	CB9615	++++	g	g	g	g	g	g	g	g	g	g	g	g	g	g	g	g	g	g	g	g	g	g	g
a	3793046	g	3903293	g	3970607	s	a	O157	++++	a	a	a	a	a	a	a	a	a	a	a	a	a	a	a	a	a	a	a	a	a	a	a
t	3800184	-	3910430	t	3977745	del	t	Sakai	++++	t	t	t	t	t	t	t	t	t	t	t	t	t	t	t	t	t	t	t	t	t	t	t
t	3803557	c	3913803	c	3981118	ns	t	O157	++++							t	t	t	t	t	t	t	t									
t	3804373	a	3914619	a	3981934	s	t	O157	++++							t	t	t	t	t	t	t	t									
c	3805497	t	3915743	t	3983058	s	c	O157	++++							c	c	c	c	c	c	c	c									
a	3806129	c	3916375	c	3983690	s	c	CB9615	++++							c	c	c	c	c	c	c	c									
t	3807023	c	3917269	c	3984584	i	t	O157	+++							t	t	c	t	t	t	t	t									
g	3807029	a	3917275	a	3984590	i	a	CB9615	+++							a	a	g	a	a	a	a	a									
g	3809055	t	3919301	t	3986616	ns	g	O157	++++							g	g	g	g	g	g	g	g									
c	3812022	t	3922268	t	3989583	s	c	O157	++++	c	c	c	c	c	c	c	c	c	c	c	c	c	c	c	c	c	c	c	c	c	c	c
g	3814526	g	3924772	a	3992087	s	g	EDL933	++++	g	g	g	g	g	g	g	g	g	g	g	g	g	g	g	g	g	g	g	g	g	g	g
c	3814789	g	3925035	g	3992350	ns	c	O157	++++	c	c	c	c	c	c	c	c	c	c	c	c	c	c	c	c	c	c	c	c	c	c	c
g	3816556	t	3926802	t	3994117	s	g	O157	++++	g	g	g	g	g	g	g	g	t	g	g	g	g	g	g	g	g	g	g	g	g	g	g
t	3820905	g	3931151	g	3998466	ns	t	O157	++++	t	t	t	t	t	t	t	t	t	t	t	t	t	t	t	t	t	t	t	t	t	t	
c	3821181	t	3931427	t	3998742	s	c	O157	++++	c	c	c	c	c	c	c	g	c	g	g	g	g	g	c	c	c		c	c	c		
t	3821395	c	3931641	c	3998956	i	t	O157	+		t	t	t	t	t	t	c	t	c	c	c	c	c	t	t	t		t	t	t		
g	3821406	a	3931652	a	3998967	i	g	O157	++++		g	g	g	g	g	g	g	g	g	g	g	g	g	g	g	g		g	g	g		
g	3821439	a	3931685	a	3999000	i	g	O157	++++		g	g	g	g	g	g	g	a	g	g	g	g	g	g	g	g		g	g	g		
a	3821791	c	3932037	c	3999352	ns	c	CB9615	++++		c	c	c	c	c	c	c	c	c	c	c	c	c	c	c	c				c		

O55 and O157 genome site details										Outgroup Strain Details[g]																						
CB9615 base[a]	CB9615 Site[b]	Sakai base[a]	Sakai Site[b]	EDL933 base[a]	EDL933 Site[b]	type[c]	Inferred ancestral base[d]	Lineage inferred to mutate[e]	Support level[f]	D1 Sd197	K-12	HS	ATCC 8739	UMN026	IAI39	SMS 3-5	E2348/69	536	ED1a	CFT073	S88	APEC O1	UTI89	E24377A	IAI1	SE11	F5 8401	F2a 2457T	F2a 301	SS Ss046	B4 Sb227	B18 BS512
c	3823768	t	3934014	t	4001329	ns	c	O157	++++	c	c	c	c	c	c	c	c	c	c	c	c	c	c	c	c	c	c	c	c	c	c	c
a	3825647	g	3935893	g	4003208	ns	g	CB9615	++++	g	g	g	g	g	g	g	g	g	g	g	g	g	g	g	g	g	g	g	g	g	g	g
t	3825951	a	3936197	a	4003512	ns	t	O157	++++	t	t	t	t	t	t	t	t	t	t	t	t	t	t	t	t	t	t	t	t	a	t	t
g	3827347	a	3937593	a	4004908	ns	g	O157	++++	g	g	g	g	g	g	g	g	g	g	g	g	g	g	g	g	g	g	g	g	g	g	g
a	3827899	g	3938145	g	4005460	ns	g	CB9615	++++	g	g	g	g	g	g	g	g	g	g	g	g	g	g	g	g	g	g	g	g	g	g	g
g	3828353	a	3938599	a	4005914	ns	g	O157	++++	g	g	g	g	g	g	g	g	g	g	g	g	g	g	g	g	g	g	g	g	g	g	g
a	3829522	c	3939768	c	4007083	s	c	CB9615	++++	c	c	c	c	t	c	c	c	t	c	c	c	c	c	c	c	c	c	c	c	c	c	c
g	3831923	a	3942169	a	4009484	ns	g	O157	++++	g	g	g	g	g	g	g	g	g	g	g	g	g	g	g	g	g	g	g	g	g	g	g
g	3832507	a	3942753	a	4010068	i	g	O157	++++		g	g	g		g				g		g	g	g									
t	3834606	a	3944852	a	4012167	i	a	CB9615	++++	a	a	a	a	a	a	a	a	a	a	a	a	a	a	a	a	a	a	a	a	a	a	a
t	3839059	a	3949305	a	4016620	ns	t	O157	++++	t	t	t	t	t	t	t	t	t	t	t	t	t	t	t	t	t	t	t	t	t	t	t
c	3844613	t	3954859	t	4022174	ns	c	O157	++++	c	c	c	c	c	c	c	c	c	c	c	c	c	c	c	c	c	c	c	c	c	c	c
c	3846017	t	3956263	t	4023578	i	t	CB9615	++	t	t	t	t	t	t	t	t	t	t	t	t	t	c	c	c	t	c	c	c	t	t	t
g	3846589	a	3956835	a	4024150	ns	g	O157	++++	g	g	g	g	g	g	g	g	g	g	g	g	g	g	g	g	g	g	g	g	g	g	g
g	3847542	t	3957788	g	4025103	ns	g	Sakai	++++	g	g	g	g	g	g	g	g	g	g	g	g	g	g	g	g	g	g	g	g	g	g	g
c	3848892	g	3959138	g	4026453	s	g	CB9615	++++	g	g	g	g	g	g	g	g	g	g	g	g	g	g	g	g	g	g	g	g	g	g	g
a	3851558	a	3961804	g	4029119	ns	a	EDL933	++++	a	a	a	a	a	a	a	a	a	a	a	a	a	a	a	a	a	a	a	a	a	a	
t	3853328	c	3963574	c	4030889	s	c	CB9615	++++	c	c	c	c	c	c	c	c	c	c	c	c	c	c	c	c	c	c	c	c	c	c	
g	3854605	a	3964851	a	4032166	ns	g	O157	++++		g	g	g	g	g	g	g	g	g	g	g	g	g	g	g	g	g	g	g	g	g	
c	3855337	g	3965583	g	4032898	ns	c	O157	++++		c	c	c	c	c	c	c	c	c	c	c	c	c	c	c	c	c	c	c	c	c	c
a	3857196	c	3967442	c	4034757	s	a	O157	++	a	a	a	a	a	a	a	c	a	c	c	c	c	c	a	a	a	a	a	a	a	a	a
t	3858887	g	3969133	g	4036448	ns	g	CB9615	++++	g	g	g	g	g	g	g	g	g	g	g	g	g	g	g	g	g	g	g	g	g	g	g
g	3859825	a	3970071	a	4037386	s	g	O157	++++	g	g	g	g	g	g	g	g	g	g	g	g	g	g	g	g	g	g	g	g	g	g	g
t	3860431	c	3970677	c	4037992	s	c	CB9615	++++	c	c	c	c	c	c	c	c	c	c	c	c	c	c	c	c	c	c	c	c	c	c	c
g	3860488	t	3970734	t	4038049	ns	g	O157	++++	g	g	g	g	g	g	g	g	g	g	g	g	g	g	g	g	g	g	g	g	g	g	g
a	3864199	g	3974445	g	4041660	s	g	CB9615	++++	g	g	g	g	g	g	g	g	g	g	g	g	g	g	g	g	g	g	g	g	g	g	g
t	3865140	c	3975386	c	4042601	ns	c	CB9615	++++	c	c	c	c	c	c	c	c	c	c	c	c	c	c	c	c	c	c	c	c	c	c	c
c	3866540	a	3976786	a	4044001	ns	c	O157	++++	c	c	c	c	c	c	c	c	c	c	c	c	c	c	c	c	c	c	c	c	c	c	c
t	3866680	g	3976926	g	4044141	i	g	CB9615	++++	g	g	g	g	g	g	g	g	g	g	g	g	g	g	g	g	g	g	g	g	g	g	g
g	3871306	a	3981552	a	4048767	s	g	O157	++	g	g	g	g	a	g	a	g	a	g	g	g	g	g	g	g	g	g	g	g	g	g	g
g	3872384	c	3982630	c	4049845	ns	c	CB9615	++	c	c	c	c	c	c	c	c	c	c	c	c	c	c	c	c	c	g	g	g	c	c	c
a	3873040	c	3983286	c	4050501	ns	a	O157	++++	a	a	a	a	a	a	a	a	a	a	a	a	a	a	a	a	a	a	a	a	a	a	a
g	3873368	a	3983614	a	4050829	s	g	O157	++++	g	g	g	g	g	g	g	g	g	g	g	g	g	g	g	g	g	g	g	g	g	g	g
-	3874322	-	3984568	c	4051784	ins	-	EDL933	++++	-	-	-	-	-	-	-	-	-	-	-	-	-	-	-	-	-	-	-	-	-	-	-
c	3881390	a	3991636	a	4058852	ns	c	O157	++++	c	c	c	c	c	c	c	c	c	c	c	c	c	c	c	c	c	c	c	c	c	c	c
a	3881607	g	3991853	g	4059069	i	g	CB9615	++++	t	g	g	g	g	g	t	t	t	t	t	t	t	t	g	g	g	g	g	g	g	g	g
a	3882174	-	3992419	-	4059635	del	a	O157	++++	a	a	a	-	a	a	a								a	a	a				a	a	a
g	3886577	a	3996822	a	4064038	ns	g	O157	++++	g	g	g	g	g	g	g	g	g	g	g	g	g	g	g	g	g	g	g	g	g	g	g
t	3887597	c	3997842	c	4065058	i	t	O157	++++	t	t	t	t	t	t	t	t	t	t	t	t	t	t	t	t	t	t	t	t	t	t	t
t	3887699	g	3997944	g	4065160	s	t	O157	++++	t	t	t	t	t	t	t	t	t	t	t	t	t	t	t	t	t	t	t	t	t	t	t
a	3887888	c	3998133	c	4065349	ns	a	O157	++++	a	a	a	a	a	a	a	a	a	a	a	a	a	a	a	a	a	a	a	a	a	a	a
c	3890140	t	4000385	t	4067601	ns	c	O157	++++	c	c	c	c	c	c	c	c	c	c	c	c	c	c	c	c	c	c	c	c	c	c	c
c	3891826	t	4002071	t	4069287	i	t	CB9615	++++	t	t	t	t	t	t	t	t	t	t	t	t	t	t	t	t	t	t	t	t	t	t	t
g	3892223	a	4002468	a	4069684	s	a	CB9615	++	a	a	a	a	a	a	a	g	g	g	g	g	g	g	a	a	a	a	a	a	a	a	a
t	3892494	a	4002739	a	4069955	ns	t	O157	++++	t	t	t	t	t	t	t	t	t	t	t	t	t	t	t	t	t	t	t	t	t	t	t
g	3893033	a	4003278	a	4070494	s	a	CB9615	++++	a	a	a	a	a	a	a	a	a	a	a	a	a	a	a	a	a	a	a	a	a	a	a
a	3893769	g	4004014	g	4071230	s	g	CB9615	++++	g	g	g	g	g	g	g	g	g	g	g	g	g	g	g	g	g	g	g	g	g	g	g
c	3896564	t	4006809	t	4074025	i	c	O157	+	a	a	a	a	c	a	t	a	a	a	a	a	a	a	a	c	c	a	a	a	a	a	a
g	3896592	a	4006837	a	4074053	i	g	O157	++++	g	g	g	g	g	g	g	g	g	g	g	g	g	g	g	g	g	g	g	g	g	g	g
c	3896991	t	4007236	t	4074452	i	c	O157	++++	c	c	c	c	c	c	c	c	c	c	c	c	c	c	c	c	c	c	c	c	c	c	c
g	3897973	a	4008218	a	4075434	ns	g	O157	+		g	g	g	g	g	g								g	g	g	a	a	a		g	g
g	3898081	g	4008326	a	4075542	ns	g	EDL933	+		g	g	g	g	g	g								g	g	g	g	a	a		g	g
t	3898133	c	4008378	c	4075594	ns	c	CB9615	++++		c	c	c	c	c	c								c	c	c	c	c	c		c	c
t	3898936	t	4009181	c	4076397	ns	t	EDL933	++++	t	t	t	t	t	t	t								t	t	t	t	t	t	t	t	t
a	3899006	g	4009251	g	4076467	i	a	O157	++++	a	a	a	a	a	a	a								a	a	a	a	a	a	a	a	a
a	3899242	g	4009600	g	4076816	ns	g	CB9615	++++	g	g	g	g	g	g	g	g	g	g	g	g	g	g	g	g	g	g	g	g	g	g	g
a	3907655	g	4018013	g	4085229	i	g	CB9615	++++		g	g	g	g	g	g	g	g	g	g	g	g	g	g	g	g		g	g			
a	3908041	c	4018399	c	4085615	ns	c	CB9615	++++		c	c	c	c	c	c	c	c	c	c	c	c	c	c	c	c		c	c			
g	3909526	a	4019884	a	4087100	ns	g	O157	++++		g	g	g	g	g	g	g	g	g	g	g	g	g	g	g	g		g	g			
a	3910124	g	4020482	g	4087698	ns	g	CB9615	++++			g	g	g	g	g	g	g	g	g	g	g	g	g	g	g		g	g			
c	3914838	t	4025196	t	4092412	s	c	O157	++++		c	c	c	c	c	c	c	c	c	c	c	c	c	c	c	c						
c	3915888	t	4026246	t	4093462	ns	c	O157	++++		c	c	c	c	c	c	c	c	c	c	c	c	c	c	c	c		c	c			
t	3916856	c	4027214	c	4094430	ns	c	CB9615	++++		c	c	c	c	c	c	c	c	c	c	c	c	c	c	c	c		c	c			
g	3917600	-	4027957	-	4095173	ins	-	CB9615	++++		-	-	-	-	-	-	-	-	-	-	-	-	-	-	-	-		-	-			
g	3922591	a	4032948	a	4100164	i	g	O157	++++	g	g	g	g											g	g	g	g	g	g	g	g	g
a	3924525	g	4035840	g	4103056	i	g	CB9615	++++	g	g	g	g	g	g	g	g	g	g	g	g	g	g	g	g	g	g	g	g	g	g	g
c	3928959	t	4040274	t	4107490	s	c	O157	++++	c	c	c	c	c	c	c	c	c	c	c	c	c	c	c	c	c	c	c	c	c	c	c
a	3930061	g	4041376	g	4108592	ns	g	CB9615	++++	g	g	g	g	g	g	g		g	g		g	g	g	g	g	g	g	g	g	g	g	g
g	3931407	a	4042722	a	4109938	ns	g	O157	++++	g	g	g	g	g	g	g	g	g	g	g	g	g	g	g	g	g	g	g	g	g	g	g
t	3935502	c	4046817	c	4114033	ns	t	O157	++++	t	t	t	t	t	t	t	t	t	t	t	t	t	t	t	t	t	t	t	t	t	t	t
t	3935603	c	4046918	c	4114134	s	c	CB9615	++++	c	c	c	c	c	c	c	c	c	c	c	c	c	c	c	c	c	c	c	c	c	c	c
a	3936139	g	4047454	g	4114670	s	g	CB9615	++	g	g	g	g	g	g	a	g	a	g	g	g	g	g	g	g	g	g	g	g	g	g	g
g	3936531	a	4047846	a	4115062	s	g	O157	++++	g	g	g	g	g	g	g	g	g	g	g	g	g	g	g	g	g	g	g	g	g	g	g
a	3941892	g	4053207	g	4120423	s	g	CB9615	++	g	g	g	g	g	g	g	a	a	g	a	g	g	g	g	g	g	g	g	g	g	g	g
t	3942628	c	4053943	c	4121159	ns	t	O157	++++	t	t	t	t	t	t	t	t	t	t	t	t	t	t	t	t	t	t	t	t	t	t	t
g	3944995	a	4056310	a	4123526	s	g	O157	++++	g	g	g	g	g	g	g	g	g	g	g	g	g	g	g	g	g	g	g	g	g	g	g
g	3948754	a	4060069	a	4127285	s	g	O157	++++	g	g	g	g	g	g	g	g	g	g	g	g	g	g	g	g	g	g	g	g	g	g	g
t	3950791	c	4062106	c	4129322	s	t	O157	++++	t	t	t	t	t	t	t	t	t	t	t	t	t	t	t	t	t	t	t	t	t	t	t
c	3951271	a	4062586	a	4129802	ns	c	O157	++++	c	c	c	c	c	c	c	c	c	c	c	c	c	c	c	c	c	c	c	c	c	c	c

O55 and O157 genome site details										Outgroup Strain Details[g]																						
CB9615 base[a]	CB9615 Site[b]	Sakai base[a]	Sakai Site[b]	EDL933 base[a]	EDL933 Site[b]	type[c]	Inferred ancestral base[d]	Lineage inferred to mutate[e]	Support level[f]	D1 Sd197	K-12	HS	ATCC 8739	UMN026	IAI39	SMS 3-5	E2348/69	536	ED1a	CFT073	S88	APEC O1	UTI89	E24377A	IAI1	SE11	F5 8401	F2a 2457T	F2a 301	SS Ss046	B4 Sb227	B18 BS512
g	3953791	a	4065106	a	4132322	i	g	O157	++	g	g	g	g	g	g	g	g	g	g	g	g	g	g	g	g	g	a	a	a	g	g	g
c	3954056	t	4065371	t	4132587	ns	c	O157	++++	c	c	c	c	c	c	c	c	c	c	c	c	c	c	c	c	c	c	c	c	c	c	c
a	3954069	g	4065384	g	4132600	s	g	CB9615	++++	g	g	g	g	g	g	g	g	g	g	g	g	g	g	g	g	g	g	g	g	g	g	g
a	3956282	c	4067597	c	4134813	ns	a	O157	++++	a	a	a	a	a	a	a	a	a	a	a	a	a	a	a	a	a	a	a	a	a	a	a
g	3965551	a	4076866	a	4144082	ns	g	O157	++++	g	g	g	g	g	g	g	g	g	g	g	g	g	g	g	g	g	g	g	g	g	g	g
t	3965812	c	4077127	c	4144343	i	t	O157	++	t	t	t	t	c	c	t	c	t	t	t	t	t	t	t	c	c	c	c	c	c	c	c
c	3965850	t	4077165	t	4144381	i	c	O157	++++	c	c	c	c	c	c	c	c	c	c	c	c	c	c	c	c	c	c	c	c	c	c	c
a	3966166	c	4077481	a	4144697	ns	a	Sakai	++++	a	a	a	a	a	a	a	a	a	a	a	a	a	a	a	a	a	a	a	a	a	a	a
t	3967415	c	4078730	c	4145946	ns	c	CB9615	++++	c	c	c	c	c	c	c	c	c	c	c	c	c	c	c	c	c	c	c	c	c	c	c
a	3967433	g	4078748	g	4145964	s	g	CB9615	++++	g	g	g	g	g	g	g	g	g	g	g	g	g	g	g	g	g	g	g	g	g	g	g
g	3970227	a	4081542	a	4148758	s	g	O157	++++	g	g	g	g	g	g	g	g	g	g	g	g	g	g	g	g	g	g	g	g	g	g	g
g	3972673	a	4083988	a	4151204	ns	g	O157	++++	g	g	g	g	g	g	g	g	g	g	g	g	g	g	g	g	g	g	g	g	g	g	g
g	3975191	a	4086506	a	4153722	s	g	O157	++++	g	g	g	g	g	g	g	g	g	g	g	g	g	g	g	g	g	g	g	g	g	g	g
c	3975543	t	4086858	t	4154074	s	c	O157	++++	c	c	c	c	c	c	c	c	c	c	c	c	c	c	c	c	c	c	c	c	c	c	c
g	3976639	t	4087954	t	4155170	s	g	O157	++++	g	g	g	g	g	g	g	g	g	g	g	g	g	g	g	g	g	g	g	g	g	g	g
g	3977087	t	4088402	t	4155618	ns	t	CB9615	++++	t	t	t	t	t	t	t	t	t	t	t	t	t	t	t	t	t	t	t	t	t	t	t
t	3980613	c	4091928	c	4159144	ns	c	CB9615	++++	c	c	c	c	c	c	c	c	c	c	c	c	c	c	c	c	c	a	c	c	c	c	c
a	3980652	t	4091967	t	4159183	ns	a	O157	++++	a	a	a	a	a	a	a	a	a	a	a	a	a	a	a	a	a	a	a	a	a	a	a
-	3981555	-	4092870	c	4160087	ins	-	EDL933	++++	-	-	-	-	-	-	-	-	-	-	-	-	-	-	-	-	-	-	-	-	-	-	-
t	3981636	a	4092951	a	4160168	nc	t	O157	++++	t	t	t	t	t	t	t	t	t	t	t	t	t	t	t	t	t	t	t	t	t	t	t
c	3981971	t	4093286	t	4160503	ns	c	O157	++++	c	c	c	c	c	c	c	c	c	c	c	c	c	c	c	c	c	c	c	c	c	c	c
t	3985323	c	4096638	c	4163855	i	c	CB9615	++++	c	c	c	c	c	c	c	c	c	c	c	c	c	c	c	c	c	c	c	c	c	c	c
a	3985393	g	4096708	g	4163925	i	g	CB9615	++++	g	g	g	g	g	g	g	g	g	g	g	g	g	g	g	g	g	g	g	g	g	g	g
g	3985974	g	4097289	t	4164506	ns	g	EDL933	++++	g	g	g	g	g	g	g	g	g	g	g	g	g	g	g	g	g	g	g	g	g	g	g
c	3986588	t	4097903	t	4165120	ns	c	O157	++++	c	c	c	c	c	c	c	c	c	c	c	c	c	c	c	c	c	c	c	c	c	c	c
g	3989281	a	4100596	g	4167813	ns	g	Sakai	++++	g	g	g	g	g	g	g	g	g	g	g	g	g	g	g	g	g	g	g	g	g	g	g
a	3993827	g	4105064	g	4172281	s	a	O157	++++	a	a	a	a	a	a	a	a	a	a	a	a	a	a	a	a	a	a	a	a	a	a	a
t	3994083	c	4105320	c	4172537	ns	t	O157	++++	t	t	t	t	c	t	t	t	t	t	t	t	t	t	t	t	t	t	t	t	t	t	t
c	3995283	t	4106520	t	4173737	s	c	O157	++++	c	c	c	c	c	c	c	c	c	c	t	c	c	c	c	c	c	c	c	c	c	c	c
3	3998968	-	4110204	-	4177421	del	3	O157	++++		3	3	3	3	3	3								3	3	3	3	3	3	3	3	3
c	3999010	a	4110244	a	4177461	ns	c	O157	++++		c	c	c	c	c	c								c	c	c	c	c	c	c	c	c
6	3999725	-	4110958	-	4178175	del	6	O157	++++		6	6	6	6	6	6								6	6	6	6	6	6	6	6	6
cg	4000104	-	4111331	-	4178548	del	cg	O157	++++	cg	cg	cg	cg	cg	cg	cg								cg	cg	cg	cg	cg	cg	cg	cg	cg
g	4002050	a	4113276	a	4180493	s	g	O157	++++	g	g	g	g	g	g	g	g	g	g	g	g	g	g	g	g	g	g	g	g	g	g	g
t	4003038	a	4114264	a	4181481	ns	a	CB9615	++++	a	a	a	a	a	a	a	a	a	a	a	a	a	a	a	a	a	a	a	a	a	a	a
g	4003612	a	4114838	a	4182055	s	g	CB9615	++++	g	g	g	g	g	g	g	g	g	g	g	g	g	g	g	g	g	g	g	g	g	g	g
t	4004276	c	4115502	c	4182719	ns	c	CB9615	++++	c	c	c	c	c	c	c	c	c	c	c	c	c	c	c	c	c	c	c	c	c	c	c
g	4005680	a	4116906	a	4184123	ns	g	O157	++++	g	g	g	g	g	g	g	g	g	g	g	g	g	g	g	g	g	g	g	g	g	g	g
a	4007407	c	4118633	c	4185850	s	c	CB9615	++++	c	c	c	c	c	c	c	c	c	c	c	c	c	c	c	t	c	c	c	c	c	c	c
g	4008579	a	4119805	a	4187022	i	g	O157	++++	g	g	g	g	g	g	g	g	g	g	g	g	g	g	g	g	g	g	g	g	g	g	g
g	4009276	a	4120502	a	4187719	i	g	O157	++++	g	g	g	g	g	g	g	g	g	g	g	g	g	g	g	g	g	g	g	g	g	a	g
a	4009361	-	4120586	-	4187803	ins	-	CB9615	++++	-	-	-	-	-	-	-	-	-	-	-	-	-	-	-	-	-	-	-	-	-	-	-
-	4010195	-	4121419	N	4188637	ins	-	EDL933	++++	-	-	-	-	-	-	-	-	-	-	-	-	-	-	-	-	-	-	-	-	-	-	-
t	4010292	g	4121516	g	4188734	ns	g	CB9615	++++	g	g	g	g	g	g	g	g	g	g	g	g	g	g	g	g	g	g	g	g	g	g	g
g	4010326	a	4121550	a	4188768	ns	g	O157	++++	g	g	g	g	g	g	g	g	g	g	g	g	g	g	g	g	g	g	g	g	g	g	g
c	4011587	t	4122811	t	4190029	s	t	CB9615	++++	t	t	t	t	t	t	t	t	t	t	t	t	t	t	t	t	t	t	t	t	t	t	t
c	4012197	t	4123421	t	4190639	ns	c	O157	++++	c	c	c	c	c	c	c	c	c	c	c	c	c	c	c	c	c	c	c	c	c	c	c
3	4012947	-	4124170	-	4191388	del	3	O157	++++	3	3	3	3	3	3	3	3	3	3	3	3	3	3	3	3	3	3	3	3	3	3	3
g	4013257	a	4124478	a	4191696	ns	g	O157	++++	g	g	g	g	g	g	g	g	g	g	g	g	g	g	g	g	g	g	g	g	g	g	g
-	4014519	6	4125741	6	4192959	ins	-	O157	++	-	6	6	6	-	-	-	6	-	-	-	-	-	-	-	-	6	-	-	-	-	-	-
c	4015465	t	4126692	t	4193910	ns	c	O157	++++	c	c	c	c	c	c	c	c	c	c	c	c	c	c	c	c	c	c	c	c	c	c	c
g	4022672	a	4133899	a	4201117	ns	g	O157	++++	g	g	g	g		g	g	g	g	g	g	g	g	g	g	g	g	g	g	g	g	g	g
g	4025174	t	4136401	t	4203619	ns	t	CB9615	++++	t	t	t	t	t	t	t	t	t	t	t	t	t	t	t	t	t	t	t	t	t	t	t
a	4028585	g	4139812	g	4207030	i	a	O157	++++	a	a	a	a	a	a	a	a	a	a	a	a	a	a	a	a	a	a	a	a	a	a	a
a	4029379	g	4140606	g	4207824	ns	g	CB9615	++++	g	g	g	g	g	g	g	g	g	g	g	g	g	g	g	g	g	g	g	g	g	g	g
g	4030313	t	4141540	t	4208758	ns	g	O157	++++	g	g	g	g	g	g	g	g	g	g	g	g	g	g	g	g	g	g	g	g	g	g	g
c	4031061	a	4142288	a	4209506	i	c	O157	++++	c	c	c	c	c		c	c							c	c	c	c	c	c	c		
c	4031963	t	4143190	t	4210408	s	c	O157	++	c	c	c	c	t	c	t	t	t	t	t	t	t	t	c	c	c	c	c	c	c	t	t
g	4032206	a	4143433	a	4210651	s	g	O157	++++	g	g	g	g	g	g	g	g	g	g	g	g	g	g	g	g	g	g	g	g	a	g	g
a	4033165	g	4144392	g	4211610	s	g	CB9615	++++	g	g	g	g	g	g	g	g	g	g	g	g	g	g	g	g	g	g	g	g	g	g	g
a	4039399	c	4150626	c	4217844	ns	a	O157	++	a	a	a	a	a	a	a	a	a	a	a	a	a	a	a	a	a	c	c	c	a	a	a
a	4040626	-	4151905	-	4219123	indel	?	O55/O157	+/-																							
g	4042581	a	4153853	a	4221071	i	g	O157	++++		g	g	g	g	g	g	g	g	g	g	g	g	g	g	g	g				g	g	g
c	4045361	t	4156633	t	4223851	nc	c	O157	++++	c	c	c	c	c	c	c	c	c	c	c	c	c	c	c	c	c	c	c	c	c	c	c
c	4045446	t	4156718	t	4223936	nc	c	O157	++++	c	c	c	c	c	c	c	c	c	c	c	c	c	c	c	c	c	c	c	c	c	c	c
a	4045524	g	4156796	g	4224014	nc	g	CB9615	++++	g	g	g	g	g	g	g	g	g	g	g	g	g	g	g	g	g	g	g	g	g	g	g
t	4045633	c	4156905	c	4224123	nc	t	O157	++++	t	t	t	t	t	t	t	t	t	t	t	t	t	t	t	t	t	t	t	t	t	t	t
g	4046017	a	4157289	a	4224507	nc	g	O157	++++	g	g	g	g	g	a	g	g	g	g	g	g	g	g	g	g	g	g	g	g	g	g	g
t	4047394	a	4158666	a	4225884	i	a	CB9615	++	a	a	a	a	a	a	a	t	a	a	t	a	a	a	a	a	a	a	a	t	a	a	a
t	4047395	a	4158667	a	4225885	i	a	CB9615	++++	a	a	a	a	a	a	a	a	a	a	a	a	a	a	a	a	a	a	a	a	a	a	a
-	4047543	-	4158815	m	4226034	ins	-	EDL933	++	-	-	-	-	c	c	-	c	c	-	c	c	c	c	-	-	-	-	-	-	-	-	-
g	4049288	a	4160560	g	4227779	nc	a	Sakai	-	a	g	g	g	a	a	a	c	c	c	c	c	g	c	g	g	a	a	a	a	g	a	a
c	4049297	t	4160569	c	4227788	nc	t	Sakai	-	t	c	c	c	t	t	t	g	g	g	g	g	c	g	c	c	t	c	c	c	c	c	t
-	4049343	-	4160615	a	4227835	ins	-	EDL933	-	-	a	a	a	a	-	-	-	-	-	-	-	a	-	a	a	-	a	a	a	a	a	-
a	4049870	g	4161142	a	4228362	nc	a	Sakai	++++	a	a	a	a	a	a	a	a	a	a	a	a	a	a	a	a	a	a	a	a	a	a	a
c	4049964	c	4161236	-	4228455	del	?	EDL933	+/-																							
-	4049975	-	4161247	a	4228456	ins	?	EDL933	+/-																							
t	4050160	t	4161432	c	4228654	nc	t	EDL933	++	t	c	c	c	c	t	t	t	c	t	t	t	t	t	c	c	c	t	t	t	t	t	t
g	4050252	c	4161524	c	4228746	nc	g	O157	++	g	g	g	g	g	c	c	c	c	c	g	c	c	c	g	g	g	g	g	g	g	g	g

O55 and O157 genome site details										Outgroup Strain Details[g]																						
CB9615 base[a]	CB9615 Site[b]	Sakai base[a]	Sakai Site[b]	EDL933 base[a]	EDL933 Site[b]	type[c]	Inferred ancestral base[d]	Lineage inferred to mutate[e]	Support level[f]	D1 Sd197	K-12	HS	ATCC 8739	UMN026	IAI39	SMS 3-5	E2348/69	536	ED1a	CFT073	S88	APEC O1	UTI89	E24377A	IAI1	SE11	F5 8401	F2a 2457T	F2a 301	SS Ss046	B4 Sb227	B18 BS512
a	4050374	a	4161646	t	4228868	nc	t	EDL933	-	t	t	a	t	a	a	a	t	a	t	t	c	t	c	a	a	a	a	a	a	a	a	a
t	4050379	t	4161651	a	4228873	nc	a	EDL933	-	a	a	t	a	t	t	t	a	t	a	a	a	a	a	t	t	t	t	t	t	t	t	t
c	4052359	t	4163631	t	4230853	nc	c	O157	++++	c	c	c	c	c	c	c	c	c	c	c	c	c	c	c	c	c	c	c	c	c	c	c
t	4052725	c	4163997	c	4231219	ns	t	O157	++++	t	t	t	t	t	t	t	t	t	t	t	t	t	t	t	t	t	t	t	t	t	t	t
g	4053577	a	4164849	a	4232071	s	g	O157	++++	g	g	g	g	g	g	g	g	g	g	g	g	g	g	g	g	g	g	g	g	g	g	g
g	4056533	a	4167805	a	4235027	s	a	CB9615	++++	a	a	a	a	a	a	a	a	a	a	a	a	a	a	a	a	a	a	a	a	a	a	a
g	4056888	a	4168160	a	4235382	ns	a	CB9615	++++	a	a	a	a	a	a	a	a	a	a	a	a	a	a	a	a	a	a	a	a	a	a	a
a	4057093	g	4168365	g	4235587	ns	g	CB9615	++++	g	g	g	g	g	g	g	g	g	g	g	g	g	g	g	g	g	g	g	g	g	g	g
a	4057929	c	4169201	c	4236423	ns	c	CB9615	++++	c	c	c	c	c	c	c	c	c	c	c	c	c	c	c	c	c	c	c	c	c	c	c
a	4058955	t	4170227	a	4237449	ns	a	Sakai	++++	a	a	a	a	a	a	a	a	a	a	a	a	a	a	a	a	a	a	a	a	a	a	a
g	4059506	c	4170778	c	4238000	ns	g	O157	++++	g	g	g	g	g	g	g	g	g	g	g	g	g	g	g	g	g	g	g	g	g	g	g
t	4060974	g	4172246	t	4239468	s	t	Sakai	++++	t	t	t	t	t	t	t	t	t	t	t	t	t	t	t	t	t	t	t	t	t	t	t
a	4061703	g	4172975	g	4240197	i	g	CB9615	++++	g	g	g	g	g	g	g	g	g	g	g	g	g	g	g	g	g	g	g	g	g	g	g
g	4063470	a	4174742	a	4241964	s	a	CB9615	++++	a	a	a	a	a	a	a	a	a	a	a	a	a	a	a	a	a	a	a	a	a	a	a
g	4068670	a	4179942	a	4247164	s	g	O157	++++	g	g	g	g	g	g	g	g	g	g	g	g	g	g	g	g	g	g	g	g	g	g	g
c	4076277	t	4187549	t	4254771	s	c	O157	++++	c	c	c	c	c	c	c	c	c	c	c	c	c	c	c	c	c	c	c	c	c	c	c
g	4078938	a	4190210	a	4257432	s	g	O157	++++	g	g	g	g	g	g	g	g	g	g	g	g	g	g	g	g	g	g	g	g	g	g	g
c	4081012	t	4192284	t	4259506	s	c	O157	++++	c	c	c	c	c	c	c	c	c	c	c	c	c	c	c	c	c	c	c	c	c	c	c
t	4083329	c	4194601	c	4261823	ns	t	O157	++++	t	t	t	t	t	t	t	t	t	t	t	t	t	t	t	t	t	t	t	t	t	t	t
c	4083761	t	4195033	t	4262255	ns	c	O157	++++	c	c	c	c	c	c	c	c	c	c	c	c	c	c	c	c	c	c	c	c	c	c	c
g	4084081	a	4195353	a	4262575	ns	g	O157	++++	g	g	g	g	g	g	g	g	g	g	g	g	g	g	g	g	g	g	g	g	g	g	g
a	4085339	g	4196611	K	4263833	ns	g	CB9615	++++	g	g	g	g	g	a	g	g	g	g	g	g	g	g	g	g	g	g	g	g	g	g	g
a	4085339	g	4196611	K	4263833	ns	g	EDL933	++++	g	g	g	g	g	a	g	g	g	g	g	g	g	g	g	g	g	g	g	g	g	g	g
t	4086163	c	4197435	c	4264657	i	t	O157	++++	t	t	t	t	t	t	t	t	t	t	t	t	t	t	t	t	t	t	t	t	t	t	t
c	4086910	g	4198182	g	4265404	ns	g	CB9615	++++	g	g	g	g	g	g	g	g	g	g	g	g	g	g	g	g	g	g	g	g	g	g	g
g	4087465	a	4198737	a	4265959	ns	g	O157	++++	g	g	g	g	g	g	g	g	g	g	g	g	g	g	g	g	g	g	g	g	g	g	g
a	4088589	g	4199861	g	4267083	ns	g	CB9615	++++		g	g	g	g	g	g	g	g	g	g	g	g	g	g	g	g	g	g	g	g	g	g
g	4089418	a	4200690	a	4267912	s	g	O157	++++	g	g	g	g	g	g	g	g	g	g	g	g	g	g	g	g	g	g	g	g	g	g	g
t	4090409	g	4201681	g	4268903	ns	g	CB9615	++++	g	g	g	g	g	g	g	g	g	g	g	g	g	g	g	g	g	g	g	g	g	g	g
c	4091034	t	4202306	t	4269528	ns	t	CB9615	++++	t	t	t	t	t	t	t	t	t	t	t	t	t	t	t	t	t	t	t	t	t	t	t
g	4093585	a	4204857	a	4272079	s	g	O157	++++	g	g	g	g	g	g	g	g	g	g	g	g	g	g	g	g	g	g	g	g	g	g	g
c	4095999	a	4207271	a	4274493	ns	c	O157	++	c	a	a	a	a	a	a	a	a	a	a	a	a	a	a	a	a	a	a	a	a	a	a
t	4099995	c	4211267	c	4278489	ns	c	CB9615	++++	c	c	c	c	c	c	c	c	c	c	c	c	c	c	c	c	c	c	c	c	c	c	c
a	4101489	g	4212761	g	4279983	s	a	O157	++	a	g	g	g	g	g	g	g	g	g	g	g	g	g	g	g	g	g	g	g	g	g	g
a	4101599	g	4212871	g	4280093	ns	g	CB9615	++++	g	g	g	g	g	g	g	g	g	g	g	g	g	g	g	g	g	g	g	g	g	g	g
a	4102152	g	4213424	g	4280646	s	g	CB9615	++++	g	g	g	g	g	g	g	g	g	g	g	g	g	g	g	g	g	g	g	g	g	g	g
t	4102712	g	4213984	t	4281206	ns	t	Sakai	++++	t	t	t	t	t	t	t	t	t	t	t	t	t	t	t	t	t	t	t	t	t	t	t
a	4109661	g	4220933	g	4288155	ns	g	CB9615	++++		g	g	g											g	g	g	g	g	g	g	g	g
a	4109735	c	4221007	c	4288229	ns	a	O157	++++		a	a	a											a	a	a	a	a	a	a	a	a
a	4110445	g	4221717	g	4288939	ns	g	CB9615	++++		g	g	g											g	g	g	g	g	g	g	g	g
t	4112240	g	4223512	g	4290734	nc	t	O157	++++		t	t	t											t	t	t	t	t	t	t	t	t
g	4113406	a	4224678	a	4291900	s	g	O157	+++		t	t	g											t	t	t	g	g	g	g	t	t
t	4114478	c	4225750	c	4292972	ns	c	CB9615	++++	c	c	c	c	c	c	c	c	c	c	c	c	c	c	c	c	c	c	c	c	c	c	c
g	4121702	a	4232974	a	4300196	ns	g	O157	++++	g	g	g	g	g	g	g	g	g	g	g	g	g	g	g	g	g	g	g	g	g	g	g
c	4122584	g	4233965	g	4301187	s	c	O157	++++	c	c	c	c	c	c	c	c	c	c	c	c	c	c	c	c	c	c	c	c	c	c	c
c	4122814	-	4234194	-	4301416	del	c	O157	++++	c	c	c	c	c	c	c	c	c	c	c	c	c	c	c	c	c	c	c	c	c	c	c
c	4125974	t	4237354	t	4304576	s	c	O157	++++	a	c	c	c	c	c	c	c	c	c	c	c	c	c	c	c	c	c	c	c	c	c	c
g	4128228	c	4239608	c	4306830	ns	g	O157	++++	g	g	g	g	g	g	g	g	g	g	g	g	g	g	g	g	g	g	g	g	g	g	g
t	4132979	g	4244359	g	4311581	ns	t	O157	++	t	t	t	t	t	t	g	g	t	t	t	g	g	g	t	t	t	t	t	t	t	t	t
c	4135350	t	4246730	t	4313952	ns	c	O157	++++	c	c	c	c	c	c	c	c	c	c	c	c	c	c	c	c	c	c	c	c	c	c	c
g	4139111	a	4250491	a	4317713	ns	g	O157	++++	g	g	g	g	g	g	g	g	g	g	g	g	g	g	g	g	g	g	g	g	g	g	g
g	4139161	t	4250541	t	4317763	s	g	O157	++++	g	g	g	g	g	g	g	g	g	g	g	g	g	g	g	g	g	g	g	g	g	g	g
g	4140853	a	4252233	a	4319455	i	g	O157	++++	g	g	g	g	g	g	g	g	g	g	g	g	g	g	g	g	g	g	g	g	g	a	g
t	4142576	g	4253956	t	4321178	ns	t	Sakai	++++	t	t	t	t	t	t	t	t	t	t	t	t	t	t	t	t	t	t	t	t	t	t	t
t	4145419	a	4256799	a	4324021	s	a	CB9615	++	a	a	a	a	a	a	a	t	t	t	t	a	a	a	a	a	a	a	a	a	a	a	a
t	4147454	g	4258834	g	4326056	s	t	O157	++++	t	t	t	t	t	t	t	t	t	t	t	t	t	t	t	t	t	t	t	t	t	t	t
g	4150440	c	4261820	c	4329042	ns	g	O157	++++	g	g	g	g	g	g	g	g	g	g	g	g	g	g	g	g	g	g	g	g	g	g	g
t	4150747	g	4262127	g	4329349	ns	t	O157	++++	t	t	t	t	t	t	t	t	t	t	t	t	t	t	t	t	t	t	t	t	t	t	t
c	4151522	t	4262902	t	4330124	s	c	O157	++++	c	c	c	c	c	c	c	c	c	c	c	c	c	c	c	c	c	c	c	c	c	c	c
g	4158154	a	4269534	a	4336756	s	g	O157	++++	g	g	g	g	g	g	g	g	g	g	g	g	g	g	g	g	g		g	g	g	g	g
g	4158563	a	4269943	a	4337165	ns	g	O157	++++	g	g	g	g	g	g	g	g	g	g	g	g	g	g	g	g	g	g	g	g	g	g	g
t	4158807	g	4270187	g	4337409	ns	g	CB9615	++++	g	g	g	g	g	g	g	g	g	g	g	g	g	g	g	g	g	g	g	g	g	g	g
g	4160443	a	4271823	a	4339045	s	g	O157	++++		g	g	g	g	g	g	g	g	g	g	g	g	g	g	g	g	g	g	g	g	g	g
t	4163991	c	4275371	c	4342593	s	c	CB9615	++++	c	c	c	c	c	c	c	c	c	c	c	c	c	c	c	c	c	c	c	c	c	c	c
t	4164972	c	4276352	c	4343574	s	c	CB9615	++++	c	c	c	c	c	c	c	c	c	c	c	c	c	c	c	c	c	c	c	c	c	c	c
c	4165486	a	4276866	a	4344088	ns	a	CB9615	++++	a		a	a		a	a	a	a	a	a	a	a	a									
t	4167562	c	4278942	c	4346164	ns	c	CB9615	++++			c	c		c	c	c							c	c	c				c	c	c
a	4167770	g	4279150	g	4346372	s	g	CB9615	++++	g		g	g		g	g	g							g	g	g				g	g	g
a	4168518	-	4279897	-	4347119	del	a	O157	++++	a	a	-	-		a	a	a							-	-	-				a	a	
g	4169276	a	4280655	a	4347877	ns	g	O157	++++	g	g	g	g	g	g	g	g	g	g	g	g	g	g	g	g	g	g	g	g	g	g	g
c	4181263	t	4292642	t	4359864	ns	c	O157	+++	c					c	c		c		c										c		
g	4181462	c	4292841	c	4360063	nc	c	CB9615	+++	c					c	c		c		c										c		
t	4181805	g	4293213	g	4360435	nc	g	CB9615	+++	a					g	g		g		g										g		
c	4182757	g	4294165	g	4361387	nc	c	O157	+++	c					c	c		c		c										c		
a	4186806	g	4298214	g	4365436	ns	g	CB9615	++++	g	g	g	g	g	g	g	g	g	g	g	g	g	g	g	g	g	g	g	g	g	g	g
g	4187659	a	4299067	a	4366289	s	g	O157	++++	g	g	g	g	t	g	g	g	g	g	g	g	g	g	g	g	g	g	g	g	g	g	g
g	4188464	t	4299872	t	4367094	ns	t	CB9615	++++	t	t	t	t	t	t	t	t	t	t	t	t	t	t	t	t	t	t	t	t	t	t	t
a	4188778	-	4300185	-	4367407	ins	-	CB9615	++++	-	-	-	-	-	-	-	-	-	a	-	-	-	-	-	-	-	-	-	-	-	-	-
t	4188862	c	4300269	c	4367491	i	c	CB9615	++++	c	c	c	c	c	c	c	c	c	c	c	c	c	c	c	c	c	c	c	c	c	c	c

O55 and O157 genome site details										Outgroup Strain Details[g]																						
CB9615 base[a]	CB9615 Site[b]	Sakai base[a]	Sakai Site[b]	EDL933 base[a]	EDL933 Site[b]	type[c]	Inferred ancestral base[d]	Lineage inferred to mutate[e]	Support level[f]	D1 Sd197	K-12	HS	ATCC 8739	UMN026	IAI39	SMS 3-5	E2348/69	536	ED1a	CFT073	S88	APEC O1	UTI89	E24377A	IAI1	SE11	F5 8401	F2a 2457T	F2a 301	SS Ss046	B4 Sb227	B18 BS512
a	4189151	g	4300558	g	4367780	ns	g	CB9615	++++	g	g	g	g	g	g	g	g	g	g	g	g	g	g	g	g	g	g	g	g	g	g	g
a	4190193	g	4301600	g	4368822	ns	?	O55/O157	+/-																							
a	4190500	-	4301906	-	4369128	indel	?	O55/O157	+/-																							
a	4190626	t	4302032	t	4369254	ns	?	O55/O157	+/-																							
a	4191169	g	4302575	g	4369797	i	?	O55/O157	+/-																							
-	4191305	-	4302711	t	4369934	ins	-	EDL933	++++	-	-	-	-	-	-			-	-	-	-	-	-	-	-	-					-	-
c	4191617	a	4303023	a	4370246	ns	c	O157	++++	c	c	c	c	c	c	c	c	c	c	c	c	c	c	c	c	c	c	c	c	c	c	c
t	4194804	g	4306210	g	4373433	s	g	CB9615	++++	g	g	g	g	g	g	g	g	g	g	g	g	g	g	g	g	g	g	g	g	a	g	g
c	4195248	a	4306654	a	4373877	ns	c	O157	++++	c	c	c	c	c	c	c	c	c	c	c	c	c	c	c	c	c	c	c	c	c	c	c
c	4196424	a	4307830	a	4375053	s	c	O157	++++	c	c	c	c	c	c	c	c	c	c	c	c	c	c	c	c	c	c	c	c	a	c	c
a	4197149	g	4308555	g	4375778	ns	g	CB9615	++++	g	g	g	g	g	g	g	g	g	g	g	g	g	g	g	g	g	g	g	g	g		g
-	4197190	-	4308596	N	4375820	ins	-	EDL933	++++	-	-	-	-	-	-	-	-	-	-	-	-	-	-	-	-	-	-	-	-	-		-
g	4204265	t	4315671	t	4382895	s	g	O157	++++	g	g	g	g	g	g	g	g	g	g	g	g	g	g	g	g	g	g	g	g	g	g	g
a	4204965	g	4316371	g	4383595	i	a	O157	++++	a	a	a	a	a	a	a	a	a	a	a	a	a	a	a	a	a	a	a	a	a	a	a
t	4205620	t	4317026	g	4384250	ns	t	EDL933	+															t	t							
c	4209055	t	4320461	t	4387685	s	c	O157	++++	c	c	c	c	c	c	c	c	c	c	c	c	c	c	c	c	c	c	c	c	c	c	c
c	4209840	t	4321246	t	4388470	ns	c	O157	++++	c	c	c	c	c	c	c	c	c	c	c	c	c	c	c	c	c	c	c	c	c	c	c
c	4211248	a	4322654	a	4389878	ns	a	CB9615	++++	a	a	a	a	a	a	a	a	a	a	a	a	a	a	a	a	a	a	a	a	a	a	a
t	4213758	t	4325164	a	4392388	ns	t	EDL933	++++	t	t	t	t	t	t	t	t	t	t	t	t	t	t	t	t	t	t	t	t	t	t	t
t	4214641	c	4326047	c	4393271	ns	c	CB9615	++++	c	c	c	c	c	c	c	c	c	c	c	c	c	c	c	c	c	c	c	c	c	c	c
t	4214826	a	4326232	a	4393456	ns	a	CB9615	++++	a	a	a	a	a	a	a	a	a	a	a	a	a	a	a	a	a	a	a	a	a	a	a
t	4215147	g	4326553	g	4393777	ns	g	CB9615	++++	g	g	g	g	g	g	g	g	g	g	g	g	g	g	g	g	g	g	g	g	g	g	g
t	4215551	c	4326957	c	4394181	s	c	CB9615	++++	c	c	c	c	c	c	c	c	c	c	c	c	c	c	c	c	c	c	c	c	c	c	c
t	4215769	c	4327175	c	4394399	ns	c	CB9615	++++	c	c	c	c	c	c	c	c	c	c	c	c	c	c	c	c	c	c	c	c	c	c	c
t	4215904	c	4327310	c	4394534	ns	c	CB9615	++++	c	c	c	c	c	c	c	c	c	c	c	c	c	c	c	c	c	c	c	c	c	c	c
a	4218146	c	4329552	c	4396776	s	a	O157	++++	g	a	a	a	a	a	a	a	a	a	a	a	a	a	a	a	a	a	a	a	a	a	a
c	4218701	t	4330107	t	4397331	ns	t	CB9615	++++	t	t	t	t	t	t	t	t	t	t	t	t	t	t	t	t	t	t	t	t	t	t	t
c	4219108	t	4330514	t	4397738	ns	c	O157	++++	c	c	c	c	c	c	c	c	c	c	c	c	c	c	c	c	c	c	c	c	c	c	c
c	4219517	g	4330923	g	4398147	ns	c	O157	++++	c	c	c	c	c	c	c	c	c	c	c	c	c	c	c	c	c	c	c	c	c	c	c
a	4222049	-	4333454	a	4400679	del	a	Sakai	+					a						a												
-	4222120	c	4333526	-	4400750	ins	-	Sakai	+					-						-												
-	4222132	a	4333539	-	4400762	ins	-	Sakai	+					-						-												
a	4222144	t	4333553	a	4400774	s	a	Sakai	+					a						g												
t	4223068	g	4334477	g	4401698	ns	t	O157	+					t						t												
g	4224563	a	4335972	a	4403193	ns	g	O157	+					g						g												
t	4228026	c	4339435	c	4406656	ns	t	O157	+					t						t												
g	4233530	a	4344939	a	4412160	ns	g	O157	+					g						g												
g	4234056	a	4345465	a	4412686	s	g	O157	+					g						g												
a	4234616	g	4346025	g	4413246	s	g	CB9615	+					g						g												
g	4237528	a	4348937	a	4416158	s	g	O157	++	g	g	g	g	g	a	a	g	g	g	g	g	g	g	g	g	g	g	g	g	g	g	g
g	4237636	a	4349045	a	4416266	s	g	O157	++++	g	g	g	g	g	g	g	g	g	g	g	g	g	g	g	g	g	g	g	g	g	g	g
a	4237716	t	4349125	t	4416346	ns	t	CB9615	++++	t	t	t	t	t	t	t	t	t	t	t	t	t	t	t	t	t	t	t	t	t	t	t
g	4239205	a	4350614	a	4417835	ns	g	O157	++++	g	g	g	g	g	g	g	g	g	g	g	g	g	g	g	g	g	g	g	g	g	g	g
g	4241134	t	4352543	t	4419764	ns	g	O157	++++	g	g	g	g	g	g	g	g	g	g	g	g	g	g	g	g	g	g	g	g	g	g	g
t	4243229	c	4354638	c	4421859	s	t	O157	++++	t				t	t	t				t	t	t	t									
t	4243490	c	4354899	c	4422120	s	t	O157	++++	t				t	t	t				t	t	t	t									
a	4245017	g	4356426	g	4423647	s	g	CB9615	+++					g	g	g				g	g	g	g									
t	4245392	c	4356801	c	4424022	ns	c	CB9615	+++					c	c	c				c	c	c	c									
c	4246978	t	4358387	t	4425608	s	t	CB9615	+++					t	t	t				t	t	t	t									
-	4247128	-	4358537	t	4425759	ins	-	EDL933	+++					-	-	-				-	-	-	-									
a	4248515	g	4359924	g	4427146	ns	a	O157	++++						a	a	a							a	a	a	a	a	a	a	a	
a	4250830	g	4362239	g	4429461	ns	g	CB9615	++++		g	g	g	g	g	g	g	g	g	g	g	g	g	g	g	g	g	g	g	g	g	g
c	4254140	t	4365549	t	4432771	ns	c	O157	++++		c	c	c	c	c	c								c	c	c	t	c	c	c	c	c
g	4254236	a	4365645	a	4432867	ns	g	O157	+		g	a	a	g	g	g								g	g	g	g	g	g	a	a	a
g	4254899	c	4366308	c	4433530	ns	g	O157	++++		g		g	g	g	g								g	g	g	g	g	g	g	g	
t	4255271	c	4366680	c	4433902	ns	t	O157	++++		t		t	t	t	t								t	t	t	t	t	t	t	t	
a	4255512	g	4366921	g	4434143	s	g	CB9615	++++		g		g	g	g	g								g	g	g	g	g	g	g	g	
-	4257603	-	4369012	c	4436235	ins	-	EDL933	++++		-		-	-	-	-								-	-	-	-	-	-	-	-	
a	4257617	g	4369026	g	4436249	i	a	O157	++++		a		a	a	a	a								a	a	a	a	a	a	a	a	
g	4258828	a	4370237	a	4437460	s	g	O157	++++		g		g	g	g	g	t	t	t	t	t	t	t	g	g	g	g	g	g	g	g	g
a	4259408	g	4370817	g	4438040	ns	g	CB9615	++++	g	g	g	g	g	g	g	g	g	g	g	g	g	g	g	g	g	g	g	g	g	g	g
-	4260712	-	4372121	c	4439345	ins	-	EDL933	++++	-	-	-	-	-	-	-	-	-	-	-	-	-	-	-	-	-	-	-	-	-	-	-
-	4262952	-	4374361	c	4441586	ins	-	EDL933	++++	-	-	-	-	-	-	-	-	-	-	-	-	-	-	-	-	-	-	-	-	-	-	-
c	4263599	t	4375008	t	4442233	i	t	CB9615	++++	t	t	t	t	t	t	t	t	t	t	t	t	t	t	t	t	t	t	t	t	t	t	t
t	4263644	c	4375053	c	4442278	i	t	O157	++++	t	t	t	t	t	t	t	t	t	t	t	t	t	t	t	t	t	t	t	t	t	t	t
g	4263947	a	4375356	a	4442581	s	g	O157	++	g	g	g	g	g	g	g	g	g	g	g	g	g	g	g	g	g	a	a	a	g	g	g
g	4264194	a	4375603	a	4442828	ns	g	O157	++++	g	g	g	g	g	g	g	g	g	g	g	g	g	g	g	g	g	g	g	g	g	g	g
g	4267318	a	4378727	a	4445952	s	g	O157	++++	g	g	g	g	g	g	g	g	g	g	g	g	g	g	g	g	g	g	g	g	g	g	g
c	4270780	t	4382189	t	4449414	i	c	O157	+	a	c	c	c	a	t	t	a	a	a	a	a	a	a	t	t	t	a	a	a	a	c	c
g	4273777	a	4385186	a	4452411	ns	g	O157	++++		g	g	g				g	g	g	g	g	g	g									
g	4273827	a	4385236	a	4452461	i	g	O157	++++		g	g	g				g	g	g	g	g	g	g									
-	4274099	-	4385508	c	4452734	ins	-	EDL933	++++		-	-	-	-	-	-	-	-	-	-	-	-	-	-	-	-	-	-	-	-	-	-
a	4274138	g	4385547	g	4452773	i	g	CB9615	++++		g	g	g	g	g	g	g	g	g	g	g	g	g	g	g	g	g	g	g	g	g	g
a	4275291	-	4386699	-	4453925	del	a	O157	++++		a	-	a	a	a	a								a	a	a	a	a	a	a	a	a
-	4275575	-	4386983	B	4454210	ins	-	EDL933	++++	-	-	-	-	-	-	-	-	-	-	-	-	-	-	-	-	-	-	-	-	-	-	-
a	4277769	g	4389177	g	4456404	ns	g	CB9615	++++	g				g	g	g	g	g	g	g	g	g	g									
t	4279263	c	4390671	c	4457898	ns	t	O157	++++	t				t	t	t	t	t	t	t	t	t	t									
t	4280572	a	4391980	a	4459207	ns	t	O157	++++	t				t	t	t	t	t	t	t	t	t	t									
g	4284212	a	4395620	a	4462847	s	g	O157	++++	g				g	g	g	g	g	g	g	g	g	g									

O55 and O157 genome site details											Outgroup Strain Details[g]																						
CB9615 base[a]	CB9615 Site[b]	Sakai base[a]	Sakai Site[b]	EDL933 base[a]	EDL933 Site[b]	type[c]	Inferred ancestral base[d]	Lineage inferred to mutate[e]	Support level[f]	D1 Sd197	K-12	HS	ATCC 8739	UMN026	IAI39	SMS 3-5	E2348/69	536	ED1a	CFT073	S88	APEC O1	UTI89	E24377A	IAI1	SE11	F5 8401	F2a 2457T	F2a 301	SS Ss046	B4 Sb227	B18 BS512
t	4285615	a	4397023	a	4464250	s	t	O157	++++	t				t	t	t	t	t	t	t	t	t	t									
g	4285767	a	4397175	a	4464402	ns	g	O157	++++	g				g	g	g	g	g	g	g	g	g	g									
t	4286670	c	4398078	c	4465305	i	t	O157	++++	t	t	t	t	t	t	t	t	t	t	t	t	t	t	t	t	t	t	t	t	t	t	t
t	4286968	c	4398376	c	4465603	ns	c	CB9615	++++	c	c	c	c	c	c	c	c	c	c	c	c	c	c	c	c	c	c	c	c	c	c	c
c	4287017	t	4398425	t	4465652	ns	c	O157	++++	c	c	c	c	c	c	c	c	c	c	c	c	c	c	c	c	c	c	c	c	c	c	c
a	4287992	t	4399400	t	4466627	ns	t	CB9615	++++	t	t	t	t	t	t	t	t	t	t	t	t	t	t	t	t	t	t	t	t	t	t	t
a	4288987	g	4400395	g	4467622	i	g	CB9615	++++	g	g	g	g	g	g	g	g	g	g	g	g	g	g	g	g	g	g	g	g	g	g	g
c	4290943	a	4402351	a	4469578	ns	c	O157	++++	c	c	c	c	c	c	c	c	c	c	c	c	c	c	c	c	c	c	c	c	c	c	c
g	4295920	a	4407328	a	4474555	s	a	CB9615	++	a	a	a	a	g	c	c	c	c	c	c	c	c	c	g	g	g	g	g	g	g	g	g
c	4296001	t	4407409	t	4474636	s	c	O157	++++	c	c	c	c	c	c	c	c	c	c	c	c	c	c	c	c	c	c	c	c	c	c	c
a	4297448	c	4408856	c	4476083	s	a	O157	++++	a	a	a	a	a	a	a	a	a	a	a	a	a	a	a	a	a	a	a	a	a	a	a
c	4302261	t	4413669	t	4480896	ns	c	O157	++++		c	c	c	c	c	c	c	c	c	c	c	c	c	c	c	c	c	c	c	c	c	c
t	4302797	c	4414205	c	4481432	i	c	CB9615	++++		c	c	c	c	c	c	c	c	c	c	c	c	c	c	c	c	c	c	c	c	c	c
a	4305311	t	4416719	t	4483946	i	t	CB9615	++++	t	t	t	t	t	t	t	t	t	t	t	t	t	t	t	t	t	t	t	t	t	t	t
g	4306749	a	4418157	a	4485384	ns	g	O157	++++	g	g	g	g	g	g	g	g	g	g	g	g	g	g	g	g	g	g	g	g	g	g	g
a	4309952	g	4421360	g	4488587	i	g	CB9615	++++	g	g	g	g	g	g	g	g	g	g	g	g	g	g	g	g	g	g	g	g	g	g	g
a	4312940	g	4424348	g	4491575	ns	a	O157	++++	a	a	a	a	a	a	a	a	a	a	a	a	a	a	a	a	a	a	a	a	a	a	a
g	4314835	a	4426243	a	4493470	ns	g	O157	++++	g	g	g	g	g	g	g	g	g	g	g	g	g	g	g	g	g	g	g	g	g	g	g
c	4315262	t	4426670	t	4493897	ns	c	O157	++++	c	c	c	c	c	c	c	c	c	c	c	c	c	c	c	c	c	c	c	c	c	c	c
g	4317112	a	4428520	a	4495747	ns	g	O157	++++	g	g	g	g	g	g	g	g	g	g	g	g	g	g	g	g	g	g	g	g	g	g	g
t	4317343	c	4428751	c	4495978	ns	t	O157	++++	t	t		t	t	t	t	t	t	t	t	t	t	t	t	t	t	t	t	t	t	t	t
c	4318922	t	4430330	t	4497557	ns	t	CB9615	++++	t	t		t	t	t	t	t	t	t	t	t	t	t	t	t	t				t	t	t
g	4319374	t	4430782	t	4498009	ns	g	O157	++++	g	g		g	g	g	g	g	g	g	g	g	g	g	g	g	g				g	g	g
10	4319495	-	4430902	-	4498129	del	10	O157	++++	10	10		10	10	10	10	10	10	10	10	10	10	10	10	10	10				10	10	10
g	4320626	a	4432024	a	4499251	s	g	O157	++++	g	g		g	g	g	g	g	g	g	g	g	g	g	g	g	g	g	g	g	g	g	g
t	4320971	c	4432369	c	4499596	s	t	O157	++++	t	t		t	t	t	t	t	t	t	t	t	t	t	t	t	t	t	t	t	t	t	t
-	4321101	c	4432500	c	4499727	ins	-	O157	++++	-	-		-	-	-	-	-	-	-	-	-	-	-	-	-	-	-	-	-	-	-	-
a	4325410	c	4436826	c	4504053	ns	a	O157	++++	a	a		a	a	a	a	a	a	a	a	-	a	a	a	a	a	a	a	a	a	a	a
g	4326520	a	4437936	a	4505163	ns	g	O157	++++	g	g		g	g	g	g	g	g	g	g	g	g	g	g	g	g	g	g	g	g	g	g
a	4326690	c	4438106	c	4505333	ns	a	O157	++++	a	a		a	a	a	a	a	a	a	a	a	a	a	a	a	a	a	a	a	a	a	a
t	4326818	c	4438234	c	4505461	ns	t	O157	++++	t	t		t	t	t	t	t	t	t	t	t	t	t	t	t	t	t	t	t	t	t	t
c	4330658	t	4442074	t	4509301	i	c	O157	++++	c	c	c	c	c	c	c	c	c	c	c	c	c	c	c	c	c	c	c	c	c	c	c
g	4331830	a	4443246	a	4510473	ns	g	O157	++++	g	g	g	g	g	g	g	g	g	g	g	g	g	g	g	g	g	g	g	g	g	g	g
c	4333247	t	4444663	t	4511890	ns	c	O157	++++	c	c	c	c	c	c	c	c	c	c	c	c	c	c	c	c	c	c	c	c	c	c	c
g	4333407	g	4444823	a	4512050	s	g	EDL933	++++	g	g	g	g	g	g	g	g	g	g	g	g	g	g	g	g	g	g	g	g	g	g	g
t	4335496	a	4446912	a	4514139	i	t	O157	++++	t	t	t	t	t	t	t	t	t	t	t	t	t	t	t	t	t	t	t	t	t	t	t
c	4336419	a	4447835	a	4515062	s	c	O157	++++	c	c	c	c	c	c	c	c	c	c	c	c	c	c	c	c	c	c	c	c	c	c	c
c	4338087	t	4449503	t	4516730	s	c	O157	++++	c	c	c	c	c	c	c	c	c	c	c	c	c	c	c	c	c	c	c	c	c	c	c
t	4339003	c	4450419	t	4517646	i	t	Sakai	++	t	t	t	t	c	t	t	t	t	t	t	t	t	t	t	c	t	c	t	t	t	c	c
a	4339682	g	4451098	a	4518325	ns	a	Sakai	++++	a	a	a	a	a	a	a	a	a	a	a	a	a	a	a	a	a	a	a	a	a	a	a
t	4340244	c	4451660	c	4518887	s	c	CB9615	+		c	c	c	c	t	t	t	t	t	t	t	t	t	c	c	c	c	c	c	c	c	c
-	4340989	-	4452405	g	4519633	ins	-	EDL933	++++		-	-	-	-	-	-		-	-	-	-	-	-	-	-	-	-	-	-	-	-	-
-	4341093	-	4452509	c	4519738	ins	?	EDL933	+/-																							
g	4341161	t	4452577	t	4519806	s	?	O55/O157	+/-																							
t	4341929	c	4453345	c	4520574	ns	?	O55/O157	+/-																							
a	4342108	t	4453524	t	4520753	ns	?	O55/O157	+/-																							
a	4342307	t	4453723	t	4520952	ns	?	O55/O157	+/-																							
t	4342371	g	4453787	g	4521016	ns	?	O55/O157	+/-																							
c	4344022	t	4455438	t	4522667	ns	c	O157	+++	c							c		c						c							
g	4344214	a	4455630	a	4522859	ns	g	O157	+++	g							g		g						g							
t	4346215	a	4457631	a	4524860	ns	?	O55/O157	+/-								g															
a	4346771	-	4458186	-	4525415	ins	-	CB9615	+								-															
c	4346785	t	4458200	t	4525429	i	c	O157	+								c															
c	4346824	t	4458239	t	4525468	i	t	CB9615	+								t															
t	4346837	c	4458252	c	4525481	i	t	O157	+								t															
g	4346883	a	4458298	a	4525527	i	g	O157	+								g															
-	4346919	a	4458335	a	4525564	ins	-	O157	+								-															
g	4347060	t	4458476	t	4525705	ns	g	O157	++++	g	g	g	g	g	g	g	g	g	g	g	g	g	g	g	g	g	g	g	g	g	g	g
g	4352263	t	4463679	t	4530908	ns	t	CB9615	++++	t	t	t	t	t	t	t	t	t	t	t	t	t	t	t	t	t	t	t	t	t	t	t
t	4352387	g	4463803	g	4531032	s	g	CB9615	++++	g	g	g	g	g	g	g	g	g	g	g	g	g	g	g	g	g	g	g	g	g	g	g
a	4353761	c	4465177	c	4532406	s	a	O157	++++	a	a	a	a	a	a	a	a	a	a	a	a	a	a	a	a	a	a	a	a	a	a	a
t	4355806	c	4467222	c	4534451	s	c	CB9615	++++	c	c	c	c	c	c	c	c	c	c	c	c	c	c	c	c	c	c	c	c	c	c	c
c	4356714	t	4468130	t	4535359	ns	t	CB9615	++++	t	t	t	t	t	t	t	t	t	t	t	t	t	t	t	t	t	t	t	t	t	t	t
t	4361353	c	4472769	c	4539998	ns	t	O157	++++	t	t	t	t	t	t	t	t	t	t	t	t	t	t	t	t	t	t	t	t	t	t	
c	4366757	t	4478173	t	4545402	s	c	O157	++++		c	c	c	c	c	c	c	c	c	c	c	c	c	c	c	c	c	c	c		c	c
g	4369109	a	4480525	a	4547754	s	g	O157	++++		g	g	g	g	g	g	g	g	g	g	g	g	g	g	g	g	g	g	g		g	g
c	4369199	t	4480615	t	4547844	s	c	O157	++++		c	c	c	c	c	c	c	c	c	c	c	c	c	c	c	c	c	c	c		c	c
g	4371621	a	4483037	a	4550266	ns	g	O157	++++	g	g	g	g	g	g	g	g	g	g	g	g	g	g	g	g	g	g	g	g		g	g
a	4372327	g	4483743	g	4550972	s	a	O157	++++	a	a	a	a	a	a	a	a	a	a	a	a	a	a	a	a	a	a	a	a		a	a
a	4374461	g	4485877	g	4553106	ns	g	CB9615	++++	g	g	g	g	g	g	g	g	g	g	g	g	g	g	g	g	g	g	g	g		g	g
g	4375323	t	4486739	t	4553968	ns	t	CB9615	++++	t	t	t	t	t	t	t	t	t	t	t	t	t	t	t	t	t	t	t	t		t	t
t	4376835	g	4488251	g	4555480	s	g	CB9615	++	g				g	a	a											g					
g	4377230	a	4488646	a	4555875	ns	g	O157	+++					g	g	g											g					
t	4377842	c	4489258	c	4556487	s	c	CB9615	+++					c	c	c											c					
t	4377973	g	4489389	g	4556618	ns	t	O157	+++					t	t	t											t					
g	4380099	a	4491515	a	4558744	s	g	O157	+++					g	g	g											g					
a	4381450	g	4492866	g	4560095	s	g	CB9615	++++		g	g	g		g	g								g	g	g	g					
c	4382099	t	4493515	t	4560744	ns	c	O157	++++		c	c	c		c	c								c	c	c	c					
t	4386104	c	4497520	c	4564749	ns	t	O157	++++		t	t	t	t	t	t	t	t	t	t	t	t	t	t	t	t	t	t	t		t	t

O55 and O157 genome site details										Outgroup Strain Details[g]																						
CB9615 base[a]	CB9615 Site[b]	Sakai base[a]	Sakai Site[b]	EDL933 base[a]	EDL933 Site[b]	type[c]	Inferred ancestral base[d]	Lineage inferred to mutate[e]	Support level[f]	D1 Sd197	K-12	HS	ATCC 8739	UMN026	IAI39	SMS 3-5	E2348/69	536	ED1a	CFT073	S88	APEC O1	UTI89	E24377A	IAI1	SE11	F5 8401	F2a 2457T	F2a 301	SS Ss046	B4 Sb227	B18 BS512
g	4386633	a	4498049	a	4565278	ns	g	O157	++++						g	g	g		g	g	g	g	g									
c	4389241	a	4500657	c	4567886	i	c	Sakai	++++		c	c	c	c	c	c	c	c	c	c	c	c	c	c	c	c	c	c	c		c	c
t	4389248	-	4500663	-	4567892	del	t	O157	++++		t	t	t	t	t	t	-	t	t	t	t	t	t	t	t	t	t	t	t		t	t
a	4390049	g	4501464	g	4568693	s	g	CB9615	++++	g	g	g	g	g	g	g	g	g	g	g	g	g	g	g	g	g	g	g	g	g	g	g
t	4390216	g	4501631	g	4568860	ns	t	O157	++++	t	t	t	t	t	t	t	t	t	t	t	t	t	t	t	t	t	t	t	t	t	t	t
a	4391540	g	4502955	g	4570184	ns	g	CB9615	++++	g	g	g	g	g	g	g	g	g	g	g	g	g	g	g	g	g	g	g	g	g	g	g
c	4393602	t	4505017	c	4572246	ns	c	Sakai	++++	c	c	c	c											c	c	c				c	c	c
t	4394300	c	4505715	c	4572944	ns	c	CB9615	++++	c	c	c	c											c	c	c				c	c	c
g	4395507	a	4506922	a	4574151	ns	a	CB9615	++++	a	a	a	a											a	a	a				a	a	a
g	4397128	g	4508543	c	4575772	ns	g	EDL933	++++	g	g	g	g											g	g	g				g	g	g
c	4399880	t	4509941	t	4578923	i	c	O157	++++		c	c	c											c	c	c				c	c	c
a	4400096	g	4510157	g	4579139	i	g	CB9615	++++		g	g	g											g	g	g				g	g	g
g	4400405	a	4510466	a	4579448	s	g	O157	++++		g	g	g											g	g	g				g	g	g
-	4400559	a	4510621	a	4579603	ins	-	O157	+		a	-	-											-	-	-				-	-	-
a	4400664	g	4510726	g	4579708	ns	g	CB9615	++++		g	g	g											g	g	g				g	g	g
t	4404604	-	4514665	-	4583647	ins	-	CB9615	++++		-	-	-	-	-	-	-	-	-	-	-	-	-	-	-	-	-	-	-	-	-	-
c	4406735	a	4516791	a	4585773	s	a	CB9615	++++		a	a	a	a	a	a	a	a	a	a	a	a	a	a	a	a	a	a	a	a	a	a
g	4408195	a	4518251	a	4587233	ns	g	O157	++++			g	g											g	g	g	g	g	g	g	g	g
c	4413488	t	4523544	t	4592526	ns	c	O157	++++	c		c	c	c	c	c	c	c	c	c	c	c	c	c	c	c	c	c	c	c	c	c
g	4416342	a	4526398	a	4595380	ns	g	O157	++++	g	g	g	g	g	g	g	g	g	g	g	g	g	g	g	g	g	g	g	g	g	g	g
t	4424489	g	4534545	g	4603527	s	t	O157	++++	c	c	c	t	t	c	c	c	t	c	c	c	c	c	t	t	c	t	t	t	t	t	t
c	4424627	t	4534683	t	4603665	s	c	O157	++++	c	c	c	c	c	c	c	c	c	c	c	c	c	c	c	c	c	c	c	c	c	c	c
g	4427826	a	4537882	a	4606864	s	a	CB9615	++++	a	a	a	a	a	a	a	a	a	a	a	a	a	a	a	a	a	a	a	a	a	a	a
g	4427962	t	4538018	g	4607000	ns	g	Sakai	++++	g	g	g	g	g	g	g	g	g	g	g	g	g	g	g	g	g	g	g	g	g	g	g
-	4430484	-	4540540	g	4609523	ins	-	EDL933	++++		-			-					-							-	-	-	-			
g	4433064	t	4543120	t	4612103	s	g	O157	++++	g	g	g	g	g	g	g	g	g	g	g	g	g	g	g	g	g	g	g	g	g	g	g
a	4433629	g	4543685	g	4612668	ns	a	O157	++++	a	a	a	a	a	a	a	a	a	a	a	a	a	a	a	a	a	a	a	a	a	a	a
a	4434958	-	4545013	-	4613996	ins	-	CB9615	+++					a					-							-	-	-	-			
c	4437171	t	4547226	t	4616209	i	c	O157	+++					c					c							c	c	c	c			
t	4437398	c	4547453	c	4616436	s	t	O157	+++					t					t							t	t	t	t			
a	4437859	c	4547914	c	4616897	ns	a	O157	+++					a					a							a	a	a	a			
c	4446295	a	4556350	a	4625333	s	c	O157	++++	c	c	c	c	c	c	c	c	c	c	c	c	c	c	c	c	c	c	c	c	c	c	c
g	4448786	a	4558841	a	4627824	s	g	O157	++++	g	g	g	g	g	g	g	g	g	g	g	g	g	g	g	g	g	g	g	g	g	g	g
a	4451003	c	4561058	c	4630041	ns	c	CB9615	++++	c	c	c	c	c	c	c	c	c	c	c	c	c	c	c	c	c	c	c	c	c	c	c
c	4454041	t	4564096	t	4633079	ns	c	O157	++++	c	c	c	c	c	c	c	c	c	c	c	c	c	c	c	c	c	c	c	c	c	c	c
g	4459673	a	4569728	a	4638711	s	g	O157	++++	g	g	g	g	a	g	g	g	g	g	g	g	g	g	g	g	g	g	g	g	g	g	g
g	4460657	a	4570712	a	4639695	s	g	O157	++++	g	g	g	g	g	g	g	g	g	g	g	g	g	g	g	g	g	g	g	g	g	g	g
g	4463106	a	4573161	a	4642144	s	g	O157	++++	g	g	g	g	g	g	g	g	g	g	g	g	g	g	g	g	g	g	g	g	g	g	g
t	4465106	a	4575161	a	4644144	ns	a	CB9615	++++	a	a	a	a	a	a	a	a	a	a	a	a	a	a	a	a	a	a	a	a	t	a	a
g	4466742	g	4576797	a	4645780	ns	g	EDL933	++++	g	g	g	g	g	g	g	g	g	g	g	g	g	g	g	g	g	g	g	g	g	g	g
g	4467471	a	4577526	a	4646509	ns	g	O157	++++	g	g	g	g	g	g	g	g	g	g	g	g	g	g	g	g	g	g	g	g	g	g	g
g	4468304	a	4578359	a	4647342	s	g	O157	++++	g	g	g	g	g	g	g	g	g	g	g	g	g	g	g	g	g	g	g	g	g	g	g
c	4468766	t	4578821	t	4647804	s	c	O157	++++	c	c	c	c	c	c	c	c	c	c	c	c	c	c	c	c	c	c	c	c	c	c	c
g	4469664	t	4579719	t	4648702	ns	t	CB9615	++++	t	t	t	t	t	t	t	t	t	t	t	t	t	t	t	t	t	t	t	t	t	t	t
g	4470582	a	4580637	a	4649620	i	g	O157	++	g	g	g	g	g	g	g	a	a	a	a	a	a	a	g	g	g	g	g	g	g	g	g
-	4472589	a	4582645	a	4651628	ins	-	O157	+++					-	-	-	-	-								-						
t	4475455	g	4585513	g	4654496	nc	g	CB9615	++++	g				g	g	g	g	g	g	g	g	g	g	g				g	g			
-	4475929	c	4588247	t	4657230	ns	t	Sakai	++++	t				t			t	t	t	t	c		t	t				t	t	t	t	t
g	4476490	a	4588998	a	4657981	i	g	O157	+								g															
g	4476607	a	4589115	a	4658098	i	g	O157	+								g															
c	4476633	t	4589141	t	4658124	i	t	CB9615	+								t															
t	4476805	g	4589313	g	4658296	ns	g	CB9615	+								g															
-	4476813	g	4589322	g	4658305	indel	?	O55/O157	+/-																							
c	4476857	t	4589347	t	4658330	s	t	CB9615	+								t															
g	4478763	t	4591253	t	4660236	s	t	CB9615	+								t															
g	4479788	a	4592278	a	4661261	s	a	CB9615	+								a															
t	4480858	g	4593348	g	4662331	ns	?	O55/O157	+/-								c															
t	4484015	c	4596505	c	4665488	ns	?	O55/O157	+/-																							
t	4484066	g	4596556	g	4665539	ns	?	O55/O157	+/-																							
-	4487906	t	4600397	t	4669380	indel	?	O55/O157	+/-																							
c	4489376	t	4601872	t	4670855	i	?	O55/O157	+/-																							
a	4491979	t	4604475	t	4673458	ns	a	O157	+								a															
c	4495788	t	4608284	t	4677267	s	c	O157	+								c															
g	4496576	a	4609072	a	4678055	s	a	CB9615	+								a															
a	4498266	c	4610762	c	4679745	s	a	O157	+								a															
a	4498936	t	4611432	t	4680415	s	t	CB9615	+								t															
c	4499965	t	4612461	t	4681444	s	t	CB9615	+								t															
t	4500082	c	4612578	c	4681561	s	t	O157	+								t															
t	4502326	c	4614822	c	4683805	s	t	O157	+								t															
t	4502473	c	4614969	c	4683952	s	c	CB9615	+								c															
a	4504190	g	4616686	g	4685669	s	a	O157	+								a															
a	4505404	c	4617900	a	4686883	ns	a	Sakai	+								a															
t	4506287	a	4618783	a	4687766	s	a	CB9615	+								a															
t	4507140	c	4619636	t	4688619	ns	t	Sakai	+								t															
c	4508319	t	4620815	c	4689798	i	c	Sakai	+								c															
-	4508362	11	4620859	11	4689842	del	11	CB9615	+								11															
a	4510254	g	4622761	g	4691744	s	a	O157	+								a															
t	4510269	c	4622776	c	4691759	s	t	O157	+								t															
t	4511885	g	4624392	g	4693375	ns	g	CB9615	++++	g	g	g	g	g	g	g	g							g	g	g					g	g

O55 and O157 genome site details										Outgroup Strain Details[g]																						
CB9615 base[a]	CB9615 Site[b]	Sakai base[a]	Sakai Site[b]	EDL933 base[a]	EDL933 Site[b]	type[c]	Inferred ancestral base[d]	Lineage inferred to mutate[e]	Support level[f]	D1 Sd197	K-12	HS	ATCC 8739	UMN026	IAI39	SMS 3-5	E2348/69	536	ED1a	CFT073	S88	APEC O1	UTI89	E24377A	IAI1	SE11	F5 8401	F2a 2457T	F2a 301	SS Ss046	B4 Sb227	B18 BS512
a	4512866	g	4625373	g	4694356	s	g	CB9615	++++	g	g	g	g	g	g	g	g	g	g	g	g	g	g	g	g	g					g	g
g	4513199	c	4625706	c	4694689	ns	g	O157	++++	g	g	g	g	g	g	g	g	g	g	g	g	g	g	g	g	g				g	g	g
g	4513884	a	4626391	a	4695374	i	g	O157	++++	g	g	g	g	g	g	g	a	g	g	g	g	g	g	g	g	g	g	g	g	g	g	g
c	4514883	g	4627390	g	4696373	s	g	CB9615	+++	a	a	a	a	a	a	a	g	g	g	g	g	g	g	a	a	a	a	a	a	a	a	a
g	4515703	a	4628210	a	4697193	i	g	O157	+++	g				g	g	g																
t	4522953	c	4635460	c	4704443	s	c	CB9615	++++	c	c	c	c	c	c	c	c	c	c	c	c	c	c	c	c	c	c	c	c	c	c	c
t	4523443	a	4635950	a	4704933	ns	t	O157	++++	t	t	t	t	t	t	t	t	t	t	t	t	t	t	t	t	t	t	t	t	t	t	t
c	4523637	t	4636144	t	4705127	s	c	O157	++++	c	c	c	c	c	c	c	c	c	c	c	c	c	c	c	c	c	c	c	t	c	c	c
g	4524282	a	4636789	a	4705772	s	g	O157	++++	g	g	g	g	g	g	g	g	g	g	g	g	g	g	g	g	g	g	g	g	g	g	g
a	4524941	t	4637448	t	4706431	s	?	O55/O157	+/-	c	c	c	c	c	c	c	c	c	c	c	c	c	c	c	c	c	c	c	c	c	c	c
c	4526593	a	4639100	a	4708083	ns	c	O157	+					t	c	c																
c	4526633	t	4639140	t	4708123	ns	c	O157	+					c	c	c																
c	4526999	-	4639505	-	4708488	ins	-	CB9615	+					-	-	-																
t	4527252	c	4639757	c	4708740	ns	c	CB9615	+					c	c	c																
c	4527590	t	4640095	t	4709078	ns	c	O157	+					c	c	c																
t	4528242	c	4640747	c	4709730	nc	t	O157	+++	t				t	t	t																
g	4528597	a	4641102	a	4710085	i	g	O157	+++	g				g	g	g																
a	4528714	c	4641219	c	4710202	i	a	O157	+++	a				a	a	a																
t	4530955	c	4643460	c	4712443	ns	t	O157	++++	t	t	t	t	t	t	t	t	t	t	t	t	t	t	t	t	t	t	t		t	t	t
g	4531815	a	4644320	a	4713303	i	g	O157	++++	g	g	g	g	g	g	g	g	g	g	g	g	g	g	g	g	g	g	g		g	g	g
t	4534717	g	4647222	g	4716205	ns	t	O157	++++	t	t			t										t	t	t	t	t	t	t		
a	4537731	c	4650236	c	4719219	ns	a	O157	++++	a	a			a	a	a	a	a	a	a	a	a	a	a	a	a	a	a	a			
a	4538937	g	4651442	g	4720425	s	g	CB9615	++++	g	g			g	g	g	g	g	g	g	g	g	g	g	g	g	g	g	g			
a	4539572	t	4652077	a	4721060	ns	a	Sakai	++++	a		a	a	a	a	a														a	a	a
a	4539573	t	4652078	a	4721061	s	a	Sakai	++++	a		a	a	a	a	a														a	a	a
g	4541258	t	4653763	t	4722746	s	g	O157	++++	g	g	g	g	g	g	g								g	g	g	a	g	g	g	g	g
t	4541755	c	4654260	c	4723243	ns	c	CB9615	++++	c	c	c	c	c	c	c								c	c	c	c	c	c	c	c	c
-	4542302	a	4654808	a	4723791	del	a	CB9615	++	a	a	a	a	a	a	a								a	a	a	-	-	-	a	-	-
g	4542466	a	4654972	a	4723955	s	g	O157	++++	g	g	g	g	g	a	g								g	g	g	g	g	g	g	g	g
a	4547678	g	4660184	g	4729167	ns	a	O157	++++	a	a	a	a	a	a	a	a	a	a	a	a	a	a	a	a	a	a	a	a	a		
a	4549647	g	4662153	g	4731136	ns	g	CB9615	++++	g	g	g	g	g	g	g	g	g	g	g	g	g	g	g	g	g	g	g	g	g		
g	4551798	a	4664304	a	4733287	s	g	O157	++	g	g	g	g	g	a	g	g	a	a	a	a	a	a	g	g	g	g	g	g	g	a	a
g	4558502	a	4671008	a	4739991	ns	g	O157	++++	g	g	g	g	g	g	g	g	g	g	g	g	g	g	g	g	g	g	g	g	g	g	g
t	4561931	t	4674437	-	4743419	del	t	EDL933	++	t																						
t	4561949	t	4674455	-	4743436	del	-	EDL933	++	-																						
t	4561954	t	4674460	c	4743441	i	t	EDL933	++	t																						
at	4562297	-	4674802	-	4743783	del	at	O157	++	at																						
c	4562581	t	4675085	t	4744066	ns	c	O157	++	c																						
t	4563020	a	4675524	a	4744505	ns	?	O55/O157	+/-																							
g	4564220	a	4676724	a	4745705	s	?	O55/O157	+/-																							
g	4564396	a	4676900	a	4745881	s	?	O55/O157	+/-																							
g	4564463	t	4676967	t	4745948	i	?	O55/O157	+/-																							
a	4566958	t	4679462	t	4748443	ns	a	O157	++++	a	a	a	a	a	a	a	a	a	a	a	a	a	a	a	a	a	a	a	a		a	a
g	4568060	a	4680564	a	4749545	ns	g	O157	++++	g	g	g	g	g	g	g	g	g	g	g	g	g	g	g	g	g	g	g	g	g	g	g
g	4570937	a	4683441	a	4752422	s	g	O157	++++	g	g	g	g	g	g	g	g	g	g	g	g	g	g	g	g	g	g	g	g	g	g	g
c	4574198	t	4686702	t	4755683	ns	c	O157	+					t	c	c																
c	4575099	a	4687603	a	4756584	s	c	O157	+					a	c	c																
g	4576599	a	4689103	a	4758084	ns	g	O157	+					g	g	g																
a	4577999	g	4690503	a	4759484	i	a	Sakai	+++	a				a	a	a																
t	4587361	c	4699863	c	4768844	ns	?	O55/O157	+/-																							
a	4588117	g	4700598	g	4769579	s	?	O55/O157	+/-																							
a	4588686	c	4701167	c	4770148	nc	?	O55/O157	+/-																							
g	4589045	a	4701526	a	4770507	nc	?	O55/O157	+/-																							
g	4589315	a	4701796	a	4770777	nc	g	O157	++++					g	g	g								g	g	g	g	g	g			g
a	4591239	a	4703720	g	4772701	s	?	EDL933	+/-																							
-	4591415	a	4703897	a	4772878	indel	?	O55/O157	+/-																							
g	4591950	a	4704432	a	4773413	s	?	O55/O157	+/-																							
g	4592933	t	4705415	g	4774396	ns	g	Sakai	++++	g	g	g	g	g	g	g	g	g	g	g	g	g	g	g	g	g	g	g	g	g	g	g
g	4596714	a	4709196	a	4778177	ns	g	O157	++++	g	g	g	g	g	g	g	g	g	g	g	g	g	g	g	g	g	g	g	g	g	g	g
g	4597735	a	4710217	a	4779198	s	g	O157	++++	g	g	g	g	g	g	g	g	g	g	g	g	g	g	g	g	g	g	g	g	g	g	g
t	4599020	c	4711502	c	4780483	ns	c	CB9615	++++	c	c	c	c	c	c	c	c	c	c	c	c	c	c	c	c	c	c	c	c	c	c	c
a	4599056	g	4711538	g	4780519	ns	g	CB9615	++++	g	g	g	g	g	g	g	g	g	g	g	g	g	g	g	g	g	g	g	g	g	g	g
g	4601089	a	4713571	a	4782552	s	g	O157	++++	g	g	g	g	g	g	g	g	g	g	g	g	g	g	g	g	g	g	g	g	g	g	g
a	4602241	c	4714723	a	4783704	ns	a	Sakai	++++	a	a	a	a	a	a	a	a	a	a	a	a	a	a	a	a	a	a	a	a	a	a	a
g	4604524	a	4717006	a	4785987	ns	a	CB9615	++++	a	a	a	a	a	a	a	a	a	a	a	a	a	a	a	a	a	a	a	a	a	a	a
t	4604682	g	4717164	g	4786145	i	t	O157	++++	t	t	t	t	t	t	t	t	t	t	t	t	t	t	t	t	t	t	t	t	t	t	t
g	4606737	t	4719219	t	4788200	i	g	O157	++++	g	g	g	g	g	g	g	g	g	g	g	g	g	g	g	g	g	g	g	g	g	g	g
c	4610143	t	4722625	t	4791606	s	c	O157	++++	c	c	c	c	c	c	c	c	c	c	c	c	c	c	c	c	c	c	c	c	c	c	c
c	4611408	t	4723890	t	4792871	s	c	O157	++++	c	c	c	c	c	c	c	c	c	c	c	c	c	c	c	c	c	c	c	c	c	c	c
a	4611994	c	4724476	c	4793457	ns	c	CB9615	++++	c	c	c	c	c	c	c	c	c	c	c	c	c	c	c	c	c	c	c	c	c	c	c
g	4612142	a	4724624	a	4793605	i	g	O157	++++	g	g	g	g	g	-	g	g	g	g	g	g	g	g	g	g	g	g	-	-	-	-	-
g	4612146	t	4724628	t	4793609	i	g	O157	++	g	t	t	t	t	t	t	t	t	t	t	t	t	t	t	t	t	c	t	t	t	t	t
t	4615359	g	4727841	g	4796822	ns	t	O157	++++	t	t	t	t	t	t	t	t	t	t	t	t	t	t	t	t	t	t	t	t	t	t	t
c	4616599	t	4729081	t	4798062	ns	t	CB9615	++++	t	t	t	t	t	t	t	t	t	t	t	t	t	t	t	t	t	t	t	t	t	t	t
g	4617554	a	4730036	a	4799017	s	g	O157	++++	g	g	g	g	g	g	g	g	g	g	g	g	g	g	g	g	g	g	g	g	g	g	g
g	4618739	a	4731221	a	4800202	ns	a	CB9615	++++	a	a	a	a	a	a	a	a	a	a	a	a	a	a	a	a	a	a	a	a	a	a	a
a	4621645	g	4734127	g	4803108	s	g	CB9615	++++	g	g	g	g	g	g	g	g	g	g	g	g	g	g	g	g	g	g	g	g	g	g	g
g	4623675	g	4736154	-	4805134	del	g	EDL933	++++	g	g	g	g	g	g	g	g	g	g	g	g	g	g	g	g	g	g	g	g	g	g	g
g	4624194	g	4736673	a	4805653	nc	g	EDL933	++++	g	g	g	g	g	g	g	g	g	g	g	g	g	g	g	g	g	g	g	g	g	g	g

O55 and O157 genome site details										Outgroup Strain Details[g]																						
CB9615 base[a]	CB9615 Site[b]	Sakai base[a]	Sakai Site[b]	EDL933 base[a]	EDL933 Site[b]	type[c]	Inferred ancestral base[d]	Lineage inferred to mutate[e]	Support level[f]	D1 Sd197	K-12	HS	ATCC 8739	UMN026	IAI39	SMS 3-5	E2348/69	536	ED1a	CFT073	S88	APEC O1	UTI89	E24377A	IAI1	SE11	F5 8401	F2a 2457T	F2a 301	SS Ss046	B4 Sb227	B18 BS512
t	4624329	a	4736808	a	4805788	i	t	O157	++	t	t	t	t	t	a	a	a	t	a	t	a	a	a	t	t	t	t	t	t	a	t	a
a	4624598	c	4737077	a	4806057	i	a	Sakai	++++	a	a	a	a	a	a	a	a	a	a	a	a	a	a	a	a	a	a	a	a	a	a	a
g	4624599	a	4737078	R	4806058	i	a	CB9615	++	a	a	g	a	a	a	a	g	a	g	a	a	a	g	a	a	g	a	a	a	a	a	a
g	4624599	a	4737078	R	4806058	i	a	EDL933	++	a	a	g	a	a	a	a	g	a	g	a	a	a	g	a	a	g	a	a	a	a	a	a
a	4624805	a	4737284	t	4806264	nc	t	EDL933	-	t	t	t	a	a	a	a	t	a	t	t	t	t	t	a	a	t	a	a	a	t	a	a
t	4624810	t	4737289	a	4806269	nc	a	EDL933	-	a	a	a	t	t	t	t	a	a	a	a	a	a	a	t	t	a	t	t	t	a	t	t
a	4625024	g	4737503	g	4806483	nc	a	O157	++	a	a	g	a	g	a	a	a	g	a	a	a	a	a	g	a	g	a	a	a	a	a	a
-	4625208	a	4737688	a	4806668	indel	?	O55/O157	+/-																							
c	4625209	-	4737701	-	4806681	indel	?	O55/O157	+/-																							
c	4626386	-	4738866	-	4807846	ins	-	CB9615	++++	-	-	-	-	-	-	-	-	-	-	-	-	-	-	-	-	-	-	-	-	-	-	-
g	4627677	t	4740157	t	4809137	nc	g	O157	++	g	g	g	g	t	t	t	t	t	t	t	t	t	t	t	g	t	g	g	g	g	g	g
g	4627883	-	4740362	g	4809343	del	g	Sakai	++++	g	g	g	g	g	g	g	g	g	g	g	g	g	g	g	g	g	g	g	g	g	g	g
t	4629998	c	4742477	c	4811458	ns	c	CB9615	++++	c	c	c	c	c	c	c	c	c	c	c	c	c	c	c	c	c	c	c	c		c	c
a	4630107	c	4742586	c	4811567	s	a	O157	++++	a	a	a	a	a	a	a	a	a	a	a	a	a	a	a	a	a	a	a	a	a	a	a
-	4634401	-	4746880	t	4815862	ins	-	EDL933	++++	-	-	-	-	-	-	-	-	-	-	-	-	-	-	-	-	-	-	-	-	-	-	-
t	4637035	c	4749514	c	4818496	s	c	CB9615	++++	c	c	c	c	c	c	c	c	c	c	c	c	c	c	c	c	c	c	c	c	c	c	c
-	4637215	-	4749694	t	4818677	ins	-	EDL933	++++	-	-	-	-	-	-	-	-	-	-	-	-	-	-	-	-	-	-	-	-	-	-	-
-	4637222	-	4749701	t	4818685	ins	-	EDL933	++++	-	-	-	-	-	-	-	-	-	-	-	-	-	-	-	-	-	-	-	-	-	-	-
-	4637225	-	4749704	g	4818689	ins	-	EDL933	++++	-	-	-	-	-	-	-	-	-	-	-	-	-	-	-	-	-	-	-	-	-	-	-
t	4638572	c	4751051	c	4820036	s	c	CB9615	++++	c	c	c	c	c	c	c	c	c	c	c	c	c	c	c	c	c	c	c	c	c	c	c
c	4638888	t	4751367	t	4820352	i	c	O157	++++	c	c	c	c	c	c	c	c	c	c	c	c	c	c	c	c	c	c	c	c	c	c	c
a	4640791	c	4753270	c	4822255	i	a	O157	++++	a	a	a	a											a	a	a				a	a	a
a	4641660	g	4754139	g	4823124	s	g	CB9615	++++	g	g	g	g	a	g	g	g	g	g	g	g	g	g	g	g	g	g	g	g	g	g	g
t	4644979	c	4757458	c	4826443	ns	c	CB9615	++++	c	c	c	c	c	c	c	c	c	c	c	c	c	c	c	c	c	c	c	c	c	c	c
c	4645142	t	4757621	t	4826606	s	c	O157	++++	c	c	c	c	c	c	c	c	c	c	c	c	c	c	c	c	c	c	c	c	c	c	c
c	4645500	t	4757979	t	4826964	ns	c	O157	++++	c	c	c	c	c	c	c	c	c	c	c	c	c	c	c	c	c	c	c	c	c	c	c
t	4652627	c	4765106	c	4834091	s	c	CB9615	++++	c	c	c	c	c	c	c	c	c	c	c	c	c	c	c	c	c	c	c	c	c	c	c
t	4653801	c	4766280	c	4835265	ns	c	CB9615	++	c	c	c	c	c	c	c	t	t	c	c	c	c	c	c	c	c	c	c	c	c	c	c
t	4654460	a	4766939	a	4835924	ns	t	O157	++++	t	t	t	t	t	t	t	t	t	t	t	t	t	t	t	t	t	t	t	t	t	t	t
a	4656974	g	4769453	g	4838438	ns	a	O157	++++	a	a	a	a	a	a	a	a	a	a	c	a	a	a	a	a	a	a	a	a	a	a	a
c	4657523	t	4770002	t	4838987	s	c	O157	++++	c	c	c	c	c	c	c	c	c	c	c	c	c	c	c	c	c	c	c	c	c	c	c
g	4657874	a	4770353	a	4839338	ns	g	O157	++++	g	g	g	g	g	g	g	g	g	g	g	g	g	g	g	g	g	g	g	g	g	g	g
a	4659121	c	4771600	c	4840585	ns	a	O157	++++	a	a	a	a	a	a	a	a	a	a	a	a	a	a	a	a	a	a	a	a	a	a	a
a	4659245	c	4771724	c	4840709	ns	a	O157	++++	a	a	a	a	a	a	a	a	a	a	a	a	a	a	a	a	a	a	a	a	a	a	a
t	4661744	c	4774223	c	4843208	i	c	CB9615	++	c	t	c	c	c	c	c	c	c	c	c	c	c	c	c	c	c	c	c	c	c	c	c
t	4663264	g	4775743	g	4844728	i	t	O157	++++	t	t	t	t	t	t	t	t	t	t	t	t	t	t	t	t	t	t	t	t	t	t	g
-	4665162	-	4777866	t	4846627	ins	-	EDL933	++	-	-	-	-	-	-	-	t	t	t	-	-	-	-	-	-	-	-	-	-	-	-	-
c	4665815	a	4778519	a	4847280	ns	a	CB9615	++++	a	a			a	a	a	a	a	a	a	a	a	a									
g	4666276	a	4778980	a	4847741	ns	a	CB9615	++++	a	a			a	a	a	a	a	a	a	a	a	a									
t	4666733	g	4779437	g	4848198	ns	t	O157	++++	t	t			t	t	t	t	t	t	t	t	t	t									
-	4667121	-	4779825	Y	4848587	ins	-	EDL933	++++	-	-	-	-	-	-	-	-	-	-	-	-	-	-	-	-	-	-	-	-	-	-	-
-	4668875	18	4781580	18	4850342	ins	-	O157	++	-	-	-	-	18	-	-	18	18	18	18	-	-	18	-	18	18	18	18	18	18	18	18
g	4671430	t	4784152	t	4852914	ns	t	CB9615	++++	t	t	t	t	t	t	t	t	t	t	t	t	t	t	t	t	t	t	t	t	t	t	t
-	4672082	-	4784804	a	4853567	ins	-	EDL933	++++	-	-	-	-	-	-	-	-	-	-	-	-	-	-	-	-	-	-	-	-	-	-	-
a	4674734	g	4787456	g	4856219	ns	a	O157	++++	a	a	a	a	a	a	a	a	a	a	a	a	a	a	a	a	a	a	a	a	a	a	a
c	4684260	t	4796982	t	4865745	ns	c	O157	++++						c	c	c	c	c	c	c	c	c	c	c	c				c		c
t	4685240	c	4797962	c	4866725	ns	c	CB9615	+++						c	g	g	g	g	g	g	g	g	c	c	c				c		
-	4686576	-	4799298	c	4868062	ins	-	EDL933	+++															-	-	-				-		
t	4686774	g	4799496	g	4868260	ns	t	O157	+++															t	t	t				t		
c	4688329	a	4801051	a	4869815	i	c	O157	++++	c	c	c	c	c	c	c	c	c	c	c	c	c	c	c	c	c	c	c	c	c	c	c
t	4692255	c	4804977	c	4873741	s	c	CB9615	++++	c	c	c	c	c	c	c	c	c	c	c	c	c	c	t	c	c	c	c	c	c	c	c
c	4692955	t	4805677	t	4874441	s	c	O157	++++	c	c	c	c	c	c	c	c	c	c	c	c	c	c	c	c	c	c	c	c	c	c	c
t	4693751	c	4806473	c	4875237	ns	c	CB9615	++++	c	c	c	c	c	c	c	c	c	c	c	c	c	c	c	c	c	c	c	c	c	c	c
a	4693866	c	4806588	c	4875352	ns	a	O157	++++	a	a	a	a	a	a	a	a	a	a	a	a	a	a	a	a	a	a	a	a	a	a	a
t	4696138	c	4808860	c	4877624	s	c	CB9615	++++	c	c	c	c	c	c	c	c	c	c	c	c	c	c	c	c	c	c	c	c	c	c	c
c	4697242	t	4809964	t	4878728	s	c	O157	++++	c	c	c	c	c	c	c	c	c	c	c	c	c	c	c	c	c	c	c	c	c	c	c
c	4697717	t	4810439	t	4879203	ns	c	O157	++++	c	c	c	c	c	c	c	c	c	c	c	c	c	c	c	c	c	c	c	c	c	c	c
c	4698266	a	4810988	a	4879752	ns	c	O157	++++	c	c	c	c	c	c	c	c	c	c	c	c	c	c	c	c	c	c	c	c	c	c	c
c	4706179	t	4818901	t	4887665	s	c	O157	++++	c	c	c	c	c	c	c	c	c	c	c	c	c	c	c	c	c	c	c	c	c	c	c
a	4712003	c	4824725	c	4893489	s	a	O157	++++	a	a	a	a	g	g	g	a	a	a	a	a	a	a	a	a	a	a	a	a	a	a	a
t	4714416	c	4827138	c	4895902	i	c	CB9615	++++	c	c	c	c	c	c	c	c	c	c	c	c	c	c	c	c	c	c	c	c	c	c	c
c	4715267	t	4827989	t	4896753	ns	c	O157	++++	c	c	c	c	c	c	c	c	c	c	c	c	c	c	c	c	c	c	c	c	c	c	c
c	4715910	t	4828632	t	4897396	s	t	CB9615	++++	t	t	t	t	t	t	t	t	t	t	t	t	t	t	t	t	t	t	t	t	t	t	t
c	4717134	c	4829856	t	4898620	s	c	EDL933	++++	c	c	c	c	c	c	c	c	c	c	c	c	c	c	c	c	c	c	c	c	c	c	c
t	4718599	c	4831321	c	4900085	i	t	O157	++++	t	t	t	t	t	t	t	t	t	t	t	t	t	t	t	t	t	t	t	t	t	t	t
g	4719073	g	4831798	a	4900562	nc	g	EDL933	++++	g	g	g	g	g	g	g	g	g	g	g	g	g	g	g	g	g	g	g	g	g	g	g
t	4719331	c	4832056	c	4900820	nc	c	CB9615	++++	c	c	c	c	c	c	c	c	c	c	c	c	c	c	c	c	c	c	c	c	c	c	c
g	4721275	g	4834000	a	4902764	nc	g	EDL933	++++	g	g	g	g	g	g	g	g	g	g	g	g	g	g	g	g	g	g	g	g	g	g	g
-	4721456	a	4834182	a	4902946	indel	?	O55/O157	+/-																							
c	4721457	-	4834195	-	4902959	indel	?	O55/O157	+/-																							
t	4724037	a	4836764	a	4905528	i	a	CB9615	++	a	a	a	a	t	t	t	t	t	t	t	t	t	t	t	a	t	t	t	t	t	t	t
-	4724086	a	4836814	a	4905578	del	a	CB9615	++	a	a	a	a	a	a	a	a	a	a	a	a	a	a	a	a	a	-	-	-	a	a	a
c	4724202	t	4836930	t	4905694	s	c	O157	++	c	t	t	t	c	c	c	c	c	c	c	c	c	c	t	t	t	t	t	t	t	t	t
g	4724815	t	4837543	t	4906307	ns	g	O157	++++	g	g	g	g	g	g	g	g	g	g	g	g	g	g	g	g	g	g	g	g	g	g	g
t	4731284	a	4844012	a	4912776	s	t	O157	++++	t	t	t	t	t	t	t	t	t	t	t	t	t	t	t	t	t	t	t	t	t	t	t
a	4732626	g	4845354	g	4914118	ns	g	CB9615	++++	g	g	g	g	g	g	g	g	g	g	g	g	g	g	g	g	g	g	g	g	g	g	g
g	4734874	a	4847602	a	4916366	s	g	O157	++++	g	g	g	g	g	g	g	g	g	g	g	g	g	g	g	g	g	g	g	g	g	g	g
a	4736541	g	4849269	g	4918033	s	g	CB9615	++++	g	g	g	g	g	g	g	g	g	g	g	g	g	g	g	g	g	g	g	g	g	g	g
c	4738369	t	4851097	t	4919861	s	c	O157	++++	c	c	c	c	c	c	c	c	c	c	c	c	c	c	c	c	c	c	c	c	c	c	c

O55 and O157 genome site details										Outgroup Strain Details[g]																						
CB9615 base[a]	CB9615 Site[b]	Sakai base[a]	Sakai Site[b]	EDL933 base[a]	EDL933 Site[b]	type[c]	Inferred ancestral base[d]	Lineage inferred to mutate[e]	Support level[f]	D1 Sd197	K-12	HS	ATCC 8739	UMN026	IAI39	SMS 3-5	E2348/69	536	ED1a	CFT073	S88	APEC O1	UTI89	E24377A	IAI1	SE11	F5 8401	F2a 2457T	F2a 301	SS Ss046	B4 Sb227	B18 BS512
c	4742230	t	4854958	t	4923722	s	c	O157	++++	c	c	c	c	c	c	c	c	c	c	c	c	c	c	c	c	c	c	c	c	c	c	c
t	4744961	c	4857689	c	4926453	s	t	O157	+		t	c	c	t	t	t		t		t				t	t	t	c	c	c			
a	4746293	g	4859021	g	4927785	ns	g	CB9615	++++		g	g	g	g	g	g		g		g				g	g	g	g	g	g			
t	4746715	c	4859443	c	4928207	ns	c	CB9615	++++		c			c	c	c			c					c	c	c						
-	4748877	3	4861606	3	4930370	del	3	CB9615	++++		3			-	3	3			3					3	3	3	3	3	3			
t	4750109	a	4862840	a	4931604	s	t	O157	+		t			a	a	a			a					t	t	t	t	t	t			
g	4751762	a	4864493	a	4933257	s	g	O157	++++		g			g	g	g			g					g	g	g	g	g	g			
a	4753302	g	4866033	g	4934797	ns	g	CB9615	+										g													
-	4757462	t	4870194	t	4938958	del	t	CB9615	++++		t			t	t	t			t					t	t	t	t	t	t			
c	4757513	a	4870245	a	4939009	i	c	O157	++++		c			c	c	c			c					c	c	c	c	c	c			
c	4758335	t	4871067	t	4939831	ns	c	O157	++++		c			c	c	c			c					c	c	c	c	c	c			
c	4763580	a	4876312	a	4945076	ns	c	O157	+++					c	c	c											c	c	c			
a	4764623	g	4877355	g	4946119	i	a	O157	++++					a	a	a	a	a						a	a	a	a	a	a			
g	4765975	a	4878707	a	4947471	i	a	CB9615	++++	a	a	a	a		a	a								a	a	a	a	a	a	a	a	a
g	4767198	a	4879930	a	4948694	s	g	O157	++++	g	g	g	g	g	g	g	g	g	t	t	t	t	t	g	g	g	g	g	g	g	g	g
a	4770243	t	4882975	t	4951739	nc	a	O157	++++	a	a	a	a	a	a	a	a	a	a	a	a	a	a	a	a	a	a	a	a	a	a	a
g	4773104	c	4885836	c	4954600	ns	g	O157	++++	g	g	g	g		g	g								g	g	g				g	g	g
a	4774055	g	4886787	g	4955551	s	a	O157	++++		a	a	a		a	a	a							a	a	a				a	a	a
c	4775098	t	4887830	t	4956594	ns	c	O157	++++		c	c	c		c	c	c							c	c	c				c	c	
-	4776461	6	4889194	6	4957958	del	6	CB9615	++++		6	6	6		6	6	6							6	6	6	6	6	6	6	6	
c	4776712	t	4889450	t	4958214	ns	t	CB9615	++++		t	t	t		t	t	t							t	t	t	t	t	t	t	t	
t	4776776	g	4889514	g	4958278	ns	g	CB9615	++++		g	g	g		g	g	g							g	g	g	g	g	g	g	g	
c	4777074	t	4889812	t	4958576	s	t	CB9615	+		t	t	t		c	c	t							t	t	t	t	t	t	t	t	
12	4777206	-	4889943	-	4958707	del	12	O157	++++		12	12	12		12	12	12							12	12	12	12	12	12	12	12	
t	4778371	c	4891097	c	4959861	s	c	CB9615	++++		c	c	c		c	c	t							c	c	c	c	c	c	c	c	
c	4778520	t	4891246	t	4960010	ns	c	O157	++++		c	c	c		c	c	c							c	c	c	c	c	c	c	c	
c	4778653	c	4891379	t	4960143	s	c	EDL933	++++		c	c	c		c	c	c							c	c	c	c	c	c	c	c	
c	4779278	t	4892004	t	4960768	ns	c	O157	++++					c	c	c	c	c	c	c	c	c	c									
g	4780398	c	4893124	c	4961888	ns	g	O157	++++	g				g	g	g	g	g	g	g	g	g	g	g								
t	4780665	c	4893391	c	4962155	ns	c	CB9615	++++	c	c	c	c	c	c	c	c	c	c	c	c	c	c	c	c	c	c	c	c	c	c	c
t	4780780	c	4893506	c	4962270	ns	c	CB9615	++++	c	c	c	c	c	c	c	c	c	c	c	c	c	c	c	c	c	c	c	c	c	c	c
t	4781673	a	4894399	a	4963163	ns	a	CB9615	++++	a	a	a	a	a	a	a	a	a	a	a	a	a	a	a	a	a	a	a	a	a	a	
g	4785628	a	4897986	a	4966750	s	g	O157	++++	g	g	g	g	g	g	g	g	g	g	g	g	g	g	g	g	g	g	g	g	g	g	g
t	4787518	c	4899876	c	4968640	nc	c	CB9615	++++	c	c	c	c	c	c	c	c	c	c	c	c	c	c	c	c	c	c	c	c	c	c	c
g	4788852	a	4901210	a	4969974	s	g	O157	++++	g	g	g	g	g	g	g	g	g	g	g	g	g	g	g	g	g	g	g	g	g	g	g
a	4788924	g	4901282	g	4970046	s	a	O157	++++	a	a	a	a	a	a	a	a	a	a	a	a	a	a	a	a	a	a	a	a	a	a	a
t	4789162	g	4901520	g	4970284	i	t	O157	++	t	g	g	g	g	g	g	g	g	g	g	g	g	g	g	g	g	g	g	g	g	g	g
g	4790525	a	4902883	a	4971647	s	g	O157	++++	g	g	g	g	g	g	g	g	g	g	g	g	g	g	g	g	g	g	g	g	g	g	g
g	4791868	g	4904226	c	4972990	ns	g	EDL933	++++	g	g	g	g	g	g	g	g	g	g	g	g	g	g	g	g	g	g	g	g	g	g	g
c	4793670	t	4906028	t	4974792	s	c	O157	++++	c	c	c	c	c	c	c	c	c	c	c	c	c	c	c	c	c	c	c	c	c	c	c
c	4794943	t	4907301	t	4976065	s	c	O157	++++	c	c	c	c	c	c	c	c	c	c	c	c	c	c	c	c	c	c	c	c	c	c	c
g	4797062	t	4909420	t	4978184	ns	g	O157	++++	g	g	g	g	g	g	g	g	g	g	g	g	g	g	g	g	g	g	g	g	g	g	g
g	4797242	g	4909600	a	4978364	ns	g	EDL933	++++	g	g	g	g	g	g	g	g	g	g	g	g	g	g	g	g	g	g	g	g	g	g	g
a	4800334	c	4912692	c	4981456	ns	c	CB9615	++++	c	c	c	c	c	c	c	c	c	c	c	c	c	c	c	c	c	c	c	c	c	c	c
c	4800963	t	4913321	t	4982085	s	c	O157	++++	c	c	c	c	c	c	c	c	c	c	c	c	c	c	c	c	c	c	c	c	c	c	c
t	4802739	c	4915097	c	4983861	ns	c	CB9615	++++	c	c	c	c	c	c	c	c	c	c	c	c	c	c	c	c	c	c	c	c	c	c	c
c	4802883	a	4915241	a	4984005	ns	c	O157	++++	c	c	c	c	c	c	c	c	c	c	c	c	c	c	c	c	c	c	c	c	c	c	c
c	4806326	t	4918684	t	4987448	s	c	O157	++++	c	c	c	c	c	c	c	c	c	c	c	c	c	c	c	c	c	c	c	c	c	c	c
a	4807126	t	4919484	t	4988248	s	a	O157	+++	a				a	a	a																
c	4816018	t	4928376	t	4997140	i	c	O157	++	c	c	c	c	c	c	c	c	c	c	c	t	t	t	c	c	c	c	c	c	c	c	c
-	4816980	-	4929338	g	4998103	ins	-	EDL933	++++	-	-	-	-											-	-	-				-	-	-
t	4817350	c	4929708	c	4998473	ns	c	CB9615	++++	c	c	c	c											c	c	c				c	c	c
t	4820063	c	4932421	c	5001187	ns	c	CB9615	++++	c	c	c	c											c	c	c				c	c	c
a	4820606	g	4932964	g	5001730	ns	a	O157	+++				a											a	a	a				a	a	a
c	4823438	t	4935796	t	5004562	s	c	O157	++	c	c	t	t	c	c	c	c	c	c	c	c	c	c	c	c	c	c	c	c	c	c	c
c	4823757	g	4936115	g	5004881	ns	g	CB9615	++++	g	g	g	g	g	g	g	g	g	g	g	g	g	g	g	g	g	g	g	g	g	g	g
c	4824622	t	4936980	t	5005746	s	c	O157	++	c	t	t	t	c	c	c	c	c	c	c	c	c	c	t	t	c	t	t	t	t	t	t
c	4825930	t	4938288	t	5007054	s	c	O157	++	c	t	t	t	c	c	c	c	c	c	c	c	c	c	t	t	t	c	c	c	t	t	t
a	4827091	g	4939449	g	5008215	i	a	O157	++++	a	a	a	a	a	a	a	a	a	a	a	a	a	a	a	a	a	a	a	a	a	a	a
g	4827144	a	4939502	a	5008268	ns	g	O157	++++	g	g	g	g	g	g	g	g	g	g	g	g	g	g	g	g	g	g	g	g	g	g	g
t	4828448	c	4940806	c	5009572	s	c	CB9615	++++	c	c	c	c	c	c	c	c	c	c	c	c	c	c	c	c	c	c	c	c	c	c	c
a	4829724	g	4942082	g	5010848	ns	g	CB9615	++++	g	g	g	g	g	g	g	g	g	g	g	g	g	g	g	g	g	g	g	g	g	g	g
c	4831762	t	4944120	t	5012886	ns	t	CB9615	++++	t	t	t	t	t	t	t	t	t	t	t	t	t	t	t	t	t	t	t	t	t	t	t
t	4833380	c	4945738	c	5014504	s	t	O157	++++	t	t	t	t	t	t	t	t	t	t	t	t	t	t	t	t	t	t	t	t	t	t	t
g	4837792	t	4950150	t	5018916	ns	g	O157	++++	g	g	g	g	g	g	g	g	g	g	g	g	g	g	g	g	g	g	g	g	g	g	g
a	4840278	g	4952636	g	5021402	ns	a	O157	++++	a	a	a	a	a	a	a	a	a	a	a	a	a	a	a	a	a	a	a	a	a	a	a
a	4841939	a	4954297	a	5023063	ns	g	O157	++++	g	g	g	g	g	g	g	g	g	g	g	g	g	g	g	g	g	g	g	g	g	g	g
g	4843144	t	4955502	t	5024268	ns	g	O157	++++	g	g	g	g	g	g	g	g	g	g	g	g	g	g	g	g	g	g	g	g	g	g	g
a	4845710	t	4958068	t	5026834	s	a	O157	++++	a	a	a	a	a	a	a	a	a	a	a	a	a	a	a	a	a	a	a	a	a	a	a
c	4849469	t	4961827	t	5030593	ns	t	CB9615	++++	t	t	t	t	t	t	t	t	t	t	t	t	t	t	t	t	t	t	t	t	t	t	t
c	4850128	t	4962486	t	5031252	s	c	O157	++++	c	c	c	c	c	c	c	c	c	c	c	c	c	c	c	c	c	c	c	c	c	c	c
c	4851435	c	4963793	t	5032559	s	c	EDL933	++++	c	c	c	c	c	c	c	c	c	c	c	c	c	c	c	c	c	c	c	c	c	c	c
a	4852468	g	4964826	g	5033592	ns	a	O157	++++	a	a	a	a	a	a	a	a	a	a	a	a	a	a	a	a	a	a	a	a	a	a	a
t	4853031	c	4965389	c	5034155	ns	c	CB9615	++++	c	c	c	c	c	c	c	c	c	c	c	c	c	c	c	c	c	c	c	c	c	c	c
t	4853976	a	4966334	a	5035100	ns	t	O157	++++	t	t	t	t	t	t	t	t	t	t	t	t	t	t	t	t	t	t	t	t	t	t	t
a	4855289	c	4967647	c	5036413	i	c	CB9615	+++	c							a	c	c	c	c	c	c									
t	4855305	c	4967663	c	5036429	i	t	O157	++++	t							t	t	t	t	t	t	t									
t	4856376	c	4968734	c	5037500	s	t	O157	++	t							c	c	c	c	c	c	c									
t	4860874	g	4973232	g	5041998	ns	t	O157	++++	t	t	t	t	t	t	t	c	t	c	t	t	t	t	t	t	t	t	t	t	t	t	t

O55 and O157 genome site details										Outgroup Strain Details[g]																						
CB9615 base[a]	CB9615 Site[b]	Sakai base[a]	Sakai Site[b]	EDL933 base[a]	EDL933 Site[b]	type[c]	Inferred ancestral base[d]	Lineage inferred to mutate[e]	Support level[f]	D1 Sd197	K-12	HS	ATCC 8739	UMN026	IAI39	SMS 3-5	E2348/69	536	ED1a	CFT073	S88	APEC O1	UTI89	E24377A	IAI1	SE11	F5 8401	F2a 2457T	F2a 301	SS Ss046	B4 Sb227	B18 BS512
g	4864990	a	4977348	a	5046111	nc	g	O157	++++	g	g	g	g	g	g	g	g	g	g	g	g	g	g	g	g	g	g	g	g	g	g	g
t	4865125	a	4977483	a	5046246	i	t	O157	++	t	a	t	a	g	a	a	a	a	a	a	a	a	a	t	t	a	t	t	t	a	a	a
t	4865163	a	4977521	a	5046284	i	a	CB9615	++++	a	a	a	a	a	a	a	a	a	a	a	a	a	a	a	a	a	a	a	a	a	a	a
g	4865334	t	4977692	t	5046455	i	t	CB9615	++++	t	t	t	t	t	t	t	t	t	t	t	t	t	t	t	t	t	t	t	t	t	t	t
a	4865601	t	4977959	t	5046722	nc	t	CB9615	++	t	t	t	t	a	a	a	t	a	t	t	t	t	t	a	t	t	a	a	a	a	a	a
t	4865606	a	4977964	a	5046727	nc	a	CB9615	++	a	a	a	a	t	t	t	a	t	a	a	a	a	a	t	a	a	t	t	t	t	t	t
g	4865728	g	4978086	c	5046849	nc	c	EDL933	-	c	c	c	c	c	g	g	g	g	g	c	g	c	g	g	g	c	c	c	c	c	c	c
a	4865820	g	4978178	g	5046941	nc	a	O157	++	a	a	g	g	a	a	a	a	g	a	g	a	g	a	g	a	g	a	a	a	a	a	a
c	4866005	-	4978362	-	5047125	indel	?	O55/O157	+/-																							
-	4866016	a	4978363	a	5047126	indel	?	O55/O157	+/-																							
g	4868472	t	4980832	t	5049595	nc	g	O157	++	g	g	t	g	g	t	t	t	t	t	t	t	t	t	t	t	t	g	g	g	g	g	g
t	4868556	c	4980916	c	5049679	nc	c	CB9615	++++	c	c	c	c	c	c	c	c	c	c	c	c	c	c	c	c	c	c	c	c	c	c	c
g	4871345	g	4983705	a	5052468	s	g	EDL933	++++	g	g	g	g	g	g	g	g	g	g	g	g	g	g	g	g	g	g	g	g	g	g	g
17	4871667	17	4984027	-	5052789	del	17	EDL933	++++	17	17	17	17	17	17	17	17	17	17	17	17	17	17	17	17	17	17	17	17	17	17	17
t	4873168	c	4985528	c	5054274	s	c	CB9615	++++	c	c	c	c	c	c	c	c	c	c	c	c	c	c	c	c	c	c	c	c	c	c	c
c	4873186	t	4985546	t	5054292	s	c	O157	++++	c	c	c	c	c	c	c	c	c	c	c	c	c	c	c	c	c	c	c	c	c	c	c
c	4886710	t	4999070	t	5067816	ns	c	O157	++++		c	c		c										c	c	c					c	c
g	4889225	a	5001585	a	5070331	s	g	O157	++	g	g	g	g	g	g	a	a	a	a	a	g	g	g	g	g	g	g	g	g	g	g	g
g	4891021	a	5003381	a	5072127	ns	a	CB9615	++++	a	a	a	a	a	a	a	a	a	a	a	a	a	a	a	a	a	a	a	a	a	a	a
a	4894246	t	5006606	t	5075352	s	a	O157	++++	a	a	a	a	a	a	a	a	a	a	a	a	a	a	a	a	a	a	a	a	a	a	a
a	4894748	g	5007108	g	5075854	ns	a	O157	++++	a	a	a	a	a	a	a	a	a	a	a	a	a	a	a	a	a	a	a	a	a	a	a
g	4896525	a	5008885	a	5077631	ns	g	O157	++++	g	g	g	g	g	g	g	g	g	g	g	g	g	g	g	g	g	g	g	g	g	g	g
c	4897223	t	5009583	t	5078329	ns	c	O157	++++	c	c	c	c	c	c	c	c	c	c	c	c	c	c	c	c	c	c	c	c	c	c	c
g	4897585	a	5009945	a	5078691	s	g	O157	++++	g	g	g	g	g	g	g	g	g	g	g	g	g	g	g	g	g	g	g	g	g	g	g
a	4900512	t	5012872	t	5081618	ns	a	O157	++++	a	a	a	a	a	a	a	a	a	a	a	a	a	a	a	a	a	a	a	a	a	a	a
t	4903215	c	5015575	c	5084321	s	c	CB9615	++++	c	c	c	c	c	c	c	c	c	c	c	c	c	c	c	c	c	c	c	c	c	c	c
a	4904439	g	5016799	g	5085545	i	a	O157	++	a	g	g	g	g	g	g	g	g	g	g	g	g	g	g	g	g	g	g	g	g	t	g
g	4906014	a	5018374	R	5087120	nc	g	EDL933	IUPAC	g	g	g	g	g	g	g	g	g	g	g	g	g	g	g	g	g	g	g	g	g	g	g
g	4906014	a	5018374	R	5087120	nc	g	O157	++++	g	g	g	g	g	g	g	g	g	g	g	g	g	g	g	g	g	g	g	g	g	g	g
t	4906149	a	5018509	a	5087255	i	t	O157	++	t	t	t	a	g	a	t	a	a	a	a	a	a	a	t	t	a	t	t	t	a	a	a
a	4906419	g	5018779	R	5087525	i	a	EDL933	IUPAC	a	a	g	g	a	a	a	g	a	g	a	g	a	g	a	g	g	a	a	a	a	a	a
a	4906419	g	5018779	R	5087525	i	a	O157	++	a	a	g	g	a	a	a	g	a	g	a	g	a	g	a	g	g	a	a	a	a	a	a
t	4906446	c	5018806	c	5087552	i	c	CB9615	++++	c	c	c	c	c	c	c	c	c	c	c	c	c	c	c	c	c	c	c	c	c	c	c
-	4906450	18	5018811	18	5087557	ins	-	O157	++	-	18	18	18	18	-	18	18	18	18	18	18	18	18	18	18	18	18	18	18	18	18	18
t	4906451	-	5018828	-	5087574	indel	?	O55/O157	+/-																							
a	4906834	g	5019204	g	5087950	nc	a	O157	++	a	g	g	g	a	a	a	a	g	a	a	a	a	a	g	a	g	a	a	a	a	a	a
-	4907018	a	5019389	-	5088134	ins	?	Sakai	+/-																							
c	4907019	-	5019402	c	5088135	del	?	Sakai	+/-																							
c	4907618	c	5019990	t	5088734	nc	c	EDL933	++++	c	c	c	c	c	c	c	c	c	c	c	c	c	c	c	c	c	c	c	c	c	c	c
t	4907989	c	5020361	c	5089105	nc	c	CB9615	++++	c	c	c	c	c	c	c	c	c	c	c	c	c	c	c	c	c	c	c	c	c	c	c
c	4911344	a	5023716	a	5092460	i	c	O157	++++	c	c	c	c	c	t	c	t	t	t	t	t	t	t	c	c	c	c	c	c	c	c	c
c	4911794	t	5024166	t	5092910	ns	c	O157	++++	c	c	c	c	c	c	c	c	c	c	c	c	c	c	c	c	c	c	c	c	c	c	c
c	4913123	-	5025494	-	5094238	ins	-	CB9615	++++	-	-	-	-	-	-	-	-	-	-	-	-	-	-	-	-	-	-	-	-	-	-	-
c	4915747	t	5028109	t	5096853	s	c	O157	++++	c	c	c	c	c	c	c	c	c	c	c	c	c	c	c	c	c	c	c	c		c	c
a	4917270	g	5029632	g	5098376	nc	g	CB9615	++++	g	g			g													g	g	g			
-	4917280	t	5029643	t	5098387	del	t	CB9615	++++	t	t			t													t	t	t			
a	4917535	t	5029898	t	5098642	nc	a	O157	++	a	a	a	a	a										a	a	a	t	t	t	a		
t	4918082	g	5030445	g	5099189	nc	g	CB9615	++	g	t	t	t	t										t	t	g	g	g	-	t		
t	4918355	g	5030718	g	5099462	nc	t	O157	++++	t	t	t	t	t										t	t	t	t	t	t	t		
t	4918467	g	5030830	g	5099574	nc	g	CB9615	++++	g	g	g	g	g										g	g	g	g	g	g	g		
g	4918730	a	5031093	a	5099837	i	g	O157	++++	g	g	g	g	g										g	g	g	g	g	g	g		
-	4918759	-	5031122	S	5099867	ins	-	EDL933	++++	-	-	-	-	-										-	-	-	-	-	-	-		
-	4919667	a	5032031	a	5100776	del	a	CB9615	++++	a	a	a	a	a	a	a	a	a	a	a	a	a	a	a	a	a	a	a	a	a	a	
a	4919690	g	5032054	g	5100799	i	g	CB9615	++++	g	g	g	g	g	g	g	g	g	g	g	g	g	g	g	g	g	g	g	g	g	g	
c	4923783	a	5036147	a	5104892	ns	c	O157	++++	c	c	c	c	c	c	c	c	c	c	c	c	c	c	c	c	c	c	c	c	c	c	c
g	4925031	a	5037395	a	5106140	ns	g	O157	++++	g	g	g	g	g	g	g	g	g	g	g	g	g	g	g	g	g	g	g	g	g	g	g
t	4926188	c	5038552	c	5107297	i	c	CB9615	++++	c	c	c	c	c	c	c	c	c	c	c	c	c	c	c	c	c	c	c	c	c	c	c
c	4930296	t	5081419	t	5111405	s	c	O157	++++					c	c	c	c	c	c	c	c	c	c				c	c	c	c	c	c
g	4932166	t	5083289	t	5113275	ns	g	O157	++++	g				g	g	g	g	g	g	g	g	g	g				g	g	g	g	g	g
c	4932874	t	5083997	t	5113983	s	t	CB9615	++++	t	t	t	t	t	t	t	t	t	t	t	t	t	t	t	t	t	t	t	t	t	t	t
c	4935166	t	5086289	t	5116275	ns	t	CB9615	++++	t	t	t	t	t	t	t	t	t	t	t	t	t	t	t	t	t	t	t	t	t	t	t
a	4937904	g	5089027	g	5119013	i	a	O157	++++	a	a	a	a	a	a	a	a	a	a	a	a	a	a	a	a	a	a	a	a	a	a	a
a	4939579	g	5090702	g	5120688	ns	a	O157	++++	a	a	a	a	a	a	a	a	a	a	a	a	a	a	a	a	a	a	a	a	a	a	a
g	4940569	c	5091692	c	5121678	ns	g	O157	++++	g	g	g	g	g	g	g	g	g	g	g	g	g	g	g	g	g	g	g	g	g	g	g
a	4942136	g	5093259	g	5123245	i	a	O157	++++	a	a	a	a	g	a	a	a	a	a	a	a	a	a	a	a	a	a	a	a	a	a	a
t	4942298	g	5093421	g	5123407	i	g	CB9615	++++	g	g		g											g		g	g	g	g	g	g	g
a	4944661	g	5095784	g	5125770	i	g	CB9615	++++	g	g	g	g											g	g	g	g	g	g	g		
c	4946770	t	5097893	t	5127879	ns	c	O157	++++		c	c	c	c	c	c	c	c	c	c	c	c	c	c	c	c	c	c	c	c		
c	4948839	t	5099962	t	5129948	i	c	O157	++++		c	c	c	c	c	c	c	c	c	c	c	c	c	c	c	c	c	c	c	c		
c	4952671	t	5103794	t	5133780	i	c	O157	++++	c	c	c	c	c	c	c								c	c	c		c	c	c	c	c
c	4952672	a	5103795	a	5133781	i	a	CB9615	++	a	c	c	c	a	c	c								c	c	c		a	a	c	c	c
g	4955045	a	5106168	g	5136154	ns	g	Sakai	++++	g	g	g	g	g	g	g	g	g	g	g	g	g	g	g	g	g	g	g	g	g	g	g
g	4960530	t	5111653	t	5141639	ns	g	O157	++++	g	g	g	g	g	g	g	g	g	g	g	g	g	g	g	g	g	g	g	g	g	g	g
a	4960875	g	5111998	g	5141984	ns	a	O157	++++	a	a	a	a	a	a	a	a	a	a	a	a	a	a	a	a	a	a	a	a	a	a	a
t	4961064	c	5112187	c	5142173	s	c	CB9615	++++	c	c	c	c	c	c	c	c	c	c	c	c	c	c	c	c	c	c	c	c	c	c	c
t	4961624	c	5112747	c	5142733	ns	c	CB9615	++++	c	c	c	c	c	c	c	c	c	c	c	c	c	c	c	c	c	c	c	c	c	c	c
t	4961721	a	5112844	a	5142830	s	t	O157	++	t	t	t	t	t	t	t	a	a	a	a	a	a	a	a	t	t	t	t	t	t	t	t
g	4962679	a	5113802	a	5143788	ns	a	CB9615	++++		a	a	a	a	a	a								a	a	a	a	a	a	a	a	a
-	4963273	a	5114397	-	5144382	ins	-	Sakai	++++		-	-	-	-										-	-	-	-	-	-	-	-	-

O55 and O157 genome site details										Outgroup Strain Details[g]																						
CB9615 base[a]	CB9615 Site[b]	Sakai base[a]	Sakai Site[b]	EDL933 base[a]	EDL933 Site[b]	type[c]	Inferred ancestral base[d]	Lineage inferred to mutate[e]	Support level[f]	D1 Sd197	K-12	HS	ATCC 8739	UMN026	IAI39	SMS 3-5	E2348/69	536	ED1a	CFT073	S88	APEC O1	UTI89	E24377A	IAI1	SE11	F5 8401	F2a 2457T	F2a 301	SS Ss046	B4 Sb227	B18 BS512
c	4963389	t	5114513	t	5144498	i	t	CB9615	+		t	t	c	c	t	t								c	t	t	t	t	t	t	t	t
a	4965275	c	5116399	c	5146384	i	c	CB9615	++++	c	c	c	c	c	c	c	c	c	c	c	c	c	c	c	c	c	c	c	c	c	c	c
c	4968684	c	5119808	t	5149793	s	c	EDL933	++++	c	c	c	c	c	c	c	c	c	c	c	c	c	c	c	c	c	c	c	c	c	c	c
c	4970818	a	5121942	a	5151927	ns	c	O157	++++	c	c	c	c	c	c	c								c	c	c	c	c	c	c	c	c
t	4972424	c	5123548	c	5153533	s	c	CB9615	++++	c	c	c	c	c	c	c	c	c	c	c	c	c	c	c	c	c	c	c	c	c	c	c
a	4977510	g	5128634	g	5158619	s	a	O157	++++		a	a	a	a	a	a	a	a	a	a	a	a	a	a	a	a	a	a	a	a	a	a
a	4977688	g	5128812	g	5158797	ns	g	CB9615	++++		g	g	g	g	g	g	g	g	g	g	g	g	g	g	g	g	g	g	g	g	g	g
t	4977692	c	5128816	c	5158801	ns	c	CB9615	++++		c	c	c	c	c	c	c	c	c	c	c	c	c	c	c	c	c	c	c	c	c	c
a	4978115	c	5129239	c	5159224	ns	a	O157	++++		a	a	a	a	a	a	a	a	a	a	a	a	a	a	a	a	a	a	a	a	a	a
g	4978461	t	5129585	t	5159570	s	g	O157	++++		g	g	g	g	g	g	g	g	g	g	g	g	g	g	g	g	g	g	g	g		g
a	4978842	g	5129966	g	5159951	s	a	O157	+		a	a	a	g	g	g	g	g	g	g	g	g	g	a	a	a	a	a	a	a		a
a	4981185	g	5132309	g	5162294	ns	g	CB9615	++++	g	g	g	g	g	g	g	g	g	g	g	g	g	g	g	g	g	g	g	g	g		g
a	4983408	c	5134532	c	5164517	ns	c	CB9615	++++	c	c	c	c	c	c	c	c	c	c	c	c	c	c	c	c	c	c	c	c	c	c	c
c	4984244	t	5135368	t	5165353	ns	c	O157	++++		c	c	c	c										c	c	c		c	c	c		
t	4984668	c	5135792	c	5165777	s	t	O157	++++		t	t	t	t										t	t	t		t	t	t		
g	4984805	a	5135929	a	5165914	ns	a	CB9615	+		g	a	a	g										a	a	a		a	a	a		
g	4985023	a	5136147	a	5166132	ns	g	O157	++++		g	g	g	g										g	g	g		g	g	g		
c	4986039	t	5137163	t	5167148	s	c	O157	++++		c	c	c	c	c	c	c	c	c	c	c	c	c	c	c	c	c	c	c	c	c	c
g	4986423	a	5137547	a	5167532	s	g	O157	++++		g	g	g	g	c	a	g	g	g	g	g	g	g	g	g	g	g	g	g	g	g	g
a	4989224	g	5140348	g	5170333	ns	g	CB9615	++++		g	g	g	g	g	g	g	g	g	g	g	g	g	g	g	g	g	g	g	g	g	g
a	4989689	t	5140813	t	5170798	i	a	O157	++++		a	a	a	a	a	a	a	a	a	a	a	a	a	a	a	a	a	a	a	a	a	a
t	4992682	c	5143806	c	5173791	ns	c	CB9615	++++		c	c	c	c	c	c	c	c	c	c	c	c	c	c	c	c	c	c	c	c	c	c
t	4992709	g	5143833	g	5173818	ns	t	O157	++++		t	t	t	t	t	t	t	t	t	t	t	t	t	t	t	t	t	t	t	t	t	t
c	4992773	t	5143897	t	5173882	s	c	O157	++++		c	c	c	c	c	c	c	c	c	c	c	c	c	c	c	c	c	c	c	c	c	c
t	4992917	c	5144041	c	5174026	s	c	CB9615	++++		c	c	c	c	c	c	c	c	c	c	c	c	c	c	c	c	c	c	c	c	c	c
c	4994631	t	5145755	t	5175740	s	c	O157	++++		c	c	c	c	c	c	c	c	c	c	c	c	c	c	c	c	c	c	c	c	c	c
t	4997948	c	5149072	c	5179057	i	c	CB9615	++++		c		c	c		c		c	c	c	c	c	c								c	
c	4999073	t	5150197	t	5180182	nc	c	O157	++++		c	c	c	c	c	c	c	c	c	c	c	c	c	c	c	c	c	c	c	c	c	
c	5002895	t	5154019	t	5184004	ns	c	O157	++++		c	c	c	c	c	c	c	c	c	c	c	c	c	c	c	c	c	c	c	c	c	
t	5003754	c	5154878	c	5184863	ns	c	CB9615	++++		c	c	c	c	c	c	c	c	c	c	c	c	c	c	c	c	c	c	c	c	c	
t	5004933	g	5156057	g	5186042	nc	g	CB9615	++++		a	g	a	g	g	g	g	g	g	g	g	g	g	g	g	g	g	g	g	g	g	
t	5007026	a	5158150	a	5188135	ns	t	O157	++++		t	t	t	t	t	t	t	t	t	t	t	t	t	t	t	t				t	t	
a	5007295	t	5158419	t	5188404	s	t	CB9615	++++		t	t	t	c	c	t	t	c	t	t	t	t	t	t	t	t				t	t	
t	5009318	c	5160442	c	5190427	s	c	CB9615	++	c																						
c	5009415	t	5160539	t	5190524	ns	c	O157	++	c																						
c	5010757	t	5161881	t	5191866	s	c	O157	++	c																						
c	5012783	t	5163907	t	5193892	ns	c	O157	++	c																						
t	5013943	c	5165067	c	5195052	ns	t	O157	++	t																						
c	5014571	t	5165695	t	5195680	s	c	O157	++	c																						
-	5015984	c	5167109	c	5197094	ins	-	O157	++	-																						
a	5016994	t	5168127	t	5198112	ns	a	O157	++	a																						
a	5017777	c	5168910	c	5198895	ns	a	O157	++++	a	a		a		a	a	a	a							a	a					a	
t	5017795	g	5168928	g	5198913	ns	g	CB9615	++++	g	g		g		g	g	g	g							g	g					g	
-	5019243	a	5170377	a	5200362	ins	-	O157	++++	-	-	-	-	-	-	-	-	-	-	-	-	-	-	-	-	-				-	-	
g	5020516	t	5171650	t	5201635	ns	g	O157	++++	g	g	g	g	g	g	g	g	g	g	g	g	g	g	g	g	g				g	g	
a	5022664	c	5173798	c	5203783	ns	c	CB9615	++++	c	c	c	c	c	c	c	c	c	c	c	c	c	c	c	c	c				c	c	
c	5022881	t	5174015	t	5204000	s	c	O157	++++	c	c	c	c	c	c	c	c	c	c	c	c	c	c	c	c	c				c	c	
c	5024199	t	5175333	t	5205318	s	c	O157	++++	c	c	c	c	c	c	c	c	c	c	c	c	c	c	c	c	c				c	c	
a	5025121	g	5176255	g	5206240	s	g	CB9615	++	g	g	g	g	g	g	g	g	g	g	a	a	a	a	g	a	g				a	g	g
g	5027617	t	5178751	t	5208736	ns	t	CB9615	++++	t	t	t	t	t	t	t	t	t	t	t	t	t	t	t	t	t	t	t	t	t	t	t
c	5027751	t	5178885	t	5208870	s	c	O157	++++	c	c	t	c	c	c	c	c	c	c	c	c	c	c	c	c	c	c	c	c	c	c	c
a	5032653	g	5183887	g	5213872	ns	g	CB9615	++++	g	g	g	g	g	g	g	g	g	g	g	g	g	g	g	g	g	g	g	g	g	g	
c	5037650	g	5188884	c	5218869	ns	c	Sakai	++++	c	c	c	c	c	c	c	c	c	c	c	c	c	c	c	c	c	c	c	c	c	c	
a	5037696	g	5188930	g	5218915	s	g	CB9615	++	g	g	g	g	g	g	g	g	a	a	a	a	a	a	g	g	g	g	g	g	g	g	
g	5037986	t	5189220	t	5219205	ns	t	CB9615	++++	t	t	t	t	t	t	t	t	t	t	t	t	t	t	t	t	t	t	t	t	t	t	
g	5038635	t	5189869	t	5219854	s	g	O157	++++	g	g	g	g	g	g	g	g	g	g	g	g	g	g	g	g	g	g	g	g	g	g	g
c	5040428	t	5191662	t	5221647	s	c	O157	++++	c	c	c	c	c	c	c	c	c	c	c	c	c	c	c	c	c	c	c	c	c	c	c
c	5041079	a	5192313	a	5222298	s	c	O157	++++	c	c	c	c	c	c	c	c	c	c	c	c	c	c	c	c	c	c	c	c	c	c	c
t	5045438	c	5196672	c	5226657	ns	c	CB9615	++++	c	c	c	c	c	c	c	c	c	c	c	c	c	c	c	c	c	c	c	c	c	c	c
a	5045536	g	5196770	g	5226755	ns	g	CB9615	++++	g	g	g	g	g	g	g	g	g	g	g	g	g	g	g	g	g	g	g	g	g	g	g
c	5048928	t	5200162	t	5230147	ns	c	O157	++++	c	c	c	c	c	c	c	c	c	c	c	c	c	c	c	c	c	c	c	c	c	c	t
c	5049708	t	5200942	t	5230927	ns	c	O157	++++	c	c	c	c	c	c	c	c	c	c	c	c	c	c	c	c	c	c	c	c	c	c	c
g	5049830	a	5201064	a	5231049	ns	g	O157	++++	g	g	g	g	g	g	g	g	g	g	g	g	g	g	g	g	g	g	g	g	g	g	g
t	5050071	c	5201305	c	5231290	ns	c	CB9615	++++	c	c	c	c	c	c	c	c	c	c	c	c	c	c	c	c	c	c	c	c	c	c	c
c	5051321	c	5202555	t	5232540	ns	c	EDL933	++++	c	c	c	c	c	c	c	c	c	c	c	c	c	c	c	c	c	c	c	c	c	c	
g	5052474	a	5203708	a	5233693	i	g	O157	++++	g	g	g	g	g	g	g	g	g	g	g	g	g	g	g	g	g	g	g	g	g	g	
a	5055429	c	5206663	c	5236648	ns	c	CB9615	++++	c	c	c	c	c	c	c	c	c	c	c	c	c	c	c	c	c				c	c	
g	5056101	t	5207335	t	5237320	i	g	O157	++++		g	g	g	g	g	g	g	g	g	g	g	g	g	g	g	g				g		
g	5059740	a	5210974	a	5240959	s	a	CB9615	++++	a	a	a	a	a	a	a	a	a	a	a	a	a	a	a	a	a				a		
g	5061372	a	5212606	a	5242591	s	a	CB9615	++	a	g	g	g	g	g	g	g	a	a	a	a	a	a	g	g	g	g	g	g	g		g
t	5064876	a	5216110	a	5246095	s	a	CB9615	++++		a	a	a	a	a	a	a	a	a	a	a	a	a	a	a	a				a		
c	5065787	t	5217021	t	5247006	nc	c	O157	++++	c	c	c	c	c	c	c	c	c	c	c	c	c	c	c	c	c	c	c	c	c	c	t
t	5066021	c	5217255	c	5247240	ns	c	CB9615	++++	c	c	c	c	c	c	c	c	c	c	c	c	c	c	c	c	c	c	c	c	c	c	c
a	5067473	g	5218707	g	5248692	ns	a	O157	++++	a	a	a	a	a	a	a	a	a	a	a	a	a	a	a	a	a	a	a	a	a	a	a
c	5067515	t	5218749	t	5248734	ns	c	O157	++++	c	c	c	c	c	c	c	c	c	c	c	c	c	c	c	c	c	c	c	c	c	c	c
g	5068162	a	5219396	a	5249381	ns	g	O157	++++	g	g	g	g	g	g	g	g	g	g	g	g	g	g	g	g	g	g	g	g	g	g	g
c	5073491	t	5224725	t	5254710	ns	c	O157	++++	c	c	c	c	c	c	c	c	c	c	c	c	c	c	c	c	c	c	c	c	c	c	c
t	5075852	c	5227086	c	5257071	s	t	O157	++++	t	t	t	t	t	t	t	t	t	t	t	t	t	t	t	t	t	t	t	t	t	t	t
c	5075858	t	5227092	t	5257077	s	c	O157	++++	c	c	c	c	c	c	c	c	c	c	c	c	c	c	c	c	c	c	c	c	c	c	c

O55 and O157 genome site details										Outgroup Strain Details[g]																						
CB9615 base[a]	CB9615 Site[b]	Sakai base[a]	Sakai Site[b]	EDL933 base[a]	EDL933 Site[b]	type[c]	Inferred ancestral base[d]	Lineage inferred to mutate[e]	Support level[f]	D1 Sd197	K-12	HS	ATCC 8739	UMN026	IAI39	SMS 3-5	E2348/69	536	ED1a	CFT073	S88	APEC O1	UTI89	E24377A	IAI1	SE11	F5 8401	F2a 2457T	F2a 301	SS Ss046	B4 Sb227	B18 BS512
t	5079468	c	5231058	c	5261043	ns	t	O157	++++	t	t	t	t	t	t	t	t	t	t	t	t	t	t	t	t	t	t	t	t	t	t	t
a	5081156	g	5232746	g	5262731	ns	g	CB9615	++++	g	g	g	g	g	g	g	g	g	g	g	g	g	g	g	g	g	g	g	g	g	g	g
c	5082530	t	5234120	t	5264105	s	c	O157	++++	c	c	c	c	c	c	c	c	c	c	c	c	c	c	c	c	c	c	c	c	c	c	c
c	5083385	t	5234975	t	5264960	ns	c	O157	++++	c	c	c	c	c	c	c	c	c	c	c	c	c	c	c	c	c	c	c	c	c	c	c
g	5084310	a	5235900	a	5265885	s	g	O157	++++	g	g	g	g	g	g	g	g	g	g	g	g	g	g	g	g	g	g	g	g	g	g	g
c	5084832	a	5236422	a	5266407	ns	a	CB9615	++++	a	a	c	a	a	a	a	a	a	a	a	a	a	a	a	a	a	a	a	a	a	a	a
c	5087620	t	5239210	t	5269195	s	c	O157	++++	c	c	c	c	c	c	c	c	t	c	c	c	c	c	c	c	c	c	c	c	c		
a	5094438	g	5246028	g	5276013	i	g	CB9615	++++	g	g	g	g	g	g	g	g	g	g	g	g	g	g	g	g	g	g	g	g	g	a	g
c	5095231	t	5246821	t	5276806	s	c	O157	++++	c	c	c	c	c	c	c	c	c	c	c	c	c	c	c	c	c	c	c	c	c	c	c
c	5096494	t	5248084	t	5278069	s	c	O157	++++	c	c	c	c	c	c	c	c	c	c	c	c	c	c	c	c	c	c	c	c	c	c	c
c	5098762	a	5250352	a	5280337	ns	c	O157	++++	c	c	c	c	c	c	c	c	c	c	c	c	c	c	c	c	c	c	c	c	c	c	c
c	5100695	t	5252285	t	5282270	s	c	O157	++++	c	c	c	c	c	c	c	c	c	c	c	c	c	c	c	c	c	c	c	c	c	c	c
g	5101184	a	5252774	a	5282759	s	g	O157	++++	g	g	g	g	g	g	g	g	g	g	g	g	g	g	g	g	g	g	g	g	g	g	g
c	5101251	a	5252841	a	5282826	ns	c	O157	++++	c	c	c	c	c	c	c	c	c	c	c	c	c	c	c	c	c	c	c	c	c	c	c
a	5102162	g	5253752	g	5283737	ns	a	O157	++++	a	a	a	a	a	a	a	a	a	a	a	a	a	a	a	a	a	a	a	a	a	a	a
t	5105762	g	5257352	g	5287337	s	t	O157	++++	t	t	t	t	t	t	t	t	t	t	t	t	t	t	t	t	t	t	t	t	t	t	t
-	5108301	-	5259891	t	5289877	ins	-	EDL933	++++	-	-	-	-	-	-	-	-	-	-	-	-	-	-	-	-	-	-	-	-	-	-	-
a	5109635	g	5261225	g	5291211	s	a	O157	++	a	a	a	a	g	a	a	a	a	a	a	a	a	a	a	a	a	a	a	a	a	g	g
g	5110680	t	5262270	t	5292256	ns	g	O157	++	g	g	g	g	g	g	g	t	t	t	t	t	t	t	g	g	g	g	g	g	g	g	g
c	5112762	a	5264352	a	5294338	ns	a	CB9615	++++		a	a	a	a	a	a	a	a	a	a	a	a	a	a	a	a					a	
a	5113491	c	5265081	c	5295067	ns	c	CB9615	++++		c	c	c	c	c	c	c	c	c	c	c	c	c	c	c	c					c	
g	5113770	a	5265360	a	5295346	ns	g	O157	++++		g	g	g	g	g	g	g	g	g	g	g	g	g	g	g	g					g	
g	5118019	g	5269609	c	5299595	s	g	EDL933	++++	a	g	g	g	g	g	g	g	g	g	g	g	g	g	g	g	g	g	g	g	g	g	g
c	5119564	t	5271154	t	5301140	ns	c	O157	++++	c	c	c	c	c	c	c	c	c	c	c	c	c	c	c	c	c	c	c	c	c	c	c
c	5119665	t	5271255	t	5301241	ns	c	O157	++++	c	c	c	c	c	c	c	c	c	c	c	c	c	c	c	c	c	c	c	c	c	c	c
t	5119958	g	5271548	g	5301534	s	g	CB9615	++++	g	g	g	g	g	g	g	g	g	g	g	g	g	g	g	g	g	g	g	g	g	g	g
c	5123180	t	5274770	t	5304756	ns	c	O157	++++	c	c	c	c	c	c	c	c	c	c	c	c	c	c	c	c	c	c	c	c	c	c	c
c	5125528	c	5277118	g	5307104	s	c	EDL933	++++	c	c	c	c	c	c	c	c	c	c	c	c	c	c	c	c	c	c	c	c	c	c	c
a	5128044	a	5279634	g	5309620	ns	a	EDL933	++++	a	a	a	a	a	a	a	a	a	a	a	a	a	a	a	a	a	a	a	a	a	a	a
t	5131074	c	5282664	c	5312650	ns	t	O157	++++	t	t	t	t	t	t	t			t					t	t	t	t	t	t	t	t	t
c	5131355	t	5282945	t	5312931	ns	c	O157	++++	c	c	c	c	c	c	c			c					c	c	c	c	c	c	c	c	c
a	5131507	c	5283097	c	5313083	ns	c	CB9615	++++	c	c	c	c	c	c	c			c					c	c	c	c	c	c	c	c	c
a	5131762	g	5283352	g	5313338	s	a	O157	++++	a	a	a	a	a	a	a			a					a	a	a	a	a	a	a	a	a
g	5132271	t	5283861	t	5313847	ns	g	O157	++++	g	g	g	g	g	g	g	g	g	g	g	g	g	g	g	g	g	g	g	g	g	g	g
c	5132551	t	5284141	t	5314127	s	c	O157	++	c	c	c	c	c	c	c	c	c	c	c	c	c	c	t	c	t	c	c	c	c	c	c
g	5134545	t	5286135	t	5316121	s	g	O157	++++	g	g	g	g	g	g	g	g	g	g	g	g	g	g	g	g	a	g	g	g	g	g	g
t	5135909	t	5287499	c	5317485	i	t	EDL933	++++	t	t	t	t	t	t	t	t	t	t	t	t	t	t	t	t	t	t	t	t	t	t	t
a	5135921	g	5287511	g	5317497	i	g	CB9615	++	g	g	g	g	g	g	g	g	g	g	g	g	a	a	g	g	g	g	g	g	g	g	g
g	5136988	t	5288578	t	5318564	i	g	O157	++++	g	g	g	g	g	g	g	g	g	g	g	g	g	g	g	g	g	g	g	g	g	g	g
t	5137189	c	5288779	c	5318765	s	c	CB9615	++++	c	c	c	c	c	c	c	c	c	c	c	c	c	c	c	c	c	c	c	c	c	c	c
t	5140559	c	5292149	c	5322135	i	c	CB9615	++++	c	c	c	c	c	c	c	c	c	c	c	c	c	c	c	c	c	c	c	c	c	c	c
a	5140709	g	5292299	g	5322285	ns	g	CB9615	++++	g	g	g	g	g	g	g	g	g	g	g	g	g	g	g	g	g	g	g	g	g	g	g
t	5141284	c	5292874	c	5322860	s	c	CB9615	++++	c	c	c	c	c	t	c	c	c	c	c	c	c	c	c	c	c	c	c	c	c	c	c
c	5141548	t	5293138	t	5323124	i	c	O157	++++		c																c	c	c	c	c	c
c	5141747	t	5293337	c	5323323	s	c	Sakai	+		c																c	c	c	c	t	t
-	5143702	17	5295293	17	5325279	del	17	CB9615	++	17	-						-	-	-	-		-	-	17						-		
c	5148270	t	5299877	t	5329863	s	c	O157	++++	c	c	c	c	c	c	c	c	c	c	c	c	c	c	c	c	c	c	c	c	c	c	c
g	5148825	c	5300432	Y	5330418	ns	g	EDL933	IUPAC	g	g	g	g	g	g	g	g	g	g	g	g	g	g	g	g	g	g	g	g	g	g	g
g	5148825	c	5300432	Y	5330418	ns	g	O157	++++	g	g	g	g	g	g	g	g	g	g	g	g	g	g	g	g	g	g	g	g	g	g	g
g	5149452	c	5301059	c	5331045	ns	c	CB9615	++++	c	c	c	c	c	c	c	c	c	c	c	c	c	c	c	c	c	c	c	c	c	c	c
g	5151521	a	5303128	a	5333114	s	g	O157	++++	g	g	g	g	g	g	g	g	g	g	g	g	g	g	g	g	g	g	g	g	g	g	g
c	5151687	t	5303294	t	5333280	s	c	O157	++++	c	c	c	c	c	c	c	c	c	c	c	c	c	c	c	c	c	c	c	c	c	c	c
a	5154071	g	5305678	g	5335664	s	g	CB9615	++++	g	g	g	g	g	g	g	g	g	g	g	g	g	g	g	g	g	g	g	g	g	g	g
a	5156027	g	5307634	g	5337620	ns	a	O157	++++	a	a	a	a	a	a	a	a	a	a	a	a	a	a	a	a	a	a	a	a	a	a	a
g	5158974	c	5310581	c	5340567	ns	g	O157	++++	g	g	g	g	g	g	g	g	g	g	g	g	g	g	g	g	g	g	g	g	g	g	g
g	5159965	a	5311572	g	5341558	ns	g	Sakai	++++	g	g	g	g	g	g	g	g	g	g	g	g	g	g	g	g	g	g	g	g	g	g	g
c	5160023	t	5311630	t	5341616	s	c	O157	++++	c	c	c	c	c	c	c	c	c	c	c	c	c	c	c	c	c	c	c	c	c	c	c
g	5164331	c	5315938	c	5345924	i	c	CB9615	++++	c	c	c	c	c	c		c	c	c	c	c	c	c	c	c	c	c	c	c	c	c	c
a	5166752	g	5318359	g	5348345	i	a	O157	++++	a	a	a	a	a	a	a	a	a	a	a	a	a	a	a	a	a	a	a	a	a	a	a
t	5167333	c	5318940	c	5348926	s	c	CB9615	++	c	c	c	c	c	c	c	c	c	c	c	c	c	c	c	c	c	t	t	t	c	c	t
g	5167675	a	5319282	a	5349268	s	a	CB9615	++++	a	a	a	a	a	a	a	a	a	a	a	a	a	a	a	a	a	a	a	a	a	a	a
c	5167694	a	5319301	a	5349287	ns	c	O157	++++	c	c	c	c	c	c	c	c	c	c	c	c	c	c	c	c	c	c	c	c	c	c	c
g	5169276	c	5320883	c	5350869	ns	c	CB9615	++++	c	c	c	c	c	c	c	c	c	c	c	c	c	c	c	c	c	c	c	c	c	c	c
c	5170276	t	5321883	t	5351869	ns	c	O157	++++	c	c	c	c	c	c	c	c	c	c	c	c	c	c	c	c	c	c	c	c	c	c	c
t	5171222	t	5322829	-	5352814	del	t	EDL933	++++	t	t	t	t	t	t	t	t	t	t	t	t	t	t	t	t	t	t	t	t	t	t	t
g	5171248	g	5322855	c	5352840	ns	g	EDL933	++++	g	g	g	g	g	g	g	g	g	g	g	g	g	g	g	g	g	g	g	g	g	g	g
g	5175296	t	5326903	t	5356888	ns	t	CB9615	++++	t	t	t	t	t	t	t	t	t	t	t	t	t	t	t	t	t	t	t	t	t	t	t
c	5176580	t	5328187	t	5358172	i	c	O157	++++	c	c	c	c	c		c	c				c			c	c	c	c	c	c	c	c	c
g	5178497	t	5330104	t	5360089	ns	g	O157	++++	g	g	g	g	g		g	g				g			g	g	g	g	g	g	g	g	g
g	5178728	t	5330335	t	5360320	i	g	O157	++++	g	g	g	g	g		g	g				g			g	g	g	g	g	g	g	g	g
a	5179489	g	5331096	g	5361081	ns	g	CB9615	+		g	g	g	g		a	g				g			a	g	g	g	g	g	a	a	a
g	5179821	a	5331428	a	5361413	ns	g	O157	++++		g	g	g	g		g	g				g			g	g	g	g	g	g	g	g	g
18	5180004	-	5331610	18	5361596	del	18	Sakai	+++		18																18	18	18			
g	5181255	t	5332754	t	5362757	i	g	O157	++++	g	g	g	g	g		g	g				g			g	g	g				g	g	g
t	5184038	g	5335537	g	5365540	ns	g	CB9615	++++	g	g	g	g	g		g					g			g	g	g	g	g	g	g	g	g
t	5184052	g	5335551	g	5365554	ns	t	O157	++++	t	t	t	t	t		t					t			t	t	t	t	t	t	t	t	t
g	5186647	t	5338146	t	5368149	s	g	O157	++++	g	g	g	g	g	g	g	g	g	g	g	g	g	g	g	g	g	g	g	g	g	g	g
c	5192616	g	5344115	c	5374118	i	c	Sakai	++++	c	c			c				c	c	c		c	c	c		c					c	c
g	5195096	a	5346595	a	5376598	ns	a	CB9615	++++	a	a	a	a	a	a	a	a	a	a	a	a	a	a	a	a		a	a	a	a	a	a

O55 and O157 genome site details										Outgroup Strain Details[g]																						
CB9615 base[a]	CB9615 Site[b]	Sakai base[a]	Sakai Site[b]	EDL933 base[a]	EDL933 Site[b]	type[c]	Inferred ancestral base[d]	Lineage inferred to mutate[e]	Support level[f]	D1 Sd197	K-12	HS	ATCC 8739	UMN026	IAI39	SMS 3-5	E2348/69	536	ED1a	CFT073	S88	APEC O1	UTI89	E24377A	IAI1	SE11	F5 8401	F2a 2457T	F2a 301	SS Ss046	B4 Sb227	B18 BS512
-	5195585	g	5349090	s	5379093	ns	g	EDL933	+			g																				
-	5195585	g	5349095	r	5379098	ns	g	EDL933	+			g																				
-	5195585	g	5349098	n	5379101	ns	g	EDL933	+			g																				
-	5195585	t	5355481	Y	5385484	ns	t	EDL933	++++																							
g	5196178	a	5357913	a	5387916	ns	g	O157	+		g					a				a											g	g
c	5196216	t	5357951	t	5387954	s	c	O157	+++		a					c				c											c	c
a	5197439	g	5359174	g	5389177	ns	a	O157	++++			a			a	a	a	a	a			a	a	a							a	
t	5198627	g	5360362	g	5390365	ns	t	O157	+				t			t																
g	5199052	a	5360787	a	5390790	s	g	O157	+				g			g																
t	5200198	a	5361933	a	5391936	s	t	O157	+				t			t																
c	5202015	t	5363750	t	5393753	i	t	CB9615	+				t			t																
g	5207132	a	5368867	a	5398870	ns	a	CB9615	+				a			a																
g	5210475	t	5372210	t	5402213	s	t	CB9615	+				t			t																
t	5213308	c	5375043	c	5405046	s	c	CB9615	+				c			c																
c	5214689	t	5376424	t	5406427	s	c	O157	+				c			c																
a	5216001	g	5377736	g	5407739	s	g	CB9615	+				g			g																
t	5218098	g	5379833	g	5409836	ns	g	CB9615	+				g																			
t	5221461	c	5383196	c	5413199	ns	c	CB9615	+				c			c																
t	5221567	c	5383302	c	5413305	ns	c	CB9615	+				c			c																
t	5221953	g	5383688	g	5413691	ns	t	O157	+				t			t																
t	5222654	c	5384389	c	5414392	s	c	CB9615	+				c			c																
a	5223426	c	5385161	c	5415164	ns	c	CB9615	+				c			c																
g	5223874	a	5385609	a	5415612	ns	a	CB9615	+				a			a																
c	5225623	a	5387358	a	5417361	ns	a	CB9615	+				a			a																
g	5228495	t	5390230	t	5420233	i	t	CB9615	+				t			t																
a	5233002	g	5394737	g	5424740	i	a	O157	++++		a	a		a	a	a	a	a	a	a	a	a	a	a	a	a	a	a	a	a	a	
g	5233141	a	5394876	a	5424879	i	a	CB9615	++++		a	a		a	a	a	a	a	a	a	a	a	a	a	a	a	a	a	a	a	a	
c	5234089	t	5395824	t	5425827	i	c	O157	++++		c	c		c	c	c	c	c	c	c	c	c	c		c	c	c	c	c		c	
c	5234660	t	5396395	t	5426398	s	c	O157	++++		c	c		c	c	c	c	c	c	c	c	c	c		c	c	c	c	c		c	
g	5235473	a	5397208	a	5427211	s	g	O157	++++		g	g		g	g	g	g	g	g	g	g	g	g		g	g	g	g	g		g	
16	5235919	-	5397653	-	5427656	del	16	O157	++++		16	16		16	16	16	16	16	16	16	16	16	16		16	16	16	16	16		16	
t	5236058	c	5397777	c	5427780	i	c	CB9615	++++		c	c		c	c	c	c	c	c	c	c	c	c		c	c	c	c	c		c	
t	5236106	c	5397825	c	5427828	i	c	CB9615	++++		c	c		c	c	c	c	c	c	c	c	c	c		c	c	c	c	c		c	
a	5236108	g	5397827	g	5427830	i	a	O157	++++		a	a		a	a	a	a	a	a	a	a	a	a		a	g	a	a	a		a	
c	5236585	t	5398304	c	5428307	ns	c	Sakai	++++		c	c		c	c	c	c	c	c	c	c	c	c		c	c	c	c	c		c	
t	5236754	c	5398473	c	5428476	s	c	CB9615	++++		c	c		c	c	c	c	c	c	c	c	c	c		c	c	c	c	c		c	
t	5236978	c	5398697	c	5428700	ns	c	CB9615	+		t	c		t	t	t	c	c	c	c	c	c	c		c	c	c	c	c		c	c
t	5236979	c	5398698	c	5428701	ns	t	O157	++++		t	t		t	t	t	t	t	t	t	t	t	t		t	t	t	t	t		t	t
g	5237030	t	5398749	t	5428752	ns	g	O157	++++		g	g		g	g	g	g	g	g	g	g	g	g		g	g	g	g	g		g	g
a	5238564	c	5400283	c	5430286	s	c	CB9615	++++		c	c		c	c	c	c	c	c	c	c	c	c		c	c	c	c	c		c	c
t	5240054	g	5401773	g	5431776	ns	t	O157	++++		t	t		t	t	t	t	t		t	t	t	t	t	t	t	t	t	t		t	t
c	5241300	t	5403019	t	5433022	s	t	CB9615	+		t	t		t	t	c	c	c		c	c	c	c	c	t	t	t	t	t		t	t
c	5242447	a	5404166	a	5434169	ns	c	O157	++++		c	c	c	c	c	c	c	c	c	c	c	c	c	c	c	c	c	c	c		c	c
a	5245543	a	5407262	t	5437265	ns	a	EDL933	++++		a	a	a	a	a	a	a	a	a	a	a	a	a	a	a	a	a	a	a	a	a	a
a	5245579	a	5407298	t	5437301	ns	a	EDL933	++++		a	a	a	a	a	a	a	a	a	a	a	a	a	a	a	a	a	a	a	a	a	a
g	5245919	a	5407638	a	5437641	ns	g	O157	++++		g	g		g	g	g	g	g	g	g	g	g	g	g	g	g	g	g	g	g	g	g
-	5245971	-	5407690	g	5437694	ins	-	EDL933	++++		-	-		-	-	-	-	-	-	-	-	-	-	-	-	-	-	-	-	-	-	-
a	5247933	g	5409652	g	5439656	s	g	CB9615	++++		g	g	g	g	g	g	g	g	g	g	g	g	g	g	g	g	g	g	g	g	g	g
t	5250828	c	5412547	c	5442551	ns	t	O157	++++		t	t	t	t	t	t	t	t		t	t	t	t	t	t	t	t	t	t		t	t
g	5252977	t	5414696	t	5444700	ns	t	CB9615	++++		t	t	t	t	t	t	t	t		t	t	t	t	t	t	t	t	t	t		t	t
t	5254071	c	5415790	c	5445794	s	c	CB9615	++++		c	c	c	c	c	c	c	c		c	c	c	c	c	c	c	c	c	c		c	c
a	5254235	-	5415953	-	5445957	del	a	O157	++++		a	a	a	a	a	a	a	a		a	a	a	a	a	a	a	a	a	a		a	a
c	5255263	t	5416981	t	5446985	s	t	CB9615	+							t					t											
-	5259803	g	5421522	g	5451526	ins	-	O157	+++	-			-			-					-											
t	5263049	g	5424816	g	5454820	ns	t	O157	+												t											
c	5263664	a	5425377	a	5455381	ns	a	CB9615	+							a					a											
t	5264374	t	5426087	-	5456090	del	?	EDL933	+/-																							
a	5264393	c	5426106	c	5456098	ns	c	CB9615	+							g					c											
c	5265381	t	5427094	t	5457086	i	c	O157	++++	c	c	c	c	c	c	c	c	c		c	c	c	c	c	c	c					c	c
a	5265456	g	5427169	g	5457161	ns	g	CB9615	++++	g	g	g	g	g	g	g	g	g		g	g	g	g	g	g	g					g	g
g	5266867	a	5428580	g	5458572	s	g	Sakai	++++	g	g	g	g	g	g	g	g	g		g	g	g	g	g	g	g					g	g
t	5267174	a	5428887	a	5458879	ns	t	O157	++++	t	t	t	t	t	t	t	t	t		t	t	t	t	t	t	t					t	t
-	5267337	-	5429050	t	5459043	ins	-	EDL933	++++	-	-	-	-	-	-	-	-	-		-	-	-	-	-	-	-					-	-
9	5267766	-	5429478	-	5459471	del	9	O157	++++	9	9	9	9	9	9	9	9	9	9	9	9	9	9	9	9	9					9	9
c	5268379	t	5430083	t	5460076	ns	c	O157	++++	c	c	c	c	c	c	c	c	c	c	c	c	c	c	c	c	c					c	c
g	5269998	a	5431702	a	5461695	i	g	O157	++++	g	g	t	g	g		g					g			g	g	g					g	g
c	5271740	t	5433469	t	5463462	ns	c	O157	++++	c	c	c	c	c		c					c				c	c						c
t	5272708	a	5434437	a	5464430	ns	t	O157	++++	t	t	t	t	t		t	t				t				t	t						t
t	5274159	g	5435888	g	5465881	nc	t	O157	++++				t		t	t		t	t	t		t	t									
g	5274227	-	5435955	-	5465948	del	g	O157	++++				g		g	g		g	g	g		g	g									
t	5274410	g	5436138	g	5466131	nc	g	CB9615	++++				g		g	g		g	g	g		g	g									
t	5275058	g	5436786	g	5466779	nc	g	CB9615	++++				g		g	g		g	g	g		g	g									
c	5276035	t	5437763	t	5467756	i	t	CB9615	++++				t		t	t	t	t	t	t	t						t	t	t			
a	5276927	g	5438655	g	5468648	ns	g	CB9615	+++				g					g	g	g							g	g	g			
a	5280327	g	5442055	g	5472048	s	g	CB9615	+++						g			g	g	g							g	g	g			
c	5280924	t	5442652	t	5472645	s	c	O157	+++						c			c	c	c							c	c	c			
g	5281161	a	5442889	a	5472882	s	g	O157	+++						g			g	g	g							g	g	g			
c	5281820	t	5443548	t	5473541	ns	t	CB9615	+++						t			t	t	t							t	t	t			
a	5282276	g	5444004	g	5473997	ns	a	O157	+++				a		a			a	a	a												

O55 and O157 genome site details										Outgroup Strain Details[g]																						
CB9615 base[a]	CB9615 Site[b]	Sakai base[a]	Sakai Site[b]	EDL933 base[a]	EDL933 Site[b]	type[c]	Inferred ancestral base[d]	Lineage inferred to mutate[e]	Support level[f]	D1 Sd197	K-12	HS	ATCC 8739	UMN026	IAI39	SMS 3-5	E2348/69	536	ED1a	CFT073	S88	APEC O1	UTI89	E24377A	IAI1	SE11	F5 8401	F2a 2457T	F2a 301	SS Ss046	B4 Sb227	B18 BS512
a	5282850	g	5444578	g	5474571	nc	g	CB9615	+++				g		g			g	g	g												
t	5283013	c	5444741	c	5474734	nc	c	CB9615	+++				c		c			c	c	c												
g	5287498	a	5449226	a	5479219	i	?	O55/O157	+/-																							
t	5290015	t	5451743	g	5481736	ns	t	EDL933	++++	t	t	t	t	t		t					t			t	t	t	t	t	t	t	t	t
-	5291361	-	5453089	g	5483083	ins	-	EDL933	++++		-	-	-	-		-					-			-	-	-	-	-	-	-	-	-
c	5294752	t	5456480	t	5486474	s	c	O157	++++	c	c	c	c	c	c	c	c	c	c	c	c	c	c	c	c	c	c	c	c	c	c	c
c	5295608	t	5457336	t	5487330	ns	t	CB9615	++	t	t	t	t	t	c	t	c	t	c	t	t	t	t	t	t	t	t	t	t	t	t	t
a	5297417	g	5459145	g	5489139	s	a	O157	++++	a	a	c	a	a	a	a	a	a	a	a	a	a	a	a	a	a	a	a	a	c	c	c
a	5298932	t	5460660	t	5490654	ns	a	O157	++++	a	a	a	a	a	a	a	a	a	a	a	a	a	a	a	a	a	a	a	a	a	a	a
g	5299069	a	5460797	a	5490791	s	a	CB9615	++++	a	a	a	a	a	a	a	a	a	a	a	a	a	a	a	a	a	a	a	a	a	a	a
t	5300139	g	5461867	g	5491861	ns	g	CB9615	++++	g	g	g	g	g	g	g	g	g	g	g	g	g	g	g	g	g				g	g	g
g	5300156	t	5461884	t	5491878	s	t	CB9615	++++	t	t	t	t	t	t	t	t	t	t	t	t	t	t	t	t	t				t	t	t
a	5302380	g	5464108	g	5494102	i	a	O157	++++	a	a	a	a	a	a	a	a	a	a	a	a	a	a	a	a	a	a	a	a	a	a	a
g	5304105	c	5465597	c	5495591	ns	g	O157	++++	g	g	g	g	g	g	g	g	g	g	g	g	g	g	g	g	g	g	g	g	g	g	g
t	5305771	c	5467263	c	5497257	ns	c	CB9615	++++	c	c	c	c	c	c	c	c	c	c	c	c	c	c	c	c	c	c	c	c	c	c	c
c	5356981	t	5469081	t	5499075	i	c	O157	++++	c	c	c	c	c	c	c	c	c	c	c	c	c	c	c	c	c	c	c	c	c	c	c
c	5357853	t	5469953	t	5499947	ns	c	O157	++++	c	c	c	c	c	c	c	c	c	c	c	c	c	c	c	c	c	c	c	c	c	c	c
a	5359368	g	5471468	g	5501462	ns	g	CB9615	++++	g	g	g	g	g	g	g	g	g	g	g	g	g	g	g	g	g	g	g	g	g	g	g
t	5359719	g	5471819	g	5501813	ns	g	CB9615	++	g	t	t	t	t	t	t	t	t	t	t	t	t	t	t	t	t	t	t	t	t	t	t
g	5360186	g	5472286	-	5502279	del	g	EDL933	++++	g	g	g	g	g	g	g	g	g	g	g	g	g	g	g	g	g	g	g	g	g	g	g
t	5360458	c	5472558	c	5502551	ns	c	CB9615	++++	c	c	c	c	c	c	c	c	c	c	c	c	c	c	c	c	c	c	c	c	c	c	c
g	5361583	-	5473682	c	5503676	del	g	Sakai	++++	g	g	g	g	g	g	g	g	g	g	g	g	g	g	g	g	g	g	g	g	g	g	g
g	5361583	-	5473682	c	5503676	ns	g	EDL933	++++	g	g	g	g	g	g	g	g	g	g	g	g	g	g	g	g	g	g	g	g	g	g	g
c	5361584	c	5473683	g	5503677	ns	c	EDL933	++++	c	c	c	c	c	c	c	c	c	c	c	c	c	c	c	c	c	c	c	c	c	c	c
g	5361585	g	5473684	a	5503678	s	g	EDL933	++++	g	g	g	g	g	g	g	g	g	g	g	g	g	g	g	g	g	g	g	g	g	g	g
g	5364082	-	5476180	g	5506175	del	g	Sakai	++++	g	g	g	g	g	g	g	g	g	g	g	g	g	g	g	g	g	g	g	g	g	g	g
t	5367951	c	5480049	c	5510044	nc	c	CB9615	++	c	c	c	c	t	c	t	t	c	c	c	c	c	c	c	c	c	c	c	c	c	c	c
t	5368845	g	5480943	g	5510938	nc	t	O157	++++	t	t	t	t	t	t	t	t	t	t	t	t	t	t	t	t	t	t	t	t	t	t	t
g	5371340	a	5483438	a	5513433	s	g	O157	++++	g	g	g	g	g	g	g	g	g	g	g	g	g	g	g	g	g	g	g	g	g	g	g
t	5372126	g	5484224	g	5514219	ns	g	CB9615	++++	g	g	g	g	g	g	g	g	g	g	g	g	g	g	g	g	g	g	g	g	g	g	g
c	5373072	t	5485170	t	5515165	ns	c	O157	++++	c	c	c	c	c	c	c	c	c	c	c	c	c	c	c	c	c	c	c	c	c	c	c
c	5373475	t	5485573	t	5515568	i	c	O157	++++	c	c	c	c			c								c	c	c	c	c	c	c	c	c
t	5375474	c	5487572	c	5517567	s	c	CB9615	++++	c	c	c	c	c	c	c	c	c	c	c	c	c	c	c	c	c	c	c	c	c	c	c
g	5378601	g	5490699	t	5520694	ns	g	EDL933	++++	g	g	g	g	g	g	g	g	g	g	g	g	g	g	g	g	g	g	g	g	g	g	g
g	5378643	g	5490741	t	5520736	s	g	EDL933	++++	g	g	g	g	g	g	g	g	g	g	g	g	g	g	g	g	g	g	g	g	g	g	g
c	5381045	t	5493143	t	5523138	s	c	O157	++++	c	c	c	c	c	c	c	c	c	c	c	c	c	c	c	c	c	c	c	c	c	c	c
t	5381292	g	5493390	g	5523385	ns	g	CB9615	++++	g	g	g	g	g	g	g	g	g	g	g	g	g	g	g	g	g	g	g	g	g	g	g
a	5381880	g	5493978	g	5523973	ns	g	CB9615	++++	g	g	g	g	g	g	g	g	g	g	g	g	g	g	g	g	g	g	g	g	g	g	g
c	5386111	t	5498209	t	5528204	ns	c	O157	++++	c	c	c	c	c	c	c	c	c	c	c	c	c	c	c	c	c	c	c	c	c	c	c

[a]Numbers in place of bases indicates number of bases where >2 bases inserted or deleted. In these cases "-" indicates absence of these bases.

[b]For indels the base indicated is the base before the insertion or deletion in the strain.

[c] s: synonymous; ns: non-synonymous; nc: in non-coding gene; i: intergenic; ins: small insert; del: small deletion; indel: the small indels that can't be allocated

[d] the base present in D1 Sd197 OR if site absent in D1 Sd197, the base in majority outgroup strains OR if base present in D1 Sd197 is not that in any of the O55 and O157 strains, the base in majority outgroup strains

[e] O157: allocated to the lineage to the ancestor of EDL933 and Sakai; Sakai/EDL933: allocated to the divergence between Sakai and EDL933 (strain not specified); O55/O157: allocated to the divergence between O55 and O157 lineages (lineage not specified).

[f] Level of support for allocation of mutation as given in previous column

++++ agreement is high - 8 or more outgroup strains with expected base and at most 1 with an alternative base, and do not present alternative base in D1 Sd197

+++ agreement good - 4 or more outgroup strains with expected base and at most 1 with an alternative base, and do not present alternative base in D1 Sd197

++ agreement in D1 Sd197 regardless of situation with other outgroup strains

+ no conflict but very limited support as either site absent in D1 Sd197, and/or support is less than required for any of the higher levels of support

+/- no conflict but no support (base not present in any outgroup OR base when present is not that in any of the O55 and O157 strains OR both alternative lineages supported equally).

- conflict data implies 2 mutations at that site - eg in the ancestor of O55 and O157 strains before isolation and again in one of the lineages.

IUPAC those insertions due to error in EDL933 sequence

[g] Base, number or "-" indicates the base type or absence of the base. Blank means the site not present.
