# Supplementary material for: Derivation of Escherichia coli O157:H7 from Its O55:H7 Precursor
Source: PLoS One. 2010 Jan 14;5(1):e8700. doi: 10.1371/journal.pone.0008700 (PMC2806823; doi:10.1371/journal.pone.0008700)
Supplement: Table S4 — Virtual outgroup analysis of the recombinant regions in the CB9651, Sakai, and EDL933 genomes. (0.29 MB PDF) [file pone.0008700.s006.pdf]

Table S4. Allocation of recombinational SNPs to lineages by virtual outgroup analysis[a]

O55 and O157 genome site details												Outgroup Strain Details[i]																						
CB9615 base[b]	CB9615 Site[c]	Sakai base[b]	Sakai Site[c]	EDL933 base[b]	EDL933 Site[c]	type[d]	Event Lineage[e,f]	Recombinant segment	Inferred ancestral base[g]	Outgroup Analysis[e]	Support level[h]	D1 Sd197	K-12	HS	ATCC 8739	UMN026	IAI39	SMS 3-5	E2348/69	536	ED1a	CFT073	S88	APEC O1	UTI89	E24377A	IAI1	SE11	F5 8401	F2a 2457T	F2a 301	SS Ss046	B4 Sb227	B18 BS512
g	272486	t	269978	t	269979	nc	O55/O157	rec01	t	CB9615	++	t	g	g	t											g	g	g				t	g	g
a	272489	c	269981	c	269982	nc	O55/O157	rec01	c	CB9615	++	c	a	a	c											a	a	a				c	a	a
g	272507	c	269999	c	270000	nc	O55/O157	rec01	c	CB9615	++	c	g	g	g											g	g	g				c	g	g
c	272519	t	270011	t	270012	nc	O55/O157	rec01	c	O157	++++	c	c	c	c											c	c	c				t	c	c
t	272540	c	270032	c	270033	nc	O55/O157	rec01	c	CB9615	+	g	c	t	t											c	t	t				c	c	g
t	272550	c	270042	c	270043	nc	O55/O157	rec01	t	O157	++	t	t	t	t											c	t	t				t	c	t
t	272570	c	270062	c	270063	nc	O55/O157	rec01	c	CB9615	+	a	c	t	c											c	t	t				c	c	g
g	272573	a	270065	a	270066	nc	O55/O157	rec01	g	O157	++++	g	g	g	g											g	g	g				a	g	g
g	272576	a	270068	a	270069	nc	O55/O157	rec01	g	O157	++	g	a	g	a											a	g	g				a	a	a
c	272591	g	270083	g	270084	nc	O55/O157	rec01	c	O157	++++	c	c	c	c											c	c	c				g	c	c
g	272693	c	270185	c	270186	nc	O55/O157	rec01	c	CB9615	++	c	g	c	g											a	c	c				a	g	g
c	272711	t	270203	t	270204	nc	O55/O157	rec01	c	O157	++	c	a	t	a											c	t	t				t	c	c
g	272723	c	270215	c	270216	nc	O55/O157	rec01	c	CB9615	++	c	g	c	g											g	c	c				g	g	g
g	272726	t	270218	t	270219	nc	O55/O157	rec01	g	O157	+	a	g	t	g											g	t	t				g	g	g
g	272736	c	270228	c	270229	nc	O55/O157	rec01	g	O157	++	g	g	c	g											g	c	c				c	g	g
c	272738	a	270230	a	270231	nc	O55/O157	rec01	c	O157	+	-	c	a	c											c	a	a				c	c	a
a	272739	c	270231	c	270232	nc	O55/O157	rec01	c	CB9615	++	c	a	c	a											a	c	c				c	a	c
c	272740	a	270232	a	270233	nc	O55/O157	rec01	a	CB9615	++	a	c	a	c											c	a	a				-	c	-
c	272741	g	270233	g	270234	nc	O55/O157	rec01	g	CB9615	++	g	c	g	c											c	g	g				-	c	-
g	272744	a	270236	a	270237	nc	O55/O157	rec01	a	CB9615	+	c	a	a	a											g	a	a				g	g	a
c	272745	a	270237	a	270238	nc	O55/O157	rec01	c	O157	++	c	c	a	c											c	a	a				c	c	a
t	272747	a	270239	a	270240	nc	O55/O157	rec01	t	O157	+	c	t	a	t											t	a	a				t	t	c
g	272759	a	270251	a	270252	nc	O55/O157	rec01	a	CB9615	++++	t	a	a	a											a	a	a				a	a	a
g	272771	a	270263	a	270264	nc	O55/O157	rec01	g	O157	++	g	a	a	a											g	a	a				g	g	g
c	272810	t	270302	t	270303	nc	O55/O157	rec01	t	CB9615	++	t	c	t	c											t	t	t				c	t	c
g	272834	t	270326	t	270327	nc	O55/O157	rec01	t	CB9615	+	c	c	t	c											c	t	t				g	c	c
g	272867	t	270359	t	270360	nc	O55/O157	rec01	t	CB9615	++++	t	c	t	c											t	t	t				t	t	t
a	272933	c	270425	c	270426	nc	O55/O157	rec01	c	CB9615	++++	g	c	c	c											c	c	c				c	c	c
c	272992	g	270484	c	270485	nc	Sakai	rec02	c	Sakai	++++	a	c	c	c											c	c	c				c	c	c
a	273010	g	270502	a	270503	nc	Sakai	rec02	a	Sakai	++++	t	a	a	a											t	a	a				a	t	t
c	273053	a	270545	c	270546	nc	Sakai	rec02	c	Sakai	++++	t	c	c	c											c	c	c				c	c	c
c	273070	g	270562	c	270563	nc	Sakai	rec02	c	Sakai	++++	c	c	c	c											c	c	c				c	c	c
a	273085	g	270577	a	270578	nc	Sakai	rec02	a	Sakai	++	a	g	a	g											g	a	a				a	g	a
g	273087	a	270579	g	270580	nc	Sakai	rec02	g	Sakai	++	g	a	g	a											a	g	g				g	a	g
a	273092	g	270584	a	270585	nc	Sakai	rec02	a	Sakai	+++	a	t	a	t											t	a	a				a	t	a
g	273100	t	270592	g	270593	nc	Sakai	rec02	g	Sakai	++++	g	g	g	g											g	g	g				g	g	g
c	273102	a	270594	c	270595	nc	Sakai	rec02	c	Sakai	+	t	a	c	c											a	c	c				c	a	c
a	273103	c	270595	a	270596	nc	Sakai	rec02	a	Sakai	+++	g	g	a	g											g	a	a				a	g	g
g	273104	t	270596	g	270597	nc	Sakai	rec02	g	Sakai	+++	c	c	g	c											c	g	g				g	c	t
g	273106	c	270598	g	270599	nc	Sakai	rec02	c	2	-	c	c	g	g											c	g	g				g	c	g
g	273135	a	270627	g	270628	nc	Sakai	rec02	a	2	-	a	a	g	a											a	g	g				g	a	a
a	273154	c	270646	a	270647	nc	Sakai	rec02	a	Sakai	++++	a	a	a	a											a	a	a				a	a	a
c	273174	t	270666	c	270667	nc	Sakai	rec02	c	Sakai	++++	c	c	c	c											a	c	c				c	c	c
g	273176	c	270668	g	270669	nc	Sakai	rec02	g	Sakai	++++	g	g	g	g											g	g	g				g	g	g
g	273182	c	270674	g	270675	nc	Sakai	rec02	g	Sakai	++++	g	g	a	g											g	a	a				g	g	g
c	412020	t	372460	t	372463	i	O55/O157	rec03	c	O157	+++			c						c		c	c	c	c			c						
a	412048	g	372488	g	372491	i	O55/O157	rec03	g	CB9615	+			a						g		g	g	g	g			a						
c	412101	g	372541	g	372544	i	O55/O157	rec03	g	CB9615	+++			g						g		g	g	g	g			g						
g	412121	a	372561	a	372564	i	O55/O157	rec03	a	CB9615	+++			a						a		a	a	a	a			a						
t	412242	-	372682	-	372684	ins	O55/O157	rec03	-	CB9615	+++			-						-		-	-	-	-			-						
t	412309	-	372748	-	372750	ins	O55/O157	rec03	-	CB9615	+++			-						-		-	-	-	-			-						
a	412337	g	372776	g	372778	i	O55/O157	rec03	a	O157	+++			a						a		a	a	a	a			a						
g	412358	a	372797	a	372799	i	O55/O157	rec03	a	CB9615	+++			a						a		a	a	a	a			a						
g	412388	a	372827	a	372829	i	O55/O157	rec03	g	O157	+++			g						g		g	g	g	g			g						
a	450717	c	411334	c	411336	nc	O55/O157	rec04	c	CB9615	+				c	c				c														
t	450735	a	411352	m	411354	nc	O55/O157	rec04	t	O157	+				t	t				c														
a	450737	g	411354	g	411356	nc	O55/O157	rec04	g	CB9615	+				a	g				g														
t	450738	c	411355	c	411357	nc	O55/O157	rec04	c	CB9615	+				c	t				c														
t	462432	t	423049	a	423051	s	EDL933	rec05	t	EDL933	++	t	a	a	a	t	t	t	t	t	t	t	t	t	t	a	a	a				a		
t	462503	t	423120	c	423122	i	EDL933	rec05	t	EDL933	++	t	c	t	t	c	t	t	c	t	t	t	t	t	t	t	t	t				t		
c	462524	c	423141	a	423143	i	EDL933	rec05	c	EDL933	++	c	a	a	a	c	c	c	c	c	c	c	c	c	c	a	a	a				a		
g	462530	g	423147	a	423149	i	EDL933	rec05	a	2	-	a	a	a	a	a	a	g	a	g	g	g	g	g	g	a	a	a				a		
g	462564	g	423181	a	423183	i	EDL933	rec05	g	EDL933	++	g	a	a	a	g	g	g	g	g	g	g	g	g	g	a	a	a				a		
t	462614	t	423231	c	423233	s	EDL933	rec05	t	EDL933	++	t	c	c	c	t	t	t	t	t	t	t	t	t	t	c	c	c				c		
g	656825	a	616828	g	616522	i	Sakai	rec06	g	Sakai	++++		g													g	g	g					g	g
t	656826	c	616829	t	616523	i	Sakai	rec06	t	Sakai	++++		t													t	t	t					t	t
t	656887	c	616890	t	616584	i	Sakai	rec06	t	Sakai	++++		t													t	t	t					t	t
c	659721	t	619723	c	619417	nc	Sakai	rec06	c	Sakai	++	c	c	c	c											t	t	t				c	c	t
g	659811	a	619813	g	619507	nc	Sakai	rec06	g	Sakai	++++	g	g	g	g											g	g	g				g	g	g
a	660075	c	620077	c	619771	nc	O55/O157	rec07	c	CB9615	++	c	a	c	a											t	a	t				c	c	c
g	660078	c	620080	c	619774	nc	O55/O157	rec07	c	CB9615	++	c	g	c	g											c	g	c				c	c	c
a	660081	g	620083	g	619777	nc	O55/O157	rec07	g	CB9615	++	g	a	g	a											g	a	g				g	g	g
t	660083	c	620085	c	619779	nc	O55/O157	rec07	t	O157	++	t	t	c	t											c	t	c				t	c	c
c	660086	g	620088	g	619782	nc	O55/O157	rec07	c	O157	++	c	c	g	c											g	c	g				c	g	g
a	660087	g	620089	g	619783	nc	O55/O157	rec07	a	O157	++	a	a	a	g											g	g	g				a	g	g
c	660096	g	620098	g	619792	nc	O55/O157	rec07	g	CB9615	++	g	c	g	a											g	a	g				a	g	g
g	660102	t	620104	t	619798	nc	O55/O157	rec07	g	O157	+	a	g	g	g											t	g	t				g	t	t
g	660105	c	620107	c	619801	nc	O55/O157	rec07	g	O157	+	t	g	g	g											c	g	c				t	c	c
a	660107	g	620109	g	619803	nc	O55/O157	rec07	a	O157	++++	a	a	a	a											a	a	a				a	a	a
g	660123	a	620125	a	619819	nc	O55/O157	rec07	g	O157	+	c	g	g	g											a	g	a				c	g	a

O55 and O157 genome site details												Outgroup Strain Details[i]																						
CB9615 base[b]	CB9615 Site[c]	Sakai base[b]	Sakai Site[c]	EDL933 base[b]	EDL933 Site[c]	type[d]	Event Lineage[ef]	Recombinant segment	Inferred ancestral base[g]	Outgroup Analysis[e]	Support level[h]	D1 Sd197	K-12	HS	ATCC 8739	UMN026	IAI39	SMS 3-5	E2348/69	536	ED1a	CFT073	S88	APEC O1	UTI89	E24377A	IAI1	SE11	F5 8401	F2a 2457T	F2a 301	SS Ss046	B4 Sb227	B18 BS512
t	660141	g	620143	g	619837	nc	O55/O157	rec07	g	CB9615	++	g	t	t	t											g	t	g				g	t	t
t	660162	c	620164	c	619858	nc	O55/O157	rec07	c	CB9615	++	c	t	c	t											c	c	c				c	c	c
c	660165	g	620167	g	619861	nc	O55/O157	rec07	c	O157	++	c	c	g	c											g	g	g				c	a	c
a	660174	c	620176	c	619870	nc	O55/O157	rec07	c	CB9615	++	c	a	t	a											c	c	c				c	c	c
a	660178	c	620180	c	619874	nc	O55/O157	rec07	c	CB9615	++	c	a	a	a											c	c	c				c	c	c
g	660192	a	620194	a	619888	nc	O55/O157	rec07	a	CB9615	++	a	g	a	g											a	a	a				a	a	a
t	660198	c	620200	c	619894	nc	O55/O157	rec07	c	CB9615	++	c	t	c	t											c	c	c				c	c	c
a	660207	g	620209	g	619903	nc	O55/O157	rec07	?	O55/O157	+/-	c	a	a	a											g	g	g				c	g	g
a	660241	g	620243	g	619937	nc	O55/O157	rec07	a	O157	++	a	a	a	a											g	g	g				a	g	a
g	660255	c	620257	c	619951	nc	O55/O157	rec07	g	O157	++	g	g	a	g											c	c	c				g	c	t
g	660256	a	620258	a	619952	nc	O55/O157	rec07	g	O157	++	g	g	a	g											a	a	a				g	a	a
g	660261	a	620263	a	619957	nc	O55/O157	rec07	a	CB9615	++	a	g	a	g											a	a	a				a	a	a
g	660267	c	620269	c	619963	nc	O55/O157	rec07	c	CB9615	++	c	g	g	g											c	c	c				c	c	g
c	660273	t	620275	t	619969	nc	O55/O157	rec07	t	CB9615	++	t	c	t	c											c	c	c				t	t	t
a	660282	g	620284	g	619978	nc	O55/O157	rec07	g	CB9615	++	g	a	g	a											g	g	g				g	g	g
g	660294	c	620296	c	619990	nc	O55/O157	rec07	c	CB9615	++	c	g	c	g											c	c	c				c	c	c
a	660306	g	620308	g	620002	nc	O55/O157	rec07	g	CB9615	++	g	a	g	a											g	g	g				g	g	g
c	660311	a	620313	a	620007	nc	O55/O157	rec07	c	O157	++	c	c	t	c											a	a	a				c	a	a
c	660315	g	620317	g	620011	nc	O55/O157	rec07	g	CB9615	+	t	c	g	c											g	g	g				t	a	g
t	660330	c	620332	c	620026	nc	O55/O157	rec07	c	CB9615	++	c	t	-	t											c	c	c				c	c	c
a	660334	g	620336	g	620030	nc	O55/O157	rec07	g	CB9615	++	g	a	-	a											g	g	g				g	g	g
c	660339	g	620341	g	620035	nc	O55/O157	rec07	c	O157	+	t	c	g	c											g	g	g				t	t	c
c	660345	t	620347	t	620041	nc	O55/O157	rec07	t	CB9615	++	t	c	t	c											c	c	c				t	t	t
g	660351	a	620353	a	620047	nc	O55/O157	rec07	g	O157	++	g	g	g	g											a	a	a				g	g	g
c	660354	t	620356	t	620050	nc	O55/O157	rec07	c	O157	++	c	c	a	c											t	t	t				c	c	c
g	660384	a	620386	a	620080	nc	O55/O157	rec07	g	O157	++	g	g	g	g											a	a	a				g	g	g
c	660502	a	620504	a	620198	nc	O55/O157	rec07	c	O157	++++	c	c	a	c											c	c	c				c	c	c
t	660514	c	620516	c	620210	nc	O55/O157	rec07	c	CB9615	++++	c	c	c	t											c	c	c				c	c	c
t	824795	c	735367	c	735149	nc	CB9615	rec08	c	CB9615	++++		c	c	c	c	c	c	c	c	c	c	c	c	c	c	c	c				c	c	c
a	824813	g	735385	g	735167	nc	CB9615	rec08	g	CB9615	++++		g	g	g	g	g	g	g	g	g	g	g	g	g	g	g	g				g	g	g
t	824867	g	735439	g	735221	nc	CB9615	rec08	g	CB9615	++++		g	g	g	g	g	g	g	g	g	g	g	g	g	g	g	g				g	g	g
a	824868	c	735440	c	735222	nc	CB9615	rec08	c	CB9615	++++		c	c	c	c	c	c	c	c	c	c	c	c	c	c	c	c				c	c	c
a	824869	g	735441	g	735223	nc	CB9615	rec08	g	CB9615	++++		g	g	g	g	g	g	g	g	g	g	g	g	g	g	g	g				g	g	g
-	824869	c	735442	c	735224	del	CB9615	rec08	c	CB9615	++++		c	c	c	c	c	c	c	c	c	c	c	c	c	c	c	c				c	c	c
a	900304	g	810840	g	810623	s	O55/O157	rec09	a	O157	++	a	g	g	g											g	g	g				a	g	g
c	900361	g	810897	g	810680	s	O55/O157	rec09	c	O157	++++	c	c	c	c											g	c	c				c	c	c
c	900364	t	810900	t	810683	s	O55/O157	rec09	c	O157	++++	c	c	c	c											t	c	c				c	c	c
c	900423	a	810959	a	810742	ns	O55/O157	rec09	c	O157	++	c	c	c	c											a	a	c				c	c	c
g	900580	a	811116	a	810899	s	O55/O157	rec09	a	CB9615	++	a	g	g	g											g	g	g				a	a	a
g	900595	a	811131	a	810914	s	O55/O157	rec09	a	CB9615	++	a	a	a	g											g	g	g				a	g	a
g	900597	c	811133	c	810916	ns	O55/O157	rec09	c	CB9615	++	c	c	c	g											g	g	g				c	g	c
c	900649	t	811185	t	810968	s	O55/O157	rec09	t	CB9615	++	t	t	t	c											t	t	t				c	t	t
t	900655	c	811191	c	810974	s	O55/O157	rec09	c	CB9615	++	c	c	c	t											c	c	t				t	c	c
g	900940	c	811476	c	813130	s	O55/O157	rec09	g	O157	++	g	c	c	g											g	g	g				g	g	g
c	900949	t	811485	t	813139	s	O55/O157	rec09	c	O157	++++	c	c	t	c											c	c	c				c	c	c
c	900951	a	811487	a	813141	ns	O55/O157	rec09	c	O157	++++	c	c	a	c											c	c	c				c	c	c
t	900961	a	811497	a	813151	s	O55/O157	rec09	a	CB9615	++++	a	a	a	a											a	a	a				a	a	a
a	900988	g	811524	g	813178	s	O55/O157	rec09	a	O157	++	a	a	g	a											a	a	a				g	g	g
t	901016	c	811552	c	813206	ns	O55/O157	rec09	c	CB9615	++++	c	c	c	c											c	c	c				c	c	c
c	901021	t	811557	t	813211	s	O55/O157	rec09	c	O157	++	c	t	t	t											t	t	t				t	c	c
c	901036	t	811572	t	813226	s	O55/O157	rec09	c	O157	++	c	t	t	t											t	t	t				t	t	t
t	901051	g	811587	g	813241	s	O55/O157	rec09	g	CB9615	++++	g	g	g	g											g	g	g				g	g	g
g	903884	a	814420	a	816074	ns	O55/O157	rec10	g	O157	++++	g	g	g	g	g				g	g		g	g	g	g	g	g				g		g
t	903930	c	814466	c	816120	s	O55/O157	rec10	t	O157	++	t	t	t	t	t				c	c		c	c	c	t	t	t				t		t
a	904195	g	814731	g	816385	ns	O55/O157	rec10	g	CB9615	++++	g	g	g	g	g				g	g		g	g	g	g	g	g				g		g
a	904288	c	814824	c	816478	ns	O55/O157	rec10	a	O157	++++	a	a	a	a	a										a	a	a				a		a
t	904289	c	814825	c	816479	ns	O55/O157	rec10	t	O157	++++	t	t	t	t	t										t	t	t				t		t
a	904293	t	814829	t	816483	s	O55/O157	rec10	t	CB9615	++	t	a	t	a	t										t	a	a				a		t
g	904308	a	814844	a	816498	ns	O55/O157	rec10	g	O157	++++	g	g	g	g	g										g	g	g				g		g
t	904350	g	814886	g	816540	s	O55/O157	rec10	t	O157	++++	t	t	c	t	t										c	t	t				t		t
g	904500	t	815036	t	816690	ns	O55/O157	rec10	g	O157	+		c	g	c	t										g	t	g				g		c
t	904551	a	815087	a	816741	ns	O55/O157	rec10	t	O157	++++		t	t	t	t				t	t		t	t	t	t	t	t				t		t
g	904657	a	815193	a	816847	ns	O55/O157	rec10	g	O157	++++		g	g	g	g				g	g		g	g	g	g	g	g				g		g
a	904703	g	815239	g	816893	ns	O55/O157	rec10	a	O157	++++		a	a	a	a				a	a		a	a	a	a	a	a				a		a
c	904789	t	815325	t	816979	ns	O55/O157	rec10	t	CB9615	++++		t	t	t	t				t	t		t	t	t	t	t	t				t		
g	904794	a	815330	a	816984	s	O55/O157	rec10	a	CB9615	++++		a	a	a	a				a	a		a	a	a	a	a	a				a		
a	983399	g	893802	g	895452	s	O55/O157	rec11	g	CB9615	++++				g	g	g			g	g	g	g	g	g	g	t	g						
a	983406	c	893809	c	895459	ns	O55/O157	rec11	c	CB9615	++++				c	c	c			c	c	c	c	c	c	c	c	c						
t	983408	c	893811	c	895461	s	O55/O157	rec11	t	O157	+				t	c	c			t	c	t	t	t	t	t	t	t						
c	983417	g	893820	g	895470	s	O55/O157	rec11	c	O157	+				c	c	g			c	g	g	c	c	c	c	c	c						
t	983472	c	893875	c	895525	ns	O55/O157	rec11	c	CB9615	++++				c	c	c			c	c	c	c	c	c	c	c	c						
a	983579	g	893982	g	895632	i	O55/O157	rec11	g	CB9615	++++				g	g	g			g	g	g	g	g	g	g	g	g						
a	983668	g	894071	g	895721	s	O55/O157	rec11	g	CB9615	++++				g	g	g			g	a	g	g	g	g	g	g	g						
t	983777	g	894180	g	895830	ns	O55/O157	rec11	t	O157	++++				t	t	t			t	t	t	t	t	t	t	t							
t	1013897	c	916867	c	918515	s	O55/O157	rec12	c	CB9615	+				t	t	c	c	c	t	c	t	c	c	c		t	c	c	c	c	c	t	t
g	1013910	t	916880	t	918528	ns	O55/O157	rec12	g	O157	++++				a	g	g	-	g	a	a	a	g	g	g		a	g	g	g	g	g	a	a
c	1014101	t	917071	t	918719	s	O55/O157	rec12	c	O157	+				t	c	c	c	c	t	g	c	t	t	c		t	c	c	c	c	c	c	c
t	1014122	c	917092	c	918740	s	O55/O157	rec12	c	CB9615	+				c	c	c	c	t	c	a	t	c	c	t		c	c	c	c	c	c	c	c
c	1014137	t	917107	t	918755	s	O55/O157	rec12	t	CB9615	+				t	t	t	t	c	t	g	c	t	t	c		t	t	t	t	t	t	t	c
a	1014155	g	917125	g	918773	s	O55/O157	rec12	g	CB9615	+				g	g	g	g	g	g	g	g	g	g	g		g	g	a	a	a	a	a	g
t	1014167	c	917137	c	918785	s	O55/O157	rec12	t	O157	+				c	c	c	t	t	t	c	t	c	c	t		t	t	t	t	t	t	t	t

O55 and O157 genome site details												Outgroup Strain Details[i]																						
CB9615 base[b]	CB9615 Site[c]	Sakai base[b]	Sakai Site[c]	EDL933 base[b]	EDL933 Site[c]	type[d]	Event Lineage[e,f]	Recombinant segment	Inferred ancestral base[g]	Outgroup Analysis[e]	Support level[h]	D1 Sd197	K-12	HS	ATCC 8739	UMN026	IAI39	SMS 3-5	E2348/69	536	ED1a	CFT073	S88	APEC O1	UTI89	E24377A	IAI1	SE11	F5 8401	F2a 2457T	F2a 301	SS Ss046	B4 Sb227	B18 BS512
t	1014227	c	917197	c	918845	s	O55/O157	rec12	t	O157	+				t	t	c	c	t	t	t	t	t	t	t		t	c	t	t	t	t	t	t
a	1014254	g	917224	g	918872	s	O55/O157	rec12	a	O157	+				g	g	g	g	t	g	t	g	a	a	t		t	g	a	a	a	a	a	a
g	1014278	a	917248	a	918896	s	O55/O157	rec12	g	O157	+				g	g	a	a	g	g	c	g	g	g	g		a	g	a	a	a	a	a	g
t	1014320	c	917290	c	918938	s	O55/O157	rec12	c	CB9615	+				c	c	c	t	c	t	c	c	c	c	c		c	t	c	c	c	c	c	c
c	1014323	t	917293	t	918941	s	O55/O157	rec12	c	O157	+				c	c	t	t	c	c	g	c	c	c	t		t	c	g	g	g	g	g	c
t	1014332	c	917302	c	918950	s	O55/O157	rec12	t	O157	+				t	t	c	c	t	t	g	t	t	t	t		c	t	g	g	g	g	g	t
c	1014342	g	917312	g	918960	ns	O55/O157	rec12	g	CB9615	++++				g	g	g	g	g	g	g	g	g	g	g		g	g	a	a	a	a	a	g
a	1014383	g	917353	g	919001	s	O55/O157	rec12	g	CB9615	++++				g	g	g	g	g	g	g	g	g	g	g		g	g	t	t	t	t	t	g
g	1014388	a	917358	a	919006	s	O55/O157	rec12	a	CB9615	++++				a	g	a	a	-	a	a	a	-	-	a		a	a	a	a	a	a	a	a
g	1014401	c	917371	c	919019	ns	O55/O157	rec12	c	CB9615	+				c	g	g	g	c	c	c	g	g	g	g		c	g	c	c	c	c	c	g
g	1014469	a	917439	a	919087	s	O55/O157	rec12	g	O157	+				g	g	g	g	g	a	a	g	g	g	g		a	g	g	g	g	a	g	g
a	1014472	c	917442	c	919090	ns	O55/O157	rec12	a	O157	+				a	a	a	a	a	c	c	a	a	a	a		c	a	a	a	a	a	a	a
a	1014481	g	917451	g	919099	s	O55/O157	rec12	g	CB9615	++++				g	g	g	g	a	g	g	g	g	g	g		g	g	g	g	g	g	g	g
t	1014496	c	917466	c	919114	s	O55/O157	rec12	t	O157	+				t	t	t	t	t	c	c	t	t	t	t		c	t	t	t	t	t	c	c
g	1014538	t	917508	t	919156	s	O55/O157	rec12	g	O157	+				t	g	g	g	g	t	t	g	g	g	g		t	g	g	g	g	g	t	g
g	1014550	a	917520	a	919168	s	O55/O157	rec12	g	O157	+				a	g	g	g	g	a	a	g	g	g	g		a	g	g	g	g	g	a	g
t	1014551	g	917521	g	919169	ns	O55/O157	rec12	g	CB9615	++++				g	g	g	g	g	g	g	g	g	g	g		g	g	g	g	g	g	g	g
g	1014561	a	917531	a	919179	ns	O55/O157	rec12	g	O157	+				a	g	g	g	g	a	g	a	a	a	a		a	g	g	g	g	g	g	a
g	1014568	a	917538	a	919186	s	O55/O157	rec12	a	CB9615	+				a	a	a	a	g	a	g	g	g	g	g		a	a	a	a	a	a	g	g
a	1014688	g	917658	g	919306	s	O55/O157	rec12	g	CB9615	++++				g	g	t	g	t	g	g	g	g	g	g		g	g	g	g	g	g	g	g
g	1014689	a	917659	a	919307	ns	O55/O157	rec12	g	O157	+				a	g	g	g	g	g	a	g	g	g	g		a	g	g	a	a	g	g	g
t	1014690	c	917660	c	919308	ns	O55/O157	rec12	c	CB9615	++++				c	c	a	c	a	c	c	c	c	c	c		c	c	c	c	c	c	c	c
t	1014721	c	917691	c	919339	s	O55/O157	rec12	c	CB9615	+				c	c	c	t	t	c	g	c	c	c	c		c	c	c	c	c	c	c	c
c	1014781	t	917751	t	919399	s	O55/O157	rec12	t	CB9615	+				t	c	c	c	t	t	c	c	t	t	c		t	c	t	t	t	t	t	c
a	1014836	c	917806	c	919454	ns	O55/O157	rec12	a	O157	+				a	c	a	a	a	c	a	a	a	a	a		a	a	c	c	c	a	c	c
g	1014899	t	917869	t	919517	ns	O55/O157	rec12	g	O157	+				t	g	g	g	g	t	t	g	t	t	g		g	g	t	t	t	g	t	g
g	1014901	a	917871	a	919519	ns	O55/O157	rec12	g	O157	+				a	g	g	g	g	a	a	g	a	a	g		g	g	a	a	a	g	a	g
g	1014904	a	917874	a	919522	s	O55/O157	rec12	a	CB9615	+				a	c	g	g	g	a	a	g	a	a	g		a	g	a	a	a	a	a	g
g	1014907	a	917877	a	919525	s	O55/O157	rec12	g	O157	+				a	g	g	g	g	a	a	g	a	a	g		g	g	a	a	a	g	a	g
a	1014919	g	917889	g	919537	s	O55/O157	rec12	g	CB9615	++++				g	g	g	g	g	g	g	g	g	g	g		g	g	g	g	g	g	g	g
c	1014976	t	917946	t	919594	s	O55/O157	rec12	c	O157	+				c	c	c	c	c	t	t	c	c	c	c		c	c	t	t	t	c	c	c
a	1015080	g	918050	g	919698	ns	O55/O157	rec12	a	O157	+				a	a	a	a	a	g	a	g	a	a	a		g	a	a	a	a	g	a	a
a	1015104	g	918074	g	919722	ns	O55/O157	rec12	a	O157	+				g	g	g	a	a	g	a	a	a	a	a		a	a	g	g	g	a	g	g
a	1015217	t	918187	t	919835	ns	O55/O157	rec12	a	O157	++++				a	a	a	a	a	a	a	a	a	a	a		a	a	a	a	a	a	a	a
a	1015260	t	918230	t	919878	s	O55/O157	rec12	t	CB9615	++++					t	t	t	t		t	c	t	t	t			t	t	t	t	t	t	a
t	1015261	g	918231	g	919879	ns	O55/O157	rec12	g	CB9615	++++					g	g	g	g		g	g	g	g	g			g	g	g	g	g	g	t
a	1015293	c	918263	c	919911	s	O55/O157	rec12	c	CB9615	+					c	c	c	c		g	g	c	c	c			c	a	a	a	a	a	c
c	1015326	t	918296	t	919944	s	O55/O157	rec12	t	CB9615	+					t	t	c	t		c	c	t	t	t			c	t	t	t	t	t	a
t	1015521	c	918491	c	920139	s	O55/O157	rec12	c	CB9615	+				c	c	c	c	t	c		c	c	c	t		t	t	c	c	c	t	c	
g	1015524	a	918494	a	920142	s	O55/O157	rec12	a	CB9615	+				a	a	a	a	g	a		a	a	a	g		g	g	a	a	a	g	a	
g	1015527	a	918497	a	920145	s	O55/O157	rec12	g	O157	++++				g	g	g	g	g	g		g	g	g	g		g	g	g	g	g	g	g	
t	1015554	c	918524	c	920172	s	O55/O157	rec12	c	CB9615	++++				c	c	c	c	c	c		c	c	c	c		c	c	c	c	c	c	c	
t	1015581	c	918551	c	920199	s	O55/O157	rec12	t	O157	+				c	t	t	c	t	c		g	c	c	t		t	t	t	t	t	t	t	
g	1015608	a	918578	a	920226	s	O55/O157	rec12	g	O157	++++				g	g	g	g	g	g		g	g	g	g		g	g	g	g	g	g	g	
t	1015617	g	918587	g	920235	s	O55/O157	rec12	g	CB9615	++++				g	g	a	g	g	g		g	g	g	g		g	g	g	g	g	g	g	
c	1015620	t	918590	t	920238	s	O55/O157	rec12	t	CB9615	+				c	t	t	a	t	t		t	c	c	a		t	t	a	a	a	a	a	
g	1015647	a	918617	a	920265	s	O55/O157	rec12	a	CB9615	++++				a	a	a	a	a	a		a	a	a	a		a	a	a	a	a	a	a	
t	1015680	c	918650	c	920298	s	O55/O157	rec12	c	CB9615	++++				c	c	c	c	c	c		c	c	c	a		c	c	c	c	c	c	c	
a	1015686	c	918656	c	920304	i	O55/O157	rec12	c	CB9615	+				c	c	c	a	c	c		c	a	a	c		c	c	c	c	c	c	c	
c	1015691	t	918661	t	920309	i	O55/O157	rec12	t	CB9615	+				c	t	t	t	t	t		t	c	c	t		t	t	t	t	t	t	t	
a	1015712	c	918682	c	920330	i	O55/O157	rec12	c	CB9615	++++				c	c	c	c	c	c		c	c	c	c		c	c	c	c	c	c	c	
c	1015730	a	918700	a	920348	i	O55/O157	rec12	a	CB9615	++++				a	a	a	a	a	a		a	a	a	a		a	a	a	a	a	a	a	a
g	1015737	a	918707	a	920355	i	O55/O157	rec12	g	O157	+				g	g	a	g	a	g		a	g	g	a		g	a	g	g	g	a	g	g
g	1015741	c	918711	c	920359	i	O55/O157	rec12	g	O157	+				c	g	c	g	c	g	c	g	g	g	c		g	c	g	g	g	c	g	g
c	1015755	a	918725	a	920373	s	O55/O157	rec12	c	O157	+				c	c	c	c	a	c	g	c	c	c	c		a	c	c	c	c	c	c	a
a	1015800	g	918770	g	920418	s	O55/O157	rec12	g	CB9615	+				g	g	a	g	g	g	a	a	g	g	g		g	g	a	a	a	g	a	g
a	1015803	c	918773	c	920421	s	O55/O157	rec12	c	CB9615	++++				c	c	c	c	c	c	c	a	c	c	c		c	c	c	c	c	c	c	c
t	1015804	a	918774	a	920422	ns	O55/O157	rec12	a	CB9615	++++				a	a	a	a	a	a	a	t	a	a	a		a	a	a	a	a	a	a	a
c	1015806	g	918776	g	920424	ns	O55/O157	rec12	g	CB9615	++++				g	g	g	g	g	g	g	c	g	g	g		g	g	g	g	g	g	g	g
a	1015810	c	918780	c	920428	ns	O55/O157	rec12	c	CB9615	+				c	c	t	t	c	t	a	a	t	t	t		t	c	t	t	t	c	t	c
c	1015818	t	918788	t	920436	s	O55/O157	rec12	t	CB9615	++++				t	t	t	t	t	t	t	c	t	t	t		t	t	t	t	t	t	t	t
a	1015824	c	918794	c	920442	s	O55/O157	rec12	c	CB9615	++++				c	c	t	c	t	c	t	a	c	c	c		c	c	t	t	t	c	t	c
c	1015827	t	918797	t	920445	s	O55/O157	rec12	t	CB9615	++++				t	t	t	t	t	t	t	c	t	t	t		t	t	t	t	t	t	t	t
a	1017194	a	920164	c	921815	ns	EDL933	rec13	a	EDL933	++++			a	a	a	a	a	a	a	a	a	a	a	a		a	a	a	a	a	a	a	a
t	1017195	t	920165	c	921816	ns	EDL933	rec13	t	EDL933	++++			t	t	t	t	t	t	t	t	t	t	t	t		t	t	t	t	t	t	t	t
a	1017200	a	920170	t	921821	ns	EDL933	rec13	a	EDL933	++++			a	a	a	a	a	a	a	a	a	a	a	a		a	a	a	a	a	a	a	a
c	1017223	c	920193	a	921844	ns	EDL933	rec13	c	EDL933	++++			c	c	c	c	c	c	c	c	c	c	c	c		c	c	c	c	c	c	c	c
c	1017262	c	920232	t	921883	ns	EDL933	rec13	c	EDL933	++++			c	c	c	c	c	c	c	c	c	c	c	c		c	c	c	c	c	c	c	c
c	1017366	c	920336	a	921987	s	EDL933	rec13	c	EDL933	+			c	c	c	c	c	a	c	c	c	c	c	c		a	c	c	c	c	c	c	c
t	1018440	c	921410	c	923061	s	O55/O157	rec14	t	O157	+++					t			t				t	t	t		g	a				g		
c	1018443	t	921413	t	923064	s	O55/O157	rec14	c	O157	++++					c			c				c	c	c		c	c				c		
c	1018569	a	921539	a	923190	s	O55/O157	rec14	c	O157	+					c			c				t	t	c		a	t				a		
g	1018571	a	921541	a	923192	ns	O55/O157	rec14	a	CB9615	+++					g			a				a	a	a		a	a				a		
t	1018575	a	921545	a	923196	s	O55/O157	rec14	t	O157	+					t			a				t	t	t		g	t				a		
g	1018651	t	921621	t	923272	ns	O55/O157	rec14	t	CB9615	+					t			g				t	t	t		g	t				g		
t	1018668	c	921638	c	923289	s	O55/O157	rec14	?	O55/O157	+/-					c			c				c	c	t		t	t				t		
c	1019133	g	922103	g	923754	s	O55/O157	rec14	c	O157	+				c	c	c	c	g		c	c	c	c	c		c	c	c	c	c	g	c	c
t	1019151	c	922121	c	923772	s	O55/O157	rec14	c	CB9615	+			c	c	c	t	c	t		t	c	c	c	c		t	t	c	c	c	t	c	c
t	1019161	a	922131	a	923782	i	O55/O157	rec14	a	CB9615	+			-	-	-	-	-	t		-	-	-	-	-		a	a	-	-	-	a	-	-
g	1019162	t	922132	t	923783	i	O55/O157	rec14	t	CB9615	+			-	-	-	-	-	g		-	-	-	-	-		t	t	-	-	-	t	-	-
a	1019172	t	922142	t	923793	i	O55/O157	rec14	a	O157	++++			a	a	-	c	c	a		a	c	a	a	c		c	c	a	a	a	c	a	a
t	1019173	a	922143	a	923794	i	O55/O157	rec14	?	O55/O157	+/-			t	t	-	a	a	t		g	a	t	t	a		a	a	g	g	g	a	t	t
-	1019177	c	922148	c	923799	ins	O55/O157	rec14	-	O157	+			-	-	-	-	-	-		-	-	-	-	-		c	c	-	-	-	c	-	-
a	1019182	g	922153	g	923804	i	O55/O157	rec14	g	CB9615	+			g	g	g	g	g	a		g	g	g	g	g		g	g	a	a	a	g	g	g

O55 and O157 genome site details												Outgroup Strain Details[i]																						
CB9615 base[b]	CB9615 Site[c]	Sakai base[b]	Sakai Site[c]	EDL933 base[b]	EDL933 Site[c]	type[d]	Event Lineage[ef]	Recombinant segment	Inferred ancestral base[g]	Outgroup Analysis[e]	Support level[h]	D1 Sd197	K-12	HS	ATCC 8739	UMN026	IAI39	SMS 3-5	E2348/69	536	ED1a	CFT073	S88	APEC O1	UTI89	E24377A	IAI1	SE11	F5 8401	F2a 2457T	F2a 301	SS Ss046	B4 Sb227	B18 BS512
t	1019193	c	922164	c	923815	i	O55/O157	rec14	c	CB9615	+			c	c	c	c	c	t		c	c	c	c	c		c	c	t	t	t	c	c	c
g	1019214	t	922185	t	923836	i	O55/O157	rec14	g	O157	++++			g	g	g	g	g	g		g	g	g	g	g		g	g	g	g	g	g	g	g
a	1019217	g	922188	g	923839	i	O55/O157	rec14	a	O157	++++			a	a	a	a	a	a		a	a	a	a	a		a	a	a	a	a	a	a	a
c	1019236	g	922207	g	923858	ns	O55/O157	rec14	g	CB9615	+			g	g	c	t	t	g		g	t	g	g	t		g	g	c	c	c	g	g	g
a	1019240	g	922211	g	923862	ns	O55/O157	rec14	g	CB9615	+			g	g	a	g	g	g		g	g	g	g	g		g	g	a	a	a	g	g	g
c	1019244	a	922215	a	923866	s	O55/O157	rec14	a	CB9615	++++			a	a	c	t	t	a		a	t	a	a	t		a	a	t	t	t	a	a	a
c	1019247	a	922218	a	923869	s	O55/O157	rec14	c	O157	+			c	c	c	t	t	c		c	t	c	c	t		a	a	c	c	c	c	c	c
t	1019253	g	922224	g	923875	ns	O55/O157	rec14	g	CB9615	++++			g	g	g	g	g	g		g	g	g	g	g		g	g	g	g	g	g	g	g
t	1019274	c	922245	c	923896	s	O55/O157	rec14	c	CB9615	+			c	c	c	t	t	c		c	t	c	c	t		c	c	t	t	t	c	t	c
c	1019285	t	922256	t	923907	ns	O55/O157	rec14	c	O157	++++			c	c	c	c	c	c		c	c	c	c	c		c	c	c	c	c	c	c	c
a	1019298	g	922269	g	923920	s	O55/O157	rec14	g	CB9615	+			g	g	g	a	a	g		g	a	g	g	a		g	g	g	g	g	g	g	g
a	1019304	g	922275	g	923926	s	O55/O157	rec14	a	O157	+			g	g	a	g	g	a		g	g	a	a	g		a	a	a	a	a	a	a	a
g	1019325	a	922296	a	923947	s	O55/O157	rec14	g	O157	+			a	a	g	c	c	g		g	c	g	g	c		g	g	g	g	g	g	g	g
g	1019328	a	922299	a	923950	s	O55/O157	rec14	g	O157	++++			g	g	g	g	g	g		g	g	g	g	g		g	g	g	g	g	g	g	g
t	1019341	a	922312	a	923963	s	O55/O157	rec14	t	O157	++++			t	c	c	c	c	c		c	c	t	t	c		t	t	t	t	t	c	c	c
c	1019342	g	922313	g	923964	s	O55/O157	rec14	c	O157	+			c	g	g	a	a	g		t	a	c	c	a		c	c	c	c	c	g	g	g
g	1019343	t	922314	t	923965	s	O55/O157	rec14	g	O157	+			g	t	g	c	t	g		g	t	g	g	t		g	g	g	g	g	g	t	t
a	1019347	g	922318	g	923969	ns	O55/O157	rec14	a	O157	+			a	a	a	g	g	a		a	g	a	a	t		a	a	a	a	a	a	a	a
t	1019351	c	922322	c	923973	ns	O55/O157	rec14	t	O157	+			t	c	t	t	t	c		c	t	t	t	t		t	t	c	c	c	t	c	c
g	1019422	a	922393	a	924044	ns	O55/O157	rec14	g	O157	+			a	a	g	a	g	g		a	g	g	g	g		g	g	g	g	g	g	a	a
g	1019424	t	922395	t	924046	ns	O55/O157	rec14	g	O157	+			t	t	g	t	g	g		t	g	g	g	g		g	g	g	g	g	g	t	t
a	1019430	c	922401	c	924052	s	O55/O157	rec14	a	O157	+			a	a	a	t	a	a		a	a	a	a	a		a	a	a	a	a	a	c	c
c	1019436	t	922407	t	924058	s	O55/O157	rec14	c	O157	+			t	t	c	t	t	c		t	t	c	c	t		c	c	c	c	c	c	t	t
t	1019480	c	922451	c	924102	ns	O55/O157	rec14	c	CB9615	++++			c	c	c	c	c	c		c	c	c	c	c		c	c	c	c	c	c	c	c
a	1019493	g	922464	g	924115	s	O55/O157	rec14	a	O157	+			g	a	a	a	a	a		g	a	a	a	a		a	a	g	g	g	a	g	a
c	1019498	a	922469	a	924120	ns	O55/O157	rec14	c	O157	++++			c	c	c	c	c	c		c	c	c	c	c		c	c	c	c	c	c	c	c
g	1019529	a	922500	a	924151	s	O55/O157	rec14	g	O157	++++			g	g	g	g	g	g		g	g	g	g	g		g	g	g	g	g	g	g	g
c	1019544	t	922515	t	924166	s	O55/O157	rec14	c	O157	++++			c	c	c	c	c	c		c	c	c	c	c		c	c	c	c	c	c	c	-
c	1019562	t	922533	t	924184	s	O55/O157	rec14	c	O157	+			c	c	c	c	t	c		c	t	c	c	c		c	c	c	c	c	c	c	c
a	1019581	g	922552	g	924203	ns	O55/O157	rec14	g	CB9615	+			g	a	a	g	g	g		g	a	g	g	a		g	g	g	g	g	g	g	g
c	1019607	t	922578	t	924229	s	O55/O157	rec14	c	O157	+			c	c	c	c	c	c		t	c	c	c	t	c	c	c	c	c	c	t	c	c
c	1019620	t	922591	t	924242	ns	O55/O157	rec14	c	O157	++++			c	a	c	c	c	c		g	c	c	c	c	c	c	c	c	c	c	a	c	c
t	1019622	c	922593	c	924244	ns	O55/O157	rec14	c	CB9615	+			c	c	c	c	c	t		g	c	t	t	c	c	t	t	c	c	c	c	c	c
t	1019775	c	922746	c	924397	s	O55/O157	rec14	t	O157	++++		t	t	c	t	t	t	t		t	t	t	t	t	t	t	t	t	t	t	t	t	t
c	1019778	t	922749	t	924400	s	O55/O157	rec14	c	O157	+		c	t	t	c	c	t	c		t	t	c	c	t	t	t	t	c	c	c	t	c	c
g	1019820	a	922791	a	924442	s	O55/O157	rec14	g	O157	+		g	a	a	g	g	a	a			a	g	g	g	a	a	a	g	g	g	g	a	a
c	1019823	t	922794	t	924445	s	O55/O157	rec14	c	O157	++++		c	c	c	c	c	c	c			c	c	c	c	c	c	c	c	c	c	c	c	c
5	1019831	-	922801	-	924452	del-5	O55/O157	rec14	5	O157	++++		5	5	5	5	5	5	5		5	5	5	5	5	5	5	5	5	5	5	5	5	5
t	1019844	c	922810	c	924461	i	O55/O157	rec14	t	O157	++++		t	t	t	t	t	t	t			t	t	t	t	t	t	t	t	t	t	t	t	t
g	1019884	a	922850	a	924501	i	O55/O157	rec14	g	O157	+		g	g	g	g	a	a	g				a	a	a	g	g	g	g	g	g	g	g	g
a	1019888	g	922854	g	924505	ns	O55/O157	rec14	a	O157	+		a	a	a	a	g	g	a				g	g	g	a	a	a	a	a	a	a	a	a
a	1019889	g	922855	g	924506	ns	O55/O157	rec14	a	O157	++++		a	a	a	a	a	a	a				a	a	a	a	a	a	a	a	a	a	a	a
-	1019896	3	922863	3	924514	ins-3	O55/O157	rec14	-	O157	+		-	-	-	-	3	3	-				3	3	3	-	-	-	-	-	-	-	-	-
a	1019900	c	922869	c	924520	ns	O55/O157	rec14	a	O157	++++		a	c	a	a	a	a	a				a	a	a	a	a	a	a	a	a	a	a	a
g	1019902	a	922871	a	924522	ns	O55/O157	rec14	g	O157	+		g	g	g	g	a	a	g				a	a	a	g	g	g	g	g	g	g	g	g
t	1019905	a	922874	a	924525	s	O55/O157	rec14	t	O157	++++		t	t	t	t	c	c	t				c	c	c	t	t	t	t	t	t	t	t	t
t	1019911	c	922880	c	924531	s	O55/O157	rec14	t	O157	++++		t	t	t	t	t	t	t				t	t	t	t	t	t	t	t	t	t	t	t
a	1019914	g	922883	g	924534	s	O55/O157	rec14	a	O157	+		a	a	a	a	g	g	a				g	g	g	a	a	a	a	a	a	a	a	a
c	1019923	t	922892	t	924543	s	O55/O157	rec14	c	O157	+		c	t	c	c	t	t	c				t	t	t	c	c	c	t	t	t	t	c	c
c	1019926	t	922895	t	924546	s	O55/O157	rec14	c	O157	+		c	c	c	c	t	t	c				t	t	t	c	c	c	g	g	g	g	c	c
a	1019927	g	922896	g	924547	ns	O55/O157	rec14	a	O157	+		a	g	a	a	g	g	a				g	g	g	a	a	a	g	g	g	g	a	a
a	1019929	g	922898	g	924549	ns	O55/O157	rec14	a	O157	+		a	a	a	a	g	g	a				g	g	g	a	a	a	g	g	g	g	a	a
g	1019938	a	922907	a	924558	s	O55/O157	rec14	g	O157	+		g	g	g	g	a	a	g				a	a	a	g	g	g	t	t	t	t	g	g
g	1019939	a	922908	a	924559	ns	O55/O157	rec14	g	O157	+		g	a	g	g	a	a	g				a	a	a	g	g	g	g	g	g	g	g	g
g	1019942	c	922911	c	924562	ns	O55/O157	rec14	c	CB9615	+		c	c	c	c	c	c	c				c	c	c	c	c	c	g	g	g	g	c	c
g	1019974	t	922943	t	924594	s	O55/O157	rec14	t	CB9615	++++		t	c	t	t	t	t	t				t	t	t	t	t	t	a	a	a	a	t	t
c	1019976	a	922945	a	924596	ns	O55/O157	rec14	a	CB9615	+		a	a	a	a	a	a	a				a	a	a	a	a	a	c	c	c	c	a	a
g	1019982	c	922951	c	924602	ns	O55/O157	rec14	c	CB9615	++++		c	c	c	c	c	c	c				c	c	c	c	c	c	c	c	c	c	c	c
a	1020025	g	922994	g	924645	s	O55/O157	rec14	a	O157	++++		a	a	a	a	a	a	a				a	a	a	a	a	a				a	a	a
c	1020028	a	922997	a	924648	s	O55/O157	rec14	c	O157	++++		c	c	c	c	c	c	c				c	c	c	c	c	c				c	c	c
t	1020031	g	923000	g	924651	s	O55/O157	rec14	g	CB9615	++++		g	g	g	g	g	a	g				g	g	g	g	g	g				g	g	g
t	1020049	c	923018	c	924669	s	O55/O157	rec14	t	O157	++++		t	t	t	t	t	t	t				t	t	t	t	t	t				t	t	t
c	1020055	t	923024	t	924675	s	O55/O157	rec14	c	O157	+		c	c	c	c	t	t	t				t	t	t	c	c	c				c	t	t
t	1020072	c	923041	c	924692	ns	O55/O157	rec14	t	O157	+		t	t	t	t	t	c	c				c	c	t	t	t	t				t	t	t
a	1020085	g	923054	g	924705	s	O55/O157	rec14	a	O157	+		a	a	a	a	a	a	a				a	a	a	a	g	g				a	g	g
g	1020097	a	923066	a	924717	s	O55/O157	rec14	g	O157	++++		g	a	g	g	t	t	t				t	t	t	g	g	g				g	g	g
a	1020116	t	923085	t	924736	ns	O55/O157	rec14	a	O157	++++		a	a	a	a	a	a	a				a	a	a	a	a	a				a	a	a
c	1020139	g	923108	g	924759	s	O55/O157	rec14	c	O157	+		c	g	c	c	a	a	g				a	a	a	c	c	c				c	c	c
t	1020196	a	923165	a	924816	s	O55/O157	rec14	a	CB9615	++++		a	a	a	a	a	a	a				a	a	a	a	a	a				a	a	a
c	1020202	t	923171	t	924822	s	O55/O157	rec14	c	O157	+		c	c	c	c	t	t	t				t	t	t	c	c	c				c	t	t
t	1020213	a	923182	a	924833	ns	O55/O157	rec14	t	O157	+		t	t	t	a	a	c	a				c	c	a	t	t	t				t	t	t
g	1020226	a	923195	a	924846	s	O55/O157	rec14	g	O157	+		g	g	g	g	a	a	g				a	a	a	g	g	g				g	g	g
t	1020228	c	923197	c	924848	ns	O55/O157	rec14	t	O157	+		t	t	t	t	c	c	c				c	c	c	t	t	t				t	t	t
g	1020229	a	923198	a	924849	ns	O55/O157	rec14	g	O157	++++		g	g	g	g	g	g	a				g	g	g	g	g	g				g	g	g
t	1020241	a	923210	a	924861	s	O55/O157	rec14	a	CB9615	+		c	c	c	c	c	c	c				c	c	t	c	c	c				c	a	a
g	1020268	a	923237	a	924888	s	O55/O157	rec14	g	O157	+		c	c	c	c			g							c	c	c				c	g	g
g	1020271	a	923240	a	924891	s	O55/O157	rec14	g	O157	++++		g	g	g	g			g							g	g	g				g	g	g
a	1020280	c	923249	c	924900	s	O55/O157	rec14	a	O157	++++		a	a	a	a			a							a	a	a				a	a	a
c	1020308	a	923277	a	924928	ns	O55/O157	rec14	c	O157	+		g	g	g	g			c							g	g	g				g	c	c
a	1020313	g	923282	g	924933	s	O55/O157	rec14	g	CB9615	+		g	g	g	g			a							g	g	g				g	a	a
a	1020318	c	923287	c	924938	ns	O55/O157	rec14	a	O157	+								a														a	a
t	1020325	c	923294	c	924945	s	O55/O157	rec14	t	O157	+								g														t	t
c	1020337	t	923306	t	924957	s	O55/O157	rec14	c	O157	+								c														c	c
g	1020348	a	923317	a	924968	ns	O55/O157	rec14	g	O157	+								g														g	g
g	1020352	t	923321	t	924972	ns	O55/O157	rec14	t	CB9615	+								g														t	t
a	1020394	t	923363	t	925014	s	O55/O157	rec14	a	O157	+								t														a	a

O55 and O157 genome site details												Outgroup Strain Details[i]																						
CB9615 base[b]	CB9615 Site[c]	Sakai base[b]	Sakai Site[c]	EDL933 base[b]	EDL933 Site[c]	type[d]	Event Lineage[ef]	Recombinant segment	Inferred ancestral base[g]	Outgroup Analysis[e]	Support level[h]	D1 Sd197	K-12	HS	ATCC 8739	UMN026	IAI39	SMS 3-5	E2348/69	536	ED1a	CFT073	S88	APEC O1	UTI89	E24377A	IAI1	SE11	F5 8401	F2a 2457T	F2a 301	SS Ss046	B4 Sb227	B18 BS512
c	1020401	a	923370	a	925021	ns	O55/O157	rec14	c	O157	+								a														c	c
t	1020412	a	923381	a	925032	ns	O55/O157	rec14	a	CB9615	+								a														g	g
g	1020427	a	923396	a	925047	s	O55/O157	rec14	g	O157	+								g														g	g
t	1020430	c	923399	c	925050	s	O55/O157	rec14	c	CB9615	+								c														c	c
c	1020435	a	923404	a	925055	ns	O55/O157	rec14	c	O157	+								a														c	c
c	1020462	t	923431	t	925082	ns	O55/O157	rec14	t	CB9615	+								t														t	t
g	1020473	a	923442	a	925093	ns	O55/O157	rec14	a	CB9615	+								g														a	a
a	1020478	t	923447	t	925098	ns	O55/O157	rec14	t	CB9615	+								t														t	t
t	1020481	c	923450	c	925101	s	O55/O157	rec14	t	O157	+								t														t	t
a	1020484	g	923453	g	925104	s	O55/O157	rec14	g	CB9615	+								g														g	g
g	1020492	a	923461	a	925112	ns	O55/O157	rec14	a	CB9615	+								a														a	a
g	1020542	t	923511	t	925162	ns	O55/O157	rec14	g	O157	+								g														g	g
g	1020562	a	923531	a	925182	s	O55/O157	rec14	g	O157	+								g														g	g
g	1020598	a	923567	a	925218	s	O55/O157	rec14	g	O157	+								g														g	g
g	1020613	c	923582	c	925233	ns	O55/O157	rec14	g	O157	+								c														g	g
c	1020623	a	923592	a	925243	s	O55/O157	rec14	c	O157	+								c														c	c
c	1020628	t	923597	t	925248	s	O55/O157	rec14	c	O157	+								c														c	c
a	1020643	g	923612	g	925263	s	O55/O157	rec14	a	O157	+								a														a	a
g	1020652	a	923621	a	925272	s	O55/O157	rec14	g	O157	+								g														g	g
a	1020666	c	923635	c	925286	ns	O55/O157	rec14	c	CB9615	+								c														c	c
g	1020670	t	923639	t	925290	s	O55/O157	rec14	g	O157	+								g														g	g
t	1020697	a	923666	a	925317	s	O55/O157	rec14	a	CB9615	+								a														a	a
g	1020703	a	923672	a	925323	s	O55/O157	rec14	a	CB9615	+								a														a	a
c	1020712	t	923681	t	925332	s	O55/O157	rec14	t	CB9615	+								t														t	
t	1020715	a	923684	a	925335	s	O55/O157	rec14	?	O55/O157	+/-								c														c	
c	1020724	t	923693	t	925344	s	O55/O157	rec14	?	O55/O157	+/-								t														c	
t	1020727	g	923696	g	925347	s	O55/O157	rec14	?	O55/O157	+/-								t														g	
t	1020729	c	923698	c	925349	ns	O55/O157	rec14	?	O55/O157	+/-								t														c	
c	1020730	a	923699	a	925350	ns	O55/O157	rec14	?	O55/O157	+/-								c														a	
c	1020742	t	923711	t	925362	s	O55/O157	rec14	t	CB9615	+								t														t	
c	1020756	g	923725	g	925376	ns	O55/O157	rec14	?	O55/O157	+/-								c														g	
t	1020760	a	923729	a	925380	s	O55/O157	rec14	?	O55/O157	+/-								t														a	
g	1020764	a	923733	a	925384	ns	O55/O157	rec14	g	O157	+								g														g	
t	1020766	a	923735	a	925386	ns	O55/O157	rec14	?	O55/O157	+/-								t														a	
c	1020778	t	923747	t	925398	s	O55/O157	rec14	?	O55/O157	+/-								c														t	
t	1020868	g	923837	g	925488	s	O55/O157	rec14	t	O157	+								t														a	
g	1020871	a	923840	a	925491	s	O55/O157	rec14	g	O157	+								g															
a	1020949	g	923918	g	925569	s	O55/O157	rec14	a	O157	+								a															
c	1020979	a	923948	a	925599	s	O55/O157	rec14	c	O157	+								c															
t	1020982	g	923951	g	925602	s	O55/O157	rec14	t	O157	+								t															
t	1020985	c	923954	c	925605	s	O55/O157	rec14	t	O157	+								t															
g	1020998	a	923967	a	925618	ns	O55/O157	rec14	g	O157	+								g															
c	1020999	t	923968	t	925619	ns	O55/O157	rec14	c	O157	+								c															
c	1021003	a	923972	a	925623	ns	O55/O157	rec14	c	O157	+								c															
a	1021022	t	923991	t	925642	ns	O55/O157	rec14	a	O157	+								a															
c	1021106	a	924075	a	925726	ns	O55/O157	rec14	c	O157	+								c															
g	1021129	a	924098	a	925749	s	O55/O157	rec14	g	O157	+								g															
g	1021144	a	924113	a	925764	s	O55/O157	rec14	g	O157	+								g															
g	1021162	a	924131	a	925782	s	O55/O157	rec14	g	O157	+								g															
a	1021195	t	924164	t	925815	ns	O55/O157	rec14	a	O157	+								a															
c	1021211	t	924180	t	925831	s	O55/O157	rec14	c	O157	+								c															
g	1021212	a	924181	a	925832	ns	O55/O157	rec14	g	O157	+								g															
a	1021214	t	924183	t	925834	ns	O55/O157	rec14	a	O157	+								a															
c	1021215	t	924184	t	925835	s	O55/O157	rec14	c	O157	+								c															
a	1021217	g	924186	g	925837	s	O55/O157	rec14	a	O157	+								a															
a	1021219	g	924188	g	925839	ns	O55/O157	rec14	a	O157	+								a															
a	1021223	g	924192	g	925843	s	O55/O157	rec14	a	O157	+								a															
c	1021233	a	924202	a	925853	ns	O55/O157	rec14	c	O157	+								c															
g	1021244	t	924213	t	925864	s	O55/O157	rec14	g	O157	+								g															
a	1021246	g	924215	g	925866	ns	O55/O157	rec14	a	O157	+								a															
c	1021263	t	924232	t	925883	ns	O55/O157	rec14	c	O157	+								c															
c	1021269	a	924238	a	925889	ns	O55/O157	rec14	c	O157	+								c															
g	1021274	a	924243	a	925894	s	O55/O157	rec14	g	O157	+								g															
g	1021275	c	924244	c	925895	ns	O55/O157	rec14	g	O157	+								g															
a	1021276	g	924245	g	925896	ns	O55/O157	rec14	a	O157	+								a															
t	1021279	c	924248	c	925899	ns	O55/O157	rec14	t	O157	+								t															
c	1021293	t	924262	t	925913	s	O55/O157	rec14	c	O157	+								c															
t	1021298	a	924267	a	925918	s	O55/O157	rec14	t	O157	+								t															
g	1021304	a	924273	a	925924	s	O55/O157	rec14	g	O157	+								g															
c	1021316	t	924285	t	925936	s	O55/O157	rec14	c	O157	+								c															
t	1021337	c	924306	c	925957	s	O55/O157	rec14	t	O157	+								t															
c	1021354	a	924323	a	925974	ns	O55/O157	rec14	c	O157	+								c															
g	1021367	a	924336	a	925987	s	O55/O157	rec14	g	O157	+								g															
g	1021388	a	924357	a	926008	s	O55/O157	rec14	a	CB9615	+								a															
t	1020796	c	923765	t	925416	s	Sakai	rec15	c	2	-								c														c	
g	1020829	a	923798	g	925449	s	Sakai	rec15	?	Sakai	+/-								g														a	
a	1020834	g	923803	a	925454	ns	Sakai	rec15	a	Sakai	+								a														c	
g	1020836	a	923805	g	925456	ns	Sakai	rec15	g	Sakai	+								g														g	
t	1020850	g	923819	t	925470	s	Sakai	rec15	t	Sakai	+								t														a	
g	1020872	a	923841	g	925492	ns	Sakai	rec15	g	Sakai	+								g															
c	1020877	t	923846	c	925497	s	Sakai	rec15	t	2	-								t															
c	1020886	a	923855	c	925506	s	Sakai	rec15	c	Sakai	+								c															
a	1020889	g	923858	a	925509	s	Sakai	rec15	a	Sakai	+								a															
a	1020904	g	923873	a	925524	s	Sakai	rec15	a	Sakai	+								a															
a	1020934	t	923903	a	925554	s	Sakai	rec15	a	Sakai	+								a															

O55 and O157 genome site details												Outgroup Strain Details[i]																						
CB9615 base[b]	CB9615 Site[c]	Sakai base[b]	Sakai Site[c]	EDL933 base[b]	EDL933 Site[c]	type[d]	Event Lineage[ef]	Recombinant segment	Inferred ancestral base[g]	Outgroup Analysis[e]	Support level[h]	D1 Sd197	K-12	HS	ATCC 8739	UMN026	IAI39	SMS 3-5	E2348/69	536	ED1a	CFT073	S88	APEC O1	UTI89	E24377A	IAI1	SE11	F5 8401	F2a 2457T	F2a 301	SS Ss046	B4 Sb227	B18 BS512
c	1020950	g	923919	c	925570	ns	Sakai	rec15	c	Sakai	+								c															
-	1258162	c	1175577	t	1264802	ns	Sakai/EDL933	rec16	c	EDL933	++++	c	c		c	c		c	c		c	c	c	c	c	c	c	c	c	c	c	c	c	c
-	1258162	t	1175578	a	1264803	ns	Sakai/EDL933	rec16	a	Sakai	++	a	t		t	t		t	a		a	a	a	a	a	a	t	t	a	a	a	a	a	a
-	1258162	t	1175581	c	1264806	ns	Sakai/EDL933	rec16	c	Sakai	++	c	t		t	t		t	c		c	c	c	c	c	c	t	t	c	c	c	c	c	c
-	1258162	c	1175596	t	1264821	ns	Sakai/EDL933	rec16	c	EDL933	++++	c	c		c	c		c	c		c	c	c	c	c	c	c	c	c	c	c	c	c	c
-	1258162	t	1175605	c	1264830	ns	Sakai/EDL933	rec16	c	Sakai	++++	c	a		a	a		c	c		c	c	c	c	c	c	a	c	c	c	c	c	c	c
-	1258162	a	1175611	t	1264836	ns	Sakai/EDL933	rec16	t	Sakai	++	t	t		t	t		a	t		t	t	t	t	t	t	t	a	t	t	t	t	t	t
-	1258162	t	1175614	c	1264839	ns	Sakai/EDL933	rec16	c	Sakai	++	c	c		c	c		t	c		c	c	c	c	c	c	c	t	c	c	c	c	c	c
-	1258162	c	1175668	g	1264893	ns	Sakai/EDL933	rec16	c	EDL933	+	t	t		t	t		t	c		t	t	t	t	t	c	t	t	t	t	t	t	t	c
-	1258162	c	1175671	g	1264896	ns	Sakai/EDL933	rec16	c	EDL933	++++	c	c		c	c		c	c		c	c	c	c	c	c	c	c	c	c	c	c	c	c
-	1258162	c	1175680	t	1264905	ns	Sakai/EDL933	rec16	c	EDL933	++++	c	g		g	g		c	c		c	c	c	c	c	a	g	c	c	c	c	c	c	c
-	1258162	a	1175683	t	1264908	ns	Sakai/EDL933	rec16	a	EDL933	++++	a	a		a	a		a	a		a	a	a	a	a	a	a	a	a	a	a	a	a	a
-	1258162	t	1175689	g	1264914	ns	Sakai/EDL933	rec16	g	Sakai	+	c	g		g	g		c	c		t	c	c	c	c	t	g	c	c	c	c	c	c	c
-	1258162	g	1175692	t	1264917	ns	Sakai/EDL933	rec16	g	EDL933	++++	g	g		g	g		g	g		g	g	g	g	g	g	g	g	g	g	g	g	g	g
-	1258162	g	1175732	a	1264957	ns	Sakai/EDL933	rec16	g	EDL933	++++	g	g		g	g		g	g		g	g	g	g	g	g	g	g	g	g	g	g	g	g
-	1258162	c	1175740	t	1264965	ns	Sakai/EDL933	rec16	t	Sakai	++	t	t		t	t		c	c		t	t	c	c	c	t	t	c	t	t	t	t	t	c
-	1258162	a	1175756	g	1264981	ns	Sakai/EDL933	rec16	a	EDL933	++++	a	a		a	a		a	t		a	a	t	t	t	g	a	a	a	a	a	a	a	t
-	1258162	t	1175757	c	1264982	ns	Sakai/EDL933	rec16	c	Sakai	++++	c	c		c	c		c	c		c	c	c	c	c	c	c	c	c	c	c	c	c	c
-	1258162	a	1175764	g	1264989	ns	Sakai/EDL933	rec16	g	Sakai	++	g	g		g	g		g	a		g	g	a	a	a	g	g	g	g	g	g	g	g	a
-	1258162	g	1175767	t	1264992	ns	Sakai/EDL933	rec16	t	Sakai	++	t	g		g	g		g	g		t	t	g	g	g	g	g	g	t	t	t	t	t	g
-	1258162	g	1175776	t	1265001	ns	Sakai/EDL933	rec16	t	Sakai	++	t	t		t	t		g	g		g	t	g	g	g	g	t	g	t	t	t	t	t	g
-	1258162	a	1175800	g	1265025	ns	Sakai/EDL933	rec16	g	Sakai	++++	g	g		g	g		g	g		g	g	g	g	g	t	g	g	g	g	g	g	g	g
-	1258162	t	1175804	c	1265029	ns	Sakai/EDL933	rec16	c	Sakai	++	c	t		t	t		t	c		t	c	c	c	c	t	t	t	c	c	c	c	c	c
-	1258162	t	1175817	c	1265042	ns	Sakai/EDL933	rec16	c	Sakai	++	c	t		t	t		c	c		t	c	c	c	c	c	t	c	c	c	c	c	c	c
-	1258162	a	1175824	g	1265049	ns	Sakai/EDL933	rec16	g	Sakai	++	g	a		t	a		g	g		a	g	g	g	g	a	a	g	g	g	g	g	g	g
-	1258162	t	1175825	c	1265050	ns	Sakai/EDL933	rec16	c	Sakai	++++	c	c		c	c		c	c		c	c	c	c	c	c	c	c	c	c	c	c	c	c
-	1258162	t	1175830	g	1265055	ns	Sakai/EDL933	rec16	g	Sakai	++	g	t		t	t		g	g		t	g	g	g	g	g	t	g	g	g	g	g	g	g
-	1258162	g	1175833	a	1265058	ns	Sakai/EDL933	rec16	a	Sakai	++	a	g		g	g		g	a		g	a	a	a	a	g	g	g	a	a	a	a	a	a
-	1258162	a	1175836	g	1265061	ns	Sakai/EDL933	rec16	g	Sakai	++	g	t		t	t		a	g		a	g	g	g	g	a	t	a	g	g	g	g	a	g
-	1258162	c	1175841	t	1265066	ns	Sakai/EDL933	rec16	t	Sakai	++	t	c		c	c		t	t		c	t	t	t	t	t	c	t	t	t	t	t	t	t
-	1258162	g	1175842	a	1265067	ns	Sakai/EDL933	rec16	a	Sakai	++	a	g		g	g		a	a		g	a	a	a	a	a	g	a	a	a	a	a	a	a
-	1258162	t	1175851	g	1265076	ns	Sakai/EDL933	rec16	g	Sakai	++	g	g		g	g		t	g		g	g	g	g	g	t	g	t	g	g	g	g	g	g
-	1258162	a	1175857	g	1265082	ns	Sakai/EDL933	rec16	g	Sakai	++	g	a		a	a		g	g		a	g	g	g	g	g	a	g	g	g	g	g	g	g
-	1258162	a	1175860	g	1265085	ns	Sakai/EDL933	rec16	g	Sakai	++	g	a		a	a		a	g		a	g	g	g	g	a	a	a	g	g	g	g	g	g
-	1258162	c	1175862	g	1265087	ns	Sakai/EDL933	rec16	g	Sakai	++	g	c		c	c		g	g		c	g	g	g	g	g	c	g	g	g	g	g	g	g
-	1258162	g	1175863	t	1265088	ns	Sakai/EDL933	rec16	t	Sakai	++++	t	a		a	a		t	t		a	t	t	t	t	t	a	t	t	t	t	t	t	t
-	1258162	c	1175966	t	1265191	ns	Sakai/EDL933	rec16	c	EDL933	++++	c	c		c	c		c	c		c	c	c	c	c	c	c	c	c	c	c	c	c	c
-	1258162	a	1176041	g	1265266	ns	Sakai/EDL933	rec16	g	Sakai	++++	g	g		g	g			g		g	g	g	g	g	g	g		g	g	g			g
-	1258162	t	1176051	g	1265276	ns	Sakai/EDL933	rec16	t	EDL933	++++	t	t		t	t			t		t	t	t	t	t	t	t		t	t	t			t
-	1258162	t	1176175	c	1265400	ns	Sakai/EDL933	rec16	c	Sakai	++++	c	c		c	c			c		g	c	c	c	c	c	c		c	c	c			c
-	1258162	t	1176316	c	1265541	ns	Sakai/EDL933	rec16	c	Sakai	++++	c	g		g	g			c		c	c	c	c	c	c	g		c	c	c			c
-	1258162	c	1176375	t	1265600	ns	Sakai/EDL933	rec16	c	EDL933	++++	c							c		c	c	c	c	c	c			c	c	c	c		c
-	1258162	c	1176509	a	1265734	ns	Sakai/EDL933	rec16	a	Sakai	++++	a							a		a	a	a	a	a	a			a	a	a	a		a
-	1258162	a	1176517	g	1265742	ns	Sakai/EDL933	rec16	g	Sakai	++++	g							a		g	g	g	g	g	g			g	g	g	g		g
-	1258162	a	1176523	g	1265748	ns	Sakai/EDL933	rec16	g	Sakai	++++	g							a		g	g	g	g	g	g			g	g	g	g		g
-	1258162	a	1176532	c	1265757	ns	Sakai/EDL933	rec16	a	EDL933	++++	a							a		a	a	a	a	a	a			a	a	a	a		a
-	1258162	a	1176535	g	1265760	ns	Sakai/EDL933	rec16	g	Sakai	++++	g							g		g	g	g	g	g	g			g	g	g	g		g
-	1258162	c	1176568	t	1265793	ns	Sakai/EDL933	rec16	t	Sakai	++++	t							t		c	t	t	t	t	t			t	t	t	t		t
-	1258162	a	1176580	g	1265805	ns	Sakai/EDL933	rec16	a	EDL933	++++	a							a		a	a	a	a	a	a			a	a	a	a		a
-	1258162	a	1176606	g	1265831	ns	Sakai/EDL933	rec16	g	Sakai	++++	g							g		g	g	g	g	g	g			g	g	g	g		g
-	1258162	a	1176607	g	1265832	ns	Sakai/EDL933	rec16	a	EDL933	++	a							g		a	a	a	a	a	g			a	a	a	a		a
-	1258162	g	1176608	t	1265833	ns	Sakai/EDL933	rec16	g	EDL933	++++	g							c		g	g	g	g	g	t			g	g	g	c		g
-	1258162	c	1176613	a	1265838	ns	Sakai/EDL933	rec16	a	Sakai	++	a							c		c	a	a	a	a	t			a	a	a	c		a
-	1258162	t	1176616	g	1265841	ns	Sakai/EDL933	rec16	g	Sakai	++++	g							a		t	g	g	g	g	c			g	g	g	a		g
-	1258162	t	1176619	c	1265844	ns	Sakai/EDL933	rec16	c	Sakai	++++	c							c		t	c	c	c	c	c			c	c	c	c		c
-	1258162	c	1176622	t	1265847	ns	Sakai/EDL933	rec16	t	Sakai	++	t							c		c	t	t	t	t	c			t	t	t	c		t
-	1258162	t	1176646	c	1265871	ns	Sakai/EDL933	rec16	c	Sakai	++	c							t		t	c	c	c	c	t			c	c	c	t		c
-	1258162	g	1176649	t	1265874	ns	Sakai/EDL933	rec16	t	Sakai	++	t							g		a	t	t	t	t	g			t	t	t	g		t
-	1258162	c	1176652	t	1265877	ns	Sakai/EDL933	rec16	t	Sakai	++	t							c		t	t	t	t	t	c			t	t	t	c		t
-	1258162	g	1176654	c	1265879	ns	Sakai/EDL933	rec16	g	EDL933	++++	g							g		g	g	g	g	g	g			g	g	g	g		g
-	1258162	t	1176661	a	1265886	ns	Sakai/EDL933	rec16	a	Sakai	++	a							t		a	a	a	a	a	t			a	a	a	c		a
-	1258162	g	1176664	a	1265889	ns	Sakai/EDL933	rec16	a	Sakai	++++	a							t		a	a	a	a	a	t			a	a	a	t		a
-	1258162	c	1176665	t	1265890	ns	Sakai/EDL933	rec16	t	Sakai	++	t							c		t	t	t	t	t	c			t	t	t	c		t
-	1258162	a	1176667	t	1265892	ns	Sakai/EDL933	rec16	t	Sakai	++++	t							t		t	t	t	t	t	t			t	t	t	t		t
-	1258162	g	1176676	t	1265901	ns	Sakai/EDL933	rec16	t	Sakai	++	t							g		g	t	t	t	t	g			t	t	t	a		t
-	1258162	c	1176682	g	1265907	ns	Sakai/EDL933	rec16	g	Sakai	++++	g									t	g	g	g	g				g	g	g	c		g
-	1293426	t	1251647	c	1336464	ns	Sakai/EDL933	rec17	t	EDL933	++++				t	t				t		t	t	t	t	t	t							
-	1293426	c	1251664	t	1336481	ns	Sakai/EDL933	rec17	c	EDL933	++++				c	c				c		c	c	c	c	c	c							
-	1293426	g	1251670	a	1336487	ns	Sakai/EDL933	rec17	a	Sakai	+				a	a				g		a	a	a	a	g	a							
-	1293426	t	1251693	c	1336510	s	Sakai/EDL933	rec17	c	Sakai	+				c	c	t			c	t	c	c	c	c	t	c	c						
-	1293426	c	1251696	t	1336513	s	Sakai/EDL933	rec17	c	EDL933	+				t	t	c			t	c	c	c	c	c	c	c	c						
-	1293426	c	1251714	t	1336531	s	Sakai/EDL933	rec17	c	EDL933	++++				c	c	c			c	c	c	c	c	c	c	c	c						
-	1293426	g	1251738	t	1336555	s	Sakai/EDL933	rec17	t	Sakai	+++				t	t	c			t	c	t	c	c	c	g	t	c						
-	1293426	t	1251746	g	1336563	s	Sakai/EDL933	rec17	g	Sakai	+				g	g	g			g	g	t	g	g	g	g	t	g						
-	1293426	c	1251809	t	1336626	ns	Sakai/EDL933	rec17	c	EDL933	+				c	c	c			c	c	c	c	c	c	t	t	c						
-	1293426	a	1251810	g	1336627	s	Sakai/EDL933	rec17	g	Sakai	++++				g	g	a			g	g	g	g	g	g	g	g	g						
-	1293426	c	1251843	t	1336660	ns	Sakai/EDL933	rec17	c	EDL933	++	c	c		c	c	c			c	t	c	c	c	c	c	c	c						
-	1293426	g	1251844	a	1336661	ns	Sakai/EDL933	rec17	g	EDL933	++++	g	g		g	g	g			g	g	g	g	g	g	g	g	g						
-	1293426	a	1251858	t	1336675	ns	Sakai/EDL933	rec17	a	EDL933	++++	a	a		a	a	a			a	t	a	a	a	a	a	a	a						
-	1293426	c	1251946	t	1336763	ns	Sakai/EDL933	rec17	c	EDL933	++	c	c		c	c	c			t	t	c	c	c	c	t	t	t						
-	1293426	c	1251955	t	1336772	ns	Sakai/EDL933	rec17	c	EDL933	++	c	c		c	c	c			t	t	y	c	c	c	t	t	t						
-	1293426	a	1251986	g	1336803	s	Sakai/EDL933	rec17	a	EDL933	++	a	g		a	a	a			a	a	a	t	t	t	a	a	g						
-	1293426	t	1251989	c	1336806	s	Sakai/EDL933	rec17	c	Sakai	++	c	c		c	c	c			c	c	c	t	t	t	c	c	c						
-	1293426	c	1252067	t	1336884	s	Sakai/EDL933	rec17	t	Sakai	++	t	c		c	c	c			t	c	t	c	c	c	t	c	c						

O55 and O157 genome site details												Outgroup Strain Details[i]																						
CB9615 base[b]	CB9615 Site[c]	Sakai base[b]	Sakai Site[c]	EDL933 base[b]	EDL933 Site[c]	type[d]	Event Lineage[ef]	Recombinant segment	Inferred ancestral base[g]	Outgroup Analysis[e]	Support level[h]	D1 Sd197	K-12	HS	ATCC 8739	UMN026	IAI39	SMS 3-5	E2348/69	536	ED1a	CFT073	S88	APEC O1	UTI89	E24377A	IAI1	SE11	F5 8401	F2a 2457T	F2a 301	SS Ss046	B4 Sb227	B18 BS512
-	1293426	a	1252142	g	1336959	s	Sakai/EDL933	rec17	g	Sakai	++	g	g		g	a	g			g	a	g	a	a	a	g	a	a						
-	1293426	a	1252181	g	1336998	s	Sakai/EDL933	rec17	a	EDL933	++	a	g		g	g	a			g	g	g	g	g	g	g	g	g						
-	1293426	a	1252218	g	1337035	ns	Sakai/EDL933	rec17	g	Sakai	++++	g	g		g	g	g			g	g	g	g	g	g	g	a	g						
-	1293426	c	1252247	g	1337064	s	Sakai/EDL933	rec17	c	EDL933	++++	c	g		c	c	c			c	c	c	c	c	c	c	c	c						
-	1293426	t	1252289	a	1337106	s	Sakai/EDL933	rec17	t	EDL933	++	t	c		c	c	c			c	c	c	c	c	c	c	c	c						
-	1293426	t	1252292	c	1337109	s	Sakai/EDL933	rec17	t	EDL933	++	t	c		c	c	t			c	c	c	c	c	c	c	t	c						
-	1293426	g	1252331	a	1337148	s	Sakai/EDL933	rec17	a	Sakai	++++	a	g		a	a	a			a	a	a	a	a	a	a	a	a						
-	1293426	a	1252334	c	1337151	s	Sakai/EDL933	rec17	a	EDL933	++	a	g		c	c	a			c	c	a	c	c	c	c	t	c						
-	1293426	g	1252385	t	1337202	s	Sakai/EDL933	rec17	g	EDL933	++	g	t		g	g	g			g	g	g	g	g	g	g	t	g						
-	1293426	c	1252391	t	1337208	s	Sakai/EDL933	rec17	t	Sakai	++	t	c		c	c	t			c	c	t	c	c	c	c	c	c						
-	1293426	c	1252400	t	1337217	s	Sakai/EDL933	rec17	c	EDL933	++++	c	g		c	c	t			c	c	c	c	c	c	c	c	c						
-	1293426	c	1252411	t	1337228	ns	Sakai/EDL933	rec17	c	EDL933	++++	c	c		c	c	c			c	c	c	c	c	c	c	c	c						
-	1293426	g	1252421	t	1337238	s	Sakai/EDL933	rec17	g	EDL933	++++	g	a		g	g	t			g	g	g	g	g	g	g	g	g						
-	1293426	t	1252451	c	1337268	s	Sakai/EDL933	rec17	c	Sakai	++	c	t		t	c	c			t	t	t	t	t	t	t	t	t						
-	1293426	c	1252460	t	1337277	s	Sakai/EDL933	rec17	c	EDL933	++++	c	a		c	t	c			c	c	c	c	c	c	c	c	c						
-	1293426	t	1252487	c	1337304	s	Sakai/EDL933	rec17	c	Sakai	++++	c	g		c	c	c			c	c	c	c	c	c	c	c	c						
-	1293426	a	1252522	t	1337339	s	Sakai/EDL933	rec17	t	Sakai	++++	t	t		t	t	t			t	t	t	t	t	t	t	t	t						
-	1293426	c	1252534	t	1337351	s	Sakai/EDL933	rec17	c	EDL933	++++	c	c		c	t	c			c	c	c	c	c	c	c	c	c						
-	1293426	a	1252539	t	1337356	ns	Sakai/EDL933	rec17	t	Sakai	++++	t	-		t	t	c			t	t	t	t	t	t	t	t	t						
-	1293426	c	1252546	t	1337363	s	Sakai/EDL933	rec17	c	EDL933	++++	c	-		c	t	c			c	c	c	c	c	c	c	c	c						
-	1293426	a	1252565	g	1337382	ns	Sakai/EDL933	rec17	a	EDL933	++	a	g		g	g	g			g	g	g	g	g	g	g	g	g						
-	1293426	c	1252570	t	1337387	s	Sakai/EDL933	rec17	c	EDL933	++	c	a		c	c	t			c	c	c	c	c	c	c	c	t						
-	1293426	g	1252603	a	1337420	s	Sakai/EDL933	rec17	g	EDL933	++++	g	g		g	a	g			g	g	g	g	g	g	g	g	g						
-	1293426	c	1252690	t	1337507	s	Sakai/EDL933	rec17	t	Sakai	++	t	c		c	c	c			c	c	c	c	c	c	c	c	c						
-	1293426	g	1252738	a	1337555	s	Sakai/EDL933	rec17	a	Sakai	++	a	g		a	a	g			a	a	a	a	a	a	a	a	g						
-	1293426	a	1252756	g	1337573	s	Sakai/EDL933	rec17	g	Sakai	++	g			g	g	g			a	a	g	g	g	g	g	a	g						
-	1293426	c	1252816	g	1337633	s	Sakai/EDL933	rec17	c	EDL933	++	c			g	g	c			c	c	g	c	c	c	c	c	c						
-	1293426	c	1252846	a	1337663	s	Sakai/EDL933	rec17	c	EDL933	++++	c			c	c	c			c	c	c	c	c	c	c	c	c						
-	1293426	g	1252879	c	1337696	s	Sakai/EDL933	rec17	c	Sakai	++++	t			c	c	g			c	t	c	c	c	c	c	t	c						
-	1293426	g	1252882	c	1337699	s	Sakai/EDL933	rec17	g	EDL933	++	g			c	c	g			c	g	c	c	c	c	c	g	c						
-	1293426	t	1252969	c	1337786	s	Sakai/EDL933	rec17	t	EDL933	++	t			c	c	t			c	c	t	t	t	t	c	t	c						
-	1293426	a	1252990	g	1337807	s	Sakai/EDL933	rec17	a	EDL933	++++	a			a	a	a			a	a	a	a	a	a	a	g	a						
-	1293426	a	1253041	g	1337858	s	Sakai/EDL933	rec17	a	EDL933	++++	a			a	a	a			a	a	a	a	a	a	a	g	a						
-	1293426	a	1253160	g	1337977	s	Sakai/EDL933	rec17	a	EDL933	++++	a			a	a	a			a	a	a	a	a	a	a	a	g						
-	1293426	t	1253275	a	1338092	s	Sakai/EDL933	rec17	t	EDL933	++	t			a	t	t			t	t	t	t	t	t	t	a	a						
-	1293426	c	1253305	t	1338122	i	Sakai/EDL933	rec17	t	Sakai	++++	t			t	t	t			t	t	t	t	t	t	t	t	t						
-	1293426	c	1253382	t	1338199	ns	Sakai/EDL933	rec17	t	Sakai	++	t			c	c	c			c	c	c	c	c	c	c	c	c						
-	1293426	g	1253463	t	1338280	ns	Sakai/EDL933	rec17	t	Sakai	++	t			t	t	t			t	t	t	t	t	t	t	g	g						
-	1293426	t	1253466	c	1338283	ns	Sakai/EDL933	rec17	c	Sakai	++++	c			c	c	c			c	c	c	c	c	c	c	c	c						
-	1293426	a	1253490	g	1338307	ns	Sakai/EDL933	rec17	a	EDL933	++	a			g	g	g			g	g	g	g	g	g	g	g	g						
-	1293426	c	1253497	t	1338314	s	Sakai/EDL933	rec17	t	Sakai	++++	t			t	t	t			t	t	t	t	t	t	t	t	t						
-	1293426	t	1253558	c	1338375	ns	Sakai/EDL933	rec17	c	Sakai	++++	c			c	c	c			c	c	c	c	c	c	c	c	c						
-	1293426	-	1253619	a	1338437	indel	Sakai/EDL933	rec17	a	Sakai	+				a	-	a			-	-	a	a	a	a	a	a	a						
-	1293426	a	1253621	g	1338439	ns	Sakai/EDL933	rec17	g	Sakai	+				g	a	g			a	a	g	g	g	g	g	g	g						
-	1293426	g	1253650	a	1338468	ns	Sakai/EDL933	rec17	a	Sakai	+				a	g	a			a	g	a	a	a	a	a	a	a						
-	1293426	g	1253673	t	1338491	s	Sakai/EDL933	rec17	g	EDL933	++++				g	g	g			g	-	g	g	g	g	g	g	g						
-	1293426	t	1253711	c	1338529	ns	Sakai/EDL933	rec17	t	EDL933	++++				t	t	t				t	t	t	t	t									
-	1293426	g	1253741	a	1338559	s	Sakai/EDL933	rec17	g	EDL933	++++				g	g	g				g	g	g	g	g									
-	1293426	c	1253744	t	1338562	s	Sakai/EDL933	rec17	c	EDL933	+++				c	c	c				t	c	c	c	c									
-	1293426	t	1253823	c	1338641	ns	Sakai/EDL933	rec17	t	EDL933	++++				t	t	t				t	t	t	t	t									
-	1293426	a	1253845	g	1338663	ns	Sakai/EDL933	rec17	a	EDL933	++++				a	a	a				a	a	a	a	a									
-	1293426	c	1254019	a	1338837	i	Sakai/EDL933	rec17	c	EDL933	+			a		c	c		a			c	c	c	c									
-	1293426	g	1254025	a	1338843	i	Sakai/EDL933	rec17	g	EDL933	+			a		g	g		a			g	g	g	g									
-	1293426	t	1254036	c	1338854	s	Sakai/EDL933	rec17	c	Sakai	+++			c		t	c		c			c	c	c	c									
-	1293426	g	1254116	a	1338934	ns	Sakai/EDL933	rec17	g	EDL933	++++			g		g	g		g			g	g	g	g									
-	1293426	a	1254245	c	1339063	ns	Sakai/EDL933	rec17	c	Sakai	++++			c		c	c		c			c	c	c	c									
-	1293426	c	1264454	a	1349779	ns	Sakai/EDL933	rec17	c	EDL933	++	c							a		a		a	a	a				a					
-	1293426	a	1264456	g	1349781	ns	Sakai/EDL933	rec17	a	EDL933	+++	a							a		a		a	a	a				a					
-	1293426	t	1264472	c	1349797	ns	Sakai/EDL933	rec17	t	EDL933	+++	t							a		c		t	t	t				a					
-	1293426	c	1264475	t	1349800	ns	Sakai/EDL933	rec17	c	EDL933	+++	c							c		c		c	c	c				c					
-	1293426	c	1264658	a	1349983	ns	Sakai/EDL933	rec17	c	EDL933	+++	c							c		a		c	c	c				c					
-	1293426	c	1264769	t	1350094	ns	Sakai/EDL933	rec17	t	Sakai	+++	t							t		t		t	t	t				t					
-	1293426	t	1264770	g	1350095	ns	Sakai/EDL933	rec17	t	EDL933	+++	t							t		g		t	t	t				a					
-	1293426	t	1264808	c	1350133	ns	Sakai/EDL933	rec17	c	Sakai	+++	c							c		c		c	c	c				c					
-	1293426	c	1264907	a	1350232	ns	Sakai/EDL933	rec17	c	EDL933	+++	c							c		c		c	c	c				c					
-	1293426	c	1264919	a	1350244	ns	Sakai/EDL933	rec17	c	EDL933	+++	c							c		c		c	c	c				c					
-	1293426	g	1264934	t	1350259	ns	Sakai/EDL933	rec17	g	EDL933	+++	g							g		g		g	g	g				g					
-	1293426	a	1264944	g	1350269	ns	Sakai/EDL933	rec17	g	Sakai	+++	g							g		g		g	g	g				g					
-	1293426	a	1264990	c	1350315	ns	Sakai/EDL933	rec17	a	EDL933	+										a													
-	1293426	c	1264993	t	1350318	ns	Sakai/EDL933	rec17	c	EDL933	+										c													
-	1293426	c	1306660	g	1390671	ns	Sakai/EDL933	rec18	?	Sakai/EDL933	+/-																							
-	1293426	a	1306676	c	1390687	s	Sakai/EDL933	rec18	a	EDL933	+										a													
-	1293426	c	1306755	t	1390766	ns	Sakai/EDL933	rec18	c	EDL933	+										c													
-	1293426	t	1306770	g	1390781	ns	Sakai/EDL933	rec18	g	Sakai	+										g													
-	1293426	-	1306813	t	1390825	indel	Sakai/EDL933	rec18	t	Sakai	+										t													
-	1293426	g	1306826	t	1390838	ns	Sakai/EDL933	rec18	g	EDL933	+										g													
-	1293426	t	1306833	a	1390845	ns	Sakai/EDL933	rec18	a	Sakai	+										a													
-	1293426	c	1306839	t	1390851	ns	Sakai/EDL933	rec18	t	Sakai	+										t													
-	1293426	t	1306861	c	1390873	s	Sakai/EDL933	rec18	c	Sakai	+										c													
-	1293426	g	1307002	a	1391014	ns	Sakai/EDL933	rec18	g	EDL933	+										g													
-	1293426	c	1307004	t	1391016	ns	Sakai/EDL933	rec18	t	Sakai	+										t													
-	1293426	c	1307008	t	1391020	ns	Sakai/EDL933	rec18	c	EDL933	+										c													
-	1293426	t	1307010	c	1391022	ns	Sakai/EDL933	rec18	c	Sakai	+										c													
-	1293426	a	1307291	g	1391032	ns	Sakai/EDL933	rec18	?	Sakai/EDL933	+/-																							
-	1293426	c	1307292	a	1391033	ns	Sakai/EDL933	rec18	?	Sakai/EDL933	+/-																							

O55 and O157 genome site details												Outgroup Strain Details[i]																						
CB9615 base[b]	CB9615 Site[c]	Sakai base[b]	Sakai Site[c]	EDL933 base[b]	EDL933 Site[c]	type[d]	Event Lineage[ef]	Recombinant segment	Inferred ancestral base[g]	Outgroup Analysis[e]	Support level[h]	D1 Sd197	K-12	HS	ATCC 8739	UMN026	IAI39	SMS 3-5	E2348/69	536	ED1a	CFT073	S88	APEC O1	UTI89	E24377A	IAI1	SE11	F5 8401	F2a 2457T	F2a 301	SS Ss046	B4 Sb227	B18 BS512
-	1293426	g	1307305	a	1391046	ns	Sakai/EDL933	rec18	?	Sakai/EDL933	+/-																							
-	1293426	t	1307306	c	1391047	ns	Sakai/EDL933	rec18	?	Sakai/EDL933	+/-																							
-	1293426	t	1307314	c	1391055	ns	Sakai/EDL933	rec18	?	Sakai/EDL933	+/-																							
-	1293426	a	1307317	c	1391058	ns	Sakai/EDL933	rec18	?	Sakai/EDL933	+/-																							
-	1293426	t	1307319	g	1391060	ns	Sakai/EDL933	rec18	?	Sakai/EDL933	+/-																							
-	1293426	g	1307325	c	1391066	ns	Sakai/EDL933	rec18	?	Sakai/EDL933	+/-																							
-	1293426	g	1307335	t	1391076	ns	Sakai/EDL933	rec18	?	Sakai/EDL933	+/-																							
-	1293426	g	1307338	a	1391079	ns	Sakai/EDL933	rec18	?	Sakai/EDL933	+/-																							
-	1293426	c	1307343	t	1391084	ns	Sakai/EDL933	rec18	?	Sakai/EDL933	+/-																							
-	1293426	t	1307344	c	1391085	ns	Sakai/EDL933	rec18	?	Sakai/EDL933	+/-																							
-	1293426	t	1307346	g	1391087	ns	Sakai/EDL933	rec18	?	Sakai/EDL933	+/-																							
-	1293426	g	1307347	a	1391088	ns	Sakai/EDL933	rec18	?	Sakai/EDL933	+/-																							
-	1293426	t	1307350	a	1391091	ns	Sakai/EDL933	rec18	?	Sakai/EDL933	+/-																							
-	1293426	a	1307353	c	1391094	ns	Sakai/EDL933	rec18	?	Sakai/EDL933	+/-																							
-	1293426	c	1307354	t	1391095	ns	Sakai/EDL933	rec18	?	Sakai/EDL933	+/-																							
-	1293426	a	1307359	g	1391100	ns	Sakai/EDL933	rec18	?	Sakai/EDL933	+/-																							
-	1293426	g	1307370	t	1391111	ns	Sakai/EDL933	rec18	?	Sakai/EDL933	+/-																							
-	1293426	c	1307371	t	1391112	ns	Sakai/EDL933	rec18	?	Sakai/EDL933	+/-																							
-	1293426	c	1307383	g	1391124	ns	Sakai/EDL933	rec18	?	Sakai/EDL933	+/-																							
-	1355153	g	1453955	K	1537727	ns	EDL933	rec19	g	EDL933	++++	g	g			g	g	g	g	g	g	g	g	g	g	g				g	g			g
-	1355153	a	1453956	R	1537728	ns	EDL933	rec19	a	EDL933	++++	a	a			a	a	a	a	a	a	a	a	a	a	a				a	a			a
-	1355153	g	1453957	K	1537729	ns	EDL933	rec19	g	EDL933	++++	g	g			g	g	a	g	g	g	g	g	g	g	g				g	g			g
-	1355153	t	1453960	W	1537732	ns	EDL933	rec19	t	EDL933	++++	t	t			t	t	t	t	t	t	t	t	t	t	t				t	t			t
-	1355153	c	1453961	M	1537733	ns	EDL933	rec19	c	EDL933	++++	c	c			c	c	c	c	c	c	c	c	c	c	c				c	c			c
-	1355153	c	1453963	Y	1537735	ns	EDL933	rec19	c	EDL933	++++	c	c			c	c	c	c	c	c	c	c	c	c	c				c	c			c
-	1355153	t	1453966	Y	1537738	ns	EDL933	rec19	t	EDL933	++++	t	t			t	t	t	t	t	t	t	t	t	t	t				t	t			t
-	1355153	g	1453967	K	1537739	ns	EDL933	rec19	g	EDL933	++++	g	g			g	g	g	g	g	g	g	g	g	g	g				g	g			g
-	1355153	a	1453968	R	1537740	ns	EDL933	rec19	a	EDL933	++++	a	a			a	a	a	a	a	a	a	a	a	a	a				a	a			a
-	1355153	a	1453969	R	1537741	ns	EDL933	rec19	a	EDL933	++++	a	a			a	a	a	a	a	a	a	a	a	a	a				a	a			a
-	1355153	c	1453970	M	1537742	ns	EDL933	rec19	c	EDL933	++++	c	c			c	c	c	c	c	c	c	c	c	c	c				c	c			c
-	1355153	t	1453971	W	1537743	ns	EDL933	rec19	t	EDL933	++++	t	t			t	t	t	t	t	t	t	t	t	t	t				t	t			t
-	1355153	g	1453972	R	1537744	ns	EDL933	rec19	g	EDL933	++++	g	g			g	g	g	g	g	g	g	g	g	g	g				g	g			g
-	1355153	a	1453973	R	1537745	ns	EDL933	rec19	a	EDL933	++++	a	a			a	a	a	a	a	a	a	a	a	a	a				a	a			a
-	1355153	t	1453974	Y	1537746	i	EDL933	rec19	t	EDL933	++++	t				t		t	t	t	t	t	t		t	t								
-	1355153	g	1453975	K	1537747	i	EDL933	rec19	g	EDL933	++++	g				g		g	g	g	g	g	g		g	g								
-	1355153	t	1453976	K	1537748	i	EDL933	rec19	t	EDL933	++++	t				t		t	t	t	t	t	t		t	t								
-	1355153	t	1453977	K	1537749	i	EDL933	rec19	t	EDL933	+++	a				t		a	t	t	t	a	t		a	t								
-	1355153	c	1453978	Y	1537750	i	EDL933	rec19	c	EDL933	++++	c				c		c	c	c	c	c	c		c	c								
-	1355153	c	1453979	Y	1537751	i	EDL933	rec19	c	EDL933	++++	c				c		c	c	c	c	c	c		c	c								
-	1355153	t	1453981	Y	1537753	i	EDL933	rec19	t	EDL933	++++	t				t		t	t	t	t	t	t		c	t								
-	1355153	c	1453982	S	1537754	i	EDL933	rec19	c	EDL933	++++	c				c		c	c	c	c	c	c		c	c								
-	1355153	a	1453983	W	1537755	i	EDL933	rec19	a	EDL933	++++	a				a		a	a	a	a	a	a		a	a								
-	1355153	c	1453984	S	1537756	i	EDL933	rec19	c	EDL933	+++	a				c		a	c	c	c	a	c		g	c								
-	1355153	c	1453985	M	1537757	i	EDL933	rec19	c	EDL933	++++	c				c		c	c	c	c	c	c		c	c								
-	1355153	c	1453986	M	1537758	i	EDL933	rec19	c	EDL933	+++	t				c		t	c	c	c	t	c		t	c								
-	1355153	g	1453987	R	1537759	i	EDL933	rec19	g	EDL933	++++	g				g		g	g	g	g	g	g		g	g								
-	1355153	g	1453988	R	1537760	i	EDL933	rec19	g	EDL933	++++	g				g		g	g	g	g	g	g		g	g								
-	1355153	a	1453991	W	1537763	i	EDL933	rec19	a	EDL933	++++	a				a		a	a	a	a	a	a		a	a								
-	1355153	a	1453992	R	1537764	i	EDL933	rec19	a	EDL933	++++	a				a		a	a	a	a	a	a		a	a								
-	1355153	c	1453993	M	1537765	i	EDL933	rec19	c	EDL933	++++	c				c		c	c	c	c	c	c		c	c								
-	1355153	g	1453994	R	1537766	i	EDL933	rec19	g	EDL933	++++	g				g		g	g	g	g	g	g		g	g								
-	1355153	a	1453995	R	1537767	i	EDL933	rec19	a	EDL933	++++	a				a		a	a	a	a	a	a		a	a								
-	1355153	g	1453996	R	1537768	i	EDL933	rec19	g	EDL933	++++	g				g		g	g	g	g	g	g		g	g								
-	1355153	t	1453997	Y	1537769	i	EDL933	rec19	t	EDL933	++++	t				t		t	t	t	t	t	t		t	t								
-	1355153	a	1453998	M	1537770	i	EDL933	rec19	a	EDL933	++++	a				a		a	a	a	a	a	a		a	a								
-	1355153	g	1454000	K	1537772	i	EDL933	rec19	g	EDL933	++++	g				g		g	g	g	g	g	g		g	g								
-	1355153	a	1454001	R	1537773	i	EDL933	rec19	a	EDL933	++++	a				a		a	a	a	a	a	a		a	a								
-	1355153	c	1454002	Y	1537774	i	EDL933	rec19	c	EDL933	++++	c				c		c	c	c	c	c	c		c	c								
-	1355153	a	1454004	R	1537776	i	EDL933	rec19	a	EDL933	+	g				g		g	a	g	a	g	g		g	g								
-	1355153	g	1454005	S	1537777	i	EDL933	rec19	g	EDL933	++++	g				g		g	g	g	g	g	g		g	g								
-	1355153	a	1454006	R	1537778	i	EDL933	rec19	a	EDL933	+	c				c		c	a	c	a	c	c		c	c								
-	1355153	t	1454007	W	1537779	i	EDL933	rec19	t	EDL933	++++	t				a		t	t	t	t	t	t		t	t								
-	1355153	g	1454008	S	1537780	i	EDL933	rec19	g	EDL933	++++	g				g		g	g	g	g	g	g		g	g								
-	1355153	c	1454011	Y	1537783	i	EDL933	rec19	c	EDL933	++++	c				c		c	c	c	c	c	c		c	c								
-	1355153	c	1454013	M	1537785	i	EDL933	rec19	c	EDL933	++++	c				c		c	c	c	c	c	c		c	c								
-	1355153	a	1454014	R	1537786	i	EDL933	rec19	a	EDL933	++++	a				a		a	a	a	a	a	a		a	a								
-	1355153	g	1454015	R	1537787	i	EDL933	rec19	g	EDL933	++++	g				g		g	g	g	g	g	g		g	g								
-	1355153	a	1454016	R	1537788	i	EDL933	rec19	a	EDL933	++++	a				a		a	a	a	a	a	a		a	a								
-	1355153	c	1454017	Y	1537789	i	EDL933	rec19	c	EDL933	++++	t				c		c	c	c	c	c	c		c	c								
-	1355153	g	1454018	K	1537790	i	EDL933	rec19	g	EDL933	++++	a				g		g	g	g	g	g	g		g	g								
-	1355153	c	1454019	S	1537791	i	EDL933	rec19	c	EDL933	++++	c				c		c	c	c	c	c	c		c	c								
-	1355153	c	1454020	S	1537792	i	EDL933	rec19	c	EDL933	++++	c				c		c	c	c	c	c	c		c	c								
-	1355153	g	1454021	S	1537793	i	EDL933	rec19	g	EDL933	++++	g				g		g	g	g	g	g	g		g	g								
-	1355153	c	1454022	S	1537794	i	EDL933	rec19	c	EDL933	++++	c				c		c	c	c	c	c	c		c	c								
-	1355153	t	1454023	W	1537795	i	EDL933	rec19	t	EDL933	++++	t				t		t	t	t	t	t	t		t	t								
-	1355153	g	1454024	R	1537796	i	EDL933	rec19	g	EDL933	++++	g				g		g	g	g	g	g	g		g	g								
-	1355153	t	1454025	K	1537797	i	EDL933	rec19	t	EDL933	++++	t				t		t	t	t	t	t	t		t	t								
-	1355153	g	1454027	S	1537799	i	EDL933	rec19	g	EDL933	++++	g				g		g	g	g	g	g	g		g	g								
-	1355153	a	1454028	R	1537800	i	EDL933	rec19	a	EDL933	++++	a				a		a	a	a	a	a	a		a	a								
-	1355153	g	1454030	K	1537802	i	EDL933	rec19	g	EDL933	++++	g				g		g	g	g	g	g	g		g	g								
-	1355153	a	1454031	M	1537803	i	EDL933	rec19	a	EDL933	++++	a				a		a	a	a	a	a	a		a	a								
-	1355153	g	1454033	S	1537805	i	EDL933	rec19	g	EDL933	++++	g				g		g	g	g	g	g	g		g	g								
-	1355153	c	1454034	S	1537806	i	EDL933	rec19	c	EDL933	++++	c				t		c	c	c	c	c	t		c	c								
-	1355153	c	1454035	Y	1537807	i	EDL933	rec19	c	EDL933	++++	c				c		c	c	c	c	c	c		c	c								
-	1355153	t	1454037	Y	1537809	i	EDL933	rec19	t	EDL933	++++	t				t		t	t	t	t	t	t		t	t								

O55 and O157 genome site details												Outgroup Strain Details[i]																						
CB9615 base[b]	CB9615 Site[c]	Sakai base[b]	Sakai Site[c]	EDL933 base[b]	EDL933 Site[c]	type[d]	Event Lineage[ef]	Recombinant segment	Inferred ancestral base[g]	Outgroup Analysis[e]	Support level[h]	D1 Sd197	K-12	HS	ATCC 8739	UMN026	IAI39	SMS 3-5	E2348/69	536	ED1a	CFT073	S88	APEC O1	UTI89	E24377A	IAI1	SE11	F5 8401	F2a 2457T	F2a 301	SS Ss046	B4 Sb227	B18 BS512
-	1355153	t	1454038	K	1537810	i	EDL933	rec19	t	EDL933	+++	g				g		t	t	g	t	g	g		t	g								
-	1355153	t	1454040	W	1537812	i	EDL933	rec19	t	EDL933	++++	t				t		t	t	t	t	t	t		a	t								
-	1355153	a	1454042	W	1537814	i	EDL933	rec19	a	EDL933	++++	a				a		a	a	a	g	a	a		a	a								
-	1355153	c	1454044	M	1537816	i	EDL933	rec19	c	EDL933	++++	c				c		c	c	a	c	c	c		c	c								
-	1355153	a	1454046	M	1537818	i	EDL933	rec19	a	EDL933	++++	a				a		a	a	a	a	a	a		a	a								
-	1355153	t	1454047	Y	1537819	i	EDL933	rec19	t	EDL933	+	c				c		c	t	c	t	c	c		t	c								
-	1355153	g	1454048	R	1537820	i	EDL933	rec19	g	EDL933	++++	g				g		g	g	g	g	g	g		g	g								
-	1355153	c	1454049	S	1537821	i	EDL933	rec19	c	EDL933	+	a				a		a	c	a	c	a	a		c	a								
-	1355153	a	1454050	W	1537822	i	EDL933	rec19	a	EDL933	++++	a				a		a	a	a	a	a	a		a	a								
-	1355153	t	1454051	K	1537823	i	EDL933	rec19	t	EDL933	++++	t				t		t	t	t	t	t	t		t	t								
-	1355153	a	1454052	R	1537824	i	EDL933	rec19	a	EDL933	++++	a				a		a	a	a	a	a	a		a	a								
-	1355153	a	1454054	M	1537826	i	EDL933	rec19	a	EDL933	++++	a				a		a	a	a	a	a	a		a	a								
-	1355153	t	1454055	K	1537827	i	EDL933	rec19	t	EDL933	+	c				c		c	t	c	t	c	c		c	c								
-	1355153	c	1454056	S	1537828	i	EDL933	rec19	c	EDL933	++++	c				c		c	c	c	c	c	c		c	c								
-	1355153	t	1454057	Y	1537829	i	EDL933	rec19	t	EDL933	++++	t				t		t	t	t	t	t	t		t	t								
-	1355153	t	1454058	W	1537830	i	EDL933	rec19	t	EDL933	++++	t				t		t	t	t	t	t	t		t	t								
-	1355153	a	1454060	W	1537832	i	EDL933	rec19	a	EDL933	++++	a				a		a	a	a	a	a	a		a	a								
-	1355153	c	1454061	S	1537833	i	EDL933	rec19	c	EDL933	++++	c				c		c	c	c	c	c	c		c	c								
-	1355153	c	1454062	M	1537834	i	EDL933	rec19	c	EDL933	++++	c				c		c	c	c	c	c	c		c	c								
-	1355153	g	1454063	S	1537835	i	EDL933	rec19	g	EDL933	++++	g				g		g	g	g	g	g	g		g	g								
-	1355153	a	1454064	R	1537836	i	EDL933	rec19	a	EDL933	++++	a				a		a	a	a	a	a	a		a	a								
-	1355153	a	1454065	W	1537837	i	EDL933	rec19	a	EDL933	++++	a				a		a	a	a	a	a	a		a	a								
-	1355153	c	1454068	Y	1537840	i	EDL933	rec19	c	EDL933	++++	c				c		c	c	c	c	c	c		c	c								
-	1355153	g	1454069	R	1537841	i	EDL933	rec19	g	EDL933	++++	g				g		g	g	g	g	g	g		g	g								
-	1355153	g	1454070	K	1537842	i	EDL933	rec19	g	EDL933	++++	g				g		g	g	g	g	g	g		g	g								
-	1355153	t	1454071	K	1537843	i	EDL933	rec19	t	EDL933	++++	t				t		t	t	t	t	t	t		t	t								
-	1439906	t	1552060	c	1637156	ns	Sakai/EDL933	rec20	c	Sakai	++++	c	c		c	c		c	c		c	c	c	c	c	c	c	c	c	c	c	c	c	c
-	1439906	a	1552061	t	1637157	ns	Sakai/EDL933	rec20	a	EDL933	++	a	t		t	t		t	a		a	a	a	a	a	a	t	t	a	a	a	a	a	a
-	1439906	c	1552064	t	1637160	ns	Sakai/EDL933	rec20	c	EDL933	++	c	t		t	t		t	c		c	c	c	c	c	c	t	t	c	c	c	c	c	c
-	1439906	t	1552079	c	1637175	ns	Sakai/EDL933	rec20	c	Sakai	++++	c	c		c	c		c	c		c	c	c	c	c	c	c	c	c	c	c	c	c	c
-	1439906	c	1552088	t	1637184	ns	Sakai/EDL933	rec20	c	EDL933	++++	c	a		a	a		c	c		c	c	c	c	c	c	a	c	c	c	c	c	c	c
-	1439906	t	1552094	a	1637190	ns	Sakai/EDL933	rec20	t	EDL933	++	t	t		t	t		a	t		t	t	t	t	t	t	t	a	t	t	t	t	t	t
-	1439906	c	1552097	t	1637193	ns	Sakai/EDL933	rec20	c	EDL933	++	c	c		c	c		t	c		c	c	c	c	c	c	c	t	c	c	c	c	c	c
-	1439906	g	1552151	c	1637247	ns	Sakai/EDL933	rec20	c	Sakai	+	t	t		t	t		t	c		t	t	t	t	t	c	t	t	t	t	t	t	t	c
-	1439906	g	1552154	c	1637250	ns	Sakai/EDL933	rec20	c	Sakai	++++	c	c		c	c		c	c		c	c	c	c	c	c	c	c	c	c	c	c	c	c
-	1439906	t	1552163	c	1637259	ns	Sakai/EDL933	rec20	c	Sakai	++++	c	g		g	g		c	c		c	c	c	c	c	a	g	c	c	c	c	c	c	c
-	1439906	t	1552166	a	1637262	ns	Sakai/EDL933	rec20	a	Sakai	++++	a	a		a	a		a	a		a	a	a	a	a	a	a	a	a	a	a	a	a	a
-	1439906	g	1552172	t	1637268	ns	Sakai/EDL933	rec20	g	EDL933	+++	c	g		g	g		c	c		c	c	c	c	c	t	g	c	c	c	c	c	c	c
-	1439906	t	1552175	g	1637271	ns	Sakai/EDL933	rec20	g	Sakai	++++	g	g		g	g		g	g		g	g	g	g	g	g	g	g	g	g	g	g	g	g
-	1439906	a	1552215	g	1637311	ns	Sakai/EDL933	rec20	g	Sakai	++++	g	g		g	g		g	g		g	g	g	g	g	g	g	g	g	g	g	g	g	g
-	1439906	t	1552223	c	1637319	ns	Sakai/EDL933	rec20	t	EDL933	++	t	t		t	t		c	c		t	t	c	c	c	t	t	c	t	t	t	t	t	c
-	1439906	g	1552239	a	1637335	ns	Sakai/EDL933	rec20	a	Sakai	++++	a	a		a	a		a	t		a	a	t	t	t	g	a	a	a	a	a	a	a	t
-	1439906	c	1552240	t	1637336	ns	Sakai/EDL933	rec20	c	EDL933	++++	c	c		c	c		c	c		c	c	c	c	c	c	c	c	c	c	c	c	c	c
-	1439906	g	1552247	a	1637343	ns	Sakai/EDL933	rec20	g	EDL933	++	g	g		g	g		g	a		g	g	a	a	a	g	g	g	g	g	g	g	g	a
-	1439906	t	1552250	g	1637346	ns	Sakai/EDL933	rec20	t	EDL933	++	t	g		g	g		g	g		t	t	g	g	g	g	g	g	t	t	t	t	t	g
-	1439906	t	1552259	g	1637355	ns	Sakai/EDL933	rec20	t	EDL933	++	t	t		t	t		g	g		t	t	g	g	g	g	t	g	t	t	t	t	t	g
-	1439906	g	1552283	a	1637379	ns	Sakai/EDL933	rec20	g	EDL933	++++	g	g		g	g		g	g		g	g	g	g	g	t	g	g	g	g	g	g	g	g
-	1439906	c	1552287	t	1637383	ns	Sakai/EDL933	rec20	c	EDL933	++	c	t		t	t		t	c		c	c	c	c	c	t	t	t	c	c	c	c	c	c
-	1439906	c	1552300	t	1637396	ns	Sakai/EDL933	rec20	c	EDL933	++	c	t		t	t		c	c		c	c	c	c	c	c	t	c	c	c	c	c	c	c
-	1439906	g	1552307	a	1637403	ns	Sakai/EDL933	rec20	g	EDL933	++	g	a		t	a		g	g		g	g	g	g	g	a	a	g	g	g	g	g	g	g
-	1439906	c	1552308	t	1637404	ns	Sakai/EDL933	rec20	c	EDL933	++++	c	c		c	c		c	c		c	c	c	c	c	c	c	c	c	c	c	c	c	c
-	1439906	g	1552313	t	1637409	ns	Sakai/EDL933	rec20	g	EDL933	++	g	t		t	t		g	g		g	g	g	g	g	g	t	g	g	g	g	g	g	g
-	1439906	a	1552316	g	1637412	ns	Sakai/EDL933	rec20	a	EDL933	++	a	g		g	g		g	a		a	a	a	a	a	g	g	g	a	a	a	a	a	a
-	1439906	g	1552319	a	1637415	ns	Sakai/EDL933	rec20	g	EDL933	++	g	t		t	t		a	g		g	g	g	g	g	a	t	a	g	g	g	g	a	g
-	1439906	t	1552324	c	1637420	ns	Sakai/EDL933	rec20	t	EDL933	++	t	c		c	c		t	t		t	t	t	t	t	t	c	t	t	t	t	t	t	t
-	1439906	a	1552325	g	1637421	ns	Sakai/EDL933	rec20	a	EDL933	++	a	g		g	g		a	a		a	a	a	a	a	a	g	a	a	a	a	a	a	a
-	1439906	g	1552334	t	1637430	ns	Sakai/EDL933	rec20	g	EDL933	++	g	g		g	g		t	g		g	g	g	g	g	t	g	t	g	g	g	g	g	g
-	1439906	g	1552340	a	1637436	ns	Sakai/EDL933	rec20	g	EDL933	++	g	a		a	a		g	g		g	g	g	g	g	g	a	g	g	g	g	g	g	g
-	1439906	g	1552343	a	1637439	ns	Sakai/EDL933	rec20	g	EDL933	++	g	a		a	a		a	g		g	g	g	g	g	a	a	a	g	g	g	g	g	g
-	1439906	g	1552345	c	1637441	ns	Sakai/EDL933	rec20	g	EDL933	++	g	c		c	c		g	g		g	g	g	g	g	g	c	g	g	g	g	g	g	g
-	1439906	t	1552346	g	1637442	ns	Sakai/EDL933	rec20	t	EDL933	++++	t	a		a	a		t	t		t	t	t	t	t	t	a	t	t	t	t	t	t	t
-	1439906	t	1552449	c	1637545	ns	Sakai/EDL933	rec20	c	Sakai	++	c	t		t	t		c	c		c	c	c	c	c	c	t	c	c	c	c	c	c	c
-	1439906	g	1552524	a	1637620	ns	Sakai/EDL933	rec20	g	EDL933	++++	g	g		g	g		g	g		g	g	g	g	g	g	g	g	g	g	g	g	g	g
-	1439906	g	1552534	t	1637630	ns	Sakai/EDL933	rec20	t	Sakai	++++	t	t		t	t			t		t	t	t	t	t	t	t		t	t	t			t
-	1439906	c	1552658	t	1637754	ns	Sakai/EDL933	rec20	c	EDL933	++++	c	c		c	c			c		a	c	c	c	c	c	c		c	c	c			c
-	1439906	c	1552799	t	1637895	ns	Sakai/EDL933	rec20	c	EDL933	++++	c	g		g	g			c		c	c	c	c	c	c	g		c	c	c			c
-	1439906	t	1552858	Y	1637954	ns	Sakai/EDL933	rec20	t	EDL933	++++	c							c		c	c	c	c	c	c			c	c	c	c		c
-	1439906	a	1552992	c	1638088	ns	Sakai/EDL933	rec20	a	EDL933	++++	a							a		c	a	a	a	a	a			a	a	a	a		a
-	1439906	g	1553000	a	1638096	ns	Sakai/EDL933	rec20	g	EDL933	++	g							a		a	g	g	g	g	g			g	g	g	a		g
-	1439906	g	1553006	a	1638102	ns	Sakai/EDL933	rec20	g	EDL933	++	g							a		a	g	g	g	g	g			g	g	g	a		g
-	1439906	c	1553015	a	1638111	ns	Sakai/EDL933	rec20	a	Sakai	++++	a							a		a	a	a	a	a	a			a	a	a	a		a
-	1439906	g	1553018	a	1638114	ns	Sakai/EDL933	rec20	g	EDL933	++	g							g		a	g	g	g	g	g			g	g	g	a		g
-	1439906	t	1553051	c	1638147	ns	Sakai/EDL933	rec20	t	EDL933	++++	t							t		t	t	t	t	t	t			t	t	t	t		t
-	1439906	g	1553063	a	1638159	ns	Sakai/EDL933	rec20	a	Sakai	++	a							a		g	a	a	a	a	a			a	a	a	g		a
-	1439906	a	1571409	g	1657548	ns	Sakai/EDL933	rec20	g	Sakai	+++							-	g		g			-									g	g
t	1445562	g	1595560	g	1679536	s	O55/O157	rec21	g	CB9615	+++						g				t	g							g	g	g	g		
g	1445568	a	1595566	a	1679542	s	O55/O157	rec21	a	CB9615	+++						a				g	a							a	a	a	a		
g	1445571	a	1595569	a	1679545	s	O55/O157	rec21	a	CB9615	+++						a				g	a							a	a	a	a		
a	1445589	g	1595587	g	1679563	s	O55/O157	rec21	g	CB9615	+++						g				c	g							g	g	g	a		
c	1445592	a	1595590	a	1679566	s	O55/O157	rec21	a	CB9615	+++						a				a	a							a	a	a	a		
t	1445604	c	1595602	c	1679578	s	O55/O157	rec21	c	CB9615	+++						c				c	c							c	c	c	a		
a	1445629	g	1595627	g	1679603	ns	O55/O157	rec21	g	CB9615	+++						g				g	g							g	g	g	g		
c	1445664	t	1595662	t	1679638	s	O55/O157	rec21	c	O157	+++						c				c	c							c	c	c	a		
g	1445691	a	1595689	a	1679665	s	O55/O157	rec21	a	CB9615	+++						a				g	a							a	a	a	a		

O55 and O157 genome site details												Outgroup Strain Details[i]																						
CB9615 base[b]	CB9615 Site[c]	Sakai base[b]	Sakai Site[c]	EDL933 base[b]	EDL933 Site[c]	type[d]	Event Lineage[e,f]	Recombinant segment	Inferred ancestral base[g]	Outgroup Analysis[e]	Support level[h]	D1 Sd197	K-12	HS	ATCC 8739	UMN026	IAI39	SMS 3-5	E2348/69	536	ED1a	CFT073	S88	APEC O1	UTI89	E24377A	IAI1	SE11	F5 8401	F2a 2457T	F2a 301	SS Ss046	B4 Sb227	B18 BS512
g	1445692	a	1595690	a	1679666	ns	O55/O157	rec21	a	CB9615	+++						a				g	a							a	a	a	t		
c	1445694	a	1595692	a	1679668	ns	O55/O157	rec21	a	CB9615	+++						a				c	a							a	a	a	a		
a	1445700	g	1595698	g	1679674	s	O55/O157	rec21	g	CB9615	+						g				a	g							g	g	g	a		
t	1445709	c	1595707	c	1679683	s	O55/O157	rec21	c	CB9615	+++						c				t	c							c	c	c	c		
a	1445712	c	1595710	c	1679686	s	O55/O157	rec21	c	CB9615	+						c				a	c							c	c	c	a		
c	1445724	t	1595722	t	1679698	s	O55/O157	rec21	c	O157	+++						c				c	c							c	c	c	c		
t	1445733	a	1595731	a	1679707	s	O55/O157	rec21	a	CB9615	+						a				t	a							a	a	a	t		
g	1445754	a	1595752	a	1679728	s	O55/O157	rec21	a	CB9615	+						a				g	g							a	a	a	g		
c	1445781	g	1595779	g	1679755	s	O55/O157	rec21	g	CB9615	+++						g				c	g							g	g	g	g		
t	1445802	g	1595800	g	1679776	s	O55/O157	rec21	g	CB9615	+++						g				t	g							g	g	g	g		
g	1445808	t	1595806	t	1679782	s	O55/O157	rec21	t	CB9615	+++						t				g	t							t	t	t	t		
t	1445811	c	1595809	c	1679785	s	O55/O157	rec21	c	CB9615	+						c				t	c							c	c	c	t		
g	1445886	t	1595884	t	1679860	s	O55/O157	rec21	t	CB9615	+++						t				t	g							t	t	t	a		
t	1445990	-	1595987	-	1679963	ins	O55/O157	rec21	-	CB9615	+++						-				-	-							-	-	-			
t	1446004	c	1596001	c	1679977	i	O55/O157	rec21	t	O157	+++						t				t	g							t	t	t			
a	1446042	g	1596039	g	1680015	i	O55/O157	rec21	a	O157	+++						a				a	a							a	a	a			
t	1446067	c	1596064	c	1680040	i	O55/O157	rec21	t	O157	+++						t				t	a							t	t	t			
g	1446179	a	1596176	a	1680152	i	O55/O157	rec21	a	CB9615	+++						a				a	a							a	a	a			
t	1446234	g	1596231	g	1680207	i	O55/O157	rec21	t	O157	+						a				a	t							a	a	a			
t	1446356	c	1596353	c	1680329	s	O55/O157	rec21	c	CB9615	+++						c				c	c							c	c	c			
a	1446552	g	1596549	g	1680525	ns	O55/O157	rec21	g	CB9615	+						g				a	a							g	g	g			
g	1446647	a	1596644	a	1680620	s	O55/O157	rec21	a	CB9615	+++						a				g								a	a	a			
g	1446652	a	1596649	a	1680625	ns	O55/O157	rec21	g	O157	+++						a				g								g	g	g			
c	1446658	a	1596655	a	1680631	ns	O55/O157	rec21	c	O157	+++						a				c								c	c	c			
c	1446680	t	1596677	t	1680653	s	O55/O157	rec21	c	O157	+++						a				c								c	c	c			
t	1446738	g	1596735	g	1680711	ns	O55/O157	rec21	g	CB9615	+						g				t								g	g	g		t	
a	1446740	t	1596737	t	1680713	ns	O55/O157	rec21	t	CB9615	+						t				a								t	t	t		a	
a	1446746	g	1596743	g	1680719	s	O55/O157	rec21	g	CB9615	+						g				a								g	g	g		a	
a	1446748	g	1596745	g	1680721	ns	O55/O157	rec21	g	CB9615	+						g				a								g	g	g		a	
t	1446804	a	1596801	a	1680777	ns	O55/O157	rec21	a	CB9615	+++						g				t								a	a	a		a	
g	1446841	a	1596838	a	1680814	ns	O55/O157	rec21	?	O55/O157	+/-										g								t	t	t		a	
g	1446846	t	1596843	t	1680819	ns	O55/O157	rec21	t	CB9615	+++										g								t	t	t		t	
c	1446893	t	1596890	t	1680866	s	O55/O157	rec21	c	O157	+++										c								c	c	c		t	
g	1447055	a	1597052	a	1681028	ns	O55/O157	rec21	g	O157	+++										g								g	g	g		g	
c	1463703	t	1617951	t	1701924	s	O55/O157	rec22	t	CB9615	++	t	t	t	t	c	t	t	t	c	t	c	t	t	t	c	c	c	c	c	c	g	g	g
c	1463754	t	1618002	t	1701975	s	O55/O157	rec22	c	O157	++	c	t	t	t	c	t	c	c	c	t	t	t	t	t	t	t	t	c	c	c	c	c	c
g	1463802	a	1618050	a	1702023	s	O55/O157	rec22	a	CB9615	++	a	g	g	g	a	a	a	a	a	a	g	a	a	a	a	a	a	a	a	a	g	g	g
a	1463820	g	1618068	g	1702041	s	O55/O157	rec22	g	CB9615	++	g	g	g	g	g	g	g	g	g	g	g	g	g	g	a	a	a	g	g	g	g	g	g
t	1463829	c	1618077	c	1702050	s	O55/O157	rec22	t	O157	++	t	a	a	a	c	t	t	c	c	t	t	c	c	c	t	t	t	t	t	t	a	a	a
c	1463844	t	1618092	t	1702065	s	O55/O157	rec22	t	CB9615	++	t	c	c	c	c	t	c	t	t	t	t	t	t	t	c	c	c	t	t	t	c	c	c
-	1463903	t	1631448	Y	1715423	ns	EDL933	rec23	t	EDL933	++++		g		c	t	c		t	t	t	c	t	t	t	c	c	c	t	t	t	c		t
-	1463903	t	1631613	K	1715588	ns	EDL933	rec23	t	EDL933	+		t		g	g	g		a	t	a	g	a	a	a	g	g	g	a	a	a	g		a
-	1463903	a	1631694	r	1715669	ns	EDL933	rec23	a	EDL933	+		a		g	a	g		c	g	c	g	c	c	c	g	g	a	c	c	c	g		c
-	1463903	g	1631706	r	1715681	ns	EDL933	rec23	g	EDL933	+		a		a	a	a		g	a	c	a	t	t	t	a	a	a	c	c	c	a		c
-	1463903	c	1631775	Y	1715750	ns	EDL933	rec23	c	EDL933	++++		g		c	c	c		c	c	c	c	c	c	c	c	c	c	c	c	c	c		c
-	1463903	t	1631817	Y	1715792	ns	EDL933	rec23	t	EDL933	+++		t		c	c	t		c	c	c	t	c	c	c	t	c	c	c	c	c	t		c
-	1463903	t	1631874	Y	1715849	ns	EDL933	rec23	t	EDL933	++++		c		c	c	c		t	c	t	c	t	t	t	c	c	c	t	t	t	t		t
-	1463903	t	1632018	Y	1715993	ns	EDL933	rec23	t	EDL933	+		c		c	c	t		a	c	a	c	a	a	a	c	c	c	a	a	a	c		a
-	1463903	g	1632042	R	1716017	ns	EDL933	rec23	g	EDL933	+++		a		g	a	g		a	a	a	a	a	a	a	a	a	g	a	a	a	g		a
-	1463903	g	1632264	t	1716239	ns	EDL933	rec23	g	EDL933	++++		a		g	g	g		g	g	g	g	g	g	g		g	g				g		g
-	1463903	t	1649126	g	1740911	ns	Sakai/EDL933	rec24	g	Sakai	+			g	g	g	g	g	g		g	g	t	a	g		t	t	g	g	g	g	t	t
-	1463903	a	1649147	g	1740932	ns	Sakai/EDL933	rec24	g	Sakai	+			g	g	g	a	g	a		g	g	a	g	g		a	a	g	g	g	g	a	a
-	1463903	g	1649165	a	1740950	ns	Sakai/EDL933	rec24	g	EDL933	+			g	g	g	g	a	g		g	a	g	g	g		g	g	g	g	g	g	g	-
-	1463903	t	1649246	c	1741031	ns	Sakai/EDL933	rec24	c	Sakai	+			c	c	c	c	c	t		g	c	t	c	c	c	t	c	c	c	c	c	c	c
-	1463903	g	1649333	a	1741118	ns	Sakai/EDL933	rec24	g	EDL933	+		a	g	a	a	a	a	a		t	g	g	g	a	g	g	a	g	g	g	g	g	g
-	1463903	c	1649356	Y	1741141	ns	Sakai/EDL933	rec24	c	EDL933	++++		c	c	c	c	c	c	c		c	c	c	c	c	c	c	c	c	c	c	c	c	c
-	1463903	t	1649420	c	1741205	ns	Sakai/EDL933	rec24	t	EDL933	++++		t	t	t	t	t	t	t		g	t	t	t	t	t	t	t	t	t	t	t	t	t
-	1463903	t	1649438	c	1741223	ns	Sakai/EDL933	rec24	t	EDL933	+		t	t	t	t	t	t	t		a	t	t	t	t	t	t	t	t	t	t	t	c	c
-	1463903	g	1649444	a	1741229	ns	Sakai/EDL933	rec24	g	EDL933	+		g	a	a	g	g	a	a		g	a	g	a	g	a	a	g	g	g	g	a	a	a
-	1463903	g	1657364	c	1749152	ns	EDL933	rec25	g	EDL933	+++	g				g				g	g	a	g			g						g		a
-	1463903	g	1657373	n	1749161	ns	EDL933	rec25	g	EDL933	++++	g				g				g	g	g	g			g						g		g
-	1463903	g	1657407	t	1749195	ns	EDL933	rec25	g	EDL933	++++	g				g				g	g	g	g			g						g		g
-	1463903	g	1657408	n	1749196	ns	EDL933	rec25	g	EDL933	+++	g				g				g	g	t	g			g						g		t
-	1463903	-	1657410	g	1749199	ins-2	EDL933	rec25	-	EDL933	++++	-				-				-	-	-	-			-						-		-
-	1463903	g	1657416	c	1749206	ns	EDL933	rec25	g	EDL933	+++	g				g				g	g	a	g			g						g		a
-	1463903	g	1657422	n	1749212	ns	EDL933	rec25	g	EDL933	++++	g				g				g	g	g	g			g						g		g
-	1463903	-	1657425	a	1749216	ins	EDL933	rec25	-	EDL933	++++	-				-				-	-	-	-			-						-		-
-	1463903	g	1657435	n	1749226	ns	EDL933	rec25	g	EDL933	+++	g				g				g	g	c	g			g						g		c
-	1463903	-	1657447	c	1749239	ins	EDL933	rec25	-	EDL933	++++	-				-				-	-	-	-			-						-		-
-	1463903	a	1657452	n	1749244	ns	EDL933	rec25	a	EDL933	++++	a				a				a	a	a	a			a						a		a
-	1463903	c	1657453	g	1749245	ns	EDL933	rec25	c	EDL933	+++	c				c				c	c	t	c			c						c		t
-	1463903	-	1657456	a	1749249	ins	EDL933	rec25	-	EDL933	++++	-				-				-	-	-	-			-						-		-
-	1463903	c	1657457	n	1749250	ns	EDL933	rec25	c	EDL933	++++	c				c				c	c	c	c			c						c		c
-	1463903	-	1657474	a	1749268	ins	EDL933	rec25	-	EDL933	++++	-				-				-	-	-	-			-						-		-
-	1463903	g	1657496	n	1749290	ns	EDL933	rec25	g	EDL933	++++	g				g				g	g	g	g			g						g		g
-	1463903	t	1657499	c	1749293	ns	EDL933	rec25	t	EDL933	++++	t				t				t	t	t	t			t						t		t
-	1463903	g	1657500	c	1749294	ns	EDL933	rec25	g	EDL933	++++	g				g				g	g	g	g			g						g		g
-	1463903	g	1657501	c	1749295	ns	EDL933	rec25	g	EDL933	++++	g				g				g	g	g	g			g						g		g
-	1463903	g	1657518	N	1749312	ns	EDL933	rec25	g	EDL933	++++	g				g				g	g	g	g			g						g		g
-	1463903	c	1657539	n	1749333	ns	EDL933	rec25	c	EDL933	++++	c				c				c	c	c	c			c						c		c
-	1463903	g	1657547	a	1749341	ns	EDL933	rec25	g	EDL933	++++	g				g				g	g	g	g			g						g		g
-	1463903	g	1657549	a	1749343	ns	EDL933	rec25	g	EDL933	++++	g				g				g	g	g	g			g						g		g

O55 and O157 genome site details												Outgroup Strain Details[i]																						
CB9615 base[b]	CB9615 Site[c]	Sakai base[b]	Sakai Site[c]	EDL933 base[b]	EDL933 Site[c]	type[d]	Event Lineage[ef]	Recombinant segment	Inferred ancestral base[g]	Outgroup Analysis[e]	Support level[h]	D1 Sd197	K-12	HS	ATCC 8739	UMN026	IAI39	SMS 3-5	E2348/69	536	ED1a	CFT073	S88	APEC O1	UTI89	E24377A	IAI1	SE11	F5 8401	F2a 2457T	F2a 301	SS Ss046	B4 Sb227	B18 BS512
g	1549440	a	1732016	a	1823952	nc	CB9615	rec26	a	CB9615	++	a	a	a	a	g		g		g	g		g	g	g	a	a	a	a	a	a	a	a	a
a	1549452	g	1732028	g	1823964	nc	CB9615	rec26	g	CB9615	++++	g	g	g	g	g		g		g	g		g	g	g	g	g	g	g	g	g	g	g	g
t	1549473	c	1732049	c	1823985	nc	CB9615	rec26	c	CB9615	++++	c	c	c	c	c		c		c	c		c	c	c	c	c	c	c	c	c	c	c	c
t	1549494	a	1732070	a	1824006	nc	CB9615	rec26	a	CB9615	++++	a	a	a	a	a		a		a	a		a	a	a	a	a	a	a	a	a	a	a	a
c	1549506	t	1732082	t	1824018	nc	CB9615	rec26	t	CB9615	++++	t	t	t	t	t		t		t	t		t	t	t	t	t	t	t	t	t	t	t	t
g	1576227	t	1758993	t	1850932	ns	CB9615	rec27	t	CB9615	++++				t	t	t	t	t		t	t	t	t	t	t	t	t	t				t	t
g	1576244	a	1759010	a	1850949	ns	CB9615	rec27	a	CB9615	+				a	a	a	g	a		a	a	g	g	g	a	a	g	g				g	a
t	1576245	c	1759011	c	1850950	ns	CB9615	rec27	c	CB9615	++++				c	c	c	c	c		c	c	c	c	c	c	c	c	c				c	c
a	1576273	g	1759039	g	1850978	s	CB9615	rec27	g	CB9615	++++				g	g	g	g	g		g	g	g	g	g	g	g	a	g				g	g
c	1576279	a	1759045	a	1850984	s	CB9615	rec27	a	CB9615	++++				a	a	a	a	a		a	a	a	a	a	a	a	c	a				a	a
c	1576282	t	1759048	t	1850987	s	CB9615	rec27	t	CB9615	++++				t	t	t	t	t		t	t	t	t	t	t	t	c	t				t	t
g	1576288	a	1759054	a	1850993	s	CB9615	rec27	a	CB9615	++++				a	a	a	a	a		a	a	a	a	a	a	a	g	a				a	a
g	1576309	a	1759075	a	1851014	s	CB9615	rec27	a	CB9615	++++				a	a	a	a	a		a	a	a	a	a	a	a	a	a				a	a
a	1576315	c	1759081	c	1851020	s	CB9615	rec27	c	CB9615	++++				c	c	c	c	c		c	c	c	c	c	c	c	t	c				c	c
c	1576335	t	1759101	t	1851040	ns	CB9615	rec27	t	CB9615	++++				t	t	t	t	t		t	t	t	t	t	t	t	t	t				t	t
c	1576379	t	1759145	t	1851084	ns	CB9615	rec27	t	CB9615	++++				t	t	t	t	t		t	t	t	t	t	t	t	t	t				t	t
g	1576384	a	1759150	a	1851089	s	CB9615	rec27	a	CB9615	++++				a	a	a	a	a		a	a	a	a	a	a	a	a	a				a	a
a	1576402	c	1759168	c	1851107	s	CB9615	rec27	c	CB9615	++++				c	c	c	c	c		c	c	c	c	c	c	c	g	c				c	c
t	1576408	c	1759174	c	1851113	s	CB9615	rec27	c	CB9615	++++				c	c	c	c	c		c	c	c	c	c	c	c	g	c				c	c
a	1576432	c	1759198	c	1851137	s	CB9615	rec27	c	CB9615	++++				c	c	c	c	c		c	c	c	c	c	c	c	a	c				c	c
c	1576450	t	1759216	t	1851155	s	CB9615	rec27	t	CB9615	++++				t	t	t	t	t		t	t	t	t	t	t	t	a	t				t	t
a	1576456	t	1759222	t	1851161	s	CB9615	rec27	t	CB9615	++++				t	t	t	t	t		t	t	t	t	t	t	t	g	t				t	t
c	1576477	t	1759243	t	1851182	s	CB9615	rec27	t	CB9615	++++				t	t	t	t	t		t	t	t	t	t	t	t	t	t				t	t
c	1576513	t	1759279	t	1851218	s	CB9615	rec27	t	CB9615	++++				t	t	t	t	t		t	t	t	t	t	t	t	a	t				t	t
g	1576515	a	1759281	a	1851220	s	CB9615	rec27	a	CB9615	++++				a	a	a	a	a		a	a	a	a	a	a	a	t	a				a	a
c	1576558	t	1759324	t	1851263	s	CB9615	rec27	t	CB9615	++++				t	t	t	t	t		t	t	t	t	t	t	t	t					t	t
a	1576567	g	1759333	g	1851272	s	CB9615	rec27	g	CB9615	++++				g	g	g	g	g		g	g	g	g	g	g	g	g					g	g
c	1576579	a	1759345	a	1851284	s	CB9615	rec27	a	CB9615	++++				a	a	a	a	a		a	a	a	a	a	a	a	a					a	a
a	1576595	g	1759361	g	1851300	ns	CB9615	rec27	g	CB9615	++++				g	g	g	g	g		g	g	g	g	g	g	g	g					g	g
c	1576596	t	1759362	t	1851301	ns	CB9615	rec27	t	CB9615	++++				t	t	t	t	t		t	t	t	t	t	t	t	t					t	t
a	1576600	c	1759366	c	1851305	s	CB9615	rec27	c	CB9615	++++				c	c	c	c	c		c	c	c	c	c	c	c	a					c	c
t	1576612	c	1759378	c	1851317	s	CB9615	rec27	t	O157	+				t	t	t	t	c		c	t	t	t	t	t	t	c					t	t
a	1576624	t	1759390	t	1851329	ns	CB9615	rec27	t	CB9615	++++				t	t	t	t	t		t	t	t	t	t	t	t	a					t	t
g	1576636	a	1759402	a	1851341	s	CB9615	rec27	a	CB9615	++++				a	a	a	a	a		a	a	a	a	a	a	a	a					a	a
g	1576642	a	1759408	a	1851347	s	CB9615	rec27	a	CB9615	++++				a	a	a	a	a		a	a	a	a	a	a	a	a					a	a
t	1576651	c	1759417	c	1851356	s	CB9615	rec27	c	CB9615	++++				c	c	c	c	c		c	c	c	c	c	c	c	t					g	c
g	1576690	a	1759456	a	1851395	s	CB9615	rec27	a	CB9615	++++				a	a	a	a	a		a	a	a	a	a	a	a	a					a	a
a	1576702	g	1759468	g	1851407	s	CB9615	rec27	g	CB9615	++++				g	g	g	g	g		g	g	g	g	g	g	g	g					g	g
g	1576714	a	1759480	a	1851419	s	CB9615	rec27	a	CB9615	++++				a	a	a	a	a		a	a	a	a	a	a	a	a					a	a
c	1576747	t	1759513	t	1851452	s	CB9615	rec27	t	CB9615	+				t	t	t	t	t		c	t	t	t	t	t	t	c					t	t
a	1576753	g	1759519	g	1851458	ns	CB9615	rec27	g	CB9615	++++				g	g	g	g	g		g	g	g	g	g	g	g	g					g	g
a	1576754	t	1759520	t	1851459	ns	CB9615	rec27	t	CB9615	++++				t	t	t	t	t		t	t	t	t	t	t	t	t					t	t
t	1576755	c	1759521	c	1851460	ns	CB9615	rec27	c	CB9615	++++				c	c	c	c	c		g	c	c	c	c	c	c	a					c	c
c	1576756	a	1759522	a	1851461	ns	CB9615	rec27	a	CB9615	+				a	a	a	a	a		c	a	a	a	a	a	a	c					a	a
c	1576758	a	1759524	a	1851463	ns	CB9615	rec27	a	CB9615	++++				a	a	a	a	a		a	a	a	a	a	a	a	a					a	a
c	1576761	t	1759527	t	1851466	ns	CB9615	rec27	t	CB9615	++++				t	t	t	t	t		a	t	t	t	t	t	t	c					t	t
t	1576762	c	1759528	c	1851467	s	CB9615	rec27	c	CB9615	++++				c	c	c	c	c		c	c	c	c	c	c	c	t					c	c
g	1576779	t	1759545	t	1851484	ns	CB9615	rec27	t	CB9615	++++				t	t	t	t	t		t	t	t	t	t	t	t	t					t	t
a	1576780	t	1759546	t	1851485	ns	CB9615	rec27	t	CB9615	+				t	t	t	t	t		a	t	t	t	t	t	t	a					t	t
t	1576782	c	1759548	c	1851487	ns	CB9615	rec27	c	CB9615	++++				c	c	c	c	c		c	c	c	c	c	c	c	c					c	c
a	1576783	t	1759549	t	1851488	ns	CB9615	rec27	t	CB9615	+				g	g	g	g	t		g	t	g	g	g	g	g	g					g	g
t	1576791	c	1759557	c	1851496	ns	CB9615	rec27	c	CB9615	++++				c	c	c	c	c		c	c	c	c	c	c	c	c					c	c
g	1576796	a	1759562	a	1851501	ns	CB9615	rec27	a	CB9615	++++				a	a		a	a		a	a	a	a	a	a	a	a					a	a
a	1576805	g	1759571	g	1851510	ns	CB9615	rec27	g	CB9615	+				g	g		g	g		a	g	g	g	g	g	g	a					a	g
g	1576808	t	1759574	t	1851513	ns	CB9615	rec27	t	CB9615	+				t	t		t	t		g	t	t	t	t	t	t	g					g	c
a	1576815	c	1759581	c	1851520	ns	CB9615	rec27	c	CB9615	++++				c	c		c	c		c	c	c	c	c	c	c	c					c	c
c	1576818	t	1759584	t	1851523	ns	CB9615	rec27	t	CB9615	++++				t	t		t	t		t	t	t	t	t	t	t	t					t	t
c	1576832	t	1759598	t	1851537	ns	CB9615	rec27	t	CB9615	+				t	t		t	t		c	t	t	t	t	t	t	c					c	t
g	1576840	a	1759606	a	1851545	ns	CB9615	rec27	a	CB9615	+				a	a		g	a		g	a	a	a	a	a	a	g					g	a
g	1576841	t	1759607	t	1851546	ns	CB9615	rec27	t	CB9615	+				t	t		g	t		g	t	t	t	t	t	t	g					g	t
a	1576843	g	1759609	g	1851548	s	CB9615	rec27	g	CB9615	++++				g	g		g	g		g	g	g	g	g	g	g	g					g	g
c	1576859	t	1759625	t	1851564	ns	CB9615	rec27	c	O157	++++				c	c		c	t		c	c	c	c	c	c	c	c					c	c
c	1576876	t	1759642	t	1851581	ns	CB9615	rec27	t	CB9615	++++				t	t		t	t		t	t	t	t	t	t	t	t					t	t
t	1576877	g	1759643	g	1851582	ns	CB9615	rec27	g	CB9615	++++				g	g		g	g		g	g	g	g	g	g	g	g					g	g
g	1576883	t	1759649	t	1851588	ns	CB9615	rec27	t	CB9615	++++				t	t		t	t		t	t	t	t	t	t	t	t					t	t
t	1576887	c	1759653	c	1851592	ns	CB9615	rec27	c	CB9615	++++				c	c		c	c		c	c	c	c	c	c	c	c					c	c
a	1576888	g	1759654	g	1851593	s	CB9615	rec27	g	CB9615	++++				g	g		g	g		g	g	g	g	g	g	g	g					g	g
t	1576911	c	1759677	c	1851616	ns	CB9615	rec27	c	CB9615	+				c	c		t	c		c	c	c	c	c	c	c	t					t	c
g	1576925	a	1759691	a	1851630	ns	CB9615	rec27	a	CB9615	++++				a	a		a	a		a	a	a	a	a	a	a	t					a	a
g	1576926	c	1759692	c	1851631	ns	CB9615	rec27	c	CB9615	++++				c	c		c	c		c	c	c	c	c	c	c	c					c	c
t	1576932	a	1759698	a	1851637	ns	CB9615	rec27	a	CB9615	+				a	a		t	a		a	a	a	a	a	a	a	t					t	a
a	1576946	g	1759712	g	1851651	ns	CB9615	rec27	g	CB9615	++++				g	g		g	g		g	g	g	g	g	g	g	g					g	g
a	1577036	g	1759802	g	1851741	ns	CB9615	rec27	a	O157	+				a	a		g	a		g	a	a	a	a	a	a	g					g	a
c	1577037	g	1759803	g	1851742	ns	CB9615	rec27	c	O157	+				c	c		g	c		g	c	c	c	c	c	c	c					g	c
c	1577050	t	1759816	t	1851755	s	CB9615	rec27	c	O157	+				t	c		t	c		t	c	c	c	c	c	c	t					t	c
t	1577056	c	1759822	c	1851761	s	CB9615	rec27	t	O157	++++				t	t		t	t		t	t	t	t	t	t	t	c					t	t
c	1577062	t	1759828	t	1851767	s	CB9615	rec27	c	O157	+				c	c		t	c		t	t	c	c	c	c	c	t					t	c
a	1577087	t	1759853	t	1851792	ns	CB9615	rec27	a	O157	++++				a	a		a	a		a	a	a	a	a	a	a	a					a	a
a	1577088	g	1759854	g	1851793	ns	CB9615	rec27	a	O157	++++				a	a		a	a		a	a	a	a	a	a	a	a					a	a
c	1577091	t	1759857	t	1851796	ns	CB9615	rec27	c	O157	++++				c	c		c	c		c	c	c	c	c	c	c	c					c	c
-	1627512	g	1796779	a	2290398	nc	Sakai/EDL933	rec28	g	EDL933	+			a	g	a	g	g	a	g	g	g	a	a	a		a	a	g	g	g	g	c	a
-	1627512	a	1796785	g	2290392	nc	Sakai/EDL933	rec28	g	Sakai	+			g	g	g	g	g	g	g	c	g	g	g	g		g	g	a	a	a	a	a	g
-	1627512	a	1796787	g	2290390	nc	Sakai/EDL933	rec28	g	Sakai	++++			g	g	g	g	g	g	a	g	g	g	g	g		g	g	t	-	-	t	-	g

O55 and O157 genome site details												Outgroup Strain Details[i]																							
CB9615 base[b]	CB9615 Site[c]	Sakai base[b]	Sakai Site[c]	EDL933 base[b]	EDL933 Site[c]	type[d]	Event Lineage[ef]	Recombinant segment	Inferred ancestral base[g]	Outgroup Analysis[e]	Support level[h]	D1 Sd197	K-12	HS	ATCC 8739	UMN026	IAI39	SMS 3-5	E2348/69	536	ED1a	CFT073	S88	APEC O1	UTI89	E24377A	IAI1	SE11	F5 8401	F2a 2457T	F2a 301	SS Ss046	B4 Sb227	B18 BS512
-	1627512	a	1796800	g	2290377	nc	Sakai/EDL933	rec28	g	Sakai	+			g	g	g	g	g	g	a	g	g	g	g	g		g	g	a	a	a	a	g	g
-	1627512	a	1796821	c	2290356	nc	Sakai/EDL933	rec28	c	Sakai	++++			t	c	g	c	t	c	c	g	c	c	c	c		c	c	g	g	g	g	a	c
-	1627512	g	1796824	c	2290353	nc	Sakai/EDL933	rec28	c	Sakai	+			c	c	g	c	c	c	c	g	c	c	c	c		c	c	g	g	g	g	g	a
-	1627512	g	1796825	a	2290352	nc	Sakai/EDL933	rec28	a	Sakai	+			a	a	g	a	a	a	a	g	a	a	a	a		a	a	g	g	g	g	g	a
-	1627512	t	1796826	c	2290351	nc	Sakai/EDL933	rec28	c	Sakai	+			c	c	t	c	c	c	c	t	c	c	c	c		c	c	t	t	t	t	t	c
-	1627512	c	1796827	g	2290350	nc	Sakai/EDL933	rec28	g	Sakai	+			g	g	c	g	g	g	g	a	g	g	g	g		g	g	c	c	c	c	a	g
-	1627512	g	1796832	a	2290345	nc	Sakai/EDL933	rec28	a	Sakai	+			a	a	g	a	a	a	a	g	a	a	a	a		a	a	a	a	a	a	g	a
-	1627512	g	1796839	c	2290338	nc	Sakai/EDL933	rec28	c	Sakai	+			g	c	g	c	g	c	g	g	c	t	t	t		c	c	t	t	t	t	g	c
-	1627512	g	1796842	t	2290335	nc	Sakai/EDL933	rec28	t	Sakai	+			t	t	g	t	t	t	t	g	t	t	t	t		t	t	a	a	a	a	g	t
-	1627512	t	1796875	c	2290302	nc	Sakai/EDL933	rec28	c	Sakai	+			c	c	c	c	c	c	c	t	c	c	c	c		c	c	t	t	t	t	t	c
-	1627512	c	1796883	a	2290294	nc	Sakai/EDL933	rec28	a	Sakai	+			c	a	a	a	a	a	a	c	a	a	a	a		a	a	a	a	a	a	c	a
-	1627512	a	1796891	g	2290286	nc	Sakai/EDL933	rec28	g	Sakai	+			a	a	a	g	g	g	g	g	g	a	a	a		a	a	g	g	g	g	g	g
-	1627512	c	1796893	g	2290284	nc	Sakai/EDL933	rec28	g	Sakai	+			c	c	g	g	g	g	g	a	g	c	c	c		c	c	g	g	g	g	a	g
-	1627512	a	1796915	g	2290262	nc	Sakai/EDL933	rec28	a	EDL933	+			a	a	t	a	a	g	a	t	a	a	a	a		a	a	a	a	a	a	t	g
-	1627512	c	1796926	t	2290251	nc	Sakai/EDL933	rec28	t	Sakai	++++			c	t	t	t	t	t	t	t	t	t	t	t		t	t	t	t	t	t	t	t
-	1627512	a	1796933	t	2290244	nc	Sakai/EDL933	rec28	a	EDL933	+			g	a	a	a	a	t	a	a	a	a	a	a		a	a	a	a	a	a	a	t
-	1627512	g	1796937	c	2290240	nc	Sakai/EDL933	rec28	c	Sakai	+			g	c	g	c	c	c	c	g	c	c	c	c		c	c	c	c	c	c	g	c
-	1627512	c	1796953	t	2290224	nc	Sakai/EDL933	rec28	c	EDL933	+			c	c	c	t	c	t	c	c	t	c	c	c		c	c	c	c	c	c	c	t
-	1627512	c	1796989	t	2290188	nc	Sakai/EDL933	rec28	t	Sakai	+			t	t	g	t	t	t	t	g	t	t	t	t		c	c	t	t	t	t	g	t
-	1627512	c	1797010	t	2290167	nc	Sakai/EDL933	rec28	c	EDL933	+			c	c	c	c	c	t	c	c	c	c	c	c		c	c	c	c	c	c	c	t
-	1627512	c	1797013	t	2290164	nc	Sakai/EDL933	rec28	c	EDL933	+			c	c	g	c	c	t	c	g	c	c	c	c		c	c	c	c	c	c	g	t
-	1627512	t	1797031	c	2290146	nc	Sakai/EDL933	rec28	t	EDL933	+			c	c	t	t	c	t	t	t	t	c	c	c		c	c	t	t	t	t	t	c
-	1627512	t	1797043	c	2290134	nc	Sakai/EDL933	rec28	t	EDL933	+			t	t	c	t	t	t	t	t	t	c	c	c		t	t	c	c	c	c	t	c
-	1627512	c	1797046	t	2290131	nc	Sakai/EDL933	rec28	t	Sakai	+			t	t	g	c	c	c	c	a	c	t	t	t		t	t	t	t	t	t	a	t
-	1627512	a	1797048	g	2290129	nc	Sakai/EDL933	rec28	?	Sakai/EDL933	+/-			a	a	a	a	a	a	a	g	a	g	g	g		a	a	g	g	g	g	g	g
-	1627512	c	1797050	g	2290127	nc	Sakai/EDL933	rec28	?	Sakai/EDL933	+/-			c	c	c	c	c	c	c	g	c	g	g	g		c	c	g	g	g	g	g	g
-	1627512	t	1797055	c	2290122	nc	Sakai/EDL933	rec28	c	Sakai	+			t	t	c	t	t	t	t	c	t	c	c	c		t	t	c	c	c	c	c	c
-	1627512	a	1797064	g	2290113	nc	Sakai/EDL933	rec28	a	EDL933	+			a	a	t	a	a	a	a	t	a	g	g	g		a	a	g	g	g	a	a	g
-	1627512	t	1797065	g	2290112	nc	Sakai/EDL933	rec28	g	Sakai	+			t	t	g	t	t	t	t	g	t	g	g	g		t	t	g	g	g	g	g	g
-	1627512	c	1797066	a	2290111	nc	Sakai/EDL933	rec28	?	Sakai/EDL933	+/-			c	c	a	c	c	c	c	c	c	a	a	a		c	c	a	a	a	a	a	a
-	1627512	c	1797067	a	2290110	nc	Sakai/EDL933	rec28	a	Sakai	+			c	c	a	c	c	c	c	g	c	a	a	a		c	c	a	a	a	a	a	a
-	1627512	c	1797070	t	2290107	nc	Sakai/EDL933	rec28	t	Sakai	+			c	c	g	c	c	c	c	c	c	t	t	t		t	t	t	t	t	t	g	t
-	1627512	t	1797073	c	2290104	nc	Sakai/EDL933	rec28	t	EDL933	+			t	t	t	t	t	t	t	t	t	c	c	c		t	t	t	t	t	t	t	c
-	1627512	g	1797094	a	2290083	nc	Sakai/EDL933	rec28	g	EDL933	+			g	g	g	g	g	g	g	g	g	a	a	a		g	g	g	g	g	g	a	a
-	1627512	a	1797097	c	2290080	nc	Sakai/EDL933	rec28	a	EDL933	+			a	a	a	a	a	a	a	a	a	c	c	c		a	a	c	c	c	c	a	c
-	1627512	c	1797106	a	2290071	nc	Sakai/EDL933	rec28	c	EDL933	+			c	c	c	c	c	c	c	g	c	a	a	a		c	c	g	g	g	g	g	a
-	1627512	t	1797115	c	2290062	nc	Sakai/EDL933	rec28	t	EDL933	+			t	t	t	t	t	t	t	a	t	c	c	c		t	t	c	c	c	c	t	c
-	1627512	t	1797122	c	2290055	nc	Sakai/EDL933	rec28	c	Sakai	+			c	c	c	c	c	t	t	c	c	t	t	t		c	c	t	t	t	t	c	c
-	1627512	t	1797130	g	2290047	nc	Sakai/EDL933	rec28	g	Sakai	++++			g	g	g	g	g	g	g	a	g	g	g	g		g	g	g	g	g	g	g	g
-	1627512	t	1797157	g	2290020	nc	Sakai/EDL933	rec28	t	EDL933	+			t	t	t	t	t	t	t	c	t	g	g	g		t	t	g	g	g	g	t	g
-	1627512	g	1797166	t	2290011	nc	Sakai/EDL933	rec28	t	Sakai	+			t	t	t	t	t	t	t	g	t	t	t	t		t	t	t	t	t	t	g	t
-	1627512	a	1797175	g	2290002	nc	Sakai/EDL933	rec28	g	Sakai	++++			g	g	g	g	g	g	g	-	g	g	g	g		g	g	g	g	g	g	g	g
-	1627512	c	1797184	t	2289993	nc	Sakai/EDL933	rec28	t	Sakai	+			t	t	t	t	t	t	t	c	t	t	t	t		t	t	t	t	t	t	c	t
-	1627512	g	1797188	a	2289989	nc	Sakai/EDL933	rec28	g	EDL933	+			g	g	g	g	g	a	g	g	g	a	a	a		g	g	a	a	a	a	g	a
-	1627512	t	1797196	c	2289981	nc	Sakai/EDL933	rec28	c	Sakai	++++			c	c	c	c	c	c	c	c	c	c	c	c		c	c	c	c	c	c	c	c
-	1627512	g	1797223	a	2289954	nc	Sakai/EDL933	rec28	a	Sakai	+			g	g	g	g	g	a	g	a	g	a	a	a		g	g	a	a	a	a	a	a
-	1627512	g	1797233	a	2289944	nc	Sakai/EDL933	rec28	g	EDL933	+			g	g	g	g	g	a	g	g	g	g	g	g		g	g	a	a	a	a	g	a
-	1627512	c	1797235	t	2289942	nc	Sakai/EDL933	rec28	c	EDL933	+			c	c	c	c	c	t	c	c	c	c	c	c		c	c	t	t	t	t	c	t
-	1627512	a	1797244	g	2289933	nc	Sakai/EDL933	rec28	g	Sakai	+			a	g	g	g	g	g	a	c	g	g	g	g		g	g	t	t	t	t	c	g
-	1627512	g	1797247	a	2289930	nc	Sakai/EDL933	rec28	a	Sakai	+			g	a	a	a	a	a	g	c	a	a	a	a		a	a	a	a	a	a	c	a
-	1627512	g	1797265	a	2289912	nc	Sakai/EDL933	rec28	?	Sakai/EDL933	+/-			g	g	g	g	g	a	g	t	g	a	a	a		g	g	a	a	a	a	c	a
-	1627512	a	1797307	c	2289870	nc	Sakai/EDL933	rec28	c	Sakai	+++			c	t	t	t	t	c	t	g	t	c	c	c		t	t	t	t	t	t	g	c
-	1627512	g	1797313	a	2289864	nc	Sakai/EDL933	rec28	g	EDL933	+			a	g	g	g	g	a	g	g	g	a	a	a		g	g	g	g	g	g	g	a
-	1627512	c	1797373	t	2289804	nc	Sakai/EDL933	rec28	t	Sakai	+			t	c	c	c	t	t	t	c	c	t	t	t		c	c	t	t	t	t	t	t
-	1627512	c	1797376	g	2289801	nc	Sakai/EDL933	rec28	g	Sakai	+			g	c	c	c	c	g	t	c	c	g	g	g		c	c	g	g	g	g	-	g
-	1627512	t	1797377	c	2289800	nc	Sakai/EDL933	rec28	c	Sakai	++++			c	c	c	c	c	c	c	c	c	c	c	c		c	c	c	c	c	c	-	c
-	1627512	t	1797379	a	2289798	nc	Sakai/EDL933	rec28	?	Sakai/EDL933	+/-			a	t	t	t	t	a	t	t	t	a	a	a		t	t	a	a	a	a	t	a
-	1627512	a	1797381	c	2289796	nc	Sakai/EDL933	rec28	?	Sakai/EDL933	+/-			c	a	a	a	a	c	a	a	a	c	c	c		a	a	c	c	c	c	a	c
-	1627512	c	1797382	a	2289795	nc	Sakai/EDL933	rec28	a	Sakai	+			a	c	c	c	c	a	c	c	c	a	a	a		c	c	a	a	a	a	t	a
-	1627512	c	1797385	a	2289792	nc	Sakai/EDL933	rec28	c	EDL933	+			a	c	c	c	c	t	t	g	c	t	t	t		c	c	a	a	a	a	c	a
t	1732193	g	1921417	g	2165749	ns	O55/O157	rec29	g	CB9615	++++	g	g						g	g	g	g	g	g	g			g	g	g	g	g	g	g
c	1732251	t	1921475	t	2165691	s	O55/O157	rec29	c	O157	++++	c	c						c	c	c	c	c	c	c			c	c	c	c	c	c	c
c	1732350	t	1921574	t	2165592	s	O55/O157	rec29	c	O157	++	c	t						c	c	c	c	c	c	c			t	c	c	c	c	c	c
c	1732365	t	1921589	t	2165577	s	O55/O157	rec29	t	CB9615	++++	t	t						t	t	t	t	t	t	t			t	t	t	t	t	t	t
t	1732467	c	1921691	c	2165475	s	O55/O157	rec29	t	O157	++	t	c						t	t	t	t	t	t	t			c	t	t	t	t	t	t
c	1732470	t	1921694	t	2165472	s	O55/O157	rec29	t	CB9615	++	t	t						c	c	c	c	c	c	c			t	t	t	t	t	t	t
a	1732473	c	1921697	c	2165469	s	O55/O157	rec29	a	O157	+		c						t	t	t	t	t	a	t			c	a	a	a	a	a	a
c	1732503	t	1921727	t	2165439	s	O55/O157	rec29	c	O157	+		t											c				t	c	c	c	c	c	c
t	1732521	c	1921745	c	2165421	s	O55/O157	rec29	c	CB9615	++++		c											c				c	c	c	c	c	c	c
a	1732584	g	1921808	g	2165358	s	O55/O157	rec29	a	O157	+		g											a				g	a	a	a	a	a	a
t	1735969	a	1925193	a	2161973	ns	CB9615	rec30	a	CB9615	+++		a											a				a						
t	1735991	c	1925215	c	2161951	ns	CB9615	rec30	c	CB9615	+++		c											c				c						
c	1736000	t	1925224	t	2161942	ns	CB9615	rec30	t	CB9615	+++		t											t				t						
g	1736038	a	1925262	a	2161904	s	CB9615	rec30	a	CB9615	+++		a								a			a				a						
a	1736044	g	1925268	g	2161898	s	CB9615	rec30	g	CB9615	+++		g								g			g				g						
g	1736056	a	1925280	a	2161886	ns	CB9615	rec30	a	CB9615	+++		a								a			a				a						
c	1736057	t	1925281	t	2161885	ns	CB9615	rec30	t	CB9615	+++		t								c			t				t						
g	1736059	c	1925283	c	2161883	s	CB9615	rec30	c	CB9615	+++		c								c			c				c						
a	1736062	g	1925286	g	2161880	s	CB9615	rec30	g	CB9615	+++		g								g			g				g						
g	1736065	a	1925289	a	2161877	ns	CB9615	rec30	a	CB9615	+++		a								a			a				a						
c	1736066	g	1925290	g	2161876	ns	CB9615	rec30	g	CB9615	+++		g								g			g				g						
a	1736071	g	1925295	g	2161871	s	CB9615	rec30	g	CB9615	+++		g								a			g				g						
g	1736080	a	1925304	a	2161862	ns	CB9615	rec30	a	CB9615	+++		a								g			a				a						
g	1736081	t	1925305	t	2161861	ns	CB9615	rec30	t	CB9615	+++		t								c			t				t						

O55 and O157 genome site details												Outgroup Strain Details[i]																						
CB9615 base[b]	CB9615 Site[c]	Sakai base[b]	Sakai Site[c]	EDL933 base[b]	EDL933 Site[c]	type[d]	Event Lineage[ef]	Recombinant segment	Inferred ancestral base[g]	Outgroup Analysis[e]	Support level[h]	D1 Sd197	K-12	HS	ATCC 8739	UMN026	IAI39	SMS 3-5	E2348/69	536	ED1a	CFT073	S88	APEC O1	UTI89	E24377A	IAI1	SE11	F5 8401	F2a 2457T	F2a 301	SS Ss046	B4 Sb227	B18 BS512
a	1736087	g	1925311	g	2161855	ns	CB9615	rec30	g	CB9615	+++		g								t			g				g						
t	1736090	a	1925314	a	2161852	ns	CB9615	rec30	a	CB9615	+++		a								t			a				a						
g	1736108	a	1925332	a	2161834	ns	CB9615	rec30	a	CB9615	+++		a								g			a				a						
g	1773669	a	1969488	a	2118993	s	CB9615	rec30	a	CB9615	+								a															
g	1773693	a	1969512	a	2118969	s	CB9615	rec30	a	CB9615	+								a															
a	1773699	c	1969518	c	2118963	s	CB9615	rec30	c	CB9615	+								c															
g	1773702	t	1969521	t	2118960	s	CB9615	rec30	t	CB9615	+								t															
c	1773705	t	1969524	t	2118957	s	CB9615	rec30	t	CB9615	+								t															
c	1773742	a	1969561	a	2118920	ns	CB9615	rec30	a	CB9615	+								a															
-	1736115	g	1941871	s	2145293	i	EDL933	rec31	g	EDL933	++++								t													t		t
-	1736115	t	1941873	r	2145291	i	EDL933	rec31	t	EDL933	+++								t													t	t	t
-	1736115	-	1941874	s	2145289	ins	EDL933	rec31	-	EDL933	IUPAC																							
-	1736115	a	1941879	k	2145284	i	EDL933	rec31	a	EDL933	+++								a													a	a	a
-	1736115	a	1942005	k	2145158	ns	EDL933	rec31	a	EDL933	+++								a													a	a	a
-	1736115	g	1942547	Y	2144616	ns	EDL933	rec31	g	EDL933	+++								g		g											g	t	g
-	1736115	a	1942758	Y	2144405	ns	EDL933	rec31	a	EDL933	+++								a		a											a	a	a
-	1736115	c	1942811	R	2144352	ns	EDL933	rec31	c	EDL933	+								t		c											t	t	t
-	1736115	a	1942877	g	2144286	ns	EDL933	rec31	g	Sakai	+								g		g											a	g	a
-	1736115	c	1943112	K	2144051	ns	EDL933	rec31	c	EDL933	+								c		c											a		a
-	1736115	c	1943150	a	2144013	ns	EDL933	rec31	a	Sakai	+								a		c											a		a
-	1736115	g	1943338	Y	2143825	ns	EDL933	rec31	g	EDL933	++++								a													a		a
-	1736115	a	1943416	c	2143747	ns	EDL933	rec31	c	Sakai	+								c													g		g
-	1736115	c	1943549	R	2143614	ns	EDL933	rec31	c	EDL933	+								c													c		c
-	1736115	a	1943570	Y	2143593	ns	EDL933	rec31	a	EDL933	+								a													a		a
-	1736115	c	1943680	R	2143483	ns	EDL933	rec31	c	EDL933	+								c													c		c
-	1736115	g	1943817	a	2143346	ns	EDL933	rec31	g	EDL933	++++					g			a			g	g	g	g				g	g	g	g		g
-	1736115	t	1943841	g	2143322	ns	EDL933	rec31	t	EDL933	++++					t			g			t	t	t	t				t	t	t	t		t
-	1736115	c	1943845	a	2143318	ns	EDL933	rec31	c	EDL933	++++					c			a			c	c	c	c				c	c	c	c		c
-	1736115	g	1968041	M	2120440	ns	EDL933	rec31	g	EDL933	++++			g	g	g	g	g	g		g	g	t	t	g		t	t	g	g	g	g	t	t
-	1736115	a	1968062	Y	2120419	ns	EDL933	rec31	a	EDL933	++++			g	g	g	a	g	a		g	g	a	a	g		a	a	g	g	g	g	a	a
-	1736115	g	1968080	Y	2120401	ns	EDL933	rec31	g	EDL933	++++			g	g	g	g	a	g		g	a	g	g	g		g	g	g	g	g	g	g	-
-	1736115	a	1968119	t	2120362	ns	EDL933	rec31	t	Sakai	++++			t	t	t	t	t	t		a	t	t	t	t		t	t	c	c	c	t	t	t
-	1736115	c	1968148	K	2120333	ns	EDL933	rec31	c	EDL933	++++			c	c	c	c	c	c		c	c	c	c	c	c	c	c	c	c	c	c	c	c
-	1736115	c	1968191	R	2120290	ns	EDL933	rec31	c	EDL933	++++		c	c	c	c	c	c	c		g	c	c	c	c	c	c	c	c	c	c	c	c	c
-	1736115	g	1968248	Y	2120233	ns	EDL933	rec31	g	EDL933	++++		a	g	a	a	a	a	a		t	g	g	g	a	g	g	a	g	g	g	g	g	g
-	1736115	c	1968271	R	2120210	ns	EDL933	rec31	c	EDL933	++++		c	c	c	c	c	c	c		c	c	c	c	c	c	c	c	c	c	c	c	c	c
-	1736115	t	1968335	R	2120146	ns	EDL933	rec31	t	EDL933	++++		t	t	t	t	t	t	t		g	t	t	t	t	t	t	t	t	t	t	t	t	t
-	1736115	t	1968353	r	2120128	ns	EDL933	rec31	t	EDL933	++++		t	t	t	t	t	t	t		a	t	t	t	t	t	t	t	t	t	t	t	c	c
-	1736115	g	1968359	y	2120122	ns	EDL933	rec31	g	EDL933	++++		g	a	a	g	g	a	a		g	a	g	g	g	a	a	g	g	g	g	g	a	a
-	1736115	g	1968513	m	2119968	i	EDL933	rec31	g	EDL933	++++		t	c	t	t	t	t	t				t	t	t	t	t	t	a	a	a	a	t	t
-	1736115	c	1968515	k	2119966	i	EDL933	rec31	c	EDL933	+++		a	a	a	a	a	a	a				a	a	a	a	a	a	c	c	c	c	a	a
-	1736115	a	1968520	y	2119961	i	EDL933	rec31	a	EDL933	++++		a	a	a	a	a	a	a				a	a	a	a	a	a				a	a	a
-	1736115	g	1968521	g	2119960	i	EDL933	rec31	g	EDL933	++++		c	c	c	c	c	c	c				c	c	c	c	c	c				c	c	c
-	1736115	c	1968609	R	2119872	i	EDL933	rec31	c	EDL933	+++		t	t	t	t	t	c	c				c	c	c	t	t	t				t	t	t
-	1736115	a	1968627	g	2119854	i	EDL933	rec31	a	EDL933	++++		a	c	a	a	t	t	t				t	t	t	c	a	a				a	a	a
-	1736115	c	1968678	g	2119803	ns	EDL933	rec31	c	EDL933	+		c	g	c	c	a	a	g				a	a	a	c	c	c				c	c	c
-	1736115	t	1968681	g	2119800	ns	EDL933	rec31	t	EDL933	++++		t	t	t	t	t	t	t				t	t	t	t	t	t				t	t	t
-	1736115	t	1968864	g	2119617	ns	EDL933	rec31	t	EDL933	+								g														t	t
-	1736115	t	1968951	M	2119530	ns	EDL933	rec31	t	EDL933	++++								a														g	g
-	1736115	a	1969104	W	2119377	ns	EDL933	rec31	a	EDL933	+								a														a	a
a	1776395	g	1972214	g	2116267	i	O55/O157	rec32	?	O55/O157	+/-																							
t	1776397	c	1972216	c	2116265	i	O55/O157	rec32	?	O55/O157	+/-																							
t	1776406	c	1972225	c	2116256	i	O55/O157	rec32	?	O55/O157	+/-																							
g	1776411	a	1972230	a	2116251	i	O55/O157	rec32	?	O55/O157	+/-																							
a	1776413	g	1972232	g	2116249	i	O55/O157	rec32	?	O55/O157	+/-																							
-	1776417	t	1972237	t	2116244	indel	O55/O157	rec32	?	O55/O157	+/-																							
t	1776427	g	1972247	g	2116234	i	O55/O157	rec32	?	O55/O157	+/-																							
t	1776433	c	1972253	c	2116228	i	O55/O157	rec32	?	O55/O157	+/-																							
g	1776434	t	1972254	t	2116227	i	O55/O157	rec32	?	O55/O157	+/-																							
g	1776435	a	1972255	a	2116226	i	O55/O157	rec32	?	O55/O157	+/-																							
g	1850625	g	2046163	c	2042310	nc	EDL933	rec33	g	EDL933	+	a	a	g	g											a	g	a				a	a	a
g	1850728	g	2046266	a	2042207	nc	EDL933	rec33	g	EDL933	++++	c	g	c	g											g	g	g				g	g	g
a	1850763	a	2046301	g	2042172	nc	EDL933	rec33	a	EDL933	++++	a	a	a	a											a	a	a				a	a	a
g	1850800	g	2046338	t	2042135	nc	EDL933	rec33	g	EDL933	++++	g	g	g	g											g	g	g				g	g	g
g	1850842	g	2046380	a	2042093	nc	EDL933	rec33	g	EDL933	+	c	g	g	g											a	g	g				g	a	a
c	1850843	c	2046381	t	2042092	nc	EDL933	rec33	c	EDL933	++++	g	c	c	c											c	c	c				c	c	c
gc	1850845	gc	2046383	-	2042091	del-2	EDL933	rec33	gc	EDL933	++++	gc	gc	-	gc											gc	gc	gc				gc	gc	gc
t	1850849	t	2046387	g	2042088	nc	EDL933	rec33	t	EDL933	++++	t	t	t	t											t	t	t				t	t	t
g	1850851	g	2046389	t	2042086	nc	EDL933	rec33	g	EDL933	++++	g	g	g	g											g	g	g				g	g	g
g	1850855	g	2046393	c	2042082	nc	EDL933	rec33	g	EDL933	++++	g	g	g	g											t	g	g				g	t	t
c	1850856	c	2046394	t	2042081	nc	EDL933	rec33	c	EDL933	++++	c	c	t	c											c	c	c				c	c	c
c	1850861	c	2046399	a	2042076	nc	EDL933	rec33	c	EDL933	++++	c	c	c	c											c	c	c				-	c	c
-	1850862	-	2046400	a	2042074	ins	EDL933	rec33	?	EDL933	+/-																							
-	1850919	-	2046457	a	2042016	ins	EDL933	rec33	?	EDL933	+/-																							
-	1850931	-	2046469	t	2042003	ins	EDL933	rec33	?	EDL933	+/-																							
-	1850942	-	2046480	g	2041991	ins	EDL933	rec33	?	EDL933	+/-																							
-	1850945	-	2046483	t	2041987	ins	EDL933	rec33	?	EDL933	+/-																							
g	1850953	g	2046491	c	2041979	nc	EDL933	rec33	g	EDL933	++++	g	g	g	g											g	g	g				g	g	g
-	1850954	-	2046492	t	2041977	ins	EDL933	rec33	?	EDL933	+/-																							
c	1850978	c	2046516	t	2041953	nc	EDL933	rec33	c	EDL933	++++	c	c	g	c											c	c	c				c	c	c
c	1850987	c	2046525	a	2041944	nc	EDL933	rec33	c	EDL933	++++	c	c	g	c											c	c	c				c	c	c
g	1851007	g	2046545	c	2041924	nc	EDL933	rec33	g	EDL933	++++	g	g	c	g											g	g	g				g	g	g
g	1851045	g	2046583	a	2041886	nc	EDL933	rec33	g	EDL933	++++	g	g	g	g											g	g	g				g	g	g

O55 and O157 genome site details												Outgroup Strain Details[i]																						
CB9615 base[b]	CB9615 Site[c]	Sakai base[b]	Sakai Site[c]	EDL933 base[b]	EDL933 Site[c]	type[d]	Event Lineage[ef]	Recombinant segment	Inferred ancestral base[g]	Outgroup Analysis[e]	Support level[h]	D1 Sd197	K-12	HS	ATCC 8739	UMN026	IAI39	SMS 3-5	E2348/69	536	ED1a	CFT073	S88	APEC O1	UTI89	E24377A	IAI1	SE11	F5 8401	F2a 2457T	F2a 301	SS Ss046	B4 Sb227	B18 BS512
g	1851065	g	2046603	a	2041866	nc	EDL933	rec33	a	2	-	a	g	g	g											g	g	a				g	g	g
g	1851088	g	2046626	t	2041843	nc	EDL933	rec33	g	EDL933	++++	g	g	g	g											g	g	g				g	g	g
g	1851100	g	2046638	c	2041831	nc	EDL933	rec33	g	EDL933	++++	g	g	c	g											g	g	g				g	g	g
c	1851101	c	2046639	t	2041830	nc	EDL933	rec33	c	EDL933	++++	c	c	c	c											c	c	c				c	c	c
g	1851104	g	2046642	c	2041827	nc	EDL933	rec33	c	2	-	c	g	c	g											g	g	g				g	g	g
g	1851307	a	2046845	a	2041624	nc	O55/O157	rec34	g	O157	+	c	g	t	g											a	a	g				g	g	g
c	1851318	g	2046856	g	2041613	nc	O55/O157	rec34	c	O157	++++	c	c	c	c											c	g	c				c	c	c
t	1851330	c	2046868	c	2041601	nc	O55/O157	rec34	t	O157	++	t	t	c	t											t	c	t				t	t	t
c	1851421	t	2046959	t	2041510	nc	O55/O157	rec34	c	O157	++++	c	c	c	c											c	c	c				c	c	c
g	1851438	c	2046976	c	2041493	nc	O55/O157	rec34	c	CB9615	++++	c	c	c	c											g	c	c				c	c	c
t	1851448	c	2046986	c	2041483	nc	O55/O157	rec34	c	CB9615	++	c	c	t	c											t	c	t				c	c	c
g	1851485	a	2047023	a	2041446	nc	O55/O157	rec34	g	O157	++	g	a	a	a											g	a	g				a	g	a
g	1851487	c	2047025	c	2041444	nc	O55/O157	rec34	c	CB9615	++	c	g	c	g											g	c	g				g	c	a
a	1851505	g	2047043	g	2041426	nc	O55/O157	rec34	g	CB9615	++	g	a	g	a											a	g	a				a	g	g
a	1851688	c	2047226	c	2041243	nc	O55/O157	rec34	c	CB9615	++++	t	c	g	c											a	c	c				c	c	c
t	1851727	c	2047265	c	2041204	nc	O55/O157	rec34	c	CB9615	++++	c	c	c	c											c	c	c				c	c	c
a	1851742	g	2047280	g	2041189	nc	O55/O157	rec34	g	CB9615	++++	g	g	g	g											g	g	g				g	g	g
g	1851943	a	2047481	a	2040988	nc	O55/O157	rec34	g	O157	+	c	g	g	g											a	g	a				g	c	a
g	1851952	c	2047490	c	2040979	nc	O55/O157	rec34	c	CB9615	++	c	g	a	g											c	g	c				g	c	t
t	1852006	g	2047544	g	2040925	nc	O55/O157	rec34	g	CB9615	+	c	g	t	g											g	g	g				g	t	g
c	1852048	t	2047586	t	2040883	nc	O55/O157	rec34	t	CB9615	++	t	t	g	c											c	c	t				c	t	t
c	1852066	t	2047604	t	2040865	nc	O55/O157	rec34	c	O157	++++	c	c	c	c											c	c	c				c	c	c
c	1852069	t	2047607	t	2040862	nc	O55/O157	rec34	c	O157	++++	g	c	t	c											c	c	c				c	g	g
t	1852070	c	2047608	c	2040861	nc	O55/O157	rec34	c	CB9615	++++	c	c	c	c											c	c	c				c	c	c
c	1852090	t	2047628	t	2040841	nc	O55/O157	rec34	c	O157	++++	c	c	g	c											c	c	c				c	c	t
c	1852099	t	2047637	t	2040832	nc	O55/O157	rec34	c	O157	++	c	c	c	t											t	t	c				t	t	c
t	1852107	g	2047645	g	2040824	nc	O55/O157	rec34	t	O157	++++	a	t	c	t											t	t	t				t	t	t
t	1852140	c	2047678	c	2040791	nc	O55/O157	rec34	t	O157	++	t	c	c	c											c	c	c				c	c	t
c	1852216	t	2047754	t	2040715	nc	O55/O157	rec34	c	O157	++++	c	c	-	c											c	c	c				c	c	c
g	1852261	a	2047799	a	2040670	nc	O55/O157	rec34	g	O157	++++	g	g	t	g											g	g	g				g	g	g
a	1852303	g	2047841	g	2040628	nc	O55/O157	rec34	a	O157	++	a	g	g	g											a	g	g				g	g	a
c	1852306	t	2047844	t	2040625	nc	O55/O157	rec34	c	O157	++++	c	c	t	c											c	c	c				c	c	c
t	1852312	c	2047850	c	2040619	nc	O55/O157	rec34	t	O157	++++	t	t	t	t											t	t	t				t	t	a
-	1963488	g	2160964	Y	1927506	ns	Sakai/EDL933	rec35	g	EDL933	++++								a															
-	1963488	g	2160982	m	1927488	ns	Sakai/EDL933	rec35	g	EDL933	++++								t															
-	1963488	a	2160991	y	1927479	ns	Sakai/EDL933	rec35	a	EDL933	+								a															
-	1963488	t	2161003	r	1927467	ns	Sakai/EDL933	rec35	t	EDL933	+								t															
-	1963488	c	2161015	r	1927455	ns	Sakai/EDL933	rec35	c	EDL933	++++								t															
-	1963488	t	2161038	V	1927432	ns	Sakai/EDL933	rec35	t	EDL933	+								t															
-	1963488	g	2161066	Y	1927404	ns	Sakai/EDL933	rec35	g	EDL933	+								g															
-	1963488	t	2161083	g	1927387	ns	Sakai/EDL933	rec35	g	Sakai	+								g															
-	1963488	a	2161092	g	1927378	s	Sakai/EDL933	rec35	a	EDL933	+								a															
-	1963488	c	2161142	k	1927328	ns	Sakai/EDL933	rec35	c	EDL933	+								c															
-	1963488	c	2161219	r	1927251	ns	Sakai/EDL933	rec35	c	EDL933	+								c															
-	1963488	t	2161230	m	1927240	ns	Sakai/EDL933	rec35	t	EDL933	+								t															
-	1963488	t	2161256	c	1927214	s	Sakai/EDL933	rec35	t	EDL933	+								t															
-	1963488	g	2161259	y	1927211	ns	Sakai/EDL933	rec35	g	EDL933	+								g															
-	1963488	t	2161269	r	1927201	ns	Sakai/EDL933	rec35	t	EDL933	+								t															
-	1963488	g	2161285	m	1927185	ns	Sakai/EDL933	rec35	g	EDL933	++++								t															
-	1963488	t	2161305	c	1927165	ns	Sakai/EDL933	rec35	c	Sakai	+								c															
-	1963488	t	2161307	c	1927163	ns	Sakai/EDL933	rec35	c	Sakai	+								c															
-	1963488	c	2161308	g	1927162	ns	Sakai/EDL933	rec35	g	Sakai	+								g															
-	1963488	g	2161322	a	1927148	s	Sakai/EDL933	rec35	a	Sakai	+								a															
-	1963488	c	2161325	a	1927145	s	Sakai/EDL933	rec35	a	Sakai	+								a															
-	1963488	t	2161328	g	1927142	s	Sakai/EDL933	rec35	g	Sakai	+								g															
-	1963488	c	2161334	t	1927136	s	Sakai/EDL933	rec35	t	Sakai	+								t															
-	1963488	c	2161358	t	1927112	s	Sakai/EDL933	rec35	t	Sakai	+								t														t	
-	1963488	g	2188677	M	1894043	i	Sakai/EDL933	rec35	g	EDL933	++++																							
-	1963488	g	2188678	S	1894042	i	Sakai/EDL933	rec35	g	EDL933	++++																							
-	1963488	c	2188896	R	1893824	i	Sakai/EDL933	rec35	c	EDL933	+										c													
-	1963488	a	2189067	Y	1893653	ns	Sakai/EDL933	rec35	a	EDL933	++++										g													
-	1963488	g	2189164	t	1893556	ns	Sakai/EDL933	rec35	g	EDL933	+										g													
-	1963488	-	2189182	c	1893537	indel	Sakai/EDL933	rec35	?	EDL933	+/-																							
-	1963488	g	2189535	S	1893184	nc	Sakai/EDL933	rec35	g	EDL933	+										g		g											
-	1963488	a	2190252	y	1892467	ns	Sakai/EDL933	rec35	a	EDL933	+												a											
-	1963488	c	2190267	r	1892452	ns	Sakai/EDL933	rec35	c	EDL933	+												c											
-	1963488	c	2190347	S	1892372	ns	Sakai/EDL933	rec35	c	EDL933	+												c											
-	1963488	c	2192509	R	1890210	ns	Sakai/EDL933	rec35	c	EDL933	++++	t	c		c	c		c	t		c	t	t	t	t	c	c	c	t	t	t	t	t	t
-	1963488	c	2193209	r	1889510	ns	Sakai/EDL933	rec35	c	EDL933	++++																							
-	1963488	a	2193225	n	1889494	ns	Sakai/EDL933	rec35	a	EDL933	++++																							
-	1963488	-	2193226	a	1889492	indel	Sakai/EDL933	rec35	?	EDL933	+/-																							
-	1963488	-	2193877	Y	1888840	indel	Sakai/EDL933	rec35	-	EDL933	IUPAC																							
-	1963488	c	2193903	R	1888814	i	Sakai/EDL933	rec35	c	EDL933	++++	c				c		c	c		c	c	c	c	c	c		c	c	c	c	c	c	c
-	1963488	a	2194158	K	1888559	i	Sakai/EDL933	rec35	a	EDL933	++++	a				a		a	c		a	a	a	c	a	a		a	a	a	a	c	a	a
-	1963488	g	2194215	Y	1888502	i	Sakai/EDL933	rec35	g	EDL933	++++	g				g		g	g		g	g	g	g	g	g		g	g	g	g	g	g	g
-	1988518	c	2231319	a	2306602	s	EDL933	rec36	c	EDL933	+								c		c													c
-	1988518	g	2231343	a	2306626	s	EDL933	rec36	g	EDL933	+								g		g													g
-	1988518	t	2231379	c	2306662	s	EDL933	rec36	t	EDL933	+								t		c													t
-	1988518	a	2231409	c	2306692	s	EDL933	rec36	a	EDL933	+								a		a													a
-	1988518	c	2231412	a	2306695	s	EDL933	rec36	c	EDL933	+								c		c													c
-	1988518	g	2231421	c	2306704	s	EDL933	rec36	g	EDL933	+								g		g													g
-	1988518	a	2231427	g	2306710	s	EDL933	rec36	a	EDL933	+								a		a													a
-	1988518	a	2231439	c	2306722	s	EDL933	rec36	a	EDL933	+								a		a													a

O55 and O157 genome site details												Outgroup Strain Details[i]																						
CB9615 base[b]	CB9615 Site[c]	Sakai base[b]	Sakai Site[c]	EDL933 base[b]	EDL933 Site[c]	type[d]	Event Lineage[ef]	Recombinant segment	Inferred ancestral base[g]	Outgroup Analysis[e]	Support level[h]	D1 Sd197	K-12	HS	ATCC 8739	UMN026	IAI39	SMS 3-5	E2348/69	536	ED1a	CFT073	S88	APEC O1	UTI89	E24377A	IAI1	SE11	F5 8401	F2a 2457T	F2a 301	SS Ss046	B4 Sb227	B18 BS512
-	1988518	g	2231444	a	2306727	ns	EDL933	rec36	g	EDL933	+								g		g													g
-	1988518	c	2231460	t	2306743	s	EDL933	rec36	c	EDL933	+								c		c													c
-	1988518	g	2231466	a	2306749	s	EDL933	rec36	g	EDL933	+								g		g													g
-	1988518	g	2231477	t	2306760	s	EDL933	rec36	g	EDL933	+								g		g													g
-	1988518	g	2231497	a	2306780	ns	EDL933	rec36	g	EDL933	+								g		g													g
-	1988518	a	2231548	g	2306831	ns	EDL933	rec36	a	EDL933	+								a		a													a
t	1990458	c	2235868	c	2311151	i	O55/O157	rec37	t	O157	+										t													
t	1990549	g	2235959	g	2311242	i	O55/O157	rec37	g	CB9615	++++	g	g	g	g	g	g	g	g		g	g	g	g	g		g		g	g	g		g	g
g	1990620	a	2236030	a	2311313	s	O55/O157	rec37	a	CB9615	++	a	a	a	a	g	a	a	a		a	a	a	g	g		a		a	a	a		a	a
a	1990626	g	2236036	g	2311319	s	O55/O157	rec37	g	CB9615	++	g	a	a	a	a	a	a	g		g	a	g	a	a		a		a	a	a		g	g
t	1990641	c	2236051	c	2311334	s	O55/O157	rec37	c	CB9615	++	c	g	a	g	t	g	g	t		c	g	t	t	t		g		g	g	g		t	t
a	1990695	t	2236105	t	2311388	s	O55/O157	rec37	t	CB9615	++	t	a	a	a	a	a	a	a		a	a	a	a	a		a		a	a	a		a	a
t	1990707	c	2236117	c	2311400	s	O55/O157	rec37	c	CB9615	++	c	c	c	c	t	c	c	t		t	c	t	t	t		c		c	c	c		c	c
a	1990743	g	2236153	g	2311436	s	O55/O157	rec37	g	CB9615	+	c	g	g	g	a	g	g	a		a	g	a	a	a		g		g	g	g		g	g
a	1990770	g	2236180	g	2311463	s	O55/O157	rec37	g	CB9615	++++	g	g	g	g	g	g	g	g		g	t	g	g	g		g		t	t	t		g	g
t	1990797	c	2236207	c	2311490	s	O55/O157	rec37	t	O157	++	t	t	t	t	c	t	t	t		c	t	c	t	t		t		a	a	a		c	c
a	1990912	c	2236322	c	2311605	ns	O55/O157	rec37	a	O157	++++	a	a	a	a	a	a	a	a		a	a	a	a	a		a		a	a	a		a	a
g	1991021	a	2236431	a	2311714	ns	O55/O157	rec37	a	CB9615	++	a	a	g	a	g	a	g	g		a	g	g	a	a		a		a	a	a		g	g
a	1991025	c	2236435	c	2311718	s	O55/O157	rec37	a	O157	++++	a	g	a	g	a	g	a	a		c	a	a	g	g		g		g	g	g		a	a
g	1991031	a	2236441	a	2311724	ns	O55/O157	rec37	a	CB9615	++	a	a	g	a	g	a	g	g		a	g	g	t	t		a		g	g	g		g	g
g	1991032	c	2236442	c	2311725	ns	O55/O157	rec37	g	O157	++	g	c	g	c	g	c	g	g		c	g	g	c	c		c		g	g	g		g	g
g	1991225	t	2236635	t	2311918	ns	O55/O157	rec37	t	CB9615	++++					t		t	t		t		t	t									t	t
t	1991227	c	2236637	c	2311920	ns	O55/O157	rec37	c	CB9615	++++					c		c	c		c		c	c									c	c
c	1991231	g	2236641	a	2311924	s	O55/O157	rec37	g	CB9615	++++					g		g	g		g		g	g									g	g
t	1991339	a	2236749	a	2312032	s	O55/O157	rec37	a	CB9615	++++					a		a	a		a		a	a									a	a
g	1991481	a	2236891	a	2312174	i	O55/O157	rec37	?	O55/O157	+/-					a			a		g		a										g	g
a	1991508	c	2236918	c	2312201	s	O55/O157	rec37	c	CB9615	+					c			c		c		c										a	a
c	1991514	t	2236924	t	2312207	s	O55/O157	rec37	c	O157	+					c			t		t		c										c	c
c	1991518	t	2236928	t	2312211	ns	O55/O157	rec37	?	O55/O157	+/-					t			t		t		c										c	c
g	1991520	a	2236930	a	2312213	s	O55/O157	rec37	g	O157	+					g			a		a		g										g	g
t	1991523	c	2236933	c	2312216	s	O55/O157	rec37	t	O157	+++					t			c		t		t										t	t
a	1991538	c	2236948	c	2312231	s	O55/O157	rec37	a	O157	+++					a			t		c		a										a	a
c	1991547	t	2236957	t	2312240	s	O55/O157	rec37	t	CB9615	+					t			c		c		t										t	t
g	1991556	c	2236966	c	2312249	s	O55/O157	rec37	c	CB9615	+++					c			g		a		c										c	c
a	1991580	g	2236990	g	2312273	s	O55/O157	rec37	g	CB9615	+++					g			a		g		g										g	g
c	1991673	t	2237083	t	2312366	s	O55/O157	rec37	t	CB9615	+++					t			t		t		t										t	t
-	2247232	t	2454766	N	2529969	ns	EDL933	rec38	t	EDL933	++++					t	t	t	t	t	t	t	t	t	t	t			t	t	-	-	t	t
-	2247232	t	2454812	n	2530015	ns	EDL933	rec38	t	EDL933	++++					t	t	t	t	t	t	t	t	t	t	t			t	t	t	t	t	t
-	2247232	t	2454814	n	2530017	ns	EDL933	rec38	t	EDL933	++++					t	t	t	t	t	c	t	t	t	t	c			c	c	c	c	c	c
-	2247232	t	2454819	n	2530022	ns	EDL933	rec38	t	EDL933	++++					t	t	t	t	t	t	t	t	t	t	t			t	t	t	t	t	t
t	2439632	t	2669844	a	2745061	s	EDL933	rec39	?	EDL933	+/-																							
c	2439671	c	2669883	g	2745100	ns	EDL933	rec39	?	EDL933	+/-																							
g	2439741	g	2669953	a	2745170	ns	EDL933	rec39	?	EDL933	+/-																							
c	2439825	c	2670037	a	2745254	ns	EDL933	rec39	?	EDL933	+/-																							
a	2439827	a	2670039	g	2745256	ns	EDL933	rec39	?	EDL933	+/-																							
t	2439829	t	2670041	c	2745258	ns	EDL933	rec39	?	EDL933	+/-																							
c	2439858	c	2670070	t	2745287	s	EDL933	rec39	?	EDL933	+/-																							
g	2439882	g	2670094	a	2745311	ns	EDL933	rec39	?	EDL933	+/-																							
g	2439922	g	2670134	a	2745351	ns	EDL933	rec39	?	EDL933	+/-																							
c	2439966	c	2670178	a	2745395	ns	EDL933	rec39	?	EDL933	+/-																							
a	2439968	a	2670180	g	2745397	ns	EDL933	rec39	?	EDL933	+/-																							
c	2439999	c	2670211	t	2745428	s	EDL933	rec39	?	EDL933	+/-																							
a	2440063	a	2670275	g	2745492	ns	EDL933	rec39	?	EDL933	+/-																							
g	2440023	a	2670235	a	2745452	ns	CB9615	rec40	?	O55/O157	+/-																							
g	2440164	a	2670376	R	2745593	ns	CB9615	rec40	?	O55/O157	+/-																							
c	2486675	g	2711967	g	2788332	ns	CB9615	rec40	g	CB9615	+++	-						g						g					-	g	g		-	g
a	2486709	g	2712001	g	2788366	i	CB9615	rec40	g	CB9615	++++	g						g						g					g	g	g		g	g
g	2486720	a	2712012	a	2788377	i	CB9615	rec40	a	CB9615	++++	a						a		a		a		a					a	a	a		a	a
g	2486724	a	2712016	a	2788381	i	CB9615	rec40	a	CB9615	++++	a						a		c		c		a					a	a	a		a	a
c	2486725	t	2712017	t	2788382	i	CB9615	rec40	t	CB9615	++++	t						t		t		t		t					t	t	t		t	t
c	2440107	a	2670319	c	2745536	ns	Sakai	rec41	?	Sakai	+/-																							
a	2440109	g	2670321	a	2745538	ns	Sakai	rec41	?	Sakai	+/-																							
c	2440140	t	2670352	c	2745569	s	Sakai	rec41	?	Sakai	+/-																							
-	2440190	t	2694827	g	2771191	ns	Sakai	rec41	g	Sakai	+								t													g		g
-	2440190	c	2694831	a	2771195	ns	Sakai	rec41	a	Sakai	+								c													a		a
-	2440190	t	2694855	c	2771219	ns	Sakai	rec41	c	Sakai	+								t													c		c
-	2440190	a	2694992	g	2771356	ns	Sakai	rec41	g	Sakai	+								g													g		g
-	2440190	c	2695102	t	2771466	s	Sakai	rec41	t	Sakai	+								t													t		t
-	2440190	g	2695560	t	2771924	ns	EDL933	rec42	?	Sakai/EDL933	+/-								g		g											t		t
-	2440190	a	2695771	g	2772135	s	EDL933	rec42	a	EDL933	+++								a		a											a	a	a
-	2440190	c	2695775	s	2772139	ns	EDL933	rec42	c	EDL933	+								c		g											g	c	g
-	2440190	g	2695777	c	2772141	s	EDL933	rec42	g	EDL933	+								g		a											a	g	a
-	2440190	c	2695783	m	2772147	ns	EDL933	rec42	c	EDL933	+								c		a											t	c	t
-	2440190	c	2695785	y	2772149	ns	EDL933	rec42	c	EDL933	+++								c		c											c	c	c
-	2440190	c	2695791	y	2772155	ns	EDL933	rec42	c	EDL933	+								c		g											c	g	c
-	2440190	a	2695801	r	2772165	ns	EDL933	rec42	a	EDL933	+								a		g											a	t	a
-	2440190	g	2695802	s	2772166	ns	EDL933	rec42	g	EDL933	+								g		g											c	t	c
-	2440190	t	2695807	d	2772171	ns	EDL933	rec42	t	EDL933	+++								t		t											t	c	t
-	2440190	g	2695861	R	2772225	ns	EDL933	rec42	g	EDL933	+								a		g											a	a	a
-	2440190	t	2695914	Y	2772278	ns	EDL933	rec42	t	EDL933	+++								t		t											t	t	t

O55 and O157 genome site details												Outgroup Strain Details[i]																						
CB9615 base[b]	CB9615 Site[c]	Sakai base[b]	Sakai Site[c]	EDL933 base[b]	EDL933 Site[c]	type[d]	Event Lineage[ef]	Recombinant segment	Inferred ancestral base[g]	Outgroup Analysis[e]	Support level[h]	D1 Sd197	K-12	HS	ATCC 8739	UMN026	IAI39	SMS 3-5	E2348/69	536	ED1a	CFT073	S88	APEC O1	UTI89	E24377A	IAI1	SE11	F5 8401	F2a 2457T	F2a 301	SS Ss046	B4 Sb227	B18 BS512
-	2440190	g	2695937	R	2772301	ns	EDL933	rec42	g	EDL933	+++								g		g											g	g	g
-	2440190	a	2695975	w	2772339	ns	EDL933	rec42	a	EDL933	+								a		t											c	c	c
-	2440190	c	2695978	y	2772342	ns	EDL933	rec42	c	EDL933	+++								c		c											c	c	c
-	2440190	g	2695990	s	2772354	ns	EDL933	rec42	g	EDL933	+								g		c											t	c	t
-	2440190	t	2706792	a	2783156	ns	EDL933	rec43	t	EDL933	+																	t						
-	2440190	t	2706796	a	2783160	ns	EDL933	rec43	t	EDL933	+																	t						
-	2440190	c	2707235	y	2783599	ns	EDL933	rec43	c	EDL933	+																	c						
-	2440190	a	2707249	r	2783613	ns	EDL933	rec43	a	EDL933	+																	a						
-	2440190	g	2707356	R	2783720	i	EDL933	rec43	g	EDL933	++++		g		g	g		g	a		g	g	g	g	g	a	g							a
-	2440190	g	2707446	R	2783810	i	EDL933	rec43	g	EDL933	++++		g		g	g		g	g		g	g	g	g	g	g	g							g
-	2440190	t	2707895	W	2784259	ns	EDL933	rec43	t	EDL933	+++								t		a		t	t	t	a								a
-	2440190	a	2708047	r	2784411	ns	EDL933	rec43	a	EDL933	+										a					-								a
-	2440190	-	2708047	g	2784412	ins	EDL933	rec43	-	EDL933	+										-					-								-
-	2440190	a	2708080	M	2784445	ns	EDL933	rec43	a	EDL933	++++										c					t								c
-	2440190	-	2708149	W	2784515	ins	EDL933	rec43	-	EDL933	+										-													-
-	2440190	c	2708974	N	2785340	ns	EDL933	rec43	c	EDL933	++++		c		c	c			c		c		c	c	c	c	c							c
-	2440190	t	2709900	y	2786266	ns	EDL933	rec43	t	EDL933	+				c	c		t	c		c	c	c	t	c	c	c	c					t	c
-	2440190	a	2709908	m	2786274	ns	EDL933	rec43	a	EDL933	++++				a	a		a	a		a	a	a	a	a	a	a	a					a	a
-	2440190	t	2709912	y	2786278	ns	EDL933	rec43	t	EDL933	+				c	c		c	c		c	c	t	c	t	c	c	c					c	c
-	2440190	g	2710396	c	2786762	ns	EDL933	rec43	c	Sakai	++++				c	c	c	c	c		c	c	c	c	c	c	c	c	c				c	c
-	2440190	t	2710405	-	2786770	ins	EDL933	rec43	-	Sakai	++++				-	-	-	-	-		-	-	-	-	-	-	-	-	-				-	-
-	2440190	c	2710431	t	2786796	ns	EDL933	rec43	t	Sakai	++++				t	t	t	t	t		t	t	t	t	t	t	t	t	t				t	t
-	2440190	g	2710732	R	2787097	i	EDL933	rec43	g	EDL933	+++							g			g			g					g				g	g
-	2514076	g	2742392	R	2818757	ns	EDL933	rec44	g	EDL933	+++					g	a	g	a	g	a	g	g	g	g	a			a	a	a	a	a	a
-	2514076	c	2742416	y	2818781	ns	EDL933	rec44	c	EDL933	++++					t	t	t	t	t	t	t	t	t	t	t			t	t	t	t	t	t
-	2514076	c	2742428	s	2818793	ns	EDL933	rec44	c	EDL933	++++					g	g	g	g	g	g	g	g	g	g	g			g	g	g	g	g	g
-	2514076	g	2742440	r	2818805	ns	EDL933	rec44	g	EDL933	++++					g	a	g	g	g	a	g	g	g	g	a			a	a	a	a	a	a
-	2514076	g	2742473	R	2818838	ns	EDL933	rec44	g	EDL933	++++					g	g	g	g	g	g	g	g	g	g	g			g	g	g	g	g	g
g	2528100	c	2748437	-	2819497	ns	O55/O157	rec45	g	Sakai	++++	g	a			g	g	g	g	g	g	c	g	g	g	g				g	g			g
a	2528205	c	2748542	-	2819497	ns	O55/O157	rec45	c	CB9615	++	c	c			a	a	a	a	a	a	a	a	a	a	a				a	a			a
c	2528220	t	2748557	-	2819497	ns	O55/O157	rec45	c	Sakai	++++	c	c			c	c	c	c	c	c	c	c	c	a	t				c	c			c
a	2528271	t	2748608	-	2819497	ns	O55/O157	rec45	t	CB9615	++	t	c			t	t	t	t	t	a	t	a	t	t	t				t	t			a
t	2528322	c	2748659	-	2819497	ns	O55/O157	rec45	t	Sakai	++	t	c			t	c	t	t	t	t	t	t	t	c	t				t	t			t
t	2528404	g	2748741	-	2819497	ns	O55/O157	rec45	t	Sakai	++	t	g			t	t	t	t	t	t	t	t	t	t	t				t	t			t
t	2540903	c	2761240	c	2831453	s	O157	rec46	c	CB9615	++	c	t	t	c	t	t	t	t	c	c	c	t	t	c	t	c	t	t	t	t	c	t	t
t	2540918	g	2761255	g	2831468	s	O157	rec46	g	CB9615	++++	g	g	g	g	t	g	g	g	g	g	g	g	g	g	g	g	g	g	g	g	g	g	g
a	2540921	g	2761258	g	2831471	s	O157	rec46	g	CB9615	++	g	g	a	g	g	g	a	a	g	g	g	g	g	g	a	g	a	a	a	a	g	a	a
g	2540966	a	2761303	a	2831516	s	O157	rec46	g	O157	++++	g	g	g	g	g	g	g	g	g	g	g	g	g	g	g	g	g	g	g	g	g	g	g
g	2540981	t	2761318	t	2831531	s	O157	rec46	g	O157	++	g	g	g	g	g	g	g	t	g	g	g	t	a	g	g	g	t	t	t	t	g	t	g
t	2541065	g	2761402	g	2831615	s	O157	rec46	t	O157	++++	t	t	t	t	t	t	t	t	t	t	t	t	t	t	t	t	t	t	t	t	t	t	t
t	2542074	c	2761968	c	2832181	i	O157	rec46	c	CB9615	++	c	t	c	c	c	t	t	c	c	t	c	c	c	c	c	t	t	c	c	c	t	c	c
c	2542190	g	2762084	g	2832297	ns	O157	rec46	c	O157	++++	c	c	c	c	c	c	c	c	c	c	c	c	-	c	c	c	c	c	c	c	c	c	c
g	2542192	a	2762086	a	2832299	ns	O157	rec46	a	CB9615	++++	a	a	a	a	a	a	a	a	a	a	a	a	-	a	a	a	a	a	a	a	a	a	a
t	2542297	c	2762191	c	2832404	i	O157	rec46	c	CB9615	++	c	c	c	c	c	t	c	c	c	c	c	c	t	c	c	c	c	c	c	c	c	c	c
a	2542425	t	2762319	t	2832532	s	O157	rec46	a	O157	++++	a	a	a	a	a	a	a	a	a	a	a	a	a	a	a	a	a	a	a	a	a	a	a
t	2542458	c	2762352	c	2832565	s	O157	rec46	c	CB9615	++	c	t	c	c	c	c	c	c	t	c	t	c	c	t	c	c	c	c	c	c	t	c	c
g	2542548	a	2762442	a	2832655	s	O157	rec46	a	CB9615	++	a	a	g	a	a	a	a	g	a	a	a	a	a	a	a	a	a	a	a	a	a	a	a
c	2542566	t	2762460	t	2832673	s	O157	rec46	c	O157	++	c	c	c	c	c	c	c	c	t	c	t	c	c	t	c	c	c	c	c	c	c	c	c
a	2542617	c	2762511	c	2832724	s	O157	rec46	c	CB9615	++	c	c	c	c	a	c	c	c	a	c	a	c	c	a	c	c	c	c	c	c	c	c	c
g	2542635	a	2762529	a	2832742	s	O157	rec46	g	O157	++	g	a	a	a	g	g	g	g	g	a	g	g	g	g	g	g	g	g	g	g	g	a	a
c	2542677	t	2762571	t	2832784	s	O157	rec46	c	O157	+	a	t	c	c	c	c	a	c	a	a	a	c	c	a	a	a	a	c	c	c	c	a	a
a	2542704	t	2762598	t	2832811	ns	O157	rec46	a	O157	++++	a	a	a	a	a	a	a	a	a	a	a	a	a	a	a	a	a	a	a	a	a	a	a
t	2542713	c	2762607	c	2832820	s	O157	rec46	t	O157	++	t	c	c	c	c	c	c	t	c	t	c	c	c	c	c	c	c	c	c	c	c	t	t
a	2542719	g	2762613	g	2832826	s	O157	rec46	a	O157	++	a	g	g	a	g	g	g	g	g	g	g	g	g	g	g	g	g	g	g	g	g	g	g
c	2542827	t	2762721	t	2832934	s	O157	rec46	t	CB9615	++	t	t	t	t	t	t	t	c	c	t	c	t	c	c	t	t	t	c	c	c	t	t	t
a	2542854	g	2762748	g	2832961	s	O157	rec46	g	CB9615	++++	g	g	g	g	g	g	g	g	g	g	g	g	g	g	g	g	g	g	g	g	g	g	g
t	2542866	c	2762760	c	2832973	s	O157	rec46	c	CB9615	++++	c	c	c	c	c	c	c	c	c	c	c	c	c	c	c	c	c	c	c	c	c	c	c
t	2542899	a	2762793	a	2833006	s	O157	rec46	a	CB9615	++	a	t	t	t	t	t	a	t	t	t	t	t	t	t	t	a	a	t	t	t	a	t	a
c	2542929	t	2762823	t	2833036	s	O157	rec46	t	CB9615	++	t	t	c	c	c	c	c	c	c	t	c	c	c	c	c	c	c	c	c	c	c	t	c
c	2542950	a	2762844	a	2833057	s	O157	rec46	a	CB9615	++	a	t	c	c	c	c	c	c	c	a	c	c	c	c	c	c	c	c	c	c	c	a	c
g	2542953	a	2762847	a	2833060	s	O157	rec46	a	CB9615	++	a	a	g	g	g	g	a	g	g	a	g	g	g	g	g	g	g	g	g	g	g	a	g
t	2542977	c	2762871	c	2833084	s	O157	rec46	c	CB9615	++	c	c	c	c	t	t	c	c	c	c	c	c	t	c	c	c	c	t	t	t	c	c	c
a	2542998	g	2762892	g	2833105	s	O157	rec46	a	O157	++	a	a	a	a	g	g	a	a	a	a	a	a	g	a	a	a	a	g	g	g	a	a	a
t	2543022	g	2762916	g	2833129	s	O157	rec46	g	CB9615	++	g	g	g	g	g	g	g	t	g	g	g	g	g	g	g	g	g	g	g	g	g	t	g
g	2543068	c	2762962	c	2833175	ns	O157	rec46	g	O157	++++	g	g	g	g	g	g	g	g	g	g	g	g	g	g	g	g	g	g	g	g	g	g	g
c	2543073	t	2762967	t	2833180	s	O157	rec46	t	CB9615	++	t	t	c	t	t	t	t	c	c	t	c	t	c	c	t	t	t	c	c	c	t	t	t
c	2543139	t	2763033	t	2833246	s	O157	rec46	t	CB9615	++	t	c	t	t	t	c	t	t	c	c	c	c	c	c	t	c	c	t	t	t	c	c	c
a	2543145	g	2763039	g	2833252	s	O157	rec46	g	CB9615	++	g	g	a	g	g	g	g	g	g	g	g	a	g	g	g	a	a	g	g	g	a	g	a
g	2543310	a	2763204	a	2833417	ns	O157	rec46	g	O157	++++	g	g	g	g	g	g	g	g	g	g	g	g	g	g	g	g	g	g	g	g	g	g	g
a	2543392	g	2763286	g	2833499	ns	O157	rec46	a	O157	++++	a	a	a	a	a	a	a	a	a	g	a	a	a	a	a	a	a	a	a	a	a	a	a
c	2543429	t	2763323	t	2833536	s	O157	rec46	c	O157	++++	c	c	c	c	c	c	c	c	c	c	c	c	c	c	c	c	c	c	c	c	c	c	c
g	2543462	a	2763356	a	2833569	s	O157	rec46	a	CB9615	++	a	g	g	g	g	g	a	g	g	a	g	g	g	g	g	g	g	g	g	g	g	a	g
a	2543469	g	2763363	g	2833576	ns	O157	rec46	g	CB9615	++++	g	g	g	g	g	g	g	g	g	g	g	g	g	g	g	g	g	g	g	g	g	g	g
t	2543496	a	2763390	a	2833603	ns	O157	rec46	a	CB9615	++++	a	a	a	a	a	a	a	a	a	a	a	a	a	a	a	a	a	a	a	a	a	a	a
a	2543585	g	2763479	g	2833692	s	O157	rec46	g	CB9615	++++	g	g	g	g	g	g	g	g	g	g	g	g	g	g	g	g	g	g	g	g	g	g	g
a	2543612	g	2763506	g	2833719	s	O157	rec46	g	CB9615	++	g	g	a	g	a	a	g	a	a	g	a	a	g	a	g	a	a	g	g	g	g	g	g
g	2543639	a	2763533	a	2833746	s	O157	rec46	?	O157	+/-	c	a	a	c	g	a	a	g	a	c	a	g	g	a	c	g	g	g	g	g	c	c	c
g	2543648	t	2763542	t	2833755	s	O157	rec46	t	CB9615	++	t	t	g	t	g	t	t	g	t	t	t	g	g	t	t	a	a	g	g	g	t	t	t
t	2543651	g	2763545	g	2833758	s	O157	rec46	g	CB9615	++	g	g	t	g	t	g	t	t	g	g	g	t	t	g	g	t	t	t	t	t	g	g	g
g	2543705	a	2763599	a	2833812	s	O157	rec46	a	CB9615	++	a	g	a	a	a	g	g	a	g	a	g	g	a	g	a	a	a	a	a	a	g	g	g
a	2543720	t	2763614	t	2833827	s	O157	rec46	t	CB9615	++++	t	t	t	t	t	t	t	t	t	t	t	t	t	t	t	t	t	t	t	t	t	t	t
c	2543742	t	2763636	t	2833849	s	O157	rec46	c	O157	++	c	c	c	c	c	t	c	c	c	t	c	c	c	c	c	c	c	c	c	c	c	c	c

O55 and O157 genome site details												Outgroup Strain Details[i]																						
CB9615 base[b]	CB9615 Site[c]	Sakai base[b]	Sakai Site[c]	EDL933 base[b]	EDL933 Site[c]	type[d]	Event Lineage[ef]	Recombinant segment	Inferred ancestral base[g]	Outgroup Analysis[e]	Support level[h]	D1 Sd197	K-12	HS	ATCC 8739	UMN026	IAI39	SMS 3-5	E2348/69	536	ED1a	CFT073	S88	APEC O1	UTI89	E24377A	IAI1	SE11	F5 8401	F2a 2457T	F2a 301	SS Ss046	B4 Sb227	B18 BS512
a	2543771	g	2763665	g	2833878	s	O157	rec46	g	CB9615	++	g	g	g	g	g	g	a	g	g	g	g	g	g	g	g	g	g	a	a	a	g	g	g
t	2543774	c	2763668	c	2833881	s	O157	rec46	c	CB9615	++	c	c	c	c	c	c	t	c	a	c	a	c	c	a	c	c	c	t	t	t	c	c	c
c	2543780	t	2763674	t	2833887	s	O157	rec46	c	O157	++	c	t	c	c	c	t	c	t	c	t	c	c	t	c	t	t	t	t	t	t	t	t	t
c	2543798	t	2763692	t	2833905	s	O157	rec46	t	CB9615	++++	t	t	t	t	t	t	t	t	t	t	t	c	t	t	t	t	t	t	t	t	t	t	t
a	2543843	g	2763737	g	2833950	s	O157	rec46	a	O157	++	a	a	g	a	g	g	g	g	g	g	g	g	a	g	a	a	a	g	g	g	g	a	a
a	2543870	g	2763764	g	2833977	s	O157	rec46	g	CB9615	++	g	a	g	g	g	g	g	g	g	g	g	g	g	g	a	a	a	g	g	g	g	g	a
a	2543894	g	2763788	g	2834001	s	O157	rec46	g	CB9615	++	g	g	g	g	a	g	g	g	g	g	g	a	a	g	a	a	a	g	g	g	g	g	a
c	2543897	t	2763791	t	2834004	s	O157	rec46	t	CB9615	++	t	t	t	t	t	t	t	t	t	t	t	t	t	t	c	c	c	t	t	t	t	c	c
a	2543915	g	2763809	g	2834022	s	O157	rec46	a	O157	++	a	a	g	a	g	a	a	a	a	a	a	g	g	a	g	g	g	a	a	a	a	a	g
c	2543924	t	2763818	t	2834031	s	O157	rec46	t	CB9615	++	t	t	t	t	t	c	t	c	c	t	c	t	t	c	c	c	c	c	c	c	t	t	c
a	2543930	g	2763824	g	2834037	s	O157	rec46	g	CB9615	++	g	g	g	g	g	a	g	a	a	g	a	g	g	a	a	a	a	a	a	a	g	g	a
c	2543985	t	2763879	t	2834092	s	O157	rec46	c	O157	++	c	c	c	c	c	c	c	c	c	c	c	c	t	c	c	c	c	c	c	c	t	t	c
t	2544005	c	2763899	c	2834112	s	O157	rec46	c	CB9615	++++	c	c	c	c	c	c	c	c	c	c	c	c	c	c	c	c	c	c	c	c	c	c	c
t	2544008	a	2763902	a	2834115	s	O157	rec46	t	O157	++	t	t	a	t	a	t	t	t	t	t	t	a	t	t	t	t	t	t	t	t	a	a	t
t	2544014	g	2763908	g	2834121	s	O157	rec46	g	CB9615	++++	g	g	g	g	g	g	g	g	g	g	g	g	g	g	a	a	a	g	g	g	g	g	a
c	2544038	t	2763932	t	2834145	s	O157	rec46	t	CB9615	++	t	c	t	t	c	t	t	t	t	t	t	c	t	t	t	t	t	t	t	t	t	t	t
c	2544041	t	2763935	t	2834148	s	O157	rec46	t	CB9615	++	t	t	c	t	c	t	t	t	t	t	t	c	t	t	t	t	t	t	t	t	t	t	t
t	2544065	a	2763959	a	2834172	s	O157	rec46	a	CB9615	++	a	a	a	a	t	a	a	a	a	a	a	t	a	a	t	t	t	a	a	a	a	a	t
a	2544083	g	2763977	g	2834190	s	O157	rec46	g	CB9615	++++	g	g	g	g	g	g	g	c	c	g	c	g	c	c	g	g	g	c	c	c	g	g	g
c	2544086	t	2763980	t	2834193	s	O157	rec46	c	O157	++	c	c	c	c	c	c	t	c	c	c	c	c	c	c	c	c	c	c	c	c	t	c	c
c	2544088	a	2763982	a	2834195	ns	O157	rec46	c	O157	++	c	c	c	c	c	a	a	a	a	c	a	c	a	a	a	a	a	a	a	a	a	c	a
t	2544089	c	2763983	c	2834196	ns	O157	rec46	t	O157	++	t	t	t	t	t	c	c	c	c	t	c	t	c	c	t	t	t	t	t	t	c	t	t
g	2544103	a	2763997	a	2834210	ns	O157	rec46	g	O157	++	g	g	g	g	g	g	a	g	g	g	g	g	g	g	g	g	g	g	g	g	a	g	g
g	2544105	c	2763999	c	2834212	ns	O157	rec46	c	CB9615	++++	c	c	c	c	c	c	c	c	c	c	c	c	c	c	c	c	c	c	c	c	c	c	c
g	2544106	a	2764000	a	2834213	ns	O157	rec46	g	O157	++	g	g	g	g	g	g	a	g	g	g	g	g	g	g	g	g	g	g	g	g	a	g	g
c	2544107	a	2764001	a	2834214	ns	O157	rec46	c	O157	++	c	c	c	c	c	c	a	c	c	c	c	c	c	c	c	c	c	c	c	c	a	c	c
a	2544110	t	2764004	t	2834217	s	O157	rec46	t	CB9615	++	t	t	a	t	a	t	t	t	t	t	t	t	t	t	t	t	t	t	t	t	t	t	t
t	2544119	c	2764013	c	2834226	s	O157	rec46	c	CB9615	++	c	c	t	c	t	t	c	c	c	c	c	t	c	c	t	t	t	c	c	c	c	t	t
g	2544134	a	2764028	a	2834241	s	O157	rec46	g	O157	++	g	g	g	g	g	g	a	g	g	g	g	g	g	g	g	g	g	g	g	g	a	g	g
g	2544140	a	2764034	a	2834247	s	O157	rec46	a	CB9615	++++	a	a	a	a	a	a	a	a	a	a	a	a	a	a	a	a	a	a	a	a	a	a	a
t	2544164	c	2764058	c	2834271	s	O157	rec46	c	CB9615	++	c	c	c	t	c	c	t	c	c	c	c	t	c	c	t	t	t	c	c	c	c	t	t
g	2544175	a	2764069	a	2834282	ns	O157	rec46	g	O157	++	g	a	a	g	a	a	g	a	a	g	a	g	a	a	a	g	g	a	a	a	a	a	a
t	2544185	c	2764079	c	2834292	s	O157	rec46	c	CB9615	++++	c	c	c	c	c	c	c	c	c	c	c	c	c	c	c	c	c	c	c	c	c	c	c
a	2544218	g	2764112	g	2834325	s	O157	rec46	g	CB9615	++	g	g	a	a	g	a	g	a	g	a	g	a	g	g	a	a	a	g	g	g	a	a	a
c	2544257	t	2764151	t	2834364	s	O157	rec46	t	CB9615	++	t	t	t	t	c	t	t	t	t	t	t	t	t	t	t	t	t	t	t	t	c	c	t
t	2544278	a	2764172	a	2834385	ns	O157	rec46	a	CB9615	++	a	a	t	a	t	a	a	a	a	a	a	a	a	a	a	a	a	a	a	a	t	t	a
c	2544332	g	2764226	g	2834439	s	O157	rec46	g	CB9615	++++	g	g	g	g	c	g	g	g	g	g	g	g	g	g	g	g	g	g	g	g	g	g	g
g	2544341	a	2764235	a	2834448	s	O157	rec46	a	CB9615	++++	a	a	a	a	g	a	a	a	a	a	a	a	a	a	a	a	a	a	a	a	a	a	a
c	2544359	a	2764253	a	2834466	s	O157	rec46	c	O157	++++	c	c	c	c	c	c	c	c	c	a	c	c	c	c	c	c	c	c	c	c	c	c	c
c	2544368	t	2764262	t	2834475	s	O157	rec46	c	O157	++	c	c	c	c	c	c	c	c	c	t	c	c	c	c	c	t	t	c	c	c	c	c	c
a	2544371	t	2764265	t	2834478	s	O157	rec46	t	CB9615	++++	t	t	t	t	a	t	t	t	t	t	t	t	g	t	t	t	t	g	g	g	t	t	t
g	2544374	a	2764268	a	2834481	s	O157	rec46	g	O157	++	g	a	g	g	g	g	g	a	a	a	a	a	g	a	a	a	a	t	g	g	g	g	g
t	2544386	c	2764280	c	2834493	s	O157	rec46	t	O157	++	t	t	t	t	t	t	t	t	c	c	c	t	t	c	t	t	t	t	t	t	t	t	t
g	2544413	a	2764307	a	2834520	s	O157	rec46	g	O157	++	g	g	g	g	g	g	a	g	g	a	g	g	g	g	g	g	g	g	g	g	g	g	g
a	2544419	g	2764313	g	2834526	s	O157	rec46	a	O157	++	a	a	a	g	a	g	g	a	a	g	a	a	a	a	a	a	a	a	a	a	a	a	a
t	2544434	g	2764328	g	2834541	s	O157	rec46	t	O157	+	c	c	t	t	c	c	g	c	c	g	c	t	c	c	c	c	c	c	c	c	t	c	c
a	2544460	t	2764354	t	2834567	ns	O157	rec46	a	O157	++	a	a	a	a	a	t	t	a	a	t	a	a	a	a	a	a	a	a	a	a	a	a	a
a	2544472	c	2764366	c	2834579	ns	O157	rec46	c	CB9615	++++	c	c	c	c	c	c	c	c	c	c	c	c	c	c	c	c	c	c	c	c	c	c	c
a	2544488	t	2764382	t	2834595	s	O157	rec46	t	CB9615	++	t	a	t	t	a	t	t	a	t	t	t	a	t	t	t	t	t	t	t	t	t	t	t
t	2544524	c	2764418	c	2834631	s	O157	rec46	c	CB9615	++	c	c	t	t	t	c	c	c	c	c	c	c	c	c	c	t	t	c	c	c	c	t	c
c	2544655	t	2764549	t	2834762	s	O157	rec46	c	O157	++	c	c	c	c	c	c	c	c	c	t	c	t	c	c	c	c	c	c	c	c	t	c	t
c	2544685	t	2764579	t	2834792	s	O157	rec46	c	O157	++	c	c	c	c	t	c	c	t	t	t	t	t	c	t	t	c	c	t	t	t	c	t	t
a	2544692	g	2764586	g	2834799	ns	O157	rec46	g	CB9615	++++	g	g	g	g	g	g	g	g	g	g	g	g	g	g	g	g	g	g	g	g	g	g	g
t	2544742	c	2764636	c	2834849	s	O157	rec46	c	CB9615	++	c	c	t	t	t	t	c	c	c	c	c	c	c	c	c	c	c	c	c	c	t	t	t
g	2544775	a	2764669	a	2834882	s	O157	rec46	a	CB9615	++	a	a	g	a	g	g	g	a	a	a	a	a	a	a	a	a	a	a	a	a	a	a	a
g	2544796	a	2764690	a	2834903	s	O157	rec46	a	CB9615	++	a	g	g	a	g	g	g	a	a	a	a	a	a	a	a	g	g	a	a	a	a	a	a
a	2544862	g	2764756	g	2834969	s	O157	rec46	g	CB9615	++	g	g	a	g	a	g	g	g	g	g	g	g	g	g	g	g	g	g	g	g	g	g	g
t	2544945	c	2764839	c	2835052	ns	O157	rec46	c	CB9615	++++	c	c	c	c	c	c	c	c	c	c	c	c	c	c	c	c	c	c	c	c	c	c	c
c	2544961	a	2764855	a	2835068	ns	O157	rec46	a	CB9615	++	a	c	c	c	c	c	a	c	c	a	c	a	c	c	c	c	c	a	a	a	c	c	c
g	2544979	a	2764873	a	2835086	s	O157	rec46	a	CB9615	++	a	a	a	a	a	g	g	a	a	a	a	a	a	a	a	a	a	a	a	a	a	a	a
g	2545042	a	2764936	a	2835149	s	O157	rec46	a	CB9615	++	a	g	g	c	g	g	g	c	c	g	c	a	c	c	g	g	g	c	c	c	g	g	g
c	2545093	g	2764987	g	2835200	s	O157	rec46	g	CB9615	++	g	c	c	g	c	c	g	c	c	c	c	g	c	c	g	g	g	c	c	c	c	c	g
a	2545111	g	2765005	g	2835218	s	O157	rec46	g	CB9615	++++	g	g	g	g	g	g	g	g	g	g	g	g	g	g	g	g	g	g	g	g	g	g	g
a	2545132	c	2765026	c	2835239	s	O157	rec46	c	CB9615	+	t	c	c	c	c	c	c	c	c	c	c	a	c	c	c	a	a	c	c	c	c	c	a
a	2545144	g	2765038	g	2835251	s	O157	rec46	g	CB9615	++	g	g	g	a	g	a	g	g	g	a	g	a	g	g	g	a	a	g	g	g	g	g	g
a	2545169	g	2765063	g	2835276	ns	O157	rec46	g	CB9615	++	g	g	g	g	a	a	g	g	g	g	g	g	g	g	g	g	g	g	g	g	a	g	a
t	2545237	c	2765131	c	2835344	s	O157	rec46	c	CB9615	++++	c	c	c	c	c	c	c	c	c	c	c	c	c	c	c	c	c	c	c	c	c	c	c
g	2545240	a	2765134	a	2835347	s	O157	rec46	a	CB9615	++++	a	a	a	a	a	a	a	a	a	a	a	a	a	a	a	a	a	a	a	a	a	a	a
t	2545274	c	2765168	c	2835381	s	O157	rec46	t	O157	++	t	c	c	c	c	c	c	c	c	c	c	c	c	c	c	t	t	c	c	c	c	c	c
c	2545288	t	2765182	t	2835395	s	O157	rec46	c	O157	++	c	c	c	t	c	c	c	c	c	t	c	c	c	c	c	c	c	c	c	c	c	t	t
g	2545351	a	2765245	a	2835458	s	O157	rec46	a	CB9615	++	a	a	g	a	g	a	g	a	a	a	a	g	a	a	a	g	g	a	a	a	a	g	g
c	2545354	t	2765248	t	2835461	s	O157	rec46	c	O157	++++	c	c	c	c	c	c	c	c	c	c	c	c	c	c	c	c	c	c	c	c	c	c	c
g	2545355	a	2765249	a	2835462	ns	O157	rec46	g	O157	++++	g	g	g	g	g	g	g	g	g	g	g	g	g	g	g	g	g	g	g	g	g	g	g
a	2545375	g	2765269	g	2835482	s	O157	rec46	g	CB9615	++	g	a	g	g	g	a	g	g	g	g	g	g	g	g	g	g	g	g	g	g	g	g	g
a	2545448	g	2765342	g	2835555	ns	O157	rec46	g	CB9615	++++	g	g	g	g	g	g	g	g	g	g	g	g	g	g	g	g	g	g	g	g	g	g	g
c	2545492	t	2765386	t	2835599	s	O157	rec46	t	CB9615	++	t	t	c	c	c	c	c	c	t	c	t	t	t	t	c	t	t	t	t	t	c	t	t
a	2545638	g	2765532	g	2835745	s	O157	rec46	g	CB9615	++	g	g	a	g	g	a	g	g	g	a	g	g	g	g	g	g	g	g	g	g	g	a	g
c	2545713	t	2765607	t	2835820	s	O157	rec46	c	O157	++++	c	c	c	c	c	c	c	c	c	c	c	c	c	c	c	c	c	c	c	c	c	t	c
g	2545753	c	2765647	c	2835860	ns	O157	rec46	g	O157	++++	g	g	g	g	g	g	g	g	g	g	g	g	g	g	g	g	g	g	g	g	g	g	g
g	2545755	a	2765649	a	2835862	ns	O157	rec46	a	CB9615	++	a	a	g	g	g	g	a	g	g	g	a	g	g	a	g	g	a	a	a	a	a	a	a
g	2545785	a	2765679	a	2835892	s	O157	rec46	g	O157	++	g	g	g	g	g	g	a	g	g	g	g	g	g	g	g	a	a	g	g	g	a	a	a
c	2545800	t	2765694	t	2835907	s	O157	rec46	t	CB9615	++	t	t	t	t	t	t	t	t	t	t	t	t	t	t	t	c	c	t	t	t	c	t	c
a	2545806	g	2765700	g	2835913	s	O157	rec46	g	CB9615	++++	g	g	g	g	g	g	g	g	g	g	g	g	g	g	g	g	g	g	g	g	g	g	g
t	2545815	g	2765709	g	2835922	s	O157	rec46	g	CB9615	++	g	t	g	t	g	g	g	t	t	g	t	g	t	t	g	g	g	g	g	g	g	t	g
t	2545845	c	2765739	c	2835952	s	O157	rec46	c	CB9615	++	c	c	t	c	c	c	t	c	c	c	c	c	c	c	t	c	c	t	t	t	c	t	c
c	2545875	a	2765769	a	2835982	s	O157	rec46	c	O157	++++	c	c	c	c	c	c	c	c	c	c	c	c	c	c	c	c	c	c	c	c	c	c	c

O55 and O157 genome site details												Outgroup Strain Details[i]																						
CB9615 base[b]	CB9615 Site[c]	Sakai base[b]	Sakai Site[c]	EDL933 base[b]	EDL933 Site[c]	type[d]	Event Lineage[ef]	Recombinant segment	Inferred ancestral base[g]	Outgroup Analysis[e]	Support level[h]	D1 Sd197	K-12	HS	ATCC 8739	UMN026	IAI39	SMS 3-5	E2348/69	536	ED1a	CFT073	S88	APEC O1	UTI89	E24377A	IAI1	SE11	F5 8401	F2a 2457T	F2a 301	SS Ss046	B4 Sb227	B18 BS512
g	2545893	a	2765787	a	2836000	s	O157	rec46	g	O157	++	g	a	g	a	a	g	g	g	a	a	a	a	g	a	g	a	a	g	g	g	a	g	g
g	2545953	a	2765847	a	2836060	s	O157	rec46	g	O157	++++	g	g	g	g	g	g	a	g	g	g	g	g	g	g	g	g	g	g	g	g	g	g	g
t	2546004	c	2765898	c	2836111	s	O157	rec46	t	O157	++	t	c	t	c	t	c	t	c	c	c	c	c	t	c	t	c	c	t	t	t	c	c	c
t	2546010	c	2765904	c	2836117	s	O157	rec46	c	CB9615	++	c	c	t	c	c	c	c	c	c	c	c	c	t	c	c	c	c	c	c	c	c	c	c
g	2546046	a	2765940	a	2836153	s	O157	rec46	a	CB9615	++++	a	a	g	a	a	a	a	a	a	a	a	a	a	a	a	a	a	a	a	a	a	a	a
g	2546049	c	2765943	c	2836156	s	O157	rec46	g	O157	++	g	g	g	g	g	g	g	g	g	g	g	g	g	g	g	c	c	g	g	g	g	a	g
g	2546052	t	2765946	t	2836159	s	O157	rec46	g	O157	++	g	g	g	g	g	g	g	g	g	g	g	g	a	g	g	t	t	g	g	g	g	a	g
t	2546082	c	2765976	c	2836189	s	O157	rec46	c	CB9615	++	c	c	c	c	c	c	c	c	c	c	c	c	c	c	c	t	t	c	c	c	c	c	c
g	2546121	a	2766015	a	2836228	s	O157	rec46	a	CB9615	++	a	a	a	a	a	a	a	a	a	a	a	a	a	a	a	g	g	a	a	a	g	a	a
t	2546127	c	2766021	c	2836234	s	O157	rec46	t	O157	++	t	c	t	t	t	t	t	t	t	t	t	t	c	t	t	t	t	t	t	t	t	c	c
a	2546129	g	2766023	g	2836236	ns	O157	rec46	a	O157	++	a	g	a	a	a	a	a	a	a	a	a	a	a	a	a	a	a	a	a	a	a	a	g
a	2546133	t	2766027	t	2836240	s	O157	rec46	a	O157	++	a	t	a	a	a	a	a	a	a	a	a	a	a	a	a	a	a	a	a	a	a	a	t
c	2546145	t	2766039	t	2836252	s	O157	rec46	c	O157	++	c	t	c	c	c	c	c	c	c	c	c	c	c	c	c	c	c	c	c	c	t	c	t
t	2546148	c	2766042	c	2836255	s	O157	rec46	t	O157	++	t	c	t	t	t	t	c	t	t	t	t	t	t	t	t	t	t	t	t	t	c	t	c
c	2546157	t	2766051	t	2836264	s	O157	rec46	c	O157	++	c	t	c	c	t	t	c	c	c	t	c	c	c	c	c	c	c	c	c	c	c	c	t
c	2546160	t	2766054	t	2836267	s	O157	rec46	c	O157	++	c	t	c	c	c	c	c	c	c	c	c	c	c	c	c	c	c	c	c	c	c	c	t
a	2546175	g	2766069	g	2836282	s	O157	rec46	a	O157	++	a	g	g	a	g	a	a	a	a	a	a	a	g	a	a	g	g	g	g	g	g	g	g
c	2546184	t	2766078	t	2836291	s	O157	rec46	c	O157	++	c	t	t	c	t	c	c	c	c	c	c	c	t	c	c	t	t	c	c	c	t	t	t
c	2546202	t	2766096	t	2836309	s	O157	rec46	t	CB9615	++	t	t	c	t	c	c	t	c	c	c	c	c	c	c	c	c	c	c	c	c	c	c	t
c	2546223	t	2766117	t	2836330	s	O157	rec46	t	CB9615	++	t	c	c	t	c	c	c	c	c	t	c	c	c	c	t	c	c	c	c	c	c	c	c
c	2546283	t	2766177	t	2836390	s	O157	rec46	c	O157	++	c	c	c	t	c	t	c	t	t	c	t	c	t	t	t	c	c	c	c	c	t	c	c
t	2546400	c	2766294	c	2836507	s	O157	rec46	c	CB9615	++	c	c	t	c	c	c	t	c	c	c	c	c	c	c	c	t	t	c	c	c	c	t	t
a	2546418	g	2766312	g	2836525	s	O157	rec46	g	CB9615	++	g	a	a	g	g	g	g	g	g	g	g	g	g	g	a	a	a	g	g	g	a	g	a
t	2546445	c	2766339	c	2836552	s	O157	rec46	t	O157	++	t	c	c	c	c	c	c	c	c	t	c	t	c	c	c	c	c	c	c	c	c	c	c
a	2546448	g	2766342	g	2836555	s	O157	rec46	a	O157	++	a	g	g	g	g	g	g	g	g	a	g	a	g	g	g	g	g	a	a	a	g	a	g
g	2546484	a	2766378	a	2836591	s	O157	rec46	a	CB9615	++++	a	a	a	a	a	a	a	a	a	a	a	a	a	a	a	a	a	a	a	a	a	a	a
a	2546490	g	2766384	g	2836597	s	O157	rec46	a	O157	++	a	g	g	g	a	a	a	a	a	a	a	a	a	a	g	g	g	g	g	g	g	a	g
g	2546502	a	2766396	a	2836609	s	O157	rec46	g	O157	++++	g	g	g	g	g	g	g	g	g	g	g	g	g	g	g	g	g	g	g	g	g	g	g
a	2546526	g	2766420	g	2836633	s	O157	rec46	a	O157	++	a	a	g	g	g	a	a	g	g	a	g	a	g	g	a	g	g	a	a	a	g	g	g
a	2546556	t	2766450	t	2836663	s	O157	rec46	t	CB9615	++	t	a	t	a	a	t	t	a	a	a	a	a	a	a	a	a	a	t	t	t	a	t	t
c	2546574	g	2766468	g	2836681	s	O157	rec46	c	O157	++	c	g	g	c	g	g	g	g	g	c	g	g	g	g	g	g	g	a	a	a	g	c	g
g	2546577	c	2766471	c	2836684	s	O157	rec46	g	O157	+++	g	a	c	g	a	g	a	a	a	g	a	g	a	a	a	a	a	a	a	a	a	g	a
c	2546607	t	2766501	t	2836714	s	O157	rec46	c	O157	++	c	t	c	c	t	t	t	c	c	c	c	t	c	c	c	t	t	t	t	t	c	c	c
t	2546634	c	2766528	c	2836741	s	O157	rec46	c	CB9615	++	c	c	c	c	c	t	c	g	c	t	c	c	c	c	c	c	c	c	c	c	c	t	c
c	2546643	g	2766537	g	2836750	s	O157	rec46	c	O157	++++	c	c	c	c	c	c	c	c	t	c	t	c	t	t	c	c	c	t	t	t	c	c	c
a	2546725	g	2766619	g	2836832	ns	O157	rec46	a	O157	++++	a	a	a	a	a	a	a	a	a	a	a	a	a	a	a	a	a	a	a	a	a	a	a
c	2546771	t	2766665	t	2836878	s	O157	rec46	t	CB9615	++	t	t	t	c	c	t	c	c	c	c	c	t	c	c	c	c	c	c	c	c	c	c	c
c	2546810	g	2766704	g	2836917	s	O157	rec46	c	O157	++	c	c	c	a	g	c	c	c	c	c	c	c	c	c	c	a	a	t	t	t	g	c	g
t	2546954	g	2766848	g	2837061	s	O157	rec46	g	CB9615	++++	g	g	g	g	g	g	g	g	c	t	c	g	c	c	g	g	g	g	g	g	g	g	g
c	2546966	t	2766860	t	2837073	s	O157	rec46	c	O157	++	c	c	t	t	c	c	c	c	c	c	c	c	c	c	c	c	c	c	c	c	c	c	c
c	2546987	t	2766881	t	2837094	s	O157	rec46	c	O157	++	c	c	t	t	t	t	c	t	c	c	c	t	c	c	c	c	c	c	c	c	c	t	c
t	2547002	c	2766896	c	2837109	s	O157	rec46	c	CB9615	++	c	c	c	c	c	c	c	c	c	c	c	c	c	c	t	c	c	c	c	c	t	c	c
c	2547062	t	2766956	t	2837169	s	O157	rec46	c	O157	++	c	c	c	c	c	c	c	t	t	c	t	t	t	t	c	c	c	c	c	c	t	c	t
t	2547110	c	2767004	c	2837217	s	O157	rec46	c	CB9615	++++	c	c	c	c	c	c	c	c	c	c	c	c	c	c	c	c	c	c	c	c	c	c	c
a	2547174	t	2767068	t	2837281	ns	O157	rec46	t	CB9615	++++	t	t	t	t	t	t	t	t	t	t	t	t	t	t	t	t	t	t	t	t	t	t	t
g	2547191	a	2767085	a	2837298	s	O157	rec46	g	O157	++++	g	g	g	g	g	g	g	g	g	g	g	g	g	g	g	g	g	g	g	g	g	g	g
t	2547197	c	2767091	c	2837304	s	O157	rec46	c	CB9615	++++	c	c	c	c	c	c	c	c	c	c	c	c	c	c	c	c	c	c	c	c	c	c	c
c	2547239	t	2767133	t	2837346	s	O157	rec46	c	O157	++++	c	c	c	c	c	c	c	c	c	c	c	c	c	c	c	c	c	c	c	c	c	c	c
t	2547322	c	2767216	c	2837429	s	O157	rec46	c	CB9615	++++	c	c	c	c	c	c	c	c	c	c	c	c	c	c	c	c	c	c	c	c	c	c	c
c	2547331	t	2767225	t	2837438	s	O157	rec46	c	O157	++	c	t	c	c	c	c	c	c	c	t	c	t	c	c	c	t	t	c	c	c	c	c	c
t	2547358	c	2767252	c	2837465	s	O157	rec46	c	CB9615	++++	c	c	c	c	c	c	t	c	c	c	c	c	c	c	c	c	c	c	c	c	c	c	c
a	2547394	g	2767288	g	2837501	s	O157	rec46	g	CB9615	++	g	g	a	a	g	g	a	g	g	g	g	g	g	g	g	g	g	g	g	g	g	g	g
t	2547412	c	2767306	c	2837519	s	O157	rec46	c	CB9615	++	c	c	c	t	c	c	c	c	t	c	t	c	t	t	c	c	c	t	t	t	c	c	c
g	2547415	a	2767309	a	2837522	s	O157	rec46	g	O157	++	g	g	g	g	g	g	a	a	g	a	g	g	g	g	g	g	g	g	g	g	g	g	g
t	2547431	c	2767325	c	2837538	s	O157	rec46	c	CB9615	++	c	c	t	t	c	t	c	t	t	t	t	c	t	t	t	c	c	t	t	t	c	t	c
c	2547437	t	2767331	t	2837544	s	O157	rec46	c	O157	++++	c	c	c	c	c	c	c	c	c	t	c	c	c	c	c	c	c	c	c	c	c	c	c
g	2547505	t	2767399	t	2837612	s	O157	rec46	g	O157	++++	g	g	g	g	g	g	g	g	g	g	g	g	g	g	g	g	g	g	g	g	g	g	g
t	2547538	c	2767432	c	2837645	s	O157	rec46	c	CB9615	++	c	c	c	c	c	c	c	t	t	c	t	c	c	t	c	c	c	t	t	t	t	c	c
g	2547577	t	2767471	t	2837684	s	O157	rec46	g	O157	++	g	g	g	g	g	g	a	g	g	g	g	g	g	g	g	g	g	t	t	t	g	g	g
c	2547580	g	2767474	g	2837687	s	O157	rec46	c	O157	++++	c	c	c	c	c	c	t	c	c	c	c	c	c	c	c	c	c	c	c	c	c	c	c
c	2547607	t	2767501	t	2837714	s	O157	rec46	c	O157	++++	c	c	c	c	c	c	c	c	c	c	c	c	c	c	c	c	c	c	c	c	c	c	c
g	2547627	t	2767521	t	2837734	ns	O157	rec46	t	CB9615	++	t	t	t	t	t	t	g	t	t	t	t	t	t	t	t	t	t	t	t	t	g	t	t
c	2547649	t	2767543	t	2837756	s	O157	rec46	t	CB9615	++	t	c	c	t	t	t	c	t	t	t	t	t	t	t	t	c	c	t	t	t	t	t	c
t	2547748	g	2767642	g	2837855	s	O157	rec46	g	CB9615	++	g	t	t	t	t	g	g	t	t	t	t	t	t	t	t	t	t	t	t	t	t	t	t
c	2547877	t	2767771	t	2837984	s	O157	rec46	t	CB9615	++	t	t	t	c	t	t	t	t	t	t	t	t	t	t	t	t	t	t	t	t	c	t	t
c	2547898	a	2767792	a	2838005	s	O157	rec46	a	CB9615	++	a	c	t	a	a	a	c	c	c	a	c	a	c	c	c	c	c	c	c	c	c	a	c
t	2547907	c	2767801	c	2838014	s	O157	rec46	c	CB9615	++	c	t	t	c	c	c	t	c	t	c	t	c	t	t	t	t	t	c	c	c	t	c	c
g	2548013	t	2767907	t	2838120	ns	O157	rec46	g	O157	++++	g	g	g	g	g	g	g	g	g	g	g	g	g	g	g	g	g	g	g	g	g	g	g
t	2548029	c	2767923	c	2838136	ns	O157	rec46	c	CB9615	++++	c	c	c	c	c	c	c	c	c	c	c	c	c	c	c	c	c	c	c	c	c	c	c
g	2548038	a	2767932	a	2838145	s	O157	rec46	g	O157	++	g	a	a	g	a	g	g	g	t	g	t	g	a	t	a	a	a	g	g	g	a	g	g
c	2548053	t	2767947	t	2838160	s	O157	rec46	t	CB9615	++++	t	t	t	t	t	t	t	t	t	t	t	t	t	t	t	t	t	t	t	t	t	t	t
g	2548068	a	2767962	a	2838175	s	O157	rec46	g	O157	++++	g	g	g	g	g	g	g	g	g	g	g	g	g	g	g	g	g	g	g	g	g	g	g
t	2548110	c	2768004	c	2838217	s	O157	rec46	c	CB9615	++	c	c	t	c	c	t	c	c	c	t	c	t	c	c	c	c	c	c	c	c	t	c	c
a	2548113	g	2768007	g	2838220	s	O157	rec46	g	CB9615	++	g	g	a	g	a	a	g	g	g	a	g	a	g	g	g	g	g	g	g	g	a	g	g
t	2548134	c	2768028	c	2838241	s	O157	rec46	c	CB9615	++	c	t	t	t	c	c	c	c	c	t	c	t	c	c	a	t	t	c	c	c	c	c	t
g	2548137	a	2768031	a	2838244	s	O157	rec46	a	CB9615	++++	a	a	a	a	a	a	a	a	a	a	a	a	a	a	g	a	a	a	a	a	a	a	a
c	2548206	t	2768100	t	2838313	s	O157	rec46	c	O157	++++	c	c	c	c	c	c	c	c	c	c	c	c	c	c	c	c	c	c	c	c	c	c	c
a	2548230	g	2768124	g	2838337	s	O157	rec46	g	CB9615	++++	g	g	g	g	g	g	g	g	g	g	g	g	g	g	g	g	g	g	g	g	g	g	g
t	2548245	c	2768139	c	2838352	s	O157	rec46	c	CB9615	++	c	t	c	c	c	c	t	t	t	c	t	c	c	c	t	c	c	t	t	t	c	c	c
t	2548248	c	2768142	c	2838355	s	O157	rec46	t	O157	++	t	t	t	c	c	t	t	t	t	t	t	t	c	t	t	t	t	t	t	t	c	c	t
g	2548326	c	2768220	c	2838433	s	O157	rec46	g	O157	++	g	t	t	c	c	c	t	t	c	c	c	c	g	c	t	c	c	c	c	c	c	g	c
t	2548341	c	2768235	c	2838448	s	O157	rec46	c	CB9615	++	c	c	c	c	c	c	c	c	c	c	c	c	c	c	t	c	c	c	c	c	c	t	c
t	2548371	a	2768265	a	2838478	s	O157	rec46	t	O157	+	c	c	c	c	t	c	c	c	c	t	c	c	c	c	c	c	c	c	c	c	c	t	c
g	2548398	t	2768292	t	2838505	s	O157	rec46	t	CB9615	++++	t	t	t	t	t	t	t	t	c	t	c	t	c	c	t	t	t	t	t	t	c	t	t
a	2548417	g	2768311	g	2838524	ns	O157	rec46	g	CB9615	++++	g	g	g	g	g	g	g	g	g	g	g	g	g	g	g	g	g	g	g	g	g	g	g
a	2548626	g	2768520	g	2838733	s	O157	rec46	g	CB9615	++	g	g	g	g	g	g	g	g	g	a	g	g	g	g	g	a	a	g	g	g	a	a	g

O55 and O157 genome site details												Outgroup Strain Details[i]																						
CB9615 base[b]	CB9615 Site[c]	Sakai base[b]	Sakai Site[c]	EDL933 base[b]	EDL933 Site[c]	type[d]	Event Lineage[ef]	Recombinant segment	Inferred ancestral base[g]	Outgroup Analysis[e]	Support level[h]	D1 Sd197	K-12	HS	ATCC 8739	UMN026	IAI39	SMS 3-5	E2348/69	536	ED1a	CFT073	S88	APEC O1	UTI89	E24377A	IAI1	SE11	F5 8401	F2a 2457T	F2a 301	SS Ss046	B4 Sb227	B18 BS512
c	2548632	t	2768526	t	2838739	s	O157	rec46	c	O157	++++	c	c	c	c	c	c	c	c	c	c	c	c	c	c	c	c	c	c	c	c	c	c	c
t	2548671	c	2768565	c	2838778	s	O157	rec46	c	CB9615	++	c	c	c	c	c	c	c	c	c	c	c	c	c	c	c	t	t	c	c	c	c	c	c
t	2548695	c	2768589	c	2838802	s	O157	rec46	c	CB9615	++	c	c	t	c	c	t	c	t	t	c	t	c	c	t	c	t	t	t	t	t	c	c	c
t	2548829	c	2768723	c	2838936	s	O157	rec46	c	CB9615	++++	c	c	c	c	c	c	c	c	c	c	c	c	c	c	c	c	c	c	c	c	c	c	c
t	2548838	g	2768732	g	2838945	s	O157	rec46	t	O157	++	t	t	g	t	g	t	t	g	g	t	g	g	g	g	t	t	t	g	g	g	a	t	t
t	2548871	c	2768765	c	2838978	s	O157	rec46	t	O157	++	t	c	c	t	c	c	c	c	c	c	c	c	c	c	t	c	c	c	c	c	c	c	t
t	2548913	c	2768807	c	2839020	s	O157	rec46	t	O157	++	t	c	c	c	c	c	c	c	c	c	c	c	c	c	t	c	c	c	c	c	c	c	c
a	2549016	g	2768910	g	2839123	ns	O157	rec46	a	O157	++	a	g	g	a	g	g	g	g	g	g	g	a	g	g	g	g	g	g	g	g	g	g	a
t	2549040	c	2768934	c	2839147	s	O157	rec46	t	O157	++	t	t	t	t	t	t	t	t	t	c	t	t	t	t	t	c	c	t	t	t	c	t	t
t	2549046	g	2768940	g	2839153	ns	O157	rec46	t	O157	++	t	g	g	t	g	g	g	g	g	g	g	t	g	g	g	g	g	g	g	g	g	t	t
g	2549048	a	2768942	a	2839155	ns	O157	rec46	g	O157	++	g	g	g	g	g	g	g	g	g	a	g	g	g	g	g	a	a	g	g	g	a	a	g
c	2549060	t	2768954	t	2839167	s	O157	rec46	c	O157	++	c	c	c	c	c	c	c	c	c	t	c	c	c	c	c	t	t	c	c	c	t	t	c
c	2549066	a	2768960	a	2839173	s	O157	rec46	c	O157	++	c	c	g	c	c	c	g	g	g	a	g	c	g	g	c	a	a	g	g	g	a	a	c
g	2549069	a	2768963	a	2839176	s	O157	rec46	g	O157	++	g	g	a	g	g	g	a	a	a	a	a	g	a	a	g	a	a	a	a	a	a	a	g
a	2549109	g	2769003	g	2839216	ns	O157	rec46	g	CB9615	++++	g	g	g	g	g	g	g	g	g	g	g	g	g	g	g	g	g	g	g	g	g	g	g
t	2549129	g	2769023	g	2839236	s	O157	rec46	t	O157	++	t	g	g	g	g	g	g	g	g	g	g	g	g	g	g	g	g	g	g	g	g	g	g
c	2549145	t	2769039	t	2839252	s	O157	rec46	c	O157	++++	c	c	c	c	c	c	c	c	c	t	c	c	c	c	c	c	c	c	c	c	c	c	c
t	2549180	c	2769074	c	2839287	s	O157	rec46	c	CB9615	++++	c	c	c	c	c	c	c	c	c	c	c	c	c	c	c	c	c	c	c	c	c	c	c
t	2549183	c	2769077	c	2839290	s	O157	rec46	c	CB9615	++	c	c	t	c	t	c	c	c	c	c	c	c	c	c	c	c	c	c	c	c	c	c	c
g	2549270	t	2769164	t	2839377	s	O157	rec46	g	O157	++++	g	g	g	a	g	g	g	g	g	a	g	g	g	g	a	a	g	g	g	g	g	a	g
a	2549303	g	2769197	g	2839410	s	O157	rec46	g	CB9615	++	g	g	a	a	a	a	a	a	a	a	a	g	a	a	g	a	a	a	a	a	a	g	a
t	2549309	c	2769203	c	2839416	s	O157	rec46	t	O157	++	t	t	t	t	c	t	t	c	c	c	c	c	t	c	t	t	t	c	c	c	c	t	c
t	2549325	c	2769219	c	2839432	s	O157	rec46	c	CB9615	++++	c	c	c	c	c	c	c	c	c	t	c	c	c	c	c	c	c	c	c	c	c	c	c
g	2549330	a	2769224	a	2839437	s	O157	rec46	g	O157	++++	g	g	g	g	g	g	g	g	g	g	g	g	g	g	g	g	a	g	g	g	g	g	g
c	2549331	t	2769225	t	2839438	s	O157	rec46	c	O157	++	c	t	c	c	c	t	t	t	t	c	t	c	t	t	c	t	t	c	c	c	c	c	c
g	2549347	a	2769241	a	2839454	ns	O157	rec46	a	CB9615	++	a	g	g	a	g	g	g	a	a	a	a	g	g	a	a	g	a	a	a	a	a	a	a
g	2549348	t	2769242	t	2839455	ns	O157	rec46	t	CB9615	++	t	g	g	t	g	g	g	t	t	t	t	g	g	t	t	g	t	t	t	t	t	t	t
g	2549360	a	2769254	a	2839467	s	O157	rec46	g	O157	++	g	g	a	g	g	g	g	a	a	a	a	g	g	a	g	a	a	g	g	g	g	g	g
g	2549387	t	2769281	t	2839494	s	O157	rec46	g	O157	++++	g	g		g	g	g	g	g	g	g	g	g	g	g	g		g	g	g	g	g	g	g
a	2549405	g	2769299	g	2839512	i	O157	rec46	g	CB9615	++++	g	g		g	c	g	g	c	c	g	c	g	g	c	g		g	c	c	c	g	g	g
g	2549420	a	2769314	a	2839527	i	O157	rec46	a	CB9615	++	a	a		a	g	a	a	g	g	a	g	a	a	g	a		-	g	g	g	g	a	g
c	2549425	t	2769319	t	2839532	i	O157	rec46	t	CB9615	++	t	t		c	a	t	t	t	t	c	t	t	t	t	t		t	t	t	t	t	t	t
c	2549457	t	2769351	t	2839564	i	O157	rec46	t	CB9615	++++	t	t		t	t	t	t	t	t	t	t	t	t	t	t	t	t	t	t	t	t	t	t
a	2549471	t	2769365	t	2839578	i	O157	rec46	a	O157	++	a	t		a	a	t	t	g	g	t	g	g	t	g	t	g	a	g	g	g	g	c	t
a	2549520	g	2769414	g	2839627	s	O157	rec46	g	CB9615	++	g	a		a	g	a	a	g	g	g	g	a	a	g	g	g	g	g	g	g	g	g	g
a	2549531	g	2769425	g	2839638	s	O157	rec46	g	CB9615	++	g	g		a	g	g	g	g	g	g	g	g	g	g	a	g	g	a	a	a	g	a	a
g	2549598	a	2769492	a	2839705	s	O157	rec46	a	CB9615	++	a	g		g	g	g	g	g	g	g	g	g	g	g	g	g	g	a	a	a	g	g	a
a	2549637	g	2769531	g	2839744	s	O157	rec46	a	O157	++	a	a		a	a	g	g	g	g	g	g	a	g	g	a	g	g	g	g	g	a	g	g
t	2549667	c	2769561	c	2839774	s	O157	rec46	c	CB9615	++	c	t		c	c	c	c	g	g	g	g	c	c	g	c	c	c	c	c	c	c	g	c
c	2549674	t	2769568	t	2839781	ns	O157	rec46	t	CB9615	++	t	c		t	c	c	c	t	t	t	t	c	c	t	t	c	t	t	t	t	t	t	t
g	2549679	a	2769573	a	2839786	s	O157	rec46	a	CB9615	++	a	g		g	g	g	g	g	a	g	a	g	g	a	g	g	a	a	a	a	a	g	g
g	2549687	a	2769581	a	2839794	s	O157	rec46	a	CB9615	++	a	g		g	g	g	g	g	g	g	g	g	g	g	g	g	a	a	a	a	a	g	g
a	2549700	t	2769594	t	2839807	s	O157	rec46	a	O157	++	a	a		a	a	a	a	a	a	a	a	a	a	a	t	a	a	t	t	t	t	a	a
g	2549802	c	2769696	c	2839909	ns	O157	rec46	c	CB9615	++	c	g		g	c	g	g	t	t	t	t	c	g	t	c	c	c	c	c	c	c	c	t
t	2549804	c	2769698	c	2839911	ns	O157	rec46	c	CB9615	++	c	t		t	c	t	t	c	c	c	c	c	t	c	c	c	c	c	c	c	c	c	c
3	2549810	-	2769703	-	2839916	del-3	O157	rec46	-	CB9615	++	-	3		3	-	3	3	-	-	-	-	-	3	-	-	-	-	-	-	-	-	-	-
t	2549820	c	2769711	c	2839924	s	O157	rec46	c	CB9615	++	c	t		t	c	t	t	t	t	c	t	c	t	t	c	c	c	c	c	c	c	c	c
t	2549825	c	2769716	c	2839929	ns	O157	rec46	c	CB9615	++	c	t		t	c	t	t	t	t	t	t	c	t	t	c	t	c	c	c	c	c	c	t
t	2549826	c	2769717	c	2839930	s	O157	rec46	c	CB9615	++++	c	c		c	c	c	c	c	c	c	c	t	c	c	c	c	c	c	c	c	c	c	c
c	2549829	t	2769720	t	2839933	s	O157	rec46	t	CB9615	++	t	c		c	t	c	c	c	c	c	c	t	c	c	t	c	t	t	t	t	t	t	c
t	2549832	c	2769723	c	2839936	s	O157	rec46	t	O157	++	t	t		t	t	t	t	c	c	c	c	c	t	c	c	c	c	c	c	c	c	c	c
t	2549837	c	2769728	c	2839941	ns	O157	rec46	t	O157	++++	t	t		t	t	t	t	t	t	t	t	t	t	t	t	t	t	t	t	t	c	t	t
a	2549892	c	2769783	c	2839996	ns	O157	rec46	a	O157	++++	a	a		a	a	a	a	a	a	a	a	a	a	a	a	c	a	a	a	a	a	a	a
c	2549913	t	2769804	t	2840017	s	O157	rec46	t	CB9615	++	t	t		c	c	c	c	c	c	c	c	c	c	c	c	c	t	c	c	c	t	t	c
a	2549965	t	2769856	t	2840069	ns	O157	rec46	t	CB9615	++++	t	t		t	t	t	t	t	t	t	t	t	t	t	t	t	t	t	t	t	t	t	t
a	2550162	t	2770053	t	2840266	s	O157	rec46	a	O157	++	a	a		a	a	a	a	a	a	a	a	t	a	a	t	a	t	a	a	a	a	t	t
t	2550189	c	2770080	c	2840293	s	O157	rec46	c	CB9615	++	c	t		t	c	t	t	t	t	t	t	a	t	t	a	t	a	t	t	t	t	a	a
t	2550201	c	2770092	c	2840305	s	O157	rec46	t	O157	++	t	c		t	c	c	c	g	c	g	c	a	c	c	a	g	a	c	c	c	t	a	a
c	2550245	t	2770136	t	2840349	ns	O157	rec46	t	CB9615	++++	t	t		t	t	t	t	t	t	t	t	t	t	t	t	t	t	t	t	t	t	t	t
t	2550270	c	2770161	c	2840374	s	O157	rec46	c	CB9615	++++	c	c		c	c	c	c	t	c	c	c	c	c	c	c	c	c	c	c	c	c	c	c
t	2550333	a	2770224	a	2840437	s	O157	rec46	t	O157	+	c	t		c	c	t	t	c	a	c	a	c	t	a	c	t	c	a	a	a	t	c	c
g	2550345	a	2770236	a	2840449	s	O157	rec46	g	O157	++++	g	g		g	g	g	g	g	g	g	g	g	g	g	g	g	g	g	g	g	g	g	g
a	2550431	g	2770322	g	2840535	ns	O157	rec46	g	CB9615	++++	g	g		g	g	g	g	g	g	g	g	g	g	g	g	g	g	g	g	g	g	g	g
g	2550486	t	2770377	t	2840590	s	O157	rec46	g	O157	++	g	t		g	t	t	t	g	t	g	t	g	t	t	g	g	g	t	t	t	t	g	g
a	2550491	g	2770382	g	2840595	ns	O157	rec46	a	O157	++	a	g		a	g	g	g	a	g	a	g	a	g	g	a	t	a	g	g	g	g	a	a
t	2550494	-	2770384	-	2840597	del	O157	rec46	t	O157	++	t	-		t	-	-	-	t	-	t	-	t	-	-	t	t	t	-	-	-	-	t	t
a	2550496	t	2770386	t	2840599	ns	O157	rec46	a	O157	++	a	t		a	t	t	t	a	t	a	t	a	t	t	a	a	a	t	t	t	t	a	a
t	2550499	c	2770389	c	2840602	ns	O157	rec46	t	O157	++	t	c		t	c	c	c	t	c	t	c	t	c	c	t	t	t	c	c	c	c	t	t
t	2550500	c	2770390	c	2840603	ns	O157	rec46	t	O157	++	t	c		t	c	c	c	t	c	t	c	t	c	c	t	t	t	c	c	c	c	t	t
g	2550506	c	2770396	c	2840609	ns	O157	rec46	g	O157	++	g	c		g	c	c	c	g	c	g	c	g	c	c	g	t	g	c	c	c	c	g	g
c	2550507	g	2770397	g	2840610	ns	O157	rec46	c	O157	++	c	g		c	g	g	g	c	g	c	g	c	g	g	c	g	c	g	g	g	g	c	c
g	2550526	c	2770416	c	2840629	ns	O157	rec46	g	O157	++	g	c		g	c	c	c	g	c	g	c	g	c	c	g	t	g	c	c	c	c	g	g
c	2550528	t	2770418	t	2840631	ns	O157	rec46	c	O157	++++	c	c		c	c	c	c	c	c	c	c	c	c	c	c	a	c	c	c	c	a	c	c
a	2550529	g	2770419	g	2840632	ns	O157	rec46	a	O157	++	a	g		a	g	a	a	a	g	a	g	a	a	g	a	g	a	g	g	g	g	a	a
a	2550547	g	2770437	g	2840650	i	O157	rec46	a	O157	++	a	g		a	g	t	t	a	g	a	g	a	t	g	a	a	a	g	g	g	g	a	a
a	2550569	g	2770459	g	2840672	i	O157	rec46	a	O157	++	a	g		a	g	a	a	a	g	a	g	a	a	g	a	g	a	g	g	g	g	a	a
a	2550571	g	2770461	g	2840674	i	O157	rec46	a	O157	++	a	g		a	g	g	g	a	g	a	g	a	g	g	a	a	a	g	g	g	g	a	a
a	2550572	g	2770462	g	2840675	i	O157	rec46	a	O157	++	a	t		a	g	c	c	a	g	a	g	a	c	g	a	c	a	g	g	g	g	a	a
g	2550576	t	2770466	t	2840679	i	O157	rec46	g	O157	++	g	t		g	t	t	t	g	t	g	t	g	t	t	g	t	g	t	t	t	t	g	g
t	2550581	a	2770471	a	2840684	i	O157	rec46	t	O157	++	t	a		t	a	a	a	t	a	t	a	t	a	a	t	a	t	a	a	a	a	t	t
g	2550586	c	2770476	c	2840689	i	O157	rec46	g	O157	++	g	c		g	c	c	c	g	c	g	c	g	c	c	g	c	g	c	c	c	c	g	g
a	2550597	t	2770487	t	2840700	i	O157	rec46	a	O157	++	a	t		a	t	t	t	a	t	a	t	a	t	t	a	-	a	t	t	t	t	a	a
c	2550602	a	2770492	a	2840705	i	O157	rec46	c	O157	++	c	a		c	a	a	a	c	a	c	a	c	a	a	c	c	a	a	a	a	a	c	c
g	2550615	a	2770505	a	2840718	i	O157	rec46	g	O157	++	g	a		g	a	g	g	g	a	t	a	a	g	a	t	g	g	a	a	a	a	g	g
a	2550626	g	2770516	g	2840729	s	O157	rec46	a	O157	++	a	g	g	a	g	g	g	g	g	g	g	g	g	g	g	g	g	g	g	g	g	a	a
t	2550650	g	2770540	g	2840753	s	O157	rec46	g	CB9615	++	g	t	t	g	t	g	g	g	g	g	g	g	g	g	g	g	g	g	g	g	t	g	g

O55 and O157 genome site details												Outgroup Strain Details[i]																						
CB9615 base[b]	CB9615 Site[c]	Sakai base[b]	Sakai Site[c]	EDL933 base[b]	EDL933 Site[c]	type[d]	Event Lineage[e,f]	Recombinant segment	Inferred ancestral base[g]	Outgroup Analysis[e]	Support level[h]	D1 Sd197	K-12	HS	ATCC 8739	UMN026	IAI39	SMS 3-5	E2348/69	536	ED1a	CFT073	S88	APEC O1	UTI89	E24377A	IAI1	SE11	F5 8401	F2a 2457T	F2a 301	SS Ss046	B4 Sb227	B18 BS512
a	2550668	c	2770558	c	2840771	ns	O157	rec46	c	CB9615	++++	c	c	c	c	c	t	t	c	c	c	c	c	t	c	c	c	c	c	c	c	t	c	c
a	2550761	g	2770651	g	2840864	s	O157	rec46	a	O157	++	a	g	a	g	g	a	a	a	g	a	g	g	g	g	a	g	g	g	g	g	g	g	g
c	2550791	t	2770681	t	2840894	s	O157	rec46	c	O157	++	c	c	a	c	g	c	c	c	c	c	c	t	c	c	c	t	c	c	c	c	c	t	t
g	2550800	a	2770690	a	2840903	s	O157	rec46	g	O157	++	g	a	a	g	g	g	g	g	g	g	g	a	g	g	g	g	g	g	g	g	g	a	a
t	2550818	c	2770708	c	2840921	s	O157	rec46	t	O157	++	t	t	c	c	t	t	t	t	c	t	c	t	t	t	t	c	t	c	c	c	c	c	c
c	2550890	t	2770780	t	2840993	s	O157	rec46	c	O157	++++	c	c	c	c	c	c	c	c	c	c	c	c	c	c	c	c	c	c	c	c	c	a	a
t	2550902	c	2770792	c	2841005	s	O157	rec46	c	CB9615	++	c	c	c	t	c	t	t	t	t	t	t	t	c	c	c	c	t	t	t	t	t	t	t
c	2550935	t	2770825	t	2841038	s	O157	rec46	t	CB9615	++++	t	t	t	t	t	t	t	t	t	t	t	t	t	t	t	t	t				c	t	t
t	2550950	a	2770840	a	2841053	s	O157	rec46	t	O157	++	t	a	a	a	a	a	a	a	t	t	t	t	a	t	t	a	a	t	t	t	t	g	a
a	2550992	g	2770882	g	2841095	s	O157	rec46	g	CB9615	++	g	g	g	g	a	g	g	g	g	g	g	g	g	g	g	a	g	g	g	g	a	g	a
g	2551028	a	2770918	a	2841131	s	O157	rec46	a	CB9615	++	a	a	g	a	a	a	a	a	a	a	a	a	a	a	a	a	a	a	a	a	g	a	a
g	2551055	a	2770945	a	2841158	s	O157	rec46	g	O157	++	g	a	g	a	a	g	g	a	a	g	a	a	a	a	a	a	a	a	a	a	g	g	a
c	2551079	t	2770969	t	2841182	s	O157	rec46	c	O157	++	c	t	c	c	c	c	c	c	c	c	c	c	t	c	c	c	c	c	c	c	c	c	c
a	2551082	g	2770972	g	2841185	s	O157	rec46	a	O157	++	a	g	a	a	a	a	a	a	a	a	a	a	g	a	a	a	a	a	a	a	a	g	a
a	2551103	t	2770993	t	2841206	s	O157	rec46	a	O157	++	a	t	a	a	a	a	a	a	a	a	a	a	t	a	a	a	a	a	a	a	a	a	a
c	2551106	t	2770996	t	2841209	s	O157	rec46	c	O157	++	c	t	c	c	c	c	c	c	c	c	c	c	t	c	c	c	c	c	c	c	c	c	t
g	2551109	a	2770999	a	2841212	s	O157	rec46	g	O157	++	g	a	c	g	g	g	g	a	g	g	g	a	a	a	g	g	a	g	g	g	g	g	g
a	2551112	g	2771002	g	2841215	ns	O157	rec46	a	O157	++	a	g	g	a	a	a	a	c	a	a	a	a	g	a	a	a	a	a	a	a	a	t	g
t	2551114	a	2771004	a	2841217	ns	O157	rec46	t	O157	++	t	a	t	t	t	t	t	t	t	t	t	t	a	t	t	t	t	t	t	t	t	a	a
t	2551160	c	2771050	c	2841263	s	O157	rec46	t	O157	++	t	c	g	t	c	t	c	t	t	t	t	t	c	t	t	t	t	t	t	t	t	c	a
c	2551175	g	2771065	g	2841278	s	O157	rec46	c	O157	++++	c	c	c	c	c	c	c	c	c	c	c	c	c	c	c	c	c	c	c	c	t	c	c
g	2551214	a	2771104	a	2841317	s	O157	rec46	g	O157	++	g	a	a	a	a	a	a	g	a	a	a	g	a	a	g	a	a	a	a	a	g	g	g
c	2551217	g	2771107	g	2841320	s	O157	rec46	g	CB9615	++++	t	g	g	g	g	g	g	t	g	g	g	t	g	g	t	g	g	g	g	g	c	t	t
a	2551220	g	2771110	g	2841323	s	O157	rec46	a	O157	++	a	g	g	g	g	g	g	a	g	g	g	a	g	g	a	g	g	g	g	g	a	g	g
g	2551226	a	2771116	a	2841329	s	O157	rec46	a	CB9615	++	a	a	a	a	a	a	a	g	a	a	a	g	a	a	a	a	a	a	a	a	g	a	a
c	2551235	t	2771125	t	2841338	s	O157	rec46	t	CB9615	++	t	c	c	t	c	a	c	c	c	t	c	c	c	c	c	t	c	c	c	c	c	t	t
c	2551268	t	2771158	t	2841371	s	O157	rec46	t	CB9615	++	t	c	c	t	c	t	c	t	c	t	c	t	c	c	c	t	c	c	c	c	c	t	t
t	2551295	c	2771185	c	2841398	s	O157	rec46	t	O157	++	t	t	a	t	t	t	t	c	t	t	t	c	t	t	t	t	t	t	t	t	t	a	g
g	2551310	t	2771200	t	2841413	s	O157	rec46	g	O157	++++	g	g	a	g	g	t	g	g	g	g	g	g	g	g	g	g	g	g	g	g	g	c	c
a	2551322	c	2771212	c	2841425	s	O157	rec46	a	O157	++	a	a	t	c	a	c	a	c	c	a	c	c	a	a	c	c	a	c	c	c	a	t	t
g	2551328	a	2771218	a	2841431	s	O157	rec46	g	O157	++	g	g	c	a	g	a	g	a	g	g	g	a	g	g	g	a	g	g	g	g	g	t	c
a	2551331	g	2771221	g	2841434	s	O157	rec46	a	O157	++	a	a	g	g	a	g	a	g	a	a	a	g	a	a	a	g	a	a	a	a	a	g	g
g	2551343	a	2771233	a	2841446	s	O157	rec46	a	CB9615	++	a	g	c	g	g	a	g	g	g	g	g	a	g	a	g	g	g	g	g	g	g	t	t
a	2551355	g	2771245	g	2841458	s	O157	rec46	a	O157	++	a	a	a	a	a	g	a	g	g	a	g	a	a	a	a	a	a	g	g	g	a	a	a
t	2551727	a	2771617	a	2841830	s	O157	rec46	t	O157	++++	t	t	c	t	t	t	t	t	t	t	t	t	t	t	t	t	t	t	t	t	t	c	c
t	2551748	c	2771638	c	2841851	s	O157	rec46	c	CB9615	++++	c	c	t	c	c	c	c	c	c	c	c	c	c	c	c	c	c	c	c	c	c	a	a
g	2551832	a	2771722	a	2841935	i	O157	rec46	g	O157	++++	g	g		g	g	g	g	g	g	g	g	g	g	g	g	g	g	g	g	g	g		
a	2551886	g	2771776	g	2841989	i	O157	rec46	a	O157	++++	a	a		a	a	a	a	a	a	a	a	a	a	a	a	a	a	a	a	a	a		
a	2551915	g	2771805	g	2842018	i	O157	rec46	g	CB9615	++++	g	g		g	g	g	g	g	g	g	g	g	g	g	g	g	g	g	g	g	g		
g	2551916	a	2771806	a	2842019	i	O157	rec46	a	CB9615	++	a	a		a	a	a	a	a	g	a	g	a	a	g	a	a	a	a	a	a	a		
c	2551948	a	2771838	a	2842051	i	O157	rec46	a	CB9615	++++	a	a		a	a	a	a	a	a	a	a	a	a	a	a	a	a	a	a	a	a		
a	2551953	t	2771843	t	2842056	i	O157	rec46	a	O157	++++	a	a		a	a	a	a	a	a	a	a	a	a	a	a	a	g	a	a	a	a		
t	2551961	c	2771851	c	2842064	i	O157	rec46	c	CB9615	++++	c	c		c	c	c	c	c	c	c	c	c	c	c	c	c	c	c	c	c	c		
t	2551980	a	2771870	a	2842083	i	O157	rec46	a	CB9615	++++	a	a		a	a	a	a	a	a	a	a	a	a	a	a	a	a	a	a	a	a	a	
t	2568186	c	2787598	c	2857812	ns	O157	rec46	c	CB9615	++++	c	c		c	c	c	c	c	c	c	c	c	c	c	c	t	c	c	c	c		c	c
a	2568187	g	2787599	g	2857813	ns	O157	rec46	g	CB9615	++++	g	g		g	c	g	g	g	g	g	g	g	g	g	g	a	g	g	g	g		g	g
g	2568188	c	2787600	c	2857814	ns	O157	rec46	c	CB9615	++++	c	c		c	c	c	c	c	c	c	c	c	c	c	c	c	t	c	c	c		c	c
c	2568189	t	2787601	t	2857815	ns	O157	rec46	t	CB9615	++++	t	t		t	c	t	t	t	t	t	t	t	t	t	t	t	t	t	t	t		t	t
g	2568192	a	2787604	a	2857818	s	O157	rec46	a	CB9615	++++	a	a		a	g	a	a	a	a	a	a	a	a	a	a	t	a	a	a	a		a	a
t	2568193	c	2787605	c	2857819	s	O157	rec46	c	CB9615	++++	c	c		c	c	c	c	c	c	c	c	c	c	c	c	c	c	c	c	c		c	c
a	2568195	g	2787607	g	2857821	s	O157	rec46	g	CB9615	++++	g	g		g	a	g	g	g	g	g	g	g	g	g	g	t	g	g	g	g		g	g
t	2568196	c	2787608	c	2857822	ns	O157	rec46	c	CB9615	++	c	c		c	c	c	t	c	c	c	c	c	c	c	c	t	c	c	c	c		c	c
g	2568198	t	2787610	t	2857824	ns	O157	rec46	t	CB9615	++++	t	t		t	t	t	t	t	t	t	t	t	t	t	t	t	t	t	t	t		t	t
a	2568202	g	2787614	g	2857828	s	O157	rec46	a	O157	++++	a	a		a	a	a	a	a	a	a	a	a	a	a	a	t	a	a	a	a		a	a
t	2568206	c	2787618	c	2857832	ns	O157	rec46	c	CB9615	++++	c	c		c	c	c	c	c	c	c	c	c	c	c	c	c	c	c	c	c		c	c
t	2568207	c	2787619	c	2857833	ns	O157	rec46	c	CB9615	++	c	c		c	t	c	c	c	c	c	c	c	c	c	c	t	c	c	c	c		c	c
c	2568208	t	2787620	t	2857834	s	O157	rec46	t	CB9615	++++	t	t	t	t	c	t	t	t	t	t	t	t	t	t	t	t	t	t	t	t		t	t
a	2568212	c	2787624	c	2857838	ns	O157	rec46	c	CB9615	++++	c	c	c	c	c	c	c	c	c	c	c	c	c	c	c	c	c	c	c	c		c	c
a	2568214	g	2787626	g	2857840	s	O157	rec46	g	CB9615	++++	g	g	g	g	g	g	g	g	g	g	g	g	g	g	g	g	g	g	g	g		g	g
t	2568217	c	2787629	c	2857843	s	O157	rec46	c	CB9615	++++	c	c	t	c	c	c	c	c	c	c	c	c	c	c	c	c	c	c	c	c		c	c
a	2568223	c	2787635	c	2857849	s	O157	rec46	c	CB9615	++++	c	c	g	c	c	c	c	c	c	c	c	c	c	c	c	c	c	c	c	c		c	c
c	2568229	t	2787641	t	2857855	s	O157	rec46	t	CB9615	++++	t	t	t	t	c	t	t	t	t	t	t	t	t	t	t	t	t	t	t	t		t	t
t	2568232	c	2787644	c	2857858	s	O157	rec46	c	CB9615	++++	g	c	c	c	c	c	g	c	c	c	c	g	g	t	c	c	c	c	c	c		g	c
g	2568234	a	2787646	a	2857860	s	O157	rec46	g	O157	++++	g	g	g	g	g	g	g	g	g	g	g	g	g	g	g	g	g	g	g	g		g	g
c	2568241	t	2787653	t	2857867	s	O157	rec46	c	O157	++	c	t	c	t	c	t	c	t	t	c	t	t	t	c	t	c	t	t	t	t		c	t
c	2568244	t	2787656	t	2857870	s	O157	rec46	c	O157	++	c	g	t	t	t	t	t	t	t	t	t	t	t	c	t	t	t	g	g	g		c	g
g	2568256	a	2787668	a	2857882	s	O157	rec46	a	CB9615	++	a	a	g	g	a	a	a	a	a	a	a	a	g	a	a	a	a	a	a	a		g	a
a	2568286	g	2787698	g	2857912	s	O157	rec46	g	CB9615	++++	g	g	g	g	g	g	g	g	g	g	g	g	g	g	g	g	g	g	g	g		g	g
g	2568295	a	2787707	a	2857921	s	O157	rec46	g	O157	++	g	a	g	g	g	g	g	g	g	g	g	a	g	a	g	g	g	a	a	a		g	g
a	2568301	g	2787713	g	2857927	s	O157	rec46	?	O157	+/-	t	g	g	a	g	a	a	g	a	a	a	c	g	c	t	a	a	g	g	g		a	a
g	2568316	t	2787728	t	2857942	s	O157	rec46	g	O157	++	g	t	c	t	g	g	g	g	g	g	g	g	g	t	g	t	g	t	t	t		g	t
c	2568328	t	2787740	t	2857954	s	O157	rec46	c	O157	++	c	t	c	c	c	c	c	c	c	c	c	c	c	t	c	c	c	t	t	t		c	c
c	2568337	t	2787749	t	2857963	s	O157	rec46	c	O157	++	c	c	c	c	c	c	c	c	c	c	c	c	c	t	c	t	c	c	c	c		c	c
t	2568343	c	2787755	c	2857969	s	O157	rec46	t	O157	++	t	c	t	t	t	t	t	c	t	t	t	c	t	c	t	t	t	c	c	c		t	t
g	2568346	a	2787758	a	2857972	s	O157	rec46	g	O157	++	g	g	g	a	g	a	g	g	a	a	a	g	g	a	a	g	a	g	g	g		g	a
a	2568352	g	2787764	g	2857978	s	O157	rec46	g	CB9615	++	g	a	g	g	a	g	g	g	g	g	g	a	g	g	g	a	g	a	a	a		g	g
c	2568373	t	2787785	t	2857999	s	O157	rec46	t	CB9615	++	t	t	g	c	c	c	c	t	c	t	c	t	c	t	c	c	t	t	t	t		c	c
t	2568388	c	2787800	c	2858014	s	O157	rec46	c	CB9615	++	c	c	c	t	t	c	c	c	c	t	c	c	c	t	c	c	c	c	c	c		c	t
g	2568394	a	2787806	a	2858020	s	O157	rec46	g	O157	++	g	a	a	g	g	g	g	a	g	g	g	a	g	a	g	g	g	a	a	a		g	g
a	2568400	c	2787812	c	2858026	s	O157	rec46	a	O157	+	t	c	c	a	a	a	a	a	a	a	a	c	c	c	t	t	a	c	c	c		a	a
g	2568433	t	2787845	t	2858059	s	O157	rec46	g	O157	++++	g	g	g	g	g	g	t	g	g	g	g	g	g	a	a	g	g	g	g	g		a	g
t	2568448	g	2787860	g	2858074	s	O157	rec46	g	CB9615	++++	g	g	c	g	g	g	g	g	g	g	g	g	g	g	g	g	g	g	g	g		g	g
t	2568481	c	2787893	c	2858107	s	O157	rec46	t	O157	++	t	t	t	t	t	t	c	c	t	t	t	t	t	t	t	t	t	t	t	t		t	t
a	2568514	g	2787926	g	2858140	s	O157	rec46	a	O157	++	a	a	g	g	a	a	a	a	a	g	a	a	a	a	a	a	a	a	a	a		a	g
a	2568568	g	2787980	g	2858194	s	O157	rec46	a	O157	++	a	a	g	a	a	a	a	a	a	a	a	a	a	g	a	a	a	a	a	a		a	a
a	2568598	g	2788010	g	2858224	s	O157	rec46	a	O157	++	a	g	g	a	g	g	g	g	g	a	g	g	g	g	a	g	a	g	g	g		g	g

O55 and O157 genome site details												Outgroup Strain Details[i]																						
CB9615 base[b]	CB9615 Site[c]	Sakai base[b]	Sakai Site[c]	EDL933 base[b]	EDL933 Site[c]	type[d]	Event Lineage[ef]	Recombinant segment	Inferred ancestral base[g]	Outgroup Analysis[e]	Support level[h]	D1 Sd197	K-12	HS	ATCC 8739	UMN026	IAI39	SMS 3-5	E2348/69	536	ED1a	CFT073	S88	APEC O1	UTI89	E24377A	IAI1	SE11	F5 8401	F2a 2457T	F2a 301	SS Ss046	B4 Sb227	B18 BS512
g	2568601	t	2788013	t	2858227	s	O157	rec46	g	O157	+	a	g	g	g	g	g	g	t	g	g	g	g	g	g	g	g	a	g	g	g		t	t
a	2568643	g	2788055	g	2858269	s	O157	rec46	g	CB9615	++	g	a	g	g	g	g	g	g	a	g	a	g	g	g	a	g	g	a	a	a		g	g
c	2568646	t	2788058	t	2858272	s	O157	rec46	c	O157	++	c	t	c	c	t	c	c	t	t	c	t	c	c	c	c	c	c	t	t	t		c	c
a	2568648	g	2788060	g	2858274	s	O157	rec46	g	CB9615	++++	g	g	g	g	g	g	g	g	g	g	g	g	g	g	g	g	g	g	g	g		g	g
-	2569292	c	2788705	c	2858919	ins	O157	rec46	-	O157	+++				c			c	c		c						c							c
c	2569808	g	2789221	g	2859435	s	O157	rec46	g	CB9615	+++				g			g	c		t						g							g
g	2569841	a	2789254	a	2859468	s	O157	rec46	g	O157	+				a			a	g		g						g							g
t	2569895	c	2789308	c	2859522	s	O157	rec46	t	O157	+++				t			t	t		t						t							t
a	2569907	g	2789320	g	2859534	s	O157	rec46	a	O157	+++				a			a	a		a						a							a
a	2569931	g	2789344	g	2859558	s	O157	rec46	?	O157	+/-				g			a	a		a						g							g
c	2570117	t	2789530	t	2859744	s	O157	rec46	t	CB9615	+++				t			t	t		c						t							t
c	2570556	t	2789969	t	2860183	s	O157	rec46	c	O157	+		t		c	c	t	c	c	t	c	t	c	t	c	c	c	c	c	c	c		t	c
c	2570560	t	2789973	t	2860187	ns	O157	rec46	t	CB9615	+		t		c	c	t	t	t	t	c	t	c	t	t	t	c	c	c	c	c		t	t
t	2570562	c	2789975	c	2860189	s	O157	rec46	t	O157	++++		t		t	t	t	t	t	t	t	t	t	t	t	t	t	t	t	t	t		c	t
t	2570606	c	2790019	c	2860233	ns	O157	rec46	t	O157	++++		t		t	t	t	t	t	t	t	t	t	t	g	t	t	t	t	t	t		t	t
c	2570619	g	2790032	g	2860246	s	O157	rec46	g	CB9615	+		a		g	g	c	g	g	c	c	c	g	c	a	g	g	g	g	g	g		g	g
g	2570625	t	2790038	t	2860252	s	O157	rec46	g	O157	++++		a		g	g	g	g	g	g	g	g	g	g	t	g	g	g	g	g	g		g	g
g	2570655	a	2790068	a	2860282	ns	O157	rec46	g	O157	++++		g		g	g	g	g	g	g	g	g	g	g	g	g	g	g	g	g	g		g	g
c	2570656	a	2790069	a	2860283	ns	O157	rec46	c	O157	++++		c		c	c	c	c	c	c	c	c	c	c	c	c	c	c	c	c	c		c	c
t	2570673	a	2790086	a	2860300	s	O157	rec46	t	O157	+		t		t	a	t	t	t	t	t	t	a	t	t	t	t	t	t	t	t		t	t
c	2570691	t	2790104	t	2860318	s	O157	rec46	c	O157	+		a		c	t	a	c	c	a	a	a	c	a	a	c	t	c	a	a	a		a	c
t	2570700	a	2790113	a	2860327	s	O157	rec46	t	O157	+		t		a	t	t	t	t	t	a	t	a	t	t	a	a	t	t	t	t		a	a
t	2570705	g	2790118	g	2860332	ns	O157	rec46	t	O157	++++		t		t	t	t	t	t	t	t	t	t	t	t	t	t	t	t	t	t		t	t
g	2570732	a	2790145	a	2860359	s	O157	rec46	a	CB9615	+		a		a	a	a	a	a	a	g	a	g	a	a	a	a	a	a	a	a		g	a
a	2570736	c	2790149	c	2860363	s	O157	rec46	c	CB9615	+		c		c	c	c	c	c	c	a	c	a	c	c	c	c	c	c	c	c		a	c
g	2570742	c	2790155	c	2860369	s	O157	rec46	c	CB9615	+		c		c	g	c	c	c	c	g	c	g	c	c	t	g	c	g	g	g		c	c
c	2570766	t	2790179	t	2860393	s	O157	rec46	c	O157	+		t		c	c	c	c	c	c	c	c	c	c	c	c	c	c	t	t	t		c	c
c	2570769	t	2790182	t	2860396	s	O157	rec46	c	O157	+		c		c	t	c	c	c	c	c	c	t	c	c	t	t	c	c	c	c		c	c
a	2570784	g	2790197	g	2860411	s	O157	rec46	a	O157	+		a		a	a	a	a	a	a	a	a	a	a	a	a	g	a	g	g	g		g	a
g	2570796	a	2790209	a	2860423	s	O157	rec46	g	O157	+		g		g	a	g	a	a	g	g	g	g	g	g	g	a	t	g	g	g		g	g
t	2570807	g	2790220	g	2860434	ns	O157	rec46	t	O157	++++		t		t	t	t	t	t	t	t	t	t	t	t	t	t	t	t	t	t		t	t
a	2570808	g	2790221	g	2860435	s	O157	rec46	g	CB9615	+		g		g	a	g	a	a	g	g	g	g	g	g	g	g	a	g	g	g		a	g
c	2570844	t	2790257	t	2860471	s	O157	rec46	t	CB9615	+		t		t	c	t	t	t	t	c	t	t	t	t	t	t	c	t	t	t		t	t
t	2570874	a	2790287	a	2860501	s	O157	rec46	t	O157	++++		t		t	t	t	t	t	t	t	t	t	t	t	t	t	t	t	t	t		t	t
t	2570886	a	2790299	a	2860513	ns	O157	rec46	t	O157	++++		t		t	t	t	t	t	t	t	t	t	t	t	t	t	t	t	t	t		t	t
c	2570982	t	2790395	t	2860609	s	O157	rec46	t	CB9615	+		t		c	c	t	t	t	t	t	t	t	t	t	t	c	t	t	t	t		t	t
c	2570985	g	2790398	g	2860612	s	O157	rec46	c	O157	+		c		g	g	g	c	c	g	g	g	g	g	g	g	c	c	c	c	c		c	c
g	2571019	t	2790432	t	2860646	ns	O157	rec46	g	O157	++++		g		g	g	g	g	g	g	g	g	g	g	g	g	g	g	g	g	g		g	g
t	2571032	g	2790445	g	2860659	ns	O157	rec46	g	CB9615	++++		g		g	g	g	g	g	g	g	g	g	g	g	g	g	g	g	g	g		g	g
g	2571078	t	2790491	t	2860705	s	O157	rec46	g	O157	+		t		g	g	g	g	g	g	g	g	t	g	g	g	g	g	g	g	g		g	g
t	2571102	c	2790515	c	2860729	s	O157	rec46	c	CB9615	++++		c		c	c	c	c	c	c	c	c	c	c	c	c	c	c	c	c	c		c	c
a	2571117	g	2790530	g	2860744	s	O157	rec46	g	CB9615	++++		g		g	g	g	g	g	g	g	g	g	g	g	g	g	g	g	g	g		g	g
g	2571138	a	2790551	a	2860765	s	O157	rec46	g	O157	+		g		g	g	g	g	g	g	a	g	a	g	g	g	g	g	g	g	g		a	g
g	2571153	a	2790566	a	2860780	s	O157	rec46	a	CB9615	+		g		g	a	a	a	a	a	a	a	a	a	a	g	a	g	g	g	g		a	a
t	2571158	c	2790571	c	2860785	ns	O157	rec46	c	CB9615	++++		c		c	c	c	c	c	c	c	c	c	c	c	c	c	c	c	c	c		c	c
g	2571162	a	2790575	a	2860789	s	O157	rec46	a	CB9615	+		a		a	a	a	g	g	a	a	a	a	a	g	g	a	g	a	a	a		a	a
a	2571165	g	2790578	g	2860792	s	O157	rec46	?	O157	+/-		g		g	a	a	a	a	a	a	a	g	a	a	a	g	a	g	g	g		g	g
g	2571186	a	2790599	a	2860813	s	O157	rec46	g	O157	+		g		g	g	a	g	g	a	a	a	a	a	g	g	a	g	g	g	g		a	a
a	2571255	g	2790668	g	2860882	ns	O157	rec46	a	O157	+		a		a	a	a	a	a	g	a	g	a	g	a	a	a	a	a	a	a		a	a
t	2571257	g	2790670	g	2860884	ns	O157	rec46	?	O157	+/-		g		t	t	t	g	g	g	g	g	t	g	t	g	g	t	t	t	t		t	t
t	2571267	c	2790680	c	2860894	s	O157	rec46	t	O157	+		t		c	c	t	c	c	t	c	t	t	t	c	c	c	c	t	t	t		c	t
g	2571288	a	2790701	a	2860915	s	O157	rec46	g	O157	+		g		g	g	a	g	g	g	a	g	g	g	g	g	g	g	g	g	g		g	a
g	2571315	a	2790728	a	2860942	s	O157	rec46	g	O157	+		a		g	a	g	g	g	a	a	a	g	a	g	g	g	g	g	g	g		g	a
t	2571336	a	2790749	a	2860963	s	O157	rec46	a	CB9615	+		a		a	a	a	a	a	a	t	a	t	a	t	t	t	a	a	a	a		t	a
g	2571384	a	2790797	a	2861011	ns	O157	rec46	g	O157	+		a		g	a	a	a	a	g	g	g	a	g	a	g	g	g	g	g	g		g	g
c	2571386	t	2790799	t	2861013	ns	O157	rec46	c	O157	++++		c		c	t	c	c	c	c	c	c	c	c	c	c	c	c	c	c	c		c	c
a	2571387	g	2790800	g	2861014	s	O157	rec46	g	CB9615	+		g		a	g	g	g	g	g	a	g	g	g	g	a	a	a	a	a	a		a	a
g	2571390	c	2790803	c	2861017	s	O157	rec46	c	CB9615	+		c		g	c	c	c	c	c	g	c	c	c	c	g	g	g	g	g	g		g	g
g	2571459	a	2790872	a	2861086	s	O157	rec46	g	O157	+		g		g	g	g	a	a	g	g	g	a	g	a	a	g	g	g	g	g		g	g
a	2571480	c	2790893	c	2861107	s	O157	rec46	c	CB9615	++++		c		c	c	c	c	c	c	c	c	c	c	c	c	c	c	c	c	c		c	c
g	2571501	a	2790914	a	2861128	s	O157	rec46	a	CB9615	+		a		a	a	a	g	g	a	g	a	a	a	g	g	a	g	a	a	a		a	g
g	2571504	a	2790917	a	2861131	s	O157	rec46	a	CB9615	+		a		g	a	g	a	a	g	a	g	a	g	g	a	a	t	a	a	a		a	a
t	2571536	c	2790949	c	2861163	ns	O157	rec46	c	CB9615	++++		c		c	c	c	c	c	c	c	c	c	c	c	c	c	c	c	c	c		c	c
a	2571591	g	2791004	g	2861218	s	O157	rec46	g	CB9615	++++		g		g	g	g	g	g	g	g	g	g	g	g	g	g	g	g	g	g		g	g
a	2571627	g	2791040	g	2861254	s	O157	rec46	g	CB9615	+		a		a	g	g	a	a	g	g	g	g	g	g	g	g	a	g	g	g		t	g
g	2571630	a	2791043	a	2861257	s	O157	rec46	?	O157	+/-		g		g	g	a	t	t	a	a	a	a	a	a	t	g	t	g	g	g		a	a
g	2571636	a	2791049	a	2861263	s	O157	rec46	a	CB9615	+		g		g	g	a	a	a	a	a	a	g	a	a	a	g	a	g	g	g		a	a
a	2571639	g	2791052	g	2861266	s	O157	rec46	g	CB9615	+		a		a	g	g	g	g	g	g	g	g	g	g	g	g	g	g	g	g		g	g
g	2571667	a	2791080	a	2861294	ns	O157	rec46	a	CB9615	++++		a		a	a	a	a	a	a	a	a	a	a	a	a	a	a	a	a	a		a	a
c	2571669	g	2791082	g	2861296	ns	O157	rec46	c	O157	+		c		c	g	c	c	c	g	c	g	c	g	c	c	g	c	g	g	g		g	g
g	2571675	a	2791088	a	2861302	s	O157	rec46	g	O157	+		g		g	g	g	g	g	g	g	g	g	g	g	g	a	g	g	g	g		a	g
g	2571687	a	2791100	a	2861314	s	O157	rec46	g	O157	++++		g		g	g	a	g	g	g	g	g	g	g	g	g	g	g	g	g	g		g	g
t	2571738	a	2791151	a	2861365	s	O157	rec46	t	O157	+		t		t	a	t	t	t	t	t	t	t	t	a	t	t	a	t	t	t		a	t
a	2571741	g	2791154	g	2861368	s	O157	rec46	g	CB9615	+		g		g	g	a	g	g	g	g	g	a	g	g	g	a	g	g	g	g		g	g
a	2571753	g	2791166	g	2861380	s	O157	rec46	a	O157	++++		a		a	a	a	a	a	a	a	a	g	a	a	a	a	a	a	a	a		a	a
g	2571756	a	2791169	a	2861383	s	O157	rec46	g	O157	++++		g		g	g	g	g	g	g	g	g	a	g	g	g	g	g	g	g	g		g	g
c	2571774	g	2791187	g	2861401	s	O157	rec46	g	CB9615	++++		g		g	g	t	g	g	g	g	g	a	g	g	g	t	g	g	g	g		g	g
g	2571801	a	2791214	a	2861428	s	O157	rec46	g	O157	+		g		a	g	g	g	g	g	g	g	g	g	a	g	g	g	g	g	g		g	g
g	2571864	a	2791277	a	2861491	i	O157	rec46	a	CB9615	+		g		g	a	g	a	a	a	a	a	g	a	g	a	a	a	a	a	a		a	g
g	2571937	a	2791350	a	2861564	s	O157	rec46	g	O157	+		a		a	g	g	g	g	g	g	g	g	g	g	g	g	g	g	g	g		g	g
a	2571952	g	2791365	g	2861579	s	O157	rec46	a	O157	+		g		g	t	a	a	a	a	a	a	a	a	a	a	t	a	a	a	a		a	g
g	2571972	a	2791385	a	2861599	s	O157	rec46	g	O157	+		a		a	g	g	g	g	g	g	g	g	g	g	g	g	g	g	g	g		g	g
t	2571999	c	2791412	c	2861626	ns	O157	rec46	c	CB9615	++++		c		c	c	c	c	c	c	c	c	c	c	t	c	c	c	c	c	c		c	c
a	2572006	t	2791419	t	2861633	s	O157	rec46	t	CB9615	+		t		t	g	t	t	t	t	t	t	t	t	t	t	t	g	a	a	a		t	g
t	2572012	c	2791425	c	2861639	s	O157	rec46	c	CB9615	+		c		c	c	c	c	c	c	c	c	c	c	c	c	c	c	t	t	t		c	c
a	2572018	t	2791431	t	2861645	s	O157	rec46	t	CB9615	++++		t		t	t	t	t	t	t	t	t	t	t	t	t	t	t	t	t	t		t	t

O55 and O157 genome site details												Outgroup Strain Details[i]																						
CB9615 base[b]	CB9615 Site[c]	Sakai base[b]	Sakai Site[c]	EDL933 base[b]	EDL933 Site[c]	type[d]	Event Lineage[ef]	Recombinant segment	Inferred ancestral base[g]	Outgroup Analysis[e]	Support level[h]	D1 Sd197	K-12	HS	ATCC 8739	UMN026	IAI39	SMS 3-5	E2348/69	536	ED1a	CFT073	S88	APEC O1	UTI89	E24377A	IAI1	SE11	F5 8401	F2a 2457T	F2a 301	SS Ss046	B4 Sb227	B18 BS512
a	2572057	g	2791470	g	2861684	s	O157	rec46	a	O157	+		g		g	a	a	a	a	a	a	a	g	a	a	g	a	a	a	a	a		g	g
g	2572060	a	2791473	a	2861687	s	O157	rec46	g	O157	+		a		a	g	g	g	g	g	g	g	g	g	g	a	g	g	g	g	g		g	a
a	2572071	g	2791484	g	2861698	s	O157	rec46	g	CB9615	++++		g		g	g	g	g	g	g	g	g	g	g	g	g	g	g	g	g	g		g	g
a	2572075	c	2791488	c	2861702	s	O157	rec46	c	CB9615	+		c		c	t	c	t	t	c	c	c	c	c	c	c	c	a	a	a	a		c	c
t	2572126	c	2791539	c	2861753	s	O157	rec46	t	O157	++++		t		t	t	t	t	t	t	t	t	t	t	t	t	t	t	t	t	t		t	t
t	2572132	c	2791545	c	2861759	s	O157	rec46	t	O157	+		t		t	t	t	t	t	t	t	t	g	t	c	t	c	t	t	t	t		t	c
t	2572135	c	2791548	c	2861762	s	O157	rec46	c	CB9615	++++		c		c	c	c	c	c	c	c	c	c	c	c	c	c	t	c	c	c		c	c
a	2572147	g	2791560	g	2861774	s	O157	rec46	a	O157	+		a		g	a	a	a	a	a	a	a	g	a	a	g	g	g	g	g	g		g	g
g	2572168	a	2791581	a	2861795	s	O157	rec46	a	CB9615	+		a		a	a	a	a	a	a	a	a	g	a	a	a	g	g	g	g	g		g	g
c	2572171	t	2791584	t	2861798	s	O157	rec46	?	O157	+/-		t		t	t	t	c	c	t	c	t	c	t	t	t	c	c	c	c	c		c	c
t	2572174	a	2791587	a	2861801	s	O157	rec46	?	O157	+/-		a		a	a	a	t	t	a	t	a	t	a	a	a	t	t	t	t	t		t	t
a	2572204	g	2791617	g	2861831	s	O157	rec46	g	CB9615	+		g		g	g	a	g	g	a	g	a	g	a	g	g	g	g	g	g	g		g	g
t	2572214	g	2791627	g	2861841	ns	O157	rec46	g	CB9615	+		g		g	g	g	g	g	g	g	g	g	g	g	g	g	g	t	t	t		g	g
a	2572249	c	2791662	c	2861876	ns	O157	rec46	c	CB9615	++++		c		c	c	c	c	c	c	c	c	c	c	c	c	c	c	c	c	c		c	c
t	2572252	c	2791665	c	2861879	s	O157	rec46	t	O157	+		c		c	c	t	t	t	t	c	t	t	t	t	c	t	t	t	t	t		t	t
g	2572285	c	2791698	c	2861912	s	O157	rec46	g	O157	+		g		g	g	g	c	c	g	g	g	c	g	g	c	g	g	g	g	g		g	g
t	2572341	c	2791754	c	2861968	ns	O157	rec46	c	CB9615	++++		c		c	c	c	c	c	c	c	c	c	c	c	c	c	c	c	c	c		c	t
a	2572360	c	2791773	c	2861987	s	O157	rec46	a	O157	+		a		c	a	a	c	c	a	a	a	a	g	a	a	c	c	a	a	a		a	a
g	2572372	a	2791785	a	2861999	s	O157	rec46	a	CB9615	+		g		g	a	g	g	g	a	a	a	g	a	a	a	a	g	a	a	a		a	g
g	2572387	a	2791800	a	2862014	s	O157	rec46	g	O157	++++		g		g	g	g	g	g	g	g	g	g	g	g	g	g	g	g	g	g		a	g
t	2572396	c	2791809	c	2862023	s	O157	rec46	c	CB9615	++++		c		c	c	c	c	c	c	c	c	c	c	c	c	c	c	c	c	c		c	t
g	2572417	a	2791830	a	2862044	s	O157	rec46	a	CB9615	+		a		a	a	a	g	g	a	a	a	t	g	a	g	a	g	t	t	t		a	g
g	2572420	a	2791833	a	2862047	s	O157	rec46	g	O157	+		a		g	g	g	g	g	g	a	g	g	g	g	g	g	g	g	g	g		a	g
t	2572423	c	2791836	c	2862050	s	O157	rec46	t	O157	+		c		t	t	t	t	t	t	c	t	t	t	t	t	t	t	t	t	t		c	t
c	2572432	t	2791845	t	2862059	s	O157	rec46	c	O157	+		c		c	c	c	c	c	c	t	c	c	c	t	c	c	c	c	c	c		t	c
c	2572450	g	2791863	g	2862077	s	O157	rec46	c	O157	+		g		c	c	c	c	c	c	g	c	c	c	c	g	c	c	c	c	c		g	c
a	2572465	g	2791878	g	2862092	s	O157	rec46	g	CB9615	+		g		a	g	g	g	g	g	g	g	g	a	a	g	g	g	g	g	g		g	a
g	2572474	a	2791887	a	2862101	s	O157	rec46	g	O157	+		a		g	g	g	g	g	g	a	g	g	g	g	a	g	g	g	g	g		a	g
c	2572477	t	2791890	t	2862104	s	O157	rec46	c	O157	+		t		c	c	c	c	c	c	t	c	c	c	c	t	c	c	c	c	c		t	c
t	2572555	c	2791968	c	2862182	s	O157	rec46	c	CB9615	+		c		c	c	t	c	c	c	c	c	c	t	c	c	c	c	c	c	c		c	c
a	2572567	g	2791980	g	2862194	s	O157	rec46	g	CB9615	+		g		g	g	t	g	g	a	g	a	g	g	a	g	g	g	g	g	g		a	g
g	2572573	a	2791986	a	2862200	s	O157	rec46	a	CB9615	++++		a		a	a	a	a	a	a	a	a	a	a	a	a	a	a	a	a	a		a	a
g	2572576	t	2791989	t	2862203	s	O157	rec46	g	O157	+		g		t	g	g	g	g	g	t	g	g	g	g	g	g	g	g	g	g		g	g
g	2572579	a	2791992	a	2862206	s	O157	rec46	a	CB9615	+		a		a	g	a	a	a	g	a	g	a	a	g	a	g	g	a	a	a		a	a
c	2572588	t	2792001	t	2862215	s	O157	rec46	t	CB9615	+		c		t	c	t	t	t	c	t	c	t	t	c	c	c	c	t	t	t		t	t
c	2572618	t	2792031	t	2862245	s	O157	rec46	t	CB9615	++++		t		t	t	t	g	g	t	t	t	t	t	t	t	t	t	t	t	t		t	t
g	2572630	a	2792043	a	2862257	s	O157	rec46	g	O157	+		g		g	t	a	g	g	g	a	g	a	g	g	g	g	g	g	g	g		a	g
a	2572639	g	2792052	g	2862266	s	O157	rec46	a	O157	+		a		a	a	g	a	a	a	g	a	g	a	a	a	g	g	a	a	a		g	a
g	2572648	a	2792061	a	2862275	s	O157	rec46	g	O157	+		g		g	g	g	g	g	g	a	g	a	g	g	g	g	g	g	g	g		a	g
c	2572667	t	2792080	t	2862294	ns	O157	rec46	c	O157	++++		c		c	c	c	c	c	c	c	c	c	c	c	c	c	c	c	c	c		c	c
g	2572687	t	2792100	t	2862314	s	O157	rec46	t	CB9615	+		g		t	t	t	t	t	t	t	t	g	t	t	t	g	g	t	t	t		g	t
t	2572708	c	2792121	c	2862335	s	O157	rec46	c	CB9615	++++		c		c	c	c	c	c	a	c	a	c	a	a	c	c	t	c	c	c		c	c
g	2572711	a	2792124	a	2862338	s	O157	rec46	a	CB9615	+		g		a	c	c	a	a	a	a	a	t	a	a	a	t	a	c	c	c		t	a
a	2572714	g	2792127	g	2862341	s	O157	rec46	g	CB9615	+		a		a	g	g	g	g	g	g	a	g	g	a	a	g	g	g	g	g		a	g
g	2572720	a	2792133	a	2862347	s	O157	rec46	a	CB9615	++++		a		a	a	a	a	a	a	a	a	a	a	a	a	a	a	a	a	a		a	a
a	2572729	g	2792142	g	2862356	s	O157	rec46	?	O157	+/-		g		a	g	a	g	g	a	a	a	g	a	a	a	a	a	g	g	g		g	a
g	2572732	c	2792145	c	2862359	s	O157	rec46	c	CB9615	+		c		g	c	g	c	c	c	g	c	c	c	c	g	g	g	c	c	c		c	g
a	2572741	g	2792154	g	2862368	ns	O157	rec46	?	O157	+/-		g		a	a	a	g	g	a	a	a	g	a	a	a	a	g	g	g	g		g	a
t	2572742	c	2792155	c	2862369	ns	O157	rec46	?	O157	+/-		c		t	t	t	c	c	t	t	t	c	t	t	t	t	c	c	c	c		c	t
g	2572746	a	2792159	a	2862373	ns	O157	rec46	a	CB9615	++++		a		a	a	a	a	a	a	a	a	a	a	a	a	a	a	a	a	a		a	a
c	2572752	t	2792165	t	2862379	ns	O157	rec46	t	CB9615	++++		t		t	t	t	t	t	t	t	t	t	t	t	t	t	t	t	t	t		t	t
a	2572754	g	2792167	g	2862381	ns	O157	rec46	g	CB9615	++++		g		g	g	g	g	g	g	g	g	g	g	g	g	g	g	g	g	g		g	g
g	2572791	a	2792204	a	2862418	ns	O157	rec46	g	O157	++++		g		g	g	g	g	g	g	g	g	g	g	g	g	g	g	g	g	g		g	g
t	2572846	a	2792259	a	2862473	s	O157	rec46	a	CB9615	++++		a		a	a	a	a	a	a	a	a	a	a	a	a	a	a	a	a	a		a	a
t	2572855	c	2792268	c	2862482	s	O157	rec46	t	O157	+		c		t	t	t	t	t	t	t	t	t	c	t	t	t	t	t	t	t		t	t
c	2572858	g	2792271	g	2862485	s	O157	rec46	g	CB9615	+		g		g	g	g	c	c	g	a	g	c	c	g	g	g	c	g	g	g		c	g
g	2572864	a	2792277	a	2862491	s	O157	rec46	g	O157	++++		g		g	g	g	g	g	g	g	g	g	g	g	g	g	g	g	g	g		g	g
g	2572870	t	2792283	t	2862497	s	O157	rec46	g	O157	+		t		g	g	g	g	g	g	g	g	a	g	g	g	g	g	g	g	g		a	g
c	2572945	a	2792358	a	2862572	s	O157	rec46	c	O157	++++		c		c	c	c	c	c	c	c	c	c	t	c	c	c	c	c	c	c		c	c
g	2572955	a	2792368	a	2862582	ns	O157	rec46	g	O157	++++		g		g	g	g	g	g	g	g	g	g	g	g	g	g	a	g	g	g		g	g
c	2572966	t	2792379	t	2862593	ns	O157	rec46	c	O157	+		t		c	t	c	c	c	t	t	t	c	t	t	c	t	c	c	c	c		c	c
c	2572968	g	2792381	g	2862595	ns	O157	rec46	g	CB9615	+		g		c	g	g	g	g	g	g	g	g	g	g	g	g	c	c	c	c		g	g
a	2572972	g	2792385	g	2862599	s	O157	rec46	g	CB9615	+		g		a	g	g	g	g	g	g	g	g	g	g	g	g	a	a	a	a		g	g
a	2572986	g	2792399	g	2862613	s	O157	rec46	g	CB9615	+		g		a	g	g	g	g	g	g	g	g	g	g	g	g	g	a	a	a		g	g
g	2572993	a	2792406	a	2862620	s	O157	rec46	a	CB9615	++++		a		a	a	a	a	a	a	a	a	a	a	a	a	a	a	a	a	a		a	a
c	2573002	t	2792415	t	2862629	s	O157	rec46	c	O157	+		c		c	c	c	c	c	c	c	c	c	c	c	c	c	c	t	t	t		c	c
t	2573008	g	2792421	g	2862635	s	O157	rec46	g	CB9615	+		t		g	t	g	g	g	t	t	t	g	g	g	t	t	t	g	g	g		g	g
g	2573026	a	2792439	a	2862653	s	O157	rec46	g	O157	+		g		a	a	g	g	g	g	g	g	g	g	a	g	g	g	g	g	g		g	g
a	2573029	g	2792442	g	2862656	s	O157	rec46	a	O157	+		a		g	g	a	a	a	a	a	a	a	a	g	a	a	a	a	a	a		a	a
a	2573035	g	2792448	g	2862662	s	O157	rec46	a	O157	+		g		g	g	a	a	a	a	a	a	a	a	g	g	a	a	a	a	a		a	a
g	2573038	a	2792451	a	2862665	s	O157	rec46	g	O157	+		a		a	g	g	g	g	g	g	g	g	g	a	a	g	g	g	g	g		g	g
g	2573041	a	2792454	a	2862668	s	O157	rec46	g	O157	+		g		a	a	g	g	g	g	g	g	g	g	a	a	g	g	g	g	g		g	g
c	2573053	t	2792466	t	2862680	s	O157	rec46	c	O157	+		t		t	c	t	c	c	c	t	c	c	c	c	c	c	c	c	c	c		c	c
g	2573073	a	2792486	a	2862700	s	O157	rec46	g	O157	+		g		g	g	a	g	g	g	g	g	g	g	g	g	g	g	a	a	a		g	g
a	2573077	g	2792490	g	2862704	s	O157	rec46	a	O157	+		a		a	a	g	a	a	a	a	a	a	a	a	a	a	a	g	g	g		a	a
g	2573098	a	2792511	a	2862725	ns	O157	rec46	a	CB9615	+		a		a	a	g	a	a	a	a	a	a	g	g	a	a	g	a	a	a		a	g
c	2573112	t	2792525	t	2862739	s	O157	rec46	c	O157	+		c		t	t	c	t	t	t	c	t	c	t	t	t	c	t	c	c	c		c	c
a	2573115	c	2792528	c	2862742	s	O157	rec46	a	O157	++++		a		a	a	a	a	a	a	a	a	a	a	a	a	a	c	a	a	a		a	a
c	2573118	t	2792531	t	2862745	s	O157	rec46	c	O157	++++		c		c	c	c	c	c	c	c	c	c	c	c	c	c	t	c	c	c		c	c
a	2573121	g	2792534	g	2862748	s	O157	rec46	a	O157	++++		a		a	a	a	a	a	a	a	a	a	a	a	a	a	g	a	a	a		a	a
t	2573124	c	2792537	c	2862751	s	O157	rec46	t	O157	+		c		t	t	t	t	t	t	t	t	t	t	t	t	c	c	c	c	c		t	t
t	2573151	c	2792564	c	2862778	s	O157	rec46	?	O157	+/-		c		t	t	c	c	c	t	c	t	c	t	t	t	c	c	t	t	t		c	t
t	2573170	c	2792583	c	2862797	ns	O157	rec46	t	O157	+		c		t	t	t	t	t	t	t	t	c	t	t	t	t	t	t	t	t		c	t
c	2573178	t	2792591	t	2862805	s	O157	rec46	t	CB9615	+		c		c	c	t	t	t	t	t	t	t	c	t	a	t	t	a	a	a		t	c
t	2573196	c	2792609	c	2862823	s	O157	rec46	c	CB9615	+		c		c	t	c	c	c	c	t	c	c	c	c	c	c	c	c	c	c		c	c
a	2573228	g	2792641	g	2862855	s	O157	rec46	g	CB9615	++++		g		g	g	g	g	g	g	g	g	g	g	g	g	g	g	g	g	g		g	g

O55 and O157 genome site details												Outgroup Strain Details[i]																						
CB9615 base[b]	CB9615 Site[c]	Sakai base[b]	Sakai Site[c]	EDL933 base[b]	EDL933 Site[c]	type[d]	Event Lineage[ef]	Recombinant segment	Inferred ancestral base[g]	Outgroup Analysis[e]	Support level[h]	D1 Sd197	K-12	HS	ATCC 8739	UMN026	IAI39	SMS 3-5	E2348/69	536	ED1a	CFT073	S88	APEC O1	UTI89	E24377A	IAI1	SE11	F5 8401	F2a 2457T	F2a 301	SS Ss046	B4 Sb227	B18 BS512
c	2573238	t	2792651	t	2862865	s	O157	rec46	t	CB9615	+		t		t	c	t	c	c	t	c	t	t	t	t	t	t	c	c	c	c		t	t
g	2573240	a	2792653	a	2862867	s	O157	rec46	a	CB9615	+		a		a	g	a	g	g	a	g	a	a	a	a	a	a	g	g	g	g		a	a
c	2573259	t	2792672	t	2862886	s	O157	rec46	t	CB9615	+		t		t	t	t	t	t	t	t	t	t	t	t	t	c	t	c	c	c		t	t
t	2573268	c	2792681	c	2862895	s	O157	rec46	t	O157	+		c		c	t	t	t	t	t	c	t	c	t	t	c	t	c	t	t	t		c	t
c	2573271	t	2792684	t	2862898	s	O157	rec46	t	CB9615	+		t		t	c	t	t	t	t	t	t	t	t	t	t	c	t	c	c	c		t	t
c	2573274	t	2792687	t	2862901	s	O157	rec46	t	CB9615	+		t		t	c	c	c	c	c	t	c	t	c	c	t	c	t	t	t	t		t	c
t	2573280	c	2792693	c	2862907	s	O157	rec46	c	CB9615	+		c		c	c	t	t	t	t	c	t	c	t	t	c	c	c	c	c	c		c	t
t	2573283	c	2792696	c	2862910	s	O157	rec46	c	CB9615	+		c		c	c	t	t	t	t	c	t	c	t	t	c	c	t	c	c	c		c	t
t	2573291	c	2792704	c	2862918	ns	O157	rec46	t	O157	++++		t		t	t	t	t	t	t	t	t	t	t	t	t	t	t	t	t	t		t	t
g	2573310	a	2792723	a	2862937	s	O157	rec46	a	CB9615	+		g		a	a	a	a	a	a	a	a	a	a	a	g	a	g	a	a	a		a	a
t	2573325	c	2792738	c	2862952	s	O157	rec46	c	CB9615	+		t		c	c	c	c	c	c	c	c	c	c	c	t	c	c	c	c	c		c	c
g	2573334	a	2792747	a	2862961	s	O157	rec46	g	O157	+		g		g	g	g	a	a	g	a	g	a	g	g	g	g	g	a	a	a		a	g
a	2573340	g	2792753	g	2862967	s	O157	rec46	g	CB9615	+		a		g	g	g	g	g	g	g	g	g	g	g	a	g	g	g	g	g		g	g
c	2573346	t	2792759	t	2862973	s	O157	rec46	t	CB9615	+		c		t	c	c	t	t	t	t	t	t	c	c	c	t	t	t	t	t		t	c
c	2573394	a	2792807	a	2863021	s	O157	rec46	a	CB9615	+		c		a	a	a	a	a	c	a	c	a	c	a	a	t	a	t	t	t		c	c
g	2573424	a	2792837	a	2863051	s	O157	rec46	g	O157	++++		g		a	g	g	g	g	g	g	g	g	g	g	g	g	g	g	g	g		g	g
g	2573445	a	2792858	a	2863072	s	O157	rec46	g	O157	+		g		g	g	a	g	g	g	g	g	a	g	g	g	g	g	g	g	g		g	g
a	2573478	g	2792891	g	2863105	s	O157	rec46	a	O157	+		g		a	a	g	g	g	a	a	a	g	a	a	a	g	a	a	a	a		g	g
t	2573487	c	2792900	c	2863114	s	O157	rec46	c	CB9615	++++		c		c	c	c	c	c	c	c	c	c	c	c	c	c	c	c	c	c		c	c
g	2573520	a	2792933	a	2863147	s	O157	rec46	g	O157	+		g		g	g	a	g	g	g	g	g	g	g	g	a	g	a	a	a	a		g	g
c	2573557	t	2792970	t	2863184	ns	O157	rec46	t	CB9615	++++		t		t	t	t	t	t	t	t	t	t	t	t	t	t	t	t	t	t		t	t
g	2573602	a	2793015	a	2863229	ns	O157	rec46	g	O157	++++		g		g	g	g	g	g	g	g	g	g	g	g	g	g	g	g	g	g		g	g
t	2573607	c	2793020	c	2863234	s	O157	rec46	t	O157	+		t		t	t	c	t	t	t	c	t	t	c	t	t	t	t	t	t	t		c	t
a	2573721	g	2793134	g	2863348	s	O157	rec46	g	CB9615	+		g		g	g	g	g	g	g	a	g	g	g	g	a	g	g	g	g	g		g	a
c	2573802	t	2793215	t	2863429	s	O157	rec46	c	O157	+		c		c	c	c	c	c	c	c	c	c	c	t	c	c	t	c	c	c		c	c
a	2573804	g	2793217	g	2863431	s	O157	rec46	?	O157	+/-		g		a	a	a	a	a	a	g	a	g	g	g	a	g	g	a	a	a		g	g
a	2573808	g	2793221	g	2863435	s	O157	rec46	a	O157	++++		a		a	a	a	a	a	a	a	a	a	a	g	a	a	a	a	a	a		a	a
g	2573828	a	2793241	a	2863455	s	O157	rec46	g	O157	++++		g		g	g	g	g	g	g	g	g	g	g	g	g	g	g	g	g	g		g	g
a	2573834	g	2793247	g	2863461	s	O157	rec46	g	CB9615	++++		g		g	g	g	g	g	g	g	g	g	g	g	g	g	g	g	g	g		g	g
a	2573838	g	2793251	g	2863465	s	O157	rec46	g	CB9615	++++		g		g	a	g	g	g	g	g	g	g	g	g	g	g	g	g	g	g		g	g
c	2573940	t	2793353	t	2863567	s	O157	rec46	c	O157	+		c		c	t	a	a	a	a	c	a	c	a	c	c	c	c	t	t	t		c	c
g	2573952	a	2793365	a	2863579	s	O157	rec46	a	CB9615	+		a		a	a	g	a	a	a	a	a	a	g	a	g	a	a	a	a	a		a	a
a	2573970	g	2793383	g	2863597	s	O157	rec46	g	CB9615	++++		g		g	g	g	g	g	g	g	g	g	g	g	g	g	g	g	g	g		g	g
a	2573973	g	2793386	g	2863600	s	O157	rec46	g	CB9615	+		a		g	g	g	a	a	g	a	g	g	g	a	g	g	a	g	g	g		a	a
c	2574030	t	2793443	t	2863657	s	O157	rec46	c	O157	++++		c		c	c	c	c	c	c	c	c	c	c	c	c	c	c	c	c	c		t	c
a	2574032	g	2793445	g	2863659	s	O157	rec46	g	CB9615	++++		g		g	g	g	g	g	g	g	g	g	g	a	g	g	g	g	g	g		g	g
a	2574072	g	2793485	g	2863699	s	O157	rec46	a	O157	+		g		a	a	a	a	a	a	g	a	a	a	a	a	a	a	g	g	g		g	g
a	2574114	g	2793527	g	2863741	s	O157	rec46	g	CB9615	+		a		g	a	g	g	g	g	g	g	a	a	a	g	g	g	a	a	a		g	g
g	2574120	a	2793533	a	2863747	s	O157	rec46	g	O157	+		g		a	g	g	a	a	g	a	g	g	a	g	a	a	a	g	g	g		a	a
c	2574189	a	2793602	a	2863816	s	O157	rec46	c	O157	++++		c		c	c	c	c	c	c	c	c	c	c	c	c	a	c	c	c	c		c	c
g	2574204	a	2793617	a	2863831	s	O157	rec46	g	O157	++++		g		g	g	g	g	g	g	g	g	g	g	g	g	a	g	g	g	g		g	g
c	2574272	t	2793685	t	2863899	ns	O157	rec46	c	O157	+		c		c	c	c	c	c	c	c	c	c	c	c	c	c	c	t	t	t		c	c
g	2574283	a	2793696	a	2863910	ns	O157	rec46	a	CB9615	++++		a		a	a	a	a	a	a	a	a	a	a	a	a	a	a	a	a	a		a	a
t	2574403	-	2793815	-	2864029	del-14	O157	rec46	?	O157	+/-																							
c	2574422	t	2793944	t	2864158	i	O157	rec46	t	CB9615	+		t		t	c	c	c	c	c	t	c	t		c	t	t	t	t	t	t		t	t
g	2574437	a	2793959	a	2864173	i	O157	rec46	g	O157	+		g		a	a	g	a	a	a	g	a	a		a	a	g	g	g	g	g		a	g
c	2574439	a	2793961	a	2864175	i	O157	rec46	a	CB9615	+		a		a	a	c	c	c	c	a	c	a		c	a	a	a	a	a	a		a	a
t	2574441	c	2793963	c	2864177	i	O157	rec46	t	O157	+		c		c	c	t	t	t	t	c	t	c		t	c	t	c	t	t	t		c	t
a	2574459	g	2793981	g	2864195	ns	O157	rec46	g	CB9615	++++		g		g	g	g	g	g	g	g	g	g	g	g	g	g	g	g	g	g		g	g
a	2574490	g	2794012	g	2864226	s	O157	rec46	a	O157	++++		a		a	a	a	a	a	a	a	a	a	a	a	a	a	a	a	a	a		a	a
t	2574538	c	2794060	c	2864274	s	O157	rec46	c	CB9615	++++		c		c	t	c	c	c	c	c	c	c	c	c	c	c	c	c	c	c		c	c
a	2574609	c	2794131	c	2864345	ns	O157	rec46	c	CB9615	++++		c		c	c	c	c	c	c	c	c	c	a	c	c	c	c	c	c	c		c	c
t	2574610	a	2794132	a	2864346	ns	O157	rec46	a	CB9615	++++		a		a	a	a	a	a	a	a	a	a	t	a	a	a	a	a	a	a		a	a
g	2574612	t	2794134	t	2864348	ns	O157	rec46	t	CB9615	++++		t		t	t	t	t	t	t	t	t	t	g	t	t	t	t	t	t	t		t	t
g	2574620	a	2794142	a	2864356	ns	O157	rec46	g	O157	+		g		a	g	g	g	g	g	g	g	a	g	g	g	a	g	g	g	g		a	g
t	2574661	c	2794183	c	2864397	s	O157	rec46	c	CB9615	++++		c		c	c	c	c	c	c	c	c	c	c	c	c	c	c	c	c	c		c	c
t	2574667	c	2794189	c	2864403	s	O157	rec46	c	CB9615	+		t		c	t	t	c	c	c	c	c	c	c	c	c	c	c	c	c	c		c	c
g	2574700	a	2794222	a	2864436	s	O157	rec46	a	CB9615	+		a		a	g	g	g	a	g	a	g	a	g	g	a	a	a	a	a	a		a	a
c	2574703	a	2794225	a	2864439	s	O157	rec46	a	CB9615	+		a		a	c	c	c	a	c	a	c	a	c	c	a	a	a	a	a	a		a	a
c	2574747	t	2794269	t	2864483	ns	O157	rec46	t	CB9615	++++		t		t	t	t	t	t	t	t	t	t	t	t	t	t	t	t	t	t		t	t
t	2574802	a	2794324	a	2864538	s	O157	rec46	a	CB9615	+		t		a	a	a	a	a	a	a	a	a	a	a	a	a	a	a	a	a		a	a
g	2574829	t	2794351	t	2864565	s	O157	rec46	?	O157	+/-		t		g	g	g	g	t	g	t	g	t	g	g	g	t	t	t	t	t		g	g
t	2574868	c	2794390	c	2864604	s	O157	rec46	c	CB9615	++++		c		c	c	c	c	c	c	c	c	c	c	c	c	c	c	c	c	c		c	c
c	2574904	t	2794426	t	2864640	s	O157	rec46	c	O157	+		t		c	c	c	c	t	c	c	c	c	c	c	c	t	c	c	c	c		c	c
t	2574907	g	2794429	g	2864643	s	O157	rec46	g	CB9615	+		g		g	t	t	t	g	g	c	g	c	t	g	g	g	g	g	g	g		g	g
t	2574916	c	2794438	c	2864652	s	O157	rec46	c	CB9615	+		c		c	c	c	c	c	c	t	c	c	c	c	c	c	c	c	c	c		c	t
c	2574919	a	2794441	a	2864655	s	O157	rec46	a	CB9615	+		a		a	c	c	c	a	c	c	c	a	c	c	a	a	a	a	a	a		a	c
a	2574925	c	2794447	c	2864661	ns	O157	rec46	c	CB9615	+		c		a	c	c	c	c	c	c	c	c	c	c	a	c	a	a	a	a		a	c
t	2574927	c	2794449	c	2864663	ns	O157	rec46	c	CB9615	+		c		t	c	c	c	c	c	c	c	c	c	c	t	c	t	t	t	t		t	c
c	2574967	t	2794489	t	2864703	s	O157	rec46	c	O157	+		c		t	c	c	c	t	c	c	c	c	c	t	t	c	c	t	t	t		t	t
a	2574970	g	2794492	g	2864706	s	O157	rec46	a	O157	+		a		g	a	a	a	g	a	a	a	a	a	g	g	a	a	g	g	g		g	g
g	2574976	t	2794498	t	2864712	s	O157	rec46	g	O157	+		g		t	g	g	g	t	g	g	g	g	g	t	t	g	t	t	t	t		t	t
g	2575087	a	2794609	a	2864823	s	O157	rec46	g	O157	+		a		g	g	g	g	a	g	g	g	a	g	g	g	g	g	g	g	g		g	g
a	2575105	g	2794627	g	2864841	s	O157	rec46	a	O157	+		g		g	a	a	a	g	a	g	a	g	a	g	a	a	a	a	a	a		g	g
c	2575108	t	2794630	t	2864844	s	O157	rec46	t	CB9615	+		t		t	c	t	t	t	t	t	t	t	t	c	c	c	c	c	c	c		t	t
g	2575123	a	2794645	a	2864859	s	O157	rec46	g	O157	+		g		a	g	g	g	a	g	g	g	a	g	g	a	g	g	g	g	g		a	a
g	2575135	c	2794657	c	2864871	s	O157	rec46	c	CB9615	+++		t		c	g	t	t	c	t	t	t	c	t	c	t	c	t	t	t	t		c	c
t	2575144	c	2794666	c	2864880	s	O157	rec46	c	CB9615	+		c		c	t	c	c	c	c	c	c	c	c	c	c	t	c	c	c	c		c	c
c	2575186	t	2794708	t	2864922	s	O157	rec46	t	CB9615	+		t		t	c	t	t	t	t	t	t	t	t	t	c	c	t	t	t	t		t	t
a	2575198	g	2794720	g	2864934	ns	O157	rec46	g	CB9615	++++		g		g	a	g	g	g	g	g	g	g	g	g	g	g	g	g	g	g		g	g
t	2575200	c	2794722	c	2864936	ns	O157	rec46	c	CB9615	+		c		c	t	c	c	c	c	c	c	c	c	c	t	c	c	c	c	c		c	c
g	2575243	a	2794765	a	2864979	s	O157	rec46	g	O157	+		a		g	g	g	a	a	g	g	g	a	a	g	g	g	g	g	g	g		g	g
t	2575300	c	2794822	c	2865036	s	O157	rec46	t	O157	+		t		t	t	t	t	c	t	t	t	t	t	t	c	t	t	t	t	t		t	t
c	2575303	a	2794825	a	2865039	s	O157	rec46	c	O157	+		c		c	c	c	c	a	c	c	c	a	c	c	a	c	c	c	c	c		c	c
c	2575306	a	2794828	a	2865042	s	O157	rec46	c	O157	+		c		c	c	c	c	a	c	c	c	a	c	c	t	c	c	a	a	a		c	c
a	2575330	g	2794852	g	2865066	s	O157	rec46	g	CB9615	++++		g		g	g	g	g	g	g	g	g	a	g	g	g	g	g	g	g	g		g	g

O55 and O157 genome site details												Outgroup Strain Details[i]																						
CB9615 base[b]	CB9615 Site[c]	Sakai base[b]	Sakai Site[c]	EDL933 base[b]	EDL933 Site[c]	type[d]	Event Lineage[ef]	Recombinant segment	Inferred ancestral base[g]	Outgroup Analysis[e]	Support level[h]	D1 Sd197	K-12	HS	ATCC 8739	UMN026	IAI39	SMS 3-5	E2348/69	536	ED1a	CFT073	S88	APEC O1	UTI89	E24377A	IAI1	SE11	F5 8401	F2a 2457T	F2a 301	SS Ss046	B4 Sb227	B18 BS512
t	2575375	c	2794897	c	2865111	s	O157	rec46	?	O157	+/-		c		t	t	t	t	c	c	t	c	t	t	c	c	t	t	c	c	c		t	t
c	2575465	t	2794987	t	2865201	s	O157	rec46	c	O157	+		c		c	c	c	c	c	c	t	c	c	c	c	c	c	t	c	c	c		c	c
c	2575513	t	2795035	t	2865249	s	O157	rec46	c	O157	+		t		c	c	t	t	c	t	c	t	c	t	t	c	t	c	c	c	c		c	c
c	2575549	a	2795071	a	2865285	s	O157	rec46	c	O157	+		t		c	c	c	c	c	a	c	a	c	c	c	c	c	c	c	c	c		c	c
g	2575582	c	2795104	c	2865318	s	O157	rec46	c	CB9615	++++		c		c	g	c	c	c	c	c	c	c	c	c	c	c	c	c	c	c		c	c
a	2575600	g	2795122	g	2865336	s	O157	rec46	a	O157	+		a		a	a	a	g	a	a	a	a	a	g	a	a	a	a	a	a	a		a	a
t	2575608	c	2795130	c	2865344	ns	O157	rec46	c	CB9615	++++		c		c	t	c	c	c	c	c	c	c	c	c	c	c	c	c	c	c		c	c
g	2575645	a	2795167	a	2865381	s	O157	rec46	g	O157	+		g		g	g	a	a	g	g	g	g	g	g	a	g	g	g	a	a	a		g	g
g	2575726	c	2795248	c	2865462	s	O157	rec46	c	CB9615	+		c		c	g	c	c	c	g	c	g	c	c	c	c	c	c	c	c	c		c	c
g	2575735	a	2795257	a	2865471	s	O157	rec46	g	O157	++++		g		g	g	g	g	g	g	g	g	g	g	g	g	a	g	g	g	g		g	g
a	2575786	g	2795308	g	2865522	s	O157	rec46	g	CB9615	+		g		g	a	g	g	g	a	a	a	g	g	g	g	g	a	a	a	a		g	g
c	2575789	a	2795311	a	2865525	s	O157	rec46	a	CB9615	+		a		a	c	a	a	a	c	a	c	a	a	a	a	a	c	c	c	c		a	a
g	2575794	a	2795316	a	2865530	s	O157	rec46	g	O157	+		g		g	g	a	g	a	a	a	a	a	g	a	a	a	a	g	g	g		g	g
g	2575795	a	2795317	a	2865531	s	O157	rec46	g	O157	+		g		g	g	a	g	a	a	a	a	a	g	a	a	a	a	g	g	g		g	g
a	2575845	g	2795367	g	2865581	s	O157	rec46	g	CB9615	++++		g		g	a	g	g	g	g	g	g	g	g	g	g	g	g	g	g	g		g	g
g	2575946	t	2795468	t	2865682	s	O157	rec46	g	O157	++++		a		g	g	g	a	g	a	g	a	g	g	g	g	g	g	g	g	g		g	g
c	2575949	t	2795471	t	2865685	s	O157	rec46	t	CB9615	+		t		c	c	t	t	t	t	t	t	t	c	t	t	t	t	c	c	c		c	c
c	2575955	t	2795477	t	2865691	s	O157	rec46	t	CB9615	+		c		t	c	t	t	t	t	t	t	t	t	t	t	t	t	t	t	t		t	t
g	2576114	a	2795636	a	2865850	s	O157	rec46	g	O157	++++		g		g	g	g	g	g	g	g	g	g	g	g	g	g	t	g	g	g		g	g
g	2576141	t	2795663	t	2865877	s	O157	rec46	g	O157	+		t		g	g	g	t	t	g	g	g	g	t	g	g	t	t	g	g	g		g	g
a	2576168	g	2795690	g	2865904	s	O157	rec46	g	CB9615	+		g		g	a	g	g	a	a	g	a	g	g	g	g	g	g	g	g	g		g	g
c	2576177	t	2795699	t	2865913	s	O157	rec46	t	CB9615	+		t		t	c	t	t	c	c	t	c	t	t	c	t	t	t	t	t	t		t	t
a	2576213	g	2795735	g	2865949	s	O157	rec46	g	CB9615	++++		g		g	a	g	g	g	g	g	g	g	g	g	g	g	g	g	g	g		g	g
t	2576249	c	2795771	c	2865985	s	O157	rec46	c	CB9615	++++		c		c	t	c	c	c	c	c	c	c	c	c	c	c	c	c	c	c		c	c
a	2576255	g	2795777	g	2865991	s	O157	rec46	g	CB9615	+		g		g	a	g	g	g	a	g	a	g	g	g	g	g	g	g	g	g		g	g
a	2576297	g	2795819	g	2866033	s	O157	rec46	?	O157	+/-		g		a	a	g	a	a	g	a	g	a	a	a	a	g	g	g	g	g		a	a
a	2576300	c	2795822	c	2866036	s	O157	rec46	c	CB9615	++++		c		c	c	c	c	c	c	c	c	c	c	c	c	c	c	c	c	c		c	c
t	2576357	c	2795879	c	2866093	s	O157	rec46	c	CB9615	+		c		c	t	c	t	c	c	c	c	c	t	c	c	c	c	c	c	c		c	c
g	2576363	a	2795885	a	2866099	s	O157	rec46	a	CB9615	++++		a		a	g	a	a	a	a	a	a	a	a	a	a	a	a	a	a	a		a	a
a	2576435	g	2795957	g	2866171	s	O157	rec46	g	CB9615	++++		g		g	a	g	g	g	g	g	g	g	g	g	g	g	g	g	g	g		g	g
c	2576534	a	2796056	a	2866270	s	O157	rec46	c	O157	+		a		c	a	c	c	a	c	a	c	c	c	c	c	c	c	c	c	c		c	c
t	2576549	a	2796071	a	2866285	s	O157	rec46	?	O157	+/-		t		a	t	t	t	a	t	a	t	t	t	t	a	a	a	a	a	a		a	a
g	2576567	a	2796089	a	2866303	ns	O157	rec46	g	O157	++++		g		g	g	g	g	g	g	a	g	g	g	g	g	g	g	g	g	g		g	g
t	2576569	g	2796091	g	2866305	ns	O157	rec46	g	CB9615	+		g		g	t	t	g	g	t	g	g	t	g	g	g	g	g	g	g	g		g	g
a	2576579	g	2796101	g	2866315	s	O157	rec46	g	CB9615	++++		g		g	g	g	g	g	g	g	g	a	g	g	g	g	g	g	g	g		g	g
a	2576585	g	2796107	g	2866321	s	O157	rec46	g	CB9615	++++		g		g	g	g	g	g	g	g	g	a	g	g	g	g	g	g	g	g		g	g
g	2576600	a	2796122	a	2866336	s	O157	rec46	?	O157	+/-		g		g	a	g	a	g	a	a	a	a	a	a	a	a	g	g	g	g		g	a
g	2576608	t	2796130	t	2866344	ns	O157	rec46	t	CB9615	++++		t		t	t	t	t	t	t	t	t	t	t	t	t	t	t	t	t	t		t	t
c	2576678	t	2796200	t	2866414	s	O157	rec46	t	CB9615	+		t		t	c	t	c	t	c	t	c	t	c	c	t	t	t	t	t	t		t	t
c	2576711	t	2796233	t	2866447	s	O157	rec46	c	O157	+		c		c	c	c	c	c	c	t	c	c	c	c	c	c	c	c	c	c		c	t
c	2576717	a	2796239	a	2866453	s	O157	rec46	c	O157	+		c		c	t	c	c	c	c	a	c	c	c	c	a	c	c	t	t	t		c	c
a	2576741	g	2796263	g	2866477	s	O157	rec46	a	O157	+		g		a	a	a	a	a	a	g	a	a	a	a	g	a	a	g	g	g		a	a
g	2576744	a	2796266	a	2866480	s	O157	rec46	g	O157	+		a		g	g	g	g	g	g	a	a	g	g	g	a	g	g	a	a	a		g	g
g	2576772	a	2796294	a	2866508	ns	O157	rec46	g	O157	++++		g		g	g	g	g	g	g	g	g	g	g	g	g	g	g	g	g	g		g	g
a	2576780	g	2796302	g	2866516	s	O157	rec46	g	CB9615	++++		g		g	g	g	g	g	g	g	g	g	g	g	g	g	g	g	g	g		g	g
a	2576786	g	2796308	g	2866522	s	O157	rec46	g	CB9615	++++		g		g	g	g	g	g	g	g	g	g	g	g	g	g	g	g	g	g		g	g
g	2576789	a	2796311	a	2866525	s	O157	rec46	a	CB9615	++++		a		a	t	a	t	a	a	a	a	a	t	a	a	a	a	a	a	a		a	t
a	2576792	g	2796314	g	2866528	s	O157	rec46	g	CB9615	++++		g		g	g	g	g	g	g	g	g	c	g	g	c	c	g	g	g	g		g	g
a	2576822	g	2796344	g	2866558	s	O157	rec46	a	O157	+		g		a	a	a	a	a	a	a	a	a	a	a	a	a	a	a	a	a		a	a
t	2576849	c	2796371	c	2866585	s	O157	rec46	t	O157	+		c		t	t	t	t	t	t	t	t	t	t	t	t	t	t	t	t	t		t	t
t	2576870	c	2796392	c	2866606	s	O157	rec46	t	O157	+		c		t	t	t	c	t	c	t	c	c	t	c	t	c	t	t	t	t		t	c
g	2576873	c	2796395	c	2866609	s	O157	rec46	g	O157	+		c		g	g	g	c	g	c	g	c	c	g	c	g	c	g	g	g	g		g	c
a	2576882	g	2796404	g	2866618	s	O157	rec46	g	CB9615	+		g		a	a	g	g	a	g	a	g	g	g	g	g	g	a	c	c	c		a	g
g	2576957	a	2796479	a	2866693	s	O157	rec46	g	O157	+		g		a	g	g	a	a	g	a	g	g	a	g	g	g	a	g	g	g		a	g
a	2576982	c	2796504	c	2866718	ns	O157	rec46	c	CB9615	++++		c		c	c	c	c	c	c	c	c	c	c	c	c	c	c	c	c	c		c	c
a	2576999	g	2796521	g	2866735	s	O157	rec46	g	CB9615	++++		g		g	g	g	g	g	g	g	g	g	g	g	g	g	g	g	g	g		g	g
g	2577067	a	2796589	a	2866803	s	O157	rec46	a	CB9615	+		a		a	g	a	a	a	a	a	a	a	g	a	a	a	a	a	a	a		a	a
a	2577193	g	2796715	g	2866929	s	O157	rec46	g	CB9615	++++		g		g	g	g	g	g	g	g	g	g	g	g	g	g	g	g	g	g		g	g
c	2577221	t	2796743	t	2866957	s	O157	rec46	t	CB9615	+		t		t	t	t	t	t	t	t	c	t	c	t	t	c	t	c	c	c		t	t
c	2577330	a	2796852	a	2867066	i	O157	rec46	a	CB9615	++++		a		a	a	a	a	a	t	a	t	a	a	a	a	a	a	a	a	a		a	a
c	2577515	t	2796956	t	2867170	s	O157	rec46	c	O157	+		c		c	c	c	c	c	t	c	t	c	c	t	c	c	c	c	c	c	a	c	c
g	2577563	a	2797004	a	2867218	s	O157	rec46	g	O157	+		g		g	a	a	g	g	a	g	a	g	g	a	g	g	g	g	g	g	g	g	g
g	2577635	a	2797076	a	2867290	s	O157	rec46	a	CB9615	++++		a		a	a	a	a	a	a	a	a	a	a	g	a	a	a	a	a	a	a	a	a
g	2577644	c	2797085	c	2867299	s	O157	rec46	c	CB9615	+		c		g	g	c	c	g	c	g	c	c	c	c	c	c	c	c	c	c	c	g	g
t	2577657	g	2797098	g	2867312	ns	O157	rec46	g	CB9615	++++		g		g	g	g	g	g	g	g	g	g	g	g	g	g	g	g	g	g	g	g	g
g	2577668	t	2797109	t	2867323	ns	O157	rec46	t	CB9615	+		t		t	g	t	t	t	t	t	t	g	t	t	t	t	t	t	t	t	t	t	t
c	2577700	a	2797141	a	2867355	ns	O157	rec46	c	O157	++++		c		c	c	c	c	c	c	c	c	c	c	c	c	c	c	c	c	c	c	c	c
a	2577719	g	2797160	g	2867374	s	O157	rec46	g	CB9615	++++		g		g	g	g	g	g	g	g	g	g	g	g	g	g	g	g	g	g	g	g	g
t	2577725	c	2797166	c	2867380	s	O157	rec46	c	CB9615	+		t		c	c	c	c	c	c	c	c	c	c	c	t	t	c	c	c	c	c	c	t
t	2577743	c	2797184	c	2867398	s	O157	rec46	t	O157	++++		t		t	t	t	t	t	t	t	t	t	t	t	t	t	t	t	t	t	t	t	t
c	2577746	t	2797187	t	2867401	ns	O157	rec46	t	CB9615	+		a		t	t	t	t	t	t	t	t	t	t	t	c	c	t	t	t	t	t	t	c
t	2577747	c	2797188	c	2867402	ns	O157	rec46	c	CB9615	+		t		c	c	c	c	c	c	c	c	c	c	c	t	t	c	c	c	c	c	c	t
a	2577821	g	2797262	g	2867476	s	O157	rec46	g	CB9615	+		g		g	g	a	a	g	g	g	g	g	g	g	g	g	g	g	g	g	g	g	g
g	2577824	a	2797265	a	2867479	s	O157	rec46	a	CB9615	+		a		a	a	g	g	a	a	a	a	a	a	a	a	a	a	a	a	a	a	a	a
a	2577872	c	2797313	c	2867527	s	O157	rec46	a	O157	+		a		c	a	a	c	c	a	c	a	a	a	a	a	a	a	c	c	c	c	c	a
c	2577914	g	2797355	g	2867569	s	O157	rec46	c	O157	+		c		a	c	g	c	g	c	g	g	c	g	g	c	c	g	c	c	c	c	g	a
a	2577920	g	2797361	g	2867575	s	O157	rec46	g	CB9615	+		a		g	g	g	g	g	g	g	g	g	g	g	a	a	a	g	g	g	g	g	g
g	2577947	a	2797388	a	2867602	s	O157	rec46	g	O157	+		g		a	g	g	g	g	g	g	g	g	g	g	g	g	a	g	g	g	g	g	g
c	2577962	t	2797403	t	2867617	s	O157	rec46	t	CB9615	+		t		t	t	t	t	c	t	c	t	t	t	t	t	t	a	c	c	c	c	c	t
g	2578019	a	2797460	a	2867674	s	O157	rec46	g	O157	+		g		g	g	g	a	g	g	g	a	g	g	a	g	g	g	g	g	g	g	g	g
a	2578040	g	2797481	g	2867695	s	O157	rec46	g	CB9615	+		a		g	g	a	g	g	a	g	g	g	a	g	g	g	a	g	g	g	a	g	g
g	2578094	a	2797535	a	2867749	s	O157	rec46	g	O157	+		a		g	g	g	g	g	g	g	a	g	g	g	g	g	g	g	g	g	a	g	g
g	2578121	a	2797562	a	2867776	s	O157	rec46	g	O157	+		g		a	g	g	g	g	g	g	a	g	g	g	g	g	g	g	g	g	g	g	g
a	2578139	g	2797580	g	2867794	s	O157	rec46	g	CB9615	++++		g		g	g	g	g	g	g	g	g	g	g	g	g	g	g	g	g	g	g	g	g
g	2578148	a	2797589	a	2867803	s	O157	rec46	a	CB9615	+		g		a	g	a	a	a	a	a	a	g	g	a	a	a	a	a	a	a	g	g	a
a	2578162	g	2797603	g	2867817	s	O157	rec46	g	CB9615	+		g		g	g	a	a	g	a	g	g	g	a	a	g	g	g	g	g	g	g	g	g

O55 and O157 genome site details												Outgroup Strain Details[i]																						
CB9615 base[b]	CB9615 Site[c]	Sakai base[b]	Sakai Site[c]	EDL933 base[b]	EDL933 Site[c]	type[d]	Event Lineage[ef]	Recombinant segment	Inferred ancestral base[g]	Outgroup Analysis[e]	Support level[h]	D1 Sd197	K-12	HS	ATCC 8739	UMN026	IAI39	SMS 3-5	E2348/69	536	ED1a	CFT073	S88	APEC O1	UTI89	E24377A	IAI1	SE11	F5 8401	F2a 2457T	F2a 301	SS Ss046	B4 Sb227	B18 BS512
t	2578163	c	2797604	c	2867818	s	O157	rec46	c	CB9615	+		t		c	c	c	c	c	c	c	c	c	c	c	c	c	c	c	c	c	c	c	c
a	2578166	g	2797607	g	2867821	s	O157	rec46	g	CB9615	++++		g		g	g	g	g	g	g	g	g	g	g	g	g	g	g	g	g	g	g	g	g
c	2578196	g	2797637	g	2867851	s	O157	rec46	c	O157	+		c		g	c	c	c	c	c	c	c	c	c	c	c	c	c	c	c	c	c	g	c
t	2578208	c	2797649	c	2867863	s	O157	rec46	c	CB9615	+		c		c	c	c	c	t	c	t	c	t	c	c	t	t	t	t	t	t	c	c	t
g	2578226	c	2797667	c	2867881	s	O157	rec46	g	O157	+		g		g	a	g	c	g	c	g	c	g	c	c	g	g	g	g	g	g	g	g	g
a	2578256	g	2797697	g	2867911	s	O157	rec46	a	O157	+		a		g	a	a	g	g	g	a	a	g	g	g	a	a	a	a	a	a	g	a	a
t	2578280	c	2797721	c	2867935	s	O157	rec46	t	O157	+		t		t	t	t	c	t	t	t	c	c	t	c	t	t	t	t	t	t	t	t	t
g	2578319	a	2797760	a	2867974	s	O157	rec46	g	O157	+		g		a	g	a	a	g	a	g	g	g	a	a	g	g	g	g	g	g	g	g	g
c	2578487	t	2797928	t	2868142	s	O157	rec46	t	CB9615	+		c		c	t	t	t	t	t	c	t	t	t	t	t	t	t	c	c	c	t	t	c
c	2578499	t	2797940	t	2868154	s	O157	rec46	c	O157	+		c		c	c	c	t	c	c	c	t	t	c	t	c	c	c	c	c	c	t	t	c
a	2578511	g	2797952	g	2868166	s	O157	rec46	g	CB9615	+		a		a	g	g	g	g	g	a	g	g	g	g	a	a	a	g	g	g	g	a	g
a	2578523	g	2797964	g	2868178	s	O157	rec46	a	O157	+		a		a	a	g	a	a	g	a	g	g	g	g	a	a	a	a	a	a	g	a	a
g	2578535	a	2797976	a	2868190	s	O157	rec46	g	O157	+		g		g	a	g	g	g	g	g	g	g	g	g	g	g	g	a	a	a	g	g	a
a	2578619	g	2798060	g	2868274	s	O157	rec46	a	O157	+		g		a	a	a	a	a	g	a	g	a	a	a	g	g	g	a	a	a	a	a	g
g	2578622	a	2798063	a	2868277	s	O157	rec46	g	O157	+		a		g	g	g	g	g	g	g	g	g	g	g	a	a	a	g	g	g	g	g	a
c	2578646	t	2798087	t	2868301	s	O157	rec46	c	O157	+		c		c	t	c	c	c	c	c	c	c	c	c	t	t	t	t	t	t	c	c	t
a	2578665	g	2798106	g	2868320	ns	O157	rec46	g	CB9615	++++		g		g	a	g	g	g	g	g	g	g	g	g	g	g	g	g	g	g	g	g	g
g	2578679	a	2798120	a	2868334	s	O157	rec46	g	O157	+		g		g	g	g	g	a	g	g	g	g	g	g	a	a	a	a	a	a	g	a	a
a	2578683	g	2798124	g	2868338	ns	O157	rec46	a	O157	++++		a		a	a	a	a	a	a	a	a	a	a	a	a	a	a	a	a	a	a	a	a
a	2578697	c	2798138	c	2868352	s	O157	rec46	c	CB9615	++++		c		c	c	c	c	c	c	c	c	c	c	c	c	c	c	c	c	c	c	c	c
g	2578733	a	2798174	a	2868388	s	O157	rec46	g	O157	++++		g		g	a	g	g	g	g	g	g	g	g	g	g	g	g	g	g	g	g	g	g
a	2578742	g	2798183	g	2868397	s	O157	rec46	g	CB9615	++++		g		g	a	g	g	g	g	g	g	g	g	g	g	g	g	g	g	g	g	g	g
g	2578751	a	2798192	a	2868406	s	O157	rec46	g	O157	+		g		a	a	a	a	a	g	a	g	a	a	a	g	g	g	g	g	g	g	g	g
g	2578777	a	2798218	a	2868432	s	O157	rec46	a	CB9615	++++		a		a	a	a	a	a	a	a	a	a	a	a	a	a	a	a	a	a	a	a	a
t	2578781	c	2798222	c	2868436	s	O157	rec46	c	CB9615	++++		c		c	c	c	c	c	c	c	c	c	c	t	c	c	c	c	c	c	c	c	c
t	2578914	g	2798446	g	2868660	i	O157	rec46	g	CB9615	++++		g		g	g			g	g	g	g	g		g	g	g		g	g	g	g	g	g
a	2578919	g	2798451	g	2868665	i	O157	rec46	g	CB9615	++++		g		g	g			g	g	g	g	g		g	g	g		g	g	g	g	g	g
t	2578922	g	2798454	g	2868668	i	O157	rec46	g	CB9615	++++		g		g	g			g	g	g	g	g		g	g	g		a	g	g	g	g	g
g	2578923	t	2798455	t	2868669	i	O157	rec46	t	CB9615	++++		t		t	t			t	t	t	t	t		t	t	t		t	t	t	t	t	t
c	2578924	t	2798456	t	2868670	s	O157	rec46	t	CB9615	++++		t		t	t			t	t	t	t	t		t	t	t		t	t	t	t	t	t
a	2579035	g	2798567	g	2868781	s	O157	rec46	g	CB9615	+		a		g	a	g	g	g	a	a	a	g	a	a	g	g	g	g	g	g	a	a	g
g	2579062	c	2798594	c	2868808	s	O157	rec46	g	O157	++++		g		g	g	g	g	g	g	g	g	g	g	g	g	g	g	g	g	g	g	g	g
g	2579098	a	2798630	a	2868844	s	O157	rec46	a	CB9615	+		a		a	a	a	a	a	g	g	g	a	g	g	a	a	a	a	a	a	a	g	a
a	2579101	g	2798633	g	2868847	s	O157	rec46	g	CB9615	+		g		g	g	a	g	a	a	a	a	g	g	a	g	g	g	g	g	g	g	a	g
t	2579119	c	2798651	c	2868865	s	O157	rec46	c	CB9615	+		c		c	c	c	c	c	c	c	c	c	t	c	c	c	c	c	c	c	c	t	c
a	2579152	g	2798684	g	2868898	s	O157	rec46	g	CB9615	+		g		g	g	g	a	g	g	g	a	g	a	g	g	g	g	g	g	g	g	a	g
a	2579248	g	2798780	g	2868994	s	O157	rec46	a	O157	+		a		a	a	a	a	a	a	g	a	g	g	a	a	a	g	a	a	a	g	a	a
c	2579254	g	2798786	g	2869000	s	O157	rec46	c	O157	+		c		c	c	c	c	c	c	g	c	c	c	c	c	c	g	c	c	c	g	c	c
t	2579353	c	2798885	c	2869099	s	O157	rec46	c	CB9615	+		c		c	c	c	t	c	t	t	t	c	c	t	c	c	t	c	c	c	c	t	c
a	2579463	g	2798995	g	2869209	ns	O157	rec46	g	CB9615	++++		g		g	g	g	g	g	g	g	g	g	g	g	g	g	g	g	g	g	g	g	g
a	2579470	g	2799002	g	2869216	s	O157	rec46	g	CB9615	++++		g		g	g	g	g	g	g	g	g	g	g	g	g	g	g	g	g	g	g	a	g
a	2579509	g	2799041	g	2869255	s	O157	rec46	a	O157	+		a		g	g	a	a	a	g	a	a	a	g	g	a	a	g	a	a	a	g	a	g
a	2579515	g	2799047	g	2869261	s	O157	rec46	g	CB9615	++++		g		g	g	g	g	g	g	g	g	g	g	g	g	g	g	g	g	g	g	a	g
a	2579518	g	2799050	g	2869264	s	O157	rec46	a	O157	+		g		g	a	a	a	a	a	a	a	a	g	a	a	a	g	a	a	a	g	a	a
t	2579569	a	2799101	a	2869315	s	O157	rec46	a	CB9615	++++		a		a	a	g	g	a	g	a	g	a	t	g	a	a	a	a	a	a	a	a	a
g	2579611	c	2799143	c	2869357	s	O157	rec46	g	O157	+		g		g	c	g	g	c	g	c	g	c	c	g	g	g	g	g	g	g	g	g	c
t	2579620	g	2799152	g	2869366	ns	O157	rec46	t	O157	++++		t		t	t	c	c	t	c	t	c	t	t	c	t	t	t	t	t	t	t	t	t
a	2579632	g	2799164	g	2869378	s	O157	rec46	a	O157	+		g		g	g	a	a	g	a	g	a	g	a	a	a	a	a	a	a	a	a	g	a
a	2579652	g	2799184	g	2869398	ns	O157	rec46	g	CB9615	++++		g		a	g	g	g	g	g	g	g	g	g	g	g	g	g	g	g	g	g	g	g
a	2579683	g	2799215	g	2869429	s	O157	rec46	a	O157	+		a		a	g	a	a	g	a	g	a	a	g	a	a	a	a	a	a	a	a	a	g
g	2579689	a	2799221	a	2869435	s	O157	rec46	a	CB9615	+		g		a	a	a	a	a	a	a	a	g	a	a	a	a	a	g	g	g	a	a	a
g	2579734	a	2799266	a	2869480	s	O157	rec46	g	O157	+		g		g	g	a	a	g	a	g	a	a	g	a	g	g	g	g	g	g	g	g	g
g	2579749	a	2799281	a	2869495	s	O157	rec46	a	CB9615	+		a		g	g	a	a	g	a	a	g	a	g	a	a	a	a	g	g	g	g	g	g
c	2579806	t	2799338	t	2869552	s	O157	rec46	c	O157	++++		c		c	c	c	c	c	c	c	c	c	c	c	c	c	c	c	c	c	c	c	c
a	2579860	c	2799392	c	2869606	s	O157	rec46	a	O157	+		a		a	a	c	c	a	c	c	a	a	a	a	a	a	a	a	a	a	a	a	a
g	2579902	t	2799434	t	2869648	s	O157	rec46	t	CB9615	++++		t		t	t	t	t	t	t	t	t	t	t	t	t	t	t	t	t	t	t	t	t
a	2579920	g	2799452	g	2869666	s	O157	rec46	g	CB9615	+		g		g	g	g	g	a	g	g	g	g	g	g	g	g	g	a	a	a	g	a	a
g	2579956	a	2799488	a	2869702	s	O157	rec46	g	O157	++++		g		g	g	g	g	g	g	g	g	g	g	g	g	g	g	g	g	g	g	g	g
a	2579959	g	2799491	g	2869705	s	O157	rec46	a	O157	+		a		a	a	a	a	a	g	a	g	a	a	a	a	a	a	a	a	a	a	a	a
a	2579974	g	2799506	g	2869720	s	O157	rec46	a	O157	+		a		a	a	a	a	a	a	a	g	g	a	a	a	a	a	a	a	a	a	a	a
c	2579980	t	2799512	t	2869726	s	O157	rec46	c	O157	++++		c		c	c	c	c	c	c	c	c	c	c	c	c	c	c	c	c	c	c	c	c
a	2580015	g	2799547	g	2869761	s	O157	rec46	a	O157	+		a		a	a	g	g	a	g	g	g	a	a	g	a	g	a	a	a	a	a	g	g
g	2580016	c	2799548	c	2869762	s	O157	rec46	c	CB9615	+		t		t	t	t	c	t	c	c	c	g	g	c	t	c	t	t	t	t	t	c	c
c	2580028	t	2799560	t	2869774	s	O157	rec46	c	O157	+		c		c	c	t	t	c	t	c	t	c	c	t	c	c	c	c	c	c	c	c	c
c	2580035	t	2799567	t	2869781	ns	O157	rec46	c	O157	+		c		c	c	t	t	c	t	c	t	c	c	t	c	c	c	c	c	c	c	c	t
a	2580088	t	2799620	t	2869834	s	O157	rec46	a	O157	++++		c		c	c	a	a	a	a	a	g	a	a	a	a	a	a	a	a	a	c	a	a
g	2580124	a	2799656	a	2869870	s	O157	rec46	g	O157	++++		g		g	g	g	g	g	g	g	g	g	g	g	g	g	g	g	g	g	g	g	g
c	2580148	t	2799680	t	2869894	s	O157	rec46	c	O157	++++		c		c	c	c	c	c	c	c	c	c	c	c	c	c	c	c	c	c	c	c	c
g	2580157	a	2799689	a	2869903	s	O157	rec46	g	O157	++++		g		g	g	g	g	g	g	g	g	g	g	g	g	g	g	g	g	g	g	g	g
c	2580193	a	2799725	a	2869939	s	O157	rec46	c	O157	++++		c		c	c	c	c	c	c	c	c	c	c	c	c	c	c	c	c	c	c	c	c
g	2580280	a	2799812	a	2870026	s	O157	rec46	g	O157	+		a		g	g	a	g	g	g	g	g	a	g	g	g	g	g	g	g	g	g	g	g
c	2580313	a	2799845	a	2870059	s	O157	rec46	a	CB9615	++++		a		a	a	a	a	a	a	a	a	a	a	a	a	a	a	a	a	a	a	a	a
g	2580495	a	2800027	a	2870241	s	O157	rec46	g	O157	++++		g		g	g	g	g	g	g	g	g	g	g	g	g	g	g	g	g	g	g	g	g
c	2580549	t	2800081	t	2870295	s	O157	rec46	c	O157	+		c		t	c	c	t	c	c	c	c	c	t	c	c	c	c	c	c	c	c	t	c
g	2580558	t	2800090	t	2870304	s	O157	rec46	t	CB9615	+		t		t	t	t	t	t	g	t	t	t	t	g	t	t	t	t	t	t	t	t	t
t	2580570	a	2800102	a	2870316	s	O157	rec46	t	O157	+		a		t	t	t	a	t	t	t	t	t	a	t	t	t	t	t	t	t	t	t	t
g	2580573	a	2800105	a	2870319	s	O157	rec46	g	O157	+		a		a	g	g	a	g	g	g	g	g	a	g	g	g	g	g	g	g	g	a	a
c	2580597	t	2800129	t	2870343	s	O157	rec46	c	O157	+		t		c	c	c	c	c	c	c	c	c	c	c	c	t	c	c	c	c	c	c	c
a	2580717	g	2800249	g	2870463	s	O157	rec46	g	CB9615	+		g		a	g	a	g	g	a	g	a	a	a	a	g	g	g	g	g	g	g	a	a
t	2580733	c	2800265	c	2870479	ns	O157	rec46	c	CB9615	+		c		c	c	c	c	c	c	c	c	t	t	c	c	c	c	c	c	c	c	c	c
a	2580762	c	2800294	c	2870508	s	O157	rec46	c	CB9615	++++		c		c	c	c	a	c	c	c	c	c	c	c	c	c	c	c	c	c	c	c	c
a	2580816	g	2800348	g	2870562	s	O157	rec46	g	CB9615	+		g		g	g	g	g	g	g	g	g	g	a	g	g	g	g	g	g	g	g	a	a
a	2580831	t	2800363	t	2870577	s	O157	rec46	a	O157	++++		a		a	a	a	a	a	a	a	a	a	a	a	a	a	a	a	a	a	a	a	a
a	2580940	g	2800472	g	2870686	ns	O157	rec46	g	CB9615	+		g		g	g	g	g	g	g	g	g	g	g	g	g	g	g	g	g	g	g	a	a
a	2580942	g	2800474	g	2870688	s	O157	rec46	g	CB9615	+		a		a	g	g	g	a	g	a	g	g	g	g	g	g	a	g	g	g	a	a	a
g	2580948	a	2800480	a	2870694	s	O157	rec46	a	CB9615	+		a		a	a	a	a	a	g	a	g	g	g	g	a	a	a	a	a	a	a	g	g

O55 and O157 genome site details												Outgroup Strain Details[i]																						
CB9615 base[b]	CB9615 Site[c]	Sakai base[b]	Sakai Site[c]	EDL933 base[b]	EDL933 Site[c]	type[d]	Event Lineage[e,f]	Recombinant segment	Inferred ancestral base[g]	Outgroup Analysis[e]	Support level[h]	D1 Sd197	K-12	HS	ATCC 8739	UMN026	IAI39	SMS 3-5	E2348/69	536	ED1a	CFT073	S88	APEC O1	UTI89	E24377A	IAI1	SE11	F5 8401	F2a 2457T	F2a 301	SS Ss046	B4 Sb227	B18 BS512
a	2580966	g	2800498	g	2870712	s	O157	rec46	a	O157	+		a		a	a	g	g	a	a	a	a	a	a	g	a	g	a	a	a	a	a	a	a
t	2580972	c	2800504	c	2870718	s	O157	rec46	c	CB9615	+		c		c	c	c	c	c	t	c	t	t	t	c	c	c	c	c	c	c	c	t	t
t	2581002	c	2800534	c	2870748	s	O157	rec46	c	CB9615	+		c		c	c	c	c	c	c	c	c	t	t	c	c	c	c	c	c	c	c	t	t
g	2581056	a	2800588	a	2870802	s	O157	rec46	g	O157	+		g		g	g	g	g	g	g	g	g	g	g	g	a	g	g	a	a	a	g	g	g
g	2581158	a	2800690	a	2870904	s	O157	rec46	g	O157	++++		g		a	g	g	g	g	g	g	g	g	g	g	g	g	g	g	g	g	g	g	g
c	2581161	t	2800693	t	2870907	s	O157	rec46	c	O157	+		c		t	t	c	c	c	c	c	c	c	c	c	c	c	c	c	c	c	c	c	c
c	2581443	t	2800975	t	2871189	s	O157	rec46	c	O157	++++		c		c	c	c	c	c	c	c	c	c	c	c	c	c	c	c	c	c	c	c	c
a	2581512	c	2801044	c	2871258	s	O157	rec46	c	CB9615	+		c		c	c	c	c	c	c	c	c	c	c	c	c	c	c	c	c	c	c	a	a
t	2581521	g	2801053	g	2871267	s	O157	rec46	g	CB9615	+		g		t	g	g	g	g	g	g	g	g	g	g	g	g	g	g	g	g	g	t	t
a	2581718	t	2801250	t	2871464	ns	O157	rec46	a	O157	+		c		c	c	a	c	c	c	c	c	t	t	c	a	a	c	c	c	c	c	a	a
a	2581727	c	2801259	c	2871473	s	O157	rec46	c	CB9615	+		c		c	c	a	c	c	c	c	c	c	c	c	a	a	c	c	c	c	a	a	a
g	2581730	t	2801262	t	2871476	s	O157	rec46	t	CB9615	+		t		t	t	g	g	t	g	t	t	t	t	t	g	g	t	t	t	t	g	g	g
t	2581841	c	2801373	c	2871587	s	O157	rec46	c	CB9615	+		c		c	c	c	c	c	c	c	t	c	c	c	c	c	c	c	c	c	c	t	t
a	2581893	g	2801425	g	2871639	ns	O157	rec46	g	CB9615	+		g		g	g	g	g	g	g	g	g	g	g	g	g	g	g	g	g	g	g	a	a
a	2582000	g	2801532	g	2871746	s	O157	rec46	g	CB9615	+		g		g	g	g	g	g	g	g	g	g	g	g	g	g	g	g	g	g	g	a	a
a	2582018	t	2801550	t	2871764	s	O157	rec46	a	O157	+		a		a	a	a	t	a	t	a	t	g	a	a	g	t	a	t	t	t	g	a	a
c	2582203	t	2801735	t	2871949	s	O157	rec46	t	CB9615	+		t		c	t	t	t	t	t	t	t	t	t	t	t	c	t	t	t	t	t	c	c
a	2582209	g	2801741	g	2871955	s	O157	rec46	g	CB9615	+		g		g	g	g	g	g	g	g	g	g	g	g	g	g	g	g	g	g	g	a	a
t	2582308	c	2801840	c	2872054	s	O157	rec46	c	CB9615	+		c		c	t	c	c	c	c	c	c	c	c	c	c	c	c	c	c	c	c	t	t
t	2582329	c	2801861	c	2872075	s	O157	rec46	c	CB9615	+		c		c	t	c	c	t	c	c	c	c	c	c	c	c	t	c	c	c	c	t	t
g	2582344	a	2801876	a	2872090	s	O157	rec46	g	O157	+		a		a	g	g	g	g	g	a	g	a	g	a	a	g	g	a	a	a	g	g	g
c	2582359	g	2801891	g	2872105	s	O157	rec46	g	CB9615	+		g		g	c	c	c	c	c	g	c	g	c	c	g	c	g	g	g	g	g	c	c
a	2582404	t	2801936	t	2872150	s	O157	rec46	a	O157	++++		g		a	a	g	g	g	g	a	g	a	g	g	a	a	g	a	a	a	a	a	a
t	2582413	c	2801945	c	2872159	s	O157	rec46	t	O157	+		t		t	t	c	t	t	c	t	c	t	t	t	t	t	t	t	t	t	t	t	t
c	2582443	t	2801975	t	2872189	s	O157	rec46	c	O157	+		c		t	c	c	t	t	c	t	c	t	t	c	t	c	t	c	c	c	t	c	c
t	2582485	a	2802017	a	2872231	s	O157	rec46	t	O157	+		t		a	t	t	a	a	t	a	t	a	a	a	t	t	a	t	t	t	a	t	t
g	2582500	a	2802032	a	2872246	s	O157	rec46	a	CB9615	+		a		a	g	a	a	g	a	a	a	a	a	g	g	a	g	g	g	g	a	g	g
a	2582509	g	2802041	g	2872255	s	O157	rec46	g	CB9615	+		g		g	a	a	g	g	g	g	g	g	g	g	a	g	g	g	g	g	g	a	a
g	2582521	a	2802053	a	2872267	s	O157	rec46	a	CB9615	+		g		a	g	g	a	a	g	a	g	a	a	a	g	a	a	a	a	a	a	g	g
t	2582524	c	2802056	c	2872270	s	O157	rec46	c	CB9615	+		t		c	c	t	c	c	t	c	t	c	c	c	t	c	c	c	c	c	c	t	t
c	2582527	g	2802059	g	2872273	s	O157	rec46	c	O157	+		c		g	c	c	g	c	c	g	c	g	g	c	c	g	c	c	c	c	g	c	c
g	2582530	a	2802062	a	2872276	s	O157	rec46	g	O157	+		g		a	g	g	a	g	g	a	g	a	a	g	g	a	g	g	g	g	a	g	g
c	2582551	t	2802083	t	2872297	s	O157	rec46	t	CB9615	+		t		t	c	t	c	t	t	t	t	t	c	t	c	t	t	a	a	a	t	c	c
a	2582569	g	2802101	g	2872315	s	O157	rec46	g	CB9615	+		g		a	g	a	a	g	g	g	g	a	a	a	g	g	g	a	a	a	g	a	a
a	2582584	t	2802116	t	2872330	s	O157	rec46	a	O157	+		t		t	a	a	a	a	a	t	a	a	a	a	a	a	a	a	a	a	t	a	a
a	2582650	g	2802182	g	2872396	s	O157	rec46	g	CB9615	+		g		g	g	g	a	g	g	a	a	g	a	g	a	g	g	g	g	g	g	a	a
a	2582704	g	2802236	g	2872450	s	O157	rec46	a	O157	+		g		g	g	g	a	a	g	a	a	a	a	a	a	a	g	g	g	g	a	a	a
g	2582788	a	2802320	a	2872534	s	O157	rec46	g	O157	+		g		g	g	a	a	g	g	a	g	g	a	a	g	g	g	g	g	g	g	g	g
c	2582842	g	2802374	g	2872588	s	O157	rec46	c	O157	++++		c		c	c	c	c	c	c	c	c	c	c	c	c	c	c	c	c	c	c	c	c
g	2582866	a	2802398	a	2872612	ns	O157	rec46	a	CB9615	+		a		g	a	a	a	g	g	a	a	a	a	a	a	g	a	g	g	g	g	g	g
t	2582868	g	2802400	g	2872614	ns	O157	rec46	g	CB9615	+		g		t	g	g	g	g	t	g	g	g	g	g	g	t	g	g	g	g	g	t	t
a	2582893	g	2802425	g	2872639	s	O157	rec46	g	CB9615	+		g		g	g	g	c	c	c	g	c	c	g	c	g	c	g	g	g	g	c	a	a
t	2583023	c	2802555	c	2872769	ns	O157	rec46	t	O157	+		t		t	t	t	t	c	t	t	t	c	t	c	t	t	t	t	t	t	t	t	t
g	2583031	a	2802563	a	2872777	i	O157	rec46	g	O157	++++		g		g	g	g	g	g	g	g	g	g	g	g	g	g	g	g	g	g	g	g	g
t	2583210	a	2802742	a	2872956	s	O157	rec46	a	CB9615	+		a		t	t	a	a	a	a	a	c	t	a	a	a	a	a	t	t	t	a	t	t
g	2583231	a	2802763	a	2872977	s	O157	rec46	g	O157	+		g		g	g	g	g	a	g	g	g	g	g	g	g	g	a	a	a	a	a	g	g
g	2583252	a	2802784	a	2872998	s	O157	rec46	a	CB9615	+		a		a	g	g	a	a	a	c	g	a	a	a	c	a	g	a	a	a	a	a	a
g	2583264	a	2802796	a	2873010	s	O157	rec46	g	O157	++++		g		g	g	g	g	g	g	g	g	g	g	g	g	g	g	g	g	g	g	g	g
a	2583300	g	2802832	g	2873046	s	O157	rec46	g	CB9615	++++		g		g	g	g	g	g	g	g	g	g	g	g	g	g	g	g	g	g	g	g	g
a	2583315	g	2802847	g	2873061	s	O157	rec46	g	CB9615	+		g		g	g	g	g	g	a	g	a	a	g	g	g	a	g	a	a	a	a	a	a
c	2583378	t	2802910	t	2873124	s	O157	rec46	c	O157	+		c		c	c	c	t	c	c	c	t	t	c	t	c	t	t	t	t	t	c	c	c
a	2583399	c	2802931	c	2873145	s	O157	rec46	c	CB9615	+		c		c	a	c	a	c	g	a	c	c	g	a	a	c	c	c	c	c	c	c	c
a	2583402	g	2802934	g	2873148	s	O157	rec46	g	CB9615	+		g		g	a	g	a	g	a	a	g	g	a	a	a	g	g	g	g	g	g	g	g
c	2583417	t	2802949	t	2873163	s	O157	rec46	c	O157	+		c		c	c	c	c	c	t	t	c	t	c	c	t	c	t	c	c	c	c	c	c
t	2583420	c	2802952	c	2873166	s	O157	rec46	c	CB9615	+		c		c	c	t	t	c	c	c	t	c	t	c	c	t	c	c	c	c	c	c	c
g	2583639	a	2803171	a	2873385	s	O157	rec46	g	O157	+		a		a	g	g	g	g	g	g	g	g	g	g	g	g	g	g	g	g	g	a	a
a	2583648	g	2803180	g	2873394	s	O157	rec46	a	O157	++++		a		a	a	a	a	a	a	a	a	a	a	a	a	a	a	a	a	a	a	a	a
t	2583696	c	2803228	c	2873442	s	O157	rec46	c	CB9615	+		c		c	t	t	t	t	c	c	c	t	c	c	c	c	c	c	c	c	c	c	c
t	2583714	c	2803246	c	2873460	s	O157	rec46	t	O157	+		c		c	c	t	c	t	c	t	c	t	t	t	t	t	c	t	t	t	t	c	c
a	2583837	g	2803369	g	2873583	s	O157	rec46	g	CB9615	+		g		g	a	a	a	a	a	g	a	g	a	a	g	g	g	g	g	g	g	g	g
t	2583846	a	2803378	a	2873592	s	O157	rec46	a	CB9615	+		a		a	t	t	t	t	t	a	t	a	a	t	a	a	a	a	a	a	a	a	a
g	2583936	a	2803468	a	2873682	s	O157	rec46	g	O157	++++		g		g	g	g	g	g	g	g	g	g	g	g	g	g	g	g	g	g	g	g	g
a	2584050	g	2803582	g	2873796	s	O157	rec46	g	CB9615	+		g		g	g	g	g	a	a	g	a	g	a	a	g	g	g	g	g	g	g	g	g
-	2584170	t	2803703	t	2873917	ins-2	O157	rec46	-	O157	++++		-		-	-	-	-	-	-	-	-	-	-	-	-	-	-	-	-	-	-	-	-
c	2584201	t	2803735	t	2873949	ns	O157	rec46	c	O157	+		c		c	c	t	t	c	t	c	t	c	c	t	c	c	c	c	c	c	c	c	c
g	2584210	c	2803744	c	2873958	ns	O157	rec46	c	CB9615	++++		c		c	c	c	c	c	c	c	c	c	c	c	c	c	c	c	c	c	c	c	c
g	2584319	a	2803853	a	2874067	s	O157	rec46	g	O157	+		g		g	a	a	a	a	g	g	g	a	g	g	g	g	a	g	g	g	g	g	g
a	2584403	c	2803937	c	2874151	s	O157	rec46	a	O157	+		a		a	c	c	c	a	c	c	c	a	a	c	a	a	a	a	a	a	a	a	a
c	2584481	g	2804015	g	2874229	ns	O157	rec46	g	CB9615	+		g		g	c	c	g	g	c	g	c	g	c	g	g	g	c	g	g	g	g	g	g
g	2584497	a	2804031	a	2874245	ns	O157	rec46	g	O157	++++		g		g	g	g	g	g	g	g	g	g	g	g	g	g	g	g	g	g	g	g	g
a	2584598	t	2804132	t	2874346	s	O157	rec46	a	O157	+		a		t	a	t	t	t	t	t	t	t	t	t	a	a	t	a	a	a	a	a	a
a	2584639	t	2804173	t	2874387	ns	O157	rec46	t	CB9615	+		t		t	t	a	a	t	t	t	t	t	t	t	t	t	a	t	t	t	t	t	t
a	2584715	g	2804249	g	2874463	s	O157	rec46	g	CB9615	+		g		g	g	a	a	g	a	g	a	a	a	a	g	a	g	g	g	g	g	g	g
t	2584787	c	2804321	c	2874535	s	O157	rec46	t	O157	+		t		c	c	t	c	t	t	t	t	t	t	t	t	t	t	t	t	t	t	t	t
t	2584811	a	2804345	a	2874559	s	O157	rec46	a	CB9615	+		a		t	t	t	t	a	a	t	a	a	a	a	t	a	t	t	t	t	a	a	a
g	2584817	a	2804351	a	2874565	s	O157	rec46	a	CB9615	+		a		g	g	g	g	a	a	g	a	a	a	a	g	a	g	g	g	g	a	a	a
c	2584838	t	2804372	t	2874586	s	O157	rec46	t	CB9615	+		t		t	t	t	c	t	c	c	c	c	c	t	t	t	t	c	c	c	t	t	t
g	2584856	c	2804390	c	2874604	s	O157	rec46	g	O157	+		c		c	g	c	g	c	g	g	g	g	g	g	g	c	g	g	g	g	c	c	c
t	2584868	c	2804402	c	2874616	s	O157	rec46	c	CB9615	++++		c		c	c	c	t	c	c	c	c	c	c	c	c	c	c	c	c	c	c	c	c
g	2584904	a	2804438	a	2874652	s	O157	rec46	g	O157	+		g		a	a	g	a	g	a	t	a	a	a	a	g	g	g	a			g	g	g
a	2584940	t	2804474	t	2874688	s	O157	rec46	t	CB9615	++++		t		t	t	t	t	t	t	t	t	t	t	t	t	t	t	t			t	t	t
g	2584949	t	2804483	t	2874697	s	O157	rec46	g	O157	++++		g		t	g	g	a	g	g	g	g	a	a	g	g	g	g	g			g	g	g
c	2584967	t	2804501	t	2874715	s	O157	rec46	t	CB9615	+		t		t	c	t	t	t	t	c	c	c	c	c	c	t	t	t			t	t	t
g	2584979	a	2804513	a	2874727	s	O157	rec46	g	O157	+		g		a	a	g	a	g	g	a	a	g	g	a	a	g	g	a			g	g	g
g	2584982	a	2804516	a	2874730	s	O157	rec46	g	O157	+		g		a	g	g	g	g	g	g	g	g	g	g	g	g	g	a			g	g	g
g	2585024	a	2804558	a	2874772	s	O157	rec46	g	O157	++++		g		a	g	g	g	g	g	g	g	g	g	g	g	g	g	g			g	g	g

O55 and O157 genome site details												Outgroup Strain Details[i]																						
CB9615 base[b]	CB9615 Site[c]	Sakai base[b]	Sakai Site[c]	EDL933 base[b]	EDL933 Site[c]	type[d]	Event Lineage[ef]	Recombinant segment	Inferred ancestral base[g]	Outgroup Analysis[e]	Support level[h]	D1 Sd197	K-12	HS	ATCC 8739	UMN026	IAI39	SMS 3-5	E2348/69	536	ED1a	CFT073	S88	APEC O1	UTI89	E24377A	IAI1	SE11	F5 8401	F2a 2457T	F2a 301	SS Ss046	B4 Sb227	B18 BS512
a	2585084	g	2804618	g	2874832	s	O157	rec46	g	CB9615	+		g		a	g	c	g	g	c	g	a	g	g	a	a	g	a	g			g	g	g
t	2585191	a	2804725	a	2874939	ns	O157	rec46	t	O157	+		t		a	t	t	t	t	t	t	t	a	a	a	t	t	t	t			t	t	t
c	2585327	a	2804861	a	2875075	ns	O157	rec46	a	CB9615	+		a		a	a	a	c	a	a	a	a	c	c	a	a	a	a	a			a	a	a
g	2585351	a	2804885	a	2875099	s	O157	rec46	g	O157	++++		g		g	g	g	g	g	g	g	g	g	g	g	g	g	g	g			g	g	g
a	2585354	g	2804888	g	2875102	s	O157	rec46	g	CB9615	+		g		g	g	g	g	g	a	g	g	g	g	g	g	g	g	a			g	g	g
g	2585360	a	2804894	a	2875108	s	O157	rec46	a	CB9615	+		g		a	a	a	a	a	g	a	g	a	a	g	g	a	a	g			a	g	g
g	2585402	t	2804936	t	2875150	ns	O157	rec46	g	O157	+		t		g	g	g	g	g	g	g	g	g	g	g	t	t	g	g			t	t	t
g	2585432	a	2804966	a	2875180	s	O157	rec46	a	CB9615	+		a		a	a	a	a	a	a	a	a	g	g	g	a	a	a	a			a	a	a
g	2585451	t	2804985	t	2875199	ns	O157	rec46	t	CB9615	+		t		t	t	g	g	t	t	t	g	t	t	t	t	t	t	t			t	t	t
c	2585572	a	2805106	a	2875320	ns	O157	rec46	a	CB9615	++++		a		a	a	a	a	a	a	a	a	a	a	a	a	a	a	a			a	a	a
t	2585703	c	2805237	c	2875451	s	O157	rec46	c	CB9615	+		c		c	c	t	c	c	c	c	c	c	c	t	t	c	c	c			t	c	c
g	2585712	a	2805246	a	2875460	s	O157	rec46	g	O157	++++		g		g	g	g	g	g	g	g	g	g	g	g	g	g	g	g			g	g	g
t	2585886	c	2805420	c	2875634	s	O157	rec46	c	CB9615	+		t		c	c	t	c	c	c	c	t	t	t	c	c	c	a	c			c	t	t
a	2585967	t	2805501	t	2875715	s	O157	rec46	a	O157	++++		a		a	a	a	a	a	a	a	a	a	a	a	a	a	a	a	a	a	a	a	a
g	2586117	t	2805651	t	2875865	s	O157	rec46	g	O157	+		t		g	g	g	g	g	g	t	g	g	g	t	g	g	g	t	t	t	g	t	t
a	2586180	g	2805714	g	2875928	s	O157	rec46	g	CB9615	++++		g		g	g	g	g	g	g	g	g	g	g	g	g	g	g	g	g	g	g	g	g
a	2586231	g	2805765	g	2875979	s	O157	rec46	a	O157	+		a		a	g	a	a	a	g	a	g	a	a	a	a	g	a	a	a	a	a	a	a
g	2586432	a	2805966	a	2876180	s	O157	rec46	g	O157	++++		g		g	g	g	g	g	g	g	g	g	g	g	g	g	g	g	g	g	g	g	g
a	2586447	g	2805981	g	2876195	s	O157	rec46	a	O157	++++		a		a	a	a	a	a	a	a	a	a	a	a	a	a	a	a	a	a	a	a	a
g	2586465	a	2805999	a	2876213	s	O157	rec46	g	O157	+		a		g	g	g	g	g	g	g	g	g	g	g	g	g	a	a	a	a	g	a	a
a	2586494	g	2806028	g	2876242	s	O157	rec46	a	O157	+		g		g	a	a	a	g	g	a	a	a	a	a	g	a	g	a	a	a	g	g	g
c	2586536	a	2806070	a	2876284	ns	O157	rec46	c	O157	++++		c		c	c	c	c	c	c	c	c	c	c	c	c	c	c	c	c	c	c	c	c
c	2586647	t	2806181	t	2876395	ns	O157	rec46	c	O157	++++		c		c	c	c	c	c	c	c	c	c	c	c	c	c	c	c	c	c	c	c	c
a	2586665	g	2806199	g	2876413	s	O157	rec46	g	CB9615	++++		g		g	a	g	g	g	g	g	g	g	g	g	g	g	g	g	g	g	g	g	g
a	2586672	g	2806206	g	2876420	s	O157	rec46	g	CB9615	+		a		g	a	a	a	g	g	g	g	g	g	g	g	g	a	g	g	g	g	a	a
a	2586754	g	2806288	g	2876502	s	O157	rec46	g	CB9615	++++		g		g	g	g	g	g	g	g	g	g	g	g	g	g	g	g	g	g	g	g	g
t	2586795	a	2806329	a	2876543	ns	O157	rec46	t	O157	++++		t		t	t	t	t	t	t	t	t	t	t	t	t	t	t	t	t	t	t	t	t
c	2586826	g	2806360	g	2876574	s	O157	rec46	c	O157	++++		c		c	c	c	c	c	c	c	c	c	c	c	c	c	c	t	t	t	c	c	c
g	2586841	c	2806375	c	2876589	ns	O157	rec46	c	CB9615	+		c		c	g	c	c	c	c	g	c	a	a	a	c	c	c	c	c	c	g	c	c
g	2586879	a	2806413	a	2876627	s	O157	rec46	a	CB9615	+		a		g	a	a	g	a	g	g	g	a	a	a	a	g	a	g	g	g	a	a	a
c	2586886	a	2806420	a	2876634	s	O157	rec46	c	O157	+		c		c	c	a	c	c	a	c	c	a	a	a	c	c	c	c	c	c	c	c	c
c	2586928	t	2806462	t	2876676	s	O157	rec46	c	O157	+		c		c	c	c	c	c	t	c	c	c	c	c	c	c	c	c	c	c	t	c	c
g	2586933	a	2806467	a	2876681	s	O157	rec46	g	O157	+		g		g	g	a	g	g	a	g	a	g	g	g	g	g	g	g	g	g	a	g	g
t	2586979	c	2806513	c	2876727	s	O157	rec46	t	O157	+		t		t	t	t	t	c	c	c	c	t	t	t	c	c	c	c	c	c	t	t	t
a	2587096	g	2806630	g	2876844	s	O157	rec46	g	CB9615	+		a		g	g	g	a	g	g	g	g	g	g	a	g	g	a	g	g	g	g	g	g
a	2587123	g	2806657	g	2876871	s	O157	rec46	a	O157	+		g		a	g	g	a	g	g	a	g	g	g	a	g	a	a	a	a	a	a	a	a
c	2587126	t	2806660	t	2876874	s	O157	rec46	c	O157	+		t		c	t	t	t	c	c	c	c	t	t	t	c	c	c	c	c	c	c	c	c
c	2587144	t	2806678	t	2876892	s	O157	rec46	t	CB9615	+		t		t	t	t	t	t	t	c	t	t	t	t	t	c	c	t	t	t	c	t	t
c	2587171	t	2806705	t	2876919	s	O157	rec46	c	O157	++++		c		c	c	c	c	c	c	c	c	c	c	c	c	c	c	c	c	c	c	c	c
g	2587219	a	2806753	a	2876967	s	O157	rec46	g	O157	+		g		a	g	g	g	g	g	g	g	g	g	g	g	g	g	a	a	a	g	a	a
t	2587234	c	2806768	c	2876982	s	O157	rec46	t	O157	++++		t		t	t	t	t	t	t	t	t	t	t	t	t	t	t	t	t	t	t	t	t
g	2587252	a	2806786	a	2877000	s	O157	rec46	g	O157	+		g		a	g	a	g	g	g	a	g	a	a	g	g	a	a	g	g	g	a	a	a
a	2587255	g	2806789	g	2877003	s	O157	rec46	a	O157	+		a		g	a	g	a	a	a	a	a	g	g	a	a	a	a	a	a	a	a	a	a
a	2587280	g	2806814	g	2877028	ns	O157	rec46	g	CB9615	++++		g		g	g	g	g	g	g	g	g	g	g	g	g	g	g	g	g	g	g	g	g
a	2587291	g	2806825	g	2877039	s	O157	rec46	a	O157	+		g		g	a	g	a	a	a	a	a	a	a	a	g	a	a	a	a	a	g	g	g
a	2587303	g	2806837	g	2877051	s	O157	rec46	g	CB9615	+		g		g	a	g	a	a	a	g	a	g	g	a	g	g	g	g	g	g	g	g	g
t	2587393	c	2806927	c	2877141	s	O157	rec46	t	O157	+		t		t	t	t	t	t	t	c	c	t	t	t	t	t	c	c	c	c	t	c	c
g	2587396	c	2806930	c	2877144	s	O157	rec46	g	O157	+		g		g	g	g	g	g	g	c	g	g	g	g	g	c	g	g	g	g	g	c	c
a	2587420	g	2806954	g	2877168	s	O157	rec46	g	CB9615	+		g		a	a	a	a	g	a	g	g	a	a	g	a	g	g	g	g	g	g	g	g
t	2587451	g	2806985	g	2877199	ns	O157	rec46	g	CB9615	++++		g		g	t	g	g	g	g	g	g	g	g	g	g	g	g	g	g	g	g	g	g
g	2587465	a	2806999	a	2877213	s	O157	rec46	g	O157	+		g		g	a	g	g	g	g	g	g	g	g	g	a	a	g	g	g	g	g	a	a
t	2587489	c	2807023	c	2877237	s	O157	rec46	t	O157	+		t		t	t	t	t	t	t	t	t	t	t	t	c	c	t	t	t	t	t	t	t
a	2587507	g	2807041	g	2877255	s	O157	rec46	g	CB9615	+		g		g	a	g	g	g	g	a	g	g	g	g	g	g	g	g	g	g	g	g	g
t	2587513	c	2807047	c	2877261	s	O157	rec46	c	CB9615	+		c		c	t	t	t	c	c	c	t	c	c	c	c	c	t	c	c	c	c	c	c
a	2587515	g	2807049	g	2877263	s	O157	rec46	g	CB9615	+		g		g	a	g	g	g	g	g	g	g	g	g	g	g	a	g	g	g	g	g	g
a	2587527	g	2807061	g	2877275	s	O157	rec46	g	CB9615	++++		g		g	a	g	g	g	g	g	g	g	g	g	g	g	g	g	g	g	g	g	g
t	2587533	c	2807067	c	2877281	ns	O157	rec46	c	CB9615	++++		c		c	t	c	c	c	c	c	c	c	c	c	c	c	c	c	c	c	c	c	c
t	2587555	c	2807089	c	2877303	s	O157	rec46	t	O157	+		t		c	t	c	t	c	t	c	t	t	t	t	c	c	c	t	t	t	t	c	c
c	2587588	t	2807122	t	2877336	s	O157	rec46	t	CB9615	+		t		t	c	t	c	t	t	c	c	c	c	t	t	t	c	c	c	c	t	t	t
a	2587590	g	2807124	g	2877338	s	O157	rec46	g	CB9615	+		g		g	a	g	a	g	g	a	a	a	a	g	g	g	a	a	a	a	g	g	g
t	2587630	a	2807164	a	2877378	s	O157	rec46	a	CB9615	++++		a		a	a	a	a	a	g	a	g	a	a	a	a	a	g	a	a	a	a	a	a
g	2587639	a	2807173	a	2877387	s	O157	rec46	g	O157	+		g		g	g	g	g	g	g	g	g	g	g	g	a	a	g	g	g	g	g	a	a
t	2587867	c	2807401	c	2877615	s	O157	rec46	t	O157	+		t		t	c	c	t	c	t	t	t	t	t	t	t	t	t	t	t	t	t	t	t
t	2588057	c	2807591	c	2877805	ns	O157	rec46	c	CB9615	++++		c		c	c	c	c	c	c	c	c	c	c	c	c	c	c	c	c	c	c	c	c
t	2588109	g	2807643	g	2877857	s	O157	rec46	g	CB9615	++++		g		g	g	g	g	g	g	g	g	g	g	g	g	g	g	g	g	g	g	g	g
a	2588127	g	2807661	g	2877875	s	O157	rec46	g	CB9615	+		a		g	g	g	g	a	g	g	g	g	g	g	g	g	g	g	g	g	g	g	g
a	2588160	c	2807694	c	2877908	s	O157	rec46	c	CB9615	++++		c		c	c	c	c	c	c	c	c	c	c	c	c	c	c	c	c	c	c	c	c
c	2588280	a	2807814	a	2878028	s	O157	rec46	c	O157	+		c		a	c	c	c	c	a	c	a	c	c	c	c	a	c	c	c	c	c	a	a
g	2588292	a	2807826	a	2878040	s	O157	rec46	g	O157	++++		g		g	g	g	g	g	g	g	g	g	g	g	g	g	g	g	g	g	g	g	g
a	2588328	g	2807862	g	2878076	s	O157	rec46	g	CB9615	+		a		a	a	g	g	a	g	a	g	g	g	g	a	g	g	g	g	g	a	g	g
a	2588331	g	2807865	g	2878079	s	O157	rec46	g	CB9615	+		a		a	g	g	g	a	g	g	a	g	g	g	a	g	g	g	g	g	a	g	g
t	2588343	a	2807877	a	2878091	s	O157	rec46	a	CB9615	+		a		a	a	a	a	a	a	a	a	a	a	a	t	a	a	t	t	t	a	a	a
c	2588382	t	2807916	t	2878130	s	O157	rec46	c	O157	++++		c		c	c	c	c	c	c	c	c	c	c	c	c	c	t	c	c	c	c	c	c
c	2588397	a	2807931	a	2878145	i	O157	rec46	c	O157	++++		c		c	c	c	c	c	c	c	c	c	c	c	c	c	c	c	c	c	c	c	c
g	2588477	a	2808011	a	2878225	s	O157	rec46	a	CB9615	+		a		a	a	g	g	g	g	a	g	g	g	a	a	a	a	a	a	a	a	a	a
g	2588522	a	2808056	a	2878270	s	O157	rec46	g	O157	+		g		g	g	g	g	a	g	g	g	g	g	a	a	g	a	a	a	a	g	g	g
g	2588528	a	2808062	a	2878276	s	O157	rec46	g	O157	+		g		g	a	g	g	c	g	a	g	g	g	g	a	g	a	g	g	g	a	g	g
a	2588534	g	2808068	g	2878282	s	O157	rec46	g	CB9615	+		g		g	g	g	g	g	g	a	g	g	g	g	g	g	g	g	g	g	a	a	a
g	2588663	a	2808197	a	2878411	s	O157	rec46	g	O157	+		g		g	a	g	g	a	g	a	g	g	g	a	a	a	g	g	g	g	g	g	g
t	2588855	g	2808389	g	2878603	s	O157	rec46	g	CB9615	+		g		g	g	g	g	t	g	t	g	g	g	g	g	g	g	t	t	t	t	t	t
g	2588870	a	2808404	a	2878618	s	O157	rec46	g	O157	++++		g		g	g	g	g	g	g	g	g	g	g	g	g	g	g	g	g	g	g	g	g
a	2588933	g	2808467	g	2878681	s	O157	rec46	a	O157	+		a		a	a	g	g	a	g	g	g	g	g	a	g	a	a	g	g	g	a	a	a
t	2588942	c	2808476	c	2878690	s	O157	rec46	t	O157	+		t		t	g	t	t	t	t	c	t	t	t	g	c	t	t	c	c	c	t	t	t
a	2588957	g	2808491	g	2878705	s	O157	rec46	g	CB9615	+		g		g	a	a	a	g	g	g	g	a	a	a	g	g	g	a	a	a	g	a	a
a	2588999	c	2808533	c	2878747	s	O157	rec46	c	CB9615	+		c		c	c	c	c	c	c	c	c	c	c	c	c	c	c	c	c	c	c	a	a
a	2589050	g	2808584	g	2878798	s	O157	rec46	a	O157	+		a		a	a	a	a	g	a	g	a	a	a	a	g	a	g	a	a	a	g	a	a

O55 and O157 genome site details												Outgroup Strain Details[i]																						
CB9615 base[b]	CB9615 Site[c]	Sakai base[b]	Sakai Site[c]	EDL933 base[b]	EDL933 Site[c]	type[d]	Event Lineage[ef]	Recombinant segment	Inferred ancestral base[g]	Outgroup Analysis[e]	Support level[h]	D1 Sd197	K-12	HS	ATCC 8739	UMN026	IAI39	SMS 3-5	E2348/69	536	ED1a	CFT073	S88	APEC O1	UTI89	E24377A	IAI1	SE11	F5 8401	F2a 2457T	F2a 301	SS Ss046	B4 Sb227	B18 BS512
g	2589053	a	2808587	a	2878801	s	O157	rec46	g	O157	+		g		g	g	g	g	a	g	a	g	g	g	g	g	g	g	g	g	g	g	g	g
t	2589246	a	2808780	a	2878994	i	O157	rec46	t	O157	++++		t		t	t	t	t	a	t	t	t	t	t	t	t	t	t	t	t	t	t	t	t
g	2589327	c	2808861	c	2879075	i	O157	rec46	g	O157	+		g		g	g	g	g	g	g		g	c	c	c	g	g	g	g	g	g	g	c	c
a	2589436	g	2808970	g	2879184	s	O157	rec46	g	CB9615	+		g		g	g	a	a	g	g	g	g	g	g	a	g	g	g	g	g	g	g	g	g
a	2589586	g	2809120	g	2879334	s	O157	rec46	a	O157	+		a		a	g	a	g	a	g	a	g	g	g	a	a	a	a	a	a	a	g	a	a
c	2589605	t	2809139	t	2879353	ns	O157	rec46	t	CB9615	+		t		t	c	t	t	t	t	t	t	t	t	t	t	t	t	t	t	t	c	t	t
t	2589634	c	2809168	c	2879382	s	O157	rec46	c	CB9615	+		c		c	c	c	t	c	c	c	c	c	c	c	c	c	c	t	t	t	c	c	c
c	2589658	t	2809192	t	2879406	s	O157	rec46	c	O157	+		c		c	t	t	c	t	c	c	t	c	c	t	c	c	c	c	c	c	c	c	c
g	2589712	t	2809246	t	2879460	s	O157	rec46	g	O157	++++		g		g	g	g	a	g	g	g	g	g	g	g	g	g	g	g	g	g	g	g	g
t	2590123	c	2809657	c	2879871	s	O157	rec46	t	O157	+		t		c	t	c	t	c	c	t	c	c	c	c	c	c	t	t	t	t	c	t	t
a	2590138	g	2809672	g	2879886	s	O157	rec46	g	CB9615	++++		g		g	g	g	g	g	a	g	g	g	g	g	g	g	g	g	g	g	g	g	g
a	2590168	c	2809702	c	2879916	s	O157	rec46	a	O157	++++		a		a	a	a	a	a	a	a	a	a	a	a	a	a	a	a	a	a	a	a	a
a	2590189	g	2809723	g	2879937	s	O157	rec46	g	CB9615	++++		g		g	g	g	g	g	g	g	g	g	g	g	g	g	g	g	g	g	g	g	g
t	2590294	c	2809828	c	2880042	s	O157	rec46	t	O157	+		c		t	t	t	t	t	t	t	t	t	t	t	t	t	t	t	t	t	t	t	t
c	2590381	t	2809915	t	2880129	s	O157	rec46	c	O157	+		c		t	t	c	c	t	t	c	t	c	c	c	t	c	c	t	t	t	c	c	c
t	2590390	g	2809924	g	2880138	s	O157	rec46	g	CB9615	++++		g		g	g	g	g	g	g	g	g	g	g	g	g	g	g	g	g	g	g	g	g
g	2590417	a	2809951	a	2880165	s	O157	rec46	g	O157	+		g		a	a	g	g	a	a	g	a	a	a	g	a	g	g	g	g	g	g	g	g
c	2590477	g	2810011	g	2880225	s	O157	rec46	c	O157	+		c		g	c	c	c	c	c	c	c	c	c	c	g	c	c	c	c	c	c	c	c
a	2590690	g	2810224	g	2880438	s	O157	rec46	g	CB9615	++++		g		g	g	g	g	a	g	g	g	g	g	g	g	g	g	g	g	g	g	g	g
c	2590957	t	2810491	t	2880705	ns	O157	rec46	c	O157	++++		c		c	c	c	c	c	c	c	c	c	c	c	c	c	c	c	c	c	c	c	c
a	2590996	g	2810530	g	2880744	s	O157	rec46	a	O157	+		g		a	g	g	g	a	g	a	a	g	g	g	a	a	a	a	a	a	a	a	g
a	2591017	g	2810551	g	2880765	ns	O157	rec46	g	CB9615	+		g		a	a	a	a	a	g	a	g	a	a	g	g	g	a	g	g	g	a	a	g
c	2591019	t	2810553	t	2880767	ns	O157	rec46	c	O157	+		t		t	c	c	c	c	t	t	c	c	c	t	c	c	t	c	c	c	t	t	t
g	2591051	t	2810585	t	2880799	ns	O157	rec46	g	O157	++++		g		g	g	g	g	g	g	g	g	g	g	g	g	g	g	g	g	g	g	g	g
g	2591065	a	2810599	a	2880813	s	O157	rec46	g	O157	++++		g		g	g	g	g	g	t	g	g	g	g	a	g	g	g	g	g	g	g	g	g
g	2591077	a	2810611	a	2880825	s	O157	rec46	g	O157	+		g		g	g	g	g	g	g	g	g	g	g	g	g	a	g	a	a	a	a	a	g
t	2591092	c	2810626	c	2880840	s	O157	rec46	c	CB9615	++++		c		c	c	c	c	t	c	c	c	c	c	c	c	c	c	c	c	c	c	c	c
a	2591101	g	2810635	g	2880849	s	O157	rec46	a	O157	+		a		a	g	g	g	a	a	a	a	g	g	a	a	a	a	a	a	a	g	g	a
g	2591179	a	2810713	a	2880927	s	O157	rec46	g	O157	++++		g		g	g	g	g	g	g	g	g	g	g	g	g	g	g	g	g	g	g	g	g
g	2591365	a	2810899	a	2881113	s	O157	rec46	g	O157	++++		g		g	g	g	g	a	g	g	g	g	g	g	g	g	g	g	g	g	g	g	g
a	2591371	g	2810905	g	2881119	s	O157	rec46	a	O157	+		g		a	a	a	a	g	a	a	a	a	a	g	a	a	a	a	a	a	a	a	a
a	2591427	g	2810961	g	2881175	s	O157	rec46	g	CB9615	++++		g		g	g	g	g	g	g	g	g	g	g	g	g	g	g	g	g	g	g	g	g
t	2591853	c	2811387	c	2881601	s	O157	rec46	c	CB9615	+		c		t	c	c	c	t	c	t	n	c	c	c	c	c	c	t	t	t	t	t	c
c	2592110	g	2811644	g	2881858	s	O157	rec46	g	CB9615	+		g		g	g	g	g	c	c	c	c	g	g	g	c	g	c	c	c	c	g	g	c
c	2592146	t	2811680	t	2881894	s	O157	rec46	c	O157	++++		c		c	c	c	c	c	c	c	c	c	c	c	c	c	c	c	c	c	c	c	c
a	2592175	g	2811709	g	2881923	s	O157	rec46	g	CB9615	++++		g		g	g	g	a	g	g	g	g	g	g	g	g	g	g	g	g	g	g	g	g
a	2592194	g	2811728	g	2881942	s	O157	rec46	g	CB9615	++++		g		g	g	g	g	g	g	g	g	g	g	g	g	g	g	g	g	g	g	g	g
a	2592224	g	2811758	g	2881972	s	O157	rec46	a	O157	+		g		a	g	g	a	a	g	g	g	g	g	g	a	a	a	a	a	a	a	a	a
g	2592230	a	2811764	a	2881978	s	O157	rec46	a	CB9615	+		a		a	a	a	a	a	a	a	a	a	a	a	g	g	g	g	g	g	a	a	g
a	2592242	g	2811776	g	2881990	s	O157	rec46	g	CB9615	+		g		a	g	g	g	g	g	g	g	g	g	g	g	a	g	a	a	a	g	g	g
a	2592248	g	2811782	g	2881996	s	O157	rec46	a	O157	++++		a		a	a	a	a	a	a	a	a	a	a	a	a	a	a	a	a	a	a	a	a
g	2592368	t	2811902	t	2882116	s	O157	rec46	t	CB9615	+		g		t	t	t	t	t	t	t	t	t	t	t	t	t	t	t	t	t	t	t	t
a	2592371	g	2811905	g	2882119	s	O157	rec46	g	CB9615	+		a		g	g	g	g	g	g	g	g	g	g	g	g	g	g	g	g	g	g	g	g
g	2592377	a	2811911	a	2882125	s	O157	rec46	a	CB9615	+		g		a	a	a	a	a	a	a	a	a	a	a	a	a	a	a	a	a	a	a	a
a	2592400	g	2811934	g	2882148	s	O157	rec46	g	CB9615	++++		g		g	g	g	g	g	g	g	g	g	g	g	g	g	g	g	g	g	g	g	g
g	2592416	t	2811950	t	2882164	s	O157	rec46	g	O157	++++		g		g	g	g	g	g	g	g	g	g	g	g	g	g	g	g	g	g	g	g	g
a	2592497	g	2812031	g	2882245	s	O157	rec46	g	CB9615	+		g		g	a	g	g	g	g	g	g	a	a	a	g	g	g	g	g	g	g	g	g
a	2592524	g	2812058	g	2882272	s	O157	rec46	g	CB9615	+		g		g	g	g	g	g	a	g	a	g	g	a	g	g	g	g	g	g	g	g	g
g	2592539	a	2812073	a	2882287	s	O157	rec46	g	O157	++++		g		g	g	g	g	g	g	g	g	g	g	g	g	g	g	g	g	g	g	g	g
g	2592554	a	2812088	a	2882302	s	O157	rec46	a	CB9615	++++		a		a	a	a	a	a	a	a	a	t	t	a	t	t	t	a	a	a	a	a	a
g	2592557	t	2812091	t	2882305	s	O157	rec46	g	O157	+		g		g	g	g	g	g	t	t	g	g	g	g	g	g	g	t	t	t	g	t	t
t	2592569	c	2812103	c	2882317	s	O157	rec46	c	CB9615	+		c		c	t	t	t	t	t	c	t	c	c	t	c	c	c	c	c	c	c	c	c
c	2592575	t	2812109	t	2882323	s	O157	rec46	t	CB9615	+		t		t	c	c	c	t	c	t	c	t	t	c	t	t	t	t	t	t	t	t	c
c	2592674	g	2812208	g	2882422	s	O157	rec46	g	CB9615	+		g		c	c	c	c	g	c	g	c	c	c	c	g	g	g	g	g	g	g	g	g
g	2592677	t	2812211	t	2882425	s	O157	rec46	t	CB9615	+		t		t	g	g	c	t	g	t	g	g	g	g	t	t	t	t	t	t	t	t	t
g	2592704	a	2812238	a	2882452	s	O157	rec46	a	CB9615	+		a		a	a	g	g	a	g	g	g	g	g	g	a	a	a	a	a	a	a	a	a
g	2592710	a	2812244	a	2882458	s	O157	rec46	a	CB9615	+		a		a	a	g	g	a	g	g	g	g	g	a	a	a	a	a	a	a	a	a	a
c	2592806	a	2812340	a	2882554	s	O157	rec46	a	CB9615	+		a		a	a	a	a	a	a	a	a	a	a	a	c	a	a	a	a	a	c	a	c
g	2592815	c	2812349	c	2882563	s	O157	rec46	c	CB9615	+		c		c	c	t	t	c	c	c	c	c	c	c	c	c	c	c	c	c	g	c	g
g	2592851	a	2812385	a	2882599	s	O157	rec46	a	CB9615	+		a		g	a	a	a	a	a	g	a	a	a	a	a	a	a	g	g	g	g	g	a
a	2593318	c	2812852	c	2883066	i	O157	rec46	c	CB9615	+		c		c	a	a	a	c	c	c	c	a	a	a	c	c	a	c	c	c	c	t	c
a	2593329	t	2812863	t	2883077	i	O157	rec46	?	O157	+/-		a		a	a	a	a	t	t	t	t	a	a	a	t	c	a	t	t	t	t	t	t
a	2593330	g	2812864	g	2883078	i	O157	rec46	g	CB9615	+		g		g	a	a	a	g	g	g	g	a	a	a	g	g	a	g	g	g	g	g	g
g	2593337	a	2812871	a	2883085	i	O157	rec46	g	O157	+		g		g	g	g	g	a	g	a	g	g	g	g	a	a	g	a	a	a	a	a	a
c	2593584	t	2813118	t	2883332	i	O157	rec46	t	CB9615	++++	t	t		t	t	t	t	t	t	t	t	t	t	t	t	t	t	t	t	t	t	t	t
a	2594420	g	2813954	g	2884169	s	O157	rec46	g	CB9615	++	g	g	g	g	g	a	g	g	g	g	g	g	g	g	g	g	g	a	a	a	g	g	g
g	2594474	a	2814008	a	2884223	s	O157	rec46	g	O157	++	g	g	a	g	g	g	g	g	g	a	a	g	g	g	g	g	g	g	g	g	g	g	g
a	2594477	g	2814011	g	2884226	s	O157	rec46	g	CB9615	++	g	g	g	g	g	g	g	g	g	g	g	g	g	g	a	g	a	a	a	a	g	a	g
a	2594528	g	2814062	g	2884277	s	O157	rec46	a	O157	++++	a	a	a	a	a	a	a	a	a	a	a	a	a	a	a	a	a	a	a	a	a	a	a
t	2594708	c	2814242	c	2884457	s	O157	rec46	c	CB9615	++++	a	a	c	a	g	a	a	a	c	c	t	a	a	a	c	c	c	a	a	a	a	c	c
a	2594711	g	2814245	g	2884460	s	O157	rec46	a	O157	++	a	a	g	a	a	a	a	a	g	g	g	a	a	a	g	g	g	a	a	a	a	g	g
c	2594759	t	2814293	t	2884508	s	O157	rec46	c	O157	++	c	c	t	c	c	t	t	c	t	c	t	c	c	t	c	c	c	c	c	c	c	c	c
c	2594762	t	2814296	t	2884511	s	O157	rec46	c	O157	++	c	c	t	c	c	t	t	c	t	c	t	c	c	t	c	c	c	c	c	c	c	c	c
t	2594765	c	2814299	c	2884514	s	O157	rec46	t	O157	++	t	t	c	t	t	c	c	t	c	t	c	t	t	c	t	t	t	t	t	t	t	t	t
a	2594774	g	2814308	g	2884523	s	O157	rec46	a	O157	++	a	a	a	a	a	g	g	a	a	a	a	a	a	g	a	a	a	a	a	a	a	a	a
c	2594811	t	2814345	t	2884560	s	O157	rec46	c	O157	++	c	c	t	c	c	t	c	c	t	c	t	c	c	c	c	c	c	c	c	c	c	c	c
g	2594819	a	2814353	a	2884568	s	O157	rec46	g	O157	++++	g	g	g	g	g	g	g	g	g	g	g	g	g	g	g	g	g	g	g	g	g	g	g
t	2594822	a	2814356	a	2884571	s	O157	rec46	a	CB9615	++	a	t	a	t	t	a	t	a	a	a	a	t	t	t	a	a	a	a	a	a	t	a	t
g	2594855	a	2814389	a	2884604	s	O157	rec46	g	O157	++++	g	g	g	g	g	g	g	g	g	g	g	g	g	g	g	g	g	g	g	g	g	g	g
g	2594930	a	2814464	a	2884679	s	O157	rec46	g	O157	++	g	g	a	g	a	a	a	a	a	g	a	g	g	g	g	g	g	g	g	g	g	a	g
g	2595173	a	2814707	a	2884922	s	O157	rec46	g	O157	++	g	g	a	g	g	a	g	g	a	g	a	a	a	a	g	g	g	g	g	g	g	g	g
g	2595182	a	2814716	a	2884931	s	O157	rec46	g	O157	++	g	g	a	g	a	a	g	g	a	a	a	a	a	a	a	a	a	g	g	g	a	a	g
a	2595902	c	2815436	c	2885651	s	O157	rec46	a	O157	+++	a				a	a	a					a	a	a									
g	2595923	a	2815457	a	2885672	s	O157	rec46	g	O157	+++	g				a	g	g					g	g	g									
t	2595975	c	2815509	c	2885724	s	O157	rec46	t	O157	+++	t				t	t	t					t	t	t									
g	2595977	a	2815511	a	2885726	s	O157	rec46	g	O157	+++	g				g	g	g					g	g	g									

O55 and O157 genome site details												Outgroup Strain Details[i]																						
CB9615 base[b]	CB9615 Site[c]	Sakai base[b]	Sakai Site[c]	EDL933 base[b]	EDL933 Site[c]	type[d]	Event Lineage[e,f]	Recombinant segment	Inferred ancestral base[g]	Outgroup Analysis[e]	Support level[h]	D1 Sd197	K-12	HS	ATCC 8739	UMN026	IAI39	SMS 3-5	E2348/69	536	ED1a	CFT073	S88	APEC O1	UTI89	E24377A	IAI1	SE11	F5 8401	F2a 2457T	F2a 301	SS Ss046	B4 Sb227	B18 BS512
a	2595996	g	2815530	g	2885745	ns	O157	rec46	a	O157	+++	a				g	a	a					a	a	a									
c	2596049	t	2815583	t	2885798	i	O157	rec46	c	O157	+++	c				c	c	c					c	c	c									
g	2596060	a	2815594	a	2885809	i	O157	rec46	g	O157	+++	g				g	g	g					g	g	g									
c	2596061	t	2815595	t	2885810	i	O157	rec46	c	O157	+++	c				c	c	c					c	c	c									
t	2596264	a	2815798	a	2886013	ns	O157	rec46	a	CB9615	++++	a	a	a	a	a	a	a	a	a	a	a	a	a	a	a	a	a	a	a	a	a	a	a
a	2596276	g	2815810	g	2886025	ns	O157	rec46	g	CB9615	++	g	g	g	g	g	g	g	a	g	g	g	g	g	g	g	g	g	g	g	g	a	g	g
t	2596277	c	2815811	c	2886026	ns	O157	rec46	c	CB9615	++++	c	c	c	c	c	c	c	c	c	c	c	c	c	c	c	c	c	c	c	c	c	c	c
a	2596282	c	2815816	c	2886031	s	O157	rec46	c	CB9615	++++	c	c	t	c	c	t	c	c	c	c	t	t	t	t	c	c	c	c	c	c	c	c	c
g	2596294	a	2815828	a	2886043	s	O157	rec46	a	CB9615	++++	a	a	a	a	c	a	a	a	a	a	a	a	a	a	a	a	a	a	a	a	a	a	a
a	2596297	c	2815831	c	2886046	ns	O157	rec46	c	CB9615	++	c	c	c	c	c	a	a	c	c	c	c	a	a	a	c	c	c	c	c	c	c	c	c
t	2596299	c	2815833	c	2886048	ns	O157	rec46	c	CB9615	++	c	c	c	c	c	t	t	c	c	c	c	t	t	t	c	c	c	c	c	c	c	c	c
c	2596300	g	2815834	g	2886049	s	O157	rec46	g	CB9615	++	g	g	g	g	g	c	c	g	g	g	g	c	c	c	g	g	g	g	g	g	g	g	g
a	2596303	c	2815837	c	2886052	s	O157	rec46	c	CB9615	++	c	c	c	c	c	a	a	c	c	c	c	a	a	a	c	c	c	c	c	c	c	c	c
c	2596333	a	2815867	a	2886082	ns	O157	rec46	a	CB9615	++	a	a	c	a	c	a	a	a	c	a	c	a	a	a	a	a	a	a	a	a	a	c	a
c	2596336	t	2815870	t	2886085	s	O157	rec46	t	CB9615	++	t	t	c	t	c	t	t	t	c	t	c	t	t	t	t	t	t	t	t	t	t	c	t
g	2596349	a	2815883	a	2886098	ns	O157	rec46	a	CB9615	++	a	a	g	a	g	a	a	a	g	a	g	a	a	a	a	a	a	a	a	a	a	g	a
a	2596351	g	2815885	g	2886100	s	O157	rec46	a	O157	++	a	g	a	g	a	g	g	g	a	g	a	g	g	g	g	g	g	g	g	g	g	a	g
a	2596354	t	2815888	t	2886103	ns	O157	rec46	t	CB9615	++	t	t	a	t	a	t	t	t	a	t	a	t	t	t	t	t	t	t	t	t	t	a	t
g	2596355	t	2815889	t	2886104	ns	O157	rec46	t	CB9615	++	t	t	g	t	g	t	t	t	g	t	g	t	t	t	t	t	t	t	t	t	t	g	t
g	2596360	t	2815894	t	2886109	ns	O157	rec46	t	CB9615	++	t	t	g	t	g	t	t	t	g	t	g	t	t	t	t	t	t	t	t	t	t	g	t
a	2596362	t	2815896	t	2886111	ns	O157	rec46	t	CB9615	++	t	t	a	t	a	t	t	t	a	t	a	t	t	t	t	t	t	t	t	t	t	a	t
t	2596374	c	2815908	c	2886123	ns	O157	rec46	c	CB9615	++	c	c	t	c	t	c	c	c	c	c	t	c	c	c	c	c	c	c	c	c	c	c	c
g	2596375	a	2815909	a	2886124	s	O157	rec46	a	CB9615	++	a	a	g	a	g	t	t	a	t	a	g	t	t	t	a	a	a	a	a	a	a	a	a
g	2596552	a	2816086	a	2886301	s	O157	rec46	g	O157	++++	g	g	g	g	g	g	g	g	g	g	g	g	g	g	g	g	g	g	g	g	g	g	g
t	2596557	c	2816091	c	2886306	ns	O157	rec46	t	O157	++++	t	t	t	t	t	t	t	t	t	t	t	t	t	t	t	t	t	t	t	t	t	t	t
g	2596638	a	2816172	a	2886387	s	O157	rec46	g	O157	++++	g	g	g	g	g	g	g	g	g	g	g	g	g	g	g	g	g	g	g	g	g	g	g
a	2596690	g	2816224	g	2886439	s	O157	rec46	g	CB9615	++	g	a	g	a	a	a	g	a	g	a	g	a	a	a	a	a	a	a	a	a	a	g	a
c	2596798	t	2816332	t	2886547	s	O157	rec46	c	O157	++++	c	c	c	c	c	c	c	c	c	c	c	c	c	c	c	c	c	c	c	c	c	c	c
t	2596948	c	2816482	c	2886697	s	O157	rec46	c	CB9615	++++	c	c	c	c	c	c	c	c	c	c	c	c	c	c	c	c	c	c	c	c	c	c	c
g	2596957	a	2816491	a	2886706	s	O157	rec46	a	CB9615	++	a	g	g	g	g	g	g	g	g	g	g	g	g	g	g	g	g	g	g	g	g	g	a
a	2596996	c	2816530	c	2886745	s	O157	rec46	c	CB9615	++++	c	c	c	c	c	c	c	c	c	c	c	c	c	c	c	c	c	c	c	c	c	c	c
t	2597020	g	2816554	g	2886769	s	O157	rec46	t	O157	++	t	g	t	g	t	t	t	g	t	t	t	t	t	t	t	t	t	g	g	g	g	t	t
t	2597071	c	2816605	c	2886820	s	O157	rec46	c	CB9615	++	c	c	t	c	t	t	t	t	t	t	t	t	t	t	t	t	t	t	t	t	t	t	c
t	2597174	c	2816708	c	2886923	ns	O157	rec46	t	O157	++++	t	t	t	t	t	t	t	t	t	t	t	t	t	t	t	t	t	t	t	t	t	t	t
t	2597236	g	2816770	g	2886985	s	O157	rec46	g	CB9615	++	g	g	g	g	t	g	g	g	g	t	g	g	g	g	t	t	t	g	g	g	g	t	g
a	2597362	g	2816896	g	2887111	s	O157	rec46	g	CB9615	++	g	g	g	g	g	g	g	g	a	g	a	g	g	g	g	g	g	g	g	g	g	g	a
t	2597995	c	2817529	c	2887744	s	O157	rec46	c	CB9615	++	c	t	t	t	t	t	t	t	t	t	t	t	t	t	t	t	t	t	t	t	t	t	t
t	2598253	c	2817787	c	2888002	s	O157	rec46	c	CB9615	++++	c	c	c	c	c	c	c	c	c	c	c	c	c	c	c	c	c	c	c	c	c	c	c
c	2598297	t	2817831	t	2888046	ns	O157	rec46	t	CB9615	++	t	c	c	c	t	t	t	c	c	c	t	t	t	t	c	c	c	c	c	c	c	c	c
c	2598298	t	2817832	t	2888047	s	O157	rec46	t	CB9615	++	t	c	c	c	t	t	t	c	c	c	t	t	t	t	c	c	c	c	c	c	c	c	c
g	2598301	a	2817835	a	2888050	s	O157	rec46	a	CB9615	++	a	g	g	g	a	a	a	g	g	g	a	a	a	a	g	g	g	g	g	g	g	g	g
c	2598424	t	2817958	t	2888173	s	O157	rec46	t	CB9615	++	t	t	c	t	c	c	c	t	c	t	c	c	c	c	t	t	t	t	t	t	t	t	t
a	2598618	g	2818152	g	2888367	i	O157	rec46	g	CB9615	++++	g	g	g	g	g	g	g	g	g	g	g	g	g	g	g	g	g	g	g	g	g	g	g
g	2599432	a	2818966	a	2889183	i	O157	rec46	a	CB9615	++	a	g	a	g	g	a	a	a	a	g	a	a	a	a	g	a	a	g	g	g	g	g	g
t	2599433	c	2818967	c	2889184	i	O157	rec46	c	CB9615	++	c	t	c	t	c	c	c	c	c	t	c	c	c	c	t	c	c	t	t	t	t	t	t
a	2599444	t	2818978	t	2889195	i	O157	rec46	a	O157	++	a	a	a	a	t	t	t	a	t	a	a	t	t	t	a	a	a	a	a	a	a	a	a
g	2599455	a	2818989	a	2889206	i	O157	rec46	a	CB9615	++++	a	a	c	a	a	a	a	a	a	a	a	a	a	a	a	a	a	a	a	a	a	a	a
t	2599665	c	2819199	c	2889416	s	O157	rec46	t	O157	++	t	t	t	t	t	c	t	t	t	t	c	c	c	c	t	t	t	t	t	t	t	t	t
g	2599776	a	2819310	a	2889527	s	O157	rec46	g	O157	++++	g	g	g	g	g	g	g	g	g	g	g	g	g	g	g	g	g	g	g	g	g	g	g
a	2599779	g	2819313	g	2889530	s	O157	rec46	a	O157	++	a	a	g	a	g	g	g	a	g	a	a	g	g	g	a	a	a	a	a	a	a	a	a
t	2599794	c	2819328	c	2889545	s	O157	rec46	t	O157	++	t	t	c	t	c	c	c	t	c	t	c	c	c	c	t	c	c	t	t	t	t	c	t
g	2599857	c	2819391	c	2889608	ns	O157	rec46	g	O157	++++	g	g	g	g	g	g	g	g	g	g	g	g	g	g	g	g	g	g	g	g	g	g	g
-	2600035	a	2819570	a	2889787	ins	O157	rec46	-	O157	++++	-	-	-	-	-	-	-	-	-	-	-	-	-	-	-	-	-	-	-	-	-	-	-
a	2600217	g	2819752	g	2889969	s	O157	rec46	g	CB9615	++++	g	g	g	g	g	g	g	g	g	g	g	g	g	g	g	g	g	g	g	g	g	g	g
t	2600301	c	2819836	c	2890053	s	O157	rec46	c	CB9615	++++	c	c	c	c	c	c	c	c	c	c	c	c	c	c	c	c	c	c	c	c	c	c	c
c	2600487	t	2820022	t	2890239	s	O157	rec46	c	O157	++	c	c	c	c	c	t	t	t	c	c	c	t	t	t	t	t	t	c	c	c	c	c	t
g	2600499	c	2820034	c	2890251	s	O157	rec46	g	O157	++	g	g	g	g	g	c	c	c	g	g	g	c	c	c	c	c	c	g	g	g	g	g	c
t	2600514	c	2820050	c	2890266	s	O157	rec46	t	O157	++	t	t	c	t	t	c	c	c	t	t	t	c	c	c	t	t	t	t	t	t	t	t	t
g	2600526	a	2820062	a	2890278	s	O157	rec46	g	O157	++	g	g	a	g	g	a	a	t	g	g	g	a	a	a	g	g	g	g	g	g	g	g	g
c	2600619	t	2820155	t	2890371	s	O157	rec46	c	O157	++	c	t	t	t	t	t	t	t	t	t	t	t	t	t	t	t	t	t	t	t	t	t	t
a	2600763	t	2820299	t	2890515	s	O157	rec46	a	O157	++++	a	a	a	a	a	a	a	a	a	a	a	a	a	a	a	a	a	a	a	a	a	a	a
c	2600847	g	2820383	g	2890599	s	O157	rec46	c	O157	++	c	c	g	c	g	g	g	c	c	c	c	g	g	g	g	g	g	g	g	g	c	g	g
g	2600865	a	2820401	a	2890617	s	O157	rec46	g	O157	++++	g	g	g	g	g	g	g	g	g	g	g	g	g	g	g	g	g	g	g	g	g	g	g
t	2600874	g	2820410	g	2890626	s	O157	rec46	t	O157	++	t	t	g	t	g	g	g	t	g	t	g	g	g	g	g	g	g	g	g	g	g	g	g
g	2600916	a	2820452	a	2890668	s	O157	rec46	g	O157	++++	g	g	g	g	g	g	g	g	g	g	g	g	g	g	g	g	g	g	g	g	g	g	g
t	2600917	c	2820453	c	2890669	s	O157	rec46	t	O157	++	t	t	c	t	c	c	c	t	c	t	c	c	c	c	t	t	t	c	c	c	t	c	c
a	2600976	g	2820512	g	2890728	s	O157	rec46	a	O157	++	a	a	g	a	g	g	g	g	g	a	g	g	g	g	a	a	a	g	g	g	a	g	g
g	2601033	a	2820569	a	2890785	s	O157	rec46	a	CB9615	++	a	g	g	g	g	g	g	g	g	g	g	a	a	g	g	g	g	g	g	g	g	g	g
t	2601036	c	2820572	c	2890788	s	O157	rec46	t	O157	++	t	t	c	t	t	c	c	t	c	t	c	c	c	c	t	t	t	t	t	t	t	t	t
c	2601054	t	2820590	t	2890806	s	O157	rec46	t	CB9615	++	t	t	c	t	t	c	t	t	t	t	t	t	t	t	t	t	t	t	t	t	t	t	t
a	2601060	g	2820596	g	2890812	s	O157	rec46	g	CB9615	++	g	g	a	g	g	a	g	g	g	g	g	g	g	g	g	g	g	-	-	-	g	g	g
g	2601069	a	2820605	a	2890821	s	O157	rec46	a	CB9615	++++	a	a	g	a	a	a	a	a	a	a	a	a	a	a	a	a	a	a	a	a	a	a	a
g	2601084	a	2820620	a	2890836	s	O157	rec46	a	CB9615	++	a	a	a	a	a	a	a	g	a	a	a	a	a	a	a	a	a	g	g	g	a	g	g
t	2601174	c	2820710	c	2890926	s	O157	rec46	c	CB9615	++	c	t	t	t	c	t	c	c	c	t	c	c	c	c	t	c	c	c	c	c	t	c	c
c	2601177	g	2820713	g	2890929	s	O157	rec46	g	CB9615	++	g	c	g	c	g	g	g	g	g	c	g	g	g	g	c	g	g	g	g	g	c	g	g
c	2601186	t	2820722	t	2890938	s	O157	rec46	t	CB9615	++	t	c	t	c	t	t	t	t	t	c	t	t	t	t	c	t	t	t	t	t	c	t	t
c	2601201	t	2820737	t	2890953	s	O157	rec46	t	CB9615	++	t	c	c	c	c	c	c	t	g	c	g	c	c	c	c	t	t	c	c	c	c	c	c
a	2601204	g	2820740	g	2890956	s	O157	rec46	g	CB9615	++	g	a	g	a	g	g	a	g	g	a	g	a	a	a	a	g	g	g	g	g	a	g	g
g	2601207	t	2820743	t	2890959	s	O157	rec46	t	CB9615	++	t	g	t	g	a	t	g	t	t	g	t	g	g	g	g	t	t	t	t	t	g	t	t
t	2601501	c	2821037	c	2891253	s	O157	rec46	c	CB9615	++	c	t	c	t	c	c	c	t	c	t	c	c	c	c	t	c	c	t	t	t	t	t	c
t	2601555	a	2821091	a	2891307	s	O157	rec46	t	O157	++	t	t	t	t	t	t	t	a	a	t	a	a	a	a	t	t	t	a	a	a	t	a	t
t	2601558	c	2821094	c	2891310	s	O157	rec46	t	O157	++	t	t	t	t	t	t	t	c	c	t	c	c	c	c	t	t	t	c	c	c	t	c	t
t	2601564	a	2821100	a	2891316	s	O157	rec46	t	O157	++	t	t	t	t	t	t	t	a	a	t	a	t	t	t	t	t	t	a	a	a	t	a	t
t	2601654	c	2821190	c	2891406	s	O157	rec46	c	CB9615	++	c	c	t	c	t	t	t	t	t	c	t	t	t	t	t	t	t	t	t	t	c	t	c
g	2601672	a	2821208	a	2891424	s	O157	rec46	a	CB9615	++	a	a	g	a	g	g	g	g	g	a	g	g	g	g	g	g	g	g	g	g	a	g	a
t	2602369	c	2821905	c	2892121	ns	O157	rec46	t	O157	++++	t	t	t	t	t	t	t	t	t	t	t	t	t	t	t	t	t	t	t	t	t	t	t

O55 and O157 genome site details												Outgroup Strain Details[i]																						
CB9615 base[b]	CB9615 Site[c]	Sakai base[b]	Sakai Site[c]	EDL933 base[b]	EDL933 Site[c]	type[d]	Event Lineage[ef]	Recombinant segment	Inferred ancestral base[g]	Outgroup Analysis[e]	Support level[h]	D1 Sd197	K-12	HS	ATCC 8739	UMN026	IAI39	SMS 3-5	E2348/69	536	ED1a	CFT073	S88	APEC O1	UTI89	E24377A	IAI1	SE11	F5 8401	F2a 2457T	F2a 301	SS Ss046	B4 Sb227	B18 BS512
t	2602527	a	2822063	a	2892279	s	O157	rec46	t	O157	++	t	t	a	t	a	a	a	a	a	a	a	a	a	a	t	a	a	t	t	t	t	t	t
a	2602539	c	2822075	c	2892291	ns	O157	rec46	a	O157	++	a	a	c	a	a	a	a	a	c	a	c	a	a	a	a	a	a	a	a	a	a	a	a
c	2602574	g	2822110	g	2892326	ns	O157	rec46	c	O157	++++	c	c	c	c	c	c	c	c	c	c	c	c	c	c	c	c	c	c	c	c	c	t	c
t	2602581	c	2822117	c	2892333	s	O157	rec46	t	O157	++++	t	t	t	t	t	t	t	t	t	t	t	t	t	t	t	t	t	t	t	t	t	t	t
a	2602587	c	2822123	c	2892339	s	O157	rec46	a	O157	++	a	a	a	a	c	c	c	c	c	a	c	c	c	c	a	a	a	a	a	a	a	a	a
g	2602590	a	2822126	a	2892342	s	O157	rec46	g	O157	++	g	g	g	g	a	a	a	a	a	g	a	a	a	a	g	g	g	g	g	g	g	g	g
c	2602591	t	2822127	t	2892343	ns	O157	rec46	c	O157	++	c	c	c	c	t	t	t	t	t	c	t	t	t	t	c	c	c	c	c	c	c	c	c
c	2602596	t	2822132	t	2892348	s	O157	rec46	c	O157	++	c	c	t	c	t	t	t	t	t	c	t	t	t	t	c	c	c	c	c	c	c	c	c
a	2602816	g	2822352	g	2892568	ns	O157	rec46	g	CB9615	++++	g	g	g	g	g	g	g	g	g	g	g	g	g	g	g	g	g	g	g	g	g	g	g
t	2602833	g	2822369	g	2892585	ns	O157	rec46	t	O157	++	t	g	t	g	t	t	t	t	t	t	t	t	t	t	t	t	t	t	t	t	t	t	t
g	2602867	a	2822403	a	2892619	ns	O157	rec46	g	O157	++++	g	g	g	g	g	g	g	g	g	g	g	g	g	g	g	g	g	g	g	g	g	g	g
t	2602982	a	2822518	a	2892734	i	O157	rec46	t	O157	++	t	a	a	a			a	a	a	a	a	a	a	a				t	t	t	a		a
c	2603012	t	2822548	t	2892764	s	O157	rec46	c	O157	++	c	t	t	t	t	t	t	t	t	t	t	t	t	t	t	t	t	t	t	t	t	t	t
t	2603171	c	2822707	c	2892923	s	O157	rec46	c	CB9615	++++	c	c	c	c	t	c	c	c	c	c	c	c	c	c	c	c	c	c	c	c	c	c	c
g	2603177	a	2822713	a	2892929	s	O157	rec46	a	CB9615	++	a	a	a	a	g	g	g	a	a	g	a	g	g	g	g	g	g	g	g	g	a	g	g
t	2603234	c	2822770	c	2892986	s	O157	rec46	c	CB9615	++	c	c	c	c	t	t	t	t	t	t	t	t	t	t	c	c	c	c	c	c	c	c	t
t	2603237	c	2822773	c	2892989	s	O157	rec46	c	CB9615	++	c	c	c	c	c	c	t	t	t	t	t	t	t	t	c	c	c	c	c	c	c	c	t
t	2603240	c	2822776	c	2892992	s	O157	rec46	c	CB9615	++	c	c	c	c	t	t	t	t	t	t	t	t	t	t	c	c	c	c	c	c	c	c	t
g	2603243	a	2822779	a	2892995	s	O157	rec46	a	CB9615	++	a	a	a	a	g	g	g	g	g	g	g	g	g	g	a	a	a	a	a	a	a	a	g
g	2603327	a	2822863	a	2893079	s	O157	rec46	a	CB9615	++	a	a	a	a	a	a	a	a	a	g	a	a	a	a	g	g	g	a	a	a	g	g	g
g	2603354	t	2822890	t	2893106	s	O157	rec46	t	CB9615	++	t	t	t	t	g	g	g	g	g	t	g	g	g	g	g	g	g	g	g	g	g	g	g
t	2603426	c	2822962	c	2893178	s	O157	rec46	c	CB9615	++++	c	c	c	c	c	c	c	c	c	c	c	c	c	c	c	c	c	c	c	c	c	c	c
c	2603498	t	2823034	t	2893250	s	O157	rec46	c	O157	++++	c	c	c	c	c	c	c	c	c	c	c	c	c	c	c	c	c	c	c	c	c	c	c
a	2603518	t	2823054	t	2893270	ns	O157	rec46	t	CB9615	++	t	a	a	a	a	t	a	a	a	a	a	t	t	t	a	a	a	t	t	t	a	a	t
c	2603556	t	2823092	t	2893308	ns	O157	rec46	t	CB9615	++	t	c	c	c	t	t	t	t	t	t	t	t	t	t	t	t	t	t	t	t	t	t	t
a	2603602	g	2823138	g	2893354	ns	O157	rec46	a	O157	++	a	a	a	a	a	a	a	a	a	g	a	a	a	a	a	a	a	g	g	g	g	a	g
t	2603630	a	2823166	a	2893382	s	O157	rec46	t	O157	++	t	t	t	t	t	a	a	t	a	a	t	a	a	a	a	a	a	a	a	a	t	a	a
a	2603639	g	2823175	g	2893391	s	O157	rec46	g	CB9615	++	g	a	a	a	a	a	a	g	a	g	a	a	a	a	g	g	g	g	g	g	a	g	g
c	2603649	t	2823185	t	2893401	ns	O157	rec46	c	O157	++++	c	c	c	c	c	c	c	c	c	c	c	c	c	c	c	c	c	c	c	c	c	c	c
c	2603748	t	2823284	t	2893500	ns	O157	rec46	t	CB9615	++	t	c	c	c	t	t	c	t	c	c	c	c	c	c	t	c	c	c	c	c	t	t	t
t	2603749	c	2823285	c	2893501	ns	O157	rec46	c	CB9615	++	c	t	t	t	c	c	t	c	t	t	t	t	t	t	c	t	t	t	t	t	t	c	c
c	2603753	a	2823289	a	2893505	s	O157	rec46	a	CB9615	++	a	c	c	c	a	a	c	c	c	c	c	c	c	c	a	c	c	c	c	c	c	a	a
g	2603771	a	2823307	a	2893523	s	O157	rec46	g	O157	++	g	g	g	g	g	g	g	g	g	a	g	g	g	g	a	a	a	a	a	a	g	a	a
a	2604018	g	2823554	g	2893770	ns	O157	rec46	g	CB9615	++++	g	g	g	g	g	g	g	g	g	g	g	g	g	g	g	g	g	g	g	g	g	g	g
g	2604020	a	2823556	a	2893772	ns	O157	rec46	g	O157	++++	g	g	g	g	g	g	g	g	g	g	g	g	g	g	g	g	g	g	g	g	g	g	g
g	2604173	a	2823709	a	2893925	s	O157	rec46	g	O157	++++	g	g	g	g	g	g	g	g	g	g	g	g	g	g	g	g	g	g	g	g	g	g	g
c	2604200	t	2823736	t	2893952	s	O157	rec46	c	O157	++++	c	c	c	c	c	c	c	c	c	c	c	c	c	c	c	c	c	c	c	c	c	c	c
g	2604278	a	2823814	a	2894030	s	O157	rec46	a	CB9615	++	a	a	a	a	a	g	a	a	a	a	a	a	a	a	a	a	a	a	a	a	a	a	g
g	2604404	a	2823940	a	2894156	s	O157	rec46	g	O157	++++	g	g	g	g	g	g	g	g	g	g	g	g	g	g	g	g	g	g	g	g	g	g	g
a	2604407	g	2823943	g	2894159	s	O157	rec46	g	CB9615	++	g	a	a	a	a	t	g	g	g	t	g	t	t	t	g	g	g	g	g	g	g	g	a
a	2604420	g	2823956	g	2894172	ns	O157	rec46	g	CB9615	++++	g	g	g	g	g	g	g	g	g	g	g	g	g	g	g	g	g	g	g	g	g	g	a
c	2604440	t	2823976	t	2894192	s	O157	rec46	t	CB9615	++++	t	t	t	t	t	t	t	t	t	t	t	t	t	t	t	t	t	t	t	t	t	t	t
t	2604467	g	2824003	g	2894219	s	O157	rec46	g	CB9615	++	g	g	g	g	t	g	g	g	g	t	g	g	g	g	g	g	g	g	g	g	g	g	g
a	2604486	g	2824022	g	2894238	ns	O157	rec46	g	CB9615	++	g	a	a	a	a	g	g	a	a	g	a	a	a	a	g	g	g	g	g	g	g	g	a
g	2604497	a	2824033	a	2894249	s	O157	rec46	a	CB9615	++	a	a	a	a	g	a	a	a	a	a	a	a	a	a	a	a	a	g	g	g	a	a	a
g	2604530	a	2824066	a	2894282	s	O157	rec46	a	CB9615	++	a	a	a	a	g	g	g	g	g	a	g	g	g	g	a	a	a	g	g	g	a	a	a
t	2604533	c	2824069	c	2894285	s	O157	rec46	c	CB9615	++	c	c	c	c	t	t	t	t	t	c	t	t	t	t	c	c	c	t	t	t	c	c	c
a	2604535	g	2824071	g	2894287	ns	O157	rec46	g	CB9615	++	g	g	g	g	a	a	a	a	a	g	a	a	a	a	g	g	g	a	a	a	g	g	g
t	2604539	c	2824075	c	2894291	s	O157	rec46	c	CB9615	++	c	c	c	c	t	c	c	t	t	c	t	t	t	t	c	c	c	t	t	t	c	c	c
a	2604569	t	2824105	t	2894321	s	O157	rec46	t	CB9615	++++	t	t	t	t	t	t	t	t	t	t	t	t	t	t	t	t	t	t	t	t	t	t	t
g	2604587	c	2824123	c	2894339	s	O157	rec46	c	CB9615	++++	c	c	c	c	c	c	c	c	c	c	c	c	c	c	c	c	c	c	c	c	c	c	c
t	2604590	c	2824126	c	2894342	s	O157	rec46	c	CB9615	++++	c	c	c	c	c	c	c	c	c	c	c	c	c	c	c	c	c	c	c	c	c	c	c
g	2604593	a	2824129	a	2894345	s	O157	rec46	g	O157	++++	g	g	g	g	g	g	g	g	g	g	g	g	g	g	g	g	g	g	g	g	g	g	a
g	2604611	a	2824147	a	2894363	s	O157	rec46	g	O157	++++	g	g	g	g	g	g	g	g	g	g	g	g	g	g	g	g	g	g	g	g	g	g	a
a	2604707	g	2824243	g	2894459	s	O157	rec46	g	CB9615	++++	g	g	g	g	g	g	g	g	g	g	g	g	g	g	g	g	g	g	g	g	g	g	g
g	2604752	a	2824288	a	2894504	s	O157	rec46	g	O157	++	g	g	g	g	g	a	a	a	a	a	a	a	a	a	c	a	a	c	c	c	c	c	a
g	2604776	a	2824312	a	2894528	s	O157	rec46	g	O157	++	g	a	g	g	g	g	g	g	g	g	g	g	g	g	a	g	g	a	a	a	a	a	a
t	2604777	c	2824313	c	2894529	s	O157	rec46	t	O157	++	t	c	t	t	t	t	t	t	t	t	t	t	t	t	c	t	t	c	c	c	c	c	c
a	2604779	g	2824315	g	2894531	s	O157	rec46	a	O157	++	a	g	a	a	a	a	a	a	g	a	a	a	a	a	g	a	a	g	g	g	g	g	g
c	2604785	t	2824321	t	2894537	s	O157	rec46	c	O157	++	c	t	c	c	c	c	c	c	c	c	c	c	c	c	t	c	c	t	t	t	t	t	t
g	2604787	t	2824323	t	2894539	ns	O157	rec46	t	CB9615	++++	t	t	t	t	t	t	t	t	t	t	t	t	t	t	t	t	t	t	t	t	t	t	t
t	2604951	c	2824487	c	2894703	s	O157	rec46	c	CB9615	++	c	t	t	t	t	t	t	t	t	c	t	t	t	t	c	c	c	t	t	t	t	t	c
a	2604995	t	2824531	t	2894747	s	O157	rec46	t	CB9615	++++	t	t	t	t	t	t	t	t	t	t	t	t	t	t	t	t	t	t	t	t	t	c	t
t	2605019	g	2824555	g	2894771	s	O157	rec46	t	O157	++	t	t	c	c	g	g	g	g	c	t	c	g	g	g	t	t	t	t	t	t	t	c	t
t	2605135	a	2824671	a	2894887	ns	O157	rec46	t	O157	++++	t	t	t	t	t	t	t	t	t	t	t	t	t	t	t	t	t	t	t	t	t	t	t
c	2605154	g	2824690	g	2894906	s	O157	rec46	g	CB9615	++	g	c	g	g	g	g	g	g	g	c	g	g	g	g	c	c	c	c	c	c	c	g	c
c	2605157	g	2824693	g	2894909	s	O157	rec46	g	CB9615	++++	g	g	g	g	g	g	g	g	g	g	g	g	g	g	g	g	g	g	g	g	g	g	g
t	2605202	a	2824738	a	2894954	s	O157	rec46	t	O157	++++	t	t	t	t	t	t	t	t	t	t	t	t	t	t	t	t	t	t	t	t	t	t	t
t	2605478	c	2825014	c	2895230	ns	O157	rec46	c	CB9615	++	c	c	t	t	c	c	c	c	c	t	c	c	c	c	t	t	t	t	t	t	c	c	c
a	2605512	c	2825048	c	2895264	ns	O157	rec46	c	CB9615	++	c	c	c	c	t	a	a	t	t	a	t	c	c	c	c	c	a	c	c	c	c	-	c
t	2605514	c	2825050	c	2895266	ns	O157	rec46	c	CB9615	++	c	c	c	c	c	t	t	c	c	t	c	c	c	c	c	c	t	c	c	c	c	-	c
g	2605530	t	2825066	t	2895282	ns	O157	rec46	g	O157	++++	g	g	g	g	g	g	g	g	g	g	g	g	g	g	g	g	g	g	g	g	g	g	t
g	2605617	a	2825153	a	2895369	s	O157	rec46	g	O157	++	g	g	g	a	a	a	a	a	a	g	a	a	a	a	g	g	g	a	a	a	g	g	a
g	2605620	a	2825156	a	2895372	s	O157	rec46	g	O157	++	g	g	g	a	a	a	a	a	a	g	a	a	a	a	g	g	g	a	a	a	g	g	a
c	2605632	a	2825168	a	2895384	s	O157	rec46	a	CB9615	++	a	c	c	c	c	c	t	t	t	c	t	t	t	t	c	c	c	a	a	a	c	a	a
g	2605644	c	2825180	c	2895396	s	O157	rec46	c	CB9615	++	c	g	g	c	g	g	g	g	g	g	g	g	g	g	g	g	g	g	g	g	g	g	c
t	2605662	c	2825198	c	2895414	s	O157	rec46	c	CB9615	++	c	t	t	c	t	t	t	c	c	t	c	t	t	t	t	t	t	t	t	t	t	c	c
t	2605665	g	2825201	g	2895417	s	O157	rec46	g	CB9615	++	g	g	g	t	c	g	c	t	t	t	t	c	c	c	g	g	g	t	t	t	g	t	t
a	2605710	g	2825246	g	2895462	s	O157	rec46	g	CB9615	++++	g	g	g	g	g	g	g	g	g	g	g	g	g	g	g	g	g	g	g	g	g	g	g
g	2605788	a	2825324	a	2895540	s	O157	rec46	a	CB9615	++	a	a	a	g	c	c	c	g	g	a	g	c	c	c	a	a	a	a	a	a	a	a	g
a	2605806	g	2825342	g	2895558	s	O157	rec46	g	CB9615	++	g	g	g	a	g	g	g	g	g	g	g	g	g	g	g	g	g	g	g	g	g	g	a
t	2605812	c	2825348	c	2895564	s	O157	rec46	c	CB9615	++	c	c	c	t	c	c	t	c	c	c	c	t	t	t	c	c	c	c	c	c	c	c	t
t	2605815	c	2825351	c	2895567	s	O157	rec46	c	CB9615	++	c	t	t	t	t	c	t	c	t	t	t	t	t	t	t	t	t	t	t	t	t	c	t
t	2605862	c	2825398	c	2895614	ns	O157	rec46	c	CB9615	++	c	t	t	t	t	t	t	c	c	c	c	c	c	c	c	c	t	t	t	t	t	c	t
g	2605981	a	2825517	a	2895733	ns	O157	rec46	g	O157	++++	g	g	g	g	g	g	g	g	g	g	g	g	g	g	g	g	g	g	g	g	g	g	g
g	2606072	a	2825608	a	2895824	s	O157	rec46	g	O157	++	g	g	g	a	a	a	a	a	a	a	a	a	a	a	a	a	g	g	g	g	g	a	g

O55 and O157 genome site details												Outgroup Strain Details[i]																						
CB9615 base[b]	CB9615 Site[c]	Sakai base[b]	Sakai Site[c]	EDL933 base[b]	EDL933 Site[c]	type[d]	Event Lineage[ef]	Recombinant segment	Inferred ancestral base[g]	Outgroup Analysis[e]	Support level[h]	D1 Sd197	K-12	HS	ATCC 8739	UMN026	IAI39	SMS 3-5	E2348/69	536	ED1a	CFT073	S88	APEC O1	UTI89	E24377A	IAI1	SE11	F5 8401	F2a 2457T	F2a 301	SS Ss046	B4 Sb227	B18 BS512
c	2606081	a	2825617	a	2895833	ns	O157	rec46	c	O157	++	c	a	a	a	a	a	a	a	a	a	a	a	a	a	a	a	a	a	a	a	a	a	a
g	2606102	t	2825638	t	2895854	ns	O157	rec46	g	O157	++	g	g	g	t	t	t	t	t	t	t	t	t	t	t	t	t	g	g	g	g	g	t	t
c	2606186	a	2825722	a	2895938	ns	O157	rec46	a	CB9615	++++	a	a	a	a	a	a	a	a	a	a	a	a	a	a	a	a	a	a	a	a	a	a	a
g	2606247	a	2825783	a	2895999	ns	O157	rec46	a	CB9615	++	a	g	g	g	g	g	g	g	g	g	g	g	g	g	g	g	g	g	g	g	g	g	g
c	2606249	t	2825785	t	2896001	ns	O157	rec46	t	CB9615	++	t	c	c	t	c	t	t	c	c	t	c	c	c	c	t	c	c	c	c	c	c	t	c
t	2606309	c	2825845	c	2896061	ns	O157	rec46	c	CB9615	++	c	c	c	c	c	c	c	c	c	c	c	c	c	c	c	c	c	t	t	t	c	t	t
g	2606325	a	2825861	a	2896077	s	O157	rec46	a	CB9615	++	a	a	a	g	a	a	a	g	a	a	a	a	a	a	a	g	a	g	g	g	a	g	g
g	2606335	c	2825871	c	2896087	ns	O157	rec46	g	O157	++	g	g	g	g	c	c	c	c	g	g	g	g	g	g	c	g	c	g	g	g	c	g	g
a	2606340	g	2825876	g	2896092	s	O157	rec46	a	O157	++	a	g	g	a	g	a	a	g	g	g	g	g	g	g	a	a	a	a	a	a	a	a	a
a	2606388	t	2825924	t	2896140	ns	O157	rec46	t	CB9615	++	t	a	a	a	a	t	a	t	t	a	t	t	t	t	a	a	a	a	a	a	a	a	a
g	2606389	a	2825925	a	2896141	ns	O157	rec46	a	CB9615	++	a	g	g	g	g	a	g	a	a	g	a	a	a	a	g	g	g	g	g	g	g	g	g
t	2606400	c	2825936	c	2896152	s	O157	rec46	c	CB9615	++	c	c	c	c	c	t	c	c	c	c	c	c	c	c	t	c	c	c	c	c	t	c	t
a	2606415	t	2825951	t	2896167	s	O157	rec46	t	CB9615	++	t	a	a	a	t	t	t	t	t	a	t	t	t	t	a	a	a	a	a	a	a	a	a
c	2606428	g	2825964	g	2896180	ns	O157	rec46	g	CB9615	++	g	c	c	c	c	c	c	g	c	c	c	c	c	c	c	c	c	c	c	c	c	c	c
t	2606436	c	2825972	c	2896188	s	O157	rec46	c	CB9615	++++	c	c	c	c	c	c	c	c	c	c	c	c	c	c	c	c	c	c	c	c	c	c	t
a	2606451	g	2825987	g	2896203	s	O157	rec46	g	CB9615	++	g	g	g	g	g	g	g	g	g	g	g	g	g	g	a	g	g	g	g	g	a	g	a
c	2606454	t	2825990	t	2896206	s	O157	rec46	t	CB9615	++	t	t	t	t	t	t	t	t	t	t	t	t	t	t	c	t	t	t	t	t	c	t	c
c	2606715	t	2826251	t	2896467	s	O157	rec46	t	CB9615	++	t	t	t	c	t	c	c	t	t	c	t	t	t	t	c	c	c	c	c	c	c	c	t
a	2606865	c	2826401	c	2896617	s	O157	rec46	a	O157	++	a	a	a	a	a	a	a	a	a	c	c	a	a	a	a	a	a	a	a	a	a	a	c
a	2606916	g	2826452	g	2896668	s	O157	rec46	g	CB9615	++++	g	g	g	g	g	g	g	g	g	g	g	g	g	g	g	g	g	g	g	g	g	g	g
g	2606955	a	2826491	a	2896707	s	O157	rec46	g	O157	++++	g	g	g	g	g	g	g	g	g	g	g	g	g	g	g	g	g	g	g	g	g	g	g
t	2606980	c	2826516	c	2896732	ns	O157	rec46	c	CB9615	++++	c	c	c	c	c	c	c	c	c	c	c	c	c	c	c	c	c	c	c	c	c	c	c
g	2607092	a	2826628	a	2896844	s	O157	rec46	a	CB9615	++++	a	a	a	a	a	a	a	a	a	a	a	a	a	a	a	a	a	a	a	a	a	a	a
t	2607099	c	2826635	c	2896851	s	O157	rec46	c	CB9615	++++	c	c	c	c	c	c	c	c	c	c	c	c	c	c	c	c	c	c	c	c	c	c	c
g	2607184	a	2826720	a	2896936	ns	O157	rec46	a	CB9615	++++	a	a	a	a	a	a	a	a	a	a	a	a	a	a	a	a	a	a	a	a	a	a	a
c	2607192	a	2826728	a	2896944	ns	O157	rec46	c	O157	++	c	c	c	c	c	c	c	c	c	c	c	c	c	c	a	a	a	a	a	a	a	c	a
c	2607194	t	2826730	t	2896946	ns	O157	rec46	c	O157	++	c	c	c	c	c	c	c	c	c	c	c	c	c	c	t	t	t	t	t	t	t	c	t
a	2607210	g	2826746	g	2896962	s	O157	rec46	g	CB9615	++	g	g	g	g	g	a	a	a	a	g	a	a	a	a	g	g	g	g	g	g	g	g	g
a	2607216	g	2826752	g	2896968	s	O157	rec46	g	CB9615	++	g	a	a	a	g	g	g	g	g	a	a	g	g	g	a	a	a	a	a	a	a	a	g
g	2607219	a	2826755	a	2896971	s	O157	rec46	a	CB9615	++	a	a	a	a	a	g	g	g	g	a	g	g	g	g	g	g	g	g	g	g	g	g	a
c	2607304	t	2826840	t	2897056	ns	O157	rec46	c	O157	++++	c	c	c	c	c	c	c	c	c	c	c	c	c	c	c	c	c	c	c	c	c	c	c
c	2607341	t	2826877	t	2897093	s	O157	rec46	c	O157	++++	c	c	c	c	c	c	c	c	c	c	c	c	c	c	c	c	c	c	c	c	c	c	c
g	2607347	c	2826883	c	2897099	s	O157	rec46	g	O157	++++	g	g	g	g	g	g	g	g	g	g	g	g	g	g	g	g	g	g	g	g	g	g	g
c	2607386	t	2826922	t	2897138	s	O157	rec46	t	CB9615	++++	t	t	t	t	t	t	t	t	t	t	t	t	t	t	t	t	t	t	t	t	t	t	t
c	2607410	t	2826946	t	2897162	s	O157	rec46	t	CB9615	++	t	t	t	t	c	t	t	t	c	t	t	t	t	t	t	t	t	t	t	t	t	t	t
a	2607422	c	2826958	c	2897174	s	O157	rec46	c	CB9615	++++	c	c	c	c	c	c	c	c	c	c	c	c	c	c	c	c	c	c	c	c	c	c	c
a	2607457	g	2826993	g	2897209	s	O157	rec46	a	O157	++	a	g	g	g	g	g	g	g	g	g	g	g	g	g	g	g	g	a	a	a	g	g	g
g	2607479	a	2827015	a	2897231	s	O157	rec46	g	O157	++	g	g	g	g	g	g	a	a	g	a	a	a	a	a	g	g	g	g	g	g	a	a	a
a	2607510	g	2827046	g	2897262	ns	O157	rec46	g	CB9615	++	g	g	g	g	g	g	g	g	g	g	g	g	g	g	a	a	a	a	a	a	g	g	g
g	2607549	a	2827085	a	2897301	ns	O157	rec46	g	O157	++++	g	g	g	g	g	g	g	g	g	g	g	g	g	g	g	g	g	g	g	g	g	g	g
g	2607737	a	2827273	a	2897489	s	O157	rec46	g	O157	++++	g	g	g	g	g	g	g	g	g	g	g	g	g	g	g	g	g	g	g	g	g	g	g
g	2607794	a	2827330	a	2897546	s	O157	rec46	g	O157	++++	g	g	g	g	g	g	g	a	g	g	g	g	g	g	g	g	g	g	g	g	g	g	g
t	2607910	c	2827446	c	2897662	ns	O157	rec46	c	CB9615	++++	c	c	c	c	c	c	c	c	c	c	c	c	c	c	c	c	c	c	c	c	c	c	c
g	2607911	a	2827447	a	2897663	ns	O157	rec46	g	O157	++	g	g	g	g	g	g	g	g	g	g	g	g	g	g	a	a	a	a	a	a	a	a	a
c	2607913	g	2827449	g	2897665	ns	O157	rec46	c	O157	++	c	c	c	c	c	c	c	c	c	c	c	c	c	c	g	g	g	g	g	g	g	g	g
a	2607914	g	2827450	g	2897666	s	O157	rec46	a	O157	++	a	a	a	a	a	a	a	a	g	a	a	a	a	a	g	a	a	g	g	g	g	g	g
t	2607917	c	2827453	c	2897669	ns	O157	rec46	t	O157	++	t	t	t	t	t	t	t	t	t	t	t	t	t	t	c	c	c	c	c	c	c	c	c
c	2607919	t	2827455	t	2897671	ns	O157	rec46	c	O157	++	c	c	c	c	c	c	c	c	c	c	c	c	c	c	t	t	t	t	t	t	t	t	t
g	2607921	a	2827457	a	2897673	ns	O157	rec46	g	O157	++	g	g	g	g	g	g	g	g	g	g	g	g	g	g	a	a	a	a	a	a	a	a	a
a	2607922	c	2827458	c	2897674	ns	O157	rec46	a	O157	++	a	a	a	a	a	a	a	a	a	a	a	a	a	a	c	c	c	c	c	c	c	c	c
a	2607925	g	2827461	g	2897677	s	O157	rec46	a	O157	++	a	a	a	a	a	a	a	a	a	a	a	a	a	a	g	g	g	g	g	g	g	g	g
t	2608072	c	2827608	c	2897824	s	O157	rec46	t	O157	++	t	c	c	c	c	c	c	c	c	c	c	c	c	c	c	c	c	c	c	c	c	c	c
t	2608114	c	2827650	c	2897866	s	O157	rec46	t	O157	++	t	c	c	c	c	c	c	c	c	c	c	c	c	c	c	c	c	c	c	c	c	c	c
g	2608141	a	2827677	a	2897893	s	O157	rec46	g	O157	++	g	a	a	a	a	a	a	a	a	a	a	a	a	a	a	a	a	a	a	a	a	a	a
a	2608162	t	2827698	t	2897914	ns	O157	rec46	a	O157	++	a	a	a	a	t	t	t	a	a	a	t	a	a	a	a	a	a	a	a	a	t	t	t
a	2608164	g	2827700	g	2897916	ns	O157	rec46	a	O157	++	a	g	g	g	g	g	g	a	a	a	g	a	a	a	g	g	g	g	g	g	g	g	g
g	2608177	a	2827713	a	2897929	s	O157	rec46	g	O157	++	g	a	a	a	a	a	a	g	g	g	a	g	g	g	a	a	a	a	a	a	a	a	a
a	2608180	g	2827716	g	2897932	s	O157	rec46	g	CB9615	++++	g	g	g	g	g	g	g	g	g	g	g	g	g	g	g	g	g	g	g	g	g	g	g
g	2608189	t	2827725	t	2897941	s	O157	rec46	g	O157	++	g	t	t	t	t	t	t	a	g	g	t	g	g	g	t	t	t	t	t	t	t	t	t
c	2608195	a	2827731	a	2897947	s	O157	rec46	c	O157	++	c	c	c	c	a	a	a	c	c	c	c	c	c	c	c	c	c	a	a	a	a	a	a
c	2608201	a	2827737	a	2897953	ns	O157	rec46	c	O157	++++	c	c	c	c	c	c	c	c	c	c	c	c	c	c	c	c	c	c	c	c	c	c	c
g	2608423	c	2827959	c	2898175	s	O157	rec46	g	O157	++	g	g	g	g	g	g	g	g	g	g	g	g	g	g	c	c	g	g	g	g	c	c	g
a	2608857	g	2828393	g	2898610	nc	O157	rec46	g	CB9615	++++	g	g	g	g	g	g	g	g	g	a	g	g	g	g	g	g	g	g	g	g	g	g	g
a	2608859	g	2828395	g	2898612	nc	O157	rec46	g	CB9615	++++	g	g	g	g	g	g	g	g	g	g	t	g	g	g	g	g	g	g	g	g	g	g	g
g	2608893	a	2828429	a	2898646	nc	O157	rec46	g	O157	++++	g	g	g	g	g	g	g	g	g	g	g	g	g	g	g	g	g	g	g	g	g	g	g
g	2608979	a	2828515	a	2898732	nc	O157	rec46	a	CB9615	+		a	a	a	g	a	a	g	g	a	g	g	g	g	a	a	a				a	a	a
c	2608987	t	2828523	t	2898740	nc	O157	rec46	c	O157	++++		c	c	c	c	c	c	c	c	c	c	c	c	c	c	c	c				c	c	c
g	2609032	a	2828568	a	2898785	i	O157	rec46	g	O157	++++		g	g	a	g	g	g		g	g	g	g	g	g	g	g	g				g	g	g
c	2609067	a	2828603	a	2898820	i	O157	rec46	c	O157	++++		c	c	c	c	c	c		c	c	c	c	c	c	c	c	c				c	c	c
a	2609070	c	2828606	c	2898823	i	O157	rec46	a	O157	++++		a	a	a	a	a	a		t	a	t	t	t	t	a	a	a				a	a	t
t	2609073	g	2828609	g	2898826	i	O157	rec46	g	CB9615	++++		g	g	a	g	g	g		g	g	g	g	g	g	g	g	g				g	g	g
a	2609086	g	2828622	g	2898839	i	O157	rec46	a	O157	+		a	a	a	a	a	a		g	a	g	g	g	g	a	a	a				a	a	g
g	2609089	t	2828625	t	2898842	i	O157	rec46	g	O157	+		g	g	g	g	g	g		t	g	t	t	t	t	g	g	g				g	g	t
c	2609090	t	2828626	t	2898843	i	O157	rec46	c	O157	+		c	c	c	c	c	c		t	c	t	t	t	t	c	c	c				c	c	t
t	2609092	c	2828628	c	2898845	i	O157	rec46	t	O157	+		t	t	t	t	t	t		c	t	c	c	c	c	t	t	t				t	t	c
a	2609094	g	2828630	g	2898847	i	O157	rec46	a	O157	+		a	a	a	a	a	a		g	a	g	g	g	g	a	a	a				a	a	g
c	2609096	t	2828632	t	2898849	i	O157	rec46	t	CB9615	+		t	t	c	c	c	c		t	t	t	t	t	t	t	t	t				c	t	t
t	2609109	a	2828645	a	2898862	i	O157	rec46	t	O157	+		t	t	t	t	t	t		a	t	a	a	a	a	t	t	t				t	t	a
c	2609110	g	2828646	g	2898863	i	O157	rec46	c	O157	+		c	c	c	c	c	c		g	c	g	g	g	g	c	c	c				c	c	g
g	2609176	a	2828712	a	2898929	i	O157	rec46	g	O157	++++		g	g	g	a	g	g		g	g	g	g	g	g	g	g	g				g	g	g
t	2609185	a	2828721	a	2898938	nc	O157	rec46	a	CB9615	++++		a	a	a	a	a	a		a	a	a	a	a	a	a	a	a				a	a	a
g	2609189	t	2828725	t	2898942	nc	O157	rec46	t	CB9615	+		t	t	t	t	t	t		g	t	g	g	g	g	t	t	t				t	t	t
a	2609191	t	2828727	t	2898944	nc	O157	rec46	t	CB9615	++++		t	t	t	t	t	t		c	t	c	c	c	c	t	t	t				t	t	t
t	2609256	a	2828792	a	2899009	nc	O157	rec46	a	CB9615	+		a	a	a	a	a	a		t	a	a	t	t	t	a	a	a				a	a	a
t	2609259	c	2828795	c	2899012	nc	O157	rec46	c	CB9615	+		c	c	c	c	g	c		t	c	c	t	t	t	g	g	g				g	g	t
t	2609263	a	2828799	a	2899016	nc	O157	rec46	a	CB9615	+		a	a	a	a	a	a		t	a	a	t	t	t	a	a	a				a	a	c

O55 and O157 genome site details												Outgroup Strain Details[i]																						
CB9615 base[b]	CB9615 Site[c]	Sakai base[b]	Sakai Site[c]	EDL933 base[b]	EDL933 Site[c]	type[d]	Event Lineage[ef]	Recombinant segment	Inferred ancestral base[g]	Outgroup Analysis[e]	Support level[h]	D1 Sd197	K-12	HS	ATCC 8739	UMN026	IAI39	SMS 3-5	E2348/69	536	ED1a	CFT073	S88	APEC O1	UTI89	E24377A	IAI1	SE11	F5 8401	F2a 2457T	F2a 301	SS Ss046	B4 Sb227	B18 BS512
a	2609268	t	2828804	t	2899021	nc	O157	rec46	a	O157	+		a	a	a	t	t	a		a	a	t	a	a	a	t	t	t				t	t	a
c	2609271	g	2828807	g	2899024	nc	O157	rec46	g	CB9615	+		c	c	c	g	g	c		c	c	g	g	g	g	g	g	g				g	g	g
g	2609294	t	2828830	t	2899047	nc	O157	rec46	g	O157	+		c	c	c	t	g	c		g	c	t	g	g	g	g	g	g				g	g	g
t	2609295	c	2828831	c	2899048	nc	O157	rec46	t	O157	+		g	g	g	c	t	g		t	g	c	t	t	t	t	t	t				t	t	t
a	2609296	c	2828832	c	2899049	nc	O157	rec46	a	O157	+		t	t	t	c	a	t		a	t	c	a	a	a	a	a	a				a	a	a
t	2609297	a	2828833	a	2899050	nc	O157	rec46	t	O157	+		g	g	g	a	g	g		t	g	a	t	t	t	g	g	g				g	g	g
t	2609324	a	2828860	a	2899077	i	O157	rec46	a	CB9615	++++		a	a	a	a	a	a		a	a	t	a	a	a	a	a	a				a	a	a
a	2609395	c	2828931	c	2899148	i	O157	rec46	c	CB9615	+		c	c	c	c	a	c		a	c	a	a	a	a	a	a	a				c	c	c
c	2609398	a	2828934	a	2899151	i	O157	rec46	c	O157	+		a	a	a	a	c	a		c	a	c	c	c	c	c	c	c				t	t	t
a	2609400	t	2828936	t	2899153	i	O157	rec46	t	CB9615	+		t	t	t	t	a	t		a	t	a	a	a	a	c	c	c				t	t	t
g	2609401	t	2828937	t	2899154	i	O157	rec46	g	O157	+		g	g	a	g	g	g		g	g	g	g	g	g	t	t	t				g	g	g
a	2609414	g	2828950	g	2899167	i	O157	rec46	a	O157	+		a	a	a	g	a	a		a	a	a	a	a	a	g	g	g				g	g	g
g	2609417	t	2828953	t	2899170	i	O157	rec46	g	O157	+		g	g	g	t	t	g		g	g	g	g	g	g	t	t	t				t	t	t
c	2609418	t	2828954	t	2899171	i	O157	rec46	c	O157	+		c	c	c	t	t	c		c	c	c	c	c	c	t	t	t				t	t	t
t	2609420	c	2828956	c	2899173	i	O157	rec46	t	O157	+		t	t	t	c	c	t		t	t	t	t	t	t	c	c	c				c	c	c
a	2609421	g	2828957	g	2899174	i	O157	rec46	g	CB9615	+		g	g	g	g	a	g		a	g	a	a	a	a	a	a	a				g	g	g
g	2609438	c	2828974	c	2899191	i	O157	rec46	g	O157	+		c	c	c	a	a	c		g	c	g	g	g	g	g	g	g				g	g	g
t	2609442	g	2828978	g	2899195	i	O157	rec46	g	CB9615	+		g	g	g	g	g	g		t	g	t	t	t	t	g	g	g				g	g	g
c	2609447	t	2828983	t	2899200	i	O157	rec46	t	CB9615	+		t	t	t	t	t	t		c	t	c	c	c	c	t	t	t				t	t	t
g	2609629	a	2829494	a	2899712	s	O157	rec46	g	O157	++++		g	g	g	g	g	g	g	g	g	g	g	g	g	g	g	g				g	g	g
a	2609768	g	2829633	g	2899851	ns	O157	rec46	g	CB9615	++++		g	g	g	g	g	g	g	g	g	g	g	g	g	g	g	g				g	g	g
c	2609857	t	2829722	t	2899940	s	O157	rec46	c	O157	++++		c	c	c	c	c	c	c	c	c	c	c	c	c	c	c	c	c	c	c	c	c	c
a	2609917	t	2829782	t	2900000	s	O157	rec46	a	O157	++++		a	a	a	a	a	t	a	a	a	a	a	a	a	a	a	a	a	a	a	a	a	a
c	2610559	a	2830424	a	2900642	s	O157	rec46	a	CB9615	+		a	a	a	c	c	c	a	c	c	c	c	c	c	a	a	a	a	a	a	a	a	a
c	2610562	a	2830427	a	2900645	s	O157	rec46	a	CB9615	+		a	a	a	c	c	c	a	c	c	c	c	c	c	a	a	g	a	a	a	a	a	a
g	2610837	a	2830702	a	2900920	s	O157	rec46	g	O157	++++		g	g	g	g	g	g	g	g	g	g	g	g	g	g	g	g	g	g	g	g	g	g
a	2610859	g	2830724	g	2900942	ns	O157	rec46	g	CB9615	++++		g	g	g	g	g	g	g	g	g	g	g	g	g	g	g	g	g	g	g	g	g	g
a	2610987	g	2830852	g	2901070	s	O157	rec46	g	CB9615	+		g	g	g	a	a	a	g	a	g	a	g	g	g	g	g	g	g	g	g	g	g	g
t	2611086	c	2830951	c	2901169	s	O157	rec46	c	CB9615	++++		c	c	c	c	c	c	c	c	c	c	c	c	c	c	c	c	c	c	c	c	c	c
g	2611137	a	2831002	a	2901220	s	O157	rec46	g	O157	+		g	g	g	g	g	g	g	a	a	g	a	a	a	g	g	g	g	g	g	g	g	a
c	2611161	g	2831026	g	2901244	s	O157	rec46	c	O157	+		c	c	c	g	g	c	c	g	c	g	g	g	g	c	c	c	c	c	c	c	c	g
a	2611182	g	2831047	g	2901265	s	O157	rec46	g	CB9615	++++		g	g	g	g	g	g	g	g	g	g	g	g	g	g	g	g	g	g	g	g	g	g
a	2611285	g	2831150	g	2901368	ns	O157	rec46	g	CB9615	++++		g	g	g	g	g	g	g	g	g	g	g	g	g	g	g	g	g	g	g	g	g	g
c	2611299	a	2831164	a	2901382	s	O157	rec46	c	O157	++++		c	c	c	c	c	c	c	c	c	c	c	c	c	c	c	c	c	c	c	c	c	c
a	2611506	t	2831371	t	2901589	s	O157	rec46	a	O157	++++		a	a	a	t	g	a	a	g	a	g	a	a	a	a	a	a	a	a	a	a	a	a
c	2611518	t	2831383	t	2901601	s	O157	rec46	c	O157	+		c	c	c	c	c	c	c	t	c	t	a	c	c	c	c	c	c	c	c	c	c	c
c	2611533	t	2831398	t	2901616	s	O157	rec46	t	CB9615	+		t	t	t	t	c	t	t	c	t	t	c	c	c	t	t	t	t	t	t	t	t	c
g	2611551	t	2831416	t	2901634	s	O157	rec46	g	O157	+		g	g	g	g	g	g	g	g	g	g	g	g	g	g	t	t	g	g	g	g	g	g
t	2611554	c	2831419	c	2901637	s	O157	rec46	c	CB9615	+		c	c	c	c	c	c	c	c	c	t	c	c	c	c	c	c	c	c	c	c	c	t
a	2611560	g	2831425	g	2901643	s	O157	rec46	g	CB9615	++++		g	g	g	g	g	g	g	g	g	g	g	g	g	g	g	g	g	g	g	g	g	a
t	2611569	c	2831434	c	2901652	s	O157	rec46	c	CB9615	++++		c	c	c	c	c	c	c	g	c	g	c	c	c	c	c	c	c	c	c	c	c	t
g	2611572	c	2831437	c	2901655	s	O157	rec46	c	CB9615	++++		c	c	c	c	c	c	c	a	c	a	c	c	c	c	c	c	c	c	c	c	c	g
a	2611578	g	2831443	g	2901661	s	O157	rec46	g	CB9615	+		g	g	g	a	g	g	g	g	g	g	g	g	g	g	g	g	g	g	g	g	g	a
t	2611588	c	2831453	c	2901671	s	O157	rec46	c	CB9615	+		c	c	c	c	c	c	c	c	c	t	c	c	c	c	c	c	c	c	c	c	c	t
g	2611590	a	2831455	a	2901673	s	O157	rec46	a	CB9615	+		a	a	a	a	a	g	a	a	a	g	a	a	a	a	a	a	a	a	a	a	a	g
t	2611593	c	2831458	c	2901676	s	O157	rec46	c	CB9615	+		c	c	c	c	c	c	c	c	c	t	c	c	c	c	c	c	c	c	c	c	c	t
c	2611641	t	2831506	t	2901724	s	O157	rec46	t	CB9615	+		t	t	t	c	c	c	c	c	t	t	c	c	c	t	c	c	t	t	t	t	t	c
c	2611686	t	2831551	t	2901769	s	O157	rec46	c	O157	++++		c	c	c	t	c	c	c	c	c	c	c	c	c	c	c	c	c	c	c	c	c	c
t	2611789	c	2831654	c	2901872	s	O157	rec46	c	CB9615	+		c	c	c	c	t	t	c	c	c	c	c	c	c	c	c	c	c	c	c	c	c	t
c	2611854	t	2831719	t	2901937	s	O157	rec46	t	CB9615	+		t	t	t	c	t	t	t	c	c	t	c	c	c	t	c	t	t	t	t	t	t	c
a	2611896	c	2831761	c	2901979	s	O157	rec46	c	CB9615	++++		c	c	c	c	c	c	c	c	c	c	c	c	c	c	c	c	c	c	c	c	c	a
c	2611950	t	2831815	t	2902033	s	O157	rec46	t	CB9615	+		t	t	t	c	c	c	c	t	c	c	c	c	c	t	c	t	t	t	t	t	t	c
c	2612941	a	2832806	a	2903024	ns	O157	rec46	c	O157	++++		c	c	c	c	c	c	c	c	c	c	c	c	c	c	c	c	c	c	c	c	c	c
c	2613039	t	2832904	t	2903122	s	O157	rec46	t	CB9615	+		t	t	t	t	c	c	c	t	t	t	t	t	t	t	t	t	c	c	c	t	t	t
g	2613075	a	2832940	a	2903158	s	O157	rec46	a	CB9615	+		g	g	g	g	a	a	a	a	a	a	a	a	a	g	g	a	a	a	a	a	a	g
c	2613114	t	2832979	t	2903197	s	O157	rec46	c	O157	++++		c	c	c	c	c	c	c	c	c	c	c	c	c	c	c	c	c	c	c	c	c	c
g	2613117	a	2832982	a	2903200	s	O157	rec46	g	O157	+		g	g	g	g	g	g	g	g	g	g	g	g	g	g	g	a	g	g	g	a	a	g
c	2613132	t	2832997	t	2903215	s	O157	rec46	c	O157	+		c	c	c	c	c	c	c	c	c	c	c	c	c	c	c	c	t	t	t	c	c	c
g	2613150	c	2833015	c	2903233	s	O157	rec46	c	CB9615	+		c	c	c	c	c	g	g	c	c	c	c	c	c	c	c	c	c	c	c	c	c	c
t	2613153	c	2833018	c	2903236	s	O157	rec46	c	CB9615	+		c	c	c	c	c	t	t	c	c	c	c	c	c	c	c	c	c	c	c	c	c	c
t	2613205	c	2833070	c	2903288	s	O157	rec46	t	O157	+		c	c	c	t	c	c	c	t	t	t	t	t	t	c	c	t	t	t	t	t	t	c
g	2613223	a	2833088	a	2903306	ns	O157	rec46	a	CB9615	+		a	a	a	a	a	a	a	a	a	a	a	a	a	a	a	g	a	a	a	g	g	a
a	2613228	g	2833093	g	2903311	s	O157	rec46	g	CB9615	+		g	g	g	g	g	g	g	g	g	g	g	g	g	g	g	a	g	g	g	a	a	g
t	2613288	c	2833153	c	2903371	s	O157	rec46	c	CB9615	++++		c	c	c	c	c	c	c	c	c	c	c	c	c	c	c	c	c	c	c	c	c	c
a	2613300	g	2833165	g	2903383	s	O157	rec46	g	CB9615	++++		g	g	g	a	g	g	g	g	g	g	g	g	g	g	g	g	g	g	g	g	g	g
g	2613333	a	2833198	a	2903416	s	O157	rec46	g	O157	++++		g	g	g	g	g	g	g	g	g	g	g	g	g	g	g	g	g	g	g	g	g	g
a	2613348	g	2833213	g	2903431	s	O157	rec46	a	O157	+		a	a	a	a	a	a	a	g	g	g	g	g	g	a	g	a	a	a	a	a	a	a
g	2613384	a	2833249	a	2903467	s	O157	rec46	g	O157	+		g	g	g	g	g	g	g	g	g	g	g	g	g	a	a	a	a	a	a	a	a	g
t	2613410	a	2833275	a	2903493	ns	O157	rec46	a	CB9615	++++		a	a	a	a	a	a	a	a	a	a	a	a	a	a	a	a	a	a	a	a	a	a
c	2613531	g	2833396	g	2903614	s	O157	rec46	c	O157	+		c	c	c	g	g	g	c	c	c	c	c	c	c	c	c	c	c	c	c	c	c	g
g	2613807	a	2833672	a	2903890	s	O157	rec46	g	O157	++++		g	g	g	g	g	a	g	g	g	g	g	g	g	g	g	g	g	g	g	g	g	g
t	2614011	c	2833876	c	2904094	s	O157	rec46	c	CB9615	+		t	t	t	c	c	c	c	c	c	c	c	c	c	c	c	c	c	c	c	c	t	c
g	2614380	c	2834245	c	2904463	s	O157	rec46	c	CB9615	++++		c	c	c	g	c	c	c	c	c	c	c	c	c	c	c	c	c	c	c	c	c	c
t	2614414	c	2834279	c	2904497	s	O157	rec46	c	CB9615	++++		c	c	c	c	c	c	c	c	c	c	c	c	c	c	c	c	c	c	c	c	c	t
c	2614467	t	2834332	t	2904550	s	O157	rec46	t	CB9615	++++		t	t	t	t	t	t	t	t	t	t	t	t	t	t	t	t	t	t	t	t	t	t
g	2614570	a	2834435	a	2904653	ns	O157	rec46	a	CB9615	++++		a	a	a	a	a	a	a	a	a	a	a	a	a	a	a	a	a	a	a	a	a	a
g	2614800	a	2834665	a	2904883	s	O157	rec46	g	O157	+		a	a	a	g	g	g	g	g	g	g	g	g	g	g	g	g	a	a	a	a	g	g
g	2614815	a	2834680	a	2904898	s	O157	rec46	g	O157	+		a	a	a	g	g	g	g	a	g	g	g	g	g	g	g	g	a	a	a	a	g	g
c	2614816	t	2834681	t	2904899	s	O157	rec46	c	O157	++++		c	c	c	c	c	c	c	c	c	c	c	c	c	c	c	c	c	c	c	c	c	c
t	2614845	a	2834710	a	2904928	s	O157	rec46	a	CB9615	+		t	t	t	a	a	a	a	a	a	a	a	a	a	a	t	t	a	a	a	t	t	t
t	2614914	c	2834779	c	2904997	s	O157	rec46	c	CB9615	+		c	c	c	t	c	c	c	c	c	c	c	c	c	t	c	c	c	c	c	c	t	c
g	2615001	c	2834866	c	2905084	s	O157	rec46	g	O157	+		g	g	g	c	g	g	g	g	g	g	g	g	g	g	g	g	c	c	c	g	g	g
c	2615103	t	2834968	t	2905186	s	O157	rec46	t	CB9615	++++		t	t	t	t	t	t	t	t	t	t	t	t	t	t	t	t	t	t	t	t	t	t
a	2615277	g	2835142	g	2905360	s	O157	rec46	a	O157	++++		a	a	a	a	a	a	a	a	a	a	a	a	a	a	a	a	a	a	a	a	a	a
t	2615302	c	2835167	c	2905385	s	O157	rec46	c	CB9615	++++		c	c	c	c	c	c	c	c	c	c	c	c	c	c	c	c	c	c	c	c	c	c
g	2615325	c	2835190	c	2905408	s	O157	rec46	c	CB9615	++++		c	c	c	c	t	c	t	t	t	t	t	t	t	c	c	c	c	c	c	c	c	c

O55 and O157 genome site details												Outgroup Strain Details[i]																						
CB9615 base[b]	CB9615 Site[c]	Sakai base[b]	Sakai Site[c]	EDL933 base[b]	EDL933 Site[c]	type[d]	Event Lineage[ef]	Recombinant segment	Inferred ancestral base[g]	Outgroup Analysis[e]	Support level[h]	D1 Sd197	K-12	HS	ATCC 8739	UMN026	IAI39	SMS 3-5	E2348/69	536	ED1a	CFT073	S88	APEC O1	UTI89	E24377A	IAI1	SE11	F5 8401	F2a 2457T	F2a 301	SS Ss046	B4 Sb227	B18 BS512
t	2615328	a	2835193	a	2905411	s	O157	rec46	a	CB9615	++++		a	a	a	a	c	a	c	c	c	c	c	c	c	a	a	a	a	a	a	a	a	a
a	2615340	g	2835205	g	2905423	s	O157	rec46	g	CB9615	+		a	a	a	a	g	g	g	g	g	g	g	g	g	a	g	g	g	g	g	g	a	g
a	2615379	g	2835244	g	2905462	s	O157	rec46	g	CB9615	++++		g	g	g	g	g	g	g	g	g	g	g	g	g	g	g	g	g	g	g	g	g	g
c	2615388	t	2835253	t	2905471	s	O157	rec46	c	O157	+		t	t	t	c	c	c	c	c	c	c	c	c	c	t	c	c	c	c	c	t	t	c
g	2615394	a	2835259	a	2905477	s	O157	rec46	g	O157	+		a	a	a	g	g	g	g	g	g	g	g	g	g	a	g	g	g	g	g	a	a	g
c	2615395	t	2835260	t	2905478	s	O157	rec46	t	CB9615	+		t	t	t	c	t	c	t	t	t	t	t	t	t	t	c	c	c	c	c	t	t	c
c	2615408	t	2835273	t	2905491	ns	O157	rec46	t	CB9615	++++		t	t	t	t	t	t	t	t	t	t	t	t	t	t	t	t	t	t	t	t	t	t
t	2615442	c	2835307	c	2905525	s	O157	rec46	c	CB9615	+		c	c	c	c	c	c	c	c	c	c	c	c	c	t	t	t	t	t	t	c	c	c
a	2615457	g	2835322	g	2905540	s	O157	rec46	g	CB9615	++++		g	g	g	a	g	g	g	g	g	g	g	g	g	g	g	g	g	g	g	g	g	g
t	2615481	a	2835346	a	2905564	s	O157	rec46	a	CB9615	+		a	a	a	a	a	a	a	t	t	t	a	a	a	a	a	a	a	a	a	a	a	a
c	2615499	t	2835364	t	2905582	s	O157	rec46	c	O157	+		c	c	c	t	t	c	t	c	c	c	t	t	t	c	c	c	c	c	c	c	c	t
c	2615502	t	2835367	t	2905585	s	O157	rec46	c	O157	++++		c	c	c	t	c	c	c	c	c	c	c	c	c	c	c	c	c	c	c	c	c	c
g	2615508	a	2835373	a	2905591	s	O157	rec46	g	O157	+		a	a	a	g	g	a	g	g	g	g	g	g	g	g	g	g	g	g	g	a	a	g
g	2615545	a	2835410	a	2905628	ns	O157	rec46	g	O157	++++		g	g	g	g	g	g	g	g	g	g	g	g	g	g	g	g	g	g	g	g	g	g
t	2615551	c	2835416	c	2905634	s	O157	rec46	c	CB9615	++++		c	c	c	t	c	c	c	c	c	c	c	c	c	c	c	c	c	c	c	c	c	c
g	2615568	t	2835433	t	2905651	s	O157	rec46	g	O157	++++		g	g	g	g	g	t	g	g	g	g	g	g	g	g	g	g	-	-	-	g	g	g
c	2615569	t	2835434	t	2905652	s	O157	rec46	c	O157	++++		c	c	c	c	c	t	c	c	c	c	c	c	c	c	c	c	-	-	-	c	c	c
g	2615583	a	2835448	a	2905666	s	O157	rec46	g	O157	+		g	g	g	a	g	g	g	g	g	g	g	g	g	g	a	a	g	g	g	g	g	g
c	2615595	t	2835460	t	2905678	s	O157	rec46	c	O157	++++		c	c	c	c	c	c	c	c	c	c	c	c	c	c	c	c	c	c	c	c	c	c
c	2615625	t	2835490	t	2905708	s	O157	rec46	?	O157	+/-		t	t	t	t	c	t	c	c	c	c	c	c	c	c	t	t	c	c	c	t	t	t
a	2615685	t	2835550	t	2905768	s	O157	rec46	t	CB9615	++++		t	t	t	c	t	c	t	t	t	t	t	t	t	c	t	t	c	c	c	t	t	t
a	2615688	g	2835553	g	2905771	s	O157	rec46	g	CB9615	+		g	g	g	g	g	g	g	g	g	g	g	g	g	a	g	g	a	a	a	g	g	g
g	2615727	a	2835592	a	2905810	s	O157	rec46	a	CB9615	+		a	a	a	g	g	g	g	g	g	g	g	g	g	a	a	a	a	a	a	a	a	a
a	2615745	c	2835610	c	2905828	s	O157	rec46	c	CB9615	+		c	c	c	c	c	a	c	c	c	c	c	c	c	c	a	a	c	c	c	c	c	c
t	2615751	c	2835616	c	2905834	s	O157	rec46	c	CB9615	++++		a	a	a	c	c	c	c	c	c	c	c	c	c	c	c	c	c	c	c	a	a	a
g	2615763	a	2835628	a	2905846	s	O157	rec46	a	CB9615	+		a	a	a	g	g	a	g	g	g	g	g	g	g	a	a	a	a	a	a	a	a	a
t	2615769	c	2835634	c	2905852	s	O157	rec46	c	CB9615	++++		c	c	c	c	c	c	c	c	c	c	c	c	c	c	c	c	c	c	c	c	c	c
g	2615820	a	2835685	a	2905903	s	O157	rec46	g	O157	++++		g	g	g	g	g	g	g	g	g	g	g	g	g	g	g	g	g	g	g	g	g	g
c	2615835	t	2835700	t	2905918	s	O157	rec46	c	O157	++++		c	c	c	c	c	c	c	c	c	c	c	c	c	c	c	c	c	c	c	c	c	c
g	2615844	a	2835709	a	2905927	s	O157	rec46	g	O157	++++		g	g	g	g	g	g	g	g	g	g	g	g	g	g	g	g	g	g	g	g	g	g
g	2615847	c	2835712	c	2905930	s	O157	rec46	c	CB9615	++++		c	c	c	c	c	g	t	t	t	t	t	t	t	c	c	c	c	c	c	c	c	c
t	2615934	c	2835799	c	2906017	s	O157	rec46	c	CB9615	+		c	c	c	c	c	c	t	c	c	t	t	t	t	c	c	c	c	c	c	c	c	c
t	2615937	c	2835802	c	2906020	s	O157	rec46	c	CB9615	+		c	c	c	c	c	c	c	c	c	c	c	c	c	t	t	c	c	c	c	c	c	c
a	2616240	g	2836105	g	2906323	s	O157	rec46	g	CB9615	+		g	g	g	g	g	g	a	a	a	a	a	a	a	g	g	g	g	g	g	g	g	g
c	2616258	a	2836123	a	2906341	s	O157	rec46	c	O157	+		t	t	t	t	t	t	t	t	t	t	t	t	t	t	t	t	t	t	t	t	t	c
t	2616261	c	2836126	c	2906344	s	O157	rec46	c	CB9615	++++		c	c	c	c	c	c	c	c	c	c	c	c	c	c	c	c	c	c	c	c	c	t
t	2616270	a	2836135	a	2906353	ns	O157	rec46	a	CB9615	++++		a	a	a	a	a	a	a	a	a	a	a	a	a	a	a	a	a	a	a	a	a	a
c	2616273	a	2836138	a	2906356	s	O157	rec46	c	O157	+		c	c	c	a	a	a	a	a	a	a	a	a	a	c	c	c	c	c	c	c	c	c
a	2616285	g	2836150	g	2906368	s	O157	rec46	g	CB9615	++++		g	g	g	g	g	g	g	g	g	g	g	g	g	g	g	g	g	g	g	g	g	g
c	2616300	t	2836165	t	2906383	s	O157	rec46	t	CB9615	++++		t	t	t	t	t	t	t	t	t	t	t	t	t	t	t	t	t	t	t	t	t	t
c	2616318	t	2836183	t	2906401	s	O157	rec46	t	CB9615	+		t	t	t	t	t	c	c	c	c	c	c	c	c	t	t	t	t	t	t	c	t	t
g	2616324	a	2836189	a	2906407	s	O157	rec46	a	CB9615	+		a	a	a	g	a	a	a	a	a	a	a	a	a	a	a	a	a	a	a	g	a	a
g	2616327	a	2836192	a	2906410	s	O157	rec46	a	CB9615	+		a	a	a	g	a	g	g	g	g	g	g	g	g	a	a	a	a	a	a	g	a	a
t	2616369	g	2836234	g	2906452	s	O157	rec46	g	CB9615	++++		g	g	g	g	g	g	g	g	g	g	g	g	g	g	g	g	g	g	g	g	g	g
g	2616387	a	2836252	a	2906470	s	O157	rec46	a	CB9615	+		a	a	a	g	g	t	g	g	g	g	g	g	g	a	a	a	a	a	a	a	a	a
a	2616414	g	2836279	g	2906497	s	O157	rec46	g	CB9615	++++		g	g	g	g	g	g	g	g	g	g	g	g	g	g	g	g	g	g	g	g	g	g
c	2616420	t	2836285	t	2906503	s	O157	rec46	t	CB9615	++++		t	t	t	t	t	t	t	t	t	t	t	t	t	t	t	t	t	t	t	t	t	t
a	2616438	t	2836303	t	2906521	s	O157	rec46	t	CB9615	++++		t	t	t	t	t	t	t	t	t	t	t	t	t	t	t	t	t	t	t	t	t	t
a	2616444	g	2836309	g	2906527	s	O157	rec46	g	CB9615	++++		g	g	g	g	g	g	g	g	g	g	g	g	g	g	g	g	g	g	g	g	g	g
t	2616450	g	2836315	g	2906533	s	O157	rec46	g	CB9615	++++		g	g	g	g	g	g	g	g	g	g	g	g	g	g	g	g	g	g	g	g	g	g
c	2616453	g	2836318	g	2906536	s	O157	rec46	g	CB9615	++++		g	g	g	g	g	g	g	g	g	g	g	g	g	g	g	g	g	g	g	g	g	g
a	2616474	c	2836339	c	2906557	s	O157	rec46	c	CB9615	+		c	c	c	c	c	c	a	a	a	a	a	a	a	c	c	c	c	c	c	a	c	c
a	2616477	g	2836342	g	2906560	s	O157	rec46	g	CB9615	+		g	g	g	g	g	g	a	a	a	a	a	a	a	g	g	g	g	g	g	a	g	g
a	2616528	c	2836393	c	2906611	s	O157	rec46	c	CB9615	++++		c	c	c	c	c	c	c	c	c	c	c	c	c	c	c	c	c	c	c	c	c	c
g	2616531	c	2836396	c	2906614	s	O157	rec46	c	CB9615	++++		c	c	c	c	c	c	c	c	c	c	c	c	c	c	c	c	c	c	c	c	c	c
t	2616603	c	2836468	c	2906686	s	O157	rec46	c	CB9615	++++		c	c	c	c	c	c	c	c	c	c	c	c	c	c	c	c	c	c	c	c	c	c
a	2616621	t	2836486	t	2906704	s	O157	rec46	t	CB9615	++++		t	t	t	t	t	t	c	c	c	c	c	c	c	t	t	t	t	t	t	t	t	t
a	2616636	g	2836501	g	2906719	s	O157	rec46	g	CB9615	++++		g	g	g	g	g	g	g	g	g	g	g	g	g	g	g	g	g	g	g	g	g	g
c	2616702	t	2836567	t	2906785	s	O157	rec46	c	O157	++++		c	c	c	c	c	c	c	c	c	c	c	c	c	c	c	c	c	c	c	c	c	c
c	2616738	g	2836603	g	2906821	s	O157	rec46	g	CB9615	++++		g	g	g	g	g	g	a	g	g	g	g	g	g	g	g	g	g	g	g	g	g	g
c	2616873	t	2836738	t	2906956	s	O157	rec46	t	CB9615	+		c	c	c	t	t	c	t	t	t	t	t	t	t	c	c	c	t	t	t	t	c	t
a	2616885	g	2836750	g	2906968	s	O157	rec46	g	CB9615	++++		g	g	g	g	g	g	g	g	g	g	g	g	g	g	g	g	g	g	g	g	g	g
c	2616898	t	2836763	t	2906981	s	O157	rec46	c	O157	+		c	c	c	c	t	c	t	t	t	t	t	t	t	c	c	c	c	c	c	c	c	c
a	2616927	g	2836792	g	2907010	s	O157	rec46	a	O157	+		a	a	a	a	c	a	c	c	c	c	c	c	c	a	a	a	g	g	g	a	a	a
c	2616933	t	2836798	t	2907016	s	O157	rec46	c	O157	++++		c	c	c	c	c	c	c	c	c	c	c	c	c	c	c	c	c	c	c	c	c	c
g	2616966	a	2836831	a	2907049	s	O157	rec46	g	O157	++++		g	g	g	g	g	g	g	g	g	g	g	g	g	g	g	g	g	g	g	g	g	g
a	2616972	c	2836837	c	2907055	s	O157	rec46	c	CB9615	++++		c	c	c	c	c	c	c	c	c	c	c	c	c	c	c	c	c	c	c	c	c	c
a	2616987	g	2836852	g	2907070	s	O157	rec46	g	CB9615	++++		g	g	g	g	g	g	g	g	g	g	g	g	g	g	g	g	g	g	g	g	g	g
c	2617009	t	2836874	t	2907092	ns	O157	rec46	c	O157	++++		c	c	c	c	c	c	c	c	c	c	c	c	c	c	c	c	c	c	c	c	c	c
t	2617119	a	2836984	a	2907202	s	O157	rec46	a	CB9615	+		a	a	a	c	a	t	t	t	t	t	t	t	t	a	a	a	a	a	a	t	a	a
t	2617182	c	2837047	c	2907265	s	O157	rec46	c	CB9615	++++		c	c	c	c	c	c	c	c	c	c	c	c	c	c	c	c	c	c	c	c	c	c
t	2617221	c	2837086	c	2907304	s	O157	rec46	c	CB9615	++++		c	c	c	t	c	c	c	c	c	c	c	c	c	c	c	c	c	c	c	c	c	c
c	2617275	t	2837140	t	2907358	s	O157	rec46	c	O157	++++		c	c	c	c	c	c	c	c	c	c	c	c	c	c	c	c	c	c	c	c	c	c
t	2617278	c	2837143	c	2907361	s	O157	rec46	c	CB9615	++++		c	c	c	c	c	c	c	c	c	c	c	c	c	c	c	c	c	c	c	c	c	c
a	2617389	c	2837254	c	2907472	s	O157	rec46	c	CB9615	++++		c	c	c	c	c	c	c	t	c	c	c	c	c	c	c	c	c	c	c	c	c	c
c	2617393	t	2837258	t	2907476	s	O157	rec46	t	CB9615	++++		t	t	t	t	t	t	t	t	t	t	t	t	t	t	t	t	t	t	t	t	t	t
t	2617395	g	2837260	g	2907478	s	O157	rec46	g	CB9615	++++		g	g	g	g	g	g	g	g	g	g	g	g	g	g	g	g	g	g	g	g	g	g
g	2617575	a	2837440	a	2907658	s	O157	rec46	a	CB9615	++++	a	a	a	a	a	a	a	a	a	a	a	a	a	a	a	a	a	a	a	a	a	a	a
c	2617576	t	2837441	t	2907659	s	O157	rec46	t	CB9615	++	t	t	t	t	c	c	c	c	c	c	c	c	c	c	t	t	t	t	t	t	t	t	t
g	2617584	a	2837449	a	2907667	s	O157	rec46	a	CB9615	++++	a	a	a	a	a	a	a	a	a	a	a	a	a	a	a	a	a	a	a	a	a	a	a
g	2617714	a	2837579	a	2907797	ns	O157	rec46	g	O157	++	g	g	g	g	a	g	g	a	g	g	g	g	g	g	g	g	g	g	g	g	g	g	g
t	2617811	c	2837676	c	2907894	ns	O157	rec46	c	CB9615	++++	c	c	c	c	c	c	c	c	c	c	c	c	c	c	c	c	c	c	c	c	c	c	c
c	2617812	t	2837677	t	2907895	ns	O157	rec46	t	CB9615	++++	t	t	t	t	t	t	t	c	t	t	t	t	t	t	t	t	t	t	t	t	t	t	t
c	2617815	t	2837680	t	2907898	s	O157	rec46	t	CB9615	++++	t	t	t	t	t	t	t	t	t	t	t	t	t	t	t	t	t	t	t	t	t	t	t
a	2617968	g	2837833	g	2908051	s	O157	rec46	a	O157	++	a	a	a	a	g	a	g	a	a	a	a	a	a	a	a	a	a	a	a	a	a	a	g
a	2617977	g	2837842	g	2908060	s	O157	rec46	a	O157	++++	a	a	a	a	a	a	a	a	a	a	a	a	a	a	a	a	a	a	a	a	a	a	a

O55 and O157 genome site details												Outgroup Strain Details[i]																						
CB9615 base[b]	CB9615 Site[c]	Sakai base[b]	Sakai Site[c]	EDL933 base[b]	EDL933 Site[c]	type[d]	Event Lineage[ef]	Recombinant segment	Inferred ancestral base[g]	Outgroup Analysis[e]	Support level[h]	D1 Sd197	K-12	HS	ATCC 8739	UMN026	IAI39	SMS 3-5	E2348/69	536	ED1a	CFT073	S88	APEC O1	UTI89	E24377A	IAI1	SE11	F5 8401	F2a 2457T	F2a 301	SS Ss046	B4 Sb227	B18 BS512
g	2617983	t	2837848	t	2908066	s	O157	rec46	g	O157	++	g	g	g	g	g	g	g	g	g	g	g	g	g	g	t	t	t	g	g	g	g	g	g
c	2618187	t	2838052	t	2908270	s	O157	rec46	c	O157	++	c	t	t	t	t	t	t	t	c	c	c	c	c	c	t	t	t	t	t	t	t	t	t
g	2618308	a	2838173	a	2908391	ns	O157	rec46	g	O157	++++	g	g	g	g	g	g	g	g	g	g	g	g	g	g	g	g	g	g	g	g	g	g	g
a	2618323	g	2838188	g	2908406	ns	O157	rec46	g	CB9615	++++	g	g	g	g	g	g	g	g	g	g	g	g	g	g	g	g	g	g	g	g	g	g	g
g	2618459	a	2838324	a	2908542	s	O157	rec46	g	O157	++	g	a	a	a	a	a	a	a	a	a	a	a	a	a	a	a	a	a	a	a	a	a	a
c	2618462	t	2838327	t	2908545	s	O157	rec46	c	O157	++	c	c	c	c	c	c	c	c	c	c	c	c	c	c	c	t	c	c	c	c	t	c	c
t	2618642	c	2838507	c	2908725	s	O157	rec46	t	O157	++	t	c	c	c	c	g	g	c	g	g	g	g	g	g	c	c	c	t	t	t	c	t	c
g	2618744	a	2838609	a	2908827	s	O157	rec46	g	O157	++	g	g	g	g	g	a	a	g	a	a	a	a	a	a	g	g	g	g	g	g	g	g	g
c	2618759	t	2838624	t	2908842	s	O157	rec46	c	O157	++++	c	c	c	c	c	g	g	c	g	g	g	g	g	g	c	c	c	c	c	c	c	c	c
a	2618789	g	2838654	g	2908872	s	O157	rec46	a	O157	++++	a	a	a	a	a	a	a	a	a	a	a	a	a	a	a	a	a	a	a	a	a	a	a
g	2618981	a	2838846	a	2909064	s	O157	rec46	g	O157	++	g	a	a	a	a	g	g	a	g	g	g	g	g	g	g	g	g	g	g	g	a	g	a
c	2618987	t	2838852	t	2909070	s	O157	rec46	c	O157	++	c	c	c	c	t	g	g	t	g	g	g	g	g	g	c	c	c	c	c	c	c	c	c
c	2618990	t	2838855	t	2909073	s	O157	rec46	c	O157	++	c	t	t	t	c	a	a	t	a	a	a	a	a	a	c	c	c	c	c	c	t	c	t
t	2619005	c	2838870	c	2909088	s	O157	rec46	t	O157	++	t	c	c	c	t	c	c	c	c	c	c	c	c	c	c	c	t	c	c	c	c	c	c
c	2619009	t	2838874	t	2909092	s	O157	rec46	c	O157	++	c	t	t	t	t	t	t	t	t	t	t	t	t	t	t	t	c	t	t	t	t	t	t
t	2619011	g	2838876	g	2909094	s	O157	rec46	t	O157	++	t	a	a	a	g	g	g	g	g	g	g	g	g	g	g	a	g	g	g	g	a	g	a
t	2619012	c	2838877	c	2909095	s	O157	rec46	t	O157	++	t	c	c	c	c	c	c	c	c	c	c	c	c	c	c	c	t	c	c	c	c	c	c
c	2619017	a	2838882	a	2909100	s	O157	rec46	c	O157	++	c	a	a	a	a	t	t	a	t	t	t	t	t	t	a	a	c	a	a	a	a	a	a
c	2619023	a	2838888	a	2909106	s	O157	rec46	c	O157	++	c	a	a	a	a	c	c	a	a	a	a	a	a	a	a	a	c	a	a	a	a	a	a
g	2619029	a	2838894	a	2909112	s	O157	rec46	g	O157	++	g	a	a	a	g	a	a	a	a	a	a	a	a	a	a	a	g	a	a	a	a	a	a
g	2619032	t	2838897	t	2909115	s	O157	rec46	g	O157	++	g	t	t	t	g	g	g	t	g	g	g	g	g	g	t	t	g	t	t	t	t	t	t
g	2619035	c	2838900	c	2909118	s	O157	rec46	g	O157	++	g	c	c	c	g	g	g	c	g	g	g	g	g	g	c	c	g	c	c	c	c	c	c
c	2619038	t	2838903	t	2909121	s	O157	rec46	c	O157	++	c	t	t	t	c	c	c	t	t	t	t	t	t	t	t	t	c	t	t	t	t	t	t
a	2619071	t	2838936	t	2909154	s	O157	rec46	a	O157	++	a	t	t	t	t	t	t	t	t	t	t	t	t	t	t	t	t	t	t	t	t	t	t
g	2619080	a	2838945	a	2909163	s	O157	rec46	g	O157	++	g	a	a	a	a	a	g	a	a	a	a	a	a	a	a	a	a	a	a	a	a	a	a
t	2619143	g	2839008	g	2909226	s	O157	rec46	t	O157	++	t	a	a	a	g	a	g	g	a	a	a	a	a	a	g	a	g	g	g	g	a	a	a
a	2619146	g	2839011	g	2909229	s	O157	rec46	a	O157	++	a	g	g	g	g	g	g	g	g	g	g	g	g	g	g	g	g	g	g	g	g	g	g
c	2619329	t	2839194	t	2909412	s	O157	rec46	t	CB9615	++	t	c	c	c	c	t	t	t	c	c	c	c	c	c	t	c	t	t	t	t	c	c	c
g	2619410	a	2839275	a	2909493	s	O157	rec46	g	O157	++++	g	g	g	g	g	g	g	g	g	g	g	g	g	g	g	g	g	g	g	g	g	g	g
a	2619413	c	2839278	c	2909496	s	O157	rec46	c	CB9615	++	c	c	c	c	c	c	t	a	a	a	a	a	a	a	c	c	c	c	c	c	c	c	c
c	2619434	t	2839299	t	2909517	s	O157	rec46	c	O157	++	c	c	c	c	c	c	c	c	c	c	c	c	c	c	t	t	t	c	c	c	c	c	c
t	2619479	c	2839344	c	2909562	s	O157	rec46	t	O157	++	t	t	t	t	t	t	t	t	t	t	t	t	t	t	t	t	t	c	c	c	t	t	t
t	2619530	g	2839395	g	2909613	s	O157	rec46	g	CB9615	++	g	g	g	g	g	g	g	g	g	g	a	g	g	g	c	c	t	g	g	g	t	t	g
a	2619587	g	2839452	g	2909670	s	O157	rec46	g	CB9615	++++	g	g	g	g	g	g	g	g	g	g	g	g	g	g	g	g	g	g	g	g	g	g	g
a	2619599	g	2839464	g	2909682	s	O157	rec46	g	CB9615	++++	g	g	g	g	g	g	g	g	g	g	g	g	g	g	g	g	g	g	g	g	g	g	g
a	2619659	g	2839524	g	2909742	s	O157	rec46	g	CB9615	++	g	g	g	g	g	g	g	g	g	g	g	g	g	g	a	a	g	g	g	g	a	a	g
t	2619698	c	2839563	c	2909781	s	O157	rec46	t	O157	++	t	t	t	t	a	a	a	c	c	c	c	c	c	c	c	c	c	c	c	c	c	c	t
t	2619723	c	2839588	c	2909806	s	O157	rec46	c	CB9615	++++	c	c	c	c	c	c	c	c	c	c	c	c	c	c	c	c	c	c	c	c	c	c	c
t	2619773	c	2839638	c	2909856	s	O157	rec46	c	CB9615	++	c	t	t	t	t	t	t	c	c	c	c	c	c	c	t	t	t	c	c	c	t	t	t
c	2619986	t	2839851	t	2910069	ns	O157	rec46	c	O157	++++	c	c	c	c	c	c	c	c	c	c	c	c	c	c	c	c	c	g	g	g	c	c	c
t	2619991	c	2839856	c	2910074	s	O157	rec46	t	O157	++++	t	t	t	t	t	t	t	t	t	t	t	t	t	t	t	t	t	t	t	t	t	t	t
a	2620003	g	2839868	g	2910086	s	O157	rec46	a	O157	++++	a	a	a	a	a	a	a	a	a	a	a	a	a	a	a	a	a	a	a	a	a	a	a
a	2620006	g	2839871	g	2910089	s	O157	rec46	a	O157	++++	a	a	a	a	a	a	a	a	a	a	a	a	a	a	a	a	a	a	a	a	a	a	a
t	2620042	c	2839907	c	2910125	s	O157	rec46	c	CB9615	++++	c	c	c	c	c	c	c	c	c	c	c	c	c	c	c	c	c	c	c	c	c	c	c
t	2620052	c	2839917	c	2910135	s	O157	rec46	t	O157	++	t	c	c	c	t	c	c	c	c	c	c	c	c	c	c	c	c	c	c	c	t	t	c
g	2620108	a	2839973	a	2910191	s	O157	rec46	a	CB9615	++	a	g	g	g	g	a	a	g	a	g	a	g	g	g	g	g	g	g	g	g	g	g	g
c	2620138	a	2840003	a	2910221	s	O157	rec46	c	O157	+++	g	g	g	g	c	c	c	g	c	g	c	g	g	g	g	g	g	g	g	g	c	c	g
a	2620141	g	2840006	g	2910224	s	O157	rec46	g	CB9615	++	g	g	g	g	a	g	g	g	a	g	a	g	g	g	g	g	g	g	g	g	a	a	g
t	2620162	c	2840027	c	2910245	s	O157	rec46	c	CB9615	++	c	t	t	t	t	t	c	c	t	c	t	c	c	c	t	t	t	c	c	c	c	c	c
t	2620165	c	2840030	c	2910248	s	O157	rec46	c	CB9615	++	c	t	t	t	t	t	c	t	t	t	t	t	t	t	t	t	t	c	c	c	c	c	c
c	2620168	t	2840033	t	2910251	s	O157	rec46	c	O157	++++	c	c	c	c	c	c	c	c	c	c	c	c	c	c	c	c	c	c	c	c	c	c	c
t	2620171	c	2840036	c	2910254	s	O157	rec46	c	CB9615	++	c	t	t	t	t	t	c	c	c	c	c	c	c	c	t	t	t	c	c	c	c	c	c
c	2620204	t	2840069	t	2910287	s	O157	rec46	t	CB9615	++	t	c	c	c	c	c	c	c	c	c	c	c	c	c	c	c	c	t	t	t	c	c	c
c	2620240	t	2840105	t	2910323	s	O157	rec46	t	CB9615	++	t	c	c	c	t	c	t	t	c	t	c	t	t	t	c	c	c	c	c	c	c	c	c
a	2620270	g	2840135	g	2910353	s	O157	rec46	a	O157	++	a	g	g	g	g	g	g	g	g	g	g	g	g	g	g	g	g	g	g	g	g	g	g
a	2620351	g	2840216	g	2910434	s	O157	rec46	g	CB9615	++++	g	g	g	g	g	g	g	g	g	g	g	g	g	g	g	g	g	t	t	t	g	g	g
c	2620441	t	2840306	t	2910524	s	O157	rec46	c	O157	++	c	c	c	c	c	c	c	t	t	t	t	t	t	t	c	c	c	c	c	c	c	c	c
t	2620528	c	2840393	c	2910611	s	O157	rec46	t	O157	++	t	c	c	c	c	c	c	c	c	c	c	c	c	c	t	t	t	t	t	t	t	t	c
g	2620602	a	2840467	a	2910685	i	O157	rec46	g	O157	++	g	g	g	g	a	a	a	c	c	c	c	c	c	c	g	g	g	g	g	g	g	g	g
a	2620795	g	2840660	g	2910878	ns	O157	rec46	a	O157	++++	a	a	a	a	a	a	a	a	a	a	a	a	a	a	a	a	a	a	a	a	a	a	a
t	2620835	g	2840700	g	2910918	s	O157	rec46	t	O157	++++	t	t	t	t	t	t	t	t	t	t	t	t	t	t	t	t	t	t	t	t	t	t	t
t	2620859	c	2840724	c	2910942	s	O157	rec46	c	CB9615	++++	c	c	c	c	c	c	c	c	c	c	c	c	c	c	c	c	c	c	c	c	a	a	c
c	2621009	t	2840874	t	2911092	s	O157	rec46	c	O157	++	c	c	c	c	c	c	c	t	t	t	t	t	t	t	c	c	c	c	c	c	c	c	c
-	2621124	g	2840990	g	2911208	ins-12	O157	rec46	?	O157	+/-																							
g	2621888	c	2841005	c	2911223	i	O157	rec46	c	CB9615	++++		c	c	c	c			c										c	c	c	c		
g	2621891	t	2841008	t	2911226	i	O157	rec46	t	CB9615	+++		-	-	-	t			-										t	t	t	-		
t	2621893	c	2841010	c	2911228	i	O157	rec46	c	CB9615	++++		c	c	c	c			c										c	c	c	c		
t	2621894	a	2841011	a	2911229	i	O157	rec46	a	CB9615	++++		a	a	a	a			a										a	a	a	a		
t	2622126	c	2841243	c	2911461	s	O157	rec46	t	O157	++++	t	t	t	t	t	t	t	t	t	t	t	t	t	t	t	t	t	t	t	t	t	t	t
c	2622327	t	2841444	t	2911662	s	O157	rec46	c	O157	++++	c	c	c	c	t	c	c	c	c	c	c	c	c	c	c	c	c	c	c	c	c	c	c
g	2622336	a	2841453	a	2911671	s	O157	rec46	g	O157	++	g	g	g	g	a	g	g	a	a	a	a	a	a	a	g	g	g	g	g	g	g	g	g
a	2622351	g	2841468	g	2911686	s	O157	rec46	a	O157	++	a	g	a	a	g	g	g	a	a	a	a	a	a	a	g	g	g	g	g	g	a	g	g
c	2622387	a	2841504	a	2911722	s	O157	rec46	a	CB9615	++	a	c	c	c	c	a	a	t	t	t	t	t	t	t	c	c	c	a	a	a	c	a	c
t	2622444	c	2841561	c	2911779	s	O157	rec46	c	CB9615	++	c	c	c	c	c	t	t	c	c	c	c	c	c	c	c	c	c	t	t	t	c	t	c
c	2622546	t	2841663	t	2911881	s	O157	rec46	c	O157	++	c	c	t	t	c	c	c	c	c	c	c	c	c	c	c	c	c	c	c	c	t	c	c
a	2622615	c	2841732	c	2911950	ns	O157	rec46	c	CB9615	++++	c	t	c	c	c	c	c	c	c	c	c	c	c	c	t	t	t	c	c	c	c	c	t
g	2622717	a	2841834	a	2912052	s	O157	rec46	a	CB9615	++	a	g	a	a	g	a	a	a	a	a	a	a	a	a	g	g	g	a	a	a	a	a	g
t	2622819	g	2841936	g	2912154	s	O157	rec46	g	CB9615	++++	g	g	g	g	g	g	g	g	g	g	g	g	g	g	g	g	g	g	g	g	g	g	g
t	2622855	c	2841972	c	2912190	s	O157	rec46	c	CB9615	++	c	t	c	c	c	t	t	c	c	c	c	c	c	c	t	t	t	t	t	t	c	t	t
c	2622861	t	2841978	t	2912196	s	O157	rec46	c	O157	++	c	c	t	t	c	a	a	c	c	c	c	c	c	c	c	c	c	c	c	c	t	c	c
c	2622900	t	2842017	t	2912235	s	O157	rec46	t	CB9615	++	t	c	c	c	c	c	c	c	c	c	c	c	c	c	c	c	c	c	c	c	t	c	c
c	2622903	t	2842020	t	2912238	s	O157	rec46	t	CB9615	++	t	c	t	t	t	t	t	t	t	t	t	t	t	t	c	c	c	c	c	c	t	c	c
t	2622939	c	2842056	c	2912274	s	O157	rec46	c	CB9615	++++	c	c	c	c	c	c	c	c	c	c	c	c	c	c	c	c	c	c	c	c	c	c	c
c	2622942	t	2842059	t	2912277	s	O157	rec46	t	CB9615	++++	t	t	t	t	t	t	t	t	t	t	t	t	t	t	t	t	t	t	t	t	t	t	t
c	2622960	t	2842077	t	2912295	s	O157	rec46	t	CB9615	++++	t	t	t	t	t	t	t	g	g	t	g	g	g	g	t	t	t	t	t	t	t	t	t
c	2623323	t	2842440	t	2912658	s	O157	rec46	c	O157	++++	c	c	c	c	c	c	c	c	c	c	c	c	c	c	c	c	c	c	c	c	c	c	c

O55 and O157 genome site details												Outgroup Strain Details[i]																						
CB9615 base[b]	CB9615 Site[c]	Sakai base[b]	Sakai Site[c]	EDL933 base[b]	EDL933 Site[c]	type[d]	Event Lineage[ef]	Recombinant segment	Inferred ancestral base[g]	Outgroup Analysis[e]	Support level[h]	D1 Sd197	K-12	HS	ATCC 8739	UMN026	IAI39	SMS 3-5	E2348/69	536	ED1a	CFT073	S88	APEC O1	UTI89	E24377A	IAI1	SE11	F5 8401	F2a 2457T	F2a 301	SS Ss046	B4 Sb227	B18 BS512
a	2623335	c	2842452	c	2912670	s	O157	rec46	a	O157	++	a	c	a	a	c	c	c	c	c	c	c	c	c	c	c	c	c	c	c	c	a	a	c
a	2623434	t	2842551	t	2912769	nc	O157	rec46	t	CB9615	++++	t	t	t	t	t	t	t	t	t	t	t	t	t	t	t	t	t	t	t	t	t	t	t
g	2623539	a	2842656	a	2912874	i	O157	rec46	g	O157	++	g	g	g	g	g	g	g	g	g		g			g	g	g	g	a	a	a	g	g	g
c	2623541	a	2842658	a	2912876	ns	O157	rec46	c	O157	++	c	a	a	a	c	a	a	c	c		c			c	c	c	c	a	a	a	c	c	a
a	2623564	g	2842681	g	2912899	s	O157	rec46	g	CB9615	++++	g	g	g	g	g	g	g	g	g		g			g	g	g	g	g	g	g	g	g	g
a	2623599	g	2842716	g	2912934	ns	O157	rec46	g	CB9615	++++	g	t	t	t	g	t	t	t	t		t			t	g	g	g	g	g	g	g	g	t
c	2623618	a	2842735	a	2912953	ns	O157	rec46	a	CB9615	++	a	a	a	a	a	a	a	a	a		a			a	a	a	a	c	c	c	a	a	a
c	2623632	a	2842749	a	2912967	ns	O157	rec46	a	CB9615	++	a	c	c	c	c	c	c	c	c		c			c	a	a	a	c	c	c	a	a	c
g	2623638	a	2842755	a	2912973	s	O157	rec46	a	CB9615	++	a	g	g	g	a	g	g	g	g		g			g	a	a	a	g	g	g	a	a	g
a	2623653	t	2842770	t	2912988	ns	O157	rec46	t	CB9615	++++	t	t	t	t	t	t	t	t	t		t			t	t	t	t	t	t	t	t	t	g
g	2623902	a	2843019	a	2913237	s	O157	rec46	a	CB9615	++	a	a	a	a	a	g	g	g	g	g	g	a	a	a	a	a	a	a	a	a	a	a	a
t	2623913	c	2843030	c	2913248	ns	O157	rec46	c	CB9615	++++	c	c	c	c	c	c	c	c	c	c	c	c	c	c	c	c	c	c	c	c	c	c	c
g	2623989	t	2843106	t	2913324	ns	O157	rec46	t	CB9615	++++	t	t	t	t	t	t	t	t	t	t	t	t	t	t	t	t	t	t	t	t	t	t	t
c	2623994	t	2843111	t	2913329	ns	O157	rec46	t	CB9615	++	t	t	t	t	t	t	t	c	c	c	c	t	t	t	t	t	t	t	t	t	t	t	t
c	2624095	a	2843212	a	2913430	i	O157	rec46	c	O157	++++	c	c	c	c	c	c	c	c	c	c	c	c	c	c	c	c	c	c	c	c	c	c	c
g	2624155	a	2843272	a	2913490	i	O157	rec46	g	O157	++++	-	g	g	g	g	g	g	g	g	g	a	g	g	g	g	g	g	g	g	g	g	-	g
t	2624180	a	2843297	a	2913515	i	O157	rec46	t	O157	++++	t	t	t	t	t	t	t	t	t	t	t	t	t	t	t	t	t	t	t	t	t	t	t
g	2624191	a	2843308	a	2913526	i	O157	rec46	g	O157	++	g	a	a	a	a	a	a	a	a	a	a	a	a	a	a	a	g	a	a	a	a	g	a
c	2624205	t	2843322	t	2913540	i	O157	rec46	c	O157	++	c	t	t	t	t	t	t	t	t	t	t	t	t	t	t	t	c	t	t	t	t	c	t
a	2624237	g	2843354	g	2913572	i	O157	rec46	a	O157	++	a	g	g	g	a	a	a	a	a	a	a	a	a	a	g	g	g	g	g	g	g	a	a
a	2624244	g	2843361	g	2913579	i	O157	rec46	a	O157	++	a	g	g	g	a	a	a	a	a	a	a	a	a	a	g	g	g	g	g	g	g	a	a
t	2624255	c	2843372	c	2913590	i	O157	rec46	t	O157	++	t	c	c	c	c	c	c	c	c	c	c	t	t	t	c	c	c	c	c	c	c	t	t
c	2624291	t	2843408	t	2913626	i	O157	rec46	c	O157	++	c	t	t	t	t	-	-	-	-	-	-	t	t	t	t	t	t	t	t	t	t	t	t
t	2624309	a	2843426	a	2913644	i	O157	rec46	t	O157	++++	t	t	t	t	t	t	t	t	t	t	t	t	t	t	t	t	t	t	t	t	t	t	t
t	2624315	c	2843432	c	2913650	i	O157	rec46	c	CB9615	++++	c	c	c	c	c	c	c	c	c	c	c	c	c	c	c	c	c	c	c	c	c	c	c
c	2624372	t	2843489	t	2913707	i	O157	rec46	c	O157	++++	c	c	c	c	c	c	c	c	c	c	c	c	c	c	c	c	c	c	c	c	c	c	c
t	2624454	c	2843571	c	2913789	s	O157	rec46	t	O157	++	t	c	c	c	t	c	c	c	c	c	c	c	c	c	c	c	c	c	c	c	c	c	c
a	2624459	c	2843576	c	2913794	s	O157	rec46	a	O157	++	a	c	c	c	a	c	c	c	c	c	c	c	c	c	c	c	c	c	c	c	c	c	c
a	2624649	g	2843766	g	2913984	ns	O157	rec46	g	CB9615	++++	g	g	g	g	g	g	g	g	g	g	g	g	g	g	g	g	g	g	g	g	g	g	g
a	2624735	g	2843852	g	2914070	s	O157	rec46	g	CB9615	++++	g	g	g	g	g	g	g	g	g	g	g	g	g	g	g	g	g	g	g	g	g	g	g
g	2624825	a	2843942	a	2914160	s	O157	rec46	a	CB9615	++	a	a	a	a	a	a	a	a	a	g	a	a	a	a	a	a	a	a	a	a	a	a	g
c	2625288	t	2844405	t	2914623	s	O157	rec46	t	CB9615	++	t	c	c	c	t	t	t	t	t		t	t	t	t	t	t	t	t	t	t	t	t	t
g	2625290	a	2844407	a	2914625	s	O157	rec46	a	CB9615	++	a	g	g	g	g	a	a	g	g		g	g	g	g	a	a	a	a	a	a	a	a	a
g	2635066	a	2844543	a	2914761	s	O157	rec46	a	CB9615	++++	a	a	a	a	a	a	a	a	a		a	a	a	a	a	a	a	a	a	a	a	a	a
c	2635072	a	2844549	a	2914767	ns	O157	rec46	a	CB9615	++	a	c	c	c	c	c	c	c	c		c	c	c	c	c	c	c	c	c	c	c	c	c
a	2635081	g	2844558	g	2914776	s	O157	rec46	g	CB9615	++	g	a	a	a	g	g	a	a	a		a	a	a	a	a	a	a	a	a	a	a	a	a
g	2635558	a	2845035	a	2915253	s	O157	rec46	g	O157	++++	g	g	g	g	g	g	g	g	g		g	g	g	g	g	g	g	g	g	g	g	g	g
g	2635612	a	2845089	a	2915307	s	O157	rec46	a	CB9615	++	a	a	a	a	g	g	g	g	g		g	g	g	g	g	g	g	g	g	g	g	g	g
t	2635645	c	2845122	c	2915340	s	O157	rec46	c	CB9615	++++	c	c	c	c	c	c	c	c	c		c	c	c	c	c	c	c	c	c	c	c	c	c
a	2635968	t	2845445	t	2915663	ns	O157	rec46	t	CB9615	++++	t	t	t	t	t	a	t	t	t		t	t	t	t	t	t	t	t	t	t	t	t	t
c	2636161	g	2845638	g	2915856	s	O157	rec46	g	CB9615	++++	g	g	g	g	g	g	g	g	g		g	g	g	g	g	g	g	g	g	g	g	g	g
c	2636170	t	2845647	t	2915865	s	O157	rec46	c	O157	++++	c	c	c	c	c	c	c	c	c		c	c	c	c	c	c	c	c	c	c	c	c	c
c	2636212	g	2845689	g	2915907	s	O157	rec46	g	CB9615	++++	g	g	g	g	g	g	g	g	g		g	g	g	g	g	g	g	g	g	g	g	g	g
c	2636263	t	2845740	t	2915958	s	O157	rec46	t	CB9615	++	t	t	t	t	c	c	c	c	c		a	c	c	c	t	t	t	t	t	t	t	t	t
c	2636277	t	2845754	t	2915972	ns	O157	rec46	t	CB9615	++	t	t	t	t	c	c	c	c	c		c	c	c	c	t	t	t	t	t	t	t	t	t
c	2636371	a	2845848	a	2916066	s	O157	rec46	a	CB9615	++	a	c	c	c	a	a	a	a	a		a	a	a	a	c	a	c	c	c	c	c	a	a
t	2636509	c	2845986	c	2916204	s	O157	rec46	t	O157	++	t	c	c	c	t	t	t	t	t		t	c	c	c	t	c	t	c	c	c	c	c	c
c	2636566	t	2846043	t	2916261	s	O157	rec46	t	CB9615	++	t	t	t	t	t	t	t	t	t		t	t	t	t	c	t	c	t	t	t	t	t	t
g	2636655	a	2846132	a	2916350	s	O157	rec46	g	O157	++	g	g	g	g	a	a	g	g	g		g	a	a	a	a	g	a	a	a	a	a	g	g
a	2636713	g	2846190	g	2916408	s	O157	rec46	g	CB9615	++	g	g	g	g	g	g	a	g	g		g	g	g	g	a	g	a	g	g	g	g	g	g
g	2636951	a	2846428	a	2916646	i	O157	rec46	g	O157	++++	g	g	g	g	g	g	g	g	g		g	g	g	g	g	g	g	g	g	g	g	g	g
g	2637004	a	2846481	a	2916699	ns	O157	rec46	g	O157	++	g	a	a	a	g	g	g	g	a		g	g	g	g	a	g	a	g	g	g	g	g	a
c	2637060	a	2846537	a	2916755	s	O157	rec46	a	CB9615	++	a	c	c	c	a	a	c	a	a		a	a	a	a	a	c	a	c	c	c	a	c	c
a	2637233	c	2846710	c	2916928	ns	O157	rec46	c	CB9615	++++	c	c	c	c	c	c	c	c	c		c	c	c	c	c	c	c	c	c	c	c	c	c
a	2637321	g	2846798	g	2917016	s	O157	rec46	a	O157	++	a	g	g	g	g	g	a	a	a		a	a	a	a	a	a	a	g	g	g	g	a	a
c	2637357	t	2846834	t	2917052	s	O157	rec46	c	O157	++	c	c	c	c	c	t	c	c	c		c	c	c	c	c	c	c	t	t	t	t	t	c
c	2637369	a	2846846	a	2917064	s	O157	rec46	c	O157	++++	c	c	c	c	c	c	c	c	c		c	c	c	c	c	c	c	c	c	c	c	c	c
c	2637372	a	2846849	a	2917067	s	O157	rec46	c	O157	++	c	c	c	c	c	a	c	c	c		c	a	a	a	a	c	a	a	a	a	a	a	a
g	2637402	a	2846879	a	2917097	s	O157	rec46	g	O157	++	g	g	g	g	g	g	g	a	a		a	g	g	g	g	g	g	a	a	a	g	g	g
c	2637567	t	2847044	t	2917262	s	O157	rec46	c	O157	++	c	t	t	t	c	c	c	c	c		c	c	c	c	t	c	t	c	c	c	c	t	t
a	2637576	c	2847053	c	2917271	s	O157	rec46	c	CB9615	++	c	c	c	c	a	a	a	a	a		a	a	a	a	c	a	c	a	a	a	a	c	c
t	2637579	c	2847056	c	2917274	s	O157	rec46	c	CB9615	++	c	t	t	t	t	t	c	t	t		t	t	t	t	t	t	t	t	t	t	t	t	t
t	2637625	c	2847102	c	2917320	ns	O157	rec46	c	CB9615	++++	c	c	c	c	c	c	c	c	c		c	c	c	c	c	c	c	c	c	c	c	c	c
t	2637732	a	2847209	a	2917427	s	O157	rec46	t	O157	++	t	a	a	a	a	a	a	a	a		a	a	a	a	a	a	a	a	a	a	a	a	a
a	2637753	g	2847230	g	2917448	s	O157	rec46	a	O157	++	a	g	g	g	g	g	g	g	g		g	g	g	g	g	g	g	g	g	g	g	g	g
g	2637756	a	2847233	a	2917451	s	O157	rec46	g	O157	++	g	a	a	a	a	a	a	a	a		a	a	a	a	a	a	a	a	a	a	a	a	a
a	2637816	g	2847293	g	2917511	s	O157	rec46	g	CB9615	++	g	a	a	a	g	g	g	g	g		g	g	g	g	a	a	a	a	a	a	a	a	a
c	2637900	t	2847377	t	2917595	s	O157	rec46	c	O157	++	c	t	t	t	c	c	c	c	c		c	c	c	c	t	t	t	c	c	c	t	c	t
g	2638178	a	2847655	a	2917873	s	O157	rec46	a	CB9615	++++	a	a	a	a	a	a	a	a	a		a	a	a	a	a	a	a	a	a	a	a	a	a
g	2638449	a	2847926	a	2918144	s	O157	rec46	a	CB9615	++++	a	a	a	a	g	a	a	a	a		a	a	a	a	a	a	a	a	a	a	a	a	a
a	2638452	c	2847929	c	2918147	s	O157	rec46	c	CB9615	++++	c	c	c	c	a	c	c	c	c		c	c	c	c	c	c	c	c	c	c	c	c	c
t	2638455	c	2847932	c	2918150	ns	O157	rec46	t	O157	++	t	t	t	t	t	c	c	c	c		c	c	c	c	t	t	c	c	c	c	t	c	t
t	2638456	c	2847933	c	2918151	ns	O157	rec46	t	O157	++	t	t	t	t	t	c	c	c	c		c	c	c	c	t	t	c	c	c	c	t	c	t
g	2638461	a	2847938	a	2918156	s	O157	rec46	a	CB9615	++++	a	a	a	a	g	a	c	a	a		a	a	a	a	a	a	a	a	a	a	a	a	a
a	2638464	g	2847941	g	2918159	s	O157	rec46	g	CB9615	++++	g	g	g	g	a	g	g	g	g		g	g	g	g	g	g	g	g	g	g	g	g	g
a	2638467	g	2847944	g	2918162	s	O157	rec46	g	CB9615	++	g	g	g	g	a	g	g	g	g		g	g	g	g	g	g	a	a	a	a	g	a	g
a	2638482	g	2847959	g	2918177	s	O157	rec46	g	CB9615	++	g	a	a	a	g	g	g	g	g		g	g	g	g	a	a	a	a	a	a	a	a	a
a	2638817	c	2848294	c	2918512	ns	O157	rec46	c	CB9615	++++	c	c	c	c	c	c	c	c	c		c	c	c	c	c	c	c	c	c	c	c	c	c
a	2638882	g	2848359	g	2918577	ns	O157	rec46	g	CB9615	++++	g	g	g	g	g	g	g	g	g		g	g	g	g	g	g	g	g	g	g	g	g	g
c	2639406	t	2848883	t	2919101	s	O157	rec46	t	CB9615	+		t	t	t	c	c	c	t	t		t	t	t	t	t	t	t	t	t	t	t	t	t
c	2639415	t	2848892	t	2919110	s	O157	rec46	c	O157	++	c	t	t	t	c	c	c	t	t		t	t	t	t	t	t	t	t	t	t	t	t	t
g	2639520	a	2848997	a	2919215	s	O157	rec46	a	CB9615	++	a	g	g	g	a	a	a	a	a		g	a	a	a	g	g	g	g	g	g	g	g	g
g	2639640	a	2849117	a	2919335	s	O157	rec46	g	O157	++++	g	g	g	g	g	t	t	t	t		t	t	t	t	g	g	g	g	g	g	g	g	g
c	2639751	t	2849228	t	2919446	s	O157	rec46	c	O157	++++	c	c	c	c	c	c	c	c	c		c	c	c	c	c	c	c	c	c	c	c	c	c
g	2640091	a	2849568	a	2919786	ns	O157	rec46	g	O157	++++	g	g	g	g	g	g	g	g	g		g	g	g	g	g	g	g	g	g	g	g	g	g
a	2640126	g	2849603	g	2919821	s	O157	rec46	a	O157	++	a	a	a	a	g	a	a	g	g		g	g	g	g	a	a	a	a	a	a	a	a	a

O55 and O157 genome site details												Outgroup Strain Details[i]																						
CB9615 base[b]	CB9615 Site[c]	Sakai base[b]	Sakai Site[c]	EDL933 base[b]	EDL933 Site[c]	type[d]	Event Lineage[ef]	Recombinant segment	Inferred ancestral base[g]	Outgroup Analysis[e]	Support level[h]	D1 Sd197	K-12	HS	ATCC 8739	UMN026	IAI39	SMS 3-5	E2348/69	536	ED1a	CFT073	S88	APEC O1	UTI89	E24377A	IAI1	SE11	F5 8401	F2a 2457T	F2a 301	SS Ss046	B4 Sb227	B18 BS512
g	2640129	a	2849606	a	2919824	s	O157	rec46	g	O157	++++	g	g	g	g	g	g	g	g	g		g	g	g	g	g	g	g	g	g	g	g	g	g
g	2640144	a	2849621	a	2919839	s	O157	rec46	g	O157	++	g	g	g	g	g	g	g	a	a		a	a	a	a	g	g	g	g	g	g	g	g	g
t	2640147	c	2849624	c	2919842	s	O157	rec46	t	O157	++	t	t	t	t	t	t	t	c	c		c	c	c	c	t	t	t	t	t	t	t	t	t
c	2640159	t	2849636	t	2919854	s	O157	rec46	c	O157	++	c	c	c	c	c	c	c	t	t		t	t	t	t	c	c	c	c	c	c	t	c	c
a	2640162	g	2849639	g	2919857	s	O157	rec46	a	O157	++	a	a	a	a	a	a	a	g	g		g	g	g	g	a	a	a	a	a	a	g	a	a
t	2640179	c	2849656	c	2919874	ns	O157	rec46	t	O157	++	t	t	t	t	c	c	c	c	c		c	c	c	c	t	t	t	t	t	t	t	t	t
t	2641036	c	2850513	c	2920731	s	O157	rec46	t	O157	++	t	t	t	t	t	c	c	c	c		c	c	c	c	t	t	t	t	t	t	t	t	t
t	2641054	a	2850531	a	2920749	ns	O157	rec46	t	O157	++++	t	t	t	t	t	t	t	t	t		t	t	t	t	t	t	t	t	t	t	t	t	t
t	2641083	a	2850560	a	2920778	ns	O157	rec46	a	CB9615	++	a	a	a	a	a	t	t	t	t		t	t	t	t	a	a	a	a	a	a	a	a	a
a	2641216	g	2850693	g	2920911	s	O157	rec46	a	O157	++++	a	a	a	a	a	a	a	a	a		a	a	a	a	a	a	a	a	a	a	a	a	a
t	2641231	c	2850708	c	2920926	s	O157	rec46	t	O157	++	t	t	t	t	t	c	c	c	c		t	c	c	c	t	t	t	t	t	t	t	t	t
t	2641275	c	2850752	c	2920970	i	O157	rec46	c	CB9615	++	c	t	t	t	t	c	t	c	c		t	c	c	c	t	t	t	c	t	t	t	t	t
t	2641285	a	2850762	a	2920980	i	O157	rec46	t	O157	++++	t	t	t	t	t	t	t	t	t		t	t	t	t	t	t	t	t	t	t	c	t	t
t	2641304	c	2850781	c	2920999	i	O157	rec46	t	O157	++	t	t	t	t	c	t	t	t	c		c	c	c	c	t	t	t	t	t	t	t	t	t
g	2641315	c	2850792	c	2921010	i	O157	rec46	g	O157	+++	a	t	t	t	g	g	g	c	t		t	t	t	t	t	g	g	t	t	t	g	t	t
g	2641368	a	2850845	a	2921063	i	O157	rec46	g	O157	++	g	a	g	g	a	a	a	a	a		a	a	a	a	g	g	g	g	g	g	g	a	g
t	2641369	a	2850846	a	2921064	i	O157	rec46	t	O157	++	t	t	t	t	a	a	a	a	a		a	a	a	a	t	t	t	t	t	t	t	t	t
c	2641371	a	2850848	a	2921066	i	O157	rec46	c	O157	++++	c	c	c	c	-	-	-	-	-		-	c	c	c	t	c	c	c	c	c	c	c	c
g	2641377	a	2850854	a	2921072	i	O157	rec46	g	O157	++	g	g	g	g	a	a	a	a	a		a	a	a	a	g	g	g	g	g	g	g	g	g
-	2641404	a	2850882	a	2921100	ins	O157	rec46	a	CB9615	++	a	a	a	a	a	a	a	-	-			-	-	-	a	a	a	a	a	a	a	a	a
t	2641414	c	2850892	N	2921110	i	O157	rec46	t	O157	++++	t	t	t	t	t	t	t	t	t			t	t	t	t	t	t	t	t	t	t	t	t
a	2641417	c	2850895	c	2921113	i	O157	rec46	c	CB9615	++++	c	c	c	c	c	c	c	c	c			c	c	c	c	c	c	c	c	c	c	c	c
g	2641418	a	2850896	a	2921114	i	O157	rec46	a	CB9615	++	a	a	a	a	a	a	a	g	g			a	a	a	a	a	a	a	a	a	a	a	a
t	2641430	g	2850908	g	2921126	i	O157	rec46	t	O157	+	a	t	t	t	t	t	t	t	t			g	g	g	t	t	t	t	t	t	t	t	t
t	2641438	c	2850916	c	2921134	i	O157	rec46	c	CB9615	++++	c	c	c	c	c	c	c	c	c			c	c	c	c	c	c	c	c	c	c	c	c
c	2641439	a	2850917	a	2921135	i	O157	rec46	c	O157	++++	c	c	c	c	c	c	c	c	c			c	c	c	c	c	c	c	c	c	c	c	c
a	2641449	g	2850927	g	2921145	i	O157	rec46	g	CB9615	++++	g	g	g	g	g	g	g	g	g			g	g	g	g	g	g	g	g	g	g	g	g
c	2641458	t	2850936	t	2921154	i	O157	rec46	c	O157	++++		c	c	c											c	c	c	c	c	c	c	c	c
t	2641471	c	2850949	c	2921167	i	O157	rec46	t	O157	++++		t	t	t											t	t	t	t	t	t	t	t	t
g	2641490	t	2850968	t	2921186	i	O157	rec46	t	CB9615	++++		t	t	t											t	t	t	t	t	t	t	t	t
a	2641525	g	2851003	g	2921221	i	O157	rec46	g	CB9615	++	g	g	g	g	a	a	a	a	a		g	g	g	g	g	g	g	g	g	g	g	g	g
g	2641534	c	2851012	c	2921230	i	O157	rec46	c	CB9615	++++	c	c	c	c	c	c	c	c	c		c	c	c	c	c	c	c	c	t	t	c	c	c
t	2641559	c	2851037	c	2921255	i	O157	rec46	t	O157	++++	t	t	t	t	t	t	t	t	t		t	t	t	t	t	t	t	t	t	t	t	t	t
9	2641563	-	2851040	-	2921258	del-9	O157	rec46	9	O157	++++	9	9	9	9	9	9	9	9	9		9	9	9	9	9	9	9	9	9	9	9	9	9
c	2642161	g	2851630	g	2921848	s	O157	rec46	c	O157	++	c	c	c	c	g	c	c	c	c		c	c	c	c	c	c	c	g	g	g	g	a	c
g	2642236	t	2851705	t	2921923	s	O157	rec46	g	O157	++++	g	g	g	g	g	g	g	g	g		g	g	g	g	g	g	g	g	g	g	g	g	g
g	2642242	a	2851711	a	2921929	s	O157	rec46	g	O157	++	g	g	g	g	g	g	g	g	g		g	a	a	a	g	g	g	a	a	a	a	a	g
g	2642260	a	2851729	a	2921947	s	O157	rec46	a	CB9615	++	a	a	a	a	g	g	g	g	g		g	g	g	g	a	a	a	a	a	a	a	a	a
a	2642263	g	2851732	g	2921950	s	O157	rec46	a	O157	++	a	a	a	a	a	a	a	a	a		a	g	g	g	a	a	a	g	g	g	g	g	a
g	2642283	a	2851752	a	2921970	s	O157	rec46	g	O157	++	g	g	g	g	g	g	g	g	g		g	g	g	g	g	g	g	a	a	a	a	a	g
c	2642299	t	2851768	t	2921986	s	O157	rec46	t	CB9615	++	t	c	c	c	c	c	c	c	c		c	c	c	c	c	c	c	t	t	t	c	t	c
a	2642539	g	2852008	g	2922226	s	O157	rec46	g	CB9615	++++	g	g	g	g	g	g	g	g	g		g	g	g	g	g	g	g	g	g	g	g	g	g
a	2642720	g	2852189	g	2922407	i	O157	rec46	g	CB9615	++++	g	g	g	g	g	g	g	g	g		g	g	g	g	g	g	g	g			g	g	g
g	2642721	a	2852190	a	2922408	i	O157	rec46	a	CB9615	++++	a	a	a	a	a	a	a	a	a		a	a	a	a	a	a	a	a			a	a	a
a	2642842	g	2852311	g	2922530	i	O157	rec46	g	CB9615	++++	g	g	g	g	g	g	g	g	g		g	g	g	g	g	g	g	g			g	g	g
g	2642897	a	2852366	a	2922585	ns	O157	rec46	g	O157	++++	g	g	g	g	g	g	g	g	g		g	g	g	g	g	g	g	g			g	g	g
t	2642963	c	2852432	c	2922651	s	O157	rec46	t	O157	++++	t	t	t	t	t	t	t	t	t		t	t	t	t	t	t	t	t			t	t	t
t	2643070	c	2852539	c	2922758	s	O157	rec46	c	CB9615	++++	c	c	c	c	c	c	c	c	c		c	c	c	c	c	c	c	c			c	c	c
g	2643113	a	2852582	a	2922801	ns	O157	rec46	g	O157	++++	g	g	g	g	g	g	g	g	g		g	g	g	g	g	g	g	g			g	g	g
t	2643123	c	2852592	c	2922811	ns	O157	rec46	t	O157	++++	t	t	t	t	c	t	t	t	t		t	t	t	t	t	t	t	t			t	t	t
c	2643139	t	2852608	t	2922827	s	O157	rec46	t	CB9615	++	t	t	t	t	t	t	t	t	c		t	c	c	c	t	t	t	t			t	t	t
t	2643253	g	2852722	g	2922941	s	O157	rec46	g	CB9615	++	g	t	t	t	g	g	g	g	g		g	g	g	g	g	g	g	g			g	g	t
c	2643307	t	2852776	t	2922995	s	O157	rec46	c	O157	++	c	c	c	c	t	t	t	t	t		t	t	t	t	c	c	c	c			c	c	c
a	2643439	g	2852908	g	2923127	s	O157	rec46	a	O157	++	a	a	a	a	g	a	a	a	g		a	g	g	g	a	a	a	g			a	a	a
c	2643652	a	2853121	a	2923340	s	O157	rec46	c	O157	++	c	c	c	c	c	c	c	c	c		c	c	c	c	a	c	a	c			c	a	c
t	2643682	c	2853151	c	2923370	s	O157	rec46	t	O157	++	t	t	t	t	c	t	t	t	c		t	c	c	c	t	t	t	t			t	t	t
a	2643722	g	2853191	g	2923410	ns	O157	rec46	g	CB9615	++++	g	g	g	g	g	g	g	g	g		g	g	g	g	g	g	g	g			g	g	g
t	2643802	c	2853271	c	2923490	s	O157	rec46	t	O157	++	t	t	t	t	t	t	c	t	c		t	c	c	c	t	t	t	t			t	t	t
t	2643811	g	2853280	g	2923499	s	O157	rec46	t	O157	++	t	t	t	t	t	g	g	g	g		g	g	g	g	t	t	t	t			t	t	t
c	2643892	t	2853361	t	2923580	s	O157	rec46	t	CB9615	++	t	t	t	t	c	c	t	c	c		c	c	c	c	c	c	c	t			t	c	t
g	2643901	a	2853370	a	2923589	s	O157	rec46	g	O157	++++	g	g	g	g	g	g	g	g	g		g	g	g	g	g	g	g	g			g	g	g
t	2644078	c	2853547	c	2923766	s	O157	rec46	c	CB9615	++	c	t	t	t	t	t	t	t	t		t	t	t	t	t	t	t	t			t	t	t
t	2644302	c	2853771	c	2923990	s	O157	rec46	t	O157	++++	t	t	t	t	t	t	t	t	t	t	t	t	t	t	t	t	t	t	t	t	t	t	t
t	2644491	g	2853960	g	2924179	s	O157	rec46	t	O157	++	t	g	g	g	g	g	g	t	g	g	a	g	g	g	g	g	g	g	g	g	g	g	t
c	2644494	t	2853963	t	2924182	s	O157	rec46	c	O157	++	c	t	t	t	t	t	t	c	t	t	t	t	t	t	t	t	t	t	t	t	t	t	c
a	2644598	g	2854067	g	2924286	ns	O157	rec46	a	O157	++	a	g	a	g	g	g	g	g	g	g	g	g	g	g	g	g	a	g	g	g	g	g	g
c	2644599	t	2854068	t	2924287	ns	O157	rec46	c	O157	++	c	t	c	t	t	c	c	t	t	t	c	t	t	t	t	t	c	t	t	t	t	c	t
g	2644618	a	2854087	a	2924306	ns	O157	rec46	g	O157	++++	g	g	g	g	g	g	g	g	g	g	g	g	g	g	g	g	g	g	g	g	g	g	g
c	2644638	g	2854107	g	2924326	s	O157	rec46	c	O157	++	c	c	c	c	c	c	c	g	c	c	c	c	c	c	c	c	c	c	c	c	c	g	c
a	2644671	g	2854140	g	2924359	s	O157	rec46	g	CB9615	++	g	a	a	a	g	g	g	a	g	g	g	g	g	g	a	a	a	a	a	a	a	a	g
c	2644707	t	2854176	t	2924395	s	O157	rec46	c	O157	++++	c	c	c	c	c	c	c	c	c	c	c	c	c	c	c	c	c	c	c	c	c	t	c
c	2644744	t	2854213	t	2924432	s	O157	rec46	c	O157	++	c	c	c	c	c	c	c	c	c	c	c	c	c	c	c	c	c	t	t	t	c	c	c
t	2644781	c	2854250	c	2924469	ns	O157	rec46	t	O157	++	t	t	t	t	t	t	t	t	t	t	t	t	t	t	c	c	t	c	c	c	t	c	t
t	2644785	c	2854254	c	2924473	s	O157	rec46	t	O157	++	t	t	t	t	t	t	t	t	t	t	t	t	t	t	c	c	c	c	c	c	t	c	c
c	2644788	g	2854257	g	2924476	s	O157	rec46	c	O157	++	c	g	c	g	c	c	c	c	c	c	c	c	c	c	g	g	g	g	g	g	g	g	c
a	2644809	g	2854278	g	2924497	s	O157	rec46	a	O157	++	a	g	a	g	a	a	a	a	a	a	a	t	t	t	g	g	a	a	a	a	g	g	a
t	2644812	g	2854281	g	2924500	s	O157	rec46	g	CB9615	++	g	g	t	g	t	t	t	t	t	t	t	t	t	t	g	g	t	t	t	t	g	g	t
c	2644962	t	2854431	t	2924650	s	O157	rec46	t	CB9615	++	t	t	t	t	c	c	c	t	c	c	c	c	c	c	t	t	t	c	c	c	t	c	c
t	2644965	c	2854434	c	2924653	s	O157	rec46	c	CB9615	++	c	c	c	c	t	t	t	c	t	t	t	t	t	t	c	c	c	c	c	c	c	t	t
c	2644998	t	2854467	t	2924686	s	O157	rec46	c	O157	++	c	c	c	c	c	c	c	c	c	c	c	c	c	c	c	c	c	c	c	c	c	t	t
c	2645013	t	2854482	t	2924701	s	O157	rec46	c	O157	++	c	t	c	t	t	t	t	t	t	t	t	t	t	t	t	t	c	t	t	t	t	t	t
g	2645028	a	2854497	a	2924716	s	O157	rec46	g	O157	++	g	a	g	a	a	a	a	a	a	a	a	a	a	a	a	a	g	g	g	g	a	a	a
t	2645031	c	2854500	c	2924719	s	O157	rec46	t	O157	++	t	t	t	t	t	t	t	t	t	t	t	t	t	t	t	t	t	t	t	t	t	c	c
g	2645050	a	2854519	a	2924738	ns	O157	rec46	g	O157	++	g	g	g	g	g	g	g	g	g	g	g	g	g	g	g	g	g	a	a	a	g	a	a
a	2645082	g	2854551	g	2924770	s	O157	rec46	a	O157	++	a	a	a	a	a	a	a	g	a	a	a	a	a	a	a	a	a	g	g	g	a	g	g
c	2645083	t	2854552	t	2924771	s	O157	rec46	c	O157	++++	c	c	c	c	c	c	c	c	c	c	c	c	c	c	c	c	c	c	c	c	c	c	c

O55 and O157 genome site details												Outgroup Strain Details[i]																						
CB9615 base[b]	CB9615 Site[c]	Sakai base[b]	Sakai Site[c]	EDL933 base[b]	EDL933 Site[c]	type[d]	Event Lineage[ef]	Recombinant segment	Inferred ancestral base[g]	Outgroup Analysis[e]	Support level[h]	D1 Sd197	K-12	HS	ATCC 8739	UMN026	IAI39	SMS 3-5	E2348/69	536	ED1a	CFT073	S88	APEC O1	UTI89	E24377A	IAI1	SE11	F5 8401	F2a 2457T	F2a 301	SS Ss046	B4 Sb227	B18 BS512
g	2645136	a	2854605	a	2924824	s	O157	rec46	a	CB9615	++	a	a	a	a	g	g	g	a	g	g	g	g	g	g	a	a	a	a	a	a	a	a	a
c	2645142	t	2854611	t	2924830	s	O157	rec46	t	CB9615	++	t	t	t	t	c	c	c	t	c	c	c	c	c	c	t	t	t	t	t	t	t	t	t
t	2645156	c	2854625	c	2924844	ns	O157	rec46	c	CB9615	++	c	c	c	c	c	c	c	t	c	c	c	c	c	c	t	c	c	c	c	c	c	c	c
t	2645159	c	2854628	c	2924847	ns	O157	rec46	t	O157	++++	t	t	t	t	t	t	t	t	t	t	t	t	t	t	t	t	t	t	t	t	t	t	t
g	2645216	t	2854685	t	2924904	s	O157	rec46	g	O157	++	g	g	g	g	g	g	g	t	g	t	g	g	g	g	g	g	g	g	g	g	g	g	g
c	2645222	g	2854691	g	2924910	s	O157	rec46	c	O157	++	c	c	c	c	c	c	c	g	c	g	c	c	c	c	c	c	c	c	c	c	c	c	c
g	2645223	a	2854692	a	2924911	ns	O157	rec46	g	O157	++	g	g	g	g	g	g	g	a	g	a	g	g	g	g	g	g	g	g	g	g	g	g	g
c	2645225	t	2854694	t	2924913	ns	O157	rec46	c	O157	++	c	c	c	c	c	c	c	t	c	t	c	c	c	c	c	c	c	c	c	c	c	c	c
g	2645234	a	2854703	a	2924922	s	O157	rec46	g	O157	++++	g	g	g	g	g	g	g	g	g	g	g	g	g	g	g	g	g	g	g	g	g	g	g
c	2645243	t	2854712	t	2924931	s	O157	rec46	t	CB9615	++	t	c	t	c	t	t	t	t	t	t	t	t	t	t	c	c	c	c	c	c	c	c	c
t	2645375	c	2854844	c	2925063	s	O157	rec46	t	O157	++++	t	t	t	t	t	t	t	t	t	t	t	t	t	t	t	t	t	t	t	t	t	t	t
g	2645396	c	2854865	c	2925084	s	O157	rec46	c	CB9615	++	c	c	c	c	c	g	c	g	g	g	g	g	g	g	c	c	c	c	c	c	c	g	g
a	2645399	g	2854868	g	2925087	s	O157	rec46	g	CB9615	++	g	g	g	g	g	g	g	g	g	g	g	g	g	g	g	g	g	g	g	g	g	a	a
g	2645438	c	2854907	c	2925126	s	O157	rec46	c	CB9615	++	c	c	g	c	g	c	c	c	c	c	c	c	c	c	c	c	c	g	g	g	c	g	g
a	2645463	g	2854932	g	2925151	ns	O157	rec46	g	CB9615	++++	g	g	g	g	g	g	g	g	g	g	g	g	g	g	g	g	g	g	g	g	g	g	g
c	2645483	t	2854952	t	2925171	s	O157	rec46	t	CB9615	++++	t	t	t	t	t	t	t	t	t	t	t	t	t	t	t	t	t	t	t	t	t	t	t
t	2645717	g	2855186	g	2925405	ns	O157	rec46	g	CB9615	++	g	t	c	t	c	t	t	t	t	t	t	t	t	t	t	t	t	c	c	c	t	c	c
t	2645735	c	2855204	c	2925423	s	O157	rec46	c	CB9615	++++	c	c	c	c	c	c	c	c	c	c	c	c	c	c	c	c	c	c	c	c	c	c	c
t	2645741	c	2855210	c	2925429	s	O157	rec46	c	CB9615	++++	c	c	c	c	c	c	c	c	c	c	c	c	c	c	c	c	c	c	c	c	c	c	c
g	2645747	a	2855216	a	2925435	s	O157	rec46	a	CB9615	++++	c	a	a	a	a	a	a	a	a	a	a	a	a	a	a	a	a	a	a	a	a	a	a
a	2645750	t	2855219	t	2925438	s	O157	rec46	t	CB9615	++++	t	t	t	t	t	t	t	t	t	t	t	t	t	t	t	t	t	t	t	t	t	t	t
c	2645751	t	2855220	t	2925439	s	O157	rec46	t	CB9615	++++	t	t	t	t	t	t	t	t	t	t	t	t	t	t	t	t	t	t	t	t	t	t	t
g	2645753	a	2855222	a	2925441	s	O157	rec46	a	CB9615	++++	a	a	a	a	a	a	a	a	a	a	a	a	a	a	a	a	a	a	a	a	a	a	a
a	2645759	g	2855228	g	2925447	s	O157	rec46	a	O157	++	a	g	a	g	a	a	a	a	a	a	a	a	a	a	g	g	g	a	a	a	g	a	a
a	2645780	g	2855249	g	2925468	s	O157	rec46	g	CB9615	++++	g	g	g	g	g	g	g	g	g	g	g	g	g	g	g	g	g	g	g	g	g	g	g
t	2645813	g	2855282	g	2925501	ns	O157	rec46	g	CB9615	++++	g	g	g	g	g	g	g	g	g	g	g	g	g	g	g	g	g	g	g	g	g	g	g
c	2645816	a	2855285	a	2925504	s	O157	rec46	a	CB9615	++++	a	a	a	a	a	a	a	a	a	a	a	a	a	a	a	a	a	a	a	a	a	a	a
g	2645819	a	2855288	a	2925507	s	O157	rec46	a	CB9615	++++	a	a	a	a	a	a	a	a	a	a	a	a	a	a	a	a	a	a	a	a	a	a	a
g	2645822	a	2855291	a	2925510	s	O157	rec46	a	CB9615	++++	a	a	a	a	a	a	a	a	a	a	a	a	a	a	a	a	a	a	a	a	a	a	a
c	2645825	t	2855294	t	2925513	s	O157	rec46	t	CB9615	++++	t	t	t	t	t	a	a	a	t	t	t	t	t	t	t	t	t	t	t	t	t	t	t
t	2645851	c	2855320	c	2925539	ns	O157	rec46	c	CB9615	++++	c	c	c	c	c	c	c	c	c	c	c	c	c	c	c	c	c	c	c	c	c	c	c
g	2645853	a	2855322	a	2925541	ns	O157	rec46	g	O157	++++	g	g	g	g	g	g	g	g	g	g	g	g	g	g	g	g	g	g	g	g	g	g	g
a	2645855	g	2855324	g	2925543	ns	O157	rec46	g	CB9615	++++	g	g	g	g	g	g	g	g	g	g	g	g	g	g	g	g	g	g	g	g	g	g	g
c	2645859	t	2855328	t	2925547	s	O157	rec46	t	CB9615	++++	t	t	t	t	t	t	t	t	t	t	t	t	t	t	t	t	t	t	t	t	t	t	t
a	2645864	c	2855333	c	2925552	s	O157	rec46	c	CB9615	++++	c	c	c	c	c	c	c	c	c	c	c	c	c	c	c	c	c	c	c	c	c	c	c
g	2645865	a	2855334	a	2925553	ns	O157	rec46	g	O157	++++	g	g	g	g	g	g	g	g	g	g	g	g	g	g	g	g	g	g	g	g	g	g	g
c	2645867	t	2855336	t	2925555	ns	O157	rec46	t	CB9615	++++	t	t	t	t	t	t	t	t	t	t	t	t	t	t	t	t	t	t	t	t	t	t	t
a	2645882	g	2855351	g	2925570	s	O157	rec46	a	O157	++	a	a	g	a	a	g	g	g	a	a	a	a	a	a	a	a	a	a	a	a	a	a	a
t	2645885	c	2855354	c	2925573	s	O157	rec46	c	CB9615	++++	c	c	t	c	c	c	c	c	c	c	c	c	c	c	c	c	c	c	c	c	c	c	c
c	2645888	a	2855357	a	2925576	s	O157	rec46	a	CB9615	++++	a	a	a	a	a	a	a	a	a	a	a	a	a	a	a	a	a	a	a	a	a	a	a
g	2645901	a	2855370	a	2925589	ns	O157	rec46	a	CB9615	++++	a	a	a	a	a	a	a	a	a	a	a	a	a	a	a	a	a	a	a	a	a	a	a
a	2645904	g	2855373	g	2925592	ns	O157	rec46	a	O157	++	a	g	g	g	g	g	g	g	g	g	g	g	g	g	g	g	g	g	g	g	g	g	g
a	2645912	t	2855381	t	2925600	s	O157	rec46	t	CB9615	++++	t	t	t	t	t	t	t	t	t	t	t	t	t	t	t	t	t	t	t	t	t	t	t
g	2645919	a	2855388	a	2925607	ns	O157	rec46	a	CB9615	++++	a	a	a	a	a	a	a	a	a	a	a	a	a	a	a	a	a	a	a	a	a	a	a
c	2645920	t	2855389	t	2925608	ns	O157	rec46	t	CB9615	++++	t	t	t	t	t	t	t	t	t	t	t	t	t	t	t	t	t	t	t	t	t	t	t
a	2645933	c	2855402	c	2925621	s	O157	rec46	a	O157	++++	a	a	a	a	a	a	a	a	a	a	a	a	a	a	a	a	a	a	a	a	a	a	a
c	2645942	t	2855411	t	2925630	s	O157	rec46	t	CB9615	++++	t	t	t	t	t	t	t	t	t	t	t	t	t	t	t	t	t	t	t	t	t	t	t
a	2645951	c	2855420	c	2925639	s	O157	rec46	c	CB9615	++++	c	c	c	c	c	c	c	c	c	c	c	c	c	c	c	c	c	c	c	c	c	c	c
c	2645957	g	2855426	g	2925645	s	O157	rec46	g	CB9615	++++	g	g	g	g	g	g	g	g	g	g	g	g	g	g	g	g	g	g	g	g	g	g	g
t	2645966	c	2855435	c	2925654	s	O157	rec46	c	CB9615	++++	c	c	c	c	c	c	c	c	c	c	c	c	c	c	c	c	c	c	c	c	c	c	c
c	2645969	t	2855438	t	2925657	s	O157	rec46	t	CB9615	++++	t	t	t	t	t	t	t	t	t	t	t	t	t	t	t	t	t	t	t	t	t	t	t
c	2645972	t	2855441	t	2925660	s	O157	rec46	t	CB9615	++++	t	t	t	t	t	t	t	t	t	t	t	t	t	t	t	t	t	t	t	t	t	t	-
g	2645987	t	2855456	t	2925675	s	O157	rec46	t	CB9615	++++	t	t	t	t	t	t	t	t	t	t	t	t	t	t	t	t	t	t	t	t	t	t	t
c	2645994	t	2855463	t	2925682	s	O157	rec46	c	O157	++++	c	c	c	c	c	c	c	c	c	c	c	c	c	c	c	c	c	c	c	c	c	c	c
a	2645999	c	2855468	c	2925687	s	O157	rec46	c	CB9615	++++	c	c	c	c	c	c	c	c	c	c	c	c	c	c	c	c	c	c	c	c	c	c	c
t	2646153	c	2855622	c	2925841	s	O157	rec46	t	O157	++	t	c	c	c	c	c	c	c	c	c	c	c	c	c	c	c	c	c	c	c	c	c	c
g	2646276	t	2855745	t	2925964	s	O157	rec46	t	CB9615	++	t	c	t	c	c	c	c	c	c	c	c	c	c	c	t	c	c	c	c	c	c	c	c
g	2646279	a	2855748	a	2925967	s	O157	rec46	a	CB9615	++++	a	a	a	a	a	a	a	a	a	a	a	a	a	a	a	a	a	a	a	a	a	a	a
g	2646291	a	2855760	a	2925979	s	O157	rec46	a	CB9615	++++	a	a	a	a	a	a	a	a	a	a	a	a	a	a	a	a	a	a	a	a	a	a	a
g	2646309	a	2855778	a	2925997	s	O157	rec46	a	CB9615	++	a	a	a	a	g	g	g	g	g	g	g	g	g	g	a	a	a	a	a	a	a	a	a
g	2646621	a	2856090	a	2926309	s	O157	rec46	g	O157	++	g	a	a	a	g	g	g	g	g	g	g	g	g	g	a	a	a	g	g	g	g	a	a
a	2646943	t	2856412	t	2926631	ns	O157	rec46	a	O157	++	a	a	a	a	a	t	t	a	a	a	a	a	a	a	a	a	a	-	c	c	a	a	a
c	2646945	t	2856414	t	2926633	s	O157	rec46	c	O157	++	c	c	c	c	c	t	t	c	c	c	c	c	c	c	c	c	c	c	-	-	c	c	c
g	2646974	a	2856443	a	2926662	ns	O157	rec46	g	O157	++++	g	g	g	g	g	g	g	g	g	a	g	g	g	g	g	g	g	g	g	g	g	g	g
g	2646981	a	2856450	a	2926669	s	O157	rec46	g	O157	++	g	g	g	g	a	a	a	a	a	a	a	a	a	a	g	g	g	g	g	g	g	g	g
t	2646996	c	2856465	c	2926684	s	O157	rec46	t	O157	++	t	t	t	t	t	c	c	t	t	t	t	t	t	t	t	t	t	t	t	t	t	t	t
g	2647020	a	2856489	a	2926708	s	O157	rec46	a	CB9615	++	a	g	g	g	g	g	g	g	g	g	g	g	g	g	a	g	g	g	g	g	g	g	g
a	2647047	g	2856516	g	2926735	s	O157	rec46	a	O157	++	a	a	a	a	a	a	a	g	g	a	a	g	g	g	a	a	a	a	a	a	a	a	a
c	2647107	g	2856576	g	2926795	s	O157	rec46	g	CB9615	++	g	g	c	c	c	c	c	g	g	g	c	g	g	g	c	g	g	g	g	g	c	g	c
t	2647139	c	2856608	c	2926827	ns	O157	rec46	t	O157	++++	t	t	t	t	t	t	t	t	t	t	t	t	t	t	t	t	t	t	t	t	t	t	t
c	2647149	t	2856618	t	2926837	s	O157	rec46	t	CB9615	++	t	c	t	c	t	c	t	a	t	t	t	a	a	a	t	t	t	t	t	t	c	t	c
t	2647190	g	2856659	g	2926878	s	O157	rec46	g	CB9615	++	g	g	g	t	g	g	g	g	g	g	g	g	g	g	g	g	g	g	g	g	t	g	t
a	2647419	g	2856888	g	2927107	s	O157	rec46	g	CB9615	++	g	a	a	a	a	a	a	a	a	a	a	a	a	a	a	a	a	a	a	a	a	a	a
a	2647425	g	2856894	g	2927113	s	O157	rec46	g	CB9615	++	g	a	a	a	g	a	g	g	g	g	g	a	a	a	a	a	a	a	a	a	g	a	g
c	2647431	t	2856900	t	2927119	s	O157	rec46	c	O157	++	c	c	c	c	t	c	t	t	t	t	t	c	c	c	c	c	c	c	c	c	t	c	t
g	2647449	a	2856918	a	2927137	s	O157	rec46	a	CB9615	++	a	g	g	g	a	g	a	a	a	a	a	g	g	g	g	g	g	g	g	g	a	g	a
g	2647536	a	2857005	a	2927224	ns	O157	rec46	a	CB9615	++	a	g	g	g	g	g	g	g	g	g	g	g	g	g	g	g	g	g	g	g	a	g	a
c	2647537	t	2857006	t	2927225	ns	O157	rec46	c	O157	++++	c	c	c	c	c	c	c	c	c	c	c	c	c	c	c	c	c	c	c	c	c	c	c
t	2647588	g	2857057	g	2927276	ns	O157	rec46	g	CB9615	++	g	g	t	g	t	t	t	t	t	t	t	t	t	t	g	g	g	g	g	g	g	g	g
t	2647632	c	2857101	c	2927320	s	O157	rec46	c	CB9615	++++	c	c	c	c	c	c	c	c	c	c	c	c	c	c	c	c	c	c	c	c	c	c	c
c	2647636	t	2857105	t	2927324	ns	O157	rec46	t	CB9615	++++	t	t	t	t	t	t	t	t	t	t	t	t	t	t	t	t	t	t	t	t	t	t	t
a	2647676	g	2857145	g	2927364	s	O157	rec46	g	CB9615	++	g	g	g	g	a	a	a	a	a	a	a	a	a	a	g	g	g	g	g	g	g	g	g
a	2647685	g	2857154	g	2927373	ns	O157	rec46	g	CB9615	++	g	g	g	g	a	a	a	a	a	a	a	a	a	a	g	g	g	g	g	g	g	g	g
g	2647701	a	2857170	a	2927389	s	O157	rec46	a	CB9615	++	a	a	a	a	g	g	g	g	g	g	g	g	g	g	a	a	a	a	a	a	a	a	a
c	2647707	t	2857176	t	2927395	s	O157	rec46	t	CB9615	++	t	t	t	t	c	c	c	c	c	c	c	c	c	c	t	t	t	t	t	t	t	t	t
a	2647746	g	2857215	g	2927434	i	O157	rec46	g	CB9615	++++	g	g	g	g	g	g	g	g	g	g	g	g	g	g	g	g	g	g	g	g	g	g	g

O55 and O157 genome site details												Outgroup Strain Details[i]																						
CB9615 base[b]	CB9615 Site[c]	Sakai base[b]	Sakai Site[c]	EDL933 base[b]	EDL933 Site[c]	type[d]	Event Lineage[ef]	Recombinant segment	Inferred ancestral base[g]	Outgroup Analysis[e]	Support level[h]	D1 Sd197	K-12	HS	ATCC 8739	UMN026	IAI39	SMS 3-5	E2348/69	536	ED1a	CFT073	S88	APEC O1	UTI89	E24377A	IAI1	SE11	F5 8401	F2a 2457T	F2a 301	SS Ss046	B4 Sb227	B18 BS512
a	2648047	g	2857516	g	2927735	s	O157	rec46	a	O157	++++	a	a	a	a	a	a	a	a	a	a	a	a	a	a	a	a	a	a	a	a	a	a	a
t	2648074	g	2857543	g	2927762	s	O157	rec46	g	CB9615	++++	g	g	g	g	g	g	g	g	g	g	g	g	g	g	g	g	g	g	g	g	g	g	g
a	2648091	c	2857560	c	2927779	ns	O157	rec46	a	O157	++++	a	a	a	a	a	a	a	a	a	a	a	a	a	a	a	a	a	a	a	a	a	a	a
g	2648149	a	2857618	a	2927837	s	O157	rec46	g	O157	++++	g	g	g	g	g	g	g	g	g	g	g	g	g	g	g	g	g	g	g	g	g	g	g
c	2648206	t	2857675	t	2927894	s	O157	rec46	c	O157	++	c	c	c	c	t	c	c	t	t	t	c	c	c	c	c	c	c	c	c	c	t	c	c
a	2648212	g	2857681	g	2927900	ns	O157	rec46	a	O157	++	a	a	a	a	g	a	a	g	g	g	a	a	a	a	a	a	a	a	a	a	g	a	a
a	2648213	g	2857682	g	2927901	ns	O157	rec46	g	CB9615	++++	g	g	g	g	g	g	g	g	g	g	g	g	g	g	g	g	g	g	g	g	g	g	g
c	2648218	t	2857687	t	2927906	s	O157	rec46	c	O157	++	c	c	c	c	t	c	c	t	t	t	c	c	c	c	c	c	c	c	c	c	t	c	c
c	2648236	t	2857705	t	2927924	s	O157	rec46	c	O157	++++	c	c	c	c	c	c	c	c	c	c	c	c	c	c	c	c	c	c	c	c	t	c	c
g	2648245	a	2857714	a	2927933	s	O157	rec46	g	O157	++++	g	g	g	g	g	g	g	g	g	g	g	g	g	g	g	g	g	g	g	g	g	g	g
g	2648359	a	2857828	a	2928047	s	O157	rec46	a	CB9615	++	a	a	a	a	a	g	g	g	g	g	g	g	g	g	g	g	g	a	a	a	a	g	g
t	2648455	c	2857924	c	2928143	s	O157	rec46	c	CB9615	++++	c	c	c	c	c	c	c	c	c	c	c	c	c	c	c	c	c	c	c	c	c	c	c
g	2648716	a	2858185	a	2928404	ns	O157	rec46	a	CB9615	++	a	g	g	g	a	g	g	g	g	g	g	g	g	g	g	g	g	g	g	g	g	g	a
g	2648736	a	2858205	a	2928424	s	O157	rec46	g	O157	++	g	g	g	g	a	a	a	a	a	a	a	a	a	a	g	g	g	g	g	g	g	g	a
t	2648819	c	2858288	c	2928507	ns	O157	rec46	t	O157	++	t	t	t	t	t	t	t	t	t	t	t	t	t	t	t	c	c	t	t	t	c	t	t
c	2648824	t	2858293	t	2928512	ns	O157	rec46	c	O157	++	c	t	t	t	t	t	t	t	c	t	t	t	t	t	t	t	t	t	t	t	t	t	t
a	2648826	g	2858295	g	2928514	s	O157	rec46	a	O157	++	a	t	t	t	t	t	t	t	a	t	t	t	t	t	t	g	g	t	t	t	g	t	c
t	2648846	c	2858315	c	2928534	ns	O157	rec46	t	O157	++	t	t	t	t	c	c	c	c	c	c	c	c	c	c	t	c	c	c	c	c	c	t	c
a	2648910	g	2858379	g	2928598	s	O157	rec46	g	CB9615	++	g	g	g	g	a	g	g	g	a	g	g	g	g	g	g	g	g	g	g	g	g	g	g
g	2648914	a	2858383	a	2928602	ns	O157	rec46	g	O157	++++	g	g	g	g	g	g	g	g	g	g	g	g	g	g	g	g	g	g	g	g	g	g	g
c	2648919	g	2858388	g	2928607	s	O157	rec46	g	CB9615	++++	g	g	g	g	g	g	g	g	g	g	g	g	g	g	g	g	g	g	g	g	g	g	g
g	2648946	a	2858415	a	2928634	s	O157	rec46	g	O157	++	g	g	g	g	g	a	g	a	a	a	a	a	a	a	a	a	a	g	g	g	a	a	a
g	2648950	t	2858419	t	2928638	ns	O157	rec46	t	CB9615	++++	t	t	t	t	t	t	t	t	t	t	t	t	t	t	t	t	t	t	t	t	t	t	t
a	2649039	g	2858508	g	2928727	s	O157	rec46	g	CB9615	++++	g	g	g	g	g	g	g	g	g	g	g	g	g	g	g	g	g	g	g	g	g	g	g
g	2649084	t	2858553	t	2928772	s	O157	rec46	t	CB9615	++++	t	t	t	t	t	c	t	c	c	c	c	c	c	c	t	t	t	t	t	t	t	t	g
g	2649114	a	2858583	a	2928802	s	O157	rec46	a	CB9615	++	a	a	a	a	g	a	g	a	a	a	a	a	a	a	g	a	a	a	a	a	a	g	g
c	2649147	t	2858616	t	2928835	s	O157	rec46	t	CB9615	++	t	t	t	t	t	c	c	c	t	c	c	c	c	c	c	t	t	t	t	t	t	c	c
t	2649236	g	2858705	g	2928924	ns	O157	rec46	g	CB9615	++++	g	g	g	g	g	g	g	g	g	g	g	g	g	g	g	g	g	g	g	g	g	g	g
a	2649288	g	2858757	g	2928976	s	O157	rec46	g	CB9615	++++	g	g	g	g	a	g	g	g	g	g	g	g	g	g	g	g	g	g	g	g	g	g	g
c	2649326	t	2858795	t	2929014	ns	O157	rec46	t	CB9615	++	t	c	c	c	c	c	c	c	c	c	c	c	c	c	c	c	c	c	c	c	c	c	t
t	2649472	c	2858941	c	2929160	i	O157	rec46	c	CB9615	++++	c	c	c	c	c	c		c	a	a	a	a	a	a	c	c	c	c	c	c	c	a	c
a	2649521	c	2858990	c	2929209	i	O157	rec46	?	O157	+/-																							
t	2649532	a	2859001	a	2929220	i	O157	rec46	?	O157	+/-																							
a	2649534	g	2859003	g	2929222	i	O157	rec46	?	O157	+/-																							
a	2649554	c	2859023	c	2929242	i	O157	rec46	?	O157	+/-																							
g	2650219	a	2859688	a	2929907	i	O157	rec46	?	O157	+/-																							
a	2650320	c	2859789	c	2930008	i	O157	rec46	?	O157	+/-																							
c	2650664	-	2860132	-	2930351	del	O157	rec46	?	O157	+/-																							
a	2650699	c	2860167	c	2930386	i	O157	rec46	?	O157	+/-																							
g	2650863	a	2860331	a	2930550	i	O157	rec46	?	O157	+/-																							
t	2650967	c	2860435	c	2930654	i	O157	rec46	?	O157	+/-																							
a	2650983	g	2860451	g	2930670	i	O157	rec46	?	O157	+/-																							
c	2651583	a	2861051	a	2931270	s	O157	rec46	?	O157	+/-																							
c	2651735	t	2861203	t	2931422	i	O157	rec46	?	O157	+/-																							
a	2651824	g	2861292	g	2931511	ns	O157	rec46	g	CB9615	++++	g	g	g	g	g	g	g	g	g	g	g	g	g	g	g	g	g	g	g	g	g		g
g	2651852	a	2861320	a	2931539	s	O157	rec46	g	O157	++++	g	g	g	g	g	g	g	g	g	g	g	g	g	g	g	g	g	g	g	g	g		g
c	2651861	t	2861329	t	2931548	s	O157	rec46	c	O157	++++	c	c	c	c	c	c	c	c	c	c	c	c	c	c	c	c	c	c	c	c	c		c
t	2651951	c	2861419	c	2931638	s	O157	rec46	t	O157	++	t	c	c	c	c	c	c	t	t	t	t	t	t	t	c	c	c	c	c	c	c		c
t	2652005	c	2861473	c	2931692	s	O157	rec46	c	CB9615	++++	c	c	c	c	c	c	c	c	c	c	c	c	c	c	c	c	c	c	c	c	c		c
c	2652102	t	2861570	t	2931789	i	O157	rec46	t	CB9615	++++	-	t	t	t	t	c	t	t	t	t	t	t	t	t	t	t	t	t	t	t	t		t
c	2652526	t	2861994	t	2932213	s	O157	rec46	t	CB9615	++	t	c	c	c	c	c	c	c	c	c	c	c	c	c	c	c	c	t	c	c	c		c
-	2652532	9	2862001	9	2932220	ins-9	O157	rec46	9	CB9615	++	9	-	-	-	-	9	-	-	-	-	-	-	-	-	-	-	-	9	9	9	-		-
t	2652538	c	2862015	c	2932234	s	O157	rec46	c	CB9615	++	c	g	g	g	g	t	g	t	g	g	g	g	g	g	t	t	g	c	t	t	-		-
g	2652541	a	2862018	a	2932237	s	O157	rec46	a	CB9615	++	a	t	t	t	t	g	t	g	t	t	t	t	t	t	a	a	t	a	c	c	-		-
-	2652549	a	2862027	a	2932246	ins	O157	rec46	a	CB9615	++	a	-	-	-	-	a	-	-	-	-	-	-	-	-	-	-	-	a	-	-	-		-
t	2652556	c	2862030	c	2932261	s	O157	rec46	c	CB9615	++	c	t	t	t	t	g	t	c	t	t	t	t	t	t	c	c	t	c	t	t	t		t
a	2652562	c	2862036	c	2932267	ns	O157	rec46	c	CB9615	++	c	a	a	a	a	g	a	t	a	a	a	a	a	a	t	t	a	a	a	a	a		a
g	2652563	c	2862037	c	2932268	ns	O157	rec46	c	CB9615	++	c	c	c	c	g	c	g	c	g	g	g	g	g	g	c	c	g	c	c	c	g		c
t	2652565	c	2862039	c	2932270	ns	O157	rec46	c	CB9615	++	c	t	t	t	t	c	t	c	t	t	t	t	t	t	c	c	t	t	t	t	t		t
c	2652566	g	2862040	g	2932271	ns	O157	rec46	g	CB9615	++	g	c	c	c	c	g	c	g	c	c	c	c	c	c	g	g	c	c	c	c	c		c
t	2652577	c	2862051	c	2932282	s	O157	rec46	c	CB9615	++	c	t	t	t	t	t	t	t	t	t	t	t	t	t	t	t	t	a	a	a	t		t
g	2652579	a	2862053	a	2932284	ns	O157	rec46	a	CB9615	++	a	g	g	g	g	a	g	g	g	g	g	g	g	g	g	g	g	g	g	g	g		g
c	2652595	t	2862069	t	2932300	s	O157	rec46	t	CB9615	++	t	c	c	c	c	c	c	c	c	c	c	c	c	c	c	c	c	c	c	c	c		c
-	2652604	9	2862079	9	2932310	ins-9	O157	rec46	9	CB9615	++	9	-	-	-	-	-	-	-	-	-	-	-	-	-	-	-	-	-	-	-	-		-
c	2652692	t	2862175	t	2932406	s	O157	rec46	t	CB9615	++	t	t	t	t	c	t	c	c	c	c	c	c	c	c	t	t	c	t	t	t	c		t
c	2652706	a	2862189	a	2932420	s	O157	rec46	a	CB9615	++	a	c	c	c	c	c	c	c	c	c	c	c	c	c	c	a	c	c	c	c	c		c
c	2652718	t	2862201	t	2932432	s	O157	rec46	t	CB9615	++	t	c	c	c	c	c	c	c	c	c	c	c	c	c	c	c	c	c	c	c	c		c
t	2652808	c	2862291	c	2932522	s	O157	rec46	c	CB9615	++	c	t	t	t	t	t	c	c	c	c	c	c	c	c	t	t	t	t	t	t	t		t
c	2652811	t	2862294	t	2932525	s	O157	rec46	c	O157	++++	c	c	c	c	c	c	c	c	c	c	c	c	c	c	c	c	c	c	c	c	c		c
g	2652817	a	2862300	a	2932531	s	O157	rec46	a	CB9615	++	a	g	g	g	g	g	g	g	g	g	g	g	g	g	g	g	g	g	g	g	g		g
t	2652818	c	2862301	c	2932532	s	O157	rec46	t	O157	++++	t	t	t	t	t	t	t	t	t	t	t	t	t	t	t	t	t	t	t	t	t		t
c	2652829	t	2862312	t	2932543	s	O157	rec46	t	CB9615	++	t	c	c	c	c	c	c	c	c	c	c	c	c	c	c	c	c	c	c	c	c		c
c	2652832	a	2862315	a	2932546	s	O157	rec46	a	CB9615	++	a	c	c	c	c	c	c	c	c	c	c	c	c	c	c	c	c	c	c	c	c		c
c	2652844	a	2862327	a	2932558	s	O157	rec46	a	CB9615	++	a	c	c	c	c	c	c	c	c	c	c	c	c	c	c	c	c	c	c	c	c		c
a	2652847	g	2862330	g	2932561	s	O157	rec46	g	CB9615	++	g	a	a	a	a	a	a	a	a	a	a	a	a	a	a	a	a	a	a	a	a		a
c	2652856	t	2862339	t	2932570	s	O157	rec46	t	CB9615	++	t	c	c	c	c	c	c	c	c	c	c	c	c	c	c	c	c	c	c	c	c		c
g	2652862	a	2862345	a	2932576	s	O157	rec46	a	CB9615	++	a	g	g	g	g	g	g	g	g	g	g	g	g	g	g	g	g	g	g	g	g		g
g	2652865	a	2862348	a	2932579	s	O157	rec46	a	CB9615	++	a	g	g	g	g	g	g	g	g	g	g	g	g	g	g	g	g	g	g	g	g		g
c	2652868	t	2862351	t	2932582	s	O157	rec46	t	CB9615	++	t	c	c	c	c	c	c	c	c	c	c	c	c	c	c	c	c	c	c	c	c		c
g	2652931	a	2862414	a	2932645	s	O157	rec46	a	CB9615	++	a	g	g	g	g	g	g	g	g	g	g	g	g	g	g	g	g	g	g	g	g		g
c	2652937	t	2862420	t	2932651	s	O157	rec46	t	CB9615	++	t	c	c	c	c	c	c	c	c	c	c	c	c	c	c	c	c	c	c	c	c		c
t	2652946	g	2862429	g	2932660	s	O157	rec46	g	CB9615	++	g	t	t	t	t	t	t	t	t	t	t	t	t	t	t	t	t	t	t	t	t		t
c	2652958	a	2862441	a	2932672	s	O157	rec46	a	CB9615	++	a	c	c	c	c	c	c	c	c	c	c	c	c	c	c	c	c	c	c	c	c		c
a	2652961	g	2862444	g	2932675	s	O157	rec46	g	CB9615	++	g	a	a	a	a	a	a	a	a	a	a	a	a	a	a	a	a	a	a	a	a		a
c	2652970	t	2862453	t	2932684	s	O157	rec46	t	CB9615	++	t	c	c	c	c	c	c	c	c	c	c	c	c	c	c	c	c	c	c	c	c		c
c	2652976	t	2862459	t	2932690	s	O157	rec46	t	CB9615	++	t	c	c	c	c	c	c	c	c	c	c	c	c	c	c	c	c	c	c	c	c		c

O55 and O157 genome site details												Outgroup Strain Details[i]																						
CB9615 base[b]	CB9615 Site[c]	Sakai base[b]	Sakai Site[c]	EDL933 base[b]	EDL933 Site[c]	type[d]	Event Lineage[ef]	Recombinant segment	Inferred ancestral base[g]	Outgroup Analysis[e]	Support level[h]	D1 Sd197	K-12	HS	ATCC 8739	UMN026	IAI39	SMS 3-5	E2348/69	536	ED1a	CFT073	S88	APEC O1	UTI89	E24377A	IAI1	SE11	F5 8401	F2a 2457T	F2a 301	SS Ss046	B4 Sb227	B18 BS512
a	2652979	t	2862462	t	2932693	s	O157	rec46	t	CB9615	++	t	a	a	a	a	a	a	a	a	a	a	a	a	a	a	a	a	a	a	a	a		a
c	2652983	a	2862466	a	2932697	s	O157	rec46	a	CB9615	++	a	c	c	c	c	c	c	c	c	c	c	c	c	c	c	c	c	c	c	c	c		c
c	2652989	a	2862472	a	2932703	i	O157	rec46	a	CB9615	++	a	c	c	c	c	c	c	c	c	c	c	c	c	c	c	c	c	c	c	c	c		c
t	2652990	a	2862473	a	2932704	i	O157	rec46	a	CB9615	++	a	t	t	t	t	t	t	t	t	t	t	t	t	t	t	t	t	t	t	t	t		t
c	2652996	a	2862479	a	2932710	i	O157	rec46	a	CB9615	++	a	c	c	c	c	c	c	c	c	c	c	c	c	c	c	c	c	c	c	c	c		c
t	2652997	c	2862480	c	2932711	i	O157	rec46	c	CB9615	++	c	t	t	t	t	t	t	t	t	t	t	t	t	t	t	t	t	t	t	t	t		t
t	2652998	c	2862481	c	2932712	i	O157	rec46	c	CB9615	++	c	t	t	t	t	t	t	t	t	t	t	t	t	t	t	t	t	t	t	t	t		t
a	2653000	g	2862483	g	2932714	i	O157	rec46	g	CB9615	++	g	a	a	a	a	a	a	a	a	a	a	a	a	a	a	a	a	a	a	a	a		a
g	2653006	-	2862488	-	2932719	del-3	O157	rec46	-	CB9615	++	-	3	3	3	3	3	3	3	3	3	3	3	3	3	3	3	3	3	3	3	3		3
t	2653009	c	2862489	c	2932720	i	O157	rec46	c	CB9615	++	c	t	t	t	t	g	t	t	t	c	t	t	t	t	t	t	t	g	g	g	g		g
t	2653011	c	2862491	c	2932722	i	O157	rec46	c	CB9615	++	c	t	t	t	t	t	t	t	t	c	t	t	t	t	t	t	t	t	t	t	t		t
-	2653015	ct	2862496	ct	2932727	ins-2	O157	rec46	ct	CB9615	++	ct	-	-	-	-	-	-	-	-	-	-	-	-	-	-	-	-	-	-	-	-		-
c	2653018	t	2862500	t	2932731	i	O157	rec46	t	CB9615	++	t	c	c	c	c	c	c	c	c	c	c	c	c	c	c	c	c	c	c	c	c		c
a	2653045	t	2862527	t	2932758	ns	O157	rec46	t	CB9615	++	t	a	a	a	a	a	a	a	a	a	a	a	a	a	a	a	a	a	a	a	a		a
t	2653046	a	2862528	a	2932759	ns	O157	rec46	a	CB9615	++	a	t	t	t	t	t	t	t	t	t	t	t	t	t	t	t	t	t	t	t	t		t
g	2653054	c	2862536	c	2932767	ns	O157	rec46	c	CB9615	++	c	g	g	g	g	g	g	g	g	g	g	g	g	g	g	g	g	g	g	g	g		g
c	2653058	t	2862540	t	2932771	ns	O157	rec46	t	CB9615	++++	t	t	t	t	t	-	t	t	t	t	t	t	t	t	t	t	t	t	t	t	t		t
t	2653059	c	2862541	c	2932772	ns	O157	rec46	c	CB9615	++++	c	c	c	c	c	c	c	c	c	c	c	c	c	c	c	c	c	c	c	c	c		c
c	2653060	t	2862542	t	2932773	ns	O157	rec46	t	CB9615	++++	t	t	t	t	t	t	t	t	t	t	t	t	t	t	t	t	t	t	t	t	t		t
t	2653061	c	2862543	c	2932774	ns	O157	rec46	c	CB9615	++	c	-	-	-	-	c	-	-	-	-	-	-	-	-	-	-	-	-	-	-	-		-
g	2653066	a	2862548	a	2932779	ns	O157	rec46	a	CB9615	++	a	g	g	g	g	g	g	g	g	g	g	g	g	g	g	g	g	g	g	g	g		g
a	2653069	g	2862551	g	2932782	ns	O157	rec46	g	CB9615	++	g	a	a	a	a	a	a	a	a	a	a	a	a	a	a	a	a	a	a	a	a		a
a	2653081	g	2862563	g	2932794	ns	O157	rec46	g	CB9615	++	g	a	a	a	a	a	a	a	a	a	a	a	a	a	a	a	a	a	a	a	a		a
g	2653082	c	2862564	c	2932795	ns	O157	rec46	c	CB9615	++	c	g	g	g	g	g	g	g	g	g	g	g	g	g	g	g	g	g	g	g	g		g
g	2653083	t	2862565	t	2932796	ns	O157	rec46	t	CB9615	++	t	g	g	g	g	g	g	g	g	g	g	g	g	g	g	g	g	g	g	g	g		g
g	2653084	a	2862566	a	2932797	ns	O157	rec46	a	CB9615	++	a	g	g	g	g	g	g	g	g	g	g	g	g	g	g	g	g	g	g	g	g		g
t	2653161	g	2862643	g	2932874	ns	O157	rec46	g	CB9615	++++	g	g	g	g	g	g	g	g	g	g	g	g	g	g	g	g	g	g	g	g	g		g
t	2653326	c	2862808	c	2933039	s	O157	rec46	c	CB9615	++	c	t	t	t	c	c	c	c	c	c	c	c	c	c	c	t	c	t	t	t	t		c
t	2653352	a	2862834	a	2933065	ns	O157	rec46	t	O157	++++	t	t	t	t	t	t	t	t	t	t	t	t	t	t	t	t	t	t	t	t	t		t
t	2653362	g	2862844	g	2933075	s	O157	rec46	t	O157	++	t	g	g	g	c	c	t	t	t	t	t	t	t	t	c	t	c	t	t	t	t		c
t	2653365	c	2862847	c	2933078	s	O157	rec46	c	CB9615	++	c	c	c	c	t	t	c	c	c	c	c	c	c	c	t	t	t	t	t	t	t		t
c	2653371	a	2862853	a	2933084	s	O157	rec46	a	CB9615	++	a	a	a	a	c	c	a	c	c	c	c	c	c	c	c	c	c	c	c	c	c		c
a	2653386	t	2862868	t	2933099	s	O157	rec46	t	CB9615	+++	t	t	t	t	c	c	t	c	c	c	c	c	c	c	c	c	c	c	c	c	c		c
a	2653419	g	2862901	g	2933132	s	O157	rec46	g	CB9615	++	g	g	g	g	a	a	a	a	a	a	a	a	a	a	a	a	a	a	a	a	a		a
a	2653434	g	2862916	g	2933147	s	O157	rec46	a	O157	++	a	g	g	g	g	g	g	g	g	g	g	g	g	g	g	g	g	g	g	g	g		g
t	2653437	g	2862919	g	2933150	s	O157	rec46	t	O157	++++	t	t	t	t	t	t	t	t	t	t	t	t	t	t	t	t	t	t	t	t	t		t
c	2653440	t	2862922	t	2933153	s	O157	rec46	c	O157	++	c	t	t	t	t	t	t	t	t	t	t	t	t	t	t	t	t	t	t	t	t		t
a	2653446	g	2862928	g	2933159	s	O157	rec46	a	O157	++	a	g	g	g	g	g	g	g	g	g	g	g	g	g	g	g	g	g	g	g	g		g
c	2653449	g	2862931	g	2933162	s	O157	rec46	c	O157	++	c	g	g	g	a	g	g	g	g	g	g	g	g	g	g	g	g	g	g	g	g		g
a	2653559	g	2863041	g	2933272	i	O157	rec46	g	CB9615	++	g	a	a	a	g	g	g	g	g	g	g	g	g	g	a	a	a	a	a	a	a		
t	2653560	g	2863042	g	2933273	i	O157	rec46	g	CB9615	++	g	t	t	t	a	a	g	g	g	g	g	g	g	g	t	t	t	t	t	t	t		
c	2653561	a	2863043	a	2933274	i	O157	rec46	a	CB9615	++	a	c	c	c	a	a	a	a	a	a	a	a	a	a	c	c	c	c	c	c	c		
a	2653562	g	2863044	g	2933275	i	O157	rec46	g	CB9615	++	g	a	a	a	g	g	g	g	g	g	g	g	g	g	a	a	a	a	a	a	a		
t	2653563	a	2863045	a	2933276	i	O157	rec46	a	CB9615	++	a	t	t	t	a	a	a	a	a	a	a	a	a	a	t	t	t	t	t	t	t		
c	2653571	a	2863053	a	2933284	i	O157	rec46	a	CB9615	++++	a	a	a	a	a	g	a		g	g	g	g	g	g	a	a	a	a	a	a	a		
g	2653586	a	2863068	a	2933299	i	O157	rec46	a	CB9615	++	a	g	g	g	a	a	g		a	a	a	a	a	a	g	g	g	g	g	g	g		
t	2653593	c	2863075	c	2933306	i	O157	rec46	c	CB9615	++	c	t	t	t	-	-	t		-	-	-	-	-	-	t	t	t	t	t	t	t		
g	2653614	a	2863096	a	2933327	i	O157	rec46	g	O157	++++	g	g	g	g	g	g	g		g	g	g	g	g	g	g	g	g	g	g	g	g		
g	2653630	a	2863112	a	2933343	s	O157	rec46	a	CB9615	++	a	g	g	g	g	g	g		g	g	g	g	g	g	g	g	g	g	g	g	g		
g	2653650	a	2863132	a	2933363	s	O157	rec46	a	CB9615	++++	a	a	a	a	a	a	a		a	a	a	a	a	a	a	a	a	a	a	a	a		
c	2653660	t	2863142	t	2933373	s	O157	rec46	t	CB9615	++	t	c	c	c	c	c	c		c	c	c	c	c	c	c	c	c	c	c	c	c		
g	2653684	t	2863166	t	2933397	ns	O157	rec46	g	O157	++++	g	g	g	g	g	g	g		g	g	g	g	g	g	g	g	a	g	g	g	g		
g	2653688	t	2863170	t	2933401	ns	O157	rec46	g	O157	++++	g	g	g	g	g	g	g		g	g	g	g	g	g	g	g	g	g	g	g	g		
t	2653689	c	2863171	c	2933402	ns	O157	rec46	t	O157	++++	t	t	t	t	t	t	t		t	t	t	t	t	t	t	t	t	t	t	t	t		
g	2653693	a	2863175	a	2933406	s	O157	rec46	g	O157	++++	g	g	g	g	g	g	g		g	g	g	g	g	g	g	g	g	g	g	g	g		
g	2653696	t	2863178	t	2933409	s	O157	rec46	g	O157	++++	g	g	g	g	g	g	a		g	g	g	g	g	g	g	g	g	g	g	g	g		
g	2653699	a	2863181	a	2933412	s	O157	rec46	g	O157	++++	g	g	g	g	g	g	g		g	g	g	g	g	g	g	g	g	g	g	g	g		
c	2654696	t	2864173	t	2934404	nc	O157	rec46	c	O157	++	c	c	c	c	c	t	c	c	t	t	c	c	c	c	c	c	c	c	c	c	c		
t	2654740	c	2864217	c	2934448	nc	O157	rec46	t	O157	++	t	t	t	t	t	c	t	c	c	c	c	c	c	c	t	t	t	t	t	t	t		
t	2654754	c	2864231	c	2934462	nc	O157	rec46	t	O157	++	t	c	c	c	c	c	c	c	c	c	c	c	c	c	c	c	c	c	c	c	c		
a	2654772	c	2864249	c	2934480	nc	O157	rec46	a	O157	++++	a	a	a	a	a	a	a	a	a	a	a	a	a	a	a	a	a	a	a	a	a		
c	2654781	t	2864258	t	2934489	nc	O157	rec46	c	O157	++	c	c	c	c	c	c	c	c	c	c	c	c	c	c	c	c	c	t	t	t	t		
a	2654802	t	2864279	t	2934510	nc	O157	rec46	a	O157	++++	a	a	a	a	a	a	a	a	a	a	a	a	a	a	a	a	a	a	a	a	a		
t	2654809	g	2864286	g	2934517	nc	O157	rec46	t	O157	++++	t	t	t	t	t	t	t	t	t	t	t	t	t	t	t	t	t	t	t	t	t		
g	2654824	a	2864301	a	2934532	nc	O157	rec46	g	O157	++	g	g	g	g	g	g	a	g	a	a	a	a	a	a	g	g	g	g	g	g	g		
a	2654830	g	2864307	g	2934538	nc	O157	rec46	a	O157	++++	a	a	a	a	a	a	a	a	a	a	a	a	a	a	a	a	a	a	a	a	a		
g	2654839	a	2864316	a	2934547	nc	O157	rec46	g	O157	++	g	a	a	a	a	a	a	a	a	a	a	a	a	a	a	a	a	a	a	a	g		
g	2654842	c	2864319	c	2934550	nc	O157	rec46	g	O157	++++	g	g	g	g	g	g	g	g	g	g	g	g	g	g	g	g	g	g	g	g	g		
t	2654875	c	2864352	c	2934583	nc	O157	rec46	t	O157	++++	t	t	t	t	t	t	t	t	t	t	t	t	t	t	t	t	t	t	t	t	t		
c	2654878	t	2864355	t	2934586	nc	O157	rec46	c	O157	++++	a	c	c	c	c	c	c	c	c	c	c	c	c	c	c	c	a	a	a	a	c		
c	2654884	g	2864361	g	2934592	nc	O157	rec46	c	O157	++++	c	t	t	t	c	c	c	c	c	c	c	c	c	c	c	c	c	c	c	c	c		
c	2654886	a	2864363	a	2934594	nc	O157	rec46	c	O157	++++	c	c	c	c	t	c	c	c	c	c	c	c	c	c	c	c	c	c	c	c	c		
c	2654893	t	2864370	t	2934601	nc	O157	rec46	c	O157	++	c	c	c	c	c	c	c	c	t	t	c	c	c	c	c	c	c	c	c	c	c		
g	2654914	a	2864391	a	2934622	nc	O157	rec46	g	O157	++++	g	g	g	g	g	g	g	g	g	g	g	g	g	g	g	g	g	g	g	g	g		
t	2654934	c	2864411	c	2934642	nc	O157	rec46	t	O157	++++	t	t	t	t	t	t	t	t	t	t	t	t	t	t	t	t	t	t	t	t	t		
t	2654941	a	2864418	a	2934649	nc	O157	rec46	t	O157	++++	t	t	t	t	t	t	t	t	t	t	t	t	t	t	t	t	t	t	t	t	t		
a	2654944	g	2864421	g	2934652	nc	O157	rec46	a	O157	++++	a	a	a	a	a	a	a	a	a	a	a	a	a	a	a	a	a	a	a	a	a		
t	2654962	g	2864439	g	2934670	nc	O157	rec46	t	O157	++	t	g	g	g	t	g	g	g	g	g	g	g	g	g	g	g	g	g	g	g	t		
c	2654967	t	2864444	t	2934675	nc	O157	rec46	c	O157	++++	c	c	c	c	c	c	c	c	c	c	c	c	c	c	c	c	c	c	c	c	c		
a	2654968	c	2864445	c	2934676	nc	O157	rec46	a	O157	++++	a	a	a	a	a	a	a	a	a	a	a	a	a	a	a	a	a	a	a	a	a		
c	2654974	a	2864451	a	2934682	nc	O157	rec46	c	O157	++++	c	c	c	c	c	c	c	c	c	c	c	c	c	c	c	c	c	c	c	c	c		
a	2654980	c	2864457	c	2934688	nc	O157	rec46	a	O157	++	a	g	g	g	a	g	g	g	g	g	g	g	g	g	g	g	g	g	g	g	g		
c	2654986	t	2864463	t	2934694	nc	O157	rec46	c	O157	++	c	t	t	t	c	t	t	t	t	t	t	t	t	t	t	t	t	t	t	t	t		
c	2654994	t	2864471	t	2934702	nc	O157	rec46	c	O157	++++	c	c	c	c	c	c	c	c	c	c	c	c	c	c	c	c	c	c	c	c	c		
g	2655021	t	2864498	t	2934729	nc	O157	rec46	g	O157	++++	g	g	g	g	g	a	a	g	a	a	a	a	a	a	g	g	g	g	g	g	g		
t	2655051	g	2864528	g	2934759	nc	O157	rec46	t	O157	++++	t	t	t	t	t	t	t	t	t	t	t	t	t	t	t	t	t	t	t	t	t		

O55 and O157 genome site details												Outgroup Strain Details[i]																						
CB9615 base[b]	CB9615 Site[c]	Sakai base[b]	Sakai Site[c]	EDL933 base[b]	EDL933 Site[c]	type[d]	Event Lineage[ef]	Recombinant segment	Inferred ancestral base[g]	Outgroup Analysis[e]	Support level[h]	D1 Sd197	K-12	HS	ATCC 8739	UMN026	IAI39	SMS 3-5	E2348/69	536	ED1a	CFT073	S88	APEC O1	UTI89	E24377A	IAI1	SE11	F5 8401	F2a 2457T	F2a 301	SS Ss046	B4 Sb227	B18 BS512
t	2655061	c	2864538	c	2934769	nc	O157	rec46	t	O157	++++	t	t	t	t	t	t	t	t	t	t	t	t	t	t	t	t	t	t	t	t	t		
t	2655070	a	2864547	a	2934778	nc	O157	rec46	t	O157	++++	t	t	t	t	t	t	t	t	t	t	t	t	t	t	t	t	t	t	t	t	t		
g	2655078	a	2864555	a	2934786	nc	O157	rec46	g	O157	++++	g	g	g	g	g	g	g	g	g	g	g	g	g	g	g	g	g	g	g	g	g		
g	2655088	a	2864565	a	2934796	nc	O157	rec46	g	O157	++++	g	g	g	g	g	g	g	g	g	g	g	g	g	g	g	g	g	g	g	g	g		
a	2655121	g	2864598	g	2934829	nc	O157	rec46	a	O157	++++	a	a	a	a	a	a	a	a	a	a	a	a	a	a	a	a	a	a	a	a	a		
t	2655127	c	2864604	c	2934835	nc	O157	rec46	t	O157	++++	t	t	t	t	t	t	t	t	t	t	t	t	t	t	t	t	t	t	t	t	t		
t	2655135	g	2864612	g	2934843	nc	O157	rec46	t	O157	++++	t	t	t	t	t	t	t	t	t	t	t	t	t	t	t	t	t	t	t	t	t		
c	2655184	a	2864661	a	2934892	nc	O157	rec46	c	O157	++++	c	c	c	c	c	c	c	c	c	c	c	c	c	c	c	c	c	c	c	c	c		
g	2655193	a	2864670	a	2934901	nc	O157	rec46	g	O157	++++	g	g	g	g	g	g	g	g	g	g	g	g	g	g	g	g	g	g	g	g	g		
a	2655196	c	2864673	c	2934904	nc	O157	rec46	a	O157	++++	a	a	a	a	a	a	a	a	a	a	a	a	a	a	a	a	a	a	a	a	a		
t	2655198	g	2864675	g	2934906	nc	O157	rec46	t	O157	++++	t	t	t	t	t	t	t	t	t	t	t	t	t	t	t	t	t	t	t	t	t		
g	2655199	t	2864676	t	2934907	nc	O157	rec46	g	O157	++++	g	g	g	g	g	g	g	g	g	g	g	g	g	g	g	g	g	g	g	g	g		
a	2655220	g	2864697	g	2934928	nc	O157	rec46	a	O157	++++	a	a	a	a	a	a	a	a	a	a	a	a	a	a	a	a	a	a	a	a	a		
g	2655221	a	2864698	a	2934929	nc	O157	rec46	g	O157	++++	g	g	g	g	g	g	g	g	g	g	g	g	g	g	g	g	g	g	g	g	g		
a	2655250	g	2864727	g	2934958	nc	O157	rec46	a	O157	++++	a	a	a	a	a	a	a	a	a	a	a	a	a	a	a	a	a	a	a	a	a		
c	2655265	t	2864742	t	2934973	nc	O157	rec46	c	O157	++	c	c	c	c	t	c	c	c	c	c	c	c	c	c	c	c	c	c	c	c	t		
a	2655268	g	2864745	g	2934976	nc	O157	rec46	a	O157	++++	a	a	a	a	c	a	a	a	a	a	a	a	a	a	a	a	a	a	a	a	c		
a	2655269	g	2864746	g	2934977	nc	O157	rec46	a	O157	++++	a	a	a	a	a	a	a	a	a	a	a	a	a	a	a	a	a	a	a	a	a		
c	2655274	t	2864751	t	2934982	nc	O157	rec46	c	O157	++++	c	c	c	c	c	c	c	t	c	c	c	c	c	c	c	c	c	c	c	c	c		
t	2655278	g	2864755	g	2934986	nc	O157	rec46	t	O157	++++	t	t	t	t	t	t	t	t	t	t	t	t	t	t	t	t	t	t	t	t	t		
t	2655286	c	2864763	c	2934994	nc	O157	rec46	t	O157	++	t	c	c	c	t	t	t	c	t	t	t	t	t	t	c	c	c	c	c	c	t		
c	2655292	g	2864769	g	2935000	nc	O157	rec46	c	O157	++++	c	c	c	c	c	c	c	c	c	c	c	c	c	c	c	c	c	c	c	c	c		
t	2655295	c	2864772	c	2935003	nc	O157	rec46	t	O157	++	t	c	c	c	t	t	c	c	c	c	c	c	c	c	c	c	c	c	c	c	t		
a	2655328	g	2864805	g	2935036	nc	O157	rec46	a	O157	++++	a	a	a	a	a	a	a	a	a	a	a	a	a	a	a	a	a	a	a	a	a		
t	2655331	c	2864808	c	2935039	nc	O157	rec46	c	CB9615	++++	c	c	c	c	c	c	c	c	c	c	c	c	c	c	c	c	c	c	c	c	c		
g	2655333	a	2864810	a	2935041	nc	O157	rec46	g	O157	++++	g	g	g	g	g	g	g	g	g	g	g	g	g	g	g	g	g	g	g	g	g		
c	2655339	t	2864816	t	2935047	nc	O157	rec46	c	O157	++++	c	c	c	c	c	c	c	c	c	c	c	c	c	c	c	c	c	c	c	c	c		
a	2655346	g	2864823	g	2935054	nc	O157	rec46	a	O157	++	a	a	a	a	g	g	g	g	g	g	g	g	g	g	g	g	g	a	a	a	a		
g	2655349	a	2864826	a	2935057	nc	O157	rec46	g	O157	++	g	g	g	g	a	a	a	a	a	a	a	a	a	a	a	a	a	g	g	g	g		
c	2655358	g	2864835	g	2935066	nc	O157	rec46	c	O157	++++	c	c	c	c	c	c	c	c	c	c	c	c	c	c	c	c	c	c	c	c	c		
a	2655361	g	2864838	g	2935069	nc	O157	rec46	a	O157	++	a	a	a	a	g	g	g	g	g	g	g	g	g	g	g	g	g	a	a	a	a		
c	2655362	t	2864839	t	2935070	nc	O157	rec46	c	O157	++++	c	c	c	c	t	c	c	c	c	c	c	c	c	c	c	c	c	c	c	c	c		
t	2655367	a	2864844	a	2935075	nc	O157	rec46	t	O157	++	t	t	t	t	a	a	a	a	a	a	a	a	a	a	a	a	a	t	t	t	t		
t	2655370	g	2864847	g	2935078	nc	O157	rec46	t	O157	++	t	t	t	t	g	g	g	g	g	g	g	g	g	g	g	g	g	t	t	t	t		
t	2655376	c	2864853	c	2935084	nc	O157	rec46	t	O157	++++	t	t	t	t	c	t	t	t	t	t	t	t	t	t	t	t	t	t	t	t	t		
c	2655385	g	2864862	g	2935093	nc	O157	rec46	c	O157	++++	c	c	c	c	c	c	c	c	c	c	c	c	c	c	c	c	c	c	c	c	c		
a	2655391	g	2864868	g	2935099	nc	O157	rec46	a	O157	++++	a	a	a	a	a	a	a	a	a	a	a	a	a	a	a	a	a	a	a	a	a		
g	2655397	a	2864874	a	2935105	nc	O157	rec46	g	O157	++++	g	g	g	g	g	g	g	g	g	g	g	g	g	g	g	g	g	g	g	g	g		
c	2655403	a	2864880	a	2935111	nc	O157	rec46	c	O157	++	c	c	c	c	a	c	c	c	c	c	c	c	c	c	c	c	c	a	a	a	c		
c	2655418	g	2864895	g	2935126	nc	O157	rec46	c	O157	++++	c	c	c	c	c	c	c	c	c	c	c	c	c	c	c	c	c	c	c	c	c		
a	2655448	t	2864925	t	2935156	nc	O157	rec46	a	O157	++++	a	a	a	a	a	a	a	a	a	a	a	a	a	a	a	a	a	a	a	a	a		
a	2655451	c	2864928	c	2935159	nc	O157	rec46	a	O157	++++	a	a	a	a	a	a	a	a	a	a	a	a	a	a	a	a	a	a	a	a	a		
a	2655459	t	2864936	t	2935167	nc	O157	rec46	a	O157	++	a	a	a	a	t	t	t	t	t	t	t	t	t	t	t	t	t	t	t	t	a		
a	2655469	g	2864946	g	2935177	nc	O157	rec46	a	O157	++++	a	a	a	a	a	a	a	a	a	a	a	a	a	a	a	a	a	a	a	a	a		
a	2655475	g	2864952	g	2935183	nc	O157	rec46	a	O157	++++	a	a	a	a	a	a	a	a	a	a	a	a	a	a	a	a	a	a	a	a	a		
a	2655505	g	2864982	g	2935213	nc	O157	rec46	a	O157	++++	a	a	a	a	a	a	a	a	a	a	a	a	a	a	a	a	a	a	a	a	a		
a	2655517	g	2864994	g	2935225	nc	O157	rec46	a	O157	++++	a	a	a	a	a	a	a	a	a	a	a	a	a	a	a	a	a	a	a	a	a		
t	2655518	g	2864995	g	2935226	nc	O157	rec46	t	O157	++++	t	t	t	t	t	t	t	t	t	t	t	t	t	t	t	t	t	t	t	t	t		
g	2655519	c	2864996	c	2935227	nc	O157	rec46	g	O157	++++	g	g	g	g	g	g	g	g	g	g	g	g	g	g	g	g	g	g	g	g	g		
a	2655520	g	2864997	g	2935228	nc	O157	rec46	a	O157	++++	a	a	a	a	a	a	a	a	a	a	a	a	a	a	a	a	a	a	a	a	a		
a	2655523	g	2865000	g	2935231	nc	O157	rec46	a	O157	++++	a	a	a	a	a	a	a	a	a	a	a	a	a	a	a	a	a	a	a	a	a		
a	2655535	c	2865012	c	2935243	nc	O157	rec46	a	O157	++	a	a	a	a	a	a	a	a	a	a	a	a	a	a	c	c	c	c	c	c	a		
t	2655568	c	2865045	c	2935276	nc	O157	rec46	t	O157	++	t	t	t	t	t	t	t	t	t	t	t	t	t	t	c	c	c	c	c	c	t		
a	2655572	c	2865049	c	2935280	nc	O157	rec46	a	O157	++	a	a	a	a	a	a	a	a	a	a	a	a	a	a	c	c	c	c	c	c	a		
g	2655573	a	2865050	a	2935281	nc	O157	rec46	g	O157	++	g	g	g	g	g	g	g	g	g	a	g	g	g	g	a	a	a	a	a	a	g		
g	2655577	a	2865054	a	2935285	nc	O157	rec46	g	O157	++	g	g	g	g	g	g	g	g	g	g	g	g	g	g	a	a	a	a	a	a	g		
a	2655591	g	2865068	g	2935299	nc	O157	rec46	a	O157	++++	a	a	a	a	a	a	a	a	a	a	a	a	a	a	a	a	a	a	a	a	a		
c	2655592	t	2865069	t	2935300	nc	O157	rec46	c	O157	++++	c	c	c	c	c	c	c	c	c	c	c	c	c	c	c	c	c	c	c	c	c		
c	2655601	t	2865078	t	2935309	nc	O157	rec46	c	O157	++++	c	c	c	c	c	c	c	c	c	c	c	c	c	c	c	c	c	c	c	c	c		
a	2655616	t	2865093	t	2935324	nc	O157	rec46	a	O157	++++	a	a	a	a	a	a	a	a	a	a	a	a	a	a	a	a	a	a	a	a	a		
t	2655631	a	2865108	a	2935339	nc	O157	rec46	t	O157	++++	t	t	t	t	t	t	t	t	t	t	t	t	t	t	t	t	t	t	t	t	t		
c	2655640	a	2865117	a	2935348	nc	O157	rec46	c	O157	++++	c	c	c	c	c	c	c	c	c	c	c	c	c	c	c	c	c	c	c	c	c		
a	2655658	t	2865135	t	2935366	nc	O157	rec46	a	O157	++++	a	c	c	c	a	a	a	a	a	a	a	a	a	a	c	c	c	a	a	a	c		
a	2655664	g	2865141	g	2935372	nc	O157	rec46	a	O157	++	a	a	a	a	g	g	g	g	g	g	g	g	g	g	g	g	g	g	g	g	a		
g	2655682	a	2865159	a	2935390	nc	O157	rec46	g	O157	++	g	g	g	g	g	g	a	a	a	a	a	a	a	a	g	g	g	g	g	g	g		
a	2655691	g	2865168	g	2935399	nc	O157	rec46	g	CB9615	++++	g	g	g	g	g	a	g	g	g	g	g	g	g	g	g	g	g	g	g	g	g		
g	2655742	a	2865219	a	2935450	nc	O157	rec46	g	O157	++++	g	g	g	g	g	g	g	g	g	g	g	g	g	g	g	g	g	g	g	g	g		
c	2655759	t	2865236	t	2935467	nc	O157	rec46	c	O157	++++	c	c	c	c	c	c	c	c	c	c	c	c	c	c	c	c	c	c	c	c	c		
g	2655766	a	2865243	a	2935474	nc	O157	rec46	g	O157	++++	g	g	g	g	g	g	g	g	g	g	g	g	g	g	g	g	g	g	g	g	g		
g	2655784	a	2865261	a	2935492	nc	O157	rec46	g	O157	++++	g	g	g	g	g	g	g	g	g	g	g	g	g	g	g	g	g	g	g	g	g		
a	2655787	t	2865264	t	2935495	nc	O157	rec46	a	O157	++	a	t	t	t	t	t	t	t	t	t	t	t	t	t	t	t	t	t	t	t	t		
a	2655793	c	2865270	c	2935501	nc	O157	rec46	a	O157	++++	a	a	a	a	a	a	t	t	t	t	t	t	t	t	a	a	a	a	a	a	a		
g	2655796	a	2865273	a	2935504	nc	O157	rec46	g	O157	++++	g	g	g	g	g	g	g	g	g	g	g	g	g	g	g	g	g	g	g	g	g		
g	2655798	a	2865275	a	2935506	nc	O157	rec46	g	O157	++++	g	g	g	g	g	g	g	g	g	g	g	g	g	g	g	g	g	g	g	g	g		
a	2655802	g	2865279	g	2935510	nc	O157	rec46	a	O157	++	a	g	g	g	g	g	a	a	a	a	a	a	a	a	a	a	a	g	g	g	g		
c	2655811	g	2865288	g	2935519	nc	O157	rec46	c	O157	++++	c	c	c	c	c	c	c	c	c	c	c	c	c	c	c	c	c	c	c	c	c		
a	2655814	g	2865291	g	2935522	nc	O157	rec46	a	O157	++	a	g	g	g	g	g	a	a	a	a	g	a	a	a	g	g	g	g	g	g	g		
t	2655836	c	2865313	c	2935544	nc	O157	rec46	c	CB9615	++++	c	c	c	c	c	c	c	c	c	c	c	c	c	c	c	c	c	c	c	c	c		
c	2655856	t	2865333	t	2935564	nc	O157	rec46	c	O157	++	c	c	c	c	c	c	c	t	t	t	t	t	t	t	c	c	c	c	c	c	c		
c	2655859	t	2865336	t	2935567	nc	O157	rec46	c	O157	++++	c	c	c	c	c	c	c	c	c	c	c	c	c	c	c	c	c	c	c	c	c		
c	2655862	t	2865339	t	2935570	nc	O157	rec46	c	O157	++++	c	c	c	c	c	c	c	c	c	c	c	c	c	c	c	c	c	c	c	c	c		
c	2655873	a	2865350	a	2935581	nc	O157	rec46	c	O157	++++	c	c	c	c	c	c	c	c	c	c	c	c	c	c	c	c	c	c	c	c	c		
g	2655883	a	2865360	a	2935591	nc	O157	rec46	g	O157	++++	g	g	g	g	g	g	g	a	g	g	g	g	g	g	g	g	g	g	g	g	g		
c	2655904	t	2865381	t	2935612	nc	O157	rec46	c	O157	++++	c	c	c	c	c	c	c	c	c	c	c	c	c	c	c	c	c	c	c	c	c		
t	2655913	c	2865390	c	2935621	nc	O157	rec46	t	O157	++	t	c	c	c	c	c	c	c	c	c	c	c	c	c	c	c	c	c	c	c	c		
g	2655919	a	2865396	a	2935627	nc	O157	rec46	g	O157	++++	g	g	g	g	g	g	g	g	g	g	g	g	g	g	g	g	g	g	g	g	g		

O55 and O157 genome site details												Outgroup Strain Details[i]																						
CB9615 base[b]	CB9615 Site[c]	Sakai base[b]	Sakai Site[c]	EDL933 base[b]	EDL933 Site[c]	type[d]	Event Lineage[e,f]	Recombinant segment	Inferred ancestral base[g]	Outgroup Analysis[e]	Support level[h]	D1 Sd197	K-12	HS	ATCC 8739	UMN026	IAI39	SMS 3-5	E2348/69	536	ED1a	CFT073	S88	APEC O1	UTI89	E24377A	IAI1	SE11	F5 8401	F2a 2457T	F2a 301	SS Ss046	B4 Sb227	B18 BS512
g	2655922	a	2865399	a	2935630	nc	O157	rec46	g	O157	++++	g	g	g	g	g	g	g	g	g	g	g	g	g	g	g	g	g	g	g	g	g		
t	2655931	c	2865408	c	2935639	nc	O157	rec46	t	O157	++++	t	t	t	t	t	t	t	t	t	t	t	t	t	t	t	t	t	t	t	t	t		
a	2655937	g	2865414	g	2935645	nc	O157	rec46	a	O157	++++	a	a	a	a	a	a	a	a	a	a	a	a	a	a	a	a	a	a	a	a	a		
c	2655943	t	2865420	t	2935651	nc	O157	rec46	c	O157	++++	c	c	c	c	c	c	c	c	c	c	c	c	c	c	c	c	c	c	c	c	c		
c	2655946	a	2865423	a	2935654	nc	O157	rec46	c	O157	++	c	a	a	a	c	c	c	t	c	c	c	c	c	c	c	c	c	c	c	c	c		
g	2655949	t	2865426	t	2935657	nc	O157	rec46	g	O157	++++	g	g	g	g	g	g	g	g	g	g	g	g	g	g	g	g	g	g	g	g	g		
g	2655958	t	2865435	t	2935666	nc	O157	rec46	g	O157	++++	g	g	g	g	g	g	g	g	g	g	g	g	g	g	g	g	g	g	g	g	g		
g	2655964	t	2865441	t	2935672	nc	O157	rec46	g	O157	++	g	g	g	g	g	g	g	g	g	g	g	g	g	g	g	g	g	t	t	t	g		
a	2655967	g	2865444	g	2935675	nc	O157	rec46	a	O157	++	a	a	a	a	a	a	a	a	a	a	a	a	a	a	a	a	a	g	g	g	a		
g	2655973	a	2865450	a	2935681	nc	O157	rec46	g	O157	++++	g	g	g	g	g	g	g	g	g	g	g	g	g	g	g	g	g	g	g	g	g		
t	2655976	c	2865453	c	2935684	nc	O157	rec46	t	O157	++	t	t	t	t	t	t	t	t	t	t	t	t	t	t	t	t	t	c	c	c	t		
a	2655979	g	2865456	g	2935687	nc	O157	rec46	a	O157	++	a	a	a	a	a	a	a	a	a	a	a	a	a	a	a	a	a	g	g	g	a		
g	2655985	a	2865462	a	2935693	nc	O157	rec46	g	O157	++	g	g	g	g	g	g	g	g	g	g	g	g	g	g	g	g	g	a	a	a	g		
a	2655988	t	2865465	t	2935696	nc	O157	rec46	a	O157	++++	a	a	a	a	a	a	a	a	a	a	a	a	a	a	a	a	a	c	c	c	a		
c	2655991	t	2865468	t	2935699	nc	O157	rec46	c	O157	++	c	c	c	c	c	c	c	c	c	c	c	c	c	c	c	c	c	t	t	t	c		
c	2655994	g	2865471	g	2935702	nc	O157	rec46	c	O157	++	c	c	c	c	c	c	c	c	c	c	c	c	c	c	c	c	c	g	g	g	c		
a	2655996	g	2865473	g	2935704	nc	O157	rec46	a	O157	++	a	a	a	a	a	a	a	a	a	a	a	a	a	a	a	a	a	g	g	g	a		
a	2655997	g	2865474	g	2935705	nc	O157	rec46	a	O157	++	a	a	a	a	a	a	a	a	a	a	a	a	a	a	a	a	a	g	g	g	a		
g	2656000	a	2865477	a	2935708	nc	O157	rec46	g	O157	++	g	a	a	a	g	g	g	g	g	g	g	g	g	g	a	a	a	a	a	a	g		
c	2656003	g	2865480	g	2935711	nc	O157	rec46	c	O157	++++	c	c	c	c	c	c	c	c	c	c	c	c	c	c	c	c	c	c	c	c	c		
g	2656006	a	2865483	a	2935714	nc	O157	rec46	g	O157	++	g	g	g	g	g	g	g	g	g	g	g	g	g	g	g	g	g	a	a	a	g		
a	2656009	g	2865486	g	2935717	nc	O157	rec46	a	O157	++	a	a	a	a	a	a	a	a	a	a	a	a	a	a	a	a	a	g	g	g	a		
c	2656018	a	2865495	a	2935726	nc	O157	rec46	c	O157	++	c	c	c	c	c	c	c	c	c	c	c	c	c	c	c	c	c	a	a	a	c		
g	2656021	a	2865498	a	2935729	nc	O157	rec46	g	O157	++	g	g	g	g	g	g	g	g	g	g	g	g	g	g	g	g	g	a	a	a	a		
a	2656029	t	2865506	t	2935737	nc	O157	rec46	a	O157	++	a	a	a	a	a	a	a	a	a	a	a	a	a	a	a	a	a	t	t	t	a		
a	2656033	g	2865510	g	2935741	nc	O157	rec46	a	O157	++	a	a	a	a	g	g	g	a	a	a	g	a	a	a	a	a	a	g	g	g	a		
g	2656051	a	2865528	a	2935759	nc	O157	rec46	g	O157	++	g	a	a	a	g	g	g	g	g	g	g	g	g	g	a	a	a	g	g	g	g		
t	2656096	c	2865573	c	2935804	nc	O157	rec46	t	O157	++	t	t	t	t	t	t	t	t	t	t	t	t	t	t	t	t	t	c	c	c	t		
c	2656105	a	2865582	a	2935813	nc	O157	rec46	c	O157	++++	c	c	c	c	c	c	c	c	c	c	c	c	c	c	c	c	c	c	c	c	c		
g	2656117	a	2865594	a	2935825	nc	O157	rec46	g	O157	++++	g	g	g	g	g	g	g	g	g	g	g	g	g	g	g	g	g	g	g	g	g		
a	2656123	g	2865600	g	2935831	nc	O157	rec46	a	O157	++	a	a	a	a	a	a	a	a	a	a	a	a	a	a	a	a	a	g	g	g	a		
c	2656126	t	2865603	t	2935834	nc	O157	rec46	c	O157	++	c	c	c	c	c	t	t	c	c	c	t	c	c	c	c	c	c	c	c	c	c		
c	2656144	t	2865621	t	2935852	nc	O157	rec46	c	O157	++++	c	c	c	c	c	c	c	c	c	c	c	c	c	c	c	c	c	c	c	c	c		
c	2656147	t	2865624	t	2935855	nc	O157	rec46	c	O157	++	c	c	c	c	c	c	c	c	c	c	c	c	c	c	t	t	t	c	c	c	c		
g	2656150	a	2865627	a	2935858	nc	O157	rec46	g	O157	++++	g	g	g	g	g	g	g	g	g	g	g	g	g	g	g	g	g	g	g	g	g		
g	2656153	a	2865630	a	2935861	nc	O157	rec46	g	O157	++++	g	g	g	g	a	g	g	g	g	g	g	g	g	g	g	g	g	g	g	g	g		
t	2656168	a	2865645	a	2935876	nc	O157	rec46	t	O157	++++	t	t	t	t	c	c	c	t	t	c	t	t	t	t	t	t	t	t	t	t	t		
c	2656171	t	2865648	t	2935879	nc	O157	rec46	c	O157	++	c	c	c	c	c	c	c	t	t	t	c	t	t	t	c	c	c	c	c	c	c		
c	2656174	t	2865651	t	2935882	nc	O157	rec46	c	O157	++	c	c	c	c	c	t	c	c	c	c	t	c	c	c	t	t	t	c	c	c	c		
t	2656177	c	2865654	c	2935885	nc	O157	rec46	t	O157	++++	t	t	t	t	t	t	t	t	t	t	t	t	t	t	t	t	t	t	t	t	t		
g	2656195	c	2865672	c	2935903	nc	O157	rec46	g	O157	++++	g	g	g	g	g	g	g	g	g	g	g	g	g	g	g	g	g	g	g	g	g		
g	2656198	a	2865675	a	2935906	nc	O157	rec46	g	O157	++++	g	g	g	g	g	g	g	g	g	g	g	g	g	g	g	g	g	g	g	g	g		
t	2656201	c	2865678	c	2935909	nc	O157	rec46	t	O157	++++	t	t	t	t	t	t	t	t	t	t	t	t	t	t	t	t	t	t	t	t	t		
c	2656204	a	2865681	a	2935912	nc	O157	rec46	c	O157	++++	c	c	c	c	c	c	c	c	c	c	c	c	c	c	c	c	c	c	c	c	c		
g	2656207	a	2865684	a	2935915	nc	O157	rec46	a	CB9615	++	a	g	g	g	a	a	a	a	a	a	a	a	a	a	g	g	g	g	g	g	a		
g	2656210	a	2865687	a	2935918	nc	O157	rec46	g	O157	++++	g	g	g	g	g	g	g	g	g	g	g	g	g	g	g	g	g	g	g	g	g		
a	2656213	g	2865690	g	2935921	nc	O157	rec46	?	O157	+/-	c	c	c	c	c	c	c	c	c	c	c	c	c	c	c	c	c	c	c	c	c		
c	2656216	t	2865693	t	2935924	nc	O157	rec46	c	O157	++++	c	c	c	c	c	c	c	c	c	c	c	c	c	c	c	c	c	c	c	c	c		
t	2656219	c	2865696	c	2935927	nc	O157	rec46	t	O157	++++	t	t	t	t	t	t	t	t	t	t	t	t	t	t	t	t	t	t	t	t	t		
c	2656222	a	2865699	a	2935930	nc	O157	rec46	c	O157	++++	c	c	c	c	c	c	c	c	c	c	c	c	c	c	c	c	c	c	c	c	c		
g	2656225	a	2865702	a	2935933	nc	O157	rec46	g	O157	++++	g	g	g	g	g	g	g	g	g	g	g	g	g	g	g	g	g	g	g	g	g		
t	2656240	c	2865717	c	2935948	nc	O157	rec46	t	O157	++	t	t	t	t	t	t	t	c	c	c	t	c	c	c	t	t	t	t	t	t	t		
t	2656243	c	2865720	c	2935951	nc	O157	rec46	t	O157	++++	t	t	t	t	t	t	t	t	t	t	t	t	t	t	t	t	t	t	t	t	t		
a	2656246	t	2865723	t	2935954	nc	O157	rec46	a	O157	++++	a	a	a	a	a	a	a	a	a	a	a	a	a	a	a	a	a	a	a	a	a		
a	2656249	g	2865726	g	2935957	nc	O157	rec46	a	O157	++++	a	a	a	a	a	a	a	a	a	a	t	a	a	a	a	a	a	a	a	a	a		
g	2656252	c	2865729	c	2935960	nc	O157	rec46	g	O157	++++	g	g	g	g	g	g	g	g	g	g	g	g	g	g	g	g	g	g	g	g	g		
c	2656255	t	2865732	t	2935963	nc	O157	rec46	c	O157	++++	c	c	c	c	c	c	c	c	c	c	c	c	c	c	c	c	c	c	c	c	c		
t	2656267	a	2865744	a	2935975	nc	O157	rec46	t	O157	++++	t	t	t	t	t	t	t	t	t	t	t	t	t	t	t	t	t	t	t	t	t		
g	2656268	c	2865745	c	2935976	nc	O157	rec46	g	O157	++++	g	g	g	g	g	g	g	g	g	g	g	g	g	g	g	g	g	g	g	g	g		
c	2656275	a	2865752	a	2935983	nc	O157	rec46	c	O157	++++	c	c	c	c	c	c	c	c	c	c	c	c	c	c	c	c	c	c	c	c	c		
g	2656282	a	2865759	a	2935990	nc	O157	rec46	g	O157	++++	g	g	g	g	g	g	g	g	g	g	g	g	g	g	g	g	g	g	g	g	g		
a	2656285	t	2865762	t	2935993	nc	O157	rec46	a	O157	++++	a	a	a	a	a	a	a	t	a	a	a	a	a	a	a	a	a	a	a	a	a		
a	2656288	t	2865765	t	2935996	nc	O157	rec46	a	O157	++++	a	a	a	a	a	a	a	a	a	a	a	a	a	a	a	a	a	a	a	a	a		
g	2656292	a	2865769	a	2936000	nc	O157	rec46	a	CB9615	++++	a	a	a	a	a	a	a	a	a	a	a	a	a	a	a	a	a	a	a	a	a		
c	2656309	g	2865786	g	2936017	nc	O157	rec46	c	O157	++++	c	c	c	g	c	c	c	c	c	c	c	c	c	c	c	c	c	c	c	c	c		
a	2656318	g	2865795	g	2936026	nc	O157	rec46	g	CB9615	++	g	a	a	a	g	g	g	a	a	a	a	a	a	a	g	g	g	a	a	a	a		
g	2656338	a	2865815	a	2936046	nc	O157	rec46	g	O157	++++	g	g	g	g	g	g	g	g	g	g	g	g	g	g	g	g	g	g	g	g	g		
a	2656351	g	2865828	g	2936059	nc	O157	rec46	a	O157	++++	a	a	a	a	a	a	a	a	a	a	a	a	a	a	a	a	a	a	a	a	a		
c	2656363	t	2865840	t	2936071	nc	O157	rec46	c	O157	++++	c	c	c	c	c	c	c	c	c	c	c	c	c	c	c	c	c	c	c	c	c		
c	2656372	g	2865849	g	2936080	nc	O157	rec46	c	O157	++++	c	c	c	c	a	c	c	c	c	c	c	c	c	c	c	c	c	c	c	c	c		
t	2656384	c	2865861	c	2936092	nc	O157	rec46	t	O157	++++	t	t	t	t	t	t	t	t	t	t	t	t	t	t	t	t	t	t	t	t	t		
a	2656393	t	2865870	t	2936101	nc	O157	rec46	a	O157	++++	a	a	a	a	a	a	a	a	a	a	a	a	a	a	a	a	a	a	a	a	a		
c	2656417	t	2865894	t	2936125	nc	O157	rec46	c	O157	++++	c	c	c	c	c	c	c	c	c	c	c	c	c	c	c	c	c	c	c	c	c		
c	2656423	a	2865900	a	2936131	nc	O157	rec46	c	O157	++++	c	c	c	c	c	c	c	c	c	c	c	c	c	c	c	c	c	c	c	c	c		
a	2656426	c	2865903	c	2936134	nc	O157	rec46	a	O157	++++	a	a	a	a	a	a	a	a	a	a	a	a	a	a	a	a	a	a	a	a	a		
t	2656429	g	2865906	g	2936137	nc	O157	rec46	t	O157	++	t	t	t	t	g	g	g	g	g	g	g	g	g	g	g	g	g	g	g	g	t		
c	2656430	g	2865907	g	2936138	nc	O157	rec46	c	O157	++++	c	c	c	c	c	c	c	c	c	c	c	c	c	c	c	c	c	c	c	c	c		
a	2656441	g	2865918	g	2936149	nc	O157	rec46	a	O157	++++	a	a	a	a	a	a	a	a	a	a	a	a	a	a	a	a	a	a	a	a	a		
a	2656450	t	2865927	t	2936158	nc	O157	rec46	a	O157	++++	a	a	a	a	a	a	a	a	a	a	a	a	a	a	a	a	a	a	a	a	a		
c	2656457	g	2865934	g	2936165	nc	O157	rec46	c	O157	++++	c	c	c	c	c	c	c	c	c	c	c	c	c	c	c	c	c	c	c	c	c		
t	2656458	c	2865935	c	2936166	nc	O157	rec46	t	O157	++++	t	t	t	t	t	t	t	t	t	t	t	t	t	t	t	t	t	t	t	t	t		
t	2656462	g	2865939	g	2936170	nc	O157	rec46	t	O157	++++	t	t	t	t	t	t	t	t	t	t	t	t	t	t	t	t	t	t	t	t	t		
c	2656477	a	2865954	a	2936185	nc	O157	rec46	c	O157	++++	c	c	c	c	c	c	c	c	c	c	c	c	c	c	c	c	c	c	c	c	c		
c	2656479	t	2865956	t	2936187	nc	O157	rec46	c	O157	++++	c	c	c	c	c	c	c	c	c	c	c	c	c	c	c	c	c	c	c	c	c		
g	2656480	a	2865957	a	2936188	nc	O157	rec46	g	O157	++	g	g	g	g	g	g	g	a	a	a	a	a	a	a	g	g	g	g	g	g	g		
a	2656483	c	2865960	c	2936191	nc	O157	rec46	a	O157	++++	a	a	a	a	a	a	a	a	a	a	a	a	a	a	a	a	a	a	a	a	a		

O55 and O157 genome site details												Outgroup Strain Details[i]																						
CB9615 base[b]	CB9615 Site[c]	Sakai base[b]	Sakai Site[c]	EDL933 base[b]	EDL933 Site[c]	type[d]	Event Lineage[ef]	Recombinant segment	Inferred ancestral base[g]	Outgroup Analysis[e]	Support level[h]	D1 Sd197	K-12	HS	ATCC 8739	UMN026	IAI39	SMS 3-5	E2348/69	536	ED1a	CFT073	S88	APEC O1	UTI89	E24377A	IAI1	SE11	F5 8401	F2a 2457T	F2a 301	SS Ss046	B4 Sb227	B18 BS512
g	2656489	a	2865966	a	2936197	nc	O157	rec46	g	O157	++++	g	g	g	g	g	g	g	g	g	g	g	g	g	g	g	g	g	g	g	g	g		
g	2656497	t	2865974	t	2936205	nc	O157	rec46	g	O157	++++	g	g	g	g	g	g	g	g	g	g	g	g	g	g	g	g	g	a	a	a	g		
g	2656501	a	2865978	a	2936209	nc	O157	rec46	g	O157	++++	g	g	g	g	g	g	g	g	g	g	g	g	g	g	g	g	g	g	g	g	g		
c	2656503	a	2865980	a	2936211	nc	O157	rec46	c	O157	++++	c	c	c	c	c	c	c	c	c	c	c	c	c	c	c	c	c	c	c	c	c		
c	2656511	t	2865988	t	2936219	nc	O157	rec46	c	O157	++++	c	c	c	c	c	c	c	c	c	c	c	c	c	c	c	c	c	c	c	c	c		
t	2656513	c	2865990	c	2936221	nc	O157	rec46	t	O157	++++	t	t	t	t	t	t	t	t	t	t	t	t	t	t	t	t	t	t	t	t	t		
g	2656525	c	2866002	c	2936233	nc	O157	rec46	g	O157	++++	g	g	g	g	g	g	g	g	g	g	g	g	g	g	g	g	g	g	g	g	g		
a	2656528	g	2866005	g	2936236	nc	O157	rec46	a	O157	++++	a	a	a	a	a	a	a	a	a	a	a	a	a	a	a	a	a	a	a	a	a		
a	2656541	t	2866018	t	2936249	nc	O157	rec46	a	O157	++++	a	a	a	c	a	a	a	a	c	a	a	a	a	a	a	a	a	a	a	a	a		
c	2656543	a	2866020	a	2936251	nc	O157	rec46	c	O157	++++	c	c	c	c	c	c	c	c	c	c	c	c	c	c	c	c	c	c	c	c	c		
t	2656546	a	2866023	a	2936254	nc	O157	rec46	t	O157	++++	t	t	t	t	t	t	t	t	t	t	t	t	t	t	t	t	t	t	t	t	t		
g	2656552	a	2866029	a	2936260	nc	O157	rec46	g	O157	++++	g	g	g	g	g	g	g	g	g	g	g	g	g	g	g	g	g	g	g	g	g		
t	2656557	c	2866034	c	2936265	nc	O157	rec46	t	O157	++++	t	t	t	t	t	t	t	t	t	t	t	t	t	t	t	t	t	t	t	t	t		
t	2656558	c	2866035	c	2936266	nc	O157	rec46	t	O157	++++	t	t	t	t	t	t	t	t	t	t	t	t	t	t	t	t	t	t	t	t	t		
a	2656564	g	2866041	g	2936272	nc	O157	rec46	a	O157	++++	a	a	a	a	a	a	a	a	a	a	a	a	a	a	a	a	a	a	a	a	a		
g	2656567	a	2866044	a	2936275	nc	O157	rec46	g	O157	++++	g	g	g	g	g	g	g	g	g	g	g	g	g	g	g	g	g	g	g	g	g		
a	2656579	t	2866056	t	2936287	nc	O157	rec46	a	O157	++++		a	a	a	a	a	a	a	a	a	a	a	a	a	a	a	a	a	a	a	a		
t	2656588	c	2866065	c	2936296	nc	O157	rec46	t	O157	++++		t	t	t	t	t	t	t	t	t	t	t	t	t	t	t	t	t	t	t	t		
c	2656594	t	2866071	t	2936302	nc	O157	rec46	c	O157	++++		c	c	c	c	c	c	c	c	c	c	c	c	c	c	c	c	c	c	c	c		
t	2656600	c	2866077	c	2936308	nc	O157	rec46	t	O157	++++		t	t	t	t	t	t	t	t	t	t	t	t	t	t	t	t	t	t	t	t		
a	2656608	g	2866085	g	2936316	nc	O157	rec46	a	O157	++++		a	a	a	a	a	a	a	a	a	a	a	a	a	a	a	a	a	a	a	a		
g	2656612	a	2866089	a	2936320	nc	O157	rec46	g	O157	++++		g	g	g	g	g	g	g	g	g	g	g	g	g	g	g	g	g	g	g	g		
t	2656624	c	2866101	c	2936332	nc	O157	rec46	t	O157	++++		t	t	t	t	t	t	t	t	t	t	t	t	t	t	t	t	t	t	t	t		
t	2656630	a	2866107	a	2936338	nc	O157	rec46	t	O157	++++		t	t	t	t	t	t	t	t	t	t	t	t	t	t	t	t	t	t	t	t		
a	2656633	g	2866110	g	2936341	nc	O157	rec46	a	O157	++++		a	a	a	a	a	a	a	a	a	a	a	a	a	a	a	a	a	a	a	a		
a	2656645	g	2866122	g	2936353	nc	O157	rec46	a	O157	++++		a	a	a	a	a	a	a	a	a	a	a	a	a	a	a	a	a	a	a	a		
c	2656651	g	2866128	g	2936359	nc	O157	rec46	c	O157	++++		c	c	c	c	c	c	c	c	c	c	c	c	c	c	c	c	c	c	c	c		
c	2656678	t	2866155	t	2936386	nc	O157	rec46	c	O157	++++		c	c	c	c	c	c	c	c	c	c	c	c	c	c	c	c	c	c	c	c		
a	2656681	g	2866158	g	2936389	nc	O157	rec46	a	O157	++++		a	a	a	a	a	a	a	a	a	a	a	a	a	a	a	a	a	a	a	a		
g	2656690	c	2866167	c	2936398	nc	O157	rec46	g	O157	++++		g	g	g	g	g	g	g	g	g	g	g	g	g	g	g	g	a	a	a	g		
a	2656705	g	2866182	g	2936413	nc	O157	rec46	a	O157	++++		a	a	a	a	a	a	a	a	a	a	a	a	a	a	a	a	a	a	a	a		
a	2656708	t	2866185	t	2936416	nc	O157	rec46	a	O157	++++		a	a	a	a	a	a	a	a	a	a	a	a	a	a	a	a	a	a	a	a		
c	2656726	t	2866203	t	2936434	nc	O157	rec46	c	O157	+		c	c	c	c	c	c	a	t	t	t	a	a	a	c	c	c	c	c	c	c		
t	2656729	c	2866206	c	2936437	nc	O157	rec46	t	O157	++++		t	t	t	t	t	t	t	t	t	t	t	t	t	t	t	t	t	t	t	t		
a	2656735	g	2866212	g	2936443	nc	O157	rec46	a	O157	++++		a	a	a	a	a	a	a	a	a	a	a	a	a	a	a	a	a	a	a	a		
g	2656738	a	2866215	a	2936446	nc	O157	rec46	g	O157	++++		g	g	g	g	g	g	g	g	g	g	g	g	g	g	g	g	g	g	g	g		
c	2656742	t	2866219	t	2936450	nc	O157	rec46	c	O157	++++		c	c	c	c	c	c	c	c	c	c	c	c	c	c	c	c	c	c	c	c		
t	2656750	c	2866227	c	2936458	nc	O157	rec46	t	O157	+		t	t	t	t	t	t	t	c	c	a	t	t	t	t	t	t	t	t	t	t		
c	2656756	a	2866233	a	2936464	nc	O157	rec46	c	O157	++++		c	c	c	c	c	c	c	c	c	c	c	c	c	c	c	c	c	c	c	c		
c	2656765	t	2866242	t	2936473	nc	O157	rec46	c	O157	++++		c	c	c	c	c	c	t	c	c	c	c	c	c	c	c	c	c	c	c	c		
g	2656771	a	2866248	a	2936479	nc	O157	rec46	g	O157	++++		g	g	g	g	g	g	g	g	g	g	g	g	g	g	g	g	g	g	g	g		
c	2656775	g	2866252	g	2936483	nc	O157	rec46	c	O157	++++		c	c	c	c	c	c	c	c	c	c	c	c	c	c	c	c	c	c	c	c		
t	2656776	c	2866253	c	2936484	nc	O157	rec46	t	O157	++++		t	t	t	t	t	t	t	t	t	t	t	t	t	t	t	t	t	t	t	t		
a	2656779	g	2866256	g	2936487	nc	O157	rec46	a	O157	++++		a	a	a	a	a	a	a	a	a	a	a	a	a	a	a	a	a	a	a	a		
g	2656783	c	2866260	c	2936491	nc	O157	rec46	g	O157	++++		g	g	g	g	g	g	g	g	g	g	g	g	g	g	g	g	g	g	g	g		
g	2656785	a	2866262	a	2936493	nc	O157	rec46	g	O157	++++		g	g	g	g	g	g	g	g	g	g	g	g	g	g	g	g	g	g	g	g		
c	2656794	t	2866271	t	2936502	nc	O157	rec46	c	O157	++++		c	c	c	c	c	c	c	c	c	c	c	c	c	c	c	c	c	c	c	c		
g	2656798	a	2866275	a	2936506	nc	O157	rec46	g	O157	++++		g	g	g	g	g	g	g	g	g	g	g	g	g	g	g	g	g	g	g	g		
t	2656809	a	2866286	a	2936517	nc	O157	rec46	t	O157	++++		t	t	t	t	t	t	t	t	t	t	t	t	t	t	t	t	t	t	t	t		
a	2656810	g	2866287	g	2936518	nc	O157	rec46	a	O157	++++		a	a	a	a	a	a	a	a	a	a	a	a	a	a	a	a	a	a	a	a		
c	2656816	t	2866293	t	2936524	nc	O157	rec46	c	O157	++++		c	c	c	c	c	c	c	c	c	c	c	c	c	c	c	c	c	c	c	c		
a	2656819	g	2866296	g	2936527	nc	O157	rec46	a	O157	+		a	a	a	a	a	a	g	g	g	g	g	g	g	a	a	a	a	a	a	a		
t	2656849	c	2866326	c	2936557	nc	O157	rec46	t	O157	++++		t	t	t	t	t	t	t	t	t	t	t	t	t	t	t	t	t	t	t	t		
c	2656852	a	2866329	a	2936560	nc	O157	rec46	c	O157	++++		c	c	c	c	c	c	c	c	c	c	c	c	c	c	c	c	c	c	c	c		
a	2656855	t	2866332	t	2936563	nc	O157	rec46	a	O157	++++		a	a	a	a	a	a	a	a	a	a	a	a	a	a	a	a	a	a	a	a		
c	2656856	t	2866333	t	2936564	nc	O157	rec46	c	O157	++++		c	c	c	c	c	c	c	c	c	c	c	c	c	c	c	c	c	c	c	c		
t	2656860	g	2866337	g	2936568	nc	O157	rec46	t	O157	++++		t	t	t	t	t	t	t	t	t	t	t	t	t	t	t	t	t	t	t	t		
g	2656861	t	2866338	t	2936569	nc	O157	rec46	g	O157	++++		g	g	g	g	g	g	g	g	g	g	g	g	g	g	g	g	g	g	g	g		
g	2656864	a	2866341	a	2936572	nc	O157	rec46	g	O157	+		g	g	g	a	a	a	a	a	a	a	a	a	a	g	g	g	g	g	g	g		
g	2656867	a	2866344	a	2936575	nc	O157	rec46	g	O157	++++		g	g	g	g	g	g	g	g	g	g	g	g	g	g	g	g	g	g	g	g		
a	2656879	t	2866356	t	2936587	nc	O157	rec46	a	O157	++++		a	a	a	a	a	a	a	a	a	a	a	a	a	a	a	a	a	a	a	a		
g	2656882	a	2866359	a	2936590	nc	O157	rec46	g	O157	++++		g	g	g	g	g	g	g	g	g	g	g	g	g	g	g	g	g	g	g	g		
g	2656894	t	2866371	t	2936602	nc	O157	rec46	g	O157	++++		g	g	g	g	g	g	g	g	g	g	g	g	g	g	g	g	g	g	g	g		
t	2656896	c	2866373	c	2936604	nc	O157	rec46	t	O157	++++		t	t	t	t	t	t	t	t	t	t	t	t	t	t	t	t	t	t	t	t		
c	2656899	t	2866376	t	2936607	nc	O157	rec46	c	O157	+		c	c	c	t	t	t	t	t	t	t	t	t	t	c	c	c	c	c	c	c		
t	2656903	a	2866380	a	2936611	nc	O157	rec46	t	O157	++++		t	t	t	c	c	c	c	c	c	c	c	c	c	t	t	t	t	t	t	t		
t	2656905	g	2866382	g	2936613	nc	O157	rec46	t	O157	++++		t	t	t	t	t	t	t	t	t	t	t	t	t	t	t	t	t	t	t	t		
t	2656906	c	2866383	c	2936614	nc	O157	rec46	t	O157	++++		t	t	t	t	t	t	t	t	t	t	t	t	t	t	t	t	t	t	t	t		
a	2656908	t	2866385	t	2936616	nc	O157	rec46	a	O157	++++		a	a	a	a	a	a	a	a	a	a	a	a	a	a	a	a	a	a	a	a		
t	2656909	c	2866386	c	2936617	nc	O157	rec46	t	O157	++++		t	t	t	t	t	t	t	t	t	t	t	t	t	t	t	t	t	t	t	t		
a	2656911	g	2866388	g	2936619	nc	O157	rec46	a	O157	++++		a	a	a	a	a	a	a	a	a	a	a	a	a	a	a	a	a	a	a	a		
a	2656912	g	2866389	g	2936620	nc	O157	rec46	a	O157	++++		a	a	a	a	a	a	a	a	a	a	a	a	a	a	a	a	a	a	a	a		
t	2656915	a	2866392	a	2936623	nc	O157	rec46	t	O157	++++		t	t	t	t	t	t	t	t	t	t	t	t	t	t	t	t	t	t	t	t		
t	2656921	a	2866398	a	2936629	nc	O157	rec46	t	O157	++++		t	t	t	t	t	t	t	t	t	t	t	t	t	t	t	t	t	t	t	t		
c	2656924	t	2866401	t	2936632	nc	O157	rec46	c	O157	++++		c	c	c	c	c	c	c	c	c	c	c	c	c	c	c	c	c	c	c	c		
g	2656930	a	2866407	a	2936638	nc	O157	rec46	g	O157	++++		g	g	g	g	g	g	g	g	g	g	g	g	g	g	g	g	g	g	g	g		
a	2656936	g	2866413	g	2936644	nc	O157	rec46	a	O157	++++		a	a	a	a	a	a	a	a	a	a	a	a	a	a	a	a	a	a	a	a		
a	2656939	g	2866416	g	2936647	nc	O157	rec46	a	O157	++++		a	a	a	a	a	a	a	a	a	a	a	a	a	a	a	a	a	a	a	a		
c	2656954	t	2866431	t	2936662	nc	O157	rec46	c	O157	++++		c	c	c	c	c	c	c	c	c	c	c	c	c	c	c	c	c	c	c	c		
g	2656957	a	2866434	a	2936665	nc	O157	rec46	g	O157	+		g	g	g	g	g	g	a	a	a	a	a	a	a	g	g	g	g	g	g	g		
c	2656966	t	2866443	t	2936674	nc	O157	rec46	c	O157	++++		c	c	c	c	c	c	c	c	c	c	c	c	c	c	c	c	c	c	c	c		
g	2656984	a	2866461	a	2936692	nc	O157	rec46	g	O157	++++		g	g	g	g	g	g	g	g	g	g	g	g	g	g	g	g	g	g	g	g		
a	2656990	g	2866467	g	2936698	nc	O157	rec46	a	O157	++++		a	a	a	a	a	a	a	a	a	a	a	a	a	a	a	a	a	a	a	a		
c	2656999	a	2866476	a	2936707	nc	O157	rec46	c	O157	++++		c	c	c	c	c	c	c	c	c	c	c	c	c	c	c	c	c	c	c	c		
g	2657005	a	2866482	a	2936713	nc	O157	rec46	g	O157	++++		g	g	g	g	g	g	g	g	g	g	g	g	g	g	g	g	g	g	g	g		
t	2657008	c	2866485	c	2936716	nc	O157	rec46	t	O157	+		t	t	t	t	t	t	t	c	c	c	c	c	c	t	t	t	t	t	t	t		

O55 and O157 genome site details												Outgroup Strain Details[i]																						
CB9615 base[b]	CB9615 Site[c]	Sakai base[b]	Sakai Site[c]	EDL933 base[b]	EDL933 Site[c]	type[d]	Event Lineage[ef]	Recombinant segment	Inferred ancestral base[g]	Outgroup Analysis[e]	Support level[h]	D1 Sd197	K-12	HS	ATCC 8739	UMN026	IAI39	SMS 3-5	E2348/69	536	ED1a	CFT073	S88	APEC O1	UTI89	E24377A	IAI1	SE11	F5 8401	F2a 2457T	F2a 301	SS Ss046	B4 Sb227	B18 BS512
a	2657026	c	2866503	c	2936734	nc	O157	rec46	a	O157	++++		a	a	a	a	a	a	a	a	a	a	a	a	a	a	a	a	a	a	a	a		
t	2657028	a	2866505	a	2936736	nc	O157	rec46	t	O157	++++		t	t	t	t	t	t	t	t	t	t	t	t	t	t	t	t	t	t	t	t		
c	2657034	t	2866511	t	2936742	nc	O157	rec46	c	O157	++++		c	c	c	c	c	c	c	a	c	a	a	a	a	c	c	c	c	c	c	c		
a	2657035	t	2866512	t	2936743	nc	O157	rec46	a	O157	++++		a	a	a	a	a	a	a	a	a	a	a	a	a	a	a	a	a	a	a	a		
c	2657038	t	2866515	t	2936746	nc	O157	rec46	c	O157	++++		c	c	c	c	c	c	c	c	c	c	c	c	c	c	c	c	c	c	c	c		
g	2657043	c	2866520	c	2936751	nc	O157	rec46	g	O157	++++		g	g	g	g	g	g	g	g	g	g	g	g	g	g	g	g	g	g	g	g		
g	2657044	a	2866521	a	2936752	nc	O157	rec46	g	O157	++++		g	g	g	g	g	g	g	g	g	g	g	g	g	g	g	g	g	g	g	g		
t	2657045	c	2866522	c	2936753	nc	O157	rec46	t	O157	++++		t	t	t	t	t	t	t	t	t	t	t	t	t	t	t	t	t	t	t	t		
a	2657056	t	2866533	t	2936764	nc	O157	rec46	a	O157	++++		a	a	a	a	a	a	a	a	a	a	a	a	a	a	a	a	a	a	a	a		
t	2657059	c	2866536	c	2936767	nc	O157	rec46	t	O157	++++		t	t	t	t	t	t	t	t	t	t	t	t	t	t	t	t	t	t	t	t		
a	2657074	g	2866551	g	2936782	nc	O157	rec46	a	O157	++++		a	a	a	a	a	a	a	a	a	a	a	a	a	a	a	a	a	a	a	a		
a	2657089	t	2866566	t	2936797	nc	O157	rec46	a	O157	++++		a	a	a	a	a	a	a	a	a	a	a	a	a	a	a	a	a	a	a	a		
a	2657097	g	2866574	g	2936805	nc	O157	rec46	a	O157	++++		a	a	a	a	a	a	a	a	a	a	a	a	a	a	a	a	a	a	a	a		
a	2657098	t	2866575	t	2936806	nc	O157	rec46	a	O157	++++		a	a	a	a	a	a	a	a	a	a	a	a	a	a	a	a	a	a	a	a		
t	2657122	g	2866599	g	2936830	nc	O157	rec46	t	O157	++++		t	t	t	t	t	t	t	t	t	t	t	t	t	t	t	t	t	t	t	t		
g	2657125	t	2866602	t	2936833	nc	O157	rec46	g	O157	++++		g	g	g	g	g	g	g	g	g	g	g	g	g	g	g	g	g	g	g	g		
t	2657140	c	2866617	c	2936848	nc	O157	rec46	t	O157	++++		t	t	t	t	t	t	t	t	t	t	t	t	t	t	t	t	t	t	t	t		
g	2657203	a	2866680	a	2936911	nc	O157	rec46	g	O157	++++		g	g	g	g	g	g	g	g	g	g	g	g	g	g	g	g	g	g	g	g		
a	2657221	t	2866698	t	2936929	nc	O157	rec46	a	O157	++++		a	a	a	a	a	a	a	a	a	a	a	a	a	a	a	a	a	a	a	a		
a	2657223	g	2866700	g	2936931	nc	O157	rec46	g	CB9615	+		g	g	g	a	g	g	g	g	g	g	g	g	g	a	a	a	a	a	a	g		
t	2657228	c	2866705	c	2936936	nc	O157	rec46	t	O157	++++		t	t	t	t	t	t	t	t	t	t	t	t	t	t	t	t	t	t	t	t		
t	2657229	c	2866706	c	2936937	nc	O157	rec46	t	O157	++++		t	t	t	t	t	t	t	t	t	t	t	t	t	t	t	t	t	t	t	t		
t	2657233	c	2866710	c	2936941	nc	O157	rec46	t	O157	+		t	t	t	c	c	c	t	t	t	t	t	t	t	t	t	t	t	t	t	t		t
c	2657243	t	2866720	t	2936951	nc	O157	rec46	c	O157	++++		c	c	c	c	c	c	c	c	c	c	c	c	c	c	c	c	c	c	c	c		c
g	2657248	t	2866725	t	2936956	nc	O157	rec46	g	O157	++++		g	g	g	g	g	g	g	g	g	g	g	g	g	g	g	g	g	g	g	g		g
a	2657249	t	2866726	t	2936957	nc	O157	rec46	a	O157	++++		a	a	a	a	a	a	a	a	a	a	a	a	a	a	a	a	a	a	a	a		a
t	2657252	c	2866729	c	2936960	nc	O157	rec46	t	O157	++++		t	t	t	t	t	t	t	t	t	t	t	t	t	t	t	t	t	t	t	t		t
c	2657258	t	2866735	t	2936966	nc	O157	rec46	c	O157	++++		c	c	c	c	c	c	c	c	c	c	c	c	c	c	c	c	c	c	c	c		c
a	2657270	t	2866747	t	2936978	nc	O157	rec46	a	O157	++++		a	a	a	a	a	a	a	a	a	a	a	a	a	a	a	a	a	a	a	a		a
a	2657271	g	2866748	g	2936979	nc	O157	rec46	a	O157	++++		a	a	a	a	a	a	a	a	a	a	a	a	a	a	a	a	a	a	a	a		a
g	2657272	t	2866749	t	2936980	nc	O157	rec46	g	O157	++++		g	g	g	g	g	g	g	g	g	g	g	g	g	g	g	g	g	g	g	g		g
g	2657275	t	2866752	t	2936983	nc	O157	rec46	g	O157	++++		g	g	g	g	g	g	g	g	g	g	g	g	g	g	g	g	g	g	g	g		g
a	2657284	g	2866761	g	2936992	nc	O157	rec46	a	O157	++++		a	a	a	a	a	a	a	a	a	a	a	a	a	a	a	t	a	a	a	a		a
a	2657287	t	2866764	t	2936995	nc	O157	rec46	a	O157	++++		a	a	a	a	a	a	a	a	a	a	a	a	a	a	a	a	a	a	a	a		a
g	2657299	c	2866776	c	2937007	nc	O157	rec46	g	O157	++++		g	g	g	g	g	g	g	g	g	g	g	g	g	g	g	g	g	g	g	g		g
a	2657305	g	2866782	g	2937013	nc	O157	rec46	g	CB9615	+		g	g	g	g	g	g	g	g	g	g	g	g	g	a	a	g	a	a	a	g		g
a	2657308	g	2866785	g	2937016	nc	O157	rec46	a	O157	++++		a	a	a	a	a	a	a	a	a	a	a	a	a	a	a	a	a	a	a	a		a
t	2657317	c	2866794	c	2937025	nc	O157	rec46	t	O157	++++		t	t	t	t	t	t	t	t	t	t	t	t	t	t	t	t	t	t	t	t		t
g	2657320	a	2866797	a	2937028	nc	O157	rec46	g	O157	++++		g	g	g	g	g	g	g	g	g	g	g	g	g	g	g	g	g	g	g	g		g
c	2657326	a	2866803	a	2937034	nc	O157	rec46	c	O157	++++		c	c	c	c	c	c	c	c	c	c	c	c	c	c	c	c	c	c	c	c		c
c	2657335	t	2866812	t	2937043	nc	O157	rec46	c	O157	++++		c	c	c	t	c	c	c	c	c	c	c	c	c	c	c	c	c	c	c	c		c
a	2657344	t	2866821	t	2937052	nc	O157	rec46	a	O157	+		a	a	a	t	t	t	a	a	a	a	a	a	a	a	a	a	a	a	a	a		a
c	2657346	g	2866823	g	2937054	nc	O157	rec46	c	O157	++++		c	c	c	c	c	c	c	c	c	c	c	c	c	c	c	c	c	c	c	c		c
t	2657347	c	2866824	c	2937055	nc	O157	rec46	t	O157	++++		t	t	t	t	t	t	t	t	t	t	t	t	t	t	t	t	t	t	t	t		t
c	2657362	g	2866839	g	2937070	nc	O157	rec46	c	O157	++++		c	c	c	c	c	c	c	c	c	c	c	c	c	c	c	c	c	c	c	c		c
c	2657365	t	2866842	t	2937073	nc	O157	rec46	c	O157	++++		c	c	c	c	c	c	c	c	c	c	c	c	c	c	c	c	c	c	c	c		c
a	2657367	c	2866844	c	2937075	nc	O157	rec46	a	O157	++++		a	a	a	a	a	a	a	a	a	a	a	a	a	a	a	a	a	a	a	a		a
t	2657368	g	2866845	g	2937076	nc	O157	rec46	t	O157	++++		a	a	a	t	t	t	t	t	t	t	t	t	t	t	t	t	t	t	t	a		t
c	2657369	t	2866846	t	2937077	nc	O157	rec46	c	O157	++++		c	c	c	c	c	c	c	c	c	c	c	c	c	c	c	c	c	c	c	c		c
c	2657370	t	2866847	t	2937078	nc	O157	rec46	c	O157	++++		c	c	c	c	c	c	c	c	c	c	c	c	c	c	c	c	c	c	c	c		c
g	2657374	a	2866851	a	2937082	nc	O157	rec46	g	O157	++++		g	g	g	g	g	g	g	g	g	g	g	g	g	g	g	g	g	g	g	g		g
a	2657377	g	2866854	g	2937085	nc	O157	rec46	a	O157	++++		a	a	a	a	a	a	a	a	a	a	a	a	a	a	a	a	a	a	a	a		a
g	2657380	a	2866857	a	2937088	nc	O157	rec46	g	O157	++++		g	g	g	g	g	g	g	g	g	g	g	g	g	g	g	g	g	g	g	g		g
t	2657384	c	2866861	c	2937092	nc	O157	rec46	t	O157	++++		t	t	t	t	t	t	t	t	t	t	t	t	t	t	t	c	t	t	t	t		t
t	2657386	c	2866863	c	2937094	nc	O157	rec46	t	O157	++++		t	t	t	t	t	t	t	t	t	t	t	t	t	t	t	t	t	t	t	t		t
c	2657389	t	2866866	t	2937097	nc	O157	rec46	c	O157	++++		c	c	c	c	c	c	c	c	c	c	c	c	c	c	c	c	c	c	c	c		c
a	2657390	c	2866867	c	2937098	nc	O157	rec46	a	O157	+		a	a	a	a	c	c	a	a	a	a	a	a	a	a	a	a	a	a	a	a		a
c	2657395	t	2866872	t	2937103	nc	O157	rec46	c	O157	+		t	t	t	c	c	c	c	c	c	c	c	c	c	c	c	c	c	c	c	t		c
g	2657404	a	2866881	a	2937112	nc	O157	rec46	g	O157	++++		t	t	t	t	t	t	g	g	g	g	g	g	g	g	g	g	g	g	g	t		g
c	2657409	t	2866886	t	2937117	nc	O157	rec46	c	O157	++++		c	c	c	c	c	c	c	c	c	c	c	c	c	c	c	c	c	c	c	c		c
c	2657431	a	2866908	a	2937139	nc	O157	rec46	c	O157	++++		c	c	c	c	c	c	c	c	c	c	c	c	c	c	c	c	c	c	c	c		c
c	2657434	a	2866911	a	2937142	nc	O157	rec46	a	CB9615	+		t	t	t	a	a	a	t	t	t	t	t	t	t	t	t	c	t	t	t	t		c
c	2657437	t	2866914	t	2937145	nc	O157	rec46	c	O157	+		c	c	c	c	c	c	c	c	c	t	c	c	c	c	c	g	c	c	c	t		c
-	2657441	a	2866919	a	2937150	ins	O157	rec46	-	O157	++++		a	a	a	a	a	a	a	a	a	a	a	a	a	a	a	a	a	a	a	a		a
t	2657445	c	2866923	c	2937154	nc	O157	rec46	c	CB9615	++++		c	c	c	c	c	c	c	c	c	c	c	c	c	c	c	c	c	c	c	c		c
t	2657448	c	2866926	c	2937157	nc	O157	rec46	c	CB9615	+		c	c	c	t	t	t	c	c	c	c	c	c	c	c	c	t	c	c	c	c		t
c	2657466	g	2866944	g	2937175	nc	O157	rec46	c	O157	++++		c	c	c	t	t	t	c	c	c	c	c	c	c	c	c	c	c	c	c	c		c
c	2657472	t	2866950	t	2937181	nc	O157	rec46	c	O157	+		c	c	c	t	t	t	c	c	c	c	c	c	c	c	c	c	c	c	c	c		c
c	2657475	t	2866953	t	2937184	nc	O157	rec46	c	O157	++++		c	c	c	c	c	c	c	c	c	c	c	c	c	c	c	c	c	c	c	c		c
g	2657477	a	2866955	a	2937186	nc	O157	rec46	g	O157	++++		g	g	g	g	g	g	g	g	g	g	g	g	g	g	g	g	g	g	g	g		g
t	2657478	c	2866956	c	2937187	nc	O157	rec46	t	O157	++++		t	t	t	t	t	t	t	t	t	t	t	t	t	t	t	t	t	t	t	t		t
a	2657487	t	2866965	t	2937196	nc	O157	rec46	a	O157	++++		a	a	a	g	g	g	a	a	a	a	a	a	a	g	g	a	g	g	g	a		a
t	2657493	a	2866971	a	2937202	nc	O157	rec46	t	O157	++++		t	t	t	t	t	t	t	t	t	t	t	t	t	t	t	t	t	t	t	t		t
g	2657495	c	2866973	c	2937204	nc	O157	rec46	g	O157	++++		g	g	g	g	g	g	g	g	g	g	g	g	g	g	g	g	g	g	g	g		g
a	2657502	g	2866980	g	2937211	nc	O157	rec46	a	O157	++++		a	a	a	a	a	a	a	a	a	a	a	a	a	a	a	a	a	a	a	a		a
t	2657508	c	2866986	c	2937217	nc	O157	rec46	t	O157	++++		t	t	t	t	t	t	t	t	t	t	t	t	t	t	t	t	t	t	t	t		c
c	2657529	a	2867007	a	2937238	nc	O157	rec46	c	O157	++++		c	c	c	c	c	c	c	c	c	c	c	c	c	c	c	c	c	c	c	c		a
g	2657541	a	2867019	a	2937250	nc	O157	rec46	g	O157	+		g	g	g	g	g	g	a	a	a	a	a	a	a	g	g	g	g	g	g	g		a
t	2657547	c	2867025	c	2937256	nc	O157	rec46	t	O157	++++		t	t	t	t	t	t	t	t	t	t	t	t	t	t	t	t	t	t	t	t		c
g	2657554	c	2867032	c	2937263	nc	O157	rec46	g	O157	++++		g	g	g	g	g	g	g	g	g	g	g	g	g	g	g	g	g	g	g	g		c
c	2657559	t	2867037	t	2937268	nc	O157	rec46	c	O157	+		c	c	c	c	c	c	t	t	t	t	g	g	g	c	c	c	c	c	c	c		t
g	2657561	a	2867039	a	2937270	nc	O157	rec46	g	O157	++++		g	g	g	g	g	g	g	g	g	g	g	g	g	g	g	g	g	g	g	g		a
c	2657568	a	2867046	a	2937277	nc	O157	rec46	c	O157	++++		c	c	c	c	c	c	c	c	c	c	c	c	c	c	c	c	c	c	c	c		a
a	2657574	g	2867052	g	2937283	nc	O157	rec46	a	O157	++++		a	a	a	a	a	a	a	a	a	a	a	a	a	a	a	a	a	a	a	a		g
g	2657580	a	2867058	a	2937289	nc	O157	rec46	g	O157	+		g	g	g	g	g	g	a	a	a	a	a	a	a	g	g	g	g	g	g	g		a
c	2657588	t	2867066	t	2937297	nc	O157	rec46	c	O157	++++		c	c	c	c	c	c	c	c	c	c	c	c	c	c	c	c	c	c	c	c		t

O55 and O157 genome site details												Outgroup Strain Details[i]																						
CB9615 base[b]	CB9615 Site[c]	Sakai base[b]	Sakai Site[c]	EDL933 base[b]	EDL933 Site[c]	type[d]	Event Lineage[ef]	Recombinant segment	Inferred ancestral base[g]	Outgroup Analysis[e]	Support level[h]	D1 Sd197	K-12	HS	ATCC 8739	UMN026	IAI39	SMS 3-5	E2348/69	536	ED1a	CFT073	S88	APEC O1	UTI89	E24377A	IAI1	SE11	F5 8401	F2a 2457T	F2a 301	SS Ss046	B4 Sb227	B18 BS512
c	2657592	t	2867070	t	2937301	nc	O157	rec46	c	O157	++++		c	c	c	c	c	c	c	c	c	c	c	c	c	c	c	c	c	c	c	c		t
g	2657598	a	2867076	a	2937307	nc	O157	rec46	g	O157	++++		g	g	g	g	g	g	g	g	g	g	g	g	g	g	g	g	g	g	g	g		a
t	2657604	g	2867082	g	2937313	nc	O157	rec46	t	O157	++++		t	t	t	t	t	t	t	t	t	t	t	t	t	t	t	t	t	t	t	t		g
c	2657608	g	2867086	g	2937317	nc	O157	rec46	c	O157	++++		c	c	c	c	c	c	c	c	c	c	c	c	c	c	c	c	c	c	c	c		g
t	2657612	c	2867090	c	2937321	nc	O157	rec46	t	O157	++++		t	t	t	t	t	t	t	t	t	t	t	t	t	t	t	t	t	t	t	t		c
t	2657616	c	2867094	c	2937325	nc	O157	rec46	t	O157	++++		t	t	t	t	t	t	t	t	t	t	t	t	t	t	t	t	t	t	t	t		c
g	2657617	a	2867095	a	2937326	nc	O157	rec46	g	O157	++++		g	g	g	g	g	g	g	g	g	g	g	g	g	g	g	g	g	g	g	g		a
c	2657619	t	2867097	t	2937328	nc	O157	rec46	c	O157	++++		c	c	c	c	c	c	c	c	c	c	c	c	c	c	c	c	c	c	c	c		t
t	2657652	c	2867130	c	2937361	nc	O157	rec46	t	O157	++++		t	t	t	t	t	t	t	t	t	t	t	t	t	t	t	t	t	t	t	t		c
t	2657661	c	2867139	c	2937370	nc	O157	rec46	t	O157	++++		t	t	t	t	t	t	t	t	t	t	t	t	t	t	t	t	t	t	t	t		c
t	2657664	c	2867142	c	2937373	nc	O157	rec46	t	O157	++++		t	t	t	t	t	t	t	t	t	t	t	t	t	t	t	t	t	t	t	t		c
a	2657666	g	2867144	g	2937375	nc	O157	rec46	a	O157	++++		a	a	a	a	a	a	a	a	a	a	a	a	a	a	a	a	a	a	a	a		g
c	2657667	a	2867145	a	2937376	nc	O157	rec46	c	O157	++++		t	t	t	c	c	c	t	t	t	t	t	t	t	c	c	c	c	c	c	t		a
a	2657673	g	2867151	g	2937382	nc	O157	rec46	a	O157	++++		a	a	a	a	a	a	a	a	a	a	a	a	a	a	a	a	a	a	a	a		g
g	2657699	t	2867177	t	2937408	nc	O157	rec46	g	O157	+		g	g	g	g	g	g	t	t	t	t	t	t	t	g	g	g	g	g	g	g		t
a	2657709	g	2867187	g	2937418	nc	O157	rec46	a	O157	++++		a	a	a	a	a	a	a	a	a	a	a	a	a	a	a	a	a	a	a	a		g
g	2657721	a	2867199	a	2937430	nc	O157	rec46	g	O157	+		g	g	g	g	a	a	g	g	g	g	g	g	g	g	g	g	g	g	g	g		a
a	2657729	g	2867207	g	2937438	nc	O157	rec46	a	O157	++++		a	a	a	a	a	a	a	a	a	a	a	a	a	a	a	a	a	a	a	a		g
c	2657730	t	2867208	t	2937439	nc	O157	rec46	c	O157	++++		c	c	c	c	c	c	c	c	c	c	c	c	c	c	c	c	c	c	c	c		t
c	2657733	t	2867211	t	2937442	nc	O157	rec46	c	O157	++++		c	c	c	c	c	c	c	c	c	c	c	c	c	c	c	c	c	c	c	c		t
a	2657739	g	2867217	g	2937448	nc	O157	rec46	a	O157	+++		c	c	c	c	c	c	c	c	c	c	c	c	c	a	a	a	a	a	a	c		g
t	2657742	c	2867220	c	2937451	nc	O157	rec46	t	O157	++++		t	t	t	t	t	t	t	t	t	t	t	t	t	t	t	t	t	t	t	t		c
a	2657748	c	2867226	c	2937457	nc	O157	rec46	a	O157	++++		a	a	a	g	a	a	g	g	g	g	g	g	g	a	a	a	a	a	a	a		c
t	2657750	g	2867228	g	2937459	nc	O157	rec46	t	O157	+		t	t	t	g	t	t	g	g	g	g	g	g	g	t	t	t	t	t	t	t		g
t	2657751	c	2867229	c	2937460	nc	O157	rec46	t	O157	+		t	t	t	c	t	t	c	c	c	c	c	c	c	t	t	t	t	t	t	t		c
a	2657766	g	2867244	g	2937475	nc	O157	rec46	a	O157	++++		a	a	a	a	a	a	a	a	a	a	a	a	a	a	a	a	a	a	a	a		g
g	2657769	a	2867247	a	2937478	nc	O157	rec46	g	O157	+		g	g	g	a	a	a	a	a	a	a	a	a	a	g	g	g	g	g	g	g		a
c	2657772	g	2867250	g	2937481	nc	O157	rec46	c	O157	++++		c	c	c	c	c	c	c	c	c	c	c	c	c	c	c	c	c	c	c	c		g
t	2657779	a	2867257	a	2937488	nc	O157	rec46	t	O157	++++		t	t	t	t	t	t	t	t	t	t	t	t	t	t	t	t	t	t	t	t		a
g	2657781	a	2867259	a	2937490	nc	O157	rec46	g	O157	++++		g	g	g	g	g	g	g	g	g	g	g	g	g	g	g	g	g	g	g	g		a
a	2657787	c	2867265	c	2937496	nc	O157	rec46	a	O157	++++		a	a	a	a	a	a	a	a	a	a	a	a	a	a	a	a	a	a	a	a		c
t	2657799	c	2867277	c	2937508	nc	O157	rec46	t	O157	++++		t	t	t	t	t	t	t	t	t	t	t	t	t	t	t	t	t	t	t	t		c
a	2657802	g	2867280	g	2937511	nc	O157	rec46	a	O157	++++		a	a	a	a	a	a	a	a	a	a	a	a	a	a	a	a	a	a	a	a		g
a	2657805	g	2867283	g	2937514	nc	O157	rec46	a	O157	++++		a	a	a	a	a	a	a	a	a	a	a	a	a	a	a	a	a	a	a	a		g
g	2657807	a	2867285	a	2937516	nc	O157	rec46	g	O157	++++		g	g	g	g	g	g	g	g	g	g	g	g	g	g	g	g	g	g	g	g		a
g	2657808	t	2867286	t	2937517	nc	O157	rec46	g	O157	++++		g	g	g	g	g	g	g	g	g	g	g	g	g	g	g	g	g	g	g	g		t
c	2657810	t	2867288	t	2937519	nc	O157	rec46	c	O157	++++		c	c	c	c	c	c	c	c	c	c	c	c	c	c	c	c	c	c	c	c		t
a	2657818	g	2867296	g	2937527	nc	O157	rec46	a	O157	+		a	a	a	g	g	g	g	g	g	g	g	g	g	a	a	a	a	a	a	a		g
a	2657820	g	2867298	g	2937529	nc	O157	rec46	a	O157	++++		a	a	a	a	a	a	a	a	a	a	a	a	a	a	a	a	a	a	a	a		g
t	2657838	c	2867316	c	2937547	nc	O157	rec46	t	O157	++++		t	t	t	t	t	t	t	t	t	t	t	t	t	t	t	t	t	t	t	t		c
a	2657861	t	2867339	t	2937570	nc	O157	rec46	a	O157	++++		a	a	a	a	a	a	a	a	a	a	a	a	a	a	a	a	a	a	a	a		t
a	2657865	t	2867343	t	2937574	nc	O157	rec46	a	O157	++++		a	a	a	a	a	a	a	a	a	a	a	a	a	a	a	a	a	a	a	a		t
a	2657867	t	2867345	t	2937576	nc	O157	rec46	a	O157	++++		a	a	a	a	a	a	a	a	a	-	a	a	a	a	a	a	a	-	a	-		t
a	2657868	g	2867346	g	2937577	nc	O157	rec46	a	O157	++++		a	a	a	a	a	a	a	a	a	-	a	a	a	a	a	a	a	-	a	-		g
-	2657868	c	2867347	c	2937578	ins-2	O157	rec46	-	O157	++++		-	-	-	-	-	-	-	-	-	-	-	-	-	-	-	-	-	-	-	-		ct
a	2657869	g	2867349	g	2937580	nc	O157	rec46	g	CB9615	++++		g	g	g	g	g	g	g	g	g	g	g	g	g	-	-	-	-	a	-	g		g
g	2657879	a	2867359	a	2937590	nc	O157	rec46	g	O157	++++		g	g	g	g	g	g	g	g	g	g	g	g	g	g	g	g	g	g	g	-		a
g	2657880	a	2867360	a	2937591	i	O157	rec46	g	O157	+		g	g	g	g	g	g	a	a	a	a	a	a	a	g	g	g	g	g	g	g		a
g	2657881	t	2867361	t	2937592	i	O157	rec46	g	O157	++++		-	-	-	g	g	g	g	g	g	g	g	g	g	g	g	g	g	g	g	g		t
a	2657884	g	2867364	g	2937595	i	O157	rec46	a	O157	++++		a	a	a	a	a	a	a	a	a	a	a	a	a	a	a	a	a	a	a	a		g
g	2657893	t	2867373	t	2937604	i	O157	rec46	g	O157	++++		g	g	g	g	g	g	g	g	g	g	g	g	g	g	g	g	g	g	g	g		t
t	2657896	a	2867376	a	2937607	i	O157	rec46	t	O157	+		t	t	t	t	t	t	a	a	a	a	a	a	a	t	t	t	t	t	t	t		a
t	2657898	c	2867378	c	2937609	i	O157	rec46	t	O157	++++		t	t	t	t	t	t	t	t	t	t	t	t	t	t	t	t	t	t	t	t		c
a	2657900	t	2867380	t	2937611	i	O157	rec46	a	O157	++++		a	a	a	a	a	a	a	a	a	a	a	a	a	a	a	a	a	a	a	a		t
c	2657901	a	2867381	a	2937612	i	O157	rec46	c	O157	+		c	c	c	a	c	c	c	c	c	g	c	c	c	c	c	c	c	c	c	c		a
g	2657905	a	2867385	a	2937616	i	O157	rec46	g	O157	+		g	g	g	g	g	g	a	a	a	a	a	a	a	g	g	g	g	g	g	g		a
g	2657907	a	2867387	a	2937618	i	O157	rec46	g	O157	++++		g	g	g	g	g	g	g	g	g	g	g	g	g	g	g	g	g	g	g	g		a
-	2657911	4	2867392	4	2937623	ins-4	O157	rec46	-	O157	++++		-	-	-	-	-	-	-	-	-	-	-	-	-	-	-	-	-	-	-	-		4
t	2657913	g	2867397	g	2937628	i	O157	rec46	t	O157	++++		t	t	t	-	-	-	-	-	-	t	-	-	-	t	-	-	-	t	-	t		g
g	2657914	a	2867398	a	2937629	i	O157	rec46	a	CB9615	+		a	a	a	-	-	-	-	-	-	g	-	-	-	a	-	-	-	g	-	a		a
c	2657915	t	2867399	t	2937630	i	O157	rec46	c	O157	++++		c	c	c	-	-	-	-	-	-	c	-	-	-	c	-	-	-	c	-	c		t
a	2657916	t	2867400	t	2937631	i	O157	rec46	a	O157	++++		a	a	a	-	-	-	-	-	-	a	-	-	-	a	-	-	-	a	-	a		t
t	2657923	a	2867407	a	2937638	i	O157	rec46	t	O157	++++		t	t	t	t	t	t	t	t	t	t	t	t	t	t	t	t	t	t	t	t		a
t	2657924	a	2867408	a	2937639	i	O157	rec46	t	O157	++++		t	t	t	t	t	t	t	t	t	t	t	t	t	t	t	t	t	t	t	t		a
-	2657926	t	2867411	t	2937642	ins	O157	rec46	-	O157	++++		-	-	-	-	-	-	-	-	-	-	-	-	-	-	-	-	-	-	-	-		t
c	2657936	g	2867421	g	2937652	s	O157	rec46	c	O157	++++		c	c	c	c	c	c	c	c	c	c	c	c	c	c	c	c	c	c	c	c		g
c	2657939	a	2867424	a	2937655	s	O157	rec46	c	O157	++++		c	c	c	c	c	c	c	c	c	c	c	c	c	c	c	c	c	c	c	c		a
g	2657953	a	2867438	a	2937669	s	O157	rec46	g	O157	++++		g	g	g	g	g	g	g	g	g	g	g	g	g	g	g	g	g	g	g	g		a
a	2657954	t	2867439	t	2937670	s	O157	rec46	a	O157	++++		a	a	a	a	a	a	g	g	g	g	g	g	g	a	a	a	a	a	a	a		t
t	2657960	g	2867445	g	2937676	ns	O157	rec46	t	O157	++++		t	t	t	t	t	t	t	t	t	t	t	t	t	t	t	t	t	t	t	t		g
a	2657961	t	2867446	t	2937677	ns	O157	rec46	a	O157	++++		a	a	a	a	a	a	a	a	a	a	a	a	a	a	a	a	a	a	a	a		t
a	2657962	t	2867447	t	2937678	ns	O157	rec46	a	O157	++++		a	a	a	a	a	a	a	a	a	a	a	a	a	a	a	a	a	a	a	a		t
t	2657963	a	2867448	a	2937679	s	O157	rec46	a	CB9615	+		c	c	c	c	c	c	c	c	c	c	c	c	c	c	c	c	c	c	c	c		a
a	2657966	t	2867451	t	2937682	ns	O157	rec46	a	O157	++++		a	a	a	a	a	a	a	a	a	a	a	a	a	a	a	a	a	a	a	a		a
t	2657967	g	2867452	g	2937683	ns	O157	rec46	t	O157	++++		t	t	t	t	t	t	t	t	t	t	t	t	t	t	t	t	t	t	t	t		g
c	2657968	t	2867453	t	2937684	ns	O157	rec46	c	O157	++++		c	c	c	c	c	c	c	c	c	c	c	c	c	c	c	c	c	c	c	c		t
t	2657972	a	2867457	a	2937688	s	O157	rec46	t	O157	++++		t	t	t	t	t	t	t	t	t	t	t	t	t	t	t	t	t	t	t	t		a
t	2657976	g	2867461	g	2937692	ns	O157	rec46	t	O157	++++		t	t	t	t	t	t	t	t	t	t	t	t	t	t	t	t	t	t	t	t		g
g	2657977	c	2867462	c	2937693	ns	O157	rec46	g	O157	++++		g	g	g	g	g	g	t	t	t	t	t	t	t	g	g	g	g	g	g	g		c
c	2657978	a	2867463	a	2937694	s	O157	rec46	c	O157	++++		c	c	c	c	c	c	c	c	c	c	c	c	c	c	c	c	c	c	c	c		a
t	2657981	a	2867466	a	2937697	ns	O157	rec46	t	O157	++++		t	t	t	t	t	t	t	t	t	t	t	t	t	t	t	t	t	t	t	t		a
g	2657982	c	2867467	c	2937698	ns	O157	rec46	g	O157	++++		g	g	g	g	g	g	g	g	g	g	g	g	g	g	g	g	g	g	g	g		c
a	2657983	c	2867468	c	2937699	ns	O157	rec46	a	O157	++++		a	a	a	a	a	a	a	a	a	a	a	a	a	a	a	a	a	a	a	a		c
g	2657984	a	2867469	a	2937700	ns	O157	rec46	g	O157	+		g	g	g	a	a	a	g	g	g	g	g	g	g	g	g	g	g	g	g	g		a
t	2657985	c	2867470	c	2937701	ns	O157	rec46	t	O157	++++		t	t	t	t	t	t	t	t	t	t	t	t	t	t	t	t	t	t	t	t		c
a	2657987	g	2867472	g	2937703	s	O157	rec46	a	O157	++++		a	a	a	a	a	a	a	a	a	a	a	a	a	a	a	a	a	a	a	a		g

O55 and O157 genome site details												Outgroup Strain Details[i]																						
CB9615 base[b]	CB9615 Site[c]	Sakai base[b]	Sakai Site[c]	EDL933 base[b]	EDL933 Site[c]	type[d]	Event Lineage[ef]	Recombinant segment	Inferred ancestral base[g]	Outgroup Analysis[e]	Support level[h]	D1 Sd197	K-12	HS	ATCC 8739	UMN026	IAI39	SMS 3-5	E2348/69	536	ED1a	CFT073	S88	APEC O1	UTI89	E24377A	IAI1	SE11	F5 8401	F2a 2457T	F2a 301	SS Ss046	B4 Sb227	B18 BS512
t	2657990	a	2867475	a	2937706	s	O157	rec46	t	O157	++++		t	t	t	t	t	t	t	t	t	t	t	t	t	t	t	t	t	t	t	t		a
g	2657995	a	2867480	a	2937711	s	O157	rec46	g	O157	++++		g	g	g	g	g	g	g	g	g	g	g	g	g	g	g	g	g	g	g	g		a
t	2657996	c	2867481	c	2937712	s	O157	rec46	t	O157	+		t	t	t	c	c	c	t	t	t	t	t	t	t	t	t	t	t	t	t	t		c
g	2658002	a	2867487	a	2937718	s	O157	rec46	g	O157	++++		g	g	g	g	g	g	g	g	g	g	g	g	g	g	g	g	g	g	g	g		a
t	2658011	c	2867496	c	2937727	s	O157	rec46	t	O157	++++		t	t	t	t	t	t	t	t	t	t	t	t	t	t	t	t	t	t	t	t		c
a	2658020	t	2867505	t	2937736	s	O157	rec46	a	O157	++++		a	a	a	a	a	a	a	a	a	a	a	a	a	a	a	a	a	a	a	a		t
a	2658023	t	2867508	t	2937739	s	O157	rec46	a	O157	++++		a	a	a	a	a	a	g	g	g	g	g	g	g	a	a	a	a	a	a	a		t
a	2658032	t	2867517	t	2937748	s	O157	rec46	a	O157	++++		a	a	a	a	a	a	a	a	a	a	a	a	a	a	a	a	a	a	a	a		t
t	2658035	a	2867520	a	2937751	ns	O157	rec46	t	O157	++++		t	t	t	t	t	t	g	g	g	g	g	g	g	t	t	t	t	t	t	t		a
c	2658038	a	2867523	a	2937754	s	O157	rec46	c	O157	++++		c	c	c	c	c	c	c	c	c	c	c	c	c	c	c	c	c	c	c	c		a
t	2658041	a	2867526	a	2937757	s	O157	rec46	t	O157	++++		t	t	t	t	t	t	t	t	t	t	t	t	t	t	t	t	t	t	t	t		a
c	2658053	t	2867538	t	2937769	s	O157	rec46	c	O157	++++		c	c	c	c	c	c	c	c	c	c	c	c	c	c	c	c	c	c	c	c		t
a	2658065	t	2867550	t	2937781	s	O157	rec46	a	O157	++++		a	a	a	a	a	a	a	a	a	a	a	a	a	a	a	a	a	a	a	a		t
a	2658071	g	2867556	g	2937787	s	O157	rec46	a	O157	++++		a	a	a	a	a	a	a	a	a	a	a	a	a	a	a	a	a	a	a	a		g
c	2658076	t	2867561	t	2937792	ns	O157	rec46	c	O157	++++		c	c	c	c	c	c	c	c	c	c	c	c	c	c	c	c	c	c	c	c		t
g	2658083	t	2867568	t	2937799	s	O157	rec46	g	O157	++++		g	g	g	g	g	g	g	g	g	g	g	g	g	g	g	g	g	g	g	g		t
a	2658086	g	2867571	g	2937802	s	O157	rec46	a	O157	++++		a	a	a	a	a	a	a	a	a	a	a	a	a	a	a	a	a	a	a	a		g
g	2658089	a	2867574	a	2937805	s	O157	rec46	g	O157	++++		g	g	g	g	g	g	g	g	g	g	g	g	g	g	g	g	g	g	g	g		a
t	2658093	c	2867578	c	2937809	ns	O157	rec46	t	O157	++++		t	t	t	t	t	t	t	t	t	t	t	t	t	t	t	t	t	t	t	t		c
a	2658098	t	2867583	t	2937814	s	O157	rec46	a	O157	++++		a	a	a	a	a	a	a	a	a	a	a	a	a	a	a	a	a	-	a	a		t
t	2658101	c	2867586	c	2937817	s	O157	rec46	t	O157	+		t	t	t	t	c	t	t	t	t	t	t	t	t	t	t	t	t	-	t	t		c
a	2658116	t	2867601	t	2937832	s	O157	rec46	a	O157	++++		a	a	a	c	c	c	a	a	a	a	a	a	a	a	a	a	a	a	-	a		t
c	2658119	a	2867604	a	2937835	s	O157	rec46	c	O157	++++		c	c	c	c	c	c	c	c	c	c	c	c	c	c	c	c	c	t	-	c		a
a	2658122	t	2867607	t	2937838	s	O157	rec46	t	CB9615	+		g	g	g	g	g	g	g	g	g	g	g	g	g	g	g	g	g	g	g	g		t
a	2658125	g	2867610	g	2937841	s	O157	rec46	a	O157	++++		a	a	a	a	a	a	a	a	a	a	a	a	a	a	a	a	a	a	a	a		g
g	2658128	a	2867613	a	2937844	s	O157	rec46	g	O157	++++		g	g	g	g	g	g	g	g	g	g	g	g	g	g	g	g	g	g	g	g		a
g	2658131	a	2867616	a	2937847	s	O157	rec46	g	O157	++++		g	g	g	g	g	g	g	g	g	g	g	g	g	g	g	g	g	g	g	g		a
c	2658139	a	2867624	a	2937855	ns	O157	rec46	c	O157	++++		c	c	c	c	c	c	c	c	c	c	c	c	c	c	c	c	c	c	c	c		a
c	2658143	t	2867628	t	2937859	s	O157	rec46	c	O157	++++		c	c	c	c	c	c	c	c	c	c	c	c	c	c	c	c	c	c	c	c		t
g	2658152	a	2867637	a	2937868	s	O157	rec46	g	O157	++++		g	g	g	g	g	g	g	g	g	g	g	g	g	g	g	g	g	g	g	g		a
g	2658159	a	2867644	a	2937875	ns	O157	rec46	g	O157	++++		g	g	g	g	g	g	g	g	g	g	g	g	g	g	g	g	g	g	g	g		a
t	2658160	g	2867645	g	2937876	ns	O157	rec46	t	O157	++++		t	t	t	t	t	t	t	t	t	t	t	t	t	t	t	t	t	t	t	t		g
t	2658161	a	2867646	a	2937877	s	O157	rec46	t	O157	++++		t	t	t	t	t	t	t	t	t	t	t	t	t	t	t	t	t	t	t	t		a
a	2658167	c	2867652	c	2937883	ns	O157	rec46	a	O157	++++		a	a	a	a	a	a	a	a	a	a	a	a	a	a	a	a	g	g	g	a		c
a	2658176	g	2867661	g	2937892	s	O157	rec46	a	O157	+		a	a	a	a	a	a	a	a	a	a	a	a	a	a	a	a	g	g	g	a		g
g	2658185	a	2867670	a	2937901	s	O157	rec46	g	O157	++++		g	g	g	g	g	g	g	g	g	g	g	g	g	g	g	g	g	g	g	g		a
a	2658194	g	2867679	g	2937910	s	O157	rec46	a	O157	++++		a	a	a	a	a	a	a	a	a	a	a	a	a	a	a	a	a	a	a	a		g
g	2658198	t	2867683	t	2937914	ns	O157	rec46	g	O157	++++		g	g	g	g	g	g	g	g	g	g	g	g	g	g	g	g	g	g	g	g		t
c	2658202	t	2867687	t	2937918	ns	O157	rec46	c	O157	++++		c	c	c	c	c	c	c	c	c	c	c	c	c	c	c	c	c	c	c	c		t
t	2658203	a	2867688	a	2937919	ns	O157	rec46	t	O157	++++		t	t	t	t	-	t	t	t	t	t	t	t	t	t	t	t	t	t	t	t		a
t	2658204	g	2867689	g	2937920	ns	O157	rec46	t	O157	+		t	t	t	t	g	t	t	t	t	t	t	t	t	t	t	t	t	t	t	t		g
c	2658205	t	2867690	t	2937921	ns	O157	rec46	c	O157	+		c	c	c	c	t	c	c	c	c	c	c	c	c	c	c	c	c	c	c	c		t
t	2658206	c	2867691	c	2937922	s	O157	rec46	t	O157	+		t	t	t	t	c	t	t	t	t	t	t	t	t	t	t	t	t	t	t	t		c
g	2658212	a	2867697	a	2937928	s	O157	rec46	g	O157	+		g	g	g	a	a	a	a	a	a	a	a	a	a	g	g	g	g	g	g	g		a
g	2658215	a	2867700	a	2937931	ns	O157	rec46	g	O157	++++		g	g	g	g	g	g	g	g	g	g	g	g	g	g	g	g	g	g	g	g		a
c	2658216	g	2867701	g	2937932	ns	O157	rec46	c	O157	++++		c	c	c	c	c	c	c	c	c	c	c	c	c	c	c	c	c	c	c	c		g
t	2658224	c	2867709	c	2937940	s	O157	rec46	t	O157	++++		t	t	t	t	t	t	t	t	t	t	t	t	t	t	t	t	t	t	t	t		c
a	2658226	g	2867711	g	2937942	s	O157	rec46	a	O157	++++		a	a	a	a	a	a	a	a	a	a	a	a	a	a	a	a	a	a	a	a		g
g	2658230	a	2867715	a	2937946	s	O157	rec46	g	O157	++++		g	g	g	g	g	g	g	g	g	g	g	g	g	g	g	g	g	g	g	g		a
g	2658245	a	2867730	a	2937961	s	O157	rec46	g	O157	++++		g	g	g	g	g	g	g	g	g	g	g	g	g	g	g	g	g	g	g	g		a
t	2658248	c	2867733	c	2937964	s	O157	rec46	t	O157	++++		t	t	t	t	t	t	t	t	t	t	t	t	t	t	t	t	t	t	t	t		c
a	2658251	g	2867736	g	2937967	ns	O157	rec46	a	O157	++++		a	a	a	a	a	a	a	a	a	a	a	a	a	a	a	a	a	a	a	a		g
t	2658252	c	2867737	c	2937968	ns	O157	rec46	t	O157	+		t	t	t	t	t	t	c	c	c	c	c	c	c	t	t	t	t	t	t	t		c
g	2658260	a	2867745	a	2937976	s	O157	rec46	g	O157	++++		g	g	g	g	g	g	g	g	g	g	g	g	g	g	g	g	g	g	g	g		a
a	2658263	t	2867748	t	2937979	s	O157	rec46	a	O157	++++		a	a	a	c	c	c	c	c	c	c	c	c	c	a	a	a	a	a	a	a		t
a	2658272	c	2867757	c	2937988	s	O157	rec46	a	O157	++++		a	a	a	a	a	a	a	a	a	a	a	a	a	a	a	a	a	a	a	a		c
t	2658281	a	2867766	a	2937997	ns	O157	rec46	t	O157	++++		t	t	t	t	t	t	t	t	t	t	t	t	t	t	t	t	t	t	t	t		a
g	2658287	t	2867772	t	2938003	s	O157	rec46	g	O157	++++		g	g	g	g	g	g	g	g	g	g	g	g	g	g	g	g	g	g	g	g		t
t	2658296	g	2867781	g	2938012	s	O157	rec46	t	O157	++++		t	t	t	t	t	t	t	t	t	t	t	t	t	t	t	t	t	t	t	t		g
t	2658320	g	2867805	g	2938036	s	O157	rec46	t	O157	++++		t	t	t	t	t	t	t	t	t	t	t	t	t	t	t	t	t	t	t	t		g
a	2658332	g	2867817	g	2938048	s	O157	rec46	a	O157	++++		a	a	a	a	a	a	a	a	a	a	a	a	a	a	a	a	a	a	a	a		g
c	2658335	a	2867820	a	2938051	s	O157	rec46	c	O157	++++		c	c	c	c	c	c	t	t	t	t	t	t	t	c	c	c	c	c	c	c		a
t	2658338	g	2867823	g	2938054	ns	O157	rec46	t	O157	++++		t	t	t	t	t	t	c	t	t	t	t	t	t	t	t	t	t	t	t	t		g
t	2658339	a	2867824	a	2938055	ns	O157	rec46	t	O157	++++		t	t	t	t	t	t	t	t	t	t	t	t	t	t	t	t	t	t	t	t		a
g	2658341	a	2867826	a	2938057	s	O157	rec46	g	O157	++++		g	g	g	g	g	g	g	g	g	g	g	g	g	g	g	g	g	g	g	g		a
t	2658345	g	2867830	g	2938061	ns	O157	rec46	t	O157	++++		t	t	t	t	t	t	t	t	t	t	t	t	t	t	t	t	t	t	t	t		g
c	2658350	t	2867835	t	2938066	s	O157	rec46	c	O157	++++		c	c	c	c	c	c	c	c	c	c	c	c	c	c	c	c	c	c	c	c		t
a	2658353	t	2867838	t	2938069	s	O157	rec46	a	O157	++++		a	a	a	a	a	a	a	a	a	a	a	a	a	a	a	a	a	a	a	a		t
c	2658368	a	2867853	a	2938084	s	O157	rec46	c	O157	++++		c	c	c	c	c	c	c	c	c	c	c	c	c	c	c	c	t	t	t	c		a
a	2658377	g	2867862	g	2938093	s	O157	rec46	g	CB9615	+		a	a	a	g	g	g	g	g	g	g	g	g	g	a	a	a	g	g	g	a		g
t	2658383	a	2867868	a	2938099	s	O157	rec46	t	O157	++++		t	t	t	t	t	t	t	t	t	t	t	t	t	t	t	t	t	t	t	t		a
a	2658395	t	2867880	t	2938111	s	O157	rec46	a	O157	++++		a	a	a	a	a	a	a	a	a	a	a	a	a	a	a	a	a	a	a	a		t
g	2658398	t	2867883	t	2938114	ns	O157	rec46	g	O157	++++		g	g	g	g	g	g	g	g	g	g	g	g	g	g	g	g	g	g	g	g		t
t	2658401	a	2867886	a	2938117	s	O157	rec46	t	O157	++++		t	t	t	t	t	t	t	t	t	t	t	t	t	t	t	t	t	t	t	t		a
a	2658407	g	2867892	g	2938123	s	O157	rec46	a	O157	++++		a	a	a	a	a	a	a	a	a	a	a	a	a	a	a	a	a	a	a	a		g
t	2658410	a	2867895	a	2938126	ns	O157	rec46	t	O157	++++		t	t	t	t	t	t	t	t	t	t	t	t	t	t	t	t	t	t	t	t		a
c	2658412	t	2867897	t	2938128	ns	O157	rec46	c	O157	++++		c	c	c	c	c	c	c	c	c	c	c	c	c	c	c	c	c	c	c	c		t
t	2658413	a	2867898	a	2938129	s	O157	rec46	t	O157	++++		t	t	t	c	c	c	c	c	c	c	c	c	c	t	t	t	t	t	t	t		a
g	2658419	a	2867904	a	2938135	s	O157	rec46	g	O157	++++		g	g	g	g	g	g	g	g	g	g	g	g	g	g	g	g	g	g	g	g		a
a	2658428	t	2867913	t	2938144	ns	O157	rec46	a	O157	++++		a	a	a	a	a	a	a	a	a	a	a	a	a	a	a	a	a	a	a	a		t
a	2658430	c	2867915	c	2938146	ns	O157	rec46	a	O157	++++		a	a	a	a	a	a	a	a	a	a	a	a	a	a	a	a	a	a	a	a		c
a	2658437	t	2867922	t	2938153	ns	O157	rec46	a	O157	++++		a	a	a	a	a	a	a	a	a	a	a	a	a	a	a	a	a	a	a	a		t
c	2658462	t	2867947	t	2938178	ns	O157	rec46	c	O157	++++		c	c	c	c	c	c	c	c	c	c	c	c	c	c	c	c	c	c	c	c		t
a	2658470	g	2867955	g	2938186	i	O157	rec46	a	O157	++++		a	a	a	a	a	a	a	a	a	a	a	a	a	a	a	a	a	a	a	a		g
a	2658473	g	2867958	g	2938189	i	O157	rec46	a	O157	++++		a	a	a	a	a	a	a	a	a	a	a	a	a	a	a	a	a	a	a	a		g
t	2658474	c	2867959	c	2938190	i	O157	rec46	t	O157	++++		t	t	t	t	t	t	t	t	t	t	t	t	t	t	t	t	t	t	t	t		c

O55 and O157 genome site details												Outgroup Strain Details[i]																						
CB9615 base[b]	CB9615 Site[c]	Sakai base[b]	Sakai Site	EDL933 base[b]	EDL933 Site[c]	type[d]	Event Lineage[ef]	Recombinant segment	Inferred ancestral base[g]	Outgroup Analysis[e]	Support level[h]	D1 Sd197	K-12	HS	ATCC 8739	UMN026	IAI39	SMS 3-5	E2348/69	536	ED1a	CFT073	S88	APEC O1	UTI89	E24377A	IAI1	SE11	F5 8401	F2a 2457T	F2a 301	SS Ss046	B4 Sb227	B18 BS512
g	2658475	a	2867960	a	2938191	i	O157	rec46	g	O157	++++		g	g	g	g	g	g	g	g	g	g	g	g	g	g	g	g	g	g	g	g		a
a	2658485	-	2867969	-	2938200	del	O157	rec46	a	O157	++++		a	a	a	a	a	a	a	a	a	a	a	a	a	a	a	a	a	a	a	a		-
a	2658488	g	2867972	g	2938203	i	O157	rec46	a	O157	++++		a	a	a	a	a	a	a	a	a	a	a	a	a	a	a	a	a	a	a	a		g
t	2658497	g	2867981	g	2938212	i	O157	rec46	t	O157	++++		t	t	t	t	t	t	t	t	t	t	t	t	t	t	t	t	t	t	t	t		g
g	2658498	t	2867982	t	2938213	i	O157	rec46	g	O157	+		g	g	g	g	t	t	g	g	g	g	g	g	g	g	g	g	g	g	g	g		t
g	2658499	t	2867983	t	2938214	i	O157	rec46	g	O157	++++		g	g	g	g	g	g	g	g	g	g	g	g	g	g	g	g	g	g	g	g		t
a	2658547	g	2868031	g	2938262	i	O157	rec46	a	O157	++++		a	a	a	a	a	a	a	a	a	a	a	a	a	a	a	g	a	a	a	a		a
a	2658575	g	2868059	g	2938290	i	O157	rec46	a	O157	++++		a	a	a	a	a	a	a	a	a	a	a	a	a	a	a	a	a	a	a	a		g
c	2658596	t	2868080	t	2938311	i	O157	rec46	c	O157	+		c	c	c	t	t	t	t	t	t	t	t	t	t	c	c	c	c	c	c	c		t
t	2658598	a	2868082	a	2938313	i	O157	rec46	t	O157	+		t	t	t	t	t	t	t	t	t	t	t	t	t	t	t	t	a	a	a	t		a
c	2658621	a	2868105	a	2938336	i	O157	rec46	c	O157	++++		t	t	t	c	c	c	c	c	c	c	c	c	c	t	t	t	c	c	c	t		a
a	2658624	c	2868108	c	2938339	i	O157	rec46	a	O157	++++		a	a	a	a	a	a	a	a	a	a	a	a	a	a	a	a	a	a	a			c
a	2658625	g	2868109	g	2938340	i	O157	rec46	a	O157	+		a	g	a	a	a	a	a	a	a	a	a	a	a	a	a	a	a	a	a			g
t	2658626	c	2868110	c	2938341	i	O157	rec46	c	CB9615	+		g	c	g	g	g	g	g	g	g	g	g	g	g	g	g	g	g	g	g			c
a	2658627	t	2868111	t	2938342	i	O157	rec46	a	O157	++++		a	a	a	c	c	c	c	c	c	c	c	c	c	a	a	a	a	a	a			t
g	2658628	a	2868112	a	2938343	i	O157	rec46	a	CB9615	+		g	t	g	a	a	a	a	a	a	a	a	a	a	g	g	g	a	a	a			a
t	2658631	a	2868115	a	2938346	i	O157	rec46	t	O157	+		t	a	t	t	t	t	t	t	t	t	t	t	t	t	t	t	t	t	t			a
t	2658632	c	2868116	c	2938347	i	O157	rec46	t	O157	+		t	c	t	t	t	t	t	t	t	t	t	t	t	t	t	t	t	t	t			c
t	2658636	c	2868120	c	2938351	i	O157	rec46	t	O157	++++		t	-	t	t	t	t	t	t	t	t	t	t	t	t	t	t	t	t	t			c
a	2658641	t	2868125	t	2938356	i	O157	rec46	a	O157	+		a	t	a	a	a	a	a	a	a	a	a	a	a	a	a	a	a	a	a			t
c	2658643	g	2868127	g	2938358	i	O157	rec46	c	O157	+		g	g	g	c	c	c	c	c	c	c	c	c	c	g	g	g	c	c	c			g
g	2658644	a	2868128	a	2938359	i	O157	rec46	a	CB9615	++++		c	c	c	a	a	a	a	a	a	a	a	a	a	c	c	c	a	a	a			a
c	2658645	a	2868129	a	2938360	i	O157	rec46	a	CB9615	+		a	a	a	c	c	c	a	a	a	a	a	a	a	a	a	a	c	c	c			a
t	2658647	c	2868131	c	2938362	i	O157	rec46	t	O157	+		c	c	c	t	t	t	t	t	t	t	t	t	t	c	c	c	t	t	t			c
c	2658648	t	2868132	t	2938363	i	O157	rec46	c	O157	+		t	t	t	c	c	c	c	c	c	c	c	c	c	t	t	t	c	c	c			t
t	2658649	g	2868133	g	2938364	i	O157	rec46	t	O157	++++		-	-	-	t	t	t	c	t	t	t	t	t	t	-	-	-	t	t	t			g
a	2658658	c	2868142	c	2938373	i	O157	rec46	a	O157	++++		a	a	a	a	a	a	a	a	a	a	a	a	a	a	a	a	a	a	a			c
t	2658659	c	2868143	c	2938374	i	O157	rec46	t	O157	++++		t	t	t	t	t	t	t	t	t	t	t	t	t	t	t	t	t	t	t			c
g	2658663	a	2868147	a	2938378	i	O157	rec46	g	O157	++++		g	g	g	g	g	g	g	g	g	g	g	g	g	g	g	g	g	g	g			a
t	2658667	a	2868151	a	2938382	i	O157	rec46	t	O157	++++		t	t	t	t	t	t	t	t	t	t	t	t	t	t	t	t	t	t	t			a
a	2658668	c	2868152	c	2938383	i	O157	rec46	a	O157	+		a	a	a	t	t	t	c	c	c	c	c	c	c	a	a	a	a	a	a			c
t	2658669	c	2868153	c	2938384	i	O157	rec46	t	O157	++++		t	t	t	t	t	t	t	t	t	t	t	t	t	t	t	t	t	t	t			c
g	2658676	a	2868160	a	2938391	i	O157	rec46	g	O157	+		g	g	g	g	g	a	g	g	g		g	g	g	g	g	g	g	g	g			a
g	2658677	a	2868161	a	2938392	i	O157	rec46	g	O157	++++		g	g	g	g	g	t	g	g	g		g	g	g	g	g	g	g	g	g			a
t	2658678	c	2868162	c	2938393	i	O157	rec46	t	O157	+		t	t	t	t	c	t	t	t	t		t	t	t	t	t	t	t	t	t			c
g	2658682	t	2868166	t	2938397	i	O157	rec46	g	O157	++++		g	g	g	a	a	t	a	a	a		a	a	a	g	g	g	g	g	g			c
t	2658683	a	2868167	a	2938398	i	O157	rec46	t	O157	+		t	t	t	t	t	a	t	t	t		t	t	t	t	t	t	t	t	t			a
a	2658686	g	2868170	g	2938401	i	O157	rec46	a	O157	++++		a	a	a	a	a	-	a	a	a		a	a	a	a	a	a	a	a	a			a
t	2658687	g	2868171	g	2938402	i	O157	rec46	t	O157	++++		t	t	t	t	t	-	t	t	t		t	t	t	t	t	t	t	t	t			g
c	2658690	g	2868174	g	2938405	i	O157	rec46	c	O157	++++		c	c	c	c	c	c	c	c	c		c	c	c	c	c	c	c	c	c			g
a	2658694	-	2868177	-	2938408	del	O157	rec46	a	O157	+		-	-	-	-	-	-	a	a	a	a	-	-	-	-	-	-	-	-	-	a		-
g	2658704	a	2868187	a	2938418	i	O157	rec46	g	O157	+		g	g	g	g	g	a	t	t	t	t	t	t	t	g	g	g	g	g	g			a
t	2658713	c	2868196	c	2938427	i	O157	rec46	?	O157	+/-		t	t	t	c	c	c	c	c	c	c	c	c	c	t	t	t	t	t	t			c
t	2658717	g	2868200	g	2938431	i	O157	rec46	t	O157	++++		t	t	t	c	c	c	t	t	t	t	t	t	t	t	t	t	t	t	t			g
a	2658718	g	2868201	g	2938432	i	O157	rec46	a	O157	+		a	a	a	g	g	g	a	a	a	a	a	a	a	a	a	a	a	a	a			g
c	2658727	t	2868210	t	2938441	i	O157	rec46	c	O157	++++		c	c	c	c	c	c	a	a	a	a	a	a	a	c	c	c	c	c	c			t
c	2658734	t	2868217	t	2938448	i	O157	rec46	c	O157	++++		c	c	c	c	c	c	c	c	c	c	c	c	c	c	c	c	c	c	c			t
g	2658740	a	2868223	a	2938454	i	O157	rec46	g	O157	++++		g	g	g	c	c	c	c	c	c	c	c	c	c	g	g	g	g	g	g			a
c	2658741	t	2868224	t	2938455	i	O157	rec46	c	O157	+		c	c	c	c	c	c	c	c	c	t	c	c	c	c	c	c	c	c	c			t
a	2658742	g	2868225	g	2938456	i	O157	rec46	?	O157	+/-		a	a	a	g	g	g	g	g	g	g	g	g	g	a	a	a	a	a	a			g
a	2658743	c	2868226	c	2938457	i	O157	rec46	a	O157	++++		a	a	a	a	a	a	a	a	a	a	a	a	a	a	a	a	a	a	a			c
t	2658750	c	2868233	c	2938464	i	O157	rec46	?	O157	+/-		t	t	t	c	c	c	c	c	c	c	c	c	c	t	t	t	t	t	t			c
c	2658751	g	2868234	g	2938465	i	O157	rec46	c	O157	++++		c	c	c	c	c	c	c	c	c	c	c	c	c	c	c	c	c	c	c			g
a	2658752	g	2868235	g	2938466	i	O157	rec46	a	O157	++++		a	a	a	a	a	a	a	a	a	a	a	a	a	a	a	a	a	a	a			g
g	2658753	t	2868236	t	2938467	i	O157	rec46	g	O157	++++		g	g	g	g	g	g	g	g	g	g	g	g	g	g	g	g	g	g	g			t
c	2658758	g	2868241	g	2938472	i	O157	rec46	c	O157	+		c	c	c	g	g	g	g	g	g	g	g	g	g	c	c	c	c	c	c	c		g
c	2658788	t	2868271	t	2938502	ns	O157	rec46	c	O157	++++		c	c	c	c	c	c	c	c	c	c	c	c	c	c	c	c	c	c	c	c		t
g	2658789	t	2868272	t	2938503	ns	O157	rec46	g	O157	++++		g	g	g	g	g	g	g	g	g	g	g	g	g	g	g	g	g	g	g	g		t
a	2658790	g	2868273	g	2938504	s	O157	rec46	a	O157	++++		a	a	a	a	a	a	a	a	a	a	a	a	a	a	a	a	a	a	a	a		g
c	2658797	t	2868280	t	2938511	ns	O157	rec46	c	O157	+		c	c	c	c	c	c	t	t	t	t	t	t	t	c	c	c	c	c	c	c		t
t	2658798	c	2868281	c	2938512	ns	O157	rec46	t	O157	+		t	t	t	t	t	t	c	c	c	c	c	c	c	t	t	t	t	t	t	t		c
a	2658799	g	2868282	g	2938513	ns	O157	rec46	a	O157	+		a	a	a	a	a	a	g	g	g	g	g	g	g	a	a	a	a	a	a	a		g
c	2658801	t	2868284	t	2938515	ns	O157	rec46	c	O157	+		c	c	c	c	c	c	t	t	t	t	t	t	t	c	c	c	c	c	c	c		t
c	2658803	t	2868286	t	2938517	ns	O157	rec46	c	O157	+		c	c	c	c	c	c	t	t	t	t	t	t	t	c	c	c	c	c	c	c		t
t	2658808	c	2868291	c	2938522	ns	O157	rec46	t	O157	++++	t	t	t	t	t	t	t	t	t	t	t	t	t	t	t	t	t	t	t	t	t		c
g	2658809	a	2868292	a	2938523	ns	O157	rec46	g	O157	++++	g	g	g	g	g	g	g	g	g	g	g	g	g	g	g	g	g	g	g	g	g		a
a	2658812	g	2868295	g	2938526	ns	O157	rec46	a	O157	++	a	a	a	a	a	a	a	g	g	g	g	g	g	g	a	a	a	a	a	a	a		g
a	2658814	t	2868297	t	2938528	s	O157	rec46	a	O157	++++	a	a	a	a	a	a	a	a	a	a	a	a	a	a	a	a	a	a	a	a	a		t
t	2658829	a	2868312	a	2938543	s	O157	rec46	t	O157	++	t	t	t	t	t	t	t	t	t	t	t	t	t	t	t	t	t	a	a	a	t		a
a	2658832	g	2868315	g	2938546	s	O157	rec46	a	O157	++++	a	a	a	a	a	a	a	a	a	a	a	a	a	a	a	a	a	a	a	a	a		g
g	2658838	t	2868321	t	2938552	ns	O157	rec46	g	O157	++++	g	g	g	g	g	g	g	a	a	a	a	a	a	a	g	g	g	g	g	g	g		t
a	2658839	g	2868322	g	2938553	ns	O157	rec46	a	O157	++++	a	a	a	a	a	a	a	a	a	a	a	a	a	a	a	a	a	a	a	a	a		g
g	2658841	a	2868324	a	2938555	ns	O157	rec46	g	O157	++++	c	g	g	g	t	t	t	t	t	t	t	t	t	t	g	g	g	g	g	g	g		a
g	2658843	t	2868326	t	2938557	ns	O157	rec46	g	O157	++++	g	g	g	g	g	g	g	g	g	g	g	g	g	g	g	g	g	g	g	g	g		t
c	2658844	a	2868327	a	2938558	s	O157	rec46	c	O157	++++	c	c	c	c	c	c	c	c	c	c	c	c	c	c	c	c	c	c	c	c	c		a
a	2658851	g	2868334	g	2938565	ns	O157	rec46	a	O157	++++	a	a	a	a	a	a	a	a	a	a	a	a	a	a	a	a	a	a	a	a	a		g
c	2658853	a	2868336	a	2938567	ns	O157	rec46	c	O157	++++	c	c	c	c	c	c	c	c	c	c	c	c	c	c	c	c	c	c	c	c	c		a
t	2658854	c	2868337	c	2938568	ns	O157	rec46	t	O157	++++	t	a	a	a	t	t	t	t	t	t	t	t	t	t	a	a	a	t	t	t	a		c
g	2658858	a	2868341	a	2938572	ns	O157	rec46	g	O157	++++	g	g	g	g	g	g	g	g	g	g	g	g	g	g	g	g	g	g	g	g	g		a
a	2658859	g	2868342	g	2938573	ns	O157	rec46	a	O157	++++	a	a	a	a	a	a	a	a	a	a	a	a	a	a	a	a	a	a	a	a	a		g
c	2658860	t	2868343	t	2938574	ns	O157	rec46	c	O157	++++	c	c	c	c	c	c	c	c	c	c	c	c	c	c	c	c	c	c	c	c	c		t
g	2658861	t	2868344	t	2938575	ns	O157	rec46	g	O157	++++	g	g	g	g	g	g	g	g	g	g	g	g	g	g	g	g	g	g	g	g	g		t
a	2658865	t	2868348	t	2938579	ns	O157	rec46	a	O157	++++	a	g	g	g	a	a	a	a	a	a	a	a	a	a	g	g	g	g	g	g	g		t
g	2658868	a	2868351	a	2938582	s	O157	rec46	a	CB9615	++++	a	a	a	a	a	a	a	a	a	a	a	a	a	a	a	a	a	a	a	a	a		a
a	2658871	t	2868354	t	2938585	ns	O157	rec46	a	O157	++	a	a	a	a	t	t	t	a	a	-	a	-	a	a	a	a	a	a	a	a	a		t
t	2658872	c	2868355	c	2938586	ns	O157	rec46	c	CB9615	+++	-	-	-	-	c	c	c	-	-	c	-	c	-	-	-	-	-	-	-	-	-		c

O55 and O157 genome site details												Outgroup Strain Details[i]																						
CB9615 base[b]	CB9615 Site[c]	Sakai base[b]	Sakai Site[c]	EDL933 base[b]	EDL933 Site[c]	type[d]	Event Lineage[ef]	Recombinant segment	Inferred ancestral base[g]	Outgroup Analysis[e]	Support level[h]	D1 Sd197	K-12	HS	ATCC 8739	UMN026	IAI39	SMS 3-5	E2348/69	536	ED1a	CFT073	S88	APEC O1	UTI89	E24377A	IAI1	SE11	F5 8401	F2a 2457T	F2a 301	SS Ss046	B4 Sb227	B18 BS512
t	2658873	g	2868356	g	2938587	ns	O157	rec46	g	CB9615	+	-	-	-	-	a	a	a	-	-	a	-	a	-	-	-	-	-	-	-	-	-		g
a	2658874	t	2868357	t	2938588	ns	O157	rec46	t	CB9615	++++	t	t	t	t	t	t	t	t	t	t	t	t	t	t	t	t	t	t	t	t	t		t
a	2658876	g	2868359	g	2938590	ns	O157	rec46	a	O157	++	a	a	a	a	g	g	g	a	a	a	a	a	a	a	a	a	a	a	a	a	a		g
c	2658877	t	2868360	t	2938591	ns	O157	rec46	t	CB9615	++++	t	t	t	t	t	t	t	t	t	t	t	t	t	t	t	t	t	t	t	t	t		t
g	2658878	a	2868361	a	2938592	ns	O157	rec46	a	CB9615	++++	a	a	a	a	t	t	t	a	a	a	a	a	a	a	a	a	a	a	a	a	a		a
t	2658879	a	2868362	a	2938593	ns	O157	rec46	a	CB9615	+	c	c	c	c	a	a	a	c	c	t	c	t	c	c	c	c	c	c	c	c	c		a
t	2658880	c	2868363	c	2938594	s	O157	rec46	t	O157	+	g	g	g	g	-	-	-	g	g	t	g	t	g	g	g	g	g	g	g	g	g		c
a	2658886	c	2868369	c	2938600	s	O157	rec46	a	O157	++++	a	a	a	a	a	a	a	a	a	a	a	a	a	a	a	a	a	a	a	a	a		c
t	2658893	c	2868376	c	2938607	ns	O157	rec46	t	O157	++++	t	t	t	t	t	t	t	t	t	t	t	t	t	t	t	t	t	t	t	t	t		c
c	2658895	t	2868378	t	2938609	s	O157	rec46	c	O157	++++	c	c	c	c	c	c	c	c	c	c	c	c	c	c	c	c	c	c	c	c	c		t
t	2658898	c	2868381	c	2938612	s	O157	rec46	t	O157	++++	t	t	t	t	t	t	t	t	t	t	t	t	t	t	t	t	t	t	t	t	t		c
g	2658902	a	2868385	a	2938616	ns	O157	rec46	g	O157	++++	g	g	g	g	g	g	g	g	g	g	g	g	g	g	g	g	g	g	g	g	g		a
t	2658904	a	2868387	a	2938618	ns	O157	rec46	t	O157	++++	t	t	t	t	t	t	t	t	t	t	t	t	t	t	t	t	t	t	t	t	t		a
c	2658908	t	2868391	t	2938622	ns	O157	rec46	t	CB9615	++	t	c	c	c	c	c	c	c	c	c	c	c	c	c	c	c	c	c	c	c	c		t
c	2658916	a	2868399	a	2938630	ns	O157	rec46	c	O157	++++	c	c	c	c	c	c	c	c	c	c	c	c	c	c	c	c	c	c	c	c	c		a
t	2658918	c	2868401	c	2938632	ns	O157	rec46	t	O157	++++	t	t	t	t	t	t	t	t	t	t	t	t	t	t	t	t	t	t	t	t	t		c
g	2658920	t	2868403	t	2938634	ns	O157	rec46	g	O157	++++	g	g	g	g	g	g	g	g	g	g	g	g	g	g	g	g	g	g	g	g	g		t
t	2658934	a	2868417	a	2938648	s	O157	rec46	t	O157	++++	t	t	t	t	t	t	t	c	t	c	c	t	t	t	t	t	t	t	t	t	t		a
g	2658939	a	2868422	a	2938653	ns	O157	rec46	g	O157	++++	g	g	g	g	g	g	g	g	g	g	g	g	g	g	g	g	g	g	g	g	g		a
g	2658943	a	2868426	a	2938657	s	O157	rec46	g	O157	++++	g	g	g	g	g	g	g	g	g	g	g	g	g	g	g	g	g	g	g	g	g		a
c	2658946	t	2868429	t	2938660	s	O157	rec46	c	O157	++++	c	c	c	c	c	c	c	c	c	c	c	c	c	c	c	c	c	c	c	c	c		t
a	2658955	g	2868438	g	2938669	s	O157	rec46	a	O157	++++	a	a	a	a	a	a	a	a	a	a	a	a	a	a	a	a	a	a	a	a	a		g
c	2658958	g	2868441	g	2938672	s	O157	rec46	c	O157	++++	c	c	c	c	c	c	c	c	c	c	c	c	c	c	c	c	c	c	c	c	c		g
g	2658967	t	2868450	t	2938681	ns	O157	rec46	g	O157	++++	g	g	g	g	g	g	g	g	a	g	g	a	a	a	g	g	g	g	g	g	g		t
c	2658969	t	2868452	t	2938683	ns	O157	rec46	c	O157	++++	c	c	c	c	c	c	c	c	c	c	c	c	c	c	c	c	c	c	c	c	c		c
c	2658973	a	2868456	a	2938687	ns	O157	rec46	c	O157	++++	c	c	c	c	c	c	c	t	t	t	t	t	t	t	c	c	c	c	c	c	c		c
t	2658976	a	2868459	a	2938690	s	O157	rec46	t	O157	++++	t	t	t	t	t	t	t	t	t	t	t	t	t	t	t	t	t	t	t	t	t		t
a	2658991	t	2868474	t	2938705	s	O157	rec46	a	O157	++++	a	a	a	a	a	a	a	a	a	a	a	a	a	a	a	a	a	a	a	a	a		a
a	2658994	t	2868477	t	2938708	s	O157	rec46	a	O157	++++	a	a	a	a	a	a	a	a	a	g	a	a	a	a	a	a	a	a	a	a	a		a
c	2658997	t	2868480	t	2938711	s	O157	rec46	c	O157	++++	c	c	c	c	c	c	c	c	c	c	c	c	c	c	c	c	c	c	c	c	c		c
a	2658999	g	2868482	g	2938713	s	O157	rec46	a	O157	++++	a	a	a	a	t	t	t	a	a	a	a	a	a	a	a	a	a	a	a	a	a		a
g	2659006	a	2868489	a	2938720	s	O157	rec46	g	O157	++++	g	g	g	g	g	g	g	g	g	g	g	g	g	g	g	g	g	g	g	g	g		g
t	2659017	c	2868500	c	2938731	ns	O157	rec46	t	O157	++++	t	t	t	t	t	t	t	t	t	t	t	t	t	t	t	t	t	t	t	t	t		t
a	2659019	g	2868502	g	2938733	ns	O157	rec46	a	O157	++++	a	a	a	a	a	a	a	a	a	a	a	a	a	a	a	a	a	a	a	a	a		a
t	2659020	c	2868503	c	2938734	ns	O157	rec46	t	O157	++++	t	t	t	t	t	t	t	t	t	t	t	t	t	t	t	t	t	t	t	t	t		t
g	2659029	a	2868512	a	2938743	s	O157	rec46	g	O157	++++	g	g	g	g	g	g	g	g	g	g	g	g	g	g	g	g	g	g	g	g	g		g
t	2659076	-	2868558	-	2938789	del	O157	rec46	t	O157	++++	t	t	t	t	t	t	t	t	t	t	t	t	t	t	t	t	t	t	t	t	-		t
a	2659079	g	2868561	g	2938792	i	O157	rec46	g	CB9615	++	g	g	g	g	g	g	g	g	g	a	g	a	g	g	g	g	g	g	g	g	a		g
a	2659084	t	2868566	t	2938797	i	O157	rec46	a	O157	++++	a	a	a	a	a	a	a	a	a	a	a	a	a	a	a	a	a	a	a	a	a		a
a	2659098	c	2868580	c	2938811	i	O157	rec46	a	O157	++++	a	a	a	a	a	a	a	a	a	a	a	a	a	a	a	a	a	a	a	a	a		a
a	2659116	g	2868598	g	2938829	i	O157	rec46	a	O157	++++	a	a	a	a	a	a	a	a	a	a	a	a	a	a	a	a	a	a	a	a	a		a
-	2659124	c	2868607	c	2938838	ins	O157	rec46	c	CB9615	++	c	c	c	c	c	c	c	c	c	c	c	c	c	c	c	c	c	-	-	-	c		c
a	2659130	g	2868613	g	2938844	i	O157	rec46	g	CB9615	++	g	a	a	a	a	a	a	a	a	a	a	a	a	a	a	a	a	a	a	a	a		a
t	2659140	a	2868623	a	2938854	i	O157	rec46	a	CB9615	++	a	t	t	t	t	t	t	t	t	t	t	t	t	t	t	t	t	t	t	t	t		t
a	2659143	g	2868626	g	2938857	i	O157	rec46	g	CB9615	++	g	a	a	a	a	a	a	a	a	a	a	a	a	a	a	a	a	a	a	a	a		a
g	2659144	a	2868627	a	2938858	i	O157	rec46	a	CB9615	++	a	g	g	g	g	g	g	g	g	g	g	g	g	g	g	g	g	g	g	g	g		g
t	2659147	g	2868630	g	2938861	i	O157	rec46	g	CB9615	++	g	t	t	t	t	t	t	t	t	t	t	t	t	t	t	t	t	t	t	t	t		t
c	2659150	g	2868633	g	2938864	i	O157	rec46	g	CB9615	++	g	c	c	c	c	c	c	c	c	c	c	c	c	c	c	c	c	c	c	c	c		c
c	2659151	t	2868634	t	2938865	i	O157	rec46	t	CB9615	++	t	c	c	c	c	c	c	c	c	c	c	c	c	c	c	c	c	c	c	c	c		c
t	2659154	c	2868637	c	2938868	i	O157	rec46	c	CB9615	++	c	t	t	t	t	t	t	t	t	t	t	t	t	t	t	t	t	t	t	t	t		t
g	2659157	a	2868640	a	2938871	i	O157	rec46	a	CB9615	++	a	g	g	g	g	g	g	g	g	g	g	g	g	g	g	g	g	g	g	g	g		g
a	2659166	c	2868649	c	2938880	i	O157	rec46	c	CB9615	++	c	a	a	a	a	a	a	a	a	a	a	a	a	a	a	a	a	a	a	a	a	a	a
g	2659174	c	2868657	c	2938888	i	O157	rec46	c	CB9615	++	c	g	g	g	g	g	g	g	g	g	g	g	g	g	g	g	g	g	g	g	g	g	g
g	2659185	t	2868668	t	2938899	i	O157	rec46	t	CB9615	++	t	g	g	g	g	g	g	g	g	g	g	g	g	g	g	g	g	g	g	g	g	g	g
g	2659189	a	2868672	a	2938903	i	O157	rec46	a	CB9615	++	a	g	g	g	g	g	g	g	g	g	g	g	g	g	g	g	g	g	g	g	g	g	g
g	2659193	a	2868676	a	2938907	i	O157	rec46	a	CB9615	++	a	g	g	g	g	g	g	g	g	g	g	g	g	g	g	g	g	g	g	g	g	g	g
a	2659195	t	2868678	t	2938909	i	O157	rec46	t	CB9615	++	t	a	a	a	a	a	a	a	a	a	a	a	a	a	a	a	a	a	a	a	a	a	a
g	2659258	a	2868741	a	2938972	i	O157	rec46	g	O157	++	g	g	g	g	g	g	g	g	g	g	g	g	g	g	g	g	g	a	a	a	g	g	a
c	2659285	a	2868768	a	2938999	i	O157	rec46	a	CB9615	++++	a	a	a	a	a	a	a	a	a	a	a	a	a	a	a	a	a	a	a	a	a	a	a
a	2659465	g	2868948	g	2939179	s	O157	rec46	g	CB9615	++++	g	g	g	g	g	g	g	g	g	g	g	g	g	g	g	g	g	g	g	g	g	g	g
a	2659468	g	2868951	g	2939182	s	O157	rec46	g	CB9615	++++	g	g	g	g	g	g	g	g	g	g	g	g	g	g	g	g	g	g	g	g	g	g	g
t	2659492	c	2868975	c	2939206	s	O157	rec46	c	CB9615	++++	c	c	c	c	c	c	c	c	c	c	c	c	c	c	c	c	c	c	c	c	c	c	c
g	2659498	a	2868981	a	2939212	s	O157	rec46	a	CB9615	++	a	a	a	a	g	g	g	g	g	g	g	g	g	g	g	g	g	a	a	a	g	g	a
c	2659507	t	2868990	t	2939221	s	O157	rec46	t	CB9615	++	t	t	t	t	c	c	c	c	c	c	c	c	c	c	t	t	t	t	t	t	t	t	t
t	2659510	a	2868993	a	2939224	s	O157	rec46	a	CB9615	++++	a	a	a	a	g	g	g	g	g	g	g	g	g	g	a	a	a	a	a	a	a	a	a
a	2659537	g	2869020	g	2939251	s	O157	rec46	g	CB9615	++++	g	g	g	g	g	g	g	g	g	g	g	g	g	g	g	g	g	g	g	g	g	g	g
c	2660886	t	2870369	t	2940600	s	O157	rec46	c	O157	++++	c	c	c	c	c	c	c	c	c	c	c	c	c	c	c	c	c	c	c	c	c	c	c
t	2661057	c	2870540	c	2940771	s	O157	rec46	c	CB9615	++	c	c	t	t	c	t	t	c	c	c	c	c	c	c	t	t	t	t	t	t	t	c	t
g	2661092	c	2870575	c	2940806	s	O157	rec46	c	CB9615	++	c	g	g	g	g	g	g	g	c	c	c	c	c	c	g	g	g	a	a	a	g	c	g
c	2661224	t	2870707	t	2940938	s	O157	rec46	c	O157	++++	c	c	c	c	c	c	c	t	c	c	c	c	c	c	c	c	c	c	c	c	c	c	c
a	2661311	g	2870794	g	2941025	s	O157	rec46	g	CB9615	++++	g	g	g	g	g	g	g	g	g	g	g	g	g	g	g	g	g	g	g	g	g	g	a
c	2661455	t	2870938	t	2941169	s	O157	rec46	c	O157	++++	c	c	c	c	c	c	c	c	c	c	c	c	c	c	c	c	c	c	c	c	c	c	t
t	2661467	a	2870950	a	2941181	s	O157	rec46	a	CB9615	++	a	t	t	t	t	a	a	t	t	t	t	t	t	t	t	t	t	t	t	t	t	t	a
t	2661507	c	2870990	c	2941221	s	O157	rec46	c	CB9615	++++	c	c	c	c	c	c	c	c	c	c	c	c	c	c	c	c	c	c	c	c	c	c	c
c	2661518	t	2871001	t	2941232	s	O157	rec46	c	O157	++	c	t	t	t	t	t	t	c	c	c	c	c	c	c	t	t	t	t	t	t	t	t	t
a	2661530	g	2871013	g	2941244	s	O157	rec46	g	CB9615	++++	g	g	g	g	g	g	g	g	g	g	g	g	g	g	g	g	g	g	g	g	g	g	g
t	2661539	c	2871022	c	2941253	s	O157	rec46	c	CB9615	++++	c	c	c	c	c	c	c	c	c	c	c	c	c	c	c	c	c	c	c	c	c	c	c
c	2661551	t	2871034	t	2941265	s	O157	rec46	c	O157	++	c	c	c	c	c	t	t	c	c	c	c	c	c	c	c	c	c	c	c	c	c	c	t
a	2661872	g	2871355	g	2941586	s	O157	rec46	g	CB9615	++++	g	g	g	g	c	g	g	c	g	c	c	c	c	c	g	g	g	g	g	g	g	g	g
t	2661881	c	2871364	c	2941595	s	O157	rec46	t	O157	++	t	c	c	c	c	c	c	c	t	c	c	c	c	c	c	c	c	c	c	c	c	c	c
g	2661941	a	2871424	a	2941655	s	O157	rec46	g	O157	++	g	g	a	a	g	a	a	g	g	g	g	g	g	g	a	a	a	a	a	a	a	g	g
c	2661962	t	2871445	t	2941676	s	O157	rec46	t	CB9615	++	t	c	t	t	c	c	c	c	c	c	c	c	c	c	c	c	c	t	t	t	c	c	c
a	2661974	g	2871457	g	2941688	s	O157	rec46	a	O157	++	a	a	g	g	a	a	a	a	g	g	g	g	g	g	a	a	a	g	g	g	a	a	a
g	2661983	a	2871466	a	2941697	s	O157	rec46	g	O157	++	g	a	a	a	g	a	a	g	a	a	a	a	a	a	a	a	a	a	a	a	a	a	a
c	2662016	t	2871499	t	2941730	s	O157	rec46	t	CB9615	++++	t	t	t	t	t	t	t	t	t	t	t	t	t	t	t	t	t	t	t	t	t	t	t
a	2662025	g	2871508	g	2941739	s	O157	rec46	g	CB9615	++	g	g	g	g	g	g	g	a	a	a	a	a	a	a	g	g	g	g	g	g	g	g	g

O55 and O157 genome site details												Outgroup Strain Details[i]																						
CB9615 base[b]	CB9615 Site[c]	Sakai base[b]	Sakai Site[c]	EDL933 base[b]	EDL933 Site[c]	type[d]	Event Lineage[ef]	Recombinant segment	Inferred ancestral base[g]	Outgroup Analysis[e]	Support level[h]	D1 Sd197	K-12	HS	ATCC 8739	UMN026	IAI39	SMS 3-5	E2348/69	536	ED1a	CFT073	S88	APEC O1	UTI89	E24377A	IAI1	SE11	F5 8401	F2a 2457T	F2a 301	SS Ss046	B4 Sb227	B18 BS512
t	2662046	c	2871529	c	2941760	s	O157	rec46	c	CB9615	++++	c	c	c	c	c	c	c	c	c	c	c	c	c	c	c	c	c	c	c	c	c	c	c
a	2662049	g	2871532	g	2941763	s	O157	rec46	g	CB9615	++++	g	g	g	g	g	g	g	g	g	g	g	g	g	g	g	g	g	g	g	g	g	g	g
t	2662052	c	2871535	c	2941766	s	O157	rec46	c	CB9615	++	c	t	t	t	t	c	c	c	c	c	c	c	c	c	c	c	c	t	t	t	c	t	c
c	2662064	t	2871547	t	2941778	s	O157	rec46	c	O157	++++	c	c	c	c	c	c	c	c	c	c	c	c	c	c	c	c	c	c	c	c	c	c	c
t	2662107	c	2871590	c	2941821	s	O157	rec46	c	CB9615	++	c	c	c	c	c	c	c	c	t	t	t	t	t	t	c	c	c	c	c	c	c	c	c
t	2662115	c	2871598	c	2941829	s	O157	rec46	c	CB9615	++	c	c	c	c	c	c	c	t	t	t	t	t	t	t	c	c	c	c	c	c	c	c	t
t	2662130	c	2871613	c	2941844	s	O157	rec46	c	CB9615	++++	c	c	c	c	c	c	c	c	c	c	c	c	c	c	c	c	c	c	c	c	c	c	c
a	2662160	g	2871643	g	2941874	s	O157	rec46	g	CB9615	++	g	g	a	a	g	g	g	a	a	a	a	a	a	a	g	g	g	a	a	a	g	g	g
a	2662184	g	2871667	g	2941898	s	O157	rec46	g	CB9615	++	g	c	c	c	g	g	g	a	a	a	a	a	a	a	g	g	g	c	c	c	g	c	g
c	2662205	a	2871688	a	2941919	s	O157	rec46	a	CB9615	++	a	c	a	a	a	a	a	c	c	c	c	c	c	c	a	a	a	c	c	c	a	c	a
g	2662217	a	2871700	a	2941931	s	O157	rec46	a	CB9615	++	a	g	a	a	a	a	a	a	a	a	g	g	g	g	a	a	a	a	a	a	a	g	g
a	2662220	t	2871703	t	2941934	s	O157	rec46	t	CB9615	++	t	a	t	t	a	t	t	a	a	a	a	a	a	a	t	t	t	t	t	t	t	a	a
g	2662229	a	2871712	a	2941943	s	O157	rec46	a	CB9615	++	a	g	a	a	a	a	a	a	a	a	a	a	a	a	a	a	a	a	a	a	a	g	g
c	2662238	t	2871721	t	2941952	s	O157	rec46	t	CB9615	++	t	t	t	t	t	t	t	t	t	t	c	c	c	c	t	t	t	t	t	t	t	t	t
t	2662277	c	2871760	c	2941991	s	O157	rec46	c	CB9615	++	c	t	c	c	c	c	c	c	t	t	c	c	c	c	a	a	a	c	c	c	a	t	c
g	2662343	a	2871826	a	2942057	s	O157	rec46	a	CB9615	++	a	g	g	g	g	g	g	g	g	g	g	g	g	g	g	g	g	g	g	g	g	g	a
c	2662403	t	2871886	t	2942117	s	O157	rec46	t	CB9615	++	t	t	c	c	t	t	t	t	t	t	t	t	t	t	c	c	c	t	t	t	c	t	t
g	2662409	a	2871892	a	2942123	s	O157	rec46	g	O157	++++	g	g	g	g	g	g	g	g	g	g	g	g	g	g	g	g	g	g	g	g	g	g	g
t	2662583	c	2872066	c	2942297	i	O157	rec46	c	CB9615	++++	c	c	c	c	c	c	c	c	c	c	c	c	c	c	c	c	c	c	c	c	c	c	c
t	2662586	c	2872069	c	2942300	i	O157	rec46	c	CB9615	++++	c	c	c	c	c	c	c	c	c	c	c	c	c	c	c	c	c	c	c	c	c	c	c
t	2662599	c	2872082	c	2942313	i	O157	rec46	c	CB9615	++++	c	c	c	c	c	c	c	c	t	c	c	c	c	c	c	c	c	c	c	c	c	c	c
g	2662644	a	2872127	a	2942358	i	O157	rec46	g	O157	++++	-	g	g	g	g	g	g	g	g	g	g	g	g	g	g	g	g	g	g	g	g	g	a
c	2662668	t	2872151	t	2942382	i	O157	rec46	t	CB9615	++	t	c	c	c	c	c	c	c	c	c	c	c	c	c	c	c	c	t	t	t	c	c	c
c	2662854	t	2872337	t	2942568	ns	O157	rec46	t	CB9615	++	t	c	c	c	c	c	c	c	c	c	c	c	c	c	c	c	c	c	c	c	c	c	c
g	2662858	a	2872341	a	2942572	s	O157	rec46	a	CB9615	++	a	g	g	g	g	g	a	g	g	g	g	g	g	g	g	g	g	g	g	g	g	g	g
c	2662866	a	2872349	a	2942580	ns	O157	rec46	a	CB9615	++	a	c	a	a	c	c	a	a	a	a	a	a	a	a	c	c	c	a	a	a	c	c	c
g	2662874	a	2872357	a	2942588	ns	O157	rec46	a	CB9615	++	a	g	g	g	g	g	g	g	g	g	g	g	g	g	g	g	g	g	g	g	g	g	g
g	2662876	a	2872359	a	2942590	ns	O157	rec46	a	CB9615	++	a	g	a	a	g	g	a	a	g	a	g	g	g	g	g	g	g	a	a	a	g	g	g
t	2662879	g	2872362	g	2942593	s	O157	rec46	g	CB9615	++	g	t	t	t	t	t	g	g	g	g	g	g	g	g	t	t	t	g	g	g	t	t	t
g	2662885	t	2872368	t	2942599	s	O157	rec46	t	CB9615	++	t	g	g	g	g	g	g	g	g	g	g	g	g	g	g	g	g	g	g	g	g	g	g
a	2662891	g	2872374	g	2942605	s	O157	rec46	g	CB9615	++	g	g	g	g	a	a	g	g	g	g	g	g	g	g	g	g	g	g	g	g	g	g	a
t	2662951	c	2872434	c	2942665	s	O157	rec46	t	O157	++	t				c	c																	t
c	2663005	g	2872488	g	2942719	s	O157	rec46	c	O157	++	c				a	a																	c
g	2663023	a	2872506	a	2942737	s	O157	rec46	g	O157	++	g				a	a																	g
c	2663026	t	2872509	t	2942740	s	O157	rec46	c	O157	+++	c				c	c																	c
g	2663031	c	2872514	c	2942745	ns	O157	rec46	g	O157	++	g				c	c																	g
c	2663033	g	2872516	g	2942747	ns	O157	rec46	c	O157	++	c				g	c																	c
t	2663050	c	2872533	c	2942764	s	O157	rec46	t	O157	++	t				t	c																	t
t	2663140	c	2872623	c	2942854	s	O157	rec46	c	CB9615	+++	c				c	c																	c
a	2663179	t	2872662	t	2942893	s	O157	rec46	a	O157	++	a				a	t																	a
t	2663214	g	2872697	g	2942928	ns	O157	rec46	t	O157	++	t				t	g																	t
g	2663219	a	2872702	a	2942933	ns	O157	rec46	g	O157	++	g				g	a																	g
a	2663223	t	2872706	t	2942937	ns	O157	rec46	a	O157	++	a				a	t																	a
g	2663226	a	2872709	a	2942940	ns	O157	rec46	g	O157	++	g				g	a																	g
a	2663242	g	2872725	g	2942956	s	O157	rec46	g	CB9615	++	g				g	a																	g
g	2663260	a	2872743	a	2942974	s	O157	rec46	a	CB9615	++	a				a	g																	a
c	2663278	t	2872761	t	2942992	s	O157	rec46	t	CB9615	++	t				t	a																	c
a	2663294	g	2872777	g	2943008	ns	O157	rec46	a	O157	+	c				c	a																	
t	2663341	c	2872824	c	2943055	s	O157	rec46	t	O157	++	t				t	t																	
c	2663355	t	2872838	t	2943069	ns	O157	rec46	c	O157	++	c				c	c																	
c	2663360	t	2872843	t	2943074	ns	O157	rec46	c	O157	++	c				c	c																	
c	2663362	t	2872845	t	2943076	ns	O157	rec46	c	O157	++	c				c	c																	
t	2663365	c	2872848	c	2943079	s	O157	rec46	t	O157	++	t				t	t																	
t	2663374	c	2872857	c	2943088	s	O157	rec46	c	CB9615	++	c				c	c																	
g	2663376	a	2872859	a	2943090	ns	O157	rec46	a	CB9615	++	a				a	a																	
c	2663409	t	2872892	t	2943123	ns	O157	rec46	t	CB9615	++	t				t	t																	
a	2663426	g	2872909	g	2943140	ns	O157	rec46	g	CB9615	++	g				g	a																	
g	2663448	a	2872931	a	2943162	ns	O157	rec46	g	O157	++	g				g	g																	
g	2663522	a	2873005	a	2943236	ns	O157	rec46	g	O157	++	g				g	g																	
g	2663524	c	2873007	c	2943238	ns	O157	rec46	g	O157	++	g				t	t																	
t	2663527	c	2873010	c	2943241	s	O157	rec46	t	O157	++	t				t	t																	
c	2663533	g	2873016	g	2943247	s	O157	rec46	g	CB9615	++	g				c	c																	
t	2663534	a	2873017	a	2943248	ns	O157	rec46	a	CB9615	++	a				t	t																	
a	2663545	c	2873028	c	2943259	s	O157	rec46	a	O157	++	a				a	a																	
g	2663552	c	2873035	c	2943266	ns	O157	rec46	c	CB9615	++	c				c	c																	
t	2663593	c	2873076	c	2943307	s	O157	rec46	t	O157	++	t				t	t																	
a	2663597	c	2873080	c	2943311	ns	O157	rec46	a	O157	++	a				a	a																	
g	2663605	a	2873088	a	2943319	ns	O157	rec46	g	O157	++	g				g	g																	
c	2663607	a	2873090	a	2943321	ns	O157	rec46	c	O157	++	c				c	c																	
g	2663608	t	2873091	t	2943322	ns	O157	rec46	g	O157	++	g				g	t																	
t	2663614	c	2873097	c	2943328	s	O157	rec46	t	O157	++	t				t	c																	
t	2663632	c	2873115	c	2943346	s	O157	rec46	t	O157	++	t				t	t																	
c	2663641	g	2873124	g	2943355	ns	O157	rec46	g	CB9615	++	g				g	g																	
t	2663653	a	2873136	a	2943367	ns	O157	rec46	a	CB9615	++	a				a	a																	
a	2663654	c	2873137	c	2943368	ns	O157	rec46	c	CB9615	++	c				c	c																	
a	2663657	g	2873140	g	2943371	ns	O157	rec46	g	CB9615	++	g				g	g																	
g	2663660	a	2873143	a	2943374	ns	O157	rec46	g	O157	++	g				g	a																	
g	2663664	c	2873147	c	2943378	ns	O157	rec46	c	CB9615	++	c				c	c																	
g	2663672	a	2873155	a	2943386	ns	O157	rec46	g	O157	++	g				g	a																	
t	2663673	c	2873156	c	2943387	ns	O157	rec46	c	CB9615	++	c				c	c																	
c	2663677	t	2873160	t	2943391	s	O157	rec46	t	CB9615	++	t				t	t																	
g	2663681	a	2873164	a	2943395	ns	O157	rec46	a	CB9615	++	a				a	a																	
c	2663682	g	2873165	g	2943396	ns	O157	rec46	c	O157	++	c				c	g																	
c	2663683	t	2873166	t	2943397	ns	O157	rec46	t	CB9615	++	t				t	t																	
t	2663685	c	2873168	c	2943399	ns	O157	rec46	c	CB9615	++	c				c	c																	
c	2663686	a	2873169	a	2943400	ns	O157	rec46	a	CB9615	++	a				a	a																	

O55 and O157 genome site details												Outgroup Strain Details[i]																						
CB9615 base[b]	CB9615 Site[c]	Sakai base[b]	Sakai Site[c]	EDL933 base[b]	EDL933 Site[c]	type[d]	Event Lineage[ef]	Recombinant segment	Inferred ancestral base[g]	Outgroup Analysis[e]	Support level[h]	D1 Sd197	K-12	HS	ATCC 8739	UMN026	IAI39	SMS 3-5	E2348/69	536	ED1a	CFT073	S88	APEC O1	UTI89	E24377A	IAI1	SE11	F5 8401	F2a 2457T	F2a 301	SS Ss046	B4 Sb227	B18 BS512
c	2663687	g	2873170	g	2943401	ns	O157	rec46	g	CB9615	++	g				g	g																	
a	2663688	t	2873171	t	2943402	ns	O157	rec46	t	CB9615	+	c				c	t																	
t	2663698	c	2873181	c	2943412	s	O157	rec46	c	CB9615	++	c				c	c																	
g	2663700	a	2873183	a	2943414	ns	O157	rec46	a	CB9615	++	a				a	a																	
t	2663701	c	2873184	c	2943415	ns	O157	rec46	c	CB9615	++	c				c	c																	
c	2663711	t	2873194	t	2943425	s	O157	rec46	t	CB9615	++	t				t	t																	
a	2663713	g	2873196	g	2943427	s	O157	rec46	g	CB9615	++	g				g	g																	
a	2663717	c	2873200	c	2943431	ns	O157	rec46	c	CB9615	++	c				c	c																	
a	2663718	g	2873201	g	2943432	ns	O157	rec46	a	O157	++	a				a	g																	
c	2663725	t	2873208	t	2943439	s	O157	rec46	t	CB9615	+	-				-	t																	
c	2663728	t	2873211	t	2943442	s	O157	rec46	t	CB9615	+	-				-	t																	
-	2663730	t	2873214	t	2943445	ins-9	O157	rec46	-	O157	++	-				-	9																	
a	2663731	t	2873223	t	2943454	ns	O157	rec46	t	CB9615	++	t				t	t																	
t	2663740	a	2873232	a	2943463	s	O157	rec46	a	CB9615	++	a				a	a																	
c	2663742	t	2873234	t	2943465	ns	O157	rec46	t	CB9615	++	t				t	t																	
c	2663747	t	2873239	t	2943470	s	O157	rec46	c	O157	++	c				c	c																	
a	2663761	g	2873253	g	2943484	s	O157	rec46	g	CB9615	++	g				g	a																	
a	2663762	g	2873254	g	2943485	ns	O157	rec46	g	CB9615	++	g				g	a																	
t	2663767	c	2873259	c	2943490	s	O157	rec46	c	CB9615	++	c				c	g																	
t	2663782	a	2873274	a	2943505	s	O157	rec46	a	CB9615	++	a				a	c																	
c	2663788	t	2873280	t	2943511	s	O157	rec46	t	CB9615	++	t				t	t																	
a	2663789	g	2873281	g	2943512	ns	O157	rec46	g	CB9615	++	g				g	g																	
c	2663792	t	2873284	t	2943515	ns	O157	rec46	t	CB9615	++	t				t	t																	
a	2663794	t	2873286	t	2943517	ns	O157	rec46	t	CB9615	++	t				t	t																	
c	2663795	a	2873287	a	2943518	ns	O157	rec46	a	CB9615	++	a				a	a																	
t	2663797	g	2873289	g	2943520	ns	O157	rec46	g	CB9615	++	g				g	g																	
t	2663799	a	2873291	a	2943522	ns	O157	rec46	a	CB9615	++	a				a	a																	
c	2663800	a	2873292	a	2943523	ns	O157	rec46	a	CB9615	++	a				a	a																	
t	2663804	c	2873296	c	2943527	ns	O157	rec46	c	CB9615	++	c				c	c																	
c	2663807	t	2873299	t	2943530	ns	O157	rec46	t	CB9615	++	t				t	t																	
c	2663809	t	2873301	t	2943532	ns	O157	rec46	t	CB9615	++	t				t	t																	
t	2663811	a	2873303	a	2943534	ns	O157	rec46	a	CB9615	++	a				a	a																	
a	2663812	c	2873304	c	2943535	ns	O157	rec46	c	CB9615	++	c				c	c																	
a	2663815	t	2873307	t	2943538	ns	O157	rec46	t	CB9615	++	t				t	t																	
t	2663816	c	2873308	c	2943539	ns	O157	rec46	c	CB9615	++	c				c	c																	
a	2663817	c	2873309	c	2943540	ns	O157	rec46	c	CB9615	++	c				c	c																	
g	2663821	t	2873313	t	2943544	s	O157	rec46	t	CB9615	++	t				t	t																	
g	2663822	t	2873314	t	2943545	ns	O157	rec46	t	CB9615	++	t				t	t																	
c	2663824	t	2873316	t	2943547	ns	O157	rec46	t	CB9615	++	t				t	t																	
t	2663827	c	2873319	c	2943550	s	O157	rec46	c	CB9615	++	c				t	t																	
a	2663831	g	2873323	g	2943554	ns	O157	rec46	g	CB9615	++	g				g	g																	
c	2663832	a	2873324	a	2943555	ns	O157	rec46	a	CB9615	++	a				a	a																	
t	2663833	a	2873325	a	2943556	ns	O157	rec46	?	O157	+/-	g				g	g																	
a	2663835	g	2873327	g	2943558	ns	O157	rec46	g	CB9615	++	g				g	g																	
a	2663839	-	2873330	-	2943561	del-3	O157	rec46	-	CB9615	++	-				-	-																	
g	2663843	a	2873332	a	2943563	ns	O157	rec46	a	CB9615	++	a				a	a																	
c	2663844	g	2873333	g	2943564	ns	O157	rec46	g	CB9615	++	g				g	g																	
a	2663846	g	2873335	g	2943566	ns	O157	rec46	g	CB9615	++	g				g	g																	
t	2663848	g	2873337	g	2943568	ns	O157	rec46	g	CB9615	++	g				g	g																	
g	2663849	a	2873338	a	2943569	ns	O157	rec46	a	CB9615	++	a				a	a																	
g	2663855	a	2873344	a	2943575	ns	O157	rec46	g	O157	++	g				g	a																	
a	2663922	t	2873411	t	2943642	ns	O157	rec46	t	CB9615	++	t				t	t																	
c	2663926	t	2873415	t	2943646	s	O157	rec46	c	O157	++	c				c	c																	
a	2663956	g	2873445	g	2943676	s	O157	rec46	a	O157	++	a				a	a																	
t	2663960	a	2873449	a	2943680	ns	O157	rec46	t	O157	++	t				t	t																	
c	2663962	t	2873451	t	2943682	ns	O157	rec46	t	CB9615	++	t				t	t																	
a	2663968	g	2873457	g	2943688	s	O157	rec46	a	O157	++	a				a	a																	
c	2663989	g	2873478	g	2943709	s	O157	rec46	c	O157	++	c				c	c																	
a	2664020	g	2873509	g	2943740	ns	O157	rec46	g	CB9615	+	-				g	g																	
t	2664025	a	2873514	a	2943745	ns	O157	rec46	a	CB9615	+	-				a	a																	
t	2664058	c	2873547	c	2943778	s	O157	rec46	c	CB9615	++	c				c	c																	
g	2664082	c	2873571	c	2943802	ns	O157	rec46	c	CB9615	++	c				c	c																	
g	2664106	a	2873595	a	2943826	s	O157	rec46	a	CB9615	++	a				a	g																	
t	2664138	a	2873627	a	2943858	ns	O157	rec46	a	CB9615	++	a				a	a																	
c	2664140	t	2873629	t	2943860	ns	O157	rec46	c	O157	++	c				t	t																	
a	2664145	c	2873634	c	2943865	s	O157	rec46	a	O157	++	a				c	c																	
c	2664196	t	2873685	t	2943916	s	O157	rec46	c	O157	++	c				c	c																	
g	2664197	t	2873686	t	2943917	ns	O157	rec46	g	O157	++	g				g	g																	
t	2664199	g	2873688	g	2943919	ns	O157	rec46	t	O157	++	t				t	t																	
a	2664202	g	2873691	g	2943922	s	O157	rec46	a	O157	++	a				a	a																	
g	2664205	c	2873694	c	2943925	s	O157	rec46	g	O157	++	g				g	g																	
g	2664209	a	2873698	a	2943929	ns	O157	rec46	g	O157	++	g				g	g																	
a	2664211	g	2873700	g	2943931	ns	O157	rec46	a	O157	++	a				a	a																	
t	2664213	c	2873702	c	2943933	ns	O157	rec46	t	O157	++	t				t	t																	
a	2664214	c	2873703	c	2943934	ns	O157	rec46	a	O157	++	a				a	a																	
t	2664217	a	2873706	a	2943937	ns	O157	rec46	t	O157	++	t				t	t																	
a	2664218	g	2873707	g	2943938	ns	O157	rec46	a	O157	++	a				a	a																	
t	2664229	a	2873718	a	2943949	ns	O157	rec46	t	O157	++	t				t	t																	
g	2664230	a	2873719	a	2943950	ns	O157	rec46	g	O157	++	g				a	a																	
t	2664231	a	2873720	a	2943951	ns	O157	rec46	t	O157	++	t				a	a																	
c	2664232	t	2873721	t	2943952	ns	O157	rec46	c	O157	++	c				t	t																	
t	2664234	c	2873723	c	2943954	ns	O157	rec46	t	O157	++	t				-	-																	
a	2664235	g	2873724	g	2943955	ns	O157	rec46	a	O157	++	a				-	-																	
c	2664238	t	2873727	t	2943958	s	O157	rec46	c	O157	++	c				c	c																	
a	2664241	g	2873730	g	2943961	s	O157	rec46	a	O157	++	a				a	a																	
t	2664248	c	2873737	c	2943968	s	O157	rec46	t	O157	++	t				t	t																	

O55 and O157 genome site details												Outgroup Strain Details[i]																						
CB9615 base[b]	CB9615 Site[c]	Sakai base[b]	Sakai Site[c]	EDL933 base[b]	EDL933 Site[c]	type[d]	Event Lineage[ef]	Recombinant segment	Inferred ancestral base[g]	Outgroup Analysis[e]	Support level[h]	D1 Sd197	K-12	HS	ATCC 8739	UMN026	IAI39	SMS 3-5	E2348/69	536	ED1a	CFT073	S88	APEC O1	UTI89	E24377A	IAI1	SE11	F5 8401	F2a 2457T	F2a 301	SS Ss046	B4 Sb227	B18 BS512
a	2664250	t	2873739	t	2943970	s	O157	rec46	a	O157	++	a				a	a																	
g	2664253	a	2873742	a	2943973	s	O157	rec46	g	O157	++	g				g	t																	
a	2664256	g	2873745	g	2943976	s	O157	rec46	a	O157	++	a				g	g																	
c	2664262	a	2873751	a	2943982	s	O157	rec46	c	O157	++	c				c	t																	
a	2664263	g	2873752	g	2943983	ns	O157	rec46	a	O157	++	a				a	g																	
g	2664269	t	2873758	t	2943989	ns	O157	rec46	g	O157	++	g				g	g																	
c	2664271	t	2873760	t	2943991	ns	O157	rec46	c	O157	++	c				c	c																	
c	2664274	t	2873763	t	2943994	s	O157	rec46	c	O157	++	c				c	g																	
a	2664275	t	2873764	t	2943995	ns	O157	rec46	a	O157	++	a				a	a																	
g	2664277	t	2873766	t	2943997	ns	O157	rec46	g	O157	++	g				t	g																	
a	2664281	g	2873770	g	2944001	ns	O157	rec46	a	O157	++	a				a	a																	
a	2664286	t	2873775	t	2944006	s	O157	rec46	a	O157	++	a				g	a																	
g	2664291	a	2873780	a	2944011	ns	O157	rec46	g	O157	++	g				g	g																	
a	2664292	g	2873781	g	2944012	ns	O157	rec46	a	O157	++	a				a	a																	
a	2664298	c	2873787	c	2944018	s	O157	rec46	a	O157	++	a				a	a																	
g	2664299	a	2873788	a	2944019	ns	O157	rec46	g	O157	++	g				g	g																	
g	2664301	a	2873790	a	2944021	ns	O157	rec46	g	O157	++	g				g	g																	
t	2664302	a	2873791	a	2944022	ns	O157	rec46	t	O157	++	t				t	t																	
t	2664303	a	2873792	a	2944023	ns	O157	rec46	t	O157	++	t				t	t																	
a	2664305	c	2873794	c	2944025	ns	O157	rec46	a	O157	++	a				a	a																	
c	2664307	g	2873796	g	2944027	ns	O157	rec46	c	O157	++	c				c	a																	
g	2664309	c	2873798	c	2944029	ns	O157	rec46	g	O157	++	g				g	g																	
t	2664310	c	2873799	c	2944030	ns	O157	rec46	t	O157	++	t				t	t																	
a	2664312	g	2873801	g	2944032	ns	O157	rec46	a	O157	++	a				a	a																	
g	2664317	t	2873806	t	2944037	ns	O157	rec46	g	O157	++	g				g	g																	
a	2664320	c	2873809	c	2944040	ns	O157	rec46	a	O157	++	a				a	a																	
-	2664321	t	2873811	t	2944042	ins-4	O157	rec46	-	O157	++	-				-	-																	
t	2664323	c	2873816	c	2944047	ns	O157	rec46	t	O157	++	t				t	t																	
g	2664327	c	2873820	c	2944051	ns	O157	rec46	g	O157	++	g				g	g																	
a	2664328	t	2873821	t	2944052	ns	O157	rec46	a	O157	++	a				a	a																	
t	2664330	c	2873823	c	2944054	ns	O157	rec46	t	O157	++	t				t	t																	
t	2664334	c	2873827	c	2944058	s	O157	rec46	?	O157	+/-	g				g	g																	
g	2664335	-	2873827	-	2944058	del	O157	rec46	g	O157	++	g				g	g																	
c	2664340	a	2873832	a	2944063	s	O157	rec46	c	O157	++	c				c	c																	
g	2664343	c	2873835	c	2944066	s	O157	rec46	g	O157	++	g				g	g																	
t	2664347	c	2873839	c	2944070	ns	O157	rec46	t	O157	++	t				t	t																	
t	2664349	g	2873841	g	2944072	ns	O157	rec46	t	O157	++	t				t	t																	
a	2664355	g	2873847	g	2944078	s	O157	rec46	a	O157	++	a				a	a																	
t	2664356	a	2873848	a	2944079	ns	O157	rec46	t	O157	++	t				t	t																	
c	2664359	a	2873851	a	2944082	ns	O157	rec46	c	O157	++	c				c	c																	
g	2664361	a	2873853	a	2944084	ns	O157	rec46	g	O157	++	g				g	g																	
c	2664367	a	2873859	a	2944090	s	O157	rec46	c	O157	++	c				c	c																	
a	2664370	g	2873862	g	2944093	s	O157	rec46	a	O157	++	a				a	a																	
t	2664379	c	2873871	c	2944102	s	O157	rec46	t	O157	++	t				c	t																	
a	2664385	g	2873877	g	2944108	s	O157	rec46	a	O157	++	a				a	a																	
t	2664387	c	2873879	c	2944110	ns	O157	rec46	t	O157	++	t				t	t																	
a	2664388	g	2873880	g	2944111	ns	O157	rec46	a	O157	++	a				a	a																	
t	2664391	c	2873883	c	2944114	s	O157	rec46	t	O157	++	t				t	t																	
g	2664392	a	2873884	a	2944115	ns	O157	rec46	g	O157	++	g				g	g																	
a	2664399	c	2873891	c	2944122	ns	O157	rec46	a	O157	+					a	a																	
c	2664411	a	2873903	a	2944134	ns	O157	rec46	c	O157	+					c	c																	
t	2664413	c	2873905	c	2944136	ns	O157	rec46	t	O157	+					t	t																	
c	2664414	a	2873906	a	2944137	ns	O157	rec46	c	O157	+					c	c																	
t	2664415	c	2873907	c	2944138	ns	O157	rec46	t	O157	+					t	t																	
c	2664418	a	2873910	a	2944141	ns	O157	rec46	c	O157	+					c	c																	
t	2664421	a	2873913	a	2944144	s	O157	rec46	t	O157	+					t	t																	
t	2664424	g	2873916	g	2944147	s	O157	rec46	t	O157	+					t	t																	
t	2664425	c	2873917	c	2944148	ns	O157	rec46	t	O157	+					t	t																	
a	2664433	g	2873925	g	2944156	s	O157	rec46	a	O157	+					a	a																	
a	2664436	g	2873928	g	2944159	s	O157	rec46	a	O157	+					a	a																	
c	2664445	a	2873937	a	2944168	s	O157	rec46	c	O157	+					c	c																	
t	2664448	g	2873940	g	2944171	s	O157	rec46	t	O157	+					t	t																	
a	2664454	t	2873946	t	2944177	ns	O157	rec46	a	O157	+					a	a																	
t	2664455	c	2873947	c	2944178	s	O157	rec46	t	O157	+					t	t																	
a	2664457	t	2873949	t	2944180	s	O157	rec46	a	O157	+					a	a																	
a	2664464	g	2873956	g	2944187	ns	O157	rec46	a	O157	+					a	a																	
t	2664466	a	2873958	a	2944189	ns	O157	rec46	t	O157	+					t	t																	
g	2664467	a	2873959	a	2944190	ns	O157	rec46	g	O157	+					g	g																	
t	2664474	g	2873966	g	2944197	ns	O157	rec46	?	O157	+/-					t	g																	
c	2664476	g	2873968	g	2944199	ns	O157	rec46	c	O157	+					c	c																	
t	2664478	g	2873970	g	2944201	ns	O157	rec46	t	O157	+					t	t																	
t	2664479	a	2873971	a	2944202	ns	O157	rec46	t	O157	+					t	t																	
a	2664483	g	2873975	g	2944206	ns	O157	rec46	a	O157	+					a	a																	
a	2664485	g	2873977	g	2944208	ns	O157	rec46	g	CB9615	+					g	g																	
t	2664487	a	2873979	a	2944210	ns	O157	rec46	t	O157	+					t	t																	
g	2664488	a	2873980	a	2944211	ns	O157	rec46	g	O157	+					g	g																	
c	2664490	a	2873982	a	2944213	ns	O157	rec46	c	O157	+					c	c																	
t	2664491	a	2873983	a	2944214	ns	O157	rec46	t	O157	+					t	t																	
c	2664494	t	2873986	t	2944217	ns	O157	rec46	c	O157	+					c	c																	
t	2664496	c	2873988	c	2944219	ns	O157	rec46	t	O157	+					t	t																	
a	2664504	t	2873996	t	2944227	ns	O157	rec46	a	O157	+					a	a																	
a	2664505	t	2873997	t	2944228	ns	O157	rec46	a	O157	+					a	a																	
t	2664506	c	2873998	c	2944229	ns	O157	rec46	t	O157	+					t	t																	
a	2664507	c	2873999	c	2944230	ns	O157	rec46	a	O157	+					a	a																	
t	2664508	g	2874000	g	2944231	ns	O157	rec46	t	O157	+					t	t																	
a	2664509	g	2874001	g	2944232	ns	O157	rec46	a	O157	+					a	a																	

O55 and O157 genome site details												Outgroup Strain Details[i]																						
CB9615 base[b]	CB9615 Site[c]	Sakai base[b]	Sakai Site[c]	EDL933 base[b]	EDL933 Site[c]	type[d]	Event Lineage[ef]	Recombinant segment	Inferred ancestral base[g]	Outgroup Analysis[e]	Support level[h]	D1 Sd197	K-12	HS	ATCC 8739	UMN026	IAI39	SMS 3-5	E2348/69	536	ED1a	CFT073	S88	APEC O1	UTI89	E24377A	IAI1	SE11	F5 8401	F2a 2457T	F2a 301	SS Ss046	B4 Sb227	B18 BS512
t	2664510	c	2874002	c	2944233	ns	O157	rec46	t	O157	+					t	t																	
a	2664511	g	2874003	g	2944234	ns	O157	rec46	a	O157	+					a	a																	
g	2664514	a	2874006	a	2944237	s	O157	rec46	g	O157	+					g	g																	
t	2664523	c	2874015	c	2944246	s	O157	rec46	t	O157	+					t	t																	
t	2664533	c	2874025	c	2944256	s	O157	rec46	t	O157	+					t	t																	
t	2664536	c	2874028	c	2944259	s	O157	rec46	t	O157	+					t	t																	
t	2664544	c	2874036	c	2944267	s	O157	rec46	t	O157	+					t	t																	
a	2664559	c	2874051	c	2944282	s	O157	rec46	a	O157	+					a	a																	
a	2664562	t	2874054	t	2944285	ns	O157	rec46	a	O157	+					a	a																	
g	2664568	a	2874060	a	2944291	s	O157	rec46	g	O157	+					g	g																	
c	2664573	t	2874065	t	2944296	ns	O157	rec46	c	O157	+					c	c																	
a	2664575	c	2874067	c	2944298	ns	O157	rec46	a	O157	+					a	a																	
a	2664577	t	2874069	t	2944300	ns	O157	rec46	a	O157	+					a	a																	
c	2664583	t	2874075	t	2944306	s	O157	rec46	c	O157	+					c	c																	
c	2664592	a	2874084	a	2944315	s	O157	rec46	c	O157	+					c	c																	
a	2664593	t	2874085	t	2944316	ns	O157	rec46	a	O157	+					a	a																	
a	2664610	c	2874102	c	2944333	s	O157	rec46	a	O157	+					a	a																	
t	2664611	a	2874103	a	2944334	ns	O157	rec46	t	O157	+					t	t																	
c	2664612	t	2874104	t	2944335	ns	O157	rec46	c	O157	+					c	c																	
g	2664616	c	2874108	c	2944339	s	O157	rec46	g	O157	+					g	a																	
t	2664619	c	2874111	c	2944342	s	O157	rec46	t	O157	+					t	t																	
t	2664626	c	2874118	c	2944349	s	O157	rec46	t	O157	+					t	t																	
a	2664628	g	2874120	g	2944351	s	O157	rec46	a	O157	+					a	a																	
g	2664631	t	2874123	t	2944354	s	O157	rec46	g	O157	+					g	g																	
a	2664634	t	2874126	t	2944357	s	O157	rec46	a	O157	+					a	a																	
g	2664643	c	2874135	c	2944366	s	O157	rec46	g	O157	+					g	g																	
a	2664649	t	2874141	t	2944372	s	O157	rec46	a	O157	+					a	a																	
c	2664655	t	2874147	t	2944378	s	O157	rec46	c	O157	+					c	c																	
a	2664661	g	2874153	g	2944384	s	O157	rec46	a	O157	+					a	a																	
a	2664665	g	2874157	g	2944388	ns	O157	rec46	a	O157	+					a	a																	
t	2664669	a	2874161	a	2944392	ns	O157	rec46	t	O157	+					t	t																	
t	2664670	a	2874162	a	2944393	ns	O157	rec46	t	O157	+					t	t																	
g	2664673	a	2874165	a	2944396	s	O157	rec46	g	O157	+					g	g																	
g	2664676	t	2874168	t	2944399	ns	O157	rec46	g	O157	+					g	g																	
c	2664677	a	2874169	a	2944400	ns	O157	rec46	c	O157	+					c	c																	
g	2664678	a	2874170	a	2944401	ns	O157	rec46	?	O157	+/-					g	a																	
a	2664679	c	2874171	c	2944402	ns	O157	rec46	a	O157	+					a	a																	
t	2664682	c	2874174	c	2944405	s	O157	rec46	t	O157	+					t	t																	
a	2664685	t	2874177	t	2944408	s	O157	rec46	a	O157	+					a	a																	
a	2664692	g	2874184	g	2944415	ns	O157	rec46	a	O157	+					a	a																	
a	2664693	g	2874185	g	2944416	ns	O157	rec46	a	O157	+					a	a																	
g	2664695	a	2874187	a	2944418	ns	O157	rec46	g	O157	+					g	g																	
g	2664697	t	2874189	t	2944420	ns	O157	rec46	g	O157	+					g	g																	
g	2664706	a	2874198	a	2944429	s	O157	rec46	g	O157	+					g	g																	
g	2664707	a	2874199	a	2944430	ns	O157	rec46	?	O157	+/-					g	a																	
t	2664718	c	2874210	c	2944441	s	O157	rec46	t	O157	+					t	t																	
a	2664719	g	2874211	g	2944442	ns	O157	rec46	a	O157	+					a	a																	
g	2664721	c	2874213	c	2944444	ns	O157	rec46	g	O157	+					a	g																	
t	2664727	a	2874219	a	2944450	s	O157	rec46	t	O157	+					t	t																	
g	2664733	a	2874225	a	2944456	s	O157	rec46	g	O157	+					g	g																	
a	2664740	g	2874232	g	2944463	ns	O157	rec46	a	O157	+					a	a																	
t	2664746	c	2874238	c	2944469	s	O157	rec46	t	O157	+					t	t																	
a	2664754	t	2874246	t	2944477	s	O157	rec46	a	O157	+					a	a																	
a	2664759	c	2874251	c	2944482	ns	O157	rec46	a	O157	+					a	a																	
t	2664760	a	2874252	a	2944483	ns	O157	rec46	t	O157	+					t	t																	
a	2664762	g	2874254	g	2944485	ns	O157	rec46	a	O157	+					a	a																	
c	2664766	t	2874258	t	2944489	s	O157	rec46	?	O157	+/-					a	a																	
g	2664772	c	2874264	c	2944495	ns	O157	rec46	g	O157	+					g	g																	
a	2664773	t	2874265	t	2944496	ns	O157	rec46	a	O157	+					a	a																	
a	2664775	c	2874267	c	2944498	ns	O157	rec46	a	O157	+					a	a																	
a	2664779	c	2874271	c	2944502	ns	O157	rec46	a	O157	+					a	a																	
c	2664782	t	2874274	t	2944505	ns	O157	rec46	c	O157	+					c	c																	
t	2664787	c	2874279	c	2944510	s	O157	rec46	t	O157	+					t	t																	
a	2664788	c	2874280	c	2944511	ns	O157	rec46	a	O157	+					a	a																	
t	2664790	c	2874282	c	2944513	ns	O157	rec46	t	O157	+					t	t																	
a	2664793	t	2874285	t	2944516	s	O157	rec46	?	O157	+/-					g	g																	
c	2664795	t	2874287	t	2944518	ns	O157	rec46	c	O157	+					c	c																	
a	2664805	g	2874297	g	2944528	s	O157	rec46	a	O157	+					a	a																	
a	2664814	g	2874306	g	2944537	s	O157	rec46	a	O157	+					a	a																	
g	2664823	a	2874315	a	2944546	s	O157	rec46	g	O157	+					g	g																	
g	2664825	a	2874317	a	2944548	ns	O157	rec46	g	O157	+					g	g																	
g	2664826	a	2874318	a	2944549	ns	O157	rec46	g	O157	+					g	g																	
a	2664841	g	2874333	g	2944564	ns	O157	rec46	g	CB9615	+					g	g																	
g	2664842	t	2874334	t	2944565	ns	O157	rec46	g	O157	+					g	g																	
c	2664847	t	2874339	t	2944570	s	O157	rec46	c	O157	+					c	c																	
a	2664848	g	2874340	g	2944571	ns	O157	rec46	a	O157	+					a	a																	
g	2664850	a	2874342	a	2944573	ns	O157	rec46	g	O157	+					g	g																	
c	2664856	g	2874348	g	2944579	s	O157	rec46	c	O157	+					c	c																	
g	2664857	c	2874349	c	2944580	ns	O157	rec46	g	O157	+					g	g																	
t	2664858	g	2874350	g	2944581	ns	O157	rec46	t	O157	+					t	t																	
t	2664859	a	2874351	a	2944582	ns	O157	rec46	t	O157	+					t	t																	
t	2664862	c	2874354	c	2944585	s	O157	rec46	t	O157	+					t	t																	
g	2664868	a	2874360	a	2944591	s	O157	rec46	g	O157	+					g	g																	
g	2664869	a	2874361	a	2944592	ns	O157	rec46	g	O157	+					g	g																	
a	2664871	t	2874363	t	2944594	ns	O157	rec46	a	O157	+					a	a																	
a	2664872	g	2874364	g	2944595	ns	O157	rec46	a	O157	+					a	a																	

O55 and O157 genome site details												Outgroup Strain Details[i]																						
CB9615 base[b]	CB9615 Site[c]	Sakai base[b]	Sakai Site[c]	EDL933 base[b]	EDL933 Site[c]	type[d]	Event Lineage[ef]	Recombinant segment	Inferred ancestral base[g]	Outgroup Analysis[e]	Support level[h]	D1 Sd197	K-12	HS	ATCC 8739	UMN026	IAI39	SMS 3-5	E2348/69	536	ED1a	CFT073	S88	APEC O1	UTI89	E24377A	IAI1	SE11	F5 8401	F2a 2457T	F2a 301	SS Ss046	B4 Sb227	B18 BS512
a	2664875	t	2874367	t	2944598	ns	O157	rec46	a	O157	+					a	a																	
c	2664877	t	2874369	t	2944600	ns	O157	rec46	c	O157	+					c	c																	
g	2664880	a	2874372	a	2944603	s	O157	rec46	g	O157	+					g	g																	
a	2664901	g	2874393	g	2944624	s	O157	rec46	a	O157	+					a	a																	
g	2664904	t	2874396	t	2944627	s	O157	rec46	g	O157	+					g	g																	
t	2664908	g	2874400	g	2944631	ns	O157	rec46	t	O157	+					t	t																	
g	2664913	c	2874405	c	2944636	s	O157	rec46	g	O157	+					g	g																	
c	2664923	a	2874415	a	2944646	ns	O157	rec46	c	O157	+					c	c																	
c	2664924	a	2874416	a	2944647	ns	O157	rec46	c	O157	+					c	c																	
g	2664925	a	2874417	a	2944648	ns	O157	rec46	g	O157	+					g	g																	
t	2664926	g	2874418	g	2944649	ns	O157	rec46	t	O157	+					t	t																	
c	2664928	a	2874420	a	2944651	ns	O157	rec46	c	O157	+					c	c																	
c	2664929	a	2874421	a	2944652	ns	O157	rec46	c	O157	+					c	c																	
a	2664932	g	2874424	g	2944655	ns	O157	rec46	a	O157	+					a	a																	
g	2664937	t	2874429	t	2944660	s	O157	rec46	g	O157	+					g	g																	
a	2664940	t	2874432	t	2944663	ns	O157	rec46	a	O157	+					a	a																	
a	2664942	g	2874434	g	2944665	ns	O157	rec46	a	O157	+					a	a																	
a	2664955	g	2874447	g	2944678	s	O157	rec46	a	O157	+					a	a																	
c	2664958	t	2874450	t	2944681	s	O157	rec46	c	O157	+					c	c																	
t	2664960	g	2874452	g	2944683	ns	O157	rec46	?	O157	+/-					g	t																	
c	2664961	t	2874453	t	2944684	ns	O157	rec46	c	O157	+					c	c																	
a	2664967	g	2874459	g	2944690	s	O157	rec46	a	O157	+					a	a																	
a	2664973	t	2874465	t	2944696	ns	O157	rec46	a	O157	+					a	a																	
c	2665006	g	2874498	g	2944729	s	O157	rec46	c	O157	+					c	c																	
t	2665010	g	2874502	g	2944733	ns	O157	rec46	g	CB9615	+					a	g																	
a	2665051	g	2874543	g	2944774	nc	O157	rec46	?	O157	+/-					g	a																	
t	2665054	c	2874546	c	2944777	nc	O157	rec46	?	O157	+/-					c	t																	
t	2665081	c	2874573	c	2944804	nc	O157	rec46	c	CB9615	+					c	c																	
a	2665084	t	2874576	t	2944807	nc	O157	rec46	?	O157	+/-					t	a																	
c	2665118	a	2874610	a	2944841	nc	O157	rec46	a	CB9615	+					a	a																	
a	2665123	g	2874615	g	2944846	nc	O157	rec46	a	O157	+					a	a																	
t	2665135	a	2874627	a	2944858	nc	O157	rec46	a	CB9615	+					a	a																	
c	2665163	t	2874655	t	2944886	nc	O157	rec46	c	O157	+					c	c																	
a	2665192	t	2874684	t	2944915	nc	O157	rec46	?	O157	+/-					a	t																	
t	2665196	c	2874688	c	2944919	nc	O157	rec46	t	O157	+					t	t																	
a	2665207	t	2874699	t	2944930	nc	O157	rec46	a	O157	+					a	a																	
t	2665220	g	2874712	g	2944943	nc	O157	rec46	g	CB9615	+					g	g																	
c	2665236	a	2874728	a	2944959	nc	O157	rec46	?	O157	+/-					c	a																	
g	2665237	t	2874729	t	2944960	nc	O157	rec46	g	O157	+					g	g																	
c	2665243	t	2874735	t	2944966	nc	O157	rec46	c	O157	+					c	c																	
a	2665245	g	2874737	g	2944968	nc	O157	rec46	a	O157	+					a	a																	
g	2665249	a	2874741	a	2944972	nc	O157	rec46	g	O157	+					g	c																	
c	2665251	t	2874743	t	2944974	nc	O157	rec46	c	O157	+					c	c																	
c	2665276	a	2874768	a	2944999	nc	O157	rec46	c	O157	+					c	c	g																
c	2665291	g	2874783	g	2945014	nc	O157	rec46	g	CB9615	+					g	g	g																
c	2665294	t	2874786	t	2945017	nc	O157	rec46	t	CB9615	+					t	t	t																
a	2665295	c	2874787	c	2945018	nc	O157	rec46	a	O157	+					a	a	a																
g	2665297	c	2874789	c	2945020	nc	O157	rec46	c	CB9615	+					c	c	c																
a	2665300	c	2874792	c	2945023	nc	O157	rec46	c	CB9615	+					a	c	c																
a	2665312	t	2874804	t	2945035	nc	O157	rec46	t	CB9615	+					t	t	t																
g	2665330	a	2874822	a	2945053	nc	O157	rec46	a	CB9615	+					a	a	g																
c	2665345	t	2874837	t	2945068	nc	O157	rec46	t	CB9615	+					t	c	t																
g	2665351	t	2874843	t	2945074	nc	O157	rec46	g	O157	+					g	g	a																
c	2665358	t	2874850	t	2945081	nc	O157	rec46	t	CB9615	+					t	t	c																
a	2665370	g	2874862	g	2945093	nc	O157	rec46	g	CB9615	+					g	g	g																
c	2665385	t	2874877	t	2945108	nc	O157	rec46	t	CB9615	+					t	t	c																
c	2665396	t	2874888	t	2945119	nc	O157	rec46	t	CB9615	+					t	t	a																
g	2665411	t	2874903	t	2945134	nc	O157	rec46	t	CB9615	+					t	g	t																
t	2665414	g	2874906	g	2945137	nc	O157	rec46	?	O157	+/-					g	t	c																
c	2665432	g	2874924	g	2945155	nc	O157	rec46	?	O157	+/-					g	c	t																
t	2665438	g	2874930	g	2945161	nc	O157	rec46	?	O157	+/-					g	t	c																
a	2665440	c	2874932	c	2945163	nc	O157	rec46	?	O157	+/-					c	a	t																
g	2665453	a	2874945	a	2945176	nc	O157	rec46	?	O157	+/-					a	g	t																
c	2665459	t	2874951	t	2945182	nc	O157	rec46	t	CB9615	+					t	c	t																
a	2665460	g	2874952	g	2945183	nc	O157	rec46	g	CB9615	+					g	a	g																
g	2665461	a	2874953	a	2945184	nc	O157	rec46	?	O157	+/-					a	g	c																
c	2665465	t	2874957	t	2945188	nc	O157	rec46	c	O157	+					t	c	c																
t	2665468	c	2874960	c	2945191	nc	O157	rec46	c	CB9615	+					c	t	c																
c	2665474	t	2874966	t	2945197	nc	O157	rec46	t	CB9615	+					t	c	t																
a	2665475	g	2874967	g	2945198	nc	O157	rec46	a	O157	+					g	a	a																
t	2665483	a	2874975	a	2945206	nc	O157	rec46	a	CB9615	+					a	a	g																
t	2665487	c	2874979	c	2945210	nc	O157	rec46	c	CB9615	+					c	t	c																
t	2665501	c	2874993	c	2945224	nc	O157	rec46	t	O157	+					t	t	c																
a	2665506	g	2874998	g	2945229	nc	O157	rec46	a	O157	+					a	a	a																
g	2665510	a	2875002	a	2945233	nc	O157	rec46	a	CB9615	+					g	a	a																
g	2665520	a	2875012	a	2945243	nc	O157	rec46	g	O157	+					g	g	a																
t	2665521	a	2875013	a	2945244	nc	O157	rec46	?	O157	+/-					t	c	a																
t	2665522	a	2875014	a	2945245	nc	O157	rec46	?	O157	+/-					t	c	a																
a	2665541	c	2875033	c	2945264	nc	O157	rec46	a	O157	+					c	a	a																
c	2665546	g	2875038	g	2945269	nc	O157	rec46	?	O157	+/-					g	c	a																
g	2665548	a	2875040	a	2945271	nc	O157	rec46	a	CB9615	+					a	a	a																
t	2665555	c	2875047	c	2945278	nc	O157	rec46	c	CB9615	+					c	t	c																
g	2665558	a	2875050	a	2945281	nc	O157	rec46	?	O157	+/-					a	g	-																
c	2665561	t	2875053	t	2945284	nc	O157	rec46	t	CB9615	+					t	c	t																
c	2665564	t	2875056	t	2945287	nc	O157	rec46	c	O157	+					t	c	c																
t	2665570	c	2875062	c	2945293	nc	O157	rec46	t	O157	+					c	t	t																

O55 and O157 genome site details												Outgroup Strain Details[i]																						
CB9615 base[b]	CB9615 Site[c]	Sakai base[b]	Sakai Site[c]	EDL933 base[b]	EDL933 Site[c]	type[d]	Event Lineage[ef]	Recombinant segment	Inferred ancestral base[g]	Outgroup Analysis[e]	Support level[h]	D1 Sd197	K-12	HS	ATCC 8739	UMN026	IAI39	SMS 3-5	E2348/69	536	ED1a	CFT073	S88	APEC O1	UTI89	E24377A	IAI1	SE11	F5 8401	F2a 2457T	F2a 301	SS Ss046	B4 Sb227	B18 BS512
c	2665573	t	2875065	t	2945296	nc	O157	rec46	c	O157	+					t	c	c																
a	2665576	g	2875068	g	2945299	nc	O157	rec46	a	O157	+					g	a	a																
a	2665582	t	2875074	t	2945305	nc	O157	rec46	a	O157	+					t	a	a																
c	2665588	t	2875080	t	2945311	nc	O157	rec46	t	CB9615	+					t	t	a																
t	2665600	c	2875092	c	2945323	nc	O157	rec46	c	CB9615	+					c	c	t																
g	2665609	t	2875101	t	2945332	nc	O157	rec46	t	CB9615	+					t	t	a																
g	2665612	a	2875104	a	2945335	nc	O157	rec46	a	CB9615	+					a	a	g																
t	2665762	a	2875254	a	2945485	nc	O157	rec46	t	O157	+					t	t	t																
a	2665768	t	2875260	t	2945491	nc	O157	rec46	a	O157	+					a	a	c																
t	2665770	a	2875262	a	2945493	nc	O157	rec46	t	O157	+					t	t	a																
a	2665783	g	2875275	g	2945506	nc	O157	rec46	a	O157	+					c	c	a																
t	2665816	a	2875308	a	2945539	nc	O157	rec46	a	CB9615	+					g	a	g																
t	2665819	c	2875311	c	2945542	nc	O157	rec46	c	CB9615	+					c	c	t																
t	2665822	c	2875314	c	2945545	nc	O157	rec46	c	CB9615	+					c	c	c																
c	2665825	a	2875317	a	2945548	nc	O157	rec46	a	CB9615	+					t	a	t																
a	2665837	g	2875329	g	2945560	nc	O157	rec46	g	CB9615	+					g	g	g																
a	2665841	g	2875333	g	2945564	nc	O157	rec46	g	CB9615	+					g	g	g																
c	2665842	a	2875334	a	2945565	nc	O157	rec46	a	CB9615	+					a	a	c																
c	2665843	t	2875335	t	2945566	nc	O157	rec46	t	CB9615	+					t	t	a																
a	2666065	t	2875557	t	2945788	nc	O157	rec46	t	CB9615	+					a	t	t																
c	2666083	a	2875575	a	2945806	nc	O157	rec46	a	CB9615	+					c	a	a																
c	2666084	t	2875576	t	2945807	nc	O157	rec46	t	CB9615	+					c	t	t																
t	2666121	c	2875613	c	2945844	nc	O157	rec46	c	CB9615	+					t	c	c																
a	2666158	g	2875650	g	2945881	nc	O157	rec46	a	O157	+					a	t	c																
t	2666161	a	2875653	a	2945884	nc	O157	rec46	t	O157	+					t	t																	
t	2666167	c	2875659	c	2945890	nc	O157	rec46	t	O157	+					t	t																	
c	2666170	t	2875662	t	2945893	nc	O157	rec46	c	O157	+					c	c																	
c	2666185	t	2875677	t	2945908	nc	O157	rec46	?	O157	+/-					c	t																	
g	2666195	a	2875687	a	2945918	nc	O157	rec46	?	O157	+/-					g	a																	
t	2666196	c	2875688	c	2945919	nc	O157	rec46	?	O157	+/-					t	c																	
c	2666197	t	2875689	t	2945920	nc	O157	rec46	c	O157	+					c	c																	
t	2666200	c	2875692	c	2945923	nc	O157	rec46	t	O157	+					t	t																	
t	2666204	c	2875696	c	2945927	nc	O157	rec46	t	O157	+					t	t																	
t	2666207	a	2875699	a	2945930	nc	O157	rec46	?	O157	+/-					t	a																	
a	2666216	c	2875708	c	2945939	nc	O157	rec46	c	CB9615	+					g	c																	
c	2666218	a	2875710	a	2945941	nc	O157	rec46	?	O157	+/-					c	a																	
t	2666224	g	2875716	g	2945947	nc	O157	rec46	?	O157	+/-					g	t																	
c	2666227	t	2875719	t	2945950	nc	O157	rec46	?	O157	+/-					c	t																	
c	2666296	t	2875788	t	2946019	nc	O157	rec46	c	O157	+					c	c																	
t	2666301	c	2875793	c	2946024	nc	O157	rec46	c	CB9615	+					c	c																	
t	2666312	a	2875804	a	2946035	nc	O157	rec46	t	O157	+					t	t																	
t	2666314	a	2875806	a	2946037	nc	O157	rec46	t	O157	+					t	t																	
t	2666321	c	2875813	c	2946044	nc	O157	rec46	t	O157	+					t	t																	
t	2666326	c	2875818	c	2946049	nc	O157	rec46	c	CB9615	+					c	c																	
c	2666328	a	2875820	a	2946051	nc	O157	rec46	c	O157	+					c	c																	
a	2666344	g	2875836	g	2946067	nc	O157	rec46	a	O157	+					a	a																	
c	2666368	t	2875860	t	2946091	nc	O157	rec46	c	O157	+					c	c																	
a	2666381	g	2875873	g	2946104	nc	O157	rec46	a	O157	+					a	a																	
t	2666388	a	2875880	a	2946111	nc	O157	rec46	t	O157	+					t	t																	
t	2666391	a	2875883	a	2946114	nc	O157	rec46	t	O157	+					t	t																	
g	2666392	c	2875884	c	2946115	nc	O157	rec46	g	O157	+					g	a																	
t	2666395	g	2875887	g	2946118	nc	O157	rec46	t	O157	+					t	t																	
c	2666398	t	2875890	t	2946121	nc	O157	rec46	?	O157	+/-					c	t																	
g	2666417	t	2875909	t	2946140	nc	O157	rec46	?	O157	+/-					g	t																	
c	2666419	g	2875911	g	2946142	nc	O157	rec46	c	O157	+					c	a																	
t	2666422	a	2875914	a	2946145	nc	O157	rec46	?	O157	+/-					t	a																	
t	2666423	g	2875915	g	2946146	nc	O157	rec46	?	O157	+/-					t	g																	
t	2666434	c	2875926	c	2946157	nc	O157	rec46	c	CB9615	+					c	c																	
c	2666451	t	2875943	t	2946174	nc	O157	rec46	?	O157	+/-					c	t																	
g	2666462	a	2875954	a	2946185	nc	O157	rec46	g	O157	+					g	g																	
g	2666531	a	2876023	a	2946254	i	O157	rec46	g	O157	+					g	g																	
a	2666594	g	2876086	g	2946317	ns	O157	rec46	g	CB9615	+					g	g																	
a	2666603	c	2876095	c	2946326	ns	O157	rec46	c	CB9615	+					c	c																	
c	2666611	t	2876103	t	2946334	s	O157	rec46	?	O157	+/-					t	c																	
c	2666620	t	2876112	t	2946343	s	O157	rec46	?	O157	+/-					t	c																	
c	2666629	t	2876121	t	2946352	s	O157	rec46	c	O157	+					c	c																	
t	2666632	c	2876124	c	2946355	s	O157	rec46	c	CB9615	+					c	c																	
t	2666662	a	2876154	a	2946385	s	O157	rec46	?	O157	+/-					t	a																	
g	2666666	a	2876158	a	2946389	ns	O157	rec46	g	O157	+					g	g																	
a	2666677	g	2876169	g	2946400	s	O157	rec46	g	CB9615	+					g	g																	
g	2666686	a	2876178	a	2946409	s	O157	rec46	g	O157	+					g	g																	
a	2666688	c	2876180	c	2946411	ns	O157	rec46	a	O157	+					a	a																	
t	2666763	c	2876255	c	2946486	ns	O157	rec46	?	O157	+/-																							
a	2666811	g	2876303	g	2946534	ns	O157	rec46	?	O157	+/-																							
a	2666856	c	2876348	c	2946579	ns	O157	rec46	?	O157	+/-																							
a	2667646	g	2877138	g	2947369	s	O157	rec46	?	O157	+/-																							
g	2667674	a	2877166	a	2947397	ns	O157	rec46	?	O157	+/-																							
t	2667677	c	2877169	c	2947400	ns	O157	rec46	?	O157	+/-																							
c	2667820	a	2877265	a	2947496	s	O157	rec46	?	O157	+/-																							
c	2667828	t	2877273	t	2947504	ns	O157	rec46	?	O157	+/-																							
g	2667877	a	2877322	a	2947553	s	O157	rec46	?	O157	+/-																							
g	2667913	t	2877358	t	2947589	ns	O157	rec46	?	O157	+/-																							
a	2668150	g	2877595	g	2947826	s	O157	rec46	g	CB9615	++++		g	g	g	g	g	g	g	g	g	g	g	g	g	g	g	g	g	g	g		g	
a	2668185	g	2877630	g	2947861	ns	O157	rec46	g	CB9615	++	g	g	g	g	g	g	g	g	g	g	g	g	g	g	g	g	g	a	a	a		g	
c	2668189	a	2877634	a	2947865	s	O157	rec46	a	CB9615	++	a	c	c	c	c	c	c	a	a	a	a	a	a	a	c	c	c	c	c	c		c	
t	2668194	a	2877639	a	2947870	ns	O157	rec46	a	CB9615	++	a	t	t	t	t	t	t	t	t	t	t	t	t	t	t	t	t	t	t	t		t	

O55 and O157 genome site details												Outgroup Strain Details[i]																						
CB9615 base[b]	CB9615 Site[c]	Sakai base[b]	Sakai Site[c]	EDL933 base[b]	EDL933 Site[c]	type[d]	Event Lineage[ef]	Recombinant segment	Inferred ancestral base[g]	Outgroup Analysis[e]	Support level[h]	D1 Sd197	K-12	HS	ATCC 8739	UMN026	IAI39	SMS 3-5	E2348/69	536	ED1a	CFT073	S88	APEC O1	UTI89	E24377A	IAI1	SE11	F5 8401	F2a 2457T	F2a 301	SS Ss046	B4 Sb227	B18 BS512
c	2668195	g	2877640	g	2947871	ns	O157	rec46	g	CB9615	++	g	c	c	c	a	c	c	c	c	c	c	c	c	c	c	c	c	c	c	c		c	
g	2668234	c	2877679	c	2947910	s	O157	rec46	c	CB9615	++	c	t	t	t	a	g	g	a	a	a	a	a	a	a	t	t	t	t	t	t		t	
a	2668325	g	2877770	g	2948001	ns	O157	rec46	g	CB9615	++++	g	g	g	g	g	g	g	g	g	g	g	g	g	g	g	g	g	g	g	g		g	
t	2668330	c	2877775	c	2948006	s	O157	rec46	c	CB9615	++++	c	c	c	c	c	c	c	c	c	c	c	c	c	c	c	c	c	c	c	c		c	
a	2668336	g	2877781	g	2948012	s	O157	rec46	g	CB9615	++++	g	g	g	g	g	g	g	g	g	g	g	g	g	g	g	g	g	g	g	g		g	
a	2668345	g	2877790	g	2948021	s	O157	rec46	g	CB9615	++++	g	g	g	g	a	g	g	g	g	g	g	g	g	g	g	g	g	g	g	g		g	
c	2668348	t	2877793	t	2948024	s	O157	rec46	t	CB9615	++	t	t	t	t	c	c	c	t	t	t	t	t	t	t	t	t	t	t	t	t		t	
t	2668369	c	2877814	c	2948045	s	O157	rec46	c	CB9615	++	c	c	c	c	t	t	t	a	c	c	c	c	c	c	c	c	c	c	c	c		c	
c	2668375	t	2877820	t	2948051	s	O157	rec46	t	CB9615	++	t	c	c	c	c	t	t	c	c	c	c	c	c	c	c	c	c	c	c	c		c	
c	2668384	t	2877829	t	2948060	s	O157	rec46	t	CB9615	++++	t	t	t	t	t	t	t	t	t	t	t	t	t	t	t	t	t	t	t	t		t	
t	2668393	c	2877838	c	2948069	s	O157	rec46	c	CB9615	++++	c	c	c	c	c	c	c	c	c	c	c	c	c	c	c	c	c	c	c	c		t	
c	2668429	t	2877874	t	2948105	s	O157	rec46	t	CB9615	++	t	t	t	t	c	c	c	c	t	t	t	t	t	t	c	t	t	t	t	t		t	
t	2668435	c	2877880	c	2948111	s	O157	rec46	c	CB9615	++	c	c	c	c	c	t	t	t	c	c	c	c	c	c	t	c	c	c	c	c		c	
t	2668495	c	2877940	c	2948171	s	O157	rec46	c	CB9615	++++	c	c	c	c	c	c	c	c	c	c	c	c	c	c	c	c	c	c	c	c		c	
c	2668513	t	2877958	t	2948189	s	O157	rec46	t	CB9615	++	t	c	c	c	c	c	c	c	c	c	c	c	c	c	c	c	c	c	c	c		c	
t	2668519	c	2877964	c	2948195	s	O157	rec46	c	CB9615	++	c	c	c	c	c	c	c	c	c	c	c	c	c	c	c	c	c	t	t	t		c	
t	2668540	a	2877985	a	2948216	s	O157	rec46	a	CB9615	++++	a	a	a	a	c	a	c	a	a	a	a	a	a	a	a	a	a	a	a	a		a	
g	2668555	a	2878000	a	2948231	s	O157	rec46	a	CB9615	++	a	a	a	a	g	a	g	g	a	a	a	a	a	a	g	a	a	a	a	a		a	
g	2668561	a	2878006	a	2948237	ns	O157	rec46	a	CB9615	++	a	a	a	a	g	a	a	g	a	a	a	a	a	a	g	a	a	a	a	a		a	
a	2668572	g	2878017	g	2948248	ns	O157	rec46	a	O157	++++	a	a	a	a	a	a	a	a	a	a	a	a	a	a	a	a	a	a	a	a		a	
t	2668579	c	2878024	c	2948255	s	O157	rec46	c	CB9615	++++	c	c	c	c	c	c	c	c	c	c	c	c	c	c	c	c	c	c	c	c		c	
t	2668585	c	2878030	c	2948261	s	O157	rec46	c	CB9615	++	c	c	c	c	c	c	t	t	c	c	c	c	c	c	t	c	c	c	c	c		c	
g	2668594	c	2878039	c	2948270	s	O157	rec46	c	CB9615	++	c	c	c	c	c	c	c	g	c	c	c	c	c	c	g	c	c	c	c	c		c	
a	2668597	g	2878042	g	2948273	s	O157	rec46	g	CB9615	++	g	g	g	g	g	a	a	g	g	g	g	g	g	g	a	g	g	g	g	g		g	
a	2668602	c	2878047	c	2948278	ns	O157	rec46	c	CB9615	++++	c	c	c	c	c	c	c	c	c	c	c	c	c	c	c	c	c	c	c	c		c	
g	2668603	a	2878048	a	2948279	ns	O157	rec46	a	CB9615	++	a	g	g	g	g	a	a	g	g	g	g	g	g	g	g	g	g	g	g	g		g	
a	2668634	g	2878079	g	2948310	ns	O157	rec46	g	CB9615	++++	g	g	g	g	g	g	g	g	g	g	g	g	g	g	g	g	g	g	g	g		g	
c	2668673	t	2878118	t	2948349	ns	O157	rec46	t	CB9615	++	t	c	c	c	c	c	c	c	c	c	c	c	c	c	c	c	c	c	c	c		c	
t	2668713	c	2878158	c	2948389	ns	O157	rec46	c	CB9615	++++	c	c	c	c	c	c	c	c	c	c	c	c	c	c	c	c	c	c	c	c		c	
c	2668726	t	2878171	t	2948402	s	O157	rec46	t	CB9615	++	t	t	t	t	t	c	t	t	c	c	c	c	c	c	c	t	t	t	t	t		c	
c	2668728	t	2878173	t	2948404	ns	O157	rec46	t	CB9615	++	t	t	t	t	c	c	c	t	c	c	c	c	c	c	c	t	t	t	t	t		c	
c	2668732	t	2878177	t	2948408	s	O157	rec46	t	CB9615	++	t	c	c	c	c	c	c	c	c	c	c	c	c	c	c	c	c	c	c	c		c	
a	2668816	g	2878261	g	2948492	s	O157	rec46	g	CB9615	++++	g	g	g	g	g	g	g	g	g	g	g	g	g	g	g	g	g	g	g	g		g	
g	2669120	a	2878565	a	2948796	ns	O157	rec46	g	O157	++++	g	g	g	g	g	g	g	g	g	g	g	g	g	g	g	g	g	g	g	g		g	
a	2669534	c	2878979	c	2949210	ns	O157	rec46	c	CB9615	++++	c	c	c	c	c	c	c	c	c	c	c	c	c	c	c	c	c	c	c	c		c	
g	2670199	a	2879644	a	2949875	s	O157	rec46	g	O157	++	g	a	a	a	a	a	a	a	a	a	a	a	a	a	a	a	a	a	a	a		a	
a	2670365	g	2879810	g	2950041	ns	O157	rec46	a	O157	++	a	g	g	g	g	g	g	g	g	g	g	g	g	g	g	g	g	g	g	g		g	
a	2670372	g	2879817	g	2950048	ns	O157	rec46	a	O157	++++	a	a	a	a	a	g	a	a	a	a	a	a	a	a	a	a	a	a	a	a		a	
g	2670395	t	2879840	t	2950071	ns	O157	rec46	g	O157	++++	g	g	g	g	g	g	g	g	g	g	g	g	g	g	g	g	g	g	g	g		g	
a	2670407	g	2879852	g	2950083	ns	O157	rec46	g	CB9615	++++	g	g	g	g	g	g	g	g	g	g	g	g	g	g	g	g	g	g	g	g		g	
a	2670425	g	2879870	g	2950101	ns	O157	rec46	a	O157	++	a	a	a	a	g	a	g	g	g	g	g	g	g	g	g	g	g	g	g	g		a	
c	2670436	g	2879881	g	2950112	s	O157	rec46	c	O157	++	c	c	c	c	g	g	c	c	c	c	c	c	c	c	c	c	c	c	c	c		c	
g	2670439	a	2879884	a	2950115	s	O157	rec46	g	O157	++	g	g	g	g	a	a	g	g	g	g	g	g	g	g	g	g	g	g	g	g		g	
c	2670448	t	2879893	t	2950124	s	O157	rec46	c	O157	++++	c	c	c	c	c	c	c	a	a	c	a	a	a	a	c	c	c	c	c	c		c	
t	2670473	c	2879918	c	2950149	s	O157	rec46	t	O157	++	t	c	c	c	c	c	c	c	c	c	c	c	c	c	c	c	c	c	c	c		c	
g	2670496	a	2879941	a	2950172	s	O157	rec46	g	O157	++	g	a	a	a	g	g	g	a	a	a	a	a	a	a	a	a	a	a	a	a		a	
a	2670504	g	2879949	g	2950180	ns	O157	rec46	a	O157	++	a	g	g	g	g	g	g	g	g	g	g	g	g	g	g	g	g	g	g	g		g	
a	2670514	g	2879959	g	2950190	s	O157	rec46	a	O157	++	a	a	a	a	g	g	g	a	a	g	a	a	a	a	g	g	g	g	g	g		a	
c	2670515	t	2879960	t	2950191	s	O157	rec46	c	O157	++	c	c	c	c	t	t	t	c	c	t	c	c	c	c	t	t	t	t	t	t		c	
g	2670517	a	2879962	a	2950193	s	O157	rec46	g	O157	++	g	g	g	g	a	a	a	g	g	a	g	g	g	g	a	a	a	a	a	a		g	
g	2670520	t	2879965	t	2950196	s	O157	rec46	g	O157	++	g	g	g	g	t	t	t	g	g	t	g	g	g	g	t	t	t	t	t	t		g	
c	2670580	t	2880025	t	2950256	s	O157	rec46	c	O157	++	c	t	t	t	t	t	t	t	t	t	t	t	t	t	t	t	t	t	t	t		t	
g	2670592	c	2880037	c	2950268	s	O157	rec46	g	O157	++	g	c	c	c	c	c	c	c	c	c	c	c	c	c	c	c	c	c	c	c		c	
c	2670673	a	2880118	a	2950349	s	O157	rec46	c	O157	++	c	a	a	a	a	a	a	a	a	a	a	a	a	a	a	a	a	a	a	a		a	
a	2670685	t	2880130	t	2950361	s	O157	rec46	a	O157	++	a	a	a	a	t	t	t	a	a	a	a	a	a	a	a	a	a	t	t	t		a	
c	2670688	a	2880133	a	2950364	s	O157	rec46	c	O157	++	c	c	c	c	a	a	a	t	t	t	t	t	t	t	c	c	c	a	a	a		c	
t	2670691	a	2880136	a	2950367	ns	O157	rec46	t	O157	++++	t	t	t	t	t	t	t	t	t	t	t	t	t	t	t	t	t	t	t	t		t	
a	2670747	c	2880192	c	2950423	ns	O157	rec46	a	O157	++	a	c	c	c	a	a	a	a	a	a	a	a	a	a	c	a	a	c	c	c		a	
g	2670751	a	2880196	a	2950427	s	O157	rec46	g	O157	++	g	a	a	a	g	g	g	g	g	g	g	g	g	g	a	g	g	a	a	a		g	
c	2670758	t	2880203	t	2950434	ns	O157	rec46	c	O157	++++	c	c	c	c	c	c	c	c	c	c	c	c	c	c	c	c	c	c	c	c		c	
a	2670759	c	2880204	c	2950435	ns	O157	rec46	a	O157	++	a	c	c	c	c	c	c	c	c	c	c	c	c	c	c	c	c	c	c	c		a	
c	2670779	a	2880224	a	2950455	s	O157	rec46	c	O157	++	c	c	c	c	c	c	c	c	c	c	c	c	c	c	a	a	a	c	c	c		a	
a	2670787	g	2880232	g	2950463	s	O157	rec46	a	O157	++	a	g	g	g	g	g	g	g	g	g	g	g	g	g	g	g	g	g	g	g		g	
t	2670793	g	2880238	g	2950469	ns	O157	rec46	t	O157	++	t	g	g	g	t	t	t	t	t	t	t	t	t	t	g	t	t	t	t	t		t	
t	2670804	c	2880249	c	2950480	ns	O157	rec46	c	CB9615	++++	c	c	c	c	c	c	c	c	c	c	c	c	c	c	c	c	c	c	c	c		c	
c	2670810	t	2880255	t	2950486	ns	O157	rec46	c	O157	++++	c	c	c	c	c	c	c	c	c	c	c	c	c	c	c	c	c	c	c	c		c	
c	2670814	a	2880259	a	2950490	s	O157	rec46	c	O157	++	c	c	c	c	c	c	c	a	a	a	a	a	a	a	c	a	a	c	c	c		c	
c	2670921	g	2880366	g	2950597	ns	O157	rec46	c	O157	++++	c	c	c	c	c	c	c	c	c	c	c	c	c	c	c	c	c					c	
c	2670934	t	2880379	t	2950610	s	O157	rec46	c	O157	++	c	t	t	t	g	g	g	t	g	t	g	g	g	g	g	t	t					t	
a	2670963	g	2880408	g	2950639	ns	O157	rec46	a	O157	++	a	a	a	a	g	g	g	g	g	g	g	g	g	g	g	a	a					a	
t	2670977	g	2880422	g	2950653	ns	O157	rec46	t	O157	++	t	g	g	g	g	g	g	g	g	g	g	g	g	g	g	g	g					g	
a	2671042	g	2880487	g	2950718	s	O157	rec46	g	CB9615	++	g	a	a	a	a	a	a	t	t	t	t	t	t	t	a	a	a					a	
c	2671091	t	2880536	t	2950767	s	O157	rec46	c	O157	++	c	c	c	c	t	t	t	c	c	c	c	c	c	c	c	c	c					c	
c	2671108	t	2880553	t	2950784	s	O157	rec46	c	O157	++++	c	c	c	c	c	c	c	c	c	c	c	c	c	c	c	c	c					c	
c	2671354	t	2880799	t	2951030	s	O157	rec46	c	O157	++	c	c	c	c	t	t	t	t	t	t	t	t	t	t	c	c	c					c	
a	2671381	g	2880826	g	2951057	s	O157	rec46	a	O157	++	a	g	g	g	g	g	g	g	g	g	g	g	g	g	g	g	g					g	
t	2671396	a	2880841	a	2951072	s	O157	rec46	t	O157	++++	t	t	t	t	t	t	t	t	t	t	t	t	t	t	t	t	t					t	
g	2671423	a	2880868	a	2951099	s	O157	rec46	g	O157	++	g	a	a	a	a	a	a	g	g	a	g	g	g	g	a	a	a					a	
a	2671445	c	2880890	c	2951121	ns	O157	rec46	c	CB9615	++++	c	c	c	c	c	c	c	t	t	c	t	t	t	t	c	c	c					c	
t	2671498	c	2880943	c	2951174	s	O157	rec46	c	CB9615	++	c	t	t	t	c	c	c	c	c	c	c	c	c	c	t	t	t		c	c		t	
t	2671551	c	2880996	c	2951227	ns	O157	rec46	c	CB9615	++++	g	c	c	c	c	c	c	c	c	c	c	c	c	c	c	c	c		g	g		c	
t	2671627	c	2881072	c	2951303	s	O157	rec46	t	O157	++	t	t	t	t	c	c	c	t	t	c	t	t	t	t	t	t	t		c	c		t	
c	2671634	t	2881079	t	2951310	ns	O157	rec46	c	O157	++	c	c	c	c	t	t	t	c	c	t	c	c	c	c	c	c	c		t	t		c	
c	2671636	t	2881081	t	2951312	ns	O157	rec46	c	O157	++	c	c	c	c	c	c	c	c	c	c	c	c	c	c	c	c	c		t	t		c	
t	2671645	a	2881090	a	2951321	ns	O157	rec46	t	O157	++	t	t	t	t	t	t	t	t	t	t	t	t	t	t	t	t	t		a	a		t	
c	2671654	t	2881099	t	2951330	s	O157	rec46	c	O157	++	c	c	c	c	t	t	t	t	t	t	t	t	t	t	c	c	c		t	t		c	

O55 and O157 genome site details												Outgroup Strain Details[i]																						
CB9615 base[b]	CB9615 Site[c]	Sakai base[b]	Sakai Site[c]	EDL933 base[b]	EDL933 Site[c]	type[d]	Event Lineage[ef]	Recombinant segment	Inferred ancestral base[g]	Outgroup Analysis[e]	Support level[h]	D1 Sd197	K-12	HS	ATCC 8739	UMN026	IAI39	SMS 3-5	E2348/69	536	ED1a	CFT073	S88	APEC O1	UTI89	E24377A	IAI1	SE11	F5 8401	F2a 2457T	F2a 301	SS Ss046	B4 Sb227	B18 BS512
g	2671678	t	2881123	t	2951354	s	O157	rec46	g	O157	++	g	g	g	g	t	t	t	g	g	t	g	g	g	g	g	g	g		t	t		g	
g	2671687	t	2881132	t	2951363	ns	O157	rec46	g	O157	++	g	g	g	g	g	g	g	a	a	g	a	a	a	a	g	g	g		t	t		g	
a	2671690	g	2881135	g	2951366	s	O157	rec46	a	O157	++	a	a	a	a	g	a	a	g	g	a	g	g	g	g	a	a	a		g	g		a	
g	2671699	a	2881144	a	2951375	s	O157	rec46	g	O157	++	g	a	a	a	g	g	a	a	a	g	a	a	a	a	a	a	a		a	a		a	
g	2671705	a	2881150	a	2951381	s	O157	rec46	g	O157	++	g	a	a	a	g	g	g	g	g	g	g	g	g	g	a	a	a		a	a		a	
c	2671711	t	2881156	t	2951387	s	O157	rec46	c	O157	++	c	c	c	c	c	c	c	c	c	c	c	c	c	c	c	c	c		t	t		c	
g	2671714	a	2881159	a	2951390	s	O157	rec46	g	O157	++	g	g	g	g	g	g	g	g	g	g	g	g	g	g	g	g	g		a	a		g	
a	2671717	c	2881162	c	2951393	s	O157	rec46	a	O157	++	a	a	a	a	a	a	a	a	a	a	a	a	a	a	a	a	a		c	c		a	
c	2671720	t	2881165	t	2951396	s	O157	rec46	c	O157	++	c	c	c	c	c	c	c	t	c	c	c	c	c	c	c	c	c		t	t		c	
c	2671723	t	2881168	t	2951399	s	O157	rec46	c	O157	++	c	c	c	c	c	c	c	c	c	c	c	c	c	c	c	c	c		t	t		c	
a	2671729	g	2881174	g	2951405	s	O157	rec46	a	O157	++	a	g	g	g	g	g	g	g	g	g	g	g	g	g	g	g	g		g	g		g	
c	2671732	t	2881177	t	2951408	s	O157	rec46	c	O157	++	c	t	t	t	t	t	t	t	t	t	t	t	t	t	t	t	t		t	t		t	
g	2671733	a	2881178	a	2951409	ns	O157	rec46	g	O157	++	g	a	a	a	a	a	a	a	a	a	a	a	a	a	a	a	a		a	a		a	
t	2671735	a	2881180	a	2951411	ns	O157	rec46	t	O157	++	t	a	a	a	a	a	a	a	a	a	a	a	a	a	a	a	a		a	a		a	
g	2671759	t	2881204	t	2951435	ns	O157	rec46	g	O157	++	g	g	g	g	t	a	a	a	a	t	a	a	a	a	g	g	g		a	a		g	
g	2671762	a	2881207	a	2951438	s	O157	rec46	g	O157	++	g	g	g	g	a	a	a	a	a	a	a	a	a	a	g	g	g		a	a		g	
t	2671765	g	2881210	g	2951441	s	O157	rec46	t	O157	++++	t	t	t	t	t	t	t	t	t	t	t	t	t	t	t	t	t		t	t		t	
a	2671777	c	2881222	c	2951453	s	O157	rec46	a	O157	++++	a	a	a	a	a	a	a	a	a	a	a	a	a	a	a	a	a		a	a		a	
c	2671783	a	2881228	a	2951459	ns	O157	rec46	c	O157	++	c	c	c	c	g	g	c	a	a	g	a	a	a	a	c	c	c		g	g		c	
g	2671829	c	2881274	c	2951505	ns	O157	rec46	g	O157	++	g	c	g	g	g	g	a	g	g	c	g	g	g	g	g	g	g		c	c		g	
c	2671830	a	2881275	a	2951506	ns	O157	rec46	c	O157	++	c	a	c	c	c	c	c	t	c	a	c	c	c	c	c	c	c		a	a		c	
c	2671838	t	2881283	t	2951514	ns	O157	rec46	t	CB9615	++++	t	t	t	t	t	t	t	t	t	t	t	t	t	t	t	t	t		t	t		t	
a	2671849	c	2881294	c	2951525	s	O157	rec46	a	O157	++	a	c	a	a	c	c	a	c	c	c	c	c	c	c	a	a	a		c	c		a	
g	2671867	a	2881312	a	2951543	s	O157	rec46	g	O157	++	g	a	g	g	g	a	g	g	g	a	g	g	g	g	g	g	g		a	a		g	
c	2671984	a	2881429	a	2951660	s	O157	rec46	c	O157	++	c	a	a	a	a	c	a	a	a	a	a	a	a	a	a	a	a		a	a		c	
t	2672089	a	2881534	a	2951765	s	O157	rec46	t	O157	++	t	a	a	a	a	a	t	t	t	a	t	a	a	a	a	a	a		a	a		a	
g	2672098	a	2881543	a	2951774	s	O157	rec46	g	O157	++	g	g	g	g	g	g	g	g	g	g	g	g	g	g	g	g	g		a	a		g	
g	2672101	a	2881546	a	2951777	s	O157	rec46	g	O157	++++	g	g	g	g	g	g	a	g	g	g	g	g	g	g	g	g	g		g	g		g	
3	2672124	-	2881568	-	2951799	del-3	O157	rec46	3	O157	++++	3	3	3	3	3	3	3	3	3	3	3	3	3	3	3	3	3		3	3		3	
t	2672151	g	2881593	g	2951824	ns	O157	rec46	g	CB9615	++++	g	g	g	g	g	g	g	g	g	g	g	g	g	g	g	g	g		g	g		g	
t	2672152	c	2881594	c	2951825	ns	O157	rec46	c	CB9615	++	c	c	c	c	t	t	t	t	t	t	t	t	t	t	c	c	c		t	t		t	
t	2672155	c	2881597	c	2951828	s	O157	rec46	c	CB9615	++	c	c	c	c	t	t	c	c	c	t	c	t	t	t	c	c	c		c	c		t	
t	2672161	c	2881603	c	2951834	s	O157	rec46	c	CB9615	++	c	c	c	c	t	t	c	c	c	t	c	t	t	t	c	c	c		c	c		t	
a	2672164	c	2881606	c	2951837	s	O157	rec46	c	CB9615	++	c	c	c	c	a	a	a	c	c	a	c	a	a	a	c	c	c		c	c		a	
t	2672171	c	2881613	c	2951844	s	O157	rec46	c	CB9615	++++	c	c	c	c	c	c	c	c	c	c	c	a	a	a	c	c	c		c	c		c	
g	2672210	a	2881652	a	2951883	ns	O157	rec46	a	CB9615	++++		a	a	a	a	a	a	a	a	a	a	a	a	a	a	a	a		a	a		g	
a	2672215	g	2881657	g	2951888	s	O157	rec46	g	CB9615	++++		g	g	g	g	g	g	c	c	g	c	g	g	g	g	g	g		g	g		a	
g	2672224	t	2881666	t	2951897	s	O157	rec46	g	O157	+		t	t	t	g	g	g	g	g	g	g	g	g	g	t	t	t		g	g		g	
t	2672231	c	2881673	c	2951904	ns	O157	rec46	c	CB9615	++++		c	c	c	c	c	c	c	c	c	c	c	c	c	c	c	c		c	c		c	
c	2672232	g	2881674	g	2951905	ns	O157	rec46	g	CB9615	++++		g	g	g	g	g	g	g	g	g	g	g	g	g	g	g	g		g	g		g	
t	2672233	c	2881675	c	2951906	ns	O157	rec46	c	CB9615	++++		c	c	c	c	c	c	c	c	c	c	c	c	c	c	c	c		c	c		c	
t	2672234	c	2881676	c	2951907	ns	O157	rec46	c	CB9615	+		c	c	c	c	c	c	t	t	c	t	c	c	c	c	c	c		c	c		c	
a	2672236	c	2881678	c	2951909	ns	O157	rec46	c	CB9615	++++		c	c	c	c	c	c	c	c	c	c	c	c	c	c	c	c		c	c		c	
a	2672245	t	2881687	t	2951918	ns	O157	rec46	t	CB9615	++++		t	t	t	t	t	t	t	t	t	t	t	t	t	t	t	t		t	t		a	
a	2672251	g	2881693	g	2951924	s	O157	rec46	g	CB9615	++++		g	g	g	g	g	g	g	g	g	g	g	g	g	g	g	g		g	g		a	
g	2672269	a	2881711	a	2951942	s	O157	rec46	g	O157	+		g	g	g	g	g	g	g	g	g	g	g	g	g	g	g	g		a	a		g	
a	2672331	g	2881773	g	2952004	ns	O157	rec46	g	CB9615	++++		g	g	g	g	g	g	g	g	g	g	g	g	g	g	g	g		g	g		g	
a	2672353	g	2881795	g	2952026	s	O157	rec46	g	CB9615	++++		g	g	g	g	g	g	g	g	g	g	g	g	g	g	g	g		g	g		g	
t	2672377	g	2881819	g	2952050	ns	O157	rec46	g	CB9615	++++		g	g	g	a	a	a	a	a	a	a	a	a	a	g	g	g		g	g		g	
t	2672404	c	2881846	c	2952077	s	O157	rec46	c	CB9615	+		c	c	c	t	t	t	c	c	t	c	c	c	c	c	c	c		c	c		c	
c	2672630	t	2882072	t	2952303	ns	O157	rec46	t	CB9615	++++		t	t	t	t	t	t	t	t	t	t	t	t	t	t	t	t		t	t		t	
c	2672638	t	2882080	t	2952311	s	O157	rec46	t	CB9615	+		t	t	t	c	c	c	c	c	c	c	t	t	t	c	c	c		t	t		t	
a	2672727	g	2882169	g	2952400	ns	O157	rec46	a	O157	++++		a	a	a	a	a	a	a	a	a	a	a	a	a	a	a	a		a	a		a	
g	2672743	a	2882185	a	2952416	s	O157	rec46	a	CB9615	+		a	a	a	a	a	a	a	a	a	a	a	a	a	g	g	g		a	a		a	
c	2672752	a	2882194	a	2952425	s	O157	rec46	a	CB9615	+		a	a	a	a	c	c	c	c	a	c	a	a	a	a	a	a		a	a		a	
t	2672761	g	2882203	g	2952434	s	O157	rec46	g	CB9615	++++		g	g	g	g	g	g	g	g	g	g	g	g	g	g	g	g		g	g		g	
t	2672770	c	2882212	c	2952443	s	O157	rec46	t	O157	+		c	c	c	t	t	t	t	t	t	t	t	t	t	t	t	t		c	c		c	
g	2672804	a	2882246	a	2952477	ns	O157	rec46	a	CB9615	+		a	g	g	a	a	a	g	g	g	g	a	a	a	a	a	a		a	a		a	
t	2672876	g	2882318	g	2952549	ns	O157	rec46	t	O157	+		t	t	t	g	a	a	g	g	g	g	a	a	a	a	a	a		t	t		t	
c	2672905	t	2882347	t	2952578	s	O157	rec46	c	O157	++++		c	c	c	c	c	c	c	c	c	c	c	c	c	c	c	c		c	c		c	
t	2672952	c	2882394	c	2952625	ns	O157	rec46	c	CB9615	++++		c	c	c	c	c	c	c	c	c	c	c	c	c	c	c	c		c	c		c	
t	2672954	g	2882396	g	2952627	ns	O157	rec46	g	CB9615	++++		g	g	g	g	g	g	g	g	g	g	g	g	g	g	g	g		g	g		g	
c	2672998	t	2882440	t	2952671	s	O157	rec46	c	O157	+		c	c	c	c	c	c	c	c	c	c	c	c	c	c	c	c		t	t		t	
c	2673092	t	2882534	t	2952765	s	O157	rec46	c	O157	+		c	t	t	c	c	c	c	c	c	c	c	c	c	c	c	c		c	c		c	
c	2673138	t	2882580	t	2952811	ns	O157	rec46	t	CB9615	++++		t	t	t	t	t	t	t	t	t	t	t	t	t	t	t	t		t	t		t	
t	2673140	a	2882582	a	2952813	ns	O157	rec46	a	CB9615	++++		a	a	a	a	a	a	a	a	a	a	a	a	a	a	a	a		a	a		a	
g	2673164	a	2882606	a	2952837	ns	O157	rec46	g	O157	++++		g	g	g	g	g	g	g	g	g	g	g	g	g	g	g	g		g	g		g	
-	2673206	a	2882649	a	2952880	ins	O157	rec46	-	O157	++++		-	-	-	-	-	-	-	-	-	-	-	-	-	-	-	-		-	-		a	
t	2673242	c	2882685	c	2952916	i	O157	rec46	c	CB9615	+		c	c	c	c	c	t	c	c	t	c	c	c	c	c	c	c		c	c		c	
a	2673411	g	2882854	g	2953085	ns	O157	rec46	g	CB9615	+		g	g	a	a	g	a	a	a	a	a	a	a	a	g	g	g		g	g		g	
c	2673413	t	2882856	t	2953087	ns	O157	rec46	c	O157	++++		c	c	c	c	c	c	c	c	c	c	c	c	c	c	c	c		c	c		c	
c	2673603	a	2883046	a	2953277	i	O157	rec46	c	O157	++++		c	c	c		c		c	c		c	c	c	c					c	c			
a	2673628	g	2883071	g	2953302	i	O157	rec46	g	CB9615	++++						g		g	g		g	g	g	g					g	g			
t	2673779	a	2883831	a	2954062	i	O157	rec46	t	O157	++++					t	t	t	t	t	t	t	t	t	t	t	t	t		t	t		t	
-	2673908	c	2883961	c	2954192	ins	O157	rec46	-	O157	++++					c	c	c	c	c	c	c	c	c	c	c	c	c		c	c		c	
t	2674098	g	2884151	g	2954382	i	O157	rec46	g	CB9615	+		g	g	g	g	g	g	g	g	g	g	g	g	g	g	g	g		t	t		g	
-	2684559	c	2897240	y	2967471	ns	EDL933	rec47	c	EDL933	+								c															
-	2684559	c	2897242	y	2967473	ns	EDL933	rec47	c	EDL933	+								c															
-	2684559	g	2897243	s	2967474	ns	EDL933	rec47	g	EDL933	+								g															
-	2684559	a	2897513	g	2967744	ns	EDL933	rec47	?	Sakai/EDL933	+/-								a														g	
-	2684559	t	2897558	Y	2967789	ns	EDL933	rec47	t	EDL933	+								t														t	t
-	2684559	-	2897584	n	2967816	ins	EDL933	rec47	-	EDL933	+								-														-	-
-	2684559	t	2897585	w	2967817	s	EDL933	rec47	t	EDL933	+								t														t	t
-	2684559	t	2897677	a	2967909	s	EDL933	rec47	t	EDL933	+								t														t	t
-	2684559	t	2897719	a	2967951	s	EDL933	rec47	t	EDL933	+								t														t	t

O55 and O157 genome site details												Outgroup Strain Details[i]																						
CB9615 base[b]	CB9615 Site[c]	Sakai base[b]	Sakai Site[c]	EDL933 base[b]	EDL933 Site[c]	type[d]	Event Lineage[ef]	Recombinant segment	Inferred ancestral base[g]	Outgroup Analysis[e]	Support level[h]	D1 Sd197	K-12	HS	ATCC 8739	UMN026	IAI39	SMS 3-5	E2348/69	536	ED1a	CFT073	S88	APEC O1	UTI89	E24377A	IAI1	SE11	F5 8401	F2a 2457T	F2a 301	SS Ss046	B4 Sb227	B18 BS512
-	2684559	t	2897723	n	2967955	ns	EDL933	rec47	t	EDL933	+								t														t	t
-	2684559	t	2897724	n	2967956	ns	EDL933	rec47	t	EDL933	+								t														t	t
-	2684559	t	2897731	a	2967963	s	EDL933	rec47	t	EDL933	+								t														t	t
-	2684559	t	2897752	Y	2967984	ns	EDL933	rec47	t	EDL933	+								t														t	t
-	2684559	a	2897885	g	2968117	ns	EDL933	rec47	g	Sakai	+								g														g	g
-	2684559	c	2897917	m	2968149	ns	EDL933	rec47	c	EDL933	+								c														a	a
-	2684559	t	2898005	N	2968237	ns	EDL933	rec47	t	EDL933	++++		t	t	t	t	t	t	t				t	t	t	t	t	t				t	t	t
-	2684559	c	2898156	t	2968388	ns	EDL933	rec47	c	EDL933	++++		c	c	c	c	c	c	c				c	c	c	c	c	c				c	c	c
-	2684559	t	2898422	y	2968654	ns	EDL933	rec47	t	EDL933	++++		c	t	t	c	c	t	t			t	c	c	c	t	t	t	c	c	c	c	t	t
-	2684559	g	2898446	R	2968678	ns	EDL933	rec47	g	EDL933	++++		a	a	a	t	a	a	t			a	t	t	t	a	a	a	a	a	a	a	a	a
-	2684559	t	2898533	Y	2968765	ns	EDL933	rec47	t	EDL933	++++		t	c	t	c	t	t	c		a	c	c	c	c	c	t	t	c	c	c	c	c	c
-	2684559	a	2898662	t	2968894	s	EDL933	rec47	a	EDL933	++++			a	a	a	a	a	a		t	a	a	a	a		a	a	g	g	g	g	a	a
-	2684559	t	2898701	c	2968933	s	EDL933	rec47	c	Sakai	+			c	c	t	t	t	t		c	t	t	t	t		c	c	c	c	c	c	c	-
-	2684559	c	2898719	t	2968951	s	EDL933	rec47	c	EDL933	+			c	c	c	c	c	c		c	c	c	c	c		c	c	c	c	c	c	t	t
-	2684559	t	2918977	g	2990240	i	EDL933	rec47	t	EDL933	+					-																		t
-	2684559	t	2918984	c	2990247	i	EDL933	rec47	t	EDL933	+					-																		t
-	2684559	c	2918994	g	2990257	i	EDL933	rec47	c	EDL933	+					t																		c
-	2684559	a	2918998	g	2990261	i	EDL933	rec47	g	Sakai	+					c																		g
-	2684559	a	2919011	t	2990274	i	EDL933	rec47	a	EDL933	+					a																		a
-	2684559	-	2919017	g	2990281	ins	EDL933	rec47	?	EDL933	+/-					g																		-
-	2684559	t	2919081	g	2990345	i	EDL933	rec47	g	Sakai	+					g																		g
-	2684559	t	2919113	c	2990377	i	EDL933	rec47	c	Sakai	+					c																		c
-	2684559	c	2919256	M	2990520	ns	EDL933	rec47	c	EDL933	+++					c		c	c		c													c
-	2684559	c	2919393	t	2990657	s	EDL933	rec47	t	Sakai	+++					t		t	t		t													t
-	2684559	a	2919429	R	2990693	ns	EDL933	rec47	a	EDL933	+					g		a	t		g													a
-	2684559	g	2919447	r	2990711	ns	EDL933	rec47	g	EDL933	+					a		g	g		a													a
-	2684559	g	2919459	k	2990723	ns	EDL933	rec47	g	EDL933	+					t		g	g		g													c
-	2684559	a	2919462	r	2990726	ns	EDL933	rec47	a	EDL933	+					g		g	a		g													g
-	2684559	c	2919465	y	2990729	ns	EDL933	rec47	c	EDL933	+++					c		c	c		t													c
-	2684559	t	2919469	w	2990733	ns	EDL933	rec47	t	EDL933	+					a		t	a		a													a
-	2684559	t	2919471	y	2990735	ns	EDL933	rec47	t	EDL933	+					c		c	t		c													c
-	2684559	g	2919515	k	2990779	ns	EDL933	rec47	g	EDL933	++++					t		t	t		t													t
-	2684559	c	2919516	s	2990780	ns	EDL933	rec47	c	EDL933	+					c		g	g		g													g
-	2684559	g	2919517	r	2990781	ns	EDL933	rec47	g	EDL933	+++					g		g	g		g													g
-	2684559	g	2919519	s	2990783	ns	EDL933	rec47	g	EDL933	+++					g		g	g		g													g
-	2684559	g	2919522	r	2990786	ns	EDL933	rec47	g	EDL933	++++					a		a	a		a													a
-	2684559	a	2919525	r	2990789	ns	EDL933	rec47	a	EDL933	+					a		a	g		g													a
-	2684559	t	2920072	N	2991336	ns	EDL933	rec47	t	EDL933	++++																							
-	2684559	a	2920469	n	2991733	i	EDL933	rec47	a	EDL933	+										a													
-	2684559	a	2920478	n	2991742	i	EDL933	rec47	a	EDL933	+										a													
-	2684559	a	2924176	M	2995440	i	EDL933	rec47	a	EDL933	++++	c							c		c		c	c	c							c	c	c
-	2684559	g	2924282	r	2995546	i	EDL933	rec47	g	EDL933	+++	g							a		g		g	a	g							g	a	g
-	2684559	g	2924286	r	2995550	i	EDL933	rec47	g	EDL933	++	g											a	-	a							a	-	a
-	2684559	g	2924291	r	2995555	i	EDL933	rec47	g	EDL933	+++	g											g	a	g							g	a	g
-	2684559	c	2924298	s	2995562	i	EDL933	rec47	c	EDL933	+++	c											c	g	c							c	g	c
-	2684559	c	2924305	y	2995569	i	EDL933	rec47	c	EDL933	+++	c											c	t	c							c	t	c
-	2684559	t	2924311	w	2995575	i	EDL933	rec47	t	EDL933	+++	t											t	a	t							t	a	t
-	2684559	c	2924325	y	2995589	i	EDL933	rec47	c	EDL933	++	c											a	t	a							a	t	a
-	2684559	a	2924329	m	2995593	i	EDL933	rec47	a	EDL933	++	a											-	c	-							-	c	-
-	2684559	a	2924330	m	2995594	i	EDL933	rec47	a	EDL933	++	a											-	c	-							-	c	-
-	2684559	g	2924332	k	2995596	i	EDL933	rec47	g	EDL933	+++	g											g	t	g							g	t	g
-	2684559	g	2924334	k	2995598	i	EDL933	rec47	g	EDL933	+++	g											g	t	g							g	t	g
-	2684559	a	2924338	m	2995602	i	EDL933	rec47	a	EDL933	+++	a											a	c	a							a	c	a
-	2684559	a	2924347	r	2995611	i	EDL933	rec47	a	EDL933	+++	a											a	g	a							a	g	a
-	2684559	c	2924349	y	2995613	i	EDL933	rec47	c	EDL933	+++	c											c	t	c							c	t	c
-	2684559	a	2924350	r	2995614	i	EDL933	rec47	a	EDL933	+++	a											a	g	a							a	g	a
-	2684559	g	2924351	r	2995615	i	EDL933	rec47	g	EDL933	+++	g											g	a	g							g	a	g
-	2684559	c	2924353	y	2995617	i	EDL933	rec47	c	EDL933	+++	c											c	t	c							c	t	c
-	2684559	c	2926313	Y	2997577	ns	EDL933	rec47	c	EDL933	++++																							
-	2684559	t	2926946	Y	2998210	ns	EDL933	rec47	t	EDL933	+										t													
-	2684559	g	2928586	N	2999850	ns	EDL933	rec47	g	EDL933	+++					g			g		g	g	g	g	g									
-	2684559	c	2928692	Y	2999956	ns	EDL933	rec47	c	EDL933	+++					c			c		c	c	c	c	c									
-	2684559	g	2928734	R	2999998	ns	EDL933	rec47	g	EDL933	+								g		g													
-	2684559	c	2929040	Y	3000304	ns	EDL933	rec47	c	EDL933	+								c		t													
-	2684559	-	2929215	c	3000480	ins	EDL933	rec47	-	EDL933	+										-													
-	2684559	g	2929451	R	3000716	ns	EDL933	rec47	g	EDL933	+										g													
-	2684559	c	2929629	y	3000894	i	EDL933	rec47	c	EDL933	++++																							
-	2684559	a	2929635	r	3000900	i	EDL933	rec47	a	EDL933	++++																							
-	2684559	t	2929645	y	3000910	i	EDL933	rec47	t	EDL933	++++																							
-	2684559	c	2929647	y	3000912	i	EDL933	rec47	c	EDL933	++++																							
-	2684559	c	2929657	y	3000922	i	EDL933	rec47	c	EDL933	++++																							
-	2684559	g	2929829	R	3001094	ns	EDL933	rec47	g	EDL933	++++																		a					
-	2684559	c	2930139	Y	3001404	ns	EDL933	rec47	c	EDL933	+++					t			t		c	t	t	t	t	c	c	c						
-	2684559	c	2930465	Y	3001730	ns	EDL933	rec47	c	EDL933	+		t			c			a		t	t	t	t	t	t	c	c						
-	2684559	c	2930635	Y	3001900	ns	EDL933	rec47	c	EDL933	++++		c			c			c		c	c	c	c	c	c	c	c						
-	2684559	c	2930858	S	3002123	ns	EDL933	rec47	c	EDL933	++++					g			g	g	g	g	g	g	g	g	g	g						
-	2684559	c	2931341	Y	3002606	ns	EDL933	rec47	c	EDL933	++++					c			c	c	c	c	c	c	c	c	c	c						
-	2684559	t	2931678	a	3002943	ns	EDL933	rec47	t	EDL933	+++					t			t		t	t	t	t	t									
-	2684559	c	2932652	t	3003917	i	EDL933	rec47	c	EDL933	+										c													
-	2684559	c	2933030	s	3004295	ns	EDL933	rec47	c	EDL933	+										c													
-	2684559	c	2933043	n	3004308	ns	EDL933	rec47	c	EDL933	+										c													
-	2684559	t	2933600	a	3004865	ns	EDL933	rec47	?	Sakai/EDL933	+/-																							
-	2684559	-	2933612	c	3004878	ins	EDL933	rec47	?	Sakai/EDL933	+/-																							
-	2684559	c	2934127	N	3005393	ns	EDL933	rec47	c	EDL933	++++																							
-	2684559	-	2935300	R	3006567	ins	EDL933	rec47	-	EDL933	+++					-						-	-	-	-									
-	2684559	t	2936553	Y	3007820	ns	EDL933	rec47	t	EDL933	++++				t	t	t				t	t	t	t	t									

O55 and O157 genome site details												Outgroup Strain Details[i]																						
CB9615 base[b]	CB9615 Site[c]	Sakai base[b]	Sakai Site[c]	EDL933 base[b]	EDL933 Site[c]	type[d]	Event Lineage[ef]	Recombinant segment	Inferred ancestral base[g]	Outgroup Analysis[e]	Support level[h]	D1 Sd197	K-12	HS	ATCC 8739	UMN026	IAI39	SMS 3-5	E2348/69	536	ED1a	CFT073	S88	APEC O1	UTI89	E24377A	IAI1	SE11	F5 8401	F2a 2457T	F2a 301	SS Ss046	B4 Sb227	B18 BS512
-	2684559	g	2936695	K	3007962	ns	EDL933	rec47	g	EDL933	++++				t	t	t				t	t	t	t	t									
-	2684559	t	2936820	Y	3008087	ns	EDL933	rec47	t	EDL933	++++				c	c	c			c	c	c	c	c	c	c	c	c						
-	2684559	a	2937124	W	3008391	ns	EDL933	rec47	a	EDL933	++++	a			t	a	a			a	a	a	a	a	a	a	t	t						
-	2684559	c	2937719	N	3008986	ns	EDL933	rec47	c	EDL933	++++	c	g		c	c	c			c	c	c	c	c	c	c	c	c						
-	2684559	a	2938470	R	3009737	ns	EDL933	rec47	a	EDL933	++++	t	g		g	g	g			g	g	g	g	g	g	g	g	g						
-	2684559	g	2939189	r	3010456	ns	EDL933	rec47	g	EDL933	+				a	g	a			a	a	a	a	a	a	a	a	g						
-	2684559	a	2939192	r	3010459	ns	EDL933	rec47	a	EDL933	+++				g	a	g			g	g	g	a	a	a	a	g	a						
-	2684559	g	2939242	R	3010509	i	EDL933	rec47	g	EDL933	+				a	a	a			a	a	a	g	g	g	a	a	a						
-	2684559	g	2939290	r	3010557	i	EDL933	rec47	g	EDL933	+++				a	g	g			a	g	a	a	a	a	g	a	g						
-	2684559	a	2939303	m	3010570	ns	EDL933	rec47	a	EDL933	++++				a	a	a			a	c	a	a	a	a	a	a	a						
-	2684559	t	2939344	Y	3010611	ns	EDL933	rec47	t	EDL933	+				c	t	c			c	c	t	c	c	c	c	c	t						
-	2684559	t	2939362	y	3010629	ns	EDL933	rec47	t	EDL933	+				c	t	c			c	c	t	c	c	c	c	c	t						
-	2684559	t	2939372	y	3010639	ns	EDL933	rec47	t	EDL933	+++				c	t	c			c	c	t	t	t	t	c	c	c						
-	2684559	a	2939378	r	3010645	ns	EDL933	rec47	a	EDL933	+				g	a	g			g	g	a	g	g	g	g	g	g						
-	2684559	c	2940615	t	3011882	ns	EDL933	rec47	?	Sakai/EDL933	+/-																							
-	2684559	a	2941626	M	3012893	ns	EDL933	rec47	a	EDL933	+										a													
-	2684559	c	2941782	t	3013049	ns	EDL933	rec47	t	Sakai	+										t							t						
c	2854935	t	3114050	t	3184006	nc	CB9615	rec48	?	O55/O157	+/-		-	-	-	-	-	-								-	-	-						-
a	2854936	t	3114051	t	3184007	nc	CB9615	rec48	?	O55/O157	+/-		-	-	-	-	-	-								-	-	-						-
t	2854938	c	3114053	c	3184009	nc	CB9615	rec48	?	O55/O157	+/-		-	-	-	-	-	-								-	-	-						-
c	2854941	t	3114056	t	3184012	nc	CB9615	rec48	t	CB9615	++++		t	t	t	t	t	t								t	t	t						t
a	2987890	g	3246820	g	3316665	s	O55/O157	rec49	g	CB9615	++	g	g	a	g	a	a	g			g					a	g	a	g	a	a	g	g	g
c	2987894	a	3246824	a	3316669	ns	O55/O157	rec49	c	O157	++++	c	c	c	c	c	c	c			c					c	c	c	c	c	c	c	c	c
g	2988052	c	3246982	c	3316827	ns	O55/O157	rec49	g	O157	++++	g	g	g	g	g	g	g			g					g	g	g	g	g	g	g	g	g
a	2988115	g	3247045	g	3316890	s	O55/O157	rec49	a	O157	++	a	a	a	g	g	g	g			a					g	a	g	a	g	a	a	a	a
g	2988268	t	3247198	t	3317043	s	O55/O157	rec49	g	O157	++	g	g	t	g	g	g	g			g					t	g	t	g	g	t	g	g	g
g	2988329	a	3247259	a	3317104	ns	O55/O157	rec49	a	CB9615	++	a	g	a	a	g	g	g			g					g	g	g	g	g	g	g	g	g
a	2988334	t	3247264	t	3317109	ns	O55/O157	rec49	t	CB9615	++	t	a	t	t	a	a	a			a					a	a	a	a	a	a	a	a	a
g	2988352	a	3247282	a	3317127	s	O55/O157	rec49	a	CB9615	++	a	g	a	a	g	g	g			g					g	g	g	g	g	g	g	g	g
t	2988355	g	3247285	g	3317130	s	O55/O157	rec49	g	CB9615	++	g	t	g	g	t	t	t			t					t	t	t	t	t	t	t	t	t
g	3230699	a	3448153	r	3517993	nc	Sakai	rec50	a	3	++	a	g	g	g	g	a	a	c	c	c	g	c	c	c	a	g	a	a	a	a	g	a	a
c	3230703	t	3448157	c	3517997	nc	Sakai	rec50	c	Sakai	++++	c	c	c	c	c	c	c	c	c	c	c	c	c	c	c	c	c	c	c	c	c	c	c
c	3230708	t	3448162	y	3518002	nc	Sakai	rec50	t	3	++	t	c	c	c	c	t	t	g	g	g	c	g	g	g	t	c	t	c	c	c	c	c	t
a	3231281	g	3448735	R	3518575	nc	Sakai	rec50	a	Sakai	++++	a	a	a	a	a	a	a	a	a	a	a	a	a	a	a	a	a	a	a	a	a	a	a
c	3231663	g	3449117	c	3518959	nc	Sakai	rec50	g	2	-	g	g	g	c	g	c	c	c	c	c	g	c	c	c	g	g	g	g	g	g	g	g	g
-	3231946	a	3449416	g	3519259	i	Sakai	rec50	-	Sakai	++++	-	g	g	g	-	-	g	g	g	g	g	g	g	g	g	g	g	g	g	g	g	g	g
a	3232188	g	3449652	a	3519495	i	Sakai	rec50	a	Sakai	++++	a	a	a	a	a	a	a	a	a	a	a	a	a	a	a	a	a	a	a	a	a	a	a
g	3232775	g	3450239	a	3520082	nc	EDL933	rec51	a	2	-	a	g	a	a	g	a	a	g	a	g	g	g	g	g	a	a	a	g	g	g	g	g	g
a	3232790	a	3450254	c	3520097	nc	EDL933	rec51	c	2	-	c	a	a	a	a	a	a	a	a	a	a	a	a	a	a	a	a	a	a	a	a	a	a
t	3232791	t	3450255	a	3520098	nc	EDL933	rec51	t	EDL933	+	c	t	a	a	t	a	a	t	a	t	t	t	t	t	a	a	a	t	t	t	t	t	t
t	3232792	t	3450256	a	3520099	nc	EDL933	rec51	t	EDL933	++++	t	t	t	t	t	t	t	t	t	t	t	t	t	t	t	t	t	t	t	t	t	t	t
c	3232793	c	3450257	t	3520100	nc	EDL933	rec51	c	EDL933	++++	c	c	c	c	c	c	c	c	c	c	c	c	c	c	c	c	c	c	c	c	c	c	c
t	3232794	t	3450258	c	3520101	nc	EDL933	rec51	t	EDL933	++++	g	t	-	-	t	-	-	t	t	t	t	t	t	t	-	-	-	t	t	t	t	t	t
a	3232803	a	3450267	g	3520110	nc	EDL933	rec51	a	EDL933	+	c	a	g	g	a	g	g	a	a	a	a	a	a	a	g	g	g	a	a	a	a	a	a
c	3232807	c	3450271	g	3520114	nc	EDL933	rec51	g	2	-	g	c	g	g	c	g	g	c	c	c	c	c	c	c	g	g	g	c	c	c	c	c	c
-	3232811	-	3450275	t	3520119	ins	EDL933	rec51	-	EDL933	++++	-	-	-	-	-	-	-	-	-	-	-	-	-	-	-	-	-	-	-	-	-	-	-
-	3233047	-	3450511	Y	3520356	ins	EDL933	rec51	-	EDL933	++++	-	-	-	-	-	-	-	-	-	-	-	-	-	-	-	-	-	-	-	-	-	-	-
-	3233114	-	3450578	g	3520424	ins	EDL933	rec51	-	EDL933	++++	-	-	-	-	-	-	-	-	-	-	-	-	-	-	-	-	-	-	-	-	-	-	-
-	3233221	-	3450685	c	3520532	ins	EDL933	rec51	-	EDL933	++	-	-	-	-	-	-	-	-	-	-	c	-	c	-	-	-	-	-	-	-	-	-	-
-	3233413	-	3450877	g	3520725	ins	EDL933	rec51	-	EDL933	++++	-	-	-	-	-	-	-	-	-	-	-	-	-	-	-	-	-	-	-	-	-	-	-
t	3259976	c	3477440	c	3547289	ns	O55/O157	rec52	?	O55/O157	+/-																							
g	3259977	t	3477441	t	3547290	ns	O55/O157	rec52	?	O55/O157	+/-																							
c	3259981	a	3477445	a	3547294	s	O55/O157	rec52	?	O55/O157	+/-																							
c	3259993	a	3477457	a	3547306	ns	O55/O157	rec52	?	O55/O157	+/-																							
t	3260007	c	3477471	c	3547320	ns	O55/O157	rec52	?	O55/O157	+/-																							
a	3260027	c	3477491	c	3547340	ns	O55/O157	rec52	?	O55/O157	+/-																							
t	3260050	c	3477514	c	3547363	s	O55/O157	rec52	?	O55/O157	+/-																							
g	3260087	a	3477551	a	3547400	ns	O55/O157	rec52	?	O55/O157	+/-																							
t	3260147	c	3477611	c	3547460	i	O55/O157	rec52	?	O55/O157	+/-																							
t	3260155	c	3477619	c	3547468	i	O55/O157	rec52	?	O55/O157	+/-																							
-	3260198	t	3477663	t	3547512	indel	O55/O157	rec52	?	O55/O157	+/-																							
a	3260205	t	3477670	t	3547519	i	O55/O157	rec52	?	O55/O157	+/-																							
t	3260235	c	3477700	c	3547549	i	O55/O157	rec52	?	O55/O157	+/-																							
c	3260243	t	3477708	t	3547557	i	O55/O157	rec52	?	O55/O157	+/-																							
g	3260320	a	3477785	a	3547634	s	O55/O157	rec52	?	O55/O157	+/-																							
t	3260362	a	3477827	a	3547676	s	O55/O157	rec52	?	O55/O157	+/-																							
g	3260375	c	3477840	c	3547689	ns	O55/O157	rec52	?	O55/O157	+/-																							
c	3260460	t	3477925	t	3547774	ns	O55/O157	rec52	?	O55/O157	+/-																							
a	3260513	c	3477978	c	3547827	ns	O55/O157	rec52	?	O55/O157	+/-																							
a	3260605	g	3478070	g	3547919	s	O55/O157	rec52	?	O55/O157	+/-																							
g	3260626	a	3478091	a	3547940	s	O55/O157	rec52	?	O55/O157	+/-																							
-	3260813	t	3478279	t	3548128	indel	O55/O157	rec52	?	O55/O157	+/-																							
c	3260816	a	3478282	a	3548131	ns	O55/O157	rec52	?	O55/O157	+/-																							
c	3260919	a	3478385	a	3548234	i	O55/O157	rec52	?	O55/O157	+/-																							
a	3260949	g	3478415	g	3548264	i	O55/O157	rec52	?	O55/O157	+/-																							
-	3260967	t	3478434	t	3548283	indel-4	O55/O157	rec52	?	O55/O157	+/-																							
t	3261009	c	3478479	c	3548328	i	O55/O157	rec52	?	O55/O157	+/-																							
g	3261021	a	3478491	a	3548340	nc	O55/O157	rec52	?	O55/O157	+/-																							
t	3261088	c	3478558	c	3548407	nc	O55/O157	rec52	?	O55/O157	+/-																							
t	3261102	c	3478572	c	3548421	nc	O55/O157	rec52	?	O55/O157	+/-																							
c	3261240	a	3478710	a	3548559	nc	O55/O157	rec52	?	O55/O157	+/-																							

O55 and O157 genome site details												Outgroup Strain Details[i]																						
CB9615 base[b]	CB9615 Site[c]	Sakai base[b]	Sakai Site[c]	EDL933 base[b]	EDL933 Site[c]	type[d]	Event Lineage[e,f]	Recombinant segment	Inferred ancestral base[g]	Outgroup Analysis[e]	Support level[h]	D1 Sd197	K-12	HS	ATCC 8739	UMN026	IAI39	SMS 3-5	E2348/69	536	ED1a	CFT073	S88	APEC O1	UTI89	E24377A	IAI1	SE11	F5 8401	F2a 2457T	F2a 301	SS Ss046	B4 Sb227	B18 BS512
c	3261308	a	3478778	a	3548627	nc	O55/O157	rec52	?	O55/O157	+/-																							
c	3261349	t	3478819	t	3548668	nc	O55/O157	rec52	?	O55/O157	+/-																							
t	3261416	c	3478886	c	3548735	nc	O55/O157	rec52	?	O55/O157	+/-																							
c	3261435	t	3478905	t	3548754	nc	O55/O157	rec52	?	O55/O157	+/-																							
a	3261442	g	3478912	g	3548761	nc	O55/O157	rec52	?	O55/O157	+/-																							
t	3261454	c	3478924	c	3548773	nc	O55/O157	rec52	?	O55/O157	+/-																							
c	3261459	t	3478929	t	3548778	nc	O55/O157	rec52	?	O55/O157	+/-																							
a	3261465	g	3478935	g	3548784	nc	O55/O157	rec52	?	O55/O157	+/-																							
g	3261472	c	3478942	c	3548791	nc	O55/O157	rec52	?	O55/O157	+/-																							
c	3261476	t	3478946	t	3548795	nc	O55/O157	rec52	?	O55/O157	+/-																							
t	3261479	c	3478949	c	3548798	nc	O55/O157	rec52	?	O55/O157	+/-																							
t	3261483	c	3478953	c	3548802	nc	O55/O157	rec52	?	O55/O157	+/-																							
a	3261486	g	3478956	g	3548805	nc	O55/O157	rec52	?	O55/O157	+/-																							
a	3261495	g	3478965	g	3548814	nc	O55/O157	rec52	?	O55/O157	+/-																							
g	3261516	a	3478986	a	3548835	nc	O55/O157	rec52	?	O55/O157	+/-																							
t	3261529	c	3478999	c	3548848	nc	O55/O157	rec52	?	O55/O157	+/-																							
-	3261532	a	3479003	a	3548852	indel-3	O55/O157	rec52	?	O55/O157	+/-																							
a	3261545	g	3479018	g	3548867	nc	O55/O157	rec52	?	O55/O157	+/-																							
a	3261565	g	3479038	g	3548887	nc	O55/O157	rec52	?	O55/O157	+/-																							
c	3261572	t	3479045	t	3548894	nc	O55/O157	rec52	?	O55/O157	+/-																							
a	3261596	g	3479069	g	3548918	nc	O55/O157	rec52	?	O55/O157	+/-																							
c	3261627	t	3479100	t	3548949	nc	O55/O157	rec52	?	O55/O157	+/-																							
t	3292159	c	3484326	c	3551648	ns	O55/O157	rec52	t	O157	++++					t			t			t	t	t	t				t	t	t	t		t
t	3292221	g	3484388	g	3551710	ns	O55/O157	rec52	g	CB9615	++++					g			g			g	g	g	g				g	g	g	g		g
t	3292596	c	3484763	Y	3552085	ns	O55/O157	rec52	t	O157	+								t													t		t
c	3293060	g	3485227	g	3552550	s	O55/O157	rec52	?	O55/O157	+/-								g													c	g	c
g	3293072	a	3485239	a	3552562	s	O55/O157	rec52	a	CB9615	+								a													c	c	c
t	3293116	c	3485283	c	3552606	ns	O55/O157	rec52	?	O55/O157	+/-								t													a	c	a
a	3293118	g	3485285	g	3552608	ns	O55/O157	rec52	g	CB9615	+								a													g	c	g
a	3293135	g	3485302	g	3552625	s	O55/O157	rec52	a	O157	+								a													a	g	a
a	3293153	g	3485320	g	3552643	ns	O55/O157	rec52	g	CB9615	+								a													g	c	g
t	3293155	a	3485322	a	3552645	ns	O55/O157	rec52	t	O157	+								t													c	c	c
c	3293174	t	3485341	t	3552664	ns	O55/O157	rec52	c	O157	+								c													c	a	c
t	3293175	g	3485342	g	3552665	ns	O55/O157	rec52	t	O157	+++								t													t	t	t
t	3293176	c	3485343	c	3552666	ns	O55/O157	rec52	?	O55/O157	+/-								t													g	c	g
a	3293212	g	3485379	g	3552702	ns	O55/O157	rec52	a	O157	+								a													a	g	a
t	3292089	g	3484256	t	3551578	ns	Sakai	rec53	g	2	-					g			g			g	g	g	g				g	g	g	g		g
c	3292093	a	3484260	c	3551582	ns	Sakai	rec53	a	2	-					a			a			a	a	a	a				a	a	a	a		a
g	3292195	t	3484362	g	3551684	ns	Sakai	rec53	g	Sakai	++++					g			g			g	g	g	g				g	g	g	g		g
g	3292822	t	3484989	g	3552312	ns	Sakai	rec53	t	2	-								t													t		t
-	3293948	t	3490148	K	3557471	ns	Sakai	rec53	t	EDL933	++++																							
-	3293948	-	3490449	g	3557773	del-6	Sakai	rec53	6	Sakai	+		6																					
c	3329489	t	3491733	c	3559062	ns	Sakai	rec53	?	Sakai	+/-																							
c	3329490	g	3491734	c	3559063	ns	Sakai	rec53	?	Sakai	+/-																							
g	3555319	t	3731319	t	3798624	ns	CB9615	rec54	t	CB9615	+					t																		
g	3555320	a	3731320	a	3798625	s	CB9615	rec54	a	CB9615	+					a																		
t	3555325	g	3731325	g	3798630	ns	CB9615	rec54	g	CB9615	+					g																		
a	3555327	t	3731327	t	3798632	ns	CB9615	rec54	t	CB9615	+					t																		
g	3678686	a	3854773	a	3922085	s	O157	rec55	g	O157	+++	g							g		g								g	g	g			
g	3678690	a	3854777	a	3922089	ns	O157	rec55	a	CB9615	+++	a							g		a								a	a	a			
a	3678693	t	3854780	t	3922092	s	O157	rec55	a	O157	+++	a							a		a								a	a	a			
g	3678694	c	3854781	c	3922093	s	O157	rec55	g	O157	+++	g							g		g								g	g	g			
c	3678701	t	3854788	t	3922100	s	O157	rec55	c	O157	+++	c							c		c								c	c	c			
g	3678713	a	3854800	a	3922112	s	O157	rec55	g	O157	+++	g							g		g								g	g	g			
g	3678714	a	3854801	a	3922113	ns	O157	rec55	g	O157	+++	g							g		g								g	g	g			
c	3678717	a	3854804	a	3922116	s	O157	rec55	c	O157	+++	c							c		c								c	c	c			
t	3678719	g	3854806	g	3922118	s	O157	rec55	t	O157	+++	t							t		t								t	t	t			
c	3678724	t	3854811	t	3922123	ns	O157	rec55	c	O157	+++	c							c										c	c	c			
t	3678725	a	3854812	a	3922124	ns	O157	rec55	t	O157	+++	t							t										t	t	t			
g	3678728	a	3854815	a	3922127	s	O157	rec55	g	O157	+++	g							g										g	g	g			
a	4051466	a	4162738	g	4229960	nc	EDL933	rec56	a	EDL933	++	a	g	g	g	a	a	a	g	g	g	g	g	g	g	a	a	a	g	g	g	a	g	g
c	4051481	c	4162753	a	4229975	nc	EDL933	rec56	c	EDL933	++++	c	-	-	-	c	c	c	c	-	-	-	-	-	-	c	c	c	-	-	-	c	c	c
a	4051482	a	4162754	t	4229976	nc	EDL933	rec56	a	EDL933	++++	c	a	a	a	a	a	a	a	a	a	a	a	a	a	a	a	a	a	a	a	a	a	a
a	4051483	a	4162755	t	4229977	nc	EDL933	rec56	t	2	-	t	t	t	t	a	a	a	t	t	t	t	t	t	t	a	a	a	t	t	t	a	t	t
t	4051484	t	4162756	c	4229978	nc	EDL933	rec56	c	2	-	c	t	t	t	t	t	t	t	t	t	t	t	t	t	t	t	t	t	t	t	t	t	t
c	4051485	c	4162757	t	4229979	nc	EDL933	rec56	c	EDL933	++++	g	c	c	c	c	c	c	c	c	c	c	c	c	c	c	c	c	c	c	c	c	c	c
g	4051494	g	4162766	a	4229988	nc	EDL933	rec56	a	2	-	c	a	a	a	g	g	g	a	a	a	a	a	a	a	g	g	g	a	a	a	g	a	a
g	4051498	g	4162770	c	4229992	nc	EDL933	rec56	g	EDL933	++	g	c	c	c	g	g	g	c	c	c	c	c	c	c	g	g	g	c	c	c	g	c	c
t	4051502	t	4162774	c	4229996	nc	EDL933	rec56	t	EDL933	++	t	c	c	c	t	t	t	c	c	c	c	c	c	c	t	t	t	c	c	c	t	c	c
a	4393486	g	4504901	g	4572130	s	O157	rec57	g	CB9615	++	g	a	a	a											a	a	a				a	a	a
c	4393528	t	4504943	t	4572172	s	O157	rec57	c	O157	++	c	t	t	t											t	t	t				t	t	t
g	4393594	a	4505009	a	4572238	s	O157	rec57	g	O157	++	g	a	a	a											a	a	a				a	a	a
g	4393621	c	4505036	c	4572265	s	O157	rec57	g	O157	++	g	c	c	c											c	c	c				c	c	c
g	4393627	c	4505042	c	4572271	s	O157	rec57	g	O157	++	g	c	c	c											c	c	c				c	c	c
c	4393671	g	4505086	g	4572315	ns	O157	rec57	c	O157	++	c	g	g	g											g	g	g				c	g	g
a	4396300	g	4507715	g	4574944	s	O55/O157	rec58	a	O157	++	a	g	g	g											g	g	g				a	g	g
c	4396357	g	4507772	g	4575001	s	O55/O157	rec58	c	O157	++	c	g	c	g											c	g	g				c	c	c
c	4396360	t	4507775	t	4575004	s	O55/O157	rec58	c	O157	++	c	t	c	t											c	t	t				c	c	c
c	4396419	a	4507834	a	4575063	ns	O55/O157	rec58	c	O157	++	c	a	c	a											c	a	c				c	c	c
g	4396576	a	4507991	a	4575220	s	O55/O157	rec58	a	CB9615	++	a	a	g	g											g	a	a				a	a	a

O55 and O157 genome site details												Outgroup Strain Details[i]																						
CB9615 base[b]	CB9615 Site[c]	Sakai base[b]	Sakai Site[c]	EDL933 base[b]	EDL933 Site[c]	type[d]	Event Lineage[ef]	Recombinant segment	Inferred ancestral base[g]	Outgroup Analysis[e]	Support level[h]	D1 Sd197	K-12	HS	ATCC 8739	UMN026	IAI39	SMS 3-5	E2348/69	536	ED1a	CFT073	S88	APEC O1	UTI89	E24377A	IAI1	SE11	F5 8401	F2a 2457T	F2a 301	SS Ss046	B4 Sb227	B18 BS512
g	4396591	a	4508006	a	4575235	s	O55/O157	rec58	a	CB9615	++	a	a	a	g											g	a	a				a	a	g
g	4396593	c	4508008	c	4575237	ns	O55/O157	rec58	c	CB9615	++	c	c	c	g											g	c	c				c	c	g
c	4396645	t	4508060	t	4575289	s	O55/O157	rec58	t	CB9615	++	t	t	t	c											t	t	t				c	t	t
t	4396651	c	4508066	c	4575295	s	O55/O157	rec58	c	CB9615	++	c	c	c	t											t	c	c				t	c	c
c	4396699	g	4508114	g	4575343	s	O55/O157	rec58	c	O157	++	c	g	c	c											g	c	g				c	c	c
c	4396843	t	4508258	t	4575487	s	O55/O157	rec58	c	O157	++++	g	c	c	c											c	c	c				g	g	g
a	4396848	c	4508263	c	4575492	ns	O55/O157	rec58	c	CB9615	++	c	a	a	a											a	a	a				a	c	c
a	4396860	g	4508275	g	4575504	ns	O55/O157	rec58	g	CB9615	++	g	a	a	a											a	a	a				a	g	g
g	4396876	a	4508291	a	4575520	s	O55/O157	rec58	a	CB9615	++	a	g	g	g											g	g	g				g	g	g
g	4397110	c	4508525	c	4575754	ns	O55/O157	rec58	g	O157	++++	g	g	g	g											g	g	g				g	g	g
c	4398299	-	4508599	t	4577342	i	O55/O157	rec58	c	EDL933	++++		c	c	c											c	c	c					c	c
a	4398300	-	4508599	g	4577343	i	O55/O157	rec58	a	EDL933	++++		a	a	a											a	a	a					a	a
g	4398338	-	4508599	a	4577381	i	O55/O157	rec58	a	CB9615	++++		a	a	a											a	a	a					a	a
g	4398346	-	4508599	a	4577389	i	O55/O157	rec58	a	CB9615	++++		a	a	a											a	a	a					a	a
a	4398368	-	4508599	g	4577411	i	O55/O157	rec58	g	CB9615	++++		g	g	g											g	g	g					g	g
t	4398401	-	4508599	g	4577444	i	O55/O157	rec58	t	EDL933	++++		t	t	t											t	g	t					t	t
t	4398463	-	4508599	a	4577506	i	O55/O157	rec58	a	CB9615	+		t	a	t											a	a	a					a	a
g	4398464	-	4508599	c	4577507	i	O55/O157	rec58	g	EDL933	++++		g	g	g											g	g	g					g	g
g	4398470	-	4508599	a	4577513	i	O55/O157	rec58	g	EDL933	++++		g	g	g											g	g	g					g	g
a	4577237	t	4689741	t	4758722	ns	O55/O157	rec59	a	O157	+++	a				a	a	a																
c	4577248	a	4689752	a	4758733	ns	O55/O157	rec59	c	O157	+++	c				c	c	c																
g	4577277	a	4689781	a	4758762	ns	O55/O157	rec59	g	O157	++	g				g	a	a																
g	4577316	a	4689820	a	4758801	ns	O55/O157	rec59	g	O157	+++	g				g	g	g																
g	4577595	a	4690099	a	4759080	ns	O55/O157	rec59	g	O157	+++	g				g	g	g																
t	4578264	c	4690768	c	4759749	nc	O55/O157	rec59	t	O157	+++	t				t	t	t																
a	4578502	c	4691006	c	4759987	nc	O55/O157	rec59	c	CB9615	++	c				a	a	a																
t	4578596	-	4691099	-	4760080	ins	O55/O157	rec59	-	CB9615	+++	-				-	-	-																
t	4578636	g	4691139	g	4760120	nc	O55/O157	rec59	g	CB9615	+++	g				g	g	g																
c	4578726	t	4691229	t	4760210	nc	O55/O157	rec59	t	CB9615	+++	t				t	t	t																
a	4578902	g	4691405	g	4760386	nc	O55/O157	rec59	g	CB9615	+++	g				g	g	g																
c	4578936	a	4691439	a	4760420	nc	O55/O157	rec59	c	O157	+++	c				c	c	c																
g	4579334	t	4691837	t	4760818	nc	O55/O157	rec59	t	CB9615	+++	t				t	t	t																
c	4579526	t	4692029	t	4761010	nc	O55/O157	rec59	t	CB9615	++	t				c	c	c																
t	4579865	c	4692368	c	4761349	nc	O55/O157	rec59	t	O157	+++	t				t	t	t																
g	4580312	a	4692815	a	4761796	nc	O55/O157	rec59	a	CB9615	++	a				t	t	t																
t	4580555	-	4693057	-	4762038	del	O55/O157	rec59	t	O157	+++	t				t	t	t																
a	4580825	c	4693327	c	4762308	i	O55/O157	rec59	c	CB9615	+++	c				c	c	c																
a	4580862	t	4693364	t	4762345	i	O55/O157	rec59	a	O157	+++	a				a	a	a																
g	4580886	a	4693388	a	4762369	i	O55/O157	rec59	a	CB9615	+++	a				a	a	a																
g	4580923	t	4693425	t	4762406	ns	O55/O157	rec59	g	O157	+++	g				g	g	g																
g	4581403	a	4693905	a	4762886	s	O55/O157	rec59	g	O157	++++	g	g	g	g	g	g	g	g	g	g	g	g	g	g	g	g	g	g	g	g	g	g	g
g	4720350	a	4833075	g	4901839	nc	Sakai	rec60	g	Sakai	++++	g	g	g	g	g	g	g	g	g	g	g	g	g	g	g	g	g	g	g	g	g	g	g
g	4720481	c	4833206	g	4901970	i	Sakai	rec60	g	Sakai	++++	g	g	g	g	t	g	c	g	g	g	g	g	g	g	g	g	g	g	g	g	g	g	g
c	4720482	t	4833207	c	4901971	i	Sakai	rec60	c	Sakai	++++	c	c	c	c	c	c	t	c	c	c	c	c	c	c	c	c	c	c	c	c	c	c	c
c	4720846	a	4833571	c	4902335	i	Sakai	rec60	a	2	-	a	a	a	a	a	a	a	a	a	a	a	a	a	a	a	a	a	a	a	a	a	a	a
a	4720847	g	4833572	a	4902336	i	Sakai	rec60	a	Sakai	++	a	a	a	a	a	a	a	g	a	a	a	a	a	a	a	g	a	a	a	a	a	a	a
a	4721272	g	4833997	a	4902761	nc	Sakai	rec60	a	Sakai	++	a	g	g	g	g	a	a	a	g	a	a	a	a	a	g	a	g	a	a	a	a	a	a
c	4816578	t	4928936	t	4997700	s	O157	rec61	c	O157	++	c	t	t	t											c	c	t				c	t	t
g	4816644	a	4929002	a	4997766	s	O157	rec61	g	O157	++	g	a	g	a											g	g	a				g	a	g
c	4816652	t	4929010	t	4997774	ns	O157	rec61	c	O157	++++	c	c	c	c											c	c	c				c	c	c
g	4816671	c	4929029	c	4997793	s	O157	rec61	g	O157	++	g	c	g	c											g	g	c				g	c	g
g	4816677	c	4929035	c	4997799	s	O157	rec61	g	O157	++	g	c	c	c											g	g	c				c	c	c
c	4816721	g	4929079	g	4997843	ns	O157	rec61	c	O157	++	c	g	c	g											g	g	g				c	g	c
c	4817596	c	4929954	g	4998719	ns	EDL933	rec62	c	EDL933	++++	c	c	c	c											c	c	c				c	c	c
c	4817601	c	4929959	t	4998724	s	EDL933	rec62	c	EDL933	++++	c	c	c	c											c	c	c				c	c	c
-	4817610	-	4929968	c	4998734	ins	EDL933	rec62	-	EDL933	++++	-	-	-	-											-	-	-				-	-	-
a	4817632	a	4929990	g	4998756	ns	EDL933	rec62	a	EDL933	++++	a	a	a	a											a	a	a				a	a	a
g	4818556	g	4930914	c	4999680	ns	EDL933	rec62	g	EDL933	++++	g	g	g	g											g	g	g				g	g	g
c	4822926	c	4935284	t	5004050	i	EDL933	rec62	c	EDL933	++++	c	c	c	c	c	c	c	c	c	c	c	c	c	c	c	c	c	c	c	c	c	c	c
a	4823405	a	4935763	c	5004529	s	EDL933	rec62	a	EDL933	++	a	a	a	a	c	a	c	a	a	a	a	c	c	c	a	a	a	a	a	a	a	a	a
a	4819350	g	4931708	g	5000474	s	O55/O157	rec63	a	O157	++	a	g	g	g											g	g	g				a	g	g
c	4819407	g	4931765	g	5000531	s	O55/O157	rec63	c	O157	++	c	g	c	g											g	g	g				c	c	c
c	4819410	t	4931768	t	5000534	s	O55/O157	rec63	c	O157	++	c	t	c	t											t	t	t				c	c	c
c	4819469	a	4931827	a	5000593	ns	O55/O157	rec63	c	O157	++	c	a	c	a											a	a	a				c	c	c
g	4819626	a	4931984	a	5000750	s	O55/O157	rec63	a	CB9615	++	a	a	g	g											g	a	g				a	a	a
g	4819641	a	4931999	a	5000765	s	O55/O157	rec63	a	CB9615	++	a	a	a	g											g	a	g				a	a	a
g	4819643	c	4932001	c	5000767	ns	O55/O157	rec63	c	CB9615	++	c	c	c	g											g	c	g				c	c	c
a	4863334	a	4975692	g	5044458	i	EDL933	rec64	g	2	-	g	g	a	g	a	a	a	a	g	g	g	g	g	g	g	a	a	g	g	g	a	g	a
a	4863375	a	4975733	g	5044499	i	EDL933	rec64	a	EDL933	++	a	g	a	a	a	a	a	a	a	a	a	a	a	a	a	a	a	a	a	a	a	a	a
a	4863381	a	4975739	t	5044505	i	EDL933	rec64	t	2	-	c	t	t	t	t	t	t	t	t	a	t	t	t	t	t	t	t	t	t	t	t	t	t
a	4863385	a	4975743	g	5044509	i	EDL933	rec64	a	EDL933	++	a	g	g	g	g	g	g	g	g	a	g	g	g	g	g	g	g	g	g	g	g	g	g
g	4863387	g	4975745	a	5044511	i	EDL933	rec64	a	2	-	a	-	-	a	a	-	a	a	-	g	-	a	-	-	-	-	-	a	a	a	a	-	g
c	4863389	c	4975747	-	5044512	del	EDL933	rec64	-	2	-	-	-	-	-	-	-	-	-	-	c	-	-	-	-	-	-	-	-	-	-	-	-	-
t	4863393	t	4975751	c	5044516	i	EDL933	rec64	c	2	-	c	c	c	c	c	c	c	c	c	t	c	c	c	c	c	c	c	c	c	c	c	c	c
a	4863398	a	4975756	c	5044521	i	EDL933	rec64	c	2	-	c	c	c	c	c	c	c	c	c	a	c	c	c	c	c	c	c	-	-	-	c	-	c
a	4863400	a	4975758	g	5044523	i	EDL933	rec64	g	2	-	g	g	g	g	g	g	g	g	g	a	g	g	g	g	g	g	g	a	a	a	g	a	g
g	4863401	g	4975759	c	5044524	i	EDL933	rec64	c	2	-	c	c	c	c	c	c	c	c	c	g	c	c	c	c	c	c	c	g	g	g	c	g	c
ct	4863406	ct	4975764	-	5044528	del-2	EDL933	rec64	-	2	-	-	-	-	-	-	-	-	-	-	ct	-	-	-	-	-	-	-	-	-	-	-	-	-
g	4863408	g	4975766	a	5044529	i	EDL933	rec64	a	2	-	-	a	a	-	-	a	-	-	a	g	a	-	a	a	a	a	a	-	-	-	-	a	-
g	4863412	g	4975770	a	5044533	i	EDL933	rec64	g	EDL933	++	g	a	g	g	g	a	g	g	a	g	a	g	a	a	g	a	a	g	g	g	g	a	g

O55 and O157 genome site details												Outgroup Strain Details[i]																						
CB9615 base[b]	CB9615 Site[c]	Sakai base[b]	Sakai Site[c]	EDL933 base[b]	EDL933 Site[c]	type[d]	Event Lineage[ef]	Recombinant segment	Inferred ancestral base[g]	Outgroup Analysis[e]	Support level[h]	D1 Sd197	K-12	HS	ATCC 8739	UMN026	IAI39	SMS 3-5	E2348/69	536	ED1a	CFT073	S88	APEC O1	UTI89	E24377A	IAI1	SE11	F5 8401	F2a 2457T	F2a 301	SS Ss046	B4 Sb227	B18 BS512
c	4863413	c	4975771	g	5044534	i	EDL933	rec64	g	2	-	g	g	c	c	c	g	c	c	g	c	g	c	g	g	c	g	g	c	c	c	c	g	c
c	4863416	c	4975774	a	5044537	i	EDL933	rec64	a	2	-	a	a	a	a	a	a	a	a	a	c	a	a	a	a	a	a	a	a	a	a	a	a	a
g	4863417	g	4975775	a	5044538	i	EDL933	rec64	a	2	-	a	a	g	g	g	a	g	g	a	g	a	g	a	a	g	a	a	g	g	g	g	a	g
t	4863418	t	4975776	g	5044539	i	EDL933	rec64	g	2	-	g	g	-	c	c	g	c	c	g	t	g	c	g	g	-	g	g	c	c	c	c	g	c
c	4863421	c	4975779	g	5044542	i	EDL933	rec64	g	2	-	g	g	g	c	c	g	c	c	g	c	g	c	g	g	g	g	g	c	c	c	c	g	c
a	4863422	a	4975780	c	5044543	i	EDL933	rec64	c	2	-	c	c	c	a	a	c	a	a	c	a	c	a	c	c	c	c	c	a	a	a	a	c	a
a	4863558	a	4975916	g	5044679	i	EDL933	rec64	a	EDL933	++	a	g	a	a	a	a	a	a	g	a	g	a	a	a	a	a	a	a	a	a	a	a	a
c	4872933	c	4985293	g	5054039	s	EDL933	rec65	c	EDL933	++++	c	c	c	c	c	c	c	c	c	c	c	c	c	c	c	c	c	c	c	c	c	c	c
g	4872942	g	4985302	c	5054048	s	EDL933	rec65	g	EDL933	++++	g	g	g	g	g	g	g	g	g	g	g	g	g	g	g	g	g	g	g	g	g	g	g
g	4872945	g	4985305	a	5054051	s	EDL933	rec65	g	EDL933	++++	g	g	g	g	g	g	g	g	g	g	g	g	g	g	g	g	g	g	g	g	g	g	g
c	4872949	c	4985309	t	5054055	ns	EDL933	rec65	c	EDL933	++++	c	c	c	c	c	c	c	c	c	c	c	c	c	c	c	c	c	c	c	c	c	c	c
c	4872960	c	4985320	t	5054066	s	EDL933	rec65	c	EDL933	++++	c	c	c	c	c	c	c	c	c	c	c	c	c	c	c	c	c	c	c	c	c	c	c
c	4872964	c	4985324	t	5054070	s	EDL933	rec65	c	EDL933	++++	c	c	c	c	c	c	c	c	c	c	c	c	c	c	c	c	c	c	c	c	c	c	c
a	4905594	a	5017954	g	5086700	nc	EDL933	rec66	a	EDL933	++	a	g	g	a	g	a	a	g	g	g	g	g	g	g	g	a	g	g	g	g	g	g	g
c	4905598	c	5017958	g	5086704	nc	EDL933	rec66	c	EDL933	++	c	g	g	c	g	c	c	g	g	g	g	g	g	g	g	c	g	g	g	g	g	g	g
c	4905602	c	5017962	t	5086708	nc	EDL933	rec66	t	2	-	g	t	t	c	t	c	c	t	t	t	t	t	t	t	t	c	t	t	t	t	t	t	t
g	4905611	g	5017971	a	5086717	nc	EDL933	rec66	g	EDL933	++++	c	g	g	g	g	g	g	g	g	g	g	g	g	g	g	g	g	g	g	g	g	g	g
a	4905612	a	5017972	g	5086718	nc	EDL933	rec66	g	2	-	g	a	a	a	a	a	a	a	a	a	a	a	a	a	a	a	a	a	a	a	a	a	a
t	4905613	t	5017973	a	5086719	nc	EDL933	rec66	a	2	-	a	a	a	t	a	t	t	a	a	a	a	a	a	a	a	t	a	a	a	a	a	a	a
t	4905614	t	5017974	a	5086720	nc	EDL933	rec66	t	EDL933	++++	g	t	t	t	t	t	t	t	t	t	t	t	t	t	t	t	t	t	t	t	t	t	t
g	4905615	g	5017975	t	5086721	nc	EDL933	rec66	g	EDL933	+++	g	-	-	g	-	g	g	-	-	-	-	-	-	-	-	g	-	-	-	-	-	-	-
t	4905630	t	5017990	c	5086736	nc	EDL933	rec66	t	EDL933	++	t	c	c	t	c	t	t	c	c	c	c	c	c	c	c	t	c	c	c	c	c	c	c
a	5179986	c	5331593	c	5361578	ns	O157	rec67	a	O157	+		a																t	t	t			
g	5179992	a	5331599	a	5361584	ns	O157	rec67	a	O157	+		g																a	a	a			
t	5180004	-	5331610	c	5361596	ns	O157	rec67	t	EDL933	+		a																t	t	t			
c	5180005	-	5331610	t	5361597	ns	O157	rec67	c	O157	+		t																c	c	c			
g	5180006	-	5331610	a	5361598	ns	O157	rec67	g	O157	+		a																g	g	g			
g	5180010	-	5331610	a	5361602	ns	O157	rec67	g	EDL933	+++		g																g	g	g			
t	5180022	a	5331611	a	5361614	ns	O157	rec67	t	O157	+		a																t	t	t			
c	5180023	t	5331612	t	5361615	ns	O157	rec67	c	O157	+		t																c	c	c			
g	5180024	a	5331613	a	5361616	ns	O157	rec67	g	O157	+		a																g	g	g			
g	5180028	a	5331617	a	5361620	ns	O157	rec67	g	O157	+		a																g	g	g			
t	5180040	a	5331629	a	5361632	ns	O157	rec67	t	O157	+		t																					
c	5180041	t	5331630	t	5361633	ns	O157	rec67	c	O157	+		c																					
g	5180042	a	5331631	a	5361634	ns	O157	rec67	?	O55/O157	+/-		t																					

[a] color in the table: white: the snps in unallocated blocks; green: the snps that supported the allocation of block; red: the snps that conflicted with the allocation of block; yellow: the unallocated snps or snps that imply 2 mutations at that site - eg in the ancestor of O55 and O157 strains before isolation and again in one of the lineages in the allocated blocks.

[b] Numbers in place of bases indicates number of bases where >2 bases inserted or deleted. In these cases "-" indicates absence of these bases.

[c] For indels the base indicated is the base before the insertion or deletion in the strain.

[d] s: synonymous; ns: non-synonymous; nc: in non-coding gene; i: intergenic; ins: small insert; del: small deletion; indel: the small indels that can't be allocated

[e] O157: allocated to the lineage to the ancestor of EDL933 and Sakai; Sakai/EDL933: allocated to the divergence between Sakai and EDL933 (strain not specified); O55/O157: allocated to the divergence between O55 and O157 lineages (lineage not specified). 2: snps that imply 2 mutations at that site

[f] The recombinational block was allocated if more than 80% of the snps in it can be allocated into the same lineage. For rec53, 56, 64, 66, there is support for 2 mutations at one site. These blocks were allocated to be the most probable lineage.

[g] The base in O55/O157 ancestor as inferred from outgroup analysis.

[h] Level of support for allocation of mutation as given in previous column

++++ agreement is high - 8 or more outgroup strains with expected base and at most 1 with an alternative base, and do not present alternative base in D1 Sd197

+++ agreement good - 4 or more outgroup strains with expected base and at most 1 with an alternative base, and do not present alternative base in D1 Sd197

++ agreement in D1 Sd197 regardless of situation with other outgroup strains

+ no conflict but very limited support as either site absent in D1 Sd197, and/or support is less than for any of the higher levels of support

+/- no conflict but no support (base not present in any outgroup OR base when present is not that in any of the O55 and O157 strains, OR both alternative lineages supported equally

- conflict data implies 2 mutations at that site - eg in the ancestor of O55 and O157 strains before isolation and again in one of the lineages.

IUPAC those insertions due to error in EDL933 sequence

[i] Base, number or "-" indicates the base type or absence of the base. Blank means the site not present.
