# Supplementary material for: Derivation of Escherichia coli O157:H7 from Its O55:H7 Precursor
Source: PLoS One. 2010 Jan 14;5(1):e8700. doi: 10.1371/journal.pone.0008700 (PMC2806823; doi:10.1371/journal.pone.0008700)
Supplement: Table S5 — A summary of mutational and recombination changes in three ExPEC genomes. The numbers of bases affected by mutation or recombination, as shown in Table S7, were separated into synonymous, non-synonymous, or non-coding sites and small inserts or deletions. (0.03 MB PDF) [file pone.0008700.s007.pdf]

Table S5. Summary of mutational and recombination changes in the 3 ExPEC genomes

	Mutational SNPs[a]							Recombination events[b]							
Lineage	NC	NS	S	ins	del	indel	total	No. events	NC	NS	S	ins	del	indel	total
UTI89	44	149	86	11	10	-	300	4	15/1	9/1	48/1	0	0	-	72/3
AS[c]	25	74	76	6	14	-	195	3	8/2	44/10	80/12	1	1/1	-	134/25
S88[d]	40/1(4)	123/2	71/2	8/2(1)	19/2(1)	-	261/9(6)	8	263/33(1)	468/56(1)	1442/193(1)	0/3	1	-	2174/285(3)
APEC 01[d]	58/6	149/4(4)	95/1	40/5(1)	21/2	-	363/18(5)	13	102/41(15)	34/14(2)	81/30	33/10	5	-	255/95(17)
UTI89/AS[c]	3	3	3	-	-	2	11	11	39	298	739	-	-	2	1078
APEC01/S88[e]	0	0	0	-	-	2	2	1	323	0	0	-	-	1	324
Total[c]	172/7(4)	498/6(4)	331/3	65/7(2)	64/4(1)	4	1134/27(11)	40	750/77(16)	853/81(3)	2390/236(1)	34/13	7/1	3	4037/408(20)

[a] Excludes SNPs in regions thought to have entered by recombination. NS, non-synonymous, S, synonymous, NC, in non-coding genes;
[b] SNPs in regions thought to have undergone recombination. NS, non-synonymous, S, synonymous, NC, non coding
[c] AS: allocated to the lineage to the common ancestor of APEC 01 and S88; UTI89/AS: allocated to the divergence between UTI89 and the common ancestor of APEC 01 and S88 (lineage not specified); APEC/S88: allocated to the divergence between APEC 01 and S88 lineages (lineage not specified)..
[d] Numbers with outgroup supported/conflict(no support)
