# Supplementary material for: Derivation of Escherichia coli O157:H7 from Its O55:H7 Precursor
Source: PLoS One. 2010 Jan 14;5(1):e8700. doi: 10.1371/journal.pone.0008700 (PMC2806823; doi:10.1371/journal.pone.0008700)
Supplement: Table S6 — Single base and small indel differences between the UTI89, S88, and APEC 01 genomes. A full list with location, nature of difference, length of indels, and name of gene affected. (0.18 MB PDF) [file pone.0008700.s008.pdf]

lineage[a]	site[b]	mutation type[c]	recomb	gene	Gene name	Type
APEC	5118	nc		intergenic		
UTI89	6015	ns		UTI89_C0007	yaaA	CDS
AS	6082	ns		UTI89_C0007	yaaA	CDS
UTI89	7680	ns		UTI89_C0008	yaaJ	CDS
APEC	31161	s		UTI89_C0035	-	CDS
S88	31393	del		intergenic		
UTI89	37126	s		UTI89_C0040	caiF	CDS
S88	39519	ns		UTI89_C0043	caiC	CDS
APEC	39679	s		UTI89_C0043	caiC	CDS
APEC	47432	ns		UTI89_C0049	fixC	CDS
UTI89	56555	s		UTI89_C0059	pdxA	CDS
APEC	58867	s		UTI89_C0061	imp	CDS
APEC	62766	ns		UTI89_C0064	hepA	CDS
APEC	65993	s		UTI89_C0065	polB	CDS
UTI89	67549	ns		UTI89_C0065	polB	CDS
APEC	73312	nc		intergenic		
APEC	73794	del		UTI89_C0071	-	CDS
UTI89	74177	del		UTI89_C0071	-	CDS
AS	74988	nc		intergenic		
APEC	76795	s		UTI89_C0075	thiP	CDS
UTI89	91573	nc		intergenic		
UTI89	91574	nc		intergenic		
AS	92765	s		UTI89_C0091	mraW	CDS
S88	97682	s		UTI89_C0095	murF	CDS
APEC	104309	ns		UTI89_C0100	murC	CDS
S88	109023	ns		UTI89_C0105	lpxC	CDS
UTI89	113567	ns		UTI89_C0108	mutT	CDS
S88	117905	ns		UTI89_C0113	hofC	CDS
S88	121009	ns		UTI89_C0117	nadC	CDS
APEC	123895	nc		intergenic		
S88	125858	ns		UTI89_C0121	usp	CDS
APEC	126423	ns		UTI89_C0122	-	CDS
APEC	127885	nc		intergenic		
AS	133966	del		UTI89_C0129	lpdA	CDS
APEC	140753	del-4		UTI89_C0132	yacL	CDS
S88	154676	ns		UTI89_C0147	panC	CDS
APEC	160783	ns		UTI89_C0153	htrE	CDS
AS	161005	s		UTI89_C0153	htrE	CDS
UTI89	164019	ns		UTI89_C0157	pcnB	CDS
AS	170142	s		UTI89_C0162	hrpB	CDS
S88	181046	s		UTI89_C0171	yadQ	CDS

lineage[a]	site[b]	mutation type[c]	recomb	gene	Gene name	Type
S88	183902	s		UTI89_C0174	yadT	CDS
APEC	184330	s		UTI89_C0175	mtn	CDS
APEC	204744	ns		UTI89_C0192	ecfK	CDS
UTI89	209338	s		UTI89_C0198	rnhB	CDS
APEC	211459	s		UTI89_C0199	dnaE	CDS
S88	216203	ns		UTI89_C0201	ldcC	CDS
UTI89	216887	ns		UTI89_C0202	yaeR	CDS
APEC	219515	ns		UTI89_C0206	yaeQ	CDS
S88	221103	ns		UTI89_C0209	yaeF	CDS
S88	221634	ns	rec	UTI89_C0209	yaeF	CDS
S88	221635	s	rec	UTI89_C0209	yaeF	CDS
APEC	228436	nc		intergenic		
APEC	228446	nc		intergenic		
APEC	228589	nc		intergenic		
APEC	228636	nc		intergenic		
UTI89	228733	nc		intergenic		
UTI89	228787	nc		intergenic		
APEC	229501	nc	rec	intergenic		
APEC	229505	nc	rec	intergenic		
APEC	229509	nc	rec	intergenic		
APEC	229518	nc	rec	intergenic		
APEC	229519	nc	rec	intergenic		
APEC	229520	nc	rec	intergenic		
APEC	229521	nc	rec	intergenic		
APEC	229522	nc	rec	intergenic		
APEC	229537	nc	rec	intergenic		
UTI89	229776	s		UTI89_C0218	-	CDS
APEC	230062	nc	rec	intergenic		
APEC	230556	nc	rec	intergenic		
APEC	230829	ns	rec	UTI89_C0222	-	CDS
APEC	230830	ns	rec	UTI89_C0222	-	CDS
APEC	231014	nc	rec	intergenic		
APEC	231016	nc	rec	intergenic		
APEC	231017	nc	rec	intergenic		
APEC	231021	nc	rec	intergenic		
APEC	231023	nc	rec	intergenic		
APEC	231025	ins	rec	intergenic		
UTI89	231135	nc		intergenic		
APEC	231319	nc	rec	intergenic		
APEC	231644	nc	rec	intergenic		
APEC	231647	ins	rec	intergenic		

lineage[a]	site[b]	mutation type[c]	recomb	gene	Gene name	Type
APEC	231651	nc	rec	intergenic		
APEC	231692	nc	rec	intergenic		
APEC	231693	nc	rec	intergenic		
APEC	231702	nc	rec	intergenic		
APEC	231703	nc	rec	intergenic		
APEC	231979	nc	rec	intergenic		
APEC	232021	nc	rec	intergenic		
UTI89	232332	nc		intergenic		
APEC	232342	del-2	rec	intergenic		
APEC	232343	nc	rec	intergenic		
APEC	232345	nc	rec	intergenic		
APEC	232678	nc	rec	intergenic		
APEC	232713	ins	rec	intergenic		
APEC	232720	ins	rec	intergenic		
APEC	232731	nc	rec	intergenic		
APEC	232733	nc	rec	intergenic		
APEC	232741	ins	rec	intergenic		
APEC	232760	nc	rec	intergenic		
APEC	232774	nc	rec	intergenic		
APEC	232776	nc	rec	intergenic		
APEC	232777	ins	rec	intergenic		
APEC	232780	nc	rec	intergenic		
APEC	232804	ins		intergenic		
APEC	232823	ins		intergenic		
APEC	232873	ins		intergenic		
APEC	232893	ins		intergenic		
S88	233418	ns		UTI89_C0223	-	CDS
UTI89	233433	ins		UTI89_C0223	-	CDS
S88	233439	ins		UTI89_C0223	-	CDS
UTI89	239048	ns		UTI89_C0231	gloB	CDS
AS	239525	s		UTI89_C0231	gloB	CDS
APEC	241861	nc	rec	intergenic		
APEC	241863	nc	rec	intergenic		
APEC	241864	nc	rec	intergenic		
S88	241945	nc		intergenic		
AS	243169	nc		intergenic		
S88	244031	ns		UTI89_C0238	-	CDS
APEC	245709	ns		UTI89_C0239	-	CDS
S88	249443	ns		UTI89_C0240	-	CDS
S88	250472	ns		UTI89_C0241	-	CDS
S88	253201	ns		UTI89_C0242	-	CDS

lineage[a]	site[b]	mutation type[c]	recomb	gene	Gene name	Type
S88	260626	ns		UTI89_C0249	-	CDS
S88	263284	nc		intergenic		
AS	263296	nc		intergenic		
APEC	266641	ns		UTI89_C0254	-	CDS
APEC	268492	ns		UTI89_C0255	-	CDS
APEC	276772	ins		intergenic		
UTI89	278807	ns		UTI89_C0266	yafL	CDS
AS	280794	ns		UTI89_C0269	fhiA	CDS
AS	311464	nc		intergenic		
UTI89	310009	ins		intergenic		
S88	300918	nc		intergenic		
APEC	315157	nc		intergenic		
S88	310135	ns		UTI89_C0309	matB	CDS
UTI89	320925	ns		UTI89_C0309	matB	CDS
UTI89	321246	del		UTI89_C0309	matB	CDS
APEC	323767	nc		intergenic		
APEC	325910	s		UTI89_C0315	-	CDS
S88	318098	nc		intergenic		
AS	331177	s		UTI89_C0319	-	CDS
AS	333894	s		UTI89_C0321	eaeH	CDS
AS	338108	nc		intergenic		
UTI89	339541	ns		UTI89_C0327	ykgC	CDS
APEC	344072	ins		UTI89_C0330	ykgF	CDS
UTI89	342623	ns		UTI89_C0330	ykgF	CDS
UTI89	344595	del		intergenic		
APEC	351257	nc		intergenic		
S88	340182	ins		intergenic		
AS	358679	s		UTI89_C0343	betT	CDS
AS	365502	s		UTI89_C0350	yahF	CDS
AS	366230	s		UTI89_C0351	yahG	CDS
APEC	368325	ns		UTI89_C0352	yahI	CDS
S88	357010	ns		UTI89_C0353	yahJ	CDS
UTI89	377150	nc		intergenic		
UTI89	378067	nc		intergenic		
S88	375592	s		UTI89_C0368	codA	CDS
UTI89	386235	s		UTI89_C0368	codA	CDS
APEC	390932	ns		UTI89_C0371	lacZ	CDS
APEC	393849	ins		intergenic		
APEC	396951	s		UTI89_C0376	adhC	CDS
AS	399882	ns		UTI89_C0381	yaiP	CDS
UTI89	399075	ns		UTI89_C0381	yaiP	CDS

lineage[a]	site[b]	mutation type[c]	recomb	gene	Gene name	Type
APEC	401910	del-5		intergenic		
APEC	406963	nc		intergenic		
APEC	409276	s		UTI89_C0389	-	CDS
S88	404881	del		UTI89_C0400	-	CDS
APEC	417669	ns		UTI89_C0402	phoA	CDS
S88	409140	s		UTI89_C0405	proC	CDS
APEC	424865	nc		intergenic		
APEC	425775	ins		UTI89_C0414	rdgC	CDS
S88	415132	ns		UTI89_C0416	araJ	CDS
APEC	432372	ns		UTI89_C0418	sbcD	CDS
UTI89	432469	ns		UTI89_C0421	phoR	CDS
APEC	434208	ns		UTI89_C0421	phoR	CDS
UTI89	433304	s		UTI89_C0421	phoR	CDS
APEC	439549	s		UTI89_C0425	malZ	CDS
APEC	441697	ns		UTI89_C0427	queA	CDS
UTI89	442626	s		UTI89_C0430	secD	CDS
APEC	456245	ns		UTI89_C0444	ispA	CDS
UTI89	456678	s		UTI89_C0446	yajK	CDS
S88	454196	ins-9		UTI89_C0456	cyoA	CDS
AS	466419	ins-9		UTI89_C0456	cyoA	CDS
AS	474663	s		UTI89_C0467	lon	CDS
AS	474886	ns		UTI89_C0467	lon	CDS
AS	483959	nc		intergenic		
APEC	485133	ns		UTI89_C0476	mdlA	CDS
APEC	493298	s		UTI89_C0484	ylaB	CDS
AS	495091	nc		intergenic		
AS	498744	s		UTI89_C0489	acrB	CDS
AS	513830	ns		UTI89_C0503	hemH	CDS
APEC	513964	s		UTI89_C0503	hemH	CDS
S88	515249	s		UTI89_C0515	ybaR	CDS
S88	517771	s		UTI89_C0517	ybaT	CDS
APEC	532997	ns		UTI89_C0521	-	CDS
S88	521184	nc		intergenic		
S88	523141	ns		UTI89_C0524	ybbK	CDS
UTI89	535190	s		UTI89_C0526	ybbM	CDS
AS	540553	ns		UTI89_C0531	ybbP	CDS
AS	540907	ns		UTI89_C0531	ybbP	CDS
AS	540920	ns		UTI89_C0531	ybbP	CDS
UTI89	543064	nc		intergenic		
AS	551199	s		UTI89_C0539	ybbW	CDS
S88	558494	nc	rec	intergenic		

lineage[a]	site[b]	mutation type[c]	recomb	gene	Gene name	Type
S88	558521	ns	rec	UTI89_C0562	intD	CDS
S88	558524	ns	rec	UTI89_C0562	intD	CDS
S88	558527	ns	rec	UTI89_C0562	intD	CDS
S88	558537	ns	rec	UTI89_C0562	intD	CDS
APEC	573656	del		intergenic		
APEC	573803	ins		intergenic		
UTI89	576235	ns		UTI89_C0568	nfrA	CDS
UTI89	576251	ns		UTI89_C0568	nfrA	CDS
APEC	586209	ns		UTI89_C0575	cusA	CDS
UTI89	586944	ns		UTI89_C0575	cusA	CDS
APEC	588572	ns		UTI89_C0575	cusA	CDS
UTI89	591673	nc		intergenic		
APEC	596254	ns		UTI89_C0584	fepA	CDS
AS	597299	ns		UTI89_C0584	fepA	CDS
UTI89	604433	ns		UTI89_C0591	fepG	CDS
AS	608911	ns		UTI89_C0593	ybdA	CDS
APEC	610399	ns		UTI89_C0595	entC	CDS
S88	657845	del		UTI89_C0603	ybdL	CDS
AS	619508	ns		UTI89_C0603	ybdL	CDS
UTI89	620352	ns		UTI89_C0605	ybdN	CDS
S88	660617	ns		UTI89_C0606	ybdO	CDS
APEC	626734	ins		intergenic		
S88	673585	ns		UTI89_C0622	citC	CDS
APEC	638418	s		UTI89_C0624	dpiA	CDS
APEC	639863	ns		UTI89_C0625	dcuC	CDS
UTI89	638644	nc		intergenic		
APEC	648599	ins		UTI89_C0635	rlpA	CDS
APEC	649809	s		UTI89_C0637	mrdA	CDS
S88	690039	s		UTI89_C0637	mrdA	CDS
UTI89	659858	ns		UTI89_C0650	gltJ	CDS
AS	663547	nc		intergenic		
S88	702738	ns		UTI89_C0653	-	CDS
AS	665560	ns		UTI89_C0653	-	CDS
APEC	668292	ns		UTI89_C0657	ybeY	CDS
AS	672546	s		UTI89_C0660	yleB	CDS
S88	720933	del		UTI89_C0676	-	CDS
UTI89	682623	ns		UTI89_C0678	-	CDS
APEC	689398	s		UTI89_C0681	-	CDS
APEC	701515	ns		UTI89_C0694	potE	CDS
UTI89	703110	ns		UTI89_C0698	kdpE	CDS
UTI89	712215	ns		UTI89_C0706	phrB	CDS

lineage[a]	site[b]	mutation type[c]	recomb	gene	Gene name	Type
UTI89	714992	nc		intergenic		
S88	766076	nc		intergenic		
APEC	732257	s		UTI89_C0723	sucC	CDS
APEC	732869	s		UTI89_C0723	sucC	CDS
APEC	736412	nc		intergenic		
APEC	741494	s		UTI89_C0734	tolR	CDS
S88	785125	nc		intergenic		
APEC	750629	s		UTI89_C0751	aroG	CDS
APEC	751717	s		UTI89_C0752	gpmA	CDS
APEC	753934	s		UTI89_C0754	galK	CDS
S88	806049	ns		UTI89_C0768	ybhI	CDS
APEC	774004	ins		UTI89_C0772	bioA	CDS
AS	774437	s		UTI89_C0773	bioB	CDS
UTI89	779702	nc		intergenic		
AS	781398	nc		intergenic		
UTI89	786791	s		UTI89_C0791	ybhP	CDS
APEC	788919	ns		UTI89_C0791	ybhP	CDS
APEC	789267	s		UTI89_C0792	ybhQ	CDS
UTI89	792683	ns		UTI89_C0796	ybhG	CDS
APEC	796678	ns		UTI89_C0798	rhlE	CDS
S88	835220	ns		UTI89_C0799	-	CDS
UTI89	799830	ns		UTI89_C0804	ybiC	CDS
APEC	804662	ns		UTI89_C0808	ybiL	CDS
S88	844082	ns		UTI89_C0808	ybiL	CDS
UTI89	806581	ns		UTI89_C0811	ybiO	CDS
APEC	809162	ns		UTI89_C0811	ybiO	CDS
UTI89	810780	ns		UTI89_C0815	-	CDS
AS	812413	ns		UTI89_C0815	-	CDS
APEC	820701	s		UTI89_C0824	ybiT	CDS
APEC	831437	ns		UTI89_C0832	-	CDS
APEC	831942	ns		UTI89_C0832	-	CDS
APEC	832915	ins		UTI89_C0833	-	CDS
APEC	835714	nc		intergenic		
UTI89	835947	s		UTI89_C0836	yliE	CDS
UTI89	843239	ns		UTI89_C0843	deoR	CDS
AS	851213	s		UTI89_C0850	ybjL	CDS
S88	896241	ns		UTI89_C0858	potG	CDS
UTI89	860894	ns		UTI89_C0863	artJ	CDS
S88	901752	del		intergenic		
AS	866914	del		UTI89_C0868	ybjP	CDS
S88	907849	ns		UTI89_C0872	ybjT	CDS
UTI89	874804	nc		intergenic		

lineage[a]	site[b]	mutation type[c]	recomb	gene	Gene name	Type
APEC	879148	ns		UTI89_C0880	ybjD	CDS
UTI89	887400	ns		UTI89_C0890	-	CDS
UTI89	898073	ns		UTI89_C0901	cydC	CDS
UTI89	899916	ns		UTI89_C0902	cydD	CDS
APEC	904128	nc		intergenic		
UTI89	905103	ns		UTI89_C0905	ftsK	CDS
AS	963391	s		UTI89_C0979	aroA	CDS
UTI89	965722	s		UTI89_C0981	cmk	CDS
APEC	968986	s		UTI89_C0984	ycaI	CDS
APEC	969657	del-5		UTI89_C0984	ycaI	CDS
APEC	969814	del		UTI89_C0984	ycaI	CDS
APEC	974947	ns		UTI89_C0989	kdsB	CDS
S88	980014	ns		UTI89_C0991	ycbC	CDS
UTI89	978577	ns		UTI89_C0993	smtA	CDS
S88	982782	ns		UTI89_C0995	mukE	CDS
S88	1000141	nc		intergenic		
UTI89	1001866	s		UTI89_C1009	ycbP	CDS
APEC	1004073	nc		intergenic		
AS	1007958	s		UTI89_C1014	uup	CDS
S88	1011855	nc		intergenic		
S88	1020061	nc		intergenic		
S88	1025569	ns		UTI89_C1028	helD	CDS
UTI89	1023423	s		UTI89_C1028	helD	CDS
UTI89	1027187	ns		UTI89_C1034	yccW	CDS
S88	1029784	del		UTI89_C1034	yccW	CDS
APEC	1035939	ns		UTI89_C1047	appB	CDS
AS	1045232	del-10		intergenic		
AS	1049569	s		UTI89_C1062	torD	CDS
APEC	1069313	s		UTI89_C1082	ycdB	CDS
AS	1070160	ns		UTI89_C1082	ycdB	CDS
UTI89	1071431	nc		intergenic		
S88	1079858	ns		UTI89_C1087	ycdS	CDS
AS	1076603	ns		UTI89_C1087	ycdS	CDS
UTI89	1158594	ns		UTI89_C1175	yceK	CDS
UTI89	1171909	ns		UTI89_C1193	mviM	CDS
APEC	1113615	ns		UTI89_C1198	flgB	CDS
S88	1120418	ns		UTI89_C1202	flgF	CDS
APEC	1119396	ns		UTI89_C1205	flgI	CDS
UTI89	1189065	s		UTI89_C1209	rne	CDS
UTI89	1197653	ns		UTI89_C1221	fabF	CDS
S88	1147584	ns		UTI89_C1230	-	CDS

lineage[a]	site[b]	mutation type[c]	recomb	gene	Gene name	Type
APEC	1146584	ns		UTI89_C1235	ycfO	CDS
UTI89	1213900	nc		intergenic		
UTI89	1214628	s		UTI89_C1241	ycfS	CDS
APEC	1152100	s		UTI89_C1241	ycfS	CDS
APEC	1154549	ns		UTI89_C1242	mfd	CDS
APEC	1170175	ns		UTI89_C1257	-	CDS
AS	1172456	s		UTI89_C1259	phoP	CDS
S88	1177079	s		UTI89_C1260	purB	CDS
AS	1179437	s		UTI89_C1267	-	CDS
UTI89	1247499	nc		intergenic		
APEC	1188415	del		intergenic		
S88	1193074	del-13		UTI89_C1290	-	CDS
UTI89	1256059	s		UTI89_C1300	ybcQ1	CDS
AS	1195284	nc		intergenic		
APEC	1198616	del		UTI89_C1310	-	CDS
UTI89	1261370	ins		UTI89_C1310	-	CDS
AS	1198962	ns		UTI89_C1311	nohA1	CDS
UTI89	1265584	s		UTI89_C1314	-	CDS
UTI89	1265659	s		UTI89_C1314	-	CDS
S88	1208449	s	rec	UTI89_C1316	-	CDS
S88	1209108	ns	rec	UTI89_C1317	-	CDS
APEC	1205714	ns		UTI89_C1317	-	CDS
S88	1209796	s	rec	UTI89_C1318	-	CDS
S88	1210104	ns	rec	UTI89_C1319	-	CDS
S88	1210334	s	rec	UTI89_C1319	-	CDS
S88	1210361	ns	rec	UTI89_C1320	-	CDS
S88	1210363	ns	rec	UTI89_C1320	-	CDS
S88	1210411	s	rec	UTI89_C1320	-	CDS
S88	1210417	s	rec	UTI89_C1320	-	CDS
S88	1210424	ns	rec	UTI89_C1320	-	CDS
S88	1210471	s	rec	UTI89_C1320	-	CDS
S88	1210479	ns	rec	UTI89_C1320	-	CDS
S88	1210526	ns	rec	UTI89_C1320	-	CDS
S88	1210573	s	rec	UTI89_C1320	-	CDS
S88	1210680	ns	rec	UTI89_C1320	-	CDS
S88	1210747	s	rec	UTI89_C1320	-	CDS
S88	1210816	s	rec	UTI89_C1320	-	CDS
S88	1210867	s	rec	UTI89_C1320	-	CDS
S88	1210947	s	rec	UTI89_C1321	-	CDS
S88	1211010	s	rec	UTI89_C1321	-	CDS
S88	1211059	ns	rec	UTI89_C1321	-	CDS

lineage[a]	site[b]	mutation type[c]	recomb	gene	Gene name	Type
S88	1211256	s	rec	UTI89_C1321	-	CDS
S88	1211328	ns	rec	UTI89_C1322	-	CDS
S88	1211335	s	rec	UTI89_C1322	-	CDS
S88	1211336	ns	rec	UTI89_C1322	-	CDS
S88	1211337	ns	rec	UTI89_C1322	-	CDS
S88	1211348	ns	rec	UTI89_C1322	-	CDS
S88	1207736	ns	rec	UTI89_C1322	-	CDS
S88	1211350	ns	rec	UTI89_C1322	-	CDS
S88	1211351	ns	rec	UTI89_C1322	-	CDS
S88	1211352	ns	rec	UTI89_C1322	-	CDS
S88	1211353	ns	rec	UTI89_C1322	-	CDS
S88	1211365	s	rec	UTI89_C1322	-	CDS
S88	1211392	ns	rec	UTI89_C1322	-	CDS
S88	1211397	ns	rec	UTI89_C1322	-	CDS
S88	1211401	s	rec	UTI89_C1322	-	CDS
S88	1211413	s	rec	UTI89_C1322	-	CDS
S88	1211429	ns	rec	UTI89_C1322	-	CDS
S88	1211430	ns	rec	UTI89_C1322	-	CDS
S88	1211453	ns	rec	UTI89_C1322	-	CDS
S88	1211503	s	rec	UTI89_C1322	-	CDS
S88	1211533	s	rec	UTI89_C1322	-	CDS
S88	1211557	s	rec	UTI89_C1322	-	CDS
S88	1211716	s	rec	UTI89_C1322	-	CDS
S88	1211746	s	rec	UTI89_C1322	-	CDS
S88	1211770	s	rec	UTI89_C1322	-	CDS
S88	1211773	s	rec	UTI89_C1322	-	CDS
S88	1211779	s	rec	UTI89_C1322	-	CDS
S88	1211785	s	rec	UTI89_C1322	-	CDS
S88	1211809	s	rec	UTI89_C1322	-	CDS
S88	1211818	s	rec	UTI89_C1322	-	CDS
S88	1211824	s	rec	UTI89_C1322	-	CDS
S88	1211827	s	rec	UTI89_C1322	-	CDS
S88	1211833	s	rec	UTI89_C1322	-	CDS
S88	1211839	s	rec	UTI89_C1322	-	CDS
S88	1211840	ns	rec	UTI89_C1322	-	CDS
S88	1211842	ns	rec	UTI89_C1322	-	CDS
S88	1211845	s	rec	UTI89_C1322	-	CDS
S88	1211848	s	rec	UTI89_C1322	-	CDS
S88	1211881	s	rec	UTI89_C1322	-	CDS
S88	1211893	s	rec	UTI89_C1322	-	CDS
S88	1211896	s	rec	UTI89_C1322	-	CDS

lineage[a]	site[b]	mutation type[c]	recomb	gene	Gene name	Type
S88	1211898	ns	rec	UTI89_C1322	-	CDS
S88	1211902	s	rec	UTI89_C1322	-	CDS
S88	1211926	s	rec	UTI89_C1322	-	CDS
S88	1212031	s	rec	UTI89_C1322	-	CDS
S88	1212032	ns	rec	UTI89_C1322	-	CDS
S88	1212055	s	rec	UTI89_C1322	-	CDS
S88	1212063	ns	rec	UTI89_C1322	-	CDS
S88	1212100	s	rec	UTI89_C1323	-	CDS
S88	1212186	ns	rec	UTI89_C1323	-	CDS
S88	1212221	ns	rec	UTI89_C1323	-	CDS
S88	1212224	ns	rec	UTI89_C1323	-	CDS
S88	1212306	ns	rec	UTI89_C1323	-	CDS
S88	1212364	s	rec	UTI89_C1323	-	CDS
S88	1212365	ns	rec	UTI89_C1323	-	CDS
S88	1212367	ns	rec	UTI89_C1323	-	CDS
S88	1212420	ns	rec	UTI89_C1323	-	CDS
S88	1212445	ns	rec	UTI89_C1323	-	CDS
S88	1212458	ns	rec	UTI89_C1323	-	CDS
S88	1212464	ns	rec	UTI89_C1323	-	CDS
S88	1212531	s	rec	UTI89_C1324	-	CDS
S88	1212582	s	rec	UTI89_C1324	-	CDS
APEC	1209027	ns		UTI89_C1324	-	CDS
S88	1212669	s	rec	UTI89_C1324	-	CDS
S88	1212721	s	rec	UTI89_C1324	-	CDS
S88	1212740	ns	rec	UTI89_C1324	-	CDS
S88	1212790	ns	rec	UTI89_C1324	-	CDS
S88	1212809	ns	rec	UTI89_C1324	-	CDS
S88	1213180	s	rec	UTI89_C1325	-	CDS
S88	1213207	s	rec	UTI89_C1325	-	CDS
S88	1213210	s	rec	UTI89_C1325	-	CDS
S88	1213216	s	rec	UTI89_C1325	-	CDS
S88	1213250	s	rec	UTI89_C1325	-	CDS
S88	1213285	s	rec	UTI89_C1325	-	CDS
S88	1213342	s	rec	UTI89_C1325	-	CDS
S88	1213394	ns	rec	UTI89_C1325	-	CDS
S88	1213399	s	rec	UTI89_C1325	-	CDS
UTI89	1274273	s		UTI89_C1325	-	CDS
S88	1216214	ns		UTI89_C1327	-	CDS
UTI89/AS	1214747	s	rec	UTI89_C1331	-	CDS
UTI89/AS	1277521	s	rec	UTI89_C1331	-	CDS
UTI89/AS	1214844	s	rec	UTI89_C1331	-	CDS

lineage[a]	site[b]	mutation type[c]	recomb	gene	Gene name	Type
UTI89/AS	1214927	s	rec	UTI89_C1331	-	CDS
UTI89/AS	1277647	s	rec	UTI89_C1331	-	CDS
UTI89/AS	1277839	s	rec	UTI89_C1331	-	CDS
UTI89/AS	1277845	s	rec	UTI89_C1331	-	CDS
UTI89/AS	1215149	s	rec	UTI89_C1331	-	CDS
UTI89/AS	1215158	s	rec	UTI89_C1331	-	CDS
UTI89/AS	1215200	s	rec	UTI89_C1331	-	CDS
UTI89/AS	1215209	s	rec	UTI89_C1331	-	CDS
UTI89/AS	1277923	s	rec	UTI89_C1331	-	CDS
UTI89/AS	1277953	s	rec	UTI89_C1331	-	CDS
UTI89/AS	1277955	ns	rec	UTI89_C1331	-	CDS
UTI89/AS	1277956	ns	rec	UTI89_C1331	-	CDS
UTI89/AS	1277986	s	rec	UTI89_C1331	-	CDS
UTI89/AS	1215293	s	rec	UTI89_C1331	-	CDS
UTI89/AS	1278046	s	rec	UTI89_C1331	-	CDS
UTI89/AS	1278118	s	rec	UTI89_C1331	-	CDS
UTI89/AS	1278166	s	rec	UTI89_C1331	-	CDS
UTI89/AS	1278337	s	rec	UTI89_C1331	-	CDS
UTI89/AS	1278346	s	rec	UTI89_C1331	-	CDS
UTI89/AS	1278361	s	rec	UTI89_C1331	-	CDS
UTI89/AS	1278376	s	rec	UTI89_C1331	-	CDS
APEC	1215695	s		UTI89_C1331	-	CDS
UTI89/AS	1278418	s	rec	UTI89_C1331	-	CDS
UTI89/AS	1278433	s	rec	UTI89_C1331	-	CDS
UTI89/AS	1278436	s	rec	UTI89_C1331	-	CDS
UTI89/AS	1278444	ns	rec	UTI89_C1331	-	CDS
UTI89/AS	1278448	s	rec	UTI89_C1331	-	CDS
UTI89/AS	1278451	s	rec	UTI89_C1331	-	CDS
UTI89/AS	1215766	ns	rec	UTI89_C1331	-	CDS
UTI89/AS	1278478	s	rec	UTI89_C1331	-	CDS
UTI89/AS	1278484	s	rec	UTI89_C1331	-	CDS
UTI89/AS	1278523	s	rec	UTI89_C1331	-	CDS
UTI89/AS	1215838	ns	rec	UTI89_C1331	-	CDS
UTI89/AS	1278550	s	rec	UTI89_C1331	-	CDS
UTI89/AS	1278556	s	rec	UTI89_C1331	-	CDS
UTI89/AS	1278604	s	rec	UTI89_C1331	-	CDS
UTI89/AS	1215954	s	rec	UTI89_C1331	-	CDS
UTI89/AS	1215959	s	rec	UTI89_C1331	-	CDS
UTI89/AS	1215963	s	rec	UTI89_C1331	-	CDS
UTI89/AS	1278691	s	rec	UTI89_C1331	-	CDS
UTI89/AS	1278733	s	rec	UTI89_C1331	-	CDS

lineage[a]	site[b]	mutation type[c]	recomb	gene	Gene name	Type
UTI89/AS	1278754	s	rec	UTI89_C1331	-	CDS
UTI89/AS	1216055	s	rec	UTI89_C1331	-	CDS
UTI89/AS	1278793	s	rec	UTI89_C1331	-	CDS
UTI89/AS	1216127	s	rec	UTI89_C1331	-	CDS
UTI89/AS	1278847	s	rec	UTI89_C1331	-	CDS
APEC	1217015	ins		UTI89_C1331	-	CDS
UTI89	1285383	ns		UTI89_C1338	sitB	CDS
AS	1222716	s		UTI89_C1338	sitB	CDS
AS	1222983	ns		UTI89_C1338	sitB	CDS
AS	1222985	ns		UTI89_C1338	sitB	CDS
APEC	1224448	ns		UTI89_C1340	-	CDS
UTI89	1290845	ns		UTI89_C1346	ycgF	CDS
S88	1241187	ns		UTI89_C1361	minC	CDS
APEC	1242532	ns		UTI89_C1370	umuC	CDS
AS	1248237	s		UTI89_C1374	ycgB	CDS
UTI89	1317484	s		UTI89_C1382	ymgE	CDS
UTI89	1318229	s		UTI89_C1383	prrA	CDS
APEC	1257278	ns		UTI89_C1383	prrA	CDS
APEC	1264192	ns		UTI89_C1389	treA	CDS
S88	1270139	ns		UTI89_C1392	ycgS	CDS
S88	1271859	ns		UTI89_C1394	-	CDS
APEC	1273071	s		UTI89_C1400	ychM	CDS
UTI89	1335889	ns		UTI89_C1400	ychM	CDS
APEC	1274989	s		UTI89_C1401	prsA	CDS
APEC	1278030	ns		UTI89_C1404	hemA	CDS
S88	1285199	s		UTI89_C1408	ychA	CDS
UTI89	1344099	s		UTI89_C1409	kdsA	CDS
APEC	1283905	ns		UTI89_C1411	chaA	CDS
S88	1288133	s		UTI89_C1411	chaA	CDS
S88	1288212	nc		intergenic		
AS	1286017	ns		UTI89_C1415	ychN	CDS
APEC	1286748	ns		UTI89_C1416	ychP	CDS
UTI89	1351411	s		UTI89_C1418	narX	CDS
S88	1293938	s		UTI89_C1419	-	CDS
S88	1298524	ns		UTI89_C1421	narG	CDS
UTI89	1356862	s		UTI89_C1421	narG	CDS
APEC	1300590	nc		intergenic		
UTI89	1383331	ns		UTI89_C1449	kch	CDS
AS	1322095	s		UTI89_C1449	kch	CDS
APEC	1334641	ns		UTI89_C1469	-	CDS
APEC	1334645	del-12		UTI89_C1469	-	CDS

lineage[a]	site[b]	mutation type[c]	recomb	gene	Gene name	Type
AS	1335515	ns		UTI89_C1470	-	CDS
UTI89/AS	1397449	ns		UTI89_C1471	-	CDS
AS	1345332	ins		UTI89_C1490	-	CDS
APEC	1347766	ins		UTI89_C1495	-	CDS
APEC	1349486	s		UTI89_C1497	-	CDS
AS	1349543	s		UTI89_C1497	-	CDS
AS	1349553	ns		UTI89_C1497	-	CDS
S88	1353928	del		UTI89_C1497	-	CDS
UTI89/AS	1350611	s	rec	UTI89_C1499	-	CDS
UTI89/AS	1350612	ns	rec	UTI89_C1499	-	CDS
UTI89/AS	1412751	s	rec	UTI89_C1499	-	CDS
UTI89/AS	1412787	s	rec	UTI89_C1499	-	CDS
UTI89/AS	1412799	s	rec	UTI89_C1499	-	CDS
UTI89/AS	1350752	s	rec	UTI89_C1499	-	CDS
UTI89/AS	1350797	s	rec	UTI89_C1499	-	CDS
UTI89/AS	1412892	s	rec	UTI89_C1499	-	CDS
UTI89/AS	1350836	s	rec	UTI89_C1499	-	CDS
UTI89/AS	1350845	s	rec	UTI89_C1499	-	CDS
UTI89/AS	1350860	s	rec	UTI89_C1499	-	CDS
UTI89/AS	1412964	s	rec	UTI89_C1499	-	CDS
UTI89/AS	1412976	s	rec	UTI89_C1499	-	CDS
UTI89/AS	1413054	s	rec	UTI89_C1499	-	CDS
UTI89/AS	1413056	ns	rec	UTI89_C1499	-	CDS
UTI89/AS	1350971	ns	rec	UTI89_C1499	-	CDS
UTI89/AS	1413063	s	rec	UTI89_C1499	-	CDS
UTI89/AS	1413064	s	rec	UTI89_C1499	-	CDS
UTI89/AS	1413066	s	rec	UTI89_C1499	-	CDS
UTI89/AS	1413069	s	rec	UTI89_C1499	-	CDS
UTI89/AS	1413114	s	rec	UTI89_C1499	-	CDS
UTI89/AS	1351040	ns	rec	UTI89_C1499	-	CDS
UTI89/AS	1351073	s	rec	UTI89_C1499	-	CDS
UTI89/AS	1413180	s	rec	UTI89_C1499	-	CDS
UTI89/AS	1351124	s	rec	UTI89_C1499	-	CDS
UTI89/AS	1351125	ns	rec	UTI89_C1499	-	CDS
UTI89/AS	1351126	ns	rec	UTI89_C1499	-	CDS
UTI89/AS	1413216	s	rec	UTI89_C1499	-	CDS
UTI89/AS	1413219	s	rec	UTI89_C1499	-	CDS
UTI89/AS	1351166	s	rec	UTI89_C1499	-	CDS
UTI89/AS	1351229	s	rec	UTI89_C1499	-	CDS
UTI89/AS	1413318	s	rec	UTI89_C1499	-	CDS
UTI89/AS	1351250	s	rec	UTI89_C1499	-	CDS
UTI89/AS	1351271	s	rec	UTI89_C1499	-	CDS

lineage[a]	site[b]	mutation type[c]	recomb	gene	Gene name	Type
UTI89/AS	1413429	ns	rec	UTI89_C1499	-	CDS
UTI89	1414911	ns		UTI89_C1500	-	CDS
APEC	1353441	s		UTI89_C1500	-	CDS
APEC	1353845	ns	rec	UTI89_C1501	-	CDS
APEC	1353852	s	rec	UTI89_C1501	-	CDS
APEC	1353867	s	rec	UTI89_C1501	-	CDS
APEC	1353890	ns	rec	UTI89_C1501	-	CDS
APEC	1353900	ns	rec	UTI89_C1501	-	CDS
APEC	1353924	s	rec	UTI89_C1501	-	CDS
APEC	1353927	s	rec	UTI89_C1501	-	CDS
APEC	1353936	s	rec	UTI89_C1501	-	CDS
APEC	1353941	ns	rec	UTI89_C1501	-	CDS
APEC	1353942	ns	rec	UTI89_C1501	-	CDS
UTI89	1416046	s		UTI89_C1501	-	CDS
S88	1358183	s	rec	UTI89_C1502	-	CDS
S88	1358189	s	rec	UTI89_C1502	-	CDS
S88	1358237	s	rec	UTI89_C1502	-	CDS
S88	1358301	ns	rec	UTI89_C1502	-	CDS
S88	1358303	ns	rec	UTI89_C1502	-	CDS
S88	1358321	s	rec	UTI89_C1502	-	CDS
S88	1358324	s	rec	UTI89_C1502	-	CDS
S88	1358330	s	rec	UTI89_C1502	-	CDS
S88	1358351	s	rec	UTI89_C1502	-	CDS
S88	1358426	s	rec	UTI89_C1502	-	CDS
S88	1358427	ns	rec	UTI89_C1502	-	CDS
S88	1358432	s	rec	UTI89_C1502	-	CDS
S88	1358438	s	rec	UTI89_C1502	-	CDS
S88	1358441	s	rec	UTI89_C1502	-	CDS
S88	1358447	s	rec	UTI89_C1502	-	CDS
S88	1358453	s	rec	UTI89_C1502	-	CDS
S88	1358498	s	rec	UTI89_C1502	-	CDS
S88	1358528	s	rec	UTI89_C1502	-	CDS
S88	1358534	s	rec	UTI89_C1502	-	CDS
S88	1358538	s	rec	UTI89_C1502	-	CDS
S88	1358564	s	rec	UTI89_C1502	-	CDS
S88	1358567	ns	rec	UTI89_C1502	-	CDS
S88	1359609	s	rec	UTI89_C1504	-	CDS
S88	1359615	s	rec	UTI89_C1504	-	CDS
S88	1359624	s	rec	UTI89_C1504	-	CDS
S88	1359636	s	rec	UTI89_C1504	-	CDS
S88	1359651	s	rec	UTI89_C1504	-	CDS

lineage[a]	site[b]	mutation type[c]	recomb	gene	Gene name	Type
S88	1359652	ns	rec	UTI89_C1504	-	CDS
S88	1359655	ns	rec	UTI89_C1504	-	CDS
S88	1361762	ns		UTI89_C1508	-	CDS
UTI89	1419789	ns		UTI89_C1508	-	CDS
AS	1360595	s		UTI89_C1510	-	CDS
S88	1365338	ns		UTI89_C1511	-	CDS
APEC	1361828	ns		UTI89_C1512	-	CDS
UTI89	1426400	s		UTI89_C1517	-	CDS
AS	1364440	s		UTI89_C1517	-	CDS
AS	1364479	s		UTI89_C1517	-	CDS
AS	1364488	s		UTI89_C1517	-	CDS
AS	1364551	s		UTI89_C1517	-	CDS
AS	1365163	s	rec	UTI89_C1517	-	CDS
AS	1365166	s	rec	UTI89_C1517	-	CDS
AS	1365169	s	rec	UTI89_C1517	-	CDS
AS	1365193	s	rec	UTI89_C1517	-	CDS
AS	1365199	s	rec	UTI89_C1517	-	CDS
AS	1365226	s	rec	UTI89_C1517	-	CDS
AS	1365229	s	rec	UTI89_C1517	-	CDS
AS	1365237	ns	rec	UTI89_C1517	-	CDS
AS	1365238	ns	rec	UTI89_C1517	-	CDS
AS	1427327	s	rec	UTI89_C1517	-	CDS
AS	1365244	s	rec	UTI89_C1517	-	CDS
S88	1371241	ns		UTI89_C1517	-	CDS
APEC	1385734	ns		UTI89_C1537	yciL	CDS
APEC	1391285	s		UTI89_C1543	topA	CDS
S88	1396291	s		UTI89_C1543	topA	CDS
S88	1400475	s		UTI89_C1547	acnA	CDS
AS	1400452	ns		UTI89_C1551	yciM	CDS
UTI89	1462662	del		intergenic		
APEC	1401489	ns		UTI89_C1552	pyrF	CDS
UTI89	1464840	ns		UTI89_C1556	-	CDS
AS	1403009	s		UTI89_C1556	-	CDS
APEC	1405685	ins		intergenic		
UTI89	1468756	ns		UTI89_C1559	rnb	CDS
AS	1410338	ns		UTI89_C1562	-	CDS
UTI89	1473133	s		UTI89_C1563	-	CDS
APEC	1412333	ns		UTI89_C1564	-	CDS
AS	1415646	ns		UTI89_C1565	-	CDS
AS	1422100	ns		UTI89_C1571	sapA	CDS
S88	1427954	nc		intergenic		

lineage[a]	site[b]	mutation type[c]	recomb	gene	Gene name	Type
S88	1429509	s		UTI89_C1574	pspA	CDS
AS	1432913	ns		UTI89_C1584	ycjQ	CDS
AS	1434346	s		UTI89_C1586	ycjS	CDS
UTI89	1500217	ns		UTI89_C1589	ycjV	CDS
AS	1439710	ns		UTI89_C1590	ompG	CDS
UTI89	1504398	ns		UTI89_C1592	ycjX	CDS
UTI89	1508902	ns		UTI89_C1596	ycjG	CDS
APEC	1457172	del		intergenic		
AS	1459783	ns		UTI89_C1610	-	CDS
APEC	1466985	ns	rec	UTI89_C1615	ydaO	CDS
APEC	1466986	ns	rec	UTI89_C1615	ydaO	CDS
APEC	1466987	s	rec	UTI89_C1615	ydaO	CDS
APEC	1466996	s	rec	UTI89_C1615	ydaO	CDS
APEC	1467000	ns	rec	UTI89_C1615	ydaO	CDS
APEC	1467002	ns	rec	UTI89_C1615	ydaO	CDS
APEC	1467004	ns	rec	UTI89_C1615	ydaO	CDS
APEC	1467050	nc	rec	intergenic		
APEC	1467055	nc	rec	intergenic		
APEC	1467095	ns	rec	UTI89_C1616	-	CDS
APEC	1467296	ns	rec	UTI89_C1616	-	CDS
UTI89	1538087	nc		intergenic		
APEC	1531299	ns		UTI89_C1624	ydbH	CDS
APEC	1533566	s	rec	UTI89_C1627	entS	CDS
APEC	1533827	s	rec	UTI89_C1627	entS	CDS
APEC	1533866	s	rec	UTI89_C1627	entS	CDS
APEC	1533893	s	rec	UTI89_C1627	entS	CDS
APEC	1533899	s	rec	UTI89_C1627	entS	CDS
APEC	1533911	s	rec	UTI89_C1627	entS	CDS
APEC	1533917	s	rec	UTI89_C1627	entS	CDS
APEC	1533932	s	rec	UTI89_C1627	entS	CDS
APEC	1533962	s	rec	UTI89_C1627	entS	CDS
APEC	1533977	ns	rec	UTI89_C1627	entS	CDS
APEC	1533985	ns	rec	UTI89_C1627	entS	CDS
APEC	1534004	s	rec	UTI89_C1627	entS	CDS
APEC	1534020	ns	rec	UTI89_C1627	entS	CDS
APEC	1534028	s	rec	UTI89_C1627	entS	CDS
APEC	1534031	s	rec	UTI89_C1627	entS	CDS
APEC	1534043	s	rec	UTI89_C1627	entS	CDS
APEC	1534057	ns	rec	UTI89_C1627	entS	CDS
APEC	1534058	ns	rec	UTI89_C1627	entS	CDS
APEC	1534067	s	rec	UTI89_C1627	entS	CDS

lineage[a]	site[b]	mutation type[c]	recomb	gene	Gene name	Type
UTI89	1544148	ns		UTI89_C1627	entS	CDS
APEC	1534106	s	rec	UTI89_C1627	entS	CDS
APEC	1534121	s	rec	UTI89_C1627	entS	CDS
APEC	1534181	s	rec	UTI89_C1627	entS	CDS
APEC	1534184	s	rec	UTI89_C1627	entS	CDS
APEC	1534202	s	rec	UTI89_C1627	entS	CDS
APEC	1534205	s	rec	UTI89_C1627	entS	CDS
APEC	1534223	s	rec	UTI89_C1627	entS	CDS
APEC	1534226	s	rec	UTI89_C1627	entS	CDS
APEC	1534229	s	rec	UTI89_C1627	entS	CDS
APEC	1534232	s	rec	UTI89_C1627	entS	CDS
APEC	1534235	s	rec	UTI89_C1627	entS	CDS
APEC	1534256	s	rec	UTI89_C1627	entS	CDS
APEC	1534262	s	rec	UTI89_C1627	entS	CDS
APEC	1534265	s	rec	UTI89_C1627	entS	CDS
APEC	1534268	s	rec	UTI89_C1627	entS	CDS
APEC	1534282	ns	rec	UTI89_C1627	entS	CDS
APEC	1534283	ns	rec	UTI89_C1627	entS	CDS
APEC	1534292	s	rec	UTI89_C1627	entS	CDS
APEC	1534322	s	rec	UTI89_C1627	entS	CDS
APEC	1534325	s	rec	UTI89_C1627	entS	CDS
APEC	1534349	s	rec	UTI89_C1627	entS	CDS
APEC	1534352	ns	rec	UTI89_C1627	entS	CDS
APEC	1534406	s	rec	UTI89_C1627	entS	CDS
S88	1492041	s		UTI89_C1627	entS	CDS
AS	1539446	ns		UTI89_C1627	entS	CDS
UTI89	1552364	ns		UTI89_C1629	-	CDS
APEC	1543645	ns		UTI89_C1632	ynbC	CDS
S88	1497829	s		UTI89_C1633	ynbD	CDS
UTI89	1570842	s		UTI89_C1645	ydcG	CDS
UTI89	1575287	ns		UTI89_C1650	tehB	CDS
AS	1567667	ns		UTI89_C1654	ydcP	CDS
S88	1526738	ns		UTI89_C1660	ydcT	CDS
S88	1530289	ns		UTI89_C1664	-	CDS
S88	1532613	ns		UTI89_C1670	yncD	CDS
UTI89	1594231	ns		UTI89_C1672	ansP	CDS
AS	1589975	del		UTI89_C1678	-	CDS
APEC	1600547	ns		UTI89_C1687	narU	CDS
APEC	1607975	ns		UTI89_C1694	-	CDS
APEC	1612991	nc		intergenic		
AS	1616188	s		UTI89_C1705	yddW	CDS

lineage[a]	site[b]	mutation type[c]	recomb	gene	Gene name	Type
APEC	1617005	ns		UTI89_C1705	yddW	CDS
S88	1577835	ns		UTI89_C1709	yddB	CDS
APEC	1629186	s		UTI89_C1711	ydeM	CDS
UTI89	1643287	ns		UTI89_C1713	-	CDS
S88	1587189	ns		UTI89_C1715	ydeP	CDS
S88	1588236	nc		intergenic		
APEC	1636388	nc		intergenic		
UTI89	1647836	ns		intergenic		
APEC	1639467	ns		intergenic		
UTI89	1648718	ns		intergenic		
APEC	1643741	nc		intergenic		
APEC	1645651	nc		intergenic		
S88	1592868	nc		intergenic		
APEC	1645926	ns		UTI89_C1738	tam	CDS
APEC	1646034	ns		UTI89_C1738	tam	CDS
S88	1594816	nc		intergenic		
UTI89	1666390	nc		intergenic		
S88	1609226	ns		UTI89_C1755	-	CDS
AS	1664685	ns		UTI89_C1758	ydeD	CDS
UTI89	1683785	ns		UTI89_C1758	ydeD	CDS
AS	1665330	ns		UTI89_C1758	ydeD	CDS
APEC	1674119	ns		UTI89_C1766	ydfI	CDS
AS	1674203	nc		intergenic		
UTI89	1695714	nc		intergenic		
APEC	1677627	s		UTI89_C1770	ynfB	CDS
UTI89	1697022	s		UTI89_C1771	speG	CDS
AS	1685262	s		UTI89_C1776	ynfG	CDS
AS	1686928	ins		intergenic		
APEC	1689456	ns		UTI89_C1781	mlc	CDS
S88	1658767	ns		UTI89_C1802	ydgA	CDS
APEC	1714212	ns		UTI89_C1803	uidC	CDS
AS	1720762	ns		UTI89_C1809	malX	CDS
APEC	1721140	ns		UTI89_C1809	malX	CDS
S88	1670223	ns		UTI89_C1810	malY	CDS
AS	1727654	s		UTI89_C1817	rnfA	CDS
APEC	1729381	ns		UTI89_C1819	rnfC	CDS
UTI89	1751902	ns		UTI89_C1823	nth	CDS
S88	1687768	ns		UTI89_C1831	ydhH	CDS
APEC	1743172	del		UTI89_C1835	ydhJ	CDS
UTI89	1768156	s		UTI89_C1842	gloA	CDS
APEC	1756863	nc		intergenic		

lineage[a]	site[b]	mutation type[c]	recomb	gene	Gene name	Type
APEC	1759829	s		UTI89_C1853	ribC	CDS
APEC	1762934	ns		UTI89_C1855	ydhQ	CDS
APEC	1765066	ns		UTI89_C1860	ydhS	CDS
AS	1766739	ns		UTI89_C1862	ydhU	CDS
S88	1715773	ns		UTI89_C1864	ydhW	CDS
UTI89	1794103	ns		UTI89_C1870	ynhG	CDS
AS	1779601	s		UTI89_C1875	sufB	CDS
APEC	1781484	nc		intergenic		
APEC	1781844	nc		intergenic		
APEC	1784095	s		UTI89_C1879	ydiJ	CDS
S88	1733401	ns		UTI89_C1880	ydiK	CDS
APEC	1787595	ns		UTI89_C1881	ydiL	CDS
APEC	1790450	ns		UTI89_C1883	ydiN	CDS
UTI89	1812687	ns		UTI89_C1886	ydiF	CDS
UTI89	1819916	ns		UTI89_C1894	ydiD	CDS
APEC	1804423	s		UTI89_C1896	ydiA	CDS
AS	1808956	nc		intergenic		
APEC	1824859	ns		UTI89_C1920	yniB	CDS
AS	1825003	ns		UTI89_C1920	yniB	CDS
AS	1825873	ns		UTI89_C1921	yniC	CDS
S88	1775469	ns		UTI89_C1923	ydjN	CDS
S88	1775781	ns		UTI89_C1924	cedA	CDS
S88	1783020	s		UTI89_C1930	celB	CDS
APEC	1839564	s		UTI89_C1936	ydjR	CDS
APEC	1844229	del		UTI89_C1941	-	CDS
S88	1800875	ns		UTI89_C1952	ynjD	CDS
AS	1860341	s		UTI89_C1958	topB	CDS
AS	1862988	ns		UTI89_C1962	sppA	CDS
APEC	1866794	ns		UTI89_C1965	ydjE	CDS
APEC	1870281	del		UTI89_C1968	ydjH	CDS
S88	1818262	s		UTI89_C1969	ydjI	CDS
S88	1819955	ns		UTI89_C1971	ydjK	CDS
S88	1829635	nc		intergenic		
APEC	1883756	nc		intergenic		
S88	1831100	ns		UTI89_C1981	yeaI	CDS
AS	1883933	ns		UTI89_C1981	yeaI	CDS
S88	1833361	ns		UTI89_C1982	yeaJ	CDS
APEC	1892910	ns		UTI89_C1994	yoaG	CDS
UTI89	1913372	ns		UTI89_C1997	-	CDS
S88	1844914	s		UTI89_C1999	fadD	CDS
APEC	1918643	s		UTI89_C2029	yebQ	CDS

lineage[a]	site[b]	mutation type[c]	recomb	gene	Gene name	Type
S88	1872194	ns		UTI89_C2034	yebS	CDS
AS	1926811	ns		UTI89_C2035	yebT	CDS
APEC	1933513	ns		UTI89_C2045	-	CDS
APEC	1938743	nc		intergenic		
S88	1887361	ns		UTI89_C2053	eda	CDS
APEC	1943379	s		UTI89_C2055	zwf	CDS
APEC	1949476	ns		UTI89_C2060	yebA	CDS
APEC	1954182	ins		UTI89_C2066	yebB	CDS
APEC	1964683	del		UTI89_C2076	bisZ	CDS
UTI89	1984824	nc		intergenic		
APEC	1966763	s		UTI89_C2078	-	CDS
S88	1917519	ns		UTI89_C2082	flhA	CDS
UTI89	1992160	ns		UTI89_C2084	-	CDS
UTI89	2015743	del		intergenic		
AS	1996984	nc		intergenic		
S88	1945561	ns		UTI89_C2114	uvrC	CDS
AS	1998815	ns		UTI89_C2114	uvrC	CDS
S88	1957323	s		UTI89_C2125	fliD	CDS
APEC	2015949	nc		intergenic		
APEC	2016022	nc		intergenic		
APEC	2021530	ins		UTI89_C2138	fliF	CDS
S88	1969712	ns		UTI89_C2139	fliG	CDS
APEC	2023662	s		UTI89_C2140	fliH	CDS
APEC	2023867	ns		UTI89_C2140	fliH	CDS
UTI89	2049617	s		UTI89_C2150	fliR	CDS
S88	1978333	del-5		intergenic		
UTI89	2050425	ns		UTI89_C2151	rcsA	CDS
APEC	2035461	nc		intergenic		
S88	1984090	ns		UTI89_C2160	yedA	CDS
UTI89	2058158	s		UTI89_C2162	dcm	CDS
S88	1988569	ns		UTI89_C2165	-	CDS
S88	1988848	ns		UTI89_C2165	-	CDS
APEC	2042646	del		intergenic		
UTI89	2061411	ins		intergenic		
APEC	2090717	nc		intergenic		
AS	2095625	ns		UTI89_C2180	-	CDS
UTI89	2080256	s		UTI89_C2183	irp2	CDS
APEC	2106175	ns		UTI89_C2184	irp1	CDS
AS	2106620	s	rec	UTI89_C2184	irp1	CDS
AS	2106656	s	rec	UTI89_C2184	irp1	CDS
AS	2106662	s	rec	UTI89_C2184	irp1	CDS
AS	2106692	s	rec	UTI89_C2184	irp1	CDS

lineage[a]	site[b]	mutation type[c]	recomb	gene	Gene name	Type
AS	2106698	s	rec	UTI89_C2184	irp1	CDS
AS	2106710	s	rec	UTI89_C2184	irp1	CDS
AS	2106746	s	rec	UTI89_C2184	irp1	CDS
AS	2106764	s	rec	UTI89_C2184	irp1	CDS
AS	2106836	s	rec	UTI89_C2184	irp1	CDS
AS	2106881	s	rec	UTI89_C2184	irp1	CDS
AS	2106887	s	rec	UTI89_C2184	irp1	CDS
AS	2106893	s	rec	UTI89_C2184	irp1	CDS
AS	2106983	s	rec	UTI89_C2184	irp1	CDS
AS	2106995	s	rec	UTI89_C2184	irp1	CDS
AS	2107022	s	rec	UTI89_C2184	irp1	CDS
AS	2107034	s	rec	UTI89_C2184	irp1	CDS
AS	2107035	ns	rec	UTI89_C2184	irp1	CDS
AS	2107036	ns	rec	UTI89_C2184	irp1	CDS
AS	2107064	s	rec	UTI89_C2184	irp1	CDS
AS	2107067	s	rec	UTI89_C2184	irp1	CDS
AS	2107073	s	rec	UTI89_C2184	irp1	CDS
AS	2107091	s	rec	UTI89_C2184	irp1	CDS
AS	2107124	s	rec	UTI89_C2184	irp1	CDS
AS	2107136	s	rec	UTI89_C2184	irp1	CDS
AS	2107145	s	rec	UTI89_C2184	irp1	CDS
AS	2107152	s	rec	UTI89_C2184	irp1	CDS
AS	2107160	s	rec	UTI89_C2184	irp1	CDS
AS	2107184	s	rec	UTI89_C2184	irp1	CDS
AS	2107190	s	rec	UTI89_C2184	irp1	CDS
AS	2107206	ns	rec	UTI89_C2184	irp1	CDS
AS	2107211	s	rec	UTI89_C2184	irp1	CDS
AS	2107220	s	rec	UTI89_C2184	irp1	CDS
AS	2107223	s	rec	UTI89_C2184	irp1	CDS
AS	2107226	s	rec	UTI89_C2184	irp1	CDS
AS	2107235	s	rec	UTI89_C2184	irp1	CDS
AS	2107256	s	rec	UTI89_C2184	irp1	CDS
AS	2107313	s	rec	UTI89_C2184	irp1	CDS
AS	2107344	ns	rec	UTI89_C2184	irp1	CDS
AS	2107361	s	rec	UTI89_C2184	irp1	CDS
AS	2107433	s	rec	UTI89_C2184	irp1	CDS
AS	2107434	ns	rec	UTI89_C2184	irp1	CDS
AS	2107452	ns	rec	UTI89_C2184	irp1	CDS
AS	2107453	ns	rec	UTI89_C2184	irp1	CDS
AS	2107466	s	rec	UTI89_C2184	irp1	CDS
AS	2107493	s	rec	UTI89_C2184	irp1	CDS

lineage[a]	site[b]	mutation type[c]	recomb	gene	Gene name	Type
AS	2107496	s	rec	UTI89_C2184	irp1	CDS
AS	2107510	ns	rec	UTI89_C2184	irp1	CDS
AS	2107535	ns	rec	UTI89_C2184	irp1	CDS
AS	2107545	ns	rec	UTI89_C2184	irp1	CDS
AS	2107553	s	rec	UTI89_C2184	irp1	CDS
AS	2107554	ns	rec	UTI89_C2184	irp1	CDS
AS	2086816	ns	rec	UTI89_C2184	irp1	CDS
AS	2107577	s	rec	UTI89_C2184	irp1	CDS
AS	2107652	s	rec	UTI89_C2184	irp1	CDS
AS	2086910	ns	rec	UTI89_C2184	irp1	CDS
AS	2088045	ns	rec	UTI89_C2184	irp1	CDS
AS	2088178	ns	rec	UTI89_C2184	irp1	CDS
AS	2088614	s	rec	UTI89_C2184	irp1	CDS
AS	2088967	ns	rec	UTI89_C2184	irp1	CDS
AS	2089126	ns	rec	UTI89_C2184	irp1	CDS
AS	2089136	s	rec	UTI89_C2184	irp1	CDS
AS	2109896	s	rec	UTI89_C2184	irp1	CDS
AS	2089151	s	rec	UTI89_C2184	irp1	CDS
AS	2089226	s	rec	UTI89_C2184	irp1	CDS
AS	2109990	ns	rec	UTI89_C2184	irp1	CDS
AS	2110508	s	rec	UTI89_C2184	irp1	CDS
AS	2090138	s	rec	UTI89_C2184	irp1	CDS
AS	2111075	s	rec	UTI89_C2184	irp1	CDS
AS	2090804	s	rec	UTI89_C2184	irp1	CDS
AS	2111603	s	rec	UTI89_C2184	irp1	CDS
AS	2091425	s	rec	UTI89_C2184	irp1	CDS
AS	2114436	ns	rec	UTI89_C2184	irp1	CDS
APEC	2114980	del-2		UTI89_C2184	irp1	CDS
AS	2096723	ns	rec	UTI89_C2187	ybtE	CDS
AS	2097028	ns	rec	UTI89_C2187	ybtE	CDS
AS	2097100	ns	rec	UTI89_C2187	ybtE	CDS
AS	2098223	nc	rec	intergenic		
UTI89	2098269	ins	rec	intergenic		
AS	2119532	s	rec	UTI89_C2188	fyuA	CDS
AS	2099057	s	rec	UTI89_C2188	fyuA	CDS
AS	2119983	ns	rec	UTI89_C2188	fyuA	CDS
AS	2099262	ns	rec	UTI89_C2188	fyuA	CDS
AS	2099300	s	rec	UTI89_C2188	fyuA	CDS
AS	2100523	nc	rec	intergenic		
AS	2121430	nc	rec	intergenic		
AS	2101147	s	rec	UTI89_C2189	-	CDS

lineage[a]	site[b]	mutation type[c]	recomb	gene	Gene name	Type
AS	2122122	ns	rec	UTI89_C2189	-	CDS
AS	2122149	ns	rec	UTI89_C2189	-	CDS
AS	2101471	s	rec	UTI89_C2189	-	CDS
AS	2122906	s	rec	UTI89_C2191	-	CDS
AS	2123830	s	rec	UTI89_C2191	-	CDS
AS	2123872	s	rec	UTI89_C2191	-	CDS
AS	2123874	ns	rec	UTI89_C2191	-	CDS
AS	2123900	ns	rec	UTI89_C2191	-	CDS
AS	2123952	ns	rec	UTI89_C2191	-	CDS
AS	2124077	ns	rec	UTI89_C2191	-	CDS
AS	2124085	nc	rec	intergenic		
AS	2124186	ns	rec	UTI89_C2192	-	CDS
AS	2124241	s	rec	UTI89_C2192	-	CDS
AS	2124289	s	rec	UTI89_C2192	-	CDS
AS	2124308	ns	rec	UTI89_C2192	-	CDS
AS	2124561	ns	rec	UTI89_C2192	-	CDS
AS	2124649	nc	rec	intergenic		
AS	2124772	ns	rec	UTI89_C2193	-	CDS
AS	2124855	s	rec	UTI89_C2193	-	CDS
AS	2124894	ns	rec	UTI89_C2193	-	CDS
AS	2124912	s	rec	UTI89_C2193	-	CDS
AS	2125029	ns	rec	UTI89_C2193	-	CDS
AS	2125107	ns	rec	UTI89_C2193	-	CDS
AS	2125152	s	rec	UTI89_C2193	-	CDS
AS	2125259	ns	rec	UTI89_C2193	-	CDS
AS	2125513	ns	rec	UTI89_C2193	-	CDS
AS	2125932	nc	rec	intergenic		
AS	2125960	nc	rec	intergenic		
AS	2126230	s	rec	UTI89_C2194	shiA	CDS
AS	2126947	s	rec	UTI89_C2194	shiA	CDS
AS	2127250	s	rec	UTI89_C2194	shiA	CDS
AS	2127307	s	rec	UTI89_C2194	shiA	CDS
AS	2127337	ns	rec	UTI89_C2194	shiA	CDS
AS	2127352	ns	rec	UTI89_C2194	shiA	CDS
AS	2127374	ns	rec	UTI89_C2194	shiA	CDS
AS	2127376	ns	rec	UTI89_C2194	shiA	CDS
AS	2127378	ns	rec	UTI89_C2194	shiA	CDS
AS	2127386	ns	rec	UTI89_C2195	amn	CDS
AS	2127413	ns	rec	UTI89_C2195	amn	CDS
AS	2127726	s	rec	UTI89_C2195	amn	CDS
AS	2127861	s	rec	UTI89_C2195	amn	CDS

lineage[a]	site[b]	mutation type[c]	recomb	gene	Gene name	Type
AS	2127876	s	rec	UTI89_C2195	amn	CDS
AS	2128104	s	rec	UTI89_C2195	amn	CDS
AS	2128252	ns	rec	UTI89_C2195	amn	CDS
AS	2128292	ns	rec	UTI89_C2195	amn	CDS
AS	2130146	s		UTI89_C2197	-	CDS
AS	2130361	nc		intergenic		
AS	2130633	s		UTI89_C2199	yeeO	CDS
UTI89	2110732	del-3		UTI89_C2199	yeeO	CDS
AS	2131875	s	rec	UTI89_C2199	yeeO	CDS
APEC	2131922	ns	rec	UTI89_C2199	yeeO	CDS
AS	2131929	s	rec	UTI89_C2199	yeeO	CDS
AS	2131953	s	rec	UTI89_C2199	yeeO	CDS
APEC	2132010	ins	rec	UTI89_C2199	yeeO	CDS
AS	2132028	ns	rec	UTI89_C2199	yeeO	CDS
APEC	2132046	ins	rec	UTI89_C2199	yeeO	CDS
APEC	2132078	ins	rec	UTI89_C2199	yeeO	CDS
S88	2039792	ns	rec	UTI89_C2199	yeeO	CDS
S88	2039831	ns	rec	UTI89_C2199	yeeO	CDS
S88	2039887	ns	rec	UTI89_C2199	yeeO	CDS
S88	2039910	ns	rec	UTI89_C2199	yeeO	CDS
S88	2040103	s	rec	UTI89_C2201	cbl	CDS
S88	2040124	s	rec	UTI89_C2201	cbl	CDS
S88	2040178	s	rec	UTI89_C2201	cbl	CDS
S88	2040262	s	rec	UTI89_C2201	cbl	CDS
S88	2040337	s	rec	UTI89_C2201	cbl	CDS
S88	2040348	ns	rec	UTI89_C2201	cbl	CDS
S88	2040359	ns	rec	UTI89_C2201	cbl	CDS
S88	2040391	s	rec	UTI89_C2201	cbl	CDS
S88	2040424	s	rec	UTI89_C2201	cbl	CDS
AS	2132790	ns	rec	UTI89_C2201	cbl	CDS
APEC	2132792	ns	rec	UTI89_C2201	cbl	CDS
APEC	2132805	s	rec	UTI89_C2201	cbl	CDS
S88	2040488	ns	rec	UTI89_C2201	cbl	CDS
APEC	2132844	s	rec	UTI89_C2201	cbl	CDS
S88	2040586	s	rec	UTI89_C2201	cbl	CDS
S88	2040592	ns	rec	UTI89_C2201	cbl	CDS
AS	2132949	s	rec	UTI89_C2201	cbl	CDS
S88	2040622	s	rec	UTI89_C2201	cbl	CDS
AS	2132967	s	rec	UTI89_C2201	cbl	CDS
AS	2133012	s	rec	UTI89_C2201	cbl	CDS
APEC	2133060	s	rec	UTI89_C2201	cbl	CDS

lineage[a]	site[b]	mutation type[c]	recomb	gene	Gene name	Type
AS	2133090	s	rec	UTI89_C2201	cbl	CDS
S88	2040787	s	rec	UTI89_C2201	cbl	CDS
AS	2133123	s	rec	UTI89_C2201	cbl	CDS
S88	2040910	s	rec	UTI89_C2201	cbl	CDS
S88	2040976	s	rec	UTI89_C2201	cbl	CDS
S88	2041055	nc	rec	intergenic		
S88	2041197	s	rec	UTI89_C2202	nac	CDS
S88	2041200	s	rec	UTI89_C2202	nac	CDS
S88	2041206	s	rec	UTI89_C2202	nac	CDS
S88	2041227	s	rec	UTI89_C2202	nac	CDS
S88	2041245	s	rec	UTI89_C2202	nac	CDS
S88	2041251	s	rec	UTI89_C2202	nac	CDS
S88	2041302	s	rec	UTI89_C2202	nac	CDS
S88	2041311	s	rec	UTI89_C2202	nac	CDS
S88	2041359	s	rec	UTI89_C2202	nac	CDS
S88	2041413	s	rec	UTI89_C2202	nac	CDS
S88	2041473	s	rec	UTI89_C2202	nac	CDS
AS	2133870	ns	rec	UTI89_C2202	nac	CDS
S88	2041563	s	rec	UTI89_C2202	nac	CDS
S88	2041608	ns	rec	UTI89_C2202	nac	CDS
S88	2041641	s	rec	UTI89_C2202	nac	CDS
APEC	2134057	ns	rec	UTI89_C2202	nac	CDS
S88	2041766	ns	rec	UTI89_C2202	nac	CDS
S88	2041824	s	rec	UTI89_C2202	nac	CDS
AS	2134182	ns	rec	UTI89_C2202	nac	CDS
S88	2041890	s	rec	UTI89_C2202	nac	CDS
S88	2041911	s	rec	UTI89_C2202	nac	CDS
AS	2134277	s	rec	UTI89_C2202	nac	CDS
S88	2041971	s	rec	UTI89_C2202	nac	CDS
S88	2042044	ns	rec	UTI89_C2202	nac	CDS
S88	2042082	nc	rec	intergenic		
APEC	2134423	nc	rec	intergenic		
S88	2042102	nc	rec	intergenic		
AS	2134439	nc	rec	intergenic		
S88	2042119	nc	rec	intergenic		
S88	2042120	nc	rec	intergenic		
S88	2042170	nc	rec	intergenic		
S88	2042176	nc	rec	intergenic		
S88	2042178	nc	rec	intergenic		
S88	2042189	nc	rec	intergenic		
S88	2042193	nc	rec	intergenic		

lineage[a]	site[b]	mutation type[c]	recomb	gene	Gene name	Type
AS	2134540	nc	rec	intergenic		
S88	2042237	del	rec	intergenic		
S88	2042251	nc	rec	intergenic		
S88	2042256	nc	rec	intergenic		
AS	2134604	nc	rec	intergenic		
S88	2042450	nc	rec	intergenic		
S88	2042567	nc	rec	intergenic		
S88	2052208	nc	rec	intergenic		
S88	2052480	nc	rec	intergenic		
S88	2052825	nc	rec	intergenic		
S88	2053177	nc	rec	intergenic		
APEC	2145532	nc	rec	intergenic		
S88	2053244	nc	rec	intergenic		
APEC	2145646	nc	rec	intergenic		
APEC	2145732	nc	rec	intergenic		
S88	2053408	nc	rec	intergenic		
APEC	2146193	nc	rec	intergenic		
S88	2054020	nc	rec	intergenic		
APEC	2146832	nc	rec	intergenic		
APEC	2146935	nc	rec	intergenic		
APEC	2146973	nc	rec	intergenic		
APEC	2147028	nc	rec	intergenic		
APEC	2147033	nc	rec	intergenic		
APEC	2147070	nc	rec	intergenic		
APEC	2147092	nc	rec	intergenic		
S88	2055203	nc	rec	intergenic		
APEC	2147678	nc	rec	intergenic		
S88	2055354	ins-3	rec	intergenic		
APEC	2147719	nc	rec	intergenic		
APEC	2147735	nc	rec	intergenic		
APEC	2147738	nc	rec	intergenic		
APEC	2147746	nc	rec	intergenic		
APEC	2147945	nc	rec	intergenic		
S88	2056361	nc	rec	intergenic		
APEC	2149052	nc	rec	intergenic		
APEC	2149719	nc	rec	intergenic		
APEC	2149814	nc	rec	intergenic		
APEC	2150121	nc	rec	intergenic		
APEC	2150230	nc	rec	intergenic		
APEC	2150632	ins	rec	intergenic		
APEC	2150633	nc	rec	intergenic		

lineage[a]	site[b]	mutation type[c]	recomb	gene	Gene name	Type
UTI89	2193784	del-7	rec	intergenic		
S88	2060851	ins	rec	intergenic		
UTI89/AS	2150697	nc	rec	intergenic		
UTI89/AS	2150875	s	rec	UTI89_C2254	-	CDS
APEC	2150877	s	rec	UTI89_C2254	-	CDS
UTI89/AS	2150893	ns	rec	UTI89_C2254	-	CDS
UTI89/AS	2195518	ns	rec	UTI89_C2254	-	CDS
UTI89/AS	2150925	s	rec	UTI89_C2254	-	CDS
UTI89/AS	2195647	nc	rec	intergenic		
S88	2061554	s	rec	UTI89_C2255	-	CDS
UTI89/AS	2151388	ns	rec	UTI89_C2255	-	CDS
S88	2061644	s	rec	UTI89_C2255	-	CDS
APEC	2151479	ns	rec	UTI89_C2255	-	CDS
UTI89/AS	2151716	nc	rec	intergenic		
APEC	2151902	nc	rec	intergenic		
UTI89/AS	2151947	nc	rec	intergenic		
AS	2152137	del	rec	UTI89_C2256	-	CDS
S88	2062555	ns	rec	UTI89_C2256	-	CDS
S88	2062561	ns	rec	UTI89_C2256	-	CDS
S88	2062572	s	rec	UTI89_C2256	-	CDS
S88	2062717	nc	rec	intergenic		
S88	2062719	nc	rec	intergenic		
S88	2062773	nc	rec	intergenic		
S88	2062787	nc	rec	intergenic		
S88	2115356	ns	rec	intergenic		
S88	2115380	ns	rec	intergenic		
S88	2115452	ns	rec	intergenic		
UTI89	2256041	s	rec	intergenic		
S88	2115473	ns	rec	intergenic		
S88	2115475	ns	rec	intergenic		
S88	2115476	ns	rec	intergenic		
S88	2115479	ns	rec	intergenic		
S88	2115483	ns	rec	intergenic		
S88	2115487	ns	rec	intergenic		
S88	2115488	ns	rec	intergenic		
S88	2115491	ns	rec	intergenic		
S88	2115492	ns	rec	intergenic		
S88	2115497	ns	rec	intergenic		
S88	2115516	ns	rec	intergenic		
S88	2115520	s	rec	intergenic		
S88	2115534	ns	rec	intergenic		
S88	2115551	nc	rec	intergenic		

lineage[a]	site[b]	mutation type[c]	recomb	gene	Gene name	Type
S88	2115553	nc	rec	intergenic		
S88	2115732	ns	rec	UTI89_C2319	wzxC	CDS
S88	2115812	s	rec	UTI89_C2319	wzxC	CDS
S88	2115815	s	rec	UTI89_C2319	wzxC	CDS
S88	2115836	s	rec	UTI89_C2319	wzxC	CDS
S88	2115839	s	rec	UTI89_C2319	wzxC	CDS
UTI89	2256495	ns	rec	UTI89_C2319	wzxC	CDS
UTI89	2256497	ns	rec	UTI89_C2319	wzxC	CDS
UTI89	2256498	s	rec	UTI89_C2319	wzxC	CDS
UTI89	2256501	s	rec	UTI89_C2319	wzxC	CDS
S88	2115941	s	rec	UTI89_C2319	wzxC	CDS
S88	2116019	s	rec	UTI89_C2319	wzxC	CDS
S88	2116031	s	rec	UTI89_C2319	wzxC	CDS
UTI89	2256649	ns	rec	UTI89_C2319	wzxC	CDS
UTI89	2256657	s	rec	UTI89_C2319	wzxC	CDS
UTI89	2256660	s	rec	UTI89_C2319	wzxC	CDS
UTI89	2256666	s	rec	UTI89_C2319	wzxC	CDS
UTI89	2256759	s	rec	UTI89_C2319	wzxC	CDS
UTI89	2256774	s	rec	UTI89_C2319	wzxC	CDS
S88	2116199	s	rec	UTI89_C2319	wzxC	CDS
UTI89	2256798	s	rec	UTI89_C2319	wzxC	CDS
S88	2116235	s	rec	UTI89_C2319	wzxC	CDS
UTI89	2256843	s	rec	UTI89_C2319	wzxC	CDS
UTI89	2256882	s	rec	UTI89_C2319	wzxC	CDS
UTI89	2256891	s	rec	UTI89_C2319	wzxC	CDS
S88	2116355	s	rec	UTI89_C2319	wzxC	CDS
S88	2116415	s	rec	UTI89_C2319	wzxC	CDS
S88	2116418	s	rec	UTI89_C2319	wzxC	CDS
S88	2116442	s	rec	UTI89_C2319	wzxC	CDS
S88	2116487	s	rec	UTI89_C2319	wzxC	CDS
S88	2116514	s	rec	UTI89_C2319	wzxC	CDS
S88	2116556	s	rec	UTI89_C2319	wzxC	CDS
S88	2116625	s	rec	UTI89_C2319	wzxC	CDS
S88	2116717	s	rec	UTI89_C2319	wzxC	CDS
S88	2116727	s	rec	UTI89_C2319	wzxC	CDS
S88	2116731	ns	rec	UTI89_C2319	wzxC	CDS
UTI89	2257335	s	rec	UTI89_C2319	wzxC	CDS
S88	2116778	s	rec	UTI89_C2319	wzxC	CDS
S88	2116952	s	rec	UTI89_C2319	wzxC	CDS
S88	2116982	s	rec	UTI89_C2319	wzxC	CDS
S88	2117160	s	rec	UTI89_C2320	wcaJ	CDS

lineage[a]	site[b]	mutation type[c]	recomb	gene	Gene name	Type
S88	2117220	s	rec	UTI89_C2320	wcaJ	CDS
S88	2117283	s	rec	UTI89_C2320	wcaJ	CDS
S88	2117289	s	rec	UTI89_C2320	wcaJ	CDS
UTI89	2257888	s	rec	UTI89_C2320	wcaJ	CDS
UTI89	2257891	s	rec	UTI89_C2320	wcaJ	CDS
S88	2117339	s	rec	UTI89_C2320	wcaJ	CDS
UTI89	2257921	s	rec	UTI89_C2320	wcaJ	CDS
UTI89	2257978	s	rec	UTI89_C2320	wcaJ	CDS
S88	2117484	s	rec	UTI89_C2320	wcaJ	CDS
S88	2117514	s	rec	UTI89_C2320	wcaJ	CDS
S88	2117520	s	rec	UTI89_C2320	wcaJ	CDS
S88	2117523	s	rec	UTI89_C2320	wcaJ	CDS
S88	2117544	s	rec	UTI89_C2320	wcaJ	CDS
S88	2117559	s	rec	UTI89_C2320	wcaJ	CDS
S88	2117601	s	rec	UTI89_C2320	wcaJ	CDS
S88	2117610	s	rec	UTI89_C2320	wcaJ	CDS
UTI89	2258245	s	rec	UTI89_C2320	wcaJ	CDS
S88	2117681	ns	rec	UTI89_C2320	wcaJ	CDS
UTI89	2258260	s	rec	UTI89_C2320	wcaJ	CDS
S88	2117685	s	rec	UTI89_C2320	wcaJ	CDS
S88	2117691	s	rec	UTI89_C2320	wcaJ	CDS
S88	2117697	s	rec	UTI89_C2320	wcaJ	CDS
S88	2117709	s	rec	UTI89_C2320	wcaJ	CDS
S88	2117790	s	rec	UTI89_C2320	wcaJ	CDS
S88	2117832	s	rec	UTI89_C2320	wcaJ	CDS
UTI89	2258422	s	rec	UTI89_C2320	wcaJ	CDS
S88	2117880	s	rec	UTI89_C2320	wcaJ	CDS
S88	2117904	s	rec	UTI89_C2320	wcaJ	CDS
S88	2118044	ns	rec	UTI89_C2320	wcaJ	CDS
UTI89	2258623	s	rec	UTI89_C2320	wcaJ	CDS
S88	2118075	s	rec	UTI89_C2320	wcaJ	CDS
UTI89	2258719	s	rec	UTI89_C2320	wcaJ	CDS
S88	2118162	s	rec	UTI89_C2320	wcaJ	CDS
UTI89	2258806	s	rec	UTI89_C2320	wcaJ	CDS
S88	2118312	s	rec	UTI89_C2320	wcaJ	CDS
UTI89	2259037	nc	rec	intergenic		
S88	2118465	ins	rec	intergenic		
S88	2118546	s	rec	UTI89_C2321	cpsG	CDS
UTI89	2259164	ns	rec	UTI89_C2321	cpsG	CDS
S88	2118594	s	rec	UTI89_C2321	cpsG	CDS
UTI89	2259198	s	rec	UTI89_C2321	cpsG	CDS

lineage[a]	site[b]	mutation type[c]	recomb	gene	Gene name	Type
UTI89	2259234	s	rec	UTI89_C2321	cpsG	CDS
UTI89	2259243	s	rec	UTI89_C2321	cpsG	CDS
UTI89	2259273	s	rec	UTI89_C2321	cpsG	CDS
S88	2118699	ns	rec	UTI89_C2321	cpsG	CDS
UTI89	2259279	s	rec	UTI89_C2321	cpsG	CDS
S88	2118743	ns	rec	UTI89_C2321	cpsG	CDS
S88	2118765	s	rec	UTI89_C2321	cpsG	CDS
UTI89	2259462	s	rec	UTI89_C2321	cpsG	CDS
S88	2118891	s	rec	UTI89_C2321	cpsG	CDS
S88	2118939	s	rec	UTI89_C2321	cpsG	CDS
S88	2118945	s	rec	UTI89_C2321	cpsG	CDS
S88	2118954	s	rec	UTI89_C2321	cpsG	CDS
UTI89	2259626	s	rec	UTI89_C2321	cpsG	CDS
UTI89	2259627	s	rec	UTI89_C2321	cpsG	CDS
UTI89	2259642	s	rec	UTI89_C2321	cpsG	CDS
S88	2119101	s	rec	UTI89_C2321	cpsG	CDS
S88	2119179	s	rec	UTI89_C2321	cpsG	CDS
S88	2119191	s	rec	UTI89_C2321	cpsG	CDS
UTI89	2259770	s	rec	UTI89_C2321	cpsG	CDS
S88	2119239	s	rec	UTI89_C2321	cpsG	CDS
S88	2119251	s	rec	UTI89_C2321	cpsG	CDS
S88	2119257	s	rec	UTI89_C2321	cpsG	CDS
S88	2119260	s	rec	UTI89_C2321	cpsG	CDS
S88	2119293	s	rec	UTI89_C2321	cpsG	CDS
UTI89	2259873	s	rec	UTI89_C2321	cpsG	CDS
UTI89	2259876	s	rec	UTI89_C2321	cpsG	CDS
UTI89	2259927	s	rec	UTI89_C2321	cpsG	CDS
UTI89	2259939	s	rec	UTI89_C2321	cpsG	CDS
UTI89	2259970	ns	rec	UTI89_C2321	cpsG	CDS
S88	2119404	s	rec	UTI89_C2321	cpsG	CDS
UTI89	2260009	ns	rec	UTI89_C2321	cpsG	CDS
UTI89	2260020	s	rec	UTI89_C2321	cpsG	CDS
S88	2119449	s	rec	UTI89_C2321	cpsG	CDS
S88	2119464	s	rec	UTI89_C2321	cpsG	CDS
S88	2119497	s	rec	UTI89_C2321	cpsG	CDS
UTI89	2260152	s	rec	UTI89_C2321	cpsG	CDS
S88	2119590	s	rec	UTI89_C2321	cpsG	CDS
S88	2119617	s	rec	UTI89_C2321	cpsG	CDS
UTI89	2260212	s	rec	UTI89_C2321	cpsG	CDS
S88	2119638	s	rec	UTI89_C2321	cpsG	CDS
UTI89	2260389	s	rec	UTI89_C2321	cpsG	CDS

lineage[a]	site[b]	mutation type[c]	recomb	gene	Gene name	Type
S88	2119854	s	rec	UTI89_C2321	cpsG	CDS
S88	2119925	ns	rec	UTI89_C2322	-	CDS
S88	2119983	s	rec	UTI89_C2322	-	CDS
UTI89	2260585	ns	rec	UTI89_C2322	-	CDS
S88	2120032	ns	rec	UTI89_C2322	-	CDS
S88	2120112	s	rec	UTI89_C2323	cpsB	CDS
S88	2120157	s	rec	UTI89_C2323	cpsB	CDS
APEC	2210287	s	rec	UTI89_C2323	cpsB	CDS
S88	2120220	s	rec	UTI89_C2323	cpsB	CDS
UTI89/AS	2210305	s	rec	UTI89_C2323	cpsB	CDS
APEC	2210323	s	rec	UTI89_C2323	cpsB	CDS
UTI89/AS	2210329	s	rec	UTI89_C2323	cpsB	CDS
APEC	2210332	s	rec	UTI89_C2323	cpsB	CDS
UTI89/AS	2260839	s	rec	UTI89_C2323	cpsB	CDS
APEC	2210356	s	rec	UTI89_C2323	cpsB	CDS
APEC	2210374	s	rec	UTI89_C2323	cpsB	CDS
APEC	2210416	s	rec	UTI89_C2323	cpsB	CDS
S88	2120337	s	rec	UTI89_C2323	cpsB	CDS
S88	2120340	s	rec	UTI89_C2323	cpsB	CDS
UTI89/AS	2210452	s	rec	UTI89_C2323	cpsB	CDS
S88	2120393	s	rec	UTI89_C2323	cpsB	CDS
UTI89/AS	2260989	s	rec	UTI89_C2323	cpsB	CDS
APEC	2210530	s	rec	UTI89_C2323	cpsB	CDS
S88	2120463	s	rec	UTI89_C2323	cpsB	CDS
UTI89/AS	2210557	s	rec	UTI89_C2323	cpsB	CDS
S88	2120541	s	rec	UTI89_C2323	cpsB	CDS
APEC	2210641	s	rec	UTI89_C2323	cpsB	CDS
APEC	2210662	s	rec	UTI89_C2323	cpsB	CDS
UTI89/AS	2210665	s	rec	UTI89_C2323	cpsB	CDS
UTI89/AS	2261190	s	rec	UTI89_C2323	cpsB	CDS
S88	2120628	s	rec	UTI89_C2323	cpsB	CDS
S88	2120631	s	rec	UTI89_C2323	cpsB	CDS
APEC	2210722	s	rec	UTI89_C2323	cpsB	CDS
APEC	2210740	s	rec	UTI89_C2323	cpsB	CDS
UTI89/AS	2210773	s	rec	UTI89_C2323	cpsB	CDS
UTI89/AS	2210809	s	rec	UTI89_C2323	cpsB	CDS
UTI89/AS	2210815	s	rec	UTI89_C2323	cpsB	CDS
UTI89/AS	2210824	s	rec	UTI89_C2323	cpsB	CDS
S88	2120754	s	rec	UTI89_C2323	cpsB	CDS
S88	2120799	s	rec	UTI89_C2323	cpsB	CDS
APEC	2210887	s	rec	UTI89_C2323	cpsB	CDS

lineage[a]	site[b]	mutation type[c]	recomb	gene	Gene name	Type
S88	2120811	s	rec	UTI89_C2323	cpsB	CDS
UTI89/AS	2261397	s	rec	UTI89_C2323	cpsB	CDS
UTI89/AS	2261403	s	rec	UTI89_C2323	cpsB	CDS
UTI89/AS	2261424	ns	rec	UTI89_C2323	cpsB	CDS
APEC	2210931	ns	rec	UTI89_C2323	cpsB	CDS
APEC	2210938	s	rec	UTI89_C2323	cpsB	CDS
APEC	2210953	s	rec	UTI89_C2323	cpsB	CDS
UTI89/AS	2261472	s	rec	UTI89_C2323	cpsB	CDS
UTI89/AS	2210983	s	rec	UTI89_C2323	cpsB	CDS
UTI89/AS	2210995	s	rec	UTI89_C2323	cpsB	CDS
S88	2120966	ns	rec	UTI89_C2323	cpsB	CDS
S88	2121012	s	rec	UTI89_C2323	cpsB	CDS
S88	2121096	s	rec	UTI89_C2323	cpsB	CDS
UTI89/AS	2211219	s	rec	UTI89_C2323	cpsB	CDS
UTI89/AS	2211220	s	rec	UTI89_C2323	cpsB	CDS
UTI89/AS	2211232	s	rec	UTI89_C2323	cpsB	CDS
UTI89/AS	2211239	ns	rec	UTI89_C2323	cpsB	CDS
UTI89/AS	2261781	s	rec	UTI89_C2323	cpsB	CDS
UTI89/AS	2261802	s	rec	UTI89_C2323	cpsB	CDS
UTI89/AS	2261826	s	rec	UTI89_C2323	cpsB	CDS
UTI89/AS	2261832	s	rec	UTI89_C2323	cpsB	CDS
UTI89/AS	2261835	s	rec	UTI89_C2323	cpsB	CDS
UTI89/AS	2261922	s	rec	UTI89_C2323	cpsB	CDS
S88	2121372	ns	rec	UTI89_C2323	cpsB	CDS
S88	2121402	s	rec	UTI89_C2323	cpsB	CDS
S88	2121578	s	rec	UTI89_C2324	wcaI	CDS
S88	2121584	s	rec	UTI89_C2324	wcaI	CDS
UTI89/AS	2262170	ns	rec	UTI89_C2324	wcaI	CDS
UTI89/AS	2262172	ns	rec	UTI89_C2324	wcaI	CDS
UTI89/AS	2211678	s	rec	UTI89_C2324	wcaI	CDS
S88	2121651	ns	rec	UTI89_C2324	wcaI	CDS
APEC	2211753	s	rec	UTI89_C2324	wcaI	CDS
UTI89/AS	2262257	s	rec	UTI89_C2324	wcaI	CDS
S88	2121687	ns	rec	UTI89_C2324	wcaI	CDS
S88	2121689	s	rec	UTI89_C2324	wcaI	CDS
APEC	2211774	s	rec	UTI89_C2324	wcaI	CDS
APEC	2211777	s	rec	UTI89_C2324	wcaI	CDS
APEC	2211828	s	rec	UTI89_C2324	wcaI	CDS
APEC	2211861	s	rec	UTI89_C2324	wcaI	CDS
UTI89/AS	2211876	s	rec	UTI89_C2324	wcaI	CDS
S88	2121842	s	rec	UTI89_C2324	wcaI	CDS

lineage[a]	site[b]	mutation type[c]	recomb	gene	Gene name	Type
UTI89/AS	2262422	s	rec	UTI89_C2324	wcaI	CDS
APEC	2211934	ns	rec	UTI89_C2324	wcaI	CDS
UTI89/AS	2211937	ns	rec	UTI89_C2324	wcaI	CDS
APEC	2211942	s	rec	UTI89_C2324	wcaI	CDS
APEC	2211951	s	rec	UTI89_C2324	wcaI	CDS
S88	2121881	ns	rec	UTI89_C2324	wcaI	CDS
APEC	2211972	s	rec	UTI89_C2324	wcaI	CDS
APEC	2212020	s	rec	UTI89_C2324	wcaI	CDS
S88	2121945	ns	rec	UTI89_C2324	wcaI	CDS
UTI89/AS	2262543	ns	rec	UTI89_C2324	wcaI	CDS
UTI89/AS	2262665	s	rec	UTI89_C2324	wcaI	CDS
UTI89/AS	2262671	s	rec	UTI89_C2324	wcaI	CDS
UTI89/AS	2262680	s	rec	UTI89_C2324	wcaI	CDS
UTI89/AS	2262689	s	rec	UTI89_C2324	wcaI	CDS
UTI89/AS	2212206	s	rec	UTI89_C2324	wcaI	CDS
S88	2122220	s	rec	UTI89_C2324	wcaI	CDS
S88	2122319	s	rec	UTI89_C2324	wcaI	CDS
S88	2122373	s	rec	UTI89_C2324	wcaI	CDS
UTI89/AS	2212461	s	rec	UTI89_C2324	wcaI	CDS
APEC	2212487	ns	rec	UTI89_C2324	wcaI	CDS
UTI89/AS	2212499	s	rec	UTI89_C2324	wcaI	CDS
UTI89/AS	2212503	s	rec	UTI89_C2324	wcaI	CDS
UTI89/AS	2212506	s	rec	UTI89_C2324	wcaI	CDS
APEC	2212536	s	rec	UTI89_C2324	wcaI	CDS
UTI89/AS	2263046	s	rec	UTI89_C2324	wcaI	CDS
S88	2122490	s	rec	UTI89_C2324	wcaI	CDS
UTI89/AS	2263089	ns	rec	UTI89_C2324	wcaI	CDS
UTI89/AS	2212611	s	rec	UTI89_C2324	wcaI	CDS
S88	2122538	s	rec	UTI89_C2324	wcaI	CDS
S88	2122544	s	rec	UTI89_C2324	wcaI	CDS
APEC	2212653	s	rec	UTI89_C2324	wcaI	CDS
UTI89/AS	2212671	s	rec	UTI89_C2324	wcaI	CDS
APEC	2212680	s	rec	UTI89_C2324	wcaI	CDS
S88	2122601	s	rec	UTI89_C2324	wcaI	CDS
APEC	2212734	s	rec	UTI89_C2324	wcaI	CDS
S88	2122785	s	rec	UTI89_C2325	wcaH	CDS
S88	2122786	s	rec	UTI89_C2325	wcaH	CDS
S88	2122823	ns	rec	UTI89_C2325	wcaH	CDS
UTI89/AS	2212922	s	rec	UTI89_C2325	wcaH	CDS
UTI89/AS	2212925	ns	rec	UTI89_C2325	wcaH	CDS
UTI89/AS	2263468	s	rec	UTI89_C2325	wcaH	CDS

lineage[a]	site[b]	mutation type[c]	recomb	gene	Gene name	Type
UTI89/AS	2263476	ns	rec	UTI89_C2325	wcaH	CDS
UTI89/AS	2212985	s	rec	UTI89_C2325	wcaH	CDS
S88	2123140	s	rec	UTI89_C2325	wcaH	CDS
S88	2123148	ns	rec	UTI89_C2325	wcaH	CDS
UTI89/AS	2263776	s	rec	UTI89_C2326	wcaG	CDS
S88	2123328	s	rec	UTI89_C2326	wcaG	CDS
S88	2123442	s	rec	UTI89_C2326	wcaG	CDS
APEC	2213548	s	rec	UTI89_C2326	wcaG	CDS
S88	2123481	s	rec	UTI89_C2326	wcaG	CDS
S88	2123520	s	rec	UTI89_C2326	wcaG	CDS
S88	2123526	s	rec	UTI89_C2326	wcaG	CDS
S88	2123550	s	rec	UTI89_C2326	wcaG	CDS
UTI89/AS	2213647	s	rec	UTI89_C2326	wcaG	CDS
UTI89/AS	2264187	s	rec	UTI89_C2326	wcaG	CDS
UTI89/AS	2264196	s	rec	UTI89_C2326	wcaG	CDS
UTI89/AS	2264199	s	rec	UTI89_C2326	wcaG	CDS
UTI89/AS	2213731	s	rec	UTI89_C2326	wcaG	CDS
UTI89/AS	2213734	s	rec	UTI89_C2326	wcaG	CDS
APEC	2213755	s	rec	UTI89_C2326	wcaG	CDS
APEC	2213824	s	rec	UTI89_C2326	wcaG	CDS
S88	2123745	s	rec	UTI89_C2326	wcaG	CDS
APEC	2213839	s	rec	UTI89_C2326	wcaG	CDS
APEC	2213854	s	rec	UTI89_C2326	wcaG	CDS
UTI89/AS	2213863	s	rec	UTI89_C2326	wcaG	CDS
UTI89/AS	2264360	s	rec	UTI89_C2326	wcaG	CDS
S88	2123784	s	rec	UTI89_C2326	wcaG	CDS
S88	2123910	s	rec	UTI89_C2326	wcaG	CDS
APEC	2214013	s	rec	UTI89_C2326	wcaG	CDS
APEC	2214097	s	rec	UTI89_C2326	wcaG	CDS
APEC	2214121	s	rec	UTI89_C2326	wcaG	CDS
APEC	2214124	s	rec	UTI89_C2326	wcaG	CDS
APEC	2214145	s	rec	UTI89_C2326	wcaG	CDS
APEC	2214148	s	rec	UTI89_C2326	wcaG	CDS
UTI89/AS	2264652	s	rec	UTI89_C2326	wcaG	CDS
S88	2124102	s	rec	UTI89_C2326	wcaG	CDS
S88	2124129	s	rec	UTI89_C2326	wcaG	CDS
APEC	2214227	ns	rec	UTI89_C2326	wcaG	CDS
APEC	2214402	s	rec	UTI89_C2327	gmd	CDS
APEC	2214408	s	rec	UTI89_C2327	gmd	CDS
S88	2124332	s	rec	UTI89_C2327	gmd	CDS
APEC	2214417	s	rec	UTI89_C2327	gmd	CDS
APEC	2214429	s	rec	UTI89_C2327	gmd	CDS

lineage[a]	site[b]	mutation type[c]	recomb	gene	Gene name	Type
APEC	2214471	s	rec	UTI89_C2327	gmd	CDS
APEC	2214474	s	rec	UTI89_C2327	gmd	CDS
APEC	2214477	s	rec	UTI89_C2327	gmd	CDS
UTI89/AS	2264978	s	rec	UTI89_C2327	gmd	CDS
S88	2124437	s	rec	UTI89_C2327	gmd	CDS
S88	2124476	s	rec	UTI89_C2327	gmd	CDS
APEC	2214582	s	rec	UTI89_C2327	gmd	CDS
S88	2124521	s	rec	UTI89_C2327	gmd	CDS
S88	2124524	s	rec	UTI89_C2327	gmd	CDS
S88	2124539	s	rec	UTI89_C2327	gmd	CDS
APEC	2214624	s	rec	UTI89_C2327	gmd	CDS
APEC	2214672	s	rec	UTI89_C2327	gmd	CDS
UTI89/AS	2265184	s	rec	UTI89_C2327	gmd	CDS
APEC	2214708	s	rec	UTI89_C2327	gmd	CDS
APEC	2214720	s	rec	UTI89_C2327	gmd	CDS
APEC	2214738	s	rec	UTI89_C2327	gmd	CDS
UTI89/AS	2214774	s	rec	UTI89_C2327	gmd	CDS
UTI89/AS	2214777	s	rec	UTI89_C2327	gmd	CDS
S88	2124701	s	rec	UTI89_C2327	gmd	CDS
APEC	2214819	s	rec	UTI89_C2327	gmd	CDS
S88	2124815	s	rec	UTI89_C2327	gmd	CDS
S88	2124818	s	rec	UTI89_C2327	gmd	CDS
APEC	2214948	s	rec	UTI89_C2327	gmd	CDS
S88	2124896	s	rec	UTI89_C2327	gmd	CDS
S88	2124959	s	rec	UTI89_C2327	gmd	CDS
APEC	2215044	s	rec	UTI89_C2327	gmd	CDS
UTI89/AS	2215050	s	rec	UTI89_C2327	gmd	CDS
S88	2124995	s	rec	UTI89_C2327	gmd	CDS
APEC	2215098	s	rec	UTI89_C2327	gmd	CDS
APEC	2215125	s	rec	UTI89_C2327	gmd	CDS
APEC	2215167	s	rec	UTI89_C2327	gmd	CDS
UTI89/AS	2265689	s	rec	UTI89_C2327	gmd	CDS
S88	2125172	s	rec	UTI89_C2327	gmd	CDS
UTI89/AS	2215405	ns	rec	UTI89_C2328	wcaF	CDS
UTI89/AS	2265919	s	rec	UTI89_C2328	wcaF	CDS
S88	2125393	s	rec	UTI89_C2328	wcaF	CDS
APEC	2215495	ns	rec	UTI89_C2328	wcaF	CDS
S88	2125417	s	rec	UTI89_C2328	wcaF	CDS
S88	2125441	s	rec	UTI89_C2328	wcaF	CDS
S88	2125447	s	rec	UTI89_C2328	wcaF	CDS
S88	2125450	s	rec	UTI89_C2328	wcaF	CDS

lineage[a]	site[b]	mutation type[c]	recomb	gene	Gene name	Type
UTI89/AS	2266056	ns	rec	UTI89_C2328	wcaF	CDS
UTI89/AS	2215607	s	rec	UTI89_C2328	wcaF	CDS
APEC	2215685	ns	rec	UTI89_C2328	wcaF	CDS
UTI89/AS	2266210	s	rec	UTI89_C2328	wcaF	CDS
APEC	2215757	s	rec	UTI89_C2328	wcaF	CDS
UTI89/AS	2266537	s	rec	UTI89_C2329	wcaE	CDS
UTI89/AS	2216093	s	rec	UTI89_C2329	wcaE	CDS
UTI89/AS	2216153	s	rec	UTI89_C2329	wcaE	CDS
UTI89/AS	2216183	s	rec	UTI89_C2329	wcaE	CDS
UTI89/AS	2216189	s	rec	UTI89_C2329	wcaE	CDS
UTI89/AS	2266696	s	rec	UTI89_C2329	wcaE	CDS
UTI89/AS	2266783	s	rec	UTI89_C2329	wcaE	CDS
UTI89/AS	2216453	s	rec	UTI89_C2329	wcaE	CDS
UTI89/AS	2216459	ns	rec	UTI89_C2329	wcaE	CDS
UTI89/AS	2216460	ns	rec	UTI89_C2329	wcaE	CDS
UTI89/AS	2216465	s	rec	UTI89_C2329	wcaE	CDS
UTI89/AS	2216468	s	rec	UTI89_C2329	wcaE	CDS
UTI89/AS	2267013	s	rec	UTI89_C2329	wcaE	CDS
UTI89/AS	2267016	ns	rec	UTI89_C2329	wcaE	CDS
UTI89/AS	2216531	ns	rec	UTI89_C2329	wcaE	CDS
UTI89/AS	2216546	s	rec	UTI89_C2329	wcaE	CDS
UTI89/AS	2216564	s	rec	UTI89_C2329	wcaE	CDS
UTI89/AS	2267074	s	rec	UTI89_C2329	wcaE	CDS
UTI89/AS	2267146	s	rec	UTI89_C2329	wcaE	CDS
UTI89/AS	2267222	nc	rec	intergenic		
UTI89/AS	2267253	nc	rec	intergenic		
UTI89/AS	2267313	s	rec	UTI89_C2330	wcaD	CDS
UTI89/AS	2267328	s	rec	UTI89_C2330	wcaD	CDS
UTI89/AS	2267448	s	rec	UTI89_C2330	wcaD	CDS
UTI89/AS	2216968	s	rec	UTI89_C2330	wcaD	CDS
UTI89/AS	2267475	s	rec	UTI89_C2330	wcaD	CDS
UTI89/AS	2267613	s	rec	UTI89_C2330	wcaD	CDS
UTI89/AS	2217163	s	rec	UTI89_C2330	wcaD	CDS
UTI89/AS	2267709	s	rec	UTI89_C2330	wcaD	CDS
UTI89/AS	2267712	s	rec	UTI89_C2330	wcaD	CDS
UTI89/AS	2217220	s	rec	UTI89_C2330	wcaD	CDS
UTI89/AS	2267823	s	rec	UTI89_C2330	wcaD	CDS
UTI89/AS	2267850	s	rec	UTI89_C2330	wcaD	CDS
UTI89/AS	2267853	s	rec	UTI89_C2330	wcaD	CDS
UTI89/AS	2267889	s	rec	UTI89_C2330	wcaD	CDS
UTI89/AS	2268069	s	rec	UTI89_C2330	wcaD	CDS

lineage[a]	site[b]	mutation type[c]	recomb	gene	Gene name	Type
UTI89/AS	2268112	ns	rec	UTI89_C2330	wcaD	CDS
UTI89/AS	2217646	s	rec	UTI89_C2330	wcaD	CDS
UTI89/AS	2217663	ns	rec	UTI89_C2330	wcaD	CDS
UTI89/AS	2217682	s	rec	UTI89_C2330	wcaD	CDS
UTI89/AS	2217691	s	rec	UTI89_C2330	wcaD	CDS
UTI89/AS	2217736	s	rec	UTI89_C2330	wcaD	CDS
UTI89/AS	2268285	s	rec	UTI89_C2330	wcaD	CDS
UTI89/AS	2217853	s	rec	UTI89_C2330	wcaD	CDS
UTI89/AS	2268372	s	rec	UTI89_C2330	wcaD	CDS
UTI89/AS	2268868	s	rec	UTI89_C2331	wcaC	CDS
UTI89/AS	2268895	s	rec	UTI89_C2331	wcaC	CDS
UTI89/AS	2268964	ns	rec	UTI89_C2331	wcaC	CDS
UTI89/AS	2218470	ns	rec	UTI89_C2331	wcaC	CDS
UTI89/AS	2218529	s	rec	UTI89_C2331	wcaC	CDS
UTI89/AS	2218532	s	rec	UTI89_C2331	wcaC	CDS
UTI89/AS	2269054	s	rec	UTI89_C2331	wcaC	CDS
UTI89/AS	2218580	s	rec	UTI89_C2331	wcaC	CDS
UTI89/AS	2218583	s	rec	UTI89_C2331	wcaC	CDS
UTI89/AS	2218646	s	rec	UTI89_C2331	wcaC	CDS
UTI89/AS	2218697	s	rec	UTI89_C2331	wcaC	CDS
UTI89/AS	2269222	s	rec	UTI89_C2331	wcaC	CDS
UTI89/AS	2269270	s	rec	UTI89_C2331	wcaC	CDS
UTI89/AS	2218793	s	rec	UTI89_C2331	wcaC	CDS
UTI89/AS	2269306	s	rec	UTI89_C2331	wcaC	CDS
UTI89/AS	2269360	s	rec	UTI89_C2331	wcaC	CDS
UTI89/AS	2269362	s	rec	UTI89_C2331	wcaC	CDS
UTI89/AS	2269666	s	rec	UTI89_C2331	wcaC	CDS
UTI89/AS	2219335	s	rec	UTI89_C2332	wcaB	CDS
UTI89/AS	2269959	s	rec	UTI89_C2332	wcaB	CDS
UTI89/AS	2269962	s	rec	UTI89_C2332	wcaB	CDS
UTI89/AS	2269965	s	rec	UTI89_C2332	wcaB	CDS
UTI89/AS	2269971	s	rec	UTI89_C2332	wcaB	CDS
UTI89/AS	2270101	ns	rec	UTI89_C2332	wcaB	CDS
UTI89/AS	2270130	s	rec	UTI89_C2332	wcaB	CDS
UTI89/AS	2270249	s	rec	UTI89_C2333	wcaA	CDS
UTI89/AS	2270294	s	rec	UTI89_C2333	wcaA	CDS
UTI89/AS	2270357	ns	rec	UTI89_C2333	wcaA	CDS
UTI89/AS	2219864	ns	rec	UTI89_C2333	wcaA	CDS
UTI89/AS	2270381	s	rec	UTI89_C2333	wcaA	CDS
UTI89/AS	2219937	s	rec	UTI89_C2333	wcaA	CDS
UTI89/AS	2270435	s	rec	UTI89_C2333	wcaA	CDS

lineage[a]	site[b]	mutation type[c]	recomb	gene	Gene name	Type
UTI89/AS	2270477	s	rec	UTI89_C2333	wcaA	CDS
UTI89/AS	2220024	s	rec	UTI89_C2333	wcaA	CDS
UTI89/AS	2220036	s	rec	UTI89_C2333	wcaA	CDS
UTI89/AS	2270603	s	rec	UTI89_C2333	wcaA	CDS
UTI89/AS	2270615	s	rec	UTI89_C2333	wcaA	CDS
UTI89/AS	2270666	s	rec	UTI89_C2333	wcaA	CDS
UTI89/AS	2270705	s	rec	UTI89_C2333	wcaA	CDS
UTI89/AS	2270714	s	rec	UTI89_C2333	wcaA	CDS
UTI89/AS	2270747	s	rec	UTI89_C2333	wcaA	CDS
UTI89/AS	2270828	s	rec	UTI89_C2333	wcaA	CDS
UTI89/AS	2271244	s	rec	UTI89_C2334	-	CDS
UTI89/AS	2271394	s	rec	UTI89_C2334	-	CDS
UTI89/AS	2271466	s	rec	UTI89_C2334	-	CDS
UTI89/AS	2220992	s	rec	UTI89_C2334	-	CDS
UTI89/AS	2271502	s	rec	UTI89_C2334	-	CDS
UTI89/AS	2221051	s	rec	UTI89_C2334	-	CDS
UTI89/AS	2221090	s	rec	UTI89_C2334	-	CDS
UTI89/AS	2271595	s	rec	UTI89_C2334	-	CDS
UTI89/AS	2271598	s	rec	UTI89_C2334	-	CDS
UTI89/AS	2221151	s	rec	UTI89_C2334	-	CDS
UTI89/AS	2221172	s	rec	UTI89_C2334	-	CDS
UTI89/AS	2272156	s	rec	UTI89_C2334	-	CDS
UTI89/AS	2272225	s	rec	UTI89_C2334	-	CDS
UTI89/AS	2221826	s	rec	UTI89_C2334	-	CDS
UTI89/AS	2221850	s	rec	UTI89_C2334	-	CDS
UTI89/AS	2221853	s	rec	UTI89_C2334	-	CDS
UTI89/AS	2221939	s	rec	UTI89_C2334	-	CDS
UTI89/AS	2221943	s	rec	UTI89_C2334	-	CDS
UTI89/AS	2272460	ns	rec	UTI89_C2334	-	CDS
UTI89/AS	2221997	s	rec	UTI89_C2334	-	CDS
UTI89/AS	2222021	s	rec	UTI89_C2334	-	CDS
UTI89/AS	2272588	s	rec	UTI89_C2334	-	CDS
UTI89/AS	2222171	s	rec	UTI89_C2334	-	CDS
UTI89/AS	2222224	ns	rec	UTI89_C2334	-	CDS
UTI89/AS	2272731	ns	rec	UTI89_C2334	-	CDS
UTI89/AS	2272801	s	rec	UTI89_C2334	-	CDS
UTI89/AS	2222318	s	rec	UTI89_C2334	-	CDS
UTI89/AS	2222330	ns	rec	UTI89_C2334	-	CDS
UTI89/AS	2222332	ns	rec	UTI89_C2334	-	CDS
UTI89/AS	2272873	s	rec	UTI89_C2334	-	CDS
UTI89/AS	2222414	s	rec	UTI89_C2334	-	CDS

lineage[a]	site[b]	mutation type[c]	recomb	gene	Gene name	Type
UTI89/AS	2222471	s	rec	UTI89_C2334	-	CDS
UTI89/AS	2272978	s	rec	UTI89_C2334	-	CDS
UTI89/AS	2273011	s	rec	UTI89_C2334	-	CDS
UTI89/AS	2222552	s	rec	UTI89_C2334	-	CDS
UTI89/AS	2273071	s	rec	UTI89_C2334	-	CDS
UTI89/AS	2273074	s	rec	UTI89_C2334	-	CDS
UTI89/AS	2273077	s	rec	UTI89_C2334	-	CDS
UTI89/AS	2222607	ns	rec	UTI89_C2334	-	CDS
UTI89/AS	2273131	s	rec	UTI89_C2334	-	CDS
UTI89/AS	2273149	s	rec	UTI89_C2334	-	CDS
UTI89/AS	2273179	s	rec	UTI89_C2334	-	CDS
UTI89/AS	2222705	s	rec	UTI89_C2334	-	CDS
UTI89/AS	2273373	s	rec	UTI89_C2335	wzb	CDS
UTI89/AS	2222968	s	rec	UTI89_C2335	wzb	CDS
UTI89/AS	2223001	ns	rec	UTI89_C2335	wzb	CDS
UTI89/AS	2273535	ns	rec	UTI89_C2335	wzb	CDS
UTI89/AS	2273537	ns	rec	UTI89_C2335	wzb	CDS
UTI89/AS	2273550	s	rec	UTI89_C2335	wzb	CDS
UTI89/AS	2273553	s	rec	UTI89_C2335	wzb	CDS
UTI89/AS	2223109	s	rec	UTI89_C2335	wzb	CDS
UTI89/AS	2223115	s	rec	UTI89_C2335	wzb	CDS
UTI89/AS	2273616	s	rec	UTI89_C2335	wzb	CDS
UTI89/AS	2273619	s	rec	UTI89_C2335	wzb	CDS
UTI89/AS	2223208	s	rec	UTI89_C2335	wzb	CDS
UTI89/AS	2273852	s	rec	UTI89_C2336	wza	CDS
UTI89/AS	2223465	s	rec	UTI89_C2336	wza	CDS
UTI89/AS	2223648	s	rec	UTI89_C2336	wza	CDS
UTI89/AS	2274227	s	rec	UTI89_C2336	wza	CDS
UTI89/AS	2274254	s	rec	UTI89_C2336	wza	CDS
UTI89/AS	2223783	s	rec	UTI89_C2336	wza	CDS
UTI89/AS	2223795	s	rec	UTI89_C2336	wza	CDS
UTI89/AS	2223837	s	rec	UTI89_C2336	wza	CDS
UTI89/AS	2223849	s	rec	UTI89_C2336	wza	CDS
UTI89/AS	2223867	s	rec	UTI89_C2336	wza	CDS
UTI89/AS	2223882	s	rec	UTI89_C2336	wza	CDS
UTI89/AS	2223888	s	rec	UTI89_C2336	wza	CDS
UTI89/AS	2274509	s	rec	UTI89_C2336	wza	CDS
UTI89/AS	2274518	s	rec	UTI89_C2336	wza	CDS
UTI89/AS	2224077	s	rec	UTI89_C2336	wza	CDS
UTI89/AS	2274689	s	rec	UTI89_C2336	wza	CDS
UTI89/AS	2274692	s	rec	UTI89_C2336	wza	CDS

lineage[a]	site[b]	mutation type[c]	recomb	gene	Gene name	Type
UTI89/AS	2224235	s	rec	UTI89_C2336	wza	CDS
UTI89/AS	2274794	s	rec	UTI89_C2336	wza	CDS
UTI89	2275405	del	rec	intergenic		
UTI89/AS	2224945	nc	rec	intergenic		
UTI89/AS	2275441	nc	rec	intergenic		
UTI89/AS	2275727	s	rec	UTI89_C2337	-	CDS
UTI89/AS	2225316	s	rec	UTI89_C2337	-	CDS
UTI89/AS	2225385	s	rec	UTI89_C2337	-	CDS
UTI89/AS	2275903	s	rec	UTI89_C2337	-	CDS
UTI89/AS	2275987	s	rec	UTI89_C2337	-	CDS
UTI89/AS	2225577	s	rec	UTI89_C2337	-	CDS
UTI89/AS	2276266	s	rec	UTI89_C2337	-	CDS
UTI89/AS	2276293	s	rec	UTI89_C2337	-	CDS
UTI89/AS	2276318	s	rec	UTI89_C2337	-	CDS
UTI89/AS	2276320	s	rec	UTI89_C2337	-	CDS
UTI89/AS	2276350	s	rec	UTI89_C2337	-	CDS
UTI89/AS	2276372	ns	rec	UTI89_C2337	-	CDS
UTI89/AS	2276382	ns	rec	UTI89_C2337	-	CDS
UTI89/AS	2276386	s	rec	UTI89_C2337	-	CDS
UTI89/AS	2225955	s	rec	UTI89_C2337	-	CDS
UTI89/AS	2276548	s	rec	UTI89_C2337	-	CDS
UTI89/AS	2276554	s	rec	UTI89_C2337	-	CDS
UTI89/AS	2226072	s	rec	UTI89_C2337	-	CDS
UTI89/AS	2226075	s	rec	UTI89_C2337	-	CDS
UTI89/AS	2226078	s	rec	UTI89_C2337	-	CDS
UTI89/AS	2276581	s	rec	UTI89_C2337	-	CDS
AS	2229281	s		UTI89_C2341	dcd	CDS
AS	2231272	s		UTI89_C2343	yegE	CDS
AS	2232346	s		UTI89_C2343	yegE	CDS
AS	2233288	s		UTI89_C2343	yegE	CDS
AS	2234367	s		UTI89_C2344	alkA	CDS
AS	2234383	s		UTI89_C2344	alkA	CDS
AS	2235060	ns		UTI89_C2344	alkA	CDS
AS	2237467	ns		UTI89_C2346	yegI	CDS
AS	2241264	s		UTI89_C2349	yegM	CDS
UTI89	2292182	ns		UTI89_C2349	yegM	CDS
S88	2152753	s		UTI89_C2350	yegN	CDS
AS	2248047	ns		UTI89_C2351	yegO	CDS
APEC	2249134	ns		UTI89_C2352	yegB	CDS
UTI89	2300902	ns		UTI89_C2353	baeS	CDS
APEC	2253699	del		UTI89_C2357	-	CDS

lineage[a]	site[b]	mutation type[c]	recomb	gene	Gene name	Type
AS	2258328	s		UTI89_C2360	yegQ	CDS
APEC/S88	2259241	nc	rec	intergenic		
APEC/S88	2259243	nc	rec	intergenic		
APEC/S88	2259394	nc	rec	intergenic		
APEC/S88	2259609	nc	rec	intergenic		
APEC/S88	2259640	nc	rec	intergenic		
APEC/S88	2259648	nc	rec	intergenic		
APEC/S88	2259649	nc	rec	intergenic		
APEC/S88	2169571	nc	rec	intergenic		
APEC/S88	2169572	nc	rec	intergenic		
APEC/S88	2259711	nc	rec	intergenic		
APEC/S88	2169720	nc	rec	intergenic		
APEC/S88	2259831	nc	rec	intergenic		
APEC/S88	2259834	nc	rec	intergenic		
APEC/S88	2259882	nc	rec	intergenic		
APEC/S88	2169825	nc	rec	intergenic		
APEC/S88	2169837	nc	rec	intergenic		
APEC/S88	2259938	nc	rec	intergenic		
APEC/S88	2259954	nc	rec	intergenic		
APEC/S88	2259969	nc	rec	intergenic		
APEC/S88	2169922	nc	rec	intergenic		
APEC/S88	2169940	nc	rec	intergenic		
APEC/S88	2169945	nc	rec	intergenic		
APEC/S88	2169951	nc	rec	intergenic		
APEC/S88	2169984	nc	rec	intergenic		
APEC/S88	2260104	nc	rec	intergenic		
APEC/S88	2260209	nc	rec	intergenic		
APEC/S88	2260211	nc	rec	intergenic		
APEC/S88	2260254	nc	rec	intergenic		
APEC/S88	2170184	nc	rec	intergenic		
APEC/S88	2260308	nc	rec	intergenic		
APEC/S88	2260314	nc	rec	intergenic		
APEC/S88	2260329	nc	rec	intergenic		
APEC/S88	2170281	nc	rec	intergenic		
APEC/S88	2170310	nc	rec	intergenic		
APEC/S88	2170338	nc	rec	intergenic		
APEC/S88	2260425	nc	rec	intergenic		
APEC/S88	2170386	nc	rec	intergenic		
APEC/S88	2170449	nc	rec	intergenic		
APEC/S88	2260753	nc	rec	intergenic		
APEC/S88	2260809	nc	rec	intergenic		
APEC/S88	2260839	nc	rec	intergenic		

lineage[a]	site[b]	mutation type[c]	recomb	gene	Gene name	Type
APEC/S88	2260886	nc	rec	intergenic		
APEC/S88	2170883	nc	rec	intergenic		
APEC/S88	2261078	nc	rec	intergenic		
APEC/S88	2171006	nc	rec	intergenic		
APEC/S88	2261111	nc	rec	intergenic		
APEC/S88	2171045	nc	rec	intergenic		
APEC/S88	2171069	nc	rec	intergenic		
APEC/S88	2261153	nc	rec	intergenic		
APEC/S88	2261162	nc	rec	intergenic		
APEC/S88	2261165	nc	rec	intergenic		
APEC/S88	2171099	nc	rec	intergenic		
APEC/S88	2171100	nc	rec	intergenic		
APEC/S88	2261215	nc	rec	intergenic		
APEC/S88	2171422	nc	rec	intergenic		
APEC/S88	2261611	nc	rec	intergenic		
APEC/S88	2261614	nc	rec	intergenic		
APEC/S88	2261620	nc	rec	intergenic		
APEC/S88	2171560	nc	rec	intergenic		
APEC/S88	2261744	nc	rec	intergenic		
APEC/S88	2171665	nc	rec	intergenic		
APEC/S88	2261755	nc	rec	intergenic		
APEC/S88	2261761	nc	rec	intergenic		
APEC/S88	2261774	nc	rec	intergenic		
APEC/S88	2261845	nc	rec	intergenic		
APEC/S88	2261881	nc	rec	intergenic		
APEC/S88	2171857	nc	rec	intergenic		
APEC/S88	2261953	nc	rec	intergenic		
APEC/S88	2171974	nc	rec	intergenic		
APEC/S88	2262058	nc	rec	intergenic		
APEC/S88	2171989	nc	rec	intergenic		
APEC/S88	2262106	nc	rec	intergenic		
APEC/S88	2172030	nc	rec	intergenic		
APEC/S88	2172031	nc	rec	intergenic		
APEC/S88	2172032	nc	rec	intergenic		
APEC/S88	2172056	nc	rec	intergenic		
APEC/S88	2262142	nc	rec	intergenic		
APEC/S88	2262148	nc	rec	intergenic		
APEC/S88	2262155	nc	rec	intergenic		
APEC/S88	2262337	nc	rec	intergenic		
APEC/S88	2262340	nc	rec	intergenic		
APEC/S88	2262349	nc	rec	intergenic		

lineage[a]	site[b]	mutation type[c]	recomb	gene	Gene name	Type
APEC/S88	2172313	nc	rec	intergenic		
APEC/S88	2262430	nc	rec	intergenic		
APEC/S88	2262451	nc	rec	intergenic		
APEC/S88	2172460	nc	rec	intergenic		
APEC/S88	2172463	nc	rec	intergenic		
APEC/S88	2172466	nc	rec	intergenic		
APEC/S88	2172469	nc	rec	intergenic		
APEC/S88	2172490	nc	rec	intergenic		
APEC/S88	2262583	nc	rec	intergenic		
APEC/S88	2262646	nc	rec	intergenic		
APEC/S88	2262700	nc	rec	intergenic		
APEC/S88	2172640	nc	rec	intergenic		
APEC/S88	2262766	nc	rec	intergenic		
APEC/S88	2172688	nc	rec	intergenic		
APEC/S88	2262898	nc	rec	intergenic		
APEC/S88	2172895	nc	rec	intergenic		
APEC/S88	2262994	nc	rec	intergenic		
APEC/S88	2172922	nc	rec	intergenic		
APEC/S88	2172931	nc	rec	intergenic		
APEC/S88	2263015	nc	rec	intergenic		
APEC/S88	2172937	nc	rec	intergenic		
APEC/S88	2172961	nc	rec	intergenic		
APEC/S88	2172970	nc	rec	intergenic		
APEC/S88	2172973	nc	rec	intergenic		
APEC/S88	2263066	nc	rec	intergenic		
APEC/S88	2263069	nc	rec	intergenic		
APEC/S88	2263090	nc	rec	intergenic		
APEC/S88	2173015	nc	rec	intergenic		
APEC/S88	2263120	nc	rec	intergenic		
APEC/S88	2173059	nc	rec	intergenic		
APEC/S88	2263147	nc	rec	intergenic		
APEC/S88	2173084	nc	rec	intergenic		
APEC/S88	2173087	nc	rec	intergenic		
APEC/S88	2263216	nc	rec	intergenic		
APEC/S88	2263225	nc	rec	intergenic		
APEC/S88	2263234	nc	rec	intergenic		
APEC/S88	2173186	nc	rec	intergenic		
APEC/S88	2173216	nc	rec	intergenic		
APEC/S88	2173258	nc	rec	intergenic		
APEC/S88	2263343	nc	rec	intergenic		
APEC/S88	2173329	nc	rec	intergenic		

lineage[a]	site[b]	mutation type[c]	recomb	gene	Gene name	Type
APEC/S88	2173336	nc	rec	intergenic		
APEC/S88	2263537	nc	rec	intergenic		
APEC/S88	2173495	nc	rec	intergenic		
APEC/S88	2263618	nc	rec	intergenic		
APEC/S88	2263624	nc	rec	intergenic		
APEC/S88	2173597	nc	rec	intergenic		
APEC/S88	2173731	nc	rec	intergenic		
APEC/S88	2173820	nc	rec	intergenic		
APEC/S88	2173826	nc	rec	intergenic		
APEC/S88	2173975	nc	rec	intergenic		
APEC/S88	2173978	nc	rec	intergenic		
APEC/S88	2264083	nc	rec	intergenic		
APEC/S88	2264096	nc	rec	intergenic		
APEC/S88	2174017	nc	rec	intergenic		
APEC/S88	2174059	nc	rec	intergenic		
APEC/S88	2174083	nc	rec	intergenic		
APEC/S88	2174121	nc	rec	intergenic		
APEC/S88	2264214	nc	rec	intergenic		
APEC/S88	2264274	nc	rec	intergenic		
APEC/S88	2264313	nc	rec	intergenic		
APEC/S88	2264382	nc	rec	intergenic		
APEC/S88	2174319	nc	rec	intergenic		
APEC/S88	2174364	nc	rec	intergenic		
APEC/S88	2264505	nc	rec	intergenic		
APEC/S88	2174429	nc	rec	intergenic		
APEC/S88	2174592	nc	rec	intergenic		
APEC/S88	2174607	nc	rec	intergenic		
APEC/S88	2174610	nc	rec	intergenic		
APEC/S88	2174763	nc	rec	intergenic		
APEC/S88	2264934	nc	rec	intergenic		
APEC/S88	2264936	nc	rec	intergenic		
APEC/S88	2264937	nc	rec	intergenic		
APEC/S88	2264938	nc	rec	intergenic		
APEC/S88	2265000	nc	rec	intergenic		
APEC/S88	2174990	nc	rec	intergenic		
APEC/S88	2265105	nc	rec	intergenic		
APEC/S88	2265141	nc	rec	intergenic		
APEC/S88	2175063	nc	rec	intergenic		
APEC/S88	2265237	nc	rec	intergenic		
APEC/S88	2265456	nc	rec	intergenic		
APEC/S88	2265480	nc	rec	intergenic		

lineage[a]	site[b]	mutation type[c]	recomb	gene	Gene name	Type
APEC/S88	2276650	nc	rec	intergenic		
APEC/S88	2276686	nc	rec	intergenic		
APEC/S88	2276725	nc	rec	intergenic		
APEC/S88	2276733	nc	rec	intergenic		
APEC/S88	2276740	nc	rec	intergenic		
APEC/S88	2276764	nc	rec	intergenic		
APEC/S88	2276767	nc	rec	intergenic		
APEC/S88	2199827	nc	rec	intergenic		
APEC/S88	2276816	nc	rec	intergenic		
APEC/S88	2199870	nc	rec	intergenic		
APEC/S88	2276825	nc	rec	intergenic		
APEC/S88	2276841	nc	rec	intergenic		
APEC/S88	2199893	nc	rec	intergenic		
APEC/S88	2276857	nc	rec	intergenic		
APEC/S88	2276920	nc	rec	intergenic		
APEC/S88	2200007	nc	rec	intergenic		
APEC/S88	2200009	nc	rec	intergenic		
APEC/S88	2200079	nc	rec	intergenic		
APEC/S88	2277037	nc	rec	intergenic		
APEC/S88	2277088	nc	rec	intergenic		
APEC/S88	2277130	nc	rec	intergenic		
APEC/S88	2277133	nc	rec	intergenic		
APEC/S88	2200202	nc	rec	intergenic		
APEC/S88	2200226	nc	rec	intergenic		
APEC/S88	2200238	nc	rec	intergenic		
APEC/S88	2200250	nc	rec	intergenic		
APEC/S88	2277211	nc	rec	intergenic		
APEC/S88	2200271	nc	rec	intergenic		
APEC/S88	2200292	nc	rec	intergenic		
APEC/S88	2277253	nc	rec	intergenic		
APEC/S88	2277256	nc	rec	intergenic		
APEC/S88	2277277	nc	rec	intergenic		
APEC/S88	2200424	nc	rec	intergenic		
APEC/S88	2200448	nc	rec	intergenic		
APEC/S88	2277409	nc	rec	intergenic		
APEC/S88	2277412	nc	rec	intergenic		
APEC/S88	2200466	nc	rec	intergenic		
APEC/S88	2200475	nc	rec	intergenic		
APEC/S88	2200478	nc	rec	intergenic		
APEC/S88	2277430	nc	rec	intergenic		
APEC/S88	2277439	nc	rec	intergenic		

lineage[a]	site[b]	mutation type[c]	recomb	gene	Gene name	Type
APEC/S88	2200526	nc	rec	intergenic		
APEC/S88	2200580	nc	rec	intergenic		
APEC/S88	2200619	nc	rec	intergenic		
APEC/S88	2277604	nc	rec	intergenic		
APEC/S88	2200664	nc	rec	intergenic		
APEC/S88	2277676	nc	rec	intergenic		
APEC/S88	2277679	nc	rec	intergenic		
APEC/S88	2277682	nc	rec	intergenic		
APEC/S88	2277698	nc	rec	intergenic		
APEC/S88	2200796	nc	rec	intergenic		
APEC/S88	2277790	nc	rec	intergenic		
APEC/S88	2277834	nc	rec	intergenic		
APEC/S88	2277907	nc	rec	intergenic		
APEC/S88	2277928	nc	rec	intergenic		
APEC/S88	2277961	nc	rec	intergenic		
APEC/S88	2201027	nc	rec	intergenic		
APEC/S88	2278099	nc	rec	intergenic		
APEC/S88	2201201	nc	rec	intergenic		
APEC/S88	2201222	nc	rec	intergenic		
APEC/S88	2278195	nc	rec	intergenic		
APEC/S88	2278210	nc	rec	intergenic		
APEC/S88	2201300	nc	rec	intergenic		
APEC/S88	2201324	nc	rec	intergenic		
APEC/S88	2278279	nc	rec	intergenic		
APEC/S88	2278321	nc	rec	intergenic		
APEC/S88	2201384	nc	rec	intergenic		
APEC/S88	2201402	nc	rec	intergenic		
APEC/S88	2201427	nc	rec	intergenic		
APEC/S88	2201440	nc	rec	intergenic		
APEC/S88	2201443	nc	rec	intergenic		
APEC/S88	2278543	nc	rec	intergenic		
APEC/S88	2201726	nc	rec	intergenic		
APEC/S88	2278744	nc	rec	intergenic		
APEC/S88	2201810	nc	rec	intergenic		
APEC/S88	2278785	nc	rec	intergenic		
APEC/S88	2201839	nc	rec	intergenic		
APEC/S88	2278844	nc	rec	intergenic		
APEC/S88	2201961	nc	rec	intergenic		
APEC/S88	2278979	nc	rec	intergenic		
APEC/S88	2202045	nc	rec	intergenic		
APEC/S88	2279099	nc	rec	intergenic		

lineage[a]	site[b]	mutation type[c]	recomb	gene	Gene name	Type
APEC/S88	2279117	nc	rec	intergenic		
APEC/S88	2279131	nc	rec	intergenic		
APEC/S88	2279158	nc	rec	intergenic		
APEC/S88	2279185	nc	rec	intergenic		
APEC/S88	2279217	nc	rec	intergenic		
APEC/S88	2279250	nc	rec	intergenic		
APEC/S88	2202439	nc	rec	intergenic		
APEC/S88	2279421	nc	rec	intergenic		
APEC/S88	2202493	nc	rec	intergenic		
APEC/S88	2279586	nc	rec	intergenic		
APEC/S88	2279589	nc	rec	intergenic		
APEC/S88	2279619	nc	rec	intergenic		
APEC/S88	2202674	nc	rec	intergenic		
APEC/S88	2202693	nc	rec	intergenic		
APEC/S88	2279780	nc	rec	intergenic		
APEC/S88	2202857	nc	rec	intergenic		
APEC/S88	2279812	nc	rec	intergenic		
APEC/S88	2279832	nc	rec	intergenic		
APEC/S88	2202943	nc	rec	intergenic		
APEC/S88	2202957	nc	rec	intergenic		
APEC/S88	2202970	nc	rec	intergenic		
APEC/S88	2279923	nc	rec	intergenic		
APEC/S88	2203005	nc	rec	intergenic		
APEC/S88	2203041	nc	rec	intergenic		
APEC/S88	2203098	nc	rec	intergenic		
APEC/S88	2203107	nc	rec	intergenic		
APEC/S88	2203113	nc	rec	intergenic		
APEC/S88	2280065	nc	rec	intergenic		
APEC/S88	2203133	nc	rec	intergenic		
APEC/S88	2203136	nc	rec	intergenic		
APEC/S88	2203137	nc	rec	intergenic		
APEC/S88	2203140	nc	rec	intergenic		
APEC/S88	2203155	nc	rec	intergenic		
APEC/S88	2203203	nc	rec	intergenic		
APEC/S88	2203206	nc	rec	intergenic		
APEC/S88	2203238	nc	rec	intergenic		
APEC/S88	2280203	nc	rec	intergenic		
APEC/S88	2203260	nc	rec	intergenic		
APEC/S88	2203311	nc	rec	intergenic		
APEC/S88	2280278	nc	rec	intergenic		
APEC/S88	2280299	nc	rec	intergenic		

lineage[a]	site[b]	mutation type[c]	recomb	gene	Gene name	Type
APEC/S88	2280302	nc	rec	intergenic		
APEC/S88	2280326	nc	rec	intergenic		
APEC/S88	2280353	nc	rec	intergenic		
APEC/S88	2280362	nc	rec	intergenic		
APEC/S88	2280380	nc	rec	intergenic		
APEC/S88	2280382	nc	rec	intergenic		
APEC/S88	2203449	nc	rec	intergenic		
APEC/S88	2280430	nc	rec	intergenic		
APEC/S88	2203514	nc	rec	intergenic		
APEC/S88	2280505	nc	rec	intergenic		
APEC/S88	2280508	nc	rec	intergenic		
APEC/S88	2280514	nc	rec	intergenic		
APEC/S88	2280529	nc	rec	intergenic		
APEC/S88	2280967	nc	rec	intergenic		
APEC/S88	2280974	nc	rec	intergenic		
APEC/S88	2204348	nc	rec	intergenic		
APEC/S88	2204425	nc	rec	intergenic		
APEC/S88	2204435	nc	rec	intergenic		
APEC/S88	2204436	nc	rec	intergenic		
APEC/S88	2204437	nc	rec	intergenic		
APEC/S88	2204520	nc	rec	intergenic		
APEC/S88	2204574	nc	rec	intergenic		
APEC/S88	2204631	nc	rec	intergenic		
APEC/S88	2204646	nc	rec	intergenic		
APEC/S88	2204740	nc	rec	intergenic		
APEC/S88	2204760	nc	rec	intergenic		
APEC/S88	2204769	nc	rec	intergenic		
APEC/S88	2281751	nc	rec	intergenic		
APEC/S88	2204916	nc	rec	intergenic		
APEC/S88	2204940	nc	rec	intergenic		
APEC/S88	2204955	nc	rec	intergenic		
APEC/S88	2204982	nc	rec	intergenic		
APEC/S88	2204985	nc	rec	intergenic		
APEC/S88	2205000	nc	rec	intergenic		
APEC/S88	2205030	nc	rec	intergenic		
APEC/S88	2282064	indel-4	rec	intergenic		
APEC/S88	2282084	nc	rec	intergenic		
UTI89	2313552	ns		UTI89_C2364	gatD	CDS
S88	2210797	ns		UTI89_C2365	gatC	CDS
S88	2211100	ns		UTI89_C2366	gatB	CDS
UTI89	2318549	s		UTI89_C2371	-	CDS
UTI89	2319019	ns		UTI89_C2371	-	CDS

lineage[a]	site[b]	mutation type[c]	recomb	gene	Gene name	Type
AS	2294676	ns		UTI89_C2374	yegV	CDS
S88	2219519	s		UTI89_C2376	yegX	CDS
UTI89	2333054	nc		intergenic		
AS	2305836	ins		intergenic		
APEC	2313982	del		UTI89_C2390	yehI	CDS
APEC	2318613	s		UTI89_C2392	yehL	CDS
AS	2327808	s		UTI89_C2398	yehU	CDS
UTI89	2359972	ns		UTI89_C2404	yehZ	CDS
UTI89	2369563	ns		UTI89_C2412	yohG	CDS
S88	2265687	del		UTI89_C2412	yohG	CDS
APEC	2345686	ns		UTI89_C2415	yohK	CDS
APEC	2350819	s		UTI89_C2421	mglC	CDS
APEC	2352523	ns		UTI89_C2422	mglA	CDS
UTI89	2380063	ns		UTI89_C2422	mglA	CDS
APEC	2354555	nc		intergenic		
S88	2281526	ns		UTI89_C2428	yeiG	CDS
APEC	2360901	del-11		intergenic		
S88	2284844	s		UTI89_C2430	lysP	CDS
APEC	2370838	ns		UTI89_C2439	yeiN	CDS
UTI89	2403611	nc		intergenic		
UTI89	2408101	s		UTI89_C2450	yeiR	CDS
APEC	2381848	ns		UTI89_C2451	-	CDS
S88	2323257	ns		UTI89_C2470	-	CDS
UTI89	2427990	s		UTI89_C2471	narP	CDS
UTI89	2430345	s		UTI89_C2474	ccmF	CDS
UTI89	2440930	ns		UTI89_C2489	eco	CDS
S88	2340255	s		UTI89_C2491	yojI	CDS
UTI89	2447839	ns		UTI89_C2495	apbE	CDS
UTI89	2449542	nc		intergenic		
APEC	2428027	ns		UTI89_C2500	rcsC	CDS
APEC	2431365	s		UTI89_C2502	atoC	CDS
S88	2355416	nc		intergenic		
APEC	2435408	ns		UTI89_C2506	atoB	CDS
UTI89	2464283	ns		UTI89_C2507	yfaP	CDS
UTI89	2465047	ns		UTI89_C2508	yfaQ	CDS
UTI89	2472015	s		UTI89_C2511	yfaA	CDS
APEC	2451215	ns		UTI89_C2514	yfaL	CDS
S88	2375780	s		UTI89_C2514	yfaL	CDS
UTI89	2486813	ns		UTI89_C2519	glpQ	CDS
S88	2391118	ns		UTI89_C2528	yfaV	CDS
APEC	2472077	s		UTI89_C2531	-	CDS

lineage[a]	site[b]	mutation type[c]	recomb	gene	Gene name	Type
S88	2395235	ns		UTI89_C2531	-	CDS
S88	2397603	nc		intergenic		
S88	2402784	ns		UTI89_C2539	yfbI	CDS
UTI89	2513997	ns		UTI89_C2548	menD	CDS
AS	2492694	s		UTI89_C2553	yfbK	CDS
S88	2416173	ns		UTI89_C2553	yfbK	CDS
APEC	2495773	s		UTI89_C2556	nuoN	CDS
APEC	2502370	s		UTI89_C2563	nuoG	CDS
APEC	2509608	s		UTI89_C2568	nuoA	CDS
S88	2433122	del		intergenic		
APEC	2511288	ns		UTI89_C2570	lrhA	CDS
AS	2512146	nc		intergenic		
UTI89	2539472	nc		intergenic		
S88	2438192	ns		UTI89_C2574	yfbS	CDS
APEC	2516773	ns		UTI89_C2575	yfbT	CDS
APEC	2517332	s		UTI89_C2576	-	CDS
APEC	2519215	s		UTI89_C2579	-	CDS
APEC	2530754	nc		intergenic		
AS	2531096	ns		UTI89_C2593	hisJ	CDS
S88	2454856	nc		intergenic		
UTI89	2560145	nc		intergenic		
S88	2462373	ns		UTI89_C2602	dedA	CDS
S88	2472491	ns		UTI89_C2611	yfcM	CDS
AS	2550514	s		UTI89_C2612	yfcA	CDS
UTI89	2577950	ns		UTI89_C2612	yfcA	CDS
UTI89	2580563	s		UTI89_C2615	prmB	CDS
APEC	2560499	s		UTI89_C2623	yfcV	CDS
S88	2485747	ns		UTI89_C2625	-	CDS
UTI89	2593016	nc		intergenic		
AS	2569930	nc		intergenic		
APEC	2574151	nc		intergenic		
UTI89/AS	2582842	s	rec	UTI89_C2644	-	CDS
UTI89/AS	2582844	s	rec	UTI89_C2644	-	CDS
UTI89/AS	2582845	ns	rec	UTI89_C2644	-	CDS
UTI89/AS	2582846	ns	rec	UTI89_C2644	-	CDS
UTI89/AS	2582848	s	rec	UTI89_C2644	-	CDS
UTI89/AS	2582855	ns	rec	UTI89_C2644	-	CDS
UTI89/AS	2609650	ns	rec	UTI89_C2644	-	CDS
UTI89/AS	2582955	ns	rec	UTI89_C2644	-	CDS
UTI89/AS	2609733	s	rec	UTI89_C2644	-	CDS
UTI89/AS	2609736	s	rec	UTI89_C2644	-	CDS

lineage[a]	site[b]	mutation type[c]	recomb	gene	Gene name	Type
UTI89/AS	2609748	s	rec	UTI89_C2644	-	CDS
UTI89	2609921	ns		UTI89_C2645	-	CDS
UTI89/AS	2586542	s	rec	UTI89_C2649	-	CDS
UTI89/AS	2613373	s	rec	UTI89_C2649	-	CDS
UTI89/AS	2613391	s	rec	UTI89_C2649	-	CDS
UTI89/AS	2613637	ns	rec	UTI89_C2650	-	CDS
UTI89/AS	2586885	s	rec	UTI89_C2650	-	CDS
UTI89/AS	2586903	s	rec	UTI89_C2650	-	CDS
UTI89/AS	2586980	s	rec	UTI89_C2651	-	CDS
UTI89/AS	2587010	s	rec	UTI89_C2651	-	CDS
UTI89/AS	2613817	s	rec	UTI89_C2651	-	CDS
UTI89/AS	2587075	ns	rec	UTI89_C2651	-	CDS
UTI89/AS	2613850	s	rec	UTI89_C2651	-	CDS
UTI89/AS	2613913	s	rec	UTI89_C2651	-	CDS
UTI89/AS	2587142	s	rec	UTI89_C2651	-	CDS
UTI89/AS	2613922	s	rec	UTI89_C2651	-	CDS
UTI89/AS	2613943	s	rec	UTI89_C2651	-	CDS
UTI89/AS	2613967	s	rec	UTI89_C2651	-	CDS
UTI89/AS	2587598	s	rec	UTI89_C2651	-	CDS
UTI89/AS	2587604	s	rec	UTI89_C2651	-	CDS
UTI89/AS	2587619	s	rec	UTI89_C2651	-	CDS
UTI89/AS	2587658	s	rec	UTI89_C2651	-	CDS
UTI89/AS	2587664	s	rec	UTI89_C2651	-	CDS
UTI89/AS	2587712	s	rec	UTI89_C2651	-	CDS
UTI89/AS	2587721	s	rec	UTI89_C2651	-	CDS
UTI89/AS	2587736	s	rec	UTI89_C2651	-	CDS
UTI89/AS	2587739	s	rec	UTI89_C2651	-	CDS
UTI89/AS	2614578	ns	rec	UTI89_C2651	-	CDS
UTI89/AS	2587808	ns	rec	UTI89_C2651	-	CDS
UTI89/AS	2614584	ns	rec	UTI89_C2651	-	CDS
UTI89/AS	2587826	s	rec	UTI89_C2651	-	CDS
UTI89/AS	2587928	s	rec	UTI89_C2651	-	CDS
UTI89/AS	2587931	s	rec	UTI89_C2651	-	CDS
UTI89/AS	2614708	s	rec	UTI89_C2651	-	CDS
UTI89/AS	2614831	s	rec	UTI89_C2651	-	CDS
UTI89/AS	2588220	ns	rec	UTI89_C2651	-	CDS
UTI89/AS	2615011	nc	rec	intergenic		
UTI89	2617087	s		UTI89_C2653	-	CDS
APEC	2595907	nc		intergenic		
S88	2519752	ns		UTI89_C2663	-	CDS
UTI89/AS	2598581	nc	rec	intergenic		

lineage[a]	site[b]	mutation type[c]	recomb	gene	Gene name	Type
UTI89/AS	2598583	nc	rec	intergenic		
UTI89/AS	2598584	nc	rec	intergenic		
UTI89/AS	2598585	nc	rec	intergenic		
UTI89/AS	2598586	nc	rec	intergenic		
UTI89/AS	2598587	nc	rec	intergenic		
UTI89/AS	2625366	nc	rec	intergenic		
UTI89/AS	2598602	nc	rec	intergenic		
UTI89/AS	2598641	nc	rec	intergenic		
UTI89/AS	2598644	nc	rec	intergenic		
UTI89/AS	2625440	nc	rec	intergenic		
UTI89/AS	2625494	ns	rec	UTI89_C2668	-	CDS
UTI89/AS	2600125	ns	rec	intergenic		
UTI89/AS	2600580	ns	rec	UTI89_C2671	-	CDS
UTI89/AS	2600581	s	rec	UTI89_C2671	-	CDS
UTI89/AS	2626690	ns	rec	UTI89_C2671	-	CDS
UTI89/AS	2600610	s	rec	UTI89_C2672	-	CDS
UTI89/AS	2626744	s	rec	UTI89_C2672	-	CDS
UTI89/AS	2626858	s	rec	UTI89_C2672	-	CDS
UTI89/AS	2627023	s	rec	UTI89_C2672	-	CDS
UTI89/AS	2600931	s	rec	UTI89_C2672	-	CDS
UTI89/AS	2600937	s	rec	UTI89_C2672	-	CDS
UTI89/AS	2600940	s	rec	UTI89_C2672	-	CDS
UTI89/AS	2627065	s	rec	UTI89_C2672	-	CDS
UTI89/AS	2627074	s	rec	UTI89_C2672	-	CDS
UTI89/AS	2627083	s	rec	UTI89_C2672	-	CDS
UTI89/AS	2627104	s	rec	UTI89_C2672	-	CDS
UTI89/AS	2601057	s	rec	UTI89_C2672	-	CDS
UTI89/AS	2627179	s	rec	UTI89_C2672	-	CDS
UTI89/AS	2601117	s	rec	UTI89_C2672	-	CDS
UTI89/AS	2601180	s	rec	UTI89_C2672	-	CDS
UTI89/AS	2601189	s	rec	UTI89_C2672	-	CDS
UTI89/AS	2627467	s	rec	UTI89_C2672	-	CDS
UTI89/AS	2627515	s	rec	UTI89_C2672	-	CDS
UTI89/AS	2601522	s	rec	UTI89_C2672	-	CDS
UTI89/AS	2601525	s	rec	UTI89_C2672	-	CDS
UTI89/AS	2627680	s	rec	UTI89_C2672	-	CDS
UTI89/AS	2601588	s	rec	UTI89_C2672	-	CDS
UTI89/AS	2627695	s	rec	UTI89_C2672	-	CDS
UTI89/AS	2601636	s	rec	UTI89_C2672	-	CDS
UTI89/AS	2601639	s	rec	UTI89_C2672	-	CDS
UTI89/AS	2627830	s	rec	UTI89_C2672	-	CDS

lineage[a]	site[b]	mutation type[c]	recomb	gene	Gene name	Type
UTI89/AS	2627862	ns	rec	UTI89_C2672	-	CDS
UTI89/AS	2601765	s	rec	UTI89_C2672	-	CDS
UTI89/AS	2601837	s	rec	UTI89_C2672	-	CDS
UTI89/AS	2627938	s	rec	UTI89_C2672	-	CDS
APEC	2603846	nc		intergenic		
APEC	2604562	ins		intergenic		
UTI89	2630681	nc		intergenic		
UTI89	2630724	nc		intergenic		
S88	2527762	ns		UTI89_C2678	-	CDS
UTI89	2630792	s		UTI89_C2678	-	CDS
AS	2606211	s		UTI89_C2684	-	CDS
UTI89/AS	2633712	s		UTI89_C2684	-	CDS
S88	2531387	del		UTI89_C2689	hkaH	CDS
UTI89/AS	2636859	indel		intergenic		
UTI89	2637847	del		intergenic		
UTI89	2638205	s		UTI89_C2695	dsdC	CDS
S88	2540223	ns		UTI89_C2700	emrK	CDS
UTI89	2644802	nc		intergenic		
S88	2544816	s		UTI89_C2702	evgS	CDS
APEC	2627766	s		UTI89_C2707	yfdX	CDS
APEC	2641323	s		UTI89_C2718	-	CDS
UTI89	2673574	s		UTI89_C2724	mntH	CDS
S88	2573088	s		UTI89_C2726	yfeA	CDS
S88	2578464	ns		UTI89_C2738	xapB	CDS
UTI89	2684105	ns		UTI89_C2739	xapA	CDS
UTI89	2692136	nc		intergenic		
UTI89	2702909	s		UTI89_C2759	ucpA	CDS
S88	2617537	del		UTI89_C2781	eutM	CDS
UTI89	2722335	ns		UTI89_C2782	eutD	CDS
S88	2619377	s		UTI89_C2783	eutT	CDS
AS	2699041	s		UTI89_C2787	maeB	CDS
APEC	2710955	s		UTI89_C2796	acrD	CDS
UTI89	2751430	s		UTI89_C2808	perM	CDS
AS	2725244	ns		UTI89_C2810	yfgC	CDS
UTI89	2753341	ns		UTI89_C2810	yfgC	CDS
APEC	2735178	ins		UTI89_C2819	yfgF	CDS
S88	2658490	ns		UTI89_C2819	yfgF	CDS
APEC	2781967	ns		UTI89_C2826	guaB	CDS
UTI89	2775794	s		UTI89_C2828	-	CDS
APEC	2790238	ns		UTI89_C2828	-	CDS
S88	2675902	ins		UTI89_C2829	sinI	CDS

lineage[a]	site[b]	mutation type[c]	recomb	gene	Gene name	Type
S88	2683288	s		UTI89_C2835	hisS	CDS
S88	2688679	s		UTI89_C2841	pbpC	CDS
APEC	2815313	s		UTI89_C2845	pepB	CDS
UTI89	2804210	s		UTI89_C2848	hscA	CDS
APEC	2819674	s		UTI89_C2851	iscU	CDS
UTI89	2812613	ns		UTI89_C2857	csiE	CDS
S88	2724157	s		UTI89_C2871	hmpA	CDS
UTI89	2829805	ns		UTI89_C2873	yfhA	CDS
AS	2849664	ins		UTI89_C2877	purL	CDS
APEC	2849665	ins		UTI89_C2877	purL	CDS
S88	2734390	ns		UTI89_C2878	yfhD	CDS
AS	2863094	ns		UTI89_C2894	rseA	CDS
UTI89	2852244	ns		UTI89_C2896	nadB	CDS
APEC	2871728	ns	rec	UTI89_C2903	yfiF	CDS
APEC	2871732	s	rec	UTI89_C2903	yfiF	CDS
APEC	2871738	s	rec	UTI89_C2903	yfiF	CDS
APEC	2871744	ns	rec	UTI89_C2903	yfiF	CDS
APEC	2871746	ns	rec	UTI89_C2903	yfiF	CDS
APEC	2871748	ns	rec	UTI89_C2903	yfiF	CDS
APEC	2871750	s	rec	UTI89_C2903	yfiF	CDS
APEC	2871754	ns	rec	UTI89_C2903	yfiF	CDS
APEC	2871755	ns	rec	UTI89_C2903	yfiF	CDS
UTI89	2863908	ns		UTI89_C2907	yfiQ	CDS
UTI89	2867376	ns		UTI89_C2910	kgtP	CDS
S88	2766612	ns		UTI89_C2913	-	CDS
UTI89	2870448	ns		UTI89_C2916	-	CDS
S88	2768769	ins		UTI89_C2916	-	CDS
APEC	2885396	del-6		UTI89_C2916	-	CDS
UTI89	2870533	ins		UTI89_C2916	-	CDS
APEC	2888816	nc	rec	intergenic		
APEC	2888819	nc	rec	intergenic		
APEC	2888820	nc	rec	intergenic		
APEC	2889094	ns	rec	UTI89_C2923	-	CDS
APEC	2889557	ins	rec	intergenic		
APEC	2889585	ins	rec	intergenic		
APEC	2889603	ins	rec	intergenic		
APEC	2889626	ins	rec	intergenic		
APEC	2889735	ins	rec	intergenic		
APEC	2889744	nc	rec	intergenic		
APEC	2889792	ins	rec	intergenic		
APEC	2889816	ins	rec	intergenic		

lineage[a]	site[b]	mutation type[c]	recomb	gene	Gene name	Type
APEC	2889832	ins	rec	intergenic		
APEC	2889956	ins	rec	intergenic		
APEC	2889979	nc	rec	intergenic		
APEC	2890003	ins	rec	intergenic		
APEC	2890027	ins	rec	intergenic		
APEC	2890120	nc	rec	intergenic		
APEC	2890174	nc	rec	intergenic		
APEC	2890247	nc	rec	intergenic		
AS	2890436	nc		intergenic		
UTI89	2875535	ins		UTI89_C2924	-	CDS
APEC	2890567	ns	rec	UTI89_C2924	-	CDS
APEC	2890618	s	rec	UTI89_C2924	-	CDS
APEC	2890620	ns	rec	UTI89_C2924	-	CDS
APEC	2890633	s	rec	UTI89_C2924	-	CDS
AS	2895044	ns		UTI89_C2927	rluD	CDS
UTI89	2885116	ns		UTI89_C2934	aroF	CDS
APEC	2904188	ins		UTI89_C2940	trmD	CDS
APEC	2904219	ins		UTI89_C2940	trmD	CDS
UTI89	2893261	ns		UTI89_C2945	yfjD	CDS
APEC	2920598	ins		UTI89_C2960	-	CDS
APEC	2924611	ns		UTI89_C2965	-	CDS
S88	2858152	ins		intergenic		
APEC	2941333	ns		UTI89_C2988	-	CDS
S88	2858799	nc		intergenic		
APEC	2943155	ns		UTI89_C2992	-	CDS
S88	2862051	s		UTI89_C2994	ydfU1	CDS
APEC	2947345	ns		UTI89_C2998	-	CDS
AS	2950761	nc		intergenic		
UTI89/AS	2942184	ns		UTI89_C3012	-	CDS
S88	2876060	nc		intergenic		
UTI89	2952549	s		UTI89_C3024	ygaP	CDS
APEC	2968781	del		intergenic		
UTI89	2954500	nc		intergenic		
APEC	2969699	s		UTI89_C3028	ygaC	CDS
APEC	2982144	ns		UTI89_C3043	ygaH	CDS
S88	2899903	ns		UTI89_C3044	mprA	CDS
AS	2983229	s		UTI89_C3045	emrA	CDS
APEC	2985767	ns		UTI89_C3046	emrB	CDS
S88	2904226	ns		UTI89_C3048	-	CDS
UTI89	2976810	nc	rec	intergenic		
AS	2991839	ins		intergenic		
UTI89	2976814	nc	rec	intergenic		

lineage[a]	site[b]	mutation type[c]	recomb	gene	Gene name	Type
UTI89	2991848	nc	rec	intergenic		
UTI89	2976823	nc	rec	intergenic		
UTI89	2976827	nc	rec	intergenic		
AS	2991856	del-2		intergenic		
UTI89	2976836	nc	rec	intergenic		
UTI89	2976840	nc	rec	intergenic		
APEC	2994313	s		UTI89_C3058	alaS	CDS
APEC	2996192	s		UTI89_C3060	oraA	CDS
APEC	3008970	s		UTI89_C3074	hypF	CDS
APEC	3013043	nc		intergenic		
UTI89	3001650	ins-9		intergenic		
S88	2936065	ns		UTI89_C3083	hycF	CDS
APEC	3020683	s		UTI89_C3084	hycE	CDS
AS	3021350	ns		UTI89_C3085	hycD	CDS
UTI89	3009573	nc		intergenic		
AS	3030593	ns		UTI89_C3094	fhlA	CDS
APEC	3036221	ns		UTI89_C3102	ygbJ	CDS
UTI89	3030738	ns		UTI89_C3112	nlpD	CDS
APEC	3049349	s		UTI89_C3119	ygbQ	CDS
APEC	3050535	s		UTI89_C3121	cysC	CDS
APEC	3063917	s		UTI89_C3135	ygcS	CDS
APEC	3063924	del-9		UTI89_C3135	ygcS	CDS
APEC	3067071	ns		UTI89_C3137	ygcW	CDS
S88	2984959	del		UTI89_C3137	ygcW	CDS
S88	2989648	del-12		UTI89_C3142	-	CDS
UTI89	3057930	ns		UTI89_C3142	-	CDS
APEC	3073363	ins		UTI89_C3144	-	CDS
UTI89	3059721	ns		UTI89_C3145	-	CDS
S88	2995493	s		UTI89_C3149	pyrG	CDS
AS	3078946	ns		UTI89_C3150	mazG	CDS
AS	3091170	nc		intergenic		
S88	3009373	s		UTI89_C3161	truC	CDS
UTI89	3079264	s		UTI89_C3163	syd	CDS
UTI89	3079927	ns		UTI89_C3164	yqcD	CDS
S88	3011922	ns		UTI89_C3164	yqcD	CDS
UTI89	3081153	s		UTI89_C3165	ygdH	CDS
UTI89	3082908	ns		UTI89_C3167	sdaC	CDS
APEC	3108534	ns		UTI89_C3177	fucR	CDS
UTI89	3098812	ns		UTI89_C3183	-	CDS
APEC	3118818	ns		UTI89_C3191	-	CDS
UTI89	3105017	ns		UTI89_C3192	-	CDS

lineage[a]	site[b]	mutation type[c]	recomb	gene	Gene name	Type
UTI89/AS	3115492	indel-10		UTI89_C3199	-	CDS
S88	3051654	s		intergenic		
UTI89	3121450	ns		intergenic		
S88	3055461	ns		UTI89_C3205	-	CDS
APEC	3146473	nc		intergenic		
UTI89	3133250	ns		UTI89_C3212	-	CDS
UTI89	3133803	s		UTI89_C3213	-	CDS
APEC	3154779	ins-3		UTI89_C3216	-	CDS
S88	3069563	ins-3		UTI89_C3216	-	CDS
UTI89	3137690	del-9		UTI89_C3216	-	CDS
UTI89	3139660	ns		UTI89_C3219	argA	CDS
APEC	3162315	ns		UTI89_C3221	recB	CDS
AS	3166588	nc		intergenic		
APEC	3172715	s		UTI89_C3230	lgt	CDS
UTI89	3166862	nc		intergenic		
AS	3190144	s		UTI89_C3245	-	CDS
APEC	3190393	s		UTI89_C3245	-	CDS
S88	3106639	nc		intergenic		
APEC	3205305	ns		UTI89_C3257	ygeY	CDS
UTI89	3188698	s		UTI89_C3257	ygeY	CDS
APEC	3211214	ns		UTI89_C3262	ygfJ	CDS
AS	3214164	s		UTI89_C3263	ygfK	CDS
APEC	3216976	s		UTI89_C3265	ygfM	CDS
AS	3226463	s		UTI89_C3271	ygfT	CDS
UTI89	3210038	ns		UTI89_C3272	ygfU	CDS
APEC	3242759	ns		UTI89_C3288	gcvP	CDS
UTI89	3229145	ns		UTI89_C3292	visC	CDS
AS	3247188	s		UTI89_C3293	ubiH	CDS
UTI89	3242026	ns		UTI89_C3306	yggA	CDS
AS	3263042	ns		UTI89_C3310	epd	CDS
UTI89	3248554	s		UTI89_C3313	-	CDS
APEC	3296792	ns		UTI89_C3349	yggL	CDS
UTI89	3280580	s		UTI89_C3351	mutY	CDS
S88	3217413	s		UTI89_C3356	speC	CDS
S88	3231571	nc		intergenic		
APEC	3324534	nc		intergenic		
S88	3255479	nc		intergenic		
APEC	3342053	nc		intergenic		
APEC/S88	3346623	indel-8		intergenic		
S88	3261650	nc		intergenic		
APEC	3349782	nc		intergenic		

lineage[a]	site[b]	mutation type[c]	recomb	gene	Gene name	Type
APEC	3353196	nc		intergenic		
S88	3268426	nc		intergenic		
AS	3360899	del		intergenic		
APEC	3361664	nc		intergenic		
APEC	3361785	nc		intergenic		
S88	3276588	nc		intergenic		
APEC	3362138	ns		UTI89_C3362	kpsF	CDS
AS	3363263	ns		UTI89_C3363	kpsE	CDS
UTI89	3298631	ns		UTI89_C3369	neuE	CDS
APEC	3375752	ns	rec	UTI89_C3372	neuB	CDS
APEC	3375755	ns	rec	UTI89_C3372	neuB	CDS
APEC	3375758	ns	rec	UTI89_C3372	neuB	CDS
APEC	3379594	ns		UTI89_C3376	gspM	CDS
APEC	3379825	s		UTI89_C3377	gspL	CDS
S88	3295499	s		UTI89_C3377	gspL	CDS
APEC	3380764	s		UTI89_C3377	gspL	CDS
S88	3296457	s		UTI89_C3378	gspK	CDS
APEC	3388503	ns		UTI89_C3385	gspD	CDS
S88	3308818	s		UTI89_C3389	yghJ	CDS
S88	3308989	s		UTI89_C3389	yghJ	CDS
S88	3313498	ns		UTI89_C3392	glcB	CDS
APEC	3402002	nc		intergenic		
APEC	3414017	s		UTI89_C3404	-	CDS
APEC	3432426	ns		UTI89_C3422	yghZ	CDS
S88	3351397	s		UTI89_C3428	metC	CDS
S88	3360716	nc		intergenic		
UTI89	3373447	ns		UTI89_C3437	-	CDS
S88	3371708	s		UTI89_C3446	parC	CDS
APEC	3458200	ns		UTI89_C3447	-	CDS
S88	3377753	s		UTI89_C3453	-	CDS
APEC	3463881	nc		intergenic		
APEC	3466306	s		UTI89_C3459	-	CDS
S88	3395334	nc		intergenic		
UTI89	3412782	nc		intergenic		
UTI89	3413113	ins		intergenic		
APEC	3493238	nc		intergenic		
UTI89	3427774	ns		UTI89_C3490	ygiF	CDS
UTI89	3444531	nc		intergenic		
AS	3519124	ns		UTI89_C3510	aer	CDS
S88	3434204	ns		UTI89_C3510	aer	CDS
APEC	3523881	ns		UTI89_C3516	ebgA	CDS

lineage[a]	site[b]	mutation type[c]	recomb	gene	Gene name	Type
APEC	3523903	ns		UTI89_C3516	ebgA	CDS
UTI89	3455665	s		UTI89_C3519	ygjJ	CDS
APEC	3543920	s		UTI89_C3530	uxaC	CDS
S88	3469668	s		UTI89_C3545	-	CDS
S88	3472293	ns		UTI89_C3547	tdcG	CDS
S88	3477691	ns		UTI89_C3551	tdcC	CDS
AS	3564880	del-6		intergenic		
AS	3572338	s		UTI89_C3559	garD	CDS
S88	3494717	nc		intergenic		
S88	3496097	s		UTI89_C3568	agaY	CDS
APEC	3586106	nc		intergenic		
UTI89	3517552	ns		UTI89_C3578	yraQ	CDS
UTI89	3525829	ns		UTI89_C3590	deaD	CDS
S88	3521232	s		UTI89_C3598	infB	CDS
UTI89	3550309	s		UTI89_C3616	yhbZ	CDS
UTI89	3550318	ns		UTI89_C3616	yhbZ	CDS
UTI89	3550306	del-12		UTI89_C3616	yhbZ	CDS
UTI89	3552525	nc		intergenic		
AS	3629597	s		UTI89_C3626	yrbB	CDS
APEC	3640398	ns		UTI89_C3641	yhbJ	CDS
S88	3557427	ns		UTI89_C3645	yhbL	CDS
APEC	3653635	ns		UTI89_C3651	yhcH	CDS
S88	3570778	s		UTI89_C3654	nanT	CDS
S88	3578425	s		UTI89_C3665	degQ	CDS
APEC	3671809	ns		UTI89_C3673	yhcR	CDS
S88	3588188	ns		UTI89_C3675	tldD	CDS
S88	3602806	s		UTI89_C3688	accB	CDS
AS	3697788	ns		UTI89_C3698	-	CDS
S88	3619445	ins		intergenic		
UTI89	3633752	s		UTI89_C3708	acrF	CDS
UTI89	3639450	ns		UTI89_C3712	yhdY	CDS
UTI89	3642556	ns		UTI89_C3718	-	CDS
APEC	3715943	nc		intergenic		
APEC	3715985	nc		intergenic		
APEC	3716261	nc	rec	intergenic		
APEC	3716262	nc	rec	intergenic		
APEC	3716271	nc	rec	intergenic		
APEC	3716272	nc	rec	intergenic		
APEC	3716280	nc	rec	intergenic		
APEC	3716313	nc	rec	intergenic		
APEC	3716318	ins	rec	intergenic		

lineage[a]	site[b]	mutation type[c]	recomb	gene	Gene name	Type
APEC	3716320	nc	rec	intergenic		
APEC	3716939	ins	rec	intergenic		
APEC	3716951	ins	rec	intergenic		
APEC	3717142	s	rec	UTI89_C3719	-	CDS
S88	3631966	ns		UTI89_C3719	-	CDS
APEC	3717350	nc	rec	intergenic		
APEC	3717409	nc	rec	intergenic		
APEC	3717514	nc	rec	intergenic		
APEC	3717532	nc	rec	intergenic		
APEC	3717917	nc	rec	intergenic		
APEC	3717923	nc	rec	intergenic		
APEC	3717926	nc	rec	intergenic		
APEC	3717927	nc	rec	intergenic		
APEC	3718201	ns	rec	UTI89_C3723	-	CDS
APEC	3719567	nc	rec	intergenic		
APEC	3719626	nc	rec	intergenic		
APEC	3719627	nc	rec	intergenic		
APEC	3719630	nc	rec	intergenic		
APEC	3719631	nc	rec	intergenic		
APEC	3719632	nc	rec	intergenic		
APEC	3719641	nc	rec	intergenic		
APEC	3719642	nc	rec	intergenic		
APEC	3741334	ns		UTI89_C3770	rplB	CDS
APEC	3745034	ins		UTI89_C3778	yheD	CDS
APEC	3745048	s		UTI89_C3778	yheD	CDS
APEC	3745049	ins-2		UTI89_C3778	yheD	CDS
APEC	3745055	ins		UTI89_C3778	yheD	CDS
APEC	3745068	ins		UTI89_C3778	yheD	CDS
APEC	3745082	ins		UTI89_C3778	yheD	CDS
APEC	3745086	ins		UTI89_C3778	yheD	CDS
APEC	3745125	ins		UTI89_C3778	yheD	CDS
APEC	3745143	ins		UTI89_C3778	yheD	CDS
APEC	3745161	ins		UTI89_C3778	yheD	CDS
APEC	3745167	ins		UTI89_C3778	yheD	CDS
AS	3745780	del-13		UTI89_C3778	yheD	CDS
S88	3660560	ins-13		UTI89_C3778	yheD	CDS
APEC	3745781	ins-19		UTI89_C3778	yheD	CDS
APEC	3745822	ins		UTI89_C3778	yheD	CDS
APEC	3749491	ns		UTI89_C3781	yheG	CDS
S88	3668804	s		intergenic		
APEC	3760585	ins-10		UTI89_C3793	yheB	CDS

lineage[a]	site[b]	mutation type[c]	recomb	gene	Gene name	Type
S88	4411761	nc		intergenic		
S88	4447035	del-4		UTI89_C3808	-	CDS
APEC	4531791	nc	rec	intergenic		
APEC	4531790	nc	rec	intergenic		
APEC	4531781	nc	rec	intergenic		
APEC	4531780	nc	rec	intergenic		
APEC	4531772	nc	rec	intergenic		
APEC	4531739	nc	rec	intergenic		
APEC	4531732	nc	rec	intergenic		
APEC	4531108	nc	rec	intergenic		
APEC	4530902	ns	rec	UTI89_C3809	-	CDS
APEC	4530878	ns	rec	UTI89_C3809	-	CDS
APEC	4530645	nc	rec	intergenic		
APEC	4530540	nc	rec	intergenic		
APEC	4530493	nc	rec	intergenic		
APEC	4529295	nc	rec	intergenic		
APEC	4528929	nc	rec	intergenic		
APEC	4528875	nc	rec	intergenic		
UTI89/AS	3698065	nc		intergenic		
S88	4443291	nc		intergenic		
APEC	4528741	nc	rec	intergenic		
APEC	4528673	nc	rec	intergenic		
APEC	4528652	nc	rec	intergenic		
APEC	4528634	nc	rec	intergenic		
APEC	4528581	nc	rec	intergenic		
APEC	4528539	nc	rec	intergenic		
UTI89/AS	3706383	ns		UTI89_C3820	yjaG	CDS
UTI89/AS	3722302	s		UTI89_C3832	rpoB	CDS
UTI89	3728156	ns		UTI89_C3841	tufA	CDS
UTI89	3728166	s		UTI89_C3841	tufA	CDS
UTI89	3728169	s		UTI89_C3841	tufA	CDS
AS	3761000	s		UTI89_C3841	tufA	CDS
UTI89	3728295	s		UTI89_C3841	tufA	CDS
AS	3761273	s		UTI89_C3841	tufA	CDS
UTI89	3728967	s		UTI89_C3841	tufA	CDS
S88	3700850	ns		UTI89_C3869	nirB	CDS
S88	3714285	s		UTI89_C3883	gph	CDS
AS	3813671	ns		UTI89_C3896	yrfF	CDS
APEC	3815788	s		UTI89_C3899	hslO	CDS
S88	3730646	s		UTI89_C3899	hslO	CDS
S88	3755234	s		UTI89_C3919	malT	CDS

lineage[a]	site[b]	mutation type[c]	recomb	gene	Gene name	Type
APEC	3844805	ns		UTI89_C3922	rtcR	CDS
AS	3845213	del		UTI89_C3922	rtcR	CDS
APEC	3847303	ns		UTI89_C3925	glpE	CDS
S88	3764277	ns		UTI89_C3927	-	CDS
UTI89	3819082	del		intergenic		
APEC	3853048	ins-8		UTI89_C3930	aufF	CDS
AS	3853399	del		UTI89_C3931	aufE	CDS
AS	3857065	s		UTI89_C3935	aufB	CDS
UTI89	3825492	ns		UTI89_C3936	aufA	CDS
UTI89	3829512	ns		UTI89_C3938	glgA	CDS
UTI89	3831347	s		UTI89_C3939	glgC	CDS
UTI89	3832427	ns		UTI89_C3940	glgX	CDS
S88	3782555	s		UTI89_C3941	glgB	CDS
AS	3876781	nc		intergenic		
S88	3793308	nc		intergenic		
APEC	3885870	ns		UTI89_C3960	ugpB	CDS
APEC	3891828	s		UTI89_C3966	-	CDS
APEC	3897280	ns		UTI89_C3974	-	CDS
APEC	3902838	ns		UTI89_C3979	ftsY	CDS
UTI89	3873522	s		UTI89_C3984	zntA	CDS
APEC	3909016	s		UTI89_C3987	yhhQ	CDS
S88	3825348	ns		UTI89_C3989	yhhS	CDS
S88	3827707	ns		UTI89_C3993	nikA	CDS
UTI89	3880907	ns		UTI89_C3993	nikA	CDS
UTI89	3881372	ns		UTI89_C3993	nikA	CDS
S88	3831612	ns		UTI89_C3996	nikD	CDS
S88	3839018	s		UTI89_C4006	yhhJ	CDS
APEC	3925162	ns		UTI89_C4006	yhhJ	CDS
UTI89	3904420	ns		UTI89_C4016	yhiQ	CDS
APEC	3941697	ns		UTI89_C4019	gor	CDS
AS	3943404	ns		UTI89_C4022	-	CDS
S88	3858728	ns		UTI89_C4023	-	CDS
UTI89	3915496	s		UTI89_C4028	chuA	CDS
S88	3865685	s		UTI89_C4034	chuW	CDS
APEC	3956382	nc		intergenic		
UTI89	3928162	ns		UTI89_C4046	yhiV	CDS
AS	3964201	nc		intergenic		
APEC	3964543	del		intergenic		
S88	3879865	ns		UTI89_C4048	yhiX	CDS
S88	3890459	ns		UTI89_C4056	yhjG	CDS
UTI89	3947345	ns		UTI89_C4059	yhjJ	CDS
S88	3897673	nc		intergenic		

lineage[a]	site[b]	mutation type[c]	recomb	gene	Gene name	Type
APEC	3983759	ns		UTI89_C4061	yhjK	CDS
UTI89	3952712	s		UTI89_C4062	yhjL	CDS
S88	3908839	del		UTI89_C4065	yhjO	CDS
S88	3911759	s		UTI89_C4068	yhjS	CDS
S88	3912625	ns		UTI89_C4070	yhjU	CDS
S88	3914910	s		UTI89_C4074	-	CDS
AS	4002418	s		UTI89_C4078	dppF	CDS
UTI89	3994180	s		UTI89_C4105	xylB	CDS
APEC	4028594	ns		UTI89_C4106	xylA	CDS
AS	4034743	del-10		UTI89_C4110	xylR	CDS
APEC	4034744	ins-10		UTI89_C4110	xylR	CDS
S88	3949483	del		UTI89_C4110	xylR	CDS
S88	3950744	ns		UTI89_C4112	-	CDS
AS	4038802	s		UTI89_C4114	avtA	CDS
UTI89	4009250	s		UTI89_C4117	yiaK	CDS
AS	4042626	ns		UTI89_C4118	yiaL	CDS
S88	3959410	ns		UTI89_C4121	yiaN	CDS
UTI89	4015231	ns		UTI89_C4124	lyxK	CDS
S88	3963881	s		UTI89_C4126	sgbU	CDS
UTI89	4017100	ns		UTI89_C4127	sgbE	CDS
APEC	4051924	ns		UTI89_C4129	aldB	CDS
S88	3971662	ns		UTI89_C4133	selA	CDS
APEC	4059675	ns		UTI89_C4135	yibH	CDS
UTI89	4038610	ns		UTI89_C4143	-	CDS
APEC	4075347	ns		UTI89_C4146	lldD	CDS
APEC	4086450	ns		UTI89_C4163	kbl	CDS
AS	4090036	ns		UTI89_C4166	rfaC	CDS
APEC	4103104	nc		intergenic		
UTI89	4072891	s		UTI89_C4184	dut	CDS
S88	4022806	ns		UTI89_C4188	yicC	CDS
UTI89	4081357	s		UTI89_C4195	spoT	CDS
UTI89	4089040	ns		UTI89_C4200	yicH	CDS
S88	4041866	s		UTI89_C4205	-	CDS
APEC	4134113	ns		UTI89_C4210	yicJ	CDS
S88	4052620	s		UTI89_C4214	yicL	CDS
UTI89	4109445	ns		UTI89_C4220	yicO	CDS
S88	4060966	ns		UTI89_C4223	uhpC	CDS
S88	4065668	s		UTI89_C4227	ilvB	CDS
APEC	4155092	ins		intergenic		
AS	4156261	ns		UTI89_C4233	yidK	CDS
AS	4156288	del-11		UTI89_C4233	yidK	CDS

lineage[a]	site[b]	mutation type[c]	recomb	gene	Gene name	Type
APEC	4156289	ins-11		UTI89_C4233	yidK	CDS
S88	4071014	del		UTI89_C4233	yidK	CDS
S88	4071738	del		UTI89_C4233	yidK	CDS
UTI89	4133561	ns		UTI89_C4243	dgoA	CDS
S88	4086477	s		UTI89_C4248	yidB	CDS
AS	4189876	s		UTI89_C4267	yieG	CDS
APEC	4190936	ns		UTI89_C4267	yieG	CDS
S88	4105945	nc		intergenic		
UTI89	4158742	ins-5		intergenic		
UTI89	4160304	ns		UTI89_C4269	yieK	CDS
S88	4116112	s		UTI89_C4277	pstB	CDS
UTI89	4172397	nc		intergenic		
APEC	4205471	ins-5		intergenic		
AS	4206407	ns		UTI89_C4281	glmS	CDS
APEC	4206508	ns		UTI89_C4281	glmS	CDS
AS	4207382	nc		intergenic		
APEC	4209772	s		UTI89_C4285	atpD	CDS
UTI89	4179841	s		UTI89_C4287	atpA	CDS
S88	4131218	nc		intergenic		
UTI89	4187124	ns		UTI89_C4297	mioC	CDS
APEC	4235385	nc	rec	intergenic		
APEC	4235420	nc	rec	intergenic		
APEC	4235609	nc	rec	intergenic		
AS	4235682	nc		intergenic		
APEC	4236086	nc	rec	intergenic		
APEC	4236087	nc	rec	intergenic		
APEC	4236331	nc	rec	intergenic		
UTI89	4204502	nc		intergenic		
AS	4237341	nc		intergenic		
S88	4152392	ns		UTI89_C4315	-	CDS
UTI89	4207259	nc		intergenic		
UTI89	4207364	nc	rec	intergenic		
UTI89	4207365	nc	rec	intergenic		
UTI89	4207367	nc	rec	intergenic		
UTI89	4207369	nc	rec	intergenic		
UTI89	4207373	nc	rec	intergenic		
UTI89	4207374	nc	rec	intergenic		
APEC	4240834	ns		UTI89_C4320	hdfR	CDS
S88	4161875	ns		UTI89_C4327	ilvD	CDS
APEC	4249690	ns		UTI89_C4328	ilvA	CDS
APEC	4251082	s		UTI89_C4329	ilvY	CDS

lineage[a]	site[b]	mutation type[c]	recomb	gene	Gene name	Type
UTI89	4239927	ns		UTI89_C4351	wzyE	CDS
APEC	4275300	nc		intergenic		
AS	4280735	del-6		UTI89_C4362	hemX	CDS
S88	4195443	del-6		UTI89_C4362	hemX	CDS
S88	4195482	s		UTI89_C4362	hemX	CDS
UTI89	4250508	s		UTI89_C4364	hemC	CDS
AS	4288878	ns		UTI89_C4370	-	CDS
UTI89	4260364	s		UTI89_C4374	xerC	CDS
S88	4208217	ns		UTI89_C4376	uvrD	CDS
S88	4210837	ns		UTI89_C4377	-	CDS
AS	4299083	ns		UTI89_C4380	-	CDS
UTI89	4267978	ns		UTI89_C4381	-	CDS
APEC	4309952	ns		UTI89_C4393	metE	CDS
AS	4314555	ns		UTI89_C4398	-	CDS
S88	4233695	ns		UTI89_C4401	-	CDS
AS	4320113	ns		UTI89_C4402	-	CDS
S88	4250212	nc		intergenic		
UTI89	4308604	ns		UTI89_C4423	tatA	CDS
APEC	4340877	ns		UTI89_C4424	tatB	CDS
UTI89	4308923	s		UTI89_C4424	tatB	CDS
UTI89	4314651	ns		UTI89_C4430	fadA	CDS
UTI89	4319323	ns		UTI89_C4433	yigZ	CDS
S88	4268259	del		intergenic		
APEC	4353787	nc	rec	intergenic		
APEC	4353789	nc	rec	intergenic		
APEC	4353821	nc	rec	intergenic		
APEC	4353822	ins	rec	intergenic		
APEC	4353857	nc	rec	intergenic		
APEC	4353858	nc	rec	intergenic		
APEC	4353859	ins	rec	intergenic		
APEC	4353950	nc	rec	intergenic		
APEC	4353953	nc	rec	intergenic		
APEC	4353964	nc	rec	intergenic		
APEC	4353967	nc	rec	intergenic		
APEC	4354123	ins	rec	intergenic		
APEC	4354137	nc	rec	intergenic		
APEC	4354139	nc	rec	intergenic		
APEC	4355438	nc	rec	intergenic		
APEC	4355571	nc	rec	intergenic		
APEC	4355572	nc	rec	intergenic		
AS	4355826	del-17	rec	intergenic		

lineage[a]	site[b]	mutation type[c]	recomb	gene	Gene name	Type
S88	4270376	ins-17		intergenic		
APEC	4355937	nc	rec	intergenic		
APEC	4356118	ns	rec	UTI89_C4441	-	CDS
APEC	4356170	ns	rec	UTI89_C4441	-	CDS
APEC	4356233	ins	rec	UTI89_C4441	-	CDS
APEC	4356241	ins	rec	UTI89_C4441	-	CDS
APEC	4356402	nc	rec	intergenic		
APEC	4356991	ins	rec	intergenic		
APEC	4357100	ins	rec	intergenic		
APEC	4357194	del	rec	intergenic		
APEC	4357232	nc	rec	intergenic		
APEC	4357276	nc	rec	intergenic		
APEC	4357361	nc	rec	intergenic		
APEC	4357403	nc	rec	intergenic		
APEC	4357724	nc	rec	intergenic		
APEC	4357726	nc	rec	intergenic		
APEC	4357729	nc	rec	intergenic		
S88	4273205	nc	rec	intergenic		
S88	4273206	nc	rec	intergenic		
S88	4273208	nc	rec	intergenic		
S88	4273210	nc	rec	intergenic		
S88	4273214	nc	rec	intergenic		
S88	4273215	nc	rec	intergenic		
UTI89	4326969	nc		intergenic		
APEC	4358993	ins		intergenic		
APEC	4358995	nc	rec	intergenic		
APEC	4358996	nc	rec	intergenic		
APEC	4358999	nc	rec	intergenic		
APEC	4359000	nc	rec	intergenic		
APEC	4359003	nc	rec	intergenic		
APEC	4359004	nc	rec	intergenic		
APEC	4359008	nc	rec	intergenic		
APEC	4359015	ins	rec	intergenic		
APEC	4359018	nc	rec	intergenic		
APEC	4359023	nc	rec	intergenic		
APEC	4359026	nc	rec	intergenic		
APEC	4359077	s		UTI89_C4443	mobB	CDS
APEC	4359434	s		UTI89_C4443	mobB	CDS
S88	4274195	ns		UTI89_C4444	mobA	CDS
APEC	4360017	ns		UTI89_C4444	mobA	CDS
APEC	4361508	ns		UTI89_C4446	rdoA	CDS

lineage[a]	site[b]	mutation type[c]	recomb	gene	Gene name	Type
APEC	4362922	s		UTI89_C4449	yihF	CDS
S88	4278408	ns		UTI89_C4450	yihG	CDS
APEC	4369802	ns		UTI89_C4455	yihI	CDS
S88	4284437	ns		UTI89_C4455	yihI	CDS
APEC	4378230	nc		intergenic		
APEC	4378233	nc		intergenic		
APEC	4382674	ns		UTI89_C4465	-	CDS
APEC	4387303	s		UTI89_C4469	yihX	CDS
AS	4388080	ns		UTI89_C4470	rbn	CDS
S88	4302820	ns		UTI89_C4470	rbn	CDS
AS	4390745	s		UTI89_C4473	-	CDS
APEC	4408714	ns		UTI89_C4490	rhaT	CDS
APEC	4410516	s		UTI89_C4492	-	CDS
UTI89	4381800	s		UTI89_C4495	cpxA	CDS
S88	4332832	ns		UTI89_C4501	sbp	CDS
APEC	4424479	ns		UTI89_C4509	glpX	CDS
APEC	4428858	ns		UTI89_C4515	menA	CDS
S88	4344750	s		UTI89_C4516	hslU	CDS
AS	4431638	s		UTI89_C4518	ftsN	CDS
S88	4362194	ns		UTI89_C4530	-	CDS
AS	4448043	ns		UTI89_C4530	-	CDS
UTI89	4418667	ns		UTI89_C4532	katG	CDS
S88	4372374	ns		UTI89_C4538	ptsA	CDS
UTI89	4432590	s		UTI89_C4545	yijO	CDS
UTI89	4439821	ns		UTI89_C4549	argC	CDS
UTI89	4451182	s		UTI89_C4558	yijC	CDS
APEC	4488334	nc	rec	intergenic		
APEC	4488517	nc		intergenic		
AS	4488585	ins	rec	intergenic		
APEC	4488591	nc		intergenic		
APEC	4488668	ins	rec	intergenic		
APEC	4488776	nc	rec	intergenic		
APEC	4488784	nc	rec	intergenic		
APEC	4488805	ins	rec	intergenic		
APEC	4488823	ins	rec	intergenic		
APEC	4488826	nc	rec	intergenic		
APEC	4488832	ins	rec	intergenic		
APEC	4488841	ins	rec	intergenic		
APEC	4489637	del	rec	UTI89_C4564	-	CDS
APEC	4489653	ins	rec	UTI89_C4564	-	CDS
APEC	4489692	s	rec	UTI89_C4564	-	CDS

lineage[a]	site[b]	mutation type[c]	recomb	gene	Gene name	Type
APEC	4489693	ins-2	rec	UTI89_C4564	-	CDS
APEC	4489719	ins	rec	UTI89_C4564	-	CDS
APEC	4489732	s	rec	UTI89_C4564	-	CDS
APEC	4489737	ins	rec	UTI89_C4564	-	CDS
APEC	4489760	ins-2	rec	UTI89_C4564	-	CDS
APEC	4489768	ins	rec	UTI89_C4564	-	CDS
APEC	4489779	del	rec	UTI89_C4564	-	CDS
APEC	4489788	ns	rec	UTI89_C4564	-	CDS
APEC	4489955	nc	rec	intergenic		
AS	4542436	ns		UTI89_C4577	metH	CDS
UTI89	4478703	ns		UTI89_C4584	-	CDS
AS	4551046	s		UTI89_C4584	-	CDS
UTI89	4481797	s		UTI89_C4589	-	CDS
AS	4564653	ns		UTI89_C4599	-	CDS
UTI89	4500681	ns		UTI89_C4607	malM	CDS
AS	4573791	s		UTI89_C4609	ubiC	CDS
UTI89	4505609	s		UTI89_C4612	dgkA	CDS
UTI89	4517245	ns		UTI89_C4623	-	CDS
APEC	4589910	nc		intergenic		
AS	4599913	s		UTI89_C4632	-	CDS
UTI89	4532922	ns		UTI89_C4636	-	CDS
S88	4522336	ns		UTI89_C4638	-	CDS
APEC	4611463	ns		UTI89_C4642	yjbQ	CDS
S88	4534435	s		UTI89_C4651	yjcD	CDS
UTI89	4548569	ns		UTI89_C4651	yjcD	CDS
UTI89	4563564	nc		intergenic		
UTI89	4575924	s		UTI89_C4676	-	CDS
UTI89	4587439	ins		intergenic		
AS	4661878	s		UTI89_C4688	phnO	CDS
S88	4579417	s		UTI89_C4692	phnK	CDS
S88	4581025	s		UTI89_C4694	phnI	CDS
APEC	4668268	ns		UTI89_C4697	phnF	CDS
APEC	4669476	s		UTI89_C4698	phnE	CDS
AS	4678848	s		UTI89_C4706	basS	CDS
UTI89	4609416	nc		intergenic		
APEC	4684017	nc		intergenic		
UTI89	4619731	ns		UTI89_C4716	dcuB	CDS
UTI89	4624459	ns		UTI89_C4721	yjdJ	CDS
AS	4696911	del		UTI89_C4722	-	CDS
S88	4619220	nc		intergenic		
UTI89	4632763	s		UTI89_C4730	cadC	CDS

lineage[a]	site[b]	mutation type[c]	recomb	gene	Gene name	Type
UTI89	4633784	ns		UTI89_C4730	cadC	CDS
APEC	4706724	nc		intergenic		
UTI89	4634670	ins		intergenic		
S88	4621567	nc		intergenic		
S88	4633514	nc		intergenic		
APEC/S88	4721636	indel		intergenic		
APEC	4727253	nc		intergenic		
APEC	4728234	nc		intergenic		
APEC	4730261	nc		intergenic		
APEC/S88	4735685	indel-9		intergenic		
S88	4657207	nc		intergenic		
S88	4666747	nc		intergenic		
S88	4671053	nc		intergenic		
AS	4795635	s		UTI89_C4759	yjeP	CDS
AS	4804012	ns		UTI89_C4769	amiB	CDS
UTI89	4685229	ns		UTI89_C4786	yjfC	CDS
S88	4735953	ns		UTI89_C4786	yjfC	CDS
S88	4738501	nc		intergenic		
APEC	4829115	s		UTI89_C4794	sgaB	CDS
AS	4830815	ns		UTI89_C4797	sgaU	CDS
APEC	4847187	s		UTI89_C4817	cycA	CDS
UTI89	4724285	ns		UTI89_C4828	ytfM	CDS
APEC	4866034	ns		UTI89_C4833	ytfR	CDS
APEC	4875107	s		UTI89_C4841	nrdG	CDS
UTI89	4748256	s		UTI89_C4847	mgtA	CDS
APEC	4888978	ns		UTI89_C4854	-	CDS
UTI89	4754373	ns		UTI89_C4854	-	CDS
APEC	4890727	ns		UTI89_C4856	-	CDS
APEC	4890774	ns		UTI89_C4856	-	CDS
APEC	4893511	ns		UTI89_C4859	yjgK	CDS
APEC	4894875	nc		intergenic		
UTI89	4759932	nc		intergenic		
APEC	4905231	ins		UTI89_C4870	yjgR	CDS
APEC	4906612	ns		UTI89_C4870	yjgR	CDS
APEC	4909847	ns		UTI89_C4873	idnO	CDS
UTI89	4775219	ns		UTI89_C4874	idnD	CDS
UTI89/AS	3318053	ns	rec	UTI89_C4889	papE	CDS
UTI89/AS	3318055	s	rec	UTI89_C4889	papE	CDS
UTI89/AS	3318071	ns	rec	UTI89_C4889	papE	CDS
UTI89/AS	3318313	ns	rec	UTI89_C4890	papK	CDS
UTI89/AS	4791459	s	rec	UTI89_C4890	papK	CDS
UTI89/AS	4791663	s	rec	UTI89_C4890	papK	CDS

lineage[a]	site[b]	mutation type[c]	recomb	gene	Gene name	Type
S88	3233390	s		UTI89_C4890	papK	CDS
UTI89/AS	4791756	ns	rec	UTI89_C4890	papK	CDS
UTI89/AS	4791765	s	rec	UTI89_C4890	papK	CDS
UTI89/AS	4791780	s	rec	UTI89_C4890	papK	CDS
UTI89/AS	4791788	s	rec	UTI89_C4890	papK	CDS
UTI89/AS	4791821	nc	rec	intergenic		
AS	3319125	s		UTI89_C4891	papJ	CDS
UTI89	4793296	s		UTI89_C4893	papC	CDS
AS	3320997	ns		UTI89_C4893	papC	CDS
UTI89	4794478	s		UTI89_C4893	papC	CDS
UTI89	4794495	ns		UTI89_C4893	papC	CDS
APEC	3321695	ns		UTI89_C4893	papC	CDS
UTI89	4795844	s	rec	UTI89_C4894	papH	CDS
UTI89	4795847	s	rec	UTI89_C4894	papH	CDS
UTI89	4795952	s	rec	UTI89_C4894	papH	CDS
UTI89	3322862	s	rec	UTI89_C4894	papH	CDS
UTI89	4795977	ns	rec	UTI89_C4894	papH	CDS
UTI89	4796117	s	rec	UTI89_C4894	papH	CDS
UTI89	4796156	s	rec	UTI89_C4894	papH	CDS
UTI89	4796177	s	rec	UTI89_C4894	papH	CDS
UTI89	4796184	ns	rec	UTI89_C4894	papH	CDS
UTI89	4796198	s	rec	UTI89_C4894	papH	CDS
UTI89	3323206	ns	rec	UTI89_C4894	papH	CDS
S88	3238107	nc		intergenic		
UTI89	4796494	nc	rec	UTI89_C4895	papA(other)	CDS
UTI89	4796512	nc	rec	UTI89_C4895	papA(other)	CDS
S88	4879115	ns	rec	UTI89_C5004	-	CDS
S88	4879119	ns	rec	UTI89_C5004	-	CDS
S88	4879144	s	rec	UTI89_C5004	-	CDS
S88	4879149	ns	rec	UTI89_C5004	-	CDS
S88	4879153	s	rec	UTI89_C5004	-	CDS
S88	4879165	s	rec	UTI89_C5004	-	CDS
S88	4879189	s	rec	UTI89_C5004	-	CDS
S88	4879193	ns	rec	UTI89_C5004	-	CDS
S88	4879228	s	rec	UTI89_C5004	-	CDS
S88	4879236	ns	rec	UTI89_C5004	-	CDS
S88	4879240	s	rec	UTI89_C5004	-	CDS
S88	4879244	ns	rec	UTI89_C5004	-	CDS
S88	4879246	s	rec	UTI89_C5004	-	CDS
S88	4879249	s	rec	UTI89_C5004	-	CDS
S88	4879256	ns	rec	UTI89_C5004	-	CDS
S88	4879257	ns	rec	UTI89_C5004	-	CDS

lineage[a]	site[b]	mutation type[c]	recomb	gene	Gene name	Type
S88	4879272	s	rec	UTI89_C5004	-	CDS
S88	4879284	s	rec	UTI89_C5004	-	CDS
S88	4879290	ns	rec	UTI89_C5004	-	CDS
S88	4879292	ns	rec	UTI89_C5004	-	CDS
S88	4879312	ns	rec	UTI89_C5004	-	CDS
S88	4879317	s	rec	UTI89_C5004	-	CDS
S88	4879474	s	rec	UTI89_C5005	yjhS	CDS
S88	4879479	ns	rec	UTI89_C5005	yjhS	CDS
S88	4879492	s	rec	UTI89_C5005	yjhS	CDS
S88	4879504	ns	rec	UTI89_C5005	yjhS	CDS
S88	4879506	ns	rec	UTI89_C5005	yjhS	CDS
S88	4879607	ns	rec	UTI89_C5005	yjhS	CDS
S88	4879704	ns	rec	UTI89_C5005	yjhS	CDS
S88	4879708	s	rec	UTI89_C5005	yjhS	CDS
S88	4879768	ns	rec	UTI89_C5005	yjhS	CDS
S88	4879799	ns	rec	UTI89_C5005	yjhS	CDS
S88	4879813	ns	rec	UTI89_C5005	yjhS	CDS
S88	4879960	s	rec	UTI89_C5005	yjhS	CDS
S88	4879974	ns	rec	UTI89_C5005	yjhS	CDS
S88	4880009	ns	rec	UTI89_C5005	yjhS	CDS
S88	4880047	s	rec	UTI89_C5005	yjhS	CDS
S88	4880074	s	rec	UTI89_C5005	yjhS	CDS
S88	4880079	ns	rec	UTI89_C5005	yjhS	CDS
S88	4880098	s	rec	UTI89_C5005	yjhS	CDS
S88	4880119	s	rec	UTI89_C5005	yjhS	CDS
S88	4880250	ns	rec	UTI89_C5005	yjhS	CDS
S88	4880266	s	rec	UTI89_C5005	yjhS	CDS
S88	4880301	ns	rec	UTI89_C5005	yjhS	CDS
S88	4880318	ns	rec	UTI89_C5005	yjhS	CDS
S88	4880332	s	rec	UTI89_C5005	yjhS	CDS
S88	4880519	s	rec	UTI89_C5006	-	CDS
S88	4880627	s	rec	UTI89_C5006	-	CDS
S88	4880671	ns	rec	UTI89_C5006	-	CDS
S88	4880768	s	rec	UTI89_C5006	-	CDS
S88	4880771	s	rec	UTI89_C5006	-	CDS
S88	4880780	ns	rec	UTI89_C5006	-	CDS
S88	4880783	s	rec	UTI89_C5006	-	CDS
S88	4880786	s	rec	UTI89_C5006	-	CDS
S88	4880818	ns	rec	UTI89_C5006	-	CDS
S88	4880826	ns	rec	UTI89_C5006	-	CDS
S88	4880972	s	rec	UTI89_C5006	-	CDS
S88	4881002	s	rec	UTI89_C5006	-	CDS
S88	4881017	s	rec	UTI89_C5006	-	CDS
S88	4881088	ns	rec	UTI89_C5006	-	CDS
S88	4881107	s	rec	UTI89_C5006	-	CDS

lineage[a]	site[b]	mutation type[c]	recomb	gene	Gene name	Type
S88	4881269	s	rec	UTI89_C5006	-	CDS
S88	4881365	s	rec	UTI89_C5006	-	CDS
S88	4881392	s	rec	UTI89_C5006	-	CDS
S88	4881455	s	rec	UTI89_C5006	-	CDS
S88	4881538	ns	rec	UTI89_C5006	-	CDS
S88	4881554	ns	rec	UTI89_C5006	-	CDS
S88	4881562	ns	rec	UTI89_C5006	-	CDS
S88	4881702	s	rec	UTI89_C5007	yjhA	CDS
S88	4881810	s	rec	UTI89_C5007	yjhA	CDS
S88	4882268	s	rec	UTI89_C5007	yjhA	CDS
S88	4882324	ns	rec	UTI89_C5007	yjhA	CDS
S88	4882431	nc	rec	intergenic		
S88	4882432	nc	rec	intergenic		
S88	4882446	nc	rec	intergenic		
S88	4882447	nc	rec	intergenic		
S88	4882449	nc	rec	intergenic		
S88	4882462	nc	rec	intergenic		
S88	4882477	nc	rec	intergenic		
S88	4882509	nc	rec	intergenic		
S88	4882538	nc	rec	intergenic		
S88	4882601	nc	rec	intergenic		
S88	4882626	nc	rec	intergenic		
S88	4882710	nc	rec	intergenic		
S88	4882721	nc	rec	intergenic		
S88	4882734	nc	rec	intergenic		
S88	4882737	nc	rec	intergenic		
S88	4882738	nc	rec	intergenic		
S88	4882797	nc	rec	intergenic		
S88	4882876	nc	rec	intergenic		
S88	4882898	nc	rec	intergenic		
S88	4882899	nc	rec	intergenic		
S88	4882900	nc	rec	intergenic		
S88	4882904	nc	rec	intergenic		
S88	4882919	nc	rec	intergenic		
S88	4882927	nc	rec	intergenic		
S88	4882938	nc	rec	intergenic		
S88	4882977	nc	rec	intergenic		
S88	4882988	nc	rec	intergenic		
S88	4883013	nc	rec	intergenic		
S88	4883048	nc	rec	intergenic		
S88	4883059	nc	rec	intergenic		
S88	4883067	nc	rec	intergenic		
S88	4883068	nc	rec	intergenic		
S88	4883085	nc	rec	intergenic		
S88	4883094	nc	rec	intergenic		

lineage[a]	site[b]	mutation type[c]	recomb	gene	Gene name	Type
S88	4883124	s	rec	UTI89_C5008	-	CDS
S88	4883125	s	rec	UTI89_C5008	-	CDS
S88	4883129	ns	rec	UTI89_C5008	-	CDS
S88	4883182	ns	rec	UTI89_C5008	-	CDS
S88	4883199	ns	rec	UTI89_C5008	-	CDS
S88	4883209	ns	rec	UTI89_C5008	-	CDS
S88	4883229	ns	rec	UTI89_C5008	-	CDS
S88	4883282	ns	rec	UTI89_C5008	-	CDS
S88	4883319	s	rec	UTI89_C5008	-	CDS
S88	4883325	ns	rec	UTI89_C5008	-	CDS
S88	4883365	ns	rec	UTI89_C5008	-	CDS
S88	4883422	ns	rec	UTI89_C5008	-	CDS
S88	4883432	ns	rec	UTI89_C5008	-	CDS
S88	4883537	nc	rec	intergenic		
S88	4883546	nc	rec	intergenic		
S88	4883548	nc	rec	intergenic		
S88	4883589	nc	rec	intergenic		
S88	4883605	nc	rec	intergenic		
S88	4883616	nc	rec	intergenic		
S88	4883622	nc	rec	intergenic		
S88	4883624	nc	rec	intergenic		
S88	4883680	nc	rec	intergenic		
S88	4883712	nc	rec	intergenic		
S88	4883847	s	rec	UTI89_C5009	fimB	CDS
S88	4883868	s	rec	UTI89_C5009	fimB	CDS
S88	4883889	s	rec	UTI89_C5009	fimB	CDS
S88	4883892	s	rec	UTI89_C5009	fimB	CDS
S88	4883931	s	rec	UTI89_C5009	fimB	CDS
S88	4884031	ns	rec	UTI89_C5009	fimB	CDS
S88	4884069	s	rec	UTI89_C5009	fimB	CDS
S88	4884072	s	rec	UTI89_C5009	fimB	CDS
S88	4884105	s	rec	UTI89_C5009	fimB	CDS
S88	4884120	s	rec	UTI89_C5009	fimB	CDS
S88	4884273	s	rec	UTI89_C5009	fimB	CDS
S88	4884475	nc	rec	intergenic		
S88	4884490	nc	rec	intergenic		
S88	4884594	nc	rec	intergenic		
S88	4884595	nc	rec	intergenic		
S88	4884602	nc	rec	intergenic		
S88	4884605	nc	rec	intergenic		
S88	4884691	nc	rec	intergenic		
S88	4884758	nc	rec	intergenic		
S88	4884911	s	rec	UTI89_C5010	fimE	CDS
S88	4885010	s	rec	UTI89_C5010	fimE	CDS
S88	4885175	s	rec	UTI89_C5010	fimE	CDS

lineage[a]	site[b]	mutation type[c]	recomb	gene	Gene name	Type
S88	4885280	s	rec	UTI89_C5010	fimE	CDS
S88	4885295	s	rec	UTI89_C5010	fimE	CDS
S88	4885447	ns	rec	UTI89_C5010	fimE	CDS
S88	4885450	s	rec	UTI89_C5010	fimE	CDS
S88	4885459	nc	rec	intergenic		
S88	4885467	nc	rec	intergenic		
S88	4885473	nc	rec	intergenic		
S88	4885515	nc	rec	intergenic		
S88	4885562	nc	rec	intergenic		
S88	4885595	nc	rec	intergenic		
S88	4885601	nc	rec	intergenic		
S88	4885639	nc	rec	intergenic		
S88	4885661	nc	rec	intergenic		
S88	4885662	nc	rec	intergenic		
S88	4885668	nc	rec	intergenic		
S88	4885697	nc	rec	intergenic		
S88	4885718	nc	rec	intergenic		
S88	4885721	nc	rec	intergenic		
S88	4885722	nc	rec	intergenic		
S88	4885736	nc	rec	intergenic		
S88	4885743	nc	rec	intergenic		
S88	4885746	nc	rec	intergenic		
APEC	4907674	nc		intergenic		
S88	4885832	nc	rec	intergenic		
S88	4885868	nc	rec	intergenic		
S88	4885918	nc	rec	intergenic		
S88	4885928	nc	rec	intergenic		
S88	4885931	nc	rec	intergenic		
S88	4885949	s	rec	UTI89_C5011	fimA	CDS
S88	4886003	ns	rec	UTI89_C5011	fimA	CDS
S88	4886108	s	rec	UTI89_C5011	fimA	CDS
S88	4886111	s	rec	UTI89_C5011	fimA	CDS
S88	4886126	s	rec	UTI89_C5011	fimA	CDS
S88	4886128	ns	rec	UTI89_C5011	fimA	CDS
S88	4886130	s	rec	UTI89_C5011	fimA	CDS
S88	4886139	ns	rec	UTI89_C5011	fimA	CDS
S88	4886197	ns	rec	UTI89_C5011	fimA	CDS
S88	4886213	s	rec	UTI89_C5011	fimA	CDS
S88	4886234	s	rec	UTI89_C5011	fimA	CDS
S88	4886247	ns	rec	UTI89_C5011	fimA	CDS
S88	4886261	s	rec	UTI89_C5011	fimA	CDS
S88	4886270	s	rec	UTI89_C5011	fimA	CDS
S88	4886300	s	rec	UTI89_C5011	fimA	CDS
S88	4886306	s	rec	UTI89_C5011	fimA	CDS
S88	4886315	s	rec	UTI89_C5011	fimA	CDS

lineage[a]	site[b]	mutation type[c]	recomb	gene	Gene name	Type
S88	4886330	s	rec	UTI89_C5011	fimA	CDS
S88	4886367	ns	rec	UTI89_C5011	fimA	CDS
S88	4886390	s	rec	UTI89_C5011	fimA	CDS
S88	4886419	ns	rec	UTI89_C5011	fimA	CDS
S88	4886425	ns	rec	UTI89_C5011	fimA	CDS
S88	4886429	s	rec	UTI89_C5011	fimA	CDS
S88	4886431	ns	rec	UTI89_C5011	fimA	CDS
S88	4886432	ns	rec	UTI89_C5011	fimA	CDS
S88	4886441	s	rec	UTI89_C5011	fimA	CDS
S88	4886574	s	rec	UTI89_C5012	fimI	CDS
S88	4886622	s	rec	UTI89_C5012	fimI	CDS
S88	4886658	s	rec	UTI89_C5012	fimI	CDS
S88	4886674	ns	rec	UTI89_C5012	fimI	CDS
S88	4886764	ns	rec	UTI89_C5012	fimI	CDS
S88	4886880	s	rec	UTI89_C5012	fimI	CDS
S88	4886952	s	rec	UTI89_C5012	fimI	CDS
S88	4887211	ns	rec	UTI89_C5013	fimC	CDS
S88	4887228	s	rec	UTI89_C5013	fimC	CDS
S88	4887345	s	rec	UTI89_C5013	fimC	CDS
S88	4887372	s	rec	UTI89_C5013	fimC	CDS
S88	4887429	s	rec	UTI89_C5013	fimC	CDS
S88	4887492	s	rec	UTI89_C5013	fimC	CDS
S88	4887551	ns	rec	UTI89_C5013	fimC	CDS
S88	4887582	s	rec	UTI89_C5013	fimC	CDS
S88	4887657	s	rec	UTI89_C5013	fimC	CDS
S88	4887711	s	rec	UTI89_C5013	fimC	CDS
S88	4887715	ns	rec	UTI89_C5013	fimC	CDS
S88	4887754	ns	rec	UTI89_C5013	fimC	CDS
S88	4887863	nc	rec	intergenic		
S88	4888014	s	rec	UTI89_C5014	fimD	CDS
S88	4888068	s	rec	UTI89_C5014	fimD	CDS
S88	4888074	s	rec	UTI89_C5014	fimD	CDS
S88	4888104	s	rec	UTI89_C5014	fimD	CDS
S88	4888158	s	rec	UTI89_C5014	fimD	CDS
S88	4888221	s	rec	UTI89_C5014	fimD	CDS
S88	4888260	s	rec	UTI89_C5014	fimD	CDS
S88	4888263	s	rec	UTI89_C5014	fimD	CDS
S88	4888362	ns	rec	UTI89_C5014	fimD	CDS
S88	4888371	s	rec	UTI89_C5014	fimD	CDS
S88	4888458	s	rec	UTI89_C5014	fimD	CDS
S88	4888515	s	rec	UTI89_C5014	fimD	CDS
S88	4888569	s	rec	UTI89_C5014	fimD	CDS
S88	4888722	s	rec	UTI89_C5014	fimD	CDS
S88	4888896	s	rec	UTI89_C5014	fimD	CDS
S88	4888980	s	rec	UTI89_C5014	fimD	CDS

lineage[a]	site[b]	mutation type[c]	recomb	gene	Gene name	Type
S88	4888989	s	rec	UTI89_C5014	fimD	CDS
S88	4889025	s	rec	UTI89_C5014	fimD	CDS
S88	4889040	s	rec	UTI89_C5014	fimD	CDS
S88	4889085	s	rec	UTI89_C5014	fimD	CDS
S88	4889145	s	rec	UTI89_C5014	fimD	CDS
S88	4889208	s	rec	UTI89_C5014	fimD	CDS
S88	4889304	s	rec	UTI89_C5014	fimD	CDS
S88	4889325	s	rec	UTI89_C5014	fimD	CDS
S88	4889331	s	rec	UTI89_C5014	fimD	CDS
S88	4889370	s	rec	UTI89_C5014	fimD	CDS
S88	4889373	s	rec	UTI89_C5014	fimD	CDS
S88	4889439	s	rec	UTI89_C5014	fimD	CDS
S88	4889613	s	rec	UTI89_C5014	fimD	CDS
S88	4889625	s	rec	UTI89_C5014	fimD	CDS
S88	4889636	ns	rec	UTI89_C5014	fimD	CDS
S88	4889637	ns	rec	UTI89_C5014	fimD	CDS
S88	4889703	s	rec	UTI89_C5014	fimD	CDS
S88	4889718	s	rec	UTI89_C5014	fimD	CDS
S88	4889730	s	rec	UTI89_C5014	fimD	CDS
S88	4889778	s	rec	UTI89_C5014	fimD	CDS
S88	4889822	ns	rec	UTI89_C5014	fimD	CDS
S88	4889883	s	rec	UTI89_C5014	fimD	CDS
S88	4889895	s	rec	UTI89_C5014	fimD	CDS
S88	4889901	s	rec	UTI89_C5014	fimD	CDS
S88	4889961	s	rec	UTI89_C5014	fimD	CDS
S88	4890019	ns	rec	UTI89_C5014	fimD	CDS
S88	4890066	ns	rec	UTI89_C5014	fimD	CDS
S88	4890075	s	rec	UTI89_C5014	fimD	CDS
S88	4890123	s	rec	UTI89_C5014	fimD	CDS
S88	4890126	s	rec	UTI89_C5014	fimD	CDS
S88	4890144	s	rec	UTI89_C5014	fimD	CDS
S88	4890237	s	rec	UTI89_C5014	fimD	CDS
S88	4890309	s	rec	UTI89_C5014	fimD	CDS
S88	4890447	s	rec	UTI89_C5014	fimD	CDS
S88	4890591	s	rec	UTI89_C5015	fimF	CDS
S88	4890672	s	rec	UTI89_C5015	fimF	CDS
S88	4890768	s	rec	UTI89_C5015	fimF	CDS
S88	4890804	s	rec	UTI89_C5015	fimF	CDS
S88	4890822	s	rec	UTI89_C5015	fimF	CDS
APEC	4912752	s		UTI89_C5015	fimF	CDS
S88	4890837	s	rec	UTI89_C5015	fimF	CDS
S88	4890927	s	rec	UTI89_C5015	fimF	CDS
S88	4890934	ns	rec	UTI89_C5015	fimF	CDS
S88	4891003	s	rec	UTI89_C5015	fimF	CDS
S88	4891059	s	rec	UTI89_C5015	fimF	CDS

lineage[a]	site[b]	mutation type[c]	recomb	gene	Gene name	Type
S88	4891132	ns	rec	UTI89_C5016	fimG	CDS
S88	4891146	ns	rec	UTI89_C5016	fimG	CDS
S88	4891153	ns	rec	UTI89_C5016	fimG	CDS
S88	4891227	s	rec	UTI89_C5016	fimG	CDS
S88	4891281	s	rec	UTI89_C5016	fimG	CDS
S88	4891407	s	rec	UTI89_C5016	fimG	CDS
S88	4891469	ns	rec	UTI89_C5016	fimG	CDS
S88	4891545	s	rec	UTI89_C5016	fimG	CDS
S88	4891554	s	rec	UTI89_C5016	fimG	CDS
S88	4891613	nc	rec	intergenic		
S88	4891616	nc	rec	intergenic		
S88	4891647	ns	rec	UTI89_C5017	fimH	CDS
S88	4891658	ns	rec	UTI89_C5017	fimH	CDS
S88	4891717	s	rec	UTI89_C5017	fimH	CDS
S88	4891723	s	rec	UTI89_C5017	fimH	CDS
S88	4891771	s	rec	UTI89_C5017	fimH	CDS
S88	4891801	s	rec	UTI89_C5017	fimH	CDS
S88	4891840	s	rec	UTI89_C5017	fimH	CDS
S88	4891855	s	rec	UTI89_C5017	fimH	CDS
S88	4891876	s	rec	UTI89_C5017	fimH	CDS
UTI89	4913801	ns		UTI89_C5017	fimH	CDS
S88	4891902	ns	rec	UTI89_C5017	fimH	CDS
S88	4891926	ns	rec	UTI89_C5017	fimH	CDS
S88	4891942	s	rec	UTI89_C5017	fimH	CDS
S88	4891945	s	rec	UTI89_C5017	fimH	CDS
S88	4891948	s	rec	UTI89_C5017	fimH	CDS
S88	4891951	s	rec	UTI89_C5017	fimH	CDS
S88	4891957	s	rec	UTI89_C5017	fimH	CDS
S88	4892026	s	rec	UTI89_C5017	fimH	CDS
S88	4892041	s	rec	UTI89_C5017	fimH	CDS
S88	4892044	s	rec	UTI89_C5017	fimH	CDS
S88	4892049	ns	rec	UTI89_C5017	fimH	CDS
S88	4892344	s	rec	UTI89_C5017	fimH	CDS
S88	4892347	s	rec	UTI89_C5017	fimH	CDS
S88	4894008	nc	rec	intergenic		
S88	4894164	nc	rec	intergenic		
S88	4894227	nc	rec	intergenic		
S88	4894337	nc	rec	intergenic		
S88	4894341	nc	rec	intergenic		
S88	4894345	nc	rec	intergenic		
S88	4894460	s	rec	UTI89_C5018	uxuA	CDS
S88	4894478	s	rec	UTI89_C5018	uxuA	CDS
S88	4894479	s	rec	UTI89_C5018	uxuA	CDS
S88	4894499	s	rec	UTI89_C5018	uxuA	CDS
S88	4894538	s	rec	UTI89_C5018	uxuA	CDS

lineage[a]	site[b]	mutation type[c]	recomb	gene	Gene name	Type
S88	4894550	s	rec	UTI89_C5018	uxuA	CDS
S88	4894574	s	rec	UTI89_C5018	uxuA	CDS
S88	4894583	s	rec	UTI89_C5018	uxuA	CDS
S88	4894634	s	rec	UTI89_C5018	uxuA	CDS
S88	4894652	s	rec	UTI89_C5018	uxuA	CDS
S88	4894676	s	rec	UTI89_C5018	uxuA	CDS
S88	4894814	s	rec	UTI89_C5018	uxuA	CDS
S88	4894817	s	rec	UTI89_C5018	uxuA	CDS
S88	4894823	s	rec	UTI89_C5018	uxuA	CDS
S88	4894862	s	rec	UTI89_C5018	uxuA	CDS
S88	4894934	s	rec	UTI89_C5018	uxuA	CDS
S88	4894937	s	rec	UTI89_C5018	uxuA	CDS
S88	4894952	s	rec	UTI89_C5018	uxuA	CDS
S88	4894985	s	rec	UTI89_C5018	uxuA	CDS
S88	4895024	s	rec	UTI89_C5018	uxuA	CDS
S88	4895039	s	rec	UTI89_C5018	uxuA	CDS
S88	4895045	s	rec	UTI89_C5018	uxuA	CDS
S88	4895072	s	rec	UTI89_C5018	uxuA	CDS
S88	4895081	s	rec	UTI89_C5018	uxuA	CDS
S88	4895105	s	rec	UTI89_C5018	uxuA	CDS
S88	4895108	s	rec	UTI89_C5018	uxuA	CDS
S88	4895135	s	rec	UTI89_C5018	uxuA	CDS
S88	4895174	s	rec	UTI89_C5018	uxuA	CDS
S88	4895483	s	rec	UTI89_C5018	uxuA	CDS
S88	4895486	s	rec	UTI89_C5018	uxuA	CDS
S88	4895576	nc	rec	intergenic		
S88	4895758	s	rec	UTI89_C5019	uxuB	CDS
S88	4895776	s	rec	UTI89_C5019	uxuB	CDS
S88	4895797	s	rec	UTI89_C5019	uxuB	CDS
S88	4895959	s	rec	UTI89_C5019	uxuB	CDS
S88	4895974	s	rec	UTI89_C5019	uxuB	CDS
S88	4896010	s	rec	UTI89_C5019	uxuB	CDS
S88	4896073	s	rec	UTI89_C5019	uxuB	CDS
S88	4896109	s	rec	UTI89_C5019	uxuB	CDS
S88	4896151	s	rec	UTI89_C5019	uxuB	CDS
S88	4896158	s	rec	UTI89_C5019	uxuB	CDS
S88	4896211	s	rec	UTI89_C5019	uxuB	CDS
S88	4896244	s	rec	UTI89_C5019	uxuB	CDS
S88	4896247	s	rec	UTI89_C5019	uxuB	CDS
S88	4896250	s	rec	UTI89_C5019	uxuB	CDS
S88	4896253	s	rec	UTI89_C5019	uxuB	CDS
S88	4896264	ns	rec	UTI89_C5019	uxuB	CDS
S88	4896280	s	rec	UTI89_C5019	uxuB	CDS
S88	4896283	s	rec	UTI89_C5019	uxuB	CDS
S88	4896297	ns	rec	UTI89_C5019	uxuB	CDS

lineage[a]	site[b]	mutation type[c]	recomb	gene	Gene name	Type
S88	4896301	s	rec	UTI89_C5019	uxuB	CDS
S88	4896313	s	rec	UTI89_C5019	uxuB	CDS
S88	4896388	s	rec	UTI89_C5019	uxuB	CDS
S88	4896436	s	rec	UTI89_C5019	uxuB	CDS
S88	4896448	s	rec	UTI89_C5019	uxuB	CDS
S88	4896463	s	rec	UTI89_C5019	uxuB	CDS
S88	4896481	s	rec	UTI89_C5019	uxuB	CDS
S88	4896487	s	rec	UTI89_C5019	uxuB	CDS
S88	4896493	s	rec	UTI89_C5019	uxuB	CDS
S88	4896499	s	rec	UTI89_C5019	uxuB	CDS
S88	4896502	s	rec	UTI89_C5019	uxuB	CDS
S88	4896508	s	rec	UTI89_C5019	uxuB	CDS
S88	4896618	ns	rec	UTI89_C5019	uxuB	CDS
S88	4896766	s	rec	UTI89_C5019	uxuB	CDS
S88	4896835	s	rec	UTI89_C5019	uxuB	CDS
S88	4896865	s	rec	UTI89_C5019	uxuB	CDS
S88	4896922	s	rec	UTI89_C5019	uxuB	CDS
S88	4896932	ns	rec	UTI89_C5019	uxuB	CDS
S88	4896937	s	rec	UTI89_C5019	uxuB	CDS
S88	4896952	s	rec	UTI89_C5019	uxuB	CDS
S88	4896970	s	rec	UTI89_C5019	uxuB	CDS
S88	4896988	s	rec	UTI89_C5019	uxuB	CDS
S88	4896994	s	rec	UTI89_C5019	uxuB	CDS
S88	4897009	s	rec	UTI89_C5019	uxuB	CDS
S88	4897030	s	rec	UTI89_C5019	uxuB	CDS
S88	4897033	s	rec	UTI89_C5019	uxuB	CDS
S88	4897078	s	rec	UTI89_C5019	uxuB	CDS
S88	4897081	s	rec	UTI89_C5019	uxuB	CDS
S88	4897154	nc	rec	intergenic		
S88	4897204	nc	rec	intergenic		
S88	4897230	nc	rec	intergenic		
S88	4897236	nc	rec	intergenic		
S88	4897239	nc	rec	intergenic		
S88	4897263	nc	rec	intergenic		
S88	4897291	nc	rec	intergenic		
S88	4897430	s	rec	UTI89_C5020	uxuR	CDS
S88	4897526	s	rec	UTI89_C5020	uxuR	CDS
S88	4897535	s	rec	UTI89_C5020	uxuR	CDS
S88	4897572	ns	rec	UTI89_C5020	uxuR	CDS
S88	4897581	ns	rec	UTI89_C5020	uxuR	CDS
S88	4897598	s	rec	UTI89_C5020	uxuR	CDS
S88	4897601	s	rec	UTI89_C5020	uxuR	CDS
S88	4897607	s	rec	UTI89_C5020	uxuR	CDS
S88	4897634	s	rec	UTI89_C5020	uxuR	CDS
S88	4897658	s	rec	UTI89_C5020	uxuR	CDS

lineage[a]	site[b]	mutation type[c]	recomb	gene	Gene name	Type
S88	4897661	s	rec	UTI89_C5020	uxuR	CDS
S88	4897670	s	rec	UTI89_C5020	uxuR	CDS
S88	4897671	s	rec	UTI89_C5020	uxuR	CDS
S88	4897706	s	rec	UTI89_C5020	uxuR	CDS
S88	4897707	ns	rec	UTI89_C5020	uxuR	CDS
S88	4897772	s	rec	UTI89_C5020	uxuR	CDS
S88	4897790	s	rec	UTI89_C5020	uxuR	CDS
S88	4898003	s	rec	UTI89_C5020	uxuR	CDS
S88	4898006	s	rec	UTI89_C5020	uxuR	CDS
S88	4898009	s	rec	UTI89_C5020	uxuR	CDS
S88	4898015	s	rec	UTI89_C5020	uxuR	CDS
S88	4898018	s	rec	UTI89_C5020	uxuR	CDS
S88	4898019	s	rec	UTI89_C5020	uxuR	CDS
S88	4898027	s	rec	UTI89_C5020	uxuR	CDS
S88	4898033	s	rec	UTI89_C5020	uxuR	CDS
S88	4898036	s	rec	UTI89_C5020	uxuR	CDS
S88	4898042	s	rec	UTI89_C5020	uxuR	CDS
S88	4898045	s	rec	UTI89_C5020	uxuR	CDS
S88	4898048	s	rec	UTI89_C5020	uxuR	CDS
S88	4898054	s	rec	UTI89_C5020	uxuR	CDS
S88	4898057	s	rec	UTI89_C5020	uxuR	CDS
S88	4899760	ns	rec	UTI89_C5021	yjiD	CDS
S88	4899761	ns	rec	UTI89_C5021	yjiD	CDS
S88	4899766	ns	rec	UTI89_C5021	yjiD	CDS
S88	4899768	ns	rec	UTI89_C5021	yjiD	CDS
S88	4899771	s	rec	UTI89_C5021	yjiD	CDS
S88	4899782	ns	rec	UTI89_C5021	yjiD	CDS
S88	4899784	ns	rec	UTI89_C5021	yjiD	CDS
S88	4899789	s	rec	UTI89_C5021	yjiD	CDS
S88	4899792	s	rec	UTI89_C5021	yjiD	CDS
S88	4899795	ns	rec	UTI89_C5021	yjiD	CDS
S88	4899800	ns	rec	UTI89_C5021	yjiD	CDS
S88	4899811	ns	rec	UTI89_C5021	yjiD	CDS
S88	4899813	ns	rec	UTI89_C5021	yjiD	CDS
S88	4899816	s	rec	UTI89_C5021	yjiD	CDS
S88	4899817	ns	rec	UTI89_C5021	yjiD	CDS
S88	4899822	s	rec	UTI89_C5021	yjiD	CDS
S88	4899823	ns	rec	UTI89_C5021	yjiD	CDS
S88	4899825	ns	rec	UTI89_C5021	yjiD	CDS
S88	4899832	ns	rec	UTI89_C5021	yjiD	CDS
S88	4899834	ns	rec	UTI89_C5021	yjiD	CDS
S88	4899835	ns	rec	UTI89_C5021	yjiD	CDS
S88	4899836	ns	rec	UTI89_C5021	yjiD	CDS
S88	4899837	ns	rec	UTI89_C5021	yjiD	CDS
S88	4899845	ns	rec	UTI89_C5021	yjiD	CDS

lineage[a]	site[b]	mutation type[c]	recomb	gene	Gene name	Type
S88	4899852	s	rec	UTI89_C5021	yjiD	CDS
S88	4899858	s	rec	UTI89_C5021	yjiD	CDS
S88	4899859	ns	rec	UTI89_C5021	yjiD	CDS
S88	4899861	ns	rec	UTI89_C5021	yjiD	CDS
S88	4899862	s	rec	UTI89_C5021	yjiD	CDS
S88	4899867	s	rec	UTI89_C5021	yjiD	CDS
S88	4899868	ns	rec	UTI89_C5021	yjiD	CDS
S88	4899869	ns	rec	UTI89_C5021	yjiD	CDS
S88	4899891	s	rec	UTI89_C5021	yjiD	CDS
S88	4899902	ns	rec	UTI89_C5021	yjiD	CDS
S88	4899909	s	rec	UTI89_C5021	yjiD	CDS
S88	4899921	s	rec	UTI89_C5021	yjiD	CDS
S88	4899923	ns	rec	UTI89_C5021	yjiD	CDS
S88	4899933	s	rec	UTI89_C5021	yjiD	CDS
S88	4899945	s	rec	UTI89_C5021	yjiD	CDS
S88	4899949	s	rec	UTI89_C5021	yjiD	CDS
S88	4899951	s	rec	UTI89_C5021	yjiD	CDS
UTI89	4919522	s		UTI89_C5021	yjiD	CDS
S88	4899957	s	rec	UTI89_C5021	yjiD	CDS
S88	4899960	s	rec	UTI89_C5021	yjiD	CDS
S88	4899963	s	rec	UTI89_C5021	yjiD	CDS
S88	4899981	s	rec	UTI89_C5021	yjiD	CDS
S88	4899990	s	rec	UTI89_C5021	yjiD	CDS
S88	4900014	s	rec	UTI89_C5021	yjiD	CDS
S88	4900017	s	rec	UTI89_C5021	yjiD	CDS
S88	4900018	ns	rec	UTI89_C5021	yjiD	CDS
S88	4900024	ns	rec	UTI89_C5021	yjiD	CDS
S88	4900025	ns	rec	UTI89_C5021	yjiD	CDS
S88	4900027	s	rec	UTI89_C5021	yjiD	CDS
S88	4900032	s	rec	UTI89_C5021	yjiD	CDS
S88	4900035	s	rec	UTI89_C5021	yjiD	CDS
S88	4900038	s	rec	UTI89_C5021	yjiD	CDS
S88	4900041	s	rec	UTI89_C5021	yjiD	CDS
S88	4900047	ns	rec	UTI89_C5021	yjiD	CDS
S88	4900062	s	rec	UTI89_C5021	yjiD	CDS
S88	4900068	s	rec	UTI89_C5021	yjiD	CDS
S88	4900083	s	rec	UTI89_C5021	yjiD	CDS
S88	4900139	s	rec	UTI89_C5037	yjiE	CDS
S88	4900193	s	rec	UTI89_C5037	yjiE	CDS
S88	4900205	s	rec	UTI89_C5037	yjiE	CDS
S88	4900214	s	rec	UTI89_C5037	yjiE	CDS
S88	4900250	s	rec	UTI89_C5037	yjiE	CDS
S88	4900253	s	rec	UTI89_C5037	yjiE	CDS
S88	4900343	s	rec	UTI89_C5037	yjiE	CDS
S88	4900388	s	rec	UTI89_C5037	yjiE	CDS

lineage[a]	site[b]	mutation type[c]	recomb	gene	Gene name	Type
S88	4900481	s	rec	UTI89_C5037	yjiE	CDS
S88	4900505	s	rec	UTI89_C5037	yjiE	CDS
S88	4900514	s	rec	UTI89_C5037	yjiE	CDS
S88	4900634	s	rec	UTI89_C5037	yjiE	CDS
S88	4900727	s	rec	UTI89_C5037	yjiE	CDS
S88	4900775	s	rec	UTI89_C5037	yjiE	CDS
S88	4900820	s	rec	UTI89_C5037	yjiE	CDS
S88	4900898	nc	rec	intergenic		
S88	4900901	nc	rec	intergenic		
S88	4900961	nc	rec	intergenic		
S88	4900979	nc	rec	intergenic		
S88	4900998	nc	rec	intergenic		
S88	4901109	s	rec	UTI89_C5038	iadA	CDS
S88	4901130	s	rec	UTI89_C5038	iadA	CDS
S88	4901325	s	rec	UTI89_C5038	iadA	CDS
S88	4901352	s	rec	UTI89_C5038	iadA	CDS
S88	4901388	s	rec	UTI89_C5038	iadA	CDS
S88	4901436	s	rec	UTI89_C5038	iadA	CDS
S88	4901460	s	rec	UTI89_C5038	iadA	CDS
S88	4901529	s	rec	UTI89_C5038	iadA	CDS
S88	4901542	ns	rec	UTI89_C5038	iadA	CDS
S88	4901547	s	rec	UTI89_C5038	iadA	CDS
S88	4901553	s	rec	UTI89_C5038	iadA	CDS
S88	4901624	ns	rec	UTI89_C5038	iadA	CDS
S88	4901670	s	rec	UTI89_C5038	iadA	CDS
S88	4901700	s	rec	UTI89_C5038	iadA	CDS
S88	4901898	s	rec	UTI89_C5038	iadA	CDS
S88	4901961	s	rec	UTI89_C5038	iadA	CDS
S88	4901973	s	rec	UTI89_C5038	iadA	CDS
S88	4902014	s	rec	UTI89_C5038	iadA	CDS
S88	4902051	s	rec	UTI89_C5038	iadA	CDS
S88	4902072	s	rec	UTI89_C5038	iadA	CDS
S88	4902156	s	rec	UTI89_C5038	iadA	CDS
S88	4902159	s	rec	UTI89_C5038	iadA	CDS
S88	4902171	s	rec	UTI89_C5038	iadA	CDS
S88	4902191	ns	rec	UTI89_C5038	iadA	CDS
S88	4902206	ns	rec	UTI89_C5038	iadA	CDS
S88	4902266	ns	rec	UTI89_C5038	iadA	CDS
S88	4902360	s	rec	UTI89_C5039	yjiG	CDS
S88	4902462	s	rec	UTI89_C5039	yjiG	CDS
S88	4902483	s	rec	UTI89_C5039	yjiG	CDS
S88	4902519	s	rec	UTI89_C5039	yjiG	CDS
S88	4902525	s	rec	UTI89_C5039	yjiG	CDS
S88	4902558	s	rec	UTI89_C5039	yjiG	CDS
S88	4902582	s	rec	UTI89_C5039	yjiG	CDS

lineage[a]	site[b]	mutation type[c]	recomb	gene	Gene name	Type
S88	4902597	s	rec	UTI89_C5039	yjiG	CDS
S88	4902618	s	rec	UTI89_C5039	yjiG	CDS
S88	4902636	s	rec	UTI89_C5039	yjiG	CDS
S88	4902648	s	rec	UTI89_C5039	yjiG	CDS
S88	4902675	s	rec	UTI89_C5039	yjiG	CDS
S88	4902681	s	rec	UTI89_C5039	yjiG	CDS
S88	4902711	s	rec	UTI89_C5039	yjiG	CDS
S88	4902735	s	rec	UTI89_C5039	yjiG	CDS
S88	4902845	s	rec	UTI89_C5040	yjiH	CDS
S88	4902917	s	rec	UTI89_C5040	yjiH	CDS
S88	4902941	s	rec	UTI89_C5040	yjiH	CDS
S88	4902950	s	rec	UTI89_C5040	yjiH	CDS
S88	4902962	s	rec	UTI89_C5040	yjiH	CDS
S88	4902977	s	rec	UTI89_C5040	yjiH	CDS
S88	4902992	s	rec	UTI89_C5040	yjiH	CDS
S88	4903007	s	rec	UTI89_C5040	yjiH	CDS
S88	4903037	s	rec	UTI89_C5040	yjiH	CDS
S88	4903049	s	rec	UTI89_C5040	yjiH	CDS
S88	4903052	s	rec	UTI89_C5040	yjiH	CDS
S88	4903058	s	rec	UTI89_C5040	yjiH	CDS
S88	4903088	s	rec	UTI89_C5040	yjiH	CDS
S88	4903115	s	rec	UTI89_C5040	yjiH	CDS
S88	4903139	s	rec	UTI89_C5040	yjiH	CDS
S88	4903142	s	rec	UTI89_C5040	yjiH	CDS
S88	4903148	s	rec	UTI89_C5040	yjiH	CDS
S88	4903163	s	rec	UTI89_C5040	yjiH	CDS
S88	4903172	s	rec	UTI89_C5040	yjiH	CDS
S88	4903178	s	rec	UTI89_C5040	yjiH	CDS
S88	4903187	s	rec	UTI89_C5040	yjiH	CDS
S88	4903208	s	rec	UTI89_C5040	yjiH	CDS
S88	4903211	ns	rec	UTI89_C5040	yjiH	CDS
S88	4903213	ns	rec	UTI89_C5040	yjiH	CDS
S88	4903220	s	rec	UTI89_C5040	yjiH	CDS
S88	4903241	s	rec	UTI89_C5040	yjiH	CDS
S88	4903244	s	rec	UTI89_C5040	yjiH	CDS
S88	4903247	s	rec	UTI89_C5040	yjiH	CDS
S88	4903292	s	rec	UTI89_C5040	yjiH	CDS
S88	4903337	s	rec	UTI89_C5040	yjiH	CDS
S88	4903346	s	rec	UTI89_C5040	yjiH	CDS
S88	4903349	s	rec	UTI89_C5040	yjiH	CDS
S88	4903361	s	rec	UTI89_C5040	yjiH	CDS
S88	4903378	ns	rec	UTI89_C5040	yjiH	CDS
S88	4903379	s	rec	UTI89_C5040	yjiH	CDS
S88	4903406	s	rec	UTI89_C5040	yjiH	CDS
S88	4903458	nc	rec	intergenic		

lineage[a]	site[b]	mutation type[c]	recomb	gene	Gene name	Type
S88	4903544	nc	rec	intergenic		
S88	4903574	nc	rec	intergenic		
S88	4903580	nc	rec	intergenic		
S88	4903583	nc	rec	intergenic		
S88	4903586	nc	rec	intergenic		
S88	4903615	nc	rec	intergenic		
S88	4903665	nc	rec	intergenic		
S88	4903690	nc	rec	intergenic		
S88	4903699	nc	rec	intergenic		
S88	4903711	nc	rec	intergenic		
S88	4903714	nc	rec	intergenic		
S88	4903747	nc	rec	intergenic		
S88	4903753	nc	rec	intergenic		
S88	4903765	nc	rec	intergenic		
S88	4903771	nc	rec	intergenic		
S88	4903813	nc	rec	intergenic		
S88	4903846	nc	rec	intergenic		
S88	4903852	nc	rec	intergenic		
S88	4903860	nc	rec	intergenic		
S88	4903861	nc	rec	intergenic		
S88	4903864	nc	rec	intergenic		
S88	4903867	nc	rec	intergenic		
S88	4903888	nc	rec	intergenic		
S88	4903911	nc	rec	intergenic		
S88	4903915	nc	rec	intergenic		
S88	4903918	nc	rec	intergenic		
S88	4903924	nc	rec	intergenic		
S88	4903938	nc	rec	intergenic		
S88	4903954	nc	rec	intergenic		
S88	4903958	nc	rec	intergenic		
S88	4903959	nc	rec	intergenic		
S88	4903963	nc	rec	intergenic		
S88	4903964	nc	rec	intergenic		
S88	4903965	nc	rec	intergenic		
S88	4903984	nc	rec	intergenic		
S88	4904014	nc	rec	intergenic		
S88	4904035	nc	rec	intergenic		
S88	4904050	nc	rec	intergenic		
S88	4904056	nc	rec	intergenic		
S88	4904059	nc	rec	intergenic		
S88	4904065	nc	rec	intergenic		
S88	4904101	nc	rec	intergenic		
S88	4904111	nc	rec	intergenic		
S88	4904122	nc	rec	intergenic		
S88	4904128	nc	rec	intergenic		

lineage[a]	site[b]	mutation type[c]	recomb	gene	Gene name	Type
S88	4904173	nc	rec	intergenic		
S88	4904215	nc	rec	intergenic		
S88	4909907	s	rec	intergenic		
S88	4909919	s	rec	intergenic		
S88	4909925	s	rec	intergenic		
S88	4909931	s	rec	intergenic		
S88	4909934	s	rec	intergenic		
S88	4909940	s	rec	intergenic		
S88	4909946	s	rec	intergenic		
S88	4909952	s	rec	intergenic		
S88	4909964	s	rec	intergenic		
S88	4909976	s	rec	intergenic		
S88	4909979	s	rec	intergenic		
S88	4909988	s	rec	intergenic		
S88	4909991	s	rec	intergenic		
S88	4909994	s	rec	intergenic		
S88	4910015	s	rec	intergenic		
S88	4910021	s	rec	intergenic		
S88	4910024	s	rec	intergenic		
S88	4910036	s	rec	intergenic		
S88	4910042	s	rec	intergenic		
S88	4910045	s	rec	intergenic		
S88	4910057	s	rec	intergenic		
S88	4910069	s	rec	intergenic		
S88	4910072	s	rec	intergenic		
S88	4910078	s	rec	intergenic		
S88	4910090	s	rec	intergenic		
S88	4910104	ns	rec	intergenic		
S88	4910117	s	rec	intergenic		
S88	4910120	s	rec	intergenic		
S88	4910123	s	rec	intergenic		
S88	4910125	s	rec	intergenic		
S88	4910129	s	rec	intergenic		
S88	4910133	ns	rec	intergenic		
S88	4910134	ns	rec	intergenic		
S88	4910144	s	rec	intergenic		
S88	4910147	s	rec	intergenic		
S88	4910149	s	rec	intergenic		
S88	4910152	s	rec	intergenic		
S88	4910156	ns	rec	intergenic		
S88	4910158	ns	rec	intergenic		
S88	4910159	s	rec	intergenic		
S88	4910162	s	rec	intergenic		
S88	4910183	s	rec	intergenic		
S88	4910192	s	rec	intergenic		

lineage[a]	site[b]	mutation type[c]	recomb	gene	Gene name	Type
S88	4910195	s	rec	intergenic		
S88	4910198	s	rec	intergenic		
S88	4910201	s	rec	intergenic		
S88	4910207	s	rec	intergenic		
S88	4910213	s	rec	intergenic		
S88	4910222	s	rec	intergenic		
S88	4910234	s	rec	intergenic		
S88	4910240	s	rec	intergenic		
S88	4910243	s	rec	intergenic		
S88	4910249	s	rec	intergenic		
S88	4910252	s	rec	intergenic		
S88	4910258	s	rec	intergenic		
S88	4910261	s	rec	intergenic		
S88	4910263	s	rec	intergenic		
S88	4910264	s	rec	intergenic		
S88	4910270	s	rec	intergenic		
S88	4910276	s	rec	intergenic		
S88	4910279	s	rec	intergenic		
S88	4910282	s	rec	intergenic		
S88	4910284	s	rec	intergenic		
S88	4910287	ns	rec	intergenic		
S88	4910288	s	rec	intergenic		
S88	4910291	ns	rec	intergenic		
S88	4910292	ns	rec	intergenic		
S88	4910293	ns	rec	intergenic		
S88	4910297	s	rec	intergenic		
S88	4910300	s	rec	intergenic		
S88	4910324	s	rec	intergenic		
S88	4910327	s	rec	intergenic		
S88	4910348	s	rec	intergenic		
S88	4910357	s	rec	intergenic		
S88	4910360	s	rec	intergenic		
S88	4910365	s	rec	intergenic		
S88	4910369	s	rec	intergenic		
S88	4910390	ns	rec	intergenic		
S88	4910391	ns	rec	intergenic		
S88	4910392	ns	rec	intergenic		
S88	4910393	s	rec	intergenic		
S88	4910400	ns	rec	intergenic		
S88	4910402	ns	rec	intergenic		
S88	4910403	ns	rec	intergenic		
S88	4910418	ns	rec	intergenic		
S88	4910419	ns	rec	intergenic		
S88	4910423	ns	rec	intergenic		
S88	4910425	ns	rec	intergenic		

lineage[a]	site[b]	mutation type[c]	recomb	gene	Gene name	Type
S88	4910429	ns	rec	intergenic		
S88	4910430	ns	rec	intergenic		
S88	4910431	ns	rec	intergenic		
S88	4910438	ns	rec	intergenic		
S88	4910444	s	rec	intergenic		
S88	4910447	ns	rec	intergenic		
S88	4910450	s	rec	intergenic		
S88	4910452	s	rec	intergenic		
S88	4910453	s	rec	intergenic		
S88	4910458	ns	rec	intergenic		
S88	4910459	s	rec	intergenic		
S88	4910462	s	rec	intergenic		
S88	4910467	ns	rec	intergenic		
S88	4910470	s	rec	intergenic		
S88	4910471	ns	rec	intergenic		
S88	4910474	ns	rec	intergenic		
S88	4910478	ns	rec	intergenic		
S88	4910486	s	rec	intergenic		
S88	4910489	s	rec	intergenic		
S88	4910495	s	rec	intergenic		
S88	4910504	s	rec	intergenic		
S88	4910513	s	rec	intergenic		
S88	4910516	s	rec	intergenic		
S88	4910523	ns	rec	intergenic		
S88	4910543	s	rec	intergenic		
S88	4910561	s	rec	intergenic		
S88	4910570	ns	rec	intergenic		
S88	4910572	ns	rec	intergenic		
S88	4910579	s	rec	intergenic		
S88	4910588	s	rec	intergenic		
S88	4910590	s	rec	intergenic		
S88	4910591	s	rec	intergenic		
S88	4910594	s	rec	intergenic		
S88	4910597	s	rec	intergenic		
S88	4910600	s	rec	intergenic		
S88	4910606	s	rec	intergenic		
S88	4910615	s	rec	intergenic		
S88	4910618	s	rec	intergenic		
S88	4910621	s	rec	intergenic		
S88	4910627	s	rec	intergenic		
S88	4910630	s	rec	intergenic		
S88	4910636	s	rec	intergenic		
S88	4910639	s	rec	intergenic		
S88	4910642	s	rec	intergenic		
S88	4910650	s	rec	intergenic		

lineage[a]	site[b]	mutation type[c]	recomb	gene	Gene name	Type
S88	4910654	s	rec	intergenic		
S88	4910669	s	rec	intergenic		
S88	4910678	ns	rec	intergenic		
S88	4910680	ns	rec	intergenic		
S88	4910687	ns	rec	intergenic		
S88	4910689	ns	rec	intergenic		
S88	4910690	ns	rec	intergenic		
S88	4910693	s	rec	intergenic		
S88	4910701	ns	rec	intergenic		
S88	4910705	s	rec	intergenic		
S88	4910709	ns	rec	intergenic		
S88	4910711	s	rec	intergenic		
S88	4910713	s	rec	intergenic		
S88	4910717	s	rec	intergenic		
S88	4910723	s	rec	intergenic		
S88	4910732	s	rec	intergenic		
S88	4910735	s	rec	intergenic		
S88	4910738	s	rec	intergenic		
S88	4910742	ns	rec	intergenic		
S88	4910744	s	rec	intergenic		
S88	4910747	s	rec	intergenic		
S88	4910753	s	rec	intergenic		
S88	4910759	s	rec	intergenic		
S88	4910765	s	rec	intergenic		
S88	4910768	s	rec	intergenic		
S88	4910780	s	rec	intergenic		
S88	4910783	s	rec	intergenic		
S88	4910786	ns	rec	intergenic		
S88	4910787	ns	rec	intergenic		
S88	4910788	ns	rec	intergenic		
S88	4910789	s	rec	intergenic		
S88	4910795	s	rec	intergenic		
S88	4910801	s	rec	intergenic		
S88	4910804	s	rec	intergenic		
S88	4910810	s	rec	intergenic		
S88	4910816	s	rec	intergenic		
S88	4910819	s	rec	intergenic		
S88	4910822	s	rec	intergenic		
S88	4910834	s	rec	intergenic		
S88	4910843	s	rec	intergenic		
S88	4910846	ns	rec	intergenic		
S88	4910847	ns	rec	intergenic		
S88	4910852	s	rec	intergenic		
S88	4910858	s	rec	intergenic		
S88	4910864	ns	rec	intergenic		

lineage[a]	site[b]	mutation type[c]	recomb	gene	Gene name	Type
S88	4910866	ns	rec	intergenic		
S88	4910873	s	rec	intergenic		
S88	4910879	ns	rec	intergenic		
S88	4910880	ns	rec	intergenic		
S88	4910881	ns	rec	intergenic		
S88	4910882	s	rec	intergenic		
S88	4910893	ns	rec	intergenic		
S88	4910900	s	rec	intergenic		
S88	4910905	s	rec	intergenic		
S88	4910909	s	rec	intergenic		
S88	4910918	s	rec	intergenic		
S88	4910921	s	rec	intergenic		
S88	4910966	s	rec	intergenic		
S88	4910968	s	rec	intergenic		
S88	4910987	s	rec	intergenic		
S88	4911005	ns	rec	intergenic		
S88	4911007	ns	rec	intergenic		
S88	4911012	ns	rec	intergenic		
S88	4911013	ns	rec	intergenic		
S88	4911020	nc	rec	intergenic		
S88	4911021	nc	rec	intergenic		
S88	4911022	nc	rec	intergenic		
S88	4911026	nc	rec	intergenic		
S88	4911033	nc	rec	intergenic		
S88	4911034	nc	rec	intergenic		
S88	4911036	nc	rec	intergenic		
S88	4911040	nc	rec	intergenic		
S88	4911041	nc	rec	intergenic		
S88	4911042	nc	rec	intergenic		
S88	4911261	s	rec	intergenic		
S88	4911264	s	rec	intergenic		
S88	4911270	s	rec	intergenic		
S88	4911273	s	rec	intergenic		
S88	4911279	s	rec	intergenic		
S88	4911284	s	rec	intergenic		
S88	4911291	s	rec	intergenic		
S88	4911293	s	rec	intergenic		
S88	4911303	s	rec	intergenic		
S88	4911307	ns	rec	intergenic		
S88	4911318	s	rec	intergenic		
S88	4911324	s	rec	intergenic		
S88	4911327	s	rec	intergenic		
S88	4911336	s	rec	intergenic		
S88	4911345	s	rec	intergenic		
S88	4911348	s	rec	intergenic		

lineage[a]	site[b]	mutation type[c]	recomb	gene	Gene name	Type
S88	4911351	s	rec	intergenic		
S88	4911354	s	rec	intergenic		
S88	4911356	s	rec	intergenic		
S88	4911357	s	rec	intergenic		
S88	4911360	s	rec	intergenic		
S88	4911363	s	rec	intergenic		
S88	4911381	s	rec	intergenic		
S88	4911386	ns	rec	intergenic		
S88	4911392	ns	rec	intergenic		
S88	4911393	s	rec	intergenic		
S88	4911396	s	rec	intergenic		
S88	4911403	ns	rec	intergenic		
S88	4911405	s	rec	intergenic		
S88	4911417	s	rec	intergenic		
S88	4911420	s	rec	intergenic		
S88	4911426	s	rec	intergenic		
S88	4911450	s	rec	intergenic		
S88	4911453	s	rec	intergenic		
S88	4911456	s	rec	intergenic		
APEC	4945745	s		intergenic		
S88	4911462	ns	rec	intergenic		
S88	4911463	ns	rec	intergenic		
S88	4911464	ns	rec	intergenic		
S88	4911465	s	rec	intergenic		
S88	4911468	s	rec	intergenic		
S88	4911471	s	rec	intergenic		
S88	4911476	s	rec	intergenic		
S88	4911477	s	rec	intergenic		
S88	4911486	s	rec	intergenic		
S88	4911489	s	rec	intergenic		
S88	4911492	s	rec	intergenic		
S88	4911495	s	rec	intergenic		
S88	4911501	ns	rec	intergenic		
S88	4911503	ns	rec	intergenic		
S88	4911512	s	rec	intergenic		
S88	4911513	s	rec	intergenic		
S88	4911522	s	rec	intergenic		
S88	4911537	ns	rec	intergenic		
S88	4911538	ns	rec	intergenic		
S88	4911549	ns	rec	intergenic		
S88	4911551	ns	rec	intergenic		
S88	4911552	ns	rec	intergenic		
S88	4911554	ns	rec	intergenic		
S88	4911556	ns	rec	intergenic		
S88	4911558	s	rec	intergenic		

lineage[a]	site[b]	mutation type[c]	recomb	gene	Gene name	Type
S88	4911579	s	rec	intergenic		
S88	4911582	s	rec	intergenic		
S88	4911585	s	rec	intergenic		
S88	4911588	s	rec	intergenic		
S88	4911594	s	rec	intergenic		
S88	4911597	s	rec	intergenic		
S88	4911606	ns	rec	intergenic		
S88	4911608	ns	rec	intergenic		
S88	4911615	s	rec	intergenic		
S88	4911627	s	rec	intergenic		
S88	4911654	s	rec	intergenic		
S88	4911657	s	rec	intergenic		
S88	4911663	s	rec	intergenic		
S88	4911674	s	rec	intergenic		
S88	4911675	s	rec	intergenic		
S88	4911678	s	rec	intergenic		
S88	4911681	ns	rec	intergenic		
S88	4911683	ns	rec	intergenic		
S88	4911687	s	rec	intergenic		
S88	4911699	s	rec	intergenic		
S88	4911704	ns	rec	intergenic		
S88	4911705	s	rec	intergenic		
S88	4911741	s	rec	intergenic		
S88	4911744	s	rec	intergenic		
S88	4911747	s	rec	intergenic		
S88	4911759	s	rec	intergenic		
S88	4911762	s	rec	intergenic		
S88	4911771	ns	rec	intergenic		
S88	4911774	s	rec	intergenic		
S88	4911779	ns	rec	intergenic		
S88	4911780	s	rec	intergenic		
S88	4911785	ns	rec	intergenic		
S88	4911789	s	rec	intergenic		
S88	4911795	s	rec	intergenic		
S88	4911798	s	rec	intergenic		
S88	4911801	s	rec	intergenic		
S88	4911804	s	rec	intergenic		
S88	4911810	ns	rec	intergenic		
S88	4911813	s	rec	intergenic		
S88	4911825	ns	rec	intergenic		
S88	4911826	ns	rec	intergenic		
S88	4911827	ns	rec	intergenic		
S88	4911831	ns	rec	intergenic		
S88	4911832	ns	rec	intergenic		
S88	4911837	ns	rec	intergenic		

lineage[a]	site[b]	mutation type[c]	recomb	gene	Gene name	Type
S88	4911839	ns	rec	intergenic		
S88	4911850	ns	rec	intergenic		
S88	4911855	s	rec	intergenic		
S88	4914690	s	rec	intergenic		
S88	4914702	s	rec	intergenic		
S88	4914801	s	rec	intergenic		
S88	4914840	s	rec	intergenic		
S88	4914842	s	rec	intergenic		
S88	4914844	ns	rec	intergenic		
S88	4914855	ns	rec	UTI89_C5044	yjiL	CDS
S88	4914856	ns	rec	UTI89_C5044	yjiL	CDS
S88	4914867	ns	rec	UTI89_C5044	yjiL	CDS
S88	4914868	ns	rec	UTI89_C5044	yjiL	CDS
S88	4914869	s	rec	UTI89_C5044	yjiL	CDS
S88	4914872	s	rec	UTI89_C5044	yjiL	CDS
S88	4914878	s	rec	UTI89_C5044	yjiL	CDS
S88	4914881	s	rec	UTI89_C5044	yjiL	CDS
S88	4914887	s	rec	UTI89_C5044	yjiL	CDS
S88	4914899	s	rec	UTI89_C5044	yjiL	CDS
S88	4914905	s	rec	UTI89_C5044	yjiL	CDS
S88	4914920	s	rec	UTI89_C5044	yjiL	CDS
S88	4914923	s	rec	UTI89_C5044	yjiL	CDS
S88	4914949	ns	rec	UTI89_C5044	yjiL	CDS
S88	4914955	s	rec	UTI89_C5044	yjiL	CDS
S88	4915070	s	rec	UTI89_C5044	yjiL	CDS
S88	4915091	s	rec	UTI89_C5044	yjiL	CDS
S88	4915100	s	rec	UTI89_C5044	yjiL	CDS
S88	4915127	s	rec	UTI89_C5044	yjiL	CDS
S88	4915136	s	rec	UTI89_C5044	yjiL	CDS
S88	4915139	s	rec	UTI89_C5044	yjiL	CDS
S88	4915154	s	rec	UTI89_C5044	yjiL	CDS
S88	4915190	s	rec	UTI89_C5044	yjiL	CDS
S88	4915193	s	rec	UTI89_C5044	yjiL	CDS
S88	4915198	ns	rec	UTI89_C5044	yjiL	CDS
S88	4915292	s	rec	UTI89_C5044	yjiL	CDS
S88	4915322	s	rec	UTI89_C5044	yjiL	CDS
S88	4915340	s	rec	UTI89_C5044	yjiL	CDS
S88	4915356	ns	rec	UTI89_C5044	yjiL	CDS
S88	4915391	s	rec	UTI89_C5044	yjiL	CDS
S88	4915400	s	rec	UTI89_C5044	yjiL	CDS
S88	4915457	s	rec	UTI89_C5044	yjiL	CDS
S88	4915463	s	rec	UTI89_C5044	yjiL	CDS
S88	4915477	ns	rec	UTI89_C5044	yjiL	CDS
S88	4915523	s	rec	UTI89_C5044	yjiL	CDS
S88	4915559	s	rec	UTI89_C5044	yjiL	CDS

lineage[a]	site[b]	mutation type[c]	recomb	gene	Gene name	Type
S88	4915582	ns	rec	UTI89_C5044	yjiL	CDS
S88	4915615	ns	rec	UTI89_C5044	yjiL	CDS
S88	4915655	s	rec	UTI89_C5045	yjiM	CDS
S88	4915823	s	rec	UTI89_C5045	yjiM	CDS
S88	4915843	ns	rec	UTI89_C5045	yjiM	CDS
S88	4915913	s	rec	UTI89_C5045	yjiM	CDS
S88	4915919	s	rec	UTI89_C5045	yjiM	CDS
S88	4915928	s	rec	UTI89_C5045	yjiM	CDS
S88	4915946	s	rec	UTI89_C5045	yjiM	CDS
S88	4916018	s	rec	UTI89_C5045	yjiM	CDS
S88	4916036	s	rec	UTI89_C5045	yjiM	CDS
S88	4916057	s	rec	UTI89_C5045	yjiM	CDS
S88	4916060	s	rec	UTI89_C5045	yjiM	CDS
S88	4916063	s	rec	UTI89_C5045	yjiM	CDS
S88	4916171	s	rec	UTI89_C5045	yjiM	CDS
S88	4916174	s	rec	UTI89_C5045	yjiM	CDS
S88	4916222	s	rec	UTI89_C5045	yjiM	CDS
S88	4916224	s	rec	UTI89_C5045	yjiM	CDS
S88	4916234	s	rec	UTI89_C5045	yjiM	CDS
S88	4916237	s	rec	UTI89_C5045	yjiM	CDS
S88	4916300	s	rec	UTI89_C5045	yjiM	CDS
S88	4916357	s	rec	UTI89_C5045	yjiM	CDS
S88	4916390	s	rec	UTI89_C5045	yjiM	CDS
S88	4916393	s	rec	UTI89_C5045	yjiM	CDS
S88	4916395	s	rec	UTI89_C5045	yjiM	CDS
S88	4916396	s	rec	UTI89_C5045	yjiM	CDS
S88	4916411	s	rec	UTI89_C5045	yjiM	CDS
S88	4916606	s	rec	UTI89_C5045	yjiM	CDS
S88	4916621	s	rec	UTI89_C5045	yjiM	CDS
S88	4916844	nc	rec	intergenic		
S88	4916898	ns	rec	UTI89_C5046	yjiN	CDS
S88	4916934	s	rec	UTI89_C5046	yjiN	CDS
S88	4916949	s	rec	UTI89_C5046	yjiN	CDS
S88	4916952	s	rec	UTI89_C5046	yjiN	CDS
S88	4916988	s	rec	UTI89_C5046	yjiN	CDS
S88	4916997	s	rec	UTI89_C5046	yjiN	CDS
S88	4917042	s	rec	UTI89_C5046	yjiN	CDS
S88	4917045	s	rec	UTI89_C5046	yjiN	CDS
S88	4917063	s	rec	UTI89_C5046	yjiN	CDS
S88	4917078	s	rec	UTI89_C5046	yjiN	CDS
S88	4917090	s	rec	UTI89_C5046	yjiN	CDS
S88	4917105	s	rec	UTI89_C5046	yjiN	CDS
S88	4917114	s	rec	UTI89_C5046	yjiN	CDS
S88	4917126	s	rec	UTI89_C5046	yjiN	CDS
S88	4917189	s	rec	UTI89_C5046	yjiN	CDS

lineage[a]	site[b]	mutation type[c]	recomb	gene	Gene name	Type
S88	4917233	ns	rec	UTI89_C5046	yjiN	CDS
S88	4917294	s	rec	UTI89_C5046	yjiN	CDS
S88	4917297	s	rec	UTI89_C5046	yjiN	CDS
S88	4917315	s	rec	UTI89_C5046	yjiN	CDS
S88	4917362	s	rec	UTI89_C5046	yjiN	CDS
S88	4917381	s	rec	UTI89_C5046	yjiN	CDS
S88	4917388	ns	rec	UTI89_C5046	yjiN	CDS
S88	4917402	s	rec	UTI89_C5046	yjiN	CDS
S88	4917498	s	rec	UTI89_C5046	yjiN	CDS
S88	4917519	s	rec	UTI89_C5046	yjiN	CDS
S88	4917570	s	rec	UTI89_C5046	yjiN	CDS
S88	4917591	s	rec	UTI89_C5046	yjiN	CDS
S88	4917594	s	rec	UTI89_C5046	yjiN	CDS
S88	4917603	s	rec	UTI89_C5046	yjiN	CDS
S88	4917678	s	rec	UTI89_C5046	yjiN	CDS
S88	4917705	s	rec	UTI89_C5046	yjiN	CDS
S88	4917756	s	rec	UTI89_C5046	yjiN	CDS
S88	4917765	s	rec	UTI89_C5046	yjiN	CDS
S88	4917777	s	rec	UTI89_C5046	yjiN	CDS
S88	4917807	s	rec	UTI89_C5046	yjiN	CDS
S88	4917810	s	rec	UTI89_C5046	yjiN	CDS
S88	4917852	s	rec	UTI89_C5046	yjiN	CDS
S88	4917876	s	rec	UTI89_C5046	yjiN	CDS
S88	4917909	s	rec	UTI89_C5046	yjiN	CDS
S88	4917978	s	rec	UTI89_C5046	yjiN	CDS
S88	4918059	s	rec	UTI89_C5046	yjiN	CDS
APEC	4949832	s		UTI89_C5046	yjiN	CDS
S88	4918177	ns	rec	UTI89_C5046	yjiN	CDS
S88	4919806	nc	rec	intergenic		
S88	4919810	nc	rec	intergenic		
S88	4919827	nc	rec	intergenic		
S88	4919829	nc	rec	intergenic		
S88	4919841	nc	rec	intergenic		
S88	4919848	nc	rec	intergenic		
S88	4919852	nc	rec	intergenic		
S88	4919857	nc	rec	intergenic		
S88	4919864	nc	rec	intergenic		
S88	4919879	nc	rec	intergenic		
S88	4919883	nc	rec	intergenic		
S88	4919926	s	rec	UTI89_C5047	-	CDS
S88	4919929	s	rec	UTI89_C5047	-	CDS
S88	4919971	s	rec	UTI89_C5047	-	CDS
S88	4919975	ns	rec	UTI89_C5047	-	CDS
S88	4920043	s	rec	UTI89_C5047	-	CDS
S88	4920127	s	rec	UTI89_C5047	-	CDS

lineage[a]	site[b]	mutation type[c]	recomb	gene	Gene name	Type
S88	4920151	s	rec	UTI89_C5047	-	CDS
S88	4920172	s	rec	UTI89_C5047	-	CDS
S88	4920181	s	rec	UTI89_C5047	-	CDS
S88	4920196	s	rec	UTI89_C5047	-	CDS
S88	4920238	s	rec	UTI89_C5047	-	CDS
S88	4920255	ns	rec	UTI89_C5047	-	CDS
S88	4920262	s	rec	UTI89_C5047	-	CDS
S88	4920301	s	rec	UTI89_C5047	-	CDS
S88	4920316	s	rec	UTI89_C5047	-	CDS
S88	4920317	s	rec	UTI89_C5047	-	CDS
S88	4920340	s	rec	UTI89_C5047	-	CDS
S88	4920346	s	rec	UTI89_C5047	-	CDS
S88	4920349	s	rec	UTI89_C5047	-	CDS
S88	4920373	s	rec	UTI89_C5047	-	CDS
S88	4920392	ns	rec	UTI89_C5047	-	CDS
S88	4920493	s	rec	UTI89_C5047	-	CDS
S88	4920496	s	rec	UTI89_C5047	-	CDS
S88	4920506	ns	rec	UTI89_C5047	-	CDS
S88	4920547	s	rec	UTI89_C5047	-	CDS
S88	4920577	s	rec	UTI89_C5047	-	CDS
S88	4920595	ns	rec	UTI89_C5047	-	CDS
S88	4920596	ns	rec	UTI89_C5047	-	CDS
S88	4920607	s	rec	UTI89_C5047	-	CDS
S88	4920611	ns	rec	UTI89_C5047	-	CDS
S88	4920623	ns	rec	UTI89_C5047	-	CDS
S88	4920634	ns	rec	UTI89_C5047	-	CDS
S88	4920668	ns	rec	UTI89_C5047	-	CDS
S88	4920725	ns	rec	UTI89_C5047	-	CDS
S88	4920727	ns	rec	UTI89_C5047	-	CDS
S88	4920730	s	rec	UTI89_C5047	-	CDS
S88	4920739	ns	rec	UTI89_C5047	-	CDS
S88	4920753	ns	rec	UTI89_C5047	-	CDS
S88	4920802	ns	rec	UTI89_C5047	-	CDS
S88	4920817	s	rec	UTI89_C5047	-	CDS
S88	4920829	s	rec	UTI89_C5047	-	CDS
S88	4920830	ns	rec	UTI89_C5047	-	CDS
S88	4920832	ns	rec	UTI89_C5047	-	CDS
S88	4920833	ns	rec	UTI89_C5047	-	CDS
S88	4920837	ns	rec	UTI89_C5047	-	CDS
S88	4920838	ns	rec	UTI89_C5047	-	CDS
S88	4920839	ns	rec	UTI89_C5047	-	CDS
S88	4920842	ns	rec	UTI89_C5047	-	CDS
S88	4920848	ns	rec	UTI89_C5047	-	CDS
S88	4920851	ns	rec	UTI89_C5047	-	CDS
S88	4920854	ns	rec	UTI89_C5047	-	CDS

lineage[a]	site[b]	mutation type[c]	recomb	gene	Gene name	Type
S88	4920855	ns	rec	UTI89_C5047	-	CDS
S88	4920868	nc	rec	intergenic		
S88	4920886	nc	rec	intergenic		
S88	4920895	nc	rec	intergenic		
S88	4922933	ns	rec	intergenic		
S88	4922966	s	rec	intergenic		
S88	4922981	s	rec	intergenic		
S88	4923001	ns	rec	intergenic		
S88	4923017	s	rec	intergenic		
S88	4923064	ns	rec	intergenic		
S88	4923066	ns	rec	intergenic		
S88	4923068	s	rec	intergenic		
S88	4923074	s	rec	intergenic		
S88	4923149	s	rec	intergenic		
S88	4923158	s	rec	intergenic		
S88	4923185	s	rec	intergenic		
S88	4923188	s	rec	intergenic		
S88	4923199	ns	rec	intergenic		
S88	4923300	nc	rec	intergenic		
S88	4923335	nc	rec	intergenic		
S88	4923336	nc	rec	intergenic		
S88	4923340	nc	rec	intergenic		
S88	4931867	s	rec	UTI89_C5055	yjiA	CDS
S88	4931887	ns	rec	UTI89_C5055	yjiA	CDS
S88	4931921	s	rec	UTI89_C5055	yjiA	CDS
S88	4931948	s	rec	UTI89_C5055	yjiA	CDS
S88	4932152	s	rec	UTI89_C5055	yjiA	CDS
S88	4932167	s	rec	UTI89_C5055	yjiA	CDS
S88	4932173	s	rec	UTI89_C5055	yjiA	CDS
S88	4932176	s	rec	UTI89_C5055	yjiA	CDS
S88	4932245	s	rec	UTI89_C5055	yjiA	CDS
S88	4932272	s	rec	UTI89_C5055	yjiA	CDS
S88	4932308	s	rec	UTI89_C5055	yjiA	CDS
S88	4932312	ns	rec	UTI89_C5055	yjiA	CDS
S88	4932353	s	rec	UTI89_C5055	yjiA	CDS
S88	4932362	s	rec	UTI89_C5055	yjiA	CDS
S88	4932449	s	rec	UTI89_C5055	yjiA	CDS
S88	4932509	s	rec	UTI89_C5055	yjiA	CDS
S88	4932563	s	rec	UTI89_C5055	yjiA	CDS
S88	4932581	s	rec	UTI89_C5055	yjiA	CDS
S88	4932587	s	rec	UTI89_C5055	yjiA	CDS
S88	4932677	s	rec	UTI89_C5055	yjiA	CDS
S88	4932725	s	rec	UTI89_C5055	yjiA	CDS
S88	4933160	ns	rec	UTI89_C5057	yjiY	CDS
S88	4933161	ns	rec	UTI89_C5057	yjiY	CDS

lineage[a]	site[b]	mutation type[c]	recomb	gene	Gene name	Type
S88	4933162	s	rec	UTI89_C5057	yjiY	CDS
S88	4933225	s	rec	UTI89_C5057	yjiY	CDS
S88	4933264	s	rec	UTI89_C5057	yjiY	CDS
S88	4933324	s	rec	UTI89_C5057	yjiY	CDS
S88	4933411	s	rec	UTI89_C5057	yjiY	CDS
S88	4933417	ns	rec	UTI89_C5057	yjiY	CDS
S88	4933419	ns	rec	UTI89_C5057	yjiY	CDS
S88	4933423	s	rec	UTI89_C5057	yjiY	CDS
S88	4933426	s	rec	UTI89_C5057	yjiY	CDS
S88	4933462	s	rec	UTI89_C5057	yjiY	CDS
S88	4933486	s	rec	UTI89_C5057	yjiY	CDS
S88	4933609	s	rec	UTI89_C5057	yjiY	CDS
S88	4933654	s	rec	UTI89_C5057	yjiY	CDS
S88	4933801	s	rec	UTI89_C5057	yjiY	CDS
S88	4933822	s	rec	UTI89_C5057	yjiY	CDS
S88	4933828	s	rec	UTI89_C5057	yjiY	CDS
S88	4933957	s	rec	UTI89_C5057	yjiY	CDS
S88	4934017	s	rec	UTI89_C5057	yjiY	CDS
S88	4934164	s	rec	UTI89_C5057	yjiY	CDS
S88	4934173	s	rec	UTI89_C5057	yjiY	CDS
S88	4934446	s	rec	UTI89_C5057	yjiY	CDS
S88	4934458	s	rec	UTI89_C5057	yjiY	CDS
S88	4934641	s	rec	UTI89_C5057	yjiY	CDS
S88	4934680	s	rec	UTI89_C5057	yjiY	CDS
S88	4934686	s	rec	UTI89_C5057	yjiY	CDS
S88	4934788	s	rec	UTI89_C5057	yjiY	CDS
S88	4935091	s	rec	UTI89_C5057	yjiY	CDS
S88	4935123	ns	rec	UTI89_C5057	yjiY	CDS
S88	4935190	s	rec	UTI89_C5057	yjiY	CDS
S88	4935312	nc	rec	intergenic		
S88	4935377	nc	rec	intergenic		
S88	4935390	nc	rec	intergenic		
S88	4935391	nc	rec	intergenic		
S88	4935392	nc	rec	intergenic		
S88	4935395	nc	rec	intergenic		
S88	4935396	nc	rec	intergenic		
S88	4935398	nc	rec	intergenic		
S88	4935427	nc	rec	intergenic		
S88	4935442	nc	rec	intergenic		
S88	4935462	nc	rec	intergenic		
S88	4935499	nc	rec	intergenic		
S88	4935501	nc	rec	intergenic		
S88	4935524	nc	rec	intergenic		
S88	4935538	nc	rec	intergenic		
S88	4935630	s	rec	UTI89_C5058	tsr	CDS

lineage[a]	site[b]	mutation type[c]	recomb	gene	Gene name	Type
S88	4935671	s	rec	UTI89_C5058	tsr	CDS
S88	4935704	s	rec	UTI89_C5058	tsr	CDS
S88	4935776	s	rec	UTI89_C5058	tsr	CDS
S88	4935836	s	rec	UTI89_C5058	tsr	CDS
S88	4935873	ns	rec	UTI89_C5058	tsr	CDS
S88	4936019	s	rec	UTI89_C5058	tsr	CDS
S88	4936131	ns	rec	UTI89_C5058	tsr	CDS
S88	4936190	s	rec	UTI89_C5058	tsr	CDS
S88	4936221	ns	rec	UTI89_C5058	tsr	CDS
S88	4936550	s	rec	UTI89_C5058	tsr	CDS
S88	4936556	s	rec	UTI89_C5058	tsr	CDS
S88	4936601	s	rec	UTI89_C5058	tsr	CDS
S88	4936622	s	rec	UTI89_C5058	tsr	CDS
S88	4936631	s	rec	UTI89_C5058	tsr	CDS
S88	4936649	s	rec	UTI89_C5058	tsr	CDS
S88	4936778	s	rec	UTI89_C5058	tsr	CDS
S88	4936826	s	rec	UTI89_C5058	tsr	CDS
S88	4936832	s	rec	UTI89_C5058	tsr	CDS
S88	4936838	s	rec	UTI89_C5058	tsr	CDS
S88	4936940	s	rec	UTI89_C5058	tsr	CDS
S88	4937078	s	rec	UTI89_C5058	tsr	CDS
S88	4937087	s	rec	UTI89_C5058	tsr	CDS
S88	4937108	s	rec	UTI89_C5058	tsr	CDS
S88	4937169	ns	rec	UTI89_C5058	tsr	CDS
S88	4937202	ns	rec	UTI89_C5058	tsr	CDS
S88	4937210	s	rec	UTI89_C5058	tsr	CDS
S88	4937249	s	rec	UTI89_C5058	tsr	CDS
S88	4938991	s	rec	intergenic		
S88	4939012	s	rec	intergenic		
S88	4939018	s	rec	intergenic		
S88	4939021	s	rec	intergenic		
S88	4939042	s	rec	intergenic		
S88	4939054	s	rec	intergenic		
S88	4939081	s	rec	intergenic		
S88	4939084	s	rec	intergenic		
S88	4939087	s	rec	intergenic		
S88	4939090	s	rec	intergenic		
S88	4939093	s	rec	intergenic		
S88	4939099	s	rec	intergenic		
S88	4939107	ns	rec	intergenic		
S88	4939117	s	rec	intergenic		
S88	4939123	s	rec	intergenic		
S88	4939129	s	rec	intergenic		
S88	4939132	s	rec	intergenic		
S88	4939134	s	rec	intergenic		

lineage[a]	site[b]	mutation type[c]	recomb	gene	Gene name	Type
S88	4939138	ns	rec	intergenic		
S88	4939140	ns	rec	intergenic		
S88	4939143	ns	rec	intergenic		
S88	4939144	s	rec	intergenic		
S88	4939153	s	rec	intergenic		
S88	4939155	s	rec	intergenic		
S88	4939159	s	rec	intergenic		
S88	4939161	s	rec	intergenic		
S88	4939165	s	rec	intergenic		
S88	4939168	s	rec	intergenic		
S88	4939174	s	rec	intergenic		
S88	4939181	ns	rec	intergenic		
S88	4939183	ns	rec	intergenic		
S88	4939184	ns	rec	intergenic		
S88	4939189	s	rec	intergenic		
S88	4939193	ns	rec	intergenic		
S88	4939198	s	rec	intergenic		
S88	4939203	s	rec	intergenic		
S88	4939205	ns	rec	intergenic		
S88	4939206	ns	rec	intergenic		
S88	4939209	s	rec	intergenic		
S88	4939210	s	rec	intergenic		
S88	4939239	s	rec	intergenic		
S88	4939246	s	rec	intergenic		
S88	4939249	s	rec	intergenic		
S88	4939254	ns	rec	intergenic		
S88	4939258	s	rec	intergenic		
S88	4939261	s	rec	intergenic		
S88	4939276	s	rec	intergenic		
S88	4939279	s	rec	intergenic		
S88	4939282	s	rec	intergenic		
S88	4939342	s	rec	intergenic		
S88	4939357	s	rec	intergenic		
S88	4939375	ns	rec	intergenic		
S88	4939393	s	rec	intergenic		
S88	4939402	s	rec	intergenic		
S88	4939416	s	rec	intergenic		
S88	4939420	s	rec	intergenic		
S88	4939423	s	rec	intergenic		
S88	4939429	s	rec	intergenic		
S88	4939435	ns	rec	intergenic		
S88	4939437	ns	rec	intergenic		
S88	4939438	s	rec	intergenic		
S88	4939441	s	rec	intergenic		
S88	4939444	s	rec	intergenic		

lineage[a]	site[b]	mutation type[c]	recomb	gene	Gene name	Type
S88	4939447	s	rec	intergenic		
S88	4939453	s	rec	intergenic		
S88	4939456	s	rec	intergenic		
S88	4939462	s	rec	intergenic		
S88	4939468	ns	rec	intergenic		
S88	4939469	ns	rec	intergenic		
S88	4939474	s	rec	intergenic		
S88	4939477	ns	rec	intergenic		
S88	4939479	ns	rec	intergenic		
S88	4939493	ns	rec	intergenic		
S88	4939498	s	rec	intergenic		
S88	4939504	s	rec	intergenic		
S88	4939513	s	rec	intergenic		
S88	4940028	s	rec	UTI89_C5064	-	CDS
S88	4940037	s	rec	UTI89_C5064	-	CDS
S88	4940038	ns	rec	UTI89_C5064	-	CDS
S88	4940058	s	rec	UTI89_C5064	-	CDS
S88	4940075	ns	rec	UTI89_C5064	-	CDS
S88	4940076	ns	rec	UTI89_C5064	-	CDS
S88	4940079	s	rec	UTI89_C5064	-	CDS
S88	4940085	s	rec	UTI89_C5064	-	CDS
S88	4940106	s	rec	UTI89_C5064	-	CDS
S88	4940137	ns	rec	UTI89_C5064	-	CDS
S88	4940141	ns	rec	UTI89_C5064	-	CDS
S88	4940142	ns	rec	UTI89_C5064	-	CDS
S88	4940143	ns	rec	UTI89_C5064	-	CDS
S88	4940144	ns	rec	UTI89_C5064	-	CDS
S88	4940145	ns	rec	UTI89_C5064	-	CDS
S88	4940148	s	rec	UTI89_C5064	-	CDS
S88	4940154	s	rec	UTI89_C5064	-	CDS
S88	4940158	ns	rec	UTI89_C5064	-	CDS
S88	4940159	ns	rec	UTI89_C5064	-	CDS
S88	4940160	ns	rec	UTI89_C5064	-	CDS
S88	4940161	ns	rec	UTI89_C5064	-	CDS
S88	4940164	ns	rec	UTI89_C5064	-	CDS
S88	4940169	ns	rec	UTI89_C5064	-	CDS
S88	4940171	ns	rec	UTI89_C5064	-	CDS
S88	4940172	ns	rec	UTI89_C5064	-	CDS
S88	4940175	s	rec	UTI89_C5064	-	CDS
S88	4940187	s	rec	UTI89_C5064	-	CDS
S88	4940190	s	rec	UTI89_C5064	-	CDS
S88	4940193	s	rec	UTI89_C5064	-	CDS
S88	4940196	s	rec	UTI89_C5064	-	CDS
S88	4940200	ns	rec	UTI89_C5064	-	CDS
S88	4940203	ns	rec	UTI89_C5064	-	CDS

lineage[a]	site[b]	mutation type[c]	recomb	gene	Gene name	Type
S88	4940205	ns	rec	UTI89_C5064	-	CDS
S88	4940235	s	rec	UTI89_C5064	-	CDS
APEC	4974091	ns		UTI89_C5064	-	CDS
APEC	4974109	s		UTI89_C5064	-	CDS
S88	4940403	s	rec	UTI89_C5064	-	CDS
S88	4940415	s	rec	UTI89_C5064	-	CDS
S88	4940419	ns	rec	UTI89_C5064	-	CDS
S88	4940493	s	rec	UTI89_C5064	-	CDS
S88	4940526	s	rec	UTI89_C5064	-	CDS
S88	4940560	ns	rec	UTI89_C5064	-	CDS
S88	4940562	ns	rec	UTI89_C5064	-	CDS
S88	4940598	s	rec	UTI89_C5064	-	CDS
S88	4940637	s	rec	UTI89_C5064	-	CDS
S88	4940676	s	rec	UTI89_C5064	-	CDS
S88	4940680	s	rec	UTI89_C5064	-	CDS
S88	4940808	s	rec	UTI89_C5064	-	CDS
S88	4940824	s	rec	UTI89_C5064	-	CDS
S88	4940838	s	rec	UTI89_C5064	-	CDS
S88	4940850	s	rec	UTI89_C5064	-	CDS
S88	4940931	s	rec	UTI89_C5064	-	CDS
S88	4940937	s	rec	UTI89_C5064	-	CDS
S88	4940971	nc	rec	intergenic		
S88	4941024	nc	rec	intergenic		
S88	4941033	nc	rec	intergenic		
S88	4941039	nc	rec	intergenic		
S88	4941044	nc	rec	intergenic		
S88	4941051	nc	rec	intergenic		
S88	4941065	nc	rec	intergenic		
S88	4941066	nc	rec	intergenic		
S88	4941156	s	rec	UTI89_C5065	mdoB	CDS
S88	4941195	s	rec	UTI89_C5065	mdoB	CDS
S88	4941276	s	rec	UTI89_C5065	mdoB	CDS
S88	4941411	s	rec	UTI89_C5065	mdoB	CDS
S88	4941438	s	rec	UTI89_C5065	mdoB	CDS
S88	4941444	s	rec	UTI89_C5065	mdoB	CDS
S88	4941483	s	rec	UTI89_C5065	mdoB	CDS
S88	4941540	s	rec	UTI89_C5065	mdoB	CDS
S88	4941603	s	rec	UTI89_C5065	mdoB	CDS
S88	4941608	ns	rec	UTI89_C5065	mdoB	CDS
S88	4941613	ns	rec	UTI89_C5065	mdoB	CDS
S88	4941617	ns	rec	UTI89_C5065	mdoB	CDS
S88	4941684	s	rec	UTI89_C5065	mdoB	CDS
S88	4941765	s	rec	UTI89_C5065	mdoB	CDS
S88	4941867	s	rec	UTI89_C5065	mdoB	CDS
S88	4941948	s	rec	UTI89_C5065	mdoB	CDS

lineage[a]	site[b]	mutation type[c]	recomb	gene	Gene name	Type
S88	4942023	s	rec	UTI89_C5065	mdoB	CDS
S88	4942086	s	rec	UTI89_C5065	mdoB	CDS
S88	4942131	s	rec	UTI89_C5065	mdoB	CDS
S88	4942167	s	rec	UTI89_C5065	mdoB	CDS
S88	4942185	s	rec	UTI89_C5065	mdoB	CDS
S88	4942272	s	rec	UTI89_C5065	mdoB	CDS
S88	4942275	s	rec	UTI89_C5065	mdoB	CDS
S88	4942317	s	rec	UTI89_C5065	mdoB	CDS
S88	4942353	s	rec	UTI89_C5065	mdoB	CDS
S88	4942356	s	rec	UTI89_C5065	mdoB	CDS
S88	4942440	s	rec	UTI89_C5065	mdoB	CDS
S88	4942509	s	rec	UTI89_C5065	mdoB	CDS
S88	4942554	s	rec	UTI89_C5065	mdoB	CDS
S88	4942560	s	rec	UTI89_C5065	mdoB	CDS
S88	4942637	ns	rec	UTI89_C5065	mdoB	CDS
S88	4942638	s	rec	UTI89_C5065	mdoB	CDS
S88	4942644	s	rec	UTI89_C5065	mdoB	CDS
S88	4942677	s	rec	UTI89_C5065	mdoB	CDS
S88	4942683	s	rec	UTI89_C5065	mdoB	CDS
S88	4942704	s	rec	UTI89_C5065	mdoB	CDS
S88	4942761	s	rec	UTI89_C5065	mdoB	CDS
S88	4942767	s	rec	UTI89_C5065	mdoB	CDS
S88	4943016	s	rec	UTI89_C5065	mdoB	CDS
S88	4943022	s	rec	UTI89_C5065	mdoB	CDS
S88	4943043	s	rec	UTI89_C5065	mdoB	CDS
S88	4943079	s	rec	UTI89_C5065	mdoB	CDS
S88	4943121	ns	rec	UTI89_C5065	mdoB	CDS
S88	4943123	ns	rec	UTI89_C5065	mdoB	CDS
S88	4943148	s	rec	UTI89_C5065	mdoB	CDS
S88	4943153	s	rec	UTI89_C5065	mdoB	CDS
S88	4943154	s	rec	UTI89_C5065	mdoB	CDS
AS	4976994	s		UTI89_C5065	mdoB	CDS
S88	4943283	s	rec	UTI89_C5065	mdoB	CDS
S88	4943292	s	rec	UTI89_C5065	mdoB	CDS
S88	4943532	nc	rec	intergenic		
S88	4943533	nc	rec	intergenic		
S88	4943539	nc	rec	intergenic		
S88	4943662	s	rec	UTI89_C5066	yjjA	CDS
S88	4943713	s	rec	UTI89_C5066	yjjA	CDS
S88	4943719	s	rec	UTI89_C5066	yjjA	CDS
S88	4943722	s	rec	UTI89_C5066	yjjA	CDS
S88	4943776	s	rec	UTI89_C5066	yjjA	CDS
S88	4943824	s	rec	UTI89_C5066	yjjA	CDS
S88	4943833	s	rec	UTI89_C5066	yjjA	CDS
S88	4943839	s	rec	UTI89_C5066	yjjA	CDS

lineage[a]	site[b]	mutation type[c]	recomb	gene	Gene name	Type
S88	4943869	s	rec	UTI89_C5066	yjjA	CDS
S88	4943911	s	rec	UTI89_C5066	yjjA	CDS
S88	4943944	s	rec	UTI89_C5066	yjjA	CDS
S88	4943948	ns	rec	UTI89_C5066	yjjA	CDS
S88	4944002	ns	rec	UTI89_C5066	yjjA	CDS
S88	4944120	ns	rec	UTI89_C5066	yjjA	CDS
S88	4944146	nc	rec	intergenic		
S88	4944202	s	rec	UTI89_C5067	dnaC	CDS
S88	4944436	s	rec	UTI89_C5067	dnaC	CDS
S88	4944601	s	rec	UTI89_C5067	dnaC	CDS
S88	4944676	s	rec	UTI89_C5067	dnaC	CDS
S88	4944712	s	rec	UTI89_C5067	dnaC	CDS
S88	4944808	s	rec	UTI89_C5067	dnaC	CDS
S88	4944820	s	rec	UTI89_C5067	dnaC	CDS
S88	4944834	s	rec	UTI89_C5067	dnaC	CDS
S88	4944853	s	rec	UTI89_C5067	dnaC	CDS
S88	4944856	s	rec	UTI89_C5067	dnaC	CDS
S88	4944960	s	rec	UTI89_C5068	dnaT	CDS
S88	4945065	s	rec	UTI89_C5068	dnaT	CDS
S88	4945095	s	rec	UTI89_C5068	dnaT	CDS
S88	4945109	ns	rec	UTI89_C5068	dnaT	CDS
S88	4945118	ns	rec	UTI89_C5068	dnaT	CDS
S88	4945128	s	rec	UTI89_C5068	dnaT	CDS
S88	4945161	s	rec	UTI89_C5068	dnaT	CDS
S88	4945182	s	rec	UTI89_C5068	dnaT	CDS
S88	4945185	s	rec	UTI89_C5068	dnaT	CDS
S88	4945194	s	rec	UTI89_C5068	dnaT	CDS
S88	4945212	s	rec	UTI89_C5068	dnaT	CDS
S88	4945226	ns	rec	UTI89_C5068	dnaT	CDS
S88	4945323	s	rec	UTI89_C5068	dnaT	CDS
S88	4945328	ns	rec	UTI89_C5068	dnaT	CDS
S88	4945547	nc	rec	intergenic		
S88	4945557	nc	rec	intergenic		
S88	4945698	s	rec	UTI89_C5069	yjjB	CDS
S88	4945738	s	rec	UTI89_C5069	yjjB	CDS
S88	4945747	s	rec	UTI89_C5069	yjjB	CDS
S88	4945765	s	rec	UTI89_C5069	yjjB	CDS
S88	4945828	s	rec	UTI89_C5069	yjjB	CDS
S88	4945843	s	rec	UTI89_C5069	yjjB	CDS
S88	4945861	s	rec	UTI89_C5069	yjjB	CDS
S88	4945872	s	rec	UTI89_C5069	yjjB	CDS
S88	4945885	s	rec	UTI89_C5069	yjjB	CDS
S88	4945914	ns	rec	UTI89_C5069	yjjB	CDS
S88	4945915	s	rec	UTI89_C5069	yjjB	CDS
S88	4945942	s	rec	UTI89_C5069	yjjB	CDS

lineage[a]	site[b]	mutation type[c]	recomb	gene	Gene name	Type
S88	4945945	s	rec	UTI89_C5069	yjjB	CDS
S88	4946070	s	rec	UTI89_C5070	yjjP	CDS
S88	4946082	s	rec	UTI89_C5070	yjjP	CDS
S88	4946112	s	rec	UTI89_C5070	yjjP	CDS
S88	4946169	s	rec	UTI89_C5070	yjjP	CDS
S88	4946190	s	rec	UTI89_C5070	yjjP	CDS
S88	4946244	s	rec	UTI89_C5070	yjjP	CDS
S88	4946289	s	rec	UTI89_C5070	yjjP	CDS
S88	4946354	ns	rec	UTI89_C5070	yjjP	CDS
S88	4946364	ns	rec	UTI89_C5070	yjjP	CDS
S88	4946366	ns	rec	UTI89_C5070	yjjP	CDS
S88	4946397	ns	rec	UTI89_C5070	yjjP	CDS
S88	4946424	s	rec	UTI89_C5070	yjjP	CDS
S88	4946529	s	rec	UTI89_C5070	yjjP	CDS
S88	4946535	s	rec	UTI89_C5070	yjjP	CDS
S88	4946625	s	rec	UTI89_C5070	yjjP	CDS
S88	4946681	s	rec	UTI89_C5070	yjjP	CDS
S88	4946682	s	rec	UTI89_C5070	yjjP	CDS
S88	4946931	nc	rec	intergenic		
S88	4946965	nc	rec	intergenic		
S88	4946978	nc	rec	intergenic		
S88	4946993	nc	rec	intergenic		
S88	4946994	nc	rec	intergenic		
S88	4947006	nc	rec	intergenic		
S88	4947009	nc	rec	intergenic		
S88	4947024	nc	rec	intergenic		
S88	4947040	nc	rec	intergenic		
S88	4947042	nc	rec	intergenic		
S88	4947047	nc	rec	intergenic		
S88	4947056	nc	rec	intergenic		
S88	4947068	nc	rec	intergenic		
S88	4947085	nc	rec	intergenic		
S88	4947101	nc	rec	intergenic		
S88	4947112	nc	rec	intergenic		
S88	4947113	nc	rec	intergenic		
S88	4947117	nc	rec	intergenic		
S88	4947129	nc	rec	intergenic		
S88	4947135	nc	rec	intergenic		
S88	4947160	nc	rec	intergenic		
S88	4947185	nc	rec	intergenic		
S88	4947356	nc	rec	intergenic		
S88	4947372	nc	rec	intergenic		
S88	4947373	nc	rec	intergenic		
S88	4947375	nc	rec	intergenic		
S88	4947391	nc	rec	intergenic		

lineage[a]	site[b]	mutation type[c]	recomb	gene	Gene name	Type
S88	4947596	s	rec	UTI89_C5071	yjjQ	CDS
S88	4947623	s	rec	UTI89_C5071	yjjQ	CDS
S88	4947632	s	rec	UTI89_C5071	yjjQ	CDS
S88	4947641	s	rec	UTI89_C5071	yjjQ	CDS
S88	4947717	s	rec	UTI89_C5071	yjjQ	CDS
S88	4947804	ns	rec	UTI89_C5071	yjjQ	CDS
S88	4947833	s	rec	UTI89_C5071	yjjQ	CDS
S88	4947974	s	rec	UTI89_C5071	yjjQ	CDS
S88	4948163	ns	rec	UTI89_C5072	bglJ	CDS
S88	4948198	s	rec	UTI89_C5072	bglJ	CDS
S88	4948273	s	rec	UTI89_C5072	bglJ	CDS
S88	4948308	ns	rec	UTI89_C5072	bglJ	CDS
S88	4948327	s	rec	UTI89_C5072	bglJ	CDS
S88	4948519	s	rec	UTI89_C5072	bglJ	CDS
S88	4948645	s	rec	UTI89_C5072	bglJ	CDS
S88	4948702	s	rec	UTI89_C5072	bglJ	CDS
S88	4948774	s	rec	UTI89_C5072	bglJ	CDS
S88	4948905	s	rec	UTI89_C5073	fhuF	CDS
S88	4948908	s	rec	UTI89_C5073	fhuF	CDS
S88	4948942	ns	rec	UTI89_C5073	fhuF	CDS
S88	4948944	s	rec	UTI89_C5073	fhuF	CDS
S88	4948947	s	rec	UTI89_C5073	fhuF	CDS
S88	4948973	ns	rec	UTI89_C5073	fhuF	CDS
S88	4948989	s	rec	UTI89_C5073	fhuF	CDS
S88	4948992	s	rec	UTI89_C5073	fhuF	CDS
S88	4949007	s	rec	UTI89_C5073	fhuF	CDS
S88	4949010	s	rec	UTI89_C5073	fhuF	CDS
S88	4949022	s	rec	UTI89_C5073	fhuF	CDS
S88	4949052	s	rec	UTI89_C5073	fhuF	CDS
S88	4949073	s	rec	UTI89_C5073	fhuF	CDS
S88	4949082	s	rec	UTI89_C5073	fhuF	CDS
S88	4949085	s	rec	UTI89_C5073	fhuF	CDS
S88	4949097	s	rec	UTI89_C5073	fhuF	CDS
S88	4949102	ns	rec	UTI89_C5073	fhuF	CDS
S88	4949142	ns	rec	UTI89_C5073	fhuF	CDS
S88	4949158	ns	rec	UTI89_C5073	fhuF	CDS
S88	4949203	ns	rec	UTI89_C5073	fhuF	CDS
S88	4949227	ns	rec	UTI89_C5073	fhuF	CDS
S88	4949229	s	rec	UTI89_C5073	fhuF	CDS
S88	4949234	ns	rec	UTI89_C5073	fhuF	CDS
S88	4949259	s	rec	UTI89_C5073	fhuF	CDS
S88	4949268	s	rec	UTI89_C5073	fhuF	CDS
S88	4949274	s	rec	UTI89_C5073	fhuF	CDS
S88	4949295	s	rec	UTI89_C5073	fhuF	CDS
S88	4949319	s	rec	UTI89_C5073	fhuF	CDS

lineage[a]	site[b]	mutation type[c]	recomb	gene	Gene name	Type
S88	4949325	s	rec	UTI89_C5073	fhuF	CDS
S88	4949334	s	rec	UTI89_C5073	fhuF	CDS
S88	4949346	s	rec	UTI89_C5073	fhuF	CDS
S88	4949360	ns	rec	UTI89_C5073	fhuF	CDS
S88	4949361	s	rec	UTI89_C5073	fhuF	CDS
S88	4949373	s	rec	UTI89_C5073	fhuF	CDS
S88	4949397	s	rec	UTI89_C5073	fhuF	CDS
S88	4949400	s	rec	UTI89_C5073	fhuF	CDS
S88	4949413	ns	rec	UTI89_C5073	fhuF	CDS
S88	4949460	s	rec	UTI89_C5073	fhuF	CDS
S88	4949504	ns	rec	UTI89_C5073	fhuF	CDS
S88	4949505	s	rec	UTI89_C5073	fhuF	CDS
S88	4949514	s	rec	UTI89_C5073	fhuF	CDS
S88	4949529	s	rec	UTI89_C5073	fhuF	CDS
S88	4949536	ns	rec	UTI89_C5073	fhuF	CDS
S88	4949537	ns	rec	UTI89_C5073	fhuF	CDS
S88	4949538	ns	rec	UTI89_C5073	fhuF	CDS
S88	4949540	ns	rec	UTI89_C5073	fhuF	CDS
S88	4949571	s	rec	UTI89_C5073	fhuF	CDS
S88	4949612	nc	rec	intergenic		
S88	4949634	nc	rec	intergenic		
S88	4949648	ns	rec	UTI89_C5074	-	CDS
S88	4949673	ns	rec	UTI89_C5074	-	CDS
S88	4949678	ns	rec	UTI89_C5074	-	CDS
AS	4983543	s		UTI89_C5074	-	CDS
S88	4949745	ns	rec	UTI89_C5074	-	CDS
S88	4949831	ns	rec	UTI89_C5074	-	CDS
S88	4949833	ns	rec	UTI89_C5074	-	CDS
S88	4949839	s	rec	UTI89_C5074	-	CDS
S88	4949866	s	rec	UTI89_C5074	-	CDS
S88	4949879	ns	rec	UTI89_C5074	-	CDS
S88	4949902	s	rec	UTI89_C5074	-	CDS
S88	4949912	ns	rec	UTI89_C5074	-	CDS
S88	4949935	s	rec	UTI89_C5074	-	CDS
S88	4949980	s	rec	UTI89_C5074	-	CDS
S88	4949984	nc	rec	intergenic		
S88	4949988	nc	rec	intergenic		
S88	4949991	nc	rec	intergenic		
S88	4949992	nc	rec	intergenic		
S88	4949995	nc	rec	intergenic		
S88	4949998	nc	rec	intergenic		
S88	4950007	nc	rec	intergenic		
APEC	4983865	nc		intergenic		
S88	4950452	ns	rec	UTI89_C5078	-	CDS
S88	4950561	ns	rec	UTI89_C5078	-	CDS

lineage[a]	site[b]	mutation type[c]	recomb	gene	Gene name	Type
S88	4950566	ns	rec	UTI89_C5078	-	CDS
S88	4950577	ns	rec	UTI89_C5078	-	CDS
S88	4950578	ns	rec	UTI89_C5078	-	CDS
S88	4950600	ns	rec	UTI89_C5078	-	CDS
S88	4950611	nc	rec	intergenic		
S88	4950613	s	rec	UTI89_C5079	yjjT	CDS
S88	4950666	s	rec	UTI89_C5079	yjjT	CDS
S88	4950684	s	rec	UTI89_C5079	yjjT	CDS
S88	4950717	s	rec	UTI89_C5079	yjjT	CDS
S88	4950749	s	rec	UTI89_C5079	yjjT	CDS
S88	4950762	s	rec	UTI89_C5079	yjjT	CDS
S88	4950765	s	rec	UTI89_C5079	yjjT	CDS
S88	4950780	s	rec	UTI89_C5079	yjjT	CDS
S88	4950801	s	rec	UTI89_C5079	yjjT	CDS
S88	4950849	s	rec	UTI89_C5079	yjjT	CDS
S88	4950861	s	rec	UTI89_C5079	yjjT	CDS
S88	4950920	ns	rec	UTI89_C5079	yjjT	CDS
S88	4950951	s	rec	UTI89_C5079	yjjT	CDS
S88	4950954	s	rec	UTI89_C5079	yjjT	CDS
S88	4950960	s	rec	UTI89_C5079	yjjT	CDS
S88	4950966	s	rec	UTI89_C5079	yjjT	CDS
S88	4950971	s	rec	UTI89_C5079	yjjT	CDS
S88	4950972	s	rec	UTI89_C5079	yjjT	CDS
S88	4950978	s	rec	UTI89_C5079	yjjT	CDS
S88	4950981	s	rec	UTI89_C5079	yjjT	CDS
S88	4950984	s	rec	UTI89_C5079	yjjT	CDS
S88	4950990	s	rec	UTI89_C5079	yjjT	CDS
S88	4951005	s	rec	UTI89_C5079	yjjT	CDS
S88	4951011	s	rec	UTI89_C5079	yjjT	CDS
S88	4951020	s	rec	UTI89_C5079	yjjT	CDS
S88	4951029	s	rec	UTI89_C5079	yjjT	CDS
S88	4951062	s	rec	UTI89_C5079	yjjT	CDS
S88	4951070	s	rec	UTI89_C5079	yjjT	CDS
S88	4951089	s	rec	UTI89_C5079	yjjT	CDS
S88	4951092	s	rec	UTI89_C5079	yjjT	CDS
S88	4951125	s	rec	UTI89_C5079	yjjT	CDS
S88	4951131	s	rec	UTI89_C5079	yjjT	CDS
S88	4951145	s	rec	UTI89_C5079	yjjT	CDS
S88	4951176	ns	rec	UTI89_C5079	yjjT	CDS
S88	4951178	ns	rec	UTI89_C5079	yjjT	CDS
S88	4951179	s	rec	UTI89_C5079	yjjT	CDS
S88	4951197	s	rec	UTI89_C5079	yjjT	CDS
S88	4951224	s	rec	UTI89_C5079	yjjT	CDS
S88	4951233	s	rec	UTI89_C5079	yjjT	CDS
S88	4951242	s	rec	UTI89_C5079	yjjT	CDS

lineage[a]	site[b]	mutation type[c]	recomb	gene	Gene name	Type
S88	4951245	s	rec	UTI89_C5079	yjjT	CDS
S88	4951266	s	rec	UTI89_C5079	yjjT	CDS
S88	4951285	ns	rec	UTI89_C5079	yjjT	CDS
S88	4951326	s	rec	UTI89_C5079	yjjT	CDS
S88	4951329	s	rec	UTI89_C5079	yjjT	CDS
S88	4951335	s	rec	UTI89_C5079	yjjT	CDS
S88	4951338	s	rec	UTI89_C5079	yjjT	CDS
S88	4951401	s	rec	UTI89_C5079	yjjT	CDS
S88	4951425	s	rec	UTI89_C5079	yjjT	CDS
S88	4951430	ns	rec	UTI89_C5079	yjjT	CDS
S88	4951446	s	rec	UTI89_C5079	yjjT	CDS
S88	4951449	s	rec	UTI89_C5079	yjjT	CDS
S88	4951482	s	rec	UTI89_C5079	yjjT	CDS
S88	4951848	ns	rec	UTI89_C5081	holD	CDS
S88	4951895	ns	rec	UTI89_C5081	holD	CDS
S88	4951902	ns	rec	UTI89_C5081	holD	CDS
S88	4951904	ns	rec	UTI89_C5081	holD	CDS
S88	4951922	ns	rec	UTI89_C5081	holD	CDS
S88	4951925	ns	rec	UTI89_C5081	holD	CDS
S88	4951931	ns	rec	UTI89_C5081	holD	CDS
S88	4951943	ns	rec	UTI89_C5081	holD	CDS
S88	4951946	ns	rec	UTI89_C5081	holD	CDS
S88	4951994	ns	rec	UTI89_C5081	holD	CDS
S88	4951996	ns	rec	UTI89_C5081	holD	CDS
S88	4952006	s	rec	UTI89_C5081	holD	CDS
S88	4952075	ns	rec	UTI89_C5081	holD	CDS
S88	4952166	s	rec	UTI89_C5082	rimI	CDS
S88	4952220	s	rec	UTI89_C5082	rimI	CDS
UTI89	4986069	s		UTI89_C5082	rimI	CDS
S88	4952424	s	rec	UTI89_C5082	rimI	CDS
S88	4952445	s	rec	UTI89_C5082	rimI	CDS
S88	4952451	s	rec	UTI89_C5082	rimI	CDS
S88	4952559	s	rec	UTI89_C5082	rimI	CDS
S88	4952575	nc	rec	intergenic		
S88	4952822	s	rec	UTI89_C5083	yjjG	CDS
S88	4952825	s	rec	UTI89_C5083	yjjG	CDS
S88	4952840	s	rec	UTI89_C5083	yjjG	CDS
S88	4952870	s	rec	UTI89_C5083	yjjG	CDS
S88	4952877	s	rec	UTI89_C5083	yjjG	CDS
S88	4952885	s	rec	UTI89_C5083	yjjG	CDS
S88	4952894	s	rec	UTI89_C5083	yjjG	CDS
S88	4952951	s	rec	UTI89_C5083	yjjG	CDS
S88	4952966	s	rec	UTI89_C5083	yjjG	CDS
S88	4953074	s	rec	UTI89_C5083	yjjG	CDS
S88	4953113	s	rec	UTI89_C5083	yjjG	CDS

lineage[a]	site[b]	mutation type[c]	recomb	gene	Gene name	Type
S88	4953179	s	rec	UTI89_C5083	yjjG	CDS
S88	4953183	ns	rec	UTI89_C5083	yjjG	CDS
UTI89/AS	4988688	nc		intergenic		
UTI89/AS	4988713	nc		intergenic		
APEC	4989741	ns		UTI89_C5086	-	CDS
UTI89/AS	4989979	s		UTI89_C5086	-	CDS
AS	4990632	ns		UTI89_C5087	-	CDS
UTI89/AS	4991962	s	rec	UTI89_C5089	-	CDS
UTI89/AS	4991968	s	rec	UTI89_C5089	-	CDS
UTI89/AS	4991977	s	rec	UTI89_C5089	-	CDS
UTI89/AS	4992003	ns	rec	UTI89_C5089	-	CDS
UTI89/AS	4992012	s	rec	UTI89_C5089	-	CDS
UTI89/AS	4992013	s	rec	UTI89_C5089	-	CDS
UTI89/AS	4992018	ns	rec	UTI89_C5089	-	CDS
UTI89/AS	4992025	s	rec	UTI89_C5089	-	CDS
UTI89/AS	4992034	s	rec	UTI89_C5089	-	CDS
UTI89	4992238	ns		UTI89_C5090	-	CDS
AS	4996702	s		UTI89_C5098	-	CDS
UTI89/AS	4997992	ns	rec	UTI89_C5099	-	CDS
UTI89/AS	4998218	ns	rec	UTI89_C5100	-	CDS
UTI89/AS	4998384	ns	rec	UTI89_C5100	-	CDS
UTI89/AS	4998539	s	rec	UTI89_C5100	-	CDS
UTI89/AS	4998542	s	rec	UTI89_C5100	-	CDS
UTI89/AS	4998617	ns	rec	UTI89_C5100	-	CDS
UTI89/AS	4998758	s	rec	UTI89_C5100	-	CDS
UTI89/AS	4998847	s	rec	UTI89_C5101	yfdN2	CDS
UTI89/AS	4998850	s	rec	UTI89_C5101	yfdN2	CDS
UTI89/AS	4998907	s	rec	UTI89_C5101	yfdN2	CDS
UTI89/AS	4998919	s	rec	UTI89_C5101	yfdN2	CDS
UTI89/AS	4998931	s	rec	UTI89_C5101	yfdN2	CDS
UTI89/AS	4999067	ns	rec	UTI89_C5101	yfdN2	CDS
UTI89/AS	4999069	ns	rec	UTI89_C5101	yfdN2	CDS
UTI89/AS	4999117	s	rec	UTI89_C5101	yfdN2	CDS
UTI89/AS	4999126	s	rec	UTI89_C5101	yfdN2	CDS
UTI89/AS	4999146	ns	rec	UTI89_C5101	yfdN2	CDS
UTI89/AS	4999176	ns	rec	UTI89_C5101	yfdN2	CDS
UTI89/AS	4999183	s	rec	UTI89_C5101	yfdN2	CDS
UTI89/AS	4999192	s	rec	UTI89_C5101	yfdN2	CDS
UTI89/AS	4999203	ns	rec	UTI89_C5101	yfdN2	CDS
UTI89/AS	4999229	ns	rec	UTI89_C5101	yfdN2	CDS
UTI89/AS	4999254	ns	rec	UTI89_C5101	yfdN2	CDS
UTI89/AS	4999255	ns	rec	UTI89_C5101	yfdN2	CDS
UTI89/AS	4999293	ns	rec	UTI89_C5101	yfdN2	CDS
UTI89/AS	4999303	ns	rec	UTI89_C5101	yfdN2	CDS
UTI89/AS	4999331	ns	rec	UTI89_C5101	yfdN2	CDS

lineage[a]	site[b]	mutation type[c]	recomb	gene	Gene name	Type
UTI89/AS	4999997	s	rec	UTI89_C5103	-	CDS
UTI89/AS	5000042	s	rec	UTI89_C5103	-	CDS
UTI89/AS	5000057	s	rec	UTI89_C5103	-	CDS
UTI89/AS	5000085	ns	rec	UTI89_C5103	-	CDS
UTI89/AS	5000105	s	rec	UTI89_C5103	-	CDS
UTI89/AS	5000183	s	rec	UTI89_C5103	-	CDS
UTI89/AS	5000186	s	rec	UTI89_C5103	-	CDS
UTI89/AS	5000199	ns	rec	UTI89_C5103	-	CDS
UTI89/AS	5000201	ns	rec	UTI89_C5103	-	CDS
UTI89/AS	5000225	s	rec	UTI89_C5103	-	CDS
UTI89/AS	5000227	ns	rec	UTI89_C5103	-	CDS
UTI89/AS	5000240	s	rec	UTI89_C5103	-	CDS
UTI89/AS	5000241	ns	rec	UTI89_C5103	-	CDS
UTI89/AS	5000267	s	rec	UTI89_C5103	-	CDS
UTI89/AS	5000270	s	rec	UTI89_C5103	-	CDS
UTI89/AS	5000280	ns	rec	UTI89_C5103	-	CDS
UTI89/AS	5000300	s	rec	UTI89_C5103	-	CDS
UTI89/AS	5000332	s	rec	UTI89_C5104	-	CDS
UTI89/AS	5000383	s	rec	UTI89_C5104	-	CDS
UTI89/AS	5000413	s	rec	UTI89_C5104	-	CDS
UTI89/AS	5000444	ns	rec	UTI89_C5104	-	CDS
UTI89/AS	5000590	s	rec	UTI89_C5104	-	CDS
UTI89/AS	5000620	s	rec	UTI89_C5104	-	CDS
UTI89/AS	5000642	ns	rec	UTI89_C5104	-	CDS
UTI89/AS	5000674	s	rec	UTI89_C5104	-	CDS
UTI89/AS	5000681	ns	rec	UTI89_C5104	-	CDS
UTI89/AS	5000738	s	rec	UTI89_C5105	-	CDS
UTI89/AS	5000774	s	rec	UTI89_C5105	-	CDS
UTI89/AS	5000795	s	rec	UTI89_C5105	-	CDS
UTI89/AS	5000807	s	rec	UTI89_C5105	-	CDS
UTI89/AS	5000831	s	rec	UTI89_C5105	-	CDS
UTI89/AS	5000861	s	rec	UTI89_C5105	-	CDS
UTI89/AS	5000863	ns	rec	UTI89_C5105	-	CDS
UTI89/AS	5000987	s	rec	UTI89_C5105	-	CDS
UTI89/AS	5001005	s	rec	UTI89_C5105	-	CDS
UTI89/AS	5001026	s	rec	UTI89_C5105	-	CDS
UTI89/AS	5001040	ns	rec	UTI89_C5105	-	CDS
UTI89/AS	5001059	s	rec	UTI89_C5105	-	CDS
UTI89/AS	5001122	s	rec	UTI89_C5105	-	CDS
UTI89/AS	5001152	s	rec	UTI89_C5105	-	CDS
UTI89/AS	5001221	s	rec	UTI89_C5105	-	CDS
UTI89/AS	5001224	s	rec	UTI89_C5105	-	CDS
UTI89/AS	5001226	ns	rec	UTI89_C5105	-	CDS
UTI89/AS	5001227	ns	rec	UTI89_C5105	-	CDS
UTI89/AS	5001239	s	rec	UTI89_C5105	-	CDS

lineage[a]	site[b]	mutation type[c]	recomb	gene	Gene name	Type
UTI89/AS	5001254	s	rec	UTI89_C5105	-	CDS
UTI89/AS	5001271	ns	rec	UTI89_C5105	-	CDS
S88	4965987	s		UTI89_C5105	-	CDS
UTI89/AS	5001293	s	rec	UTI89_C5105	-	CDS
UTI89/AS	5001319	ns	rec	UTI89_C5105	-	CDS
UTI89/AS	5001344	s	rec	UTI89_C5105	-	CDS
UTI89/AS	5001364	ns	rec	UTI89_C5105	-	CDS
UTI89/AS	5001371	s	rec	UTI89_C5105	-	CDS
UTI89/AS	5001437	s	rec	UTI89_C5105	-	CDS
UTI89/AS	5001461	s	rec	UTI89_C5105	-	CDS
UTI89/AS	5001464	s	rec	UTI89_C5105	-	CDS
UTI89/AS	5001570	s	rec	intergenic		
UTI89/AS	5001582	s	rec	intergenic		
UTI89/AS	5001585	s	rec	intergenic		
UTI89/AS	5001741	s	rec	intergenic		
UTI89/AS	5001744	s	rec	intergenic		
UTI89/AS	5001753	s	rec	intergenic		
UTI89/AS	5001849	s	rec	intergenic		
UTI89/AS	5001861	s	rec	intergenic		
UTI89/AS	5001867	s	rec	intergenic		
UTI89/AS	5001900	s	rec	intergenic		
UTI89/AS	5001903	s	rec	intergenic		
UTI89/AS	5001966	s	rec	intergenic		
UTI89/AS	5002033	ns	rec	intergenic		
UTI89/AS	5002044	s	rec	intergenic		
UTI89/AS	5002045	s	rec	intergenic		
UTI89/AS	5002095	s	rec	intergenic		
UTI89/AS	5002110	s	rec	intergenic		
UTI89/AS	5002223	ns	rec	intergenic		
UTI89/AS	5002236	s	rec	intergenic		
UTI89/AS	5002284	s	rec	intergenic		
UTI89/AS	5002293	s	rec	intergenic		
UTI89/AS	5002311	s	rec	intergenic		
UTI89/AS	5002338	s	rec	intergenic		
UTI89/AS	5002359	s	rec	intergenic		
UTI89/AS	5002374	s	rec	intergenic		
UTI89/AS	5002377	s	rec	intergenic		
UTI89/AS	5002432	ns	rec	intergenic		
UTI89/AS	5002458	s	rec	intergenic		
UTI89/AS	5002506	s	rec	intergenic		
UTI89/AS	5006849	s	rec	UTI89_C5115	-	CDS
UTI89/AS	5006854	ns	rec	UTI89_C5115	-	CDS
UTI89/AS	5006855	ns	rec	UTI89_C5115	-	CDS
UTI89/AS	5006859	ns	rec	UTI89_C5115	-	CDS
UTI89/AS	5006860	ns	rec	UTI89_C5115	-	CDS

lineage[a]	site[b]	mutation type[c]	recomb	gene	Gene name	Type
UTI89/AS	5006869	ns	rec	UTI89_C5115	-	CDS
UTI89/AS	5006871	ns	rec	UTI89_C5115	-	CDS
UTI89/AS	5006874	ns	rec	UTI89_C5115	-	CDS
UTI89/AS	5006875	ns	rec	UTI89_C5115	-	CDS
UTI89/AS	5006879	s	rec	UTI89_C5115	-	CDS
UTI89/AS	5006881	ns	rec	UTI89_C5115	-	CDS
UTI89/AS	5006887	ns	rec	UTI89_C5115	-	CDS
UTI89/AS	5006896	ns	rec	UTI89_C5115	-	CDS
UTI89/AS	5006909	s	rec	UTI89_C5115	-	CDS
UTI89/AS	5006912	ns	rec	UTI89_C5115	-	CDS
UTI89/AS	5006915	s	rec	UTI89_C5115	-	CDS
UTI89/AS	5006916	s	rec	UTI89_C5115	-	CDS
UTI89/AS	5006920	ns	rec	UTI89_C5115	-	CDS
UTI89/AS	5006926	ns	rec	UTI89_C5115	-	CDS
UTI89/AS	5006928	ns	rec	UTI89_C5115	-	CDS
UTI89/AS	5006935	ns	rec	UTI89_C5115	-	CDS
UTI89/AS	5006936	ns	rec	UTI89_C5115	-	CDS
UTI89/AS	5006938	ns	rec	UTI89_C5115	-	CDS
UTI89/AS	5006949	ns	rec	UTI89_C5115	-	CDS
UTI89/AS	5006961	ns	rec	UTI89_C5115	-	CDS
UTI89/AS	5006968	ns	rec	UTI89_C5115	-	CDS
UTI89/AS	5006969	ns	rec	UTI89_C5115	-	CDS
UTI89/AS	5006970	ns	rec	UTI89_C5115	-	CDS
UTI89/AS	5007084	ns	rec	UTI89_C5115	-	CDS
UTI89/AS	5007186	ns	rec	UTI89_C5116	-	CDS
UTI89/AS	5007534	s	rec	UTI89_C5116	-	CDS
UTI89/AS	5007537	s	rec	UTI89_C5116	-	CDS
UTI89/AS	5007776	s	rec	UTI89_C5117	-	CDS
UTI89/AS	5007887	s	rec	UTI89_C5117	-	CDS
UTI89/AS	5007947	s	rec	UTI89_C5117	-	CDS
UTI89/AS	5008007	s	rec	UTI89_C5117	-	CDS
UTI89/AS	5008544	s	rec	UTI89_C5117	-	CDS
UTI89/AS	5008704	ns	rec	UTI89_C5117	-	CDS
UTI89/AS	5008883	s	rec	UTI89_C5117	-	CDS
UTI89/AS	5008895	s	rec	UTI89_C5117	-	CDS
UTI89/AS	5009168	s	rec	UTI89_C5117	-	CDS
UTI89/AS	5009306	s	rec	UTI89_C5117	-	CDS
UTI89/AS	5009336	s	rec	UTI89_C5117	-	CDS
UTI89/AS	5009402	s	rec	UTI89_C5117	-	CDS
UTI89/AS	5009480	s	rec	UTI89_C5117	-	CDS
UTI89/AS	5009602	ns	rec	UTI89_C5117	-	CDS
APEC	5009646	ns		UTI89_C5117	-	CDS
UTI89/AS	5009661	ns	rec	UTI89_C5117	-	CDS
UTI89/AS	5009956	ns	rec	UTI89_C5118	-	CDS
UTI89/AS	5010183	s	rec	UTI89_C5119	-	CDS

lineage[a]	site[b]	mutation type[c]	recomb	gene	Gene name	Type
UTI89/AS	5010291	s	rec	UTI89_C5119	-	CDS
UTI89/AS	5010376	s	rec	UTI89_C5119	-	CDS
UTI89/AS	5010600	s	rec	UTI89_C5119	-	CDS
UTI89/AS	5010837	ns	rec	UTI89_C5119	-	CDS
UTI89/AS	5010840	ns	rec	UTI89_C5119	-	CDS
UTI89/AS	5010924	s	rec	UTI89_C5119	-	CDS
UTI89/AS	5010936	s	rec	UTI89_C5119	-	CDS
UTI89/AS	5011005	s	rec	UTI89_C5119	-	CDS
UTI89/AS	5011008	s	rec	UTI89_C5119	-	CDS
UTI89/AS	5011179	s	rec	UTI89_C5119	-	CDS
UTI89/AS	5011275	s	rec	UTI89_C5119	-	CDS
UTI89/AS	5011323	s	rec	UTI89_C5119	-	CDS
UTI89/AS	5011350	ns	rec	UTI89_C5120	-	CDS
UTI89/AS	5011498	s	rec	UTI89_C5120	-	CDS
UTI89/AS	5011558	s	rec	UTI89_C5120	-	CDS
UTI89/AS	5011621	s	rec	UTI89_C5120	-	CDS
UTI89/AS	5011669	s	rec	UTI89_C5120	-	CDS
UTI89/AS	5011775	ns	rec	UTI89_C5120	-	CDS
UTI89/AS	5011858	s	rec	UTI89_C5120	-	CDS
UTI89/AS	5011972	ns	rec	UTI89_C5120	-	CDS
UTI89/AS	5011993	s	rec	UTI89_C5120	-	CDS
UTI89/AS	5012071	s	rec	UTI89_C5120	-	CDS
UTI89/AS	5012118	ns	rec	UTI89_C5120	-	CDS
UTI89/AS	5012176	s	rec	UTI89_C5120	-	CDS
UTI89/AS	5012191	s	rec	UTI89_C5120	-	CDS
UTI89/AS	5012261	ns	rec	UTI89_C5120	-	CDS
UTI89/AS	5012264	s	rec	UTI89_C5120	-	CDS
UTI89/AS	5012266	s	rec	UTI89_C5120	-	CDS
UTI89/AS	5012437	s	rec	UTI89_C5120	-	CDS
UTI89/AS	5012443	s	rec	UTI89_C5120	-	CDS
UTI89/AS	5012455	s	rec	UTI89_C5120	-	CDS
UTI89/AS	5012521	s	rec	UTI89_C5120	-	CDS
UTI89/AS	5012530	s	rec	UTI89_C5120	-	CDS
UTI89/AS	5012650	s	rec	UTI89_C5120	-	CDS
UTI89/AS	5012761	s	rec	UTI89_C5120	-	CDS
UTI89/AS	5012893	s	rec	UTI89_C5120	-	CDS
UTI89/AS	5013022	s	rec	UTI89_C5120	-	CDS
UTI89/AS	5013082	s	rec	UTI89_C5120	-	CDS
UTI89/AS	5013286	s	rec	UTI89_C5120	-	CDS
UTI89/AS	5013301	s	rec	UTI89_C5120	-	CDS
UTI89/AS	5013400	s	rec	UTI89_C5120	-	CDS
UTI89/AS	5013406	s	rec	UTI89_C5120	-	CDS
UTI89/AS	5013427	s	rec	UTI89_C5120	-	CDS
UTI89/AS	5013621	s	rec	UTI89_C5121	-	CDS
UTI89/AS	5013660	s	rec	UTI89_C5121	-	CDS

lineage[a]	site[b]	mutation type[c]	recomb	gene	Gene name	Type
UTI89/AS	5013678	ns	rec	UTI89_C5121	-	CDS
UTI89/AS	5013753	s	rec	UTI89_C5121	-	CDS
UTI89/AS	5013786	ns	rec	UTI89_C5121	-	CDS
UTI89/AS	5014093	s	rec	UTI89_C5122	-	CDS
UTI89/AS	5014370	ns	rec	intergenic		
UTI89/AS	5014652	s	rec	UTI89_C5123	-	CDS
UTI89/AS	5014695	ns	rec	UTI89_C5123	-	CDS
UTI89/AS	5014722	ns	rec	UTI89_C5123	-	CDS
UTI89/AS	5015211	s	rec	UTI89_C5125	-	CDS
UTI89/AS	5015214	s	rec	UTI89_C5125	-	CDS
UTI89/AS	5015307	s	rec	UTI89_C5125	-	CDS
UTI89/AS	5015481	s	rec	UTI89_C5125	-	CDS
UTI89/AS	5015511	s	rec	UTI89_C5125	-	CDS
UTI89/AS	5015609	ns	rec	UTI89_C5125	-	CDS
UTI89/AS	5015610	ns	rec	UTI89_C5125	-	CDS
UTI89/AS	5015631	s	rec	UTI89_C5125	-	CDS
UTI89/AS	5015634	s	rec	UTI89_C5125	-	CDS
UTI89/AS	5015635	ns	rec	UTI89_C5125	-	CDS
UTI89/AS	5015643	s	rec	UTI89_C5125	-	CDS
UTI89/AS	5015757	s	rec	UTI89_C5125	-	CDS
UTI89/AS	5015769	s	rec	UTI89_C5125	-	CDS
UTI89/AS	5015775	s	rec	UTI89_C5125	-	CDS
UTI89/AS	5015784	s	rec	UTI89_C5125	-	CDS
UTI89/AS	5015851	ns	rec	UTI89_C5125	-	CDS
UTI89/AS	5015852	ns	rec	UTI89_C5125	-	CDS
UTI89/AS	5015871	s	rec	UTI89_C5125	-	CDS
UTI89/AS	5015912	nc	rec	intergenic		
UTI89/AS	5015957	ns	rec	UTI89_C5126	-	CDS
UTI89/AS	5016028	ns	rec	UTI89_C5126	-	CDS
UTI89/AS	5016066	s	rec	UTI89_C5126	-	CDS
UTI89/AS	5016287	ns	rec	UTI89_C5126	-	CDS
UTI89/AS	5016431	s	rec	UTI89_C5127	-	CDS
UTI89/AS	5016702	s	rec	intergenic		
UTI89/AS	5016816	s	rec	intergenic		
UTI89/AS	5016819	s	rec	intergenic		
UTI89/AS	5016930	s	rec	intergenic		
UTI89/AS	5017047	s	rec	intergenic		
UTI89/AS	5017146	s	rec	intergenic		
UTI89/AS	5017238	ns	rec	intergenic		
UTI89/AS	5017275	s	rec	intergenic		
UTI89/AS	5017371	s	rec	intergenic		
UTI89/AS	5017506	s	rec	intergenic		
UTI89/AS	5017533	s	rec	intergenic		
UTI89/AS	5017536	s	rec	intergenic		
UTI89/AS	5017600	s	rec	intergenic		

lineage[a]	site[b]	mutation type[c]	recomb	gene	Gene name	Type
UTI89/AS	5017766	ns	rec	intergenic		
UTI89/AS	5017817	ns	rec	intergenic		
UTI89/AS	5017888	ns	rec	intergenic		
UTI89/AS	5017926	s	rec	intergenic		
UTI89/AS	5017947	s	rec	intergenic		
UTI89/AS	5018025	s	rec	intergenic		
UTI89/AS	5018055	s	rec	intergenic		
UTI89/AS	5018119	ns	rec	intergenic		
UTI89/AS	5018187	s	rec	intergenic		
UTI89/AS	5018211	ns	rec	intergenic		
UTI89/AS	5018220	s	rec	intergenic		
UTI89/AS	5018395	ns	rec	intergenic		
UTI89/AS	5018660	ns	rec	intergenic		
UTI89/AS	5018814	s	rec	intergenic		
UTI89/AS	5018826	s	rec	intergenic		
UTI89/AS	5018833	ns	rec	intergenic		
UTI89/AS	5018835	ns	rec	intergenic		
UTI89/AS	5018874	s	rec	intergenic		
UTI89/AS	5018904	s	rec	intergenic		
UTI89/AS	5019058	ns	rec	intergenic		
UTI89/AS	5019062	ns	rec	intergenic		
UTI89/AS	5019063	ns	rec	intergenic		
UTI89/AS	5019357	ns	rec	UTI89_C5128	-	CDS
UTI89/AS	5019372	s	rec	UTI89_C5128	-	CDS
UTI89/AS	5019466	ns	rec	UTI89_C5128	-	CDS
UTI89/AS	5019513	ns	rec	UTI89_C5128	-	CDS
UTI89/AS	5019516	ns	rec	UTI89_C5128	-	CDS
UTI89/AS	5019535	ns	rec	UTI89_C5128	-	CDS
UTI89/AS	5019552	ns	rec	UTI89_C5128	-	CDS
UTI89/AS	5019605	s	rec	UTI89_C5128	-	CDS
UTI89/AS	5019618	ns	rec	UTI89_C5128	-	CDS
UTI89/AS	5019854	ns	rec	UTI89_C5129	-	CDS
UTI89/AS	5019899	s	rec	UTI89_C5129	-	CDS
UTI89/AS	5019938	s	rec	UTI89_C5129	-	CDS
UTI89/AS	5020180	ns	rec	UTI89_C5130	-	CDS
UTI89/AS	5020204	ns	rec	UTI89_C5130	-	CDS
UTI89/AS	5020223	s	rec	UTI89_C5130	-	CDS
UTI89/AS	5020284	ns	rec	UTI89_C5130	-	CDS
UTI89/AS	5020289	s	rec	UTI89_C5130	-	CDS
UTI89/AS	5020298	s	rec	UTI89_C5130	-	CDS
UTI89/AS	5020313	s	rec	UTI89_C5130	-	CDS
UTI89/AS	5020344	s	rec	UTI89_C5130	-	CDS
UTI89/AS	5020400	s	rec	UTI89_C5130	-	CDS
UTI89/AS	5020442	s	rec	UTI89_C5130	-	CDS
UTI89/AS	5020463	s	rec	UTI89_C5130	-	CDS

lineage[a]	site[b]	mutation type[c]	recomb	gene	Gene name	Type
UTI89/AS	5020469	s	rec	UTI89_C5130	-	CDS
UTI89/AS	5020478	s	rec	UTI89_C5130	-	CDS
UTI89/AS	5020484	s	rec	UTI89_C5130	-	CDS
UTI89/AS	5020485	s	rec	UTI89_C5130	-	CDS
UTI89/AS	5020493	s	rec	UTI89_C5130	-	CDS
UTI89/AS	5020508	s	rec	UTI89_C5130	-	CDS
UTI89/AS	5020511	s	rec	UTI89_C5130	-	CDS
UTI89/AS	5020526	s	rec	UTI89_C5130	-	CDS
UTI89/AS	5020577	s	rec	UTI89_C5130	-	CDS
UTI89/AS	5020595	s	rec	UTI89_C5130	-	CDS
UTI89/AS	5020622	s	rec	UTI89_C5130	-	CDS
UTI89/AS	5020623	ns	rec	UTI89_C5130	-	CDS
UTI89/AS	5020664	s	rec	UTI89_C5130	-	CDS
UTI89/AS	5020731	ns	rec	UTI89_C5131	-	CDS
UTI89/AS	5020813	ns	rec	UTI89_C5131	-	CDS
UTI89/AS	5020868	ns	rec	UTI89_C5131	-	CDS
UTI89/AS	5020869	ns	rec	UTI89_C5131	-	CDS
UTI89/AS	5020929	s	rec	UTI89_C5131	-	CDS
UTI89/AS	5020935	s	rec	UTI89_C5131	-	CDS
UTI89/AS	5020941	s	rec	UTI89_C5131	-	CDS
UTI89/AS	5020944	s	rec	UTI89_C5131	-	CDS
UTI89/AS	5020974	s	rec	UTI89_C5131	-	CDS
UTI89/AS	5020984	s	rec	UTI89_C5131	-	CDS
UTI89/AS	5021025	s	rec	UTI89_C5131	-	CDS
UTI89/AS	5021037	s	rec	UTI89_C5131	-	CDS
UTI89/AS	5021115	s	rec	UTI89_C5131	-	CDS
UTI89/AS	5021121	s	rec	UTI89_C5131	-	CDS
UTI89/AS	5021159	ns	rec	UTI89_C5131	-	CDS
UTI89/AS	5021230	ns	rec	UTI89_C5131	-	CDS
UTI89/AS	5021239	ns	rec	UTI89_C5131	-	CDS
UTI89/AS	5021241	ns	rec	UTI89_C5131	-	CDS
UTI89/AS	5021244	s	rec	UTI89_C5131	-	CDS
UTI89/AS	5021247	s	rec	UTI89_C5131	-	CDS
UTI89/AS	5021271	s	rec	UTI89_C5131	-	CDS
UTI89/AS	5021319	s	rec	UTI89_C5131	-	CDS
UTI89/AS	5021326	s	rec	UTI89_C5131	-	CDS
UTI89/AS	5021352	s	rec	UTI89_C5131	-	CDS
UTI89/AS	5021474	ns	rec	UTI89_C5132	-	CDS
UTI89/AS	5021487	ns	rec	UTI89_C5132	-	CDS
UTI89/AS	5021489	ns	rec	UTI89_C5132	-	CDS
UTI89/AS	5021512	ns	rec	UTI89_C5132	-	CDS
UTI89/AS	5021517	ns	rec	UTI89_C5132	-	CDS
UTI89/AS	5021519	ns	rec	UTI89_C5132	-	CDS
UTI89/AS	5021523	ns	rec	UTI89_C5132	-	CDS
UTI89/AS	5021531	s	rec	UTI89_C5132	-	CDS

lineage[a]	site[b]	mutation type[c]	recomb	gene	Gene name	Type
UTI89/AS	5021542	ns	rec	UTI89_C5132	-	CDS
UTI89/AS	5021543	ns	rec	UTI89_C5132	-	CDS
UTI89/AS	5021546	ns	rec	UTI89_C5132	-	CDS
UTI89/AS	5021555	s	rec	UTI89_C5132	-	CDS
UTI89/AS	5021561	s	rec	UTI89_C5132	-	CDS
UTI89/AS	5021585	s	rec	UTI89_C5132	-	CDS
UTI89/AS	5021590	ns	rec	UTI89_C5132	-	CDS
UTI89/AS	5021591	ns	rec	UTI89_C5132	-	CDS
UTI89/AS	5021597	s	rec	UTI89_C5132	-	CDS
UTI89/AS	5021645	s	rec	UTI89_C5132	-	CDS
UTI89/AS	5021681	s	rec	UTI89_C5132	-	CDS
UTI89/AS	5021689	ns	rec	UTI89_C5132	-	CDS
UTI89/AS	5021690	ns	rec	UTI89_C5132	-	CDS
UTI89/AS	5021699	s	rec	UTI89_C5132	-	CDS
UTI89/AS	5021801	s	rec	UTI89_C5132	-	CDS
UTI89/AS	5021807	s	rec	UTI89_C5132	-	CDS
UTI89/AS	5021831	s	rec	UTI89_C5132	-	CDS
UTI89/AS	5021834	s	rec	UTI89_C5132	-	CDS
UTI89/AS	5021838	ns	rec	UTI89_C5132	-	CDS
UTI89/AS	5021839	ns	rec	UTI89_C5132	-	CDS
UTI89/AS	5021840	ns	rec	UTI89_C5132	-	CDS
UTI89/AS	5021841	ns	rec	UTI89_C5132	-	CDS
UTI89/AS	5021844	ns	rec	UTI89_C5132	-	CDS
UTI89/AS	5021845	ns	rec	UTI89_C5132	-	CDS
UTI89/AS	5021846	ns	rec	UTI89_C5132	-	CDS
UTI89/AS	5021848	ns	rec	UTI89_C5132	-	CDS
UTI89/AS	5021849	ns	rec	UTI89_C5132	-	CDS
UTI89/AS	5021852	s	rec	UTI89_C5132	-	CDS
UTI89/AS	5021861	s	rec	UTI89_C5132	-	CDS
UTI89/AS	5021864	s	rec	UTI89_C5132	-	CDS
UTI89/AS	5021916	ns	rec	UTI89_C5132	-	CDS
UTI89/AS	5021921	s	rec	UTI89_C5132	-	CDS
UTI89/AS	5021930	s	rec	UTI89_C5132	-	CDS
UTI89/AS	5021951	s	rec	UTI89_C5132	-	CDS
UTI89/AS	5021954	s	rec	UTI89_C5132	-	CDS
UTI89/AS	5021960	s	rec	UTI89_C5132	-	CDS
UTI89/AS	5021984	s	rec	UTI89_C5132	-	CDS
UTI89/AS	5021993	s	rec	UTI89_C5132	-	CDS
UTI89/AS	5022008	s	rec	UTI89_C5132	-	CDS
UTI89/AS	5022020	s	rec	UTI89_C5132	-	CDS
UTI89/AS	5022026	nc	rec	intergenic		
UTI89/AS	5022031	nc	rec	intergenic		
UTI89/AS	5022051	nc	rec	intergenic		
UTI89/AS	5022077	nc	rec	intergenic		
UTI89/AS	5022079	nc	rec	intergenic		

lineage[a]	site[b]	mutation type[c]	recomb	gene	Gene name	Type
UTI89/AS	5022081	nc	rec	intergenic		
UTI89/AS	5022107	s	rec	intergenic		
UTI89/AS	5022113	s	rec	intergenic		
UTI89/AS	5022125	s	rec	intergenic		
UTI89/AS	5022134	s	rec	intergenic		
UTI89/AS	5022236	s	rec	intergenic		
UTI89/AS	5022247	ns	rec	intergenic		
UTI89/AS	5022272	s	rec	intergenic		
UTI89/AS	5022305	s	rec	intergenic		
UTI89/AS	5022338	s	rec	intergenic		
UTI89/AS	5022371	s	rec	intergenic		
UTI89/AS	5022389	s	rec	intergenic		
UTI89/AS	5022401	s	rec	intergenic		
UTI89/AS	5022471	s	rec	intergenic		
UTI89/AS	5022479	s	rec	intergenic		
UTI89/AS	5022515	s	rec	intergenic		
UTI89/AS	5022632	s	rec	intergenic		
UTI89/AS	5022737	s	rec	intergenic		
UTI89/AS	5022752	s	rec	intergenic		
UTI89/AS	5022782	s	rec	intergenic		
UTI89/AS	5022845	s	rec	intergenic		
UTI89/AS	5022875	ns	rec	intergenic		
UTI89/AS	5022878	s	rec	intergenic		
UTI89/AS	5022881	s	rec	intergenic		
UTI89/AS	5022950	s	rec	intergenic		
UTI89/AS	5022956	s	rec	intergenic		
UTI89/AS	5022974	s	rec	intergenic		
UTI89/AS	5023025	s	rec	intergenic		
UTI89/AS	5023034	s	rec	intergenic		
UTI89/AS	5023061	s	rec	intergenic		
UTI89/AS	5023064	s	rec	intergenic		
UTI89/AS	5023066	ns	rec	intergenic		
UTI89/AS	5023067	ns	rec	intergenic		
UTI89/AS	5023174	ns	rec	intergenic		
UTI89/AS	5023175	ns	rec	intergenic		
UTI89/AS	5023178	s	rec	intergenic		
UTI89/AS	5023370	s	rec	intergenic		
UTI89/AS	5023394	s	rec	intergenic		
UTI89/AS	5023403	s	rec	intergenic		
UTI89/AS	5023415	s	rec	intergenic		
UTI89/AS	5023421	s	rec	intergenic		
UTI89/AS	5023472	s	rec	intergenic		
UTI89/AS	5023484	s	rec	intergenic		
UTI89/AS	5023487	s	rec	intergenic		
UTI89/AS	5023505	s	rec	intergenic		

lineage[a]	site[b]	mutation type[c]	recomb	gene	Gene name	Type
UTI89/AS	5023511	s	rec	intergenic		
UTI89/AS	5023517	s	rec	intergenic		
UTI89/AS	5023529	s	rec	intergenic		
UTI89/AS	5023544	s	rec	intergenic		
UTI89/AS	5023559	s	rec	intergenic		
UTI89/AS	5023562	s	rec	intergenic		
UTI89/AS	5023634	s	rec	intergenic		
UTI89/AS	5023649	s	rec	intergenic		
UTI89/AS	5023661	s	rec	intergenic		
UTI89/AS	5023662	ns	rec	intergenic		
UTI89/AS	5023663	ns	rec	intergenic		
UTI89/AS	5023664	ns	rec	intergenic		
UTI89/AS	5023667	s	rec	intergenic		
UTI89/AS	5023688	s	rec	intergenic		
UTI89/AS	5023715	s	rec	intergenic		
UTI89/AS	5023739	s	rec	intergenic		
UTI89/AS	5023757	s	rec	intergenic		
UTI89/AS	5023770	s	rec	intergenic		
UTI89/AS	5023779	s	rec	intergenic		
UTI89/AS	5023844	s	rec	intergenic		
UTI89/AS	5023859	s	rec	intergenic		
UTI89/AS	5023865	s	rec	intergenic		
UTI89/AS	5023940	s	rec	intergenic		
UTI89/AS	5023971	ns	rec	intergenic		
UTI89/AS	5023985	s	rec	intergenic		
UTI89/AS	5024000	s	rec	intergenic		
UTI89/AS	5024009	s	rec	intergenic		
UTI89/AS	5024018	s	rec	intergenic		
UTI89/AS	5024030	s	rec	intergenic		
UTI89/AS	5024039	s	rec	intergenic		
UTI89/AS	5024040	ns	rec	intergenic		
UTI89/AS	5024097	s	rec	intergenic		
UTI89/AS	5024125	ns	rec	intergenic		
UTI89/AS	5024149	ns	rec	intergenic		
UTI89/AS	5024160	s	rec	intergenic		
UTI89/AS	5024180	s	rec	intergenic		
UTI89/AS	5024182	ns	rec	intergenic		
UTI89/AS	5024237	s	rec	intergenic		
UTI89/AS	5024252	s	rec	intergenic		
UTI89/AS	5024273	s	rec	intergenic		
UTI89/AS	5024282	s	rec	intergenic		
UTI89/AS	5024300	s	rec	intergenic		
UTI89/AS	5024336	s	rec	intergenic		
UTI89/AS	5024343	ns	rec	intergenic		
UTI89/AS	5024345	ns	rec	intergenic		

lineage[a]	site[b]	mutation type[c]	recomb	gene	Gene name	Type
UTI89/AS	5024363	s	rec	intergenic		
UTI89/AS	5024384	s	rec	intergenic		
UTI89/AS	5024422	ns	rec	intergenic		
UTI89/AS	5024423	ns	rec	intergenic		
UTI89/AS	5024441	s	rec	intergenic		
UTI89/AS	5024447	s	rec	intergenic		
UTI89/AS	5024448	ns	rec	intergenic		
UTI89/AS	5024450	ns	rec	intergenic		
UTI89/AS	5024500	ns	rec	intergenic		
UTI89/AS	5024502	ns	rec	intergenic		
UTI89/AS	5024564	s	rec	intergenic		
UTI89/AS	5024650	ns	rec	intergenic		
UTI89/AS	5024651	ns	rec	intergenic		
UTI89/AS	5024657	s	rec	intergenic		
S88	4991992	s		intergenic		
UTI89/AS	5024694	ns	rec	intergenic		
UTI89/AS	5024704	ns	rec	intergenic		
UTI89/AS	5024705	ns	rec	intergenic		
UTI89/AS	5024720	s	rec	intergenic		
UTI89/AS	5024721	ns	rec	intergenic		
UTI89/AS	5024729	s	rec	intergenic		
UTI89/AS	5024732	s	rec	intergenic		
UTI89/AS	5024741	s	rec	intergenic		
UTI89/AS	5024801	s	rec	intergenic		
UTI89/AS	5024805	ns	rec	intergenic		
UTI89/AS	5024807	ns	rec	intergenic		
UTI89/AS	5024810	s	rec	intergenic		
UTI89/AS	5024813	s	rec	intergenic		
UTI89/AS	5024816	s	rec	intergenic		
UTI89/AS	5024817	ns	rec	intergenic		
UTI89/AS	5024818	ns	rec	intergenic		
UTI89/AS	5024821	ns	rec	intergenic		
UTI89/AS	5024822	ns	rec	intergenic		
UTI89/AS	5024825	s	rec	intergenic		
UTI89/AS	5024834	s	rec	intergenic		
UTI89/AS	5024836	ns	rec	intergenic		
UTI89/AS	5024837	ns	rec	intergenic		
UTI89/AS	5024844	ns	rec	intergenic		
UTI89/AS	5024846	ns	rec	intergenic		
UTI89/AS	5024864	ns	rec	intergenic		
UTI89/AS	5024876	s	rec	intergenic		
UTI89/AS	5024877	ns	rec	intergenic		
UTI89/AS	5024878	ns	rec	intergenic		
UTI89/AS	5024879	ns	rec	intergenic		
UTI89/AS	5024891	s	rec	intergenic		

lineage[a]	site[b]	mutation type[c]	recomb	gene	Gene name	Type
UTI89/AS	5024903	s	rec	intergenic		
UTI89/AS	5024909	s	rec	intergenic		
UTI89/AS	5024920	ns	rec	intergenic		
UTI89/AS	5024939	s	rec	intergenic		
UTI89/AS	5025005	s	rec	intergenic		
UTI89/AS	5025008	s	rec	intergenic		
UTI89/AS	5025122	s	rec	intergenic		
UTI89/AS	5025140	s	rec	intergenic		
UTI89/AS	5025168	ns	rec	intergenic		
UTI89/AS	5025182	s	rec	intergenic		
UTI89/AS	5025194	s	rec	intergenic		
UTI89/AS	5025203	s	rec	intergenic		
UTI89/AS	5025207	ns	rec	intergenic		
UTI89/AS	5025212	s	rec	intergenic		
UTI89/AS	5025215	s	rec	intergenic		
UTI89/AS	5025217	ns	rec	intergenic		
UTI89/AS	5025225	s	rec	intergenic		
UTI89/AS	5025227	s	rec	intergenic		
UTI89/AS	5025231	ns	rec	intergenic		
UTI89/AS	5025235	ns	rec	intergenic		
UTI89/AS	5025236	ns	rec	intergenic		
UTI89/AS	5025241	ns	rec	intergenic		
UTI89/AS	5025245	s	rec	intergenic		
UTI89/AS	5025248	s	rec	intergenic		
UTI89/AS	5025257	s	rec	intergenic		
UTI89/AS	5025267	ns	rec	intergenic		
UTI89/AS	5025268	ns	rec	intergenic		
UTI89/AS	5025278	s	rec	intergenic		
UTI89/AS	5025296	s	rec	intergenic		
UTI89/AS	5025299	s	rec	intergenic		
UTI89/AS	5025302	s	rec	intergenic		
UTI89/AS	5025314	s	rec	intergenic		
UTI89/AS	5025325	ns	rec	intergenic		
UTI89/AS	5025326	ns	rec	intergenic		
UTI89/AS	5025329	s	rec	intergenic		
UTI89/AS	5025347	s	rec	intergenic		
UTI89/AS	5025350	s	rec	intergenic		
UTI89/AS	5025351	ns	rec	intergenic		
UTI89/AS	5025352	ns	rec	intergenic		
UTI89/AS	5025353	ns	rec	intergenic		
UTI89/AS	5025357	ns	rec	intergenic		
UTI89/AS	5025358	ns	rec	intergenic		
UTI89/AS	5025359	ns	rec	intergenic		
UTI89/AS	5025365	s	rec	intergenic		
UTI89/AS	5025377	s	rec	intergenic		

lineage[a]	site[b]	mutation type[c]	recomb	gene	Gene name	Type
UTI89/AS	5025380	s	rec	intergenic		
UTI89/AS	5025390	ns	rec	intergenic		
UTI89/AS	5025391	ns	rec	intergenic		
UTI89/AS	5025400	ns	rec	intergenic		
UTI89/AS	5025401	ns	rec	intergenic		
UTI89/AS	5025440	s	rec	intergenic		
UTI89/AS	5025458	s	rec	intergenic		
UTI89/AS	5025492	ns	rec	intergenic		
UTI89/AS	5025493	ns	rec	intergenic		
UTI89/AS	5025503	s	rec	intergenic		
UTI89/AS	5025512	ns	rec	intergenic		
UTI89/AS	5025556	ns	rec	intergenic		
UTI89/AS	5025558	ns	rec	intergenic		
UTI89/AS	5025564	ns	rec	intergenic		
UTI89/AS	5025566	ns	rec	intergenic		
UTI89/AS	5025567	ns	rec	intergenic		
UTI89/AS	5025571	ns	rec	intergenic		
UTI89/AS	5029997	nc	rec	intergenic		
UTI89/AS	5030011	nc	rec	intergenic		
UTI89/AS	5030055	ns	rec	UTI89_C5139	-	CDS
UTI89/AS	5030079	ns	rec	UTI89_C5139	-	CDS
UTI89/AS	5030085	ns	rec	UTI89_C5139	-	CDS
UTI89/AS	5030133	ns	rec	UTI89_C5139	-	CDS
UTI89/AS	5030166	ns	rec	UTI89_C5139	-	CDS
UTI89/AS	5030169	ns	rec	UTI89_C5139	-	CDS
UTI89/AS	5030172	ns	rec	UTI89_C5139	-	CDS
UTI89/AS	5030200	ns	rec	UTI89_C5139	-	CDS
UTI89/AS	5030332	ns	rec	UTI89_C5139	-	CDS
UTI89/AS	5030335	ns	rec	UTI89_C5139	-	CDS
UTI89/AS	5032799	ns	rec	UTI89_C5142	-	CDS
UTI89/AS	5032827	s	rec	UTI89_C5142	-	CDS
UTI89/AS	5032879	nc	rec	intergenic		
UTI89/AS	5032891	nc	rec	intergenic		
UTI89/AS	5032963	ns	rec	UTI89_C5143	-	CDS
UTI89/AS	5032996	ns	rec	UTI89_C5143	-	CDS
UTI89/AS	5032997	s	rec	UTI89_C5143	-	CDS
UTI89/AS	5033003	s	rec	UTI89_C5143	-	CDS
UTI89/AS	5033027	s	rec	UTI89_C5143	-	CDS
UTI89/AS	5033057	s	rec	UTI89_C5143	-	CDS
UTI89/AS	5033105	ns	rec	UTI89_C5143	-	CDS
UTI89/AS	5033106	ns	rec	UTI89_C5143	-	CDS
UTI89/AS	5033148	nc	rec	intergenic		
UTI89/AS	5033157	nc	rec	intergenic		
UTI89/AS	5033176	nc	rec	intergenic		
UTI89/AS	5033191	nc	rec	intergenic		

lineage[a]	site[b]	mutation type[c]	recomb	gene	Gene name	Type
UTI89/AS	5033213	nc	rec	intergenic		
UTI89/AS	5033214	nc	rec	intergenic		
UTI89/AS	5033348	nc	rec	intergenic		
UTI89/AS	5033413	s	rec	UTI89_C5144	prfC	CDS
S88	4999784	s	rec	UTI89_C5144	prfC	CDS
S88	4999787	s	rec	UTI89_C5144	prfC	CDS
S88	4999844	s	rec	UTI89_C5144	prfC	CDS
S88	4999877	s	rec	UTI89_C5144	prfC	CDS
S88	4999890	s	rec	UTI89_C5144	prfC	CDS
S88	4999898	s	rec	UTI89_C5144	prfC	CDS
S88	4999991	s	rec	UTI89_C5144	prfC	CDS
S88	5000036	s	rec	UTI89_C5144	prfC	CDS
S88	5000093	s	rec	UTI89_C5144	prfC	CDS
S88	5000132	s	rec	UTI89_C5144	prfC	CDS
S88	5000135	s	rec	UTI89_C5144	prfC	CDS
S88	5000198	s	rec	UTI89_C5144	prfC	CDS
S88	5000282	s	rec	UTI89_C5144	prfC	CDS
S88	5000330	s	rec	UTI89_C5144	prfC	CDS
S88	5000351	s	rec	UTI89_C5144	prfC	CDS
S88	5000390	s	rec	UTI89_C5144	prfC	CDS
S88	5000402	s	rec	UTI89_C5144	prfC	CDS
S88	5000439	s	rec	UTI89_C5144	prfC	CDS
S88	5000462	s	rec	UTI89_C5144	prfC	CDS
S88	5000486	s	rec	UTI89_C5144	prfC	CDS
S88	5000591	s	rec	UTI89_C5144	prfC	CDS
S88	5000597	s	rec	UTI89_C5144	prfC	CDS
S88	5000624	s	rec	UTI89_C5144	prfC	CDS
S88	5000642	s	rec	UTI89_C5144	prfC	CDS
S88	5000717	s	rec	UTI89_C5144	prfC	CDS
S88	5000729	s	rec	UTI89_C5144	prfC	CDS
S88	5000732	s	rec	UTI89_C5144	prfC	CDS
S88	5000816	s	rec	UTI89_C5144	prfC	CDS
S88	5000924	s	rec	UTI89_C5144	prfC	CDS
S88	5000940	s	rec	UTI89_C5144	prfC	CDS
S88	5000951	s	rec	UTI89_C5144	prfC	CDS
S88	5000954	s	rec	UTI89_C5144	prfC	CDS
S88	5001065	s	rec	UTI89_C5144	prfC	CDS
S88	5001176	s	rec	UTI89_C5144	prfC	CDS
S88	5001327	nc	rec	intergenic		
S88	5001376	nc	rec	intergenic		
S88	5001437	nc	rec	intergenic		
S88	5001499	nc	rec	intergenic		
S88	5001700	s	rec	UTI89_C5145	osmY	CDS
S88	5001856	s	rec	UTI89_C5145	osmY	CDS
S88	5001907	s	rec	UTI89_C5145	osmY	CDS

lineage[a]	site[b]	mutation type[c]	recomb	gene	Gene name	Type
S88	5001922	s	rec	UTI89_C5145	osmY	CDS
S88	5001940	s	rec	UTI89_C5145	osmY	CDS
S88	5001946	s	rec	UTI89_C5145	osmY	CDS
S88	5001949	s	rec	UTI89_C5145	osmY	CDS
S88	5002000	s	rec	UTI89_C5145	osmY	CDS
S88	5002006	s	rec	UTI89_C5145	osmY	CDS
S88	5002030	s	rec	UTI89_C5145	osmY	CDS
S88	5002060	s	rec	UTI89_C5145	osmY	CDS
S88	5002063	s	rec	UTI89_C5145	osmY	CDS
S88	5002066	s	rec	UTI89_C5145	osmY	CDS
S88	5002069	s	rec	UTI89_C5145	osmY	CDS
S88	5002105	s	rec	UTI89_C5145	osmY	CDS
S88	5002114	s	rec	UTI89_C5145	osmY	CDS
S88	5002132	s	rec	UTI89_C5145	osmY	CDS
S88	5002147	s	rec	UTI89_C5145	osmY	CDS
S88	5002150	s	rec	UTI89_C5145	osmY	CDS
S88	5002237	nc	rec	intergenic		
S88	5002241	nc	rec	intergenic		
S88	5002426	s	rec	UTI89_C5146	-	CDS
S88	5002703	s	rec	UTI89_C5147	yjjU	CDS
S88	5002706	s	rec	UTI89_C5147	yjjU	CDS
S88	5002733	s	rec	UTI89_C5147	yjjU	CDS
S88	5002840	ns	rec	UTI89_C5147	yjjU	CDS
S88	5002883	s	rec	UTI89_C5147	yjjU	CDS
S88	5002886	s	rec	UTI89_C5147	yjjU	CDS
S88	5002970	s	rec	UTI89_C5147	yjjU	CDS
S88	5002997	s	rec	UTI89_C5147	yjjU	CDS
S88	5003003	s	rec	UTI89_C5147	yjjU	CDS
S88	5003012	s	rec	UTI89_C5147	yjjU	CDS
S88	5003063	s	rec	UTI89_C5147	yjjU	CDS
S88	5003081	s	rec	UTI89_C5147	yjjU	CDS
S88	5003087	s	rec	UTI89_C5147	yjjU	CDS
S88	5003165	s	rec	UTI89_C5147	yjjU	CDS
S88	5003183	s	rec	UTI89_C5147	yjjU	CDS
S88	5003201	s	rec	UTI89_C5147	yjjU	CDS
S88	5003218	ns	rec	UTI89_C5147	yjjU	CDS
S88	5003228	s	rec	UTI89_C5147	yjjU	CDS
S88	5003231	s	rec	UTI89_C5147	yjjU	CDS
S88	5003249	s	rec	UTI89_C5147	yjjU	CDS
S88	5003282	s	rec	UTI89_C5147	yjjU	CDS
S88	5003375	ns	rec	UTI89_C5147	yjjU	CDS
S88	5003406	ns	rec	UTI89_C5147	yjjU	CDS
S88	5003444	s	rec	UTI89_C5147	yjjU	CDS
S88	5003453	s	rec	UTI89_C5147	yjjU	CDS
S88	5003459	s	rec	UTI89_C5147	yjjU	CDS

lineage[a]	site[b]	mutation type[c]	recomb	gene	Gene name	Type
S88	5003510	s	rec	UTI89_C5147	yjjU	CDS
S88	5003513	s	rec	UTI89_C5147	yjjU	CDS
S88	5003522	s	rec	UTI89_C5147	yjjU	CDS
S88	5003525	s	rec	UTI89_C5147	yjjU	CDS
S88	5003528	s	rec	UTI89_C5147	yjjU	CDS
S88	5003534	s	rec	UTI89_C5147	yjjU	CDS
S88	5003537	s	rec	UTI89_C5147	yjjU	CDS
S88	5003540	s	rec	UTI89_C5147	yjjU	CDS
S88	5003541	s	rec	UTI89_C5147	yjjU	CDS
S88	5003546	s	rec	UTI89_C5147	yjjU	CDS
S88	5003564	s	rec	UTI89_C5147	yjjU	CDS
S88	5003566	ns	rec	UTI89_C5147	yjjU	CDS
S88	5003582	s	rec	UTI89_C5147	yjjU	CDS
S88	5003597	s	rec	UTI89_C5147	yjjU	CDS
S88	5003603	s	rec	UTI89_C5147	yjjU	CDS
S88	5003606	s	rec	UTI89_C5147	yjjU	CDS
S88	5003645	s	rec	UTI89_C5147	yjjU	CDS
S88	5003821	s	rec	UTI89_C5148	yjjV	CDS
S88	5003845	s	rec	UTI89_C5148	yjjV	CDS
S88	5003849	ns	rec	UTI89_C5148	yjjV	CDS
S88	5003850	ns	rec	UTI89_C5148	yjjV	CDS
S88	5003854	s	rec	UTI89_C5148	yjjV	CDS
S88	5003866	ns	rec	UTI89_C5148	yjjV	CDS
APEC	5037621	s		UTI89_C5148	yjjV	CDS
S88	5003938	ns	rec	UTI89_C5148	yjjV	CDS
S88	5003964	ns	rec	UTI89_C5148	yjjV	CDS
S88	5004106	s	rec	UTI89_C5148	yjjV	CDS
S88	5004155	ns	rec	UTI89_C5148	yjjV	CDS
S88	5004172	s	rec	UTI89_C5148	yjjV	CDS
S88	5004211	s	rec	UTI89_C5148	yjjV	CDS
S88	5004214	s	rec	UTI89_C5148	yjjV	CDS
S88	5004241	s	rec	UTI89_C5148	yjjV	CDS
S88	5004307	s	rec	UTI89_C5148	yjjV	CDS
S88	5004325	s	rec	UTI89_C5148	yjjV	CDS
S88	5004328	s	rec	UTI89_C5148	yjjV	CDS
S88	5004343	s	rec	UTI89_C5148	yjjV	CDS
S88	5004364	s	rec	UTI89_C5148	yjjV	CDS
S88	5004370	s	rec	UTI89_C5148	yjjV	CDS
S88	5004373	s	rec	UTI89_C5148	yjjV	CDS
S88	5004379	s	rec	UTI89_C5148	yjjV	CDS
S88	5004409	s	rec	UTI89_C5148	yjjV	CDS
S88	5004412	s	rec	UTI89_C5148	yjjV	CDS
S88	5004414	ns	rec	UTI89_C5148	yjjV	CDS
S88	5004436	s	rec	UTI89_C5148	yjjV	CDS
S88	5004437	ns	rec	UTI89_C5148	yjjV	CDS

lineage[a]	site[b]	mutation type[c]	recomb	gene	Gene name	Type
S88	5004441	ns	rec	UTI89_C5148	yjjV	CDS
S88	5004457	s	rec	UTI89_C5148	yjjV	CDS
S88	5004488	nc	rec	intergenic		
S88	5004497	nc	rec	intergenic		
S88	5004512	nc	rec	intergenic		
S88	5004541	nc	rec	intergenic		
S88	5004564	nc	rec	intergenic		
S88	5004567	nc	rec	intergenic		
S88	5004615	nc	rec	intergenic		
S88	5004644	nc	rec	intergenic		
S88	5004665	nc	rec	intergenic		
S88	5004677	nc	rec	intergenic		
S88	5004889	nc	rec	intergenic		
S88	5004890	nc	rec	intergenic		
S88	5004910	s	rec	UTI89_C5149	yjjW	CDS
S88	5004925	s	rec	UTI89_C5149	yjjW	CDS
S88	5004944	ns	rec	UTI89_C5149	yjjW	CDS
S88	5004947	ns	rec	UTI89_C5149	yjjW	CDS
S88	5004948	ns	rec	UTI89_C5149	yjjW	CDS
S88	5004963	s	rec	UTI89_C5149	yjjW	CDS
S88	5004964	ns	rec	UTI89_C5149	yjjW	CDS
S88	5004965	ns	rec	UTI89_C5149	yjjW	CDS
S88	5004967	s	rec	UTI89_C5149	yjjW	CDS
S88	5004970	s	rec	UTI89_C5149	yjjW	CDS
S88	5004973	s	rec	UTI89_C5149	yjjW	CDS
S88	5004976	s	rec	UTI89_C5149	yjjW	CDS
S88	5004993	ns	rec	UTI89_C5149	yjjW	CDS
S88	5005060	s	rec	UTI89_C5149	yjjW	CDS
S88	5005072	ns	rec	UTI89_C5149	yjjW	CDS
S88	5005093	s	rec	UTI89_C5149	yjjW	CDS
S88	5005123	s	rec	UTI89_C5149	yjjW	CDS
S88	5005150	s	rec	UTI89_C5149	yjjW	CDS
S88	5005169	ns	rec	UTI89_C5149	yjjW	CDS
S88	5005183	s	rec	UTI89_C5149	yjjW	CDS
S88	5005207	s	rec	UTI89_C5149	yjjW	CDS
S88	5005209	s	rec	UTI89_C5149	yjjW	CDS
S88	5005213	s	rec	UTI89_C5149	yjjW	CDS
S88	5005261	s	rec	UTI89_C5149	yjjW	CDS
S88	5005318	s	rec	UTI89_C5149	yjjW	CDS
S88	5005321	s	rec	UTI89_C5149	yjjW	CDS
S88	5005335	ns	rec	UTI89_C5149	yjjW	CDS
S88	5005357	s	rec	UTI89_C5149	yjjW	CDS
S88	5005386	s	rec	UTI89_C5149	yjjW	CDS
S88	5005402	s	rec	UTI89_C5149	yjjW	CDS
S88	5005408	s	rec	UTI89_C5149	yjjW	CDS

lineage[a]	site[b]	mutation type[c]	recomb	gene	Gene name	Type
S88	5005411	s	rec	UTI89_C5149	yjjW	CDS
S88	5005426	s	rec	UTI89_C5149	yjjW	CDS
S88	5005435	s	rec	UTI89_C5149	yjjW	CDS
S88	5005438	s	rec	UTI89_C5149	yjjW	CDS
S88	5005454	ns	rec	UTI89_C5149	yjjW	CDS
S88	5005462	s	rec	UTI89_C5149	yjjW	CDS
S88	5005489	s	rec	UTI89_C5149	yjjW	CDS
S88	5005495	s	rec	UTI89_C5149	yjjW	CDS
S88	5005511	ns	rec	UTI89_C5149	yjjW	CDS
S88	5005531	s	rec	UTI89_C5149	yjjW	CDS
S88	5005540	s	rec	UTI89_C5149	yjjW	CDS
S88	5005543	ns	rec	UTI89_C5149	yjjW	CDS
S88	5005544	ns	rec	UTI89_C5149	yjjW	CDS
S88	5005546	s	rec	UTI89_C5149	yjjW	CDS
S88	5005550	ns	rec	UTI89_C5149	yjjW	CDS
S88	5005556	ns	rec	UTI89_C5149	yjjW	CDS
S88	5005570	s	rec	UTI89_C5149	yjjW	CDS
S88	5005600	s	rec	UTI89_C5149	yjjW	CDS
S88	5005612	s	rec	UTI89_C5149	yjjW	CDS
S88	5005615	s	rec	UTI89_C5149	yjjW	CDS
S88	5005618	s	rec	UTI89_C5149	yjjW	CDS
S88	5005624	s	rec	UTI89_C5149	yjjW	CDS
S88	5005627	s	rec	UTI89_C5149	yjjW	CDS
S88	5005630	s	rec	UTI89_C5149	yjjW	CDS
S88	5005820	s	rec	UTI89_C5150	yjjI	CDS
S88	5005949	s	rec	UTI89_C5150	yjjI	CDS
S88	5005982	s	rec	UTI89_C5150	yjjI	CDS
S88	5005991	s	rec	UTI89_C5150	yjjI	CDS
S88	5006081	s	rec	UTI89_C5150	yjjI	CDS
S88	5006099	s	rec	UTI89_C5150	yjjI	CDS
S88	5006144	s	rec	UTI89_C5150	yjjI	CDS
S88	5006231	s	rec	UTI89_C5150	yjjI	CDS
S88	5006243	s	rec	UTI89_C5150	yjjI	CDS
S88	5006261	s	rec	UTI89_C5150	yjjI	CDS
S88	5006276	s	rec	UTI89_C5150	yjjI	CDS
S88	5006342	s	rec	UTI89_C5150	yjjI	CDS
S88	5006456	s	rec	UTI89_C5150	yjjI	CDS
S88	5006492	ns	rec	UTI89_C5150	yjjI	CDS
S88	5006504	s	rec	UTI89_C5150	yjjI	CDS
S88	5006531	s	rec	UTI89_C5150	yjjI	CDS
S88	5006669	s	rec	UTI89_C5150	yjjI	CDS
S88	5006705	ns	rec	UTI89_C5150	yjjI	CDS
S88	5006749	s	rec	UTI89_C5150	yjjI	CDS
S88	5006756	s	rec	UTI89_C5150	yjjI	CDS
S88	5006768	s	rec	UTI89_C5150	yjjI	CDS

lineage[a]	site[b]	mutation type[c]	recomb	gene	Gene name	Type
S88	5006774	s	rec	UTI89_C5150	yjjI	CDS
S88	5006810	s	rec	UTI89_C5150	yjjI	CDS
S88	5006869	ns	rec	UTI89_C5150	yjjI	CDS
APEC	5040452	s		UTI89_C5150	yjjI	CDS
S88	5006963	s	rec	UTI89_C5150	yjjI	CDS
S88	5007005	s	rec	UTI89_C5150	yjjI	CDS
S88	5007044	s	rec	UTI89_C5150	yjjI	CDS
S88	5007049	s	rec	UTI89_C5150	yjjI	CDS
S88	5007122	s	rec	UTI89_C5150	yjjI	CDS
S88	5007125	s	rec	UTI89_C5150	yjjI	CDS
S88	5007155	s	rec	UTI89_C5150	yjjI	CDS
S88	5007224	s	rec	UTI89_C5150	yjjI	CDS
S88	5007227	s	rec	UTI89_C5150	yjjI	CDS
S88	5007459	nc	rec	intergenic		
S88	5007460	nc	rec	intergenic		
S88	5007621	s	rec	UTI89_C5152	deoC	CDS
S88	5007720	s	rec	UTI89_C5152	deoC	CDS
S88	5007765	s	rec	UTI89_C5152	deoC	CDS
S88	5007795	ns	rec	UTI89_C5152	deoC	CDS
S88	5007837	s	rec	UTI89_C5152	deoC	CDS
S88	5007852	s	rec	UTI89_C5152	deoC	CDS
S88	5007933	s	rec	UTI89_C5152	deoC	CDS
S88	5007936	s	rec	UTI89_C5152	deoC	CDS
S88	5007942	s	rec	UTI89_C5152	deoC	CDS
S88	5007960	s	rec	UTI89_C5152	deoC	CDS
S88	5007963	s	rec	UTI89_C5152	deoC	CDS
S88	5008032	s	rec	UTI89_C5152	deoC	CDS
S88	5008071	s	rec	UTI89_C5152	deoC	CDS
S88	5008113	s	rec	UTI89_C5152	deoC	CDS
S88	5008133	ns	rec	UTI89_C5152	deoC	CDS
S88	5008227	s	rec	UTI89_C5152	deoC	CDS
S88	5008251	s	rec	UTI89_C5152	deoC	CDS
S88	5008261	s	rec	UTI89_C5152	deoC	CDS
S88	5008284	s	rec	UTI89_C5152	deoC	CDS
S88	5008290	s	rec	UTI89_C5152	deoC	CDS
S88	5008437	s	rec	UTI89_C5153	deoA	CDS
S88	5008503	s	rec	UTI89_C5153	deoA	CDS
S88	5008533	s	rec	UTI89_C5153	deoA	CDS
S88	5008707	s	rec	UTI89_C5153	deoA	CDS
S88	5008713	s	rec	UTI89_C5153	deoA	CDS
S88	5008716	s	rec	UTI89_C5153	deoA	CDS
S88	5008758	s	rec	UTI89_C5153	deoA	CDS
S88	5008764	s	rec	UTI89_C5153	deoA	CDS
S88	5008788	s	rec	UTI89_C5153	deoA	CDS
S88	5008914	s	rec	UTI89_C5153	deoA	CDS

lineage[a]	site[b]	mutation type[c]	recomb	gene	Gene name	Type
S88	5008923	s	rec	UTI89_C5153	deoA	CDS
S88	5008938	s	rec	UTI89_C5153	deoA	CDS
S88	5008962	s	rec	UTI89_C5153	deoA	CDS
S88	5008983	s	rec	UTI89_C5153	deoA	CDS
S88	5008986	s	rec	UTI89_C5153	deoA	CDS
S88	5009025	s	rec	UTI89_C5153	deoA	CDS
S88	5009172	s	rec	UTI89_C5153	deoA	CDS
S88	5009283	s	rec	UTI89_C5153	deoA	CDS
S88	5009323	ns	rec	UTI89_C5153	deoA	CDS
S88	5009400	s	rec	UTI89_C5153	deoA	CDS
S88	5009496	s	rec	UTI89_C5153	deoA	CDS
S88	5009523	s	rec	UTI89_C5153	deoA	CDS
S88	5009533	ns	rec	UTI89_C5153	deoA	CDS
S88	5009649	s	rec	UTI89_C5153	deoA	CDS
S88	5009675	ns	rec	UTI89_C5153	deoA	CDS
S88	5009955	s	rec	UTI89_C5154	deoB	CDS
S88	5009956	ns	rec	UTI89_C5154	deoB	CDS
S88	5010000	s	rec	UTI89_C5154	deoB	CDS
S88	5010054	s	rec	UTI89_C5154	deoB	CDS
S88	5010114	s	rec	UTI89_C5154	deoB	CDS
S88	5010138	s	rec	UTI89_C5154	deoB	CDS
S88	5010222	s	rec	UTI89_C5154	deoB	CDS
S88	5010387	s	rec	UTI89_C5154	deoB	CDS
S88	5010414	s	rec	UTI89_C5154	deoB	CDS
S88	5010444	s	rec	UTI89_C5154	deoB	CDS
S88	5010486	s	rec	UTI89_C5154	deoB	CDS
S88	5010504	s	rec	UTI89_C5154	deoB	CDS
S88	5010591	s	rec	UTI89_C5154	deoB	CDS
S88	5010606	s	rec	UTI89_C5154	deoB	CDS
S88	5010651	s	rec	UTI89_C5154	deoB	CDS
S88	5010780	s	rec	UTI89_C5154	deoB	CDS
S88	5010826	s	rec	UTI89_C5154	deoB	CDS
S88	5010843	s	rec	UTI89_C5154	deoB	CDS
S88	5010846	s	rec	UTI89_C5154	deoB	CDS
S88	5010852	s	rec	UTI89_C5154	deoB	CDS
S88	5010858	s	rec	UTI89_C5154	deoB	CDS
S88	5010873	s	rec	UTI89_C5154	deoB	CDS
S88	5010876	s	rec	UTI89_C5154	deoB	CDS
S88	5010927	s	rec	UTI89_C5154	deoB	CDS
S88	5010930	s	rec	UTI89_C5154	deoB	CDS
S88	5010942	s	rec	UTI89_C5154	deoB	CDS
S88	5010948	s	rec	UTI89_C5154	deoB	CDS
S88	5010951	s	rec	UTI89_C5154	deoB	CDS
S88	5010954	s	rec	UTI89_C5154	deoB	CDS
S88	5011064	s	rec	UTI89_C5155	deoD	CDS

lineage[a]	site[b]	mutation type[c]	recomb	gene	Gene name	Type
S88	5011065	ns	rec	UTI89_C5155	deoD	CDS
S88	5011079	s	rec	UTI89_C5155	deoD	CDS
S88	5011080	ns	rec	UTI89_C5155	deoD	CDS
S88	5011102	ns	rec	UTI89_C5155	deoD	CDS
S88	5011103	ns	rec	UTI89_C5155	deoD	CDS
S88	5011112	s	rec	UTI89_C5155	deoD	CDS
S88	5011142	s	rec	UTI89_C5155	deoD	CDS
S88	5011166	s	rec	UTI89_C5155	deoD	CDS
S88	5011253	s	rec	UTI89_C5155	deoD	CDS
S88	5011289	s	rec	UTI89_C5155	deoD	CDS
S88	5011340	s	rec	UTI89_C5155	deoD	CDS
S88	5011370	s	rec	UTI89_C5155	deoD	CDS
S88	5011385	s	rec	UTI89_C5155	deoD	CDS
S88	5011517	s	rec	UTI89_C5155	deoD	CDS
S88	5011523	s	rec	UTI89_C5155	deoD	CDS
S88	5011526	s	rec	UTI89_C5155	deoD	CDS
S88	5011529	s	rec	UTI89_C5155	deoD	CDS
S88	5011541	s	rec	UTI89_C5155	deoD	CDS
S88	5011542	ns	rec	UTI89_C5155	deoD	CDS
S88	5011553	s	rec	UTI89_C5155	deoD	CDS
S88	5011556	s	rec	UTI89_C5155	deoD	CDS
S88	5011631	s	rec	UTI89_C5155	deoD	CDS
S88	5011712	s	rec	UTI89_C5155	deoD	CDS
S88	5011763	s	rec	UTI89_C5155	deoD	CDS
S88	5011790	s	rec	UTI89_C5155	deoD	CDS
S88	5013027	s	rec	UTI89_C5156	yjjJ	CDS
S88	5013036	s	rec	UTI89_C5156	yjjJ	CDS
S88	5013054	s	rec	UTI89_C5156	yjjJ	CDS
S88	5013095	ns	rec	UTI89_C5156	yjjJ	CDS
S88	5013114	s	rec	UTI89_C5156	yjjJ	CDS
S88	5013121	s	rec	UTI89_C5156	yjjJ	CDS
S88	5013146	ns	rec	UTI89_C5156	yjjJ	CDS
S88	5013171	s	rec	UTI89_C5156	yjjJ	CDS
S88	5013174	s	rec	UTI89_C5156	yjjJ	CDS
S88	5013223	ns	rec	UTI89_C5156	yjjJ	CDS
S88	5013246	s	rec	UTI89_C5156	yjjJ	CDS
S88	5013261	s	rec	UTI89_C5156	yjjJ	CDS
S88	5013296	ns	rec	UTI89_C5156	yjjJ	CDS
S88	5013325	s	rec	UTI89_C5157	lplA	CDS
S88	5013327	s	rec	UTI89_C5157	lplA	CDS
S88	5013328	s	rec	UTI89_C5157	lplA	CDS
S88	5013331	s	rec	UTI89_C5157	lplA	CDS
S88	5013340	ns	rec	UTI89_C5157	lplA	CDS
S88	5013352	s	rec	UTI89_C5157	lplA	CDS
S88	5013393	s	rec	UTI89_C5157	lplA	CDS

lineage[a]	site[b]	mutation type[c]	recomb	gene	Gene name	Type
S88	5013424	s	rec	UTI89_C5157	lplA	CDS
S88	5013436	s	rec	UTI89_C5157	lplA	CDS
S88	5013451	s	rec	UTI89_C5157	lplA	CDS
S88	5013484	s	rec	UTI89_C5157	lplA	CDS
S88	5013669	ns	rec	UTI89_C5157	lplA	CDS
S88	5013763	s	rec	UTI89_C5157	lplA	CDS
S88	5013765	s	rec	UTI89_C5157	lplA	CDS
S88	5013768	s	rec	UTI89_C5157	lplA	CDS
S88	5013769	s	rec	UTI89_C5157	lplA	CDS
S88	5013775	s	rec	UTI89_C5157	lplA	CDS
S88	5013778	s	rec	UTI89_C5157	lplA	CDS
S88	5013796	s	rec	UTI89_C5157	lplA	CDS
S88	5013799	s	rec	UTI89_C5157	lplA	CDS
S88	5013802	s	rec	UTI89_C5157	lplA	CDS
APEC	5051251	s		UTI89_C5161	nadR	CDS
S88	5017839	ns		UTI89_C5161	nadR	CDS

[a] AS: allocated to the lineage to the common ancestor of APEC 01 and S88; UTI89/AS: allocated to the SNPs at the divergence of UTI89 and AS; APEC/S88: allocated to the SNPs at the divergence of APEC 01 and S88.

[b] For indels the base indicated is the base before the insertion or deletion.

[c] ins, insertion; del, deletion; indel, insertion or deletion; nc, in non-coding genes; ns, non-synonymous; s, synonymous. number after "ins" or "del" or “indel” is number of bases if greater than 1
