# Supplementary material for: Derivation of Escherichia coli O157:H7 from Its O55:H7 Precursor
Source: PLoS One. 2010 Jan 14;5(1):e8700. doi: 10.1371/journal.pone.0008700 (PMC2806823; doi:10.1371/journal.pone.0008700)
Supplement: Table S7 — Virtual outgroup analysis for allocation of mutational single nucleotide polymorphisms and small indels to the UTI189, S88, or APEC 01 lineages. The 23 genomes used for the analysis are shown, with details of the base or bases present in both outgroup genomes and genomes under analysis, and also the final allocation and a measure of support level for that allocation. (0.08 MB PDF) [file pone.0008700.s009.pdf]

ExPEC Cluster										Outgroup Strains[g]																						
UTI89[a]	UTI89 site[b]	APEC 01[a]	APEC 01 site[b]	S88[a]	S88 site[b]	type[c]	Inferred ancestral base[d]	Lineage inferred to mutate[e]	Support level[f]	CFT073	ED1a	536	E2348/69	SMS 3-5	IAI39	UMN026	K-12	ATCC 8739	HS	D1 Sd197	CB9615	Sakai	EDL933	IAI1	E24377A	SE11	SS Ss046	F2a 301	F2a 2457T	F5 8401	B4 Sb227	B18 BS512
c	5137	a	5118	c	5137	nc	c	APEC	++++	t	c	c	c	c	c	c	c	c	c	c	c	c	c	c	c	c	c	c	c	c	c	c
t	6015	g	5996	g	6015	ns	g	UTI89	++++	g	g	g	g	g	g	g	g	g	g	g	g	g	g	g	g	g	g	g	g	g	g	g
g	6101	a	6082	a	6101	ns	g	AS	++++	g	g	g	g	g	g	g	g	g	g	g	g	g	g	g	g	g	g	g	g	g	g	g
t	7680	c	7661	c	7680	ns	c	UTI89	++++	c	c	c	c	c	c	c	c	c	c	c	c	c	c	c	c	c	c	c	c	c	c	c
g	31180	a	31161	g	31180	s	g	APEC	++++	g	g	g	g	g	g	g	g	g	g	g	g	g	g	g	g	g	g	g	g	g	g	g
1	31384	1	31375	-	31393	del	1	S88	++++	1	1	1	1	1	1	1	1	1	1	1	1	1	1	1	1	1	1	1	1	1	1	1
g	37126	a	37107	a	37125	s	a	UTI89	++++	a	a	a	a	a	a	a	a	a	a	a	a	a	a	a	a	a	a	a	a	a		
g	39520	g	39501	t	39519	ns	g	S88	++++	g	g	g	g	g	g	g	g	g	g	g	g	g	g	g	g	g	g	g	g	g		
g	39698	a	39679	g	39697	s	g	APEC	++++	g	g	g	g	g	g	g	g	g	g	g	g	g	g	g	g	g	g	g	g	g		
c	47451	t	47432	c	47450	ns	c	APEC	++++	c	c	c	c	c	c	c	c	c	c	c	c	c	c	c	c	c	c	c	c	c		
a	56555	g	56536	g	56554	s	g	UTI89	++++	g	g	g	g	g	g	g	g	g	g	g	g	g	g	g	g	g	g	g	g	g	g	g
c	58886	t	58867	c	58885	s	c	APEC	++++	c	c	c	c	c	c	c	c	c	c	c	c	c	c	c	c	c	c	c	c	c	c	c
c	62785	t	62766	c	62784	ns	c	APEC	++++	c	c	c	c	c	c	c	c	c	c	c	c	c	c	c	c	c	c	c	c	c	c	c
c	66012	t	65993	c	66011	s	c	APEC	++	c	c	c	c	c	c	c	t	t	c	c	c	c	c	c	c	c	c	c	c	c	t	t
a	67549	c	67530	c	67548	ns	c	UTI89	++++	c	c	c	c	c	c	c	c	c	c	c	c	c	c	c	c	c	c	c	c	c	c	c
c	73331	a	73312	c	73330	nc	c	APEC	+++	c	c	c		c	c																	
1	73814	-	73794	1	73813	del	1	APEC	+++	-	1	1		1	1																	
-	74177	1	74167	1	74186	del	1	UTI89	+++	1	1	1		1	1																	
c	75007	t	74988	t	75007	nc	c	AS	++++	c	c	c		c	c	c	c	c	c	c	c	c	c	c	c	c	c	c	c	c	c	c
c	76814	t	76795	c	76814	s	c	APEC	++++	c	c	c	c	c	c	c	c	c	c	c	c	c	c	c	c	c	c	c	c	c	c	c
a	91573	t	91554	t	91573	nc	t	UTI89	++++	t	t	t	t	t	t	t	t	t	t	t	t	t	t	t	t	t	t	t	t	t	t	t
t	91574	a	91555	a	91574	nc	a	UTI89	++++	a	a	a	a	a	a	a	a	a	a	a	a	a	a	a	a	a	a	a	a	a	a	a
c	92784	a	92765	a	92784	s	c	AS	++++	c	c	c	c	c	c	c	c	c	c	c	c	c	c	c	c	c	c	c	c	c	c	c
t	97682	t	97663	c	97682	s	t	S88	++++	t	t	t	t	t	t	t	t	t	t	t	t	t	t	t	t	t	t	t	t	t	t	t
c	104328	a	104309	c	104328	ns	c	APEC	++++	c	c	c	c	c	c	c	c	c	c	c	c	c	c	c	c	c	c	c	c	c	c	c
c	109023	c	109004	t	109023	ns	c	S88	++++	c	c	c	c	c	c	c	c	c	c	c	c	c	c	c	c	c	c	c	c	c	c	c
t	113567	c	113548	c	113567	ns	c	UTI89	++++	c	c	c	c	c	c	c	c	c	c	c	c	c	c	c	t	c	c	c	c	c	c	c
a	117905	a	117886	g	117905	ns	a	S88	++++	a	a	a	a	a	a	a	a	a	a	a	a	a	a	a	a	a	a	a	a	a	a	a
c	121009	c	120990	t	121009	ns	c	S88	++++	c	c	c	c	c	c	c	c	c	c	c	c	c	c	c	c	c	c	c	c	c	c	c
a	123914	g	123895	a	123914	nc	a	APEC	++	a	a	a	g	g	g	g	g	g	g	g	g	g	g	g	g	g	g	g	g	g	g	g
g	125858	g	125839	a	125858	ns	g	S88	+++	g	g	g		g																		
t	126442	g	126423	t	126442	ns	t	APEC	+++	c	t	t		t																		
c	127904	t	127885	c	127904	nc	c	APEC	+++	c	c	c		c																		
1	133986	-	133966	-	133985	del	1	AS	++++	1	1	1	1	1	1	1				1	1	1	1									
4	140774	-	140753	4	140773	del	4	APEC	++++	4	4	4	4	4	4	4	4	4	4	4	4	4	4	4	4	4	4	4	4	4	4	4
c	154677	c	154653	t	154676	ns	c	S88	++++	c	c	c	c	c	c	c	c	c	c	c	c	c	c	c	c	c	c	c	c	c	c	c
c	160807	t	160783	c	160806	ns	c	APEC	++	c	c	c	c	c	c	t	c	g			t	t	t	c	c	c	c				c	
c	161029	t	161005	t	161028	s	c	AS	++++	c	c	c	c	c	c	c	c							c	c	c	c				c	
g	164019	c	163995	c	164018	ns	c	UTI89	++++	c	c	c	c	c	c	c	c	c	c	c	c	c	c	c	c	c	c	c	c	c	c	c
a	170166	g	170142	g	170165	s	a	AS	++	a	a	a	a	a	a	g	a	g	g	a	a	a	a	a	a	a	a	a	a	a	a	a
a	181047	a	181023	t	181046	s	a	S88	++++	a	a	a	a	a	a	a	a	a	a	a	a	a	a	a	a	a	a	a	a	a	a	a
c	183903	c	183879	t	183902	s	c	S88	++++	c	c	c	c	c	c	c	c	c	c	c	c	c	c	c	c	c	c	c	c	c	c	c
g	184354	a	184330	g	184353	s	g	APEC	++++	g	g	g	g	g	g	g	g	g	g	g	g	g	g	g	g	g	g	g	g	g	g	g
t	204768	g	204744	t	204767	ns	t	APEC	++++	t	t	t	t	t	t	t	t	t	t	t	t	t	t	t	t	t	t	t	t	t	t	t
a	209338	g	209314	g	209337	s	g	UTI89	++++	g	g	g	g	g	g	g	g	g	g	g	g	g	g	g	g	g	g	g	g	g	g	g
g	211483	t	211459	g	211482	s	g	APEC	++++	g	g	g	g	g	g	g	g	g	g	g	g	g	g	g	g	g	g	g	g	g	g	g
g	216204	g	216180	a	216203	ns	g	S88	++++	g	g	g	g	g	g	g	g	g	g	g	g	g	g	g	g	g	g	g	g	g	g	g
t	216887	c	216863	c	216886	ns	c	UTI89	++++	c	c	c	c	c	c	c	c	c	c	c	c	c	c	c	c	c	c	c	c	c	c	c
g	219539	t	219515	g	219538	ns	g	APEC	++++	g	g	g	g	g	g	g	g	g	g	g	g	g	g	g	g	g	g	g	g	g	g	g
c	221104	c	221080	t	221103	ns	c	S88	++++	c	c	c	c	c	c	c	c	c	c	c	c	c	c	c	c	c	c	c	c	c	c	c
a	228460	t	228436	a	228459	nc	a	APEC	++++	a	a	a	a	a	a	a	a	a	a	a	a	a	a	a	a	a	a	a	a	a	a	a
a	228470	c	228446	a	228469	nc	a	APEC	++++	a	a	a	a	a	a	a	a	a	a	a	a	a	a	a	a	a	a	a	a	a	a	a
c	228613	a	228589	c	228612	nc	a	APEC	-	a	c	a	c	c	t	c	c	c	c	c	c	c	c	c	c	c	t	c	t	t	a	t
c	228660	t	228636	c	228659	nc	t	APEC	-	t	c	t	c	t	t	t	t	t	t	t	t	t	t	t	t	t	t	t	t	t	t	t
a	228733	c	228709	c	228732	nc	c	UTI89	++++	c	c	c	c	c	c	c	c	c	c	c	c	c	c	c	c	c	c	c	c	c	c	c
c	228787	a	228763	a	228786	nc	a	UTI89	++	a	a	a	a	c	c	c	c	c	c	c	c	c	c	c	c	c	c	c	c	c	c	c
a	229776	g	229752	g	229775	s	g	UTI89	++++	g	g	g	g	g	g	g	g	g	g	g	g	g	g	g	g	g	g	g	g	g	g	g
g	231135	c	231107	c	231134	nc	c	UTI89	++++	c	c	c	c	c	c	c	c	c	c	c	c	c	c	c	c	c	c	c	c	c	c	c
t	232332	c	232305	c	232331	nc	c	UTI89	++++	c	c	c	c	c	c	c	c	c	c	c	c	c	c	c	c	c	c	c	c	c	c	c
-	232828	1	232804	-	232827	ins	-	APEC	++++	-	-	-	-	-	-	-	-	-	-	-	-	-	-	-	-	-	-	-	-	-	-	-
-	232846	1	232823	-	232845	ins	-	APEC	++++	-	-	-	-	-	-	-	-	-	-	-	-	-	-	-	-	-	-	-	-	-	-	-
-	232895	1	232873	-	232894	ins	-	APEC	++++	-	-	-	-	-	-	-	-	-	-	-	-	-	-	-	-	-	-	-	-	-	-	-
-	232914	1	232893	-	232913	ins	-	APEC	++++	-	-	-	-	-	-	-	-	-	-	-	-	-	-	-	-	-	-	-	-	-	-	-
c	233419	c	233398	a	233418	ns	a	S88	-	a	a	a	a	a	g	a	c	a	c	a	g	g	g	a	g	a	a	a	a	a	g	g
1	233433	-	233411	-	233431	ins	-	UTI89	++	-	-	-	1	-	-	1	1	-	-	-	-	-	-	-	-	-	-	1	1	1	-	-
-	233431	-	233418	1	233439	ins	-	S88	++	-	1	-	-	-	-	-	-	-	-	-	-	-	-	1	-	1	-	-	-	-	1	1
t	239048	g	239026	g	239047	ns	g	UTI89	++++	g	g	g	g	g	g	g	g	g	g	g	g	g	g	g	g	g	g	g	g	g	g	g
c	239547	t	239525	t	239546	s	c	AS	++++	c	c	c	c	c	c	c	c	c	c	c	c	c	c	c	c	c	c	c	c	c	c	c
t	241946	t	241924	g	241945	nc	t	S88	++	t	t	t	t	t	t	t	t	t	t	g	t	t	t	t	t	t	g	t	t	t	t	g
a	243191	g	243169	g	243190	nc	a	AS	++		a	a				a																
a	244032	a	244010	c	244031	ns	a	S88	++++		a	a				a			a		a	a	a	a	a	a	a					
g	245731	a	245709	g	245730	ns	g	APEC	++++		g	g				g			g		g	g	g	g	g	g	g					
t	249444	t	249422	g	249443	ns	t	S88	++++		t	t				t			t		t	t	t	t	t	t	t					
c	250473	c	250451	t	250472	ns	c	S88	++++		c	c				c			c		c	c	c	c	c	c	c					
g	253202	g	253180	a	253201	ns	g	S88	++++		g	g				g			g		g	g	g	g	g	g	g					
c	260627	c	260605	a	260626	ns	c	S88	++		c	c				c																
t	263285	t	263263	c	263284	nc	t	S88	++		t	t				g																
a	263318	g	263296	g	263317	nc	a	AS	++		a	a				a																
g	266663	a	266641	g	266662	ns	g	APEC	++			g																				
a	268514	t	268492	a	268513	ns	a	APEC	++			a																				
-	276793	1	276772	-	276792	ins	-	APEC	++++	-	-	-	-	-	-	-	-	-	-	-	-	-	-	-	-	-	-	-	-	-	-	-
c	278807	g	278786	g	278806	ns	g	UTI89	++++	g	g	g	g	g	g	g	g	g	g	g	g	g	g	g	g	g	g				g	g

ExPEC Cluster										Outgroup Strains[g]																						
UTI89[a]	UTI89 site[b]	APEC 01[a]	APEC 01 site[b]	S88[a]	S88 site[b]	type[c]	Inferred ancestral base[d]	Lineage inferred to mutate[e]	Support level[f]	CFT073	ED1a	536	E2348/69	SMS 3-5	IAI39	UMN026	K-12	ATCC 8739	HS	D1 Sd197	CB9615	Sakai	EDL933	IAI1	E24377A	SE11	SS Ss046	F2a 301	F2a 2457T	F5 8401	B4 Sb227	B18 BS512
c	280815	t	280794	t	280814	ns	c	AS	++++	c	c	c	c	c	c	c	c	c	c		c	c	c	c	c	c	c	c	c	c		
c	309874	t	311464	t	299239	nc	c	AS	++	c		c																				
1	310009	-	311598	-	299373	ins	-	UTI89	++	-		-																				
g	311554	g	313143	a	300918	nc	g	S88	++	g		g																				
a	313568	c	315157	a	302932	nc	a	APEC	++++	a		a	a	a	a	a	a	a			a	a	a	a	a	a						
c	320771	c	322360	g	310135	ns	c	S88	++++	c	c	c	c	c	c	c	c	c			c	c	c	c	c	c					c	
t	320925	c	322514	c	310289	ns	c	UTI89	++++	c	c	c	c	c	c	c	c	c			c	c	c	c	c	c					c	
-	321246	1	322845	1	310620	del	1	UTI89	++++	1	1	1	1	1	1	1	1	1	1		1	1	1	1	1	1					-	
a	322177	g	323767	a	311542	nc	a	APEC	++	a	a	a	a	a		a	g	g	g		a	a	a	g	g	g					g	
a	324320	g	325910	a	313685	s	a	APEC	++++	a	a	a	a	a	a	a	a	a	a		a	a	a	a	a	a					a	
c	328733	c	330323	t	318098	nc	c	S88	+++	c		c	c	c	c	c																
g	329587	a	331177	a	318952	s	g	AS	+++	g		g	g	g	g	g																
c	332304	t	333894	t	321669	s	c	AS	++	c		c	c	c	c	c		t	c		c	c	c	c	t	c						
t	336518	c	338108	c	325883	nc	t	AS	++++	t		t	t	t	t	t	t	t	t		t	t	t	t	t	t	t					c
a	339541	g	341131	g	328906	ns	g	UTI89	++++	g	g	g	g	g	g	g	g	g	g		g	g	g	a	g	g	g					
-	342481	1	344072	-	331846	ins	-	APEC	++++	-	-	-	-	-	-	-	-	-	-		-	-	-	-	-	-	-	-	-	-		-
t	342623	c	344214	c	331988	ns	c	UTI89	++++	c	c	c	c	c	c	c	c	c	c		c	c	c	c	c	c	c	c	c	c		c
-	344595	1	346196	1	333970	del	1	UTI89	++++	1	1	1	1	1	1	1	1	1	1		1	1	1	1	1	1		1	1	1		1
a	349665	g	351257	a	339031	nc	a	APEC	+++	a		a							a		a	a	a			a						
-	350806	-	352407	1	340182	ins	-	S88	++	-		-							-		1	1	1			-						
g	357087	t	358679	t	346454	s	g	AS	++++	g		g	g	g	g	g	g	g	g		g	g	g	g	g	g			g			
c	363910	t	365502	t	353277	s	c	AS	++	c	c	c	c	t	t	c	c	c	c		c	c	c	c	c	c	c					
a	364638	g	366230	g	354005	s	a	AS	++++	a	a	a	a	a	a	a	a	a	a		a	a	a	a	a	a	a					
g	366733	a	368325	g	356100	ns	g	APEC	++++	g	g	g	g	g	g	g	g	g	g		g	g	g	g	g	g	g					
a	367643	a	369235	c	357010	ns	a	S88	++++	a	a	a	a	a	a	a	a	a	a		a	a	a	a	a	a	a					
t	377150	c	378742	c	366517	nc	c	UTI89	++	c	c	c	c	c	a	a	c	c	c		c	c	c	c	c	c						
a	378067	g	379659	g	367434	nc	g	UTI89	++++	g	g	g	g	g	g	g	g	g	g		g	g	g	g	g	g						
c	386225	c	387817	t	375592	s	c	S88	++++	c	c	c	c	c	c	c	c	c	c		c	c	c	c	c	c						
c	386235	t	387827	t	375602	s	t	UTI89	++++	t	t	t	t	t	t	t	t	t	t		t	t	t	t	t	t						
t	389340	a	390932	t	378707	ns	t	APEC	++++	t	t	t	t	t	t	t	t	t	t	t	t	t	t	t	t	t	t					
-	392256	1	393849	-	381623	ins	-	APEC	++++	1	-	-	-	-	-	-	-	-	-	-	-	-	-	-	-	-	-					
c	395358	t	396951	c	384725	s	c	APEC	++	c	c	c	c	c	c	c	c	a	a		c	c	c	a	a	a	a					a
g	398289	a	399882	a	387656	ns	g	AS	++++	g	g	g		g	g	g	g	g	g					g	g	g	g					g
a	399075	g	400668	g	388442	ns	g	UTI89	++++	g	g	g		g	g	g	g	g	g					g	g	g	g					g
5	400318	-	401910	5	389685	del	5	APEC	++++	5	5	5		5	5	5	5	5	5					5	5	5	5					5
c	405375	a	406963	c	394742	nc	c	APEC	++	c		c	c	t	t	t	t	t	t	c	c	c	c	t	t	t	t	t	t	t	t	t
c	407688	t	409276	c	397055	s	c	APEC	++++	c		c	c	c	c	c	c	c	c	c	c	c	c	c	c	c	c				c	c
1	415505	1	417103	-	404881	del	-	S88	-	1	-	-	-	-	-	-	-	-	-	-	-	-	-	-	-	-	-	-	-	-	-	-
c	416081	t	417669	c	405447	ns	c	APEC	++++	c	c	c	c	c	c	c	c	c	c	c	c	c	c	c	c	c	c	c	c	c	c	c
g	419774	g	421362	a	409140	s	g	S88	++++	g	g	g	g	g	g	g	g	g	g	g	g	g	g	g	g	g	g	g	g	g	g	g
t	423277	a	424865	t	412643	nc	t	APEC	++++	t	t	t	t	t	t	t	t	t	t	t	t	t	t	t	t	t	t	t	t	t	t	t
-	424186	1	425775	-	413552	ins	-	APEC	++++	-	-	-	-	-	-	-	-	-	-	-	-	-	-	-	-	-	-	-	-	-	-	-
g	425766	g	427355	c	415132	ns	g	S88	++++	g	g	g	g	g	g	g	g	g	g	g	g	g	g	g	g	g	g	g	g	g	g	g
c	430783	t	432372	c	420149	ns	c	APEC	++++	c	c	c	c	c	c	c	c	c	c	c	c	c	c	c	c	c	c	c	c	c	c	c
t	432469	c	434058	c	421835	ns	c	UTI89	++++	c	c	c	c	c	c	c	c	c	c	c	c	c	c	c	c	c	c	c	c	c	c	c
c	432619	t	434208	c	421985	ns	c	APEC	++++	c	c	c	c	c	c	c	c	c	c	c	c	c	c	c	c	c	c	c	c	c	c	c
c	433304	t	434893	t	422670	s	t	UTI89	++++	t	t	t	t	t	t	t	t	t	t	t	t	t	t	t	t	t	t	t	t	t	t	t
c	437960	a	439549	c	427326	s	c	APEC	++++	c	c	c	c	c	c	c	c	c	c	t	c	c	c	c	c	c	c	c	c	c	c	c
c	440108	g	441697	c	429474	ns	c	APEC	++++	c	c	c	c	c	c	c	c	c	c	c	c	c	c	c	c	c	c	c	c	c	c	c
t	442626	c	444215	c	431992	s	c	UTI89	++++	c	c	c	c	c	c	c	c	c	c	c	c	c	c	c	c	c	c	c	c	c	c	c
c	454656	t	456245	c	444022	ns	c	APEC	++++	c	c	c	c	c	c	c	c	c	c	c	c	c	c	c	c	c	c	c	c	c	c	c
t	456678	c	458267	c	446044	s	c	UTI89	++	c	c	c	c	c	c	c	t	t	t	c	c	c	c	t	t	t	t	t	t	t	t	t
-	464820	-	466418	9	454196	ins	-	S88	++++	-	-	-	-	-	-	-	-	-	-		-	-	-	-	-	-	-	-	-	-	-	-
-	464829	9	466419	9	454205	ins	-	AS	++	9	-	-	9	-	-	-	-	-	-		-	9	9	-	-	-	-	9	9	9	9	9
g	473065	a	474663	a	462449	s	g	AS	++++	g	g	g	g	g	g	g	g	g	g	g	g	g	g	g	g	g	g	g	g	g	g	g
t	473288	g	474886	g	462672	ns	t	AS	++++	t	t	t	t	t	t	t	t	t	t	t	t	t	t	t	t	t	t	t	t	t	t	t
c	482361	t	483959	t	471745	nc	c	AS	++	c	c	c	c	c	c	c	c	c	c	c	c	c	c	t	c	c	c	c	c	c	t	t
g	483535	a	485133	g	472919	ns	g	APEC	++++	g	g	g	g	g	g	g	g	g	g	g	g	g	g	g	g	g	g	g	g	g	g	g
g	491700	a	493298	g	481084	s	g	APEC	++	g	g	g	g	g	g	g	g	g	g	g	g	g	g	g	g	g	g	a	a	a	g	g
c	493493	t	495091	t	482877	nc	c	AS	++++	c	c	c	c	c	c	c	c	c	c	c	c	c	c	c	c	c	c	c	c	c	c	c
a	497146	g	498744	g	486530	s	a	AS	++	a	a	a	a	g	g	a	a	a	a	a	a	a	a	a	a	a	a	a	a	a	a	a
g	512232	a	513830	a	501616	ns	g	AS	++++	g	g	g	g	g	g	g	g	g	g	g	g	g	g	g	g	g	g	g	g	g	g	g
c	512366	t	513964	c	501750	s	c	APEC	++	c	c	c	c	c	c	c	c	c	c	c	c	c	c	c	c	c	c	a	a	a	t	c
g	525865	g	527463	a	515249	s	g	S88	++++	g	g	g	g	g		g	g	g	g	g	g	g	g	g	g	g	g	g	g	g	g	g
c	528387	c	529985	t	517771	s	c	S88	++++	c	c	c	c	c		c	c	c	c	c	c	c	c	c	c	c	c	c	c	c	c	c
c	531399	t	532997	c	520783	ns	c	APEC	+				c	c	c	c					c	c	c									
g	531800	g	533398	t	521184	nc	g	S88	+				g	g	g	g				g	g	g	g									
t	533757	t	535355	c	523141	ns	t	S88	++++	t	t	t	t	t	t	t	t	t	t	t	t	t	t	t	t	t	t	t	t	t	t	t
a	535190	g	536788	g	524574	s	g	UTI89	++++	g	g	g	g	g	g	g	g	g	g	g	g	g	g	g	g	g	g	g	g	g	g	g
g	538955	t	540553	t	528339	ns	g	AS	++++	g	g	g	g	g		g	g	g	g	g	g	g	g	g	g	g	g	g	g	g	g	g
g	539309	a	540907	a	528693	ns	g	AS	++++	g	g	g	g	g		g	g	g	g	g	g	g	g	g	g	g	g	g	g	g	g	g
c	539322	t	540920	t	528706	ns	c	AS	++++	c	c	c	c	c		c	c	c	c	c	c	c	c	c	c	c	c	c	c	c	c	c
t	543064	c	544662	c	532448	nc	c	UTI89	++++	c	c	c	c	c		c	c	c	c	c	c	c	c	c	c	c	c	c	c	c	c	c
c	549601	t	551199	t	538985	s	c	AS	++	c	c	c	c	t		c	t	t	t		c	c	c	t	t	c						
1	572059	-	573656	1	612093	del	1	APEC	++++	1	1	1	1		1	1	1				1	1	1			1						
-	572205	1	573803	-	612239	ins	-	APEC	++	-	1	-	-		-	1	1									-						
t	576235	g	577833	g	616269	ns	g	UTI89	++++	g	g	g	g	g	g	g	g	g		g	g	g	g	g	g	g	g	g	g	g		
t	576251	c	577849	c	616285	ns	c	UTI89	++++	c	c	c	c	c	c	c	c	c		c	c	c	c	c	c	c	c	c	c	c		
c	584611	a	586209	c	624645	ns	c	APEC	++++	c	c	c	c	c	c	c	c	c	c		c	c	c	c	c	c	c	c	c	c	c	c
t	586944	c	588542	c	626978	ns	c	UTI89	++++	c	c	c	c	c	c	c	c	c	c	c	c	c	c	c	c	c	c	c	c	c	c	c
t	586974	g	588572	t	627008	ns	t	APEC	++	t	t	t	t	t	t	t	t	t	t	t	t	t	t	t	t	t	t	t	t	t	g	g
g	591673	t	593271	t	631707	nc	t	UTI89	++++	t	t	t	t	t	t	t	t	t	t	t	t	t	t	t	t	t	t	t	t	t	t	t

ExPEC Cluster										Outgroup Strains[g]																						
UTI89[a]	UTI89 site[b]	APEC 01[a]	APEC 01 site[b]	S88[a]	S88 site[b]	type[c]	Inferred ancestral base[d]	Lineage inferred to mutate[e]	Support level[f]	CFT073	ED1a	536	E2348/69	SMS 3-5	IAI39	UMN026	K-12	ATCC 8739	HS	D1 Sd197	CB9615	Sakai	EDL933	IAI1	E24377A	SE11	SS Ss046	F2a 301	F2a 2457T	F5 8401	B4 Sb227	B18 BS512
c	594656	t	596254	c	634690	ns	c	APEC	++++	c	c	c	c	c	c	c	c	c	c	c	c	c	c	c	c	c	c	c	c	c	c	c
c	595701	a	597299	a	635735	ns	c	AS	++++	c	c	c	c	c	c	c	c	c	c	c	c	c	c	c	c	c	c	c	c	c	c	c
g	604433	a	606031	a	644467	ns	a	UTI89	++++	a	a	a	a	a	a	a	a	a	a	a	a	a	a	a	a	a	a	a	a	a	a	a
g	607313	a	608911	a	647347	ns	g	AS	++++	g	g	g	g	g	g	g	g	g	g	g	g	g	g	g	g	g	g	g	g	g	g	g
a	608801	g	610399	a	648835	ns	a	APEC	++++	a	a	a	a	a	a	a	a	a	a	a	a	a	a	a	a	a	a	a	a	a	a	a
1	617802	1	619410	-	657845	del	1	S88	++++	1	1	1	1	1	1	1	1	1	1	1	1	1	1	1	1	1	1	1	1	1	1	1
g	617910	a	619508	a	657943	ns	g	AS	++++	g	g	g	g	g	g	g	g	g	g	g	g	g	g	g	g	g	g	g	g	g	g	g
c	620352	a	621950	a	660385	ns	a	UTI89	++++	a	a	a	a	a	a	a	a	a	a	a	a	a	a	a	a	a	a	a	a	a	a	a
g	620584	g	622182	c	660617	ns	g	S88	++	g	g	g	g	g	a	g	a	a	a	a	a	a	a	a	a	a		a	a	a	a	a
-	625135	1	626734	-	665168	ins	-	APEC	++++	-	-	-	-	-	-	-	-	-	-	-	-	-	-	-	-	-	-	-	-	-	-	-
a	633552	a	635151	t	673585	ns	a	S88	++++	a	a	a	a	a	a	a	a	a	a		a	a	a	a	a	a	a	a	a	a	a	a
c	636819	t	638418	c	676852	s	c	APEC	++++	c	c	c	c	c	t	c	c	c	c		c	c	c	c	c	c	c	c	c	c	c	c
g	638264	a	639863	g	678297	ns	g	APEC	++++	g	g	g	g	g	g	g	g	g	g		g	g	g	g	g	g	g	g	g	g	g	g
c	638644	t	640243	t	678677	nc	t	UTI89	++++	t	t	t		t	t	t	t	t	t		t	t	t	t	t	t		t	t	t	t	t
-	646999	1	648599	-	687032	ins	-	APEC	++++	-	-	-	-	-	-	-	-	-	-	-	-	-	-	-	-	-	-	-	-	-	-	-
c	648209	t	649809	c	688242	s	c	APEC	++++	c	c	c	c	c	c	c	c	c	c	c	c	c	c	c	c	c	c	c	c	c	c	c
c	650006	c	651606	a	690039	s	c	S88	++++	c	c	c	c	c	c	c	c	c	c	c	c	c	c	c	c	c	c	c	c	c	c	c
t	659858	c	661458	c	699891	ns	c	UTI89	++++	c	c	c	c	c	c	c	c	c	c	c	c	c	c	c	c	c	c	c	c	c	c	c
a	661947	g	663547	g	701980	nc	a	AS	++++	a	a	a	a					a	a					a	a	a	a				a	a
g	662705	g	664305	a	702738	ns	g	S88	++++	g	g	g	g					g	g					g	g	g	g				g	g
c	663960	t	665560	t	703993	ns	c	AS	++++	c	c	c	c					c	c					c	c	c	c					c
c	666692	t	668292	c	706725	ns	c	APEC	++++	c	c	c	c	c	c	c	c	c	c	c	c	c	c	c	c	c	c	c	c	c	c	c
c	670946	t	672546	t	710979	s	c	AS	++++	c	c	c	c	c	c	c	c	c	c	c	c	c	c	c	c	c	c	c	c	c	c	c
1	680891	1	682501	-	720933	del	1	S88	++	1		1	1																			
c	682623	t	684223	t	722655	ns	t	UTI89	++	t		t	t																			
c	687798	t	689398	c	727830	s	c	APEC	++	c		c	c																			
a	699915	g	701515	a	739947	ns	a	APEC	++++	a	a	a	a	a	a		a	a			a	a	a	a	a	a	a	a	a	a	a	
t	703110	c	704710	c	743142	ns	c	UTI89	++++	c	c	c	c	c	c	c	c	c	c	c	c	c	c	c	c	c	c	c	c	c	c	c
a	712215	g	713815	g	752247	ns	g	UTI89	++++	g	g	g	g	g	g	g	g	g	g	g	g	g	g	g	g	g	g	g	g	g	g	g
g	714992	t	716592	t	755024	nc	t	UTI89	++++	t	t	t	t	t	t	t	t	t	t	t	t	t	t	t	t	t	t	t	t	t	t	t
a	726044	a	727644	g	766076	nc	a	S88	++++	a	a	a	a			a	a			a	a	a	a				a	a	a	a	a	a
t	730657	a	732257	t	770689	s	t	APEC	++++	t	t	t	t	t	t	t	t	t	t	t	t	t	t	t	t	t	t	t	t	t	t	t
t	731269	g	732869	t	771301	s	t	APEC	++++	t	t	t	t	t	t	t	t	t	t	t	t	t	t	t	t	t	t	t	t	t	t	t
g	734812	a	736412	g	774844	nc	g	APEC	++	g	g	g	a	a	a	a				a	a	a	a									
c	739894	t	741494	c	779926	s	c	APEC	++++	c	c	c	c	c	c	c	c	c	c	c	c	c	c	c	c	c	c	c	c	c	c	c
t	745093	t	746693	c	785125	nc	t	S88	++++	t	t	t	t	t	t	t	t	t	t	t	t	t	t	t	t	t	t	t	t	t	t	t
c	749029	t	750629	c	789061	s	c	APEC	++++	c	c	c	c	c	c	c	c	c	c	a	c	c	c	c	c	c	c	c	c	c	c	c
g	750117	a	751717	g	790149	s	g	APEC	++++	g	g	g	g	g	g	g	g	g	g	g	g	g	g	g	g	g	g	g	g	g	g	g
a	752334	t	753934	a	792366	s	a	APEC	++++	a	a	a	a	a	a	a	a	a	a	a	a	a	a	a	a	a	a	a	a	a	a	a
g	766017	g	767617	a	806049	ns	g	S88	++++	g	g	g	g	g		g	g	g	g		g	g	g	g	g	g		g	g	g		g
-	772403	1	774004	-	812435	ins	-	APEC	++++	-	-	-	-	-	-	-	-	-	-	-	-	-	-	-	-	-	-	-	-	-	-	-
c	772836	a	774437	a	812868	s	c	AS	++	c	c	c	c	t	c	t	c	c	c	c	t	t	t	c	c	c	c	c	c	c	c	c
t	779702	c	781303	c	819734	nc	c	UTI89	++++	c	c	c	c	c	c	t	c	c	c	c	c	c	c	c	c	c	c	c	c	c	c	c
c	779797	t	781398	t	819829	nc	c	AS	++	c	c	c	t	c	c	c	c	c	c	c	c	c	c	c	c	c	c	c	t	c	c	c
t	786791	c	788392	c	826823	s	c	UTI89	++++	c	c	c	c	c	c	c	c	c	c	c	c	c	c	c	c	c	c	c	c	c	c	c
c	787318	t	788919	c	827350	ns	c	APEC	++++	c	c	c	c	c	c	c	c	c	c	c	c	c	c	c	c	c	c	c	c	c	c	c
c	787666	t	789267	c	827698	s	c	APEC	++++	c	c	c	c	c	c	c	c	c	c	c	c	c	c	c	c	c	c	c	c	c	c	c
a	792683	c	794284	c	832715	ns	c	UTI89	++++	c	c	c	c	c	c	c	c	c	c	c	c	c	c	c	c	c	c	c	c	c	c	c
c	795077	t	796678	c	835109	ns	c	APEC	++++	c	c	c	c	c	c	c	c	c	c	c	c	c	c	c	c	c	c				c	c
c	795188	c	796789	t	835220	ns	c	S88	++++	c	c	c	c	c		c	c	c	c					c	c	c	c				c	c
t	799830	c	801431	c	839862	ns	c	UTI89	++++	c	c	c	c	c	c	c	c	c	c	c	c	c	c	c	c	c	c	c	c	c	c	c
c	803061	t	804662	c	843093	ns	c	APEC	++++	c	c	c	c	c	c	c	c	c	c	c	c	c	c	c	c	c	c	c	c	c	c	c
c	804050	c	805651	g	844082	ns	c	S88	++++	c	c	c	c	c	c	c	c	c	c	c	c	c	c	c	c	c	c	c	c	c	c	c
t	806581	c	808182	c	846613	ns	c	UTI89	++++	c	c	c	c	c	c	c	c	c	c	c	c	c	c	c	c	c	c	c	c	c	c	c
g	807561	t	809162	g	847593	ns	g	APEC	++++	g	g	g	g	g	g	g	g	g	g	g	g	g	g	g	g	g	g	g	g	g	g	g
a	810780	c	812381	c	850812	ns	c	UTI89	++	c	c	c	c	t	t	t	c	c	c	c	c	c	c	c	c	c	c	c	c	c	c	c
c	810812	t	812413	t	850844	ns	c	AS	++++	c	c	c	g	c	c	c	c	c	c	c	c	c	c	c	c	c	c	c	c	c	c	c
a	819100	g	820701	a	859132	s	a	APEC	++++	a	a	a	a	a	a	a	a	a	a	a	a	a	a	a	a	a	a	a	a	a	a	a
c	829836	t	831437	c	869868	ns	c	APEC	++++	c	c	c	c	c	c	c	c	c	c	c	c	c	c	c	c	c	c	c	c	c	c	c
t	830341	c	831942	t	870373	ns	t	APEC	++++	t	t	t	t	t	t	t	t	t	t	t	t	t	t	t	t	t	t	t	t	t	t	t
-	831313	1	832915	-	871345	ins	-	APEC	++++	-	-	-	-	-	-	-	-	-	-	-	-	-	-	-	-	-	-	-	-	-	-	-
t	834112	c	835714	t	874144	nc	t	APEC	++++	t	t	t	t	t	t	t	t	t	t		t	t	t	t	t	t	t	t	t	t	t	t
g	835947	a	837549	a	875979	s	a	UTI89	++++	a	a	a	a	a	a	a	a	a	a		a	a	a	a	a	a	a	a	a	a		a
c	843239	t	844841	t	883271	ns	t	UTI89	++++	t	t	t	t	t	t	t	t	t	t	t	t	t	t	t	t	t	t	t	t	t	t	t
c	849611	t	851213	t	889643	s	c	AS	++++	c	c	c	c	c	c	c	c	c	c	c	c	c	c	c	c	c	c	c	c	c	c	c
c	856209	c	857811	t	896241	ns	c	S88	++++	c	c	c	c	c	c	c	c	c	c	c	c	c	c	c	c	c	c	c	c	c	c	c
c	860894	t	862496	t	900926	ns	t	UTI89	++++	t	t	t	t	t	t	t	t	t	t	t	t	t	t	t	t	t	t	t	t	t	t	t
1	861711	1	863323	-	901752	del	1	S88	++	1	1	1	1	1	1	1	-	1	1	1	1	1	1	1	-	1	1	1	1	1	1	1
1	865313	-	866914	-	905343	del	1	AS	++++	1	1	1	1	1	1	1	1	1	1	1	1	1	1	1	1	1	1	1	1	1	1	1
t	867819	t	869420	a	907849	ns	t	S88	++++	t	t	t	t	t	t	t	t	t	t	t	t	t	t	t	t	t	t	t	t	t	t	t
g	874804	a	876405	a	914834	nc	a	UTI89	++++	a	a	a	a	a	a	a	a	a	a	a	a	a	a	a	a	a	a	a	a	a	a	a
a	877547	t	879148	a	917577	ns	a	APEC	++++	a	a	a	a	a	a	a	a	a	a	a	a	a	a	a	a	a	a	a	a	a	a	a
a	887400	g	888821	g	927250	ns	g	UTI89	++		g																					
a	898073	g	899494	g	937923	ns	g	UTI89	++++	g	g	g	g	g	g	g	g	g	g	g	g	g	g	g	g	g	g	g	g	g	g	g
a	899916	t	901337	t	939766	ns	t	UTI89	++++	t	t	t	t	t	t	t	t	t	t	t	t	t	t	t	t	t	t	t	t	t	t	t
g	902707	t	904128	g	942557	nc	g	APEC	++++	g	g	g	g	t	g	g	g	g	g	g	g	g	g	g	g	g	g	g	g	g	g	g
c	905103	a	906524	a	944953	ns	a	UTI89	++++	a	a	a	a	a	a	a	a	a	a	a	a	a	a	a	a	a	a	a	a	a	a	a
c	964418	t	963391	t	966974	s	c	AS	++	c	c	c	c	c	c	t	t	t	t	t	t	t	t	t	t	t	t	t	t	t	t	t
t	965722	c	964695	c	968278	s	c	UTI89	++++	c	c	c	c	c	c	c	c	c	c	c	c	c	c	c	c	c	c	c	c	c	c	c
c	970013	t	968986	c	972569	s	c	APEC	++	c	c	c	c	c	c	c	t	t	t	t	t	t	t	t	t	t	t	t	t	t	t	t
5	970685	-	969657	5	973241	del	5	APEC	++++	5	5	5	5	5	5	5	5	5	5	5	5	5	5	5	5	5	5	5	5	5	5	5
1	970847	-	969814	1	973403	del	1	APEC	++++	1	1	1	1	1	1	1	1	1	1	1	1	1	1	1	1	1	1	1	1	1	1	1

ExPEC Cluster										Outgroup Strains[g]																						
UTI89[a]	UTI89 site[b]	APEC 01[a]	APEC 01 site[b]	S88[a]	S88 site[b]	type[c]	Inferred ancestral base[d]	Lineage inferred to mutate[e]	Support level[f]	CFT073	ED1a	536	E2348/69	SMS 3-5	IAI39	UMN026	K-12	ATCC 8739	HS	D1 Sd197	CB9615	Sakai	EDL933	IAI1	E24377A	SE11	SS Ss046	F2a 301	F2a 2457T	F5 8401	B4 Sb227	B18 BS512
g	975980	a	974947	g	978536	ns	g	APEC	++++	g	g	g	g	g	g	g	g	g	g	g	g	g	g	g	g	g	g	g	g	g	g	g
c	977458	c	976425	t	980014	ns	c	S88	++++	c	c	c	c	c	c	c	c	c	c	c	c	c	c	c	c	c	c	c	c	c	c	c
t	978577	c	977544	c	981133	ns	c	UTI89	++++	c	c	c	c	c	c	c	c	c	c	c	c	c	c	c	c	c	c	c	c	c	c	c
c	980226	c	979193	t	982782	ns	c	S88	++++	c	c	c	c	c	c	c	c	c	c	c	c	c	c	c	c	c	c	c	c	c	c	c
c	997585	c	996552	a	1000141	nc	c	S88	++++	c		c	c			c								c	c	c	c	c	c	c		
t	1001866	c	1000833	c	1004422	s	c	UTI89	++++	c	c	c	c	c	c	c	c	c	c	c	c	c	c	c	c	c	c	c	c	c	c	c
a	1005106	g	1004073	a	1007662	nc	a	APEC	++++	a	a	a	a	a	a	a	a	a	a	a	a	a	a	a	a	a	a	a	a	a	a	a
c	1008991	t	1007958	t	1011547	s	c	AS	++++	c	c	c	c	c	c	c	c	c	c	c	c	c	c	c	c	c	c	c	c	c	c	c
a	1009299	a	1008266	g	1011855	nc	a	S88	++++	a	a	a	a	a	a	a	a	a	a	a	a	a	a	a	a	a	a	a	a	a	a	a
g	1017505	g	1016472	a	1020061	nc	g	S88	++	g	g	g	g	g	g	g	g	a	g	a	g	g	g	g	g	g	g	g	g	g	g	g
g	1023013	g	1021980	a	1025569	ns	g	S88	++++	g	g	g	g	g	g	g	g	g	g	g	g	g	g	g	g	g	g	g	g	g	g	g
g	1023423	t	1022390	t	1025979	s	t	UTI89	++	c	t	c	g	t	c	c	t	t	t	t	t	t	t	t	t	t	t	t	t	t	t	t
t	1027187	c	1026154	c	1029743	ns	c	UTI89	++++	c	c	c	c	c	c	c	c	c	c	c	c	c	c	c	c	c	c	c	c	c	c	c
1	1027219	1	1026196	-	1029784	del	1	S88	++++	1	1	1	1	1	1	1	1	1	1	1	1	1	1	1	1	1	1	1	1	1	1	1
t	1036972	c	1035939	t	1039527	ns	t	APEC	++++	t	t	t	t	t	t	t	t	t	t	t	t	t	t	t	t	t	t	t	t	t	t	t
10	1046266	-	1045232	-	1048820	del	10	AS	++	10	10	10	-	-	10	10	-	-	-	-	-	-	-	-	-	-	-	10	10	10	-	-
c	1050612	t	1049569	t	1053157	s	c	AS	++++	c	c	c	c	c	c	c	c	c	c	c	c	c	c	c	c	c	c	c	c	c	c	c
c	1070356	t	1069313	c	1072901	s	c	APEC	++++	c	c	c	c	c	c	c	c	c	c		c	c	c	c	c	c	c	c	c	c	c	
c	1071203	t	1070160	t	1073748	ns	c	AS	++++	c	c	c	c	c	c	c	c	c	c		c	c	c	c	c	c	c	c	c	c	c	
c	1071431	t	1070388	t	1073976	nc	t	UTI89	++++	t	t	t	t	t	t	t	t	t	t		t	t	t	t	t	t	t	t	t	t	t	
c	1077313	c	1076270	a	1079858	ns	c	S88	++++	c		c			c		c	c	c		c	c	c		c	c			c			
c	1077646	t	1076603	t	1080191	ns	c	AS	++++	c		c			c		c	c	c		c	c	c		c	c			c			
t	1158594	c	1095982	c	1099570	ns	c	UTI89	++++	c	c	c	c	c	c	c	c	c	c	c	c	c	c	c	c	c	c				c	c
g	1171909	t	1109297	t	1112885	ns	t	UTI89	++++	t	t	t	t	t	t	t	t	t	t	t	t	t	t	t	t	t	t	t	t	t	t	t
c	1176227	t	1113615	c	1117203	ns	c	APEC	++++	c	c	c	c	c	c	c	c	c	c	c	c	c	c	c	c	c	c	c	c	c	c	c
c	1179442	c	1116830	t	1120418	ns	c	S88	++++	c	c	c	c	c	c	c	c	c	c	c	c	c	c	c	c	c	c	c	c	c	c	c
c	1182008	t	1119396	c	1122984	ns	c	APEC	++++	c	c	c	c	c	c	c	c	c	c	c	c	c	c	c	c	c	c	c	c	c	c	c
a	1189065	g	1126453	g	1130041	s	g	UTI89	++++	g	g	g	g	g	g	g	g	g	g	g	g	g	g	g	g	g	g	g	g	g	g	g
a	1197653	g	1135041	g	1138629	ns	g	UTI89	++++	g	g	g	g	g	g	g	g	g	g	g	g	g	g	g	g	g	g	g	g	g	g	g
g	1206608	g	1143996	a	1147584	ns	g	S88	++++	g	g	g	g	g	g	g	g	g	g	g	g	g	g	g	g	g	g	g	g	g	g	g
c	1209196	t	1146584	c	1150172	ns	c	APEC	++++	c	c	c	c	c	c	c	c	c	c	c	c	c	c	c	c	c	c	c	c	c	c	c
t	1213900	g	1151288	g	1154876	nc	g	UTI89	++++	g	g	g	g	g	g	g	g	g	g	g	g	g	g	g	g	g	g	g	g	g	g	g
a	1214628	g	1152016	g	1155604	s	g	UTI89	++++	g	g	g	g	g	g	g	g	g	g	g	g	g	g	g	g	g	g	g	g	g	g	g
a	1214712	g	1152100	a	1155688	s	a	APEC	++++	a	a	a	a	a	a	a	a	a	a	a	a	a	a	a	a	a	a	a	a	a	a	a
t	1217161	c	1154549	t	1158137	ns	t	APEC	++++	t	t	t	t	t	t	t	t	t	t	t	t	t	t	t	t	t	t	t	t	t	t	t
c	1232787	t	1170175	c	1173763	ns	c	APEC	++++	c	c	c	c	c	c	c	c	c	c	c	c	c	c	c	c	c	c	c	c	c	c	c
g	1235068	a	1172456	a	1176044	s	g	AS	++++	g	g	g	g	g	g	g	g	g	g	g	g	g	g	g	g	g	g	g	g	g	g	g
g	1236103	g	1173491	a	1177079	s	g	S88	++++	g	g	g	g	g	g	g	g	g	g	g	g	g	g	g	g	g	g	g	g	g	g	g
c	1242049	t	1179437	t	1183025	s	c	AS	+++	c		c			c	c						c	c									
g	1247499	t	1184887	t	1188475	nc	t	UTI89	++	t											t								t			
1	1251028	-	1188415	1	1192004	del	1	APEC	++	1			-								-	1										
13	1252153	13	1189540	-	1193074	del	13	S88	+++	13	13		13			13					13	13	13									
c	1256059	t	1193446	t	1196968	s	t	UTI89	++++	t		t				t	t				t			t	t	t	t					
c	1257897	t	1195284	t	1198806	nc	c	AS	++	c		c				t	t				t			c	c	c	t	t	t	t		
1	1261320	-	1198616	1	1202229	del	1	APEC	+++	1		1			1	1					1	1										1
1	1261370	-	1198665	-	1202278	ins	-	UTI89	+++	-		-			-						1	-										-
a	1261667	c	1198962	c	1202575	ns	a	AS	+++	a		a	a		a						a	a										c
a	1265584	t	1202879	t	1206492	s	t	UTI89	+	t	a		t		a	a					t	a	a			a		a	a	a		a
t	1265659	c	1202954	c	1206567	s	c	UTI89	+	t	c		c		c	c					t	c	c			c		c	c	c		c
t	1268419	c	1205714	t	1209327	ns	c	APEC	-	t	c		c		c	t					t					c		c	c	c		c
g	1271732	a	1209027	g	1212640	ns	g	APEC	++++	g	g	g	g		g	g		g			g	g	g	g		g	g					g
g	1274273	a	1211568	a	1215181	s	a	UTI89	++	a	g	a	g		a	a		a			g	a	g	a		g	a					a
c	1275306	c	1212601	t	1216214	ns	c	S88	+	c	g	t	t	c	c	c		t			c	t	g	t		t	t	c	c	c	c	c
c	1278400	t	1215695	c	1219308	s	c	APEC	++	c	t	c	c	g	c	c		c	c		c	c	c	c		c	c	t	t	t	t	g
-	1279719	1	1217015	-	1220627	ins	-	APEC	++++	-	-	-	-	-	-	-		-	-		-	-	-	-		-	-	-	-	-	-	-
c	1285383	t	1222679	t	1226291	ns	t	UTI89	++	t	t	t		c	t	t				c							c	c	c	c	c	c
t	1285420	c	1222716	c	1226328	s	t	AS	++	t	t	t		c	t	t				c							c	c	c	c	c	c
a	1285687	g	1222983	g	1226595	ns	a	AS	++++	a	a	a		g	a	a				a							a	a	a	a	a	a
t	1285689	c	1222985	c	1226597	ns	t	AS	++++	t	t	t		c	t	t				t							t	t	t	t	t	t
t	1287152	g	1224448	t	1228060	ns	t	APEC	++	t					t	t																
t	1290845	c	1228141	c	1231753	ns	c	UTI89	++++	c	c	c	c	c	c	c	c	c	c		c			c	c	c	c					c
c	1299167	c	1237575	t	1241187	ns	c	S88	++++	c	c	c	c	c	c	c	c	c	c	c	c	c	c	c	c	c	c	c	c	c	c	c
c	1304124	t	1242532	c	1246144	ns	c	APEC	++++	c	c	c	c	c	c	c	c	c	c	c	c	c	c	c	c	c	c	c	c	c	c	c
c	1309829	t	1248237	t	1251849	s	c	AS	++++	c	c	c	c	c	c	c	c	c	c	c	c	c	c	c	c	c	c	c	c	c	c	c
t	1317484	c	1255892	c	1259504	s	c	UTI89	++++	c	c	c	c	c	c	c	c	c	c	c	c	c	c	c	c	c		c	c	c	c	c
a	1318229	c	1256637	c	1260249	s	c	UTI89	++++	c	c	c	c	c	c	c				c	c	c	c					c	c	c		
c	1318870	t	1257278	c	1260890	ns	c	APEC	++++	c	c	c	c	c	c	c				c	c	c	c					c	c	c		
c	1325784	t	1264192	c	1267804	ns	c	APEC	++++	c	c	c	c	c	c	c	c	c	c	c	c	c	c	c	c	c		c	c	c	c	c
c	1328119	c	1266527	a	1270139	ns	c	S88	++++	c	c	c	c	c	c	c	c	c	c		c	c	c	c	c	c		c	c	c	c	c
c	1329839	c	1268247	t	1271859	ns	c	S88	++++	c	c	c	c	c	c	c	c	c	c		c			c	c	c		c	c	c	c	c
g	1334663	a	1273071	g	1276683	s	g	APEC	++++	g	g	g	g	g	g	g	g	g	g	g	g	g	g	g	g	t	g	g	g	g	g	g
c	1335889	a	1274297	a	1277909	ns	a	UTI89	++++	a	a	a	a	a	a	a	a	a	a	a	a	a	a	a	a	a	a	a	a	a	a	a
c	1336581	t	1274989	c	1278601	s	c	APEC	++++	c	c	c	c	c	c	c	c	c	c	c	c	c	c	c	c	c	c	c	c	c	c	c
g	1339622	a	1278030	g	1281642	ns	g	APEC	++++	g	g	g	g	g	g	g	g	g	g	g	g	g	g	g	g	g	g	g	g	g	g	g
c	1343179	c	1281587	t	1285199	s	c	S88	++++	c	c	c	c	c	c	c	c	c	c	c	c	c	c	c	c	c	c	c	c	c	c	c
c	1344099	t	1282507	t	1286119	s	t	UTI89	++	t	t	t	t	t	t	c	c	c	c	c	c	c	c	c	c	c	c	c	c	c	c	c
g	1345497	a	1283905	g	1287517	ns	g	APEC	++++	g	g	g	g	g	g	g	g	g	g	g	g	g	g	g	g	g	g	g	g	g	g	g
g	1346113	g	1284521	a	1288133	s	g	S88	++++	g	g	g	g	g	g	g	g	g	g	g	g	g	g	g	g	g	g	g	g	g	g	g
c	1346192	a	1284600	g	1288212	nc	a	S88	++++	a	a	a	a	a	a	a	a	a	a	a	a	a	a	a	a	a	a	a	a	a	a	a
g	1347609	a	1286017	a	1289629	ns	g	AS	++++	g	g	g	g	g	g	g	g	g	g	g	g	g	g	g	g	g	g	g	g	g	g	g
g	1348340	c	1286748	g	1290360	ns	g	APEC	++++	g	g	g	g	g	g	g	g	g	g	g	g	g	g	g	g	g	g	g	g	g	g	g
t	1351411	c	1289819	c	1293431	s	c	UTI89	++++	c	c	c	c	c	c	c	c	c	c	c	c	c	c	c	c	c	c	c	c	c	c	c

ExPEC Cluster										Outgroup Strains[g]																						
UTI89[a]	UTI89 site[b]	APEC 01[a]	APEC 01 site[b]	S88[a]	S88 site[b]	type[c]	Inferred ancestral base[d]	Lineage inferred to mutate[e]	Support level[f]	CFT073	ED1a	536	E2348/69	SMS 3-5	IAI39	UMN026	K-12	ATCC 8739	HS	D1 Sd197	CB9615	Sakai	EDL933	IAI1	E24377A	SE11	SS Ss046	F2a 301	F2a 2457T	F5 8401	B4 Sb227	B18 BS512
c	1351918	c	1290326	t	1293938	s	c	S88	++++	c	c	c	c	c	c	c	c	c	c	c	c	c	c	c	c	c	c	c	c	c	c	t
t	1356504	t	1294912	c	1298524	ns	t	S88	++++	t	t	t	t	t	t	t	t	t	t	t	t	t	t	t	t	t	t	t	t	t	t	t
a	1356862	g	1295270	g	1298882	s	g	UTI89	++	g	g	g	g	g	g	g	a	a	a	a	a	a	a	a	a	a	a	g	g	g	a	a
c	1362182	t	1300590	c	1304202	nc	c	APEC	++++	c	c	c		c		c	c	c	c	c	c	c	c	c	c	c	c	c	c	c	c	c
t	1383331	g	1321739	g	1325351	ns	g	UTI89	++++	g	g	g	g	g	g	g	g	g	g	g	g	g	g	g	g	g	g	g	g	g	g	g
c	1383687	t	1322095	t	1325707	s	c	AS	++++	c	c	c	c	c	c	c	c	c	c	c	c	c	c	c	c	c	c	c	c	c	c	c
a	1396233	c	1334641	a	1338253	ns	a	APEC	+	-	a											a	a				a				a	
12	1396238	-	1334645	12	1338258	del	12	APEC	++	-	12											12	12				12				12	
c	1397119	a	1335515	a	1339139	ns	c	AS	++++	c	c		c		c	c		c			c			c	c	c	c				c	c
t	1397449	c	1335845	c	1339469	ns	?	UTI89/AS	+/-																							
-	1407410	1	1345332	1	1349440	ins	-	AS	++++	-	-		-	-	-	-	-	-	-	-	-	-	-	-				-	-	-	-	-
-	1409852	1	1347766	-	1351873	ins	-	APEC	++	-		-			-	1	1	1			1	-	1	1	1	1	1					-
c	1411572	t	1349486	c	1353593	s	c	APEC	+	t	c	a	c		c	c		a			t	c	c	a	a	t	a	c	c	c		c
g	1411629	t	1349543	t	1353650	s	g	AS	++	g	g	g	g		g	g		g			g	g	g	g	g	t	g	a	a	a		t
g	1411639	t	1349553	t	1353660	ns	g	AS	++	g	g	g	g		g	g		g			g	g	g	g	g	t	g	g	g	g		t
1	1411898	1	1349822	-	1353928	del	1	S88	++++	1	1	1	1		1	1		1			1	1	1	1	1	1	1	1	1	1		1
t	1414911	a	1352825	a	1356931	ns	a	UTI89	++	a	a		t		a	a					t	t	t			a		a	a	a		t
t	1415527	c	1353441	t	1357547	s	t	APEC	++	t	t		t		t	t					t	t	t			t		c	c	c		t
t	1416046	g	1353960	g	1358066	s	g	UTI89	++	g	g		g		g	t					g	g	g			g		g	g	g		t
g	1419751	g	1357665	a	1361762	ns	g	S88	++++	g	g	g	g		g	g		g			g	g	g	g		g	g					g
g	1419789	t	1357703	t	1361800	ns	t	UTI89	++	t	t	c	c		t	t		t			c	t	t	c		t	c					t
t	1422681	c	1360595	c	1364692	s	t	AS	++	t	g	t	t		t	t		t			g	g	g	t		t	t					t
c	1423327	c	1361241	a	1365338	ns	c	S88	++++	c	c	c	c		c	c		c			c	c	c	c		c	c					c
c	1423914	g	1361828	c	1365925	ns	c	APEC	++	c	c	c	c	t	c	c		c			c	a	a	c		c	c	c	c	c	c	c
a	1426400	g	1364314	g	1368411	s	g	UTI89	++++	g	g	g	g	g	g	g		g			g	g	g	g		g	g	g	g	g	g	a
a	1426526	g	1364440	g	1368537	s	a	AS	++	a	g	a	g	g	g	g		g			g	g	g	g		g	g	g	g	g	g	a
t	1426565	g	1364479	g	1368576	s	t	AS	++	t	c	t	c	g	t	g		g			t	c	c	t		g	t	t	t	t	t	t
c	1426574	t	1364488	t	1368585	s	c	AS	++	t	c	c	c	t	c	c		t			c	c	c	c		c	c	c	c	c	c	c
a	1426637	g	1364551	g	1368648	s	a	AS	++	a	c	a	c	a	a	a		a			a	c	c	a		a	a	a	a	a	a	a
g	1429230	g	1367144	a	1371241	ns	g	S88	++++	g	g		g	g	g	g		g	g		g	g	g	g		g	g	g	g	g	g	g
g	1447820	t	1385734	g	1389831	ns	g	APEC	++++	g	g	g	g	g	g	g	g	g	g	g	g	g	g	g	g	g	g	g	g	g	g	g
g	1453371	a	1391285	g	1395382	s	g	APEC	++++	g	g	g	g	g	g	g	g	g	g	g	g	g	g	g	g	g	g	g	g	g	g	g
g	1454280	g	1392194	a	1396291	s	g	S88	++++	g	g	g	g	g	g	g	g	g	g	g	g	g	g	g	g	g	g	g	g	g	g	g
c	1458464	c	1396378	t	1400475	s	c	S88	++++	c	c	c	c	c	c	c	c	c	c	c	c	c	c	c	c	c	c	c	c	c	c	c
c	1462538	g	1400452	g	1404549	ns	c	AS	++++	c	c	c	c	c	c	c	c	c	c	c	c	c	c	c	c	c	c	c	c	c	c	c
-	1462662	1	1400586	1	1404683	del	1	UTI89	++++	-	1	1	1	1	1	1	1	1	1	1	1	1	1	1	1	1	1	1	1	1	1	1
c	1463574	t	1401489	c	1405586	ns	c	APEC	++++	c	c	c	c	c	c	c	c	c	c	c	c	c	c	c	c	c	c	c	c	c	c	c
g	1464840	a	1402755	a	1406852	ns	a	UTI89	++++	a	a	a	a	a	a	a	a	a	a	a	a	a	a	a	a	a	a	a	a	a	a	a
g	1465094	a	1403009	a	1407106	s	g	AS	++++	g	g	g	g	g	g	g	g	g	g	g	g	g	g	g	g	g	g	g	g	g	g	g
-	1467769	1	1405685	-	1409781	ins	-	APEC	++	-	-	-	1	1	-	1	1	-	1	1	1	1	1	1	-	1	-	-	-	-	1	1
a	1468756	g	1406672	g	1410768	ns	g	UTI89	++++	g	g	g	g	g	g	g	g	g	g	g	g	g	g	g	g	g	g	g	g	g	g	g
c	1472422	t	1410338	t	1414434	ns	c	AS	++++	c	c	c	c	c	c						c	c	c									
a	1473133	g	1411049	g	1415145	s	g	UTI89	++++	g	g	g	g	g	g						g	g	g									
a	1474417	g	1412333	a	1416429	ns	a	APEC	++++	a	a	a	a	a	a					a	a	a	a									
g	1477730	t	1415646	t	1419742	ns	g	AS	++	g	g	g	g	g	g					a	a	a	a									
g	1484184	t	1422100	t	1426196	ns	g	AS	++++	g	g	g	g	g	g	g	g	g	g	g	g	g	g	g	g	g	g	g	g	g	g	g
g	1485942	g	1423858	a	1427954	nc	g	S88	++++	g	g	g	g	g	g	g	g	g	g	g	g	g	g	g	g	g	g	g	g	g	g	g
g	1487497	g	1425413	a	1429509	s	g	S88	++++	g	g	g	g	g	g	g	g	g	g	g	g	g	g	g	g	g	g	g	g	g	g	g
c	1494997	t	1432913	t	1437009	ns	c	AS	++++	c	c	c	c	c	c		c	c	c		c	c	c	c	c	c	c	c	c	c		
g	1496430	t	1434346	t	1438442	s	g	AS	++	g	g	g	g	g	g		g	g	g		g	g	g	g	g	g	g	a	a	a		
t	1500217	c	1438133	c	1442229	ns	c	UTI89	++++	c	c	c	c	c	c		c	c	c		c	c	c	c	c	c	c	c	c	c		
t	1501794	c	1439710	c	1443806	ns	t	AS	++++	t	t	t	t	t	t		t	t	t		t	t	t	t	t	t	t	t	t	t		t
g	1504398	a	1442314	a	1446410	ns	a	UTI89	++++	a	a	a	a	a	a	a	a	a	a	a	a	a	a	a	a	a	a	a	a	a	a	a
t	1508902	c	1446818	c	1450914	ns	c	UTI89	++++	c	c	c	c	c	c	c	c	c	c	c	c	c	c	c	c	c	c	c	c	c	c	c
1	1519257	-	1457172	1	1461269	del	1	APEC	++++	1	1	1	1							1								1	1	1		
g	1521868	a	1459783	a	1463880	ns	g	AS	+++		g	g	g															g	g	g		
a	1538087	g	1528329	g	1480099	nc	g	UTI89	++++	g	g	g	g	g	g	g	g	g	g	g	g	g	g	g	g	g	g	g	g	g	g	g
a	1541057	g	1531299	a	1483069	ns	a	APEC	++++	a	a	a	a	a	a	a	a	a	a	a	a	a	a	a	a	a	a	a	a	a	a	a
g	1544148	-	1534072	t	1486160	ns	t	UTI89	++	t	t	t																				
c	1550029	c	1538948	a	1492041	s	a	S88	-	a	c	a		c	c	t	g	g	g		g	g	g									
c	1550527	a	1539446	a	1492539	ns	c	AS	++++	c	c	c		c	c	c	t	c	c		c	c	c									
c	1552364	a	1541283	a	1494376	ns	a	UTI89	++	a		a																				
a	1554726	g	1543645	a	1496738	ns	a	APEC	++++	a		a		a	a	a	a	a	a	a	a	a	a	a	a	a	a	a	a	a	a	a
a	1555817	a	1544736	g	1497829	s	a	S88	++++	a		a		a	a	a	a	a	a	a	a	a	a	a	a	a	a	a	a	a	a	a
t	1570842	c	1559761	c	1512854	s	c	UTI89	++++	c	c	c	c	c		c	c	c	c	c	c	c	c	c	c	c	t	c	c	c	c	c
c	1575287	a	1564206	a	1517299	ns	a	UTI89	++++	a	a	a	a	a		a	a	a	a	a	a	a	a	a	a	a	a	a	a	a	a	a
a	1578748	c	1567667	c	1520760	ns	a	AS	++++	a	a	a	a	a		a	a	a	a	a	a	a	a	a	a	a	a					
c	1584726	c	1573645	t	1526738	ns	c	S88	++++	c	c	c	c	c		c	c	c	c	c	c	c	c	c	c	c	c					
g	1588277	g	1577196	t	1530289	ns	g	S88	++++	g	g	g	g	g		g	g	g		g	g	g	g	g	g	g	g	g	g	g	g	g
g	1590601	g	1579520	a	1532613	ns	g	S88	++++	g	g	g	g	g		g	g	g	g	g	g	g	g	g	g	g	g				g	g
t	1594231	c	1583150	c	1536243	ns	c	UTI89	++++	c	c	c	c	c		c	c	c	c	c	c	c	c	c	c	c	c				c	c
1	1601057	-	1589975	-	1543068	del	1	AS	+++	1	1	1				1																
c	1611629	t	1600547	c	1553640	ns	c	APEC	++++	c	c	c	c	c	c	c	c	c	c	c	c	c	c	c	c	c	c	c	c	c	c	c
a	1619057	t	1607975	a	1561068	ns	a	APEC	++++	a	a	a	a	a	a	a	a	a	a	a	a	a	a	a	a	a	a	a	a	a	a	a
c	1624073	t	1612991	c	1566084	nc	c	APEC	+++	c	c	c	c																			
g	1627270	a	1616188	a	1569281	s	g	AS	++++	g	g	g	g	g	g	g	g	g	g	g	g	g	g	g	g	g	g	g	g	g	g	g
g	1628087	a	1617005	g	1570098	ns	g	APEC	++++	g	g	g	g	g	g	g	g	g	g	g	g	g	g	g	g	g	g	g	g	g	g	g
a	1635824	a	1624742	g	1577835	ns	a	S88	++++	a		a	a	a	a	a	a	a	a		a	a	a	a	a	a	a	a	a	a	a	a
g	1640268	t	1629186	g	1582279	s	g	APEC	++++	g	g	g	g	g	g	g	g	g	g		g	g	g	g	g	g	g	g	g	g	g	
t	1643287	c	1632205	c	1585298	ns	c	UTI89	++++	c	c	c	c	c	c	c	c	c	c	c	c	c	c	c	c	c	c	c	c	c	c	c
c	1645178	c	1634096	a	1587189	ns	c	S88	++++	c	c	c	c	c	c	c	c	c	c	c	c	c	c	c	c	c	c	c	c	c	c	c
t	1646225	t	1635143	c	1588236	nc	t	S88	++++	t	t	t	t	t	t	-	t	t	t	t	t	t	t	t	t	t	t	t	t	t	t	t

ExPEC Cluster										Outgroup Strains[g]																						
UTI89[a]	UTI89 site[b]	APEC 01[a]	APEC 01 site[b]	S88[a]	S88 site[b]	type[c]	Inferred ancestral base[d]	Lineage inferred to mutate[e]	Support level[f]	CFT073	ED1a	536	E2348/69	SMS 3-5	IAI39	UMN026	K-12	ATCC 8739	HS	D1 Sd197	CB9615	Sakai	EDL933	IAI1	E24377A	SE11	SS Ss046	F2a 301	F2a 2457T	F5 8401	B4 Sb227	B18 BS512
c	1647470	t	1636388	-	1588975	nc	c	APEC	++++	c			c		c	c	c	c	c		c	c	c	c	c	c	c					
a	1647836	g	1638650	-	1588975	ns	g	UTI89	++++	g			g	g		g					g	g	g	g	g	g						
t	1648653	c	1639467	-	1588975	ns	t	APEC	++++	t			t	t		t					t	t	t	t	t	t						
t	1648718	c	1639532	-	1588975	ns	c	UTI89	++++	c			c	c		c					c	c	c	c	c	c						
c	1652927	g	1643741	c	1590946	nc	c	APEC	++	c		c																				
c	1664424	t	1645651	c	1592856	nc	c	APEC	++	c		c	c																			
c	1664436	c	1645663	a	1592868	nc	c	S88	++	c		c	c																			
c	1664699	a	1645926	c	1593131	ns	c	APEC	++++	c		c	c	c	c	c	c	c	c	c	c	c	c	c	c	c		c	c	c		c
t	1664807	a	1646034	t	1593239	ns	t	APEC	++++	t		t	t	t	t	t	t	t	t	t	t	t	t	t	t	t		t	t	t		t
g	1666384	g	1647611	a	1594816	nc	g	S88	++++	g	g	g	g	g	g	g	g	g	g	g	g	g	g	g	g	g	g	g	g	g		g
c	1666390	t	1647617	t	1594822	nc	t	UTI89	++++	t	t	t	t	t	t	t	t	t	t	t	t	t	t	t	t	t	t	t	t	t		t
c	1680794	c	1662021	t	1609226	ns	c	S88	+++	c				c	c				c					c	c	c						
a	1683458	c	1664685	c	1611890	ns	a	AS	++++	a	a	a	a	a	a	a	a	a	a	a	a	a	a	a	a	a		a	a	a		
a	1683785	g	1665012	g	1612217	ns	g	UTI89	++++	g	g	g	g	g	g	g	g	g	g	g	g	g	g	g	g	g		g	g	g		
c	1684103	t	1665330	t	1612535	ns	c	AS	++++	c	c	c	c	c	c	c	c	c	c	c	c	c	c	c	c	c		c	c	c		
t	1692892	g	1674119	t	1621324	ns	t	APEC	++++	t	t	t	t	t	t	t	t	t	t	t	t	t	t	t	t	t	t	t	t	t	t	t
g	1692976	a	1674203	a	1621408	nc	g	AS	++++	g	g	g	g	g	g	g	g	g	g	g	g	g	g	g	g	g		g	g	g	g	g
t	1695714	g	1676941	g	1624146	nc	g	UTI89	++	g	g	g	g	g	g	g	a	a	a	g	g	g	g	a	g	a		g	g	g		
t	1696400	c	1677627	t	1624832	s	t	APEC	++++	t	t	t	t	t	t	t	t	t	t	t	t	t	t	t	t	t		t	t	t		
t	1697022	c	1678249	c	1625454	s	c	UTI89	++	c	c	c	c	c	c	c	t	t	t	c	c	c	c	t	t	t		c	c	c	c	c
c	1704035	t	1685262	t	1632467	s	c	AS	++++	c	c	t	c	c	c	c	c	c	c	c	c	c	c	c	c	c	c	c	c	c	c	c
-	1705691	1	1686928	1	1634133	ins	-	AS	++++	-	-	-	-	-	-	-	-	-	-	-	-	-	-	-	-	-	-	-	-	-	-	-
c	1708228	t	1689456	c	1636661	ns	c	APEC	++++	c	c	c	c	c	c	c	c	c	c	c	c	c	c	c	c	c	c	c	c	c	c	c
g	1730334	g	1711562	t	1658767	ns	g	S88	++++	g	g	g	g	g	g	g	g	g	g	g	g	g	g	g	g	g	g	g	g	g	g	g
g	1732984	t	1714212	g	1661417	ns	g	APEC	++	a	g	g	g	a	a	g	g	g	g		g	g	g	g	g	g	g	g	g	g	g	
c	1739534	a	1720762	a	1667967	ns	c	AS	++++	c	c	c	c	c	c	c	c	c	c	c	c	c	c	c	c	c	c	c	c	c	c	c
g	1739912	a	1721140	g	1668345	ns	g	APEC	++++	g	g	g	g	g	g	g	g	g	g	g	g	g	g	g	g	g	g	g	g	g	g	g
g	1741790	g	1723018	a	1670223	ns	g	S88	++++	g	g	g	g	g	g	g	g	g	g	g	g	g	g	g	g	g	g	g	g	g	g	g
g	1746426	a	1727654	a	1674859	s	g	AS	++++	g	g	g	g	g	g	g	g	g	g	g	g	g	g	g	g	g	g	g	g	g	g	g
g	1748153	a	1729381	g	1676586	ns	g	APEC	++++	g	g	g	g	g	g	g	g	g	g	g	g	g	g	g	g	g	g	g	g	g	g	g
g	1751902	a	1733130	a	1680335	ns	a	UTI89	++++	a	a	a	a	a	a	a	a	a	a	a	a	a	a	a	a	a	a	a	a	a	a	a
t	1759335	t	1740563	g	1687768	ns	t	S88	++++	t	t	t	t	t	t	t	t	t	t	t	t	t	t	t	t	t	t	t	t	t	t	t
1	1761945	-	1743172	1	1690378	del	1	APEC	++++	1	1	1	1	1	1	1	1	1	1	1	1	1	1	1	1	1	1	1	1	1	1	1
g	1768156	a	1749383	a	1696589	s	a	UTI89	++	a	a	a	a	g	g	g	g	g	g	g	g	g	g	g	g	g	g	g	g	g	g	g
g	1775636	a	1756863	g	1704069	nc	g	APEC	++++	g	g	g	g	g	g	g	g	g	g	g	g	g	g	g	g	g	g	g	g	g	g	g
a	1778602	g	1759829	a	1707035	s	a	APEC	++++	a	a	a	a	a	a	a	a	a	a	a	a	a	a	a	a	a	a	a	a	a	a	a
g	1781707	a	1762934	g	1710140	ns	g	APEC	++++	g	g	g	g	g	g	g	g	g	g	g	g	g	g	g	g	g	g	g	g	g	g	g
g	1783839	a	1765066	g	1712272	ns	g	APEC	++++	g	g	g	g	g	g	g	g	g	g	g	g	g	g	g	g	g	g	g	g	g	g	g
c	1785512	t	1766739	t	1713945	ns	c	AS	++++	c	c	c	c	c	c	c	c	c	c	c	c	c	c	c	c	c	c	c	c	c	c	c
g	1787340	g	1768567	t	1715773	ns	g	S88	++++	g	g	g	g	g	g	g	g	g	g	g	g	g	g	g	g	g	g	g	g	g	g	g
t	1794103	c	1775330	c	1722536	ns	c	UTI89	++++	c	c	c	c	c	c	c	c	c	c	c	c	c	c	c	c	c	c	c	c	c	c	c
a	1798374	g	1779601	g	1726807	s	a	AS	++++	a	a	a	a	a	a	a	a	a	a	a	a	a	a	a	a	a	a	a	a	a	a	a
c	1800257	a	1781484	c	1728690	nc	c	APEC	++++	c	c	c	c	c	c	c	c	c	c		c	c	c	c	c	c	c	c	c	c	c	c
c	1800617	t	1781844	c	1729050	nc	c	APEC	++++	c	c	c	c	c	c	c	c	c	c	c	c	c	c	c	c	c	c	c	c	c	c	c
c	1802868	t	1784095	c	1731301	s	c	APEC	++++	c	c	c	c	c	c	c	c	c	c	c	c	c	c	c	c	c	c	c	c	c	c	c
g	1804968	g	1786195	a	1733401	ns	g	S88	++++	g	g	g	g	g	g	g	g	g	g	g	g	g	g	g	g	g	g	g	g	g	g	g
c	1806368	t	1787595	c	1734801	ns	c	APEC	++++	c	c	c	c	c	c	c	c	c	c	c	c	c	c	c	c	c	c	c	c	c	c	c
c	1809223	a	1790450	c	1737656	ns	c	APEC	++++	c	c	c	c	c	c	c	c	c	c	c	c	c	c	c	c	c	c	c	c	c	c	c
c	1812687	a	1793914	a	1741120	ns	a	UTI89	++++	a	a	a	a	a	a	a	a	a	a	a	a	a	a	a	a	a					a	a
t	1819916	g	1801143	g	1748349	ns	g	UTI89	++	g	g	g	g	g	g	g	a	a		a	a	a	a	a	a	a	a	a	a	a	a	a
g	1823196	a	1804423	g	1751629	s	g	APEC	++++	g	g	g	g	g	g	g	g	g	g	g	g	g	g	g	g	g	g	g	g	g	g	g
t	1827729	g	1808956	g	1756162	nc	t	AS	++	t	t	t	t	t	g	t	t	t	g	t	t	t	t	t	t	t	t	g	g	g	g	t
c	1843632	t	1824859	c	1772065	ns	c	APEC	++	c	c	c	c	c	c	c	c	c	c	c	t	t	t	c	c	c	c	c	c	c	c	c
t	1843776	c	1825003	c	1772209	ns	t	AS	++++	t	t	t	t	t	t	t	t	t	t	t	t	t	t	t	t	t	t	t	t	t	t	t
c	1844646	a	1825873	a	1773079	ns	c	AS	++++	c	c	c	c	c	c	c	c	c	c	c	c	c	c	c	c	c	c	c	c	c	c	c
g	1847036	g	1828263	a	1775469	ns	g	S88	++	g	g	g	g	g	g	g	a	a	a	a	a	a	a	g	g	g	g	g	g	g	g	g
c	1847348	c	1828575	t	1775781	ns	c	S88	++++	c	c	c	c	c	c	c	c	c	c		c	c	c	c	c	c	c	c	c	c		
a	1854587	a	1835814	g	1783020	s	a	S88	++++	a	a	a	a	a	a	a	a	a	a		a	a	a	a	a	a	a	a	a	a		
c	1858337	t	1839564	c	1786770	s	c	APEC	++++	c	c	c	c	c	c	c	c	c	c	c	c	c	c	c	c	c	c	c	c	c	c	c
1	1863003	-	1844229	1	1791436	del	1	APEC	++++	1	1	1	1	1	1	1	1	1	1	1	1	1	1	1	1	1	1	1	1	1	1	1
g	1872442	g	1853668	a	1800875	ns	g	S88	++++	g	g	g	g	g	g	g	g	g	g	g	g	g	g	g	g	g	g	g	g	g	g	g
c	1879115	t	1860341	t	1807548	s	c	AS	++++	c	c	c	c	c	c	c	c	c	c	c	c	c	c	c	c	c	c	c	c	c	c	c
g	1881762	a	1862988	a	1810195	ns	g	AS	++++	g	g	g	g	g	g	g	g	g	g	g	g	g	g	g	g	g	g	g	g	g	g	g
a	1885568	g	1866794	a	1814001	ns	a	APEC	++++	a	a	a	a	a	a	a	a	a	a		a	a	a	a	a	a		a	a	a		
1	1889056	-	1870281	1	1817489	del	1	APEC	++++	1	1	1	1	1	1	1	1	1	1		1	1	1	1	1	1		1	1	1		
g	1889829	g	1871054	a	1818262	s	g	S88	++++	g	g	g	g	g	g	g	g	g	g		g	g	g	g	g	g		g	g	g		
g	1891522	g	1872747	a	1819955	ns	g	S88	++++	g	g	g	g	g	g	g	g	g	g	g	g	g	g	g	g	g		g	g	g		
g	1901202	g	1882427	a	1829635	nc	g	S88	++++	g	g	g	g	g	g	g	g	g	g		g	g	g	g	g	g	g				g	g
c	1902531	a	1883756	c	1830964	nc	c	APEC	++	c	c	c	c	c	c	c	t	t	t		c	c	c	t	t	t	t				t	t
t	1902667	t	1883892	c	1831100	ns	t	S88	++++	t	t	t	t	t	t	t	t	t	t		t	t	t	t	t	t	t				t	t
g	1902708	a	1883933	a	1831141	ns	g	AS	++++	g	g	g	g	g	g	g	g	g	g		g	g	g	g	g	g	g				g	g
c	1904928	c	1886153	t	1833361	ns	c	S88	++++	c	c	c	c	c	c	c	c	c	c		c	c	c	c	c	c	c	c		c	c	c
g	1911685	a	1892910	g	1840118	ns	g	APEC	++++	g	g	g	g	g	g	g	g	g	g	g	g	g	g	g	g	g	g				g	g
t	1913372	c	1894597	c	1841805	ns	c	UTI89	+++	c	c	c	c																			
t	1916481	t	1897706	c	1844914	s	t	S88	++++	t	c	t	t	t	t	t	t	t	t	t	t	t	t	t	t	t	t	t	t	t	t	t
c	1937418	t	1918643	c	1865851	s	c	APEC	++++	c	c	c	c	c	c	t	c	c	c	c	c	c	c	c	c	c	c	c	c	c	c	c
g	1943761	g	1924986	t	1872194	ns	g	S88	++++	g	g	g	g	g	g	g	g	g	g	g	g	g	g	g	g	g	g	g	g	g	g	g
c	1945586	a	1926811	a	1874019	ns	c	AS	++++	c	c	c	c	c	c	c	c	c	c	c	c	c	c	c	c	c	c	c	c	c	c	c
c	1952288	t	1933513	c	1880721	ns	c	APEC	++++	c	c	c	c	c	c	c	c	c	c	c	c	c	c	c	c	c	c	c	c	c	c	c
g	1957518	a	1938743	g	1885951	nc	g	APEC	++++	g	g	g	g	g	g	g	g	g	g	g	g	g	g	g	g	g	g	g	g	g	g	g
g	1958928	g	1940153	t	1887361	ns	g	S88	++++	g	g	g	g	g	g	g	g	g	g	g	g	g	g	g	g	g	g	g	g	g	g	g
g	1962154	a	1943379	g	1890587	s	g	APEC	++++	g	g	g	g	g	g	g	g	g	g	g	g	g	g	g	g	g	g	g	g	g	g	g

ExPEC Cluster										Outgroup Strains[g]																						
UTI89[a]	UTI89 site[b]	APEC 01[a]	APEC 01 site[b]	S88[a]	S88 site[b]	type[c]	Inferred ancestral base[d]	Lineage inferred to mutate[e]	Support level[f]	CFT073	ED1a	536	E2348/69	SMS 3-5	IAI39	UMN026	K-12	ATCC 8739	HS	D1 Sd197	CB9615	Sakai	EDL933	IAI1	E24377A	SE11	SS Ss046	F2a 301	F2a 2457T	F5 8401	B4 Sb227	B18 BS512
a	1968251	c	1949476	a	1896684	ns	a	APEC	++++	a	a	a	a	a	a	a	a	a	a	a	a	a	a	a	a	a	a	a	a	a	a	a
-	1972956	1	1954182	-	1901389	ins	-	APEC	++	-	-	1	-	-	-	-	-	-	-	-	-	-	-	-	-	-	1	-	-	-	-	-
1	1983458	-	1964683	1	1911891	del	1	APEC	++++	1	1	1	1	1	1	1	1	1	1	1	1	1	1	1	1	1	1	1	1	1		1
a	1984824	g	1966049	g	1913257	nc	g	UTI89	++++	g	g	g	g	g	g	g	g	g	g	g	g	g	g	g	g	g	g	g	g	g	g	g
a	1985538	g	1966763	a	1913971	s	a	APEC	++++	a	a	a	a	a	a	a	a	a	a	a	a	a	a	a	a	a	a	a	a	a	a	a
c	1989086	c	1970311	t	1917519	ns	c	S88	++++	c	c	c	c	c	c	c	c	c	c	c	c	c	c	c	c	c		c	c	c	c	c
t	1992160	g	1973385	g	1920593	ns	g	UTI89	++++	g	g	g	g	g	g	g	g	g	g		g	g	g	g	g	g	g	g	g	g		
-	2015743	1	1996978	1	1944186	del	1	UTI89	++++	1	1	1	1	1	1	1	1	1	1	1	1	1	1	1	1	1	1	1	1	1	1	1
g	2015758	t	1996984	t	1944192	nc	g	AS	++++	g	g	g	g	g	g	g	g	g	g	g	g	g	g	g	g	g	g	g	g	g	g	g
t	2017127	t	1998353	g	1945561	ns	t	S88	++++	t	t	t	t	t	t	t	t	t	t	t	t	t	t	t	t	t	t	t	t	t	t	t
c	2017589	t	1998815	t	1946023	ns	c	AS	++++	c	c	c	c	c	c	c	c	c	c	c	c	c	c	c	c	c	c	c	c	c	c	c
c	2028889	c	2010115	t	1957323	s	c	S88	++++	c		c	c	c	c	c	c	c	c	c	c	c	c	c	c	c		c	c	c	c	c
c	2034723	t	2015949	c	1963157	nc	c	APEC	++	c	c	c				c	a				c			t	t	t	c	c	c	c		
c	2034796	t	2016022	c	1963230	nc	c	APEC	++++	c	c	c				c	c				c			c	c	c	c	c	c	c		
-	2040303	1	2021530	-	1968737	ins	-	APEC	++++	-	-	-	-	-	-	-	-	-	-		-	-	-	-	-	-	-	-	-	-	-	
g	2041278	g	2022505	a	1969712	ns	g	S88	++++	g	g	g	g	g	g	g	g	g	g		g	g	g	g	g	g	g	g	g	g	g	
c	2042435	t	2023662	c	1970869	s	c	APEC	++++	c	c	c	c	c	c	c	c	c	c		c	c	c	c	c	c	c	c	c	c	c	
g	2042640	a	2023867	g	1971074	ns	g	APEC	++++	g	g	g	g	g	g	g	g	g	g		g	g	g	g	a	g	g	g	g	g	g	
a	2049617	g	2030844	g	1978051	s	g	UTI89	++++	a	g	g	g	g	g	g	g	g	g		g	g	g	g	g	g	g	g	g	g	g	g
5	2049890	5	2031127	-	1978333	del	5	S88	++	5	5	5	5	5	5	5	5	5	5		5	5	5	5	5	5	-	-	-	-	-	5
g	2050425	t	2031652	t	1978854	ns	t	UTI89	++++	t	t	t	t	t	t	t	t	t	t		t	t	t	t	t	t	t	t	t	t	t	t
g	2054234	a	2035461	g	1982663	nc	g	APEC	++	g	g	g	g	g	g	g	g	a	a	a	g	g	g	a	g	g	g	g	g	g	g	g
c	2055661	c	2036888	t	1984090	ns	c	S88	++++	c	c	c	c	c	c	c	c	c	c	c	c	c	c	c	c	c	c	c	c	c	c	c
g	2058158	c	2039385	c	1986587	s	c	UTI89	++++	c	c	c	c	c	c	c	c	c	c	c	c	c	c	c	c	c	c	c	c	c	c	c
a	2060140	a	2041367	c	1988569	ns	a	S88	++++	a	a	a	a	a	a	a	a	a			a	a	a	a	a	a	a				a	a
g	2060419	g	2041646	a	1988848	ns	a	S88	-	a	g	a	a		a	g	g	g			g	g	g	g	g							g
1	2061420	-	2042646	1	1989849	del	-	APEC	-	-	-	-	-	-	-	-	-	-	-		-	-	-	-	-	-	-	-	-	-		-
1	2061411	-	2042646	-	1989849	ins	-	UTI89	++++	-	-	-	-	-	-	-	-	-	-		-	-	-	-	-	-	-	-	-	-		-
g	2069960	t	2090717	g	1998388	nc	g	APEC	++++	g	g	g			g	g		g										g	g	g	c	g
g	2074868	t	2095625	t	2003296	ns	g	AS	+++	g	g	g			g	g																
a	2080256	g	2101013	g	2008684	s	g	UTI89	+++	g	g	g			g	g																
c	2085418	g	2106175	c	2013846	ns	c	APEC	+++	c	c	c			c	c																
2	2094224	-	2114980	2	2022652	del	2	APEC	+++	2	2	2			2	2																
g	2109392	a	2130146	a	2037819	s	g	AS	++++	g	g	g	g	g	g	g	g	g	g		g	g	g	g	g	g	g	g	g	g	g	g
a	2109607	g	2130361	g	2038034	nc	a	AS	++	a	g	a	g	g	g	g	g	g	g		g	g	g	g	g	g	g	g	g	g	g	g
t	2109879	c	2130633	c	2038306	s	t	AS	++	t	c	t	c	c	c	c	c	c	c	c	c	c	c	c	c	c	c	c	c	c	c	c
-	2110732	3	2131496	3	2039169	del	3	UTI89	++++	3	3	3	3	3	3	3	3	3	3	3	3	3	3	3	3	-	3	3	3	3	3	3
g	2279775	a	2229281	a	2139199	s	g	AS	++++	g	g	g	g	g	g	g	g	g	g	g	g	g	g	g	g	g	g	g	g	g	g	g
g	2281766	a	2231272	a	2141190	s	g	AS	++++	g	g	g	g	g	g	g	g	g	g	g	g	g	g	g	g	g	g	g	g	g	g	g
g	2282840	a	2232346	a	2142264	s	g	AS	++	g	g	g	g	g	g	g	g	g	g	a	g	a	a	g	g	g	g	g	g	g	g	g
g	2283782	a	2233288	a	2143206	s	g	AS	++	g	g	g	a	a	a	a	g	g	g	a	a	a	a	g	g	g	a	g	g	g	g	a
g	2284861	a	2234367	a	2144285	s	g	AS	++++	g	g	g	a	g	g	g	g	g	g	g	g	g	g	g	g	g	g	g	g	g	g	g
g	2284877	a	2234383	a	2144301	s	g	AS	++++	g	g	g	g	g	g	g	g	g	g	g	g	g	g	g	g	g	g	g	g	g	g	g
g	2285554	a	2235060	a	2144978	ns	g	AS	++++	g	g	g	g	g	g	g	g	g	g	g	g	g	g	g	g	g	g	g	g	g	g	g
g	2287961	a	2237467	a	2147385	ns	g	AS	++++	g	g	g	g	g	g	g	g	g	g	g	g	g	g	g	g	g	g	g	g	g	g	g
c	2291758	t	2241264	t	2151182	s	c	AS	++++	c	c	c	c	c	c	c	c	c	c		c	c	c	c	c	c	c	c	c	c	c	c
a	2292182	t	2241688	t	2151606	ns	t	UTI89	++++	t	t	t	t	t	t	t	t	t	t		t	t	t	t	t	t	t	t	t	t	t	t
c	2293329	c	2242835	a	2152753	s	c	S88	++	t	c	t	c	c	c	c	c	c	c		c	t	t	c	c	c	c	c	c	c	c	c
g	2298541	t	2248047	t	2157965	ns	g	AS	++++	g	g	g	g	g	g	g	g	g	g		g	g	g	g	g	g	g	g	g	g	g	g
c	2299628	a	2249134	c	2159052	ns	c	APEC	++++	c	c	c	c	c	c	c	c	c	c	c	c	c	c	c	c	c	c	c	c	c	c	c
t	2300902	g	2250408	g	2160326	ns	g	UTI89	++++	g	g	g	g	g	g	g	g	g	g	g	g	g	g	g	g	g	g	g	g	g	g	g
1	2304194	-	2253699	1	2163618	del	1	APEC	++	1	1	1																				
c	2308823	t	2258328	t	2168247	s	c	AS	++++	c	c	c	c	c	c	c	c	c	c	c	c	c	c	c	c	c	c	c	c	c	c	c
t	2313552	g	2286328	g	2209375	ns	g	UTI89	++++	g		g	g	g	g	g	g	g	g	g	g	g	g	g	g	g	g	g	g	g	g	g
c	2314974	c	2287750	t	2210797	ns	c	S88	++++	c		c	c	c	c	c	c	c	c	c	c	c	c	c	c	c	c	c	c	c	c	c
c	2315277	c	2288053	t	2211100	ns	c	S88	++++	c		c	c	c	c	c	c	c	c	c	c	c	c	c	c	c	c	c	c	c	c	c
g	2318549	t	2291325	t	2214372	s	t	UTI89	++	t		t	t	g	g	g	a	g	g	a	g	g	g	a	a	g	a	g	g	g	g	g
g	2319019	t	2291795	t	2214842	ns	t	UTI89	++++	t		t	t	t	t	t	t	t	t	t	t	t	t	t	t	t	t	t	t	t	t	t
g	2321900	a	2294676	a	2217723	ns	g	AS	++++	g	g	g	g	g	g	g	g	g	g	g	g	g	g	g	g	g	g	g	g	g	g	g
g	2323696	g	2296472	a	2219519	s	g	S88	++++	g	g	g	g	g	g	g	g	g	g	g	g	g	g	g	g	g	g	g	g	g	g	g
c	2333054	a	2305830	a	2228877	nc	a	UTI89	+	-	-	-	a	a	-	-	-	-	-		-	a	a	-	-	-	-	-	a	-		a
-	2333050	1	2305836	1	2228883	ins	-	AS	++++	-	-	-	-	-	-	-	-	-	-		-	-	-	-	-	-	-	-	-	-		-
1	2341206	-	2313982	1	2237030	del	1	APEC	++++	1	1	1	1	1	1	1	1	1	1	1	1	1	1	1	1	1		1	1	1	-	
t	2345837	c	2318613	t	2241661	s	t	APEC	++++	t	t	t	t	t	t	t	t	t	t		t	t	t	t	t	t		t		t	t	
g	2355032	a	2327808	a	2250856	s	g	AS	++	g	g	g	g	g	g	g	a	g	g	g	a	a	a	g	g	g	g	g	g	g	g	g
t	2359972	g	2332748	g	2255796	ns	g	UTI89	++++	g	g	g	g	g	g	g	g	g	g	g	g	g	g	g	g	g	g	g	g	g	g	g
a	2369563	c	2342339	c	2265387	ns	c	UTI89	++++	c	c	c	c	c	c	c	c	c	c	c	c	c	c	c	c	c	c	c	c	c	c	c
1	2369854	1	2342640	-	2265687	del	1	S88	++++	1	1	1	1	1	1	1	1	1	1	1	1	1	1	1	1	1	1	1	1	1	1	1
g	2372910	a	2345686	g	2268733	ns	g	APEC	++++	g	g	g	g	g	g	g	g	g	g	g	g	g	g	g	g	g	g	g	g	g	g	g
g	2378043	a	2350819	g	2273866	s	g	APEC	++++	g	g	g	g	g	g	a	g	g	g	g	g	g	g	g	g	g	g	g	g	g	g	g
g	2379747	c	2352523	g	2275570	ns	g	APEC	++	g	a	g	g	g	g	g	g	a	a	g	g	g	g	a	g	a	a	a	a	a	a	g
g	2380063	c	2352839	c	2275886	ns	c	UTI89	++++	c	c	c	c	c	c	c	c	c	c	c	c	c	c	c	c	c	c	c	c	c	c	c
t	2381779	c	2354555	t	2277602	nc	t	APEC	++++	t	t	t	t	t	t	t	t	t	t	t	t	t	t	t	t	t	t	t	t	t	t	t
g	2385703	g	2358479	c	2281526	ns	g	S88	++	g	g	g	g	g	g	g	g	g	g	g	g	g	g	g	g	g	g	-	g	-	g	g
11	2388201	-	2360901	11	2284024	del	11	APEC	++++	11	11	11	11	11	11	11	11	11	11	11	11	11	11	11	11	11	11	11	11	11	11	11
g	2389021	g	2361711	a	2284844	s	g	S88	++++	g	g	g	g	g	g	g	g	g	g	g	g	g	g	g	g	g	g	g	g	g	g	g
t	2398148	g	2370838	t	2293971	ns	t	APEC	++++	t	t	t	t	t	t	t	t	t	t		t	t	t	t	t	t	t	t	t	t	t	t
t	2403611	g	2376301	g	2299434	nc	g	UTI89	++++	g	g	g	g	g	g	g	g	g	g	g	g	g	g	g	g	g	g	g	g	g	g	g
a	2408101	g	2380791	g	2303924	s	g	UTI89	++++	g	g	g	g	g	g	a	g	g	g	g	g	g	g	g	g	g	g	g	g	g	g	g
c	2409158	t	2381848	c	2304981	ns	c	APEC	++++	c	c	c	c	c	c	c	c	c	c	c	c	c	c	c	c	c	c	c	c	c	c	c
c	2427434	c	2400124	t	2323257	ns	c	S88	++++	c	c	c	c	c	c	c	c	c	c	c	c	c	c	c	c	c	c	c	c	c	c	c
t	2427990	c	2400680	c	2323813	s	c	UTI89	++++	c	c	c	c	c	c	c	c	c	c	c	c	c	c	c	c	c	c	c	c	c	c	c

ExPEC Cluster										Outgroup Strains[g]																						
UTI89[a]	UTI89 site[b]	APEC 01[a]	APEC 01 site[b]	S88[a]	S88 site[b]	type[c]	Inferred ancestral base[d]	Lineage inferred to mutate[e]	Support level[f]	CFT073	ED1a	536	E2348/69	SMS 3-5	IAI39	UMN026	K-12	ATCC 8739	HS	D1 Sd197	CB9615	Sakai	EDL933	IAI1	E24377A	SE11	SS Ss046	F2a 301	F2a 2457T	F5 8401	B4 Sb227	B18 BS512
a	2430345	g	2403035	g	2326168	s	g	UTI89	++++	g	g	g	g	g	g	g	g	g	g	g	g	g	g	g	g	g	g	g	g	g	g	g
a	2440930	c	2413620	c	2336753	ns	c	UTI89	++++	c	c	c	c	c	c	c	c	c	c	c	c	c	c	c	c	c	c	c	c	c	c	c
g	2444432	g	2417122	a	2340255	s	g	S88	++++	g	g	g	g	g	g	g	g	g	g	g	g	g	g	g	g	g	g	g	g	g	g	g
t	2447839	c	2420529	c	2343662	ns	c	UTI89	++++	c	c	c	c	c	c	c	c	c	c	c	c	c	c	c	c	c	c	c	c	c	c	c
t	2449542	c	2422232	c	2345365	nc	c	UTI89	++++	c	c	c	c	c	c	c	c	c	c	c	c	c	c	c	c	c	c	c	c	c	c	c
t	2455337	a	2428027	t	2351160	ns	t	APEC	++++	t	t	t	t	t	t	t	t	t	t	t	t	t	t	t	t	t	t	t	t	t	t	t
c	2458675	t	2431365	c	2354498	s	c	APEC	++++	c	c	c	c	c	c	c	c	c	c								c					
a	2459593	a	2432283	g	2355416	nc	a	S88	++++	a	a	a	a	a	a	a	a	a	a								a					
c	2462718	t	2435408	c	2358541	ns	c	APEC	++++	c	c	c	c	c	c	c	c	c	c								c					
g	2464283	a	2436973	a	2360106	ns	a	UTI89	++++	a	a	a	a	a	a	a	a	a	a	a	a	a	a	a	a	a	a	a	a	a	a	
a	2465047	t	2437737	t	2360870	ns	t	UTI89	++++	t	t	t	t	t	t	t	t	t	t	t	t	t	t	t	t	t	t	t	t	t	t	
t	2472015	c	2444705	c	2367838	s	c	UTI89	++++	c	c	c	c	c	c	c	c	c	c		c	c	c	c	c	c	c	c	c	c		
c	2478525	t	2451215	c	2374348	ns	c	APEC	++++	c	c	c	c	c	c	c	c	c	c	c	c	c	c	c	c	c	c	c	c	c	c	c
g	2479957	g	2452647	a	2375780	s	g	S88	++	g	g	g	g	g	g	g	a	a	a	g	g	g	g	a	a	a	a	a	a	a		
t	2486813	c	2459503	c	2382636	ns	c	UTI89	++++	c	c	c	c	c	c	c	c	c	c	c	c	c	c	c	c	c	c	c	c	c	c	c
c	2495295	c	2467985	t	2391118	ns	c	S88	++++	c	c	c	c	c	c	c	c	c	c	c	c	c	c				c				c	
g	2499387	c	2472077	g	2395210	s	g	APEC	++++	g	g	g	g	g	g	g	g	g	g		g	g	g	g	g	g	g	g	g	g		
g	2499412	g	2472102	a	2395235	ns	g	S88	++++	g	g	g	g	g	g	g	g	g	g		g	g	g	g	g	g	g	g	g	g		
g	2501780	g	2474470	a	2397603	nc	g	S88	++++	g	g	g	g	g	g	g	g	g	g		g	g	g	g	g	g	g	g	g	g	g	
g	2506961	g	2479651	a	2402784	ns	g	S88	++++	g	g	g	g	g	g	g	g	g	g	g	g	g	g	g	g	g	g	g	g	g	g	g
g	2513997	a	2486687	a	2409820	ns	a	UTI89	++++	a	a	a	a	a	a	a	a	a	a	a	a	a	a	a	a	a	a	a	a	a	a	a
g	2520004	a	2492694	a	2415827	s	g	AS	++	g	g			g	g		a		a													
c	2520350	c	2493040	t	2416173	ns	c	S88	++++	c	c			c	c	a	c	c	c									c		c		
g	2523083	a	2495773	g	2418906	s	g	APEC	++	g	g	g	g	g	g	g	a	a	a	g	g	g	g	g	g	a	a	g	g	g	g	g
c	2529680	t	2502370	c	2425503	s	c	APEC	++	c	c	c	c	t	t	c	t	t	c	t	c	c	c	c	c	c	c	t	t	t	t	t
g	2536918	a	2509608	g	2432741	s	g	APEC	++++	g	g	g	g	g	g	g	g	g	g	g	g	g	g	g	g	g	g	g	g	g	g	g
1	2537290	1	2509990	-	2433122	del	1	S88	++++	1	1	1	1	1	1	1	1	1	1	1	1	1	1	1	1	1	1	1	1	1	1	1
c	2538598	a	2511288	c	2434420	ns	c	APEC	++++	c	c	c	c	c	c	c	c	c	c	c	c	c	c	c	c	c	c	c	c	c	c	c
t	2539456	c	2512146	c	2435278	nc	t	AS	++++	t	t	t	t	t	t	t	t	t	t	t	t	t	t	t	t	t	t	t	t	t	t	t
c	2539472	a	2512162	a	2435294	nc	a	UTI89	++++	a	a	a	a	a	a	a	a	a	a	a	a	a	a	a	a	a	a	a	a	a	a	a
a	2542370	a	2515060	t	2438192	ns	a	S88	++++	a	a	a	a	a	a	a	a	a	a	a	a	a	a	a	a	a	a	a	a	a	a	a
t	2544083	c	2516773	t	2439905	ns	t	APEC	++++	t	t	t	t	t	t	t	t	t	t	t	t	t	t	t	t	t	t	t	t	t	t	t
g	2544642	a	2517332	g	2440464	s	g	APEC	++++	g	g	g	g	g	g	g	g	g	g	g	g	g	g	g	g	g	g	g	g	g	g	g
t	2546525	c	2519215	t	2442347	s	t	APEC	++++	t	t	t	t	t	t	t	t	t	t	t	t	t	t	t	t	t	t	t	t	t	t	t
g	2558064	a	2530754	g	2453886	nc	g	APEC	++++	g	g	g	g	g	g	g	g	g	g	g	g	g	g	g	g	g	g	g	g	g	g	g
c	2558406	t	2531096	t	2454228	ns	c	AS	++++	c	c	c	c	c	c	c	c	c	c	c	c	c	c	c	c	c	c	c	c	c	c	c
g	2559034	g	2531724	c	2454856	nc	g	S88	++++	g	g	g	g	g	g	g	g	g	g	g	g	g	g	g	g	g	g	g	g	g	g	g
t	2560145	c	2532835	c	2455967	nc	c	UTI89	++++	c	c	c	c	c	c	c	c	c	c	c	c	c	c	c	c	c	c	c	c	c	c	c
g	2566551	g	2539241	a	2462373	ns	g	S88	++++	g	g	g	g	g	g	g	g	g	g	g	g	g	g	g	g	g	g	g	g	g	g	g
g	2576669	g	2549359	a	2472491	ns	g	S88	++++	g	g	g	g	g	g	g	g	g	g	g	g	g	g	g	g	g	g	g	g	g	g	g
g	2577824	a	2550514	a	2473646	s	g	AS	++	g	g	g	g	c	c	g	g	g	g	g	g	g	g	g	g	g	g	g	g	g	g	g
c	2577950	a	2550640	a	2473772	ns	a	UTI89	++++	a	a	a	a	a	a	a	a	a	a	a	a	a	a	a	a	a	a	a	a	a	a	a
a	2580563	g	2553253	g	2476385	s	g	UTI89	++	g	g	g	g	g	g	c	c	c	g	c	c	c	c	g	g	g	g	g	g	g	g	g
g	2587809	a	2560499	g	2483631	s	g	APEC	+++	g	g	g		g	g																	
g	2589925	g	2562615	a	2485747	ns	g	S88	++++	g	g	g	g	g	g	g	g	g	g	g	g	g	g	g	g	g	g	g	g	g	g	g
g	2593016	t	2565706	t	2488838	nc	t	UTI89	++++	t	t	t	t	t	t	t	t	t	t	t	t	t	t	t	t	t	t	t	t	t	t	t
a	2597240	g	2569930	g	2493062	nc	a	AS	++++	a	a	a	a	a	a	a	a	a	a	a	a	a	a	a	a	a	a	a	a	a	a	a
-	2599715	a	2574151	g	2497248	nc	g	APEC	+						g																	
c	2609921	t	2583147	t	2506244	ns	t	UTI89	+										t									t	t	t		
a	2617087	g	2590313	g	2513410	s	g	UTI89	+										g													
g	2622681	a	2595907	g	2519004	nc	g	APEC	+				g						-										g			
c	2623429	c	2596655	a	2519752	ns	c	S88	++		c		c								c											
t	2629944	c	2603846	t	2526943	nc	c	APEC	-										c		c											
-	2630659	1	2604562	-	2527658	ins	-	APEC	+										-		-											
t	2630681	c	2604584	c	2527680	nc	c	UTI89	+										c		c											
a	2630724	g	2604627	g	2527723	nc	g	UTI89	+										g		g											
a	2630763	a	2604666	g	2527762	ns	a	S88	+										a		g											
a	2630792	c	2604695	c	2527791	s	c	UTI89	+										c		c											
a	2633676	g	2606211	g	2529307	s	a	AS	+				a																			
t	2633712	a	2606247	a	2529343	s	g	UTI89/AS	+/-				g																			
1	2635747	1	2608292	-	2531387	del	?	S88	+/-																							
-	2636859	1	2609404	1	2532499	indel	?	UTI89/AS	+/-																							
-	2637847	1	2610393	1	2533488	del	1	UTI89	+				1				1										1					
c	2638205	a	2610742	a	2533837	s	a	UTI89	++++	a	a	c		a	a		a	a	a													
g	2644591	g	2617128	a	2540223	ns	g	S88	++++	g	g	g	g	g	g	g	g	g	g	g	g	g	g	g	g	g	g	g	g	g	g	g
c	2644802	t	2617339	t	2540434	nc	t	UTI89	++++	t	t	t	t	t	t	t	t	t	t	t	t	t	t	t	t	t	t	t	t	t	t	t
c	2649184	c	2621721	t	2544816	s	c	S88	++++	c	c	c	c	c	c	c	c	c	c	c	c	c	c	c	c	c	c	c	c	c	c	c
a	2655229	c	2627766	a	2550861	s	a	APEC	++	a	c	a	a	g	g	g	g	g	g	g	g	g	g	g	g	g	g	g	g	g	g	g
c	2668786	t	2641323	c	2564418	s	c	APEC	++++	c	c	c	c	c	c	a	c	c	c	c	c	c	c	c	c	c	c	c	c	c	c	c
t	2673574	c	2646111	c	2569206	s	c	UTI89	++++	c	c	c	c	c	c	c	c	c	c	c	c	c	c	c	c	c	c	c	c	c	c	c
g	2677456	g	2649993	a	2573088	s	g	S88	++++	g	g	g	g	g	g	g	g	g	g	g	g	g	g	g	g	g	g	g	g	g	g	g
c	2682832	c	2655369	t	2578464	ns	c	S88	++++	c	c	c	c	c	c		c									c						
c	2684105	a	2656642	a	2579737	ns	a	UTI89	++++	a	a	a	a	a	a		a									a						
a	2692136	g	2664673	g	2587768	nc	g	UTI89	++++	g	g	g	g	g	g	g	g	g	g	g	g	g	g	g	g	g	g	g	g	g	g	g
a	2702909	g	2675446	g	2598541	s	g	UTI89	++	g	g	g	g	g	g	g	g	g	g	g	g	g	g	g	g	g	g	c	c	c	c	c
1	2721896	1	2694443	-	2617537	del	1	S88	++++	1	1	1	1	1	1	1	1	1	1		1	1	1	1	1	1	1				1	1
a	2722335	g	2694872	g	2617966	ns	g	UTI89	++++	g	g	g	g	g	g	g	g	g	g		g	g	g	g	g	g	g				g	g
c	2723746	c	2696283	t	2619377	s	c	S88	++++	c	c	c	c	c	c	c	c	c	c		c	c	c	c	c	c	c				c	c
g	2726504	a	2699041	a	2622135	s	g	AS	++++	g	g	g	g	g	g	g	g	g	g	g	g	g	g	g	g	g	g	g	g	g	g	g
c	2738418	t	2710955	c	2634049	s	c	APEC	++++	c	c	c	c	c	c	c	c	c	c	c	c	c	c	c	c	c	c	c	t	c	c	c
a	2751430	g	2723967	g	2647061	s	g	UTI89	++++	g	g	g	g	g	g	g	g	g	g	g	g	g	g	g	g	g	g	g	g	g	g	g
c	2752707	a	2725244	a	2648338	ns	c	AS	++++	c	c	c	c	c	c	c	c	c	c	c	c	c	c	c	c	c	c	c	c	c	c	c

ExPEC Cluster										Outgroup Strains[g]																						
UTI89[a]	UTI89 site[b]	APEC 01[a]	APEC 01 site[b]	S88[a]	S88 site[b]	type[c]	Inferred ancestral base[d]	Lineage inferred to mutate[e]	Support level[f]	CFT073	ED1a	536	E2348/69	SMS 3-5	IAI39	UMN026	K-12	ATCC 8739	HS	D1 Sd197	CB9615	Sakai	EDL933	IAI1	E24377A	SE11	SS Ss046	F2a 301	F2a 2457T	F5 8401	B4 Sb227	B18 BS512
a	2753341	g	2725878	g	2648972	ns	g	UTI89	++++	g	g	g	g	g	g	g	g	g	g	g	g	g	g	g	g	g	g	g	g	g	g	g
-	2762640	1	2735178	-	2658271	ins	-	APEC	++++	-	-	-	-	-	-	-	-	-	-	-	-	-	-	-	-	-	-	-	-	-	-	-
g	2762859	g	2735397	a	2658490	ns	g	S88	++++	g	g	g	g	g	g	g	g	g	g	g	g	g	g	g	g	g	g	g	g	g	g	g
g	2769663	a	2781967	g	2665294	ns	g	APEC	++++	g	g	g	g	g	g	g	g	g	g	g	g	g	g	g	g	g	g	g	g	g	g	g
a	2775794	g	2788098	g	2671425	s	g	UTI89	++	g	g	g		c	c	g																
a	2777934	c	2790238	a	2673565	ns	a	APEC	+++	a	a	a		a	a	a																
-	2780261	-	2792574	1	2675902	ins	-	S88	+++	-	-	-		-		-																
g	2787629	g	2799960	t	2683288	s	g	S88	++	g	g	g	g	g	g	g	g	g	g	g	g	g	g	g	a	a	a	a	a	a	g	g
c	2793020	c	2805351	t	2688679	s	c	S88	++++	c	c	c	c	c	c	c	c	c	c	c	c	c	c	c	c	c	c	c	c	c	c	c
c	2802982	a	2815313	c	2698641	s	c	APEC	++	c	c	c	c	c	c	c	c	c	c	c	c	c	c	t	c	t	c	t	t	t	c	c
a	2804210	g	2816541	g	2699869	s	g	UTI89	++++	g	g	g	g	g	g	g	g	g	g	g	g	g	g	g	g	g	g	g	g	g	g	g
g	2807343	a	2819674	g	2703002	s	g	APEC	++++	g	g	g	g	g	g	g	g	g	g	g	g	g	g	g	g	g	g	g	g	g	g	g
t	2812613	c	2824944	c	2708272	ns	c	UTI89	++++	c	c	c	c	c	c	c	c	c	c	c	c	c	c	c	c	c	c	c	c	c	c	c
t	2828498	t	2840829	c	2724157	s	t	S88	++	g	t	g	g	g	g	g	g	g	g	g	g	g	g	g	g	g	g	g	g	g	g	g
a	2829805	g	2842136	g	2725464	ns	g	UTI89	++++	g	g	g	g	g	g	g	g	g	g	g	g	g	g	g	g	g	g	g	g	g	g	g
-	2837323	1	2849664	1	2732992	ins	-	AS	++++	-	-	-	-	-	-	-	-	-	-	1	-	-	-	-	-	-	-	-	-	-	-	-
-	2837332	1	2849665	-	2732992	ins	-	APEC	++++	-	-	-	-	-	-	-	-	-	-	1	-	-	-	-	-	-	-	-	-	-	-	-
c	2838730	c	2851063	t	2734390	ns	c	S88	++++	c	c	c	c	c	c	c	c	c	c	c	c	c	c	c	c	c	c	c	c	c	c	c
t	2850761	c	2863094	c	2746421	ns	t	AS	++++	t	t	t	t	t	t	t	t	t	t	t	t	t	t	t	t	t	t	t	t	t	t	t
t	2852244	c	2864577	c	2747904	ns	c	UTI89	++++	c	c	c	c	c	c	c	c	c	c	c	c	c	c	c	c	c	c	c	c	c	c	c
t	2863908	c	2876245	c	2759568	ns	c	UTI89	++++	c	c	c	c	c	c	c	c	c	c	c	c	c	c	c	c	c	c	c	c	c	c	c
t	2867376	g	2879713	g	2763036	ns	g	UTI89	++	g	g	g	a	-	-	a	a	a	a	a	a	a	a	a	a	a	a	a	a	a	a	a
c	2868366	c	2883292	a	2766612	ns	c	S88	+++		c	c							c					c	c	c						c
c	2870448	a	2885374	a	2768694	ns	a	UTI89	++	a	a	a	a	c	a	a	c	c	c	c	c	a	a	a	a	a	c	c	c	c	c	c
-	2870517	-	2885396	1	2768769	ins	-	S88	++	-	1	-	-	-	-	-	-	-	-	-	-	-	-	-	-	1	-	-	-	-	1	1
6	2870527	-	2885396	6	2768770	del	-	APEC	-	-	-	-	6	-	-	-	-	-	6	-	-	-	-	-	-	-	-	6	6	6	-	-
1	2870533	-	2885396	-	2768775	ins	-	UTI89	++	-	-	-	1	-	-	-	-	-	1	-	-	-	-	-	-	-	-	1	1	1	-	-
c	2875411	Y	2890436	t	2773653	nc	c	AS	++	y	c	c	c	t	t	t	t	t	t	t	t	t	t	t	t	t	t	t	t	t	t	t
1	2875535	-	2890559	-	2773776	ins	-	UTI89	++	-	-	-	-	-	-	-	-	-	-	-	-	-	-	-	-	-	-	-	-	-	1	1
c	2880020	t	2895044	t	2778261	ns	c	AS	++++	c	c	c	c	c	c	c	c	c	c	c	c	c	c	c	c	c	c	c	c	c	c	c
t	2885116	c	2900140	c	2783357	ns	c	UTI89	++++	c	c	c	c	c	c	c	c	c	c	c	c	c	c	c	c	c	c	c	c	c	c	c
-	2889163	1	2904188	-	2787404	ins	-	APEC	++++	-	-	-	-	-	-	-	-	-	-	-	-	-	-	-	-	-	-	-	-	-	-	-
-	2889193	1	2904219	-	2787434	ins	-	APEC	++++	-	-	-	-	-	-	-	-	-	-	-	-	-	-	-	-	-	-	-	-	-	-	-
a	2893261	c	2908287	c	2791502	ns	c	UTI89	++++	c	c	c	c	c	c	c	c	c	c	c	c	c	c	c	c	c	c	c	c	c	c	c
-	2905562	1	2920598	-	2837908	ins	?	APEC	+/-																							
c	2909584	t	2924611	c	2841921	ns	c	APEC	+						c	c	c												c			
-	2925805	-	2940841	1	2858152	ins	?	S88	+/-																							
c	2926306	t	2941333	c	2858644	ns	?	APEC	+/-																							
g	2926461	g	2941488	t	2858799	nc	?	S88	+/-																							
g	2928128	a	2943155	g	2860466	ns	?	APEC	+/-																							
a	2929713	a	2944740	g	2862051	s	a	S88	+	g	a				a	a					a											a
g	2932318	a	2947345	g	2864656	ns	g	APEC	+++		g				t		g				g											g
a	2935734	g	2950761	g	2868072	nc	a	AS	+						a		a														g	g
g	2942184	a	2957211	a	2874522	ns	?	UTI89/AS	+/-																							
c	2943722	c	2958749	t	2876060	nc	c	S88	++++	c	c	c	c	c	c	c	c	c	c		c	c	c	c	c	c	c	c	c	c	c	c
a	2952549	c	2967576	c	2884887	s	c	UTI89	++++	c	c	c	c	c	c	c	c	c	c	c	c	c	c	c	c	c	c	c	c	c	c	c
1	2953755	-	2968781	1	2886093	del	1	APEC	++	1	1	1	1	1	-	1	1	1	1	1	1	1	1	1	1	1	1	1	1	1	1	-
c	2954500	t	2969526	t	2886838	nc	t	UTI89	++++	t	t	t	t	t	t	t	t	t	t	t	t	t	t	t	t	t	t	t	t	t	t	t
t	2954673	g	2969699	t	2887011	s	t	APEC	++	t	t	t	t	t	t	t	c	c	c	c	c	c	c	t	t	t	c	t	t	t	t	t
g	2967118	a	2982144	g	2899456	ns	g	APEC	++++	g	g	g	g	g	g	g	g	g	g	g	g	g	g	g	g	g	g	g	g	g	g	g
g	2967565	g	2982591	a	2899903	ns	g	S88	++++	g	g	g	g	g	g	g	g	g	g	g	g	g	g	g	g	g	g	g	g	g	g	g
g	2968203	t	2983229	t	2900541	s	g	AS	++++	g	g	g	g	g	g	g	g	g	g	g	g	g	g	g	g	g	g	g	g	g	g	g
g	2970741	c	2985767	g	2903079	ns	g	APEC	++++	g	g	g	g	g	g	g	g	g	g	g	g	g	g	g	g	g	g	g	g	g	g	g
c	2971888	c	2986914	t	2904226	ns	c	S88	++	c			c							c												
-	2976803	1	2991839	1	2909151	ins	-	AS	++	-	-	-	1	-	-		-	-	-		-	-	-		1		1	-	-	-		
2	2976830	-	2991856	-	2909168	del	2	AS	++	-	2	2	-	2	2		2	2	2		2	2	2	-	-		-	2	2	2		
t	2979147	c	2994313	t	2911625	s	t	APEC	++++	t	t	t	t	t	t	t	t	t	t	t	t	t	t	t	t	t	t	t	t	t	t	t
t	2981026	g	2996192	t	2913504	s	t	APEC	++++	t	t	t	t	t	t	t	t	t	t	t	t	t	t	t	t	t	t	t	t	t	t	t
c	2993804	t	3008970	c	2926282	s	c	APEC	++++	c	c	c	c	c	c	c	c	c	c	c	c	c	c	c	c	c	c	c	c	c	c	c
a	2997877	g	3013043	a	2930355	nc	a	APEC	++++			a		a	a	a	a	a	a	a	a	a	a	a	a	a	a		a		a	a
9	3001650	-	3016815	-	2934127	ins	-	UTI89	++++	-	-	-		-						-				-	-		-	-				
c	3003596	c	3018753	a	2936065	ns	c	S88	++++	c	c	c	c	c	c	c	c	c	c	c	c	c	c	c	c	c	c		c		c	c
c	3005526	g	3020683	c	2937995	s	c	APEC	++++	c	c	c	c	c	c	c	c	c	c	c	c	c	c	c	c	c	c	c	c	c	c	c
c	3006193	a	3021350	a	2938662	ns	c	AS	++++	c	c	c	c	c	c	c	c	c	c	c	c	c	c	c	c	c	c	c	c	c	c	c
a	3009573	c	3024730	c	2942042	nc	c	UTI89	++	c	c	c	c	c	c	c	t	t	t	c	t	t	t	t	t	t	c	t	t	t	c	c
g	3015436	t	3030593	t	2947905	ns	g	AS	++++	g	g	g	g	g	g	g	g	g	g	g	g	g	g	g	g	g	g	g	g	g	g	g
t	3021643	c	3036221	t	2953533	ns	t	APEC	++++	t	t	t	t				t	t	t					t	t	t	t	t	t	t	t	t
g	3030738	t	3045316	t	2962628	ns	t	UTI89	++++	t	t	t	t	t	t	t	t	t	t	t	t	t	t	t	t	t	t	t	t	t	t	t
g	3034771	a	3049349	g	2966661	s	g	APEC	++++	g	g	g	g	g	g	g	g	g	g	g	g	g	g	g	g	g	g	g	g	g	g	g
g	3035957	a	3050535	g	2967847	s	g	APEC	++++	g	g	g	g	g	g	g	g	g	g	g	g	g	g	g	g	g	g	g	g	g	g	g
c	3049339	t	3063917	c	2981229	s	c	APEC	++++	c	c	c	c	c	c	c	c	c	c	c	c	c	c	c	c	c	c	c	c	c		c
9	3049410	-	3063924	9	2981300	del	9	APEC	++++	9	9	9	9	9	9	9	9	9	9	9	9	9	9	9	9	9	9	9	9	9		9
a	3052565	g	3067071	a	2984455	ns	a	APEC	++++	a	a	a	a	a	a	a	a	a	a	a	a	a	a	a	a	a	a					
1	3053060	1	3067576	-	2984959	del	1	S88	++	1	1	-	1	-	1	1	1	1	1	-	1	1	1	1	1	1	-					
12	3057750	12	3072266	-	2989648	del	-	S88	-	-	-	-		-		-					-	-	-	-								
a	3057930	g	3072436	g	2989807	ns	g	UTI89	++++	g	g	g		g		g				g	g	g	g	g								
-	3058856	1	3073363	-	2990733	ins	-	APEC	++++	-	-	-		-		-				-	-	-	-	-								
a	3059721	g	3074228	g	2991598	ns	g	UTI89	++++	g	g	g		g		g					g	g	g									
g	3063616	g	3078123	a	2995493	s	g	S88	++	g	g	g	g	g	g	g	a	a	a	g	a	a	a	a	a	a	a	a	a	a	a	a
c	3064439	t	3078946	t	2996316	ns	c	AS	++++	c	c	c	c	c	c	c	c	c	c	c	c	c	c	c	c	c	c	c	c	c	c	c
g	3076663	a	3091170	a	3008540	nc	g	AS	++++	g	g	g	g	g	g	g	g	g	g	g	g	g	g	g	g	g	g	g	g	g	g	g
g	3077496	g	3092003	a	3009373	s	g	S88	++++	g	g	g	g	g	g	g	g	g	g	g	g	g	g	g	g	g	g	g	g	g	g	g

ExPEC Cluster										Outgroup Strains[g]																						
UTI89[a]	UTI89 site[b]	APEC 01[a]	APEC 01 site[b]	S88[a]	S88 site[b]	type[c]	Inferred ancestral base[d]	Lineage inferred to mutate[e]	Support level[f]	CFT073	ED1a	536	E2348/69	SMS 3-5	IAI39	UMN026	K-12	ATCC 8739	HS	D1 Sd197	CB9615	Sakai	EDL933	IAI1	E24377A	SE11	SS Ss046	F2a 301	F2a 2457T	F5 8401	B4 Sb227	B18 BS512
a	3079264	g	3093771	g	3011141	s	g	UTI89	++++	g	g	g	g	g	g	g	g	g	g	g	g	g	g	g	g	g	g	g	g	g	g	g
g	3079927	t	3094434	t	3011804	ns	t	UTI89	++++	t	t	t	t	t	t	t	t	t	t	t	t	t	t	t	t	t	t	t	t	t	t	t
t	3080045	t	3094552	g	3011922	ns	t	S88	++++	t	t	t	t	t	t	t	t	t	t	t	t	t	t	t	t	t	t	t	t	t	t	t
t	3081153	c	3095660	c	3013030	s	c	UTI89	++++	c	c	c	c	c	c	c	c	c	c	c	c	c	c	c	c	c	c	c	c	c	c	c
g	3082908	a	3097415	a	3014785	ns	a	UTI89	++++	a	a	a	a	a	a	a	a	a	a	a	a	a	a	a	a	a	a	a	a	a	a	a
a	3094027	t	3108534	a	3025904	ns	a	APEC	++++	a	a	a	a	a	a	a	a	a	a	a	a	a	a	a	a	a	a	a	a	a	a	a
t	3098812	a	3113319	a	3030689	ns	a	UTI89	++++	a	a	a	a	a	a	a	a	a	a	a	a	a	a	a	a	a	a	a	a	a	a	a
t	3104311	c	3118818	t	3036188	ns	t	APEC	++	t		t													t							
c	3105017	g	3119524	g	3036894	ns	g	UTI89	++	g		g													g							
10	3115492	-	3129998	-	3047368	indel	?	UTI89/AS	+/-																							
t	3119787	t	3134284	a	3051654	s	t	S88	++	t		t																				
a	3121450	g	3135947	g	3053317	ns	g	UTI89	++	g		g																				
c	3123594	c	3140677	t	3055461	ns	c	S88	++	c		c																				
a	3129390	g	3146473	a	3061257	nc	a	APEC	++	a		a																				
a	3133250	c	3150333	c	3065117	ns	c	UTI89	+++	c	c	c	c	c																	c	c
c	3133803	t	3150886	t	3065670	s	t	UTI89	+++	t	t	t	t	c																	t	t
-	3137695	3	3154779	-	3069562	ins	3	APEC	-	3	3	3	3	3																	3	3
-	3137689	-	3154781	3	3069563	ins	3	S88	-	3	3	3	3	3																	3	3
-	3137690	9	3154782	9	3069566	del	9	UTI89	+++	9	9	9	9	9																	9	9
t	3139660	c	3156755	c	3071539	ns	c	UTI89	++	c	c	c	c	c	c	c	c	c	c	c	a	a	a	a	c	c	c	c	c	c	c	c
g	3145220	a	3162315	g	3077099	ns	g	APEC	++++	g	g	g	g	g	g	g	g	g	g	g	g	g	g	g	g	g	g	g	g	g	g	g
t	3149493	a	3166588	a	3081372	nc	t	AS	++	t	t	t	t	c	c	c	c	c	c	c	c	c	c	c	c	c	c	c	c	c	c	c
g	3155620	a	3172715	g	3087499	s	g	APEC	++++	g	g	g	g	g	g	g	g	g	g	g	g	g	g	g	g	g	g	g	g	g	g	g
a	3166862	g	3183957	g	3098741	nc	g	UTI89	++	g	g	g	g	g	a	g	g	g	g	g	g	g	g	g	g	g	a	g	g	g	g	g
g	3173049	a	3190144	a	3104928	s	g	AS	++	g	g	g	g	g	g	g	a	a	a		g	g	g	a	g	a	a	g	g	g	a	a
c	3173298	t	3190393	c	3105177	s	c	APEC	++++	c	c	c	c	c	c	c	c	c	c		c	c	c	c	c	c	c	c	c	c	c	c
a	3174760	a	3191855	t	3106639	nc	a	S88	++++	a	a	a	a	a	a	a	a	a	a		a	a	a	a	a	a	a	a	a	a	a	a
g	3188210	t	3205305	g	3120089	ns	g	APEC	++++	g	g	g	g	g	g	g	g	g	g		g	g	g	g	g	g	g				g	
a	3188698	c	3205793	c	3120577	s	c	UTI89	++++	c	c	c	c	c	c	c	c	c	c		c	c	c	c	c	c	c				c	
c	3194119	a	3211214	c	3125998	ns	c	APEC	++++	c	c	c	c	c	c	c	c	c	c		c	c	c	c	c	c	c				c	
g	3197069	a	3214164	a	3128948	s	g	AS	++++	g	g	g	g	g	g	g	g	g	g		g	g	g	g	g	g	g					
g	3199881	a	3216976	g	3131760	s	g	APEC	++++	g	g	g	g	g	g	g	g	g	g		g	g	g	g	g	g	g					
g	3209368	a	3226463	a	3141247	s	g	AS	++++	g	a	g	g	g	g	g	g	g	g		g	g	g	g	g	g	g					
a	3210038	g	3227133	g	3141917	ns	g	UTI89	++++	g	g	g	g	g	g	g	g	g	g		g	g	g	g	g	g	g	g	g	g	g	
c	3225664	t	3242759	c	3157543	ns	c	APEC	++++	c	c	c	c	c	c	c	c	c	c	c	c	c	c	c	c	c	c	c	c	c	c	c
a	3229145	c	3246240	c	3161024	ns	c	UTI89	++++	c	c	c	c	c	c	c	c	c	c	c	c	c	c	c	c	c	c	c	c	c	c	c
a	3230093	c	3247188	c	3161972	s	a	AS	++++	a	a	a	a	a	a	a	a	a	a	a	a	a	a	a	a	a	a	a	a	a	a	a
t	3242026	c	3259121	c	3173905	ns	c	UTI89	++++	c	c	c	c	c	c	c	c	c	c	c	c	c	c	c	c	c	c	c	c	c	c	c
t	3245947	c	3263042	c	3177826	ns	t	AS	++++	t	t	t	t	t	t	t	t	t	t	t	t	t	t	t	t	t	t	t	t	t	t	t
t	3248554	g	3265649	g	3180433	s	g	UTI89	++	g	g	g	g	g	g	g				a	g	g	g	a	a	a	a	a	a	a	a	a
c	3279697	t	3296792	c	3211576	ns	c	APEC	++++	c	c	c	c	c	c	c	c	c	c	c	c	c	c	c	c	c	c	c	c	c	c	c
a	3280580	g	3297675	g	3212459	s	g	UTI89	++++	g	g	g	g	g	g	g	g	g	g	g	g	g	g	g	g	g	g	g	g	g	g	g
g	3285534	g	3302629	a	3217413	s	g	S88	++++	g	g	g	g	g	g	g	g	g	g	g	g	g	g	g	g	g	g	g	g	g	g	g
-	3287997	g	3316787	a	3231571	nc	g	S88	++	g					g	a																
-	3287974	a	3324534	g	3239318	nc	g	APEC	+++	g		g			g	g																
-	3287974	g	3340695	a	3255479	nc	g	S88	++	g																						
-	3287974	a	3342053	g	3256837	nc	g	APEC	++++	g	g	g	g	g	g	g					g	g	g		g		g	g	g	g	g	g
-	3287974	-	3346623	8	3261408	indel	-	S88	++	-																						
-	3287974	a	3346858	g	3261650	nc	a	S88	++	a																						
-	3287974	a	3349782	t	3264574	nc	t	APEC	++	t																						
-	3287974	a	3353196	g	3267988	nc	g	APEC	+++	g	g	g		g	g										g							
-	3287974	g	3353634	a	3268426	nc	g	S88	+++	g	g	g		g	g										g							
1	3287992	-	3360899	-	3275691	del	1	AS	++++	1	1	1	1	1	1	1	1	1	1	1	1	1	1	1	1	1	1	1	1	1	1	1
g	3288757	t	3361664	g	3276456	nc	t	APEC	-	t	a	g		g		g																
a	3288878	g	3361785	a	3276577	nc	a	APEC	+++	a	a	a		a		a																
g	3288889	g	3361796	a	3276588	nc	a	S88	-	a	a	t		a		a																
t	3289231	g	3362138	t	3276930	ns	t	APEC	+++	t	t	t		t		t																
g	3290356	a	3363263	a	3278055	ns	g	AS	+++	g	g	g		g	g	g																
a	3298631	g	3371538	g	3286330	ns	g	UTI89	+					g	g																	
c	3306687	t	3379594	c	3294386	ns	c	APEC	++++	c	c	c	c	c	c	c	c		c	c	c			c	c	c					c	c
g	3306918	a	3379825	g	3294617	s	g	APEC	++++	g	g	g	g	g	g	g	g		g	g	g			g	g	g					g	g
g	3307800	g	3380707	a	3295499	s	g	S88	++++	g	g	g	g	g	g	g			g	g	g			g	g	g					g	g
a	3307857	c	3380764	a	3295556	s	a	APEC	++	a	a	a	a	g	g	g			a	a	a			a	a	a					a	a
g	3308758	g	3381665	a	3296457	s	g	S88	++++		g	g	g	g	g	g			g	g	g			g	g	g					g	g
g	3315596	t	3388503	g	3303295	ns	g	APEC	++++		g	g	g	g	g	g			g	g	g			g	g	g					g	g
g	3321119	g	3394026	a	3308818	s	g	S88	++++		g	g	g	g	g	g	g		g		g			g	g	g						
g	3321290	g	3394197	a	3308989	s	g	S88	++		g	g	a	g	a	g	g		g		g			g	g	g						
g	3325799	g	3398706	a	3313498	ns	g	S88	++++	g	g	g	g	g	g	g	g	g	g		g			g	g	g		g	g	g		
g	3329095	a	3402002	g	3316794	nc	g	APEC	++++	g	g	g	g	g	g	g	g	g	g		g			g	g	g		g	g	g		
c	3341110	t	3414017	c	3328809	s	c	APEC	++++	c	c	c	c	c	c	c		c	c		c			c	c	c		c		c		
g	3359519	a	3432426	g	3347218	ns	g	APEC	++++	g	g	g	g	g	g	g	g	g	g	g	g	g	g	g	g	g	g	g	g	g	g	g
t	3363698	t	3436605	a	3351397	s	t	S88	++	t	t	t	t	t	t	t	t	t	t	c	c	c	c	t	t	t	t	c	c	c	t	t
t	3373017	t	3445924	c	3360716	nc	t	S88	+++	t	t	t	t	t																		
c	3373447	a	3446354	a	3361146	ns	a	UTI89	+++	a	a	a	a	a																		
g	3384009	g	3456916	a	3371708	s	g	S88	++++	g	g	g	g	g	g	g	g	g	g	g	g	g	g	g	g	g	g	g	g	g	g	g
t	3385293	c	3458200	t	3372992	ns	t	APEC	++++	t	t	t	t	t	t	t	t	t	t	t	t	t	t									
g	3390054	g	3462961	a	3377753	s	g	S88	++	g	g				a																	
g	3390974	a	3463881	g	3378673	nc	g	APEC	++++	g	g	g	g	g	g	g	g	g	g	g	g	g	g	g	g	g	g	g	g	g	g	g
a	3393399	c	3466306	a	3381098	s	a	APEC	++	g	a	g	g	g							g	g	g									
a	3407635	a	3480542	g	3395334	nc	a	S88	+++	a	a	a	a	a		a																
a	3412782	g	3485689	g	3400481	nc	g	UTI89	++++	g	g	g	g	g	g	g	g	g	g		g	g	g	g	g	g	g	g	g	g	g	
1	3413113	-	3486019	-	3400811	ins	-	UTI89	++	-	-																					

ExPEC Cluster										Outgroup Strains[g]																						
UTI89[a]	UTI89 site[b]	APEC 01[a]	APEC 01 site[b]	S88[a]	S88 site[b]	type[c]	Inferred ancestral base[d]	Lineage inferred to mutate[e]	Support level[f]	CFT073	ED1a	536	E2348/69	SMS 3-5	IAI39	UMN026	K-12	ATCC 8739	HS	D1 Sd197	CB9615	Sakai	EDL933	IAI1	E24377A	SE11	SS Ss046	F2a 301	F2a 2457T	F5 8401	B4 Sb227	B18 BS512
a	3420332	g	3493238	a	3408030	nc	a	APEC	++	a	a	a	a	a	a	a	a	a	a		a	a	a	a	a	a	a	g		g		
c	3427774	t	3500680	t	3415472	ns	t	UTI89	++	t	t	t	t	t	c	t	c	c	c	c	c	c	c	t	c	c	c	c	c	c	t	t
c	3444531	t	3517437	t	3432229	nc	t	UTI89	++	t	t	t	t	t	t	t	t	t	t	t	c	t	t	c	c	t	t	c	c	c	t	t
g	3446218	c	3519124	c	3433916	ns	g	AS	++++	g	g	g	g	g	g	g	g	g	g	g	g	g	g	g	g	g	g	g	g	g	g	g
c	3446506	c	3519412	t	3434204	ns	c	S88	++++	c	c	c	c	c	c	c	c	c	c	c	c	c	c	c	c	c	c	c	c	c	c	c
g	3450975	a	3523881	g	3438673	ns	g	APEC	++++	g	g	g	g	g	g	g	g	g	g	g	g	g	g	g	g	g	g	g	g	g	g	
a	3450997	t	3523903	a	3438695	ns	a	APEC	++++	a	a	a	a	a	a	a	a	a	a	a	a	a	a	a	a	a	a	a	a	a	a	
t	3455665	g	3528571	g	3443363	s	g	UTI89	++++	g	g	g	g	g	g	g	g	g	g	g	g	g	g	g	g	g	g	g	g	g	g	g
g	3471014	a	3543920	g	3458712	s	g	APEC	++++	g	g	g	g	g	g	g	g	g	g		g	g	g	g	g	g	g	g	g	g	g	g
g	3481970	g	3554876	a	3469668	s	g	S88	++++	g	g	g	g	g	g	g	g	g	g	g	g	g	g	g	g	g	g	g	g	g	g	g
c	3484595	c	3557501	t	3472293	ns	c	S88	++++	c	c	c	c	c	c	c	c	c	c	c	c	c	c	c	c	c	c	c	c	c	c	c
a	3489993	a	3562899	g	3477691	ns	a	S88	++++	a	a	a	a	a	a	a	a	a	a	a	a	a	a	a	a	a	a	a	a	a	a	a
6	3491975	-	3564880	-	3479672	del	6	AS	++++	6	6	6	6	6	6	6	6	6	6	6	6	6	6	6	6	6	6	6	6	6	6	6
g	3499438	a	3572338	a	3487130	s	g	AS	++++	g	g	g	g	g	g	g	g	g	g		g	g	g	g	g	g	g	g		g		
g	3507025	g	3579925	t	3494717	nc	g	S88	++	g	a	a	g	g	g	g	a	g	g		a	a	a	g	g	g						
g	3508405	g	3581305	a	3496097	s	g	S88	++++	g	g	g	g	g	g	g	g	g	g		g	g	g	g	g	g						
t	3513206	c	3586106	t	3500898	nc	t	APEC	++++	t	t	t	t	t	t	t	t	t	t	t	t	t	t	t	t	t	t	t	t	t	t	t
g	3517552	a	3590452	a	3505244	ns	a	UTI89	++++	a	a	a	a	a	a	a	a	a	a	a	a	a	a	a	a	a	a	a	a	a	a	a
c	3525829	g	3598729	g	3513521	ns	g	UTI89	++++	g	g	g	g	g	g	g	g	g	g	g	g	g	g	g	g	g	g	g	g	g	g	g
g	3533540	g	3606440	a	3521232	s	g	S88	++++	g	g	g	g	g	g	g	g	g	g	g	g	g	g	g	g	g	g	g	g	g	g	g
a	3550309	g	3623209	g	3538001	s	g	UTI89	++++	g	g	g	g	g	g	g	g	g	g	g	g	g	g	g	g	g	g	g	g	g	g	g
c	3550318	g	3623218	g	3538010	ns	g	UTI89	++	g	g	g	g	g	g	g	g	g	g	g	g	g	g	a	a	a	g	g	g	g	a	a
-	3550306	12	3623224	12	3538016	del	12	UTI89	++++	12	12	12	12	12	12	12	12	12	12	12	12	12	12	12	12	12	12	12	12	12	12	12
t	3552525	c	3625437	c	3540229	nc	c	UTI89	++++	c	c	c	c	c	c	c	c	c	c	c	c	c	c	c	c	c	c	c	c	c	c	c
g	3556685	a	3629597	a	3544389	s	g	AS	++++	g	g	g	g	g	g	g	g	g	g	g	g	g	g	g	g	g	g	g	g	g	g	g
a	3567486	g	3640398	a	3555190	ns	a	APEC	++++	a	a	a	a	a	a	a	a	a	a	a	a	a	a	a	a	a	a	a	a	a	a	a
c	3569723	c	3642635	t	3557427	ns	c	S88	++++	c	c	c	c	c	c	c	c	c	c	c	c	c	c	c	c	c	c	c	c	c	c	c
g	3580723	a	3653635	g	3568427	ns	g	APEC	++++	g	g	g	g	g	g	g	g	g	g	g	g	g	g	g	g	g	g	g	g	g	g	g
g	3583074	g	3655986	a	3570778	s	g	S88	++++	g	g	g	g	g	g	g	g	g	g	g	g	g	g	g	g	g	g	g	g	g	g	g
g	3590721	g	3663633	a	3578425	s	g	S88	++++	g	g	g	g	g	g	g	g	g	g	g	g	g	g	g	g	g	g	g	g	g	g	g
c	3598897	a	3671809	c	3586601	ns	c	APEC	++++	c	c	c	c	c	c	c	c	c	c	c	c	c	c	c	c	c	c	c	c	c	c	c
t	3600484	t	3673396	g	3588188	ns	t	S88	++++	t	t	t	t	t	t	t	t	t	t	t	t	t	t	t	t	t	t	t	t	t	t	t
c	3615102	c	3688014	t	3602806	s	c	S88	++++	c	c	c	c	c	c	c	c	c	c	c	c	c	c	c	c	c	c	c	c	c	c	c
g	3624876	t	3697788	t	3612580	ns	g	AS	+++	g	g	g			g																g	g
-	3631731	-	3704652	1	3619445	ins	-	S88	++++	-	-	-	-	-	-	-	-	-	-		-	-	-	-	-	-	-				-	-
t	3633752	c	3706664	c	3621457	s	c	UTI89	++++	c	c	c	c	c	c	c	c	c	c	c	c	c	c	c	c	c	c	c	c	c	c	c
t	3639450	c	3712362	c	3627155	ns	c	UTI89	++++	c	c	c	c	c	c	c	c	c	c	c	c	c	c	c	c	c	c	c	c	c	c	c
a	3642556	g	3715468	g	3630261	ns	g	UTI89	++++	g	g	g	g	g	g	g	g	g	g	g	g	g	g	g	g	g	g	g	g	g	g	g
c	3643031	t	3715943	c	3630736	nc	c	APEC	++	t	c	c	c	t	t	t	t	t	t	t	t	t	t	t	t	t	t	t	t	t	t	t
g	3643073	a	3715985	g	3630778	nc	g	APEC	++	a	g	g	g	a	a	a	a	a	g	a	a	a	a	a	a	a	a	a	a	a	g	g
g	3644261	g	3717176	a	3631966	ns	g	S88	++	a	g	g	g	g	a	g	g	g	g	g	g	g	g	g	g	a	g	a	a	a	a	a
g	3668419	a	3741334	g	3656124	ns	g	APEC	++++	g	g	g	g	g	g	g	g	g	g	g	g	g	g	g	g	g	g	g	g	g	g	g
-	3672118	1	3745034	-	3659823	ins	-	APEC	+++	-	-	-					-	-	-								-					
g	3672132	t	3745048	g	3659837	s	g	APEC	+++	g	g	g					g	g	g								g					
-	3672132	2	3745049	-	3659837	ins	-	APEC	+++	-	-	-					-	-	-								-					
-	3672136	1	3745055	-	3659841	ins	-	APEC	+++	-	-	-					-	-	-								-					
-	3672148	1	3745068	-	3659853	ins	-	APEC	+++	-	-	-					-	-	-								-					
-	3672161	1	3745082	-	3659866	ins	-	APEC	+++	-	-	-					-	-	-								-					
-	3672164	1	3745086	-	3659869	ins	-	APEC	+++	-	-	-					-	-	-								-					
-	3672202	1	3745125	-	3659907	ins	-	APEC	+++	-	-	-					-	-	-								-					
-	3672219	1	3745143	-	3659924	ins	-	APEC	+++	-	-	-					-	-	-								-					
-	3672236	1	3745161	-	3659941	ins	-	APEC	+++	-	-	-					-	-	-								-					
-	3672241	1	3745167	-	3659946	ins	-	APEC	+++	-	-	-					-	-	-								-					
13	3672855	-	3745780	-	3660559	del	13	AS	+++	13	13	13					13	13	13								13					
-	3672855	-	3745799	13	3660560	ins	13	S88	-	13	13	13					13	13	13								13					
-	3672867	19	3745781	-	3660559	ins	-	APEC	+++	-	-	-					-	-	-								-					
-	3672889	1	3745822	-	3660594	ins	-	APEC	+++	-	-	-					-	-	-								-					
c	3676558	a	3749491	c	3664263	ns	c	APEC	+++	c	c	c					c	c	c													
g	3681099	-	3753951	a	3668804	s	g	S88	+++	g	g	g					g	g	g													
-	3687648	10	3760585	-	3675353	ins	-	APEC	+++	-	-	-					-	-	-								-					
a	3689472	a	4497219	g	4411761	nc	t	S88	+/-	t	t	t	t	t	t	t	t	t	t	t	t	t	t	t	t	t	t	t	t	t	t	t
4	3694355	4	4532506	-	4447035	del	4	S88	++++	4	4	4	4	4	4	4	4	4	4	4	4	4	4	4	4	4	4	4	4	4	4	4
g	3698065	a	4528802	a	4443338	nc	t	UTI89/AS	+/-	t	c	c	t	t	t	t	t	t	t	t	t	t	t	t	t	t	t	t	t	t	t	t
g	3698112	g	4528755	a	4443291	nc	t	S88	+/-	c	c	c	t	c	t	c	c	c	c	c	c	c	c	c	t	c	t	t	t	t	a	t
t	3706383	c	4520477	c	4435019	ns	g	UTI89/AS	+/-	g	g	g	g	g	g	g	g	g	g	g	g	g	g	g	g	g	g	g	g	g	g	g
t	3722302	c	4504558	c	4419100	s	g	UTI89/AS	+/-	g	g	g	g	g	g	g	g	g	g	g	g	g	g	g	g	g	g	g	g	g	g	g
t	3728156	c	3760933	c	3675692	ns	c	UTI89	++++	c	c	c	c	c	c	c	c	c	c	c	c	c	c	c	c	c	c	c	c	c	c	c
t	3728166	a	3760943	a	3675702	s	a	UTI89	++++	a	a	a	a	a	a	a	a	a	a	a	a	a	a	a	a	a	a	a	a	a	a	a
t	3728169	a	3760946	a	3675705	s	a	UTI89	++	a	a	a	a	a	a	a	a	a	a	t	t	t	t	a	a	a	a	a	a	a	a	a
g	3728223	a	3761000	a	3675759	s	g	AS	++	g	a	g	a	g	g	g	g	g	g	g	g	g	g	g	g	g	g	g	g	g	g	g
a	3728295	g	3761072	g	3675831	s	g	UTI89	++++	g	g	g	g	g	g	g	g	g	g	g	g	g	g	g	g	g	g	g	g	g	g	g
a	3728496	g	3761273	g	3676032	s	a	AS	++++	a	a	a	a	a	a	a	a	a	a	a	a	a	a	a	a	a	a	a	a	a	a	a
c	3728967	a	3761744	a	3676503	s	a	UTI89	++++	a	a	a	a	a	a	a	a	a	a	a	a	a	a	a	a	a	a	a	a	a	a	a
g	3753314	g	3786091	a	3700850	ns	g	S88	++++	g	g	g	g	g	g	g	g	g	g	g	g	g	g	g	g	g	g	g	g	g	g	g
g	3766749	g	3799526	a	3714285	s	g	S88	++++	g	g	g	g	g	g	g	g	g	g	g	g	g	g	g	g	g	g	g	g	g	g	g
c	3780894	a	3813671	a	3728430	ns	c	AS	++++	c	c	c	c	c	c	c	c	c	c	c	c	c	c	c	c	c	c	c	c	c	c	c
g	3783011	a	3815788	g	3730547	s	g	APEC	++++	g	g	g	g	g	g	g	g	g	g	g	g	g	g	g	g	g	g	g	g	g	g	g
g	3783110	g	3815887	a	3730646	s	g	S88	++++	g	g	g	g	g	g	g	g	g	g	g	g	g	g	g	g	g	g	g	g	g	g	g
g	3807698	g	3840475	a	3755234	s	g	S88	++	g	g	g	g	g	g	g	g	g	g	g	a	a	a	g	g	g	g	g	g	g	g	g
g	3812028	a	3844805	g	3759564	ns	g	APEC	++++	g	g	g	g	g	g	g	g	g	g		g	g	g	g	g	g	g	g	g	g	g	g
1	3812437	-	3845213	-	3759972	del	1	AS	++++	1	1	1	1	1	1	1	1	1	1	1	1	1	1	1	1	1	1	1	1	1	1	1
t	3814527	c	3847303	t	3762062	ns	c	APEC	-	c	c	c	c	c	c	c	c	c	c	c	c	c	c	c	c	c	c	c	c	c	c	c

ExPEC Cluster										Outgroup Strains[g]																						
UTI89[a]	UTI89 site[b]	APEC 01[a]	APEC 01 site[b]	S88[a]	S88 site[b]	type[c]	Inferred ancestral base[d]	Lineage inferred to mutate[e]	Support level[f]	CFT073	ED1a	536	E2348/69	SMS 3-5	IAI39	UMN026	K-12	ATCC 8739	HS	D1 Sd197	CB9615	Sakai	EDL933	IAI1	E24377A	SE11	SS Ss046	F2a 301	F2a 2457T	F5 8401	B4 Sb227	B18 BS512
g	3816742	g	3849518	a	3764277	ns	g	S88	++++	g	g	g	g	g	g			g	g	g		g	g	g	g	g	g				g	g
-	3819082	1	3851868	1	3766627	del	1	UTI89	+++	1	1	1				1																
-	3820270	8	3853048	-	3767806	ins	-	APEC	+++	-	-	-				-																
1	3820615	-	3853399	-	3768150	del	1	AS	+++	1	1	1				1																
g	3824281	a	3857065	a	3771816	s	g	AS	+++	g	g	g				a																
a	3825492	g	3858276	g	3773027	ns	g	UTI89	+++	g	g	g				g																
t	3829512	c	3862296	c	3777047	ns	c	UTI89	++++	c	c	c	c	c	c	c	c	c	c	c	c	c	c	c	c	c	c	c	c	c	c	c
a	3831347	g	3864131	g	3778882	s	g	UTI89	++++	g	g	g	g	g	g	g	g	g	g	g	g	g	g	g	g	g	g	g	g	g	g	g
a	3832427	g	3865211	g	3779962	ns	g	UTI89	++++	g	g	g	g	g	g	g	g	g	g	g	g	g	g	g	g	g	g	g	g	g	g	g
t	3835020	t	3867804	c	3782555	s	t	S88	++++	t	t	t	t	t	t	t	t	t	t	t	t	t	t	t	t	t	t	t	t	t	t	t
g	3843994	t	3876781	t	3791529	nc	g	AS	++++	g	g	g			g	g	g	g	g					g	g	g					g	g
g	3845773	g	3878560	t	3793308	nc	g	S88	++	g	g	g			g	g	t	t	t					t	t	t					t	t
c	3853083	t	3885870	c	3800618	ns	c	APEC	++++	c	c	c	c	c	c	c	c	c	c	c	c	c	c	c	c	c	c	c	c	c	c	c
a	3859041	g	3891828	a	3806576	s	a	APEC	++++	a	a	a	a	a	a	a	a	a	a	a	a	a	a	a	a	a	a	a	a	a	a	a
g	3864493	a	3897280	g	3812028	ns	?	APEC	+/-																							
c	3870043	a	3902838	c	3817578	ns	c	APEC	++	c	c	c	c	c	c	c	c	c	c	c	c	c	c	-	-	c	c	c	c	c	-	-
a	3873522	g	3906317	g	3821057	s	g	UTI89	++++	g	g	g	g	g	g	g	g	g	g	g	g	g	g	a	g	g	g	g	g	g	g	g
t	3876221	g	3909016	t	3823756	s	t	APEC	++++	t	t	t	t	t	t	t	t	t	t	t	t	t	t	t	t	t	t	t	t	t	t	t
a	3877813	a	3910608	t	3825348	ns	a	S88	++++	a	a	a	a	a	a	a	a	a	a	a	a	a	a	a	a	a	a	a	a	a	a	a
g	3880172	g	3912967	a	3827707	ns	g	S88	++++	g	g	g	g	g	g	g	g	g	g	g	g	g	g	g	g	g	g	g	g	g	g	a
g	3880907	t	3913702	t	3828442	ns	t	UTI89	++++	t	t	t	t	t	t	t	t	t	t	t	t	t	t	t	t	t	t	t	t	t	t	t
a	3881372	g	3914167	g	3828907	ns	g	UTI89	++++	g	g	g	g	g	g	g	g	g	g	g	g	g	g	g	g	g	g	g	g	g	g	g
c	3884077	c	3916872	t	3831612	ns	c	S88	++++	c	c	c	c	c	c	c	c	c	c	c	c	c	c	c	c	c	c	c	c	c	c	c
g	3891483	g	3924278	a	3839018	s	g	S88	++++	g	g	g	g	g	g	g	g	g	g		g	g	g	g	g	g	g	g	g	g	g	g
g	3892367	a	3925162	g	3839902	ns	g	APEC	++++	g	g	g	g	g	g	g	g	g	g		g	g	g	g	g	g	g	g	g	g	g	g
c	3904420	t	3937215	t	3851955	ns	t	UTI89	++++	t	t	t	t	t	t	t	t	t	t	t	t	t	t	t	t	t	t	t	t	t	t	t
a	3908902	g	3941697	a	3856437	ns	a	APEC	++++	a	a	a	a	a	a	a	a	a	a	a	a	a	a	a	a	a	a	a	a	a	a	a
g	3910609	a	3943404	a	3858144	ns	g	AS	++++	g	g	g	g			g	g	g	g	g	g	g	g	g	g	g	g	g	g	g	g	g
t	3911193	t	3943988	c	3858728	ns	t	S88	++++	t	t	t	t	t	t	t	t	t	t		t	t	t	t	t	t	t	t	t	t	t	t
a	3915496	g	3948291	g	3863031	s	g	UTI89	++++	g	g	g	g	g	g	g				g	g	g	g									
c	3918150	c	3950945	a	3865685	s	c	S88	++++	c	c	c	c	c	c	c				c	c	c	c									
g	3923587	a	3956382	g	3871122	nc	g	APEC	++++	g	g	g	g	g	g	g	g	g	g	g	g	g	g	g	g	g	g	g	g	g	g	g
a	3928162	g	3960957	g	3875697	ns	g	UTI89	++++	g	g	g	g	g	g	g	g	g	g	g	g	g	g	g	g	g	g	g	g	g	g	g
c	3931406	t	3964201	t	3878941	nc	c	AS	++++	c	c	c	c	c	c	c	c	c	c	c	c	c	c	c	c	c	c	c	c	c	c	c
1	3931749	-	3964543	1	3879284	del	1	APEC	++++	1	1	1	1	1	1	1	1	1	1	1	1	1	1	1	1	1	1	1	1	1	1	1
a	3932330	a	3965124	c	3879865	ns	a	S88	++++	a	a	a	a	a	a	a	a	a	a	a	a	a	a	a	a	a	a	a	a	a	a	a
g	3942924	g	3975718	a	3890459	ns	g	S88	++++	g	g	g	g	g	g	g	g	g	g	g	g	g	g	g	g	g	g	g	g	g	g	g
t	3947345	g	3980139	g	3894880	ns	g	UTI89	++++	g	g	g	g	g	g	g	g	g	g	g	g	g	g	g	g	g	g	g	g	g	g	g
a	3950138	a	3982932	g	3897673	nc	a	S88	++++	a	a	a	a	a	a	a	a	a	a	a	a	a	a	a	a	a	a	a	a	a	a	a
a	3950965	g	3983759	a	3898500	ns	a	APEC	++++	a	a	a	a	a	a	a	a	a	a	a	a	a	a	a	a	a	a	a	a	a	a	a
a	3952712	c	3985506	c	3900247	s	c	UTI89	++++	c	c	c	c	c	c	c	c	c	c	c	c	c	c	c	c	c	c	c	c	c	c	c
1	3961295	1	3994099	-	3908839	del	1	S88	++++	1	1	1	1	1	1	1	1	1		1	1	1	1	1	1	1	1	1	1	1	1	1
t	3964225	t	3997019	a	3911759	s	t	S88	++++	t	t	t	t	t	t	t	t	t		t	t	t	t	t	t	t	t	t	t	t	t	t
g	3965091	g	3997885	a	3912625	ns	g	S88	++++	g	g	g	g	g	g	g	g	g		g	g	g	g	g	g	g	g	g	g	g	g	g
a	3967376	a	4000170	c	3914910	s	c	S88	-	c	c	c	c	c	c	c	c	c	c	c	c	c	c	c	c	c	c	c	c	c	c	c
g	3969624	a	4002418	a	3917158	s	g	AS	++++	g	g	g	g	g	g	g	g	g	g	g	g	g	g	g	g	g	g	g	g	g	g	g
c	3994180	a	4026974	a	3941714	s	a	UTI89	++++	a	a	a	a	a	a	a	a	a	a		a	a	a	a	a	a	a	a	a	a	a	a
g	3995800	a	4028594	g	3943334	ns	g	APEC	++++	g	g	g	g	g	g	g	g	g	g		g	g	g	g	g	g	g	g	g	g	g	g
10	4001950	-	4034743	-	3949483	del	10	AS	++++	10	10	10	10	10	10	10	10	10	10	10	10	10	10	10	10	10		10	10	10	10	10
-	4001959	10	4034744	-	3949483	ins	10	APEC	-	10	10	10	10	10	10	10	10	10	10	10	10	10	10	10	10	10		10	10	10	10	10
1	4001960	1	4034754	-	3949483	del	1	S88	++++	1	1	1	1	1	1	1	1	1	1	1	1	1	1	1	1	1		1	1	1	1	1
g	4003221	g	4036015	a	3950744	ns	g	S88	++++	g	g	g	g	g	g	g	g	g	g	g	g	g	g	g	g	g		g	g	g	g	g
c	4006008	a	4038802	a	3953531	s	c	AS	++++	c	c	c	c	c	c	c	c	c	c	c	c	c	c	c	c	c		c	c	c	c	c
a	4009250	g	4042044	g	3956773	s	g	UTI89	++++	g	g	g	g	g	g	g	g	g	g					g	g	g		g	g	g	g	g
g	4009832	a	4042626	a	3957355	ns	g	AS	++++	g	g	g	g	g	g	g	g	g	g					g	g	g					g	g
a	4011887	a	4044681	g	3959410	ns	a	S88	++++	a	a	a	a	a	a	a	a	a	a					a	a	a		a	a	a	a	a
a	4015231	g	4048027	g	3962754	ns	g	UTI89	++++	g	g	g	g	g	g	g	g	g	g					g	g	g			g			
g	4016358	g	4049154	a	3963881	s	g	S88	++++	g	g	g	g	g	g	g	g	g	g					g	g	g			a			
t	4017100	c	4049896	c	3964623	ns	c	UTI89	++++	c	c	c	c	c	c	c	c	c	c					c	c	c			c			
t	4019128	a	4051924	t	3966651	ns	t	APEC	++++	t	t	t	t	t	t	t	t	t	t		t	t	t	t	t	t		t	t	t	t	t
g	4024139	g	4056935	a	3971662	ns	g	S88	++++	g	g	g	g	g	g	g	g	g	g	g	g	g	g	g	g	g	g	g	g	g	g	g
g	4026879	a	4059675	g	3974402	ns	g	APEC	++++	g	g	g	g	g	g	g	g	g	g		g	g	g	g	g	g	g	g	g	g	g	g
c	4038610	a	4071406	a	3986133	ns	a	UTI89	++++	a	a	a	a	a	a	a		a	a	a	a	a	a	a	a	a	a	a	a	a	a	a
c	4042551	t	4075347	c	3990074	ns	c	APEC	++++	c	c	c	c	c	c	c	c	c	c	c	c	c	c	c	c	c	c	c	c	c	c	c
c	4053654	a	4086450	c	4001177	ns	c	APEC	++++	c	c	c	c	c	c	c	c	c	c	c	c	c	c	c	c	c	c	c	c	c	c	c
g	4057240	a	4090036	a	4004763	ns	g	AS	++++	g	g	g	g	g	g	g	g	g	g	g	g	g	g	g	g	g	g	g	g	g	g	g
c	4070308	t	4103104	c	4017831	nc	c	APEC	++++	c	c	c	c	c	c	c	c	c	c	c	c	c	c	c	c	c	c	c	c	c	c	c
t	4072891	c	4105687	c	4020414	s	c	UTI89	++++	c	c	c	c	c	c	c	c	c	c	c	c	c	c	c	c	c	c	c	c	c	c	c
g	4075283	g	4108079	a	4022806	ns	g	S88	++++	g	g	g	g	g	g	g	g	g	g	g	g	g	g	g	g	g	g	g	g	g	g	g
t	4081357	c	4114153	c	4028880	s	c	UTI89	++++	c	c	c	c	c	c	c	c	c	c	c	c	c	c	c	c	c	c	c	c	c	c	c
a	4089040	g	4121836	g	4036563	ns	g	UTI89	++++	g	g	g	g	g	g	g	g	g	g	g	g	g	g	g	g	g	g	g	g	g	g	g
g	4094343	g	4127139	t	4041866	s	g	S88	+++	g	g	g	g	g	g																	
c	4101317	t	4134113	c	4048840	ns	c	APEC	++++	c	c	c	c	c	c	c	c	c	c	c	c	c	c	c	c	c	c	c	c	c	c	c
g	4105097	g	4137893	c	4052620	s	g	S88	++++	g	g	g	g	g	g	g	g	g	g	g	g	g	g	g	g	g					g	g
t	4109445	c	4142241	c	4056968	ns	c	UTI89	++++	c	c	c	c	c	c	c	c	c	c	c	c	c	c	c	c	c	c	c	c	c	c	c
g	4113443	g	4146239	a	4060966	ns	g	S88	++++	g	g	g	g	g	g	g	g	g	g	g	g	g	g	g	g	g	g	g	g	g	g	g
a	4118145	a	4150941	c	4065668	s	a	S88	++	a	a	a	a	g	g	g	g	a	a	g	g	g	g	g	g	g	a	a	a	a	g	g
-	4122295	1	4155092	-	4069818	ins	1	APEC	-	1	1	1	1	1	1	1	1			1	1	1	1	1	1	1		1	1	1		
c	4123464	t	4156261	t	4070987	ns	c	AS	++++	c	c	c	c	c	c	c	c			c	c	c	c	c	c	c		c	c	c		
11	4123492	-	4156288	-	4071014	del	11	AS	++++	11	11	11	11	11	11	11	11			11	11	11	11	11	11	11		11	11	11		
-	4123502	11	4156289	-	4071014	ins	11	APEC	-	11	11	11	11	11	11	11	11			11	11	11	11	11	11	11		11	11	11		
1	4123503	1	4156300	-	4071014	del	1	S88	++++	1	1	1	1	1	1	1	1			1	1	1	1	1	1	1		1	1	1		

ExPEC Cluster										Outgroup Strains[g]																						
UTI89[a]	UTI89 site[b]	APEC 01[a]	APEC 01 site[b]	S88[a]	S88 site[b]	type[c]	Inferred ancestral base[d]	Lineage inferred to mutate[e]	Support level[f]	CFT073	ED1a	536	E2348/69	SMS 3-5	IAI39	UMN026	K-12	ATCC 8739	HS	D1 Sd197	CB9615	Sakai	EDL933	IAI1	E24377A	SE11	SS Ss046	F2a 301	F2a 2457T	F5 8401	B4 Sb227	B18 BS512
1	4124218	1	4157025	-	4071738	del	1	S88	++++	1	1	1	1	1	1	1	1			1	1	1	1	1	1	1		1	1	1		
g	4133561	a	4166358	a	4081071	ns	a	UTI89	++++	a	a	a	a	a	a	a	a	a	a					a	a	a	a	a	a	a		
a	4138967	a	4171764	g	4086477	s	a	S88	++++	a	a	a	a	a	a	a	a	a	a	a	a	a	a	a	a	a	a	a	a	a	a	a
g	4157079	t	4189876	t	4104589	s	g	AS	++	g	g	g	g	g	a	g	g	g	g	a	g	g	g	g	g	g	g	g	g	g	g	g
c	4158139	t	4190936	c	4105649	ns	c	APEC	++++	c	c	c	c	c	c	c	c	c	c	c	c	c	c	c	c	c	c	c	c	c	c	c
g	4158435	g	4191232	a	4105945	nc	g	S88	++	g	g	g	g	g	g	g	-	-	-	g	g	g	g	-	-	-	-	-	-	-		
5	4158742	-	4191538	-	4106251	ins	-	UTI89	++++	-	-	-	-	-	-	-	-	-	-	-	-	-	-	-	-	-	-	-	-	-		
c	4160304	g	4193096	g	4107809	ns	g	UTI89	++++	a	g	g	g				g	g	g					g	g	g	g	g	g	g		
g	4168607	g	4201399	a	4116112	s	g	S88	++++	g	g	g	g	g	g	g	g	g	g	g	g	g	g	g	g	g	g	g	g	g	g	g
a	4172397	g	4205189	g	4119902	nc	g	UTI89	++++	g	g	g	g	g	g	g	g	g	g	g	g	g	g	g	g	g	g	g	g	g	g	g
-	4172678	5	4205471	-	4120183	ins	-	APEC	+++	-	-	-	-				-	-	-													
c	4173610	t	4206407	t	4121115	ns	c	AS	++++	c	c	c	c	c	c	c	c	c	c	c	c	c	c	c	c	c	c	c	c	c	c	c
g	4173711	t	4206508	g	4121216	ns	g	APEC	++++	g	g	g	g	g	g	g	g	g	g	g	g	g	g	g	g	g	g	g	g	g	g	g
t	4174585	a	4207382	a	4122090	nc	t	AS	++++	t	t	t	t	t	t	t	t	t	t	t	t	t	t	t	t	t	t	t	t	t	t	t
g	4176975	a	4209772	g	4124480	s	g	APEC	++++	g	g	g	g	g	g	g	g	g	g	g	g	g	g	g	g	g	g	g	g	g	g	g
c	4179841	a	4212638	a	4127346	s	a	UTI89	++++	a	a	a	a	a	a	a	a	a	a	a	a	a	a	a	a	a	a	a	a	a	a	a
g	4183713	g	4216510	t	4131218	nc	g	S88	++	a	g	g	g	g	g	g	g	g	g	t	g	g	g	g	g	g	g	g	g	g	g	g
a	4187124	g	4219921	g	4134629	ns	g	UTI89	++++	g	g	g	g	g	g	g	g	g	g	g	g	g	g	g	g	g	g	g	g	g	g	g
c	4202885	a	4235682	a	4150390	nc	c	AS	++++	c	c	c	a	c	c	c	c	c	c	c	c	c	c	c	c	c	c	c	c	c	c	c
g	4204502	a	4237299	a	4152007	nc	a	UTI89	++	a	g	a	g	a	a	a	a	a	g	a	g	a	r	a	a	g	a	a	a	a	a	a
a	4204544	g	4237341	g	4152049	nc	a	AS	++	a	a	a	a	-	a	a	a	a	a	-	a	a	a	a	a	a	a	a	a	a	a	a
c	4204887	c	4237684	t	4152392	ns	c	S88	++	c	c	c	c	c	t	c	c	c	c	c	c	c	c	t	t	t	c	t	t	t	t	t
a	4207259	g	4240056	g	4154764	nc	g	UTI89	++++	g	g	g	g	g	g	g	g	g	g	g	g	g	g	g	g	g	g	g	g	g	g	g
g	4208037	t	4240834	g	4155542	ns	g	APEC	++++	g	g	g	g	g	g	g	g	g	g	g	g	g	g	g	g	g	g	g	g	g	g	g
c	4214370	c	4247167	t	4161875	ns	c	S88	++++	c	c	c	c	c	c	c	c	c	c	c	c	c	c	c	c	c	c	c	c	c	c	c
t	4216893	g	4249690	t	4164398	ns	t	APEC	++++	t	t	t	t	t	t	t	t	t	t	t	t	t	t	t	t	t	t	t	t	t	t	t
t	4218285	c	4251082	t	4165790	s	t	APEC	++++	t	t	t	t	t	t	t	t	t	t	t	t	t	t	t	t	t	t	t	t	t	t	t
t	4239927	c	4272724	c	4187432	ns	c	UTI89	++++	c	c	c	c	c	c	c	c	c	c	c	c	c	c	c	c	c	c	c	c	c	c	c
g	4242503	c	4275300	g	4190008	nc	g	APEC	++++	g	g	g	g	g	g	g	g	g	g	g	g	g	g	g	g	g	g	g	g	g	g	g
6	4247939	-	4280735	-	4195443	del	6	AS	++	6	6	6	6	6	-	6	-	-	-	-	-	6	6	6	6	6	6	6	6	6	6	6
6	4247935	6	4280736	-	4195443	del	6	S88	++	6	6	6	6	6	-	6	-	-	-	-	-	6	6	6	6	6	6	6	6	6	6	6
g	4247989	g	4280780	a	4195482	s	g	S88	++++	g	g	g	g	g	g	g	g	g	g	g	g	g	g	g	g	g	g	g	g	g	g	g
t	4250508	c	4283299	c	4198001	s	c	UTI89	++++	c	c	c	c	c	c	c	c	c	c	c	c	c	c	c	c	c	c	c	c	c	c	c
g	4256863	a	4288878	a	4203580	ns	g	AS	++++	g			g		g				g					g	g	g		g	g	g		
a	4260364	g	4292379	g	4207081	s	g	UTI89	++++	g	g	g	g	g	g	g	g	g	g	g	g	g	g	g	g	g	g	g	g	g	g	g
g	4261500	g	4293515	a	4208217	ns	g	S88	++++	g	g	g	g	g	g	g	g	g	g	g	g	g	g	g	g	g	g	g	g	g	g	g
t	4264120	t	4296135	g	4210837	ns	t	S88	++++	t	t	t	t	t	t	t				t	t	t	t				t				t	t
g	4267068	a	4299083	a	4213785	ns	g	AS	++++	g	g	g	g	g	g						g	g	g	g	g	g	g					
t	4267978	c	4299993	c	4214695	ns	c	UTI89	++++	c	c	c	c	c	c						c	c	c	c	c	c						
c	4277937	a	4309952	c	4224505	ns	c	APEC	++	c	c	c	c	c	c	t	t	c	c	t	t	t	t	c	c	c	t	t	t	t	t	t
g	4282540	t	4314555	t	4229108	ns	g	AS	+++	g	g	g	g																			
a	4287127	a	4319142	g	4233695	ns	a	S88	+++	a	a	a	a																			
c	4288098	t	4320113	t	4234666	ns	c	AS	+++	c	c	c	c	c																		
a	4303644	a	4335659	g	4250212	nc	a	S88	+++	a	a	a		a	a																	
t	4308604	c	4340619	c	4255172	ns	c	UTI89	++++	c	c	c	c	c	c	c	c	c	c	c	c	c	c	c	c	c	c	c	c	c	c	c
a	4308862	g	4340877	a	4255430	ns	a	APEC	++++	a	a	a	a	a	a	a	a	a	a	a	a	a	a	a	a	a	a	a	a	a	a	a
a	4308923	g	4340938	g	4255491	s	g	UTI89	++++	g	g	g	g	g	g	g	g	g	g	g	g	g	g	g	g	g	g	g	g	g	g	g
t	4314651	c	4346666	c	4261219	ns	c	UTI89	++++	c	c	c	c	c	c	c	c	c	c	c	c	c	c	c	c	c	c	c	c	c	c	c
t	4319323	a	4351338	a	4265891	ns	a	UTI89	++++	a	a	a	a	a	a	a	a	a	a	a	a	a	a	a	a	a	a	a	a	a	a	a
1	4321682	1	4353707	-	4268259	del	1	S88	++++	1	1	1	-	1	1	1	1	1	1	1	1	1	1	1	1	1	1	1	1	1	1	1
-	4323809	-	4355834	17	4270376	ins	17	S88	++	-	17	17	17	17	-	17	17	17	17	-	17	17	17	17	17	17	17	17	17	17	17	17
t	4326969	a	4358981	a	4273536	nc	a	UTI89	++	a	a	t	t	a	a	a	a	a	a	a	a	a	a	a	a	a	a	a	a	a	a	t
-	4326980	1	4358993	-	4273547	ins	1	APEC	-	1	-	1	1	-	-	-	1	-	-	-	-	-	-	1	-	-	-	-	-	-	1	1
c	4327063	t	4359077	c	4273630	s	t	APEC	-	t	t	t	t	t	t	t	t	t	t	t	t	t	t	t	t	t	t	t	t	t	t	t
g	4327420	a	4359434	g	4273987	s	g	APEC	++++	a	g	g	g	g	g	g	g	g	g	g	g	g	g	g	g	g	g	g	g	g	g	g
c	4327628	c	4359642	t	4274195	ns	c	S88	++++	c	c	c	c	c	c	c	c	c	c	c	c	c	c	c	c	c	c	c	c	c	c	c
t	4328003	c	4360017	t	4274570	ns	c	APEC	-	c	c	c	c	c	c	c	c	c	c	c	c	c	c	c	c	c	c	c	c	c	c	c
t	4329494	c	4361508	t	4276061	ns	c	APEC	-	c	c	t	c	c	c	c	c	c	c	c	c	c	c	c	c	c	c	c	c	c	c	c
c	4330908	t	4362922	c	4277475	s	c	APEC	++++	c	c	c	c	c	c	c	c	c	c	c	c	c	c	c	c	c		c	c	c	c	c
g	4331841	g	4363855	a	4278408	ns	g	S88	++++	g	g	g	g	g	g	g	g	g	g	g	g	g	g	g	g	g	g	g	g	g	g	g
c	4337788	t	4369802	c	4284355	ns	c	APEC	++++	c	c	c	c	c	c	c	c	c	c	c	c	c	c	c	c	c	c	c	c	c	c	c
c	4337870	c	4369884	a	4284437	ns	c	S88	++	c	c	c	c	c	c	t	t	t	t	c	t	t	t	t	t	t	c	c	c	c	t	t
t	4346216	g	4378230	t	4292783	nc	g	APEC	-	g	g	g	g	t	t	t	g	g	g	t	g	g	g	t	t	t	t	g	g	g	t	t
c	4346219	a	4378233	c	4292786	nc	a	APEC	-	a	a	a	a	c	c	c	a	a	a	c	a	a	a	c	c	c	c	a	a	a	c	c
c	4350660	t	4382674	c	4297227	ns	c	APEC	++++	c		c	c					c	c								c				c	t
g	4355289	a	4387303	g	4301856	s	g	APEC	++++	g	g	g	g	g	g	g	g	g	g	g	g	g	g	g	g	g	g	g	g	g	g	g
t	4356066	g	4388080	g	4302633	ns	t	AS	++++	t	t	t	t	t	t	t	t	t	t	t	t	t	t	t	t	t	t	t	t	t	t	t
g	4356253	g	4388267	a	4302820	ns	g	S88	++++	g	g	g	g	g	g	g	g	g	g	g	g	g	g	g	g	g	g	g	g	g	g	g
g	4358731	a	4390745	a	4305298	s	g	AS	+++	g	g	g	g											g	g	g						
g	4376700	a	4408714	g	4323364	ns	g	APEC	++++	g	g	g	g	g	g	g	g	g	g	g	g	g	g	g	g	g	g	g	g	g	g	g
g	4378502	t	4410516	g	4325166	s	g	APEC	++++	g	g	g	g	g	g	g	g	g	g	g	g	g	g	g	g	g	g	g	g	g	g	t
c	4381800	a	4413814	a	4328464	s	a	UTI89	++	a	a	a	a	a	a	a	c	c	c	c	a	a	a	c	c	c	c	c	c	c	c	c
c	4386168	c	4418182	t	4332832	ns	c	S88	++++	c	c	c	c	c	c	c	c	c	c	c	c	c	c	c	c	c	c	c	c	c	c	c
c	4392465	t	4424479	c	4339129	ns	c	APEC	++++	c	c	c	c	c	c	c	c	c	c	c	c	c	c	c	c	c	c	c	c	c	c	c
c	4396844	t	4428858	c	4343508	ns	c	APEC	++++	c	c	c	c	c	c	c	c	c	c	c	c	c	c	c	c	c	c	c	c	c	c	c
g	4398086	g	4430100	a	4344750	s	g	S88	++++	g	g	g	g	g	g	g	g	g	g	g	g	g	g	g	g	g	g	g	g	g	g	a
c	4399624	t	4431638	t	4346288	s	c	AS	++++	c	c	c	c	c	c	c	c	c	c	c	c	c	c	c	c	c	c	c	c	c	c	c
c	4415530	c	4447544	t	4362194	ns	c	S88	+++	c	c	c	c	c	c	c																
c	4416029	a	4448043	a	4362693	ns	c	AS	+++	c	c	c	c	c	c	c																
g	4418667	a	4450681	a	4365331	ns	a	UTI89	++++	a	a	a	a	a	a	a	a	a	a	a	a	a	a	a	a	a	a	a	a	a	a	a
g	4425710	g	4457724	a	4372374	ns	g	S88	++++	g	g	g	g	g	g	g	g	g	g	g	g	g	g	g	g	g	g	g	g	g	g	g
a	4432590	g	4464604	g	4379254	s	g	UTI89	++++	g	g	g	g	g	g	g	g	g	g	g	g	g	g	g	g	g	g	g	g	g	g	g

ExPEC Cluster										Outgroup Strains[g]																						
UTI89[a]	UTI89 site[b]	APEC 01[a]	APEC 01 site[b]	S88[a]	S88 site[b]	type[c]	Inferred ancestral base[d]	Lineage inferred to mutate[e]	Support level[f]	CFT073	ED1a	536	E2348/69	SMS 3-5	IAI39	UMN026	K-12	ATCC 8739	HS	D1 Sd197	CB9615	Sakai	EDL933	IAI1	E24377A	SE11	SS Ss046	F2a 301	F2a 2457T	F5 8401	B4 Sb227	B18 BS512
t	4439821	c	4471835	c	4386485	ns	c	UTI89	++++	c	c	c	c	c	c	c	c	c	c	c	c	c	c	c	c	c	c	c	c	c	c	c
g	4451182	c	4483196	c	4397846	s	c	UTI89	++++	c	c	c	c	c	c	c	c	c	c	c	c	c	c	c	c	c	c	c	c	c	c	c
t	4456503	c	4488517	c	4403167	nc	t	AS	++++	t	c	t	t	t	t	t	t	t	t	t	t	t	t	t	t	t	t	t	t	t	t	t
c	4456576	a	4488591	a	4403241	nc	c	AS	++++	c	c	c	c	c	c	c	c	c	c	c	c	c	c	c	c	c	c	c	c	c	c	c
a	4470134	t	4542436	t	4456964	ns	a	AS	++++	a	a	a	a	a	a	a	a	a	a	a	a	a	a	a	a	a	a	a	a	a	a	
a	4478703	g	4551005	g	4465533	ns	g	UTI89	++++	g	g	g	g	g	g	g				g	g	g	g				g	g	g	g	g	g
g	4478744	t	4551046	t	4465574	s	g	AS	++++	g	g	g	g	g	g	g				g	g	g	g				g	g	g	g	g	g
t	4481797	a	4554099	a	4468627	s	a	UTI89	++++	a	a	a	a	a	a	a				a	a	a	a				a	a	a	a	a	a
c	4492351	t	4564653	t	4479181	ns	c	AS	++++	c	c	c	c	c	c	c	c	c	c	c	c	c	c	c	c	c	c	c	c	c	c	c
a	4500681	g	4572983	g	4487511	ns	g	UTI89	++++	g	g	g	g	g	g	g	g	g	g		g	g	g	g	g	g	g	g	g	g		
g	4501489	a	4573791	a	4488319	s	g	AS	++++	g	g	g	g	g	g	g	g	g	g	g	g	g	g	g	g	g	g	g	g	g	g	g
t	4505609	c	4577911	c	4492439	s	c	UTI89	++++	c	c	c	c	c	c	c	c	c	c	c	c	c	c	c	c	c	c	c	c	c	c	c
t	4517245	a	4589547	a	4504075	ns	a	UTI89	++	a																						
a	4517608	t	4589910	a	4504438	nc	a	APEC	++	a																						
c	4527611	t	4599913	t	4514441	s	c	AS	+++	c	c	c	c																			
c	4532922	a	4605224	a	4519752	ns	a	UTI89	+++	a	a	a	a																			
g	4535506	g	4607808	a	4522336	ns	g	S88	+++	g	g	g	g																			
c	4539161	a	4611463	c	4525991	ns	c	APEC	++++	c	c	c	c	c	c	c	c	c	c	c	c	c	c	c	c	c	c	c	c	c	c	c
t	4547605	t	4619907	g	4534435	s	t	S88	++++	t	t	t	t	t	t	t	t	t	t	t	t	t	t	t	t	t	t	t	t	t		t
t	4548569	g	4620871	g	4535399	ns	g	UTI89	++++	g	g	g	g	g	g	g	g	g	g	g	g	g	g	g	g	g	g	g	g	g		g
t	4563564	c	4635866	c	4550394	nc	c	UTI89	++	c	c	c	c	c	c	c	c	t	t		c	c	c	t	t	t	c	c	c	c	c	c
t	4575924	c	4648226	c	4562754	s	c	UTI89	++++	c	c	c	c	c	c	c	c	c	c		c	c	c	c	c	c	c	c	c	c	c	
1	4587439	-	4659740	-	4574268	ins	-	UTI89	++++	-	-	-	-	-	-		-	-								-						
g	4589577	a	4661878	a	4576406	s	g	AS	++++	g	g	g	g	g	g	g	g	g	g	g	g	g	g	g	g	g	g				g	
g	4592588	g	4664889	a	4579417	s	g	S88	++++	g	g	g	g	g	g	g	g	g	g	g	g	g	g	g	g	g	g				g	
c	4594196	c	4666497	t	4581025	s	c	S88	++++	c	c	c	c	c	c	c	c	c	c	c	c	c	c	c	c	c	c				c	
c	4595967	t	4668268	c	4582796	ns	c	APEC	++++	c	c	c	c	c	c	c	c	c	c	c	c	c	c	c	c	c	c				c	c
c	4597175	t	4669476	c	4584004	s	c	APEC	++++	c	c	c	c	c	c	c	c	c	c	c	c	c	c	c	c	c	c				c	c
c	4606547	t	4678848	t	4593376	s	c	AS	++++	c	c	c	c	c	c	c	c	c	c	c	c	c	c	c	c	c	c	c	c	c	c	
a	4609416	g	4681717	g	4596245	nc	g	UTI89	++	g	g	g	g	g	g	g	g	g	g	c	c	c	c	g	g	g	g	g	g	g	g	g
c	4611716	t	4684017	c	4598545	nc	c	APEC	++++	c	c	c	c	c	c	c	c	c	c	c	c	c	c	c	c	c	c	c	c	c	c	c
a	4619731	c	4692032	c	4606560	ns	c	UTI89	++++	c	c	c	c	c	c	c	c	c	c	c	c	c	c	c	c	c	c	c	c	c	c	
t	4624459	g	4696760	g	4611288	ns	g	UTI89	++++	g	g	g	g	g	g	g	g	g	g	g	g	g	g	g	g	g	g				g	
1	4624611	-	4696911	-	4611439	del	1	AS	++	1	-	1	1	1	1	1	-	-	-	-	-	-	-	-	-	-	-				-	
a	4632392	a	4704692	g	4619220	nc	a	S88	++++	a	a	a	a	a	a	a	a	a	a		a	a	a	a	a	a	a					
t	4632763	c	4705063	c	4619591	s	c	UTI89	++++	c	c	c	c	c	c	c	c	c	c		c	c	c	c	c	c	c					
g	4633784	a	4706084	a	4620612	ns	a	UTI89	++++	a	a	a	a	a	a	a	a	a	a		a	a	a	a	a	a	a					
t	4634424	c	4706724	t	4621252	nc	t	APEC	++++	t	t	t	t	t	t	t	t		t	t	t	t	t	t	t	t	t	t	t	t		
1	4634670	-	4706969	-	4621497	ins	-	UTI89	++	-	-	1	1	1	-	1	1	1	1	1	1	1	1	1	1	1	1	1	1	1	1	1
-	4634685	t	4707039	c	4621567	nc	t	S88	++	t	t				t																	
-	4634685	g	4718986	a	4633514	nc	g	S88	++++	g	g	g		g	g							g	g		g							
-	4634685	1	4721636	-	4636163	indel	?	APEC/S88	+/-	1	-																					
-	4634685	c	4727253	t	4641780	nc	t	APEC	++	t	t																					
-	4634685	a	4728234	g	4642761	nc	g	APEC	++	g	g			g																		
-	4634685	t	4730261	a	4644788	nc	a	APEC	++	a	a																					
-	4634685	9	4735685	-	4650211	indel	?	APEC/S88	+/-	-	9																					
-	4634685	c	4742689	a	4657207	nc	c	S88	++	c	c																					
-	4634685	t	4752085	c	4666747	nc	t	S88	++	t																						
-	4634685	c	4756391	t	4671053	nc	c	S88	++	c	c																					
c	4659911	t	4795635	t	4710297	s	c	AS	++	c	c	c	c	c	t	c	t	t	c	c	c	c	c	t	t	c	t	t	t	t	t	t
a	4668288	g	4804012	g	4718674	ns	a	AS	++	a	a	g	g	g	g	g	g	g	g	g	g	g	g	g	g	g	g	g	g	g	g	g
g	4685229	t	4820953	t	4735615	ns	t	UTI89	++++	t	t	t	t	t	t	t	t	t	t	t	t	t	t	t	t	t	t	t	t	t	t	t
c	4685567	c	4821291	t	4735953	ns	c	S88	++++	c	c	c	c	c	c	c	c	c	c	c	c	c	c	c	c	c	c	c	c	c	c	c
c	4688115	c	4823839	t	4738501	nc	c	S88	++++	c	c	c	c	c	c	c	c	c	c	c	c	c	c	c	c	c	c	c	c	c	c	c
c	4693391	a	4829115	c	4743777	s	c	APEC	++++	c	c	c	c	c	c	c	c	c	c	c	c	c	c	c	c	c	c	c	c	c	c	c
c	4695091	t	4830815	t	4745477	ns	c	AS	++++	c	c	c	c	c	c	c	c	c	c	c	c	c	c	c	c	c	c	c	c	c	c	c
c	4712240	t	4847187	-	4752209	s	c	APEC	++++	c	c	c	c	c	c	c	c	c	c	c	c	c	c	c	c	c	c	c	c	c	c	c
c	4724285	a	4859232	-	4752209	ns	a	UTI89	++++	a	a	a	a	a	a	a	a	a	a	a	a	a	a	a	a	a	a	a	a	a	a	a
a	4731087	t	4866034	-	4752209	ns	a	APEC	++++	a	a	a	a	a	a	a	a	a	a	a	a	a	a	a	a	a	a	a	a	a	a	a
a	4740160	g	4875107	-	4752209	s	a	APEC	++++	a	a	a	a	a	a	a	a	a	a	a	a	a	a	a	a	a	a	a	a	a	a	a
t	4748256	c	4883203	-	4752209	s	c	UTI89	++++	c	c	c	c	c	c	c	c	c	c	c	c	c	c	c	c	c	c	c	c	c	c	c
a	4754031	t	4888978	-	4752209	ns	a	APEC	+++	a	a	a			a																	
g	4754373	a	4889320	-	4752209	ns	a	UTI89	+++	a	a	a			a																	
c	4755780	t	4890727	-	4752209	ns	c	APEC	+++	c	c	c			c																	
g	4755827	c	4890774	-	4752209	ns	g	APEC	+++	g	g	g			g																	
c	4758564	t	4893511	-	4752209	ns	c	APEC	+++	c	c	c			c																	
t	4759928	c	4894875	-	4752209	nc	t	APEC	++	t	c	t	t	t	t	t	c	c	t	c	c	c	c	t	c	t	t	c	c	c	c	c
a	4759932	g	4894879	-	4752209	nc	g	UTI89	++++	g	g	g	g	g	g	g	g	g	g	g	g	g	g	g	g	g	g	g	g	g	g	g
-	4770283	1	4905231	-	4752209	ins	-	APEC	++++	-	-	-	1	-	-	-	-	-	-	-	-	-	-	-	-	-	-	-	-	-	-	-
c	4771664	t	4906612	-	4752209	ns	c	APEC	++++	c	c	c	c	c	c	c	c	c	c	c	c	c	c	c	c	c	c	c	c	c	c	c
g	4774899	t	4909847	-	4752209	ns	g	APEC	++++	g	g	g		g	g	g	g		g								g					
a	4775219	c	4910167	-	4752209	ns	c	UTI89	++++	c	c	c		c	c	c	c		c								c					
g	4791714	g	3318606	a	3233390	s	g	S88	+++	g		g			g	g																
t	4792233	g	3319125	g	3233909	s	t	AS	+	t		g			t	t																
a	4793296	t	3320188	t	3234972	s	t	UTI89	+++	t		t			t	t																
a	4794105	c	3320997	c	3235781	ns	a	AS	+++	a		a			a	a																
a	4794478	g	3321370	g	3236154	s	g	UTI89	+++	g		g			g	g																
t	4794495	c	3321387	c	3236171	ns	c	UTI89	+++	c		c			c	c																
g	4794803	a	3321695	g	3236479	ns	g	APEC	+++	g		g			g	g																
g	4796431	g	3323323	a	3238107	nc	?	S88	+/-																							
g	4907674	t	4923903	g	4885749	nc	g	APEC	++	g	g	g	g	g	t	g	g		g		t	t	t	g		g	g	g	g		g	
g	4912752	a	4928981	g	4890828	s	g	APEC	++++	g		g	g	g	g	g	g		g		g	g	g	g	g	g	g	g	g		g	g

ExPEC Cluster										Outgroup Strains[g]																						
UTI89[a]	UTI89 site[b]	APEC 01[a]	APEC 01 site[b]	S88[a]	S88 site[b]	type[c]	Inferred ancestral base[d]	Lineage inferred to mutate[e]	Support level[f]	CFT073	ED1a	536	E2348/69	SMS 3-5	IAI39	UMN026	K-12	ATCC 8739	HS	D1 Sd197	CB9615	Sakai	EDL933	IAI1	E24377A	SE11	SS Ss046	F2a 301	F2a 2457T	F5 8401	B4 Sb227	B18 BS512
g	4913801	t	4930030	t	4891877	ns	t	UTI89	++++	t	t	t	t	t	t	t	t	t	t		t	t	t	t	t	t	t	t	t		t	t
c	4919522	t	4935751	t	4899954	s	t	UTI89	++++	t		t	t	t			t	t									t	t	t		t	t
c	4945745	t	4961974	c	4911459	s	c	APEC	++++	c		c	c	c	c	c	c	c	c		c	c	c	c	c	c					c	c
c	4949832	a	4966061	c	4918134	s	c	APEC	++++	c	c	c	c	c	c	c	c	c	c	c	c	c	c	c	c	c					c	c
t	4974091	g	4990320	t	4940268	ns	t	APEC	++++	t	t	t	t	t	t	t	t	t	t		t	t	t	t	t	t	t	t	t	t	t	t
c	4974109	t	4990338	c	4940286	s	c	APEC	++++	c	c	c	c	t	c	c	c	c	c		c	c	c	c	c	c	c	c	c	c	c	c
a	4976994	g	4993223	g	4943169	s	a	AS	++	a	a	a	a	g	a	a	g	g	g	g	g	g	g	g	g	g	g	g	g	g	g	g
a	4983543	g	4999772	g	4949716	s	a	AS	++	a	a	a	a	a	a	a	g	g	g	g	g	g	g	g	g	g	g	g	g	g	a	a
a	4983865	g	5000094	a	4950038	nc	a	APEC	++++	a	a	a	a	a	a	a	a	a	a	a	a	a	a	a	a	a	a	a	a	a	a	a
t	4986069	g	5002299	g	4952310	s	g	UTI89	++++	g	g	g	g	g	g	g	g	g	g	g	g	g	g	g	g	g	g	g	g	g	g	g
g	4988688	a	5004918	a	4954929	nc	?	UTI89/AS	+/-																							
a	4988713	g	5004943	g	4954954	nc	?	UTI89/AS	+/-																							
c	4989741	t	5005971	c	4955982	ns	?	APEC	+/-																							
g	4989979	a	5006209	a	4956220	s	?	UTI89/AS	+/-																							
c	4990632	t	5006862	t	4956873	ns	c	AS	++												c											
a	4992238	g	5007574	g	4957585	ns	g	UTI89	++++								g				g						g					
g	4996702	a	5012038	a	4962049	s	g	AS	++		g				g																t	
g	5001290	g	5015977	t	4965987	s	g	S88	++	g	g				t	g					g											t
a	5009646	g	5026944	a	4976954	ns	a	APEC	+				a		a	a					a	a	a									
g	5024687	g	5041982	a	4991992	s	g	S88	++	g	g		g	g	g	g		g	g		g	g	g	g		g	a	a	a	a	a	g
g	5037621	a	5053886	g	5003896	s	g	APEC	++++	g	g	g	g	g	g	g	g	g	g	g	g	g	g	g	g	g	g	g	g	g	g	g
c	5040452	t	5056717	c	5006930	s	c	APEC	++++	c	c	c	c	c	c	c	c	c	c	c	c	c	c	c	c	c	c	c	c	c	t	c
c	5051251	t	5067516	c	5017778	s	c	APEC	++++	c	c	c	c	c	c	c	c	c	c	c	c	c	c	c	c	c	c	c	c	c	c	c
g	5051312	g	5067577	t	5017839	ns	g	S88	++++	g	g	g	g	g	g	g	g	g	g	g	g	g	g	g	g	g	g	g	g	g	g	g

[a]Numbers in place of bases indicates number of bases where >2 bases inserted or deleted. In these cases "-" indicates absence of these bases.

[b]For indels the base indicated is the base before the insertion or deletion in the strain.

[c] s: synonymous; ns: non-synonymous; nc: in non-coding gene; i: intergenic; ins: small insert; del: small deletion; indel: the small indels that can't be allocated

[d] The base in the common ancestor of the 3 ExPEC strains as inferred from outgroup analysis.

[e] AS: allocated to the lineage to the common ancestor of APEC 01 and S88; UTI89/AS: allocated to the divergence between UTI89 and the common ancestor of APEC 01 and S88 (strain not specified); APEC/S88: allocated to the divergence between APEC 01 and S88 lineages (lineage not specified)..

[f] Level of support for allocation of mutation as given in previous column

++++ agreement is high - 8 or more outgroup strains with expected base and at most 1 with an alternative base, and at least 2 of the CFT073, ED1a and 536 support the expected base)

+++ agreement good - 4 or more outgroup strains with expected base and at most 1 with an alternative base, and at least 2 of the CFT073, ED1a and 536 support the expected base)

++ supported by at least 2 of the CFT073, ED1a and 536 regardless of situation with other outgroup strains

+ no conflict but very limited support as either site absent or conflict in all of CFT073, ED1a and 536, or conflict among them, and/or support is less than required for any of the higher levels of support.

+/- no conflict but no support (base not present in any outgroup OR base when present is not that in any of the ExEPEC strains OR both alternative lineages supported equallly.

- conflict data implies 2 mutations at that site - eg in the ancestor of 3 ExEPEC strains before isolation and again in one of the lineages.

[g] Base, number or "-" indicates the base type or absence of the base. Blank means the site not present.
