# Supplementary material for: Derivation of Escherichia coli O157:H7 from Its O55:H7 Precursor
Source: PLoS One. 2010 Jan 14;5(1):e8700. doi: 10.1371/journal.pone.0008700 (PMC2806823; doi:10.1371/journal.pone.0008700)
Supplement: Table S8 — Virtual outgroup analysis for allocation of recombinant regions to the UTI189, S88, or APEC 01 lineages. The 23 genomes used for the analysis are shown, with details of the base or bases present in both outgroup genomes and genomes under analysis, and also the final allocation and a measure of support level for that allocation. (0.32 MB PDF) [file pone.0008700.s010.pdf]

Table S8. Allocation of recombinational SNPs to lineages by virtual outgroup analysis[a]

ExPEC genome site details												Outgroup Strains Details[i]																						
UTI89[b]	UTI89 site[c]	APEC 01[b]	APEC 01 site[c]	S88[b]	S88 site[c]	type[d]	Event Lineage[ef]	Recombinant segment	Inferred ancestral base[g]	Outgroup Analysis[e]	Support level[h]	CFT073	ED1a	536	E2348/69	SMS 3-5	IAI39	UMN026	K-12	ATCC 8739	HS	D1 Sd197	CB9615	Sakai	EDL933	IAI1	E24377A	SE11	SS Ss046	F2a 301	F2a 2457T	F5 8401	B4 Sb227	B18 BS512
a	221635	a	221611	c	221634	ns	S88	rec1	a	S88	++++	a	a	a	a	a	a	a	a	a	a	a	a	a	a	a	a	a	a	a	a	a	a	a
t	221636	t	221612	a	221635	s	S88	rec1	t	S88	++++	t	t	t	t	t	t	t	t	t	t	t	t	t	t	t	t	t	t	t	t	t	t	t
g	229525	a	229501	g	229524	nc	APEC	rec2	g	APEC	++	a	g	g	g	g	a	g	a	g	g	a	a	a	a	g	g	g	a	g	g	g	g	g
g	229529	c	229505	g	229528	nc	APEC	rec2	g	APEC	++	c	g	g	g	g	c	g	c	g	g	c	c	c	c	g	g	g	c	g	g	g	g	g
t	229533	c	229509	t	229532	nc	APEC	rec2	t	APEC	++	c	t	t	t	t	c	t	c	t	t	g	c	c	c	t	t	t	c	t	t	t	t	t
a	229542	g	229518	a	229541	nc	APEC	rec2	a	APEC	++++	-	a	a	a	a	-	a	-	a	a	c	-	-	-	a	a	a	-	a	a	a	a	a
g	229543	a	229519	g	229542	nc	APEC	rec2	g	APEC	++++	g	g	g	g	g	g	g	g	g	g	g	g	g	g	g	g	g	g	g	g	g	g	g
a	229544	t	229520	a	229543	nc	APEC	rec2	a	APEC	++++	a	a	a	a	a	a	a	a	a	a	a	a	a	a	a	a	a	a	a	a	a	a	a
a	229545	t	229521	a	229544	nc	APEC	rec2	a	APEC	++	t	a	a	a	a	t	a	t	a	a	g	t	t	t	a	a	a	t	a	a	a	a	a
t	229546	g	229522	t	229545	nc	APEC	rec2	t	APEC	++++	t	t	t	t	t	t	t	t	t	t	g	t	t	t	t	t	t	t	t	t	t	t	t
c	229561	t	229537	c	229560	nc	APEC	rec2	c	APEC	++	t	c	c	c	c	t	c	t	c	c	t	t	t	t	c	c	c	t	c	c	c	c	c
a	230086	c	230062	a	230085	nc	APEC	rec2	c	2	-	c	a	c	a	c	c	c	c	c	a	t	c	c	c	t	c	t	c	t	t	t	c	c
g	230585	a	230556	g	230584	nc	APEC	rec2	a	2	-	a	g	a	g	a	a	a	a	a	a	a	a	a	a	a	a	a	a	a	a	a	a	a
a	230858	g	230829	a	230857	ns	APEC	rec2	g	2	-	g	a	g	a	a	a	g	g	a	g	a	a	a	a	a	g	g	a	a	a	a	a	a
t	230859	c	230830	t	230858	ns	APEC	rec2	c	2	-	c	c	c	t	c	c	c	c	t	c	t	c	c	c	t	c	c	t	c	c	c	t	t
c	231043	a	231014	c	231042	nc	APEC	rec2	c	APEC	++	a	c	c	c	c	c	c	a	c	c	c	c	c	c	c	c	c	c	c	a	c	c	c
c	231045	g	231016	c	231044	nc	APEC	rec2	c	APEC	++	g	c	c	c	c	c	c	g	c	c	c	c	c	c	c	c	c	c	c	g	c	c	c
t	231046	c	231017	t	231045	nc	APEC	rec2	t	APEC	++	c	t	t	t	t	t	t	c	t	t	t	t	t	t	t	t	t	t	t	c	t	t	t
t	231050	g	231021	t	231049	nc	APEC	rec2	t	APEC	++	g	t	t	t	t	t	t	g	t	g	t	t	t	t	g	g	g	g	g	g	g	g	g
g	231052	c	231023	g	231051	nc	APEC	rec2	g	APEC	++	c	g	g	g	g	g	g	g	g	g	g	g	g	g	g	g	g	g	g	c	g	g	g
-	231053	1	231025	-	231052	ins	APEC	rec2	-	APEC	++	1	-	-	-	-	-	-	1	-	1	-	1	1	1	1	1	1	1	1	1	1	1	1
t	231347	c	231319	t	231346	nc	APEC	rec2	t	APEC	++	c	t	t	t	t	t	c	c	t	t	c	t	t	t	t	t	t	t	t	t	t	t	t
a	231672	g	231644	a	231671	nc	APEC	rec2	a	APEC	++	g	a	a	a	a	a	g	g	a	a	a	a	a	a	g	g	a	g	g	g	g	g	a
-	231674	1	231647	-	231673	ins	APEC	rec2	-	APEC	++	1	-	-	-	-	-	1	1	1	-	-	-	-	-	1	1	-	1	1	1	1	1	-
t	231678	c	231651	t	231677	nc	APEC	rec2	t	APEC	++	c	t	t	t	t	t	c	c	t	t	t	t	t	t	c	c	t	c	c	c	c	c	t
g	231719	t	231692	g	231718	nc	APEC	rec2	g	APEC	++	t	g	g	g	t	t	t	t	t	t	t	t	t	t	t	t	t	t	t	t	t	t	t
c	231720	a	231693	c	231719	nc	APEC	rec2	c	APEC	++	a	c	c	c	g	a	a	a	a	g	a	g	a	a	g	g	a	g	g	g	g	g	a
g	231729	t	231702	g	231728	nc	APEC	rec2	g	APEC	++	t	g	g	g	c	t	t	t	t	c	t	c	t	t	c	c	t	c	t	t	t	t	t
t	231730	a	231703	t	231729	nc	APEC	rec2	t	APEC	++	a	t	t	t	a	a	a	a	a	a	a	a	a	a	a	a	a	a	a	a	a	a	a
c	232006	t	231979	c	232005	nc	APEC	rec2	c	APEC	++	t	c	c	c	t	t	t	t	t	t	t	t	t	t	t	t	t	t	t	t	t	c	c
g	232048	a	232021	g	232047	nc	APEC	rec2	g	APEC	++	a	g	g	g	a	a	a	a	a	a	a	a	a	a	a	a	a	a	a	a	a	a	a
2	232370	-	232342	2	232369	del	APEC	rec2	2	APEC	++	-	2	2	2	2	2	2	-	-	-	-	-	-	-	-	-	-	-	-	-	-	-	-
a	232372	c	232343	a	232371	nc	APEC	rec2	a	APEC	++	a	a	a	a	a	a	a	c	c	c	c	c	c	c	c	c	c	c	c	a	c	c	c
c	232374	a	232345	c	232373	nc	APEC	rec2	c	APEC	++	-	c	c	c	c	c	c	a	a	a	a	a	a	a	a	a	a	a	a	-	a	a	a
g	232707	a	232678	g	232706	nc	APEC	rec2	g	APEC	++	a	g	g	g	g	g	g	a	a	a	a	a	a	a	a	a	a	a	a	a	a	a	a
-	232741	1	232713	-	232740	ins	APEC	rec2	-	APEC	++++	-	-	-	-	-	-	-	-	-	-	-	-	-	-	-	-	-	-	-	-	-	-	-
-	232747	1	232720	-	232746	ins	APEC	rec2	-	APEC	++++	-	-	-	-	-	-	-	-	-	-	-	-	-	-	-	-	-	-	-	-	-	-	-
g	232758	t	232731	g	232757	nc	APEC	rec2	g	APEC	++++	g	g	g	g	g	g	g	g	g	g	g	g	g	g	g	g	g	g	g	g	g	g	g
c	232760	t	232733	c	232759	nc	APEC	rec2	c	APEC	++++	c	c	c	c	c	c	c	c	c	c	c	c	c	c	c	c	c	c	c	c	c	c	c
-	232767	1	232741	-	232766	ins	APEC	rec2	-	APEC	++++	-	-	-	-	-	-	-	-	-	-	-	-	-	-	-	-	-	-	-	-	-	-	-
a	232786	c	232760	a	232785	nc	APEC	rec2	a	APEC	++++	a	a	a	a	a	a	a	a	a	a	a	a	a	a	a	a	a	a	a	a	a	a	a
a	232800	g	232774	a	232799	nc	APEC	rec2	a	APEC	++++	a	a	a	a	a	a	a	a	a	a	a	a	a	a	a	a	a	a	a	a	a	a	a
c	232802	g	232776	c	232801	nc	APEC	rec2	c	APEC	++++	c	c	c	c	c	c	c	c	c	c	c	c	c	c	c	c	c	c	c	c	c	c	c
-	232802	1	232777	-	232801	ins	APEC	rec2	-	APEC	++++	-	-	-	-	-	-	-	-	-	-	-	-	-	-	-	-	-	-	-	-	-	-	-
g	232805	t	232780	g	232804	nc	APEC	rec2	g	APEC	++++	g	g	g	g	g	g	g	g	g	g	g	g	g	g	g	g	g	g	g	g	g	g	g
c	241883	g	241861	c	241882	nc	APEC	rec2	c	APEC	++++	c	c	c	c	c	c	c	c	c	c	c	c	c	c	c	c	c	c	c	c	c	c	c
a	241885	t	241863	a	241884	nc	APEC	rec2	a	APEC	++	a	a	a	a	a	a	a	a	a	a	a	a	a	a	a	a	a	a	g	g	a	a	a
c	241886	t	241864	c	241885	nc	APEC	rec2	c	APEC	++++	c	c	c	c	c	c	c	c	c	c	c	c	c	c	c	c	c	c	c	c	c	c	c
a	569110	a	570708	c	558494	nc	S88	rec3	a	S88	++	a	a	a	a		c	c	c				c					c						
t	569137	t	570735	a	558521	ns	S88	rec3	t	S88	++	t	t	t	t		a	a	a				a					a						
t	569140	t	570738	c	558524	ns	S88	rec3	t	S88	++	t	t	t	t		c	c	c				c					c						
t	569143	t	570741	c	558527	ns	S88	rec3	t	S88	++	t	t	t	t		c	c	c				c					c						
g	569153	g	570751	c	558537	ns	S88	rec3	g	S88	++++	g	g	g	g		c	g	g				g					g						
t	1267541	t	1204836	c	1208449	s	S88	rec4	c	2	-	t	c		c		c	c					t	c	c			g		c	c	c		c
t	1268200	t	1205495	a	1209108	ns	S88	rec4	a	2	-	t	a		a		a	a					a					a		t	t	t		a
g	1268888	g	1206183	t	1209796	s	S88	rec4	g	S88	+++	g			g			g					g											
c	1269196	c	1206491	t	1210104	ns	S88	rec4	c	S88	+++	c	c		c		c	c					c											c
c	1269426	c	1206721	t	1210334	s	S88	rec4	c	S88	++	c	a	c	a		c	c		c			t	t	t	c		c	c					a
a	1269453	a	1206748	c	1210361	ns	S88	rec4	a	S88	++++	a	a	a	a		a	a		a			c	a	a	a		a	a					a
a	1269455	a	1206750	g	1210363	ns	S88	rec4	a	S88	++++	a	a	a	a		a	a		a			g	a	a	a		a	a					a
a	1269503	a	1206798	g	1210411	s	S88	rec4	a	S88	+	a	c	g	c		g	g		g			g	g	g	g		g	g					c
t	1269509	t	1206804	c	1210417	s	S88	rec4	t	S88	+	t	g	c	g		t	t		t			c	t	t	t		t	c					g
g	1269516	g	1206811	t	1210424	ns	S88	rec4	g	S88	++	g	t	g	t		g	g		g			g	t	t	g		g	g					t
g	1269563	g	1206858	t	1210471	s	S88	rec4	g	S88	++	g	t	g	t		g	g		t			t	g	g	g		g	t					t
t	1269571	t	1206866	a	1210479	ns	S88	rec4	a	2	-	t	a	a	a		a	a		a			a	a	a	a		a	a					a
g	1269618	g	1206913	a	1210526	ns	S88	rec4	g	S88	++	g	t	g	t		g	g		g			g	g	g	g		g	g					t
a	1269665	a	1206960	g	1210573	s	S88	rec4	a	S88	+	a	c	g	c		g	g		g			g	g	g	g		a	a					c
g	1269772	g	1207067	a	1210680	ns	S88	rec4	g	S88	++++	g		g			g	g		g			a	g	g	g		g	g					
g	1269839	g	1207134	c	1210747	s	S88	rec4	c	2	-	c	c	g	c		c	g		g			g	g	g	g		a	g					c
g	1269908	g	1207203	a	1210816	s	S88	rec4	g	S88	++	g	g	a	g		g	g		g			a	g	g	g		g	g					g
g	1269959	g	1207254	a	1210867	s	S88	rec4	a	2	-	g	a	a	a		g	a		a			a	a	a	a		a	a					a
t	1270039	t	1207334	a	1210947	s	S88	rec4	t	S88	++++	t	t	t	t		t	t		t			t	t	t	t		t	t					t
t	1270102	t	1207397	c	1211010	s	S88	rec4	c	2	-	c	c	c	c		c	c		c			c	c	c	c		c	c					c
g	1270151	g	1207446	a	1211059	ns	S88	rec4	g	S88	++++	g	g	g	g		g	g		g			g	g	g	g		g	g					g
g	1270348	g	1207643	a	1211256	s	S88	rec4	g	S88	++	g	t	g	t		a	g		g			a	g	g	g		g	g					t
a	1270420	a	1207715	c	1211328	ns	S88	rec4	a	S88	++	a	a	a	a		a	a		a			a	a	a	c		c	c					a
a	1270427	a	1207722	t	1211335	s	S88	rec4	a	S88	++	a	a	t	a		t	t		t			t	a	a	t		t	t					a
a	1270428	a	1207723	g	1211336	ns	S88	rec4	a	S88	++	a	a	g	a		g	g		g			g	a	a	g		g	g					a
c	1270429	c	1207724	t	1211337	ns	S88	rec4	c	S88	++	c	c	t	c		t	t		t			t	c	c	t		t	t					c

ExPEC genome site details												Outgroup Strains Details[i]																						
UTI89[b]	UTI89 site[c]	APEC 01[b]	APEC 01 site[c]	S88[b]	S88 site[c]	type[d]	Event Lineage[ef]	Recombinant segment	Inferred ancestral base[g]	Outgroup Analysis[e]	Support level[h]	CFT073	ED1a	536	E2348/69	SMS 3-5	IAI39	UMN026	K-12	ATCC 8739	HS	D1 Sd197	CB9615	Sakai	EDL933	IAI1	E24377A	SE11	SS Ss046	F2a 301	F2a 2457T	F5 8401	B4 Sb227	B18 BS512
c	1270440	c	1207735	a	1211348	ns	S88	rec4	c	S88	++	c	c	g	c		g	g		g			a	c	c	a		a	a					c
t	1270441	g	1207736	c	1211349	ns	S88	rec4	t	S88	++	t	t	t	t		t	t		t			c	t	t	c		c	c					t
g	1270442	g	1207737	a	1211350	ns	S88	rec4	g	S88	++	g	g	a	t		a	a		a			a	g	g	a		a	a					t
g	1270443	g	1207738	a	1211351	ns	S88	rec4	g	S88	++	g	g	a	g		a	a		a			a	g	g	a		a	a					g
c	1270444	c	1207739	t	1211352	ns	S88	rec4	c	S88	+	c	a	t	a		t	t		t			t	c	c	t		t	t					a
a	1270445	a	1207740	g	1211353	ns	S88	rec4	g	2	-	a	g	g	g		g	g		g			g	a	a	g		g	g					g
g	1270457	g	1207752	t	1211365	s	S88	rec4	t	2	-	g	t	t	t		t	g		g			t	g	g	t		t	t					t
c	1270484	c	1207779	g	1211392	ns	S88	rec4	c	S88	++	c	c	c	c		g	g		g			g	c	c	g		g	g					c
g	1270489	g	1207784	a	1211397	ns	S88	rec4	g	S88	++	g	a	g	a		a	g		a			a	a	a	a		g	g					a
c	1270493	c	1207788	t	1211401	s	S88	rec4	c	S88	++	c	t	c	t		t	t		t			t	c	c	t		t	t					t
a	1270505	a	1207800	g	1211413	s	S88	rec4	a	S88	++	a	a	g	a		g	g		g			g	a	a	g		g	g					a
a	1270521	a	1207816	g	1211429	ns	S88	rec4	g	2	-	a	g	g	g		g	g		g			g	a	a	g		g	g					g
a	1270522	a	1207817	t	1211430	ns	S88	rec4	a	S88	++	a	a	t	a		t	t		t			t	a	a	t		t	t					a
a	1270545	a	1207840	g	1211453	ns	S88	rec4	g	2	-	-	g	g	g		g	g		g			g	g	g	g		g	g					g
t	1270595	t	1207890	c	1211503	s	S88	rec4	t	S88	++	t	c	t	c		c	c		c			c	t	t	c		c	c					c
c	1270625	c	1207920	t	1211533	s	S88	rec4	c	S88	++	c	c	c	t		t	t		t			t	c	c	t		t	t					c
c	1270649	c	1207944	a	1211557	s	S88	rec4	c	S88	++	c	t	c	t		t	t		t			a	c	c	t		t	t					t
g	1270808	g	1208103	a	1211716	s	S88	rec4	g	S88	++++	g	g	g	g		g	g		g			a	g	g	g		g	g					g
t	1270838	t	1208133	c	1211746	s	S88	rec4	t	S88	++	t	c	t	c		c	c		c			c	c	c	c		c	c					c
c	1270862	c	1208157	a	1211770	s	S88	rec4	c	S88	++	c	c	c	t		a	a		a			a	a	a	c		a	a					t
t	1270865	t	1208160	c	1211773	s	S88	rec4	t	S88	++	t	a	t	a		c	t		t			c	t	t	t		t	t					a
a	1270871	a	1208166	g	1211779	s	S88	rec4	a	S88	++	a	c	a	t		g	g		g			g	g	g	g		g	g					t
a	1270877	a	1208172	t	1211785	s	S88	rec4	a	S88	++	a	g	a	g		t	a		a			a	a	a	a		a	a					g
g	1270901	g	1208196	a	1211809	s	S88	rec4	g	S88	++++	g	g	g	g		g	g		g			g	g	g	g		g	g					g
t	1270910	t	1208205	c	1211818	s	S88	rec4	t	S88	++	t	a	t	g		c	c		c			c	c	c	c		c	c					g
c	1270916	c	1208211	g	1211824	s	S88	rec4	g	2	-	c	g	t	g		g	g		c			c	c	c	c		c	g					g
g	1270919	g	1208214	a	1211827	s	S88	rec4	g	S88	++	g	g	g	g		a	a		g			g	g	g	g		g	g					g
t	1270925	t	1208220	a	1211833	s	S88	rec4	t	S88	++	t	g	t	g		a	a		t			t	t	t	t		t	c					g
c	1270931	c	1208226	t	1211839	s	S88	rec4	c	S88	++	c	c	c	t		t	t		c			c	c	c	c		c	t					c
t	1270932	t	1208227	g	1211840	ns	S88	rec4	t	S88	++	t	g	t	g		g	g		a			a	a	a	a		a	g					g
c	1270934	c	1208229	t	1211842	ns	S88	rec4	c	S88	++	c	t	c	t		g	t		t			c	c	c	c		t	t					t
g	1270937	g	1208232	c	1211845	s	S88	rec4	g	S88	++	g	c	g	g		c	c		g			g	g	g	g		g	c					g
g	1270940	g	1208235	a	1211848	s	S88	rec4	g	S88	++	g	g	g	g		a	a		g			g	g	g	g		g	a					g
c	1270973	c	1208268	a	1211881	s	S88	rec4	c	S88	+	c	t	a	t		a	c		a			a	a	a	a		a	a					t
g	1270985	g	1208280	a	1211893	s	S88	rec4	g	S88	++	g	a	g	a		a	a		a			a	a	a	a		a	t					a
c	1270988	c	1208283	t	1211896	s	S88	rec4	c	S88	++	c	t	c	t		c	g		c			c	c	c	c		c	c					t
c	1270990	c	1208285	t	1211898	ns	S88	rec4	c	S88	++++	c	c	c	c		t	c		c			c	c	c	c		c	c					c
t	1270994	t	1208289	c	1211902	s	S88	rec4	t	S88	++	t	c	t	c		c	c		c			c	c	c	c		c	c					c
a	1271018	a	1208313	t	1211926	s	S88	rec4	a	S88	++	a	c	a	c		t	t		t			t	t	t	a		t	t					c
a	1271123	a	1208418	g	1212031	s	S88	rec4	a	S88	++	a	g	a	g		g	g		a			a	g	g	a		g	g					g
c	1271124	c	1208419	t	1212032	ns	S88	rec4	c	S88	++	c	t	c	t		t	t		c			c	t	t	c		t	t					t
t	1271147	t	1208442	c	1212055	s	S88	rec4	t	S88	++	t	c	t	c		c	c		c			c	c	c	c		c	c					c
a	1271155	a	1208450	c	1212063	ns	S88	rec4	a	S88	++	a	c	a	c		c	c		a			c	c	c	c		c	c					c
c	1271192	c	1208487	a	1212100	s	S88	rec4	c	S88	++	c	a	c	c		c	a		c			c	c	c	c		c	c					a
a	1271278	a	1208573	g	1212186	ns	S88	rec4	g	2	-	a	g	g	g		g	g		g			g	g	g	g		g	g					g
c	1271313	c	1208608	a	1212221	ns	S88	rec4	c	S88	++	c	c	a	c		a	a		c			a	a	a	a		a	a					c
g	1271316	g	1208611	t	1212224	ns	S88	rec4	g	S88	++	g	g	t	g		t	t		g			t	t	t	t		t	t					g
a	1271398	a	1208693	t	1212306	ns	S88	rec4	a	S88	++	a	c	a	c		t	t		t			t	t	t	t		t	t					c
c	1271456	c	1208751	t	1212364	s	S88	rec4	c	S88	++	c	t	c	t		c	c		c			c	c	c	t		c	c					t
g	1271457	g	1208752	a	1212365	ns	S88	rec4	g	S88	++	g	g	a	g		a	a		a			a	g	g	a		a	a					g
g	1271459	g	1208754	a	1212367	ns	S88	rec4	g	S88	+	g	c	a	t		g	a		a			g	g	g	a		g	a					c
g	1271512	g	1208807	a	1212420	ns	S88	rec4	g	S88	+	g		a	g		a	a		a			a	a	a	a		a	a					a
c	1271537	c	1208832	a	1212445	ns	S88	rec4	c	S88	+	c		a	a		a	a		a			a	a	a	a		a	a					a
t	1271550	t	1208845	g	1212458	ns	S88	rec4	t	S88	+	t		g	g		g	g		g			g	g	g	g		g	g					a
c	1271556	c	1208851	t	1212464	ns	S88	rec4	c	S88	++	c		c	a		c	c		c			c	c	c	c		c	c					a
a	1271623	a	1208918	t	1212531	s	S88	rec4	t	2	-	a	t	t	a		t	t		t			t	c	c	c		t	t					t
g	1271674	g	1208969	a	1212582	s	S88	rec4	g	S88	++	g	c	g	c		g	g		g			g	g	g	g		g	g					c
t	1271761	t	1209056	c	1212669	s	S88	rec4	c	2	-	t	c	c	t		c	c		c			c	c	c	c		c	c					t
t	1271813	t	1209108	c	1212721	s	S88	rec4	t	S88	++	t	c	t	c		c	c		c			c	c	c	c		c	c					c
a	1271832	a	1209127	c	1212740	ns	S88	rec4	a	S88	++	a	a	c	a		c	c		c			c	c	c	c		c	c					a
a	1271882	a	1209177	g	1212790	ns	S88	rec4	g	2	-	a	g	g	g		g	g		g			g	g	g	g		g	g					g
a	1271901	a	1209196	c	1212809	ns	S88	rec4	a	S88	++	a	g	g	g		g	g		g			g	g	g	g		g	g					g
c	1272272	c	1209567	t	1213180	s	S88	rec4	t	2	-	c	t	t	t		t	t		t			t	t	t	t		c	t					t
t	1272299	t	1209594	g	1213207	s	S88	rec4	t	S88	++	t	c	t	c		t	g		g			g	g	g	t		t	t					c
t	1272302	t	1209597	g	1213210	s	S88	rec4	t	S88	++	t	t	t	t		t	g		t			g	g	g	t		t	t					t
t	1272308	t	1209603	g	1213216	s	S88	rec4	g	2	-	t	g	g	g		g	g		g			g	g	g	t		g	t					g
c	1272342	c	1209637	a	1213250	s	S88	rec4	a	2	-	c	a	a	a		a	c		c			c	c	c	a		c	a					a
c	1272377	c	1209672	g	1213285	s	S88	rec4	c	S88	++	c	c	c	c		t	g		g			c	g	g	g		c	g					c
t	1272434	t	1209729	c	1213342	s	S88	rec4	t	S88	++	t	g	g	g		t	c		c			c	c	c	c		t	c					c
t	1272486	t	1209781	g	1213394	ns	S88	rec4	g	2	-	t	g	g	t		g	g		g			t	g	g	g		t	g					g
t	1272491	t	1209786	a	1213399	s	S88	rec4	a	2	-	t	a	a	a		a	a		a			g	g	g	a		t	a					a
c	1277452	g	1214747	g	1218360	s	UTI89/AS	rec5	c	AS	++	g	c	c	c	c	c	c		c			c	t	g	c		c	c	t	t	t	t	c
a	1277521	g	1214816	g	1218429	s	UTI89/AS	rec5	g	UTI89	++	g	g	g	g	g	g	g		g			g	g	g	g		g	a	g	g	g	g	a
c	1277549	t	1214844	t	1218457	s	UTI89/AS	rec5	c	AS	++++	t	c	c	c	c	c	c		c			c	c	c	c		c	c	c	c	c	c	c
g	1277632	a	1214927	a	1218540	s	UTI89/AS	rec5	g	AS	++	a	g	g	g	g	a	g		a			g	g	g	g		a	a	g	g	g	g	g
a	1277647	g	1214942	g	1218555	s	UTI89/AS	rec5	g	UTI89	++	g	g	a	g	g	g	g		g			g	g	g	g		g	g	g	g	g	g	a
g	1277839	a	1215134	a	1218747	s	UTI89/AS	rec5	a	UTI89	++++	a	a	a	a	a	a	a		a	a		a	a	a	a		a	a	a	a	a	a	g
c	1277845	t	1215140	t	1218753	s	UTI89/AS	rec5	t	UTI89	+	t	g	c	t	t	t	t		t	t		t	t	t	t		t	t	t	t	t	t	c
g	1277854	a	1215149	a	1218762	s	UTI89/AS	rec5	g	AS	++	a	g	g	g	g	g	g		g	a		g	g	g	g		g	a	a	a	a	a	g
c	1277863	t	1215158	t	1218771	s	UTI89/AS	rec5	c	AS	++	t	c	c	c	c	c	t		t	t		t	t	t	t		c	t	c	c	c	c	c
g	1277905	a	1215200	a	1218813	s	UTI89/AS	rec5	g	AS	++	a	g	g	g	g	a	a		a	g		a	a	a	a		a	g	a	a	a	a	g
t	1277914	c	1215209	c	1218822	s	UTI89/AS	rec5	t	AS	++	c	t	t	c	c	c	c		c	c		c	c	c	c		c	c	c	c	c	c	t
t	1277923	c	1215218	c	1218831	s	UTI89/AS	rec5	c	UTI89	+	c	g	t	c	c	c	c		c	c		c	c	c	c		c	c	c	c	c	c	t

ExPEC genome site details												Outgroup Strains Details[i]																						
UTI89[b]	UTI89 site[c]	APEC 01[b]	APEC 01 site[c]	S88[b]	S88 site[c]	type[d]	Event Lineage[ef]	Recombinant segment	Inferred ancestral base[g]	Outgroup Analysis[e]	Support level[h]	CFT073	ED1a	536	E2348/69	SMS 3-5	IAI39	UMN026	K-12	ATCC 8739	HS	D1 Sd197	CB9615	Sakai	EDL933	IAI1	E24377A	SE11	SS Ss046	F2a 301	F2a 2457T	F5 8401	B4 Sb227	B18 BS512
c	1277953	g	1215248	g	1218861	s	UTI89/AS	rec5	g	UTI89	++	g	g	c	g	t	g	g		g	c		g	c	g	c		g	c	t	t	t	t	c
g	1277955	a	1215250	a	1218863	ns	UTI89/AS	rec5	a	UTI89	++	a	a	g	a	a	a	a		a	a		a	a	a	a		a	g	a	a	a	a	a
g	1277956	c	1215251	c	1218864	ns	UTI89/AS	rec5	c	UTI89	++	c	c	g	c	c	c	c		c	c		c	c	c	c		c	g	c	c	c	c	c
t	1277986	g	1215281	g	1218894	s	UTI89/AS	rec5	g	UTI89	++	g	g	g	g	g	g	g		g	g		g	t	g	g		g	g	t	t	t	t	t
c	1277998	t	1215293	t	1218906	s	UTI89/AS	rec5	c	AS	++	t	c	c	c	c	c	c		c	c		t	c	c	c		c	c	c	c	c	c	c
g	1278046	a	1215341	a	1218954	s	UTI89/AS	rec5	a	UTI89	++	a	g	a	g	g	g	g		g	g		g	g	g	g		g	g	g	g	g	g	g
t	1278118	c	1215413	c	1219026	s	UTI89/AS	rec5	c	UTI89	++++	c	c	t	c	c	c	c		c	c		c	c	c	c		c	c	c	c	c	c	c
t	1278166	c	1215461	c	1219074	s	UTI89/AS	rec5	c	UTI89	++++	c	g	c	c	c	c	c		c	c		c	c	c	c		c	c	c	c	c	c	c
a	1278337	g	1215632	g	1219245	s	UTI89/AS	rec5	g	UTI89	++	g	a	g	a	a	a	a		g	a		a	g	a	a		a	a	a	a	a	a	a
g	1278346	a	1215641	a	1219254	s	UTI89/AS	rec5	a	UTI89	++	a	a	a	a	a	a	a		g	g		a	g	a	g		a	g	a	a	a	a	a
t	1278361	c	1215656	c	1219269	s	UTI89/AS	rec5	c	UTI89	++	c	t	c	c	c	t	c		t	t		c	t	c	t		c	t	c	c	c	c	c
t	1278376	a	1215671	a	1219284	s	UTI89/AS	rec5	a	UTI89	++	a	t	a	c	g	a	a		a	t		t	a	a	t		c	t	a	a	a	a	g
c	1278418	t	1215713	t	1219326	s	UTI89/AS	rec5	t	UTI89	++	t	t	c	c	c	c	t		c	c		c	t	t	c		c	c	c	c	c	c	c
c	1278433	g	1215728	g	1219341	s	UTI89/AS	rec5	g	UTI89	++	g	c	g	c	g	c	c		c	c		c	g	g	c		c	c	c	c	c	c	g
t	1278436	g	1215731	g	1219344	s	UTI89/AS	rec5	g	UTI89	++	g	g	t	t	t	t	t		t	t		t	g	g	t		t	t	t	t	t	t	t
c	1278444	t	1215739	t	1219352	ns	UTI89/AS	rec5	t	UTI89	++	t	c	t	c	c	c	t		c	c		t	t	t	c		t	c	c	c	c	c	c
t	1278448	c	1215743	c	1219356	s	UTI89/AS	rec5	c	UTI89	++	c	c	c	t	c	t	c		t	c		c	t	t	t		c	c	t	t	t	t	c
t	1278451	g	1215746	g	1219359	s	UTI89/AS	rec5	g	UTI89	++	g	a	g	t	g	t	g		t	g		g	g	g	t		g	g	t	t	t	t	g
g	1278471	a	1215766	a	1219379	ns	UTI89/AS	rec5	g	AS	++++	a	g	g	g	g	g	g		g	g		g	g	g	g		g	g	g	g	g	g	g
c	1278478	g	1215773	g	1219386	s	UTI89/AS	rec5	g	UTI89	++	g	c	g	c	c	c	c		c	c		c	c	c	c		c	c	c	c	c	c	c
g	1278484	a	1215779	a	1219392	s	UTI89/AS	rec5	a	UTI89	++	a	c	a	g	g	g	g		g	g		g	g	g	g		g	g	g	g	g	g	g
t	1278523	c	1215818	c	1219431	s	UTI89/AS	rec5	c	UTI89	++	c	t	c	t	t	t	t		t	c		c	t	c	t		t	c	t	t	t	t	t
t	1278543	c	1215838	c	1219451	ns	UTI89/AS	rec5	t	AS	++++	c	t	t	t	t	t	t		t	t		t	t	t	t		t	t	t	t	t	t	t
t	1278550	a	1215845	a	1219458	s	UTI89/AS	rec5	a	UTI89	++	a	-	a	a	a	a	a		a	g		g	a	a	a		a	g	a	a	a	a	t
t	1278556	g	1215851	g	1219464	s	UTI89/AS	rec5	g	UTI89	+	g	t	c	c	c	c	c		c	g		g	c	c	t		c	g	c	c	c	c	t
g	1278604	a	1215899	a	1219512	s	UTI89/AS	rec5	a	UTI89	++	a	a	g	a	a	a	g		a	a		g	a	a	g		a	a	g	g	g	g	g
c	1278659	t	1215954	t	1219567	s	UTI89/AS	rec5	c	AS	++	t	c	c	c	c	c	c		c	c		c	t	c	t		c	c	c	c	c	c	c
g	1278664	a	1215959	a	1219572	s	UTI89/AS	rec5	g	AS	++++	a	g	g	g	g	g	g		g	g		g	g	g	g		g	g	g	g	g	g	g
c	1278668	t	1215963	t	1219576	s	UTI89/AS	rec5	c	AS	++	t	c	c	c	c	c	c		c	c		c	t	c	t		c	c	c	c	c	c	c
c	1278691	t	1215986	t	1219599	s	UTI89/AS	rec5	t	UTI89	++	t	t	c	c	c	c	c		c	c		c	c	c	t		c	c	c	c	c	c	c
c	1278733	t	1216028	t	1219641	s	UTI89/AS	rec5	t	UTI89	+	t	g	c	c	t	c	c		c	c		c	c	t	t		t	c	c	c	c	c	c
g	1278754	a	1216049	a	1219662	s	UTI89/AS	rec5	a	UTI89	++	a	a	a	a	g	a	a		a	a		g	a	g	a		g	a	a	a	a	a	g
a	1278760	g	1216055	g	1219668	s	UTI89/AS	rec5	a	AS	++	g	a	a	g	a	g	a		g	g		g	a	g	a		g	a	g	g	g	g	a
t	1278793	c	1216088	c	1219701	s	UTI89/AS	rec5	c	UTI89	++	c	c	c	c	c	c	c		c	c		c	c	t	c		t	c	t	t	t	t	t
a	1278832	g	1216127	g	1219740	s	UTI89/AS	rec5	a	AS	++	g	a	a	g	g	g	g		g	g		g	g	g	g		a	g	g	g	g	g	a
c	1278847	t	1216142	t	1219755	s	UTI89/AS	rec5	t	UTI89	++	t	g	t	t	t	c	t		c	t		t	t	c	c		c	t	c	c	c	c	c
g	1412697	c	1350611	c	1354717	s	UTI89/AS	rec6	g	AS	+	g	c		g		c	g					g	g	g			g		g	g	g		c
g	1412698	t	1350612	t	1354718	ns	UTI89/AS	rec6	g	AS	+	g	t		g		t	g					g	g	g			g		g	g	g		t
g	1412751	t	1350665	t	1354771	s	UTI89/AS	rec6	t	UTI89	+	g	t		t		t	g					g	g	g			t		g	g	g		t
t	1412787	c	1350701	c	1354807	s	UTI89/AS	rec6	c	UTI89	+	t	c		c		c	c					t	c	c			c		t	t	t		c
t	1412799	c	1350713	c	1354819	s	UTI89/AS	rec6	c	UTI89	+	t	c		c		c	c					t	a	a			a		c	c	c		c
c	1412838	t	1350752	t	1354858	s	UTI89/AS	rec6	c	AS	+	c	t		c		t	c					c	c	c			c		c	c	c		t
a	1412883	g	1350797	g	1354903	s	UTI89/AS	rec6	a	AS	+	a	g		a		g	g					a	a	a			g		g	g	g		g
c	1412892	t	1350806	t	1354912	s	UTI89/AS	rec6	t	UTI89	+	c	t		t		t	t					c	c	c			c		t	t	t		t
g	1412922	a	1350836	a	1354942	s	UTI89/AS	rec6	g	AS	+	g	a		g		a	a					g	g	g			g		g	g	g		a
c	1412931	a	1350845	a	1354951	s	UTI89/AS	rec6	c	AS	+	c	a		c		a	a					c	c	c			g		a	a	a		a
c	1412946	t	1350860	t	1354966	s	UTI89/AS	rec6	c	AS	+	c	t		c		t	c					c	c	c			c		c	c	c		t
a	1412964	g	1350878	g	1354984	s	UTI89/AS	rec6	g	UTI89	+	a	g		g		g	g					a	a	a			t		g	g	g		g
c	1412976	t	1350890	t	1354996	s	UTI89/AS	rec6	t	UTI89	+	c	t		t		t	t					c	c	c			t		t	t	t		t
a	1413054	g	1350968	g	1355074	s	UTI89/AS	rec6	g	UTI89	+	a	g		g		a	g					a	a	a			a		a	a	a		g
c	1413056	g	1350970	g	1355076	ns	UTI89/AS	rec6	g	UTI89	+	c	g		g		c	g					c	c	c			g		c	c	c		g
c	1413057	t	1350971	t	1355077	ns	UTI89/AS	rec6	c	AS	++	c	c		c		c	c					c	c	c			t		c	c	c		t
g	1413063	a	1350977	a	1355083	s	UTI89/AS	rec6	a	UTI89	+	g	a		a		g	a					g	g	g			g		g	g	g		a
t	1413064	c	1350978	c	1355084	s	UTI89/AS	rec6	c	UTI89	+	t	c		c		t	c					t	t	t			t		t	t	t		c
a	1413066	g	1350980	g	1355086	s	UTI89/AS	rec6	g	UTI89	+	a	g		g		a	g					a	a	a			a		a	a	a		g
c	1413069	t	1350983	t	1355089	s	UTI89/AS	rec6	t	UTI89	+	c	t		t		c	t					c	c	c			t		c	c	c		t
t	1413114	a	1351028	a	1355134	s	UTI89/AS	rec6	a	UTI89	+	t	a		a		a	a					t	a	a			a		a	a	a		a
g	1413126	c	1351040	c	1355146	ns	UTI89/AS	rec6	g	AS	+	g	c		g		c	c					g	g	g			g		g	g	g		c
c	1413159	t	1351073	t	1355179	s	UTI89/AS	rec6	c	AS	+	c	t		c		t	c					c	c	c			g		c	c	c		t
c	1413180	t	1351094	t	1355200	s	UTI89/AS	rec6	t	UTI89	+	c	t		t		t	t					c	t	t			g		t	t	t		t
g	1413210	t	1351124	t	1355230	s	UTI89/AS	rec6	g	AS	+	g	t		g		t	g					g	t	t			g		g	g	g		t
t	1413211	c	1351125	c	1355231	ns	UTI89/AS	rec6	t	AS	+	t	c		t		c	t					t	c	c			t		t	t	t		c
t	1413212	g	1351126	g	1355232	ns	UTI89/AS	rec6	t	AS	+	t	g		t		g	t					t	g	g			t		t	t	t		g
t	1413216	c	1351130	c	1355236	s	UTI89/AS	rec6	c	UTI89	+	t	c		c		c	c					t	c	c			t		t	t	t		c
t	1413219	g	1351133	g	1355239	s	UTI89/AS	rec6	g	UTI89	+	t	g		g		g	g					t	g	g			t		t	t	t		g
t	1413252	a	1351166	a	1355272	s	UTI89/AS	rec6	t	AS	++	t	t		t		a	t					t	t	t			t		t	t	t		a
g	1413315	a	1351229	a	1355335	s	UTI89/AS	rec6	g	AS	+	g	a		g		g	g					g	g	g			a		g	g	g		a
t	1413318	c	1351232	c	1355338	s	UTI89/AS	rec6	c	UTI89	+	t	c		c		c	t					t	c	c			t		t	t	t		c
c	1413336	t	1351250	t	1355356	s	UTI89/AS	rec6	c	AS	+	c	t		a		c	t					c	c	c			c		a	a	a		t
c	1413357	g	1351271	g	1355377	s	UTI89/AS	rec6	c	AS	+	c	g		c		c	c					c	c	c			c		g	g	g		g
a	1413429	c	1351343	c	1355449	ns	UTI89/AS	rec6	c	UTI89	+	a	c		c		a	c					a	c	c			a		a	a	a		c
t	1415931	c	1353845	t	1357951	ns	APEC	rec7	c	2	-	c	t		c		t	c					c	t	t			t		t	t	t		t
c	1415938	g	1353852	c	1357958	s	APEC	rec7	c	APEC	+	g	c		c		g	g					g	c	c			t		g	g	g		c
g	1415953	a	1353867	g	1357973	s	APEC	rec7	g	APEC	+	a	g		g		g	a					a	g	g			t		g	g	g		g
g	1415976	a	1353890	g	1357996	ns	APEC	rec7	g	APEC	+	a	g		g		g	a					a	g	g			t		a	a	a		g
c	1415986	a	1353900	c	1358006	ns	APEC	rec7	a	2	-	a	c		a		a	a					a	a	a			t		g	g	g		c
a	1416010	c	1353924	a	1358030	s	APEC	rec7	c	2	-	c	c		c		c	a					c	a	a			a		g	g	g		a
t	1416013	c	1353927	t	1358033	s	APEC	rec7	c	2	-	c	c		c		c	t					c	t	t			c		t	t	t		t
c	1416022	t	1353936	c	1358042	s	APEC	rec7	c	APEC	+	t	c		c		c	c					t	c	c			g		c	c	c		c
c	1416027	g	1353941	c	1358047	ns	APEC	rec7	g	2	-	g	c		g		g	c					g	g	g			g		g	g	g		c
a	1416028	t	1353942	a	1358048	ns	APEC	rec7	t	2	-	t	a		t		t	a					t	t	t			t		t	t	t		a

ExPEC genome site details												Outgroup Strains Details[i]																						
UTI89[b]	UTI89 site[c]	APEC 01[b]	APEC 01 site[c]	S88[b]	S88 site[c]	type[d]	Event Lineage[ef]	Recombinant segment	Inferred ancestral base[g]	Outgroup Analysis[e]	Support level[h]	CFT073	ED1a	536	E2348/69	SMS 3-5	IAI39	UMN026	K-12	ATCC 8739	HS	D1 Sd197	CB9615	Sakai	EDL933	IAI1	E24377A	SE11	SS Ss046	F2a 301	F2a 2457T	F5 8401	B4 Sb227	B18 BS512
g	1416163	g	1354077	t	1358183	s	S88	rec8	g	S88	+	t	g		g		g	t					t	g	g			g		t	t	t		g
c	1416169	c	1354083	t	1358189	s	S88	rec8	t	2	-	t	c		t		c	c					t	t	t			t		t	t	t		c
c	1416217	c	1354131	t	1358237	s	S88	rec8	t	2	-	t	c		t		c	c					t	t	t			t		t	t	t		c
a	1416281	a	1354195	g	1358301	ns	S88	rec8	g	2	-	g	g		g		a	a					g	g	g			g		g	g	g		a
c	1416283	c	1354197	t	1358303	ns	S88	rec8	t	2	-	t	t		t		c	c					t	t	t			t		t	t	t		c
t	1416301	t	1354215	g	1358321	s	S88	rec8	g	2	-	g	g		g		t	g					g	g	g			g		g	g	g		t
c	1416304	c	1354218	t	1358324	s	S88	rec8	t	2	-	t	t		t		c	c					t	t	t			t		t	t	t		c
c	1416310	c	1354224	t	1358330	s	S88	rec8	t	2	-	t	c		t		c	t					t	c	c			t		t	t	t		c
a	1416331	a	1354245	g	1358351	s	S88	rec8	a	S88	+	g	a		a		a	g					g	a	a			g		g	g	g		a
g	1416406	g	1354320	a	1358426	s	S88	rec8	g	S88	+	a	g		g		g	a					a					a		g	g	g		g
g	1416407	g	1354321	t	1358427	ns	S88	rec8	g	S88	+	t	g		g		g	t					t					g		g	g	g		g
g	1416412	g	1354326	a	1358432	s	S88	rec8	g	S88	+	a	g		g		g	a					a					a		g	g	g		g
c	1416418	c	1354332	t	1358438	s	S88	rec8	c	S88	+	t	c		c		c	t					t					c		c	c	c		c
c	1416421	c	1354335	t	1358441	s	S88	rec8	c	S88	+	t	c		c		c	t					t					t		c	c	c		c
c	1416427	c	1354341	t	1358447	s	S88	rec8	c	S88	+	t	c		c		c	t					t					t		t	t	t		c
t	1416433	t	1354347	c	1358453	s	S88	rec8	t	S88	+	c	t		t		t	c					c					t		t	t	t		t
c	1416478	c	1354392	g	1358498	s	S88	rec8	c	S88	+	g	c		c		c	c					g					c		c	c	c		c
g	1416508	g	1354422	a	1358528	s	S88	rec8	g	S88	+	a	g		g		g	a					a					g		g	g	g		g
c	1416514	c	1354428	t	1358534	s	S88	rec8	c	S88	+	t	c		c		c	t					t					c		c	c	c		c
c	1416518	c	1354432	t	1358538	s	S88	rec8	c	S88	+	t	c		c		c	t					t					c		c	c	c		c
g	1416544	g	1354458	a	1358564	s	S88	rec8	a	2	-	a	a		a		g	a					a					g		a	a	a		g
t	1416547	g	1354461	c	1358567	ns	S88	rec8	c	2	-	c	c		c		t	c					c					g		g	g	g		t
t	1417598	t	1355512	c	1359609	s	S88	rec8	c	2	-	c	t		c		t	c					c											t
c	1417604	c	1355518	t	1359615	s	S88	rec8	t	2	-	t	c		t		t	t					t											c
a	1417613	a	1355527	g	1359624	s	S88	rec8	g	2	-	g	g		g		g	g					g											a
c	1417625	c	1355539	t	1359636	s	S88	rec8	t	2	-	t	t		t		t	t					t											c
g	1417640	g	1355554	c	1359651	s	S88	rec8	c	2	-	c	a		c		g	g					c											g
c	1417641	c	1355555	a	1359652	ns	S88	rec8	a	2	-	a	c		a		a	a					a											c
t	1417644	t	1355558	a	1359655	ns	S88	rec8	a	2	-	a	t		a		c	c					a											t
g	1427249	c	1365163	c	1369260	s	AS	rec9	g	AS	++	g	c	g	c	g	g	g		c	g		g	c	t	g		g	g	c	c	c	c	g
t	1427252	c	1365166	c	1369263	s	AS	rec9	t	AS	+	t	g	c	g	t	c	c		c	t		c	g	g	t		c	c	c	c	c	c	t
t	1427255	a	1365169	a	1369266	s	AS	rec9	t	AS	++	t	t	a	t	g	a	a		a	t		a	a	a	t		a	a	a	a	a	a	g
c	1427279	t	1365193	t	1369290	s	AS	rec9	c	AS	++	c	t	c	t	g	c	c		c	c		c	t	t	c		c	t	t	t	t	t	g
c	1427285	t	1365199	t	1369296	s	AS	rec9	c	AS	++	c	t	c	c	c	t	c		t	c		c	t	t	c		c	c	c	c	c	c	c
c	1427312	g	1365226	g	1369323	s	AS	rec9	c	AS	++	c	c	g	c	g	c	c		c	c		c	c	c	c		g	c	c	c	c	c	g
t	1427315	g	1365229	g	1369326	s	AS	rec9	t	AS	++	t	g	t	g	t	t	t		t	t		t	g	g	t		t	t	t	t	t	t	t
c	1427323	t	1365237	t	1369334	ns	AS	rec9	c	AS	++	c	c	t	c	c	c	t		c	c		c	c	c	c		c	c	c	c	c	c	c
c	1427324	t	1365238	t	1369335	ns	AS	rec9	c	AS	++++	c	c	c	c	c	c	c		c	c		c	c	c	c		c	c	c	c	c	c	c
t	1427327	c	1365241	c	1369338	s	AS	rec9	c	UTI89	++	t	c	c	c	c	t	c		t	c		t	c	c	t		t	t	t	t	t	t	c
t	1427330	g	1365244	g	1369341	s	AS	rec9	t	AS	+	t	a	g	a	g	t	g		t	g		t	a	a	t		g	t	t	t	t	t	g
t	1529070	a	1466985	t	1471082	ns	APEC	rec10	t	APEC	++++	t	t	t	t	t	t	t	t	t	t	t	t	t	t	t	t	t	t	t	t	t	t	t
g	1529071	t	1466986	g	1471083	ns	APEC	rec10	g	APEC	++++	g	g	g	g	g	g	g	g	g	g	g	g	g	g	g	g	g	g	g	g	g	g	g
t	1529072	c	1466987	t	1471084	s	APEC	rec10	t	APEC	++++	t	t	t	t	t	t	t	t	t	t	t	t	t	t	t	t	t	t	t	t	t	t	t
t	1529081	c	1466996	t	1471093	s	APEC	rec10	t	APEC	++	t	t	t	t	a	a	a	t	a	a	t	t	t	t	a	a	t	t	a	a	a	t	t
a	1529085	t	1467000	a	1471097	ns	APEC	rec10	a	APEC	++++	a	a	a	a	a	a	a	a	a	a	a	a	a	a	a	a	a	a	a	a	a	a	a
t	1529087	c	1467002	t	1471099	ns	APEC	rec10	t	APEC	++++	t	t	t	t	t	t	t	t	t	t	t	t	t	t	t	t	t	t	t	t	t	t	t
g	1529089	t	1467004	g	1471101	ns	APEC	rec10	g	APEC	++	g	g	g	g	c	c	c	g	c	c	g	g	g	g	c	c	g	g	c	c	c	g	g
t	1529135	a	1467050	t	1471147	nc	APEC	rec10	t	APEC	++	t	t	t	t				a			a	a	a	a			a	a	a	a	a	a	a
a	1529140	g	1467055	a	1471152	nc	APEC	rec10	a	APEC	++	a	a	a	a				g			g	g	g	g			g	g	g	g	g	g	g
g	1529180	a	1467095	g	1471192	ns	APEC	rec10	g	APEC	++++	g	g	g	g				g			g	g	g	g			g	g	g	g	g	g	g
t	1529381	a	1467296	t	1471393	ns	APEC	rec10	t	APEC	++	t	t	t	t				c				a	c	c			c	a	a	a	a	a	a
c	1543324	t	1533566	c	1485336	s	APEC	rec11	c	APEC	+++	c	c	c					c	c	c													
t	1543585	c	1533827	t	1485597	s	APEC	rec11	c	2	-	c	c	t					t	t	t													
g	1543624	t	1533866	g	1485636	s	APEC	rec11	t	2	-	t	t	t					t	t	t													
t	1543651	g	1533893	t	1485663	s	APEC	rec11	g	2	-	g	g	g					g	g	g													
c	1543657	t	1533899	c	1485669	s	APEC	rec11	c	APEC	++	c	t	c					t	t	t													
c	1543669	t	1533911	c	1485681	s	APEC	rec11	c	APEC	+++	c	t	c					c	c	c													
t	1543675	c	1533917	t	1485687	s	APEC	rec11	c	2	-	t	c	c					c	c	c													
t	1543690	c	1533932	t	1485702	s	APEC	rec11	t	APEC	++	t	t	c					g	g	g													
g	1543720	a	1533962	g	1485732	s	APEC	rec11	g	APEC	+++	g	g	g					g	g	g													
g	1543735	c	1533977	g	1485747	ns	APEC	rec11	c	2	-	c	c	c					c	c	c													
a	1543743	g	1533985	a	1485755	ns	APEC	rec11	a	APEC	+++	a	a	a					a	a	a													
t	1543762	c	1534004	t	1485774	s	APEC	rec11	c	2	-	c	c	c					t	c	c													
g	1543778	a	1534020	g	1485790	ns	APEC	rec11	g	APEC	++	g	g	g					a	a	a													
c	1543786	t	1534028	c	1485798	s	APEC	rec11	c	APEC	++	c	c	c					t	t	t													
c	1543789	t	1534031	c	1485801	s	APEC	rec11	c	APEC	+++	c	c	c					t	c	c													
g	1543801	a	1534043	g	1485813	s	APEC	rec11	a	2	-	a	a	g					a	g	g													
g	1543815	a	1534057	g	1485827	ns	APEC	rec11	g	APEC	++	a	g	g					a	a	a													
c	1543816	t	1534058	c	1485828	ns	APEC	rec11	c	APEC	++	a	c	c					a	a	a													
t	1543825	a	1534067	t	1485837	s	APEC	rec11	t	APEC	+++	t	t	t					t	t	t													
t	1545187	c	1534106	t	1487199	s	APEC	rec11	c	2	-	c	c	t					t															
t	1545202	c	1534121	t	1487214	s	APEC	rec11	c	2	-	c	c	c					t															
c	1545262	t	1534181	c	1487274	s	APEC	rec11	c	APEC	+++	c	a	c					c															
a	1545265	g	1534184	a	1487277	s	APEC	rec11	a	APEC	++	c	c	a					a															
c	1545283	t	1534202	c	1487295	s	APEC	rec11	c	APEC	+++	c	c	c					c															
c	1545286	a	1534205	c	1487298	s	APEC	rec11	c	APEC	+++	t	c	c					c															
t	1545304	g	1534223	t	1487316	s	APEC	rec11	g	2	-	g	a	t					g															
c	1545307	t	1534226	c	1487319	s	APEC	rec11	t	2	-	t	t	c					g															
c	1545310	g	1534229	c	1487322	s	APEC	rec11	c	APEC	+++	c	c	c					c															

ExPEC genome site details												Outgroup Strains Details[i]																						
UTI89[b]	UTI89 site[c]	APEC 01[b]	APEC 01 site[c]	S88[b]	S88 site[c]	type[d]	Event Lineage[ef]	Recombinant segment	Inferred ancestral base[g]	Outgroup Analysis[e]	Support level[h]	CFT073	ED1a	536	E2348/69	SMS 3-5	IAI39	UMN026	K-12	ATCC 8739	HS	D1 Sd197	CB9615	Sakai	EDL933	IAI1	E24377A	SE11	SS Ss046	F2a 301	F2a 2457T	F5 8401	B4 Sb227	B18 BS512
c	1545313	t	1534232	c	1487325	s	APEC	rec11	c	APEC	+++	c	a	c					c															
t	1545316	c	1534235	t	1487328	s	APEC	rec11	t	APEC	+++	t	c	t					t															
a	1545337	g	1534256	a	1487349	s	APEC	rec11	g	2	-	g	a	g					g															
t	1545343	c	1534262	t	1487355	s	APEC	rec11	t	APEC	+++	t	c	t					t															
c	1545346	g	1534265	c	1487358	s	APEC	rec11	c	APEC	+++	c	c	c					t															
t	1545349	c	1534268	t	1487361	s	APEC	rec11	t	APEC	++	t	c	t					c															
a	1545363	g	1534282	a	1487375	ns	APEC	rec11	g	2	-	g	g	a					g															
c	1545364	t	1534283	c	1487376	ns	APEC	rec11	c	APEC	+++	t	c	c					c															
c	1545373	t	1534292	c	1487385	s	APEC	rec11	t	2	-	t	t	c					c															
c	1545403	t	1534322	c	1487415	s	APEC	rec11	t	2	-	t	t	c					t															
c	1545406	t	1534325	c	1487418	s	APEC	rec11	t	2	-	t	t	c					t															
c	1545430	t	1534349	c	1487442	s	APEC	rec11	c	APEC	+++	c	c	c					c															
c	1545433	a	1534352	c	1487445	ns	APEC	rec11	c	APEC	++	c	t	c					a															
t	1545487	c	1534406	t	1487499	s	APEC	rec11	c	2	-	c	g	c																				
t	2085863	g	2106620	g	2014291	s	AS	rec12	t	AS	+++	t	t	t			t	t																
a	2085899	g	2106656	g	2014327	s	AS	rec12	a	AS	+++	a	a	a			a	a																
c	2085905	t	2106662	t	2014333	s	AS	rec12	c	AS	+++	c	c	c			c	c																
t	2085935	c	2106692	c	2014363	s	AS	rec12	t	AS	+++	t	t	t			t	t																
t	2085941	c	2106698	c	2014369	s	AS	rec12	t	AS	+++	t	t	t			t	t																
c	2085953	t	2106710	t	2014381	s	AS	rec12	c	AS	+++	c	c	c			c	c																
c	2085989	t	2106746	t	2014417	s	AS	rec12	c	AS	+++	c	c	c			c	c																
g	2086007	t	2106764	t	2014435	s	AS	rec12	g	AS	+++	g	g	g			g	g																
c	2086079	t	2106836	t	2014507	s	AS	rec12	c	AS	+++	c	c	c			c	c																
a	2086124	c	2106881	c	2014552	s	AS	rec12	a	AS	+++	a	a	a			a	a																
a	2086130	g	2106887	g	2014558	s	AS	rec12	a	AS	+++	a	a	a			a	a																
a	2086136	g	2106893	g	2014564	s	AS	rec12	a	AS	+++	a	a	a			a	a																
c	2086226	t	2106983	t	2014654	s	AS	rec12	c	AS	+++	c	c	c			c	c																
t	2086238	a	2106995	a	2014666	s	AS	rec12	t	AS	+++	t	t	t			t	t																
t	2086265	g	2107022	g	2014693	s	AS	rec12	t	AS	+++	t	t	t			t	t																
t	2086277	c	2107034	c	2014705	s	AS	rec12	t	AS	+++	t	t	t			t	t																
c	2086278	a	2107035	a	2014706	ns	AS	rec12	c	AS	+++	c	c	c			c	c																
a	2086279	g	2107036	g	2014707	ns	AS	rec12	a	AS	+++	a	a	a			a	a																
t	2086307	c	2107064	c	2014735	s	AS	rec12	t	AS	+++	t	t	t			t	t																
c	2086310	t	2107067	t	2014738	s	AS	rec12	c	AS	+++	c	c	c			c	c																
c	2086316	g	2107073	g	2014744	s	AS	rec12	c	AS	+++	c	c	c			c	c																
g	2086334	a	2107091	a	2014762	s	AS	rec12	g	AS	+++	g	g	g			g	g																
a	2086367	c	2107124	c	2014795	s	AS	rec12	a	AS	+++	a	a	a			a	a																
g	2086379	a	2107136	a	2014807	s	AS	rec12	g	AS	+++	g	g	g			g	g																
t	2086388	a	2107145	a	2014816	s	AS	rec12	t	AS	+++	t	t	t			t	t																
c	2086395	t	2107152	t	2014823	s	AS	rec12	c	AS	+++	c	c	c			c	c																
t	2086403	c	2107160	c	2014831	s	AS	rec12	t	AS	+++	t	t	t			t	t																
a	2086427	g	2107184	g	2014855	s	AS	rec12	a	AS	+++	a	a	a			a	a																
a	2086433	g	2107190	g	2014861	s	AS	rec12	a	AS	+++	a	a	a			a	a																
a	2086449	g	2107206	g	2014877	ns	AS	rec12	a	AS	+++	a	a	a			a	a																
g	2086454	a	2107211	a	2014882	ns	AS	rec12	g	AS	+++	g	g	g			g	g																
a	2086463	g	2107220	g	2014891	s	AS	rec12	a	AS	+++	a	a	a			a	a																
g	2086466	a	2107223	a	2014894	s	AS	rec12	g	AS	+++	g	g	g			g	g																
a	2086469	g	2107226	g	2014897	s	AS	rec12	a	AS	+++	a	a	a			a	a																
c	2086478	t	2107235	t	2014906	s	AS	rec12	c	AS	+++	c	c	c			c	c																
c	2086499	a	2107256	a	2014927	s	AS	rec12	c	AS	+++	c	c	c			c	c																
c	2086556	t	2107313	t	2014984	s	AS	rec12	c	AS	+++	c	c	c			c	c																
a	2086587	g	2107344	g	2015015	ns	AS	rec12	a	AS	+++	a	a	a			a	a																
c	2086604	t	2107361	t	2015032	s	AS	rec12	c	AS	+++	c	c	c			c	c																
a	2086676	g	2107433	g	2015104	s	AS	rec12	a	AS	+++	a	a	a			a	a																
g	2086677	a	2107434	a	2015105	ns	AS	rec12	g	AS	+++	g	g	g			g	g																
t	2086695	g	2107452	g	2015123	ns	AS	rec12	t	AS	+++	t	t	t			t	t																
c	2086696	a	2107453	a	2015124	ns	AS	rec12	c	AS	+++	c	c	c			c	c																
a	2086709	g	2107466	g	2015137	s	AS	rec12	a	AS	+++	a	a	a			a	a																
c	2086736	t	2107493	t	2015164	s	AS	rec12	c	AS	+++	c	c	c			c	c																
c	2086739	t	2107496	t	2015167	s	AS	rec12	c	AS	+++	c	c	c			c	c																
c	2086753	t	2107510	t	2015181	ns	AS	rec12	c	AS	+++	c	c	c			c	c																
g	2086778	c	2107535	c	2015206	ns	AS	rec12	g	AS	+++	g	g	g			g	g																
g	2086788	a	2107545	a	2015216	ns	AS	rec12	g	AS	+++	g	g	g			g	g																
c	2086796	t	2107553	t	2015224	s	AS	rec12	c	AS	+++	c	c	c			t	c																
g	2086797	a	2107554	a	2015225	ns	AS	rec12	g	AS	+++	g	g	g			a	g																
t	2086816	a	2107573	a	2015244	ns	AS	rec12	a	UTI89	++	a	t	a			t	t																
t	2086820	a	2107577	a	2015248	s	AS	rec12	t	AS	+++	t	t	t			t	t																
t	2086895	c	2107652	c	2015323	s	AS	rec12	t	AS	+++	t	t	t			t	t																
t	2086910	g	2107667	g	2015338	ns	AS	rec12	g	UTI89	+++	g	g	g			g	g																
t	2088045	g	2108802	g	2016473	ns	AS	rec12	g	UTI89	+++	g	g	g			g	g																
t	2088178	g	2108935	g	2016606	ns	AS	rec12	g	UTI89	+++	g	g	g			g	g																
c	2088614	t	2109371	t	2017042	s	AS	rec12	t	UTI89	+++	t	t	t			t	t																
a	2088967	c	2109724	c	2017395	ns	AS	rec12	c	UTI89	+++	c	c	c			c	c																
a	2089126	g	2109883	g	2017554	ns	AS	rec12	g	UTI89	+++	g	g	g			g	g																
t	2089136	c	2109893	c	2017564	s	AS	rec12	c	UTI89	+++	c	c	c			c	c																
c	2089139	t	2109896	t	2017567	s	AS	rec12	c	AS	+++	c	c	c			c	c																
a	2089151	c	2109908	c	2017579	s	AS	rec12	c	UTI89	+++	c	c	c			c	c																
g	2089226	a	2109983	a	2017654	s	AS	rec12	a	UTI89	++	a	g	a			a	g																
g	2089233	a	2109990	a	2017661	ns	AS	rec12	g	AS	+++	g	g	g			g	g																
c	2089751	t	2110508	t	2018179	s	AS	rec12	c	AS	+++	c	c	c			c	c																
a	2090138	g	2110895	g	2018566	s	AS	rec12	g	UTI89	+++	g	g	g			g	g																
c	2090318	t	2111075	t	2018746	s	AS	rec12	c	AS	+++	c	c	c			c	c																
t	2090804	c	2111561	c	2019232	s	AS	rec12	c	UTI89	+++	c	c	c			t	c																

ExPEC genome site details												Outgroup Strains Details[i]																						
UTI89[b]	UTI89 site[c]	APEC 01[b]	APEC 01 site[c]	S88[b]	S88 site[c]	type[d]	Event Lineage[ef]	Recombinant segment	Inferred ancestral base[g]	Outgroup Analysis[e]	Support level[h]	CFT073	ED1a	536	E2348/69	SMS 3-5	IAI39	UMN026	K-12	ATCC 8739	HS	D1 Sd197	CB9615	Sakai	EDL933	IAI1	E24377A	SE11	SS Ss046	F2a 301	F2a 2457T	F5 8401	B4 Sb227	B18 BS512
t	2090846	c	2111603	c	2019274	s	AS	rec12	t	AS	+++	t	t	t			t	t																
a	2091425	g	2112182	g	2019853	s	AS	rec12	g	UTI89	+++	g	g	g			g	g																
c	2093679	t	2114436	t	2022107	ns	AS	rec12	c	AS	+++	c	c	c			c	c																
a	2096723	c	2117478	c	2025151	ns	AS	rec12	c	UTI89	+++	c	c	c			c	c																
a	2097028	g	2117783	g	2025456	ns	AS	rec12	g	UTI89	+++	g	g	g			a	g																
a	2097100	g	2117855	g	2025528	ns	AS	rec12	g	UTI89	+++	g	g	g			a	g																
c	2098223	t	2118978	t	2026651	nc	AS	rec12	t	UTI89	+++	t	t	t			t	t																
1	2098269	-	2119023	-	2026696	ins	UTI89	rec12	-	UTI89	+++	-	-	-			-	-																
c	2098778	t	2119532	t	2027205	s	AS	rec12	c	AS	+++	c	c	c			c	c																
c	2099057	t	2119811	t	2027484	s	AS	rec12	t	UTI89	++	t	c	t			c	c																
a	2099229	g	2119983	g	2027656	ns	AS	rec12	a	AS	+++	a	a	a			a	a																
t	2099262	c	2120016	c	2027689	ns	AS	rec12	c	UTI89	+++	c	c	c			c	c																
t	2099300	c	2120054	c	2027727	s	AS	rec12	c	UTI89	+++	c	c	c			t	c																
t	2100523	c	2121277	c	2028950	nc	AS	rec12	c	UTI89	+++	c	c	c			c	c																
c	2100676	t	2121430	t	2029103	nc	AS	rec12	c	AS	+++	c	c	c			c	c																
a	2101147	t	2121901	t	2029574	s	AS	rec12	t	UTI89	+++	t	t	t			t	t																
t	2101368	c	2122122	c	2029795	ns	AS	rec12	t	AS	++++	t	t	t		t	t		t	t	t								t					
c	2101395	a	2122149	a	2029822	ns	AS	rec12	c	AS	++++	c	c	c		c	c		c	c	c								c					
c	2101471	t	2122225	t	2029898	s	AS	rec12	t	UTI89	++++	t	t	t		t	t		t	t	t								t					
c	2102152	t	2122906	t	2030579	s	AS	rec12	c	AS	++++	c	c	c		c	c	c	c	c	c		c	c	c	c	c	c	c					c
g	2103076	a	2123830	a	2031503	s	AS	rec12	g	AS	++++	g	g	g	a	g	g	g	g	g	g		g	g	g	g	g	g	g					g
t	2103118	a	2123872	a	2031545	s	AS	rec12	t	AS	++	t	a	t	a	a	t	a	a	a	a		a	a	a	a	a	a	a					a
a	2103120	c	2123874	c	2031547	ns	AS	rec12	a	AS	++	a	c	a	c	c	a	c	c	c	c		c	c	c	c	c	c	c					c
g	2103146	a	2123900	a	2031573	ns	AS	rec12	g	AS	++++	g	g	g	a	g	g	g	g	g	g		g	g	g	g	g	g	g					g
g	2103198	t	2123952	t	2031625	ns	AS	rec12	g	AS	++++	g	g	g	g	g	g	g	g	g	g		g	g	g	g	g	g	g					g
t	2103323	g	2124077	g	2031750	ns	AS	rec12	t	AS	++	t	g	t	t	g	t	g	g	g	g		g	g	g	g	g	g	g					g
a	2103331	g	2124085	g	2031758	nc	AS	rec12	a	AS	++	a	g	a	g	g	a	g	g	g	g		g	g	g	g	g	g	g					g
g	2103432	a	2124186	a	2031859	ns	AS	rec12	g	AS	++++	g	g	g	g	g	g	g	g	g	g		g	g	g	g	g	g	g					
t	2103487	c	2124241	c	2031914	s	AS	rec12	t	AS	++++	t	t	t	c	t	t	t	t	t	t		t	t	t	t	t	t	t					
g	2103535	c	2124289	c	2031962	s	AS	rec12	g	AS	++	g	c	g	c	c	g	c	c	c	c		c	c	c	c	c	c	c					
a	2103554	g	2124308	g	2031981	ns	AS	rec12	a	AS	++	a	g	a	g	g	a	g	g	g	g		g	g	g	g	g	g	g					
c	2103807	t	2124561	t	2032234	ns	AS	rec12	c	AS	++++	c	c	c	c	c	c	c	c	c	c		c	c	c	c	c	c	c					
a	2103895	g	2124649	g	2032322	nc	AS	rec12	a	AS	++++	a	a	a	a	a	a	a	a	a	a		a	a	a	a	a	a	a					
a	2104018	g	2124772	g	2032445	ns	AS	rec12	a	AS	++++	a	a	a	a	a	a	a	a	a	a		a	a	a	a	a	a	a					
a	2104101	g	2124855	g	2032528	s	AS	rec12	a	AS	++++	a	a	a	a	a	a	a	a	a	a		a	a	a	a	a	a	a					
a	2104140	c	2124894	c	2032567	ns	AS	rec12	a	AS	++	a	g	a	a	g	a	g	g	g	g		g	g	g	g	g	g	g					
g	2104158	a	2124912	a	2032585	s	AS	rec12	g	AS	++++	g	g	g	g	g	g	g	g	g	g		g	g	g	g	g	g	g					
a	2104275	c	2125029	c	2032702	ns	AS	rec12	a	AS	++	a	t	a	c	t	a	c	t	t	c		t	t	t	t	c	t	t					
g	2104353	t	2125107	t	2032780	ns	AS	rec12	g	AS	++	g	t	g	t	t	g	t	t	t	t		t	t	t	t	t	t	t					
g	2104398	a	2125152	a	2032825	s	AS	rec12	g	AS	++++	g	g	g	g	g	g	g	g	g	g		g	g	g	g	g	g	g					
t	2104505	c	2125259	c	2032932	ns	AS	rec12	t	AS	++	t	c	t	c	c	c	c	c	c	c		c	c	c	c	c	c	c					
g	2104759	t	2125513	t	2033186	ns	AS	rec12	g	AS	++++	g	g	g	g	g	g	g	g	g	g		g	g	g	g	g	g	g					
c	2105178	t	2125932	t	2033605	nc	AS	rec12	c	AS	++	c	c	c	c	c	t	c	c	c	c	c	t	t	t	c	c	c	c					
g	2105206	t	2125960	t	2033633	nc	AS	rec12	g	AS	++++	g	g	g	g	g	g	g	g	g	g	g	g	g	g	g	g	g	g					
a	2105476	g	2126230	g	2033903	s	AS	rec12	a	AS	++++	a	a	a	a	a	a	a	a	a	a	a	a	a	a	a	a	a	a					
c	2106193	a	2126947	a	2034620	s	AS	rec12	c	AS	++	c	a	c	a	a	a	c	a	a	a	a	a	a	a	a	a	a	a	a	a	a		a
g	2106496	a	2127250	a	2034923	s	AS	rec12	g	AS	++	g	a	g	a	a	a	a	a	a	a	a	a	a	a	a	a	a	a	a	a	a		a
t	2106553	c	2127307	c	2034980	s	AS	rec12	t	AS	++	t	c	t	c	c	c	t	c	c	c	c	c	c	c	c	c	c	c	c	c	c		c
c	2106583	t	2127337	t	2035010	ns	AS	rec12	c	AS	++	c	c	c	t	c	t	c	c	c	c	t	c	c	c	c	c	c	c	t	t	t		c
g	2106598	a	2127352	a	2035025	ns	AS	rec12	g	AS	++	g	g	g	g	g	a	g	g	g	g	g	g	g	g	g	g	g	g	a	a	a		g
g	2106620	t	2127374	t	2035047	ns	AS	rec12	g	AS	++	g	g	g	g	g	t	g	g	g	g	g	g	g	g	g	g	g	g	t	t	t		g
t	2106622	g	2127376	g	2035049	ns	AS	rec12	t	AS	++	t	t	t	t	t	g	t	t	t	t	t	t	t	t	t	t	t	t	g	g	g		t
a	2106624	g	2127378	g	2035051	ns	AS	rec12	a	AS	++	a	g	a	g	g	g	g	g	g	g	g	g	g	g	g	g	g	g	g	g	g		g
g	2106632	a	2127386	a	2035059	ns	AS	rec12	g	AS	++++	g	g	g	g	g	g	g	g	g	g	g	g	g	g	g	g	g	g	g	g	g		g
c	2106659	t	2127413	t	2035086	ns	AS	rec12	c	AS	++	c	c	c	c	c	t	c	c	c	c	c	c	c	c	c	c	c	c	t	t	t		c
t	2106972	c	2127726	c	2035399	s	AS	rec12	t	AS	++	t	c	t	t	c	c	c	c	c	c	c	c	c	c	c	c	c	c	c	c	c		c
c	2107107	t	2127861	t	2035534	s	AS	rec12	c	AS	++++	c	c	c	c	c	c	c	c	c	c	c	c	c	c	c	c	c	c	c	c	c		c
a	2107122	g	2127876	g	2035549	s	AS	rec12	a	AS	++	a	g	a	g	g	g	g	g	g	g	g	g	a	a	g	g	g	g	g	g	g		g
t	2107350	c	2128104	c	2035777	s	AS	rec12	t	AS	++++	t	t	t	t	t	t	t	t	t	t	t	t	t	t	t	t	t	t	t	t	t		t
c	2107498	t	2128252	t	2035925	ns	AS	rec12	c	AS	++++	c	c	c	c	c	c	c	c	c	c	c	c	c	c	c	c	c	c	c	c	c		c
g	2107538	a	2128292	a	2035965	ns	AS	rec12	g	AS	++	g	a	g	a	a	a	a	a	a	a	a	a	a	a	a	a	a	a	a	a	a		a
t	2111165	a	2131922	t	2039595	ns	APEC	rec13	t	APEC	++++	t	t	t	t	t	t	t	t	t	t	t	t	t	t	t	t	t	t	t	t	t	t	t
-	2111252	1	2132010	-	2039682	ins	APEC	rec13	-	APEC	++++	-	-	-	-	-	-	-	-	-	-	-	-	-	-	-	-	-	-	-	-	-	-	-
-	2111287	1	2132046	-	2039717	ins	APEC	rec13	-	APEC	++++	-	-	-	-	-	-	-	-	-	-	-	-	-	-	-	-	-	-	-	-	-	-	-
-	2111318	1	2132078	-	2039748	ins	APEC	rec13	-	APEC	++++	-	-	-	-	-	-	-	-	-	-	-	-	-	-	-	-	-	-	-	-	-	-	-
g	2112032	a	2132792	g	2040462	ns	APEC	rec13	g	APEC	++++	g	g	g	g	g	g	g	g	g	g	g	g	g	g	g	g	g	g	g	g	g		g
c	2112045	t	2132805	c	2040475	s	APEC	rec13	c	APEC	++	c	c	c	t	c	c	c	c	c	c	c	c	c	c	t	c	c	t	t	t	t		t
a	2112084	g	2132844	a	2040514	s	APEC	rec13	a	APEC	++	a	a	a	g	a	g	a	a	a	a	a	a	a	a	a	a	a	a	a	a	a		a
g	2112300	a	2133060	g	2040730	s	APEC	rec13	g	APEC	++	g	g	g	g	g	a	g	g	g	g	g	g	g	g	g	g	g	g	a	a	a		g
t	2113297	c	2134057	t	2041727	ns	APEC	rec13	t	APEC	++++	t	t	t	t	t	t	t	t	t	t	t	t	t	t	t	t	t	t	t	t	t	t	t
t	2113663	c	2134423	t	2042093	nc	APEC	rec13	t	APEC	++	t	t	t	t	t	t	t	t	t	t	t	t	t	t	t	t	t	t	c	c	c	t	t
-	2114000	g	2145532	a	2053201	nc	APEC	rec13	a	APEC	+++	a	a	a		a					a													
-	2114000	g	2145646	a	2053315	nc	APEC	rec13	a	APEC	++	a	g	a		g					a													
-	2114000	t	2145732	g	2053401	nc	APEC	rec13	g	APEC	++	g	t	g		t					g									g		g	g	g
-	2114000	t	2146193	c	2053862	nc	APEC	rec13	c	APEC	++	c	t	c		t					c									c		c	c	c
-	2114000	g	2146832	a	2054501	nc	APEC	rec13	a	APEC	++	a	g	a		g					a													
-	2114000	g	2146935	c	2054604	nc	APEC	rec13	c	APEC	++	c	g	c		g					c													
-	2114000	a	2146973	g	2054642	nc	APEC	rec13	g	APEC	++	g	a	g		a					g													
-	2114000	a	2147028	g	2054697	nc	APEC	rec13	g	APEC	+++	g	g	g		a					g													
-	2114000	t	2147033	a	2054702	nc	APEC	rec13	a	APEC	++	a	t	a		t					a													
-	2114000	t	2147070	c	2054739	nc	APEC	rec13	c	APEC	++	c	t	c		t					c													

ExPEC genome site details												Outgroup Strains Details[i]																						
UTI89[b]	UTI89 site[c]	APEC 01[b]	APEC 01 site[c]	S88[b]	S88 site[c]	type[d]	Event Lineage[ef]	Recombinant segment	Inferred ancestral base[g]	Outgroup Analysis[e]	Support level[h]	CFT073	ED1a	536	E2348/69	SMS 3-5	IAI39	UMN026	K-12	ATCC 8739	HS	D1 Sd197	CB9615	Sakai	EDL933	IAI1	E24377A	SE11	SS Ss046	F2a 301	F2a 2457T	F5 8401	B4 Sb227	B18 BS512
-	2114000	a	2147092	g	2054761	nc	APEC	rec13	g	APEC	+++	g	g	g		g					g													
-	2114000	c	2147678	t	2055347	nc	APEC	rec13	t	APEC	++	t	c	t		c					t													
-	2114000	t	2147719	g	2055391	nc	APEC	rec13	g	APEC	++	g	t	g		t		g			g	g					g							
-	2114000	c	2147735	t	2055407	nc	APEC	rec13	t	APEC	++	t	c	t		c		c			c	c					c							
-	2114000	c	2147738	t	2055410	nc	APEC	rec13	t	APEC	++	t	c	t		c		c			c	c					c							
-	2114000	t	2147746	c	2055418	nc	APEC	rec13	c	APEC	++	c	t	c		t		c			c	c					c							
-	2114000	t	2147945	c	2055617	nc	APEC	rec13	c	APEC	++++	c	c	c		c		c			c	c					c							
-	2114000	t	2149052	g	2056724	nc	APEC	rec13	g	APEC	++	g	t	g		t		t			t		g				g							
-	2114000	t	2149719	g	2057391	nc	APEC	rec13	g	APEC	+++	g	g	g		g		g									t							
-	2114000	a	2149814	g	2057486	nc	APEC	rec13	g	APEC	+++	g	g	g		g		g									a							
-	2114000	a	2150121	g	2060333	nc	APEC	rec13	g	APEC	+++	g	g	g		g		g			g						g							
-	2114000	c	2150230	g	2060442	nc	APEC	rec13	g	APEC	+++	g	g	g		g		g			g						g							
-	2114000	1	2150632	-	2060843	ins	APEC	rec13	1	2	-	1	-	1		1		-			1		1				1							
-	2193784	7	2150633	7	2060844	del	APEC	rec13	7	UTI89	++	7	-	7		7		-			7		7				7							
a	2195486	t	2150877	a	2061089	s	APEC	rec13	a	APEC	++	g	a	a		a		a			g		a				g							
g	2196088	c	2151479	g	2061691	ns	APEC	rec13	g	APEC	+++	g		g		g		g			g		g				g							
g	2196511	t	2151902	g	2062114	nc	APEC	rec13	g	APEC	+++	g		g		t							g											
t	2260782	c	2210287	t	2120205	s	APEC	rec13	t	APEC	++++	t	t	t	t	t	t	t	t	t			t	t	t	t	t	t	t	t	t	t	t	t
c	2260818	t	2210323	c	2120241	s	APEC	rec13	c	APEC	++	c	c	c	c	c	c	c	c	c			t	c	c	c	c	c	c	c	c	c	t	c
c	2260827	t	2210332	c	2120250	s	APEC	rec13	c	APEC	++	c	c	c	t	c	t	t	c	c			c	c	c	t	t	c	c	t	t	t	c	t
g	2260851	a	2210356	g	2120274	s	APEC	rec13	g	APEC	++	a	g	g	g	a	g	g	g	g			a	g	g	g	g	g	g	g	g	g	a	g
g	2260869	a	2210374	g	2120292	s	APEC	rec13	g	APEC	++	a	g	g	g	g	g	g	g	g			g	g	g	g	g	a	g	a	a	a	g	g
g	2260911	a	2210416	g	2120334	s	APEC	rec13	g	APEC	++++	g	g	g	g	g	g	g	g	g			g	g	g	g	g	g	g	g	g	g	g	g
c	2261025	a	2210530	c	2120448	s	APEC	rec13	c	APEC	++	c	c	c	c	c	t	c	t	c			c	c	c	c	c	c	c	t	t	t	c	t
a	2261136	g	2210641	a	2120559	s	APEC	rec13	a	APEC	++++	a	g	a	a	a	a	a	a	a			a	a	a	a	a	a	a	a	a	a	a	a
g	2261157	a	2210662	g	2120580	s	APEC	rec13	g	APEC	++++	g	g	g	g	g	g	g	g	g			g	g	g	g	g	t	g	g	g	g	g	g
a	2261217	g	2210722	a	2120640	s	APEC	rec13	a	APEC	++	a	a	a	a	a	a	a	g	g			a	g	g	a	a	g	g	a	a	a	a	a
c	2261235	t	2210740	c	2120658	s	APEC	rec13	c	APEC	++++	c	c	c	c	c	c	c	c	c			c	c	c	c	c	c	c	c	c	c	c	c
a	2261382	g	2210887	a	2120805	s	APEC	rec13	a	APEC	++	a	g	a	g	a	a	g	a	a			a	g	g	a	a	a	a	a	a	a	a	g
g	2261426	a	2210931	g	2120849	ns	APEC	rec13	g	APEC	++++	g	g	g	g	g	g	g	g	g			g	g	g	g	g	g	g	g	g	g	g	g
a	2261433	g	2210938	a	2120856	s	APEC	rec13	a	APEC	++	a	g	a	g	a	a	g	g	g			g	a	a	g	g	g	g	g	g	g	g	g
a	2261448	g	2210953	a	2120871	s	APEC	rec13	a	APEC	++	g	a	a	g	a	a	g	a	g			g	a	a	a	a	a	g	g	g	g	g	g
c	2262248	t	2211753	c	2121671	s	APEC	rec13	c	APEC	++	c	c	c	c	t	c	c	c	t			c	t	t	c	c	c	c	c	c	c	t	c
t	2262269	a	2211774	t	2121692	s	APEC	rec13	t	APEC	++	t	t	t	t	a	t	t	a	t			t	a	a	t	t	t	t	t	t	t	t	t
g	2262272	a	2211777	g	2121695	s	APEC	rec13	g	APEC	++	g	g	g	g	a	g	g	a	a			g	a	a	g	g	g	g	g	g	g	a	a
c	2262323	t	2211828	c	2121746	s	APEC	rec13	c	APEC	++++	c	c	c	c	c	c	c	c	c			c	c	c	c	c	c	c	c	c	c	c	c
c	2262356	t	2211861	c	2121779	s	APEC	rec13	c	APEC	++++	c	c	c	c	c	c	c	c	c			c	c	c	c	c	c	c	c	c	c	c	c
t	2262429	a	2211934	t	2121852	ns	APEC	rec13	t	APEC	++++	t	t	t	t	t	t	t	t	t			t	t	t	t	t	t	t	t	t	t	t	t
t	2262437	a	2211942	t	2121860	s	APEC	rec13	t	APEC	++	t	t	t	t	t	t	t	t	a			t	t	t	t	t	t	t	t	t	t	a	a
c	2262446	t	2211951	c	2121869	s	APEC	rec13	c	APEC	++	c	c	c	c	c	c	c	c	t			c	c	c	c	c	c	c	c	c	c	t	t
c	2262467	a	2211972	c	2121890	s	APEC	rec13	c	APEC	++++	c	c	c	c	c	c	c	c	c			c	c	c	c	c	c	c	c	c	c	c	c
g	2262515	a	2212020	g	2121938	s	APEC	rec13	g	APEC	++	g	g	g	g	g	g	g	g	g			a	g	g	g	g	g	g	g	g	g	a	a
g	2262982	a	2212487	g	2122405	ns	APEC	rec13	g	APEC	++++	g	g	g	g	g	g	g	g	g			g	g	g	g	g	g	g	g	g	g	g	g
c	2263031	t	2212536	c	2122454	s	APEC	rec13	c	APEC	++	c	t	c	c	c	c	c	c	t			c	c	c	c	c	c	t	c	c	c	c	c
c	2263148	t	2212653	c	2122571	s	APEC	rec13	c	APEC	++	c	c	c	t	t	t	t	t	t			c	c	c	c	c	c	c	t	t	t	c	c
g	2263175	a	2212680	g	2122598	s	APEC	rec13	a	2	-	a	g	a	g	a	a	g	g	a			a	a	a	a	a	a	a	g	g	g	a	a
t	2263229	c	2212734	t	2122652	s	APEC	rec13	c	2	-	c	t	c	t	t	c	t	t	t			t	t	t	t	t	t	t	t	t	t	t	t
a	2264043	g	2213548	a	2123466	s	APEC	rec13	g	2	-	g	a	g	g	g	g	g	a	a			g	a	a	g	a	g	g	a	a	a	g	g
t	2264250	c	2213755	t	2123673	s	APEC	rec13	t	APEC	++	t	t	t	t	c	t	c	t	t			c	t	t	t	c	t	t	a	a	a	c	c
g	2264319	a	2213824	g	2123742	s	APEC	rec13	g	APEC	++	g	g	g	a	a	g	g	a	a			g	g	g	g	g	a	g	g	g	g	g	g
g	2264334	a	2213839	g	2123757	s	APEC	rec13	g	APEC	++	g	g	g	a	a	g	g	a	a			g	g	g	g	g	a	g	g	g	g	g	g
g	2264349	a	2213854	g	2123772	s	APEC	rec13	a	2	-	a	a	g	g	a	g	g	g	g			a	g	g	g	a	g	g	g	g	g	a	a
g	2264508	c	2214013	g	2123931	s	APEC	rec13	c	2	-	g	c	c	g	c	g	g	g	g			c	c	c	c	g	g	g	g	g	g	c	c
c	2264592	g	2214097	c	2124015	s	APEC	rec13	c	APEC	++	c	g	c	c	c	g	g	g	g			a	g	g	c	g	g	c	g	g	g	a	a
c	2264616	t	2214121	c	2124039	s	APEC	rec13	c	APEC	++	t	c	c	c	t	t	c	c	t			c	c	c	t	c	c	c	t	t	t	c	c
g	2264619	a	2214124	g	2124042	s	APEC	rec13	g	APEC	++	a	g	g	g	a	a	g	g	a			g	g	g	a	g	g	g	a	a	a	g	g
t	2264640	c	2214145	t	2124063	s	APEC	rec13	c	2	-	c	t	c	t	c	c	t	t	c			t	t	t	c	t	t	t	c	c	c	t	t
c	2264643	t	2214148	c	2124066	s	APEC	rec13	c	APEC	++	c	c	c	c	t	t	c	c	c			c	c	c	c	c	c	c	c	c	c	c	c
c	2264722	t	2214227	c	2124145	ns	APEC	rec13	t	2	-	t	t	t	c	t	t	t	t	t			t	c	c	t	t	t	t	t	t	t	t	t
c	2264897	t	2214402	c	2124320	s	APEC	rec13	c	APEC	++++	c	c	c	c	c	c	c	c	c			c	c	c	c	c	c	c	c	c	c	c	c
a	2264903	t	2214408	a	2124326	s	APEC	rec13	a	APEC	++++	a	a	a	a	a	a	a	a	a			a	a	a	a	a	a	a	a	a	a	a	a
c	2264912	a	2214417	c	2124335	s	APEC	rec13	c	APEC	++++	c	c	c	c	c	c	c	c	c			c	c	c	c	c	c	c	c	c	c	c	c
a	2264924	g	2214429	a	2124347	s	APEC	rec13	a	APEC	++	a	a	g	a	a	a	a	a	a			a	a	a	a	a	g	a	a	a	a	a	a
c	2264966	t	2214471	c	2124389	s	APEC	rec13	c	APEC	++++	c	c	c	c	c	c	c	c	c			c	c	c	c	c	c	c	c	c	c	c	c
c	2264969	t	2214474	c	2124392	s	APEC	rec13	c	APEC	++++	c	c	c	c	c	c	c	c	c			c	c	c	c	c	c	c	c	c	c	c	c
g	2264972	a	2214477	g	2124395	s	APEC	rec13	g	APEC	++++	g	g	g	g	g	g	a	g	g			g	g	g	g	g	g	g	g	g	g	g	g
t	2265077	c	2214582	t	2124500	s	APEC	rec13	c	2	-	t	c	c	c	t	c	c	c	c			c	t	t	t	c	t	c	t	t	t	c	c
c	2265119	t	2214624	c	2124542	s	APEC	rec13	c	APEC	++	t	c	c	c	t	t	c	c	c			t	c	c	t	c	c	c	c	c	c	c	c
a	2265167	g	2214672	a	2124590	s	APEC	rec13	a	APEC	++	a	a	a	a	a	a	a	g	g			a	a	a	a	a	g	g	g	g	g	g	g
g	2265203	a	2214708	g	2124626	s	APEC	rec13	a	2	-	a	g	a	g	g	g	g	g	g			g	g	g	g	g	g	g	g	g	g	g	g
t	2265215	c	2214720	t	2124638	s	APEC	rec13	c	2	-	c	c	c	c	c	c	c	c	c			c	c	c	c	c	c	c	c	c	c	c	c
g	2265233	a	2214738	g	2124656	s	APEC	rec13	g	APEC	++++	g	g	g	a	g	g	g	g	g			g	g	g	g	g	g	g	g	g	g	g	g
a	2265314	g	2214819	a	2124737	s	APEC	rec13	a	APEC	++	a	g	a	a	a	g	a	a	a			a	a	a	a	g	a	a	a	a	a	a	a
a	2265443	g	2214948	a	2124866	s	APEC	rec13	g	2	-	g	a	g	a	g	g	a	a	a			a	a	a	g	a	a	a	a	a	a	a	a
g	2265539	a	2215044	g	2124962	s	APEC	rec13	g	APEC	++++	g	g	g	g	g	a	g	g	g			g	g	g	g	g	g	g	g	g	g	g	g
g	2265593	c	2215098	g	2125016	s	APEC	rec13	g	APEC	++	g	c	g	g	g	g	c	g	g			g	g	g	g	c	g	g	g	g	g	g	g
c	2265620	t	2215125	c	2125043	s	APEC	rec13	c	APEC	++	c	t	c	c	c	c	t	t	t			c	c	c	t	t	c	t	t	t	t	t	t
g	2265662	a	2215167	g	2125085	s	APEC	rec13	g	APEC	++++	g	g	g	g	g	g	g	g	g			g	g	g	g	g	g	g	g	g	g	g	g
c	2265990	t	2215495	c	2125413	ns	APEC	rec13	c	APEC	++	c	t	c	c	t	t	t	c	c			c	c	c	c	c	t	c	c	c	c	c	c
g	2266180	c	2215685	g	2125603	ns	APEC	rec13	c	2	-	c	g	c	g	g	c	c	g	g			c	g	g	g	g	c	g	g	g	g	g	g
a	2266252	c	2215757	a	2125675	s	APEC	rec13	a	APEC	++	a	a	g	a	c	a	a	a	a			a	a	a	a	a	a	a	a	a	a	a	a
g	2111362	g	2132122	a	2039792	ns	S88	rec14	g	S88	++	g	a	g	g	a	g	a	a	a	a	g	a	a	a	a	a	a	a	g	g	g	g	a
a	2111401	a	2132161	t	2039831	ns	S88	rec14	a	S88	++	a	a	a	a	a	a	a	a	a	a	a	t	t	t	a	a	a	a	a	a	a	a	a

ExPEC genome site details												Outgroup Strains Details[i]																						
UTI89[b]	UTI89 site[c]	APEC 01[b]	APEC 01 site[c]	S88[b]	S88 site[c]	type[d]	Event Lineage[ef]	Recombinant segment	Inferred ancestral base[g]	Outgroup Analysis[e]	Support level[h]	CFT073	ED1a	536	E2348/69	SMS 3-5	IAI39	UMN026	K-12	ATCC 8739	HS	D1 Sd197	CB9615	Sakai	EDL933	IAI1	E24377A	SE11	SS Ss046	F2a 301	F2a 2457T	F5 8401	B4 Sb227	B18 BS512
t	2111457	t	2132217	a	2039887	ns	S88	rec14	t	S88	++	t	t	t	t	t	t	t	t	t	t	t	a	a	a	t	t	t	t	t	t	t	t	t
t	2111480	t	2132240	g	2039910	ns	S88	rec14	t	S88	++	t	g	t	t	g	t	g	g	g	g	g	g	g	g	g	g	g	g	t	t	t	g	g
g	2111673	g	2132433	c	2040103	s	S88	rec14	g	S88	++	g	c	g	g	c	g	g	c	c	g	c	c	c	c	c	c	c	c	g	g	g		c
c	2111694	c	2132454	a	2040124	s	S88	rec14	c	S88	++	c	c	c	c	c	c	c	c	a	c	a	a	a	a	c	c	c	c	c	c	c		c
c	2111748	c	2132508	t	2040178	s	S88	rec14	c	S88	++	c	t	c	t	t	c	c	t	t	c	t	t	t	t	t	t	t	t	t	t	t		t
g	2111832	g	2132592	a	2040262	s	S88	rec14	g	S88	++	g	g	g	g	g	g	a	g	a	g	a	a	a	a	g	g	g	g	g	g	g		g
t	2111907	t	2132667	c	2040337	s	S88	rec14	t	S88	++	t	c	t	t	c	t	c	c	c	c	c	c	c	c	c	c	c	c	t	t	t		c
c	2111918	c	2132678	t	2040348	ns	S88	rec14	c	S88	++	c	c	c	c	c	c	c	c	c	c	c	t	t	t	c	c	c	c	c	c	c		c
g	2111929	g	2132689	a	2040359	ns	S88	rec14	g	S88	++	g	a	g	g	a	g	a	a	a	a	a	a	a	a	a	a	a	a	g	g	g		a
g	2111961	g	2132721	a	2040391	s	S88	rec14	g	S88	++	g	a	g	g	a	g	a	a	a	a	g	a	a	a	a	a	a	a	g	g	g		a
a	2111994	a	2132754	g	2040424	s	S88	rec14	a	S88	++	a	g	a	a	g	a	a	g	g	g	g	g	g	g	g	g	g	g	a	a	a		g
a	2112058	a	2132818	t	2040488	ns	S88	rec14	a	S88	++	a	t	a	a	t	a	a	t	a	a	a	t	t	t	a	t	t	a	a	a	a		a
g	2112156	g	2132916	a	2040586	s	S88	rec14	g	S88	++	g	g	g	g	g	g	g	g	g	g	a	a	a	a	g	g	g	g	g	g	g		g
t	2112162	t	2132922	a	2040592	ns	S88	rec14	t	S88	++	t	t	t	t	t	t	t	t	t	t	a	a	a	a	t	t	t	t	t	t	t		t
a	2112192	a	2132952	c	2040622	s	S88	rec14	a	S88	++	a	c	a	a	c	a	a	c	c	a	c	c	c	c	c	c	c	c	a	a	a		c
c	2112357	c	2133117	g	2040787	s	S88	rec14	c	S88	++	c	c	c	c	c	c	c	c	c	c	c	g	g	g	c	c	c	c	c	c	c		c
a	2112480	a	2133240	g	2040910	s	S88	rec14	a	S88	++	a	g	a	g	g	a	g	g	g	g	g	g	g	g	g	g	g	g	a	a	a		g
c	2112546	c	2133306	t	2040976	s	S88	rec14	c	S88	++	c	c	c	c	c	c	c	c	t	c	t	t	t	t	c	c	c	c	c	c	c		c
t	2112625	t	2133385	c	2041055	nc	S88	rec14	t	S88	++	t	t	t	t	t	t	t	t	c	t	c	c	c	c	t	t	t	t	t	t	t		t
t	2112767	t	2133527	c	2041197	s	S88	rec14	t	S88	++	t	c	t	t	c	t	c	c	c	c	c	c	c	c	c	c	c	c	t	t	t		c
c	2112770	c	2133530	t	2041200	s	S88	rec14	c	S88	++	c	t	c	c	t	c	t	t	t	t	t	t	t	t	t	t	t	t	c	c	c		t
t	2112776	t	2133536	c	2041206	s	S88	rec14	t	S88	++	t	c	t	c	c	t	c	c	c	c	c	c	c	c	c	c	c	c	t	t	t		c
g	2112797	g	2133557	t	2041227	s	S88	rec14	g	S88	++	g	t	g	g	t	g	t	t	t	t	t	t	t	t	t	t	t	t	g	g	g		t
t	2112815	t	2133575	g	2041245	s	S88	rec14	t	S88	++	t	g	t	t	g	t	t	g	g	g	g	g	g	g	g	g	g	g	t	t	t		g
a	2112821	a	2133581	c	2041251	s	S88	rec14	a	S88	++	a	a	a	a	a	a	t	a	c	c	c	c	c	c	a	a	a	a	a	a	a		a
a	2112872	a	2133632	g	2041302	s	S88	rec14	a	S88	++	a	g	a	g	g	a	g	g	g	g	g	g	g	g	g	g	g	g	a	a	a		g
a	2112881	a	2133641	g	2041311	s	S88	rec14	a	S88	++	a	g	a	g	g	a	g	g	g	g	g	g	g	g	g	g	g	g	a	a	a		g
g	2112929	g	2133689	a	2041359	s	S88	rec14	g	S88	++	g	g	g	g	g	g	g	g	a	g	g	a	a	a	g	g	g	g	g	g	g		g
a	2112983	a	2133743	c	2041413	s	S88	rec14	a	S88	++	a	a	a	a	a	a	a	a	c	a	a	c	c	c	a	a	a	a	a	a	a		a
c	2113043	c	2133803	t	2041473	s	S88	rec14	c	S88	++	c	c	c	c	c	c	c	c	t	c	c	t	t	t	c	c	c	c	c	c	c		c
c	2113133	c	2133893	a	2041563	s	S88	rec14	c	S88	++	c	a	c	c	a	c	a	a	c	c	c	a	a	a	c	a	a	a	c	c	c		a
g	2113178	g	2133938	t	2041608	ns	S88	rec14	g	S88	++++	g	g	g	g	g	g	g	g	g	g	g	g	g	g	g	g	g	g	g	g	g		g
a	2113211	a	2133971	g	2041641	s	S88	rec14	a	S88	++	a	a	a	a	a	a	g	a	g	g	g	g	g	g	g	a	a	a	a	a	a		a
a	2113336	a	2134096	c	2041766	ns	S88	rec14	a	S88	++	a	c	a	a	c	a	c	c	c	c	c	c	c	c	c	c	c	c	a	a	a	c	c
t	2113394	t	2134154	c	2041824	s	S88	rec14	t	S88	++	t	c	t	t	c	t	c	c	c	c	c	c	c	c	c	c	c	c	t	t	t	c	c
c	2113460	c	2134220	t	2041890	s	S88	rec14	c	S88	++	c	t	c	c	t	c	t	t	t	t	t	t	t	t	t	t	t	t	c	c	c	t	t
c	2113481	c	2134241	g	2041911	s	S88	rec14	c	S88	++	c	c	c	c	c	c	c	c	c	c	c	g	g	g	c	c	c	c	c	c	c	c	c
g	2113541	g	2134301	a	2041971	s	S88	rec14	g	S88	++	g	g	g	g	g	g	g	g	g	a	a	a	a	a	g	g	g	g	g	g	g	g	g
a	2113614	a	2134374	g	2042044	ns	S88	rec14	a	S88	++	a	a	a	a	a	a	a	a	a	g	g	g	g	g	a	a	a	a	a	a	a	a	a
a	2113652	a	2134412	c	2042082	nc	S88	rec14	a	S88	++	a	t	a	a	t	a	t	t	t	c	c	t	t	t	c	t	t	t	a	a	a	t	t
c	2113672	c	2134432	t	2042102	nc	S88	rec14	c	S88	++	c	t	c	c	t	c	t	t	t	t	t	t	t	t	t	t	t	t	c	c	c	t	t
c	2113689	c	2134449	t	2042119	nc	S88	rec14	c	S88	++	c	t	c	c	t	c	t	t	t	t	t	t	t	t	t	t	t	t	c	c	c	t	t
g	2113690	g	2134450	a	2042120	nc	S88	rec14	g	S88	++	g	a	g	g	a	g	a	a	a	a	a	a	a	a	a	a	a	a	g	g	g	a	a
c	2113740	c	2134500	t	2042170	nc	S88	rec14	c	S88	++	c	t	c	c	t	c	t	t	t	t	t	t	t	t	t	t	t	t	c	c	c	t	t
t	2113746	t	2134506	c	2042176	nc	S88	rec14	t	S88	++	t	a	t	t	a	t	t	a	a	c	c	c	c	c	a	a	a	a	t	t	t	a	a
t	2113748	t	2134508	c	2042178	nc	S88	rec14	t	S88	++	t	t	t	t	t	t	t	t	t	c	c	c	c	c	t	t	t	t	t	t	t	t	t
a	2113759	a	2134519	g	2042189	nc	S88	rec14	a	S88	++	a	g	a	a	g	a	g	g	g	g	g	g	g	g	g	g	g	g	a	a	a	g	g
a	2113763	a	2134523	c	2042193	nc	S88	rec14	a	S88	++	a	c	a	a	c	a	c	c	c	c	c	c	c	c	c	c	c	c	a	a	a	c	c
1	2113798	1	2134568	-	2042237	del	S88	rec14	1	S88	++	1	1	1	1	1	1	-	1	1	-	-	-	-	-	1	1	1	1	1	1	1	1	1
g	2113822	g	2134582	t	2042251	nc	S88	rec14	g	S88	++	g	g	g	g	g	g	g	g	g	t	c	t	t	t	g	g	g	g	g	g	g	g	g
c	2113827	c	2134587	t	2042256	nc	S88	rec14	c	S88	++	c	c	c	c	c	c	c	c	c	t	t	t	t	t	c	c	c	c	c	c	c	c	c
-	2114000	g	2134781	t	2042450	nc	S88	rec14	g	S88	++	g	t	g	g	t	g	a	t		t	t	t	t	t	t	t	t	t	g	g	g	t	t
-	2114000	c	2134898	t	2042567	nc	S88	rec14	c	S88	++++	c	c	c	c	c	c	c	c	c	c	c	c	c	c	c	c	c	c	c	c	c	c	c
-	2114000	t	2144539	g	2052208	nc	S88	rec14	t	S88	+++	t	t	t		t	t				t													
-	2114000	c	2144811	t	2052480	nc	S88	rec14	c	S88	+++	c	c	c		c	c				c													
-	2114000	a	2145156	t	2052825	nc	S88	rec14	a	S88	+++	a	a	a		a	a				a													
-	2114000	t	2145508	a	2053177	nc	S88	rec14	t	S88	+++	t	t	t		t					t													
-	2114000	g	2145575	a	2053244	nc	S88	rec14	g	S88	+++	g	g	g		g					g													
-	2114000	a	2145739	g	2053408	nc	S88	rec14	a	S88	++	a	a	a		a					a									g		g	g	g
-	2114000	g	2146351	a	2054020	nc	S88	rec14	g	S88	+++	g	a	g		g					g													
-	2114000	g	2147534	a	2055203	nc	S88	rec14	g	S88	+++	g	g	g		g					g													
-	2190930	-	2147684	3	2055354	ins	S88	rec14	3	2	-	3	-	3		-					3													
-	2114000	g	2148689	a	2056361	nc	S88	rec14	g	S88	++		g			g		a									a							
-	2195248	-	2150639	1	2060851	ins	S88	rec14	1	2	-	1	-	1		1		-			-		-				-							
g	2195951	g	2151342	a	2061554	s	S88	rec14	g	S88	+++	g		g		g		g			g		g				g							
g	2196041	g	2151432	a	2061644	s	S88	rec14	g	S88	++	g		g		g		a			a		a				a							
g	2196953	-	2152225	a	2062555	ns	S88	rec14	g	S88	++	g		g		a							a											
c	2196959	-	2152225	t	2062561	ns	S88	rec14	c	S88	+++	c		c		t							c											
t	2196970	-	2152225	c	2062572	s	S88	rec14	t	S88	++	t		t		c							c											
a	2197115	-	2152225	t	2062717	nc	S88	rec14	a	S88	++	a		a		t							t											
t	2197117	-	2152225	c	2062719	nc	S88	rec14	t	S88	++	t		t		c							c											
c	2197171	-	2152225	t	2062773	nc	S88	rec14	c	S88	++	c		c		t							t											
t	2197185	-	2152225	g	2062787	nc	S88	rec14	t	S88	++	t		t		g							g											
a	2255934	-	2152225	g	2115356	ns	S88	rec14	a	S88	++	a	a	a				g	a	g				g	g	g	a	a		g	g	g	a	a
t	2255958	-	2152225	c	2115380	ns	S88	rec14	t	S88	++	t	c	t				c	c	c				c	c	c	c	c		c	c	c	c	c
g	2256030	-	2152225	a	2115452	ns	S88	rec14	g	S88	++	g	a	g				a	a	a				g	g	a	a	a		g	g	g	a	a
a	2256051	-	2152225	g	2115473	ns	S88	rec14	a	S88	++	a	g	a				g	g	g				a	a	g	g	g		g	g	g	g	a
a	2256053	-	2152225	t	2115475	ns	S88	rec14	a	S88	++	a	t	a				t	t	t				a	a	t	t	t		t	t	t	t	t
t	2256054	-	2152225	c	2115476	ns	S88	rec14	t	S88	++	t	c	t				c	c	c				c	c	c	c	c		c	c	c	c	c
a	2256057	-	2152225	g	2115479	ns	S88	rec14	a	S88	++	a	g	a				g	g	g				g	g	g	g	g		g	g	g	g	a
c	2256061	-	2152225	a	2115483	ns	S88	rec14	c	S88	++	c	a	c				a	a	a				a	a	a	a	a		a	a	a	a	a
c	2256065	-	2152225	a	2115487	ns	S88	rec14	c	S88	++	c	a	c				a	a	a				a	a	a	a	a		a	a	a	a	a
c	2256066	-	2152225	a	2115488	ns	S88	rec14	c	S88	++	c	a	c				a	a	a				a	a	a	a	a		a	a	a	a	a

ExPEC genome site details												Outgroup Strains Details[i]																						
UTI89[b]	UTI89 site[c]	APEC 01[b]	APEC 01 site[c]	S88[b]	S88 site[c]	type[d]	Event Lineage[ef]	Recombinant segment	Inferred ancestral base[g]	Outgroup Analysis[e]	Support level[h]	CFT073	ED1a	536	E2348/69	SMS 3-5	IAI39	UMN026	K-12	ATCC 8739	HS	D1 Sd197	CB9615	Sakai	EDL933	IAI1	E24377A	SE11	SS Ss046	F2a 301	F2a 2457T	F5 8401	B4 Sb227	B18 BS512
c	2256069	-	2152225	g	2115491	ns	S88	rec14	c	S88	++	c	g	c				g	g	g				g	g	g	g	g		g	g	g	g	g
c	2256070	-	2152225	t	2115492	ns	S88	rec14	c	S88	++	c	t	c				t	t	t				t	t	t	t	t		t	t	t	t	t
c	2256075	-	2152225	t	2115497	ns	S88	rec14	c	S88	++	c	t	c				c	c	t				t	t	c	t	t		c	c	c	t	c
c	2256094	-	2152225	t	2115516	ns	S88	rec14	c	S88	++	c	c	c				c	c	t				t	t	c	t	c		c	c	c	t	c
t	2256098	-	2152225	c	2115520	s	S88	rec14	t	S88	++	t	t	t				t	t	c				c	c	t	c	t		t	t	t	c	t
c	2256112	-	2152225	t	2115534	ns	S88	rec14	c	S88	++	c	t	c	c	c	c	c	t	t			c	t	t	t	t	t		t	t	t	t	t
c	2256129	-	2152225	a	2115551	nc	S88	rec14	c	S88	++	c	a	c	c	c	c	a	a	a			c	a	a	a	a	a		a	a	a	a	a
t	2256131	-	2152225	c	2115553	nc	S88	rec14	t	S88	++	t	c	t	t	t	t	c	c	c			t	c	c	t	c	c		t	t	t	c	t
g	2256310	-	2152225	a	2115732	ns	S88	rec14	g	S88	++	g	g	g	g	g	g	g	g	a			g	a	a	a	g	g		g	g	g	a	g
g	2256390	-	2152225	a	2115812	s	S88	rec14	g	S88	++	g	a	g	a	g	g	g	a	a			g	a	a	a	a	a		a	a	a	a	a
c	2256393	-	2152225	a	2115815	s	S88	rec14	c	S88	++	c	a	c	a	c	c	c	a	a			c	a	a	a	a	a		a	a	a	a	a
a	2256414	-	2152225	g	2115836	s	S88	rec14	a	S88	++	a	g	a	g	a	a	a	g	g			g	g	g	g	g	g		g	g	g	g	g
g	2256417	-	2152225	a	2115839	s	S88	rec14	g	S88	++	g	a	g	a	g	g	g	a	a			a	a	a	a	a	a		a	a	a	a	a
g	2256519	-	2152225	t	2115941	s	S88	rec14	g	S88	++	g	t	g	t	g	g	g	t	g			g	t	t	t	g	t		t	t	t	g	g
g	2256597	-	2152225	c	2116019	s	S88	rec14	g	S88	++	g	c	g	g	t	t	t	g	g			t	g	g	g	g	g		g	g	g	g	g
c	2256609	-	2152225	a	2116031	s	S88	rec14	c	S88	++	c	c	c	a	c	c	c	a	a			c	a	a	a	a	a		a	a	a	a	c
g	2256777	-	2152225	a	2116199	s	S88	rec14	g	S88	++	g	g	g	a	g	g	g	a	g			g	a	a	g	g	g		g	g	g	g	g
g	2256813	-	2152225	a	2116235	s	S88	rec14	g	S88	++	g	g	g	a	g	g	g	g	a			g	a	a	g	a	g		g	g	g	a	a
g	2256933	-	2152225	a	2116355	s	S88	rec14	g	S88	++	g	g	g	a	a	g	g	a	g			g	a	a	g	g	g		g	g	g	g	g
c	2256993	-	2152225	a	2116415	s	S88	rec14	c	S88	++	c	c	c	a	c	c	c	c	c			c	a	a	c	a	c		c	c	c	c	c
c	2256996	-	2152225	a	2116418	s	S88	rec14	c	S88	++	c	c	c	a	c	c	c	c	c			c	a	a	c	t	c		a	a	a	c	c
g	2257020	-	2152225	a	2116442	s	S88	rec14	g	S88	++++	g	g	g	g	g	g	g	g	g			a	g	g	g	g	g		g	g	g	g	g
c	2257065	-	2152225	t	2116487	s	S88	rec14	c	S88	++	c	t	c	c	t	t	t	c	t			t	c	c	t	c	t		c	c	c	t	t
a	2257092	-	2152225	g	2116514	s	S88	rec14	a	S88	++	a	g	a	g	g	g	g	g	g			g	g	g	g	g	g		g	g	g	g	g
t	2257134	-	2152225	c	2116556	s	S88	rec14	t	S88	++	t	c	t	t	c	c	c	c	c			c	c	c	c	c	c		c	c	c	c	c
t	2257203	-	2152225	c	2116625	s	S88	rec14	t	S88	++	t	c	t	c	t	t	c	t	c			c	t	t	t	c	c		c	c	c	c	c
a	2257295	-	2152225	g	2116717	s	S88	rec14	a	S88	++	a	g	a	g	g	g	g	g	g			g	g	g	g	g	g		g	g	g	g	g
g	2257305	-	2152225	a	2116727	s	S88	rec14	g	S88	++	g	a	g	a	a	g	a	g	a			a	a	a	a	g	a		g	g	g	a	a
a	2257309	-	2152225	g	2116731	ns	S88	rec14	a	S88	++	a	g	a	g	a	a	a	a	a			a	a	a	g	a	g		a	a	a	a	a
a	2257356	-	2152225	g	2116778	s	S88	rec14	a	S88	++	a	g	a	g	g	a	a	g	g			a	a	a	g	g	g		g	g	g	g	g
c	2257530	-	2152225	t	2116952	s	S88	rec14	c	S88	++	c	t	c	c	c	c	c	c	c			c	c	c	t	t	c		c	c	c	c	c
g	2257560	-	2152225	a	2116982	s	S88	rec14	g	S88	++	g	g	g	g	g	g	g	g	g			g	g	g	a	a	g		g	g	g	g	g
c	2257738	-	2152225	t	2117160	s	S88	rec14	c	S88	++	c	t	c	t	c	c	c	c	c			c	c	c	c	c	t		c	c	c	c	c
t	2257798	-	2152225	c	2117220	s	S88	rec14	t	S88	++	t	c	t	c	c	t	c	c	t			c	c	c	c	t	c		t	t	t	t	t
c	2257861	-	2152225	t	2117283	s	S88	rec14	c	S88	++	c	t	c	c	t	c	c	c	t			c	c	c	c	c	c		c	c	c	t	t
c	2257867	-	2152225	t	2117289	s	S88	rec14	c	S88	++	c	t	c	c	t	t	c	t	t			c	t	t	t	t	t		t	t	t	t	t
a	2257917	-	2152225	g	2117339	s	S88	rec14	a	S88	++	a	a	a	a	a	a	a	a	a			a	a	a	g	g	a		a	a	a	a	a
g	2258062	-	2152225	a	2117484	s	S88	rec14	g	S88	++	g	a	g	a	g	g	g	g	a			g	g	g	a	a	g		a	a	a	a	a
c	2258092	-	2152225	t	2117514	s	S88	rec14	c	S88	++	c	a	c	t	t	c	a	t	t			a	a	a	t	a	t		t	t	t	t	a
g	2258098	-	2152225	a	2117520	s	S88	rec14	g	S88	++	g	a	g	a	a	a	a	a	a			a	a	a	a	a	a		a	a	a	a	a
c	2258101	-	2152225	g	2117523	s	S88	rec14	c	S88	++	c	g	c	g	g	g	g	g	g			g	g	g	g	g	g		g	g	g	g	g
g	2258122	-	2152225	a	2117544	s	S88	rec14	g	S88	++	g	g	g	a	a	a	g	a	a			g	g	g	a	a	a		a	a	a	a	g
g	2258137	-	2152225	t	2117559	s	S88	rec14	g	S88	++++	g	g	g	g	a	g	g	g	g			g	g	g	g	g	g		g	g	g	g	g
t	2258179	-	2152225	c	2117601	s	S88	rec14	t	S88	++	t	c	t	c	c	c	c	c	c			c	c	c	c	c	c		c	c	c	c	c
g	2258188	-	2152225	t	2117610	s	S88	rec14	g	S88	++	t	g	g	g	t	t	g	g	t			g	g	g	t	t	g		t	t	t	t	t
g	2258259	-	2152225	t	2117681	ns	S88	rec14	g	S88	++	g	g	t	g	g	t	t	g	g			t	g	g	g	g	g		g	g	g	g	g
t	2258263	-	2152225	g	2117685	s	S88	rec14	t	S88	++	t	t	g	t	t	t	g	t	t			t	t	t	t	t	t		t	t	t	t	t
g	2258269	-	2152225	a	2117691	s	S88	rec14	g	S88	++++	g	g	g	g	g	g	g	g	g			a	g	g	g	g	g		g	g	g	g	g
g	2258275	-	2152225	a	2117697	s	S88	rec14	g	S88	++++	g	g	g	g	g	g	g	g	g			a	g	g	g	g	g		g	g	g	g	g
g	2258287	-	2152225	a	2117709	s	S88	rec14	g	S88	++++	g	g	g	g	g	g	g	g	g			g	g	g	g	g	g		g	g	g	g	g
c	2258368	-	2152225	t	2117790	s	S88	rec14	c	S88	++	c	t	c	t	c	t	c	t	t			c	t	t	t	t	t		t	t	t	t	t
a	2258410	-	2152225	g	2117832	s	S88	rec14	a	S88	++++	a	a	a	a	a	a	a	g	a			a	a	a	a	a	a		a	a	a	a	a
a	2258458	-	2152225	g	2117880	s	S88	rec14	a	S88	++	a	g	a	a	g	a	g	g	a			g	g	g	g	g	a		g	g	g	a	g
g	2258482	-	2152225	c	2117904	s	S88	rec14	g	S88	++	g	g	g	g	g	g	g	g	g			a	g	g	c	c	g		g	g	g	g	g
a	2258622	-	2152225	t	2118044	ns	S88	rec14	a	S88	++++	a	a	a	a	a	a	a	a	a			a	a	a	a	a	a		a	a	a	a	a
c	2258653	-	2152225	t	2118075	s	S88	rec14	c	S88	++++	c	c	c	c	c	c	c	c	c			c	c	c	c	c	c		c	c	c	c	c
c	2258740	-	2152225	g	2118162	s	S88	rec14	c	S88	++	c	g	c	g	g	c	g	g	g			g	g	g	g	c	g		g	g	g	g	c
c	2258890	-	2152225	g	2118312	s	S88	rec14	c	S88	++	c	c	c	c	g	g	c	g	c			c	c	c	c	c	c		g	g	g	c	g
-	2259033	-	2152225	1	2118465	ins	S88	rec14	1	2	-	1	1	1	1	1	1	1	1	1				1	1	1	1	1		1	1	1	1	1
t	2259123	-	2152225	c	2118546	s	S88	rec14	t	S88	++	t	c	t	c	c	c	c	c	c			c	t	t	c	c	c	a	c	c	c	c	c
a	2259171	-	2152225	g	2118594	s	S88	rec14	a	S88	++	a	g	a	g	g	a	a	g	g			g	a	a	g	g	g	g	g	g	g	g	g
t	2259276	-	2152225	g	2118699	ns	S88	rec14	t	S88	++	t	t	t	t	t	t	g	t	t			g	t	t	t	t	t	t	t	t	t	t	t
a	2259320	-	2152225	t	2118743	ns	S88	rec14	c	S88	+/-	c	c	c	c	c	c	c	c	c			c	c	c	c	c	c	c	c	c	c	c	c
g	2259342	-	2152225	a	2118765	s	S88	rec14	g	S88	++++	g	g	g	g	g	g	g	g	g			g	g	g	g	g	g	g	g	g	g	g	g
t	2259468	-	2152225	c	2118891	s	S88	rec14	t	S88	++	t	t	t	t	t	t	t	t	t			t	t	t	c	c	t	t	t	t	t	t	t
t	2259516	-	2152225	c	2118939	s	S88	rec14	t	S88	++	t	t	c	t	t	t	t	t	t			t	t	t	t	t	t	c	t	t	t	t	c
g	2259522	-	2152225	c	2118945	s	S88	rec14	g	S88	++	g	g	c	g	c	g	c	c	a			c	g	g	c	c	g	c	c	c	c	g	a
t	2259531	-	2152225	c	2118954	s	S88	rec14	t	S88	++	t	t	c	t	t	t	a	t	c			t	t	t	t	t	t	t	t	t	t	t	c
a	2259678	-	2152225	g	2119101	s	S88	rec14	a	S88	++	a	a	a	a	a	a	g	a	a			a	a	a	a	a	g	g	a	a	a	a	a
a	2259756	-	2152225	g	2119179	s	S88	rec14	a	S88	++	a	a	a	a	a	a	g	g	a			g	a	a	a	a	a	g	a	a	a	g	a
c	2259768	-	2152225	t	2119191	s	S88	rec14	c	S88	++	t	c	c	c	c	c	c	t	c			c	c	c	c	c	c	t	c	c	c	c	c
c	2259816	-	2152225	t	2119239	s	S88	rec14	c	S88	++	c	t	c	t	c	c	c	c	c			t	c	c	t	t	t	c	t	t	t	c	t
c	2259828	-	2152225	t	2119251	s	S88	rec14	c	S88	++	c	t	c	t	c	t	t	t	t			t	t	t	t	t	t	t	t	t	t	t	t
c	2259834	-	2152225	g	2119257	s	S88	rec14	c	S88	++	c	g	c	g	c	g	a	g	g			g	c	c	g	g	g	g	g	g	g	g	g
t	2259837	-	2152225	c	2119260	s	S88	rec14	t	S88	++	t	c	t	c	t	t	t	c	t			t	t	t	c	c	c	t	c	c	c	c	c
g	2259870	-	2152225	a	2119293	s	S88	rec14	g	S88	++++	g	g	g	g	g	g	g	g	g			g	g	g	g	g	g	g	g	g	g	g	g
a	2259981	-	2152225	g	2119404	s	S88	rec14	a	S88	++	a	g	a	a	a	a	a	g	a			a	a	a	g	g	g	a	g	g	g	g	g
a	2260026	-	2152225	g	2119449	s	S88	rec14	a	S88	++	a	a	a	g	a	a	g	a	a			a	a	a	a	a	a	a	a	a	a	a	a
t	2260041	-	2152225	a	2119464	s	S88	rec14	t	S88	++	t	a	t	t	t	t	t	a	a			t	t	t	a	a	a	t	a	a	a	t	a
g	2260074	-	2152225	a	2119497	s	S88	rec14	g	S88	++	g	g	g	a	g	g	g	t	g			g	g	g	a	a	a	a	a	a	a	a	a
g	2260167	-	2152225	a	2119590	s	S88	rec14	g	S88	++++	a	g	g	g	g	g	g	g	g			g	g	g	g	g	g	g	g	g	g	g	g
t	2260194	-	2152225	c	2119617	s	S88	rec14	t	S88	++	t	c	t	c	c	c	c	c	c			c	c	c	c	c	c	c	c	c	c	c	c
g	2260215	-	2152225	a	2119638	s	S88	rec14	g	S88	++	a	g	g	a	g	a	a	a	g			a	a	a	g	g	g	a	g	g	g	a	g

ExPEC genome site details												Outgroup Strains Details[i]																						
UTI89[b]	UTI89 site[c]	APEC 01[b]	APEC 01 site[c]	S88[b]	S88 site[c]	type[d]	Event Lineage[ef]	Recombinant segment	Inferred ancestral base[g]	Outgroup Analysis[e]	Support level[h]	CFT073	ED1a	536	E2348/69	SMS 3-5	IAI39	UMN026	K-12	ATCC 8739	HS	D1 Sd197	CB9615	Sakai	EDL933	IAI1	E24377A	SE11	SS Ss046	F2a 301	F2a 2457T	F5 8401	B4 Sb227	B18 BS512
a	2260431	-	2152225	g	2119854	s	S88	rec14	a	S88	++	a	a	a	g	g	a	g	a	a			a	a	a	g	g	g	g	g	g	g	g	g
t	2260502	-	2152225	c	2119925	ns	S88	rec14	t	S88	++	t	c	t	c			t	c	c				c	c	t	t		t	c	c	c	t	t
c	2260560	-	2152225	t	2119983	s	S88	rec14	c	S88	++	c	t	c	a			c		a				a	a	c	c			a	a	a	t	c
g	2260609	-	2152225	t	2120032	ns	S88	rec14	g	S88	++++	g	g	g	g			g		g				g	g	g	g			g	g	g	g	t
a	2260689	t	2210194	c	2120112	s	S88	rec14	c	2	-	c	c	c	c	c	c	c	c	c			c	c	c	c	c	c	c	c	c	c	c	c
a	2260734	a	2210239	g	2120157	s	S88	rec14	a	S88	++	a	a	a	g	g	g	a	a	g			a	g	g	g	g	g	a	g	g	g	a	g
g	2260797	g	2210302	a	2120220	s	S88	rec14	g	S88	++	g	g	g	a	a	a	a	a	a			g	a	a	a	a	a	a	a	a	a	g	a
g	2260914	g	2210419	a	2120337	s	S88	rec14	g	S88	++++	g	g	g	g	g	g	g	g	g			g	g	g	g	g	g	g	g	g	g	g	g
c	2260917	c	2210422	t	2120340	s	S88	rec14	c	S88	++++	c	c	c	c	c	c	c	c	c			c	c	c	c	c	c	c	c	c	c	c	c
g	2260970	g	2210475	t	2120393	s	S88	rec14	g	S88	++++	g	g	g	g	g	g	g	g	g			g	g	g	g	g	g	g	g	g	g	g	g
g	2261040	g	2210545	t	2120463	s	S88	rec14	g	S88	++	g	g	g	g	g	g	a	g	g			g	g	g	g	g	g	g	g	g	g	g	t
a	2261118	a	2210623	g	2120541	s	S88	rec14	a	S88	++	a	g	a	g	g	a	g	a	g			a	a	a	g	g	g	g	a	a	a	a	g
a	2261205	a	2210710	t	2120628	s	S88	rec14	t	2	-	t	t	a	t	t	t	a	t	t			t	t	t	t	t	t	t	t	t	t	t	a
g	2261208	g	2210713	a	2120631	s	S88	rec14	a	2	-	a	a	g	a	a	a	g	a	g			a	g	g	a	a	g	g	a	a	a	a	g
a	2261331	a	2210836	g	2120754	s	S88	rec14	a	S88	++	a	g	a	g	a	a	g	g	g			a	g	g	a	a	a	a	a	a	a	g	a
a	2261376	a	2210881	g	2120799	s	S88	rec14	a	S88	++	g	a	a	a	a	a	a	a	a			a	a	a	a	a	a	a	g	g	g	a	a
a	2261388	a	2210893	g	2120811	s	S88	rec14	a	S88	++	a	a	a	a	a	a	a	g	a			g	a	a	a	a	a	a	g	g	g	a	a
t	2261543	t	2211048	c	2120966	ns	S88	rec14	t	S88	++	t	t	t	t	c	c	t	t	t			t	t	t	t	t	t	t	t	t	t	t	t
t	2261589	t	2211094	a	2121012	s	S88	rec14	a	2	-	t	a	a	a	t	t	a	a	a			t	t	t	t	a	a	a	a	a	a	a	a
a	2261673	a	2211178	g	2121096	s	S88	rec14	a	S88	++	g	a	a	a	a	a	a	a	a			a	g	g	a	a	a	a	a	a	a	a	a
a	2261949	a	2211454	t	2121372	ns	S88	rec14	a	S88	++++	a	a	a	a	a	a	a	a	a			a	a	a	a	a	a	a	a	a	a	a	a
g	2261979	g	2211484	a	2121402	s	S88	rec14	g	S88	++	g	g	g	g	g	a	g	a	g			g	a	a	g	g	g	g	g	g	g	g	g
c	2262155	c	2211660	t	2121578	s	S88	rec14	c	S88	++++	c	c	c	c	c	c	c	c	c			c	c	c	c	c	c	c	c	c	c	c	c
g	2262161	g	2211666	a	2121584	s	S88	rec14	g	S88	++++	g	g	g	g	g	g	g	g	g			g	g	g	a	g	g	g	g	g	g	g	g
g	2262228	g	2211733	c	2121651	ns	S88	rec14	g	S88	++++	g	g	g	g	g	g	g	g	g			g	g	g	c	g	g	g	g	g	g	g	g
g	2262264	g	2211769	a	2121687	ns	S88	rec14	g	S88	++++	g	g	g	g	g	g	g	g	g			g	g	g	g	g	g	g	g	g	g	g	g
t	2262266	t	2211771	c	2121689	s	S88	rec14	t	S88	++	t	t	t	c	t	t	c	t	t			t	t	t	c	c	c	c	c	c	c	t	t
a	2262419	a	2211924	g	2121842	s	S88	rec14	a	S88	++++	a	a	a	a	a	a	g	a	a			a	a	a	a	a	a	a	a	a	a	a	a
t	2262458	t	2211963	a	2121881	ns	S88	rec14	t	S88	++++	t	t	t	t	t	t	t	t	t			t	t	t	t	t	t	t	t	t	t	t	t
c	2262522	c	2212027	a	2121945	ns	S88	rec14	c	S88	++	c	a	c	c	c	c	c	c	a			c	c	c	c	c	c	c	a	a	a	c	c
c	2262797	c	2212302	t	2122220	s	S88	rec14	t	2	-	t	t	t	t	c	c	t	t	c			t	t	t	t	t	t	t	t	t	t	t	t
a	2262896	a	2212401	g	2122319	s	S88	rec14	a	S88	++	a	a	a	a	a	a	a	a	a			g	g	g	a	a	a	a	a	a	a	g	g
t	2262950	t	2212455	c	2122373	s	S88	rec14	c	2	-	c	c	c	t	c	c	c	c	c			t	t	t	c	t	c	c	t	t	t	t	t
g	2263067	g	2212572	a	2122490	s	S88	rec14	g	S88	++	g	g	g	g	g	g	g	a	g			g	g	g	g	a	g	g	g	g	g	g	g
c	2263115	c	2212620	g	2122538	s	S88	rec14	c	S88	++	c	g	c	g	c	c	g	g	c			c	c	c	g	g	g	c	g	g	g	c	c
g	2263121	g	2212626	a	2122544	s	S88	rec14	g	S88	++++	g	g	g	g	g	g	g	g	g			g	g	g	g	g	g	g	g	g	g	g	g
c	2263178	c	2212683	g	2122601	s	S88	rec14	c	S88	++++	c	c	c	c	c	c	c	c	c			c	c	c	c	c	c	c	c	c	c	c	c
a	2263362	a	2212867	g	2122785	s	S88	rec14	a	S88	++	a	g	a	g	g	a	g	g	g			g	g	g	g	g	g	g	g	g	g	g	g
c	2263363	c	2212868	t	2122786	s	S88	rec14	c	S88	++	c	t	c	c	t	c	t	c	t			c	c	c	c	c	c	c	c	c	c	c	c
t	2263400	t	2212905	c	2122823	ns	S88	rec14	t	S88	++++	t	t	t	t	t	t	t	t	t			t	t	t	t	t	t	t	t	t	t	t	t
a	2263717	a	2213222	g	2123140	s	S88	rec14	a	S88	++	t	a	t	a	t	a	a	a	a			a	t	t	t	g	a	g	t	t	t	a	a
g	2263725	g	2213230	a	2123148	ns	S88	rec14	g	S88	++++	g	g	g	g	g	g	g	g	g			g	g	g	g	g	g	g	g	g	g	g	g
a	2263905	a	2213410	g	2123328	s	S88	rec14	g	2	-	a	g	g	a	a	a	a	g	g			g	g	g	g	g	a	g	g	g	g	g	g
t	2264019	t	2213524	c	2123442	s	S88	rec14	c	2	-	c	c	c	c	t	c	c	c	c			c	c	c	c	c	c	c	t	t	t	c	c
c	2264058	c	2213563	g	2123481	s	S88	rec14	c	S88	++	c	g	c	c	c	c	c	g	g			c	g	g	c	g	g	g	g	g	g	c	c
a	2264097	a	2213602	g	2123520	s	S88	rec14	a	S88	++	a	g	a	a	a	a	a	a	g			a	a	a	g	a	a	g	g	g	g	a	a
g	2264103	g	2213608	a	2123526	s	S88	rec14	g	S88	++	g	a	g	g	g	g	a	g	a			a	t	t	a	a	g	a	a	a	a	a	a
c	2264127	c	2213632	t	2123550	s	S88	rec14	c	S88	++	c	t	c	c	c	c	c	t	c			c	c	c	t	c	c	t	t	t	t	c	c
a	2264322	a	2213827	g	2123745	s	S88	rec14	a	S88	++	a	g	a	a	a	a	a	a	a			g	g	g	a	a	a	g	g	g	g	g	g
c	2264361	c	2213866	t	2123784	s	S88	rec14	c	S88	++++	c	c	c	c	c	c	c	c	c			c	c	c	c	c	c	c	c	c	c	c	c
a	2264487	a	2213992	g	2123910	s	S88	rec14	g	2	-	g	a	g	g	a	a	g	g	g			g	a	a	g	g	g	g	g	g	g	g	g
g	2264679	g	2214184	a	2124102	s	S88	rec14	g	S88	++	g	g	g	a	g	g	g	g	g			g	g	g	g	g	g	a	a	a	a	a	a
a	2264706	a	2214211	g	2124129	s	S88	rec14	g	2	-	g	g	g	g	a	a	g	a	a			a	a	a	a	g	g	g	g	g	g	g	g
a	2264909	a	2214414	t	2124332	s	S88	rec14	a	S88	++	c	a	a	a	a	a	t	a	t			t	a	a	a	a	a	a	t	t	t	t	t
g	2265014	g	2214519	a	2124437	s	S88	rec14	a	2	-	a	g	a	g	g	g	g	g	g			a	g	g	a	g	g	a	a	a	a	a	a
c	2265053	c	2214558	t	2124476	s	S88	rec14	t	2	-	t	t	c	t	t	c	c	t	t			t	t	t	t	t	t	c	t	t	t	t	t
a	2265098	g	2214603	c	2124521	s	S88	rec14	c	2	-	c	a	g	c	a	c	a	c	c			a	c	c	c	a	c	c	c	c	c	c	c
a	2265101	a	2214606	g	2124524	s	S88	rec14	a	S88	++	g	a	a	g	a	g	a	g	g			a	g	g	g	a	g	g	g	g	g	g	g
c	2265116	c	2214621	t	2124539	s	S88	rec14	t	2	-	c	t	t	c	c	c	c	c	c			c	t	t	c	t	t	c	c	c	c	c	c
a	2265278	a	2214783	g	2124701	s	S88	rec14	a	S88	++	a	a	a	g	a	a	g	g	g			g	g	g	g	a	g	g	g	g	g	g	g
t	2265392	t	2214897	g	2124815	s	S88	rec14	t	S88	++	t	t	t	g	g	g	g	t	t			g	g	g	t	t	t	t	t	t	t	t	t
c	2265395	c	2214900	t	2124818	s	S88	rec14	c	S88	++	c	c	c	t	t	t	t	c	c			t	c	c	c	c	c	c	c	c	c	c	c
a	2265473	a	2214978	g	2124896	s	S88	rec14	a	S88	++++	a	a	a	a	a	a	a	a	a			a	a	a	a	a	a	a	a	a	a	a	a
a	2265536	a	2215041	g	2124959	s	S88	rec14	a	S88	++	a	g	a	a	a	a	a	g	g			a	g	g	g	g	g	g	g	g	g	g	g
a	2265572	a	2215077	g	2124995	s	S88	rec14	a	S88	++	a	a	a	a	a	a	a	g	g			a	a	a	g	a	g	g	g	g	g	g	g
a	2265749	a	2215254	g	2125172	s	S88	rec14	a	S88	++	a	g	a	a	g	g	g	g	g			a	g	g	g	g	g	g	g	g	g	g	g
c	2265970	c	2215475	t	2125393	s	S88	rec14	c	S88	++	c	c	c	t	c	c	c	t	t			c	c	c	t	t	t	t	t	t	t	t	t
g	2265994	g	2215499	a	2125417	s	S88	rec14	g	S88	++	g	g	g	a	g	g	g	g	g			a	a	a	g	g	g	g	g	g	g	g	g
g	2266018	g	2215523	a	2125441	s	S88	rec14	g	S88	++	g	g	g	a	a	a	a	g	g			g	a	a	g	g	a	g	g	g	g	g	g
g	2266024	g	2215529	t	2125447	s	S88	rec14	g	S88	++	g	g	g	g	t	t	g	g	g			g	g	g	g	g	t	g	g	g	g	g	g
t	2266027	t	2215532	c	2125450	s	S88	rec14	t	S88	++	t	t	t	t	c	c	t	t	t			t	t	t	t	t	c	t	t	t	t	t	t
c	2111118	t	2131875	t	2039548	s	AS	rec15	c	AS	++	c	t	c	c	t	c	t	t	t	t	c	t	t	t	t	t	t	t	c	c	c	c	t
g	2111172	a	2131929	a	2039602	s	AS	rec15	g	AS	++	g	a	g	g	g	g	g	a	g	g	g	a	a	a	g	g	g	a	g	g	g	g	a
g	2111196	a	2131953	a	2039626	s	AS	rec15	g	AS	++	g	a	g	g	a	g	g	a	a	a	g	a	a	a	a	a	a	a	g	g	g	g	a
a	2111270	c	2132028	c	2039700	ns	AS	rec15	a	AS	++	a	c	a	a	c	a	c	c	c	c	a	c	c	c	c	c	c	c	a	a	a	a	c
c	2112030	t	2132790	t	2040460	ns	AS	rec15	c	AS	++	c	t	c	t	t	c	c	t	t	c	t	t	t	t	t	t	t	t	t	t	t		t
t	2112189	c	2132949	c	2040619	s	AS	rec15	t	AS	++	t	c	t	c	c	c	t	c	c	c	c	c	c	c	c	c	c	c	c	c	c		c
c	2112207	g	2132967	g	2040637	s	AS	rec15	c	AS	++	c	g	c	g	g	g	c	g	g	c	g	g	g	g	g	g	g	g	g	g	g		g
a	2112252	t	2133012	t	2040682	s	AS	rec15	a	AS	++	a	t	a	t	t	t	a	t	t	a	a	t	t	t	t	t	t	t	t	t	t		t
g	2112330	a	2133090	a	2040760	s	AS	rec15	g	AS	++	g	a	g	a	a	a	a	a	a	a	a	a	a	a	a	a	a	a	a	a	a		a
g	2112363	a	2133123	a	2040793	s	AS	rec15	g	AS	++	g	a	g	a	a	a	a	a	a	a	a	a	a	a	a	a	a	a	a	a	a		a
c	2113110	t	2133870	t	2041540	ns	AS	rec15	c	AS	++	c	t	c	t	t	t	t	t	t	t	t	t	t	t	t	t	t	t	t	t	t		t
c	2113422	t	2134182	t	2041852	ns	AS	rec15	c	AS	++	c	t	c	t	t	t	t	t	t	t	t	t	t	t	t	t	t	t	t	t	t	t	t

ExPEC genome site details												Outgroup Strains Details[i]																						
UTI89[b]	UTI89 site[c]	APEC 01[b]	APEC 01 site[c]	S88[b]	S88 site[c]	type[d]	Event Lineage[ef]	Recombinant segment	Inferred ancestral base[g]	Outgroup Analysis[e]	Support level[h]	CFT073	ED1a	536	E2348/69	SMS 3-5	IAI39	UMN026	K-12	ATCC 8739	HS	D1 Sd197	CB9615	Sakai	EDL933	IAI1	E24377A	SE11	SS Ss046	F2a 301	F2a 2457T	F5 8401	B4 Sb227	B18 BS512
a	2113517	g	2134277	g	2041947	s	AS	rec15	a	AS	++	a	g	a	g	g	g	g	g	g	g	g	g	g	g	a	g	g	g	g	g	g	g	g
a	2113679	g	2134439	g	2042109	nc	AS	rec15	a	AS	++	a	g	a	g	g	a	g	g	g	g	g	g	g	g	g	g	g	g	g	g	g	g	g
g	2113780	a	2134540	a	2042210	nc	AS	rec15	g	AS	++	g	a	g	a	a	g	g	a	a	a	a	a	a	a	a	a	a	a	a	a	a	a	a
t	2113844	g	2134604	a	2042273	nc	AS	rec15	t	AS	++	t	a	t	g	a	t	a	a	a	g	g	a	a	a	a	a	a	a	g	g	g	a	a
g	2195306	c	2150697	c	2060909	nc	UTI89/AS	rec16	g	AS	++	t	t	g		c		c			c		c				c							
t	2195484	c	2150875	c	2061087	s	UTI89/AS	rec16	t	AS	++	c	t	t		c		t			c		t				c							
t	2195502	g	2150893	g	2061105	ns	UTI89/AS	rec16	t	AS	++++	t	t	t		g		t			t		t				t							
t	2195518	a	2150909	a	2061121	ns	UTI89/AS	rec16	a	UTI89	++++	a	a	t		a		a			a		a				a							
c	2195534	g	2150925	g	2061137	s	UTI89/AS	rec16	c	AS	++	a	c	c		g		c			t		c				t							
a	2195647	c	2151038	c	2061250	nc	UTI89/AS	rec16	c	UTI89	++++	c	c	a		c		c			c		c				c							
a	2195997	g	2151388	g	2061600	ns	UTI89/AS	rec16	a	AS	++	a		a		g		g			g		g				g							
t	2196325	g	2151716	g	2061928	nc	UTI89/AS	rec16	t	AS	++	t		t		g							g											
g	2196556	t	2151947	t	2062159	nc	UTI89/AS	rec16	g	AS	++	g		g		t							t											
1	2196747	-	2152137	-	2062349	del	UTI89/AS	rec16	1	AS	++	1		1		-							-											
a	2256041	-	2152225	c	2115463	s	UTI89	rec17	c	UTI89	++++	c	c	c				c	c	c				c	c	c	c	c		c	c	c	c	c
g	2256495	-	2152225	a	2115917	ns	UTI89	rec17	a	UTI89	++++	a	a	a	a	a	a	a	a	a			a	a	a	a	a	a		a	a	a	a	a
t	2256497	-	2152225	c	2115919	ns	UTI89	rec17	c	UTI89	++++	c	c	c	c	c	c	c	c	c			c	c	c	c	c	c		c	c	c	c	c
g	2256498	-	2152225	a	2115920	s	UTI89	rec17	a	UTI89	++++	a	a	a	a	a	a	a	a	a			a	a	a	a	a	a		a	a	a	a	a
t	2256501	-	2152225	c	2115923	s	UTI89	rec17	c	UTI89	++++	c	c	c	c	c	c	c	c	c			c	c	c	c	c	c		c	c	c	c	c
c	2256649	-	2152225	t	2116071	ns	UTI89	rec17	t	UTI89	++++	t	t	t	t	t	t	t	t	t			t	t	t	t	t	c		t	t	t	t	t
t	2256657	-	2152225	c	2116079	s	UTI89	rec17	c	UTI89	++	c	c	c	t	c	c	c	c	t			c	t	t	c	t	c		t	t	t	t	t
g	2256660	-	2152225	a	2116082	s	UTI89	rec17	a	UTI89	++	a	a	a	g	a	a	a	a	g			a	g	g	a	g	a		g	g	g	g	g
t	2256666	-	2152225	g	2116088	s	UTI89	rec17	g	UTI89	++	g	g	g	t	g	g	g	g	t			g	t	t	g	t	t		t	t	t	t	t
g	2256759	-	2152225	a	2116181	s	UTI89	rec17	a	UTI89	++	a	c	a	g	a	a	g	a	g			g	g	g	g	a	g		a	a	a	g	g
t	2256774	-	2152225	g	2116196	s	UTI89	rec17	g	UTI89	++++	g	g	g	g	g	g	g	g	g			g	g	g	g	g	g		g	g	g	g	g
c	2256798	-	2152225	t	2116220	s	UTI89	rec17	t	UTI89	++	t	t	t	t	t	t	c	t	t			c	t	t	c	c	c		c	c	c	t	t
t	2256843	-	2152225	c	2116265	s	UTI89	rec17	c	UTI89	++	c	c	c	c	c	c	c	t	c			c	c	c	c	t	c		c	c	c	c	c
a	2256882	-	2152225	t	2116304	s	UTI89	rec17	t	UTI89	++	t	t	t	t	t	t	t	t	g			t	t	t	a	t	t		t	t	t	g	g
a	2256891	-	2152225	c	2116313	s	UTI89	rec17	c	UTI89	++++	c	c	c	c	c	c	c	c	c			c	c	c	c	c	c		c	c	c	c	c
a	2257335	-	2152225	g	2116757	s	UTI89	rec17	g	UTI89	++	g	g	g	g	a	a	g	g	g			g	a	a	g	g	g		a	a	a	g	g
g	2257888	-	2152225	c	2117310	s	UTI89	rec17	c	UTI89	++	c	c	c	g	g	g	c	c	c			c	c	c	c	c	c		c	c	c	c	c
a	2257891	-	2152225	g	2117313	s	UTI89	rec17	g	UTI89	++	g	g	g	a	g	a	g	g	g			g	g	g	g	g	g		g	a	g	g	g
g	2257921	-	2152225	a	2117343	s	UTI89	rec17	a	UTI89	++	a	g	a	g	a	a	a	a	g			a	a	a	a	a	g		g	g	g	g	g
t	2257978	-	2152225	c	2117400	s	UTI89	rec17	c	UTI89	++++	c	c	c	c	c	c	c	c	c			c	c	c	c	c	c		c	c	c	c	c
a	2258245	-	2152225	g	2117667	s	UTI89	rec17	g	UTI89	++++	g	g	g	g	a	g	g	g	g			g	g	g	g	g	g		g	g	g	g	g
g	2258260	-	2152225	a	2117682	s	UTI89	rec17	a	UTI89	++	a	a	a	a	g	a	a	g	a			a	a	a	g	a	a		a	a	a	a	a
t	2258422	-	2152225	c	2117844	s	UTI89	rec17	c	UTI89	++	c	c	t	c	t	c	c	c	c			c	c	c	c	c	c		c	c	c	c	c
c	2258623	-	2152225	t	2118045	s	UTI89	rec17	t	UTI89	++	t	t	t	t	t	c	t	c	t			t	t	t	t	t	t		t	t	t	t	t
a	2258719	-	2152225	g	2118141	s	UTI89	rec17	g	UTI89	++++	g	g	g	g	a	g	g	g	g			g	g	g	g	g	g		g	g	g	g	g
g	2258806	-	2152225	a	2118228	s	UTI89	rec17	a	UTI89	++++	a	a	a	a	a	a	a	a	a			a	a	a	a	a	a		a	a	a	a	a
t	2259037	-	2152225	a	2118459	nc	UTI89	rec17	a	UTI89	++++	a	a	a	a	a	a	a	a	a				a	a	a	a	a		a	a	a	a	a
t	2259164	-	2152225	a	2118587	ns	UTI89	rec17	a	UTI89	++	a	t	a	t	a	a	a	t	t			a	a	a	a	a	a	t	t	t	t	t	a
a	2259198	-	2152225	g	2118621	s	UTI89	rec17	g	UTI89	++	g	a	g	g	g	g	g	g	g			g	g	g	a	a	g	g	g	g	g	g	a
g	2259234	-	2152225	a	2118657	s	UTI89	rec17	a	UTI89	++	a	a	a	a	a	a	g	a	a			a	a	a	a	a	g	g	a	a	a	a	a
g	2259243	-	2152225	a	2118666	s	UTI89	rec17	a	UTI89	++++	a	a	a	a	a	a	a	a	a			g	a	a	a	a	a	a	a	a	a	a	a
a	2259273	-	2152225	g	2118696	s	UTI89	rec17	g	UTI89	++++	g	g	g	g	g	g	g	g	g			g	g	g	g	g	g	g	g	g	g	g	g
t	2259279	-	2152225	c	2118702	s	UTI89	rec17	c	UTI89	++++	c	c	c	c	c	c	c	c	c			c	c	c	c	c	c	c	c	c	c	c	c
a	2259462	-	2152225	g	2118885	s	UTI89	rec17	g	UTI89	++++	g	g	g	g	g	g	g	g	g			g	g	g	g	g	g	g	g	g	g	g	g
a	2259626	-	2152225	g	2119049	s	UTI89	rec17	g	UTI89	++	a	g	g	g	a	g	g	g	g			g	g	g	g	g	g	g	g	g	g	g	g
a	2259627	-	2152225	g	2119050	s	UTI89	rec17	g	UTI89	++	a	g	g	g	a	g	g	g	g			g	a	a	g	g	g	g	g	g	g	g	g
a	2259642	-	2152225	g	2119065	s	UTI89	rec17	g	UTI89	++	a	g	g	g	a	g	g	g	g			g	g	g	g	g	a	g	g	g	g	g	g
a	2259770	-	2152225	g	2119193	s	UTI89	rec17	g	UTI89	++	g	g	a	g	a	a	g	g	g			a	g	g	g	g	g	g	g	g	g	g	g
a	2259873	-	2152225	c	2119296	s	UTI89	rec17	c	UTI89	++	c	c	c	c	c	c	a	c	a			c	c	c	c	c	c	c	c	c	c	c	c
t	2259876	-	2152225	c	2119299	s	UTI89	rec17	c	UTI89	++	c	t	c	c	c	c	t	c	t			c	c	c	c	c	c	c	c	c	c	c	c
a	2259927	-	2152225	g	2119350	s	UTI89	rec17	g	UTI89	++	g	g	a	g	a	a	g	g	a			g	a	a	g	g	g	g	g	g	g	g	g
g	2259939	-	2152225	a	2119362	s	UTI89	rec17	a	UTI89	++	g	a	a	g	g	a	g	a	g			g	g	g	a	a	a	a	a	a	a	a	a
a	2259970	-	2152225	g	2119393	ns	UTI89	rec17	g	UTI89	++++	g	g	g	g	g	g	g	g	g			g	g	g	g	g	g	g	g	g	g	g	g
c	2260009	-	2152225	g	2119432	ns	UTI89	rec17	g	UTI89	++++	g	g	g	g	g	g	g	g	g			g	g	g	g	g	g	g	g	g	g	g	g
a	2260020	-	2152225	g	2119443	s	UTI89	rec17	g	UTI89	++++	g	g	g	g	g	g	a	g	g			g	g	g	g	g	g	g	g	g	g	g	g
g	2260152	-	2152225	a	2119575	s	UTI89	rec17	a	UTI89	++	a	a	a	g	g	g	g	a	a			a	a	a	a	a	a	a	a	a	a	a	a
a	2260212	-	2152225	g	2119635	s	UTI89	rec17	g	UTI89	++	a	g	g	g	a	a	g	a	g			a	a	a	a	a	a	g	a	a	a	g	a
t	2260389	-	2152225	c	2119812	s	UTI89	rec17	c	UTI89	++++	c	c	c	c	c	c	c	c	c			t	c	c	c	c	c	c	c	c	c	c	c
c	2260585	-	2152225	t	2120008	ns	UTI89	rec17	t	UTI89	++++	t	t	t	t			t		t				t	t	t	t			t	t	t	t	t
a	2260800	g	2210305	g	2120223	s	UTI89/AS	rec18	a	AS	++	a	a	a	a	g	a	g	g	g			a	g	g	g	g	g	g	g	g	g	a	g
a	2260824	c	2210329	c	2120247	s	UTI89/AS	rec18	a	AS	++	a	c	a	c	c	c	c	a	a			a	a	a	c	c	c	a	c	c	c	a	c
a	2260839	c	2210344	c	2120262	s	UTI89/AS	rec18	c	UTI89	++++	c	c	c	c	c	c	c	c	c			c	c	c	c	c	c	c	c	c	c	c	c
a	2260947	g	2210452	g	2120370	s	UTI89/AS	rec18	a	AS	++	a	g	a	a	a	a	a	a	a			a	g	g	a	a	g	g	a	a	a	a	a
a	2260989	g	2210494	g	2120412	s	UTI89/AS	rec18	g	UTI89	++	g	g	g	g	a	a	g	g	g			g	g	g	g	g	g	g	g	g	g	g	g
t	2261052	c	2210557	c	2120475	s	UTI89/AS	rec18	t	AS	++	t	t	t	c	t	c	c	c	c			t	c	c	c	c	t	c	c	c	c	t	c
a	2261160	g	2210665	g	2120583	s	UTI89/AS	rec18	a	AS	++	a	g	a	g	a	a	g	g	a			g	g	g	g	g	g	g	g	g	g	g	a
a	2261190	g	2210695	g	2120613	s	UTI89/AS	rec18	g	UTI89	++	g	g	a	g	g	g	g	g	g			g	g	g	g	g	g	g	g	g	g	g	a
g	2261268	t	2210773	a	2120691	s	UTI89/AS	rec18	g	AS	++	g	a	g	a	g	g	a	a	a			t	a	a	a	a	a	a	a	a	a	a	a
a	2261304	g	2210809	g	2120727	s	UTI89/AS	rec18	a	AS	++	a	g	a	g	a	a	g	g	g			g	g	g	g	g	g	g	g	g	g	g	g
g	2261310	c	2210815	c	2120733	s	UTI89/AS	rec18	g	AS	++	g	c	g	c	g	g	c	g	g			g	c	c	g	g	g	g	g	g	g	g	c
c	2261319	t	2210824	t	2120742	s	UTI89/AS	rec18	c	AS	++	c	t	c	t	c	c	t	t	t			t	g	g	t	t	t	t	t	t	t	t	t
a	2261397	g	2210902	g	2120820	s	UTI89/AS	rec18	g	UTI89	++	g	g	a	g	g	g	g	g	g			g	g	g	g	g	g	a	g	g	g	g	g
c	2261403	t	2210908	t	2120826	s	UTI89/AS	rec18	t	UTI89	++	t	t	c	t	c	c	t	t	t			t	t	t	t	t	t	t	t	t	t	t	t
a	2261424	g	2210929	g	2120847	ns	UTI89/AS	rec18	g	UTI89	++++	g	g	a	g	g	g	g	g	g			g	g	g	g	g	g	g	g	g	g	g	g
a	2261472	g	2210977	g	2120895	s	UTI89/AS	rec18	g	UTI89	++	g	g	a	g	a	a	g	g	g			g	g	g	g	g	g	g	g	g	g	a	g

ExPEC genome site details												Outgroup Strains Details[i]																						
UTI89[b]	UTI89 site[c]	APEC 01[b]	APEC 01 site[c]	S88[b]	S88 site[c]	type[d]	Event Lineage[ef]	Recombinant segment	Inferred ancestral base[g]	Outgroup Analysis[e]	Support level[h]	CFT073	ED1a	536	E2348/69	SMS 3-5	IAI39	UMN026	K-12	ATCC 8739	HS	D1 Sd197	CB9615	Sakai	EDL933	IAI1	E24377A	SE11	SS Ss046	F2a 301	F2a 2457T	F5 8401	B4 Sb227	B18 BS512
c	2261478	t	2210983	t	2120901	s	UTI89/AS	rec18	c	AS	++	c	c	c	c	c	c	c	c	c			c	c	c	c	c	c	c	t	t	t	c	c
c	2261490	g	2210995	g	2120913	s	UTI89/AS	rec18	c	AS	++	a	a	c	g	g	g	g	a	g			c	c	c	g	g	g	g	g	g	g	a	g
g	2261714	a	2211219	a	2121137	s	UTI89/AS	rec18	g	AS	++	g	g	g	a	g	g	a	a	a			a	g	g	g	a	a	a	a	a	a	g	g
c	2261715	g	2211220	g	2121138	s	UTI89/AS	rec18	c	AS	++	c	c	c	t	c	t	t	t	t			g	c	c	c	t	t	t	t	t	t	c	c
t	2261727	c	2211232	c	2121150	s	UTI89/AS	rec18	t	AS	++	t	c	t	c	t	t	c	c	c			c	t	t	c	c	c	c	c	c	c	c	c
t	2261734	c	2211239	c	2121157	ns	UTI89/AS	rec18	t	AS	++	t	c	t	c	t	t	c	c	c			c	t	t	c	c	c	c	c	c	c	c	t
c	2261781	a	2211286	a	2121204	s	UTI89/AS	rec18	a	UTI89	++	a	a	c	a	c	c	a	a	a			c	c	c	a	a	a	a	a	a	a	t	c
a	2261802	c	2211307	c	2121225	s	UTI89/AS	rec18	c	UTI89	++++	c	c	c	c	c	c	c	c	c			c	c	c	c	c	c	c	c	c	c	c	a
a	2261826	g	2211331	g	2121249	s	UTI89/AS	rec18	g	UTI89	++	g	g	g	g	g	g	a	a	a			g	g	g	a	g	g	a	g	g	g	g	g
a	2261832	g	2211337	g	2121255	s	UTI89/AS	rec18	g	UTI89	++	g	g	a	g	g	g	g	g	g			g	g	g	a	g	g	g	g	g	g	g	g
t	2261835	c	2211340	c	2121258	s	UTI89/AS	rec18	c	UTI89	++++	c	c	t	c	c	c	c	c	c			c	c	c	c	c	c	c	c	c	c	c	c
a	2261922	g	2211427	g	2121345	s	UTI89/AS	rec18	g	UTI89	++++	g	g	g	a	g	g	g	g	g			g	g	g	g	g	g	g	g	g	g	g	g
g	2262170	a	2211675	a	2121593	ns	UTI89/AS	rec18	a	UTI89	++++	a	a	a	a	a	a	a	a	a			a	a	a	a	a	a	a	a	a	a	a	a
a	2262172	g	2211677	g	2121595	ns	UTI89/AS	rec18	g	UTI89	++++	g	g	g	g	g	g	g	g	g			g	g	g	g	g	g	g	g	g	g	g	g
a	2262173	g	2211678	g	2121596	s	UTI89/AS	rec18	a	AS	++	a	a	a	g	a	g	g	g	g			a	a	a	g	g	g	g	a	a	a	a	a
g	2262257	t	2211762	t	2121680	s	UTI89/AS	rec18	t	UTI89	++	t	t	g	t	t	t	t	t	t			g	t	t	t	t	t	t	t	t	t	t	t
c	2262371	g	2211876	g	2121794	s	UTI89/AS	rec18	c	AS	++	c	g	c	g	c	c	g	g	g			g	g	g	g	g	g	g	g	g	g	g	g
c	2262422	t	2211927	t	2121845	s	UTI89/AS	rec18	t	UTI89	++++	t	t	t	t	t	t	t	t	t			t	t	t	t	t	t	t	t	t	t	t	t
c	2262432	t	2211937	t	2121855	ns	UTI89/AS	rec18	c	AS	++++	c	c	c	c	c	c	c	c	c			t	c	c	c	c	c	c	c	c	c	c	c
c	2262543	t	2212048	t	2121966	ns	UTI89/AS	rec18	t	UTI89	++++	t	t	t	t	t	t	t	t	t			t	t	t	t	t	t	t	t	t	t	t	t
g	2262665	a	2212170	a	2122088	s	UTI89/AS	rec18	a	UTI89	++	a	a	a	a	g	g	a	a	a			a	g	g	g	a	a	a	a	a	a	a	a
c	2262671	t	2212176	t	2122094	s	UTI89/AS	rec18	t	UTI89	++	t	c	t	c	c	c	c	c	c			t	c	c	c	c	c	c	c	c	c	t	t
t	2262680	g	2212185	g	2122103	s	UTI89/AS	rec18	g	UTI89	++++	g	g	g	g	g	g	g	g	g			g	g	g	g	g	g	g	g	g	g	g	g
a	2262689	g	2212194	g	2122112	s	UTI89/AS	rec18	g	UTI89	++++	g	g	g	g	g	g	g	g	g			g	g	g	a	g	g	g	g	g	g	g	g
c	2262701	t	2212206	t	2122124	s	UTI89/AS	rec18	c	AS	++	c	c	c	c	c	c	c	c	c			t	c	c	c	c	c	c	c	c	c	t	t
g	2262956	a	2212461	a	2122379	s	UTI89/AS	rec18	g	AS	++	g	g	g	g	g	g	g	a	g			g	g	g	g	g	g	g	a	a	a	g	g
g	2262994	a	2212499	a	2122417	s	UTI89/AS	rec18	g	AS	++	g	g	g	g	g	g	a	g	g			a	a	a	a	g	a	g	g	g	g	a	a
a	2262998	g	2212503	g	2122421	s	UTI89/AS	rec18	a	AS	++	a	a	a	a	a	a	g	a	a			g	g	g	g	a	g	a	a	a	a	g	g
g	2263001	a	2212506	a	2122424	s	UTI89/AS	rec18	g	AS	++	g	g	g	g	g	g	a	g	a			a	a	a	a	g	a	g	g	g	g	a	a
a	2263046	g	2212551	g	2122469	s	UTI89/AS	rec18	g	UTI89	++++	g	g	g	g	g	g	g	g	g			g	g	g	g	g	g	g	g	g	g	g	g
a	2263089	g	2212594	g	2122512	ns	UTI89/AS	rec18	g	UTI89	++++	g	g	g	g	g	g	g	g	g			g	g	g	g	g	g	g	g	g	g	g	g
c	2263106	t	2212611	t	2122529	s	UTI89/AS	rec18	c	AS	++	c	t	c	t	c	t	t	t	t			t	t	t	t	t	t	t	t	t	t	t	t
c	2263166	t	2212671	t	2122589	s	UTI89/AS	rec18	c	AS	++	c	t	c	t	t	t	t	t	t			t	t	t	t	t	t	t	t	t	t	t	t
c	2263417	t	2212922	t	2122840	s	UTI89/AS	rec18	c	AS	++	c	c	c	c	c	a	c	c	c			a	t	t	a	a	c	c	c	c	c	a	a
a	2263420	t	2212925	t	2122843	ns	UTI89/AS	rec18	a	AS	++	a	t	a	t	t	t	t	t	t			t	t	t	t	t	t	t	t	t	t	t	t
a	2263468	g	2212973	g	2122891	s	UTI89/AS	rec18	g	UTI89	++++	g	g	g	g	a	g	g	g	g			g	g	g	g	g	g	g	g	g	g	g	g
a	2263476	t	2212981	t	2122899	ns	UTI89/AS	rec18	t	UTI89	++++	t	t	t	t	t	t	t	t	t			t	t	t	t	t	t	t	t	t	t	t	t
g	2263480	a	2212985	a	2122903	s	UTI89/AS	rec18	g	AS	++	g	a	g	g	g	g	g	a	g			g	g	g	a	g	g	g	g	g	g	g	g
a	2263776	g	2213281	g	2123199	s	UTI89/AS	rec18	g	UTI89	++++	g	g	g	g	g	g	g	g	g			g	g	g	g	g	g	g	g	g	g	g	g
c	2264142	t	2213647	t	2123565	s	UTI89/AS	rec18	c	AS	++	c	t	c	t	t	c	c	c	t			c	t	t	c	t	t	t	c	c	c	c	c
t	2264187	g	2213692	g	2123610	s	UTI89/AS	rec18	g	UTI89	++	g	g	g	t	g	g	g	g	g			g	g	g	g	g	t	g	t	t	t	g	g
g	2264196	c	2213701	c	2123619	s	UTI89/AS	rec18	c	UTI89	++	c	c	c	g	c	c	c	c	c			c	c	c	c	c	g	c	g	g	g	c	c
g	2264199	a	2213704	a	2123622	s	UTI89/AS	rec18	a	UTI89	++	a	a	a	g	a	a	g	a	a			g	a	a	a	g	g	a	g	g	g	g	g
c	2264226	g	2213731	g	2123649	s	UTI89/AS	rec18	c	AS	++	c	g	c	c	g	c	c	c	g			c	g	g	g	c	c	g	c	c	c	c	c
g	2264229	a	2213734	a	2123652	s	UTI89/AS	rec18	g	AS	++	g	a	g	g	a	g	g	g	a			g	a	a	a	g	g	a	g	g	g	g	g
c	2264358	t	2213863	t	2123781	s	UTI89/AS	rec18	c	AS	++	c	t	c	c	t	c	c	c	c			t	t	t	t	c	c	t	t	t	t	t	t
g	2264360	a	2213865	a	2123783	s	UTI89/AS	rec18	a	UTI89	++	a	a	a	g	a	a	a	a	a			a	a	a	a	g	g	a	a	a	a	a	a
a	2264652	g	2214157	g	2124075	s	UTI89/AS	rec18	g	UTI89	++++	g	g	g	g	g	g	g	g	g			g	g	g	g	g	g	g	g	g	g	g	g
t	2264978	c	2214483	c	2124401	s	UTI89/AS	rec18	c	UTI89	++++	c	c	c	c	c	c	c	c	c			c	c	c	c	c	c	c	c	c	c	c	c
a	2265184	g	2214689	g	2124607	s	UTI89/AS	rec18	g	UTI89	++++	g	g	g	g	g	g	g	g	g			g	g	g	g	g	g	g	g	g	g	g	g
a	2265269	g	2214774	g	2124692	s	UTI89/AS	rec18	a	AS	++	a	g	a	g	g	a	g	g	g			g	g	g	g	g	g	g	g	g	g	g	g
c	2265272	t	2214777	t	2124695	s	UTI89/AS	rec18	c	AS	++	c	t	c	t	t	t	t	t	t			t	t	t	t	t	t	t	t	t	t	t	t
t	2265545	a	2215050	a	2124968	s	UTI89/AS	rec18	t	AS	++	t	a	t	t	t	t	t	a	a			t	a	a	a	a	a	a	a	a	a	a	a
a	2265689	g	2215194	g	2125112	s	UTI89/AS	rec18	g	UTI89	++++	g	g	g	g	a	g	g	g	g			g	g	g	g	g	g	g	g	g	g	g	g
t	2265900	c	2215405	c	2125323	ns	UTI89/AS	rec18	t	AS	++	t	c	t	c	t	t	c	c	c			c	t	t	c	c	c	c	c	c	c	c	c
g	2265919	a	2215424	a	2125342	s	UTI89/AS	rec18	a	UTI89	+	g	a	t	a	g	g	a	a	a			a	a	a	a	a	a	a	a	a	a	a	a
c	2266056	t	2215561	t	2125479	ns	UTI89/AS	rec18	t	UTI89	++++	t	t	t	t	t	t	t	t	t			t	t	t	t	t	t	t	t	t	t	t	t
c	2266102	a	2215607	a	2125525	s	UTI89/AS	rec18	c	AS	++	c	c	c	a	c	c	c	a	a			a	c	c	a	a	a	a	a	a	a	a	a
c	2266210	g	2215715	g	2125633	s	UTI89/AS	rec18	g	UTI89	++	g	g	a	g	g	a	g	g	g			g	g	g	g	g	g	g	g	g	g	g	g
t	2266537	c	2216042	c	2125960	s	UTI89/AS	rec18	c	UTI89	++	c	c	c	t	c	t	t	t	t			c	t	t	t	t	t	t	c	c	c	t	t
a	2266588	t	2216093	t	2126011	s	UTI89/AS	rec18	a	AS	++	a	t	a	a	a	a	t	a	a			a	a	a	a	t	a	a		t		a	a
g	2266648	a	2216153	a	2126071	s	UTI89/AS	rec18	g	AS	++	g	g	g	g	a	g	g	g	t			g	t	t	g	g	g	g		g		g	g
a	2266678	g	2216183	g	2126101	s	UTI89/AS	rec18	a	AS	++	a	a	g	g	a	g	a	g	a			g	a	a	g	a	g	g		a		g	g
a	2266684	c	2216189	c	2126107	s	UTI89/AS	rec18	a	AS	++++	a	a	a	a	a	a	a	a	a			a	a	a	a	a	a	a		a		a	a
c	2266696	t	2216201	t	2126119	s	UTI89/AS	rec18	t	UTI89	++++	t	t	t	t	t	t	t	t	t			t	t	t	t	t	t	t		t		t	t
a	2266783	g	2216288	g	2126206	s	UTI89/AS	rec18	g	UTI89	+	a	g	c	g	g	c	g	g	a			a	g	g	g	a	a	g		g		g	g
c	2266948	t	2216453	t	2126371	s	UTI89/AS	rec18	c	AS	++	c	t	c	t	t	t	t	t	t			t	t	t	t	t	t	t		t		t	t
t	2266954	c	2216459	c	2126377	ns	UTI89/AS	rec18	t	AS	++	t	c	t	c	c	c	c	c	c			c	c	c	c	c	c	c		c		c	c
t	2266955	g	2216460	g	2126378	ns	UTI89/AS	rec18	t	AS	++	t	g	t	g	g	g	g	g	g			g	g	g	g	g	g	g		g		g	g
a	2266960	g	2216465	g	2126383	s	UTI89/AS	rec18	a	AS	++	a	g	a	g	g	g	g	g	g			g	g	g	g	g	g	g		g		g	g
t	2266963	c	2216468	c	2126386	s	UTI89/AS	rec18	t	AS	++	t	t	t	t	c	c	c	t	c			c	c	c	t	t	t	t		c		t	t
a	2267013	g	2216518	g	2126436	s	UTI89/AS	rec18	g	UTI89	++++	g	g	g	g	g	g	g	g	g			g	g	g	g	g	g	g		g		g	g
c	2267016	t	2216521	t	2126439	ns	UTI89/AS	rec18	t	UTI89	++++	t	t	t	t	t	t	t	t	t			t	t	t	t	t	t	t		t		t	t
a	2267026	c	2216531	c	2126449	ns	UTI89/AS	rec18	a	AS	++	a	a	a	a	c	a	a	a	a			c	a	a	a	a	a	a		a		a	a
a	2267041	g	2216546	g	2126464	s	UTI89/AS	rec18	a	AS	++	a	a	a	a	g	a	g	a	a			g	g	g	a	a	a	a		a		a	a
g	2267059	a	2216564	a	2126482	s	UTI89/AS	rec18	g	AS	++	g	a	g	a	a	a	a	g	a			g	a	a	a	g	a	a		g		g	g
a	2267074	g	2216579	g	2126497	s	UTI89/AS	rec18	g	UTI89	++++	a	g	g	g	g	g	g	g	g			g	g	g	g	g	g	g		g		g	g
t	2267146	c	2216651	c	2126569	s	UTI89/AS	rec18	c	UTI89	++++	c	c	c	c	c	c	c	c	c			c	c	c	c	c	c	c		c		c	c
g	2267222	a	2216727	a	2126645	nc	UTI89/AS	rec18	?	UTI89/AS	+/-																							
c	2267253	a	2216758	a	2126676	nc	UTI89/AS	rec18	?	UTI89/AS	+/-																							
g	2267313	a	2216818	a	2126736	s	UTI89/AS	rec18	a	UTI89	++++	a	a	a	a	a	a	a	a	a			a	a	a	a	a	a	a		a		a	a
c	2267328	t	2216833	t	2126751	s	UTI89/AS	rec18	t	UTI89	++++	t	t	t	t	t	t	t	t	t			t	t	t	t	t	t	t		t		t	t
t	2267448	c	2216953	c	2126871	s	UTI89/AS	rec18	c	UTI89	++++	c	c	t	c	c	c	c	c	c			c	c	c	c	c	c	c		c		c	c
c	2267463	t	2216968	t	2126886	s	UTI89/AS	rec18	c	AS	++	t	c	c	c	t	c	c	t	c			c	c	c	c	c	c	c		c		t	t

ExPEC genome site details												Outgroup Strains Details[i]																						
UTI89[b]	UTI89 site[c]	APEC 01[b]	APEC 01 site[c]	S88[b]	S88 site[c]	type[d]	Event Lineage[ef]	Recombinant segment	Inferred ancestral base[g]	Outgroup Analysis[e]	Support level[h]	CFT073	ED1a	536	E2348/69	SMS 3-5	IAI39	UMN026	K-12	ATCC 8739	HS	D1 Sd197	CB9615	Sakai	EDL933	IAI1	E24377A	SE11	SS Ss046	F2a 301	F2a 2457T	F5 8401	B4 Sb227	B18 BS512
t	2267475	c	2216980	c	2126898	s	UTI89/AS	rec18	c	UTI89	++	c	c	c	c	c	t	c	c	c			t	c	c	c	t	c	t		c		c	c
t	2267613	c	2217118	c	2127036	s	UTI89/AS	rec18	c	UTI89	++++	c	c	c	c	c	c	c	c	c			c	c	c	c	c	c	c		c		c	c
c	2267658	t	2217163	t	2127081	s	UTI89/AS	rec18	c	AS	++	t	c	c	c	c	t	c	t	c			t	c	c	c	c	a	c		c		t	t
g	2267709	a	2217214	a	2127132	s	UTI89/AS	rec18	a	UTI89	++++	a	a	g	a	a	a	a	a	a			a	a	a	a	a	a	a	a	a	a	a	a
c	2267712	t	2217217	t	2127135	s	UTI89/AS	rec18	t	UTI89	++	t	t	c	t	c	c	t	c	c			t	t	t	c	c	c	c	c	c	c	c	c
g	2267715	a	2217220	a	2127138	s	UTI89/AS	rec18	g	AS	++	a	g	g	a	g	g	a	g	g			a	a	a	g	g	g	g	g	g	g	g	g
a	2267823	c	2217328	c	2127246	s	UTI89/AS	rec18	c	UTI89	++	c	c	a	c	a	c	c	c	c			c	c	c	c	c	c	c	c	t	c	c	c
t	2267850	c	2217355	c	2127273	s	UTI89/AS	rec18	c	UTI89	++++	c	c	c	c	c	c	c	c	c			c	c	c	c	c	c	c	c	c	c	c	c
a	2267853	t	2217358	t	2127276	s	UTI89/AS	rec18	t	UTI89	++++	t	t	t	t	t	t	t	t	t			t	t	t	t	t	t	t	t	t	t	t	t
t	2267889	g	2217394	g	2127312	s	UTI89/AS	rec18	g	UTI89	++	g	t	g	g	g	g	g	t	g			g	t	t	g	g	g	g	t	t	t	t	t
a	2268069	g	2217574	g	2127492	s	UTI89/AS	rec18	g	UTI89	++	g	g	a	g	a	g	g	g	g			g	g	g	g	g	g	g	g	g	g	g	g
a	2268112	g	2217617	g	2127535	ns	UTI89/AS	rec18	g	UTI89	++++	g	g	a	g	g	g	g	g	g			g	g	g	g	g	g	g	g	g	g	g	g
g	2268141	a	2217646	a	2127564	s	UTI89/AS	rec18	g	AS	++	a	g	g	g	g	g	a	g	g			g	g	g	g	g	g	g	g	g	g	g	g
g	2268158	a	2217663	a	2127581	ns	UTI89/AS	rec18	g	AS	++++	g	g	g	g	g	g	g	g	g			g	g	g	g	g	g	g	g	g	g	g	g
c	2268177	t	2217682	t	2127600	s	UTI89/AS	rec18	c	AS	++	c	c	c	c	t	t	c	t	t			c	c	c	c	c	c	c	t	t	t	t	t
g	2268186	a	2217691	a	2127609	s	UTI89/AS	rec18	g	AS	++	g	g	g	g	a	g	g	a	a			g	g	g	g	a	g	g	a	a	a	a	a
c	2268231	a	2217736	a	2127654	s	UTI89/AS	rec18	c	AS	++++	c	c	c	c	c	c	c	c	c			c	c	c	c	c	c	c	c	c	c	c	c
a	2268285	g	2217790	g	2127708	s	UTI89/AS	rec18	g	UTI89	++++	g	g	g	g	g	g	g	g	g			g	g	g	g	g	g	g	g	g	g	g	g
c	2268348	t	2217853	t	2127771	s	UTI89/AS	rec18	c	AS	++	c	t	c	t	c	c	t	t	t			t	t	t	t	t	t	t	t	t	t	t	t
t	2268372	a	2217877	a	2127795	s	UTI89/AS	rec18	a	UTI89	++	a	a	a	a	g	g	a	a	a			a	a	a	a	a	a	a	a	a	a	a	a
a	2268868	g	2218373	g	2128291	s	UTI89/AS	rec18	g	UTI89	++	g	g	g	g	a	g	g	a	g			a	g	g	g	g	a	g	g	g	g	g	g
a	2268895	g	2218400	g	2128318	s	UTI89/AS	rec18	g	UTI89	++	g	a	g	g	a	g	g	g	a			a	g	g	a	g	a	a	a	a	a	a	a
a	2268964	g	2218469	g	2128387	ns	UTI89/AS	rec18	g	UTI89	++	g	g	g	a	g	g	g	g	a			g	g	g	g	a	g	g	g	g	g	g	g
c	2268965	t	2218470	t	2128388	ns	UTI89/AS	rec18	c	AS	++++	c	c	c	c	t	c	c	c	c			c	c	c	c	c	c	c	c	c	c	c	c
g	2269024	a	2218529	a	2128447	s	UTI89/AS	rec18	g	AS	++	g	a	g	g	g	a	g	g	a			g	a	a	a	g	a	a	g	g	g	a	a
a	2269027	g	2218532	g	2128450	s	UTI89/AS	rec18	a	AS	++	a	a	a	a	a	g	a	a	g			a	g	g	a	a	a	a	a	a	a	a	a
c	2269054	a	2218559	a	2128477	s	UTI89/AS	rec18	a	UTI89	++	a	a	a	c	a	a	a	a	a			a	a	a	c	a	c	a	c	c	c	a	a
a	2269075	g	2218580	g	2128498	s	UTI89/AS	rec18	a	AS	++	a	g	a	a	a	g	a	g	g			a	g	g	g	g	g	g	g	g	g	g	g
a	2269078	t	2218583	t	2128501	s	UTI89/AS	rec18	a	AS	++++	a	a	a	a	a	a	a	a	a			a	a	a	a	a	a	a	a	a	a	a	a
g	2269141	c	2218646	c	2128564	s	UTI89/AS	rec18	g	AS	++	g	g	c	g	g	c	g	g	g			g	g	g	g	g	g	g	g	g	g	g	g
g	2269192	a	2218697	a	2128615	s	UTI89/AS	rec18	g	AS	++	g	g	a	g	a	a	a	g	a			a	g	g	g	a	g	g	g	g	g	g	g
t	2269222	c	2218727	c	2128645	s	UTI89/AS	rec18	c	UTI89	++	c	c	c	t	c	c	c	t	c			c	c	c	c	c	c	c	c	c	c	c	c
a	2269270	g	2218775	g	2128693	s	UTI89/AS	rec18	g	UTI89	++	g	g	g	a	g	g	g	a	g			g	g	g	g	g	g	a	a	a	a	g	g
c	2269288	t	2218793	t	2128711	s	UTI89/AS	rec18	c	AS	++++	c	c	t	c	c	c	c	c	c			c	c	c	c	c	c	c	c	c	c	c	c
g	2269306	a	2218811	a	2128729	s	UTI89/AS	rec18	a	UTI89	++	a	g	a	g	a	a	a	g	g			a	a	a	g	a	g	g	a	a	a	g	g
t	2269360	c	2218865	c	2128783	s	UTI89/AS	rec18	c	UTI89	++	c	c	t	t	c	t	c	t	t			c	t	t	t	t	c	t	c	c	c	t	t
g	2269362	a	2218867	a	2128785	s	UTI89/AS	rec18	a	UTI89	++	a	a	g	g	a	g	a	g	g			a	g	g	g	g	a	g	a	a	a	g	g
g	2269666	a	2219171	a	2129089	s	UTI89/AS	rec18	a	UTI89	++	a	a	a	a	g	g	a	a	a			a	a	a	a	a	a	a	a	a	a	a	a
g	2269830	a	2219335	a	2129253	s	UTI89/AS	rec18	g	AS	++++	g	g	g	g	g	g	g	g	g			g	g	g	g	g	g	g	g	g	g	g	g
g	2269959	a	2219464	a	2129382	s	UTI89/AS	rec18	a	UTI89	++	a	a	a	a	g	g	a	a	a			a	a	a	a	a	a	a	a	a	a	a	a
a	2269962	g	2219467	g	2129385	s	UTI89/AS	rec18	g	UTI89	++	g	g	g	g	a	a	g	g	g			g	g	g	g	g	g	g	g	g	g	g	g
g	2269965	t	2219470	t	2129388	s	UTI89/AS	rec18	t	UTI89	++	t	g	t	g	g	g	g	g	g			g	g	g	g	g	g	g	g	g	g	g	g
a	2269971	t	2219476	t	2129394	s	UTI89/AS	rec18	t	UTI89	++	t	a	t	a	a	a	a	a	a			a	a	a	a	a	a	a	a	a	a	a	a
a	2270101	g	2219606	g	2129524	ns	UTI89/AS	rec18	g	UTI89	++	g	a	g	a	a	a	a	a	a			a	a	a	a	a	a	a	a	a	a	a	a
a	2270130	g	2219635	g	2129553	s	UTI89/AS	rec18	g	UTI89	++	g	g	g	g	a	a	g	g	g			g	g	g	a	g	g	g	g	g	g	a	a
a	2270249	g	2219754	g	2129672	s	UTI89/AS	rec18	g	UTI89	++	g	a	g	g	g	g	a	a	a			g	a	a	a	a	a	a	a	a	a	a	a
a	2270294	g	2219799	g	2129717	s	UTI89/AS	rec18	g	UTI89	++	g	g	g	a	g	g	g	g	g			g	a	a	g	a	a	g	a	a	a	g	g
t	2270357	c	2219862	c	2129780	ns	UTI89/AS	rec18	c	UTI89	++++	c	c	c	c	c	c	c	c	c			c	c	c	c	c	c	c	c	c	c	c	c
g	2270359	a	2219864	a	2129782	ns	UTI89/AS	rec18	g	AS	++++	g	g	g	g	g	g	g	g	g			g	g	g	g	g	g	g	g	g	g	g	g
a	2270381	g	2219886	g	2129804	s	UTI89/AS	rec18	g	UTI89	++	g	a	g	a	a	a	a	a	a			a	a	a	a	a	a	a	g	g	g	a	a
g	2270432	a	2219937	a	2129855	s	UTI89/AS	rec18	g	AS	++++	g	g	g	g	g	g	g	g	g			g	g	g	g	g	g	g	g	g	g	g	g
a	2270435	g	2219940	g	2129858	s	UTI89/AS	rec18	g	UTI89	++	g	a	g	a	g	g	a	g	g			g	a	a	a	a	g	g	g	g	g	g	g
t	2270477	c	2219982	c	2129900	s	UTI89/AS	rec18	c	UTI89	++	c	t	c	c	c	c	c	c	c			c	c	c	c	t	t	t	t	t	t	c	c
c	2270519	t	2220024	t	2129942	s	UTI89/AS	rec18	c	AS	++	c	c	c	t	c	c	c	t	t			t	t	t	t	c	t	t	t	t	t	t	t
g	2270531	a	2220036	a	2129954	s	UTI89/AS	rec18	g	AS	++	g	a	g	g	g	g	g	g	g			g	g	g	g	a	g	g	g	g	g	g	g
a	2270603	g	2220108	g	2130026	s	UTI89/AS	rec18	g	UTI89	++	g	g	g	g	g	g	a	g	g			g	g	g	g	a	a	g	g	g	g	g	g
a	2270615	g	2220120	g	2130038	s	UTI89/AS	rec18	g	UTI89	++++	g	g	g	g	g	g	g	g	g			g	g	g	g	g	g	g	g	g	g	g	g
t	2270666	a	2220171	a	2130089	s	UTI89/AS	rec18	a	UTI89	++	a	a	t	a	a	a	t	t	t			a	a	a	t	a	a	a	t	t	t	a	a
a	2270705	g	2220210	g	2130128	s	UTI89/AS	rec18	g	UTI89	++	g	g	g	a	g	g	a	a	a			a	g	g	a	g	a	a	g	g	g	a	a
g	2270714	t	2220219	t	2130137	s	UTI89/AS	rec18	t	UTI89	++	t	c	t	t	t	t	g	t	t			t	c	c	t	c	t	t	c	c	c	t	t
a	2270747	g	2220252	g	2130170	s	UTI89/AS	rec18	g	UTI89	++	g	g	g	g	g	g	a	a	a			g	g	g	a	g	g	g	g	g	g	g	g
a	2270828	g	2220333	g	2130251	s	UTI89/AS	rec18	g	UTI89	++	g	a	g	a	g	g	a	a	a			a	a	a	a	a	a	a	a	a	a	a	a
a	2271244	g	2220749	g	2130667	s	UTI89/AS	rec18	g	UTI89	++	g	g	g	g	a	a	g	g	g			a	g	g	g	g	g	g	g	g	g	g	g
a	2271394	g	2220899	g	2130817	s	UTI89/AS	rec18	g	UTI89	++	g	a	g	a	g	a	g	a	a			a	g	g	a	a	a	g	a	a	a	a	a
t	2271466	c	2220971	c	2130889	s	UTI89/AS	rec18	c	UTI89	++	t	c	c	t	c	t	t	c	c			c	t	t	c	c	c	c	c	c	c	c	c
g	2271487	a	2220992	a	2130910	s	UTI89/AS	rec18	g	AS	++++	g	g	g	g	g	g	g	g	g			g	g	g	g	g	g	g	g	g	g	g	g
c	2271502	a	2221007	a	2130925	s	UTI89/AS	rec18	a	UTI89	++++	a	a	a	a	a	c	a	a	a			a	a	a	a	a	a	a	a	a	a	a	a
g	2271546	a	2221051	a	2130969	s	UTI89/AS	rec18	g	AS	++++	g	g	g	g	g	g	g	g	g			g	g	g	g	g	g	g	g	g	g	g	g
g	2271585	a	2221090	a	2131008	s	UTI89/AS	rec18	g	AS	++++	g	g	g	g	g	g	g	g	g			g	g	g	g	g	g	g	g	g	g	g	g
a	2271595	g	2221100	g	2131018	s	UTI89/AS	rec18	g	UTI89	++++	g	g	g	g	g	a	g	g	g			g	g	g	g	g	g	g	g	g	g	g	g
a	2271598	g	2221103	g	2131021	s	UTI89/AS	rec18	g	UTI89	++	a	g	g	g	g	a	g	g	g			g	g	g	g	g	g	g	g	g	g	g	g
g	2271646	a	2221151	a	2131069	s	UTI89/AS	rec18	g	AS	++++	g	g	g	g	g	g	g	g	g			g	g	g	g	g	g	a	g	g	g	g	g
g	2271667	a	2221172	a	2131090	s	UTI89/AS	rec18	g	AS	++++	g	g	g	g	g	g	a	g	g			g	g	g	g	g	g	g	g	g	g	g	g
c	2272156	a	2221661	a	2131579	s	UTI89/AS	rec18	a	UTI89	++	c	a	a	a	c	c	c	a	a			a	a	a	a	a	a	a	a	a	a	a	a
g	2272225	a	2221730	a	2131648	s	UTI89/AS	rec18	a	UTI89	++	a	g	a	a	g	g	a	g	a			g	a	a	g	a	g	g	g	g	g	g	g
c	2272321	t	2221826	t	2131744	s	UTI89/AS	rec18	c	AS	++	c	c	t	c	t	t	t	c	c			c	c	c	c	c	c	c	c	c	c	c	c
c	2272345	t	2221850	t	2131768	s	UTI89/AS	rec18	c	AS	++	c	c	t	c	t	t	c	c	c			c	c	c	c	c	c	c	c	c	c	c	c
c	2272348	t	2221853	t	2131771	s	UTI89/AS	rec18	c	AS	++	c	c	t	g	t	t	c	c	c			g	g	g	g	c	c	c	c	c	c	c	g
g	2272434	a	2221939	a	2131857	s	UTI89/AS	rec18	g	AS	++	g	g	g	g	a	a	g	g	g			g	g	g	g	g	g	g	g	g	g	g	g
g	2272438	a	2221943	a	2131861	s	UTI89/AS	rec18	g	AS	++	g	g	g	g	a	a	g	g	g			g	g	g	g	g	g	g	g	g	g	g	g
t	2272460	c	2221965	c	2131883	ns	UTI89/AS	rec18	c	UTI89	++++	c	c	c	c	c	c	c	c	c			c	c	c	c	c	c	c	c	c	c	c	c
a	2272492	g	2221997	g	2131915	s	UTI89/AS	rec18	a	AS	++	g	a	a	a	g	g	a	g	a			a	a	a	a	a	a	a	a	a	a	a	g
c	2272516	g	2222021	g	2131939	s	UTI89/AS	rec18	c	AS	++	c	c	c	c	g	g	c	c	c			c	c	c	c	c	c	c	c	c	c	c	c

ExPEC genome site details												Outgroup Strains Details[i]																						
UTI89[b]	UTI89 site[c]	APEC 01[b]	APEC 01 site[c]	S88[b]	S88 site[c]	type[d]	Event Lineage[ef]	Recombinant segment	Inferred ancestral base[g]	Outgroup Analysis[e]	Support level[h]	CFT073	ED1a	536	E2348/69	SMS 3-5	IAI39	UMN026	K-12	ATCC 8739	HS	D1 Sd197	CB9615	Sakai	EDL933	IAI1	E24377A	SE11	SS Ss046	F2a 301	F2a 2457T	F5 8401	B4 Sb227	B18 BS512
g	2272588	a	2222093	a	2132011	s	UTI89/AS	rec18	a	UTI89	++++	a	a	a	a	a	a	a	a	a			a	a	a	a	a	a	a	a	a	a	a	a
g	2272666	a	2222171	a	2132089	s	UTI89/AS	rec18	g	AS	++	g	g	g	a	g	g	a	g	a			a	a	a	a	g	g	g	g	g	g	g	g
t	2272719	g	2222224	g	2132142	ns	UTI89/AS	rec18	t	AS	++	t	g	t	g	t	t	g	t	g			g	g	g	g	g	g	g	g	g	g	g	t
t	2272731	c	2222236	c	2132154	ns	UTI89/AS	rec18	c	UTI89	++++	c	c	c	c	c	c	c	c	c			c	c	c	c	c	c	c	c	c	c	c	c
g	2272801	a	2222306	a	2132224	s	UTI89/AS	rec18	a	UTI89	++	a	a	a	a	a	a	a	g	a			a	a	a	a	a	a	a	a	a	a	a	g
g	2272813	a	2222318	a	2132236	s	UTI89/AS	rec18	g	AS	++	g	g	g	g	a	a	a	g	g			g	g	g	g	a	g	g	g	g	g	g	g
g	2272825	a	2222330	a	2132248	ns	UTI89/AS	rec18	g	AS	++	g	a	g	a	a	a	a	g	a			a	g	g	g	g	a	a	g	g	g	a	g
t	2272827	c	2222332	c	2132250	ns	UTI89/AS	rec18	t	AS	++	c	t	t	c	c	c	c	t	t			c	t	t	c	c	t	t	c	c	c	t	t
a	2272873	g	2222378	g	2132296	s	UTI89/AS	rec18	g	UTI89	++	g	g	t	g	g	g	g	g	g			g	a	a	g	g	g	g	g	g	g	g	g
a	2272909	g	2222414	g	2132332	s	UTI89/AS	rec18	a	AS	++	a	a	a	a	g	g	g	a	a			a	g	g	a	a	a	g	a	a	a	g	a
c	2272966	t	2222471	t	2132389	s	UTI89/AS	rec18	c	AS	++	c	c	c	c	t	t	c	t	c			c	c	c	c	c	c	t	c	c	c	t	t
a	2272978	g	2222483	g	2132401	s	UTI89/AS	rec18	g	UTI89	++++	g	g	g	g	g	g	g	g	g			g	g	g	g	g	g	g	g	g	g	g	g
g	2273011	a	2222516	a	2132434	s	UTI89/AS	rec18	a	UTI89	++	a	a	g	g	a	a	a	a	a			g	g	g	g	a	a	a	a	a	a	a	a
a	2273047	t	2222552	t	2132470	s	UTI89/AS	rec18	a	AS	++	t	a	a	a	t	t	t	a	a			a	a	a	a	a	a	a	a	a	a	a	a
t	2273071	a	2222576	a	2132494	s	UTI89/AS	rec18	a	UTI89	++	a	g	a	t	a	a	a	a	g			t	t	t	a	g	g	a	g	g	g	a	g
g	2273074	a	2222579	a	2132497	s	UTI89/AS	rec18	a	UTI89	++	a	g	a	g	a	a	a	a	a			g	g	g	a	a	a	a	g	g	g	a	a
a	2273077	g	2222582	g	2132500	s	UTI89/AS	rec18	g	UTI89	++	g	a	g	a	g	g	g	g	g			a	a	a	g	g	g	g	a	a	a	g	g
c	2273102	t	2222607	t	2132525	ns	UTI89/AS	rec18	c	AS	++	t	c	c	c	t	t	t	t	t			c	c	c	t	t	t	t	t	t	t	t	t
g	2273131	a	2222636	a	2132554	s	UTI89/AS	rec18	a	UTI89	++++	a	a	g	a	a	a	a	a	a			a	a	a	a	a	a	a	a	a	a	a	a
a	2273149	g	2222654	g	2132572	s	UTI89/AS	rec18	g	UTI89	++++	g	g	a	g	g	g	g	g	g			g	g	g	g	g	g	g	g	g	g	g	g
g	2273179	a	2222684	a	2132602	s	UTI89/AS	rec18	a	UTI89	++	a	a	a	g	a	a	a	g	a			a	g	g	a	a	a	a	a	a	a	a	a
c	2273200	t	2222705	t	2132623	s	UTI89/AS	rec18	c	AS	++	c	c	c	c	t	t	c	c	c			c	c	c	c	c	c	c	c	c	c	c	c
g	2273373	t	2222878	t	2132796	s	UTI89/AS	rec18	t	UTI89	++++	t	t	t	t	t	t	t	t	t			t	t	t	t	t	t	t	t	t	t	t	t
a	2273463	g	2222968	g	2132886	s	UTI89/AS	rec18	a	AS	++	a	a	g	a	a	a	a	c	a			c	c	c	g	a	a	a	c	c	c	a	a
c	2273496	a	2223001	a	2132919	ns	UTI89/AS	rec18	c	AS	++++	c	c	c	c	c	c	c	c	c			c	c	c	c	c	c	c	c	c	c	c	c
g	2273535	a	2223040	a	2132958	ns	UTI89/AS	rec18	a	UTI89	++	g	a	a	g	g	a	g	a	g			g	g	g	g	g	g	g	g	g	g	g	g
t	2273537	g	2223042	g	2132960	ns	UTI89/AS	rec18	g	UTI89	++++	g	g	g	g	g	g	t	g	g			g	g	g	g	g	g	g	g	g	g	g	g
a	2273550	c	2223055	c	2132973	s	UTI89/AS	rec18	c	UTI89	++	c	c	c	c	c	c	a	a	t			t	t	t	a	c	c	t	t	t	t	t	c
g	2273553	t	2223058	t	2132976	s	UTI89/AS	rec18	t	UTI89	++	t	t	t	t	t	t	g	g	t			t	t	t	g	t	t	t	t	t	t	t	t
g	2273604	a	2223109	a	2133027	s	UTI89/AS	rec18	g	AS	++	a	g	g	g	a	a	g	g	g			g	g	g	a	g	g	g	g	g	g	g	g
a	2273610	g	2223115	g	2133033	s	UTI89/AS	rec18	a	AS	++	-	a	a	a	g	g	g	a	a			a	a	a	g	a	a	a	a	a	a	a	a
c	2273616	g	2223121	g	2133039	s	UTI89/AS	rec18	g	UTI89	++++	g	g	g	g	g	g	g	g	g			g	g	g	g	g	g	g	g	g	g	g	g
t	2273619	c	2223124	c	2133042	s	UTI89/AS	rec18	c	UTI89	++++	c	c	c	c	c	c	c	c	c			c	c	c	c	c	c	c	c	c	c	c	c
a	2273703	g	2223208	g	2133126	s	UTI89/AS	rec18	a	AS	++++	a	a	a	a	a	g	a	a	a			a	a	a	a	a	a	a	a	a	a	a	a
t	2273852	g	2223357	g	2133275	s	UTI89/AS	rec18	g	UTI89	++++	g	g	g	g	g	g	g	g	g			g	g	g	g	g	g	g	g	g	g	g	g
g	2273960	a	2223465	a	2133383	s	UTI89/AS	rec18	g	AS	++	g	a	g	g	g	g	g	g	a			g	g	g	g	a	a	a	g	g	g	g	g
t	2274143	c	2223648	c	2133566	s	UTI89/AS	rec18	t	AS	++	t	c	t	c	c	t	c	t	g			c	c	c	c	c	c	c	c	c	c	c	c
t	2274227	c	2223732	c	2133650	s	UTI89/AS	rec18	c	UTI89	++	c	t	c	t	c	t	c	c	t			c	c	c	t	t	t	t	t	t	t	t	t
t	2274254	c	2223759	c	2133677	s	UTI89/AS	rec18	c	UTI89	++	c	c	c	c	c	t	c	c	t			c	c	c	c	c	c	t	c	c	c	c	c
g	2274278	a	2223783	a	2133701	s	UTI89/AS	rec18	g	AS	++++	g	g	g	g	g	g	g	g	g			g	g	g	g	g	g	g	g	g	g	g	g
t	2274290	c	2223795	c	2133713	s	UTI89/AS	rec18	t	AS	++++	t	t	t	t	t	t	t	t	t			t	t	t	t	t	t	t	t	t	t	t	t
a	2274332	g	2223837	g	2133755	s	UTI89/AS	rec18	a	AS	++	a	g	a	g	g	g	g	g	g			a	g	g	g	g	g	g	g	g	g	g	g
a	2274344	g	2223849	g	2133767	s	UTI89/AS	rec18	a	AS	++++	a	a	a	a	a	a	a	a	a			a	a	a	a	a	a	a	a	a	a	a	a
a	2274362	t	2223867	t	2133785	s	UTI89/AS	rec18	a	AS	++	a	a	a	a	a	a	a	a	a			g	a	a	t	t	t	a	a	a	a	a	a
t	2274377	c	2223882	c	2133800	s	UTI89/AS	rec18	t	AS	++	t	c	t	t	t	t	t	c	c			t	c	c	c	c	c	c	c	c	c	c	c
c	2274383	t	2223888	t	2133806	s	UTI89/AS	rec18	c	AS	++	c	t	c	t	c	c	c	t	t			c	t	t	t	t	t	t	t	t	t	t	c
a	2274509	g	2224014	g	2133932	s	UTI89/AS	rec18	g	UTI89	++++	g	g	g	g	g	g	g	g	g			g	g	g	g	g	g	g	g	g	g	g	g
a	2274518	g	2224023	g	2133941	s	UTI89/AS	rec18	g	UTI89	++	g	g	g	a	g	g	a	a	a			g	a	a	a	a	a	a	a	a	a	a	a
g	2274572	a	2224077	a	2133995	s	UTI89/AS	rec18	g	AS	++	g	g	g	g	g	g	g	g	g			g	g	g	a	g	a	g	a	a	a	a	g
a	2274689	g	2224194	g	2134112	s	UTI89/AS	rec18	g	UTI89	++	g	g	g	a	a	a	a	a	a			a	a	a	a	g	a	a	a	a	a	a	a
c	2274692	g	2224197	g	2134115	s	UTI89/AS	rec18	g	UTI89	++	g	g	g	c	c	c	c	c	c			c	c	c	t	g	t	c	c	c	c	c	c
a	2274730	g	2224235	g	2134153	s	UTI89/AS	rec18	a	AS	++	a	a	a	g	a	a	a	g	g			g	g	g	g	a	g	g	g	g	g	g	g
a	2274794	c	2224299	c	2134217	s	UTI89/AS	rec18	c	UTI89	++	c	g	c	g	c	a	a	g	g			g	g	g	g	g	g	g	g	g	g	g	g
-	2275405	1	2224920	1	2134838	del	UTI89/AS	rec18	1	UTI89	++++	1	1	1	1	1	1	1	1	1		1	1	1	1	1	1	1	1	1	1	1	1	1
a	2275439	t	2224945	t	2134863	nc	UTI89/AS	rec18	a	AS	++	a	t	a	t	a	a	a	t	t		a	t	t	t	t	t	t	t	t	t	t	t	t
a	2275441	c	2224947	c	2134865	nc	UTI89/AS	rec18	c	UTI89	++	c	c	c	c	c	a	a	c	c		t	c	c	c	c	c	c	c	c	c	c	c	c
c	2275727	t	2225233	t	2135151	s	UTI89/AS	rec18	t	UTI89	++++	t	t	t	t	t	t	t	t	t	c	t	t	t	t	t	t	t	t	t	t	t	t	t
c	2275810	t	2225316	t	2135234	s	UTI89/AS	rec18	c	AS	++	c	c	t	c	t	t	c	c	c	c	c	c	c	c	c	c	c	c	c	c	c	c	c
a	2275879	g	2225385	g	2135303	s	UTI89/AS	rec18	a	AS	++	a	a	a	a	g	a	g	a	a	a	a	a	a	a	a	a	a	a	a	a	a	a	a
g	2275903	a	2225409	a	2135327	s	UTI89/AS	rec18	a	UTI89	++	a	a	g	a	a	g	g	a	a	a	a	a	a	a	a	a	a	a	a	a	a	a	a
t	2275987	c	2225493	c	2135411	s	UTI89/AS	rec18	c	UTI89	++++	c	c	c	c	c	c	c	c	c	c	c	c	c	c	c	c	c	c	c	c	c	c	c
g	2276071	a	2225577	a	2135495	s	UTI89/AS	rec18	g	AS	++++	g	g	g	g	c	g	g	g	g	g	g	g	g	g	g	g	g	g	g	g	g	g	g
c	2276266	t	2225772	t	2135690	s	UTI89/AS	rec18	t	UTI89	++++	t	t	t	t	t	c	t	t	t	t	t	t	t	t	t	t	t	t	t	t	t	t	t
a	2276293	g	2225799	g	2135717	s	UTI89/AS	rec18	g	UTI89	++++	g	g	g	g	g	g	g	g	g	g	g	g	g	g	g	g	g	g	g	g	g	g	g
t	2276318	c	2225824	c	2135742	s	UTI89/AS	rec18	c	UTI89	++++	c	c	c	c	c	c	c	c	c	c	c	c	c	c	c	c	c	c	c	c	c	c	c
a	2276320	t	2225826	t	2135744	s	UTI89/AS	rec18	t	UTI89	++++	t	t	t	t	t	t	t	t	t	t	t	t	t	t	t	t	t	t	t	t	t	t	t
t	2276350	c	2225856	c	2135774	s	UTI89/AS	rec18	c	UTI89	++++	c	c	c	c	c	c	c	c	c	c	t	c	c	c	c	c	c	c	c	c	c	c	c
a	2276372	g	2225878	g	2135796	ns	UTI89/AS	rec18	g	UTI89	++++	g	g	g	g	g	g	g	g	g	g	g	g	g	g	g	g	g	g	g	g	g	g	g
a	2276382	g	2225888	g	2135806	ns	UTI89/AS	rec18	g	UTI89	++++	g	g	g	g	g	g	g	g	g	g	g	g	g	g	g	g	g	g	g	g	g	g	g
t	2276386	c	2225892	c	2135810	s	UTI89/AS	rec18	c	UTI89	++++	c	c	c	c	c	c	c	c	c	c	c	c	c	c	c	c	c	c	c	c	c	c	c
t	2276449	c	2225955	c	2135873	s	UTI89/AS	rec18	t	AS	++++	t	t	t	t	t	t	t	t	t	t	t	t	t	t	t	t	t	t	t	t	t	t	t
c	2276548	t	2226054	t	2135972	s	UTI89/AS	rec18	t	UTI89	++	t	t	t	t	c	c	t	t	t	t	t	t	t	t	t	t	t	t	t	t	t	t	t
a	2276554	g	2226060	g	2135978	s	UTI89/AS	rec18	g	UTI89	++	g	g	g	g	a	a	g	g	g	g	g	g	g	g	g	g	g	g	g	g	g	g	g
t	2276566	c	2226072	c	2135990	s	UTI89/AS	rec18	t	AS	++	t	c	t	c	t	t	c	c	c	t	c	c	t	t	c	c	c	c	c	c	c	c	c
t	2276569	c	2226075	c	2135993	s	UTI89/AS	rec18	t	AS	++	t	c	t	c	t	t	c	c	c	t	c	c	t	t	c	c	c	c	c	c	c	c	c
c	2276572	t	2226078	t	2135996	s	UTI89/AS	rec18	c	AS	++	c	t	c	t	c	c	t	t	t	c	t	t	c	c	t	t	t	t	t	t	t	t	t
g	2276581	a	2226087	a	2136005	s	UTI89/AS	rec18	a	UTI89	++	a	a	a	a	g	g	a	a	a	a	a	a	g	g	a	a	a	a	a	a	a	a	a
a	2309736	g	2259241	a	2169160	nc	APEC/S88	rec19	a	APEC	++++	a	a	a	a	a	a	a	a	a	a	a	a	a	a	a	a	a	a	a	a	a	a	a
g	2309738	a	2259243	g	2169162	nc	APEC/S88	rec19	g	APEC	++++	g	g	g	g	g	g	g	g	g	g	g	g	g	g	g	g	g	g	g	g	g	g	g
-	2309751	g	2259394	t	2169313	nc	APEC/S88	rec19	t	APEC	+++		t			t			t								t							
-	2309751	t	2259609	c	2169528	nc	APEC/S88	rec19	c	APEC	+++		c			c			c								c							
-	2309751	t	2259640	c	2169559	nc	APEC/S88	rec19	c	APEC	+++		c			a			c								c							

ExPEC genome site details												Outgroup Strains Details[i]																						
UTI89[b]	UTI89 site[c]	APEC 01[b]	APEC 01 site[c]	S88[b]	S88 site[c]	type[d]	Event Lineage[ef]	Recombinant segment	Inferred ancestral base[g]	Outgroup Analysis[e]	Support level[h]	CFT073	ED1a	536	E2348/69	SMS 3-5	IAI39	UMN026	K-12	ATCC 8739	HS	D1 Sd197	CB9615	Sakai	EDL933	IAI1	E24377A	SE11	SS Ss046	F2a 301	F2a 2457T	F5 8401	B4 Sb227	B18 BS512
-	2309751	a	2259648	t	2169567	nc	APEC/S88	rec19	-	A/S	+/-		-			c			a								a							
-	2309751	t	2259649	a	2169568	nc	APEC/S88	rec19	a	APEC	++		a			a			t								t							
-	2309751	a	2259652	t	2169571	nc	APEC/S88	rec19	a	S88	+++		a			a			a								a							
-	2309751	a	2259653	t	2169572	nc	APEC/S88	rec19	a	S88	+++		a			g			a								a							
-	2309751	c	2259711	t	2169630	nc	APEC/S88	rec19	t	APEC	+++		t		t	t			t								t							
-	2309751	c	2259801	t	2169720	nc	APEC/S88	rec19	c	S88	+++		c		g	c			c								c							
-	2309751	t	2259831	a	2169750	nc	APEC/S88	rec19	a	APEC	++		a		c	t			t								t							
-	2309751	c	2259834	a	2169753	nc	APEC/S88	rec19	a	APEC	++		a		g	a			c								c							
-	2309751	c	2259882	t	2169801	nc	APEC/S88	rec19	t	APEC	+++		t		t	t											c							
-	2309751	g	2259906	a	2169825	nc	APEC/S88	rec19	g	S88	++		g		a	a											g							
-	2309751	c	2259918	t	2169837	nc	APEC/S88	rec19	c	S88	+++		c		c	t											c							
-	2309751	c	2259938	t	2169857	nc	APEC/S88	rec19	t	APEC	+++		t		t	t											t							
-	2309751	a	2259954	g	2169873	nc	APEC/S88	rec19	g	APEC	+++		g		g	g											g							
-	2309751	t	2259969	g	2169888	nc	APEC/S88	rec19	g	APEC	+++		g		g	g											g							
-	2309751	t	2260003	c	2169922	nc	APEC/S88	rec19	t	S88	+++		t		t	t											t							
-	2309751	g	2260021	c	2169940	nc	APEC/S88	rec19	g	S88	++		g		-	g											g							
-	2309751	t	2260026	c	2169945	nc	APEC/S88	rec19	t	S88	+++		t		t	t											t							
-	2309751	c	2260032	t	2169951	nc	APEC/S88	rec19	c	S88	+++		c		t	c											c							
-	2309751	c	2260065	t	2169984	nc	APEC/S88	rec19	c	S88	+++		c		c	t											c							
-	2309751	t	2260104	c	2170023	nc	APEC/S88	rec19	c	APEC	+++		c		c	c											c							
-	2309751	a	2260209	g	2170128	nc	APEC/S88	rec19	g	APEC	++		g		a	a											g							
-	2309751	t	2260211	c	2170130	nc	APEC/S88	rec19	c	APEC	++		c		t	t											t							
-	2309751	c	2260254	t	2170173	nc	APEC/S88	rec19	t	APEC	+++		t		t	t											t							
-	2309751	a	2260265	g	2170184	nc	APEC/S88	rec19	a	S88	++		a		g	a											g							
-	2309751	a	2260308	t	2170227	nc	APEC/S88	rec19	t	APEC	++		t		t	a											a							
-	2309751	g	2260314	a	2170233	nc	APEC/S88	rec19	a	APEC	++		a		g	g											a							
-	2309751	t	2260329	c	2170248	nc	APEC/S88	rec19	c	APEC	++		c		g	t											c							
-	2309751	g	2260362	a	2170281	nc	APEC/S88	rec19	g	S88	+++		g		a	g											g							
-	2309751	c	2260391	t	2170310	nc	APEC/S88	rec19	c	S88	+++		c		c	c											c							
-	2309751	t	2260419	a	2170338	nc	APEC/S88	rec19	t	S88	+++		t		t	t											t							
-	2309751	t	2260425	c	2170344	nc	APEC/S88	rec19	c	APEC	+++		c		g	c											c							
-	2309751	a	2260467	c	2170386	nc	APEC/S88	rec19	a	S88	+++		a		a	a											c							
-	2309751	t	2260530	c	2170449	nc	APEC/S88	rec19	t	S88	++		t		c	c											t							
-	2309751	t	2260753	c	2170672	nc	APEC/S88	rec19	c	APEC	+++		c		c	c											c							
-	2309751	t	2260809	c	2170728	nc	APEC/S88	rec19	c	APEC	++		c		a	c											t							
-	2309751	t	2260839	c	2170758	nc	APEC/S88	rec19	c	APEC	++		c		g	c											t							
-	2309751	t	2260886	c	2170805	nc	APEC/S88	rec19	c	APEC	+++		c		g	c											c							
-	2309751	c	2260964	t	2170883	nc	APEC/S88	rec19	c	S88	++		c		t	t											c							
-	2309751	a	2261078	t	2170997	nc	APEC/S88	rec19	t	APEC	+++		t		a	t											t							
-	2309751	c	2261087	t	2171006	nc	APEC/S88	rec19	c	S88	++		c		a	c											t							
-	2309751	g	2261111	t	2171030	nc	APEC/S88	rec19	a	A/S	+/-		c		a	g											t							
-	2309751	g	2261126	t	2171045	nc	APEC/S88	rec19	g	S88	++		g		a	g											a							
-	2309751	g	2261150	a	2171069	nc	APEC/S88	rec19	g	S88	++		c		g	a											g							
-	2309751	a	2261153	g	2171072	nc	APEC/S88	rec19	g	APEC	+++		g		t	g											g							
-	2309751	g	2261162	a	2171081	nc	APEC/S88	rec19	a	APEC	+++		a		c	a											a							
-	2309751	a	2261165	c	2171084	nc	APEC/S88	rec19	c	APEC	+++		c		c	c											c							
-	2309751	a	2261180	g	2171099	nc	APEC/S88	rec19	a	S88	+++		a		a	a											a							
-	2309751	a	2261181	t	2171100	nc	APEC/S88	rec19	a	S88	+++		a		a	a											a							
-	2309751	c	2261215	g	2171134	nc	APEC/S88	rec19	g	APEC	+++		g		c	g											g							
-	2309751	c	2261503	t	2171422	nc	APEC/S88	rec19	c	S88	+++		c		t	c											c							
-	2309751	a	2261611	g	2171530	nc	APEC/S88	rec19	g	APEC	++		g		a	a											g							
-	2309751	a	2261614	c	2171533	nc	APEC/S88	rec19	c	APEC	++		c		g	a											g							
-	2309751	t	2261620	c	2171539	nc	APEC/S88	rec19	c	APEC	++		c		t	t											t							
-	2309751	a	2261641	g	2171560	nc	APEC/S88	rec19	a	S88	+++		a		c	a											a							
-	2309751	c	2261744	t	2171663	nc	APEC/S88	rec19	t	APEC	+++		t		t	t											t							
-	2309751	g	2261746	t	2171665	nc	APEC/S88	rec19	g	S88	++		g		a	a											g							
-	2309751	a	2261755	g	2171674	nc	APEC/S88	rec19	g	APEC	+++		g		c	g											g							
-	2309751	a	2261761	g	2171680	nc	APEC/S88	rec19	g	APEC	+++		g		c	g											g							
-	2309751	g	2261774	t	2171693	nc	APEC/S88	rec19	t	APEC	+++		t		t	t											t							
-	2309751	c	2261845	t	2171764	nc	APEC/S88	rec19	t	APEC	+++		t		t	t											c							
-	2309751	a	2261881	c	2171800	nc	APEC/S88	rec19	c	APEC	++		c		g	a											a							
-	2309751	c	2261938	a	2171857	nc	APEC/S88	rec19	c	S88	+++		c		g	c											c							
-	2309751	a	2261953	g	2171872	nc	APEC/S88	rec19	g	APEC	+++		g		c	g											g							
-	2309751	a	2262055	g	2171974	nc	APEC/S88	rec19	a	S88	++		a		g	g											g							
-	2309751	a	2262058	g	2171977	nc	APEC/S88	rec19	g	APEC	+++		g		g	g											g							
-	2309751	g	2262070	a	2171989	nc	APEC/S88	rec19	g	S88	+++		g		a	g											g							
-	2309751	a	2262106	g	2172025	nc	APEC/S88	rec19	g	APEC	+++		g		a	g											g							
-	2309751	t	2262111	c	2172030	nc	APEC/S88	rec19	t	S88	+++		t		c	t											t							
-	2309751	g	2262112	a	2172031	nc	APEC/S88	rec19	g	S88	+++		g		c	g											g							
-	2309751	c	2262113	g	2172032	nc	APEC/S88	rec19	c	S88	+++		c		a	c											c							
-	2309751	g	2262137	a	2172056	nc	APEC/S88	rec19	g	S88	++		g		t	g											a							
-	2309751	t	2262142	g	2172061	nc	APEC/S88	rec19	g	APEC	+++		g		g	g											a							
-	2309751	a	2262148	g	2172067	nc	APEC/S88	rec19	g	APEC	+++		g		c	g											g							
-	2309751	a	2262155	g	2172074	nc	APEC/S88	rec19	g	APEC	+++		g		g	g											g							
-	2309751	t	2262337	c	2172256	nc	APEC/S88	rec19	c	APEC	+++		c		c	c											c							
-	2309751	t	2262340	g	2172259	nc	APEC/S88	rec19	g	APEC	++		g		a	g											t							
-	2309751	t	2262349	c	2172268	nc	APEC/S88	rec19	c	APEC	++		c		g	c											a							
-	2309751	g	2262394	a	2172313	nc	APEC/S88	rec19	g	S88	+++		g		g	g											g							
-	2309751	a	2262430	g	2172349	nc	APEC/S88	rec19	g	APEC	+++		g		g	g											c							
-	2309751	g	2262451	a	2172370	nc	APEC/S88	rec19	a	APEC	++		a		g	a											g							
-	2309751	g	2262541	a	2172460	nc	APEC/S88	rec19	g	S88	++		g		t	g											c							
-	2309751	g	2262544	a	2172463	nc	APEC/S88	rec19	g	S88	+++		g		g	g											a							
-	2309751	t	2262547	c	2172466	nc	APEC/S88	rec19	t	S88	+++		t		c	t											t							
-	2309751	c	2262550	t	2172469	nc	APEC/S88	rec19	c	S88	+++		c		c	c											c							

ExPEC genome site details												Outgroup Strains Details[i]																						
UTI89[b]	UTI89 site[c]	APEC 01[b]	APEC 01 site[c]	S88[b]	S88 site[c]	type[d]	Event Lineage[ef]	Recombinant segment	Inferred ancestral base[g]	Outgroup Analysis[e]	Support level[h]	CFT073	ED1a	536	E2348/69	SMS 3-5	IAI39	UMN026	K-12	ATCC 8739	HS	D1 Sd197	CB9615	Sakai	EDL933	IAI1	E24377A	SE11	SS Ss046	F2a 301	F2a 2457T	F5 8401	B4 Sb227	B18 BS512
-	2309751	a	2262571	g	2172490	nc	APEC/S88	rec19	a	S88	++		a		g	t											g							
-	2309751	g	2262583	c	2172502	nc	APEC/S88	rec19	c	APEC	++		c		a	g											g							
-	2309751	t	2262646	c	2172565	nc	APEC/S88	rec19	c	APEC	++		c		a	c											a							
-	2309751	t	2262700	g	2172619	nc	APEC/S88	rec19	g	APEC	++		g		t	g																		
-	2309751	g	2262721	a	2172640	nc	APEC/S88	rec19	g	S88	++		g		g	g																		
-	2309751	a	2262766	g	2172685	nc	APEC/S88	rec19	g	APEC	+++		g		g	g											c							
-	2309751	g	2262769	a	2172688	nc	APEC/S88	rec19	g	S88	+++		g		g	g											c							
-	2309751	g	2262898	a	2172817	nc	APEC/S88	rec19	a	APEC	++		a		a	g											c							
-	2309751	g	2262976	a	2172895	nc	APEC/S88	rec19	g	S88	+++		g		a	g											g							
-	2309751	a	2262994	t	2172913	nc	APEC/S88	rec19	t	APEC	++		t		a	c											c							
-	2309751	c	2263003	t	2172922	nc	APEC/S88	rec19	c	S88	+++		c		a	c											c							
-	2309751	g	2263012	t	2172931	nc	APEC/S88	rec19	g	S88	+++		g		g	g											g							
-	2309751	t	2263015	g	2172934	nc	APEC/S88	rec19	g	APEC	+++		g		g	g											g							
-	2309751	g	2263018	a	2172937	nc	APEC/S88	rec19	g	S88	++		g		g	a											a							
-	2309751	t	2263042	c	2172961	nc	APEC/S88	rec19	t	S88	+++		t		t	t											t							
-	2309751	c	2263051	t	2172970	nc	APEC/S88	rec19	c	S88	++		c		c	t											t							
-	2309751	g	2263054	a	2172973	nc	APEC/S88	rec19	g	S88	+++		g		c	g											g							
-	2309751	a	2263066	g	2172985	nc	APEC/S88	rec19	g	APEC	+++		g		t	g											g							
-	2309751	a	2263069	g	2172988	nc	APEC/S88	rec19	g	APEC	+++		g		g	g											g							
-	2309751	g	2263090	a	2173009	nc	APEC/S88	rec19	a	APEC	++		a		g	g											g							
-	2309751	t	2263096	c	2173015	nc	APEC/S88	rec19	t	S88	++		t		t	c											c							
-	2309751	t	2263120	c	2173039	nc	APEC/S88	rec19	c	APEC	+++		c		a	c											c							
-	2309751	g	2263140	a	2173059	nc	APEC/S88	rec19	g	S88	++		g		g	a											a							
-	2309751	a	2263147	t	2173066	nc	APEC/S88	rec19	t	APEC	+++		t		a	t											t							
-	2309751	c	2263165	a	2173084	nc	APEC/S88	rec19	c	S88	+++		c		c	c											c							
-	2309751	c	2263168	a	2173087	nc	APEC/S88	rec19	c	S88	+++		c		t	c											c							
-	2309751	c	2263216	t	2173135	nc	APEC/S88	rec19	t	APEC	+++		t		t	t											t							
-	2309751	t	2263225	g	2173144	nc	APEC/S88	rec19	g	APEC	++		g		t	t											g							
-	2309751	g	2263234	a	2173153	nc	APEC/S88	rec19	a	APEC	+++		a		g	a											a							
-	2309751	c	2263267	t	2173186	nc	APEC/S88	rec19	c	S88	+++		c		c	c											c							
-	2309751	c	2263297	a	2173216	nc	APEC/S88	rec19	c	S88	++		c		g	a											c							
-	2309751	c	2263339	t	2173258	nc	APEC/S88	rec19	c	S88	++		c		g	t											t							
-	2309751	a	2263343	t	2173262	nc	APEC/S88	rec19	t	APEC	+++		t		c	t											t							
-	2309751	c	2263410	t	2173329	nc	APEC/S88	rec19	c	S88	+++		c		c	c											c							
-	2309751	c	2263417	t	2173336	nc	APEC/S88	rec19	c	S88	+++		c		g	c											c							
-	2309751	c	2263537	t	2173456	nc	APEC/S88	rec19	t	APEC	+++		t		t	c											t							
-	2309751	g	2263576	a	2173495	nc	APEC/S88	rec19	g	S88	+++		g		c	g											g							
-	2309751	c	2263618	g	2173537	nc	APEC/S88	rec19	g	APEC	++		g		c	c											c							
-	2309751	a	2263624	g	2173543	nc	APEC/S88	rec19	g	APEC	+++		g		a	g											g							
-	2309751	g	2263678	a	2173597	nc	APEC/S88	rec19	g	S88	+++		g		g	g											g							
-	2309751	g	2263812	a	2173731	nc	APEC/S88	rec19	g	S88	+++		g		g	g											g							
-	2309751	a	2263901	g	2173820	nc	APEC/S88	rec19	a	S88	+++		a		g	a											a							
-	2309751	c	2263907	t	2173826	nc	APEC/S88	rec19	c	S88	+++		c		t	c											c							
-	2309751	t	2264056	c	2173975	nc	APEC/S88	rec19	t	S88	++		t		t	c																		
-	2309751	a	2264059	g	2173978	nc	APEC/S88	rec19	a	S88	++		a		g	g																		
-	2309751	t	2264083	c	2174002	nc	APEC/S88	rec19	c	APEC	++		c		a	c																		
-	2309751	c	2264096	t	2174015	nc	APEC/S88	rec19	t	APEC	++		t		c	t																		
-	2309751	g	2264098	a	2174017	nc	APEC/S88	rec19	g	S88	++		g		c	g																		
-	2309751	t	2264140	c	2174059	nc	APEC/S88	rec19	t	S88	++		t		c	c																		
-	2309751	g	2264164	c	2174083	nc	APEC/S88	rec19	g	S88	++		g		g	c																		
-	2309751	a	2264202	g	2174121	nc	APEC/S88	rec19	a	S88	++		a		c	a																		
-	2309751	c	2264214	t	2174133	nc	APEC/S88	rec19	t	APEC	++		t		c	t																		
-	2309751	a	2264274	g	2174193	nc	APEC/S88	rec19	g	APEC	++		g		g	g																		
-	2309751	a	2264313	g	2174232	nc	APEC/S88	rec19	g	APEC	++		g		g	g																		
-	2309751	a	2264382	g	2174301	nc	APEC/S88	rec19	g	APEC	++		g		g	a																		
-	2309751	a	2264400	g	2174319	nc	APEC/S88	rec19	a	S88	++		a		g	a																		
-	2309751	t	2264445	c	2174364	nc	APEC/S88	rec19	t	S88	++		t		c	t																		
-	2309751	c	2264505	t	2174424	nc	APEC/S88	rec19	t	APEC	++		t		t	c																		
-	2309751	c	2264510	a	2174429	nc	APEC/S88	rec19	c	S88	++		c		a	c																		
-	2309751	c	2264673	t	2174592	nc	APEC/S88	rec19	c	S88	+++		c		c	c				c														
-	2309751	t	2264688	c	2174607	nc	APEC/S88	rec19	t	S88	++		t		c	t				a														
-	2309751	a	2264691	g	2174610	nc	APEC/S88	rec19	a	S88	++		a		g	a				g														
-	2309751	g	2264844	a	2174763	nc	APEC/S88	rec19	g	S88	++		g		g	a				a														
-	2309751	t	2264934	g	2174853	nc	APEC/S88	rec19	g	APEC	++		g		c	g				a														
-	2309751	a	2264936	t	2174855	nc	APEC/S88	rec19	t	APEC	+++		t		t	t				c														
-	2309751	g	2264937	c	2174856	nc	APEC/S88	rec19	c	APEC	+++		c		c	c				a														
-	2309751	c	2264938	g	2174857	nc	APEC/S88	rec19	g	APEC	+++		g		g	g				c														
-	2309751	a	2265000	g	2174919	nc	APEC/S88	rec19	g	APEC	+++		g		g	g				g														
-	2309751	g	2265071	t	2174990	nc	APEC/S88	rec19	g	S88	+++		g		t	g				g														
-	2309751	a	2265105	g	2175024	nc	APEC/S88	rec19	g	APEC	++		g		a	g				a														
-	2309751	a	2265141	g	2175060	nc	APEC/S88	rec19	g	APEC	+++		g		g	g				g														
-	2309751	g	2265144	t	2175063	nc	APEC/S88	rec19	g	S88	+++		g		g	t				g														
-	2309751	g	2265237	t	2175156	nc	APEC/S88	rec19	t	APEC	++		c		c	t				a														
-	2309751	t	2265456	c	2175375	nc	APEC/S88	rec19	c	APEC	++		c		t	t				c														
-	2309751	c	2265480	t	2175399	nc	APEC/S88	rec19	t	APEC	++		t		g	c				a														
-	2309751	c	2276650	a	2199701	nc	APEC/S88	rec19	g	A/S	+/-					g											g							
-	2309751	t	2276686	c	2199737	nc	APEC/S88	rec19	c	APEC	+					c											c							
-	2309751	g	2276725	a	2199776	nc	APEC/S88	rec19	a	APEC	+					a											a							
-	2309751	a	2276733	t	2199784	nc	APEC/S88	rec19	t	APEC	+					t											t							
-	2309751	a	2276740	c	2199791	nc	APEC/S88	rec19	c	APEC	+					c											c							
-	2309751	t	2276764	c	2199815	nc	APEC/S88	rec19	c	APEC	+					c											t							
-	2309751	a	2276767	t	2199818	nc	APEC/S88	rec19	t	APEC	+					t											a							

ExPEC genome site details												Outgroup Strains Details[i]																						
UTI89[b]	UTI89 site[c]	APEC 01[b]	APEC 01 site[c]	S88[b]	S88 site[c]	type[d]	Event Lineage[ef]	Recombinant segment	Inferred ancestral base[g]	Outgroup Analysis[e]	Support level[h]	CFT073	ED1a	536	E2348/69	SMS 3-5	IAI39	UMN026	K-12	ATCC 8739	HS	D1 Sd197	CB9615	Sakai	EDL933	IAI1	E24377A	SE11	SS Ss046	F2a 301	F2a 2457T	F5 8401	B4 Sb227	B18 BS512
-	2309751	t	2276776	c	2199827	nc	APEC/S88	rec19	t	S88	+					t											t							
-	2309751	c	2276816	g	2199867	nc	APEC/S88	rec19	g	APEC	+					g											g							
-	2309751	c	2276819	a	2199870	nc	APEC/S88	rec19	c	S88	+					c											c							
-	2309751	c	2276825	t	2199876	nc	APEC/S88	rec19	t	APEC	+					t											t							
-	2309751	t	2276841	c	2199892	nc	APEC/S88	rec19	c	APEC	+					c											c							
-	2309751	t	2276842	c	2199893	nc	APEC/S88	rec19	t	S88	+					t											c							
-	2309751	a	2276857	g	2199908	nc	APEC/S88	rec19	g	APEC	+					g											g							
-	2309751	c	2276920	t	2199971	nc	APEC/S88	rec19	t	APEC	+					t											t							
-	2309751	g	2276956	a	2200007	nc	APEC/S88	rec19	g	S88	+					g											a							
-	2309751	c	2276958	t	2200009	nc	APEC/S88	rec19	c	S88	+					c											c							
-	2309751	a	2277028	c	2200079	nc	APEC/S88	rec19	a	S88	+					a											a							
-	2309751	c	2277037	a	2200088	nc	APEC/S88	rec19	a	APEC	+					a											a							
-	2309751	t	2277088	c	2200139	nc	APEC/S88	rec19	c	APEC	+					c											c							
-	2309751	c	2277130	t	2200181	nc	APEC/S88	rec19	t	APEC	+					t											t							
-	2309751	c	2277133	t	2200184	nc	APEC/S88	rec19	t	APEC	+					t											t							
-	2309751	g	2277151	t	2200202	nc	APEC/S88	rec19	g	S88	+					g											g							
-	2309751	c	2277175	t	2200226	nc	APEC/S88	rec19	c	S88	+					c											c							
-	2309751	a	2277187	g	2200238	nc	APEC/S88	rec19	a	S88	+					a											a							
-	2309751	g	2277199	a	2200250	nc	APEC/S88	rec19	g	S88	+					g											g							
-	2309751	g	2277211	a	2200262	nc	APEC/S88	rec19	a	APEC	+					a											g							
-	2309751	g	2277220	a	2200271	nc	APEC/S88	rec19	g	S88	+					g											g							
-	2309751	a	2277241	g	2200292	nc	APEC/S88	rec19	a	S88	+					a											a							
-	2309751	a	2277253	g	2200304	nc	APEC/S88	rec19	g	APEC	+					g											g							
-	2309751	g	2277256	a	2200307	nc	APEC/S88	rec19	a	APEC	+					a											g							
-	2309751	c	2277277	t	2200328	nc	APEC/S88	rec19	t	APEC	+					t											t							
-	2309751	a	2277373	g	2200424	nc	APEC/S88	rec19	a	S88	+					a											a							
-	2309751	g	2277397	a	2200448	nc	APEC/S88	rec19	g	S88	+					g											g							
-	2309751	a	2277409	g	2200460	nc	APEC/S88	rec19	g	APEC	+					g											a							
-	2309751	t	2277412	a	2200463	nc	APEC/S88	rec19	a	APEC	+					a											a							
-	2309751	g	2277415	a	2200466	nc	APEC/S88	rec19	g	S88	+					g											a							
-	2309751	c	2277424	a	2200475	nc	APEC/S88	rec19	c	S88	+					c											t							
-	2309751	c	2277427	t	2200478	nc	APEC/S88	rec19	c	S88	+					c											c							
-	2309751	a	2277430	g	2200481	nc	APEC/S88	rec19	g	APEC	+					g											g							
-	2309751	a	2277439	g	2200490	nc	APEC/S88	rec19	g	APEC	+					g											g							
-	2309751	g	2277475	a	2200526	nc	APEC/S88	rec19	g	S88	+					g											g							
-	2309751	t	2277529	c	2200580	nc	APEC/S88	rec19	t	S88	+					t											t							
-	2309751	g	2277568	a	2200619	nc	APEC/S88	rec19	g	S88	+					g											t							
-	2309751	a	2277604	g	2200655	nc	APEC/S88	rec19	g	APEC	+					g											a							
-	2309751	g	2277613	a	2200664	nc	APEC/S88	rec19	g	S88	+					g											g							
-	2309751	t	2277676	c	2200727	nc	APEC/S88	rec19	c	APEC	+					c											c							
-	2309751	a	2277679	g	2200730	nc	APEC/S88	rec19	g	APEC	+					g											g							
-	2309751	a	2277682	g	2200733	nc	APEC/S88	rec19	g	APEC	+					g											g							
-	2309751	g	2277698	a	2200749	nc	APEC/S88	rec19	a	APEC	+					a											a							
-	2309751	a	2277745	g	2200796	nc	APEC/S88	rec19	a	S88	+					a											a							
-	2309751	a	2277790	g	2200841	nc	APEC/S88	rec19	g	APEC	+					g											g							
-	2309751	t	2277834	c	2200885	nc	APEC/S88	rec19	c	APEC	+					c											c							
-	2309751	a	2277907	g	2200958	nc	APEC/S88	rec19	g	APEC	+					g											g							
-	2309751	g	2277928	a	2200979	nc	APEC/S88	rec19	a	APEC	+					a											a							
-	2309751	t	2277961	a	2201012	nc	APEC/S88	rec19	a	APEC	+					a											a							
-	2309751	g	2277976	t	2201027	nc	APEC/S88	rec19	g	S88	+					g											g							
-	2309751	t	2278099	g	2201150	nc	APEC/S88	rec19	g	APEC	+					g											g							
-	2309751	c	2278150	t	2201201	nc	APEC/S88	rec19	c	S88	+					c											c							
-	2309751	g	2278171	a	2201222	nc	APEC/S88	rec19	g	S88	+					g											g							
-	2309751	t	2278195	c	2201246	nc	APEC/S88	rec19	c	APEC	+					c											c							
-	2309751	a	2278210	g	2201261	nc	APEC/S88	rec19	g	APEC	+					g											a							
-	2309751	c	2278249	t	2201300	nc	APEC/S88	rec19	c	S88	+					c											t							
-	2309751	g	2278273	a	2201324	nc	APEC/S88	rec19	g	S88	+					c											g							
-	2309751	a	2278279	g	2201330	nc	APEC/S88	rec19	g	APEC	+					g											g							
-	2309751	g	2278321	c	2201372	nc	APEC/S88	rec19	c	APEC	+					c											g							
-	2309751	c	2278333	a	2201384	nc	APEC/S88	rec19	c	S88	+					c											c							
-	2309751	c	2278351	t	2201402	nc	APEC/S88	rec19	c	S88	+					c											c							
-	2309751	a	2278376	c	2201427	nc	APEC/S88	rec19	a	S88	+					a											a							
-	2309751	a	2278389	t	2201440	nc	APEC/S88	rec19	a	S88	+					a											t							
-	2309751	g	2278392	t	2201443	nc	APEC/S88	rec19	g	S88	+					g											g							
-	2309751	g	2278543	a	2201594	nc	APEC/S88	rec19	a	APEC	+					a											a							
-	2309751	g	2278675	a	2201726	nc	APEC/S88	rec19	g	S88	+					g											g							
-	2309751	a	2278744	g	2201795	nc	APEC/S88	rec19	g	APEC	+					g											a							
-	2309751	g	2278759	a	2201810	nc	APEC/S88	rec19	g	S88	+					g											g							
-	2309751	c	2278785	t	2201836	nc	APEC/S88	rec19	t	APEC	+					t											c							
-	2309751	t	2278788	c	2201839	nc	APEC/S88	rec19	t	S88	+					t											t							
-	2309751	t	2278844	g	2201895	nc	APEC/S88	rec19	g	APEC	+					g											g							
-	2309751	a	2278910	g	2201961	nc	APEC/S88	rec19	a	S88	+					a											a							
-	2309751	a	2278979	g	2202030	nc	APEC/S88	rec19	g	APEC	+					g											a							
-	2309751	c	2278994	t	2202045	nc	APEC/S88	rec19	c	S88	+					c											c							
-	2309751	g	2279099	a	2202150	nc	APEC/S88	rec19	a	APEC	+					a											a							
-	2309751	a	2279117	g	2202168	nc	APEC/S88	rec19	g	APEC	+					g											g							
-	2309751	c	2279131	t	2202182	nc	APEC/S88	rec19	t	APEC	+					t											t							
-	2309751	g	2279158	a	2202209	nc	APEC/S88	rec19	a	APEC	+					a											g							
-	2309751	g	2279185	a	2202236	nc	APEC/S88	rec19	a	APEC	+					a											a							
-	2309751	t	2279217	c	2202268	nc	APEC/S88	rec19	c	APEC	+					c											c							
-	2309751	a	2279250	c	2202301	nc	APEC/S88	rec19	c	APEC	+					c											c							
-	2309751	g	2279388	a	2202439	nc	APEC/S88	rec19	g	S88	+					g											a							
-	2309751	t	2279421	g	2202472	nc	APEC/S88	rec19	g	APEC	+					g											g							
-	2309751	c	2279442	t	2202493	nc	APEC/S88	rec19	c	S88	+					c											t							

ExPEC genome site details												Outgroup Strains Details[i]																						
UTI89[b]	UTI89 site[c]	APEC 01[b]	APEC 01 site[c]	S88[b]	S88 site[c]	type[d]	Event Lineage[ef]	Recombinant segment	Inferred ancestral base[g]	Outgroup Analysis[e]	Support level[h]	CFT073	ED1a	536	E2348/69	SMS 3-5	IAI39	UMN026	K-12	ATCC 8739	HS	D1 Sd197	CB9615	Sakai	EDL933	IAI1	E24377A	SE11	SS Ss046	F2a 301	F2a 2457T	F5 8401	B4 Sb227	B18 BS512
-	2309751	a	2279586	g	2202637	nc	APEC/S88	rec19	g	APEC	+					g											g							
-	2309751	t	2279589	g	2202640	nc	APEC/S88	rec19	g	APEC	+					g											g							
-	2309751	c	2279619	t	2202670	nc	APEC/S88	rec19	t	APEC	+					t											t							
-	2309751	c	2279623	t	2202674	nc	APEC/S88	rec19	c	S88	+					c											c							
-	2309751	c	2279642	t	2202693	nc	APEC/S88	rec19	c	S88	+					c											c							
-	2309751	a	2279780	c	2202831	nc	APEC/S88	rec19	t	A/S	+/-					t											a							
-	2309751	c	2279806	t	2202857	nc	APEC/S88	rec19	c	S88	+					c											c							
-	2309751	t	2279812	g	2202863	nc	APEC/S88	rec19	g	APEC	+					g											g							
-	2309751	t	2279832	c	2202883	nc	APEC/S88	rec19	c	APEC	+					c											c							
-	2309751	g	2279892	a	2202943	nc	APEC/S88	rec19	g	S88	+					g											a							
-	2309751	a	2279906	g	2202957	nc	APEC/S88	rec19	a	S88	+					a											a							
-	2309751	a	2279919	c	2202970	nc	APEC/S88	rec19	a	S88	+					a											a							
-	2309751	t	2279923	c	2202974	nc	APEC/S88	rec19	c	APEC	+					c											t							
-	2309751	c	2279954	t	2203005	nc	APEC/S88	rec19	c	S88	+					c											c							
-	2309751	t	2279990	c	2203041	nc	APEC/S88	rec19	t	S88	+					t											t							
-	2309751	t	2280047	g	2203098	nc	APEC/S88	rec19	t	S88	+					t											t							
-	2309751	t	2280056	c	2203107	nc	APEC/S88	rec19	t	S88	+					t											t							
-	2309751	g	2280062	a	2203113	nc	APEC/S88	rec19	g	S88	+					g											g							
-	2309751	a	2280065	g	2203116	nc	APEC/S88	rec19	g	APEC	+					g											a							
-	2309751	g	2280082	a	2203133	nc	APEC/S88	rec19	g	S88	+					g											g							
-	2309751	g	2280085	c	2203136	nc	APEC/S88	rec19	g	S88	+					g											g							
-	2309751	t	2280086	c	2203137	nc	APEC/S88	rec19	t	S88	+					t											t							
-	2309751	g	2280089	a	2203140	nc	APEC/S88	rec19	g	S88	+					g											g							
-	2309751	g	2280104	a	2203155	nc	APEC/S88	rec19	g	S88	+					g											a							
-	2309751	g	2280152	a	2203203	nc	APEC/S88	rec19	g	S88	+					g											g							
-	2309751	a	2280155	g	2203206	nc	APEC/S88	rec19	a	S88	+					a											a							
-	2309751	a	2280187	g	2203238	nc	APEC/S88	rec19	a	S88	+					a											g							
-	2309751	t	2280203	a	2203254	nc	APEC/S88	rec19	a	APEC	+					a											a							
-	2309751	t	2280209	c	2203260	nc	APEC/S88	rec19	t	S88	+					t											c							
-	2309751	c	2280260	t	2203311	nc	APEC/S88	rec19	c	S88	+					c											t							
-	2309751	g	2280278	a	2203329	nc	APEC/S88	rec19	a	APEC	+					a											a							
-	2309751	a	2280299	g	2203350	nc	APEC/S88	rec19	g	APEC	+					g											g							
-	2309751	t	2280302	c	2203353	nc	APEC/S88	rec19	c	APEC	+					c											c							
-	2309751	g	2280326	a	2203377	nc	APEC/S88	rec19	a	APEC	+					a											a							
-	2309751	c	2280353	a	2203404	nc	APEC/S88	rec19	a	APEC	+					a											a							
-	2309751	a	2280362	g	2203413	nc	APEC/S88	rec19	g	APEC	+					g											a							
-	2309751	g	2280380	a	2203431	nc	APEC/S88	rec19	a	APEC	+					a											a							
-	2309751	t	2280382	c	2203433	nc	APEC/S88	rec19	c	APEC	+					c											c							
-	2309751	a	2280398	g	2203449	nc	APEC/S88	rec19	a	S88	+					a											g							
-	2309751	t	2280430	g	2203481	nc	APEC/S88	rec19	g	APEC	+					g											g							
-	2309751	t	2280463	c	2203514	nc	APEC/S88	rec19	t	S88	+					t											t							
-	2309751	g	2280505	a	2203556	nc	APEC/S88	rec19	a	APEC	+					a											a							
-	2309751	t	2280508	c	2203559	nc	APEC/S88	rec19	c	APEC	+					c											c							
-	2309751	c	2280514	a	2203565	nc	APEC/S88	rec19	a	APEC	+					a											a							
-	2309751	a	2280529	g	2203580	nc	APEC/S88	rec19	g	APEC	+					g											g							
-	2309751	t	2280967	c	2204018	nc	APEC/S88	rec19	?	A/S	+/-																							
-	2309751	c	2280974	t	2204025	nc	APEC/S88	rec19	?	A/S	+/-																							
-	2309751	t	2281297	c	2204348	nc	APEC/S88	rec19	?	S88	+/-																							
-	2309751	a	2281374	c	2204425	nc	APEC/S88	rec19	?	S88	+/-																							
-	2309751	a	2281384	c	2204435	nc	APEC/S88	rec19	?	S88	+/-																							
-	2309751	g	2281385	a	2204436	nc	APEC/S88	rec19	?	S88	+/-																							
-	2309751	t	2281386	c	2204437	nc	APEC/S88	rec19	?	S88	+/-																							
-	2309751	c	2281469	t	2204520	nc	APEC/S88	rec19	?	S88	+/-																							
-	2309751	a	2281523	g	2204574	nc	APEC/S88	rec19	?	S88	+/-																							
-	2309751	a	2281580	g	2204631	nc	APEC/S88	rec19	?	S88	+/-																							
-	2309751	a	2281595	g	2204646	nc	APEC/S88	rec19	?	S88	+/-																							
-	2309751	g	2281689	a	2204740	nc	APEC/S88	rec19	?	S88	+/-																							
-	2309751	c	2281709	t	2204760	nc	APEC/S88	rec19	?	S88	+/-																							
-	2309751	c	2281718	g	2204769	nc	APEC/S88	rec19	?	S88	+/-																							
-	2309751	c	2281751	t	2204802	nc	APEC/S88	rec19	?	APEC	+/-																							
-	2309751	g	2281865	a	2204916	nc	APEC/S88	rec19	?	S88	+/-																							
-	2309751	a	2281889	c	2204940	nc	APEC/S88	rec19	?	S88	+/-																							
-	2309751	a	2281904	g	2204955	nc	APEC/S88	rec19	?	S88	+/-																							
-	2309751	a	2281931	t	2204982	nc	APEC/S88	rec19	?	S88	+/-																							
-	2309751	a	2281934	t	2204985	nc	APEC/S88	rec19	?	S88	+/-																							
-	2309751	t	2281949	c	2205000	nc	APEC/S88	rec19	?	S88	+/-																							
-	2309751	a	2281979	t	2205030	nc	APEC/S88	rec19	?	S88	+/-																							
-	2309751	4	2282064	-	2205114	indel	APEC/S88	rec19	?	A/S	+/-																							
-	2309751	t	2282084	c	2205131	nc	APEC/S88	rec19	?	APEC	+/-																							
t	2609616	a	2582842	a	2505939	s	UTI89/AS	rec20	t	AS	+										t									c	c	c		
a	2609618	g	2582844	g	2505941	s	UTI89/AS	rec20	a	AS	+										a									a	a	a		
c	2609619	a	2582845	a	2505942	ns	UTI89/AS	rec20	c	AS	+										c									a	a	a		
g	2609620	c	2582846	c	2505943	ns	UTI89/AS	rec20	g	AS	+										g									g	g	g		
g	2609622	t	2582848	t	2505945	s	UTI89/AS	rec20	g	AS	+										g									a	a	a		
c	2609629	g	2582855	g	2505952	ns	UTI89/AS	rec20	c	AS	+										c									c	c	c		
t	2609650	c	2582876	c	2505973	ns	UTI89/AS	rec20	c	UTI89	+										c									c	c	c		
c	2609729	t	2582955	t	2506052	ns	UTI89/AS	rec20	c	AS	+										c									c	c	c		
a	2609733	g	2582959	g	2506056	s	UTI89/AS	rec20	g	UTI89	+										g									g	g	g		
t	2609736	g	2582962	g	2506059	s	UTI89/AS	rec20	g	UTI89	+										g									a	a	a		
a	2609748	c	2582974	c	2506071	s	UTI89/AS	rec20	c	UTI89	+										c									t	t	t		
a	2613316	g	2586542	g	2509639	s	UTI89/AS	rec21	a	AS	+										a													

ExPEC genome site details												Outgroup Strains Details[i]																						
UTI89[b]	UTI89 site[c]	APEC 01[b]	APEC 01 site[c]	S88[b]	S88 site[c]	type[d]	Event Lineage[ef]	Recombinant segment	Inferred ancestral base[g]	Outgroup Analysis[e]	Support level[h]	CFT073	ED1a	536	E2348/69	SMS 3-5	IAI39	UMN026	K-12	ATCC 8739	HS	D1 Sd197	CB9615	Sakai	EDL933	IAI1	E24377A	SE11	SS Ss046	F2a 301	F2a 2457T	F5 8401	B4 Sb227	B18 BS512
t	2613373	c	2586599	c	2509696	s	UTI89/AS	rec21	c	UTI89	+										c													
a	2613391	c	2586617	c	2509714	s	UTI89/AS	rec21	c	UTI89	+										c													
c	2613637	t	2586863	t	2509960	ns	UTI89/AS	rec21	t	UTI89	+										t													
a	2613659	g	2586885	g	2509982	s	UTI89/AS	rec21	a	AS	+										a													
a	2613677	t	2586903	t	2510000	s	UTI89/AS	rec21	a	AS	+										a													
g	2613754	a	2586980	a	2510077	s	UTI89/AS	rec21	g	AS	+										g													
a	2613784	c	2587010	c	2510107	s	UTI89/AS	rec21	a	AS	+										a													
a	2613817	c	2587043	c	2510140	s	UTI89/AS	rec21	c	UTI89	+										c													
c	2613849	a	2587075	a	2510172	ns	UTI89/AS	rec21	c	AS	+										c													
a	2613850	g	2587076	g	2510173	s	UTI89/AS	rec21	g	UTI89	+										g													
t	2613913	a	2587139	a	2510236	s	UTI89/AS	rec21	a	UTI89	+										a													
c	2613916	t	2587142	t	2510239	s	UTI89/AS	rec21	c	AS	+										c													
t	2613922	c	2587148	c	2510245	s	UTI89/AS	rec21	c	UTI89	+										c													
c	2613943	t	2587169	t	2510266	s	UTI89/AS	rec21	t	UTI89	+										t													
g	2613967	c	2587193	c	2510290	s	UTI89/AS	rec21	c	UTI89	+										c													
g	2614372	a	2587598	a	2510695	s	UTI89/AS	rec21	g	AS	+										g													
c	2614378	a	2587604	a	2510701	s	UTI89/AS	rec21	c	AS	+										c													
t	2614393	c	2587619	c	2510716	s	UTI89/AS	rec21	t	AS	+										t													
a	2614432	g	2587658	g	2510755	s	UTI89/AS	rec21	a	AS	+										a													
a	2614438	t	2587664	t	2510761	s	UTI89/AS	rec21	a	AS	+										a													
g	2614486	a	2587712	a	2510809	s	UTI89/AS	rec21	g	AS	+										g													
a	2614495	g	2587721	g	2510818	s	UTI89/AS	rec21	a	AS	+										a													
a	2614510	t	2587736	t	2510833	s	UTI89/AS	rec21	a	AS	+										a													
t	2614513	c	2587739	c	2510836	s	UTI89/AS	rec21	t	AS	+										t													
t	2614578	a	2587804	a	2510901	ns	UTI89/AS	rec21	a	UTI89	+										a													
t	2614582	a	2587808	a	2510905	ns	UTI89/AS	rec21	t	AS	+										t													
c	2614584	t	2587810	t	2510907	ns	UTI89/AS	rec21	t	UTI89	+										t													
c	2614600	t	2587826	t	2510923	s	UTI89/AS	rec21	c	AS	+										c													
t	2614702	c	2587928	c	2511025	s	UTI89/AS	rec21	t	AS	+										t													
c	2614705	a	2587931	a	2511028	s	UTI89/AS	rec21	c	AS	+										c													
a	2614708	g	2587934	g	2511031	s	UTI89/AS	rec21	g	UTI89	+										g													
a	2614831	g	2588057	g	2511154	s	UTI89/AS	rec21	g	UTI89	+										g													
g	2614994	t	2588220	t	2511317	ns	UTI89/AS	rec21	g	AS	+										g													
a	2615011	g	2588237	g	2511334	nc	UTI89/AS	rec21	g	UTI89	+										g													
g	2625355	a	2598581	a	2521678	nc	UTI89/AS	rec22	g	AS	+				g																g			
g	2625357	c	2598583	c	2521680	nc	UTI89/AS	rec22	g	AS	+				g																g			
c	2625358	g	2598584	g	2521681	nc	UTI89/AS	rec22	c	AS	+				c																c			
t	2625359	g	2598585	g	2521682	nc	UTI89/AS	rec22	t	AS	+				t																t			
c	2625360	a	2598586	a	2521683	nc	UTI89/AS	rec22	c	AS	+				c																c			
a	2625361	g	2598587	g	2521684	nc	UTI89/AS	rec22	a	AS	+				a																a			
t	2625366	c	2598592	c	2521689	nc	UTI89/AS	rec22	c	UTI89	+				c																c			
c	2625376	a	2598602	a	2521699	nc	UTI89/AS	rec22	c	AS	+				c																c			
a	2625415	t	2598641	t	2521738	nc	UTI89/AS	rec22	a	AS	+				a																a			
t	2625418	c	2598644	c	2521741	nc	UTI89/AS	rec22	t	AS	+				t																c			
c	2625440	t	2598666	t	2521763	nc	UTI89/AS	rec22	t	UTI89	+																				t			
g	2625494	t	2598720	t	2521817	ns	UTI89/AS	rec22	?	UTI89/AS	+/-																							
-	2625515	t	2600125	g	2523222	ns	UTI89/AS	rec22	?	A/S	+/-																							
t	2626678	c	2600580	c	2523677	ns	UTI89/AS	rec22	t	AS	+				t																			
t	2626679	a	2600581	a	2523678	s	UTI89/AS	rec22	t	AS	+				t																			
c	2626690	t	2600592	t	2523689	ns	UTI89/AS	rec22	t	UTI89	+				t																			
c	2626708	a	2600610	a	2523707	s	UTI89/AS	rec22	c	AS	+				c																			
c	2626744	t	2600646	t	2523743	s	UTI89/AS	rec22	t	UTI89	+				t																			
a	2626858	g	2600760	g	2523857	s	UTI89/AS	rec22	g	UTI89	+				g																			
t	2627023	c	2600925	c	2524022	s	UTI89/AS	rec22	c	UTI89	+				c																			
g	2627029	a	2600931	a	2524028	s	UTI89/AS	rec22	g	AS	+				g																			
a	2627035	c	2600937	c	2524034	s	UTI89/AS	rec22	a	AS	+				a																			
c	2627038	t	2600940	t	2524037	s	UTI89/AS	rec22	c	AS	+				c																			
a	2627065	c	2600967	c	2524064	s	UTI89/AS	rec22	c	UTI89	+				c																			
t	2627074	g	2600976	g	2524073	s	UTI89/AS	rec22	g	UTI89	+				g																			
g	2627083	a	2600985	a	2524082	s	UTI89/AS	rec22	a	UTI89	+				a																			
a	2627104	g	2601006	g	2524103	s	UTI89/AS	rec22	g	UTI89	+				g																			
c	2627155	g	2601057	g	2524154	s	UTI89/AS	rec22	c	AS	+				c																			
t	2627179	c	2601081	c	2524178	s	UTI89/AS	rec22	c	UTI89	+				c																			
c	2627215	t	2601117	t	2524214	s	UTI89/AS	rec22	c	AS	+				c																			
c	2627278	t	2601180	t	2524277	s	UTI89/AS	rec22	c	AS	+				c																			
t	2627287	c	2601189	c	2524286	s	UTI89/AS	rec22	t	AS	+				t																			
c	2627467	t	2601369	t	2524466	s	UTI89/AS	rec22	t	UTI89	+				t																			
t	2627515	c	2601417	c	2524514	s	UTI89/AS	rec22	c	UTI89	+				c																			
a	2627620	g	2601522	g	2524619	s	UTI89/AS	rec22	a	AS	+				a																			
a	2627623	g	2601525	g	2524622	s	UTI89/AS	rec22	a	AS	+				a																			
a	2627680	g	2601582	g	2524679	s	UTI89/AS	rec22	g	UTI89	+				g																			
c	2627686	t	2601588	t	2524685	s	UTI89/AS	rec22	c	AS	+				c																			
c	2627695	t	2601597	t	2524694	s	UTI89/AS	rec22	t	UTI89	+				t																			
c	2627734	t	2601636	t	2524733	s	UTI89/AS	rec22	c	AS	+				c																			
a	2627737	g	2601639	g	2524736	s	UTI89/AS	rec22	a	AS	+				a																			
a	2627830	g	2601732	g	2524829	s	UTI89/AS	rec22	g	UTI89	+				g																			
c	2627862	a	2601764	a	2524861	ns	UTI89/AS	rec22	a	UTI89	+				a																			
g	2627863	c	2601765	c	2524862	s	UTI89/AS	rec22	g	AS	+				g																			
g	2627935	c	2601837	c	2524934	s	UTI89/AS	rec22	g	AS	+				g																			
a	2627938	g	2601840	g	2524937	s	UTI89/AS	rec22	g	UTI89	+				g																			
g	2859395	c	2871728	g	2755055	ns	APEC	rec23	g	APEC	++++	g	g	g	g	g	g	g	g	g	g	g	g	g	g	g	g	g	g	g	g	g	g	g

UTI89[b]	UTI89 site[c]	APEC 01[b]	APEC 01 site[c]	S88[b]	S88 site[c]	type[d]	Event Lineage[ef]	Recombinant segment	Inferred ancestral base[g]	Outgroup Analysis[e]	Support level[h]	CFT073	ED1a	536	E2348/69	SMS 3-5	IAI39	UMN026	K-12	ATCC 8739	HS	D1 Sd197	CB9615	Sakai	EDL933	IAI1	E24377A	SE11	SS Ss046	F2a 301	F2a 2457T	F5 8401	B4 Sb227	B18 BS512
ExPEC genome site details												Outgroup Strains Details[i]																						
t	2859399	c	2871732	t	2755059	s	APEC	rec23	t	APEC	++++	t	t	t	t	t	t	t	t	t	t	t	t	t	t	t	t	t	t	t	t	t	t	t
a	2859405	g	2871738	a	2755065	s	APEC	rec23	a	APEC	++++	a	a	a	a	a	a	a	a	a	a	a	a	a	a	a	a	a	a	a	a	a	a	a
c	2859411	a	2871744	c	2755071	ns	APEC	rec23	c	APEC	++++	c	c	c	c	c	c	c	c	c	c	c	c	c	c	c	c	c	c	c	c	c	c	c
c	2859413	g	2871746	c	2755073	ns	APEC	rec23	c	APEC	++++	c	c	c	c	c	c	c	c	c	c	c	c	c	c	c	c	c	c	c	c	c	c	c
a	2859415	g	2871748	a	2755075	ns	APEC	rec23	a	APEC	++++	a	a	a	a	a	a	a	a	a	a	a	a	a	a	a	a	a	a	a	a	a	a	a
a	2859417	g	2871750	a	2755077	s	APEC	rec23	a	APEC	++	a	a	a	a	a	a	g	g	g	g	a	a	a	a	a	a	g	g	g	g	g	g	g
c	2859421	g	2871754	c	2755081	ns	APEC	rec23	c	APEC	++++	c	c	c	c	c	c	c	c	c	c	c	c	c	c	c	c	c	c	c	c	c	c	c
c	2859422	a	2871755	c	2755082	ns	APEC	rec23	c	APEC	++++	c	c	c	c	c	c	c	c	c	c	c	c	c	c	c	c	c	c	c	c	c	c	c
t	2873802	c	2888816	t	2772044	nc	APEC	rec24	t	APEC	++	t	t	t	t	t	t	c	t	a	a	a	t	t	t	a	a	a	t	a	a	a	a	t
g	2873805	a	2888819	g	2772047	nc	APEC	rec24	g	APEC	++	g	g	g	g	g	g	a	g	g	g	g	a	a	g	g	g	g	g	g	g	g	g	g
c	2873806	g	2888820	c	2772048	nc	APEC	rec24	c	APEC	++	c	c	c	c	c	c	g	c	c	c	c	g	g	c	c	c	c	c	c	c	c	c	c
c	2874080	t	2889094	c	2772322	ns	APEC	rec24	c	APEC	++++	c	c	c	c	c	c	c	c	c	c	c	c	c	c	c	c	c	c	c	c	c	c	c
-	2874542	1	2889557	-	2772784	ins	APEC	rec24	-	APEC	++++	1	-	-	-	-	-	-	-	-	-	-	-	-	-	-	-	-	-	-	-	-	-	-
-	2874569	1	2889585	-	2772811	ins	APEC	rec24	-	APEC	++++	1	-	-	-	-	-	-	-	-	-	-	-	-	-	-	-	-	-	-	-	-	-	-
-	2874586	1	2889603	-	2772828	ins	APEC	rec24	-	APEC	++++	1	-	-	-	-	-	-	-	-	-	-	-	-	-	-	-	-	-	-	-	-	-	-
-	2874608	1	2889626	-	2772850	ins	APEC	rec24	-	APEC	++++	1	-	-	-	-	-	-	-	-	-	-	-	-	-	-	-	-	-	-	-	-	-	-
-	2874716	1	2889735	-	2772958	ins	APEC	rec24	-	APEC	++++	1	-	-	-	-	-	-	-	-	-	-	-	-	-	-	-	-	-	-	-	-	-	-
g	2874725	c	2889744	g	2772967	nc	APEC	rec24	g	APEC	++++	c	g	g	g	g	g	g	g	g	g	g	g	g	g	g	g	g	g	g	g	g	g	g
-	2874772	1	2889792	-	2773014	ins	APEC	rec24	-	APEC	++	1	-	-	-	-	-	-	-	-	-	-	-	-	1	-	-	-	-	-	-	-	-	-
-	2874795	1	2889816	-	2773037	ins	APEC	rec24	-	APEC	++++	1	-	-	-	-	-	-	-	-	-	-	-	-	-	-	-	-	-	-	-	-	-	-
-	2874810	1	2889832	-	2773052	ins	APEC	rec24	-	APEC	++++	1	-	-	-	-	-	-	-	-	-	-	-	-	-	-	-	-	-	-	-	-	-	-
-	2874933	1	2889956	-	2773175	ins	APEC	rec24	-	APEC	++++	1	-	-	-	-	-	-	-	-	-	-	-	-	-	-	-	-	-	-	-	-	-	-
g	2874956	c	2889979	g	2773198	nc	APEC	rec24	g	APEC	++++	c	g	g	g	g	g	g	g	g	g	g	g	g	g	g	g	g	g	g	g	g	g	g
-	2874979	1	2890003	-	2773221	ins	APEC	rec24	-	APEC	++++	1	-	-	-	-	-	-	-	-	-	-	-	-	-	-	-	-	-	-	-	-	-	-
-	2875002	1	2890027	-	2773244	ins	APEC	rec24	-	APEC	++++	1	-	-	-	-	-	-	-	-	-	-	-	-	-	-	-	-	-	-	-	-	-	-
g	2875095	t	2890120	g	2773337	nc	APEC	rec24	t	2	-	t	t	t	g	g	g	g	g	g	g	g	g	g	g	g	g	g	g	g	g	g	g	g
t	2875149	g	2890174	t	2773391	nc	APEC	rec24	g	2	-	g	g	g	g	g	g	g	g	g	g	g	a	g	g	g	g	g	g	g	g	g	g	g
g	2875222	a	2890247	g	2773464	nc	APEC	rec24	a	2	-	a	a	g	a	a	a	a	a	a	a	a	a	a	a	a	a	a	a	a	a	a	a	a
g	2875543	a	2890567	g	2773784	ns	APEC	rec24	a	2	-	a	a	g	a	a	a	a	a	a	a	a	a	g	g	a	a	a	a	a	a	a	a	a
a	2875594	g	2890618	a	2773835	s	APEC	rec24	g	2	-	g	g	a	a	a	a	a	a	g	g	a	g	g	g	g	a	g	g	g	g	g	g	a
g	2875596	a	2890620	g	2773837	ns	APEC	rec24	a	2	-	a	a	g	a	a	a	a	a	a	a	g	a	a	a	a	a	a	a	a	a	a	a	a
c	2875609	t	2890633	c	2773850	s	APEC	rec24	t	2	-	t	t	c	c	c	c	c	c	c	c	c	c	c	c	c	c	c	c	c	c	c	c	c
c	2976810	a	2991836	a	2909148	nc	UTI89	rec25	a	UTI89	++	a	-	-	a	-	c		a	a	a		-	-	-		a		a	c	c	c		
c	2976814	t	2991841	t	2909153	nc	UTI89	rec25	t	UTI89	++	t	t	t	t	t	c		c	c	c		t	t	t		t		t	c	c	c		
g	2976821	a	2991848	a	2909160	nc	UTI89	rec25	g	AS	++++	g	g	g	a	g	g		g	g	g		g	g	g		g		g	g	g	g		
a	2976823	t	2991850	t	2909162	nc	UTI89	rec25	t	UTI89	++	t	t	t	t	t	a		a	a	a		t	t	t		t		t	a	a	a		
t	2976827	a	2991854	a	2909166	nc	UTI89	rec25	a	UTI89	++	a	a	a	a	a	t		t	t	t		a	a	a		a		a	t	t	t		
c	2976836	t	2991861	t	2909173	nc	UTI89	rec25	t	UTI89	++	t	c	t	t	c	c		c	c	c		c	c	c		c		c	c	c	c		
c	2976840	t	2991865	t	2909177	nc	UTI89	rec25	t	UTI89	++	t	c	t	t	c	c		t	t	t		t	t	t		t		t	t	t	t		
c	3302845	a	3375752	c	3290544	ns	APEC	rec26	c	APEC	+					c	c																	
c	3302848	g	3375755	c	3290547	ns	APEC	rec26	c	APEC	+					c	c																	
a	3302851	t	3375758	a	3290550	ns	APEC	rec26	a	APEC	+					a	a																	
a	3643349	t	3716261	a	3631054	nc	APEC	rec27	a	APEC	++	a	a	a	a	t	t	t	t	t	t	t	t	t	t	t	t	t	t	t	t	t	t	t
c	3643350	g	3716262	c	3631055	nc	APEC	rec27	c	APEC	++	c	c	c	c	a	a	a	g	g	g	a	g	a	g	g	g	a	g	a	a	a	a	a
g	3643359	c	3716271	g	3631064	nc	APEC	rec27	g	APEC	++	g	g	g	g	t	t	t	c	c	c	t	c	t	c	c	c	t	c	c	c	c	c	t
c	3643360	a	3716272	c	3631065	nc	APEC	rec27	c	APEC	++	c	c	c	c	a	a	a	a	a	a	a	a	a	a	a	a	a	a	a	a	a	a	a
a	3643368	g	3716280	a	3631073	nc	APEC	rec27	a	APEC	++	a	a	a	a	a	a	g	g	a	g	a	a	a	a	g	g	a	g	a	a	a	a	a
a	3643401	g	3716313	a	3631106	nc	APEC	rec27	a	APEC	++	a	a	a	a	a	a	g	g	g	g	a	a	a	a	g	g	a	g	g	g	g	g	a
-	3643405	1	3716318	-	3631110	ins	APEC	rec27	-	APEC	++	-	-	-	-	-	-	1	1	1	1	-	-	-	1	1	1	-	1	1	1	1	1	-
t	3643407	c	3716320	t	3631112	nc	APEC	rec27	t	APEC	++	t	t	t	t	t	t	c	c	c	c	t	t	t	t	c	c	t	c	c	c	c	c	t
-	3644025	1	3716939	-	3631730	ins	APEC	rec27	-	APEC	++++	-	-	-	-	-	-	-	-	-	-	-	-	-	1	-	-	-	-	-	-	-	-	-
-	3644036	1	3716951	-	3631741	ins	APEC	rec27	-	APEC	++++	-	-	-	-	-	-	-	-	-	-	-	-	-	1	-	-	-	-	-	-	-	-	-
c	3644227	t	3717142	c	3631932	s	APEC	rec27	c	APEC	++++	c	c	c	c	c	c	c	c	c	c	c	c	c	c	c	c	c	c	c	c	c	c	c
c	3644435	t	3717350	c	3632140	nc	APEC	rec27	t	2	-	t	t	t	t	a	a	a	t	t	a	t	a	a	t	a	a	a	a	a	a	a	a	a
t	3644494	c	3717409	t	3632199	nc	APEC	rec27	c	2	-	t	c	c	c	c	c	t	t	t	t	t	t	t	t	t	t	t	t	t	t	t	t	t
t	3644599	g	3717514	t	3632304	nc	APEC	rec27	t	APEC	++	t	g	t	g	t	t	t	t	t	t	t	t	t	t	t	t	t	g	t	t	c	a	a
a	3644617	g	3717532	a	3632322	nc	APEC	rec27	g	2	-	g	g	g	g	g	g	g	g	g	g	g	g	g	g	g	g	g	g	g	g	g	g	g
g	3645002	a	3717917	g	3632707	nc	APEC	rec27	g	APEC	++	g	t	g	g	t	g	g	g	g	g	a	g	g	g	g	g	g	g	a	a	a	g	g
c	3645008	a	3717923	c	3632713	nc	APEC	rec27	a	2	-	t	t	t	t	a	t	t	t	t	t	t	t	t	t	t	t	t	a	a	a	a	t	t
a	3645011	g	3717926	a	3632716	nc	APEC	rec27	g	2	-	g	g	g	g	g	a	g	g	g	g	g	g	g	g	g	g	g	g	g	g	g	g	g
g	3645012	c	3717927	g	3632717	nc	APEC	rec27	c	2	-	c	c	c	c	c	g	c	c	c	c	c	c	c	c	c	c	c	c	c	c	c	c	c
t	3645286	c	3718201	t	3632991	ns	APEC	rec27	c	2	-	c	c	c	c	c	c	c	c	c	c	c	c	c	c	c	c	c	c	c	c	c	c	c
t	3646652	c	3719567	t	3634357	nc	APEC	rec27	c	2	-	c	c	t	t	t	t	t	t	t	t	t	t	t	t	t	t	t	t	t	t	t	t	t
t	3646711	g	3719626	t	3634416	nc	APEC	rec27	t	APEC	++++	t	t	t	t	t	t	t	t	t	t	t	t	t	t	t	t	t	t	t	t	t	t	t
g	3646712	c	3719627	g	3634417	nc	APEC	rec27	g	APEC	++++	g	g	g	g	g	g	g	g	g	g	g	g	g	g	g	g	g	g	g	g	g	g	g
a	3646715	c	3719630	a	3634420	nc	APEC	rec27	a	APEC	++++	a	a	a	a	a	a	a	a	a	a	a	a	a	a	a	a	a	a	a	a	a	a	a
c	3646716	t	3719631	c	3634421	nc	APEC	rec27	c	APEC	++++	c	c	c	c	c	c	c	c	c	c	c	c	c	c	c	c	c	c	c	c	c	c	c
g	3646717	t	3719632	g	3634422	nc	APEC	rec27	g	APEC	++++	g	g	c	g	g	g	g	g	g	g	g	g	g	g	g	g	g	g	g	g	g	g	g
a	3646726	t	3719641	a	3634431	nc	APEC	rec27	a	APEC	++++	a	a	a	a	a	a	a	a	a	a	a	a	a	a	a	a	a	a	a	a	a	a	a
g	3646727	t	3719642	g	3634432	nc	APEC	rec27	g	APEC	++++	g	g	g	g	g	g	g	g	g	g	g	g	g	g	g	g	g	g	g	g	g	g	g
a	3695078	t	4531791	a	4446325	nc	APEC	rec28	t	2	-	a	t	t	t	a	a	a	a	a	a	a	a	a	a	a	a	a	a	a	a	a	a	a
c	3695079	g	4531790	c	4446324	nc	APEC	rec28	g	2	-	c	g	g	g	t	t	c	c	c	c	t	c	c	y	c	c	t	c	t	t	t	t	t
g	3695088	c	4531781	g	4446315	nc	APEC	rec28	c	2	-	g	c	c	c	a	a	g	g	g	g	a	g	g	r	g	g	a	g	g	g	g	g	a
c	3695089	a	4531780	c	4446314	nc	APEC	rec28	t	APEC	+/-	t	g	g	g	t	t	t	t	t	t	t	t	t	t	t	t	t	t	t	t	t	t	t
a	3695097	g	4531772	a	4446306	nc	APEC	rec28	c	APEC	+/-	c	t	t	t	c	t	c	c	t	c	t	t	t	t	c	t	t	c	t	t	t	t	t
a	3695130	g	4531739	a	4446273	nc	APEC	rec28	c	APEC	+/-	c	t	t	t	t	t	c	c	c	c	t	t	t	t	c	c	t	c	c	c	c	c	t
t	3695136	c	4531732	t	4446267	nc	APEC	rec28	g	APEC	+/-	g	a	a	a	a	a	g	g	g	g	a	a	a	a	g	g	a	g	g	g	g	g	a
a	3695760	t	4531108	a	4445643	nc	APEC	rec28	a	APEC	++	a	a	a	a	t	a	a	a	t	a	t	t	t	t	a	a	a	a	a	a	a	a	a
t	3695966	g	4530902	t	4445437	ns	APEC	rec28	c	APEC	+/-	c	a	a	a	a	a	a	a	a	a	a	a	a	a	a	a	a	a	a	a	a	a	a

ExPEC genome site details												Outgroup Strains Details[i]																						
UTI89[b]	UTI89 site[c]	APEC 01[b]	APEC 01 site[c]	S88[b]	S88 site[c]	type[d]	Event Lineage[ef]	Recombinant segment	Inferred ancestral base[g]	Outgroup Analysis[e]	Support level[h]	CFT073	ED1a	536	E2348/69	SMS 3-5	IAI39	UMN026	K-12	ATCC 8739	HS	D1 Sd197	CB9615	Sakai	EDL933	IAI1	E24377A	SE11	SS Ss046	F2a 301	F2a 2457T	F5 8401	B4 Sb227	B18 BS512
g	3695990	a	4530878	g	4445413	ns	APEC	rec28	t	APEC	+/-	t	c	c	c	c	t	c	c	c	c	c	c	c	c	c	t	t	c	t	t	t	t	t
c	3696223	t	4530645	c	4445180	nc	APEC	rec28	g	APEC	+/-	a	g	a	g	a	g	a	a	a	a	a	a	a	a	a	a	a	a	a	a	a	a	a
g	3696328	a	4530540	g	4445075	nc	APEC	rec28	a	2	-	t	c	a	c	a	-	a	t	a	a	-	-	a	a	a	c	c	a	a	a	a	t	t
c	3696375	t	4530493	c	4445028	nc	APEC	rec28	g	APEC	+/-	a	g	a	g	a	a	a	a	g	g	a	a	g	r	g	a	g	a	a	a	a	a	a
t	3697572	c	4529295	t	4443831	nc	APEC	rec28	g	APEC	+/-	g	a	a	a	a	a	a	a	a	a	a	a	a	a	a	a	a	a	a	a	a	a	a
g	3697938	t	4528929	g	4443465	nc	APEC	rec28	c	APEC	+/-	a	a	a	c	c	c	c	c	c	c	c	c	c	c	c	c	c	c	c	c	c	c	c
t	3697992	g	4528875	t	4443411	nc	APEC	rec28	c	APEC	+/-	c	c	c	c	c	c	c	c	c	c	c	c	c	c	c	c	c	c	c	c	c	c	c
c	3698126	t	4528741	c	4443277	nc	APEC	rec28	g	APEC	+/-	a	a	a	g	g	g	g	g	g	a	a	g	g	g	a	g	g	g	g	g	g	g	g
c	3698193	t	4528673	c	4443210	nc	APEC	rec28	g	APEC	+/-	g	g	g	g	g	g	g	g	g	g	g	g	g	g	g	g	g	g	g	g	g	g	g
c	3698214	t	4528652	c	4443189	nc	APEC	rec28	g	APEC	+/-	g	g	g	g	g	g	g	g	g	g	g	g	g	g	g	g	g	g	g	g	g	g	g
c	3698231	t	4528634	c	4443172	nc	APEC	rec28	g	APEC	+/-	g	g	g	g	g	g	g	g	g	g	g	g	g	g	g	g	g	g	g	g	g	g	g
t	3698281	g	4528581	t	4443122	nc	APEC	rec28	a	APEC	+/-	a	a	a	a	a	a	a	a	a	a	a	a	a	a	a	a	a	a	a	a	a	a	a
g	3698323	t	4528539	g	4443080	nc	APEC	rec28	c	APEC	+/-	c	c	c	c	c	c	c	c	c	c	c	c	c	c	c	c	c	c	c	c	c	c	c
g	4202588	a	4235385	g	4150093	nc	APEC	rec29	g	APEC	++	g	g	a	g	a	a	a	a	a	a	a	a	a	a	a	a	a	a	a	a	a	a	a
g	4202623	a	4235420	g	4150128	nc	APEC	rec29	a	2	-	a	t	a	a	a	a	a	a	a	a	a	a	a	a	a	a	a	a	a	a	a	a	a
t	4202812	c	4235609	t	4150317	nc	APEC	rec29	c	2	-	t	c	c	c	t	t	t	t	t	t	t	t	t	t	t	t	t	t	t	t	t	t	t
c	4203289	t	4236086	c	4150794	nc	APEC	rec29	c	APEC	++++	c	c	c	c	c	c	c	c	c	c	c	c	c	c	c	c	c	c	c	c	c	c	c
g	4203290	a	4236087	g	4150795	nc	APEC	rec29	g	APEC	++++	g	g	g	g	g	g	g	g	g	g	g	g	g	g	g	g	g	g	g	g	g	g	g
g	4203534	a	4236331	g	4151039	nc	APEC	rec29	g	APEC	++++	g	g	g	g	g	g	g	g	g	g	g	g	g	g	g	g	g	g	g	g	g	g	g
c	4207364	g	4240161	g	4154869	nc	UTI89	rec30	g	UTI89	++	c	g	g	c	c	c	c	c	c	c	c	c	c	c	c	c	c	c	c	c	c	c	c
t	4207365	a	4240162	a	4154870	nc	UTI89	rec30	a	UTI89	++	t	a	a	c	t	t	t	t	t	c	t	c	c	c	t	t	t	c	c	c	c	t	t
c	4207367	t	4240164	t	4154872	nc	UTI89	rec30	t	UTI89	++	c	t	t	t	c	c	c	c	c	t	c	t	t	t	c	c	c	t	t	t	t	c	c
t	4207369	a	4240166	a	4154874	nc	UTI89	rec30	a	UTI89	++	t	a	a	t	t	t	t	t	t	t	t	t	t	t	t	t	t	t	t	t	t	t	t
a	4207373	t	4240170	t	4154878	nc	UTI89	rec30	t	UTI89	++	a	t	t	g	a	a	a	a	a	g	a	g	g	g	a	a	a	g	g	g	g	a	a
g	4207374	c	4240171	c	4154879	nc	UTI89	rec30	c	UTI89	++	g	c	c	g	g	g	g	g	g	g	g	g	g	g	g	g	g	g	g	g	g	g	g
g	4321772	a	4353787	g	4268339	nc	APEC	rec31	a	2	-	a	g	a	a	a	a	a	a	a	a	a	a	a	a	a	a	a	a	a	a	a	a	a
t	4321774	c	4353789	t	4268341	nc	APEC	rec31	c	2	-	c	c	c	c	c	c	c	c	c	c	c	c	c	c	c	c	c	c	c	c	c	c	c
t	4321806	a	4353821	t	4268373	nc	APEC	rec31	a	2	-	a	t	a	g	a	a	a	a	a	a	a	a	a	a	a	a	a	a	a	a	a	a	a
-	4321806	1	4353822	-	4268373	ins	APEC	rec31	1	2	-	1	-	1	1	1	1	1	1	1	1	1	1	1	1	1	1	1	1	1	1	1	1	1
a	4321841	t	4353857	a	4268408	nc	APEC	rec31	t	2	-	t	a	t	t	t	t	t	t	t	t	t	t	t	t	t	t	t	t	t	t	t	t	t
c	4321842	t	4353858	c	4268409	nc	APEC	rec31	t	2	-	t	c	t	t	t	t	t	t	t	t	t	t	t	t	t	t	t	t	t	t	t	t	t
-	4321842	1	4353859	-	4268409	ins	APEC	rec31	1	2	-	1	-	1	1	1	1	1	1	1	1	1	1	1	1	1	1	1	1	1	1	1	1	1
a	4321933	g	4353950	a	4268500	nc	APEC	rec31	g	2	-	g	a	g	g	a	g	g	g	g	a	g	g	g	a	a	g	g	g	g	g	g	g	g
g	4321936	a	4353953	g	4268503	nc	APEC	rec31	g	APEC	+	a	g	t	g	g	a	g	g	g	a	g	g	g	g	g	g	g	g	g	a	g	t	g
c	4321947	t	4353964	c	4268514	nc	APEC	rec31	c	APEC	+	t	c	a	c	c	t	c	c	c	t	c	c	c	c	c	c	c	c	t	t	t	a	c
t	4321950	c	4353967	t	4268517	nc	APEC	rec31	c	2	-	c	t	c	t	t	c	t	t	t	c	t	t	t	t	t	t	t	c	c	c	c	c	c
-	4322105	1	4354123	-	4268672	ins	APEC	rec31	-	APEC	++++	1	-	-	-	-	-	-	-	-	-	-	-	-	-	-	-	-	-	-	-	-	-	-
a	4322119	g	4354137	a	4268686	nc	APEC	rec31	a	APEC	++++	g	a	a	a	a	a	a	a	a	a	a	a	a	a	a	a	a	a	a	a	a	a	a
c	4322121	a	4354139	c	4268688	nc	APEC	rec31	a	2	-	a	a	a	c	c	c	c	c	c	c	c	c	c	c	c	c	c	c	c	c	c	c	c
t	4323420	a	4355438	t	4269987	nc	APEC	rec31	a	2	-	a	a	a	a	c	c	a	a	c	c	t	c	c	c	a	c	c	c	t	t	t	c	c
t	4323553	c	4355571	t	4270120	nc	APEC	rec31	c	2	-	c	c	c	c	t	c	c	c	c	t	c	c	c	c	c	c	c	t	c		c	c	c
c	4323554	g	4355572	c	4270121	nc	APEC	rec31	c	APEC	++	g	c	c	c	c	c	c	g	g	c	g	g	g	g	g	g	g	c	g		g	g	g
17	4323809	-	4355826	-	4270375	del	AS	rec31	17	AS	++	-	17	17	17	17	-	17	17	17	17	-	17	17	17	17	17	17	17	17	17	17	17	17
g	4323928	a	4355937	g	4270495	nc	APEC	rec31	a	2	-	a	g	a	g	a	a	a	a	a	a	a	a	a	a	a	a	a	a	a	a	a	a	a
g	4324109	c	4356118	g	4270676	ns	APEC	rec31	g	APEC	++	c	g	g	g	g	c	g	c	c	g	c	g	g	g	c	g	c	c	c	c	c	c	c
c	4324161	t	4356170	c	4270728	ns	APEC	rec31	c	APEC	++	t	c	c	c	c	t	c	t	c	c	c	c	c	c	t	c	t	c	t	t	t	t	t
-	4324223	1	4356233	-	4270790	ins	APEC	rec31	-	APEC	++++	1	-	-	-	-	-	-	-	-	-	-	-	-	-	-	-	-	-	-	-	-	-	-
-	4324230	1	4356241	-	4270797	ins	APEC	rec31	-	APEC	++++	1	-	-	-	-	-	-	-	-	-	-	-	-	-	-	-	-	-	-	-	-	-	-
t	4324391	a	4356402	t	4270958	nc	APEC	rec31	a	2	-	a	a	a	a	t	a	t	a	t	t	t	t	t	t	t	t	t	t	t	t	t	a	t
-	4324979	1	4356991	-	4271546	ins	APEC	rec31	-	APEC	++++	1	-	-	-	-	-	-	-	-	-	-	-	-	-	-	-	-	-	-	-	-	-	-
-	4325087	1	4357100	-	4271654	ins	APEC	rec31	-	APEC	++++	1	-	-	-	-	-	-	-	-	-	-	-	-	-	-	-	-	-	-	-	-	-	-
1	4325182	-	4357194	1	4271749	del	APEC	rec31	1	APEC	++++	1	1	1	1	1	1	1	1	1	1	1	1	1	1	1	1	1	1	1	1	1	1	1
g	4325220	t	4357232	g	4271787	nc	APEC	rec31	g	APEC	++++	t	g	g	g	g	g	g	g	g	g	g	g	g	g	g	g	g	g	g	g	g	g	g
g	4325264	a	4357276	g	4271831	nc	APEC	rec31	g	APEC	++++	a	g	g	g	g	g	g	g	g	g	g	g	g	g	g	g	g	g	g	g	g	g	g
c	4325349	t	4357361	c	4271916	nc	APEC	rec31	c	APEC	++	t	c	c	c	t	t	t	t	t	t	t	t	t	t	c	t	t	t	t	t	t	c	c
g	4325391	a	4357403	g	4271958	nc	APEC	rec31	g	APEC	++	a	g	g	g	a	a	a	a	a	a	a	a	a	a	a	a	a	a	a	a	a	a	a
g	4325712	c	4357724	g	4272279	nc	APEC	rec31	g	APEC	++	c	g	g	g	g	g	g	c	g	g	g	g	g	g	g	g	g	g	g	g	g	g	g
t	4325714	c	4357726	t	4272281	nc	APEC	rec31	c	2	-	c	c	t	t	t	t	a	c	t	-	-	a	a	a	a	a	a	a	a	a	a	-	a
c	4325717	g	4357729	c	4272284	nc	APEC	rec31	c	APEC	++	g	c	c	c	c	c	-	g	c	a	a	-	-	-	-	-	-	-	-	-	-	a	-
t	4326982	g	4358995	t	4273549	nc	APEC	rec31	g	2	-	g	t	g	g	t	t	t	t	t	t	t	t	t	t	g	t	t	t	t	t	t	-	-
c	4326983	t	4358996	c	4273550	nc	APEC	rec31	t	2	-	t	c	t	t	c	c	c	c	c	c	c	c	c	c	t	c	c	c	c	c	c	c	c
t	4326986	g	4358999	t	4273553	nc	APEC	rec31	g	2	-	g	t	g	g	a	g	t	g	g	g	g	g	g	g	g	t	t	g	t	t	t	t	t
c	4326987	a	4359000	c	4273554	nc	APEC	rec31	a	2	-	a	c	a	a	a	a	c	a	a	a	a	a	a	a	a	c	c	a	c	c	c	c	c
g	4326990	a	4359003	g	4273557	nc	APEC	rec31	a	2	-	a	g	a	a	g	g	g	g	g	g	g	g	g	g	a	g	g	g	g	g	g	a	a
a	4326991	c	4359004	a	4273558	nc	APEC	rec31	c	2	-	c	a	c	c	a	a	a	a	a	a	a	a	a	a	c	a	a	a	a	a	a	a	a
c	4326995	g	4359008	c	4273562	nc	APEC	rec31	c	APEC	++++	c	c	g	c	c	c	c	c	c	c	c	c	c	c	c	c	c	c	c	c	c	c	c
-	4327001	1	4359015	-	4273568	ins	APEC	rec31	1	2	-	1	-	1	1	-	-	-	-	-	-	-	-	-	-	1	-	-	-	-	-	-	1	1
a	4327004	g	4359018	a	4273571	nc	APEC	rec31	g	2	-	g	a	g	g	g	a	g	a	g	g	g	g	g	g	g	a	a	a	g	g	g	g	g
a	4327009	t	4359023	a	4273576	nc	APEC	rec31	t	2	-	t	c	t	t	t	c	a	c	t	t	t	t	t	t	t	c	c	c	t	t	t	t	t
a	4327012	g	4359026	a	4273579	nc	APEC	rec31	g	2	-	g	a	g	g	a	a	a	a	a	a	a	a	a	a	a	a	a	a	a	a	a	a	a
c	4326638	c	4358650	g	4273205	nc	S88	rec32	g	2	-	c	g	g	c	c	c	c	c	c	c	c	c	c	c	c	c	c	c	c	c	c	c	c
t	4326639	t	4358651	a	4273206	nc	S88	rec32	a	2	-	t	a	a	c	t	t	t	t	t	c	t	c	c	c	t	t	t	c	c	c	c	t	t
c	4326641	c	4358653	t	4273208	nc	S88	rec32	t	2	-	c	t	t	t	c	c	c	c	c	t	c	t	t	t	c	c	c	t	t	t	t	c	c
t	4326643	t	4358655	a	4273210	nc	S88	rec32	a	2	-	t	a	a	t	t	t	t	t	t	t	t	t	t	t	t	t	t	t	t	t	t	t	t
a	4326647	g	4358659	t	4273214	nc	S88	rec32	t	2	-	g	t	t	g	a	a	a	a	a	g	a	g	g	g	a	a	a	g	g	g	g	a	a
g	4326648	g	4358660	c	4273215	nc	S88	rec32	c	2	-	g	c	c	g	g	g	g	g	g	g	g	g	g	g	g	g	g	g	g	g	g	g	g
a	4456320	g	4488334	a	4402984	nc	APEC	rec33	a	APEC	++++	a	a	a	a	a	a	a	a	a	a	a	a	a	a	a	a	a	a	a	a	a	a	a

ExPEC genome site details												Outgroup Strains Details[i]																						
UTI89[b]	UTI89 site[c]	APEC 01[b]	APEC 01 site[c]	S88[b]	S88 site[c]	type[d]	Event Lineage[ef]	Recombinant segment	Inferred ancestral base[g]	Outgroup Analysis[e]	Support level[h]	CFT073	ED1a	536	E2348/69	SMS 3-5	IAI39	UMN026	K-12	ATCC 8739	HS	D1 Sd197	CB9615	Sakai	EDL933	IAI1	E24377A	SE11	SS Ss046	F2a 301	F2a 2457T	F5 8401	B4 Sb227	B18 BS512
-	4456561	1	4488585	1	4403235	ins	AS	rec33	-	AS	++++	-	-	-	-	-	-	-	-	-	-	-	-	-	-	-	-	-	-	-	-	-	-	-
-	4456652	1	4488668	-	4403317	ins	APEC	rec33	-	APEC	++++	-	-	-	-	-	-	-	-	-	-	-	-	-	-	-	-	-	-	-	-	-	-	-
g	4456760	c	4488776	g	4403425	nc	APEC	rec33	g	APEC	++++	g	g	g	g	g	g	g	g	g	g	g	g	g	g	g	g	g	g	g	g	g	g	g
g	4456768	c	4488784	g	4403433	nc	APEC	rec33	g	APEC	++++	g	g	g	g	g	g	g	g	g	g	g	g	g	g	g	g	g	g	g	g	g	g	g
-	4456788	1	4488805	-	4403453	ins	APEC	rec33	-	APEC	++++	-	-	-	-	-	-	-	-	-	-	-	-	-	-	-	-	-	-	-	-	-	-	-
-	4456805	1	4488823	-	4403470	ins	APEC	rec33	-	APEC	++++	-	-	-	-	-	-	-	-	-	-	-	-	-	-	-	-	-	-	-	-	-	-	-
g	4456808	a	4488826	g	4403473	nc	APEC	rec33	g	APEC	++++	g	g	g	g	g	g	g	g	g	g	g	g	g	g	g	g	g	g	g	g	g	g	g
-	4456813	1	4488832	-	4403478	ins	APEC	rec33	-	APEC	++++	-	-	-	-	-	-	-	-	-	-	-	-	-	-	-	-	-	-	-	-	-	-	-
-	4456821	1	4488841	-	4403486	ins	APEC	rec33	-	APEC	++++	-	-	-	-	-	-	-	-	-	-	-	-	-	-	-	-	-	-	-	-	-	-	-
1	4457618	-	4489637	1	4404283	del	APEC	rec33	1	APEC	++++	1	1	1	1	1	1	1	1	1	1	1	1	1	1	1	1	1	1	1	1	1	1	1
-	4457633	1	4489653	-	4404298	ins	APEC	rec33	1	2	-	1	-	1	-	-	-	-	-	-	-	-	-	-	-	-	-	-	-	-	-	-	-	-
a	4457672	g	4489692	a	4404337	s	APEC	rec33	a	APEC	++	t	a	t	a	a	a	a	a	a	a	a	a	a	a	a	a	a	a	a	a	a	a	a
-	4457672	2	4489693	-	4404337	ins	APEC	rec33	2	2	-	2	-	2	-	-	-	-	-	-	-	-	-	-	-	-	-	-	-	-	-	-	-	-
-	4457696	1	4489719	-	4404361	ins	APEC	rec33	1	2	-	1	-	1	-	-	-	-	-	-	-	-	-	-	-	-	-	-	-	-	-	-	-	-
g	4457709	c	4489732	g	4404374	s	APEC	rec33	c	2	-	c	g	c	g	g	g	g	g	g	g	g	g	g	g	g	g	g	g	g	g	g	g	g
-	4457713	1	4489737	-	4404378	ins	APEC	rec33	1	2	-	1	-	1	-	-	-	-	-	-	-	-	-	-	-	-	-	-	-	-	-	-	-	-
-	4457735	2	4489760	-	4404400	ins	APEC	rec33	2	2	-	2	-	2	-	-	-	-	-	-	-	-	-	-	-	-	-	-	-	-	-	-	-	-
-	4457741	1	4489768	-	4404406	ins	APEC	rec33	1	2	-	1	-	1	-	-	-	-	-	-	-	-	-	-	-	-	-	-	-	-	-	-	-	-
1	4457753	-	4489779	1	4404418	del	APEC	rec33	1	APEC	++++	1	1	1	1	1	1	1	1	1	1	1	1	1	1	1	1	1	1	1	1	1	1	1
a	4457762	c	4489788	a	4404427	ns	APEC	rec33	c	2	-	c	a	c	a	a	a	a	a	a	a	a	a	a	a	a	a	a	a	a	a	a	a	a
c	4457929	t	4489955	c	4404594	nc	APEC	rec33	t	2	-	t	a	t	c	a	c	c	t	t	a	a	c	c	c	a	a	c	c	t	t	t	c	c
t	4791161	c	3318053	c	3232837	ns	UTI89/AS	rec34	t	AS	+++	t		t			t	t																
a	4791163	g	3318055	g	3232839	s	UTI89/AS	rec34	a	AS	+++	a		a			a	a																
g	4791179	a	3318071	a	3232855	ns	UTI89/AS	rec34	g	AS	+++	g		g			g	g																
c	4791421	t	3318313	t	3233097	ns	UTI89/AS	rec34	c	AS	+++	c		c			c	c																
a	4791459	g	3318351	g	3233135	s	UTI89/AS	rec34	g	UTI89	+	g		a			g	g																
g	4791663	c	3318555	c	3233339	s	UTI89/AS	rec34	c	UTI89	+++	c		c			c	c																
c	4791756	t	3318648	t	3233432	ns	UTI89/AS	rec34	t	UTI89	+	t		c			t	t																
c	4791765	t	3318657	t	3233441	s	UTI89/AS	rec34	t	UTI89	+	t		c			c	t																
a	4791780	g	3318672	g	3233456	s	UTI89/AS	rec34	g	UTI89	+	g		t			g	g																
a	4791788	g	3318680	g	3233464	s	UTI89/AS	rec34	g	UTI89	+	g		a			g	g																
c	4791821	t	3318713	t	3233497	nc	UTI89/AS	rec34	t	UTI89	+++	t		t			t	t																
t	4795844	c	3322736	c	3237520	s	UTI89	rec35	c	UTI89	+++	c		c			c	c																
a	4795847	g	3322739	g	3237523	s	UTI89	rec35	g	UTI89	+++	g		g			g	g																
t	4795952	c	3322844	c	3237628	s	UTI89	rec35	c	UTI89	+++	c		c			c	c																
t	4795970	c	3322862	c	3237646	s	UTI89	rec35	t	AS	+++	t		t			t	t																
a	4795977	g	3322869	g	3237653	ns	UTI89	rec35	g	UTI89	+++	g		g			g	g																
a	4796117	g	3323009	g	3237793	s	UTI89	rec35	g	UTI89	+++	g		g			g	g																
g	4796156	a	3323048	a	3237832	s	UTI89	rec35	a	UTI89	+++	a		a			a	g																
t	4796177	a	3323069	a	3237853	s	UTI89	rec35	a	UTI89	+	a		t			a	a																
c	4796184	g	3323076	g	3237860	ns	UTI89	rec35	g	UTI89	+++	g		g			g	g																
a	4796198	g	3323090	g	3237874	s	UTI89	rec35	g	UTI89	+	g		a			g	g																
a	4796314	t	3323206	t	3237990	ns	UTI89	rec35	a	AS	+++	a		a			a	a																
g	4796494	a	3323386	a	3238170	nc	UTI89	rec35	a	UTI89	+						a																	
c	4796512	t	3323404	t	3238188	nc	UTI89	rec35	t	UTI89	+						t																	
c	4900894	c	4917123	a	4879115	ns	S88	rec36	c	S88	++			c	c	c	c				c		c	c	c		c		a	a	a			
g	4900898	g	4917127	a	4879119	ns	S88	rec36	g	S88	++			g	g	g	g				g		g	g	g		g		a	a	a			
t	4900923	t	4917152	a	4879144	s	S88	rec36	t	S88	++			t	t	t	t				t		t	t	t		t		a	a	a			
a	4900928	a	4917157	g	4879149	ns	S88	rec36	a	S88	++			a	a	a	a				a		a	a	a		a		g	g	g			
t	4900932	t	4917161	c	4879153	s	S88	rec36	t	S88	++			t	t	t	t				t		t	t	t		t		c	c	c			
a	4900944	a	4917173	c	4879165	s	S88	rec36	a	S88	++			a	c	c	c				c		c	c	c		c		c	c	c			
c	4900968	c	4917197	t	4879189	s	S88	rec36	c	S88	++			c	c	c	c				c		c	c	c		c		t	t	t			
g	4900972	g	4917201	t	4879193	ns	S88	rec36	g	S88	++			g	g	g	g				g		g	g	g		g		t	t	t			
t	4901007	t	4917236	c	4879228	s	S88	rec36	t	S88	++			t	c	c	t				t		c	c	c	t	t		c	c	c	c		
c	4901015	c	4917244	t	4879236	ns	S88	rec36	c	S88	++			c	c	c	c				c		c	c	c	c	c		t	t	t	t		
c	4901019	c	4917248	a	4879240	s	S88	rec36	c	S88	++			c	c	c	c				c		c	c	c	c	c		a	a	a	a		
t	4901023	t	4917252	c	4879244	ns	S88	rec36	t	S88	++			t	t	t	t				t		t	t	t	t	t		c	c	c	c		
t	4901025	t	4917254	c	4879246	s	S88	rec36	t	S88	++++			t	t	t	t				t		t	t	t	t	t		t	t	t	t		
c	4901028	c	4917257	g	4879249	s	S88	rec36	c	S88	++			c	c	c	c				c		c	c	c	c	c		g	g	g	g		
a	4901035	a	4917264	c	4879256	ns	S88	rec36	a	S88	++			a	a	a	a				a		a	a	a	a	a		c	c	c	c		
c	4901036	c	4917265	t	4879257	ns	S88	rec36	c	S88	++			c	c	c	c				c		c	c	c	c	c		t	t	t	t		
t	4901052	t	4917281	c	4879272	s	S88	rec36	t	S88	++			t	t	t	t				t		t	t	t	t	t		c	c	c	c		
c	4901064	c	4917293	t	4879284	s	S88	rec36	c	S88	++			c	c	c	t				t		c	c	c	c	t		t	t	t	t		
c	4901070	c	4917299	t	4879290	ns	S88	rec36	c	S88	++			c	c	c	c				c		c	c	c	c	c		t	t	t	t		
g	4901072	g	4917301	t	4879292	ns	S88	rec36	g	S88	++			g	g	g	g				g		g	g	g	g	g		t	t	t	t		
t	4901092	t	4917321	a	4879312	ns	S88	rec36	t	S88	++			t	t	t	t				t		t	t	t	t	t		a	a	a	a		
g	4901097	g	4917326	a	4879317	s	S88	rec36	g	S88	++			g	a	a	a				a		g	g	g	g	a		a	a	a	a		
c	4901386	c	4917615	t	4879474	s	S88	rec36	c	S88	++	c	c	c	c	c	c	t	t		c		c	c	c	c	c		t	t	t	t	c	
t	4901391	t	4917620	c	4879479	ns	S88	rec36	c	2	-	c	c	t	c	c	c	c	c		c		c	c	c	c	c		c	c	c	c	c	
t	4901404	t	4917633	c	4879492	s	S88	rec36	t	S88	++	t	t	t	c	c	c	c	c		c		c	c	c	c	t		c	c	c	c	c	
c	4901416	c	4917645	a	4879504	ns	S88	rec36	c	S88	++	c	c	c	c	c	c	a	a		c		t	t	t	c	c		c	c	c	c	c	
c	4901418	c	4917647	t	4879506	ns	S88	rec36	c	S88	++	c	c	c	c	c	t	t	t		t		a	a	a	c	c		t	t	t	t	c	
a	4901519	a	4917748	g	4879607	ns	S88	rec36	g	2	-	g	g	a	g	g	g	g	g		g		g	g	g	g	g		g	g	g	g	g	
c	4901616	c	4917845	a	4879704	ns	S88	rec36	c	S88	++	c	c	c	c	c	c	a	a		c		c	c	c	c	c		a	a	a	a	c	
a	4901620	a	4917849	c	4879708	s	S88	rec36	a	S88	++	a	a	a	a	a	a	c	c		a		a	a	a	a	a		c	c	c	c	a	
t	4901680	t	4917909	a	4879768	ns	S88	rec36	t	S88	++	a	t	t	a	a	a	a	a		a		a	a	a	a	a		a	a	a	a	a	
g	4901711	g	4917940	t	4879799	ns	S88	rec36	t	2	-	t	t	g	t	t	t	t	t		t		t	t	t	t	t		t	t	t	t	t	
g	4901725	g	4917954	t	4879813	ns	S88	rec36	g	S88	++	t	g	g	t	t	t	t	t		t		t	t	t	t	t		t	t	t	t	t	
t	4901872	t	4918101	c	4879960	s	S88	rec36	t	S88	++	c	t	t	t	t	t	c	c		c		t	t	t	t	t		c	c	c	c	t	
a	4901886	a	4918115	t	4879974	ns	S88	rec36	a	S88	++	t	a	a	t	a	t	t	t		t		t	t	t	t	t		t	t	t	t	t	
g	4901921	g	4918150	c	4880009	ns	S88	rec36	g	S88	++	c	g	g	c	c	c	c	c		c		c	c	c	c	c		c	c	c	c	c	

ExPEC genome site details												Outgroup Strains Details[i]																						
UTI89[b]	UTI89 site[c]	APEC 01[b]	APEC 01 site[c]	S88[b]	S88 site[c]	type[d]	Event Lineage[ef]	Recombinant segment	Inferred ancestral base[g]	Outgroup Analysis[e]	Support level[h]	CFT073	ED1a	536	E2348/69	SMS 3-5	IAI39	UMN026	K-12	ATCC 8739	HS	D1 Sd197	CB9615	Sakai	EDL933	IAI1	E24377A	SE11	SS Ss046	F2a 301	F2a 2457T	F5 8401	B4 Sb227	B18 BS512
t	4901959	t	4918188	c	4880047	s	S88	rec36	t	S88	++	c	t	t	c	c	c	c	c		c		c	c	c	c	c		c	c	c	c	c	
g	4901986	g	4918215	a	4880074	s	S88	rec36	g	S88	++	g	g	g	g	g	g	a	a		g		g	g	g	g	g		a	a	a	a	g	
c	4901991	c	4918220	t	4880079	ns	S88	rec36	c	S88	++	c	c	c	c	c	t	t	t		c		t	t	t	t	c		t	t	t	t	c	
a	4902010	a	4918239	g	4880098	s	S88	rec36	a	S88	++	a	a	a	a	a	g	g	g		a		g	g	g	g	a		g	g	g	g	a	
t	4902031	t	4918260	a	4880119	s	S88	rec36	t	S88	++	t	t	t	t	a	a	a	a		a		a	a	a	a	a		a	a	a	a	a	
a	4902162	a	4918391	g	4880250	ns	S88	rec36	a	S88	++	a	a	a	a	a	a	g	g		a		a	a	a	a	a		g	g	g	g	a	
t	4902178	t	4918407	c	4880266	s	S88	rec36	t	S88	++	c	t	t	c	c	c	c	c		c		c	c	c	c	c		c	c	c	c	c	
g	4902213	g	4918442	a	4880301	ns	S88	rec36	g	S88	++	g	g	g	g	a	g	g	g		g		g	g	g	g	g		a	a	a	a	g	
t	4902230	t	4918459	c	4880318	ns	S88	rec36	t	S88	++	t	t	t	t	c	c	c	c		c		c	c	c	c	c		c	c	c	c	c	
t	4902244	t	4918473	c	4880332	s	S88	rec36	t	S88	++	t	t	t	t	c	c	c	c		c		c	c	c	c	c		c	c	c	c	c	
c	4902431	c	4918660	t	4880519	s	S88	rec36	c	S88	++	c	c	c	c	t	c	c	c		c		c	c	c	c	a	c	t	t	t	t	c	
g	4902539	g	4918768	a	4880627	s	S88	rec36	g	S88	++	g	g	g	a	a	a	a	a		a		a	a	a	a	a	a	a	a	a	a	a	
c	4902583	c	4918812	t	4880671	ns	S88	rec36	c	S88	++	c	c	c	c	t	t	t	t		t		t	t	t	t	t	t	t	t	t	t	t	
t	4902680	t	4918909	a	4880768	s	S88	rec36	t	S88	++	t	t	t	t	a	a	a	a		a		a	a	a	a	a	a	a	a	a	a	a	
g	4902683	g	4918912	a	4880771	s	S88	rec36	g	S88	++	g	g	g	g	a	a	a	a		a		a	a	a	a	a	a	a	a	a	a	a	
c	4902692	c	4918921	t	4880780	ns	S88	rec36	c	S88	++	c	c	c	c	t	t	t	t		t		t	t	t	t	t	t	t	t	t	t	t	
g	4902695	g	4918924	c	4880783	s	S88	rec36	g	S88	++	g	g	g	g	c	c	c	c		c		c	c	c	c	c	c	c	c	c	c	c	
t	4902698	t	4918927	c	4880786	s	S88	rec36	t	S88	++	t	t	t	t	c	c	c	c		c		c	c	c	c	c	c	c	c	c	c	c	
c	4902730	c	4918959	a	4880818	ns	S88	rec36	c	S88	++	c	c	c	t	a	a	a	a		a		a	a	a	a	a	a	a	a	a	a	a	
g	4902738	g	4918967	t	4880826	ns	S88	rec36	g	S88	++	g	g	g	g	t	g	g	g		g		g	g	g	g	g	g	t	t	t	t	g	
a	4902884	a	4919113	t	4880972	s	S88	rec36	a	S88	++	a	a	a	a	a	c	c	c		a		a	a	a	a	c	a	c	c	c	c	c	
a	4902914	a	4919143	t	4881002	s	S88	rec36	a	S88	++	a	a	a	a	t	t	t	t		t		a	a	a	t	t	t	t	t	t	t	t	
t	4902929	t	4919158	c	4881017	s	S88	rec36	c	2	-	c	c	t	c	c	c	c	c		c		c	c	c	c	c	c	c	c	c	c	c	
t	4903000	t	4919229	c	4881088	ns	S88	rec36	c	2	-	c	c	t	t	c	c	c	c		t		c	c	c	t	c	t	c	c	c	c	c	
t	4903019	t	4919248	c	4881107	s	S88	rec36	t	S88	++	t	t	t	t	c	c	c	c		c		t	t	t	c	t	c	c	c	c	c	c	
g	4903181	g	4919410	a	4881269	s	S88	rec36	g	S88	++	g	g	g	g	g	a	g	g		g		g	g	g	g	a	g	g	g	g	g	g	
c	4903277	c	4919506	t	4881365	s	S88	rec36	c	S88	++	c	c	c	c	c	t	t	t		t		t	t	t	t	t	t	c	c	c	c	t	
c	4903304	c	4919533	t	4881392	s	S88	rec36	t	2	-	t	t	c	t	t	t	t	t		t		t	t	t	t	t	t	a	a	a	a	t	
c	4903367	c	4919596	a	4881455	s	S88	rec36	a	2	-	a	a	c	a	a	a	a	a		a		a	a	a	a	a	a	a	a	a	a	a	
t	4903450	t	4919679	c	4881538	ns	S88	rec36	c	2	-	c	c	t	c	c	t	c	c		c		c	c	c	c	c	c	c	c	c	c	c	
t	4903466	t	4919695	c	4881554	ns	S88	rec36	t	S88	++	t	t	t	t	c	c	c	c		c		t	t	t	c	t	c	c	c	c	c	c	
t	4903474	t	4919703	c	4881562	ns	S88	rec36	c	2	-	c	c	t	t	c	c	c	c		c		c	c	c	c	c	c	c	c	c	c	c	
a	4903614	a	4919843	g	4881702	s	S88	rec36	g	2	-	g	g	g	g	g	g	g	g		g		g	g	g	g	g	g	g	g	g	g	g	
c	4903722	c	4919951	g	4881810	s	S88	rec36	c	S88	++++	c	c	c	c	c	c	c	c		c		c	c	c	c	c	c	c	c	c	c	c	
g	4904180	g	4920409	a	4882268	s	S88	rec36	g	S88	++	g	g	a	g	a	a	a	a		a		a	a	a	a	a	a	a	a	a	a	a	
t	4904236	t	4920465	a	4882324	ns	S88	rec36	t	S88	++	t	t	a	t	a	a	a	a		a		a	a	a	a	a	a	a	a	a	a	a	
g	4904342	g	4920571	c	4882431	nc	S88	rec36	g	S88	++	g	g	g	g	g	g	c	c		c		c	c	c	c	c	c	g	g	g	g	c	
a	4904343	a	4920572	g	4882432	nc	S88	rec36	a	S88	++	a	a	a	a	a	g	g	g		g		g	g	g	g	g	g	g	g	g	g	g	
t	4904357	t	4920586	a	4882446	nc	S88	rec36	t	S88	++	-	-	t	-	-	-	-	t		-		-	-	-	-	t	-	a	a	a	a	a	
a	4904358	a	4920587	t	4882447	nc	S88	rec36	a	S88	++++	a	a	a	a	a	a	a	a		a		a	a	a	a	a	a	-	-	-	-	t	
a	4904360	a	4920589	t	4882449	nc	S88	rec36	a	S88	++	a	a	a	a	a	a	a	a		a		a	a	a	a	g	a	a	a	a	t	t	
g	4904373	g	4920602	t	4882462	nc	S88	rec36	g	S88	++	g	g	g	g	g	g	g	g		t		t	t	t	g	g	g	g	g	g	g	g	
t	4904388	t	4920617	g	4882477	nc	S88	rec36	t	S88	++	t	t	t	t	t	t	a	a		a		a	a	a	a	a	a	t	t	t	t	a	
a	4904420	a	4920649	g	4882509	nc	S88	rec36	a	S88	++	a	a	a	a	g	g	t	t		t		g	g	g	g	g	t	t	t	t	a	t	
c	4904449	c	4920678	a	4882538	nc	S88	rec36	c	S88	++	c	c	c	c	a	c	c	c		c		a	a	a	c	c	c	c	c	c	c	c	
a	4904512	a	4920741	g	4882601	nc	S88	rec36	a	S88	++++	a	a	a	a	a	a	a	a		a		a	a	a	a	a	a	a	a	a	a	a	
a	4904537	a	4920766	g	4882626	nc	S88	rec36	g	2	-	g	g	g	a	g	g	g	g		g		g	g	g	g	g	g	g	g	g	g	g	
g	4904621	g	4920850	a	4882710	nc	S88	rec36	g	S88	++	g	g	g	g	a	g	g	g		g		g	g	g	g	g	g	a	a	a	g	g	
t	4904632	t	4920861	a	4882721	nc	S88	rec36	t	S88	++	t	t	t	t	a	t	t	t		t		t	t	t	t	t	t	a	a	a	t	t	
g	4904645	g	4920874	a	4882734	nc	S88	rec36	a	2	-	a	a	a	a	a	a	a	a		a		a	a	a	a	a	a	a	a	a	a	a	
g	4904648	g	4920877	t	4882737	nc	S88	rec36	g	S88	++	g	g	g	g	g	g	t	t		t		g	g	g	t	t	t	g	g	g	g	t	
c	4904649	c	4920878	t	4882738	nc	S88	rec36	c	S88	++	c	c	t	c	t	t	t	t		t		t	t	t	t	t	t	t	t	t	c	t	
g	4904708	g	4920937	c	4882797	nc	S88	rec36	g	S88	++	g	g	g	g	c	c	c	c		c		c	c	c	c	c	c	c	c	c	g	c	
a	4904787	a	4921016	c	4882876	nc	S88	rec36	a	S88	++	a	a	c	a	c	c	c	c		c		c	c	c	c	c	c	c	c	c	a	c	
g	4904809	g	4921038	a	4882898	nc	S88	rec36	g	S88	++	g	g	g	g	a	a	a	a		-		a	a	a	a	a	-	a	a	a	g	a	
c	4904810	c	4921039	t	4882899	nc	S88	rec36	c	S88	++	c	c	g	a	t	t	t	t		-		t	t	t	t	t	-	t	t	t	a	t	
a	4904811	a	4921040	c	4882900	nc	S88	rec36	a	S88	++	a	t	t	t	t	a	c	c		a		c	c	c	c	c	a	t	t	t	t	c	
t	4904815	t	4921044	g	4882904	nc	S88	rec36	t	S88	++	t	t	t	t	t	t	g	g		t		g	g	g	t	g	t	t	t	t	t	g	
t	4904830	t	4921059	a	4882919	nc	S88	rec36	t	S88	++++	t	t	t	t	t	t	t	t		t		t	t	t	t	t	t	t	t	t	t	t	
g	4904838	g	4921067	a	4882927	nc	S88	rec36	a	2	-	g	a	a	a	a	a	a	a		a		a	a	a	a	a	a	a	a	a	a	a	
g	4904849	g	4921078	a	4882938	nc	S88	rec36	g	S88	++	g	g	g	g	a	a	a	a		a		a	a	a	a	a	a	a	a	a	g	a	
g	4904889	g	4921118	t	4882977	nc	S88	rec36	g	S88	++	g	g	g	g	t	t	t	t		g		t	t	t	t	t	g	g	g	g	g	t	
a	4904900	a	4921129	c	4882988	nc	S88	rec36	a	S88	++	a	a	a	a	a	a	c	c		c		c	c	c	a	c	c	a	a	a	a	c	
t	4904925	t	4921154	g	4883013	nc	S88	rec36	t	S88	++	t	t	t	t	g	g	g	g		g		g	g	g	g	g	g	g	g	g	t	g	
t	4904960	t	4921189	g	4883048	nc	S88	rec36	t	S88	++	t	t	t	t	g	g	g	g		g		g	g	g	g	g	g	g	g	g	g	g	
g	4904971	g	4921200	c	4883059	nc	S88	rec36	g	S88	++	g	g	g	g	c	c	c	c		c		c	c	c	c	c	c	c	c	c	c	c	
c	4904979	c	4921208	t	4883067	nc	S88	rec36	c	S88	++	c	c	c	c	t	t	t	t		t		t	t	t	t	t	t	t	t	t	t	t	
c	4904980	c	4921209	t	4883068	nc	S88	rec36	c	S88	++	c	c	c	c	c	c	t	t		t		t	t	t	c	c	t	c	c	c	t	t	
a	4904997	a	4921226	g	4883085	nc	S88	rec36	a	S88	++	a	a	a	a	g	g	g	g		g		g	g	g	g	g	g	g	g	g		g	
a	4905006	a	4921235	t	4883094	nc	S88	rec36	a	S88	++	a	a	a	a	t	t	t	t		t		t	t	t	t	t	t	t	t	t		t	
g	4905036	g	4921265	c	4883124	s	S88	rec36	g	S88	++	g	g	g	g	c	c	c	c		c		c	c	c	c	c	c	c	c	c		c	
a	4905037	a	4921266	t	4883125	s	S88	rec36	a	S88	++	a	a	a	a	t	t	t	t		t		t	t	t	t	t	t	t	t	t		t	
g	4905041	g	4921270	t	4883129	ns	S88	rec36	t	2	-	t	t	g	t	t	t	t	t		t		t	t	t	t	t	t	t	t	t		t	
c	4905094	c	4921323	t	4883182	ns	S88	rec36	c	S88	++	c	c	c	c	t	t	t	t		t		t	t	t	t	t	t	t	t	t		t	
t	4905111	t	4921340	g	4883199	ns	S88	rec36	t	S88	++++	t	t	t	t	t	t	t	t		t		t	t	t	g	t	t	t	t	t		t	
a	4905121	a	4921350	g	4883209	ns	S88	rec36	a	S88	++	a	a	a	a	g	g	a	a		a		a	a	a	g	a	a	g	g	g		a	
t	4905141	t	4921370	g	4883229	ns	S88	rec36	t	S88	++	t	t	t	t	t	t	t	t		t		t	t	t	g	t	t	g	g	g		t	
g	4905206	g	4921435	c	4883282	ns	S88	rec36	g	S88	++	g	g	g	g	g	c	g	g		g		g	g	g	c	c	g	c	c	c		g	
c	4905243	c	4921472	t	4883319	s	S88	rec36	c	S88	++	c	c	c	c	c	c	c	c		c		c	c	c	c	c	c	t	t	t		c	
c	4905249	c	4921478	a	4883325	ns	S88	rec36	c	S88	++	c	c	c	c	a	a	a	a		a		a	a	a	a	a	a	a	a	a		a	
a	4905289	a	4921518	g	4883365	ns	S88	rec36	a	S88	++++	a	a	a	a	a	a	a	a		a		a	a	a	a	a	a	a	a	a		a	
a	4905346	a	4921575	c	4883422	ns	S88	rec36	c	2	-	c	c	c	c	c	c	c	c		c		c	c	c	c	c	c	c	c	c		c	
a	4905356	a	4921585	g	4883432	ns	S88	rec36	a	S88	++	a	a	a	a	g	g	g	g		g		g	g	g	g	g	g	a	a	a		g	

ExPEC genome site details												Outgroup Strains Details[i]																						
UTI89[b]	UTI89 site[c]	APEC 01[b]	APEC 01 site[c]	S88[b]	S88 site[c]	type[d]	Event Lineage[ef]	Recombinant segment	Inferred ancestral base[g]	Outgroup Analysis[e]	Support level[h]	CFT073	ED1a	536	E2348/69	SMS 3-5	IAI39	UMN026	K-12	ATCC 8739	HS	D1 Sd197	CB9615	Sakai	EDL933	IAI1	E24377A	SE11	SS Ss046	F2a 301	F2a 2457T	F5 8401	B4 Sb227	B18 BS512
c	4905461	c	4921690	a	4883537	nc	S88	rec36	c	S88	++	c	c	c	c	a	a	a	a		a		a	a	a	a	a	a	a	a	a		a	
t	4905470	t	4921699	c	4883546	nc	S88	rec36	t	S88	++++	t	t	t	t	t	t	t	t		t		t	t	t	t	t	t	t	t	t		t	
a	4905472	a	4921701	c	4883548	nc	S88	rec36	a	S88	++	a	a	a	a	a	a	c	c		c		c	c	c	c	c	c	a	a	a		c	
g	4905513	g	4921742	a	4883589	nc	S88	rec36	g	S88	++	g	g	g	g	g	g	g	g		g		a	a	a	g	a	g	g	g	g		a	
a	4905529	a	4921758	c	4883605	nc	S88	rec36	a	S88	++	a	a	a	a	a	c	c	c		c		c	c	c	c	c	c	a	a	a		c	
g	4905540	g	4921769	t	4883616	nc	S88	rec36	g	S88	++	g	g	g	g	g	t	t	t		t		t	t	t	t	t	t	g	g	g		t	
a	4905546	a	4921775	g	4883622	nc	S88	rec36	a	S88	++	a	a	a	a	a	g	g	g		g		g	g	g	g	g	g	a	a	a		g	
c	4905548	c	4921777	t	4883624	nc	S88	rec36	c	S88	++	c	c	c	c	c	t	t	t		t		t	t	t	t	t	t	c	c	c		t	
a	4905604	a	4921833	c	4883680	nc	S88	rec36	c	2	-	c	c	c	c	c	c	c	c		c		c	c	c	c	c	c	c	c	c		c	
t	4905636	t	4921865	a	4883712	nc	S88	rec36	t	S88	++	t	t	t	t	t	a	a	a		a		a	a	a	a		a	a	a	a		a	
t	4905771	t	4922000	a	4883847	s	S88	rec36	a	2	-	t	a	a	a	a	a	a	a		a		a	a	a	a		a	a	a	a		a	
t	4905792	t	4922021	a	4883868	s	S88	rec36	t	S88	++	t	t	t	t	t	a	a	a		a		a	a	a	a		a	a	a	a		a	
a	4905813	a	4922042	t	4883889	s	S88	rec36	a	S88	++	a	a	a	a	t	t	t	t		t		t	t	t	t		t	t	t	t		t	
a	4905816	a	4922045	g	4883892	s	S88	rec36	a	S88	++	a	a	a	a	g	g	g	g		g		g	g	g	g		g	g	g	g		g	
c	4905855	c	4922084	t	4883931	s	S88	rec36	c	S88	++	c	c	c	c	c	t	t	t		t		t	t	t	t		t	t	t	t		t	
a	4905955	a	4922184	g	4884031	ns	S88	rec36	a	S88	++	a	a	a	a	a	g	g	g		g		g	g	g	g		g	g	g	g		g	
t	4905993	t	4922222	g	4884069	s	S88	rec36	t	S88	++	t	t	t	t	t	g	g	g		g		t	t	t	g		g	t	t	t		g	
t	4905996	t	4922225	c	4884072	s	S88	rec36	t	S88	++	t	t	t	t	t	c	c	c		c		t	t	t	c		c	t	t	t		c	
a	4906029	a	4922258	g	4884105	s	S88	rec36	a	S88	++	a	a	a	a	a	g	a	a		t		g	g	g	a		t	a	a	a		g	
a	4906044	a	4922273	g	4884120	s	S88	rec36	a	S88	++	a	a	a	a	a	g	g	g		g		g	g	g	g		g	g	g	g		g	
a	4906197	a	4922426	g	4884273	s	S88	rec36	a	S88	++	a	a	a	a	g	g	g	g		g		g	g	g	g		g	g	g	g		g	
a	4906399	a	4922628	g	4884475	nc	S88	rec36	a	S88	++	a	a	a	a	g	g	g	g		g		a	a	a	g		g	g	g	g		a	
a	4906414	a	4922643	c	4884490	nc	S88	rec36	c	2	-	c	c	c	c	c	c	c	c		c		c	c	c	c		c	c	c	c		c	
g	4906518	g	4922747	a	4884594	nc	S88	rec36	g	S88	++	g	g	g	g	t	t	t	t		t		t	t	t	t		t	t	t	t		t	
c	4906519	c	4922748	t	4884595	nc	S88	rec36	c	S88	++++	c	c	c	c	c	c	c	c		c		c	c	c	c		c	c	c	c		c	
c	4906526	c	4922755	t	4884602	nc	S88	rec36	t	2	-	c	t	t	t	c	t	c	c		c		c	c	c	c		c	c	c	c		t	
t	4906529	t	4922758	c	4884605	nc	S88	rec36	t	S88	++	t	t	t	t	c	c	c	c		c		c	c	c	c		c	c	c	c		c	
g	4906616	g	4922845	a	4884691	nc	S88	rec36	g	S88	++	g	g	g	g	g	g	g	g		g		a	a	a	g		a	g	g	g		g	
g	4906683	g	4922912	t	4884758	nc	S88	rec36	g	S88	++	g	g	g	t	t	t	t	t		t		t	t	t	t		t	t	t	t		t	
g	4906836	g	4923065	t	4884911	s	S88	rec36	g	S88	++	g	g	g	g	t	g	t	t		t		t	t	t	t		t	t	t	t		t	
t	4906935	t	4923164	c	4885010	s	S88	rec36	t	S88	++	t	t	t	t	c	t	c	c		c		t	t	t	t		t	c	c	c		t	
t	4907100	t	4923329	c	4885175	s	S88	rec36	t	S88	++	c	t	t	c	c	c	c	c		c		c	c	c	c		c	c	c	c		c	
t	4907205	t	4923434	c	4885280	s	S88	rec36	t	S88	++	t	t	t	t	c	c	c	c		c		c	c	c	t		t	c	c	c		c	
t	4907220	t	4923449	g	4885295	s	S88	rec36	g	2	-	g	g	g	g	a	g	g	g		g		g	g	g	g		g	g	g	g		g	
c	4907372	c	4923601	t	4885447	ns	S88	rec36	c	S88	++	t	c	c	c	t	t	t	t		t		t	t	t	t		t	t	t	t		t	
g	4907375	g	4923604	a	4885450	s	S88	rec36	g	S88	++++	g	g	g	g	g	g	g	g		a		g	g	g	g		g	g	g	g		g	
a	4907384	a	4923613	t	4885459	nc	S88	rec36	t	2	-	t	t	t	t	t	t	t	t		t		t	t	t	t		t	t	t	t		t	
a	4907392	a	4923621	g	4885467	nc	S88	rec36	a	S88	++++	a	a	a	a	a	a	a	a		a		a	a	a	a		a	a	a	a		a	
t	4907398	t	4923627	g	4885473	nc	S88	rec36	t	S88	++++	t	t	t	t	t	t	t	t		t		t	t	t	t		t	t	t	t		t	
t	4907440	t	4923669	c	4885515	nc	S88	rec36	t	S88	++	t	t	t	t	c	t	c	c		c		c	c	c	c		c	c	c	c		c	
a	4907487	a	4923716	t	4885562	nc	S88	rec36	a	S88	++++	a	a	a	a	a	a	a	a		a		a	a	a	a		a	a	a	a		a	
a	4907520	a	4923749	g	4885595	nc	S88	rec36	g	2	-	g	g	g	g	g	g	g	g		g		g	g	g	g		g	g	g	g		g	
t	4907526	t	4923755	c	4885601	nc	S88	rec36	t	S88	++++	t	t	t	t	t	t	t	t		t		t	t	t	t		t	t	t	t		t	
t	4907564	t	4923793	a	4885639	nc	S88	rec36	t	S88	++++	t	t	t	t	t	t	t	t		t		t	t	t	t		t	t	t	t		t	
a	4907586	a	4923815	g	4885661	nc	S88	rec36	a	S88	++	a	a	a	a	g	a	g	g		g		g	g	g	g		g	g	g	g		g	
g	4907587	g	4923816	t	4885662	nc	S88	rec36	g	S88	++++	g	g	g	g	g	g	g	g		g		g	g	g	g		g	g	g	g		g	
c	4907593	c	4923822	a	4885668	nc	S88	rec36	c	S88	++++	c	c	c	c	t	c	c	c		c		c	c	c	c		c	c	c	c		c	
c	4907622	c	4923851	a	4885697	nc	S88	rec36	c	S88	++++	c	c	c	c	c	c	c	c		c		c	c	c	c		c	c	c	c		c	
a	4907643	a	4923872	t	4885718	nc	S88	rec36	a	S88	++	a	a	a	a	t	a	t	t		t		t	t	t	t		t	t	t	t		t	
g	4907646	g	4923875	a	4885721	nc	S88	rec36	g	S88	++	g	g	g	g	a	g	a	a		a		a	a	a	a		a	a	a	a		a	
c	4907647	c	4923876	t	4885722	nc	S88	rec36	c	S88	++	c	c	c	c	t	c	t	t		t		t	t	t	t		t	t	t	t		t	
t	4907661	t	4923890	a	4885736	nc	S88	rec36	t	S88	++	t	t	t	t	a	t	a	a		a		a	a	a	a		a	a	a	a		a	
c	4907668	c	4923897	t	4885743	nc	S88	rec36	c	S88	++	c	c	c	c	t	c	t	t		t		t	t	t	t		t	t	t	t		t	
t	4907671	t	4923900	c	4885746	nc	S88	rec36	t	S88	++++	t	t	t	t	t	t	t	t		t		t	t	t	t		t	t	t	t		t	
a	4907757	a	4923986	g	4885832	nc	S88	rec36	a	S88	++++	a	a	a	a	g	a	a	a		a		a	a	a	a		a	a	a	a		a	
a	4907793	a	4924022	g	4885868	nc	S88	rec36	a	S88	++++	a	a	a	a	a	a	a	a		a		a	a	a	a		a	a	a	a		a	
t	4907843	t	4924072	g	4885918	nc	S88	rec36	t	S88	++	t	t	t	t	t	t	g	g		t		g	g	g	-		-	t	t	t		t	
c	4907853	c	4924082	t	4885928	nc	S88	rec36	c	S88	++++	c	c	c	c	c	c	c	c		c		c	c	c	c		c	c	c	c		c	
c	4907856	c	4924085	t	4885931	nc	S88	rec36	c	S88	++++	c	c	c	c	c	c	c	c		t		c	c	c	c		c	c	c	c		c	
g	4907874	g	4924103	a	4885949	s	S88	rec36	g	S88	++++	g	g	g	g	g	g	g	g		g		g	g	g	g		g	g	g	g		g	
t	4907928	t	4924157	a	4886003	ns	S88	rec36	t	S88	++++	t	-	t	t	t	t	t	t		a		t	t	t	t		t	t	t	t		-	
a	4908033	a	4924262	g	4886108	s	S88	rec36	a	S88	++	a	a	a	a	a	g	a	a		g		a	a	a	g		g	g	g	g		a	
a	4908036	a	4924265	c	4886111	s	S88	rec36	a	S88	++	a	g	g	a	g	a	a	a		c		a	a	a	c		c	a	a	a		a	
c	4908051	c	4924280	t	4886126	s	S88	rec36	a	S88	+/-	a	a	a	a	a	t	a	a		t		a	a	a	t		t	t	t	t		a	
c	4908053	c	4924282	g	4886128	ns	S88	rec36	c	S88	++	c	c	c	c	c	g	c	c		g		c	c	c	g		g	g	g	g		c	
t	4908055	t	4924284	c	4886130	s	S88	rec36	c	2	-	c	c	c	c	c	c	c	c		c		c	c	c	c		c	c	c	c		c	
g	4908064	g	4924293	a	4886139	ns	S88	rec36	g	S88	++++	g	g	g	g	g	g	g	g		a		g	g	g	g		g	g	g	g		g	
c	4908122	c	4924351	g	4886197	ns	S88	rec36	c	S88	++	a	c	c	a	c	c	a	a		g		a	a	a	c		c	c	c	c		a	
c	4908138	c	4924367	a	4886213	s	S88	rec36	a	2	-	t	a	a	a	t	c	c	c		c		c	c	c	c		c	c	c	c		c	
g	4908159	g	4924388	t	4886234	s	S88	rec36	g	S88	++	a	c	c	g	c	g	g	g		t		g	g	g	g		g	g	g	g		g	
a	4908172	a	4924401	g	4886247	ns	S88	rec36	a	S88	++	a	a	a	g	a	a	g	g		g		g	g	g	a		a	a	a	a		g	
c	4908186	c	4924415	a	4886261	s	S88	rec36	a	2	-	a	a	a	t	a	a	t	t		a		t	t	t	a		a	a	a	a		t	
a	4908195	a	4924424	g	4886270	s	S88	rec36	g	2	-	g	g	g	g	g	g	g	g		g		g	g	g	g		g	g	g	g		g	
c	4908225	c	4924454	t	4886300	s	S88	rec36	c	S88	++	c	c	c	t	a	c	c	c		c		c	c	c	c		c	c	c	c		c	
c	4908231	c	4924460	t	4886306	s	S88	rec36	t	2	-	t	t	t	t	t	c	t	t		t		t	t	t	t		t	c	c	c		t	
t	4908240	t	4924469	a	4886315	s	S88	rec36	t	S88	++	c	t	t	t	c	c	c	c		c		c	c	c	c		c	c	c	c		c	
t	4908255	t	4924484	a	4886330	s	S88	rec36	t	S88	++	g	c	c	t	t	c	t	t		t		t	t	t	t		t	c	c	c		t	
g	4908292	g	4924521	c	4886367	ns	S88	rec36	c	2	-	c	c	c	g	c	c	g	g		g		c	c	c	g		g	c	c	c		c	
t	4908315	t	4924544	c	4886390	s	S88	rec36	c	2	-	c	c	c	t	c	c	c	c		c		t	t	t	c		c	c	c	c		t	
t	4908344	t	4924573	a	4886419	ns	S88	rec36	a	2	-	a	a	a	t	t	a	t	t		t		a	a	a	t		t	a	a	a		a	
c	4908350	c	4924579	t	4886425	ns	S88	rec36	t	2	-	t	t	t	c	c	t	c	c		c		t	t	t	c		c	t	t	t		c	
t	4908354	t	4924583	c	4886429	s	S88	rec36	c	2	-	t	c	c	t	t	c	g	g		t		c	c	c	t		t	c	c	c		t	
c	4908356	c	4924585	a	4886431	ns	S88	rec36	a	2	-	c	a	a	c	c	a	c	c		c		a	a	a	c		c	a	a	a		t	

ExPEC genome site details												Outgroup Strains Details[i]																						
UTI89[b]	UTI89 site[c]	APEC 01[b]	APEC 01 site[c]	S88[b]	S88 site[c]	type[d]	Event Lineage[ef]	Recombinant segment	Inferred ancestral base[g]	Outgroup Analysis[e]	Support level[h]	CFT073	ED1a	536	E2348/69	SMS 3-5	IAI39	UMN026	K-12	ATCC 8739	HS	D1 Sd197	CB9615	Sakai	EDL933	IAI1	E24377A	SE11	SS Ss046	F2a 301	F2a 2457T	F5 8401	B4 Sb227	B18 BS512
t	4908357	t	4924586	g	4886432	ns	S88	rec36	g	2	-	c	g	g	t	c	g	c	c		c		g	g	g	c		c	g	g	g		c	
a	4908366	a	4924595	g	4886441	s	S88	rec36	g	2	-	t	g	g	g	a	g	g	g		g		g	g	g	g		g	g	g	g		t	
g	4908499	g	4924728	a	4886574	s	S88	rec36	g	S88	++	g	g	g	g	a	a	a	a		a		a	a	a	a		a	g	g	g		a	
t	4908547	t	4924776	c	4886622	s	S88	rec36	t	S88	++++	t	t	t	t	c	t	t	t		t		t	t	t	t		t	t	t	t		t	t
t	4908583	t	4924812	c	4886658	s	S88	rec36	c	2	-	c	c	c	c	c	c	c	c		c		c	c	c	c		c	c	c	c		c	c
g	4908599	g	4924828	a	4886674	ns	S88	rec36	g	S88	++	g	g	g	g	g	a	a	a		a		a	a	a	a		a	a	a	a		a	a
t	4908689	t	4924918	g	4886764	ns	S88	rec36	t	S88	++	t	g	t	g	g	g	g	g		g		g	g	g	g		g	g	g	g		g	g
a	4908805	a	4925034	t	4886880	s	S88	rec36	a	S88	++++	a	a	a	a	a	a	a	a		a		a	a	a	a		a	a	a	a		a	a
c	4908877	c	4925106	t	4886952	s	S88	rec36	c	S88	++	t	c	c	c	t	t	t	t		t		t	t	t	t		t	t	t	t		t	t
g	4909136	g	4925365	a	4887211	ns	S88	rec36	a	2	-	g	a	a	g	g	a	a	a		a		a	a	a	a		a	a	a	a		a	a
t	4909153	t	4925382	c	4887228	s	S88	rec36	t	S88	++++	t	t	t	t	t	t	t	t		t		t	t	t	t		t	t	t	t		t	t
a	4909270	a	4925499	g	4887345	s	S88	rec36	a	S88	++	g	a	a	a	g	g	g	g		g		g	g	g	g		g	g	g	g		g	g
c	4909297	c	4925526	t	4887372	s	S88	rec36	c	S88	++	t	c	c	c	t	t	t	t		t		t	t	t	t		t	t	t	t		t	t
g	4909354	g	4925583	a	4887429	s	S88	rec36	g	S88	++	g	g	g	g	a	g	a	a		g		a	a	a	g		g	a	a	a		g	g
t	4909417	t	4925646	c	4887492	s	S88	rec36	t	S88	++	t	t	t	t	c	c	c	c		c		c	c	c	c		t	c	c	c		t	t
c	4909476	c	4925705	t	4887551	ns	S88	rec36	c	S88	++++	c	c	c	c	c	c	c	c		c		c	c	c	c		c	c	c	c		c	c
t	4909507	t	4925736	c	4887582	s	S88	rec36	t	S88	++	t	t	t	t	c	t	c	c		t		t	t	t	t		t	c	c	c		c	c
g	4909582	g	4925811	t	4887657	s	S88	rec36	g	S88	++++	g	g	g	t	g	g	g	g		g		g	g	g	g		g	g	g	g		g	g
t	4909636	t	4925865	c	4887711	s	S88	rec36	t	S88	++	t	t	t	c	c	c	c	c		c		c	c	c	c		c	c	c	c		c	c
g	4909640	g	4925869	a	4887715	ns	S88	rec36	g	S88	++++	g	g	g	g	g	g	g	g		g		g	g	g	g		a	g	g	g		g	g
g	4909679	g	4925908	a	4887754	ns	S88	rec36	g	S88	++	g	g	g	a	a	a	a	a		a		a	a	a	a		a	a	a	a		a	a
t	4909788	t	4926017	a	4887863	nc	S88	rec36	t	S88	++	t	t	t	a	a	a	a	a		a		a	a	a	a		a	a	a	a		a	a
t	4909938	t	4926167	c	4888014	s	S88	rec36	t	S88	++	t	t	t	c	t	t	c	c		t		c	c	c	c		c	c	c	c		c	c
c	4909992	c	4926221	t	4888068	s	S88	rec36	c	S88	++	c	c	c	t	c	c	t	t		c		t	t	t	t		t	t	t	t		t	t
t	4909998	t	4926227	c	4888074	s	S88	rec36	t	S88	++	t	t	t	c	t	t	c	c		t		c	c	c	c		c	c	c	c		c	c
t	4910028	t	4926257	c	4888104	s	S88	rec36	t	S88	++	t	t	t	c	t	t	c	c		t		c	c	c	t		c	c	c	c		c	c
t	4910082	t	4926311	c	4888158	s	S88	rec36	t	S88	++	c	t	t	c	c	c	c	c		c		c	c	c	c		c	c	c	c		c	c
c	4910145	c	4926374	t	4888221	s	S88	rec36	c	S88	++	c	c	c	t	t	t	t	t		t		t	t	t	c		t	t	t	t		t	t
c	4910184	c	4926413	t	4888260	s	S88	rec36	t	2	-	t	t	t	c	t	t	c	c		t		c	c	c	t		t	c	c	c		t	t
c	4910187	c	4926416	a	4888263	s	S88	rec36	c	S88	++++	c	c	c	c	c	c	c	c		c		c	c	c	c		c	c	c	c		c	c
a	4910286	a	4926515	t	4888362	ns	S88	rec36	t	2	-	t	t	a	t	a	t	t	t		t		t	t	t	t		t	t	t	t		t	t
g	4910295	g	4926524	t	4888371	s	S88	rec36	t	2	-	t	t	g	t	g	t	t	t		t		t	t	t	t		t	t	t	t		t	t
c	4910382	c	4926611	t	4888458	s	S88	rec36	c	S88	++++	c	c	c	c	c	c	c	c		c		c	c	c	c		c	c	c	c		c	c
a	4910439	a	4926668	g	4888515	s	S88	rec36	a	S88	++	a	a	g	g	a	g	g	g		g		g	g	g	g		g	g	g	g		g	g
c	4910493	c	4926722	t	4888569	s	S88	rec36	t	2	-	t	t	t	t	t	t	t	t		t		t	t	t	t		t	t	t	t		t	t
t	4910646	t	4926875	c	4888722	s	S88	rec36	t	S88	++	t	t	t	c	t	t	t	t		t		t	t	t	t		t	c	c	c		c	c
a	4910820	a	4927049	g	4888896	s	S88	rec36	a	S88	++	a		a	a	a	a	a	a		a		g	g	g	a		a	a	a	a		a	a
t	4910904	t	4927133	c	4888980	s	S88	rec36	t	S88	++	t		t	t	c	c	c	c		c		c	c	c	c		c	c	c	c		c	c
t	4910913	t	4927142	c	4888989	s	S88	rec36	t	S88	++++	t		t	t	t	t	t	t		t		t	t	t	t		t	t	t	t		t	t
g	4910949	g	4927178	a	4889025	s	S88	rec36	g	S88	++	g		g	a	a	a	a	a		a		a	a	a	a		a	a	a	a		a	a
a	4910964	a	4927193	t	4889040	s	S88	rec36	a	S88	++	a		a	a	t	t	t	t		t		t	t	t	t		t	t	t	t		t	t
a	4911009	a	4927238	g	4889085	s	S88	rec36	a	S88	++	a		a	a	a	g	g	g		g		a	a	a	g		g	g	g	g		g	g
g	4911069	g	4927298	a	4889145	s	S88	rec36	a	2	-	a		a	a	a	a	a	a		a		a	a	a	a		a	a	a	a		a	a
t	4911132	t	4927361	c	4889208	s	S88	rec36	c	2	-	c		t	c	c	c	c	c		c		t	t	t	c		c	c	c	c		c	c
t	4911228	t	4927457	c	4889304	s	S88	rec36	c	2	-	c		c	t	c	c	c	c		c		c	c	c	c		c	c	c	c		c	c
g	4911249	g	4927478	a	4889325	s	S88	rec36	g	S88	++	g		g	g	g	g	a	a		g		g	g	g	g		g	a	a	a		g	g
t	4911255	t	4927484	c	4889331	s	S88	rec36	c	2	-	c		c	c	c	c	c	c		c		c	c	c	c		c	c	c	c		c	c
t	4911294	t	4927523	c	4889370	s	S88	rec36	t	S88	++	t		c	t	c	c	c	c		c		c	c	c	c		c	c	c	c		c	c
g	4911297	g	4927526	a	4889373	s	S88	rec36	g	S88	++	g		a	g	a	a	a	a		a		a	a	a	a		a	a	a	a		a	a
a	4911363	a	4927592	t	4889439	s	S88	rec36	a	S88	++	a		t	t	a	a	a	a		a		a	a	a	a		a	a	a	a		a	a
a	4911537	a	4927766	t	4889613	s	S88	rec36	a	S88	++	a		a	a	t	t	a	a		t		a	a	a	a		t	a	a	a		a	a
g	4911549	g	4927778	a	4889625	s	S88	rec36	g	S88	++	g		g	g	a	a	g	g		a		g	g	g	g		a	g	g	g		g	g
t	4911560	t	4927789	g	4889636	ns	S88	rec36	t	S88	++++	t		t	t	t	t	t	t		t		t	t	t	t		t	t	t	t		t	t
t	4911561	t	4927790	c	4889637	ns	S88	rec36	t	S88	++	t		t	t	c	c	c	c		c		c	c	c	c		c	c	c	c		c	c
t	4911627	t	4927856	g	4889703	s	S88	rec36	g	2	-	g		g	t	g	g	g	g		g		g	g	g	g	g	g	g	g	g		g	g
a	4911642	a	4927871	t	4889718	s	S88	rec36	t	2	-	t		t	t	t	t	t	t		t		t	t	t	t	t	t	t	t	t		t	t
t	4911654	t	4927883	c	4889730	s	S88	rec36	c	2	-	c		c	c	c	c	c	c		c		c	c	c	c	c	c	c	c	c		c	c
c	4911702	c	4927931	t	4889778	s	S88	rec36	t	2	-	t		t	c	t	c	c	c		c		c	c	c	c	t	c	c	c	c		c	c
c	4911746	c	4927975	t	4889822	ns	S88	rec36	c	S88	++++	c		c	c	c	c	c	c		c		c	c	c	c	t	c	c	c	c		c	c
t	4911807	t	4928036	c	4889883	s	S88	rec36	t	S88	++	t		t	t	c	t	t	t		t		t	t	t	t	c	t	t	t	t		t	t
c	4911819	c	4928048	t	4889895	s	S88	rec36	c	S88	++	c		c	c	t	c	c	c		c		c	c	c	t	t	c	c	c	c		c	c
a	4911825	a	4928054	t	4889901	s	S88	rec36	t	2	-	t		a	a	t	t	a	a		a		t	t	t	t	t	t	t	t	t		t	t
t	4911885	t	4928114	c	4889961	s	S88	rec36	t	S88	++++	t		t	t	t	t	t	t		t		t	t	t	t	c	t	t	t	t		t	t
g	4911943	g	4928172	a	4890019	ns	S88	rec36	g	S88	++++	g		g	g	g	g	g	g		g		g	g	g	g	g	g	g	g	g		g	g
t	4911990	t	4928219	a	4890066	ns	S88	rec36	t	S88	++	t		t	t	t	a	t	t		a		t	t	t	t	t	a	t	t	t		t	t
g	4911999	g	4928228	a	4890075	s	S88	rec36	g	S88	++	g		g	g	g	g	g	g		g		g	g	g	g	g	g	g	g	g		a	a
a	4912047	a	4928276	g	4890123	s	S88	rec36	g	2	-	g		a	a	a	g	g	g		g		g	g	g	g	g	g	g	g	g		g	g
a	4912050	a	4928279	g	4890126	s	S88	rec36	g	2	-	g		a	a	a	g	g	g		g		g	g	g	g	g	g	g	g	g		g	g
g	4912068	g	4928297	t	4890144	s	S88	rec36	g	S88	++	c		g	g	g	t	t	t		t		c	c	c	t	c	t	t	t	t		c	c
t	4912161	t	4928390	c	4890237	s	S88	rec36	c	2	-	c		t	t	c	c	c	c		c		c	c	c	c	c	c	c	c	c		c	c
a	4912233	a	4928462	g	4890309	s	S88	rec36	a	S88	++++	a		a	a	a	a	a	a		a		a	a	a	a	a	a	a	a	a		a	a
g	4912371	g	4928600	t	4890447	s	S88	rec36	g	S88	++	g		g	t	t	t	t	t		t		t	t	t	t	t	t	t	t	t		t	t
t	4912515	t	4928744	g	4890591	s	S88	rec36	t	S88	++	t		t	t	t	g	g	g		g		g	g	g	g	g	g	g	g	g		g	g
t	4912596	t	4928825	c	4890672	s	S88	rec36	t	S88	++	t		t	t	t	c	c	c		c		c	c	c	c	c	c	c	c	c		c	c
t	4912692	t	4928921	c	4890768	s	S88	rec36	c	2	-	c		c	c	c	t	t	t		t		c	c	c	c	c	c	c	c	c		c	c
c	4912728	c	4928957	t	4890804	s	S88	rec36	c	S88	++	c		c	c	c	t	t	t		t		c	c	c	c	t	c	t	t	t		c	c
g	4912746	g	4928975	a	4890822	s	S88	rec36	g	S88	++	g		g	g	g	a	g	g		a		a	a	a	g	a	g	a	a	a		g	g
c	4912761	c	4928990	t	4890837	s	S88	rec36	c	S88	++++	c		c	c	c	c	c	c		c		c	c	c	c	t	c	c	c	c		c	c
g	4912851	g	4929080	a	4890927	s	S88	rec36	g	S88	++++	g		g	g	g	g	g	g		g		g	g	g	g	a	g	g	g	g		g	g
g	4912858	g	4929087	c	4890934	ns	S88	rec36	g	S88	++	g		g	g	g	c	c	c		c		c	c	c	c	c	c	c	c	c		c	c
t	4912927	t	4929156	c	4891003	s	S88	rec36	t	S88	++	t		t	t	t	c	c	c		c		t	t	t	t	c	c	c	c	c		t	t
t	4912983	t	4929212	c	4891059	s	S88	rec36	c	2	-	c		c	c	t	c	c	c		c		c	c	c	c	c	c	c	c	c		c	c
t	4913056	t	4929285	g	4891132	ns	S88	rec36	t	S88	++	t		g	t	t	g	g	g		g		t	t	t	t	g	t	g	g	g		t	t
g	4913070	g	4929299	a	4891146	ns	S88	rec36	g	S88	++	g		g	g	g	a	a	a	a	a		a	a	a	a	a	a	g	g	g		a	a

ExPEC genome site details												Outgroup Strains Details[i]																						
UTI89[b]	UTI89 site[c]	APEC 01[b]	APEC 01 site[c]	S88[b]	S88 site[c]	type[d]	Event Lineage[ef]	Recombinant segment	Inferred ancestral base[g]	Outgroup Analysis[e]	Support level[h]	CFT073	ED1a	536	E2348/69	SMS 3-5	IAI39	UMN026	K-12	ATCC 8739	HS	D1 Sd197	CB9615	Sakai	EDL933	IAI1	E24377A	SE11	SS Ss046	F2a 301	F2a 2457T	F5 8401	B4 Sb227	B18 BS512
t	4913077	t	4929306	c	4891153	ns	S88	rec36	t	S88	++	t		t	t	t	c	c	c	c	c		c	c	c	c	c	c	c	c	c		c	c
a	4913151	a	4929380	g	4891227	s	S88	rec36	g	2	-	g		g	g	a	a	g	g	a	a		a	a	a	g	g	a	g	g	g		a	a
t	4913205	t	4929434	g	4891281	s	S88	rec36	g	2	-	g		c	g	c	g	t	t	t	g		g	g	g	g	g	t	t	t	t		g	g
t	4913331	t	4929560	c	4891407	s	S88	rec36	t	S88	++++	t		c	c	c	c	c	c	c	c		c	c	c	c	c	c	c	c	c		c	c
c	4913393	c	4929622	g	4891469	ns	S88	rec36	c	S88	++	c		c	c	c	c	c	c	a	c		c	c	c	c	g	c	c	c	c		c	c
c	4913469	c	4929698	g	4891545	s	S88	rec36	c	S88	++	c		g	g	g	a	a	a	a	a		g	g	g	a	g	a	g	g	g		g	g
t	4913478	t	4929707	c	4891554	s	S88	rec36	t	S88	++++	t		c	c	c	c	c	c	c	c		c	c	c	c	c	c	c	c	c		c	c
c	4913537	c	4929766	g	4891613	nc	S88	rec36	c	S88	++++	c		c	c	c	c	c	c	c	c		c	c	c	c	g	c	c	c	c		c	c
a	4913540	a	4929769	g	4891616	nc	S88	rec36	a	S88	++	a		g	a	a	g	g	g	g	g		g	g	g	g	g	g	g	g	g		g	g
c	4913571	c	4929800	a	4891647	ns	S88	rec36	c	S88	++++	c		c	c	c	c	c	c	c	c		c	c	c	c	a	c	c	c	c		c	c
g	4913582	g	4929811	a	4891658	ns	S88	rec36	g	S88	++++	g		g	g	g	g	g	g	g	g		g	g	g	g	g	g	g	g	g		g	g
t	4913641	t	4929870	c	4891717	s	S88	rec36	t	S88	++++	t	t	t	t	t	t	t	t	t	t		t	t	t	t	c	t	t	t	t		t	t
a	4913647	a	4929876	t	4891723	s	S88	rec36	a	S88	++	a	a	t	a	a	t	t	t	t	t		t	t	t	t	t	t	t	t	t		t	t
t	4913695	t	4929924	c	4891771	s	S88	rec36	t	S88	++	t	t	t	t	t	t	c	c	t	t		t	t	t	t	c	t	t	t	t		t	t
a	4913725	a	4929954	g	4891801	s	S88	rec36	g	2	-	a	g	g	g	a	a	g	g	a	a		a	a	a	a	g	a	a	a	a		a	a
a	4913764	a	4929993	g	4891840	s	S88	rec36	g	2	-	a	g	g	g	g	g	g	g	g	g		g	g	g	g	g	g	g	g	g		g	g
c	4913779	c	4930008	t	4891855	s	S88	rec36	c	S88	++	c	c	c	t	c	c	c	c	c	c		c	c	c	c	t	c	c	c	c		c	c
t	4913800	t	4930029	c	4891876	s	S88	rec36	t	S88	++	t	t	t	c	t	c	c	c	c	c		c	c	c	c	c	c	c	c	c		c	c
g	4913826	g	4930055	a	4891902	ns	S88	rec36	g	S88	++	g	g	g	a	a	a	a	a	a	a		a	a	a	a	a	a	a	a	a		a	a
a	4913850	a	4930079	g	4891926	ns	S88	rec36	a	S88	++	a	a	a	g	g	g	g	g	g	g		g	g	g	g	g	g	g	g	g		g	g
t	4913866	t	4930095	a	4891942	s	S88	rec36	t	S88	++	t	t	t	a	a	a	a	a	a	a		a	a	a	a	a	a	a	a	a		a	a
c	4913869	c	4930098	t	4891945	s	S88	rec36	c	S88	++	c	c	c	t	t	t	t	t	t	t		t	t	t	t	t	t	t	t	t		t	t
t	4913872	t	4930101	g	4891948	s	S88	rec36	t	S88	++	t	t	t	t	g	g	t	t	g	g		g	g	g	g	g	g	g	g	g		g	g
t	4913875	t	4930104	c	4891951	s	S88	rec36	t	S88	++	t	t	t	c	c	c	c	c	c	c		c	c	c	c	c	c	c	c	c		c	c
c	4913881	c	4930110	t	4891957	s	S88	rec36	c	S88	++	c	c	c	t	t	c	c	c	c	c		c	c	c	c	t	c	c	c	c		c	c
g	4913950	g	4930179	t	4892026	s	S88	rec36	t	2	-	g	t	t	t	t	t	t	t	t	t		t	t	t	t	t	t	t	t	t		t	t
g	4913965	g	4930194	c	4892041	s	S88	rec36	g	S88	++	g	g	g	c	c	c	c	c	c	c		c	c	c	c	c	c	c	c	c		c	c
a	4913968	a	4930197	g	4892044	s	S88	rec36	a	S88	++	a	a	a	g	g	g	g	g	g	g		g	g	g	g	g	g	g	g	g		g	g
c	4913973	c	4930202	t	4892049	ns	S88	rec36	c	S88	++++	c	c	c	c	c	c	c	c	c	c		c	c	c	c	t	c	c	c	c		c	c
c	4914268	c	4930497	t	4892344	s	S88	rec36	c	S88	++	c	c	c	c	c	t	t	t	a	t		a	a	a	a	t	a	a	a	a		a	a
g	4914271	g	4930500	a	4892347	s	S88	rec36	g	S88	++	g	g	g	g	g	a	a	a	g	a		g	g	g	g	a	g	g	g	g		g	a
g	4914805	g	4931034	a	4894008	nc	S88	rec36	g	S88	++	g	g	g	a	a	g	a	a	g	a		a	a	a	g	a	g	g	g	g	g	a	a
c	4914961	c	4931190	t	4894164	nc	S88	rec36	c	S88	++	c	c	c	c	c	c	c	c	c	c		c	c	c	t	t	c	c	c	c	c	t	t
t	4915024	t	4931253	c	4894227	nc	S88	rec36	t	S88	++	c	t	t	t	t	c	c	c	c	c		c	c	c	c	c	c	c	c	c	c	c	c
g	4915134	g	4931363	a	4894337	nc	S88	rec36	g	S88	++	g	g	g	g	g	a	a	a	a	a		a	a	a	a	a	a	a	a	a	g	a	a
c	4915138	c	4931367	t	4894341	nc	S88	rec36	t	2	-	t	t	t	t	c	t	t	t	t	t		t	t	t	t	t	t	t	t	t	c	t	t
a	4915142	a	4931371	g	4894345	nc	S88	rec36	g	2	-	g	g	g	g	a	g	g	g	g	g		g	g	g	g	g	g	g	g	g	a	g	g
a	4915257	a	4931486	g	4894460	s	S88	rec36	a	S88	++++	a	a	a	a	a	a	a	a	a	a		a	a	a	a	g	a	a	a	a	a	a	a
g	4915275	g	4931504	t	4894478	s	S88	rec36	g	S88	++++	g	g	g	g	g	g	g	g	g	g		g	g	g	g	t	g	g	g	g	g	g	g
c	4915276	c	4931505	t	4894479	s	S88	rec36	c	S88	++++	c	c	c	c	c	c	c	c	c	c		c	c	c	c	t	c	c	c	c	c	c	c
c	4915296	c	4931525	t	4894499	s	S88	rec36	c	S88	++++	c	c	c	c	c	c	c	c	c	c		c	c	c	c	t	c	c	c	c	c	c	c
g	4915335	g	4931564	t	4894538	s	S88	rec36	g	S88	++++	g	g	g	g	g	g	g	g	g	g		g	g	g	g	t	g	g	g	g	g	g	g
c	4915347	c	4931576	t	4894550	s	S88	rec36	c	S88	++++	c	c	c	c	c	c	c	c	c	c		c	c	c	c	t	c	c	c	c	c	c	c
t	4915371	t	4931600	a	4894574	s	S88	rec36	a	2	-	a	a	a	a	t	a	a	a	a	a		a	a	a	a	a	a	a	a	a	a	a	a
a	4915380	a	4931609	g	4894583	s	S88	rec36	a	S88	++	a	g	a	g	a	a	g	g	a	g		a	a	a	a	g	a	a	a	a	g	a	a
t	4915431	t	4931660	c	4894634	s	S88	rec36	t	S88	++	t	c	t	c	t	c	c	c	c	c		c	c	c	c	c	c	c	c	c	c	c	c
c	4915449	c	4931678	t	4894652	s	S88	rec36	c	S88	++	c	t	c	t	c	t	t	t	t	t		t	t	t	t	t	t	t	t	t	t	t	t
c	4915473	c	4931702	t	4894676	s	S88	rec36	c	S88	++++	c	c	c	c	c	c	c	c	c	c		c	c	c	c	t	c	c	c	c	c	c	c
g	4915611	g	4931840	a	4894814	s	S88	rec36	g	S88	++	g	g	g	g	g	a	a	a	a	a		a	a	a	a	a	a	a	a	a	g	a	a
t	4915614	t	4931843	c	4894817	s	S88	rec36	t	S88	++	t	t	t	t	t	t	c	c	t	t		t	t	t	t	c	t	t	t	t	t	t	t
c	4915620	c	4931849	t	4894823	s	S88	rec36	c	S88	++++	c	c	c	c	c	c	c	c	c	c		c	c	c	c	t	c	c	c	c	c	c	c
c	4915659	c	4931888	a	4894862	s	S88	rec36	c	S88	++	c	a	c	c	c	t	a	a	t	c		a	a	a	t	a	t	a	a	a	c	a	a
a	4915731	a	4931960	g	4894934	s	S88	rec36	a	S88	++	a	g	a	a	a	g	g	g	g	g		a	a	a	g	g	g	g	g	g	g	g	g
t	4915734	t	4931963	c	4894937	s	S88	rec36	t	S88	++	t	c	t	t	t	c	c	c	c	c		t	t	t	c	c	c	c	c	c	c	c	c
t	4915749	t	4931978	c	4894952	s	S88	rec36	t	S88	++	t	t	t	t	t	c	c	c	t	t		c	c	c	c	c	c	t	t	t	t	t	t
a	4915782	a	4932011	g	4894985	s	S88	rec36	g	2	-	g	g	g	a	g	g	g	g	g	g		g	g	g	g	g	g	g	g	g	g	g	g
c	4915821	c	4932050	t	4895024	s	S88	rec36	t	2	-	t	t	t	t	t	t	t	t	t	t		t	t	t	t	t	t	t	t	t	t	t	t
a	4915836	a	4932065	g	4895039	s	S88	rec36	a	S88	++++	a	a	a	a	a	a	a	a	a	a		a	a	a	a	g	a	a	a	a	a	a	a
t	4915842	t	4932071	a	4895045	s	S88	rec36	t	S88	++++	t	t	t	t	t	t	t	t	t	t		t	t	t	t	t	t	t	t	t	t	t	t
c	4915869	c	4932098	g	4895072	s	S88	rec36	g	2	-	g	c	g	g	g	g	g	g	g	g		c	c	c	g	g	g	g	g	g	c	g	g
g	4915878	g	4932107	t	4895081	s	S88	rec36	g	S88	++	g	g	g	g	g	g	t	t	g	g		t	t	t	t	t	t	g	g	g	t	g	g
g	4915902	g	4932131	c	4895105	s	S88	rec36	c	2	-	c	t	c	c	c	c	c	c	c	c		c	c	c	c	c	c	c	c	c	c	c	c
g	4915905	g	4932134	a	4895108	s	S88	rec36	g	S88	++	g	g	g	g	a	g	g	g	g	g		g	g	g	g	a	g	g	g	g	g	g	g
c	4915932	c	4932161	t	4895135	s	S88	rec36	c	S88	++	c	c	c	c	t	c	c	c	c	c		c	c	c	c	t	c	c	c	c	c	c	c
c	4915971	c	4932200	t	4895174	s	S88	rec36	c	S88	++++	c	c	c	c	c	c	c	c	c	c		c	c	c	c	t	c	c	c	c	c	c	c
t	4916280	t	4932509	c	4895483	s	S88	rec36	c	2	-	t	c	c	t	c	c	c	c	c	c		c	c	c	c	c	c	c	c	c	c	c	c
t	4916283	t	4932512	a	4895486	s	S88	rec36	a	2	-	t	a	a	t	a	a	a	a	a	a		t	t	t	a	a	a	t	t	t	a	a	a
c	4916373	c	4932602	t	4895576	nc	S88	rec36	t	2	-	c	t	t	c	t	t	t	t		t		c	c	c	c	t	t	c	c	c	c	t	t
c	4916555	c	4932784	t	4895758	s	S88	rec36	c	S88	++	c	c	c	c	c	c	c	c		c		t	t	t	c	c	c	c	c	c	c	c	c
t	4916573	t	4932802	c	4895776	s	S88	rec36	t	S88	++	t	t	t	t	c	t	t	t		t		c	c	c	t	t	t	t	t	t	c	t	t
t	4916594	t	4932823	c	4895797	s	S88	rec36	t	S88	++	t	t	t	t	c	c	c	c		c		c	c	c	c	t	c	c	c	c	t	c	c
t	4916756	t	4932985	c	4895959	s	S88	rec36	t	S88	++	t	t	t	t	c	t	t	t		c		c	c	c	t	t	t	t	t	t	t	t	t
t	4916771	t	4933000	c	4895974	s	S88	rec36	t	S88	++	t	t	t	c	t	c	c	c		c		c	c	c	c	c	c	c	c	c	t	c	c
g	4916807	g	4933036	a	4896010	s	S88	rec36	g	S88	++	g	g	g	g	g	a	a	a		a		a	a	a	a	g	a	a	a	a	g	a	a
g	4916870	g	4933099	a	4896073	s	S88	rec36	g	S88	++	g	g	g	g	g	a	a	a		a		a	a	a	a	g	a	a	a	a	g	a	a
c	4916906	c	4933135	g	4896109	s	S88	rec36	g	2	-	g	c	g	c	g	g	g	g		g		g	g	g	g	g	g	g	g	g	g	a	a
a	4916948	a	4933177	c	4896151	s	S88	rec36	a	S88	++	a	a	a	a	a	c	c	c		c		c	c	c	c	c	c	c	c	c	c	c	c
c	4916955	c	4933184	t	4896158	s	S88	rec36	c	S88	++	c	c	c	c	c	t	t	t		t		t	t	t	t	c	t	t	t	t	t	t	t
c	4917008	c	4933237	t	4896211	s	S88	rec36	t	2	-	t	t	t	t	c	t	t	t		c		t	t	t	t	t	t	t	t	t	c	t	t
t	4917041	t	4933270	c	4896244	s	S88	rec36	t	S88	++	t	t	t	c	c	c	c	c	c	c		c	c	c	c	c	c	c	c	c	c	c	c
c	4917044	c	4933273	g	4896247	s	S88	rec36	c	S88	++	c	c	c	c	g	g	g	g	g	g		g	g	g	g	g	g	g	g	g	g	g	g
t	4917047	t	4933276	a	4896250	s	S88	rec36	t	S88	++	t	t	t	t	t	a	a	a	a	t		a	a	a	a	a	a	a	a	a	t	a	a

ExPEC genome site details												Outgroup Strains Details[i]																						
UTI89[b]	UTI89 site[c]	APEC 01[b]	APEC 01 site[c]	S88[b]	S88 site[c]	type[d]	Event Lineage[ef]	Recombinant segment	Inferred ancestral base[g]	Outgroup Analysis[e]	Support level[h]	CFT073	ED1a	536	E2348/69	SMS 3-5	IAI39	UMN026	K-12	ATCC 8739	HS	D1 Sd197	CB9615	Sakai	EDL933	IAI1	E24377A	SE11	SS Ss046	F2a 301	F2a 2457T	F5 8401	B4 Sb227	B18 BS512
t	4917050	t	4933279	g	4896253	s	S88	rec36	t	S88	++	t	t	t	t	g	g	g	g	g	g		g	g	g	g	g	g	g	g	g	g	g	g
t	4917061	t	4933290	a	4896264	ns	S88	rec36	a	2	-	a	a	a	a	a	a	a	a	a	a		a	a	a	a	a	a	a	a	a	a	a	a
a	4917077	a	4933306	g	4896280	s	S88	rec36	a	S88	++	a	a	a	a	g	g	g	g	g	g		g	g	g	g	g	g	g	g	g	g	g	g
g	4917080	g	4933309	a	4896283	s	S88	rec36	g	S88	++	g	g	g	g	g	g	g	g	g	g		a	a	a	g	g	g	a	a	a	g	g	g
a	4917094	a	4933323	g	4896297	ns	S88	rec36	a	S88	++++	a	a	a	a	a	a	a	a	a	a		a	a	a	a	a	a	a	a	a	a	a	a
a	4917098	a	4933327	g	4896301	s	S88	rec36	a	S88	++	a	a	a	a	g	a	a	a	a	g		g	g	g	g	g	a	g	g	g	g	g	g
t	4917110	t	4933339	c	4896313	s	S88	rec36	c	2	-	c	c	c	c	c	c	c	c	c	c		c	c	c	c	c	c	c	c	c	c	c	c
t	4917185	t	4933414	c	4896388	s	S88	rec36	t	S88	++++	t	t	t	t	t	t	t	t	t	t		t	t	t	t	t	t	t	t	t	t	t	t
a	4917233	a	4933462	g	4896436	s	S88	rec36	a	S88	++	a	a	a	g	g	g	g	g	g	g		t	t	t	g	g	g	g	g	g	g	g	g
c	4917245	c	4933474	t	4896448	s	S88	rec36	c	S88	++++	c	c	c	c	c	c	c	c	c	c		c	c	c	c	c	c	c	c	c	c	c	c
t	4917260	t	4933489	c	4896463	s	S88	rec36	t	S88	++	t	t	t	c	t	c	c	c	c	c		t	t	t	c	t	c	c	c	c	t	c	c
c	4917278	c	4933507	g	4896481	s	S88	rec36	c	S88	++	c	c	c	c	g	g	g	g	g	g		g	g	g	g	g	g	g	g	g	g	g	g
g	4917284	g	4933513	a	4896487	s	S88	rec36	g	S88	++	g	g	g	g	a	a	a	a	a	a		a	a	a	a	a	a	a	a	a	a	a	a
t	4917290	t	4933519	c	4896493	s	S88	rec36	t	S88	++	t	t	t	t	c	c	c	c	c	c		c	c	c	c	c	c	c	c	c	c	c	c
g	4917296	g	4933525	a	4896499	s	S88	rec36	g	S88	++	g	g	g	g	a	a	a	a	a	a		a	a	a	a	a	a	a	a	a	a	a	a
t	4917299	t	4933528	c	4896502	s	S88	rec36	t	S88	++	t	t	t	t	c	c	c	c	c	c		c	c	c	c	c	c	c	c	c	c	c	c
a	4917305	a	4933534	g	4896508	s	S88	rec36	a	S88	++	a	a	a	a	g	g	g	g	g	g		g	g	g	g	g	g	g	g	g	g	g	g
c	4917415	c	4933644	a	4896618	ns	S88	rec36	c	S88	++	c	c	c	c	a	c	c	c	a	a		a	a	a	c	a	c	a	a	a	a	a	a
a	4917563	a	4933792	g	4896766	s	S88	rec36	a	S88	++	a	a	g	a	a	g	g	g	g	g		g	g	g	g	g	g	g	g	g	a	g	g
t	4917632	t	4933861	c	4896835	s	S88	rec36	t	S88	++	t	t	c	t	c	c	c	c	c	c		c	c	c	c	c	c	c	c	c	c	c	c
g	4917662	g	4933891	t	4896865	s	S88	rec36	g	S88	++	g	t	g	g	t	t	t	t	t	t		t	t	t	t	t	t	t	t	t	t	t	t
t	4917719	t	4933948	c	4896922	s	S88	rec36	t	S88	++	t	t	t	t	c	c	c	c	c	c		c	c	c	c	c	c	c	c	c	t	c	c
a	4917729	a	4933958	c	4896932	ns	S88	rec36	c	2	-	a	c	c	c	c	c	c	c	c	c		c	c	c	c	c	c	c	c	c	c	c	c
g	4917734	g	4933963	c	4896937	s	S88	rec36	g	S88	++	g	g	g	g	g	g	g	g	g	c		c	c	c	g	c	g	c	c	c	c	c	c
c	4917749	c	4933978	t	4896952	s	S88	rec36	c	S88	++	c	c	c	c	c	c	c	c	c	t		t	t	t	c	t	c	t	t	t	t	t	t
t	4917767	t	4933996	c	4896970	s	S88	rec36	t	S88	++	t	t	c	t	c	c	c	c	c	c		c	c	c	c	c	c	c	c	c	c	c	c
a	4917785	a	4934014	g	4896988	s	S88	rec36	g	2	-	g	a	g	a	g	g	g	g	g	g		g	g	g	g	g	g	g	g	g	g	g	g
g	4917791	g	4934020	a	4896994	s	S88	rec36	g	S88	++++	g	g	g	g	g	g	g	g	g	g		g	g	g	g	g	g	g	g	g	g	g	g
c	4917806	c	4934035	t	4897009	s	S88	rec36	c	S88	++	c	c	c	c	t	t	t	t	t	t		t	t	t	t	t	t	t	t	t	t	t	t
a	4917827	a	4934056	g	4897030	s	S88	rec36	g	2	-	a	g	g	g	g	g	g	g	g	g		g	g	g	g	g	g	g	g	g	g	g	g
c	4917830	c	4934059	t	4897033	s	S88	rec36	c	S88	++	c	c	c	c	t	t	t	t	t	t		t	t	t	t	t	t	t	t	t	t	t	t
t	4917875	t	4934104	c	4897078	s	S88	rec36	t	S88	++	t	t	t	t	c	c	c	c	c	c		c	c	c	c	c	c	c	c	c	c	c	c
g	4917878	g	4934107	a	4897081	s	S88	rec36	g	S88	++	g	g	g	g	a	a	a	a	a	a		a	a	a	a	a	a	a	a	a	a	a	a
a	4917951	a	4934180	g	4897154	nc	S88	rec36	a	S88	++	a	a	a	a	g	g	g	g	g	g		g	g	g	g	g	g	g	g	g	g	g	g
a	4918001	a	4934230	t	4897204	nc	S88	rec36	a	S88	++	a	a	a	a	t	t	t	t	t	t		t	t	t	t	t	t	t	t	t	t	t	t
c	4918027	c	4934256	a	4897230	nc	S88	rec36	c	S88	++	c	c	c	c		a	a	a	a	a		a	a	a	a	a	a	a	a	a	a	a	a
g	4918033	g	4934262	a	4897236	nc	S88	rec36	g	S88	++++	g	g	g	g		g	g	g	g	g		g	g	g	g	g	g	g	g	g	g	g	g
t	4918036	t	4934265	g	4897239	nc	S88	rec36	t	S88	++	t	t	t	t		g	g	g	g	g		g	g	g	g	t	g	g	g	g	t	g	g
t	4918060	t	4934289	c	4897263	nc	S88	rec36	t	S88	++	t	t	t	t		c	c	c	c	c		c	c	c	c	c	c	c	c	c	c	c	c
a	4918088	a	4934317	c	4897291	nc	S88	rec36	a	S88	++	a	a	a	a		c	c	c	c	c		c	c	c	c	c	c	c	c	c	c	c	c
a	4918227	a	4934456	g	4897430	s	S88	rec36	a	S88	++	a	a	a	a	g	g	g	g	g	g		g	g	g	g	g	g	g	g	g	g	g	g
t	4918323	t	4934552	c	4897526	s	S88	rec36	t	S88	++	t	c	t	t	c	c	c	c	c	c		c	c	c	c	c	c	c	c	c	c	c	c
a	4918332	a	4934561	c	4897535	s	S88	rec36	a	S88	++	a	c	a	a	c	c	c	c	c	c		c	c	c	c	c	c	c	c	c	c	c	c
g	4918369	g	4934598	a	4897572	ns	S88	rec36	g	S88	++	g	a	g	g	a	a	a	a	a	a		a	a	a	a	a	a	a	a	a	a	a	a
c	4918378	c	4934607	t	4897581	ns	S88	rec36	c	S88	++++	c	c	c	c	c	c	c	c	c	c		c	c	c	c	c	c	c	c	c	c	c	c
c	4918395	c	4934624	t	4897598	s	S88	rec36	c	S88	++++	c	c	c	c	c	c	c	c	c	c		c	c	c	c	c	c	c	c	c	c	c	c
c	4918398	c	4934627	t	4897601	s	S88	rec36	c	S88	++	c	c	c	c	t	c	c	c	c	t		t	t	t	c	t	t	t	t	t	t	t	t
c	4918404	c	4934633	g	4897607	s	S88	rec36	c	S88	++	c	c	c	c	g	c	c	c	c	g		g	g	g	c	g	g	g	g	g	c	c	c
g	4918431	g	4934660	t	4897634	s	S88	rec36	g	S88	++	g	g	g	g	t	g	g	g	g	t		t	t	t	g	a	t	t	t	t	t	a	a
c	4918455	c	4934684	g	4897658	s	S88	rec36	c	S88	++	c	c	c	c	g	c	c	c	c	c		c	c	c	c	c	c	c	c	c	g	g	g
g	4918458	g	4934687	a	4897661	s	S88	rec36	g	S88	++	g	g	g	g	a	g	g	g	g	g		g	g	g	g	g	g	g	g	g	a	a	a
c	4918467	c	4934696	t	4897670	s	S88	rec36	c	S88	++	c	c	c	c	t	t	t	t	t	c		c	c	c	t	c	c	a	a	a	t	c	c
c	4918468	c	4934697	t	4897671	s	S88	rec36	c	S88	++	c	c	c	c	t	t	t	t	t	c		c	c	c	t	c	c	c	c	c	t	c	c
c	4918503	c	4934732	t	4897706	s	S88	rec36	c	S88	++	c	c	c	c	t	t	t	t	t	t		t	t	t	t	t	t	t	t	t	t	t	t
a	4918504	a	4934733	c	4897707	ns	S88	rec36	c	2	-	c	c	c	c	c	c	c	c	c	c		c	c	c	c	c	c	c	c	c	c	c	c
c	4918569	c	4934798	t	4897772	s	S88	rec36	c	S88	++	c	c	c	c	c	c	c	c	c	c		c	c	c	t	c	c	c	c	c	t	c	c
c	4918587	c	4934816	t	4897790	s	S88	rec36	t	2	-	t	t	t	t	c	t	t	t	t	t		t	t	t	c	t	t	c	c	c	c	c	c
g	4918800	g	4935029	a	4898003	s	S88	rec36	g	S88	++	g	g	g	g	a	a	a	a	a	a		a	a	a	a	a	a	a	a	a	a	a	a
t	4918803	t	4935032	c	4898006	s	S88	rec36	t	S88	++	t	t	t	t	c	c	c	c	c	c		c	c	c	c	c	c	c	c	c	c	c	c
g	4918806	g	4935035	t	4898009	s	S88	rec36	g	S88	++	g	g	g	g	t	t	t	t	t	t		t	t	t	t	t	t	t	t	t	t	t	t
g	4918812	g	4935041	a	4898015	s	S88	rec36	g	S88	++	g	g	g	g	a	a	a	a	a	a		a	a	a	a	a	a	a	a	a	a	a	a
c	4918815	c	4935044	t	4898018	s	S88	rec36	c	S88	++	c	c	c	c	t	t	t	t	t	t		t	t	t	t	t	t	t	t	t	t	t	t
t	4918816	t	4935045	c	4898019	s	S88	rec36	t	S88	++	t	t	t	t	c	c	c	c	c	c		c	c	c	c	c	c	c	c	c	c	c	c
g	4918824	g	4935053	a	4898027	s	S88	rec36	g	S88	++	g	g	g	g	a	a	a	a	a	a		a	a	a	a	a	a	a	a	a	a	a	a
t	4918830	t	4935059	g	4898033	s	S88	rec36	t	S88	++	t	t	t	t	g	g	g	g	g	g		g	g	g	g	g	g	g	g	g	g	g	g
t	4918833	t	4935062	c	4898036	s	S88	rec36	t	S88	++	t	t	t	t	c	c	c	c	c	c		c	c	c	c	c	c	c	c	c	c	c	c
t	4918839	t	4935068	c	4898042	s	S88	rec36	t	S88	++	t	t	t	t	c	c	c	c	c	c		c	c	c	c	c	c	c	c	c	c	c	c
c	4918842	c	4935071	t	4898045	s	S88	rec36	c	S88	++	c	c	c	c	t	t	t	t	t	t		t	t	t	t	t	t	t	t	t	t	t	t
c	4918845	c	4935074	t	4898048	s	S88	rec36	c	S88	++	c	c	c	c	t	t	t	t	t	t		t	t	t	t	t	t	t	t	t	t	t	t
c	4918851	c	4935080	t	4898054	s	S88	rec36	c	S88	++	c	c	c	c	t	t	t	t	t	t		t	t	t	t	t	t	t	t	t	t	t	t
t	4918854	t	4935083	c	4898057	s	S88	rec36	t	S88	++	t	t	t	t	c	t	t	t	t	c		c	c	c	c	t	c	c	c	c	t	c	c
g	4919328	g	4935557	c	4899760	ns	S88	rec36	g	S88	++	g		g	g	c			c	c									c	c	c		c	c
t	4919329	t	4935558	g	4899761	ns	S88	rec36	t	S88	++++	t		t	t	t			t	t									t	t	t		t	t
a	4919334	a	4935563	g	4899766	ns	S88	rec36	a	S88	++	a		a	a	g			g	g									g	g	g		g	g
g	4919336	g	4935565	t	4899768	ns	S88	rec36	g	S88	++	g		g	g	t			t	t									t	t	t		t	t
a	4919339	a	4935568	t	4899771	s	S88	rec36	a	S88	++	a		a	a	t			t	t									t	t	t		t	t
g	4919350	g	4935579	t	4899782	ns	S88	rec36	g	S88	++	g		g	g	t			t	t									t	t	t		t	t
a	4919352	a	4935581	t	4899784	ns	S88	rec36	a	S88	++	a		a	a	t			t	t									t	t	t		t	t
a	4919357	a	4935586	c	4899789	s	S88	rec36	a	S88	++	a		a	a	c			c	c									c	c	c		c	c
c	4919360	c	4935589	t	4899792	s	S88	rec36	c	S88	++	c		c	c	t			t	t									t	t	t		t	t
t	4919363	t	4935592	a	4899795	ns	S88	rec36	t	S88	++	t		t	t	a			a	a									a	a	a		a	a
t	4919368	t	4935597	c	4899800	ns	S88	rec36	t	S88	++	t		t	t	c			c	c									c	c	c		c	c
g	4919379	g	4935608	a	4899811	ns	S88	rec36	g	S88	++	g		g	g	a			a	a									a	a	a		a	a
t	4919381	t	4935610	a	4899813	ns	S88	rec36	t	S88	++	t		t	t	a			a	a									a	a	a		a	a

ExPEC genome site details												Outgroup Strains Details[i]																						
UTI89[b]	UTI89 site[c]	APEC 01[b]	APEC 01 site[c]	S88[b]	S88 site[c]	type[d]	Event Lineage[ef]	Recombinant segment	Inferred ancestral base[g]	Outgroup Analysis[e]	Support level[h]	CFT073	ED1a	536	E2348/69	SMS 3-5	IAI39	UMN026	K-12	ATCC 8739	HS	D1 Sd197	CB9615	Sakai	EDL933	IAI1	E24377A	SE11	SS Ss046	F2a 301	F2a 2457T	F5 8401	B4 Sb227	B18 BS512
c	4919384	c	4935613	a	4899816	s	S88	rec36	c	S88	++	c		c	c	g			g	g									g	g	g		g	g
g	4919385	g	4935614	a	4899817	ns	S88	rec36	g	S88	++++	g		g	g	g			g	g									g	g	g		g	g
c	4919390	c	4935619	t	4899822	s	S88	rec36	c	S88	++++	c		c	c	c			c	c									c	c	c		c	c
c	4919391	c	4935620	a	4899823	ns	S88	rec36	c	S88	++	c		c	c	a			a	a									a	a	a		a	a
g	4919393	g	4935622	c	4899825	ns	S88	rec36	g	S88	++	g		g	g	c			c	c									c	c	c		c	c
t	4919400	t	4935629	c	4899832	ns	S88	rec36	t	S88	++	t		t	t	c			c	c									c	c	c		c	c
t	4919402	t	4935631	c	4899834	ns	S88	rec36	t	S88	++	t		t	t	c			c	c									c	c	c		c	c
c	4919403	c	4935632	a	4899835	ns	S88	rec36	c	S88	++	c		c	c	a			a	a									a	a	a		a	a
a	4919404	a	4935633	c	4899836	ns	S88	rec36	a	S88	++	a		a	a	c			c	c									c	c	c		c	c
a	4919405	a	4935634	t	4899837	ns	S88	rec36	a	S88	++	a		a	a	a			t	t									t	t	t		t	t
g	4919413	g	4935642	a	4899845	ns	S88	rec36	g	S88	++	g		g	g	a			a	a									a	a	a		a	a
c	4919420	c	4935649	t	4899852	s	S88	rec36	c	S88	++	c		c	c	t			t	t									t	t	t		t	t
a	4919426	a	4935655	g	4899858	s	S88	rec36	a	S88	++	a		a	a	g			g	g									g	g	g		g	g
c	4919427	c	4935656	g	4899859	ns	S88	rec36	c	S88	++	c		c	c	g			g	g									g	g	g		g	g
g	4919429	g	4935658	a	4899861	ns	S88	rec36	g	S88	++	g		g	g	a			a	a									a	a	a		a	a
c	4919430	c	4935659	t	4899862	s	S88	rec36	c	S88	++	c		c	c	t			t	t									t	t	t		t	t
a	4919435	a	4935664	g	4899867	s	S88	rec36	a	S88	++	a		a	a	g			g	g									g	g	g		g	g
a	4919436	a	4935665	t	4899868	ns	S88	rec36	a	S88	++	a		a	a	t			t	t									t	t	t		t	t
t	4919437	t	4935666	c	4899869	ns	S88	rec36	t	S88	++	t		t	t	c			c	c									c	c	c		c	c
t	4919459	t	4935688	c	4899891	s	S88	rec36	t	S88	++	t		t	t	c			c	c									c	c	c		c	c
g	4919470	g	4935699	t	4899902	ns	S88	rec36	g	S88	++	g		g	g	t			t	t									t	t	t		t	t
t	4919477	t	4935706	c	4899909	s	S88	rec36	t	S88	++	t		t	t	c			c	c									c	c	c		c	c
g	4919489	g	4935718	a	4899921	s	S88	rec36	g	S88	++	g		g	g	a			a	a									a	a	a		a	a
t	4919491	t	4935720	a	4899923	ns	S88	rec36	t	S88	++	t		t	t	a			a	a									a	a	a		a	a
t	4919501	t	4935730	c	4899933	s	S88	rec36	t	S88	++	t		t	t	c			c	c									c	c	c		c	c
c	4919513	c	4935742	t	4899945	s	S88	rec36	c	S88	++	c		c	c	t			t	t									t	t	t		t	t
t	4919517	t	4935746	c	4899949	s	S88	rec36	t	S88	++	t		t	t	c			c	c									c	c	c		c	c
a	4919519	a	4935748	g	4899951	s	S88	rec36	a	S88	++	a		a	a	g			g	g									g	g	g		g	g
c	4919525	c	4935754	t	4899957	s	S88	rec36	c	S88	++	c		c	c	t			t	t									t	t	t		t	t
g	4919528	g	4935757	a	4899960	s	S88	rec36	g	S88	++	g		g	g	a			a	a									a	a	a		a	a
t	4919531	t	4935760	g	4899963	s	S88	rec36	t	S88	++	t		t	t	g			g	g									g	g	g		g	g
t	4919549	t	4935778	g	4899981	s	S88	rec36	t	S88	++	t		t	t	g			g	g									g	g	g		g	g
a	4919558	a	4935787	g	4899990	s	S88	rec36	a	S88	++	a		a	a	g			g	g									g	g	g		g	g
c	4919582	c	4935811	g	4900014	s	S88	rec36	c	S88	++	c		c	c	g			g	g									g	g	g		g	g
t	4919585	t	4935814	c	4900017	s	S88	rec36	t	S88	++	t		t	t	c			c	c									c	c	c		c	c
t	4919586	t	4935815	a	4900018	ns	S88	rec36	t	S88	++++	t		t	t	t			t	a									t	t	t		t	t
g	4919592	g	4935821	a	4900024	ns	S88	rec36	g	S88	++	g		g	g	a			a	a									a	a	a		a	a
a	4919593	a	4935822	c	4900025	ns	S88	rec36	a	S88	++	a		a	a	c			c	c									c	c	c		c	c
t	4919595	t	4935824	c	4900027	s	S88	rec36	t	S88	++	t		t	t	c			c	c									c	c	c		c	c
c	4919600	c	4935829	g	4900032	s	S88	rec36	c	S88	++	c		c	c	g			g	g									g	g	g		g	g
t	4919603	t	4935832	c	4900035	s	S88	rec36	t	S88	++	t		t	t	c			c	c									c	c	c		c	c
t	4919606	t	4935835	c	4900038	s	S88	rec36	t	S88	++	t		t	t	c			c	c									c	c	c		c	c
g	4919609	g	4935838	t	4900041	s	S88	rec36	g	S88	++	g		g	g	t			t	t									t	t	t		t	t
g	4919615	g	4935844	a	4900047	ns	S88	rec36	g	S88	++	g		g	g	a			a	a									a	a	a		a	a
t	4919630	t	4935859	c	4900062	s	S88	rec36	t	S88	++	t		t	t	c			c	c									c	c	c		c	c
g	4919636	g	4935865	a	4900068	s	S88	rec36	g	S88	++	g		g	g	a			a	a									a	a	a		a	a
c	4919651	c	4935880	t	4900083	s	S88	rec36	c	S88	++	c		c	c	t			t	t									t	t	t		t	t
c	4940015	c	4956244	t	4900139	s	S88	rec36	t	2	-	t		t	t	c	c	c	c	t	c		c	c	c	c	c	c	c	c	c		c	c
a	4940069	a	4956298	g	4900193	s	S88	rec36	g	2	-	g		g	g	a	g	g	g	g	g		g	g	g	g	g	g	g	g	g		g	g
c	4940081	c	4956310	t	4900205	s	S88	rec36	c	S88	++	c		c	c	c	c	c	c	t	t		c	c	c	t	c	t	c	c	c		a	a
g	4940090	g	4956319	a	4900214	s	S88	rec36	a	2	-	a		a	a	g	a	a	a	a	a		g	g	g	a	a	a	g	g	g		g	g
a	4940126	a	4956355	c	4900250	s	S88	rec36	c	2	-	c		c	c	a	c	c	c	c	c		c	c	c	c	c	c	c	c	c		c	c
c	4940129	c	4956358	t	4900253	s	S88	rec36	t	2	-	t		t	t	c	t	t	t	t	t		c	c	c	t	t	t	c	c	c		c	c
t	4940219	t	4956448	a	4900343	s	S88	rec36	a	2	-	a		a	a	a	a	a	a	a	a		a	a	a	a	a	a	a	a	a		t	t
g	4940264	g	4956493	a	4900388	s	S88	rec36	a	2	-	a		a	a	a	a	a	a	a	a		a	a	a	a	a	a	a	a	a		g	g
c	4940357	c	4956586	a	4900481	s	S88	rec36	a	2	-	a		a	a	a	a	a	a	a	a		a	a	a	a	a	a	a	a	a		a	a
t	4940381	t	4956610	g	4900505	s	S88	rec36	g	2	-	g		g	g	g	g	g	g	g	g		g	g	g	g	g	g	g	g	g		g	g
g	4940390	g	4956619	a	4900514	s	S88	rec36	g	S88	++	g		g	g	g	a	a	a	a	a		a	a	a	a	g	a	a	a	a		g	g
g	4940510	g	4956739	a	4900634	s	S88	rec36	a	2	-	a		g	g	g	g	g	g	g	g		g	g	g	g	g	g	g	g	g		g	g
a	4940603	a	4956832	t	4900727	s	S88	rec36	a	S88	++++	a		t	t	t	t	t	t	t	t		t	t	t	t	t	t	t	t	t		t	t
c	4940651	c	4956880	a	4900775	s	S88	rec36	c	S88	++	c		c	c	c	c	c	c	c	c		a	a	a	c	a	c	a	a	a		a	a
a	4940696	a	4956925	g	4900820	s	S88	rec36	g	2	-	g		g	g	a	g	g	g	g	g		g	g	g	g	g	g	g	g	g		g	g
c	4940774	c	4957003	t	4900898	nc	S88	rec36	c	S88	++	c		c	t	t	c	c	c	c	c		c	c	c	c	c	c	t	t	t		c	c
g	4940777	g	4957006	a	4900901	nc	S88	rec36	a	2	-	a		a	a	a	a	a	a	a	a		g	g	g	a	a	a	a	a	a		g	g
a	4940837	a	4957066	g	4900961	nc	S88	rec36	a	S88	++++	a		a	a	g	a	a	a	a	a		a	a	a	a	a	a	a	a	a		a	a
a	4940855	a	4957084	g	4900979	nc	S88	rec36	g	2	-	g		g	g	g	g	g	g	g	g		g	g	g	g	g	g	a	a	a		g	g
t	4940874	t	4957103	c	4900998	nc	S88	rec36	c	2	-	c		c	c	c	c	c	c	c	c		c	c	c	c	c	c	c	c	c		c	c
a	4940985	a	4957214	t	4901109	s	S88	rec36	t	2	-	t		t	t	a	a	a	a	a	a		a	a	a	a	t	a	a	a	a		a	a
a	4941006	a	4957235	c	4901130	s	S88	rec36	c	2	-	c		c	c	c	c	c	c	c	c		c	c	c	c	c	c	c	c	c		c	c
g	4941201	g	4957430	a	4901325	s	S88	rec36	g	S88	++	g		g	g	g	g	g	g	g	g		a	a	a	g	g	g	g	g	g		g	g
t	4941228	t	4957457	c	4901352	s	S88	rec36	t	S88	++	t		t	t	c	c	c	c	c	c		c	c	c	c	c	c	c	c	c		c	c
t	4941264	t	4957493	c	4901388	s	S88	rec36	t	S88	++	t		t	t	c	c	c	c	c	c		c	c	c	c	c	c	c	c	c		c	c
a	4941312	a	4957541	g	4901436	s	S88	rec36	g	2	-	g		g	g	g	g	g	g	g	g		g	g	g	g	g	g	g	g	g		g	g
c	4941336	c	4957565	g	4901460	s	S88	rec36	c	S88	++++	c		c	c	c	c	c	c	c	c		c	c	c	c	c	c	c	c	c		c	c
g	4941405	g	4957634	a	4901529	s	S88	rec36	g	S88	++	g		g	g	a	g	g	g	a	a		g	g	g	a	g	a	g	g	g		g	g
c	4941418	c	4957647	t	4901542	ns	S88	rec36	c	S88	++++	c		c	c	t	c	c	c	c	c		c	c	c	c	c	c	c	c	c		c	c
g	4941423	g	4957652	a	4901547	s	S88	rec36	g	S88	++++	g		g	g	a	g	g	g	g	g		g	g	g	g	g	g	g	g	g		g	g
c	4941429	c	4957658	a	4901553	s	S88	rec36	c	S88	++++	c		c	c	c	c	c	c	c	c		c	c	c	c	c	c	c	c	c		c	c
a	4941500	a	4957729	t	4901624	ns	S88	rec36	a	S88	++++	a		a	a	a	a	a	a	a	a		a	a	a	a	a	a	a	a	a		a	a
a	4941546	a	4957775	g	4901670	s	S88	rec36	a	S88	++	a		a	a	g	g	g	g	g	g		g	g	g	g	g	g	g	g	g		g	g
a	4941576	a	4957805	g	4901700	s	S88	rec36	a	S88	++++	a		a	a	a	a	a	a	a	a		a	a	a	a	a	a	a	a	a		a	a
g	4941774	g	4958003	a	4901898	s	S88	rec36	g	S88	++++	g		g	g	g	g	g	g	g	g		g	g	g	g	g	g	g	g	g		g	g
g	4941837	g	4958066	a	4901961	s	S88	rec36	g	S88	++++	g		g	g	g	g	g	g	g	g		g	g	g	g	g	g	g	g	g		g	g

ExPEC genome site details												Outgroup Strains Details[i]																						
UTI89[b]	UTI89 site[c]	APEC 01[b]	APEC 01 site[c]	S88[b]	S88 site[c]	type[d]	Event Lineage[ef]	Recombinant segment	Inferred ancestral base[g]	Outgroup Analysis[e]	Support level[h]	CFT073	ED1a	536	E2348/69	SMS 3-5	IAI39	UMN026	K-12	ATCC 8739	HS	D1 Sd197	CB9615	Sakai	EDL933	IAI1	E24377A	SE11	SS Ss046	F2a 301	F2a 2457T	F5 8401	B4 Sb227	B18 BS512
g	4941849	g	4958078	a	4901973	s	S88	rec36	g	S88	++	g		g	g	a	a	a	a	a	a		a	a	a	a	a	a	a	a	a		a	a
g	4941890	g	4958119	a	4902014	s	S88	rec36	a	2	-	a		a	a	a	g	g	g	g	g		g	g	g	g	a	g	g	g	g		g	g
g	4941927	g	4958156	a	4902051	s	S88	rec36	g	S88	++	g		g	g	a	g	g	g	a	a		g	g	g	g	g	g	g	g	g		g	g
g	4941948	g	4958177	a	4902072	s	S88	rec36	g	S88	++++	g		g	g	a	g	g	g	g	g		g	g	g	g	g	g	g	g	g		g	g
g	4942032	g	4958261	a	4902156	s	S88	rec36	g	S88	++	g		g	g	a	a	a	a	a	a		a	a	a	a	a	a	a	a	a		a	a
g	4942035	g	4958264	a	4902159	s	S88	rec36	g	S88	++	g		g	g	a	g	g	g	g	g		a	a	a	g	a	g	g	g	g		g	g
a	4942047	a	4958276	g	4902171	s	S88	rec36	a	S88	++	a		a	a	g	g	g	g	g	g		g	g	g	g	g	g	g	g	g		g	g
t	4942067	t	4958296	c	4902191	ns	S88	rec36	t	S88	++	t		t	t	c	c	c	c	c	c		c	c	c	c	c	c	c	c	c		c	c
t	4942082	t	4958311	c	4902206	ns	S88	rec36	t	S88	++++	t		t	t	c	t	t	t	t	t		t	t	t	t	t	t	t	t	t		t	t
a	4942142	a	4958371	t	4902266	ns	S88	rec36	a	S88	++	a		a	a	t	t	t	t	t	t		t	t	t	t	t	t	t	t	t		t	t
a	4942236	a	4958465	g	4902360	s	S88	rec36	g	2	-	g		a	a	g	g	g	g	g	g		g	g	g	g	g	g	g	g	g		g	g
g	4942338	g	4958567	a	4902462	s	S88	rec36	g	S88	++	g		g	g	g	a	a	a	a	a		a	a	a	a	a	a	g	g	g		a	a
c	4942359	c	4958588	t	4902483	s	S88	rec36	t	2	-	t		t	t	c	t	t	t	t	c		t	t	t	c	t	c	t	t	t		t	t
g	4942395	g	4958624	a	4902519	s	S88	rec36	g	S88	++	g		g	g	a	a	a	a	a	a		a	a	a	a	a	a	a	a	a		a	
c	4942401	c	4958630	t	4902525	s	S88	rec36	c	S88	++	c		c	c	t	t	t	t	t	t		t	t	t	t	t	t	t	t	t		t	
g	4942434	g	4958663	t	4902558	s	S88	rec36	g	S88	++++	g		g	g	t	g	g	g	g	g		g	g	g	g	g	g	g	g	g		g	
g	4942458	g	4958687	c	4902582	s	S88	rec36	g	S88	++++	g		g	g	c	g	g	g	g	g		g	g	g	g	g	g	g	g	g		g	g
t	4942473	t	4958702	c	4902597	s	S88	rec36	t	S88	++	t		t	t	c	c	c	c	c	c		c	c	c	c	c	c	c	c	c		c	c
a	4942494	a	4958723	g	4902618	s	S88	rec36	a	S88	++	a		a	a	g	g	g	g	g	g		g	g	g	g	g	g	g	g	g		g	g
g	4942512	g	4958741	a	4902636	s	S88	rec36	g	S88	++	g		g	g	a	a	a	a	a	a		a	a	a	a	a	a	a	a	a		a	a
g	4942524	g	4958753	c	4902648	s	S88	rec36	g	S88	++	g		g	g	c	c	c	c	c	c		c	c	c	c	c	c	c	c	c		c	c
g	4942551	g	4958780	t	4902675	s	S88	rec36	g	S88	++++	g		g	g	g	g	g	g	g	g		g	g	g	g	g	g	g	g	g		g	g
a	4942557	a	4958786	g	4902681	s	S88	rec36	a	S88	++	a		a	a	g	g	g	g	g	g		g	g	g	g	g	g	g	g	g		g	g
a	4942587	a	4958816	g	4902711	s	S88	rec36	a	S88	++	a		a	a	g	g	g	g	g	g		g	g	g	g	g	g	g	g	g		g	g
a	4942611	a	4958840	g	4902735	s	S88	rec36	g	2	-	g		a	a	g	g	g	g	g	g		g	g	g	g	g	g	g	g	g		g	g
g	4942721	g	4958950	t	4902845	s	S88	rec36	t	2	-	t		g	g	g	g	g	g	g	g		g	g	g	g	t	g	g	g	g		g	g
g	4942793	g	4959022	a	4902917	s	S88	rec36	a	2	-	a		g	g	a	g	g	g	g	g		g	g	g	g	a	g	g	g	g		g	g
g	4942817	g	4959046	t	4902941	s	S88	rec36	g	S88	++	g		g	g	t	g	t	t	t	t		t	t	t	t	t	t	t	t	t		t	t
t	4942826	t	4959055	a	4902950	s	S88	rec36	a	2	-	a		t	t	t	t	a	a	a	a		a	a	a	a	a	a	a	a	a		a	a
a	4942838	a	4959067	g	4902962	s	S88	rec36	g	2	-	g		a	a	a	a	g	g	g	g		g	g	g	g	g	g	g	g	g		g	g
c	4942853	c	4959082	a	4902977	s	S88	rec36	a	2	-	a		c	c	a	c	a	a	c	a		a	a	a	a	a	c	a	a	a		a	a
t	4942868	t	4959097	c	4902992	s	S88	rec36	c	2	-	c		t	t	c	t	c	c	c	c		c	c	c	c	c	c	c	c	c		c	c
t	4942883	t	4959112	g	4903007	s	S88	rec36	g	2	-	g		t	t	g	t	g	g	g	g		g	g	g	g	t	g	g	g	g		g	g
a	4942913	a	4959142	g	4903037	s	S88	rec36	g	2	-	g		a	a	g	a	g	g	g	g		g	g	g	g	g	g	g	g	g		g	g
t	4942925	t	4959154	c	4903049	s	S88	rec36	c	2	-	c		t	t	c	t	c	c	c	c		c	c	c	c	c	c	c	c	c		c	c
a	4942928	a	4959157	g	4903052	s	S88	rec36	g	2	-	g		a	a	g	a	g	g	g	g		g	g	g	g	g	g	g	g	g		g	g
g	4942934	g	4959163	c	4903058	s	S88	rec36	g	S88	++	g		g	g	c	g	c	c	c	c		c	c	c	c	c	c	c	c	c		c	c
g	4942964	g	4959193	t	4903088	s	S88	rec36	g	S88	++	g		g	g	g	g	a	a	a	t		a	a	a	a	t	a	a	a	a		a	a
c	4942991	c	4959220	t	4903115	s	S88	rec36	c	S88	++	c		c	c	c	c	t	t	t	t		t	t	t	t	t	t	t	t	t		t	t
c	4943015	c	4959244	t	4903139	s	S88	rec36	c	S88	++	c		c	c	c	c	t	t	t	t		t	t	t	t	t	t	t	t	t		t	t
g	4943018	g	4959247	a	4903142	s	S88	rec36	g	S88	++	g		g	g	g	g	a	a	a	a		a	a	a	a	a	a	a	a	a		a	a
t	4943024	t	4959253	c	4903148	s	S88	rec36	t	S88	++	t		t	t	t	t	c	c	c	c		c	c	c	c	c	c	c	c	c		c	c
c	4943039	c	4959268	g	4903163	s	S88	rec36	c	S88	++	c		c	c	g	c	g	g	g	g		g	g	g	g	g	g	g	g	g		g	g
a	4943048	a	4959277	t	4903172	s	S88	rec36	a	S88	++	a		a	a	t	a	t	t	t	t		t	t	t	t	t	t	t	t	t		t	t
t	4943054	t	4959283	c	4903178	s	S88	rec36	t	S88	++	t		t	t	c	t	c	c	c	c		c	c	c	c	c	c	c	c	c		c	c
g	4943063	g	4959292	a	4903187	s	S88	rec36	g	S88	++	g		g	g	a	g	a	a	a	a		a	a	a	a	a	a	a	a	a		a	a
t	4943084	t	4959313	c	4903208	s	S88	rec36	t	S88	++	t		t	t	c	t	c	c	c	c		c	c	c	c	c	c	c	c	c		c	c
a	4943087	a	4959316	c	4903211	ns	S88	rec36	a	S88	++	t		t	a	c	a	c	c	c	c		c	c	c	c	c	c	c	c	c		c	c
t	4943089	t	4959318	c	4903213	ns	S88	rec36	t	S88	++	t		t	t	c	t	c	c	c	c		c	c	c	c	c	c	c	c	c		c	c
t	4943096	t	4959325	c	4903220	s	S88	rec36	t	S88	++	t		t	t	c	t	c	c	c	c		c	c	c	c	c	c	c	c	c		c	c
a	4943117	a	4959346	g	4903241	s	S88	rec36	a	S88	++	a		a	a	g	a	g	g	g	g		g	g	g	g	g	g	g	g	g		g	g
g	4943120	g	4959349	t	4903244	s	S88	rec36	g	S88	++	g		g	g	t	g	t	t	t	t		t	t	t	t	t	t	t	t	t		t	t
a	4943123	a	4959352	g	4903247	s	S88	rec36	a	S88	++	a		a	a	g	a	g	g	g	g		a	a	a	g	g	a	a	a	a		g	g
g	4943168	g	4959397	a	4903292	s	S88	rec36	g	S88	++	g		g	g	a	g	a	a	a	a		a	a	a	a	a	a	a	a	a		a	a
a	4943213	a	4959442	g	4903337	s	S88	rec36	a	S88	++++	a		a	a	a	a	a	a	a	a		a	a	a	a	a	a	a	a	a		-	a
a	4943222	a	4959451	t	4903346	s	S88	rec36	a	S88	++	a		a	a	t	a	t	t	t	t		t	t	t	t	t	t	t	t	t		t	t
c	4943225	c	4959454	g	4903349	s	S88	rec36	c	S88	++	c		c	c	g	c	g	g	g	g		g	g	g	g	g	g	g	g	g		g	g
g	4943237	g	4959466	a	4903361	s	S88	rec36	g	S88	++	g		g	g	a	g	g	g	a	a		a	a	a	g	a	a	a	a	a		a	a
c	4943254	c	4959483	t	4903378	ns	S88	rec36	c	S88	++	c		c	c	t	c	t	t	t	t		t	t	t	t	t	t	t	t	t		t	t
a	4943255	a	4959484	g	4903379	s	S88	rec36	a	S88	++	a		a	a	a	c	g	g	g	g		g	g	g	g	g	g	g	g	g		g	g
a	4943282	a	4959511	g	4903406	s	S88	rec36	a	S88	++	a		a	a	g	a	g	g	g	g		g	g	g	g	g	g	g	g	g		g	g
a	4943334	a	4959563	t	4903458	nc	S88	rec36	a	S88	++	a		a	a	t	a	t	t	t	t		t	t	t	t	t	t	t	t	t		t	t
t	4943420	t	4959649	g	4903544	nc	S88	rec36	t	S88	++	t		t	t	g	t	g	g	g	g		g	g	g	t	g	t	g	g	g		g	g
g	4943450	g	4959679	t	4903574	nc	S88	rec36	g	S88	++	g		g	g	t	g	t	t	t	t		t	t	t	t	t	t	t	t	t		t	t
g	4943456	g	4959685	a	4903580	nc	S88	rec36	g	S88	++++	g		g	g	a	g	g	g	g	g		g	g	g	g	g	g	g	g	g		g	g
t	4943459	t	4959688	g	4903583	nc	S88	rec36	t	S88	++++	t		t	t	g	t	t	t	t	t		t	t	t	t	t	t	t	t	t		t	t
c	4943462	c	4959691	a	4903586	nc	S88	rec36	c	S88	++	c		c	c	a	c	a	a	a	a		a	a	a	a	a	a	a	a	a		a	a
a	4943491	a	4959720	t	4903615	nc	S88	rec36	a	S88	++++	a		a	a	t	a	a	a	a	a		a	a	a	a	a	a	a	a	a		a	a
t	4943541	t	4959770	c	4903665	nc	S88	rec36	t	S88	++	t		c	t	c	t	c	c	c	c		c	c	c	c	c	c	c	c	c		c	c
a	4943566	a	4959795	g	4903690	nc	S88	rec36	a	S88	++	a		a	a	g	a	g	g	g	g		g	g	g	g	g	g	g	g	g		g	g
c	4943575	c	4959804	a	4903699	nc	S88	rec36	c	S88	++	c		c	c	a	c	a	a	a	a		a	a	a	a	a	a	a	a	a		a	a
a	4943587	a	4959816	g	4903711	nc	S88	rec36	a	S88	++	a		a	a	g	a	g	g	g	g		g	g	g	g	g	g	g	g	g		g	g
g	4943590	g	4959819	t	4903714	nc	S88	rec36	g	S88	++	g		g	g	t	g	t	t	t	t		t	t	t	t	t	t	t	t	t		t	t
t	4943623	t	4959852	c	4903747	nc	S88	rec36	t	S88	++	t		t	t	c	t	c	c	c	c		c	c	c	c	c	c	c	c	c		c	c
a	4943629	a	4959858	g	4903753	nc	S88	rec36	a	S88	++	a		a	a	g	a	g	g	g	g		g	g	g	g	g	g	g	g	g		g	g
c	4943641	c	4959870	t	4903765	nc	S88	rec36	c	S88	++	c		c	c	t	c	t	t	t	t		t	t	t	t	t	t	t	t	t		t	t
g	4943647	g	4959876	a	4903771	nc	S88	rec36	g	S88	++	g		g	g	a	g	a	a	a	a		a	a	a	a	a	a	a	a	a		a	a
c	4943689	c	4959918	t	4903813	nc	S88	rec36	c	S88	++	c		c	c	t	c	t	t	c	t		t	t	t	t	t	t	t	t	t		t	t
c	4943722	c	4959951	t	4903846	nc	S88	rec36	c	S88	++	c		c	c	t	c	t	t	t	t		t	t	t	t	t	t	t	t	t		t	t
a	4943728	a	4959957	g	4903852	nc	S88	rec36	a	S88	++	a		a	a	g	a	g	g	g	g		g	g	g	g	g	g	g	g	g		g	g
t	4943736	t	4959965	c	4903860	nc	S88	rec36	c	2	-	c		c	c	c	t	c	c	c	c		c	c	c	c	c	c	c	c	c		c	c
c	4943737	c	4959966	t	4903861	nc	S88	rec36	t	2	-	t		t	t	c	c	t	t	t	c		c	c	c	t	c	t	c	c	c		c	c
t	4943740	t	4959969	g	4903864	nc	S88	rec36	g	2	-	g		g	g	t	t	g	g	g	g		g	g	g	g	g	g	g	g	g		g	g

ExPEC genome site details												Outgroup Strains Details[i]																						
UTI89[b]	UTI89 site[c]	APEC 01[b]	APEC 01 site[c]	S88[b]	S88 site[c]	type[d]	Event Lineage[ef]	Recombinant segment	Inferred ancestral base[g]	Outgroup Analysis[e]	Support level[h]	CFT073	ED1a	536	E2348/69	SMS 3-5	IAI39	UMN026	K-12	ATCC 8739	HS	D1 Sd197	CB9615	Sakai	EDL933	IAI1	E24377A	SE11	SS Ss046	F2a 301	F2a 2457T	F5 8401	B4 Sb227	B18 BS512
c	4943743	c	4959972	t	4903867	nc	S88	rec36	c	S88	++	c		c	c	c	c	t	t	t	c		c	c	c	t	t	t	c	c	c		c	c
c	4943764	c	4959993	t	4903888	nc	S88	rec36	c	S88	++	c		c	c	t	c	t	t	t	t		t	t	t	t	t	t	t	t	t		t	t
c	4943787	c	4960016	g	4903911	nc	S88	rec36	c	S88	++	c		c	c	g	c	g	g	g	g		g	g	g	g	g	g	g	g	g		g	g
t	4943791	t	4960020	c	4903915	nc	S88	rec36	t	S88	++	t		t	t	c	t	c	c	c	c		c	c	c	c	c	c	c	c	c		c	c
t	4943794	t	4960023	c	4903918	nc	S88	rec36	t	S88	++	t		t	t	c	t	c	c	c	c		c	c	c	c	c	c	c	c	c		c	c
c	4943800	c	4960029	g	4903924	nc	S88	rec36	c	S88	++	c		c	c	g	c	g	g	g	g		g	g	g	g	g	g	g	g	g		g	g
t	4943814	t	4960043	c	4903938	nc	S88	rec36	c	2	-	c		c	c	c	t	c	c	c	c		c	c	c	c	c	c	c	c	c		c	c
c	4943830	c	4960059	t	4903954	nc	S88	rec36	c	S88	++	c		c	c	t	c	t	t	c	t		t	t	t	c	t	c	t	t	t		t	t
g	4943834	g	4960063	t	4903958	nc	S88	rec36	g	S88	++	g		g	g	t	g	t	t	t	t		t	t	t	t	t	t	t	t	t		t	t
g	4943835	g	4960064	c	4903959	nc	S88	rec36	g	S88	++	g		c	c	c	g	c	c	c	c		c	c	c	c	c	c	c	c	c		c	c
c	4943839	c	4960068	t	4903963	nc	S88	rec36	c	S88	++	c		c	c	t	c	t	t	t	t		t	t	t	t	t	t	t	t	t		t	t
a	4943840	a	4960069	g	4903964	nc	S88	rec36	a	S88	++	a		a	a	g	a	g	g	g	g		g	g	g	g	g	g	g	g	g		g	g
t	4943841	t	4960070	c	4903965	nc	S88	rec36	t	S88	++	t		t	t	c	t	c	c	c	c		c	c	c	c	c	c	c	c	c		c	c
a	4943860	a	4960089	g	4903984	nc	S88	rec36	a	S88	++	a		a	a	g	a	g	g	g	g		g	g	g	g	g	g	g	g	g		g	g
t	4943890	t	4960119	c	4904014	nc	S88	rec36	c	2	-	c		t	c	c	t	c	c	c	c		c	c	c	c	c	c	c	c	c		c	c
a	4943910	a	4960139	g	4904035	nc	S88	rec36	g	2	-	g		a	g	g	a	g	g	g	g		g	g	g	g	g	g	g	g	g		g	g
g	4943925	g	4960154	t	4904050	nc	S88	rec36	g	S88	++	g		g	g	t	g	t	t	g	g		g	g	g	g	t	g	g	g	g		g	g
c	4943931	c	4960160	t	4904056	nc	S88	rec36	c	S88	++	c		c	c	t	c	t	t	t	t		t	t	t	t	t	t	t	t	t		t	t
t	4943934	t	4960163	c	4904059	nc	S88	rec36	t	S88	++	t		t	t	c	t	c	c	t	t		t	t	t	t	c	t	t	t	t		t	t
g	4943940	g	4960169	a	4904065	nc	S88	rec36	g	S88	++	g		g	g	a	g	a	a	a	g		g	g	g	a	a	a	g	g	g		g	g
a	4943976	a	4960205	g	4904101	nc	S88	rec36	c	S88	+/-	c		g	c	g	a	g	g	g	g		g	g	g	g	g	g	g	g	g		g	g
c	4943986	c	4960215	t	4904111	nc	S88	rec36	c	S88	++++	c		c	c	c	c	c	c	c	c		c	c	c	c	c	c	c	c	c		c	c
a	4943997	a	4960226	g	4904122	nc	S88	rec36	g	2	-	g		g	g	g	a	g	g	g	g		g	g	g	g	g	g	g	g	g		g	g
t	4944003	t	4960232	c	4904128	nc	S88	rec36	c	2	-	c		c	c	t	t	t	t	c	c		c	c	c	c	t	c	c	c	c		c	c
t	4944048	t	4960277	c	4904173	nc	S88	rec36	c	2	-	c		c	t	c	t	c	c	c	c		c	c	c	c	c	c	c	c	c		c	c
g	4944090	g	4960319	t	4904215	nc	S88	rec36	g	S88	++	g		g	g	t	g	g	g	t	g		t	t	t	t	g	t	t	t	t		g	g
c	4944193	c	4960422	t	4909907	s	S88	rec36	c	S88	++	c		c	c	c	c	c	c	c	c		t	t	t	c	c	c					c	c
a	4944205	a	4960434	c	4909919	s	S88	rec36	a	S88	++	a		a	a	c	a	a	a	a	a		t	t	t	a	a	a					a	a
g	4944211	g	4960440	a	4909925	s	S88	rec36	g	S88	++++	g		g	g	a	g	g	g	g	g		g	g	g	g	g	g					g	g
a	4944217	a	4960446	g	4909931	s	S88	rec36	a	S88	++	a		a	a	g	a	a	a	a	a		g	g	g	a	a	a					a	a
c	4944220	c	4960449	g	4909934	s	S88	rec36	c	S88	++++	c		c	c	g	c	c	c	c	c		c	c	c	c	c	c					c	c
t	4944226	t	4960455	c	4909940	s	S88	rec36	t	S88	++	t		t	t	c	t	t	t	t	t		c	c	c	t	t	t					t	t
a	4944232	a	4960461	g	4909946	s	S88	rec36	a	S88	++	a		a	a	g	a	a	a	a	a		g	g	g	a	a	a					a	a
a	4944238	a	4960467	g	4909952	s	S88	rec36	a	S88	++	a		g	a	g	a	g	g	g	g		g	g	g	g	g	g					g	g
t	4944250	t	4960479	c	4909964	s	S88	rec36	c	2	-	c		c	c	c	t	c	c	c	c		c	c	c	c	c	c					c	c
g	4944262	g	4960491	a	4909976	s	S88	rec36	a	2	-	a		g	a	a	g	g	g	g	g		a	a	a	g	g	g					g	g
g	4944265	g	4960494	a	4909979	s	S88	rec36	a	2	-	a		g	a	a	g	g	g	g	g		a	a	a	g	g	g					g	g
a	4944274	a	4960503	g	4909988	s	S88	rec36	a	S88	++	a		a	a	g	a	a	a	a	a		g	g	g	a	a	a					a	a
g	4944277	g	4960506	a	4909991	s	S88	rec36	g	S88	++	g		g	g	a	g	g	g	g	g		a	a	a	g	g	g					g	g
g	4944280	g	4960509	a	4909994	s	S88	rec36	g	S88	++	g		g	g	a	g	g	g	g	g		a	a	a	g	g	g					g	g
t	4944301	t	4960530	c	4910015	s	S88	rec36	t	S88	++	t		t	t	c	t	t	t	t	t		c	c	c	t	t	t					t	t
a	4944307	a	4960536	g	4910021	s	S88	rec36	a	S88	++	a		a	a	g	a	a	a	a	a		g	g	g	a	a	a					a	a
g	4944310	g	4960539	a	4910024	s	S88	rec36	g	S88	++	g		g	g	a	g	g	g	g	g		a	a	a	g	g	g					g	g
t	4944322	t	4960551	c	4910036	s	S88	rec36	t	S88	++	t		t	t	c	t	t	t	a	a		c	c	c	a	t	a					a	a
c	4944328	c	4960557	a	4910042	s	S88	rec36	c	S88	++	c		c	c	a	c	c	c	c	c		a	a	a	c	c	c					c	c
t	4944331	t	4960560	c	4910045	s	S88	rec36	t	S88	++	t		t	t	c	t	t	t	t	t		c	c	c	t	t	t					t	t
a	4944343	a	4960572	g	4910057	s	S88	rec36	a	S88	++	a		a	a	g	a	a	a	a	a		g	g	g	a	a	a					a	a
a	4944355	a	4960584	c	4910069	s	S88	rec36	a	S88	++	a		a	a	c	a	a	a	a	a		c	c	c	a	a	a					a	a
t	4944358	t	4960587	g	4910072	s	S88	rec36	t	S88	++	t		t	t	g	t	t	t	t	t		g	g	g	t	t	t					t	t
g	4944364	g	4960593	c	4910078	s	S88	rec36	g	S88	++	g		g	g	t	g	g	g	g	g		t	t	t	g	g	g					g	g
a	4944376	a	4960605	g	4910090	s	S88	rec36	a	S88	++	a		a	a	g	a	a	a	a	a		g	g	g	a	a	a					a	a
g	4944390	g	4960619	t	4910104	ns	S88	rec36	g	S88	++	g		g	t	t	g	g	g	t	t		t	t	t	t	g	t					t	t
a	4944403	a	4960632	g	4910117	s	S88	rec36	a	S88	++	a		a	a	g	a	a	a	a	a		g	g	g	a	a	a					a	a
c	4944406	c	4960635	t	4910120	s	S88	rec36	c	S88	++	c		c	c	t	c	c	c	c	c		t	t	t	c	c	c					c	c
g	4944409	g	4960638	t	4910123	s	S88	rec36	g	S88	++	g		g	g	t	g	g	g	g	g		t	t	t	g	g	g					g	g
g	4944411	g	4960640	a	4910125	s	S88	rec36	g	S88	++	g		g	g	a	g	g	g	g	g		a	a	a	g	g	g					g	g
c	4944415	c	4960644	a	4910129	s	S88	rec36	c	S88	++	c		c	c	a	c	c	c	a	a		a	a	a	a	c	a					a	a
t	4944419	t	4960648	c	4910133	ns	S88	rec36	t	S88	++	t		t	t	c	t	t	t	t	t		c	c	c	t	t	t					t	t
c	4944420	c	4960649	t	4910134	ns	S88	rec36	c	S88	++	c		c	c	t	c	c	c	c	c		t	t	t	c	c	c					c	c
a	4944430	a	4960659	g	4910144	s	S88	rec36	a	S88	++	a		a	a	g	a	a	a	a	a		g	g	g	a	a	a					a	a
a	4944433	a	4960662	t	4910147	s	S88	rec36	a	S88	++	a		a	a	t	a	a	a	a	a		t	t	t	a	a	a					a	a
g	4944435	g	4960664	a	4910149	s	S88	rec36	g	S88	++	g		g	g	a	g	g	g	g	g		a	a	a	g	g	g					g	g
a	4944438	a	4960667	g	4910152	s	S88	rec36	a	S88	++	a		g	a	g	a	g	g	g	g		g	g	g	g	g	g					g	g
a	4944442	a	4960671	g	4910156	ns	S88	rec36	a	S88	++	a		a	a	g	a	a	a	a	a		g	g	g	a	a	a					a	a
t	4944444	t	4960673	a	4910158	ns	S88	rec36	t	S88	++	t		a	t	a	t	a	a	t	t		a	a	a	t	a	t					t	t
a	4944445	a	4960674	g	4910159	s	S88	rec36	a	S88	++	a		g	a	g	a	g	g	g	g		g	g	g	g	g	g					g	g
a	4944448	a	4960677	c	4910162	s	S88	rec36	a	S88	++	a		a	a	c	a	a	a	a	a		c	c	c	a	a	a					a	a
c	4944469	c	4960698	g	4910183	s	S88	rec36	c	S88	++	c		c	c	g	c	c	c	c	c		g	g	g	c	c	c					c	c
g	4944478	g	4960707	a	4910192	s	S88	rec36	g	S88	++	g		g	g	a	g	g	g	a	a		a	a	a	a	g	a					a	a
c	4944481	c	4960710	t	4910195	s	S88	rec36	c	S88	++	c		c	c	t	c	c	c	c	c		t	t	t	c	c	c					c	c
a	4944484	a	4960713	t	4910198	s	S88	rec36	a	S88	++	a		a	a	t	a	a	a	a	a		t	t	t	a	a	a					a	a
c	4944487	c	4960716	t	4910201	s	S88	rec36	c	S88	++	c		c	c	t	c	c	c	c	c		t	t	t	c	c	c					c	c
a	4944493	a	4960722	c	4910207	s	S88	rec36	a	S88	++	a		a	a	c	a	a	a	a	a		c	c	c	a	a	a					a	a
t	4944499	t	4960728	g	4910213	s	S88	rec36	t	S88	++	t		t	t	g	t	t	t	t	t		g	g	g	t	t	t					t	t
t	4944508	t	4960737	c	4910222	s	S88	rec36	t	S88	++	t		t	t	c	t	t	t	t	t		c	c	c	t	t	t					t	t
g	4944520	g	4960749	a	4910234	s	S88	rec36	g	S88	++++	g		g	g	g	g	g	g	g	g		g	g	g	g	g	g					g	g
a	4944526	a	4960755	t	4910240	s	S88	rec36	a	S88	++	a		a	t	t	a	a	a	t	t		t	t	t	t	a	t					t	t
c	4944529	c	4960758	g	4910243	s	S88	rec36	c	S88	++	c		c	c	g	c	c	c	c	c		g	g	g	c	c	c					c	c
t	4944535	t	4960764	c	4910249	s	S88	rec36	t	S88	++	t		t	t	c	t	t	t	t	t		c	c	c	t	t	t					t	t
t	4944538	t	4960767	c	4910252	s	S88	rec36	t	S88	++	t		t	c	c	t	t	t	t	t		c	c	c	t	t	t					t	t
g	4944544	g	4960773	a	4910258	s	S88	rec36	g	S88	++	g		g	g	g	g	g	g	g	g		a	a	a	g	g	g					g	g
t	4944547	t	4960776	c	4910261	s	S88	rec36	t	S88	++	t		t	t	c	t	t	t	t	t		c	c	c	t	t	t					t	t
a	4944549	a	4960778	g	4910263	s	S88	rec36	a	S88	++	a		a	a	g	a	a	a	a	a		g	g	g	a	a	a					a	a
c	4944550	c	4960779	t	4910264	s	S88	rec36	c	S88	++	c		c	c	t	c	c	c	c	c		t	t	t	c	c	c					c	c

ExPEC genome site details												Outgroup Strains Details[i]																						
UTI89[b]	UTI89 site[c]	APEC 01[b]	APEC 01 site[c]	S88[b]	S88 site[c]	type[d]	Event Lineage[ef]	Recombinant segment	Inferred ancestral base[g]	Outgroup Analysis[e]	Support level[h]	CFT073	ED1a	536	E2348/69	SMS 3-5	IAI39	UMN026	K-12	ATCC 8739	HS	D1 Sd197	CB9615	Sakai	EDL933	IAI1	E24377A	SE11	SS Ss046	F2a 301	F2a 2457T	F5 8401	B4 Sb227	B18 BS512
g	4944556	g	4960785	a	4910270	s	S88	rec36	g	S88	++++	g		g	g	g	g	g	g	g	g		g	g	g	g	g	g					g	g
g	4944562	g	4960791	a	4910276	s	S88	rec36	g	S88	++	g		g	g	a	g	g	g	g	g		a	a	a	g	g	g					g	g
g	4944565	g	4960794	c	4910279	s	S88	rec36	g	S88	++	g		g	g	c	g	g	g	g	g		c	c	c	g	g	g					g	g
t	4944568	t	4960797	c	4910282	s	S88	rec36	t	S88	++	t		t	t	c	t	t	t	t	t		c	c	c	t	t	t					t	t
a	4944570	a	4960799	g	4910284	s	S88	rec36	a	S88	++++	a		a	a	g	a	a	a	a	a		a	a	a	a	a	a					a	a
a	4944573	a	4960802	c	4910287	ns	S88	rec36	a	S88	++	a		a	a	c	a	a	a	a	a		c	c	c	a	a	a					a	a
t	4944574	t	4960803	a	4910288	s	S88	rec36	t	S88	++	t		t	t	a	t	t	t	t	t		a	a	a	t	t	t					t	t
t	4944577	t	4960806	c	4910291	ns	S88	rec36	t	S88	++	t		t	t	c	t	t	t	t	t		c	c	c	t	t	t					t	t
g	4944578	g	4960807	t	4910292	ns	S88	rec36	g	S88	++	g		g	g	t	g	g	g	g	g		t	t	t	g	g	g					g	g
a	4944579	a	4960808	g	4910293	ns	S88	rec36	a	S88	++	a		a	a	g	a	a	a	a	a		g	g	g	a	a	a					a	a
t	4944583	t	4960812	c	4910297	s	S88	rec36	t	S88	++	t		t	t	c	t	t	t	t	t		c	c	c	t	t	t					t	t
a	4944586	a	4960815	g	4910300	s	S88	rec36	a	S88	++	a		a	a	g	a	a	a	a	a		g	g	g	a	a	a					a	a
a	4944610	a	4960839	g	4910324	s	S88	rec36	a	S88	++	a		a	a	g	a	a	a	a	a		g	g	g	a	a	a					a	a
c	4944613	c	4960842	g	4910327	s	S88	rec36	c	S88	++	c		c	c	g	c	c	c	c	c		g	g	g	c	c	c					c	c
t	4944634	t	4960863	a	4910348	s	S88	rec36	t	S88	++	t		t	t	a	t	t	t	t	t		a	a	a	t	t	t					t	t
g	4944643	g	4960872	a	4910357	s	S88	rec36	g	S88	++	g		g	g	a	g	g	g	g	g		a	a	a	g	g	g					g	g
g	4944646	g	4960875	a	4910360	s	S88	rec36	g	S88	++	g		g	g	a	g	g	g	g	g		a	a	a	g	g	g					g	g
g	4944651	g	4960880	a	4910365	s	S88	rec36	g	S88	++++	g		g	g	g	g	g	g	g	g		g	g	g	g	g	g					g	g
a	4944655	a	4960884	c	4910369	s	S88	rec36	a	S88	++	a		a	a	c	a	a	a	a	a		c	c	c	a	a	a					a	a
t	4944676	t	4960905	g	4910390	ns	S88	rec36	t	S88	++	t		t	t	g	t	t	t	t	t		g	g	g	t	t	t					t	t
a	4944677	a	4960906	g	4910391	ns	S88	rec36	a	S88	++	a		a	a	g	a	a	a	a	a		g	g	g	a	a	a					a	a
t	4944678	t	4960907	g	4910392	ns	S88	rec36	t	S88	++	t		t	t	g	t	t	t	t	t		g	g	g	t	t	t					t	t
c	4944679	c	4960908	t	4910393	s	S88	rec36	c	S88	++	c		c	c	t	c	c	c	c	c		t	t	t	c	c	c					c	c
t	4944686	t	4960915	g	4910400	ns	S88	rec36	t	S88	++	t		t	t	g	t	t	t	t	t		g	g	g	t	t	t					t	t
c	4944688	c	4960917	g	4910402	ns	S88	rec36	c	S88	++	c		c	c	g	c	c	c	c	c		g	g	g	c	c	c					c	c
g	4944689	g	4960918	a	4910403	ns	S88	rec36	g	S88	++	g		g	g	a	g	g	g	g	g		a	a	a	g	g	g					g	g
a	4944704	a	4960933	g	4910418	ns	S88	rec36	a	S88	++	a		a	a	g	a	a	a	a	a		g	g	g	a	a	a					a	a
t	4944705	t	4960934	c	4910419	ns	S88	rec36	t	S88	++	t		t	t	c	t	t	t	t	t		c	c	c	t	t	t					t	t
t	4944709	t	4960938	c	4910423	ns	S88	rec36	t	S88	++	t		t	t	c	t	t	t	t	t		c	c	c	t	t	t					t	t
t	4944711	t	4960940	c	4910425	ns	S88	rec36	c	2	-	c		t	c	g	t	t	t	c	c		g	g	g	c	t	c					c	c
g	4944715	g	4960944	a	4910429	ns	S88	rec36	g	S88	++	g		g	g	a	g	g	g	g	g		a	a	a	g	g	g					g	g
g	4944716	g	4960945	a	4910430	ns	S88	rec36	g	S88	++	g		g	g	a	g	g	g	g	g		a	a	a	g	g	g					g	g
c	4944717	c	4960946	t	4910431	ns	S88	rec36	c	S88	++++	c		c	c	c	c	c	c	c	c		c	c	c	c	c	c					c	c
t	4944724	t	4960953	a	4910438	ns	S88	rec36	t	S88	++	t		t	t	a	t	t	t	t	t		a	a	a	t	t	t					t	t
g	4944730	g	4960959	c	4910444	s	S88	rec36	g	S88	++	g		g	g	c	g	g	g	g	g		c	c	c	g	g	g					g	g
c	4944733	c	4960962	g	4910447	ns	S88	rec36	c	S88	++	c		c	c	g	c	c	c	c	c		g	g	g	c	c	c					c	c
t	4944736	t	4960965	g	4910450	s	S88	rec36	t	S88	++	t		t	t	g	t	t	t	t	t		g	g	g	t	t	t					t	t
a	4944738	a	4960967	g	4910452	s	S88	rec36	a	S88	++	a		a	a	g	a	a	a	a	a		g	g	g	a	a	a					a	a
a	4944739	a	4960968	c	4910453	s	S88	rec36	a	S88	++	a		a	a	c	a	a	a	a	a		c	c	c	a	a	a					a	a
t	4944744	t	4960973	c	4910458	ns	S88	rec36	t	S88	++	t		t	t	c	t	t	t	t	t		c	c	c	t	t	t					t	t
a	4944745	a	4960974	c	4910459	s	S88	rec36	a	S88	++	a		a	a	c	a	a	a	a	a		c	c	c	a	a	a					a	a
t	4944748	t	4960977	a	4910462	s	S88	rec36	t	S88	++	t		t	t	a	t	t	t	t	t		a	a	a	t	t	t					t	t
t	4944753	t	4960982	g	4910467	ns	S88	rec36	t	S88	++	t		t	t	g	t	t	t	t	t		g	g	g	t	t	t					t	t
g	4944756	g	4960985	a	4910470	s	S88	rec36	g	S88	++	g		g	g	a	g	g	g	g	g		a	a	a	g	g	g					g	g
c	4944757	c	4960986	t	4910471	ns	S88	rec36	c	S88	++	c		c	t	t	c	c	c	c	c		t	t	t	c	c	c					c	c
c	4944760	c	4960989	a	4910474	ns	S88	rec36	c	S88	++	c		c	c	a	c	c	c	c	c		a	a	a	c	c	c					c	c
c	4944764	c	4960993	g	4910478	ns	S88	rec36	c	S88	++	c		c	c	g	c	c	c	c	c		g	g	g	c	c	c					c	c
c	4944772	c	4961001	t	4910486	s	S88	rec36	c	S88	++	c		c	c	t	c	c	c	c	c		t	t	t	c	c	c					c	c
c	4944775	c	4961004	t	4910489	s	S88	rec36	c	S88	++	c		c	c	t	c	c	c	c	c		t	t	t	c	c	c					c	c
g	4944781	g	4961010	a	4910495	s	S88	rec36	g	S88	++	g		g	g	a	a	g	g	g	g		a	a	a	g	g	g					g	g
t	4944790	t	4961019	c	4910504	s	S88	rec36	t	S88	++	t		t	t	c	t	t	t	t	t		c	c	c	t	t	t					t	t
c	4944799	c	4961028	t	4910513	s	S88	rec36	c	S88	++	c		c	c	t	c	c	c	c	c		t	t	t	c	c	c					c	c
g	4944802	g	4961031	a	4910516	s	S88	rec36	g	S88	++	g		g	g	a	g	g	g	g	g		a	a	a	g	g	g					g	g
t	4944809	t	4961038	g	4910523	ns	S88	rec36	t	S88	++	t		t	t	g	t	t	t	g	g		g	g	g	g	t	g					g	g
a	4944829	a	4961058	c	4910543	s	S88	rec36	a	S88	++	a		a	a	c	a	a	a	a	a		c	c	c	a	a	a					a	a
t	4944847	t	4961076	a	4910561	s	S88	rec36	t	S88	++	t		t	t	a	t	t	t	t	t		a	a	a	t	t	t					t	t
a	4944856	a	4961085	g	4910570	ns	S88	rec36	a	S88	++	a		a	a	g	a	a	a	a	a		g	g	g	a	a	a					a	a
t	4944858	t	4961087	c	4910572	ns	S88	rec36	t	S88	++	t		t	t	c	t	t	t	t	t		c	c	c	t	t	t					c	t
g	4944865	g	4961094	a	4910579	s	S88	rec36	g	S88	++	g		g	g	a	g	g	g	g	g		a	a	a	g	g	g					-	g
c	4944874	c	4961103	t	4910588	s	S88	rec36	c	S88	++	c		c	c	t	c	c	c	c	c		t	t	t	c	c	c					c	c
a	4944876	a	4961105	g	4910590	s	S88	rec36	a	S88	++	a		a	a	g	a	a	a	a	a		g	g	g	a	a	a					a	a
a	4944877	a	4961106	c	4910591	s	S88	rec36	a	S88	++	a		a	a	c	a	a	a	a	a		c	c	c	a	a	a					a	a
t	4944880	t	4961109	c	4910594	s	S88	rec36	t	S88	++	t		t	t	c	t	t	t	t	t		c	c	c	t	t	t					t	t
c	4944883	c	4961112	a	4910597	s	S88	rec36	c	S88	++	c		c	c	a	c	c	c	c	c		a	a	a	c	c	c					c	c
c	4944886	c	4961115	g	4910600	s	S88	rec36	c	S88	++	c		c	c	g	c	c	c	c	c		g	g	g	c	c	c					c	c
a	4944892	a	4961121	g	4910606	s	S88	rec36	a	S88	++	a		a	a	g	a	a	a	a	a		g	g	g	a	a	a					a	a
c	4944901	c	4961130	g	4910615	s	S88	rec36	c	S88	++	c		c	c	a	c	c	c	c	c		a	a	a	c	c	c					c	c
g	4944904	g	4961133	a	4910618	s	S88	rec36	g	S88	++	g		g	g	a	g	g	g	g	g		a	a	a	g	g	g					g	g
c	4944907	c	4961136	t	4910621	s	S88	rec36	c	S88	++	c		c	c	c	c	c	c	c	c		t	t	t	c	c	c					c	c
a	4944913	a	4961142	g	4910627	s	S88	rec36	a	S88	++	a		a	a	g	a	a	a	a	a		g	g	g	a	a	a					a	a
c	4944916	c	4961145	g	4910630	s	S88	rec36	c	S88	++	c		c	a	g	c	c	c	c	c		g	g	g	c	c	c					c	c
c	4944922	c	4961151	t	4910636	s	S88	rec36	c	S88	++	c		c	c	t	c	c	c	c	c		t	t	t	c	c	c					c	c
a	4944925	a	4961154	g	4910639	s	S88	rec36	a	S88	++	a		a	a	g	a	a	a	a	a		g	g	g	a	a	a					a	a
a	4944928	a	4961157	g	4910642	s	S88	rec36	a	S88	++	a		a	a	g	a	a	a	a	a		g	g	g	a	a	a					a	a
g	4944936	g	4961165	a	4910650	s	S88	rec36	g	S88	++	g		g	g	a	g	g	g	g	g		a	a	a	g	g	g					g	g
t	4944940	t	4961169	c	4910654	s	S88	rec36	t	S88	++	t		t	a	c	t	a	a	a	a		c	c	c	a	a	a					a	a
a	4944955	a	4961184	t	4910669	s	S88	rec36	a	S88	++	a		a	a	c	a	a	a	a	a		t	t	t	a	a	a					a	a
g	4944964	g	4961193	a	4910678	ns	S88	rec36	g	S88	++	g		g	g	a	g	g	g	g	g		a	a	a	g	g	g					g	g
c	4944966	c	4961195	a	4910680	ns	S88	rec36	c	S88	++	c		c	c	a	c	c	c	a	a		a	a	a	a	c	a					a	a
c	4944973	c	4961202	g	4910687	ns	S88	rec36	c	S88	++	c		c	c	g	c	c	c	c	c		g	g	g	c	c	c					c	c
a	4944975	a	4961204	t	4910689	ns	S88	rec36	a	S88	++	a		a	a	t	a	a	a	a	a		t	t	t	a	a	a					a	a
t	4944976	t	4961205	g	4910690	ns	S88	rec36	t	S88	++	t		t	t	g	t	t	t	t	t		g	g	g	t	t	t					t	t

ExPEC genome site details												Outgroup Strains Details[i]																						
UTI89[b]	UTI89 site[c]	APEC 01[b]	APEC 01 site[c]	S88[b]	S88 site[c]	type[d]	Event Lineage[ef]	Recombinant segment	Inferred ancestral base[g]	Outgroup Analysis[e]	Support level[h]	CFT073	ED1a	536	E2348/69	SMS 3-5	IAI39	UMN026	K-12	ATCC 8739	HS	D1 Sd197	CB9615	Sakai	EDL933	IAI1	E24377A	SE11	SS Ss046	F2a 301	F2a 2457T	F5 8401	B4 Sb227	B18 BS512
c	4944979	c	4961208	a	4910693	s	S88	rec36	c	S88	++	c		c	c	a	c	c	c	c	c		a	a	a	c	c	c					c	c
t	4944987	t	4961216	a	4910701	ns	S88	rec36	t	S88	++	t		t	t	g	t	t	t	t	t		g	g	g	t	t	t					t	t
t	4944991	t	4961220	c	4910705	s	S88	rec36	t	S88	++	t		t	t	c	t	t	t	t	t		c	c	c	t	t	t					t	t
a	4944995	a	4961224	g	4910709	ns	S88	rec36	g	2	-	g		a	g	g	g	g	g	g	g		g	g	g	g	a	g					g	g
c	4944997	c	4961226	t	4910711	s	S88	rec36	c	S88	++++	c		c	c	c	c	c	c	c	c		c	c	c	c	c	c					c	c
a	4944999	a	4961228	g	4910713	s	S88	rec36	a	S88	++	a		a	a	g	a	a	a	a	a		g	g	g	a	a	a					a	a
t	4945003	t	4961232	c	4910717	s	S88	rec36	t	S88	++	t		t	t	c	t	t	t	t	t		c	c	c	t	t	t					t	t
t	4945009	t	4961238	c	4910723	s	S88	rec36	t	S88	++	t		t	g	c	t	t	t	t	t		c	c	c	t	t	t					t	t
c	4945018	c	4961247	t	4910732	s	S88	rec36	c	S88	++	c		c	c	t	c	c	c	c	c		t	t	t	c	c	c					c	c
c	4945021	c	4961250	t	4910735	s	S88	rec36	c	S88	++	c		c	c	t	c	c	c	c	c		t	t	t	c	c	c					c	c
c	4945024	c	4961253	t	4910738	s	S88	rec36	c	S88	++	c		c	c	t	c	c	c	c	c		t	t	t	c	c	c					c	c
c	4945028	c	4961257	g	4910742	ns	S88	rec36	c	S88	++	c		c	c	g	c	c	c	c	c		g	g	g	c	c	c					c	c
c	4945030	c	4961259	t	4910744	s	S88	rec36	c	S88	++	c		c	c	t	c	c	c	c	c		t	t	t	c	c	c					c	c
c	4945033	c	4961262	g	4910747	s	S88	rec36	c	S88	++	c		c	c	g	c	c	c	c	c		g	g	g	c	c	c					c	c
a	4945039	a	4961268	g	4910753	s	S88	rec36	a	S88	++	a		a	a	g	a	a	a	a	a		g	g	g	a	a	a					a	a
c	4945045	c	4961274	t	4910759	s	S88	rec36	c	S88	++	c		c	c	t	c	c	c	c	c		t	t	t	c	c	c					c	c
a	4945051	a	4961280	g	4910765	s	S88	rec36	a	S88	++	a		a	a	g	a	a	a	a	a		g	g	g	t	a	a					a	a
t	4945054	t	4961283	c	4910768	s	S88	rec36	t	S88	++	t		t	t	c	t	t	t	t	t		c	c	c	t	t	t					t	t
c	4945066	c	4961295	t	4910780	s	S88	rec36	c	S88	++	c		c	c	t	c	c	c	c	c		t	t	t	c	c	c					c	c
t	4945069	t	4961298	g	4910783	s	S88	rec36	t	S88	++	t		t	a	g	t	t	t	c	c		g	g	g	c	t	c					c	c
c	4945072	c	4961301	t	4910786	ns	S88	rec36	c	S88	++	c		c	t	t	c	c	c	c	c		t	t	t	c	c	c					c	c
t	4945073	t	4961302	a	4910787	ns	S88	rec36	t	S88	++	t		t	t	a	t	t	t	t	t		a	a	a	t	t	t					t	t
g	4945074	g	4961303	a	4910788	ns	S88	rec36	g	S88	++	g		g	g	a	g	g	g	g	g		a	a	a	g	g	g					g	g
g	4945075	g	4961304	a	4910789	s	S88	rec36	g	S88	++	g		g	g	a	g	g	g	g	g		a	a	a	g	g	g					g	g
t	4945081	t	4961310	c	4910795	s	S88	rec36	t	S88	++	t		t	t	c	t	t	t	t	t		c	c	c	t	t	t					t	t
a	4945087	a	4961316	g	4910801	s	S88	rec36	a	S88	++	a		a	a	g	a	a	a	a	a		g	g	g	a	a	a					a	a
c	4945090	c	4961319	t	4910804	s	S88	rec36	c	S88	++	a		c	c	t	a	a	a	c	c		t	t	t	c	c	c					c	c
t	4945096	t	4961325	c	4910810	s	S88	rec36	t	S88	++	t		t	t	c	t	t	t	t	t		c	c	c	t	t	t					t	t
a	4945102	a	4961331	g	4910816	s	S88	rec36	a	S88	++	a		a	a	g	a	a	a	a	a		g	g	g	a	a	a					a	a
g	4945105	g	4961334	c	4910819	s	S88	rec36	g	S88	++	g		g	g	c	g	g	g	g	g		c	c	c	g	g	g					g	g
a	4945108	a	4961337	c	4910822	s	S88	rec36	a	S88	++	a		a	a	c	a	a	a	a	a		c	c	c	a	a	a					a	a
c	4945120	c	4961349	g	4910834	s	S88	rec36	c	S88	++	c		c	c	g	c	c	c	c	c		g	g	g	c	c	c					c	c
g	4945129	g	4961358	a	4910843	s	S88	rec36	g	S88	++	g		g	g	a	g	g	g	g	g		a	a	a	g	g	g					g	g
g	4945132	g	4961361	a	4910846	ns	S88	rec36	g	S88	++	g		g	g	a	g	g	g	g	g		a	a	a	g	g	g					g	g
c	4945133	c	4961362	g	4910847	ns	S88	rec36	c	S88	++++	c		c	c	c	c	c	c	c	c		c	c	c	c	c	c					c	c
a	4945138	a	4961367	g	4910852	s	S88	rec36	a	S88	++	a		a	a	g	a	a	a	a	a		g	g	g	a	a	a					a	a
c	4945144	c	4961373	t	4910858	s	S88	rec36	c	S88	++	c		c	c	t	c	c	c	c	c		t	t	t	c	c	c					c	c
c	4945150	c	4961379	t	4910864	ns	S88	rec36	c	S88	++	c		c	t	t	c	c	c	c	c		t	t	t	c	c	c					c	c
g	4945152	g	4961381	c	4910866	ns	S88	rec36	g	S88	++	g		g	g	c	g	g	g	g	g		c	c	c	g	g	g					g	g
t	4945159	t	4961388	g	4910873	s	S88	rec36	t	S88	++++	t		t	t	g	t	t	t	t	t		t	t	t	t	t	t					t	t
c	4945165	c	4961394	t	4910879	ns	S88	rec36	c	S88	++++	c		c	c	t	c	c	c	c	c		c	c	c	c	c	c					c	c
g	4945166	g	4961395	t	4910880	ns	S88	rec36	g	S88	++	g		g	g	t	g	g	g	g	g		t	t	t	g	g	g					g	g
a	4945167	a	4961396	c	4910881	ns	S88	rec36	a	S88	++	a		a	a	c	a	a	a	c	c		c	c	c	c	a	c					c	c
t	4945168	t	4961397	g	4910882	s	S88	rec36	t	S88	++	t		t	t	g	t	t	t	c	c		g	g	g	c	t	c					c	c
t	4945179	t	4961408	g	4910893	ns	S88	rec36	t	S88	++	t		t	t	g	t	t	t	t	t		g	g	g	t	t	t					t	t
g	4945186	g	4961415	t	4910900	s	S88	rec36	g	S88	++	g		g	g	t	g	g	g	g	g		t	t	t	g	g	g					g	g
a	4945191	a	4961420	g	4910905	s	S88	rec36	a	S88	++	a		a	a	g	a	a	a	a	a		g	g	g	a	a	a					a	a
a	4945195	a	4961424	c	4910909	s	S88	rec36	a	S88	++	a		a	a	c	a	a	a	a	a		c	c	c	a	a	a					a	a
a	4945204	a	4961433	g	4910918	s	S88	rec36	a	S88	++	a		a	a	g	a	a	a	a	a		g	g	g	a	a	a					a	a
g	4945207	g	4961436	a	4910921	s	S88	rec36	g	S88	++	g		g	g	a	g	g	g	g	g		a	a	a	g	g	g					g	g
a	4945252	a	4961481	t	4910966	s	S88	rec36	a	S88	++	a		a	a	t	a	a	a	a	a		t	t	t	a	a	a					a	a
g	4945254	g	4961483	a	4910968	s	S88	rec36	g	S88	++	g		g	g	a	g	g	g	g	g		a	a	a	g	g	g					g	g
g	4945273	g	4961502	a	4910987	s	S88	rec36	g	S88	++	g		g	g	a	g	g	g	g	g		a	a	a	g	g	g					g	g
g	4945291	g	4961520	t	4911005	ns	S88	rec36	g	S88	++	g		g	g	t	g	g	g	g	g		t	t	t	g	g	g					g	g
g	4945293	g	4961522	t	4911007	ns	S88	rec36	g	S88	++	a		a	g	t	a	a	a	a	a		t	t	t	a	g	a					a	a
g	4945298	g	4961527	t	4911012	ns	S88	rec36	g	S88	++	g		g	g	t	g	g	g	g	g		t	t	t	g	g	g					g	g
g	4945299	g	4961528	t	4911013	ns	S88	rec36	g	S88	++	g		g	g	t	g	g	g	g	g		t	t	t	g	g	g					g	g
t	4945306	t	4961535	c	4911020	nc	S88	rec36	t	S88	++	t		t	t	c	t	t	t	t	t		c	c	c	t	t	t					t	t
a	4945307	a	4961536	t	4911021	nc	S88	rec36	a	S88	++	a		a	a	t	a	a	a	a	a		t	t	t	a	a	a					a	a
a	4945308	a	4961537	g	4911022	nc	S88	rec36	a	S88	++	a		a	a	t	a	a	a	a	a		g	g	g	a	a	a					a	a
t	4945312	t	4961541	g	4911026	nc	S88	rec36	t	S88	++	t		t	t	t	t	t	t	t	t		g	g	g	t	t	t					t	t
c	4945319	c	4961548	a	4911033	nc	S88	rec36	c	S88	++	c		c	c	a	c	c	c	c	c		a	a	a	c	c	c					c	c
a	4945320	a	4961549	t	4911034	nc	S88	rec36	a	S88	++	a		a	a	t	a	a	a	a	a		t	t	t	a	a	a					a	a
t	4945322	t	4961551	a	4911036	nc	S88	rec36	t	S88	++	t		t	t	a	t	t	t	t	t		a	a	a	t	t	t					t	t
g	4945326	g	4961555	t	4911040	nc	S88	rec36	g	S88	++	g		g	g	t	g	g	g	g	g		t	t	t	g	g	g					g	g
t	4945327	t	4961556	g	4911041	nc	S88	rec36	t	S88	++	t		t	t	g	t	t	t	t	t		g	g	g	t	t	t					t	t
t	4945328	t	4961557	a	4911042	nc	S88	rec36	t	S88	++	t		t	t	a	t	t	t	t	t		a	a	a	t	t	t					t	t
g	4945547	g	4961776	c	4911261	s	S88	rec36	g	S88	++	g		g	g	c	g	g	g	g	g		c	c	c	g	g	g					g	g
t	4945550	t	4961779	g	4911264	s	S88	rec36	t	S88	++	t		t	t	g	t	t	t	t	t		g	g	g	t	t	t					t	t
a	4945556	a	4961785	t	4911270	s	S88	rec36	a	S88	++	a		a	a	t	a	a	a	a	a		t	t	t	a	a	a					a	a
t	4945559	t	4961788	c	4911273	s	S88	rec36	t	S88	++	t		t	t	c	t	t	t	t	t		c	c	c	t	t	t					t	t
a	4945565	a	4961794	t	4911279	s	S88	rec36	a	S88	++	a		a	a	t	a	a	a	a	a		t	t	t	a	a	a					a	a
g	4945570	g	4961799	a	4911284	s	S88	rec36	g	S88	++	g		g	g	a	g	g	g	g	g		a	a	a	g	g	g					g	g
a	4945577	a	4961806	c	4911291	s	S88	rec36	a	S88	++	a		a	a	c	a	a	a	a	a		c	c	c	a	a	a					a	a
g	4945579	g	4961808	a	4911293	s	S88	rec36	g	S88	++	g		g	g	a	g	g	g	g	g		a	a	a	g	g	g					g	g
t	4945589	t	4961818	c	4911303	s	S88	rec36	t	S88	++	t		t	t	c	t	t	t	t	t		c	c	c	t	t	t					t	t
t	4945593	t	4961822	g	4911307	ns	S88	rec36	t	S88	++	t		t	t	g	t	t	t	t	t		g	g	g	t	t	t					t	t
t	4945604	t	4961833	c	4911318	s	S88	rec36	t	S88	++	t		t	t	c	t	t	t	t	t		c	c	c	t	t	t					t	t
g	4945610	g	4961839	a	4911324	s	S88	rec36	g	S88	++	g		g	g	a	g	g	g	g	g		a	a	a	g	g	g					g	g
c	4945613	c	4961842	a	4911327	s	S88	rec36	c	S88	++	c		c	c	a	c	c	c	c	c		a	a	a	c	c	c					c	c
t	4945622	t	4961851	c	4911336	s	S88	rec36	t	S88	++++	t		t	t	t	t	t	t	t	t		t	t	t	t	t	t					t	t
t	4945631	t	4961860	c	4911345	s	S88	rec36	t	S88	++	t		t	t	c	t	t	t	t	t		c	c	c	t	t	t					t	t
c	4945634	c	4961863	a	4911348	s	S88	rec36	c	S88	++	c		c	c	a	c	c	c	c	c		a	a	a	c	c	c					c	c
t	4945637	t	4961866	c	4911351	s	S88	rec36	t	S88	++	t		t	t	c	t	t	t	t	t		c	c	c	t	t	t					t	t

ExPEC genome site details												Outgroup Strains Details[i]																						
UTI89[b]	UTI89 site[c]	APEC 01[b]	APEC 01 site[c]	S88[b]	S88 site[c]	type[d]	Event Lineage[ef]	Recombinant segment	Inferred ancestral base[g]	Outgroup Analysis[e]	Support level[h]	CFT073	ED1a	536	E2348/69	SMS 3-5	IAI39	UMN026	K-12	ATCC 8739	HS	D1 Sd197	CB9615	Sakai	EDL933	IAI1	E24377A	SE11	SS Ss046	F2a 301	F2a 2457T	F5 8401	B4 Sb227	B18 BS512
t	4945640	t	4961869	c	4911354	s	S88	rec36	t	S88	++	t		t	t	c	t	t	t	t	t		c	c	c	t	t	t					t	t
g	4945642	g	4961871	a	4911356	s	S88	rec36	g	S88	++	g		g	g	a	g	g	g	g	g		a	a	a	g	g	g					g	g
t	4945643	t	4961872	c	4911357	s	S88	rec36	t	S88	++	t		t	t	c	t	t	t	t	t		c	c	c	t	t	t					t	t
c	4945646	c	4961875	t	4911360	s	S88	rec36	c	S88	++	c		c	c	t	c	c	c	c	c		t	t	t	c	c	c					c	c
g	4945649	g	4961878	a	4911363	s	S88	rec36	g	S88	++	g		g	g	a	g	g	g	g	g		a	a	a	g	g	g					g	g
t	4945667	t	4961896	c	4911381	s	S88	rec36	t	S88	++	t		t	t	c	t	t	t	t	t		c	c	c	t	t	t					t	t
t	4945672	t	4961901	c	4911386	ns	S88	rec36	t	S88	++	t		t	t	c	t	t	t	t	t		c	c	c	t	t	t					t	t
t	4945678	t	4961907	g	4911392	ns	S88	rec36	t	S88	++	t		t	t	g	t	t	t	t	t		g	g	g	t	t	t					t	t
t	4945679	t	4961908	c	4911393	s	S88	rec36	t	S88	++	t		t	t	c	t	t	t	t	t		c	c	c	t	t	t					t	t
c	4945682	c	4961911	t	4911396	s	S88	rec36	c	S88	++	c		c	t	t	t	c	c	c	c		t	t	t	c	c	c					c	c
t	4945689	t	4961918	g	4911403	ns	S88	rec36	t	S88	++	t		t	t	g	t	t	t	t	t		g	g	g	t	t	t					t	t
a	4945691	a	4961920	t	4911405	s	S88	rec36	a	S88	++	a		a	a	t	a	a	a	a	a		t	t	t	a	a	a					a	a
t	4945703	t	4961932	c	4911417	s	S88	rec36	c	2	-	c		c	c	t	c	c	c	c	c		c	c	c	c	c	c					c	c
a	4945706	a	4961935	t	4911420	s	S88	rec36	a	S88	++	a		a	a	t	a	a	a	a	a		t	t	t	a	a	a					a	a
g	4945712	g	4961941	a	4911426	s	S88	rec36	g	S88	++	g		g	g	a	g	a	a	a	a		a	a	a	a	g	a					a	a
a	4945736	a	4961965	g	4911450	s	S88	rec36	a	S88	++	a		a	a	g	a	a	a	a	a		g	g	g	a	a	a					a	a
g	4945739	g	4961968	a	4911453	s	S88	rec36	g	S88	++	g		g	g	a	g	g	g	g	g		a	a	a	g	g	g					g	g
a	4945742	a	4961971	g	4911456	s	S88	rec36	a	S88	++	a		a	a	g	a	a	a	a	a		g	g	g	a	a	a					a	a
g	4945748	g	4961977	a	4911462	ns	S88	rec36	g	S88	++	g		g	g	a	g	g	g	g	g		a	a	a	g	g	g					g	g
c	4945749	c	4961978	g	4911463	ns	S88	rec36	c	S88	++	c		c	c	g	c	c	c	c	c		g	g	g	c	c	c					c	c
g	4945750	g	4961979	a	4911464	ns	S88	rec36	g	S88	++	g		g	g	a	g	g	g	g	g		a	a	a	g	g	g					g	g
a	4945751	a	4961980	g	4911465	s	S88	rec36	a	S88	++	a		a	a	g	a	a	a	a	a		g	g	g	a	a	a					a	a
a	4945754	a	4961983	g	4911468	s	S88	rec36	a	S88	++	a		a	a	g	a	a	a	a	a		g	g	g	a	a	a					a	a
g	4945757	g	4961986	a	4911471	s	S88	rec36	g	S88	++	g		g	g	a	g	a	a	a	a		a	a	a	a	g	a					a	a
a	4945762	a	4961991	g	4911476	s	S88	rec36	a	S88	++	a		a	a	g	a	a	a	a	a		g	g	g	a	a	a					a	a
t	4945763	t	4961992	c	4911477	s	S88	rec36	t	S88	++	t		t	t	c	t	c	c	c	c		c	c	c	c	t	c					c	c
a	4945772	a	4962001	g	4911486	s	S88	rec36	a	S88	++	a		a	a	g	a	a	a	a	a		g	g	g	a	a	a					a	a
g	4945775	g	4962004	a	4911489	s	S88	rec36	g	S88	++	g		g	g	a	g	g	g	g	g		a	a	a	g	g	g					g	g
a	4945778	a	4962007	g	4911492	s	S88	rec36	a	S88	++	a		a	a	g	a	a	a	a	a		g	g	g	a	a	a					a	a
g	4945781	g	4962010	a	4911495	s	S88	rec36	g	S88	++	g		g	g	a	g	g	g	g	g		a	a	a	g	g	g					g	g
g	4945787	g	4962016	t	4911501	ns	S88	rec36	g	S88	++	g		g	g	t	g	g	g	g	g		t	t	t	g	g	g					g	g
a	4945789	a	4962018	t	4911503	ns	S88	rec36	a	S88	++	a		a	a	t	a	a	a	a	a		t	t	t	a	a	a					a	a
g	4945798	g	4962027	a	4911512	s	S88	rec36	g	S88	++	g		g	g	a	g	g	g	g	g		a	a	a	g	g	g					g	g
c	4945799	c	4962028	t	4911513	s	S88	rec36	c	S88	++	c		c	c	t	c	c	c	c	c		t	t	t	c	c	c					c	c
t	4945808	t	4962037	a	4911522	s	S88	rec36	t	S88	++	t		t	t	a	t	t	t	t	t		a	a	a	t	t	t					t	t
t	4945823	t	4962052	g	4911537	ns	S88	rec36	t	S88	++	t		t	t	g	t	t	t	t	t		g	g	g	t	t	t					t	t
t	4945824	t	4962053	c	4911538	ns	S88	rec36	t	S88	++	t		t	t	c	t	t	t	t	t		c	c	c	t	t	t					t	t
g	4945835	g	4962064	c	4911549	ns	S88	rec36	g	S88	++++	g		g	g	c	g	g	g	g	g		g	g	g	g	g	g					g	g
t	4945837	t	4962066	c	4911551	ns	S88	rec36	t	S88	++	t		t	t	c	t	t	t	t	t		c	c	c	t	t	t					t	t
t	4945838	t	4962067	a	4911552	ns	S88	rec36	t	S88	++	t		t	t	a	t	t	t	t	t		a	a	a	t	t	t					t	t
g	4945840	g	4962069	c	4911554	ns	S88	rec36	g	S88	++	g		g	g	c	g	g	g	g	a		c	c	c	g	g	g					g	g
g	4945842	g	4962071	t	4911556	ns	S88	rec36	g	S88	++	g		g	g	t	g	g	g	g	g		t	t	t	g	g	g					g	g
c	4945844	c	4962073	a	4911558	s	S88	rec36	c	S88	++	c		c	c	a	c	c	c	c	c		a	a	a	c	c	c					c	c
a	4945865	a	4962094	g	4911579	s	S88	rec36	a	S88	++	a		a	a	g	a	g	g	g	g		g	g	g	g	a	g					g	g
a	4945868	a	4962097	g	4911582	s	S88	rec36	a	S88	++	a		a	a	g	a	a	a	a	a		g	g	g	a	a	a					a	a
g	4945871	g	4962100	a	4911585	s	S88	rec36	g	S88	++	g		g	g	a	g	g	g	g	g		a	a	a	g	g	g					g	g
c	4945874	c	4962103	t	4911588	s	S88	rec36	c	S88	++	c		c	c	t	c	c	c	c	c		t	t	t	c	c	c					c	c
g	4945880	g	4962109	a	4911594	s	S88	rec36	g	S88	++	g		g	g	a	g	g	g	g	g		a	a	a	g	g	g					g	g
a	4945883	a	4962112	g	4911597	s	S88	rec36	a	S88	++	a		a	a	g	a	a	a	a	a		g	g	g	a	a	a					a	a
c	4945892	c	4962121	t	4911606	ns	S88	rec36	c	S88	++	c		c	c	t	c	c	c	c	c		t	t	t	c	c	c					c	c
g	4945894	g	4962123	t	4911608	ns	S88	rec36	g	S88	++	g		g	g	t	g	g	g	g	g		t	t	t	g	g	g					g	g
g	4945901	g	4962130	a	4911615	s	S88	rec36	g	S88	++	g		g	g	a	g	g	g	g	g		a	a	a	g	g	g					g	g
g	4945913	g	4962142	a	4911627	s	S88	rec36	g	S88	++	g		g	g	a	g	g	g	g	g		a	a	a	g	g	g					g	g
c	4945940	c	4962169	g	4911654	s	S88	rec36	c	S88	++	c		c	c	g	c	c	c	c	c		g	g	g	c	c	c					c	c
a	4945943	a	4962172	c	4911657	s	S88	rec36	a	S88	++	a		a	a	c	a	a	a	a	a		c	c	c	a	a	a					a	a
t	4945949	t	4962178	g	4911663	s	S88	rec36	t	S88	++	t		t	t	g	t	t	t	t	t		g	g	g	t	t	t					t	t
a	4945960	a	4962189	g	4911674	s	S88	rec36	a	S88	++++	a		a	a	g	a	a	a	a	a		a	a	a	a	a	a					a	a
a	4945961	a	4962190	g	4911675	s	S88	rec36	a	S88	++	a		a	a	g	a	a	a	a	a		g	g	g	a	a	a					a	a
g	4945964	g	4962193	a	4911678	s	S88	rec36	a	2	-	a		g	g	a	g	a	a	a	a		g	g	g	a	g	a					a	a
a	4945967	a	4962196	c	4911681	ns	S88	rec36	a	S88	++	a		a	a	c	a	a	a	a	a		c	c	c	a	a	a					a	a
t	4945969	t	4962198	c	4911683	ns	S88	rec36	t	S88	++	t		t	t	c	t	t	t	t	t		c	c	c	t	t	t					t	t
g	4945973	g	4962202	c	4911687	s	S88	rec36	g	S88	++	g		g	g	c	g	g	g	g	g		c	c	c	g	g	g					g	g
a	4945985	a	4962214	g	4911699	s	S88	rec36	a	S88	++	a		a	a	g	a	a	a	a	a		g	g	g	a	a	a					a	a
c	4945990	c	4962219	t	4911704	ns	S88	rec36	c	S88	++	c		c	c	t	c	c	c	c	c		t	t	t	c	c	c					c	c
g	4945991	g	4962220	t	4911705	s	S88	rec36	g	S88	++	g		g	g	t	g	g	g	g	g		t	t	t	g	g	g					g	g
t	4946027	t	4962256	c	4911741	s	S88	rec36	t	S88	++	t		t	t	c	t	t	t	t	t		c	c	c	t	t	t					t	t
t	4946030	t	4962259	c	4911744	s	S88	rec36	t	S88	++	t		t	t	c	t	t	t	t	t		c	c	c	t	t	t					t	t
c	4946033	c	4962262	a	4911747	s	S88	rec36	c	S88	++	c		c	c	a	c	c	c	c	c		a	a	a	c	c	c					c	c
c	4946045	c	4962274	t	4911759	s	S88	rec36	c	S88	++	c		c	c	t	c	c	c	c	c		t	t	t	c	c	c					c	c
a	4946048	a	4962277	g	4911762	s	S88	rec36	a	S88	++	a		a	a	g	a	a	a	a	a		g	g	g	a	a	a					a	a
a	4946057	a	4962286	t	4911771	ns	S88	rec36	a	S88	++	a		a	a	t	a	a	a	a	a		t	t	t	a	a	a					a	a
c	4946060	c	4962289	a	4911774	s	S88	rec36	c	S88	++	c		c	c	a	c	c	c	c	c		a	a	a	c	c	c					c	c
t	4946065	t	4962294	c	4911779	ns	S88	rec36	t	S88	++	t		t	t	c	t	t	t	t	t		c	c	c	t	t	t					t	t
g	4946066	g	4962295	t	4911780	s	S88	rec36	g	S88	++	g		g	g	t	g	g	g	g	g		t	t	t	g	g	g					g	g
c	4946071	c	4962300	t	4911785	ns	S88	rec36	c	S88	++	c		c	c	t	c	c	c	c	c		t	t	t	c	c	c					c	c
t	4946075	t	4962304	c	4911789	s	S88	rec36	t	S88	++	t		t	t	c	t	t	t	t	t		c	c	c	t	t	t					t	t
g	4946081	g	4962310	a	4911795	s	S88	rec36	g	S88	++	g		g	g	a	g	g	g	g	g		a	a	a	g	g	g					g	g
g	4946084	g	4962313	a	4911798	s	S88	rec36	g	S88	++	g		g	g	a	g	g	g	g	g		a	a	a	g	g	g					g	g
a	4946087	a	4962316	g	4911801	s	S88	rec36	a	S88	++	a		a	a	g	a	a	a	a	a		g	g	g	a	a	a					a	a
g	4946090	g	4962319	a	4911804	s	S88	rec36	g	S88	++	g		g	g	a	g	g	g	g	g		a	a	a	g	g	g					g	g
g	4946096	g	4962325	c	4911810	ns	S88	rec36	g	S88	++	g		g	g	c	g	g	g	g	g		c	c	c	g	g	g					g	g
g	4946099	g	4962328	a	4911813	s	S88	rec36	g	S88	++	g		g	g	a	g	g	g	g	g		a	a	a	g	g	g					g	g
t	4946111	t	4962340	c	4911825	ns	S88	rec36	t	S88	++	t		t	t	c	t	t	t	t	t		c	c	c	t	t	t					t	t

ExPEC genome site details												Outgroup Strains Details[i]																						
UTI89[b]	UTI89 site[c]	APEC 01[b]	APEC 01 site[c]	S88[b]	S88 site[c]	type[d]	Event Lineage[ef]	Recombinant segment	Inferred ancestral base[g]	Outgroup Analysis[e]	Support level[h]	CFT073	ED1a	536	E2348/69	SMS 3-5	IAI39	UMN026	K-12	ATCC 8739	HS	D1 Sd197	CB9615	Sakai	EDL933	IAI1	E24377A	SE11	SS Ss046	F2a 301	F2a 2457T	F5 8401	B4 Sb227	B18 BS512
g	4946112	g	4962341	c	4911826	ns	S88	rec36	g	S88	++	g		g	g	c	g	g	g	g	g		c	c	c	g	g	g					g	g
a	4946113	a	4962342	c	4911827	ns	S88	rec36	a	S88	++	a		a	a	c	a	a	a	a	a		c	c	c	a	a	a					a	a
t	4946117	t	4962346	g	4911831	ns	S88	rec36	t	S88	++	t		t	t	g	t	t	t	t	t		g	g	g	t	t	t					t	t
g	4946118	g	4962347	a	4911832	ns	S88	rec36	g	S88	++	g		g	g	a	g	g	g	g	g		a	a	a	g	g	g					g	g
a	4946123	a	4962352	c	4911837	ns	S88	rec36	a	S88	++	a		a	a	c	a	a	a	a	a		c	c	c	a	a	a					a	a
t	4946125	t	4962354	g	4911839	ns	S88	rec36	t	S88	++	t		t	t	g	t	t	t	t	t		g	g	g	t	t	t					t	t
g	4946136	g	4962365	a	4911850	ns	S88	rec36	g	S88	++	g		g	g	a	g	g	g	g	g		a	a	a	g	g	g					g	g
a	4946141	a	4962370	g	4911855	s	S88	rec36	a	S88	++	a		a	a	g	a	a	a	a	a		g	g	g	a	a	a					a	a
a	4946388	a	4962617	t	4914690	s	S88	rec36	a	S88	++	a		a	a	t	a	t	t	t	t	a	t	t	t	t	a	t					t	t
a	4946400	a	4962629	g	4914702	s	S88	rec36	a	S88	++	a		a	a	g	a	g	g	g	g	a	g	g	g	g	a	g					g	g
g	4946499	g	4962728	a	4914801	s	S88	rec36	g	S88	++++	g		g	g	g	g	g	g	g	g	g	g	g	g	g	g	g					g	g
c	4946538	c	4962767	t	4914840	s	S88	rec36	c	S88	++	c		c	c	t	c	t	t	t	t	t	t	t	t	t	c	t					t	t
g	4946540	g	4962769	a	4914842	s	S88	rec36	g	S88	++	g		g	g	a	g	a	a	a	a	g	a	a	a	a	g	a					a	a
t	4946542	t	4962771	a	4914844	ns	S88	rec36	t	S88	++	t		t	t	a	t	a	a	a	a	t	a	a	a	a	t	a					a	a
g	4946553	g	4962782	c	4914855	ns	S88	rec36	g	S88	++	g		g	g	c	g	c	c	c	c	g	c	c	c	c	g	c					c	c
c	4946554	c	4962783	g	4914856	ns	S88	rec36	c	S88	++	c		c	c	g	c	g	g	g	g	c	g	g	g	g	c	g					g	g
t	4946565	t	4962794	a	4914867	ns	S88	rec36	t	S88	++	t		t	t	a	t	a	a	a	a	t	a	a	a	a	t	a					a	a
g	4946566	g	4962795	c	4914868	ns	S88	rec36	g	S88	++	g		g	g	c	g	c	c	c	c	g	c	c	c	c	g	c					c	c
c	4946567	c	4962796	t	4914869	s	S88	rec36	c	S88	++	c		c	c	t	c	t	t	t	t	c	t	t	t	t	c	t					t	t
c	4946570	c	4962799	t	4914872	s	S88	rec36	c	S88	++	c		c	c	t	c	t	t	t	t	c	t	t	t	t	c	t					t	t
g	4946576	g	4962805	a	4914878	s	S88	rec36	g	S88	++	g		g	g	a	g	a	a	a	a	g	a	a	a	a	g	a					a	a
c	4946579	c	4962808	t	4914881	s	S88	rec36	c	S88	++	c		c	c	t	c	t	t	t	t	c	t	t	t	t	c	t					t	t
c	4946585	c	4962814	t	4914887	s	S88	rec36	c	S88	++++	c		c	c	t	c	c	c	c	c	c	c	c	c	c	c	c					c	c
a	4946597	a	4962826	g	4914899	s	S88	rec36	a	S88	++	a		a	a	g	a	g	g	g	g	a	g	g	g	g	a	g					g	g
g	4946603	g	4962832	a	4914905	s	S88	rec36	g	S88	++	g		g	g	a	g	a	a	a	a	g	a	a	a	a	g	a					a	a
g	4946618	g	4962847	a	4914920	s	S88	rec36	g	S88	++	g		g	g	a	g	a	a	a	a	g	a	a	a	g	g	g					a	a
a	4946621	a	4962850	g	4914923	s	S88	rec36	a	S88	++	a		a	a	g	a	g	g	g	g	a	g	g	g	g	a	g					g	g
a	4946647	a	4962876	t	4914949	ns	S88	rec36	t	2	-	t		t	t	t	t	a	a	a	a	t	a	a	a	a	t	a					a	a
g	4946653	g	4962882	a	4914955	s	S88	rec36	g	S88	++++	g		g	g	a	g	g	g	g	g	g	g	g	g	g	g	g					g	g
g	4946768	g	4962997	a	4915070	s	S88	rec36	g	S88	++	g		g	g	a	g	g	t	g	g	g	g	g	g	g	g	g					g	g
t	4946789	t	4963018	g	4915091	s	S88	rec36	t	S88	++	t		t	t	g	g	g	g	g	g	g	g	g	g	g	t	g					g	g
a	4946798	a	4963027	t	4915100	s	S88	rec36	a	S88	++	a		a	a	t	t	t	t	t	t	t	t	t	t	t	a	t					t	t
a	4946825	a	4963054	g	4915127	s	S88	rec36	a	S88	++	a		a	a	g	g	a	a	g	g	g	a	a	a	g	a	g					g	g
t	4946834	t	4963063	c	4915136	s	S88	rec36	t	S88	++++	t		t	t	t	t	t	t	t	t	t	t	t	t	t	t	t					t	t
g	4946837	g	4963066	a	4915139	s	S88	rec36	g	S88	++++	g		g	g	g	g	g	g	g	g	g	g	g	g	g	g	g					g	g
g	4946852	g	4963081	c	4915154	s	S88	rec36	g	S88	++++	g		g	g	g	g	g	g	g	g	g	g	g	g	g	g	g					g	g
c	4946888	c	4963117	t	4915190	s	S88	rec36	t	2	-	t		t	t	t	c	t	t	t	t	t	t	t	t	t	t	t					t	t
t	4946891	t	4963120	c	4915193	s	S88	rec36	c	2	-	c		c	c	c	t	c	c	c	c	c	c	c	c	c	c	c					c	c
c	4946896	c	4963125	t	4915198	ns	S88	rec36	c	S88	++++	c		c	c	c	c	c	c	c	c	c	c	c	c	c	c	c					c	c
a	4946990	a	4963219	g	4915292	s	S88	rec36	a	S88	++++	a		a	a	a	a	a	a	a	a	a	a	a	a	a	a	a					a	a
a	4947020	a	4963249	c	4915322	s	S88	rec36	a	S88	++++	a		a	a	a	a	g	a	a	a	a	a	a	a	a	a	a					a	a
c	4947038	c	4963267	t	4915340	s	S88	rec36	c	S88	++	c		c	c	t	c	c	c	c	c	t	c	c	c	c	c	c					c	c
t	4947054	t	4963283	g	4915356	ns	S88	rec36	t	S88	++	t		t	t	g	t	g	g	g	g	g	g	g	g	g	t	g					g	g
c	4947089	c	4963318	t	4915391	s	S88	rec36	t	2	-	t		t	t	t	c	t	t	t	t	t	t	t	t	t	t	t					t	t
t	4947098	t	4963327	c	4915400	s	S88	rec36	t	S88	++	t		c	t	c	t	c	c	t	t	c	t	t	t	t	t	t					t	t
t	4947155	t	4963384	c	4915457	s	S88	rec36	c	2	-	c		c	c	c	c	c	c	c	c	c	c	c	c	c	c	c					c	c
c	4947161	c	4963390	t	4915463	s	S88	rec36	c	S88	++	c		c	c	t	c	c	c	c	c	t	c	c	c	c	c	c					c	c
g	4947175	g	4963404	a	4915477	ns	S88	rec36	g	S88	++++	g		g	g	g	g	g	g	g	g	g	g	g	g	g	g	g					g	g
t	4947221	t	4963450	g	4915523	s	S88	rec36	t	S88	++	t		t	t	t	t	g	g	t	t	t	t	t	t	t	t	t					t	t
a	4947257	a	4963486	t	4915559	s	S88	rec36	a	S88	++	a		a	a	t	a	t	t	t	t	t	t	t	t	t	a	t					t	t
t	4947280	t	4963509	c	4915582	ns	S88	rec36	c	2	-	c		c	c	c	t	c	c	c	c	c	c	c	c	c	c	c					c	c
t	4947313	t	4963542	c	4915615	ns	S88	rec36	t	S88	++	t		t	t	c	t	c	c	t	t	c	t	t	t	t	t	t					t	t
a	4947353	a	4963582	g	4915655	s	S88	rec36	a	S88	++++	a		a	a	a	a	a	a	a	a	a	a	a	a	a	a	a					a	a
a	4947521	a	4963750	c	4915823	s	S88	rec36	a	S88	++	a		a	a	a	a	c	c	c	c	a	c	c	c	c	a	c					c	c
g	4947541	g	4963770	t	4915843	ns	S88	rec36	g	S88	++	g		g	g	g	g	t	t	t	g	g	t	t	t	g	g	g					g	g
c	4947611	c	4963840	g	4915913	s	S88	rec36	c	S88	++	c		c	c	c	c	g	g	c	g	g	g	g	g	g	c	g					g	g
a	4947617	a	4963846	g	4915919	s	S88	rec36	a	S88	++	a		a	a	a	a	g	g	g	g	g	g	g	g	g	a	g					g	g
c	4947626	c	4963855	t	4915928	s	S88	rec36	c	S88	++	c		c	c	c	c	t	t	c	t	t	t	t	t	t	c	t					c	c
g	4947644	g	4963873	c	4915946	s	S88	rec36	g	S88	++	g		g	g	g	g	c	c	c	c	c	c	c	c	c	g	c					c	c
c	4947716	c	4963945	t	4916018	s	S88	rec36	c	S88	++	c		c	c	c	c	t	t	c	t	t	c	c	c	t	c	t					t	t
t	4947734	t	4963963	c	4916036	s	S88	rec36	t	S88	++	t		t	t	t	t	c	c	c	c	c	c	c	c	c	t	c					c	c
a	4947755	a	4963984	g	4916057	s	S88	rec36	a	S88	++	a		a	a	g	a	g	g	g	g	g	g	g	g	g	a	g					g	g
t	4947758	t	4963987	c	4916060	s	S88	rec36	t	S88	++	t		t	t	t	t	c	c	t	t	c	c	c	c	t	t	t					t	t
g	4947761	g	4963990	a	4916063	s	S88	rec36	g	S88	++	g		g	g	a	g	a	a	a	a	a	a	a	a	a	g	a					a	a
g	4947869	g	4964098	a	4916171	s	S88	rec36	g	S88	++	g		g	g	a	g	a	a	a	a	a	a	a	a	a	g	a					g	g
t	4947872	t	4964101	c	4916174	s	S88	rec36	t	S88	++	t		t	t	c	t	c	c	c	c	c	c	c	c	c	t	c					c	c
c	4947920	c	4964149	a	4916222	s	S88	rec36	c	S88	++	c		c	c	a	c	a	a	a	a	a	a	a	a	a	c	a					a	a
a	4947922	a	4964151	g	4916224	s	S88	rec36	a	S88	++	a		a	a	g	a	g	g	g	g	g	g	g	g	g	a	g					g	g
g	4947932	g	4964161	a	4916234	s	S88	rec36	g	S88	++	g		g	g	a	g	a	a	a	a	a	a	a	a	a	g	a					a	a
g	4947935	g	4964164	a	4916237	s	S88	rec36	g	S88	++	g		g	g	a	g	a	a	g	g	a	g	g	g	a	t	a					g	g
c	4947998	c	4964227	t	4916300	s	S88	rec36	c	S88	++	c		c	c	c	c	c	c	c	c	c	t	t	t	c	c	c					c	c
t	4948055	t	4964284	c	4916357	s	S88	rec36	t	S88	++++	t		t	t	t	t	t	t	c	t	t	t	t	t	t	t	t					t	t
t	4948088	t	4964317	c	4916390	s	S88	rec36	t	S88	++	t		t	t	g	t	g	g	a	c	c	t	t	t	c	t	c					c	c
c	4948091	c	4964320	t	4916393	s	S88	rec36	c	S88	++++	c		c	c	c	c	c	c	c	c	c	c	c	c	c	c	c					c	c
g	4948093	g	4964322	a	4916395	s	S88	rec36	g	S88	++	g		g	a	a	a	a	a	a	a	a	g	g	g	g	g	g					a	a
t	4948094	t	4964323	c	4916396	s	S88	rec36	t	S88	++	t		t	c	t	c	t	t	t	c	c	c	c	c	c	t	c					c	c
g	4948109	g	4964338	a	4916411	s	S88	rec36	g	S88	++	g		g	g	g	g	a	a	g	g	g	g	g	g	a	g	a					g	g
t	4948304	t	4964533	g	4916606	s	S88	rec36	t	S88	++	t		t	t	g	t	g	g	g	g	g	g	g	g	g	t	g					g	g
c	4948319	c	4964548	t	4916621	s	S88	rec36	c	S88	++++	c		c	c	t	c	c	c	c	c	c	c	c	c	c	c	c					c	c
g	4948542	g	4964771	a	4916844	nc	S88	rec36	g	S88	++	g		g	g	g	g	a	a	a	g	g	a	a	a	g	g	g					g	g
t	4948596	t	4964825	a	4916898	ns	S88	rec36	t	S88	++	t	t	t	t	a	t	a	a	a	a	a	a	a	a	a	t	a					a	a
c	4948632	c	4964861	a	4916934	s	S88	rec36	c	S88	++++	c	c	c	c	c	c	c	c	c	c	c	c	c	c	c	c	c					c	c
a	4948647	a	4964876	t	4916949	s	S88	rec36	a	S88	++	a	a	a	a	t	a	t	t	c	t	t	t	t	t	t	a	t					t	t

ExPEC genome site details												Outgroup Strains Details[i]																						
UTI89[b]	UTI89 site[c]	APEC 01[b]	APEC 01 site[c]	S88[b]	S88 site[c]	type[d]	Event Lineage[ef]	Recombinant segment	Inferred ancestral base[g]	Outgroup Analysis[e]	Support level[h]	CFT073	ED1a	536	E2348/69	SMS 3-5	IAI39	UMN026	K-12	ATCC 8739	HS	D1 Sd197	CB9615	Sakai	EDL933	IAI1	E24377A	SE11	SS Ss046	F2a 301	F2a 2457T	F5 8401	B4 Sb227	B18 BS512
t	4948650	t	4964879	c	4916952	s	S88	rec36	t	S88	++	t	t	t	t	c	t	c	c	c	c	c	c	c	c	c	t	c					c	c
g	4948686	g	4964915	a	4916988	s	S88	rec36	g	S88	++	g	g	g	g	g	g	a	a	a	g	a	g	g	g	g	g	g					g	g
g	4948695	g	4964924	c	4916997	s	S88	rec36	g	S88	++	g	g	g	g	c	g	c	c	t	c	c	c	c	c	c	g	c					c	c
t	4948740	t	4964969	c	4917042	s	S88	rec36	t	S88	++	t	t	t	t	c	t	c	c	c	c	c	g	g	g	c	t	c					a	a
g	4948743	g	4964972	a	4917045	s	S88	rec36	g	S88	++	g	g	g	g	a	g	a	a	a	a	a	a	a	a	a	g	a					a	a
g	4948761	g	4964990	a	4917063	s	S88	rec36	g	S88	++	g	g	a	g	a	g	a	a	g	a	a	a	a	a	a	g	a					a	a
a	4948776	a	4965005	c	4917078	s	S88	rec36	a	S88	++	a	a	a	a	c	a	c	c	c	c	c	c	c	c	c	a	c					c	c
g	4948788	g	4965017	a	4917090	s	S88	rec36	g	S88	++	g	g	g	g	g	g	g	g	g	g	a	a	a	a	g	g	g					g	g
a	4948803	a	4965032	g	4917105	s	S88	rec36	a	S88	++	a	a	a	a	g	a	g	g	g	g	g	g	g	g	g	a	g					g	g
t	4948812	t	4965041	c	4917114	s	S88	rec36	t	S88	++	t	t	t	t	c	t	c	c	c	c	c	c	c	c	c	t	c					c	c
g	4948824	g	4965053	c	4917126	s	S88	rec36	c	2	-	c	c	g	c	c	c	a	a	c	c	c	c	c	c	c	c	c					c	c
c	4948887	c	4965116	a	4917189	s	S88	rec36	a	2	-	a	a	c	a	a	a	a	a	a	a	a	a	a	a	a	a	a					a	a
t	4948931	t	4965160	c	4917233	ns	S88	rec36	t	S88	++++	t	t	t	t	t	t	t	t	t	t	t	t	t	t	t	t	t					t	t
g	4948992	g	4965221	a	4917294	s	S88	rec36	a	2	-	a	a	a	a	a	a	a	a	g	a	a	g	g	g	a	a	a					a	a
c	4948995	c	4965224	t	4917297	s	S88	rec36	t	2	-	t	t	t	t	t	t	t	t	t	t	t	t	t	t	t	t	t					t	t
g	4949013	g	4965242	a	4917315	s	S88	rec36	g	S88	++	g	g	g	g	g	g	g	g	a	a	g	g	g	g	a	g	a					a	a
g	4949060	g	4965289	a	4917362	s	S88	rec36	g	S88	++	g	g	g	g	g	a	g	g	g	a	a	a	a	a	a	g	a					g	g
g	4949079	g	4965308	a	4917381	s	S88	rec36	a	2	-	a	a	a	a	g	a	g	g	c	a	a	a	a	a	a	g	a					a	a
t	4949086	t	4965315	c	4917388	ns	S88	rec36	c	2	-	c	c	c	c	c	c	c	c	c	c	c	c	c	c	c	c	c					c	c
g	4949100	g	4965329	a	4917402	s	S88	rec36	g	S88	++	g	g	g	g	g	g	g	g	g	a	g	g	g	g	a	g	a					a	a
g	4949196	g	4965425	a	4917498	s	S88	rec36	g	S88	++	g	g	g	g	g	g	g	g	a	g	g	g	g	g	a	g	a					a	a
t	4949217	t	4965446	c	4917519	s	S88	rec36	t	S88	++	t	t	t	t	t	t	t	t	c	t	t	t	t	t	c	t	c					c	c
t	4949268	t	4965497	c	4917570	s	S88	rec36	t	S88	++	c	t	t	c	c	c	c	c	c	c	c	c	c	c	c	c	c					c	c
g	4949289	g	4965518	a	4917591	s	S88	rec36	g	S88	++	g	g	g	g	g	g	g	g	g	g	g	g	g	g	a	g	a					g	g
g	4949292	g	4965521	a	4917594	s	S88	rec36	g	S88	++	g	g	g	g	g	g	g	g	a	g	g	g	g	g	a	g	a					g	g
t	4949301	t	4965530	c	4917603	s	S88	rec36	t	S88	++	t	t	t	t	t	t	t	t	g	t	t	t	t	t	c	t	c					t	t
a	4949376	a	4965605	g	4917678	s	S88	rec36	g	2	-	g	a	g	g	g	g	g	g	g	g	a	g	g	g	g	a	g					g	g
g	4949403	g	4965632	a	4917705	s	S88	rec36	g	S88	++	g	g	g	g	a	g	a	a	g	a	g	a	a	a	a	g	a					a	a
g	4949454	g	4965683	a	4917756	s	S88	rec36	g	S88	++	g	g	g	g	a	g	g	g	a	a	a	a	a	a	a	a	a					a	a
g	4949463	g	4965692	a	4917765	s	S88	rec36	g	S88	++	g	g	g	g	a	g	g	g	g	g	g	a	a	a	g	g	g					g	g
t	4949475	t	4965704	c	4917777	s	S88	rec36	t	S88	++	t	t	t	t	c	t	c	c	c	c	c	c	c	c	c	c	c					c	c
c	4949505	c	4965734	t	4917807	s	S88	rec36	c	S88	++	c	c	c	c	t	c	t	t	c	t	c	t	t	t	c	t	c					c	c
c	4949508	c	4965737	t	4917810	s	S88	rec36	c	S88	++	c	c	c	c	t	c	t	t	c	t	c	t	t	t	c	t	c					c	c
a	4949550	a	4965779	g	4917852	s	S88	rec36	a	S88	++	a	a	a	a	a	a	g	g	a	g	a	a	a	a	a	a	a					a	a
t	4949574	t	4965803	c	4917876	s	S88	rec36	t	S88	++	t	t	t	t	t	t	t	t	t	c	t	t	t	t	c	t	c					c	c
g	4949607	g	4965836	a	4917909	s	S88	rec36	g	S88	++	g	g	g	g	a	g	g	g	a	g	a	a	a	a	g	g	g					a	a
c	4949676	c	4965905	t	4917978	s	S88	rec36	c	S88	++++	c	c	c	c	c	c	c	c	c	c	c	c	c	c	c	c	c					c	c
a	4949757	a	4965986	g	4918059	s	S88	rec36	g	2	-	g	g	a	a	g	a	g	g	g	g	g	g	g	g	g	g	g					g	g
t	4949875	t	4966104	a	4918177	ns	S88	rec36	t	S88	++	t	t	t	t	a	t	a	a	c	a	a	a	a	a	a	t	a					a	a
a	4949949	a	4966178	g	4919806	nc	S88	rec36	a	S88	++	a	a	a	a	g	a	a	a	g	g	a	c	c	c	c		g					-	-
t	4949953	t	4966182	c	4919810	nc	S88	rec36	t	S88	++	t	t	t	t	c	t	t	t	c	c	t	c	c	c	c		c					-	-
c	4949970	c	4966199	g	4919827	nc	S88	rec36	c	S88	++	c	c	c	c	g	c	g	g	g	g	g	g	g	g	g		g					g	g
g	4949972	g	4966201	a	4919829	nc	S88	rec36	g	S88	++	g	g	g	g	a	g	g	g	a	g	a	g	g	g	g		g					a	a
a	4949984	a	4966213	g	4919841	nc	S88	rec36	a	S88	++	a	a	a	a	g	a	g	g	g	g	g	g	g	g	g		g					g	g
a	4949991	a	4966220	t	4919848	nc	S88	rec36	a	S88	++	a	a	a	a	t	a	t	t	t	t	t	t	t	t	t		t					t	t
a	4949995	a	4966224	g	4919852	nc	S88	rec36	a	S88	++	a	a	a	a	g	a	g	g	g	g	g	g	g	g	g		g					g	g
t	4950000	t	4966229	a	4919857	nc	S88	rec36	t	S88	++	t	t	t	t	a	t	a	a	a	a	a	a	a	a	a		a					a	a
c	4950007	c	4966236	t	4919864	nc	S88	rec36	c	S88	++	c	c	c	c	t	c	t	t	t	t	t	t	t	t	t		t					t	t
a	4950022	a	4966251	c	4919879	nc	S88	rec36	a	S88	++	a	a	a	a	c	a	a	a	c	c	a	a	a	a	a		c					a	a
t	4950026	t	4966255	c	4919883	nc	S88	rec36	t	S88	++	t	t	t	t	c	t	c	c	c	c	c	c	c	c	t		c					c	c
t	4950069	t	4966298	c	4919926	s	S88	rec36	t	S88	++	t	c	t	t	c	t	c	c	c	c	c	c	c	c	c		c					c	c
g	4950072	g	4966301	a	4919929	s	S88	rec36	g	S88	++	g	g	g	g	g	g	g	g	g	a	g	g	g	g	g		a					g	g
a	4950114	a	4966343	g	4919971	s	S88	rec36	a	S88	++	a	a	a	a	c	a	g	g	g	g	g	g	g	g	g		g					t	t
t	4950118	t	4966347	c	4919975	ns	S88	rec36	t	S88	++	t	t	t	t	c	t	c	c	c	c	c	c	c	c	c		c					c	c
t	4950186	t	4966415	c	4920043	s	S88	rec36	t	S88	++	t	t	t	t	c	t	t	t	c	c	c	c	c	c	c		c					c	c
t	4950270	t	4966499	c	4920127	s	S88	rec36	t	S88	++	t	t	t	t	c	t	c	c	t	t	t	g	g	g	c		t					t	t
a	4950294	a	4966523	g	4920151	s	S88	rec36	a	S88	++	a	a	a	a	g	a	g	g	a	a	g	g	g	g	g		a					a	a
t	4950315	t	4966544	c	4920172	s	S88	rec36	t	S88	++	t	t	t	t	c	t	c	c	t	c	t	t	t	t	c		c					c	c
t	4950324	t	4966553	c	4920181	s	S88	rec36	t	S88	++	t	t	t	t	c	t	t	t	t	c	t	t	t	t	t		c					t	t
t	4950339	t	4966568	c	4920196	s	S88	rec36	t	S88	++	t	t	t	t	c	c	c	c	c	c	c	c	c	c	c		c					c	c
a	4950381	a	4966610	g	4920238	s	S88	rec36	a	S88	++	a	a	a	a	g	a	g	g	g	g	g	g	g	g	g		g					g	g
a	4950398	a	4966627	c	4920255	ns	S88	rec36	a	S88	++	a	a	a	a	c	a	c	c	c	c	c	c	c	c	c		c					c	c
a	4950405	a	4966634	g	4920262	s	S88	rec36	g	2	-	g	g	a	g	g	g	g	g	g	g	g	g	g	g	g		g					g	g
c	4950444	c	4966673	t	4920301	s	S88	rec36	c	S88	++	c	t	c	t	t	t	t	t	t	t	t	t	t	t	t		t					t	t
t	4950459	t	4966688	c	4920316	s	S88	rec36	t	S88	++	c	t	t	t	t	c	c	c	c	c	c	t	t	t	c		c					c	c
t	4950460	t	4966689	c	4920317	s	S88	rec36	t	S88	++	c	t	t	t	t	c	c	c	c	c	c	t	t	t	c		c					c	c
c	4950483	c	4966712	t	4920340	s	S88	rec36	c	S88	++	c	c	c	c	c	c	c	c	c	t	t	c	c	c	c		t					c	c
a	4950489	a	4966718	g	4920346	s	S88	rec36	g	2	-	g	g	a	g	g	g	g	g	g	g	g	g	g	g	g		g					g	g
t	4950492	t	4966721	c	4920349	s	S88	rec36	t	S88	++	t	t	t	t	t	t	t	t	t	c	c	t	t	t	t		c					t	t
c	4950516	c	4966745	a	4920373	s	S88	rec36	c	S88	++	c	c	c	c	a	c	c	c	c	a	a	c	c	c	a		a					a	a
a	4950535	a	4966764	g	4920392	ns	S88	rec36	a	S88	++	a	a	a	a	g	a	g	g	a	g	g	a	a	a	g		g					g	g
t	4950636	t	4966865	c	4920493	s	S88	rec36	t	S88	++	t	t	t	t	t	t	c	c	c	c	c	c	c	c	t		c					t	t
c	4950639	c	4966868	t	4920496	s	S88	rec36	c	S88	++	c	c	c	c	t	c	t	t	c	t	c	t	t	t	c		t					c	c
g	4950649	g	4966878	c	4920506	ns	S88	rec36	g	S88	++	g	g	g	g	g	g	g	g	g	a	g	g	g	g	g		a					g	g
t	4950690	t	4966919	c	4920547	s	S88	rec36	t	S88	++++	t	t	t	t	c	t	t	t	t	t	t	t	t	t	t		t					t	t
t	4950720	t	4966949	c	4920577	s	S88	rec36	t	S88	++	t	t	t	t	c	t	c	c	c	c	c	c	c	c	c		c					c	c
t	4950738	t	4966967	g	4920595	ns	S88	rec36	t	S88	++	t	t	t	t	t	t	t	t	g	g	t	t	t	t	g		g					g	g
g	4950739	g	4966968	a	4920596	ns	S88	rec36	g	S88	++	g	g	g	g	g	g	g	g	a	a	g	g	g	g	g		a					g	g
t	4950750	t	4966979	c	4920607	s	S88	rec36	t	S88	++	t	t	t	t	c	t	t	t	c	c	c	t	t	t	c		c					c	c
a	4950754	a	4966983	c	4920611	ns	S88	rec36	a	S88	++	a	a	a	a	c	a	c	c	c	c	c	c	c	c	c		c					c	c
c	4950766	c	4966995	a	4920623	ns	S88	rec36	c	S88	++	c	c	c	c	a	c	a	a	a	a	a	a	a	a	a		a					a	a
a	4950777	a	4967006	c	4920634	ns	S88	rec36	a	S88	++	a	a	a	a	c	a	c	c	c	c	c	c	c	c	c		c					c	c
c	4950811	c	4967040	t	4920668	ns	S88	rec36	c	S88	++	c	c	c	c	c	c	c	c	t	t	c	c	c	c	c		t					c	c
a	4950844	a	4967073	c	4920725	ns	S88	rec36	a	S88	++	a	a	a	a		a	a	a	c	c	c	a	a	a			c					c	c

Table S8. Allocation of recombinational SNPs to lineages by virtual outgroup analysis[a]

ExPEC genome site details												Outgroup Strains Details[i]																						
UTI89[b]	UTI89 site[c]	APEC 01[b]	APEC 01 site[c]	S88[b]	S88 site[c]	type[d]	Event Lineage[ef]	Recombinant segment	Inferred ancestral base[g]	Outgroup Analysis[e]	Support level[h]	CFT073	ED1a	536	E2348/69	SMS 3-5	IAI39	UMN026	K-12	ATCC 8739	HS	D1 Sd197	CB9615	Sakai	EDL933	IAI1	E24377A	SE11	SS Ss046	F2a 301	F2a 2457T	F5 8401	B4 Sb227	B18 BS512
t	4950846	t	4967075	c	4920727	ns	S88	rec36	t	S88	++	t	t	t	t		t	t	t	c	c	c	t	t	t			c					c	c
t	4950849	t	4967078	c	4920730	s	S88	rec36	t	S88	++	t	t	t	t		t	t	t	t	t	c	t	t	t			t					c	c
t	4950858	t	4967087	g	4920739	ns	S88	rec36	t	S88	++	t	t	t	t		g	g	g	g	g	g	g	g	g			g					g	g
t	4950872	t	4967101	c	4920753	ns	S88	rec36	t	S88	++	t	t	t	t		c	c	c	c	c	c	c	c	c			c					c	c
a	4950921	a	4967150	g	4920802	ns	S88	rec36	a	S88	++	a	a	a	a		g	g	g	g	g	g	g	g	g			g					g	g
g	4950936	g	4967165	a	4920817	s	S88	rec36	g	S88	++++	g	g	g	g		g	g	g	g	g	g	g	g	g			g						g
g	4950948	g	4967177	a	4920829	s	S88	rec36	g	S88	++	g	g	g	g		c	g	g	a	a	a	g	g	g			a						a
c	4950949	c	4967178	a	4920830	ns	S88	rec36	c	S88	++	c	c	c	c		c	c	c	a	a	a	c	c	c			a						a
t	4950951	t	4967180	c	4920832	ns	S88	rec36	t	S88	++	t	t	t	t		t	t	t	c	c	c	t	t	t			c						c
g	4950952	g	4967181	a	4920833	ns	S88	rec36	g	S88	++	g	g	g	g		g	g	g	a	a	a	g	g	g			a						a
c	4950956	c	4967185	g	4920837	ns	S88	rec36	c	S88	++	c	c	c	a		c	g	g	g	g	g	g	g	g			g						g
t	4950957	t	4967186	c	4920838	ns	S88	rec36	t	S88	++	t	t	t	c		t	c	c	c	c	c	c	c	c			c						c
t	4950958	t	4967187	g	4920839	ns	S88	rec36	t	S88	++	t	t	t	g		t	t	t	g	g	g	t	t	t			g						g
t	4950961	t	4967190	c	4920842	ns	S88	rec36	t	S88	++	t	t	t	t		t	t	t	c	c	c	t	t	t			c						c
c	4950967	c	4967196	a	4920848	ns	S88	rec36	c	S88	++	c	c	c	c		c	c	c	a	a	a	c	c	c			a						a
a	4950970	a	4967199	g	4920851	ns	S88	rec36	a	S88	++	a	a	a	g		a	a	a	g	g	g	a	a	a			g						g
a	4950973	a	4967202	t	4920854	ns	S88	rec36	a	S88	++	a	a	a	a		a	a	a	t	t	t	a	a	a			t						t
g	4950974	g	4967203	a	4920855	ns	S88	rec36	g	S88	++	g	g	g	g		g	g	g	a	a	a	g	g	g			a						a
g	4950987	g	4967216	t	4920868	nc	S88	rec36	g	S88	++	g	g	g			g	g	g	g	g	t	g	g	g			g						t
t	4951005	t	4967234	a	4920886	nc	S88	rec36	t	S88	++	t	t	t			t	a	a	t	t	a	a	a	a			t						a
c	4951014	c	4967243	t	4920895	nc	S88	rec36	c	S88	++	c	c	c			c	c	c	c	c	c	t	t	t			c						c
c	4953563	c	4969792	a	4922933	ns	S88	rec36	c	S88	++	c	t	c	c	t	c	c	c	a			c	c	c				t	t	t			
c	4953596	c	4969825	t	4922966	s	S88	rec36	c	S88	++	c	t	c	c	c	c	t	t	t			c	c	c				c	c	c			
a	4953611	a	4969840	c	4922981	s	S88	rec36	c	2	-	c	t	c	c	c	c	t	t	t			c	c	c				t	t	t			
t	4953631	t	4969860	c	4923001	ns	S88	rec36	t	S88	++++	t	t	t	t	t	c	t	t	t			t	t	t				t	t	t			
g	4953647	g	4969876	t	4923017	s	S88	rec36	t	2	-	t	g	t	t	t	g	t	t	g			t	t	t				g	g	g			
t	4953694	t	4969923	c	4923064	ns	S88	rec36	c	2	-	c	t	c	c	c	c	c	c	c			c	c	c				c	c	c			
g	4953696	g	4969925	a	4923066	ns	S88	rec36	g	S88	++	a	g	g	g	g	g	g	g	g			a	a	a				g	g	g			
c	4953698	c	4969927	a	4923068	s	S88	rec36	c	S88	++++	c	c	a	c	c	c	c	c	c			c	c	c				c	c	c			
a	4953704	a	4969933	g	4923074	s	S88	rec36	g	2	-	g	g	g	g	g	g	a	a	g			g	g	g				g	g	g			
a	4953779	a	4970008	g	4923149	s	S88	rec36	a	S88	++	a	g	a	g	a	a	a	a	g			a	a	a				a	a	a			
a	4953788	a	4970017	g	4923158	s	S88	rec36	a	S88	++++	a	a	a	a	a	a	a	a	a			a	a	a				a	a	a			
c	4953815	c	4970044	a	4923185	s	S88	rec36	c	S88	++++	c	c	c	c	c	c	c	c	c			c	c	c				c	c	c			
g	4953818	g	4970047	a	4923188	s	S88	rec36	g	S88	++++	g	g	g	g	g	g	g	g	g			g	g	g				g	g	g			
c	4953829	c	4970058	g	4923199	ns	S88	rec36	c	S88	++++	c	c	c	c	c	c	c	c	c			c	c	c				c	c	c			
t	4953930	t	4970159	c	4923300	nc	S88	rec36	c	2	-	c	c	c	c	c	c	c	c	c			c	c	c				c	c	c			
c	4953965	c	4970194	t	4923335	nc	S88	rec36	c	S88	++	c	c	c	c	c	c	t	t	c			t	t	t				c	c	c			
a	4953966	a	4970195	g	4923336	nc	S88	rec36	a	S88	++	a	a	a	a	a	a	g	g	a			g	g	g				a	a	a			
a	4953970	a	4970199	g	4923340	nc	S88	rec36	a	S88	++++	a	a	a	a	a	a	a	a	a			a	a	a				a	a	a			
c	4961924	c	4978153	a	4931867	s	S88	rec36	a	2	-	a	a	a	c	c	c	c	c	c	c	c	c	c	c	c	c	c	c	c	c	c	c	c
c	4961944	c	4978173	t	4931887	ns	S88	rec36	c	S88	++++	c	c	c	c	c	c	c	c	c	c	c	c	c	c	c	c	c	c	c	c	c	c	c
c	4961978	c	4978207	g	4931921	s	S88	rec36	c	S88	++	c	c	c	c	c	c	c	c	c	c	c	t	t	t	c	t	c	t	t	t	c	c	c
c	4962005	c	4978234	t	4931948	s	S88	rec36	c	S88	++	c	c	c	c	t	c	c	c	c	t	c	c	c	c	c	c	c	c	c	c	c	c	c
g	4962209	g	4978438	a	4932152	s	S88	rec36	g	S88	++	g	g	g	g	a	g	a	a	g	a	a	a	a	a	a	a	a	a	a	a	g	g	g
a	4962224	a	4978453	g	4932167	s	S88	rec36	a	S88	++	a	a	a	g	g	a	g	g	g	g	g	g	g	g	g	g	g	g	g	g	g	g	g
t	4962230	t	4978459	c	4932173	s	S88	rec36	t	S88	++	t	t	t	t	c	t	c	c	t	c	c	c	c	c	c	c	c	c	c	c	c	c	c
c	4962233	c	4978462	t	4932176	s	S88	rec36	c	S88	++	c	c	c	c	t	c	t	t	c	t	t	t	t	t	t	t	t	t	t	t	t	t	t
g	4962302	g	4978531	a	4932245	s	S88	rec36	g	S88	++++	g	g	g	g	g	g	g	g	g	g	g	g	g	g	g	g	g	g	g	g	g	g	g
a	4962329	a	4978558	g	4932272	s	S88	rec36	a	S88	++	a	a	a	a	g	a	a	g	a	a	a	a	a	a	a	a	a	g	g	g	g	g	g
t	4962365	t	4978594	c	4932308	s	S88	rec36	t	S88	++	t	t	t	t	t	t	t	t	c	t	t	t	t	t	t	t	t	c	c	c	c	c	c
g	4962369	g	4978598	a	4932312	ns	S88	rec36	g	S88	++++	g	g	g	g	g	g	g	g	g	g	g	g	g	g	g	g	g	g	g	g	g	g	g
g	4962410	g	4978639	a	4932353	s	S88	rec36	g	S88	++++	g	g	g	g	g	g	g	g	g	g	g	g	g	g	g	g	g	g	g	g	g	g	g
a	4962419	a	4978648	c	4932362	s	S88	rec36	a	S88	++	a	a	a	a	a	a	a	a	a	c	c	a	a	a	c	a	c	c	c	c	a	a	a
a	4962506	a	4978735	g	4932449	s	S88	rec36	a	S88	++	a	a	a	a	a	a	a	a	g	g	a	a	a	a	g	a	g	g	g	g	g	g	g
g	4962566	g	4978795	a	4932509	s	S88	rec36	g	S88	++++	g	g	g	g	g	g	g	g	g	g	g	g	g	g	g	g	g	g	g	g	g	g	g
a	4962620	a	4978849	g	4932563	s	S88	rec36	g	2	-	a	g	g	a	a	a	a	g	a	a	a	a	a	a	a	a	a	a	a	a	a	a	a
g	4962638	g	4978867	c	4932581	s	S88	rec36	c	2	-	g	c	c	g	c	g	c	c	t	c	t	t	t	t	c	t	c	t	t	t	t	t	t
t	4962644	t	4978873	c	4932587	s	S88	rec36	c	2	-	t	c	c	t	c	t	c	c	t	c	t	t	t	t	c	t	c	c	c	c	t	t	t
g	4962734	g	4978963	a	4932677	s	S88	rec36	g	S88	++++	g	g	g	g	g	g	g	a	g	g	g	g	g	g	g	g	g	g	g	g	g	g	g
t	4962782	t	4979011	c	4932725	s	S88	rec36	t	S88	++	t	t	t	t	c	t	t	c	t	t	t	t	t	t	t	t	t	t	t	t	t	t	t
t	4963285	t	4979514	g	4933160	ns	S88	rec36	t	S88	++	t	t	t	g	t	t	t	g	t	g	t	g	g	g	g	g	g	g	g	g	g	g	g
t	4963286	t	4979515	c	4933161	ns	S88	rec36	t	S88	++	t	t	t	c	t	t	t	c	t	c	t	c	c	c	c	c	c	c	c	c	c	c	c
c	4963287	c	4979516	a	4933162	s	S88	rec36	c	S88	++	c	c	c	a	c	c	c	a	c	a	c	a	a	a	a	a	a	a	a	a	a	a	a
g	4963350	g	4979579	a	4933225	s	S88	rec36	g	S88	++++	g	g	g	g	g	g	g	a	g	g	g	g	g	g	g	g	g	g	g	g	g	g	g
g	4963389	g	4979618	a	4933264	s	S88	rec36	g	S88	++++	g	g	g	g	g	g	g	a	g	g	g	g	g	g	g	g	g	g	g	g	g	g	g
c	4963449	c	4979678	t	4933324	s	S88	rec36	t	2	-	t	t	t	t	t	t	t	t	t	t	t	t	t	t	t	t	t	t	t	t	t	t	t
t	4963536	t	4979765	a	4933411	s	S88	rec36	t	S88	++	t	a	t	a	a	c	a	a	a	a	a	a	a	a	a	a	a	a	a	a	a	a	a
g	4963542	g	4979771	a	4933417	ns	S88	rec36	g	S88	++	g	a	g	a	a	g	a	a	a	a	a	a	a	a	a	a	a	a	a	a	a	a	a
t	4963544	t	4979773	c	4933419	ns	S88	rec36	t	S88	++	t	c	t	c	c	t	c	c	c	c	c	c	c	c	c	c	c	c	c	c	c	c	c
g	4963548	g	4979777	a	4933423	s	S88	rec36	g	S88	++	g	a	g	a	a	g	g	a	a	a	a	a	a	a	a	a	a	a	a	a	a	a	a
a	4963551	a	4979780	t	4933426	s	S88	rec36	a	S88	++	a	t	a	t	t	a	a	t	t	t	t	t	t	t	t	t	t	t	t	t	t	t	t
t	4963587	t	4979816	c	4933462	s	S88	rec36	c	2	-	t	c	c	t	c	t	c	c	c	c	c	c	c	c	c	c	c	c	c	c	c	c	c
c	4963611	c	4979840	t	4933486	s	S88	rec36	c	S88	++	c	c	t	c	t	t	c	t	t	t	t	t	t	t	t	t	t	t	t	t	t	t	t
g	4963734	g	4979963	a	4933609	s	S88	rec36	g	S88	++++	g	g	g	g	g	g	g	g	g	g	g	g	g	g	g	g	g	g	g	g	g	g	g
g	4963779	g	4980008	a	4933654	s	S88	rec36	g	S88	++++	g	g	g	g	g	a	g	g	g	g	g	g	g	g	g	g	g	g	g	g	g	g	g
a	4963926	a	4980155	c	4933801	s	S88	rec36	a	S88	++	a	a	a	a	a	a	a	c	c	a	a	a	a	a	a	a	a	a	a	a	a	a	a
t	4963947	t	4980176	c	4933822	s	S88	rec36	t	S88	++	t	t	c	t	c	c	c	c	c	t	c	c	c	c	t	c	t	c	c	c	t	c	c
a	4963953	a	4980182	g	4933828	s	S88	rec36	a	S88	++	a	a	a	a	a	a	a	g	a	a	a	a	a	a	a	a	a	a	a	a	a	g	g
t	4964082	t	4980311	c	4933957	s	S88	rec36	t	S88	++	t	t	c	t	c	c	c	c	c	c	c	c	c	c	c	c	c	c	c	c	c	c	c
g	4964142	g	4980371	a	4934017	s	S88	rec36	a	2	-	a	a	g	g	a	a	a	a	a	a	a	a	a	a	a	a	a	a	a	a	a	a	a
c	4964289	c	4980518	t	4934164	s	S88	rec36	c	S88	++++	c	c	c	c	c	t	c	c	c	c	c	c	c	c	c	c	c	c	c	c	c	c	c
g	4964298	g	4980527	a	4934173	s	S88	rec36	a	2	-	a	a	a	a	a	a	a	c	a	a	a	a	a	a	a	a	a	a	a	a	a	a	a

ExPEC genome site details												Outgroup Strains Details[i]																						
UTI89[b]	UTI89 site[c]	APEC 01[b]	APEC 01 site[c]	S88[b]	S88 site[c]	type[d]	Event Lineage[ef]	Recombinant segment	Inferred ancestral base[g]	Outgroup Analysis[e]	Support level[h]	CFT073	ED1a	536	E2348/69	SMS 3-5	IAI39	UMN026	K-12	ATCC 8739	HS	D1 Sd197	CB9615	Sakai	EDL933	IAI1	E24377A	SE11	SS Ss046	F2a 301	F2a 2457T	F5 8401	B4 Sb227	B18 BS512
a	4964571	a	4980800	g	4934446	s	S88	rec36	g	2	-	g	g	a	a	g	g	g	g	g	g	g	g	g	g	g	g	g	g	g	g	g	g	g
a	4964583	a	4980812	g	4934458	s	S88	rec36	g	2	-	g	g	g	a	g	g	g	g	g	g	g	g	g	g	g	g	g	g	g	g	g	g	g
c	4964766	c	4980995	a	4934641	s	S88	rec36	a	2	-	a	c	a	a	a	a	a	t	a	a	a	a	a	a	a	a	a	a	a	a	a	a	a
t	4964805	t	4981034	c	4934680	s	S88	rec36	c	2	-	c	c	c	c	c	c	c	c	c	c	c	c	c	c	c	c	c	c	c	c	c	c	c
a	4964811	a	4981040	g	4934686	s	S88	rec36	g	2	-	g	a	g	a	g	g	g	c	g	g	g	g	g	g	g	g	a	a	a	a	g	a	a
g	4964913	g	4981142	a	4934788	s	S88	rec36	g	S88	++	g	g	a	a	g	a	g	g	g	g	g	g	g	g	g	a	g	g	g	g	g	g	g
c	4965216	c	4981445	t	4935091	s	S88	rec36	c	S88	++	c	c	c	c	c	c	c	g	c	c	c	c	c	c	c	t	c	c	c	c	c	c	c
c	4965248	c	4981477	t	4935123	ns	S88	rec36	c	S88	++++	c	c	c	c	c	c	c	t	c	c	c	c	c	c	c	c	c	c	c	c	c	c	c
a	4965315	a	4981544	g	4935190	s	S88	rec36	g	2	-	g	g	g	g	g	g	g	g	g	g	g	g	g	g	g	g	g	g	g	g	g	g	g
c	4965437	c	4981666	t	4935312	nc	S88	rec36	c	S88	++	c	c	c	c	c	c		t			c	c	c	c		t							
c	4965502	c	4981731	t	4935377	nc	S88	rec36	c	S88	++	c	c	c	c	t	c		t			t	c	c	c		t							
g	4965515	g	4981744	t	4935390	nc	S88	rec36	g	S88	++	g	g	g	g	g	g		t			t	t	t	t		t							
a	4965516	a	4981745	t	4935391	nc	S88	rec36	a	S88	++	a	a	a	a	a	a		t			t	t	t	t		t							
a	4965517	a	4981746	t	4935392	nc	S88	rec36	a	S88	++	a	a	a	a	a	a		t			t	t	t	t		t							
g	4965520	g	4981749	c	4935395	nc	S88	rec36	g	S88	++	g	g	g	g	g	g		c			c	c	c	c		c							
c	4965521	c	4981750	g	4935396	nc	S88	rec36	c	S88	++	c	c	c	c	c	c		g			g	g	g	g		g							
g	4965523	g	4981752	t	4935398	nc	S88	rec36	g	S88	++	g	g	g	g	g	g		t			t	t	t	t		t							
c	4965552	c	4981781	g	4935427	nc	S88	rec36	c	S88	++	c	c	c	c		c		g			g					g							
t	4965567	t	4981796	c	4935442	nc	S88	rec36	t	S88	++	t	t	t	t		t		c			c					c							
c	4965587	c	4981816	t	4935462	nc	S88	rec36	c	S88	++	c	c	c	c		c		t			t					t							
g	4965624	g	4981853	a	4935499	nc	S88	rec36	g	S88	++	g	g	a	g		g		a			a					a							
g	4965626	g	4981855	t	4935501	nc	S88	rec36	g	S88	++	g	g	g	g		g		t			t					t							
c	4965648	c	4981877	a	4935524	nc	S88	rec36	c	S88	++	c	c	c	c		c		a			a					a							
t	4965662	t	4981891	a	4935538	nc	S88	rec36	t	S88	++	t	t	t	t		a	t	a	t	t	a				t	a	t						
c	4965754	c	4981983	t	4935630	s	S88	rec36	c	S88	++	c	c	c	c	t	c	t	t	t	t	t	t	t	t	t	t	t	t	t	t	t	t	t
t	4965795	t	4982024	a	4935671	s	S88	rec36	t	S88	++	t	t	t	t	a	t	a	a	a	a	a	a	a	a	a	a	a	a	a	a	a	a	a
c	4965828	c	4982057	t	4935704	s	S88	rec36	c	S88	++	c	c	c	c	t	c	t	t	t	t	t	t	t	t	t	t	t	t	t	t	t	t	t
t	4965900	t	4982129	g	4935776	s	S88	rec36	g	2	-	t	g	g	g	g	g	g	g	g	g	g	g	g	g	g	g	g	g	g	g	g	g	g
c	4965960	c	4982189	t	4935836	s	S88	rec36	c	S88	++	c	c	c	c	t	c	t	t	t	t	t	t	t	t	t	t	t	t	t	t	c	t	t
c	4965997	c	4982226	g	4935873	ns	S88	rec36	c	S88	++++	c	c	c	c	c	c	c	g	c	c	c	c	c	c	c	c	c	c	c	c	c	c	c
a	4966143	a	4982372	c	4936019	s	S88	rec36	a	S88	++	a	a	a	a	c	a	c	c	c	c	c	c	c	c	c	c	c	c	c	c	c	c	c
t	4966255	t	4982484	c	4936131	ns	S88	rec36	t	S88	++	t	t	t	t	t	t	c	c	c	t	t	t	t	t	t	c	t	t	t	t	t	t	t
a	4966314	a	4982543	g	4936190	s	S88	rec36	a	S88	++	a	a	a	a	g	a	g	g	g	g	g	g	g	g	g	g	g	g	g	g	g	g	g
c	4966345	c	4982574	t	4936221	ns	S88	rec36	t	2	-	t	c	t	c	t	c	t	t	t	t	t	t	t	t	t	t	t	t	t	t	t	t	t
a	4966674	a	4982903	g	4936550	s	S88	rec36	a	S88	++	a	a	a	a	g	a	a	g	a	a	a	a	a	a	g	a	g	a	a	a	g	g	g
t	4966680	t	4982909	c	4936556	s	S88	rec36	t	S88	++	t	t	t	t	c	t	c	c	c	c	c	c	c	c	c	c	c	c	c	c	c	c	c
c	4966725	c	4982954	t	4936601	s	S88	rec36	c	S88	++	c	c	c	c	t	c	t	c	t	t	t	t	t	t	t	t	t	t	t	t	t	t	t
c	4966746	c	4982975	t	4936622	s	S88	rec36	c	S88	++++	c	c	c	c	c	c	c	c	c	c	c	c	c	c	c	c	c	c	c	c	c	c	c
g	4966755	g	4982984	a	4936631	s	S88	rec36	g	S88	++	g	a	g	g	g	g	a	g	a	a	a	a	a	a	g	a	g	a	a	a	a	a	a
c	4966773	c	4983002	t	4936649	s	S88	rec36	c	S88	++	c	c	c	c	c	c	t	g	t	t	t	t	t	t	t	t	t	t	t	t	t	t	t
a	4966902	a	4983131	g	4936778	s	S88	rec36	a	S88	++	a	a	a	g	g	a	a	c	a	g	g	a	a	a	g	a	g	a	a	a	g	g	g
t	4966950	t	4983179	c	4936826	s	S88	rec36	t	S88	++	t	t	t	t	t	t	t	c	t	t	c	t	t	t	t	t	t	t	t	t	c	c	c
t	4966956	t	4983185	c	4936832	s	S88	rec36	t	S88	++	t	t	t	t	t	t	c	c	c	t	t	c	c	c	c	c	c	c	c	c	c	c	c
t	4966962	t	4983191	g	4936838	s	S88	rec36	t	S88	++	t	t	t	t	g	t	g	g	g	g	g	g	g	g	g	g	g	g	g	g	g	g	g
t	4967064	t	4983293	c	4936940	s	S88	rec36	t	S88	++	t	t	t	t	c	t	t	t	t	c	t	t	t	t	t	t	t	t	t	t	c	c	c
a	4967202	a	4983431	g	4937078	s	S88	rec36	a	S88	++	a	a	a	a	a	a	a	a	a	a	a	g	g	g	a	a	a	a	a	a	g	g	g
c	4967211	c	4983440	t	4937087	s	S88	rec36	c	S88	++	c	c	c	t	t	c	c	c	c	t	c	t	t	t	c	c	t	t	t	t	t	t	t
a	4967232	a	4983461	g	4937108	s	S88	rec36	g	2	-	g	g	g	g	g	g	g	a	g	g	g	g	g	g	g	g	g	g	g	g	g	g	g
g	4967293	g	4983522	a	4937169	ns	S88	rec36	g	S88	++	g	g	g	a	a	g	a	a	a	a	a	a	a	a	a	a	a	a	a	a	a	a	a
a	4967326	a	4983555	g	4937202	ns	S88	rec36	a	S88	++	a	a	a	a	g	a	a	g	g	g	g	a	a	a	a	a	g	a	a	a	g	g	g
c	4967334	c	4983563	t	4937210	s	S88	rec36	c	S88	++	c	c	c	t	t	c	t	t	t	t	t	t	t	t	t	t	t	t	t	t	t	t	t
g	4967373	g	4983602	a	4937249	s	S88	rec36	g	S88	++	g	g	g	g	g	g	g	a	g	a	a	g	g	g	g	g	a	g	g	g	a	a	a
g	4972814	g	4989043	a	4938991	s	S88	rec36	g	S88	++	g	g	g	g	a	g	a	a	a	a		a	a	a	a	a	a	a	a	a	a	a	
t	4972835	t	4989064	c	4939012	s	S88	rec36	t	S88	++	t	t	t	t	c	t	c	c	c	c		c	c	c	c	c	c	t	t	t	c	c	
a	4972841	a	4989070	g	4939018	s	S88	rec36	a	S88	++	a	a	a	a	g	a	g	g	g	g		g	g	g	g	g	g	g	g	g	g	g	
a	4972844	a	4989073	g	4939021	s	S88	rec36	a	S88	++	a	a	a	a	g	a	g	g	g	g		g	g	g	g	g	g	g	g	g	g	g	
g	4972865	g	4989094	a	4939042	s	S88	rec36	g	S88	++	g	g	g	g	a	g	a	a	a	a		a	a	a	a	a	a	a	a	a	a	a	
a	4972877	a	4989106	g	4939054	s	S88	rec36	a	S88	++	a	a	a	a	g	a	g	g	g	g		g	g	g	g	g	g	g	g	g	g	g	
c	4972904	c	4989133	g	4939081	s	S88	rec36	c	S88	++	c	c	c	c	g	c	g	g	g	g		g	g	g	g	g	g	g	g	g	g	g	
c	4972907	c	4989136	t	4939084	s	S88	rec36	c	S88	++	c	c	c	c	t	c	t	t	t	t		t	t	t	t	t	t	t	t	t	t	t	
t	4972910	t	4989139	g	4939087	s	S88	rec36	t	S88	++	t	t	t	t	g	t	g	g	t	g		g	g	g	g	g	g	g	g	g	g	g	
a	4972913	a	4989142	g	4939090	s	S88	rec36	a	S88	++	a	a	a	a	g	a	g	g	g	g		g	g	g	g	g	g	g	g	g	g	g	
t	4972916	t	4989145	c	4939093	s	S88	rec36	t	S88	++	t	t	t	t	c	t	c	c	c	c		c	c	c	c	c	c	c	c	c	c	c	
t	4972922	t	4989151	c	4939099	s	S88	rec36	t	S88	++	t	t	t	t	c	t	a	c	c	c		c	c	c	c	a	c	c	c	c	c	c	
t	4972930	t	4989159	c	4939107	ns	S88	rec36	t	S88	++	t	t	t	t	c	t	c	c	c	c		c	c	c	c	c	c	c	c	c	c	c	
g	4972940	g	4989169	a	4939117	s	S88	rec36	g	S88	++	g	g	g	g	a	g	a	a	a	a		a	a	a	a	a	a	a	a	a	a	a	
a	4972946	a	4989175	g	4939123	s	S88	rec36	a	S88	++	a	a	a	a	g	a	g	g	g	g		g	g	g	g	g	g	g	g	g	g	g	
g	4972952	g	4989181	t	4939129	s	S88	rec36	g	S88	++	g	g	g	g	t	g	t	t	t	t		t	t	t	t	t	t	t	t	t	t	t	
t	4972955	t	4989184	a	4939132	s	S88	rec36	t	S88	++	t	t	t	t	a	t	a	a	a	a		a	a	a	a	a	a	a	a	a	a	a	
a	4972957	a	4989186	g	4939134	s	S88	rec36	a	S88	++	a	a	a	a	g	a	g	g	g	g		g	g	g	g	g	g	g	g	g	g	g	
c	4972961	c	4989190	t	4939138	ns	S88	rec36	c	S88	++	c	c	c	c	t	c	t	t	t	t		t	t	t	t	t	t	t	t	t	t	t	
t	4972963	t	4989192	a	4939140	ns	S88	rec36	t	S88	++	t	t	t	t	a	t	a	a	a	a		a	a	a	a	a	a	a	a	a	a	a	
c	4972966	c	4989195	a	4939143	ns	S88	rec36	c	S88	++	c	c	c	c	a	c	a	a	a	a		a	a	a	a	a	a	a	a	a	a	a	
a	4972967	a	4989196	g	4939144	s	S88	rec36	a	S88	++	a	a	a	a	g	a	g	g	g	g		g	g	g	g	g	g	g	g	g	g	g	
a	4972976	a	4989205	t	4939153	s	S88	rec36	a	S88	++	a	a	a	a	t	a	t	t	t	t		t	t	t	t	t	t	t	t	t	t	t	
g	4972978	g	4989207	t	4939155	s	S88	rec36	g	S88	++	g	g	g	g	t	g	t	t	t	t		t	t	t	t	t	t	t	t	t	t	t	
t	4972982	t	4989211	a	4939159	s	S88	rec36	t	S88	++	t	t	t	t	a	t	a	a	a	a		a	a	a	a	a	a	a	a	a	a	a	
a	4972984	a	4989213	g	4939161	s	S88	rec36	a	S88	++	a	a	a	a	g	a	g	g	g	g		g	g	g	g	g	g	g	g	g	g	g	
a	4972988	a	4989217	c	4939165	s	S88	rec36	a	S88	++	a	a	a	a	c	a	c	c	c	c		c	c	c	c	c	c	c	c	c	c	c	
a	4972991	a	4989220	g	4939168	s	S88	rec36	a	S88	++	a	a	a	a	g	a	g	g	g	g		g	g	g	g	g	g	g	g	g	g	g	
t	4972997	t	4989226	g	4939174	s	S88	rec36	t	S88	++	t	t	t	t	g	t	g	g	g	g		g	g	g	g	g	g	g	g	g	g	g	
t	4973004	t	4989233	c	4939181	ns	S88	rec36	t	S88	++	t	t	t	t	c	t	c	c	c	c		c	c	c	c	c	c	c	c	c	c	c	
g	4973006	g	4989235	a	4939183	ns	S88	rec36	g	S88	++	g	g	g	g	a	g	a	a	a	a		a	a	a	a	a	a	a	a	a	a	a	
c	4973007	c	4989236	t	4939184	ns	S88	rec36	c	S88	++	c	c	c	t	t	c	t	t	t	t		t	t	t	t	t	t	t	t	t	t	t	

ExPEC genome site details												Outgroup Strains Details[i]																						
UTI89[b]	UTI89 site[c]	APEC 01[b]	APEC 01 site[c]	S88[b]	S88 site[c]	type[d]	Event Lineage[ef]	Recombinant segment	Inferred ancestral base[g]	Outgroup Analysis[e]	Support level[h]	CFT073	ED1a	536	E2348/69	SMS 3-5	IAI39	UMN026	K-12	ATCC 8739	HS	D1 Sd197	CB9615	Sakai	EDL933	IAI1	E24377A	SE11	SS Ss046	F2a 301	F2a 2457T	F5 8401	B4 Sb227	B18 BS512
a	4973012	a	4989241	g	4939189	s	S88	rec36	a	S88	++	a	a	a	a	g	a	g	g	g	g		g	g	g	g	g	g	g	g	g	g	g	
c	4973016	c	4989245	t	4939193	ns	S88	rec36	c	S88	++	c	c	c	c	t	c	t	t	t	t		t	t	t	t	t	t	t	t	t	t	t	
g	4973021	g	4989250	a	4939198	s	S88	rec36	g	S88	++	g	g	g	g	a	g	a	a	a	a		a	a	a	a	a	a	a	a	a	a	a	
a	4973026	a	4989255	g	4939203	s	S88	rec36	g	2	-	g	g	g	a	g	g	g	g	g	g		g	g	g	g	g	g	g	g	g	g	g	
g	4973028	g	4989257	a	4939205	ns	S88	rec36	g	S88	++	g	g	g	g	a	g	a	a	a	a		a	a	a	a	a	a	a	a	a	a	a	
t	4973029	t	4989258	g	4939206	ns	S88	rec36	t	S88	++	t	t	t	t	g	t	g	g	g	g		g	g	g	g	g	g	g	g	g	g	g	
t	4973032	t	4989261	g	4939209	s	S88	rec36	g	2	-	g	g	g	t	g	g	g	g	t	g		g	g	g	g	g	g	g	g	g	g	g	
g	4973033	g	4989262	a	4939210	s	S88	rec36	g	S88	++	g	g	g	g	a	g	a	a	g	a		a	a	a	a	a	a	a	a	a	a	a	
a	4973062	a	4989291	g	4939239	s	S88	rec36	g	2	-	g	g	g	a	g	g	g	g	g	g		g	g	g	g	g	g	g	g	g	g	g	
a	4973069	a	4989298	g	4939246	s	S88	rec36	a	S88	++	a	a	a	a	g	a	g	g	g	g		g	g	g	g	g	g	g	g	g	g	g	
g	4973072	g	4989301	a	4939249	s	S88	rec36	g	S88	++	g	g	g	g	a	g	a	a	a	a		a	a	a	a	a	a	a	a	a	a	a	
c	4973077	c	4989306	g	4939254	ns	S88	rec36	c	S88	++	c	c	c	c	g	c	g	g	g	g		g	g	g	g	g	g	g	g	g	g	g	
g	4973081	g	4989310	a	4939258	s	S88	rec36	g	S88	++	g	g	g	g	a	g	a	a	a	a		a	a	a	a	a	a	a	a	a	a	a	
a	4973084	a	4989313	c	4939261	s	S88	rec36	a	S88	++	a	a	a	a	c	a	c	c	c	c		c	c	c	c	c	c	c	c	c	c	c	
t	4973099	t	4989328	c	4939276	s	S88	rec36	t	S88	++	t	t	t	t	c	t	c	c	c	c		c	c	c	c	c	c	c	c	c	c	c	
c	4973102	c	4989331	a	4939279	s	S88	rec36	c	S88	++	c	c	c	c	a	c	a	a	a	a		a	a	a	a	a	a	a	a	a	a	a	
g	4973105	g	4989334	t	4939282	s	S88	rec36	g	S88	++	g	g	g	g	t	g	t	t	t	t		t	t	t	t	t	t	t	t	t	t	t	
c	4973165	c	4989394	t	4939342	s	S88	rec36	c	S88	++	c	c	c	c	t	c	t	t	t	t		t	t	t	t	t	t	t	t	t	t	t	
t	4973180	t	4989409	c	4939357	s	S88	rec36	t	S88	++	t	t	t	t	c	t	c	c	c	c		c	c	c	c	c	c	c	c	c	c	c	
t	4973198	t	4989427	g	4939375	ns	S88	rec36	t	S88	++	t	t	t	t	t	t	t	g	t	t		t	t	t	t	t	t	t	t	t	g	t	
a	4973216	a	4989445	g	4939393	s	S88	rec36	a	S88	++	a	a	a	a	g	a	g	g	g	g		g	g	g	g	g	g	g	g	g	g	g	
c	4973225	c	4989454	t	4939402	s	S88	rec36	c	S88	++	c	c	c	c	t	c	t	t	t	t		t	t	t	t	t	t	t	t	t	t	t	
g	4973239	g	4989468	t	4939416	s	S88	rec36	g	S88	++	g	g	g	g	t	g	t	t	t	t		t	t	t	t	t	t	t	t	t	t	t	
c	4973243	c	4989472	t	4939420	s	S88	rec36	c	S88	++	c	c	c	c	a	c	t	t	a	a		a	a	a	a	t	a	a	a	a	t	a	
t	4973246	t	4989475	a	4939423	s	S88	rec36	t	S88	++	t	t	t	t	c	t	a	a	c	c		c	c	c	c	a	c	c	c	c	a	c	
a	4973252	a	4989481	g	4939429	s	S88	rec36	a	S88	++	a	a	a	a	g	a	g	g	g	g		g	g	g	g	g	g	g	g	g	g	g	
g	4973258	g	4989487	a	4939435	ns	S88	rec36	g	S88	++	g	g	g	g	a	g	a	a	a	a		a	a	a	a	a	a	a	a	a	a	a	
t	4973260	t	4989489	c	4939437	ns	S88	rec36	t	S88	++	t	t	t	t	c	t	c	c	c	c		c	c	c	c	c	c	c	c	c	c	c	
t	4973261	t	4989490	c	4939438	s	S88	rec36	t	S88	++	t	t	t	t	c	t	c	c	c	c		c	c	c	c	c	c	c	c	c	c	c	
c	4973264	c	4989493	g	4939441	s	S88	rec36	c	S88	++	c	c	c	c	g	c	g	g	g	g		g	g	g	g	g	g	g	g	g	g	g	
t	4973267	t	4989496	c	4939444	s	S88	rec36	t	S88	++	t	t	t	t	c	t	c	c	c	c		c	c	c	c	c	c	c	c	c	c	c	
c	4973270	c	4989499	a	4939447	s	S88	rec36	c	S88	++	c	c	c	c	c	c	a	a	a	a		a	a	a	a	a	a	a	a	a	a	a	
c	4973276	c	4989505	t	4939453	s	S88	rec36	c	S88	++	c	c	c	c	t	c	t	t	t	t		t	t	t	t	t	t	t	t	t	t	t	
a	4973279	a	4989508	c	4939456	s	S88	rec36	a	S88	++	a	a	a	a	c	a	c	c	c	c		c	c	c	c	c	c	c	c	c	c	c	
g	4973285	g	4989514	a	4939462	s	S88	rec36	g	S88	++	g	g	g	g	a	g	a	a	a	a		a	a	a	a	a	a	a	a	a	a	a	
g	4973291	g	4989520	c	4939468	ns	S88	rec36	g	S88	++	g	g	g	g	a	g	a	c	a	a		a	a	a	a	a	a	a	a	a	c	a	
c	4973292	c	4989521	g	4939469	ns	S88	rec36	c	S88	++	c	c	c	c	g	c	g	g	g	g		g	g	g	g	g	g	g	g	g	g	g	
t	4973297	t	4989526	c	4939474	s	S88	rec36	t	S88	++	t	t	t	t	c	t	c	c	c	c		c	c	c	c	c	c	c	c	c	c	c	
g	4973300	g	4989529	t	4939477	ns	S88	rec36	g	S88	++	g	g	g	g	a	g	a	t	t	a		t	t	t	a	a	t	a	a	a	t	t	
c	4973302	c	4989531	g	4939479	ns	S88	rec36	c	S88	++	c	c	c	c	g	c	g	g	g	g		g	g	g	g	g	g	g	g	g	g	g	
a	4973316	a	4989545	t	4939493	ns	S88	rec36	a	S88	++	a	a	a	a	t	a	t	t	t	t		t	t	t	t	t	t	t	t	t	t	t	
a	4973321	a	4989550	t	4939498	s	S88	rec36	a	S88	++	a	a	a	a	t	a	t	t	t	t		t	t	t	t	t	t	t	t	t	t	t	
g	4973327	g	4989556	c	4939504	s	S88	rec36	g	S88	++	g	g	g	g	c	g	c	c	c	c		c	c	c	c	c	c	c	c	c	c	c	
t	4973336	t	4989565	a	4939513	s	S88	rec36	t	S88	++	t	t	t	t	a	t	a	a	a	a		a	a	a	a	a	a	a	a	a	a	a	
t	4973851	t	4990080	a	4940028	s	S88	rec36	t	S88	++	t	t	t	t	a	t	a	a	a	a		a	a	a	a	a	a	a	a	a	a	a	a
a	4973860	a	4990089	g	4940037	s	S88	rec36	a	S88	++	a	a	a	a	g	a	g	g	g	g		g	g	g	g	g	g	g	g	g	g	g	g
a	4973861	a	4990090	t	4940038	ns	S88	rec36	a	S88	++	a	a	a	a	t	a	t	t	t	t		t	t	t	t	t	t	t	t	t	t	t	t
a	4973881	a	4990110	c	4940058	s	S88	rec36	a	S88	++	a	a	a	a	c	a	c	c	c	c		c	c	c	c	c	c	c	c	c	c	c	c
c	4973898	c	4990127	g	4940075	ns	S88	rec36	c	S88	++	c	c	c	c	g	c	g	g	g	g		g	g	g	g	g	g	g	g	g	g	g	g
g	4973899	g	4990128	t	4940076	ns	S88	rec36	g	S88	++	g	g	g	g	t	g	t	t	t	t		t	t	t	t	t	t	t	t	t	t	t	t
c	4973902	c	4990131	a	4940079	s	S88	rec36	c	S88	++	c	c	c	c	a	c	a	a	a	a		a	a	a	a	a	a	a	a	a	a	a	a
g	4973908	g	4990137	a	4940085	s	S88	rec36	g	S88	++	g	g	g	g	a	g	a	a	a	a		a	a	a	a	a	a	a	a	a	a	a	a
c	4973929	c	4990158	t	4940106	s	S88	rec36	c	S88	++	c	c	c	c	t	c	t	t	g	t		g	g	g	t	t	t	g	g	g	t	t	t
a	4973960	a	4990189	g	4940137	ns	S88	rec36	a	S88	++	a	a	a	a	g	a	g	g	g	g		g	g	g	g	g	g	g	g	g	g	g	g
a	4973964	a	4990193	g	4940141	ns	S88	rec36	a	S88	++	a	a	a	a	g	g	g	g	g	g		g	g	g	g	g	g	g	g	g	g	g	g
a	4973965	a	4990194	g	4940142	ns	S88	rec36	a	S88	++	a	a	a	a	g	a	g	g	g	g		g	g	g	g	g	g	g	g	g	g	g	g
t	4973966	t	4990195	c	4940143	ns	S88	rec36	t	S88	++	t	t	t	t	c	t	c	c	c	c		c	c	c	c	c	c	c	c	c	c	c	c
c	4973967	c	4990196	t	4940144	ns	S88	rec36	c	S88	++	c	c	c	c	t	c	t	t	t	t		t	t	t	t	t	t	t	t	t	t	t	t
a	4973968	a	4990197	g	4940145	ns	S88	rec36	a	S88	++	a	a	a	a	g	a	g	g	g	g		g	g	g	g	g	g	g	g	g	g	g	g
a	4973971	a	4990200	t	4940148	s	S88	rec36	a	S88	++	a	a	a	a	t	a	t	t	t	t		t	t	t	t	t	t	t	t	t	t	t	t
c	4973977	c	4990206	t	4940154	s	S88	rec36	c	S88	++	c	c	c	c	t	c	t	t	t	t		t	t	t	t	t	t	t	t	t	t	t	t
c	4973981	c	4990210	g	4940158	ns	S88	rec36	c	S88	++	c	c	c	c	g	c	g	g	g	g		g	g	g	g	g	g	g	g	g	g	g	g
a	4973982	a	4990211	c	4940159	ns	S88	rec36	a	S88	++	a	a	a	a	c	a	c	c	c	c		c	c	c	c	c	c	c	c	c	c	c	c
c	4973983	c	4990212	t	4940160	ns	S88	rec36	c	S88	++	c	c	c	c	t	c	t	t	t	t		t	t	t	t	t	t	t	t	t	t	t	t
a	4973984	a	4990213	g	4940161	ns	S88	rec36	a	S88	++	a	a	a	a	g	a	g	g	g	g		g	g	g	g	g	g	g	g	g	g	g	g
t	4973987	t	4990216	c	4940164	ns	S88	rec36	t	S88	++	t	t	t	t	c	t	c	c	c	c		c	c	c	c	c	c	c	c	c	c	c	c
t	4973992	t	4990221	a	4940169	ns	S88	rec36	t	S88	++	t	t	t	t	a	t	a	a	a	a		a	a	a	a	a	a	a	a	a	a	a	a
t	4973994	t	4990223	a	4940171	ns	S88	rec36	t	S88	++	t	t	t	t	a	t	a	a	a	a		a	a	a	a	a	a	a	a	a	a	a	a
c	4973995	c	4990224	t	4940172	ns	S88	rec36	c	S88	++	c	c	c	c	t	c	t	t	t	t		t	t	t	t	t	t	t	t	t	t	t	t
a	4973998	a	4990227	t	4940175	s	S88	rec36	a	S88	++	a	a	a	a	t	a	t	t	t	t		t	t	t	t	t	t	t	t	t	t	t	t
g	4974010	g	4990239	t	4940187	s	S88	rec36	g	S88	++	g	g	g	g	t	g	t	t	t	t		t	t	t	t	t	t	t	t	t	t	t	t
a	4974013	a	4990242	g	4940190	s	S88	rec36	a	S88	++	a	a	a	a	g	a	g	g	g	g		g	g	g	g	g	g	g	g	g	g	g	g
t	4974016	t	4990245	c	4940193	s	S88	rec36	t	S88	++	t	t	t	t	c	t	c	c	c	c		c	c	c	c	c	c	c	c	c	c	c	c
g	4974019	g	4990248	t	4940196	s	S88	rec36	g	S88	++	g	g	g	g	t	g	t	t	t	t		t	t	t	t	t	t	t	t	t	t	t	t
a	4974023	a	4990252	g	4940200	ns	S88	rec36	a	S88	++	a	a	a	a	g	a	g	g	g	g		g	g	g	g	g	g	g	g	g	g	g	g
t	4974026	t	4990255	g	4940203	ns	S88	rec36	t	S88	++	t	t	t	t	g	t	g	g	g	g		g	g	g	g	g	g	g	g	g	g	g	g
t	4974028	t	4990257	c	4940205	ns	S88	rec36	t	S88	++	t	t	t	t	c	t	c	c	c	c		c	c	c	c	c	c	c	c	c	c	c	c
t	4974058	t	4990287	g	4940235	s	S88	rec36	t	S88	++	t	t	t	t	t	t	g	g	g	g		g	g	g	g	g	g	g	g	g	g	g	g
t	4974226	t	4990455	c	4940403	s	S88	rec36	t	S88	++	t	c	t	c	t	t	c	c	c	c		c	c	c	c	c	c	c	c	c	c	c	c
a	4974238	a	4990467	t	4940415	s	S88	rec36	a	S88	++	a	t	a	t	t	a	t	t	t	t		t	t	t	t	t	t	t	t	t	t	t	t
a	4974242	a	4990471	g	4940419	ns	S88	rec36	a	S88	++	a	g	a	g	g	a	g	g	g	g		g	g	g	g	g	g	g	g	g	g	g	g
g	4974316	g	4990545	a	4940493	s	S88	rec36	g	S88	++	g	a	g	a	a	g	a	a	a	a	a	a	a	a	a	a	a	a	a	a	a	a	a
t	4974349	t	4990578	c	4940526	s	S88	rec36	c	2	-	c	c	c	c	c	c	c	c	c	c	c	c	c	c	c	c	c	c	c	c	c	c	c

ExPEC genome site details												Outgroup Strains Details[i]																						
UTI89[b]	UTI89 site[c]	APEC 01[b]	APEC 01 site[c]	S88[b]	S88 site[c]	type[d]	Event Lineage[ef]	Recombinant segment	Inferred ancestral base[g]	Outgroup Analysis[e]	Support level[h]	CFT073	ED1a	536	E2348/69	SMS 3-5	IAI39	UMN026	K-12	ATCC 8739	HS	D1 Sd197	CB9615	Sakai	EDL933	IAI1	E24377A	SE11	SS Ss046	F2a 301	F2a 2457T	F5 8401	B4 Sb227	B18 BS512
g	4974383	g	4990612	t	4940560	ns	S88	rec36	g	S88	++	g	g	g	g	g	g	t	t	g	t	g	g	g	g	t	g	g	g	g	g	t	t	t
t	4974385	t	4990614	a	4940562	ns	S88	rec36	t	S88	++	t	t	t	t	a	t	a	a	g	a	g	g	g	g	a	g	a	g	g	g	a	a	a
g	4974421	g	4990650	a	4940598	s	S88	rec36	g	S88	++	g	g	g	g	g	g	a	a	g	a	a	g	g	g	a	g	a	g	g	g	a	a	a
t	4974460	t	4990689	c	4940637	s	S88	rec36	t	S88	++	t	t	t	t	c	t	c	c	c	c	c	c	c	c	c	c	c	c	c	c	c	c	c
a	4974499	a	4990728	g	4940676	s	S88	rec36	a	S88	++	a	a	a	a	a	a	g	g	g	g	g	g	g	g	g	g	a	g	g	g	g	g	g
c	4974503	c	4990732	t	4940680	s	S88	rec36	c	S88	++	c	c	c	c	c	c	t	t	c	t	c	c	c	c	t	t	c	c	c	c	t	t	t
c	4974631	c	4990860	t	4940808	s	S88	rec36	c	S88	++	c	c	c	c	c	c	t	t	c	t	t	t	t	t	t	t	t	c	c	c	t	t	t
t	4974647	t	4990876	c	4940824	s	S88	rec36	t	S88	++	t	t	t	t	t	t	c	c	c	c	c	c	c	c	c	c	c	c	c	c	c	c	c
g	4974661	g	4990890	a	4940838	s	S88	rec36	g	S88	++	g	g	g	g	g	g	a	a	g	a	a	a	a	a	a	a	a	g	g	g	a	a	a
c	4974673	c	4990902	t	4940850	s	S88	rec36	c	S88	++	c	c	c	c	c	c	c	t	c	c	t	t	t	t	c	t	c	c	c	c	t	t	t
g	4974754	g	4990983	a	4940931	s	S88	rec36	g	S88	++	g	g	g	g	g	g	g	a	a	g	g	a	a	a	g	g	g	g	g	g	g	g	g
g	4974760	g	4990989	a	4940937	s	S88	rec36	g	S88	++	g	g	g	g	g	g	g	a	a	g	a	a	a	a	g	g	g	g	g	g	g	g	g
c	4974794	c	4991023	a	4940971	nc	S88	rec36	c	S88	++	a	c	c	c	a	c	a	a	a	a	a	a	a	a	a	a	a	a	a	a	a	a	a
c	4974849	c	4991078	t	4941024	nc	S88	rec36	c	S88	++	c	t	c	c	t	c	c	t	c	c	t	t	t	t	c	c	c	t	t	t	c	c	c
t	4974858	t	4991087	g	4941033	nc	S88	rec36	t	S88	++	t	g	t	t	g	t	t	g	t	t	g	g	g	g	t	t	t	g	g	g	t	t	t
g	4974864	g	4991093	a	4941039	nc	S88	rec36	g	S88	++	g	a	g	g	a	g	g	a	g	g	a	a	a	a	g	g	g	a	a	a	g	g	g
g	4974869	g	4991098	t	4941044	nc	S88	rec36	g	S88	++	g	g	g	g	g	g	g	t	g	g	g	g	g	g	g	g	c	g	g	g	c	c	c
t	4974876	t	4991105	c	4941051	nc	S88	rec36	t	S88	++	t	c	t	t	c	t	t	c	t	t	c	t	t	t	t	t	t	c	c	c	t	t	t
t	4974890	t	4991119	c	4941065	nc	S88	rec36	c	2	-	c	c	c	c	c	c	c	c	c	c	c	c	c	c	c	c	c	c	c	c	c	c	c
t	4974891	t	4991120	c	4941066	nc	S88	rec36	t	S88	++	t	t	t	t	t	t	t	c	t	t	t	c	c	c	t	t	t	c	c	c	t	t	t
c	4974981	c	4991210	t	4941156	s	S88	rec36	c	S88	++	c	t	c	c	t	c	t	t	t	t	t	t	t	t	t	t	t	t	t	t	t	t	t
t	4975020	t	4991249	c	4941195	s	S88	rec36	t	S88	++	t	c	t	t	c	t	c	c	c	c	c	c	c	c	c	c	c	c	c	c	c	c	c
g	4975101	g	4991330	a	4941276	s	S88	rec36	g	S88	++	g	g	g	g	g	g	a	a	a	a	a	a	a	a	a	a	g	a	a	a	g	g	g
t	4975236	t	4991465	c	4941411	s	S88	rec36	t	S88	++	t	t	t	t	a	t	c	c	c	c	a	c	c	c	c	c	a	c	c	c	c	c	c
t	4975263	t	4991492	c	4941438	s	S88	rec36	t	S88	++	t	t	t	t	c	t	t	c	c	t	c	c	c	c	t	t	c	c	c	c	t	t	t
a	4975269	a	4991498	g	4941444	s	S88	rec36	a	S88	++	a	a	a	a	a	a	g	g	g	g	a	g	g	g	g	g	g	g	g	g	g	g	g
a	4975308	a	4991537	g	4941483	s	S88	rec36	a	S88	++	a	a	a	a	g	a	g	g	g	g	g	g	g	g	g	g	g	g	g	g	g	g	g
t	4975365	t	4991594	c	4941540	s	S88	rec36	t	S88	++	t	t	t	t	c	t	c	c	c	c	c	c	c	c	c	c	c	c	c	c	c	c	c
t	4975428	t	4991657	g	4941603	s	S88	rec36	g	2	-	g	g	g	t	g	t	g	g	g	g	g	g	g	g	g	g	g	g	g	g	g	g	g
t	4975433	t	4991662	g	4941608	ns	S88	rec36	t	S88	++	t	t	t	t	t	t	t	g	t	g	t	t	t	t	g	g	t	t	t	t	g	g	g
a	4975438	a	4991667	g	4941613	ns	S88	rec36	a	S88	++	a	a	a	a	g	a	g	g	g	g	g	g	g	g	g	g	a	g	g	g	g	g	g
c	4975442	c	4991671	t	4941617	ns	S88	rec36	c	S88	++	c	c	c	c	t	c	t	t	t	t	t	t	t	t	t	t	t	t	t	t	t	t	t
g	4975509	g	4991738	a	4941684	s	S88	rec36	g	S88	++	a	g	g	g	g	g	a	a	a	g	a	a	a	a	g	g	g	a	a	a	g	g	g
t	4975590	t	4991819	g	4941765	s	S88	rec36	t	S88	++	a	t	t	t	g	t	g	g	g	g	g	g	g	g	g	g	g	g	g	g	g	g	g
t	4975692	t	4991921	c	4941867	s	S88	rec36	t	S88	++	t	t	t	t	c	t	c	c	c	c	c	c	c	c	c	c	c	c	c	c	c	c	c
c	4975773	c	4992002	t	4941948	s	S88	rec36	c	S88	++	c	c	c	c	c	c	c	t	c	c	c	c	c	c	c	c	c	t	t	t	c	c	c
g	4975848	g	4992077	a	4942023	s	S88	rec36	g	S88	++	g	g	g	g	a	g	a	a	a	a	a	a	a	a	a	a	a	a	a	a	a	a	a
a	4975911	a	4992140	g	4942086	s	S88	rec36	a	S88	++	a	a	a	a	g	a	g	g	g	g	g	g	g	g	g	g	g	g	g	g	g	g	g
g	4975956	g	4992185	a	4942131	s	S88	rec36	g	S88	++	g	g	g	g	g	g	g	a	a	a	g	a	a	a	a	a	a	a	a	a	a	a	a
a	4975992	a	4992221	g	4942167	s	S88	rec36	a	S88	++	a	a	a	a	a	a	a	g	g	a	g	g	g	a	g	a	g	g	g	g	g	g	g
a	4976010	a	4992239	g	4942185	s	S88	rec36	a	S88	++	a	a	a	a	g	a	g	g	g	g	g	g	g	g	g	g	g	g	g	g	g	g	g
a	4976097	a	4992326	g	4942272	s	S88	rec36	a	S88	++	a	a	a	a	g	a	g	g	g	g	g	g	g	g	g	g	g	g	g	g	g	g	g
a	4976100	a	4992329	g	4942275	s	S88	rec36	a	S88	++	a	a	a	a	a	a	a	g	a	a	g	a	a	a	a	a	a	a	a	a	a	a	a
c	4976142	c	4992371	t	4942317	s	S88	rec36	c	S88	++	c	c	c	c	c	c	c	t	c	c	c	t	t	t	c	c	c	c	c	c	c	c	c
g	4976178	g	4992407	a	4942353	s	S88	rec36	g	S88	++	a	g	g	g	a	g	a	a	a	a	g	a	a	a	a	a	a	g	g	g	a	a	a
c	4976181	c	4992410	a	4942356	s	S88	rec36	c	S88	++	c	c	c	c	c	c	c	a	c	c	c	a	a	a	c	c	c	c	c	c	c	c	c
t	4976265	t	4992494	c	4942440	s	S88	rec36	t	S88	++	c	t	t	t	c	t	c	c	c	c	c	c	c	c	c	c	c	c	c	c	c	c	c
c	4976334	c	4992563	t	4942509	s	S88	rec36	c	S88	++	c	c	c	c	c	c	t	t	c	c	c	t	t	t	c	c	c	t	t	t	c	c	c
g	4976379	g	4992608	a	4942554	s	S88	rec36	a	2	-	a	g	a	g	g	a	a	a	a	a	g	a	a	a	a	a	a	g	g	g	a	a	a
t	4976385	t	4992614	c	4942560	s	S88	rec36	c	2	-	c	t	c	t	t	c	c	c	c	c	t	c	c	c	c	c	c	t	t	t	c	c	c
t	4976462	t	4992691	g	4942637	ns	S88	rec36	g	2	-	g	g	g	g	g	g	g	g	g	g	g	g	g	g	g	g	g	g	g	g	g	g	g
a	4976463	a	4992692	g	4942638	s	S88	rec36	g	2	-	g	a	g	a	g	g	g	g	g	g	a	g	g	g	g	g	g	g	g	g	g	g	g
g	4976469	g	4992698	t	4942644	s	S88	rec36	g	S88	++++	g	g	g	g	g	g	g	t	g	g	g	g	g	g	g	g	g	g	g	g	g	g	g
a	4976502	a	4992731	g	4942677	s	S88	rec36	g	2	-	g	g	g	g	g	g	a	g	g	g	g	a	a	a	g	g	g	g	g	g	g	g	g
a	4976508	a	4992737	g	4942683	s	S88	rec36	g	2	-	g	g	g	g	g	g	a	g	g	g	g	a	a	a	g	g	g	g	g	g	g	g	g
t	4976529	t	4992758	g	4942704	s	S88	rec36	t	S88	++	t	t	t	t	g	t	t	g	g	g	g	g	g	g	g	g	g	g	g	g	g	g	g
c	4976586	c	4992815	t	4942761	s	S88	rec36	c	S88	++	c	c	c	c	c	c	c	t	c	c	c	t	t	t	c	c	c	c	c	c	c	c	c
t	4976592	t	4992821	c	4942767	s	S88	rec36	t	S88	++	t	c	t	c	t	c	c	c	t	t	t	c	c	c	t	t	t	t	t	t	t	t	t
a	4976841	a	4993070	g	4943016	s	S88	rec36	a	S88	++	a	a	a	a	a	a	a	g	g	g	a	g	g	g	g	g	g	g	g	g	g	g	g
t	4976847	t	4993076	c	4943022	s	S88	rec36	t	S88	++	t	t	t	t	t	t	t	c	c	c	t	c	c	c	c	c	c	c	c	c	c	c	c
t	4976868	t	4993097	c	4943043	s	S88	rec36	t	S88	++	t	t	t	t	t	t	t	c	c	c	t	c	c	c	c	c	c	c	c	c	c	c	c
a	4976904	a	4993133	g	4943079	s	S88	rec36	g	2	-	g	g	g	a	a	g	a	g	g	g	a	g	g	g	g	g	g	a	a	a	g	g	g
c	4976946	c	4993175	t	4943121	ns	S88	rec36	c	S88	++	c	c	c	c	t	c	c	t	t	t	c	t	t	t	t	t	t	c	c	c	t	t	t
c	4976948	c	4993177	t	4943123	ns	S88	rec36	c	S88	++	c	c	c	t	t	c	t	t	t	t	t	t	t	t	t	t	t	t	t	t	t	t	t
a	4976973	a	4993202	c	4943148	s	S88	rec36	a	S88	++	a	a	a	a	c	a	c	c	c	c	c	c	c	c	c	c	c	c	c	c	c	c	c
g	4976978	g	4993207	a	4943153	s	S88	rec36	g	S88	++	g	g	g	g	g	g	g	a	g	g	g	a	a	a	g	g	g	g	g	g	g	g	g
g	4976979	g	4993208	a	4943154	s	S88	rec36	g	S88	++	g	g	g	g	a	g	a	a	g	g	g	a	a	a	g	g	g	g	g	g	g	g	g
t	4977108	t	4993337	c	4943283	s	S88	rec36	t	S88	++	t	t	t	t	c	t	c	c	c	c	t	c	c	c	c	c	c	c	c	c	c	c	c
t	4977117	t	4993346	c	4943292	s	S88	rec36	t	S88	++	t	t	t	t	c	t	c	c	t	t	c	t	t	t	t	t	t	c	c	c	t	t	t
c	4977357	c	4993586	t	4943532	nc	S88	rec36	c	S88	++	c	c	c	c	t	c	t	t	c	c	c	c	c	c	c	c	c	c	c	c	c	c	c
g	4977358	g	4993587	a	4943533	nc	S88	rec36	g	S88	++	g	g	g	g	a	g	a	a	g	g	g	g	g	g	g	g	g	g	g	g	g	g	g
g	4977364	g	4993593	a	4943539	nc	S88	rec36	g	S88	++++	g	g	g	g	g	g	g	a	g	g	g	g	g	g	g	g	g	g	g	g	g	g	g
c	4977487	c	4993716	t	4943662	s	S88	rec36	c	S88	++++	c	c	c	c	c	c	c	t	c	c	c	c	c	c	c	c	c	c	c	c	c	c	c
c	4977538	c	4993767	t	4943713	s	S88	rec36	c	S88	++	c	c	c	c	c	c	c	t	c	c	c	t	t	t	c	c	c	c	c	c	c	c	c
a	4977544	a	4993773	g	4943719	s	S88	rec36	a	S88	++	a	a	a	a	a	a	g	g	g	g	a	g	g	g	g	g	g	g	g	g	g	g	g
a	4977547	a	4993776	g	4943722	s	S88	rec36	a	S88	++	a	a	a	a	a	a	a	g	g	g	a	g	g	g	g	g	g	g	g	g	g	g	g
a	4977601	a	4993830	g	4943776	s	S88	rec36	a	S88	++	a	a	a	a	a	a	a	g	a	a	a	g	g	g	g	a	g	g	g	g	a	a	a
t	4977649	t	4993878	c	4943824	s	S88	rec36	t	S88	++	t	t	t	t	t	t	t	c	c	c	t	c	c	c	c	c	c	c	c	c	c	c	c
t	4977658	t	4993887	c	4943833	s	S88	rec36	t	S88	++	t	t	t	t	t	t	t	c	c	c	t	c	c	c	c	c	c	c	c	c	c	c	c
a	4977664	a	4993893	g	4943839	s	S88	rec36	a	S88	++	a	a	a	a	a	a	a	g	g	g	a	g	g	g	g	g	a	g	g	g	g	g	g
a	4977694	a	4993923	c	4943869	s	S88	rec36	a	S88	++	a	a	a	a	a	a	a	c	c	c	a	a	a	a	a	c	c	a	a	a	c	c	c
g	4977736	g	4993965	a	4943911	s	S88	rec36	g	S88	++	g	g	g	g	a	g	a	a	g	g	a	a	a	a	a	g	a	a	a	a	g	g	g
c	4977769	c	4993998	t	4943944	s	S88	rec36	c	S88	++	c	c	c	c	c	c	c	t	c	c	t	t	t	t	t	c	t	t	t	t	c	c	c

ExPEC genome site details												Outgroup Strains Details[i]																						
UTI89[b]	UTI89 site[c]	APEC 01[b]	APEC 01 site[c]	S88[b]	S88 site[c]	type[d]	Event Lineage[ef]	Recombinant segment	Inferred ancestral base[g]	Outgroup Analysis[e]	Support level[h]	CFT073	ED1a	536	E2348/69	SMS 3-5	IAI39	UMN026	K-12	ATCC 8739	HS	D1 Sd197	CB9615	Sakai	EDL933	IAI1	E24377A	SE11	SS Ss046	F2a 301	F2a 2457T	F5 8401	B4 Sb227	B18 BS512
t	4977773	t	4994002	c	4943948	ns	S88	rec36	c	2	-	t	c	c	c	c	c	c	c	c	c	c	c	c	c	c	c	c	c	c	c	c	c	c
t	4977827	t	4994056	c	4944002	ns	S88	rec36	t	S88	++	t	t	t	t	c	t	t	c	c	c	t	c	c	c	c	c	c	c	c	c	c	c	c
t	4977945	t	4994174	c	4944120	ns	S88	rec36	t	S88	++	t	t	t	t	c	t	c	c	c	c	c	c	c	c	c	c	c	t	t	t	c	c	c
t	4977971	t	4994200	g	4944146	nc	S88	rec36	t	S88	++	t	t	t	t	g	t	g	g	g	g	g	g	g	g	g	g	g	g	g	g	g	g	g
a	4978027	a	4994256	c	4944202	s	S88	rec36	a	S88	++	a	a	a	a	c	a	c	c	c	c	c	a	a	a	c	c	c	c	c	c	c	c	c
a	4978261	a	4994490	g	4944436	s	S88	rec36	a	S88	++	a	a	a	a	g	a	g	g	g	g	g	g	g	g	g	g	g	g	g	g	g	g	g
g	4978426	g	4994655	a	4944601	s	S88	rec36	a	2	-	a	a	a	g	g	a	a	a	a	a	g	a	a	a	a	a	a	a	a	a	a	a	a
t	4978501	t	4994730	c	4944676	s	S88	rec36	t	S88	++	t	t	t	t	c	t	c	c	c	c	c	c	c	c	c	c	c	c	c	c	c	c	c
g	4978537	g	4994766	a	4944712	s	S88	rec36	g	S88	++++	g	g	g	g	g	g	g	a	g	g	g	g	g	g	g	g	g	g	g	g	g	g	g
c	4978633	c	4994862	t	4944808	s	S88	rec36	c	S88	++	c	c	c	c	t	c	c	t	t	t	t	c	c	c	c	c	c	c	c	c	t	t	t
c	4978645	c	4994874	t	4944820	s	S88	rec36	c	S88	++	c	c	c	c	c	c	c	t	t	c	c	c	c	c	t	c	t	c	c	c	c	c	c
a	4978659	a	4994888	g	4944834	s	S88	rec36	g	2	-	g	g	g	g	g	g	g	g	g	g	g	g	g	g	g	g	g	g	g	g	g	g	g
a	4978678	a	4994907	t	4944853	s	S88	rec36	a	S88	++	a	a	a	a	c	a	a	t	t	t	c	t	t	t	t	t	t	t	t	t	t	t	t
a	4978681	a	4994910	g	4944856	s	S88	rec36	a	S88	++	a	a	a	a	a	a	a	g	g	a	a	a	a	a	g	a	g	a	a	a	a	a	a
a	4978785	a	4995014	g	4944960	s	S88	rec36	a	S88	++	a	a	a	g	a	a	a	g	a	a	a	a	a	a	a	g	a	a	a	a	g	g	g
g	4978890	g	4995119	a	4945065	s	S88	rec36	g	S88	++	g	g	g	g	a	g	a	a	a	a	a	a	a	a	a	a	a	a	a	a	a	a	a
c	4978920	c	4995149	g	4945095	s	S88	rec36	c	S88	++	c	c	c	c	g	c	g	g	g	g	g	g	g	g	g	g	g	g	g	g	g	g	g
c	4978934	c	4995163	t	4945109	ns	S88	rec36	c	S88	++	c	c	c	c	t	c	t	t	t	t	t	t	t	t	t	t	t	t	t	t	t	t	t
a	4978943	a	4995172	g	4945118	ns	S88	rec36	g	2	-	g	g	g	g	g	g	g	g	g	g	g	g	g	g	g	g	g	g	g	g	g	g	g
t	4978953	t	4995182	c	4945128	s	S88	rec36	t	S88	++	t	t	t	t	c	t	t	c	t	t	t	c	c	c	t	c	t	c	c	c	t	t	t
g	4978986	g	4995215	a	4945161	s	S88	rec36	g	S88	++	g	g	g	g	g	g	g	a	g	g	g	g	g	g	g	g	g	a	a	a	a	a	a
t	4979007	t	4995236	c	4945182	s	S88	rec36	c	2	-	c	c	c	t	c	c	c	c	c	c	c	c	c	c	c	c	c	c	c	c	c	c	c
g	4979010	g	4995239	a	4945185	s	S88	rec36	a	2	-	a	a	a	g	a	a	a	a	a	a	a	a	a	a	a	a	a	a	a	a	a	a	a
a	4979019	a	4995248	g	4945194	s	S88	rec36	a	S88	++	a	a	a	g	g	a	g	g	g	g	a	g	g	g	g	g	g	g	g	g	g	g	g
g	4979037	g	4995266	a	4945212	s	S88	rec36	g	S88	++	g	g	g	a	a	g	a	a	a	a	a	a	a	a	a	a	a	a	a	a	a	a	a
t	4979051	t	4995280	c	4945226	ns	S88	rec36	t	S88	++	t	t	t	c	c	t	c	c	c	c	c	c	c	c	c	c	c	c	c	c	c	c	c
a	4979148	a	4995377	c	4945323	s	S88	rec36	c	2	-	c	c	a	c	c	a	c	c	c	c	c	c	c	c	c	c	c	c	c	c	c	c	c
t	4979153	t	4995382	c	4945328	ns	S88	rec36	c	2	-	c	c	t	c	c	t	c	c	c	c	c	c	c	c	c	c	c	c	c	c	c	c	c
c	4979372	c	4995601	t	4945547	nc	S88	rec36	c	S88	++	c	c	c	t	t	c	t	t	c	c	t	t	t	t	c	t	c	t	t	t	t	t	t
t	4979383	t	4995612	c	4945557	nc	S88	rec36	t	S88	++	t	t	t	c	c	t	c	c	t	c	c	c	c	c	c	c	c	c	c	c	c	c	c
g	4979524	g	4995753	a	4945698	s	S88	rec36	g	S88	++	g	g	g	g	g	g	g	a	g	a	g	a	a	a	a	a	a	a	a	a	a	a	a
t	4979564	t	4995793	c	4945738	s	S88	rec36	t	S88	++	t	t	t	t	t	t	c	c	t	c	t	c	c	c	c	c	c	c	c	c	c	c	c
a	4979573	a	4995802	c	4945747	s	S88	rec36	a	S88	++	a	a	a	a	a	a	a	c	a	a	a	c	c	c	a	c	a	c	c	c	c	c	c
a	4979591	a	4995820	g	4945765	s	S88	rec36	a	S88	++	a	a	a	a	a	a	a	g	a	a	a	g	g	g	a	g	a	g	g	g	g	a	a
g	4979654	g	4995883	a	4945828	s	S88	rec36	g	S88	++	g	g	g	g	g	g	g	a	g	g	g	g	g	g	g	g	g	a	a	a	g	g	g
g	4979669	g	4995898	a	4945843	s	S88	rec36	g	S88	++	g	g	g	a	a	a	a	a	a	a	a	g	g	g	a	a	a	a	a	a	g	g	g
t	4979687	t	4995916	c	4945861	s	S88	rec36	t	S88	++	t	t	t	c	c	c	c	c	c	c	c	c	c	c	c	c	c	c	c	c	c	c	c
g	4979698	g	4995927	a	4945872	s	S88	rec36	g	S88	++	g	g	g	g	a	a	g	a	g	g	a	a	a	a	g	g	g	a	a	a	g	g	g
t	4979711	t	4995940	c	4945885	s	S88	rec36	t	S88	++	t	t	t	t	t	t	t	c	t	t	t	c	c	c	t	t	t	c	c	c	t	t	t
c	4979740	c	4995969	a	4945914	ns	S88	rec36	c	S88	++	c	c	c	a	a	c	a	a	c	c	a	a	a	a	c	c	c	a	a	a	c	c	c
a	4979741	a	4995970	g	4945915	s	S88	rec36	a	S88	++	a	a	a	g	g	a	g	g	g	g	g	g	g	g	g	g	g	g	g	g	g	g	g
t	4979768	t	4995997	c	4945942	s	S88	rec36	t	S88	++	t	t	t	c	c	t	c	c	c	c	c	c	c	c	c	c	c	c	c	c	c	c	c
a	4979771	a	4996000	c	4945945	s	S88	rec36	a	S88	++	a	a	a	a	c	a	c	c	a	a	c	c	c	c	a	c	a	c	c	c	a	a	a
t	4979896	t	4996125	c	4946070	s	S88	rec36	t	S88	++	t	t	t	c	t	t	t	c	c	c	t	c	c	c	c	c	c	c	c	c	c	c	c
a	4979908	a	4996137	g	4946082	s	S88	rec36	a	S88	++	a	a	a	g	a	a	g	g	g	g	a	g	g	g	g	g	g	g	g	g	g	g	g
t	4979938	t	4996167	c	4946112	s	S88	rec36	t	S88	++	t	t	c	c	c	t	c	c	c	c	c	c	c	c	c	c	c	c	c	c	c	c	c
t	4979995	t	4996224	a	4946169	s	S88	rec36	t	S88	++	t	t	t	a	t	t	a	a	a	a	t	a	a	a	a	a	a	a	a	a	a	a	a
c	4980016	c	4996245	t	4946190	s	S88	rec36	c	S88	++	c	c	c	c	c	c	c	t	c	c	c	t	t	t	c	c	c	t	t	t	c	c	c
a	4980070	a	4996299	g	4946244	s	S88	rec36	a	S88	++	a	a	a	a	a	a	a	g	a	g	a	g	g	g	g	g	g	g	g	g	a	a	a
g	4980115	g	4996344	a	4946289	s	S88	rec36	g	S88	++	g	g	g	g	g	g	g	a	g	a	g	a	a	a	a	a	a	a	a	a	g	g	g
c	4980180	c	4996409	t	4946354	ns	S88	rec36	c	S88	++	c	c	c	c	c	c	c	t	c	c	c	t	t	t	c	t	c	t	t	t	c	c	c
c	4980190	c	4996419	a	4946364	ns	S88	rec36	c	S88	++	c	c	a	a	a	c	a	a	a	a	a	a	a	a	a	a	a	a	a	a	a	a	a
g	4980192	g	4996421	a	4946366	ns	S88	rec36	g	S88	++	g	g	a	a	a	g	a	a	a	a	a	a	a	a	a	a	a	a	a	a	a	a	a
t	4980223	t	4996452	g	4946397	ns	S88	rec36	t	S88	++	t	t	t	t	t	t	t	g	g	g	t	g	g	g	g	g	g	g	g	g	t	t	t
g	4980250	g	4996479	a	4946424	s	S88	rec36	g	S88	++	g	g	g	a	g	g	a	a	a	a	g	a	a	a	a	a	a	a	a	a	a	a	a
c	4980355	c	4996584	a	4946529	s	S88	rec36	c	S88	++	c	c	c	c	c	c	c	a	c	c	c	a	a	a	c	a	c	a	a	a	c	c	c
t	4980361	t	4996590	c	4946535	s	S88	rec36	t	S88	++	t	t	t	c	c	t	c	c	c	c	c	c	c	c	c	c	c	c	c	c	c	c	c
g	4980451	g	4996680	a	4946625	s	S88	rec36	g	S88	++	g	g	g	g	a	g	a	a	g	g	g	a	a	a	g	a	g	a	a	a	g	g	g
a	4980507	a	4996736	g	4946681	s	S88	rec36	a	S88	++	a	a	a	a	a	a	g	g	g	g	a	g	g	g	g	a	g	g	g	g	a	a	a
c	4980508	c	4996737	t	4946682	s	S88	rec36	c	S88	++++	c	c	c	c	c	c	c	t	c	c	c	c	c	c	c	c	c	c	c	c	c	c	c
t	4980757	t	4996986	a	4946931	nc	S88	rec36	t	S88	++	t	t	t	a	a	t	a	a	a	a		a	a	a	a	a	a	a	a	a	a	a	a
t	4980791	t	4997020	c	4946965	nc	S88	rec36	t	S88	++	t	t	t	c	c	t	c	c	c	c		c	c	c	c	c	c	c	c	c	c	c	c
g	4980804	g	4997033	a	4946978	nc	S88	rec36	g	S88	++	g	g	g	a	g	g	a	a	a	a		a	a	a	a	a	a	a	a	a	a	a	a
c	4980819	c	4997048	t	4946993	nc	S88	rec36	c	S88	++	c	c	c	c	c	c	c	t	c	t		c	c	c	t	c	c	c	c	c	c	c	c
t	4980820	t	4997049	c	4946994	nc	S88	rec36	t	S88	++	t	t	t	t	t	t	t	c	c	c		t	t	t	c	t	t	t	t	t	t	t	t
g	4980832	g	4997061	a	4947006	nc	S88	rec36	g	S88	++	g	g	g	a	a	g	a	a	a	a		a	a	a	a	a	a	a	a	a	a	a	a
a	4980835	a	4997064	g	4947009	nc	S88	rec36	a	S88	++	a	a	a	g	g	a	g	g	g	g		t	t	t	g	t	g	g	g	g	g	g	g
g	4980850	g	4997079	a	4947024	nc	S88	rec36	g	S88	++	g	g	g	a	a	g	a	a	a	a		g	g	g	a	g	a	g	g	g	a	a	a
g	4980866	g	4997095	a	4947040	nc	S88	rec36	g	S88	++	g	g	g	a	a	g	a	a	a	a		g	g	g	a	g	a	g	g	g	a	a	a
t	4980868	t	4997097	c	4947042	nc	S88	rec36	t	S88	++	t	t	t	t	t	t	t	c	t	c		t	t	t	c	t	c	t	t	t	c	c	c
g	4980873	g	4997102	a	4947047	nc	S88	rec36	g	S88	++	g	g	g	g	g	g	g	a	g	a		g	g	g	a	g	a	g	g	g	a	a	a
t	4980882	t	4997111	a	4947056	nc	S88	rec36	t	S88	++	t	t	a	a	a	a	a	a	a	a		a	a	a	a	a	a	a	a	a	a	a	a
t	4980894	t	4997123	c	4947068	nc	S88	rec36	t	S88	++++	t	t	t	t	t	t	t	c	t	t		t	t	t	t	t	t	t	t	t	t	t	t
t	4980911	t	4997140	c	4947085	nc	S88	rec36	t	S88	++	t	t	t	t	t	t	t	c	t	c		t	t	t	c	t	c	t	t	t	c	c	c
a	4980927	a	4997156	g	4947101	nc	S88	rec36	a	S88	++	a	a	a	a	a	a	a	g	g	g		a	a	a	g	a	g	a	a	a	g	g	g
c	4980938	c	4997167	t	4947112	nc	S88	rec36	c	S88	++	c	c	c	-	-	c	-	-	-	-	t	c	c	c	-	c	-	c	c	c	t	t	t
c	4980939	c	4997168	t	4947113	nc	S88	rec36	c	S88	++	c	c	c	t	t	c	t	t	t	t	t	c	c	c	t	c	t	c	c	c	t	t	t
c	4980944	c	4997173	t	4947117	nc	S88	rec36	c	S88	++	c	c	t	t	t	c	t	t	t	t	t	t	t	t	t	t	t	t	t	t	t	t	t
a	4980956	a	4997185	g	4947129	nc	S88	rec36	a	S88	++	a	a	a	g	g	a	g	g	a	g	g	a	a	a	g	g	g	a	a	a	g	g	g
t	4980962	t	4997191	c	4947135	nc	S88	rec36	t	S88	++	t	t	c	c	c	c	c	c	c	c	c	c	c	c	c	c	c	c	c	c	c	c	c
a	4980987	a	4997216	t	4947160	nc	S88	rec36	a	S88	++	a	a	a	a	a	a	a	t	a	t	a	a	a	a	t	t	t	a	a	a	t	t	t
t	4981012	t	4997241	c	4947185	nc	S88	rec36	t	S88	++	t	t	t	t	t	t	t	c	t	c	t	t	t	t	c	c	c	t	t	t	c	c	c
t	4981183	t	4997412	c	4947356	nc	S88	rec36	t	S88	++	t	t	t	c	c	t	c	c	c	c	c	c	c	c	c	c	c	c	c	c	c	c	c
g	4981199	g	4997428	a	4947372	nc	S88	rec36	g	S88	++	g	g	a	a	a	a	a	a	a	a	a	a	a	a	a	a	a				a	a	a

ExPEC genome site details												Outgroup Strains Details[i]																						
UTI89[b]	UTI89 site[c]	APEC 01[b]	APEC 01 site[c]	S88[b]	S88 site[c]	type[d]	Event Lineage[ef]	Recombinant segment	Inferred ancestral base[g]	Outgroup Analysis[e]	Support level[h]	CFT073	ED1a	536	E2348/69	SMS 3-5	IAI39	UMN026	K-12	ATCC 8739	HS	D1 Sd197	CB9615	Sakai	EDL933	IAI1	E24377A	SE11	SS Ss046	F2a 301	F2a 2457T	F5 8401	B4 Sb227	B18 BS512
c	4981200	c	4997429	t	4947373	nc	S88	rec36	c	S88	++	c	c	c	t	t	c	t	t	t	t	t	t	t	t	t	t	t				t	t	t
t	4981202	t	4997431	c	4947375	nc	S88	rec36	t	S88	++	t	t	t	c	c	t	c	c	c	c	c	c	c	c	c	c	c				c	c	c
g	4981218	g	4997447	a	4947391	nc	S88	rec36	g	S88	++	g	g	g	a	g	g	a	a	g	a	g	a	a	a	g	g	a				g	g	g
g	4981423	g	4997652	a	4947596	s	S88	rec36	g	S88	++	g	g	a	a	a	a	a	a	a	a	a	a	a	a	a	a	a	a	a	a	a	a	a
c	4981450	c	4997679	t	4947623	s	S88	rec36	c	S88	++	c	c	c	t	t	c	t	t	t	t	t	t	t	t	t	t	t	t	t	t	t	t	t
g	4981459	g	4997688	a	4947632	s	S88	rec36	g	S88	++	g	a	g	a	a	g	a	a	a	a	a	a	a	a	a	a	a	a	a	a	a	a	a
c	4981468	c	4997697	t	4947641	s	S88	rec36	c	S88	++	c	t	c	t	t	c	t	t	t	t	t	t	t	t	t	t	t	t	t	t	t	t	t
t	4981544	t	4997773	c	4947717	s	S88	rec36	t	S88	++	t	c	t	c	c	t	c	c	t	t	c	c	c	c	t	t	t	c	c	c	t	t	t
t	4981631	t	4997860	g	4947804	ns	S88	rec36	t	S88	++	t	g	t	g	g	t	g	g	g	g	g	g	g	g	g	g	g	g	g	g	g	g	g
t	4981660	t	4997889	c	4947833	s	S88	rec36	t	S88	++	t	c	t	t	t	t	t	c	t	t	t	t	t	t	t	t	t	t	t	t	t	t	t
t	4981801	t	4998030	c	4947974	s	S88	rec36	t	S88	++	t	t	t	c	c	t	c	c	c	c	c	c	c	c	c	c	c	c	c	c	c	c	c
c	4981990	c	4998219	a	4948163	ns	S88	rec36	a	2	-	a	a	a	a	a	a	a	a	a	a	a	a	a	a	a	a	a	a	a	a	a	a	a
a	4982025	a	4998254	g	4948198	s	S88	rec36	a	S88	++	a	g	a	g	g	a	g	g	g	g	g	g	g	g	g	g	g	g	g	g	g	g	g
a	4982100	a	4998329	g	4948273	s	S88	rec36	a	S88	++	a	g	a	g	g	a	g	g	g	g	g	g	g	g	g	g	g	g	g	g	g	g	g
c	4982135	c	4998364	a	4948308	ns	S88	rec36	c	S88	++	c	c	c	a	c	c	a	a	a	a	c	a	a	a	a	a	a	a	a	a	a	a	a
c	4982154	c	4998383	t	4948327	s	S88	rec36	c	S88	++	c	c	c	t	c	c	t	t	t	t	c	t	t	t	t	t	t	t	t	t	t	t	t
t	4982346	t	4998575	c	4948519	s	S88	rec36	t	S88	++	t	t	t	c	t	t	c	c	c	c	t	c	c	c	c	c	c	c	c	c	c	c	c
t	4982472	t	4998701	c	4948645	s	S88	rec36	c	2	-	c	c	c	c	c	t	c	c	c	c	c	c	c	c	c	c	c	c	c	c	c	c	c
t	4982529	t	4998758	c	4948702	s	S88	rec36	t	S88	++	t	t	t	t	t	t	t	c	t	c	t	c	c	c	t	t	t	t	t	t	t	c	t
t	4982601	t	4998830	c	4948774	s	S88	rec36	t	S88	++	t	t	t	t	c	t	c	c	c	c	t	c	c	c	c	c	c	c	c	c	c	c	c
t	4982732	t	4998961	c	4948905	s	S88	rec36	t	S88	++	t	t	t	c	c	t	c	c	c	c	c	t	t	t	c	t	t	c	c	c	c	c	c
c	4982735	c	4998964	t	4948908	s	S88	rec36	c	S88	++	c	c	c	t	t	c	t	t	t	t	t	c	c	c	t	c	c	t	t	t	c	t	c
g	4982769	g	4998998	t	4948942	ns	S88	rec36	g	S88	++	g	g	g	t	t	g	t	t	t	t	t	g	g	g	g	g	g	t	t	t	t	t	t
a	4982771	a	4999000	c	4948944	s	S88	rec36	a	S88	++	a	a	a	c	c	a	c	c	c	c	c	a	a	a	a	a	a	c	c	c	c	c	c
a	4982774	a	4999003	g	4948947	s	S88	rec36	a	S88	++	a	a	a	g	g	a	g	g	g	g	g	a	a	a	a	a	a	g	g	g	g	g	g
a	4982800	a	4999029	g	4948973	ns	S88	rec36	a	S88	++	a	a	a	g	g	a	g	g	g	g	g	g	g	g	g	g	g	g	g	g	g	g	g
c	4982816	c	4999045	a	4948989	s	S88	rec36	c	S88	++	c	c	c	c	c	c	c	a	c	c	c	c	c	c	c	c	c	a	a	a	c	a	c
c	4982819	c	4999048	a	4948992	s	S88	rec36	c	S88	++	c	c	c	c	c	c	c	a	c	c	c	c	c	c	c	c	c	a	a	a	c	a	c
c	4982834	c	4999063	t	4949007	s	S88	rec36	c	S88	++	c	c	c	t	c	c	c	t	c	t	c	t	t	t	t	t	t	t	t	t	t	t	t
t	4982837	t	4999066	c	4949010	s	S88	rec36	t	S88	++	t	t	t	c	c	t	t	c	c	c	c	c	c	c	c	c	c	c	c	c	c	c	c
c	4982849	c	4999078	g	4949022	s	S88	rec36	c	S88	++	c	c	c	g	g	c	c	g	g	g	g	a	a	a	g	a	a	g	g	g	g	g	g
g	4982879	g	4999108	a	4949052	s	S88	rec36	g	S88	++	g	g	g	g	a	g	g	a	a	a	a	a	a	a	a	a	a	a	a	a	a	a	a
a	4982900	a	4999129	g	4949073	s	S88	rec36	a	S88	++	a	a	a	a	g	a	g	g	g	g	g	g	g	g	g	g	g	g	g	g	g	g	g
c	4982909	c	4999138	g	4949082	s	S88	rec36	c	S88	++	c	c	c	c	c	c	c	g	g	g	c	g	g	g	g	g	g	g	g	g	g	g	g
t	4982912	t	4999141	c	4949085	s	S88	rec36	t	S88	++	t	t	t	t	c	t	t	c	c	c	c	c	c	c	c	c	c	c	c	c	c	c	c
c	4982924	c	4999153	t	4949097	s	S88	rec36	c	S88	++	c	c	c	c	c	c	c	t	c	c	c	c	c	c	c	c	c	t	t	t	c	t	c
g	4982929	g	4999158	c	4949102	ns	S88	rec36	c	2	-	c	c	c	c	c	c	c	c	c	c	c	c	c	c	c	c	c	c	c	c	c	c	c
c	4982969	c	4999198	a	4949142	ns	S88	rec36	c	S88	++	c	c	c	c	c	c	c	a	c	c	c	c	c	c	c	c	c	c	c	c	c	a	c
a	4982985	a	4999214	g	4949158	ns	S88	rec36	a	S88	++	a	a	a	a	g	a	a	g	g	g	g	g	g	g	g	g	g	g	g	g	g	g	g
g	4983030	g	4999259	a	4949203	ns	S88	rec36	g	S88	++	g	g	g	g	g	g	g	a	a	g	g	a	a	a	g	g	g	a	a	a	a	a	a
a	4983054	a	4999283	g	4949227	ns	S88	rec36	a	S88	++	a	a	a	a	a	a	a	g	g	g	a	g	g	g	g	g	g	g	g	g	g	g	g
g	4983056	g	4999285	a	4949229	s	S88	rec36	g	S88	++	g	g	g	g	g	g	g	a	a	a	g	a	a	a	a	a	a	a	a	a	a	a	a
c	4983061	c	4999290	a	4949234	ns	S88	rec36	c	S88	++	c	c	c	c	a	c	a	a	a	a	a	a	a	a	a	a	a	a	a	a	a	a	a
t	4983086	t	4999315	c	4949259	s	S88	rec36	t	S88	++	t	t	t	t	t	t	t	c	c	c	t	t	t	t	c	c	c	t	t	t	c	c	c
a	4983095	a	4999324	c	4949268	s	S88	rec36	a	S88	++	a	a	a	a	c	a	c	c	c	c	c	c	c	c	c	c	c	c	c	c	c	c	c
c	4983101	c	4999330	t	4949274	s	S88	rec36	c	S88	++	c	c	c	c	c	c	c	t	t	t	c	t	t	t	t	t	t	t	t	t	t	t	t
t	4983122	t	4999351	c	4949295	s	S88	rec36	t	S88	++	t	t	t	t	c	t	c	c	c	c	c	c	c	c	c	c	c	c	c	c	c	c	c
c	4983146	c	4999375	t	4949319	s	S88	rec36	c	S88	++	c	c	c	c	t	c	c	t	t	t	c	t	t	t	t	t	t	t	t	t	t	t	t
c	4983152	c	4999381	t	4949325	s	S88	rec36	c	S88	++	c	c	c	c	t	c	t	t	t	t	c	t	t	t	t	t	t	t	t	t	t	t	t
a	4983161	a	4999390	c	4949334	s	S88	rec36	c	2	-	c	a	c	c	c	c	c	c	c	c	c	c	c	c	c	c	c	c	c	c	c	c	c
t	4983173	t	4999402	c	4949346	s	S88	rec36	t	S88	++	t	t	t	t	c	t	c	c	c	c	c	c	c	c	c	c	c	c	c	c	c	c	c
a	4983187	a	4999416	t	4949360	ns	S88	rec36	a	S88	++	a	a	a	a	a	a	t	t	t	t	a	t	t	t	t	t	t	t	t	t	t	t	t
a	4983188	a	4999417	c	4949361	s	S88	rec36	a	S88	++	a	a	a	a	a	a	t	c	t	t	a	t	t	t	t	t	t	t	t	t	t	t	t
g	4983200	g	4999429	a	4949373	s	S88	rec36	g	S88	++	g	g	g	g	a	g	a	a	a	a	a	a	a	a	a	a	a	a	a	a	a	a	a
g	4983224	g	4999453	c	4949397	s	S88	rec36	g	S88	++	g	g	g	g	g	g	c	c	g	g	g	c	c	c	g	g	g	c	c	c	g	g	g
t	4983227	t	4999456	c	4949400	s	S88	rec36	t	S88	++++	t	t	t	t	t	t	t	c	t	t	t	t	t	t	t	t	t	t	t	t	t	t	t
g	4983240	g	4999469	a	4949413	ns	S88	rec36	g	S88	++++	g	g	g	g	g	g	g	a	g	g	g	g	g	g	g	g	g	g	g	g	g	g	g
g	4983287	g	4999516	a	4949460	s	S88	rec36	g	S88	++++	g	g	g	g	g	g	g	a	g	g	g	g	g	g	g	g	g	g	g	g	g	g	g
c	4983331	c	4999560	t	4949504	ns	S88	rec36	c	S88	++	c	c	c	c	c	c	c	t	t	c	c	c	c	c	t	c	t	t	t	t	t	t	t
g	4983332	g	4999561	t	4949505	s	S88	rec36	g	S88	++++	g	g	g	g	g	g	g	t	g	g	g	g	g	g	g	g	g	g	g	g	g	g	g
t	4983341	t	4999570	c	4949514	s	S88	rec36	c	2	-	c	t	c	c	c	c	c	c	c	c	c	c	c	c	c	c	c	c	c	c	c	c	c
t	4983356	t	4999585	c	4949529	s	S88	rec36	t	S88	++++	t	t	t	t	t	t	t	c	t	t	t	t	t	t	t	t	t	t	t	t	t	t	t
c	4983363	c	4999592	g	4949536	ns	S88	rec36	c	S88	++++	c	c	c	c	c	c	c	g	c	c	c	c	c	c	c	c	c	c	c	c	c	c	c
c	4983364	c	4999593	t	4949537	ns	S88	rec36	c	S88	++	c	c	c	c	c	c	c	t	c	c	c	t	t	t	c	c	c	c	c	c	c	c	c
a	4983365	a	4999594	g	4949538	ns	S88	rec36	a	S88	++++	a	a	a	a	a	a	a	g	a	a	a	a	a	a	a	a	a	a	a	a	a	a	a
c	4983367	c	4999596	g	4949540	ns	S88	rec36	c	S88	++++	c	c	c	c	c	c	c	g	c	c	c	c	c	c	c	c	c	c	c	c	c	c	c
a	4983398	a	4999627	g	4949571	s	S88	rec36	a	S88	++	a	a	a	a	a	a	a	g	g	a	a	g	g	g	a	a	a	g	g	g	g	g	g
t	4983439	t	4999668	g	4949612	nc	S88	rec36	t	S88	++	t	t	t	t	c	t	g	g	g	g	g	g	g	g	g	g	g	t	t	t	g	g	g
t	4983461	t	4999690	g	4949634	nc	S88	rec36	t	S88	++	t	t	t	t	g	t	g	g	g	g	g	g	g	g	g	g	g	g	g	g	g	g	g
t	4983475	t	4999704	g	4949648	ns	S88	rec36	t	S88	++	t	t	t	t	g	t	g	g	g	g	a	g	g	g	g	g	g	g	g	g	g	g	g
a	4983500	a	4999729	g	4949673	ns	S88	rec36	g	2	-	g	a	g	g	g	g	g	g	g	g	g	g	g	g	g	g	g	g	g	g	g	g	g
c	4983505	c	4999734	a	4949678	ns	S88	rec36	c	S88	++++	c	c	c	c	c	c	c	a	c	c	c	c	c	c	c	c	c	c	c	c	c	c	c
c	4983572	c	4999801	t	4949745	ns	S88	rec36	c	S88	++++	c	c	c	c	c	c	c	t	c	c	c	c	c	c	c	c	c	c	c	c	c	c	c
c	4983658	c	4999887	t	4949831	ns	S88	rec36	c	S88	++++	c	c	c	c	c	c	c	t	c	c	c	c	c	c	c	c	c	c	c	c	c	c	c
g	4983660	g	4999889	a	4949833	ns	S88	rec36	g	S88	++	g	g	g	g	g	g	g	a	g	g	g	g	g	g	t	t	t	g	g	g	g	g	g
a	4983666	a	4999895	g	4949839	s	S88	rec36	a	S88	++	a	a	a	a	a	a	g	g	g	g	a	g	g	g	a	a	a	g	g	g	g	g	g
g	4983693	g	4999922	a	4949866	s	S88	rec36	g	S88	++	g	g	g	g	a	g	a	a	a	a	a	a	a	a	a	a	a	a	a	a	a	a	a
g	4983706	g	4999935	a	4949879	ns	S88	rec36	g	S88	++++	g	g	g	g	g	g	g	a	g	g	g	g	g	g	g	g	g	g	g	g	g	g	g
a	4983729	a	4999958	g	4949902	s	S88	rec36	a	S88	++	a	a	a	a	g	a	g	g	a	a	g	g	g	g	a	a	a	g	g	g	a	a	a
g	4983739	g	4999968	a	4949912	ns	S88	rec36	g	S88	++	g	g	g	g	g	g	g	a	g	g	g	a	a	a	g	g	g	a	a	a	g	g	g
g	4983762	g	4999991	a	4949935	s	S88	rec36	g	S88	++++	g	g	g	g	g	g	g	a	g	g	g	g	g	g	g	g	g	g	g	g	g	g	g
g	4983807	g	5000036	a	4949980	s	S88	rec36	g	S88	++	g	g	g	g	a	g	a	a	a	a	a	a	a	a	a	a	a	a	a	a	a	a	a

Table S8. Allocation of recombinational SNPs to lineages by virtual outgroup analysis[a]

ExPEC genome site details												Outgroup Strains Details[i]																						
UTI89[b]	UTI89 site[c]	APEC 01[b]	APEC 01 site[c]	S88[b]	S88 site[c]	type[d]	Event Lineage[ef]	Recombinant segment	Inferred ancestral base[g]	Outgroup Analysis[e]	Support level[h]	CFT073	ED1a	536	E2348/69	SMS 3-5	IAI39	UMN026	K-12	ATCC 8739	HS	D1 Sd197	CB9615	Sakai	EDL933	IAI1	E24377A	SE11	SS Ss046	F2a 301	F2a 2457T	F5 8401	B4 Sb227	B18 BS512
t	4983811	t	5000040	a	4949984	nc	S88	rec36	t	S88	++	t	t	t	t	t	t	a	a	a	a	t	a	a	a	a	a	a	c	c	c	a	a	a
a	4983815	a	5000044	t	4949988	nc	S88	rec36	a	S88	++	a	a	a	a	a	a	t	t	t	t	a	a	a	a	t	t	t	a	a	a	t	t	t
g	4983818	g	5000047	a	4949991	nc	S88	rec36	g	S88	++	g	g	g	g	g	g	g	a	a	a	g	g	g	g	a	a	a	g	g	g	a	a	a
t	4983819	t	5000048	g	4949992	nc	S88	rec36	t	S88	++	t	t	t	t	g	t	g	g	g	g	g	g	g	g	g	g	g	g	g	g	g	g	g
c	4983822	c	5000051	a	4949995	nc	S88	rec36	a	2	-	c	a	a	a	a	c	a	a	a	a	a	a	a	a	a	a	a	a	a	a	a	a	a
g	4983825	g	5000054	a	4949998	nc	S88	rec36	g	S88	++	g	g	g	g	a	g	a	a	a	a	a	a	a	a	a	a	a	a	a	a	a	a	a
a	4983834	a	5000063	g	4950007	nc	S88	rec36	a	S88	++	a	a	a	a	g	a	g	g	g	g	g	g	g	g	g	g	g	g	g	g	g	g	g
a	4984279	a	5000509	g	4950452	ns	S88	rec36	a	S88	++	a	a	g	g	g	a	g	g	g	g	g	g	g	g	g	g	g	g	g	g	g	g	g
c	4984311	c	5000541	a	4950561	ns	S88	rec36	c	S88	++	c	c	c	a	a	c	a	a	a	a	c	a	c	c	a	a	a	a	a	a	a	a	a
a	4984316	a	5000546	g	4950566	ns	S88	rec36	a	S88	++	a	a	a	g	g	a	g	g	g	g	a	g	-	-	g	g	g	g	g	g	g	g	g
g	4984327	g	5000557	t	4950577	ns	S88	rec36	g	S88	++	g	g	g	t	t	g	t	t	t	t	g	t	t	t	t	t	t	t	t	t	t	t	t
t	4984328	t	5000558	c	4950578	ns	S88	rec36	t	S88	++	t	t	t	c	c	t	c	c	c	c	t	c	c	c	c	c	c	c	c	c	c	t	t
g	4984350	g	5000580	t	4950600	ns	S88	rec36	g	S88	++	g	g	g	g	g	g		t	t	t	g	t	t	t	t	t	t	g	g	g	t	g	g
c	4984370	c	5000600	a	4950611	nc	S88	rec36	c	S88	++	c	c	c	c	c	c	c	a	a	a	c	a	a	a	a	a	c	c	c	c	a	c	c
c	4984372	c	5000602	t	4950613	s	S88	rec36	c	S88	++	c	c	c	c	c	c	t	t	t	t	c	t	t	t	t	t	t	c	c	c	t	t	t
g	4984425	g	5000655	t	4950666	s	S88	rec36	g	S88	++	g	g	g	g	g	g	g	t	g	g	g	t	t	t	g	g	g	g	g	g	g	g	g
a	4984443	a	5000673	g	4950684	s	S88	rec36	g	2	-	g	g	g	g	g	g	g	g	a	a	g	g	g	g	a	a	a	a	a	a	a	a	a
g	4984476	g	5000706	a	4950717	s	S88	rec36	g	S88	++	g	g	g	g	g	g	g	a	g	g	a	a	a	a	g	g	g	g	g	g	g	a	a
a	4984508	a	5000738	g	4950749	s	S88	rec36	a	S88	++	a	a	a	g	g	g	g	g	g	g	g	g	g	g	g	g	g	g	g	g	g	g	g
g	4984521	g	5000751	a	4950762	s	S88	rec36	g	S88	++	g	g	g	a	a	a	a	a	a	a	a	a	a	a	a	a	a	a	a	a	a	a	a
c	4984524	c	5000754	a	4950765	s	S88	rec36	c	S88	++	c	c	c	a	a	a	a	a	a	a	a	a	a	a	a	a	a	a	a	a	a	a	a
g	4984539	g	5000769	a	4950780	s	S88	rec36	g	S88	++	g	g	g	g	g	g	g	a	g	g	g	a	a	a	g	g	g	g	g	g	g	g	g
g	4984560	g	5000790	c	4950801	s	S88	rec36	g	S88	++	g	c	g	c	c	g	c	c	c	c	c	c	c	c	c	c	c	c	c	c	c	c	c
t	4984608	t	5000838	g	4950849	s	S88	rec36	t	S88	++	t	t	g	g	g	t	g	g	g	g	g	g	g	g	g	g	g	g	g	g	g	g	g
g	4984620	g	5000850	a	4950861	s	S88	rec36	g	S88	++	g	g	a	a	a	g	a	a	a	a	a	a	a	a	a	a	a	a	a	a	a	a	a
t	4984679	t	5000909	c	4950920	ns	S88	rec36	t	S88	++	c	t	t	c	c	c	c	c	c	c	c	c	c	c	c	c	c	c	c	c	c	c	c
t	4984710	t	5000940	c	4950951	s	S88	rec36	t	S88	++	t	t	t	t	t	t	t	c	t	t	t	t	t	t	t	t	t	c	c	c	t	t	t
t	4984713	t	5000943	g	4950954	s	S88	rec36	t	S88	++	t	t	t	c	c	t	c	g	c	c	t	c	c	c	c	c	c	g	g	g	c	c	c
a	4984719	a	5000949	g	4950960	s	S88	rec36	a	S88	++	g	a	a	g	a	a	g	g	g	g	g	g	g	g	g	g	g	g	g	g	g	g	g
a	4984725	a	5000955	g	4950966	s	S88	rec36	a	S88	++	g	a	a	g	g	a	g	g	g	g	g	g	g	g	g	g	g	g	g	g	g	g	g
g	4984730	g	5000960	a	4950971	s	S88	rec36	g	S88	++	g	g	g	g	a	g	g	a	a	a	g	a	a	a	a	a	a	a	a	a	a	a	a
a	4984731	a	5000961	g	4950972	s	S88	rec36	a	S88	++	a	a	a	a	c	a	a	g	g	g	a	g	g	g	g	g	g	g	g	g	g	g	g
g	4984737	g	5000967	a	4950978	s	S88	rec36	g	S88	++	a	g	g	a	a	g	a	a	a	a	g	a	a	a	a	a	a	a	a	a	a	a	a
g	4984740	g	5000970	a	4950981	s	S88	rec36	g	S88	++	a	g	g	a	a	g	a	a	a	a	g	a	a	a	a	a	a	a	a	a	a	a	a
c	4984743	c	5000973	t	4950984	s	S88	rec36	c	S88	++	t	c	c	t	c	c	t	t	t	t	c	t	t	t	t	t	t	t	t	t	t	t	t
t	4984749	t	5000979	c	4950990	s	S88	rec36	t	S88	++	c	t	t	t	t	t	t	c	c	c	t	c	c	c	c	c	c	c	c	c	c	c	c
a	4984764	a	5000994	g	4951005	s	S88	rec36	a	S88	++	g	a	a	g	g	a	g	g	g	g	a	g	g	g	g	g	g	g	g	g	g	g	g
g	4984770	g	5001000	t	4951011	s	S88	rec36	g	S88	++	c	g	g	c	c	g	a	t	t	t	g	c	c	c	t	t	t	t	t	t	t	t	t
t	4984779	t	5001009	g	4951020	s	S88	rec36	g	2	-	g	g	t	g	g	t	g	g	a	c	g	g	g	g	c	c	c	a	a	a	c	c	c
g	4984788	g	5001018	a	4951029	s	S88	rec36	g	S88	++	a	g	g	g	a	g	a	a	a	a	g	a	a	a	a	a	a	a	a	a	a	a	a
t	4984821	t	5001051	c	4951062	s	S88	rec36	t	S88	++	t	t	t	c	c	t	c	c	c	c	t	c	c	c	c	c	c	c	c	c	c	c	c
g	4984829	g	5001059	a	4951070	s	S88	rec36	g	S88	++	g	g	g	a	a	g	a	a	a	a	g	a	a	a	a	a	a	a	a	a	a	a	a
a	4984848	a	5001078	g	4951089	s	S88	rec36	a	S88	++	a	a	a	g	g	a	g	g	g	g	g	g	g	g	g	g	g	g	g	g	g	g	g
g	4984851	g	5001081	a	4951092	s	S88	rec36	g	S88	++	g	g	g	a	a	g	a	a	a	a	a	a	a	a	a	a	a	a	a	a	a	a	a
c	4984884	c	5001114	a	4951125	s	S88	rec36	c	S88	++	c	c	c	a	a	c	a	a	a	a	a	a	a	a	a	a	a	a	a	a	a	a	a
t	4984890	t	5001120	c	4951131	s	S88	rec36	c	2	-	t	c	c	c	c	t	c	c	c	c	c	c	c	c	c	c	c	c	c	c	c	c	c
a	4984904	a	5001134	g	4951145	s	S88	rec36	a	S88	++	a	a	a	g	g	a	g	g	g	g	g	g	g	g	g	g	g	g	g	g	g	g	g
a	4984935	a	5001165	c	4951176	ns	S88	rec36	a	S88	++	a	a	a	c	a	a	a	c	a	a	c	a	a	a	a	a	a	a	a	a	a	a	a
t	4984937	t	5001167	c	4951178	ns	S88	rec36	t	S88	++	t	t	t	c	t	t	c	c	c	c	c	c	c	c	c	c	c	c	c	c	c	c	c
g	4984938	g	5001168	c	4951179	s	S88	rec36	g	S88	++	g	g	g	g	g	g	g	c	g	g	g	g	g	g	g	g	g	c	c	c	g	g	g
c	4984956	c	5001186	t	4951197	s	S88	rec36	c	S88	++	c	c	c	t	t	c	t	t	t	t	t	t	t	t	t	t	t	t	t	t	t	t	t
g	4984983	g	5001213	a	4951224	s	S88	rec36	g	S88	++	g	g	g	a	a	g	a	a	a	a	a	a	a	a	a	a	a	a	a	a	a	a	a
g	4984992	g	5001222	a	4951233	s	S88	rec36	g	S88	++	g	g	g	a	g	g	g	a	a	a	a	g	g	g	a	a	a	a	a	a	a	a	a
t	4985001	t	5001231	g	4951242	s	S88	rec36	t	S88	++	t	t	t	c	c	t	g	g	g	g	c	g	g	g	g	g	g	g	g	g	g	g	g
c	4985004	c	5001234	t	4951245	s	S88	rec36	c	S88	++	c	c	c	t	c	c	t	t	t	t	t	t	t	t	t	t	t	t	t	t	t	t	t
g	4985025	g	5001255	t	4951266	s	S88	rec36	g	S88	++	g	g	g	t	t	g	t	t	t	t	t	a	a	a	t	t	t	t	t	t	t	t	t
a	4985044	a	5001274	c	4951285	ns	S88	rec36	c	2	-	c	c	c	c	c	c	c	c	c	c	c	c	c	c	c	c	c	c	c	c	c	c	c
c	4985085	c	5001315	t	4951326	s	S88	rec36	c	S88	++	c	c	c	c	c	c	c	t	t	c	c	c	c	c	c	c	c	t	t	t	t	t	t
g	4985088	g	5001318	c	4951329	s	S88	rec36	g	S88	++	g	g	g	g	g	g	g	c	c	c	g	c	c	c	c	c	c	c	c	c	c	c	c
c	4985094	c	5001324	t	4951335	s	S88	rec36	c	S88	++	c	c	c	c	t	c	c	t	t	c	c	c	c	c	c	c	c	t	t	t	t	t	t
a	4985097	a	5001327	c	4951338	s	S88	rec36	a	S88	++	a	a	a	c	c	a	c	c	c	c	c	a	a	a	c	c	c	c	c	c	c	c	c
g	4985160	g	5001390	a	4951401	s	S88	rec36	g	S88	++	g	g	g	a	a	a	a	a	a	a	a	a	a	a	a	a	a	a	a	a	a	a	a
a	4985184	a	5001414	g	4951425	s	S88	rec36	g	2	-	g	g	g	a	a	a	a	g	g	g	a	a	a	a	g	g	g	a	a	a	g	g	g
t	4985189	t	5001419	c	4951430	ns	S88	rec36	c	2	-	c	c	c	c	t	t	c	c	c	c	c	c	c	c	c	c	c	t	t	t	c	c	c
g	4985205	g	5001435	a	4951446	s	S88	rec36	g	S88	++	g	g	g	a	a	a	a	a	a	a	a	a	a	a	a	a	a	a	a	a	a	a	a
a	4985208	a	5001438	g	4951449	s	S88	rec36	a	S88	++	a	a	a	a	a	a	g	g	g	g	a	a	a	a	g	g	g	a	a	a	g	g	g
c	4985241	c	5001471	t	4951482	s	S88	rec36	c	S88	++	c	c	c	c	c	c	c	t	t	t	c	c	c	c	t	t	t	c	c	c	t	t	t
c	4985607	c	5001837	g	4951848	ns	S88	rec36	c	S88	++	c	c	c	g	g	c	g	g	g	g	g	g	g	g	g	g	g	g	g	g	g	g	g
c	4985654	c	5001884	t	4951895	ns	S88	rec36	c	S88	++	c	c	c	c	t	c	c	t	t	t	c	t	t	t	c	t	t	t	t	t	c	c	c
c	4985661	c	5001891	t	4951902	ns	S88	rec36	c	S88	++	c	c	c	t	t	c	c	t	t	t	t	t	t	t	t	t	t	t	t	t	t	t	t
g	4985663	g	5001893	a	4951904	ns	S88	rec36	g	S88	++	g	g	g	a	a	g	g	a	a	a	a	a	a	a	a	a	a	a	a	a	a	a	a
t	4985681	t	5001911	c	4951922	ns	S88	rec36	t	S88	++	t	t	t	c	c	t	c	c	c	c	c	c	c	c	c	c	c	c	c	c	c	c	c
g	4985684	g	5001914	a	4951925	ns	S88	rec36	g	S88	++	g	g	g	a	a	g	a	a	a	a	a	a	a	a	a	a	a	a	a	a	a	a	a
t	4985690	t	5001920	c	4951931	ns	S88	rec36	c	2	-	c	t	c	c	c	c	c	c	c	c	c	c	c	c	c	c	c	c	c	c	c	c	c
t	4985702	t	5001932	c	4951943	ns	S88	rec36	t	S88	++	t	t	t	t	t	t	t	c	t	c	t	t	t	t	t	c	c	t	t	t	t	t	t
a	4985705	a	5001935	g	4951946	ns	S88	rec36	a	S88	++	a	a	a	a	a	a	a	g	a	g	a	a	a	a	a	g	g	a	a	a	a	a	a
c	4985753	c	5001983	t	4951994	ns	S88	rec36	t	2	-	t	t	t	t	t	t	t	t	t	t	t	t	t	t	t	t	t	t	t	t	t	t	t
g	4985755	g	5001985	a	4951996	ns	S88	rec36	g	S88	++++	g	g	g	g	g	g	g	a	g	g	g	g	g	g	g	g	g	g	g	g	g	g	g
c	4985765	c	5001995	t	4952006	s	S88	rec36	c	S88	++++	c	c	c	c	c	c	c	t	c	c	c	c	c	c	c	c	c	c	c	c	c	c	c
a	4985834	a	5002064	t	4952075	ns	S88	rec36	a	S88	++	a	a	a	a	a	a	a	t	a	t	a	a	a	a	a	t	t	a	a	a	a	a	a
a	4985925	a	5002155	g	4952166	s	S88	rec36	a	S88	++	a	a	a	a	a	a	a	g	a	g	a	a	a	a	a	g	g	a	a	a	a	a	a
c	4985979	c	5002209	g	4952220	s	S88	rec36	c	S88	++++	c	c	c	c	c	c	c	g	c	c	c	c	c	c	c	c	c	c	c	c	c	c	c
g	4986183	g	5002413	a	4952424	s	S88	rec36	g	S88	++	g	g	g	a	g	g	g	a	g	a	a	a	a	a	a	a	a	a	a	a	a	a	a
c	4986204	c	5002434	t	4952445	s	S88	rec36	c	S88	++	c	t	c	c	t	c	c	t	c	t	c	c	c	c	t	t	c	c	c	c	c	t	t

Table S8. Allocation of recombinational SNPs to lineages by virtual outgroup analysis[a]

ExPEC genome site details												Outgroup Strains Details[i]																						
UTI89[b]	UTI89 site[c]	APEC 01[b]	APEC 01 site[c]	S88[b]	S88 site[c]	type[d]	Event Lineage[ef]	Recombinant segment	Inferred ancestral base[g]	Outgroup Analysis[e]	Support level[h]	CFT073	ED1a	536	E2348/69	SMS 3-5	IAI39	UMN026	K-12	ATCC 8739	HS	D1 Sd197	CB9615	Sakai	EDL933	IAI1	E24377A	SE11	SS Ss046	F2a 301	F2a 2457T	F5 8401	B4 Sb227	B18 BS512
t	4986210	t	5002440	c	4952451	s	S88	rec36	t	S88	++	t	c	t	c	c	t	c	c	c	c	c	t	t	t	t	c	c	t	t	t	t	c	c
a	4986318	a	5002548	g	4952559	s	S88	rec36	a	S88	++	a	g	a	g	g	a	g	g	g	g	g	g	g	g	g	g	g	g	g	g	g	g	g
g	4986334	g	5002564	t	4952575	nc	S88	rec36	g	S88	++++	g	g	g	g	g	g	g	t	g	g	g	g	g	g	g	g	g	g	g	g	g	g	g
a	4986581	a	5002811	g	4952822	s	S88	rec36	a	S88	++	a	a	a	g	g	a	g	g	g	a	g	g	g	g	a	a	a	a	a	a	a	a	a
g	4986584	g	5002814	a	4952825	s	S88	rec36	g	S88	++	g	g	g	g	a	g	g	a	g	a	g	a	a	a	a	a	a	a	a	a	a	a	a
g	4986599	g	5002829	a	4952840	s	S88	rec36	g	S88	++	g	g	g	a	g	g	a	a	a	t	a	a	a	a	t	t	t	t	t	t	t	t	t
t	4986629	t	5002859	c	4952870	s	S88	rec36	t	S88	++	t	t	t	t	t	t	t	c	c	t	t	c	c	c	t	c	c	t	t	t	c	c	c
t	4986636	t	5002866	c	4952877	s	S88	rec36	t	S88	++	t	t	t	c	t	t	c	c	c	t	c	c	c	c	t	c	c	t	t	t	c	c	c
g	4986644	g	5002874	c	4952885	s	S88	rec36	g	S88	++	g	g	g	c	c	g	c	c	c	c	c	c	c	c	c	c	c	c	c	c	c	c	c
c	4986653	c	5002883	t	4952894	s	S88	rec36	c	S88	++	c	c	c	t	t	c	t	t	t	t	t	t	t	t	t	t	t	t	t	t	t	t	t
c	4986710	c	5002940	t	4952951	s	S88	rec36	c	S88	++	c	c	c	c	c	c	c	t	t	c	c	t	t	t	c	c	c	c	c	c	c	c	c
a	4986725	a	5002955	g	4952966	s	S88	rec36	a	S88	++	a	a	a	g	g	a	g	g	g	g	g	g	g	g	g	g	g	g	g	g	g	g	g
a	4986833	a	5003063	g	4953074	s	S88	rec36	a	S88	++	a	a	a	g	g	g	g	g	g	g	g	g	g	g	g	g	g	g	g	g	g	g	g
t	4986872	t	5003102	c	4953113	s	S88	rec36	t	S88	++	t	t	t	c	c	c	c	c	c	c	c	c	c	c	c	c	c	c	c	c	c	c	c
g	4986938	g	5003168	a	4953179	s	S88	rec36	g	S88	++	g	g	g	g	g	g	g	a	g	a	g	a	a	a	a	g	g	g	g	g	g	g	g
a	4986942	a	5003172	c	4953183	ns	S88	rec36	a	S88	++	a	a	a	a	a	a	a	c	a	c	a	c	c	c	c	c	c	a	a	a	a	c	c
c	4991962	t	5007298	t	4957309	s	UTI89/AS	rec37	t	UTI89	+								t				t							t				
c	4991968	t	5007304	t	4957315	s	UTI89/AS	rec37	c	AS	+								a				c							a				
c	4991977	a	5007313	a	4957324	s	UTI89/AS	rec37	a	U	+								t				a							t				
t	4992003	c	5007339	c	4957350	ns	UTI89/AS	rec37	t	AS	+								t				t							t				
t	4992012	g	5007348	g	4957359	s	UTI89/AS	rec37	g	UTI89	+								g				g							g				
a	4992013	c	5007349	c	4957360	s	UTI89/AS	rec37	a	AS	+								a				a							c				
c	4992018	g	5007354	g	4957365	ns	UTI89/AS	rec37	c	AS	+								c				c							c				
a	4992025	g	5007361	g	4957372	s	UTI89/AS	rec37	g	UTI89	+								g				g							g				
c	4992034	t	5007370	t	4957381	s	UTI89/AS	rec37	c	AS	+								t				c							c				
c	4997992	t	5013328	t	4963339	ns	UTI89/AS	rec38	t	UTI89	++		t				c											c						
g	4998218	t	5013554	t	4963565	ns	UTI89/AS	rec38	?	U/AS	+/-						g											a						g
a	4998384	g	5013720	g	4963731	ns	UTI89/AS	rec38	a	AS	+						a											a						a
c	4998539	a	5013875	a	4963886	s	UTI89/AS	rec38	a	UTI89	+++		a				a											a						a
t	4998542	c	5013878	c	4963889	s	UTI89/AS	rec38	t	AS	+		t				t											t						t
c	4998617	a	5013953	a	4963964	ns	UTI89/AS	rec38	c	AS	++		c				c											a						c
a	4998758	g	5014094	g	4964105	s	UTI89/AS	rec38	g	UTI89	++		g				g																	g
g	4998847	a	5014183	a	4964194	s	UTI89/AS	rec38	g	AS	+++	g	g				g	g	g				g											g
t	4998850	c	5014186	c	4964197	s	UTI89/AS	rec38	c	UTI89	+++	c	c				c	c	c				c											t
t	4998907	c	5014243	c	4964254	s	UTI89/AS	rec38	c	UTI89	+++	c	a				c	c	c				c											c
g	4998919	a	5014255	a	4964266	s	UTI89/AS	rec38	g	AS	+++	g	g				g	g	g				a											g
t	4998931	a	5014267	a	4964278	s	UTI89/AS	rec38	a	UTI89	+	a	t				a	a	a				t											a
t	4999067	c	5014403	c	4964414	ns	UTI89/AS	rec38	c	UTI89	+++	c	c				c	-	c				t											c
t	4999069	a	5014405	a	4964416	ns	UTI89/AS	rec38	a	UTI89	+	a	t				a	-	a				t											a
c	4999117	t	5014453	t	4964464	s	UTI89/AS	rec38	t	UTI89	+	t	t				c	c	c				c											c
g	4999126	a	5014462	a	4964473	s	UTI89/AS	rec38	g	AS	+++	g	g				g	g	g				g											g
t	4999146	a	5014482	a	4964493	ns	UTI89/AS	rec38	a	UTI89	+++	a	a				a	a	a				a											a
t	4999176	a	5014512	a	4964523	ns	UTI89/AS	rec38	a	UTI89	+	a	a				a	t	a				t											a
a	4999183	t	5014519	t	4964530	s	UTI89/AS	rec38	t	UTI89	+	t	t				t	a	t				a											t
g	4999192	c	5014528	c	4964539	s	UTI89/AS	rec38	g	AS	+++	g	c				g	g	g				g											g
a	4999203	c	5014539	c	4964550	ns	UTI89/AS	rec38	c	UTI89	+++	c	c				c	a	c				c											c
c	4999229	t	5014565	t	4964576	ns	UTI89/AS	rec38	c	AS	+++	c	c				c	c	c				c											c
a	4999254	g	5014590	g	4964601	ns	UTI89/AS	rec38	g	UTI89	+++	g	g				g	g	g				a											g
t	4999255	c	5014591	c	4964602	ns	UTI89/AS	rec38	t	AS	+	t	t				t	t	t				t											t
c	4999293	t	5014629	t	4964640	ns	UTI89/AS	rec38	t	UTI89	+	t	t				t	c	c				c											t
a	4999303	t	5014639	t	4964650	ns	UTI89/AS	rec38	a	AS	+	a	t				t	a	a				a											t
g	4999331	t	5014667	t	4964678	ns	UTI89/AS	rec38	g	AS	+++	g	g				g	g	g				g											g
a	4999997	g	5014683	g	4964694	s	UTI89/AS	rec38	g	UTI89	+++	g	a				g	g					g											
c	5000042	t	5014728	t	4964739	s	UTI89/AS	rec38	?	U/AS	+/-	c	t				t	c					t											
c	5000057	t	5014743	t	4964754	s	UTI89/AS	rec38	c	AS	+++	c	c				c	c					c											
a	5000085	g	5014771	g	4964782	ns	UTI89/AS	rec38	a	AS	+++	a	a				a	a					a											
a	5000105	g	5014791	g	4964802	s	UTI89/AS	rec38	g	UTI89	++	g	g				g	a					a											
g	5000183	t	5014869	t	4964880	s	UTI89/AS	rec38	g	AS	++	g	t				g	c					g											
a	5000186	c	5014872	c	4964883	s	UTI89/AS	rec38	c	UTI89	+++	a	c				c	c					c											
g	5000199	a	5014885	a	4964896	ns	UTI89/AS	rec38	g	AS	+++	g	g				g	g					g											
g	5000201	a	5014887	a	4964898	ns	UTI89/AS	rec38	g	AS	+++	t	g				g	g					g											
g	5000225	a	5014911	a	4964922	s	UTI89/AS	rec38	g	AS	+++	g	g				g	g					g											
t	5000227	c	5014913	c	4964924	ns	UTI89/AS	rec38	t	AS	+	t	t				t	c					t											
c	5000240	t	5014926	t	4964937	s	UTI89/AS	rec38	c	AS	+++	c	c				c	c					c											
t	5000241	g	5014927	g	4964938	ns	UTI89/AS	rec38	g	UTI89	+	g	g				t	g					t											
a	5000267	c	5014953	c	4964964	s	UTI89/AS	rec38	c	UTI89	+++	c	c				a	c					c											
g	5000270	a	5014956	a	4964967	s	UTI89/AS	rec38	g	AS	+++	g	g				g	g					g											
a	5000280	g	5014966	g	4964977	ns	UTI89/AS	rec38	g	UTI89	+++	g	g				a	g					g											
a	5000300	g	5014986	g	4964997	s	UTI89/AS	rec38	g	UTI89	++	g	g				a	g					a											
t	5000332	c	5015018	c	4965029	s	UTI89/AS	rec38	t	AS	+	t	t				c	t					t											t
a	5000383	t	5015069	t	4965080	s	UTI89/AS	rec38	t	UTI89	+	t	t				t	t					t											t
a	5000413	c	5015099	c	4965110	s	UTI89/AS	rec38	a	AS	+++	a	a				a	a					a											a
g	5000444	t	5015130	t	4965141	ns	UTI89/AS	rec38	g	AS	+++	g	g				g	g					g											g
g	5000590	a	5015276	a	4965287	s	UTI89/AS	rec38	a	UTI89	+++	a	a				a	a					a											a
t	5000620	g	5015306	g	4965317	s	UTI89/AS	rec38	g	UTI89	+++	g	g				g	t					g											g
a	5000642	g	5015328	g	4965339	ns	UTI89/AS	rec38	g	UTI89	++	g	g				a	a					g											a
t	5000674	c	5015360	c	4965371	s	UTI89/AS	rec38	?	U/AS	+/-	t	c				t	c					c											c
c	5000681	a	5015367	a	4965378	ns	UTI89/AS	rec38	c	AS	++	c	g				c	a					g											a
c	5000738	t	5015424	t	4965435	s	UTI89/AS	rec38	t	UTI89	+	t					t	c																c
t	5000774	g	5015460	g	4965471	s	UTI89/AS	rec38	g	UTI89	+++	g					g	g																g
t	5000795	g	5015481	g	4965492	s	UTI89/AS	rec38	g	UTI89	+++	g					g	g																g

ExPEC genome site details												Outgroup Strains Details[i]																						
UTI89[b]	UTI89 site[c]	APEC 01[b]	APEC 01 site[c]	S88[b]	S88 site[c]	type[d]	Event Lineage[ef]	Recombinant segment	Inferred ancestral base[g]	Outgroup Analysis[e]	Support level[h]	CFT073	ED1a	536	E2348/69	SMS 3-5	IAI39	UMN026	K-12	ATCC 8739	HS	D1 Sd197	CB9615	Sakai	EDL933	IAI1	E24377A	SE11	SS Ss046	F2a 301	F2a 2457T	F5 8401	B4 Sb227	B18 BS512
c	5000807	t	5015493	t	4965504	s	UTI89/AS	rec38	c	AS	++	c					c																	
t	5000831	c	5015517	c	4965528	s	UTI89/AS	rec38	t	AS	++	t					c																	
a	5000861	g	5015547	g	4965558	s	UTI89/AS	rec38	a	AS	++	a					g																	
a	5000863	g	5015549	g	4965560	ns	UTI89/AS	rec38	g	UTI89	++	g					a																	
a	5000987	c	5015673	c	4965684	s	UTI89/AS	rec38	c	UTI89	+++	c					c	c																c
t	5001005	g	5015691	g	4965702	s	UTI89/AS	rec38	g	UTI89	+++	g					g	g																g
t	5001026	c	5015712	c	4965723	s	UTI89/AS	rec38	t	AS	+	t					t	t																c
t	5001040	c	5015726	c	4965737	ns	UTI89/AS	rec38	c	UTI89	+++	c					c	c																c
g	5001059	a	5015745	a	4965756	s	UTI89/AS	rec38	g	AS	++	g					g	g																a
t	5001122	c	5015808	c	4965819	s	UTI89/AS	rec38	?	U/AS	+/-	c	t				c	c					t											c
t	5001152	c	5015838	c	4965849	s	UTI89/AS	rec38	?	U/AS	+/-	t	c				t	t					c											t
c	5001221	t	5015908	t	4965918	s	UTI89/AS	rec38	t	UTI89	+	t	t				c	t					t											c
g	5001224	a	5015911	a	4965921	s	UTI89/AS	rec38	?	U/AS	+/-	a	g				g	a					g											g
c	5001226	a	5015913	a	4965923	ns	UTI89/AS	rec38	?	U/AS	+/-	a	c				c	a					c											c
t	5001227	c	5015914	c	4965924	ns	UTI89/AS	rec38	c	UTI89	+	c	a				t	c					a											t
c	5001239	t	5015926	t	4965936	s	UTI89/AS	rec38	?	U/AS	+/-	t	c				t	t					c											t
a	5001254	t	5015941	t	4965951	s	UTI89/AS	rec38	a	AS	+	a	g				g	a					g											g
a	5001271	g	5015958	g	4965968	ns	UTI89/AS	rec38	a	AS	+++	a	a				a	a					a											a
a	5001293	g	5015980	g	4965990	s	UTI89/AS	rec38	g	UTI89	+++	g	g				g	g					g											g
c	5001319	t	5016006	t	4966016	ns	UTI89/AS	rec38	?	U/AS	+/-	t	c				c	t					c											t
t	5001344	c	5016031	c	4966041	s	UTI89/AS	rec38	c	UTI89	+++	c	c				c	c					c											c
a	5001364	c	5016051	c	4966061	ns	UTI89/AS	rec38	c	UTI89	+++	c	c				c	c					c											c
t	5001371	c	5016058	c	4966068	s	UTI89/AS	rec38	c	UTI89	+++	c	c				c	c					c											c
g	5001437	t	5016124	t	4966134	s	UTI89/AS	rec38	t	UTI89	+	c	t				c	c					t											t
a	5001461	g	5016148	g	4966158	s	UTI89/AS	rec38	g	UTI89	+++	g	g				g	a					g											g
c	5001464	t	5016151	t	4966161	s	UTI89/AS	rec38	t	UTI89	+	t	t				t	c					t											t
c	5001570	t	5016257	t	4966267	s	UTI89/AS	rec38	c	AS	+++	t	c				c	c					c											c
a	5001582	g	5016269	g	4966279	s	UTI89/AS	rec38	g	UTI89	++	g	g				g	a					a											a
c	5001585	a	5016272	a	4966282	s	UTI89/AS	rec38	a	UTI89	+++	a	a				a	c					a											a
t	5001741	g	5016428	g	4966438	s	UTI89/AS	rec38	g	UTI89	+++	g	g				g	g					g											g
t	5001744	c	5016431	c	4966441	s	UTI89/AS	rec38	c	UTI89	+++	c	c				c	c					c											c
g	5001753	t	5016440	t	4966450	s	UTI89/AS	rec38	t	UTI89	+	t	t				t	t					t											t
a	5001849	g	5016536	g	4966546	s	UTI89/AS	rec38	g	UTI89	++	g	g				a	a					a											a
c	5001861	a	5016548	a	4966558	s	UTI89/AS	rec38	c	AS	+	a	c				c	t					c											t
c	5001867	t	5016554	t	4966564	s	UTI89/AS	rec38	c	AS	+++	t	c				c	c					c											c
c	5001900	t	5016587	t	4966597	s	UTI89/AS	rec38	t	UTI89	+	t	t				c	c					c											t
t	5001903	c	5016590	c	4966600	s	UTI89/AS	rec38	c	UTI89	+	c	c				t	t					t											c
t	5001966	c	5016653	c	4966663	s	UTI89/AS	rec38	t	AS	+	c	t				t	t					t											t
a	5002033	g	5016720	g	4966730	ns	UTI89/AS	rec38	g	UTI89	++	g	g				a	a					a											a
c	5002044	t	5016731	t	4966741	s	UTI89/AS	rec38	?	U/AS	+/-	t	c				c	c					t											t
c	5002045	t	5016732	t	4966742	s	UTI89/AS	rec38	c	AS	+++	t	c				c	c					c											c
a	5002095	g	5016782	g	4966792	s	UTI89/AS	rec38	g	UTI89	+++	g	g				a	g					g											g
g	5002110	a	5016797	a	4966807	s	UTI89/AS	rec38	g	AS	+++	a	g				g	g					g											g
a	5002223	t	5016910	t	4966920	ns	UTI89/AS	rec38	t	UTI89	+	t	t				t	t	t	t			t			t	t							t
a	5002236	c	5016923	c	4966933	s	UTI89/AS	rec38	c	UTI89	+	c	c				a	t	t	t			a			t	t							a
a	5002284	g	5016971	g	4966981	s	UTI89/AS	rec38	g	UTI89	++	g	g				a	a	a	a			g			a	a							a
t	5002293	c	5016980	c	4966990	s	UTI89/AS	rec38	t	AS	+	c	t				t	t	t	t			c			t	t							t
c	5002311	a	5016998	a	4967008	s	UTI89/AS	rec38	a	UTI89	++	a	a				c	a	a	a			t			a	a							c
t	5002338	c	5017025	c	4967035	s	UTI89/AS	rec38	t	AS	+	c	t				t	t	t	t			c			t	t							t
a	5002359	t	5017046	t	4967056	s	UTI89/AS	rec38	t	UTI89	+	t	g				a	a	a	a			t			a	a							a
c	5002374	t	5017061	t	4967071	s	UTI89/AS	rec38	t	UTI89	+	t	t				c	t	t	t			c			t	t							c
t	5002377	c	5017064	c	4967074	s	UTI89/AS	rec38	c	UTI89	+	c	c				c	c	c	c			t			c	c							t
g	5002432	a	5017119	a	4967129	ns	UTI89/AS	rec38	g	AS	++++	a	g				g	g	g	g			g			g	g							g
g	5002458	a	5017145	a	4967155	s	UTI89/AS	rec38	a	UTI89	++	a	a				g	a	a	a			g			a	a							a
g	5002506	a	5017193	a	4967203	s	UTI89/AS	rec38	a	UTI89	+	a	g				a	a	a	a			a			a	a							g
t	5006849	c	5024148	c	4974158	s	UTI89/AS	rec38	c	UTI89	+				c		c	c			c		c	c	c									
a	5006854	t	5024153	t	4974163	ns	UTI89/AS	rec38	a	AS	+				a		a	t			t		a	a	a									
g	5006855	a	5024154	a	4974164	ns	UTI89/AS	rec38	g	AS	+				g		g	a			a		g	g	g									
c	5006859	t	5024158	t	4974168	ns	UTI89/AS	rec38	c	AS	+				c		c	t			t		c	c	c									
t	5006860	g	5024159	g	4974169	ns	UTI89/AS	rec38	t	AS	+				t		t	g			g		t	t	t									
c	5006869	t	5024168	t	4974178	ns	UTI89/AS	rec38	c	AS	+				c		t	t			t		t	t	t									
a	5006871	c	5024170	c	4974180	ns	UTI89/AS	rec38	a	AS	+				a		c	c			c		c	c	c									
c	5006874	t	5024173	t	4974183	ns	UTI89/AS	rec38	c	AS	+				c		-	t			t		-	-	-									
t	5006875	g	5024174	g	4974184	ns	UTI89/AS	rec38	t	AS	+				t		-	g			g		-	-	-									
c	5006879	t	5024178	t	4974188	s	UTI89/AS	rec38	c	AS	+				c		c	t			t		c	c	c									
t	5006881	c	5024180	c	4974190	ns	UTI89/AS	rec38	t	AS	+				t		t	c			c		t	t	t									
g	5006887	a	5024186	a	4974196	ns	UTI89/AS	rec38	g	AS	+				g		g	a			a		g	g	g									
g	5006896	c	5024195	c	4974205	ns	UTI89/AS	rec38	g	AS	+				g		g	t			c		g	g	g									
c	5006909	t	5024207	t	4974217	s	UTI89/AS	rec38	c	AS	+				c		c	t			t		c	c	c									
g	5006912	c	5024210	c	4974220	ns	UTI89/AS	rec38	g	AS	+				g		g	a			a		a	g	g									
g	5006915	t	5024213	t	4974223	s	UTI89/AS	rec38	g	AS	+				g		-	g			t		-	-	-									
t	5006916	c	5024214	c	4974224	s	UTI89/AS	rec38	c	UTI89	+				c		c	c			c		c	c	c									
a	5006920	t	5024218	t	4974228	ns	UTI89/AS	rec38	a	AS	+				a		a	a			t		a	a	a									
c	5006926	t	5024224	t	4974234	ns	UTI89/AS	rec38	c	AS	+				c		c	t			t		c	c	c									
t	5006928	c	5024226	c	4974236	ns	UTI89/AS	rec38	t	AS	+				t		t	c			c		t	t	t									
g	5006935	c	5024233	c	4974243	ns	UTI89/AS	rec38	t	U/AS	+/-				t		t	c			c		t	t	t									
a	5006936	g	5024234	g	4974244	ns	UTI89/AS	rec38	g	UTI89	+				g		g	g			g		g	g	g									
c	5006938	t	5024236	t	4974246	ns	UTI89/AS	rec38	c	AS	+				-		c	c			t		c	c	c									
a	5006949	t	5024247	t	4974257	ns	UTI89/AS	rec38	a	AS	+				a		a	t			t		a	a	a									
g	5006961	a	5024259	a	4974269	ns	UTI89/AS	rec38	g	AS	+				g		g	g			a		g	g	g									
t	5006968	g	5024266	g	4974276	ns	UTI89/AS	rec38	t	AS	+				t		t	g			g		t	t	t									
g	5006969	t	5024267	t	4974277	ns	UTI89/AS	rec38	g	AS	+				g		g	c			t		g	g	g									
g	5006970	c	5024268	c	4974278	ns	UTI89/AS	rec38	g	AS	+				g		g	c			c		g	g	g									

ExPEC genome site details												Outgroup Strains Details[i]																						
UTI89[b]	UTI89 site[c]	APEC 01[b]	APEC 01 site[c]	S88[b]	S88 site[c]	type[d]	Event Lineage[ef]	Recombinant segment	Inferred ancestral base[g]	Outgroup Analysis[e]	Support level[h]	CFT073	ED1a	536	E2348/69	SMS 3-5	IAI39	UMN026	K-12	ATCC 8739	HS	D1 Sd197	CB9615	Sakai	EDL933	IAI1	E24377A	SE11	SS Ss046	F2a 301	F2a 2457T	F5 8401	B4 Sb227	B18 BS512
a	5007084	g	5024382	g	4974392	ns	UTI89/AS	rec38	t	U/AS	+/-				t		t	t					a	a	a									
a	5007186	c	5024484	c	4974494	ns	UTI89/AS	rec38	t	U/AS	+/-				t		t	t					c	c	c									
t	5007534	c	5024832	c	4974842	s	UTI89/AS	rec38	c	UTI89	+				c		c	c					t	t	t									
t	5007537	c	5024835	c	4974845	s	UTI89/AS	rec38	t	AS	+				t		t	t					t	t	t									
a	5007776	c	5025074	c	4975084	s	UTI89/AS	rec38	c	UTI89	+				c		a	a					c	c	c									
c	5007887	t	5025185	t	4975195	s	UTI89/AS	rec38	t	UTI89	+				t		c	c					c	c	c									
g	5007947	t	5025245	t	4975255	s	UTI89/AS	rec38	g	AS	+				g		t	t					g	g	g									
c	5008007	t	5025305	t	4975315	s	UTI89/AS	rec38	t	UTI89	+				t		c	c					c	c	c									
c	5008544	t	5025842	t	4975852	s	UTI89/AS	rec38	t	UTI89	+				t		t	c					t	t	t									
a	5008704	g	5026002	g	4976012	ns	UTI89/AS	rec38	a	AS	+				a		a	a					a	a	a									
t	5008883	g	5026181	g	4976191	s	UTI89/AS	rec38	g	UTI89	+				g		t	t					t	t	t									
g	5008895	t	5026193	t	4976203	s	UTI89/AS	rec38	t	UTI89	+				t		g	g					g	g	g									
g	5009168	a	5026466	a	4976476	s	UTI89/AS	rec38	g	AS	+				g		g	g					g	g	g									
a	5009306	g	5026604	g	4976614	s	UTI89/AS	rec38	g	UTI89	+				g		g	g					g	g	g									
g	5009336	a	5026634	a	4976644	s	UTI89/AS	rec38	g	AS	+				g		g	g					g	g	g									
t	5009402	g	5026700	g	4976710	s	UTI89/AS	rec38	g	UTI89	+				g		g	g					t	t	t									
t	5009480	c	5026778	c	4976788	s	UTI89/AS	rec38	t	AS	+				t		c	c					t	t	t									
g	5009602	a	5026900	a	4976910	ns	UTI89/AS	rec38	a	UTI89	+				a		a	a					g	g	g									
t	5009661	a	5026959	a	4976969	ns	UTI89/AS	rec38	t	AS	+				t		t	a					t	t	t									
g	5009956	t	5027254	t	4977264	ns	UTI89/AS	rec38	g	AS	+				g		g	g					g	g	g									
t	5010183	c	5027481	c	4977491	s	UTI89/AS	rec38	t	AS	+				t		t	t					t	t	t									
a	5010291	g	5027589	g	4977599	s	UTI89/AS	rec38	g	UTI89	+				g		g	g					g	g	g									
c	5010376	t	5027674	t	4977684	s	UTI89/AS	rec38	c	AS	+				c		c	c					c	c	c									
g	5010600	a	5027898	a	4977908	s	UTI89/AS	rec38	g	AS	+				g		a	g					a	a	a									
g	5010837	t	5028135	t	4978145	ns	UTI89/AS	rec38	g	AS	+				g		t	g					t	t	t									
t	5010840	g	5028138	g	4978148	ns	UTI89/AS	rec38	t	AS	+				t		g	t					g	g	g									
t	5010924	c	5028222	c	4978232	s	UTI89/AS	rec38	t	AS	+				t		c	t					c	c	c									
c	5010936	t	5028234	t	4978244	s	UTI89/AS	rec38	c	AS	+				c		c	c					c	c	c									
g	5011005	a	5028303	a	4978313	s	UTI89/AS	rec38	g	AS	+				g		a	g					a	a	a									
a	5011008	g	5028306	g	4978316	s	UTI89/AS	rec38	a	AS	+				a		g	a					g	g	g									
c	5011179	t	5028477	t	4978487	s	UTI89/AS	rec38	c	AS	+				c		c	t					c	c	c									
c	5011275	t	5028573	t	4978583	s	UTI89/AS	rec38	t	UTI89	+				t		t	t					t	t	t									
g	5011323	a	5028621	a	4978631	s	UTI89/AS	rec38	g	AS	+				g		g	g					g	g	g									
a	5011350	c	5028648	c	4978658	ns	UTI89/AS	rec38	c	UTI89	+				c		c	c					c	c	c									
t	5011498	a	5028796	a	4978806	s	UTI89/AS	rec38	t	AS	+				t		t	a					t	t	t									
a	5011558	g	5028856	g	4978866	s	UTI89/AS	rec38	a	AS	+				a		a	a					a	a	a									
c	5011621	t	5028919	t	4978929	s	UTI89/AS	rec38	t	UTI89	+				t		c	t					c	c	c									
g	5011669	a	5028967	a	4978977	s	UTI89/AS	rec38	g	AS	+				g		g	a					a	a	a									
a	5011775	t	5029073	t	4979083	ns	UTI89/AS	rec38	a	AS	+				a		a	t					t	t	t									
g	5011858	a	5029156	a	4979166	s	UTI89/AS	rec38	g	AS	+				g		g	a					a	a	a									
t	5011972	g	5029270	g	4979280	ns	UTI89/AS	rec38	g	UTI89	+				g		t	g					t	t	t									
g	5011993	a	5029291	a	4979301	s	UTI89/AS	rec38	g	AS	+				g		g	a					g	g	g									
c	5012071	t	5029369	t	4979379	s	UTI89/AS	rec38	c	AS	+				c		c	c					c	c	c									
a	5012118	g	5029416	g	4979426	ns	UTI89/AS	rec38	a	AS	+				a		a	a					g	g	g									
t	5012176	c	5029474	c	4979484	s	UTI89/AS	rec38	t	AS	+				t		t	t					t	t	t									
a	5012191	c	5029489	c	4979499	s	UTI89/AS	rec38	a	AS	+				a		a	a					a	a	a									
a	5012261	t	5029559	t	4979569	ns	UTI89/AS	rec38	a	AS	+				a		a	a					a	a	a									
c	5012264	t	5029562	t	4979572	s	UTI89/AS	rec38	c	AS	+				c		c	c					c	c	c									
a	5012266	g	5029564	g	4979574	s	UTI89/AS	rec38	g	UTI89	+				g		g	g					g	g	g									
t	5012437	c	5029735	c	4979745	s	UTI89/AS	rec38	t	AS	+				t		t	t					t	t	t									
a	5012443	g	5029741	g	4979751	s	UTI89/AS	rec38	a	AS	+				a		a	a					a	a	a									
c	5012455	t	5029753	t	4979763	s	UTI89/AS	rec38	c	AS	+				c		c	c					c	c	c									
g	5012521	t	5029819	t	4979829	s	UTI89/AS	rec38	g	AS	+				g		g	g					g	g	g									
g	5012530	t	5029828	t	4979838	s	UTI89/AS	rec38	g	AS	+				g		g	g					g	g	g									
t	5012650	c	5029948	c	4979958	s	UTI89/AS	rec38	c	UTI89	+				c		t	c					c	c	c									
g	5012761	a	5030059	a	4980069	s	UTI89/AS	rec38	g	AS	+				g		g	g					g	g	g									
a	5012893	g	5030191	g	4980201	s	UTI89/AS	rec38	a	AS	+				a		a	a					a	a	a									
t	5013022	g	5030320	g	4980330	s	UTI89/AS	rec38	t	AS	+				t		t	t					t	t	t									
g	5013082	t	5030380	t	4980390	s	UTI89/AS	rec38	g	AS	+				g		g	g					g	g	g									
a	5013286	c	5030584	c	4980594	s	UTI89/AS	rec38	c	UTI89	+				c		a	a					a	a	a									
a	5013301	g	5030599	g	4980609	s	UTI89/AS	rec38	g	UTI89	+				g		g	g					g	g	g									
c	5013400	t	5030698	t	4980708	s	UTI89/AS	rec38	c	AS	+				c		c	c					c	c	c									
c	5013406	t	5030704	t	4980714	s	UTI89/AS	rec38	c	AS	+				c		c	c					c	c	c									
t	5013427	c	5030725	c	4980735	s	UTI89/AS	rec38	t	AS	+				t		t	t					t	t	t									
t	5013621	g	5030919	g	4980929	s	UTI89/AS	rec38	t	AS	+				t		t	c					c	c	c									
t	5013660	a	5030958	a	4980968	s	UTI89/AS	rec38	t	AS	+				t		t	t					t	t	t									
g	5013678	a	5030976	a	4980986	ns	UTI89/AS	rec38	g	AS	+				g		g	g					g	g	g									
c	5013753	a	5031051	a	4981061	s	UTI89/AS	rec38	c	AS	+				c		c	c					a	a	a									
t	5013786	g	5031084	g	4981094	ns	UTI89/AS	rec38	t	AS	+				t		t	t					t	t	t									
c	5014093	t	5031391	t	4981401	s	UTI89/AS	rec38	c	AS	+				c		c	t					c	c	c									
c	5014370	t	5031668	t	4981678	ns	UTI89/AS	rec38	c	AS	+				c		c	c					c	c	c				c	c	c			
g	5014652	a	5031938	a	4981948	s	UTI89/AS	rec38	g	AS	+				g		g	g					g	g	g				g	g	g		g	
g	5014695	a	5031981	a	4981991	ns	UTI89/AS	rec38	g	AS	+				g		g	g					g	g	g				g	g	g		g	
t	5014722	c	5032008	c	4982018	ns	UTI89/AS	rec38	c	UTI89	+				c		c	c					c	c	c				t	t	-		-	
c	5015211	t	5032497	t	4982507	s	UTI89/AS	rec38	c	AS	+				c		t	t					t	t	t				t	t	c		t	
a	5015214	g	5032500	g	4982510	s	UTI89/AS	rec38	a	AS	+				a		g	g					g	g	g				g	g	a		g	
t	5015307	c	5032593	c	4982603	s	UTI89/AS	rec38	c	UTI89	+				c		c	c					c	c	c				c	c	c		c	
t	5015481	c	5032767	c	4982777	s	UTI89/AS	rec38	c	UTI89	+				c		t	c					c	c	c				c	c	c		t	
t	5015511	c	5032797	c	4982807	s	UTI89/AS	rec38	c	UTI89	+				c		t	c					c	c	c				c	c	c		t	
t	5015609	c	5032895	c	4982905	ns	UTI89/AS	rec38	c	UTI89	+				c		c	c					c	c	c				c	c	c		c	
a	5015610	c	5032896	c	4982906	ns	UTI89/AS	rec38	c	UTI89	+				c		c	a					t	t	t				c	c	c		c	
g	5015631	a	5032917	a	4982927	s	UTI89/AS	rec38	g	AS	+				g		g	g					g	g	g				g	g	g		g	
g	5015634	a	5032920	a	4982930	s	UTI89/AS	rec38	g	AS	+				g		g	g					g	g	g				g	g	g		g	
g	5015635	t	5032921	t	4982931	ns	UTI89/AS	rec38	g	AS	+				g		g	g					g	g	g				g	g	g		g	

ExPEC genome site details												Outgroup Strains Details[i]																						
UTI89[b]	UTI89 site[c]	APEC 01[b]	APEC 01 site[c]	S88[b]	S88 site[c]	type[d]	Event Lineage[ef]	Recombinant segment	Inferred ancestral base[g]	Outgroup Analysis[e]	Support level[h]	CFT073	ED1a	536	E2348/69	SMS 3-5	IAI39	UMN026	K-12	ATCC 8739	HS	D1 Sd197	CB9615	Sakai	EDL933	IAI1	E24377A	SE11	SS Ss046	F2a 301	F2a 2457T	F5 8401	B4 Sb227	B18 BS512
a	5015643	g	5032929	g	4982939	s	UTI89/AS	rec38	a	AS	+				a		a	a					a	a	a				a	a	a		a	
g	5015757	t	5033043	t	4983053	s	UTI89/AS	rec38	g	AS	+				g		g	g					g	g	g				g	g	g		g	
g	5015769	a	5033055	a	4983065	s	UTI89/AS	rec38	g	AS	+				g		g	g					a	a	a				g	g	g		g	
a	5015775	g	5033061	g	4983071	s	UTI89/AS	rec38	g	UTI89	+				g		g	g					g	g	g				g	g	g		g	
g	5015784	a	5033070	a	4983080	s	UTI89/AS	rec38	g	AS	+				g		g	g					g	g	g				g	g	g		g	
g	5015851	a	5033137	a	4983147	ns	UTI89/AS	rec38	g	AS	+				g		g	g					g	g	g				g	g	g		g	
t	5015852	c	5033138	c	4983148	ns	UTI89/AS	rec38	c	UTI89	+				c		c	c					c	c	c				c	c	c		c	
c	5015871	a	5033157	a	4983167	s	UTI89/AS	rec38	c	AS	+				c		c	c					t	t	t				a	a	a		a	
c	5015912	t	5033198	t	4983208	nc	UTI89/AS	rec38	c	AS	+				c		c	c					c	c	c				c	c	c		c	
g	5015957	a	5033243	a	4983253	ns	UTI89/AS	rec38	g	AS	+				g		g	g					g	g	g				a	a	a		a	
g	5016028	a	5033314	a	4983324	ns	UTI89/AS	rec38	g	AS	+				g		g	g					g	g	g				a	a	a		a	
t	5016066	g	5033352	g	4983362	s	UTI89/AS	rec38	a	U/AS	+/-				a		a	a					a	a	a				g	g	g		g	
g	5016287	t	5033573	t	4983583	ns	UTI89/AS	rec38	g	AS	+				g		g	g					g	g	g				g	g	g		g	
t	5016431	c	5033717	c	4983727	s	UTI89/AS	rec38	c	UTI89	+				c		c	c					c	c	c				c	c	c		c	
c	5016702	t	5033988	t	4983998	s	UTI89/AS	rec38	c	AS	+				c		c	t					t	t	t				t	t	t		t	
g	5016816	a	5034102	a	4984112	s	UTI89/AS	rec38	g	AS	+				g		g	g					g	g	g				g	g	g		g	
g	5016819	t	5034105	t	4984115	s	UTI89/AS	rec38	t	UTI89	+				t		t	t					t	t	t				t	t	t		t	
g	5016930	a	5034216	a	4984226	s	UTI89/AS	rec38	g	AS	+				g		g	a					a	a	a				c	c	c		c	
t	5017047	g	5034333	g	4984343	s	UTI89/AS	rec38	g	UTI89	+				g		g	g					g	g	g				g	g	g		g	
g	5017146	t	5034432	t	4984442	s	UTI89/AS	rec38	g	AS	+				g		g	g					g	g	g				g	g	g		g	
a	5017238	t	5034524	t	4984534	ns	UTI89/AS	rec38	a	AS	+				a		a	a					a	a	a				a	a	a		a	
t	5017275	a	5034561	a	4984571	s	UTI89/AS	rec38	t	AS	+				t		t	t					t	t	t				g	g	g		g	
g	5017371	a	5034657	a	4984667	s	UTI89/AS	rec38	g	AS	+				g		a	g					a	a	a				a	a	a		a	
g	5017506	t	5034792	t	4984802	s	UTI89/AS	rec38	g	AS	+				g		g	t					a	a	a				t	t	t		t	
a	5017533	g	5034819	g	4984829	s	UTI89/AS	rec38	a	AS	+				a		g	g					g	g	g				g	g	g		g	
t	5017536	c	5034822	c	4984832	s	UTI89/AS	rec38	t	AS	+				t		c	c					t	t	t				g	g	g		g	
c	5017600	t	5034886	t	4984896	s	UTI89/AS	rec38	c	AS	+				c		c	t					c	c	c				c	c	c		c	
c	5017766	t	5035052	t	4985062	ns	UTI89/AS	rec38	c	AS	+				c		c	c					c	c	c				c	c	c		c	
g	5017817	a	5035103	a	4985113	ns	UTI89/AS	rec38	g	AS	+				g		a	a					a	a	a				a	a	a		a	
g	5017888	a	5035174	a	4985184	ns	UTI89/AS	rec38	a	UTI89	+				a		a	a					a	a	a				a	a	a		a	
a	5017926	g	5035212	g	4985222	s	UTI89/AS	rec38	a	AS	+				a		t	t					g	g	g				g	g	g		g	
g	5017947	t	5035233	t	4985243	s	UTI89/AS	rec38	g	AS	+				g		t	t					t	t	t				g	g	g		g	
g	5018025	t	5035311	t	4985321	s	UTI89/AS	rec38	g	AS	+				g		g	t					t	t	t				g	g	g		g	
a	5018055	g	5035341	g	4985351	s	UTI89/AS	rec38	a	AS	+				a		a	g					g	g	g				g	g	g		g	
t	5018119	g	5035405	g	4985415	ns	UTI89/AS	rec38	t	AS	+				t		g	g					g	g	g				g	g	g		g	
g	5018187	a	5035473	a	4985483	s	UTI89/AS	rec38	g	AS	+				g		a	a					a	a	a				g	g	g		g	
g	5018211	t	5035497	t	4985507	ns	UTI89/AS	rec38	g	AS	+				g		t	t					t	t	t				t	t	t		t	
a	5018220	g	5035506	g	4985516	s	UTI89/AS	rec38	a	AS	+				a		g	g					g	g	g				g	g	g		g	
a	5018395	g	5035681	g	4985691	ns	UTI89/AS	rec38	a	AS	+				a		g	g					g	g	g				g	g	g		g	
a	5018660	c	5035946	c	4985956	ns	UTI89/AS	rec38	c	UTI89	+				c		c	c					c	c	c				c	c	c		c	
t	5018814	c	5036100	c	4986110	s	UTI89/AS	rec38	t	AS	+				t		c	c					c	c	c				t	t	t		t	
a	5018826	g	5036112	g	4986122	s	UTI89/AS	rec38	a	AS	+				a		g	g					g	g	g				g	g	g		g	
a	5018833	g	5036119	g	4986129	ns	UTI89/AS	rec38	a	AS	+				a		g	g					g	g	g				a	a	a		a	
t	5018835	c	5036121	c	4986131	ns	UTI89/AS	rec38	t	AS	+				t		c	c					c	c	c				t	t	t		t	
g	5018874	a	5036160	a	4986170	s	UTI89/AS	rec38	g	AS	+				g		a	g					a	a	a				g	g	g		g	
t	5018904	c	5036190	c	4986200	s	UTI89/AS	rec38	t	AS	+				t		c	c					c	c	c				g	g	g		g	
a	5019058	g	5036344	g	4986354	ns	UTI89/AS	rec38	a	AS	+				a		g	g					g	g	g				g	g	g		g	
c	5019062	t	5036348	t	4986358	ns	UTI89/AS	rec38	c	AS	+				c		t	t					t	t	t				c	c	c		c	
t	5019063	g	5036349	g	4986359	ns	UTI89/AS	rec38	t	AS	+				t		g	g					a	a	a				g	g	g		g	
g	5019357	t	5036652	t	4986662	ns	UTI89/AS	rec38	g	AS	+				g		t	t					t	t	t				t	t	t	t	t	
c	5019372	t	5036667	t	4986677	s	UTI89/AS	rec38	c	AS	+				c		t	t					t	t	t				c	c	c	c	c	
c	5019466	t	5036761	t	4986771	ns	UTI89/AS	rec38	t	UTI89	+				t		t	t					t	t	t				c	c	c	c	c	
t	5019513	g	5036808	g	4986818	ns	UTI89/AS	rec38	g	UTI89	+				g		g	g					g	g	g				t	t	t	t	t	
t	5019516	g	5036811	g	4986821	ns	UTI89/AS	rec38	t	AS	+				t		t	t					t	t	t				t	t	t	t	t	
g	5019535	t	5036830	t	4986840	ns	UTI89/AS	rec38	t	UTI89	+				t		t	t					t	t	t				g	g	g	g	g	
t	5019552	c	5036847	c	4986857	ns	UTI89/AS	rec38	c	UTI89	+				c		c	c					c	c	c				t	t	t	t	c	
t	5019605	c	5036900	c	4986910	s	UTI89/AS	rec38	c	UTI89	+				c		c	c					c	c	c				t	t	t	t	t	
t	5019618	c	5036913	c	4986923	ns	UTI89/AS	rec38	c	UTI89	+				c		c	c					c	c	c				c	c	c	c	c	
a	5019854	c	5037149	c	4987159	ns	UTI89/AS	rec38	a	AS	+				a		a	a					a	a	a				a	a	a	a	a	
c	5019899	t	5037194	t	4987204	s	UTI89/AS	rec38	c	AS	+				c		t	c		a			t	t	t	a		a	c	c	c	c	c	
t	5019938	c	5037233	c	4987243	s	UTI89/AS	rec38	t	AS	++	a		a	t		c	c		a			c	c	c	a		a	c	c	c	c	c	
c	5020180	t	5037475	t	4987485	ns	UTI89/AS	rec38	c	AS	++++	c	c	c	c	c	c	c		c			c	c	c	c		c	c	c	c	c	c	c
c	5020204	t	5037499	t	4987509	ns	UTI89/AS	rec38	c	AS	++++	c	c	c	c	c	c	c		c			c	c	c	c		c	c	c	c	c	c	c
a	5020223	g	5037518	g	4987528	s	UTI89/AS	rec38	a	AS	+	t	c	t	a	c	a	g		t			g	g	g	c		t	g	g	g	g	t	c
c	5020284	t	5037579	t	4987589	ns	UTI89/AS	rec38	t	UTI89	+	t	t	t	c	t	c	c		t			c	c	c	t		c	c	c	c	c	t	t
c	5020289	t	5037584	t	4987594	s	UTI89/AS	rec38	t	UTI89	+	t	c	t	c	c	t	c		t			c	c	c	t		c	c	c	c	a	t	c
g	5020298	c	5037593	c	4987603	s	UTI89/AS	rec38	c	UTI89	++	c	c	c	g	c	c	c		c			g	g	g	c		c	c	c	c	c	c	c
c	5020313	t	5037608	t	4987618	s	UTI89/AS	rec38	t	UTI89	+	t	c	t	c	t	t	t		t			c	c	c	t		t	t	t	t	c	t	t
a	5020344	c	5037639	c	4987649	s	UTI89/AS	rec38	a	AS	++	a	c	a	a	c	c	a		c			c	c	c	c		c	c	c	c	a	c	c
c	5020400	t	5037695	t	4987705	s	UTI89/AS	rec38	c	AS	+	c	g	t	c	t	t	t		t			c	c	c	t		c	c	c	c	c	t	a
c	5020442	t	5037737	t	4987747	s	UTI89/AS	rec38	c	AS	+	c	g	t	c	c	c	c		t			c	t	t	t		c	c	c	c	c	c	c
t	5020463	c	5037758	c	4987768	s	UTI89/AS	rec38	t	AS	+	t	a	c	t	c	c	c		c			t	c	c	c		c	c	c	c	c	c	c
c	5020469	g	5037764	g	4987774	s	UTI89/AS	rec38	g	UTI89	++	c	g	g	c	g	c	g		g			g	g	g	g		c	c	c	c	c	c	g
c	5020478	t	5037773	t	4987783	s	UTI89/AS	rec38	c	AS	+	c	g	t	c	t	t	t		t			c	t	t	c		t	t	t	t	t	t	c
g	5020484	a	5037779	a	4987789	s	UTI89/AS	rec38	a	UTI89	++	a	g	a	g	g	a	a		a			a	a	a	a		a	g	g	g	g	g	a
t	5020485	c	5037780	c	4987790	s	UTI89/AS	rec38	c	UTI89	++++	c	c	c	t	c	c	c		c			c	c	c	c		c	c	c	c	c	c	c
a	5020493	g	5037788	g	4987798	s	UTI89/AS	rec38	a	AS	++	a	a	g	a	a	g	g		g			g	g	g	g		g	a	a	a	a	a	g
t	5020508	c	5037803	c	4987813	s	UTI89/AS	rec38	t	AS	+	t	c	t	t	t	c	c		c			t	c	c	t		t	t	t	t	t	t	t
t	5020511	c	5037806	c	4987816	s	UTI89/AS	rec38	t	AS	+	t	g	t	t	c	c	t		t			c	c	c	c		c	c	c	c	c	c	c
g	5020526	a	5037821	a	4987831	s	UTI89/AS	rec38	g	AS	++	g	t	g	g	g	a	g		a			a	a	a	a		a	g	g	g	g	g	g
t	5020577	c	5037872	c	4987882	s	UTI89/AS	rec38	c	UTI89	+	c	t	c	t	c	c	c		c			c	c	c	c		c	t	t	t	t	t	c
t	5020595	a	5037890	a	4987900	s	UTI89/AS	rec38	t	AS	+	g	t	g	t	g	g	g		g			a	g	g	t		g	a	a	a	a	a	a

ExPEC genome site details												Outgroup Strains Details[i]																						
UTI89[b]	UTI89 site[c]	APEC 01[b]	APEC 01 site[c]	S88[b]	S88 site[c]	type[d]	Event Lineage[ef]	Recombinant segment	Inferred ancestral base[g]	Outgroup Analysis[e]	Support level[h]	CFT073	ED1a	536	E2348/69	SMS 3-5	IAI39	UMN026	K-12	ATCC 8739	HS	D1 Sd197	CB9615	Sakai	EDL933	IAI1	E24377A	SE11	SS Ss046	F2a 301	F2a 2457T	F5 8401	B4 Sb227	B18 BS512
a	5020622	g	5037917	g	4987927	s	UTI89/AS	rec38	g	UTI89	+	t	c	g	g	g	g	g		g			g	g	g	g		g	a	a	a	g	g	g
a	5020623	t	5037918	t	4987928	ns	UTI89/AS	rec38	t	UTI89	+	t	a	t	t	t	t	t		t			t	t	t	t		t	a	a	a	a	a	t
t	5020664	c	5037959	c	4987969	s	UTI89/AS	rec38	c	UTI89	+	c	g	c	c	t	t	c		c			c	t	t	t		c	g	g	g	g	g	c
a	5020731	t	5038026	t	4988036	ns	UTI89/AS	rec38	t	UTI89	+	t	t	t	t	a	t	t		t			t	t	t	t		t	a	a	a	a	a	t
a	5020813	g	5038108	g	4988118	ns	UTI89/AS	rec38	a	AS	++++	g	a	a	a	a	a	a		a			a	a	a	a		a	a	a	a	a	a	a
g	5020868	a	5038163	a	4988173	ns	UTI89/AS	rec38	c	U/AS	+/-	c	c	c	c	c	a	c		c			c	c	c	c		c	a	a	a	a	c	c
g	5020869	t	5038164	t	4988174	ns	UTI89/AS	rec38	g	AS	++	g	g	g	g	g	g	g		g			g	g	g	g		g	a	a	a	a	g	g
g	5020929	a	5038224	a	4988234	s	UTI89/AS	rec38	g	AS	++	g	a	g	g	a	g	g		g			g	g	g	g		g	a	a	a	g	a	g
t	5020935	c	5038230	c	4988240	s	UTI89/AS	rec38	c	UTI89	++++	c	c	c	c	c	c	c		c			c	c	c	c		c	c	c	c	c	c	c
c	5020941	t	5038236	t	4988246	s	UTI89/AS	rec38	t	UTI89	+	t	t	t	c	c	c	c		c			c	c	c	c		c	t	t	t	t	t	c
c	5020944	t	5038239	t	4988249	s	UTI89/AS	rec38	t	UTI89	+	t	t	c	c	c	c	c		c			c	c	c	c		c	t	t	t	t	t	c
c	5020974	t	5038269	t	4988279	s	UTI89/AS	rec38	c	AS	+	c	t	c	t	t	t	c		c			c	c	c	c		c	t	t	t	t	t	t
t	5020984	c	5038279	c	4988289	s	UTI89/AS	rec38	c	UTI89	++++	c	c	c	c	c	c	c		c			c	c	c	c		c	c	c	c	c	c	c
c	5021025	g	5038320	g	4988330	s	UTI89/AS	rec38	g	UTI89	++++	g	g	g	g	g	g	g		g			g	g	g	g		g	g	g	g	g	g	g
t	5021037	c	5038332	c	4988342	s	UTI89/AS	rec38	t	AS	+	t	c	t	t	c	t	t		t			t	t	t	t		t	c	c	c	c	c	c
c	5021115	t	5038410	t	4988420	s	UTI89/AS	rec38	t	UTI89	+	c	t	t	t	t	t	c		t			t	t	t	t		c	t	t	t	t	t	t
c	5021121	t	5038416	t	4988426	s	UTI89/AS	rec38	c	AS	+	c	c	t	t	c	c	c		t			c	t	t	t		c	t	t	t	t	t	c
a	5021159	g	5038454	g	4988464	ns	UTI89/AS	rec38	a	AS	++	a	a	a	a	g	g	g		a			a	a	a	a		g	g	g	g	g	g	g
c	5021230	g	5038525	g	4988535	ns	UTI89/AS	rec38	c	AS	++	c	c	c	c	g	c	c		c			c	c	c	c		c	c	c	c	g	c	g
g	5021239	t	5038534	t	4988544	ns	UTI89/AS	rec38	t	UTI89	+	g	t	t	g	g	g	g		t			g	t	t	g		g	t	t	t	g	t	g
g	5021241	a	5038536	a	4988546	ns	UTI89/AS	rec38	a	UTI89	++	g	a	a	g	g	g	g		a			g	a	a	g		g	a	a	a	g	a	g
g	5021244	a	5038539	a	4988549	s	UTI89/AS	rec38	a	UTI89	++	g	a	a	g	g	g	c		a			g	a	a	a		g	a	a	a	a	a	g
g	5021247	a	5038542	a	4988552	s	UTI89/AS	rec38	a	UTI89	++	g	a	a	g	g	g	g		a			g	a	a	g		g	a	a	a	g	a	g
c	5021271	t	5038566	t	4988576	s	UTI89/AS	rec38	t	UTI89	+	c	t	t	t	t	t	t		t			t	t	t	t		t	t	t	t	t	t	t
c	5021319	t	5038614	t	4988624	s	UTI89/AS	rec38	c	AS	+	c	c	c	t	c	c	c		c			c	c	c	c		c	t	t	t	c	t	t
t	5021326	c	5038621	c	4988631	s	UTI89/AS	rec38	c	UTI89	++++	c	c	c	t	c	c	c		c			c	c	c	c		c	c	c	c	c	c	c
c	5021352	t	5038647	t	4988657	s	UTI89/AS	rec38	t	UTI89	+	t	t	c	c	c	c	t		t			c	c	c	c		c	t	t	t	c	t	t
t	5021474	a	5038769	a	4988779	ns	UTI89/AS	rec38	t	AS	+	t	t	t	t	t	t	t		t			t	t	t	t		a	t	t	t	t	t	t
g	5021487	t	5038782	t	4988792	ns	UTI89/AS	rec38	g	AS	++++	g	g	g	g	g	g	g		g			g	g	g	g		t	g	g	g	g	g	g
c	5021489	t	5038784	t	4988794	ns	UTI89/AS	rec38	t	UTI89	+	t	g	t	t	c	c	t		t			c	c	c	t		c	t	t	t	t	t	t
c	5021512	t	5038807	t	4988817	ns	UTI89/AS	rec38	c	AS	+	c	t	c	c	t	c	c		c			t	t	t	c		t	c	c	c	c	c	c
c	5021517	a	5038812	a	4988822	ns	UTI89/AS	rec38	c	AS	++	c	a	c	c	a	c	c		c			a	a	a	c		a	c	c	c	c	c	c
t	5021519	c	5038814	c	4988824	ns	UTI89/AS	rec38	c	UTI89	++++	c	g	c	c	c	c	c		c			c	c	c	c		c	c	c	c	c	c	c
t	5021523	g	5038818	g	4988828	ns	UTI89/AS	rec38	g	UTI89	++++	g	g	g	g	g	g	g		g			g	g	g	g		g	g	g	g	g	g	g
c	5021531	t	5038826	t	4988836	s	UTI89/AS	rec38	t	UTI89	+	t	t	t	t	t	t	t		t			t	t	t	t		t	t	t	t	t	t	t
a	5021542	g	5038837	g	4988847	ns	UTI89/AS	rec38	g	UTI89	++	g	a	g	a	g	a	a		g			g	g	g	g		g	g	g	g	g	g	g
t	5021543	c	5038838	c	4988848	ns	UTI89/AS	rec38	c	UTI89	+	c	c	c	t	t	t	t		c			c	c	c	c		c	t	t	t	t	t	c
g	5021546	c	5038841	c	4988851	ns	UTI89/AS	rec38	c	UTI89	++	c	a	c	c	c	c	g		c			c	c	c	c		c	c	c	c	c	c	t
t	5021555	c	5038850	c	4988860	s	UTI89/AS	rec38	t	AS	+	t	c	t	t	t	t	t		t			t	t	t	t		c	t	t	t	t	t	t
g	5021561	a	5038856	a	4988866	s	UTI89/AS	rec38	a	UTI89	++	a	a	a	g	a	g	g		a			a	a	a	a		a	g	g	g	g	g	a
c	5021585	t	5038880	t	4988890	s	UTI89/AS	rec38	t	UTI89	+	t	g		t	t	t	c					t	t	t			t	t	t	t	t	t	a
a	5021590	c	5038885	c	4988895	ns	UTI89/AS	rec38	c	UTI89	++	c	c		c	c	c	a					a	a	a			c	c	c	c	c	c	c
t	5021591	c	5038886	c	4988896	ns	UTI89/AS	rec38	c	UTI89	+	c	g		c	c	c	t					t	t	t			c	c	c	c	c	c	c
a	5021597	g	5038892	g	4988902	s	UTI89/AS	rec38	a	AS	+	a	g		a	g	a	a					a	a	a			g	g	g	g	g	g	a
t	5021645	c	5038940	c	4988950	s	UTI89/AS	rec38	t	AS	+	t	t		c	c	c	c					c	c	c			c	a	a	a	a	a	c
t	5021681	g	5038976	g	4988986	s	UTI89/AS	rec38	g	UTI89	++	a	a		g	g	g	g					g	g	g			g	g	g	g	g	g	g
c	5021689	t	5038984	t	4988994	ns	UTI89/AS	rec38	t	UTI89	+	t	t		t	c	t	t					c	c	c			c	c	c	c	c	c	c
a	5021690	g	5038985	g	4988995	ns	UTI89/AS	rec38	g	UTI89	+	t	t		g	g	g	g					a	a	a			t	g	g	g	g	g	g
t	5021699	g	5038994	g	4989004	s	UTI89/AS	rec38	g	UTI89	+	a	g		g	t	g	g					t	t	t			g	g	g	g	g	g	t
t	5021801	a	5039096	a	4989106	s	UTI89/AS	rec38	t	AS	+	t		t	t	t	t	a		t			t	t	t	t		t	t	t	t	t	t	
t	5021807	g	5039102	g	4989112	s	UTI89/AS	rec38	g	UTI89	++	g		g	c	g	g	g		g			g	g	g	g		a	a	a	a	a	a	
a	5021831	g	5039126	g	4989136	s	UTI89/AS	rec38	a	AS	++	a		a	g	g	g	g		g			a	a	a	a		a	a	a	a	a	a	
t	5021834	g	5039129	g	4989139	s	UTI89/AS	rec38	t	AS	+	t		t	g	g	g	g		g			t	t	t	t		c	t	t	t	t	t	
c	5021838	a	5039133	a	4989143	ns	UTI89/AS	rec38	c	AS	++	c		c	a	a	a	a		a			c	c	c	c		c	c	c	c	c	c	
g	5021839	c	5039134	c	4989144	ns	UTI89/AS	rec38	g	AS	++	g		g	c	c	c	c		c			g	g	g	g		g	g	g	g	g	g	
c	5021840	g	5039135	g	4989145	ns	UTI89/AS	rec38	t	U/AS	+/-	t		t	a	g	g	g		g			t	t	t	t		c	t	t	t	t	t	
a	5021841	g	5039136	g	4989146	ns	UTI89/AS	rec38	a	AS	++	a		a	g	g	g	g		g			a	a	a	a		a	a	a	a	a	a	
c	5021844	g	5039139	g	4989149	ns	UTI89/AS	rec38	c	AS	++	c		c	a	g	g	g		g			c	c	c	c		c	c	c	c	c	c	
a	5021845	c	5039140	c	4989150	ns	UTI89/AS	rec38	a	AS	++	a		a	c	c	c	c		c			a	a	a	a		a	a	a	a	a	a	
g	5021846	a	5039141	a	4989151	ns	UTI89/AS	rec38	g	AS	++	g		g	c	c	c	c		a			g	g	g	g		g	g	g	g	g	g	
c	5021848	g	5039143	g	4989153	ns	UTI89/AS	rec38	c	AS	++	c		c	g	g	g	g		g			c	c	c	c		c	c	c	c	c	c	
a	5021849	t	5039144	t	4989154	ns	UTI89/AS	rec38	a	AS	+	a		a	c	t	t	t		t			a	a	a	a		a	a	a	a	a	a	
g	5021852	a	5039147	a	4989157	s	UTI89/AS	rec38	g	AS	++	g		g	g	a	a	a		a			g	g	g	g		g	g	g	g	g	g	
t	5021861	c	5039156	c	4989166	s	UTI89/AS	rec38	c	UTI89	+	c		c	t	c	c	c		c			t	c	c	t		c	c	c	c	t	c	
g	5021864	a	5039159	a	4989169	s	UTI89/AS	rec38	a	UTI89	++	a		a	g	a	a	a		a			g	a	a	g		a	a	a	a	g	a	
a	5021916	c	5039211	c	4989221	ns	UTI89/AS	rec38	c	UTI89	++++	c		c	c	c	t	c		c			c	c	c	c		c	c	c	c	c	c	
t	5021921	c	5039216	c	4989226	s	UTI89/AS	rec38	t	AS	+	g		c	t	c	t	t		c			t	c	c	t		t	t	t	t	c	t	
t	5021930	c	5039225	c	4989235	s	UTI89/AS	rec38	c	UTI89	++++	c		c	c	c	c	c		c			c	c	c	c		c	c	c	c	c	c	
a	5021951	g	5039246	g	4989256	s	UTI89/AS	rec38	a	AS	++	a		a	t	g	g	a		g			a	a	a	a		a	g	g	g	a	g	
t	5021954	g	5039249	g	4989259	s	UTI89/AS	rec38	t	AS	+	c		c	t	g	g	t		g			t	t	t	c		c	g	g	g	t	g	
a	5021960	c	5039255	c	4989265	s	UTI89/AS	rec38	t	U/AS	+/-	t		t	t	a	t	t		c			c	t	t	t		t	a	a	a	t	a	
t	5021984	c	5039279	c	4989289	s	UTI89/AS	rec38	c	UTI89	+	c		c	t	t	c	c		c			c	c	c	c		c	c	c	c	c	c	
c	5021993	t	5039288	t	4989298	s	UTI89/AS	rec38	t	UTI89	+	t		c	c	t	c	t		t			c	c	c	c		c	t	t	t	t	t	
g	5022008	a	5039303	a	4989313	s	UTI89/AS	rec38	g	AS	++++	g		g	g	g	g	g		g			g	g	g	g		g	g	g	g	g	g	
a	5022020	c	5039315	c	4989325	s	UTI89/AS	rec38	c	UTI89	++++	c		c	c	c	c	c		c			t	c	c	c		c	c	c	c	c	c	
c	5022026	a	5039321	a	4989331	nc	UTI89/AS	rec38	c	AS	++	c		c	c	a	c	c		c			a	c	c	c		c	c	c	c	c	c	
t	5022031	c	5039326	c	4989336	nc	UTI89/AS	rec38	t	AS	+	t		t	t	t	t	t		c			c	t	t	t		t	t	t	t	t	t	
g	5022051	c	5039346	c	4989356	nc	UTI89/AS	rec38	c	UTI89	++	c		g	c	g	c	c		g			c	c	c	g		g	g	g	g	g	g	
a	5022077	g	5039372	g	4989382	nc	UTI89/AS	rec38	a	AS	+	a		g	a	g	a	g		g			g	a	a	g		g	g	g	g	g	g	g
g	5022079	t	5039374	t	4989384	nc	UTI89/AS	rec38	g	AS	++++	g		g	g	g	g	g		g			g	g	g	g		g	g	g	g	g	g	g
c	5022081	g	5039376	g	4989386	nc	UTI89/AS	rec38	g	UTI89	++	g		g	c	g	c	g		c			g	c	c	g		g	g	g	g	g	g	g
a	5022107	g	5039402	g	4989412	s	UTI89/AS	rec38	g	UTI89	++++	g	g	g	g	g	g	g		g			g	g	g	g		g	g	g	g	g	g	g
c	5022113	t	5039408	t	4989418	s	UTI89/AS	rec38	c	AS	++++	c	a	c	c	c	c	c		c			c	c	c	c		c	c	c	c	c	c	c
a	5022125	g	5039420	g	4989430	s	UTI89/AS	rec38	g	UTI89	++++	g	g	g	g	g	g	g		g			g	g	g	g		g	g	g	g	g	g	g

ExPEC genome site details												Outgroup Strains Details[i]																						
UTI89[b]	UTI89 site[c]	APEC 01[b]	APEC 01 site[c]	S88[b]	S88 site[c]	type[d]	Event Lineage[ef]	Recombinant segment	Inferred ancestral base[g]	Outgroup Analysis[e]	Support level[h]	CFT073	ED1a	536	E2348/69	SMS 3-5	IAI39	UMN026	K-12	ATCC 8739	HS	D1 Sd197	CB9615	Sakai	EDL933	IAI1	E24377A	SE11	SS Ss046	F2a 301	F2a 2457T	F5 8401	B4 Sb227	B18 BS512
t	5022134	c	5039429	c	4989439	s	UTI89/AS	rec38	c	UTI89	++++	c	t	c	c	c	c	c		c			c	c	c	c		c	c	c	c	c	c	c
g	5022236	a	5039531	a	4989541	s	UTI89/AS	rec38	g	AS	++	g	g	g	g	a	g	g		g			a	g	g	g		g	g	g	g	g	a	g
g	5022247	c	5039542	c	4989552	ns	UTI89/AS	rec38	c	UTI89	++	g	c	c	g	g	c	g		g			g	g	g	c		c	g	g	g	g	g	g
t	5022272	c	5039567	c	4989577	s	UTI89/AS	rec38	t	AS	+	c	t	t	t	t	t	c		c			t	t	t	t		t	t	t	t	t	t	t
a	5022305	g	5039600	g	4989610	s	UTI89/AS	rec38	g	UTI89	++++	g	g	g	g	g	g	g		g			g	g	g	g		g	g	g	g	g	g	g
a	5022338	c	5039633	c	4989643	s	UTI89/AS	rec38	c	UTI89	+	c	a	t	c	c	c	c		t			t	t	t	c		c	c	c	c	c	c	c
c	5022371	t	5039666	t	4989676	s	UTI89/AS	rec38	t	UTI89	+	t	g	t	c	c	t	t		c			c	c	c	t		t	c	c	c	t	c	t
g	5022389	c	5039684	c	4989694	s	UTI89/AS	rec38	g	AS	++	g	c	g	g	g	g	c		g			c	c	c	c		c	g	g	g	g	g	g
t	5022401	g	5039696	g	4989706	s	UTI89/AS	rec38	g	UTI89	+	g	a	g	g	g	g	g		t			g	g	g	t		t	g	g	g	g	g	g
c	5022471	a	5039766	a	4989776	s	UTI89/AS	rec38	a	UTI89	++	a	c	a	a	a	a	c		c			c	c	c	a		c	c	c	c	c	c	a
g	5022479	a	5039774	a	4989784	s	UTI89/AS	rec38	g	AS	++	a	g	g	g	g	g	g		g			g	g	g	g		a	g	g	g	g	g	g
t	5022515	c	5039810	c	4989820	s	UTI89/AS	rec38	c	UTI89	+	c	t	c	c	c	c	t		c			t	t	t	c		c	c	c	c	c	c	c
a	5022632	g	5039927	g	4989937	s	UTI89/AS	rec38	g	UTI89	++	g	g	g	g	g	g	a		g			g	g	g	g		a	g	g	g	g	g	a
c	5022737	t	5040032	t	4990042	s	UTI89/AS	rec38	c	AS	+	c	g	c	c	c	c	c		c			t	t	t	c		c	c	c	c	c	c	c
t	5022752	c	5040047	c	4990057	s	UTI89/AS	rec38	c	UTI89	+	c	t	c	t	t	c	t		t			c	c	c	c		t	t	t	t	t	t	c
t	5022782	c	5040077	c	4990087	s	UTI89/AS	rec38	c	UTI89	+	c	c	c	c	c	c	c		c			c	c	c	c		c	t	t	t	t	t	c
a	5022845	g	5040140	g	4990150	s	UTI89/AS	rec38	g	UTI89	++	g	c	g	a	g	g	g		g			c	c	c	g		a	g	g	g	g	g	g
t	5022875	a	5040170	a	4990180	ns	UTI89/AS	rec38	t	AS	+	t	g	t	a	t	t	a		a			a	a	a	t		a	t	t	t	t	t	t
g	5022878	a	5040173	a	4990183	s	UTI89/AS	rec38	g	AS	++	g	a	g	a	a	g	a		a			a	a	a	g		a	g	g	g	g	g	g
a	5022881	g	5040176	g	4990186	s	UTI89/AS	rec38	a	AS	++	a	g	a	g	g	a	g		g			g	g	g	g		a	a	a	a	a	a	a
g	5022950	a	5040245	a	4990255	s	UTI89/AS	rec38	a	UTI89	++++	a	a	a	a	a	a	a		a	a		a	a	a	a		a	a	a	a	a	a	g
c	5022956	t	5040251	t	4990261	s	UTI89/AS	rec38	t	UTI89	+	t	g	c	t	t	t	t		t	t		t	t	t	t		t	t	t	t	t	t	c
c	5022974	t	5040269	t	4990279	s	UTI89/AS	rec38	c	AS	+	t	c	c	t	c	c	t		t	t		t	t	t	t		c	c	c	c	c	c	c
t	5023025	c	5040320	c	4990330	s	UTI89/AS	rec38	t	AS	+	c	t	t	c	c	c	c		c	c		c	c	c	c		c	c	c	c	c	c	t
t	5023034	c	5040329	c	4990339	s	UTI89/AS	rec38	c	UTI89	+	c	g	t	c	c	c	c		c	c		c	c	c	c		c	c	c	c	c	c	t
t	5023061	c	5040356	c	4990366	s	UTI89/AS	rec38	t	AS	+	t	t	t	t	c	t	t		t	t		t	t	t	t		t	c	c	c	c	c	t
c	5023064	t	5040359	t	4990369	s	UTI89/AS	rec38	c	AS	+	g	g	c	g	t	g	g		g	c		g	g	g	c		g	t	t	t	t	t	c
g	5023066	a	5040361	a	4990371	ns	UTI89/AS	rec38	a	UTI89	++++	a	a	g	a	a	a	a		a	a		a	a	a	a		a	a	a	a	a	a	a
g	5023067	c	5040362	c	4990372	ns	UTI89/AS	rec38	c	UTI89	++++	c	c	g	c	c	c	c		c	c		c	c	c	c		c	c	c	c	c	c	c
c	5023174	a	5040469	a	4990479	ns	UTI89/AS	rec38	c	AS	++++	c	c	c	c	c	c	c		c	c		c	c	c	c		c	c	c	c	c	c	c
a	5023175	g	5040470	g	4990480	ns	UTI89/AS	rec38	g	UTI89	++	g	g	a	g	g	g	a		g	a		g	g	g	a		g	g	g	g	g	g	g
a	5023178	g	5040473	g	4990483	s	UTI89/AS	rec38	g	UTI89	++	g	g	g	g	g	g	a		g	a		g	g	g	a		g	g	g	g	g	g	g
c	5023370	t	5040665	t	4990675	s	UTI89/AS	rec38	c	AS	+	c	c	c	c	t	c	t		t	t		c	c	c	t		c	c	c	c	t	c	c
c	5023394	t	5040689	t	4990699	s	UTI89/AS	rec38	c	AS	++++	c	c	c	c	c	c	t		c	c		c	c	c	c		c	c	c	c	c	c	c
c	5023403	t	5040698	t	4990708	s	UTI89/AS	rec38	c	AS	+	c	c	t	t	t	t	t		c	c		t	t	t	c		c	c	c	c	c	c	c
g	5023415	a	5040710	a	4990720	s	UTI89/AS	rec38	a	UTI89	++	a	c	a	a	g	a	a		a	a		a	a	a	a		g	g	g	g	a	g	g
c	5023421	a	5040716	a	4990726	s	UTI89/AS	rec38	c	AS	+	c	t	c	c	c	c	a		c	c		t	t	t	c		c	c	c	c	a	c	c
c	5023472	t	5040767	t	4990777	s	UTI89/AS	rec38	c	AS	+	c	t	c	c	c	c	t		t	t		c	c	c	t		c	c	c	c	c	c	c
t	5023484	c	5040779	c	4990789	s	UTI89/AS	rec38	t	AS	+	t	g	c	t	t	t	c		c	t		c	c	c	t		t	c	c	c	c	c	t
g	5023487	a	5040782	a	4990792	s	UTI89/AS	rec38	a	UTI89	+	a	t	a	t	g	t	a		a	t		a	a	a	t		g	a	a	a	a	a	g
a	5023505	g	5040800	g	4990810	s	UTI89/AS	rec38	a	AS	++	a	a	a	a	a	t	g		a	a		a	a	a	a		a	a	a	a	a	a	a
g	5023511	c	5040806	c	4990816	s	UTI89/AS	rec38	c	UTI89	+	c	t	c	c	g	c	c		c	c		c	c	c	c		g	t	t	t	t	t	g
c	5023517	t	5040812	t	4990822	s	UTI89/AS	rec38	c	AS	+	c	t	c	c	c	c	t		t	c		c	c	c	c		c	c	c	c	c	c	c
t	5023529	c	5040824	c	4990834	s	UTI89/AS	rec38	t	AS	+	t	t	c	c	c	t	c		c	c		t	t	t	c		c	c	c	c	c	c	c
g	5023544	c	5040839	c	4990849	s	UTI89/AS	rec38	g	AS	++	g	c	g	c	g	c	c		c	c		g	g	g	c		g	c	c	c	c	c	g
c	5023559	t	5040854	t	4990864	s	UTI89/AS	rec38	c	AS	+	c	c	c	c	c	t	t		t	c		t	t	t	t		c	t	t	t	t	t	c
g	5023562	t	5040857	t	4990867	s	UTI89/AS	rec38	g	AS	+	g	a	g	g	g	t	t		t	g		g	g	g	t		g	t	t	t	t	t	g
t	5023634	c	5040929	c	4990939	s	UTI89/AS	rec38	c	UTI89	+	c	t	c	c	t	t	c		t	c		c	c	c	t		t	t	t	t	t	t	t
t	5023649	c	5040944	c	4990954	s	UTI89/AS	rec38	c	UTI89	++++	c	c	c	c	c	c	c		c	c		c	c	c	c		c	c	c	c	c	c	c
a	5023661	t	5040956	t	4990966	s	UTI89/AS	rec38	a	AS	+	a	c	a	g	a	a	g		a	g		a	a	a	a		t	a	a	a	a	a	t
a	5023662	g	5040957	g	4990967	ns	UTI89/AS	rec38	a	AS	++	c	a	a	c	a	a	c		a	c		a	a	a	a		c	a	a	a	a	a	c
g	5023663	a	5040958	a	4990968	ns	UTI89/AS	rec38	a	UTI89	++	a	g	a	a	g	g	a		g	a		g	g	g	g		a	a	a	a	g	a	a
t	5023664	g	5040959	g	4990969	ns	UTI89/AS	rec38	g	UTI89	+	g	c	t	g	t	t	g		t	g		t	t	t	t		g	t	t	t	t	t	g
c	5023667	t	5040962	t	4990972	s	UTI89/AS	rec38	t	UTI89	+	g	t	c	t	c	c	t		c	g		c	c	c	t		t	c	c	c	c	c	t
t	5023688	c	5040983	c	4990993	s	UTI89/AS	rec38	c	UTI89	+	c	c	c	t	t	t	c		t	t		t	t	t	t		c	t	t	t	c	t	c
a	5023715	g	5041010	g	4991020	s	UTI89/AS	rec38	a	AS	++	a	-	g	g	a	g	g		a	a		a	a	a	g		g	g	g	g	g	g	g
t	5023739	c	5041034	c	4991044	s	UTI89/AS	rec38	c	UTI89	+	c	t	c	c	t	c	c		t	t		t	t	t	c		c	c	c	c	c	c	c
a	5023757	g	5041052	g	4991062	s	UTI89/AS	rec38	g	UTI89	++	g	g	g	g	a	g	a		a	a		g	g	g	g		g	g	g	g	g	g	g
c	5023770	t	5041065	t	4991075	s	UTI89/AS	rec38	c	AS	+	t	c	c	t	c	c	t		c	c		c	c	c	t		c	c	c	c	c	c	c
c	5023779	t	5041074	t	4991084	s	UTI89/AS	rec38	c	AS	+	t	c	c	t	c	c	t		c	c		c	c	c	t		c	c	c	c	c	c	c
c	5023844	t	5041139	t	4991149	s	UTI89/AS	rec38	t	UTI89	+	t	g	c	c	t	c	c		c	c		t	t	t	t		c	c	c	c	c	c	c
g	5023859	a	5041154	a	4991164	s	UTI89/AS	rec38	a	UTI89	++	a	a	a	g	a	a	a		g	g		g	g	g	a		a	g	g	g	g	g	a
a	5023865	g	5041160	g	4991170	s	UTI89/AS	rec38	a	AS	++	a	a	a	g	g	a	a		a	a		g	g	g	a		a	a	a	a	a	a	g
t	5023940	a	5041235	a	4991245	s	UTI89/AS	rec38	a	UTI89	++	a	g	a	a	c	a	c		a	a		a	a	a	a		a	a	a	a	a	a	a
t	5023971	g	5041266	g	4991276	ns	UTI89/AS	rec38	g	UTI89	++++	g	g	g	g	g	g	g		g	g		g	g	g	g		g	g	g	g	g	g	g
g	5023985	a	5041280	a	4991290	s	UTI89/AS	rec38	g	AS	++	a	g	g	a	a	a	a		a	g		a	a	a	a		a	a	a	a	a	a	a
g	5024000	c	5041295	c	4991305	s	UTI89/AS	rec38	g	AS	++	g	g	c	g	g	g	g		g	c		g	g	g	g		g	g	g	g	g	g	g
t	5024009	c	5041304	c	4991314	s	UTI89/AS	rec38	c	UTI89	++++	c	c	c	c	c	c	c		c	c		c	c	c	c		c	c	c	c	c	c	c
g	5024018	a	5041313	a	4991323	s	UTI89/AS	rec38	g	AS	++++	g	g	g	g	g	g	g		g	g		g	g	g	g		g	g	g	g	g	g	g
g	5024030	c	5041325	c	4991335	s	UTI89/AS	rec38	c	UTI89	+	g	c	c	-	g	c	c		c	g		t	t	t	c		c	t	t	t	t	t	c
t	5024039	c	5041334	c	4991344	s	UTI89/AS	rec38	c	UTI89	+	t	c	c	t	t	c	t		t	t		c	c	c	c		c	c	c	c	c	c	c
a	5024040	c	5041335	c	4991345	ns	UTI89/AS	rec38	a	AS	++	a	a	a	a	a	c	a		a	a		c	c	c	c		c	c	c	c	c	c	c
a	5024097	c	5041392	c	4991402	s	UTI89/AS	rec38	c	UTI89	++	c	a	c	c	a	c	c		c	c		c	c	c	c		c	a	a	a	a	a	c
g	5024125	c	5041420	c	4991430	ns	UTI89/AS	rec38	g	AS	++	g	g	c	g	g	g	c		g	c		c	c	c	c		c	g	g	g	g	g	c
c	5024149	t	5041444	t	4991454	ns	UTI89/AS	rec38	c	AS	++	g	c	g	c	c	c	g		g	c		g	g	g	g		g	g	g	g	g	g	g
a	5024160	c	5041455	c	4991465	s	UTI89/AS	rec38	c	UTI89	++++	c	a	c	c	c	c	c		c	c		c	c	c	c		c	c	c	c	c	c	c
a	5024180	g	5041475	g	4991485	s	UTI89/AS	rec38	g	UTI89	++	g	c	g	a	a	a	g		g	a		a	a	a	g		g	g	g	g	g	g	g
a	5024182	t	5041477	t	4991487	ns	UTI89/AS	rec38	a	AS	++++	a	a	a	a	a	a	a		a	a		a	a	a	a		a	a	a	a	a	a	a
g	5024237	t	5041532	t	4991542	s	UTI89/AS	rec38	g	AS	++	g	c	g	a	g	c	g		g	g		a	a	a	g		g	a	a	a	a	a	g
g	5024252	a	5041547	a	4991557	s	UTI89/AS	rec38	a	UTI89	++	a	g	a	g	g	g	g		g	g		a	a	a	g		g	a	a	a	a	a	g
t	5024273	c	5041568	c	4991578	s	UTI89/AS	rec38	c	UTI89	+	c	g	c	c	t	g	c		c	t		c	c	c	c		c	g	g	g	g	g	c
g	5024282	a	5041577	a	4991587	s	UTI89/AS	rec38	g	AS	++++	g	g	g	g	g	g	g		g	g		g	g	g	g		g	g	g	g	g	g	g
t	5024300	c	5041595	c	4991605	s	UTI89/AS	rec38	t	AS	+	t	c	t	t	t	c	t		c	t		t	t	t	c		c	c	c	c	c	c	c

ExPEC genome site details												Outgroup Strains Details[i]																						
UTI89[b]	UTI89 site[c]	APEC 01[b]	APEC 01 site[c]	S88[b]	S88 site[c]	type[d]	Event Lineage[ef]	Recombinant segment	Inferred ancestral base[g]	Outgroup Analysis[e]	Support level[h]	CFT073	ED1a	536	E2348/69	SMS 3-5	IAI39	UMN026	K-12	ATCC 8739	HS	D1 Sd197	CB9615	Sakai	EDL933	IAI1	E24377A	SE11	SS Ss046	F2a 301	F2a 2457T	F5 8401	B4 Sb227	B18 BS512
g	5024336	a	5041631	a	4991641	s	UTI89/AS	rec38	g	AS	++	g	a	g	g	g	a	g		g	g		g	g	g	g		a	g	g	g	g	g	g
a	5024343	g	5041638	g	4991648	ns	UTI89/AS	rec38	g	UTI89	++	g	g	g	t	g	a	a		a	a		g	g	g	a		g	g	g	g	g	g	a
c	5024345	g	5041640	g	4991650	ns	UTI89/AS	rec38	g	UTI89	++	g	a	g	a	g	g	c		c	c		g	g	g	c		g	g	g	g	g	g	c
g	5024363	a	5041658	a	4991668	s	UTI89/AS	rec38	a	UTI89	++++	a	a	a	c	a	a	a		a	a		a	a	a	a		a	a	a	a	a	a	a
t	5024384	c	5041679	c	4991689	s	UTI89/AS	rec38	c	UTI89	+	c	t	c	t	t	g	c		c	c		t	t	t	c		t	c	c	c	c	c	t
a	5024422	g	5041717	g	4991727	ns	UTI89/AS	rec38	a	AS	++	a	a	a	a	a	a	a		a	a		a	a	a	a		a	g	g	g	g	g	a
a	5024423	g	5041718	g	4991728	ns	UTI89/AS	rec38	a	AS	++	a	g	a	g	a	g	a		a	a		a	a	a	a		a	g	g	g	g	g	a
c	5024441	t	5041736	t	4991746	s	UTI89/AS	rec38	t	UTI89	+	t	g	t	c	t	g	c		t	t		c	c	c	c		t	t	t	t	t	t	t
t	5024447	c	5041742	c	4991752	s	UTI89/AS	rec38	c	UTI89	+	c	t	c	t	c	c	t		c	c		t	t	t	t		c	c	c	c	c	c	c
a	5024448	g	5041743	g	4991753	ns	UTI89/AS	rec38	a	AS	++	a	g	a	g	a	g	a		g	g		a	a	a	a		g	g	g	g	g	g	g
c	5024450	t	5041745	t	4991755	ns	UTI89/AS	rec38	c	AS	+	c	g	c	t	t	g	c		t	t		c	c	c	c		t	t	t	t	t	t	t
a	5024500	g	5041795	g	4991805	ns	UTI89/AS	rec38	a	AS	++	a	g	a	a	a	a	g		a	a		g	g	g	a		a	g	g	g	g	g	g
c	5024502	g	5041797	g	4991807	ns	UTI89/AS	rec38	c	AS	++	c	g	c	c	c	c	g		c	c		g	g	g	c		c	g	g	g	g	g	g
c	5024564	t	5041859	t	4991869	s	UTI89/AS	rec38	g	U/AS	+/-	g		g	g	g	g	c		g	g		c	c	c	g		g	c	c	c	c	c	g
a	5024650	g	5041945	g	4991955	ns	UTI89/AS	rec38	a	AS	+				a			a					a	a	a									
c	5024651	t	5041946	t	4991956	ns	UTI89/AS	rec38	c	AS	+				c			c					c	c	c									
a	5024657	g	5041952	g	4991962	s	UTI89/AS	rec38	a	AS	+				a			a					a	a	a									
g	5024694	a	5041989	a	4991999	ns	UTI89/AS	rec38	a	UTI89	++	c	a		a	c	c	a		c	c		a	a	a	c		c	a	a	a	a	a	a
g	5024704	a	5041999	a	4992009	ns	UTI89/AS	rec38	g	AS	+	g	t		a	g	g	a		g	g		a	a	a	g		g	g	g	g	g	t	t
g	5024705	a	5042000	a	4992010	ns	UTI89/AS	rec38	a	UTI89	++	c	a		a	c	c	a		c	c		a	a	a	c		c	c	c	c	c	a	a
g	5024720	t	5042015	t	4992025	s	UTI89/AS	rec38	t	UTI89	+	t	t		t	t	t	t		t	t		t	t	t	t		t	t	t	t	t	t	t
c	5024721	a	5042016	a	4992026	ns	UTI89/AS	rec38	a	UTI89	++++	a	a		a	a	a	a		a	a		a	a	a	a		a	a	a	a	a	a	a
t	5024729	c	5042024	c	4992034	s	UTI89/AS	rec38	c	UTI89	++	c	g		c	c	c	c		c	c		c	c	c	c		c	c	c	c	c	g	g
t	5024732	c	5042027	c	4992037	s	UTI89/AS	rec38	t	AS	+	t	g		c	t	t	c		t	t		c	c	c	t		t	t	t	t	t	g	g
t	5024741	c	5042036	c	4992046	s	UTI89/AS	rec38	c	UTI89	+	c	t		c	t	c	c		c	t		c	c	c	c		c	t	t	t	t	t	t
c	5024801	t	5042096	t	4992106	s	UTI89/AS	rec38	c	AS	+	t	c		c	t	t	c		t	t		c	c	c	t		t	t	t	t	t	c	c
g	5024805	t	5042100	t	4992110	ns	UTI89/AS	rec38	g	AS	++++	g	g		g	g	g	g		g	g		g	g	g	g		g	g	g	g	g	g	g
a	5024807	t	5042102	t	4992112	ns	UTI89/AS	rec38	a	AS	++	g	g		a	g	g	a		g	g		a	a	a	g		g	g	g	g	g	g	g
c	5024810	t	5042105	t	4992115	s	UTI89/AS	rec38	c	AS	+	t	c		c	t	t	c		t	t		c	c	c	t		t	t	t	t	t	c	c
g	5024813	c	5042108	c	4992118	s	UTI89/AS	rec38	c	UTI89	++	c	c		g	c	c	g		c	c		g	g	g	c		c	c	c	c	c	c	c
t	5024816	c	5042111	c	4992121	s	UTI89/AS	rec38	c	UTI89	+	c	c		t	c	c	t		c	c		t	t	t	c		c	c	c	c	c	c	c
g	5024817	a	5042112	a	4992122	ns	UTI89/AS	rec38	a	UTI89	++	a	a		g	a	a	g		a	a		g	g	g	a		a	a	a	a	a	a	a
a	5024818	c	5042113	c	4992123	ns	UTI89/AS	rec38	c	UTI89	++	c	c		a	c	c	a		c	c		a	a	a	c		c	c	c	c	c	c	c
c	5024821	g	5042116	g	4992126	ns	UTI89/AS	rec38	c	AS	++	a	c		c	a	a	c		a	a		c	c	c	a		a	a	a	a	a	c	c
g	5024822	a	5042117	a	4992127	ns	UTI89/AS	rec38	g	AS	++++	g	g		g	g	g	g		a	g		g	g	g	g		g	g	g	g	g	g	g
g	5024825	a	5042120	a	4992130	s	UTI89/AS	rec38	g	AS	++++	g	g		g	g	g	g		g	g		g	g	g	g		g	g	g	g	g	g	g
g	5024834	t	5042129	t	4992139	s	UTI89/AS	rec38	g	AS	+	t	g		g	t	t	g		t	t		g	g	g	t		t	t	t	t	t	g	g
c	5024836	g	5042131	g	4992141	ns	UTI89/AS	rec38	c	AS	++	g	c		c	g	g	c		g	g		c	c	c	g		g	g	g	g	g	c	c
g	5024837	t	5042132	t	4992142	ns	UTI89/AS	rec38	g	AS	+	t	g		g	t	t	g		t	t		g	g	g	g		g	t	t	t	t	g	g
a	5024844	g	5042139	g	4992149	ns	UTI89/AS	rec38	a	AS	++	g	a		a	g	g	a		g	g		a	a	a	g		g	g	g	g	g	a	a
c	5024846	t	5042141	t	4992151	ns	UTI89/AS	rec38	t	UTI89	+	t	t		c	t	t	c		t	t		c	c	c	t		t	t	t	t	t	t	t
a	5024864	c	5042159	c	4992169	ns	UTI89/AS	rec38	a	AS	+	a	t		c	a	a	c		c	c		c	c	c	a		a	c	c	c	c	t	t
g	5024876	a	5042171	a	4992181	s	UTI89/AS	rec38	a	UTI89	+	a	g		a	a	a	g		a	a		g	g	g	a		a	a	a	a	a	g	g
t	5024877	c	5042172	c	4992182	ns	UTI89/AS	rec38	c	UTI89	+	t	c		c	t	t	c		c	c		c	c	c	c		c	c	c	c	t	c	c
a	5024878	g	5042173	g	4992183	ns	UTI89/AS	rec38	g	UTI89	+	a	g		g	a	a	g		g	g		g	g	g	g		g	g	g	g	g	g	g
t	5024879	c	5042174	c	4992184	ns	UTI89/AS	rec38	t	AS	+	t	a		c	t	t	c		c	c		c	c	c	c		c	c	c	c	c	g	g
c	5024891	g	5042186	g	4992196	s	UTI89/AS	rec38	c	AS	++	c	g		c	c	c	c		c	c		c	c	c	c		c	g	g	g	g	g	g
t	5024903	c	5042198	c	4992208	s	UTI89/AS	rec38	c	UTI89	++	c	g		c	c	c	c		c	c		c	c	c	c		c	c	c	c	c	g	g
c	5024909	t	5042204	t	4992214	s	UTI89/AS	rec38	c	AS	+	t	c		c	t	t	c		a	t		c	a	a	t		t	t	t	t	t	c	c
t	5024920	c	5042215	c	4992225	ns	UTI89/AS	rec38	c	UTI89	+	c	c		c	c	c	t		c	c		t	t	t	t		t	c	c	c	c	c	c
c	5024939	t	5042234	t	4992244	s	UTI89/AS	rec38	c	AS	++	c	g		c	c	c	c		c	c		c	c	c	c		c	c	c	c	c	g	g
t	5025005	c	5042300	c	4992310	s	UTI89/AS	rec38	c	UTI89	++++	c	c		c	c	c	c		c	c		c	c	c	c		c	c	c	c	c	c	c
t	5025008	c	5042303	c	4992313	s	UTI89/AS	rec38	c	UTI89	+	c	c		c	c	c	c		c	t		t	c	c	c		c	c	c	c	c	c	c
g	5025122	a	5042417	a	4992427	s	UTI89/AS	rec38	g	AS	++	a	g		g	a	a	a		g	g		a	a	a	g		a	g	g	g	g	g	g
t	5025140	c	5042435	c	4992445	s	UTI89/AS	rec38	c	UTI89	++++	c	c		c	c	c	c		c	c		c	c	c	c		c	c	c	c	c	c	c
t	5025168	c	5042463	c	4992473	ns	UTI89/AS	rec38	c	UTI89	++	c	c		c	c	c	a		c	c		a	a	c	c		c	c	c	c	c	c	c
a	5025182	g	5042477	g	4992487	s	UTI89/AS	rec38	g	UTI89	++	g	g		g	g	g	g		a	g		g	g	g	g		g	a	a	a	a	a	a
a	5025194	t	5042489	t	4992499	s	UTI89/AS	rec38	g	U/AS	+/-	g	g		g	g	g	g		a	c		g	g	g	c		g	c	c	c	c	g	g
t	5025203	a	5042498	a	4992508	s	UTI89/AS	rec38	a	UTI89	++	a	a		a	a	a	a		a	g		a	a	a	g		a	g	g	g	g	a	a
t	5025207	a	5042502	a	4992512	ns	UTI89/AS	rec38	a	UTI89	++	a	a		a	a	a	c		t	c		c	c	a	c		a	c	c	c	c	a	a
t	5025212	c	5042507	c	4992517	s	UTI89/AS	rec38	c	UTI89	+	c	c		c	c	c	t		c	c		t	t	c	c		c	t	t	t	t	c	c
c	5025215	t	5042510	t	4992520	s	UTI89/AS	rec38	t	UTI89	+	t	t		t	t	t	t		t	t		t	t	t	t		t	t	t	t	t	t	t
g	5025217	a	5042512	a	4992522	ns	UTI89/AS	rec38	a	UTI89	++++	a	a		a	a	a	a		a	a		a	a	a	a		a	a	a	a	a	a	a
c	5025225	a	5042520	a	4992530	s	UTI89/AS	rec38	a	UTI89	++	a	a		a	a	a	a		c	c		a	a	a	c		c	c	c	c	c	a	a
g	5025227	a	5042522	a	4992532	s	UTI89/AS	rec38	a	UTI89	++	a	a		a	a	a	g		g	g		a	a	a	g		g	g	g	g	g	a	a
a	5025231	c	5042526	c	4992536	ns	UTI89/AS	rec38	c	UTI89	++	c	c		c	c	c	a		a	a		c	c	c	a		a	a	a	a	a	c	c
g	5025235	c	5042530	c	4992540	ns	UTI89/AS	rec38	c	UTI89	++	c	c		c	c	c	g		a	a		c	c	c	a		a	a	a	a	a	c	c
c	5025236	t	5042531	t	4992541	ns	UTI89/AS	rec38	t	UTI89	+	t	t		t	t	t	c		c	c		t	t	t	c		c	c	c	c	c	t	t
c	5025241	t	5042536	t	4992546	ns	UTI89/AS	rec38	t	UTI89	+	t	t		t	t	t	c		t	t		t	t	t	t		t	t	t	t	t	t	t
t	5025245	c	5042540	c	4992550	s	UTI89/AS	rec38	c	UTI89	+	c	c		c	c	c	t		t	t		c	c	c	t		t	t	t	t	t	c	c
g	5025248	c	5042543	c	4992553	s	UTI89/AS	rec38	c	UTI89	++	c	c		c	c	c	g		g	g		c	c	c	g		g	g	g	g	g	c	c
c	5025257	t	5042552	t	4992562	s	UTI89/AS	rec38	t	UTI89	+	t	t		t	t	t	c		c	c		t	t	t	t		c	c	c	c	c	t	t
a	5025267	c	5042562	c	4992572	ns	UTI89/AS	rec38	c	UTI89	++	c	c		c	c	c	c		a	a		c	c	c	a		c	a	a	a	a	c	c
a	5025268	g	5042563	g	4992573	ns	UTI89/AS	rec38	g	UTI89	++	g	g		g	g	g	g		a	a		g	g	g	a		g	a	a	a	a	g	g
g	5025278	t	5042573	t	4992583	s	UTI89/AS	rec38	t	UTI89	+	t	t		t	t	t	t		t	t		t	t	t	t		t	t	t	t	t	t	t
a	5025296	t	5042591	t	4992601	s	UTI89/AS	rec38	t	UTI89	+	t	t		t	t	t	t		t	t		t	t	t	t		t	a	a	a	a	t	t
g	5025299	c	5042594	c	4992604	s	UTI89/AS	rec38	c	UTI89	++	c	c		c	c	c	c		c	c		c	c	c	c		c	g	g	g	g	c	c
t	5025302	g	5042597	g	4992607	s	UTI89/AS	rec38	g	UTI89	++	g	g		g	g	g	g		g	g		g	g	g	g		g	c	c	c	c	g	g
t	5025314	g	5042609	g	4992619	s	UTI89/AS	rec38	g	UTI89	++++	g	g		g	g	g	g		g	g		g	g	g	g		g	g	g	g	g	g	g
c	5025325	g	5042620	g	4992630	ns	UTI89/AS	rec38	g	UTI89	++	g	g		g	g	g	g		g	c		g	g	g	c		g	c	c	c	c	g	g
g	5025326	t	5042621	t	4992631	ns	UTI89/AS	rec38	t	UTI89	+	t	t		t	t	t	t		t	a		t	t	t	a		t	g	g	g	g	t	t
t	5025329	c	5042624	c	4992634	s	UTI89/AS	rec38	c	UTI89	+	c	c		t	c	c	t		t	t		c	c	c	t		t	t	t	t	t	c	c
a	5025347	c	5042642	c	4992652	s	UTI89/AS	rec38	c	UTI89	+	c	c		c	c	c	c		t	t		c	c	c	t		c	c	c	c	c	c	c

ExPEC genome site details												Outgroup Strains Details[i]																						
UTI89[b]	UTI89 site[c]	APEC 01[b]	APEC 01 site[c]	S88[b]	S88 site[c]	type[d]	Event Lineage[ef]	Recombinant segment	Inferred ancestral base[g]	Outgroup Analysis[e]	Support level[h]	CFT073	ED1a	536	E2348/69	SMS 3-5	IAI39	UMN026	K-12	ATCC 8739	HS	D1 Sd197	CB9615	Sakai	EDL933	IAI1	E24377A	SE11	SS Ss046	F2a 301	F2a 2457T	F5 8401	B4 Sb227	B18 BS512
c	5025350	t	5042645	t	4992655	s	UTI89/AS	rec38	t	UTI89	+	t	t		t	t	t	a		t	t		t	t	t	t		t	t	t	t	t	t	t
a	5025351	c	5042646	c	4992656	ns	UTI89/AS	rec38	c	UTI89	++++	c	c		c	c	c	c		c	c		c	c	c	c		c	c	c	c	c	c	c
g	5025352	a	5042647	a	4992657	ns	UTI89/AS	rec38	a	UTI89	++	a	a		a	a	a	a		a	g		a	a	a	g		a	a	a	a	a	a	a
t	5025353	c	5042648	c	4992658	ns	UTI89/AS	rec38	c	UTI89	++++	c	c		c	c	c	g		c	c		c	c	c	c		c	c	c	c	c	c	c
g	5025357	c	5042652	c	4992662	ns	UTI89/AS	rec38	c	UTI89	++++	c	c		c	c	c	a		c	c		c	c	c	c		c	c	c	c	c	c	c
t	5025358	a	5042653	a	4992663	ns	UTI89/AS	rec38	a	UTI89	++++	a	a		a	a	a	c		a	a		a	a	a	a		a	a	a	a	a	a	a
a	5025359	c	5042654	c	4992664	ns	UTI89/AS	rec38	c	UTI89	++++	c	c		c	c	c	g		c	c		c	c	c	c		c	c	c	c	c	c	c
a	5025365	g	5042660	g	4992670	s	UTI89/AS	rec38	a	AS	++	g	a		a	g	g	a		g	g		g	g	g	g		a	g	g	g	g	g	g
t	5025377	g	5042672	g	4992682	s	UTI89/AS	rec38	g	UTI89	+	g	g		g	g	g	c		g	g		g	g	g	g		g	t	t	t	t	g	g
a	5025380	g	5042675	g	4992685	s	UTI89/AS	rec38	g	UTI89	++	g	g		g	g	g	a		g	a		g	g	g	a		g	a	a	a	a	a	a
t	5025390	c	5042685	c	4992695	ns	UTI89/AS	rec38	c	UTI89	++++	c	c		c	c	c	g		c	c		c	c	c	c		c	c	c	c	c	c	c
g	5025391	t	5042686	t	4992696	ns	UTI89/AS	rec38	t	UTI89	+	t	t		t	t	t	g		t	t		t	t	t	t		t	t	t	t	t	t	t
g	5025400	a	5042695	a	4992705	ns	UTI89/AS	rec38	a	UTI89	++	a	a		a	a	a	a		a	a		a	a	a	a		a	g	g	g	g	a	a
t	5025401	c	5042696	c	4992706	ns	UTI89/AS	rec38	c	UTI89	++++	c	c		c	c	c	c		c	c		c	c	c	c		c	c	c	c	c	c	c
t	5025440	g	5042735	g	4992745	s	UTI89/AS	rec38	g	UTI89	+	g	g		g	g	g	g		g	g		g	g	g	g		g	t	t	t	g	g	g
t	5025458	c	5042753	c	4992763	s	UTI89/AS	rec38	c	UTI89	+	c	t		c	c	t	a		c	a		a	c	c	a		t	a	a	a	t	c	c
c	5025492	a	5042787	a	4992797	ns	UTI89/AS	rec38	a	UTI89	++	c	a		a	c	c	a		a	a		a	a	a	a		a	a	a	a	a	a	a
a	5025493	t	5042788	t	4992798	ns	UTI89/AS	rec38	a	AS	+	a	g		t	a	a	g		t	t		t	t	t	t		a	g	g	g	g	t	t
t	5025503	c	5042798	c	4992808	s	UTI89/AS	rec38	c	UTI89	+	t	c		c	t	t	t		c	c		c	c	c	c		c	c	c	c	c	c	c
a	5025512	g	5042807	g	4992817	ns	UTI89/AS	rec38	g	UTI89	+	a	g		g	a	a	a		g	g		g	g	g	g		g	g	g	g	g	g	g
t	5025556	g	5042851	g	4992861	ns	UTI89/AS	rec38	g	UTI89	+	t	g		g	t	t	c		g	g		c	c	c	c		t	c	c	c	g	g	g
a	5025558	t	5042853	t	4992863	ns	UTI89/AS	rec38	t	UTI89	+	a	t		t	a	a	t		t	t		t	t	t	t		a	t	t	t	t	t	t
t	5025564	a	5042859	a	4992869	ns	UTI89/AS	rec38	a	UTI89	+	t	a		a	t	t	c		a	a		c	c	c	c		t	t	t	t	a	a	a
t	5025566	c	5042861	c	4992871	ns	UTI89/AS	rec38	c	UTI89	+	t	c		c	t	t	c		c	c		c	c	c	c		t	c	c	c	c	c	c
t	5025567	c	5042862	c	4992872	ns	UTI89/AS	rec38	c	UTI89	+	t	c		c	t	t	c		c	c		c	c	c	c		t	c	c	c	c	c	c
c	5025571	t	5042866	t	4992876	ns	UTI89/AS	rec38	t	UTI89	+	c	t		t	c	c	t		t	t		t	t	t	t		c	t	t	t	t	t	t
c	5029997	t	5046927	t	4996937	nc	UTI89/AS	rec38	t	UTI89	+	t	t	t			t	t										t						
t	5030011	c	5046941	c	4996951	nc	UTI89/AS	rec38	c	UTI89	+++	c	c	c			c	c										c						
c	5030055	t	5046985	t	4996995	ns	UTI89/AS	rec38	c	AS	+++	c	c	c			c	c										c						
c	5030079	t	5047009	t	4997019	ns	UTI89/AS	rec38	t	UTI89	+	t	t	t			t	t										t						
t	5030085	c	5047015	c	4997025	ns	UTI89/AS	rec38	t	AS	+	c	t	t			t	c										c						
t	5030133	a	5047063	a	4997073	ns	UTI89/AS	rec38	a	UTI89	++	g	a	a			a	a										t						
t	5030166	g	5047096	g	4997106	ns	UTI89/AS	rec38	g	UTI89	+++	g	g	g			g	g										g						
t	5030169	c	5047099	c	4997109	ns	UTI89/AS	rec38	t	AS	+	c	t	t			c	c										c						
a	5030172	g	5047102	g	4997112	ns	UTI89/AS	rec38	g	UTI89	+	g	t	t			g	a										a						
t	5030200	g	5047130	g	4997140	ns	UTI89/AS	rec38	g	UTI89	+++	g	g	g			g	g										g						
t	5030332	c	5047262	c	4997272	ns	UTI89/AS	rec38	c	UTI89	++++	c					c	c	c	c	c		c			c	c	c						
c	5030335	t	5047265	t	4997275	ns	UTI89/AS	rec38	t	UTI89	+	t					t	t	t	t	t		t			t	t	t						
g	5032799	a	5049064	a	4999074	ns	UTI89/AS	rec39	a	UTI89	++++	a					a	a	a	a	a		a	a	a	a	a	a	a	a	a	a	a	-
c	5032827	t	5049092	t	4999102	s	UTI89/AS	rec39	t	UTI89	+	t					t	t	t	t	t		t	t	t	t	t	t	c	c	c	c	c	c
a	5032879	c	5049144	c	4999154	nc	UTI89/AS	rec39	c	UTI89	+	c					t	t	t	t	t		c	c	c	t	t		t	t	t	t	c	a
c	5032891	t	5049156	t	4999166	nc	UTI89/AS	rec39	t	UTI89	+	t					t	t	t	t	t		t	t	t	t	t		c	c	c	c	c	c
t	5032963	c	5049228	c	4999238	ns	UTI89/AS	rec39	c	UTI89	++++	c					c						c	c	c				c	c	c	c	c	c
t	5032996	g	5049261	g	4999271	ns	UTI89/AS	rec39	g	UTI89	++++	g					g						g	g	g				g	g	g	g	g	g
c	5032997	g	5049262	g	4999272	s	UTI89/AS	rec39	g	UTI89	++++	g					g						g	g	g				g	g	g	g	g	g
a	5033003	g	5049268	g	4999278	s	UTI89/AS	rec39	g	UTI89	++++	g					a						g	g	g				g	g	g	g	g	g
t	5033027	c	5049292	c	4999302	s	UTI89/AS	rec39	c	UTI89	++++	c					c						c	c	c				c	c	c	c	c	c
c	5033057	t	5049322	t	4999332	s	UTI89/AS	rec39	t	UTI89	+	t					t						t	t	t				t	t	t	t	t	t
g	5033105	a	5049370	a	4999380	ns	UTI89/AS	rec39	g	AS	++++	g					a						a	a	a				a	a	a	a	a	a
t	5033106	g	5049371	g	4999381	ns	UTI89/AS	rec39	g	UTI89	++++	g					g						g	g	g				g	g	g	g	g	g
t	5033148	a	5049413	a	4999423	nc	UTI89/AS	rec39	c	U/AS	+/-	c					t						a	g	g				a	a	a	a	g	g
t	5033157	c	5049422	c	4999432	nc	UTI89/AS	rec39	t	AS	+	t					t						c	t	t				c	c	c	c	c	c
a	5033176	g	5049441	g	4999451	nc	UTI89/AS	rec39	a	AS	++	a					c						g	a	a				a	a	g	a	a	a
g	5033191	a	5049456	a	4999466	nc	UTI89/AS	rec39	g	AS	++	g					g						a	a	a				a	a	a	a	a	a
g	5033213	a	5049478	a	4999488	nc	UTI89/AS	rec39	a	UTI89	+						a						a											
t	5033214	c	5049479	c	4999489	nc	UTI89/AS	rec39	c	UTI89	+						c						c											
g	5033348	a	5049613	a	4999623	nc	UTI89/AS	rec39	a	UTI89	+						a						a											
c	5033413	t	5049678	t	4999688	s	UTI89/AS	rec39	t	UTI89	+	t	t	t	t	t	t	t	t	t	t	t	t	t	t	t	t	t	t	t	t	t	t	t
c	5033509	c	5049774	t	4999784	s	S88	rec40	c	S88	+++	c	c	c	t	t	c	t	t	c	t	t	t	t	t	t	t	t	t	t	t	t	t	t
t	5033512	t	5049777	c	4999787	s	S88	rec40	t	S88	+++	t	t	t	c	c	t	c	c	t	c	c	c	c	c	c	c	c	c	c	c	c	c	c
c	5033569	c	5049834	t	4999844	s	S88	rec40	c	S88	+++	c	c	c	t	t	c	t	t	t	t	t	t	t	t	t	t	t	t	t	t	t	t	t
c	5033602	c	5049867	t	4999877	s	S88	rec40	c	S88	+++	c	c	c	t	t	c	t	t	t	t	t	t	t	t	t	t	t	t	t	t	t	t	t
t	5033615	t	5049880	c	4999890	s	S88	rec40	t	S88	+++	t	t	t	c	c	t	c	c	c	c	c	c	c	c	c	c	c	c	c	c	c	c	c
t	5033623	t	5049888	c	4999898	s	S88	rec40	t	S88	+++	t	t	t	c	c	t	c	c	c	c	c	c	c	c	c	c	c	c	c	c	c	c	c
a	5033716	a	5049981	t	4999991	s	S88	rec40	a	S88	+++	a	a	a	a	t	a	t	t	t	t	t	t	t	t	t	t	t	t	t	t	t	t	t
t	5033761	t	5050026	c	5000036	s	S88	rec40	t	S88	+++	t	t	t	t	t	t	c	c	c	c	t	c	c	c	c	c	c	c	c	c	c	c	c
a	5033818	a	5050083	g	5000093	s	S88	rec40	a	S88	++	a	a	a	a	a	a	g	g	a	a	a	a	a	a	a	a	a	a	a	a	a	a	a
c	5033857	c	5050122	t	5000132	s	S88	rec40	c	S88	++	c	c	c	c	c	c	c	t	c	c	c	c	c	c	c	c	c	c	c	c	c	c	c
a	5033860	a	5050125	g	5000135	s	S88	rec40	a	S88	++	a	a	a	a	a	a	a	g	a	a	a	a	a	a	a	a	a	a	a	a	a	a	a
t	5033923	t	5050188	c	5000198	s	S88	rec40	t	S88	+++	t	t	t	t	t	t	t	c	c	c	t	t	t	t	t	t	c	t	t	t	c	c	c
c	5034007	c	5050272	t	5000282	s	S88	rec40	c	S88	++++	c	c	c	c	c	c	c	t	t	c	c	t	t	t	t	t	c	t	t	t	c	c	c
g	5034055	g	5050320	a	5000330	s	S88	rec40	g	S88	++	g	g	g	g	a	g	a	a	a	a	a	a	a	a	a	a	a	a	a	a	a	a	a
t	5034076	t	5050341	c	5000351	s	S88	rec40	t	S88	+++	t	t	t	t	c	t	c	c	t	c	c	c	c	c	c	c	c	c	c	c	c	c	c
c	5034115	c	5050380	t	5000390	s	S88	rec40	c	S88	+++	c	c	c	t	t	c	t	t	t	t	t	t	t	t	t	t	t	t	t	t	t	t	t
t	5034127	t	5050392	c	5000402	s	S88	rec40	t	S88	+++	t	t	t	c	t	t	c	c	c	c	t	c	c	c	c	c	c	c	c	c	c	c	c
c	5034164	c	5050429	t	5000439	s	S88	rec40	c	S88	++	c	c	c	c	c	c	c	t	c	c	c	c	c	c	t	c	c	c	c	c	c	c	c
g	5034187	g	5050452	a	5000462	s	S88	rec40	g	S88	++	g	g	g	t	c	g	c	a	t	t	c	g	g	g	a	a	t	g	g	g	t	c	c
c	5034211	c	5050476	t	5000486	s	S88	rec40	c	S88	++++	c	c	c	t	t	c	t	t	t	t	c	c	c	c	t	t	t	c	c	c	t	t	t
c	5034316	c	5050581	t	5000591	s	S88	rec40	c	S88	++++	c	c	c	t	t	c	t	t	c	c	t	t	t	t	c	t	c	t	t	t	c	c	c
a	5034322	a	5050587	g	5000597	s	S88	rec40	a	S88	++	a	a	a	g	g	a	g	g	a	a	g	g	g	g	a	g	a	g	g	g	a	a	a
g	5034349	g	5050614	a	5000624	s	S88	rec40	g	S88	++	g	g	g	g	g	g	g	a	g	g	g	g	g	g	g	g	g	a	a	a	g	g	g
c	5034367	c	5050632	t	5000642	s	S88	rec40	c	S88	+++	c	c	c	t	t	c	t	t	t	t	t	t	t	c	t	t	t	t	t	t	t	t	t

Table S8. Allocation of recombinational SNPs to lineages by virtual outgroup analysis[a]

ExPEC genome site details												Outgroup Strains Details[i]																						
UTI89[b]	UTI89 site[c]	APEC 01[b]	APEC 01 site[c]	S88[b]	S88 site[c]	type[d]	Event Lineage[ef]	Recombinant segment	Inferred ancestral base[g]	Outgroup Analysis[e]	Support level[h]	CFT073	ED1a	536	E2348/69	SMS 3-5	IAI39	UMN026	K-12	ATCC 8739	HS	D1 Sd197	CB9615	Sakai	EDL933	IAI1	E24377A	SE11	SS Ss046	F2a 301	F2a 2457T	F5 8401	B4 Sb227	B18 BS512
t	5034442	t	5050707	g	5000717	s	S88	rec40	g	2	-	g	t	g	t	t	g	g	g	g	g	t	g	g	g	t	g	g	g	g	g	g	g	g
t	5034454	t	5050719	c	5000729	s	S88	rec40	c	2	-	c	t	c	t	t	c	c	c	c	c	t	c	c	c	c	c	c	c	c	c	c	c	c
t	5034457	t	5050722	c	5000732	s	S88	rec40	c	2	-	c	t	c	t	t	c	c	c	c	c	t	c	c	c	c	c	c	c	c	c	c	c	c
g	5034541	g	5050806	a	5000816	s	S88	rec40	g	S88	++	g	g	g	g	g	g	g	a	g	a	g	a	a	a	a	a	a	a	a	a	a	a	a
g	5034649	g	5050914	a	5000924	s	S88	rec40	g	S88	++	g	g	g	g	g	g	g	a	a	g	g	a	a	a	g	g	g	a	a	a	g	g	g
t	5034665	t	5050930	c	5000940	s	S88	rec40	c	2	-	c	t	c	c	c	c	c	c	c	t	c	c	c	c	c	t	t	c	c	c	t	t	t
c	5034676	c	5050941	t	5000951	s	S88	rec40	c	S88	+++	c	c	c	c	t	c	t	t	t	t	t	t	t	t	t	t	t	t	t	t	t	t	t
g	5034679	g	5050944	a	5000954	s	S88	rec40	g	S88	++	g	g	g	g	a	g	g	a	g	t	a	g	g	g	g	a	t	g	g	g	t	t	t
g	5034790	g	5051055	a	5001065	s	S88	rec40	g	S88	++	g	g	g	g	g	g	g	a	a	g	g	a	a	a	g	g	g	a	a	a	g	g	g
g	5034901	g	5051166	a	5001176	s	S88	rec40	g	S88	++	g	g	g	g	g	g	g	a	g	g	g	a	a	a	a	g	g	a	a	a	g	g	g
a	5035052	a	5051317	c	5001327	nc	S88	rec40	a	S88	+++	a	a	a	a	c	a	a	c	c	c	a	c	c	c	c	c	c	c	c	c	c	c	c
a	5035101	a	5051366	g	5001376	nc	S88	rec40	a	S88	++	a	a	a	a	a	a	a	g	a	a	a	g	g	g	a	a	a	g	g	g	a	a	a
a	5035162	a	5051427	c	5001437	nc	S88	rec40	c	2	-	c	c	c	c	c	c	c	c	a	c	c	c	c	c	c	t	c	c	c	c	c	c	c
c	5035224	c	5051489	t	5001499	nc	S88	rec40	c	S88	+++	c	c	c	c	c	c	c	t	c	c	c	t	t	t	c	c	c	c	c	c	c	c	c
a	5035425	a	5051690	g	5001700	s	S88	rec40	a	S88	++	a	a	a	a	g	g	g	g	g	g	a	g	g	g	g	g	g	g	g	g	g	g	g
c	5035581	c	5051846	a	5001856	s	S88	rec40	c	S88	++	c	c	c	c	c	c	c	a	c	c	c	c	c	c	a	c	c	c	c	c	c	c	c
t	5035632	t	5051897	c	5001907	s	S88	rec40	t	S88	++++	t	t	t	t	c	t	c	c	t	t	c	c	c	c	c	c	t	c	c	c	t	t	t
g	5035647	g	5051912	a	5001922	s	S88	rec40	g	S88	++	g	g	g	g	a	g	a	a	a	a	a	a	a	a	a	a	a	a	a	a	a	a	a
t	5035665	t	5051930	c	5001940	s	S88	rec40	t	S88	++++	t	t	t	t	c	t	c	c	c	c	c	t	t	t	c	c	c	c	c	c	c	c	c
g	5035671	g	5051936	a	5001946	s	S88	rec40	g	S88	++	g	g	g	g	a	g	a	a	a	a	a	a	a	a	a	a	a	a	a	a	a	a	a
c	5035674	c	5051939	g	5001949	s	S88	rec40	c	S88	++	c	c	c	c	c	c	g	g	g	g	c	g	g	g	g	c	g	c	c	c	g	g	g
t	5035725	t	5051990	g	5002000	s	S88	rec40	t	S88	++	t	t	t	t	t	t	t	g	t	t	t	t	t	t	t	t	g	g	g	g	t	g	g
a	5035731	a	5051996	g	5002006	s	S88	rec40	a	S88	++	a	a	g	g	a	a	g	g	g	g	g	g	g	g	g	g	g	g	g	g	g	g	g
t	5035755	t	5052020	c	5002030	s	S88	rec40	t	S88	++	t	t	c	c	c	t	c	c	c	c	c	c	c	c	c	c	c	c	c	c	c	c	c
a	5035785	a	5052050	g	5002060	s	S88	rec40	a	S88	++	a	a	a	g	g	a	g	g	g	g	g	g	g	g	g	g	g	g	g	g	g	g	g
t	5035788	t	5052053	c	5002063	s	S88	rec40	t	S88	+++	t	t	t	c	c	t	c	c	c	c	c	c	c	c	c	c	c	c	c	c	c	c	c
c	5035791	c	5052056	t	5002066	s	S88	rec40	c	S88	+++	c	c	c	t	t	c	t	t	t	t	t	t	t	t	t	t	t	t	t	t	t	t	t
t	5035794	t	5052059	c	5002069	s	S88	rec40	t	S88	+++	t	t	t	c	c	t	c	c	c	c	c	c	c	c	c	c	c	c	c	c	c	c	c
t	5035830	t	5052095	c	5002105	s	S88	rec40	t	S88	++++	t	t	t	t	t	t	c	c	c	c	t	t	t	t	c	c	c	t	t	t	c	c	c
c	5035839	c	5052104	t	5002114	s	S88	rec40	c	S88	+++	c	c	c	c	c	c	t	t	t	t	t	t	t	t	t	t	t	t	t	t	t	t	t
a	5035857	a	5052122	c	5002132	s	S88	rec40	a	S88	++++	a	a	a	a	a	a	c	c	c	c	a	a	a	a	c	c	c	a	a	a	c	a	a
g	5035872	g	5052137	a	5002147	s	S88	rec40	g	S88	++	g	g	g	g	g	g	g	a	g	g	a	a	a	a	g	g	g	a	a	a	g	a	a
c	5035875	c	5052140	t	5002150	s	S88	rec40	c	S88	++++	c	c	c	c	c	c	c	t	c	c	t	t	t	t	c	c	c	t	t	t	c	t	t
a	5035962	a	5052227	t	5002237	nc	S88	rec40	a	S88	+++	a	a	a	a	a	a	t	t	t	t	a	t	t	t	t	t	t	t	t	t	t	t	t
c	5035966	c	5052231	t	5002241	nc	S88	rec40	c	S88	++	c	c	c	c	c	c	c	c	c	c	c	c	c	c	c	c	c	c	c	c	c	c	c
t	5036151	t	5052416	c	5002426	s	S88	rec40	t	S88	+++	t	t	t	t	t	t	c	c	c	c	t	c	c	c	c	c	c	c	c	c	c	c	c
g	5036428	g	5052693	a	5002703	s	S88	rec40	g	S88	++	g	g	g	g	g	g	a	a	a	a	g	a	a	a	a	a	a	a	a	a	a	a	a
t	5036431	t	5052696	a	5002706	s	S88	rec40	t	S88	+++	t	t	t	t	t	t	a	a	a	a	t	a	a	a	a	a	a	a	a	a	a	a	a
a	5036458	a	5052723	g	5002733	s	S88	rec40	a	S88	++	a	a	a	g	g	a	g	g	g	g	g	g	g	g	g	g	g	g	g	g	g	g	g
a	5036565	a	5052830	t	5002840	ns	S88	rec40	a	S88	++++	a	a	a	a	a	a	a	t	t	a	a	a	a	a	t	a	a	t	t	t	t	t	t
t	5036608	t	5052873	c	5002883	s	S88	rec40	t	S88	+++	t	t	t	t	c	t	c	c	c	c	c	c	c	c	c	c	c	c	c	c	c	c	c
c	5036611	c	5052876	t	5002886	s	S88	rec40	c	S88	+++	c	c	c	c	t	c	t	t	t	t	t	t	t	t	t	t	t	t	t	t	t	t	t
c	5036695	c	5052960	t	5002970	s	S88	rec40	c	S88	++	c	c	c	c	c	c	t	t	c	c	c	c	c	c	c	c	c	c	c	c	c	c	c
c	5036722	c	5052987	t	5002997	s	S88	rec40	c	S88	++	c	c	t	t	t	t	t	t	t	t	t	t	t	t	t	t	t	t	t	t	t	t	t
a	5036728	a	5052993	c	5003003	s	S88	rec40	a	S88	+++	a	a	a	t	c	a	c	c	c	c	c	c	c	c	c	c	c	c	c	c	c	c	c
a	5036737	a	5053002	g	5003012	s	S88	rec40	a	S88	++	a	a	a	a	g	a	g	g	g	g	g	g	g	g	g	g	g	g	g	g	g	g	g
t	5036788	t	5053053	c	5003063	s	S88	rec40	t	S88	+++	t	t	t	t	c	t	t	c	c	c	c	c	c	c	c	t	c	c	c	c	c	c	c
g	5036806	g	5053071	a	5003081	s	S88	rec40	g	S88	++	g	g	g	g	a	g	a	a	a	a	a	a	a	a	a	a	a	a	a	a	a	a	a
t	5036812	t	5053077	c	5003087	s	S88	rec40	t	S88	+++	t	t	t	t	c	t	c	c	c	c	c	c	c	c	c	c	c	c	c	c	c	c	c
t	5036890	t	5053155	c	5003165	s	S88	rec40	t	S88	+++	t	t	t	t	c	t	c	c	c	c	c	c	c	c	c	c	c	c	c	c	c	c	c
t	5036908	t	5053173	c	5003183	s	S88	rec40	t	S88	+++	t	t	t	t	c	t	c	c	c	c	c	c	c	c	c	c	c	c	c	c	c	c	c
c	5036926	c	5053191	t	5003201	s	S88	rec40	c	S88	+++	c	c	c	c	c	c	t	t	t	t	c	t	t	t	t	t	t	t	t	t	t	t	t
t	5036943	t	5053208	c	5003218	ns	S88	rec40	c	2	-	c	c	c	c	c	c	c	c	c	c	c	c	c	c	c	c	c	c	c	c	c	c	c
a	5036953	a	5053218	c	5003228	s	S88	rec40	a	S88	++++	a	a	a	a	a	a	a	c	c	c	a	c	c	c	c	c	c	c	c	c	c	c	c
a	5036956	a	5053221	t	5003231	s	S88	rec40	a	S88	++++	a	a	a	a	a	a	a	t	t	t	a	t	t	t	t	t	t	t	t	t	t	t	t
c	5036974	c	5053239	t	5003249	s	S88	rec40	c	S88	+++	c	c	c	c	c	c	c	t	c	c	c	c	c	c	c	t	c	c	c	c	t	t	t
a	5037007	a	5053272	g	5003282	s	S88	rec40	a	S88	++	g	a	a	g	g	g	g	g	g	g	a	g	g	g	g	g	g	g	g	g	g	g	g
g	5037100	g	5053365	c	5003375	ns	S88	rec40	g	S88	++	g	g	g	g	g	g	c	c	c	c	g	c	c	c	c	c	c	c	c	c	c	c	c
c	5037131	c	5053396	a	5003406	ns	S88	rec40	c	S88	+++	c	c	c	c	c	c	a	a	a	a	c	a	a	a	a	a	a	a	a	a	a	a	a
g	5037169	g	5053434	a	5003444	s	S88	rec40	g	S88	++	g	g	g	g	g	g	g	a	a	a	g	a	a	a	a	a	a	a	a	a	a	a	a
t	5037178	t	5053443	c	5003453	s	S88	rec40	c	2	-	c	c	t	c	t	c	c	c	c	c	t	c	c	c	c	c	c	c	c	c	c	c	c
c	5037184	c	5053449	t	5003459	s	S88	rec40	c	S88	+++	c	c	c	c	c	c	t	t	t	t	c	t	t	t	t	t	t	t	t	t	t	t	t
t	5037235	t	5053500	c	5003510	s	S88	rec40	t	S88	+++	c	t	t	c	t	c	c	c	c	c	t	c	c	c	c	c	c	c	c	c	c	c	c
g	5037238	g	5053503	t	5003513	s	S88	rec40	g	S88	++	g	g	g	g	g	g	t	t	t	t	g	t	t	t	t	g	t	t	t	t	t	t	t
t	5037247	t	5053512	c	5003522	s	S88	rec40	t	S88	+++	t	t	t	t	t	t	c	c	c	c	t	c	c	c	c	c	c	c	c	c	c	c	c
g	5037250	g	5053515	c	5003525	s	S88	rec40	g	S88	++	g	g	g	g	g	g	g	c	c	c	g	c	c	c	c	c	c	c	c	c	c	c	c
a	5037253	a	5053518	c	5003528	s	S88	rec40	a	S88	++++	a	a	a	a	a	a	a	c	c	c	a	c	c	c	c	c	c	c	c	c	c	c	c
c	5037259	c	5053524	t	5003534	s	S88	rec40	c	S88	+++	c	c	c	c	c	c	t	t	t	t	c	t	t	t	t	t	t	t	t	t	t	t	t
t	5037262	t	5053527	c	5003537	s	S88	rec40	t	S88	+++	t	t	t	t	t	t	c	c	c	c	t	c	c	c	c	c	c	c	c	c	c	c	c
a	5037265	a	5053530	g	5003540	s	S88	rec40	a	S88	++	a	a	a	a	a	a	g	g	g	g	a	g	g	g	g	g	g	g	g	g	g	g	g
t	5037266	t	5053531	c	5003541	s	S88	rec40	t	S88	+++	t	t	t	t	t	t	c	c	c	c	t	c	c	c	c	c	c	c	c	c	c	c	c
g	5037271	g	5053536	a	5003546	s	S88	rec40	g	S88	++	g	g	g	g	g	g	a	a	a	a	g	a	a	a	a	a	a	a	a	a	a	a	a
g	5037289	g	5053554	t	5003564	s	S88	rec40	g	S88	++	g	g	g	g	g	g	a	t	t	t	g	t	t	t	t	t	t	t	t	t	t	t	t
g	5037291	g	5053556	c	5003566	ns	S88	rec40	g	S88	++	g	g	g	g	g	g	c	c	c	c	g	c	c	c	c	c	c	c	c	c	c	c	c
g	5037307	g	5053572	a	5003582	s	S88	rec40	g	S88	++	g	g	g	g	g	g	a	a	a	a	g	a	a	a	a	a	a	a	a	a	a	a	a
g	5037322	g	5053587	a	5003597	s	S88	rec40	g	S88	++	g	g	g	g	g	g	a	a	a	a	g	a	a	a	a	a	a	a	a	a	a	a	a
c	5037328	c	5053593	t	5003603	s	S88	rec40	c	S88	++++	c	c	c	c	c	c	t	t	c	c	c	t	t	t	c	c	c	t	t	t	c	c	c
t	5037331	t	5053596	c	5003606	s	S88	rec40	t	S88	++++	t	t	t	t	t	t	c	c	t	t	t	c	c	c	t	t	t	c	c	c	t	t	t
t	5037370	t	5053635	c	5003645	s	S88	rec40	c	2	-	c	c	c	c	c	c	c	c	c	c	c	c	c	c	c	c	c	c	c	c	c	c	c
g	5037546	g	5053811	a	5003821	s	S88	rec40	g	S88	++	g	g	g	g	g	g	g	a	g	g	g	a	a	a	g	g	g	g	g	g	g	g	g
c	5037570	c	5053835	t	5003845	s	S88	rec40	c	S88	+++	c	c	c	c	c	c	t	t	c	c	c	c	c	c	c	c	c	t	t	c	c	t	t
c	5037574	c	5053839	t	5003849	ns	S88	rec40	c	S88	++	c	c	c	c	c	c	t	t	c	c	c	c	c	c	c	c	c	c	c	c	c	c	c

ExPEC genome site details												Outgroup Strains Details[i]																						
UTI89[b]	UTI89 site[c]	APEC 01[b]	APEC 01 site[c]	S88[b]	S88 site[c]	type[d]	Event Lineage[ef]	Recombinant segment	Inferred ancestral base[g]	Outgroup Analysis[e]	Support level[h]	CFT073	ED1a	536	E2348/69	SMS 3-5	IAI39	UMN026	K-12	ATCC 8739	HS	D1 Sd197	CB9615	Sakai	EDL933	IAI1	E24377A	SE11	SS Ss046	F2a 301	F2a 2457T	F5 8401	B4 Sb227	B18 BS512
a	5037575	a	5053840	t	5003850	ns	S88	rec40	a	S88	++	a	a	a	a	a	a	t	t	a	a	a	a	a	a	a	a	a	a	a	a	a	a	a
g	5037579	g	5053844	a	5003854	s	S88	rec40	g	S88	++	g	g	g	g	g	g	a	a	g	g	g	g	g	g	g	g	g	g	g	g	g	g	g
a	5037591	a	5053856	t	5003866	ns	S88	rec40	a	S88	++++	a	a	a	a	a	a	t	t	a	a	a	t	t	t	a	a	a	t	t	t	a	a	a
t	5037663	t	5053928	g	5003938	ns	S88	rec40	t	S88	++	t	t	t	t	t	t	t	g	t	t	t	t	t	t	t	t	t	g	g	g	t	g	g
a	5037689	a	5053954	g	5003964	ns	S88	rec40	a	S88	++	a	a	a	a	a	a	g	g	g	g	g	g	g	g	g	g	g	g	g	g	g	g	g
a	5037831	a	5054096	g	5004106	s	S88	rec40	a	S88	++	a	a	a	a	g	a	g	g	g	g	g	g	g	g	g	g	g	g	g	g	g	g	g
t	5037880	t	5054145	c	5004155	ns	S88	rec40	t	S88	++++	t	t	t	t	c	t	c	c	t	c	c	c	c	c	t	c	c	c	c	c	t	c	c
t	5037897	t	5054162	c	5004172	s	S88	rec40	t	S88	+++	t	t	t	t	c	t	c	c	c	c	c	c	c	c	c	c	c	c	c	c	c	c	c
g	5037936	g	5054201	a	5004211	s	S88	rec40	g	S88	++	g	g	g	g	a	g	a	a	a	a	a	a	a	a	a	a	a	a	a	a	a	a	a
a	5037939	a	5054204	g	5004214	s	S88	rec40	a	S88	++	a	a	a	a	g	a	g	g	g	g	g	g	g	g	g	g	g	g	g	g	g	g	g
g	5037966	g	5054231	t	5004241	s	S88	rec40	g	S88	++	g	g	g	g	t	g	c	t	t	t	t	t	t	t	t	t	t	t	t	t	t	t	t
g	5038032	g	5054297	a	5004307	s	S88	rec40	g	S88	++	g	g	g	g	g	g	g	a	g	g	g	g	g	g	g	g	g	g	g	g	g	g	g
a	5038050	a	5054315	g	5004325	s	S88	rec40	a	S88	++	a	a	a	g	a	a	g	g	g	g	g	g	g	g	g	g	g	g	g	g	g	g	g
a	5038053	a	5054318	g	5004328	s	S88	rec40	a	S88	++	a	a	a	g	a	a	g	g	g	g	g	g	g	g	g	g	g	g	g	g	g	g	g
t	5038068	t	5054333	c	5004343	s	S88	rec40	t	S88	+++	t	t	t	c	t	t	c	c	c	c	c	c	c	c	t	c	c	c	c	c	c	c	c
t	5038089	t	5054354	c	5004364	s	S88	rec40	t	S88	+++	t	t	t	c	t	t	c	c	c	c	c	c	c	c	c	c	c	c	c	c	c	c	c
t	5038095	t	5054360	g	5004370	s	S88	rec40	t	S88	++	t	t	t	g	t	t	g	g	g	g	g	g	g	g	g	g	g	g	g	g	g	g	g
a	5038098	a	5054363	g	5004373	s	S88	rec40	a	S88	++	a	a	a	a	a	a	a	g	a	a	a	a	a	a	a	a	a	a	a	a	a	a	a
c	5038104	c	5054369	t	5004379	s	S88	rec40	c	S88	++++	c	c	c	c	a	c	c	t	t	t	t	t	t	t	c	t	t	t	t	t	t	t	t
a	5038134	a	5054399	g	5004409	s	S88	rec40	a	S88	++	a	a	a	a	a	a	a	g	g	a	g	g	g	g	g	a	g	a	a	a	g	g	g
a	5038137	a	5054402	c	5004412	s	S88	rec40	a	S88	+++	a	a	a	c	a	c	c	c	c	t	c	c	c	c	c	t	c	c	c	c	c	c	c
c	5038139	c	5054404	g	5004414	ns	S88	rec40	c	S88	++	c	c	c	t	c	c	c	g	c	c	c	c	c	c	c	c	c	c	c	c	c	c	c
c	5038161	c	5054426	g	5004436	s	S88	rec40	c	S88	++	c	c	g	c	c	c	c	g	c	c	c	c	c	c	c	c	c	c	c	c	c	c	c
g	5038162	g	5054427	c	5004437	ns	S88	rec40	g	S88	++	g	g	c	g	g	g	g	c	g	g	g	g	g	g	g	g	g	g	g	g	g	g	g
t	5038166	t	5054431	c	5004441	ns	S88	rec40	t	S88	++	t	t	c	t	t	t	t	c	t	t	t	t	t	t	t	t	t	t	t	t	t	t	t
c	5038182	c	5054447	g	5004457	s	S88	rec40	c	S88	++	c	c	c	g	g	c	a	g	g	g	g	g	g	g	g	g	g	g	g	g	g	g	g
c	5038213	c	5054478	t	5004488	nc	S88	rec40	c	S88	++++	c	c	c	t		c	t	t	c	c	t	t	t	t	t	c	c	t	t	t	t	t	t
t	5038222	t	5054487	c	5004497	nc	S88	rec40	t	S88	++++	t	t	t	c		t	t	c	t	t	c	t	t	t	t	t	t	c	c	c	c	c	c
c	5038237	c	5054502	a	5004512	nc	S88	rec40	c	S88	++	c	c	c	a			a	a	a	a	a	a	a	a	a	a	a	a	a	a	a	a	a
g	5038266	g	5054531	t	5004541	nc	S88	rec40	g	S88	++	g	g	g	g			g	a	g	g	t	a	a	a	g	g	g	t	t	t	t	t	t
a	5038288	a	5054553	g	5004564	nc	S88	rec40	a	S88	++	a	a	a	a			a	g	g	a	g	g	g	g	g	g	g	g	g	g	g	g	g
c	5038291	c	5054556	t	5004567	nc	S88	rec40	c	S88	+++	c	c	c	c			c	t	t	t	t	t	t	t	t	t	t	t	t	t	t	t	t
c	5038339	c	5054604	t	5004615	nc	S88	rec40	c	S88	+++	c	c	c	c		c	t	t	t	t	t	t	t	t	t	t	t	t	t	t	t	t	t
a	5038368	a	5054633	t	5004644	nc	S88	rec40	a	S88	+++	a	a	a	a		a	a	t	a	t	a	a	a	a	t	t	t	a	a	a	a	t	t
g	5038389	g	5054654	a	5004665	nc	S88	rec40	g	S88	++	g	g	g	g		g	t	a	a	a	a	a	a	a	g	g	g	a	a	a	a	a	a
t	5038401	t	5054666	a	5004677	nc	S88	rec40	t	S88	+++	t	t	t	t		t	a	a	a	a	a	a	a	a	a	a	a	a	a	a	a	a	a
a	5038411	a	5054676	g	5004889	nc	S88	rec40	a	S88	++	a	a	a	g	a	g	g	g	g	g	g	g	g	g	g	g	g	g	g	g	g	g	g
c	5038412	c	5054677	t	5004890	nc	S88	rec40	c	S88	+++	c	c	c	c	c	t	t	t	c	t	t	t	t	t	t	t	t	t	t	t	t	t	t
t	5038432	t	5054697	g	5004910	s	S88	rec40	t	S88	++	t	t	t	t	t	t	t	g	c	c	g	c	c	c	g	g	g	g	g	g	g	g	g
t	5038447	t	5054712	c	5004925	s	S88	rec40	t	S88	++++	t	t	t	t	t	t	t	c	c	t	c	t	t	t	c	c	c	c	c	c	c	c	c
t	5038466	t	5054731	a	5004944	ns	S88	rec40	t	S88	++++	t	t	t	t	t	t	a	a	a	a	a	t	t	t	a	a	a	a	a	a	a	a	a
c	5038469	c	5054734	t	5004947	ns	S88	rec40	c	S88	++++	c	c	c	c	c	c	t	t	t	t	t	c	c	c	t	t	t	t	t	t	t	t	t
g	5038470	g	5054735	t	5004948	ns	S88	rec40	g	S88	++	g	g	g	g	g	a	t	t	t	t	t	g	g	g	t	t	t	t	t	t	t	t	t
g	5038485	g	5054750	a	5004963	s	S88	rec40	g	S88	++	g	g	g	g	g	g	a	a	a	a	a	a	a	a	a	a	a	a	a	a	a	a	a
t	5038486	t	5054751	c	5004964	ns	S88	rec40	t	S88	+++	t	t	t	t	t	t	t	c	c	c	c	c	c	c	c	c	c	c	c	c	c	c	c
t	5038487	t	5054752	g	5004965	ns	S88	rec40	t	S88	++	t	t	t	t	t	t	g	g	g	g	g	g	g	g	g	g	g	g	g	g	g	g	g
t	5038489	t	5054754	c	5004967	s	S88	rec40	t	S88	+++	t	t	t	t	t	t	c	c	c	c	c	c	c	c	c	c	c	c	c	c	c	c	c
c	5038492	c	5054757	a	5004970	s	S88	rec40	c	S88	+++	c	c	c	c	c	c	a	a	a	a	a	a	a	a	a	a	a	a	a	a	a	a	a
a	5038495	a	5054760	g	5004973	s	S88	rec40	a	S88	++	a	a	a	a	a	a	g	g	g	g	g	g	g	g	g	g	g	g	g	g	g	g	g
c	5038498	c	5054763	t	5004976	s	S88	rec40	c	S88	+++	c	c	c	c	c	c	t	t	t	t	t	t	t	t	t	t	t	t	t	t	t	t	t
g	5038515	g	5054780	c	5004993	ns	S88	rec40	g	S88	++	g	g	g	g	g	g	g	c	c	c	g	c	c	c	c	c	c	c	c	c	c	c	c
a	5038582	a	5054847	g	5005060	s	S88	rec40	a	S88	++	a	a	a	a	a	a	a	g	a	a	a	g	g	g	a	a	a	a	a	a	a	a	a
t	5038594	t	5054859	a	5005072	ns	S88	rec40	t	S88	+++	t	t	t	t	t	t	a	a	a	a	t	a	a	a	a	a	a	a	a	a	a	a	a
a	5038615	a	5054880	g	5005093	s	S88	rec40	a	S88	++	g	a	a	g	g	g	g	g	g	g	g	g	g	g	g	g	g	g	g	g	g	g	g
t	5038645	t	5054910	c	5005123	s	S88	rec40	t	S88	+++	t	t	t	t	t	t	t	c	t	t	t	t	t	t	t	t	t	c	c	c	t	t	t
a	5038672	a	5054937	g	5005150	s	S88	rec40	a	S88	++	a	a	a	a	a	a	g	g	g	a	a	a	a	a	a	a	a	g	g	g	g	g	g
c	5038691	c	5054956	t	5005169	ns	S88	rec40	c	S88	+++	c	c	c	c	c	c	c	t	c	c	c	c	c	c	c	c	c	t	t	t	c	c	c
g	5038705	g	5054970	a	5005183	s	S88	rec40	g	S88	++	g	g	g	g	g	g	a	a	a	a	g	a	a	a	a	a	a	a	a	a	a	a	a
t	5038729	t	5054994	g	5005207	s	S88	rec40	t	S88	++	t	t	t	t	t	t	t	g	t	t	t	t	t	t	t	t	t	t	t	t	t	t	t
a	5038731	a	5054996	g	5005209	s	S88	rec40	a	S88	++	a	a	a	a	a	a	a	g	a	t	a	a	a	a	a	a	a	a	a	a	a	a	a
c	5038735	c	5055000	t	5005213	s	S88	rec40	c	S88	++++	c	c	c	c	c	t	c	t	t	t	c	c	c	c	t	c	c	t	t	t	t	t	t
a	5038783	a	5055048	g	5005261	s	S88	rec40	a	S88	++	a	a	a	a	a	a	g	g	a	g	a	g	g	g	g	g	g	a	a	a	a	a	a
g	5038840	g	5055105	c	5005318	s	S88	rec40	g	S88	++	g	g	g	g	g	g	c	c	c	c	g	c	c	c	c	g	g	c	c	c	c	c	c
g	5038843	g	5055108	a	5005321	s	S88	rec40	g	S88	++	g	g	g	g	g	g	a	a	a	a	g	g	g	g	a	g	g	a	a	a	g	g	g
a	5038857	a	5055122	g	5005335	ns	S88	rec40	a	S88	++	a	a	a	a	a	a	a	g	g	g	a	g	g	g	g	g	g	g	g	g	g	g	g
g	5038879	g	5055144	a	5005357	s	S88	rec40	g	S88	++	g	g	g	g	g	g	g	a	a	a	g	a	a	a	g	g	g	a	a	a	a	a	a
a	5038908	a	5055173	g	5005386	s	S88	rec40	a	S88	++	a	a	a	a	a	a	g	g	g	g	a	g	g	g	g	g	g	g	g	g	g	g	g
g	5038924	g	5055189	a	5005402	s	S88	rec40	g	S88	++	g	g	g	g	g	g	g	a	g	g	g	g	g	g	g	g	g	g	g	g	g	g	g
g	5038930	g	5055195	a	5005408	s	S88	rec40	g	S88	++	g	g	g	g	g	g	g	a	g	a	g	a	a	a	g	g	g	g	g	g	g	g	g
t	5038933	t	5055198	c	5005411	s	S88	rec40	t	S88	+++	t	t	t	t	t	t	t	c	t	c	t	c	c	c	t	t	t	t	t	t	t	t	t
a	5038948	a	5055213	g	5005426	s	S88	rec40	a	S88	++	a	a	a	a	a	a	a	g	a	g	a	g	g	g	a	a	a	a	a	a	a	a	a
a	5038957	a	5055222	c	5005435	s	S88	rec40	a	S88	+++	a	a	a	a	c	a	c	c	c	c	c	c	c	c	c	c	c	c	c	c	c	c	c
c	5038960	c	5055225	t	5005438	s	S88	rec40	c	S88	+++	c	c	c	c	c	c	c	t	c	c	c	t	t	t	c	c	c	c	c	c	c	c	c
t	5038976	t	5055241	c	5005454	ns	S88	rec40	t	S88	+++	t	t	t	t	t	t	c	c	c	c	t	c	c	c	c	c	c	c	c	c	c	c	c
c	5038984	c	5055249	t	5005462	s	S88	rec40	c	S88	++++	c	c	c	c	c	c	c	t	t	t	c	c	c	c	c	c	c	t	t	t	t	t	t
t	5039011	t	5055276	g	5005489	s	S88	rec40	t	S88	+++	t	t	t	t	t	t	c	g	c	c	t	t	t	t	t	t	t	t	t	t	t	t	t
a	5039017	a	5055282	g	5005495	s	S88	rec40	a	S88	++	a	a	a	a	g	a	g	g	g	g	g	g	g	g	g	g	g	g	g	g	g	g	g
a	5039033	a	5055298	c	5005511	ns	S88	rec40	a	S88	++++	a	a	a	a	a	a	a	c	c	c	a	c	c	c	c	a	c	a	a	a	c	a	a
t	5039053	t	5055318	c	5005531	s	S88	rec40	t	S88	++++	t	t	t	t	t	t	t	c	c	t	t	t	t	t	c	c	c	t	t	t	c	c	c
g	5039062	g	5055327	a	5005540	s	S88	rec40	g	S88	++	g	g	g	g	a	g	a	a	g	g	a	g	g	g	a	a	a	g	g	g	a	g	g
a	5039065	a	5055330	c	5005543	ns	S88	rec40	a	S88	+++	a	a	a	a	a	a	c	c	c	c	a	c	c	c	c	c	c	c	c	c	c	c	c
g	5039066	g	5055331	a	5005544	ns	S88	rec40	g	S88	++	g	g	g	g	g	g	a	a	g	a	g	a	a	a	a	a	a	a	a	a	a	a	a
c	5039068	c	5055333	a	5005546	s	S88	rec40	c	S88	+++	c	c	c	c	c	c	a	a	a	a	c	a	a	a	a	a	a	a	a	a	a	a	a
c	5039072	c	5055337	t	5005550	ns	S88	rec40	c	S88	+++	c	c	c	c	c	c	t	t	t	t	c	t	t	t	t	t	t	t	t	t	t	t	t

ExPEC genome site details												Outgroup Strains Details[i]																						
UTI89[b]	UTI89 site[c]	APEC 01[b]	APEC 01 site[c]	S88[b]	S88 site[c]	type[d]	Event Lineage[ef]	Recombinant segment	Inferred ancestral base[g]	Outgroup Analysis[e]	Support level[h]	CFT073	ED1a	536	E2348/69	SMS 3-5	IAI39	UMN026	K-12	ATCC 8739	HS	D1 Sd197	CB9615	Sakai	EDL933	IAI1	E24377A	SE11	SS Ss046	F2a 301	F2a 2457T	F5 8401	B4 Sb227	B18 BS512
g	5039078	g	5055343	a	5005556	ns	S88	rec40	g	S88	++	g	g	g	g	g	g	a	a	a	a	g	a	a	a	a	a	a	a	a	a	a	a	a
g	5039092	g	5055357	a	5005570	s	S88	rec40	g	S88	++	g	g	g	g	g	g	a	a	a	a	g	a	a	a	a	a	a	a	a	a	a	a	a
c	5039122	c	5055387	t	5005600	s	S88	rec40	c	S88	++++	c	c	c	c	c	c	c	t	t	t	c	t	t	t	t	t	t	t	t	t	t	t	t
t	5039134	t	5055399	c	5005612	s	S88	rec40	t	S88	++++	t	t	t	t	t	t	t	c	t	c	t	c	c	c	c	c	c	c	c	c	c	t	t
g	5039137	g	5055402	a	5005615	s	S88	rec40	g	S88	++	g	g	g	g	g	g	g	a	g	a	g	a	a	a	a	g	a	a	a	a	a	g	g
a	5039140	a	5055405	g	5005618	s	S88	rec40	a	S88	++	a	a	a	a	a	a	g	g	a	g	a	g	g	g	g	a	g	g	g	g	g	a	a
a	5039146	a	5055411	g	5005624	s	S88	rec40	a	S88	++	a	a	a	a	a	a	g	g	a	g	a	g	g	g	g	g	g	g	g	g	g	g	g
g	5039149	g	5055414	a	5005627	s	S88	rec40	g	S88	++	g	g	g	g	g	g	g	a	g	a	g	a	a	a	a	g	a	a	a	a	a	g	g
c	5039152	c	5055417	t	5005630	s	S88	rec40	c	S88	++++	c	c	c	c	c	c	t	t	c	t	c	t	t	t	t	t	t	t	t	t	t	t	t
t	5039342	t	5055607	g	5005820	s	S88	rec40	t	S88	++	t	t	t	t	t	t	t	g	g	g	t	g	g	g	g	g	g	g	g	g	g	g	g
a	5039471	a	5055736	g	5005949	s	S88	rec40	g	2	-	g	g	g	g	g	g	g	g	g	g	g	g	g	g	g	g	g	g	g	g	g	g	g
c	5039504	c	5055769	t	5005982	s	S88	rec40	c	S88	++++	c	c	c	c	c	c	t	t	c	t	c	c	c	c	c	t	t	t	t	t	c	c	c
a	5039513	a	5055778	g	5005991	s	S88	rec40	a	S88	++	a	a	a	a	a	a	g	g	a	g	a	g	g	g	g	g	g	g	g	g	a	a	a
a	5039603	a	5055868	g	5006081	s	S88	rec40	a	S88	++	a	a	a	a	a	a	a	g	g	g	a	g	g	g	g	g	g	g	g	g	g	g	g
a	5039621	a	5055886	g	5006099	s	S88	rec40	g	2	-	g	g	a	g	g	a	a	g	g	g	g	g	g	g	g	g	g	g	g	g	a	g	g
t	5039666	t	5055931	g	5006144	s	S88	rec40	t	S88	++	g	t	t	g	g	t	g	g	g	g	g	g	g	g	g	g	g	g	g	g	g	g	g
a	5039753	a	5056018	g	5006231	s	S88	rec40	a	S88	++	g	a	a	g	g	a	g	g	g	g	g	g	g	g	g	g	g	g	g	g	g	g	g
c	5039765	c	5056030	t	5006243	s	S88	rec40	c	S88	+++	c	c	c	c	c	c	c	t	c	c	c	t	t	t	c	c	c	c	c	c	c	c	c
c	5039783	c	5056048	t	5006261	s	S88	rec40	c	S88	++	t	c	c	t	t	c	t	t	t	t	t	t	t	t	t	t	t	t	t	t	t	t	t
t	5039798	t	5056063	c	5006276	s	S88	rec40	t	S88	+++	c	t	t	c	t	t	c	c	c	t	c	c	c	c	c	c	c	c	c	c	c	c	c
c	5039864	c	5056129	t	5006342	s	S88	rec40	c	S88	+++	c	c	c	c	t	c	t	t	t	t	t	t	t	t	t	t	t	t	t	t	t	t	t
t	5039978	t	5056243	c	5006456	s	S88	rec40	t	S88	+++	t	t	t	t	t	t	c	c	c	c	c	c	c	c	c	c	c	c	c	c	c	c	c
c	5040014	c	5056279	a	5006492	ns	S88	rec40	c	S88	+++	c	c	c	a	c	a	a	a	a	a	c	a	a	a	a	a	a	a	a	a	a	a	a
a	5040026	a	5056291	g	5006504	s	S88	rec40	a	S88	++	a	a	a	a	a	g	a	g	g	g	a	g	g	g	g	g	g	g	g	g	g	g	g
t	5040053	t	5056318	a	5006531	s	S88	rec40	t	S88	+++	a	t	t	a	t	a	a	a	a	a	t	a	a	a	a	a	a	a	a	a	a	a	a
t	5040191	t	5056456	c	5006669	s	S88	rec40	t	S88	+++	t	t	t	t	c	t	t	c	c	c	c	c	c	c	c	c	c	c	c	c	c	c	c
a	5040227	a	5056492	t	5006705	ns	S88	rec40	a	S88	+++	a	a	a	a	t	a	t	t	t	t	t	t	t	t	t	t	t	t	t	t	t	t	t
g	5040271	g	5056536	a	5006749	s	S88	rec40	g	S88	++	g	g	g	g	g	g	g	a	a	a	a	a	a	a	a	a	a	-	-	-	a	a	a
g	5040278	g	5056543	a	5006756	s	S88	rec40	g	S88	++	g	g	g	g	a	g	a	a	g	g	g	g	g	g	g	g	g	g	g	g	g	g	g
t	5040290	t	5056555	g	5006768	s	S88	rec40	t	S88	++	t	t	t	t	g	t	g	g	g	g	g	g	g	g	g	g	g	g	g	g	g	g	g
a	5040296	a	5056561	g	5006774	s	S88	rec40	a	S88	++	a	a	a	a	a	a	a	g	a	g	g	g	g	g	g	g	g	g	g	g	g	g	g
a	5040332	a	5056597	g	5006810	s	S88	rec40	a	S88	++	a	a	a	a	g	a	g	g	g	g	g	g	g	g	g	g	g	g	g	g	g	g	g
t	5040391	t	5056656	c	5006869	ns	S88	rec40	t	S88	++++	t	t	t	t	t	t	t	c	t	c	c	c	c	c	c	c	c	c	c	c	c	c	c
a	5040485	a	5056750	g	5006963	s	S88	rec40	g	2	-	a	g	g	a	g	a	g	g	g	g	g	g	g	g	g	g	g	g	g	g	g	g	g
g	5040527	g	5056792	a	5007005	s	S88	rec40	a	2	-	g	a	a	g	a	g	a	a	a	a	a	a	a	a	a	a	a	a	a	a	a	a	a
g	5040566	g	5056831	c	5007044	s	S88	rec40	g	S88	++	c	g	g	g	c	c	c	c	c	c	c	c	c	c	c	c	c	c	c	c	c	c	c
a	5040571	a	5056836	g	5007049	s	S88	rec40	a	S88	++	g	a	a	a	g	g	g	g	g	g	g	g	g	g	g	g	g	g	g	g	g	g	g
c	5040644	c	5056909	a	5007122	s	S88	rec40	c	S88	+++	c	c	c	c	a	c	a	a	a	a	a	a	a	a	a	a	a	a	a	a	a	a	a
c	5040647	c	5056912	t	5007125	s	S88	rec40	c	S88	+++	c	c	c	c	c	c	c	t	c	c	c	t	t	t	c	c	c	t	t	t	c	c	c
c	5040677	c	5056942	a	5007155	s	S88	rec40	c	S88	+++	c	c	c	c	c	c	a	a	c	a	a	a	a	a	a	a	a	a	a	a	a	a	a
t	5040746	t	5057011	c	5007224	s	S88	rec40	t	S88	+++	t	t	t	t	t	t	t	c	c	c	c	c	c	c	c	c	c	c	c	c	c	c	c
c	5040749	c	5057014	t	5007227	s	S88	rec40	c	S88	+++	c	c	c	c	c	c	c	t	t	t	t	t	t	t	t	t	t	t	t	t	t	t	t
a	5040981	a	5057246	g	5007459	nc	S88	rec40	a	S88	++	a	a	a	a	a	a	a	g	a	a	a	a	a	a	a	a	a	g	g	g	a	a	a
c	5040982	c	5057247	t	5007460	nc	S88	rec40	c	S88	+++	c	c	c	c	c	c	t	t	t	t	t	t	t	t	t	t	t	t	t	t	t	t	t
t	5041143	t	5057408	c	5007621	s	S88	rec40	t	S88	+++	t	c	t	t	t	t	c	c	c	c	c	c	c	c	c	c	c	c	c	c	c	c	c
g	5041242	g	5057507	t	5007720	s	S88	rec40	g	S88	++	t	g	g	t	t	g	g	t	g	g	a	a	a	a	g	g	g	t	t	t	g	g	g
t	5041287	t	5057552	c	5007765	s	S88	rec40	t	S88	+++	t	t	t	t	t	t	c	c	c	c	c	c	c	c	c	c	c	c	c	c	c	c	c
a	5041317	a	5057582	c	5007795	ns	S88	rec40	c	2	-	c	a	c	a	a	a	c	c	a	a	a	a	a	a	a	a	a	a	a	a	a	a	a
c	5041359	c	5057624	t	5007837	s	S88	rec40	c	S88	++++	c	c	c	c	c	c	c	t	a	t	c	t	t	t	t	t	t	t	t	t	t	t	t
g	5041374	g	5057639	t	5007852	s	S88	rec40	g	S88	++	g	a	g	g	g	a	g	t	t	t	g	t	t	t	t	t	t	g	g	g	t	t	t
t	5041455	t	5057720	g	5007933	s	S88	rec40	t	S88	++	t	t	t	t	t	t	a	g	g	g	g	g	g	g	g	g	g	g	g	g	g	g	g
g	5041458	g	5057723	a	5007936	s	S88	rec40	g	S88	++	g	g	g	g	g	g	g	a	a	a	a	a	a	a	a	a	a	a	a	a	a	a	a
c	5041464	c	5057729	t	5007942	s	S88	rec40	c	S88	+++	c	c	c	c	c	c	c	t	t	t	t	t	t	t	t	t	t	t	t	t	t	t	t
t	5041482	t	5057747	c	5007960	s	S88	rec40	t	S88	+++	t	t	t	t	t	t	c	c	c	c	c	c	c	c	c	c	c	c	c	c	c	c	c
t	5041485	t	5057750	c	5007963	s	S88	rec40	t	S88	+++	t	t	t	t	t	t	c	c	c	c	c	c	c	c	c	c	c	c	c	c	c	c	c
t	5041554	t	5057819	g	5008032	s	S88	rec40	t	S88	++	t	t	t	t	t	t	t	g	g	g	g	g	g	g	g	g	g	g	g	g	g	t	t
t	5041593	t	5057858	c	5008071	s	S88	rec40	t	S88	+++	t	t	t	t	t	t	c	c	c	c	c	c	c	c	c	c	c	c	c	c	c	c	c
c	5041635	c	5057900	t	5008113	s	S88	rec40	c	S88	+++	c	c	c	c	c	c	c	t	t	t	t	t	t	t	t	t	t	t	t	t	t	t	t
t	5041655	t	5057920	c	5008133	ns	S88	rec40	c	2	-	c	c	c	c	c	c	c	c	c	c	c	c	c	c	c	c	c	c	c	c	c	c	c
t	5041749	t	5058014	a	5008227	s	S88	rec40	t	S88	++++	t	t	t	t	t	t	t	a	t	a	a	t	t	t	t	a	a	t	t	t	a	a	a
t	5041773	t	5058038	c	5008251	s	S88	rec40	t	S88	++	t	t	t	t	t	t	c	c	t	t	t	t	t	t	t	t	t	t	t	t	t	t	t
t	5041783	t	5058048	c	5008261	s	S88	rec40	t	S88	+++	t	t	t	t	t	t	c	c	c	c	c	c	c	c	c	c	c	c	c	c	c	c	c
t	5041806	t	5058071	g	5008284	s	S88	rec40	t	S88	++	t	t	t	t	t	t	g	g	g	g	g	g	g	g	g	g	g	g	g	g	g	g	g
c	5041812	c	5058077	t	5008290	s	S88	rec40	c	S88	+++	c	c	c	c	c	c	c	t	c	c	c	t	t	t	c	c	c	c	c	c	c	c	c
a	5041910	a	5058175	t	5008437	s	S88	rec40	a	S88	+++	a	a	a	a	a	a	a	t	t	t	t	t	t	t	t	t	t	t	t	t	t	t	t
t	5041976	t	5058241	c	5008503	s	S88	rec40	t	S88	+++	t	t	t	t	t	t	c	c	c	c	c	c	c	c	c	c	c	c	c	c	c	c	c
c	5042006	c	5058271	t	5008533	s	S88	rec40	c	S88	++++	c	c	c	c	c	c	c	t	t	t	c	t	t	t	t	t	t	t	t	t	c	c	c
g	5042180	g	5058445	c	5008707	s	S88	rec40	g	S88	++	g	g	g	g	g	g	c	c	c	c	c	c	c	c	c	c	c	g	g	g	c	c	c
c	5042186	c	5058451	t	5008713	s	S88	rec40	c	S88	+++	c	c	c	c	c	c	t	t	c	c	c	t	t	t	c	c	c	c	c	c	t	c	c
t	5042189	t	5058454	c	5008716	s	S88	rec40	t	S88	+++	t	t	t	t	t	t	c	c	c	c	c	c	c	c	c	c	c	c	c	t	c	c	c
t	5042231	t	5058496	a	5008758	s	S88	rec40	t	S88	+++	t	t	t	t	t	t	a	a	a	a	a	a	a	a	a	a	a	a	a	a	a	a	a
t	5042237	t	5058502	c	5008764	s	S88	rec40	t	S88	+++	t	t	t	t	t	t	c	c	c	c	c	c	c	c	c	c	c	c	c	c	c	c	c
c	5042261	c	5058526	t	5008788	s	S88	rec40	c	S88	+++	c	c	c	c	c	c	c	t	c	c	c	t	t	t	c	c	c	c	c	c	t	c	c
c	5042387	c	5058652	t	5008914	s	S88	rec40	c	S88	++++	c	c	c	c	t	t	t	t	t	c	t	t	t	t	c	c	c	t	t	t	t	t	t
c	5042396	c	5058661	t	5008923	s	S88	rec40	c	S88	+++	c	c	c	c	c	c	c	t	c	c	c	c	c	c	c	c	c	t	t	t	c	c	c
g	5042411	g	5058676	t	5008938	s	S88	rec40	g	S88	++	g	g	g	g	g	g	g	t	g	g	g	g	g	g	g	g	g	t	t	t	g	g	g
c	5042435	c	5058700	t	5008962	s	S88	rec40	c	S88	++	c	c	t	c	t	t	t	t	t	t	t	t	t	t	t	t	t	t	t	t	t	t	t
t	5042456	t	5058721	c	5008983	s	S88	rec40	t	S88	+++	t	t	t	t	c	c	c	c	c	c	c	c	c	c	c	c	c	c	c	c	c	c	c
a	5042459	a	5058724	c	5008986	s	S88	rec40	a	S88	+++	a	a	a	a	c	c	c	c	c	c	c	c	c	c	c	c	c	c	c	c	c	c	c
g	5042498	g	5058763	t	5009025	s	S88	rec40	g	S88	++	g	g	g	g	g	g	g	t	g	g	t	g	g	g	g	g	g	g	g	g	t	g	g
t	5042645	t	5058910	c	5009172	s	S88	rec40	t	S88	++++	t	t	t	t	t	t	c	c	c	t	c	c	c	c	t	t	t	c	c	c	c	c	c
a	5042756	a	5059021	g	5009283	s	S88	rec40	a	S88	++	a	a	a	a	g	g	g	g	g	g	g	g	g	g	g	g	g	g	g	g	g	g	g
a	5042796	a	5059061	g	5009323	ns	S88	rec40	g	2	-	g	g	g	g	g	g	g	g	g	g	g	g	g	g	g	g	g	g	g	g	g	g	g

ExPEC genome site details												Outgroup Strains Details[i]																						
UTI89[b]	UTI89 site[c]	APEC 01[b]	APEC 01 site[c]	S88[b]	S88 site[c]	type[d]	Event Lineage[ef]	Recombinant segment	Inferred ancestral base[g]	Outgroup Analysis[e]	Support level[h]	CFT073	ED1a	536	E2348/69	SMS 3-5	IAI39	UMN026	K-12	ATCC 8739	HS	D1 Sd197	CB9615	Sakai	EDL933	IAI1	E24377A	SE11	SS Ss046	F2a 301	F2a 2457T	F5 8401	B4 Sb227	B18 BS512
c	5042873	c	5059138	a	5009400	s	S88	rec40	a	2	-	a	a	a	a	a	a	a	a	a	a	a	a	a	a	a	a	a	a	a	a	a	a	a
g	5042969	g	5059234	t	5009496	s	S88	rec40	g	S88	++	g	g	g	t	g	t	t	t	t	t	t	t	t	t	t	t	t	t	t	t	t	t	t
a	5042996	a	5059261	g	5009523	s	S88	rec40	g	2	-	g	g	g	g	g	g	g	g	g	g	g	g	g	g	g	g	g	g	g	g	g	g	g
a	5043006	a	5059271	g	5009533	ns	S88	rec40	g	2	-	g	g	g	g	g	g	g	g	g	g	g	g	g	g	g	g	g	g	g	g	g	g	g
a	5043122	a	5059387	g	5009649	s	S88	rec40	a	S88	++	a	a	a	a	a	a	g	g	g	t	g	g	g	g	g	g	g	g	g	g	g	g	g
g	5043148	g	5059413	a	5009675	ns	S88	rec40	g	S88	++	g	g	g	g	g	g	g	a	g	g	g	g	g	g	g	g	g	g	g	g	g	g	g
c	5043428	c	5059693	t	5009955	s	S88	rec40	t	2	-	t	t	c	t	t	t	t	t	t	t	t	t	t	t	t	t	t	t	t	t	t	t	t
c	5043429	c	5059694	a	5009956	ns	S88	rec40	a	2	-	a	a	c	a	a	a	a	a	a	a	a	a	a	a	a	a	a	a	a	a	a	a	a
t	5043473	t	5059738	g	5010000	s	S88	rec40	g	2	-	g	g	t	g	g	g	g	g	g	g	g	g	g	g	g	g	g	g	g	g	g	g	g
t	5043527	t	5059792	c	5010054	s	S88	rec40	c	2	-	c	c	t	c	c	c	c	c	c	c	c	c	c	c	c	c	c	c	c	c	c	c	c
c	5043587	c	5059852	t	5010114	s	S88	rec40	t	2	-	t	t	c	t	t	t	t	t	t	t	t	t	t	t	t	t	t	t	t	t	t	t	t
c	5043611	c	5059876	t	5010138	s	S88	rec40	c	S88	+++	c	c	c	t	c	c	c	t	c	c	c	t	t	t	c	c	c	c	c	c	a	c	c
t	5043695	t	5059960	c	5010222	s	S88	rec40	t	S88	++++	t	t	t	c	c	t	c	c	c	t	c	c	c	c	c	t	t	c	c	c	t	c	c
c	5043860	c	5060125	t	5010387	s	S88	rec40	c	S88	+++	c	c	t	t	c	c	t	t	t	t	t	t	t	t	t	t	t	t	t	t	t	t	t
t	5043887	t	5060152	c	5010414	s	S88	rec40	t	S88	+++	t	t	c	c	t	t	c	c	c	c	c	c	c	c	c	c	c	c	c	c	c	c	c
c	5043917	c	5060182	t	5010444	s	S88	rec40	c	S88	+++	c	c	c	c	c	c	c	t	c	c	c	t	t	t	c	c	c	t	t	t	c	c	c
t	5043959	t	5060224	c	5010486	s	S88	rec40	t	S88	+++	t	t	t	c	c	c	c	c	c	c	c	c	c	c	c	c	c	t	t	t	c	c	c
t	5043977	t	5060242	c	5010504	s	S88	rec40	t	S88	+++	t	t	t	t	c	c	c	c	c	c	c	c	c	c	c	c	c	c	c	c	c	c	c
g	5044064	g	5060329	t	5010591	s	S88	rec40	g	S88	++	g	g	g	g	t	t	t	t	t	t	t	g	g	g	t	t	t	t	t	t	t	t	t
a	5044079	a	5060344	g	5010606	s	S88	rec40	a	S88	++	a	a	a	a	g	c	a	g	a	a	a	a	a	a	a	a	a	a	a	a	a	a	a
c	5044124	c	5060389	t	5010651	s	S88	rec40	c	S88	+++	c	c	c	c	t	c	t	t	t	t	t	t	t	t	t	t	t	t	t	t	t	t	t
t	5044253	t	5060518	c	5010780	s	S88	rec40	t	S88	++	t	t	t	t	t	c	t	c	t	t	t	t	t	t	t	g	g	t	t	t	t	t	t
t	5044299	t	5060564	c	5010826	s	S88	rec40	t	S88	++++	t	t	t	t	c	t	c	c	t	t	c	c	c	c	t	c	c	c	c	c	c	t	t
t	5044316	t	5060581	c	5010843	s	S88	rec40	t	S88	+++	t	t	t	t	c	c	c	c	c	c	c	c	c	c	c	c	c	c	c	c	c	c	c
t	5044319	t	5060584	c	5010846	s	S88	rec40	t	S88	+++	t	t	t	t	c	c	c	c	c	c	c	c	c	c	c	c	c	c	c	c	c	c	c
t	5044325	t	5060590	c	5010852	s	S88	rec40	t	S88	+++	t	t	t	t	c	c	c	c	c	c	c	c	c	c	c	c	c	c	c	c	c	c	c
t	5044331	t	5060596	c	5010858	s	S88	rec40	t	S88	++++	t	t	t	t	c	t	c	c	t	t	t	c	c	c	t	t	t	c	c	c	t	t	t
t	5044346	t	5060611	c	5010873	s	S88	rec40	t	S88	+++	t	t	t	t	t	t	t	c	t	t	t	t	t	t	t	t	t	c	c	c	t	t	t
c	5044349	c	5060614	t	5010876	s	S88	rec40	c	S88	+++	c	c	c	c	t	t	t	t	t	t	t	t	t	t	t	t	t	t	t	t	t	t	t
t	5044400	t	5060665	a	5010927	s	S88	rec40	t	S88	+++	t	t	t	t	a	t	t	a	t	t	t	t	t	t	t	t	t	a	a	a	t	t	t
c	5044403	c	5060668	t	5010930	s	S88	rec40	c	S88	+++	c	c	c	c	c	c	c	t	c	c	c	c	c	c	c	c	c	t	t	t	c	c	c
g	5044415	g	5060680	a	5010942	s	S88	rec40	g	S88	++	g	g	g	g	a	a	a	a	a	a	a	a	a	a	a	a	a	a	a	a	a	a	a
a	5044421	a	5060686	g	5010948	s	S88	rec40	a	S88	++	a	a	a	a	g	g	g	g	g	g	t	g	g	g	g	g	g	g	g	g	g	g	g
t	5044424	t	5060689	c	5010951	s	S88	rec40	t	S88	++++	t	t	t	t	c	t	c	c	t	t	c	c	c	c	t	c	c	c	c	c	c	t	t
g	5044427	g	5060692	a	5010954	s	S88	rec40	g	S88	++	g	g	g	g	a	a	a	a	a	a	a	a	a	a	a	a	a	a	a	a	a	a	a
a	5044537	a	5060802	g	5011064	s	S88	rec40	a	S88	++	a	a	a	a	g	g	g	g				g	g	g				g	g	g			
g	5044538	g	5060803	t	5011065	ns	S88	rec40	g	S88	++	g	g	g	g	t	t	t	t				t	t	t				t	t	t			
a	5044552	a	5060817	g	5011079	s	S88	rec40	a	S88	++	a	a	a	a	g	g	g	g				g	g	g				g	g	g			
a	5044553	a	5060818	t	5011080	ns	S88	rec40	a	S88	+++	a	a	a	a	t	t	t	t				t	t	t				t	t	t			
t	5044575	t	5060840	c	5011102	ns	S88	rec40	c	2	-	c	t	c	c	c	c	c	c	c	c	c	c	c	c	c	c	c	c	c	c	c	c	c
g	5044576	g	5060841	a	5011103	ns	S88	rec40	g	S88	++	g	g	g	g	a	a	a	a	a	a	a	a	a	a	a	a	a	a	a	a	a	a	a
t	5044585	t	5060850	c	5011112	s	S88	rec40	t	S88	+++	t	t	t	t	c	c	c	c	c	c	c	c	c	c	c	c	c	c	c	c	c	c	c
t	5044615	t	5060880	c	5011142	s	S88	rec40	t	S88	+++	t	t	t	t	c	c	c	c	c	c	c	c	c	c	c	c	c	c	c	c	c	c	c
t	5044639	t	5060904	c	5011166	s	S88	rec40	c	2	-	c	t	c	c	c	c	c	c	c	c	c	c	c	c	c	c	c	c	c	c	c	c	c
t	5044726	t	5060991	c	5011253	s	S88	rec40	t	S88	+++	t	t	t	t	c	c	c	c	c	c	c	c	c	c	c	c	c	t	t	t	c	c	c
c	5044762	c	5061027	t	5011289	s	S88	rec40	c	S88	+++	c	c	c	c	g	t	t	t	t	t	t	t	t	t	t	t	t	t	t	t	t	t	t
t	5044813	t	5061078	c	5011340	s	S88	rec40	t	S88	+++	t	t	t	t	c	c	c	c	c	c	c	c	c	c	c	c	c	c	c	c	c	c	c
t	5044843	t	5061108	g	5011370	s	S88	rec40	t	S88	++	t	t	t	g	a	a	a	g	a	g	g	g	g	g	g	g	g	t	t	t	g	g	g
t	5044858	t	5061123	a	5011385	s	S88	rec40	t	S88	++++	t	t	t	t	t	t	t	a	t	a	a	a	a	a	a	a	a	a	a	a	a	a	a
g	5044990	g	5061255	a	5011517	s	S88	rec40	g	S88	++	g	g	g	g	g	g	a	a	g	g	g	a	a	a	g	g	g	a	a	a	g	g	g
c	5044996	c	5061261	t	5011523	s	S88	rec40	c	S88	++++	c	c	c	c	t	c	t	t	c	c	c	t	t	t	c	c	c	t	t	t	c	c	c
g	5044999	g	5061264	a	5011526	s	S88	rec40	g	S88	++	g	g	g	g	a	g	a	a	g	g	g	a	a	a	g	g	g	a	a	a	g	g	g
a	5045002	a	5061267	t	5011529	s	S88	rec40	a	S88	+++	a	a	a	a	t	t	t	t	t	t	t	t	t	t	t	t	t	t	t	t	t	t	t
c	5045014	c	5061279	t	5011541	s	S88	rec40	c	S88	++	c	c	c	c	c	c	c	t	c	c	c	c	c	c	c	c	c	c	c	c	c	c	c
g	5045015	g	5061280	a	5011542	ns	S88	rec40	g	S88	++	g	g	g	g	a	g	a	a	g	a	g	a	a	a	a	a	a	g	g	g	a	a	a
t	5045026	t	5061291	c	5011553	s	S88	rec40	c	2	-	c	t	c	c	c	c	c	c	c	c	c	c	c	c	c	c	c	c	c	c	c	c	c
t	5045029	t	5061294	g	5011556	s	S88	rec40	t	S88	++	t	t	t	t	t	t	t	g	t	t	t	t	t	t	t	t	t	g	g	g	t	t	t
c	5045104	c	5061369	t	5011631	s	S88	rec40	c	S88	+++	c	c	c	c	c	c	c	t	c	c	c	t	t	t	c	c	c	c	c	c	c	c	c
g	5045185	g	5061450	a	5011712	s	S88	rec40	g	S88	++	g	g	g	g	g	g	g	a	g	g	g	g	g	g	g	g	g	g	g	g	g	g	g
c	5045236	c	5061501	t	5011763	s	S88	rec40	c	S88	++	c	c	c	c	c	c	c	t	c	c	c	c	c	c	c	c	c	c	c	c	c	c	c
g	5045263	g	5061528	a	5011790	s	S88	rec40	g	S88	++	g	g	g	g	a	a	a	a	a	a	a	a	a	a	a	a	a	a	a	a	a	a	a
c	5046500	c	5062765	t	5013027	s	S88	rec40	c	S88	++	c	c						t															
a	5046509	a	5062774	g	5013036	s	S88	rec40	a	S88	++	a	a						g															
a	5046527	a	5062792	g	5013054	s	S88	rec40	a	S88	++	a	a						g															
c	5046568	c	5062833	t	5013095	ns	S88	rec40	c	S88	++	c	c						t															
c	5046587	c	5062852	t	5013114	s	S88	rec40	c	S88	++	c	c						t															
t	5046594	t	5062859	c	5013121	s	S88	rec40	t	S88	++	t	t						c															
g	5046619	g	5062884	a	5013146	ns	S88	rec40	g	S88	++	g	g						a															
t	5046644	t	5062909	c	5013171	s	S88	rec40	t	S88	++	t	t						c															
a	5046647	a	5062912	t	5013174	s	S88	rec40	a	S88	++	a	a						t															
g	5046696	g	5062961	a	5013223	ns	S88	rec40	g	S88	++	g	g						a															
a	5046719	a	5062984	g	5013246	s	S88	rec40	a	S88	++	a	a						g															
a	5046734	a	5062999	g	5013261	s	S88	rec40	a	S88	++	a	a						g															
g	5046769	g	5063034	a	5013296	ns	S88	rec40	g	S88	++	g	g						a															
t	5046798	t	5063063	c	5013325	s	S88	rec40	t	S88	++	t	t					c	c			c							c	c	c	c	c	c
g	5046800	g	5063065	t	5013327	s	S88	rec40	g	S88	++	g	g					g	t			t							t	t	t	t	t	t
c	5046801	c	5063066	t	5013328	s	S88	rec40	c	S88	++	c	c					t	t			t							t	t	t	t	t	t
c	5046804	c	5063069	a	5013331	s	S88	rec40	c	S88	++	c	c	c	c	c	c	c	a	c	c	c	c	c	c	c	c	c	c	c	c	c	c	c
t	5046813	t	5063078	c	5013340	ns	S88	rec40	t	S88	++	t	t	c	t	t	c	t	c	t	t	t	t	t	t	t	t	t	t	t	t	t	t	t
c	5046825	c	5063090	t	5013352	s	S88	rec40	c	S88	++	c	c	c	c	c	c	t	t	c	c	c	c	c	c	c	c	c	c	c	c	c	c	c
g	5046866	g	5063131	a	5013393	s	S88	rec40	g	S88	++	g	g	g	a	a	g	a	a	a	a	a	a	a	a	a	a	a	a	a	a	a	a	a
c	5046897	c	5063162	t	5013424	s	S88	rec40	c	S88	++	c	c	c	c	c	c	a	t	c	c	c	c	c	c	c	c	c	c	c	c	c	c	c
a	5046909	a	5063174	g	5013436	s	S88	rec40	a	S88	++	a	a	a	g	g	a	g	g	g	g	g	g	g	g	g	g	g	g	g	g	g	g	g

ExPEC genome site details												Outgroup Strains Details[i]																						
UTI89[b]	UTI89 site[c]	APEC 01[b]	APEC 01 site[c]	S88[b]	S88 site[c]	type[d]	Event Lineage[ef]	Recombinant segment	Inferred ancestral base[g]	Outgroup Analysis[e]	Support level[h]	CFT073	ED1a	536	E2348/69	SMS 3-5	IAI39	UMN026	K-12	ATCC 8739	HS	D1 Sd197	CB9615	Sakai	EDL933	IAI1	E24377A	SE11	SS Ss046	F2a 301	F2a 2457T	F5 8401	B4 Sb227	B18 BS512
c	5046924	c	5063189	t	5013451	s	S88	rec40	c	S88	++	c	c	c	c	c	c	t	t	c	c	c	c	c	c	c	c	c	c	c	c	c	c	c
a	5046957	a	5063222	g	5013484	s	S88	rec40	a	S88	++	g	a	a	g	g	a	g	g	g	g	a	g	g	g	g	g	g	g	g	g	g	g	g
c	5047142	c	5063407	t	5013669	ns	S88	rec40	c	S88	++	c	c	c	c	c	c	c	t	c	c	c	c	c	c	c	c	c	c	c	c	c	c	c
t	5047236	t	5063501	c	5013763	s	S88	rec40	t	S88	++	c	t	t	c	c	c	c	c	c	c	c	c	c	c	c	c	c	c	c	c	c	c	c
g	5047238	g	5063503	a	5013765	s	S88	rec40	g	S88	++	g	g	g	g	g	g	g	a	g	g	g	g	g	g	g	g	g	g	g	g	g	g	g
a	5047241	a	5063506	g	5013768	s	S88	rec40	a	S88	++	a	a	a	a	g	a	a	g	g	g	g	g	g	g	g	g	g	g	g	g	g	g	g
t	5047242	t	5063507	c	5013769	s	S88	rec40	t	S88	+++	t	t	t	t	c	t	t	c	c	c	c	c	c	c	c	c	c	c	c	c	c	c	c
c	5047248	c	5063513	g	5013775	s	S88	rec40	c	S88	++	g	c	c	c	g	c	c	g	g	g	g	g	g	g	g	g	g	g	g	g	g	g	g
a	5047251	a	5063516	g	5013778	s	S88	rec40	a	S88	++	g	a	a	a	g	a	a	g	g	g	g	g	g	g	g	g	g	g	g	g	g	g	g
c	5047269	c	5063534	t	5013796	s	S88	rec40	c	S88	+++	t	c	c	t	t	t	t	t	c	c	c	c	c	c	c	c	c	c	c	c	c	c	c
t	5047272	t	5063537	c	5013799	s	S88	rec40	t	S88	+++	c	t	t	c	c	c	c	c	t	t	t	t	t	t	t	t	t	t	t	t	t	t	t
g	5047275	g	5063540	c	5013802	s	S88	rec40	g	S88	++	g	g	g	g	c	g	c	c	g	g	g	a	a	a	g	g	g	a	a	a	g	g	g

[a] color in the table: white: the snps in unallocated blocks; green: the snps that supported the allocation of block; red: the snps that conflicted with the allocation of block; yellow: the unallocated snps or the snps that implies 2 mutations at that site - eg in the ancestor of O55 and O157 strains before isolation and again in one of the lineages in the allocated blocks.

[b] Numbers in place of bases indicates number of bases where >2 bases inserted or deleted. In these cases "-" indicates absence of these bases.

[c] For indels the base indicated is the base before the insertion or deletion in the strain.

[d] s: synonymous; ns: non-synonymous; nc: in non-coding gene; i: intergenic; ins: small insert; del: small deletion; indel: the small indels that can't be allocated

[e] AS: allocated to the lineage to the common ancestor of APEC 01 and S88; UTI89/AS: allocated to the divergence between UTI89 and the common ancestor of APEC 01 and S88 (strain not specified); APEC/S88: allocated to the divergence between APEC 01 and S88 lineages (lineage not specified).

[f] The recombinational block was allocated if more than 80% of the snps in it can be allocated into the same lineage.

[g] The base in the common ancestor of the 3 ExPEC strains as inferred from outgroup analysis.

[h] Level of support for allocation of mutation as given in previous column

++++ agreement is high - 8 or more outgroup strains with expected base and at most 1 with an alternative base, and at least 2 of the CFT073, ED1a and 536 support the expected base)

+++ agreement good - 4 or more outgroup strains with expected base and at most 1 with an alternative base, and at least 2 of the CFT073, ED1a and 536 support the expected base)

++ supported by at least 2 of the CFT073, ED1a and 536 regardless of situation with other outgroup strains

+ no conflict but very limited support as either site absent or conflict in all of CFT073, ED1a and 536, or conflict among them, and/or support is less than required for any of the higher levels of support.

+/- no conflict but no support (base not present in any outgroup OR base when present is not that in any of the ExEPEC strains OR both alternative lineages supported equallly.

- conflict data implies 2 mutations at that site - eg in the ancestor of 3 ExEPEC strains before isolation and again in one of the lineages.

[i] Base, number or "-" indicates the base type or absence of the base. Blank means the site not present.
