# Supplementary material for: Derivation of Escherichia coli O157:H7 from Its O55:H7 Precursor
Source: PLoS One. 2010 Jan 14;5(1):e8700. doi: 10.1371/journal.pone.0008700 (PMC2806823; doi:10.1371/journal.pone.0008700)
Supplement: Table S9 — Large Indels. Large indels in affecting one or two of the genomes are shown with the length (bp), if thought to be an insertion or deletion, the strain(s) affected and the gene or genes affected. (0.02 MB PDF) [file pone.0008700.s011.pdf]

Indel[a]	Lineage[b]	Type[c]	Site[d]	Length	Name or range
1	CB9615	del	248790	28	putative macrophage toxin
2	CB9615	ins	269801	2565	rhsG
3	CB9615	del	273929	36	hypothetical protein
4	CB9615	ins	288595	88	intergenic
5	O157	ins	300076	4904	CP-933H & Sp1 part 1
6	Sakai	ins	304980	1830	inversion between Sp1 and CP-933H
7	EDL933	ins	304981	1830	inversion between Sp1 and CP-933H
8	O157	ins	306810	3788	CP-933H & Sp1 part 3
9	CB9615	ins	302638	48949	Ep1
10	O157	ins	345357	2450	ISEc8
11	O157	del	368438	1037	autotransporter
12	O157	ins	411141	188	part of O-island #18
13	O157	del	433522	303	intergenic
14	O157	del	589397	300	hypothetical protein
15	Sakai	ins	596503	309	hypothetical protein
16	O157	del	598209	318	hypothetical protein
17	CB9615	ins	697268	49415	Ep3
18	EDL933	ins	670387	87	macrophage toxin
19	CB9615	ins	865971	35	hypothetical protein
20	EDL933	ins	810985	1871	rhsC
21	CB9615	del	935055	60	putative symport protein
22	CB9615	ins	950886	81	tolA
23	O157	del	865671	109	tRNA
24	CB9615	ins	985624	7432	Ep4 part
25	CB9615	del	1019441	27	lomK
26	O157	del	922439	27	Ail/Lom family protein
27	EDL933	ins	1058635	87563	SpLE1 copy
28	O157	ins	1161156	15541	CP-933M & Sp4 part 1
29	Sakai	ins	1176695	32577	Sp4 part 2
30	EDL933	ins	1265920	28169	CP-933M part 2
31	O157	ins	1209272	1534	CP-933M & Sp4 part 3
32	CB9615	ins	1290089	45	yccE
33	O157	ins	1246025	8400	BP-933W & Sp5 part 1
34	Sakai	ins	1254424	10013	Sp5 part 2
35	EDL933	ins	1339242	10520	BP-933W part 2
36	O157	ins	1264437	5926	BP-933W & Sp5 part 3
37	Sakai	ins	1270363	1314	IS629 in Sp5
38	O157	ins	1271677	35340	BP-933W & Sp5 part 4
39	Sakai	ins	1307016	271	zinc finger protein in Sp 5
40	O157	ins	1307287	1299	BP-933W & Sp5 part 5
41	EDL933	ins	1392327	31	intergenic
42	O157	ins	1308586	147	BP-933W & Sp5 part 5
43	O157	del	1309217	66	intergenic
44	CB9615	del	1312390	81	intergenic

Indel[a]	Lineage[b]	Type[c]	Site[d]	Length	Name or range
45	O157	ins	1370470	84306	SpLE1
46	EDL933	ins	1538545	1313	IS629
47	O157	ins	1454773	1946	SpLE1
48	O157	ins	1541482	11589	CP-933N & Sp6 part 1
49	Sakai	ins	1553071	18327	Sp6 part 2
50	EDL933	ins	1638167	19370	CP-933N part 2
51	O157	ins	1571398	1907	CP-933N & Sp6 part 3
52	Sakai	ins	1573305	2756	Sp6 part 4
53	EDL933	ins	1659442	594	CP-933N part 4
54	O157	ins	1576061	13846	CP-933N & Sp6 part 5
55	O157	ins	1597058	12576	CP-933C & Sp7
56	CB9615	ins	1447061	8328	Ep5
57	O157	ins	1618152	23624	CP-933X & Sp8 part 1
58	Sakai	ins	1641773	7321	Sp8 part 2
59	EDL933	ins	1725749	15130	CP-933X part 2
60	O157	ins	1649094	13861	CP-933X & Sp8 part 3
61	Sakai	ins	1662945	389	hypothetical protein in Sp8
62	O157	ins	1663334	1306	CP-933X & Sp8 part 4
63	CB9615	ins	1463904	14477	elbA-ypjA
64	O157	ins	1678776	33	intergenic
65	O157	del	1681748	1868	ipaH
66	O157	ins	1685462	1313	IS629
67	O157	del	1700128	3154	dhaR-ycgV
68	EDL933	ins	1806889	529	hypothetical protein
69	CB9615	del	1549532	178	hypothetical protein
70	CB9615	del	1577103	21	exoO
71	O157	del	1762254	85	intergenic
72	O157	ins	1771044	3644	CP-933O-1 and Sp9 part 1
73	Sakai	ins	1774688	22068	Sp9 part 2,3
74	EDL933	ins	1866628	5717	CP-933O-1 part 2
75	CB9615	ins	1588344	39169	Ep6 part
76	O157	ins	1796756	18845	CP-933P & Sp9 part 4
77	CB9615	ins	1627513	169	yciD
78	O157	ins	1909167	1313	IS629
79	O157	ins	1925340	18516	CP-933R and Sp10 part 1
80	Sakai	ins	1943853	24157	Sp10 part 2
81	EDL933	ins	2143310	22839	CP-933R part 2
82	O157	ins	1968010	1399	CP-933R and Sp10 part 3
83	CB9615	ins	1736116	37474	Ep7 part
84	CB9615	ins	1776450	348	intergenic
85	O157	del	1974047	20	ompN
86	O55/O157	indel	1984517	33	hypothetical protein
87	O55/O157	indel	1984550	57	hypothetical protein
88	CB9615	del	1851898	37	rhsE
89	O157	del	2047473	37	rhsE
90	CB9615	del	1877926	178	intergenic

Indel[a]	Lineage[b]	Type[c]	Site[d]	Length	Name or range
91	O157	del	2116465	619	hipAB
92	O157	ins	2158572	2864	CP-933O-2 & Sp11 part 4
93	Sakai	ins	2161436	27173	Sp11 part 3
94	EDL933	ins	1927034	32923	CP-933O-2 part 3
95	O157	ins	2188609	15631	CP-933O-2 and Sp11 part 2
96	Sakai	ins	2204236	1313	IS629 in Sp11
97	O157	ins	2205549	114	CP-933O-2 and Sp11 part 1
98	O157	ins	2206518	2456	IS in CP-933O-1 & Sp12
99	Sakai	ins	2211676	7589	Sp12 part 3
100	EDL933	ins	2290422	4126	CP-933P part 3
101	CB9615	ins	1967051	21468	Ep8
102	O157	ins	2219265	14664	CP-933P & Sp12 part 2
103	O157	ins	2237104	17757	CP-933P & Sp12 part 1
104	CB9615	ins	1991694	18166	Ep9
105	EDL933	del	2383841	96	rsxC
106	CB9615	ins	2152246	35751	Ep10
107	O157	ins	2454184	1313	IS629
108	CB9615	del	2252700	778	putative transport protein
109	O157	ins	2593022	21120	CP-933T & Sp13
110	O157	ins	2641063	62	fliI
111	Sakai	ins	2670403	24417	Sp14 part 2
112	EDL933	ins	2745620	25564	CP-933U part 2
113	O157	ins	2694820	17138	CP-933U & Sp14 part 1
114	CB9615	ins	2440191	46474	Ep11
115	O157	ins	2735504	2450	ISEc8
116	O157	ins	2741819	1314	IS629
117	Sakai	ins	2743133	86	hypothetical protein
118	O157	del	2743218	8805	EpLE1
119	EDL933	del	2819497	6068	yafZX-yeeS
120	O157	ins	2761713	210	hypothetical protein
121	O157	del	2761922	653	yoeB-yefM
122	O157	ins	2771874	55	gnd
123	CB9615	ins	2551984	3919	O-antigen
124	O157	ins	2773347	2139	O-antigen
125	CB9615	ins	2557321	4812	O-antigen
126	O157	ins	2777741	60	manC
127	O157	del	2777800	60	manC
128	O157	ins	2778783	985	O-antigen
129	CB9615	ins	2565428	163	gmd
130	O157	ins	2780874	6716	O-antigen
131	CB9615	ins	2566697	1481	wbgM
132	O157	ins	2793816	123	intergenic
133	O55/O157	indel	2796854	35	intergenic
134	O55/O157	indel	2577332	116	intergenic
135	O157	del	2798333	20	intergenic
136	O157	ins	2798334	111	intergenic

Indel[a]	Lineage[b]	Type[c]	Site[d]	Length	Name or range
137	O157	ins	2828986	330	putative membrane protein
138	O157	del	2841001	760	Plasmid stabilization system protein
139	O157	ins	2844410	88	hypothetical protein
140	CB9615	ins	2625293	9728	ribitol gene cluster
141	O157	ins	2863193	971	yehA
142	O157	del	2864163	976	yehA
143	CB9615	ins	2667743	47	molR_C
144	O157	ins	2883087	609	hypothetical protein
145	O157	ins	2890776	1313	IS629
146	O157	ins	2895926	2848	CP-933V & Sp15 part 3
147	Sakai	ins	2898773	20176	Sp15 part 2
148	EDL933	ins	2969005	21207	CP-933V part 2
149	O157	ins	2918949	24861	CP-933V & Sp15 part 1
150	CB9615	ins	2797724	1450	IS-yfaS
151	Sakai	ins	3088840	1313	IS629
152	CB9615	ins	2897983	182	intergenic
153	EDL933	del	3314859	115	ala-tRNA
154	CB9615	ins	3161159	41483	Ep13
155	O157	ins	3479106	1101	hypothetical protein
156	Sakai	ins	3480207	4042	Sp17 part 1
157	CB9615	ins	3261633	30449	Ep14 part 1
158	EDL933	ins	3550056	1515	IS1203
159	O157	ins	3486116	5157	CP-933Y and Sp17 part 2
160	CB9615	ins	3293949	35074	Ep14 part 2
161	Sakai/EDL933	indel	3559164	24	hypothetical protein
162	Sakai/EDL933	indel	3491860	24	hypothetical protein
163	O157	del	3557253	204	norV
164	CB9615	del	3436264	94	intergenic
165	CB9615	ins	3436265	94	intergenic
166	CB9615	ins	3462172	122	intergenic
167	O157	ins	3715004	14073	type III secretion apparatus
168	O157	ins	3839916	91	intergenic
169	O157	ins	3869332	3420	SpLE3-IS629
170	O157	del	3872751	8430	EpLE2-lifA
171	O157	ins	3875152	340	SpLE3-IS3
172	O157	del	3875491	61164	T2SS and glc operon
173	EDL933	del	4038200	100	intergenic
174	CB9615	del	3899115	113	rnpB
175	O157	ins	4033206	1311	IS629
176	O157	del	4034516	353	yraK
177	O157	del	4103490	78	yhcG
178	O157	ins	4151853	53	acrF
179	CB9615	del	4121921	109	yrfAB
180	O157	ins	4293021	29	DNA helicase
181	CB9615	ins	4397185	1093	rhsA

Indel[a]	Lineage[b]	Type[c]	Site[d]	Length	Name or range
182	EDL933	ins	4575829	1492	rhsA
183	Sakai	del	4508599	261	yibJ
184	O157	ins	4585988	2450	SpLE4-ISEc8
185	O157	del	4589334	31	espF
186	O55/O157	indel	4700552	21	lpfD
187	CB9615	del	4622584	39	intergenic
188	O157	del	4735105	42	intergenic
189	Sakai	ins	4776204	225	tRNA
190	CB9615	del	4718739	46	intergenic
191	CB9615	ins	4718740	43	intergenic
192	CB9615	ins	4781953	368	intergenic
193	Sakai	ins	5040843	38759	Sp18
194	O157	ins	5180729	100	intergenic
195	CB9615	del	5077101	356	yjeJ
196	CB9615	ins	5180103	90	yjgL
197	O157	ins	5347085	10236	SpLE5
198	O157	ins	5424452	48	hypothetical protein
199	CB9615	ins	5263174	54	hypothetical protein
200	CB9615	del	5271158	25	hypothetical protein
201	O157	del	5464526	236	intergenic
202	CB9615	ins	5306033	49392	Ep15

[a] The extent of the indel events is shown in table S2

[b] O55/O157: Indel allocated to the O55/O157 divergence; Sakai/EDL933: Indel allocated to the divergence between Sakai and EDL933

[c] ins, insertion; del, deletion; indel: deletion or insertion

[d] For indels the base indicated is the base before the insertion or deletion.
