# Supplementary material for: Derivation of Escherichia coli O157:H7 from Its O55:H7 Precursor
Source: PLoS One. 2010 Jan 14;5(1):e8700. doi: 10.1371/journal.pone.0008700 (PMC2806823; doi:10.1371/journal.pone.0008700)
Supplement: Table S10 — The orthologs in the E. coli CB9651, Sakai, and EDL933 genomes. Genes present in two or three of the genomes are listed with the gene tag numbers, gene name, and product. For those absent in one or two of the genomes due to one of the indel events, the indel number from Table S3 is shown in place of the locus tag. (0.49 MB PDF) [file pone.0008700.s012.pdf]

Locus_tag[a]				
CB9615	Sakai	EDL933	Gene	Product
G2583_0001	ECs0001	Z0001	thrL	thr operon leader peptide
G2583_0002	ECs0002	Z0002	thrA	Aspartokinase I, homoserine dehydrogenase I
G2583_0003	ECs0003	Z0003	thrB	Homoserine kinase
G2583_0004	ECs0004	Z0004	thrC	Threonine synthase
G2583_0005	ECs0005	Z0005	yaaX	conserved hypothetical protein
G2583_0006	ECs0006	Z0006	yaaA	UPF0246 protein yaaA
G2583_0007	ECs0007	Z0007	yaaJ	inner membrane transport protein
G2583_0008	ECs0008	Z0008	talB	Transaldolase 1
G2583_0009	ECs0009	Z0009	mog	Molybdopterin biosynthesis mog protein
G2583_0010	ECs0010	Z0010	yaaH	membrane protein, GPR1/FUN34/yaaH family
G2583_0011	ECs0012	Z0011	yaaW	hypothetical protein
G2583_0012	ECs0011	Z0012	htgA	Positive regulator for sigma 32 heat shock promoters
G2583_0013	ECs0013	Z0013	yaaI	UPF0412 protein yaaI precursor
G2583_0014	ECs0014	Z0014	dnaK	Chaperone protein dnaK
G2583_0015	ECs0015	Z0015	dnaJ	Chaperone protein DnaJ
G2583_0016	ECs0016	Z0016	mokC	Gef protein
G2583_0017	ECs0017	Z0018	nhaA	Sodium/proton antiporter nhaA
G2583_0018	ECs0018	Z0019	nhaR	DNA-binding transcriptional activator
G2583_0019	ECs0019	Z0020	-	hypothetical protein
G2583_0019	ECs0020	Z0020	-	hypothetical protein
G2583_0019	ECs0021	Z0021	-	hypothetical protein
G2583_0020	ECs0022	Z0022	yehB	Putative usher protein
G2583_0021	ECs0023	Z0023	yehC	Gram-negative pilus assembly chaperone
G2583_0022	ECs0024	Z0024	stcA	Putative type-1 fimbrial protein
G2583_0023	ECs0025	Z0025	-	hypothetical protein
G2583_0024	ECs0026	Z0027	rpsT	30S ribosomal protein S20
G2583_0025	ECs0027	Z0028	yaaY	hypothetical protein
G2583_0026	ECs0028	Z0029	ribF	Riboflavin biosynthesis protein ribF
G2583_0027	ECs0029	Z0030	ileS	Isoleucyl-tRNA synthetase
G2583_0028	ECs0030	Z0031	lspA	Lipoprotein signal peptidase
G2583_0029	ECs0031	Z0033	fkpB	FKBP-type 16 kDa peptidyl-prolyl cis-trans isomerase
G2583_0030	ECs0032	Z0034	ispH	4-hydroxy-3-methylbut-2-enyl diphosphate reductase
G2583_0031	ECs0033	Z0035	rihC	Non-specific ribonucleoside hydrolase rihC
G2583_0032	ECs0034	Z0036	dapB	Dihydrodipicolinate reductase
G2583_0033	ECs0035	Z0037	carA	Carbamoylphosphate synthase small subunit
G2583_0034	ECs0036	Z0038	carB	Carbamoyl-phosphate synthase large chain
G2583_0035		Z0039	-	hypothetical protein
G2583_0036	ECs0037	Z0040	caiF	DNA-binding transcriptional activator CaiF
G2583_0037	ECs0038	Z0041	caiE	Carnitine operon protein caiE
G2583_0038	ECs0039	Z0042	caiD	Crotonobetainyl CoA hydratase
G2583_0039	ECs0040	Z0043	caiC	Putative crotonobetaine/carnitine-CoA ligase
G2583_0040	ECs0041	Z0044	caiB	Crotonobetainyl-CoA:carnitine CoA-transferase
G2583_0041	ECs0042	Z0045	caiA	Crotonobetainyl-CoA dehydrogenase
G2583_0042	ECs0043	Z0046	caiT	L-carnitine/gamma-butyrobetaine antiporter

Locus_tag[a]				
CB9615	Sakai	EDL933	Gene	Product
G2583_0043	ECs0044	Z0047	fixA	conserved hypothetical protein
G2583_0044	ECs0045	Z0048	fixB	hypothetical protein
G2583_0045	ECs0046	Z0049	fixC	PAPS (adenosine 3'-phosphate 5'-phosphosulfate) 3'(2'),5'-bisphosphate nucleotidase
G2583_0046	ECs0047	Z0050	fixX	Ferredoxin-like protein fixX
G2583_0047	ECs0048	Z0051	yaaU	Major facilitator family transporter
G2583_0048	ECs0049	Z0052	kefF	Glutathione-regulated potassium-efflux system ancillary protein kefF
G2583_0049	ECs0050	Z0053	kefC	Glutathione-regulated potassium-efflux system protein kefC (K(+)/H(+) antiporter)
G2583_0050	ECs0051	Z0055	folA	Dihydrofolate reductase
G2583_0051	ECs0052	Z0056	-	putative antitoxin of gyrase inhibiting toxin-antitoxin system
G2583_0052	ECs0053	Z0057	-	CcdB protein
G2583_0053	ECs0054	Z0058	apaH	Bis(5'-nucleosyl)-tetraphosphatase, symmetrical
G2583_0054	ECs0055	Z0059	apaG	ApaG
G2583_0055	ECs0056	Z0060	ksgA	Dimethyladenosine transferase (S-adenosylmethionine-6-N', N'-adenosyl(rRNA) dimethyltransferase)
G2583_0056	ECs0057	Z0061	pdxA	4-hydroxythreonine-4-phosphate dehydrogenase
G2583_0057	ECs0058	Z0062	surA	Chaperone surA precursor
G2583_0058	ECs0059	Z0063	imp	LPS-assembly protein precursor
G2583_0059	ECs0060	Z0064	djlA	putative oxidoreductase Fe-S binding subunit
G2583_0060	ECs0061	Z0065	yabP	hypothetical protein
G2583_0061	ECs0062	Z0066	rluA	Ribosomal large subunit pseudouridine synthase A
G2583_0062	ECs0063	Z0067	hepA	RNA polymerase-associated protein rapA
G2583_0063	ECs0064	Z0068	polB	DNA polymerase II
G2583_0064	ECs0065	Z0069	araD	L-ribulose-5-phosphate 4-epimerase
G2583_0065	ECs0066	Z0070	araA	L-arabinose isomerase
G2583_0066	ECs0067	Z0072	araB	Ribulokinase
G2583_0067	ECs0068	Z0073	araC	arabinose operon regulatory protein
G2583_0068	ECs0069	Z0074	yabI	hypothetical protein
G2583_0069	ECs0070	Z0075	thiQ	Thiamine import ATP-binding protein thiQ
G2583_0070	ECs0071	Z0076	thiP	Thiamine/thiamine pyrophosphate ABC transporter, permease protein
G2583_0071	ECs0072	Z0077	tbpA	Thiamin/thiamin pyrophosphate ABC transporter, periplasmic thiamin/thiamin pyrophospate-binding
G2583_0072	ECs0073	Z0078	-	hypothetical protein
G2583_0073	ECs0074	Z0079	sgrR	putative transport protein
G2583_0074			-	ncRNA
G2583_0075	ECs0075	Z0080	leuD	3-isopropylmalate dehydratase small subunit
G2583_0076	ECs0076	Z0081	leuC	3-isopropylmalate dehydratase large subunit
G2583_0077	ECs0077	Z0082	leuB	3-isopropylmalate dehydrogenase
G2583_0078	ECs0078	Z0083	leuA	2-isopropylmalate synthase
G2583_0079	ECs0079	Z0084	leuL	leader; Amino acid biosynthesis: Leucine

Locus_tag[a]				
CB9615	Sakai	EDL933	Gene	Product
G2583_0080	ECs0080	Z0086	leuO	leucine transcriptional activator
G2583_0081	ECs0081	Z0087	ilvI	Acetolactate synthase
G2583_0082	ECs0082	Z0088	ilvH	Acetolactate synthase III, valine sensitive, small subunit
G2583_0083	ECs0083	Z0089	fruL	fruR leader peptide
G2583_0084	ECs0084	Z0090	fruR	Fructose repressor
G2583_0085	ECs0085	Z0091	mraZ	hypothetical protein
G2583_0086	ECs0086	Z0092	mraW	S-adenosyl-L-methionine-dependent methyltransferase mraW
G2583_0087	ECs0087	Z0093	ftsL	Cell division protein ftsL
G2583_0088	ECs0088	Z0094	ftsI	Peptidoglycan synthetase ftsI precursor
G2583_0089	ECs0089	Z0095	murE	UDP-N-acetylmuramoyl-L-alanyl-D-glutamate--2, 6-diaminopimelate ligase
G2583_0090	ECs0090	Z0096	murF	UDP-N-acetylmuramoyl-tripeptide--D-alanyl-D-alanine ligase
G2583_0091	ECs0091	Z0097	mraY	Phospho-N-acetylmuramoyl-pentapeptide-transferase
G2583_0092	ECs0092	Z0098	murD	UDP-N-acetylmuramoylalanine--D-glutamate ligase
G2583_0093	ECs0093	Z0099	ftsW	Cell division protein ftsW
G2583_0094	ECs0094	Z0100	murG	UDP-N-acetylglucosamine--N-acetylmuramyl-(pentapeptide) pyrophosphoryl-undecaprenol N-acetylglucosamine transferase
G2583_0095	ECs0095	Z0101	murC	UDP-N-acetylmuramate--L-alanine ligase
G2583_0096	ECs0096	Z0102	ddlB	D-alanine--D-alanine ligase B
G2583_0097	ECs0097	Z0103	ftsQ	Cell division protein FtsQ
G2583_0098	ECs0098	Z0104	ftsA	Cell division protein ftsA
G2583_0099	ECs0099	Z0105	ftsZ	Cell division protein ftsZ
G2583_0100	ECs0100	Z0106	lpxC	UDP-3-O-[3-hydroxymyristoyl] N-acetylglucosamine deacetylase
G2583_0101	ECs0101	Z0107	secM	Secretion monitor protein
G2583_0102	ECs0102	Z0108	secA	preprotein translocase subunit SecA
G2583_0103	ECs0103	Z0109	mutT	7,8-dihydro-8-oxoguanine-triphosphatase
G2583_0104	ECs0104	Z0110	-	hypothetical protein
G2583_0105	ECs0105	Z0111	yacG	Uncharacterized protein conserved in bacteria
G2583_0106	ECs0106	Z0112	yacF	UPF0289 protein yacF
G2583_0107	ECs0107	Z0113	coaE	Dephospho-CoA kinase
G2583_0108	ECs0108	Z0114	guaC	GMP reductase
G2583_0109	ECs0109	Z0115	-	hypothetical protein
G2583_0110	ECs0110	Z0116	hofC	Type IV pilus assembly protein PilC
G2583_0111	ECs0111	Z0117	hofB	GspE family protein HofB
G2583_0112	ECs0112	Z0118	ppdD	Prelipin peptidase dependent protein
G2583_0113	ECs0113	Z0119	nadC	Nicotinate-nucleotide pyrophosphorylase
G2583_0114	ECs0114	Z0120	ampD	N-acetylmuramoyl-L-alanine amidase
G2583_0115	ECs0115	Z0121	ampE	regulatory protein AmpE
G2583_0116	ECs0116	Z0122	aroP	Aromatic amino acid transport protein
G2583_0117	ECs0117	Z0123	pdhR	transcriptional regulator, GntR family

Locus_tag[a]				
CB9615	Sakai	EDL933	Gene	Product
G2583_0118	ECs0118	Z0124	aceE	Pyruvate dehydrogenase E1 component
G2583_0119	ECs0119	Z0125	aceF	Dihydrolipoyllysine-residue acetyltransferase
G2583_0120	ECs0120	Z0126	lpd	Pyruvate/2-oxoglutarate dehydrogenase complex, dihydrolipoamide dehydrogenase (E3) component, and related enzymes
G2583_0121	ECs0121	Z0127	yacH	hypothetical protein
G2583_0122	ECs0122	Z0128	acnB	Aconitate hydratase 2
G2583_0123	ECs0123	Z0129	yacL	hypothetical protein
G2583_0124	ECs0124	Z0130	speD	S-adenosylmethionine decarboxylase proenzyme
G2583_0125	ECs0125	Z0131	speE	Spermidine synthase
G2583_0126	ECs0126	Z0132	yacC	hypothetical protein
G2583_0127	ECs0127	Z0133	cueO	Copper oxidase CueO
G2583_0128	ECs0128	Z0134	gcd	Quinoprotein glucose dehydrogenase
G2583_0129	ECs0129	Z0136	hpt	Hypoxanthine phosphoribosyltransferase
G2583_0130	ECs0130	Z0137	can	Carbonic anhydrase
G2583_0131	ECs0131	Z0138	yadG	ABC transporter, ATP-binding protein
G2583_0132	ECs0132	Z0139	yadH	hypothetical protein
G2583_0133	ECs0133	Z0140	yadI	PTS system IIA component domain protein
G2583_0134	ECs0134	Z0141	yadE	Polysaccharide deacetylase domain protein
G2583_0135	ECs0135	Z0142	panD	Aspartate 1-decarboxylase precursor
G2583_0136	ECs0136	Z0143	yadD	hypothetical protein
G2583_0137	ECs0137	Z0144	panC	Pantothenate synthetase
G2583_0138	ECs0138	Z0145	panB	3-methyl-2-oxobutanoate hydroxymethyltransferase
G2583_0139	ECs0139	Z0146	yadC	Putative fimbrial protein
G2583_0140	ECs0140	Z0147	yadK	Putative fimbrial protein
G2583_0141	ECs0141	Z0148	yadL	Putative fimbrial protein
G2583_0142	ECs0142	Z0149	yadM	Putative fimbrial protein
G2583_0143	ECs0143	Z0150	htrE	Probable outer membrane porin protein involved in fimbrial assembly
G2583_0144	ECs0144	Z0151	ecpD	Gram-negative pili assembly chaperone
G2583_0145	ECs0145	Z0152	yadN	Putative fimbrial protein
G2583_0146	ECs0146	Z0153	folK	2-amino-4-hydroxy-6-hydroxymethyldihydropteridine pyrophosphokinase
G2583_0147	ECs0147	Z0154	pcnB	Poly(A) polymerase
G2583_0148	ECs0148	Z0155	yadB	Glutamyl-Q tRNA(Asp) synthetase
G2583_0149	ECs0149	Z0156	dksA	RNA polymerase-binding protein DksA
G2583_0150	ECs0150	Z0157	sfsA	Sugar fermentation stimulation protein A
G2583_0151	ECs0151	Z0158	ligT	hypothetical protein
G2583_0152	ECs0152	Z0159	hrpB	ATP-dependent helicase HrpB
G2583_0153	ECs0153	Z0160	mrcB	Penicillin-binding protein 1B
G2583_0154	ECs0154	Z0161	fhuA	Ferrichrome-iron receptor
G2583_0155	ECs0155	Z0162	fhuC	Ferrichrome transport ATP-binding protein FhuC
G2583_0156	ECs0156	Z0163	fhuD	Ferrichrome-binding periplasmic protein FhuD
G2583_0157	ECs0157	Z0164	fhuB	Ferrichrome ABC transporter, permease protein FhuB

Locus_tag[a]				
CB9615	Sakai	EDL933	Gene	Product
G2583_0158	ECs0158	Z0165	hemL	Glutamate-1-semialdehyde 2,1-aminomutase
G2583_0159	ECs0159	Z0166	clcA	H(+)/Cl(-) exchange transporter clcA
G2583_0160	ECs0160	Z0167	yadR	Iron-sulfur cluster insertion protein erpA
G2583_0161	ECs0161	Z0168	yadS	UPF0126 inner membrane protein yadS
G2583_0162	ECs0162	Z0169	btuF	Vitamin B12-binding protein precursor
G2583_0163	ECs0163	Z0170	mtn	MTA/SAH nucleosidase
G2583_0164	ECs0164	Z0171	dgt	Deoxyguanosinetriphosphate triphosphohydrolase
G2583_0165	ECs0165	Z0173	degP	Protease do precursor
G2583_0166	ECs0166	Z0174	cdaR	hypothetical protein
G2583_0167	ECs0167	Z0175	yaeH	UPF0325 protein yaeH
G2583_0168	ECs0168	Z0176	dapD	2,3,4,5-tetrahydropyridine-2,6-dicarboxylate N-succinyltransferase
G2583_0169	ECs0169	Z0177	glnD	[Protein-PII] uridylyltransferase
G2583_0170	ECs0170	Z0178	map	Methionine aminopeptidase
G2583_0171			-	ncRNA
G2583_0172	ECs0171	Z0180	rpsB	30S ribosomal protein S2
G2583_0173	ECs0172	Z0181	tsf	Elongation factor Ts
G2583_0174	ECs0173	Z0182	pyrH	UMP kinase
G2583_0175	ECs0174	Z0183	frr	Ribosome recycling factor
G2583_0176	ECs0175	Z0184	dxr	Putative ATP-binding component of a transport system
G2583_0177	ECs0176	Z0185	ispU	Undecaprenyl pyrophosphate synthetase
G2583_0178	ECs0177	Z0186	cdsA	Phosphatidate cytidylyltransferase
G2583_0179	ECs0178	Z0187	rseP	Regulator of sigma E protease
G2583_0180	ECs0179	Z0188	yaeT	Outer membrane protein assembly factor yaeT
G2583_0181	ECs0180	Z0190	skp	Chaperone protein skp precursor
G2583_0182	ECs0181	Z0191	lpxD	UDP-3-O-[3-hydroxymyristoyl] glucosamine N-acyltransferase
G2583_0183	ECs0182	Z0192	fabZ	(3R)-hydroxymyristoyl-(acyl carrier protein) dehydratase
G2583_0184	ECs0183	Z0193	lpxA	Acyl-[acyl-carrier-protein]--UDP-N-acetylglucosamine O-acyltransferase
G2583_0185	ECs0184	Z0194	lpxB	Lipid-A-disaccharide synthase
G2583_0186	ECs0185	Z0195	rnhB	Ribonuclease HII
G2583_0187	ECs0186	Z0196	dnaE	DNA polymerase III subunit alpha
G2583_0188	ECs0187	Z0197	accA	Acetyl-coenzyme A carboxylase carboxyl transferase subunit alpha
G2583_0189	ECs0188	Z0198	ldcC	Lysine decarboxylase, constitutive
G2583_0190	ECs0189	Z0199	yaeR	Putative lactoylglutathione lyase
G2583_0191	ECs0190	Z0200	tilS	tRNA(Ile)-lysidine synthase
G2583_0192	ECs0191	Z0201	rof	Rof protein
G2583_0193			yaeP	hypothetical protein
G2583_0194	ECs0192	Z0202	yaeQ	hypothetical protein
G2583_0195	ECs0193	Z0203	yaeJ	hypothetical protein
G2583_0196	ECs0194	Z0204	nlpE	Copper homeostasis protein CutF
G2583_0197	ECs0195	Z0205	yaeF	hypothetical protein

Locus_tag[a]				
CB9615	Sakai	EDL933	Gene	Product
G2583_0198	ECs0196	Z0206	proS	Prolyl-tRNA synthetase
G2583_0199	ECs0197	Z0207	yaeB	hypothetical protein
G2583_0200	ECs0198	Z0208	rcsF	Regulator in colanic acid synthesis
G2583_0201	ECs0199	Z0209	metQ	D-methionine-binding lipoprotein metQ precursor
G2583_0202	ECs0200	Z0210	metI	D-methionine transport system permease protein metI
G2583_0203	ECs0201	Z0211	metN	Methionine import ATP-binding protein metN
G2583_0204	ECs0202	Z0212	gmhB	D,D-heptose 1,7-bisphosphate phosphatase
G2583_0205	rrsH	RNA001	rrs	16S ribosomal RNA
G2583_0206	ileV	RNA002	ileV	Ile tRNA
G2583_0207	alaV	RNA003	alaV	Ala tRNA
G2583_0208	rrlH	RNA004	rrl	23S ribosomal RNA
G2583_0209	rrfH	RNA005	rrf	5S ribosomal RNA
G2583_0210	aspU	RNA006	aspU	Asp tRNA
G2583_0211	ECs0203	Z0229	dkgB	2,5-diketo-D-gluconic acid reductase B
G2583_0212	ECs0204	Z0230	yafC	putative transcriptional regulator LYSR-type
G2583_0213	ECs0205	Z0232	yafD	hypothetical protein
G2583_0214	ECs0206	Z0233	yafE	Methyltransferase, UbiE/COQ5 family
G2583_0215	ECs0207	Z0235	mltD	Membrane-bound lytic murein transglycosylase D precursor
G2583_0216	ECs0208	Z0236	gloB	Hydroxyacylglutathione hydrolase
G2583_0217	ECs0209	Z0237	yafS	hypothetical protein
G2583_0218	ECs0210	Z0239	rnhA	RNase HI
G2583_0219	ECs0211	Z0241	dnaQ	DNA polymerase III, epsilon subunit
G2583_0220	aspV	RNA007	aspV	Asp tRNA
G2583_0221		Z0243	yafT	hypothetical protein
G2583_0222	ECs0212	Z0244	-	hypothetical protein
G2583_0223	ECs0213	Z0245	-	hypothetical protein
G2583_0224	ECs0214	Z0246	-	hypothetical protein
G2583_0224	ECs0215	Z0247	-	hypothetical protein
G2583_0225	ECs0216	Z0248	yhhZ	hypothetical protein
G2583_0226	ECs0217	Z0249	ImpA	ImpA domain protein
G2583_0227	ECs0218	Z0250	-	putative macrophage toxin
	ECs0219		-	hypothetical protein
G2583_0228	ECs0220	Z0251	ImpA	ImpA domain protein
G2583_0228	ECs0221	Z0252	ImpA	ImpA domain protein
G2583_0229	ECs0222	Z0253	-	Type VI secretion-associated protein, VC_A0118 family
G2583_0230	ECs0223	Z0254	clpB	type VI secretion ATPase, ClpV1 family
G2583_0231	ECs0224	Z0255	-	hypothetical protein
G2583_0232	ECs0225	Z0256	-	Uncharacterized protein conserved in bacteria
G2583_0233	ECs0226	Z0257	-	Type VI secretion lipoprotein, VC_A0113 family
G2583_0234	ECs0227	Z0258	-	hypothetical protein
G2583_0235	ECs0228	Z0259	-	hypothetical protein
G2583_0236	ECs0229	Z0260	-	hypothetical protein
G2583_0237	ECs0230	Z0261	-	hypothetical protein

Locus_tag[a]				
CB9615	Sakai	EDL933	Gene	Product
G2583_0238	ECs0231	Z0262	-	hypothetical protein
G2583_0239	ECs0232	Z0263	-	hypothetical protein
G2583_0240	ECs0233	Z0264	-	hypothetical protein
G2583_0241		Z0265	-	hypothetical protein
G2583_0242	ECs0234	Z0266	-	Hcp
G2583_0243	ECs0235		-	hypothetical protein
G2583_0244	ECs0236	Z0267	VgrG	VgrG
G2583_0245	ECs0237	Z0268	rhsG1	rhsG-1
G2583_0246	Indel-2	Indel-2	-	Ankyrin repeat protein
G2583_0247	ECs0237	Z0268	rhsG2	RhsG-2
G2583_0248	ECs0238	Z0269	yibG	hypothetical protein
G2583_0249	ECs0239		-	hypothetical protein
G2583_0250	ECs0240		-	Hypothetical membrane protein
G2583_0251	ECs0241	Z0271	yhhI	ISEc3, transposase
G2583_0252	ECs0242	Z0272	-	unknown protein associated with Rhs element
G2583_0253	ECs0243		ydcD	hypothetical protein
		Z0273	-	hypothetical protein
G2583_0253	ECs0244	Z0274	ydcD	hypothetical protein
G2583_0254	ECs0245	Z0275	yncI	Putative transposase yncI
G2583_0255	ECs0246	Z0276	yafV	Hydrolase, carbon-nitrogen family
G2583_0256	ECs0247	Z0277	ivy	Inhibitor of vertebrate lysozyme precursor
G2583_0257	ECs0248	Z0278	fadE	Acyl-coenzyme A dehydrogenase
G2583_0258	ECs0249	Z0280	lpcA	Phosphoheptose isomerase
G2583_0259	ECs0250	Z0281	yafJ	Glutamine amidotransferase, class II
G2583_0260	ECs0251	Z0282	yafK	hypothetical protein
G2583_0261	ECs0252	Z0284	yafQ	Addiction module toxin, RelE/StbE family
G2583_0262	ECs0253	Z0285	dinJ	Addiction module antitoxin, RelB/DinJ family
G2583_0263	ECs0254	Z0287	yafL	NlpC/P60 family protein
G2583_0264	ECs0255	Z0288	yafM	Putative transposase
G2583_0265	ECs0257	Z0290	FhiA	Type III secretion protein, FHIPEP family
G2583_0266	ECs0256	Z0291	mbhA	Putative motility protein
G2583_0267	ECs0258	Z0292	dinB	DNA polymerase IV
G2583_0268	ECs0259	Z0293	yafN	Prevent-host-death family protein
G2583_0269	ECs0260	Z0294	yafO	putative toxin YafO
G2583_0270	ECs0261	Z0295	yafP	Acetyltransferase, GNAT family
G2583_0271	ECs0262	Z0296	ykfJ	hypothetical protein
G2583_0272	ECs0263	Z0297	prfH	Probable peptide chain release factor
G2583_0273	ECs0264	Z0298	pepD	Aminoacyl-histidine dipeptidase
G2583_0274	ECs0265	Z0299	gpt	Xanthine phosphoribosyltransferase
G2583_0275	ECs0266	Z0300	frsA	Esterase frsA
G2583_0276	ECs0267	Z0301	crl	Sigma factor-binding protein crl
G2583_0277	ECs0268	Z0302	phoE	Outer membrane protein (porin)
G2583_0278	ECs0269	Z0303	proB	Glutamate 5-kinase
G2583_0279	ECs0270	Z0304	proA	Gamma-glutamyl phosphate reductase

Locus_tag[a]				
CB9615	Sakai	EDL933	Gene	Product
G2583_0280	thrW	RNA008	thrW	Thr tRNA
Indel-5	ECs0271	Z0307	intH	putative integrase
Indel-5	ECs0272		-	putative transcription antitermination protein
Indel-5	ECs0273	Z0308	-	hypothetical protein
Indel-5	ECs0274	Z0309	-	repressor protein CI
Indel-5	ECs0275		-	Cro repressor
Indel-5	ECs0276	Z0310	-	regulatory protein cII
Indel-5	ECs0277	Z0311	-	replication protein
Indel-5	ECs0278	Z0312	-	replication protein
Indel-5	ECs0279	Z0313	-	replication protein
Indel-5	ECs0280	Z0314	-	putative tail fiber protein
Indel-6	ECs0281	Indel-6	-	hypothetical protein
Indel-6	ECs0282	Indel-6	-	hypothetical protein
Indel-6	ECs0283	Indel-6	-	putative tail fiber protein
Indel-7	Indel-7	Z0315	-	unknown protein from prophage CP-933H
Indel-7	Indel-7	Z0316	-	unknown protein from prophage CP-933H
Indel-7	Indel-7	Z0317	-	putative tail fiber protein from prophage CP-933H
Indel-8	ECs0284	Z0318	pinH	DNA-invertase
Indel-8	ECs0285	Z0319	-	hypothetical protein
Indel-8	ECs0286		-	hypothetical protein
Indel-8	ECs0287	Z0321	-	putative transcription regulator
Indel-8	ECs0288	Z0322	-	hypothetical protein
G2583_0281			-	Site-specific recombinase, phage integrase family
G2583_0282	Indel-9	Indel-9	-	hypothetical protein
G2583_0283	Indel-9	Indel-9	eaa	hypothetical protein
G2583_0284	Indel-9	Indel-9	-	ORF8
G2583_0285	Indel-9	Indel-9	-	hypothetical C4-type zinc finger protein TraR-family
G2583_0286	Indel-9	Indel-9	-	hypothetical protein
G2583_0287	Indel-9	Indel-9	-	unknown protein encoded by prophage CP-933K
G2583_0288	Indel-9	Indel-9	-	hypothetical protein
G2583_0289	Indel-9	Indel-9	-	Exonuclease
G2583_0290	Indel-9	Indel-9	bet	Bet protein
G2583_0291	Indel-9	Indel-9	gamW	Gam protein
G2583_0292	Indel-9	Indel-9	CIII	antitermination protein
G2583_0293	Indel-9	Indel-9	ral	Lambda ant-restriction protein
G2583_0294	Indel-9	Indel-9	-	Superinfection exclusion protein B
G2583_0295	Indel-9	Indel-9	-	Antitermination protein
G2583_0296	Indel-9	Indel-9	-	Gene 38 protein
G2583_0297	Indel-9	Indel-9	ymfK	SOS-response transcriptional repressors (RecA-mediated autopeptidases)
G2583_0298	Indel-9	Indel-9	cro	gene 40 protein
G2583_0299	Indel-9	Indel-9	-	Phage regulatory protein
G2583_0300	Indel-9	Indel-9	-	Putative replication protein O of bacteriophage
G2583_0301	Indel-9	Indel-9	-	putative replication protein P of bacteriophage BP-933W

Locus_tag[a]				
CB9615	Sakai	EDL933	Gene	Product
G2583_0302	Indel-9	Indel-9	-	Ren protein
G2583_0303	Indel-9	Indel-9	ninB	Unknown protein encoded within prophage
G2583_0304	Indel-9	Indel-9	-	Putative DNA N-6-adenine-methyltransferase of bacteriophage
G2583_0305	Indel-9	Indel-9	ninE	NINE Protein
G2583_0306	Indel-9	Indel-9	NinG	hypothetical protein
G2583_0307	Indel-9	Indel-9	-	Serine/threonine-protein phosphatase 1
G2583_0308	Indel-9	Indel-9	-	putative antitermination protein
G2583_0309	Indel-9	Indel-9	-	conserved hypothetical protein
G2583_0310	Indel-9	Indel-9	YjhS	YjhS
G2583_0311	Indel-9	Indel-9	-	hypothetical protein
G2583_0312	Indel-9	Indel-9	-	Putative lysis protein S of prophage CP-933V
G2583_0313	Indel-9	Indel-9	ybcS	Phage-related lysozyme (muraminidase)
G2583_0314	Indel-9	Indel-9	-	Putative Rz endopeptidase from lambdoid prophage DLP12
G2583_0315	Indel-9	Indel-9	-	unknown protein encoded within prophage CP-933R
G2583_0316	Indel-9	Indel-9	-	Putative transcriptional regulator
G2583_0317	Indel-9	Indel-9	ynfO	unknown protein encoded within prophage CP-933R
G2583_0318	Indel-9	Indel-9	nohA	conserved hypothetical protein
G2583_0319	Indel-9	Indel-9	-	Prophage Qin DNA packaging protein NU1 homolog
G2583_0320	Indel-9	Indel-9	-	Putative terminase large subunit of prophage CP-933O
G2583_0321	Indel-9	Indel-9	-	Head-stabilizing protein
G2583_0322	Indel-9	Indel-9	-	Putative capsid protein of prophage
G2583_0323	Indel-9	Indel-9	-	Head-tail preconnector protein GP5
G2583_0324	Indel-9	Indel-9	-	Head decoration protein
G2583_0325	Indel-9	Indel-9	-	Major head protein
G2583_0326	Indel-9	Indel-9	-	Uncharacterized 13.5 kDa protein in GP7-GP8 intergenic region
G2583_0327	Indel-9	Indel-9	-	Putative head-tail joining protein of prophage
G2583_0328	Indel-9	Indel-9	-	Prophage minor tail protein Z
G2583_0329	Indel-9	Indel-9	-	Minor tail protein U
G2583_0330	Indel-9	Indel-9	-	Putative tail component of prophage CP-933X
G2583_0331	Indel-9	Indel-9	-	Putative tail component of prophage
G2583_0332	Indel-9	Indel-9	-	Minor tail protein T
G2583_0333	Indel-9	Indel-9	-	Phage-related minor tail protein
G2583_0334	Indel-9	Indel-9	-	minor tail protein
G2583_0335	Indel-9	Indel-9	-	Phage-related protein
G2583_0336	Indel-9	Indel-9	-	Tail assembly protein
G2583_0337	Indel-9	Indel-9	-	Tail assembly protein I
G2583_0338	Indel-9	Indel-9	-	Phage-related protein, tail component
G2583_0339	Indel-9	Indel-9	lomK	Enterobacterial Ail/Lom family protein
G2583_0340	Indel-9	Indel-9	-	Putative tail fiber protein
G2583_0341	Indel-9	Indel-9	nleG	hypothetical protein
G2583_0342	Indel-9	Indel-9	-	PotB, trcA, ORF2, ORF3, ORF4 genes,

Locus_tag[a]				
CB9615	Sakai	EDL933	Gene	Product
G2583_0343	Indel-9	Indel-9	-	PotB, trcA, ORF2, ORF3, ORF4 genes,
G2583_0344	Indel-9	Indel-9	IpgB	Putative chaperone protein
G2583_0345	Indel-9	Indel-9	-	Tail fiber assembly protein
G2583_0346	Indel-9	Indel-9	intR	Putative integrase for prophage CP-933R
G2583_0347	Indel-9	Indel-9	-	NleB
G2583_0348	Indel-9	Indel-9	-	hypothetical protein
G2583_0349	Indel-9	Indel-9	gogB	conserved hypothetical protein
G2583_0350	Indel-9	Indel-9	-	conserved hypothetical protein
G2583_0351	ECs0289	Z0324	-	Integrase protein for prophage CP-933I
G2583_0352	ECs0290	Z0325	-	unknown protein encoded in prophage CP-933I
G2583_0353	ECs0291	Z0326	-	unknown protein encoded in prophage CP-933I
G2583_0354	ECs0292	Z0327	-	unknown protein encoded in prophage CP-933I
G2583_0355	ECs0293	Z0328	-	hypothetical protein
G2583_0356	ECs0294	Z0330	-	unknown protein encoded in prophage CP-933I
G2583_0357	ECs0295	Z0331	-	unknown protein encoded in prophage CP-933I
G2583_0358	ECs0296	Z0332	ogrK	Putative activator encoded in prophage CP-933I
G2583_0359	ECs0297	Z0333	psuI	Putative polarity suppression protein encoded in CP-
G2583_0360	ECs0298	Z0334	sidI	Putative capsid morphogenesis protein encoded in CP-933I
G2583_0361		Z0335	-	unknown protein encoded in prophage CP-933I
G2583_0362	ECs0299	Z0336	alpA	Phage DNA binding protein
G2583_0363	ECs0300	Z0337	-	putative CI repressor
G2583_0364	ECs0301		-	hypothetical protein
G2583_0365	ECs0302	Z0338	-	unknown protein encoded in prophage CP-933I
G2583_0366	ECs0303	Z0339	-	Alpha replication protein of prophage CP-933I
G2583_0367	ECs0304	Z0340	-	unknown protein encoded in prophage CP-933I
		Z0341	-	unknown protein encoded in prophage CP-933I
G2583_0368	ECs0305	Z0342	yagP	putative LysR-like transcriptional regulator
G2583_0369	ECs0306	Z0343	-	Clavaldehyde dehydrogenase
G2583_0370	ECs0307	Z0344	-	hypothetical protein
	ECs0308		-	hypothetical protein
G2583_0371		Z0345	-	hypothetical protein
G2583_0372	ECs0309	Z0346	-	LysR substrate binding domain protein
G2583_0373	ECs0310	Z0347	-	Hydrolase of the alpha/beta superfamily
G2583_0374	ECs0311	Z0348	-	Purine ribonucleoside efflux pump NepI
	ECs0312		-	hypothetical protein
G2583_0375	ECs0313	Z0349	yagQ	Putative xanthine dehydrogenase accessory factor
G2583_0376	ECs0314	Z0350	yagR	Putative xanthine dehydrogenase yagR molybdenum-binding subunit
G2583_0377	ECs0315	Z0351	yagS	FAD binding domain in molybdopterin dehydrogenase
G2583_0378	ECs0316	Z0352	yagT	Putative xanthine dehydrogenase yagT iron-sulfur-binding subunit precursor
G2583_0379	ECs0317	Z0353	yagU	Inner membrane protein yagU
G2583_0380	ECs0318	Z0354	ykgJ	putative ferredoxin

Locus_tag[a]				
CB9615	Sakai	EDL933	Gene	Product
G2583_0381	ECs0319	Z0356	yagV	hypothetical protein
G2583_0382	ECs0320	Z0357	yagW	putative receptor
G2583_0383	ECs0321	Z0358	yagX	putative enzyme
G2583_0384	ECs0322	Z0359	matC	hypothetical protein
G2583_0385	ECs0323	Z0360	matB	hypothetical protein
G2583_0386	ECs0324	Z0361	matA	MatA
G2583_0387		Z0362	-	hypothetical protein
	ECs0325		-	hypothetical protein
G2583_0388	ECs0326	Z0363	ykgL	hypothetical protein
G2583_0389			ykgO	50S ribosomal protein L36
G2583_0390	ECs0327	Z0364	ykgM	Ribosomal protein L31
Indel-10	ECs0328	Z0365	-	hypothetical protein
Indel-10	ECs0329	Z0366	-	hypothetical protein
Indel-10	ECs0330	Z0367	-	hypothetical protein
G2583_0391	ECs0331	Z0369	-	Oxidoreductase, FAD/FMN-binding
G2583_0392	ECs0332	Z0370	ycjY	hypothetical protein
G2583_0393	ECs0333	Z0371	ycaN	Putative LysR-like transcriptional regulator
G2583_0394	ECs0334	Z0372	-	hypothetical protein
G2583_0395		Z0373	-	hypothetical protein
G2583_0396	ECs0335	Z0374	-	2,5-diketo-D-gluconic acid reductase A
G2583_0397	ECs0336	Z0375	eaeH	Attaching and effacing protein homolog precursor
G2583_0398	ECs0337	Z0376	ykgA	Putative AraC-like transcriptional regulator
G2583_0399	ECs0338	Z0377	-	Putative dehydrogenase
G2583_0400	ECs0340	Z0378	ykgB	protein of unknown function DUF417
G2583_0401	ECs0339	Z0379	-	hypothetical protein
G2583_0402	ECs0341	Z0380	ykgI	hypothetical protein
G2583_0403	ECs0342	Z0381	ykgC	Pyridine nucleotide-disulphide oxidoreductase
G2583_0404	ECs0343	Z0382	ykgD	Hypothetical transcriptional regulator ykgD
G2583_0405	ECs0344	Z0384	ykgE	Cysteine-rich domain protein
G2583_0406	ECs0345	Z0385	ykgF	Iron-sulfur cluster binding protein
G2583_0407	ECs0346	Z0386	ykgG	hypothetical protein
G2583_0408	ECs0347	Z0387	-	hypothetical protein
G2583_0409	ECs0348	Z0388	ykgH	hypothetical protein
G2583_0410	ECs0349	Z0389	-	Putative autotransporter
G2583_0410	ECs0350	Z0390	-	Putative autotransporter
G2583_0411	ECs0351	Z0391	-	hypothetical protein
G2583_0412	ECs0352	Z0392	-	hypothetical protein
G2583_0412	ECs0353		-	hypothetical protein
		Z0393	-	hypothetical protein
G2583_0413	ECs0354	Z0394	fimX	Site-specific recombinase, phage integrase family
G2583_0413	ECs0355	Z0395	fimX	Site-specific recombinase, phage integrase family
	ECs0356		-	hypothetical protein
G2583_0414		Z0397	-	hypothetical protein
G2583_0415	ECs0357	Z0398	betA	Choline dehydrogenase

Locus_tag[a]				
CB9615	Sakai	EDL933	Gene	Product
G2583_0416	ECs0358	Z0399	betB	Betaine aldehyde dehydrogenase
G2583_0417	ECs0359	Z0400	betI	Transcriptional regulator
G2583_0418	ECs0360	Z0401	betT	High-affinity choline transport protein
	ECs0361		-	hypothetical protein
G2583_0419	ECs0362	Z0402	AidA-I	AidA-I adhesin-like protein
G2583_0420	ECs0363	Z0403	yahA	LuxR-family transcriptional regulator/cyclic diguanylate phosphodiesterase (EAL) domain protein
G2583_0421	ECs0364	Z0404	yahB	Uncharacterized HTH-type transcriptional regulator
G2583_0421	ECs0365	Z0405	yahB	Uncharacterized HTH-type transcriptional regulator
G2583_0422	ECs0366	Z0406	yahC	Uncharacterized protein yahC
G2583_0423	ECs0367	Z0407	yahD	Ankyrin repeat protein
G2583_0424	ECs0368	Z0408	yahE	hypothetical protein
G2583_0425	ECs0369	Z0409	yahF	Bacterial FdrA protein
G2583_0426	ECs0370	Z0410	yahG	hypothetical protein
	ECs0371		-	hypothetical protein
G2583_0427			yahH	hypothetical protein
G2583_0428	ECs0372	Z0412	yahI	Carbamate kinase family protein
G2583_0429	ECs0373	Z0413	yahJ	Amidohydrolase family protein
G2583_0430		Z0414	-	hypothetical protein
G2583_0431	ECs0374	Z0415	-	Sugar ABC transporter, periplasmic sugar-binding
G2583_0432	ECs0375	Z0416	-	Sugar ABC transporter, ATP-binding protein
G2583_0432	ECs0376	Z0417	-	Sugar ABC transporter, ATP-binding protein
G2583_0433	ECs0377	Z0418	-	Putative permease component of transport system, probably ribose specific
G2583_0434	ECs0378	Z0419	-	Putative permease component of transport system
G2583_0435	ECs0379	Z0420	yahK	Oxidoreductase, zinc-binding dehydrogenase family
G2583_0436	ECs0380	Z0421	yahL	hypothetical protein
G2583_0437		RNA009	-	Xaa tRNA
G2583_0438	ECs0381	Z0423	yahM	hypothetical protein
G2583_0439	ECs0382	Z0424	yahN	Putative homoserine/threonine efflux protein
G2583_0440	ECs0383	Z0425	yahO	hypothetical protein
G2583_0441	ECs0384	Z0426	prpR	Propionate catabolism operon regulatory protein PrpR
G2583_0442	ECs0385	Z0427	prpB	Methylisocitrate lyase
G2583_0443	ECs0386	Z0428	prpC	2-methylcitrate synthase
G2583_0444	ECs0387	Z0429	prpD	2-methylcitrate dehydratase
G2583_0445	ECs0388	Z0430	prpE	Propionate--CoA ligase
G2583_0446			nrdB	mannitol-1-phosphate 5-dehydrogenase
		Z0431	-	hypothetical protein
G2583_0447	ECs0389	Z0432	codB	Cytosine permease
G2583_0448	ECs0390	Z0433	codA	Cytosine deaminase and related metal-dependent hydrolases
G2583_0449	ECs0391	Z0434	cynR	DNA-binding transcriptional regulator CynR
G2583_0450	ECs0392	Z0435	cynT	Carbonic anhydrase 1
G2583_0451	ECs0393	Z0436	cynS	Cyanate hydratase

Locus_tag[a]				
CB9615	Sakai	EDL933	Gene	Product
G2583_0452	ECs0394	Z0437	cynX	Cyanate transport
G2583_0453	ECs0395	Z0438	lacA	Galactoside O-acetyltransferase LacA
G2583_0454	ECs0396	Z0439	lacY	Lactose permease
G2583_0455	ECs0397	Z0440	lacZ	Beta-D-galactosidase
G2583_0456	ECs0398	Z0441	lacI	lac repressor
G2583_0457	ECs0399	Z0442	-	putative AraC-like transcriptional regulator
G2583_0458	ECs0400	Z0443	-	Beta-lactamase fold protein
G2583_0459	ECs0401	Z0444	mhpR	DNA-binding transcriptional activator, 3HPP-binding
G2583_0460	ECs0402	Z0445	mhpA	3-(3-hydroxy-phenyl)propionate/3-hydroxycinnamic acid hydroxylase
G2583_0461	ECs0403	Z0446	mhpB	2,3-dihydroxyphenylpropionate/2,3-dihydroxicinnamic acid 1,2- dioxygenase
G2583_0462	ECs0404	Z0447	mhpC	2-hydroxy-6-oxononadienedioate/2-hydroxy-6-oxononatrienedioate hydrolase
G2583_0463	ECs0405	Z0448	mhpD	2-keto-4-pentenoate hydratase
G2583_0464	ECs0406	Z0450	mhpF	Acetaldehyde dehydrogenase
G2583_0465	ECs0407	Z0452	mhpE	4-hydroxy-2-oxovalerate aldolase
G2583_0466	ECs0408	Z0453	mhpT	Putative transport protein
G2583_0467	ECs0409	Z0454	yaiL	Nucleoprotein/polynucleotide-associated enzyme
G2583_0468	ECs0410	Z0455	frmB	putative esterase
G2583_0469	ECs0411	Z0456	frmA	S-(hydroxymethyl)glutathione dehydrogenase
G2583_0470	ECs0412	Z0457	frmR	regulator protein FrmR
G2583_0471	ECs0413	Z0458	afuC	Fe(3+) ions import ATP-binding protein fbpC
G2583_0472	ECs0414	Z0459	afuB	Binding-protein-dependent transport systems inner membrane component
G2583_0473	ECs0415	Z0460	afuA	Periplasmic ferric iron-binding protein
G2583_0474	ECs0416	Z0461	UhpC	putative permease; hexosephosphate transport
G2583_0475	ECs0417	Z0462	-	Integral membrane sensor signal transduction histidine kinase
G2583_0476	ECs0418	Z0463	-	Putative response regulator
G2583_0477	ECs0419	Z0464	tauA	Taurine transport system periplasmic protein
G2583_0478	ECs0420	Z0465	tauB	Taurine import ATP-binding protein tauB
G2583_0479	ECs0421	Z0466	tauC	ABC-type nitrate/sulfonate/bicarbonate transpo rt system, permease component
G2583_0480	ECs0422	Z0467	tauD	Taurine dioxygenase
G2583_0481	ECs0423	Z0468	hemB	Delta-aminolevulinic acid dehydratase
G2583_0482	ECs0424	Z0469	yaiU	Putative flagellin structural protein
G2583_0483	ECs0425	Z0470	yaiV	putative DNA-binding transcriptional regulator
G2583_0484	ECs0426	Z0472	ampH	Penicillin-binding protein ampH
G2583_0485	ECs0427	Z0473	sbmA	Inner-membrane transport protein, Microcin 25
G2583_0486	ECs0428	Z0474	yaiW	hypothetical protein
G2583_0487	ECs0429	Z0475	yaiY	Inner membrane protein yaiY
G2583_0488	ECs0430	Z0476	yaiZ	hypothetical protein
G2583_0489	ECs0431	Z0477	ddlA	D-alanine--D-alanine ligase A

Locus_tag[a]				
CB9615	Sakai	EDL933	Gene	Product
G2583_0490	ECs0432	Z0478	iraP	Anti-adapter protein iraP
G2583_0491	ECs0433	Z0479	phoA	Alkaline phosphatase
G2583_0492	ECs0434	Z0480	psiF	Phosphate starvation-inducible protein
G2583_0493	ECs0435	Z0481	adrA	MASE2 domain/diguanylate cyclase
G2583_0494	ECs0437	Z0482	proC	Pyrroline-5-carboxylate reductase
G2583_0495	ECs0436	Z0483	yaiI	hypothetical protein
G2583_0496	ECs0438	Z0484	aroL	Shikimate kinase 2
G2583_0497	ECs0439	Z0485	yaiA	hypothetical protein
G2583_0498	ECs0440	Z0486	aroM	AroM protein
G2583_0499	ECs0441	Z0487	yaiE	UPF0345 protein yaiE
G2583_0500			ydbD	hypothetical protein
G2583_0500	ECs0442	Z0489	ydbD	hypothetical protein
G2583_0501	ECs0443	Z0491	-	hypothetical protein
G2583_0502	ECs0445	Z0492	rdgC	Recombination-associated protein rdgC
G2583_0503	ECs0444	Z0493	mak	Possible NAGC-like transcriptional regulator
G2583_0504	ECs0446	Z0494	araJ	protein AraJ
G2583_0505	ECs0447	Z0495	sbcC	Nuclease SbcCD, C subunit
G2583_0506	ECs0448	Z0496	sbcD	Nuclease sbcCD subunit D
G2583_0507	ECs0449	Z0497	phoB	Positive response regulator for pho regulon
G2583_0508	ECs0450	Z0498	phoR	Signal transduction histidine kinase
G2583_0509	ECs0451	Z0499	brnQ	Branched-chain amino acid transport system 2 carrier protein
G2583_0510	ECs0452	Z0500	proY	Proline-specific permease proY
G2583_0511	ECs0453	Z0501	malZ	Maltodextrin glucosidase
G2583_0512	ECs0454	Z0502	-	hypothetical protein
G2583_0513	ECs0455	Z0503	acpH	Acyl carrier protein phosphodiesterase
G2583_0514	ECs0456	Z0504	queA	S-adenosylmethionine:tRNA ribosyltransferase-
G2583_0515	ECs0457	Z0505	tgt	Queuine tRNA-ribosyltransferase
G2583_0516	ECs0458	Z0506	yajC	UPF0092 membrane protein yajC
G2583_0517	ECs0459	Z0507	secD	Protein-export membrane protein secD
G2583_0518	ECs0460	Z0508	secF	Protein-export membrane protein SecF
G2583_0519	ECs0461	Z0509	-	UPF0156 protein Z0509/ECs0461
G2583_0520	ECs0462	Z0510	-	Plasmid stabilization system protein, RelE/ParE family
G2583_0521	ECs0463	Z0511	yajD	hypothetical protein
G2583_0522	ECs0464	Z0512	tsx	Nucleoside-specific channel-forming protein, Tsx
G2583_0523	ECs0465	Z0513	yajI	hypothetical protein
G2583_0524	ECs0466	Z0514	nrdR	ATP-cone domain protein
G2583_0525	ECs0467	Z0515	ribD	Bifunctional deaminase-reductase, C-terminal:Riboflavin biosynthesis protein RibD
G2583_0526	ECs0468	Z0516	ribE	6,7-dimethyl-8-ribityllumazine synthase
G2583_0527	ECs0469	Z0518	nusB	N utilization substance protein B homolog
G2583_0528	ECs0470	Z0519	thiL	Thiamine-monophosphate kinase
G2583_0529	ECs0471	Z0520	pgpA	Phosphatidylglycerophosphatase A
G2583_0530	ECs0472	Z0521	-	hypothetical protein

Locus_tag[a]				
CB9615	Sakai	EDL933	Gene	Product
G2583_0531	ECs0473	Z0522	yajO	Oxidoreductase, aldo/keto reductase family
G2583_0532	ECs0474	Z0523	dxs	1-deoxy-D-xylulose-5-phosphate synthase
G2583_0533	ECs0475	Z0524	ispA	Geranyltranstransferase
G2583_0534	ECs0476	Z0525	xseB	Exodeoxyribonuclease 7 small subunit
G2583_0535	ECs0477	Z0526	thiI	Thiamine biosynthesis protein thiI
G2583_0536	ECs0478	Z0527	yajL	4-methyl-5(Beta-hydroxyethyl)-thiazole monophosphate synthesis
G2583_0537	ECs0479	Z0528	panE	2-dehydropantoate 2-reductase
G2583_0538	ECs0480	Z0529	yajQ	UPF0234 protein yajQ
G2583_0539	ECs0481	Z0530	yajR	Hypothetical transport protein YajR
G2583_0540	ECs0482	Z0531	cyoE	Protoheme IX farnesyltransferase
G2583_0541	ECs0483	Z0532	cyoD	Cytochrome O ubiquinol oxidase protein CyoD
G2583_0542	ECs0484	Z0533	cyoC	Cytochrome o ubiquinol oxidase subunit 3
G2583_0543	ECs0485	Z0534	cyoB	Ubiquinol oxidase subunit 1
G2583_0544	ECs0486	Z0535	cyoA	Cytochrome o ubiquinol oxidase, subunit II
G2583_0545	ECs0487	Z0536	ampG	Regulates beta-lactamase synthesis
G2583_0546	ECs0488	Z0537	yajG	hypothetical protein
G2583_0547	ECs0489	Z0539	bolA	transcriptional regulator BolA
G2583_0548	ECs0490	Z0541	tig	Trigger factor
G2583_0549	ECs0491	Z0542	clpP	ATP-dependent Clp protease proteolytic subunit
G2583_0550	ECs0492	Z0543	clpX	ATP-dependent Clp protease ATP-binding subunit clpX
G2583_0551	ECs0493	Z0545	lon	DNA-binding ATP-dependent protease La
G2583_0552	ECs0494	Z0547	hupB	DNA-binding protein HU-beta
G2583_0553	ECs0495	Z0548	ppiD	Peptidylprolyl isomerase
G2583_0554	ECs0496	Z0549	ybaV	Competence protein ComEA
G2583_0555	ECs0497	Z0550	ybaW	Thioesterase family protein
G2583_0556	ECs0498	Z0551	queC	Queuosine biosynthesis protein queC
G2583_0557	ECs0499	Z0552	ybaE	Bacterial extracellular solute-binding protein, family 5
G2583_0558	ECs0500	Z0553	cof	Predicted hydrolases of the HAD superfamily
G2583_0559	ECs0501	Z0555	ybaO	Putative transcriptional regulator YbaO
G2583_0560	ECs0502	Z0557	mdlA	Multidrug resistance, ATP-binding protein mdlA
G2583_0561	ECs0503	Z0559	mdlB	Multidrug resistance-like ATP-binding protein mdlB
G2583_0562	ECs0504	Z0562	glnK	Glutamine synthetase regulation protein
G2583_0563	ECs0505	Z0563	amtB	Ammonia channel precursor
G2583_0564	ECs0506	Z0564	tesB	Acyl-CoA thioesterase II
G2583_0565	ECs0507	Z0565	ybaY	Glycoprotein/polysaccharide metabolism precursor
G2583_0566	ECs0508	Z0566	ybaZ	hypothetical protein
G2583_0567	ffs	RNA010	ffs	ncRNA
G2583_0568	ECs0509	Z0568	ybaA	hypothetical protein
G2583_0569	ECs0510	Z0569	ylaB	hypothetical protein
G2583_0570	ECs0511	Z0570	ylaC	hypothetical protein
G2583_0571	ECs0512	Z0571	maa	Maltose O-acetyltransferase
G2583_0572	ECs0513	Z0573	hha	Haemolysin expression modulating protein
G2583_0573	ECs0514	Z0574	ybaJ	hypothetical protein

Locus_tag[a]				
CB9615	Sakai	EDL933	Gene	Product
G2583_0574	ECs0515	Z0576	acrB	Acriflavine resistance protein B
G2583_0575	ECs0516	Z0578	acrA	Acriflavine resistance protein A
G2583_0576	ECs0517	Z0579	acrR	DNA-binding transcriptional repressor
G2583_0577	ECs0518	Z0581	kefA	Potassium efflux system KefA
G2583_0578	ECs0519	Z0583	ybaM	hypothetical protein
G2583_0579	ECs0520	Z0584	priC	Primosomal replication protein N''
G2583_0580	ECs0521	Z0585	ybaN	Inner membrane protein ybaN
G2583_0581	ECs0522	Z0586	apt	Adenine phosphoribosyltransferase
G2583_0582	ECs0523	Z0587	dnaX	DNA polymerase III, tau subunit
G2583_0583	ECs0524	Z0588	ybaB	UPF0133 protein ybaB
G2583_0584	ECs0525	Z0589	recR	Recombination protein recR
G2583_0585	ECs0526	Z0590	htpG	Chaperone protein htpG
G2583_0586	ECs0527	Z0591	adk	Adenylate kinase
G2583_0587	ECs0528	Z0592	hemH	Ferrochelatase
G2583_0588	ECs0529	Z0593	aes	Acetyl esterase
G2583_0589	ECs0530	Z0596	gsk	Inosine-guanosine kinase
G2583_0590	ECs0531	Z0597	ybaL	Transporter, monovalent cation:proton antiporter-2
G2583_0591	ECs0532	Z0598	fsr	Fosmidomycin resistance protein
G2583_0592	ECs0533	Z0599	ushA	UDP-sugar hydrolase/5'-nucleotidase
G2583_0593	ECs0534	Z0600	ybaK	hypothetical protein
G2583_0594			sroB	ncRNA
G2583_0595	ECs0535	Z0601	ybaP	GumN family protein
G2583_0596	ECs0536	Z0602	ybaQ	Addiction module antidote protein, HigA family
G2583_0597	ECs0537	Z0604	copA	Copper-transporting P-type ATPase
G2583_0598	ECs0538	Z0606	ybaS	Glutaminase 1
G2583_0599	ECs0539	Z0607	ybaT	Amino acid permease family protein
G2583_0600	ECs0540	Z0608	-	Putative outer membrane export protein
G2583_0601	ECs0541	Z0609	-	hypothetical protein
G2583_0601	ECs0542	Z0615	-	hypothetical protein
G2583_0602	ECs0543	Z0634	-	Putative cytoplasmic membrane export protein
G2583_0603	ECs0544	Z0635	-	Membrane spanning export protein
G2583_0604	ECs0545	Z0636	cueR	DNA-binding transcriptional activator of copper-responsive regulon genes
G2583_0605	ECs0546	Z0638	-	hypothetical protein
	ECs0547		-	hypothetical protein
G2583_0606	ECs0548	Z0639	-	hypothetical protein
G2583_0607	ECs0549	Z0640	-	hypothetical protein
	ECs0550		-	hypothetical protein
G2583_0608	ECs0551	Z0641	ybbJ	Nodulation efficiency family protein
G2583_0609	ECs0552	Z0642	qmcA	putative protease
G2583_0610	ECs0553	Z0643	ybbL	ABC transporter, ATP-binding protein
G2583_0611	ECs0554	Z0644	ybbM	Putative metal resistance protein
G2583_0612	ECs0555	Z0645	ybbN	putative thioredoxin-like protein

Locus_tag[a]				
CB9615	Sakai	EDL933	Gene	Product
G2583_0613	ECs0556	Z0646	ybbO	Oxidoreductase, short chain dehydrogenase/reductase family
G2583_0614	ECs0558	Z0647	tesA	Acyl-CoA thioesterase I
G2583_0615	ECs0557	Z0648	ybbA	Uncharacterized ABC transporter ATP-binding protein ybbA
G2583_0616	ECs0559	Z0649	ybbP	putative oxidoreductase
G2583_0617	ECs0560	Z0651	rhsD	RHS Repeat family protein
G2583_0618	ECs0561	Z0653	ybbD	hypothetical protein
G2583_0619			-	ncRNA
G2583_0620	ECs0562	Z0654	-	hypothetical protein
G2583_0621		Z0655	-	hypothetical protein
G2583_0622	ECs0563	Z0656	ylbG	hypothetical protein
G2583_0623	ECs0564	Z0657	ybbB	tRNA 2-selenouridine synthase
G2583_0624	ECs0565	Z0658	allS	DNA-binding transcriptional activator of the allD operon
G2583_0625	ECs0566	Z0659	allA	Ureidoglycolate hydrolase
G2583_0626	ECs0567	Z0660	allR	Putative regulator
G2583_0627	ECs0568	Z0661	gcl	Glyoxylate carboligase
G2583_0628	ECs0569	Z0662	hyi	Glyoxylate-induced protein
G2583_0629	ECs0570	Z0663	glxR	2-hydroxy-3-oxopropionate reductase
G2583_0630	ECs0571	Z0664	ybbV	hypothetical protein
G2583_0631	ECs0572	Z0665	ybbW	Cytosine/uracil/thiamine/allantoin permeases
G2583_0632	ECs0573	Z0666	allB	Dihydroorotase and related cyclic amidohydrolases
G2583_0632	ECs0574	Z0667	allB	Dihydroorotase and related cyclic amidohydrolases
G2583_0633	ECs0575	Z0668	ybbY	Putative purine permease ybbY
G2583_0634	ECs0576	Z0669	glxK	Glycerate kinase
G2583_0635	ECs0577	Z0670	ylbA	hypothetical protein
G2583_0636	ECs0578	Z0671	allC	Allantoate amidohydrolase
G2583_0637	ECs0579	Z0672	allD	Ureidoglycolate dehydrogenase
G2583_0638	ECs0580	Z0673	fdrA	Bacterial FdrA protein
G2583_0639	ECs0581	Z0674	ylbE	hypothetical protein
G2583_0640	ECs0582	Z0675	ylbF	putative carboxylase
G2583_0641	ECs0583	Z0676	ybcF	Carbamate kinase
G2583_0642	ECs0584	Z0677	purK	Phosphoribosylaminoimidazole carboxylase, ATPase subunit
G2583_0643	ECs0585	Z0678	purE	Phosphoribosylaminoimidazole carboxylase catalytic subunit
G2583_0644	ECs0586	Z0679	lpxH	UDP-2,3-diacylglucosamine hydrolase
G2583_0645	ECs0587	Z0680	ppiB	Peptidyl-prolyl cis-trans isomerase
G2583_0646	ECs0588	Z0681	cysS	Cysteinyl-tRNA synthetase
G2583_0647	ECs0589	Z0682	ybcI	hypothetical protein
G2583_0648	ECs0590	Z0683	ybcJ	hypothetical protein
G2583_0649	ECs0591	Z0684	folD	Bifunctional protein folD [Includes: Methylenetetrahydrofolate dehydrogenase
G2583_0650	ECs0592	Z0686	sfmA	Putative fimbrial-like protein

Locus_tag[a]				
CB9615	Sakai	EDL933	Gene	Product
G2583_0651	ECs0593	Z0688	sfmC	Chaperone protein FimC homolog
G2583_0652	ECs0594	Z0689	sfmD	Outer membrane usher protein SfmD
G2583_0653	ECs0595	Z0690	sfmH	Involved in fimbrial asembly precursor
G2583_0654	ECs0596	Z0691	sfmF	Putative fimbrial protein
G2583_0655	ECs0597	Z0693	fimZ	Fimbrial Z protein
G2583_0656	argU	RNA011	argU	Arg tRNA
G2583_0657	Indel-17	Indel-17	intD	Prophage DLP12 integrase
G2583_0658	Indel-17	Indel-17	-	Hypothetical phage protein
G2583_0659	Indel-17	Indel-17	-	Hypothetical phage protein
G2583_0660	Indel-17	Indel-17	-	Unknown protein encoded within prophage
G2583_0661	Indel-17	Indel-17	-	protein of unknown function DUF1382
G2583_0662	Indel-17	Indel-17	-	hypothetical protein
G2583_0663	Indel-17	Indel-17	ybcC	Putative exonuclease encoded by prophage CP-933K
G2583_0664	Indel-17	Indel-17	betW	Bet protein
G2583_0665	Indel-17	Indel-17	gam	Host-nuclease inhibitor protein gam
G2583_0666	Indel-17	Indel-17	kil	Prophage Kil protein
G2583_0667	Indel-17	Indel-17	-	Hypothetical phage associated protein
G2583_0668	Indel-17	Indel-17	-	hypothetical protein
G2583_0669	Indel-17	Indel-17	-	Putative repressor protein
G2583_0670	Indel-17	Indel-17	-	hypothetical protein
G2583_0671	Indel-17	Indel-17	cII	hypothetical protein
G2583_0672	Indel-17	Indel-17	-	Phage replication protein O
G2583_0673	Indel-17	Indel-17	-	replication protein P
G2583_0674	Indel-17	Indel-17	renD	Ren protein
G2583_0675	Indel-17	Indel-17	emrE	EmrE SMR transporter
G2583_0676	Indel-17	Indel-17	ybcK	DLP12 prophage; predicted recombinase
G2583_0677	Indel-17	Indel-17	ybcL	Putative phosphatidylethanolamine-binding protein
G2583_0678	Indel-17	Indel-17	ybcM	DLP12 prophage; predicted DNA-binding transcriptional regulator
G2583_0679	Indel-17	Indel-17	ylcH	hypothetical protein
G2583_0680	Indel-17	Indel-17	ybcN	hypothetical protein
G2583_0681	Indel-17	Indel-17	ninE	NinE
G2583_0682	Indel-17	Indel-17	ybcO	hypothetical protein
G2583_0683	Indel-17	Indel-17	rusA	Crossover junction endodeoxyribonuclease rusA
G2583_0684	Indel-17	Indel-17	ylcG	hypothetical protein
G2583_0685	Indel-17	Indel-17	ybcQ	Phage antitermination Q type 1 family
G2583_0686	Indel-17	Indel-17	nmpC	Outer membrane porin protein C
G2583_0687	Indel-17	Indel-17	essD	hypothetical protein
G2583_0688	Indel-17	Indel-17	ybcS	Lysozyme
G2583_0689	Indel-17	Indel-17	rzpD	Putative Rz endopeptidase from lambdoid prophage DLP12
G2583_0690	Indel-17	Indel-17	borD	Lambdoid prophage DLP12 Bor-like protein
G2583_0691	Indel-17	Indel-17	nohB	Bacteriophage DNA packaging protein
G2583_0692	Indel-17	Indel-17	-	Bacteriophage tail assembly protein

Locus_tag[a]				
CB9615	Sakai	EDL933	Gene	Product
G2583_0693	Indel-17	Indel-17	-	Lambda prophage-derived head-to-tail joining protein W
G2583_0694	Indel-17	Indel-17	-	Putative capsid structural protein of prophage
G2583_0695	Indel-17	Indel-17	-	Minor capsid protein C
G2583_0696	Indel-17	Indel-17	-	Putative head-DNA stabilization protein of prophage
G2583_0697	Indel-17	Indel-17	-	phage major capsid protein E
G2583_0698	Indel-17	Indel-17	-	Putative DNA packaging protein of prophage
G2583_0699	Indel-17	Indel-17	-	Phage Head-Tail Attachment
G2583_0700	Indel-17	Indel-17	-	prophage minor tail protein Z
G2583_0701	Indel-17	Indel-17	-	Permeases of the major facilitator superfamily
G2583_0702	Indel-17	Indel-17	-	Putative tail component of prophage
G2583_0703	Indel-17	Indel-17	-	phage minor tail protein G
G2583_0704	Indel-17	Indel-17	-	Minor tail protein T
G2583_0705	Indel-17	Indel-17	-	Minor tail protein H
G2583_0706	Indel-17	Indel-17	-	Minor tail protein M
G2583_0707	Indel-17	Indel-17	-	Phage-related protein
G2583_0708	Indel-17	Indel-17	-	Putative tail fiber component K of prophage
G2583_0709	Indel-17	Indel-17	-	Putative tail component of prophage CP-933K
G2583_0710	Indel-17	Indel-17	-	Host specificity protein J
G2583_0711	Indel-17	Indel-17	-	hypothetical protein
G2583_0712	Indel-17	Indel-17	ydfN	PPE-repeat proteins
G2583_0713	Indel-17	Indel-17	ynaC	Tail fiber assembly protein
G2583_0714	Indel-17	Indel-17	ybcY	unknown protein encoded by prophage CP-933X
G2583_0715	Indel-17	Indel-17	ylcE	tail fiber assembly protein
G2583_0716	Indel-17	Indel-17	-	Putative protease encoded within prophage CP-933X
G2583_0717	Indel-17	Indel-17	ompT	Protease VII
G2583_0718	Indel-17	Indel-17	-	Helix-turn-helix domain protein
G2583_0719	Indel-17	Indel-17	yjaB	Hypothetical acetyltransferase YjaB
G2583_0720	ECs0598	Z0696	envY	envelope protein; thermoregulation of porin biosynthesis
G2583_0721	ECs0599	Z0697	ybcH	hypothetical protein
G2583_0722	ECs0600	Z0698	nfrA	Bacteriophage N4 adsorption protein A
G2583_0723	ECs0601	Z0699	nfrB	Bacteriophage N4 adsorption protein B
G2583_0724	ECs0602	Z0700	yhhI	ISEc4, transposase
G2583_0725		Z0701	-	hypothetical protein
G2583_0726	ECs0603	Z0702	-	YD repeat
G2583_0727	ECs0604		-	hypothetical protein
G2583_0728	ECs0605	Z0705	rhsI	Rhs core protein with extension
G2583_0729	ECs0606	Z0706	-	conserved hypothetical protein
G2583_0730	ECs0607	Z0707	-	Type VI secretion system Vgr family protein
G2583_0731	ECs0608	Z0708	cusS	Sensor kinase cusS
G2583_0732	ECs0609	Z0709	cusR	DNA-binding response regulator in two-component regulatory system with CusS
G2583_0733	ECs0610	Z0711	cusC	Cation efflux system protein cusC precursor
G2583_0734	ECs0611	Z0712	cusF	Cation efflux system protein cusF precursor
G2583_0735	ECs0612	Z0713	cusB	Cation efflux system protein CusB

Locus_tag[a]				
CB9615	Sakai	EDL933	Gene	Product
G2583_0736	ECs0613	Z0714	cusA	Cation efflux system protein cusA
G2583_0737	ECs0614	Z0715	pheP	Phenylalanine-specific permease
G2583_0738	ECs0615	Z0716	ybdG	Transporter, small conductance mechanosensitive ion channel (MscS) family
G2583_0739	ECs0616	Z0717	nfsB	Oxygen-insensitive NAD(P)H nitroreductase
G2583_0740	ECs0617	Z0718	ybdF	hypothetical protein
G2583_0741	ECs0618	Z0719	ybdJ	putative membrane protein YbdJ
G2583_0742	ECs0619	Z0720	ybdK	Carboxylate-amine ligase ybdK
G2583_0743	ECs0620	Z0721	mokC2	Hok/Gef family protein
G2583_0744	ECs0621	Z0722	hokE	Hok/Gef family protein
G2583_0745	ECs0622	Z0723	entD	4'-phosphopantetheinyl transferase entD
G2583_0746	ECs0623	Z0724	fepA	Ferrienterobactin receptor
G2583_0747	ECs0624	Z0725	fes	Enterochelin esterase
G2583_0748		Z0726	ybdZ	MbtH-like protein
G2583_0749	ECs0625	Z0727	entF	Enterobactin synthetase component F
G2583_0750	ECs0626	Z0728	fepE	Ferric enterobactin transport protein fepE
G2583_0751	ECs0627	Z0729	fepC	Ferric enterobactin transport ATP-binding protein
G2583_0752	ECs0628	Z0731	fepG	Ferric enterobactin transport protein
G2583_0753	ECs0629	Z0732	fepD	ABC-type Fe3+-siderophore transport system, permease component
G2583_0754	ECs0630	Z0733	entS	Enterobactin exporter entS
G2583_0755	ECs0631	Z0734	fepB	Ferrienterobactin ABC transporter, ferrienterobactin-binding periplasmic protein FepB
G2583_0756	ECs0632	Z0735	entC	Isochorismate synthase entC
G2583_0757	ECs0633	Z0736	entE	Enterobactin synthetase component E (Enterochelin synthase E) [Includes: 2,3-dihydroxybenzoate-AMP
G2583_0758	ECs0634	Z0737	entB	Isochorismatase
G2583_0759	ECs0635	Z0738	entA	2,3-dihydroxybenzoate-2,3-dehydrogenase
G2583_0760	ECs0636	Z0739	ybdB	Esterase ybdB
G2583_0761	ECs0637	Z0740	cstA	Carbon starvation protein A
G2583_0762			ybdD	hypothetical protein
G2583_0763	ECs0638	Z0742	ybdH	Alcohol dehydrogenase, iron-containing
G2583_0764	ECs0639	Z0743	ybdL	Aminotransferase, classes I and II
G2583_0765	ECs0640	Z0744	ybdM	Immunoglobulin-binding regulator family protein
G2583_0766	ECs0641	Z0746	ybdN	Phosphoadenosine phosphosulfate reductase family protein
G2583_0767	ECs0642	Z0747	ybdO	putative transcriptional regulator LYSR-type
G2583_0768	ECs0643	Z0748	dsbG	Thiol:disulfide interchange protein DsbG
G2583_0769	ECs0644	Z0749	ahpC	Alkyl hydroperoxide reductase subunit C
G2583_0770	ECs0645	Z0750	ahpF	Alkyl hydroperoxide reductase subunit F
G2583_0771	ECs0646	Z0751	uspG	Universal stress protein UspG
G2583_0772	ECs0647	Z0752	ybdR	Oxidoreductase, zinc-binding dehydrogenase family
G2583_0773			-	hypothetical protein
	ECs0648	Z0753	-	hypothetical protein

Locus_tag[a]				
CB9615	Sakai	EDL933	Gene	Product
G2583_0774	ECs0649	Z0754	rnk	Regulator of nucleoside diphosphate kinase
G2583_0775	ECs0650	Z0755	rna	Ribonuclease I
G2583_0776	ECs0651	Z0756	citT	Citrate carrier
G2583_0777	ECs0652	Z0757	citG	2-(5''-triphosphoribosyl)-3'-dephosphocoenzyme-A synthase
G2583_0778	ECs0653	Z0758	citX	Apo-citrate lyase phosphoribosyl-dephospho-CoA transferase
G2583_0779	ECs0654	Z0759	citF	Citrate lyase, alpha subunit
G2583_0780	ECs0655	Z0760	citE	Citrate lyase beta chain
G2583_0781	ECs0656	Z0761	citD	Citrate lyase acyl carrier protein
G2583_0782	ECs0657	Z0762	citC	Citrate lyase synthetase (Citrate (Pro-3S)-lyase ligase)
G2583_0783	ECs0658	Z0764	dpiB	Sensor histidine kinase DpiB
G2583_0784	ECs0659	Z0765	dpiA	transcriptional regulatory protein DpiA
G2583_0785	ECs0660	Z0766	dcuC	C4-dicarboxylate transporter
G2583_0786	ECs0661	Z0767	pagP	Antimicrobial peptide resistance and lipid A acylation protein PagP
G2583_0787	ECs0662	Z0769	cspE	hypothetical protein
G2583_0788	ECs0663	Z0770	crcB	camphor resistance protein CrcB
G2583_0789	ECs0664	Z0771	ybeM	UPF0012 hydrolase ybeM
G2583_0790	ECs0665	Z0772	tatE	Sec-independent protein translocase protein tatE
G2583_0791	ECs0666	Z0773	lipA	Lipoyl synthase
G2583_0792	ECs0667	Z0774	ybeF	putative DNA-binding transcriptional regulator
G2583_0793	ECs0668	Z0775	lipB	Octanoyltransferase
G2583_0794	ECs0669	Z0776	ybeD	Putative cytoplasmic protein
G2583_0795	ECs0670	Z0777	dacA	D-alanyl-D-alanine carboxypeptidase dacA precursor
G2583_0796	ECs0671	Z0778	rlpA	Rare lipoprotein A precursor
G2583_0797	ECs0672	Z0780	mrdB	rod shape-determining protein RodA
G2583_0798	ECs0673	Z0781	mrdA	Penicillin-binding protein 2
G2583_0799	ECs0674	Z0782	ybeA	UPF0247 protein ybeA
G2583_0800	ECs0675	Z0783	ybeB	hypothetical protein
G2583_0801	ECs0676	Z0785	cobC	Alpha-ribazole phosphatase
G2583_0802	ECs0677	Z0786	nadD	Nicotinate-nucleotide adenylyltransferase
G2583_0803	ECs0678	Z0787	holA	DNA polymerase III, delta subunit
G2583_0804	ECs0679	Z0788	rlpB	LPS-assembly lipoprotein rlpB precursor
G2583_0805	ECs0680	Z0789	leuS	Leucyl-tRNA synthetase
G2583_0806	ECs0681	Z0790	ybeL	hypothetical protein
G2583_0807	ECs0682	Z0791	ybeQ	FOG: TPR repeat, SEL1 subfamily
G2583_0807		Z0793	ybeQ	FOG: TPR repeat, SEL1 subfamily
G2583_0808	ECs0683	Z0794	ybeR	hypothetical protein
G2583_0809	ECs0684	Z0795	djlB	DnaJ domain protein
G2583_0810	ECs0685	Z0796	ybeT	Sel1 domain protein repeat-containing protein
G2583_0811	ECs0686	Z0797	ybeU	putative tRNA ligase
G2583_0812	ECs0687		ybeV	DnaJ domain protein
G2583_0812	ECs0688		ybeV	DnaJ domain protein

Locus_tag[a]				
CB9615	Sakai	EDL933	Gene	Product
G2583_0813	ECs0689	Z0800	hscC	DnaK family protein HscC
G2583_0814	ECs0690	Z0801	rihA	Pyrimidine-specific ribonucleoside hydrolase rihA
G2583_0815	ECs0691	Z0802	gltL	Glutamate/aspartate transport ATP-binding protein gltL
G2583_0816	ECs0692	Z0803	gltK	Glutamate/aspartate transport system permease protein gltK
G2583_0817	ECs0693	Z0804	gltJ	Glutamate/aspartate ABC transporter, permease protein GltJ
G2583_0818			sroC	ncRNA
G2583_0819	ECs0694	Z0805	gltI	Glutamate/aspartate periplasmic binding protein
G2583_0820	ECs0695	Z0806	lnt	Apolipoprotein N-acyltransferase
G2583_0821	ECs0696	Z0807	ybeX	Magnesium and cobalt efflux protein corC
G2583_0822	ECs0697	Z0808	ybeY	conserved hypothetical protein
G2583_0823	ECs0698	Z0809	ybeZ	PhoH family protein
G2583_0824	ECs0699	Z0810	miaB	UPF0004 protein yleA
G2583_0825	ECs0700	Z0811	ubiF	2-octaprenyl-3-methyl-6-methoxy-1,4-benzoquinol hydroxylase
G2583_0826	ECs0701		-	hypothetical protein
G2583_0827	glnX	RNA012	glnX	Gln tRNA
G2583_0828	glnV	RNA013	glnV	Gln tRNA
G2583_0829	metU	RNA014	metU	Met tRNA
G2583_0830	ECs0702		-	Putative RNA
G2583_0831	glnW	RNA015	glnW	Gln tRNA
	ECs0703		-	putative RNA
G2583_0832	glnU	RNA016	glnU	Gln tRNA
G2583_0833	leuW	RNA017	leuW	Leu tRNA
G2583_0834	metT	RNA018	metT	Met tRNA
G2583_0835	ECs0704	Z0821	asnB	Asparagine synthase
G2583_0836	ECs0705	Z0822	nagD	UMP phosphatase
G2583_0837	ECs0706	Z0823	nagC	N-acetylglucosamine repressor
G2583_0838	ECs0707	Z0824	nagA	N-acetylglucosamine-6-phosphate deacetylase
G2583_0839	ECs0708	Z0825	nagB	Glucosamine-6-phosphate deaminase
G2583_0840	ECs0709	Z0826	nagE	PTS system, N-acetylglucosamine-specific IICBA component
G2583_0841	ECs0710	Z0827	glnS	Glutaminyl-tRNA synthetase
G2583_0842	ECs0711	Z0828	ybfM	Outer membrane porin, OprD family
G2583_0842	ECs0712	Z0829	ybfM	Outer membrane porin, OprD family
G2583_0843	ECs0713	Z0830	ybfN	hypothetical protein
G2583_0844	ECs0714	Z0831	fur	Ferric uptake regulation protein
G2583_0845	ECs0715	Z0832	fldA	Flavodoxin
G2583_0846	ECs0716	Z0834	ybfE	CopG domain protein DNA-binding domain protein
G2583_0847	ECs0717	Z0835	ybfF	Esterase YbfF
G2583_0848	ECs0718	Z0836	seqA	Negative modulator of initiation of replication
G2583_0849	ECs0719	Z0837	pgm	Phosphoglucomutase
G2583_0850	ECs0720	Z0838	potE	Putrescine-ornithine antiporter

Locus_tag[a]				
CB9615	Sakai	EDL933	Gene	Product
G2583_0851	ECs0721	Z0839	speF	Ornithine decarboxylase, inducible
		Z0840	-	hypothetical protein
G2583_0852	ECs0722	Z0841	kdpE	KDP operon transcriptional regulatory protein KdpE
G2583_0853	ECs0723	Z0842	kdpD	sensor protein KdpD
G2583_0854	ECs0724	Z0843	kdpC	Potassium-transporting ATPase C chain
G2583_0855	ECs0725	Z0844	kdpB	Potassium-transporting ATPase B chain
G2583_0856	ECs0726	Z0845	kdpA	Potassium-transporting ATPase A chain
G2583_0857	ECs0727		kdpF	K+-transporting ATPase, F subunit
G2583_0858	ECs0728	Z0846	ybfA	hypothetical protein
G2583_0859	ECs0729	Z0847	rhsC	RHS repeat protein
Indel-20	Indel-20	Z0848	-	rhsC protein in rhs element
Indel-20	Indel-20	Z0849	ybfB	orf, hypothetical protein
G2583_0859	ECs0729	Z0851	rhsC	RHS repeat protein
G2583_0860	ECs0730	Z0853	ybfC	hypothetical protein
		Z0855	-	hypothetical protein
G2583_0861		Z0856	yhhI	Transposase IS4 family protein
G2583_0861	ECs0731	Z0854	yhhI	Transposase IS4 family protein
G2583_0862	ECs0732	Z0858	ybgA	hypothetical protein
G2583_0863	ECs0733	Z0859	phr	Deoxyribodipyrimidine photolyase
G2583_0864	ECs0734	Z0860	ybgH	Amino acid/peptide transporter
G2583_0865	ECs0735	Z0861	ybgI	NIF3 family protein
G2583_0866	ECs0736	Z0862	ybgJ	putative carboxylase
G2583_0867	ECs0737	Z0863	ybgK	Putative carboxylase
G2583_0868	ECs0738	Z0864	ybgL	UPF0271 protein ybgL
G2583_0869	ECs0739	Z0865	nei	Endonuclease VIII (DNA glycosylase/AP lyase Nei)
G2583_0870	ECs0740	Z0867	abrB	Putative transport protein
G2583_0871	ECs0741	Z0868	ybgO	hypothetical protein
G2583_0872	ECs0742	Z0869	ybgP	Periplasmic pilus chaperone family protein
G2583_0873	ECsp001	Z0870	ybgQ	truncated outer membrane protein
G2583_0873	ECs0743	Z0871	ybgQ	truncated outer membrane protein
G2583_0874	ECs0744	Z0872	ybgD	Putative fimbrial-like protein
G2583_0875	ECs0745	Z0873	gltA	Citrate (Si)-synthase
G2583_0876	ECs0746	Z0875	sdhC	Succinate dehydrogenase cytochrome b-556 subunit
G2583_0877	ECs0747	Z0876	sdhD	Succinate dehydrogenase hydrophobic membrane anchor subunit
G2583_0878	ECs0748	Z0877	sdhA	Succinate dehydrogenase flavoprotein subunit
G2583_0879	ECs0749	Z0878	sdhB	Succinate dehydrogenase, iron-sulfur subunit
G2583_0880	ECs0750	Z0879	-	hypothetical protein
G2583_0881	ECs0751	Z0880	sucA	2-oxoglutarate dehydrogenase E1 component
G2583_0882	ECs0752	Z0881	sucB	Dihydrolipoyllysine-residue succinyltransferase, E2 component of oxoglutarate dehydrogenase (Succinyl-transferring) complex
G2583_0883	ECs0753	Z0882	sucC	Succinyl-CoA ligase [ADP-forming] subunit beta
G2583_0884	ECs0754	Z0883	sucD	Succinyl-CoA ligase [ADP-forming] subunit alpha

Locus_tag[a]				
CB9615	Sakai	EDL933	Gene	Product
G2583_0885		Z0884	-	conserved domain protein
G2583_0886	ECs0755	Z0885	-	putative LysR-like transcriptional regulator
G2583_0887	ECs0756	Z0886	cobO	Cob(I)yrinic acid a,c-diamide adenosyltransferase
G2583_0888	ECs0757	Z0887	fumA	Hydro-lyase, Fe-S type, tartrate/fumarate family
G2583_0889	ECs0758	Z0888	-	Transporter, dicarboxylate/amino acid:cation family
G2583_0890	ECs0759	Z0890	-	hypothetical protein
G2583_0891	ECs0760	Z0891	-	hypothetical protein
G2583_0892	ECs0761	Z0892	-	Putative methylaspartate ammonia-lyase
G2583_0893	ECs0762	Z0893	mutE	Methylaspartate mutase, E subunit
G2583_0894	ECs0763	Z0894	-	Putative glutamate mutase mutL
G2583_0895	ECs0764	Z0895	mamA	Methylaspartate mutase S chain
G2583_0896	ECs0765	Z0896	-	hypothetical protein
G2583_0897	ECs0766	Z0897	-	hypothetical protein
G2583_0898	ECs0767	Z0898	-	hypothetical protein
		Z0899	-	hypothetical protein
G2583_0899	ECs0768	Z0900	cydA	Cytochrome d ubiquinol oxidase subunit 1
G2583_0900	ECs0769	Z0901	cydB	Cytochrome d ubiquinol oxidase subunit 2
G2583_0901			ybgT	hypothetical protein
G2583_0902	ECs0770	Z0903	ybgE	hypothetical protein
G2583_0903	ECs0771	Z0904	ybgC	Putative esterase YbgC
G2583_0904	ECs0772	Z0905	tolQ	Inner membrane protein
G2583_0905	ECs0773	Z0906	tolR	hypothetical protein
G2583_0906	ECs0774	Z0907	tolA	TolA colicin import membrane protein
G2583_0907	ECs0775	Z0908	tolB	Protein tolB precursor
G2583_0908	ECs0776	Z0909	pal	Peptidoglycan-associated lipoprotein precursor
G2583_0909	ECs0777	Z0910	ybgF	Tol-pal system protein YbgF
G2583_0910	lysT	RNA019	lysT	Lys tRNA
G2583_0911	valT	RNA020	valT	Val tRNA
G2583_0912	lysW	RNA021	lysW	Lys tRNA
G2583_0913	valZ	RNA022	valZ	Val tRNA
G2583_0914	lysY	RNA023	lysY	Lys tRNA
G2583_0915	lysZ	RNA024	lysZ	Lys tRNA
G2583_0916	ECs0778	Z0919	nadA	Quinolinate synthetase A
G2583_0917	ECs0779	Z0920	pnuC	Nicotinamide mononucleotide transporter PnuC
G2583_0918	ECs0780	Z0922	zitB	Zinc transporter zitB
G2583_0919	ECs0781	Z0923	ybgS	hypothetical protein
G2583_0920	ECs0782	Z0924	aroG	Phospho-2-dehydro-3-deoxyheptonate aldolase, Phe-sensitive
G2583_0921	ECs0783	Z0925	gpmA	2,3-bisphosphoglycerate-dependent phosphoglycerate mutase
G2583_0922	ECs0784	Z0926	galM	Galactose mutarotase
G2583_0923	ECs0785	Z0927	galK	Galactokinase
G2583_0924	ECs0786	Z0928	galT	Galactose-1-phosphate uridylyltransferase
G2583_0925	ECs0787	Z0929	galE	UDP-galactose-4-epimerase

Locus_tag[a]				
CB9615	Sakai	EDL933	Gene	Product
G2583_0926	ECs0788	Z0930	modF	ABC transporter, ATP-binding protein
G2583_0927	ECs0789	Z0931	modE	transcriptional regulator ModE
G2583_0928	ECs0790	Z0932	ybhT	hypothetical protein
G2583_0929	ECs0791	Z0933	modA	Molybdate ABC transporter, periplasmic molybdate-binding protein
G2583_0930	ECs0792	Z0934	modB	ABC-type molybdate transport system, permease component
G2583_0931	ECs0793	Z0935	modC	Molybdenum import ATP-binding protein modC
G2583_0932	ECs0794	Z0936	ybhA	Phosphatase YbhA
G2583_0933	ECs0795	Z0938	pgl	6-phosphogluconolactonase
G2583_0934	ECs0796	Z0939	ybhD	putative transcriptional regulator LYSR-type
G2583_0935	ECs0797	Z0940	ybhH	hypothetical protein
G2583_0936	ECs0798	Z0941	ybhI	Anion transporter
G2583_0937	ECs0799	Z0942	ybhJ	aconitase family protein
G2583_0938	ECs0800	Z0943	ybhC	Pectinesterase
G2583_0939	ECsp002	Z0946	intE	Phage integrase family
G2583_0940	ECs0801		-	Excisionase
G2583_0941	ECs0802	Z0947	-	unknown protein encoded by prophage CP-933K
	ECs0803		-	hypothetical protein
G2583_0942	ECs0804	Z0948	-	unknown protein encoded by prophage CP-933K
G2583_0943	ECs0805		-	hypothetical C4-type zinc finger protein TraR-family
G2583_0944	ECs0806	Z0949	-	hypothetical protein
G2583_0945	ECs0807	Z0950	-	unknown protein encoded by prophage CP-933K
	ECs0808		-	hypothetical protein
G2583_0946	ECs0809	Z0951	-	Exonuclease
G2583_0947	ECsp003	Z0952	bet	Bacteriophage recombination protein
G2583_0948	Indel-24	Indel-24	gamW	Gam protein
G2583_0949	Indel-24	Indel-24	CIII	antitermination protein
G2583_0950	Indel-24	Indel-24	-	Lambda prophage-derived protein ea10
G2583_0951	Indel-24	Indel-24	-	Gene 34 protein
G2583_0952	Indel-24	Indel-24	-	N protein
G2583_0953	Indel-24	Indel-24	-	Hypothetical protein
G2583_0954	Indel-24	Indel-24	-	CI protein
G2583_0955	Indel-24	Indel-24	CRO	hypothetical protein
G2583_0956	Indel-24	Indel-24	-	regulatory protein CII
G2583_0957	Indel-24	Indel-24	-	hypothetical protein
G2583_0958	Indel-24	Indel-24	-	putative replication protein P of bacteriophage BP-933W
G2583_0959	Indel-24	Indel-24	-	Ren protein
G2583_0960	Indel-24	Indel-24	ninB	Unknown protein encoded within prophage
G2583_0961	ECs0810		ninE	NinE protein
G2583_0962	ECs0811		NinF	hypothetical protein
G2583_0963	ECs0812	Z0953	NinG	Bacteriophage Lambda NinG protein
G2583_0964	ECs0813	Z0954	-	Serine/threonine-protein phosphatase 1
G2583_0965	ECs0814	Z0955	-	Putative outer membrane protein

Locus_tag[a]				
CB9615	Sakai	EDL933	Gene	Product
G2583_0966	ECs0815	Z0956	-	Putative antiterminator Q protein of prophage CP-933K
G2583_0967	ECs0816	Z0957	-	unknown protein encoded by prophage CP-933K
G2583_0968		Z0958	-	unknown protein encoded by prophage CP-933K
	ECs0817		-	hypothetical protein
		Z0959	-	unknown protein encoded by prophage CP-933K
G2583_0969	ECs0818		-	Putative holin protein
G2583_0970	ECs0819	Z0960	ybcS	putative lysozyme protein R of prophage CP-933K
G2583_0971	ECs0820	Z0961	-	Bacteriophage lysis protein
	ECs0821		-	putative lipoprotein Rz1 precursor
G2583_0972	ECs0822		-	hypothetical protein
G2583_0973	ECs0823		-	hypothetical protein
		Z0962	-	unknown protein encoded by prophage CP-933K
G2583_0974	ECs0824	Z0963	-	unknown protein encoded by prophage CP-933K
G2583_0975	ECs0825	Z0964	-	Phage terminase large subunit
G2583_0976	ECs0826		-	hypothetical protein
G2583_0977	ECs0827	Z0965	-	Putative capsid protein
G2583_0977	ECs0828	Z0966	-	Putative capsid protein
G2583_0978	ECs0829	Z0967	clpP	Clp protease domain protein
G2583_0979	ECs0830	Z0968	-	hypothetical protein
G2583_0980	ECs0831	Z0969	-	unknown protein encoded by prophage CP-933K
G2583_0981	ECs0832	Z0970	-	prophage minor tail protein Z
G2583_0982	ECs0833	Z0971	-	putative tail component of prophage CP-933K
G2583_0983	ECs0834	Z0972	-	Putative tail component of prophage CP-933K
G2583_0984	ECs0835	Z0973	-	ATPase components of ABC transporters with duplicated ATPase domains
G2583_0985	ECs0836	Z0974	-	Putative tail component of prophage
G2583_0986	ECs0837	Z0975	-	Putative tail component of prophage CP-933K
G2583_0987	ECs0838	Z0976	-	putative minor tail protein
G2583_0988	ECs0839	Z0977	-	putative minor tail protein
G2583_0989	ECs0840	Z0978	-	Putative tail fiber component K of prophage
G2583_0990	ECs0841	Z0979	-	Putative tail component of prophage CP-933K
G2583_0991	ECs0842	Z0980	-	Host specificity protein J
G2583_0992	ECs0843	Z0981	-	Enterobacterial Ail/Lom family protein
G2583_0993	ECs0844	Z0982	-	hypothetical protein
G2583_0994	ECs0845	Z0984	-	putative prophage tail fibre C-terminus family protein
G2583_0995	ECs0846	Z0985	nleB	NleB
G2583_0996	ECs0847	Z0986	nleC	Non-LEE encoded type III effector C
G2583_0997	ECsp004		-	tail fiber assembly proteins
G2583_0998	ECs0848	Z0989	nleH	non-LEE-encoded type III effector H
G2583_0999	ECs0849		-	hypothetical protein
G2583_1000	ECs0850	Z0990	nleD	hypothetical protein
G2583_1001	ECs0851	Z0992	ybhB	UPF0098 protein ybhB
G2583_1002	ECs0852	Z0993	bioA	Adenosylmethionine-8-amino-7-oxononanoate transaminase

Locus_tag[a]				
CB9615	Sakai	EDL933	Gene	Product
G2583_1003	ECs0853	Z0994	bioB	Biotin synthase
G2583_1004	ECs0854	Z0995	bioF	8-amino-7-oxononanoate synthase
G2583_1005	ECs0855	Z0996	bioC	Biotin biosynthesis protein BioC
G2583_1006	ECs0856	Z0997	bioD	Dethiobiotin synthase
G2583_1007	ECs0857	Z0998	uvrB	UvrABC system protein B
G2583_1008	ECs0858	Z0999	ybhK	hypothetical protein
G2583_1009	ECs0859	Z1000	moaA	Molybdenum cofactor biosynthesis protein A
G2583_1010	ECs0860	Z1001	moaB	Molybdenum cofactor biosynthesis protein B
G2583_1011	ECs0861	Z1002	moaC	Molybdenum cofactor biosynthesis protein C
G2583_1012	ECs0862	Z1003	moaD	Molybdopterin biosynthesis
G2583_1013	ECs0863	Z1004	moaE	Molybdopterin converting factor, subunit 2
G2583_1014	ECs0864	Z1005	ybhL	hypothetical protein
G2583_1015	ECs0865	Z1006	ybhM	hypothetical protein
G2583_1016	ECs0866	Z1007	ybhN	hypothetical protein
G2583_1017	ECs0867	Z1008	ybhO	Putative cardiolipin synthetase ybhO
G2583_1018	ECs0868	Z1009	ybhP	hypothetical protein
G2583_1019	ECs0869	Z1010	ybhQ	Inner membrane protein ybhQ
G2583_1020	ECs0870	Z1012	ybhR	hypothetical protein
G2583_1021	ECs0871	Z1013	ybhS	ABC-2 type transporter, permease protein
G2583_1022	ECs0872	Z1014	ybhF	Putative ATP-binding component of a transport system
G2583_1023	ECs0873	Z1015	ybhG	UPF0194 membrane protein ybhG precursor
G2583_1024	ECs0874	Z1016	ybiH	Hypothetical transcriptional regulator ybiH
G2583_1025	ECs0875	Z1017	rhlE	ATP-dependent RNA helicase RhlE
		Z1018	-	hypothetical protein
G2583_1026	ECs0876	Z1019	sopA	hypothetical protein
G2583_1027	ECs0877	Z1020	dinG	ATP-dependent DNA helicase DinG
G2583_1028	ECs0878	Z1021	ybiB	Glycosyl transferase family protein
G2583_1029	ECs0879	Z1022	ybiC	Uncharacterized oxidoreductase ybiC
G2583_1030	ECs0880	Z1023	ybiJ	hypothetical protein
G2583_1031	ECs0881	Z1024	ybiI	C4-type zinc finger protein, DksA/TraR family
G2583_1032	ECs0882	Z1025	ybiX	Putative enzyme
G2583_1033	ECs0883	Z1026	fiu	Catecholate siderophore receptor fiu precursor
G2583_1034	ECs0884	Z1027	ybiM	hypothetical protein
G2583_1035	ECs0885	Z1028	ybiN	hypothetical protein
G2583_1036	ECs0886	Z1030	ybiO	Transporter, small conductance mechanosensitive ion channel (MscS) family
G2583_1037	ECs0887	Z1031	glnQ	Glutamine ABC transporter, ATP-binding protein
G2583_1038	ECs0888	Z1032	glnP	Glutamine transport system permease protein glnP
G2583_1039	ECs0889	Z1033	glnH	hypothetical protein
G2583_1040	ECs0890	Z1034	dps	Global regulator, starvation conditions
G2583_1041	ECs0891	Z1035	rhtA	Inner membrane transporter rhtA
G2583_1042	ECs0892	Z1036	ompX	Outer membrane protein X precursor
G2583_1043	ECs0893	Z1037	ybiP	Sulfatase family protein
G2583_1044		Z1038	yliL	hypothetical protein

Locus_tag[a]				
CB9615	Sakai	EDL933	Gene	Product
G2583_1045	ECs0894	Z1039	mntR	manganese transport regulator MntR
G2583_1046	ECs0895	Z1040	ybiR	Citrate transporter family protein
G2583_1047	ECs0896	Z1041	ybiS	hypothetical protein
G2583_1048	ECs0897	Z1042	ybiT	Uncharacterized ABC transporter ATP-binding protein ybiT
G2583_1049	ECs0898	Z1043	ybiU	hypothetical protein
G2583_1050	ECs0899	Z1044	ybiV	Sugar phosphatase SupH
G2583_1051	ECs0900	Z1045	ybiW	Pyruvate-formate lyase
G2583_1051	ECs0901	Z1046	ybiW	Pyruvate-formate lyase
G2583_1052	ECs0902	Z1047	ybiY	Glycyl-radical enzyme activating protein family
G2583_1053	ECs0903	Z1048	fsaA	Fructose-6-phosphate aldolase 1
G2583_1054	ECs0904	Z1049	moeB	Molybdopterin biosynthesis
G2583_1055	ECs0905	Z1050	moeA	Molybdopterin biosynthesis protein MoeA
G2583_1056	ECs0906	Z1051	iaaA	Putative asparaginase
G2583_1056	ECs0907	Z1052	iaaA	Putative asparaginase
G2583_1057	ECs0908	Z1053	gsiA	Glutathione ABC transporter, ATP-binding protein GsiA
G2583_1058	ECs0909	Z1054	gsiB	Glutathione-binding protein gsiB precursor
G2583_1059	ECs0910	Z1055	gsiC	Glutathione transport system permease protein gsiC
G2583_1060	ECs0911	Z1056	gsiD	Glutathione transport system permease protein gsiD
G2583_1061	ECs0912	Z1057	yliE	cyclic diguanylate phosphodiesterase (EAL) domain protein
G2583_1062	ECs0913	Z1058	yliF	Uncharacterized membrane protein yliF
	ECs0914		-	hypothetical membrane protein
G2583_1063		Z1059	-	Helix-turn-helix DNA-binding domain protein
G2583_1064		Z1060	-	hypothetical protein
G2583_1065	ECs0915	Z1061	yliG	UPF0004 protein yliG
G2583_1066	ECs0916	Z1062	bssR	Biofilm regulator bssR
G2583_1067	ECs0917	Z1063	yliI	Glucose / sorbosone dehydrogenase protein
G2583_1068	ECs0918	Z1064	yliJ	putative transferase
G2583_1069	ECs0919	Z1066	dacC	Penicillin-binding protein 6
G2583_1070	ECs0920	Z1067	deoR	Deoxyribose operon repressor
G2583_1071	ECs0921	Z1068	ybjG	Undecaprenyl-diphosphatase
G2583_1072	ECs0922	Z1069	cmr	Multidrug translocase MdfA
G2583_1073	ECs0923	Z1070	ybjH	hypothetical protein
G2583_1074	ECs0924	Z1071	ybjI	Cof-like hydrolase
G2583_1075	ECs0925	Z1072	ybjJ	putative DEOR-type transcriptional regulator
G2583_1076	ECs0926	Z1073	ybjK	putative DEOR-type transcriptional regulator
G2583_1077			rybB	ncRNA
G2583_1078	ECs0927	Z1074	ybjL	Putative transport protein ybjL
G2583_1079	ECs0928	Z1075	ybjM	Inner membrane protein ybjM
G2583_1080	ECs0929	Z1076	grxA	Glutaredoxin 1
G2583_1081	ECs0930	Z1077	ybjC	hypothetical protein
G2583_1082	ECs0931	Z1078	nfsA	Oxygen-insensitive NADPH nitroreductase
G2583_1083	ECs0932	Z1079	rimK	Ribosomal protein S6 modification protein

Locus_tag[a]				
CB9615	Sakai	EDL933	Gene	Product
G2583_1084	ECs0933	Z1080	ybjN	Putative sensory transduction regulator
G2583_1085			potF	Putrescine ABC transporter, periplasmic putrescine-binding protein
G2583_1085	ECs0934	Z1081	potF	Putrescine ABC transporter, periplasmic putrescine-binding protein
G2583_1086	ECs0935	Z1082	potG	Putrescine transport ATP-binding protein PotG
G2583_1087	ECs0936	Z1083	potH	Putrescine transport protein
G2583_1088	ECs0937	Z1084	potI	Putrescine ABC transporter, permease protein PotI
G2583_1089	ECs0938	Z1085	ybjO	Inner membrane protein ybjO
G2583_1090	ECs0939	Z1086	rumB	23S rRNA (uracil-5-)-methyltransferase rumB
G2583_1091	ECs0940	Z1087	ulaA	Ascorbate-specific PTS system enzyme IIC
G2583_1092	ECs0941	Z1088	-	hypothetical protein
G2583_1093	ECs0942	Z1089	-	putative sulfatase
G2583_1094	ECs0943	Z1090	artJ	Arginine 3rd transport system periplasmic binding
G2583_1095	ECs0944	Z1091	artM	Arginine transport system permease protein artM
G2583_1096	ECs0945	Z1092	artQ	Arginine ABC transporter, permease protein ArtQ
G2583_1097	ECs0946	Z1093	artI	Arginine ABC transporter, periplasmic arginine-binding protein ArtI
G2583_1098	ECs0947	Z1094	artP	Arginine transport ATP-binding protein ArtP
G2583_1099	ECs0948	Z1095	ybjP	Putative lipoprotein YbjP
G2583_1100		Z1096	-	hypothetical protein
G2583_1101	ECs0949	Z1097	-	hypothetical protein
G2583_1101	ECs0950	Z1098	-	hypothetical protein
G2583_1102	ECs0951		-	hypothetical protein
G2583_1103	ECs0952	Z1099	ybjQ	UPF0145 protein ybjQ
G2583_1104	ECs0953	Z1100	ybjR	N-acetylmuramoyl-L-alanine amidase AmiD
G2583_1105	ECs0954	Z1102	ybjS	NAD dependent epimerase/dehydratase family
G2583_1106	ECs0955	Z1103	ybjT	NAD dependent epimerase/dehydratase family protein
G2583_1107	ECs0956	Z1104	ltaE	Low specificity L-threonine aldolase
G2583_1108	ECs0957	Z1105	poxB	Pyruvate dehydrogenase
G2583_1109	ECs0958	Z1106	hcr	NADH oxidoreductase hcr
G2583_1110	ECs0959	Z1107	hcp	Hydroxylamine reductase
G2583_1111	ECs0960	Z1108	ybjE	Putative surface protein
G2583_1112	ECs0961	Z1109	aqpZ	Aquaporin Z
G2583_1113	ECs0962	Z1110	ybjD	hypothetical protein
G2583_1114	ECs0963	Z1112	ybjX	putative enzyme
G2583_1115	ECs0964	Z1115	macA	macrolide transporter subunit MacA
G2583_1116	ECs0965	Z1116	macB	Macrolide-specific ABC-type efflux carrier protein MacB
G2583_1117	ECs0966	Z1117	cspD	Cold shock proteins
G2583_1118	ECs0967	Z1118	clpS	ATP-dependent Clp protease adapter protein clpS
G2583_1119	ECs0968	Z1119	clpA	ATP-dependent clp protease ATP-binding subunit clpA
Indel-27	Indel-27	Z1120	-	putative P4-family integrase
Indel-27	Indel-27	Z1121	-	hypothetical protein
Indel-27	Indel-27	Z1122	-	hypothetical protein

Locus_tag[a]				
CB9615	Sakai	EDL933	Gene	Product
Indel-27	Indel-27	Z1123	-	unknown in IS1N
Indel-27	Indel-27	Z1124	-	putative prophage regulatory protein
Indel-27	Indel-27	Z1125	-	hypothetical protein
Indel-27	Indel-27	Z1126	-	unknown
Indel-27	Indel-27	Z1127	-	hypothetical protein
Indel-27	Indel-27	Z1128	-	hypothetical protein
Indel-27	Indel-27	Z1129	-	putative helicase
Indel-27	Indel-27	Z1130	-	hypothetical protein
Indel-27	Indel-27	Z1131	-	unknown protein encoded in ISEc8
Indel-27	Indel-27	Z1132	-	unknown protein encoded in ISEc8
Indel-27	Indel-27	Z1133	-	partial putative transposase
Indel-27	Indel-27	Z1134	-	unknown in IS600
Indel-27	Indel-27	Z1135	-	putative complement resistance protein
Indel-27	Indel-27	Z1136	-	hypothetical protein
Indel-27	Indel-27	Z1137	-	hypothetical protein
Indel-27	Indel-27	Z1138	-	hypothetical protein
Indel-27	Indel-27	Z1139	-	putative diacylglycerol kinase
Indel-27	Indel-27	Z1140	-	unknown
Indel-27	Indel-27	Z1141	-	hypothetical protein
Indel-27	Indel-27	Z1142	ureD	putative urease accessory protein D
Indel-27	Indel-27	Z1143	ureA	putative urease structural subunit A (gamma)
Indel-27	Indel-27	Z1144	ureB	putative urease structural subunit B (beta)
Indel-27	Indel-27	Z1145	ureC	putative urease structural subunit C (alpha)
Indel-27	Indel-27	Z1146	ureE	putative urease accessory protein E
Indel-27	Indel-27	Z1147	ureF	putative urease accessory protein F
Indel-27	Indel-27	Z1148	ureG	putative urease accessory protein G
Indel-27	Indel-27	Z1149	-	hypothetical protein
Indel-27	Indel-27	Z1150	-	unknown in IS
Indel-27	Indel-27	Z1151	-	hypothetical protein
Indel-27	Indel-27	Z1152	-	putative ribosomal protein
Indel-27	Indel-27	Z1153	-	hypothetical protein
Indel-27	Indel-27	Z1154	-	hypothetical protein
Indel-27	Indel-27	Z1155	-	hypothetical protein
Indel-27	Indel-27	Z1156	-	hypothetical protein
Indel-27	Indel-27	Z1157	-	hypothetical protein
Indel-27	Indel-27	Z1158	-	unknown in ISEc8
Indel-27	Indel-27	Z1159	-	unknown in ISEc8
Indel-27	Indel-27	Z1160	-	unknown in ISEc8
Indel-27	Indel-27	Z1161	-	unknown in ISEc8
Indel-27	Indel-27	Z1162	-	unknown in ISEc8
Indel-27	Indel-27	Z1163	-	unknown in ISEc8
Indel-27	Indel-27	Z1164	terW	unknown associated with putative tellurite resistance
Indel-27	Indel-27	Z1165	-	hypothetical protein
Indel-27	Indel-27	Z1166	-	hypothetical protein

Locus_tag[a]				
CB9615	Sakai	EDL933	Gene	Product
Indel-27	Indel-27	Z1167	-	hypothetical protein
Indel-27	Indel-27	Z1168	-	hypothetical protein
Indel-27	Indel-27	Z1169	-	hypothetical protein
Indel-27	Indel-27	Z1170	-	hypothetical protein
Indel-27	Indel-27	Z1171	terZ	putative phage inhibition, colicin resistance and tellurite resistance protein
Indel-27	Indel-27	Z1172	terA	putative phage inhibition, colicin resistance and tellurite resistance protein
Indel-27	Indel-27	Z1173	terB	putative phage inhibition, colicin resistance and tellurite resistance protein
Indel-27	Indel-27	Z1174	terC	putative phage inhibition, colicin resistance and tellurite resistance protein
Indel-27	Indel-27	Z1175	terD	putative phage inhibition, colicin resistance and tellurite resistance protein
Indel-27	Indel-27	Z1176	terE	putative phage inhibition, colicin resistance and tellurite resistance protein
Indel-27	Indel-27	Z1177	terF	partial putative phage inhibition, colicin resistance and tellurite resistance protein
Indel-27	Indel-27	Z1178	-	putative receptor
Indel-27	Indel-27	Z1179	-	hypothetical protein
Indel-27	Indel-27	Z1180	-	hypothetical protein
Indel-27	Indel-27	Z1181	-	hypothetical protein
Indel-27	Indel-27	Z1182	-	hypothetical protein
Indel-27	Indel-27	Z1183	-	hypothetical protein
Indel-27	Indel-27	Z1184	-	hypothetical protein
Indel-27	Indel-27	Z1185	-	hypothetical protein
Indel-27	Indel-27	Z1186	-	hypothetical protein
Indel-27	Indel-27	Z1187	-	hypothetical protein
Indel-27	Indel-27	Z1188	-	unknown
Indel-27	Indel-27	Z1189	-	hypothetical protein
Indel-27	Indel-27	Z1190	-	putative glucosyltransferase
Indel-27	Indel-27	Z1191	-	hypothetical protein
Indel-27	Indel-27	Z1192	-	IS1 protein InsB
Indel-27	Indel-27	Z1193	-	hypothetical protein
Indel-27	Indel-27	Z1194	-	hypothetical protein
Indel-27	Indel-27	Z1195	-	hypothetical protein
Indel-27	Indel-27	Z1196	-	hypothetical protein
Indel-27	Indel-27	Z1197	-	hypothetical protein
Indel-27	Indel-27	Z1198	-	putative transposase
Indel-27	Indel-27	Z1199	-	unknown in IS
Indel-27	Indel-27	Z1200	-	hypothetical protein
Indel-27	Indel-27	Z1201	-	hypothetical protein
Indel-27	Indel-27	Z1202	-	hypothetical protein
Indel-27	Indel-27	Z1203	-	unknown

Locus_tag[a]				
CB9615	Sakai	EDL933	Gene	Product
Indel-27	Indel-27	Z1204	-	unknown
Indel-27	Indel-27	Z1205	-	hypothetical protein
Indel-27	Indel-27	Z1206	-	hypothetical protein
Indel-27	Indel-27	Z1207	-	partial transposase
Indel-27	Indel-27	Z1208	-	unknown in putative ISEc8
Indel-27	Indel-27	Z1209	-	hypothetical protein
Indel-27	Indel-27	Z1210	-	putative histone
Indel-27	Indel-27	Z1211	-	putative adhesin
Indel-27	Indel-27	Z1212	-	unknown
Indel-27	Indel-27	Z1213	-	unknown
Indel-27	Indel-27	Z1214	-	hypothetical protein
Indel-27	Indel-27	Z1215	-	unknown
Indel-27	Indel-27	Z1216	-	unknown
Indel-27	Indel-27	Z1217	-	putative DNA repair protein, RADC family
Indel-27	Indel-27	Z1218	-	orf, hypothetical protein
Indel-27	Indel-27	Z1219	-	hypothetical protein
Indel-27	Indel-27	Z1220	-	putative structural protein
Indel-27	Indel-27	Z1221	-	putative transposase
Indel-27	Indel-27	Z1222	-	unknown in IS
Indel-27	Indel-27	Z1223	-	hypothetical protein
Indel-27	Indel-27	Z1225	-	hypothetical protein
Indel-27	Indel-27	Z1226	-	unknown
G2583_1120	serW	RNA025	serW	Ser tRNA
G2583_1121	ECs0969	Z1228	infA	hypothetical protein
G2583_1122	ECs0970	Z1229	aat	Leucyl/phenylalanyl-tRNA--protein transferase
G2583_1123	ECs0971	Z1230	cydC	ABC transporter, CydDC cysteine exporter (CydDC-E) family, permease/ATP-binding protein CydC
G2583_1124	ECs0972	Z1231	cydD	ABC transporter, CydDC cysteine exporter (CydDC-E) family, permease/ATP-binding protein CydD
G2583_1125	ECs0973	Z1232	trxB	Thioredoxin reductase
G2583_1126	ECs0974	Z1234	lrp	Leucine-responsive regulatory protein
G2583_1127	ECs0975	Z1235	ftsK	DNA translocase FtsK
G2583_1128	ECs0976	Z1237	lolA	Outer-membrane lipoprotein carrier protein precursor
G2583_1129	ECs0977	Z1238	rarA	Replication-associated recombination protein A
G2583_1130	ECs0978	Z1239	serS	Seryl-tRNA synthetase
G2583_1131	ECs0979	Z1240	dmsA	Anaerobic dimethyl sulfoxide reductase, A subunit
G2583_1132	ECs0980	Z1241	dmsB	Anaerobic dimethyl sulfoxide reductase chain B
G2583_1133	ECs0981	Z1242	dmsC	Anaerobic dimethyl sulfoxide reductase subunit C
G2583_1134	ECs0982	Z1243	ycaC	Protein ycaC
G2583_1135	ECs0983	Z1244	ycaD	putative MFS family transporter protein
G2583_1136	ECs0984	Z1245	ycaM	Amino acid permease family protein
G2583_1137	ECs0985	Z1246	pflA	Pyruvate formate lyase-activating enzyme 1
G2583_1138	ECs0986	Z1248	pflB	Formate acetyltransferase
G2583_1139	ECs0987	Z1250	focA	FocA formate FNT transporter

Locus_tag[a]				
CB9615	Sakai	EDL933	Gene	Product
G2583_1140	ECs0988	Z1251	ycaO	hypothetical protein
G2583_1141	ECs0989	Z1252	ycaP	hypothetical protein
G2583_1142	ECs0990	Z1253	serC	Phosphoserine aminotransferase
G2583_1143	ECs0991	Z1254	aroA	3-phosphoshikimate 1-carboxyvinyltransferase
G2583_1144	ECs0992	Z1255	ycaL	Peptidase, M48B family
G2583_1145	ECs0993	Z1256	cmk	Cytidylate kinase
G2583_1146	ECs0994	Z1257	rpsA	30S ribosomal protein S1
G2583_1147	ECs0995	Z1258	ihfB	Integration host factor subunit beta
G2583_1148	ECs0996	Z1259	ycaI	DNA internalization-related competence protein ComEC/Rec2
G2583_1149	ECs0997	Z1260	msbA	Lipid A export ATP-binding/permease protein msbA
G2583_1150	ECs0998	Z1261	lpxK	Tetraacyldisaccharide 4'-kinase
G2583_1151	ECs0999	Z1262	ycaQ	hypothetical protein
G2583_1152	ECs1000	Z1263	ycaR	UPF0434 protein ycaR
G2583_1153	ECs1001	Z1264	kdsB	3-deoxy-manno-octulosonate cytidylyltransferase
G2583_1154	ECs1002	Z1265	ycbJ	hypothetical protein
G2583_1155	ECs1003	Z1267	ycbC	hypothetical protein
G2583_1156	ECs1004	Z1268	smtA	SmtA protein
G2583_1157	ECs1005	Z1269	mukF	Chromosome partition protein mukF
G2583_1158	ECs1006	Z1270	mukE	MukE
G2583_1159	ECs1007	Z1271	mukB	Uncharacterized protein involved in chromosome partitioning
G2583_1160	ECs1008	Z1272	ycbB	Putative peptidoglycan binding domain
G2583_1161	ECs1009	Z1273	ycbK	hypothetical protein
G2583_1162	ECs1010	Z1274	ycbL	Metallo-beta-lactamase family protein
G2583_1163	ECs1011	Z1275	aspC	Aspartate transaminase
G2583_1164	ECs1012	Z1276	ompF	Outer membrane protein F
G2583_1165	ECs1013	Z1278	asnS	Asparaginyl-tRNA synthetase
G2583_1166	ECs1014	Z1279	pncB	Nicotinate phosphoribosyltransferase
G2583_1167	ECs1015	Z1280	pepN	Aminopeptidase N
G2583_1168	ECs1016	Z1281	ssuB	ABC transporter, ATP-binding protein
G2583_1169	ECs1017	Z1282	ssuC	Putative sulfonate ABC transporter, permease protein
G2583_1170	ECs1018	Z1283	ssuD	Alkanesulfonate monooxygenase
G2583_1171	ECs1019	Z1284	ssuA	ABC transporter, periplasmic substrate-binding protein, aliphatic sulfonates family
G2583_1172	ECs1020	Z1285	ssuE	NAD(P)H-dependent FMN reductase
G2583_1173	ECs1021	Z1286	ycbQ	Putative fimbrial-like protein
G2583_1174	ECs1022	Z1287	ycbR	Putative chaperone
G2583_1175	ECs1023	Z1288	ycbS	PapC-like porin protein involved in fimbrial biogenesis
G2583_1175	ECs1024	Z1289	ycbS	PapC-like porin protein involved in fimbrial biogenesis
G2583_1176	ECs1025	Z1290	ycbT	Putative fimbrial protein
G2583_1177	ECs1026	Z1291	ycbU	Uncharacterized fimbrial-like protein ycbU precursor
G2583_1178	ECs1027	Z1292	ycbV	Putative fimbrial-like protein
G2583_1179	ECs1028	Z1293	ycbF	hypothetical protein

Locus_tag[a]				
CB9615	Sakai	EDL933	Gene	Product
G2583_1179			ycbF	hypothetical protein
G2583_1180	ECs1029	Z1294	pyrD	Dihydroorotate dehydrogenase
G2583_1181	ECs1030	Z1295	ycbW	hypothetical protein
G2583_1182	ECs1031	Z1297	ycbX	MOSC domain protein
G2583_1183	ECs1032	Z1298	rlmL	Putative RNA methylase family UPF0020
G2583_1184	ECs1033	Z1299	uup	ABC transporter, ATP-binding protein
G2583_1185	ECs1034	Z1300	pqiA	Paraquat-inducible protein A
G2583_1186	ECs1035	Z1301	pqiB	Paraquat-inducible protein B
G2583_1187	ECs1036	Z1302	ymbA	putative lipoprotein
G2583_1188	ECs1037	Z1303	rmf	Ribosome modulation factor
G2583_1189	ECs1038	Z1304	fabA	3-hydroxydecanoyl-ACP dehydratase
G2583_1190	ECs1039	Z1305	ycbZ	Peptidase, S16 (Lon protease) family
G2583_1191	ECs1040	Z1306	ycbG	UPF0268 protein ycbG
G2583_1192	ECs1041	Z1307	ompA	OmpA domain protein transmembrane region-containing protein precursor
G2583_1193	ECs1042	Z1308	sulA	Suppressor of lon
G2583_1194	ECs1043	Z1309	sxy	conserved protein
G2583_1195	ECs1044	Z1311	yccS	hypothetical protein
G2583_1196	ECs1045	Z1312	yccF	hypothetical protein
G2583_1197	ECs1046	Z1313	helD	DNA helicase IV
G2583_1198	ECs1047	Z1314	mgsA	Methylglyoxal synthase
G2583_1199	ECs1048	Z1315	yccT	UPF0319 protein yccT precursor
G2583_1200	ECs1049	Z1317	yccU	hypothetical protein
G2583_1201	ECs1050	Z1318	hspQ	Heat shock protein hspQ
G2583_1202	ECs1051	Z1319	yccW	putative oxidoreductase
G2583_1203	ECs1052	Z1320	yccX	Acylphosphatase
G2583_1204	ECs1053	Z1321	yccK	Sulfurtransferase tusE
G2583_1205	ECs1054	Z1322	yccA	Inner membrane protein yccA
Indel-28	ECs1055	Z1323	-	putative integrase
Indel-28	ECs1056		-	hypothetical protein
Indel-28	ECs1057	Z1324	-	hypothetical protein
Indel-28	ECs1058	Z1325	-	hypothetical protein
Indel-28	ECs1059	Z1326	-	putative division inhibition protein
Indel-28	dicF1	RNA026	dicF1	DicF antisense RNA
Indel-28	ECs1060		-	hypothetical protein
Indel-28	ECs1061	Z1328	-	hypothetical protein
Indel-28	ECs1062		-	hypothetical protein
Indel-28	ECs1063	Z1329	-	hypothetical protein
Indel-28	ECs1064	Z1330	-	hypothetical protein
Indel-28	ECs1065	Z1331	-	hypothetical protein
Indel-28	ECs1066		-	hypothetical protein
Indel-28	ECs1067		-	hypothetical protein
Indel-28	ECs1068	Z1332	-	hypothetical protein
Indel-28	ECs1069	Z1333	-	putative regulatory protein

Locus_tag[a]				
CB9615	Sakai	EDL933	Gene	Product
Indel-28	ECs1070	Z1334	-	hypothetical protein
Indel-28	ECs1071	Z1335	-	hypothetical protein
Indel-28		Z1336	-	unknown protein encoded by cryptic prophage CP-
Indel-28	ECs1072		-	hypothetical protein
Indel-28	ECs1073	Z1337	-	hypothetical protein
Indel-28	ECs1074	Z1338	-	putative DNA replication protein
Indel-28	ECs1075	Z1339	-	hypothetical protein
Indel-28	ECs1076	Z1340	-	hypothetical protein
Indel-28	ECs1077	Z1341	-	hypothetical protein
Indel-28	ECs1078		-	hypothetical protein
Indel-28	ECs1079		-	hypothetical protein
Indel-28	ECs1080	Z1342	-	prophage maintenance protein
Indel-28	ECs1081		-	hypothetical protein
Indel-28	ECs1082	Z1343	-	hypothetical protein
Indel-28	ECs1083	Z1344	-	putative crossover junction endodeoxyribonuclease
Indel-29	ECs1084	Indel-29	-	putative antitermination protein
Indel-29	ECs1085	Indel-29	-	hypothetical membrane protein
Indel-29	ECs1086	Indel-29	-	hypothetical protein
Indel-29	ECs1087	Indel-29	-	putative transcriptional regulator
Indel-29	ileZ1	Indel-29	ileZ1	Ile tRNA
Indel-29	argN1	Indel-29	argN1	Arg tRNA
Indel-29	argO1	Indel-29	argO1	Arg tRNA
Indel-29	ECs1088	Indel-29	-	hypothetical protein
Indel-29	ECs1089	Indel-29	-	putative transposase OrfA protein of insertion sequence IS629
Indel-29	ECs1090	Indel-29	-	putative transposase OrfB protein of insertion sequence IS629
Indel-29	ECs1091	Indel-29	-	putative transcriptional regulator
Indel-29	ECs1093	Indel-29	-	putative endopeptidase
Indel-29	ECs1092	Indel-29	-	putative lipoprotein Rz1 protein precursor
Indel-29	ECs1094	Indel-29	-	hypothetical protein
Indel-29	ECs1095	Indel-29	-	hypothetical protein
Indel-29	ECs1096	Indel-29	-	putative endolysin
Indel-29	ECs1098	Indel-29	-	hypothetical protein
Indel-29	ECs1097	Indel-29	-	hypothetical protein
Indel-29	ECs1099	Indel-29	-	hypothetical protein
Indel-29	ECs1100	Indel-29	-	putative holin protein
Indel-29	ECs1101	Indel-29	-	hypothetical protein
Indel-29	ECs1102	Indel-29	-	hypothetical protein
Indel-29	ECs1103	Indel-29	-	hypothetical protein
Indel-29	ECs1104	Indel-29	-	hypothetical protein
Indel-29	ECs1105	Indel-29	-	putative terminase small subunit
Indel-29	ECs1106	Indel-29	-	putative terminase large subunit
Indel-29	ECs1107	Indel-29	-	putative portal protein

Locus_tag[a]				
CB9615	Sakai	EDL933	Gene	Product
Indel-29	ECs1108	Indel-29	-	putative head-tail preconnector protein
Indel-29	ECs1109	Indel-29	-	putative head decoration protein
Indel-29	ECs1110	Indel-29	-	putative major head protein
Indel-29	ECs1111	Indel-29	-	hypothetical protein
Indel-29	ECs1112	Indel-29	-	putative minor tail protein
Indel-29	ECs1113	Indel-29	-	putative minor tail protein
Indel-29	ECs1114	Indel-29	-	putative tail length tape measure protein
Indel-29	ECs1115	Indel-29	-	putative minor tail protein
Indel-29	ECs1116	Indel-29	-	putative minor tail protein
Indel-29	ECs1117	Indel-29	-	putative tail assembly protein
Indel-29	ECs1118	Indel-29	-	putative tail assembly protein
Indel-29	ECs1119	Indel-29	-	hypothetical protein
Indel-29	ECs1120	Indel-29	-	putative copper/zinc-superoxide dismutase
Indel-29	ECs1121	Indel-29	-	putative host specificity protein
Indel-29	ECs1122	Indel-29	-	putative outer membrane protein
Indel-29	ECs1123	Indel-29	-	putative tail fiber protein
Indel-29	ECs1124	Indel-29	-	hypothetical protein
Indel-31	ECs1125		-	hypothetical protein
Indel-29	ECs1126	Indel-29	-	EspF-like protein
Indel-30	Indel-30	Z1345	-	antitermination protein Q homolog of cryptic prophage CP-933M
Indel-30	Indel-30	Z1346	-	unknown protein encoded by cryptic prophage CP-
Indel-30	Indel-30	Z1347	-	unknown protein encoded by cryptic prophage CP-
Indel-30	Indel-30	Z1348	-	unknown protein encoded by cryptic prophage CP-
Indel-30	Indel-30	Z1349	-	conserved hypothetical protein similar to yjhS for cryptic prophage CP-933M
Indel-30	Indel-30	Z1350	-	putative holin protein of cryptic prophage CP-933M
Indel-30	Indel-30	Z1351	-	unknown protein encoded by cryptic prophage CP-
Indel-30	Indel-30	Z1352	-	putative endolysin of cryptic prophage CP-933M
Indel-30	Indel-30	Z1353	-	putative antirepressor protein of cryptic prophage CP-933M
Indel-30	Indel-30	Z1354	-	putative endopeptidase of cryptic prophage CP-933M
Indel-30	Indel-30	Z1355	-	unknown protein encoded by cryptic prophage CP-
Indel-30	Indel-30	Z1356	-	unknown protein encoded by cryptic prophage CP-
Indel-30	Indel-30	Z1357	-	unknown protein encoded by cryptic prophage CP-
Indel-30	Indel-30	Z1358	-	unknown protein encoded by cryptic prophage CP-
Indel-30	Indel-30	Z1359	-	unknown protein encoded by cryptic prophage CP-
Indel-30	Indel-30	Z1360	-	unknown protein encoded by cryptic prophage CP-
Indel-30	Indel-30	Z1361	-	unknown protein encoded by cryptic prophage CP-
Indel-30	Indel-30	Z1362	-	unknown protein encoded by cryptic prophage CP-
Indel-30	Indel-30	Z1363	-	unknown protein encoded by cryptic prophage CP-
Indel-30	Indel-30	Z1364	-	unknown protein encoded by cryptic prophage CP-
Indel-30	Indel-30	Z1365	-	unknown protein encoded by cryptic prophage CP-
Indel-30	Indel-30	Z1366	-	unknown protein encoded by cryptic prophage CP-

Locus_tag[a]				
CB9615	Sakai	EDL933	Gene	Product
Indel-30	Indel-30	Z1367	-	unknown protein encoded by cryptic prophage CP-
Indel-30	Indel-30	Z1368	-	unknown protein encoded by cryptic prophage CP-
Indel-30	Indel-30	Z1369	-	unknown protein encoded by cryptic prophage CP-
Indel-30	Indel-30	Z1370	-	putative tail component encoded by cryptic prophage CP-933M
Indel-30	Indel-30	Z1371	-	putative tail assembly chaperone encoded by cryptic prophage CP-933M
Indel-30	Indel-30	Z1372	-	unknown protein encoded by cryptic prophage CP-
Indel-30	Indel-30	Z1373	-	unknown protein encoded by cryptic prophage CP-
Indel-30	Indel-30	Z1374	-	unknown protein encoded by cryptic prophage CP-
Indel-30	Indel-30	Z1375	-	putative tail component encoded by cryptic prophage CP-933M; partial
Indel-30	Indel-30	Z1376	-	putative tail component encoded by cryptic prophage CP-933M; partial
Indel-30	Indel-30	Z1377	-	putative tail component encoded by cryptic prophage CP-933M
Indel-30	Indel-30	Z1378	-	putative tail component encoded by cryptic prophage CP-933M; partial
Indel-30	Indel-30	Z1379	-	putative tail component encoded by cryptic prophage CP-933M; partial
Indel-30	Indel-30	Z1380	-	putative tail component encoded by cryptic prophage CP-933M; partial
Indel-30	Indel-30	Z1381	-	putative outer membrane protein Lom precursor encoded by cryptic prophage CP-933M
Indel-30	Indel-30	Z1382	-	putative tail component encoded by cryptic prophage CP-933M; partial
Indel-30	Indel-30	Z1383	-	unknown protein encoded by cryptic prophage CP-
Indel-31	Indel-31	Z1386	-	unknown protein encoded by cryptic prophage CP-
Indel-31	ECs1127	Z1387	-	hypothetical protein
G2583_1206	serT	RNA027	serT	Ser tRNA
G2583_1207	ECs1128	Z1389	hyaA	Nickel-dependent hydrogenase 1, small subunit
G2583_1208	ECs1129	Z1390	hyaB	Hydrogenase-1 large subunit
G2583_1209	ECs1130	Z1391	hyaC	Probable Ni/Fe-hydrogenase 1 B-type cytochrome
G2583_1210	ECs1131	Z1392	hyaD	hydrogenase 1 maturation protease
G2583_1211	ECs1132	Z1393	hyaE	Hydrogenase-1 expression protein HyaE
G2583_1212	ECs1133	Z1394	hyaF	Hydrogenase-1 operon protein HyaF
G2583_1213	ECs1134	Z1395	appC	Cytochrome bd-II oxidase, subunit I
G2583_1214	ECs1135	Z1396	appB	Cytochrome bd-II oxidase, subunit II
G2583_1215	ECs1136	Z1397	appA	Phosphoanhydride phosphorylase
G2583_1216	ECs1137	Z1398	etk	Tyrosine-protein kinase etk
G2583_1217	ECs1138	Z1399	etp	Putative phosphatase
G2583_1218	ECs1139	Z1400	gfcE	Putative polysaccharide export protein gfcE precursor
G2583_1219	ECs1140	Z1401	gfcD	Group 4 capsule (G4C) polysaccharide, lipoprotein
G2583_1220	ECs1141	Z1402	gfcC	Group 4 capsule (G4C) polysaccharide, YmcB

Locus_tag[a]				
CB9615	Sakai	EDL933	Gene	Product
G2583_1221	ECs1142	Z1403	gfcB	Group 4 capsule (G4C) polysaccharide, lipoprotein
G2583_1222	ECs1143	Z1404	ymcD	putative inner membrane protein
G2583_1223	ECs1144	Z1405	cspH	Cold shock-like protein cspH
G2583_1224	ECs1145	Z1406	cspG	Cold shock-like protein cspG
G2583_1225	ECs1146	Z1408	ymcE	cold shock gene
G2583_1226	ECs1147	Z1409	yccM	4Fe-4S binding domain protein
G2583_1227	ECs1148	Z1410	torS	hybrid sensory histidine kinase TorS
G2583_1228	ECs1149	Z1411	torT	Periplasmic protein torT precursor
G2583_1229	ECs1150	Z1412	torR	TorCAD operon transcriptional regulatory protein torR
G2583_1230	ECs1151	Z1414	torC	Cytochrome c-type protein TorC
G2583_1231	ECs1152	Z1415	torA	Trimethylamine-N-oxide reductase 1 precursor
G2583_1232	ECs1153	Z1416	torD	Chaperone protein torD
G2583_1233	ECs1154	Z1417	cbpM	Chaperone modulatory protein cbpM
G2583_1234	ECs1155	Z1418	cbpA	Curved DNA-binding protein
G2583_1235	ECs1156	Z1419	yccE	conserved hypothetical protein
G2583_1235	ECs1157	Z1420	yccE	conserved hypothetical protein
G2583_1236	ECs1158	Z1421	agp	Glucose-1-phosphatase
G2583_1237	ECs1159	Z1422	yccJ	hypothetical protein
G2583_1238	ECsp005	Z1423	wrbA	Flavoprotein wrbA
Indel-33	ECs1160	Z1424	intW	putative integrase
Indel-33	ECs1161	Z1425	xisW	putative excisionase
Indel-33	ECs1162	Z1426	-	hypothetical protein
Indel-33	ECs1163	Z1428	-	hypothetical protein
Indel-33	ECs1164	Z1429	-	hypothetical protein
Indel-33	ECs1165	Z1430	-	hypothetical protein
Indel-33	ECs1166		-	hypothetical protein
Indel-33		Z1431	-	unknown protein encoded by bacteriophage BP-933W
Indel-33	ECs1167		-	hypothetical protein
Indel-33	ECs1168		-	hypothetical protein
Indel-33	ECs1169	Z1432	-	hypothetical protein
Indel-33	ECs1170		-	C4-type zinc finger protein (TraR family)
Indel-33	ECs1171	Z1433	-	hypothetical protein
Indel-33	ECs1172	Z1434	-	hypothetical protein
Indel-33	ECs1173		-	hypothetical protein
Indel-33	ECs1174	Z1435	exoW	exonuclease
Indel-33	ECs1175	Z1437	betW	recombination protein Bet
Indel-33	ECs1176	Z1438	gamW	host-nuclease inhibitor protein Gam
Indel-33	ECs1177	Z1439	kilW	Kil protein
Indel-33	ECs1178		-	regulatory protein cIII
Indel-33	ECs1179	Z1440	ssbW	single strand binding protein Ea10
Indel-34	ECs1180	Indel-34	-	hypothetical protein
Indel-34	ECs1181	Indel-34	-	putative anti-termination protein N
Indel-34	ECs1182	Indel-34	-	hypothetical protein
Indel-34	ECs1183	Indel-34	-	hypothetical protein

Locus_tag[a]				
CB9615	Sakai	EDL933	Gene	Product
Indel-34	ECs1184	Indel-34	-	hypothetical protein
Indel-34	ECs1185	Indel-34	-	putative cI repressor protein
Indel-34	ECs1186	Indel-34	-	putative regulatory protein
Indel-34	ECs1187	Indel-34	-	regulatory protein CII
Indel-34	ECs1188	Indel-34	-	hypothetical protein
Indel-34	ECs1189	Indel-34	-	replication protein O
Indel-34	ECs1190	Indel-34	-	replication protein P
Indel-34	ECs1191	Indel-34	-	hypothetical protein
Indel-34	ECs1192	Indel-34	-	hypothetical protein
Indel-34	ECs1193	Indel-34	-	hypothetical protein
Indel-34	ECs1194	Indel-34	-	hypothetical protein
Indel-34	ECs1195	Indel-34	-	hypothetical protein
Indel-34	ECs1196	Indel-34	-	putative DNA methylase
Indel-34	ECs1197	Indel-34	-	NinE protein
Indel-34	ECs1198	Indel-34	-	hypothetical protein
Indel-34	ECs1199	Indel-34	-	putative antirepressor protein
Indel-35	Indel-35	Z1441	-	unknown protein encoded by bacteriophage BP-933W
Indel-35	Indel-35	Z1442	-	putative antitermination protein N of bacteriophage BP-933W
Indel-35	Indel-35	Z1443	-	unknown protein encoded by bacteriophage BP-933W
Indel-35	Indel-35	Z1444	-	putative serine/threonine kinase encoded by bacteriophage BP-933W
Indel-35	Indel-35	Z1445	-	unknown protein encoded by bacteriophage BP-933W
Indel-35	Indel-35	Z1446	-	unknown protein encoded by bacteriophage BP-933W
Indel-35	Indel-35	Z1447	-	putative repressor protein CI of bacteriophage BP-933W
Indel-35	Indel-35	Z1448	-	regulatory protein Cro of bacteriophage BP-933W
Indel-35	Indel-35	Z1449	-	putative regulatory protein CII of bacteriophage BP-
Indel-35	Indel-35	Z1450	-	putative replication protein O of bacteriophage BP-
Indel-35	Indel-35	Z1451	-	putative replication protein P of bacteriophage BP-933W
Indel-35	Indel-35	Z1452	-	unknown protein encoded by bacteriophage BP-933W
Indel-35	Indel-35	Z1453	-	unknown protein encoded by bacteriophage BP-933W
Indel-35	Indel-35	Z1454	-	putative DNA N-6-adenine-methyltransferase of bacteriophage BP-933W
Indel-35	Indel-35	Z1456	-	unknown protein encoded by bacteriophage BP-933W
Indel-36	ECs1200	Z1457	-	DNA-binding protein
Indel-36	ECs1201	Z1458	-	hypothetical protein NinG
Indel-36	ECs1202		-	hypothetical protein
Indel-36	ECs1203	Z1459	-	antitermination protein Q
Indel-36	ECs1204	Z1460	-	hypothetical protein
Indel-36	ileZ2	RNA028	ileZ2	Ile tRNA
Indel-36	argN2	RNA029	argN2	Arg tRNA
Indel-36	argO2	RNA030	argO2	Arg tRNA
Indel-36	ECs1205	Z1464	stx2A	Shiga toxin 2 subunit A
Indel-36	ECs1206	Z1465	stx2B	Shiga toxin 2 subunit B

Locus_tag[a]				
CB9615	Sakai	EDL933	Gene	Product
Indel-36	ECs1207	Z1466	-	hypothetical protein
Indel-37	ECs1208	Indel-37	-	putative transposase OrfB protein of insertion sequence IS629
Indel-37	ECs1209	Indel-37	-	putative transposase OrfA protein of insertion sequence IS629
Indel-38	ECsp006	Z1466	-	unknown protein encoded by bacteriophage BP-933W
Indel-38	ECs1210		-	hypothetical protein
Indel-38	ECs1211	Z1467	-	hypothetical protein
Indel-38	ECs1212	Z1468	-	putative holin protein
Indel-38	ECs1213	Z1469	-	putative endolysin
Indel-38	ECs1214	Z1471	-	putative antirepressor protein
Indel-38	ECs1215	Z1473	-	putative endopeptidase
Indel-38	ECs1216		-	putative lipoprotein Rz1 precursor
Indel-38	ECs1217	Z1474	borW	Bor protein precursor
Indel-38	ECs1218		-	hypothetical protein
Indel-38	ECs1219	Z1475	-	putative small subunit terminase
Indel-38	ECs1220	Z1476	-	putative terminase large subunit
Indel-38	ECs1221	Z1477	-	putative portal protein
Indel-38	ECs1222	Z1478	-	hypothetical protein
Indel-38	ECs1223	Z1479	-	hypothetical protein
Indel-38	ECs1224	Z1480	-	hypothetical protein
Indel-38	ECs1225	Z1481	-	hypothetical protein
Indel-38	ECs1226		-	hypothetical protein
Indel-38	ECs1227	Z1482	-	hypothetical protein
Indel-38	ECs1228	Z1483	-	putative tail fiber protein
Indel-38	ECs1229	Z1484	-	hypothetical protein
Indel-38	ECs1230	Z1485	-	hypothetical protein
Indel-38	ECs1231		-	putative outer membrane protein
Indel-38	ECs1232	Z1486	-	hypothetical protein
Indel-38	ECs1233	Z1487	-	putative tail tip fiber protein
Indel-38	ECs1234		-	putative outer membrane protein
Indel-38	ECs1235	Z1488	-	hypothetical protein
Indel-38	ECs1236	Z1489	lomW	putative outer membrane precursor
Indel-38	ECs1237	Z1490	-	hypothetical protein
Indel-38	ECs1238	Z1491	-	hypothetical protein
Indel-38	ECs1239	Z1492	-	hypothetical protein
Indel-38	ECs1240	Z1493	-	hypothetical protein
Indel-38	ECs1241	Z1494	-	hypothetical protein
Indel-38	ECs1242	Z1495	-	hypothetical protein
Indel-38	ECs1243		-	hypothetical protein
Indel-38	ECs1244	Z1498	-	hypothetical protein
Indel-38	ECs1245		-	MokW protein
Indel-38	ECs1246	Z1500	-	hypothetical protein
Indel-38	ECs1247	Z1501	-	hypothetical protein

Locus_tag[a]				
CB9615	Sakai	EDL933	Gene	Product
Indel-38	ECs1248	Z1501	-	hypothetical protein
Indel-38	ECs1249		-	hypothetical protein
Indel-39	ECs1250	Indel-39	-	C4-type zinc finger protein (TraR family)
Indel-40	ECs1251	Z1503	-	putative anti-repressor protein
G2583_1238		Z1504	wrbA	Flavoprotein wrbA
G2583_1239	ECs1252	Z1505	rutG	Putative purine permease ycdG
G2583_1240	ECs1253	Z1506	rutF	Flavin reductase domain protein FMN-binding
G2583_1241	ECs1254	Z1507	rutE	Putative NADH dehydrogenase/NAD(P)H nitroreductase rutE
G2583_1242	ECs1255	Z1508	rutD	putative acetyltransferase
G2583_1243	ECs1256	Z1509	rutC	hypothetical protein
G2583_1244	ECs1257	Z1510	rutB	Putative synthetase
G2583_1245	ECs1258	Z1511	rutA	Putative monooxygenase rutA
G2583_1246	ECs1259	Z1512	rutR	putative tet operon regulator
G2583_1247	ECs1260	Z1513	putA	Bifunctional protein PutA
G2583_1248	ECs1261	Z1515	putP	Sodium/proline symporter
G2583_1249	ECs1262	Z1516	-	hypothetical protein
G2583_1250		Z1517	-	hypothetical protein
G2583_1251		Z1518	-	hypothetical protein
G2583_1252	ECs1263	Z1519	ycdN	Ferrous iron permease efeU
G2583_1253	ECs1264	Z1520	ycdO	UPF0409 protein ycdO precursor
G2583_1254	ECs1265	Z1521	ycdB	Tat-translocated enzyme
G2583_1255	ECs1266	Z1522	phoH	hypothetical protein
G2583_1256	ECs1267	Z1523	pgaD	Biofilm PGA synthesis protein pgaD
G2583_1257	ECs1268	Z1524	pgaC	Biofilm PGA synthesis N-glycosyltransferase pgaC
G2583_1258	ECs1269	Z1525	pgaB	Biofilm PGA synthesis lipoprotein pgaB
G2583_1259	ECs1270	Z1526	pgaA	Biofilm PGA synthesis protein pgaA precursor
G2583_1260	ECs1271	Z1527	ycdT	diguanylate cyclase (GGDEF) domain protein
G2583_1261	ECs1272	Z1528	rtn	Rtn-like protein
G2583_1262	ECs1273	Z1530	fidL	hypothetical protein
G2583_1263	ECs1274	Z1531	marT	hypothetical protein
		Z1532	-	hypothetical protein
G2583_1264	ECs1275	Z1533	ygcW	2-deoxy-D-gluconate 3-dehydrogenase
G2583_1265	ECs1276	Z1534	-	Putative chaperone protein
G2583_1266	ECs1277	Z1535	mrkD	hypothetical protein
G2583_1267	ECs1278	Z1536	mrkC	Putative outer membrane usher protein
G2583_1268	ECs1279	Z1537	-	Gram-negative pili assembly chaperone
G2583_1269	ECs1280	Z1538	mrkA	Fimbrial protein
G2583_1270	ECs1281	Z1539	-	hypothetical protein
G2583_1271		Z1540	-	hypothetical protein
G2583_1272		Z1541	-	hypothetical protein
G2583_1273	ECs1282	Z1542	-	Hemagglutinin/hemolysin-related protein
G2583_1274	ECs1283	Z1543	-	Putative outer membrane transporter of ShlA/HecA/FhaA exoprotein family

Locus_tag[a]				
CB9615	Sakai	EDL933	Gene	Product
G2583_1275	ECs1284	Z1544	-	Probable holo-[acyl-carrier-protein] synthase 2
G2583_1276	ECs1285	Z1545	fabG	NAD dependent epimerase/dehydratase family
G2583_1277	ECs1286	Z1546	cylZ	Putative fatty acyl chain dehydrase
G2583_1278	ECs1287	Z1547	-	Putative acyl-carrier protein
G2583_1279	ECs1288	Z1548	gcvT	Putative aminomethyltransferase
G2583_1280	ECs1289	Z1549	fabF	Putative beta-ketoacyl-[acyl carrier protein] synthase
G2583_1281	ECs1290	Z1550	olmA	Beta-ketoacyl synthase, C-domain protein
G2583_1282	ECs1291	Z1551	-	hypothetical protein
G2583_1283	ECs1292	Z1552	-	Lipoprotein-releasing system ATP-binding protein LolD
G2583_1284	ECs1293	Z1553	-	hypothetical protein
G2583_1285	ECs1294	Z1554	-	hypothetical protein
G2583_1286	ECs1295	Z1555	-	hypothetical protein
G2583_1287	ECs1296	Z1556	-	hypothetical protein
G2583_1288	ECs1297	Z1557	-	Malonyl CoA-acyl carrier protein transacylase
G2583_1289	ECs1298	Z1558	ycdU	hypothetical protein
Indel-45	ECs1299	Z1559	-	putative integrase
Indel-45	ECs1300	Z1560	-	putative membrane protein
Indel-45	ECsp007	Z1561	-	hypothetical protein
Indel-45	ECs1301	Z1562	-	transposase
Indel-45	ECs1302	Z1563	-	putative regulatory protein
Indel-45		Z1564	-	hypothetical protein
Indel-45	ECs1303	Z1565	-	hypothetical protein
Indel-45		Z1566	-	hypothetical protein
Indel-45	ECs1304	Z1567	-	hypothetical protein
Indel-45	ECs1305	Z1568	-	hypothetical protein
Indel-45		Z1569	-	hypothetical protein
Indel-45	ECs1306		-	hypothetical protein
Indel-45	ECsp008		-	IS30 transposase
Indel-45	ECs1307	Z1570	-	hypothetical protein
Indel-45	ECs1308	Z1571	-	hypothetical protein
Indel-45	ECs1309		-	hypothetical protein
Indel-45	ECs1310	Z1572	-	IS30 transposase
Indel-45	ECs1311	Z1573	-	putative transposase
Indel-45	ECs1312	Z1574	-	putative complement resistance protein precursor
Indel-45	ECs1313	Z1575	-	hypothetical protein
Indel-45	ECs1314	Z1576	-	hypothetical protein
Indel-45	ECs1315		-	hypothetical protein
Indel-45		Z1577	-	hypothetical protein
Indel-45	ECs1316	Z1578	-	putative diacylglycerol kinase
Indel-45	ECs1317	Z1579	-	putative outer membrane protein
Indel-45	ECs1318		-	hypothetical protein
Indel-45	ECs1319	Z1580	-	hypothetical protein
Indel-45	ECs1320		-	hypothetical protein
Indel-45	ECs1321	Z1581	ureD_2	urease-associated protein UreD

Locus_tag[a]				
CB9615	Sakai	EDL933	Gene	Product
Indel-45	ECs1322	Z1582	ureA_2	urease gamma subunit
Indel-45	ECs1323	Z1583	ureB_2	urease beta subunit
Indel-45	ECs1324	Z1584	ureC_2	urease alpha subunit
Indel-45	ECs1325	Z1585	ureE_2	urease accessory protein UreE
Indel-45	ECs1326	Z1586	ureF_2	urease accessory protein UreF
Indel-45	ECs1327	Z1587	ureG_2	urease accessory protein UreG
Indel-45	ECsp009		-	autosecreted serine protease
Indel-45		Z1588	-	hypothetical protein
Indel-45	ECs1328	Z1589	-	hypothetical protein
Indel-45	ECs1329		-	hypothetical protein
Indel-45		Z1590	-	hypothetical protein
Indel-45	ECs1330	Z1591	-	ribosomal protein L31-like protein
Indel-45		Z1592	-	hypothetical protein
Indel-45	ECs1331		-	hypothetical protein
Indel-45		Z1593	-	hypothetical protein
Indel-45	ECs1332		-	putative colicin immunity protein
Indel-45	ECs1333	Z1594	-	putative membrane protein
Indel-45	ECs1334		-	hypothetical protein
Indel-45		Z1595	-	hypothetical protein
Indel-45		Z1596	-	hypothetical protein
Indel-45	ECs1335		-	hypothetical protein
Indel-45	ECs1336	Z1597	-	hypothetical protein
Indel-45	ECs1337	Z1598	-	hypothetical protein
Indel-45	ECs1338	Z1599	-	hypothetical protein
Indel-45	ECs1339	Z1600	-	hypothetical protein
Indel-45	ECs1340	Z1601	-	hypothetical protein
Indel-45	ECs1341	Z1602	-	hypothetical protein
Indel-45	ECs1342		-	hypothetical protein
Indel-45	ECs1343	Z1603	terW_2	TerW protein
Indel-45	ECs1344	Z1604	-	hypothetical protein
Indel-45	ECs1345	Z1605	-	hypothetical protein
Indel-45	ECs1346	Z1606	-	hypothetical protein
Indel-45	ECs1347	Z1606	-	hypothetical protein
Indel-45	ECs1348	Z1607	-	hypothetical protein
Indel-45	ECs1349	Z1608	-	hypothetical protein
Indel-45	ECs1350	Z1609	-	hypothetical protein
Indel-45	ECs1351	Z1610	terZ_2	putative tellurium resistance protein TerZ
Indel-45	ECs1352	Z1611	terA_2	putative tellurium resistance protein TerA
Indel-45	ECs1353	Z1612	terB_2	putative tellurium resistance protein TerB
Indel-45	ECs1354	Z1613	terC_2	putative tellurium resistance protein TerC
Indel-45	ECs1355	Z1614	terD_2	putative tellurium resistance protein TerD
Indel-45	ECs1356	Z1615	terE_2	putative tellurium resistance TerE
Indel-45	ECs1357		-	hypothetical protein
Indel-45	ECs1358	Z1616	terF_2	putative tellurium resistance protein TerF

Locus_tag[a]				
CB9615	Sakai	EDL933	Gene	Product
Indel-45	ECs1359		-	hypothetical protein
Indel-45	ECs1360	Z1617	-	Iha adhesin
Indel-45		Z1618	-	hypothetical protein
Indel-45		Z1619	-	hypothetical protein
Indel-45	ECs1361		-	hypothetical protein
Indel-45	ECsp010		-	ShiA
Indel-45	ECs1362	Z1620	-	hypotheical protein
Indel-45		Z1621	-	hypothetical protein
Indel-45	ECsp011	Z1622	-	hypothetical protein
Indel-45	ECsp012	Z1622	-	hypothetical protein
Indel-45	ECs1363		-	hypothetical protein
Indel-45	ECsp013		-	transposase OrfB
Indel-45		Z1623	-	hypothetical protein
Indel-45	ECs1364	Z1624	-	hypothetical protein
Indel-45	ECs1365		-	hypothetical protein
Indel-45	ECs1366	Z1625	-	hypothetical protein
Indel-45	ECs1367	Z1626	-	hypothetical protein
Indel-45	ECs1368		-	hypothetical protein
Indel-45		Z1627	-	hypothetical protein
Indel-45	ECs1369		-	hypothetical protein
Indel-45		Z1628	-	hypothetical protein
Indel-45	ECs1370	Z1629	-	putative glucosyl-transferase
Indel-45	ECs1370	Z1630	-	putative glucosyl-transferase
Indel-45	ECs1371	Z1631	-	ferric enterochelin esterase
Indel-45	ECs1372	Z1632	-	transposase
Indel-45	ECs1373		-	InsA protein of insertion sequence IS1
Indel-45	ECs1374	Z1633	-	putative membrane protein
Indel-45	ECs1375		-	hypothetical protein
Indel-45	ECs1376	Z1634	-	hypothetical protein
Indel-45	ECs1377	Z1635	-	hypothetical protein
Indel-45	ECs1378	Z1636	-	hypothetical protein
Indel-45	ECs1379		-	hypothetical protein
Indel-45		Z1637	-	hypothetical protein
Indel-45	ECs1380	Z1638	-	putative transposase OrfB protein of insertion sequence IS629
Indel-45	ECs1381	Z1639	-	putative transposase OrfA protein of insertion sequence IS629
Indel-45	ECs1382	Z1640	-	HecB-like protein
Indel-45	ECs1383	Z1641	-	hypothetical protein
Indel-45	ECs1384	Z1642	-	hypothetical protein
Indel-45	ECs1385		-	hypothetical protein
Indel-45	ECs1386	Z1643	-	hypothetical protein
Indel-45	ECs1387	Z1644	-	hypothetical protein
Indel-45	ECs1388		-	putative transcriptional regulator

Locus_tag[a]				
CB9615	Sakai	EDL933	Gene	Product
Indel-45	ECs1389	Z1645	-	hypothetical protein
Indel-45	ECs1390	Z1646	-	hypothetical protein
Indel-45	ECs1391		-	BfpM-like protein
Indel-45		Z1647	-	partial transposase
Indel-45	ECs1392		-	hypothetical protein
Indel-45	ECs1393	Z1648	-	hypothetical protein
Indel-45	ECs1394	Z1649	-	hypothetical protein
Indel-45	ECs1395	Z1650	-	hypothetical protein
Indel-45	ECs1396	Z1651	-	AidA-I adhesin-like protein
Indel-45	ECs1397	Z1652	-	hypothetical protein
Indel-45	ECs1398	Z1653	-	hypothetical protein
Indel-45	ECs1399	Z1654	-	hypothetical protein
Indel-45	ECs1400		-	hypothetical protein
Indel-45	ECs1401	Z1655	-	hypothetical protein
Indel-45	ECs1402	Z1656	-	hypothetical protein
Indel-45	ECs1403	Z1657	-	putative DNA repair protein
Indel-45	ECs1404		-	hypothetical protein
Indel-45	ECs1405	Z1658	-	hypothetical protein
Indel-47	ECs1406		-	hypothetical protein
Indel-46	Indel-46	Z1660	-	transposase for IS629
Indel-46	Indel-46	Z1661	-	unknown protein encoded by IS629
Indel-47	ECs1407	Z1662	-	hypothetical protein
Indel-47	ECs1408	Z1663	-	hypothetical protein
Indel-47	ECs1409	Z1664	-	hypothetical protein
G2583_1290	serX	RNA031	serX	Ser tRNA
G2583_1291	ECs1410	Z1666	ghrA	Putative dehydrogenase
G2583_1292	ECs1411	Z1667	ycdX	Putative hydrolase ycdX
G2583_1293	ECs1412	Z1668	ycdY	Chaperone, TorD family
G2583_1294	ECs1413	Z1669	ycdZ	hypothetical protein
G2583_1295	ECs1414	Z1670	csgG	Curli production assembly/transport component csgG precursor
G2583_1296	ECs1415	Z1671	csgF	Curli production assembly/transport component csgF precursor
G2583_1297	ECs1416	Z1672	csgE	Curli production assembly/transport component csgE precursor
G2583_1298	ECs1417	Z1673	csgD	CsgD
G2583_1299	ECs1418		-	hypothetical protein
G2583_1300	ECs1419	Z1675	csgB	Minor curlin subunit
G2583_1301	ECs1420	Z1676	csgA	Major curlin subunit precursor
G2583_1302	ECs1421	Z1677	csgC	CsgC
G2583_1303	ECs1422	Z1678	ymdA	hypothetical protein
G2583_1304	ECs1423	Z1679	ymdB	UPF0189 protein ymdB
G2583_1305	ECs1424	Z1680	ymdC	Putative synthase
G2583_1306	ECs1425	Z1681	mdoC	Glucans biosynthesis protein C

Locus_tag[a]				
CB9615	Sakai	EDL933	Gene	Product
G2583_1307	ECs1426	Z1683	mdoG	Glucans biosynthesis protein G precursor
G2583_1308	ECs1427	Z1684	mdoH	Periplasmic glucans biosynthesis protein MdoH
G2583_1309	ECs1428	Z1685	yceK	hypothetical protein
G2583_1310	ECs1429	Z1686	msyB	Acidic protein MsyB
G2583_1311	ECs1430	Z1687	-	hypothetical protein
G2583_1312	ECs1431	Z1688	mdtG	Multidrug resistance protein mdtG
G2583_1313	ECs1432	Z1690	lpxL	Heat shock protein
G2583_1314	ECs1433	Z1691	yceA	UPF0176 protein yceA
G2583_1315	ECs1434	Z1692	yceI	hypothetical protein
G2583_1316	ECs1435	Z1693	yceJ	Nickel-dependent hydrogenase b-type cytochrome
G2583_1317	ECs1436	Z1695	yceO	hypothetical protein
G2583_1318	ECs1437	Z1696	solA	N-methyl-L-tryptophan oxidase
G2583_1319	ECs1438	Z1697	bssS	hypothetical protein
G2583_1320	ECs1439	Z1698	dinI	DinI
G2583_1321	ECs1440	Z1699	pyrC	Dihydroorotase
G2583_1322	ECs1441	Z1700	yceB	Putative lipoprotein yceB
G2583_1323	ECs1442	Z1701	grxB	Glutaredoxin-2
G2583_1324	ECs1443	Z1702	mdtH	Multidrug resistance protein mdtH
G2583_1325	ECs1444	Z1703	rimJ	Ribosomal-protein-alanine acetyltransferase
G2583_1326	ECs1445	Z1704	yceH	UPF0502 protein yceH
G2583_1327	ECs1446	Z1705	yceM	Oxidoreductase family, NAD-binding
G2583_1328	ECs1447	Z1707	yceN	Virulence factor mviN homolog
G2583_1329	ECs1448	Z1708	flgN	Flagellar biosynthesis/type III secretory path way chaperone
G2583_1330	ECs1449	Z1709	flgM	Negative regulator of flagellin synthesis
G2583_1331	ECs1450	Z1710	flgA	Flagellar biosynthesis
G2583_1332	ECs1451	Z1711	flgB	Flagellar basal-body rod protein FlgB
G2583_1333	ECs1452	Z1712	flgC	Cell-proximal portion of basal-body rod
G2583_1334	ECs1453	Z1713	flgD	Flagellar hook protein FlgE
G2583_1335	ECs1454	Z1714	flgE	Flagellar hook protein FlgE
G2583_1336	ECs1455	Z1715	flgF	Flagellar basal-body rod protein FlgF
G2583_1337	ECs1456	Z1716	flgG	Flagellar basal-body rod protein flgG
G2583_1338	ECs1457	Z1717	flgH	Flagellar L-ring protein precursor
G2583_1339	ECs1458	Z1718	flgI	flagellar basal body P-ring protein
G2583_1340	ECs1459	Z1719	flgJ	Peptidoglycan hydrolase flgJ
G2583_1341	ECs1460	Z1720	flgK	Flagellar hook-associated protein FlgK
G2583_1342	ECs1461	Z1721	flgL	Flagellar hook-associated protein 3
G2583_1343	ECs1462	Z1722	rne	Ribonuclease, Rne/Rng family
G2583_1344	ECs1463	Z1724	yceQ	hypothetical protein
G2583_1345	ECs1464	Z1725	rluC	Ribosomal large subunit pseudouridine synthase C
G2583_1346	ECs1465	Z1726	yceF	Septum formation protein Maf
G2583_1347			sraB	ncRNA
G2583_1348	ECs1466	Z1727	yceD	hypothetical protein
G2583_1349	ECs1467	Z1728	rpmF	50S ribosomal protein L32

Locus_tag[a]				
CB9615	Sakai	EDL933	Gene	Product
G2583_1350	ECs1468	Z1729	plsX	fatty acid/phospholipid synthesis protein
G2583_1351	ECs1469	Z1730	fabH	3-oxoacyl-[acyl-carrier-protein] synthase 3
G2583_1352	ECs1470	Z1731	fabD	Malonyl CoA-acyl carrier protein transacylase
G2583_1353	ECs1471	Z1732	fabG	3-oxoacyl-[acyl-carrier-protein] reductase
G2583_1354	ECs1472	Z1733	acpP	Acyl carrier protein
G2583_1355	ECs1473	Z1734	fabF	3-oxoacyl-[acyl-carrier-protein] synthase 2
G2583_1356	ECs1474	Z1735	pabC	Aminodeoxychorismate lyase
G2583_1357	ECs1475	Z1736	yceG	Putative thymidylate kinase
G2583_1358	ECs1476	Z1737	tmk	Thymidylate kinase
G2583_1359	ECs1477	Z1738	holB	DNA polymerase III, delta' subunit
G2583_1360	ECs1478	Z1739	ycfH	Hydrolase, TatD family
G2583_1361	ECs1479	Z1740	ptsG	hypothetical protein
G2583_1362	ECs1480	Z1741	fhuE	Outer membrane receptor for ferric iron uptake
G2583_1363	ECs1481	Z1742	hinT	hypothetical protein
G2583_1364	ECs1482	Z1743	ycfL	Putative outer membrane lipoprotein YcfL
G2583_1365	ECs1483	Z1744	ycfM	hypothetical protein
G2583_1366	ECs1484	Z1745	thiK	Thiamine kinase
G2583_1367	ECs1485	Z1746	nagZ	Beta-hexosaminidase
G2583_1368	ECs1486	Z1747	ycfP	hypothetical protein
G2583_1369	ECs1487	Z1748	ndh	NADH dehydrogenase
G2583_1370	ECs1488	Z1749	ycfJ	Surface antigen domain protein
G2583_1371	ECs1489	Z1750	ycfQ	hypothetical protein
G2583_1372	ECs1490	Z1751	bhsA	Putative outer membrane protein
G2583_1373	ECs1491	Z1752	ycfS	hypothetical protein
G2583_1374	ECs1492	Z1754	mfd	transcription-repair coupling factor
G2583_1375	ECs1493	Z1756	ycfT	hypothetical protein
G2583_1376	ECs1494	Z1757	lolC	outer membrane-specific lipoprotein transporter subunit LolC
G2583_1377	ECs1495	Z1758	lolD	ABC transporter, ATP-binding protein
G2583_1378	ECs1496	Z1759	lolE	Lipoprotein releasing system, transmembrane protein
G2583_1379	ECs1497	Z1760	nagK	N-acetyl-D-glucosamine kinase
G2583_1380	ECs1498	Z1761	cobB	NAD-dependent deacetylase
G2583_1381	ECs1499	Z1762	potD	Spermidine/putrescine-binding periplasmic protein precursor
G2583_1382	ECs1500	Z1763	potC	Spermidine/putrescine transport system permease
Indel-48	ECs1501	Z1764	intN	putative integrase
Indel-48	ECs1502	Z1765	xisN	putative excisionase
Indel-48	ECs1503	Z1766	-	hypothetical protein
Indel-48	ECs1504	Z1768	-	hypothetical protein
Indel-48	ECs1505	Z1769	-	hypothetical protein
Indel-48	ECs1506	Z1770	-	putative phage repressor
Indel-48	ECs1507	Z1771	-	hypothetical protein
Indel-48	ECs1508	Z1772	-	hypothetical protein
Indel-48	ECs1509	Z1773	-	hypothetical protein

Locus_tag[a]				
CB9615	Sakai	EDL933	Gene	Product
Indel-48	ECs1510	Z1774	-	putative replication protein
Indel-48	ECs1511	Z1775	-	hypothetical protein
Indel-48	ECs1512	Z1776	-	hypothetical protein
Indel-48	ECs1513	Z1777	-	hypothetical protein
Indel-48	ECs1514	Z1778	-	hypothetical protein
Indel-48	ECs1515	Z1779	-	hypothetical protein
Indel-48	ECs1516	Z1780	-	hypothetical protein
Indel-48	ECs1517		-	hypothetical protein
Indel-48	ECs1518	Z1781	-	hypothetical protein
Indel-48		Z1782	-	unknown protein encoded by prophage CP-933N
Indel-48	ECs1519		-	hypothetical protein
Indel-48	ECs1520	Z1783	-	prophage maintenance protein
Indel-48	ECs1521		-	hypothetical protein
Indel-48	ECs1522	Z1784	-	hypothetical protein
Indel-48	ECs1523	Z1785	-	putative crossover junction endodeoxyribonuclease
Indel-49	ECs1524	Indel-49	-	putative antitermination protein
Indel-49	ECs1526	Indel-49	-	hypothetical protein
Indel-49	ECs1525	Indel-49	-	hypothetical protein
Indel-49	ECs1527	Indel-49	-	hypothetical protein
Indel-49	ECs1528	Indel-49	-	hypothetical protein
Indel-49	ECs1529	Indel-49	-	hypothetical protein
Indel-49	ECs1530	Indel-49	-	putative holin
Indel-49	ECs1531	Indel-49	-	hypothetical protein
Indel-49	ECs1532	Indel-49	-	putative endolysin
Indel-49	ECs1533	Indel-49	-	antirepressor protein
Indel-49	ECs1534	Indel-49	-	putative endopeptidase
Indel-49	ECs1535	Indel-49	-	putative lipoprotein Rz1 precursor
Indel-49	ECs1536	Indel-49	-	hypothetical protein
Indel-49	ECs1537	Indel-49	-	hypothetical protein
Indel-49	ECs1538	Indel-49	-	hypothetical protein
Indel-49	ECs1539	Indel-49	-	hypothetical protein
Indel-49	ECs1540	Indel-49	-	putative Dnase
Indel-49	ECs1541	Indel-49	-	putative terminase small subunit
Indel-49	ECs1542	Indel-49	-	putative large terminase subunit
Indel-49	ECs1543	Indel-49	-	putative major head protein/prohead proteinase
Indel-49	ECs1544	Indel-49	-	putative portal protein
Indel-49	ECs1545	Indel-49	-	hypothetical protein
Indel-49	ECs1546	Indel-49	-	putative head-tail adapotor
Indel-49	ECs1547	Indel-49	-	hypothetical protein
Indel-49	ECs1548	Indel-49	-	hypothetical protein
Indel-49	ECs1549	Indel-49	-	putative major tail subunit
Indel-51	ECs1550		-	putative tail assembly chaperone
Indel-50	Indel-50	Z1786	-	putative Q antiterminator of prophage CP-933N
Indel-50	Indel-50	Z1787	-	unknown protein encoded by prophage CP-933N

Locus_tag[a]				
CB9615	Sakai	EDL933	Gene	Product
Indel-50	Indel-50	Z1788	-	unknown protein encoded by prophage CP-933N
Indel-50	Indel-50	Z1789	-	putative envelope protein encoded within prophage CP-933N
Indel-50	Indel-50	Z1793	-	unknown protein encoded by prophage CP-933N
Indel-50	Indel-50	Z1794	-	putative holin protein
Indel-50	Indel-50	Z1795	-	unknown protein encoded by prophage CP-933N
Indel-50	Indel-50	Z1796	-	putative endolysin of prophage CP-933N
Indel-50	Indel-50	Z1797	-	putative antirepressor of prophage CP-933N
Indel-50	Indel-50	Z1798	-	putative endopeptidase of prophage CP-933N
Indel-50	Indel-50	Z1799	-	partial tonB-like membrane protein encoded within prophage CP-933N
Indel-50	Indel-50	Z1800	-	unknown protein encoded by prophage CP-933N
Indel-50	Indel-50	Z1802	-	unknown protein encoded by prophage CP-933N
Indel-50	Indel-50	Z1803	-	putative terminase encoded by prophage CP-933N
Indel-50	Indel-50	Z1804	-	unknown protein encoded by prophage CP-933N
Indel-50	Indel-50	Z1805	-	unknown protein encoded by prophage CP-933N
Indel-50	Indel-50	Z1806	-	unknown protein encoded by prophage CP-933N
Indel-50	Indel-50	Z1807	-	unknown protein encoded by prophage CP-933N
Indel-50	Indel-50	Z1808	-	unknown protein encoded by prophage CP-933N
Indel-50	Indel-50	Z1809	-	unknown protein encoded by prophage CP-933N
Indel-50	Indel-50	Z1810	-	unknown protein encoded by prophage CP-933N
Indel-50	Indel-50	Z1811	-	putative tail component encoded by prophage CP-933N
Indel-51	ECs1551	Z1813	-	putative phage tail protein
Indel-51	ECs1552	Z1814	-	putative tail length tape measure protein
Indel-51	ECs1553	Z1814	-	putative tail length tape measure protein
Indel-52	ECs1554	Indel-52	-	putative minor tail protein
Indel-54	ECs1555		-	putative minor tail protein
Indel-54	ECs1556		-	putative regulatory protein
Indel-54		Z1816	-	unknown protein encoded by prophage CP-933N
Indel-54		Z1817	-	unknown protein encoded by prophage CP-933N
Indel-54	ECs1557	Z1818	-	putative antirepressor protein
Indel-54	ECs1558	Z1819	-	putative tail assembly protein
Indel-54	ECs1559	Z1820	-	putative tail assembly protein
Indel-54		Z1821	-	unknown protein encoded by prophage CP-933N
Indel-54	ECs1560	Z1822	-	putative secreted effector protein
Indel-54	ECs1561	Z1823	-	hypothetical protein
Indel-54	ECs1561	Z1824	-	hypothetical protein
Indel-54	ECs1562		-	hypothetical protein
Indel-54	ECs1563		-	hypothetical membrane protein
Indel-54	ECs1564	Z1825	-	putative transposase OrfB protein of insertion sequence IS2
Indel-54	ECs1565	Z1826	-	putative transposase OrfA protein of insertion sequence IS2
Indel-54		Z1827	-	putative IS encoded protein

Locus_tag[a]				
CB9615	Sakai	EDL933	Gene	Product
Indel-54	ECs1566		-	hypothetical protein
Indel-54	ECs1567		-	hypothetical protein
Indel-54	ECs1568	Z1829	-	hypothetical protein
Indel-54	ECs1569		-	hypothetical protein
G2583_1383	ECs1570	Z1830	potB	Spermidine/putrescine ABC transporter, permease protein PotB
G2583_1384	ECs1571	Z1831	potA	Spermidine/putrescine import ATP-binding protein potA
G2583_1385	ECs1572	Z1832	pepT	Peptidase T
G2583_1386	ECs1573	Z1833	ycfD	YcfD protein
G2583_1387	ECs1574	Z1835	-	Site-specific recombinase, phage integrase family
		Z1836	-	unknown protein encoded by prophage CP-933C
G2583_1388	ECs1575		-	putative DNA binding protein
G2583_1389		Z1838	-	unknown protein encoded by prophage CP-933C
Indel-55		Z1839	-	unknown protein encoded by prophage CP-933C
Indel-55	ECs1577		-	Icd-like protein
Indel-55	ECs1578	Z1840	-	hypothetical protein
Indel-55	ECs1579		-	hypothetical protein
Indel-55	ECs1580	Z1841	-	hypothetical protein
Indel-55	ECs1581		-	hypothetical protein
Indel-55	ECs1582	Z1842	-	hypothetical protein
Indel-55	ECs1583	Z1843	-	hypothetical protein
Indel-55	ECs1584	Z1843	-	hypothetical protein
Indel-55	ECs1585		-	hypothetical protein
Indel-55	ECs1586	Z1844	-	hypothetical protein
Indel-55	ECs1587	Z1845	-	putative single stranded DNA-binding protein
Indel-55	ECs1588		-	putative transcriptional activator
Indel-55	ECs1589	Z1846	-	hypothetical protein
Indel-55	ECs1590	Z1847	-	putative major head protein
Indel-55	ECs1591	Z1848	-	putative prohead protease
Indel-55	ECs1592	Z1849	-	putative head portal protein
Indel-55	ECs1593		-	putative head-tail adaptor
Indel-55		Z1850	-	unknown protein encoded by prophage CP-933C
Indel-55	ECs1594	Z1851	-	hypothetical protein
Indel-55	ECs1595	Z1852	-	hypothetical protein
Indel-55	ECs1596		-	hypothetical protein
Indel-55	ECs1597	Z1853	-	putative terminase small subunit
Indel-55	ECs1598	Z1854	-	putative terminase large subunit
Indel-55	ECs1599	Z1855	-	hypothetical protein
Indel-55		Z1856	-	unknown protein encoded by prophage CP-933C
Indel-55	ECs1600	Z1857	-	hypothetical protein
G2583_1390	Indel-56	Indel-56	-	conserved hypothetical protein
G2583_1391	Indel-56	Indel-56	-	hypothetical protein
G2583_1392	Indel-56	Indel-56	-	hypothetical protein
G2583_1393	Indel-56	Indel-56	-	hypothetical protein

Locus_tag[a]				
CB9615	Sakai	EDL933	Gene	Product
G2583_1394	Indel-56	Indel-56	-	hypothetical protein
G2583_1395	Indel-56	Indel-56	-	hypothetical protein
G2583_1396	Indel-56	Indel-56	YabA	Bacteriophage P4 DNA primase
G2583_1397	Indel-56	Indel-56	-	hypothetical bacteriophage protein
G2583_1398	Indel-56	Indel-56	-	Single-stranded DNA-binding protein
G2583_1399	Indel-56	Indel-56	-	hypothetical protein
G2583_1400	Indel-56	Indel-56	-	Major capsid protein
G2583_1401	Indel-56	Indel-56	-	Capsid protein small subunit
G2583_1402	Indel-56	Indel-56	-	hypothetical protein
G2583_1403	Indel-56	Indel-56	-	Phage DNA packaging protein NU1-like protein
G2583_1404	Indel-56	Indel-56	-	Hypothetical bacteriophage protein
G2583_1405	ECs1601	Z1858	phoQ	sensor protein PhoQ
G2583_1406	ECs1602	Z1859	phoP	DNA-binding response regulator in two-component regulatory system with PhoQ
G2583_1407	ECs1603	Z1860	purB	Adenylosuccinate lyase
G2583_1408	ECs1604	Z1861	hflD	hypothetical protein
G2583_1409	ECs1605	Z1862	mnmA	Predicted tRNA(5-methylaminomethyl-2-thiouridylate) methyltransferase, contains the PP-loop ATPase
G2583_1410	ECs1606	Z1863	nudJ	Phosphatase nudJ
G2583_1411	ECs1607	Z1864	rluE	Ribosomal large subunit pseudouridine synthase E
G2583_1412	ECs1608	Z1865	icd	Isocitrate dehydrogenase [NADP]
Indel-57	ECs1609	Z1866	-	intergrase
Indel-57	ECs1610	Z1867	-	excisionase
Indel-57	ECs1611	Z1868	-	putative replication protein
Indel-57	ECs1612	Z1869	-	replication protein P
Indel-57	ECs1613		-	Ren protein
Indel-57	ECs1614	Z1870	-	integral membrane drug resistance protein EmrE
Indel-57	ECs1615	Z1871	-	hypothetical protein
Indel-57	ECs1616	Z1872	ybcN	hypothetical protein
Indel-57	ECs1617		-	NinE protein
Indel-57	ECs1618		-	hypothetical protein
Indel-57	ECs1619	Z1873	rus	putative crossover junction endodeoxyribonuclease
Indel-57	ECs1620	Z1874	-	antitermination protein
Indel-57	ECs1621	Z1875	-	putative holin protein
Indel-57	ECs1622	Z1876	-	putative endolysin
Indel-57	ECs1623	Z1877	-	putative endopeptidase
Indel-57	ECs1624		-	lipoprotein Rz1 precursor
Indel-57	ECs1625	Z1878	-	Bor protein precursor
Indel-57	ECs1626	Z1879	-	hypothetical protein
Indel-57	ECs1627	Z1880	-	hypothetical protein
Indel-57	ECs1628	Z1881	-	hypothetical protein
Indel-57	ECs1629	Z1882	-	terminase small subunit
Indel-57	ECs1630	Z1883	-	terminase large subunit
Indel-57	ECs1631	Z1884	-	head-to-tail joining protein

Locus_tag[a]				
CB9615	Sakai	EDL933	Gene	Product
Indel-57	ECs1632	Z1885	-	portal protein
Indel-57	ECs1633	Z1886	-	minor capsid protein
Indel-57	ECs1634	Z1887	-	major capsid protein
Indel-57	ECs1635	Z1888	-	major capsid protein
Indel-57	ECs1636	Z1889	-	DNA packaging protein
Indel-57	ECs1637	Z1890	-	minor capsid protein
Indel-57	ECs1638	Z1891	-	minor tail protein
Indel-57	ECs1639	Z1893	-	minor tail protein
Indel-57	ECs1640	Z1894	-	major tail protein
Indel-57	ECs1641	Z1895	-	minor tail protein
Indel-57	ECs1642	Z1896	-	minor tail protein
Indel-57	ECs1643	Z1898	-	tail length tape measure protein precursor
Indel-58	ECs1644	Indel-58	-	minor tail protein
Indel-58	ECs1645	Indel-58	-	minor tail protein
Indel-58	ECs1646	Indel-58	-	tail assembly protein
Indel-58	ECs1647	Indel-58	-	tail assembly protein
Indel-58	ECs1648	Indel-58	-	host specificity protein
Indel-60	ECs1649		-	putative membrane protein precursor
Indel-59	Indel-59	Z1901	-	unknown protein encoded by prophage CP-933X
Indel-59	Indel-59	Z1902	-	putative head-tail adaptor of prophage CP-933X
Indel-59	Indel-59	Z1903	-	unknown protein encoded by prophage CP-933X
Indel-59	Indel-59	Z1905	-	unknown protein encoded by prophage CP-933X
Indel-59	Indel-59	Z1906	-	unknown protein encoded by prophage CP-933X
Indel-59	Indel-59	Z1908	-	putative tail component of prophage CP-933X
Indel-59	Indel-59	Z1910	-	unknown protein encoded by prophage CP-933X
Indel-59	Indel-59	Z1912	-	unknown protein encoded by prophage CP-933X
Indel-59	Indel-59	Z1913	-	putative tail component of prophage CP-933X
Indel-59	Indel-59	Z1914	-	putative minor tail fiber protein of prophage CP-933X
Indel-59	Indel-59	Z1915	-	putative tail protein (partial) of prophage CP-933X
Indel-59	Indel-59	Z1916	-	putative tail protein (partial) of prophage CP-933X
Indel-60		Z1919	-	unknown protein encoded by prophage CP-933X
Indel-60	ECs1650	Z1918	-	putative tail fiber protein
Indel-60	ECs1651	Z1920	-	tail fiber assembly protein
Indel-60	ECs1652	Z1921	-	putative catalase
Indel-60	ECs1653	Z1922	-	hypothetical protein
Indel-60	ECs1654	Z1923	-	hypothetical protein
Indel-60	ECs1655	Z1924	-	hypothetical protein
Indel-60	ECs1656	Z1925	-	hypothetical protein
Indel-60	ECs1657	Z1926	-	hypothetical protein
Indel-60	ECs1658	Z1927	-	hypothetical protein
Indel-60	ECs1659	Z1928	-	hypothetical protein
Indel-60	ECs1660	Z1929	-	hypothetical protein
Indel-60	ECs1661		-	BfpA-like protein
Indel-60	ECs1662	Z1930	-	hypothetical protein

Locus_tag[a]				
CB9615	Sakai	EDL933	Gene	Product
Indel-60	ECs1663	Z1931	-	outer membrane protease precursor
Indel-60	ECs1664	Z1932	-	hypothetical protein
Indel-62	ECs1665	Z1933	-	putative transposase OrfA protein of insertion sequence IS629
Indel-62	ECs1666	Z1934	-	putative transposase OrfB protein of insertion sequence IS629
G2583_1413	Indel-63	Indel-63	elbA	Anti-adapter protein iraM
G2583_1414	Indel-63	Indel-63	ycgX	hypothetical protein
G2583_1415	Indel-63	Indel-63	ycgE	transcriptional regulator mlrA
G2583_1416	Indel-63	Indel-63	ycgF	BLUF domain/cyclic diguanylate phosphodiesterase (EAL) domain protein
G2583_1417	Indel-63	Indel-63	ycgF	BLUF domain/cyclic diguanylate phosphodiesterase (EAL) domain protein
G2583_1418	Indel-63	Indel-63	ycgZ	hypothetical protein
G2583_1419	Indel-63	Indel-63	-	hypothetical protein
G2583_1420	Indel-63	Indel-63	ymgA	hypothetical protein
G2583_1421	Indel-63	Indel-63	ymgB	hypothetical protein
G2583_1422	Indel-63	Indel-63	ymgC	hypothetical protein
G2583_1423	Indel-63	Indel-63	ycgG	Cyclic diguanylate phosphodiesterase (EAL) domain protein
G2583_1424	Indel-63	Indel-63	ymgF	hypothetical protein
G2583_1425	Indel-63	Indel-63	-	hypothetical protein
G2583_1426	Indel-63	Indel-63	-	Porin, autotransporter (AT) family
G2583_1427	Indel-63	Indel-63	-	Porin, autotransporter (AT) family
G2583_1428	Indel-63	Indel-63	ymgD	hypothetical protein
G2583_1429	Indel-63	Indel-63	-	hypothetical protein
G2583_1430	Indel-63	Indel-63	ymgI	hypothetical protein
G2583_1431	Indel-63	Indel-63	ymgJ	hypothetical protein
G2583_1432	Indel-63	Indel-63	ypjA	Putative ATP-binding component of a transport system
	ECs1667	Z1935	-	hypothetical protein
G2583_1433	ECs1668	Z1936	minE	Cell division topological specificity factor
G2583_1434	ECs1669	Z1937	minD	Septum site-determining protein minD
G2583_1435	ECs1670	Z1938	minC	Septum site-determining protein minC
G2583_1436	ECs1671	Z1939	ycgJ	Putative fels-1 Prophage Protein
G2583_1437	ECs1672	Z1940	ycgK	Bacterial pre-peptidase C-terminal domain protein
G2583_1438	ECs1673		-	hypothetical protein
G2583_1439	ECs1674	Z1941	ycgL	hypothetical protein
G2583_1440	ECs1675	Z1942	ycgM	Fumarylacetoacetate hydrolase family protein
G2583_1441	ECs1676	Z1943	ycgN	conserved hypothetical protein
G2583_1442	ECs1677	Z1944	hlyE	Hemolysin E
G2583_1443			-	ncRNA
G2583_1444	ECs1678	Z1946	umuD	DNA polymerase V subunit UmuD
G2583_1445	ECs1679	Z1947	umuC	DNA polymerase V subunit UmuC
G2583_1446	ECs1680	Z1948	dsbB	Disulfide bond formation protein B

Locus_tag[a]				
CB9615	Sakai	EDL933	Gene	Product
G2583_1447	ECs1681	Z1949	nhaB	Na(+)/H(+) antiporter nhaB
G2583_1448	ECs1682	Z1950	fadR	Fatty acid metabolism regulator protein
G2583_1449	ECs1683	Z1951	ycgB	SpoVR family protein
G2583_1450	ECs1684	Z1952	dadA	D-amino acid dehydrogenase small subunit
G2583_1451	ECs1685	Z1953	dadX	Alanine racemase, catabolic
G2583_1452	Indel-65	Indel-65	ipaH	hypothetical protein
G2583_1453	Indel-65	Indel-65	-	hypothetical protein
G2583_1454	ECs1686	Z1954	cvrA	potassium/proton antiporter
G2583_1455	ECs1688	Z1955	ldcA	Muramoyltetrapeptide carboxypeptidase
G2583_1456	ECs1687	Z1956	emtA	Membrane-bound lytic murein transglycosylase E
Indel-66	ECs1689	Z1957	-	putative transposase OrfB protein of insertion sequence IS629
Indel-66	ECs1690	Z1958	-	putative transposase OrfA protein of insertion sequence IS629
G2583_1457	ECs1691	Z1959	ycgR	hypothetical protein
G2583_1458	ECs1692	Z1960	ymgE	hypothetical protein
G2583_1459	ECs1693	Z1961	prrA	Putative TonB dependent outer membrane receptor
G2583_1460	ECs1694	Z1962	modD	Putative pyrophosphorylase modD
	ECs1695		-	hypothetical protein
G2583_1461	ECs1696	Z1963	-	Putative methyltransferase
G2583_1462	ECs1697	Z1964	-	Putative iron compound ABC transporter, ATP-binding protein
G2583_1463	ECs1698	Z1965	-	Putative iron compound ABC transporter, permease protein
G2583_1464	ECs1699	Z1966	-	Putative iron compound ABC transporter, periplasmic-binding protein
G2583_1465	ECs1700	Z1967	-	hypothetical protein
G2583_1466	ECs1701	Z1968	treA	putative trehalase
G2583_1467	ECs1702		treA	trehalase
G2583_1468	ECs1703	Z1969	dhaM	Putative PTS system enzyme I
G2583_1469	ECs1704	Z1970	dhaL	Putative dihydroxyacetone kinase
G2583_1470	ECs1705	Z1971	dhaK	Dihydroxyacetone kinase, N-terminal domain
	ECs1706		-	hypothetical protein
G2583_1471	Indel-67	Indel-67	dhaR	PTS-dependent dihydroxyacetone kinase operon regulatory protein
G2583_1472	ECs1707	Z1972	ycgV	putative outer membrane autotransporter
G2583_1473			-	hypothetical protein
G2583_1474	ECs1708	Z1974	ychF	GTP-dependent nucleic acid-binding protein engD
G2583_1475	ECs1709	Z1975	pth	Peptidyl-tRNA hydrolase
G2583_1476	ECs1710	Z1976	ychH	predicted inner membrane protein
G2583_1477	ECs1711	Z1977	ychM	Putative sulfate transporter ychM
G2583_1478	ECs1712	Z1978	prs	Ribose-phosphate pyrophosphokinase
G2583_1479	ECs1713	Z1979	ispE	4-diphosphocytidyl-2-C-methyl-D-erythritol kinase
G2583_1480	ECs1714	Z1980	lolB	Outer membrane lipoprotein LolB

Locus_tag[a]				
CB9615	Sakai	EDL933	Gene	Product
G2583_1481	ECs1715	Z1981	hemA	Glutamyl-tRNA reductase
G2583_1482	ECs1716	Z1982	prfA	Peptide chain release factor 1
G2583_1483	ECs1717	Z1983	prmC	Protein-(Glutamine-N5) methyltransferase, release factor-specific
G2583_1484	ECs1718	Z1984	ychQ	Putative Invasion gene expression up-regulator SirB
G2583_1485	ECs1719	Z1985	ychA	putative transcriptional regulator
G2583_1486	ECs1720	Z1986	kdsA	2-dehydro-3-deoxyphosphooctonate aldolase
G2583_1487		Z1987	ldrB	hypothetical protein
Indel-68	Indel-68	Z1989	-	hypothetical protein
G2583_1488	ECs1721	Z1991	chaA	Calcium/proton antiporter
G2583_1489	ECs1722	Z1992	chaB	Cation transport regulator chaB
G2583_1490	ECs1723	Z1993	chaC	Cation transport regulator
G2583_1491	ECs1724	Z1994	ychN	hypothetical protein
G2583_1492	ECs1725	Z1995	ychO	Putative invasin
G2583_1493	ECs1726	Z1996	narL	Nitrate/nitrite response regulator protein narL
G2583_1494	ECs1727	Z1998	narX	Nitrate/nitrite sensor protein NarX
G2583_1495			-	hypothetical protein
G2583_1496	ECs1728	Z2000	narK	Nitrite extrusion protein 1
G2583_1497	ECs1729	Z2001	narG	Nitrate reductase, alpha subunit
G2583_1498	ECs1730	Z2002	narH	Nitrate reductase, beta subunit
G2583_1499	ECs1731	Z2003	narJ	Respiratory nitrate reductase 1 delta chain
G2583_1500	ECs1732	Z2004	narI	Respiratory nitrate reductase, gamma subunit
G2583_1501	ECs1733	Z2005	ychS	hypothetical protein
G2583_1502			-	ncRNA
G2583_1503	tyrV	RNA034	tyrV	Tyr tRNA
G2583_1504			-	ncRNA
G2583_1505	tyrT	RNA035	tyrT	Tyr tRNA
G2583_1506	ECs1734	Z2008	purU	Formyltetrahydrofolate deformylase
G2583_1507	ECs1735	Z2009	ychJ	SEC-C motif domain protein
G2583_1508	ECs1736	Z2010	rssA	Phospholipase, patatin family
G2583_1509	ECs1737	Z2011	rssB	response regulator of RpoS
G2583_1510	ECs1738	Z2012	galU	UTP--glucose-1-phosphate uridylyltransferase
G2583_1511	ECs1739	Z2013	hns	DNA-binding protein H-NS
G2583_1512	ECs1740	Z2015	tdk	Thymidine kinase
G2583_1513	ECs1741	Z2016	adhE	Aldehyde-alcohol dehydrogenase
G2583_1514	ECs1742	Z2017	ychE	Integral membrane protein, MarC family
G2583_1515	ECs1743	Z2019	oppA	Periplasmic oligopeptide-binding protein
G2583_1516	ECs1744	Z2020	oppB	Oligopeptide transport system permease protein oppB
G2583_1517	ECs1745	Z2021	oppC	Oligopeptide transport system permease protein oppC
G2583_1518	ECs1746	Z2022	oppD	Oligopeptide ABC transporter, ATP-binding protein
G2583_1519	ECs1747	Z2023	oppF	Oligopeptide ABC transporter, ATP-binding protein
G2583_1520	ECs1748	Z2024	yciU	hypothetical protein
G2583_1521	ECs1749	Z2026	cls	Cardiolipin synthetase
G2583_1522			yciY	hypothetical protein

Locus_tag[a]				
CB9615	Sakai	EDL933	Gene	Product
G2583_1523	ECs1750	Z2028	kch	Potassium channel protein kch
G2583_1524	ECs1751	Z2029	yciI	Uncharacterized protein conserved in bacteria
G2583_1525	ECs1752	Z2030	tonB	outer membrane receptor-mediated transport energizer protein TonB
G2583_1526	ECs1753	Z2031	yciA	Acyl-CoA thioester hydrolase yciA
G2583_1527	ECs1754	Z2032	yciB	Probable intracellular septation protein
G2583_1528	ECs1755	Z2033	yciC	UPF0259 membrane protein yciC
G2583_1529	ECs1756	Z2034	ompW	Outer membrane protein W
G2583_1530	ECs1757	Z2036	intE	Putative integrase for prophage CP-933O
G2583_1531	ECs1758		xisE	Excisionase
G2583_1532	ECs1759	Z2037	exoO	putative exonuclease
G2583_1533	ECs1760	Z2038	ydfD	unknown protein encoded by prophage CP-933O
G2583_1534	ECs1761	Z2039	dicB	Putative regulator of cell division encoded by prophage CP-933O
G2583_1535	dicF4		dicF4	ncRNA
G2583_1536	ECs1762	Z2040	-	unknown protein encoded by prophage CP-933O
G2583_1537	ECs1763	Z2041	-	unknown protein encoded by prophage CP-933O
G2583_1538	ECs1764	Z2042	ydfC	unknown protein encoded by prophage CP-933O
G2583_1539		Z2043	-	hypothetical protein
G2583_1540	ECs1765	Z2045	dicA	Similar to DicA, regulator of DicB encoded by prophage CP-933O
G2583_1541	ECs1766	Z2046	dicC	DNA-binding transcriptional regulator DicC
G2583_1542	ECs1767	Z2047	-	unknown protein encoded by prophage CP-933O
G2583_1543	ECs1768	Z2048	-	unknown protein encoded by prophage CP-933O
G2583_1544	ECs1769	Z2049	-	unknown protein encoded by prophage CP-933O
G2583_1545	ECsp014	Z2051	-	hypothetical protein EcolO15_16731
G2583_1546	ECs1770		papH	putative fimbrial minor pilin protein precursor
	ECs1771		-	fimbrial minor pilin protein precursor
		Z2052	-	unknown protein encoded by prophage CP-933O
G2583_1547	ECs1772	Z2053	acfC	Porcine attaching-effacing associated protein
G2583_1548	ECs1773	Z2054	gef	Putative killer protein encoded by prophage CP-933O
G2583_1549	ECs1774	Z2055	rem	hypothetical protein
G2583_1550	ECs1775		-	hypothetical protein
G2583_1551	ECs1776	Z2056	-	hypothetical protein
Indel-72	ECs1777	Z2057	-	endonuclease
Indel-72	ECs1778	Z2058	-	hypothetical protein
Indel-72	ECs1779	Z2059	-	hypothetical lipoprotein
Indel-72	ECs1780	Z2060	-	putative DNA methylase
Indel-72	ileZ3	RNA036	ileZ3	Ile tRNA
Indel-72	argO3	RNA037	argO3	Arg tRNA
Indel-72		Z2063	-	unknown protein encoded by prophage CP-933O
Indel-73	ECs1781	Indel-73	-	hypothetical protein
Indel-73	ECs1782	Indel-73	-	putative holin protein
Indel-73	ECs1783	Indel-73	-	hypothetical protein

Locus_tag[a]				
CB9615	Sakai	EDL933	Gene	Product
Indel-73	ECs1784	Indel-73	-	putative endolysin
Indel-73	ECs1785	Indel-73	-	antirepressor protein
Indel-73	ECs1786	Indel-73	-	endopeptidase
Indel-73	ECs1787	Indel-73	-	lipoprotein Rz1 precursor
Indel-73	ECs1788	Indel-73	-	hypothetical protein
Indel-73	ECs1789	Indel-73	-	putative Dnase
Indel-73	ECs1790	Indel-73	-	hypothetical protein
Indel-73	ECs1791	Indel-73	-	putative terminase small subunit
Indel-73	ECs1792	Indel-73	-	putative terminase large subunit
Indel-73	ECs1793	Indel-73	-	major head protein/prohead protease
Indel-73	ECs1794	Indel-73	-	hypothetical protein
Indel-73	ECs1795	Indel-73	-	putative portal protein
Indel-73	ECs1796	Indel-73	-	hypothetical protein
Indel-73	ECs1797	Indel-73	-	putative head-tail adaptor
Indel-73	ECs1798	Indel-73	-	hypothetical protein
Indel-73	ECs1799	Indel-73	-	hypothetical protein
Indel-73	ECs1800	Indel-73	-	putative major tail subunit
Indel-73	ECs1801	Indel-73	-	putative tail assembly chaperone
Indel-73	ECs1802	Indel-73	-	putative tail assembly chaperone
Indel-73	ECs1803	Indel-73	-	putative tail length tape measure protein
Indel-73	ECs1804	Indel-73	-	minor tail protein
Indel-73	ECs1805	Indel-73	-	minor tail protein
Indel-74	Indel-74	Z2065	-	similar to conserved hypothetical phage protein YjhS encoded within prophage CP-933O
Indel-74	Indel-74	Z2066	-	unknown protein encoded by prophage CP-933O
Indel-74	Indel-74	Z2068	-	unknown protein encoded by prophage CP-933O
Indel-74	Indel-74	Z2069	-	putative holin protein of prophage CP-933O
Indel-74	Indel-74	Z2070	-	unknown protein encoded by prophage CP-933O
Indel-74	Indel-74	Z2071	-	putative endolysin of prophage CP933-O; partial
Indel-74	Indel-74	Z2072	-	putative IS encoded proteinen coded by prophage CP-933O
Indel-74	Indel-74	Z2073	-	putative transposase within CP-933O
Indel-74	Indel-74	Z2074	-	putative IS encoded protein within CP-933O
G2583_1552	Indel-75	Indel-75	-	Holliday junction resolvase
G2583_1553	Indel-75	Indel-75	-	Antitermination protein Q
G2583_5267	Indel-75	Indel-75	-	Met tRNA
G2583_5268	Indel-75	Indel-75	-	Arg tRNA
G2583_1554	Indel-75	Indel-75	-	hypothetical protein
G2583_1555	Indel-75	Indel-75	-	hypothetical protein
G2583_1556	Indel-75	Indel-75	-	hypothetical protein
G2583_1557	Indel-75	Indel-75	-	hypothetical protein
G2583_1558	Indel-75	Indel-75	-	Putative lysis protein S of prophage CP-933V
G2583_1559	Indel-75	Indel-75	ybcS	lysozyme-like protein
G2583_1560	Indel-75	Indel-75	ybcT	Bacteriophage lysis protein

Locus_tag[a]				
CB9615	Sakai	EDL933	Gene	Product
G2583_1561	Indel-75	Indel-75	-	hypothetical protein
G2583_1562	Indel-75	Indel-75	-	Putative transcriptional regulator
G2583_1563	Indel-75	Indel-75	ynfO	unknown protein encoded within prophage CP-933R
G2583_1564	Indel-75	Indel-75	-	unknown protein encoded by prophage CP-933N
G2583_1565	Indel-75	Indel-75	-	Phage terminase, small subunit
G2583_1566	Indel-75	Indel-75	-	Phage terminase-like protein, large subunit
G2583_1567	Indel-75	Indel-75	-	Phage head maturation protease
G2583_1568	Indel-75	Indel-75	insN	unknown protein encoded by IS911 within prophage CP-933L
G2583_1569	Indel-75	Indel-75	-	putative transposase
G2583_1570	Indel-75	Indel-75	-	Phage head maturation protease
G2583_1571	Indel-75	Indel-75	-	conserved hypothetical protein
G2583_1572	Indel-75	Indel-75	-	conserved hypothetical protein
G2583_1573	Indel-75	Indel-75	-	Bacteriophage head-tail adaptor
G2583_1574	Indel-75	Indel-75	-	Phage protein, HK97 gp10 family
G2583_1575	Indel-75	Indel-75	-	unknown protein encoded by prophage CP-933N
G2583_1576	Indel-75	Indel-75	-	Putative major tail subunit
G2583_1577	Indel-75	Indel-75	-	Phage tail assembly chaperone
G2583_1578	Indel-75	Indel-75	-	putative tail protein
G2583_1579	Indel-75	Indel-75	-	putative tail length tape measure protein
G2583_1580	Indel-75	Indel-75	-	minor tail protein
G2583_1581	Indel-75	Indel-75	-	Phage-related protein
G2583_1582	Indel-75	Indel-75	-	Putative tail fiber component
G2583_1583	Indel-75	Indel-75	-	putative tail assembly protein
G2583_1584	Indel-75	Indel-75	-	putative host specificity protein
G2583_1585	Indel-75	Indel-75	insF	IS911 transposase orfB
G2583_1586	Indel-75	Indel-75	insN	unknown protein encoded by IS911 within prophage CP-933L
G2583_1587	Indel-75	Indel-75	-	Opacity protein and related surface antigens
G2583_1588	Indel-75	Indel-75	-	hypothetical protein
G2583_1589	Indel-75	Indel-75	-	conserved hypothetical protein
G2583_1590	Indel-75	Indel-75	-	NleA8-2 protein
G2583_1591	Indel-75	Indel-75	-	putative integrase fragment
G2583_1592	Indel-75	Indel-75	nleH	Non-LEE-encoded type III effector H
G2583_1593	Indel-75	Indel-75	nleF	hypothetical protein
G2583_1594	Indel-75	Indel-75	-	Putative transposase within CP-933O; partial
Indel-76	ECs1806	Z6029	-	putative host specificity protein
Indel-76	ECs1807	Z6028	lomP	putative outer membrane protein precursor
Indel-76	ECs1808	Z6027	-	putative tail fiber protein
Indel-76	ECs1809	Z6026	-	hypothetical protein
Indel-76	ECs1810		-	hypothetical protein
Indel-76	ECs1811	Z6025	-	hypothetical protein
Indel-76	ECs1812	Z6024	-	hypothetical protein
Indel-76		Z6023	-	unknown protein encoded by cryptic prophage CP-933P

Locus_tag[a]				
CB9615	Sakai	EDL933	Gene	Product
Indel-76	ECs1813	Z6022	-	integrase
Indel-76	ECs1814	Z6021	-	hypothetical protein
Indel-76	ECs1815	Z6020	-	hypothetical protein
Indel-76	ECs1816	Z6019	-	hypothetical protein
Indel-76	ECs1817	Z6017	-	hypothetical protein
Indel-76	ECs1818	Z6016	-	hypothetical protein
Indel-76	ECs1819	Z6015	-	hypothetical protein
Indel-76	ECs1820	Z6014	-	hypothetical protein
Indel-76		Z6012	-	hypothetical protein
Indel-76	ECs1821		-	hypothetical protein
Indel-76	ECs1822		-	hypothetical protein
Indel-76	ECs1823	Z6011	-	hypothetical protein
Indel-76	ECs1824	Z6010	-	hypothetical protein
Indel-76	ECs1825	Z2565	-	bfpT-regulated chaperone-like protein
Indel-76	ECs1826	Z2563	-	hypothetical protein
Indel-76	ECsp015	Z2562	-	putative transposase (partial)
Indel-76	ECs1827	Z2561	-	IS630 hypothetical protein
Indel-76	ECs1828	Z2560	-	hypothetical protein
Indel-76	ECsp016		-	insertion sequence IS3
Indel-76		Z2558	-	hypothetical protein
G2583_1595		Z2557	yciD	Putative outer membrane protein
G2583_1596	ECs1829	Z2555	yciE	YciE protein
G2583_1597	ECs1830	Z2554	yciF	YciF protein
G2583_1598	ECs1831	Z2553	yciG	hypothetical protein
G2583_1599	ECs1832	Z2551	trpA	Tryptophan synthase alpha chain
G2583_1600	ECs1833	Z2550	trpB	Tryptophan synthase beta chain
G2583_1601	ECs1834	Z2549	trpC	bifunctional indole-3-glycerol phosphate synthase/phosphoribosylanthranilate isomerase
G2583_1602	ECs1835	Z2548	trpD	Anthranilate synthase, component II
G2583_1603	ECs1836	Z2547	trpE	anthranilate synthase component I
G2583_1603	ECs1836	Z2546	trpE	anthranilate synthase component I
G2583_1604	ECs1837	Z2545	trpL	trp operon leader peptide
G2583_1605	ECs1838	Z2544	trpH	Putative phosphoesterase
G2583_1606	ECs1839	Z2543	yciO	hypothetical protein
G2583_1607	ECs1840	Z2542	-	conserved hypothetical protein
G2583_1608	ECs1841	Z2541	rluB	Ribosomal large subunit pseudouridine synthase B
G2583_1609	ECs1842	Z2540	btuR	Cob(I)yrinic acid a,c-diamide adenosyltransferase
G2583_1610	ECs1843	Z2539	yciK	Oxidoreductase, short chain dehydrogenase/reductase family
G2583_1611	ECs1844	Z2538	sohB	Peptidase, S49 (Protease IV) family
G2583_1612	ECs1845	Z2537	yciN	hypothetical protein
G2583_1613	ECs1846	Z2536	topA	DNA topoisomerase I
G2583_1614	ECs1847	Z2535	cysB	transcriptional regulator CysB
G2583_1615		Z2534	ymiA	hypothetical protein

Locus_tag[a]				
CB9615	Sakai	EDL933	Gene	Product
G2583_1616	ECs1848	Z2533	yciX	hypothetical protein
G2583_1617	ECs1849	Z2532	acnA	Aconitate hydrase 1
G2583_1618	ECs1850	Z2531	ribA	GTP cyclohydrolase-2
G2583_1619	ECs1851	Z2529	pgpB	Phosphatidylglycerophosphatase B
G2583_1620	ECs1852	Z2528	yciS	hypothetical protein
G2583_1621	ECs1853	Z2526	yciM	hypothetical protein
G2583_1622	ECs1854	Z2525	pyrF	Orotidine 5'-phosphate decarboxylase
G2583_1623	ECs1855	Z2524	yciH	Putative translation initation factor SUI1
G2583_1624	ECs1856	Z2523	osmB	hypothetical protein
G2583_1625	ECs1857	Z2521	yciT	putative DEOR-type transcriptional regulator
G2583_1626		Z2519	yciZ	UPF0509 protein yciZ
G2583_1627	ECs1858	Z2516	gmr	sensory box-containing diguanylate cyclase/cyclic diguanylate phosphodiesterase
G2583_1628	ECs1859	Z2514	rnb	Exoribonuclease 2
G2583_1629	ECs1860	Z2513	yciW	Putative oxidoreductase
G2583_1630	ECs1861	Z2512	fabI	Enoyl-[acyl-carrier-protein] reductase [NADH]
G2583_1631		Z2511	-	hypothetical protein
G2583_1632	ECs1862	Z2510	eefR	Putative transcriptional repressor
G2583_1633	ECs1863	Z2509	eefA	Acriflavine resistance protein A
G2583_1634	ECs1864	Z2508	acrB	putative multidrug-efflux transport protein
		Z2507	-	hypothetical protein
G2583_1635	ECs1865	Z2506	eefC	Multidrug efflux outer membrane protein EefC
G2583_1635	ECs1865	Z2504	eefC	Multidrug efflux outer membrane protein EefC
G2583_1636	ECs1866	Z2503	eefD	Multidrug efflux transport protein EefD
G2583_1636	ECs1866	Z2501	eefD	Multidrug efflux transport protein EefD
G2583_1637	ECs1867	Z2500	sapF	Peptide transport system, ATP-binding protein SapF
G2583_1638	ECs1868	Z2499	sapD	Peptide transport system ATP-binding protein sapD
G2583_1639	ECs1869	Z2498	sapC	Peptide ABC transporter, permease protein SapC
G2583_1640	ECs1870	Z2496	sapB	Peptide transport system permease protein sapB
G2583_1641	ECs1871	Z2494	sapA	Homolog of Salmonella peptide transport periplasmic protein
G2583_1642	ECs1872	Z2493	ymjA	hypothetical protein
G2583_1643	ECs1873	Z2492	puuP	Putative amino acid/amine transport protein
G2583_1644	ECs1874	Z2491	puuA	Putative glutamine synthetase
G2583_1645	ECs1875	Z2490	puuD	Gamma-glutamyl-gamma-aminobutyrate hydrolase
G2583_1646	ECs1876	Z2489	puuR	DNA-binding transcriptional repressor
G2583_1647	ECs1877	Z2488	puuC	Gamma-glutamyl-gamma-aminobutyraldehyde dehydrogenase
G2583_1648	ECs1878	Z2487	puuB	Gamma-glutamylputrescine oxidoreductase
G2583_1649	ECs1879	Z2486	puuE	4-aminobutyrate transaminase
G2583_1650	ECs1880	Z2484	pspF	Psp operon transcriptional activator
G2583_1651	ECs1881	Z2482	pspA	Phage shock protein
G2583_1652	ECs1882	Z2480	pspB	Phage shock protein B
G2583_1653	ECs1883	Z2479	pspC	Phage shock protein C

Locus_tag[a]				
CB9615	Sakai	EDL933	Gene	Product
G2583_1654	ECs1884	Z2478	pspD	Phage shock protein
G2583_1655	ECs1885	Z2477	pspE	Phage shock protein E
G2583_1656	ECs1886	Z2476	ycjM	Alpha amylase family protein
G2583_1656	ECs1887	Z2475	ycjM	Alpha amylase family protein
G2583_1657	ECs1888	Z2474	ycjN	Sugar-binding periplasmic protein
G2583_1657	ECs1889	Z2473	ycjN	Sugar-binding periplasmic protein
G2583_1658	ECs1890	Z2472	ycjO	Putative sugar ABC transporter, permease protein
G2583_1659	ECs1891	Z2471	ycjP	Binding-protein-dependent transport system inner membrane component
G2583_1660	ECs1892	Z2470	ycjQ	Oxidoreductase, zinc-binding dehydrogenase family
G2583_1661	ECs1893	Z2469	ycjR	AP endonuclease, family 2
G2583_1662	ECs1894	Z2468	ycjS	Gfo/idh/mocA family
G2583_1663	ECs1895	Z2467	ycjT	glycosyl hydrolase, family 65
G2583_1664	ECs1896	Z2465	ycjU	Beta-phosphoglucomutase
G2583_1665	ECs1897	Z2463	ycjV	Uncharacterized ABC transporter ATP-binding protein ycjV
G2583_1666	ECs1898	Z2462	ompG	Outer membrane protein G
G2583_1667	ECs1899	Z2461	ycjW	putative LACI-type transcriptional regulator
G2583_1668	ECs1900	Z2458	ycjX	putative EC 2.1 enzyme
G2583_1669	ECs1901	Z2456	ycjF	UPF0283 membrane protein ycjF
G2583_1670	ECs1902	Z2454	tyrR	DNA-binding transcriptional dual regulator, tyrosine-binding
G2583_1671	ECs1903	Z2452	tpx	Thiol peroxidase
G2583_1672	ECs1904	Z2450	ycjG	Mandelate racemase/muconate lactonizing enzyme family protein
G2583_1673	ECs1905	Z2448	mpaA	murein peptide amidase A
G2583_1674	ECs1906	Z2446	ymjC	NmrA family protein
G2583_1675	ECs1907	Z2445	-	hypothetical protein
G2583_1675	ECs1908	Z2444	-	hypothetical protein
G2583_1676	ECs1909	Z2442	ycjY	hypothetical protein
G2583_1677	ECs1910	Z2439	ycjZ	putative transcriptional regulator LYSR-type
G2583_1678	ECs1911	Z2438	mppA	Putative transport periplasmic protein
G2583_1679	ECs1912	Z2437	ynaI	MscS family inner membrane protein ynaI
G2583_1680	ECs1913	Z2436	ynaJ	hypothetical protein
G2583_1681	ECs1914	Z2435	uspE	Universal stress protein E
G2583_1682	ECs1915	Z2433	fnr	Fumarate and nitrate reduction regulatory protein
G2583_1683	ECs1916	Z2432	ogt	Methylated-DNA-[protein]-cysteine S-methyltransferase
G2583_1684	ECs1917	Z2431	abgT	Aminobenzoyl-glutamate transport protein
Indel-78	ECs1918	Z2430	-	putative transposase OrfB protein of insertion sequence IS629
Indel-78	ECs1919	Z2429	-	putative transposase OrfA protein of insertion sequence IS629
G2583_1684	ECs1920	Z2428	abgT	Aminobenzoyl-glutamate transport protein
G2583_1685	ECs1921	Z2427	abgB	Aminobenzoyl-glutamate utilization protein B

Locus_tag[a]				
CB9615	Sakai	EDL933	Gene	Product
G2583_1686	ECs1922	Z2425	abgA	Aminobenzoyl-glutamate utilization protein A
G2583_1687	ECs1923	Z2423	abgR	putative DNA-binding transcriptional regulator
G2583_1688			-	ncRNA
G2583_1689	ECs1924	Z2422	ydaL	Smr domain protein
G2583_1690	ECs1925	Z2421	ydaM	hypothetical protein
G2583_1691	ECs1926	Z2419	ydaN	Zinc transport protein zntB
G2583_1692			-	ncRNA
G2583_1693	ECs1927	Z2417	dbpA	ATP-independent RNA helicase DbpA
G2583_1694	ECs1928	Z2416	ttcA	C32 tRNA thiolase
G2583_1695	ECs1929	Z2415	intR	Putative integrase for prophage CP-933R
G2583_1696	ECs1930	Z2414	ydaQ	hypothetical protein
G2583_1697	ECs1931	Z2413	ydaC	hypothetical protein
G2583_1698	ECs1932	Z2412	lar	Restriction alleviation and modification enhancement protein
G2583_1699	ECs1933	Z2410	recT	Recombinase, DNA renaturation protein encoded by prophage CP-933R
G2583_1700	ECs1934	Z2409	recE	Putative exodeoxyribonuclease VIII of prophage CP-
Indel-79	ECs1935	Z2408	racC	racC protein
Indel-79	ECs1936	Z2406	ydaD	hypothetical protein
Indel-79	ECs1937	Z2404	sieB	phage superinfection exclusion protein
Indel-79	ECs1939		-	hypothetical protein
Indel-79	ECs1938	Z2403	-	hypothetical protein
Indel-79	ECs1940	Z2402	-	hypothetical protein
Indel-79	ECs1941	Z2400	-	putative transcriptional regulator
Indel-79	ECs1942	Z2399	-	regulatory protein
Indel-79	ECs1943	Z2398	-	hypothetical protein
Indel-79	ECs1944	Z2397	-	hypothetical protein
Indel-79	ECs1945	Z2396	-	putative DNA replication factor
Indel-79	ECs1946	Z2395	-	hypothetical protein
Indel-79	ECs1947		-	hypothetical protein
Indel-79	ECs1948	Z2394	-	hypothetical protein
Indel-79	ECs1949	Z2393	-	hypothetical protein
Indel-79	ECs1950	Z2392	-	hypothetical protein
Indel-79	ECs1951	Z2391	-	hypothetical protein
Indel-79	ECs1952	Z2390	-	hypothetical protein
Indel-79	ECs1953	Z2389	-	putative methyltransferase
Indel-79	ECs1954	Z2387	-	hypothetical protein
Indel-79	ECs1955	Z2386	-	hypothetical protein
Indel-79	ECs1956	Z2385	-	hypothetical protein
Indel-79	ECs1957		-	hypothetical protein
Indel-79	ECs1958	Z2384	-	putative antitermination protein
Indel-79	ECs1958	Z2382	-	putative antitermination protein
Indel-79	ileZ4	RNA039	ileZ4	Ile tRNA
Indel-79	tRNA25		tRNA25	-

Locus_tag[a]				
CB9615	Sakai	EDL933	Gene	Product
Indel-79	argO4	RNA038	argO4	Arg tRNA
Indel-79	ECs1959	Z2379	-	hypothetical protein
Indel-79	ECs1960	Z2378	-	hypothetical protein
Indel-79	ECs1961	Z2377	-	hypothetical protein
Indel-80	ECs1962	Indel-80	-	putative holin protein
Indel-80	ECs1963	Indel-80	-	hypothetical protein
Indel-80	ECs1964	Indel-80	-	putative endolysin
Indel-80	ECs1965	Indel-80	-	putative antirepressor protein
Indel-80	ECs1966	Indel-80	-	putative endopeptidase
Indel-80	ECs1967	Indel-80	-	hypothetical protein
Indel-80	ECs1968	Indel-80	-	putative DNase
Indel-80	ECs1969	Indel-80	-	hypothetical protein
Indel-80	ECs1970	Indel-80	-	putative terminase small subunit
Indel-80	ECs1971	Indel-80	-	putative terminase large subunit
Indel-80	ECs1972	Indel-80	-	putative phage major head protein/prohead protease
Indel-80	ECs1973	Indel-80	-	hypothetical protein
Indel-80	ECs1974	Indel-80	-	putative portal protein
Indel-80	ECs1975	Indel-80	-	hypothetical protein
Indel-80	ECs1976	Indel-80	-	hypothetical protein
Indel-80	ECs1977	Indel-80	-	putative head-tail adaptor
Indel-80	ECs1978	Indel-80	-	hypothetical protein
Indel-80	ECs1979	Indel-80	-	hypothetical protein
Indel-80	ECs1980	Indel-80	-	major tail protein
Indel-80	ECs1981	Indel-80	-	putative tail assembly chaperon
Indel-80	ECs1982	Indel-80	-	hypothetical protein
Indel-80	ECs1983	Indel-80	-	putative tail length tape measure protein
Indel-80	ECs1984	Indel-80	-	putative minor tail protein
Indel-80	ECs1985	Indel-80	-	putative minor tail protein
Indel-80	ECs1986	Indel-80	-	putative tail assembly protein
Indel-80	ECs1987	Indel-80	-	putative tail assembly protein
Indel-80	ECs1988	Indel-80	-	hypothetical protein
Indel-80	ECs1989	Indel-80	-	putative copper/zinc-superoxide dismutase
Indel-80	ECs1990	Indel-80	-	putative host specificity protein
Indel-81	Indel-81	Z2343	-	partial putative outer membrane protein Lom precursor encoded by prophage CP-933R
Indel-81	Indel-81	Z2344	-	putative tail fiber protein encoded by prophage CP-
Indel-81	Indel-81	Z2346	-	partial putative phage tail protein encoded by prophage CP-933R
Indel-81	Indel-81	Z2347	-	putative copper-zinc superoxide dismutase encoded within prophage CP-933R
Indel-81	Indel-81	Z2348	-	partial putative phage tail protein encoded by prophage CP-933R
Indel-81	Indel-81	Z2350	-	partial putative phage tail protein encoded by prophage CP-933R

Locus_tag[a]				
CB9615	Sakai	EDL933	Gene	Product
Indel-81	Indel-81	Z2351	-	putative tail component of prophage CP-933R
Indel-81	Indel-81	Z2352	-	putative tail component of prophage CP-933R
Indel-81	Indel-81	Z2353	-	putative tail component of prophage CP-933R
Indel-81	Indel-81	Z2354	-	partial putative tail component of prophage CP-933R
Indel-81	Indel-81	Z2355	-	partial putative tail component of prophage CP-933R
Indel-81	Indel-81	Z2356	-	putative tail component of prophage CP-933R
Indel-81	Indel-81	Z2357	-	putative tail component of prophage CP-933R
Indel-81	Indel-81	Z2358	-	putative tail component of prophage CP-933R
Indel-81	Indel-81	Z2359	-	partial putative capsid structural protein of prophage CP-933R
Indel-81	Indel-81	Z2360	-	putative capsid protein of prophage CP-933R
Indel-81	Indel-81	Z2361	-	putative capsid assembly protein of prophage CP-933R
Indel-81	Indel-81	Z2362	-	putative capsid protein of prophage CP-933R
Indel-81	Indel-81	Z2363	-	putative DNA packaging protein of prophage CP-933R
Indel-81	Indel-81	Z2364	-	putative DNA packaging protein of prophage CP-933R; terminase large subunit
Indel-81	Indel-81	Z2365	-	putative DNA packaging protein of prophage CP-933R; terminase small subunit
Indel-81	Indel-81	Z2366	-	unknown protein encoded within prophage CP-933R
Indel-81	Indel-81	Z2367	-	unknown protein encoded within prophage CP-933R
Indel-81	Indel-81	Z2368	-	unknown protein encoded within prophage CP-933R
Indel-81	Indel-81	Z2369	-	putative endopeptidase Rz of prophage CP-933R
Indel-81	Indel-81	Z2370	-	unknown protein encoded within prophage CP-933R
Indel-81	Indel-81	Z2371	-	putative lysozyme R of prophage CP-933R
Indel-81	Indel-81	Z2372	-	unknown protein encoded within prophage CP-933R
Indel-81	Indel-81	Z2374	-	putative holin protein of prophage CP-933R
Indel-81	Indel-81	Z2375	-	orf; hypothetical protein in IS629 within prophage CP-
Indel-81	Indel-81	Z2376	-	putative IS629 transposase within prophage CP-933R
Indel-82	ECs1991	Z2342	-	putative outer membrane protein
	ECs1992	Z2340	-	putative tail fiber protein
G2583_1701	Indel-83	Indel-83	racC	Putative bacteriophage protein
G2583_1702	Indel-83	Indel-83	ydaE	Putative bacteriophage protein
G2583_1703	Indel-83	Indel-83	kilR	Putative cell division inhibitor protein
G2583_1704	Indel-83	Indel-83	-	conserved hypothetical protein
G2583_1705	Indel-83	Indel-83	racR	Rac prophage repressor
G2583_1706	Indel-83	Indel-83	ydaS	putative tail fiber protein
G2583_1707	Indel-83	Indel-83	ydaT	conserved hypothetical protein
G2583_1708	Indel-83	Indel-83	ydaU	hypothetical protein
G2583_1709	Indel-83	Indel-83	ydaV	Putative DNA replication factor encoded by prophage CP-933R
G2583_1710	Indel-83	Indel-83	ydaW	conserved hypothetical protein
G2583_1711	Indel-83	Indel-83	renP	hypothetical protein
G2583_1712	Indel-83	Indel-83	-	unknown protein encoded by cryptic prophage CP-933P
G2583_1713	Indel-83	Indel-83	-	conserved hypothetical protein

Locus_tag[a]				
CB9615	Sakai	EDL933	Gene	Product
G2583_1714	Indel-83	Indel-83	-	hypothetical protein
G2583_1715	Indel-83	Indel-83	mokP	Putative cell killing protein encoded within cryptic prophage CP-933P
G2583_1716	Indel-83	Indel-83	-	Putative bacteriophage cohesive ends
G2583_1717	Indel-83	Indel-83	-	hypothetical protein
G2583_1718	Indel-83	Indel-83	-	Putative antitermination protein
G2583_1719	Indel-83	Indel-83	-	unknown protein encoded by prophage CP-933O
G2583_1720	Indel-83	Indel-83	-	YjhS
G2583_1721	Indel-83	Indel-83	-	hypothetical protein
G2583_1722	Indel-83	Indel-83	-	Putative lysis protein S of prophage CP-933V
G2583_1723	Indel-83	Indel-83	-	putative endolysin
G2583_1724	Indel-83	Indel-83	-	putative endolysin
G2583_1725	Indel-83	Indel-83	-	Anti-repressor protein Ant
G2583_1726	Indel-83	Indel-83	-	Endopeptidase
G2583_1727	Indel-83	Indel-83	-	hypothetical protein
G2583_1728	Indel-83	Indel-83	-	hypothetical protein
G2583_1729	Indel-83	Indel-83	-	conserved hypothetical protein
G2583_1730	Indel-83	Indel-83	-	Phage terminase large subunit
G2583_1731	Indel-83	Indel-83	-	hypothetical protein
G2583_1732	Indel-83	Indel-83	-	Peptidase S14, ClpP
G2583_1733	Indel-83	Indel-83	-	unknown protein encoded within prophage CP-933U
G2583_1734	Indel-83	Indel-83	-	hypothetical protein
G2583_1735	Indel-83	Indel-83	-	Putative tail fiber component Z
G2583_1736	Indel-83	Indel-83	-	putative tail fiber component U of prophage CP-933U
G2583_1737	Indel-83	Indel-83	-	hypothetical protein
G2583_1738	Indel-83	Indel-83	-	Minor tail protein G
G2583_1739	Indel-83	Indel-83	-	Minor tail protein T
G2583_1740	Indel-83	Indel-83	-	Minor tail protein H
G2583_1741	Indel-83	Indel-83	-	putative tail component of prophage CP-933O
G2583_1742	Indel-83	Indel-83	-	Phage-related protein
G2583_1743	Indel-83	Indel-83	-	Putative tail fiber component
G2583_1744	Indel-83	Indel-83	-	putative tail assembly protein
G2583_1745	Indel-83	Indel-83	-	Phage-related protein, tail component
G2583_1746	Indel-83	Indel-83	-	Putative outer membrane protein Lom of prophage CP-933O
G2583_1747	Indel-83	Indel-83	-	putative tail fiber protein
	ECs1993		-	hypothetical protein
G2583_1748	ECs1994	Z2339	-	unknown protein encoded by prophage CP-933R
G2583_1749	ECs1995	Z2338	-	unknown protein encoded by prophage CP-933R
G2583_1750	ECs1996	Z2337	-	unknown protein encoded by prophage CP-933R
G2583_1751	ECs1997	Z2335	uspF	Universal stress protein F
G2583_1752	ECs1998	Z2334	ompN	Outer membrane protein N
G2583_1752	ECs1999	Z2333	ompN	Outer membrane protein N
G2583_1753			-	ncRNA

Locus_tag[a]				
CB9615	Sakai	EDL933	Gene	Product
G2583_1754	ECs2000	Z2332	ydbK	Pyruvate-flavodoxin oxidoreductase
G2583_1755			ydbJ	hypothetical protein
G2583_1756	ECs2001	Z2330	hslJ	Heat shock protein HslJ
G2583_1757	ECs2002	Z2329	ldhA	D-lactate dehydrogenase
G2583_1758	ECs2003	Z2328	ydbH	hypothetical protein
G2583_1759	ECs2004	Z2327	ynbE	hypothetical protein
G2583_1760	ECs2005	Z2326	ydbL	hypothetical protein
G2583_1761		Z2325	entS	hypothetical protein
G2583_1762	ECs2006	Z2323	-	putative BigA-like protein
G2583_1763	ECs2007	Z2322	entS	putative outer membrane protein
G2583_1764	ECs2008	Z2321	ydbC	Oxidoreductase, aldo/keto reductase family
G2583_1765	ECs2009	Z2320	ydbD	hypothetical protein
G2583_1766	ECs2010	Z2319	ynbA	Phosphatidylglycerophosphate synthase
G2583_1767	ECs2011	Z2318	ynbB	Phosphatidate cytidylyltransferase
G2583_1768	ECs2012	Z2317	ynbC	hypothetical protein
G2583_1769	ECs2013	Z2316	ynbD	hypothetical protein
G2583_1770	ECs2014	Z2315	azoR	FMN-dependent NADH-azoreductase
G2583_1771	ECs2015	Z2313	hrpA	HrpA-like helicases
G2583_1772	ECs2016	Z2312	-	hypothetical protein
G2583_1773	ECs2017	Z2311	-	hypothetical protein
G2583_1774	ECs2018	Z2310	-	hypothetical protein
G2583_1775	ECs2019	Z2309	-	hypothetical protein
G2583_1776	ECs2020	Z2308	ydcF	hypothetical protein
G2583_1777	ECs2021	Z2306	aldA	Aldehyde dehydrogenase A
G2583_1778	ECs2022	Z2304	gapC	Glyceraldehyde-3-phosphate dehydrogenase C
G2583_1779	ECs2023	Z2303	cybB	Nickel-dependent hydrogenase, b-type cytochrome subunit
G2583_1780			-	ncRNA
G2583_1781	ECs2024	Z2302	ydcA	hypothetical protein
G2583_1782	ECs2025	Z2301	mokB	hypothetical protein
G2583_1783	ECs2026	Z2300	trg	Methyl-accepting chemotaxis protein III, ribose sensor receptor
G2583_1784	ECs2027	Z2299	ydcI	putative transcriptional regulator LYSR-type
G2583_1785	ECs2028	Z2298	ydcJ	hypothetical protein
G2583_1785	ECs2028	Z2297	ydcJ	hypothetical protein
G2583_1785	ECs2028	Z2296	ydcJ	hypothetical protein
G2583_1786	ECs2029	Z2294	mdoD	Glucans biosynthesis protein D precursor
G2583_1787	ECs2030	Z2293	-	hypothetical protein
G2583_1788	ECs2031	Z2292	ydcH	hypothetical protein
G2583_1789	ECs2032	Z2291	rimL	Ribosomal-protein-serine acetyltransferase
G2583_1790	ECs2033	Z2290	ydcK	hypothetical protein
G2583_1791	ECs2034	Z2289	tehA	Tellurite resistance protein tehA
G2583_1792	ECs2035	Z2288	tehB	Tellurite resistance protein TehB
G2583_1793	ECs2036	Z2287	ydcL	Uncharacterized lipoprotein ydcL precursor

Locus_tag[a]				
CB9615	Sakai	EDL933	Gene	Product
G2583_1794	ECs2038	Z2286	ydcO	hypothetical protein
G2583_1795	ECs2037	Z2285	ydcN	DNA-binding protein
G2583_1796	ECs2039	Z2284	ydcP	Peptidase, U32 family
G2583_1797	ECs2040	Z2283	yncJ	hypothetical protein
G2583_1798	ECs2041	Z2282	-	hypothetical protein
G2583_1799	ECs2042	Z2281	ydcQ	Predicted DNA-binding transcriptional regulator
G2583_1800	ECs2043	Z2280	ydcR	multi modular; putative transcriptional regulator; also putative ATP-binding component of a transport system
G2583_1801	ECs2044	Z2279	ydcS	ABC transporter, periplasmic substrate-binding protein
G2583_1802	ECs2045	Z2278	ydcT	ABC transporter, ATP-binding protein
G2583_1803	ECs2046	Z2277	ydcU	ABC transporter, permease protein
G2583_1804	ECs2047	Z2276	ydcV	Putative transport system permease protein
G2583_1805	ECs2048	Z2275	ydcW	Gamma-aminobutyraldehyde dehydrogenase
G2583_1806	ECs2049	Z2274	ydcX	hypothetical protein
G2583_1807	ECs2050	Z2273	ydcY	hypothetical protein
G2583_1808	ECs2051	Z2272	ydcZ	hypothetical protein
G2583_1809	ECs2052	Z2271	yncA	Acetyltransferase, GNAT family
G2583_1810	ECs2053	Z2270	yncB	Putative NADP-dependent oxidoreductase yncB
G2583_1811	ECs2054	Z2269	yncC	putative DNA-binding transcriptional regulator
G2583_1812	ECs2055	Z2268	yncD	TonB-dependent receptor
G2583_1813	ECs2056	Z2267	yncE	Uncharacterized conserved protein
G2583_1814	ECs2057	Z2266	ansP	L-asparagine permease
G2583_1815	ECs2058	Z2265	yncG	putative transferase
G2583_1816	ECs2059	Z2264	yncH	conserved hypothetical protein
G2583_1817		Z2263	-	hypothetical protein
G2583_1818	ECs2060	Z2262	vgrE	unknown protein associated with Rhs element
G2583_1819	ECs2061	Z2261	rhsE	protein rhsE
G2583_1819	ECs2061	Z2259	rhsE	protein rhsE
G2583_1819	ECs2061	Z2257	rhsE	protein rhsE
G2583_1820		Z2256	-	DsORF-e4
G2583_1821		Z2255	-	unknown protein associated with Rhs element
G2583_1822		Z2254	ydcC	H repeat-associated protein
G2583_1822	ECs2062	Z2253	ydcC	H repeat-associated protein
G2583_1822	ECs2063		ydcC	H repeat-associated protein
G2583_1823	ECs2064	Z2252	pptA	Probable tautomerase ydcE
G2583_1824	ECs2065	Z2251	yddH	hypothetical protein
G2583_1825	ECs2066	Z2250	nhoA	Putative N-hydroxyarylamine O-acetyltransferase
G2583_1825		Z2249	nhoA	Putative N-hydroxyarylamine O-acetyltransferase
G2583_1826	ECs2067	Z2248	yddE	Uncharacterized isomerase yddE
G2583_1827	ECs2068	Z2247	narV	Nitrate reductase 2, gamma subunit
G2583_1828	ECs2069	Z2246	narW	Nitrate reductase molybdenum cofactor assembly chaperone 2
G2583_1829	ECs2070	Z2245	narY	Nitrate reductase 2, beta subunit
G2583_1830	ECs2071	Z2244	narZ	Nitrate reductase 2, alpha subunit

Locus_tag[a]				
CB9615	Sakai	EDL933	Gene	Product
G2583_1831	ECs2072	Z2243	narU	Nitrite extrusion protein 2
G2583_1832	ECs2073	Z2242	yddJ	Leucine-rich repeat protein
G2583_1833	ECs2074	Z2241	-	hypothetical protein
G2583_1834	ECs2075	Z2240	-	hypothetical protein
G2583_1835	ECs2076	Z2239	-	Gram-negative porin family
G2583_1836	ECs2077	Z2238	yddG	hypothetical protein
G2583_1837	ECs2078	Z2236	fdnG	nitrate-inducible formate dehydrogenase-N alpha
G2583_1838	ECs2079	Z2235	fdnH	Formate dehydrogenase, nitrate-inducible, iron-sulfur subunit
G2583_1839	ECs2080	Z2234	fdnI	Formate dehydrogenase-N gamma subunit
G2583_1840	ECs2081	Z2233	yddM	hypothetical protein
G2583_1841	ECs2082	Z2232	adhP	Zn-dependent alcohol dehydrogenases
G2583_1842	ECs2083	Z2231	maeA	NAD-dependent malic enzyme
G2583_1843	ECs2084	Z2230	sra	Stationary-phase-induced ribosome-associated protein
G2583_1844	ECs2085	Z2229	bdm	biofilm-dependent modulation protein
G2583_1845	ECs2086	Z2228	osmC	Peroxiredoxin OsmC
G2583_1846	ECs2087	Z2227	ddpF	Putative ABC transport system ATP-binding protein
G2583_1847	ECs2088	Z2226	ddpD	ABC transporter, ATP-binding protein
G2583_1848	ECs2089	Z2225	ddpC	Inner membrane ABC transporter permease protein
G2583_1849	ECs2090	Z2224	ddpB	Binding-protein-dependent transport systems inner membrane component
G2583_1850	ECs2091	Z2223	ddpA	Putative ABC transporter periplasmic-binding protein
G2583_1851	ECs2092	Z2222	ddpX	D-alanyl-D-alanine dipeptidase
G2583_1852	ECs2093	Z2221	dos	FOG: PAS/PAC domain
G2583_1852	ECs2094	Z2220	dos	FOG: PAS/PAC domain
G2583_1853	ECs2095	Z2219	yddV	Diguanylate cyclase yddV
G2583_1854	ECs2096	Z2217	yddW	Uncharacterized lipoprotein yddW precursor
G2583_1855	ECs2097	Z2216	gadC	Probable glutamate/gamma-aminobutyrate antiporter
G2583_1856	ECs2098	Z2215	gadB	Glutamate decarboxylase beta
G2583_1857	ECs2099	Z2214	pqqL	Putative peptidase
G2583_1858	ECs2100	Z2213	yddB	TonB-dependent receptor
G2583_1859	ECs2101	Z2212	yddA	Inner membrane ABC transporter ATP-binding protein
G2583_1860	ECs2102	Z2211	ydeM	Putative enzyme
G2583_1861	ECs2103	Z2210	ydeN	Putative sulfatase
G2583_1862	ECs2104	Z2209	ydeO	transcriptional regulator YdeO
G2583_1863	ECs2105	Z2208	-	Two-component-system connector protein yneN
G2583_1864	ECs2106	Z2207	ydeP	putative oxidoreductase major subunit
G2583_1865	ECs2107	Z2206	ydeQ	transcriptional regulator BolA
G2583_1866	ECs2108	Z2205	ydeR	Uncharacterized fimbrial-like protein ydeR precursor
G2583_1867	ECs2109	Z2204	ydeS	biofilm-dependent modulation protein
G2583_1868	ECs2110	Z2202	fimD	Fimbrial usher family protein
	ECs2111		-	hypothetical protein
G2583_1868		Z2203	fimD	Fimbrial usher family protein
G2583_1869	ECs2112	Z2201	FimC	P pilus assembly protein, chaperone PapD

Locus_tag[a]				
CB9615	Sakai	EDL933	Gene	Product
G2583_1870	ECs2113	Z2200	fmlA	Putative Fml fimbriae subunit
G2583_1871	ECs2114	Z2199	yneL	Putative ARAC-type regulatory protein
G2583_1872	ECs2115	Z2197	hipA	regulator with hipB
G2583_1873	Indel-91	Indel-91	hipB	transcriptional regulator HipB
G2583_1874	ECs2116	Z2196	ydeU	Outer membrane autotransporter barrel domain protein precursor
G2583_1875	ECs2117	Z2195	ydeK	hypothetical protein
G2583_1876	ECs2118	Z2194	lsrK	Carbohydrate kinase FGGY
G2583_1877	ECs2119	Z2193	lsrR	putative transcriptional regulator, sorC family
G2583_1878	ECs2120	Z2192	lsrA	Autoinducer-2 ABC transporter, ATP-binding protein
G2583_1879	ECs2121	Z2191	lsrC	Autoinducer-2 ABC transporter, permease protein LsrC
G2583_1880	ECs2122	Z2190	lsrD	Autoinducer-2 ABC transporter, permease protein LsrD
G2583_1881	ECs2123	Z2189	lsrB	Autoinducer-2 ABC transporter, periplasmic autoinducer-2-binding protein LsrB
G2583_1882	ECs2124	Z2188	lsrF	Uncharacterized aldolase yneB
G2583_1883	ECs2125	Z2187	lsrG	autoinducer-2 (AI-2) modifying protein LsrG
G2583_1884	ECs2126	Z2186	tam	Trans-aconitate 2-methyltransferase
G2583_1885	ECs2127	Z2185	yneE	hypothetical protein
G2583_1886	ECs2128	Z2184	uxaB	Altronate oxidoreductase
G2583_1887	ECs2129	Z2182	yneF	Diguanylate cyclase (GGDEF) domain protein
G2583_1887		Z2181	yneF	Diguanylate cyclase (GGDEF) domain protein
G2583_1888	ECs2130	Z2180	yneG	hypothetical protein
G2583_1889	ECs2131	Z2179	yneH	Glutaminase 2
G2583_1890	ECs2132	Z2178	yneI	NAD-dependent aldehyde dehydrogenases
G2583_1891	ECs2133	Z2177	yneJ	putative transcriptional regulator LYSR-type
G2583_1892	ECs2134	Z2176	yneK	hypothetical protein
G2583_1892		Z2175	yneK	hypothetical protein
G2583_1893	ECs2135	Z2173	ydeA	Probable sugar efflux transporter
G2583_1894	ECs2136	Z2172	marC	MarC
G2583_1895	ECs2137	Z2171	marR	MarR
G2583_1896	ECs2138	Z2170	marA	Multiple antibiotic resistance protein marA
G2583_1897	ECs2139	Z2169	marB	Multiple antibiotic resistance protein MarB
G2583_1898	ECs2140	Z2168	eamA	Transporter, 10 TMS drug/metabolite exporter (DME) family
G2583_1899	ECs2141	Z2166	ydeE	Transporter, major facilitator family
G2583_1900		Z2165	-	Rhodanese domain protein
	ECs2142		-	hypothetical protein
G2583_1901	ECs2143	Z2164	ftrA	Transcriptional activator FtrA
G2583_1902	ECs2144	Z2163	ydeH	Diguanylate cyclase
G2583_1903	ECs2145	Z2162	ydeI	hypothetical protein
G2583_1904	ECs2146	Z2161	ydeJ	Competence/damage-inducible protein CinA C-terminal domain protein
G2583_1905	ECs2147	Z2160	dcp	Peptidyl-dipeptidase Dcp
G2583_1906	ECs2148	Z2158	ydfG	L-allo-threonine dehydrogenase, NAD(P)-binding

Locus_tag[a]				
CB9615	Sakai	EDL933	Gene	Product
G2583_1907	ECs2149	Z2157	ydfH	Hypothetical transcriptional regulator ydfH
G2583_1908	ECs2150	Z2156	ydfZ	Putative selenoprotein ydfZ
G2583_1909	ECs2151	Z2155	ydfI	Mannitol dehydrogenase family protein
G2583_1910	ECs2152	Z2153	ydfJ	Inner membrane metabolite transport protein ydfJ
G2583_1911	ECs2153	Z2152	-	unknown protein encoded within CP-933O
Indel-92	ECs2154	Z2151	-	hypothetical protein
Indel-92	ECs2155	Z2150	-	hypothetical protein
Indel-92	ECs2156	Z2149	-	hypothetical protein
Indel-92	ECs2157	Z2148	-	hypothetical protein
Indel-92	ECs2158		-	putative tail fiber protein
Indel-93	ECs2159	Indel-93	-	putative tail fiber protein
Indel-93	ECs2160	Indel-93	-	putative outer host membrane protein precursor
Indel-93	ECs2161	Indel-93	-	putative host specificity protein
Indel-93	ECs2162	Indel-93	-	putative tail assembly protein
Indel-93	ECs2163	Indel-93	-	putative tail assembly protein
Indel-93	ECs2164	Indel-93	-	putative minor tail protein
Indel-93	ECs2165	Indel-93	-	putative minor tail protein
Indel-93	ECs2166	Indel-93	-	putative tail length tape measure protein precursor
Indel-93	ECs2167	Indel-93	-	putative minor tail protein
Indel-93	ECs2168	Indel-93	-	minor tail protein
Indel-93	ECs2169	Indel-93	-	hypothetical protein
Indel-93	ECs2170	Indel-93	-	putative minor tail protein
Indel-93	ECs2171	Indel-93	-	putative minor tail protein
Indel-93	ECs2172	Indel-93	-	putative tail attachment protein
Indel-93	ECs2173	Indel-93	-	putative DNA-packaging protein
Indel-93	ECs2174	Indel-93	-	putative major capsid protein
Indel-93	ECs2175	Indel-93	-	putative head decoration protein
Indel-93	ECs2176	Indel-93	-	putative minor capsid protein precursor
Indel-93	ECs2177	Indel-93	-	putative portal protein
Indel-93	ECs2178	Indel-93	-	putative head-to-tail joining protein
Indel-93	ECs2179	Indel-93	-	putative terminase large subunit
Indel-93	ECs2180	Indel-93	-	putative terminase small subunit
Indel-93	ECs2181	Indel-93	-	hypothetical protein
Indel-93	ECs2182	Indel-93	-	putative transcriptional regulator
Indel-93	ECs2184	Indel-93	-	putative endopeptidase
Indel-93	ECs2183	Indel-93	-	putative lipoprotein Rz1 precursor
Indel-93	ECs2185	Indel-93	-	putative antirepressor protein
Indel-93	ECs2186	Indel-93	-	putative endolysin
Indel-93	ECs2187	Indel-93	-	hypothetical protein
Indel-93	ECs2188	Indel-93	-	putative holin protein
Indel-93	ECs2189	Indel-93	-	hypothetical protein
Indel-94	Indel-94	Z2108	-	hypothetical phage protein similar to YjhS encoded within prophage CP-933O

Locus_tag[a]				
CB9615	Sakai	EDL933	Gene	Product
Indel-94	Indel-94	Z2109	-	hypothetical phage protein similar to YjhS encoded within prophage CP-933O
Indel-94	Indel-94	Z2110	-	putative transposase encoded within prophage CP-
Indel-94	Indel-94	Z2111	-	putative transposase encoded within prophage CP-
Indel-94	Indel-94	Z2112	-	putative ClpP-like protease encoded within prophage CP-933O
Indel-94	Indel-94	Z2113	-	unknown protein encoded within prophage CP-933O
Indel-94	Indel-94	Z2114	-	unknown protein encoded within prophage CP-933O
Indel-94	Indel-94	Z2115	-	unknown protein encoded within prophage CP-933O
Indel-94	Indel-94	Z2116	-	unknown protein encoded within prophage CP-933O similar to terminase large subunit of phage lambda
Indel-94	Indel-94	Z2117	-	unknown protein encoded within prophage CP-933O
Indel-94	Indel-94	Z2118	-	putative endopeptidase Rz of prophage CP-933O
Indel-94	Indel-94	Z2119	-	unknown protein encoded within prophage CP-933O
Indel-94	Indel-94	Z2120	-	putative endolysin of prophage CP-933O
Indel-94	Indel-94	Z2121	-	unknown protein encoded within prophage CP-933O
Indel-94	Indel-94	Z2122	-	putative holin protein of prophage CP-933O
Indel-94	Indel-94	Z2123	-	unknown protein encoded within prophage CP-933O
Indel-94	Indel-94	Z2124	-	unknown protein encoded within prophage CP-933O
Indel-94	Indel-94	Z2127	-	putative IS encoded protein encoded within prophage CP-933O
Indel-94	Indel-94	Z2130	-	putative IS encoded protein encoded within prophage CP-933O
Indel-94	Indel-94	Z2131	-	putative terminase large subunit of prophage CP-933O
Indel-94	Indel-94	Z2132	-	putative head completion protein of prophage CP-933O
Indel-94	Indel-94	Z2133	-	putative capsid assembly protein of prophage CP-933O
Indel-94	Indel-94	Z2134	-	putative head-tail preconnector protein of prophage CP-933O
Indel-94	Indel-94	Z2135	-	putative capsid protein small subunit of prophage CP-933O
Indel-94	Indel-94	Z2136	-	partial putative major capsid protein of prophage CP-
Indel-94	Indel-94	Z2137	-	putative tail component of prophage CP-933O
Indel-94	Indel-94	Z2138	-	putative tail component of prophage CP-933O
Indel-94	Indel-94	Z2139	-	putative tail component of prophage CP-933O
Indel-94	Indel-94	Z2140	-	putative tail component of prophage CP-933O
Indel-94	Indel-94	Z2141	-	putative tail component of prophage CP-933O
Indel-94	Indel-94	Z2142	-	putative tail component of prophage CP-933O
Indel-94	Indel-94	Z2143	-	putative tail component of prophage CP-933O
Indel-94	Indel-94	Z2144	-	putative tail component of prophage CP-933O
Indel-94	Indel-94	Z2145	-	putative tail component of prophage CP-933O
Indel-94	Indel-94	Z2146	-	putative outer membrane protein Lom precursor of prophage CP-933O
Indel-92	Indel-92	Z2147	-	putative tail fiber protein of prophage CP-933O
Indel-95		Z2107	-	unknown protein encoded within prophage CP-933O

Locus_tag[a]				
CB9615	Sakai	EDL933	Gene	Product
Indel-95	ECs2190	Z2106	-	hypothetical protein
Indel-95	ECs2190	Z2105	-	hypothetical protein
Indel-95	argO5		argO5	Arg tRNA
Indel-95	tRNA99		tRNA99	-
Indel-95	ileZ5		ileZ5	Ile tRNA
Indel-95	ECs2191	Z2104	-	putative transcriptional regulator
Indel-95	ECs2192	Z2103	-	hypothetical protein
Indel-95	ECs2193	Z2102	-	putative ante-terminator protein
Indel-95	ECs2194	Z2101	-	putative crossover junction endodeoxyribonuclease
Indel-95	ECs2195	Z2100	-	hypothetical protein
Indel-95	ECs2196		-	hypothetical protein
Indel-95	ECs2197	Z2099	-	hypothetical protein
Indel-95	ECs2198		-	MokW
Indel-95	ECs2199	Z2098	-	hypothetical protein
Indel-95	ECs2200		-	hypothetical protein
Indel-95	ECs2201	Z2097	-	hypothetical protein
Indel-95	ECs2202		-	hypothetical protein
Indel-95	ECs2203		-	hypothetical protein
Indel-95	ECs2204	Z2096	-	hypothetical protein
Indel-95	ECs2204	Z2095	-	hypothetical protein
Indel-95	ECs2205	Z2094	-	putative replication protein
Indel-95	ECs2206	Z2093	-	hypothetical protein
Indel-95	ECs2207	Z2092	-	hypothetical protein
Indel-95	ECs2208	Z2091	-	putative regulatory protein
Indel-95	ECs2209	Z2090	-	putative repressor protein
Indel-95	ECs2210	Z2089	-	hypothetical protein
Indel-95	ECs2211	Z2088	-	hypothetical protein
Indel-95	ECs2212		-	hypothetical protein
Indel-95	ECs2213		-	hypothetical protein
Indel-95		Z2087	-	unknown protein encoded by prophage CP-933O
Indel-95	dicF2		dicF2	DicF antisense RNA
Indel-95	ECs2214	Z2086	-	putative cell division inhibitor
Indel-95	ECs2215		-	hypothetical protein
Indel-95	ECs2216	Z2085	-	putative exonuclease
Indel-95	ECs2217	Z2084	-	putative integrase
Indel-95	ECs2218	Z2083	-	hypothetical protein
Indel-96	ECs2219	Indel-96	-	putative transposase OrfB protein of insertion sequence IS629
Indel-96	ECs2220	Indel-96	-	putative transposase OrfA protein of insertion sequence IS629
G2583_1912	ECs2221	Z2082	tnpA	Transposase and inactivated derivatives
Indel-98	ECs2222		-	hypothetical protein
Indel-98	ECs2223	Z2081	-	hypothetical protein
Indel-98	ECs2224	Z2080	-	hypothetical protein

Locus_tag[a]				
CB9615	Sakai	EDL933	Gene	Product
Indel-98	ECs2224	Z2079	-	hypothetical protein
G2583_1912	ECs2225	Z2078	tnpA	Transposase and inactivated derivatives
G2583_1913	ECs2226	Z2077	-	unknown protein encoded by prophage CP-933O
G2583_1914	ECs2227		-	hypothetical protein
G2583_1915	ECs2228	Z2076	-	conserved hypothetical protein
G2583_1916	ECs2229	Z2075	-	hypothetical protein
	ECs2230		-	hypothetical protein
Indel-99	ECs2231	Indel-99	-	putative tail fiber protein
Indel-99	ECs2232	Indel-99	-	putative outer membrane protein
Indel-99	ECs2233	Indel-99	-	putative host specificity protein
Indel-99	ECs2234	Indel-99	-	putative host specificity protein
Indel-99	ECs2235	Indel-99	-	putative host specificity protein
Indel-99	ECs2236	Indel-99	-	putative tail assembly protein
Indel-99	ECs2237	Indel-99	-	putative tail assembly protein
Indel-100	Indel-100	Z6030	-	putative tail component of cryptic prophage CP-933P
Indel-100	Indel-100	Z6031	-	putative tail assembly protein of cryptic prophage CP-933P
Indel-100	Indel-100	Z6032	-	putative tail assembly protein of cryptic prophage CP-933P
G2583_1917	Indel-101	Indel-101	-	putative tail fiber protein encoded by prophage CP-
G2583_1918	Indel-101	Indel-101	-	Enterobacterial Ail/Lom family protein
G2583_1919	Indel-101	Indel-101	-	Host specificity protein J
G2583_1920	Indel-101	Indel-101	-	Host specificity protein
G2583_1921	Indel-101	Indel-101	-	Putative tail fiber component K of prophage
G2583_1922	Indel-101	Indel-101	-	minor tail protein
G2583_1923	Indel-101	Indel-101	-	Minor tail family protein
G2583_1924	Indel-101	Indel-101	-	putative tail component of prophage CP-933O
G2583_1925	Indel-101	Indel-101	-	hypothetical protein
G2583_1926	Indel-101	Indel-101	-	phage minor tail protein G
G2583_1927	Indel-101	Indel-101	-	COG5492: Bacterial surface proteins containing Ig-like domains
G2583_1928	Indel-101	Indel-101	-	hypothetical protein
G2583_1929	Indel-101	Indel-101	-	hypothetical protein
G2583_1930	Indel-101	Indel-101	-	hypothetical protein
G2583_1931	Indel-101	Indel-101	-	conserved hypothetical protein
G2583_1932	Indel-101	Indel-101	-	phage major capsid protein E
G2583_1933	Indel-101	Indel-101	-	Bacteriophage lambda head decoration protein D
G2583_1934	Indel-101	Indel-101	-	COG0616: Periplasmic serine proteases (ClpP class)
G2583_1935	Indel-101	Indel-101	-	COG5511: Bacteriophage capsid protein
G2583_1936	Indel-101	Indel-101	-	putative head completion protein
G2583_1937	Indel-101	Indel-101	-	Putative terminase large subunit of prophage CP-933O
G2583_1938	Indel-101	Indel-101	-	Prophage Qin DNA packaging protein NU1 homolog
G2583_1939	Indel-101	Indel-101	nohA	conserved hypothetical protein
Indel-102	ECs2238	Z6033	-	minor tail protein

Locus_tag[a]				
CB9615	Sakai	EDL933	Gene	Product
Indel-102	ECs2239		-	putative minor tail protein
Indel-102	ECs2240	Z6034	-	putative tail length tape measure protein
Indel-102	ECs2241	Z6035	-	putative tail protein
Indel-102	ECs2242	Z6036	-	putative tail assembly chaperone
Indel-102	ECs2243	Z6037	-	putative major tail subunit
Indel-102	ECs2244	Z6038	-	hypothetical protein
Indel-102	ECs2245	Z6039	-	hypothetical protein
Indel-102	ECs2246	Z6040	-	putative head-tail adaptor
Indel-102	ECs2247	Z6041	-	hypothetical protein
Indel-102	ECs2248	Z6042	-	putative portal protein
Indel-102		Z6043	-	unknown protein encoded by cryptic prophage CP-933P
Indel-102	ECs2249		-	hypothetical protein
Indel-102	ECs2250	Z6044	-	putative major head protein/prohead protease
Indel-102	ECs2251	Z6045	-	putative terminase large subunit
Indel-102	ECs2252	Z6046	-	putative terminase small subunit
Indel-102	ECs2253		-	hypothetical protein
Indel-102	ECs2254	Z6047	-	putative Dnase
Indel-102	ECs2255	Z6048	-	hypothetical protein
G2583_1940			-	conserved hypothetical protein
G2583_1941	ECs2257	Z6049	-	Putative endopeptidase Rz
	ECs2256		-	lipoprotein Rz1 precursor
G2583_1942	ECs2258	Z6050	antU	Antirepressor protein
G2583_1943	ECs2259	Z6051	-	putative endolysin
G2583_1944	ECs2260	Z6052	-	unknown protein encoded by cryptic prophage CP-933P
G2583_1945	ECs2261	Z6053	-	Putative lysis protein S of prophage CP-933V
Indel-103	ECs2262	Z6054	-	hypothetical protein
Indel-103	ECs2263		-	hypothetical protein
Indel-103		Z6055	-	unknown protein encoded by cryptic prophage CP-933P
Indel-103	ECs2264		-	hypothetical protein
Indel-103	ECs2265	Z6056	-	hypothetical protein
Indel-103	ECs2266		-	hypothetical protein
Indel-103	argO6	RNA040	argO6	Arg tRNA
Indel-103	argN4	RNA041	argN4	Arg tRNA
Indel-103	ileZ6	RNA042	ileZ6	Ile tRNA
Indel-103	ECs2267	Z6060	-	putative antitermination protein
Indel-103	ECs2268	Z6061	-	putative crossover junction endodeoxyribonuclease
Indel-103	ECs2269	Z6062	-	hypothetical protein
Indel-103		Z6063	mokP	putative cell killing protein encoded within cryptic prophage CP-933P
Indel-103	ECs2270		-	hypothetical protein
Indel-103	ECs2271	Z6064	-	hypothetical protein
Indel-103	ECs2272	Z6065	-	hypothetical protein
Indel-103	ECs2273	Z6066	renP	hypothetical protein
Indel-103	ECs2274	Z6067	-	hypothetical protein

Locus_tag[a]				
CB9615	Sakai	EDL933	Gene	Product
Indel-103		Z6068	-	unknown protein encoded by cryptic prophage CP-933P
Indel-103	ECs2275	Z6069	-	putative DNA replication protein
Indel-103	ECs2276	Z6070	-	putative replication protein
Indel-103	ECs2277	Z6071	-	hypothetical protein
Indel-103	ECs2278	Z6072	-	hypothetical protein
Indel-103	ECs2279	Z6073	-	hypothetical protein
Indel-103	ECs2280	Z6074	-	hypothetical protein
Indel-103	ECs2281		-	hypothetical protein
Indel-103	ECs2282		-	hypothetical protein
Indel-103	ECs2283	Z6075	-	hypothetical protein
Indel-103		Z6076	-	unknown protein encoded by cryptic prophage CP-933P
Indel-103	dicF3	RNA043	dicF3	DicF antisense RNA
Indel-103	ECs2284	Z6078	-	inhibitor of cell division
Indel-103	ECs2285	Z6079	-	hypothetical protein
Indel-103	ECs2286	Z6080	exoP	hypothetical protein
Indel-103	ECs2287	Z6081	xisP	excisionase
Indel-103		Z2566	intP_1	integrase fragment, cryptic prophage CP-933P
		Z2568	intP_2	integrase fragment, cryptic prophage CP-933P
G2583_1946	Indel-104	Indel-104	-	hypothetical protein
G2583_1947	Indel-104	Indel-104	-	hypothetical protein
G2583_1948	Indel-104	Indel-104	-	hypothetical protein
G2583_1949	Indel-104	Indel-104	-	hypothetical protein
G2583_1950	Indel-104	Indel-104	-	hypothetical protein
G2583_1951	Indel-104	Indel-104	-	DnaJ-class molecular chaperone with C-terminal Zn finger domain
G2583_1952	Indel-104	Indel-104	rusA	Putative endonuclease of cryptic prophage CP-933M
G2583_1953	Indel-104	Indel-104	ydfU	conserved hypothetical protein
G2583_1954	Indel-104	Indel-104	-	hypothetical protein
G2583_1955	Indel-104	Indel-104	hokD	Putative cell killing protein encoded within cryptic prophage CP-933M
G2583_1956	Indel-104	Indel-104	-	Eaa protein
G2583_1957	Indel-104	Indel-104	-	unknown protein encoded within prophage CP-933R
G2583_1958	Indel-104	Indel-104	-	hypothetical protein
G2583_1959	Indel-104	Indel-104	-	unknown protein encoded within prophage CP-933R
G2583_1960	Indel-104	Indel-104	-	Putative bacteriophage protein
G2583_1961	Indel-104	Indel-104	-	Unknown protein encoded within prophage
G2583_1962	Indel-104	Indel-104	-	conserved hypothetical protein
G2583_1963	Indel-104	Indel-104	-	hypothetical protein
G2583_1964	Indel-104	Indel-104	cro	hypothetical protein
G2583_1965	Indel-104	Indel-104	cII	SOS-response transcriptional repressors (RecA-mediated autopeptidases)
G2583_1966	Indel-104	Indel-104	ydaF	unknown protein encoded by prophage CP-933N
G2583_1967	Indel-104	Indel-104	-	unknown protein encoded by prophage CP-933N

Locus_tag[a]				
CB9615	Sakai	EDL933	Gene	Product
G2583_1968	Indel-104	Indel-104	dicB	Putative regulator of cell division encoded by prophage CP-933O
G2583_1969	Indel-104	Indel-104	-	conserved hypothetical protein
G2583_1970	Indel-104	Indel-104	ydfE	Exonuclease family protein
G2583_1971	Indel-104	Indel-104	xisP	Putative phage excisionase protein
G2583_1972	Indel-104	Indel-104	intQ	Integrase family protein
G2583_1973	Indel-104	Indel-104	YdfJ	Inner membrane metabolite transport protein ydfJ
G2583_1974	Indel-104	Indel-104	rspB	Starvation sensing protein RspB
G2583_1975	Indel-104	Indel-104	rspA	Starvation-sensing protein rspA
G2583_1976	ECs2288	Z2569	ynfA	UPF0060 membrane protein ynfA
G2583_1977	ECs2289	Z2570	ynfB	UPF0482 protein ynfB precursor
G2583_1978	ECs2290	Z2571	speG	Spermidine N(1)-acetyltransferase
G2583_1979	ECs2291	Z2572	ynfC	hypothetical protein
G2583_1980	ECs2292	Z2573	ynfD	hypothetical protein
G2583_1981	ECs2293	Z2575	ynfE	Anaerobic dimethyl sulfoxide reductase, A subunit YnfE
G2583_1982	ECs2294	Z2576	ynfF	Anaerobic dimethyl sulfoxide reductase, A subunit, DmsA/YnfE family
G2583_1983	ECs2295	Z2577	ynfG	Probable anaerobic dimethyl sulfoxide reductase chain ynfG
G2583_1984	ECs2296	Z2579	ynfH	Oxidoreductase, membrane subunit
G2583_1985	ECs2297	Z2581	dmsD	Twin-arginine leader-binding protein dmsD
G2583_1986	ECs2298	Z2583	clcB	Putative chloride channel
G2583_1987	ECs2299	Z2585	ynfK	Putative dethiobiotin synthetase
G2583_1988	ECs2300	Z2587	dgsA	putative NAGC-like transcriptional regulator
G2583_1989	ECs2301	Z2589	ynfL	putative transcriptional regulator LYSR-type
G2583_1990	ECs2302	Z2590	ynfM	Major facilitator family transporter
G2583_1991	ECs2303	Z2591	asr	acid shock protein precursor
G2583_1992	ECs2304	Z2592	ydgD	V8-like Glu-specific endopeptidase
G2583_1993	ECs2305	Z2593	mdtI	multidrug resistance protein MdtI
G2583_1994	ECs2306	Z2594	mdtJ	Spermidine export protein mdtJ
G2583_1995	ECs2307	Z2595	tqsA	Putative permease, PerM family
G2583_1996	ECs2308	Z2597	pntB	NAD(P) transhydrogenase subunit beta
G2583_1997	ECs2309	Z2600	pntA	NAD(P) transhydrogenase, alpha subunit
G2583_1998	ECs2310	Z2603	ydgH	predicted protein
G2583_1999	ECs2311	Z2605	ydgI	Arginine/ornithine antiporter
G2583_2000	ECs2312	Z2606	folM	Dihydrofolate reductase folM
G2583_2001	ECs2313	Z2608	ydgC	Inner membrane protein ydgC
G2583_2002	ECs2314	Z2609	rstA	DNA-binding response regulator in two-component regulatory system with RstB
G2583_2003	ECs2315	Z2610	rstB	Sensor histidine kinase RstB
G2583_2004	ECs2316	Z2611	tus	DNA replication terminus site-binding protein
G2583_2005	ECs2317	Z2614	fumC	Fumarate hydratase class II
G2583_2006	ECs2318	Z2615	fumA	Fumarate hydratase class I, aerobic
G2583_2007	ECs2319	Z2616	manA	Mannose-6-phosphate isomerase, class I

Locus_tag[a]				
CB9615	Sakai	EDL933	Gene	Product
G2583_2008	ECs2320	Z2617	ydgA	hypothetical protein
G2583_2009	ECs2321	Z2618	uidC	membrane-associated protein
G2583_2009	ECs2322	Z2619	uidC	membrane-associated protein
G2583_2010	ECs2323	Z2620	uidB	Glucuronide permease uidB
G2583_2011	ECs2324	Z2621	uidA	Beta-glucuronidase
G2583_2011	ECs2325	Z2622	uidA	Beta-glucuronidase
G2583_2012	ECs2326	Z2623	uidR	repressor for uid operon
G2583_2013	ECs2327	Z2624	hdhA	7-alpha-hydroxysteroid dehydrogenase
G2583_2014	ECs2328	Z2625	malI	Maltose regulon regulatory protein MalI
G2583_2015	ECs2329	Z2626	malX	PTS system, maltose and glucose-specific IIABC component
G2583_2016	ECs2330	Z2627	malY	Maltose regulon modulator MalY
G2583_2017	ECs2331	Z2628	add	Adenosine deaminase
G2583_2018	ECs2332	Z2629	ydgJ	Oxidoreductase, NAD-binding
G2583_2019	ECs2333		blr	Beta-lactam resistance protein
G2583_2020	ECs2334	Z2631	cnu	OriC-binding nucleoid-associated protein
G2583_2021	ECs2335	Z2632	ydgK	hypothetical protein
G2583_2022	ECs2336	Z2633	rsxA	Electron transport complex protein rnfA
G2583_2023	ECs2337	Z2634	rsxB	Electron transport complex protein rnfB
G2583_2024	ECs2338	Z2636	rsxC	Electron transport complex protein RnfC
G2583_2025	ECs2339	Z2639	rsxD	Electron transport complex protein rnfD
G2583_2026	ECs2340	Z2640	rsxG	Electron transport complex protein rnfG
G2583_2027	ECs2341	Z2642	rsxE	Predicted NADH:ubiquinone oxidoreductase, subu nit RnfE
G2583_2028	ECs2342	Z2644	nth	Endonuclease III
G2583_2029	ECs2343	Z2646	tppB	Tripeptide permease tppB
G2583_2030	ECs2344	Z2647	gst	Glutathione S-transferase
G2583_2031	ECs2345	Z2648	pdxY	Pyridoxamine kinase
G2583_2032	ECs2346	Z2650	tyrS	Tyrosyl-tRNA synthetase
G2583_2033	ECs2347	Z2652	pdxH	Pyridoxine/pyridoxamine 5'-phosphate oxidase
G2583_2034	ECs2348	Z2653	ydhA	predicted lipoprotein
G2583_2035	ECs2349	Z2654	anmK	Anhydro-N-acetylmuramic acid kinase
G2583_2036	ECs2350	Z2655	slyB	Outer membrane lipoprotein slyB precursor
G2583_2037	ECs2351	Z2657	slyA	Transcriptional regulators
G2583_2038	ECs2352	Z2658	ydhI	hypothetical protein
G2583_2039	ECs2353	Z2659	ydhJ	hypothetical protein
G2583_2040	ECs2354	Z2660	ydhK	Uncharacterized transporter ydhK
G2583_2041	ECs2355	Z2661	sodC	Superoxide dismutase [Cu-Zn]
G2583_2042	ECs2356	Z2664	ydhF	Oxidoreductase ydhF
G2583_2043	ECs2358	Z2665	ydhL	hypothetical protein
G2583_2044	ECs2357	Z2666	ydhM	hypothetical protein
G2583_2045	ECs2359	Z2668	nemA	N-ethylmaleimide reductase
G2583_2046	ECs2360	Z2669	gloA	Lactoylglutathione lyase
G2583_2047	ECs2361	Z2671	rnt	Ribonuclease T

Locus_tag[a]				
CB9615	Sakai	EDL933	Gene	Product
G2583_2048	ECs2362	Z2673	lhr	DEAD/DEAH box helicase family protein Lhr
G2583_2049	ECs2363	Z2676	grxD	Glutaredoxin-4
G2583_2050	ECs2364	Z2677	ydhO	NlpC/P60 family protein
G2583_2051	ECs2365	Z2678	sodB	Superoxide dismutase [Fe]
G2583_2052	ECs2366	Z2679	ydhP	predicted transporter
G2583_2053	ECs2367	Z2681	purR	DNA-binding transcriptional repressor PurR
G2583_2054	ECs2368	Z2682	ydhB	putative DNA-binding transcriptional regulator
G2583_2055	ECs2369	Z2685	ydhC	Drug resistance transporter, Bcr/CflA subfamily
G2583_2056	ECs2370	Z2686	cfa	Cyclopropane-fatty-acyl-phospholipid synthase
G2583_2057	ECs2371	Z2688	ribC	Riboflavin synthase alpha chain
G2583_2058	ECs2372	Z2690	mdtK	Multidrug resistance protein mdtK
G2583_2059	ECs2373	Z2691	ydhQ	hypothetical protein
G2583_2060	valV	RNA044	valV	Val tRNA
G2583_2061	valW	RNA045	valW	Val tRNA
G2583_2062	ECs2374	Z2694	ydhR	Protein ydhR
G2583_2063	ECs2375	Z2695	ydhS	hypothetical protein
G2583_2064	ECs2376	Z2696	ydhT	hypothetical protein
G2583_2065	ECs2377	Z2697	ydhU	Nickel-dependent hydrogenase, b-type cytochrome subunit
G2583_2066	ECs2378	Z2698	ydhX	Uncharacterized ferredoxin-like protein ydhX
G2583_2067	ECs2379	Z2700	ydhW	hypothetical protein
G2583_2068	ECs2380	Z2701	ydhV	hypothetical protein
G2583_2069	ECs2381	Z2702	ydhY	Uncharacterized ferredoxin-like protein ydhY
G2583_2070	ECs2382	Z2703	ydhZ	hypothetical protein
G2583_2071	ECs2383	Z2704	pykF	Pyruvate kinase I
G2583_2072	ECs2384	Z2705	lpp	Major outer membrane lipoprotein precursor
G2583_2073	ECs2385	Z2706	ynhG	LysM domain/ErfK/YbiS/YcfS/YnhG family protein
G2583_2074	ECs2386	Z2707	sufE	Cysteine desulfuration protein sufE
G2583_2075	ECs2387	Z2708	sufS	Cysteine desulfurase
G2583_2076	ECs2388	Z2709	sufD	FeS assembly protein SufD
G2583_2077	ECs2389	Z2710	sufC	FeS assembly ATPase SufC
G2583_2078	ECs2390	Z2711	sufB	cysteine desulfurase activator complex subunit SufB
G2583_2079	ECs2391	Z2712	sufA	FeS assembly scaffold SufA
G2583_2080			-	ncRNA
G2583_2081	ECs2392	Z2713	ydiH	hypothetical protein
G2583_2082	ECs2393	Z2714	ydiI	Esterase YdiI
G2583_2083	ECs2394	Z2715	ydiJ	Oxidoreductase, FAD-binding
G2583_2084	ECs2395	Z2716	ydiK	Inner membrane protein YdiK
G2583_2085			rprA	ncRNA
G2583_2086	ECs2396	Z2717	ydiL	hypothetical protein
G2583_2087	ECs2397	Z2718	ydiM	Major facilitator family transporter
G2583_2088	ECs2398	Z2719	ydiN	Transporter, major facilitator family
G2583_2089	ECs2399	Z2720	ydiB	Quinate/shikimate dehydrogenase
G2583_2090	ECs2400	Z2721	aroD	3-dehydroquinate dehydratase

Locus_tag[a]				
CB9615	Sakai	EDL933	Gene	Product
G2583_2091	ECs2401	Z2722	ydiF	Propionate CoA-transferase
G2583_2092	ECs2402	Z2723	ydiO	Acyl-coA dehydrogenase
G2583_2093	ECs2403	Z2724	ydiP	putative ARAC-type regulatory protein
G2583_2094	ECs2404	Z2726	ydiQ	camphor resistance protein CrcB
G2583_2095	ECs2405	Z2727	ydiR	iron donor protein CyaY
G2583_2096	ECs2406	Z2728	ydiS	putative sulfate transport protein CysZ
G2583_2097	ECs2407	Z2729	ydiT	Iron-sulfur cluster-binding protein
G2583_2098	ECs2408	Z2730	fadK	Putative ligase/synthetase
G2583_2099	ECs2409	Z2731	pps	Phosphoenolpyruvate synthase
G2583_2100	ECs2410	Z2732	ydiA	Putative phosphotransferase ydiA
G2583_2101	ECs2411	Z2733	aroH	Phospho-2-dehydro-3-deoxyheptonate aldolase, Trp-sensitive
G2583_2102	ECs2412	Z2734	ydiE	hypothetical protein
G2583_2103	ECs2413	Z2735	ydiU	UPF0061 protein ydiU
G2583_2104	ECs2414	Z2736	ydiV	hypothetical protein
G2583_2105	ECs2415	Z2737	nlpC	Lipoprotein, NlpC/P60 family
G2583_2106	ECs2416	Z2738	btuD	Vitamin B12 import ATP-binding protein btuD
G2583_2107	ECs2417	Z2739	btuE	Vitamin B12 transport periplasmic protein btuE
G2583_2108	ECs2418	Z2740	btuC	Vitamin B12 import system permease protein btuC
G2583_2109	Indel-106	Indel-106	-	hypothetical protein
G2583_2110	Indel-106	Indel-106	intT	integrase
G2583_2111	Indel-106	Indel-106	cI	gpC
G2583_2112	Indel-106	Indel-106	-	hypothetical phage DNA-binding protein
G2583_2113	Indel-106	Indel-106	-	unknown protein encoded by prophage CP-933T
G2583_2114	Indel-106	Indel-106	-	hypothetical protein
G2583_2115	Indel-106	Indel-106	-	hypothetical protein
G2583_2116	Indel-106	Indel-106	-	hypothetical protein
G2583_2117	Indel-106	Indel-106	-	hypothetical protein
G2583_2118	Indel-106	Indel-106	-	hypothetical protein
G2583_2119	Indel-106	Indel-106	-	conserved hypothetical protein
G2583_2120	Indel-106	Indel-106	-	hypothetical protein
G2583_2121	Indel-106	Indel-106	-	hypothetical protein
G2583_2122	Indel-106	Indel-106	-	putative phage replication protein
G2583_2123	Indel-106	Indel-106	-	Molecular chaperone
G2583_2124	Indel-106	Indel-106	-	hypothetical protein
G2583_2125	Indel-106	Indel-106	clpX	ATP-dependent protease Clp, ATPase subunit
G2583_2126	Indel-106	Indel-106	-	hypothetical protein
G2583_2127	Indel-106	Indel-106	gpq	Putative phage protein
G2583_2128	Indel-106	Indel-106	gpP	Putative phage protein gpP
G2583_2129	Indel-106	Indel-106	-	putative capsid scaffolding protein
G2583_2130	Indel-106	Indel-106	gpn	Predicted major capsid protein
G2583_2131	Indel-106	Indel-106	gpm	Putative phage terminase
G2583_2132	Indel-106	Indel-106	gpl	phage head completion protein
G2583_2133	Indel-106	Indel-106	-	Probable phage tail protein

Locus_tag[a]				
CB9615	Sakai	EDL933	Gene	Product
G2583_2134	Indel-106	Indel-106	-	Phosphotransferase system IIC components, glucose/maltose/N-acetylglucosamine-specific
G2583_2135	Indel-106	Indel-106	-	Phage capsid scaffolding protein
G2583_2136	Indel-106	Indel-106	-	hypothetical protein
G2583_2137	Indel-106	Indel-106	-	Predicted tail completion phage protein
G2583_2138	Indel-106	Indel-106	gps	Predicted tail completion phage protein
G2583_2139	Indel-106	Indel-106	-	Phage P2 baseplate assembly protein gpV
G2583_2140	Indel-106	Indel-106	-	Phage baseplate assembly protein
G2583_2141	Indel-106	Indel-106	-	probable phage baseplate assembly protein
G2583_2142	Indel-106	Indel-106	gpI	phage tail protein I
G2583_2143	Indel-106	Indel-106	-	DNA inversion product
G2583_2144	Indel-106	Indel-106	-	Alternative bacteriophage tail fiber C-terminus
G2583_2145	Indel-106	Indel-106	tfaE	Tail fiber assembly protein homolog
G2583_2146	Indel-106	Indel-106	-	Sc/SvM1 protein
G2583_2147	Indel-106	Indel-106	ycfK	putative tail fiber protein
G2583_2148	Indel-106	Indel-106	pin	DNA-invertase
G2583_2149	Indel-106	Indel-106	-	Phage protein U
G2583_2150	Indel-106	Indel-106	-	Phage-related tail protein
G2583_2151	Indel-106	Indel-106	-	Putative phage tail protein
G2583_2152	Indel-106	Indel-106	-	ABC-type antimicrobial peptide transport syste m, ATPase component
G2583_2153	Indel-106	Indel-106	-	Putative tail fiber component of prophage CP-933T
G2583_2154	Indel-106	Indel-106	-	Phage tail sheath protein FI
G2583_2155	Indel-106	Indel-106	gpd	Phage protein D
G2583_2156	Indel-106	Indel-106	-	hypothetical protein
G2583_2157	Indel-106	Indel-106	-	putative phage transcriptional activator, Ogr/Delta
G2583_2158	Indel-106	Indel-106	-	conserved hypothetical protein
G2583_2159	ECs2419	Z2741	ihfA	Integration host factor subunit alpha
G2583_2160	ECs2420	Z2742	pheT	Phenylalanyl-tRNA synthetase beta chain
G2583_2161	ECs2421	Z2743	pheS	Phenylalanyl-tRNA synthetase alpha chain
G2583_2162	ECs2422	Z2744	pheM	phenylalanyl-tRNA synthetase (pheST) operon leader peptide
G2583_2163	ECs2423	Z2745	rplT	50S ribosomal protein L20
G2583_2164	ECs2424	Z2746	rpmI	50S ribosomal protein L35
G2583_2165	ECs2425	Z2747	infC	Translation initiation factor IF-3
G2583_2166	ECs2426	Z2748	thrS	Threonyl-tRNA synthetase
G2583_2167	ECs2427	Z2749	arpA	Ankyrin repeat protein B
G2583_2168	ECs2428	Z2751	ydiY	hypothetical protein
G2583_2169	ECs2429	Z2752	pfkB	6-phosphofructokinase II
G2583_2170	ECs2430	Z2753	ydiZ	hypothetical protein
G2583_2171	ECs2431	Z2754	yniA	hypothetical protein
G2583_2172	ECs2432	Z2755	yniB	hypothetical protein
G2583_2173	ECs2433	Z2756	yniC	Phosphatase yniC
G2583_2174	ECs2434	Z2757	ydjM	Inner membrane protein ydjM precursor

Locus_tag[a]				
CB9615	Sakai	EDL933	Gene	Product
G2583_2175	ECs2435	Z2758	ydjN	Sodium:dicarboxylate symporter precursor
G2583_2176	ECs2436	Z2759	ydjO	hypothetical protein
G2583_2177	ECs2437	Z2760	cedA	Cell division activator cedA
G2583_2178	ECs2438	Z2761	katE	Catalase HPII
G2583_2179	ECs2439	Z2763	chbG	UPF0249 protein chbG
G2583_2180	ECs2440	Z2764	chbF	Phospho-beta-glucosidase
G2583_2181	ECs2441	Z2765	chbR	ChbR
G2583_2182	ECs2442	Z2766	chbA	N,N'-diacetylchitobiose-specific phosphotransferase enzyme IIA component
G2583_2183	ECs2443	Z2767	chbC	N,N'-diacetylchitobiose permease IIC component
G2583_2184	ECs2444	Z2768	chbB	PTS system, lactose/cellobiose family IIB subunit precursor
G2583_2185	ECs2445	Z2769	osmE	Osmotically-inducible lipoprotein E precursor
G2583_2186	ECs2446	Z2770	nadE	NAD synthase
G2583_2187	ECs2447	Z2771	cho	Excinuclease cho
G2583_2188	ECs2448	Z2774	ves	Uncharacterized protein conserved in bacteria
G2583_2189	ECs2449	Z2775	spy	Spy
G2583_2190	ECs2450	Z2776	astE	Succinylglutamate desuccinylase
G2583_2191	ECs2451	Z2777	astB	N-succinylarginine dihydrolase
G2583_2192	ECs2452	Z2778	astD	N-succinylglutamate 5-semialdehyde dehydrogenase
G2583_2193	ECs2453	Z2779	astA	Arginine N-succinyltransferase
G2583_2194	ECs2454	Z2780	astC	Succinylornithine transaminase
G2583_2195	ECs2455	Z2781	xthA	Exodeoxyribonuclease III
G2583_2196	ECs2456	Z2782	ydjX	hypothetical protein
G2583_2197	ECs2457	Z2783	ydjY	hypothetical protein
G2583_2198	ECs2458	Z2784	ydjZ	hypothetical protein
G2583_2199	ECs2459	Z2785	ynjA	Carboxymuconolactone decarboxylase family protein
G2583_2200	ECs2460	Z2786	ynjB	hypothetical protein
G2583_2201	ECs2461	Z2787	ynjC	ABC transporter, permease protein
G2583_2202	ECs2462	Z2788	ynjD	Putative ATP-binding component of a transport system
G2583_2203	ECs2463	Z2789	ynjE	Putative thiosulfate sulfurtransferase YnjE
G2583_2204	ECs2464	Z2790	ynjF	Putative cytochrome oxidase
G2583_2205	ECs2465	Z2791	nudG	CTP pyrophosphohydrolase
G2583_2206	ECs2466	Z2792	ynjH	hypothetical protein
G2583_2207	ECs2467	Z2793	gdhA	NADP-specific glutamate dehydrogenase
G2583_2208	ECs2468	Z2795	ynjI	hypothetical protein
G2583_2209	ECs2469	Z2796	topB	DNA topoisomerase III
G2583_2210	ECs2470	Z2797	selD	Selenide, water dikinase
G2583_2211	ECs2471	Z2798	ydjA	Protein ydjA
G2583_2212	ECs2472	Z2799	sppA	Protease 4
G2583_2213	ECs2473	Z2800	-	hypothetical protein
G2583_2214	ECs2474	Z2801	ansA	L-asparaginase 1
G2583_2215	ECs2475	Z2802	pncA	hypothetical protein
G2583_2216	ECs2476	Z2803	ydjE	Inner membrane metabolite transport protein ydjE

Locus_tag[a]				
CB9615	Sakai	EDL933	Gene	Product
Indel-107	ECs2477	Z2804	-	putative transposase OrfA protein of insertion sequence IS629
Indel-107	ECs2478	Z2806	-	putative transposase OrfB protein of insertion sequence IS629
G2583_2217	ECs2479	Z2808	ydjF	transcriptional regulator, DeoR family
G2583_2218	ECs2480	Z2809	ydjG	Oxidoreductase, aldo/keto reductase family
G2583_2219	ECs2481	Z2810	ydjH	Kinase, pfkB family
G2583_2220	ECs2482	Z2811	ydjI	Fructose-bisphosphate aldolase, class II family
G2583_2221	ECs2483	Z2812	ydjJ	Sorbitol dehydrogenase
G2583_2222	ECs2484	Z2813	ydjK	major facilitator family transporter
G2583_2223	ECs2485	Z2815	ydjL	Oxidoreductase, zinc-binding dehydrogenase family
G2583_2224	ECs2486	Z2816	yeaC	hypothetical protein
G2583_2225	ECs2487	Z2817	msrB	Methionine sulfoxide reductase B
G2583_2226	ECs2488	Z2818	gapA	Glyceraldehyde-3-phosphate dehydrogenase A
G2583_2227	ECs2489	Z2820	yeaD	Aldose 1-epimerase family protein
G2583_2228	ECs2490	Z2821	yeaE	Oxidoreductase, aldo/keto reductase family
G2583_2229	ECs2491	Z2822	mipA	MltA-interacting protein precursor
G2583_2230	ECs2492	Z2823	yeaG	hypothetical protein
G2583_2231	ECs2493	Z2824	yeaH	UPF0229 protein yeaH
G2583_2232	ECs2494	Z2825	yeaI	Diguanylate cyclase (GGDEF) domain protein
G2583_2233	ECs2495	Z2826	yeaJ	hypothetical protein
G2583_2234	ECs2496	Z2827	yeaK	hypothetical protein
G2583_2235	ECs2497	Z2828	-	hypothetical protein
G2583_2236	ECs2498	Z2829	yeaL	Predicted membrane protein
G2583_2237	ECs2499	Z2831	yeaM	Putative AraC-type regulatory protein
G2583_2238	ECs2500	Z2833	yeaN	inner membrane transport protein yeaN
G2583_2239	ECs2501	Z2834	yeaO	hypothetical protein
G2583_2240	ECs2502	Z2835	yoaF	hypothetical protein
G2583_2241	ECs2503	Z2836	yeaP	GAF domain/diguanylate cyclase (GGDEF) domain protein
G2583_2242	ECs2504	Z2837	yeaQ	hypothetical protein
G2583_2243	ECs2505	Z2838	yoaG	Protein yoaG
G2583_2244	ECs2506	Z2839	yeaR	hypothetical protein
G2583_2245	ECs2507	Z2841	leuE	Leucine efflux protein
G2583_2246	ECs2508	Z2842	yeaT	putative transcriptional regulator LYSR-type
G2583_2247	ECs2509	Z2843	yeaU	Tartrate dehydrogenase/decarboxylase
G2583_2248	ECs2510	Z2844	yeaV	Transporter, betaine/carnitine/choline transporter (BCCT) family
G2583_2249	ECs2511	Z2845	yeaW	Putative dioxygenase subunit alpha yeaW
G2583_2250	ECs2512	Z2846	yeaX	Putative diogenase beta subunit
G2583_2251	ECs2513	Z2847	rnd	Ribonuclease D
G2583_2252			-	ncRNA
G2583_2253	ECs2514	Z2848	fadD	Long-chain-fatty-acid--CoA ligase
G2583_2254	ECs2515	Z2849	yeaY	Outer membrane protein Slp

Locus_tag[a]				
CB9615	Sakai	EDL933	Gene	Product
G2583_2255	ECs2516	Z2850	yeaZ	Glycoprotease family protein
G2583_2256	ECs2517	Z2851	yoaA	Helicase c2
G2583_2257	ECs2518	Z2852	yoaB	hypothetical protein
G2583_2258	ECs2519	Z2853	yoaC	hypothetical protein
G2583_2259	ECs2520	Z2854	yoaH	UPF0181 protein yoaH
G2583_2260	ECs2521	Z2855	pabB	p-aminobenzoate synthetase, component I
G2583_2261	ECs2522	Z2856	nudL	Uncharacterized Nudix hydrolase nudL
G2583_2262	ECs2523	Z2857	sdaA	L-serine ammonia-lyase 1
G2583_2263	ECs2524	Z2858	yoaD	hypothetical protein
G2583_2264	ECs2525	Z2859	yoaE	UPF0053 inner membrane protein yoaE
G2583_2265	ECs2526		-	putative cytoplasmic protein
G2583_2266	ECs2527	Z2860	manX	Putative PTS system, mannose-specific component
G2583_2267	ECs2528	Z2861	manY	PTS enzyme IIC, mannose-specific
G2583_2268	ECs2529	Z2862	manZ	Mannose permease IID component
G2583_2269	ECs2530	Z2863	yobD	UPF0266 membrane protein yobD
G2583_2270	ECs2531	Z2864	yebN	hypothetical protein
G2583_2271	ECs2532	Z2866	rrmA	23S rRNA methyltransferase A
G2583_2272	ECs2533	Z2868	cspC	hypothetical protein
G2583_2273	ECs2534	Z2869	yobF	hypothetical protein
G2583_2274	ECs2535	Z2871	yebO	hypothetical protein
G2583_2275	ECs2536	Z2872	mgrB	hypothetical protein
G2583_2276		Z2873	yobH	hypothetical protein
G2583_2277	ECs2537	Z2874	kdgR	putative regulator
G2583_2278	ECs2538	Z2875	yebQ	Putative transport protein
G2583_2279	ECs2539	Z2876	htpX	Probable protease htpX
G2583_2280	ECs2540	Z2877	prc	C-terminal processing peptidase
G2583_2281	ECs2541	Z2878	proQ	ProP effector
G2583_2282	ECs2542	Z2879	yebR	GAF domain protein
G2583_2283	ECs2543	Z2880	yebS	hypothetical protein
G2583_2284	ECs2544	Z2881	yebT	Mce-related protein
G2583_2285	ECs2545	Z2882	rsmF	Ribosomal RNA small subunit methyltransferase F
G2583_2286	ECs2546	Z2883	yebV	hypothetical protein
G2583_2287	ECs2547	Z2884	yebW	hypothetical protein
G2583_2288	ECs2548	Z2885	pphA	Protein phosphatase 1 modulates phosphoproteins, signals protein misfolding
G2583_2289			-	ncRNA
G2583_2290			-	ncRNA
G2583_2291	ECs2549	Z2887	yebY	hypothetical protein
G2583_2292	ECs2550	Z2888	yebZ	Copper resistance protein D
G2583_2293	ECs2551	Z2889	yobA	Copper resistance protein CopC
G2583_2294	ECs2552	Z2891	holE	DNA polymerase III subunit theta
G2583_2295	ECs2553	Z2893	yobB	Hydrolase, carbon-nitrogen family
G2583_2296	ECs2554	Z2894	exoX	Exodeoxyribonuclease 10
G2583_2297	ECs2555	Z2896	ptrB	Oligopeptidase B

Locus_tag[a]				
CB9615	Sakai	EDL933	Gene	Product
G2583_2298	ECs2556	Z2897	yebE	hypothetical protein
G2583_2299	ECs2557	Z2899	yebF	hypothetical protein
G2583_2300	ECs2558	Z2900	yebG	hypothetical protein
G2583_2301	ECs2559	Z2901	purT	Phosphoribosylglycinamide formyltransferase 2
G2583_2302	ECs2560	Z2902	eda	KDPG and KHG aldolase
G2583_2303	ECs2561	Z2903	edd	Phosphogluconate dehydratase
G2583_2304	ECs2562	Z2904	zwf	Glucose-6-phosphate 1-dehydrogenase
G2583_2305	ECs2563	Z2905	yebK	DNA-binding transcriptional regulator HexR
G2583_2306	ECs2564	Z2906	pykA	Pyruvate kinase II
G2583_2307	ECs2565	Z2907	lpxM	Lipid A biosynthesis (KDO)2-(Lauroyl)-lipid IVA acyltransferase
G2583_2308	ECs2566	Z2908	yebA	Uncharacterized metalloprotease yebA
G2583_2309	ECs2567	Z2909	znuA	Putative adhesin
G2583_2310	ECs2568	Z2910	znuC	Zinc import ATP-binding protein znuC
G2583_2311	ECs2569	Z2911	znuB	Zinc uptake ABC transporter, permease protein ZnuB
G2583_2312	ECs2570	Z2912	ruvB	Holliday junction ATP-dependent DNA helicase ruvB
G2583_2313	ECs2571	Z2913	ruvA	Holliday junction ATP-dependent DNA helicase ruvA
G2583_2314	ECs2572	Z2914	yebB	hypothetical protein
G2583_2315	ECs2573	Z2915	ruvC	Crossover junction endodeoxyribonuclease ruvC
G2583_2316	ECs2574	Z2916	yebC	UPF0082 protein yebC
G2583_2317	ECs2575	Z2917	nudB	dATP pyrophosphohydrolase
G2583_2318	ECs2576	Z2919	aspS	Aspartyl-tRNA synthetase
G2583_2319	ECs2577	Z2920	yecD	hypothetical protein
G2583_2320	ECs2578	Z2921	yecE	hypothetical protein
G2583_2321	ECs2579	Z2922	yecN	hypothetical protein
G2583_2322	ECs2580	Z2923	cmoA	tRNA (cmo5U34)-methyltransferase
G2583_2323	ECs2581	Z2924	cmoB	tRNA (mo5U34)-methyltransferase
G2583_2324	ECs2582	Z2925	torZ	Trimethylamine N-oxide reductase III, subunit TorZ
G2583_2325	ECs2583	Z2926	torY	Cytochrome c-type protein torY
G2583_2326	ECs2584	Z2927	cutC	Copper homeostasis protein cutC
G2583_2327	ECs2585	Z2928	yecM	hypothetical protein
G2583_2328	ECs2586	Z2929	argS	Arginyl-tRNA synthetase
G2583_2329	ECs2587	Z2930	yecT	hypothetical protein
G2583_2330	ECs2588	Z2931	flhE	hypothetical protein
G2583_2331	ECs2589	Z2932	flhA	Flagellar biosynthesis protein flhA
G2583_2332	ECs2590	Z2934	flhB	Flagellar biosynthetic protein FlhB
G2583_2333	ECs2591	Z2935	cheZ	Chemotaxis phosphatase CheZ
G2583_2334	ECs2592	Z2936	cheY	Chemotaxis protein cheY
G2583_2335	ECs2593	Z2937	cheB	Chemotaxis response regulator protein-glutamate methylesterase
G2583_2336	ECs2594	Z2938	cheR	Chemotaxis protein methyltransferase CheR
G2583_2337	ECs2595	Z2939	tap	Methyl-accepting chemotaxis protein IV
G2583_2338	ECs2596	Z2940	tar	Methyl-accepting chemotaxis protein II
G2583_2339			-	ncRNA

Locus_tag[a]				
CB9615	Sakai	EDL933	Gene	Product
G2583_2340	ECs2597	Z2941	cheW	Chemotaxis protein cheW
G2583_2341	ECs2598	Z2942	cheA	Sensory transducer kinase between chemo-signal receptors and CheB and CheY
G2583_2342	ECs2599	Z2943	motB	Chemotaxis protein motB
G2583_2343	ECs2600	Z2944	motA	Chemotaxis protein MotA
G2583_2344	ECs2601	Z2945	flhC	Flagellar transcriptional activator flhC
G2583_2345	ECs2602	Z2946	flhD	Transcriptional activator
		Z2947	-	hypothetical protein
G2583_2346	ECs2603	Z2948	uspC	Universal stress protein C
G2583_2347	ECs2604	Z2949	otsA	Alpha,alpha-trehalose-phosphate synthase [UDP-
G2583_2348	ECs2605	Z2950	otsB	Trehalose-phosphatase
G2583_2349	ECs2606	Z2951	araH	ABC-type arabinose transport system, permease component
G2583_2349	ECs2607	Z2952	araH	ABC-type arabinose transport system, permease component
G2583_2350	ECs2608	Z2953	araG	Arabinose import ATP-binding protein araG
G2583_2351	ECs2609	Z2954	araF	L-arabinose-binding periplasmic protein
G2583_2352	ECs2610	Z2956	ftnB	Ferritin-like protein 2
G2583_2353			yecJ	hypothetical protein
G2583_2354	ECs2611	Z2958	-	hypothetical protein
G2583_2355	ECs2612	Z2959	yecR	hypothetical protein
G2583_2356	ECs2613	Z2960	ftnA	Ferritin-1
G2583_2357	ECs2614	Z2962	yecH	hypothetical protein
G2583_2358	ECs2615	Z2963	tyrP	Tyrosine-specific transport protein
G2583_2359	ECs2616	Z2964	yecA	SEC-C domain protein
Indel-109	ECs2617	Z2966	intT	putative integrase
Indel-109	ECs2618	Z2967	-	hypothetical protein
Indel-109	ECs2619	Z2968	-	putative membrane protein
Indel-109	ECs2620	Z2969	-	putative transcriptional regulator
Indel-109	ECs2621		-	hypothetical protein
Indel-109	ECs2622	Z2970	coxT	putative DNA-binding protein
Indel-109	ECs2623	Z2971	-	hypothetical protein
Indel-109	ECs2624	Z2972	-	hypothetical protein
Indel-109	ECs2625	Z2973	-	hypothetical protein
Indel-109	ECs2626	Z2974	-	hypothetical protein
Indel-109	ECs2627		-	hypothetical protein
Indel-109	ECs2628		-	hypothetical protein
Indel-109	ECs2629	Z2975	-	hypothetical protein
Indel-109	ECs2630		-	hypothetical protein
Indel-109	ECs2631	Z2976	-	putative derepression protein
Indel-109	ECs2632	Z2977	-	hypothetical protein
Indel-109	ECs2633	Z2978	-	putative phage replication protein
Indel-109	ECs2634	Z2979	-	putative plasmid partition protein
Indel-109	ECs2635	Z2980	-	putative plasmid partition protein

Locus_tag[a]				
CB9615	Sakai	EDL933	Gene	Product
Indel-109	ECs2636	Z2981	-	putative transposase OrfB protein of insertion sequence IS629
Indel-109	ECs2637	Z2982	-	putative transposase OrfA protein of insertion sequence IS629
Indel-109	ECsp017	Z2983	-	putative tail fiber assembly protein of prophage CP-
Indel-109	ECs2638	Z2984	-	hypothetical protein
Indel-109	ECs2639	Z2985	-	putative tail protein
Indel-109	ECs2640	Z2986	-	phage tail protein
Indel-109	ECs2641	Z2987	-	putative tail protein
Indel-109	ECs2642	Z2988	-	putative phage tail protein
Indel-109	ECs2643	Z2989	-	putative tail protein
Indel-109	ECs2644	Z2990	-	putative tail tube protein
Indel-109	ECs2645	Z2991	-	putative tail sheath protein
Indel-109	ECs2646	Z2992	-	putative tail protein
Indel-109	ECs2647	Z2993	tra8_2	transposase of insertion sequence IS30
Indel-109	ECs2648		-	hypothetical protein
Indel-109	ECs2649	Z2994	-	hypothetical protein
G2583_2360	leuZ	RNA046	leuZ	Leu tRNA
G2583_2361	cysT	RNA047	-	Cys tRNA
G2583_2362	glyW	RNA048	glyW	Gly tRNA
G2583_2363	ECs2650	Z3000	pgsA	CDP-diacylglycerol--glycerol-3-phosphate 3-phosphatidyltransferase
G2583_2364	ECs2651	Z3001	uvrC	UvrABC system protein C
G2583_2365	ECs2652	Z3002	uvrY	Response regulator uvrY
G2583_2366	ECs2653	Z3003	yecF	hypothetical protein
G2583_2367	ECs2654	Z3004	sdiA	DNA-binding transcriptional activator
G2583_2368	ECs2655	Z3005	yecC	Amino acid ABC transporter, ATP-binding protein
G2583_2369	ECs2656	Z3006	yecS	putative transport system permease protein
G2583_2370	ECs2657	Z3008	dcyD	Putative 1-aminocyclopropane-1-carboxylate
G2583_2370	ECs2658	Z3008	dcyD	Putative 1-aminocyclopropane-1-carboxylate
G2583_2371	ECs2659	Z3010	fliY	Cystine-binding periplasmic protein
G2583_2372	ECs2660	Z3011	fliZ	protein FliZ
G2583_2373	ECs2661	Z3012	fliA	RNA polymerase sigma factor for flagellar operon
G2583_2374	ECs2662	Z3013	fliC	Flagellin
G2583_2375	ECs2663	Z3014	fliD	Flagellar capping protein
G2583_2376	ECs2664	Z3015	fliS	Flagellar protein FliS
G2583_2377	ECs2665	Z3016	fliT	Flagellar protein fliT
G2583_2378	ECs2666	Z3017	amyA	Alpha-amylase, cytoplasmic
G2583_2379	ECs2667	Z3018	yedD	hypothetical protein
G2583_2380	ECs2668	Z3019	yedE	predicted inner membrane protein
G2583_2381	ECs2669	Z3020	yedF	UPF0033 protein yedF
G2583_2382	ECs2670	Z3021	yedK	hypothetical protein
G2583_2383	ECs2671	Z3022	yedL	Acetyltransferase, GNAT family
G2583_2384	ECs2672	Z3023	yedM	putative secreted protein

Locus_tag[a]				
CB9615	Sakai	EDL933	Gene	Product
G2583_2385	ECs2673	Z3025	yedL	acetyltransferase, gnat family
G2583_2386	ECs2674	Z3026	yedM	putative secreted protein
G2583_2387	ECs2675	Z3024	int	Putative lambdoid prophage defective integrase
G2583_2388	ECs2676	Z3027	fliE	Flagellar hook-basal body complex protein fliE
G2583_2389	ECs2677	Z3028	fliF	Flagellar M-ring protein FliF precursor
G2583_2390	ECs2678	Z3029	fliG	Flagellar motor switch protein fliG
G2583_2391	ECs2679	Z3030	fliH	Flagellar biosynthesis
G2583_2392	ECs2680	Z3031	fliI	Flagellum-specific ATP synthase
G2583_2393	ECs2681	Z3032	fliJ	Flagellar fliJ protein
G2583_2394	ECs2682	Z3033	fliK	Flagellar hook-length control protein Flik
G2583_2395	ECs2683	Z3034	fliL	Flagellar fliL protein
G2583_2396	ECs2684	Z3035	fliM	Flagellar motor switch protein FliM
G2583_2397	ECs2685	Z3036	fliN	Flagellar motor switch protein FliN
G2583_2398	ECs2686	Z3037	fliO	Flagellar biogenesis protein
G2583_2399	ECs2687	Z3038	fliP	Flagellar biosynthetic protein fliP precursor
G2583_2400	ECs2688	Z3039	fliQ	hypothetical protein
G2583_2401	ECs2689	Z3040	fliR	Flagellar biosynthetic protein FliR
G2583_2402	ECs2690	Z3041	rcsA	Colanic acid capsular biosynthesis activation protein A
G2583_2403	ECs2691	Z3042	dsrB	putative oxidoreductase Fe-S binding subunit
G2583_2404	ECs2692	Z3043	yodD	hypothetical protein
G2583_2405	dsrA	RNA049	dsrA	ncRNA
G2583_2406	ECs2693	Z3045	yedP	Putative mannosyl-3-phosphoglycerate phosphatase
G2583_2407	ECs2694	Z3047	yedQ	Cellulose synthesis regulatory protein
G2583_2408	ECs2695	Z3048	yodC	hypothetical protein
G2583_2409	ECs2696	Z3049	yedI	putative membrane protein
G2583_2410	ECs2697	Z3050	yedA	Uncharacterized inner membrane transporter yedA
G2583_2411	ECs2698	Z3053	vsr	Very short patch repair protein
G2583_2412	ECs2699	Z3054	dcm	DNA-cytosine methyltransferase
G2583_2413	ECs2700	Z3055	yedJ	Putative metal-dependent phosphohydrolase
G2583_2414	ECs2701	Z3056	yedR	hypothetical protein
G2583_2415	ECs2702	Z3057	ompS	Outer membrane protein
G2583_2415	ECs2703	Z3058	ompS	Outer membrane protein
G2583_2416	ECs2704		-	hypothetical protein
G2583_2417	ECs2705	Z3059	hchA	Chaperone protein hchA
G2583_2418	ECs2706	Z3060	yedV	Heavy metal sensor histidine kinase
G2583_2419	ECs2707	Z3061	yedW	Heavy metal response regulator
G2583_2420	ECs2708	Z3062	yedX	Transthyretin-like protein precursor
G2583_2421	ECs2709	Z3063	yedY	UPF0190 protein yedY precursor
G2583_2422	ECs2710	Z3064	yedZ	UPF0191 membrane protein yedZ
G2583_2423	ECs2711	Z3065	yodA	Ribulose-phosphate 3-epimerase precursor
G2583_2424		Z3066	-	hypothetical protein
G2583_2425	ECs2712	Z3067	yodB	putative cytochrome
G2583_2426	ECs2713		-	hypothetical protein
G2583_2427		Z3069	-	unknown protein encoded within prophage CP-933U

Locus_tag[a]				
CB9615	Sakai	EDL933	Gene	Product
G2583_2428	ECs2714	Z3071	-	unknown protein encoded within prophage CP-933U
G2583_2429	ECs2715	Z3072	-	Tir-cytoskeleton coupling protein
Indel-111	ECs2716	Indel-111	-	hypothetical protein
Indel-111	ECs2717	Indel-111	-	putative tail fiber protein
Indel-111	ECs2718	Indel-111	-	putative outer membrane protein precursor
Indel-111	ECs2719	Indel-111	-	host specificity protein
Indel-111	ECs2720	Indel-111	-	putative host specificity protein
Indel-111	ECs2721	Indel-111	-	putative tail assembly protein
Indel-111	ECs2722	Indel-111	-	putative tail assembly protein
Indel-111	ECs2723	Indel-111	-	putative minor tail protein
Indel-111	ECs2724	Indel-111	-	putative minor tail protein
Indel-111	ECs2725	Indel-111	-	putative tail length tape measure protein precursor
Indel-111	ECs2726	Indel-111	-	putative tail length tape measure protein precursor
Indel-111	ECs2727	Indel-111	-	putative minor tail protein
Indel-111	ECs2728	Indel-111	-	putative minor tail protein
Indel-111	ECs2729	Indel-111	-	hypothetical protein
Indel-111	ECs2730	Indel-111	-	putative major head protein
Indel-111	ECs2731	Indel-111	-	putative head decoration protein
Indel-111	ECs2732	Indel-111	-	putative head-tail preconnector protein
Indel-111	ECs2733	Indel-111	-	putative portal protein
Indel-111	ECs2734	Indel-111	-	putative head completion protein
Indel-111	ECs2735	Indel-111	-	putative terminase large subunit
Indel-111	ECs2736	Indel-111	-	putative terminase small subunit
Indel-111	ECs2737	Indel-111	-	putative transcriptional regulator
Indel-111	ECs2739	Indel-111	-	putative endopeptidase
Indel-111	ECs2738	Indel-111	-	putative lipoprotein precursor
Indel-111	ECs2740	Indel-111	-	hypothetical protein
Indel-111	ECs2741	Indel-111	-	putative endolysin
Indel-111	ECs2742	Indel-111	-	hypothetical protein
Indel-111	ECs2743	Indel-111	-	putative holin protein
Indel-111	ECs2744	Indel-111	-	putative transposase OrfA protein of insertion sequence IS629
Indel-111	ECs2745	Indel-111	-	putative transposase OrfB protein of insertion sequence IS629
Indel-112	Indel-112	Z3073	-	unknown protein encoded within prophage CP-933U
Indel-112	Indel-112	Z3074	-	putative tail fiber protein of prophage CP-933U
Indel-112	Indel-112	Z3075	lomU	putative outer membrane protein of prophage CP-933U
Indel-112	Indel-112	Z3077	-	putative tail fiber component J of prophage CP-933U
Indel-112	Indel-112	Z3079	-	putative tail fiber component I of prophage CP-933U
Indel-112	Indel-112	Z3081	-	putative tail fiber component K of prophage CP-933U
Indel-112	Indel-112	Z3082	-	putative tail fiber component L of prophage CP-933U
Indel-112	Indel-112	Z3083	-	putative tail fiber component M of prophage CP-933U
Indel-112	Indel-112	Z3084	-	putative tail fiber component H of prophage CP-933U
Indel-112	Indel-112	Z3085	-	putative tail fiber component T of prophage CP-933U

Locus_tag[a]				
CB9615	Sakai	EDL933	Gene	Product
Indel-112	Indel-112	Z3086	-	putative tail fiber component G of prophage CP-933U
Indel-112	Indel-112	Z3087	-	putative tail fiber component V of prophage CP-933U
Indel-112	Indel-112	Z3088	-	putative tail fiber component U of prophage CP-933U
Indel-112	Indel-112	Z3089	-	putative tail fiber component Z of prophage CP-933U
Indel-112	Indel-112	Z3090	-	unknown protein encoded within prophage CP-933U
Indel-112	Indel-112	Z3091	-	unknown protein encoded within prophage CP-933U
Indel-112	Indel-112	Z3092	-	unknown protein encoded within prophage CP-933U
Indel-112	Indel-112	Z3093	-	unknown in IS629 encoded within prophage CP-933U
Indel-112	Indel-112	Z3095	-	putative transposase encoded within prophage CP-
Indel-112	Indel-112	Z3097	-	putative peptidase encoded within prophage CP-933U
Indel-112	Indel-112	Z3098	-	putative head-tail preconnector protein of prophage CP-933U
Indel-112	Indel-112	Z3099	-	putative DNA packaging protein of prophage CP-933U
Indel-112	Indel-112	Z3100	-	unknown protein encoded within prophage CP-933U
Indel-112	Indel-112	Z3101	-	putative endopeptidase of prophage CP-933U
Indel-112	Indel-112	Z3103	antU	putative antirepressor protein of prophage CP-933U
Indel-112	Indel-112	Z3104	-	putative endolysin of prophage CP-933U
Indel-112	Indel-112	Z3105	-	unknown protein encoded within prophage CP-933U
Indel-112	Indel-112	Z3106	-	putative holin protein of prophage CP-933U
Indel-113	ECs2746	Z3107	-	hypothetical protein
Indel-113	ECs2747		-	hypothetical protein
Indel-113	ECs2748	Z3108	-	hypothetical protein
Indel-113	ECs2749	Z3109	-	hypothetical protein
Indel-113	argO7	RNA050	argO7	Arg tRNA
Indel-113	argN5	RNA051	argN5	Arg tRNA
Indel-113	ileZ7	RNA052	ileZ7	Ile tRNA
Indel-113	ECs2750	Z3114	-	antiterminator
Indel-113	ECs2751	Z3115	-	putative crossover junction endodeoxyribonuclease
Indel-113	ECs2752	Z3116	-	hypothetical protein
Indel-113	ECs2753		-	hypothetical protein
Indel-113		Z3117	-	unknown protein encoded within prophage CP-933U
Indel-113	ECs2754		-	prophage maintenance protein
Indel-113	ECs2755	Z3118	-	hypothetical protein
Indel-113	ECs2756	Z3119	-	hypothetical protein
Indel-113	ECs2757	Z3120	-	hypothetical protein
Indel-113	ECs2758		-	hypothetical protein
Indel-113	ECs2759	Z3121	-	hypothetical protein
Indel-113	ECs2760		-	hypothetical protein
Indel-113	ECs2761	Z3122	-	hypothetical protein
Indel-113	ECs2762	Z3123	-	putative phage replication protein
Indel-113	ECs2763	Z3124	-	hypothetical protein
Indel-113	ECs2764	Z3125	-	hypothetical protein
Indel-113	ECs2765		-	putative cell division control protein
Indel-113	ECs2766	Z3126	-	putative repressor protein

Locus_tag[a]				
CB9615	Sakai	EDL933	Gene	Product
Indel-113	ECs2767	Z3127	-	hypothetical protein
Indel-113	ECs2768	Z3128	-	putative cell division inhibition protein
Indel-113	ECs2769		-	hypothetical protein
Indel-113	ECs2770	Z3129	exoU	hypothetical protein
Indel-113	ECs2771	Z3129	exoU	hypothetical protein
Indel-113	ECs2772		-	hypothetical protein
	ECs2773	Z3130	intU	putative integrase
G2583_2430	Indel-114	Indel-114	-	Tail fiber protein
G2583_2431	Indel-114	Indel-114	-	Enterobacterial Ail/Lom family protein
G2583_2432	Indel-114	Indel-114	-	Putative host specificity protein
G2583_2433	Indel-114	Indel-114	-	Superoxide dismutase [Cu-Zn]
G2583_2434	Indel-114	Indel-114	-	hypothetical protein
G2583_2435	Indel-114	Indel-114	-	Cell wall-associated hydrolases (invasion-associated proteins)
G2583_2436	Indel-114	Indel-114	-	Phage-related protein
G2583_2437	Indel-114	Indel-114	-	Putative tail component of prophage CP-933R
G2583_2438	Indel-114	Indel-114	-	putative tail component of prophage CP-933O
G2583_2439	Indel-114	Indel-114	-	Phage tail assembly protein T
G2583_2440	Indel-114	Indel-114	-	hypothetical protein
G2583_2441	Indel-114	Indel-114	-	hypothetical protein
G2583_2442	Indel-114	Indel-114	-	Putative tail component of prophage
G2583_2443	Indel-114	Indel-114	-	prophage minor tail protein Z
G2583_2444	Indel-114	Indel-114	-	Phage Head-Tail Attachment
G2583_2445	Indel-114	Indel-114	-	conserved hypothetical protein
G2583_2446	Indel-114	Indel-114	-	phage major capsid protein E
G2583_2447	Indel-114	Indel-114	-	Bacteriophage lambda head decoration protein D
G2583_2448	Indel-114	Indel-114	-	Periplasmic serine proteases (ClpP class)
G2583_2449	Indel-114	Indel-114	-	Bacteriophage capsid protein
G2583_2450	Indel-114	Indel-114	-	putative head completion protein
G2583_2451	Indel-114	Indel-114	-	Putative terminase large subunit of prophage CP-933O
G2583_2452	Indel-114	Indel-114	-	Prophage Qin DNA packaging protein NU1 homolog
G2583_2453	Indel-114	Indel-114	-	hypothetical protein
G2583_2454	Indel-114	Indel-114	-	hypothetical protein
G2583_2455	Indel-114	Indel-114	-	putative endopeptidase
G2583_2456	Indel-114	Indel-114	-	conserved hypothetical protein
G2583_2457	Indel-114	Indel-114	-	Phage-related lysozyme (muraminidase)
G2583_2458	Indel-114	Indel-114	-	hypothetical protein
G2583_2459	Indel-114	Indel-114	-	Putative holin protein
G2583_2460	Indel-114	Indel-114	-	YjhS
G2583_2461	Indel-114	Indel-114	-	Putative envelope protein encoded within prophage CP-933N
G2583_2462	Indel-114	Indel-114	-	CAAX amino terminal protease family
G2583_2463	Indel-114	Indel-114	ybcQ	Phage antitermination Q type 1 family
G2583_2464	Indel-114	Indel-114	rusA	Crossover junction endodeoxyribonuclease RusA

Locus_tag[a]				
CB9615	Sakai	EDL933	Gene	Product
G2583_2465	Indel-114	Indel-114	ydfU	hypothetical protein
G2583_2466	Indel-114	Indel-114	-	hypothetical protein
G2583_2467	Indel-114	Indel-114	rem	hypothetical protein
G2583_2468	Indel-114	Indel-114	-	Helix-turn-helix domain protein
G2583_2469	Indel-114	Indel-114	-	hypothetical protein
G2583_2470	Indel-114	Indel-114	-	Putative cell killing protein encoded within cryptic prophage CP-933M
G2583_2471	Indel-114	Indel-114	-	conserved hypothetical protein
G2583_2472	Indel-114	Indel-114	-	LygF
G2583_2473	Indel-114	Indel-114	-	Phage O protein family
G2583_2474	Indel-114	Indel-114	ydfX	hypothetical protein
G2583_2475	Indel-114	Indel-114	-	hypothetical protein
G2583_2476	Indel-114	Indel-114	dicA	Similar to DicA, regulator of DicB encoded by prophage
G2583_2477	Indel-114	Indel-114	ydfA	hypothetical protein
G2583_2478	Indel-114	Indel-114	-	unknown protein encoded by prophage CP-933N
G2583_2479	Indel-114	Indel-114	DicB	Putative regulator of cell division encoded by prophage CP-933O
G2583_2480	Indel-114	Indel-114	-	hypothetical protein
G2583_2481	Indel-114	Indel-114	recE	Exodeoxyribonuclease VIII
G2583_2482	Indel-114	Indel-114	-	hypothetical protein
G2583_2483	Indel-114	Indel-114	intU	Site-specific recombinase, phage integrase family
G2583_2484	serU	RNA053	serU	Ser tRNA
G2583_2485	ECs2774	Z3132	mtfA	hypothetical protein
G2583_2486	asnT	RNA054	asnT	Asn tRNA
G2583_2487	ECs2775	Z3135	yeeJ	Putative invasin
		Z3136	-	hypothetical protein
G2583_2488	ECs2775	Z3135	-	putative factor
G2583_2488	ECs2776	Z3135	-	putative factor
G2583_2489	ECs2777	Z3137	-	hypothetical protein
G2583_2490	ECs2778	Z3138	shiA	Shikimate transporter
G2583_2491	ECs2779	Z3139	amn	AMP nucleosidase
G2583_2492	ECs2780	Z3140	yeeN	UPF0082 protein yeeN
G2583_2493	asnW	RNA055	asnW	Asn tRNA
G2583_2494	ECs2781	Z3143	yeeO	MATE efflux family protein
G2583_2495	ECs2781	Z3143	yeeO	MATE efflux family protein
G2583_2495	ECs2782	Z3144	yeeO	MATE efflux family protein
G2583_2496	asnU	RNA056	asnU	Asn tRNA
G2583_2497	ECs2783	Z3146	cbl	transcriptional regulator Cbl
G2583_2498	ECs2784	Z3147	nac	Nitrogen assimilation regulatory protein Nac
G2583_2499	asnV	RNA057	asnV	Asn tRNA
G2583_2500	ECs2785	Z3150	erfK	Conserved protein with NAD(P)-binding Rossmann-fold domain
G2583_2501	ECs2786	Z3151	cobT	Nicotinate-nucleotide--dimethylbenzimidazole phosphoribosyltransferase

Locus_tag[a]				
CB9615	Sakai	EDL933	Gene	Product
G2583_2502	ECs2787	Z3152	cobS	cobalamin synthase
G2583_2503	ECs2788	Z3153	cobU	cobalamin synthase
Indel-115	ECs2789	Z3154	-	hypothetical protein
Indel-115	ECs2790	Z3155	-	hypothetical protein
Indel-115	ECs2791	Z3156	-	hypothetical protein
G2583_2504	ECs2792	Z3159	yoeE	Putative outer membrane receptor for iron compound or colicin
	ECs2793		-	hypothetical protein
Indel-116	ECs2794	Z3161	-	putative transposase OrfA protein of insertion sequence IS629
Indel-116	ECs2795	Z3162	-	putative transposase OrfB protein of insertion sequence IS629
G2583_2505	Indel-118	Indel-118	ibrB	putative ParB-like nuclease
G2583_2506	Indel-118	Indel-118	ibrA	Immunoglobulin-binding regulator A homolog
G2583_2507	Indel-118	Indel-118	insN	unknown protein encoded by IS911 within prophage CP-933L
G2583_2508	Indel-118	Indel-118	-	putative transposase
G2583_2509	Indel-118	Indel-118	-	Putative transposase subunit
G2583_2510	Indel-118	Indel-118	-	hypothetical protein
G2583_2511	Indel-118	Indel-118	-	hypothetical protein
G2583_2512	Indel-118	Indel-118	-	hypothetical protein
G2583_2513	Indel-118	Indel-118	yfjP	hypothetical protein
G2583_2514	ECs2796	Indel-119	-	hypothetical protein
G2583_2515	ECs2797	Indel-119	-	hypothetical protein
G2583_2516	ECs2798	Indel-119	-	putative esterase
G2583_2517	ECs2799	Indel-119	-	hypothetical protein
G2583_2518	ECs2800	Indel-119	-	hypothetical protein
G2583_2519	ECs2801	Indel-119	yafZ	conserved hypothetical protein
G2583_2520	ECs2802	Indel-119	yafX	Antirestriction protein
G2583_2521	ECs2803	Indel-119	yeeS	DNA repair protein, RadC family
G2583_2522	ECs2804	Z3163	yeeT	hypothetical protein
G2583_2523	ECs2805	Z3164	yeeU	Putative structural protein
G2583_2524	ECs2806	Z3165	yeeV1	hypothetical protein
G2583_2525	ECs2807	Z3166	yeeW	hypothetical protein
G2583_2526	ECs2808	Z3167	yoeF	hypothetical protein
G2583_2527	ECs2809	Z3168	yeeX	hypothetical protein
G2583_2528	ECs2810	Z3169	yeeA	hypothetical protein
G2583_2529	ECs2811	Z3170	sbmC	DNA gyrase inhibitory protein
G2583_2530	ECs2812	Z3171	dacD	Serine-type D-Ala-D-Ala carboxypeptidase
G2583_2531	ECs2813	Z3173	sbcB	Exonuclease I
G2583_2532	ECs2814	Z3174	yeeD	hypothetical protein
G2583_2533	ECs2815	Z3175	yeeE	Membrane protein, YeeE/YedE family
G2583_2534	ECs2816	Z3176	yeeF	Hypothetical transport protein YeeF
G2583_2535	ECs2817	Z3177	yeeY	Predicted DNA-binding transcriptional regulator

Locus_tag[a]				
CB9615	Sakai	EDL933	Gene	Product
G2583_2536	ECs2818	Z3178	yeeZ	NAD dependent epimerase/dehydratase family protein
		Z3179	-	orf; hypothetical protein
G2583_2537	Indel-121	Indel-121	yoeB	Toxin yoeB
G2583_2538	Indel-121	Indel-121	yefM	Antitoxin yefM
G2583_2539	ECs2819	Z3180	hisL	his operon leader peptide
G2583_2540	ECs2820	Z3181	hisG	ATP phosphoribosyltransferase
G2583_2541	ECs2821	Z3182	hisD	Bifunctional histidinal dehydrogenase and histidinol dehydrogenase
G2583_2542	ECs2822	Z3183	hisC	Histidinol-phosphate aminotransferase
G2583_2543	ECs2823	Z3184	hisB	Fused histidinol-phosphatase/imidazoleglycerol-phosphate dehydratase
G2583_2544	ECs2824	Z3185	hisH	Imidazole glycerol phosphate synthase
G2583_2545	ECs2825	Z3186	hisA	1-(5-phosphoribosyl)-5-[(5-phosphoribosylamino)methylideneamino] imidazole-4-carboxamide isomerase
G2583_2546	ECs2826	Z3187	hisF	Imidazole glycerol phosphate synthase subunit hisF
G2583_2547	ECs2827	Z3188	hisI	Fused phosphoribosyl-AMP cyclohydrolase/phosphoribosyl-ATP pyrophosphatase
G2583_2548	ECs2828	Z3189	cld	O-antigen chain length determinant Wzz
G2583_2549	ECs2829	Z3190	ugd	UDP-glucose-6-dehydrogenase
G2583_2550	Indel-123	Indel-123	ydcC	Transposase IS4 family protein
G2583_2551	Indel-123	Indel-123	WbdK	Putative pyridoxamine 5-phosphate-dependent
G2583_2552	Indel-123	Indel-123	wbcJ	WbdJ
G2583_2553	ECs2830	Z3191	gnd	6-phosphogluconate dehydrogenase, decarboxylating
Indel-124	ECs2831	Z3192	wbdR	acetyltransferase
Indel-124	ECs2832		-	H repeat-associated protein
Indel-124	ECs2833		-	H repeat-associated protein
Indel-124	ECs2834		-	H repeat-associated protein
G2583_2554	Indel-125	Indel-125	rfpA	Putative glycosyltransferase WbgP
G2583_2555	Indel-125	Indel-125	wblT	Putative glycosyltransferase WbgO
G2583_2556	Indel-125	Indel-125	wzx	O-antigen flippase Wzx
G2583_2557	Indel-125	Indel-125	-	O-antigen polymerase Wzy
G2583_2558	Indel-125	Indel-125	fucT	Putative fucosyltransferase WbgN
G2583_2559	ECs2835	Z3194	cpsG	Phosphomannomutase ManB
G2583_2560	ECs2836	Z3195	manC	Mannose-1-P guanosyltransferase ManC
G2583_2561	ECs2837	Z3196	wcaH	GDP-mannose mannosyl hydrolase
G2583_2562	ECs2839	Z3198	gmd	GDP-mannose dehydratase Gmd
Indel-128	ECs2838	Z3197	fcl	fucose synthetase
Indel-130	ECs2840	Z3199	wbdP	putative glycosyl transferase
Indel-130	ECs2841	Z3200	per	perosamine synthetase
Indel-130	ECs2842	Z3201	wzx	O antigen flippase
Indel-130	ECs2843	Z3202	wbdO	putative glycosyl transferase
Indel-130	ECs2844	Z3203	wzy	O antigen polymerase
Indel-130	ECs2845	Z3204	wbdN	putative glycosyl transferase

Locus_tag[a]				
CB9615	Sakai	EDL933	Gene	Product
G2583_2563	Indel-131	Indel-131	wbgM	Putative galactosyltransferase WbgM
G2583_2564	ECs2846	Z3205	galF	UDP-glucose pyrophosphorylase GalF
G2583_2565	ECs2847	Z3206	gne	UDP-N-acetylglucosamine 4-epimerase
G2583_2566	ECs2848	Z3207	wcaM	Predicted colanic acid biosynthesis protein WcaM
G2583_2567	ECs2849	Z3208	wcaL	Predicted glycosyl transferase WcaL
G2583_2568	ECs2850	Z3209	wcaK	Predicted pyruvyl transferase WcaK
G2583_2569	ECs2851	Z3210	wzxC	Colanic acid exporter WzxC
G2583_2570	ECs2852	Z3211	wcaJ	Predicted UDP-glucose lipid carrier transferase WcaJ
G2583_2571	ECs2853	Z3212	cpsG	Phosphomannomutase CpsG
G2583_2572	ECs2854	Z3213	cpsB	Mannose-1-phosphate guanyltransferase CpsB
G2583_2573	ECs2855	Z3214	wcaI	Predicted glycosyl transferase WcaI
G2583_2574	ECs2856	Z3215	gmm	GDP-mannose mannosyl hydrolase
G2583_2575	ECs2857	Z3216	fcl	Bifunctional GDP-fucose synthetase
G2583_2576	ECs2858	Z3217	gmd	GDP-mannose 4,6-dehydratase
G2583_2577	ECs2859	Z3218	wcaF	Predicted acyl transferase WcaF
G2583_2578	ECs2860	Z3219	wcaE	Predicted glycosyl transferase WcaE
G2583_2579	ECs2861	Z3220	wcaD	Putative colanic acid polymerase
G2583_2580	ECs2862	Z3221	wcaC	Predicted glycosyl transferase WcaC
G2583_2581	ECs2863	Z3222	wcaB	Predicted acyl transferase WcaB
G2583_2582	ECs2864	Z3223	wcaA	Predicted glycosyl transferase WcaA
G2583_2583	ECs2865	Z3224	wzc	Protein-tyrosine kinase Wzc
G2583_2584	ECs2866	Z3226	wzb	Low molecular weight protein-tyrosine-phosphatase wzb
G2583_2585	ECs2867	Z3227	wza	Putative polysaccharide export protein wza precursor
G2583_2586	ECs2868	Z3229	yegH	Putative transport protein
G2583_2587	ECs2869	Z3230	-	hypothetical protein
G2583_2588	ECs2870	Z3231	-	Putative DNA-binding protein
G2583_2589	ECs2871	Z3232	asmA	Predicted assembly protein AsmA
G2583_2590	ECs2872	Z3233	dcd	Deoxycytidine triphosphate deaminase
G2583_2591	ECs2873	Z3234	udk	Uridine/cytidine kinase
G2583_2592	ECs2874	Z3235	yegE	Predicted diguanylate cyclase
G2583_2592	ECs2875	Z3236	yegE	Predicted diguanylate cyclase
G2583_2592	ECs2876	Z3236	yegE	Predicted diguanylate cyclase
G2583_2593	ECs2877	Z3237	alkA	3-methyl-adenine DNA glycosylase II
G2583_2594	ECs2878	Z3238	yegD	Predicted chaperone
G2583_2595	ECs2879	Z3239	yegI	conserved predicted protein
G2583_2596	ECs2880	Z3240	yegK	conserved predicted protein
G2583_2597	ECs2881	Z3241	yegL	Uncharacterized protein encoded in toxicity protection region of plasmid R478, contains von Willebrand factor (vWF) domain
G2583_2598			-	ncRNA
G2583_2599			-	ncRNA
		Z3242	-	hypothetical protein
G2583_2600	ECs2882	Z3243	mdtA	hypothetical protein
G2583_2601	ECs2883	Z3244	mdtB	Multidrug resistance protein mdtB

Locus_tag[a]				
CB9615	Sakai	EDL933	Gene	Product
G2583_2602	ECs2884	Z3245	mdtC	Multidrug resistance protein mdtC
G2583_2603	ECs2885	Z3246	mdtD	Multidrug resistance protein mdtD
G2583_2604	ECs2886	Z3247	baeS	Sensor histidine kinase BaeS
G2583_2605	ECs2887	Z3248	baeR	DNA-binding response regulator in two-component regulatory system with BaeS
G2583_2606	ECs2888	Z3249	yegP	hypothetical protein
G2583_2607	Indel-138	Indel-138	-	Putative addiction module antidote protein, CC2985
G2583_2608	Indel-138	Indel-138	-	Plasmid stabilization system protein, RelE/ParE family
G2583_2609	ECs2889	Z3250	yegQ	hypothetical protein
G2583_2610			-	ncRNA
G2583_2611	ECs2890		-	hypothetical protein
G2583_2612	ECs2891	Z3251	yegR	hypothetical protein
G2583_2613	ECs2892	Z3252	yegS	Lipid kinase yegS
G2583_2614	Indel-140	Indel-140	-	hypothetical protein
G2583_2615	Indel-140	Indel-140	rbtT	D-arabitol membrane transporter
G2583_2616	Indel-140	Indel-140	-	hypothetical protein
G2583_2617	Indel-140	Indel-140	-	Arabitol dehydrogenase
G2583_2618	Indel-140	Indel-140	-	D-arabitol repressor
G2583_2619	Indel-140	Indel-140	-	hypothetical protein
G2583_2620	Indel-140	Indel-140	-	Ribitol dehydrogenase
G2583_2621	Indel-140	Indel-140	rtlK	Ribitol kinase
G2583_2622	ECs2893	Z3253	gatR	Galactitol utilization operon repressor
G2583_2623	ECs2894	Z3254	gatD	Galactitol-1-phosphate 5-dehydrogenase
G2583_2624	ECs2895	Z3255	gatC	PTS system, galactitol-specific IIC component
G2583_2625	ECs2896	Z3256	gatB	Galactitol-specific phosphotransferase enzyme IIB component
G2583_2626	ECs2897	Z3257	gatA	Galactitol-specific phosphotransferase enzyme IIA component
G2583_2627	ECs2898	Z3258	gatZ	Tagatose 6-phosphate kinase
G2583_2628	ECs2899	Z3259	gatY	Class II aldolase, tagatose bisphosphate family
G2583_2629	ECs2900	Z3260	fbaB	Fructose-bisphosphate aldolase class 1
G2583_2630	ECs2901	Z3261	yegT	Nucleoside transporter
G2583_2631	ECs2902	Z3262	yegU	ADP-ribosylglycohydrolase family protein
G2583_2632	ECs2903	Z3263	yegV	Kinase, PfkB family
G2583_2633	ECs2904	Z3264	yegW	transcriptional regulator, GntR family
G2583_2634	ECs2905	Z3266	yegX	hypothetical protein
G2583_2635	ECs2906	Z3267	thiD	Hydroxymethylpyrimidine/phosphomethylpyrimidin e kinase
G2583_2636	ECs2907	Z3268	thiM	Hydroxyethylthiazole kinase
G2583_2637	ECs2908	Z3269	-	hypothetical protein
G2583_2638		Z3270	-	hypothetical protein
G2583_2639	ECs2909	Z3271	-	hypothetical protein
G2583_2640	ECs2910	Z3272	-	hypothetical protein
G2583_2641	ECs2911	Z3273	rcnR	hypothetical protein

Locus_tag[a]				
CB9615	Sakai	EDL933	Gene	Product
G2583_2642	ECs2912	Z3274	rcnA	Nickel/cobalt efflux system rcnA
G2583_2643	ECs2913	Z3275	yohN	hypothetical protein
Indel-141	ECs2914	Z3276	-	putative type-1 fimbrial protein
G2583_2644			yehA	predicted fimbrial-like adhesin protein
G2583_2645	ECs2915	Z3277	yehB	fimbrial usher protein
G2583_2646	ECs2916	Z3278	yehC	gram-negative pilus assembly chaperone
G2583_2647	ECs2917	Z3279	yehD	Fimbrial protein
G2583_2648	ECs2918	Z3280	yehE	hypothetical protein
G2583_2649	ECs2919	Z3281	mrp	Putative ATPase
G2583_2650	ECs2920	Z3282	metG	Methionyl-tRNA synthetase
G2583_2651	ECs2921	Z3283	molR_A	putative molybdate metabolism regulator
G2583_2651	ECs2922	Z3284	molR_A	putative molybdate metabolism regulator
G2583_2652	ECs2922	Z3284	molR_B	putative molybdate metabolism regulator
G2583_2653	ECs2923	Z3285	molR_C	interrupted molybdate metabolism regulator
G2583_2653	ECs2924	Z3286	molR_C	interrupted molybdate metabolism regulator
G2583_2654	ECs2925	Z3287	yehI	Molybdate metabolism regulator MolR homolog
G2583_2655	ECs2926	Z3288	yehK	hypothetical protein
Indel-144		Z3289	-	hypothetical protein
		Z3290	-	hypothetical protein
G2583_2656	ECs2927	Z3291	yehL	hypothetical protein
G2583_2657	ECs2928	Z3292	yehM	hypothetical protein
G2583_2657	ECs2929	Z3293	yehM	hypothetical protein
G2583_2658	ECs2930	Z3294	yehP	hypothetical protein
G2583_2659	ECs2931	Z3295	yehQ	hypothetical protein
Indel-145	ECs2932	Z3297	-	putative transposase OrfB protein of insertion sequence IS629
Indel-145	ECs2933	Z3299	-	putative transposase OrfA protein of insertion sequence IS629
G2583_2660	ECs2934	Z3300	yehR	Hypothetical lipoprotein YehR
G2583_2661	ECs2935	Z3301	yehS	hypothetical protein
G2583_2662	ECs2936	Z3302	yehT	putative two-component response-regulatory protein
G2583_2663	ECs2937	Z3303	yehU	Sensor histidine kinase
G2583_2664	ECs2938		mlrA	DNA-binding transcriptional regulator
Indel-146	ECs2939	Z3305	-	hypothetical protein
Indel-146	ECs2940	Z3306	-	hypothetical protein
Indel-146	ECs2941	Z3307	-	putative tail fiber protein
Indel-146	ECs2941	Z3309	-	putative tail fiber protein
Indel-146	ECs2942	Z3310	-	putative outer membrane protein Lom precursor
Indel-147	ECs2943	Indel-147	-	putative host specificity protein
Indel-147	ECs2944	Indel-147	-	putative host specificity protein
Indel-147	ECs2945	Indel-147	-	putative tail assembly protein
Indel-147	ECs2946	Indel-147	-	putative tail assembly protein
Indel-147	ECs2947	Indel-147	-	putative minor tail protein
Indel-147	ECs2948	Indel-147	-	putative minor tail protein

Locus_tag[a]				
CB9615	Sakai	EDL933	Gene	Product
Indel-147	ECs2949	Indel-147	-	putative tail length tape measure protein precursor
Indel-147	ECs2950	Indel-147	-	putative minor tail protein
Indel-147	ECs2951	Indel-147	-	putative minor tail protein
Indel-147	ECs2952	Indel-147	-	hypothetical protein
Indel-147	ECs2953	Indel-147	-	putative minor tail protein
Indel-147	ECs2954	Indel-147	-	putative minor tail protein
Indel-147	ECs2955	Indel-147	-	hypothetical protein
Indel-147	ECs2956	Indel-147	-	hypothetical membrane protein
Indel-147	ECs2957	Indel-147	-	hypothetical protein
Indel-147	ECs2958	Indel-147	-	putative transposase OrfA protein of insertion sequence IS629
Indel-147	ECs2959	Indel-147	-	putative transposase OrfB protein of insertion sequence IS629
Indel-147	ECs2960	Indel-147	-	putative protease/scaffold protein
Indel-147	ECs2961	Indel-147	-	putative portal protein
Indel-147	ECs2962	Indel-147	-	hypothetical protein
Indel-147	ECs2963	Indel-147	-	putative terminase large subunit
Indel-147	ECs2964	Indel-147	-	hypothetical protein
Indel-148	Indel-148	Z3311	-	putative tail fiber protein of prophage CP-933V
Indel-148	Indel-148	Z3312	-	putative superoxide dismutase
Indel-148	Indel-148	Z3313	-	putative tail component of prophage CP-933V
Indel-148	Indel-148	Z3314	-	putative tail component of prophage CP-933V
Indel-148	Indel-148	Z3315	-	putative tail component of prophage CP-933V
Indel-148	Indel-148	Z3316	-	unknown protein encoded within prophage CP-933V
Indel-148	Indel-148	Z3318	-	putative tail component of prophage CP-933V
Indel-148	Indel-148	Z3319	-	unknown protein encoded within prophage CP-933V
Indel-148	Indel-148	Z3320	-	unknown protein encoded within prophage CP-933V
Indel-148	Indel-148	Z3322	-	putative major tail subunit encoded within prophage CP-933V
Indel-148	Indel-148	Z3323	-	unknown protein encoded within prophage CP-933V
Indel-148	Indel-148	Z3325	-	unknown protein encoded within prophage CP-933V
Indel-148	Indel-148	Z3326	-	unknown protein encoded within prophage CP-933V
Indel-148	Indel-148	Z3327	-	unknown protein encoded within prophage CP-933V
Indel-148	Indel-148	Z3328	-	putative portal protein for prophage CP-933V
Indel-148	Indel-148	Z3329	-	unknown protein encoded within prophage CP-933V
Indel-148	Indel-148	Z3331	-	unknown protein encoded within prophage CP-933V
Indel-148	Indel-148	Z3332	-	unknown protein encoded within prophage CP-933V
Indel-148	Indel-148	Z3333	-	unknown protein encoded within prophage CP-933V
Indel-148	Indel-148	Z3334	-	unknown protein encoded within prophage CP-933V
Indel-148	Indel-148	Z3335	-	unknown protein encoded within prophage CP-933V
Indel-149	ECs2966	Z3336	-	putative endopeptidase
Indel-149	ECs2965		-	lipoprotein Rz1 precursor
Indel-149	ECs2967	Z3337	antV	putative antirepressor protein
Indel-149	ECs2968	Z3339	-	putative endolysin

Locus_tag[a]				
CB9615	Sakai	EDL933	Gene	Product
Indel-149	ECs2969	Z3340	-	putative holin protein
Indel-149	ECs2970	Z3341	-	hypothetical protein
Indel-149	ECs2971		-	hypothetical protein
Indel-149	ECs2972	Z3342	-	hypothetical protein
Indel-149	ECs2973	Z3343	stx1B	Shiga toxin I subunit B precursor
Indel-149	ECs2974	Z3344	stx1A	Shiga toxin I subunit A precursor
Indel-149	ECs2975	Z3345	-	antitermination protein
Indel-149	ECs2976		-	hypothetical protein
Indel-149	ECs2977	Z3346	-	hypothetical protein
Indel-149	ECs2978	Z3347	-	hypothetical protein
Indel-149	ECs2979	Z3348	-	hypothetical protein
Indel-149	ECs2980		-	hypothetical protein NinE
Indel-149	ECs2981	Z3349	-	putative DNA methylase
Indel-149	ECs2982	Z3351	-	hypothetical protein
Indel-149	ECs2983	Z3352	-	hypothetical protein
Indel-149	ECs2984	Z3353	-	hypothetical protein
Indel-149	ECs2985	Z3354	-	Ren protein
Indel-149	ECs2986	Z3355	-	phage replication protein P
Indel-149	ECs2987	Z3356	-	phage replication protein O
Indel-149	ECs2988	Z3357	-	regulatory protein CII
Indel-149	ECs2989		-	putative regulatory protein
Indel-149	ECs2990	Z3358	-	putative prophage repressor CI
Indel-149	ECs2991	Z3359	-	hypothetical protein
Indel-149	ECs2992	Z3360	-	putative membrane protein
Indel-149	ECs2994		-	hypothetical protein
Indel-149	ECs2993	Z3361	-	putative regulatory protein
Indel-149	ECs2995	Z3362	-	putative superinfection exclusion protein
Indel-149	ECs2996	Z3363	-	putative single-stranded DNA binding protein
Indel-149	ECs2997		-	regulatory protein cIII
Indel-149	ECs2998	Z3364	-	Kil protein
Indel-149	ECs2999		-	host-nuclease inhibitor protein gam
Indel-149	ECs3000	Z3365	-	putative host-nuclease inhibitor protein Gam
Indel-149	ECs3001	Z3366	-	recombination protein Bet
Indel-149	ECs3002	Z3367	-	exonuclease
Indel-149	ECs3003		-	hypothetical protein
Indel-149	ECs3004	Z3368	-	hypothetical protein
Indel-149	ECs3005	Z3369	-	hypothetical protein
Indel-149	ECs3006		-	putative C4-type zinc finger protein
Indel-149	ECs3007	Z3370	-	hypothetical protein
Indel-149	ECs3008		-	hypothetical protein
Indel-149	ECs3009	Z3371	-	hypothetical protein
Indel-149	ECs3010	Z3372	-	hypothetical protein
Indel-149	ECs3011	Z3373	-	hypothetical protein
Indel-149		Z3374	-	unknown protein encoded within prophage CP-933V

Locus_tag[a]				
CB9615	Sakai	EDL933	Gene	Product
Indel-149	ECs3012		-	putative excisionase
Indel-149	ECs3013	Z3375	intV	putative integrase
G2583_2664	ECs3014	Z3376	mlrA	DNA-binding transcriptional regulator
G2583_2665	ECs3015	Z3377	yehW	ABC transporter, quaternary amine uptake (QAT) family, permease protein
G2583_2666	ECs3016	Z3378	yehX	ABC transporter, quaternary amine uptake (QAT) family, ATP-binding protein
G2583_2667	ECs3017	Z3379	yehY	ABC transporter, quaternary amine uptake (QAT) family, permease protein
G2583_2668	ECs3018	Z3380	osmF	Substrate-binding region of ABC-type glycine betaine transport system precursor
G2583_2669	ECs3019	Z3381	bglX	Periplasmic beta-glucosidase
G2583_2670	ECs3020	Z3382	dld	D-lactate dehydrogenase
G2583_2671	ECs3021	Z3383	pbpG	Penicillin-binding protein 7
G2583_2672	ECs3022	Z3384	yohC	hypothetical protein
G2583_2673	ECs3023	Z3385	yohD	hypothetical protein
G2583_2674	ECs3024	Z3386	yohF	Putative 3-oxoacyl-[acyl-carrier-protein] reductase
G2583_2675	ECs3025	Z3387	yohG	Multidrug resistance outer membrane protein mdtQ precursor
G2583_2676		Z3388	-	hypothetical protein
G2583_2677	ECs3026	Z3389	dusC	tRNA-dihydrouridine synthase C
G2583_2678	ECs3027	Z3390	-	FAD dependent oxidoreductase
G2583_2679	ECs3028	Z3391	maiA	Putative glutathione-S-transferase
G2583_2680	ECs3029	Z3392	-	Fumarylacetoacetate hydrolase family protein
G2583_2681	ECs3030	Z3393	gtdA	Gentisate 1,2-dioxygenase
G2583_2682	ECs3031	Z3394	-	Putative transporter
G2583_2683	ECs3032	Z3395	-	putative regulator
G2583_2684	ECs3033	Z3396	yohJ	UPF0299 membrane protein yohJ
G2583_2685	ECs3034	Z3397	yohK	Putative seritonin transporter
G2583_2686	ECs3035	Z3398	cdd	Cytidine deaminase
G2583_2687	ECs3036	Z3399	sanA	hypothetical protein
G2583_2688	ECs3037	Z3400	yeiS	hypothetical protein
G2583_2689	ECs3038	Z3401	yeiT	Uncharacterized oxidoreductase yeiT
G2583_2690	ECs3039	Z3402	yeiA	putative oxidoreductase
G2583_2691	ECs3040	Z3403	mglC	Galactose ABC transporter, permease protein
G2583_2692	ECs3041	Z3404	mglA	Galactose/methyl galactoside import ATP-binding protein mglA
G2583_2693	ECs3042	Z3405	mglB	Galactose ABC transporter, periplasmic galactose-binding protein
G2583_2694	ECs3043	Z3407	galS	DNA-binding transcriptional repressor
G2583_2695	ECs3044	Z3408	yeiB	hypothetical protein
G2583_2696	ECs3045	Z3409	folE	GTP cyclohydrolase 1
G2583_2697	ECs3046	Z3410	yeiG	S-formylglutathione hydrolase yeiG
G2583_2698	ECs3047	Z3411	cirA	Colicin I receptor

Locus_tag[a]				
CB9615	Sakai	EDL933	Gene	Product
G2583_2699	ECs3048	Z3413	lysP	Lysine-specific permease
G2583_2700	ECs3049	Z3414	yeiE	putative DNA-binding transcriptional regulator
G2583_2701	ECs3050	Z3415	yeiH	UPF0324 inner membrane protein yeiH
G2583_2702	ECs3051	Z3416	nfo	Endonuclease 4
G2583_2703	ECs3052	Z3417	yeiI	Kinase, pfkB family
G2583_2704	ECs3053	Z3418	nupX	Nucleoside permease
G2583_2705	ECs3054	Z3419	rihB	Pyrimidine-specific ribonucleoside hydrolase rihB
G2583_2706	ECs3055	Z3420	yeiL	DNA-binding transcriptional activator of stationary phase nitrogen survival
G2583_2707	ECs3056	Z3421	yeiM	Nucleoside transporter, NupC family
G2583_2708	ECs3057	Z3422	yeiN	Indigoidine synthase A like protein
G2583_2709	ECs3058	Z3423	yeiC	Kinase, pfkB family protein
G2583_2710	ECs3059	Z3425	fruA	fructose-specific PTS system IIBC component
G2583_2711	ECs3060	Z3426	fruK	1-phosphofructokinase
G2583_2712	ECs3061	Z3427	fruB	Pts system mannitol-specific eiicba component
G2583_2713	ECs3062	Z3428	setB	Sugar efflux transporter B
G2583_2714	ECs3063	Z3430	yeiP	Elongation factor P family protein
G2583_2715	ECs3064	Z3431	yeiQ	Mannitol dehydrogenase family protein
G2583_2716	ECs3065	Z3432	yeiR	CobW/P47K family protein
G2583_2717	ECs3066	Z3433	yeiU	hypothetical protein
G2583_2718	ECs3067	Z3434	spr	Lipoprotein spr precursor
G2583_2719	ECs3068	Z3435	rtn	Putative cyclic diguanylate phosphodiesterase
G2583_2720	ECs3069	Z3436	yejA	ABC transporter, periplasmic solute-binding protein
G2583_2721	ECs3070	Z3437	yejB	Inner membrane ABC transporter permease protein
G2583_2722	ECs3071	Z3438	yejE	ABC transporter, permease protein
G2583_2723	ECs3072	Z3439	yejF	ABC transporter, ATP-binding protein
G2583_2724	ECs3073	Z3440	yejG	hypothetical protein
G2583_2725	ECs3074	Z3441	bcr	Bicyclomycin resistance protein
G2583_2726	ECs3075	Z3442	rsuA	Pseudouridine synthase
G2583_2727	ECs3076	Z3443	yejH	Putative helicase
G2583_2728	ECs3077	Z3444	rplY	50S ribosomal protein L25
G2583_2729	ECs3078	Z3445	yejK	Nucleoid-associated protein ndpA
G2583_2730	ECs3079	Z3446	yejL	UPF0352 protein yejL
G2583_2731	ECs3080	Z3447	yejM	Inner membrane protein yejM
G2583_2732	proL	RNA058	proL	Pro tRNA
G2583_2733	ECs3081	Z3449	yejO	Putative autotransporter, IS5K-containing
G2583_2734	ECs3082	Z3450	narP	Nitrate/nitrite response regulator NarP
G2583_2735	ECs3083	Z3451	ccmH	Cytochrome c-type biogenesis family protein
G2583_2736	ECs3084	Z3452	ccmG	Thiol:disulfide interchange protein dsbE
G2583_2737	ECs3085	Z3453	ccmF	Cytochrome c-type biogenesis protein CcmF
G2583_2738	ECs3086	Z3454	ccmE	Cytochrome c-type biogenesis protein ccmE
G2583_2739	ECs3087	Z3455	ccmD	heme exporter protein D
G2583_2740	ECs3088	Z3456	ccmC	Heme exporter protein C
G2583_2741	ECs3089	Z3457	ccmB	Heme exporter protein B

Locus_tag[a]				
CB9615	Sakai	EDL933	Gene	Product
G2583_2742	ECs3090	Z3458	ccmA	Cytochrome c biogenesis ATP-binding export protein ccmA
G2583_2743	ECs3091	Z3459	napC	Cytochrome c-type protein napC
G2583_2744	ECs3092	Z3460	napB	Cytochrome c-type protein
G2583_2745	ECs3093	Z3461	napH	Polyferredoxin
G2583_2746	ECs3094	Z3462	napG	Ferredoxin-type protein napG precursor
G2583_2747	ECs3095	Z3463	napA	Periplasmic nitrate reductase precursor
G2583_2748	ECs3096	Z3464	napD	Periplasmic nitrate reductase
G2583_2749	ECs3097	Z3465	napF	Ferredoxin-type protein napF
G2583_2750	ECs3098	Z3467	eco	Ecotin precursor
G2583_2751	ECs3099	Z3468	mqo	Malate:quinone oxidoreductase
G2583_2752	ECs3100	Z3469	yojI	ABC transporter ATP-binding protein
G2583_2753	ECs3101	Z3470	alkB	alkylated DNA repair protein
G2583_2754	ECs3102	Z3471	ada	O6-methylguanine-DNA methyltransferase; transcription activator/repressor
G2583_2755	ECs3103	Z3472	apbE	Thiamine biosynthesis lipoprotein apbE precursor
G2583_2756	ECs3104	Z3473	ompC	Outer membrane protein C precursor
G2583_2757	micF		micF	ncRNA
G2583_2758	ECs3105	Z3475	rcsD	Sensor histidine kinase YojN
G2583_2759	ECs3106	Z3476	rcsB	Capsular synthesis regulator component B
G2583_2760	ECs3107	Z3477	rcsC	Sensor histidine kinase/response regulator RcsC
G2583_2761	ECs3108	Z3478	yfaP	hypothetical protein
G2583_2762	ECs3109	Z3479	yfaQ	hypothetical protein
G2583_2762	ECs3110	Z3480	yfaQ	hypothetical protein
G2583_2763	ECs3111	Z3481	yfaSR	alpha-2-macroglobulin family protein
G2583_2764	Indel-150	Indel-150	-	hypothetical protein
G2583_2765	Indel-150	Indel-150	ISSfl2	ISSfl2 ORF
G2583_2766	ECs3111	Z3481	yfaS	alpha-2-macroglobulin family protein
G2583_2767	ECs3112	Z3482	yfaT	hypothetical protein
G2583_2768	ECs3113	Z3483	yfaA	hypothetical protein
G2583_2769	ECs3114	Z3484	gyrA	DNA gyrase, A subunit
G2583_2770	ECs3115	Z3486	ubiG	3-demethylubiquinone-9 3-methyltransferase
G2583_2771	ECs3116	Z3487	yfaL	Putative ATP-binding component of a transport system
G2583_2772	ECs3117	Z3489	nrdA	Ribonucleoside-diphosphate reductase, alpha subunit
G2583_2773	ECs3118	Z3491	nrdB	Ribonucleoside-diphosphate reductase 1, beta subunit,
G2583_2774	ECs3119	Z3492	yfaE	Ferredoxin
G2583_2775	ECs3120	Z3493	inaA	pH-inducible protein involved in stress response
G2583_2776	ECs3121	Z3494	-	transporter, major facilitator family
G2583_2777	ECs3122	Z3495	yfaH	putative regulator
G2583_2778	ECs3123	Z3496	-	hypothetical protein
G2583_2779	ECs3124	Z3497	glpQ	Glycerophosphoryl diester phosphodiesterase
G2583_2780	ECs3125	Z3498	glpT	Glycerol-3-phosphate transporter
G2583_2781	ECs3126	Z3499	glpA	Anaerobic glycerol-3-phosphate dehydrogenase subunit
G2583_2782	ECs3127	Z3500	glpB	Anaerobic glycerol-3-phosphate dehydrogenase subunit

Locus_tag[a]				
CB9615	Sakai	EDL933	Gene	Product
G2583_2783	ECs3128	Z3501	glpC	Anaerobic glycerol-3-phosphate dehydrogenase subunit
G2583_2784	ECs3129	Z3502	yfaD	hypothetical protein
G2583_2785	ECs3130	Z3503	yfaU	putative aldolase
G2583_2786	ECs3131	Z3504	yfaV	Putative transport protein
Indel-151	ECs3132	Indel-151	-	putative transposase OrfA protein of insertion sequence IS629
Indel-151	ECs3133	Indel-151	-	putative transposase OrfB protein of insertion sequence IS629
G2583_2786	ECs3134	Z3504	yfaV	Putative transport protein
G2583_2787	ECs3135	Z3505	yfaW	Putative racemase
G2583_2788	ECs3136	Z3506	yfaX	putative regulator
G2583_2789	ECs3137	Z3507	yfaY	CinA family protein
G2583_2790	ECs3138	Z3508	yfaZ	hypothetical protein
G2583_2791	ECs3139	Z3509	nudI	Nucleoside triphosphatase nudI
G2583_2792	ECs3140	Z3510	ais	protein induced by aluminum
G2583_2793	ECs3141	Z3511	arnB	UDP-4-amino-4-deoxy-L-arabinose--oxoglutarate aminotransferase
G2583_2794	ECs3142	Z3512	arnC	Undecaprenyl-phosphate 4-deoxy-4-formamido-L-arabinose transferase
G2583_2795	ECs3143	Z3513	arnA	Bifunctional polymyxin resistance protein arnA [Includes: UDP-4-amino- 4-deoxy-L-arabinose
G2583_2796	ECs3144	Z3514	yfbH	hypothetical protein
G2583_2797	ECs3145	Z3515	arnT	Undecaprenyl phosphate-alpha-4-amino-4-deoxy-L-arabinose arabinosyl transferase
G2583_2798		Z3516	yfbW	Sucrose-6 phosphate hydrolase
G2583_2799	ECs3146	Z3517	yfbJ	putative transport/receptor protein
G2583_2800	ECs3147	Z3518	pmrD	Polymyxin resistance protein B
G2583_2801	ECs3148	Z3520	menE	O-succinylbenzoate-CoA ligase
G2583_2802	ECs3149	Z3521	menC	o-succinylbenzoate synthase
G2583_2803	ECs3150	Z3522	menB	Naphthoate synthase
G2583_2804	ECs3151	Z3523	yfbB	2-succinyl-6-hydroxy-2,4-cyclohexadiene-1-carboxylate synthase
G2583_2805	ECs3152	Z3524	menD	2-succinyl-5-enolpyruvyl-6-hydroxy-3-cyclohexene-1-carboxylate synthase
G2583_2806	ECs3153	Z3525	menF	Isochorismate synthase, menaquinone-specific
G2583_2807	ECs3154	Z3526	elaB	regulatory protein AmpE
G2583_2808	ECs3155	Z3527	elaA	Acetyltransferase, GNAT family
G2583_2809	ECs3156	Z3528	rbn	ribonuclease Z
G2583_2810	ECs3157	Z3529	elaD	putative sulfatase / phosphatase
G2583_2811	ECs3158	Z3531	yfbL	Peptidase, M28 family
G2583_2812	ECs3159	Z3533	yfbM	hypothetical protein
G2583_2813	ECs3160	Z3534	nuoN	NADH-quinone oxidoreductase subunit N
G2583_2814	ECs3161	Z3536	nuoM	NADH-quinone oxidoreductase subunit M
G2583_2815	ECs3162	Z3537	nuoL	NADH-quinone oxidoreductase, L subunit

Locus_tag[a]				
CB9615	Sakai	EDL933	Gene	Product
G2583_2816	ECs3163	Z3538	nuoK	NADH-quinone oxidoreductase subunit K
G2583_2817	ECs3164	Z3539	nuoJ	NADH-quinone oxidoreductase subunit J
G2583_2818	ECs3165	Z3540	nuoI	NADH-quinone oxidoreductase subunit I
G2583_2819	ECs3166	Z3541	nuoH	NADH-quinone oxidoreductase subunit H
G2583_2820	ECs3167	Z3542	nuoG	NADH-quinone oxidoreductase
G2583_2821	ECs3168	Z3543	nuoF	NADH-quinone oxidoreductase, F subunit
G2583_2822	ECs3169	Z3544	nuoE	NADH-quinone oxidoreductase, E subunit
G2583_2823	ECs3170	Z3545	nuoC	NADH-quinone oxidoreductase, C/D subunit
G2583_2824	ECs3171	Z3546	nuoB	NADH-quinone oxidoreductase subunit B
G2583_2825	ECs3172	Z3547	nuoA	NADH-quinone oxidoreductase subunit A
G2583_2826	ECs3173	Z3549	lrhA	Probable HTH-type transcriptional regulator lrhA
G2583_2827	ECs3174	Z3551	yfbQ	Uncharacterized aminotransferase yfbQ
G2583_2828	ECs3175	Z3552	yfbR	5'-nucleotidase yfbR
G2583_2829	ECs3176	Z3553	yfbS	Putative transport protein
G2583_2830	ECs3177	Z3554	yfbT	Sugar-phosphatase, YfbT
G2583_2831	ECs3178	Z3555	yfbU	UPF0304 protein yfbU
G2583_2832	ECs3179	Z3556	yfbV	UPF0208 membrane protein yfbV
G2583_2833	ECs3180	Z3558	ackA	Acetate kinase
G2583_2834	ECs3181	Z3559	pta	Phosphate acetyltransferase
G2583_2835	ECs3182	Z3560	yfcC	C4-dicarboxylate anaerobic carrier protein
G2583_2836	ECs3183	Z3561	yfcD	Uncharacterized Nudix hydrolase yfcD
G2583_2837	ECs3184	Z3562	yfcE	Phosphodiesterase yfcE
G2583_2838	ECs3185	Z3563	yfcF	Glutathione S-transferase domain protein
G2583_2839	ECs3186	Z3564	yfcG	Glutathione S-transferase
G2583_2840	ECs3187	Z3565	folX	D-erythro-7,8-dihydroneopterin triphosphate epimerase
G2583_2841	ECs3188	Z3566	yfcH	NAD-binding domain 4 protein
G2583_2842	ECs3189	Z3567	yfcI	hypothetical protein
G2583_2843	ECs3190	Z3568	hisP	ATP-binding component of histidine transport
G2583_2844	ECs3191	Z3569	hisM	Histidine transport system permease protein hisM
G2583_2845	ECs3192	Z3570	hisQ	ABC-type arginine transport system, permease component
G2583_2846	ECs3193	Z3571	hisJ	Histidine-binding periplasmic protein precursor
G2583_2847	ECs3194	Z3572	argT	Lysine-arginine-ornithine-binding periplasmic protein precursor
G2583_2848	ECs3195	Z3573	ubiX	3-octaprenyl-4-hydroxybenzoate carboxy-lyase
G2583_2849	ECs3196	Z3574	purF	Amidophosphoribosyltransferase
G2583_2850	ECs3197	Z3575	cvpA	CvpA protein
G2583_2851	ECs3198	Z3576	dedD	Sporulation and cell division repeat protein
G2583_2852	ECs3199	Z3577	folC	Tetrahydrofolate synthase/dihydrofolate synthase
G2583_2853	ECs3200	Z3578	accD	Acetyl-coenzyme A carboxylase carboxyl transferase subunit beta
G2583_2854	ECs3201	Z3579	dedA	hypothetical protein
G2583_2855	ECs3202	Z3580	truA	tRNA pseudouridine synthase A
G2583_2856	ECs3203	Z3581	usg	USG-1 protein

Locus_tag[a]				
CB9615	Sakai	EDL933	Gene	Product
G2583_2857	ECs3204	Z3582	pdxB	Erythronate-4-phosphate dehydrogenase
G2583_2858	ECs3205	Z3583	flk	Cell division protein
G2583_2859	ECs3206	Z3585	yfcJ	Permeases of the major facilitator superfamily
G2583_2860	ECs3207	Z3586	fabB	3-oxoacyl-[acyl-carrier-protein] synthase 1
G2583_2861	ECs3208	Z3587	mnmC	UPF0209 protein yfcK
G2583_2862	ECs3209	Z3588	yfcL	hypothetical protein
G2583_2863	ECs3210	Z3589	yfcM	putative transporting ATPase
G2583_2864	ECs3211	Z3590	yfcA	Inner membrane protein yfcA
G2583_2865	ECs3212	Z3591	mepA	Penicillin-insensitive murein endopeptidase precursor
G2583_2866	ECs3213	Z3592	aroC	Chorismate synthase
G2583_2867	ECs3215	Z3593	prmB	Putative adenine-specific methylase
G2583_2868	ECs3214	Z3594	yfcN	UPF0115 protein yfcN
G2583_2869	ECs3216	Z3595	yfcO	hypothetical protein
G2583_2870	ECs3217	Z3596	yfcP	Fimbrial protein
G2583_2871	ECs3218	Z3597	yfcQ	Fimbrial subunit
G2583_2872	ECs3219	Z3598	yfcR	Putative minor fimbrial subunit
G2583_2873	ECs3220	Z3599	yfcS	Chaperone protein PapD
G2583_2874	ECs3221	Z3600	yfcU	Fimbrial usher family protein
G2583_2875	ECs3222	Z3601	yfcV	Fimbrial protein
G2583_2876	ECs3223	Z3603	sixA	Phosphohistidine phosphatase SixA
G2583_2877	ECs3224	Z3604	fadJ	Fatty oxidation complex, alpha subunit FadJ
G2583_2878	ECs3225	Z3605	fadI	3-ketoacyl-CoA thiolase
G2583_2879	ECs3226	Z3606	yfcZ	hypothetical protein
G2583_2880	ECs3227	Z3608	fadL	Transport of long-chain fatty acids
G2583_2881	ECs3228	Z3609	yfdF	hypothetical protein
G2583_2882	ECs3229	Z3610	vacJ	Lipoprotein, VacJ family
G2583_2883	ECs3230	Z3611	yfdC	putative transport
G2583_2884	argW	RNA059	argW	Arg tRNA
G2583_2885	ECs3231	Z3613	intS	putative prophage integrase
G2583_2886	ECs3232	Z3614	-	Putative prophage DNA injection protein
G2583_2887	ECs3233	Z3615	-	Phage DNA transfer protein
G2583_2888	ECs3234	Z3616	-	hypothetical protein
G2583_2889	ECs3235	Z3617	-	Gene 9 protein
G2583_2890	ECs3236	Z3618	-	hypothetical protein
	ECs3237		-	hypothetical protein
		Z3619	-	hypothetical protein
G2583_2891	ECs3238	Z3620	-	hypothetical protein
G2583_2892	ECs3239	Z3621	-	hypothetical protein
G2583_2893	ECs3240	Z3622	-	Resolvase domain protein
G2583_2894	ECs3241	Z3623	lacY	Oligosaccharide:H+ symporter
G2583_2895	ECs3242	Z3624	cscK	Fructokinase
G2583_2896	ECs3243	Z3625	-	Sucrose-6-phosphate hydrolase
G2583_2897	ECs3244	Z3626	-	Sugar binding transcriptional regulator, LacI family
G2583_2898		Z3627	dsdX	Putative uncharacterized dsdX-like protein

Locus_tag[a]				
CB9615	Sakai	EDL933	Gene	Product
G2583_2899	ECs3245	Z3628	dsdA	D-serine ammonia-lyase
G2583_2900	ECs3246	Z3629	emrY	Multidrug resistance protein Y
G2583_2901	ECs3247	Z3630	emrK	Drug resistance MFS transporter, membrane fusion protein (MFP) subunit EmrK
G2583_2902	ECs3248	Z3631	evgA	Positive transcription regulator evgA
G2583_2903	ECs3249	Z3632	evgS	hybrid sensory histidine kinase in two-component regulatory system with EvgA
G2583_2904	ECs3250	Z3633	yfdE	hypothetical protein
G2583_2904	ECs3251	Z3634	yfdE	hypothetical protein
G2583_2905	ECs3252	Z3635	yfdV	Uncharacterized transporter yfdV
G2583_2906	ECs3253	Z3637	oxc	Thiamine pyrophosphate-dependent enzyme
G2583_2907	ECs3254	Z3639	frc	Formyl-coenzyme A transferase
G2583_2908	ECs3255	Z3640	yfdX	Protein yfdX precursor
G2583_2909	ECs3256	Z3641	ypdI	hypothetical protein
G2583_2910	ECs3257	Z3642	yfdY	hypothetical protein
G2583_2911	ECs3258	Z3643	lpxP	hypothetical protein
G2583_2912	ECs3259	Z3644	yfdZ	Aminotransferase, classes I and II
G2583_2913	ECs3260	Z3645	ypdA	Inner membrane protein ypdA
G2583_2914	ECs3261	Z3646	ypdB	Uncharacterized response regulatory protein ypdB
G2583_2915	ECs3262	Z3647	ypdC	helix-turn-helix- domain containing protein AraC type
G2583_2916	ECs3263	Z3648	fryA	Putative phosphoenolpyruvate-protein
G2583_2917	ECs3264	Z3649	ypdE	Aminopeptidase
G2583_2918	ECs3265	Z3651	ypdF	Aminopeptidase YpdF
G2583_2919	ECs3266	Z3652	fryC	Fructose-like permease IIC component
G2583_2920	ECs3267	Z3653	fryB	Fructose-like phosphotransferase enzyme IIB component 1
G2583_2921	ECs3268	Z3654	glk	Glucokinase
G2583_2922	ECs3269	Z3655	yfeO	Putative ion-transport protein yfeO
G2583_2923	ECs3270	Z3656	ypeC	conserved hypothetical protein
G2583_2924		Z3657	-	hypothetical protein
G2583_2925	ECs3271	Z3658	mntH	Manganese transport protein mntH
G2583_2926	ECs3272	Z3659	nupC	Nucleoside transporter NupC
G2583_2927	ECs3273	Z3660	yfeA	hypothetical protein
G2583_2928	alaX	RNA060	alaX	Ala tRNA
G2583_2929	alaW	Indel-153	alaW	Ala tRNA
G2583_2930	ECs3274	Z3662	yfeC	hypothetical protein
G2583_2931	ECs3275	Z3663	yfeD	hypothetical protein
G2583_2932	ECs3276	Z3664	ydcM	IS605 family transposase orfB
G2583_2933	ECs3277		-	Putative transposase TnA
G2583_2934	ECs3278	Z3665	gltX	Glutamyl-tRNA synthetase
G2583_2935	valU	RNA061	valU	Val tRNA
G2583_2936	valX	RNA062	valX	Val tRNA
G2583_2937	valY	RNA063	valY	Val tRNA
G2583_2938	lysV	RNA064	lysV	Lys tRNA

Locus_tag[a]				
CB9615	Sakai	EDL933	Gene	Product
G2583_2939	ECs3279	Z3672	flxA	hypothetical protein
G2583_2940	ECs3280	Z3673	yfeR	transcriptional regulator, LysR family protein
G2583_2940	ECs3281	Z3674	yfeR	transcriptional regulator, LysR family protein
G2583_2941	ECs3282	Z3675	yfeH	putative cytochrome oxidase
G2583_2942		Z3676	ypeB	hypothetical protein
G2583_2943	ECs3283	Z3677	ligA	DNA ligase
G2583_2944	ECs3284	Z3678	zipA	Cell division protein ZipA
G2583_2945	ECs3285	Z3679	cysZ	putative sulfate transport protein CysZ
G2583_2946	ECs3286	Z3680	cysK	Cysteine synthase A
G2583_2947	ECs3287	Z3681	ptsH	PTS system, phosphocarrier protein
G2583_2948	ECs3288	Z3682	ptsI	Phosphoenolpyruvate-protein phosphotransferase
G2583_2949	ECs3289	Z3683	crr	Glucose-specific phosphotransferase enzyme IIA component
G2583_2950	ECs3290	Z3684	pdxK	pyridoxal kinase
G2583_2951	ECs3291	Z3685	yfeK	hypothetical protein
G2583_2952	ECs3292	Z3686	cysM	Cysteine synthase
G2583_2953	ECs3293	Z3687	cysA	Sulfate/thiosulfate import ATP-binding protein cysA
G2583_2954	ECs3294	Z3688	cysW	Sulfate ABC transporter, permease protein CysW
G2583_2955	ECs3295	Z3689	cysU	Sulfate ABC transporter, permease protein CysT
G2583_2956	ECs3296	Z3690	cysP	Sulfate/thiosulfate ABC transporter, periplasmic sulfate/thiosulfate- binding protein
G2583_2957	ECs3297	Z3691	ucpA	Oxidoreductase ucpA
G2583_2958	ECs3298	Z3692	yfeT	hypothetical protein
G2583_2959	ECs3299	Z3693	murQ	Predicted sugar phosphate isomerase
G2583_2960	ECs3300	Z3694	murP	Phosphoenolpyruvate-dependent sugar phosphotransferase system EIIABC, probable beta-glucoside-specific
G2583_2961	ECs3301	Z3695	yfeW	Putative hydrolase/beta lactamase fusion protein
G2583_2962	ECs3302	Z3696	yfeX	hypothetical protein
G2583_2963	ECs3303	Z3697	yfeY	hypothetical protein
G2583_2964	ECs3304	Z3698	yfeZ	hypothetical protein
G2583_2965	ECs3305	Z3699	ypeA	Acetyltransferase ypeA
G2583_2966	ECs3306	Z3700	amiA	N-acetylmuramoyl-L-alanine amidase
G2583_2967	ECs3307	Z3701	hemF	Coproporphyrinogen III oxidase, aerobic
G2583_2968	ECs3308	Z3702	eutR	AraC-type DNA-binding domain-containing protei ns
G2583_2969	ECs3309	Z3703	eutK	Ethanolamine utilization protein EutK
G2583_2970	ECs3310	Z3704	eutL	Putative ethanolamine utilization protein EutL
G2583_2971	ECs3311	Z3705	eutC	Ethanolamine ammonia-lyase light chain
G2583_2972	ECs3312	Z3706	eutB	Ethanolamine ammonia-lyase heavy chain
G2583_2973	ECs3313	Z3707	eutA	Ethanolamine utilization protein EutA
G2583_2974	ECs3314	Z3708	eutH	EutH
G2583_2975	ECs3315	Z3709	eutG	EutG
G2583_2976	ECs3316	Z3710	eutJ	Ethanolamine utilization protein EutJ
G2583_2977	ECs3317	Z3711	eutE	Ethanolamine utilization

Locus_tag[a]				
CB9615	Sakai	EDL933	Gene	Product
G2583_2978	ECs3318	Z3712	eutN	Ethanolamine utilization protein
G2583_2979	ECs3319	Z3713	eutM	Detox protein
G2583_2980	ECs3320	Z3714	eutD	Ethanolamine utilization protein EutD
G2583_2981	ECs3321	Z3715	eutT	Ethanolamine utilization cobalamin adenosyltransferase
G2583_2982	ECs3322	Z3716	eutQ	Ethanolamine utilization protein EutQ
G2583_2983	ECs3323	Z3717	eutP	hypothetical protein
G2583_2984	ECs3324	Z3718	eutS	hypothetical protein
G2583_2985	ECs3325	Z3719	maeB	Malate dehydrogenase (Oxaloacetate-decarboxylating) (NADP(+))
G2583_2986	ECs3326	Z3720	talA	Transaldolase A
G2583_2987	ECs3327	Z3721	tktB	Transketolase 2
G2583_2988	ECs3328	Z3722	ypfG	hypothetical protein
G2583_2989	ECs3329	Z3723	nudK	GDP-mannose pyrophosphatase nudK
G2583_2990	ECs3330	Z3724	aegA	putative oxidoreductase Fe-S binding subunit
G2583_2991	ECs3331	Z3726	narQ	Nitrate/nitrite sensor histidine kinase NarQ
G2583_2992	ECs3332	Z3727	acrD	Probable aminoglycoside efflux pump
G2583_2993	ECs3333	Z3729	yffB	ArsC family protein
G2583_2994	ECs3334	Z3730	dapE	Succinyl-diaminopimelate desuccinylase
G2583_2995		Z3731	ypfN	UPF0370 protein ypfN
G2583_2996	ECs3335	Z3732	ypfH	Esterase, AB hydrolase 2 family
G2583_2997	ECs3336	Z3733	ypfI	hypothetical protein
G2583_2998	ECs3337	Z3734	ypfJ	hypothetical protein
G2583_2999	ECs3338	Z3735	purC	Phosphoribosylaminoimidazole-succinocarboxamide synthase
G2583_3000	ECs3339	Z3736	nlpB	Lipoprotein-34
G2583_3001	ECs3340	Z3737	dapA	Dihydrodipicolinate synthase
G2583_3002	ECs3341	Z3738	gcvR	Glycine cleavage system transcriptional repressor
G2583_3003	ECs3342	Z3739	bcp	Putative peroxiredoxin bcp
G2583_3004	ECs3343	Z3741	hyfA	Hydrogenase 4 Fe-S subunit
G2583_3005	ECs3344	Z3742	hyfB	Hydrogenase 4 membrane subunit
G2583_3006	ECs3345	Z3743	hyfC	Hydrogenase 4 membrane subunit
G2583_3007	ECs3346	Z3744	hyfD	Hydrogenase-4 component D
G2583_3008	ECs3347	Z3745	hyfE	Hydrogenase-4 component E
G2583_3009	ECs3348	Z3746	hyfF	Hydrogenase 4 membrane subunit
G2583_3010	ECs3349	Z3747	hyfG	Hydrogenase-4, G subunit
G2583_3011	ECs3350	Z3748	hyfH	Iron-sulfur cluster-binding protein
G2583_3012	ECs3351	Z3749	hyfI	Hydrogenase-4, I subunit
G2583_3013	ECs3352	Z3750	hyfJ	putative protein processing element
G2583_3014	ECs3353	Z3751	hyfR	Hydrogenase-4 transcriptional regulator
G2583_3015	ECs3354	Z3753	focB	Formate/nitrite transporter
G2583_3016	ECs3355	Z3755	yfgO	Putative permease perM
G2583_3017	ECs3356	Z3757	yfgC	Peptidase, M48 family
G2583_3018	ECs3357	Z3758	yfgD	Arsenate reductase
G2583_3019	ECs3358	Z3759	hda	DnaA-homolog protein hda

Locus_tag[a]				
CB9615	Sakai	EDL933	Gene	Product
G2583_3020	ECs3359	Z3760	uraA	Uracil permease
G2583_3021	ECs3360	Z3761	upp	Uracil phosphoribosyltransferase
G2583_3022	ECs3361	Z3762	purM	Phosphoribosylformylglycinamidine cyclo-ligase
G2583_3023	ECs3362	Z3763	purN	Phosphoribosylglycinamide formyltransferase 1
G2583_3024	ECs3363	Z3764	ppk	Polyphosphate kinase
G2583_3025	ECs3364	Z3765	ppx	exopolyphosphatase
G2583_3026	ECs3365	Z3766	yfgF	Putative cytochrome C-type biogenesis protein
G2583_3027		Z3767	-	hypothetical protein
G2583_3028	ECs3366	Z3768	yfgG	hypothetical protein
G2583_3029	ECs3367	Z3769	yfgH	Uncharacterized lipoprotein yfgH precursor
G2583_3030	ECs3368	Z3770	yfgI	hypothetical protein
G2583_3031	ECs3369	Z3771	guaA	GMP synthase [glutamine-hydrolyzing]
G2583_3032	ECs3370	Z3772	guaB	Inosine-5'-monophosphate dehydrogenase
G2583_3033	ECs3371	Z3773	xseA	Exodeoxyribonuclease 7 large subunit
G2583_3034	ECs3372		yfgJ	hypothetical protein
G2583_3035	ECs3373	Z3774	der	GTP-binding protein engA
G2583_3036	ECs3374	Z3775	yfgL	Outer membrane assembly lipoprotein YfgL
G2583_3037	ECs3375	Z3776	yfgM	UPF0070 protein yfgM
G2583_3038	ECs3376	Z3777	hisS	Histidyl-tRNA synthetase
G2583_3039			-	ncRNA
G2583_3040	ECs3377	Z3778	ispG	4-hydroxy-3-methylbut-2-en-1-yl diphosphate synthase
G2583_3041	ECs3378	Z3779	yfgA	Helix-turn-helix DNA-binding domain protein
G2583_3042	ECs3379	Z3780	yfgB	Radical SAM enzyme, Cfr family
G2583_3043	ECs3380	Z3781	ndk	Nucleoside diphosphate kinase
G2583_3044	ECs3381	Z3782	-	Putative polyferredoxin
G2583_3045	ECs3382	Z3783	ynfH	Putative dimethyl sulfoxide reductase subunit C
G2583_3046	ECs3383	Z3784	-	Dimethylsulfoxide reductase, chain B
G2583_3047	ECs3384	Z3785	dmsA	putative anaerobic dimethyl sulfoxide reductase chain A precursor
G2583_3048	ECs3385	Z3786	pbpC	Penicillin-binding protein 1C
G2583_3049	ECs3386	Z3787	yfhM	Alpha-2-macroglobulin domain protein
G2583_3050	ECs3387	Z3788	sseA	Putative thiosulfate sulfurtransferase
G2583_3051			-	ncRNA
G2583_3052	ECs3388	Z3789	sseB	Enhanced serine sensitivity
G2583_3053	ECs3389	Z3790	pepB	Peptidase B
G2583_3054	ECs3390	Z3791	iscX	hypothetical protein
G2583_3055	ECs3391	Z3792	fdx	2Fe-2S ferredoxin
G2583_3056	ECs3392	Z3793	hscA	Chaperone protein hscA
G2583_3057	ECs3393	Z3794	hscB	Fe-S protein assembly co-chaperone HscB
G2583_3058	ECs3394	Z3795	iscA	Iron-binding protein iscA
G2583_3059	ECs3395	Z3796	iscU	NifU-like protein
G2583_3060	ECs3396	Z3797	iscS	Cysteine desulfurase
G2583_3061	ECs3397	Z3798	iscR	DNA-binding transcriptional repressor
G2583_3062	ECs3398	Z3799	trmJ	tRNA (cytidine/uridine-2'-O-)-methyltransferase trmJ

Locus_tag[a]				
CB9615	Sakai	EDL933	Gene	Product
G2583_3063	ECs3399		suhB	Inositol-1-monophosphatase
G2583_3064	ECs3400	Z3802	yfhR	putative enzyme (3.4.-)
G2583_3065	ECs3401	Z3804	csiE	Transcriptional antiterminator
G2583_3066	ECs3402	Z3807	hcaT	3-phenylpropionic acid transporter
G2583_3067	ECs3403	Z3808	hcaR	Hca operon transcriptional activator
G2583_3068	ECs3404	Z3809	hcaE	3-phenylpropionate/cinnamic acid dioxygenase subunit alpha
G2583_3069	ECs3405	Z3810	hcaF	3-phenylpropionate/cinnamic acid dioxygenase subunit beta
G2583_3070	ECs3406	Z3811	hcaC	3-phenylpropionate/cinnamic acid dioxygenase ferredoxin subunit
G2583_3071	ECs3407	Z3813	hcaB	3-phenylpropionate-dihydrodiol/cinnamic acid-dihydrodiol dehydrogenase
G2583_3072	ECs3408	Z3814	hcaD	3-phenylpropionate/cinnamic acid dioxygenase ferredoxin--NAD(+) reductase component
G2583_3073	ECs3409	Z3815	yphA	Predicted inner membrane protein
G2583_3074	ECs3410	Z3816	yphB	Aldose 1-epimerase family protein
G2583_3075	ECs3411	Z3817	yphC	Hypothetical zinc-type alcohol dehydrogenase-like protein yphC
G2583_3075		Z3818	yphC	Hypothetical zinc-type alcohol dehydrogenase-like protein yphC
G2583_3076	ECs3412	Z3819	yphD	Putative transport system permease protein
G2583_3077	ECs3413	Z3821	yphE	Putative sugar ABC transporter, ATP-binding protein
G2583_3078	ECs3414	Z3823	yphF	Periplasmic binding protein/LacI transcriptional regulator precursor
G2583_3079	ECs3415	Z3825	yphG	hypothetical protein
G2583_3080	ECs3416	Z3826	yphH	ROK family protein
G2583_3081	ECs3417	Z3827	glyA	Serine hydroxymethyltransferase
G2583_3082	ECs3418	Z3828	hmp	Flavohemoprotein
G2583_3083	ECs3419	Z3829	glnB	Nitrogen regulatory protein P-II 1
G2583_3084	ECs3420	Z3830	yfhA	putative 2-component transcriptional regulator
G2583_3085	ECs3421	Z3831	yfhG	hypothetical protein
G2583_3086	ECs3422	Z3833	yfhK	putative 2-component sensor protein
G2583_3087			-	ncRNA
G2583_3088	ECs3423	Z3835	purL	Phosphoribosylformylglycinamidine synthase
G2583_3089	ECs3424	Z3838	yfhD	Predicted soluble lytic transglycosylase fused to an ABC-type amino acid-binding protein
G2583_3090	ECs3425	Z3839	tadA	tRNA-specific adenosine deaminase
G2583_3091	ECs3426	Z3840	yfhB	HAD hydrolase YhfB
G2583_3092	ECs3427	Z3841	yfhH	hypothetical protein
G2583_3093	Indel-154	Indel-154	-	hypothetical protein
G2583_3094	Indel-154	Indel-154	pinE	DNA invertase from prophage CP-933H
G2583_3095	Indel-154	Indel-154	yfdL	putative tail fiber protein
G2583_3096	Indel-154	Indel-154	yfdK	tail fiber assembly protein

Locus_tag[a]				
CB9615	Sakai	EDL933	Gene	Product
G2583_3097	Indel-154	Indel-154	tfaE	hypothetical protein
G2583_3098	Indel-154	Indel-154	-	Phage-related tail fibre protein
G2583_3099	Indel-154	Indel-154	-	putative bacteriophage protein
G2583_3100	Indel-154	Indel-154	-	Uncharacterized homolog of phage Mu protein gp 47
G2583_3101	Indel-154	Indel-154	-	putative bacteriophage protein
G2583_3102	Indel-154	Indel-154	-	Phage P2 baseplate assembly protein gpV
G2583_3103	Indel-154	Indel-154	-	Putative secreted protein precursor
G2583_3104	Indel-154	Indel-154	-	hypothetical protein
G2583_3105	Indel-154	Indel-154	-	Putative bacteriophage protein
G2583_3106	Indel-154	Indel-154	-	putative bacteriophage protein
G2583_3107	Indel-154	Indel-154	-	Putative bacteriophage protein
G2583_3108	Indel-154	Indel-154	-	Putative bacteriophage protein
G2583_3109	Indel-154	Indel-154	-	Putative bacteriophage protein
G2583_3110	Indel-154	Indel-154	-	Hypothetical prophage protein
G2583_3111	Indel-154	Indel-154	-	Putative bacteriophage protein
G2583_3112	Indel-154	Indel-154	-	Putative bacteriophage protein
G2583_3113	Indel-154	Indel-154	-	Putative bacteriophage protein
G2583_3114	Indel-154	Indel-154	-	hypothetical protein
G2583_3115	Indel-154	Indel-154	-	Putative bacteriophage protein
G2583_3116	Indel-154	Indel-154	-	Putative bacteriophage protein
G2583_3117	Indel-154	Indel-154	-	Putative bacteriophage protein
G2583_3118	Indel-154	Indel-154	-	Uncharacterized protein conserved in bacteria
G2583_3119	Indel-154	Indel-154	-	Uncharacterized protein, homolog of phage Mu protein gp30
G2583_3120	Indel-154	Indel-154	-	Uncharacterized protein conserved in bacteria
G2583_3121	Indel-154	Indel-154	-	hypothetical protein
G2583_3122	Indel-154	Indel-154	-	Phage terminase, small subunit
G2583_3123	Indel-154	Indel-154	-	conserved hypothetical protein
G2583_3124	Indel-154	Indel-154	-	Lysozyme
G2583_3125	Indel-154	Indel-154	-	phage holin, lambda family
G2583_3126	Indel-154	Indel-154	-	hypothetical protein
G2583_3127	Indel-154	Indel-154	-	Transcriptional regulator
G2583_3128	Indel-154	Indel-154	-	Predicted P-loop ATPase and inactivated derivatives
G2583_3129	Indel-154	Indel-154	-	hypothetical protein
G2583_3130	Indel-154	Indel-154	-	hypothetical protein
G2583_3131	Indel-154	Indel-154	-	hypothetical protein
G2583_3132	Indel-154	Indel-154	-	Bbp38
G2583_3133	Indel-154	Indel-154	-	hypothetical protein
G2583_3134	Indel-154	Indel-154	-	hypothetical protein
G2583_3135	Indel-154	Indel-154	dpoL	DNA polymerase I - 3""-5"" exonuclease and polymerase domains
G2583_3136	Indel-154	Indel-154	-	hypothetical protein
G2583_3137	Indel-154	Indel-154	-	Phage associated protein
G2583_3138	Indel-154	Indel-154	-	hypothetical protein

Locus_tag[a]				
CB9615	Sakai	EDL933	Gene	Product
G2583_3139	Indel-154	Indel-154	-	Superfamily II DNA/RNA helicases, SNF2 family
G2583_3140	Indel-154	Indel-154	-	hypothetical protein
G2583_3141	Indel-154	Indel-154	-	Integrase
G2583_3142	ECs3428	Z3842	yfhL	hypothetical protein
G2583_3143		Z3843	-	hypothetical protein
G2583_3144	ECs3429	Z3844	acpS	Holo-[acyl-carrier-protein] synthase
G2583_3145	ECs3430	Z3845	pdxJ	Pyridoxine 5'-phosphate synthase
G2583_3146	ECs3431	Z3846	recO	DNA repair protein recO
G2583_3147	ECs3432	Z3847	era	GTP-binding protein era
G2583_3148	ECs3433	Z3848	rnc	Ribonuclease 3
G2583_3149		Z3849	-	hypothetical protein
G2583_3150	ECs3434	Z3850	lepB	Signal peptidase I
G2583_3151	ECs3435	Z3851	lepA	GTP-binding protein lepA
G2583_3152	ECs3436	Z3852	rseC	Sigma-E factor regulatory protein RseC
G2583_3153	ECs3437	Z3853	rseB	Sigma-E factor regulatory protein rseB precursor
G2583_3154	ECs3438	Z3854	rseA	Sigma-E factor negative regulatory protein
G2583_3155	ECs3439	Z3855	rpoE	DNA-directed RNA polymerase specialized sigma subunit, sigma24 homolog
G2583_3156	ECs3440	Z3856	nadB	L-aspartate oxidase
G2583_3157	ECs3441	Z3857	yfiC	Putative enzyme
G2583_3158	ECs3442	Z3859	srmB	ATP-dependent RNA helicase SrmB
G2583_3159	ECs3443	Z3860	yfiE	putative transcriptional regulator LYSR-type
G2583_3160	ECs3444	Z3861	eamB	Cysteine/O-acetylserine efflux protein
G2583_3161	ECs3445	Z3862	yfiD	Autonomous glycyl radical cofactor
G2583_3162	ECs3446	Z3864	ung	Uracil-DNA glycosylase
G2583_3163	ECs3447	Z3865	yfiF	rRNA methylases
G2583_3164		Z3866	-	hypothetical protein
G2583_3165	ECs3448	Z3867	trxC	Thioredoxin-2
G2583_3166	ECs3449	Z3868	yfiP	DTW domain protein
G2583_3167	ECs3450	Z3869	yfiQ	CoA binding domain/acetyltransferase domain protein
G2583_3167	ECs3451	Z3869	yfiQ	CoA binding domain/acetyltransferase domain protein
G2583_3168	ECs3452	Z3870	pssA	CDP-diacylglycerol--serine O-phosphatidyltransferase
G2583_3169	ECs3453	Z3871	yfiM	hypothetical protein
G2583_3170	ECs3454	Z3872	kgtP	Alpha-ketoglutarate permease
G2583_3171	rrfG	RNA065	rrf	5S ribosomal RNA
G2583_3172	rrlG	RNA066	rrl	23S ribosomal RNA
G2583_3173	gltW	RNA067	gltW	Glu tRNA
G2583_3174	rrsG	RNA068	rrs	16S ribosomal RNA
G2583_3175	ECs3455	Z3886	clpB	Heat shock protein
G2583_3176	ECs3456	Z3887	yfiH	hypothetical protein
G2583_3177	ECs3457	Z3888	rluD	Ribosomal large subunit pseudouridine synthase D
G2583_3178	ECs3458	Z3889	yfiO	predicted lipoprotein
G2583_3179	ECs3459		-	hypothetical protein
G2583_3180	ECs3460	Z3890	raiA	Ribosome-associated inhibitor A

Locus_tag[a]				
CB9615	Sakai	EDL933	Gene	Product
	ECs3461		-	leader peptide of chorismate mutase-P-prephenate dehydratase
G2583_3181	ECs3462	Z3891	pheA	P-protein [Includes: Chorismate mutase
G2583_3182	ECs3463	Z3892	tyrA	Chorismate mutase/prephenate dehydrogenase
G2583_3183	ECs3464	Z3893	aroF	Phospho-2-dehydro-3-deoxyheptonate aldolase, Tyr-sensitive
G2583_3184	ECs3465	Z3895	yfiL	hypothetical protein
G2583_3185	ECs3466	Z3897	yfiR	hypothetical protein
G2583_3186	ECs3467	Z3898	yfiN	GGDEF domain protein
G2583_3187	ECs3468	Z3899	yfiB	OmpA family protein
G2583_3188	ECs3469	Z3900	rplS	50S ribosomal protein L19
G2583_3189	ECs3470	Z3901	trmD	tRNA (guanine-N(1)-)-methyltransferase
G2583_3190	ECs3471	Z3902	rimM	16S rRNA-processing protein rimM
G2583_3191	ECs3472	Z3903	rpsP	30S ribosomal subunit protein S16
G2583_3192	ECs3473	Z3904	ffh	Signal recognition particle protein
G2583_3193	ECs3474	Z3905	ypjD	hypothetical protein
G2583_3194	ECs3475	Z3906	yfjD	CBS/transporter associated domain protein
G2583_3195	ECs3476	Z3907	grpE	heat shock protein GrpE
G2583_3196	ECs3477	Z3908	nadK	Probable inorganic polyphosphate/ATP-NAD kinase
G2583_3197	ECs3478	Z3909	recN	recombination and repair protein
G2583_3198	ECs3479	Z3910	smpA	Lipoprotein, SmpA/OmlA family
G2583_3199	ECs3480	Z3911	yfjF	hypothetical protein
G2583_3200	ECs3481	Z3912	yfjG	Polyketide cyclase/dehydrase family protein
G2583_3201	ECs3482	Z3913	smpB	SsrA-binding protein
G2583_3202	ssrA	RNA069	ssrA	tmRNA
G2583_3203	ECs3483	Z3916	-	DinI-like protein Z3916/ECs3483
	ECs3484		-	hypothetical protein
		Z3917	-	orf, hypothetical protein
G2583_3204	ECs3485	Z3918	IpgB	Putative chaperone protein
G2583_3205	ECs3486	Z3919	-	PotB, trcA, ORF2, ORF3, ORF4 genes,
Indel-155	ECs3487	Z3920	-	hypothetical protein
Indel-155	ECs3488	Z3921	-	hypothetical protein
Indel-156	ECs3489	Indel-156	-	hypothetical protein
Indel-156	ECs3490	Indel-156	-	putative transposase OrfA protein of insertion sequence IS629
Indel-156	ECs3491	Indel-156	-	putative transposase OrfB protein of insertion sequence IS629
Indel-156	ECs3492	Indel-156	-	hypothetical protein
Indel-156	ECs3493	Indel-156	-	hypothetical protein
Indel-156	ECs3494	Indel-156	-	hypothetical protein
Indel-156	ECs3495	Indel-156	-	putative endolysin
Indel-156	ECs3496	Indel-156	-	hypothetical protein
Indel-156	ECs3497	Indel-156	-	holin protein
G2583_3206	Indel-157	Indel-157	-	conserved hypothetical protein

Locus_tag[a]				
CB9615	Sakai	EDL933	Gene	Product
G2583_3207	Indel-157	Indel-157	-	hypothetical protein
G2583_3208	Indel-157	Indel-157	-	putative tail fiber protein encoded by prophage CP-
G2583_3209	Indel-157	Indel-157	-	Opacity protein and related surface antigens
G2583_3210	Indel-157	Indel-157	-	Phage-related protein, tail component
G2583_3211	Indel-157	Indel-157	-	putative tail assembly protein
G2583_3212	Indel-157	Indel-157	-	Cell wall-associated hydrolases (invasion-associated proteins)
G2583_3213	Indel-157	Indel-157	ant	Antirepressor protein
G2583_3214	Indel-157	Indel-157	-	hypothetical protein
G2583_3215	Indel-157	Indel-157	-	putative tail fiber component L of prophage CP-933U
G2583_3216	Indel-157	Indel-157	-	Putative minor tail protein
G2583_3217	Indel-157	Indel-157	-	Phage-related minor tail protein
G2583_3218	Indel-157	Indel-157	-	Gp14
G2583_3219	Indel-157	Indel-157	-	Putative tail assembly chaperone encoded by prophage CP-933N
G2583_3220	Indel-157	Indel-157	-	Putative tail component of cryptic prophage CP-933P
G2583_3221	Indel-157	Indel-157	-	Gp11
G2583_3222	Indel-157	Indel-157	-	Phage protein, HK97 gp10 family
G2583_3223	Indel-157	Indel-157	-	Bacteriophage head-tail adaptor
G2583_3224	Indel-157	Indel-157	-	unknown protein encoded by cryptic prophage CP-
G2583_3225	Indel-157	Indel-157	-	Portal protein (GP3)
G2583_3226	Indel-157	Indel-157	-	Portal protein (GP3)
G2583_3227	Indel-157	Indel-157	-	hypothetical protein
G2583_3228	Indel-157	Indel-157	-	Phage head maturation protease
G2583_3229	Indel-157	Indel-157	-	Phage terminase-like protein, large subunit
G2583_3230	Indel-157	Indel-157	-	unknown protein encoded by prophage CP-933N
G2583_3231	Indel-157	Indel-157	-	unknown protein encoded by prophage CP-933N
G2583_3232	Indel-157	Indel-157	ynfO	conserved hypothetical protein
G2583_3233	Indel-157	Indel-157	tonB	Partial tonB-like membrane protein encoded within prophage CP-933N
G2583_3234	Indel-157	Indel-157	-	conserved hypothetical protein
G2583_3235	Indel-157	Indel-157	-	Putative endopeptidase Rz
G2583_3236	Indel-157	Indel-157	antU	Antirepressor protein
G2583_3237	Indel-157	Indel-157	-	putative endolysin
G2583_3238	Indel-157	Indel-157	-	hypothetical protein
G2583_3239	Indel-157	Indel-157	-	putative holin protein of prophage CP-933U
G2583_3240	Indel-157	Indel-157	insN	unknown protein encoded by IS911 within prophage CP-933L
G2583_3241	Indel-157	Indel-157	-	putative transposase
Indel-158	Indel-158	Z3922	-	putative transposase
Indel-158	Indel-158	Z3923	-	hypothetical protein
Indel-158	Indel-158	Z3924	-	partial putative transposase
Indel-158	Indel-158	Z3925	-	partial putative transposase
G2583_3242	ECs3498	Z3926	-	YjhS

Locus_tag[a]				
CB9615	Sakai	EDL933	Gene	Product
G2583_3242	ECs3498	Z3927	-	YjhS
Indel-159	ECs3499		-	hypothetical protein
Indel-159		Z3929	-	unknown protein encoded by prophage CP-933Y
Indel-159	ECs3500	Z3931	-	hypothetical membrane protein
Indel-159	ECs3501	Z3932	-	putative antitermination protein
Indel-159	ECs3502	Z3933	-	putative serine/threonine protein phosphatase
Indel-159	ECs3503	Z3934	-	hypothetical protein
Indel-159	ECs3504	Z3935	-	hypothetical protein
	ECs3505	Z3936	tra8_3	transposase of insertion sequence IS30
G2583_3243	Indel-160	Indel-160	-	hypothetical protein
G2583_3244	Indel-160	Indel-160	-	unknown protein encoded within prophage CP-933R
G2583_3245	Indel-160	Indel-160	-	DNA modification methylase
G2583_3246	Indel-160	Indel-160	ybcQ	Antitermination protein Q
G2583_3247	Indel-160	Indel-160	ninH	hypothetical protein
G2583_3248	Indel-160	Indel-160	NinG	protein ninG
G2583_3249	Indel-160	Indel-160	roi	hypothetical protein
G2583_3250	Indel-160	Indel-160	ant	Putative antirepressor
G2583_3251	Indel-160	Indel-160	ninE	hypothetical protein
G2583_3252	Indel-160	Indel-160	-	Putative DNA N-6-adenine-methyltransferase of bacteriophage
G2583_3253	Indel-160	Indel-160	ninB	hypothetical protein
G2583_3254	Indel-160	Indel-160	-	Ren protein
G2583_3255	Indel-160	Indel-160	-	P protein
G2583_3256	Indel-160	Indel-160	-	phage replication protein O
G2583_3257	Indel-160	Indel-160	-	CII protein
G2583_3258	Indel-160	Indel-160	cro	Cro
G2583_3259	Indel-160	Indel-160	ymfK	P22 repressor protein c2
G2583_3260	Indel-160	Indel-160	-	hypothetical lipoprotein
G2583_3261	Indel-160	Indel-160	-	hypothetical protein
G2583_3262	Indel-160	Indel-160	-	Gp45
G2583_3263	Indel-160	Indel-160	-	Lambda prophage-derived protein ea10
G2583_3264	Indel-160	Indel-160	CIII	Lambda phage regulatory protein CIII
G2583_3265	Indel-160	Indel-160	kilW	putative Kil protein of bacteriphage BP-933W
G2583_3266	Indel-160	Indel-160	bet	Recombination protein Bet
G2583_3267	Indel-160	Indel-160	-	Putative exonuclease encoded by prophage CP-933K
G2583_3268	Indel-160	Indel-160	-	hypothetical protein
G2583_3269	Indel-160	Indel-160	-	hypothetical protein
G2583_3270	Indel-160	Indel-160	-	hypothetical protein
G2583_3271	Indel-160	Indel-160	-	C4-type zinc finger protein
G2583_3272	Indel-160	Indel-160	-	Enterohemolysin 2
G2583_3273	Indel-160	Indel-160	-	Valyl-tRNA synthetase
G2583_3274	Indel-160	Indel-160	-	hypothetical protein
G2583_3275	Indel-160	Indel-160	-	Putative bacteriophage protein
G2583_3276	Indel-160	Indel-160	-	hypothetical protein

Locus_tag[a]				
CB9615	Sakai	EDL933	Gene	Product
G2583_3277	Indel-160	Indel-160	-	hypothetical protein
G2583_3278	Indel-160	Indel-160	intA	phage integrase family protein
G2583_3279	Indel-160	Indel-160	-	hypothetical protein
G2583_3280	Indel-160	Indel-160	-	hypothetical protein
G2583_3281	Indel-160	Indel-160	-	hypothetical protein
G2583_3282	Indel-160	Indel-160	-	hypothetical protein
G2583_3283	Indel-160	Indel-160	-	hypothetical protein
G2583_3284	Indel-160	Indel-160	intA	Prophage integrase
G2583_3285	Indel-160	Indel-160	-	conserved hypothetical protein
G2583_3286	Indel-160	Indel-160	-	Hypothetical purine NTPase
G2583_3287	Indel-160	Indel-160	-	hypothetical protein
G2583_3288	Indel-160	Indel-160	-	hypothetical protein
G2583_3289	Indel-160	Indel-160	-	hypothetical protein
G2583_3290	ECs3506	Z3937	-	hypothetical protein
		Z3938	-	hypothetical protein
G2583_3291	ECs3507	Z3939	-	hypothetical protein
G2583_3292	ECs3508	Z3940	-	hypothetical protein
G2583_3293	ECs3509	Z3941	-	hypothetical protein
G2583_3294	ECs3510	Z3942	-	Conserved DNA-binding protein
G2583_3295	ECs3511	Z3943	-	Site-specific recombinase, phage integrase family
G2583_3296	ECs3512	Z3945	-	Putative site specific recombinase
G2583_3297	ECs3513	Z3946	alpA	Putative DNA binding protein
G2583_3298	ECs3514	Z3947	-	hypothetical protein
G2583_3299	ECs3515	Z3948	ypjA	putative ATP-binding component of a transport system
G2583_3300	ECs3516	Z3949	-	Putative DNA-invertase from prophage CP4-44
G2583_3301	ECs3517	Z3950	ypjB	hypothetical protein
G2583_3302		Z3951	-	hypothetical protein
G2583_3303	ileY	RNA070	ileY	Met tRNA
G2583_3304	ECs3518	Z3954	ygaR	hypothetical protein
G2583_3304	ECs3519	Z3955	ygaR	hypothetical protein
G2583_3305	ECs3520	Z3956	csiD	alpha amylase family protein
G2583_3306	ECs3521	Z3958	ygaF	hypothetical protein
G2583_3307	ECs3522	Z3959	gabD	Succinate-semialdehyde dehydrogenase (NAD(P)(+))
G2583_3308	ECs3523	Z3960	gabT	4-aminobutyrate transaminase
G2583_3309	ECs3524	Z3961	gabP	GABA permease
		Z3962	-	hypothetical protein
G2583_3310	ECs3525	Z3963	csiR	DNA-binding transcriptional regulator CsiR
G2583_3311	ECs3526	Z3964	ygaU	Phospholipid-binding protein
G2583_3312	ECs3527	Z3965	yqaE	UPF0057 membrane protein yqaE
G2583_3313	ECs3528	Z3966	ygaV	hypothetical protein
G2583_3314	ECs3529	Z3967	ygaP	hypothetical protein
G2583_3315	ECs3530	Z3968	stpA	DNA-binding protein H-NS
G2583_3316	ECs3531	Z3970	ygaW	hypothetical protein
G2583_3317	ECs3532	Z3971	ygaC	hypothetical protein

Locus_tag[a]				
CB9615	Sakai	EDL933	Gene	Product
G2583_3318	ECs3533	Z3972	ygaM	hypothetical protein
G2583_3319	ECs3534	Z3973	-	transcriptional regulator, GntR family
G2583_3320	ECs3535	Z3974	-	Carboxymuconolactone decarboxylase family protein
G2583_3321	ECs3536	Z3975	nrdH	Glutaredoxin-like protein nrdH
G2583_3322	ECs3537	Z3976	nrdI	ribonucleotide reductase stimulatory protein
G2583_3323	ECs3538	Z3977	nrdE	Ribonucleoside-diphosphate reductase, alpha subunit
G2583_3324	ECs3539	Z3978	nrdF	Ribonucleoside-diphosphate reductase 2 subunit beta
G2583_3325	ECs3540	Z3979	proV	ATP-binding component of transport system
G2583_3326	ECs3541	Z3980	proW	Glycine betaine/L-proline ABC transporter, permease protein
G2583_3327	ECs3542	Z3981	proX	Glycine betaine-binding periplasmic protein precursor
G2583_3328	ECs3543	Z3982	ygaX	Putative transport protein
G2583_3329	ECs3544	Z3983	ygaZ	Transporter, branched chain amino acid exporter (LIV-E) family
G2583_3330	ECs3545	Z3984	ygaH	predicted inner membrane protein
G2583_3331	ECs3546	Z3985	mprA	transcriptional repressor MprA
G2583_3332	ECs3547	Z3986	emrA	Multidrug resistance protein A
G2583_3333	ECs3548	Z3987	emrB	Probably membrane translocase
G2583_3334	ECs3549	Z3988	luxS	S-ribosylhomocysteine lyase
G2583_3335			sraD	ncRNA
G2583_3336	ECs3550	Z3989	gshA	Glutamate--cysteine ligase
G2583_3337	ECs3551	Z3990	yqaA	hypothetical protein
G2583_3338	ECs3552	Z3991	yqaB	HAD-superfamily hydrolase, subfamily IA
G2583_3339	argQ	RNA071	argQ	Arg tRNA
G2583_3340	argZ	RNA072	argZ	Arg tRNA
G2583_3341	argY	RNA073	argY	Arg tRNA
G2583_3342	argV	RNA074	argV	Arg tRNA
G2583_3343	serV	RNA075	serV	Ser tRNA
G2583_3344	ECs3553	Z3998	csrA	Carbon storage regulator homolog
G2583_3345	ECs3554	Z3999	alaS	Alanyl-tRNA synthetase
		Z4000	-	hypothetical protein
G2583_3346	ECs3555	Z4001	recX	regulatory protein RecX
G2583_3347	ECs3556	Z4002	recA	RecA/GFP fusion protein
G2583_3348	ECs3557	Z4003	ygaD	Competence/damage-inducible protein CinA domain protein
G2583_3349	ECs3558	Z4004	mltB	Membrane-bound lytic murein transglycosylase B
G2583_3350	ECs3559	Z4005	srlA	Glucitol/sorbitol permease IIC component
G2583_3350	ECsp018	Z4006	srlA	Glucitol/sorbitol permease IIC component
G2583_3351		Z4007	srlE	Phosphotransferase system sorbitol-specific component IIBC
G2583_3351	ECsp019	Z4009	srlE	Phosphotransferase system sorbitol-specific component IIBC
G2583_3352	ECs3560	Z4011	srlB	sorbitol-6-phosphate dehydrogenase
G2583_3353	ECs3561	Z4012	srlD	Sorbitol-6-phosphate 2-dehydrogenase

Locus_tag[a]				
CB9615	Sakai	EDL933	Gene	Product
G2583_3354	ECs3562	Z4013	gutM	Glucitol operon activator protein
G2583_3355	ECs3563	Z4014	srlR	Glucitol operon repressor
G2583_3356	ECs3564	Z4015	gutQ	D-arabinose 5-phosphate isomerase
G2583_3357	ECs3565	Z4017	norR	Anaerobic nitric oxide reductase transcription regulator norR
G2583_3358	ECs3566	Z4018	norV	Uncharacterized flavoproteins
G2583_3359	ECs3567	Z4019	norW	Nitric oxide reductase FlRd-NAD(+) reductase
G2583_3360	ECs3568	Z4020	hypF	Carbamoyltransferase HypF
G2583_3361	ECs3569	Z4021	hydN	Electron transport protein hydN
G2583_3362	ECs3570	Z4022	ascG	transcriptional regulator AscG
G2583_3363	ECs3571	Z4023	ascF	PTS system enzyme II ABC (Asc), cryptic, transports specific beta- glucosides
G2583_3364	ECs3572	Z4024	ascB	6-phospho-beta-glucosidase
G2583_3365	ECs3573	Z4025	hycI	Hydrogenase 3 maturation protease
G2583_3366	ECs3574	Z4026	hycH	Formate hydrogenlyase maturation protein hycH
G2583_3367	ECs3575	Z4027	hycG	Formate hydrogenlyase, subunit G
G2583_3368	ECs3576	Z4028	hycF	Formate hydrogenlyase, subunit F
G2583_3369	ECs3577	Z4029	hycE	Formate hydrogenlyase, subunit E
G2583_3370	ECs3578	Z4030	hycD	Membrane-spanning protein of hydrogenase 3
G2583_3371	ECs3579	Z4031	hycC	Formate hydrogenlyase, subunit C
G2583_3372	ECs3580	Z4032	hycB	Formate hydrogenlyase, subunit B
G2583_3373	ECs3581	Z4033	hycA	Formate hydrogenlyase regulatory protein hycA
G2583_3374		Z4034	-	hypothetical protein
G2583_3375	ECs3582	Z4035	hypA	Hydrogenase nickel insertion protein HypA
G2583_3376	ECs3583	Z4036	hypB	Hydrogenase isoenzymes nickel incorporation protein hypB
G2583_3377	ECs3584	Z4037	hypC	Hydrogenase isoenzymes formation protein hypC
G2583_3378	ECs3585	Z4038	hypD	Hydrogenase expression/formation protein HypD
G2583_3379	ECs3586	Z4039	hypE	Hydrogenase maturation factor
G2583_3380	ECs3587	Z4040	fhlA	Formate hydrogenlyase transcriptional activator
G2583_3381	ECs3588	Z4041	ygbA	hypothetical protein
G2583_3382		Z4042	-	hypothetical protein
G2583_3383	ECs3589	Z4043	mutS	DNA mismatch repair protein mutS
G2583_3384	ECs3590	Z4044	pphB	O218 protein
G2583_3385	ECs3591	Z4045	kpdD	4-hydroxybenzoate decarboxylase, subunit D
G2583_3386	ECs3592	Z4046	kpdC	YclC protein
G2583_3387	ECs3593	Z4047	padI	Probable aromatic acid decarboxylase
G2583_3388	ECs3594	Z4048	-	putative regulator
G2583_3389	ECs3595	Z4049	rpoS	RNA polymerase sigma factor
G2583_3390	ECs3596	Z4050	nlpD	Lipoprotein
G2583_3391	ECs3597	Z4051	pcm	Protein-L-isoaspartate O-methyltransferase
G2583_3392	ECs3598	Z4052	surE	Multifunctional protein surE [Includes: 5'/3'-nucleotidase
G2583_3393	ECs3599	Z4053	truD	tRNA pseudouridine synthase D
G2583_3394	ECs3600	Z4054	ispF	2-C-methyl-D-erythritol 2,4-cyclodiphosphate synthase

Locus_tag[a]				
CB9615	Sakai	EDL933	Gene	Product
G2583_3395	ECs3601	Z4055	ispD	2-C-methyl-D-erythritol 4-phosphate cytidylyltransferase
G2583_3396	ECs3602	Z4056	ftsB	Cell division protein ftsB homolog
G2583_3397	ECs3603	Z4057	ygbE	hypothetical protein
G2583_3398	ECs3604	Z4058	cysC	Adenylyl-sulfate kinase
G2583_3399	ECs3605	Z4059	cysN	Sulfate adenylyltransferase subunit 1
G2583_3400	ECs3606	Z4060	cysD	Sulfate adenylyltransferase subunit 2
G2583_3401	ECs3607	Z4061	iap	Alkaline phosphatase isozyme conversion peptidase
G2583_3402	ECs3608	Z4062	ygbF	CRISPR-associated protein Cas2
G2583_3403	ECs3609	Z4064	ygbT	CRISPR-associated protein Cas1
G2583_3404	ECs3610	Z4065	cse	CRISPR-associated protein, Cse3 family
G2583_3405	ECs3611	Z4066	ygcI	CRISPR-associated protein Cas5
G2583_3406	ECs3612	Z4067	ygcJ	CRISPR-associated protein, Cse4 family
G2583_3407	ECs3613	Z4068	cse	CRISPR-associated protein, Cse2 family
G2583_3408	ECs3614	Z4069	ygcL	CRISPR-associated protein, Cse1 family
G2583_3409	ECs3615	Z4070	ygcB	CRISPR-associated helicase Cas3
G2583_3410	ECs3616	Z4071	small	Small toxic membrane polypeptide
G2583_3411			-	ncRNA
G2583_3412	ECs3617	Z4072	cysH	Phosphoadenosine phosphosulfate reductase
G2583_3413	ECs3618	Z4073	cysI	Sulfite reductase [NADPH] hemoprotein beta-
G2583_3414	ECs3619	Z4074	cysJ	Sulfite reductase [NADPH] flavoprotein alpha-
G2583_3415	ECs3620	Z4075	sscR	Putative 6-pyruvoyl tetrahydrobiopterin synthase
G2583_3416	ECs3621	Z4076	ygcN	hypothetical protein
G2583_3417	ECs3622	Z4077	ygcO	hypothetical protein
G2583_3418	ECs3623	Z4078	ygcP	Glycerol-3-phosphate responsive antiterminator
G2583_3419	ECs3624	Z4079	ygcQ	Electron transfer flavoprotein
G2583_3419	ECs3625	Z4080	ygcQ	Electron transfer flavoprotein
G2583_3420	ECs3626	Z4081	ygcR	Electron transfer flavoprotein
G2583_3421	ECs3627	Z4082	ygcS	Permease (major facilitator superfamily)
G2583_3421	ECs3628	Z4083	ygcS	Permease (major facilitator superfamily)
G2583_3422	ECs3629	Z4084	ygcU	Uncharacterized flavoprotein ygcU
G2583_3423	ECs3630	Z4085	ygcW	Hypothetical oxidoreductase ygcW
G2583_3424	ECs3631	Z4086	yqcE	putative transport protein
G2583_3425	ECs3632	Z4087	ygcE	Carbohydrate kinase, FGGY family protein
		Z4088	-	hypothetical protein
G2583_3426	ECs3633	Z4089	ygcF	hypothetical protein
G2583_3427	ECs3634	Z4090	LemA	LemA family protein
G2583_3428	ECs3635	Z4091	-	hypothetical protein
	ECs3636		-	hypothetical protein
G2583_3429	ECs3637	Z4092	-	hypothetical protein
G2583_3430	ECs3638	Z4093	ygcG	hypothetical protein
G2583_3431	ECs3639	Z4094	eno	Enolase
G2583_3432	ECs3640	Z4095	pyrG	CTP synthase
G2583_3433	ECs3641	Z4096	mazG	Nucleoside triphosphate pyrophosphohydrolase
G2583_3434	ECs3642	Z4097	chpA	PemK-like protein 1

Locus_tag[a]				
CB9615	Sakai	EDL933	Gene	Product
G2583_3435	ECs3643	Z4098	chpR	PemI-like protein 1
G2583_3436	ECs3644	Z4099	relA	GTP diphosphokinase
G2583_3437	ECs3645	Z4100	rumA	23S rRNA (uracil-5-)-methyltransferase rumA
G2583_3438	ECs3646	Z4101	barA	Signal transduction histidine-protein kinase barA
G2583_3439	ECs3647	Z4102	gudD	Glucarate dehydratase
G2583_3440	ECs3648	Z4103	gudX	Glucarate dehydratase
G2583_3440		Z4104	gudX	Glucarate dehydratase
G2583_3441	ECs3649	Z4105	gudP	Glucarate permease
G2583_3442	ECs3650	Z4106	yqcA	flavodoxin
G2583_3443	ECs3651	Z4107	truC	tRNA pseudouridine synthase C
G2583_3444	ECs3652	Z4108	yqcC	hypothetical protein
G2583_3445	csrB		csrB	ncRNA
G2583_3446	ECs3653	Z4110	syd	SecY interacting protein Syd
G2583_3447	ECs3654	Z4111	queF	NADPH-dependent 7-cyano-7-deazaguanine reductase
G2583_3448	ECs3655	Z4112	ygdH	hypothetical protein
G2583_3449	ECs3656	Z4113	sdaC	Serine transporter family protein
G2583_3450	ECs3657	Z4114	sdaB	L-serine ammonia-lyase 2
G2583_3451	ECs3658	Z4115	xni	Uncharacterized exonuclease xni
G2583_3452	ECs3659	Z4116	fucO	Lactaldehyde reductase
G2583_3453	ECs3660	Z4117	fucA	L-fuculose phosphate aldolase
G2583_3454	ECs3661	Z4118	fucP	L-fucose:H+ symporter permease
G2583_3455	ECs3662	Z4119	fucI	L-fucose isomerase
G2583_3456	ECs3663	Z4120	fucK	L-fuculokinase
G2583_3457	ECs3664	Z4121	fucU	Fucose operon fucU protein
G2583_3458	ECs3665	Z4122	fucR	L-fucose operon activator
G2583_3459	ECs3666	Z4123	ygdE	Putative RNA 2'-O-ribose methyltransferase ygdE
G2583_3460	ECs3667	Z4124	ygdD	UPF0382 inner membrane protein ygdD
G2583_3461	ECs3668	Z4125	gcvA	Glycine cleavage system transcriptional activator
G2583_3462	gcvB		gcvB	ncRNA
G2583_3463	ECs3669	Z4126	ygdI	hypothetical protein
G2583_3464	ECs3670	Z4127	csdA	Cysteine desulfurase, catalytic subunit CsdA
G2583_3465	ECs3671	Z4128	csdE	Uncharacterized sufE-like protein ygdK
G2583_3466	ECs3672	Z4129	ygdL	Putative enzyme
G2583_3467	ECs3673	Z4130	mltA	Membrane-bound lytic murein transglycosylase A
G2583_3468	metZ	RNA076	metZ	Met tRNA
G2583_3469	metW	RNA077	metW	Met tRNA
G2583_3470	metV	RNA078	metV	Met tRNA
G2583_3471	ECs3674	Z4134	amiC	Putative amidase
G2583_3472	ECs3675	Z4135	argA	Amino-acid acetyltransferase
G2583_3473	ECs3676	Z4136	recD	Exodeoxyribonuclease V, alpha subunit
G2583_3474	ECs3677	Z4137	recB	Exodeoxyribonuclease V, beta subunit
G2583_3475	ECs3678	Z4138	ptrA	Protease III
G2583_3476	ECs3679	Z4139	recC	Exodeoxyribonuclease V, gamma subunit
G2583_3477	ECs3680	Z4140	ppdC	Prepilin peptidase-dependent protein C

Locus_tag[a]				
CB9615	Sakai	EDL933	Gene	Product
G2583_3478	ECs3681	Z4141	ygdB	hypothetical protein
G2583_3479	ECs3682	Z4142	ppdB	Prepilin peptidase dependent protein B
G2583_3480	ECs3683	Z4143	ppdA	Prepilin peptidase-dependent protein A
G2583_3481	ECs3684	Z4144	thyA	Thymidylate synthase
G2583_3482	ECs3685	Z4145	lgt	Prolipoprotein diacylglyceryl transferase
G2583_3483	ECs3686	Z4146	ptsP	Phosphoenolpyruvate-protein phosphotransferase
G2583_3484	ECs3687	Z4147	nudH	RNA pyrophosphohydrolase
G2583_3485		Z4148	ygdT	hypothetical protein
G2583_3486	ECs3688	Z4149	mutH	DNA mismatch repair protein mutH
G2583_3487	ECs3689	Z4150	ygdQ	UPF0053 inner membrane protein ygdQ
G2583_3488	ECs3690	Z4151	ygdR	Uncharacterized lipoprotein ygdR precursor
G2583_3489	ECs3691	Z4152	tas	predicted oxidoreductase, NADP(H)-dependent aldo-keto reductase
G2583_3490	ECs3692	Z4153	lplT	hypothetical protein
G2583_3491	ECs3693	Z4154	aas	2-acyl-glycerophospho-ethanolamine acyltransferase
G2583_3492			-	ncRNA
G2583_3493			-	ncRNA
G2583_3494	ECs3694	Z4155	galR	DNA-binding transcriptional repressor
G2583_3495	ECs3695	Z4156	lysA	Diaminopimelate decarboxylase
G2583_3496	ECs3696	Z4157	lysR	DNA-binding transcriptional regulator LysR
G2583_3497	ECs3697	Z4160	ygeA	Putative resistance proteins
G2583_3498	ECs3698	Z4161	araE	Arabinose-proton symporter
G2583_3499	ECs3699	Z4162	kduD	2-deoxy-D-gluconate 3-dehydrogenase
G2583_3500	ECs3700	Z4163	kduI	4-deoxy-L-threo-5-hexosulose-uronate ketol-isomerase
G2583_3501	ECs3701	Z4164	yqeF	Acetyl-CoA acetyltransferase
G2583_3502	ECs3702	Z4165	yqeG	putative transporter protein
G2583_3503	ECs3703	Z4166	yqeH	hypothetical protein
G2583_3504	ECs3704	Z4167	yqeI	putative sensory transducer
G2583_3505	ECs3705	Z4168	yqeJ	hypothetical protein
G2583_3506	ECs3706	Z4169	yqeK	hypothetical protein
G2583_3507		Z4170	-	hypothetical protein
G2583_3508	ECs3707	Z4171	ygeF	hypothetical protein
G2583_3509	ECs3708	Z4172	ygeG	Tetratricopeptide repeat protein
G2583_3510			ygeH	transcriptional regulatory protein
Indel-167	ECs3709	Z4173	ygeH	putative invasion protein
Indel-167	ECs3710	Z4174	-	hypothetical protein
Indel-167	ECs3711	Z4175	-	hypothetical protein
Indel-167	ECs3712	Z4176	-	hypothetical protein
Indel-167		Z4177	-	hypothetical protein
Indel-167	ECs3713	Z4178	-	hypothetical protein
Indel-167	ECs3714	Z4179	-	hypothetical protein
Indel-167	ECs3715		-	hypothetical protein
Indel-167	ECs3716	Z4180	-	type III secretion system lipoprotein precursor EprK
Indel-167	ECs3717	Z4181	-	type III secretion protein EprJ

Locus_tag[a]				
CB9615	Sakai	EDL933	Gene	Product
Indel-167	ECs3718		-	type III secretion protein EprI
Indel-167	ECs3719	Z4182	-	type III secretion protein EprH
Indel-167		Z4183	-	hypothetical protein
Indel-167	ECs3720	Z4184	-	putative transcriptional regulator
Indel-167	ECs3721	Z4185	-	type III secretion protein EprS
Indel-167	ECs3722	Z4186	-	type III secretion protein EpaR2
Indel-167	ECs3723	Z4187	-	type III secretion protein EpaR1
Indel-167	ECs3724	Z4188	-	type III secretion protein EpaQ
Indel-167	ECs3725	Z4189	-	type III secretion protein EpaP
Indel-167	ECs3726	Z4190	-	type III secretion protein EpaO
Indel-167	ECs3727	Z4191	-	type III secretion protein EivJ
Indel-167	ECs3728	Z4192	-	hypothetical protein
Indel-167	ECs3729	Z4193	-	type III secretion protein EivI
Indel-167	ECs3730	Z4194	-	type III secretion protein ATP synthetase EivC
G2583_3511	ECs3730	Z4194	-	flagellum-specific ATP synthase
G2583_3512	ECs3731	Z4195	EivA	Type III secretion apparatus protein
G2583_3513	ECs3732	Z4196	EivE	Putative secreted protein
G2583_3514	ECs3733	Z4197	EivG	Putative type III secretion apparatus protein
G2583_3515	ECs3734	Z4198	invF	Putative type III secretion apparatus regulatory protein
G2583_3516	ECs3735	Z4199	-	hypothetical protein
G2583_3517	ECs3736	Z4200	-	hypothetical protein
G2583_3517	ECs3737	Z4201	-	hypothetical protein
G2583_3518	glyU	RNA079	glyU	Gly tRNA
G2583_3519	ECs3738	Z4203	ygeR	Hypothetical lipoprotein YgeR
G2583_3520	ECs3739	Z4205	xdhA	Xanthine dehydrogenase
G2583_3521	ECs3740	Z4206	xdhB	Xanthine dehydrogenase FAD-binding subunit
G2583_3522	ECs3741	Z4207	xdhC	Xanthine dehydrogenase iron-sulfur-binding subunit
G2583_3523	ECs3742	Z4208	ygeV	Putative transcriptional regulator
G2583_3524	ECs3743	Z4209	ygeW	hypothetical protein
G2583_3525	ECs3744	Z4210	ygeX	Putative diaminopropionate ammonia-lyase
G2583_3526	ECs3745	Z4211	ygeY	peptidase
G2583_3527	ECs3746	Z4212	hyuA	D-phenylhydantoinase
G2583_3528	ECs3747	Z4213	yqeA	Carbamate kinase arcC homolog
G2583_3529	ECs3748	Z4214	yqeB	Putative xanthine dehydrogenase accessory factor
G2583_3530	ECs3749	Z4215	yqeC	Probable selenium-dependent hydroxylase accessory protein YqeC
G2583_3531	ECs3750	Z4216	ygfJ	Molybdenum hydroxylase accessory protein, YgfJ family
G2583_3532	ECs3751	Z4217	ygfK	putative oxidoreductase, Fe-S subunit
G2583_3533	ECs3752	Z4218	ssnA	Cytosine deaminase and related metal-dependent hydrolases
G2583_3534	ECs3753	Z4219	ygfM	hypothetical protein
G2583_3535	ECs3754	Z4220	xdhD	Probable hypoxanthine oxidase xdhD
G2583_3536	ECs3755	Z4221	ygfO	Putative purine permease ygfO
G2583_3537	ECs3756	Z4222	guaD	guanine deaminase

Locus_tag[a]				
CB9615	Sakai	EDL933	Gene	Product
G2583_3538	ECs3757	Z4223	ygfQ	hypothetical protein
G2583_3539	ECs3758	Z4224	ygfS	Putative electron transport protein ygfS
G2583_3540	ECs3759	Z4225	ygfT	putative oxidoreductase Fe-S binding subunit
G2583_3541	ECs3760	Z4226	ygfU	Putative xanthine permease
G2583_3542	ECs3761	Z4227	idi	Isopentenyl-diphosphate Delta-isomerase
G2583_3543	ECs3762	Z4228	lysS	Lysyl-tRNA synthetase
G2583_3544	ECs3763	Z4229	prfB	Peptide chain release factor 2
G2583_3545	ECs3764	Z4230	recJ	Single-stranded-DNA-specific exonuclease RecJ
G2583_3546	ECs3765	Z4231	dsbC	Thiol:disulfide interchange protein dsbC precursor
G2583_3547	ECs3766	Z4232	xerD	Tyrosine recombinase xerD
G2583_3548	ECs3767	Z4233	fldB	Flavodoxin-2
G2583_3549	ECs3768	Z4234	ygfX	hypothetical protein
G2583_3550	ECs3769	Z4235	ygfY	UPF0350 protein ygfY
G2583_3551	ECs3770	Z4236	ygfZ	tRNA-modifying protein ygfZ
G2583_3552	ECs3771	Z4237	yqfA	UPF0073 inner membrane protein yqfA
G2583_3553	ECs3772	Z4238	yqfB	Uncharacterized protein conserved in bacteria
G2583_3554	ECs3773	Z4239	bglA	6-phospho-beta-glucosidase BglA
G2583_3555	ECs3774	Z4240	gcvP	Glycine dehydrogenase [decarboxylating]
G2583_3556	ECs3775	Z4241	gcvH	Glycine cleavage system H protein
G2583_3557	ECs3776	Z4242	gcvT	Glycine cleavage complex protein T, aminomethyltransferase, tetrahydrofolate-dependent
G2583_3558	ECs3777	Z4243	visC	Ubiquinone biosynthesis hydroxylase, UbiH/UbiF/VisC/COQ6 family
G2583_3559	ECs3778	Z4244	ubiH	2-octaprenyl-6-methoxyphenol 4-monoxygenase
G2583_3560	ECs3779	Z4245	pepP	Xaa-Pro aminopeptidase
G2583_3561	ECs3780	Z4246	ygfB	UPF0149 protein ygfB
G2583_3562	ECs3781	Z4247	zapA	Z-ring-associated protein
G2583_3563	ssrS	RNA080	ssrS	ncRNA
G2583_3564	ECs3782	Z4249	ygfA	Putative ligase
G2583_3565			-	ncRNA
G2583_3566	ECs3783		-	hypothetical protein
		Z4250	-	hypothetical protein
G2583_3567			-	ncRNA
G2583_3568	ECs3784	Z4251	serA	D-3-phosphoglycerate dehydrogenase
G2583_3569	ECs3785	Z4252	rpiA	Ribose 5-phosphate isomerase
G2583_3570	ECs3786	Z4253	argP	Chromosome initiation inhibitor
G2583_3571	ECs3787	Z4254	scpA	Methylmalonyl-CoA mutase
G2583_3572	ECs3788	Z4255	argK	LAO/AO transport system kinase
G2583_3573	ECs3789	Z4256	scpB	Methylmalonyl-CoA decarboxylase
G2583_3574	ECs3790	Z4257	scpC	Succinate CoA transferase
G2583_3575	ECs3791		ygfI	Uncharacterized HTH-type transcriptional regulator ygfI
G2583_3575	ECs3792	Z4258	ygfI	Uncharacterized HTH-type transcriptional regulator ygfI
G2583_3576	ECs3793	Z4259	yggE	hypothetical protein
G2583_3577	ECs3794	Z4260	argO	arginine exporter protein

Locus_tag[a]				
CB9615	Sakai	EDL933	Gene	Product
G2583_3578	ECs3795	Z4261	mscS	Small-conductance mechanosensitive channel
G2583_3579	ECs3796	Z4263	fbaA	Fructose-bisphosphate aldolase
G2583_3580	ECs3797	Z4265	pgk	Phosphoglycerate kinase
G2583_3581	ECs3798	Z4266	epd	D-erythrose-4-phosphate dehydrogenase
G2583_3582	ECs3799	Z4267	-	hypothetical protein
G2583_3583	ECs3800	Z4268	-	hypothetical protein
G2583_3584	ECs3801	Z4269	-	hypothetical protein
G2583_3585	ECs3802	Z4270	-	ABC transporter, ATP-binding protein
G2583_3586	ECs3803	Z4271	-	putative ATP-binding protein of ABC transport system
G2583_3587	ECs3804	Z4273	yggC	putative fructose transport system kinase
G2583_3588	ECs3805	Z4274	yggD	Mannitol operon repressor
G2583_3589	ECs3806	Z4275	yggF	Fructose-1,6-bisphosphatase, class II
G2583_3590	ECs3807	Z4276	yggP	L-sorbose 1-phosphate reductase
G2583_3591	ECs3808	Z4277	cmtA	PTS system mannitol-specific EIICB component (EIICB-Mtl) (EII-Mtl) [Includes: Mannitol permease IIC component (PTS system mannitol- specific EIIC
G2583_3592	ECs3809	Z4278	cmtB	hypothetical protein
G2583_3593	ECs3810	Z4279	tktA	transketolase
G2583_3594	ECs3811	Z4280	yggG	hypothetical protein
G2583_3595	ECs3812	Z4281	speB	agmatinase
G2583_3596	ECs3813	Z4282	-	hypothetical protein
G2583_3597	ECs3814	Z4283	speA	Biosynthetic arginine decarboxylase
G2583_3598	ECs3815	Z4284	yqgB	hypothetical protein
G2583_3599	ECs3816	Z4285	yqgC	hypothetical protein
G2583_3600	ECs3817	Z4286	yqgD	hypothetical protein
G2583_3601	ECs3818	Z4287	metK	S-adenosylmethionine synthetase
G2583_3602	ECs3819	Z4288	galP	Galactose-proton symporter
G2583_3603	ECs3820	Z4289	yggI	hypothetical protein
G2583_3604	ECs3821	Z4290	endA	Endonuclease I
G2583_3605	ECs3822	Z4291	rsmE	hypothetical protein
G2583_3606	ECs3823	Z4292	gshB	Glutathione synthetase
G2583_3607	ECs3824	Z4293	yqgE	UPF0301 protein yqgE
G2583_3608	ECs3825	Z4294	yqgF	Putative Holliday junction resolvase
G2583_3609	ECs3827	Z4295	yggR	Twitching motility family protein
G2583_3610	ECs3826	Z4296	yggS	UPF0001 protein yggS
G2583_3611	ECs3828	Z4297	yggT	putative resistance protein
G2583_3612	ECs3829	Z4298	yggU	conserved hypothetical protein
G2583_3613	ECs3830	Z4299	rdgB	Nucleoside-triphosphatase rdgB
G2583_3614	ECs3831	Z4300	yggW	Putative oxygen-independent coproporphyrinogen III oxidase
G2583_3615	ECs3832	Z4301	yggM	putative alpha helix chain
G2583_3616	ECs3833	Z4302	ansB	L-asparaginase 2
G2583_3617	ECs3834	Z4303	yggN	hypothetical protein
G2583_3618	ECs3835	Z4304	yggL	hypothetical protein

Locus_tag[a]				
CB9615	Sakai	EDL933	Gene	Product
G2583_3619	ECs3836	Z4305	trmI	tRNA (Guanine-N(7)-)-methyltransferase
G2583_3620	ECs3837	Z4306	mutY	A/G-specific adenine glycosylase
G2583_3621	ECs3838	Z4307	yggX	Probable Fe(2+)-trafficking protein
G2583_3622	ECs3839	Z4308	mltC	Membrane-bound lytic murein transglycosylase C precursor
G2583_3623	ECs3840	Z4309	nupG	Transport of nucleosides, permease protein
G2583_3624	ECs3841	Z4310	speC	Ornithine decarboxylase isozyme
G2583_3625	ECs3842	Z4311	yqgA	putative transport protein
G2583_3626	pheV	RNA081	pheV	Phe tRNA
G2583_3627	ECs3843	Z4313	intB	Site-specific recombinase, phage integrase family
G2583_3628	ECs3844	Z4314	-	unknown protein encoded by ISEc8
	ECs3845		-	hypothetical protein
G2583_3629	ECs3846	Z4315	ISSfl4	ISSfl3 OrfA
G2583_3630	ECs3847	Z4316	ISSfl4	ISSfl4 ORF2
G2583_3631	ECs3848	Z4317	ISSfl4	Transposase and inactivated derivatives
G2583_3632	ECs3849	Z4318	-	DNA helicase II
		Z4320	-	hypothetical protein
G2583_3633	ECs3850	Z4321	PagC	PagC-like membrane protein
	ECs3851		-	hypothetical protein
G2583_3634	ECs3852	Z4322	-	hypothetical protein
G2583_3635			-	ISEc13 transposase
		Z4323	-	hypothetical protein
G2583_3635	ECs3853	Z4324	-	ISEc13 transposase
G2583_3636	ECs3854		-	ST51 protein
		Z4325	-	hypothetical protein
G2583_3637	ECs3855	Z4326	ospD	Ent protein
	ECs3856		-	hypothetical protein
		Z4327	-	hypothetical protein
G2583_3638	ECs3857	Z4328	-	NleB
G2583_3639	ECs3858	Z4329	NleE	ST47 protein
G2583_3640	ECs3859	Z4330	-	Putative transposase
		Z4331	-	hypothetical protein
G2583_3641	ECs3860	Z4332	lifA	EHEC factor for adherence
G2583_3641	ECs3861	Z4333	lifA	EHEC factor for adherence
Indel-169	ECs3862	Z4334	-	putative transposase OrfB protein of insertion sequence IS629
Indel-169	ECs3863	Z4335	-	putative transposase OrfA protein of insertion sequence IS629
Indel-169	ECs3864		-	hypothetical protein
Indel-169	ECs3865	Z4336	-	hypothetical protein
Indel-169	ECs3866	Z4337	-	hypothetical protein
G2583_3642	Indel-170	Indel-170	-	Transposase and inactivated derivatives
G2583_3643	Indel-170	Indel-170	-	Transposase and inactivated derivatives
	ECs3867		-	hypothetical protein

Locus_tag[a]				
CB9615	Sakai	EDL933	Gene	Product
G2583_3644	ECs3868	Z4338	-	IS66 family transposase orfB
G2583_3645	ECs3869	Z4340	-	Transposase and inactivated derivatives
G2583_3646	ECs3870		-	IS3
Indel-171	ECs3871		-	hypothetical protein
G2583_3647	Indel-172	Indel-172	yagK	hypothetical protein
G2583_3648	Indel-172	Indel-172	-	hypothetical protein
G2583_3649	Indel-172	Indel-172	-	DNA-directed RNA polymerase, beta subunit/140 kD subunit
G2583_3650	Indel-172	Indel-172	-	hypothetical protein
G2583_3651	Indel-172	Indel-172	yagK	hypothetical protein
G2583_3652	Indel-172	Indel-172	-	hypothetical protein
G2583_3653	Indel-172	Indel-172	-	hypothetical protein
G2583_3654	Indel-172	Indel-172	-	Predicted transcriptional regulator
G2583_3655	Indel-172	Indel-172	-	hypothetical protein
G2583_3656	Indel-172	Indel-172	-	ORFa1203
G2583_3657	Indel-172	Indel-172	-	IS629 transposase orfB
G2583_3658	Indel-172	Indel-172	-	hypothetical protein
G2583_3659	Indel-172	Indel-172	-	hypothetical protein
G2583_3660	Indel-172	Indel-172	yfjP	hypothetical protein
G2583_3661	Indel-172	Indel-172	flu	Type V secretory pathway, adhesin AidA
G2583_3662	Indel-172	Indel-172	insN	unknown protein encoded by IS911 within prophage CP-933L
G2583_3663	Indel-172	Indel-172	-	putative transposase
G2583_3664	Indel-172	Indel-172	ykfF	hypothetical protein
G2583_3665	Indel-172	Indel-172	yafZ	hypothetical protein
G2583_3666	Indel-172	Indel-172	yfjX	hypothetical protein
G2583_3667	Indel-172	Indel-172	yeeS	DNA repair protein, RadC family
G2583_3668	Indel-172	Indel-172	yeeT	hypothetical protein
G2583_3669	Indel-172	Indel-172	yeeU	Putative structural protein
G2583_3670	Indel-172	Indel-172	yeeV	hypothetical protein
G2583_3671	Indel-172	Indel-172	yeeW	hypothetical protein
G2583_3672	Indel-172	Indel-172	tnpA	Transposase for ISEc12
G2583_3673	Indel-172	Indel-172	tnpA	TnpA
G2583_3674	Indel-172	Indel-172	-	hypothetical protein
G2583_3675	Indel-172	Indel-172	-	hypothetical protein
G2583_3676	Indel-172	Indel-172	-	hypothetical protein
G2583_3677	Indel-172	Indel-172	yghD	Putative general secretion pathway protein M-type yghD
G2583_3678	Indel-172	Indel-172	gspL	Type II secretory pathway, component PulL
G2583_3679	Indel-172	Indel-172	gspK	General secretion pathway protein K
G2583_3680	Indel-172	Indel-172	gspJ	General secretion pathway protein GspJ
G2583_3681	Indel-172	Indel-172	gspI	General secretion pathway protein GspI
G2583_3682	Indel-172	Indel-172	gspH	General secretion pathway protein H
G2583_3683	Indel-172	Indel-172	gspG	General secretion pathway protein G
G2583_3684	Indel-172	Indel-172	gspF	Hypothetical type II secretion protein

Locus_tag[a]				
CB9615	Sakai	EDL933	Gene	Product
G2583_3685	Indel-172	Indel-172	gspE	Type II secretory pathway, ATPase PulE/Tfp pil us assembly pathway, ATPase PilB
G2583_3686	Indel-172	Indel-172	gspD	General secretion pathway protein D
G2583_3687	Indel-172	Indel-172	gspC	General secretion pathway protein C
G2583_3688	Indel-172	Indel-172	yghG	Hypothetical lipoprotein
G2583_3689	Indel-172	Indel-172	pppA	Putative prepilin peptidase A
G2583_3690	Indel-172	Indel-172	yghJ	Putative lipoprotein acfD homolog precursor
G2583_3691	Indel-172	Indel-172	-	hypothetical protein
G2583_3692	Indel-172	Indel-172	-	hypothetical protein
G2583_3693	Indel-172	Indel-172	glcA	Glycolate permease glcA
G2583_3694	Indel-172	Indel-172	glcB	Malate synthase G
G2583_3695	Indel-172	Indel-172	glcG	hypothetical protein
G2583_3696	Indel-172	Indel-172	glcF	Glycolate oxidase iron-sulfur subunit
G2583_3697	Indel-172	Indel-172	glcE	Glycolate oxidase, subunit GlcE
G2583_3698	Indel-172	Indel-172	glcD	Glycolate oxidase subunit glcD
G2583_3699	Indel-172	Indel-172	glcC	Glc operon transcriptional activator
G2583_3700	Indel-172	Indel-172	yghO	hypothetical protein
G2583_3701	Indel-172	Indel-172	-	AMP-binding enzyme
G2583_3702	Indel-172	Indel-172	-	hypothetical protein
G2583_3703	Indel-172	Indel-172	-	Phosphopantetheine-binding
G2583_3704	Indel-172	Indel-172	-	7-keto-8-aminopelargonate synthetase and relat ed enzymes
G2583_3705	Indel-172	Indel-172	-	Permease YjgP/YjgQ family protein precursor
G2583_3706	Indel-172	Indel-172	-	Permease YjgP/YjgQ family protein precursor
G2583_3707	Indel-172	Indel-172	ytfJ	Protein ytfJ
G2583_3708	Indel-172	Indel-172	-	hypothetical protein
G2583_3709	Indel-172	Indel-172	yghQ	Putative polysaccharide biosynthesis protein
G2583_3710	Indel-172	Indel-172	yghR	Thymidylate kinase
G2583_3711	Indel-172	Indel-172	yghS	Uncharacterized ATP-binding protein yghS
G2583_3712	Indel-172	Indel-172	yghT	Uncharacterized ATP-binding protein yghT
G2583_3713	ECs3872	Z4341	pitB	Probable low-affinity inorganic phosphate transporter 2
G2583_3714	ECs3873	Z4342	gsp	Glutathionylspermidine amidase/glutathionylspermidine synthase
G2583_3715	ECs3874	Z4343	yghU	putative glutathione S-transferase YghU
G2583_3716	ECs3875	Z4344	hybG	Hydrogenase-2 operon protein hybG
G2583_3717	ECs3876	Z4345	hybF	Probable hydrogenase nickel incorporation protein hybF
G2583_3718	ECs3877	Z4346	hybE	Hydrogenase-2 operon protein hybE
G2583_3719	ECs3878	Z4347	hybD	predicted maturation element for hydrogenase 2
G2583_3720	ECs3879	Z4348	hybC	Hydrogenase-2 large chain precursor
G2583_3721	ECs3880	Z4349	hybB	Probable Ni/Fe-hydrogenase 2 b-type cytochrome
G2583_3722	ECs3881	Z4350	hybA	Hydrogenase-2 operon protein hybA precursor
G2583_3723	ECs3882	Z4351	hybO	Hydrogenase-2 small chain precursor
G2583_3724	ECs3883	Z4352	yghW	hypothetical protein
G2583_3725	ECs3884	Z4353	yghYX	Putative enzyme

Locus_tag[a]				
CB9615	Sakai	EDL933	Gene	Product
G2583_3726	ECs3885	Z4354	yghZ	aldo-keto reductase
G2583_3727	ECs3886	Z4355	yqhA	UPF0114 protein yqhA
G2583_3728	ECs3887	Z4356	yghA	Uncharacterized oxidoreductase yghA
	ECs3888	Z4357	-	hypothetical protein
G2583_3729	ECs3889	Z4358	exbD	Biopolymer transport protein exbD
G2583_3730	ECs3890	Z4359	exbB	Biopolymer transport protein exbB
G2583_3731	ECs3891	Z4360	-	hypothetical protein
G2583_3732	ECs3892	Z4361	metC	Cystathionine beta-lyase
G2583_3733	ECs3893	Z4362	yghB	Inner membrane protein yghB
G2583_3734	ECs3895	Z4363	yqhC	Putative ARAC-type regulatory protein
G2583_3735	ECs3894	Z4364	yqhD	Alcohol dehydrogenase yqhD
G2583_3736	ECs3896	Z4365	dkgA	2,5-diketo-D-gluconic acid reductase A
G2583_3737	ECs3897	Z4367	yqhG	hypothetical protein
G2583_3737	ECs3898	Z4368	yqhG	hypothetical protein
G2583_3738	ECs3899	Z4369	yqhH	predicted outer membrane lipoprotein
G2583_3739	ECs3900	Z4370	ygiQ	UPF0313 protein ygiQ
G2583_3740	ECs3901	Z4371	sufI	repressor protein for FtsI
G2583_3741	ECs3902	Z4372	plsC	1-acyl-sn-glycerol-3-phosphate acyltransferase
G2583_3742	ECs3903	Z4373	parC	DNA topoisomerase 4 subunit A
G2583_3743	ECs3904	Z4374	ygiS	Bacterial extracellular solute-binding protein, family 5
G2583_3744	ECs3905	Z4375	ygiV	hypothetical protein
G2583_3745	ECs3906	Z4376	ygiW	Protein ygiW
G2583_3746	ECs3907	Z4377	qseB	DNA-binding response regulator in two-component regulatory system with QseC
G2583_3747	ECs3908	Z4378	qseC	sensor protein QseC
G2583_3747	ECs3909	Z4378	qseC	sensor protein QseC
G2583_3748	ECs3910	Z4379	mdaB	Modulator of drug activity B
G2583_3749	ECs3911	Z4380	ygiN	Protein ygiN
G2583_3750	ECs3912	Z4381	-	Iron transport system regulatory protein FitR
G2583_3751	ECs3913	Z4382	-	Putative iron compound-binding protein of ABC transporter family
G2583_3752	ECs3914	Z4383	-	Putative iron compound permease protein of ABC transporter family
G2583_3753	ECs3915	Z4384	-	Iron ABC transporter, permease protein FitC
G2583_3754	ECs3916	Z4385	FepC	Iron ABC transporter, ATP binding protein FitB
G2583_3755	ECs3917	Z4386	-	TonB-dependent outer membrane ferric coprogen receptor FitA
G2583_3756	ECs3918	Z4387	parE	DNA topoisomerase IV, B subunit
G2583_3757	ECs3919	Z4388	yqiA	Esterase yqiA
G2583_3758	ECs3920	Z4389	cpdA	cyclic 3',5'-adenosine monophosphate
G2583_3759	ECs3921	Z4390	yqiB	hypothetical protein
G2583_3760	ECs3922	Z4391	nudF	ADP-ribose pyrophosphatase
G2583_3761	ECs3923	Z4392	tolC	Outer membrane protein TolC
G2583_3762	ECs3924	Z4393	ygiA	hypothetical protein

Locus_tag[a]				
CB9615	Sakai	EDL933	Gene	Product
G2583_3763	ECs3925	Z4394	ygiB	UPF0441 protein ygiB
G2583_3764	ECs3926	Z4395	ygiC	putative synthetase/amidase
G2583_3765	ECs3927	Z4396	ygiD	Aromatic ring-opening dioxygenase
G2583_3766	ECs3928	Z4397	zupT	Zinc transporter zupT
G2583_3767		Z4398	-	hypothetical protein
G2583_3768	ECs3929	Z4399	ribB	3,4-dihydroxy-2-butanone 4-phosphate synthase
G2583_3769	ECs3930	Z4400	yqiC	hypothetical protein
G2583_3770	ECs3931	Z4401	glgS	Glycogen synthesis protein glgS
G2583_3771	ECs3932	Z4402	yqiJ	Putative oxidoreductase
G2583_3772	ECs3933	Z4403	yqiK	SPFH/band 7 domain protein
G2583_3773			-	ncRNA
G2583_3774	ECs3934		-	hypothetical protein
G2583_3775			-	ncRNA
G2583_3776	ECs3935	Z4405	rfaE	Bifunctional protein hldE [Includes: D-beta-D-heptose 7-phosphate kinase
G2583_3777	ECs3936	Z4406	glnE	bifunctional glutamine-synthetase adenylyltransferase/deadenyltransferase
G2583_3778	ECs3937	Z4407	ygiF	Adenylate cyclase
G2583_3779	ECs3938	Z4408	htrG	putative signal transduction protein
G2583_3780	ECs3939	Z4409	cca	TRNA nucleotidyltransferase
G2583_3781	ECs3940	Z4410	bacA	Putative undecaprenol kinase
G2583_3782	ECs3941	Z4411	folB	Dihydroneopterin aldolase
G2583_3783	ECs3942	Z4412	ygiH	UPF0078 membrane protein ygiH
G2583_3784	ECs3943	Z4413	ttdR	transcriptional activator TtdR
G2583_3785	ECs3944	Z4414	ttdA	L(+)-tartrate dehydratase subunit alpha
G2583_3786	ECs3945	Z4415	ttdB	L(+)-tartrate dehydratase subunit beta
G2583_3787	ECs3946	Z4416	ttdT	L-tartrate/succinate antiporter
G2583_3788	ECs3947	Z4417	ygjD	Probable O-sialoglycoprotein endopeptidase
G2583_3789	ECs3948	Z4418	rpsU	Ribosomal protein S21
G2583_3790	ECs3949	Z4419	dnaG	DNA primase
G2583_3791	ECs3950	Z4420	rpoD	RNA polymerase sigma factor RpoD
G2583_3792	ECs3951	Z4421	mug	G/U mismatch-specific DNA glycosylase
G2583_3793	ileX	RNA082	ileX	Met tRNA
G2583_3794	ECs3952	Z4423	yqjH	Siderophore-interacting protein
G2583_3795	ECs3953	Z4424	yqjI	transcriptional regulator, PadR family
G2583_3796	ECs3954	Z4425	aer	Aerotaxis receptor
G2583_3797	ECs3955	Z4426	ygjG	Ornithine/acetylornithine aminotransferase
G2583_3798	ECs3956	Z4427	ygjH	T-RNA-binding domain protein
G2583_3799	ECs3957	Z4428	ebgR	DNA-binding transcriptional repressor
G2583_3800	ECs3958	Z4429	ebgA	Evolved beta-D-galactosidase, alpha subunit
G2583_3801	ECs3959	Z4430	ebgC	Evolved beta-galactosidase subunit beta
G2583_3802	ECs3960	Z4431	ygjI	Amino acid permease family protein
G2583_3803	ECs3961	Z4432	ygjJ	hypothetical protein
G2583_3804	ECs3962	Z4433	ygjK	predicted glycosyl hydrolase

Locus_tag[a]				
CB9615	Sakai	EDL933	Gene	Product
G2583_3805	ECs3963	Z4434	fadH	2,4-dienoyl-CoA reductase
G2583_3806	ECs3964	Z4435	ygjM	Helix-turn-helix DNA-binding domain protein
G2583_3807	ECs3965	Z4436	ygjN	hypothetical protein
G2583_3808	ECs3966	Z4437	rlmG	Putative enzyme
G2583_3809	ECs3967	Z4438	ygjP	hypothetical protein
G2583_3810	ECs3968	Z4439	ygjQ	Putative SanA protein
G2583_3811	ECs3969	Z4440	ygjR	Oxidoreductase, NAD binding
G2583_3812	ECs3970	Z4441	alx	Inner membrane protein alx
G2583_3813	ECs3971	Z4442	sstT	Sodium:serine/threonine symporter
G2583_3814	ECs3972	Z4443	ygjV	Putative inner membrane protein
G2583_3815	ECs3973	Z4444	uxaA	Altronate dehydratase
G2583_3816	ECs3974	Z4445	uxaC	Uronate isomerase
G2583_3817	ECs3975	Z4446	exuT	Hexuronate transporter
G2583_3818	ECs3976	Z4448	exuR	Exu regulon transcriptional regulator
G2583_3819	ECs3977	Z4449	yqjA	hypothetical protein
G2583_3820	ECs3978	Z4450	yqjB	hypothetical protein
G2583_3821	ECs3979	Z4451	yqjC	hypothetical protein
G2583_3822	ECs3980	Z4452	yqjD	hypothetical protein
G2583_3823	ECs3981	Z4453	yqjE	ORF_o157; overlaps o101 by 64 bases, no frameshift found, other starts possible
G2583_3824	ECs3982	Z4454	yqjK	hypothetical protein
G2583_3825	ECs3983	Z4455	yqjF	Putative inner membrane protein YqjF
G2583_3826	ECs3984	Z4456	yqjG	putative transferase
G2583_3827	ECs3985	Z4457	yhaH	putative inner membrane protein YhaH
G2583_3828	ECs3986	Z4458	yhaI	Inner membrane protein yhaI
G2583_3829	ECs3987	Z4459	yhaJ	putative transcriptional regulator LYSR-type
G2583_3830	ECs3988	Z4460	yhaK	Pirin-like protein yhaK
G2583_3831	ECs3989	Z4461	yhaL	hypothetical protein
G2583_3832	ECs3990	Z4462	yhaM	UPF0597 protein yhaM
G2583_3833	ECs3991	Z4463	yhaO	putative transport system permease protein
G2583_3834	ECs3992	Z4464	tdcG	L-serine ammonia-lyase TdcG
G2583_3835	ECs3993	Z4465	tdcF	TdcF protein
G2583_3836	ECs3994	Z4466	tdcE	Keto-acid formate acetyltransferase
G2583_3837	ECs3995	Z4467	tdcD	Putative kinase
G2583_3838	ECs3996	Z4468	tdcC	Threonine/serine transporter tdcC (H(+)/threonine-serine symporter)
G2583_3839	ECs3997	Z4469	tdcB	Threonine dehydratase catabolic
G2583_3840	ECs3998	Z4470	tdcA	DNA-binding transcriptional activator
G2583_3841	ECs3999	Z4471	tdcR	Threonine dehydratase operon activator protein
G2583_3842	ECs4000	Z4472	yhaB	hypothetical protein
G2583_3843	ECs4001	Z4473	yhaC	hypothetical protein
G2583_3844			-	hypothetical protein
		Z4474	-	hypothetical protein
G2583_3845	rnpB	RNA083	rnpB	ncRNA

Locus_tag[a]				
CB9615	Sakai	EDL933	Gene	Product
G2583_3846	ECs4002	Z4476	garK	hypothetical protein
G2583_3847	ECs4003	Z4477	garR	2-hydroxy-3-oxopropionate reductase
G2583_3848	ECs4004	Z4478	garL	2-dehydro-3-deoxyglucarate aldolase
G2583_3849	ECs4005	Z4479	garP	Probable galactarate transporter
G2583_3850	ECs4006	Z4480	garD	Galactarate dehydratase
G2583_3851	ECs4007	Z4481	sohA	HtrA suppressor protein
G2583_3852	ECs4008	Z4482	yhaV	hypothetical protein
G2583_3853	ECs4009	Z4483	agaR	Putative aga operon transcriptional repressor
G2583_3854	ECs4010	Z4484	kbaZ	D-tagatose-bisphosphate aldolase, class II, non-catalytic subunit
G2583_3855	ECs4011	Z4485	agaV	N-acetylgalactosamine-specific PTS system enzyme IIB component
G2583_3856	ECs4012	Z4486	agaW	Putative phosphotransferase system enzyme subunit
G2583_3857	ECs4013	Z4487	agaE	PTS system N-acetylgalactosamine-specific, IID component
G2583_3858	ECs4014	Z4488	agaF	PTS system, mannose/sorbose-specific, IIA component
G2583_3859	ECs4015	Z4489	agaA	N-acetylgalactosamine-6-phosphate deacetylase
G2583_3860	ECs4016	Z4490	agaS	Putative sugar isomerase, AgaS family
G2583_3861	ECs4017	Z4491	kbaY	Tagatose-1,6-bisphosphate aldolase kbaY
G2583_3862	ECs4018	Z4492	agaB	PTS system, D galactosamine-specific, IIB component
G2583_3863	ECsp020	Z4493	agaC	N-acetylgalactosamine permease IIC component 1
G2583_3864	ECs4019	Z4494	agaD	N-acetylglucosamine transport enzyme IID component 1
G2583_3865		Z4495	agaI	putative galactosamine-6-phosphate isomerase
G2583_3865	ECsp021	Z4497	agaI	putative galactosamine-6-phosphate isomerase
G2583_3866	ECs4020	Z4498	yraH	Putative type 1 fimbrial protein
G2583_3867	ECs4021	Z4499	yraI	Gram-negative pili assembly chaperone protein
G2583_3868	ECs4022	Z4500	yraJ	Fimbrial usher family protein
G2583_3869	ECs4023	Z4501	yraK	Putative fimbrial protein
Indel-175	ECs4024	Z4502	-	putative transposase OrfA protein of insertion sequence IS629
Indel-175	ECs4025	Z4503	-	putative transposase OrfB protein of insertion sequence IS629
G2583_3869	ECs4026	Z4504	yraK	Putative fimbrial protein
G2583_3870	ECs4027	Z4505	yraL	UPF0011 protein yraL
G2583_3871	ECs4028	Z4506	yraM	hypothetical protein
G2583_3872	ECs4029	Z4507	yraN	UPF0102 protein yraN
G2583_3873	ECs4030	Z4508	diaA	DnaA initiator-associating protein diaA
G2583_3874	ECs4031	Z4509	yraP	hypothetical protein
G2583_3875	ECs4032	Z4510	yraQ	Putative permease
G2583_3876	ECs4033	Z4511	yraR	hypothetical protein
G2583_3877	ECs4034	Z4512	yhbO	hypothetical protein
G2583_3878	ECs4035	Z4514	yhbP	UPF0306 protein yhbP
G2583_3879	ECs4036	Z4516	yhbQ	UPF0213 protein yhbQ
G2583_3880	ECs4037	Z4517	yhbS	Uncharacterized acetyltransferase yhbS

Locus_tag[a]				
CB9615	Sakai	EDL933	Gene	Product
G2583_3881	ECs4038	Z4518	yhbT	hypothetical protein
G2583_3882	ECs4039	Z4519	yhbU	Peptidase, U32 family
G2583_3883	ECs4040	Z4520	yhbV	hypothetical protein
G2583_3884	ECs4041	Z4521	yhbW	Monooxygenase, luciferase family
G2583_3885	ECs4042	Z4522	mtr	Tryptophan-specific transport protein
G2583_3886	ECs4043	Z4523	deaD	Cold-shock DEAD-box protein A
G2583_3887	ECs4044	Z4524	nlpI	Lipoprotein nlpI precursor
G2583_3888	ECs4045	Z4525	pnp	Polynucleotide phosphorylase/polyadenylase
G2583_3889			-	ncRNA
G2583_3890	ECs4046	Z4526	rpsO	30S ribosomal protein S15
G2583_3891	ECs4047	Z4527	truB	tRNA pseudouridine synthase B
G2583_3892	ECs4048	Z4528	rbfA	Ribosome-binding factor A
G2583_3893	ECs4049	Z4529	infB	Translation initiation factor IF-2
G2583_3894	ECs4050	Z4530	nusA	Transcription elongation protein nusA
G2583_3895	ECs4051	Z4531	yhbC	hypothetical protein
G2583_3896	metY	RNA084	metY	Met tRNA
G2583_3897	ECs4052	Z4534	argG	Argininosuccinate synthase
G2583_3898	ECs4053	Z4535	yhbX	Outer-membrane protein yhbX
G2583_3899	leuU	RNA085	leuU	Leu tRNA
G2583_3900	ECs4054	Z4537	secG	Preprotein translocase IISP family, auxillary membrane component
G2583_3901	ECs4055	Z4538	glmM	Phosphoglucosamine mutase
G2583_3902	ECs4056	Z4539	folP	7,8-dihydropteroate synthase
G2583_3903	ECs4057	Z4540	hflB	Cell division protease ftsH
G2583_3904	ECs4058	Z4541	rrmJ	Ribosomal RNA large subunit methyltransferase J
G2583_3905	ECs4059	Z4542	yhbY	hypothetical protein
G2583_3906	ECs4060	Z4543	greA	Transcription elongation factor greA
G2583_3907	ECs4061	Z4544	dacB	D-alanyl-D-alanine carboxypeptidase/D-alanyl-D-alanine-endopeptidase
G2583_3908	ECs4062	Z4545	obgE	GTP-binding protein Obg/CgtA
G2583_3909	ECs4063	Z4546	yhbE	Uncharacterized inner membrane transporter yhbE
G2583_3910	ECs4064	Z4547	rpmA	50S ribosomal protein L27
G2583_3911	ECs4065	Z4549	rplU	50S ribosomal protein L21
G2583_3912	ECs4066	Z4550	ispB	hypothetical protein
G2583_3913	ECs4067	Z4551	sfsB	Sugar fermentation stimulation protein B
G2583_3914	ECs4068	Z4552	murA	UDP-N-acetylglucosamine 1-carboxyvinyltransferase
G2583_3915	ECs4069	Z4553	yrbA	Predicted transcriptional regulator, BolA superfamily
G2583_3916	ECs4070	Z4554	yrbB	hypothetical protein
G2583_3917	ECs4071	Z4555	yrbC	Toluene tolerance protein Ttg2D
G2583_3918	ECs4072	Z4556	yrbD	hypothetical protein
G2583_3919	ECs4073	Z4557	yrbE	UPF0393 inner membrane protein yrbE
G2583_3920	ECs4074	Z4558	yrbF	Uncharacterized ABC transporter ATP-binding protein yrbF
G2583_3921	ECs4075	Z4559	yrbG	Sodium/calcium exchanger protein

Locus_tag[a]				
CB9615	Sakai	EDL933	Gene	Product
G2583_3922	ECs4076	Z4560	kdsD	Arabinose 5-phosphate isomerase
G2583_3923	ECs4077	Z4561	kdsC	3-deoxy-D-manno-octulosonate 8-phosphate
G2583_3924	ECs4078	Z4562	yrbK	hypothetical protein
G2583_3925	ECs4079	Z4563	lptA	Protein yhbN precursor
G2583_3926	ECs4080	Z4564	lptB	Uncharacterized ABC transporter ATP-binding protein yhbG
G2583_3927	ECs4081	Z4565	rpoN	RNA polymerase sigma-54 factor
G2583_3928	ECs4082	Z4566	hpf	Probable sigma(54) modulation protein
G2583_3929	ECs4083	Z4567	ptsN	PTS IIA-like nitrogen-regulatory protein PtsN
G2583_3930	ECs4084	Z4568	yhbJ	UPF0042 protein yhbJ
G2583_3931	ECs4085	Z4569	npr	Phosphocarrier protein NPr
G2583_3932	ECs4086	Z4570	yrbL	hypothetical protein
G2583_3933	ECs4087	Z4571	mtgA	Monofunctional biosynthetic peptidoglycan transglycosylase
G2583_3934	ECs4088	Z4573	elbB	Sigma cross-reacting protein 27A
G2583_3935			-	ncRNA
G2583_3936	ECs4089	Z4574	arcB	Aerobic respiration control sensor protein arcB
G2583_3937	ECs4090	Z4575	yhcC	hypothetical protein
G2583_3938	ECs4091	Z4576	gltB	Glutamate synthase (NADPH), large subunit
G2583_3939	ECs4092	Z4577	gltD	Glutamate synthase (NADPH), small subunit
G2583_3940	ECs4093	Z4578	yhcG	conserved hypothetical protein
G2583_3941	ECs4094	Z4579	yhcH	hypothetical protein
G2583_3942	ECs4095	Z4580	nanK	N-acetylmannosamine kinase
G2583_3943	ECs4096	Z4581	nanE	Putative N-acetylmannosamine-6-phosphate 2-
G2583_3944	ECs4097	Z4582	nanT	Sialic acid transporter
G2583_3945	ECs4098	Z4583	nanA	N-acetylneuraminate lyase
G2583_3946	ECs4099	Z4584	nanR	Transcriptional regulators
G2583_3947	ECs4100	Z4585	dcuD	Putative C4-dicarboxylate carrier protein
G2583_3948	ECs4101	Z4586	sspB	Stringent starvation protein B
G2583_3949	ECs4102	Z4587	sspA	Stringent starvation protein A
G2583_3950	ECs4103	Z4588	rpsI	30S ribosomal protein S9
G2583_3951	ECs4104	Z4589	rplM	50S ribosomal protein L13
G2583_3952	ECs4105	Z4591	yhcM	hypothetical protein
G2583_3953	ECs4106	Z4592	yhcB	hypothetical protein
G2583_3954	ECs4107	Z4593	degQ	Serine peptidase DegQ
G2583_3955	ECs4108	Z4594	degS	Protease degS precursor
G2583_3956	ECs4109	Z4595	mdh	Malate dehydrogenase
G2583_3957	ECs4110	Z4596	argR	Arginine repressor
G2583_3958	ECs4111	Z4597	yhcN	hypothetical protein
G2583_3959	ECs4112	Z4598	yhcO	hypothetical protein
G2583_3960	ECs4113	Z4599	aaeB	p-hydroxybenzoic acid efflux pump subunit aaeB
G2583_3961	ECs4114	Z4600	aaeA	p-hydroxybenzoic acid efflux pump subunit aaeA
G2583_3962	ECs4115	Z4601	aaeX	hypothetical protein
G2583_3963	ECs4116	Z4602	aaeR	putative DNA-binding transcriptional regulator

Locus_tag[a]				
CB9615	Sakai	EDL933	Gene	Product
G2583_3964	ECs4117	Z4603	tldD	suppresses inhibitory activity of CsrA
G2583_3965	ECs4118	Z4604	yhdP	hypothetical protein
G2583_3966	ECs4119	Z4605	rng	Ribonuclease G
G2583_3967	ECs4120	Z4606	yhdE	Maf-like protein yhdE
G2583_3968	ECs4121	Z4607	mreD	Rod shape-determining protein
G2583_3969	ECs4122	Z4609	mreC	Rod shape-determining protein MreC
G2583_3970	ECs4123	Z4610	mreB	Rod shape-determining protein mreB
G2583_3971	ECs4124	Z4611	csrD	Cyclic diguanylate phosphodiesterase (EAL) domain
G2583_3972	ECs4125	Z4612	yhdH	Quinone oxidoreductase, YhdH family
G2583_3973	ECs4126	Z4613	-	hypothetical protein
		Z4614	-	hypothetical protein
G2583_3974	ECs4127	Z4615	accB	acetyl-CoA carboxylase, biotin carboxyl carrier protein
G2583_3975	ECs4128	Z4616	accC	Acetyl CoA carboxylase, biotin carboxylase subunit
G2583_3976	ECs4129	Z4617	yhdT	hypothetical protein
G2583_3977	ECs4130	Z4618	panF	Sodium/pantothenate symporter
G2583_3978	ECs4131	Z4619	prmA	Ribosomal protein L11 methyltransferase
G2583_3979	ECs4132	Z4620	dusB	tRNA-dihydrouridine synthase B
G2583_3980	ECs4133	Z4621	fis	DNA-binding protein fis
G2583_3981	ECs4134	Z4622	yhdJ	DNA methylase, N4/N6-methyltransferase family
G2583_3982	ECs4135	Z4623	yhdU	hypothetical protein
G2583_3983	ECs4136	Z4624	envR	Probable acrEF/envCD operon repressor
G2583_3984	ECs4137	Z4625	acrE	Acriflavine resistance protein E
G2583_3985	ECs4138	Z4626	acrF	Transporter, hydrophobe/amphiphile efflux-1 (HAE1) family precursor
G2583_3985	ECs4139	Z4627	acrF	Transporter, hydrophobe/amphiphile efflux-1 (HAE1) family precursor
G2583_3986	ECs4140	Z4628	yhdV	hypothetical protein
G2583_3987	ECs4141	Z4629	yhdW	Putative amino-acid ABC transporter-binding protein yhdW precursor
G2583_3988	ECs4142	Z4630	yhdX	Amino acid ABC transporter, permease protein, His/Glu/Gln/Arg/opine family
G2583_3989	ECs4143	Z4631	yhdY	general L-amino acid transport system permease protein AapM
G2583_3990	ECs4144	Z4632	yhdZ	Amino acid ABC transporter, ATP-binding protein
G2583_3991	rrfF	RNA086	rrf	5S ribosomal RNA
G2583_3992	thrV	RNA087	thrV	Thr tRNA
G2583_3993	rrfD	RNA088	rrf	5S ribosomal RNA
G2583_3994	rrlD	RNA089	rrl	23S ribosomal RNA
G2583_3995	alaU	RNA090	alaU	Ala tRNA
G2583_3996	ileU	RNA091	ileU	Ile tRNA
G2583_3997	rrsD	RNA092	rrs	16S ribosomal RNA
G2583_3998	ECs4145	Z4650	yrdA	hypothetical protein
G2583_3999	ECs4146	Z4651	yrdB	hypothetical protein
G2583_4000	ECs4147	Z4652	aroE	Shikimate dehydrogenase

Locus_tag[a]				
CB9615	Sakai	EDL933	Gene	Product
G2583_4001	ECs4148	Z4653	rimN	Sua5/YciO/YrdC/YwlC family protein
G2583_4002	ECs4149	Z4654	yrdD	predicted DNA topoisomerase
G2583_4003	ECs4150	Z4655	smg	hypothetical protein
G2583_4004	ECs4151	Z4656	smf	hypothetical protein
G2583_4005	ECs4152	Z4657	def	Peptide deformylase
G2583_4006	ECs4153	Z4658	fmt	Methionyl-tRNA formyltransferase
G2583_4007	ECs4154	Z4659	rsmB	Ribosomal RNA small subunit methyltransferase B
G2583_4008	ECs4155	Z4660	trkA	Trk system potassium uptake protein trkA (K(+)-uptake protein trkA)
G2583_4009	ECs4156	Z4661	mscL	Large-conductance mechanosensitive channel
G2583_4010	ECs4157	Z4662	zntR	zinc-responsive transcriptional regulator
G2583_4011	ECs4158	Z4663	yhdN	hypothetical protein
G2583_4012	ECs4159	Z4664	rplQ	50S ribosomal protein L17
G2583_4013	ECs4160	Z4665	rpoA	DNA-directed RNA polymerase subunit alpha
G2583_4014	ECs4161	Z4666	rpsD	30S ribosomal protein S4
G2583_4015	ECs4162	Z4667	rpsK	30S ribosomal protein S11
G2583_4016	ECs4163	Z4668	rpsM	30S ribosomal protein S13
G2583_4017	ECs4164	Z4669	rpmJ	ribosomal protein L36
G2583_4018	ECs4165	Z4670	secY	Preprotein translocase subunit secY
G2583_4019	ECs4166	Z4671	rplO	50S ribosomal protein L15
G2583_4020	ECs4167	Z4672	rpmD	50S ribosomal protein L30
G2583_4021	ECs4168	Z4673	rpsE	30S ribosomal protein S5
G2583_4022	ECs4169	Z4674	rplR	50S ribosomal protein L18
G2583_4023	ECs4170	Z4675	rplF	50S ribosomal protein L6
G2583_4024	ECs4171	Z4676	rpsH	30S ribosomal protein S8
G2583_4025	ECs4172	Z4677	rpsN	30S ribosomal protein S14
G2583_4026	ECs4173	Z4678	rplE	50S ribosomal protein L5
G2583_4027	ECs4174	Z4679	rplX	50S ribosomal protein L24
G2583_4028	ECs4175	Z4680	rplN	50S ribosomal protein L14
G2583_4029	ECs4176	Z4681	rpsQ	30S ribosomal protein S17
G2583_4030	ECs4177	Z4683	rpmC	50S ribosomal protein L29
G2583_4031	ECs4178	Z4684	rplP	50S ribosomal protein L16
G2583_4032	ECs4179	Z4685	rpsC	30S ribosomal protein S3
G2583_4033	ECs4180	Z4686	rplV	50S ribosomal protein L22
G2583_4034	ECs4181	Z4687	rpsS	30S ribosomal protein S19
G2583_4035	ECs4182	Z4688	rplB	50S ribosomal protein L2
G2583_4036	ECs4183	Z4689	rplW	50S ribosomal protein L23
G2583_4037	ECs4184	Z4690	rplD	50S ribosomal protein L4
G2583_4038	ECs4185	Z4691	rplC	50S ribosomal protein L3
G2583_4039	ECs4186	Z4692	rpsJ	Ribosomal protein S10
G2583_4040	ECs4187		-	hypothetical protein
G2583_4041	ECs4188	Z4693	gspO	Peptidase, A24 (Type IV prepilin peptidase) family
G2583_4042	ECs4189	Z4695	bfr	Bacterioferritin
G2583_4043		Z4696	bfd	Bacterioferritin-associated ferredoxin

Locus_tag[a]				
CB9615	Sakai	EDL933	Gene	Product
G2583_4044	ECs4190	Z4697	tufA	Elongation factor Tu 1
G2583_4045	ECs4191	Z4698	fusA	Elongation factor G
G2583_4046	ECs4192	Z4699	rpsG	30S ribosomal protein S7
G2583_4047	ECs4193	Z4700	rpsL	Ribosomal protein S12
G2583_4048	ECs4194	Z4701	yheL	sulfur relay protein TusD/DsrH
G2583_4049	ECs4195	Z4702	yheM	hypothetical protein
G2583_4050	ECs4196	Z4703	yheN	Sulfurtransferase tusD
G2583_4051	ECs4197	Z4704	yheO	hypothetical protein
G2583_4052	ECs4198	Z4705	fkpA	FKBP-type peptidyl-prolyl cis-trans isomerase fkpA precursor
G2583_4053	ECs4199	Z4706	slyX	slyX protein
G2583_4054	ECs4200	Z4707	slyD	FKBP-type peptidyl-prolyl cis-trans isomerase SlyD
G2583_4055		Z4708	yheV	hypothetical protein
G2583_4056	ECs4201	Z4710	kefB	Glutathione-regulated potassium-efflux system protein kefB (K(+)/H(+) antiporter) (NEM-activatable K(+)/H(+) antiporter)
G2583_4057	ECs4202	Z4712	kefG	Glutathione-regulated potassium-efflux system ancillary protein kefG
G2583_4058	ECs4203	Z4713	yheS	Uncharacterized ABC transporter ATP-binding protein yheS
G2583_4059	ECs4204	Z4714	yheT	Hydrolase, alpha/beta fold family
G2583_4060	ECs4205	Z4715	yheU	UPF0270 protein yheU
G2583_4061	ECs4206	Z4716	prkB	Phosphoribulokinase/uridine kinase family protein
G2583_4062	ECs4207	Z4717	yhfA	Protein yhfA
G2583_4063	ECs4208	Z4718	crp	Catabolite gene activator
G2583_4064	ECs4209	Z4719	yhfK	integral membrane protein, YccS/YhfK family
G2583_4065	ECs4210	Z4720	argD	Acetylornithine/succinyldiaminopimelate
G2583_4066	ECs4211	Z4721	pabA	Aminodeoxychorismate synthase, component II
G2583_4067	ECs4212	Z4722	fic	Cell filamentation protein Fic
G2583_4068	ECs4213	Z4723	yhfG	hypothetical protein
G2583_4069	ECs4214	Z4724	ppiA	Peptidyl-prolyl cis-trans isomerase
G2583_4070	ECs4215	Z4725	tsgA	hypothetical protein
G2583_4071	ECs4216	Z4726	nirB	Nitrite reductase [NAD(P)H], large subunit
G2583_4072	ECs4217	Z4727	nirD	Nitrite reductase [NAD(P)H] large subunit
G2583_4073	ECs4218	Z4728	nirC	Response regulator receiver protein
G2583_4074	ECs4219	Z4729	cysG	Siroheme synthase [Includes: Uroporphyrinogen-III C-methyltransferase
G2583_4075	ECs4220	Z4730	yhfL	putative lipoprotein
G2583_4076	ECs4221	Z4731	frlA	Amino acid transporters
G2583_4077	ECs4222	Z4732	frlB	SIS family protein
G2583_4078	ECs4223	Z4733	frlC	predicted isomerase
G2583_4079	ECs4224	Z4734	-	hypothetical protein
G2583_4079		Z4735	-	hypothetical protein
G2583_4080	ECs4225	Z4736	frlR	GntR-family transcriptional regulator FrlR

Locus_tag[a]				
CB9615	Sakai	EDL933	Gene	Product
G2583_4081	ECs4226	Z4737	trpS	Tryptophanyl-tRNA synthetase
G2583_4082	ECs4227	Z4738	gph	Phosphoglycolate phosphatase
G2583_4083	ECs4228	Z4739	rpe	Ribulose-phosphate 3-epimerase
G2583_4084	ECs4229	Z4740	dam	DNA adenine methylase
G2583_4085	ECs4230	Z4741	damX	Uncharacterized protein conserved in bacteria
G2583_4086	ECs4231	Z4742	aroB	3-dehydroquinate synthase
G2583_4087	ECs4232	Z4743	aroK	Shikimate kinase
G2583_4088	ECs4233	Z4744	hofQ	predicted fimbrial transporter
G2583_4089	ECs4234	Z4746	hofP	hypothetical protein
G2583_4089	ECs4235	Z4747	hofP	hypothetical protein
G2583_4090	ECs4236	Z4748	hofN	PilN family protein
G2583_4091	ECs4237	Z4749	hofM	hypothetical protein
G2583_4092	ECs4238	Z4750	mrcA	Penicillin-binding protein 1A
G2583_4093	ECs4239	Z4751	nudE	ADP compounds hydrolase NudE
G2583_4094	ECs4240	Z4752	yrfF	hypothetical protein
G2583_4095	ECs4241	Z4753	yrfG	Putative phosphatase
G2583_4096	ECs4242	Z4754	hslR	Heat shock protein 15
G2583_4097	ECs4243	Z4755	hslO	Disulfide bond chaperones of the HSP33 family
G2583_4098	ECs4244	Z4756	yhgE	putative transport
G2583_4099		Z4757	-	hypothetical protein
G2583_4100	ECs4245	Z4758	pck	Phosphoenolpyruvate carboxykinase [ATP]
G2583_4101	ECs4246	Z4759	envZ	Osmolarity sensor protein envZ
G2583_4102	ECs4247	Z4760	ompR	hypothetical protein
G2583_4103	ECs4248	Z4761	greB	Transcription elongation factor greB
G2583_4104	ECs4249	Z4762	yhgF	protein yhgF
G2583_4105	ECs4250	Z4763	feoA	Ferrous iron transport protein A
G2583_4106	ECs4251	Z4764	feoB	Ferrous iron transport protein B
G2583_4107	ECs4252	Z4765	feoC	conserved hypothetical protein
G2583_4108	ECs4253	Z4766	yhgA	hypothetical protein
G2583_4109	ECs4255	Z4767	bioH	Carboxylesterase bioH
G2583_4110	ECs4254	Z4768	gntX	gluconate periplasmic binding protein
G2583_4111	ECs4256	Z4769	gntY	Fe/S biogenesis protein nfuA
G2583_4112	ECs4257	Z4770	gntT	High-affinity gluconate transporter
G2583_4113	ECs4258	Z4771	malQ	4-alpha-glucanotransferase
G2583_4114	ECs4259	Z4772	malP	Maltodextrin phosphorylase
		Z4773	-	hypothetical protein
G2583_4115	ECs4260	Z4774	malT	transcriptional regulator MalT
G2583_4116		Z4775	-	hypothetical protein
G2583_4117	ECs4261	Z4776	-	hypothetical protein
G2583_4118	ECs4262	Z4777	-	Acetyltransferase, gnat family
G2583_4119	ECs4263	Z4778	rtcA	RNA 3'-terminal phosphate cyclase
G2583_4120	ECs4264	Z4779	rtcB	RtcB protein
G2583_4121	ECs4265	Z4780	rtcR	Sigma-54 dependent transcriptional regulator RtcR
G2583_4122	ECs4266	Z4781	glpR	Glycerol-3-phosphate regulon repressor

Locus_tag[a]				
CB9615	Sakai	EDL933	Gene	Product
G2583_4123	ECs4267	Z4784	glpG	Rhomboid protease glpG
G2583_4124	ECs4268	Z4785	glpE	Thiosulfate sulfurtransferase glpE
G2583_4125	ECs4269	Z4786	glpD	Glycerol-3-phosphate dehydrogenase
G2583_4126	ECs4270	Z4787	-	hypothetical protein
G2583_4127	ECs4271	Z4788	-	hypothetical protein
G2583_4128	ECs4272	Z4789	yzgL	Hypothetical membrane protein
G2583_4129	ECs4273	Z4790	glgP	Glycogen phosphorylase
G2583_4130	ECs4274	Z4791	glgA	Glycogen synthase
G2583_4131	ECs4275	Z4792	glgC	Glucose-1-phosphate adenylyltransferase
G2583_4132	ECs4276	Z4794	glgX	Glycogen debranching enzyme
G2583_4133	ECs4277	Z4796	glgB	1,4-alpha-glucan-branching enzyme
G2583_4134	ECs4278	Z4797	asd	Aspartate-semialdehyde dehydrogenase
G2583_4135	ECs4279	Z4798	yhgN	UPF0056 inner membrane protein yhgN
G2583_4136	ECs4280	Z4799	-	Putative DNA processing protein
G2583_4137	ECs4281		-	putative ATP-dependent DNA helicase
G2583_4137	ECs4282	Z4801	-	putative ATP-dependent DNA helicase
G2583_4137	ECs4283	Z4802	-	putative ATP-dependent DNA helicase
G2583_4137	ECs4284	Z4803	-	putative ATP-dependent DNA helicase
G2583_4137	ECs4283		-	putative ATP-dependent DNA helicase
G2583_4138	ECs4285	Z4804	gntU	H+/gluconate symporter and related permeases
G2583_4139	ECs4286	Z4805	gntK	Thermoresistant gluconokinase
G2583_4140	ECs4287	Z4806	gntR	regulator of gluconate operon
G2583_4141	ECs4288	Z4807	yhhW	hypothetical protein
G2583_4142	ECs4289	Z4808	yhhX	Oxidoreductase, NAD-binding
G2583_4143			-	ncRNA
G2583_4144	ECs4290	Z4809	yhhY	Acetyltransferase, GNAT family
G2583_4145	ECs4291	Z4810	-	hypothetical protein
G2583_4146	ECs4292	Z4811	-	hypothetical protein
G2583_4147	ECs4293	Z4813	ggt	Gamma-glutamyltranspeptidase
G2583_4148	ECs4294	Z4815	yhhA	hypothetical protein
G2583_4149	ECs4295	Z4817	ugpQ	Glycerophosphodiester phosphodiesterase, cytosolic
G2583_4150	ECs4296	Z4818	ugpC	sn-glycerol-3-phosphate import ATP-binding protein
G2583_4151	ECs4297	Z4819	ugpE	sn-glycerol-3-phosphate transport system permease protein ugpE
G2583_4152	ECs4298	Z4820	ugpA	sn-glycerol-3-phosphate transport system permease protein ugpA
G2583_4153	ECs4299	Z4822	ugpB	sn-glycerol-3-phosphate-binding periplasmic protein ugpB precursor
G2583_4154	ECs4300	Z4823	-	hypothetical protein
G2583_4155	ECs4301	Z4824	livF	ATP-binding component of leucine transport
G2583_4156	ECs4302	Z4825	livG	High-affinity branched-chain amino acid transport ATP-binding protein livG
G2583_4157	ECs4303	Z4826	livM	High-affinity branched-chain amino acid ABC transporter, permease protein LivM

Locus_tag[a]				
CB9615	Sakai	EDL933	Gene	Product
G2583_4158	ECs4304	Z4827	livH	High-affinity branched-chain amino acid transport system permease protein livH
G2583_4159	ECs4305	Z4829	livK	High-affinity branched-chain amino acid ABC transporter, periplasmic leucine-specific-binding protein
G2583_4160	ECs4306	Z4831	yhhK	Acetyltransferase, GNAT family
G2583_4161	ECs4307	Z4832	-	hypothetical protein
G2583_4162	ECs4308	Z4833	-	hypothetical protein
G2583_4163	ECs4309	Z4834	livJ	High-affinity amino acid transport protein, periplasmic binding protein
G2583_4164	ECs4310	Z4835	rpoH	RNA polymerase sigma factor
G2583_4165	ECs4311	Z4836	ftsX	Cell division protein ftsX
G2583_4166	ECs4312	Z4837	ftsE	Cell division ATP-binding protein ftsE
G2583_4167	ECs4313	Z4838	ftsY	Cell division protein FtsY
G2583_4168	ECs4314	Z4839	rsmD	16S rRNA methyltransferase RsmD
G2583_4169	ECs4315	Z4840	yhhL	hypothetical protein
G2583_4170	ECs4316	Z4841	yhhM	putative receptor
G2583_4171	ECs4317	Z4842	yhhN	Uncharacterized membrane protein yhhN
G2583_4172	ECs4318	Z4843	zntA	Cadmium-translocating P-type ATPase
G2583_4173	ECs4319	Z4844	sirA	Sulfurtransferase tusA
G2583_4174	ECs4320	Z4845	yhhQ	hypothetical protein
G2583_4175	ECs4321	Z4846	dcrB	hypothetical protein
G2583_4176	ECs4322	Z4847	yhhS	Transporter, major facilitator family
G2583_4177	ECs4323	Z4848	yhhT	hypothetical protein
G2583_4178	ECs4324	Z4849	-	hypothetical protein
G2583_4179	ECs4325	Z4850	-	O-methyltransferase, family 2
G2583_4180	ECs4326	Z4851	-	Beta-ketoacyl synthase domain protein
G2583_4181	ECs4327	Z4852	-	Putative phospholipid biosynthesis acyltransferase
G2583_4182	ECs4328	Z4853	-	Acyl carrier protein
G2583_4183	ECs4329	Z4854	acyl	acyl carrier protein
G2583_4184	ECs4330	Z4855	-	DNA gyrase subunit B
G2583_4185	ECs4331	Z4856	-	Putative surfactin synthetase
G2583_4186	ECs4332	Z4857	-	hypothetical protein
G2583_4187	ECs4333	Z4858	-	Glycosyl transferase, family 2
G2583_4188	ECs4334	Z4859	-	Thioesterase superfamily protein
G2583_4189	ECs4335	Z4860	-	Outer membrane lipoprotein carrier protein LolA
G2583_4190	ECs4336	Z4861	-	hypothetical protein
G2583_4191	ECs4337	Z4862	-	hypothetical protein
G2583_4192	ECs4338	Z4863	-	3-oxoacyl-(acyl carrier protein) synthase I
G2583_4193	ECs4339	Z4864	-	FabA-like domain protein
G2583_4194	ECs4340	Z4865	fabG	Putative 3-oxoacyl-(Acyl-carrier-protein) reductase
G2583_4195	ECs4341	Z4866	-	Beta-ketoacyl synthase
G2583_4196	ECs4342	Z4867	acpT	4'-phosphopantetheinyl transferase acpT
G2583_4197	ECs4343	Z4868	nikA	Nickel ABC transporter, periplasmic nickel-binding protein NikA

Locus_tag[a]				
CB9615	Sakai	EDL933	Gene	Product
G2583_4198	ECs4344	Z4869	nikB	Nickel transport system permease protein nikB
G2583_4199	ECs4345	Z4870	nikC	Nickel transport system permease protein nikC
G2583_4200	ECs4346	Z4871	nikD	Nickel import ATP-binding protein nikD
G2583_4201	ECs4347	Z4872	nikE	Nickel import ATP-binding protein nikE
G2583_4202	ECs4348	Z4873	nikR	Nickel-responsive regulator
G2583_4203	ECs4349	Z4874	-	putative regulator
G2583_4204	ECs4350	Z4875	-	PEP-dependent sugar transporting PTS family, IIA component
G2583_4205	ECs4351	Z4876	-	PEP-dependent sugar transporting PTS family, IIB component
G2583_4206	ECs4352	Z4877	-	PEP-dependent sugar transporting PTS family, IIC component
G2583_4207	ECs4353	Z4878	-	Carbohydrate kinase, FGGY family
G2583_4208	ECs4354	Z4879	-	Phosphocarrier, HPr family
G2583_4209	ECs4355	Z4881	gatY	Fructose-bisphosphate aldolase, class II
G2583_4210	ECs4356	Z4882	hicB	HicB family protein
G2583_4211	ECs4357	Z4883	HicA	HicA-like protein
G2583_4212	ECs4358	Z4884	yhhJ	hypothetical protein
G2583_4213	ECs4359	Z4885	rbbA	ABC transporter, ATP binding protein
G2583_4214	ECs4360	Z4886	yhiI	Auxiliary transport protein, membrane fusion protein (MFP) family
G2583_4215	ECs4361	Z4887	yhiJ	hypothetical protein
G2583_4216	ECs4362	Z4888	yhiL	hypothetical protein
G2583_4217	ECs4363	Z4890	yhiM	Inner membrane protein yhiM
G2583_4218	ECs4364	Z4891	yhiN	Pyridine nucleotide-disulfide oxidoreductase family
G2583_4219	ECs4365	Z4893	pitA	Low-affinity inorganic phosphate transporter 1
G2583_4220	ECs4366	Z4894	uspB	Universal stress protein B
G2583_4221	ECs4367	Z4895	uspA	Universal stress protein A
G2583_4222	ECs4368	Z4896	yhiP	Hypothetical transporter YhiP
G2583_4223	ECs4369	Z4897	yhiQ	UPF0341 protein yhiQ
G2583_4224	ECs4370	Z4898	prlC	Oligopeptidase A
G2583_4225	ECs4371	Z4899	yhiR	DNA utilization protein YhiR
G2583_4226	ECs4372	Z4900	gor	Glutathione-disulfide reductase
G2583_4227	ECs4373	Z4903	arsR	Arsenical resistance operon repressor
G2583_4228	ECs4374	Z4904	arsB	Arsenical pump membrane protein
G2583_4229	ECs4375	Z4905	arsC	Arsenate reductase
G2583_4230		Z4906	-	hypothetical protein
G2583_4231	ECs4376	Z4907	yhiS	hypothetical protein
G2583_4232	ECs4377	Z4908	slp	Outer membrane lipoprotein, Slp family
G2583_4233	ECs4378	Z4909	dctR	hypothetical protein
G2583_4234	ECs4379	Z4910	chuS	Hemin transport protein HmuS
G2583_4235	ECs4380	Z4911	chuA	Outer membrane heme/hemoglobin receptor ChuA
G2583_4236	ECs4381	Z4912	-	hypothetical protein
G2583_4237	ECs4382	Z4913	chuT	Putative periplasmic binding protein

Locus_tag[a]				
CB9615	Sakai	EDL933	Gene	Product
G2583_4238	ECs4383	Z4914	chuW	Putative coproporphyrinogen III oxidase
G2583_4239	ECs4384	Z4915	chuX	hypothetical protein
G2583_4240	ECs4385	Z4917	chuY	hypothetical protein
G2583_4241	ECs4386	Z4918	chuU	Putative permease of iron compound ABC transport system
G2583_4242	ECs4387	Z4919	shuV	Hemin import ATP-binding protein hmuV
G2583_4243	ECs4388	Z4920	yhiD	putative Mg2+ transporter-C (MgtC) family protein
G2583_4244	ECs4389	Z4921	hdeB	acid-resistance protein
G2583_4245	ECs4390	Z4922	hdeA	Chaperone-like protein hdeA precursor
G2583_4246	ECs4391	Z4923	hdeD	acid-resistance membrane protein
G2583_4247	ECs4392	Z4925	gadE	hypothetical protein
G2583_4248	ECs4393	Z4926	mdtE	Multidrug resistance protein mdtE precursor
G2583_4249	ECs4394	Z4927	mdtF	Multidrug resistance protein mdtF
G2583_4250	ECs4395	Z4928	gadW	putative ARAC-type regulatory protein
G2583_4251			-	ncRNA
G2583_4252	ECs4396	Z4929	gadX	DNA-binding transcriptional dual regulator
G2583_4253	ECs4397	Z4930	gadA	Glutamate decarboxylase alpha
G2583_4254	ECs4398	Z4931	yhjA	Di-haem cytochrome c peroxidase family protein
G2583_4255	ECs4399	Z4932	treF	Cytoplasmic trehalase
G2583_4256	ECs4400	Z4933	yhjB	Putative HTH-type transcriptional regulator yhjB
G2583_4257	ECs4401	Z4934	yhjC	putative transcriptional regulator LYSR-type
G2583_4258	ECs4402	Z4935	yhjD	Putative ribonuclease
G2583_4259	ECs4403	Z4936	yhjE	Inner membrane metabolite transport protein yhjE
G2583_4260	ECs4404	Z4937	yhjG	hypothetical protein
G2583_4261	ECs4405	Z4939	yhjH	Cyclic diguanylate phosphodiesterase
G2583_4262	ECs4406	Z4940	kdgK	Ketodeoxygluconokinase
G2583_4263	ECs4407	Z4941	yhjJ	Predicted Zn-dependent peptidases
G2583_4264	ECs4408	Z4942	dctA	C4-dicarboxylate transport protein
G2583_4265	ECs4409	Z4943	yhjK	Putative diguanylate cyclase
G2583_4266	ECs4410	Z4944	bcsC	Cellulose synthase operon protein C
G2583_4266		Z4945	bcsC	Cellulose synthase operon protein C
G2583_4267	ECs4411	Z4946	bcsZ	Endoglucanase precursor
G2583_4268	ECs4412	Z4947	bcsB	Cyclic di-GMP binding protein
G2583_4269	ECs4413	Z4948	bcsA	Cellulose synthase catalytic subunit [UDP-forming]
G2583_4270	ECs4414	Z4949	yhjQ	ATPases involved in chromosome partitioning
G2583_4270		Z4950	yhjQ	ATPases involved in chromosome partitioning
G2583_4271	ECs4415	Z4951	yhjR	hypothetical protein
G2583_4272	ECs4416	Z4952	bcsE	Putative protease
G2583_4273	ECs4417	Z4953	bcsF	hypothetical protein
G2583_4274	ECs4418	Z4954	bcsG	hypothetical protein
G2583_4275		Z4955	ldrD	hypothetical protein
G2583_4276	ECs4419	Z4956	yhjV	Serine transporter family protein
G2583_4277	ECs4420	Z4957	dppF	ABC-type oligopeptide transport system, ATPase component

Locus_tag[a]				
CB9615	Sakai	EDL933	Gene	Product
G2583_4278	ECs4421	Z4958	dppD	Dipeptide transport ATP-binding protein dppD
G2583_4279	ECs4422	Z4959	dppC	Dipeptide transport system permease protein dppC
G2583_4280	ECs4423	Z4960	dppB	Dipeptide transport system permease protein dppB
G2583_4281	ECs4424	Z4961	dppA	Dipeptide ABC transporter, periplasmic dipeptide-binding protein
G2583_4282	proK	RNA093	proK	Pro tRNA
G2583_4283	ECs4425	Z4964	eptB	hypothetical protein
G2583_4284	ECs4426	Z4965	lpfE	putative fimbrial subunit
G2583_4285	ECs4427	Z4966	lpfD	putative fimbrial protein
G2583_4286	ECs4428	Z4967	fimD	PapC-like porin protein involved in fimbrial biogenesis
G2583_4286	ECs4429	Z4968	fimD	PapC-like porin protein involved in fimbrial biogenesis
G2583_4287	ECs4430	Z4969	lpfB	Putative fimbrial chaperone
G2583_4288	ECs4431	Z4971	lpfA	Putative major fimbrial subunit
G2583_4289	ECs4432	Z4972	yhjX	Inner membrane protein YhjX
G2583_4290	ECs4433	Z4973	yhjY	Putative lipase
G2583_4291	ECs4434	Z4974	tag	3-methyladenine DNA glycosylase I
G2583_4292	ECs4435	Z4975	yiaC	Acetyltransferase, GNAT family
G2583_4293	ECs4436	Z4976	bisC	Biotin sulfoxide reductase
G2583_4294	ECs4437	Z4977	yiaD	Inner membrane lipoprotein yiaD precursor
G2583_4295	ECs4438	Z4978	ghrB	2-ketogluconate reductase
G2583_4296	ECs4439	Z4979	yiaF	hypothetical protein
G2583_4297	ECs4440	Z4980	yiaG	putative transcriptional regulator
G2583_4298	ECs4441	Z4981	cspA	Cold shock protein cspA
G2583_4299		Z4982	hokA	small toxic polypeptide
G2583_4300	ECs4442	Z4983	glyS	Glycyl-tRNA synthetase beta subunit
G2583_4301	ECs4443	Z4984	glyQ	Glycyl-tRNA synthetase alpha subunit
G2583_4302		Z4985	ysaB	Uncharacterized lipoprotein ysaB precursor
G2583_4303	ECs4444	Z4986	wecH	Acyltransferase family protein
G2583_4304	ECs4445	Z4987	yiaA	Inner membrane protein yiaA
G2583_4305	ECs4446	Z4988	yiaB	hypothetical protein
G2583_4306	ECs4447	Z4989	xylB	Xylulokinase
G2583_4307	ECs4448	Z4990	xylA	Xylose isomerase
G2583_4308	ECs4449	Z4991	xylF	Xylose binding protein transport system
G2583_4309	ECs4450	Z4992	xylG	Xylose import ATP-binding protein xylG
G2583_4310	ECs4451	Z4993	xylH	Xylose transport system permease protein xylH
G2583_4311	ECs4452	Z4994	xylR	Xylose operon regulatory protein
G2583_4312	ECs4453	Z4995	bax	hypothetical protein
G2583_4313	ECs4454	Z4996	malS	Alpha-amylase, periplasmic
G2583_4314	ECs4455	Z4997	avtA	Alanine-alpha-ketoisovalerate (Or valine-pyruvate) transaminase, transaminase C
G2583_4315	ECs4456	Z4998	ysaA	4Fe-4S binding domain protein
		Z4999	-	hypothetical protein
G2583_4316	ECs4457	Z5000	-	AraC-type DNA-binding domain-containing protei ns
G2583_4317	ECs4458	Z5001	-	Sugar transporter, glycoside-pentoside-hexuronide

Locus_tag[a]				
CB9615	Sakai	EDL933	Gene	Product
G2583_4318	ECs4459	Z5002	-	hypothetical protein
G2583_4319	ECs4460	Z5003	yiaT	Putative outer membrane protein yiaT precursor
G2583_4320	ECs4461	Z5004	yiaU	Transcriptional regulator
G2583_4321	ECs4462	Z5006	yiaV	Auxiliary transport protein, membrane fusion protein family
G2583_4322	ECs4463	Z5007	yiaW	Inner membrane protein yiaW
G2583_4323	ECs4464	Z5008	aldB	Aldehyde dehydrogenase B
G2583_4324	ECs4465	Z5009	-	Fic family protein
G2583_4325	ECs4466	Z5010	yiaY	Alcohol dehydrogenase II
G2583_4326	ECs4467	Z5011	selB	Selenocysteine-specific translation elongation factor
G2583_4327	ECs4468	Z5012	selA	L-seryl-tRNA(Sec) selenium transferase
G2583_4328	ECs4469	Z5013	yibF	Glutathione S-transferase
G2583_4329	ECs4470	Z5014	rhsA	Rhs family protein
Indel-182	Indel-182	Z5015	yibA	hypothetical protein
G2583_4330	ECs4470	Z5017	yibJ	Rhs family protein
G2583_4331	ECs4471	Z5018	yibG	hypothetical protein
G2583_4332	ECs4472		yibV	hypothetical protein
G2583_4333	ECs4473	Z5021	yibH	Inner membrane protein yibH
G2583_4334	ECs4474	Z5022	yibI	hypothetical protein
G2583_4335	ECs4475	Z5023	mtlA	PTS system, mannitol-specific IIABC component
G2583_4336	ECs4476	Z5024	mtlD	Mannitol-1-phosphate dehydrogenase
G2583_4337	ECs4477	Z5025	mtlR	Mannitol operon repressor
G2583_4338	ECs4478	Z5026	yibL	conserved hypothetical protein
G2583_4339		Z5027	-	hypothetical protein
G2583_4340	ECs4479	Z5028	-	putative lipoprotein
G2583_4341	ECs4480	Z5029	-	Putative adhesin
G2583_4342	ECs4481	Z5030	lldP	L-lactate permease
G2583_4343	ECs4482	Z5031	lldR	Putative L-lactate dehydrogenase operon regulatory protein
G2583_4344	ECs4483	Z5032	lldD	L-lactate dehydrogenase [cytochrome]
G2583_4345	ECs4484	Z5033	yibK	RNA methyltransferase, TrmH family, group 2
G2583_4346	ECs4485	Z5034	cysE	Serine acetyltransferase
G2583_4347	ECs4486	Z5035	gpsA	Glycerol-3-phosphate dehydrogenase [NAD(P)+]
G2583_4348	ECs4487	Z5036	secB	Protein-export protein secB
G2583_4349	ECs4488	Z5037	grxC	Glutaredoxin-3
G2583_4350	ECs4489	Z5038	yibN	hypothetical protein
G2583_4351	ECs4490	Z5039	gpmM	Putative 2,3-bisphosphoglycerate-independent phosphoglycerate mutase
G2583_4352	ECs4491	Z5040	envC	hypothetical protein
G2583_4353	ECs4492	Z5041	yibQ	Uncharacterized protein conserved in bacteria
G2583_4354	ECs4493	Z5042	yibD	Putative regulator
G2583_4355	ECs4494	Z5043	tdh	L-threonine 3-dehydrogenase
G2583_4356	ECs4495	Z5044	kbl	2-amino-3-ketobutyrate CoA ligase
G2583_4357	ECs4496	Z5045	htrL	Involved in lipopolysaccharide biosynthesis

Locus_tag[a]				
CB9615	Sakai	EDL933	Gene	Product
G2583_4358	ECs4497	Z5046	rfaD	ADP-L-glycero-D-manno-heptose-6-epimerase
G2583_4359	ECs4498	Z5047	rfaF	Lipopolysaccharide heptosyltransferase II
G2583_4360	ECs4499	Z5048	rfaC	Lipopolysaccharide heptosyltransferase I
G2583_4361	ECs4500	Z5049	waaL	Lipid A-core:surface polymer ligase WaaL
G2583_4362	ECs4501	Z5050	waaD	Lipopolysaccharide 1,2-N-acetylglucosaminetransferase
G2583_4363	ECs4502	Z5051	rfaJ	Lipopolysaccharide 1,2-glucosyltransferase
G2583_4364	ECs4503	Z5052	rfaY	lipopolysaccharide core biosynthesis protein
G2583_4365	ECs4504	Z5053	rfaI	Lipopolysaccharide 1,3-galactosyltransferase
G2583_4366	ECs4505	Z5054	rfaP	Lipopolysaccharide core biosynthesis protein RfaP
G2583_4367	ECs4506	Z5055	rfaG	Lipopolysaccharide core biosynthesis protein RfaG
G2583_4368	ECs4507	Z5056	rfaQ	Lipopolysaccharide core biosynthesis protein
G2583_4369	ECs4508	Z5057	waaA	Kdo transferase WaaA
G2583_4370	ECs4509	Z5058	coaD	Phosphopantetheine adenylyltransferase
G2583_4371	ECs4510	Z5059	mutM	Formamidopyrimidine-DNA glycosylase
G2583_4372	ECs4511	Z5060	rpmG	50S ribosomal protein L33
G2583_4373	ECs4512	Z5061	rpmB	50S ribosomal protein L28
G2583_4374	ECs4513	Z5062	yicR	DNA repair proteins
G2583_4375	ECs4514	Z5063	dfp	Dfp
G2583_4376	ECs4515	Z5064	dut	Deoxyuridine 5'-triphosphate nucleotidohydrolase
G2583_4377	ECs4516	Z5065	slmA	HTH-type protein slmA
G2583_4378	ECs4517	Z5066	pyrE	Orotate phosphoribosyltransferase
G2583_4379	ECs4518	Z5068	rph	Ribonuclease PH
G2583_4380	ECs4519	Z5069	yicC	Uncharacterized stress-induced protein
G2583_4381	ECs4520	Z5070	dinD	Pyridoxine biosynthesis enzyme
G2583_4382	ECs4521	Z5071	yicG	hypothetical protein
G2583_4383	ECs4522	Z5073	ligB	DNA ligase B
G2583_4384	ECs4523	Z5074	gmk	Guanylate kinase
G2583_4385	ECs4524	Z5075	rpoZ	DNA-directed RNA polymerase, subunit K/omega
G2583_4386	ECs4525	Z5076	spoT	Guanosine-3',5'-bis(diphosphate) 3'-
G2583_4387	ECs4526	Z5077	trmH	tRNA guanosine-2'-O-methyltransferase
G2583_4388	ECs4527	Z5078	recG	ATP-dependent DNA helicase recG
G2583_4389	ECs4528	Z5079	-	hypothetical protein
G2583_4390	ECs4529	Z5081	gltS	Glutamate transport protein
G2583_4391	ECs4530	Z5082	yicE	Putative purine permease yicE
G2583_4392	ECs4531	Z5083	yicH	hypothetical protein
G2583_4393	ECs4532	Z5084	yicI	Alpha-xylosidase
G2583_4394	ECs4533	Z5085	yicJ	Sugar transporter, glycoside-pentoside-hexuronide (GPH):cation symporter family
G2583_4395	selC	RNA094	selC	Sec tRNA
G2583_4396	ECs4534	Z5087	intL	CP4-like integrase
G2583_4397	ECs4535	Z5088	insN	unknown protein encoded by IS911 within prophage CP-933L
	ECs4536		-	hypothetical protein
G2583_4398	ECs4537	Z5089	-	putative transposase

Locus_tag[a]				
CB9615	Sakai	EDL933	Gene	Product
G2583_4399	ECs4538		YeeU	hypothetical protein
G2583_4400	ECs4539	Z5091	yeeV	unknown protein encoded within prophage CP-933L
G2583_4401	ECs4540	Z5092	-	unknown protein encoded within prophage CP-933L
G2583_4402	ECs4541	Z5093	-	hypothetical protein
G2583_4403	ECs4542	Z5094	-	hypothetical protein
G2583_4403	ECs4543		-	hypothetical protein
G2583_4403	ECs4544	Z5095	-	hypothetical protein
Indel-184	ECs4545	Z5096	-	hypothetical protein
Indel-184	ECs4546	Z5097	-	hypothetical protein
Indel-184	ECs4547	Z5098	-	hypothetical protein
G2583_4404	ECs4548		-	hypothetical protein
	ECs4549		-	hypothetical protein
G2583_4405	ECs4550	Z5100	espF	espF
G2583_4406	ECs4551	Z5102	-	hypothetical protein
G2583_4407	ECs4552	Z5103	EscF	Type III secretion apparatus needle protein
G2583_4408	ECs4553	Z5104	-	hypothetical protein
G2583_4409	ECs4554	Z5105	espB	Secreted protein EspB
G2583_4410	ECs4555	Z5106	espD	secreted protein EspD
G2583_4411	ECs4556	Z5107	espA	espA
G2583_4412	ECs4557	Z5108	sepL	SepL
G2583_4413	ECs4558	Z5109	EscD	Pas
G2583_4414	ECs4559	Z5110	eae	Gamma intimin
G2583_4415	ECs4560	Z5111	CesT	Tir chaperone
G2583_4416	ECs4561	Z5112	tir	Translocated intimin receptor Tir
G2583_4417	ECs4562	Z5113	ipgB	hypothetical protein
G2583_4418	ECs4563	Z5114	-	hypothetical protein
G2583_4419	ECs4564	Z5115	-	hypothetical protein
G2583_4420	ECs4565	Z5116	SepQ	sepQ
G2583_4421	ECs4566	Z5117	-	hypothetical protein
G2583_4422	ECs4567	Z5118	-	hypothetical protein
G2583_4423	ECs4568	Z5119	escN	escN
G2583_4424	ECs4569	Z5120	escV	escV
G2583_4425	ECs4570	Z5121	-	hypothetical protein
G2583_4426	ECs4571	Z5122	sepZ	SepZ
G2583_4427	ECs4572	Z5123	-	hypothetical protein
G2583_4428	ECs4573	Z5124	EscJ	EscJ
G2583_4429	ECs4574	Z5125	SepD	hypothetical protein
G2583_4430	ECs4575	Z5126	EscC	escC
G2583_4431	ECs4576	Z5127	cesD	Type III secretion low calcium response chaperone LcrH/SycD
G2583_4432	ECs4577	Z5128	-	hypothetical protein
G2583_4433	ECs4578	Z5129	-	hypothetical protein
G2583_4434	ECs4579	Z5131	-	transglycosylase SLT domain
G2583_4435	ECs4580	Z5132	escU	secretion system apparatus protein SsaU

Locus_tag[a]				
CB9615	Sakai	EDL933	Gene	Product
G2583_4436	ECs4581	Z5133	EscT	escT
G2583_4437	ECs4582	Z5134	EscS	EscS
G2583_4438	ECs4583	Z5135	escR	Type III secretion system EscR protein
G2583_4439	ECs4584	Z5136	-	hypothetical protein
G2583_4440	ECs4585	Z5137	-	hypothetical protein
G2583_4441	ECs4586	Z5138	-	hypothetical protein
G2583_4442	ECs4587	Z5139	-	type III secretion system protein, YseE family
G2583_4443	ECs4588	Z5140	-	hypothetical protein
	ECs4589		-	hypothetical protein
G2583_4444	ECs4590	Z5142	EspG	hypothetical protein
G2583_4445	ECs4591	Z5143	-	hypothetical protein
G2583_4446	ECs4592		-	conserved hypothetical protein
G2583_4447	ECs4593		-	hypothetical protein
G2583_4448	ECs4594	Z5146	yicL	YicL
G2583_4449	ECs4595	Z5147	nlpA	Lipoprotein 28 precursor
G2583_4450		Z5148	yicS	hypothetical protein
G2583_4451	ECs4596	Z5149	nepI	Major facilitator family transporter
G2583_4452	ECs4597	Z5150	-	hypothetical protein
G2583_4453	ECs4598	Z5151	-	Putative DNA-binding protein
G2583_4454	ECs4599	Z5152	yicN	hypothetical protein
G2583_4455	ECs4600	Z5153	yicO	Inorganic anion transporter, sulfate permease (SulP) family
G2583_4455	ECs4601	Z5154	yicO	Inorganic anion transporter, sulfate permease (SulP) family
G2583_4456	ECs4602	Z5155	ade	Adenine deaminase
G2583_4457	ECs4603	Z5156	uhpT	Hexose phosphate transport protein
G2583_4458	ECs4604	Z5157	uhpC	Regulator of uhpT
G2583_4459	ECs4605	Z5158	uhpB	sensory histidine kinase UhpB
G2583_4460	ECs4606	Z5159	uhpA	transcriptional regulatory protein UhpA
G2583_4461	ECs4607	Z5160	-	hypothetical protein
G2583_4461	ECs4608	Z5161	-	hypothetical protein
G2583_4462	ECs4609	Z5162	-	hypothetical protein
G2583_4463	ECs4610	Z5163	-	hypothetical protein
G2583_4464	ECs4611	Z5164	ilvN	Acetolactate synthase isozyme 1 small subunit
G2583_4465	ECs4612	Z5165	ilvB	Acetolactate synthase, large subunit, isozyme I
G2583_4466			-	hypothetical protein
G2583_4467	ECs4613	Z5166	ivbL	ilvBN operon leader peptide
G2583_4468	ECs4614	Z5168	emrD	Multidrug resistance protein D
G2583_4469	ECs4615	Z5169	yidF	putative transcriptional regulator
G2583_4470	ECs4616	Z5170	yidG	Inner membrane protein yidG
G2583_4471	ECs4617	Z5171	yidH	Inner membrane protein yidH
G2583_4472	ECs4618	Z5172	yidI	hypothetical protein
G2583_4473	ECs4619	Z5173	yidJ	Sulfatase
G2583_4474	ECs4620	Z5174	yidK	Transporter, solute:sodium symporter (SSS) family

Locus_tag[a]				
CB9615	Sakai	EDL933	Gene	Product
G2583_4475	ECs4621	Z5175	yidL	putative ARAC-type regulatory protein
G2583_4476	ECs4622	Z5177	glvA	Maltose-6'-phosphate glucosidase
G2583_4477	ECs4623	Z5178	glvC	PTS system arbutin-like IIC component
G2583_4478	ECs4624	Z5179	yidP	transcriptional regulator, GntR family
G2583_4479	ECs4625	Z5181	yidE	Putative transport protein yidE
G2583_4480	ECs4626	Z5182	ibpB	Small heat shock protein ibpB
G2583_4481	ECs4627	Z5183	ibpA	Small heat shock protein ibpA
G2583_4482	ECs4628	Z5184	yidQ	hypothetical protein
G2583_4483	ECs4629	Z5185	yidR	hypothetical protein
G2583_4484	ECs4630	Z5186	cbrA	conserved hypothetical protein
G2583_4485	ECs4631	Z5187	yidX	Putative replicase
G2583_4486	ECs4632	Z5188	yidA	Phosphatase yidA
G2583_4487	ECs4633	Z5189	yidB	hypothetical protein
G2583_4488	ECs4634	Z5190	gyrB	DNA gyrase subunit B
G2583_4489	ECs4635	Z5191	recF	DNA replication and repair protein recF
G2583_4490	ECs4636	Z5192	dnaN	DNA polymerase III subunit beta
G2583_4491	ECs4637	Z5193	dnaA	Chromosomal replication initiator protein dnaA
G2583_4492	ECs4638	Z5194	rpmH	hypothetical protein
G2583_4493	ECs4639	Z5195	rnpA	Ribonuclease P protein component
G2583_4494	ECs4640	Z5197	yidC	Preprotein translocase subunit YidC
G2583_4495	ECs4641	Z5198	mnmE	tRNA modification GTPase trmE
G2583_4496	ECs4642	Z5199	-	ShET2 enterotoxin, N-region family
G2583_4496	ECs4643	Z5200	-	ShET2 enterotoxin, N-region family
G2583_4497		Z5201	-	hypothetical protein
G2583_4498	ECs4644	Z5202	tnaL	tryptophanase leader peptide
G2583_4499	ECs4645	Z5203	tnaA	Tryptophanase
G2583_4500	ECs4646	Z5204	tnaB	Low affinity tryptophan permease
G2583_4501	ECs4647	Z5205	mdtL	Multidrug resistance protein mdtL
G2583_4502	ECs4648	Z5206	yidZ	DNA-binding transcriptional regulator YidZ
G2583_4503	ECs4649	Z5207	yieE	hypothetical protein
G2583_4504	ECs4650	Z5208	yieF	hypothetical protein
G2583_4505	ECs4651	Z5209	yieG	Putative membrane transport protein
G2583_4506	ECs4652	Z5210	yieH	HAD hydrolase, IA family
G2583_4507	ECs4653	Z5211	-	hypothetical protein
G2583_4508	ECs4654	Z5212	-	hypothetical protein
G2583_4509	ECs4655	Z5213	-	hypothetical protein
G2583_4510	ECs4656		-	hypothetical protein
G2583_4511	ECs4657	Z5214	-	hypothetical protein
G2583_4511	ECs4658		-	hypothetical protein
G2583_4512			-	hypothetical protein
G2583_4513	ECs4659		-	hypothetical protein
G2583_4514	ECs4660	Z5215	phoU	Phosphate transport system regulatory protein PhoU
G2583_4515	ECs4661	Z5216	pstB	Phosphate import ATP-binding protein pstB
G2583_4516	ECs4662	Z5217	pstA	Phosphate ABC transporter, permease protein PstA

Locus_tag[a]				
CB9615	Sakai	EDL933	Gene	Product
G2583_4517	ECs4663	Z5218	pstC	Phosphate transport system permease protein pstC
G2583_4518	ECs4664	Z5219	pstS	Phosphate ABC transporter, periplasmic phosphate-binding protein PstS
G2583_4519	ECs4665	Z5220	lpfD	Fimbrial family protein
G2583_4520	ECs4666	Z5221	lpfD	Putative fimbrial protein
G2583_4521	ECs4667	Z5222	lpfC	fimbrial usher protein
G2583_4522	ECs4668	Z5223	stgB	Putative fimbrial chaperone
G2583_4523	ECs4669	Z5224	stgB	Putative fimbrial chaperone
G2583_4524	ECs4670	Z5225	lpfA	Fimbrial protein
		Z5226	-	hypothetical protein
G2583_4525	ECs4671	Z5227	glmS	Glucosamine--fructose-6-phosphate aminotransferase [isomerizing]
G2583_4526	ECs4672	Z5228	glmU	Bifunctional protein glmU [Includes: UDP-N-acetylglucosamine pyrophosphorylase
G2583_4527	ECs4673	Z5229	atpC	ATP synthase epsilon chain
G2583_4528	ECs4674	Z5230	atpD	ATP synthase subunit beta
G2583_4529	ECs4675	Z5231	atpG	ATP synthase gamma chain
G2583_4530	ECs4676	Z5232	atpA	ATP synthase subunit alpha
G2583_4531	ECs4677	Z5233	atpH	ATP synthase delta chain
G2583_4532	ECs4678	Z5234	atpF	ATP synthase B chain
G2583_4533	ECs4679	Z5235	atpE	ATP synthase C chain
G2583_4534	ECs4680	Z5236	atpB	ATP synthase A chain
G2583_4535	ECs4681	Z5238	atpI	Membrane-bound ATP synthase
G2583_4536	ECs4682	Z5240	gidB	Ribosomal RNA small subunit methyltransferase G
G2583_4537	ECs4683	Z5241	mnmG	tRNA uridine 5-carboxymethylaminomethyl modification enzyme gidA
G2583_4538	ECs4684	Z5243	mioC	flavodoxin
G2583_4539	ECs4685	Z5244	asnC	DNA-binding transcriptional regulator AsnC
G2583_4540	ECs4686	Z5245	asnA	Aspartate--ammonia ligase
G2583_4541	ECs4687	Z5246	viaA	hypothetical protein
G2583_4542	ECs4688	Z5247	ravA	ATPase ravA
G2583_4543	ECs4689	Z5248	kup	Low affinity potassium transport system protein kup
G2583_4544	ECs4690	Z5249	rbsD	D-ribose high-affinity transport system
G2583_4545	ECs4691	Z5250	rbsA	Ribose import ATP-binding protein rbsA
G2583_4546	ECs4692	Z5251	rbsC	Ribose transport system permease protein rbsC
G2583_4547	ECs4693	Z5252	rbsB	D-ribose-binding periplasmic protein
G2583_4548	ECs4694	Z5253	rbsK	Ribokinase
G2583_4549	ECs4695	Z5254	rbsR	Regulator for rbs operon
G2583_4550	ECs4696	Z5255	hsrA	Drug resistance MFS transporter, drug:H+ antiporter-1 (DHA2) family
G2583_4551	ECs4697	Z5258	yieP	hypothetical protein
G2583_4552	rrsC	RNA095	rrs	16S ribosomal RNA
G2583_4553	gltU	RNA096	gltU	Glu tRNA
G2583_4554	rrlC	RNA097	rrl	23S ribosomal RNA

Locus_tag[a]				
CB9615	Sakai	EDL933	Gene	Product
G2583_4555	rrfC	RNA098	rrf	5S ribosomal RNA
G2583_4556	aspT	RNA099	aspT	Asp tRNA
G2583_4557	trpT	RNA100	trpT	Trp tRNA
G2583_4558	ECs4698	Z5275	hdfR	transcriptional regulator HdfR
G2583_4559	ECs4699	Z5276	yifE	UPF0438 protein yifE
G2583_4560	ECs4700	Z5277	yifB	Putative 2-component regulator
G2583_4561	ECs4701	Z5278	ilvL	ilvGMEDA operon leader peptide
G2583_4562	ECs4702	Z5279	ilvG	Acetolactate synthase, large subunit, isozyme II
G2583_4563	ECs4703	Z5280	ilvM	Acetolactate synthase isozyme 2 small subunit
G2583_4564	ECs4704	Z5281	ilvE	Branched-chain-amino-acid aminotransferase
G2583_4565	ECs4705	Z5282	ilvD	Dihydroxy-acid dehydratase
G2583_4566	ECs4706	Z5283	ilvA	Threonine dehydratase biosynthetic
G2583_4567	ECs4707	Z5284	ilvY	DNA-binding transcriptional regulator IlvY
G2583_4568	ECs4708	Z5285	ilvC	Ketol-acid reductoisomerase
G2583_4569	ECs4709	Z5286	ppiC	Peptidyl-prolyl cis-trans isomerase C
G2583_4570	ECs4710	Z5287	yifO	hypothetical protein
G2583_4571	ECs4711	Z5288	rep	ATP-dependent DNA helicase Rep
G2583_4572	ECs4712	Z5289	gpp	Guanosine-5'-triphosphate,3'-diphosphate pyrophosphatase
G2583_4573	ECs4713	Z5290	rhlB	ATP-dependent RNA helicase RhlB
G2583_4574	ECs4714	Z5291	trxA	thioredoxin TrxA
G2583_4575	ECs4715	Z5292	rhoL	rho operon leader peptide
G2583_4576	ECs4716	Z5293	rho	Transcription termination factor rho
G2583_4577		Z5294	-	hypothetical protein
G2583_4578	ECs4717	Z5295	rfe	Undecaprenyl-phosphate alpha-N-acetylglucosaminyl 1-phosphate transferase
G2583_4579	ECs4718	Z5296	wzzE	Lipopolysaccharide biosynthesis protein
G2583_4580	ECs4719	Z5297	rffE	UDP-N-acetylglucosamine 2-epimerase
G2583_4581	ECs4720	Z5298	rffD	UDP-N-acetyl-D-mannosamine dehydrogenase
G2583_4582	ECs4721	Z5299	rffG	dTDP-glucose 4,6-dehydratase
G2583_4583	ECs4722	Z5300	rffH	Glucose-1-phosphate thymidylyltransferase
G2583_4584	ECs4723	Z5301	rffC	TDP-D-fucosamine acetyltransferase
G2583_4585	ECs4724	Z5302	rffA	TDP-4-oxo-6-deoxy-D-glucose transaminase
G2583_4586	ECs4725	Z5303	wzxE	Polysaccharide biosynthesis protein
G2583_4587	ECs4726	Z5304	rffT	TDP-Fuc4NAc:lipid II Fuc4Nac transferase
G2583_4588	ECs4727	Z5305	wzyE	Putative ECA polymerase
G2583_4589	ECs4728	Z5306	rffM	Probable UDP-N-acetyl-D-mannosaminuronic acid transferase
G2583_4590	ECs4729	Z5307	yifK	Probable transport protein yifK
G2583_4591	argX	RNA101	argX	Arg tRNA
G2583_4592	hisR	RNA102	hisR	His tRNA
G2583_4593	leuT1	RNA103	leuT1	Leu tRNA
G2583_4594	tRNA51	RNA104	-	Pro tRNA
Indel-189	leuT2	Indel-189	leuT2	Leu tRNA

Locus_tag[a]				
CB9615	Sakai	EDL933	Gene	Product
Indel-189	proM2	Indel-189	proM2	Pro tRNA
G2583_4595	ECs4730	Z5313	aslB	Arylsulfatase-activating protein AslB
G2583_4596	ECs4731	Z5314	aslA	Arylsulfatase precursor
G2583_4597			-	ncRNA
G2583_4598	ECs4732	Z5316	hemY	putative protoheme IX biogenesis protein
G2583_4599	ECs4733	Z5317	hemX	Uroporphyrinogen III methylase
G2583_4600	ECs4734	Z5318	hemD	Uroporphyrinogen-III synthase
G2583_4601	ECs4735	Z5319	hemC	Porphobilinogen deaminase
G2583_4602	ECs4736	Z5322	cyaA	Adenylate cyclase
G2583_4603	ECs4738	Z5323	cyaY	iron donor protein CyaY
G2583_4604	ECs4737	Z5324	yzcX	hypothetical protein
G2583_4605		Z5325	yifL	Uncharacterized lipoprotein yifL precursor
G2583_4606	ECs4739	Z5326	dapF	Diaminopimelate epimerase
G2583_4607	ECs4740	Z5327	yigA	hypothetical protein
G2583_4608	ECs4741	Z5328	xerC	Tyrosine recombinase xerC
G2583_4609	ECs4742	Z5329	yigB	HAD-superfamily hydrolase
G2583_4610	ECs4743	Z5330	uvrD	DNA helicase II
G2583_4611	ECs4744	Z5331	orf	hypothetical protein
G2583_4612	ECs4745	Z5332	yigE	hypothetical protein
G2583_4613	ECs4746	Z5333	corA	Magnesium transport protein corA
G2583_4614		Z5334	-	putative membrane protein
G2583_4615	ECs4747	Z5335	-	hypothetical protein
G2583_4616		Z5336	corA	hypothetical protein
G2583_4617		Z5337	-	hypothetical protein
G2583_4618	ECs4748	Z5339	-	Hypothetical membrane protein
G2583_4619	ECs4749	Z5340	rarD	hypothetical protein
G2583_4620	ECs4750	Z5341	yigI	Thioesterase family protein
G2583_4621	ECs4751	Z5342	pldA	Phospholipase A1 precursor
G2583_4622	ECs4752	Z5343	recQ	ATP-dependent DNA helicase RecQ
G2583_4623	ECs4753	Z5344	rhtC	Threonine efflux protein
G2583_4624	ECs4754	Z5345	rhtB	Homoserine/homoserine lactone efflux protein
G2583_4625	ECs4755	Z5346	pldB	Lysophospholipase L2
G2583_4626	ECs4756	Z5347	yigL	Putative hydrolase
G2583_4627	ECs4757	Z5348	yigM	Uncharacterized membrane protein yigM
G2583_4628	ECs4758	Z5349	metR	regulator for metE and metH
G2583_4629	ECs4759	Z5351	metE	5-methyltetrahydropteroyltriglutamate-homocysteine S-methyltransferase
G2583_4630	ECs4760	Z5352	ysgA	Putative enzyme
G2583_4631	ECs4761	Z5353	udp	Uridine phosphorylase
G2583_4632	ECs4762	Z5354	rmuC	DNA recombination protein rmuC
G2583_4633	ECs4763	Z5355	ubiE	Ubiquinone/menaquinone biosynthesis methyltransferase ubiE
G2583_4634	ECs4764	Z5356	yigP	hypothetical protein
G2583_4635	ECs4765	Z5357	ubiB	Probable ubiquinone biosynthesis protein ubiB

Locus_tag[a]				
CB9615	Sakai	EDL933	Gene	Product
G2583_4636	ECs4766	Z5358	tatA	Sec-independent twin-arginine translocase subunit TatA
G2583_4637	ECs4767	Z5359	tatB	sec-independent translocase
G2583_4638	ECs4768	Z5360	tatC	TatABCE protein translocation system subunit
G2583_4639	ECs4769	Z5361	tatD	MttC
G2583_4640	ECs4770	Z5362	rfaH	transcriptional activator RfaH
G2583_4641	ECs4771	Z5364	ubiD	3-octaprenyl-4-hydroxybenzoate carboxy-lyase
G2583_4642	ECs4772	Z5365	fre	Oxidoreductase
G2583_4643	ECs4773	Z5366	fadA	Beta-ketoadipyl CoA thiolase
G2583_4644	ECs4774	Z5367	fadB	Fused 3-hydroxybutyryl-CoA epimerase/delta(3)-cis-delta(2)-trans- enoyl-CoA isomerase/enoyl-CoA hydratase/3-hydroxyacyl-CoA dehydrogenase
G2583_4645	ECs4775	Z5369	pepQ	Xaa-Pro dipeptidase
G2583_4646	ECs4776	Z5370	yigZ	hypothetical protein
G2583_4647	ECs4777	Z5371	trkH	Trk system potassium uptake protein trkH
G2583_4648	ECs4778	Z5372	hemG	Protoporphyrinogen oxidase
G2583_4649	rrsA	RNA105	rrs	16S ribosomal RNA
G2583_4650	ileT	RNA106	ileT	Ile tRNA
G2583_4651	alaT	RNA107	alaT	Ala tRNA
G2583_4652	rrlA	RNA108	rrl	23S ribosomal RNA
G2583_4653	rrfA	RNA109	rrf	5S ribosomal RNA
G2583_4654	ECs4779	Z5388	mobB	Molybdopterin-guanine dinucleotide biosynthesis protein
G2583_4655	ECs4780	Z5389	mobA	Molybdopterin-guanine dinucleotide biosynthesis protein
G2583_4656	ECs4781	Z5390	yihD	hypothetical protein
G2583_4657	ECs4782	Z5391	rdoA	predicted kinase
G2583_4658	ECs4783	Z5392	dsbA	Thiol:disulfide interchange protein dsbA precursor
G2583_4659	ECs4784	Z5393	yihF	Putative GTP-binding protein
G2583_4660	ECs4785	Z5394	yihG	Acyltransferase domain protein
G2583_4661			-	hypothetical protein
G2583_4662	ECs4786	Z5398	polA	DNA polymerase I
G2583_4663	spf	RNA110	spf	ncRNA
G2583_4664	ECs4787	Z5400	yihA	Probable GTP-binding protein engB
G2583_4665		Z5401	-	ncRNA
G2583_4666	ECs4788	Z5402	yihI	UPF0241 protein yihI
G2583_4667	ECs4789	Z5403	hemN	Oxygen-independent coproporphyrinogen III oxidase
G2583_4668	ECs4790	Z5404	glnG	Nitrogen regulation protein NR
G2583_4669	ECs4791	Z5405	glnL	Signal transduction histidine kinase, nitrogen specific
G2583_4670	ECs4792	Z5406	glnA	Glutamine synthetase
G2583_4671	ECs4793	Z5407	typA	GTP-binding protein typA/bipA
G2583_4672	ECs4794	Z5408	yihL	putative transcriptional regulator
G2583_4673	ECs4795	Z5409	yihM	AP endonuclease, family 2
G2583_4674	ECs4796	Z5410	yihN	Transporter, major facilitator family
G2583_4675	ECs4797	Z5411	ompL	hypothetical protein
G2583_4676	ECs4798	Z5412	yihO	Sugar (Glycoside-Pentoside-Hexuronide) transporter family protein

Locus_tag[a]				
CB9615	Sakai	EDL933	Gene	Product
G2583_4677	ECs4799	Z5413	yihP	Sugar transporter family protein
G2583_4678	ECs4800	Z5414	yihQ	Glycosyl hydrolase, family 31
G2583_4679	ECs4801	Z5415	-	hypothetical protein
G2583_4680	ECs4802	Z5416	yihR	Aldose-1-epimerase family protein
G2583_4681	ECs4803	Z5417	yihS	N-acylglucosamine 2-epimerase
G2583_4682	ECs4804	Z5418	yihT	Uncharacterized aldolase yihT
G2583_4683	ECs4805	Z5420	yihU	3-hydroxyisobutyrate dehydrogenase family
G2583_4684	ECs4806	Z5421	yihV	Kinase, PfkB family
G2583_4685	ECs4807	Z5422	yihW	Putative DEOR-type transcriptional activator
G2583_4686	ECs4808	Z5424	yihX	Phosphatase YihX
G2583_4687	ECs4809	Z5425	yihY	tRNA-processing ribonuclease BN
G2583_4688	ECs4810	Z5426	dtd	D-tyrosyl-tRNA(Tyr) deacylase
G2583_4689	ECs4811	Z5427	yiiD	putative acetyltransferase
G2583_4690	ECs4812	Z5428	-	hypothetical protein
G2583_4690	ECs4813	Z5429	-	hypothetical protein
G2583_4691	ECs4814		-	hypothetical protein
G2583_4692		Z5430	-	hypothetical protein
G2583_4693	ECs4815	Z5431	yiiE	Ribbon-helix-helix protein, copG family
G2583_4694	ECs4816	Z5432	yiiF	hypothetical protein
G2583_4695	ECs4817	Z5433	fdhE	protein AraJ
G2583_4696	ECs4818	Z5434	fdoI	Formate dehydrogenase, cytochrome b556(fdo) subunit
G2583_4697	ECs4819	Z5435	fdoH	Formate dehydrogenase-O, iron-sulfur subunit
G2583_4698	ECs4820	Z5436	fdoG	formate dehydrogenase-O major subunit
G2583_4699	ECs4821	Z5438	fdhD	ApaG
G2583_4700	ECs4822	Z5439	yiiG	hypothetical protein
G2583_4701	ECs4823	Z5440	frvR	Putative PTS system, IIA component
G2583_4702	ECs4824	Z5441	frvX	Aminopeptidase
G2583_4703	ECs4825	Z5442	frvB	Phosphotransferase system, fructose-specific I IC component
G2583_4704	ECs4826	Z5443	frvA	PTS system, fructose family, IIA component
G2583_4705	ECs4827	Z5444	rafY	Glycoporin RafY
G2583_4706	ECs4828	Z5445	rhaM	L-rhamnose 1-epimerase
G2583_4707	ECs4829	Z5446	rhaD	Rhamnulose-1-phosphate aldolase
G2583_4708	ECs4830	Z5447	rhaA	L-rhamnose isomerase
G2583_4709	ECs4831	Z5448	rhaB	Rhamnulokinase
G2583_4710	ECs4832	Z5449	rhaS	positive regulator for rhaBAD operon
G2583_4711	ECs4833	Z5450	rhaR	positive regulator for rhaRS operon
G2583_4712	ECsp022	Z5452	rhaT	rhamnose transport
G2583_4712			rhaT	rhamnose transport
G2583_4713	ECs4834	Z5453	sodA	Superoxide dismutase
G2583_4714	ECs4835	Z5454	kdgT	2-keto-3-deoxygluconate permease
G2583_4715	ECs4836	Z5455	yiiM	MOSC domain protein
G2583_4716	ECs4837	Z5456	cpxA	two-component sensor protein

Locus_tag[a]				
CB9615	Sakai	EDL933	Gene	Product
G2583_4717	ECs4838	Z5457	cpxR	DNA-binding response regulator in two-component regulatory system with CpxA
G2583_4718	ECs4839	Z5458	cpxP	P pilus assembly/Cpx signaling pathway, periplasmic inhibitor/zinc-resistance associated protein
G2583_4719	ECs4840	Z5459	fieF	Ferrous-iron efflux pump fieF
G2583_4720	ECs4841	Z5460	pfkA	6-phosphofructokinase isozyme 1
G2583_4721			-	hypothetical protein
G2583_4722	ECs4842	Z5462	sbp	Sulfate-binding protein
G2583_4723	ECs4843	Z5463	cdh	CDP-diacylglycerol pyrophosphatase
G2583_4724	ECs4844	Z5464	tpiA	Triosephosphate isomerase
G2583_4725	ECs4845	Z5465	yiiQ	hypothetical protein
G2583_4726	ECs4846	Z5466	yiiR	hypothetical protein
G2583_4727	ECs4847	Z5467	yiiS	hypothetical protein
G2583_4728	ECs4848	Z5468	uspD	Universal stress protein D
G2583_4729	ECs4849	Z5469	fpr	Ferredoxin--NADP(+) reductase
G2583_4730	ECs4850	Z5470	glpX	Fructose-1,6-bisphosphatase, class II
G2583_4731	ECs4851	Z5471	glpK	Glycerol kinase
G2583_4732	ECs4852	Z5472	glpF	glycerol uptake facilitator protein
G2583_4733	ECs4853	Z5473	yiiU	Cell division protein zapB
G2583_4734	ECs4854	Z5474	-	hypothetical protein
G2583_4735	ECs4855	Z5475	-	hypothetical protein
G2583_4736	ECs4856	Z5476	rraA	Regulator of ribonuclease activity A
G2583_4737	ECs4857	Z5477	menA	1,4-dihydroxy-2-naphthoate octaprenyltransferase
G2583_4738	ECs4858	Z5478	hslU	ATP-dependent hsl protease ATP-binding subunit hslU
G2583_4739	ECs4859	Z5479	hslV	ATP-dependent protease hslV
G2583_4740	ECs4860	Z5480	ftsN	Cell division protein FtsN
G2583_4741	ECs4861	Z5481	cytR	Transcriptional regulators
G2583_4742	ECs4862	Z5482	priA	Primosomal protein N'
G2583_4743	ECs4863	Z5484	rpmE	50S ribosomal protein L31
G2583_4744	ECs4864	Z5485	rhsF	RhsH core protein with extension
G2583_4744	ECs4864	Z5487	rhsF	RhsH core protein with extension
G2583_4744	ECs4864	Z5488	rhsF	RhsH core protein with extension
G2583_4745		Z5489	-	hypothetical protein
G2583_4746		Z5490	insB	hypothetical protein
G2583_4747		Z5491	-	IS1N transposase
G2583_4748	ECs4865		-	hypothetical protein
G2583_4749	ECs4866	Z5492	yiiX	hypothetical protein
G2583_4750	ECs4867	Z5493	metJ	MetJ
G2583_4751	ECs4868	Z5494	metB	O-succinylhomoserine (Thiol)-lyase
G2583_4752	ECs4869	Z5495	metL	Aspartokinase/homoserine dehydrogenase II
G2583_4753	ECs4870	Z5496	metF	5,10-methylenetetrahydrofolate reductase
G2583_4754	ECs4871	Z5497	katG	Peroxidase/catalase HPI
G2583_4755	ECs4872	Z5498	yijE	Hypothetical transport protein yijE
G2583_4756	ECs4873	Z5499	yijF	hypothetical protein

Locus_tag[a]				
CB9615	Sakai	EDL933	Gene	Product
G2583_4757	ECs4874	Z5500	gldA	Glycerol dehydrogenase,
G2583_4758	ECs4875	Z5501	fsaB	Fructose-6-phosphate aldolase 2
G2583_4759	ECs4877	Z5502	ptsA	PEP-protein phosphotransferase system enzyme I
G2583_4760	ECs4876	Z5503	yijI	hypothetical protein
G2583_4761	ECs4878	Z5504	frwC	PTS system, fructose-like-2 IIC component
G2583_4761	ECsp023	Z5505	frwC	PTS system, fructose-like-2 IIC component
G2583_4762	ECs4879	Z5506	frwB	PTS system fructose-like IIB component 1
G2583_4763	ECs4880	Z5507	pflD	Formate C-acetyltransferase 2
G2583_4764	ECs4881	Z5508	pflC	Glycyl-radical enzyme activating protein family
G2583_4765	ECs4882	Z5509	frwD	Predicted enzyme IIB component of PTS
G2583_4766	ECs4883	Z5512	yijO	putative ARAC-type regulatory protein
G2583_4767	ECs4884	Z5513	yijP	Membrane protein
G2583_4768	ECs4885	Z5514	ppc	Phosphoenolpyruvate carboxylase
G2583_4769	ECs4886	Z5515	argE	Acetylornithine deacetylase
G2583_4770	ECs4887	Z5516	argC	N-acetyl-gamma-glutamyl-phosphate reductase
G2583_4771	ECs4888	Z5517	argB	Acetylglutamate kinase
G2583_4772	ECs4889	Z5518	argH	Argininosuccinate lyase
G2583_4773	oxyS		oxyS	ncRNA
G2583_4774	ECs4890	Z5519	oxyR	Transcriptional regulator
G2583_4775	ECs4891	Z5521	sthA	Soluble pyridine nucleotide transhydrogenase
G2583_4776	ECs4892	Z5522	-	Hippuricase
G2583_4777	ECs4893	Z5523	-	Major facilitator superfamily
G2583_4778	ECs4894	Z5524	fabR	Transcriptional regulator
G2583_4779	ECs4895	Z5525	yijD	Inner membrane protein yijD
G2583_4780	ECs4896	Z5526	trmA	tRNA (uracil-5-)-methyltransferase
G2583_4781	ECs4897	Z5527	btuB	Vitamin B12 transporter btuB precursor
G2583_4782	ECs4898	Z5528	murI	Glutamate racemase
G2583_4783	rrsB	RNA111	rrs	16S ribosomal RNA
G2583_4784	gltT	RNA112	gltT	Glu tRNA
G2583_4785	rrlB	RNA113	rrl	23S ribosomal RNA
G2583_4786	rrfB	RNA114	rrf	5S ribosomal RNA
G2583_4787	ECs4899	Z5543	murB	UDP-N-acetylenolpyruvoylglucosamine reductase
G2583_4788	ECs4900	Z5544	birA	Bifunctional protein BirA
G2583_4789	ECs4901	Z5545	coaA	Pantothenate kinase
G2583_4790	ECs4902	Z5547	-	hypothetical protein
G2583_4791	thrU	RNA115	thrU	Thr tRNA
G2583_4792	tyrU	RNA116	tyrU	Tyr tRNA
G2583_4793	glyT	RNA117	glyT	Gly tRNA
G2583_4794	thrT	RNA118	thrT	Thr tRNA
G2583_4795	ECs4903	Z5553	tufB	Elongation factor Tu 2
G2583_4796	ECs4904	Z5554	secE	Preprotein translocase
G2583_4797	ECs4905	Z5555	nusG	Transcription antitermination protein nusG
G2583_4798	ECs4906	Z5556	rplK	50S ribosomal protein L11
G2583_4799	ECs4907	Z5557	rplA	50S ribosomal protein L1

Locus_tag[a]				
CB9615	Sakai	EDL933	Gene	Product
G2583_4800	ECs4908	Z5558	rplJ	50S ribosomal protein L10
G2583_4801	ECs4909	Z5559	rplL	50S ribosomal protein L7/L12
G2583_4802			-	ncRNA
G2583_4803	ECs4910	Z5560	rpoB	DNA-directed RNA polymerase subunit beta
G2583_4804	ECs4911	Z5561	rpoC	DNA-directed RNA polymerase subunit beta'
G2583_4805	ECs4912	Z5563	yjaZ	Heat shock protein C
G2583_4806			-	ncRNA
G2583_4807	ECs4913	Z5564	thiH	Thiazole biosynthesis protein ThiH
G2583_4808	ECs4914	Z5565	thiG	Thiamin biosynthesis, thiazole moiety
G2583_4809		Z5566	thiS	Thiamine biosynthesis protein ThiS
G2583_4810	ECs4915	Z5567	thiF	Thiazole biosynthesis adenylyltransferase ThiF
G2583_4811	ECs4916	Z5568	thiE	Thiamine-phosphate pyrophosphorylase
G2583_4812	ECs4917	Z5569	thiC	Thiamine biosynthesis protein thiC
G2583_4813	ECs4918	Z5570	rsd	Regulator of sigma D
G2583_4814	ECs4919	Z5571	nudC	NADH pyrophosphatase
G2583_4815	ECs4920	Z5572	hemE	Uroporphyrinogen decarboxylase
G2583_4816	ECs4921	Z5574	nfi	Endonuclease V
G2583_4817	ECs4922	Z5575	yjaG	hypothetical protein
G2583_4818	ECs4923	Z5576	hupA	DNA-binding protein HU-alpha
G2583_4819	ECs4924	Z5577	yjaH	hypothetical protein
G2583_4820	ECs4925	Z5578	zraP	Zinc resistance-associated protein
G2583_4821	ECs4926	Z5579	zraS	sensor protein ZraS
G2583_4822	ECs4927	Z5580	zraR	transcriptional regulatory protein ZraR
G2583_4823	ECs4928	Z5582	purD	Phosphoribosylamine--glycine ligase
G2583_4824	ECs4929	Z5583	purH	IMP cyclohydrolase / phosphoribosylaminoimidazolecarboxamide formyltransferase
G2583_4825	rrsE	RNA119	rrs	16S ribosomal RNA
G2583_4826	gltV	RNA120	gltV	Glu tRNA
G2583_4827	rrlE	RNA121	rrl	23S ribosomal RNA
G2583_4828	rrfE	RNA122	rrf	5S ribosomal RNA
G2583_4829	ECs4930	Z5598	yjaB	hypothetical protein
G2583_4830	ECs4931	Z5599	metA	Homoserine O-succinyltransferase
G2583_4831	ECs4932	Z5600	aceB	Malate synthase A
G2583_4832	ECs4933	Z5601	aceA	Isocitrate lyase
G2583_4833	ECs4934	Z5602	aceK	Isocitrate dehydrogenase kinase/phosphatase
G2583_4834	ECs4935	Z5608	arp	ShET2 enterotoxin, N- region family
G2583_4835	ECs4936	Z5609	iclR	Repressor of aceBA operon
G2583_4836	ECs4937	Z5610	metH	Methionine synthase
G2583_4837	ECs4938	Z5611	yjbB	hypothetical protein
G2583_4838	ECs4939	Z5612	pepE	Peptidase E
G2583_4839	ECs4940		-	hypothetical protein
G2583_4840	ECs4941	Z5613	sorE	Putative L-sorbose-1-P-reductase

Locus_tag[a]				
CB9615	Sakai	EDL933	Gene	Product
G2583_4841	ECs4942	Z5614	sorM	PTS system, mannose/fructose/sorbose family, IID component
Indel-193	ECs4943	Indel-193	-	putative regulatory protein
Indel-193	ECs4944	Indel-193	-	putative DNA-binding protein
Indel-193	ECs4945	Indel-193	-	phage transposase
Indel-193	ECs4946	Indel-193	-	putative DNA transposition protein
Indel-193	ECs4947	Indel-193	-	hypothetical protein
Indel-193	ECs4948	Indel-193	-	hypothetical protein
Indel-193	ECs4949	Indel-193	-	hypothetical protein
Indel-193	ECs4950	Indel-193	-	hypothetical protein
Indel-193	ECs4951	Indel-193	-	hypothetical protein
Indel-193	ECs4952	Indel-193	-	putative host-nuclease inhibitor protein
Indel-193	ECs4953	Indel-193	-	hypothetical protein
Indel-193	ECs4954	Indel-193	-	hypothetical protein
Indel-193	ECs4955	Indel-193	-	hypothetical protein
Indel-193	ECs4956	Indel-193	-	hypothetical protein
Indel-193	ECs4957	Indel-193	-	hypothetical protein
Indel-193	ECs4958	Indel-193	-	hypothetical protein
Indel-193	ECs4959	Indel-193	-	hypothetical protein
Indel-193	ECs4960	Indel-193	-	hypothetical protein
Indel-193	ECs4961	Indel-193	-	putative transcription regulator
Indel-193	ECs4962	Indel-193	-	putative endolysin
Indel-193	ECs4963	Indel-193	-	hypothetical protein
Indel-193	ECs4964	Indel-193	-	hypothetical protein
Indel-193	ECs4965	Indel-193	-	C4-type zinc finger protein (TraR family)
Indel-193	ECs4966	Indel-193	-	hypothetical protein
Indel-193	ECs4967	Indel-193	-	hypothetical protein
Indel-193	ECs4968	Indel-193	-	hypothetical protein
Indel-193	ECs4969	Indel-193	-	putative portal protein
Indel-193	ECs4970	Indel-193	-	hypothetical protein
Indel-193	ECs4971	Indel-193	-	hypothetical protein
Indel-193	ECs4972	Indel-193	-	putative virion morphogenesis protein
Indel-193	ECs4973	Indel-193	-	putative protease protein
Indel-193	ECs4974	Indel-193	-	putative major head subunit
Indel-193	ECs4975	Indel-193	-	hypothetical protein
Indel-193	ECs4976	Indel-193	-	hypothetical protein
Indel-193	ECs4977	Indel-193	-	hypothetical protein
Indel-193	ECs4978	Indel-193	-	hypothetical protein
Indel-193	ECs4979	Indel-193	-	putative tail sheath protein
Indel-193	ECs4980	Indel-193	-	hypothetical protein
Indel-193	ECs4981	Indel-193	-	hypothetical protein
Indel-193	ECs4982	Indel-193	-	putative tape measure protein
Indel-193	ECs4983	Indel-193	-	putative DNA circulation protein
Indel-193	ECs4984	Indel-193	-	putative tail protein

Locus_tag[a]				
CB9615	Sakai	EDL933	Gene	Product
Indel-193	ECs4985	Indel-193	-	hypothetical protein
Indel-193	ECs4986	Indel-193	-	hypothetical protein
Indel-193	ECs4987	Indel-193	-	hypothetical protein
Indel-193	ECs4988	Indel-193	-	hypothetical protein
Indel-193	ECs4989	Indel-193	-	tail fiber
Indel-193	ECs4990	Indel-193	-	putative tail fiber assembly protein
Indel-193	ECs4991	Indel-193	-	putative tail fiber protein
Indel-193	ECs4992	Indel-193	-	putative DNA-invertase
Indel-193	ECs4993	Indel-193	-	hypothetical protein
Indel-193	ECs4994	Indel-193	-	hypothetical protein
Indel-193	ECs4995	Indel-193	-	hypothetical protein
Indel-193	ECs4996	Indel-193	-	hypothetical protein
Indel-193	ECs4997	Indel-193	-	translational regulator
Indel-193	ECs4998	Indel-193	-	putative DNA modification protein
G2583_4841	ECs4999	Z5614	sorM	PTS system, mannose/fructose/sorbose family, IID component
G2583_4842	ECs5000	Z5615	sorA	PTS system, mannose/fructose/sorbose family, IIC component
G2583_4843	ECs5001	Z5616	sorB	Putative sorbose PTS component
G2583_4844	ECs5002	Z5617	sorF	PTS system, mannose/fructose/sorbose family, IIA component
G2583_4845	ECs5003	Z5618	sorD	Sorbitol-6-phosphate 2-dehydrogenase
G2583_4846	ECs5004	Z5619	-	Putative transcriptional regulator of sorbose uptake and utilization genes
G2583_4847	ECs5005	Z5620	rluF	Ribosomal large subunit pseudouridine synthase F
G2583_4848	ECs5006	Z5621	pagB	hypothetical protein
G2583_4849	ECs5007	Z5622	lysC	Lysine-sensitive aspartokinase 3
G2583_4850	ECs5008	Z5623	pgi	Glucose-6-phosphate isomerase
G2583_4851	ECs5009	Z5624	yjbE	hypothetical protein
G2583_4852	ECs5010	Z5625	yjbF	hypothetical protein
G2583_4853	ECs5011	Z5626	yjbG	hypothetical protein
G2583_4854	ECs5012	Z5627	yjbH	hypothetical protein
G2583_4855	ECs5013	Z5628	psiE	phosphate-starvation-inducible protein PsiE
G2583_4856	ECs5014	Z5629	xylE	D-xylose-proton symporter
G2583_4857	ECs5015	Z5630	malG	Part of maltose permease, inner membrane
G2583_4858	ECs5016	Z5631	malF	Maltose transport system permease protein malF
G2583_4859	ECs5017	Z5632	malE	Maltose-binding periplasmic protein precursor
G2583_4860	ECs5018	Z5633	malK	Maltose/maltodextrin import ATP-binding protein malK
G2583_4861	ECs5019	Z5634	lamB	Maltoporin precursor
G2583_4862	ECs5020	Z5635	malM	Maltose operon periplasmic protein precursor
G2583_4863	ECs5021	Z5636	yjbI	hypothetical protein
G2583_4864	ECs5022	Z5638	ubiC	Chorismate--pyruvate lyase
G2583_4865	ECs5023	Z5639	ubiA	4-hydroxybenzoate octaprenyltransferase
G2583_4866	ECs5024	Z5640	plsB	Glycerol-3-phosphate acyltransferase

Locus_tag[a]				
CB9615	Sakai	EDL933	Gene	Product
G2583_4867	ECs5025	Z5641	dgkA	Diacylglycerol kinase
G2583_4868	ECs5026	Z5642	lexA	LexA repressor
G2583_4869	ECs5027	Z5643	dinF	MATE efflux family protein
G2583_4870	ECs5028	Z5644	yjbJ	UPF0337 protein yjbJ
G2583_4871	ECs5029	Z5645	zur	Putative zinc uptake regulation protein
G2583_4872	ECs5030	Z5646	yjbM	hypothetical protein
G2583_4873	ECs5031	Z5647	dusA	tRNA-dihydrouridine synthase A
G2583_4874	ECs5032	Z5648	pspG	hypothetical protein
G2583_4875	ECs5033	Z5649	qor	Quinone oxidoreductase
G2583_4876	ECs5034	Z5650	dnaB	Replicative DNA helicase
G2583_4877	ECs5035	Z5651	alr	Alanine racemase, biosynthetic
G2583_4878	ECs5036	Z5652	tyrB	Aromatic-amino-acid transaminase
G2583_4879			-	hypothetical protein
G2583_4880	ECs5037	Z5654	aphA	Diadenosine tetraphosphatase
G2583_4881	ECs5038	Z5655	yjbQ	UPF0047 protein yjbQ
G2583_4882	ECs5039	Z5656	yjbR	hypothetical protein
G2583_4883	ECs5040	Z5657	uvrA	UvrABC system protein A
G2583_4884	ECs5041	Z5658	ssb	Single-stranded DNA-binding protein
G2583_4885	ECs5042	Z5659	yjcB	hypothetical protein
G2583_4886	ECs5043	Z5660	yjcC	Cyclic diguanylate phosphodiesterase (EAL) domain protein
G2583_4887	ECs5044	Z5661	soxS	DNA-binding transcriptional dual regulator
G2583_4888	ECs5045	Z5662	soxR	Redox-sensitive transcriptional activator soxR
G2583_4889	ECs5046	Z5663	yjcD	Inorganic anion transporter, sulfate permease (SulP) family
G2583_4890	ECs5047	Z5664	yjcE	Na+/H+ antiporter
G2583_4891	ECs5048	Z5665	yjcF	hypothetical protein
G2583_4892	ECs5049	Z5666	actP	Cation/acetate symporter actP
G2583_4893	ECs5050	Z5667	yjcH	hypothetical protein
G2583_4894	ECs5051	Z5668	acs	Acetyl-coenzyme A synthetase
G2583_4895	ECs5052	Z5669	nrfA	Cytochrome c-552 precursor
G2583_4896	ECs5053	Z5670	nrfB	NrfB, formate-dependent nitrite reductase
G2583_4897	ECs5054	Z5671	nrfC	formate-dependent nitrite reductase; Fe-S centers
G2583_4898	ECs5055	Z5672	nrfD	NrfD protein
G2583_4899	ECs5056	Z5673	nrfE	Cytochrome c-type biogenesis protein NrfE
G2583_4900	ECs5057	Z5674	nrfF	Formate-dependent nitrite reductase complex subunit nrfF precursor
G2583_4901	ECs5058	Z5675	nrfG	Formate-dependent nitrite reductase complex NrfG subunit
G2583_4902	ECs5059	Z5676	gltP	Sodium:dicarboxylate symporter
G2583_4903	ECs5060	Z5677	yjcO	hypothetical protein
G2583_4904	ECs5061	Z5678	fdhF	selenopolypeptide subunit of formate dehydrogenase H
G2583_4905	ECs5062	Z5680	mdtP	Multidrug resistance outer membrane protein mdtP precursor

Locus_tag[a]				
CB9615	Sakai	EDL933	Gene	Product
G2583_4906	ECs5063	Z5681	mdtO	Multidrug resistance protein mdtO
G2583_4907	ECs5064	Z5682	mdtN	multidrug resistance protein MdtN
G2583_4908	ECs5065		ytcA	hypothetical protein
G2583_4909	ECs5066	Z5683	yjcS	hypothetical protein
G2583_4910	ECs5067	Z5684	-	Putative transcriptional regulator
G2583_4911	ECs5068	Z5686	PfkB	PfkB domain protein
G2583_4912	ECs5069	Z5687	-	Fructose-bisphosphate aldolase
G2583_4913	ECs5070	Z5688	-	hypothetical protein
G2583_4914	ECs5071	Z5689	-	Putative periplasmic ribose-binding protein of ABC transport system
G2583_4915	ECs5072	Z5690	-	Putative permease of ribose ABC transport system
G2583_4916	ECs5073	Z5691	-	Ribose import ATP-binding protein rbsA 2
G2583_4917	ECs5074	Z5692	-	Integral membrane sensor hybrid histidine kinase
G2583_4917	ECs5074	Z5693	-	Integral membrane sensor hybrid histidine kinase
G2583_4918		Z5694	yjdP	hypothetical protein
G2583_4919	ECs5075	Z5695	phnP	Phosphonate utilization protein
G2583_4920	ECs5076	Z5696	phnO	predicted acyltransferase with acyl-CoA N-acyltransferase domain
G2583_4921	ECs5077	Z5697	phnN	Phosphonate metabolism protein/1,5-bisphosphokinase (PRPP-forming) PhnN
G2583_4922	ECs5078	Z5698	phnM	Phosphonate metabolism protein PhnM
G2583_4923	ECs5079	Z5699	phnL	Phosphonate C-P lyase system protein PhnL
G2583_4924	ECs5080	Z5700	phnK	Phosphonate C-P lyase system protein PhnK
G2583_4925	ECs5081	Z5701	phnJ	PhnJ protein
G2583_4926	ECs5082	Z5702	phnI	Phosphonate metabolism protein PhnI
G2583_4927	ECs5083	Z5703	phnH	Bacterial phosphonate metabolism protein PhnH
G2583_4928	ECs5084	Z5704	phnG	Phosphonate C-P lyase system protein PhnG
G2583_4929	ECs5085	Z5705	phnF	Phosphonates metabolism transcriptional regulator
G2583_4930	ECs5086	Z5706	phnE	Phosphonate ABC transporter, permease protein
G2583_4931	ECs5087	Z5707	phnD	Phosphonate ABC transporter, periplasmic phosphonate-binding protein
G2583_4932	ECs5088	Z5708	phnC	Phosphonates import ATP-binding protein phnC
G2583_4933	ECs5089	Z5709	yjdN	Putative phnB protein
G2583_4934	ECs5090	Z5710	yjdM	Alkylphosphonate utilization operon protein PhnA
G2583_4935	ECs5091	Z5711	yjdA	hypothetical protein
G2583_4936	ECs5092	Z5712	yjcZ	hypothetical protein
G2583_4937	ECs5093	Z5713	proP	Proline/betaine transporter
G2583_4938	ECs5094	Z5714	basS	Sensor histidine kinase BasS
G2583_4939	ECs5095	Z5715	basR	DNA-binding response regulator BasR
G2583_4940	ECs5096	Z5716	eptA	Putative sulfatase
G2583_4941	ECs5097	Z5717	adiC	Putative amino acid permease
G2583_4942	ECs5098	Z5718	adiY	putative ARAC-type regulatory protein
G2583_4943	ECs5099	Z5719	adiA	Biodegradative arginine decarboxylase
G2583_4944	ECs5100	Z5720	melR	Melibiose operon regulatory protein

Locus_tag[a]				
CB9615	Sakai	EDL933	Gene	Product
G2583_4945	ECs5101	Z5721	melA	Alpha-galactosidase
G2583_4946	ECs5102	Z5722	melB	Melibiose carrier protein
G2583_4947	ECs5103	Z5723	yjdF	hypothetical protein
G2583_4948	ECs5104	Z5724	fumB	Fumarase B
G2583_4949	ECs5105	Z5725	dcuB	Anaerobic C4-dicarboxylate transporter DcuB
G2583_4950	ECs5106	Z5726	dcuR	DNA-binding response regulator in two-component regulatory system with DcuS
G2583_4951	ECs5107	Z5727	dcuS	sensory histidine kinase DcuS
G2583_4952	ECs5108	Z5728	yjdI	hypothetical protein
G2583_4953	ECs5109	Z5729	yjdJ	hypothetical protein
G2583_4954	ECs5110	Z5730	yjdK	hypothetical protein
G2583_4955		Z5731	yjdO	hypothetical protein
G2583_4956	ECs5111	Z5732	lysU	Lysyl-tRNA synthetase, heat inducible
G2583_4957	ECs5112	Z5733	yjdL	Amino acid/peptide transporter
G2583_4958	ECs5113	Z5734	cadA	Lysine decarboxylase, inducible
G2583_4959	ECs5114	Z5735	cadB	Probable cadaverine/lysine antiporter
G2583_4960	ECs5115	Z5736	cadC	Transcriptional activator cadC
G2583_4961	pheU	RNA123	pheU	Phe tRNA
G2583_4962	ECs5116	Z5740	yjdC	YjdC
G2583_4963	ECs5117	Z5741	dipZ	Thiol:disulfide interchange protein DsbD
G2583_4964	ECs5118	Z5742	cutA	Divalent-cation tolerance protein cutA
G2583_4965	ECs5119	Z5743	dcuA	Anaerobic C4-dicarboxylate transporter dcuA
G2583_4966	ECs5120	Z5744	aspA	aspartate ammonia-lyase
G2583_4967	ECs5121	Z5745	fxsA	FxsA
G2583_4968	ECs5122	Z5746	yjeH	Amino acid-polyamine-organocation (APC) permease family protein
G2583_4969	ECs5123	Z5747	groS	hypothetical protein
G2583_4970	ECs5124	Z5748	groL	60 kDa chaperonin 1
G2583_4971	ECs5125	Z5749	yjeI	hypothetical protein
G2583_4972	ECs5126	Z5750	yjeJ	conserved hypothetical protein
G2583_4973	ECs5127	Z5751	yjeK	KamA family protein
G2583_4974	ECs5128	Z5752	efp	Elongation factor P
G2583_4975		Z5753	ecnA	Predicted small secreted protein
G2583_4976		Z5754	ecnB	Entericidin B
G2583_4977	ECs5129	Z5755	sugE	SugE
G2583_4978	ECs5130	Z5756	blc	Outer membrane lipoprotein blc precursor
G2583_4979	ECs5131	Z5757	ampC	Beta-lactamase
G2583_4980	ECs5132	Z5758	frdD	Fumarate reductase subunit D
G2583_4981	ECs5133	Z5759	frdC	Fumarate reductase subunit C
G2583_4982	ECs5134	Z5760	frdB	Fumarate reductase iron-sulfur subunit
G2583_4983	ECs5135	Z5762	frdA	Fumarate reductase, anaerobic, flavoprotein subunit
G2583_4984	ECs5136	Z5763	poxA	Putative lysyl-tRNA synthetase
G2583_4985	ECs5137	Z5764	yjeM	Hypothetical transporter YjeM
G2583_4986	ECs5138	Z5765	yjeP	Mechanosensitive ion channel family protein

Locus_tag[a]				
CB9615	Sakai	EDL933	Gene	Product
G2583_4987	ECs5139	Z5766	psd	Phosphatidylserine decarboxylase proenzyme
G2583_4988	ECs5140	Z5767	rsgA	Probable GTPase engC precursor
G2583_4989	ECs5141	Z5768	orn	Oligoribonuclease
G2583_4990	glyV	RNA124	glyV	Gly tRNA
G2583_4991	glyX	RNA125	glyX	Gly tRNA
G2583_4992	glyY	RNA126	glyY	Gly tRNA
G2583_4993	ECs5143	Z5773	yjeS	hypothetical protein
G2583_4994	ECs5142	Z5774	yjeF	hypothetical protein
G2583_4995	ECs5144	Z5775	yjeE	UPF0079 ATP-binding protein yjeE
G2583_4996	ECs5145	Z5776	amiB	N-acetylmuramoyl-L-alanine amidase AmiB
G2583_4997	ECs5146	Z5777	mutL	DNA mismatch repair protein MutL
G2583_4998	ECs5147	Z5778	miaA	tRNA delta(2)-isopentenylpyrophosphate transferase
G2583_4999	ECs5148	Z5779	hfq	RNA-binding protein Hfq
G2583_5000	ECs5149	Z5780	hflX	GTP-binding protein HflX
G2583_5001	ECs5150	Z5781	hflK	FtsH protease regulator HflK
G2583_5002	ECs5151	Z5782	hflC	FtsH protease regulator HflC
G2583_5003	ECs5152	Z5783	yjeT	hypothetical protein
G2583_5004	ECs5153	Z5784	purA	Adenylosuccinate synthetase
G2583_5005	ECs5154	Z5785	nsrR	transcriptional repressor NsrR
G2583_5006	ECs5155	Z5786	rnr	Putative enzyme
G2583_5007	ECs5156	Z5787	rlmB	23S rRNA (guanosine-2'-O-)-methyltransferase rlmB
G2583_5008	ECs5157	Z5788	yjfI	hypothetical protein
G2583_5009	ECs5158	Z5789	yjfJ	PspA/IM30 family protein
G2583_5010	ECs5159	Z5790	yjfK	hypothetical protein
G2583_5011	ECs5160	Z5791	yjfL	hypothetical protein
G2583_5012	ECs5161	Z5792	yjfM	hypothetical protein
G2583_5013	ECs5162	Z5793	yjfC	Glutathionylspermidine synthase domain protein
G2583_5014	ECs5163	Z5794	aidB	Putative acyl coenzyme A dehydrogenase
G2583_5015	ECs5164	Z5795	yjfN	hypothetical protein
G2583_5016	ECs5165	Z5796	yjfO	hypothetical protein
G2583_5017	ECs5166	Z5799	yjfP	Esterase yjfP
G2583_5018	ECs5167	Z5800	ulaR	putative DEOR-type transcriptional regulator
G2583_5019	ECs5168	Z5801	ulaG	Predicted Zn-dependent hydrolases of the beta-lactamase fold
G2583_5020	ECs5169	Z5802	ulaA	ascorbate-specific PTS system enzyme IIC
G2583_5021	ECs5170	Z5803	ulaB	Ascorbate-specific phosphotransferase enzyme IIB component
G2583_5022	ECs5171	Z5804	ulaC	Ascorbate-specific phosphotransferase enzyme IIA component
G2583_5023	ECs5172	Z5805	ulaD	Putative hexulose-6-phosphate synthase
G2583_5024	ECs5173	Z5806	ulaE	L-ribulose-5-phosphate 3-epimerase ulaE
G2583_5025	ECs5174	Z5807	ulaF	L-ribulose-5-phosphate 4-epimerase ulaF
G2583_5026	ECs5175	Z5808	yjfY	UPF0379 protein yjfY precursor
G2583_5027	ECs5176	Z5809	rpsF	Ribosomal protein S6

Locus_tag[a]				
CB9615	Sakai	EDL933	Gene	Product
G2583_5028	ECs5177	Z5810	priB	Primosomal replication protein n
G2583_5029	ECs5178	Z5811	rpsR	30S ribosomal protein S18
G2583_5030	ECs5179	Z5812	rplI	50S ribosomal protein L9
G2583_5031	ECs5180	Z5813	yjfZ	hypothetical protein
G2583_5033		Z5814	-	hypothetical protein
G2583_5032	ECs5181		-	conserved hypothetical protein
G2583_5034	ECs5182	Z5815	-	Transposase, family
G2583_5035	ECs5183	Z5816	ydcM	IS605 family transposase orfB
G2583_5036	ECs5184	Z5817	ytfB	hypothetical protein
G2583_5037	ECs5185	Z5818	fklB	Peptidyl-prolyl cis-trans isomerase
G2583_5038	ECs5186	Z5819	cycA	D-serine/D-alanine/glycine transporter
G2583_5039	ECs5187	Z5820	ytfE	Regulator of cell morphogenesis and NO signaling
G2583_5040	ECs5188	Z5821	ytfF	Putative transmembrane subunit
G2583_5041	ECs5190	Z5822	ytfG	NmrA family protein
G2583_5042	ECs5189	Z5823	ytfH	hypothetical protein
G2583_5043	ECs5191	Z5824	cpdB	2',3'-cyclic-nucleotide 2'-phosphodiesterase
G2583_5044	ECs5192	Z5825	cysQ	PAPS (adenosine 3'-phosphate 5'-phosphosulfate) 3'(2'),5'-bisphosphate nucleotidase
G2583_5045	ECs5193	Z5826	ytfI	hypothetical protein
G2583_5046	ECs5194	Z5827	ytfJ	hypothetical protein
G2583_5047	ECs5195	Z5828	ytfK	hypothetical protein
G2583_5048	ECs5196	Z5829	ytfL	UPF0053 inner membrane protein ytfL
G2583_5049	ECs5197	Z5830	msrA	Peptide methionine sulfoxide reductase msrA
G2583_5050	ECs5198	Z5831	ytfM	hypothetical protein
G2583_5051	ECs5199	Z5832	ytfN	hypothetical protein
G2583_5052	ECs5200	Z5833	ytfP	UPF0131 protein ytfP
G2583_5053	ECs5201	Z5834	yzfA	Ile repressor (ileR)
G2583_5054	ECs5202	Z5835	chpS	PemI protein 2
G2583_5055	ECs5203	Z5836	chpB	PemK protein 2
G2583_5056	ECs5204	Z5837	ppa	Inorganic pyrophosphatase
G2583_5057	ECs5205	Z5838	ytfQ	Putative sugar ABC transporter, periplasmic sugar-binding protein
G2583_5058	ECs5206	Z5839	ytfR	Uncharacterized ABC transporter ATP-binding protein
G2583_5059	ECs5207	Z5840	ytfT	Putative transport system permease protein
G2583_5060	ECs5208	Z5841	yjfF	Putative sugar ABC transporter, permease protein
G2583_5061	ECs5209	Z5842	fbp	Fructose-1,6-bisphosphatase
G2583_5062	ECs5210	Z5843	mpl	UDP-N-acetylmuramate:L-alanyl-gamma-D-glutamyl-meso- diaminopimelate ligase
G2583_5063	ECs5211	Z5844	yjgA	UPF0307 protein yjgA
G2583_5064	ECs5212	Z5845	pmbA	Putative peptide maturation protein
G2583_5065	ECs5213	Z5846	cybC	Soluble cytochrome b562 precursor
G2583_5066	ECs5214	Z5847	nrdG	Anaerobic ribonucleoside-triphosphate reductase-activating protein
G2583_5067	ECs5215	Z5848	nrdD	Anaerobic ribonucleoside-triphosphate reductase

Locus_tag[a]				
CB9615	Sakai	EDL933	Gene	Product
G2583_5068	ECs5216	Z5849	treC	Trehalase 6-P hydrolase
G2583_5069	ECs5217	Z5850	treB	PTS system, trehalose-specific IIBC component
G2583_5070	ECs5218	Z5851	treR	Trehalose operon repressor
G2583_5071		Z5852	-	hypothetical protein
G2583_5072	ECs5219	Z5853	mgtA	Magnesium-transporting ATPase, P-type 1
G2583_5073	ECs5220	Z5854	yjgF	hypothetical protein
G2583_5074	ECs5221	Z5855	pyrI	Aspartate carbamoyltransferase regulatory chain
G2583_5075	ECs5222	Z5856	pyrB	Aspartate carbamoyltransferase
G2583_5076	ECs5224	Z5857	pyrL	PyrBI operon leader peptide
G2583_5077	ECs5223	Z5858	yjgG	hypothetical protein
G2583_5078		Z5859	yjgG	ORF_o110
G2583_5079	ECs5225	Z5860	yjgH	Endoribonuclease L-PSP family protein
G2583_5080	ECs5226	Z5861	yjgI	Oxidoreductase, short chain dehydrogenase/reductase family
G2583_5081	ECs5228	Z5862	yjgJ	hypothetical protein
G2583_5082	ECs5229	Z5864	yjgK	hypothetical protein
G2583_5083	ECs5230	Z5865	yjgL	hypothetical protein
G2583_5084	ECs5231	Z5866	argI	Ornithine carbamoyltransferase
G2583_5085	ECs5232	Z5867	rraB	hypothetical protein
G2583_5086	ECs5233	Z5868	yjgM	Acetyltransferase, GNAT family
G2583_5087	ECs5234	Z5869	yjgN	hypothetical protein
G2583_5088	ECs5235	Z5870	valS	Valyl-tRNA synthetase
G2583_5089	ECs5236	Z5871	holC	DNA polymerase III, chi subunit
G2583_5090	ECs5237	Z5872	pepA	Cytosol aminopeptidase
G2583_5091	ECs5238	Z5873	yjgP	Inner membrane protein yjgP
G2583_5092	ECs5239	Z5874	yjgQ	Inner membrane protein yjgQ
G2583_5093	ECs5240	Z5875	yjgR	hypothetical protein
G2583_5094	ECs5241	Z5876	yjgB	Alcohol dehydrogenase
G2583_5095	leuX	RNA127	leuX	Leu tRNA
Indel-197	ECs5242	Z5878	-	putative integrase
Indel-197	ECs5243	Z5879	-	putative transposase OrfA protein of insertion sequence IS629
Indel-197	ECs5244	Z5880	-	putative transposase OrfB protein of insertion sequence IS629
Indel-197	ECs5245	Z5881	-	hypothetical protein
Indel-197	ECs5246	Z5882	-	hypothetical protein
Indel-197		Z5883	-	hypothetical protein
Indel-197	ECs5247		-	hypothetical protein
Indel-197	ECs5248	Z5884	-	hypothetical protein
Indel-197	ECs5249	Z5885	-	putative resolvase
Indel-197	ECs5250	Z5886	-	hypothetical protein
Indel-197	ECs5251	Z5887	-	hypothetical protein
Indel-197	ECs5252	Z5888	-	putative transcription regulator
Indel-197		Z5889	-	hypothetical protein

Locus_tag[a]				
CB9615	Sakai	EDL933	Gene	Product
G2583_5096	ECs5253	Z5890	-	Prophage P4 integrase
G2583_5097		Z5891	-	hypothetical protein
G2583_5098	ECs5254	Z5892	-	hypothetical protein
G2583_5099	ECs5255	Z5893	yjhT	N-acetylneuraminate epimerase 2
G2583_5100	ECs5256	Z5894	-	hypothetical protein
G2583_5101	ECs5257	Z5895	-	hypothetical protein
G2583_5102	ECs5258	Z5896	-	hypothetical protein
G2583_5103	ECs5259	Z5897	-	hypothetical protein
G2583_5104	ECs5260	Z5898	-	hypothetical protein
G2583_5105	ECs5261	Z5899	-	Helicase family protein
G2583_5106	ECs5262	Z5900	-	hypothetical protein
G2583_5107	ECs5263	Z5901	-	DEAD/DEAH box helicase domain protein
G2583_5108	ECs5264	Z5902	-	ATP-dependent DNA helicase, UvrD/REP family
		Z5903	-	hypothetical protein
	ECs5265		-	hypothetical protein
G2583_5109	ECs5266	Z5904	-	hypothetical protein
G2583_5109	ECs5267		-	hypothetical protein
G2583_5110	ECs5268	Z5905	yjhS	hypothetical protein
G2583_5111	ECs5269	Z5906	yjhT	N-acetylneuraminate-epimerase precursor
G2583_5112	ECs5270	Z5907	nanC	hypothetical protein
G2583_5113	ECs5271	Z5910	fimB	Type 1 fimbriae regulatory protein fimB
G2583_5114	ECs5272	Z5911	fimE	Type 1 fimbriae regulatory protein FimE
G2583_5115	ECs5273	Z5912	fimA	Major type 1 subunit fimbrin
G2583_5116	ECs5274	Z5913	fimI	FimI fimbrial protein
G2583_5117	ECs5275	Z5914	fimC	Chaperone protein FimC
G2583_5118	ECs5276	Z5915	fimD	Export and assembly outer membrane protein of type 1 fimbriae
G2583_5119	ECs5277	Z5916	fimF	Fimbrial protein FimF
G2583_5120	ECs5278	Z5917	fimG	FimG
G2583_5121	ECs5279	Z5918	fimH	Adhesin
G2583_5122	ECs5280	Z5919	gntP	High-affinity gluconate transporter
G2583_5123	ECs5281	Z5920	uxuA	D-mannonate dehydratase
G2583_5124	ECs5282	Z5921	uxuB	Fructuronate reductase
G2583_5125	ECs5283	Z5922	uxuR	DNA-binding transcriptional repressor
G2583_5126	ECs5284	Z5923	yjiC	hypothetical protein
G2583_5127		Z5924	-	hypothetical protein
G2583_5128	ECs5285	Z5925	yjiD	Anti-adapter protein iraD
G2583_5129	ECsp024	Z5926	yjiE	transcriptional regulator, LysR family
G2583_5130	ECs5286	Z5927	iadA	Beta-aspartyl peptidase
G2583_5131	ECs5287	Z5928	yjiG	putative membrane protein
G2583_5132	ECs5288	Z5929	yjiH	hypothetical protein
G2583_5133	ECs5289	Z5930	yjiI	Putative uncharacterized protein yjiI
G2583_5133			yjiI	Putative uncharacterized protein yjiI
G2583_5134	ECs5290	Z5932	yeeJ	Putative invasin

Locus_tag[a]				
CB9615	Sakai	EDL933	Gene	Product
	ECs5291		-	hypothetical protein
G2583_5134	ECs5292		yeeJ	Putative invasin
G2583_5135	ECs5293	Z5933	yjiJ	Transporter, major facilitator family
G2583_5136	ECs5294	Z5934	yjiK	SdiA-regulated protein
G2583_5137	ECs5295	Z5935	-	hypothetical protein
G2583_5138	ECs5296		-	hypothetical protein
G2583_5139	ECs5297	Z5936	yjiL	(R)-2-hydroxyglutaryl-CoA dehydratase activator
G2583_5140	ECs5298	Z5937	yjiM	hypothetical protein
G2583_5141	ECs5299	Z5938	yjiN	conserved hypothetical protein
G2583_5142	ECs5300	Z5939	mdtM	Multidrug resistance protein mdtM
G2583_5143	ECs5301	Z5940	yjiP	hypothetical protein
G2583_5144	ECs5302	Z5941	yjiR	transcriptional regulator, GntR family/aminotransferase, classes I and II
G2583_5145	ECs5303	Z5942	yjiS	YjiS
G2583_5146	ECs5304	Z5943	-	hypothetical protein
G2583_5146	ECs5305	Z5944	-	hypothetical protein
G2583_5147		Z5945	yjiW	HSP20-like domain protein
G2583_5148	ECs5306	Z5946	hsdS	type I restriction-modification system, S subunit
G2583_5149	ECs5307	Z5947	hsdM	Type I restriction-modification system, M subunit
G2583_5150	ECs5308	Z5948	hsdR	Type I restriction-modification system, R subunit
G2583_5151	ECs5309	Z5949	-	protein of unknown function DUF262
G2583_5152	ECs5310	Z5950	orf	protein of unknown function DUF262
G2583_5153	ECs5311	Z5951	yjiA	Putative GTPase
G2583_5154	ECs5312	Z5952	yjiX	hypothetical protein
G2583_5155	ECs5313	Z5953	yjiY	Carbon starvation family protein
G2583_5156	ECs5314	Z5954	-	hypothetical protein
G2583_5157	ECs5315	Z5955	tsr	Methyl-accepting chemotaxis protein I
G2583_5158	ECs5316	Z5956	yjjL	Transporter, major facilitator family
G2583_5159	ECs5317	Z5957	yjjM	transcriptional regulator, GntR family
G2583_5160	ECs5318	Z5958	yjjN	Putative oxidoreductase
G2583_5161	ECs5319	Z5959	mdoB	Phosphoglycerol transferase I
G2583_5162	ECs5320	Z5960	yjjA	hypothetical protein
G2583_5163	ECs5321	Z5961	dnaC	DNA replication protein DnaC
G2583_5164	ECs5322	Z5962	dnaT	Primosomal protein 1
G2583_5165	ECs5323	Z5963	yjjB	Uncharacterized conserved protein
G2583_5166	ECs5324	Z5964	yjjP	putative structural protein
G2583_5167	ECs5325	Z5966	yjjQ	hypothetical protein
G2583_5168	ECs5326	Z5967	bglJ	transcriptional regulator, LuxR family
G2583_5169	ECs5327	Z5968	fhuF	Ferric iron reductase protein FhuF
G2583_5170	ECs5328		yjjZ	hypothetical protein
G2583_5171	leuQ	RNA128	leuQ	Leu tRNA
G2583_5172	ECs5329	Z5972	rsmC	16S RNA G1207 methylase RsmC
G2583_5173	ECs5330	Z5973	holD	DNA polymerase III, psi subunit
G2583_5174	ECs5331	Z5974	rimI	Ribosomal-protein-alanine acetyltransferase

Locus_tag[a]				
CB9615	Sakai	EDL933	Gene	Product
G2583_5175	ECs5332	Z5975	yjjG	5'-nucleotidase yjjG
G2583_5176	Indel-202	Indel-202	-	Phage integrase
G2583_5177	Indel-202	Indel-202	-	hypothetical protein
G2583_5178	Indel-202	Indel-202	yfdT	hypothetical protein
G2583_5179	Indel-202	Indel-202	yfdS	hypothetical protein
G2583_5180	Indel-202	Indel-202	yfdR	hypothetical protein
G2583_5181	Indel-202	Indel-202	yfdQ	hypothetical protein
G2583_5182	Indel-202	Indel-202	yfdP	hypothetical protein
G2583_5183	Indel-202	Indel-202	-	hypothetical protein
G2583_5184	Indel-202	Indel-202	rpc	Putative regulatory protein
G2583_5185	Indel-202	Indel-202	-	hypothetical protein
G2583_5186	Indel-202	Indel-202	ymfL	hypothetical protein
G2583_5187	Indel-202	Indel-202	ymfM	e14 prophage; predicted protein
G2583_5188	Indel-202	Indel-202	yfdO	Unknown protein encoded by cryptic prophage
G2583_5189	Indel-202	Indel-202	yfdN	hypothetical protein
G2583_5190	Indel-202	Indel-202	yfdM	putative DNA adenine methylase
G2583_5191	Indel-202	Indel-202	-	hypothetical protein
G2583_5192	Indel-202	Indel-202	rusA	Holliday junction resolvase
G2583_5193	Indel-202	Indel-202	KilA-N	KilA-N domain family
G2583_5194	Indel-202	Indel-202	ydfU	hypothetical protein
G2583_5195	Indel-202	Indel-202	ydfT	Antitermination protein Q
G2583_5196	Indel-202	Indel-202	cspF	Cold shock-like protein cspF
G2583_5197	Indel-202	Indel-202	cspB	Cold shock-like protein cspB
G2583_5198	Indel-202	Indel-202	essQ	Lysis protein S-like from lambdoid prophage Qin
G2583_5199	Indel-202	Indel-202	ydfR	hypothetical protein
G2583_5200	Indel-202	Indel-202	-	hypothetical protein
G2583_5201	Indel-202	Indel-202	ydfQ	phage lysozyme
G2583_5202	Indel-202	Indel-202	ydfP	hypothetical protein
G2583_5203	Indel-202	Indel-202	cspI	Cold shock-like protein cspI
G2583_5204	Indel-202	Indel-202	ynfN	hypothetical protein
G2583_5205	Indel-202	Indel-202	gnsB	GnsB protein
G2583_5206	Indel-202	Indel-202	ybcW	conserved hypothetical protein
G2583_5207	Indel-202	Indel-202	ylcI	hypothetical protein
G2583_5208	Indel-202	Indel-202	nohA	Prophage Qin DNA packaging protein NU1-like protein
G2583_5209	Indel-202	Indel-202	-	Putative DNA packaging protein of prophage
G2583_5210	Indel-202	Indel-202	-	Head-stabilizing protein
G2583_5211	Indel-202	Indel-202	-	Putative capsid protein of prophage
G2583_5212	Indel-202	Indel-202	-	Head-tail preconnector protein GP5
G2583_5213	Indel-202	Indel-202	-	Head decoration protein
G2583_5214	Indel-202	Indel-202	-	Major head protein
G2583_5215	Indel-202	Indel-202	-	Uncharacterized 13.5 kDa protein in GP7-GP8 intergenic region
G2583_5216	Indel-202	Indel-202	-	Putative head-tail joining protein of prophage
G2583_5217	Indel-202	Indel-202	-	Prophage minor tail protein Z

Locus_tag[a]				
CB9615	Sakai	EDL933	Gene	Product
G2583_5218	Indel-202	Indel-202	-	Permeases of the major facilitator superfamily
G2583_5219	Indel-202	Indel-202	-	Putative tail component of prophage
G2583_5220	Indel-202	Indel-202	-	Putative tail component of prophage
G2583_5221	Indel-202	Indel-202	-	Minor tail protein T
G2583_5222	Indel-202	Indel-202	-	Putative tail length tape measure protein
G2583_5223	Indel-202	Indel-202	-	Minor tail protein
G2583_5224	Indel-202	Indel-202	-	Phage-related protein
G2583_5225	Indel-202	Indel-202	-	Putative tail fiber component K of prophage
G2583_5226	Indel-202	Indel-202	-	Putative tail component of prophage CP-933K
G2583_5227	Indel-202	Indel-202	-	hypothetical protein
G2583_5228	Indel-202	Indel-202	-	Phage-related protein, tail component
G2583_5229	Indel-202	Indel-202	-	putative membrane protein precursor
G2583_5230	Indel-202	Indel-202	-	PPE-repeat proteins
G2583_5231	Indel-202	Indel-202	tfaQ	Tail fiber assembly protein homolog from lambdoid prophage Qin
G2583_5232	Indel-202	Indel-202	pinR	Putative DNA-invertase from lambdoid prophage
G2583_5233	Indel-202	Indel-202	ydfK	hypothetical protein
G2583_5234	Indel-202	Indel-202	-	hypothetical protein
G2583_5235	ECs5333	Z5976	prfC	Peptide chain release factor RF-3
G2583_5236	ECs5334	Z5977	osmY	Osmotically inducible protein Y
		Z5978	-	hypothetical protein
G2583_5237	ECs5335	Z5979	yjjU	hypothetical protein
G2583_5238	ECs5336	Z5980	yjjV	Hydrolase, TatD family
G2583_5239	ECs5337	Z5981	yjjW	Putative activating enzyme
G2583_5240	ECs5338	Z5982	yjjI	hypothetical protein
G2583_5240	ECs5339	Z5982	yjjI	hypothetical protein
G2583_5241	ECs5340	Z5983	deoC	Deoxyribose-phosphate aldolase
G2583_5242	ECs5341	Z5984	deoA	Thymidine phosphorylase
G2583_5243	ECs5342	Z5985	deoB	Phosphopentomutase
G2583_5244	ECs5343	Z5986	deoD	Purine nucleoside phosphorylase
G2583_5245	ECs5344	Z5987	-	Helix-turn-helix domain protein
G2583_5246	ECs5345	Z5988	lplA	protein smp precursor
G2583_5247	ECs5346	Z5989	serB	Phosphoserine phosphatase
G2583_5248	ECs5347	Z5990	radA	Predicted ATP-dependent serine protease
G2583_5249	ECs5348	Z5991	nadR	bifunctional DNA-binding transcriptional repressor and NMN adenylyltransferase
G2583_5250	ECs5349	Z5993	yjjK	Uncharacterized ABC transporter ATP-binding protein
G2583_5251	ECs5350	Z5994	slt	Soluble lytic murein transglycosylase
G2583_5252	ECs5351	Z5995	trpR	Trp operon repressor
G2583_5253	ECs5352	Z5996	yjjX	conserved hypothetical protein
G2583_5254	ECs5353	Z5997	ytjC	Probable phosphoglycerate mutase gpmB
G2583_5255	ECs5354	Z5999	rob	Right origin-binding protein
G2583_5256	ECs5355	Z6000	creA	hypothetical protein
G2583_5257	ECs5356	Z6001	creB	DNA-binding response regulator CreB

Locus_tag[a]				
CB9615	Sakai	EDL933	Gene	Product
G2583_5258	ECs5357	Z6002	creC	sensory histidine kinase CreC
G2583_5259	ECs5358	Z6003	creD	Inner membrane protein CreD
G2583_5260	ECs5359	Z6004	arcA	Aerobic respiration control protein arcA
G2583_5261	ECs5360	Z6005	yjjY	hypothetical protein
G2583_5262	ECs5361	Z6006	yjtD	RNA methyltransferase, TrmH family, group 1

[a] rRNA and tRNA genes in Sakai and EDL933 lack locus_tags, gene name is given as a substitute.

Some genes were not predicted in one or two of the genomes and lef as a blank cell. All insertion or deletion of genes are described in the number of the indels.

In some cases a gene in one genome was annotated as 2 or more genes in other genomes, and the same gene name appears 2 or more times for a genome. This may be due to mutation to stop codon, frameshift mutation or sequencing errors.
