# Supplementary material for: Derivation of Escherichia coli O157:H7 from Its O55:H7 Precursor
Source: PLoS One. 2010 Jan 14;5(1):e8700. doi: 10.1371/journal.pone.0008700 (PMC2806823; doi:10.1371/journal.pone.0008700)
Supplement: Table S11 — Comparison of the phages in the O55:H7 strain and the two O157:H7 strains. (0.02 MB PDF) [file pone.0008700.s013.pdf]

**Table S11.** Comparison of the phages in the O55:H7 and the 2 O157:H7 strains

| CB9615 | | | | Sakai | | | | EDL933 | | | | Comparisons | |
|---|---|---|---|---|---|---|---|---|---|---|---|---|---|
| name | site | Type[c] | length | name | site | Type[c] | length | name | site | Type[c] | length | CB9615 / Sakai[d] | EDL933 / Sakai[d] |
| Ep1 | 302638-351615 | Lambda-like | 48,949 | Sp1 | 300041-310626 | Lambda-like | 10,586 | CP-933H | 300042-310627 | Lambda-like | 10,586 | replacement | 100% 2kb inv |
| Ep2 | 351616-364486 | P4-like | 12,871 | Sp2 | 310627-323513 | P4-like | 12,887 | CP-933I | 310628-323514 | P4-like | 12,887 | 12.9kb rec | 100% |
| Ep3 | 697268-746682 | | 49,415 | - | - | | | - | - | | | ins | - |
| Ep4 | 980716-1026738 | Lambda-like | 46,023 | Sp3 | 891123-929708 | Lambda-like | 38,586 | CP-933K | 892772-931359 | Lambda-like | 38,588 | 99.17% 4.4kb rec, 7.4kb ins | 99.97% 0.3kb rec |
| - | - | | | Sp4 | 1161091-1210740 | Lambda-like | 49,650 | CP-933M | 1250314-1294565 | Lambda-like | 44,252 | - | 99.97% 1.1kb rec, 28.2k ins, 32.6k del |
| - | - | | | Sp5 | 1246012-1308719 | Lambda-like | 62,708 | BP-933W | 1330829-1392491 | Lambda-like | 61,663 | - | 99.99% 5.4kb rec, 10.6k ins, 11.6k del |
| - | - | | | Sp6 | 1541470-1589892 | Lambda-like | 48,423 | CP-933N | 1626566-1673868 | Lambda-like | 47,303 | - | 99.96% 1.1kb rec, 20k ins, 21.1k del |
| Ep5 | 1444732-1455388 | P4-like | 10,657 | Sp7 | 1594570-1610032 | | 15,463 | CP-933C | 1678546-1694005 | | 15,460 | replacement | 99.95% |
| elbA-ypjA[a] | 1463904-1478380 | | 14,477 | Sp8 | 1618153-1665049 | Lambda-like | 46,897 | CP-933X | 1702126-1756455 | Lambda-like | 54,330 | replacement | 99.96% 1.3kb rec, 15.1kb ins, 7.7kb del |
| Ep6 | 1574754-1627761 | Lambda-like | 53,008 | Sp9 | 1757506-1815680 | Lambda-like | 58,175 | CP-933O-1 & CP-933P[b] | 1849445-1868506 2292538-2271497 | Lambda-like | 40,104 | 99.78% 1.3kb rec, 40.7kb ins, 27.8~45.9kb del | 99.99% 0.7kb rec, 4k ins, 22.1kb del |
| Ep7 | 1732190-1777066 | Lambda-like | 44,877 | Sp10 | 1921414-1972525 | Lambda-like | 51,112 | CP-933R | 2165752-2115956 | Lambda-like | 49,797 | 99.83% 1.3kb rec, 37.8kb ins, 43~44kb del | 99.99% 1.4kb rec, 22.8kb ins, 24.2kb del |
| - | - | | | Sp11 | 2158174-2203951 | Lambda-like | 45,778 | CP-933O-2 | 1930297-1878765 | Lambda-like | 51,533 | - | 99.97% 6.1kb rec, 32.9kb ins, 27.2kb del |
| Ep8 | 1967051-1988518 | Lambda-like | 21,468 | Sp12 | 2203952-2250093 | Lambda-like | 46,142 | CP-933O-1 & CP-933P[b] | 1878764-1872344 2280925-2325378 | Lambda-like | 50,875 | replacement | 99.96% 0.2k rec, 4.1kb ins, 8.9kb del |
| Ep9 | 1988519-2009859 | Lambda-like | 21,341 | - | - | | | - | - | | | ins | - |
| Ep10 | 2152246-2187996 | P2-like | 35,751 | - | - | | | - | - | | | ins | - |
| - | - | | | Sp13 | 2592901-2614020 | P2-like | 21,120 | CP-933T | 2668112-2689231 | P2-like | 21,120 | - | 99.96% |
| Ep11 | 2437795-2486743 | Lambda-like | 48,949 | Sp14 | 2668007-2712035 | Lambda-like | 44,029 | CP-933U | 2743224-2788400 | Lambda-like | 45,177 | replacement | 99.97% 5kb rec, 25.6kb ins, 24.4 kb del |
| - | - | | | Sp15 | 2895926-2943804 | Lambda-like | 47,879 | CP-933V | 2966157-3015071 | Lambda-like | 48,915 | - | 99.97% 17.2kb rec, 21.2kb ins 20.2kb del |
| Ep12 | 2934049-2942599 | P22-like | 8,551 | Sp16 | 3192983-3201533 | P22-like | 8,551 | CP-22 | 3262939-3271489 | P22-like | 8,551 | 99.94% | 99.96% |
| Ep13 | 3161159-3202641 | | 41,483 | - | - | | | - | - | | | ins | - |
| Ep14 | 3258501-3337894 | Lambda-like | 79,394 | Sp17 | 3475965-3500163 | Lambda-like | 24,199 | CP-933Y | 3545814-3567453 | Lambda-like | 21,640 | 99.85% 8.7kb rec, 35.1kb ins, 7.8~10.3kb del | 99.97% 0.1kb rec, 1.5kb ins 4kb del |
| - | - | | | Sp18 | 5040843-5079601 | Mu-like | 38,759 | - | - | | | - | - |
| Ep15 | 5306033-5355424 | Lambda-like | 49,392 | - | - | | | - | - | | | ins | - |
| - | - | | | SpLE1 | 1370456-1456704 | CP4-like | 86,249 | SpLE1 | 1454228-1541789 | CP4-like | 87,562 | - | 99.97% 0.1kb rec, 1.3kb ins |
| - | - | | | - | - | | | SpLE1 copy | 1058621-1146183 | CP4-like | 87,563 | - | 99.98% |
| EpLE1 | 2510337-2531200 | | 20,864 | SpLE2 | 2738079-2751537 | | 13,459 | SpLE2 | 2814443-2821749 | | 7,307 | replacement | 99.99% 0.4kb rec, 6.1kb del |
| EpLE2 | 3675949-3704076 | | 28,127 | SpLE3 | 3852036-3875489 | | 23,454 | SpLE3 | 3919348-3942802 | | 23,455 | 99.92% 0.04kb rec, 8.4kb ins, 3.4kb del | 99.97% |
| EpLE3 | 4470809-4511806 | | 40,998 | SpLE4 | 4580864-4624313 | | 43,450 | SpLE4 | 4649847-4693296 | | 43,450 | 99.93% 2.4kb del-ISEc8 | 99.99% |
| - | - | | | SpLE5 | 5347085-5357319 | CP-4 like | 10,235 | SpLE5 | 5377088-5387322 | CP-4 like | 10,235 | - | 99.99% 9bp rec |
| EpLE4 | 5195636-5229783 | | 34,148 | SpLE6 | 5357371-5391518 | | 34,148 | SpLE6 | 5387374-5421521 | | 34,148 | 99.94% | 99.98% |

[a] This region is not the phage and was deleted in O157 lineage.

[b] These 2 phages at the ends of the inversion in EDL933.

[c] The phage type in Sakai was based on the Hayashi *et al.*[25]. The phage types in other 2 genomes were based on similarity to known phages in GenBank.

[d] Replacement: the phages are inserted at the same site but different in sequence; The percentage ratio shows the divergence in conserved region. rec: recombination region. ins: insert in phages; del: deletion in phages compared to phages in Sakai
