# Supplementary material for: Derivation of Escherichia coli O157:H7 from Its O55:H7 Precursor
Source: PLoS One. 2010 Jan 14;5(1):e8700. doi: 10.1371/journal.pone.0008700 (PMC2806823; doi:10.1371/journal.pone.0008700)
Supplement: Table S12 — Type III secretory system effectors of CB9651, Sakai, and EDL933. The effectors are shown with their distribution among the genomes and the level of amino acid identity where shared by two or three of the strains. (0.06 MB PDF) [file pone.0008700.s014.pdf]

Table S12. Summary of the T3SS effectors in Sakai, CB9615 and E2348/69

Effector[a]	Family[b]	Sakai ID	identities[c]	CB9615 ID	identities[c]	E2348/69 ID	identities[c]
EspX1	PPR	ECs0025	22.14	G2583_0023	22.14	-	
EspY1	SopD-N	ECs0061	33.93	G2583_0060	33.93	-	
EspY2	SopD-N	ECs0073	-	G2583_0072	-	-	
EspY3	SopD-N;PRR	ECs0472	23.48	G2583_0530	23.48	-	
NleB2-1	NleB	ECs0846	61.54	G2583_0995	61.54	E2348_C_1041	61.54
NleC	NleC	ECs0847	90.3	G2583_0996	90.3	E2348_C_1042	90.61
NleH1-1	NleH	ECs0848	83.28	G2583_0998	83.28	E2348_C_1043	85.19
NleD	NleD	ECs0850	75.74	G2583_1000	76.6	E2348_C_1044	76.6
EspX2	PPR;LRR	ECs0876	25.74	G2583_1026	25.41	-	
EspF2-1	EspF	ECs1126	35.85	-		-	
EspV│	AvrA	ECs1127	46.3	-		-	
EspX7	PPR	ECs1560	33.39	-		-	
EspN	CNF	ECs1561	37.57	-		-	
NleB2-2	NleB	ECs1566	72.97	G2583_0347	66.03	-	
EspO1-1	OspE	ECs1567	44.64	G2583_0348	41.51	-	
EspK	LRR	ECs1568	31.44	G2583_0349/50	31.44	-	
NleG2-1	NleG	ECs1810/1	60.91	-		-	
NleA	NleA	ECs1812	81.26	pO55_0114	81.72	E2348_C_1442	81.22
NleH1-2	NleH	ECs1814	81.19	G2583_1592	81.19	E2348_C_1444	81.19
NleF	NleF	ECs1815	85.19	G2583_1593	85.19	E2348_C_1445	85.19
EspO1-2	OspE	ECs1821	37.74	-		E2348_C_1447	37.74
NleG-1	NleG	ECs1824	39.22	G2583_0343	40	-	
EspM1	IpgB	ECs1825	32.94	G2583_0344	25	-	
NleG9	NleG	ECs1828	38.32	-		-	
NleG2-2	NleG	ECs1994	60	G2583_1748	59.09	-	
NleG6-1	NleG	ECs1995	41.12	G2583_1749	39.25	-	
NleG5-1	NleG	ECs1996	48.45	G2583_1750	48.45	-	
EspR1	LRR	ECs2073	25.64	G2583_1832	24.07	-	
EspR2	LRR	ECs2074/5	31.91	G2583_1833/4	31.91	-	
NleG5-2	NleG	ECs2154	49.48	-		-	
NleG6-2	NleG	ECs2155	42.71	-		-	
NleG2-3	NleG	ECs2156	59.09	-		-	
NleG7	NleG	ECs2226	38.1	G2583_1913	37.01	-	
NleG3	NleG	ECs2227/8	40.48	G2583_1914/5	40.48	-	
NleG2-4	NleG	ECs2229	67.01	G2583_1916	46.02	E2348_C_1040	44.33
EspL1	AR	ECs2427	21.47	G2583_2167	21.47	E2348_C_1849	
EspR3	LRR	ECs2672	30.28	G2583_2384	30.28	-	
EspR4	LRR	ECs2674	30.99	G2583_2386	30.99	-	
EspJ	EspJ	ECs2714	22.83	G2583_2428	22.83	E2348_C_0723	34
TccP	EspF	ECs2715	34.67	G2583_2429	32.29	-	
EspM2	IpgB	ECs3485	25	G2583_3204	25	-	
NleG8-2	NleG	ECs3486	40	G2583_3205	36.25	-	
EspW	HopW	ECs3487	30.35	G2583_0342	35.19	-	
NleG6-3	NleG	ECs3488	40.37	-		-	
EspL2	AR	ECs3855	38.98	G2583_3637	38.79	E2348_C_3230	38.79
NleB1	NleB	ECs3857	100	G2583_3638	100	E2348_C_3231	97.87
NleE-1	NleE	ECs3858	86.16	G2583_3639	86.16	E2348_C_3232	85.71
EspF1	EspF	ECs4550	100	G2583_4405	95.67	E2348_C_3930	100
EspB	EspB	ECs4554	30.77	G2583_4409	30.77	E2348_C_3934	26.32
Tir	Tir	ECs4561	100	G2583_4416	99.64	E2348_C_3941	100
Map	IpgB	ECs4562	28.37	G2583_4417	28.37	E2348_C_3942	22.76
EspH	EspH	ECs4564	-	G2583_4419	-	E2348_C_3944	-
EspZ	EspZ	ECs4571	100	G2583_4426	100	E2348_C_3951	100
EspG1	EspG	ECs4590	100	G2583_4444	100	E2348_C_3970	100
EspL3	AR	ECs4642/3	23.38	G2583_4495/6	23.38	-	
EspY4	SopD-N	ECs4653	26.09	G2583_4507	26.09	-	
EspX3	PPR	ECs4654/5	26.09	G2583_4508/9	26.09	-	
EspY5	SopD-N	ECs4657	22.93	G2583_4511	22.93	-	
EspL4	AR	ECs4935	23.05	G2583_4834	23.05	-	
EspX4	PPR	ECs5021	22.95	G2583_4863	22.95	-	
EspX5	PPR	ECs5048	24.1	G2583_4891	24.62	-	
EspX6	PPR	ECs5295	22.57	G2583_5137	20.9	-	

Effector[a]	Family[b]	Sakai ID	identities[c]	CB9615 ID	identities[c]	E2348/69 ID	identities[c]
NleG-2	NleG	-		G2583_0341	45.23	-	
IpaH	IpaH	-		G2583_1452	35.5	-	
PSPTO0836		-		G2583_2614	32.5	E2348_C_2227	33.33
NleG-3	NleG	-		G2583_3207	45.69	-	
Hpa2	BapC/Hpa2	-		pO55_0014	41.41	E2348_P1_077	41.41
Ipx53-1		-		-		E2348_C_0568	25.89
NleH	NleH	-		-		E2348_C_0718	-
Cif	Cif	-		-		E2348_C_0719	-
TC0438		-		-		E2348_C_1079	27.47
NleE-2	NleE	-		-		E2348_C_1080	59.38
NleB	NleB	-		-		E2348_C_1081	-
EspL5	AR	-		-		E2348_C_1082	42.93
MxiC		-		-		E2348_C_1530	23.6
IncC		-		-		E2348_C_2912	23.68
EspG2	EspG	-		-		E2348_C_2916	42.46
Ipx53-2		-		-		E2348_C_3003	44.36
BapC	BapC/Hpa2	-		-		E2348_P1_012	36

[a] Named according to Tobe *et al.* [44]

[b] LRR, leucine-rich repeats; AR, ankyrin repeats; PPR, pentapeptide repeats; SopD-N, SopD N-terminal domain.

[C] Based on the top hit in the blastp result
