# Supplementary material for: Derivation of Escherichia coli O157:H7 from Its O55:H7 Precursor
Source: PLoS One. 2010 Jan 14;5(1):e8700. doi: 10.1371/journal.pone.0008700 (PMC2806823; doi:10.1371/journal.pone.0008700)
Supplement: Table S13 — A comparative proteomic analysis of the CB9615 and EDL933 genomes. All proteins with expression levels that differed by twofold or more in one or both of the genomes are shown, with details of the protein involved and the direction of change. (0.11 MB PDF) [file pone.0008700.s015.pdf]

Table S13. The regulated proteins in the different growth stages

| CB9615 | EDL933 | Gene | Product | COG | Category | Description | CB9615 | EDL933 |
|---|---|---|---|---|---|---|---|---|
| G2583_4753 | Z5496 | metF | 5,10-methylenetetrahydrofolate reductase | COG0685 | E | Amino acid transport and metabolism | down | down |
| G2583_0771 | Z0751 | uspG | hypothetical protein | COG0589 | T | Signal transduction mechanisms | down | down |
| G2583_1094 | Z1090 | artJ | arginine 3rd transport system periplasmic binding protein | COG0834 | ET | Amino acid transport and metabolism/ Signal transduction mechanisms | down | down |
| G2583_1856 | Z2215 | gadB | glutamate decarboxylase isozyme | COG0076 | E | Amino acid transport and metabolism | down | down |
| G2583_4253 | Z4930 | gadA | glutamate decarboxylase isozyme | COG0076 | E | Amino acid transport and metabolism | down | down |
| G2583_4629 | Z5351 | metE | tetrahydropteroyltriglutamate methyltransferase | COG0620 | E | Amino acid transport and metabolism | down | down |
| G2583_1040 | Z1034 | dps | global regulator, starvation conditions | COG0783 | P | Inorganic ion transport and metabolism | down | down |
| G2583_1845 | Z2228 | osmC | osmotically inducible protein | COG1764 | O | Posttranslational modification, protein turnover, chaperones | down | down |
| G2583_2171 | Z2754 | yniA | orf, hypothetical protein | COG3001 | G | Carbohydrate transport and metabolism | down | |
| G2583_1042 | Z1036 | ompX | outer membrane protein X | COG3637 | M | Cell envelope biogenesis, outer membrane | down | |
| G2583_1164 | Z1276 | ompF | outer membrane protein 1a (Ia;b;F) | COG3203 | M | Cell envelope biogenesis, outer membrane | down | |
| G2583_1601 | Z2549 | trpC | N-(5-phosphoribosyl)anthranilate isomerase and indole-3-glycerolphosphate synthetase | COG0134 | E | Amino acid transport and metabolism | down | |
| G2583_3175 | Z3886 | clpB | heat shock protein | COG0542 | O | Posttranslational modification, protein turnover, chaperones | down | |
| G2583_3576 | Z4259 | yggE | putative actin | COG2968 | S | Function unknown | down | |
| G2583_0014 | Z0014 | dnaK | chaperone Hsp70; DNA biosynthesis; autoregulated heat shock proteins | COG0443 | O | Posttranslational modification, protein turnover, chaperones | down | |
| G2583_0585 | Z0590 | htpG | chaperone Hsp90, heat shock protein C 62.5 | COG0326 | O | Posttranslational modification, protein turnover, chaperones | down | |
| G2583_3311 | Z3964 | ygaU | orf, hypothetical protein | COG1652 | S | Function unknown | down | |
| G2583_3870 | Z4505 | yraL | orf, hypothetical protein | COG0313 | R | General function prediction only | down | |
| G2583_4200 | Z4871 | nikD | ATP-binding protein of nickel transport system | COG0444 | EP | Amino acid transport and metabolism/ Inorganic ion transport and metabolism | down | |
| G2583_4611 | Z5331 | orf | orf; conserved hypothetical protein | COG1912 | S | Function unknown | down | |
| G2583_4622 | Z5343 | recQ | ATP-dependent DNA helicase | COG0514 | L | DNA replication, recombination, and repair | down | |
| G2583_4870 | Z5644 | yjbJ | orf, hypothetical protein | COG3237 | S | Function unknown | down | |
| G2583_5241 | Z5983 | deoC | 2-deoxyribose-5-phosphate aldolase | COG0274 | F | Nucleotide transport and metabolism | down | |
| G2583_2417 | Z3059 | hchA | hypothetical protein | COG0693 | R | General function prediction only | down | |
| G2583_4715 | Z5455 | yiiM | orf, hypothetical protein | COG2258 | S | Function unknown | down | |
| G2583_1751 | Z2335 | uspF | hypothetical protein | COG0589 | T | Signal transduction mechanisms | down | |
| G2583_3560 | Z4245 | pepP | proline aminopeptidase P II | COG0006 | E | Amino acid transport and metabolism | down | |

| CB9615 | EDL933 | Gene | Product | COG | Category | Description | CB9615 | EDL933 |
|---|---|---|---|---|---|---|---|---|
| G2583_0531 | Z0522 | yajO | putative NAD(P)H-dependent xylose reductase | COG0667 | C | Energy production and conversion | down | |
| G2583_0598 | Z0606 | ybaS | putative glutaminase | COG2066 | E | Amino acid transport and metabolism | down | |
| G2583_1108 | Z1105 | poxB | pyruvate oxidase | COG0028 | EH | Amino acid transport and metabolism/Coenzyme metabolism | down | |
| G2583_1134 | Z1243 | ycaC | orf, hypothetical protein | COG1335 | Q | Secondary metabolites biosynthesis, transport and catabolism | down | |
| G2583_1238 | Z1423 | wrbA | trp repressor binding protein | - | - | - | down | |
| G2583_1599 | Z2551 | trpA | tryptophan synthase, alpha protein | COG0159 | E | Amino acid transport and metabolism | down | |
| G2583_2348 | Z2950 | otsB | trehalose-6-phosphate phophatase, biosynthetic | COG1877 | G | Carbohydrate transport and metabolism | down | |
| G2583_2807 | Z3526 | elaB | orf, hypothetical protein | COG4575 | S | Function unknown | down | |
| G2583_2986 | Z3720 | talA | transaldolase A | COG0176 | G | Carbohydrate transport and metabolism | down | |
| G2583_2987 | Z3721 | tktB | transketolase 2 isozyme | COG0021 | G | Carbohydrate transport and metabolism | down | |
| G2583_4609 | Z5329 | yigB | putative phosphatase | COG1011 | R | General function prediction only | down | |
| G2583_5260 | Z6004 | arcA | negative response regulator of genes in aerobic pathways | COG0745 | TK | Signal transduction mechanisms/Transcription | down | |
| G2583_2831 | Z3555 | yfbU | orf, hypothetical protein | COG3013 | S | Function unknown | up | down |
| G2583_0849 | Z0837 | pgm | phosphoglucomutase | COG0033 | G | Carbohydrate transport and metabolism | up | up |
| G2583_3939 | Z4577 | gltD | glutamate synthase, small subunit | COG0493 | ER | Amino acid transport and metabolism/ General function prediction only | up | up |
| G2583_4803 | Z5560 | rpoB | RNA polymerase, beta subunit | COG0085 | K | Transcription | up | up |
| G2583_4859 | Z5632 | malE | periplasmic maltose-binding protein | COG2182 | G | Carbohydrate transport and metabolism | up | up |
| G2583_0102 | Z0108 | secA | preprotein translocase; secretion protein | COG0653 | U | Intracellular trafficking, secretion, and vesicular transport | up | up |
| G2583_3032 | Z3772 | guaB | IMP dehydrogenase | COG0516 | F | Nucleotide transport and metabolism | up | up |
| G2583_3345 | Z3999 | alaS | alanyl-tRNA synthetase | COG0013 | J | Translation, ribosomal structure and biogenesis | up | up |
| G2583_0273 | Z0298 | pepD | aminoacyl-histidine dipeptidase (peptidase D) | COG2195 | E | Amino acid transport and metabolism | up | up |
| G2583_4754 | Z5497 | katG | catalase; hydroperoxidase HPI(I) | COG0376 | P | Inorganic ion transport and metabolism | up | up |
| G2583_5250 | Z5993 | yjjK | putative ATP-binding component of ABC transport system | COG0488 | R | General function prediction only | up | up |
| G2583_0034 | Z0038 | carB | carbamoyl-phosphate synthase large subunit | COG0458 | EF | Amino acid transport and metabolism/ Nucleotide transport and metabolism | up | |
| G2583_0646 | Z0681 | cysS | cysteine tRNA synthetase | COG0215 | J | Translation, ribosomal structure and biogenesis | up | |

| CB9615 | EDL933 | Gene | Product | COG | Category | Description | CB9615 | EDL933 |
|---|---|---|---|---|---|---|---|---|
| G2583_4525 | Z5227 | glmS | L-glutamine:D-fructose-6-phosphate aminotransferase | COG0449 | M | Cell envelope biogenesis, outer membrane | up | |
| G2583_0118 | Z0124 | aceE | pyruvate dehydrogenase (decarboxylase component) | COG2609 | C | Energy production and conversion | up | |
| G2583_0258 | Z0280 | lpcA | phosphoheptose isomerase | COG0279 | G | Carbohydrate transport and metabolism | up | |
| G2583_0278 | Z0303 | proB | gamma-glutamate kinase | COG0263 | E | Amino acid transport and metabolism | up | |
| G2583_0835 | Z0821 | asnB | asparagine synthetase B | COG0367 | E | Amino acid transport and metabolism | up | |
| G2583_0841 | Z0827 | glnS | glutamine tRNA synthetase | COG0008 | J | Translation, ribosomal structure and biogenesis | up | |
| G2583_1351 | Z1730 | fabH | 3-oxoacyl-[acyl-carrier-protein] synthase III | COG0332 | I | Lipid metabolism | up | |
| G2583_4670 | Z5406 | glnA | glutamine synthetase | COG0174 | E | Amino acid transport and metabolism | up | |
| G2583_1130 | Z1239 | serS | serine tRNA synthetase; also charges selenocystein tRNA with serine | COG0172 | J | Translation, ribosomal structure and biogenesis | up | |
| G2583_1143 | Z1254 | aroA | 5-enolpyruvylshikimate-3-phosphate synthetase | COG0128 | E | Amino acid transport and metabolism | up | |
| G2583_2160 | Z2742 | pheT | phenylalanine tRNA synthetase, beta-subunit | COG0072 | J | Translation, ribosomal structure and biogenesis | up | |
| G2583_2194 | Z2780 | astC | acetylornithine delta-aminotransferase | COG4992 | E | Amino acid transport and metabolism | up | |
| G2583_2571 | - | cpsG | Phosphomannomutase CpsG | COG1109 | G | Carbohydrate transport and metabolism | up | |
| G2583_3769 | Z4400 | yqiC | hypothetical protein | COG2960 | S | Function unknown | up | |
| G2583_3906 | Z4543 | greA | transcription elongation factor: cleaves 3' nucleotide of paused mRNA | COG0782 | K | Transcription | up | |
| G2583_5062 | Z5843 | mpl | putative ligase | COG0773 | M | Cell envelope biogenesis, outer membrane | up | |
| G2583_0122 | Z0128 | acnB | aconitate hydrase B | COG1049 | C | Energy production and conversion | up | |
| G2583_0276 | Z0301 | crl | transcriptional regulator of cryptic csgA gene for curli surface fibers | - | - | - | up | |
| G2583_0717 | Z1931 | ompT | outer membrane protein 3b (a), protease VII | COG4571 | M | Cell envelope biogenesis, outer membrane | up | |
| G2583_0883 | Z0882 | sucC | succinyl-CoA synthetase, beta subunit | COG0045 | C | Energy production and conversion | up | |
| G2583_2650 | Z3282 | metG | methionine tRNA synthetase | COG0073 | R | General function prediction only | up | |
| G2583_4738 | Z5478 | hslU | heat shock protein hslVU, ATPase subunit, homologous to chaperones | COG1220 | O | Posttranslational modification, protein turnover, chaperones | up | |
| G2583_2203 | Z2789 | ynjE | putative thiosulfate sulfur transferase | COG2897 | P | Inorganic ion transport and metabolism | up | |
| G2583_3892 | Z4528 | rbfA | ribosome-binding factor A | COG0858 | J | Translation, ribosomal structure and biogenesis | up | |
| G2583_3901 | Z4538 | glmM | similar to phosphoglucomutases and phosphomannomutases | COG1109 | G | Carbohydrate transport and metabolism | up | |

| CB9615 | EDL933 | Gene | Product | COG | Category | Description | CB9615 | EDL933 |
|---|---|---|---|---|---|---|---|---|
| G2583_4488 | Z5190 | gyrB | DNA gyrase subunit B, type II topoisomerase, ATPase activity | COG0187 | L | DNA replication, recombination, and repair | up | |
| G2583_5075 | Z5856 | pyrB | aspartate carbamoyltransferase, catalytic subunit | COG0540 | F | Nucleotide transport and metabolism | up | |
| G2583_0154 | Z0161 | fhuA | outer membrane protein receptor for ferrichrome, colicin M, and phages T1, T5, and phi80 | COG1629 | P | Inorganic ion transport and metabolism | up | |
| G2583_0756 | Z0735 | entC | isochorismate hydroxymutase 2, enterochelin biosynthesis | COG1169 | HQ | Coenzyme metabolism/Secondary metabolites biosynthesis, transport and catabolism | up | |
| G2583_1033 | Z1026 | fiu | putative outer membrane receptor for iron transport | COG4774 | P | Inorganic ion transport and metabolism | up | |
| G2583_1360 | Z1739 | ycfH | orf, hypothetical protein | COG0084 | L | DNA replication, recombination, and repair | up | |
| G2583_2207 | Z2793 | gdhA | NADP-specific glutamate dehydrogenase | COG0334 | E | Amino acid transport and metabolism | up | |
| G2583_2504 | Z3159 | yoeE | putative outer membrane receptor for iron compound or colicin | COG1629 | P | Inorganic ion transport and metabolism | up | |
| G2583_2542 | Z3183 | hisC | histidinol-phosphate aminotransferase | COG0079 | E | Amino acid transport and metabolism | up | |
| G2583_2702 | Z3416 | nfo | endonuclease IV | COG0648 | L | DNA replication, recombination, and repair | up | |
| G2583_2726 | Z3442 | rsuA | 16S pseudouridylate 516 synthase | COG1187 | J | Translation, ribosomal structure and biogenesis | up | |
| G2583_3954 | Z4593 | degQ | serine endoprotease | COG0265 | O | Posttranslational modification, protein turnover, chaperones | up | |
| G2583_4234 | Z4910 | chuS | putative heme/hemoglobin transport protein | COG3720 | P | Inorganic ion transport and metabolism | up | |
| G2583_4300 | Z4983 | glyS | glycine tRNA synthetase, beta subunit | COG0751 | J | Translation, ribosomal structure and biogenesis | up | |
| G2583_4662 | Z5398 | polA | DNA polymerase I | COG0258 | L | DNA replication, recombination, and repair | up | |
| G2583_4781 | Z5527 | btuB | outer membrane receptor for transport of vitamin B12, E colicins, and bacteriophage BF23 | COG4206 | H | Coenzyme metabolism | up | |
| G2583_4878 | Z5652 | tyrB | tyrosine aminotransferase, tyrosine repressible | COG1448 | E | Amino acid transport and metabolism | up | |
| G2583_4998 | Z5778 | miaA | delta(2)-isopentenylpyrophosphate tRNA-adenosine transferase | COG0324 | J | Translation, ribosomal structure and biogenesis | up | |
| G2583_5001 | Z5781 | hflK | protease specific for phage lambda cII repressor | COG0330 | O | Posttranslational modification, protein turnover, chaperones | up | |
| G2583_5027 | Z5809 | rpsF | 30S ribosomal subunit protein S6 | COG0360 | J | Translation, ribosomal structure and biogenesis | up | |
| G2583_2866 | Z3592 | aroC | chorismate synthase | COG0082 | E | Amino acid transport and metabolism | | down |
| G2583_5172 | Z5972 | rsmC | putative enzyme | COG2813 | J | Translation, ribosomal structure and biogenesis | | down |

| CB9615 | EDL933 | Gene | Product | COG | Category | Description | CB9615 | EDL933 |
|---|---|---|---|---|---|---|---|---|
| G2583_0921 | Z0925 | gpmA | phosphoglyceromutase 1 | COG0588 | G | Carbohydrate transport and metabolism | | down |
| G2583_3735 | Z4364 | yqhD | putative oxidoreductase | COG1979 | C | Energy production and conversion | | down |
| G2583_1010 | Z1001 | moaB | molybdopterin biosynthesis, protein B | COG0521 | H | Coenzyme metabolism | | down |
| G2583_2062 | Z2694 | ydhR | orf, hypothetical protein | - | - | - | | down |
| G2583_3748 | Z4379 | mdaB | modulator of drug activity B | COG2249 | R | General function prediction only | | down |
| G2583_4356 | Z5044 | kbl | 2-amino-3-ketobutyrate CoA ligase (glycine acetyltransferase) | COG0156 | H | Coenzyme metabolism | | down |
| G2583_1107 | Z1104 | ltaE | putative arylsulfatase | COG2008 | E | Amino acid transport and metabolism | | down |
| G2583_2008 | Z2617 | ydgA | orf, hypothetical protein | COG5339 | S | Function unknown | | down |
| G2583_2846 | Z3571 | hisJ | histidine-binding periplasmic protein of high-affinity histidine transport system | COG0834 | ET | Amino acid transport and metabolism/ Signal transduction mechanisms | | down |
| G2583_3749 | Z4380 | ygiN | orf, hypothetical protein | COG1359 | S | Function unknown | | down |
| G2583_4771 | Z5517 | argB | acetylglutamate kinase | COG0548 | E | Amino acid transport and metabolism | | down |
| G2583_1153 | Z1264 | kdsB | CTP:CMP-3-deoxy-D-manno-octulosonate transferase | COG1212 | M | Cell envelope biogenesis, outer membrane | | up |
| G2583_2541 | Z3182 | hisD | L-histidinal:NAD+ oxidoreductase; L-histidinol:NAD+ oxidoreductase | COG0141 | E | Amino acid transport and metabolism | | up |
| G2583_3043 | Z3781 | ndk | nucleoside diphosphate kinase | COG0105 | F | Nucleotide transport and metabolism | | up |
| G2583_4134 | Z4797 | asd | aspartate-semialdehyde dehydrogenase | COG0136 | E | Amino acid transport and metabolism | | up |
| G2583_0882 | Z0881 | sucB | 2-oxoglutarate dehydrogenase | COG0508 | C | Energy production and conversion | | up |
| G2583_2693 | Z3405 | mglB | galactose-binding transport protein; receptor for galactose taxis | COG1879 | G | Carbohydrate transport and metabolism | | up |
| G2583_0492 | Z0480 | psiF | induced by phosphate starvation | - | - | - | | up |
| G2583_0642 | Z0677 | purK | phosphoribosylaminoimidazole carboxylase | COG0026 | F | Nucleotide transport and metabolism | | up |
| G2583_3334 | Z3988 | luxS | orf, hypothetical protein | COG1854 | T | Signal transduction mechanisms | | up |
| G2583_3031 | Z3771 | guaA | GMP synthetase (glutamine-hydrolyzing) | COG0518 | F | Nucleotide transport and metabolism | | up |
| G2583_4599 | Z5317 | hemX | uroporphyrinogen III methylase | COG2959 | H | Coenzyme metabolism | | up |
| G2583_4966 | Z5744 | aspA | aspartate ammonia-lyase (aspartase) | COG1027 | E | Amino acid transport and metabolism | | up |
| G2583_1911 | Z2083 | - | unknown protein encoded within CP-933O | - | | - | | up |
| G2583_0188 | Z0197 | accA | acetylCoA carboxylase, carboxytransferase component, alpha subunit | COG0825 | I | Lipid metabolism | | up |
| G2583_0279 | Z0304 | proA | gamma-glutamylphosphate reductase | COG0014 | E | Amino acid transport and metabolism | | up |
| G2583_1321 | Z1699 | pyrC | dihydro-orotase | COG0418 | F | Nucleotide transport and metabolism | | up |
| G2583_1628 | Z2514 | rnb | RNase II, mRNA degradation | COG4776 | K | Transcription | | up |

| CB9615 | EDL933 | Gene | Product | COG | Category | Description | CB9615 | EDL933 |
|---|---|---|---|---|---|---|---|---|
| G2583_1777 | Z2306 | aldA | aldehyde dehydrogenase, NAD-linked | COG1012 | C | Energy production and conversion | | up |
| G2583_2049 | Z2676 | grxD | orf, hypothetical protein | COG0278 | O | Posttranslational modification, protein turnover, chaperones | | up |
| G2583_2328 | Z2929 | argS | arginine tRNA synthetase | COG0018 | J | Translation, ribosomal structure and biogenesis | | up |
| G2583_3447 | Z4111 | queF | orf, hypothetical protein | COG0780 | R | General function prediction only | | up |
| G2583_3557 | Z4242 | gcvT | aminomethyltransferase of glycine cleavage system | COG0404 | E | Amino acid transport and metabolism | | up |
| G2583_3950 | Z4588 | rpsI | 30S ribosomal subunit protein S9 | COG0103 | J | Translation, ribosomal structure and biogenesis | | up |
| G2583_4262 | Z4940 | kdgK | ketodeoxygluconokinase | COG0524 | G | Carbohydrate transport and metabolism | | up |
| G2583_5056 | Z5837 | ppa | inorganic pyrophosphatase | COG0221 | C | Energy production and conversion | | up |
| G2583_5235 | Z5976 | prfC | peptide chain release factor RF-3 | COG4108 | J | Translation, ribosomal structure and biogenesis | | up |
| - | Z2386 | - | unknown protein encoded within prophage CP-933R | - | - | - | | up |
| G2583_0058 | Z0063 | imp | organic solvent tolerance | COG1452 | M | Cell envelope biogenesis, outer membrane | | up |
| G2583_0138 | Z0145 | panB | 3-methyl-2-oxobutanoate hydroxymethyltransferase | COG0413 | H | Coenzyme metabolism | | up |
| G2583_1162 | Z1274 | ycbL | orf, hypothetical protein | COG0491 | R | General function prediction only | | up |
| G2583_1842 | Z2231 | maeA | NAD-linked malate dehydrogenase (malic enzyme) | COG0281 | C | Energy production and conversion | | up |
| G2583_2536 | Z3178 | yeeZ | putative enzyme of sugar metabolism | COG0451 | MG | Cell envelope biogenesis, outer membrane/ Carbohydrate transport and metabolism | | up |
| G2583_2546 | Z3187 | hisF | imidazole glycerol phosphate synthase subunit in heterodimer with HisH | COG0107 | E | Amino acid transport and metabolism | | up |
| G2583_3331 | Z3985 | mprA | regulator of plasmid mcrB operon | COG1846 | K | Transcription | | up |
| G2583_3336 | Z3989 | gshA | gamma-glutamate-cysteine ligase | COG2918 | H | Coenzyme metabolism | | up |
| G2583_4884 | Z5658 | ssb | ssDNA-binding protein | COG0629 | L | DNA replication, recombination, and repair | | up |
| G2583_5030 | Z5812 | rplI | 50S ribosomal subunit protein L9 | COG0359 | J | Translation, ribosomal structure and biogenesis | | up |
