# Supplementary material for: Derivation of Escherichia coli O157:H7 from Its O55:H7 Precursor
Source: PLoS One. 2010 Jan 14;5(1):e8700. doi: 10.1371/journal.pone.0008700 (PMC2806823; doi:10.1371/journal.pone.0008700)
Supplement: Table S14 — Virtual outgroup analysis of the large indels in the CB9651, Sakai, and EDL933 genomes. (0.02 MB PDF) [file pone.0008700.s016.pdf]

O55 and O157 Details								Outgroup Strain Details[e]																							
event	CB9615 block[a]	Sakai block[a]	EDL933 block[a]	type	Event Lineage[b]	Inferred ancestral[c]	Support level[d]	D1 Sd197	K-12	HS	ATCC 8739	UMN026	IAI39	SMS 3-5	E2348/69	536	ED1a	CFT073	S88	APEC O1	UTI89	E24377A	IAI1	SE11	F5 8401	F2a 2457T	F2a 301	SS Ss046	B4 Sb227	B18 BS512	Note[f]
1	-	+	+	del	CB9615	+	+++			+												+	+	+				+			
2	+	-	-	ins	CB9615	-	+++			-													-	-				-			
3	-	+	+	del	CB9615	+	+				+												+								
4	+	-	-	ins	CB9615	-	++	-	+	-	-	-	-	-	-	-	-	-	-	-	-	-	-	-	+	+	+	-		-	
5	-	+	+	ins	O157	-	++++	-				-	-	-	-		-	-	-	-	-	-	-	-		-	-	-	-	-	
6	-	+	-	ins	Sakai	-	++++		-			-	-		-	-	-	-	-	-	-	-	-	-							
7	-	-	+	ins	EDL933	-	++++		-			-	-		-	-	-	-	-	-	-	-	-	-							
8	-	+	+	ins	O157	-	+++		-		-				-		-		-	-	-										
9	+	-	-	ins	CB9615	-	++++	-	-	-	-	-	-	-	-	-	-	-	-	-	-	-	-	-	-			-			
10	-	+	+	ins	O157	-	++++		-	-	-	-	-	-	-	-	-	-	-	-	-	-	-	-					-		
11	+	-	-	del	O157	+	+			+					-	+	+	+	+	+	+	-		+							
12	-	+	+	ins	O157	-	++++		-	-	-	+	-	-	-	-	-	-	-	-	-	-	-	-							
13	+	-	-	del	O157	+	+		+		+						-														
14	+	-	-	del	O157	+	+					+																			
15	-	+	-	ins	Sakai	-	++	-				-																			
16	+	-	-	del	O157	+	+					+																			
17	+	-	-	ins	CB9615	-	++++	-	-	-	-	-	-	-								-	-	-	-	-	-	-	-	-	
18	-	-	+	ins	EDL933	-	+++				-											-	-	-				-			
19	+	-	-	ins	CB9615	-	++++	-	-	-	-	-	-	-	-	-	-	-	-	-	-	-	-	-	-	-	-	-	-	-	
20	-	-	-	ins	EDL933	-	++++	-	-	-	-											-	-	-				-	-	-	
21	-	+	+	del	CB9615	+	+					+	+	+																	
22	+	-	-	ins	CB9615	-	++	-	+	+	+	+	-	-	-	+	+	+	-	-	-	-	+	+	-	-	-	-	-	-	
23	+	-	-	del	O157	+	++	+	-	+	+	-	-	+	-	+	+	+	+	+	+	-	-	-	-	-	-	-	-	-	
24	+	-	-	ins	CB9615	-	++++	-			-	-	-			-	-	-	-	-	-	-	-	-							
25	-	+	+	del	CB9615	+	+				-	-			+								+					+	+	+	
26	+	-	-	del	O157	+	+		-		+	+			+				+	+		-	+	+	-	-	-	+	+	+	
27	-	-	+	ins	EDL933	-	++++	-	-	-	-	-	-	-	-	-	-	-	-	-	-	-	-	-	-	-	-	-	-	-	
28	-	+	+	ins	O157	-	++++	-	-	-	-	-	-	-	-	-	-	-	-	-	-	-	-	-	-	-	-	-	-	-	
29	-	+	-	ins	Sakai	-	+								-		-					-									
30	-	-	+	ins	EDL933	-	++++	-				-			-		-	-	-	-	-	-			-	-	-	-	-	-	
31	-	+	+	ins	O157	-	++++	-	-	-	-	-	-	-	-	-	-	-	-	-	-	-	-	-	-	-	-	-	-	-	
32	+	-	-	ins	CB9615	-	++++	-	-	-	-	-	-	-	-	-	-	-	-	-	-	-	-	-	-	-	-	-	-	-	
33	-	+	+	ins	O157	-	++++	-	-	-	-	-	-	-	-	-	-	-	-	-	-	-	-	-	-	-	-	-			
34	-	+	-	ins	Sakai	-	++++			-	-	-	-		-	-	-	-	-	-	-	-	-	-							
35	-	-	+	ins	EDL933	-	++++			-	-	-	-		-	-	-	-	-	-	-	-	-	-							
36	-	+	+	ins	O157	-	++++	-				-	-	-	-	-	-	-	-	-	-	-			-	-	-	-	-	-	
37	-	+	-	ins	Sakai	-	++	-																					-	-	
38	-	+	+	ins	O157	-	++++					-	-	-	-	-	-	-	-	-	-	-			-	-	-	-	-	-	
39	-	+	-	ins	Sakai	-	+						-		-						-										
40	-	+	+	ins	O157	-	++++	-	-	-	-	-	-	-	-	-	-	-	-	-	-	-	-	-	-	-	-	-			
41	-	-	+	ins	EDL933	-	++++	-	-	-	-	-	-	-	-	-	-	-	-	-	-	-	-	-	-	-	-	-			
42	-	+	+	ins	O157	-	++++	-	-	-	-	-	-	-	-	-	-	-	-	-	-	-	-	-	-	-	-	-			
43	+	-	-	del	O157	+	++	+	+	+	+	+	+	+	+	+	+	+	+	+	+	+	+	+	-	-	-	-			
44	-	+	+	del	CB9615	+	+		+	+	+	+	-	+	+	+	+	+	+	+	+	+	+	+	-	-	-	+	+	-	
45	-	+	+	ins	O157	-	++++	-	-	-	-	-	-	-	-	-	-	-			-	-		-	-	-	-				
46	-	-	+	ins	EDL933	-	++++	-	-	-			-	-	-	-	-	-	-	-	-	-				-	-			-	
47	-	+	+	ins	O157	-	++	-	-	-	-	-	-	-	+	-	-	-	-	-	+	-	-	-	-	-	-	-	-		
48	-	+	+	ins	O157	-	++++	-	-	-	-	-	-	-	-	-	-	-	-	-	-	-	-	-	-	-	-	-	-		
49	-	+	-	ins	Sakai	-	++++	-				-			-		-	-	-	-	-	-			-	-	-	-	-	-	
50	-	-	+	ins	EDL933	-	+++								-		-					-		-					-	-	
51	-	+	+	ins	O157	?	+/-								+		+												-	-	part of phage
52	-	+	-	ins	Sakai	-	+++								-		-												-	-	
53	-	-	+	ins	EDL933	-	+++								-		-												-	-	
54	-	+	+	ins	O157	-	++++	-	-	-	-	-	-	-	-	-	-	-	-	-	-	-	-	-	-	-	-	-	-	-	
55	-	+	+	ins	O157	-	++++	-	-	-	-	-	-	-	-	-	-	-	-	-	-	-	-	-	-	-	-	-	-	-	
56	+	-	-	ins	CB9615	-	++++	-	-	-	-	-	-	-	-	-		-	-	-	-	-	-	-	-	-	-	-	-	-	
57	-	+	+	ins	O157	-	++++	-	-	-		-		-	-	-	-	-	-	-	-	-	-	-	-	-	-	-	-	-	
58	-	+	-	ins	Sakai	-	++++		-	-	-	-	-		-	-		-	-	-	-	-	-	-				-			
59	-	-	+	ins	EDL933	-	++++		-		-		-		-	-	-	-	-	-	-	-	-	-				-			
60	-	+	+	ins	O157	-	++++			-		-	-	-	-	-	-	-			-	-	-		-	-	-	-	-	-	
61	-	+	-	ins	Sakai	-	++++		-			-	-	-	-				-	-	-	-		-	-	-	-	-	-	-	
62	-	+	+	ins	O157	-	++++	-	-	-	-	-	-	-	-	-	-	-	-	-	-	-	-	-	-	-	-	-	-	-	
63	+	-	-	ins	CB9615	-	++	-	-	-	-	-	-	+	-	-	-	-	-	-	-	+	+	+	-	-	-	-	-	-	
64	-	+	+	ins	O157	-	++++	-	-	-	-	-	-	-	-	-	-	-	-	-	-	-	-	-	-	-	-	-	-	-	
65	+	-	-	del	O157	+	++	+	-	-	-	+		+	-	-	-	-	-	-	-	-	-	-	+	+	+	-	-	-	
66	-	+	+	ins	O157	-	++++	-	-	-	-	-	-	-	-	-	-	-	-	-	-	-	-	-	-	-	-	-	-	-	
67	+	-	-	del	O157	+	++	+	+	+	+	+	-	-	-	-	-	-	-	-	-	+	+	+	+	+	+		+	+	
68	-	-	+	ins	EDL933	-	++	-	+	+	+	+	+	+	-	-	-	-	-	-	-	+	-	+	-	-	-	+	+	+	
69	-	+	+	del	CB9615	+	++++	+	+	+	+	+	+	+	+	+	+	+	+	+	+	+	+	+	+	+	+	+	+	+	
70	-	+	+	del	CB9615	+	+		-		+	+	-	+	+		+	+	+	+	+	+	+		-				+	+	
71	+	-	-	del	O157	+	+		+		+	+		-	+		-	-	+	+	+	+	+								
72	-	+	+	ins	O157	-	++++	-				-		-	-		-	-	-	-	-			-	-	-	-	-	-	-	
73	-	+	-	ins	Sakai	?	+/-																								part of phage
74	-	-	+	ins	EDL933	?	+/-																								part of phage
75	+	-	-	ins	CB9615	?	+/-																								part of phage
76	-	+	+	ins	O157	-	++++	-	-	-	-	-	-	-	-	-		-	-	-	-	-	-	-	-	-	-			-	
77	+	-	-	ins	CB9615	-	++	-	-	-	-	-	-	-	+	-		-	+	+	+	+	-	-	-	-	-				
78	-	+	+	ins	O157	-	++++	-	-	-	-	-	-	-	-	-	-	-	-	-	-	-	-	-	-	-	-	-	-	-	
79	-	+	+	ins	O157	-	++++		-			-					-	-	-	-	-			-					-	-	
80	-	+	-	ins	Sakai	-	+++		-			-			-							-						-	-		
81	-	-	+	ins	EDL933	-	++++					-	-	-	-	-	-	-	-	-	-	-			-	-	-	-		-	
82	-	+	+	ins	O157	-	+++					-			+								-	-				-			
83	+	-	-	ins	CB9615	-	+++		-						-					-				-							
84	+	-	-	ins	CB9615	-	++++	-	-	-	-	-	-	-	-	-	-	-	-	-	-	-	-	-	-	-	-	-	-	-	
85	+	-	-	del	O157	+	++++	+	+	+	+	+	+	-	+	+	+	+	+	+	+	+	+	+	+	+	+	+	+	+	
86	-	+	+	indel	O55/O157	?	+/-																								
87	-	+	+	indel	O55/O157	?	+/-																								
88	-	+	+	del	CB9615	+	+		+		+											+	+	+				+	-	-	
89	+	-	-	del	O157	+	+		+		+											+	+	+				+	-	-	

O55 and O157 Details								Outgroup Strain Details[e]																							
event	CB9615 block[a]	Sakai block[a]	EDL933 block[a]	type	Event Lineage[b]	Inferred ancestral[c]	Support level[d]	D1 Sd197	K-12	HS	ATCC 8739	UMN026	IAI39	SMS 3-5	E2348/69	536	ED1a	CFT073	S88	APEC O1	UTI89	E24377A	IAI1	SE11	F5 8401	F2a 2457T	F2a 301	SS Ss046	B4 Sb227	B18 BS512	Note[f]
90	-	+	+	del	CB9615	+	++	+	+	+	+	+	-	-	-	+	+	+	+	+	+	-	-	-	+	+	+	+	-	-	
91	+	-	-	del	O157	+	+		+	+	+	+	-	-	+	-	+	+	+	+	+	+	+	+				+		-	
92	-	+	+	ins	O157	-	++++	-	-	-	-	-	-	-	-	-	-	-				-	-	-	-	-	-	-	-	-	
93	-	+	-	ins	Sakai	-	+								-		-														
94	-	-	+	ins	EDL933	-	++++		-			-	-		-				-	-		-	-	-					-	-	
95	-	+	+	ins	O157	-	++++					-	-	-	-	-	-	-	-	-	-	-			-	-	-	-	-	-	
96	-	+	-	ins	Sakai	-	+++		-		-	-	-				-					-	-								
97	-	+	+	ins	O157	-	++++					-	-	-	-	-	-	-	-	-	-	-			-	-	-	-	-	-	
98	-	+	+	ins	O157	-	+								-																
99	-	+	-	ins	Sakai	?	+/-																								part of phage
100	-	-	+	ins	EDL933	?	+/-																								part of phage
101	+	-	-	ins	CB9615	?	+/-																								part of phage
102	-	+	+	ins	O157	-	+++					-	-		+		-	-	-										-	-	
103	-	+	+	ins	O157	-	++++	-	-	-	-	-	-	-	-	-	-	-	-	-	-	-	-	-	-	-	-		-	-	
104	+	-	-	ins	O55	-	++++	-	-	-	-	-	-	-	-	-	-	-	-	-	-	-	-	-						-	
105	+	+	-	del	EDL933	+	++++	+	+	+	+	+	+	+	+	+	+	+	+	+	+	+	+	+	+	+	+	+	+	+	
106	+	-	-	ins	CB9615	-	++++	-	-	-	-	-	-	-	-	-	-	-	-	-	-	-	-	-	-	-	-	-	-	-	
107	-	+	+	ins	O157	-	++++	-	-	-	-	-	-	-	-	-	-	-	-	-	-	-	-	-	-	-	-				
108	-	+	+	del	CB9615	+	++++	+	+	+	+	+	+	+	+	+	+	+	+	+	+	+	+	+	+	+	+				
109	-	+	+	ins	O157	-	++++	-	-	-	-	-	-	-	-	-	-	-	-	-	-	-	-	-	-	-	-	-	-	-	
110	-	+	+	ins	O157	-	++++		-	-	-	-	-	-	-	-	-	-	-	-	-	-	-	-	-	-	-	-	-		
111	-	+	-	ins	Sakai	-	+								-																
112	-	-	+	ins	EDL933	-	+								-																
113	-	+	+	ins	O157	-	++++	-	-	-	-		-	-				-		-	-		-	-	-	-	-	-	-	-	
114	+	-	-	ins	CB9615	-	++++	-	-	-	-	-	-		-	-	-	-	-		-	-	-	-	-	-	-	-		-	
115	-	+	+	ins	O157	-	++++	-	-	-	-	-	-	-	-	-	-	-	-	-	-	-	-	-	-	-	-	-	-	-	
116	-	+	+	ins	O157	-	++++						-	-	-	-		-	-	-	-										
117	-	+	-	ins	Sakai	-	+					-	+	+		-	-	+	+	+	-	-			-	-	-	-	-	-	
118	+	-	-	del	O157	+	+						+	+	-	-		+	-	+	+										
119	+	+	-	del	EDL933	+	++	+	-			-	-	+		+	-	+	+	+	+	-			-	-		-	-	-	
120	-	+	+	ins	O157	-	+		-	-	+	+	+	+	-	-	+	-	-	-	-	-	-	-	-	-	-	-	-	-	
121	+	-	-	del	O157	+	+		+	+	-	-	-	-	+	+	-	+	+	+	+	+	+	+	-	-	-	+	+	+	
122	-	+	+	ins	O157	+	-	+	+		+	+	+	+	+	+	+	+		+	+	+	+	+	+	+	+	+	+	-	O-antigen
123	+	-	-	ins	CB9615	-	++++	-	-		-	-	-	-	-	-	-	-	-	-	-	-	-	-	-	-	-	-			
124	-	+	+	ins	O157	-	++++	-	-		-	-	-	-	-	-	-	-	-	-	-	-	-	-	-	-	-	-	-	-	
125	+	-	-	ins	CB9615	?	+/-																								O-antigen
126	-	+	+	ins	O157	?	+/-																								O-antigen
127	+	-	-	del	O157	?	+/-																								O-antigen
128	-	+	+	ins	O157	?	+/-																								O-antigen
129	+	-	-	ins	CB9615	?	+/-																								O-antigen
130	-	+	+	ins	O157	-	++++	-	-		-	-	-	-	-	-	-	-	-	-	-	-	-	-	-	-	-		-	-	
131	+	-	-	ins	CB9615	-	++++	-	-		-	-	-	-	-	-	-	-	-	-	-	-	-	-	-	-	-		-	-	
132	-	+	+	ins	O157	-	+		-		-	-	-	-	-	-	-	-	-	-	-	-	-	+	+	+	+		-	-	
133	-	+	+	indel	O55/O157	?	+/-																								
134	+	-	-	indel	O55/O157	?	+/-																								
135	+	-	-	del	O157	+	+		+		-	-	+	+	-					+	-	-		+				+			
136	-	+	+	ins	O157	-	+		-		+	+	-	-	+					-	+	+		-				-			
137	-	+	+	ins	O157	-	++	-	-	-	-	+	+	-	-	-	-	-	-	-	-	+	+	-	-	-	-	+	+	+	
138	+	-	-	del	O157	+	++	+	-	-	-	-	+	+	-	-	-	-	-	-	-	+	+	+	-	-	-	-	-	+	
139	-	+	+	ins	O157	-	++++	-	-	-	-	-	-	-	-	-	-	-	-	-	-	-	-	-	-	-	-	-	-	-	
140	+	-	-	ins	CB9615	-	++++	-	-	-	-	-	-	-	-	-	-	-	-	-	-	-	-	-	-	-	-	-	-	-	
141	-	+	+	ins	O157	-	++++	-	-	-	-	-	-	-	-	-	-	-	-	-	-	-	-	-	-	-	-	-		-	
142	+	-	-	del	O157	+	++++	+	+	+	+	+	+	+	+	+	+	+	+	+	+	+	+	+	+	+	+	+		-	
143	+	-	-	ins	CB9615	-	+++	-														-			-				-		
144	-	+	+	ins	O157	-	+		-	-	-	+	-	-	-	-	-	+	-	-	-	+	+	+		+	+		-		
145	-	+	+	ins	O157	-	++++	-	-	-	-	-	-	-	-	-	-	-	-	-	-	-	-	-	-	-	-	-	-	-	
146	-	+	+	ins	O157	-	++++	-	-	-	-	-	-	-		-	-	-	-	-		-	-	-	-	-	-	-	-	-	
147	-	+	-	ins	Sakai	-	+++		-						-		-					-						-	-	-	
148	-	-	+	ins	EDL933	-	+++		-			-			-		-					-							-	-	
149	-	+	+	ins	O157	-	++++	-	-	-	-	-	-	-	-	-	-	-	-	-	-	-	-	-	-	-	-	-	-	-	
150	+	-	-	ins	CB9615	-	++++	-	-	-	-	-	-	-	-	-	-	-	-	-	-	-	-	-	-	-	-	-	-	-	
151	-	+	-	ins	Sakai	-	++++	-	-	-	-	-	-	-	-	-	-	-	-	-	-							-	-		
152	+	-	-	ins	CB9615	-	++++	-	-	-	-	-	-	-	-	-	-	-	-	-	-	-	-	-	-	-	-	-	-	-	
153	+	+	-	del	EDL933	+	+		+	+	+	+	+	+	+	+	-	+	+	+	+	+	+	+	-	+	+	+	+	+	
154	+	-	-	ins	CB9615	-	++++	-	-	-	-	-	-	-	-	-	-	-	-	-	-	-	-	-	-	-	-	-	-	-	
155	-	+	+	ins	O157	-	++++					-	-	-	-	-	-	-	-	-	-	-			-	-	-	-	-	-	
156	-	+	-	ins	Sakai	-	+								-																
157	+	-	-	ins	CB9615	-	+								-																
158	-	-	+	ins	EDL933	-	+								-																
159	-	+	+	ins	O157	-	+								-																
160	+	-	-	ins	CB9615	-	+								-																
161	+	+	-	indel	Sakai/EDL933	?	+/-																								
162	+	+	-	indel	Sakai/EDL933	?	+/-																								
163	+	-	-	del	O157	+	++++	+	+	+	+	+	+	+	+	+	+	+	+	+	+	+	+	+	+	+	+	+	+	+	
164	-	+	+	del	CB9615	+	+++					+		+								+	+	+							
165	+	-	-	ins	CB9615	-	+		+	+	+	+	-	+	-	-	-	-	-	-	-	+	-	+	-	-	-	-	-	-	
166	+	-	-	ins	CB9615	-	++	-	+	+	+	+	-	-	-	-	-	-	-	-	-	+	+	+	-	-	-	-	-	-	
167	-	+	+	ins	O157	-	+		-	-	-	+	-									+	+	+				-			
168	-	+	+	ins	O157	-	++++	-	-	-	-	-	-	-	-	-	-	-	-	-	-	-	-	-	-	-	-	-	-	-	
169	-	+	+	ins	O157	-	++++			-		-		-	-		-	-	-										-	-	
170	+	-	-	del	O157	+	+								+																
171	-	+	+	ins	O157	-	++++	-	-	-	-	-	-		-	-	-	-				-	-	-		-	-	-			
172	+	-	-	del	O157	+	++++	+	+	+	+	+	+	+	+	+	+	+				+	+	+		+	+	-			
173	+	+	-	del	EDL933	+	++++		+	+	+		+		+							+	+	+					+	+	
174	-	+	+	del	CB9615	+	++	+	+	+	+	-	-	+	-	-	-	-	-	-	-	+	+	+	+	+	+	+	+	+	
175	-	+	+	ins	O157	-	++++	-	-	-	-	-	-	-	-	-	-	-	-	-	-	-	-	-	-	-	-	-	-	-	
176	+	-	-	del	O157	+	++	+	+	+	+	-	-	-	-	-	-	-	-	-	-	+	+	+	+	+	+	+	+	+	
177	+	-	-	del	O157	+	++	+	+	+	+	+	+	-	-	+	-	-	-	-	-	+	+	+	+	+	+	+	+	+	
178	-	+	+	ins	O157	-	++++	-	-	-	-	-	-	-	-	-	-	-	-	-	-	-	-	-				-	-	-	

O55 and O157 Details								Outgroup Strain Details[e]																							
event	CB9615 block[a]	Sakai block[a]	EDL933 block[a]	type	Event Lineage[b]	Inferred ancestral[c]	Support level[d]	D1 Sd197	K-12	HS	ATCC 8739	UMN026	IAI39	SMS 3-5	E2348/69	536	ED1a	CFT073	S88	APEC O1	UTI89	E24377A	IAI1	SE11	F5 8401	F2a 2457T	F2a 301	SS Ss046	B4 Sb227	B18 BS512	Note[f]
179	-	+	+	del	CB9615	+	++++	+	+	+	+	+	+	+	+	+	+	+	+	+	+	+	+	+	+	+	+	+	+	+	
180	-	+	+	ins	O157	-	+++	-					-	-		-		-										-			
181	+	-	-	ins	CB9615	-	++++	-	-	-	-											-	-	-				-	-	-	
182	-	-	+	ins	EDL933	-	++	-	+	+	+											-	-	-				-	-	-	
183	+	-	+	del	Sakai	+	+++		+	+	+											+	+	+					+	-	
184	-	+	+	ins	O157	-	+								-																
185	+	-	-	del	O157	+	+								+																
186	+	-	-	indel	O55/O157	?	+/-																								
187	-	+	+	del	CB9615	+	++++	+	+	+	+	+	+	+	+	+	+	+	+	+	+	+	+	+	+	+	+	+	+	+	
188	+	-	-	del	O157	+	++++	+	+	+	+	+	+	+	+	+	+	+	+	+	+	+	+	+	+	+	+	+	+	+	
189	-	+	-	ins	Sakai	-	++++	-	-	-	-	-	-	-	-	-	-	-	-	-	-	-	-	-	-	-	-	-	-	-	
190	-	+	+	del	CB9615	+	++++	+	+	+	+	+	+	+	+	+	+	+	+	+	+	+	+	+	+	+	+	+	+	+	
191	+	-	-	ins	CB9615	-	++	-	-	-	-	-	-	-	-	-	-	+	-	+	-	-	-	-	-	-	-	-	-	-	
192	+	-	-	ins	CB9615	-	++	-	+	-	+	-	-	-	-	-	-	+	-	-	-	-	-	-	-	-	-	-	-	-	
193	-	+	-	ins	Sakai	-	++++	-				-	-	-	-	-	-	-	-	-	-				-	-	-	-	-	-	
194	-	+	+	ins	O157	-	++++	-	+	-	-	-	-	-	-	-	-	-	-	-	-	-	-	-	-	-	-	-	-	-	
195	-	+	+	del	CB9615	+	+		+	-	+	+	+	+	+	+	+	+	+	+	+	-	-	-	+	+	+	-	+	+	
196	+	-	-	ins	CB9615	-	++++	-	-	-	-	-		-	-				-			-	-	-	-	-	-	-	-	-	
197	-	+	+	ins	O157	-	++++	-	-	-	-	-	-	-	-	-	-	-	-	-	-	-	-		-	-	-	-	-	-	
198	-	+	+	ins	O157	-	+							-					-												
199	+	-	-	ins	CB9615	-	+							-					-												
200	-	+	+	del	CB9615	+	++++	+	+	+	+	+		+	+				+				+	+					+	+	
201	+	-	-	del	O157	+	++++	+	+	+	+	+	+	+	+	+	+	+	+	+	+	+	+	+	+	+	+	+	+	+	
202	+	-	-	ins	CB9615	-	++++	-	-	-	-	-	-	-	-	-	-	-	-	-	-	-	-	-	-	-	-	-	-	-	

[a] "+" indicates the block present, "-" indicates the block absent

[b] O157: allocated to the lineage to the ancestor of EDL933 and Sakai; Sakai/EDL933: allocated to the divergence between Sakai and EDL933 (strain not specified); O55/O157: allocated to the divergence between O55 and O157 lineages (lineage not specified).

[c] presence or absence of indel block in O55/O157 ancestor as inferred from outgroup analysis.

[d] Level of support for allocation of indel blocks as given in previous column

++++ agreement is high - 8 or more outgroup strains with expected type and at most 1 with the opposite, and D1 Sd197 agrees or site not present

+++ agreement good - 4 or more outgroup strains with expected type and at most 1 with the opposite, and D1 Sd197 agrees or site not present

++ agreement in D1 Sd197 regardless of situation with other outgroup strains

+ no conflict but very limited support as either the block and nearby sequence absent in D1 Sd197, and/or outgroup support less than given above

+/- no conflict but no support (the block and its nearby sequence not present in any outgroup OR both alternative lineages supported equally).

- not independent indel as in O-antigen related recombination event

[e] +/- indicates presence or absence of the block. Blank means site not present.

[f] Special reasons affecting allocation: "O antigen" means within major O-antigen related recombination; "part of phage" means within a phage that is inserted
